# Development of new genetic resources for faba bean (*Vicia faba* L.) breeding through the discovery of gene-based SNP markers and the construction of a high-density consensus map

**DOI:** 10.1038/s41598-020-63664-7

**Published:** 2020-04-22

**Authors:** E. Carrillo-Perdomo, A. Vidal, J. Kreplak, H. Duborjal, M. Leveugle, J. Duarte, C. Desmetz, C. Deulvot, B. Raffiot, P. Marget, N. Tayeh, J. P. Pichon, M. Falque, O. C. Martin, J. Burstin, G. Aubert

**Affiliations:** 1 Agroécologie, AgroSup Dijon, INRAE, Univ. Bourgogne, Univ. Bourgogne Franche-Comté, F-21000 Dijon, France; 2 Université Paris-Saclay, INRAE, CNRS, AgroParisTech, GQE - Le Moulon, 91190 Gif-sur-Yvette, France; 3 Biogemma, Chappes, France; 4 Terres Inovia, Thiverval-Grignon, France

**Keywords:** Agricultural genetics, Genetic linkage study, Genetic markers, Plant breeding, Plant genetics, Genetics, Sequencing

## Abstract

Faba bean (*Vicia faba* L.) is a pulse crop of high nutritional value and high importance for sustainable agriculture and soil protection. With the objective of identifying gene-based SNPs, transcriptome sequencing was performed in order to reduce faba bean genome complexity. A set of 1,819 gene-based SNP markers polymorphic in three recombinant line populations was selected to enable the construction of a high-density consensus genetic map encompassing 1,728 markers well distributed in six linkage groups and spanning 1,547.71 cM with an average inter-marker distance of 0.89 cM. Orthology-based comparison of the faba bean consensus map with legume genome assemblies highlighted synteny patterns that partly reflected the phylogenetic relationships among species. Solid blocks of macrosynteny were observed between faba bean and the most closely-related sequenced legume species such as pea, barrel medic or chickpea. Numerous blocks could also be identified in more divergent species such as common bean or cowpea. The genetic tools developed in this work can be used in association mapping, genetic diversity, linkage disequilibrium or comparative genomics and provide a backbone for map-based cloning. This will make the identification of candidate genes of interest more efficient and will accelerate marker-assisted selection (MAS) and genomic-assisted breeding (GAB) in faba bean.

## Introduction

Legume crops serve as a source of food and feed. They also play an important role in sustainable agriculture because of their ability to improve soil fertility by fixing atmospheric nitrogen and increasing crop yield when used in crop rotation with cereals or intercropping^1^. In particular, faba bean (*Vicia faba* L.; Vf) is a primary ingredient of daily meals in both developing and industrialized countries due to its high content in proteins, carbohydrates, dietary fibers and micronutrients^2,3^. It is the most yielding pulse crop after field pea. However, its yield is still about half that of wheat, indicating that great breeding efforts are still needed^4^. Faba bean yield is greatly affected by environmental conditions, especially extreme temperatures, drought and acidity^5,6^. In addition, diseases such as chocolate spot (*Botrytis fabae* S. or *B. cinerea* P.) or ascochyta blight (*Ascochyta fabae* S.), viruses such as faba bean necrotic viruses, parasitic weeds of *Orobanche* genus and pests such as leaf weevil (*Sitona lineatus* L.), aphids (*Aphis fabae* S., *A. craccivora* K., *Acyrthosiphon pisum* H., *Myzus persicae* S.) or seed weevils (*Bruchus rufimanus* B.) considerably reduce its yield and affect the commercialization of the grains^5,7^. Other factors limiting the production of faba bean include the overproduction of flowers resulting in a variable fertilization rate and abortion of ovules^2^, the need for pollinators for outcrossing and fertilization of ovules^8^ and the strong influence of symbiosis for optimal concentration of nitrogen (N) in the grain and for N soil fertility^9^.

Faba bean is a diploid outcrossing species (2n = 12) with a “giant genome”^10^ of approximately 13 Gb distributed on six chromosomes. The high content in transposable elements^11^ complexifies the faba bean genome assembly and map-based cloning. Most of the linkage maps generated so far have a low to medium saturation and are based on morphological, isozyme, restriction fragment length polymorphism (RFLP), random amplified polymorphic DNA (RAPD), sequence characterized amplified region (SCAR), intron targeted amplified polymorphism (ITAP), simple sequence repeat (SSR) and low-density single-nucleotide polymorphism (SNP) markers^12–21^. SNP-based genetic maps have been recently developed^21^. To date, the most saturated map is the one reported by Webb *et al*.^21^ consisting of 687 SNPs. This map provided a first glimpse of synteny of faba bean with other legumes such as barrel medic (*Medicago truncatula* L.; Mt), lupine (*Lupinus albus* L.), soybean (*Glycine max* (L.) M.; Gm) or lentil (*Lens culinaris* M.)^16,21^. In fact, the SNPs used to construct the consensus map of Webb *et al*.^21^ were designed based on orthologous sequences in *M. truncatula* with the objective of physically anchoring the faba bean consensus map to the Medicago genome. Webb *et al*.^21^ also took advantage of the macrosynteny between these two species to discern the level of conservation of the genetic organization between faba bean and lentil, one of its most closely related crop species. A high conservation of genomic blocks between *V. faba* and these legume species was reported.

The development of dense and robust genetic maps that involve multiple populations and gene-based markers is a prerequisite for marker-assisted selection (MAS) and paves the way to *V. faba* genome assembly. Now that pulse genomes are becoming available, it is important to implement more accurate comparative genomic approaches that will reinforce faba bean breeding programs through a faster and more efficient identification of candidate genes. Transcriptome sequencing has been intensively used in the development of SNP markers for genetic mapping and diversity panel structuration of model species and crops with large genomes^21–28^. In addition, Illumina MiSeq.^29^ has made possible the identification, location and functional characterization of genes that control traits of interest and has been used to provide a more comprehensive view of diversity and gene function in plants^30–32^. The design of SNP markers is strongly recommended for the construction of genetic linkage maps, as they stand out for their uniform distribution throughout the genome, for being numerous and for their tendency to be biallelic and codominant^33,34^. Therefore, we chose to exploit next-generation sequencing (NGS) technologies and transcriptome sequencing to specifically address the expressed gene fraction (exome) for the discovery of gene-based SNPs. The objective of our work was to develop high-resolution genetic linkage maps in three interconnected faba bean recombinant line populations and build a high-density consensus map. Our work also took advantage of macrosynteny among legume relatives to locate faba bean genomic regions conserved in different sequenced species. A comparative alignment and mapping between the faba bean consensus map developed herein and the genomes of barrel medic, birdsfoot trefoil (*Lotus japonicus* L.; Lj), chickpea (*Cicer arietinum* L.; Ca), common bean (*Phaseolus vulgaris* L., Pv), cowpea (*Vigna unguiculata* (L.) W.; Vu), pea (*Pisum sativum* L.; Ps) and soybean has been performed. The identification of highly syntenic and collinear areas between faba bean and already sequenced species will facilitate candidate gene discovery. In this way, SNP markers developed in genes resulting from the present work will be very useful in MAS and in map-based isolation of candidate genes.

## Materials and Methods

### Plant material

Three connected bi-parental mapping populations have been built using cultivar (cv.) HIVERNA as a common female parent, and the accessions NOVA GRADISKA, SILIAN, and QUASAR as male parents for Pop1, Pop2 and Pop3, respectively. The winter type cv. HIVERNA of German origin was selected as a common parent between the three populations. The parents NOVA GRADISKA (originated in Croatia) and SILIAN (originated in Northern Sudan) are both minor type landraces sown in late and early spring, respectively; while QUASAR (originated in United Kingdom) is a winter type cultivar adapted to oceanic climate (cool winters with abundant rainfall). NOVA GRADISKA and QUASAR have previously been reported as resistant to the faba bean weevil (*Bruchus* spp.) attack^35^. All segregating recombinant populations used for mapping were made of F_3_ plants produced by single seed descent (SSD) of F_2_ plants. Pop1 includes 102 F_3_ individuals, Pop2 147 F_3_ plants derived from two F_1_s and Pop3 96 F_3_ plants. Samples were collected from the leaves of an F_3_ plant for each recombinant line. Tissues were flash-frozen in liquid nitrogen and stored at −80 °C until DNA extraction for genotyping.

For SNP discovery, the parental lines were grown in a growth chamber (photoperiod of 16 h light/day, 15 °C night, 20 °C day, hygrometry 60% min) for 15 days. Samples of whole plant per parental line were collected. Tissues were flash-frozen in liquid nitrogen and stored at −80 °C until RNA extraction.

### Transcript assembly and SNP Discovery

SNP discovery was carried out as described by Duarte *et al*.^28^ with slight modifications. Total RNAs were extracted from the parental lines and checked for quality and integrity. RNAs were then converted to full-length double-stranded cDNA and normalized with the Mint-2 and Trimmer-2 kits (Evrogen, Moscow, Russia), respectively. Later, q-RT-PCR assays were developed on a set of genes of different abundances to assess the efficiency of normalization. Normalized double-stranded cDNAs were sheared into 450 bp fragment size using the Covaris E220 system (Covaris Inc., Massachusetts, USA). Individual indexed NGS libraries were then produced with the SPRIworks HT reagent kit (Beckman Coulter, Indianapolis, USA). An equimolar pool of the four libraries was sequenced on the Illumina MiSeq platform (V2 chemistry, PE 2 × 250 nt, 12 million of clusters) (Illumina, California, USA). FastQC was used to check raw data quality. Then, sequencing adaptor removal and quality trimming were performed using trim_galore and SMART oligos (normalization primers) that were masked in the raw sequence readings using an in house script. MIRA 4.0 (http://mira-assembler.sourceforge.net) software was used to perform a *de novo* assembly of all samples at once. Reads of parental genotypes were remapped on the assembly using BWA^36^ and only contigs with more than 10x coverage were kept for further analysis. Transdecoder^37^ was used to predict open reading frames in contigs. BUSCO v3.0.1^38^ was used on viridiplantae odb10 database to assess completeness of the dataset. Functional annotation was performed using eggNOG-mapper^39^ on eggNOG database 5.0^40^.

The SNP discovery was then performed with SAMtools mpileup^36^ and BCFtools call^41^. Only homozygous SNPs were retained.

### Genotyping F_3_ populations

DNA samples were extracted from leaf tissues of the parents and the individuals of the three populations using the NucleoSpin Plant II Mini kit (https://www.mn-net.com/, Hoerdt, France) following the manufacturer’s protocol. DNAs were normalized before being fragmented with Adaptive Focused Acoustics Technology (Covaris Inc., Massachusetts, USA). A 250 bp target size value was obtained by using a Covaris E220 system, according to the manufacture’s instructions. Then DNA fragments underwent a NGS library preparation procedure consisting in end repair and Illumina adaptor ligation using the KAPA HTP kit (Roche, Basel, Switzerland). Individual index sequences were added to each library for identifying reads and sorting them according to their initial origin. Two thousand SNPs were targeted to design capture probes. The Sequence Capture was done by using SeqCap EZ Developer kit from Roche according to the manufacture’s instructions. The sequence capture reaction efficiency was evaluated by measuring, with a qPCR assessment, a relative fold enrichment and loss of respectively targeted and non-targeted regions before and after the sequence capture reaction.

The captured samples were sequenced on HiSeq sequencing platform (Illumina, California, USA) with a Paired End sequencing strategy of 2 reads of 100 bases. The objective was to produce around 3 Million sequencing clusters per sample.

Raw reads were trimmed for adaptor sequence using cutadapt 1.8.3^42^ and then aligned on the targeted regions with Novoalign (http://www.novocraft.com, Selangor, Malaysia). Genotype at each position of interest was determined using SAMtools mpileup^36^ and in house Perl scripts to filter out low quality positions, call the SNPs, and to produce a genotyping matrix for all 2000 selected markers.

### Genetic maps construction for each population

Markers were first filtered for deviation from Mendelian segregation (37.5:25:37.5) using the following index $Distortion=\frac{1}{2}\cdot \frac{{\sum }_{i=1}^{{N}_{genotypicclasses}}|{f}_{i,observed}-{f}_{i,expected}|}{1-min({f}_{expected})}$ with threshold of 0.8. Then, markers were assigned to linkage groups by estimating the pairwise recombination frequencies within each population using the maximum likelihood procedure with the forward-backward algorithm and a LOD score of 5.0 as the threshold for significant linkage in the software JoinMap V5.0^43^. In a second stage, markers were ordered and assigned to their positions in each linkage group by likelihood maximization. Candidate orders’ likelihoods were computed as for F_2_ populations using Spell-QTL Bayesian inference^44^ and heuristic local maximization was performed using custom R scripts implementing the algorithm described earlier in Ganal *et al*.^45^. This heuristic was based on the serial inference of marker orders, proceeding according to the following steps. First, 10 “seed” markers were randomly chosen in each chromosome, and one statistically robust scaffold map replicate was constructed from each seed marker, by iteratively choosing the most strongly linked neighbour with at least 10 cM between adjacent markers. Then given these scaffold maps, marker density was increased to produce framework maps containing as many markers as possible while keeping a LOD score >3.0 for the robustness of marker orders. Finally, the complete maps were obtained by placement of additional markers using bin-mapping^45^. As a post-processing step, it was necessary to calculate the distance between pairs of markers taking into account that the recombination rates estimated by the algorithm assumed that the populations were F_2_ whereas they were in fact F_3_. For that, we first determined the correspondence between *r*F_2_, the recombination rate estimated assuming an F_2_ population, and the true recombination rate, *r*. That correspondence was obtained in two steps. In the first step we used the explicit formula relating *r*F_2_ to the two-locus genotype frequencies *f*AaBb*, f*AaBB *= f*AABb, *f*AABB *= f*aabb, and *f*AAbb*=f*aaBB where the two parental types are denoted A (respectively a) and B (respectively b) and the labels refer to unphased genotypes. Then in the second step we inferred *r* from those 7 frequencies by maximum likelihood using the formulas for those frequencies in F3 as a function of *r*. Finally, centiMorgan (cM) distances were calculated using Haldane’s mapping function^46^.

### Consensus genetic map construction

SNP sequences anchored to the sequences of the SNPs previously mapped by Webb *et al*.^21^ were used to assign each linkage group (defined by the initial seed used in the map construction) to one chromosome number. For each chromosome, we produce a consensus map that summarizes the recombination information within the three populations. Our method is quite general and in particular it does not assume collinearity of the individual maps. Its key feature is the minimization of an index *I*_*D*_ which measures the differences between the (unknown) consensus map M* and all of the individual maps *M1, M2*. We forced our consensus map to include all the markers present in the individual maps. M* is to be specified by assigning a genetic position for each marker and of course that will also define the marker ordering in M*. Without loss of generality, the genetic position of the first marker of M* can be considered as the origin of genetic coordinates. The task is then to estimate the genetic positions of the rest of the markers.

In our framework based on the minimization of the index *I*_*D*_, we first define a “distance” between two maps via the formula:

$${D}_{(M,M{\prime} )}=\sum _{i}\sum _{j(i)}{({d}_{({M}_{(i,j)})}-{d}_{({M{\prime} }_{(i,j)})})}^{2}$$

where *i* labels the markers that are common in the two maps and *j(i)* labels the markers that are not only in common in the two maps but also meet a criterion for their distance to marker *i*. Specifically, if marker *j(i)* is quite far from marker *i*, that corresponding interval does not provide much information, so it is better to exclude it from the sum. In general, there are many of these markers and thus it is also computationally efficient to exclude them. Inversely, if a marker *j(i)* is very close to *i*, the corresponding distance is often not very well determined and so again it is better to exclude it. Therefore, our criterion imposes both a minimum and maximum distance between *j(i)* and *i*. In addition, to avoid having many *j(i)* markers for some *i* and only a few for others, we keep only a subset of the possible markers *j(i)* for a given *i*. As a result, all the *i*s are treated on an equal footing and have the same importance in *D(M, M*′). Given this definition for the distance *D(M, M*′) between two arbitrary maps, we numerically search for the positions of the markers in M* to minimize the index

$${I}_{D}=\sum _{n}{D}_{({M}^{\ast },{M}_{n})}$$

where *n* runs through all the individual maps from which the consensus is being built. If the maps have been determined using very different population sizes, we weight each term of this sum by the corresponding population size. In this way, if a map has a much smaller population than the other maps, it will have little influence on the construction of M* which is justified since its marker positions are not very accurate. On the contrary, if a map has a much larger population than the other maps, it will have a strong influence on the construction of M*, which is again justified since its marker positions are rather precisely determined.

### Synteny with other legume crops

The flanking sequences of the SNP markers placed in the consensus map were searched against *C. arietinum* (v1)^47^ (https://www.ncbi.nlm.nih.gov/assembly/GCF_000331145.1/), *P. sativum* (v.1)^48^ (https://urgi.versailles.inra.fr/Species/Pisum/Pea-Genome-project), *M. truncatula* (Mt5.0)^49^ (https://medicago.toulouse.inra.fr/MtrunA17r5.0-ANR/), *L. japonicus* (v 2.5)^50^ (http://www.kazusa.or.jp/lotus/), *G. max* (v2.0)^51^ (http://www.plantgdb.org/GmGDB/), *Phaseolus vulgaris* (v2.1)^52^ (https://phytozome.jgi.doe.gov/pz/portal.html#!info?alias=Org_Pvulgaris) and *Vigna unguiculata* (v1.1)^53^ (https://phytozome.jgi.doe.gov/pz/portal.html#!info?alias=Org_Vunguiculata_er) genomes assemblies using the BLASTn function in order to identify the corresponding orthologous genes and their position in those genomes.

## Results

### Transcriptome assembly and gene annotation

A total of 1.1 million RNA-seq reads from 4 faba bean accessions, i.e. HIVERNA, NOVA GRADISKA, SILIAN and QUASAR, were assembled into 164,529 contigs (Table S1). After filtering on coverage (see Materials and Methods section), a transcriptome resource of 39,423 high-quality contigs with a N50 of 1460 bp (Table S2) and a BUSCO completeness score of 84.8% was built (Table S3). Functional annotation was obtained for 24,507 contigs (Fig. S1). Each contig of the unigene set was assigned, if possible, to the categories of biological processes, molecular functions and cellular components (Fig. S1). Among the biological processes, the metabolism of nucleobase, nucleoside, nucleotide and nucleic acid (10.26%), biosynthesis (9.67%) and cell organization and biogenesis (8.02%) were the main classes represented (Fig. S1A). Catalytic (36.49%) and transferase (13.17%) activities contributed in greater proportion to the category of molecular function (Fig. S1B). The cytoplasmic (31.68%) and nucleus (15.91%) cellular components were the most represented classes within the annotation (Fig. S1C).

### SNP discovery, selection, genotyping and individual genetic linkage of the three F3 populations

A total of 105,828 homozygous SNPs (Fig. S2) were detected on 19,190 contigs (5.5 SNP/contig) with an average coverage of 47.7×. In total, 64.77% of the SNPs were transitions while 35.23% were transversions. Out of these robust SNPs, 2,000 were selected based on the following parameters: SNP quality score, polymorphism in more than one population (see Materials and Methods section), potential synteny with pea and *Medicago truncatula* and 1 SNP maximum per contig. Capture probes were then designed to allow large-scale targeted genotyping. Of the 2,000 gene-based SNP markers selected above, 1,911 markers were successfully scored on the progenies of three F_3_ inbred populations, *i.e*., Pop1-3 (Table S4). Of them, 95.2% (1,819 SNPs) were polymorphic in at least one population. Altogether, 1,446 SNPs were polymorphic in Pop1, 1,499 in Pop2 and 1,409 in Pop3 (Table S4).

### Linkage mapping of the three F3 populations

**Figure 1:**
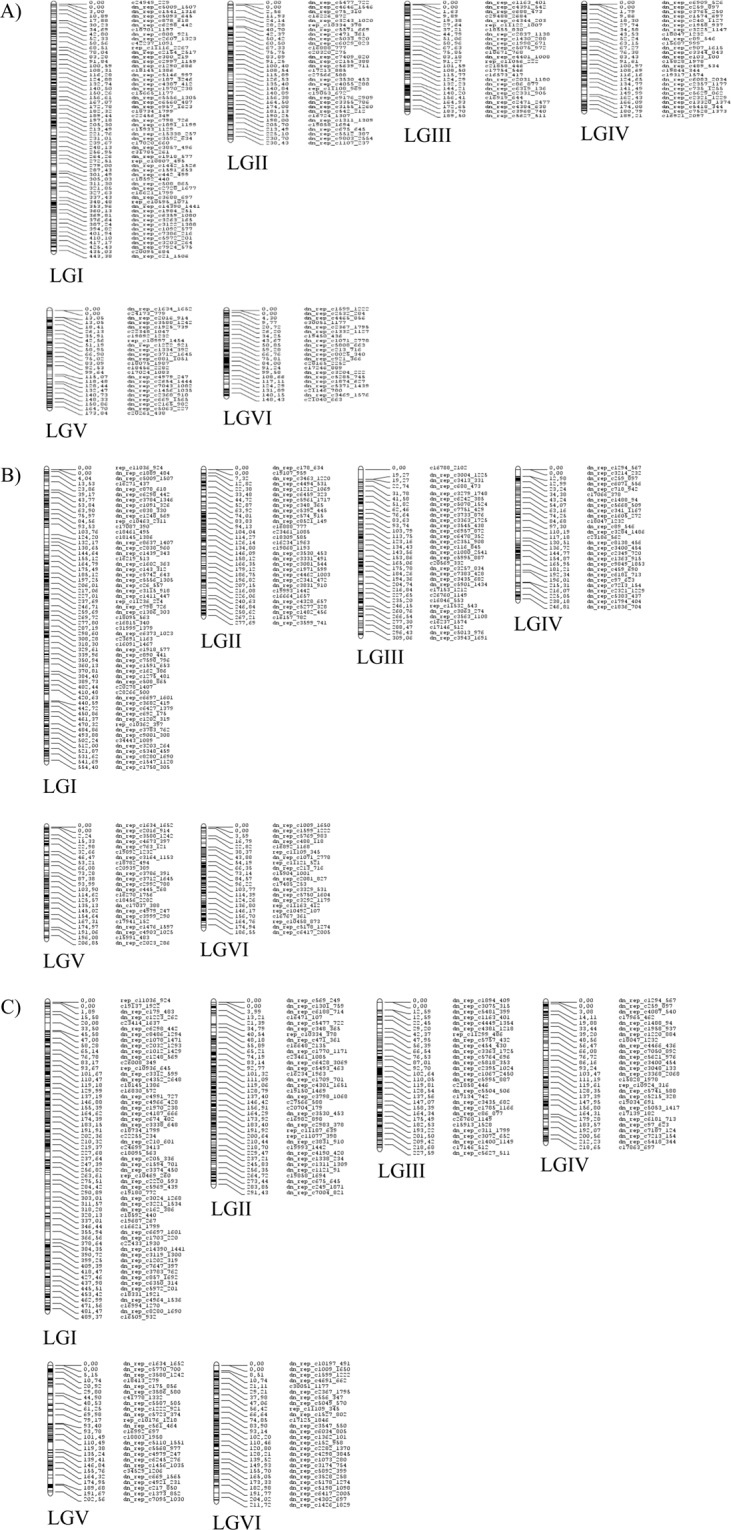
Faba bean individual genetic linkage maps constructed from three populations of (**A**) 102 F_3_ recombinant lines derived from the cross between *Vicia faba* cv. Nova Gradiska and Hiverna; (**B**) 147 F3 derived from the cross between cv. Silian and Hiverna and **(C**) 96 F_3_ derived from the cross between cv. Quasar and Hiverna.

**Figure 2:**
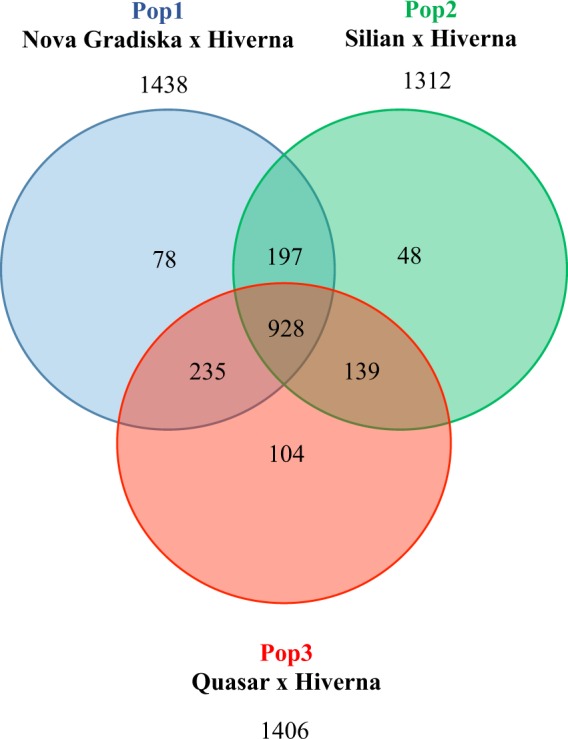
Venn diagram showing the SNP markers shared between the individual genetic maps of the three populations presented in this study.

**Figure 3:**
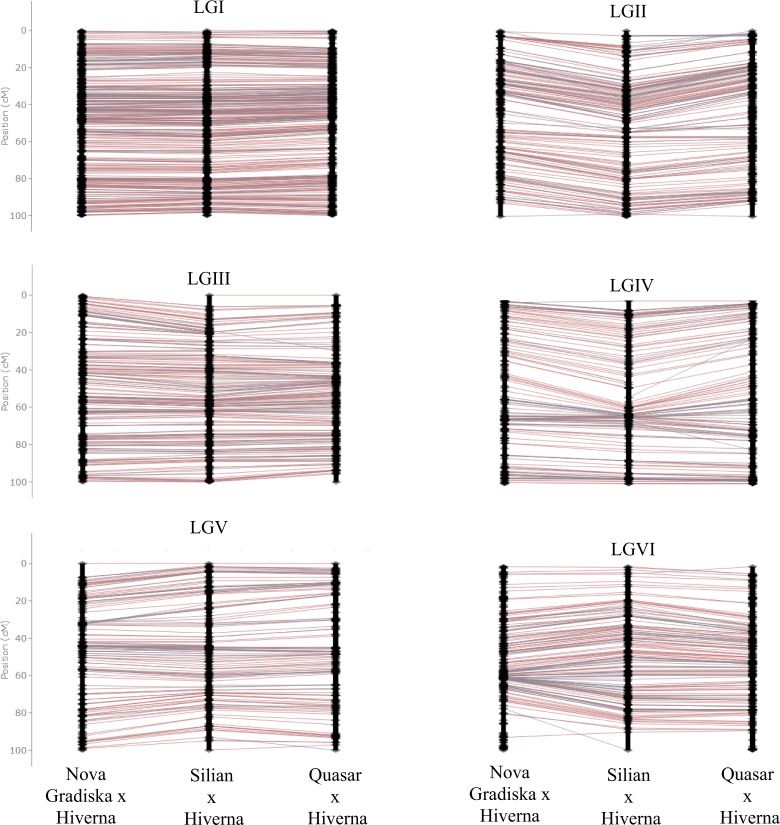
Order comparison of the marker assignments by linkage group between the individual linkage maps of the threee recombinant populations presented in this study. Marker positions are normalized.

Individual genetic maps were constructed for the three F_3_ populations after filtering markers for distortion and missing data (see Materials and Methods section). Two hundred thirty-three (Pop1), 189 (Pop2) and 175 (Pop3) SNP markers were placed on the individual scaffold maps (Fig. S3). The framework maps included 350 (Pop1), 209 (Pop2) and 326 (Pop3) markers (Fig. S4). The full maps had 1,438 markers for Pop1, 1,312 markers for Pop2 and 1,406 markers for Pop3 that covered all six faba bean LGs (Table 1; Figs. 1, 2 and S5, Tables S5–S7). The number of markers per LG ranged between 141 (Pop3, LGV) and 401 (Pop1, LGI) SNP markers (Table 1). Total map lengths were: 1,426 (Pop1), 1,832 (Pop2) and 1,697 (Pop3) cM. The density of markers was high for all LGs in the three populations. Pop1 had an average marker density of 1.01 markers per cM, while in Pop2 this density was 0.72 markers/cM and in Pop3 it was 0.83 markers/cM. The average gap size between pairs of non-colocalized markers was 1.16 cM in Pop1, 1.47 cM in Pop2 and 1.36 cM in Pop3 (Table 1). Only a few large gaps (>10 cM) were observed: three gaps on the linkage map for Pop1 (LGII and LGV), nine gaps on the map from Pop2 (LGI, LGIII, LGIV, LGV and LGVI) and eight gaps on the map from Pop3 (LGI, LGII, LGIII and LGV) (Table 1). Nine hundred twenty-eight markers were common to the three populations (Fig. 2). One thousand five hundred and four SNPs were mapped onto at least two of the three linkage maps. Pairwise comparisons of the positions and the orders of common markers were performed among the three populations to assess synteny and collinearity (Fig. 3). The positions of the marker were consistent in the LGs of the three populations. High positive correlations (Spearman test) between map orders were obtained: Pop1-Pop2, r = 0.98; Pop1-Pop3, r = 0.99 and Pop2-Pop3, r = 0.98; P < 0.001).

**Table 1:** Map features of the three individual genetic linkage maps and the consensus map of faba bean presented in this study.

Map	LG	Markers	Distance (cM)	Unique positions	Average marker density (marker/cM)	Average gap size (cM)	Gaps >10 cM	Biggest gap size (cM)
**Pop1**^**a**^								
1		401	444.99	360	0.90	1.24	0	9.51
2		228	262.44	191	0.87	1.38	2	18.94
3		226	194.59	199	1.16	0.98	0	7.90
4		180	192.88	150	0.93	1.29	0	6.92
5		164	176.20	136	0.93	1.31	1	13.05
6		239	154.81	211	1.54	0.74	0	7.26
*Total*		1438	1425.91	1247	1.01	1.16	3	18.94
**Pop2**^**b**^								
1		376	555.35	367	0.68	1.52	2	13.66
2		219	278.87	204	0.79	1.37	0	7.80
3		231	315.56	221	0.73	1.43	1	19.27
4		168	247.25	153	0.68	1.62	2	12.98
5		152	222.56	147	0.68	1.52	2	11.20
6		166	212.34	160	0.78	1.34	1	20.78
*Total*		1312	1831.93	1252	0.72	1.47	8	20.78
**Pop3**^**c**^								
1		384	493.68	350	0.78	1.41	2	12.08
2		275	312.25	257	0.88	1.22	2	20.82
3		223	242.73	201	0.92	1.21	2	12.59
4		168	223.30	147	0.75	1.53	0	8.50
5		141	208.45	126	0.68	1.67	2	12.45
6		215	216.13	190	0.99	1.14	0	8.51
*Total*		1406	1696.53	1271	0.83	1.36	8	20.82
**Consensus**								
1		467	481.74	375	0.97	1.29	3	18.44
2		334	252.46	219	1.32	1.16	2	17.05
3		271	216.51	231	1.20	0.88	3	11.03
4		220	204.06	168	1.08	1.22	0	13.65
5		183	196.69	141	0.93	1.4	0	6.98
6		253	168.94	156	1.35	1.09	1	20.51
*Total*		1728	1520.40	1290	1.12	1.17	9	20.51

^a^Recombinant population derived from the cross Nova Gradiska × Hiverna.

^b^Recombinant population derived from the cross Silian × Hiverna.

^c^Recombinant population derived from the cross Quasar × Hiverna.

### Segregation distortion of the individual maps

There was a significant distortion of segregation (χ^2^ test, *P* < *0.05*) with respect to the expected Mendelian segregation ratio (37.5:25:37.5) for a minority of markers in the three populations (5.57% in Pop1, 9.14% in Pop2, 8.52% in Pop3). Only a few markers exceeded the index distortion threshold of 0.8 used for mapping (see Materials and Methods section).

**Figure 4:**
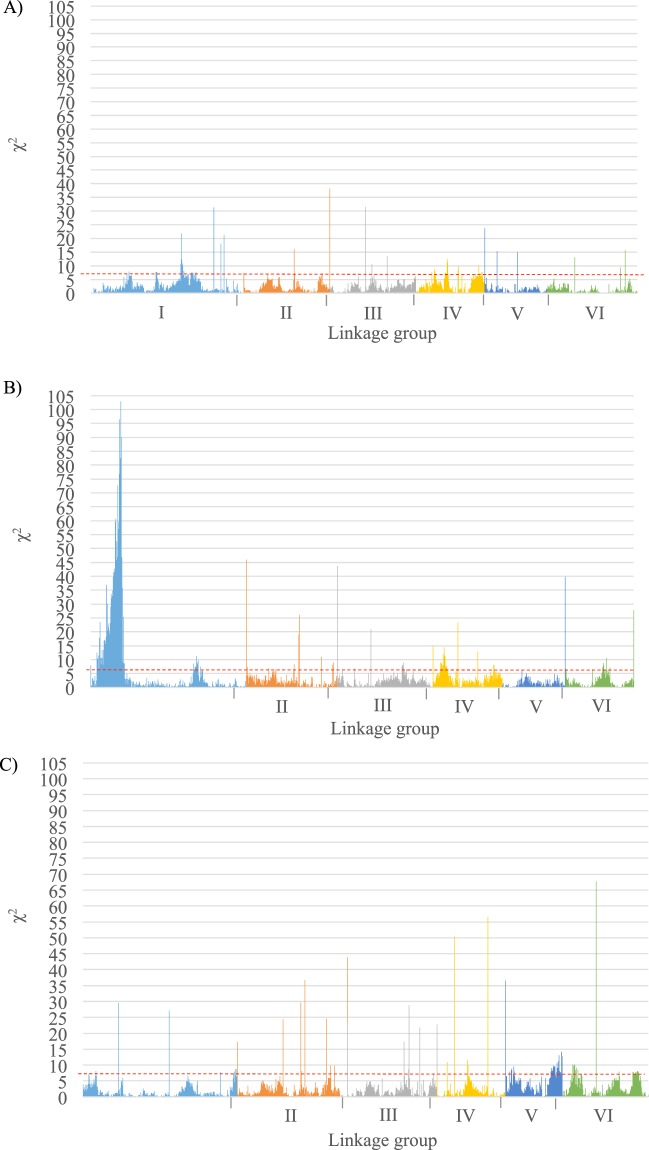
Distribution of the segregation distortion (χ^2^ test, *P = 0,05*) of the SNPs used to contruct the three faba bean individual maps in the recombinant populations studied throughout the six linkage groups. (**A**) Pop1; (**B)** Pop2 and; (**C**) Pop3. *Red dashed line* indicates the threshold above which segregation distorsion is significant.

Pop2 presented a region with segregation distortion towards SILIAN alleles at the bottom of LGI (Figs. 4 and S6). Although with less intensity, LGI of Pop1 also displayed a region in which segregation favoured the alleles of the male parent NOVA GRADISKA (Figs. 4 and S6). Outside of this region, the segregation distortion slightly favoured NOVA GRADISKA alleles in Pop1 (except in LGIII where HIVERNA alleles were over-represented) and SILIAN alleles in Pop2 (except in LGVI where HIVERNA alleles were favoured) while in Pop3 HIVERNA alleles were favoured (except in LGIII and LGIV where QUASAR alleles were more frequent) (Fig. S6).

### Integration of the individual maps into a consensus map of faba bean

**Figure 5:**
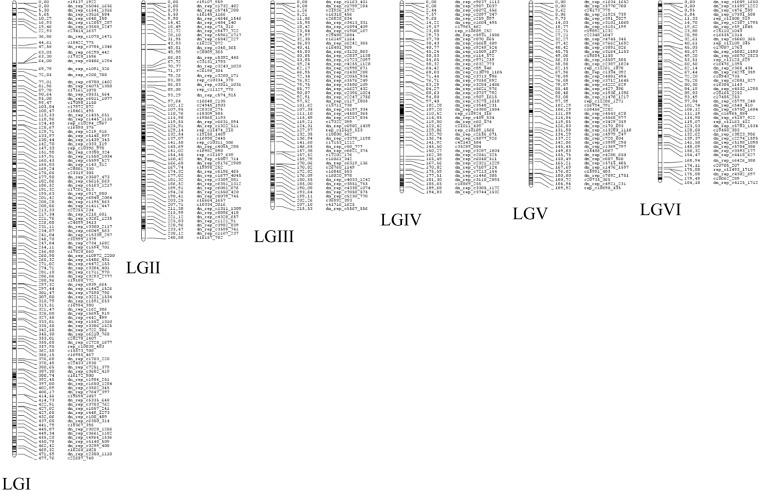
Faba bean consensus genetic linkage map generated after the integration of three individual maps originated from three populations consisting of (**A**) 102 F_3_ recombinant lines derived from the cross between *Vicia faba* cv. Nova Gradiska and Hiverna; (**B**) 147 F_3_ derived from the cross between cv. Silian and Hiverna and (**C**) 96 F3 derived from the cross between cv. Quasar and Hiverna.

**Figure 6:**
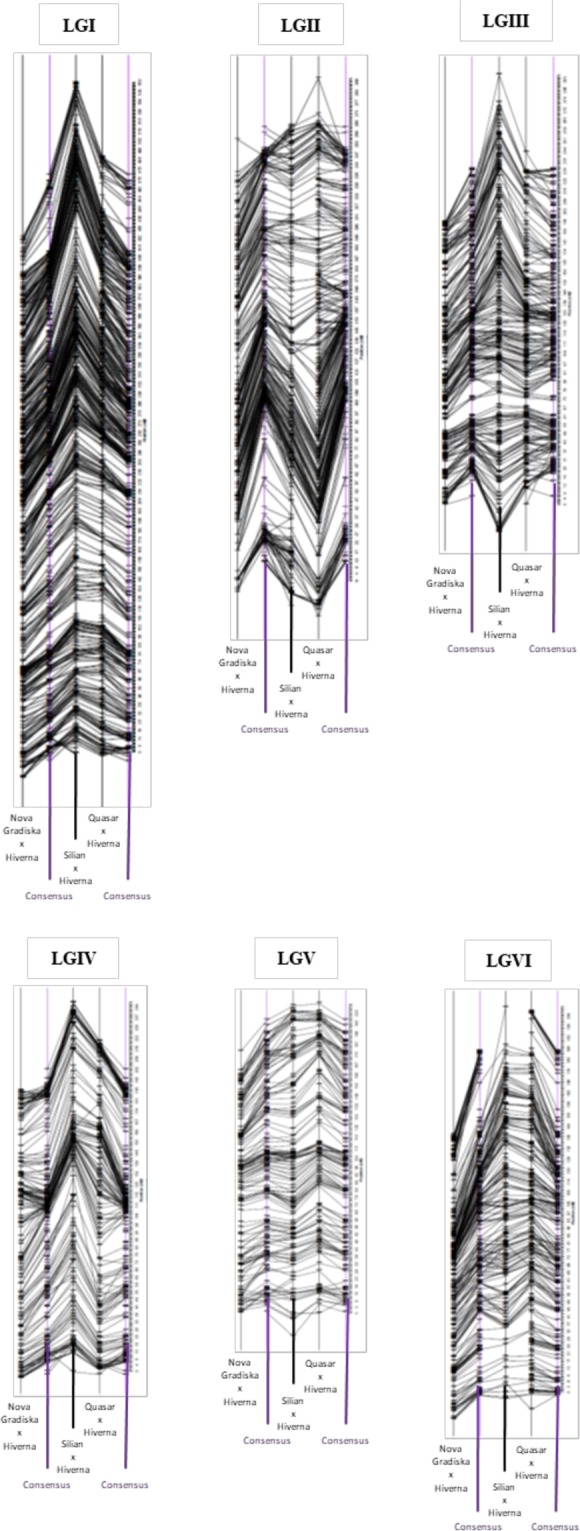
Comparison of the marker assignments by position between the individual maps of the three populations and the consensus map.

General collinearity among the individual genetic linkage maps (Fig. 3) helped to construct a dense consensus map of 1,547.71 cM that included 1,728 markers (95% of the polymorphic SNPs) (Table 1, Fig. 5, Table S8). The number of markers per LG varied from a minimum of 153 (LGV) to a maximum of 375 (LGI) SNPs (Table 1). The density of markers in this consensus map was high in all LGs, with an average density of 1.12 markers/cM. The average distance between two markers was 1.17 cM (Table 1). Nonetheless, 9 intervals were found with a distance between two markers greater than 10 cM, the largest gap being 21.51 cM (Table 1). The position and order of markers on the individual and consensus maps was overall conserved (r = 0.99 for Pop1-consensus, Pop2-consensus Pop3-consensus, P < 0.001) (Figs. 6 and S7). A few local inversions of the order of markers were observed (Fig. S7).

Comparison between the faba bean consensus map SNP marker sequences reported here and those presented in the Webb *et al*.^21^ consensus map using BLASTn search highlighted markers located in the same genes. Eighty-eight common markers (e.g. markers corresponding to the same *Medicago truncatula* gene sequence) were found. The distribution and position of these common markers between both maps showed highly conserved collinearity between them in the six LGs, although a few marker inversions were also observed (Fig. S8), which is another evidence of the reliability of the data presented here.

### Macrosynteny between the faba bean consensus map and the genomes of related legume species

**Figure 7:**
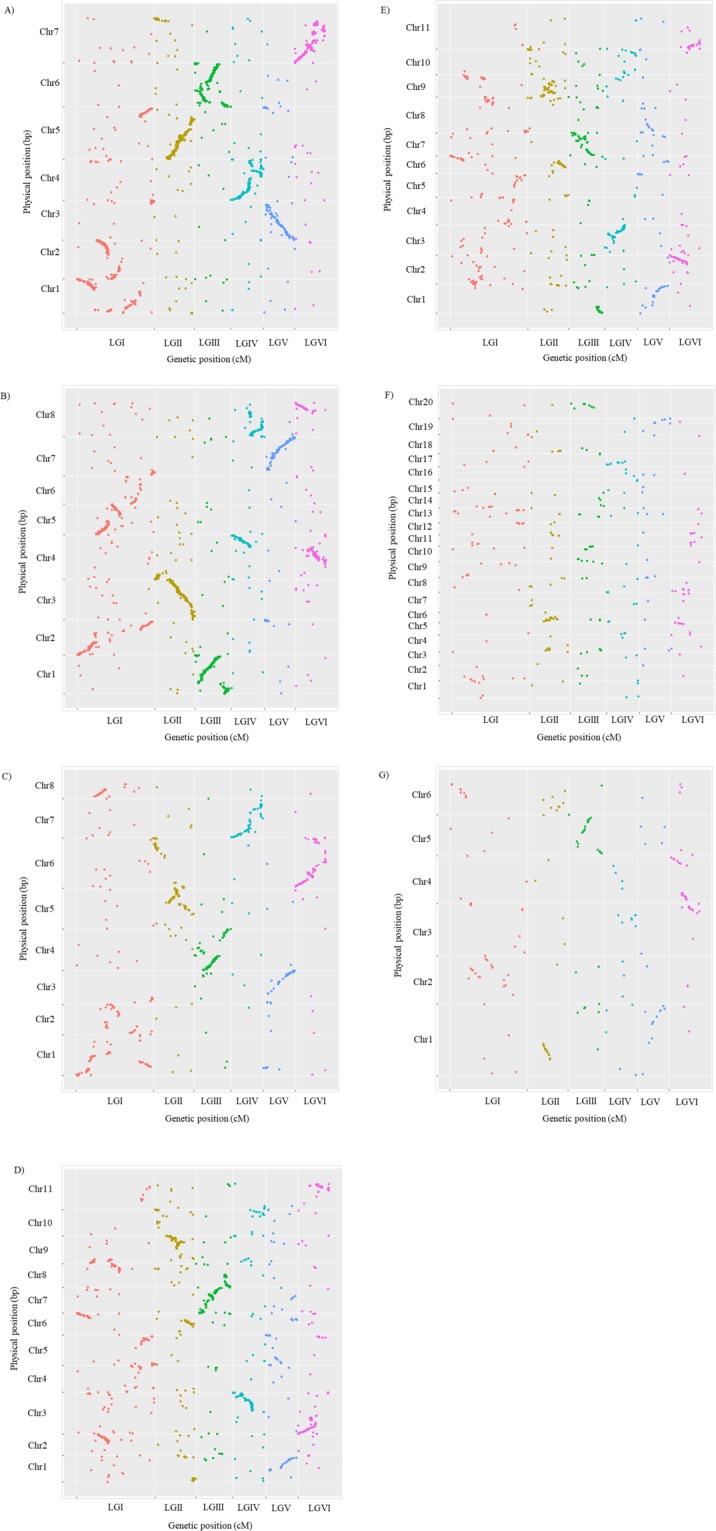
Dot-plots for synteny and collinearity comparisons between the faba bean consensus map (cM) and the genomes (bp) of (**A**) *Pisum sativum*; (**B**) *Medicago truncatula*; (**C**) *Cicer arietinum*; (**D**) *Vigna unguiculata* (**E**) *Phaseolus vulgaris;* (**F**) *Glycine max* and (**G**) *Lotus japonicas*.

Syntenic and collinear relationships between the faba bean consensus map presented in this study and the genomes of related legume species are summarized in Fig. 7. As expected, the degree of synteny and collinearity between faba bean and the legume species compared here increased when the phylogenetic distance decreased and vice versa. The genomes of *P. sativum*, *C. arietinum* and *M. truncatula* showed high levels of macrosynteny with our consensus map (Fig. 7). The best blast hits on the *M. truncatula* and *P. sativum* genomes for the faba bean markers’ flanking sequences are described in Table S4, including their annotations and positions. There was a high conservation of synteny and collinearity between LGs II, IV, V and VI of faba bean and PsChr5, PsChr4, PsChr3 and PsChr7 of *P. sativum* and LGs II, III, IV and V of faba bean and MtChr3, MtChr1, MtChr4 and MtChr7 of *M. truncatula*, respectively (Fig. 7). LGs III, IV, V and VI of faba bean turned out to be almost completely collinear to the chromosomes CaChr4, CaChr7, CaChr3 and CaChr6 of *C. arietinum*, respectively (Fig. 7). However, syntenic regions were associated with several chromosomes of the sequenced legumes in the rest of the LGs of faba bean. The most striking case was that of LGI, which was the longest LG. Syntenic blocks corresponding to *V. faba* LGI were found in CaChr1, CaChr2 and CaChr8, in PsChr1, PsChr2 and PsChr5, and also in MtChr2 and MtChr5 (Fig. 7). Substantial collinear blocks were also found between *V. faba* and *P. vulgaris, V. unguiculata* and *L. japonicus*, despite their greater phylogenetic distance from faba bean (Fig. 7). The same was observed between faba bean and *L. japonicus*: VfLGI-LjChr2 and -LjChr6, VfLGII-LjChr1, VfLGIII-LjChr5, VfLGV-LjChr1, VfLGVI-LjChr3 and -LjChr4; faba bean and *P. vulgaris*: VfLGI-PvChr6, VfLGII-PvChr6 and -PvChr9, VfLGIII-PvChr7, VfLGIV-PvChr3, VfLGV-PvChr1 and -PvChr8, VfLGVI-PvChr2 and -PvChr11; faba bean and *V. unguiculata*: VfLGI-VuChr2, -VuChr7 and -VuCVhr9, VfLGII-VuChr6 and -VuChr9, VfLGIII-VuChr7 and -VuChr8, VfLGIV-VuChr3, VfLGV-VuChr1 and VfLGVI-VuChr3 (Fig. 7). In the case of soybean, conservation patterns can be intuited between VfLGI-GmChr13, VfLGII-GmChr4 and -GmChr6, VfLGIII-GmChr10, -GmChr14 and -GmChr20, VfLGIV-GmChr17, VfLGV-GmChr19 and VfLGVI-GmChr5, -GmChr8, -Gm12 and -Gm13 (Fig. 7).

## Discussion

### New faba bean genetic resources: transcriptome, gene-based SNPs, gene-based SNP markers and a consensus map

Europe suffers a significant deficit of plant proteins that makes it necessary to import up to 70% of the plant-based proteins consumed^54^. Grain legumes including faba bean are good candidates to boost EU plant protein production due to the high protein content of their seeds. Despite this potential and the important environmental services related to grain legume production, these crops represent only 3–4% of the arable land. The low investment in breeding programs has limited the development of stable high-yielding varieties, resistant to biotic and abiotic stresses^55^. The future of the faba bean crop depends on efficient breeding programs including MAS and/or genomic-assisted breeding (GAB), in which the development of improved varieties is accelerated. For this, new faba bean genetic resources are needed. Fortunately, the progress and cheapening of NGS and assembly technologies in recent years are allowing the development of new genetic resources. The suitability and effectiveness of the transcriptome sequencing approach for the generation of gene-based SNP markers resulted in a transcriptomic resource of 39,423 faba bean transcripts obtained after *de novo* assembly (File S1), of which 24,507 contigs were annotated. Although this amount is lower than that of other studies^56,57^, it is a sufficiently high number for the discovery of robust SNP markers well distributed throughout the faba bean genome. These data are also available for use in transcriptome comparisons with other faba bean genotypes and between faba bean and other species. In addition, the faba bean transcriptome was used to identify 105,828 gene-based SNPs (File S2). The present work makes this new source of SNPs available to the faba bean community, who can develop additional SNP markers useful in other genetic backgrounds. Two thousand non-redundant *loci* (Table S4) from the 105,828 SNPs were selected to develop the molecular markers for genotyping 245 recombinant lines from three populations and the four parental lines that originated them. One thousand nine hundred eleven SNP markers (95.5%) were validated after a successful genotyping (Table S4). Our set of 1,819 polymorphic gene-based SNPs is a valuable tool for the faba bean community and particularly for breeders. Since the markers were designed in genes, they are highly informative and allow establishing syntenic relationships with other species^32,58,59^. The high quality of this set of markers was confirmed after the construction of three individual genetic maps derived from three different populations.

High collinearity between individual maps led to the construction of the densest faba bean consensus map known to date. The map includes 1,728 well-distributed markers along six LGs that correspond to the six faba bean chromosomes and covers 1,547.71 cM with a dense marker placement (0.89 cM between adjacent pairs of markers on average) (Fig. 5). In accordance with previous cytogenetic studies LGI, corresponding to chromosome 1, was the largest linkage group^60,61^. The total map size is consistent with the 1,403.8 cM size of the consensus map previously published by Webb *et al*.^21^. Despite having different genetic backgrounds, both consensus maps showed a good collinearity (Fig. S8), which confirming the good quality of both maps. Consensus gene-based genetic linkage maps are useful in meta-QTL analysis, phylogenetic and comparative genomic studies, map-based cloning and GAB, especially in the absence of a genome sequence. The phenotypic characteristics of the parents of the recombinant lines make these populations and maps useful resources. The next steps in our research will be to perform Quantitative Trait Locus (QTL) analyses to identify potential candidate genes for resistance to faba bean seed weevils in Pop1 and Pop3. In a previous work^35^, we reported that the male parents NOVA GRADISKA and QUASAR present partial resistance to the attack of bruchids. Differences in parental responses to bruchids attacks suggested distinct resistance mechanisms in the two accessions. This would be of great advantage in breeding since different genes could be pyramided and introgressed simultaneously in cultivars, making the resistance to faba bean weevils more durable and contributing to agriculture with less need for pesticides. In the case of Pop2, a distorted region was located on the top of LGI (Figs. 4 and S6). Knowledge about this area is of great importance for MAS because the genes located in this part of LGI of Pop2 seem to segregate together in favour of the genetic background of SILIAN. If favourable alleles of a gene of interest were located in this area of the LGI but at the same time there were nearby genes that carried unfavourable alleles, most likely, they would all segregate together. Thus, the introgression of favourable agronomic traits will be quite difficult in such a situation. Other previously published faba bean maps have also noted the presence of distorted regions throughout the different linkage groups^62–64^.

### Syntenic regions shared between faba bean and other legumes will facilitate future comparative genomic studies

Exploitation of the syntenic relationships between the faba bean consensus map developed in this work and available legume genomes will make the identification of candidate genes of important traits easier in the future and will enable forthcoming synteny-based gene cloning approaches. As expected, robust macrosyntenic blocks that sometimes nearly cover a complete chromosome were found between faba bean LGs and pea, barrel medic and chickpea chromosomes since their phylogenetic proximity is greater than that of the rest of the compared sequenced legumes (Fig. 7). In accordance with our results, Webb *et al*.^21^ also reported good levels of synteny between their consensus map, the genome of *M. truncatula* and the genetic map of lentil developed by Sharpe *et al*.^27^. In addition, we have been able to locate abundant blocks of macrosynteny between faba bean and common bean, cowpea or birdsfoot trefoil despite their greater evolutionary distances (Fig. 7), providing further evidence of the mapping accuracy. By contrast, the number of markers in our consensus map may not be enough to clarify the macrosynteny between faba bean and soybean due to the extensive chromosomal rearrangements and polyploidization of the soybean genome. Hopefully, gene conservation with soybean will be more evident once the faba bean genome is available, as has happened in the case of the pea^48^. Although duplication of the soybean genome and chromosomal rearrangement are a limitation for translational genomics with faba bean, synteny in duplicate regions would be a good resource to exploit. Despite the large size of the faba bean genome, synteny data reflects a globally conserved organization with respect to the genome of the legumes studied here. There is of course a certain amount of reorganization that can be easily observed, for example, in the condensed LGI of faba bean that gathers the genes located on chromosomes 1, 2 and 5 of pea. These results include *V. faba* as an additional syntenic species in the paleogenomic scheme described in Kreplak *et al*.^48^.

In conclusion, this work provides to faba bean researchers and breeders a new faba bean exome assembly originated from transcriptome data of four accessions (HIVERNA, NOVA GRADISKA, SILIAN and QUASAR), a set of 105,828 gene-based SNPs and 1,819 mapped SNP markers on three individual linkage maps and one consensus map. The high quality of the assembly resulted in the identification of a large number of SNPs of the most informative type due to their location in genes. The molecular markers designed from this set of SNPs were validated in three recombinant populations, resulting in the densest faba bean consensus map to date. The SNP markers designed here are available for genotyping other inbred populations that could be integrated later into our consensus map. These robust resources will be useful for trait mapping, genetic diversity and linkage disequilibrium studies or map-based cloning, and will enable faba bean MAS and GAB as well as the identification of candidate genes of agronomic interest through synteny-based approaches.

## Supplementary information

Supplementary Information.

Supplementary Information 1.

Supplementary Information 2.

Supplementary Information 3.

## References

[1] Stagnari, F., Maggio, A., Galieni, A. & Pisante, M. Multiple benefits of legumes for agriculture sustainability: an overview. *Chemical and Biological Technologies in Agriculture* **4** (2017).

[2] O’Sullivan DM, Angra D (2016). Advances in Faba Bean Genetics and Genomics. Front. Genet..

[3] Mulualem T, Dessalegn T, Dessalegn Y (2012). Participatory varietal selection of faba bean (Vicia faba L.) for yield and yield components in Dabat district, Ethiopia. Wudpecker. J. Agric. Res.

[4] Food and Agriculture Organization of the United Nations (FAO). FAOSTAT. Available at, http://www.fao.org/faostat (2017).

[5] Kharrat M, Le Guen J, Tivoli B (2006). Genetics of resistance to 3 isolates of Ascochyta fabae on Faba bean (Vicia faba L.) in controlled conditions. Euphytica.

[6] Cernay C, Ben-Ari T, Pelzer E, Meynard J-M, Makowski D (2015). Estimating variability in grain legume yields across Europe and the Americas. Sci. Rep..

[7] Maalouf, F. *et al*. Development of faba bean productivity and production in the Nile Valley, Red Sea and Sub-Saharan region. (2009).

[8] Nayak GK (2015). Interactive effect of floral abundance and semi-natural habitats on pollinators in field beans (Vicia faba). Agric. Ecosyst. Environ..

[9] Denton MD, Pearce DJ, Peoples MB (2013). Nitrogen contributions from faba bean (Vicia faba L.) reliant on soil rhizobia or inoculation. Plant Soil.

[10] Cooper JW (2017). Enhancing faba bean (Vicia faba L.) genome resources. J. Exp. Bot..

[11] Negruk V (2013). Mitochondrial Genome Sequence of the Legume Vicia faba. Front. Plant Sci..

[12] Patto MCV, Torres AM, Koblizkova A, Macas J, Cubero JI (1999). Development of a genetic composite map of Vicia faba using F 2 populations derived from trisomic plants. TAG. Theor. Appl. Genet..

[13] Román B, Torres AM, Rubiales D, Cubero JI, Satovic Z (2002). Mapping of quantitative trait loci controlling broomrape (Orobanche crenata Forsk.) resistance in faba bean (Vicia faba L.). Genome.

[14] Avila CM (2004). Isolate and organ-specific QTLs for ascochyta blight resistance in faba bean (Vicia faba L). TAG. Theor. Appl. Genet..

[15] Gutierrez MV (2005). Cross-species amplification of Medicago truncatula microsatellites across three major pulse crops. Theor. Appl. Genet..

[16] Ellwood, S. R. *et al*. Construction of a comparative genetic map in faba bean (Vicia faba L.); conservation of genome structure with Lens culinaris. BMC Genomics **9**, (2008). 10.1186/1471-2164-9-380 PMC2533332 18691425

[17] Zeid M (2009). Simple sequence repeats (SSRs) in faba bean: new loci from Orobanche -resistant cultivar ‘Giza 402’. Plant Breed..

[18] Díaz-Ruiz R (2009). Confirmation of QTLs controlling Ascochyta fabae resistance in different generations of faba bean (Vicia faba L.). Crop Pasture Sci..

[19] Satovic, Z. *et al*. A reference consensus genetic map for molecular markers and economically important traits in faba bean (Vicia faba L.). *BMC Genomics* **14** (2013). 10.1186/1471-2164-14-932 PMC3880837 24377374

[20] Kaur S (2014). SNP discovery and high-density genetic mapping in faba bean (Vicia faba L.) permits identification of QTLs for ascochyta blight resistance. Plant Sci..

[21] Webb A (2016). A SNP-based consensus genetic map for synteny-based trait targeting in faba bean (Vicia faba L.). Plant Biotechnol. J..

[22] Barbazuk WB, Emrich SJ, Chen HD, Li L, Schnable PS (2007). SNP discovery via 454 transcriptome sequencing. Plant J..

[23] Ma Y (2011). Development and characterization of 21 EST-derived microsatellite markers in Vicia faba (fava bean). Am. J. Bot..

[24] Galeano CH (2011). Saturation of an Intra-Gene Pool Linkage Map: Towards a Unified Consensus Linkage Map for Fine Mapping and Synteny Analysis in Common Bean. PLoS One.

[25] Kaur S (2012). Transcriptome sequencing of field pea and faba bean for discovery and validation of SSR genetic markers. BMC Genomics.

[26] Loridon K (2013). Single-nucleotide polymorphism discovery and diversity in the model legume Medicago truncatula. Mol. Ecol. Resour..

[27] Sharpe AG (2013). Ancient orphan crop joins modern era: Gene-based SNP discovery and mapping in lentil. BMC Genomics.

[28] Duarte J (2014). Transcriptome sequencing for high throughput SNP development and genetic mapping in Pea. BMC Genomics.

[29] Kim C (2016). Application of genotyping by sequencing technology to a variety of crop breeding programs. Plant Sci..

[30] Davey JW (2011). Genome-wide genetic marker discovery and genotyping using next-generation sequencing. Nat. Rev. Genet..

[31] Rothberg JM (2011). An integrated semiconductor device enabling non-optical genome sequencing. Nature.

[32] Tayeh N (2015). Development of two major resources for pea genomics: The GenoPea 13.2K SNP Array and a high-density, high-resolution consensus genetic map. Plant J..

[33] Schlötterer C (2004). Opinion: The evolution of molecular markers — just a matter of fashion?. Nat. Rev. Genet..

[34] Gupta PK, Rustgi S, Mir RR (2008). Array-based high-throughput DNA markers for crop improvement. Heredity (Edinb)..

[35] Carrillo-Perdomo E (2019). Identification of Novel Sources of Resistance to Seed Weevils (Bruchus spp.) in a Faba Bean Germplasm Collection. Front. Plant Sci..

[36] Li H (2009). The Sequence Alignment/Map format and SAMtools. Bioinformatics.

[37] Haas BJ (2013). De novo transcript sequence reconstruction from RNA-seq using the Trinity platform for reference generation and analysis. Nat. Protoc..

[38] Waterhouse RM (2018). BUSCO Applications from Quality Assessments to Gene Prediction and Phylogenomics. Mol. Biol. Evol..

[39] Huerta-Cepas J (2017). Fast Genome-Wide Functional Annotation through Orthology Assignment by eggNOG-Mapper. Mol. Biol. Evol..

[40] Huerta-Cepas J (2019). eggNOG 5.0: a hierarchical, functionally and phylogenetically annotated orthology resource based on 5090 organisms and 2502 viruses. Nucleic Acids Res..

[41] Li H (2011). A statistical framework for SNP calling, mutation discovery, association mapping and population genetical parameter estimation from sequencing data. Bioinformatics.

[42] Martin M (2011). Cutadapt removes adapter sequences from high-throughput sequencing reads. EMBnet.journal.

[43] Van Ooijen, J. W. In Kyazma BV 33, 1371 (2006).

[44] Leroux, D. & Jasson, S. Spell-QTL, a New Tool for QTL Analysis on Modern Datasets. in PAG XXV - Plant and Animal Genome Conference (2017).

[45] Ganal MW (2011). A Large Maize (Zea mays L.) SNP Genotyping Array: Development and Germplasm Genotyping, and Genetic Mapping to Compare with the B73 Reference Genome. PLoS One.

[46] Haldane J (1919). The combination of linkage values, and the calculation of distances between the loci of linked factors. J. Genet..

[47] Varshney RK (2014). Genetic dissection of drought tolerance in chickpea (Cicer arietinum L.). Theor. Appl. Genet..

[48] Kreplak J (2019). A reference genome for pea provides insight into legume genome evolution. Nat. Genet..

[49] Pecrix Y (2018). Whole-genome landscape of Medicago truncatula symbiotic genes. Nature Plants.

[50] Sato S (2008). Genome structure of the legume, Lotus japonicus. DNA Res..

[51] Schmutz J (2010). Genome sequence of the palaeopolyploid soybean. Nature.

[52] Schmutz J (2014). A reference genome for common bean and genome-wide analysis of dual domestications. Nat. Genet..

[53] Lonardi S (2019). The genome of cowpea (Vigna unguiculata [L.] Walp.). Plant J..

[54] Rubiales D, Mikic A (2015). Introduction: Legumes in Sustainable Agriculture. CRC. Crit. Rev. Plant Sci..

[55] Magrini, M.-B. et al. Pulses for Sustainability: Breaking Agriculture and Food Sectors Out of Lock-In. Front. Sustain. *Food Syst*. **2** (2018).

[56] Khan MA (2019). Transcriptome profiling of faba bean (Vicia faba L.) drought-tolerant variety hassawi-2 under drought stress using RNA sequencing. Electron. J. Biotechnol..

[57] Gao, B. *et al*. Comprehensive transcriptome analysis of faba bean in response to vernalization. *Planta* **251** (2020). 10.1007/s00425-019-03308-x 31781953

[58] Choi H, Mun J, Kim D (2004). … H. Z.-P. of the & 2004, U. Estimating genome conservation between crop and model legume species. Natl. Acad Sci..

[59] Aubert G (2006). Functional mapping in pea, as an aid to the candidate gene selection and for investigating synteny with the model legume Medicago truncatula. Theor. Appl. Genet..

[60] Lucretti S, Doležel J, Schubert I, Fuchs J (1993). Flow karyotyping and sorting of Vicia faba chromosomes. Theor. Appl. Genet..

[61] Doležel J, Lucretti S (1995). High-resolution flow karyotyping and chromosome sorting in Vicia faba lines with standard and reconstructed karyotypes. Theor. Appl. Genet..

[62] Ellwood SR (2008). Construction of a comparative genetic map in faba bean (Vicia faba L.); conservation of genome structure with Lens culinaris. BMC Genomics.

[63] Cruz-Izquierdo S (2012). Comparative genomics to bridge Vicia faba with model and closely-related legume species: Stability of QTLs for flowering and yield-related traits. Theor. Appl. Genet..

[64] Khazaei H, O’Sullivan DM, Sillanpää MJ, Stoddard FL (2014). Use of synteny to identify candidate genes underlying QTL controlling stomatal traits in faba bean (Vicia faba L.). Theor. Appl. Genet..

