# Supplementary material for: Development of new genetic resources for faba bean (Vicia faba L.) breeding through the discovery of gene-based SNP markers and the construction of a high-density consensus map
Source: Sci Rep. 2020 Apr 22;10:6790. doi: 10.1038/s41598-020-63664-7 (PMC7176738; doi:10.1038/s41598-020-63664-7)
Supplement: Supplementary file 1 — Supplementary Information. [file 41598_2020_63664_MOESM1_ESM.pdf]

Development of new genetic resources for faba bean (*Vicia faba* L.) breeding through the discovery of gene-based SNP markers and the construction of a high-density consensus map

Carrillo-Perdomo E.1*, Vidal A.2, Kreplak J.1, Duborjal H.3, Leveugle M.3, Duarte J.3, Desmetz C.1, Deulvot C.1, Raffiot B.4, Marget P.1, Tayeh N.1, Pichon J. P.3, Falque M.2, Martin O.C.2, Burstin J.1, Aubert G.1

1Agroécologie, AgroSup Dijon, INRAE, Univ. Bourgogne, Univ. Bourgogne Franche-Comté, F-21000 Dijon, France ; 2Université Paris-Saclay, INRAE, CNRS, AgroParisTech, GQE - Le Moulon, 91190 Gif-sur-Yvette, France; 3Biogemma, Chappes, France; 4Terres Inovia, Thiverval-Grignon, France.

Supplementary figures:

Fig. S1
Fig. S2
Fig. S3
Fig. S4
Fig. S5
Fig. S6
Fig. S7
Fig. S8

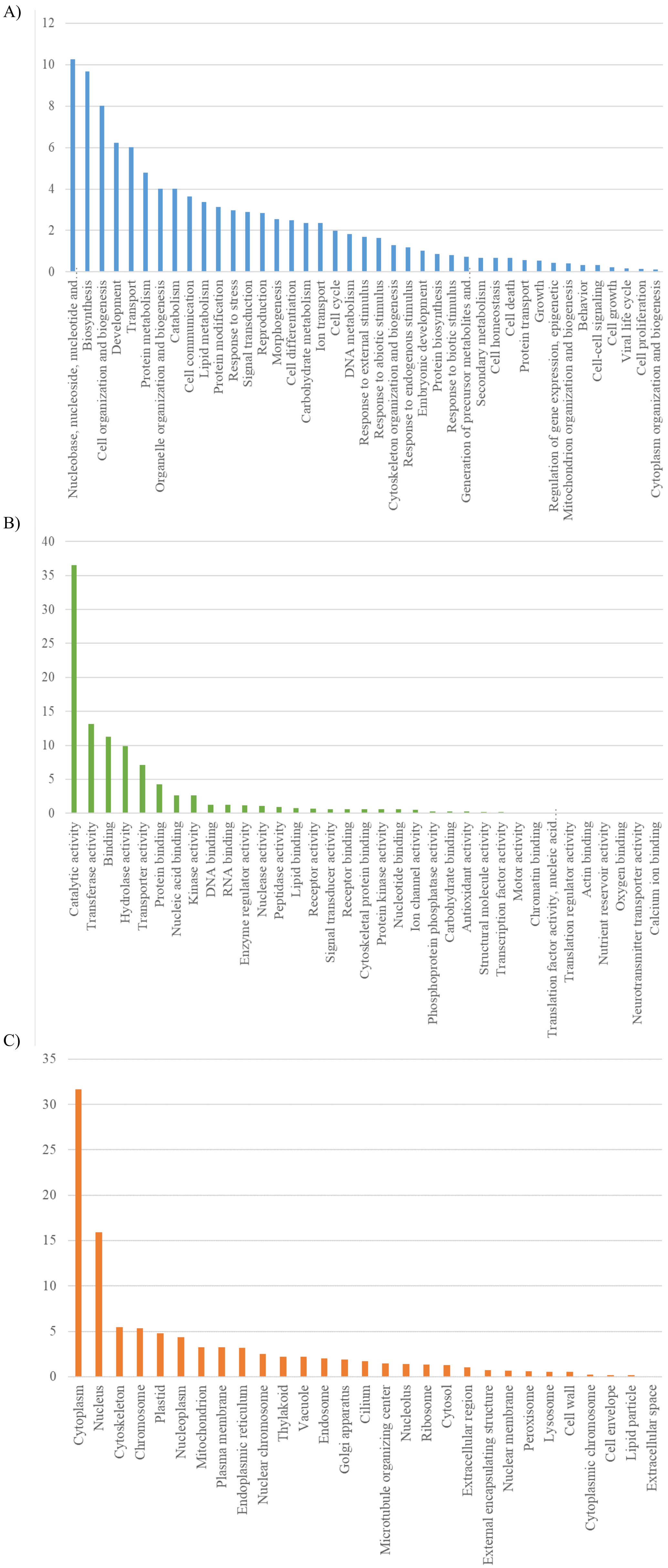

Fig. S1 Classification of the results of the annotation of the gene ontology (GO) terms of the faba bean transcripts. A) Biological process; B) molecular function and C) cell component.

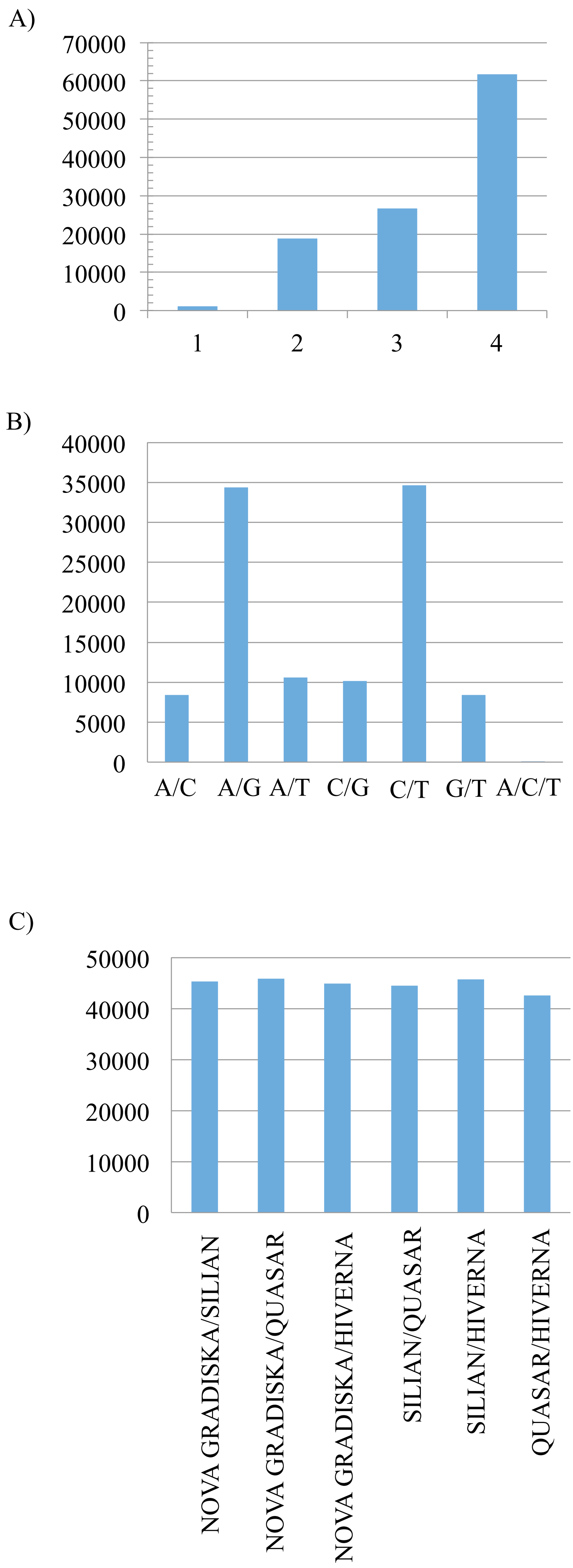

Fig. S2 Faba bean gene-based SNP calling statistics. A)Number of genotypes per SNP; B) SNP types and C) number of polymorphisms.

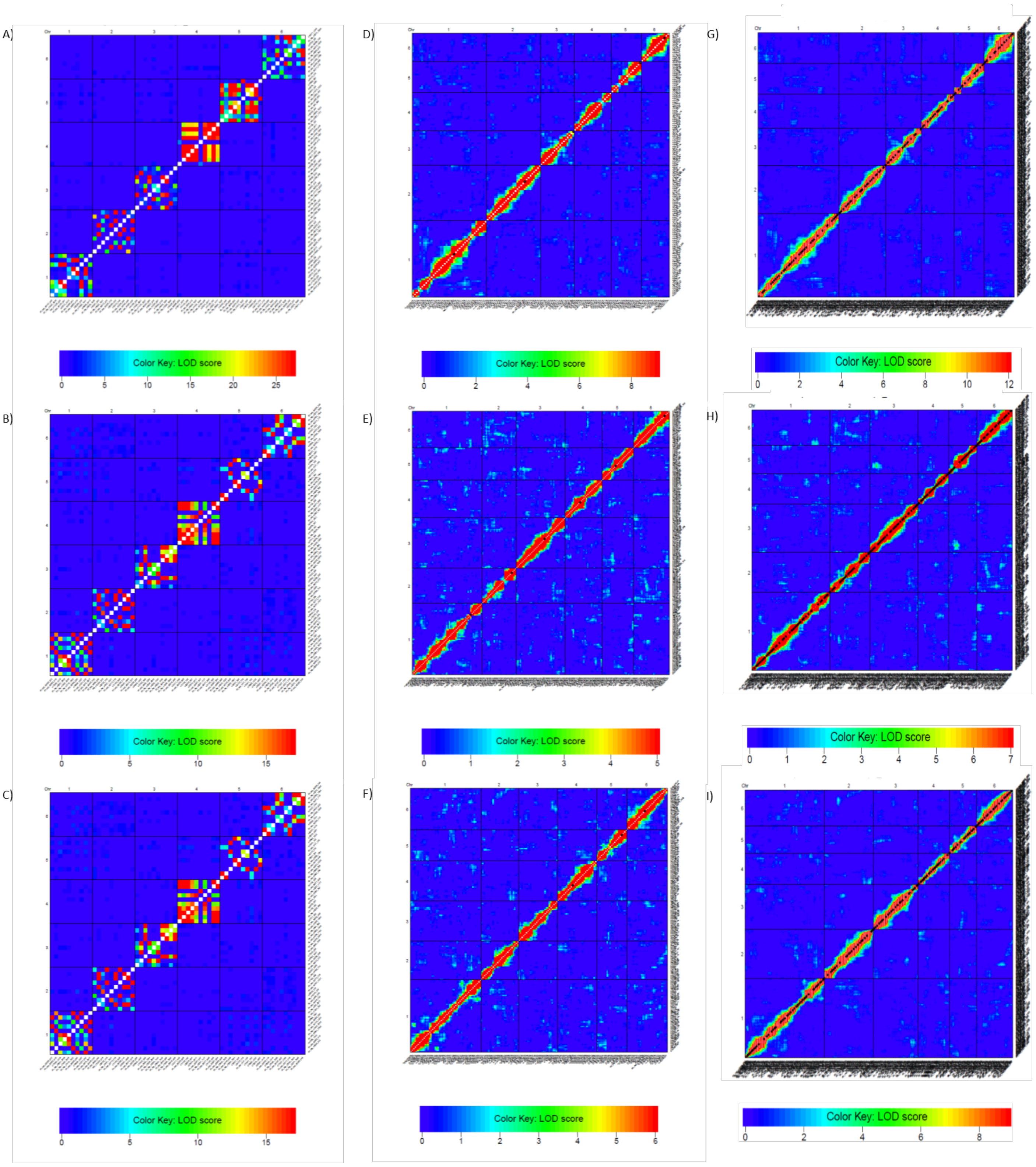

Fig. S3 Two-point LOD score of the seed (left column), scaffold (middle column) and framework (right column) SNP markers used in the construction of the three faba bean individual genetic maps of the three recombinant populations presented in this study. A, D and G) Pop1; B, E and H) Pop2 and; C, F and I) Pop 3

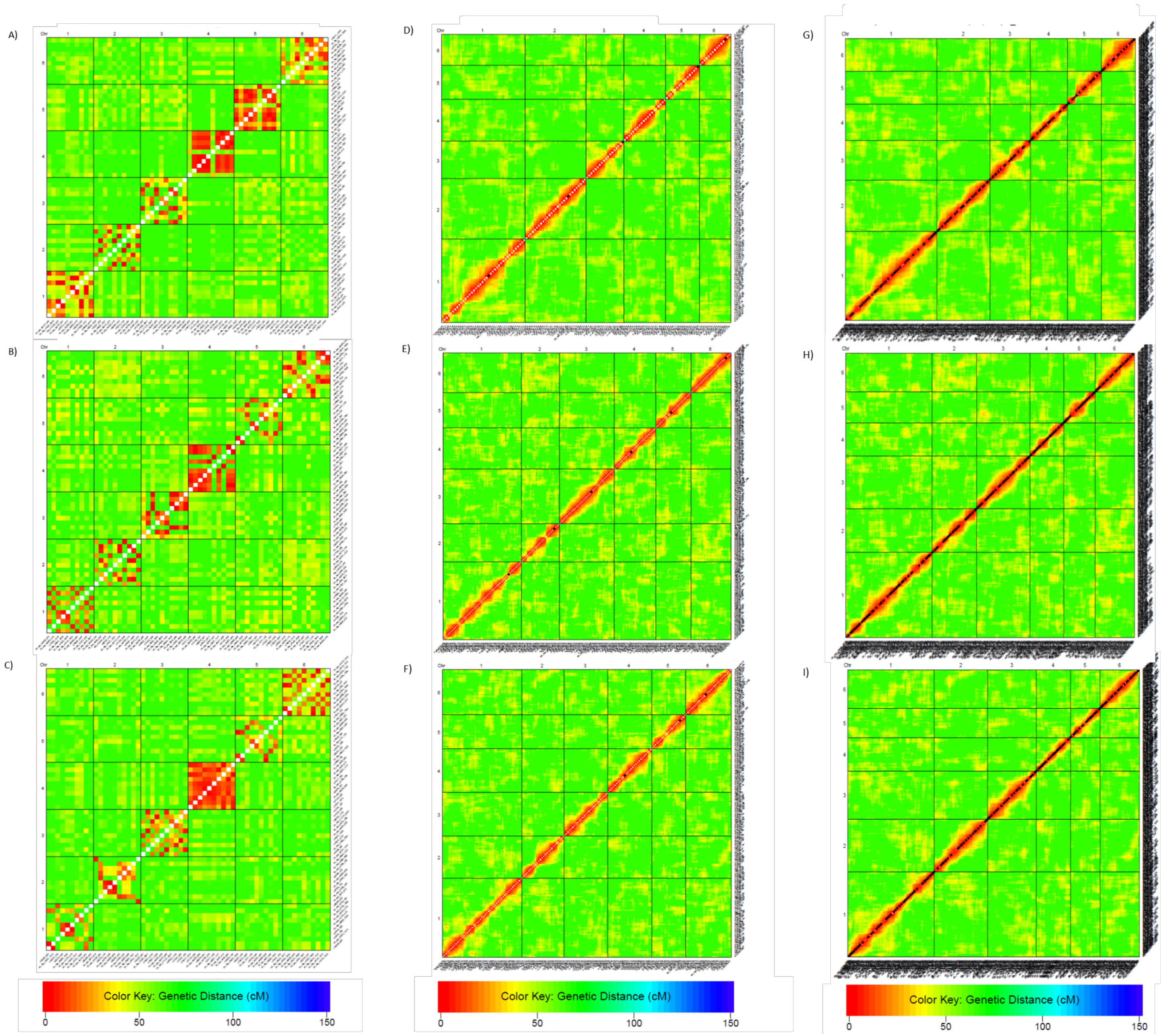

Fig. S4 Two-point genetic distance (cM) of the seed (left column), scaffold (middle column) and framework (right column) SNP markers used in the construction of the individual genetic maps of the three recombinant populations presented in this study. A, D and G) Pop1; B, E and H) Pop2 and; C, F and I) Pop 3

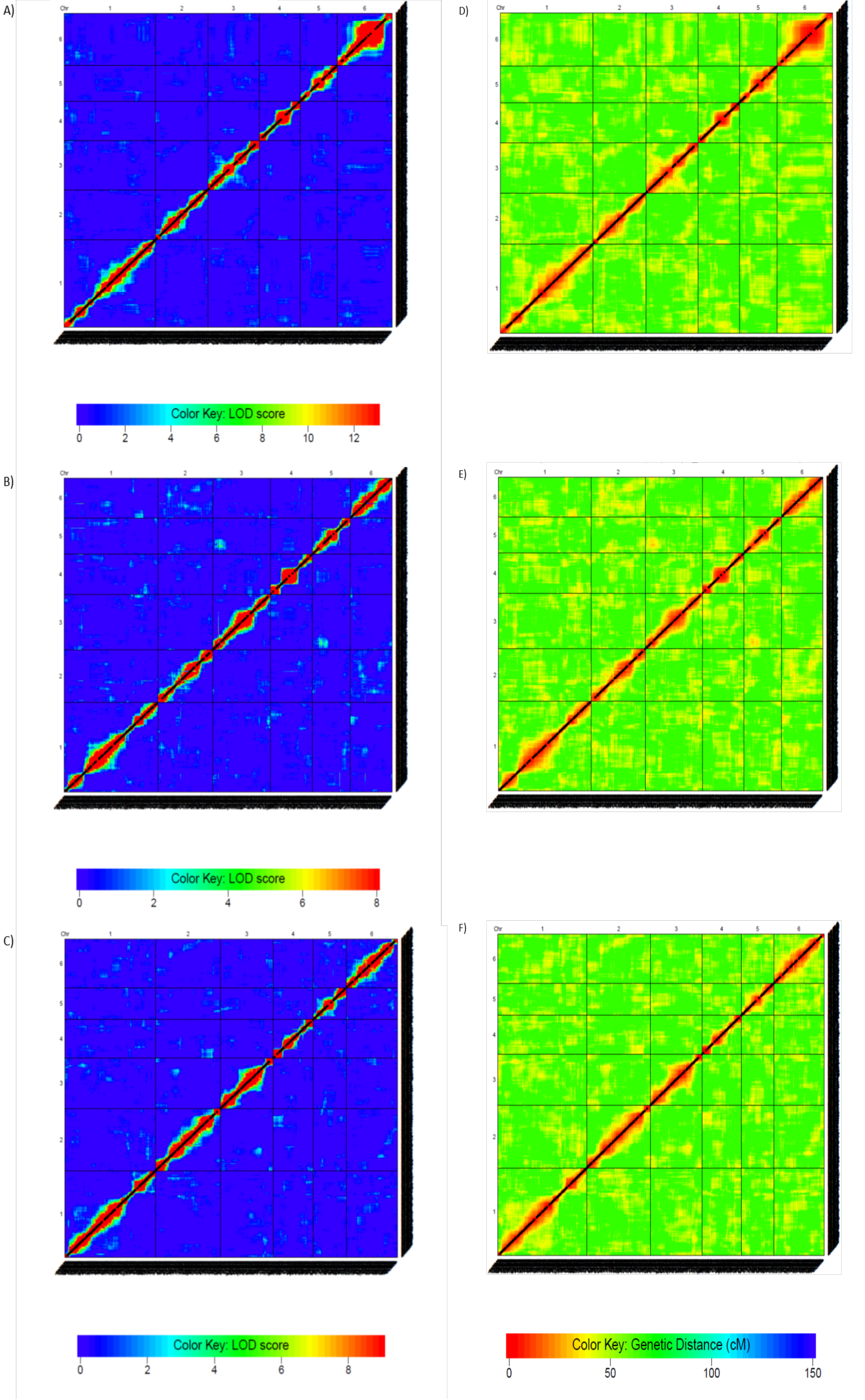

Fig. S5 Two-point LOD score (left column) and genetic distance (cM) of the full individual genetic maps of the three recombinant populations presented in this study. A and D) Pop1; B and E) Pop2 and; C and F) Pop3

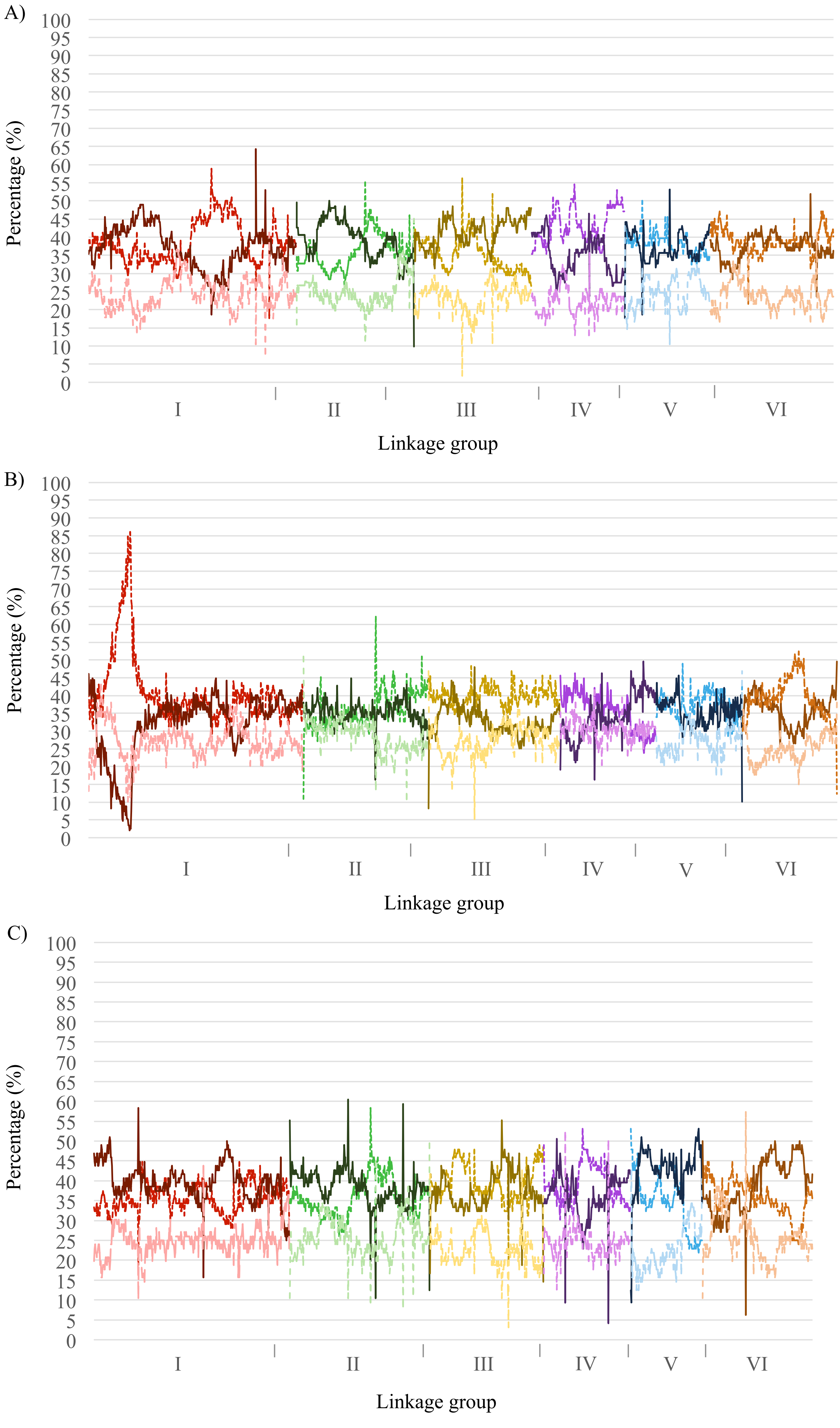

Fig. S6 Allele frequency of the SNPs placed in the individual maps of the three faba bean recombinant populations presented in this study throughout the six linkage groups. A) Pop 1; B) Pop 2 and; C) Pop 3. D*otted lines* indicate that the allele derives from the homozygous parent « NOVA GRADISKA » (A), « SILIAN » (B) or « QUASAR » (C); *solid lines* indicate that the allele derives from the homozygous parent « HIVERNA » and *dashed lines indicate* heterozygosity

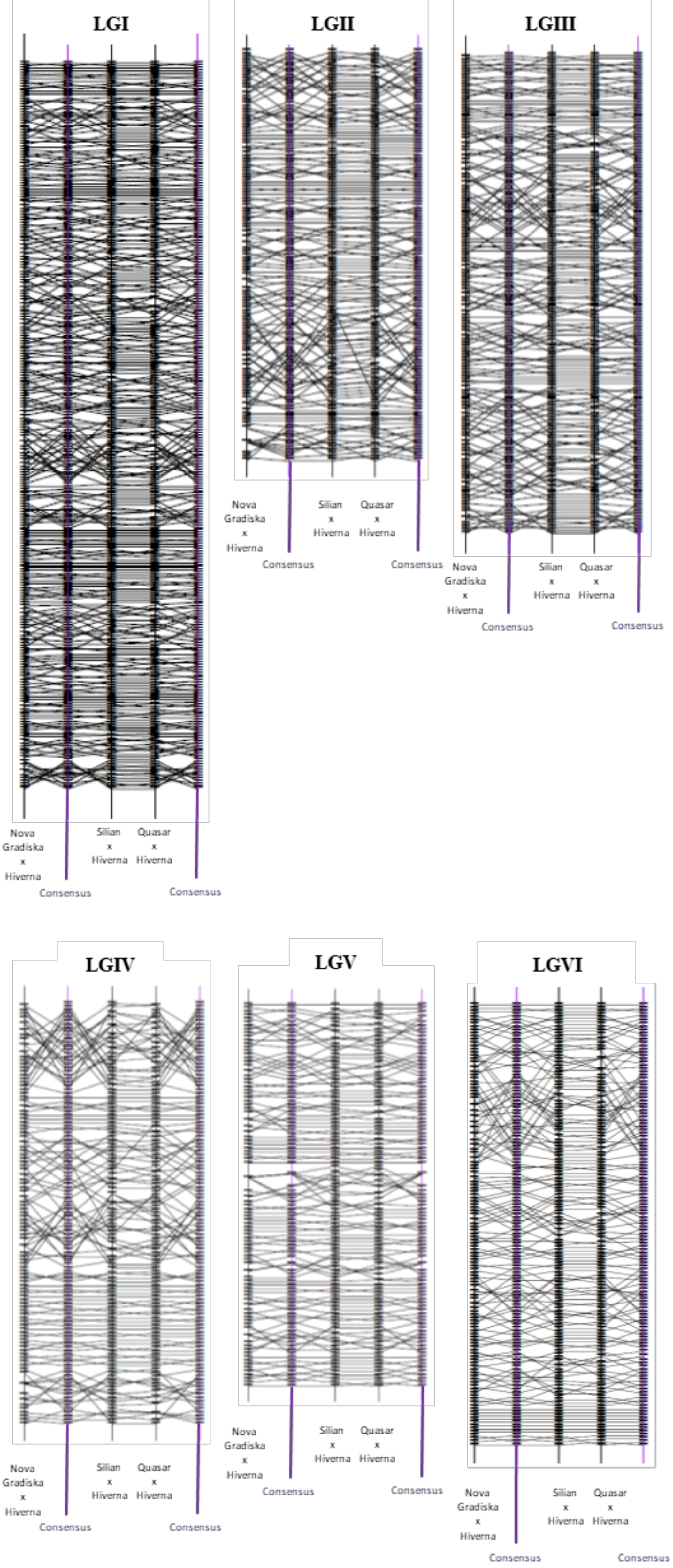

Fig. S7 Comparison of the marker assignements by order between the individual maps of the threee populations and the consensus map

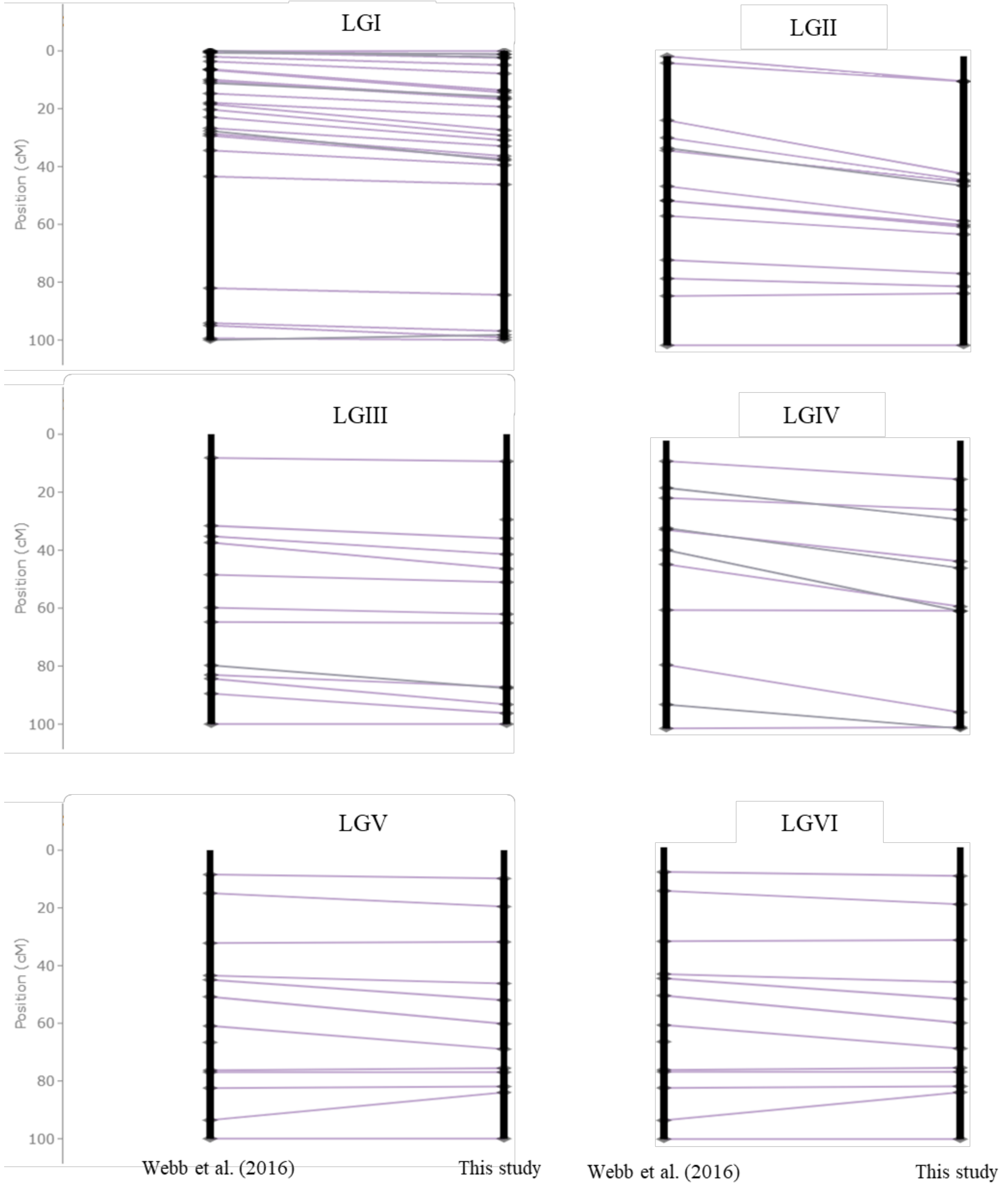

Fig. S8 Order comparison of the marker assignments by linkage group between the faba bean consensus map reported in Webb et al.[21] and the faba bean consensus map derived from the integration of the individual genetic linkage maps of the three recombinant populations presented in this study. Marker positions are normalized
