# Supplementary material for: Development of new genetic resources for faba bean (Vicia faba L.) breeding through the discovery of gene-based SNP markers and the construction of a high-density consensus map
Source: Sci Rep. 2020 Apr 22;10:6790. doi: 10.1038/s41598-020-63664-7 (PMC7176738; doi:10.1038/s41598-020-63664-7)
Supplement: Supplementary file 2 — Supplementary Information 1. [file 41598_2020_63664_MOESM2_ESM.doc]

Development of new genetic resources for faba bean (Vicia faba L.) breeding through the discovery of gene-based SNP markers and the construction of a high-density consensus map

Carrillo-Perdomo E.1*, Vidal A.2, Kreplak J.1, Duborjal H.3, Leveugle M.3, Duarte J.3, Desmetz C.1, Deulvot C.1, Raffiot B.4, Marget P.1, Tayeh N.1, Pichon J. P.3, Falque M.2, Martin O.C.2, Burstin J.1, Aubert G.1

1Agroécologie, AgroSup Dijon, INRAE, Univ. Bourgogne, Univ. Bourgogne Franche-Comté, F-21000 Dijon, France ; 2Université Paris-Saclay, INRAE, CNRS, AgroParisTech, GQE - Le Moulon, 91190 Gif-sur-Yvette, France; 3Biogemma, Chappes, France; 4Terres Inovia, Thiverval-Grignon, France.

File S2. Faba bean transcripts.

>fava_dn_rep_c1

ttgggatgaacaagcctttaatcaactatggagtataatcgcggggaaccatgacataag

atagatatcatattcagagaatatgattccctttaaggatgccttggtggtgaaatggta

gacacgcgagactcaaaatctcgtgctaaacagcgtggaggttcgagtcctcttcaaggc

ataatattgagatctcttattgaaattgaattggaataagttcgccagcagatcacgaag

ttttggtgatcttatctatctaatgaatggagagtacattttgaaatcgtcccacctgca

atccccctaccctaaaccaacggataaagtacattatatatttgattttggagattggtg

acttacccattcaatgactttggcactcgacattcaaaaaaatgggtactgtacgctcgg

gtcgggtgaattccaaaatagccgcctatgtcaaccttctttctcctttcaggacaggac

ctatccgaaaagaaagaaaattctgtacttattggtcgtgaatatctgaataggacaaat

taccctgtggatctctttatttgcttcaaaacaagtaggctcggtcaacggcaatatgga

ttatcaatattaggggatctttcaattgagaagatcccctaatatcgacttgagacgacg

agaaagaaagtatctattttatttcatttcattattcagttaaaccaatcgttcgttatt

ggaacagatagtcacaaccatctcgtctgggaaaagccatttttttctcaacaatgtctt

tgtgagttgagccaatagggttccgttagataggaacagatttgataaatattgataact

ctcgtatagagtattagaacgaaaaaattcatttgatactgaactattggttctaaacca

tctctggcgatcaatcaacaatttgaaattctgttcttgcatattcttcctaaaccaggg

tttatttagatatttccaatcaattttttgggtttgggaagtcaaaagtatccttgacat

ctcttcatctggagaaaattcttgatgtgaaaatacagatccaaaaggatcctcttggaa

tagcaaaaacagtggatctgcggggtcccaaatgaattggcttattcgaaaaaagccctg

ttcgttggaaaatctcgctcgtgtctggtcctgcatgattccatcctgcaagaactgcga

atcattatcattctcttgaagcttaccctcttcatcataatcatcaaggatcggtttgtc

ctgaaatgacccttcccaatagggaaatctcaattcattggacccttcgtcaaagagaaa

gccccaagggcgccacagtctcggagcccaaagtatgtgattgaataaatcctcctctag

cggttgcgggtcgaagactccttccccttcttcgaaccccgattcatatttttcatagag

aaatctctgatcaacgatagaagaagacccattttgaatcatatttaagggatttcttgg

ttcggaccgaagaagcaatgtcacgccatcattatcaaaccgacttcctgtctgtgaggg

ttccagcagagcaccttctatttctaatagcccatgaactagatcagaatcattctcaag

gagtctataagaagtgatcccatcatttgtttcattaggtccgggtagagaccaaagatc

ttgagcgaccgatccggcagaacaactcaaaagataaagaagtatggttaatttcttcat

gcttgttccaagttggaagtaccatttgtacaaataagaatccccctctgcacatgattt

cttcttcatatagatagatataggatcgatagagcaattacttagaagtagattttgtga

aacagccctccctatctgataaaaaaaggatcccatgatcctgaatcgatcttatctgag

atctaaaatcccaagtttgtctatgaagagcagatctaattatattagtgtctataatag

atttcttttgtataatactaatcgatagcgcctcattagtaagtgctacaagatctcgta

cattagaacccagagttatagaccccaatccattagtatcgaatatttgcttttccaagt

gaaatcccctagtatatgaaagagtaaaaaagtgctttctttgttgtggaataagaagcc

ttcgtatcttaatgcatgtatttaatttattcggagctattaaagcagggtctacttttt

ttggaatatgagtagaagcaataactagaatatttctagtagaacctttttccgaatcgc

tggaaagagagttcactaatatacccagcgataagtcattcgactcattcctatccagat

catgaatgtttggaatccaaattatgcaaggagacattgcttttgctaattcgaattgaa

gcgtgagataaaaccggtctatttccatcagatcgctagataccggatcctccatactta

gaaacttcaactggctatcaagcttacggtcgagatcgtcactatcatccctttcataat

tctcggaattatagcaactaccctcgttattaggtaaaagactgtaaatggtaccctctc

tttcatcaaacatgtcgtcctcactatcatcactatcaaaacccttaggaaagttctcca

ggaacttgtttacaaatactgtaatgaaaggaacataggtggttgtcgctagatatttga

gcaaataggatcgtccagttcctatcgaacctatcactaaaatacccctagcaggggata

gggctaagcgtagcgaaaagggtttcccatgagatgataaatccaaacaactagccccac

acgggatttctgaataagtgattgtctgataatgagcgaggaatacccgtctttctgcta

agcaggatgtattgaactcataattcatcagatcctttttatgaatgtcaattaaatatc

gtaagtaaattgttcctggttgctcaatcatttgataaccagagttattctttgataact

gatcactattagtcagactcaataaaatttgatccagccttttttctgtcgttaaggtag

agaactgaaccaagaattctctttctttatcagcaatgaaatcactagtcgagatccagg

attctattttatcatcaatccaattactattcatgttttttcttttccttatcaatgaat

agattgctttacttgtatgacttaaatgtctcgtatttctcaaaaacgcgattcgattga

tgggatttggtataatacttatgagatccatgagattaaaaccgttcttcttcgaacgta

tggatttgaccccatcagcgaaaccaccaccccttgggatgtccccgccagaaggccaac

ctcgcctttcttctaaaaaatttcctaattgttctcgagcaactccaaagatatccttta

accagaaagaattttgttctgatagaggatacctatccagaagttttcgcaattcaatga

tgtaggatggaatcatcaaagatttgacctgttcgaactctgtctgtaactcattagaga

agcgagaaacaaagaaaagatgtgtataaacaagatatccagtaacaagaagaagtaaaa

ggattgaaaccaggaactcctcaatatttcgtgatctcagatgtgtcgatatgaatggtg

actcattatttgtgtgaaaaagatctttgtacatgtctacaagaaaattatggaaagact

tactcgaaatctcacttatacgattccgttgtgaaagacacagttttttccgaagaattc

cccacagttttttccgaagacttcccttggatccatgcataatattctgccaaatagata

caaattttttaaattgaggactcctccttagtatcgcaaataggtctgaaagagtatcta

aaaatatgaaattgatatatttgtgccctgtccaggtaaggaacaacggtattatatacg

ggcgtaatcgcttcaatttggagagaattgaagaaatactctttctttttctttttcttt

gaaagtttctatacatctgcccttttccaaggaatttttttagaggttttttcctctttg

cggatggtataaatttttcagaatatagagtgtcaagcaaacccaaacccatgtttgaat

cgaaatcaagatactgatgcaagttcttcctttttgaatcagataaattaataggggaaa

gaggttgacaagaagttctttccaaattcactatttcttcctctgttagaggtgttccag

aaatgtccgcgatcgagtaaatagatctaggaacacgagttgaaaaatggaaagatttgt

acaagtcatatccgtcatcaccactttgcggaaaattcttcggtctcaatatcttagaga

cctctaactcgcttggtgaaatagtatctctgtccaaaaaagcatgtgtttttttaccgc

cgcacaaagaaaatcttttctttcgaatgaacaagacattgaggaattgttcatacataa

aatcataattataggacctttccccataaaaagagaatcttttgttacaatcgaaagaga

agttatattgattattcaagaatcgaagtcgatttactttataaaaagaggaaatcaatg

aacttctatgaaaacatttcacaggattcagccaattgtcttggtttggtatcttattga

acaaaaatggtaatattgttccttcattgatcaagaattttgatttttgagaagtataat

agtcatccaataatggggatttcctttttttcaaatgaaccgtttgcagacctattgatt

ctaacaactgattctcgagtgcatcattcatacgtttcaattcatacatatggctctggg

acctatgaacgggcatactcccgaaactcacaaagaaaaaaggaagtgagttagacaaaa

agggaagaagcttggacaaaaagaaacaaagtgacttagaaaaatcttttttgtcgataa

cctcagcccaatcaatcgaatatgcattcatatgtaatcgatcgaacactactttaaaac

gactattctgctcagaaatgaaatggtccaaatgttcctggaaatttgtgctccccttgg

accatttgtatttatatgcatttggatccttattcacggatctctcggttcgataaataa

aaataagacgatcaaagcatttcttctgactctttttcaaatttgagaaacgttgattga

tcgtgtgtttccttataggtctatgattcagaatatcattttgatacctttgaagtacat

attcgaagttgcgattgaatcgattaccttgaaaataattcccacaagaggtctggaccc

atttttttatgatctttggataaaaagattcattcgcttcgtcaaaaagccgcggcagaa

ccaataaagattttttttttgattccggttcagaaaacccaatagaaaaagagaatccat

tacgatagaccagatccggcttagttagtgatagattaaatagatttgccatttcttgaa

atctctcttctgattgaaaattgcgatgtaacaagtacccccccctattctggtcatgga

atagatgaaataaaccaaaaactagatttttggtcaagaatgaattattattttgaactg

tcaaaagtttctcctttgcaatcctatcacatcctccatcggatgaaataggatgaattg

agacattattttgtaaatacatgattatcttgactatattggctagttctttcttttcca

atttcctccaaagaacaaaagaaacatcttcttctttctttaataattgaagatcttgag

cggacttctcagtaggattcaaatccagatgaagttgggaccatctgtccgagaaaaaag

agcgatcagagatctttttttcttttggaccagacaggagcggaacaatcaacctattga

tattgaacgaatcttttgagtcttccaatctatcattgccggatccaatggaatttattt

gtataggaagaagccctgtcaaataaatattttttttttcgatcatccttcgattgttaa

tacgatatagaaggatcgctactacaaaaagtactacattcttgatcgtgaaatatcgat

ttcttgttaaaccctgtgactgacgtgaaactaggatactccaaattctggagtctaaga

cttttatcaaactctcttgatgcaaaaaaatgtgaatgacagatatcgctgaattgaatt

gcgtccaggaatctaagaaataggcacaattcttgctctctctaaaaatttctcgcaatt

cgaaaatccaaaagtggaattgattgttcttcataggatcctccttaaaaaattaaaaat

aatttatctctttatgtatgtatttctattaagttatttatttatgtatgtatttctatt

aagttatttctttatgtatgtatttctattaagttatttatttatgtatgtatttctatt

aagttatttatttatgtatgtatttctattaagttatttctttatgtatgtatttctatt

aagttatttatttatgtatgtatttctattaagttatttctttatgtatgtatttctatt

aagttatttctttatgtatgtatttctattaagttatttctttatgtatgtatttctatt

gatttatccaaaagatttcacttcaattgaaatttggttattcatcaagtcggagggttg

ttccctcgaagcatccatggctgaatggttaaagcgcccaactcataattggcgaagtcg

taggttcaattcctactggatgcatgccaatagaaatatctcccctaaaaactatatttt

taatcatgaattattattttgaactgtcaaaagtttctcctttgcaatcctatcacatcc

tccatcggatgaatataggatgaattgagacattattttgtaaatacatgattatcttga

ctatattggctatttctttcttttccaatttcctccaaagaaaaaaagaaacatcttctt

ctttctttaataattgaatttgaaaataatagaaacttcaagaattgatcatttctaaat

aaagaaagaatttctttatttattctatttaaataatttctgatctgtagtagacctctt

agcttagtagtatttgaatcgagacttggatctgtatcaatggaatctcatcatccatac

ataacaaattggtgtggtatattcaaattttaataaatgaacagtaaaaaaactagtatt

tttagtgagactagaactcatcgggaagaaagaataaaatagatttatgaatggaataaa

atctgcagtatttacagacactaaattttcgttatttcgaaaaaatcaatatacttttca

tgtcaaatcaggattaactaggacagaaagaaaacattgggtcgaactcttctttggtgt

caaggtaagaactatgaatcaactcgcaatcccaataaagggtagaagaatacgacctat

tatgggacatacaaggcattacaaacgtatgatcattacgcttcaaccgggttattctat

tccacctcttagaaagaaaataacttaaataaaaatacacttcataacatggcgatacat

ttatccaaaacttctagcccgaacacacgcaatggagccgtaaacagtcaagtgaaatcc

aattcacgaaatcgtttgatttctggacagcatcattgtggtaaagggcgtaatgccaga

ggaatcattaccgcagggcatagagggggaggtcataagcgtctataccgtaaaattgat

tttcgacgcaatgaaaaagacatatatggtagaattgtaactatcgaatacgaccctaat

cgaaatgcacacatttgtctcatacactatggggatggtgagaaaagatatattttacac

cccagaggggctataattggagataccattatttatggtacaaaagttcctataaaaatg

ggaaatgccctacctttgagtgcggtttgaactatagatttacgtaattggaagtaacca

attaggtttaaggcgaaacctagaaatcgatcactgatcctatttgagtacctctacagg

atagacttcaacagaaaactgaagagtaacagcagcaagtgattgagttcagtagttcct

aatataaaattattgaccctagagatatagtaatatggagaagacaaaattgtttcaagc

accgacagaacacgaagtgctcctcgtttcaaaaaacaaaggggggaagacacagattat

tcacatttcatttgatggtcaaaggcaaattgaaagctaagcagtggtaattctaaggat

tcccccgggaaaaaatagaaatgtctcctacgttacccgtactatgtctaagtagcaacg

taatttcatagagtcattcggtctgaatgctacatgaagaacataagccagatgaaggaa

cagaaagacctaggatgtagaagatcataacatgagtcattcagaatatttggattcgta

tattatatatccactcatgtggtatggtacttcgttatgtcatatataatatacgaaata

aacgatatataagatgtatctgtatagatattctaatctacatccagaaagccgtatgct

ttggaagaagcttgtacagtttgggaaggggttttgattgatcaaaaagacgaatctact

tcaaccgatatgcccttaggcacggccatacataacatagaaataacacttggaaagggt

ggacaattagctagagcagcgggtgctgtagcgaaactgattgcaaaagagggtaaatca

gcaacattaaaattaccttctggagaggtccgtttgatatcaaaaaactgctcagcaaca

gtcggacaagtggggaatgttggggtaaaccagaaaagtttgggcagagccggagctaaa

cgttggcgaggcaagcgtcctgtagtaagaggggtagttatgaaccctgtagaccatcca

catgggggtggtgaagggagggccccaattggtagaaaaaaaccctcaactccttggggg

tatcctgcacttggaagaagaactagaaaaaggaagaaatatagtgataatttgattctt

cgtcgccgtagtcaatagtggatagtagagaaaatagaatttgtttcttcgtctttacaa

aagaaaagagcgattgactatgacattctatgattttcttttttgagagattctatattt

taaaaagagattctatattttaaaattggaaataaaataaaaaatataagagaaaaaatt

catttttttagaaattttaagaggaataattaactatgacacgttcactaaaaaaaaatc

cttttgtagcgaatcatttattaagaaaaataaataagcttaacacaaaaggagaaaaag

aaataataataacttggtccagaagatctaccattctacctacaatgattgggcatacca

ttgctatccataatggaaaagagcatttacctatttatataacagatcgtatggtaggcc

ataaattgggagaattttcacctactcgaaacttccgaggctttgcgaaaaatgataata

gatctcgtcgttaacattcattttaacataaatttagtaatagtcattaataaatattaa

ttaataaattactcaaaaagaattgtattattctgcaatcaaaaaaacagattttatttt

ttcgtgaaaatagaaccaatacagagaaatataaccctttgttcaaaaaatatttttgtc

tattcacaacacaaagcgtgaatggtaattaataatattcaaaaattgaaagggttatta

taatagaactaatgctttgcagaaaaaaaaagatatatcaagatattaaaaaaaatatat

atatatagataaaaaagtagacaaatcagacgtattattttatatagagtagtgagggat

tatgggacaaaaaatacatccgcttggtttcagacttggtacaactcaaagtcatgattc

tatttggtttgcacaaccaagaatttattccgagaatgtaaaagaagataaaataatacg

agattgtatcaagaattatatacaaaaaatacaaaaaacaacaaccgctgtcgatggcga

gggaattggacgaataaagattaaaaaaacacgcgatctgattcacgtgataatcaatat

gggacttccaaacccaaacttagtaatagaggataaccctgaaagaatggaagaattaca

tactaatgtgcaaaaaaaaataaattgtgtgaaccgacaaatcaacattgaaattacaag

aattccaaatgtttatagagacccgaatattcttgcagaatttctagccagacaattaaa

gaatagaattccgtttcgtaaagcaatgcaaaaaactataaaaaaattcgctgaagaggc

aggtgcaggtacaaaaggagttcaaatacaaattgcgggacgcatcggcggaaacgaaat

tgcacgtgtcgaatggatcagagaaggtaggattcctctacaaaccattcgagctaaaat

tgattattgttcctatccagttcgaactatttatggggtcttaggaatcaaagtttggat

atttataaacaacgactaataaccttttccttataacgttccatcttttgagaaaacaaa

aaatgacgaattcttactacgttcttattctcaaagaagaaatgaaaaattttaaaagat

taaataaaaaaaaattaattaatcattttataatgaaggagttgctatgcttagtgtgtg

actcgttgggtttttttttaagggtattaattttctacaaaaattcgtcgaattaattac

taaataataaataacctactcatcgcttcccattatctggatataaagaaccgccgaaaa

tctaaaatcaaaataaggatctatgtgatgatataattatagcaactgaaataaaaaaaa

aaataagattattagttgtaaagcaataagaatatacagataaatgaaggggtgaaagaa

agatagaaaaaggatatcaatgatatagaattctcctatgtaaaggtctatgggtcatct

cataaaaagtagtgtaataaagcatcaatcttcaaaattcatccatatatggatgaatat

attcctagaataaacgaatcaagagccacgaatcaagaaaactgagaaggttgattcaac

aaaaaaaaaaataagaaaataaaaaaaatggaatcttcttaattaaagaggctccattgt

agaatttagacctaaccataaattgaaaagcgatgggaacgacggaacctgtgaatacaa

aagattatattaaaatttgaaatcttactgattcattagatgggatggcggaaggaacta

agaattcattcttttttgaggagttgtggactaaccctacattacatcttcaatttaatc

aatttataatgagagagtaaatattcgcccgcgaaaatgtggatttttttggaattgata

aatttttgccaatccaatatgacaaaaaaaaaaagaaaaatactgattcgttagaacctt

gcctccaaagagagatttgacgaggtttgtgaaatggcctaagactgttcaaatccagag

gaagaagaggatcctcaagcagaggttgaaggttccacctgcactcaaccagttcaccaa

gacactcgataaaaaccttgccacaaacctatttaagatgctccttaagtataggcctga

ggacaaagctgagaagaaagagcgtctattgaaaagggctcaagctgaatctgagggcaa

aacagtcgagacaaagaagcctattaatgtgaaatacggtctcaaccatgttacctatct

tattgagcagaacaaggcacaacttgttgtgattgctcacgatgtcgacccaattgagct

tgttgtatggctcccagcattgtgcaggaagatggaaattccctactgcattgtcaaggg

caaagcacgccttggaacaatagttcacaagaaaactgcagctgttttgtgcttgaccac

tgtcaagaacgaagacaaaatggaattcagcagaatcctagaagccattaaggcgaactt

caatgacaagtatgaagagtacaggaagaagtggggtggcggtatcatggggtccaaatc

tcaagcaaaaaccaaggccaaggaaagactcattgccaaggaagctgcccaaagaatggc

ctagaagaagtttcacatgcttttggttattttagaactttgaggtgttattcttctata

tttttgactgaggttttcttttcattctggtagttgtaatctataattactggttcaata

cctgcactttttgctgatttatttcagtagttgttttagacttaatgatggaatatgtta

ttatttttgatttccatttgtttctctaattgatttatttatgttgcttaaatctatttc

aactacccttacttatacttggtataagtctgatc

>fava_dn_rep_c2

ttggccgcgggaactcaattataacaacaatggcttctatgatttcctcttcagctgtga

ctacagttaaccgtgcctctacggtgcaatccactgcaggggctctatttggtggccgca

aatccatgtctggattcccagttaagaaggtcaacactgacattacttccattacaagca

atggtggaagagtaaagtgcatgcaggtgtggcctccaattggaaagaagaagtttgaga

cactttcatatttgccaccattgaccagagatcaattgttgaaagaagttgaataccttc

tcaggaagggatgggttccttgcttggaatttgagttggagaaaggatttgtgtaccgtg

agcacaacaagtcaccaggatactatgatggaagatactggacaatgtggaagcttccta

tgtttggtaccactgatgctgctcaagtcttgaaggagcttgatgaagttgttgcggctt

accctcaagctttcgttcgtatcattggtttcgacaacgttcgtcaagttcaatgcatca

gtttcattgcacacacacctgaatcctactaagttctctgcattggagcttcctatttat

atgttatgctttaagttccttttgttgtgtatttttataatttctgtttttggatttcaa

attgcaaatgggatgtataagagttaatgaatgatatggttactttattccaaaaaaaaa

aaag

>fava_dn_rep_c3

aggaacaccaacggcgaaagcactctgctgggccgacactgacactgagagacgaaagct

aggggagcaaatgggattagagaccccagtagtcctagccgtaaacgatggatactaagc

ggctgtgcgtatcgacccgtgcaacgctgtagctaacgcgttaagtatcccgcctgggga

gtacgttcgcaagaatgaaactcaaaggaattgacgggggcccgcacaagcgatggagca

tgtggttcaattcgatacaaagcgaagaaccttaccagggtctgacatgccgcgaatcct

cttgaaagagaggagtgccttcgggaatgcggacacaggtggtgcatggctgtcgtcagc

tcgtgccgtgaggtgttgggttcagtcccgcaacgagcgcaaccctcgtgtttagttgcc

aacgtttagtttggaactctgaacagactgccggtgataagccggaggaaggtgaggatg

acgtcaagtcatcatgccctttggccctgggcgacacacgtgttacaatggacgggacaa

aggatcgcgaccccgcaagggtaagctaacttcaaaaacccgtcctcagttcggattgta

ggctgcaactcgcctacatgaagtcggaatcgctagtaatcgccggtcagccatacggcg

gtgaattcgttcccgggccttgtacacaccgcccgtcacactagggaagctggccatgtc

cgaagtccttaccttaaccgcaaggagggggatgccgaaggcacggttagtgactggagt

gaagtcgtaacaaggtagccgtactggaaggtgcgactggatcacctccttttcagggag

agctaatgctttttgggtaattagttgttacactgcttcacaccccaataaagtaataaa

gaagtaaactacatctcattaaataggtagatagccgttatttttcgtttcttgacggtg

aagttacacctacctagcccttgagctagctcctgggagagctcacgctttttgggtgat

tgggtttcatactgttgcacaccgctgaagcgaaggtggtcgagtcaaggttaatcgatg

gaagtcttctttcgtttcacgagctcgagggcgaagtgatgacagtgagactaatctcat

gaactagcttattatcctaggtcggaacaacttgataggagctcctcttttttgccccat

gttgccccacggggcgacatgaggcaaaaggggggaaagagagggatggagtttctctca

ctttctttttttgacatagtaggcggccccgcccctccgggaggcccacacattagtggg

ccccgcccctccgggaggccgacacatttgacatagtgggcccctccgggaggcccacac

aacgggctattagctcagtggtagagcgcgcccctgataattgcgtcgttgtgcctggac

tgtgagggctatcagccacatagatagttcaatgtgttcatcggcgcctgaccctgagat

gtggaccatccaaggcacattagcatggcgtacttctcctgtttgaaccggggtttgaaa

ccaaacttctcctcaggaggatagatggggcgattcaggtgagatccaatgtagatccaa

ctttctattcactcgtgggatccgggcggtccaggggggaccatcatggcgcctctcctc

tcgagaattcatacatccttatcagtatatggacagctatctctcgagcacaggttgagg

tttggcctcaatggaaaaagaaaaacggagcacctaacaacgtatcttcacagaccaaga

actacgagatcatcccttttattctggggtgacggcgggatcgtaccattctcttgactc

gaaatgggagcaggtttgaaaaaggatcttagagtgtcccttaacgtcttcttttttctt

ctcataagagttattccacaaagacttgtcatgataaggaagaagggggaacaagcacac

ttggagagcgcagtacaatggatagttgtatgctgcgttcgggaaggatgaatcgctccc

gaaaaggaatctattgattctctcccaattggatggaccgtaggtgcgatgatttacttc

acgggcgaggtctctggttcaagtccaggatggcccagctgcgccaaggaaaagaataaa

agaaagacggatttgactcctttatgcatgctccaacttggctcggggggatatagctca

gttggtggagctccgctcttgcaattgggtcgctgcgattacggtttggatggctaattg

tccaggcggtaatgatagtatcttgtatctgaaccggtgattaactttttctaagtaatg

ggaaagaggaccgaaacatgccactgaaaggccctaccgggacaaagatgggctgtcaaa

aacgtagaggaggtaggatgggaagttggtcagatctagtatggatcatacatggacggt

agttggagtcggtggctctcctagggtttcctcatctgaaatcctggggaagaggatcaa

gctggcccttgcgaacagcttgatgcactatctcccttcaaccctttgagcgaaatgcgg

gaaaacaaaaggaaagaaaatccatggaccgaccccatcgtctccaccccgtaggaacta

cgagatcaccccaaggacgccttcggcatccaggggtcgcggaccgaccatagaaccctg

ttcaacctgaaaagggataactaatgcgttctcaggttgaacagttaagggtcggagaag

tgcaattactcattcttaacttaaaacctacattcttaagagcaaagagtcggacggaaa

aggggggagaagctcttcgttctcggttctcctgtagctggatcctccggaaccacaaga

atccttgtttgaatgggattctaactcagcaccctttgatattttgagaagagttgctct

ttggagagcacagtacgatgaaagttgtaagctgtgttcggagggttattgtctatcgtt

ggcctctatggtagaatcagttggggcttgagaggcggtgatttaccctgtggcggatgt

cagcggttcgagtccgcttatctccaactcgttaatttttatgatataaaactatatgat

agcatttatagcactctatttttccgattcggtagtttagtttaagctatgttatgattt

atcatttatggacgttgataagatccttccatctagcagcaccttaggatggcatagagt

tgcaattacggccgcgggtcaaacgaggaaaggcttatggtggatacctaggcacccaga

gacgaggaagggcgtactaagcgacgaaatgcttcggggagttgcaaagaatcaaagatc

cgaggattcccgaataggtcaacctttcgaactgctgctgaattcacgagcaggcaagag

acaacctggcgaactgaaacatcttagtagccagaggaaaagaaagcaaaagcgattccc

gtagtagcggcgagcgaaatgggagcagcctaaaccgtgaaaacggggttgtgggagagc

aatacaagcgtcgtgctgctaggcgaagcagtcgaatgttgcaccctagatggtgaaagt

ccagtagccgaaagcatcactagcttatgctctgacccgagtagcatggggcacgtgaaa

tcccgtgtgaatcagcaaggaccaccttgcaaggctaaatactcctgggtgaccgatagt

gaagtagtaccgtgagggaaaggtgaaaagaacccccggcggggagtgaaatagaacatg

aaaccgtaagctcccaagcagtgggaggagctgagggctctgaccgcgtgcctgttgaag

aatgagccggcgactcataggcagtggcttggttaagggaaaccaccggagccgtagcga

aagcgagtcttcatagggctattgtcactgcttatggacccgaacctgggtgatctatcc

atgaccaggatgaagcttaggtgaaactaagtcgaggtccgaaccgactgatgttgaaaa

atcagcggatgagttgtggttagcggtgaaatgccaatcgaacccagagctagctggttc

tccccgaaatgcgttgaggcgcagcagttgactgtacatctaggggtaaagcactgtttc

ggcgcgggccgcgagagcggtaccaaatcgaggcaaactctgaatactagatatgacccc

gacgggtcaaggtcggccagtgagacgatgggggataagcttcatcgtcgagagggaaac

agcccagatcaccagctaaggcccctaaatgatcgctcagtgataaaggcggtaggagtg

cagagacagccaggaggtttgcctagaagcagccacccttcaaagagtgcgtaatagctc

actgatcgagcgctcttgcgccgaagatgaacggggctaagcgatctgccgaagctgtgg

gatgtaaaaatgaatcggtaggggagcgttccgccttagaaagaagtacccgcgcgagca

gggttggacgaagcggaagcgagaatgtcggcttgagtaacgaaaacattggtgagaatc

caatgccccgaaaacctaaggattcctccgccaggttcgtccacggagggttagtcaggg

cctaagatcaggccgaaaggcgtagtcgatggacaacaggttaacattcctgtactgccc

cttgttggtcccgagggacggaggaggctaggttagccgaaagatggttatcggttcaag

aacataaggtgactccctgttttttcagggtaagaaggggtagagaaaatgcctcgagcc

caaatttgagtacgaggcgctccggcgcttaagtaattgatgccatactcccaggaaaag

ctcgaacgacctacaacaagagggtacctgtaccggaaaccgacacaggtgggtaggtag

agaatacctaggggcgcgagacaactctctctaaggaactcggcaaaatagccccataac

ttcgggagaaggggtgcctcctcacaaagggggtcgcagtgactaggcccgtgcgactgt

ttaccaaaacaacagggctccgcaaagtcgtaagaccatgtatgggggctgacgcctgcc

cagtgccggaaggttaaggaagttggtgacctgatgacagggaagccggcgaccgaagcc

ccggtgaacggcggccgtaactataacggtcctaaggtagcgaaattccttgtcgggtaa

gttccgacccgcacgaaaggcgtaacgatctgggcactgtctcggagagagactcggtga

aatagacatgtctgtgaagatgcggactacctgcacctggacagaaagaccctatgaagc

ttcactgttccctgggattggctttgggcttttcctgcgcagcttaggtggaaggcaaag

aaggcccccttccgggggggctcgagccatcagtgagataccactctggaagagctagaa

ttctaaccttgtcccaagacagaccaagggacagtctcaggtagacagtttctatggggc

gtaggcctcccaaaaggtaacggaggcgtgcaaaggtttcctcgggccagacggagattg

gccctcgagtgcaaaggcagaagggagcttgactgcaagacccacccgtcgagcagggac

gaaagccggccttagtgatccgacggtgccgagtggaagggccgtcgctcaacggataaa

agttactctagggataacaggctgatcttccccaagagctcacatcgacgggaaggtttg

gcacctcgatgtcggctcttcgccacctggagctgtagtaggttccaagggttgggctgt

tcgcccattaaagcggtacgtgagctgggttcagaacgtcgtgagacagttcggtccata

tccggtgtgggcgttagagcattgagaggacctttccctagtacgagaggaccgggaagg

acgcacctctggtgtgccagttatcgtgccaacggtaaacgctgggtagccaagtgcgga

gcggataactgctgaaagcatctaagtagtaagcccaccccaagatgagtgctctcctat

tctgacttccccagagcctccgcgtaggggcagagaaccctcccaccccggagggtgggt

tctgcccctacggggacggagcaaaagccctacggggacggagcaaaagccctacgggga

cggagcaaaagccctacggggacggagcaaaaaaaaaagatagaatagagggtcacgnna

gagaggagggtgcttttattgttgactcattgttgatcttataaaaacttatttatgtgc

tcatattgtaaatttggactagttcaggtatttgcaggttttgtgatagcaaatctttca

gattggatgacaaagaggttgggattatagcacatgatgttttgttgattagtttgtgga

caacacatattctttttttggttgatttatccttccacttttctgatttagtgtcatagc

atgagtcaatatatgttgaaaaccaaagccttaatcctgttttgttttttgaaattttta

aggaaagcaacaaaagagtgttgtttccatgatgtatttgttttgtgtatatgttttttt

tgtgttttgttttagagtgagtacgtctttgaatgaggtggtagcacaatttgtgactac

accagtaaatgttgtttttttaatggaagaaattgaaaattacttgagagcgatatagaa

atgtaccatgaacaagaacaactatagttatccgaatatctatatgttttatcaattacg

tatacgaaaaataagtatattacttttgtgtttgtctaaaattatgaaaaggaaattttg

tattt

>fava_dn_rep_c4

tcgaactgattttatgttagtctaggaatgaaaaacatgaaacataagtttaaaacttgg

taagaaaaaacgcatcacctagtattagtgaacctatgcatagagacatgataatgataa

taattcacattcaaatgggaagaacattacaacaacgtatacttgacagaaaacataaag

tgttcactttccgggaacaaagttggtggcatatgaccaggcgttgttgttgactgggtc

ggcaagatgatcggcaaggttctccaaaggaccctttccggtcacaatagcttggacaaa

gaatccaaacatggagaacatagctaatctaccgttcttgagttccttcaccttcaactc

tgcgaatgcttctgggtcgtcagctaagcctaatggatcaaagctgccacctgggtaaag

tgggtcaacaacctcaccaagaggaccaccggcaatacggtaaccttcaacagctcccat

caagataacttgagtggcccatatggctaggatgctttgtgcatggaccaagcttgggtt

tcccaagtaatcgagtccaccctcgctaaagatttgagatccagctttaaaccatacagc

ttcaccgaatttaacaccgttgcgagacaatagctctgggaaaacacatcccaaagctcc

caacatagcccatctggagtggataacttcaagctcacggttcttggagaatgtctctgg

gtcagcagagagtccagcagtgtcccaaccgtagtcaccggggaattctccagtcaagta

tgacggagactcaccggagaatgggcctaagtacttaacacggtctggtccgtaccatgg

gcttccagaggaggctactttcttggtggtagcagacttcctcatggtgaaccttgcagc

tcccaattcttggcttgatgggttcagcttgactggcttgccagtcaaggttggggaaga

gagagccatggatgatgcggccatcgtgtgtattgcaatgaaattgaaaagattgataag

cttttgataattgtattcttatgaatggtcccgcggcct

>fava_dn_rep_c5

aatttctgtacataccagatgcaccatncttttttttttttactcacgaatttattactt

cacattgataaaaggaactcaattacatccattgaactaatgaattgaaagaagaagaag

aaaaaaaatacaagaaaataggttgaatgaaataacaactattataagcataagcattac

ttagaaaataggttgaatgaaatagcaactattataaataaggcttaaccgcttccacca

aggacactcttgagtttcttgataagaacaggatcaaggaaagtggtctttgtaatcaaa

ggagtagagaaattgcttgcaaaaagtgcaaaatcaagaatctgaagaccaggactagca

ctactaaaaacaggaaaagcaacagcatttctcttaccagcagtagcttgaaaatgcaac

aaaccttgtggaaaaaccatgagttcacctttcttcaatgttttaatataaacagtgtta

gctgaagaaacaaatccagcagtgatgtgaccttgtgtcacaataagaagttcacttgca

cccgggtgactgtgtagagggataacacctgctggaccaaggtctaacctagctgctgag

agaccaagaccattaaggcctggaaattgtgcgacgaatgcaggcgtaacggcagcgttg

ataatgtttgttacgtttgctggctcaattaggcctgaatatgcaaagtcttccacggtt

actgttgtggctgacatgcaagggaagcctgaaggggtgtctgagcctttgagatctgct

acacagaagtcttggactgaggcattggaaatggggaggaaaagtgagaggaggaaaatg

atttgaaacatgtttggttgtgtttgatgaattgttggtttggagtgtcccgcggccgta

atggccactctggttgataggattcttcggtgctatatggcttctacaatcatctgaaat

cttcaacttttctggtaaacttccatgtaactcagcatttttttcgattgatgatttaaa

atctgtttgtcttcaaaactctttcgatttctagcccaccagatgtagtaagagatggta

gttatcattttagagcttctacaggagcatgcagaatcgacctttgaatctgcaacttat

tttgtatattcttatttctcaattttctatgatatcatgattctcaacatggtatcagag

tcatagtttcctccttgaatcacaactccgctacattttcttgagaaaaatcaattccgc

tacattttccaaatttcttgattcacgtttcttgattattcgttgtcaagaaaatatttc

aaaacctaattggttattcat

>fava_dn_rep_c6

ttttagaatttcagtattttattttagnnnttttttcggtttaaatgtgagatcctcaac

ataactcatgttgcatttaaacatgtgagaatccaaattcgattaacaccataattatgt

ttacactataatgataagctttaaacatgctttgaacatcataaacagaaatggtgctag

cttatagaataaatatgattttcttttgctcggtacatgtaatatttccattactaacaa

acaatgaaaaacaacaagagataaatttaaaagggaaaattaagatcctacgtcacttct

tcttccctgcctttccttcctttctccttacaacttcatcttcatatttgatacccttac

ctttgtagggttccgggggtctccattttctaattgaagccgcaaactgaccaatatccg

acttgtcatatccactaactgtgattctggtgttttcgtctaccttcacttttagaccat

tagggattggcatcctaacaggatgagaaaatccaagattcaacactacttcatttcctt

ctaccgaagcacgataaccgacaccgataagttggagtgttttttcaaaacctttagata

ctccaaccaccaggttgtctgtaagtgtcctgaaaagcccatgcatttcgttcgcccttc

tggtttcgactgccttactgaccttcaaaatcccttcctcttgcctctcaaccttaactt

ctcgaggataagttaacgaaagttctcctaaaggacctttcacttgtatatgctgccctt

ccaatttgatagtaacattggatggaacaggaattggctgtttcccaattctcgattctt

tacattctatgatctttctagagaaaccaacatgagtagcaggagcacttgaaactctta

gcctacttctctcgccgataaaagcagacttaaaattgctgatttgaaaagaagaggtaa

tggaagaagccattgttacaaagcacaaaggcttttgagagtgtgttggaaaatccttat

ccaatgaagatcccgcggccg

>fava_dn_rep_c7

tcgagaaaaataccgttattttatatcaaagattagaaagcaacgtgactagatgaattc

tttatttaagctgaacacagaataacaataagttgttgccactcagaacatacatagaca

atacaacaattattcgtactaaagacaaatattcaacaaaacacatggaatgagatgatg

agacaatcttagaaaataacatcaacatgtcttatgactcaaaaataaccacctgaacta

ataacaacatagagaatgtttttgaggtgaataattttcaacaggtagctgtgattacta

ctggttctactaaagactttgccccaccggcaactccattttccgctctatgaacctcct

cggccgccatgacaacaaagtcctcgaaacaatcctccatgctcctgaacgattctcccg

gatacaaactgctaatctcaatatcatgaattccatctatcttgtaattaacttgacatc

cattgataaatatatcatgagtgaatgctgcaacaatactctctggtattatattttctg

cggctattgctagaagatcgttttcagagacgacgagtctaggaatttttcgagcaattt

tattctcccacaaagaagcaagttcattcatgttataacagttgatcgaaggacgaaaat

gaatatttttattaatcgctctatcatcatcaactatcttcatagtgaactttccaatat

caatgccatcaacaaagtaagctttgatgttgccgttaccatagacgtgcaactgatcca

acggtggaggaagcgaggatggatggcaattgttatagtaaggccaagatgcgatggaat

tgcaacacacgtaggtgtagggtacaccggattcctcaagcgcgcgcctaaccaaacgct

tctgtttgtacatttctagacctggttctacaggttctgctctgtccacatcgtgaccaa

attctgaaggcaaaaacctcttaatggtcttaattgatttcatggcctccaccaaagtaa

tctgatccagcaaaccatcagctcctattgcagaaattactatatctatttcatactttc

tcaaaatcttctccatgaactccttgttatctactccaccgtaaatgataacggctccat

tatcttggaaagtcttaataatggaagacttggaagaaataagagatcctccgggtcgga

tcaacaaaaaggtaggatgttgtgaggagagacttgcctcagctaagaattttcctatga

aacctgttgcaccaatgattagaactcgagtcttgggagcaagagtggttggtggtgatg

atagtgccattgttttgcttttggggagtgataataattgtaacaatttgggtatggaat

gaccaaatcaaagggtgctaagtttgtttatatagaataaagaaatgttaccgttacatg

aatattatttatttactataaatggaagtaatattggtagggaggggttgctatattgtt

ttacatcatctactcttcaacacatgtaacgtgtt

>fava_dn_rep_c8

cgcaacattgaagtctaagccataatgagaaatgataggtaggagattcaatccgccatt

caccacttgtaactccatctccattcacctacctaccaccacctccatttctatatatca

cacttcacaacacaagaaactgcattgcaattaacttaccatccaatgacaacaccgtcg

gtaaccgctttgaagatcgagaactaccaattccctcccaccgtcaaaccaccaggatcc

gacaacaacttcttcctcgccggtgcaggagacagaggtcttcagattcaagacaaattc

gtcaagttcaccgctattgctgtttatttgcagactattgctgttccgttcctcgccgac

aaatggaaggggaaggatgctcatgagttgacggaaactgttcccttcttcagggacatc

gttacgggtcctttcgagaagtttatgcaggtgacaatgatcttgccgttgacagggcaa

caatactccgagaaagtttcagaaaactgtgtcgccatttggaagtctctcggaatttac

acagaagaagaaggcaaagcaattgacaagtttgtttctattttcaaagacgaaacattc

ccaccaggctcctctattctcttcacagtatcacccaaagaatcaggatcattatcgata

agtttctctaaagatggatcaattccagaggctgagtctgcagttatagagaataaacta

ctttcacatgccgtgcttgagtcgatgataggcgcgcacggtgtctcccctgcagcaaaa

cagagtctggctgccagattatctgagttattcaaagaagctggcgatgctaaaaactga

ttatatcactaaacagaagataaaagtctgttctgcaataaagaccaagcgcaaaattta

tcttaagtccacttttatatcacctcttgaagacttttagtgttctaatcaagaatgtat

tgtatttgtttgaagctatataatattagttaaaaaaattaaggtaagaaatggaacatt

ctatcattttattaagccttttatttcaaataatggcataaaagtacacaaaatgcagag

acaagacaatttatttaaaaagaaaataacattaaacctatgacacatagatgacacatg

gattattcaaagattatgaactttgtatatagacaactgctaacaattctcagtgttcaa

ttttgaagttgtcattctgcataaactcatacaatcttgtagccaaactctctttcaatg

aaggcgaaaccggaatttcgccgatcatagtctccaacaccgcctccgaaaccggcttat

tgtgaataattgtatgctcatgttcaggtattgtctcatctttggagaaactaagagcta

atgttccatttggtgtttgtctgtaaaaagcagcagtccttggtggaaaattttgatcct

tgaaagcttgttttaattcttgaatggctttttcttcttcttcaccatataatccatcag

attttatatgggctatgcagttttctgagactttactcacatattttggacctgatagtg

ttttcagctttacacttcgaatcagtttctcaaagggacctttgatgatgtctctgtaga

agtcaagggattcaatgagttcaggtggagtctttcccttccacttaggagataatgatg

atacagcgctctcttccaaataaactcctattgtagtgaacttcacaaactctccatcaa

tgtctaaacctctcactcctgcgccaccgaggaaataagacttggtggtggccggagatg

tcaacagtgccgggaattcaacaagctccaccgtcactccagtgatagatgatactgtcg

ccatagctacctacacctttgttctactggtggtttttgaagtccccgcggctnnnnnnn

nngttttttccttttttttttttatcttggagattttgtaatgcttactctaaaactatt

tgtttacacaatagtgatattctttgtttctcttttcatcttcggattcctatctaatga

tccaggacgtaatcctggacgtgaagaataagaaaatctttgttcttttccgtacttatg

ctggatttttaaatatttttcgtttttctatctattttacatctttaactagtaaaaaat

gaaacaaacagggaataaaaaaaaaagaaattccagaagttcaaaagaaaaaaatgccaa

atcatcaataaacggaaagagagggattcgaaccctcggtacaaaaattcgtacaacgga

ttagcaatccgacgctttagtccactcagccatctctccccaattcaaaaaattgaatta

ttatgcttatgatacctcggataaacaaaaagaacaaaaagtagggctcaaaaaagcctt

ctatatatcttatttgatattgatcgtcatttgatatatcttatctatctatcttttttc

tatttgattatcattttatttaattactattcataattctatattaactcataaatatta

gttatattttcttatacctatactaaagttatattctatatatacgcaatataaaatttt

ttcttttcttttttttttagtttgtttttttatccaaccagagtccagctaggtactggc

acggctcttttttcccatgaatcacaatgatttatttttctgtatcagatttgaaagaaa

cttgtaattaaggaaaacattttaatacgatcaaacaaaaataaaaataactttttgatc

gtatatcatacgaattaatcagttaacgtatctaaaaaaaaaaaaagnnnnnttacactg

ccactttatattgaaataaatgttgatttgtatcatagtttaattaaagatgacttataa

ttgttgtacactttctactacctcctgcacattttagcctaacttatatggttgaagatg

aaactgaaagcatgagctttcgtcataagataaattgtctttgactaaggttatatatga

aattatcaatgtaacactcatcgtagggctattcccgcaaaattgttgctttcatgcatt

taatgcatgagcct

>fava_dn_rep_c9

gaaggatatatcaaaaaaaataaacatttctcaaatatcaaatgtaaagagaacaaacag

taagtaaaccataacaaaatatcacattaaacatcaattcaagattgtcttcaaaacaga

cataaaaacagctcagtttaacaatataataaaaggcagagaaaaaaacaaattcaaaat

ccatgtagcaaaactaaagtaatcaaaagtctaatatttcaaaactaaagataagcctct

aagcattgcagcttgcattgataaaagctcttccaggttgacgattggccagaggtccat

ggcatccaagaagcttagcattcacaccatcattcagaatcctgttctctgacttcaact

tcaagtaatcatttctgtcaatcttggtgaatccccacttcctactgacaatgatcttct

gacgaccagggaacttaaacttagccctacgaagagcctcctgtgcattctgtccattgc

tgtccttgcagcgaacagagagaagcacctgtccaatagcaaccctagcacaaacacctt

gtggctttccaaaagcaccacgcataccagtctgaagcctatcagctccagcacaggaaa

gcatcttgttaatcctaagaacatggaaaggatgaaccctgaccctcaagtggaatgcat

cctttccagcaaacttagtcatgtacttgttgcaagcaatacgagccgcctcaagtgccc

cgcttgaaacattctccttctcccaactgacaagatggacacagaaaggaaactcatcaa

cacctttcttcttcatgccaacatcgtagatcctgatcttgggatcgggaacaccacgac

agaatcgggactttgggtatggcttgttcttaatctgacggtaacatcttgctggtcttc

ttcccatggcggcggcgcgatttgttctctgcgacccgcggccgtaatggccactcttcg

tcttcccacatcaatggctctaaatctctgcacctcccctaaatggattcgcaccaccgt

cttcgatagtgcttcttcatcgaagccatcacttgctgcttcttcttcttcttctacaac

ttcgttttcttcttcaattctaagttcgaagagtgttggtctacagagattaagcttgaa

gaggaatgttggtgtcagtgttagatctagcttggaaactgcgggacccactgtgacggt

agggaaagtaactgaggtcaataaggatacgttttggcccatcgttaatgccgccggtga

taaaaccgtcgtcctcgatatgttcaccaaatggtgtggtccttgcaaagtgattgctcc

gttatacgaagaattatctcaaaagtatttggatgttgtatttctaaagcttgattgcaa

ccaagataacaagtccttggcaaaagaactaggaattaaggtggttcccacttttaaaat

tctgaaggacagcaagattgtaaaagaagtaactggtgctaaatttgatgatttgctcgc

tgccattgacagtgttcggtctagttagacggtagtattataactattctgtttctacgt

aaaaatatcttgtttgatactttctgtaagtatttctactgtaattgtgagcttgctgag

gggaagacaacgaaatgtcatcatgttgttggaagctgtaattgattgttccctacgtat

agggaaaagttgctcaaaataatttctgttaccaacttgatcatgactatctataaaggt

ggctctgattgcttatatttttggaagaaaataaaactgcattagataaaaaagatttga

gtctgtgctgtcaaggaatatctaatctagcggggtattatagtatgccaaaacactcta

cacttaataatgtctagcagttcaatacacttcaaatcaagttgatgcagttagaaatcc

tgcacatataaaaatccaagcaagaaaatgtggagaagttacttaaactttgtttgagag

ctaggagggaatgggaaatagaggactttgaaaaag

>fava_dn_rep_c10

atctatttccattataacaggaactgttaatatatgtcaaccccataatatcacttttca

ttgttacacattatataattgggtattttctatgtaattatggattacagggaattttca

ataaattctcgtactttcatttttttgccaatttttcatgaaatagattgatgaatgaat

agaaaaaaaatgaacacaacatattatgtcccgaacaaataggactgcaataacgtaata

gacatatatagatagctatagctgaagttagatctatgcatgtttcataaatccaagtct

ttttatgcactgcaatcctctcattctaaaaaaaaaaaaagggtcaaaatatctctcttc

tttgttaattttcattcttttttgaacacttgaaatggtaaagggctcaaaaaaattgta

tattttttctgattattttctttttaaaatctttctctcatggagccaatcaaatcctga

atttatttttggggtatcaatcgaattggaatatgtaatatcatactctactaaaagtct

atttgtttgttttgttgcaaattctaatttgagtagcaaaattcctcttttcataatgcg

tatttcatagacgtagtgaggtaaaatttcatgtgattcagtaaagtaaaaagaacataa

attccattattgctaaatatattcatgtgattcgatgaagaatgacagcaaatcatggga

tattttctataaaataaaaaaggggtaatcaaaaatgcttggggttgggaatgaaggaat

gtctacactaccgggattgaatcaaatacaatttgaagggttttgtagattcattgatcg

gggcttaacagaagaacttttgaagtttccaaaaattgaagatacagatcaagaaattga

atttcagttatttgcggaaacatatcaattggtagaaccctcaataaaagaaaaagatgc

tgtatatgaatcacttacatattcctctgaattatatatatccgcgggagtaatttggaa

aaacagtcaggatatccaagaacaaattatttttattggaaacattcctctaatgaattc

cctgggaacttttatagtaaatggaatatacagaattgtaatcaatcaaatattgcaaag

ccccggtatctattatcggtcagaattggaccataacggaatttcagtctatactggcac

aataatatcagattggggaggaagattagagttagaaattgatagaaaagcaaggatatg

ggctcgtgtaagtaggaaacagaaaatatctattctagttctatcatcagctatgggttc

gaatttaagtgaaattcttgagaacgtttgttaccccgaaattttcttgtctttcctcaa

tgaaaaggaggaaaaaaaaataaggtcaaaagaaaatgccattttggagttttatcgaca

atttgcttgtgtaggcggagatcccatatttcctgaatctttatgtacggaattacaaaa

aaaattttttcaacaaagatgtgaattaggaggaattgggcggcgaaatatgaaccgaag

gctgaatattgatatacccgagaacaatacatttttgttaccgcgagatatattgacagc

tgcggatcatttgattggaatgaaatttggaatgggtacacttgacgatatgaatcattt

gaaaaataaacgtattcgttccgtagcagatctcttacaagatcaatttggattggctct

cgttcgtttagaaaatctaattataggaactatatgtggagcaattagatataaattgat

accgactcctcagaatttagtgacttcaacgccattaacaaccacttatgaatctttttt

tggattacatccattatctcaagttttagatcaaaccaatccattgacccaaatagttca

tggaaggaaattgagttctttgggtcctggaggattgacggggcgaactgctagttttcg

gatacgggatatccatcctagtcactatggacgcatttgtccaattgacacctctgaagg

aatcaatgtcggacttattggatccttggcaattcacgcaaggattggtcgttgggggtc

tatagaaagtccattttatgaaatttctgagagatcaaaaagaatacgaatgctttcttt

atcaccaagtagagatgaatactatatggtagcgacgggaaattttttagcactgaatcg

aggtattcaggaggagcagattgttccagctcgataccgtcaagaatttcttactattgc

atgggaacaggttcatcttcgaagtatttttcccttccaatatttttctattggagcttc

cctcattccttttatcgagcataatgatgcgaatagagctttaatgagttctaatatgca

acgtcaagcagttcctctttctcggtccgaaaagtgcattgttggaactggattggaacg

ccaagtggccttagattcgggagttcccgctatagccgaacacgagggaaagatcgttta

taatgatactgagaagattattttatttggaagtgggaatggtctaagtattccattaat

tatatatcaacgttccaacaaaaatacttgcatgcatcaaaaatcccaggttcagaaggg

taaatgcgtaaaaaagggacaaattttagcagatggtgctgctacagttggtggtgaact

cgctttgggaaaaaacgtattagtagcttatatgccatgggaaggttacaattttgaaga

tgctgtactcattagtgagcgtctggtctatgaagatatttatacttcttttcacatacg

gaaatatgaaattcagactcatgtgacaagccatggtcctgaaagaatcactaataaaat

tccacatctagaagcgcatttactccgaaatttagacaaaaatggaattgtaattctggg

atcttgggtagaaacgggcgatattttagtgggtaaattaacgcctcaaatggcaaaaga

atcctcatatgccccggaagatagattattacgagctatacttgggattcaggtatccac

ctcaaaggaaacttgtctaaaactacctataggtggtaggggccgagttattgatgtgag

atggctccacaaaaaagcggattctagctataatccagaaacaattcatatatatatttc

acagaaacgtgaaatcaaagtaggtgataaagtggccgggagacatggaaataaaggtat

cgtttcaaagattttgtctagacaggatatgccttatttgcaagatggaagacccgttga

tatggtcttcaatccattaggggtcccttctcgaatgaatgtaggacagatattagaatg

ttcgctcggattagctgggagtatgctaaatagacattatcgaatagcaccttttgatga

gagatatgaacaagaggcttccagaaaacttgtgttttctgaattatatgaggccagtaa

agaaacatcgaatccatggatatttgaacccgagtatccgggaaagagcagaatatttga

tggaagaacggggaatccttttgaacagcccgttataataggaaagccttatatcttgaa

attaattcatcaagttgatgataaaatacatggacgttccagtggaccttatgcacttgt

tacacaacaaccccttaaaggaagggccaaacaaggaggacaacgggtaggcgaaatgga

agtttgggccctcgagggattcggtgttgctcatattttacaagagatgcttacttataa

atctgatcatattaaagctcgtcaagaagtacttggaactacaattagtggaggaacaat

accgaaacctgtggatgctccagaatcttttcgattgctcgttcgcgaactacgatcttt

ggctctggaactgaatcatttccttgtctctgagaaagactttcagattcaaaggaagga

agtttaattggactgaatccgaaattttattttatgattgatcagtataaacatcaacac

cttcgaattggattagtttcctctgaacaaataagtgcttgggcaaaaaaaatcctacct

aatggagaaacagttggggaggtaacaaaaccctatactcttcattataaaaccaataag

cctgaaaaagatggattattttgtgaaagaatttttgggcctataaaaagtgggatttgt

gcttgtggaaattatcgagtaatcggagataaaaaagaccaacctcaattttgtgaacaa

tgcggagtagaatttgttgattctcggatacgtagatatcaaatggggtatatcaaatta

gcatgtccagtaacccatgtgtggtatttgaaacgtcttcctagttatatcgcgagcctt

ttagataaacctcttaaagaattagagggtctagtatactgcgatgtgtgatttgataaa

aattcttattgtacagatttcgaatgaaaaactgtcattccattcaattaaattggaatg

tcctatatctggcatgtctcttgggatgagtaacatgaagctcagaattcaattcatggg

tgtattgaatactcccaataagggagttggtctatggtcaattcagtaacaaaaaactct

ttatactgtatgtaccttgcgaaaaaaaaaatacttttttgtggaattcattattccatc

tcttttttttttattttaaaagaactaaaattatgttcaaagaataaaatatatcatgat

tgcagaagtttatctatcgcatataggctttaagacatcgtggcatacccgtcgaggtga

cgtcttgacctaaaagatcaaacggtatgatacagagacaaagaaatctcttatgaagtc

gaagatattccttttttattaagaatttcattttagaggattcctcgttcgaagggaggt

agactactcaagaattttacattttatttctgtcggaatttcgaattgcataaaaaattc

agaaaaaccgaagaatgtatcaatgaaatgttgcttggtctttattgataacttgagtca

agagtaaaccttttttattttatattttagattagaggttttcaaaaatatgaaagcgga

aacccctttctttatctttaattgtgtgtaaatactttagccggatgagaggaaaccctc

atgtccgattttcaaagggggagatccatcttattggatcctatccaaatttttcttttg

ctaggcccgtagttaaaaaaccaactttcttacgattacgaggttcattcgactatgaaa

tccaatcctggagatacagtctcccactcttttttgctactcaaggtttggatacatttc

gaaatagagaaatttctagtggagcgggtgctatacgagaacaattagttgatctggatt

taagaatgattatggattcttcgttgctagaatggacagaattaggagaagaggggtcca

ctgacaatgaaaatgaatgggaagatcaaaaagttggaagaagaaagaattttttggttc

gacgcatggaattagctaagcattttatccgaacaaatatagaaccagaatggatggtat

tatgtctcttaccagtgcttcctcccgaattgagaccaattattcaaatagatggcggta

aactaatgagttcggatattaatgaactctatagaagagttatctatcggaacaatactc

ttattgatctattaacaacaagtagatctacaccagaggaattagtaatgtgtcaagaaa

aattggtacaagaagccgtggatacacttcttgataatggaatccgcggacaaccaatga

gggacggtaataataaagtttacaaatcgttttccgatataattgagggcaaagagggaa

gatttcgagagactcttcttggaaaacgagttgattattcggggcgttctgttattgtcg

taggtccatcactttcattacatcgatgtggattgccccgcgaaatagcaatagagctat

ttcagacgtttctaattcgtggtttaattcaaaaccattttgcttcgaacataggagttg

ctaagaggaaaatccgggaaaaagaacccattgtatgggaaatacttcaagaagttatgc

gggggcatccagtattgctgaatagagcgcctactttgcatagattgggcatacaggcat

ttcaacccattttagtcgaaggacgtgctatttgtttacatccattggtgtgtaagggat

tcaatgcagactttgatggggatcaaatggctgttcatgtgcctttatctttggaggctc

aagcagaagctcgtttacttatgttttctcatacgaatctcttgtctccggctattgggg

atcccatttgtgtaccaactcaagatatgcttattggactttacgtattaactagcggaa

atcgtcgaggtatttgtgcaaacaggtataattcgtttaattcccgaaattctcacaatg

aaaaaattagcaataataacttgaagtatatgaaaaaaaaagaaccctttttttgcaatt

cctatgatgcaattggagcttatagacagaaaagaataaatttcgatagtcctttttggc

tccggtggcgaatagatcaatgcattatgtcttcaagagaagttcctatcgaagttcact

atgaatcttttggtacctattatgagatttatggggattatttagtaataagaagtataa

aaaaagaaattcgttgtatatacattcgaaccactattggtcatatttctttttatcgag

aaatcgaagaagctatacaaggtttttctcgagccgattcatatggtatctaatcatata

aatggttagtgttggtatccacgctaagtcaatttatgatactggtgtaaattcaggtca

actagcatcttttcaattttctacgtgaataaacataaagaaaaggaagttttccaatca

ttaactcaaatccattgtcgaaccgaataacactgagtggaatatggaggtacttatggc

agaacgggccgatctagtctttcacaataacgtgataggtggaactgccattaaacgact

tattagcagattaatagatcatttcggaatggcatatacatcacacatcctagatcaagt

aaagactctaggattccatcaagctactgctacatctatttcattaggaattgatgatct

tttaacaataccttcaaaaggatggctagtccaagatgctgaacaacaaagttccatttt

ggaaaaacataatcattatggaaatgtccatgcggtagaaaaattacgccaatccattga

aatatggtatgctacaagcgaatatttgcgacaacaaatgaattccaattttaggatgac

tgacccctttaatccagtccatatcatgtctttttcaggagctagaggaaatgcatctca

agtgcaccaattagtcggaatgagaggattaatgtcagatccacaaggacaaatgattga

tttacccattcaaagcaatttacgtgaaggactgtctttaacagaatatataatttcttg

ctacggagcccgtaaaggggttgtagatactgctgtacgaacatcagatgctggatatct

tacacgtcgacttgttgaagtggttcaacacattgttgtacggcgaacagattgcggtac

catccgtgggatttatgtaaacacccgaaatggaatgatgccagaaagaattttgataca

aacattaattggtcgtgtagtagcagacgatatatatataggttcacggtgcattgtcgt

tagaaatcaagatattggaattggacttatcaatcgattcataacttttcaaacacaacc

aatatttattcgaaccccctttacctgtaggaatacgtcttggatctgccgattgtgtta

tgggcggagtcctattcatggggacctggtagaattgggagaagctgtagggattattgc

tggtcaatctattggagaaccgggaactcaactaacattaagaacttttcatactggtgg

agtattcacagggggtactgctgaatatgtgcgagccccctcgaatggaaaaataaaatt

taatgaggatttagttcaccctacacggacacgtcatggatatcctgcttttatatgtaa

tatagacttctatgtaactattgaaagtgacgatattatacataacgtcattattccacc

aaaaagttttctattagttcaaaataatcaatatgtaaaatcagaacaagtgattgcgga

gatccgcgcgggaacatctactttaaatttgaaagagagggttcaaaaacatatttattc

tgactcagaaggggaaatgcattggagtaccgatgtgtaccatacatcacaatttatata

tagtaatgtacatatcttaccaaaaacaagtcatttatggatattgtcaggaaagtcgtg

caggtctaatacaatccattttttacttcgcaaggatcaagatcaagttaccatggattc

actttctaatggaaaaacaaatatttcgaatcttttagaaaggaatgatcaagtaaaaca

taaattcaatacttttggtacaaaagaaaagtggattagcgattcttcaatattgaatca

aatcatatgtacagatcattctgatttaatgtatcctgctattttgaacgatacttttta

tttcttggcgaaaaggcgaagaaatagatttcttattccatttccattccaatcgattca

agaacgaaagaacgaactaatgtccccttccggtgtctccattgaaatacctatcaatgg

tatttttcatgaaaatagtatttttgcttatttcaatgatcctcaatacataagacagag

ttcaggaattactaaatatacaactataggaattcattctatttttcaaaaagaagattt

cagtgagtatcaaggaatcgaagaagtaaagccaaaataccaaattcaagtagataaaat

tttttttattcccgaagaagtgcatattttacccgaatcttcttccctaatggtacggaa

taatagtcttgttggaataggaacaccaatcactttcaatataagaagtcgagtaggcgg

attggtccgattagaaaagaaaaaaaaaatagaattaaaaatattttctggaaatatcca

ttttcccggagagatggataagatatcccgacacagtgccatcttgagaccgcccggaac

ggtaaaaaaaaaaaaatgcaaggaatcaaaaaaatttaaaaattggatttatgtccaatg

gatcgcaactaccaagaaaaagtattttgttttggttcgacctgtaattttatatgaaat

accggacagtatcaattttgtaaaactttttccccaagatctattccaggaaaaggataa

tctggaactaaaagttgtcaattatattctttatggaaatggaaaatccattcggggaat

ttccgacacaaggattcaattagttcggacttgtttagtcttcaattgggatcacgggaa

aaaaagttcttctattgaggaggcccccgcttcctttattgaagtaagtacaaatggttt

gattgagtattttctaagaattgacttagtcaaatctaatacttcatatattagaaaaag

aaatgacccgtctggtttaggattgatcagggataatcaatcggatcgaatcaatccctt

tttttctattcattccaagggaaaaattcaacaatcacttagccaaaatcacggaactat

tcgtatgttgttgaataaaaagaaagaatgccgatcttggataattttgtcatcatctaa

ttgttttcaaatgagaccattcaacaatgtaaaatatcacaatggaataaaaaaagatcc

tataatttcaattaataataataattcgttcggccctttaggaatagcccttcaagttgc

gaatttttattcactttatcatttaataactcataataagatctcaatcattaaaaattt

gcaacttgacaaattcactgaaatttttcaagtaattaaatattatttaatggacgaaaa

tgagaaaatttttaaacccgatctatacagtaatattgttttgaatccattccatttgaa

ttggttttttctccatcatttttattgtgaaaaaacgtttacaataataagtcttggaca

attgatttgtgaaaatatatgtatagctaaaatgaaaaatggaccacacctaaaactaaa

atcgggtcaagttataacagttcaaatggattctgtaataataagattggctaatcctta

tttggcgactccagaagcaaccattcacggccattatggggagatcctttctcaaggaga

tattttagttacattcatatatgaaaaatcgagatccggtgatataacacaaggtcttcc

aaaagtggaacagatattagaaatacgttcgattgattcaatatccatgaatctagaaaa

aagaattgatgcttggaacgagtgtataacaaaaattctcggcatgccttggggattctt

gattggtgctgagctaactatagcgcaaagtcgtatttctttggttaataaaatccaaaa

ggtttatcgatcccagggagtacatatccataatagacatatcgagattattgtgcgtca

aataacatccaaagtcttggtttcagaagatggaatgtctaatgtatttttacctggcga

actaattggattattgcgggcagaacgaatggggcgtgccttggaagaacccatttgtta

ccgagctttattattgggaataacaaaaacatctctgaatactcaaagtttcatatctga

agcgagttttcaagaaactgttagagttttagcaaaagccgctcttcggggtcgtattga

ttggttgaaaggtcttaaagagaatgttgttttaggggggatgatacccgttggtaccgg

attcaaaagaataatgcaccgttcacggtcaaggcaacataacaagattacccgaaaaaa

aaaaagg

>fava_dn_rep_c11

aaaaaaatagttaagaaccatagcatttcgcgattcattgggaaatctactttgattctc

tatcaaccaagaattttggaacattaccatggttaaagctaaccagtttgaagtttcgac

gcagtctagtattctttctaccactatgttaatagtgaaattcgaattttttcgatatag

aacactcatgtcgaaaaactgacttgaattctctttatgatggatgaaaaataggaaaaa

ggtgttgtgtttaacgcctccgtttctacttttctacaatgcagaattgatgacctacgt

ataaattaatgagaattctttggatttcaagaaaaaaaaaaaacaactttgctgacaatt

atttgtttggtcagaagagtcctccaaatattggggtcttgtattgataatcattttcaa

gatttttcgaaatagagaaaagaggataggctcattccataataaatatggggaaatttc

ctataaggaattgagtgtgagagccaaatgaattgaaagattcatgtttggttcgggaag

agatcatagacattttgaaatgaatggaaagagaatctactttcattaagtgatttatta

gataatcgaaaacagagaatcttgagaactattcgaaattcagaagaactacgggaaaca

gccattgaacagctggaaaaagcccggtcccgtttaaggaaagtagaaatggaagcagat

cggtttcgagtgaatggttattccgagatagaacgagaaaaattgaatttaattaattca

atttatacaactttggaacaattcgaaaattacaaaaatgaaaccattcattttgaacaa

caaagggcaattaatcaagtgcaacaaagggttttacaacaagcattacaaggagctctg

ggaactttgaatagttgcttaaacaacgagttacatttacgtacgatcagtgctaatatt

ggtatgtttggggcgatgaaagaaaaaaataactgattagttgttctactataattgaga

gtataggcaggcaggccttctttttgtttttcttcgaaaaagaatcaaattcagaaatat

tcatggtaaccattcgtgcagatgaaattagtaaaattatccgtgaacgtattgagcaat

ataataccgaagtaaaaattgtaaatacaggtaccgtacttcaagtaggcgatggcattg

ctcgtatttatggtcttgatgaagtaatggcgggtgaattagtagaatttgaagaaagta

cagtaggcattgctttgaatttggaatcaaaaaatgttggtgttgtattaatgggtgatg

gtttgatgatacaagagggaagttccgtaaaagcaacaggaagaattgctcagataccgg

taagtgagggttatttgggtcgtgttgtaaatgccctagctaaacctattgacggtcgag

gagaaatttcagcttcggaatctcggttaatcgaatctcccgctcccggtattatttcca

gacggtccgtgtatgagccccttcaaacgggacttattgctattgattctatgatcccta

tagggcgtggccagcgagaattgattattggggacagacaaactggtaaaacagcagtag

ctacagatacgattctcaatcaacagggacaaaatgtagtatgtgtttatgtagctattg

gtcaaaaagcatcttctgtggctcaagtcgtaacttctttacaagaaagaggagcaatgg

aatacactattatagtggctgaaacagcaaattccccagctacattacaatacctcgcgc

cttatacaggagcagctctggctgaatattttatgtaccgtgaacgccacactttaatca

tttatgatgatctctccaaacaagcacaggcttatcgccaaatgtctcttctattacgaa

gaccaccaggtcgcgaagcttatcccggagatgttttttatttacattcgcgtcttttgg

aaagagccgctaaattaagttctcagttaggtgaaggaagtatgactgctttaccaatag

ttgagacccaatcaggagatgtttcagcttatattcctactaatgtaatttctattacag

acggccaaatattcttatctgccgatttattcaatgctgggatcagacccgcaattaatg

tgggtatttccgtttccagagttggatcggcggctcaaataaaagccatgaaacaagtag

ctggtaaattaaaattggaattggcacaattcgcagaattagaagcttttgcacaattct

cttcagatctcgataaagccacgcaaaatcaattggcaagaggtcaacgattgcgcgagt

tgcttaaacaatcccaatcagctcctcttactgtggaagaacagataataactatttata

ctggaacgaacggctatcttgattcattagaaattgaacaggtaaggaaatttctccttg

agttccgggcttatttaaaaacgaataaacctaaattcaacgaaatcatatcttctacca

agacattcactggggaagcagaagccattttgaaggaagctattcaggaacagatggaac

tctttttacttcaggaacaagtagaaaaaaattgattaaaattttcataccgttaaaaaa

tttctatcattagtctcttaaaaaaaaaaagaacaaaaaagaattctatactaaaactaa

atgaagaaatttactaaataaaaaatagaatataagcgggtagcgggaatcgaacccgca

tcgttagcttggaaggctaagggttatagtcgacgtttatgaatgaacgtctctaattca

aaactgaacgtgaaacttttgtttcattcagctcctttacagaataatttaagagataga

tggaatacatgaaaaaaagaatccataacatctatgtcagcctttcggtatgaatacaat

taacgttgctttttagatgatccctatagaaaaggagtattcgaacaatctctttttttc

tagttacttcgttctctatttctatttgagagaatctttaggaaaagaataaaatttccg

tcgagctaaaaaaatatgttgatgtttctagtaaactaaaaaaatcatttcatagctatt

ttgcttcaatctctcctatacaaaacaaataataaaaaaattggaagatttagttacgat

tggaaacgaattttatatatctttctccctggatcctttacttatattcaaaaattggga

ttctggatccaattgcaaaaacttatgtttcataattttagaatcaattctaaattgtat

acaaattatgataatgtatattattcctcgaattgctccttgagaggtataaaggattaa

atacctttaagtaagaaataaagtttttgatcgtgatatgaatcacggaagggacccctt

aactattactattaaggggtcgatagaacgaatcgcgcttttaccactaaactatacccg

ctaccccgcggcctaatgcttcggcattgtggctgctcgatcacagaacaaaaaaaaa

>fava_dn_rep_c12

ggtgttttactgtgggccaccggcaattactaaaatcgggtcaagttataacagttcaaa

tggattctgtaataataagattggctaatccttatttggcgactccagaagcaaccattc

acggccattacggggagatcctttctcaaggagatattttagttacattcatatatgaaa

aatcgagatccggtgatataacacaaggtcttccaaaagtggaacagatattagaaatac

gttcgattgattcaatatccatgaatctagaaaaaagaattgatgcttggaacgagtgta

taacaaaaattctcggcatgccttggggattcttgattggtgctgagctaactatagcgc

aaagtcgtatttctttggttaataaaatccaaaaggtttatcgatcccagggagtacata

tccataatagacatatcgagattattgtgcgtcaaataacatccaaagtcttggtttcag

aagatggaatgtctaatgtatttttacctggcgaactaattggattattgcgagcagaac

gaatggggcgtgccttggaagaacccatttgttaccgagctttattattgggaataacaa

aaacatctctgaatactcaaagtttcatatctgaagcgagttttcaagaaactgctagag

ttttagcaaaagccgctcttcggggtcgtattgattggttgaaaggtcttaaagagaatg

ttgttttaggggggatgatacccgttggtaccggattcaaaagaataatgcaccgttcac

ggtcaaggcaacataacaagattacccggaaaaaaaaattattcgaagtagaaattagaa

atcttttgttccatcacagaaaattctttgatttttatcatttcaaagaatttatgtgat

acattattagaaatataggattaaatggttcctaaaagcggattcgactcagccccttaa

aatctttaaatgcagtcatttgatttggtaataaagtagaataaaaaataataataaata

gaaataaaagaatggcctgattctatcttaacggccaatcgtctataaaagagaaggttc

catcggaacaattatttattgctatttcaggatactgggtctctctttattttttttcaa

tttttttttttttaaaaaagtgtggggataaatgacaaaaagatattggaacatcacttt

tgaagagatgatggaagctggggttcattttggccatgagcctaggaactggaatcctcg

aatggcaccttttatatcgggaaaacgtaaaggtattcatattataaatcttactaaaac

tgctcgttttttatcagaagcttgtgatttggcttttgatgcagcaagcacaggaaaaca

atttttaattgttggtacaaaaaaaaaagcagccgatttagtaacacgggctgcaataag

agctcgatgtcattatgttaataaaaaatggctcagaggtatgttaacgaattggtatac

tacagaaacgagacttcgtgagttcagggacttgagaacggagcaaaagacggggaaact

gaactctcttccaaaaagagatgccactatgttgaagagacaattatctcatttggaaag

atatctaggcggtataaaatatatgacaagattacctgatattgtaataatccttgatca

gcaagaagaatatacggctcttcaagaatgtatcactttgggaattccaacgatttcttt

aattgatacaaattctgacccagatcttgcagatatttcgattccggctaatgatgatgc

tatagcttccatcggattaattcttaacaaattagtttttgcaatttgtgagggtcgttc

tagctatatacaaaaaaattgattaataataagataaatcaatttttcgatttggttggg

cgtgcggtcatagatttttggaattggttattatagcattacaaaattgtgtaaaaagaa

atattttgtgattagttaggtattcaaaatagaaaatcaaagtaaaataaggaaatggtt

gaatcaaaataattccctttcaaggtatatttttttattttagaggacagggcaatatga

atgttctattatgttacatcaacacattaaacagattctatgatatatctgctgtggaag

taggtcaacatttctattggcaaataggggattttcaagtgcatgctcaagtacttatta

cctcttgggttgtaattgctatcttattaatttcaaccattctagttgttagaaatccgc

aaactattcccacgtctggtcagaatttttttgaatatgtccttgaattcattcgagacg

tgagcaaaactcagattggagaagaatatggtccatgggttccgtttattggaactctgt

ttctatttatttttgtttcgaattggtcaggggcccttttgccttggaaaattataaagt

tacctcatggagagttagctgcacccacaaatgatataaatactactgctgcattagctt

tacttacgtcagtagcctatttctatgcgggtatttcaaaaaagggattggcctattttg

gtaaatacatccaaccaacgccaatccttttaccgattaacatcttagaagatttcacaa

aacccttatcgcttagttttcgactttttggaaatatattagcggatgaattagtagttg

ttgttcttgtttcgttagtacctttagtagttcctatacctgttatgttccttggattat

ttacaagtggtattcaagctcttattttcgctactttagctgcggcgtatataggtgaat

ccatggaagggcatcattgactagttatcaaaatcgtgttttagcccgcaacaaagtatg

tgtggctcgcgataatatacttaaggaatattaataaaaaaatacaaagaaattttaaaa

aggtttaataacaatcaaaaagtagaaagaaaattaaaacaattaccagggaattgtaaa

aa

>fava_dn_rep_c13

tatggaacaaacacatgtatatgtcatagtagatattcattagttatatatgacgatcta

tctaaatttgttctgctactactctaaatttaggtagggattcaaaaaaaagattccatc

tcttgtaattctgaattgaatataaaaaaatgactaaaaaaatgaaatcaaaaaaaaaaa

aagtttaaaatgtaagataagaccaaaaattgtatgtattcacaaaaaactacaagggta

gaaacgaaaaaagtgaatatcgaagtaattgggataattcaataaaaattattaagtttg

aaattttgaattacacttcgactagataaagagaaatatgaagaatgaaataattaagtc

ataaattttttgttgattatattattaactattttttattttctttggaataggagaaac

ttatcatgaatccactgattgctgctgcttctgttatcgctgctgggttggccgtcgggc

ttgcttctattggacctggagttggtcaaggcacagctgcagggcaagctgtagaaggga

ttgcgagacaaccggaagcagaggacaaaatacggggtactttattacttagtctggctt

ttatggaagctttaactatttatgggctggttgtagcattagctcttttatttgccaatc

cttttgtttaataaaaaaaatacatcttgttttttccatggactttcgttgctgctcttg

ctttttttttcattctatttagattttactcctacaattttttcttcattcggattaaca

tactgattcgccttcttccgtcccttttttttagtccactgaccccttaattttgattta

gaaagtgttgaaaccaactaagtattttgcaattgacataagaactagtatttccttcta

agaagtatcattcttatagggactctttcaattgactaaccaattttagtatatttttat

tcttactaatatactcattttagtaagaataaaaatatactaatttgatatattcatatt

aaataattaaaaaattagattatagaataaactatcataaacaaataaaaaaaacttgga

atcgtaaaaagaaattttgtctatccataagaggagatgcgatcatatgaaaaatataac

cgattcttttctttgcttcatttactggccatccgccggaagtttcgggtttgataccga

tattttagcaacaaatctaataaatctaagtgcagtgctaggtgtattgattttttttgg

aaagggagtgtgtgcgagttgtttatttcaaagaatagattggatacaaccaactgcact

ttttttgttataactaggaaaatgtgcatgatccggcgaatgacttcttacaaaataacg

aaaa

>fava_dn_rep_c14

aagattccatctcttgtaattctgaattgaatataaaaaaatgactaaaaaaatgaaatc

aaagaaaaaaagtttaaaatgtaagataagaccaaaaattgtatgtattcacaaaaaact

acaagggtagaaacgaaaaaagtgaatatcgaagtaattgggataattcaataaaaatta

ttaagtttgaaattttgaattacacttcgactagataaagagaaatatgaagaatgaaat

aattaagtcataaattttttgttgattatattattaactatttttttattttctttggcg

cgggagaaacagagagaaatattttgaagcaaaataatgggttactggaaaacaaaagtt

cttcctaaactcaagaaagtttttgagaagaatggtactaagaaagctgctgctgaggcc

tctaagtcctttgatgaatcaaaggagggaatcaatacagagtttgaggaaaagaaaagt

gaacttgaaacaaaagttcttgcaatatatgaggcttcttcaactgaaatcaagggtgtg

atcaaaaagcgagatgaagcagggctaaagaagaattcaacagaagttcagaaattcata

gatgaacttgttaaaattgattttcctggatcaaaagcagtatcagaagcatcttcaaaa

tttggaccagccttagtttcaggtccagttctctttgtttttgagaaagtatcaactttc

atagtgacagaagaaaaaactgaagaagcaactaaaacagaagaggaaacaagtggtgta

aaagatagggaaattgtggttgaagaaggaaaaaaagaagaaacaagtgaatctgcagca

gagaaggatgaagaaacggctaaggctgcaaatgactctcttcctccgccgccgccaccg

gcagaggagaaacaggaagagaaaccggttgaagcaactccggtggaaaaagtggaacca

ccaaaggcttgaaaagttgttgatgaattgaagatcatgaatatgttgtatatgtgtaat

gtaatagtttgcatatactcaaaaaaaaaaaatgttacatattaatctcttattctttta

tatgtgtaattgaatttgctttgcttagtttgatgcatgtgaatgtgtattgtgaatctc

tcagtttgtaattttcatttttcattttttcattttaatgtcaaagaattgtgactttgg

attggatcaatttgtttttttaatcctttatgtttgttgtttattcaatccatatgtttt

gggttcatttcgcaataaatttatatatggtgatcaaatggcaactttaatatttatttt

gataaaaaatcggtactgttgatcctgacacgaagaaaaaatcaactaagaaaaagcaaa

gaaaggatgacaagccctcggacaaggctttggctcttgaggagaatgtcaatagcattg

agaagaaaaagaagaagaatcaaaagcctgagaagcgaagtcaatttgatattattgatg

atccagaaatttcatttgtggacctctttaacatccaggatgaacacagtttgaatcatg

gtagtgcaatgaaactaaaggacaaagtggctaaggatttgaaaccgttgggcagcatag

agacacctgtcaagcataagagcgcggaaatgcataacctggagtctctatcatctccaa

taactgaaatcggaatgggagggcaatcaacatgggacgactagtgatatatatcatgtc

gagatatgccaatatatttaaaacctacaattttttattagatcgttgttcaaggtgagc

tcagttgttttctgctgcagttcttgactttttgatcagtaagaatcgaacttgaacttg

gtactccggggtttacaatgcacattgcgcatcaaccacttgctctacattccagatgtt

gctaatatgtgaattgcacgtcagattcaggtctgacttgtgatggtggtctctttagat

ccaaccaacacctttcttgctctttccgttatttaatgagttgtatgaaaccagttactt

caaatgaacttattcgtgtatgaaatgcggtgtgtttcatgcccctctcccatttttgca

tggctactgtgaaatgcagttagcaattgttgttactttccaaagagagtttaaattttt

gttactcttccaatgtaccatgaacttcaattatattctgtaatactatcttgacatcca

tatataattaaaagatacttctggtaa

>fava_dn_rep_c15

agaaagttagtttcaaggctatggctgctaacctcctcaaggattggtaatggattaata

agtnnnnnnnnnnnnnnnnnnntttttttgaatatgtccttgaattcattcgagacgtga

gcaaaactcagattggagaagaatatggtccatgggttccgtttattggaactctgtttc

tatttatttttgtttcgaattggtcaggggcccttttgccttggaaaattataaagttac

ctcatggagagttagctgcacccacaaatgatataaatactactgttgcattagctttac

ttacgtcagtagcctatttctatgcgggtatttcaaaaaagggattggcctattttggta

aatacatccaaccaacgccaatccttttaccgattaacatcttagaagatttcacaaaac

ccttatcgcttagttttcgactttttggaaatatattagcggatgaattagtagttgttg

ttcttgtttcgttagtacctttagtagttcctatacctgttatgttccttggattattta

caagtggtattcaagctcttattttcgctactttagctgcggcgtatataggtgaatcca

tggaagggcatcattgactagttatcaaaatcgtgttttagcccgcaacaaagtatgtgt

ggctcgcgataatatacttaaggaatattaataaaaaaatacaaagaaattttaaaaagg

tttaataacaatcaaaaagtagaaagaaaattaaaacaattaccagggaattgta

>fava_dn_rep_c16

aaaaatagagattaaataaattaacaaaggttgagggggtcaaactaggtgtatatataa

tctaatcgttataagacagctgggggatttccaagaatgattgattgaatctaagataca

actcattggtttacggtatggaacaaacacatgtatatgtcatagtagatattcattagt

tatatatgacgatctatctaaatttgttctgctactactctaaatttaggtagggattca

aaaaaaagattccatctcttgtaattctgaattgaatataaaaaaatgactaaaaaaatg

aaatcaaaaaaaaaaaagtttaaaatgtaagataagaccaaaaattgtatgtattcacaa

aaaactacaagggtagaaacgaaaaaagtgaatatcgaagtaattgggataattcaataa

aaattattaagtttgaaattttgaattacacttcgactagataaagagnnnnnnnnnnnn

nnggttgtggtagaggaggaagaaaaagaaaatgaagaaggacaaggagagaaggagaat

ggagaggagattggtgcataagaagttgcagagaggatgaagaatgtcattttcgcagtg

ttgttgaaggatgaaggtgatggagtgaagaatgaaaaattaatggtgtagattgaatgg

ttctggaagctgagaaattggtggtgagtgtgaaggttatggagaatctcagagataaag

aaaggatgaaagggtagagaatgaaatcgtgaagtggtgaatgaagatggagatgtggtt

atataggagtttgaagatgatgaatgcagtcgtgggagaatcaatctaagccactaattt

gaatagagcttgttatagattgaagggtgagattaaaagttagttacaaggtggttatgt

tagttgtagctgttatttagtttgagagttctgttaggattcaattattgttagtttgga

aatcttgaagttc

>fava_dn_rep_c17

aaacccttatcgcttagttttcgactttttggaaatatattagcggatgaattagtagtt

gttgttcttgtttcgttagtacctttagtagttcctatacctgttatgttccttggatta

tttacaagtggtattcaagctcttattttcgctactttagctgcggcgtatataggtgaa

tccatggaagggcatcattgactagttatcaaaatcgtgttttagcccgcaacaaagtat

gtgtggctcgcgataatatacttaaggaatattaataaaaaaaatacaaagaaattttaa

aaaggtttaataacaatcaaaaagtagaaagaaaattaaaacaattaccagggaattgta

aaaaaaaaaatagagattaaataaattaacaaaggttgagggggtcaaactaggtgtata

tataatctaatcgttataagacagctgggggatttccaagaatgattgattgaatctaag

atacaactcattggtttacggtatggaacaaacacatgtatatgtcatagtagatattca

ttagttatatatgacgatctatctaaatttgttctgctactactctaaatttaggtaggg

attcaaaaaaaagattccatctcttgtaattctgaattgaatataaaaaaatgactaaaa

aaatgaaatcaaaaaaaaaaaaaactatctttttgaagtttttcatttagcctattttac

caacaaatttcaaaaatggaagagaacattaagtgtgaactaagacaaggtaaggaacta

gcaaagcaactttgtggtcaatttttgtctttaacatcatcatcaaattcatcacatgaa

gcaaatgaagtgttaattgataaaatacttttggcttttgagaaagcaattaccatggtg

aaagataatgtgggacttgatgaatgggatgacgaagaggaagaggaagaggaagatggc

tatgatgacgatgacggagtaatcattgtagacgatgatgatttgggaatgttgacctga

aaattaaaataatgaggattttgagattgtaggtaggtaggtaggttttttcttttgcct

tgaattttttgttccttcgttttcgtaagaatataaagcacttttgaaagttatgactct

acatgaatgaagatcataattgttatcaaaaaaaaaaa

>fava_dn_rep_c18

aaatacgacgtcgttccatgcgaattcgaggcaaaagcaaccagtatcatagattcaaca

caccactgtgactgaatctgaaacaaaaccctcacaaaaccctaaccaccgtctttccga

ttttccaatactcaaacaaatggcgttacgatctggatctctatctcgctccttcatctc

tacagccagaaattccctccgcccggtcgctccttctccccttccccgcatccgtccacc

gcctctcgccgctcctcgcatccagtcccgcctcttttccatgcctgccaccacttccag

gaatttggggcaattgggttgcgtacagtcacttttgccgctgcatagcacgatggcggt

agcttgtctcggttctcacattgtcgttagtgcacgagcttgcagcgagctgtcgaatgt

tacttaagcaagctgctggattttcatggctgcttgggccaataattatagggagtattc

ttggaagaattggttgtaagaatcttgaacgtgactctccacctaatttgtttttcttta

actccagaagaaagaacctgttttattatagttaaactgccacaacattcaagattagtt

attgattcttgttgtatctggttttggttaaatgttcattcatcgcatacctttcaagtt

aaaaaaaagggaaattgatttgaattaaattcttattgttgaaaataatacagatttgca

tggaatacataaacacacatacaatattaaaagaaagataattcaacaatgcgagaaaat

aaatacaagaaatacataaacacacatacaatatcaaaagaaagataattcaacaatcca

ataaacaaaagatagggtcttcaacattccgagtcttaagaagtagtgtttgatcggagg

ttaagtagaaacttctgtaaagagaaagtcttgggagccttgctagagattggaaaaccc

caaaaaacaatgccaggaagtaattcgagatcgaagagttcccctattccgtcagaaagt

acggggtcacaataaatgttaatttcaggaaggactagagctggagcgtaattatgtgct

aattctttatagagctcaacaactttttctttggttgagaaactctgtttggagaaccca

tagtcaacatagtcaataatgtctttgttgtccaaatacaatttgtgatagtaaggttga

aggggttttgatggagcaatagatacgagcttgattgatgatagttgtttgagttgtctt

ataacttttccaatacagaaggaaaaatcatcctcacttgaatagatggcctcataatga

atatcaataccatcaattatatttttgggggtgccatataaatcaatgatttggttgata

gattttacagcattgtcgatccaaacatttttttcaattggattgaacggaaattcagaa

gaaaaacccccgatacttataaccacccttgtatctgcataatctttcttgatatttttc

acccattcaatactttgactatcagtttcccaagtttgcttgaaaacaccattgcctttt

ccatactcgtcataatgctcggtggcaaagctcaaaatgcagtcttctttctcctcactc

tcctcactctcactcttgaaattgttttcaggttttggtatatgcggtacatatacacga

aaaatggttgcctcaacattttcagacattgtgacttatgaaatttggaggaggaaataa

ttatatcacttaattggcttctttttttttattttatgttcccgcggccgctaaaacatt

acaacacacatgagaataggagatggactggaggagagacgacatggaaaaagttcacaa

attcatcgttatgatcacaaccatggtgagaaagagcacacagactagtcaaaatccaaa

acataaggaaataggaaatcaaaataaaacttcattagttcttttggcccccaaatagag

tacaacatgaaactggttgggattgggtttgtgagcagtcagaaaggcctctgacaatca

catctggaccgcccaaccgggtgggggacaatttgtttagtcatatctatcgggtgtttt

tgtagaagtagtaaaagcatggctttattggtccagcagcaaaggacatcaacctcttca

gttgcaaaagacatatatgtattatttatatacacaatgacgagtctgatgcaagaagtt

ccatctgatgagtaagatggtccatatgttgtttcttatccatattttcttgtggaatct

ctttttctaatgaaaattcctggtccttggtcaaaccatatcctagattgctacctttaa

cttcatctgtgtctccggtaagtgagcttggcataaaaaacagatcccagaactcttcgt

tgattccttgaagtttagaaatcccactcggatcaggagaaaaagcatcagtttccaaag

actgtgttccatcaaaaacagctgatatcacatccagttcctcattattctcagtgacag

accccacaaaattttgattagaattcatgaagctgctctctgtagccagtccttgaactt

caccaaaattcaaaacagtgtcttggaagttatgtgctgttaattcaggaaattcagctg

ctttgacacagtcagttaacacagaaggtgaagattgtacctcagacatgctattagtca

tacaacctacaggaaattgagattctactgccatacatgactgcccagaaataggtggaa

cctcagaaagcgttacaccagaaacttgggtggacgaactactgctgtctaacggaatgg

ctgaaggagtatcgtcaataaggaatgcatcggggttcttaatggaggactccgtccttg

tagagttgttcatttgcaacatctggttatataatgcttttgcagtctcattaattgaag

gctgatacttgacaacacgtccatcaagaggattatggagattcttggtggctaagtttt

cttcttcctgcctctggagccgcctctttttacccccggtgacatgtctattactttcag

tctgctgctgtgaaaattgagccatgaagccaggactatgcatagcctttgctaggaacg

acatcatctgttgctgacgctgctccatcacttgcacacgttgcccaacagtttgcaatt

ggttatcagtcgtttgttgttgctgtcttaacctaacaagttccgtcataagaacatttt

tatcccttttcagtctttcaacctcttcgtcaatcccaaacttccccacctcaacacatg

ccccaacaggtgcgtcctgtaccttaggcggttgttgactattaccatttgcttgagtag

atttccgcctattaatagttttcaacaactctttttgaccccttaaaaatccttcatgcg

caaattcccagtggtcagggtcaacctttctaaaaccatatgtattcaactgcctaacaa

agctggagaagttgttatgcttaaaatacttaggcaagatatacttagcgaaatcggtag

cattcaaaacaacaaaagtgttattgtttttcccccacgaaacgatcgcgtcagttgaag

agtcatcaaccatgtcataggttttgctcaagaatggcggtaacacatagctcgcgttcc

cagctgtcatattcaatccaaactcgatgatgatggttatacgaaaacactgaaaacaaa

acgatgaagaagatgaaagagagttgagaggaacaacactgatggtgaaat

>fava_dn_rep_c19

aaacttcaagaaaatatactagaacattgtatatcagtctaccacaccaaatatcatccc

acaaacaaatataccaaccatttcaagttgaacaacacatacagaatatgcaatgcattg

aacccaacaccatatagacaacatatgaaaacactatttttttcttctctgaactaacat

atgctaaaaagattacataactttatgaatctatatgctcagaaatagttttattttcaa

tatatccaagcagtagttcataggattaattactaataaactttgtttgagcatccacaa

caacaaagtcgcatccaatttaacaaaaacacaaaaacaattgcataatccaaaacattg

gggtttttttcttacacaagtgaaagtgactaaatcaaaagagtgctaaatatttacttt

ttcttcttgccagcaacagtaaccttcttacccttcaatgtcctgcaattgttcttcgtt

cgctgtcctctacaaggaagcccccgaatatgccttattcctctataacactgaatctcc

ttcaacctcttaatgttaagcgcattgaaccgtttcaaatctccttcaatcatgtatttg

gaaacttcacgcctaagaataataagctcttcctcactcaattcctttgtaactttgtta

tccattttgatatcagttagaatctgtctcgatcggctccttccaactccatgaatgtac

tgcaacgagaattcaattcgtttgttgtttggaatttcaacaccaccgacacgaacgcac

tggatgcttaatcgttgaaccttaggcttgggagtgttgaggatagggagagaaagtctg

ttggagaagtggccattggagatgattgagagagaaggtgccacaggcattgcaagcgtt

tgtaccattcttgttcttctcttctctatcctctaactgtttctgtgaaaaatgaagtgt

accccatagccatatccttatcgtt

>fava_dn_rep_c20

aaaaatcttactattctattcgtttagaagaaaaacaaaaattgcgttttcattatggtc

ttccagaacgccaattgctgaaatatgttcgtatcgctggaaaagcgaaagggtcaaccg

gtcaagttttacttcaattacttgaaatgcgtttggataacatactttttcgattgggta

tggctgtgactattcctcaagcccgtcaattagttaaccacagacatgttttagttaatg

gtcgtatagtaaatataccaagttatcgttgtaaacctgaagatattattacagccaagg

atgaacaaaaatctagaactctgattcaaaattctcttcaatcagccccccgcgaaaaat

tgccaacccatttgactcttgatccatcccaatataaaggattagtcaatcaaataatag

atagtcaatgggttggtttgaaaattaaggaattgctggttgtagaatattattctcgtc

agactaaaacctagttcaaataacaagaattttgatccgagtattttcccccattcgtgt

atagaagtgggtataggaaaattgttattccttgcccactttatccacaattttctatat

tacataaacaaaaatcttatctattattatttcaaacattgaggtcagtaacattgatga

acttggtaagggtgcggaaagagagggattcgaaccctcggtaaacaaaaagcctacata

gcagttccaatgctacgccttgaaccactcggccatctctcctacataatgattatgccc

ctaaaccgagtgaatagtgaggcatttatatcccatttgcgtacgcgcacacaatcaatt

gaaactaaaaaaatagaaatagaaagaatttttttttttttgaaaaaaaaacctacttca

aggcctaccgtagaataaagtaatttgattaagaagtccatttttacgttatttcgtact

gtattgtacaattcctctgtttggggaattattttatcatgctaaaattttagaatggat

tgctacctatgactagatctcggataaatggaaattttattgataagacctttaccattg

tagccaatatcttattacgaataattccgacaacttctggagaaaaagaggcattcactt

attacagagatgggatgtcggctcaatctgaaggaaattatgcggaagctttacagaatt

attatgaggctatgcgactggaaattgatccctatgatcgaagttatatactttataaca

taggtcttatccacacaagtaacggagaacatacaaaagctttggaatactattttcggg

cactcgaacgaaaccctttcttacctcaagcttttaacaatatggccgtgatctgtcatt

acgtgcgactatctccactatagaaagaaaaaagaaaagaacctccactaatagtaagaa

atactagacaaaaaaagaaaaataggctttctacatatatatccgttcgaaacaacgctt

tttatgagctgtagcaaagaaagaaacttcatagaagtaaaaatctgaagaaatagaata

tatatgcctagatacttttttctatggataaaagatctaattgatagaggaagcaccgta

aagatcaattcacaaggtttttggccgatacaacaagaactgcttacttatatgatgata

gattaggaatcgacttatgtaataaagtggatcccctaaggttttgagcagcggtgtagt

atcagatcccaaagagagtaactcttttcttatgaagaaaagtctttctcaaggattcta

tagaaatatatatatcatatatgaaatatatgaaatgatatatgaaatcgagatagttac

ctttcagaaaattcgaataagaacgttagtgtgaatgtcgatgctttattcttcagaagg

taggaaaaaagataaaactaaaaacaaagggttaaattgaattattaatccaaattcaag

tttaaaactcatgtaattaacctcttttcttgttattaactccagaaaaaatagaagatc

aaaagaattgactgttgagccgtatgagtcaggaaactctcaagtacggttctaagggaa

ggattttactcacctattccgaccgcggagaacaggccattcgacagggagattctgaaa

tttcagaatcttggtttgatcaagctgctgaatattggaaacaagctatagcccttaccc

ctggtaattatattgaggcacagaattggttgaagatcacagggcgttttgaataagaac

agtctttttattattttattttttttattcgaattctaatattattctctatattttatt

ctttaatagagaataaaatatagagagaatccattatatatattgaattttttatagttt

attgcccccatcagtcaaataaaagggtccgttgagcacctcactgctatgtcataatag

atccgaacacttgccctggattaacttccggatcataattgttctagtgaataactaaag

aaaatatagaatagatatatgggagatagaagagaaagatattcaatataaacatctctc

ataagttcattaattatgttttctgttggcgggtctctttgtatgtgttgtccggaaaga

ggaggactcaatgattattcgttcgccggaaccaaaagtcaaaattttggtagatccaga

agtcaaaattttggtagatagggatcccataaaaacttctttcgagcaatgggcaaaacc

tggtcatttctcaagaacaatagctaagggacctgatactactacttggatctggaacct

acatgctgatgctcatgatttcgatagccatactagtgatttagaggaaatttcccgaaa

agtatttagtgctcatttcggccaactctccattatctttctttggctgagtggcatgta

tttccatggtgctcgtttttccaattatgaagcttggctaaatgatcctactcacattcg

acctagtgcccaggtggtttggccaatagtgggccaagaaatattgaatggtgatgtagg

cggaggtttccgcggaatacaaataacctctggtttttttcagatttggcgagcatctgg

aataactagtgaattacaactctattgtactgcaattggtgcgttggtctttgcagcctt

aatgctttttgctggttggtttcattatcacaaagctgctccaaaattggcttggtttca

agatgtagaatccatgttgaatcaccatttggcaggtctactaggacttgggtctctttc

ttgggcggggcatcaagtacatgtatctttaccaattaaccaatttctaaatgctggagt

agatcccaaagagatcccacttcctcatgaatttatcttgaatcgggatcttttggctca

actttatccaagttttgctgagggagcaaccccattttttaccttgaattggtcaaaata

cgcggactttcttacttttcgtggaggattagacccactaactggtggtctatggctgac

tgatattgcacatcatcatttagctattgcaattctttttctcattgctggtcatatgta

tcgaactaactggggtattggtcatggtataaaagatattttagaagcccataaaggtcc

atttacaggccagggtcataaaggtctatatgagatcctaacaacgtcatggcatgctca

attatctattaacctagctatgttaggctctttgaccattattgtagctcaccatatgta

tgctatgcctccttatccatacctagctactgactatggtacacaactgtcattgttcac

acatcacatgtggattgggggctttctcatagttggtgctgctgcacatgcagccatttt

tatggtaagagactatgatccaactactcgatacaacgatctattagatcgtgttcttag

acatcgtgatgcaatcatatcacatctcaactgggtgtgtatatttttaggctttcacag

ttttgggttgtatattcataatgataccatgagtgctttagggcgccctcaagatatgtt

ttcagataccgctatacaattacaacctgtctttgctcaatggatacaaaatactcatgc

tttagcacctggcacaacagcccctggtgcaacaacaagcaccagtttgacttggggcgg

tggtgatttagtggcagtgggcggtaaagtagctttgttacctattccattaggaactgc

agattttttggtacatcatattcatgcatttactattcatgtaacggtattgatactcct

aaaaggtgttctatttgctcgtagctcacgattgataccggataaagcaaatctcggttt

tcggttcccttgtgatggccctggaagaggggggacatgccaagtatccgcttgggatca

tgtcttcctaggattattttggatgtacaatgcaatttctgtagtaatattccatttcag

ttggaaaatgcagtcagatgtttggggaagtataaatgatcaaggagtagtaactcatat

cacaggaggaaactttgcccagagttcgattaccattaatgggtggcttcgcgatttctt

atgggcgcaggcatcccaggtaattcagtcttatggttcctcattatcggcatatggtct

ttttttcttaggcgcccattttgtatgggcttttagtttaatgtttctattcagcgggcg

tggttattggcaagaacttattgaatccatcgtttgggctcataataaattaaaagttgc

tcctgctactcagcctagagccttgagcattgtacagggacgtgctgtaggagtaacaca

ttaccttctgggtggaattgccacaacgtgggcattcttcttagcaagaattattgcagt

aggataatggctaggaggatttgaaaagcattatggcattaagatttccaaggtttagcc

aaggcttagctcaggaccccactactcgtcgtatttggtttggtattgctaccgcacatg

acttcgagagtcatgatgatattactgaggaacgtctttatcagaatatttttgcttctc

atttcggacaattagcaataatttttctgtggacttccgggaatctcttccatgtagctt

ggcaaggaaattttgaggcatgggtacaggatcctttacatgtaagacctattgctcatg

caatttgggatccccattttggtcaaccggctgtagaagcttttactcgagggggtgctc

ttggcccagtgaatattgcctattccggtgtttatcagtggtggtatacaattggtttac

gtactaatgaggatctttatactggagctatttttctattatttctttctttcatatcct

tattagcgggctggttacacctacaaccaaaatggaaaccgagcgtttcgtggtttaaaa

atgccgaatcgcgcctcaatcatcatttgtcaggactatttggagtcagttccttagctt

gggcagggcatttagtccatgtggctattccaggatccagaggagaatatgttcgatgga

ataatttcttaagtgtattgccccaccctcaaggattagggccacttttgacgggtcagt

ggaatctttatgctcaaaaccccgattctagtaatcatttatttagtacttcgcaaggcg

cgggaactgccattctaacacttctcgggggattccatcctcaaacgcaaagtttatggc

ttaccgatatggcccatcatcatttagctattgcaattctttttctcattgctggtcata

tgtatagaactaacttcggtattgggcacagtataaaagatattttagaagcgcatattc

ctccggggggtagattggggcgtggacataagggtctttatgacacaatcaataattcaa

ttcattttcaattaggccttgctctagcctcgttaggggtcattacttctttggtagctc

aacacatgtactctttacctgcttatgcgtttatagcacaagactttactactcaagctg

cgttatatactcatcatcaatacattgcaggatttattatgacaggggcttttgctcatg

gagctatcttttttattagagattacaatcccgaacagaatgaggataatgtattggcaa

gaatgttagagcacaaagaagctatcatatcccatttaagttgggccagcctatttctgg

ggttccatactttgggactttatgtccataatgatgtcatgcttgcttttggcactccgg

agaaacaaatcttgatcgaacctatatttgcccaatggatacaatctgctcatggtaaaa

cttcatatggtttcgatgtacttttatcttcaacgaatagtccggcgctcaatgcggggc

gaagcatatggttgccgggttggttaaatgctattaatgagaatagtaattctctattct

taacaatagggcctggagacttcttggttcatcatgctattgctcttggtttacatacaa

ctacattgatcttagtaaaaggtgctttagatgcacgtggttccaagttaatgccggata

aaaaggattttggttatagttttccttgcgatggtccgggacgaggtggtacttgtgata

tttctgcttgggacgcattttatttggctgttttctggatgttaaataccattggatggg

ttactttttattggcattggaagcacattacattatggcagggtaatgtttcacaattta

atgaatcttcaacctatttgatgggatggttaagagattatctatggttaaattcttcac

aacttatcaatggatataacccctttggtatgaatagtttatccgtctgggcgtggatgt

tcttatttggacatcttgtttgggctactggatttatgttcttaatctcatggcgcggat

attggcaggaattgattgaaactttagcatgggctcatgaacgcacacctttggcgaatt

tgattcgatggagagataaacctgtggctctttccattgtccaagcaagattggttggat

tagtccacttttcggtaggttatatatttacttatgcagctttcttgattgcctctacat

ccggtaaatttggttaatttggttaatttgattctttattgtatctgcaaaaattgaatc

tctttgtatgaagaaggggaaccaccttcttcaattttcgacatcttaggatccgacttc

tatcattgatacaggaaatgaatcagcatggcaaagaaaagtgtaattgagagggagaag

aagaggcaaaaattggaacaaaaatatcatttgattcgacgatctctaaaaaaagaaatg

agcaaagctcaatcgttaagtgagaaatgggaaattcagggaaagttggaagtactaccg

cgtaatagtgcacctacacgtcttcatcgacgttgttttgcgaccggaagaccacgaggt

aattatcgacactttggattatctggacacgtacttcgtgaaatggttcacgaatgtttg

ttgcccggtgcaacaagatcgagttggtaagaataaaaaattatcttcattttttttatt

cattttgatgatccgcgatcataggacggtccctttaccattctgtatcaatggtttatt

ctatttgtacagatatggtggaggggtacattcaatctctgttttccattcattgtcatt

tgttcttcttatggcggagtagagcaacttggtagctcgcaaggctcataaccttgaggt

cacgggttcaaatcctgtctccgcaacatttttgataaaaattggaattcactattttct

atttttgaaatagatagataaatgtactattcaatacataagcaacggcggatagcggga

attgaacccgcgtcttctccttggcaaagagaaattataccattaaactatatccgcgtt

tttctttttattgtactttatactttctatattgtactttttacttgatatatcaattcg

atttgtgcagagcggtgtttgatatctattaggagtgatcaagaaaagtcgaaatccata

tatattttatagataatagattatggaatatctatatctttctattcgaaatatacgatt

ttctattaatattcgtttctattaagaattcttaatattctattcttatttttttctaga

atagaattcaaattagtatattctatattaaaaaaataatatgaatattttcgttttact

atatatcatattcaggataagatataagctttttccaatagaagaagtttgacccctccc

attaataataaaaatgttttagttttctttctgtgtggggtcttatatattccattttaa

tgtgcctcacaacccgaaagaattcggggggaggggtcatttcgttttttgatctagaat

gaatagtttcaagagatgagagaattaagaatacccactagaaatactaatccaatccat

aatgatgtaccggaaaatacaacatttttgttacttgaccaaccatcaggagaagcaaat

acaacaggtacactaattaataaaatggatgaagtaacaattaatgcaaaaacagctaat

tgaaaagcaatagtcatgtttataatcttctaattgaccaacaaaagaactataccattt

gatcccccaaccccttttttttgacccctttcaaaaaagaaataaaaaacacattatcat

tatggagggttttttcatgaatccattgattttagataacaccccttattgaggcatgga

tcgaggggtagttttttttatttcacaaaagagcatgctggatcatgtatatataatata

tacggatagagaactagaccaatagattcacactagatatctttctcatagatagataac

agggtatgaattcgtatggtcatgttctcttgagtgaaataaagataaaatagaaattag

cttttggagagatggctgagtggttgatagctccggtcttgaaaaccggtatagtttgaa

acaaataactatcgagggttcgaatccccctctctcctttttcttggcgaatggattttt

ttattttattgattcttgatttaggttggctcggttttttgggctcggctaagtggtgat

atagccgagcccgaaaaaagattagatataaatgaattgaaaaacaaaatattttttcaa

tatgatctgctcgactcaacccaatatgtatactaaaatcaaagtgattcttacgtcaag

cattggatttcatgtctcaattaagaggagtcatggaaagaacaggttcaaaatctctat

caattcctttttcaaatcctgcggcagctgcgcgagctcgtcccgcgtgccataaatgac

ctacgaataggaagaatcctagaacaaaatgagaagtagctaaccaacttctaggagata

cataattgactgcattaatctctgtagctacgccaccgacggaatttaaagaacctaaag

gagcatgagtcatatattccgcggaacgacgttcttgccaaggctgtatgtcttttttca

gtctactcaagtccaaaccattaggacctcttagaggttctaaccagggagcacgcagat

cccaaaaacgcatagtttctcctccaaaaatgacttctccggtcggagaacgcattagat

atttacctaaaccggtaggcccttgagcggagcctacattagctccaagacgttggtctc

taactagaaaagtaaatgcttgagcttgagaagcttccggtccagtgggaccataaaact

cactaggataagcagtattattaaaccagacaaagcaacaagctataaaaccaaaaacgg

ctaaagcacctaaactataagataagtaagcttctccagaccatactagtgcacgccgag

cccatgcaaaaggtttggttaagatatgccagattccaccaagtatacaaatggaaccca

accatacatgtcccccaattatatcttccaaatcgtctacactaacaatccaaccttctc

ccccaaaaggcgattttagtaaataaccaaatataatgcttggactaagggttaagttgg

taatttttcttacatctccccctcctggagcccaggtatcatatagacctccaaaataaa

aagccttaaatactaaaagaaaagcccctatacctaacaaaattaagtgaatacctaaaa

tagtagtcattttatttctatctttccatacataaccaaagaatggaaaagattcttcaa

gagtctcaggtcccagaagtgcatgataaataccgccaaagcccaatactgcagaggaaa

ttaagtgaagtactccagacacaaagtatggaaaggtgtcgataacttcccctcccggac

caaccccccaacctagagtagctagatggggaagtaaaattaagccttgttcatacatgg

gcttctcgggtacgaaatgagcaacttcaaataggttcattgctccggcccagaatacta

ttaatccggcatgggctacatgggcccccagtagtttaccggataaattgataagtcggg

cattcccggcccaccaagcaaaaccagtagtttcttggtcacgaccagttaaagctaaag

ttccattaaagagcgtttccacgtggtagaacctcctcagggaatataaggttttcatga

ggctgatcttgagccgccatccacgcacgaataccttcgtttaagagaatatttttggtg

tagaaagtctcaaattcaggatcttctgctgcgcggatttcttgagaaacaaagtcatag

gcacgcaggttcagggccagaccgactactccaagagcactcatccataaaccagttact

ggtacaaataacataaagaaatgtaaccaacgtttattggaaaaagcaaccccaaagatt

tgggaccaaaagcggttagcagtaaccattgaataagtctcttctgcttgggttgggtta

aaagcacggaatgtatttgcgccatcaccatcttcaaataaggtattttctacggtagcg

ccatgaatagcgcatagtagggcagcgcccaatacaccagcaactcccatcatatgaaaa

gggttcaatgtccaattatgaaacccttggaaaaagagaatgaatcgaaatatagctgct

acaccaaaactaggcgcaaagaaccaaccagactggcccagtggataaatcaggaagaca

gaaacaaaaacagcaattggaccggagaatgcgattgcattataaggccgcaattgaaca

gatcgagcaagttcaaattgacgtaacataaaacctattaatccgaaagcaccgtgaaga

gcaacaaaagtccacagaccacctaattgacaccaacgggtaaaatctccctgtgcttca

ggaccccacagtaacaacaaagagtgtgctaaactattagcaggagtagagactgctgca

gttaagaagttacaaccttccaaataggaacttgccaatccatgagtataccatgaagtt

acaaaggtggtacctgtgaaccaaccccccacggcaaaataggcgcaaggaaagagcaat

agaccggaccaacccacaaaaacaaaacggtccctccgtaaccagtcatccataatatca

aataaatcattttgatctttggtaaatttaccaagaactatagtcatagtgatcctccta

ttcaactacttcaaaccatttccgaacacctactagtattttcagggatgcaaccttcga

ggcttttttcattttatatatgatatgatttctcgtagactgtcccctcttttctactgg

gtttcgaatagaaaaattatttttttgtcgatggatatctaatctaggtcaaatcaattg

gcttctttctttatattctaaaaaaattccctaagtcatgactcgcaaattgtcttatga

ctcataaatcatagaaataaaatttttaaagaactattccataaacaaatgatcgatcgc

ttttattactctcgttaccagcagatccccagacatactactctgcttttatcaaataag

attcttcttgaatcttgttcgtgaaataaaaaaaaagagttttctatattcttcgaattt

gtatgtctaaaaaactatttgtatgtctaaaaaactactagaggatcctatatttgactt

tctattttacatttctttctttttttgaatctttttctctatttcttttttcatctatct

gtcatattctttgaatactctatggaattttagtaataataatagattctaagtgaaagt

ttcgtccattaattgccttaaaaatctcgtatgcatagatatcaaaaccaaatgtttgat

actcaaagtcatgattttatggatttttctattattttgatagacatatcttcacgaaat

aatagaaatcaaatttgatcttttcttgtattcggaaaaaatagatttttaagtcaaatg

acttgtatgttggaccttagaatagaataagcccttccactggaggagaggagtagcaat

tcattcctacgaacaataagattttatccgaaatatcgacagattcttgcagagttagaa

ccaaaaaggtattaaaaagaaaaaaagatccaccgttcaaatttcatttctatccaattt

gaatatcagaatagtggatatagttatgattcaatagcttaggtccacttactttttttt

agaatcttttccttttttgattcaagaaaatatcatattctaaaaaaataaatatatata

tatatatagaaaagaataccattttactttctaactcgaatgagtttactctattcgaat

gataacaagaacttaatagagttgaaattcgattttttaatgaaattcaactattaatta

gtttttttat

>fava_dn_rep_c21

agctaaaatctcaactaccaatataatcaatcacaaaaacatatatatgaaatataggat

tttacaaaattacaataaattgaaatatcgaattgcatgacttgtttttttaaaaataaa

ttagagatatcatctgcgtgcaattgtagagattaatacaacaaaattctttattaaatt

ttacacaaataattttgtgctttccacatcatctactatgaatccaccataacaaattac

tcaaagagtttccatcatcaaaacctttcaaagttctaatattcttcaatgcattttcct

tatcataaacaccttgcaaagcataagtaaaccctttccacccttccttactcaaagttg

gcaatgtccattccacaagctccttagcataaacaccatcattgaacaaattctcagtga

taggcaacaatggcaaaacttgtatactaagccgacattcacgacacgccgacgaagccc

accaaagactactatctctcttgttcgcccacaaaactccgacaatcggattttgtttag

caaaatcggtaccatacacattgttctcggctttcacatgccaccaagattgcgccgcgt

taatttccaacgctaaaagtgtcgatccaaccacaaccagatcttcgtcaccataagcta

aacctattaacgtaactgaataatacgcattcacagcttcgctcgtactttcttgattcc

ttccgtctctgaattcggtcaaccccgaagcccaagaatgtaacttgtaaggatcaaaac

tccttaaagttggatagtttggattatcccctagacccaagttcataaaatctttcaaaa

gtgaataaactattggcttatacttttgtccccaatcttgatcaatctttgcaagaactc

caattccataaagaaaataacctaaatgataatgatgatcattataaacaccaaaaccaa

aatcagcaccagcatcattaaccccttgttgagtaaccaatccaccccaagttttctcat

acaaaaaaccattccctttgaactttccatctaaccatggctcaatagtttgcttcaaaa

aatccttaacaaccggaatcactttctcataagaaacttcttcagctatcaaagcaaacc

ttgcagctcttgcaacaatcttaccataaaaatacgacgaagtcgtcgtaatcgacgcaa

cattcaactcattaacatctttctcaagagcagaaacaacttcctcgtatgattcttttg

taacacctttactagaatgccatgttacattaaaagaatcggttttcaacaaccatgaat

ttccaacaacaccaacaagatcaccatcaatgcttctatacttaaaatcattcaaaatag

taaccttatgatctttactattagacaaaagcttagcatgaagagggtgtgctagcatga

gtaaatcaccggaattttgagtctcccatttataaactaaaccgaatttattctcgatct

tcgcatagcccgaaaccggataactcgagctgaattcatcgagaattttctcattattcg

aattagaaacacttgcaatacgaattataccggaaaatggatcggatataacctcggaac

cgagatggttgaagttaattggtgaagaagtgtatatgatccatttctgagtattgttga

gtgaaagtgtatatttggttttcttgttatcgaatgtagagaaagaaacaatggtgtgaa

tagttttgattgaaagaggtgttggttttgtgactagagcagttacaaaaggacttcctc

taacgagaaaaaatcgtagattcgaagtgggaatatcaagagtcacaccaagatcactgt

acgaggatataacatggtttatgtttgtttgaggttgttgtgtggatgagatagtgagat

ctggtgagaaaacctggtagagcaatgctgttgagaagaggagtaaagggtatgaaactg

agagggaagagtttgaggattggatgaggtaagggtgaatgtattcaggttggtcaccat

ttttgagagcaaagttttggaagaaagagtttgtaccgcggggtgtgactcgttggtttt

tttttagagtggttaattttctacaaaaattcgtcaaattaattactaaagaataaataa

cctactcatcgcttccaattatctggatataaagaaccgccgaaaatctaaaatcaaaat

aaggatctatgtgatgatataattatagcaactgaaataaaaaaaaaaataagattatta

gttgtaaagcaataagaatatacagataaatgaaggggtgaaagaaagatagaaaaagga

tatcaatgatatagaattctcctatgtaaaggtctatgggtcatctcataaaaagtagtg

taataaagcatcaatcttcaaaattcatccatatatggatgaatatattcctagaataaa

cgaatcaagagccacgaatcaagaaaactgagaaggttgattcaacaaaaaagaagaaaa

taagaaaataaaaaaaatggaatcttcttaattaaagaggctccattgtagaatttagac

ctaaccataaattgaaaagcgatgggaacgacggaacctgtgaatacaaaagattatatt

aaaatttgaaatcttactgattcattagatgggatggcggaaggaactaagaattcattc

ttttttgaggagttgtggactaaccctacattacatcttcaatttaatcaatttataatg

agagagtaaatattcgcccgcgaaaatgtggatttttttggaattgataaatttttgcca

atccaatatgataataataaaaaaagaaaaatactgattcgttagaaccttatatccaaa

aatattagctagttagatacggatagcaaaaatggtctataagaatacatatttcgttat

atatcaaagcaagattaaaaaatttctaatagatcaatagattctattaaatgtaaaaaa

atcccgcgattatattctgttgtatcttattgtagtaattattattattttctcgattca

atatttttcaatcgatttattcgcgaggagccgtatgaggtgaaaatttcatgtacggtt

ctgtaatagagatggaattgagaaataaccatcaactataacccaaaaagaacgagattc

cgcaaacatcatagaggaagaatgaaaggaaaagcctctcgtggtagtcaaatttgcttc

ggaaaatatgctcttcaggcacttgagcccgcttggatcacatctagacaactagaagcg

ggtcgacgggcaatgtcacgaaatgttcgccggggtggacaaatatgggtacgcatattt

ccagacaaacctgttacagtaagacctaccgaaacgcgtatgggttcgggaaaaggatcg

cccgaatattgggtagctgttgttaaacccggcaaaatactttatgaaatgggaggtgtt

ccagaaaatatcgctagaaaggcgatttcaatagcggcatccaaaatgcctatacgaact

caatttattctttccgaataatcattagaacgaaagagatctttagtatgaaaaaaggca

attctccgttttttttttgaacacaaaatattttttttacttcaactttttgactgaaac

tgaaagataagaaaaaaggatatgattcaacctcaaacctatttaaatgtagccgataat

agtggagcgcgcgaattgatgtgtattcgaatcataggagctagtaatcgaaggtatgct

tatattggtgacattgttgttgctgtaatcaaaaaagcagtgccaaattcatctttaaaa

agatctcaagtcatcagagctgtaattgtacgtacttgtaaagaactaaaacgtcgtaat

ggtataataataaagtatgatgataatgcggcggttctcattgataaagaaggaaatcca

aaaggaactcgaattttttccgcaatagcccgagaattgagacagttaaatttcactaaa

atagtttcattagcacctgaggtattataataagagatcatgatatagatatattattta

tgacttgaatgaataggaaatagattagaatacgaatgaaatatatattctatattcaag

tcaatatattcaaaataaaaattcgaattctatttgtataaaaaaatagaattcgaattc

aatataagattatttatatagtttcgaataggtcattcgttcattttatttctatctttt

tttagttttagaatattctatattcggatatttagattatcctaattacaatcaaatttt

ctatacatatagagtattattctattctcatattcaatatgagagatttgattgaatcaa

aaaataaaaaatcaaaaaacgggagcacgttgattatatcgaaagtaaggagcaaaaatc

tatcgtgggtaaagatactatcgctgatatactaaccttgatacgcaatgctgacatgaa

tagaaaaagaactgttaaaataccacttactaatatcaccgaaaacattgtaaaaattct

tttacgagagggttttgttgaaaacgtcagaaaacatcgagaaagcaacaaatacttttt

agttttaactctacggtatagaagaaataggaaaggatcctataaaacttttttaaattt

aaaaaggatcagtacaccttgtctacgaatatattctaagtatcaaaaaattccgaaagt

tttaggagggatggggattgtaattctttctacttctaggggtataatgacagatcgaga

ggctcgattagaaaaaatcgggggagaagttttatgttatatatggtaatgctaattttg

ccgatatctgaattatatgaaaaaaacttagagccaacagtattgtgaatggagggagta

gagaaaaaacagatttctaatattattcatgatacattagtgaatatgggaagaggtttt

aactagaatagaaatgaaaattaataaagattaaattaatcaacaacttctgagggtatg

ttgcaggttgaggttgctttagagaaatagaattcaaatcagattctaggttatgtttcc

actggatacgaccaaaaaaggaagtatagctgactccattcgattagactattttataac

ttcaacatattttttaggaggaacaatttgaagttcaaaatgtaaggaaaccctattttc

ttgcaatctataaaattggtattcaatttaatttaaggagtgaaaaatatgaaagtggcg

gcctctgttcgtaaaatttgtgaaaaatgtcgattgatccgtaggcgaggtcgacttata

gtaatttgttccaatccaaaacataaacaaagacaaggataatattaaaagaaaaaaggt

ctttctattcataagattttttatttgaattgtaatagaattcaaataaaaaatcttatg

aatattccattttattaaatactctatctaaaaatatagattctatattcgaatagagtc

tatagttataagtattctcataaatataattgcttattccttgatatttgaaaatggtat

tctatattaattgaattatagaatacaatagaataactagttagaaaatagtcaagaatc

gttttttgacaggaaatggatatatccatatatttcggacttatatttttttaaataata

aaacatggcaaaatctataccaaaaattggttcacgtaaaactggacgtattggttcgcg

taagcatcctcgtaaaataccaaagggggttatttatattcaagctagtttcaacaatac

cattgtgactgttacagatgtaagaggtcgggtgatttcttggtcctccgccggttcatg

tggattcaagggtacacgaagggggacaccatttgccgctctaaccgcagcgggaaatgc

tattcaaacagtagtcgatcaaggcatgcaacgagcagacgtcatgataaaaggtcccgg

tctaggaagagaggcggcattaagagctatttctggaagtcggatacgagtaagggttat

acgggatgtaacccctatgtcacataatggatgtagggctcctaaaa

>fava_dn_rep_c22

aagcatatggatgattgttatataccaagttaagagaacgaatactgtctaacagcggtt

aattctgatcaagtaataatggtggaggataataataaacttcaaaattaactcagtaat

acacttcataatgagcaaaccacaaaaataacttctgaaataatcatatctagtattcaa

caacacagagaactaaacataaatagaattcaactggatatgtttgaaaaacaaaatatt

aaaatgctaccaaagccaactcagtacttgacaggagcaagtatttcgagttgggagcca

agcttctcttcgacaattttctcaaccttcacccttaatttattgagctgcctctttctc

tcataaaccaatgctgatctttcctttctcttcttttccaattcctttatggtatcatag

tagttccatcctacttcagatgaaagttgtccaagatgacagtacttgtgacctttctga

agcctcaataccttaagtgcatctgggacgaccattctcttgatcttgtcatatggaggt

ggaataccttcataaacctttaggcgagcaagagcagcttccccacgcttcgtcttatgt

gggatcattcctcgaagagtcctccagaaaatcttcgacggagcgcggaaatgaatagga

ctgtgagacggcctcgtgttcatccgtttacgaagaaacctcatgtacttcattttccgc

ctgacgaggccaccggagatgcatatctcttcgcatctaacaaccacaactttctgtccg

tttagaagctccttcgctacgatcgatgccaagcgaccgagcatgtggtggcgagcatcc

accacgactcgcttcgcgcatgcgccggaacctgaaaccatttttttcttctctccggct

tgtttctatgcttcacaaggttttgcagatgcggcagcccgcggcc

>fava_dn_rep_c23

actcaattgttaatattgaacccaaaacaatttaacagttaactaattcaattagcacaa

tgacataaaaaaggcagtgaaactatgtaacaagaagcataaagaacataagagcaataa

gtttaaataagatattctgaaagacaaaaccttgataccaaaaacatccaaactcaaaaa

acaaaccgtcttgaagtacccaacaaaagagtaagcaataatccataaacaatgaaaagc

tcaaatgaaactacaaaaaaagataagctttaagagtatcacagtctattcttcaacaac

tacagctcccttctcactaacataaataccatcaagaaacttcctgatatccttcttttt

aacatggcatttctgattaataagagcacatgatctagaaacaagttcaatgtcatttcc

atccaaaacaacctcgtccttcaccttctcagatcgaacaattgaaactccatcaagcaa

atccactttcctcaccttcttctcaccgagaaagttacggatctcgatggacttgttctc

attggtaatgctagcgttgattgggaaatgagcatacacaaacctcatcttgtagcggaa

acccttggtgacaccagtgatgagattctcgacgtggctgagagcggttctgatggcggc

ggaggttttcctggaaccgaaccaggcgtcgatcttgagcttcttctttccgttctcatc

ggtgatgagatcaaaatcgaggttgagatgcttgaagtcacgaactagtttaccgcgtgg

gccttcgacctcgatcaccttggcgttaaccttgatgctaacgccgtcagggatgttcat

ggtctcggaagatagaatggtcttcattgttgctactgtgtgatcaaacaaactgtgtct

acttcagggcctctctgtgtcgctcaccactctctagaatgcgattccaaaaacaacctt

aacctccactagttcaccagattctagatttgctgtaatactttgccaatcaccatcctc

aaaggagattagtgtatctcttttatagacctgaatggttgtctttgtgtaatttacgac

caacataccatcgcaaccttctgatattatgttgtctggggaagagtaatagcggacaaa

caacatcatactcttcaggatacaccctttgnnnnnnnnnnnnnnnnnnnnnnnnnnnnn

nnnnnnnnnnnnnnngtttaaccaatcagatatattgtcacaggggagcaaaaatgaatc

ccaagtcccgtctgctgtctgtataatgttgtcttcggcaatattggatgcttggcattt

cgttcccatttgaattaagaa

>fava_dn_rep_c24

tttttcatacatcaaacagttgttatttttattgataaatatttaacaatatagaattaa

ttacatatacatacataagggctcccttagcttcgaccttgaatcattaatacaacggca

gcaacaacaaataatttgtgatacaaacatacaaaaagaaattcatagtaaaattacagt

atacatgaatattcaaatgtcaccgtctcttttgcattcagagggaatttgaggtaaacg

ttttctatcagtgcagtaattgtaaatggtgtatttctctctcacccactttaagctacg

ccattgagctgcatcaagatcacgaaactcagcttgatcccaccatctctttccttgtgt

tgcacagaattttgcattcacagatgcttcgcagccatcaatgtggaaacttttgtaacc

tgctatgaatggtgcttttgaccaatctgttttctctaatccacctcttgttgcccaatc

atctgcattccataaactgttgtatatcttcattggttggtcaaatgggaatttcactcc

caaatccttgctgttcttgaacactcttattggaacatcatccacatagaacacaatctg

gtacatgttccatagaatggagtatctgtgaaactcttttgttggatcaaaccaaagaaa

gatcctttgttctttgttaccttgtccacccgtgaacacatttgtttgtaagatgtaagg

ttgtccagttctgttgcccaagaactcgaaatcaatctcatcatgttcagaattttgtga

agataaatagaaagcagtaactgttccagcagaatcaccaggaaccatcttaatgttcat

actgaaatgaccaaacaagtatgaacccttggactgaaaaccagtaccggttgatttatc

aagatgaagctgaatctcagaaccgccattgaagtacttgatatgatcaaaagcccaagt

tggataatagtttctgccgaatggtacatccactggcttccttgggagagcacagaatga

agaagaaacaagtgatgcaagaatcaaacacagagtccaaaatgaagaacccatgttgtg

tgttgttgtgttcaagtagtggctaagacttgggtatatataagcccaaagacccttgag

ttagatggtaatagacccatgttcctctctctc

>fava_dn_rep_c25

tcaaaattaaactactatagtttgccttcaaaatttacatccaacttatgaatctatcga

taatatgacaccataaaaggaggggggaagaaactcaaaaaaatgaagaaaaaaatcaaa

gtaaacctttttttgtcgacaagattgaaaattaaaggtgataccgtgattattttcacc

gtttacttcacgacacactcttgttccgtgcccaaacaacgtatgcgaacaatgacagta

tcaaaatctttctccagtcgaggtcgaggaggctcttcatgttttcttcagaaaatgcag

gcctgttgtggaacaagtgttccatgcaatatcagattctttcaattgagcttctctgag

tgaataataatctttcatgcttctgttaaagcttgtttcatcatcatttttcaaacttaa

accacatccaatttgcataaagtgcttcattttcaaagggtaaatcactagttggcaaag

gtcctcttgtagcgacataaggagaaggtagcttctgcttatatttctctgccatcatat

cagcttccagaaatactctctctttattccaaagaaaaaggccaggttcactttcaaggg

ctgcagtgcttagagaaattttatcggggaacattgacttcaaaacaagaaccttaacct

cctctttaacggaaagtatgtcgctgacagaagtaacttcagcttgagtgatattagaaa

catgcagcaatccatatctgtcactttttcctatcctaatcagtgctccatacggataga

tcttttttactgttccgtctataactgtcccttcacgaagatatagcttttcccaagctt

gtttttcactaagtaccaaactgtttttagcttcatctatttgagtaatttctacataca

ttcggcgccccacattttctcttaagtcagtaaagctatttactcttgtcataagctcag

ttttagataggaaagctcgcaaaccctcaatcattgttatcaagccggcagtattccact

ctgtaattctaacctcaataggttcattgagctgttgtatctgcctcactcgacgccaag

caatccggcgaaagagtcttctagtagaaagcaatggcctaccactaagtgttctaccta

aaacctcggcaaacaaaacggttccagtctctacaataggccttccaggcactgatactc

cacccattacttcatcattctttacaattcccatcttccctcgaacagtaaaagcctctg

catccctattcatatcacataacaaattctccatttctttaccattcaaaggaagcactt

ccttggttaacattgtacctagtaagtctgccccaacattcacatcaagcttgttttcat

taccagaaacaacaacaccaaccacaaaatccccgggtttcggctcatagtactcaacat

taacctccttcttatcttcttcttgttcattgtcttgttctttttcatcattttcctttt

cattctcatcactcacattatctttttcctcagaagcttccactacctcattatcttctt

caacttctttcacataatcctcacccctttggaaaatctcgaaaaacgatgctaaaggct

cctcttccgaaggcttctcttgctcaatatcaacttttaaagcaggtgaaggctttttca

acaacttaatgccttcaagctcttgaatttcctcatcaacggcttctcgagatacatcac

tggaaaccccatcaaacccactattcctagagcaaaacttgaagtgggcaccactcaaat

tcgaaactttcttagtaaccattgaaccatgaggagtggaaagaataatattgggaaaat

aacaaaatttgtggggagttagagatacttgtttggttgagtttgttagtggcttcttgc

tgaaaatgacggagttgcaggtaaagaataaggaagacttacaaggttgaagaagaagaa

atggcatattggtaggtgaagctgaacaaagcattacctttggttttcaggaagataacg

ttgattttggccgcggggcttctttttgttccaacgaatagaatgttcctttgtttctaa

aaaactagaacaaatataaattatttcatgcgatataatttactaataaggggaagggcc

atagcatagttttttacagtgcaataaagttacatagtgtctattttttgttgatataag

gggtattttcatgggtttgccttggtatcgtgttcataccgttgtattaaatgatcccgg

ccgtttactttctgtccatataatgcatacagctctggttgctggttgggccggttcgat

ggctttatatgaattagcagtttttgatccctctgacccggttcttgacccaatgtggag

acagggtatgttcgttatacccttcatgactcgtttaggaataaccaattcgtggggtgg

ttggaatatcacaggaggcactataacgaatccgggtatttggagttacgaaggtgtggc

cgcggcacatattgtgttttcgggcttgtgctttttggcggctatttggcattgggtcta

ttgggatctagaaatcttttgtgatgaacgtactggaaaaccttctttggatttaccaaa

aatatttggaattcatttatttcttgcaggggtggcgtgttttggttttggcgcctttca

tgtaacaggattatttggtcctggaatatggatatccgatccttatggactaacaggaag

ggtacaatctgtaaatcccgcatggggtgtggacggttttgatccttttgttccgggggg

aatagcctctcatcatattgcagcagggacattgggcatattagcgggtcttttccatct

tagtgtgcgtccacctcaacgtttatacaaaggattgcgtatgggaaatattgaaaccgt

cctttccagtagtatcgctgctgtattttttgcagcttttgttgttgctggaactatgtg

gtatggttcagcaactaccccgattgaattatttggtcctactcgttatcaatgggatca

gggatacttccagcaagaaatatatcgaagagttggtgctgggctagtcaaaaatcaaag

tttatcagaagcttggtctaaaattcctgaaaaattagctttttatgattacatcggtaa

taatccggcaaaagggggtttgtttagagcgggctcaatggacaatggagatggaatagc

ggttggttggttagggcatcctatctttagagacaaagaggggcgtgaactttttgtacg

tcgtatgcctactttttttgaaacatttccggttgttttggtagacggagacggaattgt

tagagcggatgttccttttcgaagggcagaatcaaagtatagtgtcgaacaagtcggtgt

aattgttgaattctatggtggtgaactcaatggagtcagttatagtgatcctgctactgt

gaaaaaatatgctcgacgtgctcaattgggtgaaatttttgaattagatcgtgctacttt

aaaatcagatggtgtttttcgtagcagtccaaggggttggtttacttttggacatgcttc

gtttgctctgctcttctttttcgggcacatttggcatggtgctagaaccttgttcagaga

tgtttttgctggtattgatccagatttggatgcgcaagtagaatttggagcattccaaaa

acttggagatccaactacaaaaaaacagggagtctgatagaaataggtttgttccttttc

gattcgactttttttgttgttatttgaatttgatatagggaactagagaaatattgattt

caatcattccattttgttttactctggttctttctttttctttatccaggagataattcc

cccaaaacaggtatgaaagctataattgtaaaccacgatcaaatctatggaagcattggt

ttatacattcctcttagtctcgactttaggaatcatttttttcgctattttttttagaga

accccctcaagttccaactaaaaacacaaaatgatttttaattatctcaattgaagtaac

gagcctccaatattacgatattgggggctcattacttcaactagtccccatgttcttcga

atggatctcttagttgttgagagggttgcccaaaagcagtatataaggcatacccagtaa

aacttacaattaaaccggatatagagatggcaattagggttgctgtttccatgattatag

aatatcaagactacaatggatctatagggtacgatcctttatctacagcggaatggtata

caaagtcaacagatctcaataaaaaaagatatataggatttatggctacacaaaccgttg

agggtagttctagatctggtccaagacgaactgttgtaggagatttattgaaaccattga

attcagaatatggtaaagtagctccggggtggggaactacccctttgatggggattgcaa

tggccctatttgcggtatttctctctattattttagagatttataattcgtcccttttac

tggaccaaatttctatgaattagagaattagattcacaagaaccaactaactagcttttc

aatcgaaaagtcaagtttagcatttaaatcgttaatttttagcccattccattttgattt

ggtagttcgaccgcgaaacttctttgtttctgtatttccggaatatgagtgtgtgacttg

ttataatggatcctattgatagtacagaacagaaaatggatacgtcatcttatcttgata

gatggctttcccccgtcagatatttattctagtatctggagcacggaatatagaaaataa

atagattctaaagtgttagaactatgattcatacttcatatttatattgatatttagacc

tcgcaaccggacgaaaaaaaatgaaagaattataagaattcatttagaaaaagaaatgga

attttattctttaaaactgccaccgcttatcatttctttgtctttttttttttcattcta

tctattaattgaataaatgatgatccagcagttcttactcagggaatttatggacttcga

ttaaaggttttttggggaaatcatcgtggttctggtatgaatctcaggtttttaaattga

ttctatagggtcttaacaagaaaattcttattaatcataaaa

>fava_dn_rep_c26

cctgcgcacaaaacactactttcaatctcgtttaaaaaaaaaaccacttccaaactcggt

gctccaccgttcccaccacgacgcagaatcaccgcgccctctctactccgtcacaaactc

caccatacctctcatttcaattccacctcctgaatctcaaacccctttccattccattac

ccacagaagcttttattcttcttcaaattcgcagaaactaaattagggttagggttagga

caacacttcaataccgcttggtctgcttcaaatcgtttctgttcatgcctcgttttcgag

ttggtatcatatcgtagtagctgcagaaatttccgcttaattgacaatttagggtttgtg

ctgttgatatgtctaattcttcgaggggccgagttaccattactcttggacgcagcggtc

aggttgtgaagagggatgtatctgcggcagatgttgcttactcttcttctatgtcttcag

ctgggagtaagcggtctgtgagagacagaattggaaataatgcagatagttccatgtggc

atgggaatgggctcggcggcaataaaagacagcgaggagatgtaagcttgcaaaagggac

aagatgatcggcgtattggaaaagacgatctccgattgaaactcatgcaaaaaagtgcag

ggaagaggactgaaagtaatggcaataagaggaatgttgatctccgtgaaaaattgtcga

agaccgcttatcctccgacaaacagttttaattcaaagcagcgcatgcctgaaccaaggg

aaacaagctcgtacaggctagttccctcagctagaagttctgaggatttaatgcgtatgg

aatccatgagaagctcatactctccttggacactggatcaaattcggcaaagatcacccg

acgggtttccaagttcttccagggggatttccccccaaagaaatgttggagaccctcaga

gaaggcctttaaacaggacatacgatggagtcagaccagtttcttatgcaggtagggatg

ttcttgaaacttcaaggcctccaagtgctgcaccatcatcttttatgtcaagatccggaa

tatcgtcattgcctccagtaactgccaaaccggtagcatctcgtcctggccagcatcctc

catcaagcagcgttgcgcaaagggttccatttgtgggtgaagaacagcaaccacaaactg

tcgatggcctgttgcaagcactgggactacaaaaatatgtcatactcttcaaggccgagg

aagtggatatgacagcactgaagcagatgggagaaaatgaccttaaagagcttggaattc

ctatgggtccaaggaaaaaacttcttcttgcactcttgcctcgtaaacgacagcagtgtt

gattttcttcttcgctggatgctaaaattgacacattgttgtatcccagagttttataaa

acaattattaggtgatgtgaaatacatatctgagaaatttgactagtatgaatgtcttgt

tcagtgtgggattcggacgtgccttgtattgactctcacatttttgcatttgagaacaag

tatcagttgaagttttgttggaagaaagtattggtctgtaacatattctgtccaaactta

taactgtaagaaataggaaccagaatcaatttatccctaatcgggacatattaacgcctt

ctcggttttcacatcatcaaaaaattagggttgctgtttccatgattatagaatatcaag

actacaatggatctatagggtacgatcctttatctacagcggaatggtatacaaagtcaa

cagatctcaataaaaaaaggatatataggatttatggctacacaaaccgttgagggtagt

tctagatctggtccaagacgaactgttgtaggagatttattgaaaccattgaattcagaa

tatggtaaagtagctccggggcggggaactacccctttgatggggattgcaatggcccta

tttgcggtatttctctctattattttagagatttataattcgtcccttttactggaccaa

atttctatgaattagagaattagattcacaagaaccaactaactagcttttcaatcgaaa

agtcaagtttagcatttaaatcgttaatttttagcccattccattttgatttggtagttc

gaccgcgaaacttctttgtttctgtatttccggaatatgagtaaagtctatgattggttc

gaagaacgtcttgagattcaggcgattgccgatgatataactagtaaatatgtacctcct

catgtcaatatattttattgtttaggaggaattacacttacttgttttttagtccaagta

gcgacgggtttcgctatgactttttattatcgtccaaccgttacggaggcttttgcttct

gttcaatatataatgactgaggctaactttggttggttaatccgatcagttcatcgatgg

tcagcaagtatgatggtcttaatgatgatcttgcacgtatttcgcgtgtatctcactggt

ggttttaaaaaacctcgcgaattgacttgggttacaggtgtagttttgggtgtattgact

gcatcctttggtgtaacaggttattccttaccttgggaccaaatcggttattgggcagtc

aaaattgtaacgggtgtacccgacgctattcctgtaataggatcctcggtggtagagtta

ttgcgcggcagtgctagtgtgggacaatctaccttgactcgtttttatagtttacatact

tttgtattacctcttcttactgccgtattcatgttaatgcactttctaatgatacgtaag

caaggaatttctggtcctttatagagaatatgaatcatagagatttgtaatcaatcattt

tggggaggagtaatagtatttcattgctacaaatatgcattatttttctttaataagaca

tgtatttggatatttcccttcaacttaacaaaataaactggattctttatttgacataca

caaatagttgaagggaactctccgaaaaaagaatggattatgggagtgtgtgacttgaac

tattgattgggccatgcagatatatgacttgatcttatctgccacatttgaattcacaat

gaaatgtgtctttgttccaaccaccgcgtcagtcctctacagaggatagtccggttcgct

tgaagataatcttttctatgatcagactcgatcatatcctgcatgaacaggctccgtaat

atccagtaaaaaaaggaatatgatataatctagattatgtcgtatgtatttcattaaact

tacatagtatggaaatgcattcatttcctatgcattgactcaatttatgatactatcgga

gttaaacaagtagatctaaagaaaaaggggggttgtattctattagtaacaagtaaatcc

tttctatgtcaaaaattccaaaattcatttttcgaggtataacaggcgaaaggtttgaga

tcacccagaaagaacttttgatttttattttaaaacttacttgggtgaaaagcatttact

taaaaataagaacttaattttttgcaatggatagttgtaatttcggaaaaaaaagaattc

aacccgttttattacatagaatcatgatatatgagtgactcacatctacaaaatttctat

gtctgcattcagttggtttgattcgtgctcgagccggatgatgaaaaattatcatgtccg

gttctttcggaggatggatctataagaattcacctatcccaataacaaaaaaacctgact

tgaatgatcctgtattaagagcaaaattggccaaagggatgggtcataattattatggcg

aacccgcatggcctaatgatcttttatatatttttccagtagttattctgggtactattg

cctgtaacgtaggcttagcggttctcgaaccatcaatgattggggaacccgcagatccgt

ttgcaactcctttggaaatattgcccgaatggtatttttttcccgtatttcaaatacttc

gtaccgtacccaataaattattgggcgttcttttaatggtttcagtaccggcgggattat

taacagtaccttttttggagaatgtgaataagttccaaaacccatttcggcgtccagtag

caacaaccgtctttttgattggtactgtcgtggctctttggttgggtattggagcaacat

tacctattgaaaaatccctaacgttaggtcttttttaaattgattctattgtaaaatatc

atgacaggtgtattgtgttgtgtatctagggaaaattcactttgaaatgactaatcccta

gatacatctatttcattttagacctattctgaatatattgatttgtgctaaagattcaaa

tttcatatttgaacctttctttttataaaaatgaaagattcaaaaaaatccaatagattt

tcaatttataaaaaatgcttttcgatttgtaaaaagttgaataatatattttgtaaatct

tctacgcgaaaatgttcaattttcagaaggtcttcttgagtcttattcaaaagttcaaat

aatgtatgtatattggactttttaagacaattatagatccggggaggcaattctaattgg

tcaataaaagtagatttcaatttcatttcttttttttttttccttagtttatccttaacc

aatctatcatgaaaagtaaaaggggcaaagtaattttgtgttgattgttttcaaaattta

agttttcttcttctgcatgtaaaaaaggaataaataaatcaatcaaattccgggaggctt

cataaagtgcttccttaggagttaaacttccatttgtccatatttcgagaactagtatct

cttgcttttcatttccattcacataagaatgaatactatgatttgcattcctaacaggca

tgaatacagcatctatagtataacttccatcttgaatgttgtttagtgtttttatacgat

atccccgctttctatcaatttctaattcaatacacaaattaataggttctgttaggttag

ctatatgttgtgtattatcaatgacttccaccgaaggtggtaaaatgatgtcttgagcag

ttacatatccaggacctttgaaacaaatagacgcgtcccgagttccatacagattacttc

tcaatacaacttcttttaaattcattaaaatttcatgtattgattcttgaatacctatta

tggtagaatattcatgtggtattttctcagattttgcacgtgtgatacatgttccctcta

tttctccgagcaaaattcttcgcattgcaatgcctattgtatctgcttgacctttcataa

gtggagacataataaagcgtccataataaagacgcttactgtctacccttgattcaacac

acttccactgtagtgtctgagtagatacttttagtttttctcgaatcatagtaatatatt

tttatgaaactcttgatttaattgtttatttctcttgaaatctttttctttaatgtttat

tgttagttttacacacgtctttttttaggagccctacatccattatgtgacataggggtt

acatcccgtataacccttactcgtatccgacttccagaaatagctcttaatgccgcctct

cttcctagaccgggaccttttatcatgacgtctgctcgttgcatgccttgatcgactact

gtttgaatagcatttcccgctgcggttagagcggcaaatggtgtcccccttcgtgtaccc

ttgaatccacatgaaccggcggaggaccaagaaatcacccgacctcttacatctgtaaca

gtcacaatggtattgttgaaactagcttgaatataaataaccccctttggtattttacga

ggatacttacgcgaaccaatacgtccagttttacgtgaaccaatttttggtatagatttt

gccatgttttattatttaaaaaaatataagtccgaaatatatggatatatccatttcctg

tcaaaaaacgattcttgactattttctaactagttattctattgtattctataattcaat

taatatagaataccattttc

>fava_dn_rep_c27

ttttttttttttttttcacaaaatacacagcttttttttattaatatttgggataataat

taaccattaaccagtttacatagccatcaagaagcaacaaacaaagaagcagaagataca

gaacagttgaacaccaatctccaaagcaacaaacaactcgtagaaatgtcattattcaga

tacatagtttttgcaattctcaaacaaactgatcaaggtattcatcaacagtagtgtact

taacatctgggtacaactcaaaagcctcaaccccaaaagatggctcaatcacaaagttag

tgtgatcacccttcacaaaaacagagtggttaattgataatatcacattgattggaagag

gtgcctcttcaatatccttgagaagtttatcttctggaatataagttttctcaagagtct

tcccaatcttcttctcccacaatgcgacaagctcgttgaatgagtaaatgttcttagggg

gtttaatgtatagaatcttgttcaatgtccttggatcatccacagctctaatggtgaacg

ttccaatgtcatcttccttgttaaacaccgctttgggatttccatcaccatagatgaaaa

ctttgtcttttggtggaggtggagcaaattgtcctgcctgagctaatgtggggagaaaat

atccagcaaagtagttgctagagacatatgtatagggtattccttccgcttcaatagcac

gtcgaatttggactttggtttcaaatgcagactttgctggctctactgcatggacacggt

cgacatcgtttccaaattctgaagggaaaaatctcttaatgttaccggcctccttaatag

cagcgatgatcttgacctgatccgcaagctgaacgtgacctaccgtggaaatcaccacat

ccacctccttaactgctttcaccaacttctcatgatcatacaaatccccagggaccaaat

tgacgcctaatgatttgaaattattgagaagatcagctttcgctggatctgaaagagtgg

attctctgatcagtgcaaaagtaggatgtccagctttagcacttgcttctactatgtgtt

ttcctatgtaacctgttcctccgatgatcaaaatcttgcttttctcacccatggatttgt

tatgtgttaactaatgctttcttgagttttttctttttcctctgtcttaccagcacttca

tttatagcccaaagaatttttttttaattacaattacacccttactttagtgtcttaaac

tatactccaacaattaacgtaaaaaa

>fava_dn_rep_c28

cttttttttttttacaagtacaaatacgggatacaacagtttttataaaattaaaatctt

aaagcttttaacaattttaatcaaaaccaataacagaaacagtgcaaagaaatatatggt

atatgatgatggagaagttggatacaattcaaagagagcaacaacactaggcaaataaaa

tgttgtaatttttcaccatttccagaaaacattagaaccacacttgaatttgtcttttcc

ttcgcattcaagttctaaatcttcagaaatgaatggtactttcttctgtttagcaagatc

ttggcaaccagtgaaattctcagggaacttgcgtgtaccaaattgcactgtatatgctct

tgcaaaatttgctccactagtggctaaccttttcttgtcattcaattccttgttggcctt

gctcttttcaagatattcatcaaatacactagcactagcagaagaattggaggcagaaac

agcagtggtaaaaagagtagctgctaataaggcaagagcgtttctccttccttctgattg

tttggctttggggattctggcttgctgggccttgatggatggcaacttggaacctgagac

tccgactggtgatgcaacagagggcatggaaatgagctttgcgttgaggtcagaaccaga

tatggcgtagctacaagccaatacacttgagttcattgcagccatgtatgtggattgggt

tgttggatagtttagttgtggtgatctgtgatgtgtatcagagtccaatgatgagagaaa

aatgaagtgaagtttaagttgtagggaggaaaaagattgtggttggcgtgtggataaggt

gagattgcattgatttcctattggcaaatcttgttctctacatttcacatagtt

>fava_dn_rep_c29

cataacataatcggattcacatatgtttggtaaaagtaaaaataataaatttctcctttg

agaataaaataaagggaaagtgtttaattgaaacatgaaaaaaaacgtgactgacttaat

tagttctcgttcatttgggggaagaagtggagattatcgaacgaggaaaaggatctaatt

acgtcgaaagaattgaacgaggagccgtatgcggtgaaaatctcaggtacggttctgtag

agtggcagtaagaatgacttatctgtcaacttttccaatattacccctaaaaaaccaaac

tctgcgttacgtaaagttgccagagtacgcttaacctctggatttgaaatcactgcttat

atacccggtattggccataatttacaaatacattctgtagttttagtaagagggggaagg

gttaaggatttacctggtgtgagatatcacattgttcgaggaaccctagatgctgccgca

gtaaaggatcgtcgacaagggcgttcaaaatatggaaccaaaaagccaaaataaaatatt

ttagcccctataagttataaaaaaatggattattacgctcatgtcacgacgaggtactgc

agaaaaaaaaatcgcaaaatccgatccaatttatcgtaatcgagtagttaacatgttggt

gaaccatattatgaaacacggaaaaaaatcattggcgtatctaattatctatcgagctct

gaaaaggattcaacaaaagacaaaaacaaatccactatacgttttacgtcaagcaatacg

tggggtaactcccaatataggagttaaagcaagacgcgtgagcgaatcgactcatcaagt

tcccattgaaataggatcgacacaaggaaaagcacttgccattcgttggttattagcggc

atcccgaaaacgtccgggtcgaaatatggctttcaaattaagttccgaattagtggatgc

tgccaaagggagtggtgatgccatacgcaaaaaggaagagactcagagaatggcggaggc

aaatagaattcttgcacattttcgttaatccatgaacaggatctatatacacacatggac

ctatggatccatacataaattgggatttttttggagattcaatggaattattaattcatg

atttgacatgtacagaatgaaaatatcattctcgagtctacgagaatttttatgaaagcc

tttcatttgcttcttttcgatggaagttttatttttccggaattgatcctaattttgggc

ctaattcttcttctgatgattgatttaacttcggatcacaaagagatatcttggttctat

tttctctcttcaacaagtttagtaatgagcataacggcgctgttgttccgatggagagaa

gaacccatgattagtttttcgggaaatttccaaacaaacaatttcaacgaaatctttcaa

tttcttattttactatcttcaactctatgtattcctctatccgtagagtacattgaatgt

acagaaatggctataacggagtttctgttattcatattaaccgctactctaggaggaatg

tttttatgcagcgctaacgatttaataactatctttgtagctctagaatgtttcagttta

tgctcctatctactatctggatataccaagaaagatgtacggtctaatgaggctactatg

aaatatttactcatgggtggggcaagctcttctattctggtttatggtttctcttggcta

tatggttcatccgggggagagatagagcttcaagaaatagtgaatggtcttatcaataca

caaatgtataactctccaggaattttaattgcgcttatatgcatcactgtaggaattggg

ttcaagctttccctagccccttctcatcaatggactcctgacgtatatgaaggagtgtgg

ttcgttcgagaaattccgaccgacctctctatctatttctgagatgtttggatttttcaa

aactctatggacatgcagaagagatatgctattcccactcggaccaagacataactttac

ttgttaaaataacaattaaggtgaagcagagtcaggaacaacgaatccctttatgataaa

catgataaacaaattctttttgcaagtaggttattaggggtagttcctacaaaggatcat

actaatgacgtatacaatacttgaattttcgatgtagatgctacatagttggttctcatc

cttcagagactacaagtataataggagcatccgtcaacaaagtgatcgccctaagatgat

catctcgtggctattgagaacgaatcaaatcagatggttctattttttaatcttttcaac

ctaggttccgtaggagcaagtcataaagattgagaaaaatccgtcattcacaaccattga

tgaaggattcctcgaaaagttcaggattactaatccttttgaaaagtcaaaatcgaatgg

attcggtcttatacatacgcgaggaaggaaatactaaaaaaaaaataaagaagaaacaat

cttcttatttcttttatcacttaggagccgtgcgagatgaaagtctcaagcacggttttg

aatgagagaaagaagtgaggaatcctcttttcgactctgactctcccactccagtcgttg

cttttctttctgttacttcaaaagtagctgctttagctttagccactagaattttggata

ttcctttttatttctcatcaaacgaatggcatcttcttctagaaatcctagctattctta

gcatgatattagggaatctcattgctattacccaaacaagcatgaaacgtatgcttgcct

attcgtccataggtcaaatcggatatgtaattattggaataattgttggagactcaaatg

gtggatatgcaagcatgataacttatatgctgttctatatctccatgaatataggaactt

ttgcttgcattgtgttatttggtctacgtaccggaactgataacattcgagattatgcag

gattatacacgaaagatccttttttggctctctctttagccctatgtctcttatccttag

gaggtcttcctccactagcaggttttttcggaaaactttatttattctggtgtggatggc

aagcaggcctatatttcttggtttcaataggacttcttacaagcgttgtttctatctact

attatctaaaaataattaagttattaatgactggacgaaaccaagaaataactcctcacg

tgcgaaattatagaaggtctcctgtaagatcaaacaattcgatcgaatttagtatgatta

tatgtgtgatagcatctactatactaggaatatcaatgaaccctattattgaaattgctc

aggatacccttttttagctttagcttctacaatttctttcttagttgacctcttatctta

ctaactgaaatgaaataattagtagatctgttccaccccaaaatgggaatgggctgaggt

tatgaacttataatctataatctgatgatcgaatcgattccaggattataagttcattcc

atttggggaatttggagtcttcgaatttgggaacttagaatttggaaatgacttgacttg

gattagaattggagtataataatattgcacctgactatgcatggttgcaattggattaga

attggagtataataatattgcaccctccgataattcggacaaagggaggtaatatatatt

atattctagcaaactagtcctagtcagggggtcgatttttcattgatgtctccatatcct

tcgggttgtggagaagaaaaagggaaagggaaagggaaagggaaagggaaagggaaaggg

aatttcagaaattaatgggaagtaaaaattgtaaactggaggataaccccgaatgtcttt

tcggaaattacgcaaaatttgcgtagcatatttgatgtcaaaactgcgcaagatactacg

atcgtactataagatggttcagcgtatcgcagttttagataaacttttgatgtcaaaact

gcgcaagatactaagatcgtactataatatggttcagcgtatcgcagttttagataaact

tgagagacgaggaagagctcgatttaataagctggttgaattcatcgagcaaataaatgt

attttcttatatggtactattcgacattgtattgcgaatcagagcgcactatggaaacag

tgattatttgcattttgtattgaaatatattcggaatagaataatatcttctaaagtaaa

tttatattacgttcgccaccaaggtcttatagttttgtatattttctttatacagctcta

tgtgtatatcttagtggaatatacaatcattttttatacttttctagctacttttggacg

gtctctgacctaccgaaacatgtttctctatctcggaattacttatttcctatttgatac

ctatgtatcggggatctatagagtctatcaagagttatcaaaatatgtgacgttttctta

tataattatcactattgaatattatgtaatgataaccatcactagcaatagatttctacc

tctacttatggtgacattttatctcttgtattttttattgagaaagtaaagtactagata

tataagagatagagaaatcggctagatagataagagatagataaatcgggatgtgatttt

taaatttggttcgatctaatctttcctattttttcgaataggaaagattctatactaaac

taaaaaaaatagaatcttactatatgatatatgaattcaaattcttgtttatcaaataag

ttttttatggatcaaaaaatatttgagaaattatgaattattagagaaatgaaaaaaata

gtattctaggtaactggtaattcaaataattgagatccgtatcttaaaattattccgatt

gtgattatatgttatatactagaatatcaaagaaatatagaatattctatatttctttga

tattctagtatttatttgattagattatgatatagtaattgtgttatgaagagctaatag

taaatagtaaatagttaataactaggatcccggtagtttaggaaagaattctgagaaatg

cacaatttgtgttctgagagcccgcttagctcagaggttagagcatcgcatttgtaatgc

gatggtcatcggttcgattccgatagccggcttttttctctctattttttttttttcatt

tttgacagaagggataatctctttttttttaggaaaaaaaaagaatagggtgttcctttt

ctgtagaacaaatcaagaaaataaccgccttttttttatttatatagatatacaatagaa

ttaaattcggatcctgatagaatctttccaattcttctttgtactacactatatt

>fava_dn_rep_c30

gtggtagtcttgacaacaaatcacgatatgacatagaacaatttggtcacaaaatgacat

ggaacaaattagtctcattaaaaatatcagtgtttacttgtaggctttttgaataacaaa

ctagttgcaaaagacaaatttgaaactatgacttgaatattatactaatctttctaacat

caaaatacttttttcatgtgcatggaaacttacaatgccatgaatcaaacaattattagt

ctcccttttctctgagtcattctcatctttcactttgagttgcccttcatcttgagtatc

aaaactggacagtacaataccacagttttacaatttatgacattcgttaataccctttgt

ttcctctaatccttgctctcatatcaatctggcctcttttgcaattgaaataggtttccc

tgagtcctacacactcacttgatattgatggacacttttctccagcacactccctgtagg

atctattctcaaccttaacacaatcagattcactgagacacttcaccagctccatggcca

atcccttgcaagatttggccatcgtcctttacataagaaagattgaaccttgaaacgctt

tttttttttttcgggaaaaaaagttcatgacccgtaggccttctacctccacgcggcatt

gctccgtcaggctttcgcccattgcggaatattccccactgctgcctcccgtaggagtct

gggccttgtctcagtcccagtgtggctgatcatcctctcggaccagctactgatcatcgc

cttggtaagctattacctcaccaactagctaatcagacgcaagcccctccttgggtggat

tcctcctttcgctcctcagcctacggggtattagcagccgtttccagctgttgtccccct

cccaagggcaggttcttacgcgttactcacccgtccgccactggaaacaccacttcccgt

ccgacttgcatgtgtaaagcatgccgccagcgttcatcctgagccaggatcaaactctcc

atgagattcctagttgcattccttataacttccttgtttctagacaaaactgattcggaa

ttttattttctttgctgatgctgatgctgagaagacataacttgtatccatgggcttact

attcgcccggagttgcttcccaaaaatataatacagccctctgtccccttttgtcaaccc

ccaggcctcttatccatttggattgaatcttgtcatcaaaatcgaacaatttatattgaa

tcgaacaattgatattgggtttagggataatcagattcgaaccgatgacttccaccacgt

caaggtgacactctaccgctgagttataccccctcccttgcccctatcgagaaaaaagac

tgcctaatcctacgtcaaaaaaatcaacgcgactatttttgaaacttaaacaaggccttc

ttttcacactcttacatagttgtatattggaaagaggttgatctacttctttatatgatg

atacaatcctcttgttgcttttctcctcggcccacagcgacaaaatgtaggtagttcatc

acgaaagaaaggactcgctgaaccgagatcattaactaaactaatagataataatctaat

cgtatacaatagaaactgtcttttccatctccatatattctctctggttccgttgctact

acacgctttacacaattaatcgtatcataatagattacggccgcgggcaacataggtcat

cgaaaggatctcaacgacccaccaaagcacgaaagccggtatttttcacaaaatagattc

ctattcaaagagtgcataaccgcatggataagctcacactaacccgtcaatttaagatcc

aattcggtattttctttgggaaatattggaaaggaattggaatgtaataatattgattca

tacagaagaaaaggttttctattgatattgatttaaacagtatacttacaagaaaggcat

atagaaagagaaaaaaccgaagatttcatatagtatgatcaaataaagaaatcttcttta

ttacgtaccttccttaccttttgatgaataagaaaatgggtcagatcttacaggatgaaa

cgaaggaatagaagtaaaaaaaaaaaagaattagaagattacaaaacaacaaattcaaac

ttgattgaaaagcatcttttttgattctcgaagaatcagctgtaaaaggattaatcgaga

aagatttattcttctgatttctgattataagatcataacataatcggattcacatatgtt

tggtaaaagtaaaaataataaatttctcctttgagaataaaataaagggaaagtgtttaa

ttgaaacatgaaaaaaaacgtgactgacttaattagttctcgttcatttgggggaagaag

tggagattatcgaacgaggaaaaggatctaattacgtcgaaagaattgaacgaggagccg

tatgcggtgaaaatctcaggtacggttctgtagagtggcagtaagaatgacttatctgtc

aacttttccaatattacccctaaaaaaaccaaactctgcgttacgtaaagttgccagagt

acgcttaacctctggatttgaaatcactgcttatatacccggtattggccataatttaca

aatacattctgtagttttagtaagagggggaagggttaaggatttacctggtgtgagata

tcacattgttcgaggaaccctagatgctgccgcagtaaaggatcgtcgacaagggcgttc

aaaatatggaaccaaaaagccaaaataaaatattttagcccctataagttataaaaaaat

ggattattacgctcatgtcacgacgaggtactgcggaaaaaaaatcgcaaaatccgatcc

aatttatcgtaatcgagtagttaacatgttggtgaaccatattatgaaacacggaaaaaa

atcattggcgtatctaattatctatcgagctctgaaaaaaaaaaagaaaaga

>fava_dn_rep_c31

gccgcggggatcaacctttgtttcttctctttcatctcgtagtttttcttttcaaaaatg

gtgctcttgggtaaacttataactgaacttggtatcaaaacaactgctgaaaagttctac

aaactatttcaatcagaacttcacgaagttcaaaaccattgtgaaagaattcatcacacc

aagttgcatgaaggtgaagactggcatgacactgaaacagttaaacactggacatatgtc

ataggtgatgaggtacacacatgttatgagagtattgaagaagttgatgaagaaaaaaat

aaaataatatggaagctgtttggtggagacattgataagcactataaggtgtttaagctg

atccttgaagcaagtgataaggctgatggtaccgctgcagttaaatggacaattgaatat

gagaaaatcaatgaggacattgagcctccaaatggatggatggagtaccttggcaaatgt

actagagacattgatgttaatctttccaaagcaagggccgctcgaaaggattgaagaagt

acaatggcttagtgcaaccttttggggttggcttaactaagtggtcacctttataattaa

ataagtgtgatgtttgaataatgatgttgttgtgtatgcatgttggcttatgttgttgtt

tcctttgtatgtgtcttgtgaagactgcctcttgaattgtgttatgcatatcaataaaat

atatatatgtgcttttttagtatctagtaaaaaaatgtttatgc

>fava_dn_rep_c32

attcaccacacatgaaatgcaataatactaatcaggaagatatggtaaaatgcaaatagt

agaaacgacaatgatataaatacaaattgttattacatgatacaggaaaggcaacatttt

cacgagccactgatatatttcaagtggcagaaactatctcaattcatctgtaaaattaaa

caactttcaaatactgtaaaaagatattggagatctcacaattcatcagtctaaacaggg

ctagattcatgactcaagtttgcaatcacagcatttatttatgtagcttgtctgtttact

ttcagatacagcaatgctccaattacattgtcatagtcacttctgttgacctggaccatt

ttggagagacgcaatttcctctttttatttttcttgtatagcaaatgttgcttccctgct

ctcctccgcacaattttaccggcaccggtaaccctaaatcgcttggcggaagccttgtga

gttttcaatttgtagcctttagcggatacaattgtgagagaacgaggaggagaagtaagg

gtggaatggttttggtgaagaattggggcggaaatgctaccggaccaattgagtttgagg

gaattgacattgtttgagaaagggagtttaaccgatgcgagagagacgcgagaagaagaa

gatggtgggagtcttaaggcgacggaggagactgtggcgattgcggaggccaaagccgcc

atgttgagagttttcttttttttttgtgtgagattactaggtggttgtggataaagaaga

tagttgaaacataattgagtggtagcgacaatgtagattttgtggatagaaagtcataga

aagggcttggttctaccatgtaattatcacattgaggatttgtgactttggatatcattt

taatgacctatatacccttcacataggtgagactttaatatgatctttgtcgatgaatat

gatgaca

>fava_dn_rep_c33

tttttgaaacaaaaatataccgattttataaaatcattaaaaaaaaatccaaatgtattg

aaaacaattctaaatcatataatataggctttgatacaaaatagacttgtcacacaaaga

ataatcttatccgccatagagcaattgtgtggtggagctacattgtattaataaagcgta

gtggttccaataacaactcaataagaacacaaaacatatgaatgtgtacattgaaactca

ttttgaggatgcaaaaatcatcaatctttctttaactagtcggtgaaaccagcaataaca

gcatgaggctcacaggctttagcagctggactaggctcaacacttaggctaggctcaaca

tgcttagcaacaagctcatgaggcaaatccttaatacctttaagataaaaggtataaaat

agcttaaatggaaacacaatttgatttaatttcacttgaccaaacacaccttcaaatatt

ccagttccaccagtgacagccaaataactatcctcataagttaaataagaaccttgcaca

gaaatatgaccatagtctccaaagtgaaaactgaatattgcctcatacatgtcccctttt

ctttcagccacgttttgaatcaaaatgcagactccagctgttatcccaacgcgcttttgt

aaacatcctgtgtacaacttgttgctaaagggaactaaatcaccaagtgaattcacatct

tttttactaaacggaagataagcaggacttccacgatctctttcattaatctcataaaca

caaagttcttgaacttttactgtttcagattgttcagcttggctttgtttggtttgggat

aaagcttgatcttggcttttgcaaataaaagacttgtttaggttgagtgttgttgttgtt

gtgagtgaagggaagtgtaaaagagatgatttggatgaagtgtttagtattgatgatttt

gagttataaggtttcatagaaatggaagaaattggtctcatagcaaaacatgaggtagac

atgattgttgaagatctagtttgatcattcaagagatcgcttgaacgtgataaggatttg

ttgaaattgagattgagaga

>fava_dn_rep_c34

ttttttttttaatgaaacaactcaatttattgaccttgcggttcagttatattattggca

tatgttattacaagtacaacatgtaactcagctatacccctctcatattttgtaaaattt

gagtacacaaatatacatataaaactatagaatatactattatacacgtacgtataaaat

acaatacaagaaaatatagagatgatttaagctcaaaggctaacagagcaacgtcgatca

atctgatgcttaacgacgttagctccaatataagcgccgttaacccccatgccaacgccg

gtaccgtcgttacgaactgagatataagcaggacactgaacatagagatgataatgacca

gaaataaaagtaccaaccttccaacgaactcttccgtcaactttaacgatagcgtagaaa

tttccgttagattgatcttgtgagagtgtctgaaagttgaaaggagcaacagggacgtca

gtgccgtagacccccggcgaccagacgtcgacttctttgtggccttggtaggaaggtggt

atggaggtacggtaagtgatttgttggctccggtaggtgatataggtgtcgagacggtcg

tagtagataccgatgtggtcgttggggtttcttgacattagggttatttggaaattggtg

gttaaaagattgggctgcgttgcgttgaaggcggagacggttatgtcggagaggatggag

gttggtttggttggtcttaggattgcccatatggggagaattgtgagtaagactaagaaa

aggaacactatgattccccagaagattctttggaagagtattctttttcttcctgtgtgg

tgatcacactcctttaccgacatgatgttgagagtgtgaagaggagtgtgagagatgaaa

aatatgcgtaaacaagaataatttgtgataatattttggaggaaggtgtgagagtggaag

tgttgttgtcaggtgaggtgaggtgagagagggaaatatagaatggtttttgagagtgga

caaggaattatgaggaggtaacgtgtggatgacgttttttgtgtttgtttgtttgtgttt

ggtgttgattagatttttggaaagtttgagtattttatttagaggttagatgtatgataa

gattatgaaatatatggtaatgggtagtgtttggaaatttggaggtatgtaaatgtgaat

at

>fava_dn_rep_c35

caaaacttattgagtgtagatttgttgatttaacacaatgagaacaaagctgttagcaaa

ggcatccatttaagtttcgaaatctcttgcatgtaaattgtttcgatgagtgcttgaatg

tgacgcaatcggtgaatcatggttttgataaccctgtctctttccttacaaagtacaaga

ggcataaaacatttaaggcaggaataaatgtaggttactgcaaacaagatactattacac

tacacaatgagatgacaacgtccggcaaaaacataaaaacaaaagactaacaacactttt

aaccaattaactctataacttgcatatttaagctgttcatgcagccttttcaacaatgct

tctcttccactttttatctagctttctggacaacctcttactaataccctcctcaatctc

tctttcccgctttacagtaagcaagcgagcaatcgtcttgcgcattctcgaaaactcact

agatttaaactcagtaccagcagatttctcaagacgaagcatggccaagccgccttgaag

gttaacaacctcttcgttgatttgttcagtacttttagttctaatttccttcaattgttc

ttctctctttcccatcatcactgggaccaaagcggcgcgtttcgtcgctggaattgtgca

agtgttaggttgaagaagccgaatgccatcgaaggaggttttgaattgtaatggtttcgg

aagaaaggaaagtgttgaagaagatggtgaagtaacggagaggctcagcattttggttat

gtgttctcttggttgtttattgtatgaaccaataatgtgtaatgtttcaagataatatcc

acccctatctaatctatctccttcatcttttatcagtgccacttct

>fava_dn_rep_c36

gggctagatagacagatgcctcttcctatctngccgcggggacacaaaaagagaagagct

catcagaagaaaatttctaagaatgcctttcagaaagccacatcagacatcagaccctaa

gtctatttacaagaataacacggggtacgagataaagcccagaggactcaacattatctg

gggaaatgaccttcgctactggaaaataacatccgatgaccatgcagaactaatacaagt

ttcatggttggaggtatctggtaaagtagttatagaaggagggaaaacatataaggtgaa

atttgatgtggaggttaaggaaaacggtttcggttgggataacacaccagtacttgtcat

ggctaaaattgggaaaaagggagcatatctatataaggaagtcaaattagtttgtaataa

atcacaaacaattccagatgataatgaatcgcttacaataactgttggacaacaatcaaa

tgacctcgaacttcattttggattgtatgaagtttggagtggtaaatggaaaggtggctt

ggtaatcaaaaaagcacaagttctgcagatcaactaattattaatacttgttcatgttta

atctatgtcatatgagtctttgaaaagagaaccaatacaaaaataaaggggattattact

accctaatatttctaataaattaaaggtgtgcgttttattgtatgttgtaactttgtcct

tattagacaaatataaaatccattacttggcctcgaaaaaaaaaagctnnnnnnnnnagc

caacagtattgtgaatggagggagtagagaaaaaacagatttctaatattattcatgata

cattagtgaatatgggaagaggttttaactagaatagaaatgaaaattaataaagattaa

attaatcaacaacttctgagggtatgttgcaggttgaggttgctttagagaaatggaatt

caaatcagattctaggttatgtttccactggatacgaccaaaaaaggaagtatagctgac

tccattcgattagact

>fava_dn_rep_c37

ataatcagatgaatgcacagtgttatttcaaaacctttttaaattaccaatcatgaattg

aaatgaccaagtatgtgtttattagaacgtaatcacttgccacatatcacatacaaagtt

acacccgagtttgaatacatttccaaccatcacagatagcataatgcacaatcatgtgac

ctaacaatggcttttttattttctgttggatgtagccacaacttgaaattatcataattt

atatgtctaagttggagaaagttgaacttagtaataatttcaatttataaattatgagtc

tgcatcacaacttgcttttgggaacaccaaccggttcacttctggatttcttttctagtg

cacgtgatacttgcctcactgcctccttcggtatatgacgagggctgctcagccaaagtc

caccatcaattatcatagtgtcgccattaatgaattttcctgcatctgatactaggtaga

gagcagccattgcaatatcccatttctccccaattttaaatagaggcatttcttctctgg

ctttgctacctaattcttcaggagccagcttactcatgccaggagtgccacttattggac

ctggtgcaatcccattgactctaatatcataatctgttccccattccagtgccaagtttc

ttgttgtggcatcaacagctgcctttgctgcagatacatgaatttgataccaagaagctg

tataatgcaaggtagcactaatgttaattattgctccaccactagaagagtcccgtcctg

gtccccctttcttgagatatttaagtgcttcatggcacatagtgaatgtgccaacagaat

caatatccaaaacggttcgaaatccatttggggagagatcctcagccgcagcaaggaaat

taccagcagcagcattaacaagaatatcgatcttaccgaaacgcttgaaagtggattcga

gaactctggcggcatcttcttgcttccttacatcaccttcaaaagcaacggctgggattg

aaagtgattggagaacagaaacagcggattgaagaacttgtttacgacgacccatgagag

cgacggaagcaccatgtttccctaattgtgttgatatttcaaacccgattcccgatgctc

cgcctgttatgagcgctacttttcctttcaatatgtcggatttgaacggtgactccgttg

aagccatctttgatctctttgccgttgcgctcgatcttcgactcaaacgccaatgtggcg

gctttaacacctcttgtgtccttttttttnnnnnnttacatgcttcgtgggtccctctgc

ca

>fava_dn_rep_c38

agaaacaaatcctgacttcatttttgattttgtttttccttttgtcatattacttatagc

agcacttacaacaatactacaaatattttttacatttttctctctcttctccttctctct

atttaccatagatttgggtaactaaagaaggaacgtcgaaaccttgcctatacaaaagtt

acagaaagttaaaactgccgtggtacattatctaaatgacccaaagccatcgcattcagc

ctctttggtagtaagatatattgcaacctccatcttcgcctcatccagtaaatttctttg

caaaagcttcttcttgagcttcagaagacttgataaactcttcaagctgcgcttcaatag

aaagatcatcatcatcatcatcattcacatcaagatcaaatgcatcaggaagcaaatcaa

ccacgtcgttgccgtcaaattcatcgaagaagccttcatcatcatgctgctggttaagcc

agtagtcatggtaccatgtcagtgtcgtcaccagctgccaccattccggagaaaaatcct

ccacctgacgaaaagcagccggaatgtaaagaggtgcattcgggtttagggttgatcttc

ctccagaaactaatgccatcttgattttgttcctttgtctgagaaattttcgttaccgag

agattcacgcgcccgcggccgtctatcctctcaatttgcactttggatttttccaaatga

tccttcaatctcataaccacgccagaaagccttttgatataatctgataatacatgctcc

gatataccgacgaggttggcggagcaagcaacaatggtagcaacagtccaatacacaaca

agaggaatctcttgcattgcatcagacaaagaatgaacttcctctaaatcataacccata

gcagaccatgttgcccactgtataatgtgttgaaacacttgcagtaaaaacttgacaatc

tctgtgatatctgaaggaacctttctgaactgaacttggttaagttgcttcaatgagttt

cctaatgtgtctgatgattgaatcctataaaggtgcatgaattcaccatactccaaacag

aatgcagctagagttatcaatgcttttgcatcccatgagaagcctctgagatgctgaaaa

atccacattgttgtttggtgtagacactctgctttgccacgtgtcattatcatctgacaa

gaaatcctcttcaaggcaggaaagtctggtttgaaagaaatctgagtatgattagttcca

gaaatttgagcagaagaagcagagatgatggtggagacaaggttgaaaagcacatcctta

tcatatttttcatcatcattgacatgagtgagataaactttgttgagaatatgatagtcg

tcaaattcaaaggggttagggagcttagatttctgttgctgttggaaggtaccattctgt

tggatcttagcaccatttggtgcaactgaggacaatgcaatggacatgatgatgattgta

tctaattaacaaatgattagtagaaaattaactatcacaccttgcttc

>fava_dn_rep_c39

gaaatattggaaatatataatgatcaaaagtcatcatgtaaactagtcttactctgataa

gttgtgacacaccattaaatttgagtcacctcaggaaaaaaaaatgtggaacacaattag

aagtgattacaatttataatgaaaaattgagaaggaaaacatgttttacaaagatcaaaa

ttactcataattgcaggggtattttacatgattaaatattttatctaatttgcatcattc

atgtttactaattgtttatcaaataaaactaatttgcatcactcatttatgtagctcatg

gagcattaagcccaagctcttttccttgggacaagatctgatgtctttattgcatctatc

tttgcaactacaatgaaatcattcatactcaatccaccaatggatgttgtccatagttct

gctctgactagatttggttgttcaatgtggatatttgggaaatggcctgcagtctctaca

acattagagattctgttaatgagctcaactccgcatttaaaatctctcaatttccataag

catcgaagtttaagttctccttcctcgttcacaactctccaacccaaaacctttcgaata

agctggttagcatcttctatggaaagaggagactgtaaaggggaaatgggaagacactcc

tgctgagaaagagaaagagcagagatattggggatgaggattttgtgttcagtgttaaaa

ctacctaaaactttatctccgaatttactttccacttcagctgggaaagggtctctggct

ccaaaatcccccaagaagtcgttggccaaggagcggagtggggagaatccattaggagtg

tgacggtgctggaaattgaatgaaggattggttttggtgttggtggtgagcagttgctgt

tgcaagacagggaatgataatagaagattggttgtagccattgatttatggagagtgcta

gtttcttcctgtttgggttgggtggattgtgaagttgaattgaattcgatcggtcttgtt

ttgtttagtggttatcaatagccttttgtgctagtggtggagcaacttctaatttgttta

tttgcttctatttcttgattttct

>fava_dn_rep_c40

cggcgtaggcaccctggcaaaaaaataaaatcacaaataataatatatgaacacaaactc

aacttcatagtgctaaacacgttgtaatagattttcattattcattcaatacacgacaaa

gggaacattacaaagtaaaatatgaaattattcttttaatgataacataaacaatacaag

tagtaactaacacaaaaacaaaatacgaaaatttgaccagtactaaacatgttccggttt

tattaactttggcaaaccatgcttcttatatatacctcaaacgaatgcattcaaatactc

gtcgacagtagtatatttcacctcagggtaaagctcagtaacttccactccaaaagaaga

ttcaatctcaaaatttgcagcatcaccctttactagcatcgaatgacctaaagccaacat

caaatttgcagggaagggagattcttgaatagtcttaagaatttgatcttctggaagata

gattttctgaagggtactcttaatcttattctcccaaatggagacaagttcgttgaaagt

cacaacgttgccacgaggcctaaagtatacagttttgttcaaagttcttggatcgtccac

tgttttaatagtaaaagttccaatgtcttcctccttcacatatattgctgggacgtttcc

gtctccaagaatgatcactttgtctctgggaggagcagtgacattttgttgtgctaatgt

tgggaggaagtatccggcaaaggcgttcgagctgatgtaagtgtaaggaattccggaagt

ttcaacggctcttcggattttcactttttggttaaagaagctggttacaggctcaacggc

gtggtgacgatcaacgtccatcccaaattcagatgggagaaacctcttgatgtttccggc

ttcttttatggcagctatgagcttaacttgatcacttatttgtcctcctccaagtgtaga

aataacaacatcaacttgcttaaccgccttaacaagactttcatgatcagctagatcacc

atagaccaaagtaactccagagcttttgaaactgtcaataagcttagatttttcaggatg

agaaactgtgctttctctaactaaagcaaacgtggggtgtcctgcttttgcacttgcctc

aactatgaattttcctatgtaacctgttcctcctaggactaggattttgctcttctgtgc

catgtccttgattaaagattaaaagtttgaagtagaaattgaaagaagttgcgagtttgt

gattaacttaattgtggatgtggctgtcccgcggaaagggagggagggctatttgtagga

caacataattcctaaatggacataaatgtccttagttaatggtcattggttcaattcaat

attgtatgccacaaattcgtctcttcta

>fava_dn_rep_c41

ttattttttttaataagtgtggttttatttttttcttttatattttgagaacagggaata

cattatacatggcaaataataataagtacttcatataagtttttgcatagttattggaaa

tcataaggtcccgttattattacatgtattacaaaatgatccagtcagcacttatttctt

agtaggaacaaaacccactaaacatgctacttatttcaaatgcataataaagacaataaa

cttaaccagaaactaaactgattaaacactgcctttaatagcaggactgtagccactttg

tccatcgttgaatctatcccaaatcataacaccaccatatttaggagaactcttaatagc

aggaagaacttgagagatcaaaacatcagaaggaataaaaccaccacttggagcagcagc

atcattagcaggaacacctaagaacacttgttttgcttgacttgaagtccattgattcca

agcattaagaagattgttagtattcccatttgcgtattgacactgaggattattgtaaaa

ctgaacccaaacatagtcaaataaaccagtattaatagctgaatttaaatgagcatcagg

gaaaggacactgtggtgctgcagacaagtaaactctcctttgtgcactaaaaccattaag

tgcttttgcaagttcatcaaaatggtcaccaccggcttcaatatcaaaatcaattccatc

caaaaccgcatcacctagtggtcttgaagttgacgtgccgcccaagaaattgttccatag

ataatttgcaacttgtgtagcatcatcagcggagctaagagagtagcttccggcgccacc

tccgagagagagtagaactttgattccatttgcttggcatgcttgaatttcggagctaaa

ctttgtgcactcattggcacttggattacaatggccagcaaggtttagtactggggtttg

gccattgccgaaactggatagaaaagctatgtttacaaattggtagttgtttgtgttgca

agcatctgccagggaaccttcacctccattttggccccaatagactgcaattccagcagc

atgagagtatttgaatagggaaataaggaaaagagggaacaataggattgaaacttgttt

taaagatgccatgttttttaaggttgttgttttgagttgtgatcccgcggcctagttttc

gtttcacgccgagcaagt

>fava_dn_rep_c42

ttttagacaaaatagaagtgactcactagtcagaaagtacattttcagattttatgggca

tttttggtgtggaggcagtgagcaatcacctagtttttattgaatttcagtacccaataa

caagaaaagcaaaactagattggaatttatcattcaaactgagaaaatgtttcaacagaa

caacatagatctaacatccaaaccaaccaagacaacaaccaaactttaattaatttcaaa

accatagtagggcagatccacaatcaaacagatctttactcatagatccaagagtgaagg

ctgctttgccagttagcagcaccctcagggaaccacaaagcaatttccttgttagcactt

tcaacagcatcacttccgtgaataacatttctgccaatgtcaatggcaaagtcaccacgg

atagttccgggctcagattgagcagggtttgtggcaccgataatctttcttccggttgtc

acaacattcttaccctcccagatcatagcaacaacagggccagagataatgtaatccacc

aatccactaaagaaaggctttgcagacaaatctgcatagtgcttctcagcaaaagcacgc

tccacattcacaaatttcaaacctttcaagtagaaacccttcttctcaaacctgctaata

atctcaccaacgaggcctctttgaacgccatctggcttgatcatgatgaaggtttgctcg

gccatcttggtttctgtttggtattgagttcagttactgtggctgtgtgagagagggtga

gaaatgaggaagaacgtgtgggagagaaaggggggtttatatatggctgtagaggaaccc

taaacctcatttca

>fava_dn_rep_c43

gtagagaaaaaccttgagagagtatcttcgatccaaacaatacaacataaacttatttaa

tttcattctatcacaattgaatcaaatgtcacatgaagtgttatgaagatgtaaaagaat

taatttaacattctctcttaacttaaaatttgtgttctcccttgtgaaagaaaagccatc

ccccttttctcctctcaaccaccaaacatataatatttcatttagtccctttcctccatt

aaaatgcatcttcttatttgaataatgaccatttgaggcactacataaccaagccaccca

ccttccttgattttgaagtctttaactgcttctcttagcctttaaattaggaccctcggc

ctagaaagtgtgtctttgtaggtttcaaacatgatcgtagaaaggttacctcttacaaat

cttgattgtaagaaaatacgaatgtgttaaaggacaattttattttgctaacatgtggaa

cttaaatgaaaaaacatattttgcatagttaaataacatattcaataaaattgtcaaccc

aaaaatggtacaaactgttaacatatacaaaacttactataaactacaaacaattgtatg

tgatccaccatcatgcaacaggcttccgctttgaacgcttacttcttctttccctagaaa

aactttgagccacttggctaactttagaagtgttcgactcttctgtaacttcttgttcct

tgttgttttctacatctgagccatcattagaagctgaagcagacgccaaaggctgaactt

cctccactgggagtgccttaagtagctttttcttcttctcctcttcttttaactgtgcaa

acctttcaccacccttttctggcttagaagctgatctcttagctcgtccagcagtaataa

ttccaccagcattttcattaacagcgagctttgcacctcctagtttgcgcaaccatattt

gttgagctgtgctaaggacattatttgtaaaccagtaaattgttagtccagatgggacag

ataacgagaagtagccgatcatgagtgggagaaactttaaaataacaagtgtactctttt

gattaggatcatttgtctgagggggtttcatgacttccattgaaacatattgagaaacaa

taagaagaataggtaaaacaagatatgctgcagtgttcggccaacccaaaggtggatggc

catccacaaacggaaaaagccaagaaattccagatccactttgtctagcagcaatgctag

tgggaccacccagagaagggatccataagaaaccttctgttaacaatccctcatttgcca

cattagataaagcttggtatagaccaatccagactggaatagtagccaaggttggtaaac

aacctgccaacgggttaaccccagcctgggtatatagccttgaagtctcaagttgtattc

tttcctgattgccggcatatctttcttgaatggccttaattttaggttgaaggttttgca

tagctagtgctgattcaacctgttgctttgtcaagggaagtgtagcagccttaacaataa

cagtgagtaatatgatagcaaatccatatgagtaaggcacatgcacggcagacaaaccat

cctttaacaccttgagaacaaactccattccatcagaaataaacccgaaccaacctccgc

tcttctgcacggcaacatcactagaaccaacagcatccgccgcaacagtagcatcggcga

gcgtgtaaagtaaaccttcagctctagcgaagattccggacaagtcaaccgaatgactta

cggattgaattggagggagttggtgtaaactgaattgaactttggtgaacagcctggtgc

gattcggggagaaactgcgatgaagtgaaggaagtggagtaccgatgaatgatggagaag

aaatcagtgtcttcgccatggatgatgagaaggttcctgtgcgagacgcttcaagcttga

aggctttaggttctcgtcacagaattgagatgagaggatgagaggatattcttttttttt

tcgaattattgttgttattattatccaaattcgtttaattttttgcgaggtagagacaag

gtgcacgatatgggtagtggtggatttttagccagcggctgagtgacacgtcataagcta

>fava_dn_rep_c44

ctctttttttttttccatcagaaattaaactttattagggaagaaacacccacctgactg

catgatgaaactatctagtaaacgactgccgtgttccgcaatctcgaggacattaagctc

tatagtttacattgctagaaaacaaaatatcaggaaagcttcatgggggtgattaatttc

tgaaaggtgattaatatcaagcttcataaaacaaggataactgaagaaatcaaacatcag

ataaaataacaaggctggtacgaaagcatttgaagcatttacactggagtcaagccaacc

acaccacatgctaatcttccaccagcatttccagtgctcaaactaagttcatgtccaccc

tttccaagatcatcttcaagctcgtgaaccactaaggctctcccaacaactgaattgggg

ccagtgagtggtatctgattgtccacgattgttgcttctgcaactccttcagcattagca

actatgtttcccaggtcacccgcatgacgggtttcatcttcaggagcaccatgtgtcaac

ttgttgggattaaaatgtggtcctgttgagatacacccatttgtggtatcaccatactca

tgtagatgaaaaccatgaagccctggagtaaggccagtgatacgaacattaactgttgtt

ggaccttcatcgtcttgagtgagagtgacaacaccttcaacagcgtaggttcccttaagg

acagcgacggctttcttggcggcagcaacgacggtgagaggtttgaaattggaagttgta

agggttgagaattgaggagcgagcttgacggagacgccggagaaagatgttcggagaaga

gagtgagaagagagcggtgaaggtgagacgagagtttgtgaagccattgttagttgcata

gtgtgatagagattgagtttgaggtgtttgtgataatgcaacagaatgtgtgaaataaaa

ttgtgctggcttgtgttgctcacatcacaccccgcgg

>fava_dn_rep_c45

ttatggccgcgggatagcgattagggtttttgctgatacctgttgctataccttcctccg

tagcctccgagttcaacacagaagaccacagccatgggtaagacacgtggaatgggagct

gctcgcaagctgaagtctcaccgcaggaggcaaaggtgggccgacaagtcatacaagaag

tctcatcttggtaatgaatggaagaagccttttgctggatcttcccacgccaagggaatt

gtccttgaaaagataggtattgaggctaagcagcctaactctgccattcgtaagtgtgcc

agggttcaacttatcaagaatgggaagaagattgcagcttttgtccctaatgatggttgc

ttgaactacatcgaagaaaatgatgaagttttgattgctggatttggaagaaagggtcac

gctgtgggagatattcctggagttaggttcaaggttgtgaaggtttctggtgtttctctt

cttgcccttttcaaggagaagaaggagaagccaagatcttaagtttgttgaaggcacttt

catttatttagtttccttaaagtatgaacaattttgtttcgtgttgaatgagttttatgg

acagatatgtatttctgattattttgtggaacttatttttcacttaggaatatttactgc

ctttcttaattgtgtttctgctcttttctcttttctgttttgctatttatttttgttata

catgaatatcagtaatttttttacaaaaaaaaaag

>fava_dn_rep_c46

aatagttggatcaggtttgtgttttgtgggcatgtcatggtgtgtcaagaagagaggtcc

cgtnnnnngccgcgggtaacatatttctttatcttatcctctagtctaaactactttgaa

gggttcccctagaaacttctaaacaaaatggctggcatagcatttggaaggtttgatgat

tctttcagttttgggtcaattaaggcctatattgctgaattcatctcaactttactcttt

gtttttgctggtgttggttctgccatagcctatggtaagttgacatcagatgcagctctt

gatccagctggtttactagcagttgctgtttgccatggttttgcactgtttgttgctgtt

tcagttggtgctaacatttctggtggtcatgttaaccctgctgtcacttttggtttggct

cttggtggacagatcactatcatcactggtatcttctactggattgcacagcttcttggc

gccattgttgcatgttttcttctcaagtttgtcaccggaggattggaaactccaatccat

agtgtggcagctgaagtaggaattattggaggagtagttacagaaataatcataacgttt

ggtttggtgtacacagtttatgcaacagcagcagatcctaagaaaggatcaataggcaca

attgcacccattgctattggtttcattgttggtgctaacatattagcagcaggcccattc

tctggtgggtcgatgaacccagcaagatcttttgggcctgcagttgttagtggtgatttt

catgacaactggatctattgggttgggcctcttattggtggtgggctggctgggcttgtc

tatggcaatgtcttcatgcgttcttctgaacatgcacctcttactaatgatttttaattt

tttttgtgagcttatttaaattatgattattattagtgttgtttatgtgatgtatgtgat

tcatgttggatttatgattattgttttcttttttgttttttatttattttttatttttag

tttgattgtaaagttgtaagagttaatcattgtttggtaagaaagttggattaattgaga

gactttttatgttgtcgcaaaaaaaaaaag

>fava_dn_rep_c47

tcttttttttttactaggaaatttctttgcattatttatcagagaaatagtacagatagt

acaaaacctcagcatcaacccatagatttttgtatttaaacatgaaatatccaaccacac

tcaaaccgacacttagttcacaacctcacacatgtaaattaaacaaaccatatcccaaca

ctttaatcattattcccataacaaaacacaaccaagtagaagattatacaattaatcagt

aatcaggattagccaaaacgtaaccctcaatagccttgataagtccagttcctttggcct

tagtttcatcacgaactgcatcagatagaacttcatcacctttggtgtgatatttcacag

aaatcttaacaatggacccaccatcagaaccagccacaataattgtctcgaatgtaactt

tttccaaactttcatgtagccctggtcctcctactaagctgtagttgtaccccaagtttg

cttcatcaattgcatctagtttgtgtagcagatagtcgatttttccatcttcaagaatgg

atagcttcttgatggttcctggacctccatttccttcgataatttcgactccttgtgctt

ccttgatcacctttgggacgatttcatcagcgtcttttgcaagagctttgtagagtttag

gtggtgccacagttgaaacgtattcatcatcgaaaacaaagacacccatgataaaaagga

taaattagtatgtaagaagataaagagggaatggtatgtgtatatgaatacaagtaattt

ataagtttgtgatgtcccgcggcc

>fava_dn_rep_c48

acatcaatattaacattatttgaggtgcattgtttttatgttaccatattccaaggtcaa

gaaaaaacatcattttatttccttccactaagattgagaatcacattcaaacactgatga

tggaatctttaacctcatcttcttcttcaacaacgctaccttctttctccatcttcccat

caacaacaacaagaacatcatctctttcttccaagaagacccttcacttccgtatacctg

cctccaaacgtgacgatgctaaggattccgatgagatcaacctcaacatcaacctcccag

ttctcagtaatcaatgcttctccatttcccctctttctaatgatgcggcgatggggctgg

tgttgagtgctgcaactggtagaggatggacaacgggttctgggatggaagggccgcctg

ttcctgctgttggaaaggatgatgaattggggacggggaacatttcaacttttccgtggt

cgcttttcacgaaatctccaaggaggaggatgcttattgcttttacttgtaccatttgtg

gccagcgaactacacgggctattaatcctcatgcgtataacgacggaactgtttttgttc

agtgctgtggatgcaatgcataccataagcttgtggatcacttgaacttgtttcaggaga

caaattgctatctgaattcaagctttaattataaaggtcctggatgggatgatcttaagt

tcaggttcatggacgttgatagcgacaacaatgatgatgatatattcccggttacatgag

tgaattaccagcttatggagtatatattttctgcctctgatcttaagaaattttagatta

gtttgtcttgtatattgtttatgttttcatgcttaatcattaaatatgtcagaggattgc

tttgtatatagtatagcttcgcaaccgaattgtacaatcatgtacaatcatgtacaatac

tttgcataactatttcctctgcttgtgacttgccattgctccttggccactctctgaatg

ctctactataatttaaaacctttttaattagtgcaataaaaaaataaaaaaacaatatgg

atatgatcttttctcctatcaatatataaattatgcagataacaaaaaatcctatattta

tggatataaatcaccattgcaagcaaacaaaaaccaaacgattaattacaacatacctaa

aaataaaaaagaattatttgatataatgggcgatagctttatccaaaattatatagcaga

agatggtattatagatatggatatggaaacaatggaaaaaaatctgaatagaaagtatct

aaattggatgggaaaaagaaaaaagaattccaaaacgaatcagaaattattgactccgag

atttgggttcttttcaaaattaagtgcatataagaagaatccttggatcttaccaatgaa

tttcttttttgtgcagttttatggaaacaaaaacattactgatgtagccaagttaaagtt

agaaaagaaagaatacgggaagcaagcagatctcaaatcatatctctcaaactattatag

ggacactcggtatagtataactagatacaggaaggatctaaatggcgaacaaaatttctt

actagataaatattttggtttttatttgcactgtaagactcccgtccaagaaacactaat

ggataatatcaacttatattgtctcctggttagattgaaaaatctaaaacaatttttcat

aatgtctataaaaaaggcagacctcgattttgaaattacggcgcgtctgcagatgaagga

cacttgttatacagaatgtagggacacagaggaattgagggacaacctaatgttttttat

tgaacctattcgtatttctagaaaaaactatgaacaattttttatatatcaaaccataag

catatcattgaggcacaaacgtaaaattttgaaaagaaactatgatttacttgttcctga

aaatcttttgtccactagacgccgtagagaattgagaattctaatttgtttgaacccaca

gaatagaaatactgtgcatagaaacacaataaatgacactgagaaaaaacgaaataattc

ttctgaagttttgactcaaaatgaagatattgatagcgatacaaaaaaactaatgacttt

aaaattctttctttggccaaattatcgattcgaagatttagcttgtatgaaccgctattg

gtttgatacacataatggaagccgtttcagtatattaagaatacatatgtatcctcgatt

gnnnnnnnnnnnnnnnnnnnnatttatcattatcatgttcacannnnnnnnnnnnnnnna

ataaataaaataaacgcgcacacgcattgtgggattgatacgacttcaatgatgtctcca

ttagatagaaataaatcaatagaaaaatgaataaatcaaaattgtctttttttttttatt

ttttaaatacaagacaatagaattttttattttttgaatacataaatatagtatttattc

tctatttgaattacattccttaataaggaatttgaaaaaaaaaaattcagaattgtgaaa

attgttaaataaattaaggtaaaatttgatataaaaaattcaaaatgagtgtcattacta

ttactgatcaataaaaatatctaccaaaaaggagggggaaagaaaagctttcattttatg

ataaaaaattcatttatacctgttatttcacaaaaaaaaagaagaagaaaacccgggatc

ggttgaatttcaagtattcaacttcactaataaaatacaaagacttacttcacattttaa

attacacccaaaagacgatttatctcaaggaggtttacgtataattctgggaaaacggaa

acgactattgtcgtatttgtcaaagaaaaacaaaatacgttatcaaaatttaataaatcg

gttgggtattcgggattcacaaattaggtaatttaaagaatcattttcaattattcgatt

tattagatactgaattttgatgaactttttttttgaacgattcatggaagaatgaatcga

ggaaaaacctatgaatatcccagctacaagaaaagacctcatgatagtaaatatggggcc

tcaacacccatcgatgcatggtgttcttcgacttattgttactctggatggtgaagatgt

tattgattgtgaaccaatattgggttatttacacagaggtatggaaaaaatagcggaaaa

ccgaacaattatacaatatctgccttatgtaacacgttgggattatttagcgactatgtt

cacagaagcaataactgtaaacgggccggaacaattgggaaatattcaagtacctaaaag

agccagctatatacgagtaattatgttggagttaagtcgtatagcttctcatctactatg

gttgggaccttttatggcagatattggtgcacaaaccccttttttctatatttttagaga

aagagaattaatatatgatctattcgaagctgcgacaggtatgagaatgatgcataattt

ttttcgtattgggggggtagccgctgatctaccttatggttggatagataaatgttttga

tttctgcaattattttttaacaagggttattgaatatcaaaaacttattacgcgaaatcc

aatttttttagaacgggttgaaggagtaggttttgttggtagagaggaagttataaattg

ggggttatcaggaccgatgcttcgggcttccggaatacaatgggatctacgtgaagttga

taattatgagtgttacgaggaattggattgggaagttcaatggcaaaaagaaggagattc

attagctcgttatttagttcgaattggtgaaatggtggaatccataaaaataattcaaca

ggctttggaaggaattccaggggggccttatgaaaattttgaaatccgctgctttgatag

agaaaaggagccagaatggaatgattttgaatatcgattcattggtaaaaaatcgtctcc

tacttttgaattgccgaaacaagaactttatgtgagagttgaggcccccaagggagaatt

aggaatttttctaataggagatcagaatggttttccttggagatggaaaattcgtccacc

tggttttatcaatttgcaaattcttcctcaattagttaaaagaatgaaattggctgatat

tatgacaatactaggtagcatagatatcattatgggagaagttgatcgttgaaatgagaa

ttgatacaacggaagtccaagatataaactccttttccggattggaatctttcaaagagg

tctatggaattctatggatacttgtaccaattttgattcttatcttgggaatcacaataa

gtgtactagcaattgtatggttagaaagagaaatatcagcaggggtacaacagcgtattg

gacctgaatacgctggtccttttggaattcttcaagctctagcagacggaacaaaactac

tattcaaagagaatcttattccatctaggggagatattcgtttattcagtattggaccat

ccatatcaataatatcaattctaataagttatttagtaattccctttggctataactttg

ttttatctgatttcaagataggtgtttttttatggattgctatttcgagtattgctccca

ttggacttcttatgtcaggatatggctcaaataataaatattcctttttgggtggtctac

gagctgctgctcaatcgattagttatgaaataccattaactctatgtgtcttatcaatat

ctctacgtgcgattcgttgaaaccaaaaaagttattcgatgctattgctatgctcctctc

tttatagtactatataggaaaggaggcgaatcaattgaatagaaaccttcattatcttta

tttttctccgattgaagaactaaattagatagttatatgagtgaaataaaacagcttata

ttgaatagaatttcagaatactttcttacttctagttaatagatagtatgatgattttta

atggcagtaaaaatattgagtctcattttctatgtataagaatgaaataaaattataaac

agaagaggccatttaacccaagattggataatttgtcatcctgttttgaagcgggctcta

aagaccaaccttattgagttttagttaccatttttatgaattctcatagatatattaaaa

taaataagggtgattaataaaaaagagtgggcggttagaaacaccaagatgcgcaaaagg

ttagtaacacggattttgtaaattatacaaaaggcaatttacgcaaaaaaaaaaaaaaat

actaaatgggactttaagatggtagaaaaaataaaacggtactccccacaaccccgatcc

agagtatgcttccatccactcattaaataaatgactatcaggaacgaaaaaatcctttaa

aattttttaagtccctttttcatagcacaaaaaaaaaagaggaacgtaaaacaattacca

gggaattg

>fava_dn_rep_c49

ttgcattttcaacaatatcatgatcacannnnnnnnnnnnnnnnncgattaagaaacatt

ttataaagatatactaaacacattatgggaaaaaaacaagatattctttatcatgattac

atgatatgaaacagaattactttatcatgatatgaaacagaactacctttaaccatgtgt

tgaagggcataagtttgcactatcaataaccaggatagttagattgttgatacttatgac

tgtcatccttgcattggcatttaatatgagctttttagaaaaagtctagtttttttcatt

tttgcaaatgcaattacacagaaaatagatcaaggcaaatataaaagatcaaccactatt

tcaaaccgaacaagagcaaaaaatttgaccaacaaagaaaaatagctcacaatccaaaat

ctaaaacactgccaattctgagcattaattggtaagctccataatggcagcaacaatgtc

tccactggtagctttgagagctttaacggctttagatcttgggacaccagcttgagtcac

caccaactcaatatccttgggatcgacaccagtctcgtctacatcttcctcttcggtaac

aataccagaggattctggcttcgctccaacattgctcaagttaggagccttgaactgctc

tgctgcctgagtttgtagctgtgaactcaagtcctcaatcttagcttctccaaaaataat

gtaggtgtcagaagttgggctcttgaaaacatctggctttgagatgacgaacaagatatt

cttgctcttcttgactgtcacacggctgacaccggtaacaggtttcattccaagtttaag

catcgccttgcggctcttcttttcacttctggtttgtttggacctaccagatgcatcacc

ttctagtccttctatgtcatcatcatcctcgtcatcatcttcatcctcatcttcctcatc

atcgtcgtcctcaactacaggctcatcatgatggatcttttgctgttcgagatgctgagc

aagaagctcttcttgagtttgagcagtcatggtggatttctgagaagttgagtttggggc

ggtaatagatccaacacagcacaagcacgagcaacaacctcccctgtcccattcccttcg

aagagcttcatcgcgaagctatgatgtcgttgaaacctgagctttttgagggtatgcgtt

ttgatttcaccaaaatgctaaatcagaaattctctctcaatcacagcgtgtcgatgggac

ctactgaggttccttctcagtctgcggagattattaaaattccaactgctaactatgagt

ttggtgcaactgtcattgatcatcctaagatgttgcttatggggagagctttgactgatg

gcagggtaactgctagagtcaagtatgacttgactgagaatctcactgtaaaagccaatg

ctcagctatcaagtgagccacatgcgtcgcaagggattgtcaactttgattacaagggaa

aggattataggacccagattcaactaggaaatggatctttgctaggagcaagttatattc

agagtgtcaccaatcatttatcattgggtggggaggtattttggactggtcagcatcgga

agtctggtgttggttatgctgctcgctacaacactgataaaatggttgccactggacaaa

ttgccagcactggaatggcccttctaagctatgttcagaaggtatctgataaggtttctc

tggctactgatttgatgtgcaacttcaactccatgtcaagagacgtcactgctagctttg

gttatgactacatccttagacagtgccgccttaggggaaaggttgattccaatggcgtct

gtgctgcatttttggaggagcggttaaacatgggtctgaacttcattctttctgcagagc

ttgatcacaagaaaaaagactacaaatttggatttggtttgactgttggagaataaaata

gcaggactagagctgtatttccttttctttagccagcctagcctagcaagttttcttgct

tcattttacttgttggagatttttctttttgggtaaaagaaaaacaggaggaatatatca

tttgtgtcggcaaacatcatttaatccacaagttggcctaaaaatacttcaaatttgttt

tgaacccttgccctgttttccctggagggagatgtagagtgataatagcagaccatgcat

attataagattcatttcaacataatgctcgtgtttgctaatgatctataagattcatttc

aacatattataagatccatttcaacatattataagatccatttcaacataatgcttgatt

ttgcagacgtctaatgatctatggaacctctgttgcaagaaggatgacttaaaaaagtcc

ttgcataatttggtggttatgtttatgattgggatgtttccacagattcatacacaagtt

ctattatgtagaaataatcaagttaatatagcgattaatactgatatctagtattcataa

ggttttagataccacttgtttttcttatacacctcaaataagctgatttagcgcatgctc

ttgttgaaattcggatccataatatattgaatggaaagtgttgtggactccctt

>fava_dn_rep_c50

gtaaatgtcactactttattatatcttataacattttatatgatttacccttttttttaa

ccttttgcatcatacaaagttgttttgatcattaaatcaaatgaaatatgatacaactag

ttgattcaatactgaaaacatttttgtcaatagtgcaaatctattttactagtgcataat

tgacactaaagagtgactcatagataaacttctcactttatcaaccttcttgagaagaaa

tcttttaatgcaataaattgtgcaataaattcaaacaaagctaccccaacataattgtag

tttcagaaaacaaactaatgtcctataacttgtttcctttccttattttcctaaaatgat

tcaataagttacacaaacaccaacaacaaagtttcacaataaccagctacttgacacata

accaaagcaagcatatagaaaaactaataaaatgtgaaaatcacttagtagagtgcttcc

tccttgtgagtgtaaatcacggtgtctgatttcgggtacgaaacacatgtgagaagatag

cccttctcaatttgctggtcatcgagaaaagactgatcagactgatccaccgaaccctta

tccaattgtccggcacaagtagagcatgcaccagctctgcatgagtaaggtagctccact

ccagcctcctcagctgaatccagaatgtaaacgtcgtcgggggcgtcaaatgtattctct

ttaccatctggtccaatcagtgtcactttgtatgttgccattgcagatactctaaacgag

gaagattttagtccaaatgatttggacacattcttcacagatctcaaagttgatggactt

ttcataagcgcacaagacgcccgagggggcgcttttccgtacaatgaagctgttggaagt

gtcatggtcgacatattcacaaccgacattttaaaacagttagttgatgaagaagtgaaa

gagaaagaagtgagggggcggagttatattggagagtggaaagtggccttttgagtgttc

ggcgggacacgacacaaagggagagagttgtgtttatattgtctttcgttttcacctacc

ctttcaagggttcaatgaaactaactagtttgttttttc

>fava_dn_rep_c51

atttagttataaatatttctgctttctatacagaactttgtggacgattaatctatgcat

tttcaatgatcaaacagatatattttcaaggtatataaatgcaaagtatatatactatga

ttctacatataagaacagacataaggtaaatgcatccaatattagaacatagaatttaat

ttgtcatggattaacagacttaggaaaaagcttcatttgcaattctctagcatctcagtt

caattatctacatgaaatgttttcgattacaagccaagagcgccgagtggagtcttccct

tgtccagcttcaaagtagaaaataagcatagctaacatagcgagcctagcatgcttaatt

tcagcgagtttcaatctctctaacttgtctgtatctggaatgtaaactccattttggatg

gtaccagcgagtgacaatggatcgaagaattttccacctgggtagccttgttctccggtt

gaattgacgaagttctcagctgttttagaccatggagtggcccattcgaccgactgtgag

tccgggttgaagaaatcaacccatctcttgctctctacccatcccatgagaatcaactgt

gtgcctagaagagtaccgaatgagaatggagcaatcgcgttggggtcagctccagcctcg

aaccatggaactccactccatgcctgaccaacaaatatacctagaactgcagccattgcc

catctaccatgaatgagctcggcttctctgtaccatttgagaaatgccgggtccttgcca

agccctagagggtcgaaaccaaagtcacctggtagcgatccatcgagccattcggggtcg

acgaggtttccaccgaatctaacaccagggatccatgatttctttggtgcagaagcagca

gcagcaacaataagtcttttaggactagcagctgcaacaccaactttgcttccaatggca

gtagctagcaaggtttggctcctctttccaccagataagaaggaagatcccaaaccattc

aacacagcaccagatgttgcagctgccattgctattctcttcacactctaaagaagtttt

ctctatgaagaagtataatgagaaaatagtaatagaagtagctagatagtattttatgtg

aagtgggagataatgtgaggggttttcatagaagaccaagta

>fava_dn_rep_c52

tggaggtaagtccttgtgttatcacaacacaacataataataagaggaaatgagacacac

acttcaaacttcacactaacacaagtatggctagattggtggcattccaacagaatcaac

tgtctttccctcctttagcttcctctctttctgatttcaatggaacaagacttcaaactc

acattcagttgaaaagaaaaacatggcagccaaaaggatccttatatgtctcagcatcaa

gcacaaagaaaattctcatcatgggaggtactagattcattggtgtgtttttgtctaggc

aacttgtcaaagaaggtcaccaggtaactttatttacaagaggaaaagcacctatcactc

aacaattgccaggtgaatcagacactgattttgctgatttttcttcaaagatcttgcatt

tgaaaggggacaggaaggactatgattttgtaaaatctagtctctcagctgaaggatttg

atgttgtttatgacataaatggacgagaggcggaagaagtcgagccaatacttgatgctc

tccccaatctagaacagttcatttactgttcttctgctggtgtctatctcaaatctgatt

tattacctcacgccgagattgatgcagttgaccctaagagcagacacaagggaaagcttg

aaacagaaagcttgttgcaatcaaaggatgttaactggacatctataaggccagtttaca

tttatggacctttgaactacaaccctgttgaagagtggttcttccacagactgaaagccg

gtcgcccgattcctattcccggctcaggaatacagataactcaacttggtcatgttaagg

atttggcgacagcttttgtgaaggttcttggaaacgataaggccagcaagcagatattca

acatctcaggagacaaatatgtcacatttgatggactagcaagagcatgtgctaaggccg

gtgggttccctgaacccgagctcattcactacaatcctaaagattttgattttgggaaaa

agaaatcgtttccatttcgcgaccagcatttctttgcatcagtggagaaagcaaaaagtg

tgcttgggttggaacctgaatacgggcttgtagaaggtcttacagattcgtataacctcg

actttggtagaggaactttccgcaaagaagctgatttttcaacagatgatataattcttg

gcaagagtcttgttagtgtatgatgattattacaaattactcttataatttatgtatgta

tttcacatattatataatacttgctataatatcatctcataaagttctgaaatattcata

agctactcattcagtgattttttttcttcctaaaataaaagaatgttcctgcttacaatt

taacaaaaaattttggcatacagagatgtttttatggtttagccgtatgaatctctgtca

agtcagcttgattgaaatggacaaaaagatgtttttgctgttattcttagttgaacagtt

tgaggaccattcttaataattcatatgaacaaatactagaggttgttacacacaattcat

aaatttaacaatcttacacaagcagctctcaaatttgaatcactgcaattgtttacaaag

caatgtctgttgtatacactatattgtaattgtattatgtactttacgaaatgtaaaccc

atgcaacccaatactgcgacttaagcgccttccaagtgagtcatggtgctcttacaaaca

tactcattttgatgaaatggtttcaaacccactccttaatttccttgatgtctttgcgtg

gtggtgcacgccgtggctcagaagttgaatgttggatccttatgtttccagcgtcatagg

cgcggatgccattcccaatggaagagagcttaaactgtccattttgtttttttacacgac

gagttggtctgcaaagtgaccctttcggaatattatgaactttcaccctgcatacttttt

ccaagttgggaatagcatgaagataggatgggaggtccaccataaaatctccaaattcat

cagttacaccttttcctaccattgattttccttttctttcactgccatggctttggcagt

taacagcaaccaaagctcctggtattggccatgcatgtggatgatgatgaagatgatgat

tatgttcacaatgaagtgagccagtaataagaacagttgaaagcttctcttctccatagc

cagcagtttccaccacttcttttctgctagataataaatccaacaaagagtgaccatctt

cttcaatttctgatgataatggaaatccaaccacaatgttattaagcaatgataataaag

ggaggaaaaacaatatcacaagaacgttgttggcgtgaaagtagctcatggtttttgtgt

ttgtttatcaacttacgtaagatatataat

>fava_dn_rep_c53

atcaagtagatcaagtctataattaatatcgacatagttataaaaatgtaaatactttaa

atttgacacctaatgtccaacatatactaacaatctatatagtagatgaagttaaggaat

tttaacttccacaatcattttgactggatatatgaaacttaagataacttcgaacaagtc

aatataagagtgcacaattacaatgcttttacatgttttttcttttcgggtttaacataa

acacattactttaacaatacagacagtaacctctatgatgccatagcaatcaatcataag

atggtatatgtaatatagctacaatgcagcttcagcggacacaacaaacatttatttagc

acatagctcaaactctcgacaaacatgttacctccccaacttatacaaagatgatccgcc

aaacactactttccaagcttacgcttcatggaaaatctgccaacaacacacagcaggcac

actgaaacctatgtattattttgcatgatggattatttgcttagactgcagaaccagctt

gttcacataggtccattagatccagggtctctggaactgagagctcaaggcgaaactttg

gaccatcataatgatgcaatactggcctgaatgaatcttcttccagtgccttgcaaattt

ttctgacacggattcttacctgagctgggaaccttgactcgcctttgcattttttatcct

cccatgcagttggatctatgttagagcccccaaaacatgttgcctcaaaaattccatgca

gctgatgggtagtataattataaagaaatagaggtaaccccggtgttattgccctgaccg

aatctcgataccttggaggcagaccaaatagctgacgttttaagtcttcctgcatcgtat

cattgttacaaacaaagatataacctccaagcacctcattgcgtgggagtgtctctgctg

caggcaaagtcttgaaccttttgtccgtagcatttgtgttgttttcattgttgttattgt

tattgctatgtggatctttgttgagctgaacattaccactatacttgttactgttcatgt

tacccaacatgtttgcattgtttttctggtacacagcattcatattgtaaacaccattcc

gaagcgagcttttgttcatattttcaataaatttcgattctaggtttaacatgctcaagt

taagagtgtcaaatttgttctcctcctgaaaaccaaatttatctcttggccttgtttcaa

tgggaccctttgaaagatcaaggttgttaaagcgctctgcctttgatctctgctcagcta

acttagaagcaaccattaaccatttgtgatcctcagatgcttttgagtgtccgcggagct

catcccctaactgccagaagctgtgcatgttgtccatcttcttttacgacttcgctatgt

aactcgaagacgccagagtgagggcccgcgccgtaa

>fava_dn_rep_c54

aatttacattggccaaaatagtttttgtataataagctaaattaagctgagaggaaaagc

aaaaatagtgagagaagctgatcgagttcgttccgtcgccaagagtgaaaaatgtcacac

agcgacaccatccccctccacgcttcctcccaatctgacatcgacgagatcgagaatctc

atctatgcatctccggccaccgttcttccggcgagacctccaagtccaccgcgcgcttca

attcccgtttcatcctcttctcctttcatcaactccaatgcttctctttttcagctctct

gcttctctttttcagctttctctgcttcttttctatttctatccaactgtttaatcagca

atttttcttcttggcttgcttttctcttatccatgtcttcactgtttttctgcaacaagc

cttcaactatcaagtggatgtcagccccggtagaagttttggccagcttttttgaggcct

tagttaagtcttgactataattaggctcacttacatttttcttgagtgacactatcattt

cagttacagccataatcctctcattgatctttttccgcattgtgcgccggataagaagct

cccctcggacagattttggaagcaacttaacatccctagtctcccaacaccaaagacagg

aatcagaatgatcctctagtatatcagcatcagcattaggcacaccatacatcatcctct

gcccaacaaacaacacactactcttcaccaacgcctcattcccaaacctttgttgagaga

gcttcacagtttccaccaagatgcagatctttgtgaaaaccctaacggggaagacgataa

ctctcgaggttgagtcttccgacaccatcgacaacgtcaagaccaagatccaagacaagg

aggggattcctccagatcagcagcgtctcatcttcgccgggaagcagttggaagacggcc

gcactctcgccgactacaacatccagaaggagtcaactctccacctcgtcctccgtctcc

gcggtggcgcgaagaagcgtaagaagaagacctataccaagccgaagaagatcaagcata

agcacaggaaggtgaagcttgcggttctccagttttacaaggtggatgattccggaaagg

tgcagaggctgaggaaggagtgtcctaatgctgagtgtggtgccgggactttcatggcga

atcatttcgatcgtcattattgtggtaagtgcgggcttacctatgtttaccagaaggctg

aagcatgattggaatttctaagtattttgatttcttgcttttcttcaagttttgaatgtt

ttgaattcaatagcttcattctcagacttttattatcttgcttagttgattaaatataaa

tcagattattctctgttttgaattatgattttgtgttatttagtatgttgatgaggttct

gaatggttagttagtgtatttggtttcgtgtttggcttatctaaatgactttattcaaaa

ctgcgctcatgccttttgaaagagcttttatttctgtatgaaacctcatgtaagcttatt

aaacatggaagaatctgtttttcaatgaaaatttggccactttatctcaaaaaaaaa

>fava_dn_rep_c55

gaatctctgcgttaaaactttgacgtattctcacaatcaatatacataatcaattcattc

aaaaatcaatttcttttacccaaaaccaaacatacaataaaatgataaaatagatacaaa

atcaatccatgcatgtttgtagttagatttacaaaatcatcatggagttggatggaagtt

tgttactaatatcaagtgtgtgtctcagagggaattataattcaaaacaagttgatatgt

tgaccatttcacacaattttcaaatatgaaaagtcttccattttcagatgatgattgatg

atgatggcgacgccgccacgtaatattcaaacaggggctggttcagaagcagtttcaccg

cgcttcttggcaaagtattcatcggcacgtgcaaggtttttggcaactgatggcttaacc

catttcttacggccgggaagaacaatgagggaacagccagcagactcgagtttctccttt

gcagacgtggaaaaagcgcaggcttttatcgttaacttcttgcttagttctccatcagcc

aaaatcttcaaaggaagtttccgatctcttcctgaagggttgatcacgcgtttctccttc

agtgtctccaaagacacctcctccccatcttgaaatccagcgttttcaatgtccgtaaga

tttacattgacatattttggcaatcccgcgctcatacctccagcaattcctctaagttta

ggtagtcttctataaagcggcatttgacccccttcaaaacccctcataatccccggtccg

gatctcgacttctgccccctcattccaaacccacaactcgctccttgtcccgcagctata

cctctcccttttctcttggccttctttctcgaaccaggctgaggccccaaattgtccaac

ctaaacctaacattctcaccgactggtttcacgtaagcagaaccagccttgttaactaca

gaaacacggtgttttggtctctgaattttcaaacacggacataaacatggttttgccaac

aaagttttcacatttcccttgaaaggtgaagcgagagagggtaacaaagatacaggaatg

agagaagcagcactcatgtctatctatctactcttcactatgtcaagcaataagataact

ggttatagataagatgatcccgcgc

>fava_dn_rep_c56

aaagtttaaataatgattaaaattagagaccaaataaaaaataggttaccaaatgattat

tctttagcacaattgctagtcacaagataagtttcaattacaaaatataaaatacaaaca

ctttacttgtcaacaccaacaccaatagttacattgttatattcatattaaaagaaaaat

acacaagcccaaaaactcattttatttaatactacaatcctttttatagaagacatatga

gtcaaacaaaagttaatatatcaatttttgtcaggttcatatgtctcttatgataaaaat

gaagtgtctttctatgaatcttattcaatttgaaaaagaaaggattttaagaggaagctt

tcatctcagatttgattgcatcaaccaactgatcataagcttctccccatgcattcttca

tagctggtgaccaaatttcaggtactgcttcttttattgtctccaatagtgcaaactttg

tcacctcaaaatgctcatctactacaccatttttaaaatgtgtagcacctatttttttca

agctagattctcttactgtgactttcccagcttttcttagttgaactgctgattcacgtg

tcatgagaaagacagacatggcatgaggcttgagcttggtgttttgctccaaaggaacat

ttgagtctttcaagaaagagaacagtttttgagctgatggagcaatctcaaatattttca

agaaaaacttgagagctagttctgcagaattcttcttcattacactccatgacttgacaa

caagagcttcttgctcttccgtgaaacattttgtatccaaagtgctcatattttgtgaat

tgtgatgggagtgagaaaatggttgatgtttcccgcggacagaagaaattctctctacac

>fava_dn_rep_c57

agacagtgaaaaactcataattttaaggttgttcctagagttatcacattgcatactcat

agtagttacaaaacatcatgtcaacaatatgatgataaagaaaatgaaaaatgaatcgtc

caagatgtcaacaataagaaaagtacttaacccttttttattaagaacaatacattcttt

acatgaatatttcacaagacataaacatagctgaacttccaaacatgacatattgatcgc

tgaatgtatgaaaatacacaagtttagttggaatgtgatgtaaactggtaagaagcataa

gagagagttcccatttcaatactactatggataagtttagcctttgaatttccaccagcc

gcaccaattgcaacatgcaatggataaaaatgatctggatgtggatgagcctttctggca

tgcggcgccttttgttcataacgattcacatcttcatatcttccatcaagaagagcgtct

ttcaaccaattatcgaattccaaggcccaaggagcaacaccgccacgagggttaattgct

cccaaattatgaacagcacttccggaacctattatcaatacaccttcatctttaagaggt

gccaatgcttttcctatgttgtaatggtaagttccatccatgcgagattgaacagaaagc

tgacaaacaggaatgtctgcttctggatacattaacatgagtggaacccaagcaccatga

tcaagtcctcgctttttgtcttcgtcgacgcggttgatacccgagtttttgagtagttcc

ttcactcttttggctaaatctggagctccaggtgctggatatttaagctggtacataggt

ttgggaaaaccatagaagtcatagatggtatcattgtttttctgaataacgttaacggtc

ggaacagtcgtatcccaatgaccagaaataatcaaaatcgatttaggtttctccccatac

acgtccttcttccatgattgtagaaacttcctcgccacaatcgattcatctatcgacaac

g

>fava_dn_rep_c58

tttttgatgataaagaaaatgaaacccttttttattaagaacaatacattctttacatga

atatttcacaagacataaacatagccgaacttccaaacatgacatattgatcgctgaatg

tatgaaaatacacaagtttagttggaatgtgatgtaaactggtaagaagcataagagaga

gttcccatttcaatactactatggataagtttagcctttgaatttccaccagccgcacca

attgcaacatgcaatggataaaaatgatctggatgtggatgagcctttctggcatgcggc

gccttttgttcataacgattcacatcttcatatctacaaaatcgattcaaatcaaacatt

caaaatcgttaatgcagaaccaaacagacgctaaaaagcacgtaccttccatcaagaaga

gcgtctttcaaccaattatcgaattccaaggcccaaggagcaacaccgccacgagggtta

attgctcccaaattatgaacagcacttccggaacctattatcaatacaccttcatcttta

agaggtgccaatgcttttcctatgttgtaatggtaagttccatccatgccagattgaaca

gaaagctgacaaacaggaatgtctgcttctggatacattaacatgagtggaacccaagca

ccatgatcaagtcctcgctttttgtcttcgtcgacgcggttgatacctgagtttttgagt

agttccttcactcttttggctaaatctggagctccaggtgctggatatttaagctggtac

ataggtttgggaaaaccatagaagtcatagatggtatcattgtttttctgaataacgtta

acggtcggaacagtcgtatcccaatgaccagaaataatcaaaatcgatttaggtttctcc

ccatacacgtccttcttccatgattgtagaaacttcctcgccacaatcgattcatctatc

gacaacgttggcgatccatgtgagatgtaaaacgtatccttcaaagccatttttgttgat

taattgttgcttctctatgtatctctatgcttttgattttttttgggtcccgcggccnnn

acctccgccacaatctccaactccgccg

>fava_dn_rep_c59

gccgcggggcaaccacaaacctaaataagctcataacaaacatatcaaagaatggcttac

gttaaggttgctactttggctatcttcgggcttgctatattctgtgtcttttcaatgaag

gaggtaggagctgtagtttgttcgggtgcatgttccccctttgagatgccaccatgtggc

tcttctagctgtcgttgtatccccattttaatattttttggttattgcacatatccatca

tctatagatgttaagaatattgctgaaaaaaatcctaatttatgccagtctcatgatgat

tgcattaagaaggaaagtgggagcttttgtgctcgttatcttaattctgatatcgaatat

ggttggtgttttgcctcaagctctgaagcaaaagacacgttcttcaaaatttactccaac

actacattcgcgaacgaattgaagatgtctgccactctttaatattgacatgttcaacat

aaaataattaatcatgcaatacacgaatcgtctactttttaaataatcattcttcgtttc

caacttgattgttggatttcttagatgtccatcttaatttcaattaaataatgttgcatg

tgtatgagtttgactatgtaacttatgttgttacttctccatcttaatatcaattaaata

atgttgcatgtgtatgagtttgactatgtaacaaatgttgttacttctgcaagtaatgta

taaatatgtatttctattgttaaatacatggtattagtgttatcattaaaaatacatgaa

acaataaaaaagcagtaaaaagaaagaaaaaaagtctatacagcgaaccggcgcatcgat

gattcaaacagggaggttggcgacgtcgtttgtgttctgaacggaggtattaggattatt

gacggcggtggtagcggttgtgctgacggagtaatcgtcgtcgtcgtcttcgtcgttgta

agggtcagaagtagcgacgttgacgcaggaaaagcgttccaaggaagagaagaaagcgga

ggagacggtggaccccagccaggtagccatctgatcgatcttctctctggatataagatt

gccgttgttctccatcgtcgccgcccgatcagatccgaatttttgctctgagattttgta

acaaccgaaacggttttctttgttttttttgcttgagatttacgagaggaataatcagcg

aaattaccaattgcccttataactaattattagtaattgcacgtaaccgctaatgtaatt

tcaaatcattgcgtcattggtcccttgccggggtgcatagatggaattcaaaacatgagt

ttttgttatcaaaaaataaaaataaaaaacatgagttttgaaatttaggttcgttgatag

acgttataagatctattcctttt

>fava_dn_rep_c60

ctttttttttttccatataagctctcatttatttatttcacaatattgcattggcgagaa

attatttatgaataatttcaagtcttaattaagcagtattgttgtgaacttctacactaa

ataataaccagaatcagcaacgcagtttaacaagttataaaagaaataaatattgcataa

aagatttgttgacaaaattgtaaattataaataaccaaaatagtttaactcaaacaacat

actattgtcaaaaacattaaaagatgctaaaatgtctgaaaagcacaaatcatctttgtt

ggaaactagtctcaaaacaaataaagcaaaataataacttggaaagtcatcaagctgaag

caccctttcccttcttcttctggagcttcttcatgtaaaattccaattctctaccttcca

aaatgtagccatcagctctgccacattgtccagggcgagaagaaatgcatgcaagcagcc

ttccaccaccaaattgttcttcaatatgtggatccaattggcgatccttttggcgctgtt

ctaatttcctctgaacatggctgctctttttaacttcttcagtaacagcttcagcctcct

cggagtctttcttgccagcagttttctttttccttccaatatcaacaccatagtgttgaa

ggtaccactgcttgaaaggagcagcatcaacctggacaatggcactcttcacaagggtct

gagtacgcaccaactcattgttggaggcattgtaaaccacatcaagtagacgggttttgc

gggtggcagtttcacttggccaggaaaggttaccagtatccaacctcaaagctctccact

tgacatttccacctctgacgcgaattctcctgatggttttgttgcttgaaagctttgtgt

gagcagcttgacgaccaagctcatactttctcttcttcctccatgccttcttctttcctc

cagtctcacgcctcttgtgcatagaatccctggaaatacccatgatgaatgatgctggtg

aaacgaggcgcagccctctctctctctctctctaggtttttgtctcgctgccacactgag

actcccgcgcctcttactttcatttcacaaaaaaagtcagatttcaaatgctaatcatgg

gcctatttgaagtctattgaaaatcacaaacaaaattggtccacaaaaaggaaaattcaa

aagaggttcaaaaatacaaacgaaagtcgc

>fava_dn_rep_c61

gccgcgggatttctcgcgtcacactcatcgaatcagtctagcaccgaagagtgagatcac

agaacaaatcaaccaaacgatgggaaaaacgacgtcgttgcttctctgtgcactgtgtct

ctttggaactctagctcttatccaggcaaagaagccgaaggaggatctgaaggaggtgac

tcacaaggttttctttgatgttgagattgatggaaaggctgcaggtcgtgttgtaattgg

actttttgggaaggcagtaccaaaaactgcagaaaacttccgagctctttgcacagggga

aaagggaactggtaaaagtggaaagcctcttcattacaaaggtagctcattccataggat

tatacctagctttatgatccaaggaggtgattttacacacggcaatggtatgggcggaga

atcaatttacggagagaagtttgctgatgagaacttcaagctaaagcataccggaccagg

atttttatcaatggcaaatgctggtcctgataccaatggttcacaatttttcattacaac

tgtaacaactagctggttggatggaaggcatgttgtttttggaaaggtgctttcaggaat

ggatgttgtttacaaggttgaagctgaaggaactcaaagcggaactcccaagagcaaggt

tgtaattgctgatagtggtgaactacctctgtaaatgcattcatgcttgttgaagatgtt

acagttttcatttccattccacctatgtcaacagttagttctgtctttatgatttctaga

agatgaaatttcttgtcttaatgatatgagaaaaaagtggcatttgttaacatgaaattt

gtagtaagaaatcaacttgaacgtggcaatctagtatattcagttgttaacatgaaattt

gtagtaagaaatcaacttgaacgtggccaaaaaaaaa

>fava_dn_rep_c62

ctactaatttgttaccaataatttcggaaataccgttatccaactttcacatcacttttc

cccttcacaaattcacttcaaaatcaaatttcccaaacaattgagactaatctcagtctc

tgcacttgtcaaccacacaaatggcttccatgtctttctccacaaccttcttcactcctc

ttcgtgatttcaaccaacctagaaccaattcaaccccttcaacttctttcccttttacca

aatcctctattgcttcttccaattcaccgttttttcaactagggttttctcaacaagcag

cttcctccaatttttccattgttccgtctaagactcggtctttctctgtcaacgctaaag

ctattaaagataaaaccatttatgattttactgttaaggatattgataagaaggatgttt

ctcttagcaaattcaaggggaaggttctcttgattgtcaatgttgcttcacgatgtggct

tgacatcatcaaattacacagaactctcgcacttgtatgaaaattttaaagatagaggtt

tggaggttctagctttcccttgcaatcaatttggcatgcaggagcctggatcaaatgaag

aaattaagcaatttgcttgcactaagttcaaagcagaatttccaatttttgataaggttg

atgtaaatggaccatttactgctccagtataccagtttctgaaatcaagttccgggggct

ttttcggtgatcttgtcaagtggaatttcgagaaattcttggttgataaaaacggtaaag

ttgttgaaagatacccaccaacaacatctccttttcaaattgagaaggatatccagaagt

tacttgctgcataagtatattcaggggaataaggaaaatcaaagccatgattgcacaagg

agttaaagtttatcaaaaacaataaacattattattatgtaaattcgtttggcatgaaga

gaagaaaaatattactatactttagcgctaaaggatttttgttaatacgttggagttcta

tatggtcagaatttttgttaaatttcagactttacattttttttaacattattcattttc

cttcttcaaccatttgaattccatcacatccctagagacgnnnnnnnnnnnnnnnnnnnn

nnnnnnnnnnnnnnnnnnatgtatcaagtgaatacttggaatgata

>fava_dn_rep_c63

agtaaaatttccaatagtgttggcgcagtttgttgtccgtnnnnnnnnnnnnnnnnnnnn

nnnnnnnacaaatgcctcaacacatgtacaaatactcaaatagatctaaacaatagccac

aaattactcataaaaaaatagtattgagtatttatcagtcagatacaagcaaaaattgag

ttgcaggtttaacattatgatacaaagttacaatcatactgaaactatattttgcagtaa

tctctgcaattattttacttcattatttcttatattgacaggcaattacaagagtgcaac

ctcccaatcgaaaaaactgaaatgtcaaaatttaattgagaaaccttgatggtgcaaaca

tgcagcttacattggattaaattggataggataccacggagggatttcacacagcaactg

gttcaggtcttttgctagctttgatccctcttatagcttcagcataatctttagctccaa

acacagcagaaccagcaactagtgcattagctccagcctcaatcaccttgtaagcatttg

ctggggtaaccccaccatcgacttcaatccatggattcacacccttctccacgcacaatc

ttcttaactcagcaattttctttacttgactctcaataaaactttggcctccaaagccag

ggtttacggacataattaagaccaaatcaatctcctcaaggacatattctattgcactta

gtggggtaccagggtttaggacaactccagctttcgctcccaaacttttcacttgattaa

cagcacgatgcaaatggatagtggaagactgttcacaatgaacactaattatgtcagctc

ctgctttgatgaaatctggaacccgcaggtctggttctacgatcatcagatgtacatcca

aaggtaaatctgttacagggcgtaacgcgtcaaccacaagaggtccaattgtaatgtttg

gaacaaagcgaccgtccataacatcaatatgaatccaatcacaaccagccaactccactg

cttttacctgttctcccaattttgcaaagttagcggaaagaatagacggagaaacaatga

tatcgtttttggaaaacttgtcaacgcgagatgaggccttaaccacggttgaaacttttc

tcctggagaaagtgagagaagggggatggagaagagaagttttgcgaagggagagtccat

tgatctgagattggagggtaggtgagcatagagaagatgtagctgaagccatcttgtgcc

tctcaaaccttgagagtgtatcgataaagcgaaggttttgaataaagttgttgttgttat

tgttgtaatagtgaatgaatgaacgatgttaaaactcaagaaaggaaagtggtgtggatg

actcgtgttgagttggttggtggggatgtgcttatctgcaatggttggttccttgccgct

ctacttctccccctcccgtttgttcttgtca

>fava_dn_rep_c64

gccgggggggcagtagaagcaaaagttgagaaggaaaaatggccttgatttcctccgccg

caatcaccgccgttaaccgagcctcccccgtacaagccagcttgggtacaaccctaaccg

gtcccaaattctcgaccggcttcccggttaccaagaaggtcaacaatgacattacctcta

ttacaagcaatggttcaagagtgaactgtatgcaggtgtggccaccaattggcaagaaga

agtttgagacactttcctaccttccacctctcactcaggaacaattggctaaggaagtag

aataccttataaggaagggatgggttccttgcttggaatttgagttggagaaaggatttg

tgtaccgtgagcacaacaaatcaccaggatactatgatggacgttactggacaatgtgga

agctacctatgtttggtaccactgatgcatctcaagtgttgaaggagcttgaagaagcta

aaactgcataccctaatgcattcatcagaatcattggattcgacaatgtccgtcaagtgc

aatgcattagtttcattgctcacacaccaaaggagtactaagtttcatatttgcaccacc

ctttattactgttttgcttgtattatttgaccatttgtaagaactatatttcctaccatt

tgtttttgttatatgaatgtatcatcatccttgtattttttttttttaaactgcaaagcc

caaatatccatttatttaatttgcattacataataacaatataagatacgatcatcaata

catggccgacacattcattcatacaaaggaagcataagagaagcaaacatgatgcatgct

tatatataatatgatacatgcatgccattacaaacatcacaataattataaagatgcaat

attttataatatccagaaaaccactactcttttatttaatatataatgtctttatgactt

ataatgcaaccttttctcccttgataagatgaccatcaatgtctctagtggctttgctaa

ggtaatccatccagccatttggagtatcaacatcttcattgatttttacatagtcaatag

tccatctaacagcagcagtgccatcagctttattgattacttccagagtcagcttaaaaa

cagtgaagttgtcaatatctccaccaaacagtttataagtgattttttttgttctcccca

tcaacttcttcaatactttcattacatgtgtgaacctcaccacctatttcgtaagtccag

tgtttaacagattcattgtgatgccagtcttcaccttcatgcagcttggcatgatgaact

ctttcacaaatgttttgaacttcgtgaagttctgatgcaaacagtttgaagaacttgtca

gcgggtgttttgaccccaagttctgtaataagtttgccagctagaaccatttttaagaag

aaaatgtaaatatataataaaagaaagaaaagagatgttatgatatccccgcggccc

>fava_dn_rep_c65

ttttgcttagatcgtgtttcaagtccaaagaaaaaaacagagttgaatttcaacacctag

atatctatattctctctctctctctctctctctctctctctctctctctagattctcatc

aaactctacttcttctccctcttcactactttccttttccattcctcttctcattcaggt

gtgttaatcttgtgatttggagaagactaagttaatcatgataaagaggcttccgagtag

taggaatcaaagatccaaaggtgttaagataaagcatgttctgcaagttattctgctgct

tggtgtgtgtttctggttgatctaccaggttaagcacaatcacgacaagaagaaggaatt

tgataagaatgattcaacactatcatcggtcagaactgagacggataaggttgtgaatat

cggtagaaaggacctgaaaccagttaaggatgaagtagatcaaaacggaaagcacgaaga

agaggaagacgaacctgttgtagaggatttggaaaataaacgcgaacacggtgaacaaca

agaagaagaagggaacaagcacgaaagtgaagttaacgtgcatgaagggagagaagaaca

ggatgaagaagagaataagcatggaacagaagaacaggaggaaggtgaaaataaaatcga

agagattgaagatgaaggaggagatgctgaggtagatgaaaatgatcatgagaaatcaga

tgttgataatgatcaagatgatgatgaggatgcagatgaattgaaagacaaagtagatga

taatgatgagacggagaatgaagacaaggaggacgaggaaaaggggggttcggctgaaaa

cgatgagagtcatgaagctcgggaggaacattacaagggggatgacgcgtctagtgctgt

ggctcatgacactcaagcaactagcactgaaactgaaccgattaatttgacacaggctgg

tctaagcgtacaaatgaatatcgaacaaccagacaacaagacaacttttcattctgatga

gaataacaggaatgaaaatgattctgatttgaaggatagagaaggtgaagcggctgatgc

aatttcttctaatgtgactgccggtaaagagactggaaataccagtttgtctaccgaaac

agccgaaacaaacgcggatagccacttcgatgtaagaagtaatctgacagcagtgattac

tgaagcaagcagtaactcaacaggtgccggtgatgacacatcgagttcatctgaacaaat

taaaacagttgtcgtatccaagtctgataattctcaaaatgcgacagctaatacaacagt

tgctggagatataaagcaaacagagggattagagcagcctgtcaacaaaacttctgaagg

aaacctgcctgataatgatgcaacagtctctgttaaaccagataatcgtgactctgctcc

gcaagaatctcctactctagtgggtagtgcattagagaaaacaatcgattttgcagcttc

aaatgaaaccgatgacatttctggtaatttgaataataatgcgacctctggtaccaccga

aagtgacaagtccaaaggtatcaccgaaactagtgaagcaaaagaaactaagaacatcta

cgccactgtggatgagatgttcaagggcaatactcaaacaggcgaaactgatgaaaaatc

agactcttcctcaaccaatgaaattctagactcggttatacacgacgctattgatctttc

tgatactcagaacatacacgaggaggatatggcaacggctcaaactgatctcgatacgtt

gccagatattcaaaacgaaggaaacgatggcgatgaaaattcagcagagtgaacagaaac

ttttgcttgattcaaaattgtatcaagagaagatgttgaaaggtacataaggtgtatgat

tgtgtctgggtctggatatgtataatttatttgtactttccacttttctgctactattac

ttcttttatttttatgctagcttcttattctttcattgacatgtggcatattgtttttca

taacattgacataaaatattacaaccaactcaactaaacatattgcagcatatatacaca

agattctcctaatgaagcatctgtgcttattacaaatagttcaaagtttagttgattatc

cttattctcaaacagacacacagctaaaagtaggcacaacaacaaactcaacactgacac

cactatatatagcagcaatacttcttcatgtgtttcctttatcttcatggcagatttgaa

gccggtgctgctgatccttgatagaactgatacgaagtttcaaatctcaaattacagctt

ggcctaacaattctagcaccaatcctattgctacaacatcttgaaacttcaacgatggat

gaaatcaaacaatcatcacactgtggaccactcaaatcaggtgtacactgaacaagacca

tatattatgtcaccattactaggacctggcaatgttcccacaacatacttacgattcgag

tcaccttttatagctttgttttttaaatcattcaacaattgtttcacagcaacgttaaat

tgatctgccaatgttgcgttgttcatgttccatgcaaaataagccggtccaatttcattt

atgtttaaaatcgaacggtcggaataacgtagcatgcatttctcgtcttcgtaccatcca

attgcttcttttttgtttggacatgcttgtgtgagattgggtttagaaccaatgagacat

ttgcgacaatcttgagaattaatgtctcctctacatattcctatcaggtgtattttgttg

ttgttttcgccgaaaatggtattggtgaaaccgttatcaagttttgagttgtaagtgaga

tgcgtaaaagcaattttgaggttgttgtcgtaggtatcaccggttatgaaattacctctg

tcgttgttttggtcacagaagtagtggaatttttggtcgttgtggtcgttgttgtctgct

ttggatttagacgtgagaaggatagtgatgagaaagagattacagaaaaaggaaaatgga

gttaaagagagagtagacatggttgattgcagtttagggaagaaattactcagatgatgt

cccgcggcc

>fava_dn_rep_c66

ggatcttaacaccttcaggtactcaatacttcttagcaaatgtgtcatatatagaaaaga

aaatgcaaatcaatataagattttgagtgagacaaggaaacgaagcaatttaacatagcc

aatgtgctaaaatttcaatacaataagatatcatttgggtcagcatatcttatgaaaatg

ggaggaagtaatttatacaccacaagaaatgaaaaggaacttctcataaaggtgaagcaa

gtgcatcatggtaggtacatggccatcctcttctcatttctttggcgcacaattgttgaa

catctaagagctttttccgcgtttctatcgaccttacgagcgttttccttgagccaaact

tacgcgcaatgcccgtccttctagttccacatcattcatgattgtaagggcagcttccat

ttctgatttattggcataggaaacaaagccatagccacgagaatttccagtttcgccatc

atatatcacccgagctccaaccacagttccatgctcttgaaaggcttctgtcagaatctc

agaagttactttccaagacaagttcccaataaagagcttgtattcggtttcagggtacaa

cgattcttttggcttcggtttgtctgagaaattcactcgcaaggttcgccccatgaactc

ttttccatcaagattttcaattactgctttacaatcttcaacacaagtcatggtggcaaa

tgcaaagcctctactttttccagtgtctctgtcgtaaagaacctcaactagttctgcgct

accatattcctcaattaaccctgcaagttttgcactatcaacactataaggcaaattccc

aaaataaagcttagtcctagtgtcttcttctgctacaatctcctctccattctccttctc

tccttgttcttcttcattttctttttcttccccctctaccacaaccttctcttcatcatc

aacagccacaacagcctcttcctgagcaacagcaacataaaatctaggcatccataactt

ttgtgatgagattgttgtaccaatgcacaaaggttcaactgaggattgtggtataacaac

agatgcaaacatgcggtttgaggaacgtgaagatttgacagtgttgatggaagatgaaaa

caaggaggctacgcctgcagcagcagtagccatgttgtggtgattgtgggagtggtgagt

ggtgtgaaacttgaactattagaagttggagagtttaacaagtatgatgtgagagaaagg

gagattttttgtggtgattattgatgaagcgg

>fava_dn_rep_c67

ataagtaaaataattatatttaagatgcctccaatcccaacatcacacctactaatagaa

tcatatgctaacaatacaaaattaaatttaattcagtatccattgatcatagtccataga

agcttttatccaaataaaattgctttaatccaaggaacttaacatgttggatatctcata

caaataaactatacatcatcatcatcgcattacataaaaggaacaataaaagacaaatta

aaacaacataaatgaagaaccccatgaacaacatagtaatctcaattaaccaaagaaact

aggatcataaccattgctagaagtggccaagatataataagcaacaatatgacctaaaga

accccaagccaaaacatcaacaaggttaaacccaacaggatcatttgacttaagcaagct

aacatattccttagcacgtgagtcaccagcttcaaagtgtgtcacaccattttgttcagg

taacccttgttttgcaacattctctctttggaagttgaaaaacacaaaccttcctaagaa

caatgagaggccagtgcttaagcttatcacaagtgaagggttgagttcagctttgacaac

aagagaagagaatctctttttggtttgtgtgagttttgtggatcttgttaagggtttgaa

gccttggaaacatagatttgatggtttgaggttgtgtggttggtgtttttggagtgtggt

tgttagggcgtttgttgagatcatggacgaagctgaagctgccattgtttttggtttgat

gagtaattactattgatgaatcccgcggccnnnnnaaattaagtcaatattctattttca

tggtttgtaaattaaatataaaaaaatcaatttctaaaaaaaaaaaaaggaatctttatt

cggatatacaatttgtattgaaataggtatatatcttgaatagatatgatctgggacgga

aggattcgaacctccgaataacgggaccaaaacccgctgccttaccgcttggccacgccc

cattttgattcgacataaatagtattatgagttgttcgtaaattacacttcgaaatttat

tctatagaataaattgttgctagaattttgatatgtatcgatatcgaattaaacgaaatt

tattgatcattccatagaattcaattaagatattgtttgaacttattcagttcaaattgt

atatccgaataaagattcctnaaaccggcaagatcgagactgaggctgagactgatac

>fava_dn_rep_c68

agatatactttcactttagaggttgtaatcaacggtttgtgtccagttgtagccacatgg

atcaccgacaaagccagctacactaactacaataattattactatatttacaccgccact

gtttttatcctctactctttatctttnnnnnnnccaccatcctctgttacatctaacctt

aagattcaataatcatgaggtggcacgttactttacgatacactccaccgcaggtgaata

tttcacacgcgttttgttttatcttttccattcctccaactttactccactaattgaaaa

ataaaatccaactacataaataaaccacttctcattctttctacatcttcatcctctctc

actatggcttcaagagttgctttgttgttgtttgcttttgttcttccagctatggtttct

gccattcgcccagcgaagaaccctctctgcttgaaaggtcacgtcgtatgtgacctttgc

cgtgctggttatgaaacctccgctattactcacattgctggggctgaggttgccttggaa

tgcaaggacaggataagcaacaaagtagtgtacacaaagctagggaagactgactcaaat

ggagaatataccatgtatgttgacgaggatcgcgttgacaatgtgtgtgatgtgaagcta

gtaagcagtcctcaaaacagttgtaatgaagctacaccagggcgcgaccaagcacgtgtt

attcttacccgttacaatgggattgcttctaatgacaggtttgccaatgccatgctattc

atggcaaatgaagttgcttctggatgtgttgggatactccagaaaatgcatgaattggat

gatgatgagaattaattactgtattagtttatttacttggatttgggttttctcatgtct

atttgattcaatcttgatggagtgttaactcaacagtcagtttattatatgcctatgtat

gttattggatttggacaatgctaaattatattattcttatctctcttgtagtagaggatt

tagtctcagttctattactatgttttttttatatatgaatgaattcatcatagaatgatt

ttcgtttggtgaatagctttttattatatcagtagtaatgatttgttgcgattctttgtg

agtgttaaataatcagggaaggactgacaatggacattttagtg

>fava_dn_rep_c69

atgattattataagcaaaagggaacaagaacaaaaaccatgggaagaatgaagaagggaa

atgacaatgttattttggcggtaatagacagaaattctgttgcctatgttctaaagcaaa

ccaactagcactgcttctgtccggatcaaacaaatccgaagcatcatgaacaagcctcca

cgagaagatgaccgcaacaacactgtttctactctcgactccagattcagccaaacccta

cgaaatgttcaagggttactcaaaggtcgtagcatgcccggtaaaatattgttgagcaga

aggtcagagccatccgataactctatctcgaaggtatcctcaacattttacaaaaggagt

ttctcgcagaatgatgctgggacgagcaatcacccgtctggtgcagtagaggaggaagtt

cagagtacaagcaaatcagtcagtgttgctaatgttagtaagttgaaaatatcaacctcc

cttggggaaagagataacttgtccgaagatattcgcaagtatactatgggtgctagagct

actgattcggcaagagttacaaagtttactaaagtgctttcaggaccagtggttatatta

gacaaattgcgggaattagcttggagtggtgttccagattatatgcgtcctaaagtgtgg

agactcctcttgggatatgaaccacctaattcagatagaaaggagggagttctgagaagg

aagcgtggagagtatcttgattgtatatctcagtattatgatattcctgcttcagaacgt

tcggatgatgaggtcactatgcttcgccagattgcggttgattgtccaagaactgtacct

gatgttcctttcttccagcaacagcaagttcagaaatcattggaacgtattctttatgca

tgggccattcgacatcctgcaagtggatatgttcaggggataaatgatctcgttacacca

tttttagttgtttttatatcagaatacttggaaggaggtatagatgattggtccatgtct

gatttatcttccgacaaaatctctaatatagaggctgactgctacgggtgtttgtcaaag

ttgcttgatggtatgcaagaccattacacatttgctcaacctggaattcaaaggcttgtt

tttaaattgaaggaactggtcaggaggatagatgagcctgtttcacaacatatagaggat

cagggacttgaatttcttcaatttgctttccgctggttcaactgtcttctaatccgggag

ataccatttgagctcatcacccgcctttgggacacatatctagctgaaggagatgcctta

ccagacttcctcgtgtatatatttgccagcttccttctaacgtggtcagacaagctaccg

agcttggaattccaggagttggtaatgttccttcaacaccttccaactcagaagtggact

caccaggaccttgatatggtgctttcccgggcatttatgtggcacaccatgttcaacaac

tctccaagtcatttctctagctgagcatgcaattgtggttttctcaatttagtccttctt

caatttctactcctctctgaactccactagtgttgctcttgcttagtgtatggtgcttta

ttgtcggtgaatgatttttttcaaccgattgaatatttttgtaaaggagagacaaataaa

tgattgtctcagttttgttgtatttatatatttttcagaatatcaacaaatatccagttt

gtatccataagtgttcttttctaatgtatgccagtttgccggtacacatctctcaaagat

atgctaatacagtgtttggttggaggaaaacgaatatgctaaattgctaacaaactaatc

tccagttattaatatggcttaaaattccactgtttgaccaaaagctcaataccattttag

gtatgaaaattatatgcaaagaagattggttcaatatacttgatatggtaaacttcaaag

aatattactttacatagccataagttttaagctcattcctcaaaaaattaacaattgtat

aagatagtgaaatgatggcgattgattgatttcgcgtgttgagcgcgtaaaatcaaggag

gaacggataacctaattttattcctcttccacttcaagtccaggtacatattccaagctt

acatcaaatataactggactatttggagccacccttggtcttcctggagcagaattaagc

ccttttggaaatgccaatgatccaggtatgtatagccgacgtttcccgcctactttcatg

cttaaaagaccttcatcgagtccttttatcacctgaccagatccaactcgaaaaatgtaa

agctggcctttctccaatgaactatcaaatatctgtccagatggaaccattgcaacataa

ttagcagccacctgaaacccaactggtggactagggccttgcccaaccttaatatccttg

tattgcaaacctgattcagtagttaccataggtacattttcaagctctttctcacaagtg

ctgtcacatagtttaggtttttcttctggtggtaaaccagctccattggcagcatcaaat

gaatggatgaaaagtgtagaaactcctcctaatgctaatccaagaaagtctctacgtgaa

ttggtattaaacatggcattatcattagcattagaagtggattctcttgtctctgaggac

ttcactttaagaacaaatcttctaacagaaatgccttgaggagcagcattaagagaagga

attttaccacaagtagaataaaatggaaggagaagagaggaagccatgctaggcatatcc

gaggatgtaatgttgtgtcatgtgatggtgatgatgttattttttgcaaatggataaacc

aaatctagtttgaacaataaacatggattattggatt

>fava_dn_rep_c70

ggatttctgnnnnnnntcattgaattaaaaaggggttacaagatgttttatcccattggc

atcacaaaattgttagaaacacgtcacattgcacaaggataattacatgataacatcata

aaacacctaatgatctaagcatctaaagatcctcatgtcctctccatgctaagacattca

tgacttatcatcctcttatccacaattcaagcttcttgtcttaaacatgattctccatgc

tatctccatgtgttcaactaccttggaacattcttaaatatgacaccatgattaatatgc

aacaattccaaccgttcatcattctaacatccatatgcaatacaaggtcagttagatttc

aagttcataacacttacccaagttcactacctagatgccataccaaatgcaaaattacat

caacatctaacatgtttgctacatagggcctgcattgtaatcctagataattatcgctct

gcagtgacttccattgcagtatctgaagatggatgggcacttctcagtgccggaagagat

aagattgtaacattgtgggatcttcggaattataatatcaagaagactgtcatcataaat

gaggcagttgaatttgtttgtcctattagtactgggagtccttttgcttaatcattagat

tcctatcggcaaaattcaaaaaaaaaatgttgtttctcaagcaatactttttgttactgt

tggtaaacaaggcattgtacgcatatggagttctgaagttgcagttgcttgtttgggcaa

aaggcttcggatgttacaaacaacacagacgacaatgtgaaaaatggagaatttataaaa

gcagaaaatatcatcatgaactcccagaaactaccaggggatatgcattctcctaggatg

aaatttaagcagcatgaggacagcataatcaacctgggaggttccagtaatcatattctt

gataaaataacctcttcaacacttcagccttgccatattcttaaacacaataaacagttc

aacagttcaaaagtttaaaaaaaagggatagcttaatagtctacataaaagaaccaacag

gtttccaacttgcagctaactgattaactgagttgtaacagaaatttgccagctcatcaa

catataactaactctctaacacttctaatggtttcggagctaattgttaacaacaataaa

ggaattgtgagaaaaagtgctaacaaattcgttacagaattgggagagagaagtgctaat

agattcttaacagaattgggagatgcaggtgctaacagattggtaacagatttccggggg

gggggattaataaaaaagagtggatggttagaaacaccaagatacacaaaggattagtaa

cacagattttgtaaattatccaaaagacaatttacgcagaatataaaaagaatactaatt

ggggctttaagttggtagaaaaaataaagcagtactccccacaactccgatccagagtat

gcttccatccactcattaaataaatgactatcaggaacgaaaaaatcctttaaaattttt

taagtccctttttcatagcacaaaaaagaagaataggaacgaaaaaaaaaacataagaaa

tcaaaaacgaaagagaaatctatttttcgtttttgatttcttatatccatatccatattc

atggagatcaaatcataattaagaaacgaatgtttaattctttttcgtaattcaaaatca

tgagggctattgagaattataaaagagtattttattgaagaattcagttattactcaaca

aatttttatttttgagggatgagatcaattcagaagtgccttacattggatcttttatta

aactatatttctctttcttatttcgttatttctagaacagacagaattgaattggtctaa

ttcagaaccgttcgatctattttcatcttctatatcttagggatatagcaagagagaaat

aattgacccggtccttagatttacttaaggctttccaggagccgtatgaggtgaaaattt

catgtacggttctgtaatagagatgggaacagtaatgttatcaccgactaggattatcta

acagtttaagtacagttgatatagttgatgcgcaagcaaaatatggtttttggggttgga

atttgtggcgtcaacctatgggtttcatcgtttttctaatttcttcgctagcagaatgtg

aaagattaccttttgatttaccagaagcagaagaagaattagtagctggttatcaaacag

aatactcaggtattagatttggtttattttacgttgcttcctatttaaaccttttaattt

cttcattatttgtaacagttctttacttgggcggttcaaatatctctattccgtacatat

ttgtttctgaattttttgaaataaataaaacatacggagtttttggaactacaattgatc

tctttattacattagctaaaagctattttttcttatttgtttctatcataacaagatggt

ctttgcctaggctaagaatggatcaattattaaatcttggatggaaatttcttttaccta

tttctctcggtaatctattattaacaacttcgtttcaattgttttcgctgtaaaagatgg

atcttctatattcattacttgtctcaaataagagaaagaaataaaacaaaactattcata

gatatttacgatatgttccttatggtaactgggttcatgaattatggtcaacaaacagtc

cgagctgcaaggtacattggtcaaagtttcatgattatcttatctcacgcaaatcgttta

cctgtaactattcaatatccttatgaaaaattaatcacatcagaacgtttccgcggtcga

atccattttgaatttgataaatgcattgcttgtgaagtatgtgttcgtgtatgtcctata

gatttacccgttgttgattggaaaatggaaacggatattagaaagaaacgattgctaaat

tacagtattgatttcggaatctgtattttttgtggtaactgtattgagtattgtccaaca

aattgtttatcaatgactgaagaatatgaactttcaacttatgatcgtcatgaattgaat

tataatcaaattgctttgggccgtttaccaatgtcaataattgatgattatacaattcga

acaattcaaatcaaataaaaaaagacaattttttataatttaataaaatacaattctaaa

aaaaaaaagagtgaaaagggagtggcacatagcttgctagatgcaaataacagaaacaag

gtctatctctctcccattgtgatatgttgaatgcaactaaggtcaaataattccatcaaa

acacaatctcagcaattcagttagtcttgacaagaagctgagatctattgtatctgttct

tcctgtgtgagttgaactgaattgaagatatcttgcgcgcaacttcctgccaaatcatgt

ccactgcatccccgggccgggatggatattgatactcgtaatgatgtgtaatctccttca

atctttcccacatcttgatccattcctcgcgcttgattgaatgaaggagattcaacaggt

agcctttctttatggcatcggcggcgcgaacaaacaaggaaaactctgagtagtccaaaa

catcttcgtatggtagctcgatttcatcactgatgatcacgggaacacaatggcttacta

cggcatcaaagagtcgattggaagatggagtatctccagctatgttcaggcagaatttgg

acgtcgacatgccctggcttgcctgattaaccccatttcctcctatgcttccgaatgcaa

aatgcacgtctttctcatctttgagaaggtaatacagttcttggcgaataactcctccat

cttttctgtatatcgccccttggaaatacacgagtgtggaacgttcctcatatgaaggtg

actctgcttttggtatagtacctaccaaatgtttatagggagctatgatatctttcttga

tatttgctaactcaacaggataccttccgaaatccgcaagcacaagcatagcagaagcca

agtttcttcgcgcatccaacaagctgttaggatggtgagctacaattagatgattcttac

cacccgcacgtttccattctttgcgccctctcaagaaacgtaccaatctgtcttgcaaca

ttttgttaacactaacttgtccctccccttgagtcttggaatgccgattgtaactcagag

aagagaaaaacggcacaaaaataatatctgcttgacttgaattctgcactctaattacag

tacaaggccgaccagctttagcattttttgatgctaaaagatcaagtgtgagccaatatt

ccatactgtgctgcaaattcagtcccccaggataacgtgggacacgactggggttatcca

catcaggccatgtttgatttacacttcccttccaacccaataacccaaagtgaaattccg

gaggcaagtcatacatgaatactttcaataaagcccgattctggtcacatgccatattta

tctcttgttctacagaatgttctctctcgctcgaattggaaacattcccatcagattctc

taggttttaacgtttgaaatcttaaatcctcagaagattcagtaggtagcgcaatttgtt

ccctcttgaaattgggtgtaaagtaaaacgacgagttattgacaataataagctccagaa

ctgatctaggtataaaagaatgactgctaaactgaagtaggaaaagggaagacaaaacca

aaaggaacatagaaataaggatcacacaaaacattaacctagagggaaccatactcttct

ccgacatagctaaagattaagctccaacaaaaccaaccttaaatccaatactaaattaag

aagataaacacagaacacaacaatcgatcacgaagtttggccaattgtgcctgcgtctct

cagatacacaaaataagataaatttcctcaaacacacagcacatatagttcctcaatctg

tatccatagctcaaatccctcaaatcaaatgactatacccactaacctactattgactta

tttaccttaaatattattgaaaagatctcaaaaaggcctttgaaattcaatctgctgacc

caattcaaagctgatgatgaaaactgagattgatgcaaagcgttcacttcatcaaagtac

agtaaaaaaggaagaaactttatattgataagaggattatgtt

>fava_dn_rep_c71

atgcaatttcgtatttgtattcgtcaacaagaccaaaaagaaaaaacaaagaaaacgcaa

ggacaaattcgtcatttcaatcccattctttttctacagaacctcgaccaagaagaaacg

cagcggagacagtgacagagactctgtgaaagagagagagatttcgcaattcgcaaaccc

taatcaacgagatcggcgatggcttccaccaaggatcgcgagaacttcgtctacatcgcc

aagctcgccgagcaagctgaacgatacgaagagatggtggattcgatgaagaatgttgcg

aatctagatgttgaactgactattgaagagaggaatttgctttctgttggttataagaat

gtgatcggtgctcgcagagcgtcttggaggattctgtcttcgattgagcaaaaggaggag

tcgaagggaaatgatgtaaatgcgaaacggattaaggagtataggcataaggttgaaact

gagctttcgaacatctgtattgatgttatgagagttattgatgaacatctaataccttcg

gctgcagctggcgaatcaactgtgttttactataagatgaaaggagattattatcgttac

cttgcggaatttaagacagggaatgagaagaaggaggctggtgatcagtcaatgaaagca

tatgagtctgctactactgcagcagaggctgaattaccccccactcatcccattcggttg

ggtttggctttgaatttctcagttttctattacgagatcttgaattcaccagaaagagcc

tgccatcttgcaaagcaggcatttgatgaagctatttcggagctcgacactttgaatgag

gagtcctacaaagatagtacattgatcatgcaacttctcagggacaacctgactctctgg

acttctgacatcccagaagatggagaagattcccagaaagcgaatggcactgccaagttc

ggcggaggtgatgatgcagagtgaggctggtgtttggtgggaacttctttaggatttgat

gcaggtgcttgagtatgaagcattagggctgattacttatttgtgtactagctagagtta

gggttccattgtttctctttctctgttttgtgtcaattggccttacattcctatagagga

aaacaaaactccatttgtgcttatattatgattatgtttttatttgtgtggtgtcttgaa

atggtctgtttcactcctcagcacctccttctgtctttgggagattgattttatgaggat

tgttcttgatgtatttccagttccaatgaaatgcttcacctttatatggagattgatcac

ttgattattattctattatgacttgaatccacggtttaatcagaatcagaaaaactcaga

ggaggtgtaagctcataacagaataaatcccagccatccagatctctaacagaaattcaa

atcaatagttcagatcaattcaatctctaactaactttctaatgtctaatcaactatata

acagaactcaccagatttaccaaggggattcttcaattctctaaagaatcgcagaaaaaa

ccatcaaattctctctgcttcatcttcattcttctacgcgcgttctagagaatcaccatc

gaaatctgaagagaattatttcttatctctcgtgatcttcattgactttcctctgcaaat

ctcacaaaaatgcaaaatcatcaaccttcactctcaactcaacaacctgcatcattaaca

caaaccacacgctccattcttcaatcatcatcacgttcatccttcaatatatttaatctt

catcgccctcgattcatcatgtgaatcttattgcagcaagtgcggtattctgcaagaacg

gagatgaatcgcagtatcaagattcactgagaatcgtgatggaagatgcaaacaaaagca

aagggtgaagataaaagagaggacagaaaacacctgagattaacttcatcaaaggtacat

caatcttcctcgctttcggtcgttcttcatacggttgtggaaagtgagaagagaacaatc

gattccccatggattcaaatgctgaagctcagggatgagcatctaatttaagatatgaag

acttagggttccattttgttcttccgatttggccgttctctgaaccttcgaaagagagtg

gcaatgaatttgaggttaggagatcatctgagactgaggtccacgtctgaatgaaggacg

agttgaggagtacggcgaggatgagaagttgatgggtatcggaattgccgtgaagtgaaa

tgctttatcacgttttgtgaagatgattcgtcacagtctgag

>fava_dn_rep_c72

actacaatctaacaagtaaaaccctaaatcaacaaaactcatcagctcagtcaagttcga

acaactatatcatatacaacgcaggcggagtttcgagcaagtcatcttccaggaagccga

tatcaactcttatctccttgtttgttgccattttgaaagaaagaaaaaactattaaccct

aaccctgcaagtacttttactggaaattaatccaaaaacatgagtcgaggaagtggaggt

ggatacgatcgtcacatcactattttctcccctgaggggcgtctctttcaagtcgagtat

gctttcaaggctgttaaggctgcagggataacctccattggtgtccgaggaaacaattct

gtatgtgtcgttactcagaaaaaagttccggacaaacttttggaccagacaagtgtaaca

cacctgtttcccatcacaaagtacctcggtttactggcaactggcatgacagctgatgct

aggacactggttcaacaagcaagaaatgaagcggctgagtttcgccataaatatggatat

gagatgcccgtggatgtgttggctaaatggattgcagacaaatcacaagtctatactcaa

catgcttatatgagaccacttggagttgttgctatggtcttgggtattgatgacgaaaat

gggcctcagctttacaaatgtgaccctgctggtcattactttggtcacaaggccacaagt

gctggattgaaggatcaagaggcaattaatttcttggagaagaagatgaagaacgaccct

tcatttacctatgaggagacagttcagactgcaatttctgccttgcaatcagttcttcaa

gaggattttaaggcgactgagattgaggttggagtggtgcaaaaggacaagccagaattt

agagttttgtcgactgaggaaattgatgagcacttgactgctatcagcgagcgtgattga

gttcaaaataaggatatgactgttcaaactttgtgatgttcaattaaatcataaacttat

tgtctaaaattcactaaattaatatccttatgatatttgagtatatcactgatgttgaag

attgtatttgcttctgttacaagttggagccttccaatcaatcatgtttacatcttctat

ccttgaaacgttttacaatgtctgtgtttgaagaaactagtgtttgaactgaacagagta

atggtggtcctatcataatttcaatacagttttttcttaaaaa

>fava_dn_rep_c73

ttcctttcaaggagcaagcctttcaattatttaggtatgtgaccatacaagtcttgctcc

ctttcaagttcccattgtcaaaagaaaaggcacaccgtatcaatttcaatttgtgcttct

gtttaggcataaacctgtaatataacttatggcacaatatggataacaatttatggatga

taaaactaaaactacattattatgctttcaatgaagtaaagcagatagatataagtcata

acataatgtataatctgaaatacggaaacagaagatgatctacaagagatcaagtacaat

gcaagaagaaacagaacattccatatttaaatgccatcacaaattgttcatctttacttg

ctgctggtgcggatattatccttgacaatagtcacagggcaaggagcattagtcatcaca

aagttgcttacacttcccattatgatcctttgaattgtgctgagacccctgcttcccata

acaagagaatccagcttcagatcttcaacagcatcaagaagtttctctcttgcatcaccc

caatacacttttgaaacaatattcacctctttttgtcttgaaaaagtgtcaagcaaatca

agaacctcaatatcaatttgaacaccatactttttcatgatctcgggttctctgaactct

ttcaaaggaatcaaaggagaaccggatttggaccagagctgattgcgagattcatcaaga

ctctcttggctgatgtggatgatgtaaatgttgtcacctttgtcagctaaattttcaagt

gcccattttagagcattcttgctgcttttggagaaatccaaagctataccgatggttcta

tctttcggcatttttgtgaagaggaaatgggtttgacaagggcttgaaagctattgagaa

agtgtatatttattgcaaaaaaatatgctacatgcttttggcgaggaaagagataga

>fava_dn_rep_c74

ttttcaacttgaatcaaaagatctcacatttgtcatgcatatatcacacaactttttatc

aatttcttttctatccatgtttaactgatttgtgttaattttcgtctaactatttccgta

ttttatatccaaataatatatcatcatctttctagcacgttctttttttccgatcacttt

ttacttcctcgcacaaaatcctccctgtaaatatcattcacacataaactttactcacca

ctctcacaatttctatacacgaaaccctcattttaatcatattgtggtacatatcactac

aattttaaaccaacctgcataaatccaagagcaatgtgtgactccattaaacctccatga

gcttaatacacatacaaacctgttatttctatcaagctacaaataatccaatctacaatt

tttattcaaagttgtacaaaatcatgaatgtatcaccattgtttcaatatacataaactt

gtataattttccccctaaattaacataacaacacaatatcaatattggtagaaagattta

tttttttttatttaccaagaactcaatgcattgatcatagataaacaaacgtacaagttt

tcttttaatacaaataaactagtcactctctaatctaatcttcttcgtcatcagttacaa

actggccagttccaggattcaaagccgcaatgaagaagaaagcaacattgaacaacacta

gtggttcaatctcattgattggtacaccggtttcaatgttgagttgagctagtgctccct

ttccggtaataatctctccgatcaaagagaaagcaaatcccaattgagccaatcttccaa

caaacagttcattggatttggtgaatccgaataaaggacctccttcgctgagaccgagag

ctgctctaaagccttttccaggaggaataacgcctccggtagtaggttcatcatcgacga

atttaccacggtcacctagagctccaatggctcctagaagtgtgaaaatgatgaaaaata

agaggagaggctcagcttcataaattggaataccagtttccaaattcaattgtgatagaa

ttcctttgccggttactgcttcacccaaaattgatgccgcaaaaccgatcatagcaacac

gaccaacaaaaagttcattctgtttagtgaagccaaatccaccagaagttccaaacacac

catcttcaacctttggttttggtttaggtacaaccttagcaggggctttggtctttgatt

tgaatagagccagagttttaaatgtgcgagatgaaaaagactttgaattagaagaaagtg

ggttgaatgagatatcagaaaacctaggcttcaatctttgacactgtaattgaagcaaag

gatctttgttcaaggtaacagaataggtgctggagacactagacatgagcaacatagttt

gagccattgctatgttacctttgccttgtgttgtgtgttgtgtgttgtcgtgtgatatga

aatgtgtgtgtggtattatgctagagttttactgaaacatgggtggagaatggaaatatt

atcttt

>fava_dn_rep_c75

ggccgcgggatatcctttgatttcatctattctattttcttcttcaaacctcttttaaaa

tattcaaaaaaagaaaatctaaacccattagtatttaatagtgttttggagtttggacag

attccccatcaatatcaaaatcaacttgccgtgcccattgttccaattctcaaaaacaat

atcttccttcattaataaaaccacatcaaagtttgtttctttacaacaaaaccatttctg

gctcattcttaatttgtgttggaaaccttatagtttggtcctttataacaaaaactcttc

agatcttttgtgttggagatttttgtgatttttaagaatggctgctactactgctgttgg

tgttattggtggtaatggatcttctgtttcatggatgcaatttggaagaaaagttaagaa

acagaataagatgaacagagctagggtttcttgttcttcttcttctgtgatagatcctta

taagacattgaggattcaaccaggtgcttctgaatctgatattagaaaggcttttagaca

acttgctttgcagtatcatccagatgtttgcaaaggaaaagattgtggtgtgcagtttca

tctaatcaatgaggcttatgatgttgcaatgtcaaatttgagaaaagaaacaaaaaaggc

agaaacatataaggcaacttatgataatgaatcatttagaggaaagaatgatccaggttg

ggattattgggaagaatggatgggttgggaaggagcaggaattcgtgactacacttctca

tattaatccttacatttgataaagtttatgttatgtaaattagtaaattaatcctttaaa

ttaatatgtatgtgcattattattagttaagatgggtctaatctagacctactacttgtc

cccatttgtccccatctgaaactggttggtgctttgtatatagtccagtccatgtttgtg

tcaaatttttctctacataattgaagtttaggacaatatttggcttgtcattattgttaa

aaacaaataagaactatttcacaatgaatgtattattatgttttttttagattaaataag

ttttttattattttaaaatatctcaaatttcgttgctaaataaaaatcgtcattaaaatt

gttg

>fava_dn_rep_c76

tagcttcttcaatttatttcagtgaacaatttctttcccctctgatctatctgcttataa

atctacactaagcacacaaaaaaaatgtcaatggatgtgatggcaaatgaacgtgtttgt

tatgttcactgcaacttctgcaacactactcttgcggttagtgttccatgcagtagcttg

ctgactatagtgacagttagatgtgggcattgtgccaatttgctttcggttaacatggga

gcatcacttcaatcttttccacctcaacaagaccctcagaaacagcagcttgtaagctgt

caccaagaaccaattagcaggaaggaagttgtaggatcatcatcatcttcttcttcaaaa

tgcaaagcatttcagcctcatgagcaacctaggactcctcccattcgccctcctgagaag

agacaacgtgtaccttctgcttataatcggttcattaaggaggaaatacaaagaattaag

gctagtaatccagatatcagtcacagggaagcttttagcacagcagccaaaaactgggca

cattttcctcacattcactttgggttaaagttggatggtagcaagcaagcaaagttggac

catggggttggggacagtactactcaaaagtctaacggattctattgatacgtataaaat

ggaattaataagtctataattaattatcaatatcagttggttttttcttctttttttttc

ctggaagtttcaaccctgttgactatgacaacagtcgcgactataaacagactcgcaact

atggcagggcggtacgctctgctctctcgcgacccctactctaacgagtaagcaagtaaa

ctaccttttgaagcgccagtcgcgtagggcgacctggccaggccgagtcagaaagctttg

atgactcaaggctaatcttagggaatgagagtgaggggaagagggggcagccctcggccc

gatcatccaattcactccaacagacaggcatggttctgtagtaaagcagcccgtcacttt

gtgtaccccggacggcagccctttcggggcttccttcgggaccgattaactctgcgtaga

ttgactgaacgcagaaagccttccactggcaggcgatcgtgtttttcacaggatttctcg

ttactcatgtcagcattctcacttctgatatctccaggtgttgtcaccaaaaaccttccc

cgattgacagaacgtcccgctactgacacttgaacaactttcaaggtctcgtcgcttcgg

tgaatcacttgagccctgatacattttcggtgccatggagctagaccagtgagctattac

gctttcttcaaaggatggctgcttccaagcccacctcctggttgtcatcgctcgatcact

tccttttccactaagtgattgcttagggaccttagcgtacgatctgggctgtttccctct

cgactttggatcttagcaccctaaaagtctgtctgtacaaacgatctaggcctgtattcg

gagtttccctggggttggtaaggcgaaatggggccaccctagcccattgagtgctctacc

tcgggccatcgacatcatacgctctactgaaatagatttcgcggaaaaccagctatatcc

gatcttggttggcctttcacccctagccacaagtcatccccgtattttgccacatacgtg

ggttcggtcctccaaggcctgttagagctctcttcaacctgctcatggctagatcgatcg

gtttcgggtcaaataggaagaactagaagattccacctctgaaaggcgcctacacctaat

ggcttaagccgctgttcccattttctcgctgacccatcatgcaaaaggtacgccgttaga

gtgagtgcgctttactaatagaatcccaagtaaaggctctaagctccttcgactgattgt

tcgcatcggatctcaggttctctattgcactccctaaatagggttcttttcacctttccc

tcacggtacttgtacgctatcggtcattgaggaatacttaggcttagagggtggtccccc

tttctcgcgtaaaagcgatcagaattcgaacacgccgcgttttactgggaaggatcgaac

catgggaacgaatctacagggctatcaccttctttggccagatcttccaaccttttcaca

attacagttcactgcgccctaaaaattaaatgaaaggggcttgctggtttttccatcatc

caatccacaacaaatcgaatgaaacctggcgaaaaataagtgaacactttgctgctgagg

aatttctcatactcgaaagagaaacgaagcttcgtgtttgtttttcttcaaaccccaatc

ccctcccgctcgccgctactaacggggtctcggttgatttcccttcctttagctactaag

atgtttcagttcgctaagtttgaaaagtccaaagagcgcagactagccgcggagcttgga

tacggtttcccgatcggagatccatggatcaaagacggtatctccccatggcctttcgcc

cctgaaagcgtccttccttctcaatgcccggacctccatccaatgtattccccgggccgt

aatggccactctgattcatcgcagatgtggagtgcaaatgcattgctaaacgatagacga

atctcaactacgttgatgcgccaaaagtttatcgtgattttgtctaaattaccggcaaag

atataagttaaatacaatttacatgaaaagaattaatggtttatttcatcatcttctcca

cttgtcaatttctcttgatgttgccttgataagctttttagtgtttatgacaatgtcatc

cacaagtgcagtaatatcatcttctgaaataacgcttcgagcacccagaaaaggacttcc

ggaaccacgaaaaggatttccgacattagagtaccttctatactcatcttgtgcaattcc

cttccctatgccatagccaaatcctaaaccaacaccacatccagcaccaaatccgaaacc

aacttgcatgccaggaattccaggaccaaaaccaactcctccgagaagaccaaggccgaa

tccaacgccacagcctgcaccaaccccgaatccggggcccatttttccgaaactgctgga

cctcaccaccggaagcttccacagcaaacccttgtcatcgtcgttcattttttccttcaa

tttttcttgtgtattttgtgatatatacgcaagccaaattctagaagattggattgctct

gattctaacacactgccactgtgttcaccaaatatggagtcctagttgaatttcaaatta

attttacatgttttctatgggttttttctttgcgtttgtgttttagcttcttttttttct

gagttttttcaaaaaagtaaatcgtatttttttaaaaatattatgatgaagggaaaaag

>fava_dn_rep_c77

aaaaacgagtgtaggtaaaaacaggttccttatctagaatgagaattgtggttgtatcct

cgtgcagttcagttcgttctcaccttcgtaagctttaaggttttcaaaatcatcgttcaa

tcgaatccgaatcgaatttaacaatgaacatgttagcttcatcatcaccatcaaagatgc

ttcttcttcccaccacatcaattccatcgattcgacccatcaaaaccaactacatcaaga

tgaaaacaaaccgttttactgcttctgctgttgtggctgaagatgccgtttctccacagt

cctctccttctccccagtctccgcctcaatctcagaagctaggagttgttgtgaaaccaa

cagtcagaccaagacttgtgcttaagttcatttggatggagaagaacatcggtattgcac

ttgaccaaatggtacctggccacggaaccattcctctaagcccttactacttttggccaa

ggaaagacgcttgggaagagctcaaggagttgctagagagtaagccatggatatctcaaa

agcagatgattattcttctcaatcaagccactggtattatcaatttgtggtaacaaagtg

gtggtaatttgtcctcctaaggctcgaatctaggatatttcaaacaatgtttttgttttt

gtatcaatagaatgtttcacctttttcatgttgtggctgatctgttgtattctatttgct

gttactatgtacaccagacaagtaccaaattcttttagatgaatgattacttttcggtct

attgtagttttatcttatcaatgaaatttctgcatgacgaaaattctatttgctgattgc

tgatcaaacaaactgtgtctacttcagagcctctctgtgtcgctcaccactctctctcgc

gg

>fava_dn_rep_c78

acaaattaaacaatgaaattatcaaacagcataataaacaattaagattttttttcacaa

taaacaacaacatcaacattcatttctcaattccaatttcttcataatagtacaaaataa

ctgcaaccttcattatgaaagttacaatgcaaattacataacctaaaaacaccgaaaaaa

attgcgataaaattaaatagcaagagtacgagattgcaaggctaatccttggactgtttc

tctccctcttgctgagactgaagcggaatgtcaagctcaccggaagagttgcccttttta

gagttccgagcaggagcaggaagagcgagaaagcacttgttcttgctaataggcttaatt

ttatgaagctgaacaatgtccccaactttgaaaccgttttcagggtcatgagcctggtac

ttcttcttaatcctgattagctttttgtatttcctatcagttgccaaacgagtaaccccc

gccgccaccgtcttgttgttggtggtgcatacgacctttccttgcattgtttgcacggct

ttgattgccggcaacggatgggaacgggtttgggtaagggagattgaggaagtggggttt

gagaaacggttgacggcgattccattgccggtgaggaatggggttgagagcttagcgggg

agttgcagaagcgacatggttgaagggataagaaacaagataaaactatatttattttat

ttgattctctttcttgtgtgacttatggtgcatcttaatacaattccaatggcaatgcaa

atgcaaatgcaaatgcaatcaaaggaaaatgacgaggacggtctaggcgtggacgaggaa

gatgaggaagacgagcaagacatggaagatgacgaagagcacggagatactttgtgtggg

gcatgcggagagagctatgaaactaaccagttctggatttgctgtgacatttgtgaggta

tggtttcatggaaaatgtgtgaagattactccagcgagagctgatcatatcaaacagtac

aaatgtccatcttgcagcagcaagagagctcgccctagtggattgatataggagtttgtg

tagattggtgactctaatcttctcggttttatgactactagtttgttcagttaatcaggc

ttataagactcccctttaattttaaggtatgttatattcggtaaatatgctactatcgaa

ttattcagaatcaggatatttattgcagaaacagactttgaaataatattctgtcatgtg

ttttcttagattctaatgttctgtacttggtgttatattttagtaaatttcatgttatct

ctc

>fava_dn_rep_c79

actatcagtaaaagaaaaatggcttctttcttctttcctgtatatttcttttccattgtt

tacaaatcatcaaccacaaaaatcccaaaatgaatatactctcaaattaacaccataatc

aaacatcataaataaaaaaacaaaatcaagaaaattgttaccggatccggatccggatcc

ggatctgtgcggtggtacgatggttcttgcatgcggccgcgggaacatcaaaacacacaa

aacaacacattagtgtacacttcataacaatggcttccaacaagcttagtgcactcattg

tattgttttctcttctttcttataccacattttcacatgcttgtggttcatgcaaaccac

ctacacctgaggctagcccacctccttcaaaatctcaaaaatgtccaagggacaccttaa

agctaggtgtttgtgctgatgttctaggacttgttaatgttgctcttggatcacctgctt

caagcaagtgttgtgcattgattcaaggtttggctgatttagatgcagcactttgtcttt

gcactgctattaaggctgatgttcttggtatcaacttgaatgttcctatcacactcagtc

tcttacttagtgcttgtcaaaaatctgttccttctggattccaatgctcgtaagttcaag

ctatactagtactgcatgcattggaactataataattttaacgaaatcacagtgacattg

taacagatcacggtgtcactgagattatgttaaatttataatcaattcaaatatgaagta

gtactatattataacttgtgtgggatgtgtgtcactgtaatagtggggtaagtcttattt

acaaacttttttatgttgatttgatgattgtgcttgtcatgcaatgcatggattattata

atttttattttggatttataatattggagttgtgaaaattggatttgagtatctatgatg

cttaattagtggaattgaattgtattattgctaaaccatatgagtattaattttggtgca

cactattgaacattatcaatcacgtgaaggctatattgtgattttgtatggaccacacgt

tgtcttgcatgttgtcatttacaaaagtgcactatcacactcacatgtatattaagtatc

aggatttcttaacccctcccatatatatcacactctatgtaattttgaatttatctatgt

gaaagttggaaatatatttttggttcgtaaaacattgtgctatggaaaaaataatatcac

aaatatactttcgattaataattgaaaatttgtttccggttct

>fava_dn_rep_c80

aaattgtgagagcaacattcatttgagacctaggataataagataagggtttagaaaact

tgattccacacaaaattaaatttttgctataattatataaaattcaaaatacattggaca

tatcatatagtaactcttcaacttccttacacaccacttctccctctttccctcaatgga

agcacacgcaggagccagccagaggattgcaacaatctctgctcatctccacccttcaaa

tttccaggaaggaggtgatgctgctattaacaaagctaactgcagagcaaaaggtggagc

acctggattcaaagtagcaatcttgggggctgctggtggaattggtcaaccccttgcttt

gttgctgaagatgaatccattggtttcagttcttcatctttatgatgttgtcaacactcc

tggtgtcactgctgatgttagtcacatggacactggtgctgtggtccgtggcttcctagg

gcagccacaacttgaaaatgcactctcaggagtggacttggtagttatacctgctggtgt

gccgaggaaacctggaatgacgagggacgatttatttaagataaatgctggaattgtgag

gacactttgtgaaggaattgccaaaagctgtcccaatgcaattgtcaacttgattagcaa

tccagtgaattccactgtgccaattgctgctgaggtcttcaaaaaagctggtacatatga

tccaaaaagacttttaggagttacaacccttgatgttgtgagggcaaatacttttgtggc

agaggtacttggagttgatccaagagaggttgatgttccagtggtaggaggtcacgcagg

agtcacaatattgcctcttttgtcacaggttaagcctcctagtagcttcagtgctgaaga

agccgaatacctgacaaatcgcattcaaaatggtggaacagaagttgttgaggcaaaagc

tggtgctggttctgcaacactatcaatggcatatgcggctgccaagtttgctaactcatg

cctccatggcttgaaaggagaagctggggtggtggagtgtgcttttgtggattctcaggt

tacggatcttcctttctttgcaaccaaggttcgtcttggtcgcggtggagcagaagagat

atatcaactcggtcccctgaatgaatatgagaggatcgggttggaaaaagcgaagagaga

gttagcaggaagcatccagaagggagtagaattcatcaaaaaataagtcagttaagacaa

aaatagtt

>fava_dn_rep_c81

attacattcaatcttttctctctcttcttctattctctctcgtttctctcttcttcaaca

caagagcaacaactatgtacaaaccctcaacaatgaaccccacccaaaataaccccaggc

agcgacaaccctaagcaacatcaaccctaaatccaaccccagaatcacaacaaaaaatta

caacaataaaccctaagctaagtgttatttcagactacctctatgaccgtgaatcttcaa

gttcttcctgatcctctttctagatcatctgcaatgatccttccgcaggttcacctacgg

aaaccttgttacgacttctccttcctctaaatgataaggttcagtggacttctcacaacg

tcgcgggcagcggaccgcccacgtcgccgcaatccgaacacttcaccggaccattcaatc

ggtaggagcgacgggcggtgtgtacaaagggcagggacgtagtcaacgcgagctgatgac

tcgcgcttactaggaattcctcgttgaagaccaacaattgcaatgatctatccccatcac

gatgaaatttcaaagattacccgggcctgtcggccaaggctatagactcgttgaatacat

cagtgtagcgcgcgtgcggcccagaacatctaagggcatcacagacctgttattgcctca

aacttccgtggcctaagcggccatagtccctctaagaagctggccgtggagggttacctc

cacatagctatttagcaggctgaggtctcgttcgttaacggaattaaccagacaaatcgc

tccaccaactaagaacggccatgcaccaccacccatagaatcaagaaagagctctcagtc

tgtcaatccttactatgtctggacctggtaagtttccccgtgttgggtcaaattaagccg

caggctccactcctggtggtgcccttccgtcaattcctttaagtttcggccctgcgacca

tactccccccggaacccaaagactttgatttctcataaggtgccagcggagtcctaaaag

caacatccgctgatccctggtcggcatcgtttatggttgagactaggacggtatctgatc

gtcttcgagcccccaactttcgttcttgattaatgaaaacatccttggcaaatgctttcg

cagttgttcgtctttcataaatccaagaatttcacctctgactatgaaatacgaatgccc

ccgactgtccctgttaatcattactccgatcccgaaggccaacacaataggatcagaatc

ctgtggtgttatcccatgctaatgtatccagagcgtaggcttgctttgagcactctaatt

tcttcaaagtaactgcgccggaggcacgacccggccaattaaggccaggagcgcatcgcc

ggcagaagggacgagccaaccggtgcacaccagaggcggaccgatcgacccaacccaagg

tccaactacgagctttttaactgcaacaacttaaatatacgctattggagctggaattac

cgcggctgctggcaccagacttgccctccaatggatcctcgttaagggatttagattgta

ctcattccaattaccagactcaatgagcccggtattgttatttattgtcactacctcccc

gtgttaggattgggtaatttgcgcgcctgctgccttccttggatgtggtagccgtttctc

aggctccctctccggaatcgaaccctaattctccgtcacccgtcaccaccatggtaggcc

actatcctaccatcgaaagttgatagggcagaaatttgaatgatgcgtcgccagcacaaa

ggccgtgcgatccgacgagttatcatgaatcatcaaagcaacaggcagagcctgcgttga

ccttttatctaataaatgcatcccttccaaaagtcggggtttgttgcacgtattagctct

agaattactacggttatccgagtagtagataccatcaaacaaactataactgatttaatg

agccattcgcagtttcacagtctgaattagttcatacttacacatgcatggcttaatctt

tgagacaagcatatgactactggcaggatcaaccaggtacccgcggcctaatggcccctc

acacattgcaagaattgtgccgcccgaa

>fava_dn_rep_c82

ctacaattaaaccatgataaatttattaacgatgtcagaaaacttaggaggttgtcctta

gaaaagnnnnnnnnnnnnnnnnnnnnnnnnnnnnnnnnnnnnnnnnnnnnnnntgtctcc

gaaaacttaagacatattactactatatcgatcccttgatcattattgtttctacaatga

agccaaaaattgtctcagagactgaatttcatattataaaataaaacattcatcattctt

atttctacaatgaagccaaaaagtgtctcaaaagaaacttgatttcatattttaaacatg

caaacgaaacaacaaaggtgatacattacgtcaaacaatggcagagtatttgacatcaac

atccgacaaatgaatgactacagaacacaacagactcgaacttaacaatcaaattcaaac

gcaggtacctaaaaaattataacgagaagcgcccagatggggtaaactagcagaaggatg

atgccaatggaattcgacacgccatttgctctagtgaaggagtttctgaaaccaccagac

gcacgaatatgttcctgtagcaaataacatgcaatgacaagacacacaatcactcccaca

atgttttctctaaaagtgattgcaactagatttggagctacaacagccagaagtaccaaa

gcatctggtaaagccaaccattcagggtaatgttttggaataaaaagctttaacacaaca

gtaactaaagcaatccattttccaatctctcctctgaaagcaccgaaaactagttgcggc

aaactgaagaaaatgtatggaatgagtagtgatgtaagaatgttggtcttccagtttgtg

cgatccaaaaccaacaaataaatagcagcaacagcagcaaagaaaccaaagaaagaagct

ccaaatcccaaaccaccgagtttaagaacatgatcagcgagtttatgagcagcatcaccc

aagtctctgaaatccgagttgatcaactgaactgcctcatcttgaaaattcatcctgttc

cacattttcttcctaatttgtgatcacaagttagaagaaagaagaaaataaataaacaat

tatgaaaattatctagcaagagaggacccagaaatttagtgctctcatgtgcgcactatg

atctgtccccgcggccnnntggccgctctcctgttatgtgtcnnnnnnnngaagaactct

tgaaactttgcaaagctta

>fava_dn_rep_c83

tttgttcgtaagcataaaaaatcgacttaaagagtagacagcagnnnnnntttttttttt

ttgacagaatacatgaattaagcagaaatcaaatgagctacattcaaatattgtaccaac

atcttcgaagttagttcaagaagacaaaaacaattgatattttaacaacaagatgacaaa

taaattcaagagagagtgcaacatttgatcaaagtattgccattgcactggacgacatac

tcacaacacagacgatcttctccaaacatattgttggcatccccttcaaacttgtaagcc

aatagaaagaacagcttctggagatataccatcagcttcattggcatcggcttcaactgg

ctgcttgggatacttaccacctttacgtttcgtttgtaaaccagctcgaaataaaagact

atccacagtctctgaaggttgagcaccaacagatagccagtacttcactctttcaagttt

gatactcactcttttctcatcatctttggctgcaacaggattgtagtaacctacaacctc

aatgttcttgccatctcttgcacatctgctatcagtaacaacaaccctatagaaagggtt

gtgtttgcaaccaattctggccaatcgaatagtcaccaccattttcacctgtcttccaaa

ggtagttttcttgtagaatggttcgctgctctctatccctttatgcgtgcgtgcgctgtt

accgttccttctcaatcttcttatcttataccgtgttttctcttatctctctctctctct

cttttaaatactagattttaatgt

>fava_dn_rep_c84

atcagttatttgcaatatttattataaatattttctattaacttatttttttaagatcta

tttcacatcctaccgccaatagaaataattactctgttcattttgttgatgtgttccttg

cgtcctattttccaagcagcaatgtaaatgtgacaattctcatccaaaaacggatcttgt

cagtgtataccatagctgtttctgtaccacacctcattgcaagtgaaaattgggtacttg

gagaactaggctcctgtcttttagaggactattttgatatcattgatacaccaatggatt

ttggaactaaatgcagcaaccttgaaaagaatggcaagtatatgatgtcggaaaatgtct

ataatgatgtaaaatacatttggaagaactgctataagtataacaatgagggagactata

ttgtggatcttatgaggcgagtaaagaagaactttatgaagtactagactgctggttctt

ggaacataatgggccattgttgaaagtaactataattcctcacggaacaacagctcttgc

gtttgctcagtatgttccaaatgagaaccttctgatgacaaaggaacaaatttatgatag

gacttgtatgatccttggtggcagggcttcttaaaaggttcgtattggagcaatctatac

aggcgcacaaaatgacttggaggattcatatgagatgtctaaaccgtacatcggtaaaac

tggtacaatcattgattcggaagagcgataatgggtgaacaaagcatatgaacatacaat

ataacttattgaggaacacaaggagaatgtggctcgaatagtagagttactgctcgaaaa

attagtacttcaccaagatgacttacttcaagtcttaggcacacgaccctttaagtgtgc

caaagtcactaattatgatagatttgagctaggattttatgatgacgagaaggttttaga

aaccactgtcgatggagtaaggttgttctctcatagttgattaagtttatgttcattata

tttctgttactactatacatgaatttggggtgttaatgatacctgtaaatatgatagatt

gatgatgtgataagaaatgcatttaaaatagaagtaaaaaaagacccagtagctcctagt

agcccgaatcagtcaatgcaaatcaatttggcaacaatgcaactaagaccgctgtgtcac

acatgctaattcattcaaggtgttgtactcaatagtagattcatgaattaacaactaatg

gcagtgaaagtaggggaaattcattctagtcttgtactcattcatataaataaaaagaac

ccaaaaaaaggtaaaacataaatgcaaaacacctaaatccgacgtcattcattacattgt

ctcaattgaaataagcaaaatcttgtgagtaaactaaaactatagctagaaccacaaatg

tctaacaagtaaaccacttagtcgaataagctgaatcccatgtcatcatcggattcttcc

ttttcttccaccttcttctcctccttagcttcagcagctggagcagcagctgcagcacct

cctccagatgcagcagaaagagcaacagcaccaccaccaccaccagatggaactgaagcc

aacttttctcttccactggcaatgagctcagcaaaatccttgcccttgacttcggttatg

agcaactcaatgtttgagtcctcagcctcaattccaactgatgcgagaatcttcttaata

tcagcagctgaaggggctgcgttgcctcccaaaacagccaacaagtaagcagcgataacc

ttcatttttgatgtagagggaagaggaatgagaactcgatttagggtttcgccagagaga

tattgaggcacactgccccgcggcc

>fava_dn_rep_c85

cgaaccaagaaattgacatttctaagttannnnnnnnnnnnnnnnnnnnnnnnnnnnnaa

taaaaaacaaaagttatacattgatatctcatcttgaaatctatcatttcaaggatacaa

agtacaaaacatttagcagtcatatactataaacaaaagctgctttttttctctatatat

ataaaaataaaaaaggctaatgccatgaagaaatgtatcttctccaaaatacatcatgca

tcatccatttaggtgactttatcaatagattagagaaacattattcatgttcattcatgt

tcttgaacaaaaagaccagctcaaaatttgtctcaaaaatccacaaacattgcttggtcc

tcctcgttactttaagacattggtttaacataataattttcttgtccactagcatatttg

cctgcctccttcatgttgcaactccaattaactttagaatgttgttatgaggacttcccc

aataaaatccttggatgaaaattgggaaaatgttgcaagctgctaggacatatatcatca

atgcatgcatgcccatccattccaaaatcaatgtcatgcgactatatccacatacatcaa

ccatcaagtaaattccaacaaagaggatgccagcagcaccagcagtgagacaagtataac

tcaatgagtagagaactttatttacatgcattccaaataaatccaagataagaccagaag

ctacaagacaagaggttggaatcatccagtatatgattctaattctatggtccttaaaat

ggagaataatgtgaccataatgcaagccaaccaagcaggtaacaattgccatcactgaac

ttaagagtccttcaggatcaaaaggggcttgacaccaagaaggagcatcaggaggcaatg

gtccatagtcaggagaattgatactgcattcaggcatccgcgcatatattggtctccggt

agagatgttgtatacccaataagctacgatcgatcattccaacaacattgcaagctggtc

cagtgtcacccctcactccacatttaacagaaaatatcaaaggtgctttagaaggttctg

ttggaacttcatactcccaatcaggaacgtacatcccatacagcaagcaaagataaatga

cagatagaaacaaagccaatgcccattgatatcgatactttctcaaaagggtcgatccac

aattaacgatatcttcacgctttagccaaatttcacagagagctgtaatcaaatacgcta

ctgcgatcctttgcaatatccccatccatcgtatctgcttcaaatccactccgaatgaca

aatcatttacgcgatgaacataacctccttggagaaaaatgcctaacgcgagaagcttaa

gcgcccgtaaaattgactttctagttgcatcaactttgcaagacggtttcttgtatgtga

atgctagtgaaacaccaacaatgaatagaaaaaatggcattacaaaatctgctattgtta

atccattccatggagaatgattgagtgctggaatgagtcctccagcatcatccaccaaaa

tcataagcgcaacggtgaggccacgaaaaacatcaagagaaagcaaacggtgagatttag

gttgttgttcatcaacaacaatcttgttcttgagtttggttgttgttgttgttgaatcag

agatctgttcttgtttttttttttaagaactcaacattttttattaacaaacttccaaat

ttcaccacataaaaggcccacatcatttgagcctagaacttaaagtacaaagtgactcca

acttttaaatcacattactcttatttttgaaatccatttattttctccatatttaaatat

aagcaaaagaaagtatatgtatttatacatgtaccattttgtttatacttaaaatcgaag

agaatatttcttaacagaagacaaaatacaaattcttcaaaatacatatatcaaattaaa

acacaaatttatctgacccttctgcaattgattcggatctcaccttgtgtcccggtaaga

ggcttcatgttccccatcttgatcaaagaactcttaaaatctctagcaaaagccaaagga

cttttactataaagttgcaccaatttgtcactttctctaccttttccctcaaacaattgt

tgatcagaatgaagaagaccctttttgtatatcaaatctctataatatgttgtgtcaact

tttgtaggagtggcatcaagtggtgataaattgctgtctccgccttttctaggacatgtc

tttcttaaagatgctgcaaaactaggatcaatgtttgtttcattgtaaattcggtttcta

aatgtattgcactttgcaaatccaatggtgtggccaccagaaagtgctacaaggtctttt

acatctaatccatgtgatttgaagttggaaataagctgtgagaagctgaatgttggagga

gggagatttgtgtttgcagctgatttgcttgctgttcttgcatctcttcttcccaataac

acattatagaaaagttgtggacctcccaatatggcaacagaatcacgagcagcaatggct

aaaatatcagcacatgaaaccacagggcgtttacatgctttatcaacagctgctttgatt

tcatcaatcacagaaaatcctcggattgaattaatatttggaagagctgtcttctcccct

gtgaagttcttagtgtcatctagcagaactgatccatcacatccattaacgaagcaatca

tggaaatgcaggcgtaggagtgatgccccaatgcgtttttccttaagaattgcttcctta

acaactgactttatgactggcaatgcttgagggcaaaccttatcataaaaatgaggagtg

agcaatgcattggaagagatcaatagtgagaccaaagtcaccataacaaatatttgacta

gaagtcattgtgtgtcttttggctaagatatggatcccgcggcc

>fava_dn_rep_c86

tttttttttttaatgcaaattagttgcatgtcatattgctagttcattagccaaattgtt

tcaagtattacaacacaacgattttattcacatatccaaaccatgttttacatttctaaa

ttccaatccattctgaaaacaagcaaactgacttaatacaaatgcaaacagactcgaaat

aaataaagttttgttcatatgacccttcaagcactagcaacagctgcatgcttgttaatt

gtcaatagcagctcctccttcttagcacccacaactttgtccacaacttttccttctttg

aagaacaggaatgttggcatagcttcaattccccactcctcggaaacagtcttcaattca

tcaacatcaaccttaaggaaggtgacatgtggaagctttttagcaatctctgccaaaatt

ggagcaatgaaacggcatggaccgcaccaagaagcagtgaaatcaaccacaatcagtttc

ttagaggcatttcccttctggagctgttccttccatgcctccacgctgtgaacaccgatc

acttgtccctcttctgccattttcttctttcttcctcgaaaataacgcttcttctttcaa

atacagtacttttctctttgattttctatgatcccgcttgacaaatcccccaaataagct

gcgacaagcactgcctgatccttgccttgcaatagcagatagctggctttcatcttcttt

gacattcattagcttcccaagggcataaacaagacaagcgaaacccgcagctgatgaagc

caatccggccgcagtggggaagttgttgtaggaagcaatatgtacatgaaatttgctcca

atcctctttactgatcttaacaccctttttatcatcctcaacatcacaagcgcgggcacg

aatctcccttaaacaactctggaatcttcccccagaaagagaaatctcctttccattgag

ccacatacgatcctgttgaaaggagggactaacagaaaccgtggtggttgtgcaaaggtg

agtgggatcaagagtgacgctgatactatcattgacaggtaagattagggtttcatctcg

cttaccccaatacttgatgacagcaatgttggtgggagtctgagccgtaaccatcactac

ccaattctgctgagaatcacccgccattttccaatcaatcgatgaaatttttcaaattca

acgagaagatgaaatggtaatagatctatgatctaattcaatctttaattagtttgctgt

aataatgaactgaaacaagaagaatgaacaaggtatttatattgctaacaactaacacta

cccttttatggttttggattgttttatttttttatttttttattcctttcgttttggatt

cactt

>fava_dn_rep_c87

tatgaaattattgatgcagttcaatatataaatgagggggggaaaagtaaattttacagg

tagaaccaaaccatgacaaatagtaaactaaacaatagaaaagcaaacaccagacaatgt

tataccagacgatgatacagcatacgagaatgaaaaggtaataagaaaattatgttaata

cgaacatcaagaaattccatcatgaaaatctaaaagctctacaaaagataaacacctctt

aactagagttggccttaaaattacaaataaaagcttgctccctcattttaaactattcca

agttttgaacagacaaaacatcaacaaagagcagcaaaatattctggtagcaagatatgt

ttatcgggaaatgaccaaatcatctacataagatgatcaaatcatcaacatagcatccta

cataaatgttatcatcatccaaatgtctattcgtcatcctcctcctcttcactttcttcc

tcatcatcattctcatcatgcacctcagattctgatttcccagattcttcttcttcttca

acagcagctgggccttcagcctgtttcttggtataggacttcatgctcttctcatattca

gccttccttttctctgccttggcagcataaggtgctttttcagcatcagtcagtgacttc

cattttactccagctgctttaccaactgcagacacagctttgttatcagggttttccttt

ttgaactgcttcctaaagtcctccatgaaaacgaagaaagcactcggaggcctctttggt

ttgttagggtcctttggttcctttcccttggctggtttctttccgcccttcgtggcggca

gcacccttcttattcaccgcaagcttggtttcagctttcttcgattcacccttggacttg

cctcctttcatgatgaaaccgaacggtgaagagagatatgagagactagggtttgggaga

gaaggagagagagagagcgtgcttttgagatatgcgtttgttgtgaatgagactatgacg

taaggctaggcacaaatgcgggtagatattaaaggccttaaagggaaattgaaatgaaat

gaaatgaaaaatgaagggaatggtaggggtttaggcgggaatttaaatttaatgccatgt

cattgatccgtgtgagggtgcgttaatgctaagcttgtttctattttgtgttttctatgt

gctttctcattcc

>fava_dn_rep_c88

tttttttggtacttataacatggagtagttttttacttaaaaaacttgcaaggttcaaac

aaacctaattacattctatagcatcaaaaaaacatgttcataccaaatgacagactcaga

aaacaatacaagcaccagtgaacaatctgaaatacaacccaagagagagcaaggatgtaa

aggaacagctggtttaacatcatttatgcttacataatttaggtacccatcaatctataa

gcctcagtctcaatttcagtacttatatatttatttatgaaccctgggcatcacatccat

ccagtggattacattgcacaaaactaagaattgactggtgcaaatttcagtgaaatgcta

ttgacacaatgccgttcgttagtgggtgttggaaatccttcacctttaaatacatgacca

agatgtcccccacatgcagcgcatgttatttcgatcctccttccatcggggtcaggatgg

cgatttatggctccaggaagaccctcaaagaaggctggccagccacaaccggaattgaat

tttgtcgtagacttgtagagtggagtttcacaacctgcacaactgtagactccctcctca

aagaacttgtcatattctcctgatccggcatactcagtgcctttctgcctcagaattcga

aactgctctggagagagaatagcttgccattcttcctccgatttgtttgcagatccatgt

ccaggtgcagccatggcgacaattccagcgcggaaaccacgcttgggttgacgaatggaa

acgttgggaggagtaaaagcgaattgggtgttgaagagaagtgagagagggtttgaggaa

atgggtttggtagtgttgaaacaacaaagagatgtggttcttagaatattgagcctcgtc

accgtcatcttgttcgtgcgtgtgtttatcactcgcgcaagcaaagtgaggtctttgttt

ggataaataaatgttttttccaacaaatactctattaaattaccaaactctcattaaatt

gtatgcctcacttgcatcaatttttaatgtctctttt

>fava_dn_rep_c89

tagttataaccattagcttttaatatccttcaaaagcttgaaagttattttgagcttcat

tatttgatgaacaataatgcatgtagtcaaattatatcaattacattgcaattaaaatta

caactgtgagacgattcacacaagctcaaacttcatgagctgtgaaagatgagtatgttg

gtctttcctagacaccacaatttggtgacccagaagggaaaattaatggaattttctata

tctctaaagatgaaataaaatttcttgtagcttcttcactcaatgttcaaagcccattca

gtttgccaaccagccctgtcaaggaggtccacttctcaaatgcggcggctgaagacacaa

aggcaattttggaggacttcacatcattctgagcaactgatgaaaccaatgaagcaagtg

aggaattgaatgtgtccacctcagttttctcttcaggactggaactcagcttattcaaag

ctatgttggctttttggacccaatcagtagtatccttgccactccctaagacagaaccag

ctgtggttagatcattcttttcaattgctttaccaacttcactcaatttgtacaccacat

cctgaagataccctgtttctttggtggaggaactgataataccttgtttctcaagtctct

tgattctatccttcttcactttttccaagagctcaatatcatcatctgcttcaagtattg

cagcattagcatcaaacaagcctttaccagtcaatgaaaggaccatactggtaacaaaac

taatggatatgcttcttttggtcacacttggattcactactttgcaaagtgatgaatctc

ttttcacattgcatttgatgatttggttgttttgaacataagttggtgttgctagatgaa

aagggtttgctgttgagagaaaatttgttgccatttttctttgtttctctctctctctct

ctctcttgttttttgtttattttttggccccgcggccatttagaagacatggcagtttgg

aacatttggaggatc

>fava_dn_rep_c90

ttaccaaattgcagtagaacaatttgtggcacattcatagaatatcagactacaaccatg

gccatggtatatatatatataatacagaacaaattaacacagagaactctctgaatatta

tttatacaaatctgttattacatacatatatattatttagaatgaaacacaaaaactgaa

caaaaagtgagcccttttcggattaaattgcagttttcttagtgcacaaagtttgcacat

taagaaaagacaataaaattgcaacaattgcatgcatagcataacagtcttatacaggca

tatataaaatgagagggaaattttcattataattaccatataatacaactctatatcaag

gcatcagcaccagcaacgatttcgagaatttcgccagtgatctttgcttgacgttgccta

ttgtaaacccttgacaaatccttcttcaactcaacagcattatcataagcactactcata

gcactcattctagcagctaactcgctagcaagcgattcctgcaatgccctcaagatttga

ctgttcaaataaagtggcaagagtgcatcaagaatctgaacaggatcctgctcaaactgc

aaaatcggcgaaaaatcaaccgtcttagatcgaatagcatctctttcaacggttaacttt

ccttcttttgttgttaacctgaaaagctcatcctcagcagcatcgacacagtttccattg

atatcacagatttcaccttttggtgagagtggaagcaaagtatgaattataggatctgat

ttcactagtgacacaaacttagtgtacaaaagttcgactttatcgacttcttcactaaca

aaaagtgagaaaacatcatcagcaatagtctgtgcttctttagcagtaggaagtgatcca

ccttcaagaaacctatcaacaggtatataaggtctacgagtgaaataagagttacccttt

ttaccaacactgataacagtgtactcaagaccaagacctttcaactcagcaattctagct

tcagcttttttcaatattgcattgttgaatccaccacagagacctcgatcacccgtgcag

acgacaagagcgactttcttcaccggtctaagcttagttaaaggcgcttcgatatcgtcg

gtttgaagctgttcattgatgctgtaaagaacttcaacaagtgtctgtgagaaaggtctg

ccattcacaacagcctcttgagctcttctgactttagcagcagcgacgagcttcattgcc

tcggtgattttctgagtgtttttgacggaatcaatacggtttctgagttctctgagacca

cactgaattgaggtgggtcttgaaggggtgcaagaggatgatgagttttgtgaaggaagc

tgaaaagggttgaaagctgaacggaaggagaggttggaagcatcaggtaaggaaggtttg

gaggagactaacatggtgacattggagcaagacattgttgctgtgatgaagttctgaaga

aaacagcagagaaagagagagtggtgtgtgtgaatttggttgatattttggttttgtgat

gtcttatcttgtacttctcatgttaatgtgtttgtgtgtgtgttatttttctgatctgta

ttaggatgaaagtatgaggtggtgaatgagtggagggaatgaaaggaggtggatgaggtt

ttgagat

>fava_dn_rep_c91

accaagatgttcatgattacaagataagaatgtaagtttcatacaaattttgaatcatgt

taaaaagttttcaagtttcctaaagcctaatctgcaaagttacacgagaattccaattct

tggtttcttctaaaggcttcttgaatcaatctttacttcatgataagcttcaacattctt

caatcattcttgcatcctccaaatccttgatagtagctacagtttcatcatggctccttg

aaaatattttcaaactcctgaacttagcccgaccaccacgccagaaatattatcccctgt

atcataagcataataattcaattttttgaattggggagagatggctgagtggactaaagc

gtcggattgctaatccgttgtacgaatttttgtaccgagggttcgaatccctctctttcc

gtttattgatgatttggcatttttttttttgaacttctggaatttcttttttttttattc

cctgtttgtttcattttttactagttaaagatgtaaaatagatagaaaaacgaaaaatat

ttaaaaatccagcataagtacggaaaagaacaaagattttcttattcttcacgtccagga

ttacgtcctggatcattagataggaatccgaagatgaaaagagaaacaaagaatatcact

attgtgtaaacaaatagttttagagtaagcattacaaaatctccaagataataaaaaaaa

aaaacgacagagaaagagaatagattctcttgtatttcatattatgtttcctttgatact

cagtttataagaaatgaagttatttcaaaaaaactaaaaaaattgatttgtagtaagagt

tgagcctttgttttaccattaccaacaattcaattttctttcatatccacaagatccacg

tattggtggtagactcaaatcgaataatagaataatataatttttatttttgaatggaaa

aaaaggaaatgatgagatgatccatatttttattgtctatccaacaatttcaagatttat

aatcacggattcacactgtaagacttatcttatttttaaaaatgttaaaatttgagtttt

ctaataaattctgacttttttttcggacattattcaaattaaagatcttatcgaaaactt

acagcagcttgccaaacaaaagctaagagaaaaaagagtaagggtattactggcataaaa

tctacaattggattcaaaaaagcgtaagcttctggtaatttccccaagaaaaaactactt

gaataaagggcagagtgaatacaaatacagaccgagctaaagatattaagcataacaaaa

atgtttttctttaagaaaatgaattgtcttttttcttttgtttttaattctaattttgat

tgtattttttttattattatatagatgatttattctatatatgatatgatttattctata

tatgatcccgcggcagtaatggccactctgnnnnnnnncgacaaagacaaatatatatta

accctttatttaattacacacgtttcctatgatatttatattttgttgaagtagaaagca

aagagagtactcacttatttattacattaagagaatgaatcaaacaacacacattttaca

taatgttgtgaaaacaacagaagaaaaccaccacagttatggagcaaacaattacctgcc

acaacttaattattaccacaaacatgatgtttacattgtgtaaaaggaaaaagtaaacat

taaaacacacgagtttcttcacgaatcttagtcaacaattcttcattttctttaagtgac

acgagattagggatagtaatgtaatatgtcataaagagcaggccaaacatcattaattaa

ctttattctaccacccacaataaaatcaccatcagttttgtttggtgcaaaatttatacg

tgcaaaatattctcctttgaaaatgtaagcttcatctttaacgtgagaagaaaaagcagc

atcaattccatactcaaataccgtaccaaccaaactacgaaaaccatttccaatagagtc

atgagatataatatcctcagagtgaaagtctataacgtaatattcagctcctttgaataa

gaaaacatgtttctcagtagatcttattgcagcatctattccatttataagcggtgtatc

cttcagataaggaaacatgacagggattggaatattacctgcagtaagtaaactgcactc

agatgaatgtgcggctaccttcattttggcacattgatttccagaaaaaatgtatccatc

atttgcttcagcgttaaaggcacaatctattacatttgcaaaaggtatttgagcgatcat

tgatcctttagacataagactcggcgcttcaataacgtctttttttgtctctccaggagc

ataattcaaaactataaacttttctttcacgaaaatgtaacattcattcaacttgggtga

ccgaaatgcagcatctatgttatgatatggcatttttttctttatctaaggtttatatat

ttatttatttatttggaaagggatatgccatttttttcctgatcagtggaatgtgtg

>fava_dn_rep_c92

ctgagtggactaaagcgtcggattgctaatccgttgtacgaatttttgtaccgagggttc

gaatccctctctttccgtttattgatgatttggcatttttttcttttgaacttctggaat

ttcctttttttttttccctgtttgtttcattttttactagttaaagatgtaaaatagata

gaaaaacgaaaaatatttaaaaatccagcataagtacggaaaagaacaaagattttctta

ttcttcacgtccaggattacgtcctggatcattagataggaatccgaagatgaaaagaga

aacaaagaatatcactattgtgtaaacaaatagttttagagtaagcattacaaaatctcc

aagataaaaaaaaaaaaacgacagagaaagagaatagattctcttgtatttcatattatg

tttcctttgatactcagtttataagaaatgaagttatttcaaaaaaactaaaaaaattga

tttgtagtaagagttgagcctttgttttaccattaccaacaattcaattttctttcatat

ccacaagatccacgtattggtggtagactcaaatcgaataatagaataatataattttta

tttttgaatggaaaaaaaaaaagtgatgagatgatccatatttttattgtctatccaaca

atttcaagatttataatcacggattcacactgtaagacttatcttatttttaaaaatgtt

aaaatttgagttttctaataaattctgacttttttttcggacattattcaaattaaagat

cttatcgaaaacttacagcagcttgccaaacaaaagctaagagaaaaaagagtaagggta

ttactggcataaaatctacaattggattcaaaaaagcgtaagcttctggtaatttcccca

agaaaaaactacttgaataaagggcagagtgaatacaaatacagaccgagctaaagatat

taagcataacaaaaatgtttttctttaagaaaatgaattgtcttttttcttttgttttta

attctaattttgattgtattttttttattattatatagatgatttattctatatatgata

tgatttattctatatatgatatcccgcggccattaaggaatatatgcaaatgtaattgcc

atgaatttggcaatacaaaattaataatctaatcttctattaaataaatttgtgtttatg

cctgtatgagtgaaacacaattaatacaatacaacgatgatgttgtgacattctgtgctt

aaaggagtttaaatataacttttgtttggcaaaaaa

>fava_dn_rep_c93

cgctgctacgatttttggtcctaggtttatcgattaaacaattgaaagaacattacaaat

tgtattgtatcctctatatcacatatatgatgggattctttaaaacgctaattaattaca

acataattatttatctcaatgagtcgatcccaagatgagagaagactggtgttaatcatg

aattaccgtactatacagataatatagcgtgcttcaatgcagcatcacaaactttatttt

cgagatccaaaaaccaagtcacctaaagaaaccaaaggtgaattgtgaccatgcactaca

ctgtctctccagaaagtcattcctatccatgaaaccagaccgacagttgctgccaagaag

aattccttgctgataatgtttaaaaggaataggcatgcaagattcccatatccatagaat

atggtagcccatgttgacccagtgaaccacatgaaatcacgaatgaatagagcacgtgga

agcatgagtccatttccaagcatcgcaagcaacatagaaaaagcagacaagcctttcatg

ttctcaggattgagaaaatttgtccacatctgggaaactggcatccacatgaagagtaac

gtagctgtccatccagatattccaccaacaaattgaacacctttctcagaaagctttcca

cttcttgccatggtaacagccaagagagctatgagaaaggaaagtgccccaggcaagata

ctgttaggtagatagggaacaaatgtagaccacattatttgaggaagcacggataaacct

ccaattgtaatgaaatcttcccaaaaacgccagatttgagcattaagtatgccaaagtaa

ttgatgaaattgagaaaactttttttttttacaccaagtataaagctttgtcttccacat

ataatataattctgatacatatgatccaaaggcagaagaaagatgcacaaaatccataac

aaaagataataaaacaaacaaaaaaacaagatttggaagctaattcccttatctcttttg

tttcatattaatgcagtgcacatgacatttgtttcagttttttaaaagaaccgctgaatc

agttggaataaataacccttggtaccacagcttccaaatctctgttcctagttgctgaat

tttgctttattctcattataacacagtagaagttgcaaactattccaaacaagttactcc

acaacattaacaagttcatattcaataagggagaggttgttgtcttccttaacagtgaaa

tcagcaggttctgttacttcgacacggccccatttatccacagcaagcctcattgatcct

ttaaacatgtcgattttcgcatttcgcaaaatgatggtcgaaccctctttcagcagatca

acttgatcatttctagctgtaaatatgatcatcccagtctcatcacccaccaaactctca

gcaattctcatttggcgcgattgaggaccatccggacgacccttctgcatcaccatctta

gtattcaccactttcacagttagagtatgaccactagtccccggtcgaagctgttcaacc

ttagtgaaaaccggctttctcaatccaggttttgcctctgccatatcttaacctaaaatc

ctgaaattgcaaaggcaaaaatgaaaagggagatctgaaacgagaaatgtacccgcgagg

ttagcttttaaggtatatgttcgtgctcactgatgtcgggtc

>fava_dn_rep_c94

ttttttttgaatggagactaaaaaattatcaaatagacataaggagaccaaaactccatt

taagcctaaaaacaaacatatcatccaagtatctgaaatgttaaatgaagtcttataaaa

ctctattaaactcctttgatacatattattgaagcaaatataacataatgtcataaaatc

cataataccacataacaattgttcaaaagactatatcacaataaatacgacaatcactca

caaatacccagctcgatgttcgatgtgagccagatacaaacaactgaacactttcaagtt

tccagctgcttagaaaccatgaattttaatatgctccttcttcacgattccagcctggac

taggaaggtagaaacattcttcctctggtcgccttgaagttgaataacctgtccttgttc

tgggtcctggacaactgtaccattacagcaaaactccttcttaaggtctttaagaatctt

gttgtaactgaattctttcttcaatccctggacagttgtcaagctttttctaccatttcg

ctgctgtatacgaatatgcacgtactcctttgatgatccagcgccggcgtcctcagcatt

tgcctcagcaaaaggatcaaaggtagtaggaatttgtgcgtctaattcagacatgaacct

taactctccgaaacagaatgtcccaccaaccagatagctcagacctgatcctagtttagg

gtttcgatgcgtaaagatctgggcgattagcgaagagtagaaaccctaaaattggataag

gaaggacctcctctcgctgagatttgctttgtttgggccgagagaaagtggtgccagatg

aatctttgagagaagaaaatatagagacaaatgtgaggggtaaattcggtgaggtttgtt

>fava_dn_rep_c95

aaaatcacccataagattttcgcctagaattagataaaatatagggaatgatttaagaaa

attatttcaaactcatcgaatatacacaagctaaaaaatactagtctagatagcacgtac

aaagaacaaagccagagtggctacatacccagtaagaacaagaacatttaacatattcaa

cgtcatcgaaagcaaatacaacccaaaatctaaaattaagatagtatattcctatcatca

caattaatgagagtctacaaataaaagatatttccctaaacaactaagcatttcaataac

aaaacactatcagcagccacccctctaaacagcaccaattccattatccttaagcacgtt

caccacggatcctgcgtgccaactgaatatccttgggcataatagtaacacgcttagcat

ggatggcacacaagttggtatcctcaaagagtccaacaaggtacgcctcagcagcctctt

gcaatgcaagaacagcatggctctggaaacgaaggtcagtcttgaagtcctgagcaattt

cacgaaccagtctctggaaggggagcttcctgatcaaaagctcagtactcttctgatatt

tcctaatttcacgaagagcaacagttccagggcgataacgatgtggcttcttgacaccac

cagtagttggtgcagacttacgagcagccttggttgcaagttgcttccttggcgcctttc

caccagtagacttacgtgcagtttgctttgtacgtgccatcttgagacttttgctttctt

cttctcttcaagaactcgttcgctgaatgtcgggatatagaatctaaagttttcccgcgg

cttcatggcctctcgcaatttcttcacaccctcttccttccgtcttcattcttatcctca

ttcatcttcatctcttcctctttccactctctctaaacccaggaagagaatttctcttag

gaagtttgcagttcctagtttggtgtcaaatgcagtcactccaagggtgtatatgtcttc

gttatctgcggctgaagatattcataagactgaaggttctaaagcttatggttctgatca

aattcaggtacttgaaggcttggaccctgtaagaaaaaggcctgggatgtatattggaag

cactggcatgcgtgggttgcaccatttggtctatgaaattttggataatgctgttgatga

agctcaagctgggtttgcttcaaagattgccgttgttttacatgaagacaatgcagtgag

cattactgataatggtcgcgggattcctactgacatgcatccaactacaaagaaatctgc

cttagagacagtgttgacgatcttgcatgctggtggaaaatttggtggcatcaatagtgg

ctactctgtttctggaggtttacatggtgttggtttatctgttgtgaatgccttgtctga

ggtactagaggtaaccgtttggcgtgaagggttggaatatacgcaagcatattctcgcgg

gaagccggtgacaactttgaattgtgtcatgcttccttttgaaagtaaagaccgtcaagg

aacgcggatcaaattttggcctgacaaagaagtattcaccactgctattcagtttgaata

caacacaatagctggacgcattagggagttggctttccttaacccagagcttacaattgt

gcttcggaaagaagacaatgatccagaaaaggtccaatataatgagtatttctatgctgg

gggattggccgaatatgtgaaatggcttaacacggataagaaagctgttcatgatgttct

gagtttcagaagagaaatagatggaatcacagttgatgtagctttacaatggtgtgaaga

tgcatattccgatacgatactaggatatgctaatagtatacggactgtcgatggcggcac

ccatattgatggcatgaaggcttctttaacaagaacactcaatagtctcggaaagaaatc

aaaagttatcaaggaaaaggacattactttaagtggagagcatgtgagagaaggcttaac

atgtgttgtctctgtcaaggtcccaaatccagagtttgaaggacaaacaaagacaaggtt

gggaaatccagaggtgcggaaagtggttgaccaatctattcaagaatatcttactgagta

tttggaactgcatccagatgttcttgatgcagttctctctaaagctcttaatgctttcaa

ggcagctttggctgctaaaagagcaagggagttggtgagacaaaagagtgtattgagatc

ttcatctcttccgggcaaactcgctgattgttcatcatcaaatcccgaagactgtgaaat

ttttatagttgaaggtgattcagctgggggaagtgctaagcaaggtcgcgacaggcgctt

tcaggccattctcccattgaggggtaagattctgaatattgaaaggagggatgaagcagc

aatgtacaaaaatgaagagatccaaaatttaatcctcggtctcggtctaggagtgaaggg

agaggattttaaaaaagatgcacttcgatatcataagattatcattttaactgatgccga

tgtggatggcgcccatatccgtactctgctgctgacatttttctatagatatcagagagc

tttgtttgatgaaggttacatatatgttggtgttccgccactttacaaggttgttagggg

aaaacaagtgcactattgttatgatgaagccgatcttaaaaaacttaagagttcgttccc

tcctaatgcatcatacaacatgcaaaggttcaaagggctgggagagatgatgcctttaca

actatgggagacaaccatggatccagagcagaggttgttgaagaagttgacggttgagga

tgcagctgaagcaaatattgttttctcatcccttatgggtgttcgggtggatgttcgaaa

ggagctgatacgaaatgctgccaacatgattgatcttgatcagctagatatttgatgatg

ctacaggctatggaagaaatgtgattttttcatctttgtattaacaaccatacattacat

cttagtcaagcacagattactctatataagatggaaggtctttcttaattgaagcctggc

gcgcccgttatatttggaagcatttttctttacactggctagagtggagtggtcatttgc

acttggtggtaaagtttttggaggcttggttgaaatctaatttgatttctacaaatctga

caagtgtacatttttctgatccaacattatgtgaagttttgtgtaatgttgttgatacac

attcaaaagctaccagcttctgaactgtgtttgattgtgccattttagaaggttaagtta

tagggtaatcaatcaaaataacttcaataattttgactgcattcagcaactttattaagg

agatagttgtctttatttattattattttaatgcagtgtgttaatagacaatgcactctt

atttcactt

>fava_dn_rep_c96

ggagtactagtaccaaataggtaaccttcctaagaataacaggtatcaaagacttagacc

gttgttggtcacattcatttagatttgctctttttcctcccccatttccactttacaaaa

atcagtacattatgttccaaaaaattaaactgtcagacatttaatggaggaaaactttca

taaaacttcaggacacaagaaaatatattcattaaggaatctaacactcgaagcatacaa

atatatgtattgatttttacatgacatgtatctgctatcacaaattgaaaaatcctttta

atacttgttggaggtcatagatctgtttccaagtatgatagcatgcaatgctatccttgt

ttgtcccctaactcatacagcagttggtgcttcctctgagggcagtttcttcttaccttt

ggtgttttctttaatttcccataaatccctcatctgagttttcggaggtggtgttgcatc

tgcaattggagcaactaatcctggttttccgtcatcaataacctcatcaaccaaaccgta

ttccttcgcttcccaagcactcataaaattatcacgttccgtgtccaattcaatctgcga

ttcggcctttcctgtaattcttgatagtatcttgtttattttaaccttgtgatatgccat

ttctctcacacggatgtacatttctatagccgctccgccaacagttccgagcggttggtg

gatcataactctagaattcggcatacaatatctctttccttttgtaccagcagcaaggag

aaatgcacccatggatgctgcaagtccaaggcaaactgttgacacatccgccttgcataa

cttcatggcatcataaattcccattccagcagtaatagaaccaccgggtgaatttataaa

taacttgatttctttcttaggatcatcagcatccaaaaataaaagctgactgattacaaa

atctgcagtcatatcatccacctgagaacccaaaaagacaatcctttgacggagaagcat

gttggtagtatcgagttcttcgaatttgggaagccatggagaagcagttttggcagataa

atcatgggaaacgagccagttggaggataaagtgggtttgattgcgttagagattgttgt

tctgtgtttggttgaaagattgagggtgggaatttgagagtggaaaaggttagtattgtt

atgcttcaagaacgtattgttcgatttgaagcttagtggtatacatgttgaggataaact

cacttccattttcttcctcttttcttcttctttgtttttgtaactatataacgaatgggt

gaaggatcccgctttgtttgtttggtttgaaaatgaagtgaatgtattgggattagatgt

tgggctcaaaatttataaccacctttgggattgc

>fava_dn_rep_c97

tttttttttttaagaaaactatgcaaatagcttgtatctttttcttgatttacttacata

ttgtccatttttaaacaaaaaataaatcatgtactactagagatgattaagaaaataaga

tagtcttaaattgtaatgtagattcatatttataagatacaatattttttccagaaaata

ttaaattacatgggataaggtttggcctaaaagcatatgagtacaaaatgaatcatggtg

aaatcttcaacagaatcctgcaagatagaactctatcatgtcaacagcattttagcattt

gccaactcttttatttgctcccaattatttcattaactgtttcctttactttctgtataa

gttcttccctttctggtttgaaaactaggttcttgtatgtaaaccaccccgagtagccaa

tgcctattaattcaagaactccaggaatcaagggaagcttatcaattgctgaaatcaaac

cagttgagctccataatgcaatcacactagctacaaccaatgtactcactgcatatttat

catcaactttgtcccaagttgcttgcagactcttaacaaactcaggtagttcagttgatt

caacatctgctggtgcatctccagtggccattaccataacctcacgaccaatctttcgat

aatgagaacttgtcttcactgcatgattctgagacaacaaagggagtgatggaagagagg

ttagtctcatacattgaggagaagaaggtgatggttgaaaaggagctttgcaatcaaaaa

ggttagtagtgagagaagaagatgagattgaggtcattgttattaattagtgagcacttt

ttcaaggtgtaattttatttttctatgaaaatttgagtccactaagttgttgatatgttt

gtaatgtattagtttcaattgtgtttgatgatttgcaatgtggttgtggagatcctgttg

ctatagcttgaagtgaagcagaaccacttatttatttttcatttagataatgttgtgat

>fava_dn_rep_c98

ttacggcgcgggagccacagaaaacacccagttgcagaccgacacacaccacaaaatccc

gccggaaaacccatctccggtgaccatcgtcctctctaactccggcaaacccttgaagtt

cgaaactgaagcaaagagcagaccaccggaagcggtggttttgatcttactgtgcatgat

gggatcttgcatatgtccgttagaaactccggcgaggttgctctggacaactagtttctt

ccgtcataagttaatgatcttttaatccattcccaacaaagataattcaataaaaatacc

aactttgaattagaattagaatttgaaagaaacaaagaatcgtttctggttctgaaaaat

tgtaaaaagtgaaattttagcataccctttaacgtgaagtttgaatctttgaagttgaaa

aaatggagtctggtcgtctttactttgatacctctacttgccgtggtaacaacaacatga

acatgctcttccttggaaatgctgatcttggttttcgaggaaggtcaatgatgagcatgg

aggaaggctcgaagaggagaccttttttcagctcgccggatgaactgtatgatgaggagt

attacgaggaacagtcgccggagaagaagcgccgcctcacttccgagcaggtccatctat

tggaaaagagctttgaagaagagaacaaacttgagccagagaggaaaactcaattggcca

agaagctagggttgcaacctagacaggtggctgtatggtttcagaaccgtagggctagat

ggaagacaaaacaacttgaaagagattatgatgttcttaagtcttcctatgagtcccttc

tttcaacatatgattccattgtcaaggagaatgagaaactcaaatctgaggtggtttgct

taaatgagaagtttcaagttcaaggtaaggatatgcttgaagagcctttgtcggagaaga

aagtcgatccacttccggtggatatcactcagattttcggcatgaagttggatgaacacg

tgagttctggaagcgttggaagcgcggtggtggacgagggtagtcctcgtgttgttattg

acagtgttgattcgtactttccagctgacgactatggtggatgtgtggcccctatcgaaa

gagttcaatcagaggaagatgatggaagtgatgatgggaggtgttgttttgatgtgtttg

ttacatctgaaactgagcaacaaaaccatgaaggagaagggttgaattggtggggtaata

tgtattatgttgcatgatgcaacattgctttcttattaagttacataagaaagagaaaga

acgctgtataattagagatgcatcatgtaatttggacgcatacgatatgtccctaagcca

agcttagtaaataaaaagtccagtaatttgctgattggcatcctgtattgtattttcaaa

tttacaataaagtattcatctttcttaatgccaagtctcgaaccgtgtttctcaaggtta

aaggaattactcctaaccaagtgacttcacaggaaacaggccccgcggcctttttttttt

tggaaaaacaataaatggcttaagaaattatcataaagttccttgaaatagactaacagc

aaaattgttcataacaagttgattcaacaaaactgtgaaaaataaccagcaaaaggtttt

acaccatgttttaaaacaacaatgtcaaacaaatgtaatacaaaaaccaatataaaatat

tcctcatcactaagcctacttcgaaatactgcttaacgattagccttggcaactctctct

atctcatcctttttcttgatagcatagctgttggatgaacctttggcagcattaataagt

tcatcagctaagcattcagcaatagtcttaatgttcctaaatgcagcttcacgggcacct

gttgtaagaagatagatggcctgattaacacggcgaagaggggagatatcaacagcctgc

ctcctaacaactccagcagacccgattctagttgcatcttctctgggaccactgttgaca

acagcatcaacaatcacttgaattggattcagatcagttagcaagtgaatgatctccatg

gcatgcttaataatcctaacagccttgagtttctttccattgttcctcccgtgcatcata

agagagttggtgagcctctcaacaatggggcactgagccttcctgaaacgcttcactgag

tacctaccagcagtgtgaggcacatatgtagcatgtttcgatggttgaaccccaatgtaa

tcactaagagacacatcactaagctgaatatcgtcgaagctccatctgttgaagagcttg

acttcgatttgattgggatcgacttcgacggcgacgggggcagcaacgacggtttcagcc

atgatcgagtagagagggtgcagccgtaaacacttccaatgctctggttgtaagcgggtt

cttgattaatgaaaacatccttggcaaatgctttcgcagttgttcttctttcataaatcc

aagaatttcacctctgannnggggtttgttgcacgtattagctctagaattactacggtt

atccgagtagtagataccatcaaacaaactataactgatttaatgagccattcgcagttt

cacagtctgaattagttcatacttacacatgcatggcttaatctttgagacaagcatatg

actactggcaggatcaaccaggtagcatccattaatgactccgcacacattgcaagaatt

gtgccgcccgaagggtgcaaaacttgcaaaagtgcggggccttatggctccgcacacatt

gcaagaattgtgccgcccgaagggtgcaaaacttgcaaaagtgcggggccttatggctcc

gcacacagtgcaagaattgtgccgcccgaagggtgcaaaacttgcaaaagtgcggggcct

tatgactccgcacacagtgcaagaattgtgccgcccgaagggtgcaaaacttgcaaaagt

gcggggcataatttagagcaaccattagtaagggacgcaatgggataaaccaaatgtcat

ctcaagtaccgcctccaagaaatcaatgacccgcgcgtaccgcaagagacgccacacgtg

acatctaagacggcacatacgtatcacttcaagagccaccacaacaccaagcaatgggat

gggatggttttaaaactaaccagccaaatgtttaccgttttataggcaagacaaacagga

acaagaaaccaacaccgattacgcaagcaagcgatgaaaattgatagggaatgttgtgtt

caccgttcggtgcaaaagcatggagccagcaaacacaaacatccgcaatgccactcacac

gcccccgcgtcagcaagctccaccatatgtctacaaatcaccaccccaatcttatccttc

accaactgcattttatccttcaccaactccttacatttataagtctccacctacaccata

caatccatatttatatagttcacctcctcctcccttgtattaaacttaatatgtattttg

attttcttcaataattttactatgatcaatattgtgtggtttgtaataatgtcttcattg

taataaagacttaaattttaataaaagatgtatatgaactctatgtgtatcattatgaat

cagaattaattttacagttgatttatttatttatt

>fava_dn_rep_c99

ttcatctcagaaaaattaagtactttgtggaatcagagacaatgatgattcgcgaaatct

caaaatctcattctaaattgggaggtgatagtgttactattttgaccttattttaatttt

attattattaattttttaaaataaaattaaaaaacagaatgtcaaaatctaactatccca

ataatacacgcgttaaatatgcgcacgaatctccagcaaacactactcctccgcttgttg

ctagtgtgcaccattctcaccttacgtacgccggttgcgcaacctcctaacgtgcgcatc

cctaaccaatccatcgaatctaatagcaccactatcgtttatcatcaccaccacaaagct

cagatctcagattccaatcccaacgaaatgtccaaagctcgcgtctacaccgatgtcaat

gttattcgccccaaagagtattgggattatgaatctctcactgttcaatggggtgatcaa

gatgattacgaggttgtaaggaaagtgggaagggggaaatatagtgaggtgtttgaaggc

gtgaatgtgaacagcaatgagcgatgtgttatcaagattctcaagcctgtcaagaaaaag

aagattaaaagagagataaaaatacttcagaacctttgtgggggcccaaatattgtcaag

cttcttgacattgtcagagatcagcactctaaaactccaagcttaatatttgagtatgtc

aacagtacagactttaaagttttgtatccaaccttgactgattatgacatacgctattac

atatatgagcttcttaaggctttggattactgccattctcaaggcataatgcatagagat

gtcaaaccacacaatgttatgatagatcatgaattgcgaaaactccgcttgatagattgg

ggtcttgctgaattttatcatcctggaaaggaatataatgtccgtgtggcttcaagatac

tttaagggtcctgaacttctagttgatttgcaagactatgattactcattggacatgtgg

agccttggttgcatgtttgctggaatgatttttcggaaggaaccgttcttttatggtcat

gataaccatgaccagcttgtgaaaatagctaaggtgcttgggactgatgaactgagtgca

tatctgaataagtatcatcttgagctagatcctcaacttgatgcactggttggaaggcac

agtcgcaaaccttggtctaaatttatcaatgcggacaatcagcatcttgtatctccagag

gcaatcgattttcttgataagctccttcgttatgaccaccaggacaggctaacagcaaga

gaagcaatggcacatccctatttctctcaggtaagggcagcagaaagtagcagaatgagg

acatagtaacttcatttatggatccataaacctaagttcgtttatgtggaattgttttta

tataccttaacatgtggtatggttgtcttctgttaattacagtcggttacttttcttgtg

gaatgtaacgatatcatatcttgttcggatgaataatttgtcttctgtttttgaaaatta

aaatttttacattaaagttacttaaagtctcaactttaggcaaatgtttggtcatttaaa

ttagttcttttaattttaaaaattattgtgatacaaattttttggag

>fava_dn_rep_c100

tttttttttttcgctcataaacacttgttcaagttatatatcatcaaatatgtgacaatt

aacaaaaaaataaaaataagttatgggaaaagtttagtaacttctggttcctcttcacct

tatacaaacttcaattgacataatattaatttgattacaaaatgtgtttatgcattaata

caaaacagatatgtttgaatttaagatttgaatatatattacatgtaggaacaattttaa

gaagtctaagaccaccatggagcatcacctgttctgaaatctgtttcaacccaaggaacg

aacacaaccttcccatcatccaaaccaacaccacaatgtgccaatgccaaagacaaaggc

gcaggtcctctcacaactcttccttggtcattgtactgagatccatggcatgggcagatg

aacttgttttcagcttgattaaacggcacgacacaccctagatgagtgcacacggcatta

atcgcaaatgttgcaagtgttctgtctttctctaccacaaggtaggtaggatcacccttt

aatccttgtgtaagagtacggtcgccaggtccatgagacttgagccattccgctgcaact

acatcgttgccaacggcatccttggcaacggtaccaccagttgcagaacctgagcctgga

ggtacaaggaaggaaccatatggaacaagcattccagcagtaggaagtgaaggagcacca

agaagaagcaaattcaaggtcttcctcttacccaaatcaggcacacgatcagcaggaatg

ctggtagcttggcaagtaattttcattcccttattacccttcccaatcatctgtgtcctt

gttggtttcacaagaaaagctattgagggacatgaaattccactctttccagaacatagc

agagatggggttgtgggagatagagtggtggaagacatggttgaagctcttgatgaagat

tcgttcccttgagaatgagagaggtgaatgtaataataatatgatgtttttgtttggttt

ttttaaaatgttaaacgaccaccacaactgtcgatggtttggttaagtggataaggggct

aaggtaatgtatgtaggtgcc

>fava_dn_rep_c101

tttttttttattaggaagcattgcctggtatatatacggtggaaaacattccgactccac

ttaagtaaatgaattacattacacaagttcatctcattcaatagtaattccataacaaca

acgattaattaagctctattacaagaacaaaattaacatgaacaaaccatattataaaga

tgatacaaagcattttaatttaattaacagtgacttgtccaaccataccagctccttggt

gaggtgagcagtagaatttgtaggtacccttagcatccaacttaacgctgtaagtctcac

caggagcgttgagaagatcttcttcaggcatggaaattttcgctgcatcaaccccggaag

gaatctcgtcttcatcaaagacaacgttgtgaggaaaaccagcattgttcttgaatacaa

tcgtgtctccagcagacacttcgaaactgtttggaacaaaagtcaaacccccatcactag

caccaagcaagacttcaacagccaaggcattgctagctagcactgcacttgcagcagtgg

caacaagagcaactccaaagtccttgagtgaagctctgacacaaagccttggtgattggg

aagatgaagttggaattttagccatggcactaactttggtggctgcgtttgccttaagac

ctgtgaacgatggaatagcaacggtggtggaagtgactgtggccatttttttctcaagag

taattggtttcttctctactgaatttgtatatgcttgaagggaatcgaaagagagacaca

gagggtgattttttatagaatggatgataaagatgtggattgggtttttgtgtggctaag

atgcc

>fava_dn_rep_c102

tttttttttttagaaaaaaatttcctaaacattttcatacatccatcatcaaagaaagat

gcaaacaaaaaagtaaaaaaaataaatagtaactctattccacacttaaaaattatcaca

aagctccatattatatataattatagatgtaattacacattacacacaacataaattaaa

acaaaacaaaaaaggaaccaaccacatttaagggattgccaacaacctcaaaagcaaaaa

ctttgactccttgtgtttaatttctgcctagtgattagcagcatattgattaatgagtga

aagagcattactagtaacttgagcaacattaaccattctagatctaatagaggctttgat

ttttccatcaagtgcttttccagcaaatccatcaccacaagtgctttcatcagtcaaagc

tgagctaacccatgtctcaacattacttatatgccaattgaaatcttcacccttgatctt

gcaaagtttaagctctttcaatgaacggctaagcctatcaacactatcactaatctcctc

agcacaatcatgaagagcagcatattctcttggtttaagatttttgaagcttttgcacat

tgtaacaaagccttttgtggattgagttttgttgaggctaagtgataaagctgtttggac

taattggtgagggtcttgttggattgtggatgcatagacagagagagattgaacacaaag

tgttggatattgagttgtgctacacgaggatttaatgaaacttgttggtgttgatgttgc

aaatgagagtagagttaggagaaggaaaagaagagcaaatttagttgccatgtttgaggg

ttgagatttggtttggtagagagaacaaaacaggttttgttttgaggtttggttgtcccg

cggccgctgcggatttgattccggtaaaatcaacgttcaagagttacctcggtcgtcctt

ggaaaaaatattcaagaacagtttatttaaattctgttatagaggatttgatagatccag

caggatggttagagtggaatggaacgtttgcgttggacacattgtactatggcgagtatc

agaatcggggtccgggttcgacgtagagttgtccctcatttttttaaatcggtccagaca

atttgaatgcctataacttgtatcacagacgtaagatctgcagcccttatcgtgagagct

gcaaagtaggagaacagcattatgtggatggtccatgcagataggacatgaaacttcatc

caattctttgtgaagagcatggatatctgaatcactacataatcttcgcttaaaaccagc

catcttcctcgaaccgtgttaacaagacaataagctctagctatattccctaccagagga

ttctgaatctcggtttgtaattataggacctttacttgacttcacaaaagaatcacagga

aatcacttaagcaacgctccaaagtacgatataaataaggaatcaatgtacattacaatc

ttggacatatgtaattttatcgaacacttctgtacaacagaatcaccgacaagccataaa

gtaaagggtgttgaatttaattatttttaatattattttttgcccttctttcttctgggt

tggtctctgcaaaagatcccaaggatgtagagaagaaacaagacacggaaggaaatcgga

tttccaaaataaacggtttttttttttatgtttatatatatggtaggaaatggagaataa

ataaacagatgataagggagtgtttgttgtggtttttattttaatggtaagatgaaaatg

cactatgaacctatcaaacaagat

>fava_dn_rep_c103

ggccgcgggacacttgagttgctcattccctttattgtgcccattctgaaaagatgagat

agtgagagaggatagaaagatagcctaagaaacacaaacagaaattgaaaactcagaaga

gagaatacccaaaccacacattcattctcacactatcatcatcaatgcaattgtaaaact

atgtcaactaccttctccatggcttctttctctcctctaactccaatctcaaccactcat

tcttcttcacactgcaaaccccatcaccacaacactctctccactctcactctcagacca

aaaaaccatcctttctttaccctctctcccttgagagtttctgctggaacttctgattca

cctgaaactgctatcaaacaacctccagaaaacatctctgtcggtacaaatggctctgct

tctgctgttactgtgccagaagaaattaaggtttccaatggttttgttgaccctagatgg

gttgctgggacttgggacttgatgcagtttaggaaaaacggtaccaccgattgggatgct

gttattgatgctgaggcgaggaggagaaaatggctcgagactaacccagaatcatccagc

aatgacagtcctgtcgtgtttgacacctccatcgtcccttggtgggcatggataaaaagg

tttcatctccctgaagctgaactacttaatggtcgcgcagcaatgataggattctttatg

acatatctggtcgatagcttgacaggagtaggtctagttgatcaaatgaacaacttcttt

tgcaaaactcttctgtttgtagcagtgggaggagtacttctcatccgaaagaatgaggat

gttgatactttcaagaagctattggaggagactacattttatgacaagcaatggcaagca

acttggcaggatgagaactcaggcaattccaacaaatgattagtgggattgttgttatgt

agtctcttaatatagtgtgctctctattattaatcttgctagtaaagaaaaaatacatga

atatgatttaatcattgtatttcaattccttttatgatcctaatgcagaatgatagagtt

ttttgcgtttgcaatgcaggatttttcttaagttgatgtgaaattgggttgttagtttga

gtaactcttaattgagttagatacacctaatttgacaaaaataacaaatggtttgaagtt

gaacttgtcaaatgaatcaatcctagaaaaaagcatacaatcagttgtacttgtttgaat

tatcatcaatagttttccatttgttgaagataatggcatctaatacaaaatgagtatttt

ccagttttcatctaatcaaaagtctgataccaataggtcattatcctttaaccgactaca

atatttattagaaagtttcacataactatctcaattagccgactaacactaagataccaa

aggccttgttgaaaggtaaaaaactaagacaaaaattctacttctgcagtagtaagcaat

ggtgtttctgatataactaaacaatagcaaaaatggttgaatcacttcttaagcttccca

tccccttaagcttcccatcccctcaaaagtgttgcacgggcgacgcggttcactgccaaa

gtaaaggctcccattcttagatcacattcatgaatcttgcacattgccttgatgtccaag

aaagctctgctcatgtacctcttcagctcacggttcactttttcttcatcccacataaaa

ccttgaatgttctgaacccattcaaagtagctcacggtcactccaccggcattagcataa

atatcaggtatgatagtgactcctctcttagacaagatctcatgagcttcagggtcagtg

gggtgatttgcagcttctacaacaagtttagctctaatatcagcagcattttccttgctt

atgactccacccaaagcgcatgggataagaatatcacattcatgggtgagcacttcatct

gcatccatgacatctgctccttgaaactcagtcaagtttccattggtttccttgtgtttc

agaagagcagcgatatcaattccattgggattttttatggcaccggtgatgtcactaaca

gcaaccaccttaccacctctatcaaagatagacttagcagcccaagttcccacatttcca

aagccctggatgacaaatgtgtgatcagaaattgacttcccattatcggctaaccaagcc

tcagtagcaaaaatcactccaagacccgtggctgcctctcttcccaaagaacccccaaga

tcaataggcttcccggtcacaacagcaggtgagtgtccgtgaaactttgaatactcatcc

agaatccatgccatggtctgagcattggttcccatatcaggagcaggaacatctcgttga

acaccaattagatcgtgaatcttttgggtgaaaactcgagttagacgttctaactcactg

atgcttagatccctcgggttgcaaccaataccacccttagcacctccatagggaatgtca

gttacagcagtcttccatgtcatgagctgagctagagcattcacttcatcaggttcaacc

tcgggatgataacgaatccctcccttcataggacctcgagcattgtcgtgttggatccta

aaacccacgtaagaggccaaagttccatcatcttttgggatcgtgcattcaaccttgatc

tctctgaagggaatgagaagactcttctcaagtttggaatccaaaccgagaatcctagcg

gcacgttggaaattgcggtttgttgctgctaaagcattcatagtgatgaattacaagaga

acagtgatgaagagtgaagtgttgttgatagagaaaaacaacaagattgagaatgaatga

atacaagaaacacaataactcggagttttata

>fava_dn_rep_c104

gtaataacgcagaagagataagagaagaaaccaagaacacatttcgcgtggcgagttttc

aaaacaacaaacaatggcatcatcaacgttatcttctctctcctcaaccttttcactacc

gttgcatcacagtttatccgcaaacttgaaaacttgttctcaatttcccaacaaaacttc

gggttttatggtttttgctcaaaagaaaacaaagaaaactcgaaagataatactgaagga

agatatggttaatattggaaagaaagggcaacttcttgatgtgagggctgggtttttcag

aaatttcctctttccaactggcaaggctcaacttgtaactcctgatatactcaaggaaat

gaagatagaagaagaaagaattgaggctgagaaacaacgggtaaaagaagacgcacagca

acttgctataatttttgaatctgttgggactttcaaggtgaagcgtaaaggtggtaaagg

aaaacagatctttggaagtgttacggctcaagatcttgtggacataataaaggcacaact

tcaaagggaggtggacaagagaattgttgatcttccggagatcagagaaacaggagaata

tattgcagagttaaaacttcacccagatgttacagctaaagtgaaggtgaatgttatagc

taactaagcagcactaaaatgtttcaaagttccatatgctaaatgcacaattgggagtca

caaacttgcatagcttcaaggtgcataacaaccatcttatgaactatgatgagaaatgtt

cagacttaattcaagcacgacgtagtagtcttgtgactttggccgaaggctcggacgagg

aggatgtgtagattgtttcttatttgaacattttgttttttggtatctccttgaatgtgt

attcaattttcaggacaatttttgtaatgttcttatttatgatggtaaaagagatatttc

ttgtcagttcattaacataacgaggaaaagaaaaatgaacctctagtttttctagtatgt

cttacttcaatgatcaatttgtataaacttaagtgtgaaaatctttgaaagcaatatttt

tggactttgttacatatcaataaacttgcttaagattagt

>fava_dn_rep_c105

ctttttttttttaaaacccaattaaaattttctaatatatatagagtgatagtacaacac

aagcagtaagagaatccagtccagtctagtgcaacaagaggataggaccacatataacat

gagcctaaaacactcactcattacacgttattcttaataatagcattaaatggcactctc

atatactgagaagtttccataaccttgaaaccgcagcatgttcttcaaaagttaatggtg

ttacagttggtggaggtactgatcttataaggaatgttgacaccacatttgccagggaga

gcagcagcattgttagcattcaaattagaaatagcaccagcggttgatttcaagcagtta

caggcagcacgacgatcaggaacggtgggggcggcagcaagaagcttcttcactcctgca

cagcatggtggtgaaggaccaggaccacctgtaagataagcaaggcacggagcaagatca

gcagttacagctccacattggaatgcagcttctgccataggtgcaactaacatgcacata

gccaaaacaacacatgctaccttcatgcttcttgccatttcttttctttcttttgaattt

tttttttcttggtttgtttgattgggtgtccccgcggccctaagttcatgtaatgttaaa

cttttttagttgggctacacttgcaaaataaaatcattaggaattgatatggcttttgaa

atgagtgtataacttatctgtcatgaatcctataaacttcgagagctctcgggcattagt

gttagtgcatgagatacttttagtgaggagaaaaagagagaataataaatgagataaaaa

tttgtattattgaattgtacaaataatgatagacactatgactattt

>fava_dn_rep_c106

agaaagaaacgtttttagctgcagggcagtgactattgggaaagttgaagtatgtgttac

aaagatgcaaattaagatagactgaaaattgttaagcaaatcaaacatatcacggcggca

ttaaatatcaaactggaaatattatctatatatagaaatcagcacaaggaaacaaaaatt

gcatagacaaaattactcaatgcctcaatcaactggatatcagaaaaaccgagcattgta

cagagttttacaactagaaatactgttaacaatgtctagaaaattgaaaagaaaacacca

tgcaaattctttactacttttactgatcaccaggaattgacttaatgatgtcggaatcac

caggatcaacaatgctgaggcagcacactctgtagtactttccacatgcagtgcccaagt

caacattgtttccattgtaatgatgaactccaaccttggccaacatagcatagtattcaa

tttcagatttcctcaaaggaggacagttgttagcaatgataatcaacttccctttggaac

tccttagggacttgagaacggttttgtaccctagggtatatttgccactcttcatgacaa

gagcgagcctgttgttgatgctctcatgagtcttcttggtcttcttggccgcaaccattg

tcgatggattgggtggcgatttacagacaagatgcggctgcagagatgaaaatggatgca

ctgccactaggcccgcggcctcctattttgaagattaaacttttctttgtaaacttaaac

ttgaatgagaaatggtgtattatattgt

>fava_dn_rep_c107

attcagaaaggactgttatcttataggtatagcaatataaaaccaagtatgcttattgtt

aattcatccacattcgataagagagtctctctgtggtggaggggttttatgatagtatga

ggagggacaaaactggttttctagtaatattgcttgcaaactaggagatggaagagatat

tgacttttggaggagtaaatggattgaaaagagcatcttagtaacttgtttccggaacta

ttttttgtttcaaattagcaggatgagaaagtaacaaaaatgagcagcttgatcgattag

aggtgggagtgggatctttgagtggcagaaaatgtattgacagatgtcacacgaaaacaa

ttggaggaactgaaattaatactagttggagtagagccaagaagttaggctaaagataga

tttgtgtggatctgtgaccaacaaggattttctctgaaagattgttatttgttgttgtct

aaacgtgtgtgtgcatgtacaaacatggaggaaaccatattgagatcgttggatacaata

tgaaaatatagtgtcccatctagaaaaagagtttttgatggagattgattatcaacaagc

tcccaactagagtggaatcgtctaaaagagatgtaatcatcggaagccatggcgtggtat

gcgcgatttgccttatagaagaagaatttgcatcacatctatttctgtcatgcctattcg

ccggaggtgtggaaattagtgcttgaatggattggcatggaaggttagtgtcctgctgta

acagtttcagaaatcttcatgttgttccaaaccagtttgaaggattaaattaaaagtagt

ctaaggagcttactatggttatcaacgcagagtggccattacggccgcgggctcttcact

ctcagatcgctccgaacctcacttttacatccctaaatctgtaaccaggagcgatcagag

acagaatttccaatcatgttggtgtaccaggatctccttacaggtgacgagcttttgtcg

gactcttacccggccaaggaaattgagaatggaatgttgtgggaagttgaggggaagtgg

gttgtcaagggagctgttgatgtgaacattggtgcaaacccttctgtcgaaggcggagaa

gatgagggtgttgatgacactgctgttaaggtggttgacattgttgatgtattcagactt

caggaacaacctccttttgacaagaagcagtttcttggttttgttaagaagtatattaag

ttgttgactcccaaactagatgctgagaaacaagagcatttcaaaaagaatattgaggga

gcaaccaaatacctccttggcaagctcaaggaccttcagttctttgttggtgagagcatg

catgatgatggtagcttggtctttgcctactacaaggatggtgctgctgatccaacattt

ctctattttgctcatgctttgaaggagatcaagtgctaagtgttgtattgcacttgtttt

ctatcttaatttatcatgtttttgttgttctaagttattttgagagttggagttaagact

aatttggtaatttttgataatgatgtgatggttttgcacttagtatcattcccatataat

ttaatcaggttggtaataattcataacatttgattttaagtgtttaatgaaacatgccac

acgatttctgtgtttcaagatttatttggtttgttggagaatcttaatttgtgtgtacct

aagtagttaagtttatggtggaaactttatgctggccaataatttagaggagtgttattt

gaacaccttatatttaacaccgaataccaatgtanntacctctcgtattttttttatttc

>fava_dn_rep_c108

caacagccacaaccttgaatttgcattgaactaaaatgaaagtacttacctagccactcc

ggtcctcatttcaacaagttgtgaaataatcatcaaaacttcagccatggcagctgcttc

tcctatggcaagccaactcaagtccaccttcactagacctttggtcacacctaagggact

ttgtggctcttcaccacttcatcaatttcctagaaggcaatttaacttcaccgttaaggc

catccaatccgaaaagcccacataccaagtggttcagcccatcaacggcgatccattcat

cggaagcctggaaactccggtgacatcaagcccattggttgcatggtatctctccaatct

accaggctatagaactgcagtaaacccactactcagaggtattgaagtgggtttagccca

tgggttcttcctcgtaggcccatttgtgaaagccgggcctttaaggaacaccgaatacgc

cggaagtgcaggctctctagcagctgctgggcttgttgttatccttagcatttgtcttac

aatctatggagtttcatccttcaacgaaggcgatgcatcaactgctccagcattgacttt

gacgggccggaagaagcagcccgatcggctccagacagcagatggatgggccaagtttac

tggtgggtttttctttggtggaatttctggtgtcacttgggcttttttttcttctttatg

ttttggatcttccatactttgttaagtaaacagattaactatctttcttagtttctgttg

cttttgtgtggtaatgagatgatggttgtaattacatgctgtattttattaatctcagtt

tatcattataacaatgttctaatgttttattgaactaaatattaatggaaattgatcatt

gaaaaggataaaaaaaaaagtgatgatgtggatggaattaagcaaagtaacacagaaggt

gagcttgttcaagaagcttcagatgctgtggttgaggaggcaatagtagaatcggaaaag

gaaaagtcttctgcagaagaactgaatgccgatccaatggatgctgatagcaatatcgat

gataaaacaaagagtaatgatgcagaggaaattaagcaaagtaatacagaaggtgaactt

gttcaagaagctgtggttgaggaggcaatagtagaatcggaaaaggaaaagtcttctgca

gaagaactgaatgccgatccaatggatgctgatagcaatatcgatgataaaacaaagagt

aatgatgcagaggaaattaagcaaagtaatacagaaggtgaacttgttcaagaagcttca

aatgctgtggttgaggaggcaatagttgaatcggaaaaggaaaagtcttatgcagaagaa

ctgaatgccgatccaatggatgctgatagcaatatcgaagataaaacaaagagtaatgat

gcagaggaaattaagcaaagtaatacagaaggtgaacttgttcaagaagcttcaaatgct

gtggttgaggaggcaatagttgaatcgggcaaggaaaatccttctgtggaagaaccgaat

gccgatccaatcgatacagatagcaacgttgatgataaaacaaagagtaatgatgcagag

gtaattaagcaaagtaacacagaaggtgaacttgttccagaagcttcaaatgctgtggtc

gaagaagcaatagttgaatcagaaaaggaaaatccttctgcagaaaaactgaatgcagat

ccaatggatgcagataacaacttcgatgataaaacaaagagtagtgatgcagaggaaatt

aagcaaagtaatacagtaggtgaacttgttcaagaagctacgaatgctgtgatcgaggag

gcaatagttgaatcggaaaaggaagtgtcttctgcagaagaaccaaaagtcgtcggttca

gttgatgctgataacataatcattgataacttaaagagtgataatgcagaggaaattaag

caaagtaatgtggaagtggaagctgaaaatttggctgttgagaaccctcaagcggaagaa

acaccaatggcagaaccagaggaacaacttgttgaagaagcttcagaggccatggtggtt

actgaagaacctcaagaggaggccccagttgaattggaaaatgaaaacggttcaattgac

agtagtaagcaggaaaaattaggtgctgaggaaaccgaaggagctgagaatgcgagtctc

aacgttgaagaaatcaatgacacggatgaaattccagcaagtgatgagaaacaaacaatt

caagacgaggaaacattaggtgctgaggaaaccgaaggagctgaggaagtgagtttcaac

tttgaagaaatcaatggcaagaatgaaattccagcaagtgatgagaaacaaacaattcaa

ggtgaggaaaaattaggtgctgaggaaaccgaaggagctgagaaagtgagtctcaatgtt

gaagaaactaatggcaagaatgaaattccagcggcaagtggtgagaaacaaacaattcaa

ggtgaggagcaagtgaagatggttggcaatgaaagtgttatttggagcagtgctcaatga

aagtatattattaccattactatcacttagtgtaagctactgatatgcactttattaata

cctagtatccttaattttttgtaatgctatttaagatgtttagcactgacattttagatt

gaaacacattgacacaacaccgactcatgtatcacagtagtgtctggtgtatgtgtccgt

gtggtttaaacagctttaaaggtttatttattttgttaccttatggatgactggatgact

gtaatcttgcattctgtttcaacattttgttaaattaggagattaaacttgtatgcctct

ttaagatttgatcttttggttttccgtagttcatattgaaactagattgtagacttttgt

cacatttttatgtgaacaaaattcggaattgatttaatttc

>fava_dn_rep_c109

agagaacttttagggcgaaactcaaaataaaacgacacctaattaagtcttttcctcctc

gattattccagaatcaaacaaactgcaacgaaaaaaagcgagtgagagagaacaaagctc

aacaatggcgtcgttgctacaagcgattgtagatccaaagaaaaactggttcgccgctca

gcacatgaaagccgtttccaaacgccttcgcaaatacggtctccgatacgacgatctgta

cgatccgtattacgatgttgatatcaaggaggctctgaatcggcttccgaaggaggttgt

tgatgctcgccatgcgcgtctcaagcgcgctattgacctttccatgaagcacgagtatct

ccctgagaatcttcaggcaatgcaaacaccattcaggagctaccttcaggagatgctgac

ctttgtgaagagggaacggacagagcgggaatcattgggaggtttgcccctatatcaacg

aaccattccataagtgcattaagtactgcatgaagctatattttgattggaattggatgt

tgaaatgtattagtattgtggtagtatgttttcgttgaagaagaaatcttagacagcttt

gatgaaaccttatatggaccacaattttttactgctataaactttgaccattttctaata

agtggcaacaataaactgggttgccttcataatttattgtttgcttattcttttagcctt

gtctttattggaatactttcttgtttctagataaatccgttgcatactttttgaacatac

cttgcattcctatttgtaacttaaaaataaaagtattttt

>fava_dn_rep_c110

aaaaaaaacnnnnnnnnnnnnnnnnnncacaaaattcatgttagtgtaaagtggacaact

acattcccattagacaagcgaacagtcctcaatcaaaacaaattgaaggaaaaaaaaaaa

ctatatttaatctgattaattaattttaaaatgaataatagtatttatatagtttcagcc

tataaaacccagtacaaatattcatatagtaaaatcccattacaaataatatcccatttg

gttaggaagagacaaagaagtaatgataaaatacatttttcaacttgaatctcttcaaaa

caaacactagctcaatagttgtttcctatctgaccactttgtaatatgcaattttgcttt

tgctgcctctttctcccaatcacacttaagtgtaatccaacacaatatcaatgtctgagt

tgctgttcctccaaatatcatccctgcccatattcccataactccttgcttaaagtacca

acccattataaatccaagaggaactccaataatatagtagcaacccaaatttatgtatgc

aacatatgattgccatcccgatccgatggccactcctgatagaactggctgaatgctatt

gagaagaatagtgaatgccaatagaagagaaagcttgcttacttcatcaagaacaggttt

actagtagtaaatatgtaaccaaatttattgtgcaatgccaaaatcagtatccagaagaa

aattcctattgttaatgatgtcaacacagctacaatagtagcaaattttgctccttttcc

atttccagctccaagctcatttgcaacccttactcctgttgcagcaaagaaggccaaagg

aatcatcatctccataccatttactgtcatacatatggacaaagcatctactgcaatctc

tgcgttaggtagattcccagtcatcaatatcagaattctataataccaattctccaaaca

tagcataacaccagaagcagcagagagtttaagaaattggccattacggccgcgggaatc

attttcttctttacacttttctctttcacccttgtttcattttcttctcctagagagaaa

gaagaaaaagaagaagaaaatgtctacattgattttacctccaattcctccatctcctag

agatgatgccatgcaactctaccgtgctttcaaaggatttgggtgtgacacaactgctgt

gatcaatattcttgctcatcgtgactctacgcagcgtgcgtacattcaacacgaatacaa

agtaacatattctgaggagctttcaaaacgcctagtttccgagctaagtggaaagttaga

gactgcggttctgctttggttgcatgaccctgccagccgtgatgcagaaattattaggaa

gtctctaattgtggacagtagccttgtagctgctaccgaagtaatatgttcgcgcactcc

atcccagctacaacatctaaaacagctctaccattcaaagtttggtgtttatctcgagca

tgaaatcgaagcaaacacttctggggatcttcaaaagatcttgcttaaatatataagcac

ccctcgccacgaaggccccgaggttaatagagaaatagctcagaaggacgcaaaggctct

ctacaaagcaggagagaagaaactaggaacggacgaaaagacttttgttcacatattcag

tgaacgaagtggcgcgcatttggctgctgtcagttcttattatcatgacatgtatggtca

ctcattgaagaaggcagtaaagaatgaagcatctgggaatttcggtttcgcacttttgac

aataactgaatgtgctaataatccagcaaagtattttgcaaaggttttgtataaggcaat

gaaaggtatggggactaacgacgacacactcataagggtaattgtatcaaggactgagat

tgatatgcagtatatcaaagctgaatattcgaagaaatacaagaagacattgaatgatgc

ggttcactctgaaacatccggcaactatagggcttttcttctcgcacttttgggtcctaa

tcattagtcaattgatgttcaattatgtttcattttggatattttcaaactttgatgttt

aaatgttttgaaatttggttgtttgttgtcctttgtaagggatttgtaagcatcatcatg

aagactcttggtatatccaaccctccttaaaacatttcttgaaggctcttggtttgcttc

ttagatccatgaatttggcactagaaagtgtcttagtctaaatgtatgcctattgctatt

tagaattgcaatgcaacaattgatcatctcttttctttcttttcagtttctcttatagca

tcaaatttgacaatgatatgcttttttcacccatgttgaattggattattggcagttgat

atgtctgatttgttgtcacactatagcaatatagaagtagcttttgtagcc

>fava_dn_rep_c111

ctttttttttttaaatcacaacacattaattttcattgtcacaatttgaggaaccagtgg

cacatagtattcacaacataaagtgccataatcatagtcatagtcatcagtgaattcaat

aaaggatcaatctacacatccttcactaaaattcaaagagactcctacacggatagcaaa

tatgcactaagcagtgttagtggaactaatcaaacaaacaaccgaaacaagtaatgaaga

ttgcgagttagttcaaggaaagaaaaggtagtaaataatcaacaacaaaagtcattactt

agatacatatcaatatatcatcacagaaaataactgaagtacttttctacattccatgat

tgatcatttcaagtaaatttaaagggacaaaccagactcttcatcctcatcaagacctga

agcatagataaaatagagagcccaaataagaccaaacacaccaaaaaggatccaaccaag

aagattgttactgagtccaaagggaagaccagttccttcagtgctcattctttcatccac

taatgccatggctgctgggcttgacatggctgcagcacatgcagctgataacaaagatgc

tgtcattcccatctttgagttgctttcattcactgaagaaggctttccttccattgaaca

cctcactcttccaaccttagccattgttggtaatcctagaacaggagaggatgagactgc

aagaggtcgcttctgtgcaagacatgcacgggagattgatgcggttgtggtggtagctga

gatggttgccatttttatgttttgtttgtgtagctgagatgattatgaagtgaagtgaag

tgagtatgaacaagttacttccaagtttaggtgaagtttgtgaaaattaataataaggtc

agtcccgc

>fava_dn_rep_c112

aactattgaataacaatttccttgatatttacatgattttaaaaagcacaacatattggc

aattcatattgtcacagttaacaacaaactaatagtactaactagttttaaatgaaatac

atgaatactactcaatatatagtttattaaatatcctcgcatatcaaactttaaaacaaa

tcacagctcaagaggctaataaatttgcaaatatgatctagataacacagattttacatt

atccagaatcgttatcagaagcagaagcaaaaagaacaagaggttacatacaacaacaac

gacaacaacaatggtagctatggctatggcttctcttcagagttccatgacctctctctc

cctctcccccaactctttcttcggccagtctctctctcccatcactctctccccttttct

tccgggcaagacaactgagaagaaatgtcttattgtaatgaagcttaatcgatgggagcg

aaaggaatgtaaaccaaacagccttcctgtgttacacaaattgcatgttaaagttggaga

tacagtgaaagtcatatctggacatgaaaagggtgaaataggggagattactaaggtcct

caaacacaacagcactgtcattgttaaagacataaacttgaagacaaagcatgtgaaaag

tagcgaagagggaaaaccaggcgagataattacggttgaagctcctatccacagttcaaa

tgtgatgctttactccaaagaaaaggatgtagctagccgcgtaggtcataaagttcttga

caatggtaaaagagtccgctacctgatcaaaactggagagataattgatagtgcagagaa

ttggaagaaactgaaggaagctaataagaaaactgctgctacctagatttactctattac

ttcatgtactcagcagttatgtaattttatattttttgctcattttattgttaactaact

gtatattatgttattgttttcgttgcctatgttgatcttcatatacaacttagtaattga

attaatttgttgtccttttaaagagagaaaatgttcttgactctctctacctgctggaga

atagtctcattcacttgagtctggttcccacacgaaagcgatacattggaggaattatag

tggggctatatgcttcaaaattatgtactcaatcaatccttcctcttgagaggataagcc

ttgataatgtgagttagatgtgttataatattataatattattatcttgttacg

>fava_dn_rep_c113

ttggtagcatatccgatctggatatgtgagaagaaagtggtaagtattagtgaaaaaaac

gaagttaaattaatttgcattaaaattaggtacagcttctccttttcttagatatcatcg

atccatttcaacactatttcttcttcctctcctctccgaggcaacaacaactcatctcat

ctcttgtctcaccatggcttccgccaccgccgccgtgtcatcttctttcctcctaaccaa

ttccggtgtatccaaaaacaagatggatcactctattctcaagatcaaaccatccacttt

ctcccttcctcaacgcagaatgcacctttcttcaactacaaagcccctaattgttcaagc

tgcttatagtgatggtggaaggcccaacagtgctggcatctttgttggcggctttatctt

gggaggattaatcgttggaactcttggctgcgtgtatgcacctcagatcagcaatgctat

aactggacctgacaggagggagttaatgagaaaactgccaaagtttatatatgatgaaga

aaaggctttagagaaaacaaggaaagtattggctaataagattgaacaattgaactctgc

tatagatgaaatttcttcccagcttcggtcaggggacaccccaaatggggtagccgtgaa

atctggtgaagttgaagctgccaaatgagagactgttgctttgatgttgtctgttacact

gtttgccgaaaatatcgttggtacaaaatccatttcgaacttgtaatatctttccatttc

ttcatatagtaataaactaagttgatggcagcctctactaggtaaatttgtcatttcaat

tagaggcttcttgtgtcctgtttctatctgtcctttttagtgaaagaatatggaaataac

tcttagttaactgttttatggtttgtatcgtatgaataatcaggtatccaattgcttaga

agggtgaacaatgttactttggattctccatattaggatgttgtcacttttactgcaaac

atggaatagatgataactatgtattcatattttatgatgtttgtcctatgtatgttatgt

tgctaataactctgttcactgtgcagtcttcatgtattataatgcataagtttctgaaaa

tgatttttagagcatctctttgcaatctataataactgatgatatcatccatttctaaag

agagacaaatgtaacatgagttttcatgtattatatgtttgagagaagatgaaatttata

cttggttcgggttt

>fava_dn_rep_c114

aaggatcgagtttagttataattaactcctcaaaattaataaggtattttagctcaatct

tttatcaatagaatggattataccttactaaaaatataagctccaaagagtaaggtcaac

agggttgccaccacacaaacaagctataacttttttacataatcatatcacacattaaag

atcacaaatgcatcttatggagaactgtgaatttggaaacataggtatccaacttttccc

agaacactcgtgtgagttgggcgctataaagaaaagacacagtcccttcaaggaaatgac

cccaatgaattataaagcacgataaagcaaagtgatttcaaaacattaatgcagaaaagt

agactgaattacaatagctgctcactaattcaaaaattgaagaatccaaggggatgggag

tctgcaagatgttgctgcaactatctcaaacttgcacatcttctgaaaagaaaatgtaca

gtccactacagaaaactcaactaagaatagggaagatgcgagtaatattctccaatggta

cactttcagcagtgtttttcttcaaaattggatcattatattcatttctgggaaggacga

tgagttgacccctaccctggcttttctcaattacccaaccagagtttgctacttggtgtt

caaggaatttgtccaaagctaaaccttctatgttgatagcctcagcaagcacagttctag

gaaccttttggtaagtcaatgataagacatgaactgcatagccttggattgcctgctcaa

atcctggcacagtatcaagtatatgacggttttttgctgcttcgtcccagaactgacgaa

accttccggtctccaaatagtgagacagaacaatcaatgtcttgaactggtcctccattt

gcacacgttcgggaatcaaaaagagacatagactgaagtctggacccggcatagccatga

gtgccttaaccaacatgcgagtcacaatctgggaactcagtttctcaggttcaaactggt

agaggcgaagaaggcagagatttgattcgaggctgtaagtctgttcattgacggagtttt

cgagatctggaaaaatatcgggattgtaacgattgaatgcgacgagctgatcgacggtgt

aatcggtttcttgaccttgttgcggctttgctggttctcttcccatctctgacgaactca

ctctcagttgatatgcgcaaaaccctaatttgctactatcttagagagtgtggggacgaa

aaatacatattcaaaattgtttaat

>fava_dn_rep_c115

attgtgccaatagcatatttagtgtttggttggttgttttgttaatctaaccccagctca

caagcaaatagctgatgcataggtttgatcttcattttcagatctcatctcatttaatat

ggaactcttaatagatactgaatattcacatttaaatttcaggaaatcgataaagtcggt

ttcatctatactgcatgatcctcaaaattatttcttgcaacgattccgtaaacaatattg

ttcacaagcaaggtataattttttgtcataccaaaattacagggttgtaatgtaacctat

ctacagaatacaggagggtgatatagataagatgctgcacaaagtggaacttgttatcaa

tgcaagtgaaaagaaattaatagacttcaacattccattgctccgccttcttcaacgttc

tcttgtactcgatccaatcgataccgtctttggcagcgggtggcaccattatgtcatcaa

ttcccttttccattcctttcagtccacacatgtaaacaaaagtgttgtctttcttcagta

attcccacaactcttctgcatattgagccattctggtttggatgtacattttctctcctt

tatcatttacttgttctctgctcacagcaaagtcaagcctgaagttctcaggtgctttct

ccttcatcttttcaaattcctccttataaagcagtgagctgcttgtagggacacctagga

agagccatgccaaaccattgaacttgtaatcttcatgcttctcaaagaacattttccata

aaaatgagcgaaatggggcgattccagttccagttcccaacatgatgacagtggcattag

gatcttttggcataagcatttctttaccaactggtccagttatctttacttctgatccgg

gcttcaaatcgcacaagaaatttgagcaaactcccttaacaacttctccggcatcattcg

tgtacacaagacgtttcacacatagagaaacagttttggagtctccaaaatcaccaatgg

cactgctagcaattgaatataatctcagcttgtgaggcttgccattcttgtcaataccat

ctggaactatcccaattgattgtccttctctgtaaggaacctctccctcagtggtgaaaa

ccatgtgccaagtttcaccaggtgcatcatcaccagtaatctttgtgttgagaagacacc

tcccaacgtatggctccttgggtttgaacttgttcacaacaataccttcatcttgtttct

ttgaatgctttactaccttcgtaacaggtgcctctgcctcagtagtaatctgagctctga

tggtacctacccttccacttatagaaacactgttcaatgaaacctttttgaagacaagtc

tctctggtgcaacaatagatgtcctgatcggaagagaagtggagttggagtatggcaagg

acactgcggctgttactgcagcagccatgattgttgttgatggaagaatctagaagaagg

agaaagagagaataatgttgattaattaagtgaagatatgtatatgcagtgatgagtgtt

ctataggaaagtgtcgttttttttttctggttaaacagaagatattatgattgagtggat

gaaatttgtaagacaaagaagtggtagatatcatgtaaggataagtctatctctatgtat

gtatgattcttaaacttgcgattaccattttagcctgaggtt

>fava_dn_rep_c116

gttcactcaattttttttgataaaaaagtattacactataagaacccatttaaatttttt

catgtgtannnnnnnnnnnnnnnnnnnnnnnnnnnnnnnnnnnnnnntgcttcataatag

aatgaaagaaaacttgagattttcaaatctacaacacaaattttagtgtaaagctgtata

aacccctttgaaacaaaattcatatttgtgaaacactcttcttaaacccatttcgtattt

gaaaatcagcaaattcgtgataggtttataacacaagcatgcatcacctaactaccaaat

ttttgttaagaacaaaaaacaaatgtagcctaccaaaatgataggcataacaaaacttct

acataaaattagggagcaaggaataaaatctccatactgaaaaattcatcaaacataaaa

taacaacaatgtttaggatgtaggaaaaattcggaatttcagatactaataccagcaaaa

aactactatcacatcaaactctgaatcctcggctgtttctctttcctctagccttctcaa

acttccttccctttgctcttacataaggtttggtgtggctgtgtggaacaccaggagcac

gaccaaagtgcttcactgcttcacgagaattctttggacctctaagaaggaccgtgtttt

gtcccagaggggccatactagccaagagatcgaatgtcaagcattctcctccagcctttt

caatccttgcccttgcagtctctgtaaacctaagtgctacaactttgatggctggaactt

cgtacacacggatatcatcagttacagcacccacaactacagcaattctaccttccttcc

cctcagtatacttgatcaactttgacaaagatagaggaggcctgttaaccttgctcatga

acaacctcttgagaataacagcgttgaacttgctgccagttcttcgaacaaggaacttgt

aaagcttgaccaagagcttgaggtagatatcattggactttggtgcagttctcttggact

tcttgttcttacctcctgctttcagatcgatacccatggctgcttagggtcttcaacacc

tgcgcgactgctacaactgctacaccttctgcaccttcaaattccccgcggccccgctct

gccaaccttgcagataccaactctttcaacatctccttatattccccagcatgatgctcc

agagtccatcaacatcagcagccgggccacaaatcttgtgaagccttctttttttattcc

tcccccttcttctgcatcaatgatgttaccccctgtatcttcatccggacctactgctcc

tccgcttcatcctactggtacagtacagcgtccttatggtactccaatgctacagccatt

tccgcctccgactccaccaccttcactaactcctggttcttctccccttccaaactatgc

tccagttatttctagagaaaaggtccgggatgctcttctggtgctcgttcaggacaatca

gttcattgatatggtatatagagcgctgctgaatgctcatcaataatgactttcaagagg

tgtttaatcatcaattcactggagaaattgttttatgggctgagttacattttctataag

ttttaggccaggctttaatcatgtcctaaacttttaaatctttcagtcataaccataaca

atttgtaaaatcgattctacattgtgactaccattttgatcttgctagtttcctcagtgt

cataattcagtttgctatagttaaacggggatatatatggcagcataacatggcaggaaa

tgacaaagatctggatgagtagttatagcaatgcggaattgattttacaaatcattatgg

cgattggttgaaacccatcttaaggttataggagtggtaaggttgttattaagtcttgtt

tattgtgtactttattaaattgttatatggtaagacgactttgcatagcaccttttgtta

gtgtttcaatgacatggactctgctgtataatgcaagtagatagataggcctttttatta

atggattacgctgcagttaagaagttttttttatggattatgtgtaggatacatacatga

aaaaattctaataaatatatatttagtttggccacg

>fava_dn_rep_c117

gattttggttactgaaatttttatgcagattcaaatctcttcaagatgagtagttgcagg

atttctggagtcatatatgagtgcttgctttttgatatggatgacactctctatccattg

agcttagggatcaatttggcatgtcgcaagaatattatagactatatgttagaacacttg

catattgaagaaagtaaagtatcaaagatgtgtctggatttgtatctcgaacatggaaca

actatggcaggaatgaaggctcttggttacgagtttgataatgatgagtttcatgctttt

gtacatggaagattaccatatgagaaactaaagcatgatttagtgttaaggaaccttctt

ctttccatgcctcagcgtaaaataatattcaccaatgcagatcacgcgcatgcaattgaa

gttctcagcagacttggcttggaggactgttttgaaggcatcatatgctttgaaacgctt

aatcctctcaactcttgccgacaaatcctctgcaaaccatctgttgaagcctttgaggct

gctattagaatcgcgaaagtggatccaaagaaaacgattttctttgacgacagtgttaga

aatgttgcaagtggaaaagtagctggatttcacactgtgattgtcggtcgttcagattta

gtgccgggtgcagaccatgctctcaacagcattcataatatcagagaagcattacctgaa

atatgggaggttgaagaaggcaaccaacagcaaagaatccaatcgcttgcagttgaagct

acggtccatgcataggttttgcttcgataaaatatttcactaagcttatcaggcaaatct

agagttttacctcagcagaattttgtctatctaaattacaataatgttgcaaactgtctc

cacagcaaagtggtgcttaaatgtatgaatcttgcaaatgacatcacgaaatggatgcga

gactattaaatggaaaagtcttaagcataaatttatcatagaaaattacaattactgttc

aaggattttggtacaagtcgagcattacaaaatataggtcttgcactctcttaacaatat

tattcaccaaataccctttcagtaacattaaattttaactgcggcagagagcaatgacaa

ctcaaacacatgaatcgaacaactccgttgcccaaacaccctccaaaactgaattgcact

cgacagccagaagaatgtacagaactttggagtaaagatcaaccatctaaaggcagttct

ccagacttggcaattttgcacggtttcttgggactattgtctagcttgcttgtctcttgt

gattcaagttgcttcacaacatccagtccttcaatgacatgtccaaacaccacatggcga

ttgtctaaccatggagtcggtacagtgcaaataaaaaattgactgccattggtattagga

cctgcattcgccatgctcaaaactccaggaccaacatgcttcaagtcaaaactctcatct

tcaaatttagaaccatagatactgactccaccagttccatttccttcggtgaaatcgcct

ccctgaatcatgaaatttggtattatacgatggaagaagcttccttggtaaccatatcct

ttcgctcctgtagacaatgttttgaaattctcaacagttttaggaacagcatctccaaat

agaccaattacaatccttccagcagattcacccccaatttctatatcaaagaaaattttg

cttgtcactttggcttgcagctctgcaacctctccctgagcaccactagcacatgttatt

ctccaagaagtgattgatttggctttagattgttgtgaaacagaaagccttggtgcataa

tgatatgttgatgacaatttactgcatccaatgctagaaactacatgtggcttcccctgg

acagcatgaaagcgggggagtaggttctgactctgcaccagctgagttgagaatgaagat

gccatggatggatagtgaagaagaaaaatgagttttttgggtgggaaatatggcgcaaca

gtttctggatctcacacacaatcttctttgcactcaggtttatcttgttacttctctcac

ttaaactgaaacctttaaagctcacaacggcattgtgttatcttaaaggataaagtgatt

>fava_dn_rep_c118

cttttttttttctaataatgcacctttattccaaaaagatgtatcagactcaaaaaaacc

aagtaacaaactccataaaccaggtttggagttttacattcagtttccttcaaaatcaaa

taaaaagacagaaaacaaaaaacaaaacaaaaaactaacaagcagatcacttatgtctca

tagatccacacacttgcaaacaaatattgaataccatcataacatcaaaagcatgagaga

tatcagaaacatgtagttcataaaacgaaacatgagaactggaaaatttctgagacttaa

aagactctttcatcatgtttcagccgcaacttgttcgccgttgatttttgcatgtttctc

atgatcaccttcaagctggcttaaatttcccttttccttaatcagttgaacatggtgatg

tttgcagtaaagttttccctcatgtgctatgtaattggaaggactaataacacatcctcc

atgggagcatttgaaacaactcttatggtaaggagttccattcacagtaaccttctcagt

tggataaactgttttctgacaaccagaacatttatctcttgttccaccaaacatactcga

ggctttaaccgccgcaggtttctcattatcactacttctttctggttttgcaatttttgg

tgtcccttcaaagcttttctcaagacttccagttctcttgaagagttgatcaaagtgtgg

cttgcagtaaagaactccctcaaaagagttgtagttgctcaacttgagggttcctttgca

gtgatgacatctgaaacaagctttgtgaaagattctattatcagcagtcaatttatcaac

aagataaactgttttggtacaagccatacatttctgagtagttcctgcaaatgccattct

tctttttcttacctcaccaactttgaacaagtctatgaagatgagagaaagaaaaaagtg

aaggagaaatttggacaataaagcaattatattgtcattacaatcatttttatttttgat

gaaatgttaagcacctaactatacttttacttttttaaattataataagtggaatttatt

aggtcattaaaaaaattggatattttggttaaagaaattgtgtgtttattagaaagctac

tttgttaatttgtggggtaggatgaattgaaaatcacaactgccaaccagacagcagctt

atcattcaacatccagctggctagctttcattcttggatctcattttttcaaacacaaat

gaaatgaatacatt

>fava_dn_rep_c119

tttttttttgtgaccaaatggttgctcttatttattatgtctcagttggttccgcggtga

cgagaattgattctgagttaattgctttatttaatagacattgataaagaaacttgcatt

gaaaggtaaaaacatgtatctcatgatttattcttgattttgatacaaaagcataaactt

agaactataaaggattataaaaacaatctaccataaagtttccttcttatgccactcaaa

ttcaatcactgcttctgtgactttcccttgctcttctcgatatagaagaacacgcccaag

gacaataccgagaaagcactgaaacaaatagagcaaatgtcaagtgtggcgtggcgtcca

ctgatcgcgttgatttcaaacaaattagtactcttatcagcattcgtgctttgtccgtaa

gcaacttccacatcattggagtcaaatgcgtaggcgcgtacaaaatatgtggcctgaggc

acggcgcgctcgatcaaccagtcataggtttgcaccgtcttgttagaagtgtcgtagtgc

attgcttccattttgtgttggcatgtcttgtcccttgagagatcgtctactgtctttctc

cacgcgcggtcttgttggcttattcgcgcataacataacttcagttttatggttttgtaa

gaagagtctgttcctgctgggaaggtcttgtttagagaccatgttccactgattttgtcc

accccagatgacaaaacttgtccttgtttgggggaagcagtgacaacaagggttcttttc

aaggaagaaaagagaatctttccataacaaatttcagctaaacagcagagaaaaagtgat

gctaccacaagcttgtgtgctgccattctcaaacaacaaaagcacttttgtaaagaaaag

tcaacttgtgagggggagaagacttgagagaacacccaatgagttggaattggaaagata

tgatgagaagaatctgaatcccgcggccgtaat

>fava_dn_rep_c120

aatctacagtgagaaatggatggaagggttccgaccattatacccactaggccactagaa

aagaaaaaaagataacacaaagactttccaaacctcaaaggcttctagatcagttatcat

catcagggttcagaacttgtcttgttcaccaaatggtgagtgataggaaaatcggtgtag

ctttagatttttccaagggaagcaagatcgctctcaaatggactattgataatcttctcc

gccacagtgacactctctatatcgttcacatcaatcattcccaaggcacagaatctcgta

atcttctatggtcaaccactggttctcctcttatcccgttgtcggagtttcgtgagaaga

acgtgattcatcagtatgaggtggaacctgatgctgaggttttggatatacttgatactg

cttctaaacagaaacaggtaactgtggtggccaaggtatattggggagatgcaagagaga

aaatttgtgagtcggttggtgatttgaagcttgatgctttggtcatgggtagcagaggtt

tgggtgccatacaaagggtattgctggggagtgtgtctactcatgtgactttaaatgcaa

cttgtccagtaacaattgtgaaagaatgagtctcttctactgtctgatatgcagctgtgt

ttggttgatgtctttgcaaatggatgttcactttgcgcttacatgtacttatttttggtc

tactgagacttggtttttaagactctatttttagtttatataagtgcttaagttaaagcc

aaaagtgtgttaataattaagctttgtgctggatctgattcaaatttgctacctaacatg

atggagtgaattgacatgtttcatatttgattcttactgtatttgtgtgataaataatca

tcaaagtgttgctttatgcaaaaaaaaaaagaaaattaaattatctagttcttcttttcc

ttccctctaataaggagggaaaggaataataaaagggtcaagaagaagaaaaagaagaag

aaagataggagaaggatttgtgccttataaacatgaacagaaaaagttaaattaataatt

tctttgtgtaatcaacgttatatgttgttacaacttgttacaaccaggaata

>fava_dn_rep_c121

cagtaacattgatgaacttggtaagggtgcggaaagagagggattcgaacccccggtaaa

caaaaagcctacatagcaattccaatgatacgacttgaaccactcggccatctctcctac

ataatgattatgcccctaaaccgagtgaatagtgaggcatttatatcccatttgcgtacg

cgcacacaatcaattgaaactaaaaaaatagaaatagaaagaattttttttttttttgaa

aaaaaacctacttcaaggcctaccgtagannnnnnnnnnnggccgcgggtccatttttac

gttatttcgtactgtattgtacaattcctctgtttggggaattattttatcatgctaaaa

ttttagaatggattgctacctatgactagatctcggataaatggaaattttattgataag

acctttaccattgtagccaatatcttattacgaataattccgacaacttctggagaaaaa

gaggcattcacttattacagagatggtgcgatttgattattattattctctttttttttt

accgatctcagtcttaggagaaagatacattcttcagagagaaataaaaaaattttgaaa

aaaaacctacgcttgctgaaggtgaagtttggaaataaaacgattaatcccttctgtcgt

gtattcccgattaatgcatcctcaatatgcttcaattttagtatagtattaagcgggaag

cgaagaaatcaagatcgatagagttgtagatagatatggctgcgaaacttatgcaagctg

ttcagtacacttcctatggcggaggatcctccggattgaagcatgccgaaattcctgttc

caactccaaaaaccaatgaagttttgctcaaattggaagctgctagcattaatccagttg

actggaagattcagaagggtgatcttcgggttatgttcttgcctcgaaaatttccccata

caccttgtactgatgtagcaggagaggtagtagaggttggaccgcaagttaaggatttta

aagctggtgataaagttattgctaaactcacacatcaatatggaggtggactagctgagt

ttgcagtggcaagcgagagcttaacagctgccagaccatctgaagtctcagccgctgaag

ctgcgggtttacctatagccggtctcacagctcgtgacgcactcactgaaattggaggaa

ttaagcttgacgggactggcgagcagaagaatgttttggtaactgctgcttcaggtggtg

taggtgcatacgctgttcaacttgccaaactagggaacaaccatgtgacagccacttgtg

gcgctcgtaacgttgactttgttaaaggcttaggcgctgatgaagttctcgactacaaga

ctccagaaggagtagcactgaagagtccatccggtaggaaatatgatgcagtgatacatt

gcaccactggaataccgtggtcaactttcgatcctaatttggctgaaaaaggggttgtag

tggatttaacacctggcccgagttcaatacttacttttgctctgaagaaacttacttttt

cgaagaagcggttggtgccgtttattgtaaatgtcaagcgtgagggcttggaacatcttg

ctcaattagtaaaggatggaaaactgaagacaatcatcgactccaagtttcctttgagca

aagctgaagatgcttgggctaagagcattgatggtcatgctactggaaaaatcatcgtcg

agccataggcacagtttgcttgctggtgttgtgtttgttccttccacttttctactcaaa

agtttgtatgtgtttatatagaaatataaatgaaataataaccttcaatttatgtatgtg

ccaagtctcttttgctgggttgtatgtgatatattggtacagaattgttctcttctttga

tgagtgatttggaaataaagacttatttgatataagagtgactctttccttttaaaaaaa

aataccatatagaggcaatagtgaagagacagattcatatacctggaagacaggtgttgt

ttcttccatccaatcccaggaagaatgcactgtcagtttattgttgtttttttgtatgca

cattttcattttatatctattgtataagtggtacaaattgatatttaaaataaatatgat

aattctttttccctaaaaaaaaacacatcttaccttgctctcatgctcttatcccaatat

atcaatagaggataacaaacattataaaaatggtaattaaaaaaaaaacaaagatcctca

accatttatagtatttcttagtaaaaaaaatctgcatccttagaagcatttcctgtgaac

aattgaagggcctgattcatcatactccgcctttgcaatccacatctgttggaatgtgct

gagagatgccaaaatggagcctccaatccagacactgtacttcctctccggcggtgcaac

caccttaattttcatgctacttggtgccaaagccgagatctccttgttcatcctatcagc

aatcccggggaacatagtcgaaccaccactcaatacaatgtttccgtagagatccttcct

aatgtcaacatcacacttcatgattgaattgtatgttgtttcatgtataccaggagcttc

cataccgatcatcgatggctggaagagtacttcgggacaacggaacctttccgcgcctat

tgtgatcacctgtccgtcgggcaactcatagctcttctcaacagctgagctagtctttga

ggtttctagctcttgttgataatcaagagcaatgtaggctagcttttccttcacgtctct

cacgatttctcgctcggctgtagtggtgaaagagtatccacgctcggttaagattttcat

cagggcgtctgtaagatcacgacccgcgagatccaaacgcaggattgcgtgcgggagagc

atagccttcgtagataggaactgtgtggctgacaccatctccggaatcaagaacaatacc

agtcgttcggccactcgcgtaaagcgaaagcacggcctgaatagcaacatacatagcagg

agtattgaaggtctcaaacataatctgagtcattttctcgcgattagccttcggattaag

cggtgcttccgtgagaagaacaggatgttcttccggagccacacgcagctcattgtagaa

agtatgatgccagatcttttccatatcatcccagttactaactataccatgctcaatcgg

atacttcagagttaaaatacctcttttcgattgtgcttcatcaccaacatatgcatcctt

ttgtcccataccaaccatcacaccagtgtggcgcggacgaccaacaatgctagggaacac

cgccctcggcgcatcatctcccgcaaatccagccttgaccattccagttccattatcaca

aacaagaggttgaatatcttcagcatctgccatttcttaataacttagaatctccgaaat

gaatgaagcaacgtgtgtgagtgatgattagttatgtgttgtgttttctatttttaacga

acatgtcttgttatgtccnnnnnnnnnttttgctgttttgggaacacttgctagtctcct

cactgttcctgtttagaattacccccac

>fava_dn_rep_c122

ctacgaaattcttccaaaatcaattttataaataatgggatactaaacatcttttgcaag

ggattaagcaaatagagaaagcatgtaaacatagttgcaaaggagagacaactagcaaag

tataacattagtgtttccgcaacaaaattcataacaaaataaaagcaaaacaccatttga

aatagagactattataaattcatacacctgacaaacgaaaacaaaacaatgtcttaaacc

aacaaaatgactgtcaaaactactttagaacagtaactgaggccctactattctgccaag

caacttcaagctgccactgccttctttgatttctttgatggttgggtttgctttgctact

tcggctggtggagcagcagtaggagctgctgctggcttctctcctcctccaggaccttga

gcaaaacgctcctccctacgagcaaacttcctttctcggctggccttgttcttggcacgc

ttggcctcaaactggtcagataaggtcttctctcttgccttctcggctttagacttgtgg

atgctctccattagaaccctcttattcttaaacacattacccttaaccttcatgtacatg

tcatggtacatatgcttgtcaatcttcttggcctcacggtacttgcggagcaaacgcctg

agcacacgcattctcctcatccaaaggatctttgttggaagccttgcctccctggtaccc

ttacgcttaccatatccagagtgtcttcctttcctcttcgcttccttcatcctacgagca

cgtgatctggagtgaatcttggtaggtttcctgatgataaacccatccttaacaagcttc

cttatgttttggcgagagttagccatagaaatctcgtttccctcatttggatctagccag

acctttccccttccgcacttgaggacactggcggcgagacgcttttggagtttgagcgaa

accatgtttccctgtgtgtagaagctaaggctatcgcagaatggtggaagagatgcnnnn

nnnnnnnnnnacctagggtttggaattttgtaat

>fava_dn_rep_c123

atgactatacaatattgtcatcttttattagtagaaggtatattagaaaaaatatcatta

atgttgtactgtgattactcaaactgtccctaattatactgtccctaattattgtttttt

tcaactcttttatacagggtatctaattatgagggaaattaccctaaaatcatacataat

ttatcttttccatatcataataattaaataattaaatttcttaatttaattattctattt

cttaattgtattataaagcagaaacaggactaactgcacaaaggaataaagttcaagaca

gaagaacacttctttacagacaactgttattggtatctcactattttccaaagattttaa

ggagaatcgtcaaacaagcttgattgctaaaaaaaattaatctcttctcttcttcttttt

ctcagtcctctccccagaattcttcaacatgataactagaatccggacaaagattgcagc

caagtcagtaaataaggtcagagcatgcttgacataatccagatcacccaagtgtgcctt

ctcaactatttcttgtgtgtcgactacaatgtaacccacaaacaccagaagtccaaaata

caactcaaatttaaagagagccatcgaacctccaaagatggaagaagcaaagtgcaacca

gagaagaatggacaacccagaagaaaccaaaccaccgagatacaggtactccctacgctt

agcaaccaaagctgctccagagaaacacccaaacgccaaggcagttgccacaaatgaact

gaagatgaggcttgaatcaacttgaatagccaaatcaatcaagggtccaatagaggcacc

ctgaaacattgccgcggccatcaacaaacccaacctctttctctcttcataaggaggtgt

tgaaagtaaccaaacactggttcccacacacgcaatcgtggtgagaatcccgccaaggtt

caagagaacatgaaggtaagctccaacagcagcagcagccacagcaaaacacaaggtcaa

gtaaacctgtttaagatgattcctgacaacgggagaaatttgaccgaaattcttgagagt

atcaaaattccatcggtttgttgaatcgaagagcgatgtgaaagaagaagccattgatag

attcgtaacaaggggtgagtagaaagagaaatatgaatcccgcgg

>fava_dn_rep_c124

ggcgcccttttgtttttcctttttttttttgagcgtggttgattatttctttttctaatt

catatgaattctattttatcatacaaatctattttattctaccaccttcccggagttaag

tctccggggaatttttatttttgaatgatatcgttggcaatcataaaaagacaattcccc

tttaatctagctatttgtgcaactattttacgattaagaagcaattgattcttgtacaga

ttatacattaaattactatagcgatagtatattttttctttattctggcttattattgca

tttattcgactgatccacaaactgcgaaagtccctttttttcttatctctatcccgttga

gacgaaaccaaagcttttattctctgttggaaaataattcgaataagttttgaatgagct

ccgcgaaagcctgatgtaaaggaactcaattttgtcctccgttttctcgcgatagatcct

cgtttaattctgttcattggataaatgagactttgattaataactatttaatttttttct

ttcagttattcttttatcctttctagtctattacgaacaacatggattcttccaatgtgg

aaaatataaattccaatggcttttgctactataaccttcccaaccactattttttttttt

tcgttttttctaagtataagtattttaactcttaataataacttctattgatactaggta

ttcaaaaaaacatagtaaataaaatagaaataaacaaagaaatcgtgggttccatcgttt

ctatgattacttgttaaacggtgaggtcctctctatacaccggagccccttccttcattg

aatcttgattcaatcaatgttattgtgaacttgtacagttcacacttcttagctctaccc

attgaatatatagtaataggtctttcacaataaaatctagctatacagtaacggtattta

agtatgaagattagctgggtagttgaccctcttagtccgttattgctaaaattagggcat

aatttgtctttatttcaaagaggatttttcccgcttaatggatcactttttgttaccaat

gggaaaagtatatttcatcttaaattcaaaattaaggtaatttggtttgcaccaattgag

aaagcataaattttatacacaatagaattatatatatacaattcttattaattgaatata

ttttttaatttcagtatatgatctaaacgagtcgcacatacaccttaatacatgttcctc

ggcgttgagggcatccccgaagagcgggagattttgttacagtccggattggctgtcttg

tgtttctaataagttggtttatagttggcatggggagtcatatacataatgagctggttt

ggatcaatcctaacctgataattttcaaattgataaggactctataaaatcgggtgatta

aaaagataaaagacaacatcaaattataattctgcatttagtcggtacgttaggaaagaa

gcatcagaaatagtagaaagaaaatattctcatttattaggatcaaaaagggaataaaga

ttttatgcaaattttggatttacaaagaatccgctactgtatcaattattccgtaggctt

gggcttcttctgctgacataaaaaaatccctttccatgtctttatatatctgccaggaag

gtttgcctgttctttggcaataaatatttatcatagttgtttgcattttcattatttccg

tcacttccatcatagattcgcccgtgtttccctcataaaaagaactagcaggttgatgca

gcattaccctagcatgagggaatgctagacgtttggtaatttctcctcctaacaaaataa

gagatcccattgaagcggctaatcctacgcatactgtctgtacttctgcttctacaaatt

gcataacatcaaaaattgccattccagatagaatccctccgcctggagaatttataaaca

tatacaaatctttgttcttgttctctaaacttagatataccattataccgacgagttgat

ttgatatttcactgctaacctctttacctaaaaaaagtagtctttcgtgaaaaagtcgat

agtataagtcaatccaagaagcttcatcctcgccgggaacatcaaaggctactttgggaa

cacctaccggcataaactggaatggaaattgtcgaccggattttgctttggtgtgagaac

atccggagacagaatgagttgattttgttcaaaatcatttttatttcgccaaataacatc

aaaaaagaagactaaaggaaagttttgatgaataggtcgttcttggatatgtctctactg

ctattcttgttacgtgttactggacgactaatgaattgaattctttaaaatgaaagaggg

gattctaacaaaaaggatctccgacaaccaaaaaaaagtatatttctatatttcctatcg

aattgaattctagaaatattcttctttgcttcatgaattatattattagaaaccaattaa

tcctattcacattttcattgatcctcagtttatagtccgaagctaataatgaatgtaaag

aaccctcagcttctagttctagtataagaaagtcttctatatatatagaaagctctatct

ttgtaattaaaccaagaaagacccgtagactttttttttgtgtctaatacacgaacaacg

aatctttcttttgacgaaaaaagggatttgttgttcttgtattagtatccagtgtctgca

ttcactgatataaacacgcatataaaaaaaaaaaactaaaccccaccaccattgccattg

cgtattgttacttatcggatatagaatagatctgcttctttttgttccaacgaatagaat

gttcctttgtttctaaaaaactagaacaaatataaattatttcatgcgatataatttact

aataaggggaagggccatagcatagttttttacagtgcaataaagttacatagtgtctat

tttttgttgatataaggggtattttcatgggtttgccttggtatcgtgttcataccgttg

tattaaatgatcccggccgtttactttctgtggaatttggagatggggacacgggctaaa

ggttgggccgatttgactatggttggaagtagttgggttagggttaggcccgttgattgg

cccatcgattttgcatctgatgactctattatgaatctgggtttctccattttttttcac

acttttttcaaaaattaaaattattttaatttaaaagcgtgtgtgagagagaaagaggaa

gagaggggggaagattaaggatggtttcatgctaatttatagacgggatgaagaggcaat

atgccagctattaatactagtactaccaataatttaagattgttttctttctatatttct

ttaatgcatttaaatttctgataatacttgtggaaagatatcagatacaaaaatgtagtt

ttgattttgcaaatttaaatcaattgtgatatgaatctctggaattaggtga

>fava_dn_rep_c125

tttttttaaagttttaaaattacaaatgttttataaagttttcagacaaatttttggtag

atagaacagtacactgtttatttctagcatgatctgatcaatattataacctaataatgt

atatcacatatgttgttccataaactcagaagataataaagtttataattattttctagt

tcatttacctgctatgctgccatggctaagggataaacatcaccatggtaacaggaagac

ataattaccaactactgaaataacatgatcgtggcagtctcttgatactacttaagagat

agttttgccataactagaagatacattcacaacttcatgataatatatttacacaaattc

aactaacttagtgtcacatgagatgaagatagacttagaatcagcatccctgtatttaat

tgttggattgagctgcagctacagttgaaaccattgagtcgacaccacatacatttctac

ctctgcagatcttgtagtatccctgctctccccaattctgcccccaagagttcttaacga

tccagtaaggcttttccttcaatcgaatgggggcataagcaccctttccaaaccgactag

aagaacaccatgatccaaacgtgattttgcacatacgtatgggcatgaaacgccactcat

gtatgtctgcatccaggctgcattaataccaactgcaagagggccattctttactagatt

tgcagcaatttgttcttcgtctaaggaaaccacactgaaattagacacggaagcaacaac

tttgcttttgtcaaatttgcaggagccatctcttcctgtgtaagcatagtccttttcttg

cactactcccccagactggagtaaatattcaaatgcattgttcatcaatccaccattaca

gcccgagtcacatgatccagcttgctctggatcacacacatgatcgcaatccacaagctg

ttgttcgctaaggctaactagtttcccagtagccagatagtgtgctccttctaaagctcc

agttgtgctaaacgcccaacatgagccgcatgaaccctgatccttgacgggagtaacggc

gcctttctcacgccaatcaaaatcctctgggaggttggtggtaggaaggatcggagcctt

ctgagcatgagctggcaaacggagacgtttcttgagtccgagaaactgacggcgaaactc

agacgccgttaaatcggagaatttagttatcccgtgctccgcggttggatcgagcttctg

gtggagcttcgctttgatgagattcgacttgaacacgccgaatcggtaatcgtgttcctc

ttttgtcgagtagcttttgctgaactttgacttgaaggaagtgaagtgatgttcggcgtt

tagtaaatggtcttcttcgttgtcgacgacttgacggattatgaagtcgtcggtgttggt

gtcgtcggtggaggaggttgctacggcggcgaagaggaagagggcgaagatgaaacggcg

gtccatggtgggggagagagggtttgttgggagagaagagaaagtggattgggttcccgc

ggtcgagaaaaaatcacagaaagtgattgttaatgaagatgctgagggatgtacaagggg

aaggttgaagacgaagatgaagagtgcttcagaaaaatattcgagagattcagagggctg

ccatggtaaaatatttttgaaggaagcatgaagaagtttaggcatagtgcaagcacggtt

gaagtgagaagaaacagaagtaggatggaagattagaactaagttctaagaggagttttt

atgaaattttctttgtaattttgaaatgtcatatgatgtatggttttggaatggtatcat

acatcctcttggatatagagtcttgtattggtaatgagcttaaaatgtcttcttatcatg

tacttatgtaaggattcacattgtaaacggtcctgacttttggaaaccttgtgatatttt

aaggttttaatgatgataatcaaagtattcttcattgttagcgact

>fava_dn_rep_c126

tggtccctgcataattattatttttccatttgttggttatgttgttgataagtttagccc

tacattctttcaaagattgtccacttatacttatgatcattattgctccataacccaact

tgtctttcatcattcatcattcatcattcatctacacccaaacaatgacaaaaattaatg

tacatagattaagacattaaccaataaatatttattttctccactccactccaatccaac

aatattattatcattattttaaaacatttcataatattttctgcacgttacaaggaataa

catcacatcacatcactcactatataaataaacaaaactatatatactaatttgagagac

taatacacttcactttaccctttttttactttgcttgtttccccttaaacaaacatgttt

acatatatatttaaatatttatttatagtaaaacataattaaactgttgcaattggtccc

aggttaatgtttgattccttaaatgcttcaaatcttggttctttggcttgaaactttaac

cctgcatagagtttcatgtaatcctcaaacacaaattttgggtaaacttcattgttttct

tcaatcaatgttggtgctgggtagataacagcatcactaccagggttgtagaatgaggct

atggacattcttgttccatcattttgtgctatcactcggtgctctatgcttttgtactta

ccattggttatcacctcgagctggtcaccgaggttgatcacaatggagtgatgcatggga

ggaacatctacccagatgccatctttgagaagctgaaggccactgactttgtcgtcttgg

aaaaggaggatgattccacctgcatcggtgtgtgcgcggagacccttcactaggtctggt

tttgggcatggagggtaatttgcaatcttggtgccaaaagttggtccttttgatccatag

aaggcctttttgaggtaccctttttctaatccaagattctcgcataacaagtctagtagc

tcctctgctagtttctctagtctcaaagcaaattccttcattgatttcctgtattcatca

ctaagatcagggatctctgaaatgtttgactcaggtaagtgacgtaagtggaaggtactc

tcccaatccatgtctttgacttcagtttgaacagcttcaagccctttctcagccactaat

tcattgaatctttgttccatgcatttcctgtagtgttcttttgtcaacctctccacagtg

cccatcaagtcatgaggtatgccatgattcaccagctcaaagaatccccagttttcacaa

gcatctttgattttctccatagtagcttctctcttctcaccattgatgttctctaagttg

ataactgggaaattctccatatctcactcaaactcttagcttcaacttttttctatgctt

ttcgttcagtttattgtaggttgcttgcttgctttaggatcccgcggccgt

>fava_dn_rep_c127

aagaacatgannnnnnnnnnnnnnnnnnnnnnngatctttttaatatcttcaatttaaaa

ctccaaaataagcattcactttacacaaataaaagtcatgataacaattccatatcaaat

aactcatgcatggtctttatatagaatgataaaaagatatagatgaaattaaatatatta

caaacaataactgatttatgccacattaataaatgatacaaatcacagtcaacaatatat

gttgtagtaataaaaacacacccaaacaacatagaaattgaacacaacatgtgcctcaat

atgacacacaatcataaagtagaaacgaaaagcctcaaataacagaaacaaactttaaca

cactcatttactaatagttggaatatatagtatatatatttgatgaagttttgaagtatt

tatgctttagggccagatggaacagtaggcaactctggcttaggaatctgaggaatttca

ggaaccttagggacagttggtacaacagggatatcagggattggaacacctaggtttgga

atttctggcaattgtggctgtggtggcaatgcaggtagagtaggtagtgttggtagagtt

ggtattggtagtagtgttggcaattcaactttggaaacttcatgcatgttttgaaggtta

cgtgctccttcaacatttgtcattgatgaaaaagttacgagtacaagaagtggtatcatg

aatgacaatacattaatggaagccattttttgaggtagtgagatgaatactagtgtatgt

gatgattgttttggtgtgtttgaggtcccgcggccgtaa

>fava_dn_rep_c128

ctagaataacgagagtatcatatcatccaagaaacagtaatttttttttttaaactatca

attattcttgtgcattgaaattgaaattcacgatatcatcatcactgatatcccataata

gagtacaacataaaaccccaaacattacatataatcatgttcatcatcaacacatataac

gagactaataataattaaaacccaacttttgaacttgaaacaaacaaaaagatgaattca

aatcttatcaatatcctcatcctcaaactcaatataatcattagcatccccgtcatcctc

ctcatcaaggccagccccaatcccttcattcaatctagtattatcaggaagctcaccgta

cgctttcaagagcctagcctcatccggcatgtatttcagaatcacatccgccttatcatc

ctggtaatcacggagtccgacgaggatgatatcgccggcggcaatccagaccttcttgtg

catcttgccgcggatgtgacagagacgctttgtgccgtcgatgcacatggcttcgcatct

tccgttacccaacatgcggaggacttgagcgtactcttggccgtcttccttgaaaacaag

ctcgcgtttctcgtcgtcggcttcgttctttcctctctttcgattctttcctccctttcc

cttgttcttcggcatgattcagctcaatttgcagcaaaatccactccttttgatctccac

aacgaaaccctagttttgggaacttgttcttattcttatttttctccactcggccgtaat

gacactctgcgttgataccactgctta

>fava_dn_rep_c129

agtagtacccttttcaagcggactttctcatctctttgaaaaaataaaattcacgcttca

caacacacttctcaacaacaacacactctctctctctttcactcactcactctctcaaca

aacaatggcaccaccttccatcgtaagtaacggaaccgttactcccaccgtcacatctcg

cttcgcttccgtctacagcgaggtccaaaacagccgcgtcgatcacaaactccgtcttcc

ttccgttctccaagcaccgttcgccattgtcgacggtcctaaaagctccgccgccgggaa

tcctgatgagatcgcgaaactgtttccgtttttgtttggacaaccttccgctgctttggt

gccggctgattcagccaatgcgcttccgcatcggaagttgaagattggcgttgttctctc

cggtggtcaggctcccggaggtcacaatgttatatctggaatttacgattatcttcaaca

acgtgcaagtggaagcacattgtatggtttcaatggtggtcctgctgggattatgaaatg

caaatacactgaactcaattctgactatatttatccttacagaaatcagggtggttttga

catgatttgcagtgggagggacaagattgaaactccagagcagtttaaacaagctgagga

aacagtgaataagctagatttggatgggcttgttgttattggtggagatgactccaacac

caatgcatgcctccttgccgaaaacttcagaagcaaaaatttaaaaacacaggtgcttgg

atgccccaaaaccattgatggtgatctgaaaagtaaagaagttcccacaagctttgggtt

tgatactgcttgcaagatatatgcagaaatgattggaaatgttatgatagatgctcgatc

aactggaaaatattatcattttgtgcggcttatggggcgtgcagcttcccacattacgct

ggaatgtgctctacaaactcatccaaacattactattattggagaagaggtggctgcaaa

gaagctgacattgaaaaatgtcacagactatattgtcgatgttatctgtaaaagagctga

agttaattataactatggggtcattcttatccctgaaggtctaattgatttcattcccga

ggtccagcatcttattgcagaactcaatgaaattctggctcatgatactgtagatgaggc

tggtttgtggaaaaagaaactcactgatcagtcattggagctttttgactttttacctca

agcaattcaagaacaattgttgcttgaaagagatccacatggaaatgttcaggttgccaa

aattgaaacagagaaaatgcttatcgaaatggctgaaactgagttgggaaagagaaagca

agaaggaaagtataaaggagaattcaaagggcagtctcactttttcggttatgaaggtag

atgtgggttgccaacaaattttgacgctaattattgctatgctcttggttatggtgctgg

agccctccttcatagtggcaagactggactaatatcatcagttggaaatttatgtgctcc

agtagaagagtggacagttggtggaactgcactcacctcactcatggatgtggagaggag

acatggtaaatttaagcctgtgatcaagaaggcaatggtggagcttgaaggggaaccttt

cagaaagtttgcctccttgcgtgatgaatgggccctgaagaattgctacatcagtccagg

tccaattcaattctctggcccaggatctgatgcagttagtctcactctactcttggagtc

tggatttcaaatttaagcataattggggccttgttttaccggaggctattcatttttgtg

gcatcttacgaatatttgaacggatattagttagattttttttcttttctttcttttggt

ttacttttgaagttttccagaaaattgtttatttgaatgtcaaaattttgaaaggaataa

aatttgataaagtgatacaatgacattacaccttgattttatttcatgcttaataatctt

gatcttcctttagaaaaattgcaaaactaaaagtggggagttgataagcaacgaatagat

acaatacaagaataataatatacaacattgattcatcatgaaagcgatgttacaccaatt

ttttccagtgcaatggaaaaggtaccagtgttatagatggtagattacaaggcagaaaat

aacttaaaaggagaagcaattaataacagaccatacgttattattatgaatcagaagtac

tcctcagtccttcccttgaaaccaatttgacccaaagtgagggagatgaacagccccaag

tgccaacagatcagccataaccaaaactgtgaagagagagcaggaggagtagggaagtga

gcaagttcagttccgatgctatcaaagaaaagtccagtcagacttttaccgtcaatagcc

ggtatgctagacggtgccaaccaacctatcaacccaaatccaatcacattgaaatccctc

cttagccaatccctctggaagcatgtaacccttccaccatttcctcttgccatagcttgc

ctgaaaggattcctagcagttacttgaggtttcacaaatcctgaggtgagacgtgatgca

ccacccattcctacgacggttgcaaaggttgttgttgccattctagtatgtgattttggg

gtggacaatgcaagagatttgagttagatatgtgcggaatctagtggtgatggtagtttt

gtgttccaattattattattgttggattctatgaaaaaaattgaatcc

>fava_dn_rep_c130

attgaggggtatcatgccatacccatgtctccccattacgtctgcctctatgtaccgact

gttcactacctcggttaattacaatgaannnnnnnnnnnnnnnnnnnnnnnnngccaata

gtatctttcacttactctttacaccatcaaaatggtgtatgacactcgagcaatatgtca

aatagattgaaatatgcattatatatatgggaatagtctatttcatgcctccttccttgg

gcaagtaggagcggtacaagtacaaaacatactcaaaagtgaaattttacatagtgccca

attccttactaaatagggcttcattattctgtaagatagcaacaatgtcacttaggatcc

gatttcacaatattcaaaattggaaacacaaacttgatagagagcgaaacaaaggtttag

atacacatatattcccaacagtgactcaatttcaattacagcatatattgacagccttgg

aatcagatctcatgatacaaatcaacccaaacaagtagccaatttgaataacaaaacgtt

tccaaaaagcacacccaaggttagctacataacacaagagtgtttcaggccaaaagaaaa

acaagcataactaggttaattgattgatccacttgaagaagccgaaacaaaagaggaaaa

taaacaagcatcacaaattaaacatagaaaatagcgaaacactagaaaaagcaattgaaa

tagaagaatctgggtctgggctcacttagcaaccattctagcgatgaaatcctcgtagcg

aatcttgccatcagaaccaacatcaacttccctgatccattcatcaaactcagcaggctc

aagcttctcaccgatacttgtcagaatatgacgaagctcgttaacggagacgaatccggt

ggaatccttgtcgagaaccttgaacgcatcgcgtaactgacgatcgaacggctcaggttt

catgtgtttggacattagatccagaaaccgattgaaatcgaacggcgatgtgagattctc

ttctgcgacgatggatttgagttgagcttgggtcgggtttccgccgagagatcgcatgag

gattccgagctccgatggagcgatcttgccgtcgccgtcggtgtcgaatagagtgaaagc

ttctttcatggaagagatttgatcgtcgctcagatctttgcccatttcggtttcgtttcg

ggttttcgggtttggggattttctcttctggttttattagaagaagatgaaactgaaaat

gttttcccactctgtttttcagtttgtgttctcagattgatggttacggatttacggtgg

aatgtacaccattgagagttttacttacgacgtcgtatgggagggtggattatttgggcc

taagcccattaagaaagggtggtatcgagtagtgtggggacgggtgcaggagtaggctgg

tgatcaattatttctgtgagg

>fava_dn_rep_c131

atcataattaacttaattttaagctaaggaggtaaaagctatatcatagatggaacaaat

aacagttcaacaagtgtcaatgactaactaaagtttgattggttgcatcaaaaactttcg

tattaaacaatgaaaaacatcaaaatttcatttctgcaagttcaagtaggtttgcatcaa

gctgatagtaagtttccagcctcaagagtataaatagagagaatgggtcacaaacaagtt

cagtctcaacaaatgagaattgggcaaagtattaaaccatcaaaatcactacgcaaataa

tccataaaaaccaaaatcttagtacttatacaaaaacttcatatctgagacccacaaatc

taggctaactgcaactcagttagatttcacatgttgcaaaacaacattatatgcttcgtg

ttcctcgccaaaatccttgacaaccacgcaggagcaaccagttaccttcctagcttttcc

ttctgaatcaatcttgcacagaccagcccattctccgagggtctttgcatttggaacagt

caacaaactaacattgtgttcggcacaaagggccttgaccaacttaacataatcaggttg

gtcacaatcctcagctaggacacaaagctgtgcagtatgcttctcaatcactttagcact

ctcatgaagacctcttgcaagaccaccataagcacgcgactttctcagcacaagctgaac

agcagtcatgatgtccattggctcccctggaattccgggtgcagcaacaacaggctcggc

tgcaacaacgggctcttcaccagacataattgctatttgatttcaaagagatttctcttc

tttcttcttcctttgaggcgatgaagcagccgaaatcactaggttttctgatgtagctgg

tgtgcccgcggaacggctctgtagactcttgttcagtccttattcaacccaaaccctaaa

accctagccgtcttcttccctttctcaatcaacaatgacaggtgaagcagttaaccctaa

agcataccctttggctgatgctcagcttacgattacgattatggatcttgttcaacaagc

cgctaactacaagcaactcaagaagggtgctaatgaagcgactaagactctgaatagagg

tatttctgagtttgttgttatggcggctgatgccgagcctctcgagattcttcttcatct

tcctctacttgctgaggataagaatgttccctatgtgtttgtcacatcaaaacaagcact

tggccgtgcttgtggagtaacccgaccagtgattgcttgttctgttacaactaatgaagg

aagtcaattgaaatcccaaattcagcaactcaaggatgctattgagaagttgctgatctg

aaaacttcatattgcgaatgatggtatgatgggcttcttgagttgtgctgaaagcgcctt

tagaggctttgactggacgacttgttgtgctatcctatatactctttgttaacctctact

ttaatttttctttctactgcaaaaggttgtttttgtatggtgttttggttctagtatgag

ttatgttttgaactgttcttataaaaattgtttagtgatattttgatgagtagaattttc

agtttaatatcaaaaatgtgtttagtgcagttctcttgtaatactacttttactttggtt

attatagattagagaactatcgcatgagtgtaagtgtctttgatgaaaattgctttgtgg

ttacccatttcccacgttacataatttacgtttaaaatgaataatttgttggtaa

>fava_dn_rep_c132

ccctaaaaagaaggggaaatacattgtatttcatttcccacgcgaccactgtgttgtttg

ttgttgtgtgacctgtttatacttcacaacacaacaaattcctactacaactgattcata

ctataaacaaagatcacaactttgcaaagagagagagaaaacttaggtgggttcttcttc

aatttcaagaaacatggatgtgggtcagatgcggagacaatggatggactacatcaaatc

catgttcgaggagaggttcttagatggtcagtttctgcaacttcaacagctccaagatga

gaataaccctgagtttgtttttgaagttgtttctcttttctttgatgattctgagaggat

tctcaaagatctgtcttttgctctggagcagcaaagtgctgacttcaaaaagttgatgct

catgtgcatcagtttaagggtagcagtgcaagcataggtgcacaaggggtgaaaaatgcc

tgcattgctttccgcaacttctgtgaggaacaaaacatagatgcttgtcgcagatgttta

ctgcaagtgaaacaagagtactttgttgttaagaataagcttgaagcattgttgaggctt

gagcagcagatagttgcaactggcggctcgatccctataatggaactgggtttttaaagt

gagtgatatgtaaaaatgcttgtattatgatcctcttgtctgtcgaggatcatctatttc

tcctatgtttcttgtgaatgtatgaatttttatgtgggtagaagttttcatccaatgtac

cttgttcttcacaagaaaacactttcataatctgtaatatttttcatgctttaatgaagg

ttaatggcaccaatataagatagaaaagtgaattaggagagagaaagaaagaaacatcat

gaagccaaggacatgacagagagtctactaggtgtcggtttgaaattcaaccatctaagt

gcaaaccgtttcggcgctggcatcaaagctcttctcaaaatctctttgctcgcaacgcgc

ttcttcatcctttcccctggtacaacatacccatgacgcgcacattgaactgaaccgggt

cggctcgagcacgtgcaagccggtctgaaccgttgagacatgggcaagtaaccctcgttc

ttgttttgtccggaaaccaacatctttggtggaagttgcgaactttctttcgtttgttgt

tgactaacgtttgtgtcttgcatatcttcggagaaaactatgttgttttgttttgaggtt

ttgagattttatatgaaaaaactgaggttggttaaggttaggaaagatgaggaagaggaa

ggttcatgaattgtttttgggttgtttgaatgattgaccaaattgaggacttggtagcaa

gactttgtattagtaaaggactaatatttggtcaactttatagtgctagaaacacagatt

tgaaattcgaacgtttgaggattggttctttaatattttttatttggaaaatcttgtttg

tcacgttttggaggtggagttggtcggaagagaagaacgttcggaatgggccatgaagcc

cacttggcattgatggagccacatgaatcttttatgtgtactgat

>fava_dn_rep_c133

gggataataaagacggacctcaaaattaagtgaagagactaaataagcgataaagcttta

aaacagagcttaacacttcacatataatcattgaatgaagcacgggaggaaaatagagaa

tctatttaccaattagagttattattaccattacaaccgcaatgcttggagcattattca

tgcattttcgatggtagaaaatgacataaaataaaacatagtagacacgatagatttcta

tttaccatcagaacattcatcacaaaatatgttaactcaatattgtggcaacaacaaaag

gcagtggaaaatgataaagttaaacaaacatggagcagccacatccaccaatacaatgat

agaattgtagtggctcatactcatcactttatcctaacgaaaaccaacagaaaaaatcta

gccaaagcactgatcagcagggagaggaccaggaatgtacccttgttcaaaatactgcaa

accaaaaacaacaacaattgcaaccaatgccaaatatgacagcccttcaacggcaccaag

taacccaaaaggaccattaggtaaaccagaaccagtttttgattttgtatatatagacca

agcaacaattcccacaacaacaagataacttattccttctaatgcaccaattgatccgcc

tggtcctggtggaaggccacaaccagttgttttcagtgtgtagagagaccaacctataac

tggagatgagattaaaccacctgctattgctgctttttctgctatgccgatgtttgtgtc

ggtttcttcttttgccatgctcgtgatcttgagttttagtctcatttgatgagattttag

ctttctaagtgggtgatttgaatggtttgtgtgtaactttgaggttgatatcgaggagaa

tagtaatatctttgaggcttccattgttagagaacttgatctatgtccgagagcttttga

gaggataagatactcgcaggaagataatttcagaataaataatccatttataatgtgtaa

attgtgtaag

>fava_dn_rep_c134

taaaagtattcagagaaaaatgaaataagtaaaataaagtacagtactttagtaaatgcg

aagaacaaaaactattttcagtttaaaatttaacgtcgaactgcgagttggaatttttag

ggaacaagggtccaactaatttttggttgtattgaaaatgacatgctagctgattctaga

accttccaggttcaatgcaaacaacaacacccaattcttaccctactaagtagaatcaac

tccatggatcaattttcattgtaaggttcaatggaaataacagaacagaagcaaatatca

tatatctgtgtccgtaaaatcaaagaagcatatacggacttaaaagccatctgcagttta

acatcaccaagagaagtttactggttttaacataaaaataaacttggaacagttcctgac

atatatcctgtaacaaagcttcggaaatgtccataagtaacctacccatgacatgatgga

acagcaagttaagcagtttctgttgctggttgggtcaccccagacttcaccaacagctta

gtcaagttctctggggagcaaacttgatacctcacttttccagagggtgcaagaaaaacc

tctgcaagttcaagcttatctgaggtaagactagtggtgtccatagttttgctcagtact

ttcagtgcaagctgaactgcttcttcccttgtgatatcatccttgtagtcctgttttaga

attgactgtgctgcctggttgttggcaccaatagcaccggctttccaacctccataattt

ccactaggatcactcatgtaaagttgaaagccgaagttcttgtcccatcctgcaaacagg

aaagagactccaaaaggacggagaccaccaaattgagtgtaaccttgtttggtatcacaa

agagattgaaccaactgctcaacaggcattggctcttggtaagcaaatgagaaacgctgt

gcttggatcctagcagtattgattaggatgtttgcatcagacatgatcccagcaacagca

catgcaagatgatcatcaaccttgtacattttctcagttgaagttgaggtctgcagaagc

ttggatgtcaccttcttttcgccaaccagaacgaccccgtcttttgacaagattcctatg

gcagtaccagcatttccaatagcctccattgcatattccacttggtaaagacgcccttca

ggagagaagattgttgtacggctatcatatcttcgagacattttgatctgaacgagtttc

tagttgcagacgacaaacactggtttcgttttccggctagagttttctttcaaataacaa

ggtgtttctgaaagccgatgaagcgtctgattttcgttgttttgcttttatttgtttttt

atttttttcaattttttcatttagccgacggatttttttttagatctaactat

>fava_dn_rep_c135

aaaacgaaagaaagcgaagcaacaaggaaataaagatgaaacaacaacaataatattaaa

gttcataactttacataaatatatatacccattcttcttccttttaggcttcaccaacat

tttttgtttagcattcttaatctttgtgaaatttgtttgtagttactctctctcagaata

tggccgagttttccattaccgattctctttctagctcttccggtacagattttgctgaga

ttattgtggtgcgtcatggtcaaacaatttggaattttcaaggaaaatgccagggacaat

tggatattgaattgaatgaagtgggaaaacagcaagcagctgctgttgctgatagactat

caaaggaaccgaagatctcagctgtatattcatctgacttgaaaagagctttagaaacag

cagaaacaattgcatccagatgtggaggtctagaggttgtgaaggatttggacctgcgag

aaagacacatggggagtctccaaggagttggttttagtgaatcagaaaaaactaatccca

taggttacaatattttgaaatccaggaatgaaaatcaagaaattccaggtggtggagaaa

gtcttgatcaactgtatgaacgttgcaaatctgcattgctgagaattggcagaaaacata

taggagaacgtgttgttgttgtttctcatggagcatcgatcgaagtactttacaaatggg

catgtgcagatggaaagtatgcagggaatatacgcaatgcatctatcactgtttttcact

tatatgcggacgacaaatgggctgtcaaagtgtgggctgatattactcatcttagcccat

attgatttctccactccaaacacttatatatttacttgcatcatgtgaaatcatttgtat

tttgtatcgttgtgcttaaaagtattgtccttatttaaataacgaggtaaatacctgaga

tcacgtgtttgtgtttgatatattgttagtttaaatgagatgtactgacacacgtgtttg

tgtttgatatattgttagtttaaatgagatgtaccgacacagtgtaaaaaaattttacat

tttcattcaataaaagtatattgttttgtagtattattcattcattcactaaaaaatcct

caacattcattcattcaccatggcaatggcaacacaagcctctctcttcacaccaccact

ttcatctccaaaaacatccaaacaaccatcaactctatctttcatctctctcaaacccgt

caaattcaccccaagaacaacaaaaattgctgcttcagaagacaaaaccgatgtagcaac

a

>fava_dn_rep_c136

cctctttttcttcctcactctctctnnnctacgcgggattattcattcattcactaaaaa

atcctcaacattcattcattcaccatggcaatggcaacacaagcccctctgttcacacca

ccactttcatctccaaaaacatccaaacaaccatcaactctatctttcatctctctcaaa

cccgtcaaattcaccccaagaacaacaaaaattgctgcttcagaagacaaaaccgatgta

gcaacaaaaaaagaagcaccagttggattcaccccacctgagctagacccaaacacacct

tccccaattttcggtggaagcaccggtggactattgcgtaaggcccaagttgaagaattc

tatgttatcacatgggaatcacctaaagaacagatttttgaaatgccaactggtggtgca

gctataatgagagagggtcctaatcttctcaaattggctaggaaagaacagtgtttggct

ttaggaactaggttaaggtcaaagtacaagattaagtatcagttttatagggcttttcct

aatggtgaagttcaatatttgcatcctaaggatggtgtttatccagagaaggttaatccg

ggtcgtcaaggagttggtcagaatttcaggtctattgggaagaatgttagcccaattgag

gttaagtttactggtaagcaaccttatgatgtttaagggatgattctaaccctcaagatg

tttgcttaattgcgcatgtaatcatgttatgtacttattataatcacatttatttctgtc

agaaattatgcttaaactctttacctgtatttaattttatgatcactgcttttctcaaac

atattttgctttttggagtatatgttcgctttccattttggtatacgtagaattgattct

gatcgtgatttcgttgaataagaaaaaaattatagtctg

>fava_dn_rep_c137

aaaacaaaaaaccatgaattctccttggaatgagtaacaaaaacaaagacatttcaaaaa

aaaaaatcaacaacaaaataccatagaaatttaattaacctcgagattataagcatattg

taattgaacatggatataatatataaacaagataatcctctcagctcttgaaagttgttt

agtcaaaggataatgcatcaacatgttgaagtttacaacaaaacaaagcataacaaagaa

gctatctatgattcaagctgagcataccaaacaccttggatcttaacatcctttggagct

tttgctcccaaatcagtatcagatggctgaatactctcaaacacaccgataacctcaccg

gtctcgggcttagtctgagtaacactcaatgtgattttcccttttgatgatgcagcactc

ttgttgttttcctttccaagttcttcttcatcacctcttccaccagctggcaatgcaacc

gcgttgtcataaccggtagatgcacctcttccctttgggtccaagaaagagcttcctctg

tatgatggcaccaaaaactcaccgctgaagctatcaggtttccctgatgctaccaactgc

ttgatggtgaaaaggaatggcacacgctcgcctccaggaagctgaacggtgactgcagcg

taatcaattccgtctttctcctcgaatttcacactcccgtctgctgaaacctcgaatggc

ccttcaatctcgtcaagagtgtaggttaaacgtgtcatgagcttggtgttttggaatgcg

agtggagtgtttttggttacgccttctgattttactgtgaatgaagttggttcaaggcag

agtttcttggcattgtattttccttccttgaaggcgaatgtgtctactcctccatcaatg

gttggacattggtttgcagttcctgttcccttgacttccaagtatgttttgctttggatt

tcgtcgaaagttagcctctttggagcaccttctgcacttgctccagagacaacaagagca

gaagtggcaagagcaaatcctgcaatcttagaagcctcaacacacttgtaagccaactcc

ttgaaatcagactgaagggaacaagtaaccttagctccatagtgttccaaaccaaaggcc

ttagaaacagattggttagacttaagctgcaaagtgttgctacgcaactttgtaggttgc

atgagagtagcagctgcttgaagtgaggctgccatgactcctgttaactattcctcgtgt

tctccacaaactaactagttttcaagagtttttgtgttggttgagaatgtgaaggttgca

attatttggtggctatggtatgagtaagattttggat

>fava_dn_rep_c138

agtattaccaatacaaatctaaaataaaaacaagttcatgcattttcattcctcataatc

caaacatatgcaagttactaatgagtaaaagctttggaacacttgtttcatgatttgaaa

ataatacttacagtgagtagtaagtagttagttattcaatatgagactgtaccgaagcaa

ttaacacataaccaaacacagttcaagcaatagaaagcatggaagaggattcacacaaac

ttcaaatttaaactcaaatttactccacagcaaaatctaaataataactgaaatcttcca

aaataagtttcgacaagacaacaaaatacaggattgatccaacagataaataatcaaagc

aaaatcttcaaaacgctactcgagctatctaaaaatccaatacaccaggtgctcagcgct

tcccgcctccaccaccaagcttaggaccttttgatgcagcacccttggtaacatttcctt

ttcctccggtcttttgtgccttagcttgactttctgccttctttgctttcttttcatcct

tagttttcttcaccctctccttaatttcacgaagagcagcttctctggcagcatcgcgaa

cctctggcttctctgatctcttcttctgaataacttccaaagtagcaccaacaatagacc

ttgagtaaggcttcttggtagctctacgcctcttcttcgcagcttcttgtgcagtgtcct

tcttatgttgcttccggtacatggcagtccaggtaagcttagatggcttcaacttgttat

ggaagtaccttttgcatttcgaattgacgaaaaggaaaacctgtgaatcactccggataa

atctgattcctctcccaggatagatcttggcaccactgaaacggcacagctcggtcttga

gaaccattttgttgatgcttccgctgttgcctgccttctcttgtgcccaaaactctcccg

cggccgtaatggccactctgcttgaaccactgcttcatgtggaaatatttcattttacac

ataaattttaaggtactcatccacgcgtgtgtaattcacctcagggtagagctcagatgc

ttcttctccatcttccccaattccaaagtttgtcaaacaaccctcatagaaaata

>fava_dn_rep_c139

aaaaaacagaataataagtagagtaatattttatattgatcaaatgtgaataaagtatac

acatacatagtacatagatacaaacaaacataaacataaaactatacatacaaattttgt

gtcttaaccttaacatctactaacaacaaaagagaataattttcttgagctaagaaattt

atgaagaagacccaacaaataatgaattccctaatctatatgatcatcttataattaaga

attaacaaggcaaaagatcatcaccaaagagataaagaagtctctcaacagcccaactag

gttgactaggagaaacacaagcagtcaaaacctcggaagaaacataatcagtagtagtaa

tcaaaggagctctacaaagaggacaagtctgattacaattgtcattattctcataaccat

gttcgaaccatttatcaatacattctctatgaaacagatgagaacagttcatcaattctc

gaacctcgtcttccattttcattttgttcaaacaaacagcacatgtagtagaagaatcct

gtgtttcgggatgtctttctatgatttcgccgaagcttgtgagaggtaacatttgacaac

attgttgttggtgttgtctatgttgttttttgattgttttgaaaatgaaacataggatcc

atcttaggagtgcgaggattaaagctgcttcgtatatggattgggttagggtgaaattgg

agatgggttgttgggtggagaaagacatgttgaagaagatgagaaggggattagggtttg

agggagaagggggcgcgatagggatggtggcggacgtgaaagggatggtggaggtcgtga

tagggatggtggtgggcgtgatagggatggtggcggtcgtgacagggatcgtgctggacc

atatgagcgtcgtggatcatctggaagttaccgttgatacatttagcttcattgagtttt

cgaattgaagggttgctgttactgcctcaactttttcttttgatgacaatgttttggatt

tttttttcaaagattgagaagttggaaattttgaattcaagttcaaacctttaataatgt

ctttacaaccatgattttaaattgtgattattgcaattgcaacctcaatattgttggtat

ggtagcttctgcaacagcatcaaaccacaaccataactacttagttgtatttatcttaaa

acgaatgctaccataccacataacattattgaatacaaaatgatatctagtagaaaatct

accaacaataagctgataaaccatggggagttgcagaaagcagataatcaaaatcaactt

gcagcatgcaaaattgtattacatacataccaacaattaattttcatctatctacatacg

aatgtatatatctagtttccataatcataactaaaatcagaaatcccaaccatagcagaa

agaacttgagaaacaatataaaacccacctccaaaaatagccacaatcaaaatagcagtg

attggattcttgagaagctcaaatataggcaccatgattgtataaatagcacctgtacta

acagtcagtgcatacaatccataccttctaacattgtcccaaaactcctcaacaagtggc

ccttcaggtcccaaagccaatgccttatgttcatccattccacccaaaagcaacaaaact

ccaaaagaaactgctcctattatcataacccttgaatttccattttcaagattttcatga

acttgaagtttcattggtgcactgtccacaattggagtttgttttctgagttttacagca

gcagattttgaaggtgtgtgtttcatgtttttgatgcgtagcactttgtttggtttgatg

aatagttgatggttttttggagtgaaaagagaacaagaagggtgaggggaatttggaaga

tgaagctttaatttcaggctaagattttccatctttttttttcctccttagttttcttag

agctatggctctcacatatgcagataatctttaaagtttattcaacttgtgtggatgtaa

atgaaggtgtttagagcaatccataccttatcttcatttgtttatattttaagccttgtt

atctt

>fava_dn_rep_c140

taaagtgattttgttataaaaataggcgcgtgtgaattttaaaacnnnnnnngtaaaaat

tacgttttcatatttactcatgtactacacgagggtgtagtaagcaccacccatagttta

aatcgattgttagagataattataaaaacacttggtggagagatctaaatccataggagt

ggtggtataaactgctcaaacaagccatggcccgggcccgtcccaacctgaattttagaa

tattggaaataatctattaagcatgatatttctcaaagtactcatctatgttctgaagag

ctaaagaaaacaggccaattatgtcccaaataccaaaactgtcaaaatacatagggtgaa

agaagaaatggaagacataacaagttaaaattgagataaaaatcaagcatctgcaatggt

cacttcaacttcgacaccaggttcaatggtgatggaggtgatttgcttaacaacatccgg

agaactgtataggtcaatgactctcttgtgcacacggagttcaaatctatcccaagtgtt

ggtaccttctccacaaggggacttcctggtagtgatgtgaagaactttagtgggcatcct

aacaggtcccttcactctaagacgcttatccttagcaccacgaaccaaatcaccacaaac

cttttcgagattctgcacgtgcttcgaggaaagggtgatcctgatcttgtggatctgttc

ctgagactcctccaaaccaggcttcgtgggtttaggagctgcgtacgccatttttgtttc

ttcagagtacaaactgagtggagatgaaactagggcgccgccccgagacgtgaaagaggg

tttaaggggggtagagggttgaattgtgtt

>fava_dn_rep_c141

tttttttttttgaagcatatgaagtaattttgcattcatttccattttgggaagagttct

atttaccttggaaagaaaggtatgacatgttacattcaaccttctgaagtatgagtcatt

aaccacaaagactccatcgatgaatagcgctattaacaaattacaaacagataaaatctc

aaaaatgaacattcttgccgacgaagagtttattatctctaatattacaacagttgattt

tcatcaaacattgaaaagtttgaaggattctactatagtctgcaacctgtctttcatctt

cccccatcttctctcgtttgctcccgtcgtcaacgtgtaaaacttaccattgccaataga

gactgcgctgagagcgtgacgagtgaagtttggagcctgagcagtgaactcaaattgata

atatacttttccatcaatatcacgctcggttgctttaattatctttgttttctggtttgg

aggtgccaaaacctttttgatcagagttgcagcaacctcttcaggggatccgaattcttt

aatatcttgttttccagttgggatcgtagttacgctgacgttttccaacggttcgataac

atctttgaacaccttatcttgaccttcaatcactacttcctgccacccgaaagggtagac

aaacgaatatccgtctttcctatcgacaaccggttgaaatcctgattctgcggcaaaaga

aattgatcgttggtcgaacaaaaagactaacggagcagtagctccaatcgccatcatttg

gcgcctacccggttcatcttgagaaagtaacccgtttgaagaaagtgaaggttcttgaga

agctttgacaatgaaggaaatgccatctcttctagaagaacgaaaagcactaccatgttt

ctgagggaaagagtcgtgaaagagtgttctatgaagtgtaggtgaattctgaagggacgc

cattgttggcttggattttgcaaagaaaatgagactttttttcttattgtgttgttttgt

tgtatctatgtggtggaggacactcttgtgaatttgacttatccattatcctaaaagtgg

aaccacaat

>fava_dn_rep_c142

ttcaataatgatgttatccaatcaaaaaggtcgagattaggaaaggaagcataaataaag

acacagacagacaaaagttaccaacagcctagcattcaagacaataatcccatttgtaaa

ccgaaagaacataatttgcattaagaaaagaaaacaaaaggtgaacagagatataaatat

gaaagctttctgccagatagataagaatctgcaaagaattcaaaaatatgccacaaccat

tataccatagaatcagaatttaaaaccactttaatgtatagggtacccactaaaaaagta

caaattcaaagattatagttatcatttctcaagcaccaaacacttcttcgccacaccaga

cccaatatccaaaataattattacactgcaatcaagcatatcccattcgagatcgacgca

aaaaccttgtacagaagccaatgaaatatgaactataataagatatgttgaggggtttag

agaccctgttcaacgagataatcaccaagcctttcaactaccatgttgcactgaccaggg

gtcattggttcatcataaataccaatgaccagagccagagtggtcttcttaacagtaaca

ccaccaggacccttcttccctcgaataacagctccatgttcaccttgaatcaccatatat

ttagttccaccaatgtgtaatccagttggagcaagtgttccagactcagcaaaatcatta

ttgatagcagtaatttcctcaggcttaaactgagggaaattggcgctctgagcccaaacg

ctgccgtcttgaccgaggatagctgcatgagtgagatggttaccttcgatttcacagaga

aggtgatcatcgacgtaagtttgccacgacatggttgaagatttgagagattttcagaaa

agcgattatttctcggaaaagagattattgagtttgagtttgtgtgaagctgcgtcgaaa

tgacggagatagctatcctttaaaaatgagtctaaaagatgaagtgagggaggaagttac

gaga

>fava_dn_rep_c143

gcaaattgtttttaaccttgttaagaggtgtagagattatcacgaaaaattaagctgcct

tattgaattcaatgaacctcacataccaaacacacgtttttgctcatcaatcatcataaa

caactattacttacatttcagactgcacttagttcataaaggaagataaagctgttcagt

gaatatagttaacactcagcacaaataatccaaaagataaacaagtacttaacatataac

atagagaaatttacattgcggatcttccatgaataaacaaataagcataacaaatccaca

ttgcaaatcttcgttttttaagagcatcattcaaatgcccattggctttccctgcggact

tcctcctcttcctcaggactgaagtcattcttaatgttaaatgtcttgcggatttcctct

ggtgtctttcccttgatcatgtcggctaccgtctggcatgtcagatcaagaaggctcttg

atgttcaaatagtttgcagccagaatgagatcgaacaacgttgcctggtcaaccttgaca

aactcagcatcccaagccttgagttcatcttcagagggtttttcatcagaactcaccgca

tcaacgtgtttcttacagtattcaattaccttagcaaggatcttgctggtaacgttcgga

agaggaattccgctgtcggcacaatcatcttcgatcatgtgcttgattgtttgcgattcc

aaagccaccgcctcgtcaacctcaaaagcttcgccgtcggagctcttgagagtgattttt

cttggggaagccatggtggtgattagggttttgaggtgaaaattgggaaatggcgaacgc

tctttttaaaattgtgatcttgaaattgtgaatgaccccgcggccgtaatggccactctg

cgttgacac

>fava_dn_rep_c144

ttttcacgtgtaacactgatgacgtccatttttcttttgccaaggttgttagcagcatca

gaatcccaaaccctagcttcttccaatctcggaatcgcaatttcaaccatgtcgtcaaag

agaaaggttagggagccaaaggaagagacagtgactttgggtccagcggttaaggacggg

gaacatgttttcggtgttgctcgcatttttgcttcctttaacgacaccttcatccatgtc

acggatttgtctggcagggaaacacttgttcgcatcactggtggaatgaaggttaaagct

gacagagatgagtcatctccttatgctgctatgcttgcagcccaggatgttgctaccaga

tgcaaggagctgggtattactgcacttcacatcaagctacgtgccactggtggaaacaag

accaagacacctggccctggtgctcaggctgctctccgtgctcttgctcggtctggaatg

aaaattggccgtatagaggatgttactcccattccttctgacagcacccgtagaaagagc

ggtagaagaggtagaaggctctaggttatctcttatggtctatggaaagctggagttgta

tcttttactttccatgcaaaacataattgtggcattttgaaatttgatatttagccaatt

ttaatttggaacattaaaacatcaagtatttcttgtgttatgtttaagaatgagttttat

ccgtattaattttgaaagactgttgaaatttttgaattttgctccattgcttggaatgtt

gcccccttctaacggataggattcattcttttgttgttttactattttgctcctttataa

ttctgaaaccaataatacttagcaacac

>fava_dn_rep_c145

taatttaatttactccacaattaatataagatagagtaagatcaagacaagtcaaaatga

cttaaacaacctttcattcccctatccactaccactttattccagccatggtcgttcact

tcaacatggcgatttcaccttacgccgccgtttcaacacctcgtgtttattctcgaccta

gtgtttccactaaaatctactctggattgaaactccaatcttcacgtccttttggagcgt

cttcagtttcatctaacgtaaatgttcagtttttcggtgaagttaacaaagttctcaatt

tccggtatgctaaccagaaaccagttagggcacaaattcaaatgatgtcctttggaactc

ctagagtcatctataaaacacccggtgaaggaacttggcaatgggttgatatatggaatg

ccctttatcgagagcgtgttgtcttcattggagaagagatagatgaagaatttagtaatc

aattattggcaactttgctgtatcttgacagtttagataactccaagaagctctatttgt

acattaatggtcctggtggggatcttacaccatgcatggctctctatgataccatgcaga

gcttgcagagtcctatcgccactcattgtattggccaagcttataatcttgcagcgtttc

ttcttgcagctggagataagggagatcgtagtacaatgcctcttgcaagaattactctcg

aatctccagcaggagctgctcgtggtcgggctgatgacattcgaaatgaagcaaatgaac

ttcttagaatcagagattacctattcaaggagttagctagcaaaacaggccagccttttg

agaagattaccgaagacttgaaagtaacaaaacggtttgatgcacaagaggcccttgact

atgggatcattgacaaaattgtcaggccacgccgcataaaagcagatgcacctcgcaggg

attctggcacaggcattggttaagaccttattttacactctgtttatatttgcagaatca

gaatgtttcacattttatttccttagtcaactttgggaatatgtataatcttcgggtagt

catattatgttgtccatatcttgatgctcgttgcttgaattccaactgtattgtgaaact

ttacaatgtcctttattttctcttgcaaatttatcacatctcaggacattataaatatga

cgattttctatatagttttcagactagcgcaatattgtgggttttataactgcaacaaac

ttttgtttacttttgatcattacttttctatagtaagttaatctttaagta

>fava_dn_rep_c146

cccttcaacacaacacaatacattcttagttaataactactatttactttctctctgatc

tcttgctacatctgaaagactgttcttttttctactttggtttggtacgaggatgttgtt

ctttatcatgttcttctatcccacagtttttttatgagagatttatgaatggggtttgtt

atcaaaagaaggaagaaagaattagaagaaaaagaaaatagagaaatcatgccaaagtct

ttcaaggattccctcaaagctcttgaagctgacattcattttgccaatacactagcttct

gattatccaagtgatccagatggtgcttgccttcaaatgagattgtcctatagcccagct

gcccatttcttcctcttttttgttcagtggactgactgttacctcgccggagccttaggg

ttgcttaaaattcttatctacaaggcatataaagatgggaagacaaccatgtctatttat

gaaagaaatgccagtttgaaggagttctatggtgtggtgtttccgtctttattgcaactt

catcgaggaatcactgatgttgatgataggaaacaaaagcatttgtgcgctactaagtac

aagcgtaaagaattaataaacaacggaaagagcactgaaattgatacagaagaagatgaa

gaagagtgtgatatttgcatggagatgaacactatggttgtattgcccagttgcaatcat

tcaatgtgtatgaaatgctatcgagactggcatggaagatcggaatcttgcccgttctgt

cgggacagtcttaaacgcgtaaactccggtgatctttgggtatacataagcaccaatgag

atagatgatctagctacaatcaacaaggagaatttgaaaagactatttatgtatatagaa

aagttgcctcttgttgctagaccctatattcatctcatttcatcacagattatggttgcc

tcatagtggaatcaaaacctcattcaaatctattgcttgtaaataatatatgttccactg

tcactgtattcctttgattttattcattgaagcagtaacattgcatttgggttgtgattt

ttcaagtacgcaaacaatataacagaagatagtaaaagcattgatcaaaatgacatttat

aatatttaaagcctacacattagaggcacaatgcacaaaggagaatttaagtaccaacaa

caatttgagggaagctcgagtagctggcaacaaagtaacaagacaacagatattcaacca

acacagatatcgaaaagtagcctactacaaaatacataatagcatccctccaccaatgaa

acattgtattacaagttatgcacactgatttcaatcatcgtcttcttcatcatccccatc

ttcctcgtcttcatcatcgtcatcttggttgtcgtcctctccacttgcttcatcttcgtt

attaacttcagatttggacttgtctgattcctcctcgtcatcctcgactgaacttgccgc

tttgttattataagcgttcagcagtttttcatattcaactttcctttttgcagccttggc

ttcatatggtgctttttcagccttagtcaaggatttccatttctctcctccagcctttcc

aacagctgatacagccttcacattagggttttctgccttgaaagtctttctgaaatcctc

aaggaagacaaagaaagcgctgggaggcctttttggtttgttgggatctttcttggcctt

ctccttcttaggcttcggtgcactcttttcgggtttagaaacagcctttcgctttcctac

ttttcggtcatcaactggtttcagtacttccctcgaagtctttccagttcccttgccctt

tgtcatcgtctaagctcctcgactaatacgatcttgaaatagatgaagggaagaagagag

gatcttcaacaagcgaagcagaaccgaacacagcaagctgagaatcaaagcgacaaaggg

ccccgcggcc

>fava_dn_rep_c147

ttttttttttttagcatatcaacaaatcacatttaatcgaatcaaacttgtaacagcaac

agccaagtgtagacaattgaatttataaacaaatactccattactttaagaatctacaca

atatatctagaaatatgaaagaacatatacatagaacaaacagaatgcttagaaaagcaa

agacagaacttgtgcataatttaatcatatatatgcaagagctattttccaaaaatggat

ttaaaccaccaaatgcctttgttgtcttgggggccttcttcctccggcggtgtatcagta

tgaggtgttttgcggagtttggtgacatcatatccctcttcctttgccttttcaaccagt

tcattatagatatcatcatctaaacgagtctccctgcataatatccaaagggatttcttg

gttggttcaccaatcaaagcataatgataatcctgagcaatagccaaaacccaatagtta

cctgtaacaggaataattggaagaaatggaggaaggtaaaacttgactttgaatttagcc

tcatcattattagggttagccttaaaagcagatccttgaataaaacctcttttgccacca

ctccaagtctcgttaagaacatcaacagttccatcactgttgagtttgtatgttgctcgt

gtgttggatccatcctttggctgatcccttgatggaaaagaagctatctcgtaccatctt

cccatatacctttcaagatctaaaccttcaaccactttcatctctttttcttctcccatg

cttttgctgcctctactctttctttgttgaagattgaaatgatcccgcggccgtaatggc

gactctgcgttgataccnnnnnnnnnnnnnnnnntaacaacctctctaaatcctaaccaa

ttaacagaataacttcctaacagaatttctgttaaccaccctgctaacctcctaactaat

ttctaaccttatcctaactaac

>fava_dn_rep_c148

gttctatggaaaagaagaatgttcctattgcaaaatataggatatgcttcacatcaggca

taaacaagcgtcaaaagcattaggggacttgtattccccggagcaaaatcagatatcaaa

actatacaattatcatccacaattctacatagcattcaaagtcttcacaaactattgtgc

tcagacctacattttacataacaccaccagcaatgatatcacagttttctgcataccagg

gtgaaagtatgataagctatgtattatatataaaagacaacattaaattcaaagttaaaa

taacacatcccaaaggagattcttatatcacacgtaaatacaattgtaaagctacaaatt

ttccatttcaccaaaagtgctgtctatcagctgtaaaggaatactagagaagcattgtat

atctgcataatgcttactacagcatcacaaagatgcgacacaacaaagtagtttcactta

tttttggtcaagatcaatttgtgaaggatgactaattgtttgcctccctcagaatcctca

agatggaaaggaacaggttcaggatgtccagataaagagtgacagaggccccaatgtatt

catcataagtgaagcgcttgatcaggttgtcagtgtcatagacaatgtaagcagagaaaa

tcatagcaccaattccaccatagatagcatgtgctgctggtccaagagggaagaacatct

gcatcatgccggtgagaacaagagtaaacagacaggtaaacaaaagaggaccaaggtagc

taaaatctttccccttcttggaagcccaaaaggcataaccagttaaagaagaaaccacag

cagaggtcaaaatcaatgcctcgagaacaatttttccgtcggtgttagcacaggtaactc

caacggtgaagctaatcgacaaggtaaaaagtccaagcaaaatataattatgaggatgct

tttgttggtacctcaacaaaggaatcaaaaagataaatgggaggaagacgaggaacagga

gaagaggagaatttcccctgaggagaagattaagaggagcatagaaaacggttacgacgg

aaacgacggtggttagaaggatctgagcgcagaggattccgtagactttacggatgaaac

cccaacggagttgattttcgccgacgctgagaccggggtagagggtttctccggattcga

tgtcgaattcttgatcgtttttgacgcttgtgtagcctcgcttgtcctgctgcgcctgtg

gcgattcatcgaacatcatcatcttcgtctttatcgctcgattcctcttcttctttcttc

tgttttttttttatcttgaagatttggtactttgtttatatgatgatgacagaaattaat

gaatgcgcgttgttcagttttnnnnggtgacaggcttttttcaagccgttggattagaga

ttagagtttacttcatcatgtcatttgtaatatttatttaaaactttactaaaa

>fava_dn_rep_c149

gatgagtctagtttttttcatttaataatcgatgaacatatacttcaaaataaaaaagat

aaacatacaaattatgttctagactacttcaaaatagaaaatacatcatcaacaatagga

gtaataaaaaaggaacttatacgcaatagaaaattaagttaaaacacattaacccaactc

agtcaaaagattaaccagaaataggccaaaataaaagtatccaaaattttaacctgagaa

acaagtaatgcaggcaacaaaaatttcaagaaaatgttgatctaagtgcaaattcttaaa

aagttctagatatctgtctcttggatgcatacaaatgcatttcactcatcatcctcgtca

tcttcatcaactgcagagttgagattttccaagcgttggatcaacttagctcttctctcc

tcgtaggtaagctttttcagattgtatctcttgtgctgtgttggagccttcttctctgct

ttcttaatggtggggtctgcacggatggcagcatgaaccttcttgtacagctcctcaagt

ccatcagcctcaattcctttcttaatatactcgctgaaatgagtttggtatttctcaggc

tcatcttcagtcaaagtcttcatataggcagcaacgtgtccaccaaagatatatttgcgg

tgaacctcagcatcaagctccttcttttccttgtcaaaaccagcaaaccttttgtcactg

tgaggaatatccaaacccccatccagtgctcccttcagggcaccaaagacacggtttcca

gttgtggtctttacaagaccaacatcaaggagagcacggaatggcctcctggaatcagca

ggctccacagaataatcttctccagtagcctcaacatttccctcatattcttcatccatc

tcaagtgttttgagaacacgacgggccaacaggagtccagtgcaataagctgcagcataa

ttggtaagacctacttcaagaccataatgtggcagctcatgtgcatatgcagctgcaaga

acaatatcaccagcaatactagcagatactatttgcgcaacaatatctttgttggtaaat

cgcacaacaaaccgatacttcggagtgttgtacttgttcttatcctgattaatcaaccga

atccgagcacggtaatcggtcttaccctctctccttctcttaaacttgacttggtatctc

ttgaagtaagcctttgacttctgagacttgacaaaaaccattgttgcggaaggtgagaag

gtcgatccacaagagtggtgcaagagtgataaccagagctcccgcggc

>fava_dn_rep_c150

agtcattaaaacatacaacaacaaattgaaaagaaaaaaaattaagataaaaccgaggga

aaatttggttaccttctcgtacatgatataaaaccaaaatactagttccaagaattttac

aagaaagtattgagccaataaggtgaattaagaaaattactgacaaggacaacaataaca

tattgagattattttcaaacatgacaattacaatctacatgatgtctttctagagtagga

aagatgtccataatgcccaacatgagccagtacctagtagtagattgctaaaatgcttct

gtttttgtcagaattaaacttgttgacaaattgaataaggtttaagatttgactgcttgc

gaatcatctttctttggaataggaatatgaccttgctcttgcagactcttaacggtttca

tataaacattgactcactggagtgaattccaaccctaaatctttcagttttttgtttgag

aagatatgtggtttcactcgaggattcttttcatctgaacgcttggtaggaattgggtat

tcaggaaaatacttggccagaatttcaacaagctctccacggtgcaatgagctttcagca

cataagtatctaccagaagcagaaggcttttcataaacaagtatgtgtgctaatgccaca

tccctaacatgaacataagcttgtgttgcatttgcatatgtttttgcagagccggttaag

tactttagtatgtgaattgtgcttgcattgattgtgggttgtagtaatggtccaagaacc

aaaactggattcactacaaccaaatctacttctttctcttttgctatatcccatgctgct

tgttctgccactgctttcccatagcaataccaattcttggtgttcttgcaaaagtccaaa

tcactccagcaagactcatcaacttcaacatcaacacttctttttgggtccatatagaca

gcgccaattgaggaagtgaaaaccacgcgcctcacatttgcttctgctgcagctataata

acattctttgctccatttactgcaggctccaccatctcttcggggttatcagtaacggga

gaagcagtatgaaaaacaccgtgacaaccgttaacgacggctttaacggagttaagatcc

agtagatcaagtttatgcagagttagcctttccttggctccttccaactccatcaagtgt

ccatttttcgcatcatctgggtttcgcaaggttcctctgacggtgtagcctttttggagg

aggagtttaaccatccatgaagcgatgaatccgccggcgccggtgacgcatatggtttgg

ccggaaagcggtgaaggttcggcagaaggcatattcgtgattggaaagggacaaaggaag

gtgaatgagtgagtgggatagaaggtgatgggaaagttgttgtgatgactagtgagtggc

gtggggatgaataagtggaaatatcccgcgg

>fava_dn_rep_c151

tttttttttttataaaaaaatgtaattatattgaaatttgtgtaaacaaatcattttaaa

tccaatctcaaagcattgtacaaacctcaagcaaataatatatcgacactggtaataaaa

aggttacacatgtatacaaacttgaaatagccatccatgggaattgcaacgcagatcaat

gttcatgtcaatacttgatttttctctttcttttctattttacaaattcaacttaacaac

aataaagatggaacatagacataaactataagatcacacttgaatcgcaccaaacatttc

ttcattccttactttgtggcttgtgcaataatattactcaaggaactggcttcttcctta

gcagtctccaccaatgaatcctgtgcagctatcaaacgggcaacagtttttttgctcgac

tcctgaagctccccggcaattagaagttcatcaagtatataataggccttatggaagttg

aatatcaagtccaattcacagacactgccgaaataccgatcaagaatctccacaaaatga

tgaatcatttcaaggacttccaattcgttgtcagcatcatcaatacacatacagaaataa

agactagcataccttttataaacaactttatgtcctctccactctacaaagttacatagc

ttgggagcacgactaagaatcactccgctgagctcacggattaccttacttctttccttc

tgagtgtaaggtgaataccattttgtgagcctgaccttcccttgacgactaatgagcagc

acaaagtgtatcattgaaattggggaaagagatctcaatgttgcagagatagaaatggaa

acgaagattgttgacatgtacgattacgaatgaagagagttttgtttttgttttttgtca

aatgaaaatgtggg

>fava_dn_rep_c152

ttttttttttttcaataaaaactgaatattataagttggaatgataatacaaaaaccatg

ataaaaaaataccaagtagtacacaataactacacacatttactataacatcaccacata

aaaaacatgatctcaaagaaaaaggtaacaattaccaagttcatgttattaacaacaaca

ctgtaattgcttaatttgaattaactttttattctgttggagattataggaactggaaac

tgaatgagatgcacaacacaacactcttaatgtataaaaatcactgagatcctttgagct

tctttgcaatggcagcaaagtacttcccctgatggaatcaacggggtagtctcttggcca

cctccttgagaaccagtgctgtaaaagattcctgcaggttttcctgcaagtgcccgcggg

gatcgcaccatcacttgcttcttccttttcctcattcacagcgttacacaaccatcatcc

atcaatggctgccactaaagtttacattgtttactactctacgtacggacatgttgagaa

gctagctgaagagattaaaaaaggggcggcttctgtcgaaggagtagaagcaaaactgtg

gcaggtacctgaaacacttccagaggatgttcttgggaagatgggagcacctccaaagag

tgatgtgccgattattacaccgaatgagctaactgaggctgatggtttgttgctcggttt

cccgacaagatttggaatgatggcttctcaatttaaagcattcttagatgcaactggagg

cctatggcgcacacaggcacttgcaggaaaacctgcaggaatcttttacagcactggttc

tcaaggaggtggccaagagactaccccgttgacttccattacccagcttgttcaccacgg

aatgattttcgtgccaattggctacacattcggtgctgggatgttcgagatggagaacgt

gaaaggtggctccccatacggtgcaggtacctacgcaggggacggctcgagacagcctac

cgatttagaattggctcaagcattccatcaggggaagtactttgctgccattgcaaagaa

gctcaaaggatctcagtgatttttatacattaagagtgttgtgttgtgcatctcattcag

tttccagttcctataatctccaacagaataaaaagttaattcaaattaagcaattacagt

gttgttgttaataacatgaacttggtaattgttacctttttctttgagatcatgtttttt

atgtggtgatgttatagtaaatgtgtgtagttattgtgtactacttggtatttttttatc

atggtttttgtattatcattccaacttataatattcagtttttattgataaactgtctca

attttctatgaataaaagtatttgaaaggaatataattttgctgtattccaactcg

>fava_dn_rep_c153

gattatgaaatgagaaattcacattttcttctttaaaaaaacattctatacccaattgag

atacttttcaccttttagatcttcattcacacaaaaaaaatccactttggatcaacaatc

ttttcattcaaataacacacaatattgtattccattcatcatacgcagagatttatgatt

caatcttttagctacactttacacaagacaaccaaacaaaacacaaagcaaccacaattc

tcataacctttcaacaaaatcaaacaacatttcaatctgctgaggagagatccatgacca

catcatattattcttaaaaagccaacacaaatgcttcttgtagatattaaacgtgtactc

atacacacaattacacataatggttccaatatctctgcttcaagctcttatatgtcacac

ttcattaacagttacaaggatcacaagtgcacttatcaccacacttgcagccttcttcct

tagaagcaacactcatttcagcaccatcacaatggatcttcgccggaccgaagccaagaa

tcacagtttctttggtttctttggtttccatttcggagtagctcaatccactagacctct

tgttgcatttgcagctatcaccacagttgcaactgcttccacaaccacagccagacatat

tttgagatactaagattttttgcaaagaacaattgatattacttggttgctgttatctta

ttggtgtcccgcggcc

>fava_dn_rep_c154

gaatggggagataggaagaaggagaaacagaaaggataaaggtgagagagagagaggaag

ttctctcctatctccataatccaaaatggcttctgcaaccgctactctttccactctctc

ttcttctttccaaacccattgctcaatttcccaaacctcaacaaaatcccatttctcaat

ttctcaacgcttcccttctcaatcttacaacctctctttccaatccactttctcacaacg

cttccctctatcacccattcccaaatccactgaatcttcagtcgcagtcgttgagtccaa

gtctgaagcttctccaactcagccgaagtctgattccactcaaattgttcaatctccgtc

atgggagaagggtcttttcgcagttgtaatgatcggtggacgtcaatatattgttcaccc

tggccgttggcttgttgttcagaggctcaaagatgctaaggttaacgacaagattgccct

gcataaggttttactggttggcacagatacatcctgctacatagggaaacctatagtaac

aaatgcggtggtatatgcaaccgtggaagatcagggtttagacaacaaagtaattgtatt

caaatacaaaagaaagaagcactaccgcagaaacatcggtcacagacagccaaatacacg

catacggataaatagcattatgggctatgaggactacccaaaagttactatggatgatat

aaatttggaatcatcatgaatattttgtgcactatgccagtattatatcttctttcatct

ataactattagcaaacattttgccatgtttagtttttcttaatggagttgtttgcatgtt

tatgtagctactgtatggcatattgtaatattttgtttccaaatacattgctatttttcc

tgtctccagtttccattccaattcctactatgaagagcttgtttgctctgaattgttaca

cgggttctccttaccttgcatggaatggtttattggaacgagtttaatttcaaaatgtca

ctttgaattggttccttttatcttattggagcaagtaaacatctattttaaaccttagtt

agcc

>fava_dn_rep_c155

ttttttttctttactgaatataaccaaaaataaaccccaaacacttaacaattatctaaa

attccagattaaacaagtgtaagaggggtggctcaagaaggtaagaaatacacacgtttg

agaagggggtgggtttcagctttatttctacttcaaacaaaaaacaagacatgattttgt

gtaactccaacgaatttgacatgatcagaattggcaaaattattgccagtaataataatc

cttttcataaataaaaacgaagcttgaaggcagctagttgacaacatcaagaaccaactt

gcgggacttgagaacagaccatcgaagacggcgataatatgcagcttgaagtaatgccaa

tgaaagtacaaatatccatttaaatcccatcagttttctctcttccatctcaggttctgc

agcccaagacaaaaatgacacgacatctttccccatctgagcttctgtagcaggggtacc

atcttcatattcaacagcaccatcattaagcatcttaggcatggcaatggctccacccgg

aaaataaggattgtaatgcaatccctctctgatcgaaacgccagcaggggggtcacgata

accggttaggagggcaaacacatagttctgaccattgtgacgagctttggtaataagact

tagatctggaggataggcccctccattagcaaacctagcagctgattcatttgcatatgg

ctgaggaaagcggtcactgagtttaccagggcgtgaaaacatctcaccctcatcattagg

gccatcttccacctcaatctcagcagccatggcctttacctcttcttctgtgtaagccac

accaaccaaatcacggtatgatatcaaagacatggaatggcaggaagcacagacttgtgt

ataaacttgatgaccacgacgaatcgaagcatgatcatatgaactgaggatgccctcgtg

aggccatggatagtgcggagatgcaaggccatgctcagcttcatcagcagatgctgttgt

tgcaaaacccaaaagacctgagacaccagctcctattaaagcaaggcctcttaaggaact

gctgccagtagagccagaagaactccgagaatgagattggagtttcctcctcaacaattg

ttgaataacacctccagccatctcaattcagagaaatggaagcgcgattgggaagtgagg

atcgaatcgaatggagaagaagcagcagagagagaaagaggctgctgattgaataagagt

caagaggggaggtgtttggggacccaatcggattaacgattgtagcgttc

>fava_dn_rep_c156

tgatatttcaggttgaaaaatgaacggctgagatgagatctatgttactcaagttttcta

tttagagatagaaatggaagtcgccgggacttttgcttttacaaacacacacttgtgaag

agggagtttcttattccttctagaccattctagcggaatcttgaaattagggttttttta

tcgatcggcgattgaaaactgagatttagatttttgaggagaagttatggcagatcatca

ggagcatgaggaagtaaagggtgaatcattgttggagaagatttcggggaagaatcatga

ccatgattcgtcttcgtcgtcttcggattcggataacgagaaggagaagaagagttcgtc

gccgaattcactgaagaataaggtgtataggctttttggcagggagaagccgcttcacaa

tgttcttggtggtggaaaacctgctgatgtttttctatggagaaataagaagatatcagc

aacaactcttggtgttgcgacggctttctgggttctgtttgagttacttgaatatcatct

cctgactttggtttcccacttagcgatacttgctctggctgtgttgttcttatggtcaaa

tgcatccactttcatcaacaagactccaccaaagatcccacatgtgcacattcctgagga

acctgttttacagattgcctctgctattagaattgagatcaaccgggcttttactctatt

aagggatattgcttctggaagggatctcaaaaagtttctctcggtcattgctgggttatg

ggtcttgtctattgttgggagttgggccaacttcttgacattgttctatatagcttttgt

tttgctccacactgtacctgtgctttatgagaagtacgaagatcatgttgactcttttgg

tgagaaggcacatgctgagttgaagaaacagtatgcagtgtttgatgagaaggttttgag

caagattcccaaaggacctttgaaagacaaaaagaaagattgaagggctgacaagtatgt

tgtagtcgggtttcaccgatgctgttatattctgattcatcatcagtttaaaccctgtga

tgttataattttatttttttagggtttgttagtagacatgacattgtcggaaggaatgct

ttatatgtttttgtggcgcttacatgtaagccttggaatatcttatatgtttatggttga

ctcgctgttgtgttgtatgttcttgatgaatatagtactttctgctcttgaatgttttca

taggagtctgtactggtacttatgtcactttacactggcaatctcagttgttagacaggt

ttcccatctagttcacatgatctgcctagattcaaaaatgggcatattgcctttcccagc

ccatgttattttgctccaacctggaagaacatgggcacatggcatttttctcctcggact

tctagttctcttgcgaatgtaaggcttctttttgtacaatcaaaagttgctgcattttag

catttctatctctcaaaccactatttaggtattctgtatgtttatttgatctggcactgt

ttgcattggaatggtccgagtgttgatccttttaaagttgtttggattgcatataaacaa

cgttactattcaaaaattgatattgatttaaattgaagcttgattttggactttcaatta

ggtcattttaaaattgaaagtcgttgcataagtgactttttaaggcacatgtataat

>fava_dn_rep_c157

ccgcgggaagtttttcctttaccttcatcttcttcccacctttgcgctctgtggtctttc

catgactcgtatccgctgaagtagtaaccctcttctcaaaccataatctccctcttcatc

catggcttctcaagtttttcttcagactctgtttgctcctactcaatctcttccgaacaa

gctatgtttctcttcttgctccgcttccacattcggttcccgtaacttgccgttgatttc

aacaacctcaatttcacgacgttgcagtcctctcattgctaaacctagttctgttgttag

agctgattccaatcccgacgctgcttcggcttcggatgttgtcaatgaagttccggaaac

tgatgctgaagaggtttcgggatctgatcctgagattgagcaattgaaatcacccagaca

aactcgggtcaagcttggtgatgttatggggatattgcataagagggcaattaaggcttc

ggatgaagtgagggtcactcctgacattaggactggagatattatagaaatcagattgga

agttcctgagaacaagcgtaggttatctctttacagaggtattgtaatctcaagacaaaa

tgctggtattcacacaactattcgagtccgaagaattattgctggcacaggtgttgagat

aaccttcccactttactcgccaaacatcaaagaaatcaaagtggtacagcaccgaaaggt

caggcaggcaagattgtactatctaaaacagaagctccccagattctccactttcaaaaa

attataaggtcatacattgttcatcatgttcttagtcaaagtaatctatggatcccggac

atgttatagatgggccacaatttgattatgttgatttgttgtaatgtagattgtataatt

tttcttccctttaactatcctcttgaggaatggaattgtacgttgtttttgataatattg

agaaatttgaagtgtctatactgcatgcttatatcctatagaaagccaattgctgctaaa

taaatacgggtcccatttgaaatagtaagacctaatcgttgtatttttcatgtgttgtga

tgtgcctgatcaaagcatgattgcggatcttggaataaaggctatacattttttt

>fava_dn_rep_c158

tttttttttttacaaaactgagaattttattaaggggaaaacatttagtcacacacaaga

catgtttcctcaaataaaataagaaaagtacatatggtatgattatatgctaacacagtt

gaaatataaatagttgtaggcttttattgaaaggaacacaacttttcaagcatggtactt

caatggaacacttgggatatttttttttttcataaaaagatatttctttggattcatatt

tctaacatataatcagccacatacaggttcttctgctagagtaattacattgatgaaaat

gggagtttaacacaacagaaatgaaaacgggagcagatgattgcagctgaagagacaaca

ttattattgtccggggacaaagttagtagcgaaagcccatgcgttgtttgcgacagggcc

agcaacgtggtcatacaggttctgaatagggcacttgccagtaacaatggcttgaacaag

gaaaccaaacgtggggaacatagccaaccgaccgttcttgagttccttgaccttcaactc

agcaaatgaatcagggtcatcagcaagaccaagagggtcaaaagcaccaccagggtaaag

tgggtctagtccttcaccaaggggtcctccaccaattctgtacccttcaacaaatcccat

gagcacaacctgagtagcccagattgcaagaatgctctgagcatgaatcaggtttgggtt

tcccaaatagtcaaggccaccctcagcaaagatctgggaaccggctttgaaccacactgc

ctcgccgaatttaacaccattcttttcaaggagttcagggaaggtacatcccaacgcacc

aagcatagcccatctgctgtgaattacttcaagctcacggttccttgcaaatgtttcagg

gtcagctgataatccagctgtgtcccatccataatcaccaggaaactcaccagtaaggta

tgatggaatttgttcagagaatggacccaagtacttgggacggtcaggaccataccaaat

gctttcaggagcactcttcacggtacgacgcatggtgaagcgactttggccaaggttacc

aactttcctgatgaattcatttccctgcctcaaatcagtcttaccagtgaatgctgattg

ttggatagcagaggcggccatgagggcgttgagacttcttgcactttgatgttgttccct

ttgatgctagtgttgttgtcccgcggccaaaaatcttaattgtttg

>fava_dn_rep_c159

caaaccataaatgcagctgggaatattatttgataataggaaccaactactacgagtaat

tagtagcaactgtcccaatttcatttaccagattattggcattgtcattccgagtctggg

caaaaacaccaaaccaatagaataggatctcaatacaacaaacatatcaaacagagtttg

catcaacaggaccacaggatgcatttgaatatccatacccgaaaaataacatgaacatga

gccagtgaccaagtgtcggtattttaaaaaaatgaagcccatttgttgtccgatcacaca

aaacagttacaaattccacctaataaggaaacaggactaaaatgccactcaaacacagat

tgtatatatcacaaatcataacaaaatacacaggaacaagagaagacactgtcgaaggtg

caatacactttatgccacaacttggaaaattgagagtttgagaccataacctagtcaaat

tgaccaacccacaaactatggatatctcaaataagagcaactaaggcttaaggggagaga

tctcccaccagaaaagcctatactattcataatcattatttaattggcacggcttttgaa

cacatcaagacccatttcagtaggatcagttgcctgcaactctatttggatcccatccag

ctccctcttgaacctgtctttggagcttgcataaatcatcttgcttctcacccttgctgt

atcaggtgaccagccaatgaaaaaaattctgctcttggggacattcccttcggtcagata

ctcaaaatcataaaccgcataacgacactcatcagccggaagggaagcagtgaaatcctc

atagccttggattggttcaccaagcttctccacaatcacttgcttctgattctcctcaat

cttataaacaatgaaccggtgtgtcctttttgccttgagctccataaaccttaacttgca

gtcatcatggactgccatacctgatgctgcatttgccatgagttagttgtggttgttgga

gatttgattggaagcgatggtggtggagaagatataggtgaagatgagtttgttttctcg

attgagggttcgatgcagagagagaaaaatgagaaatgagaaatgtcccgaaaaaggtcc

ccgcggccaccatttgggttttctgttgctgatcagccttttcaaagctttccaagggca

caagtctccaaccgaatggatcaagctcaagcttatgcatctgctgctccttttggtgga

agaccgggctctgtctcaattccaccccaacaacaccctgcatttccctttgctggtcaa

ccttcaccaagatctcaagtcccacaaatgggtatgaggaacttcattcctacccctcaa

atgccaaacttgcccagtcctggggtccaaagaggtatgcacaatcaacaatgctatcct

gctcagggaacatggcctatgaatcagaagtttggcagcaacccatccttggcatccagt

aagccagcgtatcatgcagaccaaatttatgatcccttttcacccacatctgttgcatct

ccacaccagaaaggtaatcttggaaaatgaggaaattggatatggctacagatatgaaat

agtgacaaaatctcagttatagctgcagtttttggcagacggaggcgatacaaatttcat

atgggtacagttgtaatttgcaaattcatattgtttctaaccagagaaggttcctttagc

tccctttgatgactcgttgtattcctttttaagtctacctttcttttatgaatgcccatt

attgatgctttgattaggaattgagttttatgaaactatgtcaaaacaaaatcattgtag

caattatgatagcatcttatttggtacattgtttagcatgcctaagactaaacctgtaat

ttacaaattattgtatcacaataaaggccgtttacattggtaatttggaaaannnnnnnn

taacaggtcatggaagagtataattttgtttagtaaagaaaagaagagctattttg

>fava_dn_rep_c160

aggctttggaagcagacgtttttcatccaaccatctgtgaccacattggataagtgggcc

acatgttttatttttcttaactaaaacaacaataatacatgtggttgtgaaagagtagca

attttgaacaattgatttgtcgtcctagaataggaacagataattagagaaacaatggtc

gcacttgcagctatagcaagaagcttccattttcagaaacattctttaatcaacatcaat

accattggtaaaccttcagcagcaggactttactggctcaggcctgctgctgcagaaaca

ggagttgcaccacgttataagcaagtttgtgcagcagcacaacagcgaccgacatggctc

cctggacttgattctccaacttatctcgatggaactttggctggagattttgggttcgac

ccacttggacttggagaggatcctgaaagcttaaagtggtatgtgcaagcagaattggtt

cattctcgctttgcaatgcttggtgtcttgggaattctagtcacagatctacttcgcgtc

gcaggagttagtaccataccaatttggtttgaagctggtgcagtaaaatacgagattgcg

aacacagggacgctcgttgctgttcaactacttttgatggggtatgctgaaacaagaagg

tacatggattttgtaagtcctggatctcaagctaaagaaggttccttctttggattggaa

gcttcactggaaggcttagagccaggatatcctgggggtcctctgtttaaccctcttggt

ctagctaaagacagtaaaaatgctcatggctggaagcttaaagagatcaagaatgggcga

cttgcaatggtggcaatacttggcatctttgtacaagcttcagtgactcatgttggacca

attgataaccttgtggagcatctctccaatccatggcataaaactattattcagaccctt

gcaagttctagttcttagttctgacacaaatacatgtactagttatatggaacttggttt

gatccataaaaccatattgtttcttttagctttggggaaatgactcaaattctgattcct

aaaatattttctctttcaagttttgtttcagttcctatgttgactaaatatctaacacat

ttaactaacataaattattactcgattaaataagtttgt

>fava_dn_rep_c161

ggcaattcacttctagtagatttattaaaaacaactacaattctctcattttaatggtcc

aaatcttcaaaagacaacacagaatccaaataacacacattaacagacttaaacggcgat

gaaaagtaaagtcaactaaaacaaacgcatttaacatattatttactgaccaaatgtcat

ggcagtacactatttctgcataaaaacttaaccaaaaacccaattcatcgcccaccgatg

aggtaaaaggaaatcatgtttattcagagtacttactaaacacaagttcaaagcattgtt

acaaatacgaatcataactcaacagaaatttccgaagctgcttcaaaagcagcaaatcaa

ttcctacttacgaatttcagttcttcttcacagtcgcggagttgatgatgtcaatgaact

ccggcttcaaggaagcaccaccaaccaaaaatccatcaatgtcaggctgtgatgccaatt

ctttgctgtttcctccatttacagagcctccatagatgattcttagagaggcagcaactt

cagcacccacgttgtcgtgaacccatttccttaaagcagcatggacctcttgtgcctgag

caggagttgcaacctttccagttccaatggcccaaactggctcataggccaaaacaacat

tgtcccagtttgatactttgtctgcaattgcctttgtttgctcagaaacaacatccagag

tagtaccagcttcacgctgttcaagagtctccccaatgcaagcgataactttcctcccta

gtgagagtgcatatgcaactttgtctcccacaaactcatttgattcatttagaagctgcc

ttcgctcagagtgcccaagaataacataagggatatccaaatttacaagcatttcagcac

taatctccccagtataagcaccacctttgcgaacccaactgttttgagcagcaacatgga

agtcagagcgaagtaagcttttcacgaagggaaggaacacgtaaggggggctcacaacaa

cttcaacaacatcttctccagggactttagcttcgttcaaagtaccaacaatcttcttca

cctcttcagttgttccattgcatttccagttaccgccgacgaagaattttctgcccatgt

tttggttgatgttttcgatttagacgaggaagtgaaaaacccttgttgttgttgctgttc

tgaagtgtttgttgttcaccctttatattgtagataaaccgaagaatatgctttaaaca

>fava_dn_rep_c162

ttattgatgagtatctacattttgttatttaatcctatgacaagtacatgttttccgagt

ataccatacctgtattaaaatctctcaannnnnnncttttttttttaaacaaaacgaggc

cagaaatgaataattcttttttcaaccatctcaatagaaacacaaaaacaggtgatatat

aaaaaacgtctcatgtctaagcaaaatgagcactttaacaatcttaaaacaataacttaa

tataataaagcaaaaaaactagatatatgtttctccaggcttcaaaacagatagatagat

aaatgataaatcaatcttcatcatctatgaccttggtgcccaaaccttttagctcctcct

taggaatttcaacaactgtaacaagggagtataattcctcctttgcatcttcatcgtcgt

tcctcttgcgcgcaatgcgtactctgactctcctgggaacacttctaattccttggctcc

agatagccttgttcagcttgacatcgaccctaacatcgttagtgcccatggctttttgag

caaacttccttatctccttgatagcttttggtgccttcttcttgaatgtgcagccatgaa

gacgcttgtgaaggttgatcgtgtactccctggtaacaacctcctcttttctaccacctg

cacccttctccaccattttcaacttctgataaccctaaaccctgaagactgtgcaaggtt

ttcggcagtgtttggcacaaaacgaaagaggcccgcggnnnnnnnnnnnnntgcaaataa

tttatcaatcgagaatt

>fava_dn_rep_c163

aaatgagcacattatataaagtctacactcacaaaccctaaaaattttttcgcgttctca

agtggccacagtgatacacagcctcgccggcaaccatccctcttcaacaaatcctccccg

gctaggcgaaaatgtcattggtggcgaacgaagagttccagcacatcctgcgtgtgctga

acaccaacgtcgatggaaagcagaagataatgtttgcaatgacctccatcaagggtattg

gaaggcgattttctaacatttgctgtaagaaagctgatattgacatgaacaagagggctg

gggagttgagtgctgcagagttggataatttgatgacagtggttgccaaccctcgtcaat

tcaagatacctgattggttcttgaataggaagaaggattacaaagatggcaaattctctc

aagttgtctccaatggactcgacatgaagttgagggatgatttggagagactcaagaaaa

tcagaaaccaccgtggtttgaggcactactggggcctccgtgttcgaggtcagcatacca

agactactggccgtagaggaaagactgttggtgtctcaaagaagcgttgagtggttatat

tttgacatggagattagatattttaaggaatgcatattttgagttcatttagacatgttt

cttagaccgctgttaaacgcattttcaatttatttcaattttactatatgatattggtat

ttctctgctaaaaaaaaaag

>fava_dn_rep_c164

cggctaaggggtgtctgtcatatttggatcacacgttgaaacaaatcaaccatggcttcc

cttgctagcttagcttctgttcaaccagcttctctcaatgttaagggtcttgctggtagt

tcccttaatggaactaagcttgctttcaaaccctctcgtcagagtttcaaatccaagaat

ttaaggagtggcgctgtggtagcaaagtatggtgacaagagtgtttactttgatttggag

gatattggcaacactacaggtcagtgggatttgtatggctcagatgcaccatcaccctac

aatcctcttcagagcaagttctttgagacatttgcggcaccattcacaaaaagaggacta

ttactcaagtttttgatccttggaggtggttcaactcttgcatacttcagtgtcacagcc

tcaggtgacattctaccaatcaagaaaggtccacagcttccaccaaagctcggtccccgc

ggcaagatctaatttagtttccgaattcaatctatttacttttctcactttgaaaccgaa

catgtatatctatctcgtatcgcatgatgtgtaagtatgattacatcttacttctaatca

atgatgcttattagtcattttcagtctatatatacagcgtcattgccgctattttcgatt

gaatcgaagataatgatgtctacataaagtttattaccacaacaatagaaaagatactgt

ggtatcataaaaaattctcaagttggtagctacctaccagactacaaatccttcagagga

agatatatctaggacaatgtttcgaatgattccgccattcctatgtacaa

>fava_dn_rep_c165

ataaaaggcaccactctcttatttggctgcattgcacataacatgagttctacgtatgtt

gttgcattacaagttgtgttatcttgcatctgtttggttttggcaaattctgttgacttc

cattatccggcagttttcaactttggtgattcaaactcagacacaggcgaacttactgca

gctaagggatttaaactcgccccgccttacggacaaaattacttcaagacttcatctggg

agattctgtgatggccgtctcatagtcgacttcttaatggatgcgatgcatttgccattt

ttaaatgcctatttggattcagtgggtttaccaaatttccatcaaggatgcaactttgca

gcagcaggttcgactatccttccagcaaatgcagcgtcgattagtccattcggctttggc

gttcaagtatctcagtttcagctattcaaagcccgggttcttgagtttcttgcaggcaag

aaatttcataaatatgtcccagccgaagaatatttccagaaggggttatacatgtttgac

atagggcagaatgatattggcggcgcattttattcaaaagacttggaccaaatactttct

ttaattccaacaattctactggaattcgaaaatggaataaagagactgtatgacagtggc

gcgaggaatttttggatacataacacgggtccgctgggatgcttggctcagtatgttgcc

accttgggcaatgatacaacgaagctcggtgaacaaggatgtcttcgagcacacaatcaa

gctgctaaagcctttaatctacaactgcaagaattttgtactaaattgcagggccagtat

ctagatgtaaatgttacatatgttgatatctttactataaaatatgacctcattgcaaac

tcctcaaaacatgggtttgaacaacctattatggcttgctgtggatatggaggtccgccg

ttgaactgtgacagtcgcatttcttgtggactaacaaagatcttgaacgggactacaatt

gcagcaaaaggttgcaatgatagtagtgtgtatgtaagctgggatgggactcattacact

gagtctgcaaatcaatatgtcgcatcacaaattctcactggaaactattccaacactcat

ttgtagtagaaggtgtcattccttctctagctcaagttctaatccaactactgattgtgt

aacctcgaatgacattcaagctctttaaacaaatcttatttaagaccgaatcaattgaca

ttatgcaaagttttatgcttgtttggagttgtatgacacataacaggcgataagccgata

accttttttttgggtaggtacacgtgataacatgaacccttctgataactgataaatgtt

tgcatcgagtacgtaagaactggtatataaaaatagtcgaacaaacatattttcgaatta

catttacaagtaaaaaaaaaaatctgatcttatttgtcatttttctcaaactcaatcgta

acatgagctcgtttactggcttacagctgcacattatacatctgaattcaaagattgata

attcggaagacagatctagatgtgtggccattattcccttattttttggctatataagca

tagtattgtaccataggaacaacaaatgcaatgaccaacaaacctccaaccacaagagag

aattgtccgcgcttctcatctgtctcttgttttgatttgaagttagactcccgtttgtcg

tctttaactaacggcccgccaggatctggaagtccgtcaatggcagcaactagccgttta

gccgtgctataaatcgcttcgttgtacttctcatccgtagccaatactggaagattctct

gaaacggtagcatcgaggattttttctccgacggcttggacaaaagcaggtccaccagta

actgctccttctttctgacttgttacaagaaccacaacacccttttcatttccctcctcc

acagaaggataccatcgctccaaaatttggtcagcaaactcaaaagcatcagctttactc

gtgagttttctgagtgtaataaaattgatgtgaaaattcttccttgattccaaatcagac

aacaaccgtttcaaatcagacttggtcaccctactaagaactccagcatcatccaccaca

tatgaatctttggctggcctctcattgatcacatcaaattcagacgccaatgcattgcca

ctatgcagaactgggctgaagttaagtgctaaggtgatagctaaagctgcaagaccctgt

tgagcatgagagaaccaagatgttgatgaaggaagggatggtggttttagagataaagtt

tgattctttttgagagatgaaacaatgggtttgtaaggaaagggggaatttggtttgggt

tgaagggaaggtgaaggtttgatgttgagaagtggagagagggagttgggaggaaaaagg

gttcccatgaggaagatgaacaagggttggagtgaggaaaggtgaagttgttcttcctat

gttgttccacatccctggctttgaatgttgtttgtatttgagttattatctgtatgtcaa

caataatggatcttttacatgtatacttcatcacaaataatataacattataagatcttt

atgttatattgcataccaccaaatttgtgcatgttttaattctcattaggtttggtaaaa

tgtgttaggttctactaagatcaatttgagtttgtatatgtgttatacaaaattactttt

ataaataaaaatagatgagaagaaatagagaaga

>fava_dn_rep_c166

gatagttattagttaaaacgaaaaagagttaaacaagtgaacggttggattggagataga

tatggtgtgagttgaaagaagacattgtttgggtatctttaatttattccattctattct

tttcttgtgttcctcatcgattcaccatccacaacacaagtaccgactcaactccattca

ttcccatccatggcttcttcaattcgacctactctttccctcttcaatcgctctcctcgc

ctctctttctcccatttttcaaccgtagctgtagttccacaatccaggagttttgggttg

aaagcgtgtagaatgttgagaaaaagtggaaactccgttagagttatggcatctgggaac

gtgccttcatcactcactgatgcttcacaagaaaatctactggaatgggtcaaacaagat

aaaagaagaatgcttcatgttgtttatcgtgttggcgacttggatagaaccattaaattc

tatactgagtgcctcggaatgaagctccttagaaagcgcgatatacccgaggagaaatat

actaatgcttttcttggatatggacctgaagactcacactttgttattgaactcacttac

aattacggagttgagaagtatgatattggtactggatttggccattttggtattgctgtt

gatgatattacaaaaacagtggaactaataagagctaaggggggcaaaatatctagagaa

cctggtcctgtcaaaggaggcaaaacagtaattgcatttattgaggatcctgatggttac

aaatttgaacttttggaaagaggccccactcctgagccattgtgccaagtgatgcttcga

gtgggtgatcttaaccgttcaatcgagttttatgagaaggcttttggtatggagctgctt

cgaacacgagataatccagagaacaagtataccatagcaatgctgggttatggtccagaa

gataaaactactgttctggagttgacatacaattatggagtcacggaatatgataaagga

aatgcttatgctcagattgcaataggtacagatgatgtgtacaaaactgccgaagcgatt

aaactatccactggaaaggttactcgggaacctggaccattgcctggtatcaacacaaaa

attactgcatgcctggatcccgatggatggaagacggtatttgtagataatattgatttt

cttaaggagttggagtagaagagtatttaggtaggtcgtcatgacagcaatcttgtaaca

tctgtatcaaacctctgccaaagaactggtatgtcttatatgaagaaagaagattgagaa

agcatcatatcatatccgattttgtagaaaatgtaatattaactctagctatggtgcttg

tagggtaaatggttgaattacaacaaagttatttattcaatggattgtttcatggtctat

atatacaacacaagtaattttatacgcatgcatttactataataaattcatattttattt

aaaaaaataggttaccatgtta

>fava_dn_rep_c167

ataaacaatttgaatgaaaacgtttttcccttttaccaatttaagcttctttgagcggtt

ccttttgccctgtattatctttcaaagaaacaaaacaacttgttcatattctcagccctt

tgcgcatttccacaaacacctcaactgcacgatcgatggaagctcttacttcacttcacc

accgtcaccaacctctttgttcctccatctacccttctctatcatcatcaaccacttctt

cactctttctctcacaatcttcaccatcatcatcttccatcaaagcttcatcggcttcct

cagattcccttcaatacccagagcccaaaacccctcaattttccaaccctcttcaccaaa

ttccaaatcttgaccctcctcttcatgaatcagaacccaaatctccccaatttttcaacc

ctaatcgtaaacttccaaacgttgacccagaacccaaatctcctcaatttcctaacccta

ttcgtaaatttcctcttttatcttctagttgaaacattggatccaaaatccattttccca

ccaccaatttctctccgcatagaatctaaaccttctcttcttctctttcccatatcaacc

cacacacactaaccattcttcctccacacaaacaaaaatgtctctcaccattccaactaa

cctctccaaacccacctcaaccctaagacccaaactcaccctaaaaccaaaactatccac

aaacataatccactgcagcacaaaccaaaccccaaacaacctagaaaaaaccgaccatgc

aaaccaaaacctaaaagccttctcagctgccctagccctctcatcaatcctcatctcctc

cccactccccgctgtcgccgacatctccggcttaaccccatgcaaagaatcaaaacagtt

cgccaaacgcgagaaacagtccatcaaaaaactcgagtcctctctcaagctttacgctcc

agacagcgctccggcgctggctataaacgcgacgatcgagaaaacaaagagaaggtttac

caattatgcgaaacaaggcttgttgtgcggtgctgatgggttacctcatttgattgtgag

cggtgatcagagacattggggtgagttcatcactccggggattctgtttctgtatattgc

gggatggattggatgggtgggaaggagttacttgattgcgatccgtgatgagaagaaacc

tactcagaaagagattattattgatgttccacttgcttctaggttggtttttagaggttt

cagttggcctattgctgcttacagagagttgttgaatggtgaactcgttgccaaggatgt

ttagtttcttttgtttccatcaaggaaagggaggatgatattgatcatggatggaggaag

ggtgtgacaagagaggggggtgcttttattgttgactcattgttgatcttataaaaactt

atttatgtgctcatattgtaaatttgaactagttaatgtattgtatgaaatgcaatcaaa

ctgatctgagtttttgttcaaaaaaaaaaag

>fava_dn_rep_c168

agaatgagaagatgagggattggctatccaccattcatcaaagataatatttggagaaaa

ggaaaacacacactcacatacatgaccactatctcatcatttcatttcattgatattttt

atttgttctacacactactcacaaacacaagcctaacaacggtactcaggatgtgggctt

cttccactgccaatgcactcaaactctcctcttcctctagattacatctctcccctacct

tttccatctccagatgtttctccactgtattggatggactcaagtacgcaccttcacatg

aatgggtcaagcatgaaggatcagtagccactattggtatcactgcccatgcccaggacc

gtcttggagaggtagtgtttgtggagcttccagaacctggtgtttcagttacaaagggta

aaggtttcggagctgttgaaagtgttaaggcaacaagtgatgttaactctccaatttctg

gtgagattattgaggttaacactgagctcactggaaagccaggcctgatcaattcaagcc

catatgaagatggatggatgataaaaataaagccaagtagtccagatgaattagaatccc

tgttgggtccaaaagagtacacaaaattttgtgaggaagaagatgctgcacattgatttt

aatttcattatattggactgactgtttttaatgttgtaatactatctattattttgttgt

ctgctttcttcaagttattgttctttccatgctgttattgtcgccgacacttcagtgtgt

cactctatctagcttgtcatgtaactaagaagtaaatttctctttcacatctatccatta

ttatttcacttaaaaaaaaaaattattatgttgtgaacttgtgttgcnnnnnnnnnnnnn

nnnnnnnnataatgtaattaatatatagttgtatacattaatgattctgtcaaaaaaaa

>fava_dn_rep_c169

cataagctttttcatgttcagtttgcattttggctttttcggatatgaaaatcattaaaa

acttttaaatttatcatctcaacaatgactagcacttctcacatcataggaatggataat

ttcgtgaattctccaatcacaaaagttggaggcatgccagacgcaataggatcaattcaa

tgcctaaaataaataaattatgctttacttttaagagattgcgaagtttatatggatcag

aacattcatagcatccagacataggcaaacacagtctacaacaaatgtgcaagttccttg

gaccctttacttctaatgagctcaatagaaatactggtttcttagaacagaaattacagt

aaaacttaattatataagacttgggtagaccggtcctttagatatcccaaggcgaacttc

cattccatgatgcatatccaatggccggaaagtctctgattcgcgagggtcatttagaaa

atcctcgaaaccaaagtgatcaagacttcctgtaattgtctccaaacccagcattgaaga

aggaattaccccaggaggcctctggaaccgagaaagtatctgcctatcaagctcctcctt

cagtggaaaagcagctccatacaggttcactttgcattgtctcttcatagcttcttccac

tccccttacagattggagagatgattcgagagggtgagagccaacgatgtcgcttttgac

tccggggagtccgaatcggagcacgtcgttttggatacctccgatttggtgtgcaatggt

ttttgatgattcctccatttttgggtttcgatcttcaattctctttcagtttcaagcttc

aacagtgcacaatcccgcggccggtcaaattaagccgcaggctccactcctggtggtgcc

cttccgtcaattcctttaagtttcagctttgcaaccatacttcccccggaattcaaaaac

tttggtttcccggaagctgcccgataaggcataaaaagaacctttatcggatggctagtc

aacatcgtttatggttagaactagggcggtatctgatcgccttcgaacctctaactttcg

ttcttgatcaacgagaacatccttggcaaatgctttcaatctgtcaatccttccagtgtc

cgggcctggtgaggtttcccgtgttgggtcaaattaagccgcag

>fava_dn_rep_c170

acagataaaagacagtttttgaaaacacaaacccccttcaattgccttccacaccatcat

tggtgagaacagttaaaacaaacagttcaaattagaaatgtagtacataccaacattgaa

atatgggttgtaagaagttgataagatactgtcctgataagataagatgtagccattatc

tgcactgtacaatagaatctcattctaaccaggacaccaaataagatagggtgttgaaga

atgtcatggtcaaaacttttacttaactaaatgaaaggtaaacatgcaagccctaattga

acacaacaggaagggaggaacacgttaagtagagctctagatattaatcaatcaagtctt

ccttgctaaaattatatctcgaatgaatcagtattgtcttgtcagttcaactctgattca

gcagaacataaggtttattcatctgtccttggccgcttagaggcttgatggctgtcttca

ttatcattatgcttaagatcgtcagaagcttgggctttgaaggtatcctgcttcccacct

gcttcctgcgagctatcctcaactgtggtactgccattagcttgatcagatcctggcctg

caaaggtccgatttagatgcttccgattcattttttgtaataggatcttttatggtgtca

ggagacactccagatggattctcagtggccgatgatgatgccttaaaaggttcaacacca

gatttgcattgtttctcgaacatcatctggaggtacctgccttgctcttctattcgcaac

tgtagatttctttgaatctcaagctgttcatgcaaccgtttctgaacctccatttgcagt

cgtagagcttctgtgatctcaataccagtttttaaatctagagatgaaatatcttcaatg

gggcttagttttttctctccagctccttcagatgactctggtctatatctagctgtcctg

tatttttgtaagtggcttttaacatgatatatagttagtccttcaactttcatgagcttc

agcacacccttaggggtagctctttcactaccaccaagttggctgacagcctccacaaat

gcctcatgaagctcaggagtccaacgcatccgtggcttggcagatgcagaatttgctgag

gaagatggagcagtaccaacatggttttctccggatagagctggaagttgttgattgcct

tgggattggtgtcctgccgaaaattgtgaagatgactttgcaatgggatccaccgccttt

ggctcgagatcttgaatattatcagcaagaatatcattccaattagaagtcaaagtatcg

tcgtcactgattagctggtcagcccactcctgccaatcattccgcttagaaaactcctca

gtagccataatattgcagtcactgctttctaatgaattattattaattgaggcattcgct

gagaaatcaagaaagctaggcagggaatctgcatgccacggagcactgtttccattagaa

taatgtgttgatgtggtagaaggtactggtccattatttgaagaatacggtaatggcact

ggagctacgctactcaatggctgtacaataaaatgagcatttctagagggtttttcatga

ggtgaaagagatgagtgatgaaggtctgtcgaatatccaggggaagatgaaaacatatgc

ccaactgctccactggaacttatatgacctgcatgattaaaagttcttgtcttaagatct

ttttccacaaaagcctgctgcgaatcagataatctggggtatgtctcgtctagaggtgtt

ggatggatggacatcgatgaaggaagagctccagacattcctatgttattcagctgccgg

gcacttgatctttcaattgagaaagcaggacgagcttccattattttccagcaaaatact

acagaggtctctttttttttttccaataaaattgagattattatctgagtatactaatac

tcatattattctatccactattaatctattcgaatagaaattactttattcattctttct

attacaaaaagtattgaaagtatatttatactagtagtattactagtatggagtattcaa

tatagtactaatctagtacatatagtattagtacatatatatttgtaatgacagatatta

gataagaaatctaaatctgagtagtatcggtagtaatattagggctatacggactcgaac

cgtaggccttctcggtaaaacacatcaaactattatttattattatcaaaatgatttgaa

ttgtttgaaagacccaacgtgcattttttttgcattgggctttttcattaactgatagaa

atatcaattagtctaccatcttttttctttacacaaagaaaaaggaaatgactccctgtg

ctctgatttcttatttagatcaaaatctaagagcactaccaaagtttttcaaagaagaag

gttatcctgacgtaggtctgcttctggcctggatcaacttaagttaaatggactctctat

caacctacttcaataaaaatatcaaataggaaatgaaacttcatacaccttaaagttcat

aagataggaagaaaagagattttttttgaggtccctagactcattgcctagcattgaata

gattaagtattcaccgtatcaatatctcaaatccattatggattccatttgacgtctaaa

tcctttgtcaggctattgttctcttattttgttccgggagtaagacatcgatttatcaat

taagatcaactcttttaattacatggtgggctcctatgaaaaaacattggcgcatgtgta

aacgaggtgctctacctaactgagctatagcccttgtgctttggatacatatttgatcat

attcgataaataaattcttgtcaagataaatatgacatgatccaatctctttgatttata

ttgcgtttgtattgtttataagtaatataacatttataatccattgatctgatagacagc

tactttttttatttaggatgataaatgacctacttaactcagtggttagagtattgcttt

catacggcaggagtcattggttcaaatccaatagtaggtaaggtattggtagaacttatt

agataccattaattccggtatctaataagtttttctactcaccattgttttattgtaatt

ttctatcttttttctattttttggctgcaccaacttgttatgagatcatattgatagcct

ctactcgtgtcctagctcgccggagagctagatttgcttcaattgtttgtctcttgcctt

cagccttattcaagttagcttctgctatttgaagagtttgttgtgcttcttgtggattaa

tgtcactacccctttctgcatcatttactaaaatagtgatctcattattgcctattctag

caaaaccgcccatcagagccatcgttaaccatcggtcgttaaggcgtattctcaaaatac

ctatatctaaagatgtggcaataggcgcatgattttttaataccccaatttgtccactat

tagtagataaaataatttctttcacttctgaatcccaaacaattcgattgggggtcagta

cacaaagatttaaagtcatttcttgaatttgttctccatttctaagttaagttcgtagcc

ttcgcagtagcttcatcgatattacccaccaaataaaaggcttgttcaggaagactatct

aattctccagaaagaatcaatttaaatcctcgaattgtttctgctagaccaacatatttc

cctggggaaccggtaaatacttctgctacgaaaaaaggttgtgataagaaacgttcaatt

tttcgtgctcttgctacggttaagcgatcctcttcggataattcgtccaacccaagaata

gctataatgtcctgaagttctttgtaacgttgtaacgtttgtttcactctttgcgcagtt

tcataatgttcttcaccgacgatgcgaggttggagcatggttgacgttgaatctaaagga

tctactgctggatagatacctttggcagctaatcctcttgatagtacagtagttgcatcc

aaatgtgcaaatgtcgtggcaggagcaggatcagtcaaatcgtctgcaggtacataaact

gcttgaatagaagttatagacccttctttggtagaagtaattctttcttgtaaagtaccc

atttcagtacccagggtgggttgataacccacagcggaaggcattcggcccaataaggcg

gatacttcggatccggcttggacaaaacggaagatattgtcgataaatagaaggacgtcc

tgctcattgacatctcggaaatattctgccatagttagggcagttaaaccaactctcata

cgagctccgggcggttcattcatttgaccgtagactagagctacttttgattccgcaata

tttttttcattaattactccggattctttcatttccatataaagatcatttccctcacga

gtacgctcacctactccgccaaatacggatacacctccatgagctttggcaatgttattg

atcaattccataatgagaactgttttacctaccccagctccaccgaaaagtcctattttt

ccgccacggcgataaggagctaaaagatcgactactttaattcctgtttcaaaaatggat

aattgtgtatctaactgtataaaggcaggcgcagatctatgaataggagatgttgtgcga

gtatctacaggacccaaattatcaataggctctccaagcacgttgaaaattcgacctagg

gttgctccaccgactggaacacttagagcaactcccgtgtcaatcacttccattcctcta

tttagaccatctgtagcactcatagctacagctctaattctattatttcctaataattgc

tgtacttcacacgttacgttaatttgttggccaaccgtatctcgaccttgaactatcaga

gcgttgtaaatataaggcatcttccctggtggaaaaactacatcgagtaccggaccgatt

atttgcgtgatacgccccagattttttttttcaagtgcagaaacctcagtatcagaagga

ggaggagttattgtcatattaaaaaatatccatttttttgaaaaaaaaaaattgaaatta

agaaaaaactgtttgataacaaagcaagttgatcggttaatattcttaattttcttaata

tttaataatatataaaagaataaatgtaagttaataatcgattttcttggtaccatccaa

ccaattcaattgtttttaaatttcaatgagtgaattttcaaggtcaacccagtcattatg

aaaattttaattggatgaaatcttttgaaagtctttcatttgtttatcattctagattat

agacaatactatctatattatctatgtaattcgaacctgaactttgtttgtttatgattc

cgtttttctatctcattggtccttcttttttctttcctcagcataggatttatactatgc

agattctttttaccaatttttctttttattgggcaaattctgccgattttgacatctagg

atttacatatacaacatatatcactgtcaaggtcaagagtatattttttattatttagat

tagaaaatgagattccaaaattcaaaaaaaaaagtaagagactcaaaaaaaaaacggttg

ggttgcgccatacatatgaaacagtatagaataatgatgtatttgccaaatcaaatatca

tggtaaaattgaacaatgacccattcggattaattgataatattagttgatggatcattt

gtaaaaaggttttcttaactcctaagttatgtcgagtagaccttgttcttgctataattc

ttaattcatgcgttgtaggggggggatttatgtcaccacaaacagaaactaaagcaaagg

ttgggttccaagctggtgttaaagattataaattgacttattatactcctgaatatcaaa

ccaaagatactgatatcttggcagcattccgagtaactcctcaaccaggagttccggctg

aagaagcaggtgcagcggtagctgcagaatcttccactggtacatggacaactgtgtgga

ccgatggacttaccagccttgatcgttataaaggacgctgctacgagatagagcctgttc

ctggagaagataatcaatttattgcttatgtagcttatcccttagacctttttgaagaag

gttctgttactaacatgtttacctccattgtaggtaatgtatttgggttcaaggctttac

gcgctctacgtctggaagatttgcgaatccctaatgcttatgttaaaactttccaaggtc

ctcctcacggaatccaagttgagagagataaattgaacaagtatggacgtcccctattgg

gatgtactattaaaccaaaattgggtttatccgctaagaattatggtagagcagtttatg

aatgtctccgcgggggacttgattttaccaaagatgatgaaaatgtgaactcccaaccat

ttatgcgttggagagaccgtttcttattttgtgccgaagcaatttataaatcacaggccg

aaacaggtgaaatcaaaggacattatttgaatgctactgcgggtacatgtgaagaaatgc

taaaaagagctgtatttgctagagaattgggcgttcctatcgtaatgcatgactacttaa

caggtggattcactgcaaatactaccctgtctcactattgccgcgataatggtctacttc

ttcatatccaccgtgctatgcatgcagttatcgatagacaaaaaaatcatggtatgcact

ttcgtgtattagctaaagccttacgtatgtctggtggagatcatattcacgctggtactg

tagtaggtaaacttgaaggagagagggagattacgttaggttttgttgatttactacgtg

atgattatattgaaaaagatagaagtcgcggtatttatttcactcaggattgggtttctt

taccaggtgttatccctgttgcttcagggggtattcacgtttggcatatgcctgctctga

ccgagatatttggagatgattctgtactccaattcggtggaggaactttaggacaccctt

ggggaaatgcacctggtgccgtagcgaatcgagtagctctggaagcatgtgtacaagctc

ggaatgagggacgtgatcttgctcgcgagggtaatgcaattatccgtgaagcttgcaaat

ggagtcctgaattagctgctgcttgtgaagtctggaaggaaatcaaatttgaattcccag

caatggatactttgtaatccagtaataatcattcgttctattaatttccattaaactcgg

cccaatcttttactaaaaggattgagccgaatacaatacacaatacaaaaatacttgttg

tatctagaatgaatcctatagatctttcggattgatctattttttttctagttcattctt

cttttttgatttggaatagaatgaattaaaatagaaatattttctatttaaaaatgagaa

tagaaatatttttttatttgttttctcgattgtattctttttttcaacactaatattgac

aacagtgtatcaaacaaatataatctgatcgtgaatcaatggatggattgactcatgtca

cataggttttttacttcatcaaatggtggattcattgtattttgattttgtttgatacaa

acaagaagttttttttcttgaaatagaaatatgaaaaaaataatactaatttaaaactat

attaatagacaaataactacagtactgtactaaacaagtcgtctatcatttttgattctc

attttttctttttttcattgtattgtaatataatgtaatatgtaacatttgattctatta

tttatagaattccatcttttttttattgcgattggatatacagatccttgtttgtaattt

tattagggttgctaactcaatggtagagtactcggcttttaagtgcgactccatttttta

cacatttctatgaactaattggttcatccataccatcggtagggtttgtaagaccacgac

tgatccagaaaggaatgaatggaaaaagcagcatgtcgtatcaacggtgaattcaaaaaa

tttttcattcatattggatccgaccaatctttttctttgaattgttggctgggaacaaat

gaattccgttgggttgaattaataaagggatagagcttagctgctcgaattctagggaaa

gaaaaagcaacgagctttcattttttattggaatgattaccacatctagttatacgttaa

aaaaggattagtgcttgctgtggaaaaagtttttcccacgaatggattaaggatttttgt

tatgagtcctaattattagctattccccattatgttatggggtagcaagaaatgtgtaga

agaaagagtatattgataaagatatttttttccaaaatcaaaagagcgattggtccccaa

aaaataaaggatttctaactagcttgttgtcctagaaaaattagaaggaacaagcgaggg

gagatagtccattaatgtgttttaactcgttttctaggtatctattcatatttgtttttt

atttcaattcgaatatagatatagaataccttgttttgactgtatcgcactatgtatcat

ttgataatccaatgaatctctgatcctttgaccaaatatcatttctaaaatgaaggaata

taaagtctatttagaacgagctagatctcgccaacagcacttcctatacccacttatttt

tagggagtatatttatggacttgcttatagtcataatttgaatagatccatttttgtgga

aaatataggttatgacaagaaatatagtttactaattgtaaaacggttaattactcgaat

gtatcaacagaatcatttaatcatttgggctaatgattctaacaaaaatccattttgggg

gtataagaataattattattctcaaataatatcagagggttttgccatcgtcgtggaaat

tccatttttcctacaattaagctcttccttagaggaggaagaaatcatcaaatcttataa

aaatttgagatcaattcattccatttttccctttttggaggataaattgacatatttgaa

ttatgtgtcagatatacgaataccctatcctatccatctggaaatcttagttcaaatcct

tcgatactgggtgaaagatgcccctttttttcatttcttacggctgtttctttggaattg

gaatagttttattactaccaaaaaatcaatttctactttttcaaaaagtcatcgaagatt

cttcttgttcctctataatttttatgtatgtgaatatgaatctatcttcgtttttctacg

taataaatcctctcatttacgattaaaatcttttagcgttttttttgagcgaattttttt

ttatgcaaaaagagagcatcttgtcaaagtttttgctaaagatttttcgtatactttaac

attcttcaaggatcctaacattcattatgttcgatatcaaggaaaatgcattctggcttc

aaagaatgcgccttttttgatgaataaatggaaacacttttttatccatttatggcaatg

tttttttgatgtttggtctcaacccagaatgatcaatataaacccattatccgaacattc

atttcagctgttaggctatttttcaaatgtgcgactaaatcgttcagtggtacggagtca

aatgctgcaaaatacatttctaatagaaattgttatcaaaaaacttgatataatagttcc

aattattcctctaattagatcattggctaaagcgaaat

>fava_dn_rep_c171

ccgcggggtgaatcaatggatggattgactcatgtcacataggttttttacttcatcaaa

tggtggattcattgtattttgattttgtttgatacaaacaagaagttttttttcttgaaa

tagaaatatgaaaaaaataatactaatttaaaactatattaatagacaaataactacagt

actgtactaaacaagtcgtctatcatttttgattctcattttttctttttttcattgtat

tgtaatataatgtaatatgtaacatttgattctattatttatagaattccatcttttttt

attgcgattggatatacagatccttgtttgtaattttattagggttgctaactcaatggt

agagtactcggcctttaagtgcgactccattttttacacatttctatgaactaattggtt

catccataccatcggtagggtttgtaagaccacgactgatccagaaaggaatgaatggaa

aaagcagcatgtcgtatcaacggtgaattcaaaaaatttttcattcatattggatccgac

caatctttttctttgaattgttggctgggaacaaatgaattccgttgggttgaattaata

aagggatagagcttagctgctcgaattctagggaaagaaaaagcaacgagctttcatttt

ttattggaatgattaccacatctagttatacgttaaaaaaggattagtgcttgctgtgga

aaaagtttttcccacgaatggattaagggggtataagaataattattattctcaaataat

atcagagggttttgccatcgtcgtggaaattccatttttcctacaattaagctcttcctt

agaggaggaagaaatcatcaaatcttataaaaatttgagatcaattcattccatttttcc

ctttttggaggataaattgacatatttgaattatgtgtcagatatacgaataccctatcc

tatccatctggaaatcttagttcaaatccttcgatactgggtgaaagatgcccctttttt

ttcatttcttacggctgtttctttggaattggaatagttttattactaccaaaaaaatca

atttctactttttcaaaaagtcatcgaagattcttcttgttcctctataatttttatgta

tgtgaatatgaatctatcttcgtttttctacgtaataaatcctctcatttacgattaaaa

tcttttagcgttttttttgagcgaattttttttttatgcaaaaagagagcatcttgtcaa

agtttttgctaaagatttttcgtatactttaacattcttcaaggatcctaacattcatta

tgttcgatatcaaggaaaatgcattctggcttcaaagaatgcgccttttttgatgaataa

atggaaacacttttttatccatttatggcaatgtttttttgatgtttggtctcaacccag

aatgatcaatataaacccattatccgaacattcatttcagctgttaggctatttttcaaa

tgtgcgactaaatcgttcagtggtacggagtcaaatgctgcaaaatacatttctaataga

aattgttatcaaaaaaacttgatataatagttccaattattcctctaattagatcattgg

ctaaagcgaaattttgtaatgtattagggcagcccattagtaagccggtctgggccgatt

catctgattttgatattattgaccgatttttgggaatatgcagaaatctttctcattatt

ataatggatcctcaaaaaaaaaaagtttgtatcgaataaagtatatacttcggctttctt

gtattaaaactttggcttgtaaacataaaagtactgtacgcgcttttttgaaaagatcag

gttcagaagaattcttgcaagaattctttacagaggaagaagagattcttgcttttcttt

ttccaagagattcctctactttgcagcggttacatagaaatcgcatttggtatttggata

ttcttttcagtaacgatctggtccatgatgaatgattggttatgttatgatacttaaata

tctatttcaatatatatatagaaaggattttgaaatgttcatgtagtttagagttgagtt

tcaagattcctctgaagtaactcaggtttagatgtatacatagggaaagccgtgtgcaat

gaaaaatgcatgcacggcttggggagggattttttattgttttatttgattaaacaatga

attttcctccatccgacaggaaaaggaccatttgataacctgttgcagcacatttctgat

ccatggcacaacaccattgttcaaacactgggtggaaactaaaatgtttgtattattatt

gtccaatgacatgaaagcctatattgttcttctcgtgtgaatcctgaatgggtgtatttt

gaaagacatttgggcatgattgttaagaatccttaatctattttcatgtacagaagaact

gagatggttttatattgttctttatattctcgagcctattgacagaaattaagggtccct

ctactttgcatgtttcatgtttttgttttcatttcaagtaataaacataagagaatcctt

tcaccagaaaaaaaaaaacagaagaagaaagaagaacagcagagacttgatgaagaaggt

gctgcaattgcggaagctgttgctctacatgttctactcggtgaagactcagatgattcc

tataaagttgaatgcaagacttgggatgattataatcgtaatcttgatttcttcatgggt

gggaaaagagcttgctttccaaatctagatggaggcacatggtccgtcactgccgaaaat

ggcaagtggtctatctcgtccgggcctttcgaaaaccgcaatgtgcacgctccaccttat

ggagaagcagcatgggatcctacaagattttctgtggaccttatagcagcacaagcggtt

agatcactccagattgcagagaatgcagatgaggataggattctattctaggggaatgct

tacatatcggattaatcacgcgtctttgctgtggactggagtctttttttatttattttg

tttgaggtctttcatgtgtatataactatatttcatattatatatcacgagtcttgtgtt

tgtcaaacaacactttctgcttcagctgtgattgaacatgttggtagtgtccccatgttt

tattctgattttctttacctgtatgtgaggatgctaattttctttgtggagtgatgactt

tcatactcaagtattgtagggaactatacttttttgagctgattatcaatataaatgggg

acctcatgatttatataaaaaa

>fava_dn_rep_c172

tccctataatagtttgagagatatgatttgagatctgcttgcttcccgtattcttttttt

tctaactttaacttggctacatcagtaatgtttttgtttccataaaactgcacaaaaagg

aaattcattggtaagatccaaggattcttcttatatgcacttaattttgaaaagaaccca

aatctcggagtcaataatttctgattcgttttggaattcttttttctttttcccatccaa

tttagatactttctattcagattttttccattgtttccatatccatatctataataccat

cttctgctatataattttggataaagctatcgcccattatatcaaataattcttttttat

ttttaggtatgttgtaattaatcgtttggtttttgtttgcttgcaatggtgatttatatc

cataaatataggattttttgttatctgcataatttatatattgataggagaaaagatcat

atccatattgtttttttatttttttttttttttctaataaatctacttgttgtttttttt

gttttttgtaaaaaatgaattggtttttttcagatgaatcatattcaactaaatttttct

tttgagccacgcgatgttcatggattctcttcctccacttttgtggtactaattttgacc

atccgctctgaggtaaagcatattgataatgaacctttaaccaatttatccattgattca

ttatagaatcgggacggttcttatgtcttaatttggaatttaatcttcctccttgtattc

taaaaaaataatcttttatttcattcttaagaaacaaagatcttccatgagattcaaaaa

tagatcttaacttatatttatatccattactaacttgtgataatttaaaaaatacatagg

cttgtgacaaagaagatacatcacaagaattctgtgaattcatatctgtaatattccaag

atttgtctataatcgacataaatggaattatactttcatttgttttatgaactctttgtt

catttttttttttagtgtaaatggaaataaatacatttacaattttgtctgttaattcaa

taatattaagaaaacgttgtccagacatcataataatacgagtgatagagaaaacgatct

ctatgtatatccttcccattaaaatttgaaaaaaaaaataggatttacggattaatcgaa

cggttcttctttttaatattttcaaactattttttagtaattctaatcttttagcatcat

aagtagttttggtagaattcaaattaatctctaaagttataaccccctcttttttttctt

ctgtcattttttctagttgttttaagattgtctttgttttaacattaagatcttttatcc

ttttttctgtgaaatttgtccaattcatagattgaattagaacgggcgattcctcaatca

ttggattactcttactgattgttgaattttttttgctttcactcaattcatctctttttt

tgaacctattttcgatttcttctaacacgattcgagaccccttttttctttcattaaaaa

cttttagaactagaaaaaaacgatttttcaaatcttttttcaattgtttttttatttttt

taaaatgggacccaaaaataaaaaagggtttgggacatgatcatcaaggtacgattcgac

ttgtgttccataacctgttaaaaaccataagtttttttctttcagtggatcttttttttt

ttcagtagatttttttttttttcagtagatcgtaccttattgtgccacggtttcagaaca

aaagggaataagatccttatctgaataccctcccagaaccagtttgttggcaacgcggtg

tgagatactggaacgccctgataggtgcagtaaatatggacttcttttctccaatccctc

aaatcttctgaccattcaggattttgaaataatagtatacgaatgatattttttgtgatt

atcaatgaaggtaatagaatatattttctaaatattgattggaatagtaatacaaaactt

ctgattattagaccatagagaatgctctcccaggcttcttcgatttgtctaagtcttatt

tcagactcgtctttgttttcctctatctctttctgttctatcgttttctcaaacgccaaa

aattccgaattatcagtacagaaaaagcaacgagctttcattttttattggaatgattac

aacacctagtnnnnngttaaaaaatccattttgggggtataagaataattattattctca

aataatatcagagggttttgccatcgtcgtggaaattccatttttcctacaattaagctc

ttccttagaggaggaagaaatcatcaaatcttataaaaatttgagatcaattcattccat

ttttcccttttggaggataaattgacatatttgaattatgcgtcagatatacgaataccc

tatcctatccatctggaaatcttagttcaaatccttcgatactgggtgaaggatgcccct

ttttttcatttcttacggctgtttctttggaattggaatagttttattactaccaaaaaa

tcaatttctactttttcaaaaagtcatcgaagattcttcttgttcctctataatttttat

gtatgtgaatatgaatctatcttcgtttttctacgtaataaatcctctcatttacgatta

aaatcttttagcgtttttttgagcgaattttttttatgcaaaaagagagcatcttgtcaa

agtttttgctaaagatttttcgtatactttaacattcttcaaggatcctaacattcatta

tgttcgatatcaaggaaaatgcattctggcttcaaagaatgcgccttttttgatgaataa

atggaaacacttttttatccatttatggcaatgtttttttgatgtttggtctcaacccag

aatgatcaatataaacccattatccgaacattcatttcagctgttaggctatttttcaaa

tgtgcgactaaatcgttcagtggtacggagtcaaatgctgcaaaatacatttctaataga

aattgttatcaaaaaacttgatataatagttccaattattcctctaattagatcattggc

taaagcgaaattttgtaatgtattagggcagcccattagtaagccggtctgggccgattc

atctgattttgatattattggccgatttttgggaatatgcagaaatctttctcattatta

taatggatcctcaaaaaaaaaaagagtatcgaataaagtatatacttcggctttcttgta

ttaaaactttggcttgtaaacataaaagtactgtacgcgctt

>fava_dn_rep_c173

ctcgtaataataccatttgattgtttcaaaattttggaatctcactcttgtattagcaaa

taataaagagccctaaacaaaatagtttgcatcaaaaactcaaaattagcaaagttatca

agcaaataaaaccttttaatgcaagcaatgaatatgaactaaacatttcaatatgctcag

cattcaaaataacacaacaaaaatcttttaaagcaaaactggaattaacaaaaacaaatg

tcttaaagctggataactggataatgaaccttagcagaaccatgagccaaaatctaattc

atccttctaatgagttcgttgatgtattcttccctgttgccggcatcaccaccttcaaca

taatgatttcttttcttcttcatgccacccaagggagctttgagcttgaaaggccatagg

aagttgtttgcctctctgaagtgaggtccaacagtcaagatctcgtgaatcagatcttca

atgcaaatgattccgtgcttcccaaggacctgttcgatgattgagttgtcagtcaaagca

attctctgcctgtcaacctttccatagcccctcttgtagatcaattccctgacactcttc

agattaggatacccgtaggtgacatatggctctaccctgtggagcatgttcacggtggct

ttgttaactttgagaaagacaccattaaagatctgtctcaaacgcaaaagctgtaaaatc

ttccttgattttgggtccatggcattgatacctctgatcctgacaatgaacaagagctta

gcttcaggatcaacataaaacccacccttcagtttagcttcacgcttcaagctaatcaac

tccttttgctggtcatcatactccttagcgtactgcttggctctgctccaaatgagcttc

cttgtttcagatctcttcttcttcgctgactcttgttcctgcttcttcaccaaagcccat

tcctcgttcctcttctgcttcttcaaaacagattcaggaaccacggtttttgcttcctct

ttggccatttcgcgttgttgcggtgagaattgtagacggcggaggaagctctcccagcga

tgaaatggagtcatatattaattaggatattttgaattttctgctatttcatgacg

>fava_dn_rep_c174

gaacctgttgtgttcttgagagtaattagatatcatcccaagcaatcatcaatttgaaag

taaacagcattcaaacattgaaagaagtctgtttatccaatgtaaactttacaaaatgat

gaaaaataaaagaaatatacaaggattattcaatgtgttgaatccatagattgaaacata

acccttacatatagtgatgataaagatacaaactaccttttgccttgtaagacatggaaa

aaagaatgtgataaaatgaaaatcagcaatgtcacatttttcatttcttccatcccattc

catccatttctgatattataatgcatgtcataagagtatcattttttattctcagttcat

gcatcaatatacttcgcaactttttcagacagagtagccttaggaactgcaccgattaca

ctgtcttttttctctccattcttgaaaaacagaacagttggtatgcttctgatgccatac

tttgttgcagtgtttggagattcatctgtgttgagcttgtaacacttaatttttccagca

tattctttggctaattcatcgattataggcgctatcattctgcatggaccacaccatggt

gcccaaaaatctacaagcacaggcgtttcacctccaatcacaagttcatcccagcttgag

tcattcactacttgaacttcatttacagcttcacgagcattgcaaacaaaacgagatttt

ctataagcaacaccagatggaagtgaaactgttgagttcagcacagatttcttgaatcct

accttttgagttgagaaaacaagctttcctcttgaagatgaaaacagtggaggaagacaa

tgaggtcttggtgtggaaactcttaaacaactctcaagggccatgttgttaccttttttt

gtgtgtgtgtgtgtaactgagtagtttgtggaaaatgaagtgagggttttcagtacttgt

tgtggataagcttctagtgttgtggtggtttttaatttgtttggtatgaaattgtgaagt

gtggatattgttggagattgtgaggttatgagatggcttggattcatgtggatggcattt

aggttttcaaacctctctctactttttgtcccactaaaa

>fava_dn_rep_c175

tttacaaataattttatgaacaatttccaaatccaaagttacattttctctcctgcacat

agttttctaaaacaataatttttcacacacacccatcttacattaaaaattcacggtaga

cttacaatgcacagtacatatgcttgtatagatctccccttgtcaagtttcacggtcggc

tttaccaacaaaaaaaaaacattgttgtcaatgttttcctcaagctatctacacgatttt

tctccttctttttacataaaccacaccgatttcattacaaagtaaaaccatacatctaaa

ataatattacaccatataccaacataaccttacaccacctttttcacgttgttgttttta

caacaacaaactagaccatgttaaaccaaaaatgaagaatcatcaaattttcacaaaatt

aagttctgaaataatactgttgagttttttggaagatcaaagaaagaaaggagatgagtt

tgacactatctgcactgtcaaaagcataaagttgaatgtcgcaacacttcggatctgcat

gtgtgtcgttgcacaaccgtcggatctccgcaaccacgtccctttgaagctcagcccgtc

accaccgcgaggagcctccgtcttcgaccgcaacatgctcagaccgcagccacaaatcca

ctcaacgacacagaaacaaaaaagaacaacaacaaagagcagcttccacaatcttatcat

tttcagcaacacatcaatggctatctcaacctccttctccaacttcttctctttcctctc

cccccaaacaaagccacaacctctcctttctcactttccatctcaccacaacaaccagcg

caacaagataatcccccttgttctcagccgcaatgaaacctcttcttcagttacatcttc

accaccagaggacatgtcttccgatttcacctcatctctctctacttccagctccatgct

cttaaattccaactacaatgttcaagttgttgttgaagacgatgagccgggagagagatt

gcttactaggttccgtagagaggttatgagggctggtattattcaggagtgtaagagaag

aaagttttttgagaatactcaggatgagaagaaacggagagttcgtgatgctgctaggcg

taggcgtaggtacaggtttcctaggcgtccgatgacagggaaacctccaggagaacggta

caacaactacaggcaggacgatccaaaacctgcgaaggaagaggatggtgataactggga

tatacctgaagaggacgtgctacaattttgactatacaatttcttgcgttccgtatgatt

cttcactttttactctgagaggctttgtctgcatgtaggctcttttatgtctactattgt

attgttttatatacatttcatatgatcatgatatttatatttatacaatatgttcatttg

tgcatcaaaacttccatctgccattgatttttctctcaaatcttacaccagtattttttt

taaaaaa

>fava_dn_rep_c176

ctcaaacattgcgattttatgcatcagttcttcattgattctgtaatttgtctcaactct

caaatcaatagttcagcaacaaaatatagtgtgtacttttcacacatgaagatctagaaa

aataagttgaacttgcataagatttgagcatctaaatattttagaaatgaaaaataatca

acaaaaaattaactgaaatttactgtaggttccagtcattttttataaatgaaaattgaa

atatactgcaatttcttaaacagcaatttcataccaacaacacctccaattcataagtac

agaaacaaacttaaaagatatatgagataccaagccatctcttactaaaggactatttga

tgtattgagaaacccatttgaaaccttcaccataacccatcttcttcacaatactgcaca

tgaatacctccatgggacggacatttgagtctgacaaatttaccttacccttgccagttg

tgaagttgctcaggcccaaatggtaacgtaactcttcttctgaggcagcatatggaatat

ctattttgtttccaagaacaaggaaagggacatttgccaatgattcatcagagaggagag

catccagctccttttttgactcggcaaatctttccttgtcataagcatccaccaagtaga

ccactgcatctacctgggcatagtagtctttccagactctacgagcaatctgatgacctc

ccaaatcaaaagccttaaacttgatcttcccaatactcaactcttctgatgttggatact

gtgttggctgatgctggactaatctctcgtctttgagcatgtgaagcaaggtggttttac

cagcgttatcgagacctaagaacaagatcttagcctctttctgccataacccaagcgatg

ctagaacgccgtaaaaccaatcgacgagaaacattgtttttcacgatcgagttgaattgg

aaaggggagaaaagtatccaagtgtgagacaaaattgatgtattgagtgttgaatgacct

aacctaaccccaaaaccagagagagaagagaagtttttatattcgcgttctgtttggttt

tgttgcaacttgttgtgggacttg

>fava_dn_rep_c177

ggctcaaaacaattttatttaattcaatcataattactaatattatgacaaatcacatga

ataaacaacacataaaaaatctggatatacaaaagaaaaccaaagaacatcaatggtgaa

tgaattatcacaattcacaagatggccaaaaccaattaaataaaccccaactcagagatc

catataataaatgtgaaacattaaaatcatgtagcataacttaagtcatacaaaagcaga

tataaaatcctcaaagtagaaattatgaaatcccaaatacaaatccaaaatcaaattcta

aaaagacaaaacgacaaaacgattaagcaagaaaaattaaaacagcttccttcaaaacct

gagcttggtaaagaaccacctgttcttcccggtcttgaacctctcctcaaacctcttctt

ggcctccttcaaagcagtaaccttcttgtccttactcaagagcgcgtcattggtaaccac

ctccttaagatcaacatccaaagtgtaacgggtaggcatgagatgctggtagttaacaag

cttcacaaacgccttcacacgagacttcttggcggttttctttgcggaattcttttttat

aaccttgctgggatatttcttgatcccagccaccaaagcatgaccataagcacgttcacg

ggttccgtcgtcaaagttcttcacgatcacagctttcttccctgcatatcgaccctgaag

gacgatcacagctttgttcggcttcagaaatttcaccattttcgcctttgcttcctttct

ctctcactgaatcgccgtttcagggttagaaatgaaaaccctagtttgttctgatgtata

t

>fava_dn_rep_c178

gagaaatttcgtttgtatataaacatgttttgcaaaacatgaatataaaacgcagtttgc

aaaacttgatgcttgcgcaaatacataaaaaagtaaatttgcttggtaacagagagcaga

tgattaaattcttagaaatatcagagcagaacaaaagtgagtaactaaattagagtaaca

ataacaactggaagcagcaaaatcatattaaataattgaaatccctaggagaactccagc

tagaagcataattcattcggatagaaaagctacaaaatgaaaagaaacagacactcaaag

tgatcctgcaacttattgagctttcacgtgctgcagaacaacattgtaggcttcatgccc

ctccccgaaatccttgacaaccacacgggagcagccagtaacttttctagcttttccctc

ggaatcaatcttacacaaaccagcccattctccaagtgtctttgcactgggaactgtcag

caggctaacattgtgctcagcacaaagagccttgactagtttaacatagtcaggttggtc

acagtcttctgcaagaacacaaagttgtcctgcgtgcttctcaattacttttgcaccttc

gtgaaggccgcgtgcaagacctccatatgccagtgacttcctgagcacaagttgcaaggc

agtcatgatgtccattggctcacctggaattccaggtgcggcagcagcaacaggctcagc

tacaacaacagcttcctcacctgacattttcctagtagatttgaaaaaacaagctttctt

ttgtttctggctgccgacggcgttaagttcagtgagactcaaatttctatccaatttgct

tctgatgcaaaggaggcgaagtaatggccactccccgcggccgtaatggctcaccttctt

cgcaatcccacacccacttcctcccttctttcaccatcatcactctctcatcacctctca

acacaaccacgccaggttgggaataaaggttatgctaatgggtcaattatgctgtatcag

agacttttatctggtactgctaatcaagataaaccaccagctgataatagcaattcatca

aaagatgattctggtcaaggaaaagagtctggtggagaacaggaacagaaacgtgatacc

ggaaaatccattcgcggatcgccggtttcttggttgagcttggtttttctggttctaacc

ggagctggattggtgtattactatgatcgcgaaaagaaacgacatatagaagaaatacgt

aatgcttcagaggaagttaagcagggaccttctgcgggaaaagctgcgatcgggggtcca

tttgagctcaccaaccaccacgggaaacgagtaactgataaagacttcttgggtagatgg

acattgatgtattttggattcactcactgcccagatatttgtcccgaggaactacagaaa

ttagctgcagcagttgataaaataaaggagaaatctggaattcaaactgtgccggttttt

atctcggttgatcctgagagggatactgttgaacaagtggctgaatacgtcaaagagttt

catccaaagttgatcggactaactggtagcacagatgagataaaaagtgttgctcgtgca

tatcgtgtttattacatgaaaacagcggaggaagactcggattatcttgttgatcactcc

atagtcatatacttgatggcacctgacatgggattcgtaaagtttttcggcaagaacaat

gatgttgactcacttactgatggagttattaaggagataaagcagtataagaagaagtaa

catgcaccactttgctattggtagcaatttttcttcattatttgaaagaaattatcattg

agtttaggcttatgctagaaaactacacaagtctaaatataatttgaaggctagttcctt

tttaggtgatgttggagatttttgacacgtttataaagagaaacttaattgatgtcattg

tttcaatggttgttgtacttgaaaaaatattatggaattcttgagatgcaattatacttc

aactacaatgttctttgaacattttacttaatcttaatatggtattatgaaatgaatgtt

gcacttgctattg

>fava_dn_rep_c179

gcctattgccacaagctcctttattttattcattcattctctatcttattatcaccaaca

caaacacaaatcagtgtttgttttgaaaccatagtagtaatggtaaccacaccagctttg

tatggaacttccgtcagcacctccttcctcaggactcagccaaagccaatgtcatccaca

acaacaaaggcattttccaatgctttcattggcttaaaaaccactctcaaacgtggagat

cttgcacttgccatggcttcttacaaagtgaaactagttacgccagatggaactcaagag

tttgaatgtccagatgatgtttacattcttgaccatgctgaagaagttggaattgatctc

ccttactcatgcagagctggttcttgttcttcatgtgctggtaaagttgttggtggtgaa

gttgatcaatctgatggttctttccttgatgatgaccagattgaaggtggttttgttctt

acctgtgttgcttatcctaagtctgatgttgttattgagactcacaaggaagaagaactc

actgcttaattttccaattctcatgttgctactatcatatctatcatgttatgtttgtct

cttcaatttttacattttctgtttctacaatgagtagtaactctattaatttcttcacct

gtttgtagtaacatgtgaagttaataatgttatgtttctgccatttatcttgtttttttg

ctcacatttccccacttttcttgggttgagtcttatatgaattttgatatttttcaaaaa

aaaaaag

>fava_dn_rep_c180

ctttttttttttgaagccaaaaccaaacaaaatatgaaataggattgattaacatatctg

tacatgctgacatatgcagcagaattaagaaaaacataaacaaataaggggtgaaagatc

ttggttcaagcaataatagagtggacaacacaactatcttaccttacatattacattcat

catcacacaacactatatcctatcatatattaattatatgacaagataacaacaatactc

aaaggaactggaaataaagtgtccttattaatgaagccagttaagacactgaagcaaaat

cagataattttcatttaaagaaacctccagcaaccttagcaaaatctccacctagtccac

caagtccacctgatcctcctccatcttcggatttgacagcttcttcagatttgggtgcaa

catcatcacctttgggttgatcaggtttggaagttggtggagtagcagcagtggtggtgg

tgttggtggactgatactgatgcagataatcagcagccttatcaggatgctctcctgcgc

ccttattatcatccaatttagcatactgaccaactgcatctagaagatcacctgcagctt

gtgccgctttcgctttgtctgcctctgattctttcccgaaacctgtctgtgctgcctctg

ctaccacctttgcgcttgccataagctcgcttgttgaatacttcttctcttctccacttg

ctgccatcgtttttgttgtttgtttttgagtaatgttgattggttctgcatcccgcggcc

gagattaacgaagtttgtgcagagaataaannnnnccttattatcatccaatttagcat

>fava_dn_rep_c181

caaaaacattaattcatagtttggtagttaccacctaacattcataaaacgtggtaaatt

ctccatcacaaactttaccaaactcatctataacaatataaagaatccctacccaatctg

aaccaaaattcacatccacattttcttatcctagaaagtttcttcacttgcaatggctga

agttcaattgttgggtgtaagaggaagcccatttgtccaaagagtgcaatatgctctaag

actaaagggcattgagtacagtttcattgaagataatctaagaaacaaaagtgaacttct

tcttaagtataatcctattcataagaaggtgcctgtttttgttcacaatggaaaaccatt

atctgagtcacttgtgatcattgaatacattgatgaaacttgggggaattatcacatttt

tcctcaacaaggttatgagaaagccttagctagattttggtccaagttcatcgatgacaa

gatcttgcctccaatattcaaggcagcatggacagtgaacaaagaggaacgagagaaaaa

cactgaagaagcattgcagggtctacaatttcttgaaaatgaactcaagaacaaattctt

tggtggagaaaacattggcattgtggacattgctgcttcctacttagctttctggcttcc

tgctgtggaagaagctgttggactgaagttgcttagtggtgacaaatttcctaagctcct

taaatggagtcaagagttcatcaatcaacctattgtgaaagagattcttccttctagaga

aggactactagggtttttcaaatctcgatatgctgcgattagtgcttctgcaaaatagat

catcgctcgacgagtaataaataaaaatagattcaaccgtgtggcgcgtgcgcggaagag

ttggatttagagaaaaataagttagttctagactaatcctagaaataagaaatgttgaag

ctaagtcattttagtttagatgttatcatgtgagaagtgttttgagattattatgacttg

tgaggacaaatttgatatttataattagtttaagtatcctattttgtatggttgatagct

tagttatgagtgaatcaaatatgaaatctttgtgtgcaatctctctgtcattcttattac

tttgattgaatgtttgaaatttgcatcatctttgaacaagtttga

>fava_dn_rep_c182

agacctcaaacaatatcactcatctatattcccaaacttcatgagctgtagaaatagtga

aaacagatacaatgagaaatgaagatatatcattctcacggattttcatcaaatcatcag

acatgaagtcatagcttcaaaaaaaacacaacatgattcatgaactattttgacaacaaa

gcatcgggcaatccatatttgctcaatttaaatggaaacagaagccaattaaaacctcaa

caggccaaaataaaaatatatccgagcacaagaaacagcaactaagtatataacaaaaac

ttggaaaatgtggctttttatttgcaaaatgatggcatattccatcccaaataaatccat

attcaaacaattttatattaatcaataatgtaacatttctttcatcaaacgatgaggatg

cagaccgtcaggagaaaattgttgtcttctaaatggaatagtttgccggttcccggagga

tcctctccatctgttgtgccctagactttatcgacatgtcaaagacttttggctgaactc

cagcactagaccaccaagtatgcttggatcaatcttctgttcaagcttaacctttgcccc

agaacctagcatttcctgaacagcctgcttcaatgcattctcctcctcagggggaagagc

aatgacagtagtcacggttgctttcacttctcccttatatgccattgccaactctgcaaa

cctctttgcaatggtatctacgtttttaagtctgccattctcagcaaggagaagaaggaa

gttctttgtgacttcagagaacttagcttgggtggcaatatcttggatgacttttactct

aacatccttagccacagatatatcctttataaattgtgaggttatgggactaccctttac

tccctcaacaaactgaagaagctcagagtcaaccttgtcgactgcattaactttcactgc

cgcaatatacaaagcagaggcatagtttcctgaacctccaaacattgcaatgggaacctt

aattttggtttccttttggcccggcacatcagcaaaatttctgaaagcctgggaaatgga

aggcgcgatgagagagcgttgaagagtagatctctgggaagaaagaagacctaatttgtt

gcagagagagattccagatttgacgcgaccgtaaaacgccatcaccgcgattcgaaaccc

taatttcgttttctgaagattctgcgttttctgtttgagggagtgggtaacgaagtgatt

ccaatcccgcgg

>fava_dn_rep_c183

tatagaaactttttttttcaagcattaatccacaattcggatttaatatattataaatag

atgattattgatgttatacaaactaaaaacatacataataattataaagcactcacaaac

acattattattattcaaacaaaacaaactcattaaaaacacacataataaatttatccca

ataaaacaagtcaaactaaatagtaggaggaatgagatttgtgttttctgtggccttaga

tataaaaagcttttgaaacttagagtccacattgaaagacacttcttgcaccaaaggtga

taaggctccccaaactgatgtcttgccagctaactcttcaaacaaaggcttggttgttgt

cacaattgaatagttctccattccttcatcacctgcaatttgagcaactacaaaaaaatg

tggcacaacaaccaaatgaccagccttaacttgagaatccaatacacgttttccactcaa

tcctacaatttcaatttttccacttcctctagctatgtaaattaactgaatcccaggggt

aattaaatttgatggcgccttaatagcattaggttcaagtttaactctaatcacactcaa

cccaacatccttaataaaaggaaaatctttctcagttaaggttgtcactaatcccccatt

ttggacctcagtttctggaactttagtatcaatgtcataaataaagtctttagttaggtc

caattgtggcttaggcatttgttggcctttttctaacttgattatcacaacccccttttg

gctttgtgttaacttagtcacctcatctttattaaaattgtatactttgcttattaattc

actagagaaacttcctaaaagtccttgaagaccagttaagaaaaaataagtgaattgtcc

tggaacatgagctgttgaagtttcaccaagaaatataatattaaggtttgagtgaccatc

attgaaccaccatgaaatagctccaataggcacaggtatgacatctccttgtttaagttt

caaaacaacttcttttccagttttggggagcaccattccaactacaccatcagtcccttg

aacaacataaccaactttagatgaatctgcataatgaggaagagcaaagcctcgtggttg

aagaacaagttgaccagcaccaacatttgtttgagccaaaacttgaacttgagagcttga

ccaaatatagtaaccaccaccatctccttcaagcattggttgtgctctctttggtgttag

gtctaactccattgaattcttctcctctgtgatgctggagcttccacaacgcttaattaa

gaagctaagaaaaagcaaaaatggtttgggatctatcaatgcgtccccgcggtaa

>fava_dn_rep_c184

tgatatttcttttagaaatcgaaccagacatcaaaccggcgaggttcaataagttgaact

gtccggtttggtttttaaaacactgttttatattcttcaacaataacttggctattctaa

agtacttaaaaacttcatataagtgttcattgttcttgcagactacgtatgagaacttca

gataccgatatgatcgacgagccaacccatataacagaggagtgttcgataatttcaaag

agatattcttcaccagcattccaccgtcaaagaataatttcagggcaaaggtcccaatcg

aaccagtgttacctgcccgatcagtgggtggaggctttatgagtccaagtatgggaaaag

ctgttgacgacatagagatgggaaggaaaacagtctggggtgacgtgggtagcaacatcg

atcattgtgaaggacaattaaacaatcgcgtggctgtaaaggatggagagtttggcgagt

tgtctccagaaattaggacaacagttgacgagacgagcgaccgtgttggaatacatccta

ggcgatctagctggggaagaaaaagcggaagctgggagatgtcccctgaagttcttgctt

tggcagccagggttggagaaccaaatcgcgttggcggaggtagcagcagtttgagcactg

agagcagacatacatagcaactaaacgagcgactgtgaccgtgacactaatttaaaaatg

ttaaatctacagctaaattaattgtaactgaagtgtgagggattgaaaggcttctaatac

attcttgtttaacttgctttctggattcaatattaaggattgtgcattgaaattcataca

acaaaatctaataaccaaggatataactggttcacttaagcttcaactcgtttgatacca

acatgtatctaatattcgaaagtttaattacaaagacagtgaaaacaacaccatagagag

agatatgattcatcacaaaacaaaaacaatgtatttggtggagttttacaagtagctgct

aatatataaagaaagattgaggttgaaaactattgtggtggaataagaacatttccaatg

ttgttgtgccatggatcagccaagtgagttgccaaattttccaaaggtccagtccctggg

tatgctgattgttgaatacagattccaacaaaagccaacaaagcgagacggccattctta

acttctttgattttgtattcatggaactttttagggtctttagagtaacccaaaggatca

aaagcaccaccagggtatttcttcttttcagggtctttctccatacttctttggtgttca

acaaaagcaatagaaaggaactcaatgaccaaaatggtgggtagggtaccccatggaaca

gggtttcctaagtaggttgcttgtcctccaggaagtgctgcccattcctgtgcttttacc

cagttccctaaccccaatgcctctggtaccaaaatccctggaacagcaagcattgcccat

ctgcagtgaatgagttcagactctttgaatctctcaagattctctggtactgaaccaaga

ccaagaggatcaaatccaaagtcacctggagctgaaccatcaagataggaaggtctaggc

tgaccaggcatccaatcagaagtcatggtgaatctagaagcattggtagaagcaacacaa

cgaagtggaattgaagttgcaaatcttgattttgatgaagaaagaagagatggaaaagct

gagatagctgaactcatcaatgtgttacaagtcatatttgtgattcttgttacacacaat

gagaatagtttgtgagcgactcttcttgcaatagtattatgtcaatgaatgagtgaaagt

ttcccgagaatatagaagagtggtaaggatataagcaaatccaaaattagatttccat

>fava_dn_rep_c185

gatgaggcaagatgttattaaaaaaacttagggggttggagttgtaaagtaacgagtaat

tcctccataatttgttcattttcacatcttgggttttaagccgtcactacaattcaacca

cgctcctcattcatcttccttcctccttgcgatggattccgtatccgaaagccagagaac

tctgtatccgtatgtaactggatcttctgtagttgctatcaaattcaaagatgggattct

tatggctgctgatatgggaggttcttatggatctactctgcgttacaagagtgttgagcg

tttgaagcctattggaaagcactcccttcttggtgctagtggggaaataagtgactttca

ggagattatgcgctaccttgatgagcttatccttaatgataacatgtgggatgatgggaa

ttctttggggcctaaggaggtgcacaactatttaactcgggtgatgtataataggcgtaa

caagttcaacccattgtggaactcacttgttctcggtggtattaaaaaggggcagaagta

ccttggcacggttaacatgattggcattaattatgaagacaaccatgtggcaactgggct

tggaaatcatcttgctaggccaattcttcgtgatgagtggaatgaaaacttgtcatttga

agaaggtgtcaggttacttgaaaaatgcatgagagtgctcttataccgtgatagatctgc

tgtcaacaagattcagatatctagaattactgaagaaggtgctactgtttttccaccata

ctctttgaaaacatactgggagttctcggccttcaagaatccaacagttggtgctgaagg

ttcatggtaacttgcttgttgtgattgaaactaaaacagaactttaaatggatttcagta

gaactatttaccgggttgtatggatatgaaatgaaatgagacacaaccttctgaagttaa

ctgagactgagttgggcgtttatttcttaaattattaatggttgtttggattgttgatat

atcttcaaaatttctatttctattcagttttctttcagtgacattatcttggttattatt

tttattgaattgtttggatttagccatgagctttgaatcctctaaaccaggggtggagtt

tggcttaagacataatgactaagtgacaaaaactaaagtgttaatattttcttgttatat

cacccttccaaaatctaagtgacaaaaactaaagtgttaatattttctt

>fava_dn_rep_c186

cgaacttgcaattaataaatttttatactaaatttctttaaaatcaggaaaagcaaaaac

aaataataatactgtgtattgctctgctgctataagcagcactatattatatggatcagg

attattgtatgccaacacagaagtacatacatatacatatatggtcttttatgttataca

tacatagcagttcttaatttctgcggataccgaaaaaattattgacaattttaacaagga

gtgaataacattgagccttatagacgatagagaagatattgagctaaaagggtttcatta

tgtatataatcatgtcactggttgaaggtagcaatgaaaccaataggtccttttcctgta

acagcagcctggattgcaaagacgaggaagaccaccatagccaagcgagaatgcttaatc

tctgccagttgaagtctttctttctcttcagggtcattggccaaaccaagaggatcaaag

aattttccacctgggtataaccttttctccgggtcaagttcagcattcctttgaaactca

atgtacccaattactaatacttcaatccatatcaatgtagtcaatgaaaaaggaagtgaa

aatccaaagtaggatggcccttccaccagctccacctttccagcatcttgccatgcgaca

ccagtgaaagcttcaacagccaaagcaccaagagcaccaagcatggcccaccttccatga

ataagttcacactcacgaaacctctcaatcccaaaaacctctgaatacggctgaaacggc

gtcggtttcacctctacagcttccaccctagtcccgatcacatcaccagcaaggttctta

gctaagttctggtcaagcgagtcaaggtcaaactgcagatactcagcaggctttgcaagt

cccaatggatcaaaaccacggtcgccgatcatagttccgtcgagccactccggcggctgc

gcgccgggaaaccataccagtccatcactttcgggctgtctcactttttttacttccttt

ttctttggtagtgcttttttgaagccgatgttgaaaagggcgcggaatcttccaatgttt

gaggttgttttgcggagatgggtgccgaaaaagtgtgacgtgacggcggcggttgcggtg

gtggccatagtgagtatgtttggggtttagagggagaaagttggaaatttatatttgtga

tggagtgtgtatgtgaggctcaaacacaagggtgtctggcatatcttggtgccttatctg

>fava_dn_rep_c187

gtctcccatttgcgccgccgacaggatcctcgtacacgcgtctttaacctcgtacgggct

tacaggaatacgcggagttgggattctttcaactccattggaacggtgttgaacgcacca

atcttgaatcattctacgaagtgagtgatttggaatgatttcaaaggatgttaactcggt

ttttgtaaccggacacgttttgttaccagactcgatccatttctcgatgctgtccctgtc

gtaggttatgccggttgaaagtgtaacgggatctttcattaaatctagtgttactgggcn

nnnnnnnnnnccacaactcattctccttcatcatactcacaacaaatctcattcaacaca

aacaacaacaacaacaacatcatcatagtcaaaaacaaaaacatggttttatcatggaca

aaaggtagtagagttttccgtcgtgcaagaaaaggcaaagagttattatcaaactcatgc

aacgatttacaagtggagatagcggttccaactcattttcgttgcccagtaacactagat

ttaatgaaagatcccgttacactttcaaccggcataacctacgacagggacagcatcgag

aaatggatcgagtctggtaacaaaacgtgtccggttacaaaaaccgagttaacatccttt

gaaatcattccaaatcactcacttcgtagaatgattcaagattggtgcgttcaacaccgt

tcctatggagttgaaagaatcccaactccacgtattcctgtaagcccgtacgaggttaaa

gacgcgtgtacgaggatcctgtcggcggcgcaaatgggagacgagaacaaggttgttgaa

ttggtgaagaagataaaaggatggggaaaagagagtgagaggaacaagaaatgtatagtt

tcaaatggtgctgctctcgttctttcgaatgtttttgattctttctcacgtggttcaatt

gagaagaattttgttgtcttggaagagattttggaggtgctgacgtggatgcgtcctatt

cctgaagagggtagattcctctatttgggatcttcaaattctttgagttgtttggtttgg

ttcttaaatgataaacaacaagtttcaactagacaaaatgcttctttgttgcttaaggaa

attcatgttgagtcattggtgaaagttgaaggagttcttgaatctttggtgaacatggtt

aaggttaatgttggtgatgtttctacaaaagcatgtttgtcaacaatttttcacttggtt

tactcatcaaagagtaaaaaagtgattatagagaggtttgttgaactgggtttagtttca

attttattggagattctagttgatgctgaaaaagggacatgtgagaaagctttaggcgtt

ttgagttgtctttgtgatagtgaaaatggggtgcaaatagcaaagtcaaatgctttgact

ttgcctcttgttattaagaagcttttgagggtttctgaattgagctcaagttttgttgtt

tctattgtctacaagatttgtgataaggctgaggaaggtgttttgattgaggcaattcaa

gttgggatgtttcagaaactacttgttttgttgcaagttggttgtgctgatagtacaaag

gagaaagcaactgaattgttgaagttattgaatggttacaagagcaaagcagagtgtgtt

gattcatcattggatctcatgcatctaaagaagccactctagttttatttattcatgaag

ttgagatttgaattcatttccattctttttttttttgaagagaataaattttataattat

atatattcagtctttaagtaaaccgccccatgagagcagcataatcaaagattcaaatat

aacaagcattgctgtaaagcaagaaatggaaaggattacaatgtattgaaagttgaacac

gacacaacgagattttaataaacttattttaatggatatgcatacttcagaatcagattc

agacctctttgacttcaacaggtgcagcagcagcaacaggcacagtttctttgacttgat

ctttgttttcttccttcacatccttaaccccctcttcaatttcaaaagccgaggccggga

atgtcctgaaaagccctgtaacagccttgaaggtgattttgccagtaggtggttcatcca

taaggatttcatgaagaggaagccaaatcaagagctccttcaccttcacaccagttagct

tcttgatcttaccaacctcaacagtggcagtgacttcagtaccataaatcacatgcctat

caactttcacaaacttgtggttatagcttgctttctgcttaagccacgcaaaaccactct

ctctatggtatccacattcttctatgtctttcaatggcaaaaggccattgggcatgctta

tttccttgagcaattccttggatttgatttggcagatctcatctccatagtatacctctg

attgtgctttgatctcgtcggtgaccagagccatcttgtgttttggtatgtttgaacttt

gaatagtaagttgctacttgattgtgaatgaatgaatcccgcggccgtaatggcgactct

gcgttgctctccttcttactattttcttcttcgatcattgattccagaagttccttttct

agaatcgggtggctctgcttcattgctttccatccttctgactccgaaacagttttgaag

ttttttagtattttacgatgacaaatgagactgagccgtggtgcatcacacagtaatgca

agttgaaatacatccactacattatctatggtgagtaagcccaattctagcttcagttca

cattcacgcttcaagtgagggactgcgtatacatgtgacagcaccaacaaatgtaggaca

aattccttcatttcttcgttctcgtaacaggaagagtacaagaatcgaataaaaactcgt

actgcatcatgtggaaccccaaaggttgagattgatcgccatcgattggaacgatttgct

tgcttcagcatacctcccaatacaggagaagccaccgcaataatgttagaatgagcataa

acaatgccaccgttatcagtattaatgcgaacatcggctttataaccttcattgaaaaga

cgctcccacaaatctcttgttgcagtagggacacagctgcttcgccctctcggtgctaaa

ttattcatcaccacatactcgcaaggataacctgttgctgctgattgaggcggaggaggt

ggtataggaatggatttcttcctactcaggagtgatttgccatccatgttagccatcagt

ttattcttcactgctcttaatcagaggacgttgggcggagaaacgacgacgtttattgga

agtcgggaaagagagaggaaagagagaaaacaagggtttggggaatgagagtcacccttc

ttcttctctctctttgagagatatctacggaaagataaataaatataaagggttagaaga

aaaaaaaatgttcaagtctattatctgtcactggccgctgggtgcttgcttcctagctaa

aaatatcaattactgtcacgtc

>fava_dn_rep_c188

ctttttttttttggaaaaattcagagagcaatctctgaaggaaaaatcaaataatatgga

ttttctaaacatgaaaaaagaaacttctgaactgtactaaatacattacaaactgaatgc

aaatataagacttgaataagttcaagctcattttgttactaaactcttacataaaatcat

acaaaagtacttcacaaaaccatatagttgaaggcataactcaccttttatatgtataaa

ttcatatataaagttgctcaaaaacaaacatctgatgtaaaagattcataagcctttaga

accaggaagatcaatccagtgcaaactgatttcaacttcaccacattccacattttgcaa

tctaagaatcatatcttggataatctttccactgttgtaatagatacagctctcctcagt

aagacagttgtgtttgcttggttgaattcgtttaagtacggtgccgtttgaaaggccggt

tgtgttcatcctcaaggcttctatataggacgagacgtcaaattccgcatctcccatttt

gtcatctttgctaaaagtgtcatgatcatacacagtaattgaaacgatgtgattaggatc

tatgacagaaagagtaagatcttcattccactcaggattaacatcccttttaatcactcg

agtcttgagtttctgattatacatcttgatgacaagataaggatcgctgctacgaacatc

acgaacagcaagattaacaccacgtttgacacgaaccctcaaaagacctaacagattctc

catcagagattttggaacagctgctgccgctgctgctgctgctgctgaatcctccatttt

ttccatcttctttttcccttttattcctaatcttaatcttaattaattattattattatg

ataatttgaatgagttttttttattattcttaattatgttattactatatatcaaattga

cgagtaaaattttccttttctgaaagt

>fava_dn_rep_c189

ttgttttttgattaacttgtgatgcactcttcccctgtttctgaaatcctgtgtcaaaat

tatgacagactttcaggaaaataagtagataaaataaatataatttattcaatccaggtg

aaatacataataaccaagctagttgaaccttgttggaaccatcatcattcccaaacttca

gtggctataactctatataatatgataaaccttaaaaagccatatccaaagactttacct

tcacaacaagatcaatggaaaaaaggaaaaagaaaccaaacaaataaaatagaaaagttc

tcatcaatgcgaaaaaggtcgacaacaattgtctatgcttctgtggtgacttctgcggcg

acttcttccactgcagtttcagctgctttgttctcttcaactggtgctgctgctggttct

gtttcagtagtttctttagcctcctcagttgttgcttcctctttcacttcttcaactttt

tcggttgtctggttctctacctctggcttggcttcttctgctacttctgtagtctccact

tcaactggtacttcagtttctgcttctttctctactgctgcttctacggcagcttcttct

tttggttgttccgtagtgactggttctgagactaaaacctcggaggcgggtgttgtttcg

tccaccttggttacttcaactgtttcattttctgtcacaattgttggtactacttgctgt

gcttcaacctaaacagtggccatttgggctagctgctgttgcacaagaaggaaagatgag

ggaaagaatgatagttgtcccgcggccnnnnnnnnnnnnnnnnnnnnnnnnnnnnnnnnn

nnnncctaaaagtgcggactttgaaattgctgctttactataaaattttccacgctggca

cataaaaaa

>fava_dn_rep_c190

aattggttgagataagagggttgcggtgtgtggtagtaatggaagacgtggcgaaattat

gatcatgagtattggattgaagagttagaaagagaagaatgaagcatttgacggtaatat

gaaaatggcgggtttggcggttcccactgctactggtacatgcagtaaggtgagactatt

tggtgttgcagcgacgaggaggagagatggggtttatggaaggagtaggagagtatctat

tagagtgggtggtgtgaaatgtagtattagtggagatgttgctgatagatcagccattaa

tcttgggacgggaacacctgttaggtcaaccaacatacttgtcgtgggagccaccggcac

tcttggaaggcagattgtgagaagagcactcgacgaaggttacgacgtcaggtgccttgt

aaggcccagacccgctcctgctgatttccttcgtgattggggagcaactgttgtcaacgg

cgaccttagtaaaccagacactatccccgctactttggttggtgttcacactattattga

ttgcgctactggtcgtccagaagagcccatcaaaactgttgattgggaaggtaaagttgc

tctcatacaatgtgccaaagctatgggaattcagaaatatgttttctattccatccacaa

ctgcgataaacatcctgaggttccacttatggagatcaagtattgcaccgagaacttcct

ccgtgattctggactcaatcacgttgtcatccgcttatgtggtttcatgcaaggtcttat

cggccagtatgcagttcctatcctcgaagaaaaatctgtttggggaactgatgctcccac

cagaatcgcttacatggatactcaggatatagctcgcttgacttttatagcattgcgaaa

cgagaaaatcaatggtaaacttcttacttttgccggtccgcgtgcatggacaacacaaga

ggtgataacattgtgtgagaggcttgctggtcaagatgctaatgtaactacagttcctgt

ctctgttttaagatttactcgtcagttaactcgcttctttgagtggacaaacgatgttgc

tgacagattggcattttcagaggtgcttacaggtgacacagttttctctgttccgatggc

ggagacatacaatctgctaggtgtggatacaaaagatataattacattggagaagtatct

gcaggactacttcactaacatattgaagaagttgaaggatcttaaagcccagtcaaaaca

atcagatattttcttctaaaatatgattttgtaattgcgtagtacaaggtaatgttgtgg

gaatttagagttttattttcaaatatgctttccattgtatatcgttttatattaaaattg

attttgtaaccggtgtattcacccaaaaacattcgtctatagtatatataatattaagat

ccataagcttaaaaaaaaaaaggctccacacaataaaacccactattctgttactcaaac

tttagtcattatccacttgaaagctccattttcataggaagcttcacttcttcaatcaga

tcttcatgctccaagaccactggtcagaatggtgttgatctctccgattatattaactgg

ccttccatcaaacatggatgaaagcgtgttctcacaaagttttttcagttcctttgtttt

tgccacagcagcgtcttgctttggccataatgaagataatgaattttgtagctccgtgcg

cttagtagccaagtccttcagcccctctttcaacttgacttcatgatgtttcttttcatc

caaagagttcactagtctgtccagaaatcttttggagttgagaatcataatcagatccct

tgtctttctatttgtcagcaacgaaattgccaaagaaatatcactttgcatcgtgtctat

agtatcagcagcatactgctgtagtacaaagggagaaaccgcctgtacttgattttgcag

cgacaaagtttcctcattcttcaactcagctaaccgttgatttaagaaggatttcatctc

atatagatcatcgagaatcttatttctgtactccgtgtctaaaagctggctccttccttc

cttgctctctgtcaactgagataaggcatctggaagagaagtctgattgtccagccctgc

atttgatgcattcgcattgtcaatcacatcaacatgagcagactcaacactaatatccca

gcatatatctgcatgtgcgcctagttcttcatttgatattgttggatcagattcaacatc

ctcagttgatgaagaagtctgtatggcctcagaggcattaatgatttcaaatggacccat

gccatttccggaatcctcggtttcttctaaggttccgatatcccaatcaatttgtgaact

ttccactgaaatatcccagtcaatgtcaggagcagcaacttccacactgcctactgcagg

atttgtctccttgtcacttgagtgtacaccaacaatattaataacctcagagtcaacagc

aatatttaaagatggaggattttcacgcatgttcctcaagttttgtagtacagcttccga

agatctatctttttcagtgtgagcatctctgacaaaattgcaatagtactctattgcctg

tgaaagattgtcactattcacaacatccaatatcctgctaaatgtagttggaagtgattt

tgctgtctccaaaagttctaatctaacatttttcccctgtaggccaagctcctgacaagc

ttcaacatatttggctgccgagagtgcagcacttctttttatatcagtctccttgcgatc

aagttctgccagctgctgctgaatcttatgtacctgtttcctctgataaggaatttcata

attcacattttgggtgataataagagcagcctcgccaaggaaaacatggtctttctcaaa

agatcgtacaattgcttcccatgcacccgcagaaccagataggcgaccaaatatattcct

gctctccgtattagacttcaagagaatatcatatatctgtttggcctctacataaccaat

cccttcagggtctagggtttgaaaaaggggatgagaatccttagggagggaaccaaattc

gttggaaattcgagccctgattgcggcgacgcgcttcctccaatcagaaggtactctctt

gcgatcgaccaaccattctccgagacgggaaaaggtgatgtcgattgggagtttgcggat

atcgtcgggagtttgcattgggaatgaagatcaaaacaacaacaagagagagatgttctt

tcgcaccactgtgttcctccgacaacccacagtttcagcttcaactcaattcaacttcgc

gttttttcatcgggttatgatcattttttttcctttttttcttttcacttttattaactt

attgttttcgaatcgtttcctttgtggtcataaaagaatgatgaagttggacattcttgg

atacaaaacatatctgttgtgttgagatgaaaaacaaggccttgtttcaagtttgttttg

gttgggatgagtttcatttttctaaggaatctgcttctcattccttacacgatatcttca

aaaagtggcttatactacacatgctatgatcctcttgaagagtgaggaaaacaaagagga

tgagattttgagaatgagttctatggttgaagataagttctctctttcactagtttctag

tgactatataaagtagaaatt

>fava_dn_rep_c191

acaaacaaagaccgtatactgaatgaaatgcgcgtttcatatgagcgtataaataggagc

cagacctaaaccctaatttctgccgtctatcttttccttcgcagcgtgcagttcttctgt

cttctcttcaggttcctagaagcaaaaatgggtcgagtagatgcagctggttccaagggt

aagaagaagggatctacaaccttcaccatcgactgcgctaagcctgtagaggacaagatc

atggacattgcttctctcgagaagtttcttcaagagaggattaaggttggtggtaaggcc

ggtgctctgggtgacgctgttactgtttctcgcgagaagagcaagatcactgttacctct

gatagcaacttctccaaaaggtatctcaagtacttgaccaaaaagtacttaaaaaaacac

aatgtccgggactggctccgtgtcattgcttctaacaaagacagaaatgtttatgagttg

cgatacttcaacatcgcagagaatgaaggagaagaagaggattagttgtgtgttcatctt

atttattttagaacctatgttaaaccatttacttgtcttatcttctcttttgcctgagta

ttgtttgagatatgttgctttggttgcatcagttgcaatttaacaattttgtttaattaa

gagttgtgatgccacttttgtttgaaagggaaatatagttctctattatccaatcaagca

ccaccttcattacaattcttatgtggtaatcttttaccttaaatctcattttattttctt

agtagttctaaaaaaaaaaa

>fava_dn_rep_c192

tttttttttttagcataataacttttgtcattgattggtttaaacaaaacatattaacaa

aagatctcccatctttaaaatgaaccttagtccttaagaaccaagaaacaagtgcactaa

tccaaccaacaatgatcatgatcataatctacataacaatgccaagtctcatcattcaca

caaatgcaatcatagataacataaaatgtttaagcaccaggcacaaactttgtagcataa

acccaagcattgttagcaacagggttatcaaggtgatccaaaagattctccaaaggtcct

ttaccagtcacaatagcttgaacaaagaaaccaaacatagagaacatagccaatcttcca

ttcttgatttccttcactttgagttcggcgaaagtgactggatcgtcggcaagaccaaga

ggatcaaagtattggccaccagggtaaagatcattgccttctccaacatcgggaagaccg

ttgatgcggaagccttctacgagccccattagaacaatttggaaacctagtactgctaag

atactttgtgcatgaacaaggttagggtttcctaaatagtcaagtccaccttctgagaat

atctgtgatcctgctttgaaccaaactggttctttgaagtcaactctaacccatttttga

agtacttctggtgttatgcatcctagcgctcctagcatagcccatcttccatggatcacc

tcaagagccctgttcttggcaaatgcttctggatcagcagataaaccagcagtgtcccat

ccatagtcaccagggaattctccagttaagtatgaaggagtctgagctgaaaaaggtcct

aaatatttcactctgtctggtccataccacaaatcatttcccatggtgaatttggaagtt

cccatggcaaccacatctctaagaggattagcactctttctttgaccaaggaaaggagtt

tgcttaacaacagtggctgagcttgctgtggctgccatcaatgccatgattttcttcttg

ttgagtggaaagcttactcagtttactcacaagctgttgtcactagaaatgtgtcccgcg

gccgtaatggccactctgctttaataccacaattttttaacacaatattaacactaatga

ccattaaacagttacactttgacaaaagagaatcaacagtttcttgcactttcgcaaggg

aaaaacacacaaaccccttttcttttactagccaccaagacaaacatagacgcatttttt

tatattaaagtcacaattcatataatatgatatagtgtatcgacatgccatcccctaatg

ttcagggtttaagatgaggaaaacatgtgaagagatcagatttagggtgtctcgcctcag

catgctccttgcacttgacttctgatgtggtgcagataaatgtttgcatgcacactttgc

actggatcgacatggctttcttgttggtttcaagttggctccccttgccgcccttctgct

tctcgaggttcctctcccgagccatctttgccttttggccgttgcctcctcccatgactg

atcgggtaggacgaaacgagagacccctcaaaaccctaatttccttatcaaggaggagag

actgatgtgagcggaaaactagaatcccgcggcc

>fava_dn_rep_c193

atttaatttacactataatctcaaacttcaatcagagttcttataaacaagcaacaacca

gtataaactaacaaacaacaatattttctacttttacataaaacacaataatattctaat

agaaacatcattaagtaacgttcatttatatctcaaagatcatcaaatctgttggagcaa

aacttaatacttggaaaagaaaacttcaaaaacaacacatctcaggtaaattgtaagtaa

tgcataacaaataatctaaaaagtctaatacagatttaaaaaggtgggtgcaaggaacta

agcaaaagaaaatcacaacttaatcttcgtcctcctcatctccatcaccgccagaagcct

tcttagctgcagccttaaggttctttcgcttcacgcgaccaggacgaccaccaccaagag

gacttgtgagtgaaaagtcaatgtgcttctgtgaatcaaccctcaccatgaatgatggga

tgttcaccacttgcctcccaaccctgatgtgcctttgcctaataagaaccctagcatgat

ggatggacttagccatgccagatttgaagacaagtgtctgaagacggcgctcgagaaagt

tctcaacagtgagagccaagacataatcgagcttgttctgagtttcatcaaggagtccgt

aacgaaacattctccgcaaaagagcttcaccttcaaagatccggcgaggattcttctcat

ccagtgtcaagagattccttgcattgttacggatacggctcagagcgtactgaaccctcc

acagctctctcttgcaccgaagtccgtactctcccaccagcttcaactctgcgtccaatc

gctccttctcgtatggacgacgaggcttcttgaacgttttcccatagttccggtaaaacg

aaacatgcaccatagctgcttgtctcgccgccggttctctctggtaggttttagcttttg

tttctctccccgcggca

>fava_dn_rep_c194

tggctgtgaaaaattaatctaattaatctgaaaacaaaacatttttttttcggaaaacat

ttgcatattaccaacttgtcaatgaaaaattaaacaaatcattatagatgatttgacaga

agaagtgccatcataacaagcaacgttgttatgtgacactcactatttctacattgcttc

atagagaaagttacaacaagggatataacatcttgttcatgactttccattatgacaaac

acatcactgcttgaaacacaaagtttacttttcttatcaaatacaacaccgccttttctt

tcccacaacaaacataatctcaccacaagaaacataagcaaaccagaaacagttttcata

aatgttatttatacatggtctaaaactccaaatcagcccatggcatacttctgagtccag

ctgcgtgcagttgcttcatacttggtcctgtcggttttgtacatgtgagcaatctccggt

acaagagggtcatcaggatttggatcagtcagcagtgagcagatagaaagcagtacctta

gagatggtcagtgcagggctccattgctccttcaggatatcaagacaaatgctaccgttg

ctattgatattgggatgaaaaaccttagtcctgaatgcaaccttaggaggcttgaaagga

taatccggaggaaaatgaatggtaaccaaaaaaacacctccagcatacggactatccgcc

ggacccataatagttgcttgccaatgaaacatgtcttcagcaaccggacctgcgctgcag

gacgtaggtggatccttctgcaagtccttgagctccttcaagatgcgcttggaagccatg

gaaacgactctggagtttcagatcttaaggtgaggctatgggaggatacggaatgagaga

tccaacaaagaacagacgcagcgtgaatccctcactcgccttcccaatcccaaccccttc

ttttttatatttcaacttttcttcacaaaaacaacaacaaaagctgctcgataatacccc

gcggcagatttctcacaaacaaaacctttcaattttcttgggtcattgacacatagttcc

aaaactcttaaatgagttgaagataatcctacaaaaaaagaaaagaaacaagagaagaga

agtttggtttcaaaccaatattctttccctttaaccaagtatatccgaaaaagaaagcga

aaatggatagtaatttaagtcgaagtataagtaggagtttgagtagatcaagttggaaaa

tggaagaagtttttgcaagtggaagatattcaagaagaacttcccaagttgatgaagatg

aagaagcacttaaatgggctgcaattgagaaattaccaacctatgataggcttagaacaa

gtattatgcaaacatttacagaggaacctcaacaaggaaacaggatgcaacacaaagaag

ttgatgttaggaaacttgatatgaatgaaagacaacaaatcattgaccaaattttcaagg

ttgctgaagaagataatgaaaaatatctcaagaaattcagaaacagaattgaaaaggttg

gaataagacttccaacagttgaagtaaggtttaagaatttaacgattgaagctgattcct

atgttggaagcagagctctaccaacattaccaaatgttgcattaaacattgttgaatcta

ctcttggaatgtttggaattagtacagctaaaagaacaaaactcaccattcttaaaaatg

cttctggcattattaaaccatcaaggatggcccttttacttggtcccccttcatcaggta

aaacaaccctattgctggcattggctggaaaattggatgctgaattgagggtgaaaggag

aaattacttacaatggacacaaacttaatgaatttgtgccgaggaaaacttcagcatata

ttagtcaaaatgatgttcatgttggagaaatgactgttaaagaaactttggatttctcag

ctagatgtcaaggagttggaactagatatgatctattaagtgaacttgctagaagggaaa

aagaagctggtatatttcctgaggcagaacttgaccttttcatgaaggctactgcagtga

aaggaacagaaagtagtctcattactgattatactttaaaaatattagggcttgatattt

gtaaggacaccatagtgggagatgagatgcatagaggtgtatctggtggccaaaagaaac

gtgtcaccacaggtgaaatgattgttggacctactaaaacactat

>fava_dn_rep_c195

acatttaacgagtgacactcattttaataaatgtcaattaatctcttttcttaatcaagc

ttaaaataaataaaaaatttgtttaggacacatttgtagccacactctcaccaccagcct

atataactagggttttacattcggaattggcacaactcatttcaattcccaattttcaac

tacagactcaagttcttcatcaatggctccaatcactgtaggagatgcgattccagatgg

caccttagcctatctcgatgaagaaaacaaaccccaatccgtttcaattcattccctctc

cgccggcaaaaaagtcatcatctttggtgttcccggtgccttcacacccacttgcagttt

gaagcatgtgcctggattcattgaaagagcagaagaactgaaaggaaagggtgttgatga

aatcatctgcatcagtgtgaatgatccatttgtgttgaattcatgggccaaaacattccc

agagaacaagcatgtcaagttccttgctgatggctcagccaaatacactcatgctcttgg

gttggagcttgacctctctgagaaaggcctcggcattcgctcgaggaggtttgctctttt

ggtggaagacttgaaggtgaaggttgcaaatgttgaaggtggaggagaattcaccatttc

aagtgctgaagagatcatcaaggctctttgaagctcttaatgaatgtttttagtactttt

gaatccagaacatgataatggatgtattttatgttatggagaaataaaattgcttgtttg

tttcaagtatgtgttattttgctgttgaattagttaatttttcatctcataaaagaacag

aaaaaaagttgcatagcaaacatcatttacatttttattttatttaaatttgtgttttta

tgagatgtctatgtcacatcgagtaatatgaaatgttgggttgagtatttaaaaaatttc

>fava_dn_rep_c196

aaattattttttttttggaagaaaattaattatattttaaacttctacactaaggtcttc

aaaaattccccttttgcaaaaatcaaccatgttttgaaaaagagaaagatacatatatta

aaaccgatcttatttgaaacaagtagtgtttcaactttatgtaatacaaaattatagctt

catcatccatggcaaacaaactaaagtaaaaatactaaactgcatgtccctccagcatta

ttattgacatgtgtttgctgaagaagctaaatagatatactactgaaatcggagcttaaa

gaactgcaatgcagtcgatttcaatcttggagttcaatggtaatgcagctacttgtaaac

tcacgcgggctgggaaaggtgcagtgaaatatttagcataaaccccgttgactgtcgtag

cgtccttcaagtcagctaacagaactgttgttttaacaactgatgcgtagcttgcaccac

ctgctttaaggatttcccccatgtttttgagaatctgttctgtttgttcatcaactttat

cagagatgaacttccccgtctcgggaatacgaccagaaacaccagaaacaaagagaaggt

tattgactttaattgcttgggaatatggtcccaatgcaggaggtgcttttttcgtttcaa

cagcttccttagctcgtcttctggcatcagaagaaccgctcaggcatgtgaacgacaaag

accgcttcgatatgccgccggtaactgagacacactctcttccggtgctcaatgaagggc

ggttgcggagtacgccgccgttgatcgccggaatctggaagctcgttgcagtacaaaacg

aagtcatttctaagtttcccagtttctctgtttgaccattttatacaagttcatctgtat

tttagttcacagaaaatgggtcggtgcaatctacaccacacaatacaatgacacgtttta

aat

>fava_dn_rep_c197

ttttttttttagagattaaaatgatcaaacttttattttccccataacaaagcataaaca

aagttgcaactaagaactatatcaaattaaagagaaccatagtagaaacgttagggtctg

atagataagcacataattaaatgttacagactagtagccaatataacttcttcacagcat

aaaatagtcttggtggttctagtattgatctcagaccacaaaaaataagtggtcaaagac

tcaataaactcaaccattaccatatcttgtaagtttgtaagaattgcacaaatataatag

caaacacgcagaaatattaaatatatcatctcaattccaacagcattcatggatttatta

cttagggccaacgtccttaagaccacaaatctgctcctcgcccatggcagacatgacaga

cacaatcagatctttaccttcgttaaatccatccttgatctgcgaaagcagactatcatc

ggacgggagctttaggtcatcctttgtaccaccactctctgtcagcagactcacaaatcc

atcctcagagatatcaatcagctggtaatcagtacgattaacatgaggaacatcacagtt

atgagaagaaggaacaatatcttcaagctttttcccagtgaagatatcaatagcaacaaa

gtgacacttagcatgtccatgtttcccagtttttgaagttgaaacctcaactaccttgca

gggtctgtttttgataacaatgtgaccatttttgcgaatggttccagcttgttgtggata

tgtcttggatgctccagcatcagctttggactcgaaatggtgttcctcgtctgacatgtt

tgtttcacacttcttgttcttcacaccacgcaatacttttgttttcgcgttttcttttat

acacaaatatatagtgtaacaactcgctattttttagtgat

>fava_dn_rep_c198

taatcgtcaaatgaaactcaataaaccaattaaattttacaaaaccaaatttattcaaaa

taacgtataacaatcaccaaatattgccccggacagatatctgtccaataaaaacaatca

accatacaattcgcattgagcatctgaattatgggcgtctaaaaaattatgaatattgta

ctttctttggtagtatcttacaaaaatgaaaatctccatccaatactaaatatgaaaagg

tataaatgaccaaaacaaaggtataaatcagaacctgatggcgcatagaattagtatctc

cattacagttgaaggctttgcatccaaacccattagaaagctcaaaagtattaaacatcc

catgaaacaaattcctactacaactgtagccaccaatatgtcctaaatcataatacatga

acacaaattcaagttgttttggaagaggactcggaaataaaagctagataatattcaccc

ctgcatccagaatgaccaatccaaaagactcccaaaatatcattatgccttattggctat

attgttggagagccagtccaaaccttcgtaaagaccctccccagaggttgcacaagtgct

ctgaatgtaccagtggcgctgtctgagagagtggagacccagcttgtcagtaatctcagc

agcattcattgcattgggaagatcttgtttgttggcaaacacaagcaatactgcatctct

cagttcatcctcattcaacattctatgtagctcatctctggcctcagcaactctgtccct

gtcattgctgtcaacgacaaaaataagaccctgagtgttctggaagtagtgcctccacaa

gggacgaatcttgtcctggcctccgacatcccaaacggtgaagctaatgttcttgtattc

cacagtctcaacattgaacccaatggtagggatggtggtgacaatctctccaagcttgag

cttgtagaggatagtggtcttaccagcagcatcgagacccaccatcaaaattctcatttc

cttctttgcaaataaccgactgaacagcttcgtgaatgttaaccccatgtttccttccct

tacttgattgatcctcctttgaagaagaaaagttgatgtagcagaaaccgcagagagaga

gagagagaggtgaagaatgctttgcagcaacgcggaatgggaagagagatgggtatgaga

attgattttattacgccaaaagggtaccaccacaattgattctttttattt

>fava_dn_rep_c199

ggccgcgggattcaaagacgcgctctttcataatcatcatagaaaaagaaacaccgattt

ttcttttctctctctatggcagatcaactcacagacgatcagatctcggagtttaaggaa

gctttcagcctattcgataaggatggcgatggttgcatcaccaccaaggagcttggaact

gttatgcgatcattgggacaaaacccaactgaggcagagcttcaagacatgatcaatgaa

gtagatgctgatgggaatggtactattgacttccctgagtttcttaacctcatggcaagg

aagatgaaggacacggactctgaggaggagctgaaagaggcatttcgggttttcgacaag

gatcagaatggattcatctctgctgctgagcttcgccatgtgatgaccaaccttggcgag

aagctcactgatgaagaagtcgatgagatgattcgagaggctgatgttgatggtgatggc

caaataaactacgaggaattcgttaaggtgatgatggccaagtgattgatctctctctca

cttcatttctattacttgtataacagtgctttgtagtagccgttgtcgtttcttcttaca

tttttatttcatggttaaatgttaattagtggtaagtgttgctactcatgattgttgtcc

aatggtgtctttactagtttttcttgtaattttattccttaaaaagatgtaatgttgatt

tcacttcagattctaccacttctcattttttggtgaatggttattacatatgcgattttg

taagtttcattattagatttatcctttcaagttttttcttttagctat

>fava_dn_rep_c200

cggagaacaaattcttaggaagattttattaagaaacataataagatgataaattgttgg

gacaaagacaagaatacaaaataaatattatttattcaaatctagagagaaacacagtaa

cacaccatgacaaaccttcttaagaactatagccccttatcacttcaatacacaactcat

tgaatagagtttcacatgtccaccactcagcatgtatatatatgctgatgtggagccaag

tgccatatcccaaagccttaccataataacaataataaactttaataaaggaaaggaaac

taaagaaatatttaatagaaaagcttttcaaccttgatgacatgcaaaatggtagacccg

atcccaacaactagaaccattaatttaaacttccttttttttaccagttccattcacacc

ccttccattggtttaagcttctggattctcaactggaacttcctctgctgctggagcttc

cactgattcaactgatttgttctcaatcacatccgctgcagttgctgctggtggtgctgc

tgactcattttcagtagttgctattggctcctccactactgcttctgcttcttccttcac

ttcttcaaccgattcctcagtttctttaggtgcttccgagttctcctcctttgcatcctc

tgttaccacttccttgctctcaacttcaagtggatcttgggtttctggagcatctacagc

ttcttctgctgcagctgtttctgttggtttttcggttgtttcttccgttggtacttcggt

tgcggttgcaggaacttcggattctggttgttctgtagcaggaacttcagtggctggttg

ttgttctggaattgtttcctctgtcttgatgatctcgggtgttgtttcattttcttgcac

tgttgttggtggagcttggtgtgcaacctcaacactggccatttggaaaggtgagaggga

ttgaaaaaaaagctgaagggtgtgtgaatgaagaatgaaaagagaaattaagagtggtag

tgagtgagatcccgcggc

>fava_dn_rep_c201

tttgaggttgtaactatttgaagtgattattttgataagcacttatgctataaactgcaa

ataagtttttatctaaacaaggggaaaaaaagcaacggagaaaaacattgctattattaa

tttgtacaacttgcaagtaaaataaaaaatgtgctgcaggaactaacctactacacatac

tatttgattcactatccacaactatagttataatacatactataattattacactattca

aacatcaaagctaatagtgtaacaaaggccagatttgattagtcaaaagagttaagttaa

actcttttattgaaattcaaattaaatccttcaactacatcatcaatttcttcttcccct

ttagcaatttgaaaccaactttgttttagtagattcacaatttcaatatcccttgtttgt

tggattggtcttgaaatttgtctataagaaggcccttcatcacatccttcacaaattatt

tctccactctcatcttggtagcaaactatgccttgagattcatcctcataaaccttgttc

ttgatggatgctgtgactttaaacactacttgaccaactgatgactttttgtttgttgca

tagcatcgtttggacatcaacaaattgtttgaatatttcacaaatgaatgatcatatttc

tgaattgggatttgagctagagaattgcagtgaatagccatgaatctaatacttgaacaa

caagaaaaataaggaggagagaaattgaagaggaaaaagaaaatgaaatggatgttaatg

taatgatagtcccgcggcc

>fava_dn_rep_c202

atccaacatgttagataaggtaatgactcacaatcacacacnnnacactcatcgtatcca

taaacccttctttattactattgtcaccaatatcatcatcaaacattcaagttcaagaaa

gaaagaaactattatggctcaagctatggcatcatcaatggctggttgtttacgtggttc

ttcatctcaagctgtgttagaaggaagtcttcaattgagtggctcaaacaggttgagttt

gttgcatggtaacattaacagtaacaaggtttcaacaacacgttcattggtgacagttag

agctcaacaacaggaaagtagtcgaagggctgttattggtcttgttgctactggtttggt

ttcttcctcttttgttcaagctgtttttgtcgaggctattcctattaaagttggtggccc

tcctccaccttctggtggccttcctggaacattgaactctgatgaagcaagagaccttaa

gctaccattgaaagaaaggttctttcttcaaccattggcaccaactgaagcagctgcaag

aactaaggaatcagcaaaggaaattgttgctgctaagaagttcattgaccagaaggcttg

gccatttcttcagaacgatctccgtctcagggctggttatcttcgatatgaccttaaaac

tatcatctcttcaaagcctaaggaccagaaacaatccatcaaggaactcaccgataagct

cttccgggatatcgacaatttggatcatgcagcaaaaataaagagcccagcagaagcaga

gaagtactatgctatagctgtatctactctgaatgatgttcttagcaaaattgcttaatg

tgaatgatttcttgctttcttgtgtaagctttgtctatggttttttttaagacattttat

gtttaataactactattttgaacgcattcaaggctctcaagttgcaagaagcagtcatca

actgtttcagtatgttctgcccaaagtgacatcctatatccatggatctacatggtatag

tttccaaaaaatttctgtcagatttagaatacatgttgacggattcaaacatatatgcag

gaaaacaaaaatcaaagaaacgccaatcaaaactgtaaggccaaatcattaacataaatg

tcagtgacctatctcctaacgccaatgaagacttggttcaaagaagaatatttttaataa

ccggaagtgcatttttggaaatttttgaaaatgtattttcgaaataaattttgtcgtaaa

ataaggtgaatctcaaataccaatgaattatgtgtgtgcgcgcatcagaaatatatttat

aatttctgaagggtattttagaaaatgcataggtatttctttattctatagggtgaaaaa

cgaaatttccaatattaaaatattgtatttgggtctgaaataaattcttatgatttagat

ttgtnnnnnnnnnnnnnnnnnnnnnnnnnataataataatcattctacg

>fava_dn_rep_c203

gtaagaggtaatataacctgaatgcccatttaatttacccatgcatctactcattggata

gtttctaaacacaaaatctgatctcattgaaatttgtagcagtaaccaaattcttgttct

aacaataacaaagcacaatactccattcaacaacactataataattaaattattctccaa

ctcttttcccactttatgttacacatgttatttagttaaactacatgataagtagacaca

caatatgcaatgtaagataaaaattattgtgatcccttgagcttctttgcaattccagca

aagtacttgccttgatggaaagcttgagccaattccaactcactaggttgacctacatca

tgctccccaaaagttcctgaaccatatggtgatccacccttcaccttctctatctcaaac

attccagcaccaaatgtgtaaccaattggcacataaagcattccatggtgaacaagctga

gtaattgatgtaaacgctgtttcctcctggccacttccttgagaagcagtagaaaaaaag

aagcctgcaggctttccagcaagtgcttgtttgttccataaacttccagttgcatccaaa

aaaaaatttgaattgagctgacatcattccatatcttgctggaaaaccaaacaaaaaacc

atctgcttctgtaagttcttttggtgaaatgtctggaacttcactccttgctggtgcttt

catcttcctaagtatctcttcagacaatatttcaggtacctgccaaagttttgcttctac

accttctacagatgaagcaccctttttaatctcttcagctagcttctcaacatgtccata

cacagaatggtaaataatgtaaactttagtggccattgatttaatttgcagggcctgtct

gtatagaaaataagtttgtgaatcccgcggccgtaatggccactctgcgttgatacccgt

gcctacttcaccgctgctaccatgatcatagctgtccccactggaattaaaatctttagt

tggatcgctaccatgtgggggggttcgatacaatacaaaacacccatgttatttgctgta

gggttcatctttttgttcaccataggaggactcactggaatagtcctggcaaattcaggg

ctagacattgctctacatgatacttattatgtggttgcacatttccattatgtactttct

atgggagccgtttttgctttatttgcaggattttactattgggtgggtaaaatctttggt

cggacataccctgaaactttaggtcaaatccatttttggatcacttttttcggggttaat

ctgaccttctttcccatgcatttcttagggctttcgggtatgccacgtcgcattccagat

tatccagatgcttacgctggatggaatgcccttagcagttttggctcttatatatccgta

gttgggatttgttgtttcttcgtggtcgtaacaatcactttaagcagtggaaataacata

acaagggcgaacattccttgggctgttgaacaaaattcaaccacactggaatggctggta

caaagtcctccagcttttcatacttttggagaacttccagctatcaaggagacgaaaagc

tatgtgaagtaaaagaaggaagaaaaggtcgccgactgctactaagaacctaacagaact

tttcaaaatgtgggttccaacgaagaagagttgaggaccaacttcgacctaatttaagag

ttaagaaagcaagctcagttcagaatggctacttaccaacagaggatagcgaggtactac

aaggcctgagttaaattttgaaatttgatcgagctgagccggagctgctcctactgacta

tgactggacagcagtggactctttctcaaagtctgaccctgccacctatatttgatgaag

caaccatcactgaagccattttttttgtagccagctcaactccatttaacaggttagtag

cgaaaggtaaggcttcattcccgagtggacgagaagagggagcaagccctattaaggtta

agggaagaggctattttactagactagctaccgagctgactcaaggaagccggaaagaaa

gggcctcaagacagcttcggcaacataaagtcgagctgaggaatacggaagagaagcgga

ttcaactcaatcttagcctaagagctgttcagagaagagaatcaaaggtcaacggccaat

ggaggatttccctcccccggattcatcttaaaccgaccgatatgccgagatgacatccag

cacgagagcaagaaaaggaaatggtctccgtgcagcttcaagtcacgccactctcaccaa

gatagaatcatggtcacatctgtagtatatatatagtttatagaagcggaatgccaatcg

acactggtagccatcgggattgagttcagctgctttgacgatactgacgaatggaagaat

gaattaatcagatgagctcaagtctggactctcattggtcaaacaggcgcaacgggccaa

gagataataaaaggacacctgaagagcaagaaaaagagctgtatgataagagtccttatc

ctacccaccctatttctttagtttatgggagtagcaagaacagctccatttaaaaggtta

gccgcgtaccaaagagctcgatcagctattctatttttcagtccggttgacgccactcga

taggaagaaaagagcataaaggtttttcgaaggcttcaagccctgtttcagctgagagca

ttgattcccctggcacctctcttccacagcttggcacgtttaaagaaggtgcctacacta

aagtgtacaaagtggcataaagttattcttttcggatttggtcaggagggacaaaagaga

gatgctttctatcagtcaaaagcggatatcgccccccc

>fava_dn_rep_c204

ggcagcaaatcattctctgatcatttgacataaatctatcaaaatatattattccacaaa

cataatgtaccatttccatactttttttttttgggacaaagtttcgaataataataatat

tgttccaaaaacaaccacaagaattcagacaaagtacattctcaaaacagaaccaaacat

gaacaaaacaaaaagtataaaagttaatattgataaactaaggacataatcatagatgca

aaaacaaaatttaacttcactagagaatgcatagaatgcaactaaaacacagtagtttaa

tcctcatcctcctcatcaccatcaccaccagaagccttcttggcagcagcctttaggtta

tttcgcttcacccttcctggacgtccaccaccaaggggacttgtaagtgaaaagtcaata

tgcttctgtgagtcaaccctcaccatgaatgatgggatgttaacaacctgcctcccaacc

ctgatatgcctctgtctgatgagaaccctagcatgatgaatagacttagccataccagat

ttaaaaaccagcgtctgaagacgacgttccagaaaattctcaacagttaaagcaagaaca

taatcaagcttattctgagtctcatcaagtaatccataacggaacattcttcgaagcaaa

gcttcaccttcaaaaatcctacgaggactcttctcatcaagagttagaaggtttctagca

ttgttcctgattctactgagagcgtactgcactctccagagctcacgtttgcacctgagc

ccgtactcaccgacgagcttcaactcggcgtctaaacgctccttctcgtatggacgacgg

ggtttcttgaaggtttttccatagttgcggtaaaatgatacatgaaccatagttctggag

ctgttgggcggagaagagggttgctcttcgttgatctctagcgcctgtgagcagaagccc

gcggcctaactgcttcttgtttgttgctgccacggaaca

>fava_dn_rep_c205

caacgcagagtggccattaccgccgcgggagactcacctcactcacaaacacaacaaaag

cacaaacaagaaacaatggaagctaaggaacaggatgtgtccttgggaggcaacaagttt

ccagagagacagccacttgggattgcggctcagagccaagacgatccaaaggactatcaa

gagccaccaccggcaccactttttgagccgtcggagctcacttcatggtctttctacaga

gccgggatagccgagttcatagccactttccttttcctgtatataaccgttttaaccgtt

atgggtgttgttcgagaaagttccaaatgcaaaacggttggtattcaaggaatcgcttgg

gcttttggtggcatgatcttcgctcttgtttactgcaccgctggaatctcagggggtcat

ataaatcctgctgtgacgtttgggctatttttggcgaggaaattatcgttaccaagagca

ctattctacatggtgatgcaagtgttgggtgctatttgtggtgctggtgtggtgaaaggt

tttgaaggtaaacaaaggtttggagatctgaacggtggtgccaactttgttgctcctggt

tacaccaaaggtgatggacttggtgctgaaattgttggcacgtttattcttgtatacacc

gttttctcagccactgatgctaaacgtagcgccagagactctcatgttcctattttggca

ccgttgccaattggattcgctgtgttcttggtgcatttggcaactattccaattactgga

acgggtattaaccctgctcgtagtcttggtgctgctattgtcttcaacaagaaaattggt

tggaatgatcactggattttctgggttggaccatttattggagcaggtcttgcagcacta

taccatcaagttgtaatcagagccattcccttcaagtctaagtgatcgaatggaatcaaa

cggttcttgatcaatcatgtcgtgtttgtggattttgtttcatcaacatgaaatgaaata

atcttatgtatgatatttttctgaattttcaaatgtttgttttgtaatttctatgtaaag

atgtaatatttattatgattattaggtgtagcatgtgtgttgtgtaatgggtccagctcg

tgtgtaattgtgtactctgccaaatcttaaatatcactcgagtcatcatttctgaatttt

ttgttatcttcccaagagtatatggcaagtttgggtttctttatccgtacttttcgtttg

ttatcacttgttaaggttgagggaaaggaagttgtgtagggatggggctttgttgttacc

taacactaaatgggaccatcctcttcattaataatggatttcttgttttatggtatatgc

aactagaatgtgaagacacacattgagaaacaaaagttataaatttatgggccaaacaca

ttatatagtttaaagtttgatggttataattattagaatcgatcttggttgaacaagatt

caatcgctttaagattttcgttttttatttgnnnnnnnnnnncatactttagttgtaaga

tgattctcattaaaatgagaaagaagaatataattgagagaatatgatttattgaagt

>fava_dn_rep_c206

aagaccagtataatatctgatgcaaactcaagtacagggtcttgtgacttatggcaattt

tttaccattgttaacttactattattaaactaccattacttcggaatcaaaacaaatata

tttaccggcacaacaatttattaacagaaccacaccattttactgaaagttggtgtctga

actgaaaccataataaaacagcagtttctaaactacaacagcaactccataaaattttga

caactttgaaaatggaaatgtgaaccttttaaaccgatgaggagaaccaagttacaaaaa

aaatcaatgtagttattatttattaaaccagcaagtaaaacatcaacttcggatgttttg

ctacactttgaatgtctgaaaatcaatagccccattgatcgttcctaatctcatcatcaa

atttcttcttcagctcaggataatcggcgaagtgctcgtctgctgtcatggtactcatat

ttttcttcaacttttgtacatcagcaatttccttttccaagcgctcagactccttccaag

atttctcttcggcttctttaagctccacatacagtgcctcaaatttaggcttatattgag

gagttactgtgtcaacaaacttggggatctcaatgccctcataagcctccttgtacatat

ccaccaaacgaggaccaattccttttctataatattcccagcctatcggctcaggctcct

ggctgaactttgtttgaagttggctgttaacctcgtcgaaagcgcgacggagaccggaga

attcacggcgagcttcatcggagacgagaagcttagccataccttcccagtcaatgttct

tgccggctttgaatgcaacatccaccacttttttggctggtccgctcattttcgctttct

cttcttccaaaacgcgattccgatttgtttctctgcttaccctcccaaatcaccttcaca

cctagccactatcactaccaatgttac

>fava_dn_rep_c207

aaaaagctaaaagattaagcatgtgcttaatcaccttccaagatacacaacgtttatata

acctggagctgcatagccacattttttacagaatgaaatacataaacaagatagctctcg

ccaattccattgaatatatttaaagttcctgtttgtttctcaaatattgctgctcaccat

acaagatcttatctaataactgacaaaagagagttgatacatgaattcaaactcactaac

acaacattcagatgcctggaaaaccagtagcaccttatttgaataatttggtgatcaaaa

caaggtgaaaactagtaacaaaatgtagtgcctactgaaagagtgtatcatgcgtgttca

tcttacgattgtattcctaagagagaatagccatgacatctgaactcctcaatgttatgt

actcgaaaccatcttttcctttgaagtcattaccagcgtatttggagtacaaaactgtgt

tcccaggagtgacagacaaaggttttctgttgccttcctcgtccacaagccctggaccaa

ctgctacgactgttccaaacgaatgtttctccttggttgcctctgtgagaaacaaaccac

cagaggttttttcctcggctttttcaatctttatcaggactctatcgttcaatggtttaa

gatccttgatgtcaccagtttcaaggatgccaacaatgtcatcatccttcacgataagat

gcttctcaccattgaaatccacctcagtacctgcatactttgaatacacaacttgtgcac

ctggcttcacactaatatcaacttggttctttccaagtgtctttccctctccaacagcaa

ccacctcaccaccttgaggctttgtttgagcagttgatggaagcaaaattccaccctctg

ttttctcttctgcgtccttaacttttacaagtactctgtcacccagaggcttgattgtgg

tgtacttgggggcaacgacagtggctgctttgacaaccacacccttgaaggaccttcgtg

cagggttaccaattctaacattttgagcaccatgaaactgaactcctgaaggtcgaagtc

cttgaaaggatgcaacgttcctggtagaaattgacgatgctgtcaactgagaagaagcca

tttacacttaatttgttgttggaagaggaagaagataatggaggattgggttttgtgtgg

gagagtggcgttgaaattgaaatgaattgaagataataaaaaccctagaaatgaagattg

ggagggttttatctgtaacggactattctagaattaactagcacactcggagtgtggtcg

tgtcactcacgcgcctacccaatcaagtttgggaatgtgtttggagaaagtcttgtcgag

tc

>fava_dn_rep_c208

aagtataagatctctgtgcaaggtgacaatggtgcatgcttcacaaagctggtaaattag

tcttccaattttcactgaataaattactcaaacatttcactaaaaaataataatcacaac

aacaaaattcattattagttataaatgaatgagcccacaaggtacaacaatacaatacaa

tatagaggcaaaagcctcgagtataaaattgatttttctatatctagcatcaaaatgcaa

ggcaaaagaacaagcagccaacactaatgtgacctcttaaaaatccagcatgaaatcaag

ggtgatatactcaatcagccttctcattttttggaccaaaaattgctttgactgaatcaa

atacctctacaacaggccttgcagcatcaatcttgcggacttttcccttagcatcataat

aattaataacagggagactagagtccaagaaaaccttgaatcgcttcctaattgtttcaa

tgttgtcgtcttctctaccctggttcctactaagaagccgtctctccatctcttcctccg

ggcaatcaaagtataagacaaatgttggctctattcctgtcactttctcaaaggctgcac

ggttttcctcattgcgaggaaaaccatcaataagaaatttgtcattgccattttcctgca

ttgctcgttgcagaagcttaattgttacctcagatggaacaatctttccttctttaatca

tattctggatcattgtgccattttcagagccagatttgatttcggcacgcagaagatcac

cagcactgagatgagtaaacccaaaatgttcaacaaggtttgcacattgggtaccctttc

cactgcctgggccacctagcacaaacacaactgtggggtttttatttaggatgcttccat

ttgtatccttgtttgcagcttcaagaactgtccccatgagtagtatcaactaaactgtct

ttatctgtgcgtgaaagagaagaagaaaaaaagtgaatctgaaaattgttgctatccccg

cggatggttaggttcgcttatggtatttatagcgaagtaagaaagttcgtttattagtcg

gtaggggtaaaattggaaagttaagttcatggagtgagggaaggcaagagaggtggttgc

gtgattgtaacggctacgatggacgtttttcacggctttgattgtgaggatgcttcaaca

acaagggagaaaggtggtaagattcttgtgtaagtgtcattaccaaaatca

>fava_dn_rep_c209

aaacaaccatgttaggcccacataataaatgacattagannnnaaaagtgagtgcgagaa

gaggccgcgggaatcggaaagagtggttcgaggaggaagaataacagagaagaagagaag

agatggctcgtagtatctcctacgtcactggctcgcagcttgtctctctcaaacgacttc

cgaccatcgccgtcgtcgatgtcagagacgacgagaggactcacgacggtcacatatccg

gttctcttcattacgctagcgacggtttcgaccaaaacatctccaagcttcttcatgatg

ttaagggaaaagataccctcgtctttcactgcgctctaagtcaggttcgaggcccaacat

gtgcaaggaagcttgtcaattatcttgaagagaacaaggaagatgttggtataaagaaca

ttatggtcttggaacgtggcttcaacgggtgggaggcatctggtaggcctgtttgccgct

gcaacaatgttccttgcaaggagcatgaaaatgcttaataggtgggaagcttcgggtaga

cctatctttctcctgtactatctgcaaacatttgtatactaaagaaaatgtatgactatt

ataactgcatcattatataaatcataggtaagctgaatacctagtttctattttcttaac

cactttatgatgtaataaatccttgaatggagattgacaaaaataacaaaagtactcttt

cgaaatttcattctgaacttctctacataaccataaaatgcaatttgatggttatagata

atagattgtttggtttagatttctatggaagtaaacaagaaaatgctccttaaagcactt

tttatcgtctgcaatgtgggatgtgcttctgaaatgctagttgtgtctcatcctcatagt

tcatttgtcagctaaacaaaacatgtcaaatttgatctccatagtccatattctttagcc

atcctgttgataggtnnnnnnnnnnnnnnnaacccattttgacctacctgcactgcataa

c

>fava_dn_rep_c210

tgacgcgtgacagaatttcatatctctatttttcttttttttctcatatgtacagtacaa

acatgtgtgagatcttcactaattctgagagaaaaaacaatatgtttaacaaatcggaga

tgtatataaagttatgaacgatgatgtttataaaatatatatgcagaattaaacatgtta

atcaaaagcaaaaactttaaacagtataataatacatatgctaaaatgcatccatccaga

agcttaatacttatagtccttaacatacaagctctcagaagccaatccagtttcacttcc

accaccatactttcccaaagtagcttcagaattggccttgcatcttgccagaaacacatc

ttgtgctttgccaacattctctttctttccagaccatgtcttgagtgtgctttgctgcag

tgctctcccaaatgagaatgaaagtgtccatggcttcacaacatcaaacttgttcattgc

attcaagttcagtgtagcttgttcttcactttgtcctcctgacagaaatacaatccctgg

taccgcagctgggacagttctgcgcaacgcagtaactgtgtactcgccaatcacctcagg

tgatacctttgggctatcagaaccgggggtgaccatgttgggcttgagaagagttccttc

aagaaggacatgttgatcattcagagccttgtaacaggctgcaagcacagtttcagtaac

agcagcacatttggcgatgtcatgagatccatcagttaaaatctcgggctcaacaatagg

cacgaggccattctcctggcaaatgatggcgtagcgagccaacccctgtgcgttttgctg

gatagacaactcagagggttcattggggccaatcttgaggactgcacgccacttggcaaa

gcgcgctccagccttgtagtactgctggcatctagctccaagagagtcaaagccttgtgt

tgttgtttcaccatttgttccagccaattcaacaacacccttgtcaactttgatgcctgg

tatgacattgttctcttggaggatttcaacaaaaggcttcccttcagaggacttctggta

aagagtttcctcaaagaggatgacaccagagaggtattggagtgcattaggagaagtgaa

aagaagttcgcgaagagcttgacggttggcctcgatgttctcaacgttgatgcttgctag

acgcttgccaatggtccctgtgctttcatctgctgccaagatacccttgccaggggtagc

tatgtacttggcattcttgataagctcatctgcatactttccaacaaaggcagacatgct

ttgaaattgatattggatggcgggaaacaaacacgacagcaccccgcggccgta

>fava_dn_rep_c211

gacaagaaaattaaactttagaagtagagaatttgaaagcaaggcttaagaaataagcat

attttaccaataaagtacaaactcatgaaataaacaaccaacgacaaatatatacacaaa

ccacgttggttggtataagttggcaacaggtgcatgaaatttagcgaaaacaaagtgatg

taaaaaatcataaccgataatgtcaacattgtatcttttaatcggtagttttctctgtac

aaaagcaaaatgcagcacatgaaatatacaacagatctgcgtgaatattcagtttctttc

tggttgagtattgtttccatttgcatttgaactagaagacgacgatgatgatgaaaataa

tccgttgaaaggccatgggaatgagaatcttctacccccgcttggacttctcccttcact

atctccttcagcattggcatgtccaatattatcattctccctttgatttctgttattcct

tgacacattagagttttgcttaggttcatccactcgcaattgagatctacaaaccggaca

agaactatgaagctccaaccagggaaggatacaagcagtatgaaagctatgtttacatgg

catctctttagcatcggaaccaacttcaaaatcatccaaacagacagaacactgcatatt

ctcattgatttttacagttggtaaagcttcaacggcttcctttggtgcaggtggagttcc

atacctattagggtcattatcagacaaatgttgcaacaatagatccaaaccaggccctac

gaaatagtcacccaaagatcctatggggttatgattatcactttgatcccggtttgaatc

ataagaaccttgaacgataatagtctgattaaaagggttaatcaagattacatgctctct

ttctctttccctaacacgatcctccctatcagtattctcatgtgactcagatgtcagtcc

agctcttatgccttggagcaattgctgaatagtagcagaacttcttctcctccttcgaag

gatagactcaagctctcgatcataatgtcctacaccaccattacccctatcatcatcgtg

attcccactatcatagtcctcttcaagttccattcgtctcaatcttcgacgaagccgcgg

aaacaacccatcacaaacaaccctatcaccaacaaaatgaacatgtttctcactatctcc

ttcttcattttctctctactttcactctctcatgcatcagtagtagatttctgcgtagca

gattacaatgcaccaaacggcccagccggatattcatgcaaaacacccaaaaaaagtaac

cgccgatgacttcgtcttccacggcctagccacaagtggaaacaccacaaacattatcaa

agccgctgtcacaccagcattcgacgcacaatttcctggcgtaaacggacttggaatttc

cattgcgcgtttagatttagctggaggtggagtcattccacttcacactcaccccggtgc

ttccgagatattggttgttgttcaaggaacaatctgtgctggttttgtttcatctgacaa

tgttgtttaccttgaaacactttacaaaggtgatgttatggtttttcctcaagggttgtt

gcatttccaaattaatagtggtggttctaatgcacttgcttttgttagctttagtagtgc

taatcctggtttacagatattggatttcgctttgtttaaaagtgattttcctactgagct

gattacacaaactactttcattgatgctgttgtggtgaagaagcttaagggtgttcttgg

aggtagtggttaaaaaaaaaacatgttattatccatgaaagttattatccatgaaagagt

gaggattaaggattagtgtgttgtgtttcaattgttgttggatttgtttcgttgggtcaa

gtttttttgttcgtttgaaaatttgtgacaagagatgaataaattgtatgtgtggtgttt

ttttttaatatttgaataattttgtgtgcgcaactaattatagtggtaaaagaatagtaa

tataattgtgggttcttatatttgaaatagagaaaatgtt

>fava_dn_rep_c212

ccgcggcatccttccttgttacattccaggagcagctcttcaaacaggtaagatggccgt

tcctctgctaaccaagaagattatcaagaagcgtgtcaagaggttcatcaggcctcaaag

tgacaggagaatatgtgtcaaggaaagttggcgcagacccaagggtattgattcccgtgt

taggagaaagttcaaaggtgttacgttgatgcccaacatcggttacggttccgacaagaa

gacccgtcactatctgcctaatggattcaagaagtttgttgtccacaatgtcaaggattt

agaactcttgatgatgcacaacaggacttactgtgctgagattgcacacaatgtctcaac

aaggaagagaaaggacattgttgaacgtgcagcacaattggacgttgttgtcaccaacaa

acttgccagacttcgcagtcaagaggatgaatgatttctttttgcagtatgttgaactat

ttttgctttggttggttttttatgatcatggaagaaactcttctgaaatgttgtttgatg

agttaatctgtttgaatcctagatatttttgtatttggctactctaagttatattttgaa

ttttggtattttgacacaagcgctatatgatgaaatatttcagtatctgttatttttgtg

gtcctggttttgatgtgtgataatcatagattcatgctgatttttttagtnnnnnnnnng

gtatactgtctttgttcttgcactcttgtatatacataaagt

>fava_dn_rep_c213

ccgcgggaccttttcttcatctatttgattgactcaactcaacgcaacccaaagcaacat

ttgcgttctcagttctttgatccaaagattctctccctatatctcctcctccgcttccac

cggtcactcctctctttcatttcaactatggcgaatgcggcatcaggtatggcggtccat

gatgaatgcaagttgaggtttatggagctcaaggcgaaaaggacacacaggtttgtagtt

tacaagattgaggaggctcagaaacaagtcattgtggagaagcttggtgagccctccaaa

ggctatgaagatttcgctgcttttcttccggctgatgagtgtcgctatgctgtttatgat

tttgagtttatcactgaaggcaatgtccctaaaagcaggatttttttcattgcatggtct

cctgacacagcaagggttagaagcaagatgatttatgcaagctccaaggatagattcaag

agagagcttgatggtattcagattgagttgcaggcaactgaccctactgagattggtctt

gatgtgttcaaaagccgtgccaactaaatgatcttcaagaggggcagcatttgtactggg

gtgagccccttaatgctttgttacttttattgaaatttctagtttgtggagtggtcataa

tatctcatgatttgtgatttatgcctcctctatgttggagtggcattttagtcttatgct

tatctatcagttgaatttgtaactgtttggtgtgatgaaacaaaaattggggtcctgttt

tacttaaaaaggctattatatcctttttttttgtatggataggaaaagaaaagaaatccc

atctattattacttacaacatttattttattgtttggtgttatcagatcctattatattg

gttgattattattcttcaaagttttacaagtctaatagttagagaagttatagctcttgt

cgaagattgttttgtatcatttcttttgcttttaattataggaggatgttttgtgaattc

aaa

>fava_dn_rep_c214

aaaaaacaggccaagacatgattatcctaactcagttgctcttcatctctcaatgtattt

tccatagagctaagcttcatctttcttttgaatcagtttctctatcaaatcagctgcttc

ttgaatggttgtgatgttttcagagttatcatcctcaatattaagcccaaactcttcctc

taaacccatcactatttcaactgtgtcaagggaatcagcaccaagagctgaaaacttggt

ttctggagtaagctcagtttcaggagtcaaagctagttgtttcctcacaattttgctcac

tttctgcactgtctcaggttgtgcctgaactgcagaaatacgaaaaccggaggatctgat

agaaggaaagcatgtccttgtccatcctacagaaactgctccaaaactagaagttctgta

agaaacctggttagtccttgtagacaaatggaaagagggttggaaggtgagagaagtggt

tgaaaaagaggccattgtgttgaagataggtagagaggaatgaagagtaagtgatgtgta

ttgggttgatgaatgaaacggtgttatctagttggtgcttagttggaaagaaaaggcatg

tcaggaacaaaattggattatatggatgattattgtatgaataatgagtggt

>fava_dn_rep_c215

agctgacaaagtctatttattataaataaaaaattataaacaattttatttatttgagag

tactagagatattttaatccaaatataaaagaacagtaggaaatcaaaatcattacacgt

ttttaatacagtccaacgggttttggcatcattcagaaagatgaatactttctactagca

ctatgagatcgaaaaacagtgaaagccaccacttattacaagatacagacatatattatt

gagatatgatggtctttcaagaacttgtaacaagcatttgagttttttgataggctgaat

tagtaattgcagagttttgagcatgcttcactgggtaataaggccatattgcgatgccac

acttgcctgcccttgagtttctataattacgctgaatcttaaagtaaccatcttcacccc

agttagttccccatgagttcgtcgccaaccaataatcaacaccattttcagaaccatatc

caactaccaccacagcatgatctaagtttgtcccacaataaccagtaaatacccccgatt

tataaagtttgaaagaattttcataagcctcaatctgaacgacgacaggttgatgagcca

cagctctcttcaatgcattttcgttatatctcggaacaagctcatagtcatcaatgctca

ataccttgctatttttttttccttcgtcacatgtgccgtcttgttcttggtatggatagt

cttcctctgaatcaagcccaccattattcacaacgaactccaaggcttcgtatatgtatc

caccttcgcacccactatttcttattctatcacaatccacaagctcttgtacagacaaat

tcttagaactccctgtcactatcttgttaatcgcttcgattgctgctactgtagaaaacg

cccaacaagctccacaacttccttggtttttgatactaccaacagcccatctccaatcaa

cagacttaggcaatatatcattatcatctgtcttataagcatatcgttcactgctgatct

tatttttaatattattactctttgtacccaaatacatacgatattcttcatcactcatat

ctgcaaactcgttcaatccaactttataagtgtggttttcagcattgtgcttatcaatga

attttaaattatctttaaatgtttgaaatctttcgtctttctctcctaatccgttatata

ctctttggtgttttattaaccatttctggtacatggtcataacctccttgttactacgtc

cacttgacatgtccaatgcaaatgacaaagtgatcaaagaaaataagagcaagaaggata

gtattgctgttgaagccatgaatagtgtttggataagaggagaaagagtggttaagcttt

gaacaagtttgatatttttggtgtcccgcggccgtaatggccactctgcgttga

>fava_dn_rep_c216

ttttttttatagcttaatacttacctgctattattattattattcatattagagagctgt

ctcattgaaatggaaagtaaataaagaatgacacataaaacttagaatacaaaagcaata

tagatgtattcaatattttcaactagtgcctaaccaaaagggcattgatgtcaaaaacat

aaccggaaagctaaacaaatataacagtttgctacttcataaatagaatcataaaaaagc

attgtcatgaataactgatactcttttgccaatgcatttacacaagtgacagctctcctg

caggaattgatctcttcacaagtttactccaagactcgttcgtctctgtgattattttca

gcgcataatccttatttactgccttgttgccaagaccaaatctattagcaggcttcccgt

caggtatcttgtagtctctgaaccagtccctaattgcagtgagggttcctggaaaatgct

tctcaacatcatcgacgtcattcacaagtgaagctttaggatcatctaatgaaattgcta

ctattttccaatcaagttctccttcatcaatcatggctaatgcagctaagggtttgactc

tgagaacctctcctattttcctttggctttcaccgatctcaaccacatcaactggatcat

tatctccaaatgctccttcaacttcatggttagcaagagttggatcttcccatgtttgag

gaagtaagccatagttccaatgtatattataggggtaaaaacgaagcttcccctttttta

tatcctgttttataggagtgaaaggctcgtcggtagcaacctccatctttgcactggatt

cttttggtatttcaacaatgaagttgaagacaccatcacctagctgtaatggaacatcat

gccatggagaaacctttttgccagatttgtcatgaaagaagactctataatcaagggttt

gtggctgaccatcctccttgacagtaacctcagggtaatagatagccttgcaagtataag

acctagtagttctagaagaaaccttgagattgttaagacgaagagcacgggcaccaagaa

agggcttattggtggtgagaagagagtaggttgagctgctaatcgctctggtagtggctg

ccatggttgtttcgtttcaaaaacgtgaaaagagagtgttgttatttgtgtgagacttgt

ttctgataggtttttgatttgttttgtgtggcactgtctcattgtt

>fava_dn_rep_c217

ttttttttttttagtcatttttttatattcaattcagaattacaagagatggaatccttt

ttttgaatccctacctaaatttagagtagtagtagaacaaatttagatagatcgtcatat

ataactaatgaatatctactatgacatatacatgtgtttgttccataccgtaaaccaatg

agttgtatcttagattcaatcaatcattcttggaaatcccccagctgtcttataacgatt

agattatatatacacctagtttgaccccctcaacctttgttaatttatttaatctctatt

tttttttttatcaagatcaccatactgtattattatcctggtttacagaagatggtctaa

tatgaagtctattaaaaactcaactccttaagaagtaagatcatactgcaaactgaaaac

aaacacgacaataaccaaaggcacagacttcttgcaaagttatagattatgcaccagtca

tatagcctttgagatttcaatacaaacaaaaaacacattaaccagcagtacggatcagac

ctaaacaagcaagagatggatgagtgcaatttattcttctaaagcttggacaccacacac

atttcaaacttcatgtatcccgaacatggagatggagagtagcaatctatccttgcaacc

caatgataccacaagctactctgccaccggcatttccagtagttttgctaagctcgtgac

cacctttcccaagatcatcaggatcagcatggacaacaacagcccttcctatgatggagt

ttgttccagtgagagggatatggttgtcagtaatggtgaagcttacagttccatcatcac

caacattgacatttcctaaatcaccagcatgtctagtctcatcctcgggtgcaccatgtt

ccttcccattaggattgaaatgtggtccagttgaaatgcaaccgtttgtggtgtcaccca

aggcatggatatggaatccgtggaggccaggcttaagaccagcaagagttccagttacag

tggttggaccatttccttcctgactaaagttaatagtaccggagacttcgttactgttac

taagaactgccacggccttcaccattgttcaatgtgatctcaggacaccccagcagcgag

tacagagagagagaagagaagtgaaatggaaaagtctagaagtaggtgatgaatcaagcg

gcttgttcgttttggttttttatatgcgagccccgcg

>fava_dn_rep_c218

gaagtcaatttttgtatttgtggtaaagctccattgcaagtcgcaattgaaaaattaaca

gttgtcactatagaaacatattttataaaacgaaatggcaggctatgtaattttgtccat

tatgtacacaaggattcacacaaaattatgatagctaacatatgaaccttcagtttaaag

agtttatagaatgcagttatccttactaaaagtaataaaaaatacaattcaagtaactac

cttcatctcttgatgatgatgaatcaataattactatgtatatacttaaaaatgaaaaca

caaataaagttacattcttctctatgcattcagcaggctttattattgtaaactttgtcc

tgaactctatcagaaactatagtctgttttgagcatattctgagctgatgtctttgatga

atcttttaaccctttgaatgctccatttcctccaaatgaaacttcaacttcatcttggac

tgcatcaaaagctggatggctaaaaatattactcagcaactgtgttcctctgagtgcatt

tgtcaaaggtaaatgaccctctggtgtatcatcattgagttcccatataaactctgtagg

aaaagacctatagttatattgctccatctcagtgtcaagtttcttcatccaaccaacctt

aatgaagaagttggtgaagtcctttttagccttaccaaatagtttcttctgaacactgta

cccaaacttgttgtcactatgctccctccacagcttgtcaattgtttggaggtcggtttc

tgagatgaactggacctctgagaagaaaacatatccgcgcttctgagccgcttcgcctgc

taagacgatgaggaggcgccttgtttcatcgtcagcttctcggaagtttttggctgagag

aagctgctgaaggagttcaaaggaggaggattgagaggttgatgatggtgtggaagatgt

tgtttgagatagcgaaaatgtggtaaaagtgtttgaagttgaagagtgagagaggcttat

gctggtgctggttggtttgaggaaaagtgaggatgaaagtgtggatggaggagctgtttc

agaagggtagtagtattggcgtttgatgagagaatggtgttgaattgaatgaagagagtt

agttgccataatgcaatattctataattagtttaggatacaagaaaggataagggttata

gaaagtgttgaaggagaagatgagtgaatatgatgtgaagaatttcaaggctttgatatt

gtgcagtgttgctggtgtcccgcggc

>fava_dn_rep_c219

gccttgccatgcaaatnnnnnnnnnnnnnnnnnnnnnnnnnnnnnnnngaatcacactgg

ccaaccctacaaacccctaccaaccgcaaatcactggccagccctacagagtactcccaa

acctattcaatatttgatattttcaaccaaaatacattttaataaaatattgcaacgttt

tcactcaagaaaaaacaaaaaaactcagacaatttagggcatgacaaggtaaggttacca

ataagtaaaactcaacacacaatttaagagtatgataaggtaaggctgaagcttaggtcc

aaaattgtaccaattcttgataatgaattaaaaaagatccagaaataattgtgaataaaa

actttagtcaacaaaactgggggaaaacagcatacatcctaaacaaacaaaaatgcataa

ctagttggatcaagctccaacaatttcgggggttccctcaaccactgtgtcatcaactgg

tctttccaccttggtgccgacatcttctgagtaatcaccatgaacctccatcagtttccc

aagatcaaacttaggagctttcaggatcttgactttacggatgaaaacattctggagagg

gtagatgccacatgtgtgcttctcaatctcctttccaatcatctcagggataaatttgcg

cacaagctctttcaggtcacaggatgttgcctggttgaccataatctccctcatcttcct

acggatctgtctgatctggcttgattgggcataacaagtcctcttaacttggtttgctcg

cctcttagtgaacccaatgcaaaacatcctaagagtataattatcagttgttttaacatc

aacatgggcttctataagagtttgccatttgcgaaccaatgacctgagcttgtcagttgt

gaaatccataccccagaagttggtgaggaggtttttcccctgaacatcttcagccctaag

cctgatcttcctgaaagcattgtcttcatctgcttgaagatcagccaatgacacctcaaa

cacacgatgtttgagtccatcagaagcaatcttggtaccctgagtacgggagacaagtgt

tttgccaacatttttgacctggaagacagaaggggctttgatgtcataccagtccttctt

tgcaaagggatcagcggccttcttcttgccacccttctttccctttgaaatccgtttgtt

cttccctactgccatggctgcttaccttcagatgctgccgactgacaaaacttcaaaacc

ctagtatcgccccgcggccataa

>fava_dn_rep_c220

aatctaacctgataaatctctatataaacaatcatgtacatgacttaagaaaaagacaac

acagacatcataaaccacactatcaaaaacattcaagaaacaatgaagttcattccacta

tgtttcctaatttctgtattggtgttgttcaacacaaaaccactacaaggagctgaacca

gaagcagtagtggacaaacaaggcaaccctcttcaaccaggtgaaggttactatgtattc

ccactctgggccgacaatggaggcatcacattgagtccaacaagaaacagaacatgtcct

ctcgacgtcattcgcaacccggccgcactcggctcgccggtgtacttctatgcaccaggt

cttgattacattccaactcaaactgatctcactattgagattccaatcctgggaagtcct

tgcaaagagccaaaaatatggaagctttcaaaggagggagctggattctggtttgtgagc

acaggtggtgcggctgggaaccttgtcagcaagttcaagattgagagacttgaaggtgaa

catgcatatgagatttatagcttcaagttctgtccttcagttcctggtgttctttgtgct

cctgttggaacttttgaagatagcgatggaactaaagttatggctgttggtgatggtatt

gatgaaccttattatgttaggtttcagaaagtttccacttttgctcaagatttgagcagt

gtttagtttgataagtgggatatgaagtaataaggcagaaa

>fava_dn_rep_c221

ttttttttaagttaagctgttactccttattatttatgagatactcaaatattaagttca

aataacaacatttctcttatcaatcatactaatatcaattgaaaagaactctctcacatt

acaaaaaaaaacataaaactctccattatgtatattatattaaattatatatttataaag

tgaccagcgggtgctacataaccacctcagctactttacattacaatcaaaacagaacgc

ataagcaatttccaaaacttaacggcatggaaagataaacaaaaaagtctagcagatatt

caatcaatctctaggatgtaagaatttgtcaacattcaatcaaaaatggcaattaactaa

atcactctattattgatgaaacatcgtctacaaccaaaaagattatccgcacacaaatct

ggaaatactactcaggatgagagatttaaaagacttagaaagcattaagcaacatgttga

atatcggagaggcatctctcatgcaaattgctagtttgctatacagttcaaacaacatat

ttgtttttagcatttctgtctagcttccttgcatcatcatgaaatataaagcccatggtc

tcattataatcaagaaactccttccactgttttccaatgctcacctgagcagcttcattt

gaagcattcttagtccaaatagaaatcttctcagccctaccccttacattcacaaccgct

ccgcaaatttcatctccatgatcaaattgttctccaatcatagccaacaacgtatacaac

caactggtatcagattttcccctctgatagttcgcagtccatttcccaccattagcacaa

atgggatcctcccatttcggttcaatcttatgcttgaaacaatagaaatccgctcccaca

accaacttaccaggatgatgaatgttattgtaaatgctccaaaactcttcgacagtggaa

aaagtgtagatgggtcgcattgaactaccccaagaggcttgtttggattttgctgtagga

gtatcaaaccagaaagtccaggaattctcgagggggtgagattggtggacgacgggtttg

gaagtggcggaggagtcgtcttcatcgaggatctctccttcttcgagattgttgtcgtct

tcgatggcccgattagggtttgctgtgatttgatcgtcggtgatgatggattttgggatg

tcttctacaaccatttttgccctcaatttccaattacaaatgagttggggatttactcgt

ttagtggttttaatgaaagaatagatggtgaagt

>fava_dn_rep_c222

ttttatcaaataatagtaaaactaaaataccatagtaaaacttaatatttttttttttaa

tcaaaaagaaaaattgtcataaatctctctttaacaaacaaaatgagtttccaagcagta

gacaatacaaaaacttgataattaaattatcccaacaaaatataaagcattagaaaaatg

cagataatgaagcaaaacaaaaaagttgcagaaatcaacggtatcgacggagagaaacag

tattgttcttcttccaggttgccctgcgggagggacggttcttgtggttgcgatgaccct

ttcccctcaaacccctgttttctttccctgcagaagtgaggccacgaagttccctatgct

tgtggacagggttaacaagccagttgattcttgggtcatttctaatagcagtgtgtgcaa

catcaaccaaaatgacctcaaaatatttgaaggtggaatcctcattgacccagtaggaat

tgagaaccctgagaccaccaagcttacgtccagcacgctcctcagcaagtgacctcttgc

ttctctgaaacttcaattgggttactccctggtttgttggcttaccatacacaataccct

tgggaacagacctcttccttccacctctacggacacgaactcggtaaacaacataaccct

gtttggccttgtagcccaaacggcgagccttgtcagggcgagtaggcctggtgagacgaa

caatggaagattgctgacggtactcccagcacctcacacgctgcataaatctcatcacgt

cggactgcttcttgcgccatagctcagaaacgtacttgtaagcacccatttcctctttct

gactctgaagagagggaagcagctcacaaaaccagtgacaggatttagcggtcacacatt

tccacagatccacaatcttctttctctttccagcacttcattctctctctctaaaatgtc

tcctataactctccttaccattctcttcctcatcacatctctccaaacccaagcctccgt

ccacaattacaaaagcgaaactttctccgtcaaaggcaacgcctttgtcgtccatggagg

cagcgaaggaatttactcatctaccctcaacgaaacttccatcacttcaattcccgactc

tttcattaggtttgataaagtcacattccgtagaaacaaggaattatcaaactttagctc

atggccgatacaagcggttgtttttgaagttgaggatagagagatgattggtggttcggc

ttatgggggtcaaagggctgtttgttgcactggtgatctggctaaactcggtgtgtgcaa

tgaaggacaggtcattcatcgtccatctactgtgaattcggattggcctcaagtttatgg

cgttgcttttgagatggacgatgaagtagcagagcttccattgaagtctattcaaatcac

gaaaaccgggatgtacaatttgtatttcattcattgtgatccgaggcttaaggagttggt

tgttgaagggaaaactgtatggaagaatccgtcgggttatctaccgggtagaatggcacc

tatgaagattttctttcagttcatgtcttttgcgtatgtgttgcttggtattttttggtt

ctttcaatatcttagattttggaaggaagtgtttccactgcaaaactgcatcactctagt

gataacactaggaatgtttgagatggctttttggtactttgactatgctgagtttagcga

aactggagtcaggccaactgggacgactatatgggctgttacgtttggaactgttaagag

aactattgcacggttgatcatcttgattgtgtcaatgggctacggtgttgtgagaccgac

tcttggaggccttacatcgaaggttgttatgcttggaggaaccttctttgttgcatctca

agtgcttgaattggtggaacatgtcggcgcaataagtgatctttctggtaaagctaaact

ttttttggttcttcctgccgcagttttagatgtgttcttcattctttggattttcacttc

actgtctgcgactttaaataaacttcaggcaagaaggatgatgataaaattggatatgta

taggaagttcactaatgctttggcagtagctgtggtggtatctgtaggatggatatgtta

tgagctgtatttcaaatcaaatgatatatacaatgagcaatggcaaaatgcttggatcat

cccagccttttggcaagttctatcctactctctcctgtgtgtcatttgtgttctctgggc

accgtctcaaaattcgacaagatacgcttaccgtgatgatgggagcgaagagtttgacag

ggatgatactactttgacgctcataaaaccatcatctatttcaacaaaggatgttagaag

tgttccagatgtaagaccaggacaagacaacaatggaacttcaaatgatgatttagaaga

agataaaagagaataggctagaagtagccttgtttctataattccattgtatcattagca

ctaaaactattttcttttattgaaaattagctctaaaactcttttcttttatttattatt

tttctgattatcttctctgttctcaatctattagcattttgagccagcatttacttgtga

gtttggattccattataaaagtatagttcagttcagtttaccaatacaaagaaactatct

tcaattatgaagtgatttatgtatattattgttatccatgtgcatgcaagcatacatgtg

ggggatattttcaagtttcc

>fava_dn_rep_c223

tgtatttataaatagagacaatctatttaatctatttcttacaaactgaatttgtcataa

tgaattatgtccgatccacatattctaagcatttcaaatgcttagaatgagcaaaaacaa

taaatataggagagaaaagcaataagagaattgttaaaaagtagaaagtaaagcaaaaag

taaagtagcaagtagaaagtaaagtagcatggaataatatagcatattacaaatgtaaca

tccaaactgaataaaaatattcattgtgcaatacaactaaatacaactatagacgtaaga

actcgcaaagatggagccctcgttgccctttgtttattctcattagataactctccagct

agtgctggtattactgtagtagaaagaagtgccatgctacctgcttcttcctcttcctct

catccaaactatatttagatcccgatagcattacagttggtagaggggctaatcttgtaa

ggaagagtaacaccacatttgccagggagagtagctgcggcgttgagattcaaaccacga

atagaaccagcagcacctttcaagcagttgcaaacatccttacggtcgggggtactttgt

gcctgcgcgttaagtctcctcactccaccacagcattgtggtgaaagactagaacgaccc

tcaagataattaaggcacggagcaagggctccagctaccactccgcacgagattgcagcc

tctgacatgggtgcagtaataacgaccatgcacatcaatgcaaccacacaagaaactctc

aagcttgccattgcttctctaggaaatagattgaaatagaaatattgtaatttgcttttg

cttggtgaatataagtgattcaaggtgtggctttatatagaacataaaagagatgatttt

tggtatgtgtttggttttgaaatttatgagcaatataacatgctcatttnnnnnnnnnnn

nnnnnnnnnnnnnnnnnnnatatactttcaaatattaataacaacaaaaagtc

>fava_dn_rep_c224

ggccgcggggtatactggaacgagacccttggtacacgattgacgatcgtaacaaagatt

gaaaatttcagtaaatttctacttactgatctgatcttttatggaatcgatccccctttg

actgtataagaatatgtggagctcggcatgtctggaagcacaggagaacgttcttttgct

gatattattaccagtattcgatactggattattcatagcattactataccctccctattc

attgcgggttggttatttgtcagcacgggtttagcttacgatgtatttggaagcccccgt

ccaaacgagtattttacagaaagccgacaaggaattcctttaataaccggccgttttgat

tctttggaacaactcgatgaatttagtagatctttttaggaggctccaatgaccatagat

aggacctatcccatttttacagtacgatggttggctgttcacggactagcggtacctacc

gttttttttttgggatcaatatcagcaatgcagttcatccaacgataaacctaatccgaa

ttatagagctatgacacaatcaaacccaaatgaacaaaatgttgaattgaatcgtaccag

tctatactggggtttattactcatttttgtacttgctgttttattttcaaattatttttt

caattaattgaagaaaagagaatagtatttatatattaggaattctcttatcccattcgg

aaggatatcatttcataattatctatgactgtttaggtctctagtatgactgactacttg

atcaaatgtggaggaaagtgggataaatggccgatactactggaagaattcctctttgga

taataggtactgtagctggtattgttgtgatcggtttaattggtattttcttttatggtt

catattccggattgggatcatccctgtaataatcggataaatttagttttcgaaatgaaa

gcataagaactcaacggggatccactctttctttgatgaattcgaaggggtcccgttgag

ttcttcataatcagaatatttttgtcagtgagtcaatagataactttattgtatgtttca

aaaaattccatttcctttttttaggatcttccctgtaagaatagggaactatatactata

tttaatactctattaaacataaaaaagattctgtagaaccaaacctttttgatactaaca

gaatcttactagaaatctatttctaatatagactcatgattaaattctttttttgaagtg

aattaagaaagttgtatttgctgaattcaattcccggtcttttttgtttgtccattcctt

aactacgtatcgatttcgacataaacaagaaggaattggactctaggaaaagacaaatta

tccatcctagaatcctgttttccctttttctactttacttaatattatctacctacctat

ttttttaggtttagtatcgagtcgaatttcattctattacaatataaattctaatatttc

tattctaggacatgaaaatgtgaaaacagcgacataatcagcgacataatcatatggttc

gaattaattgtgcagttgaaaaaaaaccaagaaccaaaggtctttacacagaatttatca

acaaaaatggagttctttttaccagcttaatatgttgataaattgacattcctaaaaatt

catttcagacaattgcaccttctcaaattgtttctttttaagaaccaaaaaaatttgtgc

caaaataatagatgccaagaagagcaagagaccttggacacgtaacggatcttgaagcac

tatttctgcatccccctgaccaaatccacccacattaggattactcgttaatggttgatc

aagtttgatagattcaccctctgaaacaagaagttctggtcctggcggtataatatcaat

cacttcacgtccatctgatgcatctactatcgttatttcgtatccacctttttcttttcg

tattattttgtttactacacccgttgctgtagcattataaacattattattactcttgct

tccgtcgggataaatctgaccccttcccctgttcccgcctacgtatataggatattttaa

gaagtaaacatctctcttagtagcgggatctggagaaagaataggaaaggtaatttcact

atatttttttccaggaacggggcctatcacaagaatatttttttttgtgggacgatagct

ttgaaaagacaaattacctatcttttctttaatctcgggcgaaagacgatcaggaggggc

caattcaaaaccctctggtaaaataagaacagctcccacattcaaagccccctttttacc

attagcaagaacttgtttcacttgcatatcataaggaattcgaacaactgcttcaaatac

tgtatcgggaagtatcgcttgtggaacctcaatatctacgggcttattagctaaatggca

attagcacatacaatccggccggtagcttctcgaggattttcataaccttgttgggcaaa

aataggatatgcatttgaaatgggtgctcgagttattatatatatcatgagcaatacgga

aatggatcgggtaatctctttctttatccaagaaaaagcatttctagtttgcatggtcca

atcattgatcctgaaaatttctacaataagattaggtaggttactggcacagttccctat

ccatgattctgctatttactccaattcttttcctataatataggaagaatacgagtaaaa

cgaaaacgaataagttcaattttaaatgtttagcttttctatacaaaaaacaaattttag

aattggatcagtgggtcgttttttcattccttcattgaatgataaatgactacaagcgac

ggagatacacgatttaaataacggaaaatagaatatttaaaaatagtatctaaaatgact

ggaaaaatcgaaacaagacaagatagaatctgatcattcggagtaaatccaaaatcttta

tagacagacccaatcattagttcccaaccctgagtcgaatggaatcctatacaaaaatca

gttaataaaagaatagaaaaagcttttattgtgtcacttaagttatatagaaattcttga

acccaagagttaagaataatgagttcttgattacctcgaatagaataaccacttagaata

aggaaacagattatatttgtacagaaatggaaaatcgtatggatacgatctaggttgttc

gtctcgatcaattggatggtctctttgtagatttcgatacgaaggttttgtaaacgtgtt

tccgggtattcctttagcatttcatccaagaggaacagttcctcgaactctagcaatttc

tttaaaatacttttttcttgaatagtattcaagacaatttcggattctttcatattccac

caattagtaatccaagattccagacttttctgaaatgtaaaagagatgcaccagggcaaa

aagactatagatgtaagacatagaaggggaatgcatgctttcttttttgccatttttcgt

ctttaatgaactcagtttcatctcatttttcaactatatataataataatctttctatca

agcataagttctattacaagtaaatacaagtaatagagcttagcctagaatatatcaatt

tataattttttcatagaaattgagaagctattctttttatttgtaattcggaggttagtt

tctttttccttcaatttgaaaaaaagaatccaaaagaatacagaaatcaaataagaaaac

gaaataagccattgccaatttgaatgaagaagaaaaaaagtgtttcaaaagctatgaatt

aaaaatgaaattgtttggatacagcgatttccattggtatactcaagaatatggaacgat

ttccattggtacactcaagaatctggacaagtcccaagctttttgtataacgttgcatgg

agtcctatcatcaacaggagtcaagggaatggtcccctgttctctggtttgcatataaag

gacaccactactaggttctgggttagcttgtagtatgagggactgaatatcttccatacg

aactcggagaaaaatacgacgatatgttccaggaaatccgtaacgaaaaaaacataccat

tcgattttttttatcgaaaaaattatagccactccccacattccaaaaaattagaagcca

ccaatgtaaacttagaaagagaccggcaattccatagaaagtcagcgtgactccttgtgt

caaaaaaggaatgacaaattcggacgaaatgaaagagaaaaaatccctcccataataact

ggaaactgaaatatataaaactcctaatgaacctaaaagagtgaaagacgcccagaataa

attgataatttttcgggaccccggtacaatgtcaagccatgcgtcttgtgaacgacaacc

aagtttttctgacccccaactaatgaattttggaacatgaaccaataaagcagacattac

aattaatacaaggcccactaaaaacacataaacgtttatcataaaatgggtgcctcaatt

tcatatttgtacctgttcttttttcttgattgttagattaatatttatgttgttatatga

aaacctccttatcttattaaggcttatataatattagtacttttctttttttttattgaa

ggcaatagttattcaaaaatggaacaaaagaacaaatccaaactaaaaaatttaacccta

ttttcaaaaatatatgaaacttgtggtatctaaaaaatattgtttttttgaacataaaga

aataaagaaaccattgcaactgccggaaatactaggcctactaaaggaacaaaaagggat

ggtaagtttatcataaaatggggtacctcaatttcatatttgtacctgttcttttttgtt

gattgttagattaatatttggattcttcttatattggaataaagaaagtctataatcact

agaattcatatagacttatactaagtctatttttttaattctactatgtagagctaaata

aatagatatgaatatatatatatattctatatggagtatataaagagaataaacccataa

gaaaaaaatggaataagatttattaagaatcaaaatcgttaattttaataatttgaatat

taaattaaatataaggaatgtcgaacgaaataactggatttattttgctgtctatttcta

caagaaatatgtccatgataattaatctttattttttattcttatcttattactttactc

ttgtgtgttctcattttctttttatatcatttttatataataagaagatttctttatgat

attcattttatgatattaatgaaaagttaaaaattgaaaaacataaataaaatgatagtc

tgcattagttttcattttcaatccaaggaaagcaaccatggaattcaaataactcactta

aaaagtcctttaaagaatcacgtgatacgattgcatccaataagcctttttcgaaaaaaa

cttcagccgattgggaaccttcaggcacttccttattcaacgtttgttcaattactcttt

tacccgcaaatgcaaggtaagcttttggttcggcaataatgatatcccccaacatcccaa

aactagctgttaccccaccagtagtaggcgatgtaagtatcgctacatagagtaactttt

gattgatttgataattatataaagcagaagatattttagccatttgcattaagctcaaac

ttccttcttgcatacgcgctcctccggacgcacatactataataagaggtaaaagttgat

tggtagcatattcgatcaaccgagttattttctcacccactacggatcccatactacccc

ccagaaactgaaaatccataatacctattgctacaggaataccatttagttgacctgtgc

ctgtttgaaccgcctccagtaatcctgtactttcttgataaaaatcaagacgatctatat

aaggttcgtcttccgtagaagagtcgtattcgtcttccgtagaaggttcgtcttccggag

aagattcgtcttccggagaagattcgtcttccggagaagattcgtcttccggagaagatt

cgtcttccgtataaggtttgtcttccgtcgaaggttcgttagaaggtttgtcttccgtcg

aaggttcgttagaaggtttgtcttccgtcgaaggttcgttagaaggtttgtcttccgtag

aaggttcgttagaaggtttgtcttccgtagaagattcgtattcgtcttccgtagaagagt

cgtattcgtcttccgtagaaggttcgtcttccgtataaggtttgtcccattcaatgggat

ccagagaaaacatgtcttcatccataggattccaagtacctggatcaatcaaaagttcga

ttctatctgaactgctcattttcaaatgatctccacagtgttcgcaaatattcattttgg

atttaaaaaatttcttataatttaatccaaagcaactttcgcattcaacccataaatgct

taaatttttcagtataagaagtatcttttggatcatcaggatattgtattggagtaaatt

tactattatttgtatcattagaatattctattggagtaaaatcactatcatttgtatcat

tagaattttctatttcatacaaatcactatcatttgtatcatctaaaatgtaactattca

agtcactgatattggcattgtagttgtcactatcatctaaataacgaagataactctcaa

tccaactatgaatgtcattatccatatcagttaaaatagatgggtcatcagttaaaatag

atgagtcatcagttaaaatagatgggtcatcagttaaaatatatgggtcatcagttaaaa

tagatgggtcttcatttacaccgttattttcaataggataaaaactatcttcaataggat

caaaactatctagggatttacttaaaccacacctatattctaaatccctattaaacagca

acattggattgaaccactttaaaatacaatagatgaatagtcattcgatgatcatttttt

atcgacttaagtatagaaatagaattgcgaggattaataacttataataataatgagcga

agaagtattgaacaaatgtatcatttatatttgaatatcaaatcaataactatttctaat

ataaaaatgaatgaatagaataaaaacaaagaagtaaaaaggctcatttggtctacggta

tttatgcaccaaattagtttatttcttgactttttcctaatatgaggtaggttgagcacc

gaattttggaataaatagatcactcaaatcgaaaaatcgaggatccctatttctagatac

tagaaactagatagtataaagaaaatagggcatgtaagactccgcagctaatatagtcac

gtttttcaagtcccacgctgctttttactacatcgtttcaaacgaggttttaccataaca

ttattttttagtttcaatctggtatgtaattgattccattttgaaatcgtgcatagtcat

tgactcaatcaatagataataatatatacttttcacttctaggttttaggttttctgact

atacaattttatttctaaagtcaagaagtcgaaattaagtcaatattctattttcatggt

ttgtaaattaaatataaaataatcaatttctaaaaaaaaaaaaaagaatctttattcgga

tatacaatttgtattgaaataggtatatatcttgaatagatatgatctgggacggaagga

ttcgaacctccgaataacgggaccaannnnnnnnnnnnngctacggactgcattctcata

tgtcatgttaattcaagttgatgttgcgaccat

>fava_dn_rep_c225

tgaaaatataaaattccgaattaaatatcatttaaggagtgaaaataaaaaaaaaattac

atatatgtaagaaaaaattttacacttatcatatttttattggaaaataaaaacaaccta

aggaattgtattttaattttctccgtatataaggctttgtgacttggttctttttttaaa

acctctagacaaaaccatcgattcctcgcgcaaaaataattcagtgaatttttaacgata

tggctacaacgataatacacaatgactgtataccgtataattcccaattgtacaagacta

atcatttcaaagcaccttaggaaagggaataataaaccaatctgagagttagacatgcaa

atgccactagaaaatccagaaatgagtcgcagggatattacatcagagttcaaaacacag

tagtttctcagctctttgatttggaatgcttcacaagccagtctataacagaatcaatgt

tggtggaatttttgcaagagatcatgaagcagcaaacttctctatcagtaatggatttca

gatccatttggtcagtcaaagcttgtttagacagcgctcctgccttgtcaatcttgttcc

ccaacaccaatagagggataccactcaatgatggtttgctcagcaaatcatgaagttcac

ttcttgatatgctaaggttatctggatcagcagcatcaacgacgtaaacaatagcagaaa

ctgcacggcagtaacgttcccacatgctacggaacctaggttgccctccaagatcccata

acttgattgtaacattgccttttgttactttcctcatattgaatcccactgtagggatca

tgtcctcgctgtatccaccggtagcaacaacatttacaagggaagtttttccagcattct

gaagtcctataagagataactccatttcctgcttgaagaaaaggctgcgtaaccaattca

gaaaagcctcccacaatcccattgctttttcttctgatcacgaaacaatcgattgaaaga

aagaagaaa

>fava_dn_rep_c226

gggaatgaaataaaattattaacaggatgagatagaaccttgtttaggagtttgtcctta

tccgttgaggaaaataatgtaacattgtctgctgtcattttatttagtatccgtgtttat

tttaagactgtcttcatcttcagctgcagatagtaaacagtgaaaaaaacactttctttt

gttgttgttgttcttttctatatagatcaattcatctagaagttgaaggaaggtgataat

ggaaactattgagcaaaacaagcctaaggtggtggtgattgattccttgcaatcatggga

atcctatgtcaaccaagcttctaaccagaattcccatattgttgttcactttactgcttc

atggtgcatgccatcagtggctatgatttcgtttttttgaagaacttgcattagattatc

cggatcttctctttctctctgttgatgttgatgaagttaaggaggtggccaccaagaatg

acataaaggcaatgccgacatttttgttgctgaaagatggtgctgcattggagaagatag

ttggtgcgaatccagaagagttaaagaaaaggatagatgggtttgttcagacaactcgcg

catcggttgcgtagtatacgcatctgtacgatgttttaacttgcaagttgtgagaagtgt

gtttgtggtcaaatccttttcatttgtgatgttttatgtcaagtcttcaagtgtaaattt

cctagttccattagcttaatggttttgttaatggttactactttccacagtttttatggc

accaaggatttgtatattttatcagttttcaatgttgtggatttttattactatgttgga

agtgatgtttatttatcatggttaacttgttcatattcttttgatttgttacatttcaga

cacgaaataaagaatgccgtcattacttagtttttgttcatagattgtttttttatatgt

tgtggaaatcctacatcagaaggatataagttaagattagcttaagtgcttcataaataa

aataagcattctttgatgtagtagcttcagtttcaagttaataaagtttgttatgtttct

aattttcttgttgatacttttagaatttttggtctgtatcttctattcaatcaagtttct

ttactaggtctgtttggattgacttgtgaaactatttggaagagtttattatggc

>fava_dn_rep_c227

atgggcgagtccttatctcaacccttttcctattaataaatatcgaagtttatcgctccc

aaccccaaacacactttctttcctcttctccgttagggttttctccaccctcagcgttag

ggttttcttcccccaaatctattttaaaaccacaaatctctctcttttctcaccctatca

cacaacaaatcgaaattcgaaacgatgactggaaaagcgaagccaaagaagcacacggcg

aaggagatccaagccaaggtggacgcagcgctgaccaaccgcggcggaggcaaagccggt

ttgcttgatcggatcggtgttgagaagggaggacacgccaagtgggagtgccctcactgc

aaaacaaccgtgcctgatgtgaaatcgatgcagattcatcacgatgctcgtcatccaaag

atcccctttgaggaagacaagcttgtgaatcgtcatgcaactgcggctgctgttccggaa

tcttccaagcctcgccctggtgttcgtggtagcctcaaaaagtgatcgttgaatcctcat

cgttgaatcgtcgtcgttgaatcgcagcctttcattcatggctttttactatattttcta

ttttgtactttttctgttcaagtttttctttttaggttgttattgaataattcgtgtcat

gggtatgtttagttattttatgttttttttttttaatatcaagatgatgaattttaggta

ttaatgttgtgattgaagtgttttgcgatgaaaggtttttgatgggaatgggattgagtt

gtgctgttattattaatttggcccttcttaagccttgaatgtaacatattgttcccttga

tgccagttaatatgatcgtgctactacttactgtaaaaaaaaaaagcgtaaagataataa

attcacaatatatttttataaatatacgaattcaattaatatatatgcaattgcaatcaa

ttcaatatgtaatcaatttgcgtccaataggattcgaacctataccaaagatttagaaga

cctctgtcctatccattagacaatggacgcttttctttttttctttcccttttcacgaaa

atttcgtgaaaagggaaagaaaaaagaattctatactaaaactaaatgaagaaatttact

aaataaaaaatagaatataagcgggtagcgggaatcgaacccgcatcgttagcttggaag

gctaagggttatagtcgacgtttatgaatgaacgtctctaattcaaaactgaacgtgaaa

cttttgtttcattcagctcctttacagaataatttaagagatagatggaatacatgaaaa

aaagaatccataacatctatgtcagcctttcggtatgaatacaattaacgttgcttttta

gatgatccctatagaaaaggagtattcgaacaatctctttttttctagttacttcgttct

ctatttctatttgagagaatctttaggaaaagaataaaatttccgtcgagctaaaaaaat

atgttgatgtttctagtaaactaaaaaaatcatttcatagctattttgcttcaatctctc

ctatacaaaacaaataataaaaaaaattggaagatttagttacgattggaaacgaatttt

atatatctttctccctggatcctttacttatattcaaaaattgggattctggatccaatt

gcaaaaacttatgtttcataattttagaatcaattctaaattgtatacaaattatgataa

tgtatattattcctcgaattgctccttgagaggtataaaggattaaatacctttaagtaa

gaaataaagtttttgatcgtgatatgaatcacggaagggaccccttaactattactatta

aggggtcgatagaacgaatcgcacttttaccactaaactatacccgctacctcgcggccg

ta

>fava_dn_rep_c228

tgcacctgacttttatcataaaataaatatttaattgtttttataaaggctatggataaa

tcgaaacatcaataaaattcaattccaaaatctataaattaaaaaaacctatattttatc

actatagacaacactttgagtttaccagttaaaacttttctttactcacatctcaaagtg

ttttgacataattataacatgaatattacatagtgaaacacaatacacatttcatatatg

caaataaataagaaaatatagaaaatcacactctccaaaccatagctcaaagtttattta

accacccaacatcacataacaaaccctatgtaatctttctgagcttgattataattatat

caccctcatcaaaagagatggtggtgcttctttccatgagcttcttcttcttctttctta

gtttcttttttctcatggtgttcatggtaggtaaaaccaccagatcccacggcggccgcc

gccgcaatctcctcctctatcttgtgcctgtgagcatgctcaggatctttctttgactca

tgcttctcatgcaaggcataggcaccagcagctgcagttccaagctcaccaaggtgctca

agatgtttatggtgtttctcctcttttttatagtcaacctcttccacataaccagactta

tcttcatggtggaaaaggtgatggtgcttctcctcagccatgattattgaatctaggaat

gaattaaagaagtgagtttatggtggaatgggaaagttgtgaaggatttgttcgcaagcg

gagattccgcagagagcctgaccttaatcctgagcttgtctctcgtgttgagaaagatct

tctcaaaatcattgctggagga

>fava_dn_rep_c229

aaataagaannnnnnnnnnnnnnnnnnnnnnnnnnnnnnnnnnnnnntttggcaaaatat

taaattctcctgtctttgtatagacgattttggttccattgtagtcaattttaaagtgtt

cattttaactgccacagatattgaagaaaatataaatctcaaacttacttagcaacagag

tattcaacaattttaaacaatacgcaataagcctagttccttagagaaagaaactaacaa

aatagagaaattctaataacttgagcaaggaaacctcaggtctgataaagaaaaaactag

ataacacaaacatgcaaatacaaaagtagaattatcaaaacttgtcaactatgaagaaag

taaaatctccaatagagatagatatggttgttctagagtttaagctttgatggcatactt

cctcaatgggaagtatgtctcctttttcttctcccgctcagtcttcaaagatgcctggtg

cttggtgagccttcggcggatagccctggtcttcttagggcgcagatcaagaggcaagta

cttcttattcttgtaaacttccctcaatgcagccttctgcttctgagaaatcactgtcaa

aacctgagcaatcgacagcctaacaaccttgatcttggagagcttgttgggtgcaccacc

agtgaccttagcaacacggagcaaagcgagctcagccttgaggtctttgagctgactcaa

cagatcagctttggctttctgcctcaactcatgaaccttgattctggccattgttgaatt

tcacgacggctgctgctcctcctctccttgctacaaaccctagaaaacctgaaatgggag

aggcagagaaagcctttggatattataaat

>fava_dn_rep_c230

gaatcaattatatttttaataaaccaaacacctcaaaatcaatccaaacatactaaaaat

ttctctgtataaccaatgaatgtgctaacttacttgctaagtaactggagtttgcaatat

gtaattttcttttttgaaagatattacaatccaaaacaactccctggcagatacacaatt

cctccagtgcttaaaatgagaaataatatgaaagttcggttgtttagaaaggaataaaca

acgcatttctacttcatacacattcaacgaaatggtattcactcctcatacatttgttac

atcagcaatacaaataaacatgtctttgaattttgaattgtttatccgaacaaacacgaa

attatttatttgaatacatccaataaagattttaaataatgataatgttgtccacgactt

aatatctagatccaaaaataaacttgcgatatgatagttaacataatttaggggtccact

cagctgcaccaacatatcatccaaggttcctattttttcacagttgttacatacttgagg

agcatggcaacaacatcctccgaaattcttgcagcctcttctttcaggtgatggattttt

tcttcggtttcttgatcaagtcgcttaacattagctccagagtcaccactgctatctgaa

actttcttttgaaactcagactcaagcttggcacgatattcagcaatctccttttcagcc

tcttctttggcctgtttcaacctagccgatttttcgtttttcgcagcatttacaatccgt

tgtgcttcttgttcagcagccaacaattgctgaattcctccttgacccctattggatgcc

attctctatgtatctatcgggagtgattagattcagaggaaatcggagggagatatcacg

aaactgctagcgagggatttgcgtacttgtagtggactgggcagttgaataatagagaca

cggatgaagaataggagaatgatttcagatttt

>fava_dn_rep_c231

tgttttaaagcatagtatactacaaaagtactgggcgacgctttcttggttacttgccac

atccaaatccaatttcgaaattcacaacgcaaagaggtgaaggagagatggaagccatgg

ccgtgttttccggaagcaatatctgtgttacatcctctctcctacgcacaaccaactcca

aaaccagatgctctcaattacgcaccacccaacacctatctgcatttccttctaaatctc

atctcttcacttctactactttttcttatcctaaaaccttccgtaccagaagcagcaccg

aatccggcatcttccttccccacctaattgcttctctcaaacaagttgaccagacttaca

ttatggtcaaacccgatggtgtgcaacgtggtgttgtcggtgaaattatttctaggtttg

agaaaaaggggtttaagttaactggcttgaagcttttccaatgctctaaagaattagctg

aggagcattacaaggaactaagccaaaagtctttcttccctaagctgattgagtatatta

cttcaggtcccgttgtgtctatggcatgggagggtgttggagtagtggcatcagcacgta

aacttataggggctactgatcctcttcaagctgaaccaggcacaataagaggcgactttg

ctgttcaaacaggaaggaatattattcatggaagtgacagccctgaaaatggcgagcgtg

aaatagctctatggttcaaggaaggtgaattatgcgaatggactcctgtccaacaaccat

ggctaagagaataataaatcaaagatgcacaatgacaacgaaacatgctgtttaatgtca

tatactttattcatttgttttcacttttcggttttcaatttcactgtagcactcctttaa

cctctctgtccaaacagttacttggatatttttaaataaaattttgcagttgagtaaaaa

aaaaa

>fava_dn_rep_c232

acaaagcaaaagtaaagagaataatagaatttcaattagcctacaatatatataagagaa

aaaccaaaattatattcctgccatttaaattgtcacaagtgactttaggaaattttaagc

aagacaataatgctaaacactcaatactaatatcacttgagagaaacctttcaaccaaaa

ggtcttcttaatggatttgggtccatattcctatcagaaatagttaattttatttgagaa

aatattatagctaattcataatagattacaagataggaatatttcaagtagaaaaataaa

attcatccggcaagtagccaattattttaaccatggataaagcttagttaattaagcgat

gtagtagatgggatcagggagcttgaaagtcctatcgccagtgttggttccaagaatatc

actccattgatcaccaatgtttccgacgattctgtaaccttcttcctcaagcttctttct

ctcacttgatttgtatgtaactgctgttttaccatgatatattgataaatccttcaaaat

aagcttttcccatgtgtgatagccagcagttctcaagttcgtatcagttataacagtttg

tttgaggggtcttcctgttaagaacacaatcttgacaccaagagacaacaattttttgta

taatttctgtgactctggcagtgctggtgcttttccaaggtcaacccattcattaaacga

tgtatcattgtatggattcaccccaaatccatgtttagcataatatggaagattagatag

tgaagtttcatcaatatcaaacacccatatgtcttttccatctccacttatattaagtgt

cttagcatagtaataaccttcacgctcaacagttttagaatcagctctatattgatcacc

taacatataattaccaacatatttttcacattctagtgggacagttttccagttaataac

gttgtgcgcttcaacaccaagtctccaactagaacatgacacttctggaatgtagtgtcc

acctggaccagttttcatccggagtgggaatattttgttggttgaaccatggtcatggct

tggaacatcaccaagacatgttgctaaaagtgtaaccaaaaataaaaggaagatcttcat

tcttataaatgattgatttgctacttagttgatcccgcggccgtaa

>fava_dn_rep_c233

gaaaaatagatcttgttaaatataaatttgtcataacaacttatttgtgtttgtgaagac

aaaatcaagacagacactaaataaaatttagaaataagcaagtaattttaatcagataat

gtcaaaagctatgttgcttaactaggccacaaagtacttttttaaagccaccaaacaaaa

tccagattaaaggcaacttgaacaaaatttaactggcaacattcatcatatgcaattcat

gaaacaaattcaaaactgggaaacagaacctaagaatctaagcctcggtaatgggatact

caaacacaacatctttgccagacaatttcctataaactgcagaaaaggtctccaacttgt

actcggtgttgttcctctccttggggtccaaaaacaccttcatgatcttggatccatcaa

ctcggtacctagtacgtttcccaacaatctcagcaggcaatacaacatcctccagcattg

cctcatgaacagcagtgagtgttctgctgcgggggcgctgagcagctgaacccttctttg

ggggtcttacaattctcctggtggcaatcaggattacatccttgccgctaaacttcttct

caagctccctaacaagcctgacatgaattttgcggaaacccttcctcaatctgtatggga

cgtggataacaacagccttgcggttgccagaaacatcaacttgaactgcagagtttatgt

ataaatctttcaactcactcttcaactcatgattggtattttcaagatcaaacaaagcct

gtccaacagattcctcaaattcagttggctcagcacccttatccttgctgattttcttcc

ttgatgtaaacatcttcctaactgaatcgaacctcagctgaagaaggttgaagctccgca

gcaaaatcaaatccctctctgtcactgagtgtatgaagcagaaaccctagagcccgcggc

gg

>fava_dn_rep_c234

ctcacaaatactgttacttcgtacagaagtttgcttgccgacttaaatttcagtcagctt

catttcaaattttcagcaatggcacataatcatgaatttcaaatctgaccacaacacata

aatgctcttctattatgaaatggtcattgtactactgtctaagcagcatttagatctaag

cttgcatccaatatgaaaaaaatccacaaatgcatacaagttatattcccaactaaatca

ttacacgcactagtttcagcagcagcaaaaacaaagtaggctcatataatacgacacgac

acgacacataagcttaaaaagctttattaagcaaagatagactaacttatacaaacacca

tcaaataacatagcaggtggagctattgctttcaaagtatttgttaaatactgcaattgg

tgttcaaacatcactaaccgcacaactaaaaattcacccttcaccctcttaataagaagg

tttgtggtcaaagaatattttgcaacatagctccttacttattcggctctagactggcca

gcacgtgaagacaagataataccaacaatgagaccataaagagcaagagcttcagcaaag

ataaggataagaatcattccaacgaaaagttttggctgctgtgcattggctctaacacca

gcatcaccaacaatcccaatagccataccagcagagaggccggcaaggccacaagcaaga

ccagatgagagatgtgcataaccatcaaaaaggtaataagacttagcctttgggttaatt

ccagtactgataataacagcaataataagaccataaatacccaaaacaccagccataaca

acaggcacaatcgacttcatcaccagctccggtctcatcacacccatcgaagccactccc

acaccactctttgctgtgccatacgcagctcccatacatgagaaaacgagagcagctgcg

gctccaaggaagccaaaaaacggtgccgtttcatcgccgctgaatggagccattcctact

gaaacgacgtcgaatagatccgatctaagataaacggttttacgattttctcagagagag

aagttccgatttgcgatctcaattgtcgtagagaaaaatggttagtgaccgtggatgatt

gtttgcctttatttatagttagttaataattcattta

>fava_dn_rep_c235

ttttccatcaataattaataactgacataaaatatctaaatcaaaattactttttttttc

caacctcaaaatcactttttgtcatagattctcttcactcgaaataatcttattcgggac

attattccattaaaccagcttaattaatagttgtacaagaacacgaatttaaactcacga

catttcacttattcatctgtaaatcatactgatccaaaccaacgtataatgtcaagttct

gtggatactattaaatatcaaatacaaactgagtccaaccatgtaaacaaactatcatat

ttccttagtccaagtacaaaccaacaacaacaaacataacattacattaattagctgata

tgggtgcatagtcatcagtagctggtggagcttgagatatggcagataatacaacaaaag

taccaaataagattgtgattattgcagtgaagttgacaagtaaagcctcagaaccatatt

ttgcaactcctgaactctccaagaatgtgagcttttccagaaacccaagagaagcagtgg

caagagctaaaatatacacaaacagcccaaataatacatgccatggaactgactcacgtc

ttatctctgaactcccaccagggtagaaaaataccacaaacccaaatatccactgaatgc

cataaaaggaaataaccccaattccaagccatgaatgcaagctgtacagattggcaatcc

cactttcattatgattcttgaacgcagcaagaattccaattattccaagtattaatgcaa

tcgcgtggagaacaaggtgtatcagtttcttcacttccttcttcaatggaagagatttgt

aacttataattgcttcacccccaaatattattaagccaataagcataagaacaggatgaa

gattgaagatgagagctttgttgtgagaactccaagccaacccacctctgaagtgtatat

tccataccaacaccaacacggcggcgacaacaccaagaacatgaacaacgtacgtgaacg

gagcagcgggtacacccagcgccatcgtttaacgttattagcgtttgttcgtgtgaacgt

gctgttgttgttgttgttgggatttatatgaagaaaaaggcacgcgagagacacgagttt

cgtcgatgtagttgttaagatatatacggtgtc

>fava_dn_rep_c236

gacaaaaaccagacatcaatcaatcatagatataactattatggcatcatattaataagc

atcaagacaagtatttcatctggcaatcatgtttaattgatcatgcatcatcaatacaaa

tgtgcaataaaaatggcgcccgataatgtttgttcaaaataacgataagcacacataatc

accatttgtccatacaattgaccaaattgacttgcatgactcgcttaggccacaagaaac

cccaagtaatgttacgacatcaaatatagacataaaacagaaacaaaacataatgaagac

ttcatccaacattcaaggtaccatgagtaaccaaactaacatggaaatcagaatgtagaa

ccgttccatccaaagttggaacccttggtgtcatagaatttcaagaagaaacgtgacttg

ggcacggacaacttggtttcgataattgcagcaattgcagcactgagtttcttgttcaca

tccgggttaagaccgccaatggaaaccagttctccataagctgctggctgctcagtccca

ccaaaagatatgggtaccgatcctttcagtacaatcatcacataggcctcaggttttccg

atgatgttagcgacggtggaggtggcttcggagaggatggaagaggtgtcgataccgtca

aggttaacgttggtggagagattgaggcacggcatcgtttcgtttcgatatctctctcca

aatcagagtgaaagtaagagctacttctaccaaccagttgtagaaattctaaacccttta

taagataaacaatattt

>fava_dn_rep_c237

tctaatttgttaacgtaagcccatttattgccagagtacaatttatcattcatcaagcat

gtgcttggaacacttaagtcacgaaggagacaaattgaaattactcaaaaacaaagcaaa

ctcttaattaccagctttccaactaaatatacattagtcatactagtatttcaaaattta

caattcatttgccatgaatcatgatataaagaaatctgctgcatgcctgtgacaactcag

ttctgtagtgttgtagatagtatattttgatacaaattatcatcggcacagttttacaca

aatagtaaaaaaacaaaacgtactatatgaactaaactgtaatgtatttgagaacagccc

cacggagcttttgcatgaattctgcagcttgtgtctggttggcaccatgtgcagaagata

tgttttcatcgagaagggtcaacaaagttcgattaatctcattgcgttcaaccatatcca

gcaaagttgctttcacatccttcgatgttaagattttggttagattttccttggcttttg

tgagatcagtttgcatcttatcataggcttcttttccttcttggatggctttctctagcg

cctccagctcaaccaatctatcctccatatccgttgttttgttaacagcaaaccgaatct

gtcctaactcagatttcagttccgaaaagaattcctcgtttaatcgcggtctcattctcg

caatctcaaattcaaaatcctgagcttcagtgtcaagaaagtattcaatcagctcttttg

gtgtatcaggaacttcgcgtgattcgcgaagggcttgaaggcgttccttctcttcacgga

cttgacgctcgaaggcttcacgagcgtcggagtctttttcgaggcttttacgaaactgct

ggcgggcgatgtggccgccactgggtggggccagtatgccttcaggatcccatccaatgc

agacaattgtggatttgcgttggttgaatttgggtgtggtgcgaaaaccagaagcggaat

tcacgcgtggtgtgactatgccgaagttgagcgtagccattttaagttgaaggataggcc

acactgaataaactttccaactagttctatgttactatcttcaatcagatcccatcggcc

ttgaaaacaactatttgaaaccctaaatcgtaagatttcccatattcgaaaccaaaaccc

taaatctttccccaaatttcaattgttttatatcgcaatccgtgtttagtctcgtttcat

tctccatcgttcactttgatttcaatctcgtttctgccaaaatcattgaaatcgctcgaa

tagctcaaactacaacctaatcgttcgaataactcaaactatagcttgaccattcgaatc

tcattagtttcagcatcaaagtgttagattcgacgacaatagttcattgacgaatattcc

tcttgcttcactgattcgatccttcatcattatatgtgtttattccatttgtgatggcac

tactctttcacacagcccttttcttggaactaggactctcttctttttcctcaattgttc

ttctttcagtcaaaacttgtgttttcactgtaagttcccatattgaagttgaagcttcag

tagtttctttaaaaaggtagagacttcatttgaaggaatcatagggaatgcctctttcgt

ctgttgtgtttgcccttggccattattattgctgcttacagaaccgactgtagagcacag

agaaagcccttgtttcatcgcgttgactctgaagttgtaattttggaattgttatattca

gtttatgtaatttcttaggctggtttatatgttgcttctttggtttaactttttttcaat

ataaagttgttgtaatactacaagatggcttgaagaaaatttatgaaattctcaatactt

tttgttggtggctcatacgagttaaaactgcataagtttatttatgtttttttaccc

>fava_dn_rep_c238

cacactctcatccttactaaatcacaccattcaatatccaccacaagaatctccacgttg

catgtatctagtggcttaaacttcttcatatcttcttccatttggcttattagttactgc

gatatccatcattcattctaaccatctactacaacattcttgaagaagagaaaaaaactc

cattggtgtaacaacgagggaaaggaaatggctacacaagctctagtgtcgtcatcatct

cttacttttgcagccgaggctgtgagacagagttttggagcaagatcacttccttcatca

gttggatgttctagaaaaggttcctttcttgtcaaggcagctgctactcctcctgtcaag

caaggtggagttgatagaccattgtggtttgcatcaaagcaaagcctttcttacttggat

ggaagcctaccaggtgactatggatttgatcctctaggactatcagacccagaaggaaca

ggaggtttcatcgagccaagatggctagcatacggtgaggtaatcaacggtcgctttgca

atgttgggagcagtcggagcaatagcgcctgaaatactcggaaaagctggtttaatcccc

caggaaaccgcccttgcttggttccaaaccggcgtgatcccacctgcaggaacatacagc

tactgggcagacaattacacactcttcgtcttggaaatgtcactcatgggattcgcggaa

cacagaagattccaagattgggccaaacctggctccatgggaaaacagtacttcttaggg

cttgaaaaaggtcttggaggctctggtaatcctgcttaccctggtggacctttctttaac

cctctcggtttcggtaaagacgagaagtctttgaaggagttgaagcttaaggaagtgaaa

aatggaaggttagctatgttggcaatattgggatacttcattcaaggtcttgttacagga

gttggaccataccaaaacttgttagatcatttggctgatcctgtcaacaacaatgtcttg

acaagtctcaagtttcattgagtgaatgtattatattatatatatgttgttaattacata

tattcttttctttgctctttggttgttgtaagactattattacaaa

>fava_dn_rep_c239

ttacggcgcgggagacctcaggtgccgcgggctctttctggtttgtttgtttgttcacaa

tctcaagcaaaccctcctcaactctcaaaactctctttctcttttcgtttccatggcctc

ccaagcaagccttcttcttcaaaaacagcttaaagatctttgcaaacatcccgtcgatgg

cttttccgctggtttggtcgacgaaaccaacatttttgaatggagtgtaaccataattgg

accacctgatactctttacgagggaggatttttcaatgccattatgagctttccgccgaa

ctaccctaacagtccaccatctgtgaagttcacctcagagatatggcatcctaatgtata

tcctgacgggcgggtttgcatatctattcttcatcctcctggcgatgacccaaatggtta

tgagcttgcaagtgagcgatggacacctgttcatactgtagagagtatagtgttgagtat

catatcgatgctttctggtcccaatgatgaatctcctgcaaatgttgaagctgcgaagga

gtggagagacaggagggacgatttcaagaaaaaggttagccgatgtgtgcggaagtcaca

ggaaatattgtgattcatatctggcttctcataacttgcttttgtgaagtgaagaacata

caacttaaatgtaaggattggttcgccttttaccattgatctgtgtagacttgctcctgg

aagttgacagcatgcttctttcttcagttgcgtgttattgttttcttatctacatggtca

tttgtagcaactttggaaaacaaactgtattctaagtgtttttggctagtgctctgggga

ctagcaattcattggaaatttagtttgttcatttgcataacctttgctgaaatttgatat

taggcgcctgagttttgctttttgcttatacaatattgtgatatggatgttgcattcaga

ttttaccattttgccttctttacatgtattattgccagttttggtagcatatattatttg

cttttaatcatgcagttttatcgaatctattaatgtgt

>fava_dn_rep_c240

gtttaagtttagttatttttgaattagattgtaaaaggctaaaatattataggcaggaaa

caaaacgaagtacgctataggtactctacattacaacaaagccctatagaaagaacaaat

aagacaacaatacaagaacccaagcgaaccatgtgggaggtgaaaaataccataaataaa

aaatgaaccctgctgagagagaggtcgaacaaaagtgcgcaagaagatcctgaaatctta

gccagaataccatttccaagaagagaattgaatctaattcatcacattcacatcaacgaa

atctttgtaagagaacatcatttctagatctccaaatcacccacttctgtaaatatcaca

ccaaagctaagctatcattgaatcttgaatcttaaatctaaccatagtgcaaaaagggga

aaaaaacccgcaaaacatcggccgcggcagtctccaatcatctcttcaacacatagcaga

agctgaaaaggttgaagaagaagaaaaggaaacataaacagtacaagaatataacaatgg

caaccaacgaggatcaaaagcaaactgaatctggaagacactcagaggttggtcacaaga

gtcttctccaaagtgatgctctttaccagtatattctagagaccagtgttttcccaagag

aacatgaagccatgaaagaattgagagagatcacagcaaaacacccatggaacctcatga

caacctctgcagacgaaggacaatttttgagcatgctccttaaactcatcaatgctaaga

acaccatggaaattggtgtctacactggctactcccttcttgccactgcccttgccattc

ctgaagacggaaagattttagctatggatattaacaaggagaattacgaactgggtcttc

ctgtgataaaaaaagctggagttgatcacaaaattgatttcagagaaggtcccgctctac

cagttcttgatgaaatggttaaagatgaaaagaatcatgggagttacgatttcatttttg

tggatgcggataaggacaattacatcaactaccataagaggttgattgatcttgtgaaag

tgggaggtgtgatcggctacgacaacaccctatggaatggatctgtggtcgcacctcctg

atgctcctctcagaaagtatgttaggtactacagggattttgttttggagcttaacaagg

ctttggctgtggaccctaggattgaaatctgtatgcttccggttggtgatggaatcacta

tctgccgtcggatcaagtgattgatttgcgtgtgcatattatgtaccgctccacatgatt

atgtcggagtttatgcatctatttcaaagttatcaagtttttttttttttaatttcaaaa

tatttgtgtttgtaattttctcttgccattggcattgagatggcgaataattgaaattat

atatatgtttggccttttagtaccaaaaaaaaaag

>fava_dn_rep_c241

aaactctaagccatttaaaagaaaaactggcagacaacgttttgagtattnnnnnnnnnn

nnnnaaacaaaaagattcattagtttcactagaaaacgtttattcctacctcttgaaagt

gctgaacttttgtctaaccagaattgtttttcaaagttcttgagatttttatgaaacact

cttttcaatcctacctcaacaaccctttttaccaacaccatccatgatctattatccaaa

caaaatcaatcaacataatactaagcaaaaacattcagttcaaaatcttcattgcatgaa

taactatgaattaaaataaaatgtcaggggaaatactataaagtctaacgacagccaaaa

ctgcatgaactggagcagaaatcataacatttaagcatcatttgaactggttggtgctcc

aaagccaccagaaccacgaccaaagccaggtcttccaccaggacccccaaaagaaggcct

gtagtcagctggagctccacccttgtctcctccaaactcaccaccaggtcctctggaacc

tggaccaccacggtatccatcacggtcaccaccgaatctcctctctccctcgaatctagg

tgggcctctagggcgatcaccaggtggaccaccgaatggtctaccagctggcttagcctg

cttcttcaaagtagctggtacaatctcagatggaagattgagataagtcctgaggaactc

aattccatcattagtcaaaaaccagtaatagtgcatccaagcaaaagtctccctcacata

ttccttggacttaaagctctgcatcagcttaatcacttgcagatttggaacatcaatctc

tgggtgcttcgccaaattgaaatccttcttagcatagcaaactccctcttggaagaggta

tttggagatctctctacggtttttctcggatatgatcatggttgaagtgtggaggcggca

caagagaaggttcagcagcaaaaattcccaatcctctctatcgctcagctgctgcaatct

gaactaccgcgcgga

>fava_dn_rep_c242

aaattttaaatgcatttttatattcagacgatttgaatcaaggctaactcaaattgtgga

gttttaaacaagaaattattctgagtgtaatctaatttcagaactagttgatactcaaca

caatatcaacaactaactggaccatactaattcaaacaactctaatatgctcaaaacttt

taataaatgtctttgccaatcaaaacaagtataacagtacattccggtacaatcaaaaat

atatagaagaaattggataaattatactgaataactaggtttcccaaaataaaatcacat

aacaaaaacagctcctggaattactatggacaaggatcatccattacatccggttgaaac

cacaaaattctcagtagaagaaaccaaggaccttaccaccaacattctttctcctagcct

cttcgtgatccataatgccagcagaagtggtcaaaacaatgtaaccaaactgtctagatg

ggagaagtctggcagtccaaccttcaatctccttgaccccaacatcaaaacgaggactaa

taaccccacatttgttcaacctgccattcaactcaacaacaatctttccagatctgtgat

catcaacatactcaaactccccaatatatccatgcttctgcataacaataaggaacttga

taatgactttggatgatggccttatcatgacttggcgctttcctcttttctcagcattgt

acatactctttagagcatcgttcaatacactgactctcaccatggttacgacaaagaaga

gatgggtggctgaatcaacctcgccgtcaattcacgctctgaaatcgaaaagagttgaaa

tgtaaatcgcggctgcgtgg

>fava_dn_rep_c243

ctctctctttaaaactcattctttttatttgtttgttttccatggtctttcaaccattga

atcataaagggattgctggatatatgggcaaaagaggagaagacatccattgattagacc

caaaaaaaagtttatcatcgccattcaaaagtcaccatactaataattcaaatttgacat

atagtaacaacaaataacatacaactaaagcacattcacacacacatacacttaacattc

ttcatcttcaaaaagatgaaatccttattgctgaaacatcttcattcttcaagatcattc

tgctgccttaaccggcttaacaagtgtcgatggaaaatccagtagcacaatgttttcaat

ggccgctgaaaatttttcagaaaactcaacatggtttggatggctctgaaatgcagcaaa

atcttctttcttgttgaatgtcatcaagaatgcatgagtgaaaccttgtctcaacacatc

taggctttcaatatcttgtccccattcaaaggatttgacaagatcaatctctgagactag

cttctccattccttttgtgaggtcttctactactacaccttctttgaacttaacaatcac

aaaatgcttgaattcctccatttttgtcccgcggccnnnntaggaggctaatatatatgc

acaatttatgcttcaaagttcaacttacctcactgtctaaatctgtcaaaactgtcacaa

ccccacgacccatttcatgacatgttggtactaatcaattagataaatactcatctcttt

taaaacttaaagtcagtgttattttaacaaataaaatttattttaaaattgagcagatta

atattttaagtcactattctcaatcatgtcttttacgtctcatttctttttctttttcta

gatttttatagaacgaatctaa

>fava_dn_rep_c244

atatagatactaacgtgtaatatctagcacctggaatcatcgaagcaagacagtaagcat

gtcaggtccagaatgttgctcaaatccaccaaaactgaatccgagtgcaggatctggtca

cgttgacaagatcggtgctctcgatgcctatgtcgccggttctcctaattccaactccgc

cgttcttctcgtctccgatatttttggatatgaagcaccaaatttaaggaagcttgcaga

caaagttgcagctgcgggttattacgtcgttgtcccggacttccttaatggggaacccta

caatcctgagaaccttaataggcccttgcctatttggataaaagatcatggaacggacaa

gggttttgaagatgcaaaactcataatcgaagccataaaaagtaaaggtgtttcatttgt

cggggctgccggattttgttggggtggtaaggttgctactgaacttgcaaaattcaaact

tattccagctgttgtgctattgcatccatcatttgtctctgtggatgacataaagggtgt

tgacattccaatttctatacttggagctgagattgacagactttctcctcctgagcttgt

caaacagtttgaagaaatccttactgccaattctgtgacaagttttgtgaaaatatttcc

caaagtttcacatggttggactgtgagatacaaaccggaggatgcagaagaagtgaaggc

tgcagaggaggcacatcaggatttgctggactggtttgctaaacatctcaagtgagaaat

ctttgtcagttgatgttgtctgtatctatgtgtgtacttgaagtgcagtatgcaacatgc

tgcacattccacaaagcttcagtttaccacaaaataaatgctgtattgtttcgtattgga

cacgaattatagataagttcagcagcgtgtatagtaaaaaaatgtaatgtttcttcttgt

ggacagatgtaatttgtgatatcaagaaatcatattttaagacttatattttgaatatcg

ggttttaatcgaacttatatgttctttgtttattctatggtgaatgaaaggaaactctgc

aaaggtgctgaaa

>fava_dn_rep_c245

tgtgagaccctttggcctttcaaccttgattgttggctttgatcctaacactagatcaca

gtgtgtttcttcacaaaacaaaaaagagaaagcttcttcatataggttttgttcaaatcg

gagaagtagaagaaagaagaatatcaaaatggcgagatacgacagagcgatcacagtctt

ctcccccgatggccatctcttccaagtcgagtacgctctcgaagccgttcgcaaaggtaa

cgccgccgtcggtgttcgcggtaccgataacgtcgttctcggcgtcgagaagaaatccac

cgccaaactccaagactctagatcagtgaggaagattgttaatctggacgatcacattgc

acttgcgtgcgctggactaaaagccgatgctcgtgttcttattaaccgggcgcgagttga

atgtcaaagtcacaggcttaccgttgaggatccttgcactgtggagtatataacccgtca

cattgcaggtcttcagcagaagtacacacagagtggtggtgtgagaccctttggcctttc

aaccttgattgttggctttgatccttacactggatcgccgtccctttaccaaacagaccc

ttctggcacattttcagcttggaaagctaatgctactggaagaaactctaattcaatccg

ggag

>fava_dn_rep_c246

tttttaaaagttaacttgaattaaagtttaaaatcgcaacgcaatgaaaagaatatcaac

ataaattgaatttatttacaagtgaattaaataaaatataaaatcaaaagtactatacat

cattagatattaaattgaaatagtaaaaataaatacaataaaaatgtaaatgaagaaaaa

gtatagatgctctaagagaaaatgtatgtttggaccatccttgaatgccttcacaataac

aaactagttagtagcataagagggataaatgaaatcaaatgaaattgcaacaatttagaa

catgtgttagtaatcacgaggtataaaatcgaaatgaaatttgcatgatgcacatggaca

attcaagaaccaaacaatcgattattcaagtatttcaattcacaaatgaactaaaaccaa

agaaactcatgccattattcaaaccaaaatcaaaacatagaaaattgattttgaaacaag

ctaagatcaaacaaaatcaaatccggacagcaactaatgcatatgaaacaatgttcaaaa

actcaatctcaaaagtattcaaaacaagtaaaatacgcaacgatgcttatcacaatcaaa

ggcataacatcaacaaacttcagaaaaccaaaacatcacaacttcaacaatcatcgtaaa

taaaaacaagtatttcaacaaaagctcataaccaagttatacaggaacaaattaacaaca

tcacaacaatattacatccacattgacataccataacttcatatcaattatcacaagacc

aaacatagtgtcaatttcaatattcaaattaaccagaatagtaccctaaatatcaacatc

aattccatcttatttcagaaattcaaccaactacataatagaagtaatgaatacaagaga

taccaaacttacaaaatgagacaaaagtggaattgctggaacagtaactcgcggccggtt

tgctgttgttgtgtagtaggcagcagctgtgttaacagtagggagtaacgagaaccggat

gatagttggtgaaagaggaggaaacaaccgattgataagaggaagttgcagtaggcggaa

ggaaggttcgggcgcgaagctgtttggcgacggtgatgatagtgaatcagatacgcggag

ccggaaggagtatgttgcagaatcgtgagattacagagggatttcagaaggtgttcgaag

gttcggttgttgtggctagtaatcgacggagggagtgtatatctcagacggagggagtgt

atatctcagacggagggagttgaatgtcagagtcacaggcttaccgttgaggatccttgc

actgtggagtatataacccgtcacattgcaggtcttcagcagaagtacacacagagtggt

ggtgtgagaccctttggcctttcaaccttgattgttggctttgatccttacactggatcg

ccgtccctttaccaaacagacccttctggcacattttcagcttggaaagctaatgctact

ggaagaaactctaattcaatccgggagtttttggagaagaactttaaagaaacttctggg

caagaaactgtcaaattggctatccgggccttgcttgaagttgttgagagtggaggcaag

aacattgaagttgctgtgatgaccaaggagcatggtctgcgtcaacttgaggaagctgaa

attgacgccattgttgctgagattgaagcagagaaagctgctgctgaggccgccaagaaa

gctccccccaaggatacatgattttaggtttcttttgctttgtactaccaagcaccactc

tgataggtgttggctctacttctacttattggaagctaaaattgatgccatcatatctga

gattgaaacaaaaaaaaaaggcctactgaggctacacaagagatctttagacatgttttt

catttatatttgtttagatcgttaaatgtatttgctgggtgatggcctaacttaaaaatg

atttcttttactgttttttttgggtcatgtcatggagttattttcaatttaatagcatgc

cgttattattcaagtttgtaaatgttaatttttaaatatttcatgccaagactttattaa

aaaaattgagttttattttaaatattctaatatgaaagtgctatacttaacaaaaaa

>fava_dn_rep_c247

gggtactaannnnnnnnnnnnnnnnnngagagcagtgaaatacaattttaatgtgaaaat

acaattttaatgtgaaaaaacaaaaccgcgggaaatttcgtgactcaatataaaataaac

ccagttggtttggtttttagaggcgaccgcatagctcagctctcgacccaggaatatgat

taaactatttaaagtaaaggaaaagcaaagagaacttgcagaaaataccagtaatggagt

gcctgttaagaagcaaagtgctggcgagctgcgtcttcacaaagatatcagtgagctgaa

tctaccaaagtcttgtaccatgcaattccccaatggcaaagatgacctcatgaactttga

agttttaattcgacctgatgatggatattatctggggggcacttttttgttttccttcca

cgtgtctcccatctatccacatgaagcacccaaggtgaagtgcaagacaaaggtctacca

tccaaatattgatttggaagggaatgtgtgtctgaacatcttaagagaggactggaaacc

tgttttgaatataaacactgttatctatggcttgtatcatctctttacggagccaaatta

cgaggatcccctgaatcatgatgcagctgctgtgttgagagataacccaaagatatttga

gtctaatgtgaggagggcaatgtctggtgggtatgtgggtcaaacctttttcccacgctg

tatgtaaaccatgctcgacagcttccaagcccttgattgaaatatgttatcaatgctttg

atggcccctggtttgtaaaatttatttatgaaaattgcattttacttttgtcagtatgtt

atttttcttggtggggggagcatcaggttccttagaccaccaaacgcatctaaaagccaa

acaagtggaggaatgttgaatgtatcttttgtgcaatgttgacatactattctaaacaat

caaaaatgggttcagcggtcaaaaaaaaaaa

>fava_dn_rep_c248

gattcattcattattaatgaaaatacaatttaaattcactaaaatttgaagataagtcaa

tacacatgcccgttgatagtataaagtaatttgatcaaattgttaataatcaggaagtaa

aattaaaaaataaccacaatgaatataaagagttgatttaacatgacatatattcagagg

atgataaatttatatatttatgatataaccgaatcagaaacttaacaaaatggtttttga

aaccaactccaaacagggtcttgtttctactaagatgaaaaccataccaattataattta

tttaacaacaaccaagcatgctgggagccagattattcttccatgctcacaatgttttcg

acttcgcagaagcaggaggaagcaactgatagcaagatataagagacgaagcaaatccaa

acgccccggtaacacggggagtgattttctgaggtgccaactgaagtagtccagcagcta

ccactacatcaatgcctgctttgataagtgctagagtcctttcatttgacttttttagct

tggcttggtattgctcattatcatatttattactgttcttgatttctttctctaacttct

tcatggatccagagagtcttccgagctccccgagctcaaccaaggtggtacacactgatg

aagccatccaacagaagagagaaatccggccaagtagttcggtacgttctttgttctcaa

cgataccggttctaccaagccacacgaattgatcaagaaacaagaaagtggacagcaatg

cattcttggactttcccaacagaattagagggaggggagtaccctgtggtgttggactta

tcagagcatgtaggtcattgacaaacttaaatagacgaaaaacttttcgtgctaagctgg

ttgttttgtctacattctgcgccttaccaggttcaccattactcaaaaatttagaaccat

attgtattgctctgcaaatcttgtcccttgcctcagccttgttcaaatacaaaacaagaa

ggccaagttctgccctagttgcatccaatgaactcattttctatgctagtgcgagatcac

gaaagaaatggccgaagagaaaaaaactagtgttgagaatcagagagcatgagaagaaag

aggtagctatgaattgggcctgtgaaagatagaatagtgataagggcttgtttggttgtg

tatttcccgaaagaaagaaacaaagaaacaaaaatggattatatcctatggaaaaatgtt

gaaccgt

>fava_dn_rep_c249

aatttccaaatgaaattttatatatacaaaatattaaaagcatacaaattctcttatcct

cgtctctaatatcatcctttcccaaactcactcctgtggcctcttatctctcacaatctt

caccacaacagcaaccaaccatggctatatgcatgggtttcaacatagcaaccgcattca

aatccctttccttatcttcttcatcgtcttcatcttcatcttcctttttatccggttcct

ccactcttcgtttcactccatcactatctctccctcggattcacaggtcggtgcttacca

tccagaatgctcacaagaaaggagccggaagtaccaaaaacggccgtgactccgccggga

aacggctcggagtcaaggtttatggtgaccaggtcgctaaacctggttccatcatcgttc

gccaacgcggaaccaagtttcatgcaggaaagaatgtggggattggtaaggaccatacaa

ttttctccctgattgatggagttgtaaaatttgagaaatatgggcccgacaagaaaaaga

tcagtgtttacccacgagaagttacgcctccgaaccccgatagctatagagcaaggaaga

tagagtacttcaggatgaggcgtgagcgcaaaaaagctgcacaagaaagaagagcgtttc

aaccgcagctggtgcttgcttcagttgatgatgccgctgatgacgccgcattaactaatg

tagattgttgattctatacttcattcacttatgtgtaaaatctagccgtcttctgatgtt

tttcttttgtggatctgttaaacctttagcattgtaatatcgggtgtttgttggacagag

aaattgattatggttaaaagtgatttgaaattaatgtgatctgaaattagtgtaaaaatc

acgttctcaaaagttatagatcctactttcaattagaatcaataaggtgtttttcacata

cacaatatggcacctgagttgcataaagacaatttcttatttgatcaaatttgaaatgca

tcttgatctaagtctactaaaattcaagggtagtaactttcttttacacttcagagacaa

acacacattcttactcaaccaattaatggaaaaactatagtgaatttgtttattaacaag

catcgaaaactactaatcatcatcatcgaaaaactttgagtgagtgatttgttcgggtga

atcctccatcaaccataagattctctccacttatatactttgcatcatcacttgcaagaa

aaagaacagcattagccacatcatcagcagttaactctacaccctgcaagtttgcgtttt

tcccaacgaaagaacgaaagccttccaacgcatcctcggttctctcgtcctcgggcaaat

gagccaaagccaaacctgttgcaacaccataaggcgacacgcagttcactcttattccat

ggttccccaattcagcagcaacattctttgttatcccccacacagcatgcttggaacctg

tatatgcatgaggccctagtccccctaaggagcttgcaacacttgagatagaaattattg

agccacttttctttgggatcagaaaatgagcagcatgtttcattccatggaaaacacctt

tcacatttatgtcaaatactttgtcgaattcggacatgtccacgttgcggatatcaggac

aaggtgctccggaaattccagcattgttaaccatgatgtcaagagtaccgaacttaccaa

ctgttatgtatattgcatcggaaacatcagtctctaccgcaacatcacaatgaacaaaat

aaacattttccggatcatggaaagaatcacagagcttctgtccaaggtcgtcttggacat

cggctatacagactttagcaccgtgggaatggaacaagcgaacgatactttctccaatcc

cggaggctccaccggtgaccacagccactttgcctaataacctttgtgatggagcaggag

ttgaagcattgaaggaagttgacatggttaagatgtgtgaagtttctctctcttgttcaa

cagtttgttgatgatagtgttggtggctcacaatatttcatttttctaccttctattttc

ttttcttctttctttttcatgaattctacattctattctatttgagtgggtacttttaaa

ttgtgacgtttttgcttttcattaaaattggaataatgcatgataaagtgaagtattgat

ctcagattt

>fava_dn_rep_c250

gaggctgtaagcaatttcttcatctaacttttacaagatatcagtagttgtacatgctca

tctaggtatgtattttatttccaacaatcaaagataacattataaccccttgtgtgaaac

aaaaagaaatgctaaattcaaaatcaatcttgtaccagtacatcaaagacagaaaaataa

atgacaacataacaggtaaacaaaatactcaacaaataacaaactaggaaaaatgcaagc

tatgtaagagtttgcagggaatgaagttttaataaaacacgattatcttcagtgagctct

ttaatgtgctaactgataataaaataaagcaactccaatatgagaaaggaaatatgacta

ctgaatcacataaacaggttgggcctgttagccttgtaggtggtttttaggctcctggat

ggtggcctaatcttcttgaacaccaaggggaacttgattttagagttgtggaactgctta

gtgctctcccttttgcaaagactggctggaatggtggcagtcttgatgatttgaatgcat

ggaaacctgaccctgtgacgagatgccatttcattgtacattgtctcaacagcgccgttt

agagtggtatcacgatattccttgtacatattatgataaccagtacggctttggtatctc

agccatataccatagttttttatcttggtgggatttttctcaaagatctcattgatggcc

agcacttgtccattacttttcttaaccttcttcaacttcctcaagaaataccagaacttg

gatttggcgcgaacctcattggtggcccaaagcttcatgcggtaaatcttagggtgctca

tcggtttccgtgggaagaccccttccgacgacctggtattggtgaaactttgcgttaacc

attttttacaacgagcagcgattccgattgtgactcgccgccgattcaagtcgtgctgtt

gtgtaaaggatcacgcg

>fava_dn_rep_c251

ttttttttttagaggttagaaatgttgaagtaatttagtcaaattattccaccttttttt

atttttagtttattaataatcttataactttagagaacacacagatgaagggaaagataa

aagctcctacctacaacttcatttctgtttcacaagttacagtctcacatttctttcggt

acatctttgtaacaaaagcctaaaaaagactattaacataatcctatttatatgcataac

attcttcaacttatttgcatatgaaatacttattcagacctggaccccgatcctctgtgc

tacctccttaaaagagtgaacatcacttccccaaagccaggcatcacagcctgcatctct

agcaccccatacatcgttacggcggtcatccccgacatgaacagcatcctcaggttttac

atccaatagttcgcacgctttaagaaatatagttggatttggtttctctgctgcaacctc

agctgatactgctacagcatcaaaccaatcatcacagtttaatgcccgcaggagaggtct

taaccgagtgtcaaaatttgacacaacagccaatttcacacctgattttctaagagcttt

gaacacttctcctgcattagggtcacataggtgccatgccttgtctgtcacatagtagtt

ataaagttcctcaaagtattgagaatcatcacatccggtagaattactaactatgtattg

ccagaaaggtctaccatcattaacatatctgagacgagatttaccccaaggctgactata

agctcttctgtatctatgcaaaatctcttcctctgaatactccactccatatttctcccc

aatctttctatatatctgagccataggttgagaaggaagaacaagagtcccaacagcatc

aacgagaagagccttatgcgtaatctcaccgtacagagatcttctataatcagcataatc

cttcaaatacgtaccgctatcaaaaccatcatccgccacaaccgccgcggaggaggagga

ggaggagtacaacgccgaccgccgtgatttaacgagtggcgcaactgacctcgatttgga

agatagaagcctgaaaccactcataacggccattttcatagctctttcttttcttcacat

tctatttatcactcagttagttgctcacatgaatcacacggaaaggaaatgaaacggaaa

atgactctttgttgcgg

>fava_dn_rep_c252

acaagtaaagtcagaaaacctaatttcctctccttccttcgccgttccgtctcttctcac

tcttccctctttgcagctatcgaagatgagtaagctttcaagcgaggctgtcagagaggc

tattaccggaatcatggctgattccaaggagaaaaaccgtaaatttgttgagactattga

actccagattggccttaagaactacgatccccaaaaggataagcgtttcagtggctctgt

gaaattgcctcacattcctagacccaagatgagaatttgcatgctcggagatgctcagca

tgttgaagaggctgagaaaattggattggactcgatggatgttgaagctttgaagaagct

aaacaaaaacaagaaactggtgaagaaattggcaaagaagcaccatgcttttttagcttc

tgaagctgttatcaagcagattccacgtttgttgggtcctggtctaaacaaggcaggaaa

gtttcctacacttgtttcccaccaagaatctcttgaagccaaggttaatgagactaaggc

tatggtcaaattccagcttaagaaagtgctttgcatgggagtagctgttggtaatgtcag

tatggatgaaaaacagatcttccaaaatgtacaattgagcgtcaacttccttgtctcctt

gttgaagaagaactggcaaaatgttaggtgcttgtatctgaagagctcgatgggcaaatc

ttaccgcgtcttctaaatgttttggccatttcaaaggttcttaaatttggtgtacttgaa

tttgggattaatattttggtcgctgtaatagctgagacaagagttttcctctttctaaaa

ttttgtttttgataagattgagtatttctattttgtattattgaatggtatttaattgct

tagcaaggttgaactccttaaaaaaaaaag

>fava_dn_rep_c253

tttcccttttccctttctctgcttccctattggtacaattccaacccatctcttgtctct

ctctcttacaaagagagagagagagagaatatgtgtcacaaagcaaagcaactcgcaaag

tagcaaatgccaaattatactagtaataccaatatcaaataccattcttgtgtaatagct

ttcccattcttgtatagtgagaaacaaaaacgaataaccatccattaagctcaacttgcc

ctttaatcgcaaagttgctgctactacgcctgcatatgcaaaaagcctcttcaattttga

gctaaagtttggatttttaagagtttttgattgtggggttcttcttatttcatctgggtc

atcagcaaatttgttttatcagctatggttgtttgaatagaatctaaagtaaagattttg

atcttggttggttgattggagttggatacagtgagtcagtgaatttggtgaagggttttt

gctttttttgttttgaactgtaacagaattgaatcacacttcattgcttttagaatcgtt

acaacagacccatatataggcctaacagaaatgccagctcagcaaagctgaactaactaa

acaatctatatatataggcctaatagaagagtaatctatatttatctctaatagttccat

aatttcaattgtttaccgcaaatgggataacattagaatatggcattttattgaaatcca

ttaaaatatatgcatggaaagttacattcattttctgactagattacattacatagtaca

aagtatagtcagaatcatttagatatcgatcccagtaatttctttaacagttccttttat

tttctgtaccaaaacttccctgtctggcttgaaaagtatgtttttgtatgcaaaccaccc

agtgtaaccaatgcctacaacttcaagagctccaggaactaaaggaagcctatcaattgc

cgagatcacaccagctgagccccataatgctattgcaccagctacagctattgtactcac

tgcatatttgtcttcaactttctcccatgtttcttgaatagtcttgagaaattctggaga

atcactaatactgagctgctcagctgaggtagcaactggatcaaccggtgctgtggctgt

ctctgtggtggtactggaaaccttggacataacgttgcgagcaatctttcgacagaatgc

tggggttttccatggacgagtttgagtttgagactgcacaggtggaggaagagttggaag

gctcacacagtgcggagatgtagattgtctacaaggaggtttggcatcaataagggtgga

agaagaagatatagaaagagtggaggatgaggttgaagaagccataacttgttttagtga

tgaacctctttgatttgaggtcaccaatatagatcatgttaaggttgaaatggagattct

tctgctgccacagtttgaattgaaatgctgagccactcaatttgtttcatctggatagtg

tatgtgctgacttggctacaagatatggcttttatggtatttcattggataggagatttc

tggttcacctttttcttatatcaattttgggacaaaacatgttagaatcatcaagtctaa

ccttagtctnnnnnnnnnnnnnnnnnnnnnngcattttataagttatgagactttgtcct

tctatctttcnnnnnnnnnnnnnnnnntgaccttggatgaggttgtatggttgttccat

>fava_dn_rep_c254

tttttttttgttgatatgcttaaattcaggcctccgccattgttgctgttgctcagtaat

ctcagccgtcactagtagttgctgttatctgcaaacagtgagctatggctgtaggaaaaa

acaagaggatctccaaggggaagaagggtggaaagaagaaagctgctgatccctttgcta

aaaaggattggtatgatatcaaagctccttcagttttccagatcaaaaatgttggcaaaa

ccctcgtttctcgtactcaaggaactaagattgcatcagatggactcaaacatagagtgt

ttgaggtttctctggctgatctccaagcggatgaagaacatgctttcaggaagattaggc

taagggctgaggatgttcaagggaagaatctcttgacaaatttctgggggatgaatctca

ccactgacaaattgaggtcattggtgcgtaagtggcaaacactaattgaagcccatgttg

atgtcaagacatctgacaactacacattaaggatgttctgcatcggtttcaccaagagga

gaggtaaccaggttaagagaacctgttatgctcagtctagccaagttaaacagatccgta

agaagatgagagaaatcatgaccaaccaagcatcatcctgtgatttgaaggaattggtca

ggaagttcattcccgagatgattggaaaagaaattgagaaggctaccactggtatttacc

cgttgcagaatgtgttcatccgtaaagtgaagatccttaaagctcccaagtttgatcttg

gaaaattgatggaggttcatggagattactctgaagacattggcacaaaggttgacagag

ctgctgaagaaacagttgcagaggaacctactgaaattgttggagcttgaattagtgtta

atgttgttctttgtgtttgatttaggttttctgtttcttatgtttttaagtgatattaag

tttaaacttaatccgatcttttaatttaagcagagcatacaacattttttgactcaatta

tgtatggattatgttgatactgaatgcttgtttatgctgggaaaaatttgtatcagtaat

taaactaatttttatacatttcagatatcacaacaaatcaagcttgaaagtcatttgata

aaaaactatacataacaatatct

>fava_dn_rep_c255

atctagcaagccttaccactcttaaaattaaaagtagaaaaaagtcatacaagtctccag

tttaggtctgcaaaaaaatggaacggagagggcaaagtagaggtggcggatcagaaacca

ggcttcatccggcaaaaatgacggaaaacagacacgccggcgtgctatgcacattttgcc

acctctaaatcacacaaaaatatttcatacaaagactatataacttcacatttattcaaa

tatcaattgttcaaaccttaaccaccacaacataaaatcatcaatacatatcagccagca

catttcaaatcaactggagtccatactctagaatgtgtcatgatcacttattatattcaa

attcaaaatacatggctgataaaaaccaagacaagatttgaaccaagtcattagccatag

caccttatgggatcagtgcaacccttttcagcagactgactgaggccttggcctccaccc

ttctccaaatattgctgatgatattcctctgctctataaagcctctttgctggaagtatt

tctgttacaattttgttcttatgttccaactgtttagcatcttttgattcttgagctaaa

cgagcctgggtttcattgtagtagtaaattcctgatctatattgtacacccacatcacct

ctctggcgattgagggtagtggggtcatgtcgtgaccaaaagagatccaagagattagtg

taaggacatactttcgggtcaaactgagcccttaccacttcaacatggttagtacttccg

gtgcagaccaatttgtaggttggatccggtgtatgaccctgtgaatatccaacttcagtc

ttcacaactcctacaactcgctgaaaagccagctccacaccccagaaacaaccggcacca

aactcagcaaactcgtgacccgtgatggccggagtatccaaatccgggtcaaaagcaggg

ctgtggtttccgtcgccgctttcactcgtagccattgtagctcccactcctcgtatgtga

ttcgcttaagttgatattaatattgaagtgaattattagttgccttcacaggcaaagacc

aatgaaaagtgaactacacttataatattggttggattcattctcatatcttatgaggct

nnnnnnnnnnnaccaatccattcttgacctttggtgattatggaaaataatttttttaat

atttttaatttcagaaaaatatatcttctacctttatttatttttaattttttttactaa

tttagtggtaaaagtaaagaatataatcaattaaacatt

>fava_dn_rep_c256

ctttttttttttaaataaaagcctaatgattcattcatgaaatctccatcaatattaaaa

aaatggagattgagttcaaacatacaaattacaaaccaacaatttcatattattttacaa

aaagacactaccattaattaaacccataataaatccataatacttgcaaagaaactttat

ggaaatcccttattctagctttaaaaaaaaagcaacaatctcttaattgaaataaacata

gcaaattttcctaatgttaatgatccttgtacttattcttaacactttcagtgtttgagt

tactcttagtagcttcttcagctgcttcaaatgctttatcaacactttgacttttcactt

ttggtgtttcatttgctctttcctctgccactcttaagaattcttcagctgttgatgatg

ccctctcttttatcaaatgactctccttttccttgatttcttgcccataattcttgatgg

tgttgtaaaaaaatggtgtagagggtttgagaaaggaagatcttgaagatgagtgaacaa

gagtgaggaatttgctaccaccggccatagtgttgttgtgatagggtgctgcttaattat

tcaattcaatgtttctctctctgtcttctatgaggttggttttgagagagaggctaaaaa

aatgtggatgtgttggaaggtggaagctagtgatatacacgtggagttgttgaagtggag

cacttcatgcaacctaaaaagacaactttgaacactcaccactctatctcttcccgcgg

>fava_dn_rep_c257

cgcggggcctttccgttgtttctgaagctacactaagagagtaagccgcggctggatcag

aacgagattgcaacatgaagttcaacgttgcgaatcccaccactggatgccagaagaagc

tggaaatcgatgacgacacgaagcttcgtgcattttgggacaagaggatctcacaggagg

ttcttggtgatgctttgggcgaggagtttaaaggatatgttttcaagattactggtgggt

gtgacaaacaaggattcccaatgaagcagggtgtgttgacacctggccgtgttagactct

tgctccacagaggcactccttgtttccgtggacacggaaggcgtaatggagagcgtagaa

gaaagtctgttcgtggatgcattgtcagccccgacctatctgttttgaacttggtaattg

tgaagaaaggtgacaatgatttaccaggtctaactgatgttgagaagccaaggatgagag

gtcctaagagagcatccaaaatccgcaagctgttcaacctgtcaaaggaagatgatgtgc

ggaaatatgtcaacacctatcgtcgtacttttaccaccaaagctgggaaaaaggagagca

aagctcccaagatacaacgtcttgtcacacctttgacactccaaaggaagcgagcaagaa

ttgcagataagaaaaagagaattgccaaggctaaatcagaggcagcagactatcagaaac

tccttgcatccagattgaaggagcagagggaccgccgaagtgagagcttagcgaagaaga

ggtctagactatccactgctaccaagccaactgccacagcataggcagcttacagatatg

gtgaagtttgaactttgccttagttttctgtagtcttgtttcatgagggaggttatgata

aacatttatgaattttcctatttaaatacttttgagttgattggcttatttttaaatgtc

tgtttggaatttcaatcgaattttgaaggatttttgcttagttgaaatttatctatttga

tcttatgtt

>fava_dn_rep_c258

tgatggaagtggaacatgttttgaggttgnnnnnnnnnnnnnnnnnnccggcgcagagtg

gaaggctgctcgcggcaaggtgttgtgtgtgtggttatgctaggtggcgtgaagggtgat

ggtgcatagtagcggtgcgacaatgaacatgacgcgtagtagctttgacactggatgcaa

caatgttttcgtggactgagtcggtgattgcttcagctttgttttccactttattttttt

tttttaaggttcaatattaaactcaataaaagcataaagttataaggtacatttatgaca

aatccaaccagccaaaactgcagggcttaataacttaaagagacaaagataaataaagtt

cctatttcccacctcaagctcaaggcagcttcttttcaagtttattgcgcacacatgcat

atgaaaaattactacctgttagcaacataagaataaaatatgtgctatcacccaacagcc

agagtattactaaagcaaactagactttgcttatgtccttcaaagaacatatctgctctt

ctcccattgcagacatgacagaaacaacaagatcttttccttcagcaaatccatctttaa

tctgagtaaggagtgcatcatcagtgggaagtttcaaatcatccttagtacttccattat

cagtcaacagactcacaaaaccatcttcagaaatatcaatcagctgatagtcagtacggt

tcacatgaggaacatcacaattgtgagatggaggaacaatatcttcaagctttttcccat

tgaaaatatcaatggcaacaaagtgacactttgcatgtccatgctttcctgtctttgaag

tagaaacttcaacaaccttgcatggcctggccttgatgacgatgtacccgttcttgcgaa

tggtaccggcctgctgaggataggttttggaggctccggcgtcggcggcggcttcgaatt

ggtgctcttcgtcggacatggtggatgatttctcttttcttctgtgtgtgagcgaaacgc

tggaggagttcgcggaagaagaaagagttaggagacttatgggggtgcggtggtccctct

cttattctaaacctaatcacacgtgcaat

>fava_dn_rep_c259

ttttttttttttgacccagtaaaacataatcatttttgttgattgattaagacagagctt

gctttcataattgtcagtggattgagaaagggaagagtagtatacataaattttacaaca

gttatgagacaagctaaccaaagataacaggaagtaagaaaaaaccttacaagagaaaga

gactaattcttgggaatgttcttgccaacaatcttagccggagctatccgaattagattc

ccaggcttaccaggcagagcacctttgatcatgataacattaagctctttatcaatttta

acaatcttaagctttctgatcttggtcttggatccacccatcctcccaggcatctttttt

cccttgtatactcgaccaggtgtagttccggcaccaattgaacccagctgtctatgactc

ttggaaccatggctcatttgacccctcttgaagttatgacgcttgattccaccttggaaa

cccttgccaatggtggtgccagagatatcaacaagatcaccctctttgaaaagttcatca

aagacaaggcgctgattaggttgaaagccgtcaacagtttgaagacggaactcctgaaga

tggcgcaacggaatggctccagcctttttaagatgacccatttctgctgtgttcaatttc

ctgtccctaaccctacggtaaccgacttggacagcgttgtaaccgtcagtggcctcggtt

ttgatctgggtgacaatgttgccttctttgaaaccgacgatggtgacgggaacaacttct

ccggtagattcgaaaaaagtcatcattccgagtttggtgcccatgactccaacgcctgcg

tccaaactcatgacaaccactcttcgtttgggtctatcgtgttgttgttgaaatttgagg

aattgagaagagggattcttggaaatagaagttgagaatgaaagtgaagtagtagaacgc

gaggaaggtaatgaaattgagaatgaggagaatgcggaagaaattgaagctaccgacatg

atggatggatggttagttaagtttgaatgctcatatgctttcaaaccgagtcttatcttc

tttctttctgctacggattacggatttcattttacgctc

>fava_dn_rep_c260

ccgctgaatttaaacaaacaaaaaggaaactaataagaaacaatggtgccacgtggaaga

tgaaagcatgacaatgtggttgtgacatctccagaacaccactgaatggatgatgtttgg

agtttcatttcattccctcatcacaacaacaacatctctaacattcattcaacactaagt

tgaaaaatgactgtatcttcttctctttctctccctcacttccattcctcctttctttct

tcccctctcaaatctttctctcccactccatcctctagaaatttcattattcaaccaaaa

cctacattttatccacgtattagagctcttgaacttgatcaaaacacggttatagctata

actgttggtgttttgagtgtggctgttggaattggcattccagtgttctatgaaacacaa

attgataatgcagctaagagagataacacacagccatgcttcccatgcaatggatctgga

gctcagaaatgcagattttgtttgggaactggcaatgtgacagttgaacttggtggggct

gagacggaggtttctcgttgtataaactgtgatggtgctggttcactgacatgcacaact

tgtcaaggttcaggaattcaacctcgttaccttgaccgtagagagttcaaggatgatgac

tgagacaagctaagattgagaggcgctgattttgtatttaatcgccatgttattgtactc

attcaattttttcgatctctgtatacaccagatacaactaaaagaatcgtgttgatgttt

acatttggtttgcattttaaccagtgatattaggccactcccttccatgacctatctttg

taaaattttggtatttgtaaacattatatattgaatatccatttgtaaagaatggccccg

agaaactatgcctaaattagtttcattgatgttaattctgtttctgtttttgtttattaa

agtattagaaggatgataggctttagtagtgtggatagggtgagaggtttcactaaatta

ctcaaggttattggttccaa

>fava_dn_rep_c261

atggtagagtactcggtttttaagtgcgactccatttttttcttatttctacgaactgat

tggtttatccatacaattaagttcttccttagaggaggaagaaatcatcaaatcttataa

aaatttgagatcaattcattccatttttccctttttggaggataaattgacatatttgaa

ttatgtgtcagatatacgaataccctatcctatccatctggaaatcttagttcaaatcct

tcgatactgggtgaaagatgccccttttttcatttcttacggctgtttctttggaattgg

aatagttttattactaccaaaaaatcaatttctactttttcaaaaagtcatcgaagattc

ttcttgttcctctataatttttatgtatgtgaatatgaatctatcttcgtttttctacgt

aataaatcctctcatttacgattaaaatcttttagcgttttttttttgagcgaatttttt

tttatgcaaaaagagagcatcttgtcaaagtttttgctaaagatttttcgtatactttaa

cattcttcaaggatcctaacattcattatgttcgatatcaaggaaaatgcattctggctt

caaagaatgcgccttttttgatgaataaatggaaacacttttttatccatttatggcaat

gtttttttgatgattggtatcaacgcagagtgatcaatataaacccattatccgaacatt

catttcagctgttaggctatttttcaaatgtgcgactaaatcgttcagtggtacggagtc

aaatgctgcaaaatacatttctaatagaaattgttatcaaaaaacttgatataatagttc

caattattcctctaattagatcatttgctaaagcgaaattttgtaatgtattagggcagc

ccattagtaagccggtctgggccgattcatctgattttgatattattgaccgatttttgg

gaatatgcagaaatctttctcattattataatggatcctcaaaaaaaaaaagtttgtatc

gaataaagtatatacttcggctttcttgtattaaaactttggcttgtaaacataaaagta

ctgtacgcgcttttttgaaaagatcaggttcagaagaattcttgcaagaattctttacag

aggaagaagagattcttgcttttctttttccaagagattcctctactttgcagcggttac

atagaaatcgcatttggtatttggatatttttttcagtaacgatctggtccatgatgaat

gattggttatgttatgatacttaaatatctatttcaatatatatatagaaaggattttga

aatgttcatgtagtttagagttgagtttcaagattcctctgaagtaactcaggtttagat

gtatacatagggaaagccgtgtgcaatgaaaaatgcatgcacggcttggggagggatttt

ttattgttttatttgattaaacaatgaattttctactccatccgactagttccgggttcg

agtcccgggcaacccattataatgaatagataaattgtacttaatatagcgaaataatga

atctatcactattacatatcatagcgaagtcatatctagagaatatagaaaacacttttt

cttttttttgaatggatggtgaaatgaggtaaaaaaacacaatttgtctgaatctagatc

aataacaggatacagtggatagtggtattggttgacacccgtatataagtcatgttatac

tgttttataacaagcccttaattctatagttctagagaattcgtgtgcttgggagtccct

gataattaaatttcaataaacccaagattttaccatgactgcaattttagagagacgcga

tagcgaaaacctatggggtcgcttctgtaactggataaccagcactgaaaaccgtcttta

cattggatggtttggtgttttgatgatccctaccttattgaccgcaacttctgtatttat

tatcgctttcattgctgcccctccagtagatattgatggtattcgtgagcctgtttctgg

atctctactttacggaaacaatattatttctggtgccattattcctacttctgcggctat

cggtttgcacttttacccgatatgggaagctgcatccgttgatgaatggttatacaatgg

tggtccttatgaactaattgttctacacttcttacttggtgtagcttgttacatgggtcg

tgagtgggaacttagttttcgtctgggtatgcgcccttggattgctgttgcatattcagc

tcccgtcgcagctgctactgcagttttcttgatctacccaattggtcaaggaagcttttc

agatggtatgcctctaggaatctctggtactttcaactttatgattgtatttcaggctga

gcataatattcttatgcacccatttcacatgttaggtgtagctggtgtattcggcggctc

cctattcagtgctatgcacggttccttggtaacttctagtttgatcagggaaaccacaga

aaatgaatctgctaatgaaggttacagattcggtcaagaggaagaaacctataatattgt

agctgctcacggttattttggccgattgatcttccaatatgctagtttcaacaattctcg

ctctttacatttcttcctagctgcttggcctgtagtaggtatctggtttaccgcgttagg

tatcagcactatggctttcaatttaaatggtttcaattttaaccaatctgtagttgatag

tcaaggtcgtgtaattaatacctgggctgatattattaaccgtgctaaccttggtatgga

agttatgcatgaacgtaatgctcataatttccctctagacctagctgcagtcgaggctcc

atctataaatggataatagtttggtttaaggatacgagtttttgaaagctaagggagtaa

tatcaacattgtggatattactcccttacttttagtattctttttcagtctacacataca

gaaattaataaataatttattaacttccatattctttagaattagcattctttatttcaa

caaaatcaatttgtaaattttgagttttattattattatttttttgataaataaatgaat

gttttcttattttctaatattttagaagattcgtaagaacttagaagaaaagaataatga

aaaggtataaaaagttatgtaatttagacatagtgtaatttaaccatacttatagggcg

>fava_dn_rep_c262

agtacaccctgtcttatccacaaaaccatgagaaatcttgagatatatgacatggcctta

tccaaataatttccaaaccaagctacgtgtcatcttcatatccaacctctaatctgataa

taacttcaaaaaaaaaaaacacaattgttcattattcttagttctcacgaactgtttctt

cgattgttttctgttacaaacatgggatccatgactcctttgcagacaatccttcccact

gtcacaatttccaacaacacaaaactctcatttggaggctcaacaagtttgtcctgcaac

aagattcacaataaccacaagaacaagaacagatcttcatcaattgttgtttcagcagtt

ggtgatgtatcttctgatggcacaatttttcttgtcgctggtgctattggaattgctctc

attggaactgcattccccatcatcttctctcgtaaagacacatgccctgaatgtgatgga

gcagggtttgttaggaaaagtggggtgacattgagagcaaatgcagcaagaaaggatcaa

actcaaattgtttgtgctcgttgtgatggtttgggaaaacttaaccaagttgacaaatag

aaagatgtgaatgcttttcttttcccttttatgtaatgcaaggagggaatgacacaagtc

tgatttgttgcattggaaatttgatttgtgttattctctttatatcatgcatgctacatt

gctctatgttcttatgaaattgaaacctattttgtgtattttaagcaaaatgtgttcaat

agttgttatagtgaagcaagcaagtttcaatgatcctttatagttattgacgctactagt

acagtagattttcctatgttccataatttgattatggaaatagatatatataataacaaa

ttagtaaaaagattagtacaaaaaagaaaatatacgaagaaattcgcccccctcccacat

atttgatagcctctcctataaaaaaattggaaataccaactccattcggaattccatcaa

ttagctgtctatcaaaaaaataattgaatttagctaactttcttacactcgcaattaaag

atacttcaaaaaaagcatctatataaccacgattatatgaccaatcatatagaatattga

ttattttatcagaaataatttttttgggaacatttttttgaaataaattaaataagttca

aattttgtaaagaagaaaaaaccggtttatagaaaaaagacgctataaatagtccgaaaa

aagctatacttaccgaaaaagttgcatttataaaaaattcataccaatccacagaattct

ttgaattttgatgtaaaagatctatagacggaattaacaatttagataatatatccaaat

ctattccttcttggctgaaagaaattccaatggacccaacgaagaaagtaaatagtacta

atactagcatagaaaagagcatagtattgtctgattcatgaggataaaaaaaaggaagat

ttttagtaccaaaatgggtactatcaagaaaaagccgtcttatgcttttgacatttcgat

ttattcgatgtgaatatgtcctctcccgaaaaaaagaagtcctttcattattattcattg

ttaagaaacctaataaatgaattttcttttttaattttttttttcttctttgccccataa

agatattgaatagaaggaactatttttctttccattgaaattttgaaaatgaacgtttaa

atatccttcaaaaacaagtaaataaattcgaaacatataaaatgcagttaatcctgcggt

ggaacaagctattattgcgaaaattggtgaatacaaccaactatcattaagaatctcatc

cttggaccaaaaacaagcaaaaggtgggataccacaaagagaaagtgtacctattaaaaa

agacatttttgtaattggcgcatgttttgttaaaccgcccataagaaccatattttgact

tttatctggagaatatccaacaatagcttccattgaatgaataatggatccagatcctaa

aaacaacaatgcttttgaataagcatgagtaatcaaatgaaataaagcggctcgataaga

gcccatacctaaagctaacatcatataacccaattgagacattgtagaataggctaaatt

tttcttaatatctttttgagcaatagctaaagtagctcctaatactactgttattatacc

tatcaaagctattccactcattatcgagggtataactatgaaaagaggaagaagtcgagc

tacaagaaaaattcctgctgctaccatagtagcagcatgtataagagcagaaataggagt

aggaccttccatagcatctggtaaccatacatgaagagggaattgggcagattttgcaac

tgaaccacaaaataacaagagggcacacaaagtaacaaaaagaatatttacctcattctt

ataaattaagttcttgaatatttgaaataaatccctaaattccaaactaccggttatcca

ataaaaacctaaaattcctaataataaaccaaagtcccctacacgattcgttacaaacgc

tttttgacaagcatttgccgcaataggacgtgtgaaccaaaaacctattaatagataaga

acacattccaaccaattcccaaaaaatataaacttgtatcaaatttgaactagtaactaa

tcctaacattgaagtattaaaaaaactcatataagtaaaaaatctcaaatacccttgatc

atgagacatgtaactatcactataaattagaaccagaataccaacagtagtgattaatat

taacataatagaagtaagtgaatcgatcaagtagccaaattctaaagaaagatcattatt

tatagtccaagaccatacatattgatagatagaactattctcgatttgctgaatagataa

atcgatcgaaaaaatcataactatggttaacaagaaaatactaggaaaagcccacatacg

gcgaagattttttgttgccgtgggaaaaagtagaagtcctacccctattaaaataggaac

tggaagagggatgaaaggtatgatccatgaagattgatgtgtatattccatacaaaaaga

aagaataaaaaatatttagattcttgatgaattttgtttcttattcgtttgttttatatc

ttttgtaaaggggctcggttaataaaaaagtggatatagaaatagaattggatattctta

attgttctgaatctttgcacaatggtccccatattggaaagtacaaagtcaaaactggcc

aataacaaaattcctagtgaaaaaaataaataataattattattttcagtatttcagtaa

tatattcattaattaatattactattaattcctatgttaggttagttattttttaattta

tatatatattaagaaaaatgactattaataattgtaaaataaaaatttttatctcttatc

tatagagcgaggatgaggataaatcagtacaccaaaactaacaacatttgtttattttga

tatacattagaaaaataatataatataaaataatactttttttttattcagaaacattag

ttcctttttgaactaattgattcttttacattacaataaaaagagatccatatatatcta

ttatctatgaaaaatttggaaaaggtttataatattcaatgtttttactttacctttcga

tggaaaaaaagagaaagagttaatttagatagataagacatacggctaattacagaataa

ttcgttttcatttacagaacaaatgtctttcacatcaaactatagcaacgaaaaaactat

actaattgtatgccagttccaaaaaaacgcacttctatatcag

>fava_dn_rep_c263

agttacattacatggatctataccatacaaaccattggttgctttctcatccaaaaacca

aaaccagataagaagataaggccacatggcagaatctcaaactcactacttcatcctttc

tctccatcacaacacaaaatctctccaaatttctctccaattctcccaacaccaccatgg

cttcaccaacactcataaccccaacctccacacccaaatcactcccacccatcaaaacca

aacccaccaccatctccgccaccttatcaccaccaaccacctccaccaccgtacacaacc

gccgccgtgagtttctctccaccacagcctccatcctcaccaccacatttcttgtccatg

tcacccctgcattcgccgcttcagatgaagaatacgtgaaagaaacagaggaggttataa

gcaagctgagaacaaccataacaatggataagaatgaccctaatgtgtctactgcagttg

ctgaactaagagagaattcaaactcatgggttgctaagtatagaagagaaaaagcacttc

ttgcaagaacttcttttagagatatgtactctgcgctcaatgctgtttcagggcattaca

ttagttttggaccaactgctcctattcctgcaaaacgaagggcaaggattttggaagaag

ttgaagttgctgagaaatcgcttaaaagaggaagataaggaattaaggaagaaaatggca

ctgtatttggaggaaatgaattttggttttcattcctatgtttctgttattagattctta

ctgttttttctttgttgttcaatgtttgtattgttgtatgtatcgaaaactttaattaca

ttaagaggctcactttttctgttaataaaaggagaagattatgactaaaaaaaaaaa

>fava_dn_rep_c264

gcacgtgaaaagactcaacaaaccttgttgcgtgggcttcaaatcatatcacaagaaaat

gaagaaaatgaagaaaatcataatccaattacataatagataaaacagtaatacatggat

ttaaacaaattttcttctagtacataaattactttgactctccacatacgatacttctaa

tacagatgatggtaacaagcttatttatatacaaaacaaattatcaccagttgtttaaaa

aacacttagaaaaataacatatttaattaatacattcgaaatttttccttagttttctca

aacacttcttcagcaataatggcctctttgccatcagcccttttaatttccaaatgtgct

ttttgataaaatccagttcctcgtaatgcatcagctataaaatcatcaaaaccaatcgca

attgctgcttctgcttttgctccataaaccaacctcttaacacgtgaaaggtgaatagca

ccaaagcacattgggcatggctcacaagaagcatatatttcacaatcagaaagttctatc

tgcttcagtttcttacaagcctctcttattgcagtgacttcagcatgagcagtagggtca

ttgtttctgagaaccatattgtgacaactagcaacaacttcatcattaaaaacaataaca

gcaccaaaaggacctccatcttcacaatctacacctttatatgcttcttcaactgctttt

cttaaaaatttatggtctctatcctttatagcttgcttgtggccagaaaaagcagaagct

actgcaacaacagttccatccttagtttgcaccattttttaataaaataaagttcaataa

cttgctagtgctctttatgtcccgcggcc

>fava_dn_rep_c265

ttttttttttttgaacaaaccacacatttattatagaagagaacaacatactaattaaac

cgttcaaaataataagttacaacatactactcttatttcttaaccttcaatcacagtgca

tgtctggtgttgttcattaaactcatagtttcgagctacttcatggattgacctttggca

accatcctgcaatgacgtatttctcttcggccttggttttctcaaatgattcacgagcga

agagcggcttgatgtagttgtgaacatgtgtcaaactctcaggaatagtccatttcttga

agtggccaagggcaagcactagatggtaaagttttggtgccaaactcaaatcaacagcag

agaccttctcaccagcaacatatggaccattggccttaagatgttcctccaaagcattga

gttcagcaaccaaagcttgctctgttccatcatttgcatccttgctcttcacaaagctca

caaaagtcccaaatatgtttgatccaacggaggaaaactgaggaggagtaacaagagatg

gttcagggtatttttcttcaagaatcttcacaataacatcagagtcaggaatccattttc

catccaacttaatcactggaaccttcccttccggattcacttccaaaaaccattggggtt

tgttagagacatcaattaggtggatagtgtatgggaccttcttctcttccaaagttaaca

gtgctctttgggaaaatggacagtctccgaggatagtcggagcaccaacggcggctttga

cggcgatctcgaaagccatatgcgatttgcgatgaatgaatggaagaaatgcggagagag

aatgtgaattggtgaatggtgtgtcccgcggccgtaatggccactctgcgttgatacca

>fava_dn_rep_c266

gattttgtattaatgaataaattggagaatcatagaaaaaagatcaaacaagcaaatgaa

taatgtaattcagattgaacaaacagcatttgaagttgtttactttccaagaaactttca

aaaaaaaattggaatactttctgaggtactaacaccagttctacatgacaatgattgttg

aatgaaaattacattggcttcacaaatttcatttacttccataactcttctataggaatc

ccacgctcttcggagacctgagtgtactgcttcattttctgcattgcagctacagttgct

ctggcattgttgagtggattgtcacttcctagttgtttccccaaagcattctcaacacct

gccatttcaagaacaattctcacagatccaccagcaataactccagaaccggaaaaagca

ggtctaagcatcacttttgctgctccatactctccgtctgatctatgaggaaaagtgaag

ttcttagtcaagggaactttaataagattcctcctaccattaatagcagctttctgaata

gcagacacaacttccttagccttcccaacaccaataccaacttgtcctttcctatcacca

acaacaacaatagccctaaagctcattcgtttccctcctttaacaaccttactaaccctg

ttcacttgcacaactctttcttgaaacccatctctaagcttctctgtcttccgtcgagta

cgaagcccactcgccttcctcgtctttgaatccatcgcatatacatcatttccaagcaca

ctcaccccactgtacgccgtcccgtaaatatcttcatatgcagcagtaatttcatctttt

tcttcgtctggtccctcgaatatggaaggtggggcgatgaagccttccgggacttcgggt

gggttgaccggaagaatttcttcggagacgaatgagttgtcgtcgaagggggtggctttc

gtggtgaaggagagagtggtggttttggtgaggagagggaatgtcagtttgttggttgtg

gggaggagagagaaacgagaggaagaagaagaggaacggaaagagagagaagagagagaa

ttgagagctgaggaatgtgcagaagccattgaattgaagagatgaagatgattcctcact

ctcagaggaaactattgttgaacggttgttgatgacatcaaatgaaaagatggataaaag

ggatacaaaaattttccagagataaggtttttggttttagataccgc

>fava_dn_rep_c267

aatgaaggttgtnnnnnnnnggatataatcatattttcaataaccaaaccttgtgtaaca

tacatcaggtctcaaagaaggaacaaatgtacaattaccaaccaacattaatcgaggaca

tcgagcagtatgttcaagaattgatgctaacaacttcaattgcaatgcacctgctgcagt

ttatgttccagtaatataataccacatcctagtttcaataccttgcttgggttcacaaga

ctacagatctagggtcggtatttcagtatttcatatgtaatagcaagtgttttcatatgg

tattttaaagcatacctgaacacaaaattctgcaatatgatacgacaattaaaagacacc

tactaataccagagggaaacatcaaagaaactatgtgctatgttttactttcttgacctt

ttcagctcatccagagaatacatttgtagactacagttgtattcaaatataatccaagaa

taattaggaaacccaaattgtaaataataacttgtctccttaacatgctaagattaacca

gaaggtccaaaataacaactctagaagacaacagtgtcaagcatcattccaagagtttga

acgatgttagaagacatcaaattggagacatgctcttcgaggtgcttctttgcatcttgt

ctacccacatttgcaattgcaagcttctcagggggcagttcatcttcctcctggacagcc

ttgttgtatttggtagctaagctcagcatttcctgaacagtctgttcgttagttttggaa

tgtgtatcaaatcgcctaagtgtcaaaccatcggtccatttcttcttgtgaagattaagc

agcatcttctcctcaagttcatttttcctataattgatggctatggaataataatgccgg

ttcaacccatggatcaaagcctgaatggatggtttattcagatgccccagattggatgtt

gtctgccttggttcttgcccaagcatcattgtctgtggattgatcaaccgaaaagcatca

atcaccacttttcctttaacactttgaattggatccacaactacagccactgctcgctga

ttcaaagcctcaaaactctgctgtgtattgatgtcaacaccagacaaccaacatccaaat

ccaggatgtgaatggtaccaaccaacaaccatctcaggtcgtccagtttgtttaagcata

tcaagcatattagtctgaaaaacatgatcaacagcttcaacactaacaccagttccactc

tgcggcattgcaaacacatcaacgacacgaacagtatactcatccacaaattctccaagc

atcaaacccataacctccatgggaacaccagccctaccgtgtttaagcatcttgagaaga

gcgagggatgagatgtagacttgctcggaggaatctagggttggggaatctggaggtgga

tgacctagggctccaccggcgccggcgaacattcgttgaagcctttccatggtgattcgc

aaaatctacgtttggagaagtttcggatttgaaattagaactacgaagtggtttcagtga

gatttttgaaggtggaacaaagagaggagaaagaatatgaactactgctacttgatctc

>fava_dn_rep_c268

aaaccaatttcatttaataataattttctatcaaattgatctattttctgttcaaattct

ggatcattactattaatagaaagaaggagaccatgaatcttatttatcaaaatgtagttt

gttgtgtcagtttcatttggaatgaagggtgaaaagcctttttggatttctccacgaacg

cgtccattcaagaaaggatcatatattttagttaagtattttgttttcctattatcatca

gacaatctaattcgattttcaaatacatccacaggactaaatttaatagatttattatct

atcatttttgccctttgaaaaaattcattgattagtttatttcctttttctttattaaca

gagctccaataattagacaattcattatcattataggaggttttatctcttctgaagaga

tccatttttttttccatgattttcagaaaagtagataaatgaggtggatacgtgaaagat

attcgtttttttccatcactttgacatacctgaaaaaaaaattgtgaattttcatctctt

acaaccctttcaaagtgatcattttttatatatcgcaaaggcctattccattttcgataa

tcgaaaagagtagttacaagagatttttccaacggggcgagttttttgttgaaagtattc

tcttttttatcagaaagataaacagtaatatcttcttcttcttcgttagtttcttccttt

tcccaatctattgcagctttgacttcctccaggtccctgtcatcgtatcgcatcatttcc

tcaatataataatatgggagcggtgttctgcctaaatagtgtgcaaagctaacaaatgaa

aaaacaacaaatatttgccccacataatttcgcaattgtaggagaatgtatttatcaaat

ttcatagttatcttatatttgatcgactttttgtgctgtaaccagactaataccaatcca

atacatttcattaaaaagatgtgacctattaaccacccaacaaaactacttgttaagaat

aacaatttattgttggatcgaaacagataaatgttcattaatcttattaagattgaactt

ggcaaaagaaaggggtttaataactgaaaaaaaaagattgttaaagaatactttaacaat

cctaaaatgacgtattgaatttggattcttgtattcagaatccagtttggatcccgaacc

caaatagtagtgtttgtcatttttgtggacaaaattaaagaaaagatacggtagagttaa

gactgttattgtatgaggtctactcaatgccaaatgcaaaggcgcataatagatcgatat

aaacatcatgagctgtcccgtaataaacccggttgttgctgatattttcgtttcagatcc

ctttcctaagatttgccctcgaagaaggaagagataagacggtcctatggagaatgtagt

cagaaatccataatagaatccgatcacaacggctgaattcagtattttaacgcagaaacc

aacgagataatttccccaaactaagctataaatcataatacctctatgagtgttcgattt

gtttttctttgatatactttgttttgtttttctatgttatttattatatcttctatgtta

atgttattgattatattttctatgttatttattatatcttctatgttaatgttattgatt

atattaaaatcaattcttctgatctaaagaacaaaagaaacatcttcttcttgttaattt

gaaaataattaatgatataaacttcaatacttcatcatttctaaataaataactaatttc

tttatttattcttttctgtgttctttttttttctttgcttcgttcttctatgttattctt

ttctgtgttctttttctttgttcttctatgttattcttttctgtgttagtatctgatatt

tcctatgtttgttaagcatataatataaatatgttaagcatataatataaatatgttaag

catataagtttgttaaggatattcttttctgtgttagtatctgatatttcctatgtttgt

taagcatataatataaatatgttaagcatataagtttgttaaggatattctatgttttat

tatgtgatattattctttctatgtcttagtataatatattattagatatcttattatatg

ataatatagatgggatagaatttgccgttgttcctcagtagctcagtggatagcggtcag

gtttggtgtttggaccatgggatgttaggttatggtggagttatattatgattgaataat

agatgactattatggtaaggctgatgatggtgaggattgaaagtggtaatggtttggtgg

ggttaagttagaaagtttgaaggttagtttgttcaatggagttacacattggtgatggtg

tatatcagtatgttgtgtgggaaaaggtgagttgaaggtttcgtgagtgtgttaatggtt

gggttacatattgtgtttcctacagagagtataagtgaggtattgatagaggttaaagta

gtttgaggactgcaatgtcattttgttatcattttctctcctttgatggtggctgcccca

tttgtgtagaatgtgttggtttatataggctcaggattagggtgagtttgataggaattg

gggggagtttttgagccataaagtggtgaattaaaagtaatttcatactaatttgttgtt

ttttcttgatgcatgaatgtttagctactttggacaacttttggacagcatggttaaagt

ggaaagcaggaggtttttttatcaatattttgtactgatttggcttaaataatgatggaa

tttggagcttgaaatttttgtttagcatgaatgctagtaaatgagttttatacttatgaa

tttgaatgtaacttgtgatttttcttggaggtatcttgaaattgaacaatagaacttgaa

tatgagagatgtaacttgcaatttg

>fava_dn_rep_c269

aataagcggaggaaaagaaactaacaaggattcccttagtaacggcgagcgaaccgggat

aagcccaccatgagaatcggtcgccctcggcgttcgaattgtagtctggagaagcgtcct

cagcggcggaccgggcccaagtcccctggaagggggcgccggagagggtgagagccccgt

tgtgctcggaccctgtcgcaccacgaggcgctgtcggcgagtcgggttgtttgggaatgc

agccccaatcgggcggtaaattccgtccaaggctaaatattggcgagagaccgatagcga

acaagtaccgcgagggaaagatgaaaaggactttgaaaagagagtcaaagagtgcttgaa

attgtcgggagggaagcggatgggggccggcgatgtgtctcggtcggatgtggaacggtt

agtgccggtccgccaatcgactcgggacatcgaccgacgcggattgtgatggtggcccaa

gcctgggttgttgaaatgctcgcggagacgtcatcatcacgattgtggacggcagcgcgc

gctgtttcggcgtgcttcggcacttgcgtgctcctggcgtcggcctgtgggctccccatt

cgacccgtcttgaaacacggaccaaggagtctgacatgtgtgcgagtcaacgggcgagta

aacccgtaaggcgcaaggaagctgattggtgggatcctcttgtgggttgcaccgccgacc

gaccctgatcttttgtgaagggttcgagtgagagcatacctgtcgggacccgaaagatgg

tgaactatgcctgagcggggcgaagccagaggaaactctggtggaggcccgcagcgatac

tgacgtgcaaatcgttcgtatgacttgggtataggggcgaaagactaatcgaaccgtcta

gtagctggttccctccgaagtttccctcaggatagctggagcccgagggcgagttctatc

gggtaaagccaatgattagaggcatcgggggcgcaacgccctcgacctattctcaaactt

taaataggtaggacggtgtggctgctctgttgagccatgccatggaatcgagagctccaa

gtgggccatttttggtaagcagaactggcgatgcgggatgaaccggaagctgggttacgg

tgcccaactgcgcgctaacctagaccccacaaagggtgttggtcgattaagacagcagga

cggtggtcatggaagtcgaaatccgctaaggagtgtgtaacaactcacctgccgaatcaa

ctagccccgaaaatggatggcgctaaagcgcgcgacctatacccggccgtcggggcaagg

gccaggccctgatgagtaggagggcgcggcggtcgctgcaaaacccggggcgcgagcctg

ggcggagcggtcgtcggtgcagatcttggtggtagtagcaaatattcaaatgagaacttt

gaaggccgaagaggggaaaggttccatgtgaacggcacttgcacatgggttagtcgatcc

taagggacgggggaagcccgtctgatagcgctctaagcgcgtacaccgaaagggaatcgg

gttaaaattcctgaaccgggacgtggtggttgacggcaacgttagggagtccggagacgt

tggcgggggccccggaaagagttatcttttctgtttaacagcctgcccaccctggaaacg

gctcagccggaggtagggtccagcggctggaagagcaccgcacgtcgcgtggtgtccggt

gcgcccctggcggcccttgaaaatccggaggaccgagtgccttccatgcccggtcgtact

cataaccgcatcaggtctccaaggtgaacagcctctggtcgatggaacaatgtaggcaag

ggaagtcggcaaaatggatccgtaacctcgggaaaaggattggctctgagggctgggctc

gggggtcccagttccgaacccgtcggctgttggtgaactgcttgagctgctcccgcggcg

agagcgggttgccgcgtgccggtcgggggacggattgggaacgggccctttggggcctct

tccccgggcatcgaacagtcaactcagaactggtacggacaaggggaatccgactgttta

attaaaacaaagcattgcgatggtccctgcggatgttgacgcaatgtgatttctgcccag

tgctctgaatgtcaaagtgaagaaattcaaccaagcgcgggtaaacggcgggagtaacta

tgactctcttaaggtagccaaatgcctcgtcatctaattagtgacgcgcatgaatggatt

aacgagattcccactgtccctgtctactatccagcgaaaccacagccaagggaacgggct

tggcggaatcagcggggaaagaagaccctgttgagcttgactctagtccgactttgtgaa

atgacttgagaggtgtaggataagtgggagctggaaacagcgaaagtgaaataccactac

ttttaacgttattttacttattccgtgaatcggaggcggggcgctgcccctctttttgga

tccaaggccgacttttgttggtcgatccgggcggaagacattgtcaggtggggagtttgg

ctggggcggcacatctgttaaaagataacgcaggtgtcctaagatgagctcaacgagaac

agaaatctcgtgtggaacaaaagggtaaaagctcgtttgattctgatttccagtacgaat

acgaaccgtgaaagcgtggcctatcgatcctttagaccttcggaatttgaagctagaggt

gtcagaaaagttaccacagggataactggcttgtggcagccaagcgttcatagcgacgtt

gctttttgatccttcgatgtcggctcttcctatcattgtgaagcagaattcaccaagtgt

tggattgttcacccaccaatagggaacgtgagctgggtttagaccgtcgtgagacaggtt

agttttaccctactgatgacagtgtcgcaatagtaattcaacctagtacgagaggaaccg

ttgattcgcacaattggtcatcgcgcttggttgaaaagccagtggcgcgaagctaccgtg

cgttggattatgactgaacgcctctaagtcagaatccgggctagaagcgatgcgtgcgcc

cgtcgttcgcttgccgaccagcagtagggggctttggccccccagaggcacgtgccgttg

gtgtaccccgtaaggtggatgagccttgcgggacactttgaaacgcaattcctattgagc

ggcgggtagaatcctttgcagacgacttaaatacgcgacggggtattgtaagtggcagag

tggccttgctgccacgatccactgagattcagcccttgtcgcttcgattcgtactggaaa

tcagaatcaaacgagcttttacccttttgttccacacgagatttctgttctcgttgagct

catcttaggacacctgcgttatcttttaacagatgtgccgccccagccaaactccccacc

tgacaatgtcttccgcccggatcgaccaacaaaagtcggccttggatccaaaaagagggg

cagcgccccgcctccgattcacggaataagtaaaataacgttaaaagtagtggtatttca

ctttcgctgtttccagctcccacttatcctacacctctcaagtcatttcacaaagtcgga

ctagagtcaagctcaacagggtcttctttccccgctgattccgccaagcccgttcccttg

gctggtttcgctggatagtagacagggacagtgggaatctcgttaatccattcatgcgcg

tcactaattagatgacgaggcatttggctaccttaagagagtcatagttactcccgccgt

ttacccgcgcttggttgaatttcttcactttgacattcagagcactgggcagaaatcaca

ttgcgtcaacatccgcagggaccatcgcaatgctttgttttaattaaacagtcggattcc

ccttgtccgtaccagttctgagttgactgttcgatgcccggggaagaggcccccaagggc

ccgttcccaatccgtcccccgaccggcacgcggcaaccc

>fava_dn_rep_c270

catggttggactgtgagatacaaaccggaggatgcagaagaagtgaaggctgcagaggag

gcacatcaggatttgctggactggtttgctaaacatctcaagtgagaaatctttgccagt

tgatattgtctgtatctatgtgtgtacttgannnnnnnnatgcagcatgctgcacattcc

acaaagcttcagtttaccacaaaataaatgctgtattgtttcgtattggacacgaattat

agataagttcagcagcgtgtatagtaaaaaaatgtaatgtttcttcttgtggacagatgt

aatttgtggtatcaacgcagagtgccattacggccgcgggagacctcaggtcaggcgggg

ctacccgctgaatttaagcatatcaataagcggaaaaaaaaaaagcgggagaggacagga

tagtagagtgcaggatcggatcggattttataagagatactgtgacatttttggaattgg

ttacggagataatcttgactgttactctcagaggccattcggatcttcactctctttttc

atccaccctgctcgactccatcgccgccgcagcctagattgcctttttctttcttttaaa

ttaataaataaatccgaatcactgttcactcatctatatgtgagtgtgacacgtgtataa

ccaccttattcgcttcccatctatatgtactaccatcaactttactcatgcgcacatttc

aaatcaaataaataacactatccttatcttctaaaaaaaaaaagatgtctaaccaaaaaa

tcaatatacaaagcatcaaaatagaaagagtcaaggtcttgctagcaccgaacaccatgg

atactaatttcccaatctcatgataatatgaccagtccaaggtacaaagatgcgcaaaga

aatacaaatgatatcatcccccaaataactccccacccaaaaccattacaagggcatgga

cagacttctgcaaacttctcagcatcactagagttgactcttctagatattaaatcatca

taaatatcataaactgaaaacaaactattcatcacaccaatgaagagaatgacatagcga

aggatatggactttggttttctcttgcagaagccaaatcacagcaataaaaataataaat

ccaatacagagtcctcggagtgtccaatttttagcaacaaagagcacaataaccagagca

gcaaggaagcaaccagcagcaatccttgcagtgagaatgtttgtggaagcaagaatcaaa

gccattccccaaaatgacgaaccaagatatccagcaggcaagataatccagtatattcca

ccacgcgtctgtgttaccccgccctcgtttccatgaacctggataccctccaccttgcca

caagtaagcaaacaggcaatcgcatgactcgcttcatgcagaaacacagtaataagctta

aacggcgtaagcagaaaagtcctccaaagcaggaggatgacaacggtgtaaacaccaacg

aaagcaatgaagagcttctgatcgtgatcacagcagttcttaagctcccagtttgccatt

gtttcctgtgattgaactgcagagattggaagaactcaaactttgaagaacaaattgaga

atgatttttgagttggtggaaaaggtaagagaaaatagcaaagaaagataatttttgctt

ccc

>fava_dn_rep_c271

agcatgaaaatgatttgattcatcacagagttggagccctctcaacatttccagcaatga

cagtgagcaaattgttgccaaaagggtcactgagatgtttcgcgaaattctcaactggtc

cttctcctgtgacataggcttgaatgaagaaaccaagcatggcaaacatagcgagtctgc

cattcttaatctccttcacttttaggattgcagcttggtcgggatcgtttgctagaccta

atgggtcgaatggaccacctggatgaagcttgtcttccaagtccagtccattggtgattc

tgtagtactcagcacctcccaagagaacaacctcagcaacaactgcgagaataaggttga

tggggattggtttaccgaagtagttcaacgtgcctccgtcaagaagaagtgctccggtct

tgaaccaaacagcctcgggaccacagttggctccatatttgttgaatgcctcgggaatga

tgaatccggcagcaccaagcattgcccatcttgcatgaatcaattcatatccctgatatt

tggcaaagtcttcaggcttcttgcttagaccaaaaggatcataaccatagtctccgggga

cttctccggtcaagtactcgggaatctccgacctgtccaagagaccgtcaggcaagaaga

tccttctgtcaggaccataccacttggcgagttcctcgttggtcggcgcaaccaccttct

gcttcgccggagctgccttcttcttcgagaaaagagcaacagtcttaaaactagaaacac

ttgaggaagttgaagcagaccttgttgcaccactgaatttaatttgacttccaagcattt

ctgacattccaagtgaagcagctgcagttgaagcagcaatggaagccatgtttttggaat

cttcaactcagatgaatcaatgtgttgctgaattcacaatcctccatcataccatcttta

cccaaatcatcagcaccagcaggtggggttgcaaatccaagacccttaacagcctcttta

gtttgctcccttaacttattaagagcctcagaaacatgagcatctttagaaaccttctgt

ttcccttttctcttgcttctcaaatcaggtaaccctaatcccaatccttgaacccctgaa

ggtaaagaaggagactcttttgtgttcctactttgttctccggttcttggaagggaagaa

tttaggttgagggtttggaaatcatccaaagcactgtcaaggagttggttgaggtcgtcg

gactggtcgaccgtggttgtctgtcaccgccagacagactcttccttgttggaacgaaca

aaaattgggacagcctcaatgtcccgcgaactgtcacagctctgcatcctaccactgcta

atgctactgcttcccatgctacaccacaaccctcgaagaatttcaacaacaatcacaatc

acaaatgatgctcaactttccatattttctaagaatatattttttgtggctatttagatt

ttgtatgtttcttttggtggtttgattcatttgtttatgattggtaaatctcatttgtct

tgtgcatagttagtttatatctcttgtattgtaatttgtaagcttacctctagggggtta

actgtaatcaatacaaactttagtgtgatccaatgaaggataaaatattttaacagggac

aattctgttttcttc

>fava_dn_rep_c272

tttagaaaaacacataaatatgagcttaataatattattttgaatcaagttgaaataaga

caattttgaaatgtctcaactcacaactgcacgccatagataagcacgagtttatacagt

gaaaaaacaaggtgcgtcccaatcccaaccaaacagatttagttagttagacattcattc

accacaatcacattcagttttttgattcaatatataagtccaaaactacttatcaagcac

agaattaataaataatgacacaatgaaatcgttaaaaccacaagtcagcatcatgattat

catacaagtaatttcttcttgaataatcttcttcccaccaacaacttgtaaaatgttatc

ttcaaccacaacatgctgacttttgtgcagcctgtccagctcccgctgcagagtcttggt

tgattttgattgttgtcggctcggccttgttgtcagtgtcagcaagcctttgttttatgt

ctcttgctatggaaaagaaaacttcctccacatttagatttgtctttgcactagtttcaa

agaatttgattccatattcatcagccagtgcttggcctttggacgttggcacagcccttt

tgctttcgtccatgtctgctttgttcccaaccagtattttgttaacattatctgaagcat

gttgctcaatgttgcgaatccaattcctgatattgttaaacgatgcttcatctgtaacat

catagactagcaatatccccatagcaccacgatagtaagcggtcgtaattgtccgaaacc

gctcctgccctgcagtatcccagatttggagcttgatccttttggcatcaagctcaatgg

ttcttatcttaaaatcaatgccaatagttgtgatgaaactggttgtgaaagagccatcag

aaaaacgcaaaagaagacaactctttccaacaccgctatcgccgatcaagagaagcttga

tgaggtaatcgtaatcggcacgagcccttgccggtggagcagccatgaaaacggaagaat

gtaacgaaaatggtgttgtttttttctgtgtgaaaaaattgaaaaatggtgtgagagaga

gaagtggatctcctccaaagttctaagagtaaaggaacatggagattttagagagaaaat

aacggtgcttattttttggaaaaataaaaataaaaataaaaataaaagtaattaagattg

acgagaagagaagacaaaacagaggtgattacaacctt

>fava_dn_rep_c273

tgggcccaaaacagttaacccttccttacatttccgttttaacaatatgagggagtcgtt

ttgatggtaatcgtgttggtgctgccgagtagagtgcgacgacatgtatttctctcttca

actcaatttttagggttccatttcattcaaatttcgttatcaacagccgcacaagattag

attagagtgtatcactatggaaattacaaactccaatgtctccgacattcaaattccgcc

ggaaaaccaacccgacgccggcgccaaaatagcgcaatccgtgaacaaactcaacgaatt

gtcgattttcatacaagctttcaaaaacagatatgacgaactgcaaaagcatcttgattt

catcgaccaagccatctccacaaggatcagtgaactcgaagccctaggcgctaccgccgc

cgcagaagcagtcattgttcaattggaatcaaagaccgatgacaaaggagccgaacaaca

acaatcagaagaagatccagaagaagagccagaagaggaaaaagcaaaagaagaggaaaa

aaaggaagaaaaggaagaaggtgatgagtttgtcttgctttgcaaaacgatgaataaccg

tggtttgcgtaggtatattctcacgcatctctctgaaacggcgttgctcaaggaacaaat

ccctgcggctctgaaagtcgcgcagaacccggcgaaattggtgtttgaatgtattggaag

attttaccttcagggaagcaaagcttatactgtaaactcgccgatgattacggcaaggcg

agcttcggttttggttttggaatactatctgatatctggttgtattgtaagtgaagcaca

aatggaacgttcactgaaggaagaagctgaagctgcagcggttgcatggaggaagagact

cattgttgaaggtggtgtgggtgctgccggtgaaatggatgcaaggggtttgattctttt

ccttgcgtgctttgggattcctggtatttttaggaatgaggatattgcaaatttggttcg

attgagcaaaccttgggaaatgaagaacaatataaaaccatctcagttcttctgtacatg

aaaatagattaaggattcttaacaatcatgcccaaatgtctttcaaaatacacccattca

ggattcacacgagaagaacaatataggctttcatgtcattggacaataataatacaaaca

ttttagtttccacccagtgtttgaacaatggtgttgtgccatggatcagaaatgtgctgc

aacaggttatcaaatggtccttttcctgtcacattgtgctgaattataaatcccaagaat

gccaacatcgccaatctcccatttgcaatttccttctctttagcctccaaagttggtgca

aagttcaaaggattaaagatcccaccagggtaaccgacctcaccttttggcaagctgtat

tgcttaaagataggatcttggttaacactgccagggttcttaatgtcttgccatcttcta

atctcaacgtaatggaacaaaatgaactcaatcacaaagagtgttgaagatgaagcaaag

tattcttctttccctgcatcgtaccattttggaacattgatgattccaatgcttgtgaat

acctcagggagtaacatccctgcaacacccaacatagcccaacgcccgttcactagctca

gcttgaacaaaccacctcaaattctctggatcctccgcgagtcccaatggatcaaatcca

ttgtctccgggaagactgccagtgaggtaacctggggaggccaaacctggcaaccactct

cctttcttagcttcaactttaaaagaagcagaaggtgaacacccaactgacctaaaagcc

acttctctgttcaacttccctgaagaaccaccaagaaacctcgatttcaaaccacatggc

cggaaaaccgcggcggaagcttgtgtagtaacggtagccatctaaaaccactagtggtta

tggtggtgacaaagaaaaaaaagagtgaaaaatggaagatgaaaatgttgttgctgttgt

tggttttgtttttagcatgggttgttgtttcatatgtatttagcttcggaaccattcatg

tggttgttgttttctgtttatgatctaccgttggtttgcgcgatatttgggatctaa

>fava_dn_rep_c274

cagttgaaaccgaaggaataaaaaaaccctactctcttctaacgctcttccccgtcaccg

ccacaacacttcccttactcaccaccatgtcggacgaagagcatcactttgaatccaagg

ccgatgccggtgcctccaaaacctacccccagcaggccggtaccatccgtaaaaatggtc

acatcgtcatcaagaatcgtccatgcaaagttgtggaagtttccacatctaagactggaa

agcatggtcatgcaaagtgtcattttgttgctattgatatttttactggcaagaaacttg

aagatattgtgccttcttctcataattgtgatgttccccatgtgaatcgtactgattacc

agctcattgatatttctgaggatggatttgttagtctgcttactgaaaatggcaacacta

aggatgatctcaagcttccaaccgatgacagtctgctcactcagatcaaggatggatttg

ctgagggaaaggaccttgtggtgtctgtgatgtctgctatgggtgaggaacagatctgcg

ccttgaaggatattggtcccaagaactagatttctgttatttggttgtttcatttaaggt

tttatttgtttttgtcaaagatttccatatagattcttgttgatttagtggtgtttatgg

attttgcttggtttatttatttttgctatgttatcatgtgcttttattataaacaagggt

ttttactatattgcttgtttatgttataatctttactgttagaatagtctgcagtaatgt

agaaaacttgtgtatttctcactgcaagcgtgagatgaacctattgtgcgacacacaatc

acaagatttcttattgtctcttcagtttctccttgaaaataaaataaacctattgtgcga

cacacgacacacaatc

>fava_dn_rep_c275

agaggaaaaaaaatcaattctagatgaaaaatacaaatgcaaaaaaacagcagttgccat

catgtgcagtttgagatttctattagtcttggaacatcttaaaaaccaaaatatgcaatc

aaccccaaacaaaaggaaagttcgaaagatctgaaaacaatatattaaaaataagtgcct

ttctacgatgtcactcaatcattatattgaatctcataaaacaaacaggtgtactaatat

caaaataaattcaccagaaaattcaaaattttatcactaactaatgcctaagctgcagtc

ttcttcagcttagcaagctcttgcctgacaccagcagccctcttgatctttgccaacatt

atcttgaacctatcaaaatcattgagtgctgctctagtcttcttcacaatcagttttcta

ccccatgagctctttgcccacttgttctgaacatcagcagcctccaaagctttgacgaga

tccttcttcttgggaaccctcttaatatcaatcttgagatcagtgagtgaaagcctcttg

aaatttataggggacctctccatatcaggagcatcaacaagagctctggtttggtcgatg

acatcaacgataacgacgagtctaccatagtctttaccgtagttgatcaaagcaaccctc

ccaatctcaacgaacctcttgaaaggcattttttctagggtttctgagattcaagcttca

agcacggagagcccgcgg

>fava_dn_rep_c276

cgttcattgtttaaaatttccacgtcaatttatttatttttaatattagacatcnnnnnn

nnnnatgaagcatatgtttggattttgagaaaattctgcaaatctacataagcttgcatt

tgtggtttaaaaaaatgccaatacattatgcgcaaaagaacgaaatattctgtagaaatg

tactcgtatttgtgtagaggagcatttctgatttgtcttgagaatacaaatttctcaaca

ttataatcaaatggttttgattacttcacaattgatggaggaattagtttttttttttaa

aaacataaaaagcaaatttattgaagacatcggtaactagttaaatctaaaaacataaaa

taagggtgcatctgataaaaactatacaggagcgtaaatgagtatattaagtaagcaaaa

tatggaaaaaagatagctccttagttccaggagattaagacattggttaaatttaaagct

tataagattttttctatggtggatgttagtgttgacttgggcacagctccaacaacagtg

tctttcttttcgccgtctttaaagatcataacagttgggatacttctaattccgtagcgg

gtagcagttgaaggactctcatctgtattgagtttgtaacacttgagtttcccagcatat

tgttttgctagttcatcaagtatagggtggatcattctgcagggaccacaccatggagcc

cagaactcaaccaaaacgggtgaatcagattcaagtacaagtgattgccaatttgcttcc

gttataggagccacatccacagcggtatcctgggcctcgcacactacacgtgaagtgcgg

gtagcgtttctgaaagcaggcgaagcagtgaaaatgggcctgaatttgggattgggcttc

agttttagggccacgtaacgtgagaacttgagagaactccgggtgacagaagaagggtga

ggaacgatggccatggattctatgacggtggccatggagtgcggctggtgttctgatgaa

ggtgaagagagttggatattgttatgtggatgaggatggggtgagagtgtttgttgtccg

gttgcattttcatgttactgtcatatcagctccaccaatgatttcacgccatgtcaccac

tgcttctaaccaataacatcttttccnnnnnnnnnnnnnnnnnnnnnnnnnnnnnnnctt

taattttacattcttaaatgccattt

>fava_dn_rep_c277

gcaaatttttatgtgtttgtataaggtaacctatatctttaaaaataggttcataacaaa

taataaaccatcaaccaaacaaaaggaaaatcatatatcagtcaaccaaataaaagcatt

taaatgttcagctgaataatcaaaattggtcactgctggtactcaatctcaacacataaa

agttaaaatacaaaaaacaactagaaagctataagactataaaatagcctcaagaaatac

caacagcaaaataactttaatttcataacaaaatatctgaaagcttatttcttctttcca

gccttggcagcatcaccagccttggtcttcttaactccgcgaatcttcttggccctgttc

tttctttccttcatttgcttccttgacttctcaaccttggtatcaagcccattcctgata

agtctgtattttggttcatacttcttagcattctcaacagaatcatatatcaaaccaaaa

ccagtagacttgccgcctccaaaatgagttctgaacttaaagacgaaaacagtgtttggg

tctttcacgtcataaattcttgccaacttctctttcaactcagccttggagacattggcc

ctccctggatgaagaacatcaatgacaaattgcttccttgagagaagcctgttagtcatg

aacttcctggttctgatggtgactgccttgtccgccatgttgttgttcagcgccggttac

aatctaaacctaggaggcggcaatttcagacgcagcgcacaagactaaggttgaagcccg

cgg

>fava_dn_rep_c278

taagttacaatcatcataaacttgaattggacattttttgctcattttacaatcatgatg

aactactgatgacaattgtactttgctcatgacatgttttatctattacaatcatgataa

attagtttcaaaacatcaactgataaatgcaggttttgaaattattacagataccagaac

atgataagttatcaaattaataacaatggattttagtatctaaaacatatggattcataa

attcaaactcatacagtacagtcaagcagactggaatttacaacagtaactgcttaaagc

ttaagcccatcatcggtctccagaatccaaattcatattactggaggtacatttttgtgg

ctagatgggttttgtaacttataaagcaaggaaggataattgattaaaagtaaagggttt

tgatgagaacatcaataacttgattaaactatcagtgtgctcggtccctcagcacttcaa

gatccatttctgtgggatcagtagcctggatttcataatggacaccatctagctcacgtc

taaacctttctttagttgtggcatagagcatcttggcgcggattcgagaagttgaaggag

accatgcaatgaaaaagattttgctcttttgacaattatcagaagtaacaaaatcataat

caaacacagcatatctgcaatcattgtcaggcaaagaagcagcaaaatcatcataactct

cagcaggaccaccagtcttctcaactacaacctctcttttcttctcatcaaccttaaaga

taacataacgatgaaccttcttttgcttcaattccaagaacgtgctcttgctgttctcat

caacacccatgccagatgaagcatttggccggctgagacctctgaaagacatggaaaatt

gagttgaaagaagagaaggagtgaaattgagtgttgtattttagggaagagaggttttat

aaataaaatagttaagaaaaaataaaattgataagagtggggcggtgcgttgaggcaacg

ctaacgaaaacagaatgaatt

>fava_dn_rep_c279

caaaaatttatctacataaaatttcatattattatgtaatgagcataaaagagaggcatt

aagagagtcaacttataagtggttgcatggtagatccataatttaagggtctttatagat

cagaaccaatttccaaaccatgaagaaagacaaataatgtccaaaccatattgcatgtat

gacatatatcaaatattttacacaaaggacacataattttgtctaacggttcacaatctg

taaataaacaagcatattcgtccctttagtcactgtagaattccacagattcatcttcat

agaattcttcgtcaacatcatcatcccactcgtcatatagaagagatcttagctgttgtg

ccttcagctgttcttgaattctgtctctgattgccgttcccattttgtggcatccttctt

caaacatctcgaggaatccagcaactaatcgatcagcattttcgacccattcagtacggt

tcatgccagcaatttttttcactgttcctatcttctttcccacagtctcttgctgtttct

tcactctctcctgcaactttttaagtcccatgttcattctcaatcttttctccttgacat

agctaacacctaggtcttttcttgaataaccacgatccaaattacgcattacatactggt

tataatctttgatgattctcattatgatatcagaagtagaaattccttcagttcgctgtg

tttccttaaattttccaatagccttaacaaattcatagacatcccttccagctccacttg

tatcagcataaggaagagaatcatgagccacgtagtcaatttgatgcttgtcaatgaact

cctgattgattacccatggcacatctggaataacttcgtcaacccacttgcagtggcgaa

gagactcgtagcgttcggcctcagtcataacagtttttcccttatacttatgagtggtct

catcattgcagcatccaacaaggagataagtgtttggaaacgatttcttggcttgttcaa

gagaacgggcatgaccgaagtgaaagagatcgtaaattccatcggcataaactcgaacag

gtttatcgttttggtgaattgcacactccttgtgatcgttcatgatcactctgcttgtgt

gtttgtgctgttgcaatgtgcagagtcaaaacggaattcaaaacccagtcacacggtttt

ctttcctgtttttgttttttcctattttactttatcatttttttttttgcttttttttta

attatagtttcatcatttcatgttttgctatatttatttaca

>fava_dn_rep_c280

aaatgtaacctttcaaatttgagtttcaaggaaaaaaagtttctcttataatctcataaa

taacatttatcaaaatatgacatctacaacaatcctataaaccaaacctgaaatcctagt

aacaataaaatatgatataaatccattgacaaatatgtgtagcaactaaattcatgtaaa

aattcttcaggatcgacaaaaaccgtttcaagacttgcacacggaagcataaagccagaa

acaaaaaccataagctcttttctaattgataaagaagttggtaaaagcagaaatgtttca

gaccagtggaattttatttagattgatgttcaactcaaccagagtagatatcattcatag

tgcaaggataccattaaacatgtccaaggaaatagaggatactcaactcatactaataag

ttaccatgggaaagactaaaaaagttttcattgtgcccttaagacctcaatctcaaactc

caagacagagttgggctgtattccccaggcaggaaaaccaccagcaccataggcataatc

aggagagcaccgaagacgagcaacttcgccgagttgcatgccaaggacaccttcatccca

tcctttgatgacagaaccttggccgattttgaaagtgaatggattctggccaggatcctt

cgtgctccagaacttctgagataggtcaccgtttttaccgtaaccggtgcagtgaacggt

gacgttctggccgcggctagggttgggaccggttccagctcttatgatttgcttctcaac

acccatttttcttcttcgccaacgaatcacgcttctgcttttcttttcttttattcctcc

acataatacaaaatggcttttcttttcttttattcctccaaataatacaaaatggctttt

ctttttaatattccgttttttaaattaaaagata

>fava_dn_rep_c281

cattgatgagtgagaaatacgaatggccacggtttcatcatcacttaattgaaaacaaaa

tagaaagagataacagatactacaatcttgcaattccattgttcattacagaaacttcac

catcaaatccaatccatggcacttgctcaagctccaccttctgcttctctttccgtctct

cttcgcgatgctcgtgttttcgccacaagaaactcccctgcagcctctgcgaagttttct

aaatccagtttggctccagttcgagcatccttcgtcactggctcccctctaatgattagg

aaaaatcatggaaggaagtcagcatatagagcaatgccagtttcgataagatgtgaacaa

aacactaaggatgggaacggtttggatgtatggttaggtcgggtagccatggttggcttt

gctgttgccattactgttgaaattacatctggaaaaggacttctagagaactttggactc

acaagcccactgcctacagttgccctggccgtaacaggactggtgggtgtcttgacagca

gttttcatcttccagtcagcctcagagaattgatcacaaatttagactgattctgtaaat

tctcaagttccaaatgatatttcatttttcaagtaacatttttattagtattttgttata

atatttgtattggtgaaaataatttgcataattgatccgaaaaggttttagtaatgagag

ctttagatggctgaatccaaagccggtgttcaaaaaaaaaaaagtaaaaacaatgaaagt

tctgtctgtaatttgtaattgtaatcttaatttcgatattattagtgtttttttcttgct

ctaaattaaataattttttaattcatgagctagattagatgtatctgtttttcttaatat

ttctcttaatcatgcattaactttaattgaaa

>fava_dn_rep_c282

cttttttttttgacaatttaaaatattgtgttgaactttaaccaatcaaacaaaatactt

ataaaacaaaacaaaatatagcaggtatacactcacccactaaaccattataaattcagg

tataatccaaaaaaaaatttttagaaattcaaacaaaatcttattgccacaatagctcag

aatacataaatgaataattcgcattacaaaaagactaaaaacacatgaacaagtggatgt

cgacttcctttttctctttgttcaaagctaagaacaatcttcaagattcaatatagccaa

aatcttctcctaatcatcaaatgtgctcatgaatccaccaataaagccagctccattaca

gcttccacacaaaatatcctttttacctctgcatagccagcataatccaccagctttaaa

tcgtccgttgtagtgatctatcaaattaactccaccaccttcgcattgagtacataatat

ggcaccatttccatcacaatcaggacaaacaatgctccttgtttttgtagctggagtact

atctcctgtagccttaacttccaaagactcaaagtttctaactttagaattcagacaaac

atccttcaccgaaaaagcctttcgggtaactgaattaccaataagaactccaggtgtgtt

tgaagatttcaaggaacagataggagcaaaagacaaagagtgcgccattatcagaagggt

agtgaagctttgtctctgaactgaactgaacagaaggagtaagtaaactgttgtaataaa

ggagttgtggaaaagataagacccttgaatgaatatgaatccgt

>fava_dn_rep_c283

aattctaattcggccgcggaatctttcagagaatcagtgaagaagaggtttcaatttcaa

tggcggtttcttctctctctacaacctttgttccttctctttctctctcctccaaaccaa

acgtccaatctccatgctttctctccggtaacactttcaggcttccgaaccccaaacctc

ttcattcatcaaccgtggtttatgctgctccggaagtgttgaattccgctgaaaccaaag

atgttgagtctggctctcaggttgatgacttggaaactccaagcacttcgaaaattagca

ttggagctgatgcagacatgatggcaccaaagctaagaatcagaatcaagcttagatctt

actttgtgcccttgatagaggattcttgcaagcagatattggatgcagcaagaactacca

atgcaaaaacaatgggtcctgttcctttaccaaccaagaagctagttttttgtactctta

ggtctccacacgtacacaaagatgctcggttccatttcgagatcagaactcatcagcgtc

tcattgatattctctttcctactgctcagacaatagattctcttatgcaactggatcttc

ctgctggtgttgatgtcgaggtcaagctttagagatatttgcttcgggttgataagcatc

ttgattatgagtgaacttcaacttgctagtaatagcaaaagaggggataattggcagata

tcatgtatttcagtctctgaacttatttctttgaacaatgcaaatgatccttttggttgc

tttagatttttagaaagttcactccttttgttgcatagatatttgcatattctattcaac

aatgtaatgtaagttttgtattacgatgtgttattcttctaaattatgcacatgaattta

tatttactgatctaaaaaaaaaaagtctgtagcaaacgttttcctaatgatacatccaaa

attgtttgggtttttttgctatttttaactttttgttattgtcccttttattgcatcggt

tttgccaaataannnnnntatgtttcg

>fava_dn_rep_c284

tttttttttttgacaatcaacaaccttcttcaataaataaatacaactagttagttacat

tattctaaatacaactagttagttacgtgtctgtatacaaacacacctataataaggtgc

agagcttaaagccccataaaatggaactgaactagctttgcgtgccaagagcttgatctt

accaagtgaataaccttgctttcctcttgtagaagttgatattagaagctattttctttt

ttcttatctttttttttttttaaacggaacttaacttatttacattcatacaatagtaat

aagtcaagtacacagtgtcatgaatacgaactaaaatcagagatacaacataacaactgt

tatggagaaaattgcaccaacatctgtctgcaatacattcctttattcacatacaaaata

gtaaagttctcatcatccatattcatttattccaggttctaacattcataacacttcatg

catcaagcaacttcaacaagtccttctcaatgctaagaggtgatgttgtgggtgcataac

gctctacaacattgccatttttatcaacaaggaacttggagaagttccatttgataccgt

ccccaaagagtccacctttgcttgactttagatacttgtagattggagcagcattggcac

catttacatcaaccttgtcaaaaacgggaaactcagctttgaagcgagtacagacaaagt

cttgtatctcttcaacagatccaggctcctgtgccccaaactgattgcatgggaatgcaa

gaatttccaaacctttctgtttgtatttctcatacaactgactcagctctgtgtaattgg

agttagtcaagccacattgtgatgcaacattgacaatgataaggacctttcccttgtagt

caccaagattaacatccttacccttagcatccttaacggtgaaatcgtaaattgattggg

ggtttgatggagaagccatggtgtgatttgttcttagagcgaagaacgaaggggttagag

gagtagtgaaaattggtttaggtttagaaaaagatttgtttggaagagaagaaactgaga

ttggacaatttgtggagaaaaagtgaaaggaagaagaagaagcagctaaaggtagttttg

ttattgttgaaaagaggtttcgagttgaagtacaaaacattgggatttgagaagaaaatt

gatattgaatttttatagtgtgaactgattgatatctagaacttgggtggtccccactcg

gttgaatgccaccgacagagttgtgaatgtgaa

>fava_dn_rep_c285

catgatccaacggacactaatatttcaaatgcatgaatgcgtgcgtagttgtttgactac

aacgaattcgatttcaataatcaccgatttaaaaaattactaaattacaatttcattttt

atgcattatcccttcctcataaaacacgcctttcacttccattcattcccccaaataatt

caaatatgcagtttaatttggtggccaggcaacacgcccaaaatttcaaagtaaaacata

tttcacaacccactcactcataaagtacttcgtctgtaaaatacatacataacaaagcat

taaatcattagccaagaaaccagttaagagagggtgaaagctaatcatcatcctcgagaa

cacttcgacgcctctgtattgtaacttttgtttgctttgctgtttggctgttaatctgct

ccagcagagttatgagcctttcttcagtaaccttttcagctatctgacccatttgagcag

ctctcaatataacatcttcaacaccttttgctttctctggtttcaccaaagcaattcgag

caagtctttctcgtgcctcagctgacagtatctgactaagcatcatttgtcttctttccc

ctgcctccctctttgcatcctcctgagcgctctgctgttcagggttgggttgatttcccg

cgccatgcagacccataagctcctgcatccttctctgtctgattgcttccagctcaggat

cagccatttggaagggttaacagaagaaagataccctagcgggtgtgtgaatgggggttt

tggttttgcctttgatgtttctgcagcagcaaaagcaccaccacagatcgcgtttggaat

caagcgcttgtttacgaaccaaaaactgtatttcaataaattccc

>fava_dn_rep_c286

agaaaaataaaattgcattttcaatgcatattatagttagcctcatcctttattctcaac

ttccacccatataccaatcgtatgagcatttaatttaattccatcaacacaaatgcatat

atctctatgaaactcataacatataccagaaattagtgtacaatggaagagattatgggc

tatctaatctcaaaatacaaatttgtgaaagtgaaaaacaatggtctacatagttcagga

cagcatttccctcgactttccttcgcagaccagctgtgacagcaaccttttttgtaccaa

ggaaggactgcagtatgaaccatattttttttttttttcccactaaataatatcataatt

tattctcgtgcgttgcatgtgtaaagacattaggataattctacacaactcaatttattt

tacatattttattcaatggttataatacatcataactggattatcatttggataaactta

tagcatacaattttcttttaacacatgtcatgttcttatttattcaatcattatacttgg

tagcacttggcctaggttcaaactcatcattcacggatgatttcatatttttatgaccct

catcaccatcatactttgtagcacttggtcttggttcaaactcaccattcactggtagtt

tcatagttttataactctcatcaccatcatattttgtgagacttggtatgggctcaaact

caccgttcaccggtagtttcatatttttataacccccatcaccatcatattttgtaacac

ttggtatgggttcaaattcttcagtgaccacttttttttctttgaaatttagtatttttg

gtatgggatgctgatcacacttacctttggcatgttgttcttgcgccttggagtttgtta

agggtgcaatctcagatttgagttgaagaagcccctgaattccttctggcatctcttcat

ctttcataacatttttccaatattcttttagatcttttcttgattccgtagtagccacaa

acaagaaaaggatgagaagaggaagcagagcaagagcgtgcctcatattcaaagtaaaat

gaatctgatttctcttcttttatgtctctattgatttatgagatgcccgcggcgnnnnnn

nnactccatatttatagactctgtaacag

>fava_dn_rep_c287

ccactcaccaccacctcacacattttttgtgaaatttgaggaacaagtcaagtgtcctgt

ctgtgtccatacgcctcctcacctcaaccattctacccggaaccttctccctctctctct

cactcattctctgacagctaaaatgcaaggagcattgcgatcatttttgtcgaatggaaa

cgtcataaaaaatgctgttttgcaacgagtccgcatggtgaatcccctgttgcagccagc

ttccttttcacggtttgaatctgttacacctgctcgtattgaggagcatggttttgagag

caccacaatttcagacatcttgcaaggcaaaggtaaaggcgcagatggttcctggctttg

gtgcactacggacgacactgtctatgaggctgtgaaatcgatgacgcaaaacaacgtcgg

agctttggtggtcgtgaaaccaggcgaggagaaatcaattgctggaattataacagaaag

agattacctgaggaagatcattgtgcagggaaggtcatccaagtccaccaaggtcggcga

tattatgactgaagagaacaaacttatcacagtcacccctgataccaaagttcttcgagc

aatgcagctgatgacagataacaggatcagacacatccctgtcataagcgacaaagacat

gctaggtatggtgtccattggagacgtcgttcgtgctgtcgtgggcgagcatcgtcaaga

gcttgaccgcttgaatgcttacatacaaggggggtattagtctattgttagacgacgata

aaaatccccggaacaacactcaagctgactcgaatcatgtaataagctcgagtgtatgta

aatatgttttggtcagagagccatctttgtctgtttcgttgtaatttcgggtttcagttc

tatggaacattaatctttccataatccatggagtagcatatgttaataatataactactg

gatgcatcttgtttctgttgcttaactagtaaaaagatgcaatgatctttaactttcaaa

aaaaaaaagnnnnnnaactaaactaaatgacaaaaaatattccccc

>fava_dn_rep_c288

ggataagaaaacacgacgaagaagaacactgattattattaatattggtacaaagcaggc

acaagtaaaatcagaaatacaattccgatttgcacgaaatacacatataattcatttgtt

tttgaaactcactttcaaaaatttatatatattactaacatgcacaatgttagctgtcaa

aagtggaacatgcacaaaattaacaagtctaaaagtgagatggaatgtgttgcaccatca

taagtatattttcggaaatttccgaaagtgcatttccgaaataaattttgttgtaaaatg

agacgaatctcaaaaacaaattaattatatgtgtgtggagtgcgtaccaaaaatacattt

ctgattttcagatgtatttttgaaatttcgcgtggtgcgctagaaaactatgagaggtta

agaaatcccctttaataaaacaaaatatattactcgttccctgggtactgccgtttacat

ataaacggggaggatcatggttcaaaataatcaacagatgcaggtgaatagttatcaaca

tgacataggaaagctaagacaaaggcagttcagtcccatttattttaaatcagaaattca

cattttaactttctccctcccaggaaaggatgcagagaaaagactaagaaactcctgaag

ataaaacgcatgaacaatacaaacagtaaatcactgttcagcagcctttggcggaccctt

ccatttaaagttgtcagcatataacttaaccttcagcaatggtgtgcggtgcttcttgac

cgcatattcccaaccatgtctctctgcaaattcctttgctgcttcttcagtatcaaaatc

caaggcagaatcaccaacatgggcatacgggtccccggtggatgtccaccccatcagtgg

attttcccacttttgggttgataagaagttgatcctccatcttccaacattcccagatcc

ttgttgaccagcggttctagcaggagagtaaatcaaaacccttctacgaaggtgttcttg

agggattccggaaaccaatccgatctcgcctggtttggcctcgaccaatgcgtcggtgga

aaaccgtctggtcaaggtggcgcgtagggaaccaccacgagtcgcggcgcgtagaaggat

gttcgtcattgtacgatcagaattttgattctgcggaggatgctagctaaagccaaaatg

aacctcccaaacctttctcgcccaactttgttcccccccc

>fava_dn_rep_c289

tttttttttagaaataaaattgcaatattcgtctattcatttataagcatgaaaaagctt

atgagaacactcactcttagacagaaattgtagcaatcttaagtttgttatagaaatagg

gaatatacacatcttgattcaacataaaacagaaaaagcaacaactttataaagatgtgc

ttgatattcactatatatttgtaagaattgtcatctacatataaacaagatcaacatgaa

acaattaatcatcttttcattatgtcactggcatccttcttgattgtaaaatcccaaacg

tcaccatatttttcattgaaatcccatatgctcatagaattatagtcatttatcaacttg

tctctagctcctctttccatgttgtatatttgaggttcaaagtggtgtggttgatcaggt

ttatccatctcaataatgcaaaatacgattgtaaatatgcccgctccaatgaacaagtac

tcacccggaacattgaagaataccccagcagcaatggctggtgggaagagccatgaaatt

agaccctgaaatccagtggggggtcctgctcctactacctcatccttaagtgcaccccaa

aatgttcctttattttcttctccttcatagaaagtatggtgaccatcaaatattccaaac

tcaaactcctcaggtggaatgccattagtacgaaattggtctctaggatcttcattgagg

tcaggctcaataacattgaaaccagcattcaatgaaaattgtgttgagtttttgttagat

ttggtgaagaggcaatggaagttgggtggtgttagagatgagaaggatttggaagtgagt

ttgggtggagaagtggtgatgggagggatgagtgagagtgaacatagcgccatgtgttgg

aattggttggttctctttctaatgttcccgcggccgtaatggccactctgcgttggtacc

acagcttatttggctcgagtggaacaattgttgcaatatcaatctgatgttacaagagct

gaggattggttgaagcctaatgtagtgattgaagcatttgaagctagggctgccaggatg

aatgtggctgttgctcaaaatctcagcaagttcactaatcctgaagagggttttcaagaa

ctctcggctgacttagttgaggcagcagctgctcactgccagttaattgttgtttccaaa

tttatagagaagttgcagcaagatatacctggaaaaggagtaaaacagcaactacaagtt

ctttgcagcatttatgctctatttcttcttcataagcatttgggagattttctttccact

ggctccatcaatcaaaaacaaggatcactcgcgaacgagctgttgaggtccttgtattca

caggttcgtcctaatgcaattgcgctcgtagatgcatttaatcactctgatcacttactt

ggttcgactcttggtcgctatgacggaaatgtgtatcctaagttgtatgaagaggcttgg

aaagatcctttgaacgattcagttgttcctgatggctttcatcagtatgttcaaccaatt

cttaggcaacaactacgtaatgccagactctagagtgttttataattgagatactctttt

gttgaaaatcatcaataacgttgaatggaagaataaataaatgtagatgataagtttatt

tccatggttaatattttatgagaggaagttgcaattcggtgtatctggaatgtaaactaa

aacattattatttatttatgtatacttggcccctcatgtttcatttcatcgatcttgttt

cttgaaatatttgatttttacaatccatattgttaaattcatatagtcacattgttaatt

tcaacactgttattctcatgactattgaaaatataannnnnnnnnnnnnnnnnnnnnnnn

nnnnnnnnnnnnnnnnnnnnnnnnnnnnnaaatttgacc

>fava_dn_rep_c290

acacgaagtgaggtgctttggtgtcttcatatccatccaccaccagacaaatattatctt

tttttccaaaataaaaataaaaatattctctcttccaaccccaactgaaccaagcttgtg

tctaccacacacgaaaaaaaccatgtcatctatggttgcaggtacctcatcttcatgttc

tgccaccttcaccacaatcaaaaactctcatctcacaccttccaagacactcactcctcc

atgccagaaaacttctttgctaggactctcacttcatgaagccaaaaggggtgtttcagg

atctttctatagtgacaacaaaaatggctcttcctccattgctagaaggagactcgagat

cactgcaaaaactgctggagcttctaagacaattgaggctgaagttgataagccacttgg

tctcactttaggccaaaagaatggtggtggtgttatcataactgcagttgaaggtggggg

aaatgcagcaagagcagggttaaagtcaggggatcaagttctttataccagcagtttctt

tggagatgaattatggccagcagataagcttggattcactaaaactgcaatccaagctaa

gcctgattctgtctactttgttgtcagcaggggtgctgatgtagatgttaaaaggcttcc

gaagcgtcctgctcctcctcgcttcgggagaaaattgacagaatctcaaaaggctagagc

tactcatatttgccttgactgtggatacatatacttcttaccaaaatcctttgatgacca

gccggacacatatggttgtccacaatgtcaagcgccgaaaaagaggtttgctccatatga

tgttaacaccggaagagcagtcgggggtggagggttgcctccaattggagttattattgg

acttgtggctggtttaggtggtgttggagctttgcttgtttttggtcttcagtgataaaa

tattattatactacattttagtgcaaactgaagtttgtagcttacaaactcatttattgt

atatgtgcaagttgaagctatacgtatgagattgagcatgttctgcaaaatgaatttcaa

actattagcaatttgcagagctaaatgtgatgattatctacttgataacgttgtccattg

aagttgacactattatcttgttcactattcagattggctcccaattgatcctaaagggaa

aataatacacaaataaccaggagaatggaccataatgcctgttgatactatggtaaagta

actattgccaactcatatcgttttgtttgcttgaattcaacaagtaaactcaagcaaaaa

aatgtatcaaatcactgtaataaagattcacattgataaccttgtagttgtgtcaaacaa

actaagttatgcctccaaaaagaatacatcttaattggttttttaaatttagtaactgct

cttcagttttttaattagttatagtgttgtaatgtggtagttattggaagttttagcttt

gtgaacctatgtaaaagagggtgtgtatgttaagtttgtctatcagaccaattatatgaa

tcaaagcatttattttctcg

>fava_dn_rep_c291

aaaaagaaannnnnnnnnnnntaaattaacaatataagcaataatttttggttttgtaca

acatcatattagttgtttaacaatttccagcaatgttttaagaggaacatttccagtagc

agcaaactcataatacatccaaaaaacacggtttcaatttgatttaaaaaggtagtaacc

gaccaccattctctagataatctggttctagatgtgatcatatctacaagttattaatca

aaatcagatcatcccagttttcacttttgggatatcaaatactgagatgggagcagataa

catccatgaaattaataagaataaaccgggaaacaacttcacaaaaaaatagaacatttg

attacttcaacttccaaatttaactaggaatttgaattaaaacgaccttgaaaacacaag

aatttcaaaatcattggtgtctacttattgaagaaacatggcctcaccaagtgcaatatt

ttataatgtataaaaatatgatgaagacattttaaaggaagttccggatgtccaaagatt

tgacatcaactgccccagcttcatccttgaattcctcctttttctgttgagtttctattg

cctcaacaacatcttttttcaactcctccagctgtgctttggctattttaagctccagct

cctcttctaactttggcaattcttgcttttgaattccaagtttcttattaatcttatcaa

actctgccaaaagaaggtccattcctttcttgtcatttctcaataggggtttttttaaat

ccttttcaattttgtccaatgcatcaatcatcaaagcctcagctccaagattatctataa

gacctctcttggttcttatttccttcaatgtcaaaagataagttcgagcatcaggaatat

cgtgcgtcgctagttcaatggtttctgcgatatcttgggactctgacaagaggtttgcct

tctctctgatcgttttcatgacctttgcataatgagaaacagcagctggatcctctggag

ctagagtgatcttctccctttttaatattccgatagctgtctcaaatttgttcttcacct

ccagaaaaatattcttcagcatctcatctcccttaagggcaggaagttcagaccctttag

cataatgacgaaccggaacagcaaactccctttgcagaagaatttgactaccatacagca

atctggatttggatgcgatgcgagaagcgagtgccatggctgatgagttttctctctctg

aaatgcgattagggctttctctgtgtttcactttgatgccgttgttctctcttagcgtgt

cttgaaattgagaagagattcacaaggtaaggtgcactctgttcccgatgaataggcttt

accttatctcagccgttcgatattttgtttatgcttattctaaatttttatttt

>fava_dn_rep_c292

aaccatcatagtagttttctgctaagttaacttgtgcgtagctcacacttttttgccata

tgtaaagcaagaattgtgcataatgaaaaaggataatcacattattgttatctaacttca

taataaagcaagcttgcttgctacatatgaagatgaaacaaattgataaactttgtttac

acgttcaaaagcaacaagaaactattgacaacatattatataaaattattatattcattc

caaatctaatatcccaaaattggagaagaggatcttcacttgatcaacaaagtctgagcc

cgtcgcaaactcggttagcaacccaacagcaaaaccaaacattgcccaccttccattact

aatctcatttttggcaatgaatccaaaaaagggaccttgatctgcagcttgttcaagctt

cttcgatttgaaatactcctgcaattccttagccttttgtctctgaaactctaatgtaac

aacagttctatcatccccaacaacaagagaactagctttctgaggttgtggtggagactg

agaaggaggtgagggtttcaagggttgaggaagattagatggttctttcacagaaggagc

cattggtcttctcaaaggaccttcactctgagagtttcttacagtaacagcatagggttt

gagagaagagaatctaaaagaataagatgaagttgatgaagatgcacacgaagatgttgt

tgggattttgatgcatgggattgaagatgttacagacattttttgaggggattgatgata

ctaaaaatttctgaaattgatgtgttttgtgttgaagttattgtgaacatgtgttatcaa

aatcaatggataagtgtgtgtggacaaggtatgtccaagtttcctttggaaagtggtttg

agttaaatggagtggagatttgaagtgcaacatcaaccattggattttggctactgtttt

gacttgtcagaacatattatctctctctcggatatctattacgatgtttggat

>fava_dn_rep_c293

ggcacaagaaagaaaaatgccatttcatattgagaaaatacaaatttcaatcaggataga

ctaaaatagaggcaaaaaaacatccaagtgaaatacaatgttgggaaaaccgaaatgact

acaaattcattttgttgttggactaaccacttaaaccttttagagtttggactctaaatg

gtctgaattatgttttcttcaacactttaatgaaagaaaaataatgtactgcaatcaaat

ccaaccctcctgaggaggggaaaattttaaaagaagggaaagtgcttattatttgaattg

tagtgcaatagcatgaaaaacagatcaaagtaaatgatcttcatagacaatttacaaata

tattttaatatgtctttgcaatttgcttgctaatcagtttccaaagaagtcacttcctta

agtttccaactacataagcagcatacaggtccatggagtgttcgatagcctgaacagcag

attcagctggtagaaagtcttcaacattgactattttgttctcattcttcttgtagtcct

caaagaatcttctgatttcagcaagcctatgtggaggaagctccttgatgtcctggtagt

ggcggaactcggggtcatcagcacaaactgctatgatcttgtcatccttctctccctggt

caatcatgggcatcaacccaatagcacgggcacgaaggaaacaaccaggtatcacaggct

cctgcatcagaaccaggacatccaagggatcttcatcttcacagagggttcttggaataa

aaccatagttatgtgggtagacaactgatgagtaaagaatgcgatcaacctttataagtc

cacttttcttgtccagttcatacttaaccttactccctttgccaatttcaaccacacaat

tgaaaactgatggagctcctggtccaatttctaagtcatgccatggatgagcagcagcat

cttttttatctgccatggaagaaagtatcctttcattgagcgcaaaaaccggaggcgaat

cagaaggattcgtcgatttagaatcttgatcattggtgttggtagccattttgtgaaaaa

gtgttactagtaattagagtgatgaatgaagtagtaaagttagattaattaagaagatga

agaagttgaatggaggtagctgagaagagaatttgtgtgaggtgggtgagactgagaggt

acgttagtgactgagatacttcttgagttgtggaatga

>fava_dn_rep_c294

atgaatggacaataagcaatgggaatagtggtggatgagtctcatttccacgtgtcatta

tgaattttatcattcactcattcactcattgcgatggaaccaaacatgaaaaagataagg

aaccaatctaagaaaaagcgctaaccaaaaaatgagctgctcattcagcctccatgttcc

caaagctctacctcctctcccctattattcatcatcatcaccttctctcttcatttcttc

caaacacaaaccatacaacaacactacaactccttccaaccaattgcatatagtaaaatg

caacagccaaaagccaaatgatcatgtcactctaaagaaaccaaatttggcactgcccat

aggagcacttctcttggccttggctgagcatccagcagcattagcagtgactggagataa

taataatccacaagaactctcatggattttgatacaactgggaattgtttcttttctcta

cttcatcactgcaccaccattgattttgtattggctttggaaaagatggtacagaagaaa

gcttgttgaaatgtatttccagttcatgtttgtcttcattttcttcccagcgctgcttct

ttgggtaccgtttctaaacatcaggaagtttccaagggatccagacatgaagtatccttg

gtctatacctgaagatccttcaaaagtaagaaatgcatactataagtatccttttgctga

tcctgaggattatgactagccatcaaactaccaacaacaaatcatatgtatcattttatg

cctaaactacattattattgatcaatagggtttggttataccttttttctgcttttgatc

tttgaatggccattcagttgtaattttgtaaaaattaaaggaatctttaaattcaaagtt

ggcactagattttcacttttggtgttcaagagtgtcatactgccaattggattgtgttaa

agaaaccaacttatctttgttgaaacttcaaagggagtttcattgggttggatcaaaaca

ttaggttaaaagcaaccacataagtcattgacacc

>fava_dn_rep_c295

ttttttttttcgaacaaatggttgaaacttgaaattactcaaaattaagtagcaatgaca

taagtgtatatttgctaatgtttcatcagaagtgacaatatatagcccatcttgaagatc

tataacaggattttaaattataaccaaagagtagaaattattcaagctactcagaactca

tgaatttcaagagaagattattgaccataatgtacaaagaaaagtaacaaacactcaaaa

agctccaaccaaaggaccattcagcgtctatcagatgccactgattcattagagaagaga

tacttcactcgtgaaagaactgaatcatgggtgggatcataaggatggtcttttgaaggg

atttcaagtattgcaggaacaggcttgttgtaactatcaactaaaaaccttatcatattt

gcaacaaattgactaatcagcacaatagcaatatcctcccgtgatgtgaattctttgaat

gcgtcctcaatttgtttgacagttgtttttgaatccacaataaggtaatttgtcttccta

cgtatgtcaacatttcccactccagccaaaagaaacccaactatggtatcctcgtctgca

atcatggcaataagtgatgaattgttggtaggaatttgaactctgttagccatttggaat

cacagatgggaagaacagcgacggatgtaatggagacagcggggcgtgacggagcaaggg

aggaattcggtctgttaatccttcacgcacgatactaattgaacctctccactctttatc

tttttcttt

>fava_dn_rep_c296

ttcaaaatatctgatttatttgtaagtaaacctctactaatgaaaaactcttttattggc

cctatttttctttcccctgcgtttcacttgatcgattttgcttttgaaatcagcaacaat

aacttctttacaacagtgtttagaaatactgaggccaagcaattccaaaacattgctaac

aagggaaattctgtgatagcgataaaaaccataataatatacaatctactcatgtctatg

gatccttcatgaatcaactgtcaattgcacataccattttgacatgtactattacagaga

tatatagatagggaacaaaataaaagccttataatgcttacaccaccaacttccaccaaa

ctagcagagatttaaagacttaaaaaacactggggcatatattttcgccaatcacatgaa

atatgcagtctctctcaaactgcagagataatactccaagtcccccatcacatataaaaa

tataataacaacatcaataaaatatgcaggaaactcggatcatcccatgatgacaaagag

cataacagcagctcgagacatgaaacttttaagacaccaaatctatatcttgtcaagctt

ctcagctttgacaaccgggtttgctttggctatcgcatcccgtccggcagtgcggtaatc

aaatggacaatcatgcttgtctgagtagcgatgtacagcacagaaaaggttaccacatcg

acaattaaatcctgttaagccaacccgcttattgcagttggtgcaccgtttaggaccatc

cttgggcttcttttccagattctcctctgagcccgaagcaactaatggttgtgcagagat

ggttttgggctccactgaatcaactgagatttccacattggcggcaatagcaggttcaac

tccactgctgcttgatgaaccattcataatgttgccgatggaagatgctgcaagtgtggc

ctgctcctgtttcagcatcatgtctttgtggcacttggaacacatgttcatggtagctgc

acttccgaaaaacccacagttgttgacgcagagcataggaccttcgggggctgcttcgca

tccagtctggtcatggtccatcttttttcaacttttctgatagatttgggatcgacgagg

agagagaaagcgaatcggagaaggagaagaggagagagaaagaaattgaaataagtgttg

tttcagtgtttgtttctgtttctctctctttctctctttctctctctccgcttgaaaaag

tttctttctctttgtgttgccaacgaacgttgtttactgattgggggcgacgtgggtaat

cgttattgg

>fava_dn_rep_c297

attttcaatttagggatttttccatttccaaaacaaaacccaaagcatggattcaacacc

ttccgcaaaacgacgtcgtcagagcaaagacgacgagactgaagcagcatcaacgctgcc

aaagcaaatgtctccggtggttatcttcgctcacggtgccggtgctccttcctcttccga

ttggatgcaaaggtggaagagtatgttgatggaaaccctacacgctgctgatgttgtcac

gttcgattacccttacatgtctggtgggaaaaagagggctcctccaaaggcagaaaagtt

ggtggagtttcattcaggtattgtgaaagaaacggcaactaaataccctggtcatcctct

catactggctggcaaatcaatgggttcaagagttggatgcatggtggcgagcatggaaga

catcaatgtttctgctgttttgtgcttgggctacccattaaagggaattaatggtgcggt

tagagatgaaacactgttacagctaacagttcctacaatgtttgtacagggcagcaagga

tagtctctgtccacttgataagttagagaccacgcggaagaagatgaaagcgcctactga

gctgcatgtcattgatggtggcgatcactcttttaagattggtaagaagcatctacaagc

aaacagttccacccagactgatgctgaagttgctgctatgaaggccgttgctgctttcat

ttccagattccttgaaggttgattctacagtgtagcaaggtttgtgttattgtatataca

ctctgctgtatctagatttgcagttatctaggtcttgttcttgcaagtgggctgtaattt

tattttgggtgatttggtcaagtaatttttgctagcggagtagacatgtatgaatttttt

gttctggcagttttagaaacctacttggtgagaatgggtttgtttaacttaatgcagttt

tacatattaatgttagacttggtaacaaaggcatgtaagatcctcatggaacattccagt

gaaaataggcaaagttcaatatctattatttatcacaaggtacacacagacaagtgaaat

atgggcatttatttacccctattattgagtacaagaatgacatgataaaattataaatac

actttatttaaaagctttgtttagttgcatccgtttgcagctcttagttctgtgcatcaa

ttctttggctttggacatttcttctcatcttcaagtgcacctacctttggctttgtcatc

ggacatttcttctcattgtcagttgcacccacctttgtctttgtctttgaacttttcttc

tcatcatcgctttcgctgtcactgctgctgctgctgtttccttcaccgttatggcgggac

attccattcttaatcctccggaacaaattgatatctttcttattgttgagctctctgttt

ttcttaggagttccaaagcatgttgtagttgcaccataaggagaaggaccctctttggtt

tcagtgatttctgcttgaactacacaaactgtgatatgggttttggtgacaccatgtttc

ttgtcatgttgggtctgagtatgaccatactggtgagtttctgttttggtgccattgtgg

ccatttgattcaacctgactttgggagctgtagctttggacttgattttgagtactgcca

tggcgagcatgatgtcctttgaaagctttgttagttatctcagatattttttggccgaac

gagctgtgttgggtttttggttggcaggcttcaatggcagtagagggtttcacagactga

gctgaagccattgttgtgtcctcactctgatttgtttctttgatgttgctgggtaattgc

gcattaagggccttttattggcctaagaatgagtgaagctttctctcctcctcccatagt

ggagaaataaaataactattcaattatgctattggaaat

>fava_dn_rep_c298

cagaatgtctcctcccaatgctccaaatccagcctctgacccactctccactgccactca

tctcacccatcgtgagcttctgtgccgtcgtcttcacaacactaaacgtcttatcaaaca

ctacagacgtatgtattgggctctcatggaacaagtcaggatcaaacgtagacaatatgt

tattgacactggtcttagccctttccaggatgaacctgatgttgtcgatgacaattacac

taacgtttgtgcttttcatggttgtatgtccaagcctatgccttgcacttccttttgctt

tacgcatgtcctctctgaacccaaacaagtgctttataagccttgtacttttgtcatcgc

aaggtcagaggctggacccgtaacatgccagaaacctataataagttctacgactccgtc

gtactgcactgtccacatgcagaaggcggagatgcatctcgcaatggcattgaagaaaga

aggaatgaatatctccccaatggacaaagtgagtcctaattttcatgaactggtgcctga

atttgttcgtcaaattcaggagaaaagaagggcaatgagggcaaaggaatgtgaaactgt

ccttaagaaggaggaggatgcagagaaatgtgaaaactgagattaagaaggaagatgaca

ttgctgaggggtgaggaagggatacaaacataaataataatccttgttataaacctgttt

caattggagcagtgtgaataacagaagacaccaaataaacttgtttcaatttgagagaat

aataaatcgaatctttagaataaaaacagcctggtttaaaataactggttcgtatacaga

gtttggtttcgttttcggtaatgtgaattgtgcaatggcttgctttttctcaatttcctc

tgctcgtgttaacacctgatctgctcgtgttatttgaatggtaatatctatcattttcaa

tctgcttaattacatttactgaatggaaactgaagcttaaaatgtcaaacttttgcagta

gtttatataccaaacacagttacaattataatcagctacaacaagaaaaaattatttctg

ctctagattttagtgccttataaaaaagatgcaaacaacaacatctaaaacataaataag

tcaataaaatttcaagttatccaaagtaattagatagcacactttgcttgatttcacaaa

aaatatcaaaatgataatactctatgtagcaccgacacctttaaaaaatagtttatcaat

atccatgtacacgtcctgacacttatgataactttttattcattatcttttaaattaata

tcggtgtcgtagtgtggatgtttcatagacttacggaggaggaaacaactaaatcagatt

aatttccttgactctacctttcttacaattggctctgaaataacacaggagcataattga

cagagttagcattataacttactactgtcactaatttggaagaaagaaaaaagtgcagtt

aagttaggttagattagttacctcagcaaatacaaatggtgttcatcaatacaagcagct

ctcataatctactgaacaatgaaacctaaaatcagtagatgaattcggaattcattgcag

ccaaatataaataaggaatgtaaaattgaattttttttgtaatcagaataaattcataat

actttattagatcaattaataacctggatgatacaataatgaacaacatatatgaacatg

aaattaatagctgttaagttccgaagctagcttaacctctctagagagatgcatagagtt

ggatgaacacaacacctccttcttctattcattagtacctattccagagaatcatacata

attcatttatgaatcaacaattagatacaattagttacaaagtttaaccgtacaagcaac

agcttgtgcccagtatttcaacctagctttaacatcttcaggactatcacctggactcga

gatcttccaattcgcaaacgacggagaagaatccgacattgaatagcataactccaatgc

tgggagagtgttgcatagttctggaatctcatcgtaactgaatccgaatcctagatctaa

acatcctttcaactcgtcaagatcctcgtcggttaagcttttcgtccttggaagatcttc

actgacgaagccttctaacaacactcccttctccttcttctttttcatctttcttttcca

ttcacagcttccacttctttcaatacttttcattttttttgtattgtattatctcttctc

ttggcacccactatttatatttattttgtgactgagtttatctattcgatacaaccaaca

tcatcatgtatgtcattttatgcatgtctatctatccatctcatgtatgtatgcttattt

tgt

>fava_dn_rep_c299

gctcggaagatcctacttaatcatttttgtaagttcgaggagagttaacaagattgtaaa

ttaaaagttcgtaacaaaatttataagatgatactaaaatgcaaatgtatcttttgtaaa

atgataattgttgcgccgctacatttggcagatcatgcccagacttggcacaatcgcaca

aaatcaatagttttaggattttttttataacttggcaacaacatacaattctacttaaga

tttgaatggtttgggtgagtgggatagcaaaatcgtagtaaaattttaaaatttagatct

gagattttggctaccttgattacaactagtgcatacatctaagcatctaaaaaatggata

gatattaaatagaagatgttgagccataaaaaagtcacttggaagcgaggaaatggcatt

aaaaaaaaattgtttttccaatttaaacatgaaatacaaagagagtgcgcattttagaag

tccaggccatgttcgcgagccgaatcagcgattgctttaacacggccatggtacgggtaa

ccaccacgatcaaagacaacttttgtgattcccttcgccagacaggatctcgcaatgatc

tcacccaccctctttgctacttcaattgtagggccggaggtgtagttgaactcttcagca

agtttcttctgcattgtggaagctgaagcaagtgtatgcatcttggtgtcatcaatcacc

tggacaaagaggtgcttgttggatcggaagacagacaatcttggtcgttcggttgttcct

tcaaccttcttccggatacgaacatggcgagcagttctgtcttcccttcgggaacttgat

ttgacttcaaccacaaaggaaagagaagacggagaattgggatttggagatggaattgtg

aacgaagaacaagaggggaaagtgaaagagtgaaggaatgagagcgaagaagataacatt

gttgcaaaacccaaacgttttctttatctatcgctaccttatccttctactatcatcact

ccccgcggccgtaa

>fava_dn_rep_c300

acgcggcgaatcaagcaatattagtcccatctaccaaacaaacaaacaaagagagaatgt

tgagatcccaggactaccacaaccacattgctggagttagaagggagccaagtgttctct

ctgagttcgaccgctacccaaatgctgaaatggccttcaacaagagggttgtttacgatg

agatcgaaggatctcaccgccaccaccaccaccacaaccctgaagtcgtcgagagggttg

aagtggaggagtatgaacgagtacctgaggttaggccatatggtggtgaagtgatctatg

aagagtacatggatgttgaaaccagtcagtataacccacgaaggaacaggccaaatggtc

ttggactgcaaaaatgggaggcataaagagcataagcttgtgctttttgtgttgttgcta

atttgctaggcaggatgaaccctaacatatgatcactgtgtgtggttgtattaaattaaa

taaattcctgcttagttagcttgttgttattatgttaagaatcatgtatgaatgattaat

aaagcatatctatagcatgtatttgaattaattaatatgtcatgggttattattgcactg

tttatttaaactattgttgtttagagttcttgttcaaagtttaaactcaacacgataaaa

ctcgttttagccattctggtatatactcaactggtgcagggaataaaaatttgtattttg

ttaaaaaaaaaaa

>fava_dn_rep_c301

tttttttttgctgcatcaaaccatcatatagggtgaaatataataaatactatagctcaa

atgctacaagaacagacaaatccataccatatcaactaaaataataaacactcaatttaa

aactacacgcagaaacaaatactaaaatagaaagtatcatctagtatctcttcaagcatt

ctacttattataaactagaaaagcaaatactgcaaactactgagattcaagcaacccaac

cagtcccattgtgtatgaacgtcagtcgtgagtgactcctttgctgttgaaagaaatcat

tgtaagctcatgttttaagaataacaagccggggaacacctattcacatattactaaatt

tatttgtaaactatagtcatggctttcaacaaattcactttcataattttagtaatttct

cttctttcttactcaacattcactaatgctaataattgtccaacaccttcaccaactgct

tctccaccatcatcacaaaattcctgccccaaggacgccttaaagttaggggtttgtgcc

gaccttttagggttggttaatattgttattggagaccctccttcgggtagtagttgttgc

gcattgatcaaaggtttggctgatttggaggccgcactatgtctttgtactgcacttaag

gccaatgtgcttggaatcaacctgaatgtacctatctcgcttagtttaatccttagtgct

tgtcagaaaaccgttccttctggttttcaatgtgtctaagaggttttgtgtggttgttga

ataaatatatatgtgcattataaataatggtttctcgtacaagagaattgtttatttttt

taaagaattgttagcaacacattctaatgcttatcgattgaagagcgtgttactatcaat

tttttaaaattactataattatttaatgtcgatgttaatgtggagtttgttcaaacatta

atttgtgtagatttgttaaggtgttttgtcaaaataaaaattgggcaagcatatttgccc

aaattattctcttatattttcagtgatttattatgaaatattatgtttgtttcttgagta

cattgagcagtaacactttactttatgaattcaatttcatgatgtgaataataattattc

aattattc

>fava_dn_rep_c302

ttcattttacatcacacatatatttcttctatttttcacactcttttttatttcttcttc

tttctccttgtgcatttgataaattcttcatcaacctttaattaacacaaatcactcttt

gtgttttgattatttgatcatacacaacaaaaaaacatatttagaaacttgaacttgtgt

ttttgttgtgttggttcctaacctttgtgtctcttcaactcaaccatggcttctaccttg

tgttacaactctctaaatccctcaacagcatctttctcaagaacccatttctcaattccc

gttaacaaagacttttcaccagaaaactcttcatcttttcatagttatgcaagaaccaga

acaagaaaacataggaagaatgtttttctgatgcatgtgaaagtgaaagctgcagtggct

gaatctactgcaccaccttcctcatcaagacaatctgagaagaagaatcttagggtactt

gtggcaggtggtggaattggtggattggtttttgctttggctgcaaagagaaaagggttt

gaggtgatggtgtttgagaaggatttgagtgctgtgagaggagagggacagtatagaggt

ccaattcagatacagagtaatgctttggctgctttggaagctattgattcagatgttgct

gatgaggttatgagagttggttgtattactggtgatagaatcaatggccttgttgatgga

gtttctggttcttggtacgttaagtttgatacattcactcctgcagtggaacgcgggctt

ccagtcacacgagttattagtcgaatggttttgcaagggattcttgctcgtgcagttgga

gaagatatcgttatgaatgccagtaatgttgttagttttgtagatgatggaaacaaggta

acagtagagcttgagaatggtcaaaaatatgaaggagatctattggttggagcggacggc

atatggtccaaggtaaggacacagttatttggacaaacagaagctgtttactccggctat

acttgttataccggtattgcagattttgtgcctgctgacattgattctgtcgggtaccga

gtattcttgggacacaaacaatactttgtatcttcagatgttggagctggaaagatgcaa

tggtatgcgtttcacaaagaagcacccggtggtgacgatgaaccaaatagaaaaaaggaa

aggttgctcaagatatttaagggctggtgtgataatgcggtagatctcatagttgccaca

gacgaagaagcaattctgcgacgagacatatatgataagataccgacatttaattgggga

aagggtcgtgtaactttgcttggcgattctgttcatgccatgcagccaaatatgggccaa

ggaggatgcatggccattgaggacagttatcaacttgcatgggaattagacagtgcatgg

gaaaaaagtattaaatcagggaacccaattgaagttgattcatctctgaggagatatgaa

agtgaaagaaaaattcgagttgcggttattcatggaatggctagaatggcagctctgatg

gcatccacctacaaggcatatctaggtgttggtcttggtcctttggagtttttgaccaac

tttcgtatacctcatcccggaagagttggagggaggtttttcgtcgacattttgatgccg

tctatgctgaattggatattaggtggaaatagttcaaaacttgaaggtagaccaataagt

tgcaggctctccgacaaagcaaacggacaattgcgcgaatggtttgaagatgatgatgcg

cttgagcgtgctattaacggagagtggtttttattaccatgtggagatgaaacaggtctt

tcaaaaccgatacgtttaacacaaaatgagatgaagcccttcataattgggagtgcagtg

caagaagattctccagacaattcaattaccgtttcttcacccgaggtttctccaacacat

gctcgaatttactataaggatggtgccttcttcgtaactgatttgcgaagtgaacatggc

acctggatcgttgacattgaaggaaagcgatatcggatacctccaaattaccctgctcgt

gtccgcccatttgatgtacttgcgtttggttctgataaggtttcatttcgagttaaggtg

aaaagttcggctccaagcatctctacgaatgaagagacacaagttttgcaagaagcgtga

ttggttgtgttgaaacagaatattgaagttacaggagataaatttgtagctgtagaacat

tatctgcaatcttacaggggcagggtattatacaaagttgccaattattttggtgatgat

attgggtaggaattgtttcctacaagcactccattcaattataatgtgactcataaactt

gtacatgttgaatggagtgaccatgtatgtagctaagaatttctgagacttttgtgattt

acaaaatggaaatgtatatttatcaatgtaaaaagagcaatttcaattctttggatatgt

gtcatgccatgatatcctacaaaatcaagtgtcctaaacacatttgaacgatccaactat

gaggagtttgtttcataaactgttttcagttgatttttttaaattcttctgaataaaaat

agtttatac

>fava_dn_rep_c303

aactcgctaacatttcagggagatttgtatatttaacccatgtttttatacttgcaaaac

aaggcaactataaacaataatactaataataataatttgagagggtgatgttactcccac

tttagcatctgtcaaaaaatgttaatgccttgttcacattcaaaggtacaaaaggaaagt

ccttacaaagtttgacaatggacattggatacacctacagacaatataaacatcaaatga

aatacattcaagtagcagctaatgcaagaagctcccatgttcagttaaatagacaagaat

gatcaatgtttgtcatatccaacatctttgactttatctctgcaactgtattctcttatc

cctaattggcaaatcaatcggctgctgcgcctcatgtggatgattcgggccactataaac

cttcaggcgtgtgaatagagtcctgccaagcctcccttttggaagcatgccacgaacagc

atgttcaacaattctttcaggaattctgttctgcagctgatcaaatgtctcgactgtcat

accaccaggtcgacctgagtgcctccgataaagcttttgtgtcctctttttcccacttac

agcaaccttttctgcatttaccacaatcacaaatgcacccatgtccacactaggcgtata

agtcactagattcttgcctcggatgtgattcgcaatagttgatgccagtctcccaagaat

tttatcttccgcatcaacaacataccatgttttctccgtgttgacatgatccgaagcttt

tggataccacgtcgtgttccaaatgtccgggccttgaaatcggggctcgggttcaacagt

gatgaagcgtggatcttgttctacgagggaagtgtcctgttgagaacggacaatgaaaga

gggagagaaatgggtagtacgagttgacaatgaaagggttgggagaggaaattgggaagc

aataagtgaagatttggttgttgcgtttttgtgatgggtgaatgaagatgaagggaagga

gaccgacggtgaaccacgcagtaaggccattgttgctctctcgcgctgcgttctgcgcta

tatctaactattatccttttcttcggaccaagataatgtacaagacggagactcggataa

gagatatttcgttaccactccactagtgttacttcttttttttttaa

>fava_dn_rep_c304

agaaaaaccacattattatatcgtttaacaataatgtaggctcaataatttcacaatcca

tgttaatttcacaatccatgtgaaaatgctttttgtaaccacattctaaaaaacgtaaac

ccaaaattcttaaacataaaactatttagactaacataaatgtatttgggcaaaaagaat

atataaccaattataaaatgtaaaataactaaagggatataattttagaaaacaaataaa

atctgtgtagccaaaaacatagcatccctcaaaagtatacttgcaacaactcaaggaata

acataaagccgggcataaaataaatctaagacagactttaaaccctataaagaaaaaagc

tagttgaaacagaattccactcaaacaatggcattggcagcacttgcgattctaggccat

agatcagcacactccttaccaataggaccagtaatagcagaacctttcatttctccctta

ggattcacaatgacaccagcgttatcttcaaagtacatgaagacaccgtcctttcggcgc

catggcttacgttgacggacgatgacagcaggcaaaacctttttcctcagatctggcttt

cccttcttcacggtggccatcaccatgtctccgacgcaagcagaaggaagacggttaagt

ctacccttgatccccttcaccgaaatgatgtacagattcttcgctccggtgttgtcagca

caattaaccgtcgcagctactggaagacccagcgacatgcggaacttgcttccagcggaa

ccaccgcgacctctcttcgacatcttgtcgtctctcttaactccccgcggctccgatcca

gagtatgcttccatccactcattaaataaatgactatcaggaacgaaaaaatcctttaaa

attttttaagtccctttttcatagcacaaaaaagaagaataggaacgaaaaaaaaataag

aaatcaaaaacgaaagagaaatctatttttcgtttttgatttcttatatccatatccata

ttcatggagatcaaatcataattaagaaacgaatgtttaattctttttcgtaattcaaaa

tcatg

>fava_dn_rep_c305

tggaacctttaatggagttttattaagcnnnnnnncaacaagtttttcatatggatcata

tgagaaaagagacaaatttgattaaaacatttttgtccaactctctttacaaatcataac

cgtcctttaccgattagacacaaacgtcttggtttgagataatttctcattcacaaacac

caaacatggattcacagggcaatggatttgacttctcttagaagagtgagattattaaaa

ttgataaacgataaatagctgttattgaataattccacagctcaatattataaaatacaa

gcgttagaattttcaattaatcagctttgattccacgctttccctttaaaagggcactca

ctcttcccttaagagaagccaaatacatggtactaaagacaaaccaagagaaatgcttat

tgaaaagtatcagtagagaatcccaaattacatttagctgttgaagatggagttttaaat

ttttgacacaacatcccatatattcatacaagttccaatagacatgtaaccagaaactag

gaggaatatagactccaaaatagtattagacaattcctctaaaattttccatttcaattt

gatagttaatagcatacatttccactatttctatttgagaccagaagtatccacccaatt

cgtcaagcaccactggcatataatcgggtccactccttggctgtctcaacagcctcagcc

tcattagatttccaatgcttggcaatgttctcagaaagtggatcatcagggtttggtgca

ctcagaagagcttgaatactcaaaagaacagtgcgaatctgaagggcaggactccactta

tctttcagaatatcaagacatattctaccaagcttatcaatgttagggtgatatattttt

gtcaggaacctaaccttcggagcagccattggatattcttcaggcaaaaacagttctagc

ttgaaaaccccaccttcataaggagactgagttgggccaaggatcatcacattgaaatac

cgcatattgtcttccgaaggagatgcactaattcccggcgctggttcactgagcaaacgc

tgagtttccttgatgattcttcggggaaggttgctgttggccattactaggtcgattgaa

aaacacgctataggggtttgagagttgcagagagagagagagagatgaaggtttctctca

ctaacgcaaatgaaatgaaaaataacgttttgggcttnnnnnnnnnntgattacttaata

gttttacctcttttgtgactatcaatcttaatatatataaatatgaagttatcact

>fava_dn_rep_c306

cacgaacaacaactttcaagatttcatcacagattcaaatgtttataacgcgaaaacaaa

atatatcaatcgtttcaaacaaaatatgatactaacaatatcgaaattattcctcaacag

aaaactcgtcatagtaattcaaactgaaattactatattatcttcaaagaagctatcacc

atattcacaacatcacagtttacgatcataagaaacgctaacacgatcgaaccaatatta

ttatagagttctcacaacatcataacattttaactcaaacaaacacaacctgaaaaggaa

tcaaagtttactgaaaagtggaaaagttgtggcgtcaagttgaagcttgtaatgagaacg

gagcagagannnnnnnnctttttttttttttatgcaaacatagaatgacattaaaactga

ttacatatgcaacacaagttcacacattaataatttccttttttacaatgcaccaatgtt

cttaagaccaagaacaacaccaacaccaatgatgtgtccaactgttccacaagccaaagt

atcagcaagggtgaatccagctgggtcaccagtctgcaaacctgagtcccttgcctccag

ctttagtccagcagttgctttcctgtttgctgagggagccaatccaaatcttccagcaaa

caacattagggttgtagatgccaccataatcacattggtggatgaaccaatgaagtcaca

acgaactcccaaagcacccttccccttgcgctttgtcatagattgggaagcaaccaaacc

ctgcacaggagaagatttcaattgtggtctaagaccagagaattgtgggagagttgtcat

catagaggttgccatttcttcaagctatgagagcaaatgaaaaataatttgaatcacttt

ttcttgctatgtttgagcttcacaccgtttttcaaggtttatgataatgatattatgatg

tagcaagtaaacagaatctaatattagccaccaaagtaacttgtggcttcagaatgttga

tattagatggccatagatatgttactccaacgtggcattatctgatttgatttcattttg

tgggattggataatgtcttagctcctcgtttgttt

>fava_dn_rep_c307

tagattttgagagtgcacgcaatcaaacaatgaacaaattttttggcacggacagagagg

acttgggtctaatgaatctgcaattaggcaaacagcgccatttgtgcaaatgagcaaaca

actcattgtaatattaacaaaaccatcagtttttaccaagggacaagaacgccccttcag

tgagaatccaaatctgcattaaacaagcaccaaaaccaattaccaaaatataaaggcaca

catccaaattgatacttcccattttccatatagctccagcttacttacaaggcagaaaga

gagtaaagcaaaaggatgcaagactaaaattgaaaatagcattgtgataaaactgaaaga

aaaatactacttaaaagagttgctttcaatatttgacacacacaatttcatcatcaaaca

gcatcccttcagcacagttgacaaagtaagacaacaaaacaactctgaagccatagagat

ggaaataggaagctttttagggcattccccaacatgttcttgaattcagaaggatccgtg

aagcattacacactataaattgcatactgcaaatgcaatatgatccaacaactatatcat

cttggatgttaaaagtaagttccttctggaggctggacaagcttacggttatgaggagcg

gccatttctttcttcagctgggtcagtatgtcctccatgctgtactctctttgccaattt

gcaagaagcccaaatttctttggttcaactgataccacgccagtttcatggttaacacaa

gtcatattgattcgtgaatgaaaccgaacacttggaggcttttctgggtagtctttatca

caaaacagcttcagttgatagattcttccttcatgtacagtattatggggtccaatgatg

gctccagtccaagaacgcatgtaaatgtcatctccgtcatccataccatagctaactgag

ccatcaccaatacctttttctcccctttcaagttcctccagcaatctgaagttccttgga

acgacgacactggatcctgctgagccaagcgtcatggatgattatcagaggtttttctcg

gagtgagagttgcggatctgatcggaaaattgaaaacagttaagagaaacgataaagatg

aagaatcccgggaaagagcaaacaagaacgcgatttgtagggaataataaaggaaaaaca

aagggtaaagagggatttggtggaacgaaataggaaatggtgattgggtt

>fava_dn_rep_c308

ggaagaaaaaggcgcaacacatgtttttatttttaacagtgtcagccatcatgtttgaat

taatcataaatggattgaaaaatcaaattggaacactgatgatcataatcttcatactaa

taagaaatgaacttcattagtacatctctgaagtattgactttattggcgagcccaaata

aatacacatttttttatttataaatgaatcaacactaatgattcagtacttcaaaaaaac

taaaatttaatcaacagagtgataaatcggatgacacacaggcccttctataacaactca

ttggtagataatacagcctacagttgtaatggacgcaagctatcatgttggtttttacat

tcctgctaaaagggatgataagcagagaagacttaatcactaaaagtacacaaaacagag

tgtgctctaccataaccgcattctcacacattgatgatacaaggcaaactaaaattttga

gggccatgttgcttgagccgacataataattccaacaataactccaaataggccaagtgc

actgccaaagatctcaatcacaagaattttcacaaagagggaggagttctgagcatctga

caatgcacagctgcttccaattattcctacacacaacccgcaaacaagatttgcgaagcc

cacgataagcccagaagcaaagattgcatatccagctctaagagattctggttcatagat

acttgcttttggaacgctttctagttttgtctgcaggataatagccacaataacaccata

tatagcaacagcttcacagaagataacgctgatgagattttttgacgtgattcgaggagc

cttaatcgcagcaccgatcaagctactaccggtgatataaattccccaagcggcgccgag

aacggagacgccgattgaaacagcgataccgattgcggaaaaggtgtaaggagagatcct

aacgagcgccgcggcccaggagctagaatgcgccgcaatgattgcagagcctgacatggt

gatggtgttcgttgtgaaggatcggatcttagagattttgttttgttgttaaccacgtag

ttggagagaaagaattaaacgcagtgtcgtttta

>fava_dn_rep_c309

aaatttaaatttaaattttataaccgcacagaatatcgaccgtaacaactaacagacgtt

gtggcaagggggcaactggtaatttaatggcatccatgtccccggagaaacccgccggag

caccgtcttccgccgccgaaccatctcaacctcaaattcaaactcaaccgccgacctcca

ctccgcctctgcctcctacaaaaccaccaccaccaccactaccaccgccgacgcctaaat

cggaagcctctttcccagactccaaaccctctcccgacgctactttaatccacatcccat

ctcactctcgatggttctcatgggattccattcacgaatccgaactccgtaacattcccg

attcttccaagaaccctagggtttacaagtactacagaaactccatcgtcaagtttttca

gattcaatcccaaccgaaaaattaccttcaccgatgttcgcaagacgctcgtcggtgacg

ttggttcgatcaggagggtcttcgattttctcgaagcttgggggttaatcaattatcatc

cttcttcttcgcttgcaaaacctttgaaatgggaagataaagacaccaaaacagaatcag

cttcgaattccactgattctcctccggtgcctatcaaagaaactgctaagagaatatgta

gtaactgcaaaaatctctgtgccattgcttgctttgcctgtgataagaataatatgactc

tatgtgcgaggtgttttgttcgtggaagctataaagttgggacgagtaatactgatttca

agagagtggagataagtgaggagaccaaacctgattggactgagaaggaaacactgaaac

ttcttgaatgtattacgaattttggcgatgattggaagagggtttctcatcatgttattg

gtaggactgataaggaatgtgttgctcgttttctcaaacttccttttggtgatcagttca

tgcattctcaacgctccgagtctgctcatcttattggtgatggttgttctgatctgttga

agccctctgttgatgctgaatgtgaatcagaaactgctggattgggtaaatctagtaaga

ggatgcgcctcacgcctcttgccgatgcaagcaatcctatcatggctcaggctgctttcc

tttctgctttggcagggacagaggttgcacaagctgcagctcaagctgctctgacaagtt

tgtccgatgtttacaagtcaactagaattaattatcggtcatttccaaagcaagaggctg

gtgttgcatcaaatggcattaatgcttcagattctatccaaggatctctactgcgtgcaa

atttagagcgtgagaaggaagaatcagatgtagaaaaagcaatttatgagattgtagaag

ttcagatgaaaaatatccaggacaagcttatcaattttgaagatttagacctgctaaggg

aaaaagaatgtcagcagttagagcaagtgaagagtttgtttttccttgatcagcttaatc

ttttatttcgcaaaacatccacatcaacaaccggagaaggcaatcatgtaacgaggaatt

gatgcctgactagtcttaattaagctatttatcttaggttaagctgaatgtattcaattt

ccatttggttgcaaagtcactgagtgtctgtttctccaatattgttagttaaaactgcat

atgtcatccatggcaggattttcattatatattgtcagcctacttacacattccggccta

aaaacagctttaaagtttccattatgttctgttcgcactgcatttcgctactgcaattgt

aaataggggtagtgaaatgagaaaaagtggcattaatagtactttaaaattcttgaggat

gtgttttctagtttcaaacatccatttcaaggaaggaacttttaaaatttatctatacta

cattgttattgcttgctttgagaagagtattttcaatcgttcttgaagttgtgatgttca

ctgcaaaatatctttgtccattacatcaatttatgtaattcatttatactgctttggtct

atcagtattgaagtaaatatcatactaataggatttagacagacatttctagccaataaa

taatgtcatgaagacaccatgtgggaggccgtcacagaaagcaacggaaaaaatttctca

cgtcgtgaacatttctctatatgaacttgaaagatcgaaccgccttctgaattatggaag

catacttgtctgtttcctcatctccatactgtcctgttactgtatacagtctgttatacc

agccattcgttaacatcccaatagctgaatatagatgcttgcgactctcgccaggacttt

gcagtgtatactcaatgaaataaaatcctttagatgatttacagtctatgagtttagcag

ccacaccaggtggttttttccagcttctgtccaatccactaaccagagtgtcagcaaatt

cttcaactttgccaaacgattccatcttagtgaaatccggtcctacccccgtgatcacaa

cgctcacattggaattagattgctcttttggatagaaagccgttagtgatttaaatccag

cagattctcctgttcctgattgccactcttgaggaatctctatcttgaacttgttctcat

catcggtgtagacactaaaatcctcagaaacatcattgagtgccaatgcatttgagacag

tagatgaagctgcaattgctgctgttgaagtgtaaagaatcagttgtcttctattaacag

catgtccttgttctaaggaagaactgatagaattttgtggtcttgttttatgatgttccg

cggttacggaagatgataaacctttgttgtctgcagaggctgttggtcggatgtgaagac

agctgaaccatgaaatggatgccatttctttaagatggatgaccttcacttctcaccctt

catttcctttattatctgtttaattacattagtatcctttgcaaagagactatttttatc

aatatttttaaatggttaaatannnnnnnnnnnnnnngaagattaataaatttagttttt

cataaataaaaaaaatgacccttctttcctctatacaaaatttttatgtgtacagttttc

taaataccctctcccaccacttctcaatcgcagcttcttccactcccttcttccatgact

ccagtaacaatgattttgtggatttggttttatatgcggattttgtgtgtgtggatttca

tactgttattcagtacagttttactgttttctgtaaattgctcaactttatatgacagta

tgcggattgaaacgacttcaaattaacctttggtgcaatcctatagttatcacttatgga

gtaacgaaaaaacttctccaaatcaatgaaaataatctaattttcttca

>fava_dn_rep_c310

ttgaacgtcagtttctcctagtttttagtttatttcgactatagcatgaacacaatgcaa

gactaatcatgaacatagtgcaaatggagcatatcatgttacaaatgacttaaaccatta

tataactaatcaaagttgtaaatcatgtaacaaaaagttattgaaattcaattacaataa

acatatgtataatgattctacttgtttcatttgaatggagtggtaacattgagattttgt

tatggaaactgatatcctgatgcatatacattacactgttacagtgcggcgaacatgtaa

cgatcgatggttaccaccgccgtactaatgatgtgagaagctcaaaaagttcaaagggat

ttccattgcgatagacgtcgttcttcttactattgtcgacgggagtaatggcgtatagta

atgcccggactgcgatgagtaaaacagctgtgcttccgagactcgcaaatacaaaaccat

agttaggcaagtggcctgattttatagcccttattgctattcctatagggatcccaacag

cccatgattttgcagtagcaatgagacctttgggtagaccgttcatcccacgcccatctt

cactaaaacctccaaggaagtaagcccctaaaaaccacccagctataaaaggatcagcag

tatgcagggtttcaaagtcaaagatagagagtccatgactatatcttccaattgtcgaaa

acaaaagcaaacccaacacatcgccaccagcaaacaaagctacacgcccccatttttcaa

tacgagctgatgagttgggtttatcgaactgaatgacaccctccaatggaacatccttat

caccaacaaaaacagtttgatcattggaagagggtgttgtgggtgaagttgagtctgcat

ttcttaaggctttggctagtgtaagatgaaagtttctaatggagcttgttctagtgaaga

atttttgtggagagtttgaagtgatgaatttggttgaagatgagatggaacagtttgcaa

acatggttctaaaaggaggttggatgtggctaacaagtatcattattatttacaaaactc

actaacgtttgtgttggttgattctcaatatttaaggttcatttgttctatctatggttt

ctcaaccacataacatgat

>fava_dn_rep_c311

tctctctctctctccttcaatcgaaagtgataggccacaaaacaggccctttcctttggt

ttggttcggtttgattttcataacataaaacaccttcatggcgatcttgaacaacgtgtc

actcctgcagcttctttccaactgggtcatgtttctgggccatgaaggctttgcttgaaa

ttctcaaaactctagtaaaaaacaaaactttacttcttttttcctgttctactgtcaagg

gttgttttaatttgtttgggatgttttggcgctgtgtggattttgggtttgtttgatttt

gttttttgggagttgggttttgtttnccttttttttttaccagaaaagaggaatcatata

tattgtgaagtttacaatgaatacacaagtagttttttattacaaaaaagatttgactgt

acaagttctacagtacagatcctttggggttttacagagacaatccagaatcgtcatcct

cgtcaagggaggaagcatagataaaatagagagcccaaataagaccgaagacaccaaaca

agatccaaccaagaagattattgctaagaccaaagggaagtccagttccttcagtgctca

ttcgctcatctaccaaagccatggctgctgggcttgacaaggttgcagcagagatagctg

ctatcaaggatgctcccattcctatgcttgagctgctttcttgcacatcaggctttcctt

caatggaacacctcactcttccaaccttagccattgatggcaatccaataacggtctgga

atgagactccaaaaggttgcttgtgcacaagacctgtgcggatgattgaagatgttgagg

tgccagcaataatggttgccatttttctttaatatcgattcagctttccaagtatgggta

gccgtttatttcttacaaattggaaagtgtgtcagtctatttaccacacaccgtggtgga

cgatagattgaaaaatgaaaacccgtttatagatatccatatctacagatatccaattaa

gtttatccacgtgtcatacgatcagtttggtttgctgcccaacacttccttgcacaacca

ttgcttctctatcgctcctcagcctacggggtattagcagccgtttccagctgttgtccc

cctcccaagggcaggttcttacgcgttactcacccgtccgccactggaaacaccacttcc

cgtccgacttgcatgtgtaaagcatgccgccagcgttcatcctgagccaggatcaaactc

tccatgagattcctagttgcattccttataacttccttgtttctagacaaaactgattcg

gaattcttttttttttgctgatgctgatgctgagaagacataacttgtatccatgggctt

actattcgcccggagttgcttccccccgcggccgtaatgtgcatgtcagttaatacaatc

catataaataaaagaaaatatcaaattatctaaaggtaactcaatcagtcaaaccaaagc

agcgacgaaattatccacaaatcaagttatatcctcaacaaatttacattgaaatccatc

tttttccgcgctgacttgggcaaactactaaccttcactcctttgaggacttgttgataa

gagacttgtggatatgaggaataacaccacctccggcaattgttcctttgatgagagtgt

cgagttcttcgtctccacggatagcaagctgtagatgtctcggtgtaatacgcttcacct

tcagatccttgcttgcattaccagccaactccagcacctctgcagttaaatactccagga

tagcagcagaataaactgcagctgttgccccgactcttccatgcgccattgtccttgact

tcagaagacgatggatacgtccaacagggaactgaagaccagctctggaggaacgagaag

tgggtttcttcttgtccttgtctttgagggatgatggggttttaccggttgtcaaaccct

ttgcgcctttccccgacattgtttcgaacttcgatcgcttcagagagagagttttggttt

ttgagatttcgcttctatgttccctagtgtccctttttatactcaaaaatatctaattca

>fava_dn_rep_c312

ggccgcgagacacaactaagcaagcaatttcttagttatttgttatctttcatacgagct

agcattataaatcatcattatcatgggtgttttcaattttgacgatgaaatcacttctgt

tgtagcacctgctatacttttcaaagctcttgttattgacgctgacactcttactacaaa

gattattgatgatatcaaaagtgttgaaattgttgaaggaaacggtggtgctggaaccat

aaaaaaaatcacttttgttgaagatggtgaaaccaagcatgtgttacacagagtggagtt

ggtagatgttactaactgggttcacaactacagcatagttggtggtgttgggcttccaga

cacaattgaaaagatctcatttgaggctaaattgtctgaaggaccaaatggaggatccat

tggaaagctgaatgtgaaatacttcacaaaaggtgatgatgctcctagtgaagagcaact

caagaaggacaaaactaagggtgatggtcttttcaaggcccttgaaaattactgtattgc

tcatcctgattacaactaaagcatataatcgtcatcggatttttgctttatacattgtgt

gtcgctatactatatatccggtgtgcttaatttggctacacatttgtaattctctgtttt

attttgcccctttccttttgttgtggaagtaaaaatgtgagattgtaagtcgtgtgtcta

cctctctttcaataaattataataatacttcttatttttgctaaaaaaaaaaag

>fava_dn_rep_c313

cgggggtttttcaataaacaaaaagtatttgggcaaggtgcatcccccttagtcctctgc

tcttcaaattcaatccaccaaaaaccctcgcacgcaacttttcccatttgctccttcacc

tctgcactttccaccatgggaaggggaaaattcaaggccaagcccactggtcgccgcaac

ttttctagccatgaagacatggttgctggcacctccacccgtcctaggacttttagacag

aaagaagctgagaatgaagaggagccatctaaagaagtttccgaggacgagtctgaagaa

gaatctgaagatgaaaatactaaggcaaagggtgttcaaggcgttattgagattgaaaat

cctaacttggtaaagcccaagaacattaaggctagagacattgatattgggaaaaccagt

gagctttcaaggcgtgaaagagaggagatagagaaacagagagctcatgagcgctacatg

aaacttcaagagcaaggaaaaacagaacaatccaggaaagatctagatcgtttagctctg

atacgccaagaaagagctgaagctgctaaaaagcgagaagaagagaggttggccaaagaa

caaaagaagaccgaagctcgcaagtaaatttcatggagttttactccaatttgctggagg

aatatttgcacttgctatattttaacattttatatgtccattctccttatattgaatatg

aacattccatgcctggtagtgtgtttctcttcacttatgacttgaaattttctgtatgtg

gctttaggtgatctaaattcagctttaagcataatttttgccctgcctagcacatgtttc

tgttgagagttttaaatcgatttttttggcataagcctaactttgttagggcaatatcta

tctttcaggagtgttgaaaaaagtgttgaaaaagagtgttcgttcttcgttacattctct

tatcttgaatatgctaaaggataatgcaagtcaaaccatcgaggttgtgaacagtcacaa

tttgtcattatcattagacaaaataatttactacaaaatttgttttattttatttaaatt

tttagtttctc

>fava_dn_rep_c314

aaatgggattggaaatgtacacaagttcatttttttcttgacatggagcttcaccggcat

tantttttttttttgagaaaatattaatgaaatagatgccaaatgaagagatatgaagtt

cacaatccaacattattaggttcctcagtcacaatgacaccagtaccatttaccaaagaa

agttgaaaaacattatcatctagttattgctcaagacaccaagaagaaactaaacactgt

cctttaaatgacccactataaatagctaacagacccccctcacaagggtgcaacatgata

aagaaggaactactctttggtgtagtttctgacaacaaaggctaagcccaaaatcaggag

aggcaccaggaactgcaaaatcttgatcacgaattccgaagtcttgtcaggattgtactg

agattgctgaggcggaatgtaggagcgttttagtggcacagttgctggatcaatgtcacc

gatgtagtattggtccatcatttctctagcagaatcgctgtgtcccacatcttcaaaatc

tgtggttgcatctttccctgttgcagacaacagaacctcgtcgcctccaggatgatcctc

cataaaagggctgacatcatacacctttccagaaagaataagccaacaatctttggtctt

gctatgttttgccacctcttcgaaagtgtgaatctttggatctgaagccattttttagtt

ctttcacacttccggttcttggtagattcttgagtgtcaaatctcttcactcagtggttg

gtcccgcggccgtaa

>fava_dn_rep_c315

ctataaccagttaaggccaaaggagatgctctaacaattacacaaccgcgggaatctctg

atccatatctagagaagaagaaacttctcaaaaccgaaaaccaaaccaaccatcatcata

actagagaagaagaaaaaatggcaacaatagctggtctgagtctctcaaaccctaggctt

ctcttcaactcaccaggattggcacaaaccatcaagatctcaccagcatcacggcaaagc

ttaacaggatcaggacggatgaagatagttcaacctcttcgtgctgcaccagaacaaata

tctaagaaggtggaggagagcataaagagcgcgcaagagacttgtgctgatgatccggtt

agtggagaatgcgtggctgcatgggatgaggtggaggaactcagcgcggctgcgagccat

gcaagggataggaagaaggagtctgacccgttggaggattactgtaaggataacccggag

accgatgagtgtaagacttatgataactgagtctgagagagttgtaaaagtgaaactgtt

gctttatatgatgagtatgatgatgaacatgatgagatttgagtagtattgtacttgaat

tgtgtgtgtgtgtgtttatggatgtttaccttagcatcatagattatatagcatcttaga

agataaatgtaatttacattgaaagatttctgcaacagtgcttttacttgatttttatga

actttgttatgataaggagggggcttggatccactaagaaccttgttatgatagtgtcgt

aggttatagagaattccaacatggtgataaggtgaatatttattagtatgtgtcccattg

aagatctacacacatttatgtataaagttgccaaaagaatctaaaacaccttatgttaag

tacttaaatataccttcctcaacacctagtttcttcactactaattttgaaagccacttt

catagtctcaactcatgaaacctactatgcttcagttggagacatagccatcatgaattg

agttgatgttttttagaaaataggtctacttgttatgtgagttgtattcataaccactat

gaattacactatggattgaattgatgtttttttagaaaataagtctacttgtaagttgta

tacatagccattatgaattgaatcgatgatttttttagaaaataaatctacttgtaagag

ttaagacatatccttttgtagacttcttatcatccgaatcacatattggttctttcaagt

ttttggaaatccacttgatcgaaaatataacatcggatct

>fava_dn_rep_c316

agacttcccccacttcaacgtgcttttgccttctctattattgctacaaagctatcaaat

cagtgaatgcttagagagagagagagagagagaaagagagttttagagagagaaaattca

tggaagttgcaggtctgaaaaaggaagcgaagatggtgtttgatgaaactgaactgacgt

tgggattgcctggaaatggaagcaaagtaacagaagtagtagtaaggaagagaggtttca

gtgaaactgagagtgagagtgacaatgaaagcgaaataaccactgtagatttgaagctta

atctctctcccaaagaaggtgcagcagatccgcagtttaagcctaaggagatagctcttc

tggtttctgattctcgcaccaagcctcctgctaaggcacaagtggtgggatggccaccgg

tgaggtcgttcaggaagaacctgtttgcagggcaaaagagcagtgaaggatcagaagaaa

ccaagaacaaaagcattgacggttctaatgcaataacctttgtgaaagttagcatggatg

gagcaccttatcttcgcaaaatagacctcaaaatgtacaagagttacccggagctttctg

atgccttagccaaaatgttcaactccttcaccacaggaaattgtgaatcccaaggaatca

aggatttcatgaatgagagtaacaagttgatggatctattgaatagctctgactatgttc

caacttatgaagacaaagatggtgactggatgcttgtcggtgatgtaccatgggagatgt

tcatggaatcatgcaagcgtttacgtatcatgaaaggaaaggaagctattggactggcac

caagagccatggaaaagtgcaagaacagaagctagctagactagtctttacagtccatag

cctgctccatcctcagtgctactactgcctaagctgaagcatggttaagaagaaaggtgt

tgaaaatatatagataatctctgtttgtgtgtgtgtgtaacattatggttattaatttgt

aatattataatactacttcaaacaaattaagctttgagaagtttaagttatgaacagaag

cgataatcactttatattatgtgtttctcttgcaaggttttatttatatttacttgttgt

gtgtttgcatgattcataataagaataataatgcgcttgtttgttgttaaaaaaaaaaa

>fava_dn_rep_c317

tttttttttgaaagagaagaacactctgatcatttggatttgtcattggattcaaaagct

cacgaggcgacaagatccattactacttcaaatgtcacaatacatgttcattacaatttc

aatcccatcattacaagaacttcgcattatcaaaagtcataagaaaacctaaacctacaa

aagaaactgaaaacaatcttcaagacctcttcagattttccccatgccatctctatgttg

caaaccatgtcatgattaagtcttcatgcccttatgtcttaatctcatcaccatgcttca

tgttctcacattcacatcatccatgaccaagtcattcaccaatcttcatgccatgacttt

gtccaaactcgctttaaacaatgacactcaagagttgttgcataaaacagggaccaggcc

cgtcaggtatccaaaaaaaaatcatatgtgccataaaataacctgcatattcattaggaa

ggaacagaaagaaaaaaaacagaaaacaagttctgcaggaaaaaacccaaaacagttcac

acagtcagcttgctaaaacagccctgcaccttgtcaaaacagatcccaaactcacacttc

agaagatcacacatactatgcgcattaataagaaaataccaactataatacacatttaat

tgatcatcaaagcaagcctaatgtagcatacatgaacctgcactacacaactatgtacat

acagttcaaaacagagaggtgagaaagtaaaactctacaaaaaaactaacaagaactaac

ctatgacagctcccacaacgatatccttaatcttttgatcctcgaagttgggcaaaatta

atgaaatccatgatcaggataggtttggtgttgagactttcgaatttcatcatgtccggc

gcggcggcgctttccgttcgaatggtgagaagagagagttcaggacgagagggtagagtc

tgagatggttgcgaacaaagacgaatcgacacatgccaaacgcgtcgtttttattaatcc

cc

>fava_dn_rep_c318

tggtatcaactcagagtggccattatcgttgccgcgctaatcgtcttcctttcagcctcc

tccgaaatcaattctttgcagaagggtagccatggtgaagtactcccaagaggccgacaa

tgccaccaaatcaaccaaggccagaggcgccgaccttagggttcatttcaagaacactag

ggaaaccgcgtttgccatcaggaagttgccactcaccaaagccaaaagatacttggaaga

tgttttggcccacaaacaggccattcctttccgacgcttctgtcgtggtgttgggaggac

agcccaagccaagaacaggcattccaatgggcaaggacgttggcctgccaagtctgctaa

gtttattcttgatttgctcaagaatgctgagagtaacgctgaagtgaaaggtttggatgt

ggatgctctctatgtttctcatattcaggtcaatcaagctcaaaagcaaagacgtcgtac

ttacagggctcatggaagaatcaatccctacatgtcatccccatgccacattgagctcat

tttatctgaaaaggaagaggctgtccagaaagagcccgagtcccagttggcaacaaacaa

gaagaagtctcaagctcttcgaagtggtgcttcgtcttagattgttgtttgagctaatag

ttgagggcccttcatttctagattatgtcatttttgttgcactttctttgttatgagctg

tttttgaagtttcaacagcagtttttgattcacacagtatggtttatgttaattgacatg

tttgttaaattttatggtggtaattgcatgattgatttgagttttatttaaaaaaaaaaa

aacctaaacctacaaaagaaactgaaaacaatcttcaagacctcttcagattttccccat

>fava_dn_rep_c319

tacactttacaaggtgattgggttccaatatcattgttggccagcatccaagtaaagcta

taggccacgtattatcaacaatctatcacattctcttcttatatcatccaaccatactca

tctcaccttatccttttctcccatttaatccaaaaacacaaatctcccttatcacaaact

ccacacagatttcaaggagaaaaaaaaggttattgcatttattttccttatagtatcatt

catcacttcaatatgtttctctaacctctttctcatatttaacagataataaaaatggcc

acagcctcagcaacatctccatctacaatcactgttgtaggaagcacaatcggttctaag

tcaaggaacaacaggaagggtaacaaagtgaagttcatcacaagactcaatgctttaaac

ggattgaaagctcagaacactgtggcatcactaggagttcctgttagcactgaacaagct

tttgcaaaggtgatgaattcaatatataagggaggaagaagaagtggtcgaggtggtggt

gctgcttcttctacatgtaatgctgcaggtgagatttttcagattgcggctattatgaat

ggacttactcttgttggtgttgctgttgggtttgttcttcttcgaattgaagcttttgtt

gaagaagctgagtaagaatttttattatatatgtatgtaatgtaatgaaatgcatgtttg

ttatatcactttaattcttgatgtgatgatgtagaactcaatgttaatgttcttgaattt

ttattttgagatgcataattgtacacaaatgctgtctggttttgtacatcaatattggtt

gggttatctatgtttagaataatcatattttgatacacaattttgaaatttgaaattttc

atcaaaaggttctgccg

>fava_dn_rep_c320

aaaccgttcttttacatttctgtactattcagaaannnnnnnnncgctggataatccatg

atatatttagattttgagtcttacattcataccattacaaacattaattagggttatgtt

tgaaaaaaataataataaagcattaattctttaacaccaacgataaatttttataaaaac

acgagagaaagtaaatttttattaataacataccattaatgagaaatagcactttctgga

gagcagtaaaattaaattaacaaggcccaatatactagataatatgtcaactatatcttc

aagcaccattcaagagatacttttctaggcagccaagataaaataaagtggattccgatg

tcttggtaggtttgaccttttcttctatgtcggattcatgatcataagagatcttcacgt

tgaccaaagtcttatcttgtgttactgctgtgagtttaaatgttgtcttgtagtatgaca

aaccttgattcagatagcctccttcaatcacttgcagcccaatttcatgggataactcat

caaactcagtgatctcttccctctgatagtttactggggatataccaggcaaaaaattaa

agatgaggatggtgccaacccctccatccccttgaactacttgcacatctttcacaatat

ttggcagaacctttggaacaatgaaaatgaaatctctggactgagctgcccatagggtct

ccagccctacattgagtgttgtttgggtgttgaattcctttatcatgattcttcaatgtg

cgtttcaacaaagtagcatcatatattgatttatatactatatggaatttgataatccca

catcttataacactaattaaggagaaaaattacgaaacagcatcttccactatgccattg

actta

>fava_dn_rep_c321

tttaaattagtttgaaggggtggacaaaagcatactagtattggttttatcagattgggt

tggataagaagaaagtcacagtgaaaacgaaaaacaagagcaatggcgggttccgcaatt

tcgatcggaaacactttcgtcttggcttctatgcgcaccgtttcaccaaccaagtcttac

aaactttcatccctctcaccccaattagggttcctgaattctcaattaactggcctcaga

atctcctctcagacttcattgaatacggctacgcctatcgtcgcttctccttttctaccc

attgttgctaaaagagtgtgccccttcactgaaaaaaaaagcaaacaaggccaacaaagt

atctttctcaaaccataagacaaagaagttgcaatttgtaaatctccagtacaagagact

ttggtgggaagctggaaaacgctttgtaaagcttcgtttgtcgactaaggcattgaagac

gatagataagaatggacttgatgcagttgcaaaaaaggctggaattgatctcagtaagaa

ataagagcctaattcgccttgtaagtgagtgaaatgataaatccatgttgattaactttt

gttgtagaatttctttatttatgtattatttttagggactacggttgactttctctatgg

tcaaaagaattataagttatgtagaaacgcatatgatgagttgataaaatttacttgcca

taaatttcgaatggaatgtctttgataatcccgttcatattgaatagtttgaatcatatg

aaactaggttgtttgcatattgattattgtattatatactagatatattttatcagtgca

tctaacttataatatcgttcatt

>fava_dn_rep_c322

ccgcggggtatatatataatataaataatataaagtccatatatatagtcttaaactttg

aggaattttcgattcagtacatcgaaacaaacaaccaggaaaaaaagaaattttcttttg

tagtactcgatctcgtcacttattgtgaaatttcgttgctgtgttagaagaaggatagct

atactgatttggtatactggaacgagacccttggtacacgattgacgatcgtaacaaaga

ttgaaaatttcagtaaatttctacttactgatctgatcttttatggaatcgatccccctt

tgactgtataagaatatgtggagctcggcatgtctggaagcacaggagaacgttcttttg

ctgatattattaccagtattcgatactggattattcatagcattactataccctccctat

tcattgcgggttggttatttgtcagcacgggtttagcttacgatgtatttggaagccccc

gtccaaacgagtattttacagaaagccgacaaggaattcctttaataaccggccgttttg

attctttggaacaactcgatgaatttagtagatctttttaggaggctctaatgaccatag

ataggacctatcccatttttacagtacgatggttggctgttcacggactagcggtaccta

ccgttttttttttgggatcaatatcagcaatgcagttcatccaacgataaacctaatccg

aattatagagctatgacacaatcaaacccaaatgaacaaaatgttgaattgaatcgtacc

agtctatactggggtttattactcatttttgtacttgctgttttattttcaaattatttt

ttcaattaattgaagaaaagagaatagtatttatatattaggaattctcttatcccattc

ggaaggatatcatttcataattatctatgactgtttaggtctctagtatgactgactact

tgatcaaatgtggaggaaagtgggataaatggccgatactactggaagaattcctctttg

gataataggtactgtagctggtactgttgtgatcggtttaattggtattttcttttatgg

ttcatccaacgataaacctaatccgaattatagagctatgacacaat

>fava_dn_rep_c323

ttttcttcatacttttatgttttatgcttgcaaaggattcaaagtttttgcaccattcaa

gtgattctctcaaagaatggtcactcacataggatagtacaaccattaatatcctagaaa

tttaacgtggatggatgcttttcttatatcgactaaaatttacaccaatattataaacta

ataatcacagaacatgaagaagaaaaaacacagttgccttaaaatgatgctaacagaaac

atcagattctatatattttgttactatccaagtataattgtataataaatccccaagttc

agaaaaaaagggcggtcctgctagctagagactccacctttgagatgaattttaagttca

atctatttgtagatccattcgtttgctcttgcaccagactcgtccatatcaataaagatg

cttacagccttccctatccgaggccgttccttcgactttgctaggctgctcctaatatgt

gagacggctccccagcattttaatgagtttgcaacatcatcaagcctcaaaatatactct

tcttcagtcaatggaaaagatctctgctccatgtacttccacatgacattcatagcaagg

agatttcttcccatgaattccttcttaatcaactgaacatcataagatttcccatatttt

ttcctgataagagtagcaagatcaaatccactaaacctcgaatcatctgtaccaactatt

ttctcgagactttctctgagaatttcgctattcacccctctaacttcatcattactctga

gatttgttcttctcactctcatctgaatcatatggatccatattgtcactcactgtactg

gaaaaaaaccggcctacaatttctggaactacggcgacatttatgagcaacattagggaa

gtccaatcctttggaaggagaatgatatcctgaaaatggagccttattaataccacttcc

tttgtcaagatgatcctgaattttcaatggccaaccacactggttggtggacataatatt

cattgtgtcctgtgaaagacagaagcagagacagattcagtttgtgttttttcttataaa

tactgttgttattgttttctgggtgaagagattaataaaataagttccactttaatataa

atatgatgatgaaaaaaagatttgagatagagtagaaaagaaaaagataataacaatgaa

caagtagaataattgtggatattaaaaaattgtt

>fava_dn_rep_c324

acgtgtaatgaacatgtaccacatgacttttgatggatatgcttttttatcattcccatt

cttcttcctactcacttgtctcttcattcccaatccttagctctctcatccatggcttca

atcacccagtgcagcaatttcatatgcaaaccctcaatcccttcttcaatcagaaccctc

cctaagttccttccccttcattcccttggttcttctctaccaacttttcatgctccatcg

ctactctctcacacaaagactcgtgttgttatcacaaaagccacagcagctccaggtgca

aagaaaaaaagatgaacgagttcaaaaagtccacagcatacaagagttcgaccaagcact

tgaatcggcaaaagacaaactggtagtagtggaatacgccaccaaggagaatccagaaag

caccgagatataccctttcttagtcgacctcagtcgaacctgcaacgacgttgagttcat

cctcgtcatgggagacgaatcagagcaaacaaaagaactctgcaaaagagaaaaagtaga

caaagtacctcacttcactttctacaaaagcaaagtaaaaatccacgaagaagatgctat

tggacccgatatgcttgtcggagatgtgctatactacggtgacagccactcagctgtggt

gcaacttcacaacagagaagatgttgagaaacttattgaagatcataaggctgatcataa

gcttattgtgcttgatgttggattgaaacattgtggtccttgtgttaaggtttatccgac

agtgataaaactgtcgagacaaatgattgatacggttgtttttgcgaggatgaatggtga

tgagaatgaaagctgtatgcagtttttgggtgacatgaaagtgattcaggttcctacgtt

tttgttcataagagatgggaacatttgtggaaggtatgttggttctgggaaaggggagct

tatcggtgaaattcttaggtaccaaggagttcgtgttacttattagtaaatctttcatta

ttcaattcatcaatactgtctttagatcattggtgagataggaatatatatatagttaag

gattgtctcaccggttcgaaagatatatatccttaattgtattgtatccttatgtatctt

tttttattgatttcactcgattgctctattttgttgttaacaaaatcaattaaactgtta

cagataaaattgcttgtttggggtcaagaggaagataattaaacatggaaaa

>fava_dn_rep_c325

aaacaaatagatatcctacggtttgattttggcagtaaactaaaggtttttgtacaatca

tatttagtgacacatgagtaattgaaacaatcatataccagtcaacatgtaaaagcacaa

attgcatgcatcccattcatgaatataataatccataaagaaaagatgaaaattgagtac

aaatgcatggtaaaacaactaattcaaactacttaaacaaagggacaaaccaaacaagta

gaatactaataatgacaagtagcaataatttaagcaaacaaaccacccaacaaaaaacta

ataacccaaacaaaacatgtcctaatgaaaaatagaatagtaataataatcctcatcaaa

actcattagctttgtcccttttctgttcatatgctttttcaaattttagttatggttttg

attgatccattcacttagccatcatgactttgacaaactcctcgtagttgatctgaccat

caccatcaacatcagcttcgcggatcatctcgtcaacctcttcatcagtcagcttctcgc

cgagatttgtcataacatgacggagctcagctgcagagatgaaaccattctgatccttat

cgaacacacggaaagcttccttaagctcttcctcggaatcagtatccttcatcttgcggg

ccatcaggttgaggaattcagggaaatcaatggtaccgtttccatcagcatccacctcgt

ttatcatgtcctgtaactcagcctcagttgggttctggccaagtgatcgcataacagtcc

cgagctccttggtagtaatgcaaccatcgccatccttgtcgaatagactgaaagcttcct

tgaactcagagatctgttcgtcggtgagttgatcggccattggaagggaagaagatttgg

ttgtggaaaggggaaagaggagaagaagaagagaatgatgtgtgtgatgtggaaaatgta

acccgcggccgtaatggccactctgcgttgatgagttgagatctgttttggtgtcgttgg

gattgaaacagggaagaactgtggaagattgtaagaagatgatagggaaggttgatgttg

atggaggagctgtttcagaagggtagtagtattggcgtttgatgagagaatggtgttgaa

ttgaatgaagagggttagttgccataatgcaatattctataattagtttaggatacaaga

aaggataagggttatagaaagtgttgaagaa

>fava_dn_rep_c326

atatgtgaagggtcggctataagtgtggggtgtcaggtacatctatagggtgccaagtgc

aagtgtgggggtgtcaggtagatgtgtgggggtgcaaggtagatttttccacattctaaa

aggagaattcagaaactcacaaaatataaccaaacaggcccaaacaaccaagccaggatg

aaaggaaccaagttatatataatttcctaacttgcacaaaacacctgcacttattaaaac

acgagaagtttttcctgctatatctaatcattttctgtcaaaattgaaacaactagacca

gactcaaaatgaaaacacattgttgggaacttttattttgcgtcaaccatgagaaagaag

acaaacatgaaaaatatcatgccatgtgaagttctagatcacaattcaataaacaatacg

aaacaaaaataatcacttttttccgaggctacaaacatcagtgcccataaacaaccttca

tgaaaccactaatactcctagtggtgctcttccttgatttcaaatagcccatcaggaccc

catgggaattccttattgcggatatgcaaatatgggtatgcaggaggtgcgtcatagtga

tggtgaccctttgagagaatataagctgctaaagcggtgcacgaaacaatgcctagatat

gtgatcttctcccatttggccgtttcgtatgcatcatcgtgatgagcggaggaagaaaag

cttcgcttgggagcggaggagctgccggagccgccgcgtcgtaagacgttgcgaagatat

ccagatctcaccaaagttgtcgccatttactcttctagaaattgttagctttttttgttt

ttcaaccccgcggccatatttttaaagttaaataatcacgtgtaataaaatgattgtggt

tttgttaggacacatattttcttactctttgaaatacctttgactaataagaaaatatta

atgttgtcttgcccaaattaagaaatatgtttgaacccgggaaaaaaaagatggaaaatt

gggaagggggaagtgattttataatttgtttgttccagaattttccatagaccctcttcc

cccct

>fava_dn_rep_c327

aattgatgatgtaataacatttgttgttttcgtggtactttgattcattttggtgttgga

attagatgtagatggatcggtttgtaatttaatagtaattgtaaatagataccagaagtg

cattgttatcaatatacattacaatgatacttatgaataattgttacaccaaaactcatt

attggccacagataaaagtaaacttaaagttaattcctcagacatcaaataacacttgtt

tgtctgaacaatttcttcttttttctcatatgaatgaaatcatacgttgataacaaaagg

aaaaaacaaatggcttgctggtagaggtaataatcaagtcagaaaatgattctcctctat

tcaaaaactttgcattccttatcagccggactctcctcgcagaagtcctccaatggatct

ccactcccaactttaggggtgcctggccatttgtttgctacaagatgtgccaaatccacc

actctttgactgtaaccccattcattgtcataccaagcaaccactttaaccatatcatct

cccatgaccatagtcaaggaagagtcaatagttgttgaaacatcggagcagcggaaatca

acagacacgagcggaacatcacagacatccaatatacctttcattggcccctcagctgcc

tttctgaatgctgcattgacatcttcagctgaaatacccttcttcgcaacattcaccaca

aggtcaacaactgaaacattaggtgtaggcacacgcagggcgattccgttgagctttccc

ttgagctgtggcaacactagagatacagccttggctgctcctgtgctggtgggaacaatg

ttcagtgctgcagccctagctcttctcaagtccctatgtgaagcatccaaaagcctctgg

tctcctgtgtaggaatgtgtggttgtcatggttcccttaacgattccgaactcttcatcc

aggaccttagcaaagggagcaagacagtttgtggtgcaagaagcattacttatgatgtcg

gctacttcatggccgtagtcttgttcattcactccaataacataagttggaatatcagca

ccctttgcaggagcagtgatgataactttcttggcacctgcttggaggtgcttgcccgcg

ccagggccgtccacaaacactcctgttccctcaataacaatgtcaattccgagttcggcc

caaggaagcttaagaggatctctgctagagacaaccttgatgggtttaccatcaactgta

atagtctcattgtttagtatcttcacttctgctttaaaagttccaagcatagaatcatat

ttcaacaaatgtgaagcattcttgacacctccactgtcattgacaacaatgacttcaagt

ggcgagtcctttcgaccgtgccaacatcgaaggaagtttctaccaatgcgtccgaagcca

ttgattgctaccttcaacttagccacagcctctcccctcacagcagttgatccagttctc

ttggaagagagttgagcagctacaacatcataaaaagaagactctctagcattaagagca

taggtaatacatgaagttgattttactccagagaattcaggtacctcaagtctcttagag

gcacattgagttgggaaagagtgagaggtcttagatggaaaccttgtgtttgttgggatt

cttgtagaagctaaagcagcatgagtagccatagctaaagctgcaagcaagtttaaacaa

gaagtccttggtgagtgtttatattcagaatgaaaattatggttgatgaatgagtggtta

atatgtttctttttagagatttttatgaagttggttgacaaaaagaaaataatggttgct

aggaagtaatggaat

>fava_dn_rep_c328

gaaaacgattctaaaaaaaacccaaaaaaaaccattttcatattaaaaaacccacacttg

tccttacgtgcctcttcgggcagcccgtaattcgatttttaagcttctgaaatctgccct

aactgcccgaaacttggctgagaaattacgagcagaacctgtgtctccttctctctttct

ctcactcacctctgatatttctatatttcgaagctcagtgtttcatcgatcaagatccaa

cggtccataatgtacgagctttcttctcctatttttcagaggttttagtgcttcaatctc

tcagatcttgcttttcactctctagatctttgttcctggataaaaattttctggtttgag

aaaggacttgtttgtttgtcacacgaaatggagactgagcacagttcgaagatggtcacg

ctccgttacggcgcgttaacgtacgccgcggaagtgcttgtaactttgttagcttctgct

gtcgtgaaagccacgcttttggctccggaactctatgagctaatgctaaccaacatgccg

tcaacaaaaggagagagtaaacccccaagtcaagacaagcaactcgacgctgaaggagaa

gatggcaatgacgatgaagatgaagaagatgacgatggaaacggtgcatttggagaagga

gaagatgaattgtcatctgaagatggtggtggatatggcaacaattctaacaataagagc

aactcaaagaaggctcctgaaggcggtgctggtggggcagatgaaaacggagaagaggaa

gacgatgaagatggcgatgatccagatgaagacgatgatgaggacgaggatgatgatgat

gatgaagaaggtggagaagaggatgaggaggaaggtgttgatgaagaagagaatgaagag

gatgaggaggatgaagacgaggaagctttgcagccaccgaagaaaaggaagaagtgaatt

taggttttagttacgcctccttcctgtattgttgtgtttggatttggcttctaacttccc

atgttgttttattcagttctaaacttttatgttgttatttacattataagggaggcctat

gtagtaggacatgttttaattgatatctgtgtttaccattgcgtaattagaatgctacat

tatattaagcagacagtcttaaattaacgagagaaaactcatgttccttgttttatcttg

acttttccttc

>fava_dn_rep_c329

gaacgataatacatccttgtgaattcattattgacaaccagattcacaagaagatgctgt

acagaatgaaattttaagaaattttatccttatatcacttccagaaatacatagaattta

gtagaaatcgagtgcaaataagtaatacttgaattagacataggtaaacagattaaattc

aagtattcggtctgtttaaagtatatgacaggagaacaaaagaacctaaaatacaaaatt

gaaaatcatgattttctttgttgaaaaaacacaagatggcacacaataacactctaacta

tccttggcctcctcagcagctgtctcaaaagtctctcccgggacaagctccggaacgtcg

tcatcattctcttcttgaactgtggctgagccagcacctgcttcaggaacctgcttattg

aactgctcagctatcttcttcaggttttccaaattatccggccctaattggtggataatg

ctaggaagtatgtcctgcaacttcttggtttgaggagcaccactaacaacccatgtatta

gcagcaatggatgcttgaactttggggtttgagaattggatgacaacgtcatccttaaag

atattgacctcctcaatttgagggatggcattcaccccaattctcttcagggtgctctga

agccttttgtcatctgtagttgttgtcttgtggacggccttcttctttcttctgacagta

ccctttccaccagttcgaactgaaccagccatcttaattaacttttcacgattcatcttt

gtgaaatcactgatttgggaaacagggtaaacctcaccgtgaagaagaagaacgaaacgg

cc

>fava_dn_rep_c330

caccaccaatgagttactaaatatatattccaatatacaacaaaagaaaacaacaagcat

tcattaccaccaaagtaagcataagaatgaagacaatcaaatataaacatattatcaatg

tcaacacataatcataacaatctcgcttaatgttaaagctaaacatccaaattcagttaa

atatacacaaaaccactccataaagcataaaatagcaacgtaataacaatactatataac

cagagtgtatcatcattcttcaacgagaaaacaagaggaacattaacctctagctttaag

ctccgcaagccgcctcgacaaatcatcctcatcaaccttctcagcatccttctccttaag

cagaacagcatgagcagcaggctgaggaagcccaaccgacacctcaagtccataatcatc

agcaacctgttgcatcaaactgttaacctcaccttccggcgtggaaagcgaggtggatcc

agccatagcactctccataaactccgcctgcacctccatgttcacaaactgtttctcgaa

ctgatccatcgtctccgacatcttctgtaaatttccggtagcgagagaagattccaacga

tttcacgatgttccccatcgatttgctgatcgtcgacatcttcgcctgcgtatcgagacg

agcgctgacggcgtcgagtcgggaggataatcggaggtagttcatctgctccgttcgttt

tcggatcgcgttttcggcatagatccgtgcgccgtccatgtttcctttttcgattgcttt

ctttactttgagtttctctgatttctcttccttttcgcactttcttgcttggcgttgaag

cgatttcgaggtgaatttcaattccatgatttgattcagaagcttttctgtgtttcccat

ggttgattatcgcactagggttttgaattttcttgcacaatttgggaattttctttcgaa

tagaaatagcgatgaaaagagagaataattcacagcgttgttggggtcttcatcactcga

ggagttgggg

>fava_dn_rep_c331

cactaaaacatttctaactccaaagaaattagtaaggactaaaattcacaacatgtcata

tacaagtcattttaggcttcactaggccaaacccttggggttagaatgctactacaattg

ttcatagagaatcacatacaacatagcctaacaagttgtaaattaattgagggcaaaaca

aatgcaaggagagggatcacaattcaattatattactactatattcaacattatgaaggt

tcatcacttcacacgtggactctgggaacatgaaccttctctctctcttggttgcaaacg

tttacgtcctcttgcaccaaagatttctgataacaatccttctctttcttttgatctcaa

gaccttcattagaccagaatcttcttcttctgatcatgataacaagaaagatccaccttc

accacaaagccaggttgaaacgcatgttccaggagggacaaggtggaatccaacacaaga

acaaataggaatattggagatgttatacagaggtggaatgagaacaccaaatgcacaaca

aatagaacagataactcttcaacttagcaagtacggtaaaattgaagggaaaaatgtgtt

ttattggttccaaaaccacaaagctctcaaattcacaagaagaaaaaaaaactatagcat

actctaagaaaaatggcatctacacaatgtttcttgcaccaccaatatgctattacaact

ccaactagaaccttgtctcaacgccaagttgttagcaccaaaccaaaccatattgtttgc

aaggcacagaaacaagatgatgttgttgatgctgttgtttctcgtaggttagcactttct

gttctcattggtgctgctgctgttggctccaaggtttcacctgctgatgctgcttatgga

gaagctgctaatgtgtttggaaaggcaaagacaaacacagactacttgccatacaatgga

gatggattcaaactgttggtaccagcaaagtggaatccaagcaaagagagagagttccct

ggtcaggttcttagatatgaggacaattttgatgcaacaagcaatgtttctgttttagtc

caaacaactgataagaagtccatcactgactatggttcacctgtagagttcctatctaag

gtggactatttgcttggaaaacaagccttctttggacaaactgattcagagggaggtttc

gacacaaacgctgtagctgttgcaaacatcttagagagctcagaaccagtgattggtggg

aaacagtattacaacatttcagtattgacaaggactgctgatggagatgaaggaggaaag

catcagctgattacagcaactgtaaatgatggaaaactatacatttgcaaggctcaagct

ggtgacaagaggtggtttaagggagcaagaaagtttgtggaggacactgcaagttcattc

agtgttgcctaattaagaactttggtgaagggctgttattttgcatgtaaatcttattct

taagtaagcaagcacttggagttttaatttataatggcttttttaacctgctctttgatt

aaaaatttcaagttgttacaagccttttaagaagcactataatatgaaaaatcatttgtt

ggaagaaatcagcctcttaaatggataaaacttaaaagtttctctagttcttggcagaat

atgctatggatttcttttgggtgaagatatttaccatggatttcattaaaaaaaaacatt

ttaatatgtttaataagtggttcatttgtatataaaatggttacaatgtttttattagga

agtgtattgaaaagagtgtaattgatctttattcatggaatagtgtgttagtagtgtttt

t

>fava_dn_rep_c332

gaatccacaaaaagctttcgctaattttacatgcactttattgatttgcacgataaacat

ataaataagaatatagttgctcttctatcacatcatgacagcaatgaatatccaatatat

ttgctacttcatcattagagaggctaacaggagcaataagctaaagcaatctttatttgg

cagacaacatttatcgctataactttaaagtgcgacgcaaaaaaatgaagtaactacaga

acaataatactctgtttacaagagcaatggcaccacatatcttccgctcacacaatctct

gcagtaaattataaatattattgtaggtgaaactaaataatacaaccaaagtcttcaaac

agcaacttgtccaaagagctccttgcggatcgctgggagtttgttacggaacaatacatc

caaacgtgcatcaagtgtattttcaaacacaatcttcccatcatgagaagccaataccac

acctccagagcagtggagatcatgaggattgtgatggctgggggcaggtggaagatagac

atccttatcaacaaggatctctggtacatgaagatttgccttttcagcatactcttgagc

agcagactccaacacatcctctaccaaaggcaggtccacttcccgacacctcaatagaac

agaaggttctttcagcctcagcaaactctgaataatgagttcttttaagaggtttccgta

cacttcgggatcaccactcacattcaagagctccttggctgcagcctccttcattttatt

aaccacatcatcttgagcttgaagaacttttaaccgtgaagcattcaactgcatagagta

ctcaattttcttccgaacatcaacttgtttctctttgcgttcatactcttgacggatctt

cttcttatcagcctcaaccaactgcaacttctcgatgttgaattcttcttcggcggaaac

ggagatctcgttggccttctcctcggcttcttggcggataaacctcaccatctgctggat

ttgcttggagacatctgcgtcgttcatgatgacggggattgtggaatcgaatcggaggag

atcgagagggagaagggaaactgtttcggaggttttgcgagagcttcgtcgatcgtgctt

tgctttgtttgtggatcagaaataagattgggacaacccggtcttttttactatacacgt

cggcaatag

>fava_dn_rep_c333

tagcgttgatctctggaacaaggcgtgttcgctccgcacgtggtgtaggtagttagtgcc

gtgatcgtgaatttggaagaaaagggacagagcgcggaaggagagagcgtgagagtgaaa

gaaggagcgagcatggccgatctatttggatggctcgtctccttcttcctcctcgtcgcc

ttactcgtcctcatcacttaccagctaatgtgcctcgcagacctagaatttgattatata

aatccttacgacttcgcgtcgcgaattaattctgtggttttggttgaatttgttcttcaa

ggtgtcctttgctgtttctaccttgttactggccattggtttatgtcattgttatgcgtt

ccttatctgtacttcaatatcagatcgtatagacaaaaaaagcatttaattgatgttaca

gagatattcagcatgctctcatgggaaaagaagcaacgactcgtgaaactcttctatctt

gttataacccttttcctctctgtattttggctgatctatacaacattggatgagcatgat

gcgtaattgcagtggatacttgttttgtggagtgctttctgaaagtgagctccacagtta

atttccagatgtcggtggttgggtgcatttttgttgacactaactgtattttgtcagtta

gatatctttatattgggctagcattttatgcttatcttattcatgatattatgtatatgg

gtaaggagctaaaggcatcaaaaggaaagtgctggtggctgatgaggggtttgggttatt

tgcatagtgttgttggtccatacgtggatatgtaccaaaaagattatgtatttattcata

atgagttgtttaccaaactttgtagtctgcagttttaactttatcagcaaatctatggtt

cattccttcaaagaggatgtaccatacaacaaaattcttgaacttctgaagggattgaat

aattttttattagtgttttatggagggaatgagtatcaagaaactttgtgttttgaacga

tacaatatcgataattcgtagataagcgtggatatgggtctgaaaagtaatataattttt

taagnnnnnnnnnnnnnncacgtgatatatag

>fava_dn_rep_c334

atcaataaatctcaaattttatatannnnnnnnnnnnnnnnnnnnnnnnnnnnnnnnnnn

nnnnnnnnnnnnnnnnnnnnnnnnnnnnnnnnnnnnnnnnnnngtaaacttaaaattttt

tctttattttcttattcaattataaatgatgacttcatatgtgtttatgtgatatacgat

tcctaattttaggatgataggagtgagaaatggagactacaggcagtggcgatttcacct

gctagatttgagagcagaaaaccactaccatatctttggaggggtttggaaaatgacagg

ctatctgaggttgctggaatcccaggttgtacttttgtgcacatgagtggttttattgga

ggaaataaaagttatgacggtgctctagcaatggcaaaaacatctttaaatgcttaggaa

attgcaggttaagcaaatacctttctcctctgcatggtttttcttctttagaattgggtt

atggacataaggattagatggcaatggaaccataaatgcttgatcactaaacttgtgttt

tagttcaatcggtatgctatagtggttccaaagcatgtttcacgtgcatcagtgaattgc

aaactgccgacaagtgataatacgatgctgataaatagaccctctcatgccaaagctatg

gaggcaatctttagaaaatgtaacatggcattttttctcatgctaaatatctacaccagt

tcaatgattacatttcaactcactcgttctgttctttgtatgcatttatcagcatcctca

ttatggtcaaaactaggtccaggattctctgcatgcacaatgttagcaatgaattcactc

ctctgaaacagttggtagatcctctttaaaccacctgtaaagcccaccttctagatggaa

gacattggtgtaaccatttaggaccaacaagtaagctgctattagagacctagactgttg

accttctggcagattttgtgttggtttcattgttccacctgccgagcaagctattattat

ctttgcatttttatctaatttatcctcaacagacttgataaactcggggttctcttctgt

tccagagaaaataccaaaaaatgcaaatgcagcacgtctggcaatgtcccaagctgtcca

ctcttttataagcctgtatatttgcacattaattgcatctgcgggatgagcctccttgaa

ctctgcttctggccttacatcaagaatcacaaaattattttctttctgaaggcgcagagc

ttcctttggttccacactttttacccttttttgcagcagtaattctcgcttaaccttcca

ctcttcttcagctggtgatttcgcttgttttgtggctgcacactggattgttagtcctct

tgcaatgttctgttgttgaaatctttgagaagtgagtttgtacgatctcactctgacaca

ccatgaattatgatcacgagttccttctatagatggcacccaagattgtaaatatgatgt

ggatgaatactgtagagcagttggtgtaaaagccgtcatgcttcggagctctctctgttt

ccacagaaaaatcaaatatttaagggtaaatttgaaaggaagtacttgggttccgcccgt

tgtcaagtgaactctttggttagttagtgagaaatgtgcatctgagtatagagatatttt

gggtacaaaatctgaatttgtggtcgatgttttaggagtcttgtgctatttggatatcct

tgttatcatcattttgtcttctttt

>fava_dn_rep_c335

gccgcgggatcccatcacaagcggtgtaataaaccaattgaaaaaggacaagttattttt

atttaatttaaaaaatgttggggatcttcagtagctccgtcgtgtctcctccggaagagc

tggtggcagccggtagcagaacaccgtctccaaagacgaaggcgagtttgcttctgaaga

ggttcgttgagaagaaggcgtcggcggtgtcgctgcaggttggggacgacgtgcagttgg

cttatacacaccacgaggagtcaccgtggcaacccagatcatttgcagtgaaggatgata

tattctgcttgtttgaaggagctctagacaatttagggagcttgagacagcaatatggac

ttgcgaaatctgcgaatgaagtcgttcttatgatcgaagcttacaaagctttacgcgatc

gagcaccgtatcctgcgaatcatgttgttggtcatcttagtggtagttttgccttcatag

tttatgacaaatccacttccaccctctttgttgcttctgaccaatctggaaaggttcctc

tgtattggggaataactgctgatgggtatgtagcatttgctgatgatgctgatttgctta

aaggtgcttgtggaaaatcacttgcttcttttcctcaaggatgtttctactcgaccgcag

ttggaggattaagatgctacgagaatccaaagaataagattaccgcaataccagctaacg

aggaagaaatttggggtgcaacattcaaggtggaagggccagcagttgttgcagccacag

aatagatatgttttcaaatggagtgtcatattctggatgcatttatggagtaatttatgt

gtttatgtgtttgtgatgactgtaatacatgcatgaaggggtcataatgtaaatatcttt

actcataaatgtgaagagtgtgagactaattatgtagagtttattatgttgcaggttttg

tgcagtattgcatagggttttctacttttctgtgtttcaaaaaatgtccagttgcatttt

ctatgtaataaaaatatatacatgcgtctggcttgagttctgtattgtagaatgtgtaga

tacaaactttcatctttcaactaataaattaatgatacactttgttcaaaaaaaaaaaag

attaccacggtaaaatcacatcaaagtca

>fava_dn_rep_c336

tttttttttttgcagaaatacgaaaattgtttaaaataactttaagctgtttcatttgtt

acttgaaaacaattacaacacactaaatgtaaaataatatctctgcttaatcaaaggttc

tcaacaaaataagaaattgcatggtccatctaaactctggactacatctaaatgtcccag

cagattgacagttgagcaaccagaacccatcaaacatgagcacatgcaatctttaagcaa

ctagagtgctggcagttgttgattcatatttccacacagggggaagcttcttagttttct

tgtaatagcgagcaagcctatggattctgctctcaacaagaatgagcctgaacttagagt

ccttgtccttcctgttcctctccaaatgcttgcgaattgaaactgccttcttgatcaaat

ggtatagatcctcgggaatctcaggagcaagtccatgagctttgagaatgcggaggattt

tgctaccggtaacactcttgacctgagcaataccatgagaatcacggagaataacaccga

tctgagaaggagtcaaacccttcttagcgaacttgcaaatattttcatcaacatcttgtg

aagagatcttgagccagctaggagaagttcgcctgtacggcaaagcggaagaggatatac

ccttaccgctactatgcatacgccccatggtttctggttttggttgcaactctaccgcgc

cggtcaagtattggctatgcgatcgccgcgg

>fava_dn_rep_c337

tgaagtctattattactatccgattgtccaatttgtacattatctctctctctctctcta

tcctatctagtttctaagtcagttatcatcacaagaactgattctgagagagaaaatcca

aacgcatggaactaggaatgtctctgaacaccctacttcgacctcctctctcaacttcct

cctcctcctccttcaaccattctctcttctccattccaaaacaattcccaacaaaaactc

aacaccaccaccaccatgtcttggtggttgaagcaaagggtaaaaaaggaatgatgtatc

gccaataccagcgccgtactcctgctcctttacccaaagtcgaagacgacggtaacccta

agttcgtcgtcttcattcgctcggctgatgtttacctttggtatcctcttagtattgtcg

ccggcggcaccgcagccaaaatcatggtggctgctaaagataactttgtcggaaaatatc

tcttcaaggatactcttgatagaaatattgctgctgttatctatagagatgagaaggaga

taaagaaaaatgcattcgaacaacatcgagtgctgaagtcagctactaaattcagatatg

gctataaaattattgttaatggcaatgtcaaatctgcactttcttcccgtgatgttattg

agcttcctacaccagataaactaaaaccctttgttgatcaagtgaaagactttttcgaag

atgtaaaggatacctttaggcagataacgtcattagacgatactacaaccgaggagccag

aggaagataagaaagcaaaatcaaaatctaaatctaaatctaaggttaaaggatgatgaa

agtatagcaggaaacgatttgcgtgaattgttatgtgctgcttgggggcagcatgagctt

tagaaagtaagcttcacgctgtttgtagattggttaatggctgctttcattacacaacaa

atgctacttgtactttgttcttatacctgtactgcacccaactaaattatgttctaatga

gtgagcattacttttatggtgctgtttactcatttatgttttgattcataatgcattggg

atggatagtttgttgagtgactataatggattcagttttacttcagcaaattttggttct

gctgtacaaattatcagttgtaatattcatggcttttatttcacaggaattcattctgtt

gatcaggaatattatgtattacggtactttttgcaatatatgtatgtataaaacaagaaa

aacaacttaaaacgagttgccacaaaaaaaa

>fava_dn_rep_c338

aggaccattcttccaattgcaattgagaaaaagctgtaatcataatttgttctagttatt

gaatatttaagtgaagtgagctatggaaaattataattttattttgtaaaaaaagaaaaa

gaaaaaccattgtaaataccatattgtcccaacgtgattgattcgtaaccaaagaaacaa

agcgataagataaaccaaatcaacccattttcagtttctaccatttctcaacaatggaag

ctactgcatctctcttcaccttctctcttccttccaccaaaacccaaacccgtcaattcc

taacccgacccgttaacccattcaccataccctcaaaacccgttttcaaaacccaccgtt

tcctccctaccatcaccgccgcaatctcccgcacaaagaaagaagaaaccgtcgaaaccg

tcagagaccagctcgaaaactgctacctcctggccggaatcaactacaaaggcttcaccg

tgaaacaattccaagacctccgaaaatctctcccggaaacaacgaaactcatcgtcgcga

agaacaacttggtttataaagcaattgaaggaacgcaattcgagacgctgaagccgtgta

tgaaaggtatgaacgtgtggctgtttgttcacacggaagagattccgtctgctctaaagc

cttacagagattttcagaaagaggagaagcttgaggagaacgatttcactggtgctgttt

ttgaaggaaagttttatgggcctgatgactttaagacacttgaaacattgccgactcggg

cggagatttatgcgaatttgttggggtctttgaaaacccctgcttccagtttggttacga

cgattcaggctccggcgagggagcttcttatggttcttaaggcacatattaagaatcttg

aagaaaaggaacaagggaatgctgtttcatagataagtttttttcattttgtttgtaact

ttgttttatagtttttatgttacaatgtttctttttgtttcttacgtttgagtatgtatg

tttgtttatgttcattctcatattatgtttacaaatgtagattgtatgattgatgctttg

aggttctatcacaaaatgtagaatgattcatttgtaggtcaaaaaaaaaaag

>fava_dn_rep_c339

actcaaatgagtcgagtgactccagcaattttgttttaannnnnnnnnnnnnnnacctaa

acttaaaataattaagtgaccccagcatttgttttgagaaagagaatcccaaaatcacac

aaaaggaattgattattcgtctaaagcagaaccagcttcagttgctacatgaacccactg

gcccaaacaaacttttttttaacaagattaatctgaaaataataaaatataaactccaaa

tgtctcattcaaaatactcaataaaagaaaagaacgtaaataacttaacaactatttgat

gtcccacaacttataaaaccaacatataactgagcacaaggttcagcaaacaacaaaatt

tggtaacagcaattaacaaacagaaatcatagcaatagcaacaatgtctaaacgcggatt

aaaaatttgttctctgattcggcaaacaaaaacacttcatcgcttcttagagacaccaac

agtctttcccctgcgaccagtagtcttggtatgttgaccacggacacgaagaccccagta

gtgcctcaaaccacggtggtttctgatcttcttcaaccgctccaaatcatccctcaactt

catgtccaatccattagacacaacctgagaaaacttcccatccttataatccttcttcct

attcaaaaaccaatcagggatcttaaactgcctagggtcggcaaccacagtcataacatt

ctccaacccagcagcactcaattcgccagccctcttgttcatgtcaacatcagctttctt

gcagcaaatgttagcgaatcttctcccaatacctttgattgaggtaagagcaaacatgat

cttctgctttccatcaacgttggtgttcaacacacggagaatgtgttggaactcttcgtt

tgctaccaaagacatcttgtgttgattttggatgcgaggtggttggagagagaggaagaa

gatggagaatgccgccgaagatctaacctatgcttgtcccgcggccgtaatggccacccg

cggccgtaatggccact

>fava_dn_rep_c340

cttttttttttatgaaaacaagtactaattgataacttgataataggaatagcttgcact

agtcaatcacaataggcatttcaaataacaaaatatatctatcagaaatcaataatccaa

caaaacaaaacatattccaacacaggaaatataacatttaaaacagcaaatcctaattct

ccacctagaagagagatgttccttttcccttcttgtctccaccaatctcaaaatgcttgc

acctcttgatggcatgttgagaaacatgtttgcaaccctggcattgcaacctcagaacaa

tcttcttagtggttttagccttcttgtggaagacaggcttagtctgaccaccataaccgg

actgtttccgatcgtaacgacgctttccttgagcggcgatactgtccttacccttcttgt

attgggtaaccttgtggagtgtgtgcttcttgcattccttgttcttgcagtaggtcttct

tggtcttcggaacgttaaccattgtggcggcgcgtggatcggatctggaagatgaagttg

tgcgccgtgaaaatgccgttgtgaggt

>fava_dn_rep_c341

agtcttaaaatacctctgcctttctctctcctttatctcagatctggaacggaaaaaaaa

cataggggtttcttttccctcacgttctcttatcttcttcaaaataaacaacccaaaact

caaatctcatacttcacaaatcaattcttcttgaagaacatctagatggatttgacggtg

aagatggcggagatcgaggaacgtggttcggaagtttgttggtgagtgtggtggttcggt

attaaacaacgtcggtagttgcggtggcgacgagtgtgttgaagctcgctgggtgggtgg

cttgcaacggttggtgaggggtgatctcgcgtcgagtttgagttgttgatgtcagtattg

tgaatttcagtgtttctcctcagtgctcttccactatagattgtgatatacctgttataa

aaacctgaaaaattgaggagaatgaatgataaaagtagaaatcagaaatgttatgatgag

agaggtaggaggattctggtttcaatctgcttcttgatatttgttgttgctggttttgta

acttcatatttatatatattaagattgatagtcacaaaagaggtaaaactattaagtaat

caaatgataaacacctacatgacatccactaacgaaattatgtctctgtgataatgatac

atgatgtacaatcttattcagtaatgtggtatatggctaatagaagaaattgcatctcaa

ttccaaacttattttacagattaacatcaacaagcgtacaagatggaaagtcaccaagga

atccttttgatcacaaccctattttccatttaggccacaacgggataggaggaacacgtt

gcaacaccgcacatattcttccccatttccatcttgaagtagccatggtcaccccaatca

cctccccacgaatttttaatgagccaatatggtatgccgtcttcaattccatacccaaca

gcaagaacagcgtgattcacatccattggcgtgctgccacaagttgtactagtgtaaact

ccactcttgtataatttgaagccactcaccacctcaaatgccacactaacgggccgagca

aaagcaactgcgtgtttcaattcatcctcagcacccaaggaaatgttgacagagccaagg

actttaacagcaacgttttcagatgtaaatttgcagagaccattacttccagtgtaggga

tatgtttcctctgtctgaaggccaccgttgtatttaatgtattcaaaggcttgtgacggc

aacccaccgctgcatccaaagttattgaaagcaccagcacaatccactagctgctgctca

gaaagagagacattctttccgaatgcctgtgcgtaagctgattccaatgctccagttgtg

ctgaatgtccagcatgatccacatctgccttgatctttaacttcgctgactataccttct

ttcctccagtctttctcgtcgggaagattagcatcagtaatcttatgattccccttaaga

gtagcagagcaattttgagcagcaccaagtctatgccttctaaactcctcccaggtccaa

tcagcaaaacgattaacaccgagcttgtaactgagtcctcttttattagtcgatctgata

acttcaagattctccaaaaagatcttaaaccgaagcttcatctcatcaaccgtagcgtat

cgcttaccgtatcggttagcgaaacgggcgaaagagacagcgtgacgactttctccgatc

acgtgaagtagctgctcctccgcgtcggagaccattcggatagggtttgagtcgtggaag

gtgaatccggccgcggcggtggccgcgcagaagaaaaggatgagtaacgaccactgtgcc

atctcactctcccaaaactctgctatgaaaagtttctaacttctactcttcccgcggcca

taatttgattgttcgcctgcggcataatgtcatcaggacaggtttacgtaacatcagcat

ctcatattctcgcatctcactagttgatgttgctaaaaaattgaggttggactcgccaaa

tcctgttgctgatgctgagagtattgtatcaaaggctattcgtgatggggcaattgatgc

tacattagaccattcaagtgggtggatggtatccaaggaaactggagatatttactccac

aaatgagccccagcaggcttttaattctcggattgccttctgtcttaacatgcacaatga

agctgttagagcacttcgttttccaccaaacacacacaaagaaaaagaaagtgctgaaaa

gagaagagagagacaacagcaagagcaagaactggccaagcatattgcagaggaggatga

cgatgatttttgatgagattatcttttctcaccctaccagttgcaaacaagacctgttta

attgcttgtatttccattctgaagaacttccattagcatttccctttgacaagatgaaat

tttcattttctacatggaagacctcctatagtcttttaccttatggaaatctctacaatt

tgttttcattcattatgactgttatgctttgtttctgttgggataattgtgttttaggat

cttggaaaactaaagtcagttatttagttctgttctgaagtttttttttcttttatattt

ttaattaaaagatgttactctgagtgtcttgttgaattaagcattgcttgttagtaaggc

aatgtagaaaatgaaaaaggaagagagtcgggactcaggcgtgtatcttctgtcctggtt

gaaatagcagattgt

>fava_dn_rep_c342

acttggatgaaaatatcaaatgctttttaacactattgtaacagtttgatacatttttca

actgtcaaagattacattttacttaataacccaagataacaattatacatactatttgag

gcccttcggttagccataggtggcaaatgaaaactcgaattcacttctcttatattcatg

ctctttttcagtgtaacttgtgacaacattcttgttataagctattggatattgaacaaa

agaacctaaatagaactgttctaaagtgttgatgaaccaaataaacaaaagaacatgaat

acattacaggtatcattttcgaacataaaagcataatgaggtaaacattcagctaaaatg

tgctgttgagatagaagtataatcccacaagagctacaactcctacggcaactgcaatag

gaagtgctttcccaattgcttcatctctttggagttcagcttttcgtgcctttctaacat

ctttttcatttgcacttttgttgacaggtggagcatagggcttaaaccttcgttttggag

caccacaaacggggcagaagtatttatcaggcaatttctcaaaggggctcttctcattgt

aaatatacccgcagtcacgacagatataagcttgtttggaagctagacgcatggtaaacc

taggaggtccataaagaaaactatgttgttgattaggatgaagtacgaggttaagggaac

gagagaagaaagatgatttcaaagaagaactggatttggagagtgttggagataataacg

aaggtcgtgtggatcttgtagtgtgaagtgtggcctgcacttgcaaggccattttgatct

gtgtttgattcttcttcttcctgtggttctgaggtgaggaagtgagaagaaggatgaaga

taagagaatagtagttcgtgtgttgtggaaagttctggtgaatggtgattgaatcctacg

tggaaagcgtggcatgcatactccccacacaaccttttttctcactttttggtttcaact

ttatcatctcaa

>fava_dn_rep_c343

aaaaaactannnnttaagttaatcctcttttatactattattatatactcgagtgttttt

accagacatgaccatttttaacaagatttaaaacgaaaatacatccttgtgaattcatta

ttgcaaaccagattcacaacaaggtgctgtactgaataaaattttaagaaatttcattct

cttgtcacttcaaaaaatacatagaatttagtagaaatcgagtgcaaggaaataatactt

gaattagacaaaggaaaacagattaaattcaagtattcggactgtttaaagtatatgaca

tgagaacaaaacaacctaaaatacttaaataaaaatcaagattttctttgttgaaaaaac

acaaatggcacacaataacactctaactatccttagcctcctcagcagctgtctcaaaag

tctctcctgggacaagctctggaacatcatcatcattctcttcttgcgctgtggctgcgc

cagcacctgcttcaggaacctgcttattgaactgctcagctatcttcttcaggttttcca

aattatcaggccctaattggtggataatgctaggaagaatgtcttgcaacttcttggttt

gaggagcaccactaacaacccatgtattagcagcaatggatgcttgaactttggggtttg

agaactggatgacaacgtcatccttaaagatattgacctcctcaatttgagggatggcat

tcaccccaattctcttcagggtgctctgaagccttttatcatctgtagttgttgtcttgt

ggacagccttcttctttcttctgacggtaccctttccaccagttcgaactgaaccggcca

tcttcatcaacttttcacgattcatctttgtgaaatcagtgattttgaacacagggtaag

cctcagcgtgaagaagaacgaaacaacgcaacgagataaggatccttcttataccacctg

ctgtgtttaatgaatcttttggtcctgtcattagttcgtaatacttattgattgcatttt

agtacctatattaaggtttttaatggattaaatatactatgaacaaatatgacatgtaga

ttcaatggtcatggttataaatagatgcgtattgactttatattgaaaatgttgagctga

tttttttgtgccttttgtcatctagtagtataatgattaattacttccatcttggggctg

gagtttgagaattaagacattgtaatttattgtctataatttatgtggtagttcattttt

gaggtattttcaatcaaatcaatggttagtataatcttaactattttactttaaacttta

gagacaactttattttgggagaattttatatttaattatgtaccaaaacttatatcaaca

aaatgagttctcaaagaagactctttaacatgcataagtaagtatttattgtatggtgta

tttttttt

>fava_dn_rep_c344

atattcaaatacatattaaaaaatatttttaaaaagtgttgtcaacaccatcgcacgcaa

gctccatcgctgccagaatcatcaccaccttcaactccttcttctccttactcgaaagta

gagtttttgatcaagaacaggaaacatgtcgtacatgttgcctcacttgcattcggggtg

ggctgtggatcaggctatccttgcggaggaagagcgtctcgttgtcattcgttttggaca

cgattgggacgagacttgtatgcagatggatgaggtgctgtcatcagttgcagagacaat

aaagaactttgcggtgatatacctggtggacatcacagaagtgcctgattttaacactat

gtatgagctgtacgatccttcaacagttatgttcttctttaggaacaagcacatcatgat

tgaccttggaactggaaacaacaacaagattaattgggctatgaaggacaaacaagagtt

cattgacattattgaaactgtctacagaggtgcaaggaaggggcgtggtcttgtcattgc

tcccaaagattactcaaccaagtatcgttactagaatatctcaatttctattgggatgtt

gcctcatttggttattggggtgttacctcaatctatcttttattcatgtatggattgtgc

aaatgtcactaaactggtttacacacattcttgtctgtcatcttaaactctaataagtaa

taactgctatcatgtgtgtatgcttaatttggactttattctctcaacaaggatttatat

tttattatagaagtgataaaaggaatagaaaggtagtataatgtggaggtaaagaatcaa

acaaataaaaaaggaatgtctggtaagagtaaaaatttaaaatacaattttggttctctt

atttgcg

>fava_dn_rep_c345

tatcccaaatctgagctgcaatacataattgaagaatatcccaaatctgggctgcaatac

ataattgaagaataaacatttttaaaacaaagaataattctgaagaggataatggcgcta

tcatcttcatcatcgccatgttcatgcttttcagctcctactacaacacttgttcctcct

acacgccttttcactactcaattccacagcaacaccaaaccctacatccgtttccggcga

acaattgccgccaccgccaccgattccactccgccactctactctaccaccagaaaaggg

cttgacggcgtcaacatcgccgaagatgtcactcagttgattggaaatacaccgatgata

tatctgaacaaagttacagagggatctgaagcaaaggttgctgcaaaacttgaatcaatg

gagccttgcagaagtgtcaaggacagaattggctatagtatgttatgtgatgccgaagag

aatggagcaattacgcccggaaagactgttttagttgaaccaacaactggaaataccgga

cttggccttgcttttgttgcagcaactaaggggtataaactgatagtcacaatgcctgcc

tccgttaatattgagaagagaattcttttacgctcgtttggtgcagagattgtgttgact

gatgcagagaaagggctgaaaggggctgttgacaaagccaaagaaattgtcaacgccaca

cccgatacttacatgtttcggcagtttgaaaacagtaataatgcaaagatccacttcaaa

actacagggccagagatatggaaggataccaagggtaaagttgatatattggtggcttct

attggaactggcggcactattgaagggactggaagttatctaaaatcgatgaacactaac

ataaaggtggttggggtggcacctgcggatggaagtatagtctatacaagtacaatcgta

ggagacaatccaggttccgtaccaagcattttggatgtcaaactacttgatgaggtcatt

aaggttaccaatgctgaatctaatgaaatggcaaggaaattagcattggaagaaggatta

ctggttgggatttcttcaggaactgcggcagcagctgccataactttggcaaaacggcct

gaaaataatggaaaacttatagcggttatatttcctagttttggtgagaggtatcttagt

actcccctcttcagttccatctatgaagaggctcagagaatgtaacaagtagattttccc

ttgatgtgttaaatgccattcgaaaattcaagaaagtggttgacttatgatttgtaatgg

tatcatcacacagtatgtgacagattgaaactagtactatgtatgtagctgctgtaagtt

ttttaaccattgaaggttttgcatcagatgtatccatttggacaatgaactgttcttgaa

gtaagttcatattatgggtgtttaaattcaaataaaaaattataaagtgaattaaatatt

attgaatgttttttaatgaaaatcacccgg

>fava_dn_rep_c346

ttgtgtttgtgacacacaagacattttgtggaccacaattttacttactctatcttgtga

ttttatggcaactagagaggcaaggtgctgagaatcacttctctcttttccttgcaatgg

caactatgcactctcttgcattatcctctccactctacaactcagtacagaagccacgct

catactcagtttcttctgcaattgttcatggaagtttgaactttaactcctcattcaatg

gtcaacatttacatgtgccaagtttgagggtaccaatgataactcaaaaaactcccatgc

acacgccggttatcatgatggcggggaaaccgaaaatacagttcattcaaggaactgatg

agctgacaataccagatgtgaagcttacaaaatcaaaggatggaaccaatggcatggcta

tcttcaggtttgatcaaccctcagtttttgattcttcaggagaagtaggtgacattactg

gtttttacatgattgatgaggaaggagtccttcaatcagttgatgtaaatgctaaatttg

tcaatggaaagccttcaggaattgaagcaaagtatataatgcggactccacgcgactggg

atcggttcatgagattcatggagcgatactcgaatgcaaatgatttgcagttcgtaaaat

attgagttaatgtgctaacttttttccttctattcttttgtcatctgtcaagaagtttgt

tttctgtaatttagttaccagcatgtaactaattttctatacatctcaaagcttattgta

ctaaggctttctttcattatgtttaaaaaaaggtaagaaattttgttagaaaatactatt

gtaataccagtatgcacctgcattaaggacaaaagtatcatttgccagtcatgtctacca

aaaataatggctttaaaatttgtctgtaacgaaacttctggtatcaaagaccatgtgatg

aatcatttcacaggccaatctcattatcaatatggaggagtaagtatttcatgcg

>fava_dn_rep_c347

gaggaaatannnnnnnnnnctttttttttttgaatttcaatgaaaaacatatgaaatatt

ttttatttctcatttttcgacatctagtatcacatggttgttattgatgaaatacaaata

tataataaaacatgtcaaacaatctctaagtacaacatgaaactattaacacataggtta

taacacattgaacaaacttttaattgagctacattttcttgttttcaaagtagcattttc

cttttgcacaatacaaacataacaaattctccccatttcattctcaaatgaagggatctt

taatggaagacaacagtttttcatacaaaggctgttaggcctagacacttgaattaaaaa

tggatctccctcacaaataccatattaacccatagcatatttctgggtccagcttcttgc

ggttgactcatacttgttcttgtctgtcttgtacatgtgagcaatctcgggaaccaaagg

atcgtcaggatttgggtccgtcaacagggagcagatagagagcaacaccttggaaatagt

aagagcagggctccactgctcctttagtatatcaagacagatgcttccattactattaat

gtttgggtgaaatacctttgtcctgaatgcaaccttgggtggcttaaatggataatctgg

agggaaatggatagtaacaagaaaaacacctccggaataaggactgtctggaggacccat

gattgttgcttgccaatgaaacatatcttcagcaacaggaccagcactgcacgaagttgg

aggatccttctgcaaatccttgagttccttcaagatccgcttcgaagccatagcgtaaac

ctagggagatcctccttcgaatttgcaacgaataaggcgaagacgatggaaggaatctga

gaattgaagaacgaaaacgagttgttgtggctttggagcgttttcggaatttaagggctt

tttttcttttggtctgtgattggaatattattaattggtgttcctatatgttgttcatta

ttgtatcatccaggttattaattgatctaataaagtattatgaatttattcggattaaaa

aaaa

>fava_dn_rep_c348

ccgcggtccttctgatacttgcgcaggcactgcatagttagggtttgttgttttcttcct

ctccatcactcatcatgggtcgcttgcactccaaaggtaagggtatttccagttcagctc

ttccatacaagaggactgctccttcttggctcaagatctcttcccaggatgttgatgaaa

ccatctgcaagtttgccaagaaaggtttaactccatcccaaattggtgttattcttcgtg

actcccatggaattgcccaggttaagagtgtaactggaagcaagattctccgcattttga

aggctcatggtcttgctcctgaaattcctgaagatctgtatcacttgatcaagaaggctg

tctccatcaggaagcatttggagaggaacaggaaggataaggattcaaagttcgggctca

ttttggttgagagcaggatccatcgtcttgctcgttactacaagaagacaaagaagcttc

caccagtatggaaatatgaatcaacaactgccagtaccctcgttgcttaagagaagattg

gagaatgaattagtttgttgagattttgaaattttgggttatttcaagtttttgttgaga

ctcatttatgatgttggatcttcttcctttatttgatatactccttttctctactcaata

atcgtttttaatttggtgttgtggtaaaaaaattgatatttaagtgactcggtataagat

ctaattagtgccctataatctaaaagttaattagtcaaattaattagtcttgatttgcct

gttgtgttttattattcaaatgaagattctttgctgtannnnnnnnnnnnnnnnnnnnnn

nnnnnnnnnnnnnnnnnnnngataaatgagttttagaaaacatgagttgtggactgattt

ggaaaatatacttcaataatagaaatttgaaacctgtgtatcagatt

>fava_dn_rep_c349

tgggaattacgccgcgggatctctctctattcacaacgaattcatttcgttgaaccctga

agcgataaaacaaattgacactgtaagaacgagcgataatggcgaagaagggaaaaaagc

aactgatgtcttcggcgccatggagaggcgaagacgaaaccgaccaattccccgacgcaa

acctcaaagtcacccgtcaacaggatgatacttcaactatgcacgttcctcgctccaaga

caaacaatcacaatcatgatgacgatttcattgaaattgatcccgaacttcgttacagtt

tccaacgcaattttcagtttattcagagagtttttagcattgacactgtggtgaaacctc

tcccacccgtcatggcttacaatgtctctcgcaacttgaacttcttcacgcgcattttca

ctcaattctttgatcctgaaggcattgctgctgcccagaaatccatgggcatgggacagg

aaaacagagatcgcagagttcgttgaagataatcgtggaagacataaaataaataagtaa

taatgtaagaactttgaagatgatattggacaatcatttatgagtaaaattttgtcctta

cgttattaattggtataggcttatctgtatagtaattgtattgtattttgagtgtcgcac

tatgttaatttgtcggacccttgattttttgccttctcgataataggtgattttgcatga

atagtagaatgtgtggttgatatctctagcagacaatgagcatgaaagattgtctgttta

cgtgcatgaggaagttgaggcttccatctatctagaatcatgtttggttggaccttttct

cagtattagattgatcatgtggtatattacgaaggagaaactcctgtgtattttgtgttc

gatgaaggctttatgcattatccaccaaataacatacttctataaaatagatgtatgcag

ttaaatatgacaatttatgaacatgaaacagaatattgaaatttattaagataaggatta

agaatattgaaatttaaaatagttttttagatttatatattattaaccatttttacacac

tacgatattgaggtta

>fava_dn_rep_c350

attactctccctatcttaaattataagtttaaaaaaaaacaattatcttaaattataagt

cactttataataccaatcacaacagtggcgtgcaatgtcatttttgtaaactcataactt

catttctcaccagattgtctaccaaaaatctctccatgcataatctctcgtgcatcgcac

atatacctatactagcaaatttattcaatgtaatacaaggtactcctccagctttttcac

aaccaaactaaaacagtttgtaataatttactaaagaagagagaataacagacggtatgg

aaaggtcggagttacaaacagacgataagaaagacagcgtacctatcggaatgtccatat

aaaaagctgctggaaagtagggtgggggacatggaactatcctgtgaaaattggaagact

atcatcacattttataaatgaaaactttagctgtgccaaagtgtaatcatatcatgccct

ttatgccaaaaaattcatattgtcacaaaatacaatcttgccatcgcatgaaagttcccc

cttatcaaaatccattacatttttttcctcaataataatctgattcaaagatcatcatga

tcgtcggtctcaattcctagaagtttagccaaaagcccgctctcatatgcttcgacagta

tcatctgatccgccaaggtgtttcccgttgatgaaaacttgtggcacagtacgcttacca

acaatgttagtcaaaacatcctgaatctttgaaccatcatctctctcatcaagctccaca

acaaacggaacttggttcaattctttgaaaacagcttttgcttttctacagtacggacaa

taggtcttggaaaagatgacgatcttgtgggaattaatggtcttgtcaacaaactcaccc

acagatgaagctgaagcagaatcccaataaagagagattgctagagctatcatcaccatc

atcattgttacgttgattcttgcagatgcagccatgatttgagcaaggcgattgtgcagc

acgagtgggagattgtgaaacggatctgatgtctgccctcggaaattgtgatcagtagaa

attttggttgatttgtgtgcttaatacgacgccgttttgattcaattgtttttcttctat

acaaacattaagc

>fava_dn_rep_c351

agccgcgggtgttgagatttggccgaatttgcttcagctgccgtgaagatggtgaaagga

cgtcaaggcgagcgtgttaggctttatgtgaggggcacaattcttggatacaagagatcc

aaatcaaaccagtacccaaacacatctctggtccaaatcgaaggagttaacaccaaggag

gaggtagcttggtatgccggcaagcgattggcatatatctacaaggccaaggtgaagaag

aatggaacccattaccgctgcctatggggaaaggttacaaggccacacggaaacagtggt

attgttcgtgccaaattcacatcaaatcttcctcccaaatcaatgggatcaaggattaga

gtatttatgtatcccagtaacatctgagatgcaggtgatatggccagtagatttgtgggt

gatttctagcctctgaattttgtttgattttaacttttgaggaattgttttttatgttag

taatctgcttgtgtactttgttatctctaatgattacactgtttgttatctctaatgata

caatgtttgtttttcttgcattttattcattaatctttaagaaacttaagttaatgaaat

tattactgttcgagaaaagtcttaagctttagttactacacataaagattcaaattagaa

attacagaaatagaaattaaaaaaaaaaaaactcaaaagaatgataataacaactattgt

atgaagtaaccactaagaaaaagtgcaaagaaaaacaacaaagtaccataaataagcaca

gttttctgaccagaagtaagagaatttccaccaagtccatactgttgattctgagcaaag

ccagcaatctcaacacttttactctcaaagaaactctgcgccgcgccgcaagtaggacat

ctccaatcctccggaagcttctcgaactgatagccaggtggaatgggatacgaagggtca

cctacaccttcgtcgtatttgtaaccacatgaacgacactcgtatattcccgtgttcaac

acagcgaatttctcttccaaccgaagcttttcggagtcgggtgaagtttgttgttcttcg

tttgagatgtcggtggagttgatggtgaattttcgagggtgaatgggtttgagattgtta

tgggttttggagaggaggaagagggagggattggtgaaatggagtttgggtttgagaggg

tggagttgagagaagggagttgagagatggaaggtggaagccattgttagaggttagttt

taccgacggatggtgggttgtagtgacagggaagatgaactgaggagtagagagtagagg

atagtgtatcattgtaatgtgccgttgggatggaaacannnnnnnnnnnnnnnnnnnnnn

nnnnngttgttttgatctatgcggaaagggccaaagt

>fava_dn_rep_c352

tgattgtttctttgtagttttaagaccaggtaagttgtgcaattaaaacataattagtac

gtcaaaattttatacaaatacttatatgcaaactaatctgaagatccttgaaatatttat

ttcccttctttttctttggaacattgaaataaaatgggaggaataaaataaaaagaactc

ttccttaaaagcacttgattgtgaccagtttcattgagattaagattcttatacaaaaca

actttctttgcatcagtatgtcatttaagctaagtacttttcaaccttctccacctcttc

tgtgctcccaatgtaaagtggaacccgttgatgaatttctgtgggttgaatgtcaagtac

tctctgatgaccatctgaaccttttccaccagcctgttcaacaatgaagctcattggagc

acactcatataaaagcctaagcttcccattcttactcttcttgtccctaggatatccata

aatgccaccatataacagtgtcctgtggaaatcaccaaccaaactaccaatataccttgc

tgaataaggcttgccactaggacctggttccttaagatcatcaatatatttcttcaagtt

ttcatcccacagcttgtaattcccttcattgaaagaatagattttccccgatttcggtat

ttggagattctcctgagtcaaaacgaattctccgtacattggatctaatgtgaatacaaa

cactcctttgcctatggtaagaacaaagattactgaactcgaatacatgcagtaaccagc

tgctagaaggttgcttccaggttgacacacattcacaatgcacctttgttcttctgtgcc

aagtgtgttgacatcagagtcgtcaccaaagtcaggaagacactcgtcattggggctata

aatcccgaaaatcgaaccagttgagactgcagcatcgagattggatgaaccatcaagtgg

atcaaatacaacaatgtagtttcctgaataactctcttctactgccacgggcacgtcctc

ttcctccgacgctattatccctgtcctcccacttgatctcaagcaattcgagaatacctc

atttgagataacatcaagttttttctggtcttccccttgaatatttacagcaccttgagt

accagtgaggttagaaatattagctctttgaaccaaagaagcaatttgtttacatgccaa

agaaatactagaaagtacaatagtaagttctgcatcaataatccctttttgttcttgctg

caacaaccaactagtgagtgttataatatcatatccacttctcttctttgtctgagtagt

tgcttccttcacagccatacatttaacaccattgccactcacatgctttctctttgaact

tgataacactgattttgtatcaaagacacatagttggaagggacatagacgtgaaggagt

gtaaggttttgagaatattaactgagatgaggctgttgctgctgccattgcaaccatggt

tactgcaaaattgtggattattttgttgttgatattttgctgagtgagttttgcatggat

ggattgtgttgtaaggcactgcggaaaagagattgtgaggttgataat

>fava_dn_rep_c353

tgagatttttagggttgatcttgtgaataaagttttnnnnnnnnnnnnnnnnnnnnnnnn

nnnnnnnnnnnnnnnnnnnnnnnnnnnnnnnnnnnnnnnnnnnnnnnnnnnnnnntagct

aaaagcctaagactacaattcctcttcaaaaagaagactgcttaactccaaatttcccta

taatatccattcatttactattccaaatttccctataatgtccattcatttcatttacta

tttcaaatgcaacaactaacaccaacatacaaaacaaggttcaaaaattagaaatcgttc

atctagacagcactcaaaacagagtgaagagtactgcactcaaaaaagcatgcccgtttc

taaaaagagtgaagagcagtcttcacaaacacattatgatgaagcttgatagaaatttca

agctttggaataaacacattattaaaccaattgcatatgatagaaatttgagtgaaattt

agaaaacaggcaattcagaaacacgtacagaaaaggaaggacaaactaaaaaagcccagt

gcatccatatatcataaaatagaccatgaacgcctaaaatctcagtagcttcaacattat

caatcagcaccttctccctttaaaaacatgatagaagagaagtttaataactaattagca

gtcacacattttactgtggttcatcattgcctttaggtgcagcttctttgatttcatcag

caccgtcatcctgcatatcagaggtccaaagggtgaggttatcacggagaagttgcataa

tcaaagtgctatccttgtatgactcctctcccaatgtatccaactcagcaatagcttcat

caaaagcctgttttgcaaggccgcaagccctgtcaggagagttgagaatctcatagtaaa

acacggagaagttcagagcaagaccaagcctgataggatgagttggaggcaactcagtgt

ttgcaatgtcctgagcagatttataagcagtgagagtgctttcggcggcatctttacgtt

cagcgccgctcttaaactcagcaagatacctatgataatcacctttcatcttcaggtaga

aaaccttcgaatcaccgagcgcagcagacgggatgagacgcgaatcaaggagtttgagaa

taccgttacagatgttggagagttcagtctcgatcttagatctgtaatcacggatggccg

acacgtgttcgtcgttgcctctgctttcttccttctgctcgatggaggagatgatcctcc

acgaagcgcgtcgtgctccgatcacgtttttgtaagcaacggggaggaggttccgttctt

cgacggtgagttcctcgctctcgacggcggcggttactttttccatgaactccaccatct

cttcgtaacgctcggcttgttcggcgagtttcgccatgtagacgaactcttcacgcggtg

ttggtgcggtggccatcggggaatctggttttgggaacggagagggaaagtagatagaga

aagagaaagagatagaaagagagatgttatcgatgtgaatgagaagaagacatgctttta

ttttttctttttacttttaaaaaaagaaaagggatttatggcg

>fava_dn_rep_c354

tgacgatcccaattcttcaatccgcaagcattcctcttcttcttcacgtagttctcttct

tctctttaaagtgcgccgctgcaacaccatttctggtagatcgcccccatttctttccga

tctcaagcttctccttcatcaagttcacggattggacacctcatctccgttcataatctt

ctgcgtccctcttttcatcaccgattttttttcaaactttgaaataggtaacaagaatca

cacgtagaaatgaagagaaataagattttgaagcttattgatcggagatgctctgatggt

gcttcgaaactgctaacgcagaagcttcagataatgttattcaatgcttcagttcttgta

gtttcttcttctctcgtttgtgattctaaaaaaatcttatgctcgtgattcttcttctca

cttgcgattctggtgtttttgtgaattgtgtggtgttgttcgttgaagttgttttgtgat

atcgttggtgtgcattatgaagcaggtgaagatgaaggtgcaattgttgaagattcaagt

tgatgaaggttgttgcaacaatgaagctgaagtttgcaatgaagaacgattatggtgatg

gagtgttcaaagggagatagagaattgaaggtgaagttggtggtgattctccagaattgt

tgaagaatccgattgaaagaggcgagtttctgtttttatatgttatgagtttgattgaag

ttatctgagcccttgatttttgattttctgttatggaatagagggataggatgagagaag

ttagtaggtttttttacagttagagatttggatcagttagctgttaattatattttggat

tcagttatggacagttagaaaattggttatggagtttagactttctgttataacagttag

ggggttaattccgacttattgctatttttccattattttcctgatgcaaattcttcctat

gtattttctgttatgctgcacagtttcctctctccatctatatgattcccctgatttttt

tctgatttatgttgaagctaaacaagaatatgtgagagttttaggtagggactgatttgg

atttctgaattggaattgaactgctgattggttgttaattgcagggctgcattatttgat

gtaagcacaagtttggaggatgctgaaactgacttttcttcctgtttcttttttattctg

tttgatgcaagctaaggttttggaactgtttttattgtgtatgattcatgtttggatatg

gaaacaggttaatgatttgtacctggttctgttttatgcaggttgcttgatattttgatg

gcgtaaagatgaaaggagaggaaggagaggtctcgtggtgaaaggaattagtataggttt

aggaatgatttagacgtaaagttctcttatgggcttaggatagaaatatgtacattcgtt

tggtgtaattttcttgaaattgtaatgtaatgggaatattacggccgcgggatcatcaac

cattccttgaaaacaatggcatcactaaccggaaataccgatagcgatcttgtgaagaat

gcaaatgagacagcagcatcagcaaagacagctgcgagtgaacagactgagaaggttatc

gccgctgctggggaagctaagaagacagtaggagagtacgttgacaaggctgctgatcat

gttcattccaaacctgaaccagaaccaggtgtttttagtggtgttgcaaaggctgccggt

gatgctcagaagaccgtaggagatttcgttaccaaggctgttgattatgttactcccact

cccaaaccagaccctgtcgctaaaactgaagctgcggttactgatgctacaaaggctgct

ggagacgtgtttaaaaaatgaaatatcatcttgtgattcgttaataaggatactaaactt

gtcattgctttcagtattaattgttgtgtgtcttatcatcgtgtatatggtaggatatac

tccgttagcactgtgttatgatggattgaatctgtaaggttccatttcattgtctttgaa

ccaacatcatgcctctctagatttgattatgtttttcgtaattctgttgcatataccatt

caatcacatgcttcccttttcgtatgttgttgttctcttctggctttaattaatattttt

gctcttccaacgggtttaactaatttgtctttgtgtattttggtcttatattttgactaa

aatcaataaaattcatcttattttagagagataagctgcatgtggtaataattgtttttg

caggtggagt

>fava_dn_rep_c355

aatctaaatttgttatcaccgatagtttttcacaactttacaggagcaatataactatta

atccaaaaataatttgtgaaacattcatagatcattttggataattgggcattcgataaa

aaccaaagttttaaaatatactcaataattcaaaatagtatataaccttaacaatttgaa

aggattatcctacaaatatctcttacaaataatggataaattaaaacatggcaacaaaat

tgttctctcgacataaactaagttcaagacaaagtgccacttgatagagttttaagctct

cttcttgctagaagccaactgggtctcaggctctttcttaacagcttcttccttttcaga

taatatcaattctatatggcatggggatgacatgtaagggttgattcttccatgtgccct

gtatgttcggcgtctttgcttctgtgcttgattgacttggatgtgagaaatatatagagc

atcaacatccaaacctttcacttcagcgttactctctgcattcttcaacaaatcaaggat

gaacttagcagatttggctggccaccttccttgtccgttggagtgtctgttcttagcctg

ggctgtcctcccgacaccacggcagaaacgtctaaatggaatggcttgtttgtgggcaag

aacatcttccaagtaccttttagccttgatcaagggcatcttcctgatggaaaacgctgt

ttccctagtgttcttgaaatgaactctaaggtctgcacctcgtgccttggtgggcttggt

ggaattatcgggctctctcgagtacttaaccatgacgacggcttgataggtcaagataca

gaagagctgcccgcggccaccaagtccaccaaggcacgaggtgcagaccttagagttcat

ttcaagaacactagggaaacagcgttttccatcaggaagatgcccttgatcaaggctaaa

aggtgcttggaagatgttcttgcccacaaacaagccattccatttagacgtttctg

>fava_dn_rep_c356

ttactaatacaggttttagaaactaagctgaaccataaaaatgtgtgtaggacaacnnnn

nnnnnnnnnnnnnnnnnnnnnnnnnnnnnnnnnnnnnnnnnnnnnnnntaatgggaaaaa

agacagtcaaaatctaaattcagctatgatcattctatccgcaactcatgctttgctttg

atgtcaagcacagacaaaagttaagttcgccattataaaacatatctaaccagttctatc

ttcccttcctccatagtttattatatatataaaagttgaaaaatttaaactagtcgtcct

catgtaaccaaacacaaactgcagccaacagtagtttcataataataaccaaaaaaagat

taaaagtaagtggtgagagacaactagcacaaactacttaaacaagtaggaaactaataa

ggagcagtaccaattaaagcaaacaataaaatagaccaacaaaaactaaagatatcctga

tggaaacataattcacatcaaaactaatctgccctgtcccttttcttttttatatatttt

tccaatttgggttagggttgattggtcctcacttggccatcataactttgacaaactcct

catagttgatctgcccatcaccgtcaacatcagcctcgcgaatcatctcatcaacttcct

cgtctgtgagcttctcgccaagatttgtcataacatggcggagctcagccgcagaaataa

aaccattttgatccttatcaaagacgcggaaggcttccttgagttcctcctctgaatcag

tgtctttcatcttgcgggccataaggttgaggaattcagggaaatcaatggtaccattgc

catcggcatcaacttcatttatcatgtcttgcagctctgcctcagttggattttgcccaa

gagaccgcattacagtcccgagttccttggttgtaatacaaccatcgccgtccttgtcga

ataggctgaaagcctccttgaactcggcgatctgatcatcggtgagctggtcggccattg

gagctgcaaaagaatgagaaaaagagaaatgaaacggagaaaacaaaatgtagaaagtcc

ccgcgg

>fava_dn_rep_c357

ccccttttgaaatccaacctctctggcgctctcttttctcattagtctttgttctatctc

tgaccgggaaagaaaaacaaacaccacactaaattccactcaaccctagtttctctctcc

tctttttctctctctagaatctcaacaatggcgttcgcttctttcctcggaagagtcctc

ttcgcctccgtcttcatcctctctgcttaccaagaatttcatgactatggagttgatggg

ggacctgcagcaaaagcaatcagaccaaagtttgatgcctttattaataaagttgattca

catgttggcttccaacttccagaaattgacatgaaatttttagttgctggggctattgct

ctcaagggcgttggagggatttttttcatacttggcagttcacttggagctacacttctg

cttttgcatcagttgattaccactccaatattgtatgatttctataattatgacactgag

gacagagaatatattaaacttttcatcaaatttacccagaatatggccctctttggggct

ttattgttttttattgggatgaagaactccattcctagaaggcaacccaagaagaaggtt

cccaaaacaaaaacctattagcagctgtaatttgaagtctcattttgcaagaaggttccc

tggttgttcactattcccattcccaaatttcccaggacctggcttgagtgtttgtctggc

acatcacaaatctcccagcattagtctcatggattacacataatttatgtatgcatatat

tttgcccttttatttgacatgatgaactatctttcaattgctaaacttgtccaccaagag

ggtttaaaatgcatgattggagcctgtttgctcaaacttagagtgttatatcattttgtt

aatggtcctaaaagtgcggactttgaaattgctgctttactataaaattttccacgctgg

caataattctgtgtttccaatttgaattctttggcccatttcaccttaatgtttatttgt

actgtggcgaattcgaaaagaattggcttgatttccaac

>fava_dn_rep_c358

aatgtttgcgattattgcggacagcacaagacgttaaaaaaatagataagaaaagaggaa

gagtaggcgtacacgctaacgacatggcatagagtgttattttgaaaagtgcaattgcag

gtaacagcattctcagaaccatgtagttgagttggcgatatccagttgttccttccacta

catggatctacactgaagttccaagaatgtctcccaaacccatggattaataacggcacc

aaatatgcagtttttttatgcaaggctcttcagttcaatgcaaaaccattgttatcgctg

tttctatattcttaaaccagcatagtctttggactgatatacattgcaatttgcaagtga

agtgttgtgataatctatccgtcattatatcttagagtaaaaaactacatatgaacgaag

aaattgaagcaatttaacaagtcagcacaaattccaccaactaaatgagaaggaaaataa

acagttaaaaatataaactttaaaacacgacgtctgaagttgggagatagagagggacaa

atacatgctcccacgaatattgtcatcagaataacattagcaccccaaatgagcactact

gtagaacctgcatcctcttactgcaggtattattcttttcactaaccgcagcaagctgat

tttgttgcagcttgaccgccattcgctgccggatcctgttggttaatcttgatagtctga

ggctcggatctagaatccgtatctgcaagcctttgcttgatgtctcttgctattgaaaag

aaaacttcctccacgttcatgtttgtttttgcactagtttcaaagaacttgataccatac

tcatcagcaagagcttgacctttggaggtaggtacagcccttttactttcatccatatca

gccttgtttcccacaagtatcttgtttacattgtcagaagcatgttgttcaatattgcga

atccaattcctaatattattgaaagatgcttcatcagtaacatcataaaccagcaagata

cccatggctccacggtagtaagcagtagtaatagttctgaatctctcctgaccagctgta

tcccagatttgcaacttaatgcgtttgccatcaagttcaatggttcttatcttgaaatca

ataccaatggtggtgataaaactggttgtgaaggaaccatcagaaaatctcaaaagcaga

caactcttccccacaccactatcgccgataagaagaagcttgatgaggtaatcgtaatcg

gcacgagctctagcgggtgctgctgccatggctatcgttgaaaaaaatgaagagttcttg

ttcggtgttatcctattctatgccactaccagtaagcacagagatcggaatcgaaacagt

tagaagcgattcgttggcgacatggaaacagatcacgaacgaaacgacgaatctcttttt

gagtttgagttttctttttctctctctctctctgtgtaataagatgattgtgaatgcagt

ggcccgc

>fava_dn_rep_c359

taacaaactctaaccaattcctaactaacttctnnnnnnnnnnnnnnnnnnnnnnggccg

cggggactccttgctgctacttcaactttgctctctctctcaacccaaagaaaaattaac

aaaaatggcagtcacattgtataaccctaaatctgaatctgggttgaagaaacttgatga

gtaccttctaacccgcagttacatcactgggtaccaagcatcaaaggatgatatcactgt

ctattcagctttggcatcagttccatcggaggaatatgtcaatgttgctaggtggtacaa

gcacattgatgctttgttgagaatttctggtgtttctggcgagggatctggtgtcattgt

ggaatcttctcttgtggcggaagaggctattgccactcctccagttgccgacacaaaggc

cactgaagctgaggatgatgatgacagtgatgtggatttgtttggtgaagagactgagga

agagaagaaggcagctgaggaacgggcagcagccgtgaaggcttctggcaaaaagaaaga

gagtggaaaatcatctgtattgttggatgttaagccatgggatgatgaaactgacatgaa

aaagcttgaagaagcagtgagatctgttaagttggatggcttgttatggggtgcatccaa

acttgttcctgttgggtacggtatcaagaaacttcaaattatgatgaccattgtggacga

cctggtttccgttgacaatatggttgaggattatcttaccgttgagcccatcaatgagta

tgtccagagttgtgacattgttgccttcaataaaatataatctgctatttatgtgacatg

aagatcatgatagtgggggagaatttggaattattagagatgacaatcaagggttactgt

tatattaagttgagttttgtgttttggaaacttacctgttactgttgacatttgaagaat

tttgtcttgtgatttcatcactgttatatggattgtgttataattatttaagttttgaga

attgaagttgtgttatgtttttagaattttgattctttgttgagattttgttgataatag

agcttgccactctctttggactgagccacctctaattttggtggcttttctataccgtaa

tgcaaagactagggaggaatttgttttgacaagccacac

>fava_dn_rep_c360

caatgataaatatacggtgcaaaatgaattagttaggtagtactattaatagctgttagt

aaaactcattaaattcagcaaccaaaagttagaaccaaaatttccaagtcaagcccctct

atttatgagaatttatttactccaactacaccacacctatattcataacacaaaacccaa

tttaacctcttatcctattgaacaaacattttcaaaacacatcacagaaaaaccaaaatg

ggcactacaccattagtactactagcaatttctttactctttgcatcactaccatatcat

ggagtatctgctcctactcctagggctgaatctccggtaccggagtccgtcggagctcca

tcgcctgctgctggccagggatgcttgaatgctctaacaaatatgtcggattgcttaaca

ttcgtggaagatgggagtaaattgagaaagccggataaaggttgttgtcctgagttagca

gggttggttgatagtaaccctatttgtttgtgtcagcttttaggaaactctgattctatt

gggattaagattaatgttaataaagctctcaagcttccttctctttgtggtgttactact

cctcctgttagtacttgctcagctgctggagttcctgtgtccttgcctggcccatcaagt

caagattctatgccaccaaatatggcaatgacaccaaaaggatcagctcccagtccttca

aattcagctgatagttcagtagctggtgttccaagtccttctgatagcaaaaagggagga

gcctcaagcattcatgcctctctcctcactttcatttttgcaatgtccactctctctttt

tctatattcttctgattttagaatttacattttatcctatttgaatattgagtgctttga

tttagtgaattggacacttttaattaggaactgcattacaacaccattgaattcttcatt

cttggtgatgtgctattttttcttctcaactacattcttgctatatttgtaataatatat

ttccattttcaatatgatgtttcattttgttactggatgatttaaaaaaaaaannnnaaa

aaagaagaagaatgaaatgaaataattgcct

>fava_dn_rep_c361

tttttttttgaactatgatctgaaatccagttgttcaagaacaattcatttattgacaac

aacacaagttcacataaatataattaacatataacttttacatctttgcataaaaattcc

aacacaatcatataataatataaaaacataaacagaaacatgtcccagaacattacaatt

ttacaaaacatcatgaaaagataagccaagaatcaattttcttaacaaactctattataa

gtaccacatctattgttaggtccaccaccattttgagagggcaagcaagtacggtagccg

ttaacaattggacatataacagcagcaccattgttgttagctgttctacctgtttgagtt

tgactctgatccaaaagcatccttgctacatgagaactcatcaagaattccgattccaaa

tcatcgttgatcagacaatccccagagttacaacctccaatcttcaatgaagttgttggg

tttagatgtagttggatcaataagataaggaacaatagtgaaagactacccttgttcatt

ttctgtcttttttcgatctctattttctcttcacaccaacaaaaatgaatttcgctagtt

cattgaatagccccaacaatttgtattgtatttgtaactaaataataataaaataacaaa

ggaatcttttcttttctttatatataattaaaagtttagacgactt

>fava_dn_rep_c362

tttttttttaatagaaaactgaaaagtaacataataaatcacaacacaaataaatatctc

ataatcaaacatttaaccagttgtcttaacgatacaaaacactcaaacatcaaaagttgt

actaaatagacaaaatccaaaacaaaatttgaaaaatgtatcatgaattactgtctaagc

tgctgtcttccttagcttagcaagttcttgcctaacactagcagccctcttaatctttgc

caacataatcttgaacctgtcaaaatcattgagggcagcccttctcttcctaacaatgag

tttccttccccaagaactcttctcccacttgttcttcacatctgcggcttccatggcctt

gatgagatccttcttcttagggactctcttgatatcaatcttgatatcagtaagagaaag

cctcttgaaattaattgggatcctctccatgtcaggtgcatcaacaagagccctagtttg

gtcgataacatcgacgataacaactagctttccatagtctttgccatagttgacaagtgc

aactctcccgatctcaacaaacctcttgaacggcattttcgagtttcggcttgaagagaa

gctaacgcagacaacgtaggtttcagagtgttcccgcggc

>fava_dn_rep_c363

aacaaagaagtaaaaaggctcattttgtctaccgtattaatacaccaatgtagtttattt

cttgcttttttccttatctgaggtatgtgttgcaccaaattttgtaataaatagatcact

caaatcgaaaaatcgaggatccctatttctagatactagaaactagatagtataaagaaa

atagggcatgtaagactccgcagctaatatagtcacgtttttcaagtcccgcgctgcttt

ttactacatcgtttcaaacgaggttttaccataacnntttttttttataggaataaaatc

taatttattgaaggtagctcatcattttaatgttcagcataacaagactcaactactaat

ttcatacggatagtaacagtaacagaaaattaaaaagggagggttcaataggatccatag

agcaccacaaccacaaaccctaacacgagaaaagtaaaacgacgcgaatcagaaaaacac

aactagaatttgagatcaaattaaaaatcagagccaccagtttaaaaaacgccaaaacag

tatgatctacagttgtccacaattggcgatcacaacaggcttagaggtcttgccagagcc

agatccaaccttctcaatatccctcacaacgttcaatccatcgacaacctgaccgaacac

gacgtgtttcccgtcgagccagtcagtcttggcagtgcagatgaagaactgagatccgtt

ggttccgggtccagcgttcgccatggataagattcctggaccggtgtgcttcttgatgaa

gttctcgtcagcgaacttggaaccgtagatcgattctcctccggtgccgtttccggcggt

gaagtcacctccctggcacatgaagttagggatcacacggtggaaggaggatcccttgaa

gtggggtggcttgccgctacgaccgactcctttctcgccggtgcagagagcacggaaatt

ctcagcggttctgggagtgacatcggcaaaaagctcgaagatgatgcgtccagcgttttg

gccgccgacggtcatatcgaagaaaactttggggtttgacgtggcttagggtttgagaag

tggttgaatcagatcgtaatgggtaaaatggttagggtttgtggcccgcgg

>fava_dn_rep_c364

tcgccaattaggctatttagataagagaagaatctacatatcacaatataactggttctc

acttctccaacgtggcttttcccatctatattctaaacaaactctagtccccattcttca

atagaaaagaccagttccctaccactcttgaagaatctgtgtcaatctcaacatagtatt

catctatggcagcaacaatggcaatgatgatcaactccaagtgtctaaacacagccatac

caaagttgcaaaacccagcaactagcactactagagtaacaacatgcaaaaccaccaccg

ttcagaacctaccaaaggctcttacctctattgacaccaacgttgtgtcaccttccattg

caattgcaggggcaatattttcttccctcgcaacctgtgatgctgctttcgccgctcaac

aaatagcagaaatagcagaaggtgataacagaggattagcactgttgttgccattggttc

cagctatagggtgggttttgtacaacatattgcaaccagctttgaaccaaatcaaccgta

tgcgtagcacgaggggagtgattgttgggcttggactcgggcttggcgggcttgcggtat

caggtatggtgtcagaagcatctgctagtgagataggtatgatcgctgaggctgctcgaa

gtggcaacagggggacgcttctgctgtttgtggttgcaccagctatactttgggtgcttt

acaatattttgcagcctgctcttaaccagatcaataagatgagatctgagtaaacagggg

tgggtgatttttgtgtaatgtctgtctgtaacacttgcttgtaacttatatctaatactc

tttctttctctattttgtgttatatatcaaactcggttttctacttatccttagcagcca

tttcattttctgatttggcttactttgttctttatttgagatcatttcactattggacat

cactcatttttgtttttacagattaaacatttcatctctctaaaaaaaa

>fava_dn_rep_c365

ccctgttctnntttttttttgaaaagcatgtgatattaataaatgtgaaaaataggtgaa

gttgctctccaactagtatgcgaagtatcaactactatgttacatttttttcaattctgc

ttcaccaatcattagacaaaattattactggtaaaactcttaagaacagaagccacagaa

atattcaactgataatgaaactaaaaaaataaacatgaaggcacacaaaaagtgattgca

agagttccaattgtagctaaaagtattagctcatttaagcaatcatacaaacccaagtaa

cactcaagaggataatattcctaacaatcattcctctattacaacattcacttgagagga

aaagcatccttcttcaagaacgaccagactgacaggaaatgatcagatacacccaaaaat

tcaaatgtctccttatgccttgcttgcaatgttgttagaaagccagtccaaaccctcgta

tagaccctctccagaagttgcacaggtgctctgaatataccagtggcgttggcggagtga

atgaagaccaagcttgtctgttatttctgcagcattcattgcattgggaagatcttgttt

gttggcgaaaacaagcaaaacagcatctctaagttcgtcctcattcaacatcctgtgcaa

ctcatcccttgcctcaaccactcggtctctatcattgctatcaaccacaaaaataagacc

ctgagtgttctggaagtagtgcctccacaatgggcggatcttgtcctgacccccgacatc

ccacacggtgaagctgatgttcttgtactccacggtttcaacattaaacccaatggttgg

gatggtggtgacgatttctccaagcttgagtttatacaaaatagttgtcttaccagccgc

atcgagaccaaccatcaaaatcctcatttccttcttcgcgaataacctactgaacagctt

cgtgaacgttaaccccatcttcaccaacacacaaatcgctcttcttttcgatctagagat

tttttggagaagagcgaatcgcgatttgttcttgagaggaaagagagagaaagacc

>fava_dn_rep_c366

attcaaattacaagacaaaaatcaagctaaccaagttcaaagtctgtacattcaaaatca

ttcaagtatgtatattcaagtgagtgccatgactttgtttttttttttcaaaatcaagaa

aaaacagatatagcattataggtacatgttacaagagaattgaatggtatatagtatatc

caacaacagaaacaacagaaacaagaacaacaacaacaaaactaattttattttattata

agaaaaagtttcagtctgatctcattttgttgatttggttaagagcaggctgcaaaatat

tatagagaacccaaagaatagcaggtgctacaacaaacaacagaagctgacccctattat

cactaccagcttcagcaatagcagaaatttcagtcgccattgcatcttgtgttgacacaa

accctgaagctgttaatccaccaagacccaaaccaatgataacccctttcgagttacgca

tacggttgatttggttaagtgcaggttgaagaatgttgaagagaacccaagctatggctg

gaattataggtaacaaaagtgctaaaccacggttatctgatgatgcttcagctatgtcag

ctatttgttgtgctgcaaaagcggggttacaggctccaagggttgggaatattgctcctg

caagcgctgttcctgctatggaagatgatagtttggtgttttctgttgtgggggttcctt

ttggaagaaggttcttgagggaaaggagtgaagtgggttttgtaatgagttttgttgagt

tgtggaactttggtgagcttgtagtgttgaagcactttgtgttcatcatggtcattgcaa

ctatggttgatgccattgattgatgaaggagtgaaaatgtttgtgttgagtgtgtgaatg

atttctggatttgcaggtaaggagaaatttttggtttggtttggaattatatttggtaaa

gttaagttgaagaaatgagaaccaatggaattgtggtattaagattcttttcttat

>fava_dn_rep_c367

cagcaaataatcatctcgtctattactttccactcactttctatcttgttacactctttc

ctttcacattcaattttgatccaaaaatcataaaatgaaccgatttatcatggaactagg

ttgattagaatttgacaatagaatgtcccttttgaattggctgcatagacagcccaaaac

acaagagcagacaaactaacaaaacttagagacaagaaagtagggtccaaatcgtcacga

gaagctgcgtctctgaatttgatacgtgccccattcacacccaatatccgattattttga

catttcaacttgagaattttaaacaaatgcacatggtttctatagtctactagccctgaa

ataatattcaagaagaatttgtgacaataaattaattaacaaaaacaacagttattggaa

aaagaaattacacgcaatcatatactagctcagtagccaatcaatgtatagaattacata

gataaattaaatacaatacaagtttcctaattgaacagcatggatgctaataagagtaca

aaagaaaacccagtgaatgagactctgcctgcacctcctttatcacccgattcaccacca

cctttagccgatggggacccgccggcagttggagctggagctgacccagcttgtttgcag

ctggagataccaccgggaataccacacttaccagcgagtccaagagcctgagtaatattg

acgtgaaaggtttcaagcaaaccaggagtatagaagagattacagagacaagtgagttga

gtttcaacggttttcttgagtggatcacagcaagagcttggtggtttggtggaattcaag

aaatcagcgcaaggaattaaattagaagcgcaagaagttgtgtcttgtgcctctgtactg

ttgatcatcattatcaacaccatgcctaccatcaatgatgctaaacatgcgatgcttctt

cgaccacccattttgttgtttttgaggtttggttctgtgttctggatctgctttttttct

ctggttttgcttggtgacatgtctgatttatagtaatgtactgtgaatacttaatacgtt

acagtgaagtcaagccggtaaccgcg

>fava_dn_rep_c368

aaatactctttcttgcctctcatttttgctcccccttcacctttcatctttcttcattac

cattcttaaattctcttgttgtttcttaaggttttgttcttgaaatggaatatgagaatc

ggttcagacaagctcatagacaaaaatatgattgtcttctttttgatttagatgatactt

tgtatccactaagctgtggtattgctaaagcatgtggccaaaacattaaagattacatgg

ttgagaagcttggcatagagagaaacataattgatgacatgtccaatcatctttacaaaa

actatggaacaactatggctggtttaagggcaattggatatgactttgactatgatgaat

atcatagttttgttcatgggaaattaccatatgagaatttgaaaccagacccagttttaa

gaaacctcttgctgagtctaccttatagaaaactaatcttcacaaatgcaggcaaagttc

atgcagttaaggcactaaacaagcttggattagaagattgttttgaaggaattatatgtt

ttgagacacttaatcctatccacaagaacactgtctctgatgatgatgaagatgacattg

actttgtaggttcaagaagaaccaaccatacaacaagtactagtgctagtaacttgcaaa

tatttgacattattggccattttgcacaatccaatccaactcaagctttgccaaaaacac

caattatttgcaaaccttcagaatttgccattgagctggctctcaagattgctaatctgg

acccacaaagaactttattctttgaggatagtgctagaaacatacaagctggaaaacgtg

taggacttgacacagtgctggttggaaaatcacagagaattaaaggtgcagattatgcct

tagaaagtatccacaacttaagggaggcagtgccagaattatgggaatctgaacttaaat

cagaagttgcttatcctagcaaccttgctgtggagacatctgtgactgcttaattaggat

taattaactatttaattacaaggaattaagctacttaattattgatgtcaaaaaaatttc

agaagaaaaaaaatgaaaataaaaagagattccttttgttgaattgtagtgtagtaattg

tagttgatgtagatgtaaatgttttgatattataataaaataatataaagtttatgttta

tgtttatgttatgatcactaataataataaatataataatgttgaatgtta

>fava_dn_rep_c369

atccaatatctagtgaatcattaaatggctgccacatattcaagtgacaagtccatctcc

catgcaaccaaaagcaaactactatttttcatttttttccttttccattcactaagcaaa

caattctagcaacaatggaagccctcacaaaatctatctacctaaaaaaaattcccaagt

ctcaaacctactctatttcttcatcaaaatcaccactttcattacactccaaaaccactt

cagtaactttcaattcatcaacacaccatttccacaaatgctttctctttcatcaacctg

gtggtggtgttataaatcccatagcaaaagaaaaggggaaactaggagctatacatgcat

cagaggctgcaaccacagtcacagatgctactggaagatggattcttgaaccaattggag

atggtgatacaaggcatataggttacaaggttgcgatgccaggtgcatatgaaattactt

ctaatgaagtcactgtaggtcgtgttccagagaaagctgatctagtgattcaagttgcta

cagtgtcgggtgcgcatgcacgcattcggaaaaaagaagacaatcttctagttatggatt

tggatagcacgaatgggacatacgttaatgagaaacggctgaaacctggagtagttacct

ctgtatctcctgggagttttattacttttggggataccaatttggctatgttccgtgtgt

caaagataaaagacgagaaagctgcagatacaactggagaaacagaaggagaaactgaag

gtgaatcagacaatggtgacaaatctgatattactgaaacaagttgagcttgtacttgta

atgcatagtactttgttttgtatagttaagatatttgcattgtttcgcaatgccgttttg

atacaaaacatattctaaggttgatagttgatattctgttcaatgttgtcactgccaaac

aataggaaaagataagtttgcactgtgagaagaaactgtagtgttgtgttattacctatt

ttcagttattatgctagtttgattttcacctgttaacacaatattttattaaaatatgtc

tttctgtattgttaatcttagactaagttaaaatttagggttaatagtcacttatccctg

taaa

>fava_dn_rep_c370

ataacatacccggaatgcttacaactatgagcacaacaaatcaaccataattcttcaaag

attagggaatttttttgacatccctattacatgtcttttttttttgatagaaaacctcaa

taaattttataacatttcatgtatctaaactctaaagaacaaaataagacagcaattata

taagaatgacaattcaaaactagagaacatttcctagtttacattctgctgtcaattgaa

aaaccatcatcaagtttcaacatgaaagtttataacattgtaattacaaaatgttgaaca

tatgttaagtttatcatgcaagaaagccaagccattctccattcttcttagccaacctga

tcagaccttgtctctgttttgcatctgctcctattgatttacagaattctgtaattacct

gagagtgtaaagcaatggctttgcctctaagctgattgaaacgtttctttccaacaatat

tacgagtaacaacaacaattggagcaaacacaccttttccatcattaacattcttcatca

taggttgcaacctcactggcttactccctcttccaattcgaacttgcgatgaaactgact

tagccaacatagtataatcttcaccaactatagaagttccccaacttccatagaacgagg

aaccaacacaccctgttgcagaaattgaagtagccattttagttactatgatgttttttt

cctatgtttttgtttttgttttgtgtgaatataaaactagaaggctgtttgtttgaggtt

gttagaaacattagcttgaatgcatggtttataatatttgtatgtgaaggaagggatatt

tgggtgtaaaatggagaaccagtggtttagaattggactagagtgtgaatcatagcttag

aaggattaggagccacatatattgatggctttgatttttatttatttttgtttatcccgc

ggc

>fava_dn_rep_c371

cattaggccgcgggattccaattccaaaccaactcattcttcttcgtgagactgctccga

tttcgatcacaaatggctaccaaagcaggaggcgtcttcgattcccccggttctcaaaac

tcggccgaaatcgaatcccttgctcgattcgccgtcgatcaacacaacaccaaacagaat

tctcttctggagtttacaagggtggtcaaagcacgggaacaggttgttgctggtacactg

catcaccttacgattgaggctgttgatgcgggtgagaagaagatctatgaagctcaagta

tgggtcaaaccatggctgaattttaaagaacttcaagagttcaagcatgccggtggtgat

tagaagatgcttgtccgtttctgtaaggaattgtatacccttacttttactactttcata

ctctcgagttaaataagtggattgtctgtactttcatactctcaagtttaagtaagtgga

ttgtcttgtgtgtatgtgtttctgatatcctatgtttaagtaaaagtttaattgtacctc

aagttttaaaaagttcctatttagagattagagtatcaaacgtataactacacattttac

ttaaaaaaaaaaa

>fava_dn_rep_c372

gaattcaagtttctaaacaaactcatcttgtcatgcagtgactgaaaaacaacattaaat

tcacatttaattgtacataattttcacaataattcacaattgctatcacataatcaaaaa

ttgttgcatcattgattgttcattgttagtttaatttctgatattgtgtttgattttttg

agaattctttccatatatcggaagacaggttcctaacaatgactctcaaatagaagatcc

attatgtaacaacactgtaatgcacacaaattatagaaactactactgaaatgaatgtcc

aacagaagcaagtgatgaaccttccttgagttcaatatcaccaactagagaggttcttga

gccattcaaaaattgcttataccattttgcggagagctttgggtatcttctcaactctgg

atcattcctatcaacagagtatagcccaaatatcgatttatatccatccagcaactcgaa

tgcatcaaggaaagaccacacaaaatacccctttacgtttgatccatttctcaacgaatc

gagcacgctaccaatgtatccatgcaagtactgcaccctagatacatcctggagtgatgc

attgcttactgtccgttggccatttccatgaatgaacataggtggattgccataaagggt

cttgaatgtttccagcacttcttgtaaagcccatggtgccattgggtactctgcgtctcc

gagtatatcctctacaccgagtatttttgcacctgagtctgcacttatatctcttagtgt

tgtcttcagggcatcagaattgtctgatacattaaacttgctgtagtgaataattccaat

aaagtcgtaggaacccttaacttgttcagattcacgagttgtgaatgccggtattcttgt

gcctgcattttccttcatcaaattgggatagtctccgtgcaacaagggttccataaacca

accaaaattaaaagtacggattcgttcacatgctgccctgtctttctctgtatttgtctg

aggaactgtcccaaatgtatatagagagataccaacaaatccatgttgttgctccctgta

ctttcttttatacaatctcacagccgaagaatgagctaacaaaatatgatgtgcagccag

gtacggttcaaatgttgagtttcccttagaactcggtatcatacaaaatggaggagaaca

tcgttgaggtgggctaattcccgtaccgtaactacccttggcaaagacatttggctcgtt

cacagttgtccaatatttgactctatctccgaattctctaaaacacacgtctgcatagtt

cgtgaagtcctttatgacatgacgactaagccatccttcatagtcatcttcaagtgcttg

tggaagatcatagttgtgtagggtgacatgtggctggataccattggtaatgagttcatt

gatgagattgttgtagtagtgaagtcccttgggattgacgggtcctctaccatttggtat

caatctggaccaagaaatggaaaatctataggcatcaaggcctgtttcaaccatgagctg

cacatcttccttgtatttgtggtacgcgtcgcaggctacatcaccatttccaccatgtgc

aaaaccagcatgagcaaaggtatcccaaatgctaggagttcttccatcttcatcagcagc

tccttccacttgataagcagatgtacctgaaccaaaaacaaagtcaactggaaaatcact

tctgttataatcaccaccaccactcacaagaactcccacaacaaaactgagaatacaaag

taacaaagaaaaggcaaaacatgcttctggtttcattattatcaataatagtagtatttg

cgttttcaattttgtaagaacctctaatgtatattgcactaatcagaggattgtcatgtt

tcttt

>fava_dn_rep_c373

ggaatttggatctgatatacacaacaacaatcataccaattacaaatacgtcaccgtatt

tatttccctttccacgcgtttctcatctcgtcattcaaaacactcaacaacccaaccaaa

cttcacattcccttctgatatcccaaggttaaccaccgccaccgtgccaatcaccgacca

accctccggcgacaactcaccgatctccgtctaatcaactcttccttatactctttatca

gagatgaatcctgagtatgattatctgttcaagcttcttcttattggagactctggtgtc

ggcaaatcatgccttcttctgagatttgctgatgattcttacattgatagctacataagc

accattggggttgattttaaaatccgtaccgttgagcaggatgggaagactattaagctc

cagatttgggatacagctggccaagaacgatttaggacaatcaccagtagctactaccgc

ggggcacatggaatcattattgtttatgatgttacagatgaagagagcttcaataatgtg

aagcaatggctcagtgaaattgaccgctatgctagtgataatgttaacaagctcttggtt

ggcaacaagtgtgatctgacagaaaacagagctgtgccatatgaaacagctaaagcattt

gccgatgaaataggcataccttttatggagactagtgcaaaggattctacaaatgtcgaa

caggcatttatggcaatggcttcttccatcaaggaaagaatggcaagccaacctacaaac

aatgcaaggcctccaacagtgcagataaggggacagccagttgggcagaaaagtgggtgc

tgctcatcttaaccacgagatatcttctaatctatttcatttggtctactgatcattgct

tgtaagattttttttttgctatgttattatatgactaaaatagtggaccaatgtaagaat

ttgtcttgtggtggtttggaactgacctagatgatcccattctttgaatatacttaatga

tatggtttgtgcaaactacaatattcattaatgctaaatctatatattcccaaaaaaaaa

aagtcaaaaatatggtgtcatgcaactgtatattcaagcgataatctgttgatctgtttc

ctt

>fava_dn_rep_c374

tatatagaacagaaagagggcgttagggtttgtttgttttcggtgatcgagcagccacaa

tgtcgaagcgaggtcgcggaggttccgcgggtaacaagttcaggatgtcactcggtctgc

cggtggctgcgacggtgaactgcgccgataacaccggagctaagaatctttacatcattt

ccgtcaaaggtatcaagggaagattgaaccgtttgccctctgcttgcgtcggtgacatgg

ttatggctactgtgaagaagggaaagccagatctccgtaagaaggttctgccggctgtta

tcgttcgtcagcgcaaaccatggcgccgaaaggacggtgttttcatgtactttgaagata

atgctggtgtgattgtgaatcctaagggtgaaatgaaaggatctgctattactggtccaa

ttgggaaggagtgtgctgatctgtggcctaggatcgcaaatgccgctaatgttattttat

aagtcgaattttctagtattcaaaaagattttttttctgatttttggggttgttttacgt

tggatattttgcattttgataagtgacaaaagctctttaattctctgtaatgaaaagcgt

tttgaagatattattgcttagctataaaaattgtttgtgcttatgcatgtgtttttattt

attttgtggtattaagatgcttctttacatttgcaatattgggttagtgtgtaaattgat

tctactatgttgtgaatgaatggaagcttttcccaagttttcattcatgtgtttattgat

ctttaattagtgtggtcgttgaaaatctgaaacaaccattctagtagttttgatgatatt

aatgcttagctataggagttgtttgtttatatgcatgtgttttta

>fava_dn_rep_c375

tttcgcaattttggtccacagtagtcagtcatagattctatagaatggtaactcggcacc

tgtttatctttcattataaaaattctatatatactagtaagcatttatgtgataaatgct

tgcattacaatcaatttattgagctatttttccgggttgttactccacaaagtggtcaaa

atcaccaaacctgcacaatgctacagaatactgatttttcaaacaaaaaccaacaagatc

atcatcatggatgtatgtagtttgacatgctatcttcattttatgtgaaagcacaataaa

tgttccttttgaacacaattaaattacaagcatgttccaactttcaaaatctcatctaag

ttattatgtttgaagaggaattgatttgttctgtcctgcatcatgcatttcttcagaggc

atttagatgatcaatggtaaagattcatacaaacaaagaaaagatcaaatgaatttggga

aaacaggcgctcaactccaaattgcctgtgatattattttaagcaatagcagtgtgaatg

atttcattccaaagaacattttttctcaaatgcttcttgctctttgcggcataagtcaaa

ttcatttgtatggtagtacatgcaaccaacataatcgtccatctctttcgtgcacttttg

gtgtaaatccttcagaagtccaagaacacatcgagtaacatcgcggcctttatcgagaca

cttctcagggttctgatccttcttcttacatttcagaaactccagattttccgaatgaca

ccttagtccaatgtgcttcgacgatgccaccaacaccgccgatgtagggatcgggtttcc

agaagcatccacagcactcgccgcactagccatgtctgaatcagtgcttcactggaatag

tactgagtgttttttggatcgatgtgggaatgaagagagaagatcaagattagggcagtg

tatgtgtacaacgttttttttgcttttggttcagttttattatttattcttaatggtaaa

aaaaattgtaaacccaagattaatttaagaaatatgggtgttattt

>fava_dn_rep_c376

atttcatgaactcagcttgcaaagttggagtcctacgttttcgtttaacgggttcatatt

gtgatttttcaagcactttgtaattgaagatttgaagcacggcggtgggtggttgggtta

tcgtgaaatgggttggaaagcagctgagaaactcattaggcattggaaagttgtcagagg

ggataatgttatgattataaggggcaaagataaaggccagactggggttattaagcgtgt

tgttcgctctcagaatcgcgttattgttgaaggcaaaaatctggtaaagaagcatatcaa

gcaaggccaaggtcatgaagggggtatcttcacggttgaagccccacttcatgcttccaa

tgtgcaacttctcgatccactcacagggaagccttgcaaggttggaactaagtatcttga

agatggtaccaaagtcagagtgtccaggggatttggagcttccggtcttatcttaggttt

tccagtcagtcattcattcatcatcccaatggctctttctttagccattaacgttcctcc

tgttcgcgataaccaactccgaccccaacaattccaactccaattccgtccaaacctagt

caaattcccgataccctcctccgcccgaattcgatgttcctcctctttcaacgacatttc

actcaaaacggttacccccaaccgcaacaactctttcgcatgtcacgctacccagttcga

agttcaggagagcaacaaagcattcgcagaagcctatgctgtagggagaggcattcgtat

gtctgctgacaaagctcgaagagtaatcgatcagattcgtggacgtaaatacgaggatac

acttatgatattggaactcatgccttaccgtgcctgtgaacccattcttaagattgtctt

ttctgctggagcaaatgcaagcaataacttgggtctaagcaaatcgagtttagttattag

tagagcagaggttaatgaaggcaaaactttgaaaagagttagacctcgagctcaggggcg

ggctaaccaaattctaaagcgcacttgtagtattaccattacggtgaaaggtttacctga

gcctgagcctgagcctgaggctgaggctgtggtggaagcaaatccctcttgatcatgttt

gaagacaagaaaaatatgtatccctattatgaatctcttccagccttggaaatttctgca

ttttgatattatgtagtttctttagtgacttctacatgagtgttcagaatctgataaatg

tacttatgttcaacttaatactagtatgaaatgttttagcattgacatttgatattgttg

tgaaaacaatgcctttgaagtaaaatataaactgtttattatcaggaaaaaaaaa

>fava_dn_rep_c377

gaaatttctgaaacataatgtctattcaaagattctattaagaggtttttagacacaaaa

tcaatctttctcctaaaaattgttccttttacttagacacaaagtcatcaacaaaattgc

tcctttctacttaggcaccaactcagtatcactattatgcagcatgattctttcaactct

actaaacaaatctatgcacattcacaattatcctcacatcacatccatgtttagaaaata

aaaatggcatttgcacacatcttattgttacaaagttctaactactatccatttacaaat

agttgagtgttatatccaacctcattaagtgaaatgaattttgaatttgcagcggttaca

aattcacgtattcatgtagtcaacacatcaataacggttggatcgagatcagacaagtca

ttgatgtgttaacggcgtgaatgtatgaaatttgttattgtagcaaatccaaatccaagt

aaaattagataagaaaatggattaaactacaatgcacactcataaaattcaccactttgt

cgatttacatttcgctgattttgcatttccttgaaagtgaggctttaaggttcctacagc

ttcttcataagttggtcttttgtagtccacagcctgctcaataacatcttcagtgtttgc

ccatttccactctgcgaattccgggtctgcttcaccagtagctaagttgatttcgccttc

atcttttgtaaatctcatgagaaaccatttctgtgcctgtccgtgccattcgcctcccca

gagacgattgaccttagccttcacagcaggaggaaaatcatatgtcaaccatttaccaat

ctcagtgattatctcggctgatactattccagtttcttctcgaagttctctaatcgcagc

agatttgggttcttcaccgtcctcaatgccaccctgaggcatttgccatgctcctggaac

attcaatcttgaagccacaaatatctgatcatcggagttgatgagacagacaccaacgtt

aggccggtaaccttgtggaagatcctccatgagaaagaagtcgatgaagataatggatga

ttattgggaagaaagaatagaaagaaagaacacaagtggtggtatatgatgaatattgta

atgtgaaagtgcgttgaggaat

>fava_dn_rep_c378

gttgcgtataaatatgcttttatagtctctctaatgactcaccaaacaccatccatcaaa

accaaacttttctcaacatggtaatgccaatgacctcacttctccttcctctcatggtta

tcctaatactatccacattaacacaaatctcatatgctcaaaactcaccacaagactacc

tcaacattcacaacaaagcacgttctcaagttggtgttggccctatatactgggacacaa

aacttgcctcctatgcacaaaactatatcaaccaattaaaagcaaattgcaaaatggtac

actcaaagggtccatatggtgagaacctagcatggagcagtggtgacataacaggagcag

gtgctgttaacatgtgggtcggagagaagaaatactatgattacaagagtaactcatgtg

ctgttggttataagtgtggacactatactcaggttgtttggcgcgattcggttcgggttg

gatgtgctaaggtaaggtgtaatgatggtagcagcaccattattagttgtaactatgatc

cacctggtaattatattggtcagagaccttttgatatttttgaagtttcattgagcttca

accatggtagtttttaggagaaataaggtgagattgataagaagtgtggtgaattgaatt

aaataagttgagattgataagaagtgtggtgaattgaattaaaaaaaaagttgtaaaaaa

aaaatacagaggatagaagaaatcattacagtaaaagaaaaataa

>fava_dn_rep_c379

ccacgtgtgaagcactgaaaagcagtgcctcaaaacacattagagtgtggataacaaacc

aagtttcatctcaaattcaatctaaagaaacgaagcagagaaaggataacagaaaataat

ggcaatggcagcaagcacaagcacagtgttagttcctcctctccctcgtctcttgaccac

caccactcgctgctctgctttcccttatctccctcctcctcgcctctccaccacaaccac

tccttcttcttcccctttccaaaaacacttttcagagtcgcggaagttttctctgcatca

gactagagcttcctcttctgatgatacatccacttctctcgataccaatgagttgtttac

cgaattgaaggagaagtgggatggtcttgaaaacaagtccacagtgcttctgtatggtgg

tggtggtttagttgctgtttggttatcttcaattcttgtaggtgccatcaactctgtccc

cttgcttccaaagattatggagttggtaggactagggtacaccggatggtttgtctaccg

gtaccttctgtttaagtctagcagaaaggaactagcaacggatattgaggcactgaaaaa

gaaaattgccggaactgaatagaactgtattagttgcaagtcaagttgatgttccttgct

catcatttgttttttttgctgtatgtcttgttatatgagaggcatttgtattccttgata

tagaatagaattgctcaatctctatcagtatcaactttgtattaatccatttcattgttg

aaaataatgcagcaaatcttatatatatgtttatgattctgaatgaaaaccattcatatt

tttacttctgagacaaaaaa

>fava_dn_rep_c380

accggtagtatatattgccccccaaaagaaaacaatataatttctaacaaaccgtgtctc

tgagaaagatagcgtgaacgtgaaaattgaatctagggtttgctctgcgtagagaatcaa

caatggctttgaggggattctacaaccaaatcaagggaaagaaggtgaccgaggtccctg

gacatgtcaaaccgatgttttcattcgcttacatcaaggattccgtcaaacgaggcttgg

acaattactcggagaagtatatcgaaacagactcgattcagccgctgcttcatgttcttt

atggtggcatgatcttctcctacctcgtcgctcttcccaacgagcgtcgccatctcgctc

acaaggagcatgctgaatctcatcactgattcaggatccttgttgatttggagatatggt

tggtttaggcagaaggatattgacattaaataagtttcttgtattttaattgttgttaaa

tgtgcgcaaaacatgcttgtacactttggttctcgtgatttgatgatttcctcttgcacc

tacccatttagttgggatagtttcatttatatgcagaacattgaaatcaatgtttttcaa

gacaaaaagtatgccctttgtttcttactaagggggtaagtggttcatatatccaaattt

ccacaataagaaagaatctagaacacatgtcttatatttttaggctttcctttgattgaa

ccaataagctaagcaatggaagttagtcattaatggatggcataccctgcaaaaactgta

gagttaaagggcttaccactttatctaaatttttttatctattaaaaagatttgcggca

>fava_dn_rep_c381

tttttttaatttcaatagggttcatgctttatatatatgatgatttgagcataagttaga

tatgcagaataacattagtagcaacaatgacaactgaacaaaggaagtcaaattttcaat

ataaccacattaatacagtaccatagctttgaaaagaaacaacaatttggggccttattg

tagcacaccaagaatacatcacaggacatattctaaccattcaaacccatcaaatagtat

ttaatttacaaaactacttatctcacatagaaaccgagccaaatattttagtaacaggtt

acttagtctcagatgatgcttcgccttcccaaaaggcagttggcttcccagtcttggaaa

ctgtcttcagaactgtatctggttcaacatttcctttcactaccactttctgttccttca

aatcaatgtcatatgattccacgccatccaattttccaagaaccctcttcactgctccaa

cacatccttcacatgacataccaactttgagaacaacagtctgagacatggttatgacta

aattgagacgcgaaacgaagaaataaaagtcaaaataaaatatagatgcagattcgatgt

agatagcgtttgagggtgttttggtttttatagggataagaaaaaacaatttgcccgaga

cggtgagagagagaaaagaaagagacaacaacgttgggttaaattgaacacagcatagct

taaactacacgcaaaaaagacgcgct

>fava_dn_rep_c382

accgcttatatttatcaaaaacaaaaacccgaagcgcgatagaacgaaagcgattttcag

tgtttttctgctttttttgataatcgaaaaaaaaacctcgccgctaaagcgatacaaagt

gcggcaattttttcggaatggggagtgaaagtaataatgctcgcagtggaaagacggcgg

cggtggatgttgttgcgacggtgaaggagatcgttaactgtacggaacaggagatctatg

acgttcttaaggactgtgatatggaccctaacctcgccgtcgagaagcttctcgctcaag

gttttttgttttttgattactttttctttcacttctgtttcttgattcggttacgtaact

gacgtttcgttgttgcattggagtttgttgttttctttttttcttttttcaaccgttttg

tggtttgttacagggaagggagtttagtgatttttttgaaactcatagaaacttagataa

ttttgtattatacgaaagaaagaaatacattttatttaattttgcaaaagtgagattgaa

accctctttcaattttcactattgttgcgagcaacaaaaaagggagagtccaaatactct

gcattattatgacataaagaaaatcatataatacgacatcaaattccaacaaggtgttca

agtactgaaaacctattaactcctaggctcatacttgaaggacttgtaacgaagagggtg

atcttcatttaccaattcagatggcaccttaatgagtgtttcttgatttggcacaccaaa

aagaataaatgacaatctctctttgtctcctcttgtcaccactctgtgttttggtgcttg

gattctcccattgctccatgccttcaacataccaccaacaagtacaataaaaccatttgg

aggaatatttacattagcccaattgcccgattttgttaacacctgcaaaccttggacttc

attgtggaatattatggccatggtgcctctgtcagtgtggggcaccaaagcaatttcaga

atctttgttttcttcaggaagatggtaccttgtcattcttgaatcactatggctattcaa

cttttcaacttcagaaatgtacttttctggaaggccaaagccctccacaaccatctttag

gataagtaagcttagttcccttgtttttgagatcaaagaacttagtgtctcacaaaaatt

tggatttccttctgaccacatgagattagtgaagttttcaattgtatcttgcttaagagc

atcataaatcctaaagctttcacaatgaccaataacatgattcttggcatagcctcttga

gttagagaacttcaactttgtctcctcaggtagttcaaacaaagatttcatgcatgtgaa

cattttttcacgtaattcatttgatatttcatcacaccataaatgaaaagcaccatgatt

ctctaatgcttctcgcactttcttactcatttctttccatccttcacttccttcttctaa

tgttactccactgctctttcgaaaatctaaaattggaatttcactctcactatccatttt

tctctctctttttcttcttttgcactatacttgtttatgaattattctggtcggccttat

ttataagtaaaaaagaaatgatgaaaacacgtgagttaatgcacaagattcaaatataat

ctcaaccatgcattttgtgtgtactttctgtcaagtgctagctgtatgataagaaatgat

aatattttttatgaaaccgagtaacccaaatcaaccaacttgtacatgagtttgttgggt

cggacggttagtaccgattcggaaaatcttgtatattct

>fava_dn_rep_c383

cacttactctctttaaaggatggccattaagaaaaaagttactaatgttttaattaacat

ttgcactcaagtcaaaaaataattaatactatttacttaagaaaatcaagcattatctca

atccgttcattgcgtgagtttaacgaatagcacttttatgtatttctcttcctttcacaa

gaaaactaatttggcctctctctcaaccaatagacacaaaatcaatacgcaatttcatgt

atttcaccactcaaacaagagaacaattgcaatcgtaatcctccacttctggaaattaaa

gaagcacagtatttcctcaacagaggcataataattcatcaaagataaacttaaatttat

tttctcataaactataacacaattaaacaagactacaacttcgcaccaagtgctgtgggc

acttctttgatgtgtgatccataactataagtatcgagacacaactcatattaatttatt

ttgataagaacttcaacttccataactatatacagtttttcaccagctcaaaaaaagaac

acttactctggactctttcccggagtttcaaccggaacataaagcaggcaagggactcgc

acgtggtgacctgagagggaattcgcttacctgcttccctttactatcgtagtaaatcac

gccggagaaatccaacggctcgcacttatctcccatcatgatcgctttctgccacacacc

gccgtctccccaatcatccttccctttcttcttcttcttcatcttctcgataaacggcat

cgctttgttgcttatgttcttcagaagcttcttcggtgactttatctcgattctccgatt

ctcgctcgccggcgccgccttcagcttcaacggtaaacgacttgctcgcttcacaaccat

catcatcagcgttgttaggttgatgataacacggttcgctttgcgcttcggcgattctcg

ggtgattatgtcgaccatggtttcgttaaaacttagtgaggctttcacggagccccgcga

atgtgaaatactccgatatttatagttaccaaacagtctacgcttctttcaattcactcg

ttttt

>fava_dn_rep_c384

tactctatccccttctaaattacattttcactatcttagtttcaatagattgaaaacttc

ttatgagaattgtgttgtagtgatgttgtgctaaaattgttcttattcaagagttattca

agagtgagattctcttccttttcaactgcttctctctttctttcaatccgtcaaaatata

atacatggaagaaaaaaaataatctataccttttaattttatacatatttagtaggacct

aagtcttatgattaacttaaccaagcaacatggaataggaggataaaatggtcaagaaca

tcatgaagaagttgcattagggtctgtactggaaataagtcgaacctcgtataaatcatt

ttcgcccttaccaacttctgctgtaattccaggcccaaagaatgaagtaaatttagcctg

tctcttttcaaaatctgacaattccagagtcttagcctcaaaaaccaagaccaaactata

tttcccatcttgtgtgacctcttcacgaatcaattgtagaacaggtgcacttcttctggg

gattcctcctcctagaccatactgaaaaatccgtttcaatgcttcttctaaatgctgttc

atccccataacgaaatctggtaacatcactcttcacctgtttaagaataggagttgcaca

tttttcctgcaatttgaaagcatctgaatttgtgaggcatggcactggcttgagttctgc

atacttgagcgccatgccaataatagcaagagggaacccatatgttaacattatagctga

ccattcagatccagggagaatattaaagtatgctccaaaaccataggacaagagtgagac

tccaagacttagtccaatcgtaccaaacgaaatcttggtaatggagatttcgtcgtcggg

aacaagcgtgatgttgttgttgtttggaacgtcagaagatcgggaagttgtggtggttga

agaatcagcagctcttgtgggagtaacacaaaaccgattggagttgaagaaaagctttgt

gtgaaaacatgaaattgattggggatgaacagaaacgagtcgagaccgagtcgaattggt

gagctggggacgagttgagtgtggaagaagaggaagacattgaaaccggtgaccggagca

gacggtggctccacctggcatcgcaaacggtacggttttattccttttggtgtttccgac

tgaacgaaagagttgcaaaagcttacaagagagtgtgaagttgtggttgttgcttctcaa

ccgccaaatttttggttacgtgtcttcca

>fava_dn_rep_c385

ctatctttaagttcaaataaaaaacatgtacattgatcaatagacgcgatcataatatat

atatatctaattgcaagttgatcaaaacaacactttatataccatataatgcatagtcaa

tgagccaagaaatattgaactttatgctacatcatttgcaaccaaaaagaaaattaatca

atctttccatacaggaataatcccctgtgctgccaaacgtttttcaacccaaaagacaat

gtgttttgaagtaattttcttctcatcaacgagaaacggttcaacagtgcttgcccctga

gttataagatgacagtgaaaacccctttggaccagtaattcctgtgggaaaaaaatgccc

aaaatgaataaggattcccttttttcagaaaggaaccttccaacttattgtcctgaaaaa

ccaactcaatatcagagagatctttattggattctaagattgttttcaatgaaggaatca

catcctcttccatcatttgaggtaaaggcttgaccggagctttagcaggaggagccttct

tctctgttgctgcctttggagctgctgcctttacctcctcctttgtttcagcagtagctg

atgtactagtagtagatgattcttgaactgcagagcaaattatcttgtttctacttgccg

ttttaaatctacttgcaatcaaagatagtgttggtgatgatgatgagttgggtatggcaa

aattttgagagacatgtaaagaagatgttgaagttaaagaacacaaacgaaaggtagaat

ctgcaagtgctccacctcttaacaaaagtgccatttgcattatgattccagtgttttttt

tcttgttattttttattccattttctgtcactggtttgttgatgatgttagtgagaagca

tacgttggaggaacaaaatgaatgagccttgtgatccacatggttcctcagacaatatgt

accactcccgcg

>fava_dn_rep_c386

ggcaaaaacaaggctctaaggcacttggtaaaaattataaatgtgaaaatttgactttta

ctagagaaattttcacttttaaaaagtaaaatgctccacaaacattaatcttaacacaga

aatagcacacaataacttgtaaaataacaaaatactcgttttagatgagtttgtgcttga

acaaaattgtagggcagtcaggttcattcattttccccttggggagaaggaaatataaaa

acaaaacaaattgtcccaacaccacaaagggagaaaacaatacattacgataacgaaata

aaaggaaaggcccatgtttgagctccaaaactcaaactctaaagcctctgctcttcctct

ttcctctggccttctcaaacttgcgtcctttagaacggatgtagggcttggtgtgactgt

gaggcacaccaggagctcttccaaagtgcttgacagcctcacgagcatttttgggacctc

ttaaaagaaccgacttctcccctaaaggtgctctaacagctagctcatcaaaggtaaggc

actccccaccagccttcacaattctagctctggcagtctctgtgaaccttaaagcagtca

ccttaatcgcaggcacatcataaacacgaatatcatcggtcacagtacccacaactacag

caatcttaccctcctttcccttcgtgaagcgaatcaacctagagagagatagcggaggtc

tgttaactttgctcatgaaaagacgcttaaggataaccttattgaatctactgggagtcc

tccgaacaaggaaacggtaaagcctgacgagaagcttgaggtagatatcattggattttg

gtgcggttcgtttggtcttcttggacttacccccggccttcaaatcgatacccatgttgg

atggagcttctctggctgctgcgagtgaaagggggctaggccgcggttagggttcagtgg

atttatttatggctttggatttcgtcgctcc

>fava_dn_rep_c387

ataaatcaccctgaaacacccttttgtgcctttttccacttccctattggttgaatttca

aatccatctctttcttctttctctctctaactctctctctagatagagaaaaagcaagtt

tcttttttcctaattattaatcgagaaaagtaaaaagtatcaaatgctaaatgggtagta

gtgccactgccactgcactttcaacaacaacattcagaaagaaaaaggtacaaccgtcac

caattatgcctcacacacaaaatttctaactagaaaacaaaagggttgtctttgaatttt

ctgtagctgaaaattttatttgggtttttttggtttgagatttaggacttgttttgaggt

aaagtttggatttttatttgattttgataaaggggttttgtttttttagctgaatctgtg

ttgttgctgagggtttgtgtgttgtagttgaatttagcaagattctgtttttgttttttt

gtttttttttttttaaagataatgagagaagccttttcattgcatattgtattccattta

gagtatacatagtgctaagtataataagcacacgaaacctactgtccatacattaattaa

tacatcatggacatacacaaacttgaaaccatataacataattaaacagtgttgcataat

gcatctctcataatttaaacctttaaagtagctgctatagtgtaatttattattcaagga

taaccctatattgatcaaactgttactgttttaatcacttggaaacccaaacaacatgat

cctgtggaaggaagtgacaaactggaacagttcctggtttaactttaagcacttgaaaag

ccaaatgtttaggattccattgtgatgtatcagtgtgacaaaccgcaacagcttgaactc

tatttccatcaacaccttccagtggcaccgagtaaactttagttgtgtctgttttatgac

agtaaaaaactgcatatggataattctctttgtggcaaacaacagctttgttcttctctc

ccattttcttcactcctttagctattacatattcttgtaactcactactttcttttttca

cttctgttgaaacagcttcaacattgtttccgagttttgaagtagtgaaatcaaccatag

attccaatgattttacacaaagcttttcttcacctttgataccgttttcttcgcactcac

ttatcgtgttcttcacaatttcagcttcctctgagcctttcttgatggagaacttgttaa

ggatgctttccacttggtttgatgaaaaggggattgagtcagcaacttctttagttaaga

agtttgctttgttgtttgaggttgtttttgtgaactgcaggttcaactttgttccataat

gcaaattcctttctaagaagaaaagtgctacatttgatttatcctgtaactgtgttgcag

ttgcagcataattgtataaaaatggagaactttttccaactacaacatgtactggttttc

ctcttccttttcctgtgttaacattgacaccaccttttccaacgttaaccgctgttccac

ctgaaggttttccttttccagtattaacatggactccaccttttcctacgttaaccgcgg

ttcctcctggttttggttttcctgcatcaacattgacaccaccttttcctacagctaccg

atgtgcttttctcttctgtcaaatcagttgggttcaggagatctgtgatggcttttggca

tttgagttgttggaagtttggacttccaatacaattcagggggcagtgcagcattggttg

ctacaataagtgcaaaactgagtgctataaagatggacaaatgaggaaactccatagtgg

aaatgaacttaaagctccaatgaaatggtaagtggaagtaaagctagattgagaaataga

tgaactgaatgcaagatagaatcccgcggcccctaggtggt

>fava_dn_rep_c388

tattacaattttgttagtatgtactattatttttaataaaaaatcattaacaataatagt

gatgctaataaatacttataactactatataaaattgataatagtgatgctgataattat

ttatattgaaagaaataaaccatagatttgaagcataagaatttactcctgtgatctagt

tatatacataataatgacttaatggatggaattataggcatgcatttttacgataaatca

tttaacaagttcttcctctttctttgctgcttcttcaagttctctcaaccctccaacttt

ttgttctatttcctcaatcaactcctctcgccacagagcttctcttgcctctgcttccat

cgtctcttgcttctcatagatccagtttccagtgccatctccaccatctcctctatatgt

ttctgaatcataatatgggtccttcatagaaccaaaaaataagactccccatacagtgaa

cattatgtaccaagcataaacattttttatttgctgatcactaagatgccaaattgtttt

ctcagttataagatctctctgtccttccaattgttctactagaattgccatcaatggaat

atctggaaatccattaactttacacaaagatcctctttttgtcttcccatctaacacatg

cgatggcttgtgaaaaggtgtagaacttgaagggagcttaggcaatcttgaagatgaatt

gacttcaaatctttttgtttgaagacatgagctatgaatatgtgagttgaaaacagtgaa

accaatgatagcttcagccattgttttgaagaagaagaaaaaaagtggttaaatgaaaag

ggtgttatatgttggttcttacatatatgggatagagtggataagagtagaatgaagaac

aacccttcaagtatctggttgcgcggtattctataatatgattcatc

>fava_dn_rep_c389

actgtcaaaacatagttccatggaatggaatatcattatttatctgcatcttattctatt

ccactttaatggccaccattctcagatggcttctacaataagactnnnnnnnnnnnnnnn

nnnnnngtattgtcatttagttttgaattttagaaaaaaagtgttttcaaagaaatttcg

atttctgcaatgaactttcaaacccgacttacaaaaaaaaaacacattgcacaaggtcaa

ggtagaataaaatcaacaatatagatgttacaacataacaaaatgtttaaaatgagaatg

ttggtgttaatacaagtatttatgggccgagcaaaatcctcacctgcaccatattagatg

aaatatcataactagaggtttgcctggcttcggttacatgccattctaagtcacaagatg

ctcaacacacaactcaaaccaatgattctataatgactgcctcataatttctcattcaaa

actcagtaggaggcttcaaccttatcaatctcttgattctatctttttcagtattttcca

aatatcccttccatacaatttcatccaaacacaagcaaattcttccatacttatcaagga

aacggcgctcagttgggggcttcccacatacatcctttacagctgaggttattgtaaaga

tcacctctgacaaagcaagttcgtcgtactcgtccttccccacaacgtatatactgacat

ctccaagtattgtgtatacaatataaactgacttgtggcaagcaactaaaagttcttcat

tttttacacccttaagattgtccgctcctagtttgaccaagaaagaacgccagtgtagac

gctcctccgcaggaacaccatgaaaacgttcgatgaggatattgccctcggcgttcgcaa

acagcaccgcaaggatcatattcttctcgctctgaaattgaactgaagtttgtttaccgg

cgtcgttaacttgatgagtttaaaattacttacgtgcccttagcaaacaggggagggtta

atttgtcttttgctcaaacttgagttgtgactcag

>fava_dn_rep_c390

aacaaattctaaaatacctccaacaaaataagtctcaataaattcaacaccacaaaacaa

ataaaaaattttaaaaatgcaaaggtgaaacacagatgatatattgtaactacgagagtt

acaacactaatcgaaaagtatcatataatctaagaaaatgtcacagacaagataggagat

accaagtgattaaatataagccaagcttatttagtagagctgaaaagttacagtcaccaa

aacgaaggtgagtttgtggcggtggagtatgaacagcctagagtacaattatacaaaata

atattctttatcaaaagaacacatcctcatagcaatatagcatcactgttttcatgtttc

tagaggcaaaataaataactcgagttttttaattttgaccatatcacttacctttcttgt

tttgcttaagcattttttcaacttctccgctatatggtgaagcatgataagcatactgtt

gaagaacagaaaataccaccatttcaatacaaaccaaaatattctgcattgcttcctcaa

tgtgctccacgtctaatttcaaatgacgagattgaatcacgccaactgctactaaaccgt

ccagcaccattccctgccaaaagcagaagaaaacaattcctttgatgcacaggaatttcg

ccagaggtttgtgtggttcaagttctttagcaaacaagtggtaaaagatgaccaaagaat

acaaggccaatgaaacagaaatattgagaatgatggtgaaggtccagctcaaccaattcg

gatagatcccaagtagctgaaatgtaatcatcaaaatagaacacaccgggcgggtaaaaa

caaactgccatgtccaatatttcagaagcttcagattatggtggttcagccgaaccgaat

gaggctgaaaaagtgtcataggaaaggagtgatgaatttcccttcctttaatttcatcag

gcacaatattcctactaatagatatattcaagtaactatacatcaaagccaaaaacttag

caatgacaagagcttcatagcattctttaacagactccaaaagagtgaaaaactctttac

ttcctctaatatccaagagacccacaaatgaaacaatagcgtaaataggagccatgagga

ttataataacaatagccttttgttcttttggattcttccaatagaaaagatgctgagaaa

gtagttgcagagtgaaatgcatagaaagcatgacacagaaagcagatccatacacagtaa

tctgtgcaggactgagttgagttagatccatcacaaacacaactcaatagacgaaagctt

gaatctttcaaccttaatgtaatcgccgctgaggtgaaccgaggttgtttgtttgactca

aaactttgtttcaatggcagtagcagtgaggagaacggtcaataatgatgtttgataatt

tatcaa

>fava_dn_rep_c391

tagtagggttgcacgctgcagtgtcacttgagaacaaaaacgccatccagaatctcaatc

cacttcaaaatcactctcaccataactctcaccataaccacaatcacaaaccccttactt

aactcgtttcgctcactcgctcttcccaactcaccctgcggcggcggcggcggcggctac

gacaacaatgggtgctactcttcctcccaaagaggccaacctcttcaaactcatcgtgaa

atcctatgaaaccaaacaatacaaaaagggtctcaaagcagccgatgccattttgaaaaa

atatccagatcatggagaaactttatcgatgaagggtttgacattaaattgcatggatcg

taaatctgaagcatatgaacttgttcgccaaggattgaagaatgaccttaagagtcatgt

ttgctggcatgtttatggtcttctctatcggtcggatagagaatacagggaagcaatcaa

gtgctatcgaaatgcactaaggatagatcctgacaatattgaaatcttacgtgacctatc

tctcctacaggctcaaatgcgagatttagctggctttgttgagacgcggcaacaacttct

gactctcaagtcaaatcatcgcatgaactggattggctttgctgtttcacatcatttgaa

ctctaatgcatcaaaagctattgaaattctggaagcatatgaagggacattagaagatga

ttatcctccagaaaatgaacggattgagcatggtgaaatgcttttatataaggtctcgtt

gttggaggaatgtggattttttgagagagcttttgaggagttgcagaagaaggagtcaaa

aattattgataagcttggatataaagaacaagaggtttcactgttagtcaagcttggtcg

cttagaagaaggcgagaaattgtaccagacattgctttccatgaatccagacaattatag

atattatgaaggccttcaacaatgtgtaggattatattctgcaaatggccatttttctcc

tgatgaaatagatcggttagataccttgtacaaaacacttgggcagcaatttaagtggtc

atctgctgttaagagaataccactagattttcttcaaggtgataggtttcgagaagctgc

ggatagttatattagacctcttctaactaagggagttccatctcttttctcggatctttc

ttctttatacaatcaccctgggaaggcagacattttggaacaaattattcttgagttaga

gaattctattaggataactggtcaataccctgggagagtagaaaaagagcccccatcaac

ttttatgtggactttgttcttgttggctcagcattatgacaggaggggtcaatatgaaat

cgcactttctaaaatcaatgaggctatagaacatacccctaccgtcattgatctatactc

tgcaaagagtcggatactgaagcatgctggtgatttggctgctgctgctgcattggcaga

tgaagctaggtgtatggatctcggcgatcgttatgttaatagtgactgtgtgaagcgcat

gctacaggctgatcagatggttttggccgaaaaaactgctgtgctattcacaaaagacgg

ggatcaacacaacaacctccatgacatgcagtgcatgtggtatgagcttgcctctgctga

aagccacttccgccaaggggatcttggactgtccctcaaaaaattcttagcagtggagaa

gcattatgcagatattactgaagaccaatttgattttcattcatactgcttaaggaaaat

gacattgcgtacatatgtggaaatgcttcaatttcaagacagattgcattcacatgcata

ttttcgtaaagcggctgcaggatctatcagatgttatataaaattgcatgattctcctcc

aaagtcatctgacgaggaggatagtgaaatgtctaagttgcttcctgctcagaaaaagaa

attgaggcaaaaacagagaaaggcagaagcaagagctaaaaaagaggcagaagagaaaaa

tgaagaatcaagtgctagtggtatatcaaagtctgggaagcgtcatacaaaacccgtaga

tccagatcctcggggtgaaaagttgttgcaggttgaagatccattgttggaagccacaaa

atacttgaaattgctgcagaagaattcacctgattcgttggagactcacttgctctcctt

tgagttatacatgagaaaacagaagattttgctagccttccaggctttaaagcaattgct

gaggttggatgctgaacaccctgattctcattgttgcttgattaaattcttccataaagt

gggatcgatgaatactcccgtgactgatggtgagaaactcgtttggagtgttttagaagc

tgagcgccaaactataagtcagctgcacggaaaatctttgcttgaggcaaataatctttt

ccttgaaaaacatgaaggttctttgatgcatagagctgctgttggagaaatggtgtacct

tttagatcccaatagaagatctgaggctgtcaagttgattgaagggtcaacaaataacct

cgtgtcaagaaatggcgcacttggaccaatcccggagtggagacttaaagactgtattgc

agttcataaacttctcggatcagttcttgaggatcaggatgctgcattgagatggaaagt

acgctgtgccgagtttttcccatactctacatactttgagggtagtcaaagctctgcctc

acccaactctgctcttagtcaaatatgcaagaccgccgcaaatggtacttctagccattc

actgggcgatcacgttgtggatcatgtcacatcaaacggaaaactcgacacatttaaaga

tctcactatctgacagtaaagttccgacttcccagcaagggtcactcagttttggtgcag

gttatatcaaaatttgggaacaagtcaaaagatatattttccttgcacgagttctccctc

taaacagatttgtattccttttgaaaatgtgtataattttatctcttgggttaaattttt

tatttgatcacccttttttcccaagggatggtaaagagcagagtcaatggagttgcatca

tagtccaaggtagcagggagagctactgaaattttaagtttctggtcctgcacgaggccc

agtatacatggagagcatgttgtacttttccatctgttggcttcttcatcatgtaaaaat

atttaactaagtgaaaattttggcttagtaatcttacattaatttatgtgaaaaccgttg

gttgagttctagcaaaaaaaatattatcaattatgttttgaattaagtgggttaactcat

gctggtttttcatcttttctacaacataaaaataaaaaggggcctcactacacaaataaa

gaagaaaccctgtccaaatcgtctaagcgagggtttctgaacgactcaaaacgctccatc

tctctcaccgttttgattctgggactgaatcgtaaatttcataaccatgtctggcgaaga

ggaggaaaacgccgccgagctcaaaatcggagacgagtttttgaaagcaaagtgcttgat

gaactgtgaagtttcattaattctggagcacaaatatgagcagcttcagcagtcatctga

tgatgccgtgaatcaagtttctcaggtatttgaaaagtcattgcagtatgtaaagcgttt

cagccgctataaaaatcctgatgctgttagacaagttcgagaaattctggcaagatatca

gttggctgagtttgagctgtgtgtcctaggaaatctctgcccagaaactgtggaggaagc

tattgctatggttccatctatcaagacaagaggacgtgcccaggatgacgaagcaattga

gaagatgttaaatgacttatcactgattaagaaatttgaataatggctattcctgagttg

tatgtaatgtaagtggtactatatgacatatcacttgactgcaagcaaatgcgttgtgtg

gttgttacaaaactcaacaatgtaatatagtagcaactttaccgcgactttgatcaaagg

gggatttgatagagatccttgatagtgtgacttgctatgttaatgaaaaaacacgaatat

tattttgcaaaaaaaaaaag

>fava_dn_rep_c392

cgattatgaatggattaacattataaaaagtgttacactattaatcaaatagatgtttag

tgacaattttcaaaaaaaataataacaaattagcacaatagtaaatcaggtgacagaata

cggtcaatacatatccatttaactctaaaaagtaatggcctatcaactataggtatatta

ccgaaagtaatcatcaacttcttccttctctcactcaaagcaacatatatcccgtccttt

atcaaccatatgacaacctcaaaaatgttgttcaattaaaagggggaaaatgaagaacca

agtaaagacggttgataggacacaggccagtgcaaatagtgcaaaagctataaaaaaact

tggcatagtatcatcaaaactcctttgattcaactaaattaacttcataatacaggtgta

gttgcctcattggcattttgccagcatggtaatgtaaaggcataggacactgaaatttta

agactcaacaagcaaaaaacataaaataaattataaaatggtcaaactaaagtaaacctc

aaagtagcattgacctcaactattctgtgacatctgaacaggttttgagctaagtgtaca

acctccgcattacgcactttattacgaggcaattgttttgctgtacccattgtggatgat

gtgagctgaagagaaaaccgctatttgatgcaaccaactgccagtaatgcagaacattct

ccaagtactcagaactgggagttgaaaagtacagagagtttattgagatccacagattcc

atgttggggtacaaaacagcagcaccaacagccaaagaaagaacaacaaaggtcaaacaa

gccttgcccccgtggccttgagaaactcttcctgagattatcttacagtatttatctgca

tccttgaagaatcgagcagcatcggtctcagatgccaaagctttctcattcttttgtcta

aaattccttataatctccctaagtggctcataaggagacaattttgtagcttgcaccttc

caatcatcagaaagcttctttaaaactgcaacgcttgcgacaatgttgtccagataaact

ttttcccactgcttgtagcattcagagctttggctaaaacaccaaattgaaataccagct

gcttcttttgataactcaggattgtcttctcctgctgctatgatagcgaaattgaatatc

tgctgggagacttgtttcattgccttacttccagtagaaccaccaagagccacctctctc

agagcgggatatatggcctcaaatctttctgtagccttcagtctagctgaagacggaaaa

gtaacacgcagcaaagtctcaaatgcgggaggaggaattaatcgttccccttttcttaca

gcaccattaaccaaaataggccgtgctttgggggtagacaaaattttttccacaagctgc

aaaactaaatccctggattgcgggttcccacttttgctaacaactataggaagaaggttc

cgcgaccaagcatacaaacctactgagagatctcctactgaagcctgagcaaccatccat

acaatcactggaagtttatcttgtccttgatattttgtattctccctcaatgtgggcaat

acagcaataagagcatcaggctttcggcgcaatacaatcgctaaaccaacaaatattgca

acctgagactttgaagttaatggttgcacagcctttttggaacccttggtcacaatttgc

tggctacccaagtcagcaagaatactatccaaagaccaaagaataaagaaactgagtgcc

tcaggagatcgatgatttatccagtcagctgatgtcttgtatacagcatccgatacgtga

gaaagtggcacatcaacaatcttagctacagctgactccctgaacaatttcacccaggga

aactgagaagcgctcactgaagaaaaagcacgtccaaaataatccgcaaaccgcatcatc

tgtatatcctccttctgatcgaaggatgccgatatttcaacgagaaaagactccagatca

gccgcatcaatttttgccgcagcctcagccaccgtcaccttaggcttcttcggcttcttc

tccttcttcaccttcttcgcctcctccactttcccgttctccgctgatcgatccacatcc

tcatcatcctcatcaaagtcatcatcatctcgacgcgacctctgcttcgatctgaccgga

gcgtcatcgaaatcggcatccgccgcagactgcgcctcgagaatgcgccgacgtcgatcc

tccgagtgcagctcgagcgaccggaaggcagcatcgttgccgttaaacgtcagtttgtta

gaaccggcgacgccattagcttccttcttcttatgcttcttagcgtaggtaaccttctgc

cagccgtgatcggcggtgcgagatactccgttagtttcggcggcgaaggcggagtcgaag

ggagcgtaggtttcatccatggcggtaactgtatctgaatattcgagaaaatcaaaatca

aaatcgaaatcgaaatcgaaatcgaaaacgagggagaaattgattgaaaggaagagagaa

agtgagaaacgaaattggagaagaaggtgaaagggaaatttggatgttagaaagagttga

cttgttggaattgacctcactctcaccacgaaattaccgcaatg

>fava_dn_rep_c393

agcacaaatcaaaagataacagattaaaaaaaatcaatcctggggaatttcaacatcgcc

atcattgatctcctgctgcaaatccttgggatccttcccatcaacagtacatccaaccga

aacacaggtccccagaatctccttcacagtccccgccagctccttcgccatcgacctcgg

cttcataatcttggcgatctcaataacatcatccagcgaaatattcccattatgcttaat

attcttcgtcttcttcctgtcccgctccggctccttcaacgccttgataaccagcgccgc

cgcagacggaacaacagcaaccttcgcctgacgattctgaacagtcagcttgacggtaac

cctaagacccttccagtccttagcggtttccttagcgatgtcttctccgattttctttgg

agagagaccgaggggaccgatcttgggagcgagggaactcgctgctccgacttcacctcc

ggtgacgcggacgtaaacttcaacgacttgggatgggtcgagcttgggagacattttgga

gaggcttttggaggtagggtttcagacggccaaaaatgatgatgaagaagaagccgtaac

tgagtgccgcggccgcgacaatgaaaatttttaagaactgataatttttaaaa

>fava_dn_rep_c394

gaaatcaatttgcaactgcaattgtgactactattgttatttcatacataactccttgtt

agatattgaggttctattcattgcttttcttcatgataactgtagtaatatatctgtaaa

tataattgaaatgccattaacaagagaaaatgcaacgagtgcacttcaagaatgacatag

ttctacttcagggcctccttatactgttttataagtaactttcatgctctaattttacta

acattgcatacacatccacatacaactatattatttcattcaagcagaaacactaataga

ttcggaacaacaaaatgcactagttgtatatatcagcagtaagatggaacgtccaccggt

ttgtaaacaaaggttctgacagtgtgtgaataccctgatggatgattgtaactgaacttc

acccctacgctaccactcccaagatttgtgcagcgatcatggaaactactgattggtcga

gagagacatgcatcccaacgatcactctcaggcattgtctcggctactgtgtatatcccc

ttagcatttgtaaatgcctggtagtttagaacctcgccggatttggtgatgcaaagaaca

gcaacttccgcaccttcaagaacatgatcttcaggtccaagagaagaatccccacaaata

tcacaaacaactcttccatgaatttcaccagtccaaccacttgcacccaaaacaaacaac

atcaacgacaacacaatcaaatccatcaccatcttctccttcttcgctcccatcgctgaa

atcgaaaaaaaaagccaaaaaataacactcacctcaattcacactgattttaggtttatg

attttttttcaccgacactggctgagtcactgagtccattccccgagtcagacaaactat

aaggactaacatccc

>fava_dn_rep_c395

tgaaaggaccatactggtaacaaaactaatggagatgcttcttttggtcacacttggatt

cactactttgcaaagtgatgaatctcttttcacattgcatttgatgatttggttgttttg

aacataggttggtgttgctagatgaaaagggtttgctgttgagagaaaatttgttgccat

ttttctttgtttctctctctctctctcttgttttttgtnnnnnnnnnnttaaaaacttcc

acaaacaatagtgatcaagtggaactgaatcatctatatggaattgaatatttatttatg

acataatgtttgtacattacatatgtgaaaaagtaaataaattatttactcaaaatcttg

acaagttcttgtaattaagtgcttaaaataaaattggtgagaatgaagatatggtgaaca

cgacagaactttaaggaagtgtttggtttgatcttatctaataggtaccttaatccaacc

atttgcaaaagcaatggctaagattacaaaaggagttgacattccaaatatgataatgta

aggaagtggtgtcttcattggattctctccctccctctctcctgctgattgccaatattg

ttcaggttcttgttctttggttatgaatttctttttttctcctttctttgtttcttcatc

tcgaccacctaattgagcatgaactttgagaccaagattagttgttttgggagagacaat

ggtgtgagaaagtgaaggtgatttaggaagaaatgaagttttgaaggtaagaggagaata

aagaagaatcactttggtgattgaagccatttttttgtgttgaattgaatggtttgttct

tgtgtgctctatgagtcctcatctttctactatctatca

>fava_dn_rep_c396

atgtacaactgcatcttgttctaaatgtctattggctagcctcgtgacaggataatgata

attaaaagggatgagaaaaatataaccacacaacacattatcttggtcaccatatttcat

tctaccaagaaaagagatgggaactacctccctctcttcaaactcaccttctctcatcaa

catcattctcactagacgttcgacttcactaccatcaaccaggcacaacaatttcctcac

acttcaacttcacacttccctcacttttcattccatcacatgcaacagcagcagcaacag

caaccctgccaatacagaccaggatgaaacaggctcaattcaagcaccaactacaacacc

agctgaaccggttgagctaaggctcaagagaagattaagaagacaagcgaaacggcagag

agagaatggaactgttacaaatagcattgagcaacccagtaaggcaaaggctgctcctaa

gaaatgggaggaaatgaatttgacagagaaggcgttggagctttatgtgggagagaaagg

tgctctcttttggcttaataaatttgcatacgcttcgatctatatcatgattggagcttg

gatagtatttaggtttgtgggtcctgcacttaatgtttatcagttggattcacctctact

gtcacctgctgatgtattgaagggataacagtttcaacttatgtttgtattgtaatttta

gcatatgtacaatatactcaagatcacaaaaacctatcatttttgttaaatgagttgtaa

tgataaagtcttttatgttcctttgttcactacattagttgatatttagcaatgtataaa

tacagaacaaaaacatgactannnnnnnnnnnnnnnnnctcaattgcaatcgcagccata

nnnnnnnnnnnnnnncccacacacctttactaggtgatcacaaaccggaatt

>fava_dn_rep_c397

tggttcccaacatcaaattcaaaatgggttatattgctgatgtatacagaatgtttgtcc

taaaattatttttttagggaaattaactttttattaaagaacattgaattgtcaaagaaa

agtaaaaacagagcatagcattcaagtttcacacatttaaaaaatcaaaattatgtcatc

gcatataacaaacaatagcataactgatatcaaagtgtctcaaaccaaaacaaaatcatg

aacaaaaacggtcagttcaacttccaacaaacttcctacaaaactaagaagctaattatc

gaaagacattctccagtaatccatgacatcaaaaaaatccttcctgcgatcagaacagtc

tacttgatcttcttcttgggcctcaactggttgctgtgtccacacttctttttcctgcag

ttgacagcacgagggtgcagacgtgcgtagcacttgcggcagatagtcttatcctggttg

tatttcctggccaatgccatcaaagatggctcaataattccaccacgaagcctcaaaaca

agatgcaatgttgactctttctggatgttatagtcagcaagggtacggccatcctccaac

tgctttccggcaaagatcaacctctgctgatccggaggaatcccttctttgtcctgaatc

tttgctttgacattgtcgatggtgtcgcttgactccacctcaagggtgatagtcttgccg

gtaagggttttcacaaagatctgcatcttcttccgcctgttactctttccaagctgctgt

cacagcgttaattgttaatagcttttgtgcaa

>fava_dn_rep_c398

ttttccaaacaacaaatttgttaaacaatttcatttaaccgagcatgaggattatcatag

gaaaaagaagattgcaacgaatataatttagtttgatctcccattaggataacagattca

aagcatatcagacataaaaaacaagacaggttcatatgacactataccacagattgccgg

taaatttataaactaggcgattcttaatcaacacatataaatgacattcatagcccacct

ccttcagagggaagaatacgactcgtcaacctatgagagaaatcatggctcatctagctg

gtcctggacatcagaagtccaaagggtgaggttgtctctcaaaagttgcattataagcgt

gctgtctttgtaagattcttctcccaatgtgtccagctcagcaattgcttcctcaaaagc

ctgtttcgccatggcacaagctttgtcagactggttaagaatctcataatagaacacaga

gaaattgagagccaaacccaatcttataggatgagtagatggaagatcagcagcagcaat

atcctgtgcagccttgtacgacaacatagtatcctcagcagcagatttcctctcctcccc

aatcttaaactcagccaaatacctatgataatcccctttcatcttataataaaaaacctt

actctcactcgaagaccccgaaggaatcaaattcttgtccaacagctccaaaatactagc

acaaacattcgacaactccgcttcaaccttagatctataatccttaacaagcacaacatg

gtcatcattctttctaccttcctccttctgctcaatcgacgagacaatcctccacgccgc

acgtagcgacccgatcacgttcttgtaagcgacggatatcaggttcctctcttcgacgct

gagctcagaggatggtgtggacactagcactagcttctgcatgaaggataccatctcttc

ataacgctctgcttgctctgcgagctttgcgaggtacacgtactgttctctgtttaggtt

ttctggaaccgtgacacccagttttgttaggttttctggaaccgtgatacccatttttgt

tgttcggtttttttgtgtgtgttctgtgtgtggtgtttttctgaggtttgtgtttgactg

tctttgagacgataccgatatgaatggatgaatcgc

>fava_dn_rep_c399

caaaaaaataaaccataagttttcattaacaaaacaaaaaatctgcaattaaagaacatg

ctccaacaaattatagataataacaaaagatttcttttaacatatcacagcacatacata

ctgattggtaacagtatatagaaaatgtttaaatttcccaatgaatttgtctgctatcac

agctaataattcgtttattgttaaaaaagtaaaaactgttttccacttcatgaaatgaat

tcaaacgatttgtcaggaggaaagaaaaacaaacatccaacccaatacatctgcaactca

aagctgaaccatattcacggtttggtcgtggattcccatggttggtacctggtccagttt

ctctgccctggattgagagcatgtcccttggaatgatagataaactcgtcaccttctcca

gacaaattttctttatggtcaagaccgtacctttttggtctcagtaaaagaagctcatca

ccggtgtgatcagttatgaagtggagccagccatgccattcagcaggaacttgagaagcg

ttgtacctagtcttctctgcatattcaacccacctgtgccttccatactgtgtgttttca

gttttctcgtaatacttgttaccatatttatcaacaccaacaagtgttgccccaatattg

tggatcttggtttgcaataggtttccatcaggaaggcatctccggtatccttcatccttg

aggtatctgaagaagccgccgagacccttctccctcaccgtgcttatgatgctcttcacc

accgacgacgccatgatcccaccgccgagttcgcttcgctttatgtcgtttgtatgtaag

gctgcgtttggtccccgcggcctt

>fava_dn_rep_c400

tttcaattcaatagattgcctttattgctgtttgaggataaatagagctttcttttcata

ttccaaaaacaaattcaagtacataaaactaccacaaaggttactggcaaactcaataag

aaaatgaaatgacaatcattgagcaatgtcaacattattgatgtcacaatacaatcaaag

ccactactacatctgttttatttagctcaagaggagtcaataccatgcatatttagatat

acacatgcaagaaattaaataatttgacaatgcaattgtttttataaaaaaaagtaccaa

accaggagtatgctactaagtagcacggcaacttagttaatactgcactacaacagcaat

aaatactcttctataacagcaatactgacggtcaatactcttgtggttaatacaccaaag

aataaacgccaaaatagaagagagaaattaagcggataaaccattgaccatccctgcaag

ctggttgaggagattcagtcccaaaggtgtttctggtgaataaaatcagcagaaagactg

ccacaaattctaaatcagatactaataacctagcttgagaaaaatccactgttataacat

actggggtcatcttcctgtcttggcacgcttcattccattactattgaaaggtgacatac

tcggactcatactagggctcatactgggactttcctccctcagagtaccattaagcatag

ctagctcccgcagttgctgcttcttatagtgatcaagtgattcatccacaggcttcaaaa

gattttctagtactgcaacagcatggtccaagcgtgaattgattatatcctctggaaact

ctgcctccaccaacaagtgcaatggctcgttaaggtgctcatacccaggtttatctttaa

gcttctcttccttaatagaatccttcacagatccacagcctctgatgtaaaccctacatt

ctgtcatcgcttccactctttttaatgagttcccgcgaggtcccagaatccgaccgacaa

aattatactgatttggatatttatcgacagggacgtcaagcctgataactctcttcacta

caggagtcgtcggaattccttgtgccccatgccaacccactagtggtgcttgaaacggtc

ccattctttggagatttccattgtcctctctaggtacagcaggccatccctccatatcca

ttggcctactatttggatgttgacctgaaggcctaaatggactgtctggctcaaatcttt

catgatccggaaaaccttgattaaatccggatatccgtctgatttcttgagttaagagcc

tggtacattgtgggagaacttgcaaaaagggacccaatttctgcctctcagccaatagtt

cagccaaaaatctttcacgatgatcggaagacctgataggagaaggaccgggaggaggga

actgaaaataacttccattcccatttccgttcccagatgggatcctctctcccatcttct

tagagttacaaagtcggaggagagaaagggagagggtgagccacgacactacagtcggtg

actacactaccagtaccaaccaaagtccaaaggacgaacaacaaagagcccgcgc

>fava_dn_rep_c401

taaaagggaagtaaaactagattcaatatgtattctaaaagtatctttatctcattttca

aaatgaaatgagattttattcgaattctaagagttgaagagttgttactgtcaatcactt

cagcctcaatctcactactgcaaataagatcggtgcaacaagagatataacgctctacaa

agacacattaacaacaataaccaagtcttatcttactaagtagaatcggttacatggatc

aactttcgccactctacaaagacacatgccactaaaaactacaacaaaataccatgctta

actcaccacatggaaaacccaacaactatgatagcattaattatatctattgtacaaaac

agaaacgaaggaatatcatatcttattttggaccaagaaatcagtagctcgttctgccat

caccctccgtgatcacatccttgaaagatccattacttggaatcattggcaagacatgtt

cttgatgaggtgtaatgacatcaagaagataagggccaggagtgtccaacattttctgaa

tagcttcacgaagctcttctttccttgtcacacgagccgccggtatcccacaagcatcag

caaatccaagcatgttagggaaaatctcttcctccctagaaggatcaccaagataagtat

gacctctatttgacttatagaatctatcctcccattgaacaaccatacccaaatgttgat

tattcaacaacaatatcttaattggaagattctccactctaatagtagccaactcttgaa

cattcataataaagctaccatccccatcaatatcaacaacgatcgcatcagggttagcaa

tagaagcaccaatagcagccggcaatccaaaacccatagcaccaagtccacccgaagtta

accactgcctaggtctcttgtactcataaaactgagcagcccacatttgatgttgtccaa

caccagtactaataatagcatctccattcgtcaattcatcaagcacctgaatagcatact

gaggcgaaatcgcattctcaaacgtcttaaacccaagaggaaacttcaatttctgaacat

tcaactcctgcctccatgcttcaaaatcaagtttccccttatctcctttactctccaaaa

ccctattaagaccttccaatgccaccttcatatcagcacaaattgacaaatgtggaatct

tattcttcccaatctcagccgaatcaatatcaatatgaacgatcttagccctactcgcaa

aagcttctaactttcccgtcacacgatcatcaaacctaaccccgaaagcaagcagcaaat

cactactatcaacagcataattagcataaacagtaccatgcatacccaacatatgaaggg

aatgttcacctccaatagggtaactaccaagtcccattaaagtactagcaacaggaatac

cggttagctcaacaaaccggttcaattcctcactggaattcaaacaaccacctccaacat

acaaaacaggttttttagattctaatattaacctcaaaacctgttcaaattgggcttcat

caggaatcttgggtagcctggaaacatatccagtgagcttgatgggctcatcccaattcg

gaactgctaactgttgttgaatgtctttgggtacatcgatgagaaccgggccgggcctac

cagaagttgcaaggaaaaaagcctctttcacaaccctaggaatatcatcaacatcgagaa

tgagataattatgctttgtgatagatctcgttacttcaacgattggggtttcttgaaaag

catcagttccgatcattctccgggggacctgaccggtaatggcgatgagagggacactgt

ccatcaaagcatcggcgaggccgctgactaagttggtggcgccgggaccggaggtagcga

tgcagacaccggggagaccggaggagcgtgcgtagccttcagcagcgaagactccgcctt

gttcgtggcgggggaggatgtttcggattgttttggagcgcgttagagcttggtggatct

ccatggaagctccgccggggtaagcgaatacgttggtgacgccttgacgctcgagagctt

cgacgaggatgtcggcgccttttcgtggctcggtggaggagaaacgggagatgtgttggt

catcggaggtggagatggtggacggtagtgcggtcggcgagttggagagggtggatgaga

tagtgaagggacgagtgtgaacggattgtggtttgttgaaaatggaagaaaaagggaatg

tgagagtagagtttggtttaaggaaagtggaataggaagaagaagatgaagtgaacggag

atctggaaggagtggtggtggtggtggcggcggcggccattgttgaatgcaaatagagtt

tgagtttcctagtgtgatgatac

>fava_dn_rep_c402

ttttttttttttagcatagaaacataacacaaattattttaatattaataactctccaat

agtctaactaacaagcataagagcatatcccaaaggaaacattacaacacatagaagttc

aaatcatcaaaagcaaccaactaaataaagacaacaccttaacaaaccatattgtaatca

tcacataaccataacttaaacataatccaaactcattattaatatgcccttagattaagt

acttttactcttgcttccccaaaagagcaagaatgaacttcttgtaatctcctgaagttt

ccttggagactgcatcttcaagatgaacactgtttctcttgtagtatagctccttgatgt

ccctcaaatccttctcagccctagtaacaatcacgcggcttagtccatcctcatcggttc

caaactttttgatcgcattgcgcaaaaccttctcgtagtactttttgtgatcattgatcg

aacgaatagccgtgtgcaatgccttgtggaaatcatcagatgtttgatccaacaatttct

tactgacggagacgccatgttcgtctctatagcggttgaaagtggccacaagttgagtct

tgcttcttgtagtgagaatcctaatgacttcttcttcattgtttcctttcttttctttga

gactttcatgaatgatactagcttcggtttgtgctaatttcggattgatttcgtctcctt

cgtacctatatgagctcaccaatccaaccaaaagctgacgaagatgaccggtggtgttag

cagacacgtcttcttcgatggaacgcttgtagcggatacgataggcacgcctcacggcca

aaagctcttcggctgatagcacagaagcgatttcagcaacaatgtgatagtctttgctgc

catgcttggttgctacattgatcagaactgcatcgcggtctgcaggttccaacatccagc

ggtacacagctttctcaaaatcaccagagatctctgactcaagacgtttgatgagatctt

cttcgaaaggctgctcataagcctttctaatttgttgcctttgatgaacatttctatgac

ccaatattgctataatggtcttctcatcagcaccaaaccctttgaaagcaagtcgtagag

cttcagcatcttcaacaggagaagtttggctaggagcaacaatggtagccatttgagtgc

ttaagtttttctttctctctcaaattttgtgtctatgaaagcaataagtcccgcggcg

>fava_dn_rep_c403

gttattagtccttccaattacatagcacatgggggaaaactttactgcaaacatcaccat

gttcaactgattaaggaaaagggaaatttaagccagcttgaaggtgatcatgagaaacat

gcaaaaatcaacggcgaacaagttgcggctgaaacatgatgaaagagtcttttaagtctc

agaaattttccagttctcatgtttcgttttatgaactacatgtttctgatatctctcatg

cttttgatgttatggtggtattcaatatttgtttgcaagtgtgtggatctatgagacata

agtgatctgcttgttagttttttgttttgttttttgtctttttttatatagaaactatag

aatttcattcaactccaaacatttttccattacattacatcattgtttttttgggataaa

agtttcacaggttgatgcataatatacacagattaatgtttctataagtttagtccaatt

gataacacaaatgctagcacaatccatcccactaggaagtatccaatcagtctggttggt

ccaacatcctcaggttcctgtggtggaggatctccttttggaatctctgggacatcaacc

tcatatggccaaataaatttttctgtgttaccttctctggccaaactccagtcatacatt

cttctttcatctggcgatgacatgatggtgtatgattctctgagaagttccaatttcttg

ttgagtatgtctttctctaaaccttgactctcgatttctcgaaccttgttgttatacgca

actggcaccatgtcataggagcatcctcttggtatgccgagtctaccatagtgatctgca

tctgtaattggtataccacgaagagctctttctacattgagagtagaaattaaagaggat

gattcctttggaagttcaatagaactctccgaatctacctctgtagctcccgcagagttt

tcactggagcttcttatgcgtaatctgggtgggaaattgagaggaaaaagaaggttagtt

tggaatctaaaatagggtggaagaatgttgttagttggaaatggaatgcatgatgatgta

gtggtagaggacaaggccattggtaagtcgagagtagtattatttttaatgtaacattat

gaaatgaaacaaaaagcagtgatggatgagagagataatgtntaaggaatgtttgccaca

aacgtgtt

>fava_dn_rep_c404

tttgtttttttagattgataacgagggactgacgtaatacgggtataacaaaatcccaat

ataacgaatgatatgcaacaaaacacaaggaaaagaaataaattaagaaaactagaagta

ttatgggtacaacaaaatcccaatagtaacaggaaactcaaataaccagagaattcaagt

ttatcaaaaaaagtctaatagacaagttccaacaaaacataaagataagttccaacaatt

caatccaactaacaaaactaagctttgaggcgtccatagaacttttgcttctcttgtgtt

gtctggaaacgaccatggccaaacttggaagaggtatcaataaacttgagcttgatatcc

tcaagtgcaagacgagaggtctgcttaagaagggattggcgcaaagtaatgactctcttc

ttgggaccaacacagcctcccttgatcatcagatagtcctccttcacaacaccatagtga

gggaaaccacccatgggggtgatgtctttctcagtcctatcaaactcagtctgggcatca

tgggtctcttcaccagttttgccaagcttgtaaatcttcttgttaagctcagtacggtgg

tggtaaccgttctgaccagccctggcaacagtgaaggaaactctggcaggatgccatgca

ccaatacaagcaaccttcctcaaacccctgtgagtcttacgggggagacgagtaacaccc

caacgggtaacaacaccttcataacccttaccctttgtcactccaatgatgtcaatcatt

tcatccttctggaacacagcatcaataggaacctgcttctcaaagaagttgtatgcaaag

tcaaccttctgagcaatagtgccaccgttaatctgaatctccataatatgggctttcttt

tgcttcaatcccttcattttcctgatctgagtgtgagccaacacacggataacagttgca

tacttcttcagtttctcaagctgagtctgaatgttcttcttgccttcatcactttcatac

tgctttgagtacttgctgaaagccttcttcttggacttgcaccagttcttgtagaaccta

cgcttaatttcctcactcaaatgctgagtccatacagtgttcaatgtacgaagaccacgt

ggagtcttcacataaccaacaacaccaacaacaaccattggaggagtctcaatgattgta

acaggctcacaagtctccttcttgtgaagcttagatcctggcttctcgacctctctcaca

atgtgggtcattccagccttgtaacccaagaaggcagtaaactttggtggcttagttggg

tcatccttggggaaggccttcacttttcctctgtgacgagcagcacgctttcttgggaga

aatcccagggaaccgtgtcttgggtgctcgaacttcctgtgagacattcttctcctacct

gaggctgctgtgccgctgcaatggaagtgcgttcggaagagatcccgcgg

>fava_dn_rep_c405

aggatatggaattaaccaataaagtaggggagtgtaactagtatttttgcacctcaaacc

cttttttccatcgtgcttgtgacaagttcctctaacacagtcgtttgctagagagagaga

gagagagggagatcgaaagagaagaaaaatggcactgagagcagccatactccgccatgt

tcgcgtgccactcgaagctactcctaaattgcaaccatggaatggttccattcgctctat

gtcttcacacgacgatcatatcaccaaagaagaggtcgtcgatcgagtactctctgtcat

aaaagatttccccaaagtcgatccttccaaggtgactccagaagtgcattttcagaagga

tttgggtttggacagcttagacaccgtggaaatcgtaatggcactagaagaagagttcaa

gcttgaaatcccagacaaggaagctgataaaattgactcttgtccgcttgctattgagta

catttctaaccacccaatgtctagttaagtgaatcaaaatgtccctttcaaattgaagtg

aaggggtaggttgttgaaaataatatgaattgttctgaaaggagtttgtcagtgaagaac

agatacgggtttcttaaagtgttttaaattcagctgtggtgttttgattttctttctgtg

actgagaaatagtatggatggaacaaataccacccttttcttgttgcataaaccattatt

gtttacaagtttttccttaaataatatttgaactattgcatcgccttcaaaacgttccgc

catggtcaaaagcaacctatcattgtcttctctcttc

>fava_dn_rep_c406

ggtcggctatgtccctggacctcctggcttccctgtcacgtccgaacgtaatcagagaag

acctgcccaagcaccgcaccaccttgtgatcataaagaagaatatccaatacaattacag

gaaagnnnnnnnnnnactatatgaaatccacacgggtcgtacgcctggaacagtcgtaca

atttcggcgattcaaaaaggagtatatccctctcccttagtgtctttccacttccctcgt

tcaggaacaaacccttcgcctaattcttttgagtcccggcctggtttttccccactaacc

aattacgttacgaccactgaagaaacttggttgacaaacatagtttatgcgccgctaatg

tagcggcttgtcgagcatttgacaaactcacaccatccatctcaaataggacttgtcccg

cggacacacgagcaatccaacccgtaggatttccttttcctcttcccattcttacttctg

taggtttcccggtaatagggatatctgcgaaaactcttacccatatcttaccattttttc

ggaattgtccgctcatagcacgatggaagtgcccgattatagcccgacgcgctgcttcaa

tggctcgatatgaaagacgaccagctctacaactttgaatgccatatcttccaaaaccta

gttttgttccgtctggtttgcaacccctactacatctgcctttacgatatttactaaatt

tcgtacgtttcggatatagcacgtcccttttttttttactatatgaaatccacactttga

cacctaagattccgtaacgagtagatacttccgcagaagcataatctattttctggttaa

atacattacaagatgtttttccatactttccgcattcagttctagctatttctgcgcctt

ctaatcgacctgaacaacatatacggatcccctcaaccccttttttcatttctaatggaa

tatccttaactattctactaaaaatggaacgaaatgatcttgttttctttctcagttgaa

aagagatgtcttgagcaatcagagaagcactttgataaacagatttgatcttgaccgact

caattaaggtattagtgtttgttctattagacaacaaagatcgcatttttttcacttcgt

tcaaataagagttgtacccgtacggaatttttctgtttctcagtatgatctctatcatca

tctctattccctttatccactctcctatcctacctaggtctatgaatttatctatcaatt

ccattaccttatccttacccatgaggttccaacatttgatccttaattgacctaggagtg

gttcccgggcatcctcaaaaaaaaggttattataaatcccaactctatcccttagaaaga

aaaaggtagcaccgaagaaaggaaagagcgagatggtggtttttgttgttcccgcgaagc

gaagatcattcgccaagcgaattaagtgggtcaacctctttttggcttcggccaaacact

ttttctcgctggtcaagctttctacaaaaaaagcgatgcgagaacgtattctcttatcta

agcttcttccctgcgcattcgctccccccatcgtagatggttcagccacgcccggtgcca

cgaaatgattgagaactacgacggggtcgaaatgcatttggttctttctacaaaaaaagt

attgcatgaccaaataattcaaggaggggcgcactgcagggagggtcctttgaaatagat

gactcgtcgggccgtcggagcgggacttcttggggaaaaagaacttaaaaaacttcgttt

ttctgaaggagtcgtaattttctatcaggaacgctatgtcatttacagccccggcgtatt

tcggatgcttgaaggccccgctgacccgaagtaatttagaaagattcttctttatcgatg

gtgatcggtcatggtatccatagcgttgcttctttttcggccaaatcctaatttcgtttt

gcttctcccggtcgtcgagcctgatcgactcgactcttttccctgccctccggcctctca

cttcgtttcgttcttcttctttcttgttgctggaatgaagacacccgatcggcccgactt

tcccaaatgcccaccaccggcccttctcctttccgggtctggatttatcgcgtcgtttca

gtcgtcgtggtcgacggggaagaaagaaatgaatgaatgttcttttgggaaaatgtataa

gaatacacctaccgagacgaaagccaaaggttagtctcgcaggtggacgtatcgaaccaa

aataagatctcagattgacatcttgataaactaatttaccataataataatcactgaacc

aacttgaatctgaactacgattcagatcgagtcttaccgaaatcggatttccttttcgtg

ccatatgcgccttattgttactttctttactttttccccttttttcttcccggtttaata

tttgttctcgaaagtcttcgtttcgcaaactctcaaaattgttgaccaagcaaggtcgca

ctgagactgtgagaacactaacggaacactctctgacccccactgaccagctagtctgaa

ctgacgaccggcccgcaagctactcgatttttttttcttagctgctctggattcgattct

agatgctacttacttacatatgatgttttctttcacaaggtagcgcatctatatagcata

gctgaaagcggacaaaaaaagctgttcccatagcgtgacgtaggcttagcctctttctct

atctacatttatatatgataatctcactagtggacagcgaacaaaaagatgccacactaa

gaaagcaatccaatcaggaacaaggtagttagggacatgcggactaactgctctgacatt

cctattttttgaaatgaatcttcggaccttctgccagcgctaacaccatccgagcttgca

ctgccgctcgaagagagatgcctgtgagaccccctccagttctgtaattcgaaggaggtt

ctgagaatgaaattcaaacccctcgtcacttactagtgagccggaaagcttagggacgcc

tctcgttcctcttctcccatttgacaaccgtatctcagaatgttcggatgattctctgga

actaatatatgctttcttttttttttagaatggggatatatcgttgggaaaaagcagaag

aaaaggatttaacgggcagaagaagggaagttcggaaaagcctagcagacgggactatca

actcctttctattacgatctcggcaccttccttacccccctacgaaaaatttacccctat

tttgaagaggaaagctcccaggttccgcatcttaaaaaagtgcatgaccagattggaaga

attcgcaatgggccaactcacccagccatgatgcgcatgacagaagagagcgagcacaaa

atggagtttccatcttggatgaggctcacgtccggaaataatctttttcaagactttctc

ccctatgttgaactttctctatattacctgattttggaatgcactggaattttctggtag

acttggacatgttccatggcgcgaagtctcttctcatctaatagctctaattgcggggat

atctaaaaaagatgctccccctctcatccacatcaaaatctttgtcaagctgggccctga

tgagggatgggaatggctgaatacgtctgcccgtacgcaagtaagcaactggccaacaat

tgtatcgagctacaggcccttctcataagatgttgtaagctacgggccaaaaaatgtccg

acgaagcttaaagcagacataaagagctaactcagaagggggccaacaagcccctttaaa

gcaaatgcctgtgagaagccttagaagttacaggacatagaagcaggcctaagcagcacc

taagaagctggtcaagaagtggatttaatcttttccagtctagagactggcggattaccc

tcgaaagaaaggattcaaatcttccttctgctttgaacaagataagtggacagatgagtt

gaataatacagggacagataagctaggcgaggttcccctttgatatctcccaacctaaag

gtttattcagaggtgggtctttcaaccgcctactctttcgatgaagggtgcactacaaga

ccatcggtactaaaaagataaaatgggggggtttcgttgatattgagtcgactataaact

actcatcttgcttggaaccgtcgttgtgtatgggaaagaggggttctacctatgcttgga

aaagggtgaatggtcccaaaagggacgggataaaacccactagaaagaacggggggtcat

ccactccgtcgctgggtagtcaactttatccgtgactctgagtacaagatttccgggatc

aagaaactagcaaacaaatatgctcggctggactcttttctcgtatgcccggaaaatcaa

attgcggtacaactcagcttgctgcaaagcctttctttctcgtccaaacactccagatct

gcacttcccttttctccatagggccgaagctttactaagaagggctttttttagagagag

cgtaaggggcgatctatattgcttgccacagctctattcccgactctaaatccctaaagg

actttaagagaggatgaacattcccgttctataggtggcactgcccctattagagaaggg

tgagggcctactgattctctgctagcactgttaattaccttagacctacgcagcaggaac

gatatggagcacctactctaccggtcgtctgctctctcgaggtcgtccttctctaggtac

aaaaaggacattacgggatatctaaaaaattcgatgtacttcttaaagtgtgggacacat

ctcatgtttgccgaatcgaaaaactatcgcctttgcatcctcacagcggctatgatcaga

tcccagtgttggttcactttggactttatcctccacacaatcttatggtctcctgttcta

aattatgttaaacagctcctgatcgcatcaatcatgccttcgaaggtacgctttttttag

gggggccttgcctcttg

>fava_dn_rep_c407

tttggtaaaattttgataaaaattgattaattaaatgggatacatgtatcggatttgtca

aacacgagaaatatcttcaatcagaagtgttaacaagacatatcttgaatcagaagtgtt

aagacaacaaattcatgaaaaacatatataatgaatttaagtagaaacaattaaacaagt

atggtaggtatgtttcttcttgtattagaaacaattacgcataaattacaagaagcataa

aatgtaaacttttaagattgagacagcacagttgaattaagttctcttaacaggatcaaa

gttggaaacaccaataacagcacctaatatagctgtaagaacaattccaccagcagcaat

actaagtaaaaagtttttaagagaaggtgaaaagtcatttccagctgcatgagcaacatc

tggaatgaccatagaagctgtcaaagcaaaagctgttagacccttgactgtttggtcttt

caatgacgcttgaactccaacaagattggattttgaagttgaattgttgactagtgtctt

ctttgcttgtggaaagggtaaggctttcaagaagggtgtggtgaggtttttgtgtgtggc

ttgaggtagtggtaaagacattgaaacagaagctgaagccattattaattattggtttct

tttaaacaagtgttgctcatgccaggcttggatatagtatgaggcttagatattagtggt

tcaagaaatggatgagaggtggaaaatggagattttcttcttgattggagataaggttat

cctcaaaaaaatgtaca

>fava_dn_rep_c408

ctttttttttttaactaagatgaggcttctgatgaaattcaagatcaagcaattaaggtc

caaagtataaatagctttaagtaaactacatagaacatttcaagtttctagccactgcat

ctgtctacagacattttcagtagtagtgataaagttaaccgaactcattgcaacacatct

aattcaatacagaacaatttagattatagattaaagttttcgatcaaacaccaaacactg

caactgttttattttccaaatacttagaaacttttagcgtagaaatagaaagctagacat

gacaatgttgttaagcaggaagaccagcttcttccagcagctgtttgaccttagtgatgt

aatcactcattgcttcatccttggattttccttcaactgccttccatgcatcccatttgg

ctctgtccttctggctgaaaattccaggacgagcggtggtaacaggtccaacagtggctt

gcttgtacaatccataaaggataagcaagttttcatttgatggactctcttttagagtct

tgactttggcagcatgctcctcaaaatcctcctgcaaacccatgatgtgtgtgttttggt

tgggagattgaaagagagtgaagtgagagatatgaagtatggtccccgcgccgtaatgaa

atactttgcatataagcatctaatgcttcctttgtacttggaaatgatttgtaatcagaa

aatggaaagttatacatg

>fava_dn_rep_c409

aggagattgaaaaaaatgagtaaaaaacaattggttggagcaaaaagaatctctctcatt

cttatttatctctcgtgagcttatcgccatggaagaagcactttttttataagaaaaaag

ttaaattccgcgtttccacaatcattcactttcttatataacttgttcacacccacaatt

attcattctctttctaagcttcaattttctctcgtctctcagtcttcatcgtctctctca

accttctccactcgcaagtggtgaagatgtcgactcctgctaggaagagactgatgagag

attttaagaggttgcaacaagatcctcctgctggtatcagtggggcaccccaagacaaca

atatcatgctttggaatgctgtgatctttgggccagatgacaccccttgggatggaggta

cgttcaagttgacgcttcagttcacagaggattatcctaataaaccacctactgtgcgct

ttgtttctcggatgtttcatccaaacatttatgcagatggaagtatatgcttggatattt

tacaaaatcagtggagtccaatttatgacgtggctgctatacttacctcaattcagtcat

tgttgcgtgatccaaacccaaaccctccagcaaattctgaagctgctagaatgttcagcg

aaaacaagcgtgaatacaaccgtagaatacgtgggattgtcgagcagagttggactgctg

attaaccatatcatatttggcattgactagacaatggctgtgataaaaatggatatgatt

gaataactaccaacttctataaacaactatgttttattttacttgcccttgctggactat

tatgtgtttgctcaaacttcatatcaattaagcattcttccctttgttgtgcgaaaaaaa

aaaagattccagaagttcaaaagaaaaaaatgccaaatcatcaataaacggaaagggagg

gattcgaaccctcggtacaaaaattcgtacaacggattagcaatccgacgcttt

>fava_dn_rep_c410

tttttttcttcttagtttacgttttattcatggcatgagagtttcgtttcttctatagat

aaatttaaaatgacaattgaaatttataataacaaaaacatagcacaaaaatcatccaac

attaactacatattttgtgtttgtcgtacatgccacacatgacaatttgttaaaactaac

tcacacaagcaacaatatattggtagtgtgacatgaaatgtccactaaattattattctg

caaagccttcttccattactgagggatcttattaagcttacaataagcataatccttata

tttcttggtcttaaactttggtggattttctttgttaatcaactttgggtgaggaccaac

ttcaaactctccttgtggctcaatgaaaacaggccaagacattctagtctcctctttgtt

cactgttgttctgtgcaacacagccttatatttcccattgcttagtatctccatttggtc

accaatgtgaatgacaagagcattagggacatatttaacatcataccattgaccatctct

agaggcttgaaggccctgcacttcattagggacaagaatggtgacatgagacatatctgt

gtgtggtggcacacctaagacaagatcaggacttggacaaggtgggtagtagtttatttt

caatagatgaatcatgttatctccacctgcagcttccttaagttcatgttctttaagccc

aagcccaattgataagcttttgaatagtttgtctatcactccacgtagatacttcccata

ttcctcattcacctctctgtaagaagcaggtttgttaggccaaatacgataattaacaga

agaaggtggccaaatgatatgaaacaaatgatccacccaacctttcttcccattcacctc

tttctgaagactagtcccatacccttcaatcgaattcgacccaacaggtttagcaataac

ctctttttcctcttgtggtaactcaaagaactctttaccaacactttgcaactttcttat

aacttcatttggaatctcatggttcagaatttgaaacattccccactctttactcgcttc

cgctatctccttttgtaatgtttctccatctggattactgaaatcgattatcggcacctc

cagtttcacaccgtggacggttgttgttcctggttgctcgctttctgctctaacgaagat

ggaggggactgctgtgtctttcgattgatgagatattgtttggactcttagtacctccat

tcttgcttttttcagatttggttttcaaacctttttttacatgttctcccgcggtggcca

ctctgcgttgatacccgtgcctacttcaccgctgctaccatgatcatagctgtccccact

ggaattaaaatctttagttggatcgctaccatgtgggggggttcgata

>fava_dn_rep_c411

agattttacaaatttatttctgcaaatagaacggatatgagtcctgctataacccccata

caattgtttaaattacaaataatatttatttaacttataaatcgatattatgcattcctc

ttataaaagagacatgataatatcctggacaaaatttagaagttgacaaaaagtaaaata

tcacttaaacttcaatttaaaaaatgagatcaaaattctgttatcattttttatctctaa

atagcactttgatactgtgaccattttagtaagtcatcttcactatcatcagattgctct

ggaattggatatgccatagaatcttcttgatttgtaacatctatgtgatcctgctccagc

tcctgcttagttgttgcaacctcttcattagtttccgatgtcagaaggccattgaaaagg

ccaagaggaagcttagaggtttcatcgctgagaagaaatgcgctcctctaattctccgtt

tggcgtaagttttattannnnnnnnnnnnnnnnnnnnnnnnnnnnnnnnnnnnnnnnnnt

tttttttttagctaaaaacgaaggtggcattgatcatcgaactggaggtatggcaaaact

acagggaaatgtaaatgacagacgtacaaaaaaaattaatacctgcattatgccattact

caaaatacatcgctattattttcagcatgcacaacaattaagcaaaacatcacgttctca

aattaagccatgaataataatgaaagtaacagtttgatctgaaacaaacagtcttaactt

catcttgtattgatccacataacatttaagacagttctttatttgaggaaacagagaaca

cgtctcattatcaaacttcaagagtaattgctgttgtagaaattcttcaggaaatttgag

atgccctgctcttttgcagccaactcctgctgctccagtccacgcctgccgaccacatat

tgaccaactatatggtgcctttgctttgaatggtttacaacattcttcaactcttccaat

cccttgaaaagcagcaaatcaattaccttgggattggtgatgtggtcgtttttgcggatc

tcggcggcgatggtggaacggagctgggagatagtagcgacatcgtacaggttgtaaact

tccatgacggtaggtagcgatctgcaagcgcttctgaagaattcgaagactcgttgacgc

gcttcttccagattcactgagttcgggggcactttcgcgtttcgaattgcttgcgccatt

tccgattcgactcaactcaactcaactcgggtttttcttttttctgtttttgtttatgat

gatggttcctttgatcttcaaggtgagaaaaagaaaactggagcgtgagtgtggaggaca

cgggccatacgtcgtcgttttgaatgtgcgtttcacctt

>fava_dn_rep_c412

agacaatgtgtatataaatcatctcacttataaaatattcaacttctatttttctataca

atgtggaacttcacaattgtattcatgcctcaacatataaaaaccatacgtttttttttt

ttagtcttcaaattttcgtgatactgaagaattttttttatgaagctacagaaattgttg

gattaaacaaataatagtaatagtagtggctgcatctagtgctagcttaatacatcaaat

tctatttaactgaactatgcaaaagcatacgcaacaactcctcacaacacaaatcaatct

caattccaaacaaattcctttgcctcagactgaatcgcatgaaaaacaacaaaattcgtc

aatagaacgaagatacacaaaggaaagatgtgtcagcaattggagctaacacagatcatc

tcacttccgaacacttgtatcacgagtctcccatgctggttttttttcattagcaaggtc

actcttgattagtccttctattggcttcatgagaaagaagtattgtagagcaaatatgag

aggaatccataattttcctctctgatttggatttggtccatcaatcaaattcttgtcaac

aaaaatagactcacttttgttcaatgcaacttctgtgattgttttgactagatgaggaat

ccaagcaatcccatttggaagaaccttttcatcactgtcatttttgttgcatctcccact

attctcgatcaaaacaacaggaatctcagaagcctgagcatccttctttaaggaggcacc

tgatcttacaacttgcaagagagcctcggatctttttgagaagaagtcgtcataaggcaa

tccatctggtggagagaattgggcatgtgtgagtgctactatagccttgttccatattcc

tttgccaaaactgtcggtgatagctttggcgactaacttatccaagttgtctactcgata

tgcatctaagcggtccacgtaaagcaaaacatctatggtcttgtccagaaggaaattttt

tattatattaagcgccatatcattaatgtatcccccttcaataagaccaggagtatcgat

aatgttcagtgtaaaacctgcccttgatcgcgacaccataacaggtcttggcccttcaga

ctgaaagggactaattgaaaccaccctttccccaatgatggagttcacagttgaagactt

cccaacgccaccttttcccatcacaagtatggtcaaggagttcacatcctcttgtttaag

ttttcccaagagttcaagcaacttagtctgtgtagcaggagcgaatgtattgattcctga

ccattcacgaacagtttgttgctgggaagccataacgttccacggaaaggaaagagagat

tgtggtagggaggcgcaacgataggaagaaccgcccttagcctctccttatccaactagt

cttccccgcggcc

>fava_dn_rep_c413

gagaaaaacatcaagaactcaccagaaaactttcttttctttctatgctttctctagnnn

nnnnnnnnnnnnnnnnnaagacagaaacacaacaacttgattcattatccagttgcatac

aattctgtggacttactcccaccacccatcactccacacatgcagatgctttctttctcc

aacaacactttccttctcacaacctccatccatggctgccaccaaccctctatccctttc

ctgctcctcccaaacacacacttctccccattttctcctccctcgtaaaccctttcttct

cctttccacccgtcactcaaaccgctatgcacgcccaaagtctctccgactcaaaaccac

ctgtgaagccacacaagtgtccgtcactgaagagtcgtccccatccggaaactgggttcc

ggtagtgccagtgtcagctcttcctagaggagagcggcgcgtgattattcaagaagggga

aaccatattgcttctttggtataaagatcaaatatttgcgattgaaaataggtcacctgc

tgaaggtgcatattccgaaggattgaaaaatgccaagctcactcaggatggttgtatagt

ttgtccaacaactgatagcacatttgatctaagaacaggagagatcaaggagtggtatcc

aaacaatcctgttctgagagttctaacacctgctttgaggagtctctttgtatatcctgt

caaaacagatgaaaaaaatatttatatcagcatcacaggaggtttcaaatctgatgctgc

tactgaaattgtctttagcgggaaggcgcaacccggtataaccgcatctgatgtaaatgt

cgatgaggttaaaatggtggttgatgagactcaattgggatttggcttcagtagaaagaa

cgaaataataaacggtaaagcggctgttattggattccttctgttgttggattttgaact

tttaacaggtaagggactcttaaaaggaacaggtttcttggatttcatatactccgcttc

taatgctctcaattagacgtcacgtcggacatgtttggactgtgcttggaattgcggtag

cattctttccaatgctggtgaactgatcagtagcaaagagtttcgcttctccgaaatgca

tttattggtgagaatgctgcagattttattctgtgaaatcacagtatgttctatcagtaa

tcacatttactttttgtatgctttgtatctatctagtagtgtaaggttgtgagtttagtt

tgtggctaatgtacaatcttttctgcattttgtttcagtatactatacccaatttttata

gttcacatttttataaatggcttgtcaattatgagctcccaagaaagcttgcttgtgttt

gttttgttaggttagcatatatgtttggctgcacatatccttggtgacaagttgtaaaat

ttatttccaggaaagtatcactattttgac

>fava_dn_rep_c414

agttaaagatcaaaataatatagaactaaaaaaatattaaacctaaataaaattagcaat

tgggcctgttattcatctgcccaaattttaaaacattgactaaaatagcagtatatacta

atcgaattaattgattaaaaaaatactaatcgaattagtaatataaacgacaaaatgttt

tacatcacatgagtcacgcatgtatattacaatttccaaaagttgaaaacaaagaagata

aaaaatatgttgaagttattgttgaaggacctcttccaaccacaattgagacccctccat

gagttcagggctcaatctaaacatcaaaacacaaaactccatttggttaagagcaccatc

tccatcaaaatcaccttccattaccatggaaaaaagatcttcatcgctcaaaccatttaa

tcccaacaaagctgaattcctcttcaggctttcaaaagtgatgacctctttatcacaatc

cttcaagaggtttgagtatgtggcgagaaatgcattgaacttgaaattttgtgagcgaca

catctccacccaaaacgacatttcccttcgtttaaagcttgtgctaccaaaaccaaaacg

acatttcccttcgtttaaagcttgtgctacgtgtttctctctctatcactcggttaattc

aatggcttcactgatgctcagtggaaccggaaagctcgcagtgttgaataacagaacccc

aaacggattaggtttcacaacctcagattttcacggtaagaccttaagtttccccaaatc

gagtatcaatgcaacttctagggtttcatattcgagaaccaatttagtacctaagtgtac

tagttctagtgtatcatcatcatcttcgaggccaagctcacaacctaggtttatacagca

caaaaaagaggcgtattggttttacaggtttctgtcaattgtgtatgaccatgttgtaaa

ccctggtcattggactgaggatatgagagatgaggctcttgaaccggctgaactcactca

taggaatatgcttgttgttgatgttggtggtgggactggttttaccacgttgggtattgt

gaagcatgtggacgctaagaatgttaccattcttgatcagtctccgcatcagcttgctaa

agctaaggaaaaggaaccgcttaaggattgtaagataattcaaggggatgctgaagatct

tccctttaaaactgattatgccgatagatatgtgtctgcgggaagtattgagtactggcc

agatccacagcgtggtatcaaggaagcgtacagggttttgaaatttgggggaaaggcatg

tttaattggacccgtccacccaacattttggctgtcgcgtttctttgcagatgtgtggat

gctttttcctacagaagaggagtatattgaatggtttacgaaggcagggtttaagaatgt

ccaactaaaaaggattggcccaaaatggtaccgtggagttcgccgtcatggcttgattat

gggttgttcggtgactggtgttaagcctgcatctggggattctcctttgcagcttggtcc

aaaggaagaagatgttgaaaagcctgtagatccttttgtcttcttaccgcggtttatttt

gggtgtcctggcagctgcatggttttcattggttcctatttacatgtggctcaaagatca

aattgttcccaaaggtcagccaatatgagagatttgtggttagtgtacgctttgatacta

tcaatgtgtctgttcaaagttttttcaaatgtaagattattacatatgcagttgagaaat

attatatcaaacctcgtaatcggtagtttcttttcctagttgagctctgtttttctgcat

gttttttataggccatgctaattttatcggtgcagtgtctaataaaaccttgttttataa

gtacacttgttattctgttattgctcaacatcaacattagacattcaacattctaacatt

gcggttaatatgacatgttattagaggtcattttctccgtttttctccagaccagtcaat

ttgaagttgaagctttcggttaatagtgtaagagacaaattgatttgcacaaataagggt

tccaggaacgtttgattaaagtgttttctgatcttatttttgtttacaaaacatatac

>fava_dn_rep_c415

tgatactcaactctctatttttcttttttttgaatattaatgtgtttcatttatattaat

ctctttggtgggtgatttttaaatacatttataaacgctctatgagtcttgaggtcaacc

ctgattctgatttttgtatttctaaatacatttataagcgctccatcgagtatttctctt

ctcttcatatatcataatacaataaaattacaaagaactaatataattatatatcaaacc

aaaagagataaatcaaacatcaaggccgtctctgagagcgtgtaacgttaaatagagtct

cagtaaaatatttgtcacggttaagacggccttgtcaagcatcataaatttattttagtt

tctgcaaattagacacaaaacttgatgcagcaaaaccagcagaaccaccaacaacactga

tcaaagcaaccacaacctgtaatccaatctcaccaaagttatcttcaggaacaaggtaaa

caaacaaaggtcccaaaacaagtaaaacaaagctgagtgtgaagagagatcctggtgttg

ttggatcagtagcggcagataaaacaccgagttcttctgcttttgaaaggaggccaagct

tctctattgttgaaagagatagcccgaatttctcggcggccgataatagccccgctttct

cggcttttgttaacagtctcagttgttccattcttgtgagaagttttattgatggtgatg

ctgttacagcactcctaggtttttcaccaagtggaaatacagttgaattctttttaggag

ctacagttctagagacagatgctggaggaggtttgggagtagccatggatttgacaagaa

aaggtgagaaagtgtgagagttttgagttgagggagaagaacagaagatgaagctgttac

tacaaatggatggtgaggttttcattgtcatcatcataagtttctttgtttctttctttt

ttggtttctcttatctcttgttgttgagatgaacaagagaatcctaagagattgtagtga

gtggatgaagataatgatgatgaggcagaacccagtccatggattggtttttgtttattt

>fava_dn_rep_c416

tttttttagggaaagtcaactttttagtattttaagtgtttagtctttaactatggaaca

agtggtcgtaaaataaagatatttttttttcatttacacatcaagatgaagataagatat

acatctcaaatcattaaataaaagtaaaccaaattcaacaaaaaccaataacacataaaa

ttaaaatgatcaataacaataattgcactgtctcttccctagcccatgaaacccaaaacc

acaaacaaagaacccatcaaaataccaacaccatttgaaacactaacagctccactatct

tttggttgtgatggagaagttgaattagtctcagaagatggagttgatggagtagttgat

ggtgttgttgatggagcaggagtttttgttgttggaggggtgccacttggagttggggtg

gtgctagcagcaacaacatcgacttcaagcttcattccaacagtacaatggcctgttgtt

ggacatatgaagtaaatttttcctgcttttgttaatggaacctttgaatctccatcgttg

taattctttatggtgttgcttgaactgcaacttttgtaatcactctcatcaacttcattc

acttggtgagttcctccatatttgaacactaaattatcaccaactttgaaagtttttcca

gaagtccatgtggtgtaatcaacaccttgagtccattggttagcatcaccaacagtgtaa

tcagttgcaaatacacaaggaaaagccaacaacaaaaccaagaaagaagcagtcattgat

gtgttcatgtttatgaactcactttgctctcaaaatacaaaatatatgtaggaaaatgtt

cttgtgccgatcccgcggccataa

>fava_dn_rep_c417

gaggtggccttttgttttttctctttttttttttgctgattcctcccaattaaatttgcc

atgttgcactaagttacttacgtatgtatgcatgcagtccaggaacactttggggtaaac

acccatccaaacaactccaacaagaaaaggtataaatatgaaaacttctctgccatttag

atcggagaatttatggaggaaatcggcttttaaatttccagaaaccacacgattatatag

ccaaagggaatacgccgcgcctaaaatcatcccaagcgctgctaatgtggctactaagct

atttctttggaaagctcctactaagataagaaattccccgataaagctgctagtacctgg

taaactcatattggccaaagtgaaaaagaagaaaatggtagagaaattcggcatggtgct

cactaaccctccgtaatatctaacaagtcgagtcttatgtcggtcatatagaacaccaac

acatagaaaaagggctgaagaaaccagtccatgacttaacatcagtaaaatgctacctcc

aattccctgtatgttcagactaaacataccaatagtcaccagattcatatgggctactga

ggagtaagcaatgatcttcttaagatcgatctgtcttaaagtggtcaaggaagtatatat

tatagcaatcgcgcttaaagtataaatgaaaggagtgaaacaaagtgttgcttcgggaaa

catgggtattgaaaatcttaaaaacccgtaggctcccaattttaaaagaattcctgccaa

gatgacggatcctgccgtaggtgcctctacatgagcttcgggtaaccaaatatgaactgg

taccataggcactttgacggcgaaagcggcgaaaaaagcaatccatagaaagatttggcg

ccgctcactaaattctgtggttaataatatttgtaaatcggcggtccctgtttggaaaaa

aatcaacagaatagctaatagcataaaaacagatccaagtaaagtataaaggaaaaactg

atatgctgccttgatctttctttgtctcgaaccccatacccctataataatgaacatagg

gattagcacgctttcgaaaagaacatagaatagtagaagatccagcatgcagaacacggc

gatcattagaaattcacaaattagaaatgctataatatactctttcccataacttctcat

accagaccaacccactaaaatacaaatagggataagaaatgtggtcaatatcacgaagaa

aaaagagagaccgtctatacccaaattaaaattgatgttttcataaggaagccatcgaag

gctttccacaaattgaaatttggccgtagaaggatcgaattgtatccaaagaacaaggga

atacaaaaaagtaataagagaggcacacagaccaatcaatcgtatcagtcgtattcttga

atttggaatgaaaagaagaataatgcttcctagcacgggacacagaataagaccacttag

atcaaaatagcattcacagaaatgttctaacatagagtagaatcgaacattgaagagatt

agttgacaacagtcaaaagatgggcgcctaattccaatacaataggggtctatcagtgca

agtcagctgaacaccgtgattgatgagtgggtaggtgggttaggtgcgcctctcgtccag

tgggaagtcttgaggcgagtctcctccccccttcctttttttacttttcctctctccttt

tgcttgtgcttctacccgggcttttcatttttatagattttgagagacccgaggatattt

ttataagattcattgctcgattttgcatgctctgctcccaaaatgcagagatactttcga

gggcagatccgggttggtccggggagaggcgggctaagtgaaagaaaaaaaagaatatga

atataaatgaagctatgtcgacttacgtacgtctcgttgaccaaacaatcaggtatacca

cgccacgtatcatcccctctttctatagaggagagataaagaaaaataaaagagatagcg

atcgtgccgtaagaccttgttcgtttctacttgacgttattttcctcggttttttatttg

acattaaggcctttagttttgagagacccgaggatatttttataagattcattgctcgat

tttgcat

>fava_dn_rep_c418

gaaatcaaaaggatgaatcaaaaaattgtaaggatttctgaaagatgggtagatcctttt

gttcgaatcctatcattccattattttgacattttcaacaaattgttcatactatgaaca

tagtagaatggaaatcgggcaaggaggcccccatcgtctagcggtttaggacatctctct

ttcaaggaggcaacggggattcgacttcccctgggggtagggtactacgaaaggaaatgg

atgagggattgtcattcatttcattgatacatggacccctaattccaatatttccgaatc

tttgataaatggattctatcctgtccctcaactcaattcttattgatcttcatccttttt

tactcaatttccggattcattcgcatttttgtatttcccaacttagtgtgaaatacaaat

atacccaatttcatcttcttaacactgaatgaaagtgaaagaaataaggtgttaatgccc

ccctgttccagcaaatcaattctttccacaaagtcttctatgtttattttggtttctttc

gcattacttcgttttattttctattttgttgaattctatagattggtgattggaatgaac

aatttagaaatcaaaaggatgaatcaaaaaattgtaaggaattctgaaagatgggaagat

ccttttgatcgaatcatatcattccattatattgacaatttcaaaaaactgttcatacta

tgaacatagtagaatggaaatcgggcaaggaggcccccatcgtctagcggtttaggacat

ctctctttcaaggaggcaacggggattcgacttcccctgggggtagggtactacgaaagg

aaatggatgagggattatcaagaaagactaaaatgaattcttcctgggtcgatgcccgag

cggttaatggggacggactgtaaattcgttggcaatatgtctacgctggttcaaatccag

ctcggcccaaaaatatttctggatccaccatgaaatgatataacccatttggtgttctct

aaaaatcaccaatgggttcgcatacataagattataatcttgagtcctatgtcttttttc

cagattacttacatatttttttccacaaattcggattttgcaaaacacctattgggatct

gtaagtataaagtcgaaggaaagggttcaagtcaaaaaagaaaaaaatagtcttttttgt

ttccttgattgattttttaatcaagttggcaataacgaacggattgcgagtcatccgtgt

cgctaaagcgcagacccataaaaatatgaatatatatatatagaagtccgcctttaaaag

aggcgttctcgaatctcaaatagaaaaacaaagagttagaatgaaattgtaagaatatgt

gtgtatactagacgactttttccctgggattgtagttcaattggtcagagcaccgccctg

tcaaggcggaagctgcgggttcgagtcccgtcagtcccgatggatacaaatccaaaaaat

atcaattgatttcgtgtatgataagggaataaaactaaaaaaatatcatttcaaacaggt

atccccctttttctttcttttttagtttaagggaaaggttcggacaaagaaagaaaaagg

actttttgattgcatcagcaagtcattttcttatttggtttggtatggataaaacatctt

ccgatgttatactatgaaattctcgaccatgaattattgatttgatagctcagatattgc

tattcgtgatattgatccgatttgatatcatcgagatccgatttatcccattgaatttag

tgacgaaagatttttcatttctatgtgattaaatcccgaagtatttattagaaattaaag

agaaataaaacatagagattatggaagtaaatattctcgcatttatagctactgcactct

tcattttagttcctactgcctttttacttataatttatgtaaaaacggtaagtcaaagtg

actaaattttactgaaacatgacttcttatttcttatcaatgcaatgatgaagaaaaaaa

tgaaaaaataatttctatttcgggtcttttgttttttaagaaaatgatcagactacaacc

agcagaattctattaaatcctagtatagtagaaagaaatcaaatctatttctttctacta

tattatctattacagtagaactagaatgttttactaaaaaaaaaagaggtttaatatgga

ggaaccaatctagaattttgttttcatatggatagatggatatctatctatagatatgga

atgtcaattccatggcgtccacatttttcttgatttcatctaaatctattctatttgtat

tggttccaatcaatctgaaataattaaaaagcaacgcaatttttattcttgttattttca

ttttttgatttcagattcttttcagaatcccggtaactaataaataaaaagaaataatct

tttgagtattccaaaattcaaattgatttaattgaatagaaatagtaaaaagaatttggt

ttggaatagtaatccgtttcccaattatctgtattcccgggacatcaaaatggcaacaat

taaatatatttggttgattcaaagagtttttttttcaatattctctttcttatacatgga

atatagtaaaccactggaatacaatagaatttctaaatccagatataattttcattaatt

agtattaataaaataactaaaatagttcaaaactggaagaacccagctagaaaacaattg

agacagtttgatcccttaactcaatgagtattaccgttagagtccacttcttccccatac

tacaagggaaagggaaaatgtaaaaactaccattaaagcagcccaagcaagacttactat

atccatgtaaattatgtctcctatctatatcaatatgaagaaattcttttattaattctt

tttttattgtttactaatttttcttgtattctttttgtttttatttttcactaaaaattt

gataatatcttataataatagtggaatcgctagtacagagtcaaaaaagtattccattcc

gtcataacaccattgatgaaacatccaattagaacatctagcaagttcttatcagattcg

gagtagaattgagaaattttcagcataaataaaaaatatccttagaagtagtaaggtaat

gaatcatactaataggttggaatacaaagacctatttttctttttttctcccccccccca

tttcattttatttaagtaaaaaataaagaatcaagatagattcctttgcatactcttatt

tgcatctcttagttcaatttgatctaataatccttattgtatcccgattcgttctctaaa

acaattggaaaacagcctattaaattcttttactatcgattacccaaaagaaagagtttt

ctttttcttagaatggaaatgaaatcaaatggaatggaattttttttgaattggatgaaa

tgaaaatgaaattcatagtaaaaaaaaaataatatgaatatcatgaaattcaataaataa

aaaaaattcaataaataaaaaaaaagaaatcgaattagatattcaatttcattaatctaa

caaatatggaaaaaatgcagaagcgttcatatattttacatacgtttgtatagacggttt

ttcttcaatcccttcgccaactaaaaatggaattatattccctgtcctacctatccttat

ccaatctaattcaagacccaatcagtagacggacactacagcagtagtagaatgaaaaga

ctttcatttcaagattgatctattccttttcatttccctactctaattcatttttccttg

aatccgagacgcaaagattgaaagcattttagagaacaaatctagcgatgcgtagaattt

tgaatcaaacaagtctcaagaactctgtccctacacttgcttccacatggaaaaaatact

aagtttctgtatcaacaaaatggaagaaaccctttttgattctcttgtcgatcaggcgac

acccggatttgaactggggaaaaaggatttgcagtcccccgccttaccactcggccatgc

cgccaaaatgatacaaaaaaatagatcagaatatcctcctctcaaaaaaaccaacgtata

ccttttcagtcaaaaaaaaaaaagtaaaaatttcgggacaaatagcaactgttaactaca

aattccggaagaattcaggaatctgaatttcttaatgagaaaaaaatcgaaattgataca

taacaaat

>fava_dn_rep_c419

aaagcaatataaatcaatgatcttacaattagaaatatatacttgtaaattacatgatac

ctataaaaatggatttataaaactggtcaattattcgtagctattattcaatttaccact

tttccatcattcaaattccaaaacatcattatttcattaaaaaaaatcaaaccatgtccc

aaaacagaagatcacatagagtctcacagcactgaactatttcatcaggcattaaaatct

ttaatttctcattacaaaaaccatatcctactcaaaatctcaagattctgactacagaag

aaatagacatcacaagaatgaaagaaatgactccttggtttctctcatccaatgccttta

aaggtcttgaacactcaaacctggaggaagtgattgcttcaacttgtcggctttctcaac

atcaaacacacaaagtgtgtaaaggtacttggaacatctgaccttgaacttaacgacatc

cttgctcctcttgattctcactgacctagcatccttccttctggcagtgagaaggaaatc

cttaatctcgttgatttgcttaggcatggttccaggcgacaatcagtcccaagctgtaga

agaaagaaaagtggcgctgctgataaaccctaattctaatcgctctcttatttcttgaac

ttgattcacatgaaagcccgtatcatatttgggcctgtggccctcttctnnnnnnnnnnn

nnnnnnnnnnnnnnnnnnagcaagaaaaggataagtggggtggga

>fava_dn_rep_c420

accaatgttttatagtattaattaatattaagatgaagaccaacattaaagagatgtttt

ggaaaatatataatacatcaaacatttcgaagccactttttccacgaaacagaatctaag

gtgtttatgaaaatcatgcacactttcatgctttctcaatcttttctcgtgttgcaaaaa

aaatgcatactttaattctgtcgtcgcactaaacattctcattgtatacacaataaatga

acaaaaaacagtacggtggcaacaatcagattcgtggccaaaccaccgcggggtatgcaa

aggaaaagctaaggctatggccatggcatcagcaaatagcatcatctccatatcctctgc

ttcattgttgaatcgaactcctcaatcaaaagcaacatcactaattagcctcctaataca

caaccaagatcatgaacaacgttgtacttcaaaatcctctttcaccaccgctcttaagat

atcaactccaacaaccacaaggcgtaggttgagttgctcagcctcagtttctgaagcttc

aacgctgccctctgctcttctttttgactgcgatggagttcttgttgacactgagaaaga

tggccaccgcatttctttcaatgacaccttccaagagaaagaactcggagttacatggga

cgtggaattgtacggtgaattgctcaaaatcggaggcggaaaagaaagaatgacagcgta

ctttaataagacgggctggccagcgaacgctccgacaggtgaacaagaaaggaaagattt

catagcttcacttcacaagcgaaagacagagttattcatggtgcttattgagaaaaaact

gttgcctcttcgcccaggtgttgcaaagcttattgatcaggctttaactcaaggagtcaa

tgttgcagtttgcagcacttccaatgagaaggcggtctctgctatagtatcgtttttact

gggacccgagcgagcagaaaaaatccagatatttgcaggagacgtagttccccgcaaaaa

gcctgatccggccatttatttattagcagccagcactctcggtgtcgaaccttcaagatg

tgtcgtggtcgaagacagtgccataggcctagcagctgctaaaggagctgggatgaagtg

tatagtaacaaagagcggatatacagcagacgaagatttcctgaatgcagatgctgtatt

cgacttcattggtgatcctcctgaggagagattcgatttagcattttgtggtaaccttct

tgagaagcaatttgtcagctagaaaagtgtgaagtgatcatctggaaattgtatgattca

agtcttgttcactatttctatcaatgttcaattctaaatttgttttcagaaccacccttt

taaggcaatgatgaaaagattaatgtttatattgattgtgtaaagaagcttgtttgttgt

taaacttcgtttatgtttgattttattatttttcttttcattattgttatttacatgaag

agaacaaatggagcttatagttttttctgaacacttacacgcttcatttttgtcatacca

ataagtagttacatttca

>fava_dn_rep_c421

ccgcgggatctatatagtttgtatggctttgcgtttctcttcatggctttctgtgtgaat

ctcagctactacccctccttcccttctctttcacatttttcaaaaacgttgagaaggaaa

catggccattgttctttcccgttgattcgagcggttcaagccggtgaaccgggtggcagc

tcacagccacagactaataagagtgatacttcctcgtcctcatctagtactgctcagtct

tcaactgctgcccctaagactcttcccaagaaacctgtttattcaatgaagaaaggtcag

attgtgagagtggacaaggagaaatatctcagtagcatcaattatctttctgttggacac

ccgccttattacaaaggattggactacatttatgaagaccgtggtgaggttctggatatg

cgaatatttgaaacaggagagcatgcacttgttgcatgggtaggagtcccaacagctcca

gcttggcttcctacatacatgcttatcaagtcagagaagctcaattatgagaggctataa

cctcagttggagtgcagaactatgtaatatatacaatttcatgtttgtctagttttgtat

gattcaactggattaatttgaaatatcacctgtgacacaaataggtcaaataaagcacta

aaacaagatttttttttgttagtattgtttacagtgaaatatcacctgtgacacaaatag

gtcaaataaagcactaagacaatattttcttttgttagtattgtttacagcgatgcaact

tatgccaatataattttattggagccttaaa

>fava_dn_rep_c422

gggtatccaaagcacaagggctatagctcagttaggtagagcacctcgtccgcgggatgc

gccaatgttttttcataggagtccatcatgtaattaaaagagttgatcttaattgataaa

tcgatgtcttactcccggaacaaaataagagaacaatagcctgacaaaggatttagacgt

caaatggaatccataatggatttgagatattgatacggtgaatacttaatctattcaatg

ctaggcaatgagtctagggacctcaaaaaaaatctcttttcttcctatcttatgaacttt

aaggtgtatgaagtttcatttcctatttgatatttttattgaagtaggttgatagggagt

ccatttaacttaagttgatccaggccagaagcagacctacgtcaggataaccttcttctt

tgaaaaactttggtagtgctcttagattttgatctaaataagaaatcagagcacagggag

tcatttcctttttctttgtgtaaagaaaaaagatggtagactaattgatatttctatcag

ttaatgaaaaagcccaatgcaaaaaaatgcacgttgggtctttcaaacaattcaaatcat

tttgataataataaataatagtttgatgtgttttaccgagaaggcctacggttcgagtcc

gtatagccctaatattactaccgatactactcagatttagatttcttatctaatatctgt

cattacaaatatatatgtactaatactatatgtactagattagtactatattgaatactc

catactagtaatactactagtataaatatactttcaatactttttgtaatagaaagaatg

aataaagtaatttctattcgaatagattaatagtggatagaataatatgagtattagtat

actcagataataatctcaattttattagaaaaaatgtaaacttaagaaaatacaaagcat

ttctatttagattctattaatgaatctaagaatttgaaaatttcaattcaaatcgactca

gaattaatatgaatccagtatattttccccattcctaggaggacatttatgtttttgctt

tatgaatatgatattttttggacatttctaataatatcgatctttattcctattttggca

tttctaatttctggaattttagctccaattaggaaagggccagaaaaactttctagttat

gaatccggaatagaaccgatgggcgatgcttggttacaatttcaaatccgttattatatg

tttgctctagtttttgttgtttttgatgttgaaacagtctttctttacccgtgggcaatg

agttttgatgtattgggggtatccgtatttatagaagctttcatcttcgtgcttatccta

attgttggtttagtttatgcatggcgaaagggagcattagaatggtcttaattcttgaat

atttagacaattaaacaaaacattgagacagttatgaattccattgagtttcccttactt

gatcaaaaaaccaaaaattcagttatttctactacattaaatgatctttcaaattggtca

agactatctagtttatggccgcttctctatggtactagttgttgcttcattgaatttgct

tcattaataggatcgcgatttgactttgatcgttatggactggtaccacgctctagtcct

aggcaggcagacctcattttaacagcgggcaccgtaactatgaaaatggctccttcttta

gttcgattatatgagcaaatgcccgaaccaaaatatgttattgctatgggagcctgtaca

attacaggaggtatgttcagtaccgattcttatagtactgttcggggcgtggataagcta

attcctgtggatgtatatttgccgggctgtccacctaaaccggaagccgttatagatgct

ataacaaaacttcgtaagaaaatatctcgagaaatctatgaagatcaatttagttatcaa

cgagagaataggtgttttactaccaaccacaaattttatgttggacgcggtactcatacc

ggcaattataatcaaggattattctatcaaccatcatctatttcagagataacctctgac

acatttttgaaatataaaaaagttcagtatcctccgacgaacgaaaaagtgaattaaaca

ggatccttttgtacaaaatacaataagaaatagcaagcaagtcaatcttcaaaaatttca

ttgtaatgtaaataaatggtaaatacttataccaatcttataaaaaactcgggagaagta

aaaaaatgcaaggtaatttgtctgcttggctagtcaaacacggaatagttcatagatctt

taggttttgattatcaaggaatagagactttacaaataaagcccgaggattggcattcga

ttgctgttattttatatgtatatggttacaattatctacgttcccaatgtgcctatgatg

tagcaccaggaggactattagcgagtgtctatcatcttacgagaatagagtatagtctag

atcaacccgaagaggtatgcataaaagtatttaccccaaggcaaaatccgagaattcctt

ctattttctgggtttggaaaagtgcagattttcaagaaagggaatcttatgatatgttag

gaatctattatgataatcatccgcgtctgaaacgtattttaatgcccgaaagttggatag

gatggcctttgcgtaaagattatatcgcccccaatttttatgaaatacaagatgctcact

aaatgataaaaatcagaaagtaatctgaacttcgacaaccccagatattaaagaaatatc

tgtatgtacattttctttttcttatagattagacagaataatagaggtttcattcctaat

cttattctagattaggatggtataaataagaaaattgagaaataaaaaagacaataatat

agggattaattgagattccaaaattggatggagctatacttatttaggacgagccaatag

gatgaactgaaaaagttctgcatcattaactatgtaagatgcacatcagcatcaattcat

accttttttattttcattatagatatttttattttcattatatatatgatatttacaact

ttagagcatatattaactcatatatcgttttccgtgatatcaattgttatttcaattcat

ttgataaccttattaggcaatgaaatcgtaggattatatgatttgtccaaaaaagccatg

ataataagttttttctgtgtaacgggattgttaattactcgttggtttttttcgggacat

ttaccatttagtgatttatatgaatccctaatctttctttcatggggtttttcaattttt

catatggttcctttttttaaaaaggagaaaaaccttttaagtacaataattgcaccaagt

gttatttttacccaaggctttgcgacttcgggtcttttaaccaaaatgcatcaatccgta

atattagtacccgctctacaatcccattggctaatgatgcacgtaagtatgatgatactg

ggctatgcagctcttttatgtggatcattattatcagtggctattttagtcattacgttt

caagaagtcatccaaattcttgcttttaccaagaatttggattctttaaaaaaatcagtt

gattttgttgaaatcaaatacatgaataggaatgaaagaaacaatgttttacgaaaaact

tctttttcttcttctataaattattacaggtctcaatttattcaacaattggatcgttgg

ggttatcgtattattagtctgggttttctctttttaacgataggtattctttcaggagca

gtatgggctaacgaggcatggggttcctattggaattgggatccaaaggaaacttgggcc

tttattacttggaccatattcgcgatttatttacatactagaaaaaataaaaaattagaa

ggtgtaaattcttcaatagtggcctctataggatttcttataatttggatatgttatttg

gggatcaatctattaggaataggactacatagttatggttcatttacatctaattgacta

aaaatgagtaaagaacctgagaaagaaaaatatggaaagcatataaatatatacgttcca

taaatcgttgagaaccctttcaatcaagtaatgtaatgattcaaggggttctcacaaaca

acaaacatattcgattatcatttcaaattttgaagtgaatctaacggaaaaatctttttt

ttatctttttggttcatttattcacaaaaatcatccatcaaaaattagatagaatagctt

caaccttgtcaaccgaaaatgagaaaatgaaatctggataaataccaatacctattatgg

gtataagaattgaaatcgaaataaataattctctgggccctgaatcaaaaaaagaagagt

ttggtgtattaaaaaatttgtatccatagaacatctgccgtaagatagataataaataaa

taggagttaatataattccaattgctgttacaaaagtaattagtattttcatcatgaaaa

gatatttttgactagtaattattccaaaaaaaactatcaattctgcaacaaaaccgctca

tgccaggcaatgcaagagaagccattgataagatagtgaaaatagtgaatatttttggca

ttgggatagccattccgcccatttcgtcaagataaagaagacgcagtctatcataactag

ttcctgccaagaaaaaaagggcagcgccaataaatccatgagatattatttgtaaaatgg

ccccgttgagaccagtatcacttatagaatttattcctataattataaaacccatatgag

ataccgaagaataagctattcttttttttaaattacgttgaccaaaagatgttaaagcgg

catagattatttgaatagaacctaatatcattaaccaggggcaaaatatggaatgagcgt

gggaaaaaaattccatattaattcgaaccaacccatacgctcccatttttaataagattc

cggctaaaagcatacaagtgctgtaatgtgcctctccgtgggtatctggtaaccatgtat

gtaagggtataatcggcaatttgacagcaaaagcaataagaaatgccgtatataatatta

tttctagtgccacgggatacgattgattaattaatgtttcaaaatttaatgttggttcat

tagagccatataaaccgatacccagaattcccattaataaaaaaatagaagctcctccag

tgtacaaaataaactttgtagctgaatacaaacgtttctttcccccccacatggataaaa

gtagataaacgggaattaattccaattcccacatgatgaaaaaaagtaaaatgtctcgag

aagaaaatagtccgatttgaccgctatacattgctaacatcaggaaatagaataattggt

attctcgagtaaccggctgagccgctaaagtggctaaagtagttataaatcccgtcagta

aaataggtcctatagagagtccatctattcctaatctccagtaaaaatcaaaaaaattga

tccatttagaattctccgtcagttgaattagtggatcatccaattggaaatgataacaaa

atgcataggttgttagaaggagatcgattaagcatatacaaatgctataccacctaatta

ccttatttcccctatgaggaaataagaaaatgaaggaacctgcggctattgggaaaacaa

caactattgttaaccaaggaaaatcattcgtgataaaaataagatacacttgggccagaa

aagcccgtgctcaaaatcaaatagaaaatttttttctatttgattttgagcacgagtttt

tgccagtaaaaaacgtattcgtgtggtgttttttgaaacgtatcaataagctagacccat

acttcgagttgtttcatgccataaataaactcgaatacttaagaaatctgtcggacaagc

ggactcacacctcttacaaccaacacagtcctctgttcttggggcagaagctatttgctt

agctttacatccatcccaaggtatcatttctaatacatctgtgggacaggctcggacaca

ttgagtacatcctatacatgtatcataaatctttactgaatgtgacattatatctatagt

aatttttgatgttcaaaaatgaaaattatcgatctagtaaactcgtaaatgaatcgtata

tttatataccagatgaatcaatgatttgtgataatattgttaactgttaataaaaaaggc

gtctagataaatttagaagaggaaatagaagaggaccaggatactttgatttcttatgat

ttagggaatgcaaacatgatatgatcataagttgtgtcgaataaatactaaaatgaattg

attaatattacactatttgaaattctactttgtttcttttttattaattatgatatgatt

catgctatttattcaacaaattcgattgattgatacgggttgattttctgttacgagcaa

ttgaggaaacaatagccagtccgatagccgcttcagcggctgcaatagctataacaaaaa

ttgaaaaaatatttccttttaattggcgattatcaaaaaaatcagaaaaagtgactagat

ttatattcactgcattcaatataagttcaagacacataagggctctaaccatatttcggc

tcgtgatcaatccatagataccaatagaaaataaataggcactcaaaacaagtacatgtt

cgagcatcattgaccaactccttatcaattttgattcatttcaatatgaacaacaattca

acagattcaattgactaaagaatataacaagtctgcaacaaaagaatatattggcaataa

aaaaaaagaatttctacctaaaaactctttcacttcacattgaaaaaaaaaaatacatat

atatatatagtcttgatttatattagtagttctctaaagatttcttactggcgagctacg

acaattgcacctatcaaagaaactaaaagaattattgaaattagttcaaatggaagaaaa

aaatctgttgataaatgaattccaatttgttgactagtacttatcaaatcttgttcaata

atctgatttggtcttgtagtccaaataattccgtaccatgatgtatctaaaataatagtt

attagtgaaacaaaaatacttgtacaaactatcaaagtaattccatcccctatggtccaa

agatgaaaatcttggtaatattcggaactattcatgaacatcacagcaaatatgattaaa

atgttaatagctcccacgtaaataagtagctgtgatgcagctacaaaatgcgagtttgct

aaaatatataagaaagagatacaaacaagaaccagtcccaacgaaaaagcagaaaaaatg

gggttggtaagtaatactactcctaaacttcctaatataagacccgatcccagaaagact

aaaagaaaatcgtgcagcgtttcaggcaaatccattatatgtaaaaaaaaaaagacaaag

aaatcgaaatttcatgaccttattgatatgaccaggaaaaaattgttatatacttaaatt

tttctttgcattgaaaaaacctaagtagtttaaattgatatagttacggttatataatca

atgtcattccacttaaactactctttatacgaaagagtagtttgaaacgatattaaaact

aaagtataaaggtagatagaacatatataaccatttgaactagtctttaaattaaagaat

atttcaaatttcgataatatatttctctattctatttaagaatatatttctctaataatt

agagaatatttctaatccaatatcgattatatgattcattttttatacaatttttgaaaa

tattttatttcgattattctatatattctatatatattctat

>fava_dn_rep_c423

aatggaaacttaggaaagtgctttataaacatatgtataaaaagaatatatttcatctag

tttccttatgttcactctagccagttatttcggttttctactagcggttttaactataac

cgcaggcatttttatcagtctgaataagatacgacttatttgatttgcaattttgagaga

aatttggaaattgtagatattctatagttccttatcatatttccaattcttggtcattga

gattcatggtcaatataaattcatatttaaggatcgatattacccccaccttttttactt

tcaaccaaactcaaatgattgaagtttttctatttggaatcgtgttaggtctaattccta

ttactttggctggattatttgtaacggcctatttacaataccgacgtggcgatcagttgg

atatttgatttagtactgtctcttttgtttattgacttcctatttttttgtttgaatcct

aattcacaggagatcaaattaacacttttgattagacttagactgttatcccattatttt

agtctcattctcagaagaatggtatcacgctctgtaggatttgaacctacgacatcgggt

tttggagacccgcgttctaccgaactgaactaagagcgctttcttttcctaaataatttg

atgtgatggcaatatatcttgcatgcatgcatacaaactaaatatggagaactattcata

ttgagtatgtatgtacaatttcaattgctcaaaattgatctcttgttactgctgatatga

tattagggatagggatgacaggatttgaacctgtgacattttgtacccaaaacaaacgcg

ctaccacgctgcgctacatccctttacatcagtttccattgtcattctaaagaatttttg

tcttattttccacatttttccattatctatatcctgccattttgtttgtttttttacttt

cttatatcgatcgtagaaataagatatcgaatcaaatattcttatagaatatttatattc

aattaatgaaaaacagagaatatatagaatctataaataataagatagaaaaagtataat

aacaagaacaaagaatatacaaataatatagaaaatagaattgatatatgaaaaatagaa

taatttcaaatattattttaatatttcatagaattcataatttcaaaaaatatataataa

tatagttattagaagataacaatttgaataaaaaaaattaagaatataaaaatagtac

>fava_dn_rep_c424

ttatattcaattaatgaaaaacagagaatatatagaatctataaataataagatagaaaa

agtataataacgggaacaaagaatatacaaataatatagaaaatagaattgatatatgaa

aaatagaataatttcaaatattattttaatatttcatagaattcataatttcaaaaaata

tataataatatagttattagaagataacaatttgaataaataaaattaagaatataaaaa

tagtactctataaaaaaagatctaattcattgttgtacaattcaaattggattcggactt

cccccgagaaatccaagtggttttattaagaaaaaaaacattggatcatattcctatatg

taattctgtattattatgtattacaaattacaagaaaaaacgaaggaggatttgaaatgc

gagatctaaaaacatatctctctgtggcaccagtggcaagtactttatggttcgcggctc

tagcaggtctcttgatagaaatcaatcgtttattcccggatgcattgacattcccttttt

tttgattctagttattggcacgggaaggggtgacgaagattagagatccaataaaatatc

tgtgattaaatccctcttcgttcgtttttttttcgaattatactagaataagttcaaaaa

aaaaaagaggaaattaagaaaggtggatttggcctcagtgaaatttggaatttttcccac

gtttcgtttcaatggaattgaattaatacttcaattccattgctattcttaattattaag

agtaagaccagaaatggaattgttagggtaggggcaataatatcatacaagatatttcaa

taaaaactgaaatactagactctggattcgaaatctatttcgaaagcattgtagtagtga

attattactgtactatataaaattaattggaacaaaatatttcataatttgattttgaat

tatcaacttatcttttttttcgttccttttttcttcttcccttcgaatcttaaaatcgaa

gagttaagtgaataaaaaaaccaaaggaggtttatggctaagggtaaaaatatccgaata

actgttattttagaatgtactagttgtgataaaaagagtgttaataaggaatctagaggt

atttctcgatatattactcaaaagaatcgacacaatacgcctagtcgattggaattgaga

aaattctgtcccttttgttgcaaacatatgattcacgcagagatcaagaaatagagaaaa

tggattacttgtgtgtcacccttaaggaggtaaattgaaaaaatacataaaaattaatta

tatagataccagataaattatcttatctatattatataaataacattctataacataaaa

ttatatacatagatcattatatagcatatattttccaaatatattacaaaaaaaaataag

aatataactaaaacaaatcctttttttataaaacaaacgggattttcggtataggaataa

acaaaccatggagaaatctaagcgactgtttgttaaatcgaagcaatcaattcgtaggag

ttcgcccctaatccaatctggggatcgaattgattataaaaacttgagtttacttttcga

atttatcacgctacaaggaaaaattatacctagatgtgtgaataaattgaccttaaaaca

acaacgattgattacgattgctataaaacaagctcgtattttatctttgttaccttttgt

aaattcgtcaccttttgttaaaaatgcaaaaaaaaaatatgaaaaaatcaagtcgattac

tagagctactcctcctgttcttaaaaaaaaaaagag

>fava_dn_rep_c425

ttttttttttcgatgaggcaaggaactttataacttataataaaataacaattgtatttc

tcaaccaaatgaaccatatccataccaaaattggcaaaacaaaataccaaatacaaaatt

gtacttgtacaactgaataatcagataaaattaaagctcaaatgaaatacttgatctact

tcttggatttctttgatggttgggcaggctgggttgctgttgcagctggtggtgctgcag

gtgccttctctccgggaccttgagcaaaacgctcttctcttcgagcatgttttctttccc

tgcttgccttgttcttagccctcttagcctcaaactgatcagacaatgtcttctctcttg

ccttctcagccttggacttgtgaatactctccataaggactctcttgttcttgaatacat

tacccttcaccttcatgtacatatcatggtacatgtgcttgtcaattttctttgcttctc

ggtacttacgaagcaaacgcctaaggacacgcatcctcctcatccataggatcttggtgg

ggagccttgcctctcttgtacccttccgcttaccatatccagaatgacgacccttcctct

tagcctcctccatacgcctagcacgggagcgggagtggatcttagttggcttcctgataa

taaagccatccttaaccaacttcctgatgttttggcgtgaattggccatggagatttcat

tgccttcatttggatcgagccacacctttcctctgccgcacttgaggacgctggcggcaa

gcctcttctgaagtttcagtgacaccatcgttatcctcactctctctctggtttgataag

ctgccggctgaatagccttatatatttctaagaaaccctagttttta

>fava_dn_rep_c426

acaatcctcatagaggatttgccatgtctatcctccacataattaacataaacttcttga

gaacaattatctaagaactccaattccatcctattgttaattctcatttttgcaagtgaa

ccaggacagcaatttgcttcacgtaacacatgtactatgcgggtctccataacccttcct

agaggccagaaaacaaacatgttgagtagcaaaagttaatttctttcaaaagaaaaagaa

aaactttaaaagtgagatctcatttctaaaggttcaggtcttgttagattgtactgatga

taacaagcttgaaccgggattaccaattgcaatttatttcacaccagtttcaacaagcag

actcactagacagcatcatgacttcataaaacttcagcaacaattaacacaacaaacagg

gtattcttattctcaaccacagcatacatgctgttcaggcagagagatcaaacacaatta

tgttttctaaatgcatgacaataataccgagtttttgacacaacatatgtcacataagaa

ttcaaccactgttattaatccaaagcaacttaattgcgacttgggtcagtaaagaagccg

ttgatgccaggcataatctcaggtgacctgttgcgtgaaaacttcctcaacaaatgttct

gtatacttctctccctctgaaaagttctccaacactcctgcagctcggctctgcttgttc

accctgacttcagggttgatgcacatgtgacgaaccatagaaaacccacagatcccaaca

gcagctccaaccgcaaagaacagcgggtatacctcaggcctgacccagcgattggccatg

gattgcagtagaacagaagataaacgagaaagggaggagagtggttgcaatgaaaatgga

gtgagagataaagaagtgtaggttaagagttactactaatatattatatgctcttcttac

aaactaaataaaatgttttttttttttggctaaaaaattaaaataagcatagacgcaacc

ttgt

>fava_dn_rep_c427

tattctctttaagatttttaacctcaagcaagtgtgatttgaacctactatgaaactaat

tctctaattgttctaactaatatttagagtaattttatttgtatagtttagttttccacc

ccttatccatggtctcttatcatcttgtccctcatctatactcttctcaaacttggtaaa

taaggttcaaaacaagggcactttgtaacacggtaataaacccccttccattttgcttca

aacaacgctatccatatttcctcaatccacaacaatggctacagctccttcgcttctcca

ctcctccggctcctctttcctcgctcaatttcgaactcttccaacccaattttcttcttc

ttcattgttttcccatggtaacaaccgccacggaagtgcagtgctgtccgtgaaggccac

ggtttctggggttgtgttggtggggaagtctgaagcagagaaggtttatagactcaaaac

aacttacaatgagaaaattgttcctttgctcacggaagagttctcttacaccaatattca

tcaggtgcctaaagttgataagattgtggtgaactgtggtattggagaagctgcacagaa

tgcaaaaggtttggatgcggcgattaatgatctagcacttatcaccggacagagacctgt

taagactagggcgcgaatagctgttgccaccttcaagatcagggaaggtcaaccacttgg

aattgctgtgacactccgaggaaaaataatgtactcatttctagaccgcgttatcaactt

aggacttcctaggacaagagattttcaaggtttgaatttaagcagctttgatgggaatgg

gaattacaacattggtattcaggaccaaaccgtgttccccgagctgaaatctgctattgg

tacacctagaggaatggatgtatgcatctcaacaactgctaaaactgatcaagaaggaca

gaaattactagctctcatgggcatgcccttcaaagagggagttgaagttactcaaatagt

tcgcaaaaagaagttgaagtctcatcattttgatgcaaaatcaaagggtagaggagatag

agcaaagaagtagaatcatttttatgaatgcataatcagaggaataatgaactaaggttg

taaccagaagagacgctaagttgtaccacttgttttgttcagatgtaatggaaagattat

tgattctttttgtaagagatatgaatgttttcatattgtcatgttattacaaggttgtat

gtattatggccatgcagattatgcttgcttactaagccccaaggattgaatttgatcaat

taattaatttatatatta

>fava_dn_rep_c428

catcaataatcaataagttaggtaattaattacttaacttaatccaatttgattctcctc

ttcttttgcatcttctgtttggtgtgatccatcattcatcactcttctcgttcgttgctc

aggagctttcattacttccttcaactttactgttattagatcacacgagtgctagggttt

tggtttcatcgtttctctttttcaatctcgcgatcaggaattgaagtttttcagaggttc

ttgcaatggctgcgaattcttctaatggagatcaaacaacagctaagcaacctccattgc

catctcccctgcgtttctccaaattctttcagtctaacatgagaatcttggttactggag

gagctggattcattggttctcaccttgtcgatagattgatggaaaatgaaaagaatgagg

tcgttgttgctgataactacttcactggatcgaaggacaacctcaaaaaatggattggtc

atccaagatttgagcttatccgtcatgatgtcactgagcctttgatgcttgaggtggatc

agatctaccatctcgcatgtcctgcatctcctattttctacaaatataatcctgtgaaga

caatcaagacaaatgtgattggcactctgaacatgcttgggcttgcaaaacgagttggag

caaggattttgcttacgtcaacttcagaggtatatggggatcctcttgagcatcctcaac

ctgaaacctattggggcaatgttaaccctattggagttcgtagctgctatgatgagggga

aacgtgtggctgagactttgatgtttgattatcatagacagcatggaatagaaatacgtg

ttgcaagaatcttcaacacatatgggccgcgcatgaatattgatgatggacgtgttgtca

gtaacttcatcgctcaagcattacgtggtgaatccttgacagtccaatctccaggaacgc

aaactcgcagtttctgttatgtctctgacctggttgatggccttatccgtctcatgggag

gatcagacaccggtccaatcaatcttggaaacccaggtgaattcactatgcttgaacttg

ctgaaacagtgaaggagcttattaatccaaatgtggagataaagatagtggagaacactc

ctgatgatccaagacagagaaaaccagacataaccaaagctcaagaattacttggctggg

aaccaaaggtcaagctgcgcgacggtcttcctcttatggaaggagatttccgtttgaggc

ttggagttgagaagagcatctaaagcgtttgagttctttttattacatgtcaatagataa

taagtgggatggattgctctcacttttcatatttttttactttacataattatggtactg

aggaagtttgggtgcctaaaatttagcttccaagagtatcataggcgtgctagctcttgt

ttgacaaaacaataccgcatctctgtccaatttttgcttgtgaattgttattgagtttac

atttatctaaaaggcttctgctttatgtttctctttacagttttatgtgattttctatta

aggaacttcattttataaaacttaaaccattgtaggacatttctaatggtcaagatttaa

acaatccaatttgagttcttatctcatt

>fava_dn_rep_c429

tatatagacaaggtagcgttgttagcctcgtcattattcttaccacgtatgctctcgtca

ctcatcatccttcgttatttctgaggtttttgataccatggctgtgcaaagatctggaat

tgctaatgatgttacagaattgattggaaaaactccattagtgtatctaaataaacttac

tgatggttgtgttgctcgagtcgctgccaagctcgaattgatggagccgtgctctagtgt

taaagacaggattggttatagtatgattgctgatgcagaagagaaggggcatatcacacc

tggacagagtgttctcattgaaccaacaagtggtaatactggcgttggattagccttcat

ggcagcggccaagggttacaagctcataattacgatgcctgcttccatgagtcttgaaag

aagaattattcttttagcttttggagctgaattggttttgacagaccctgcaaagggaat

gaaaggagctgttcagaaagcggaagagatattggccaagacacctaatgcctacttact

tcagcaatttgaaaaccctgctaatcccaagatacattatgaaactaccggtccagagat

atggaaaggcacagatgggaaaattgatgcatttgtttctggggtaggtactggtggaac

cataacaggaactgggaaatatctcaaggagcaaaattccaatataaagctgattggcgt

ggaaccagtggagagtccagtgctctcagggggaaaacctggtcctcacaagattcaagg

gattggtgctggctttatccctggtgtcttagacgttggtcttattgatgaagtcattca

aatatcaagcgatgaagcaattgaaactgcaaagcttcttgcacttaaagaaggcctatt

tgtgggaatatcttctggagctgcagccgccgctgccatcaagatagccaaaagaccaga

aaatgctggaaagcttattgttgtcatttttccaagctttggggagaggtacctatcctc

tgtgctatttgagtcggtgagacgtcaagctgaaaccatgaccttcgagccctgaattat

tgtttccggctttgaattgatttacttctgttttttcttaagaattttagtggaaatgac

tctcatatttactgttgttttgtgattctgaaacaaaacttgctttagaatatttggatg

gaataaatttggtatttgagagtacatatgtatactgtttttcttatggttaatttatgg

aaagaaacaataattgttaatttttaggaaaaa

>fava_dn_rep_c430

ttttttttggctgagaattgaatccaattatttaataataataatgtttcctcatcaagt

tgctgggaactctacaagagctgtttgtttcaaactttttaagaacaaccaaagtacaaa

attggaagagaattgagaagtatcataacatcgactttaagtacaggataatgatacaat

gatagtgaagataaaattaggattgttcaccactatctaaccctctagaaaagacaataa

aaatccataaactatcttaaaaataacaacgcattagtaaacttcccaacatattataag

ccaatctaaacataacatatgtttccccaaccttcctaagcttgtcagaaccttcttcct

tattatcatttttaatcatcatcttcatcctcctcatcctcctcatcttcatcctcatca

ttgacttcagattttgacttgtcagaaccctcttcatcatctttcccctccagtttctta

ttataggccttaagagttatctcatactcctctttcttcttcaaagccctatcaacataa

ggagccttctcctcttcagacaaggatttccattcctcaccgcaagcttttccaacagcg

gcaacagatttgttgttaggatgatccttcttatactgttctctaaaatcagccatgaaa

acgaagaaagcactgggaggcctcttaggcttgtttggatccttagcagctttcttcgtc

tgcttccttccagttccagcgcctttgcgtttcaaccggctatcagcggctttggagtcg

gatttggctttcggcatgacgatcgaagaaaatcaagcaaaaacgagttaccgattcgga

gcgagtgagagtgacgagttaggagcgtgttgcgaagcgttgttgtgaggattctgaagc

gagggagaaggggataagaagggttagaagcgaagggtataggagagaaaacgctcataa

aaactgaaaagagaaaaaattgaagtttagggtttacgcgggatttcgtttatattttgc

cacgtcatcgatccgtgtgatttgatttgattggctattgggattgtttgtgttttcttt

gtgtgtgtgtttggtggactagggagttgggttggaatgtaaggcggagaagaatcggaa

gaggaattggaatcactagcttccgtgccactcttgttgctattcttcttgttgttgtgg

ttgttgtttaggtttttgtttttggtggatgaggaagaagaatgcttatttggtttgaca

ttggaggcgctacagatgataggttggaggcgaggacttcgaattgagaatgatgagaag

cgtggttgattgaactttgaatgaggatagcaaaattgaagtctcattgaagagggataa

acctgtgccgccatttttgttgtaaatttcaagttcagtttcactatcatcttcttatat

catttgaaaaccgctatttacaacactactcgtaaattttttttttttctggcttaaaca

gtgatctcgtcactcgtaagttagctggattttaaatttagtttct

>fava_dn_rep_c431

tcagttagcgtgaataatttgaaataggcaaggcatcaatcaatgcaacaaaaaatatat

atacctatcacaatatacccaatgtttgtttttaagaaatacaatgtgggacaaaccagt

gatccatatgaagtatacctctaaacactgaaaaacttgagctagcaatcccttaaaaca

aaaaaaattttcaagaaaatcatatcattaaatctgtataaaaacaacggtacagcctca

tgcccgtaaagatctcaaagtcgtttgaggcataagaggctgccaatttatactattgga

cagaagcagcaacttaaaactcaagaaacaaatagcaatctgtaccaaatagaaccaaca

acgaacgattccataaccttcagatacacacctagccctgtctcctcagctaccataatg

atgactccgcgcagcaatattagcaacctgagcctgcaactgagcagctctaacttttgc

agccgtagcccggtgaaagaactgctccctcgacaacaccctcacactcctcaagtactg

atcaaacggcacagctcccacctgaacacccttatccaatgcataaagagtatcttcaat

agccaaatcagcagcagtacaatcaagcatctgtttagaaaggacatccacaaacaaaaa

accatccttaatcacctaaccatgaaacctctttcaccactcaccctttccttcttcctc

tttgttttcatcaccaatctctcactagctttctcaaatgaagatgttgaacaagtattg

gacataaatgataaccccattttcccaggtggccaatactacattttgcctgccatccgt

ggccctcccggcggaggagtaagactgggaagaacaggtaatctaacctgtccagtcacc

gtcctacaagaccgccgagaagtaaaaaaatggtataccagtgaagttcgttataccagg

aatatctcctggtataattttcactggtacaccaattgacattgagtacattaggaaacc

tagttgtgcaaaatcatcgaaatggttagtatttgttgacaatgttattcaaaaagcttg

tgttggtattggtggtcctgagaattaccctggtgtgcaaacatttagtggaacatttag

gattcagaagcatgaatctggatttggttataagcttggattttgtatcaagggttctcc

tacttgtttggatgttgggaggtttgataatgatgaagctggaagaaggttgaacttgac

tgagcatgagtcttttcatgttgtgtttgttgaagctgaaactaatgatgctgactttat

taagtctgttgtttgaattttagttcagcaaactagattatgtaactttagctttttttg

tgttttgcacaatttaagctttaaataagtggaatatgatactttagtgtgttgaatttt

cccaaaaaaaaaaaccattacactacttctgatccactaagaannnnnnnnnnaacaaaa

tccccaaaatcaaaacacgaaatgcaacaaaaacaaaatcaaaaaagcagaagcaaaatt

ggaaacagaaaagtgattaacgaa

>fava_dn_rep_c432

aaaagaataataattaaaaaacattttacaaaatacacattatatatataaagaatcaat

tccctacaaggtgtacaagaattgacgttaaaaatacaattacacaaggaacaaaagact

aaacccaccacacataaaacaaaaagatgcataacaagaagaaaagctgcaaaattgcat

atgaaaatatacaaaattgccagtcatccatttatttttcacactgataataacataaac

tgttcaatggtattgaacatcttgaatcacttgtcatcatgtttggtgaagctcaacggt

atttcaaaaggactattctcaaaaggtctctcaccaatccagttgcctggaggatcatag

ttacagctaataatggtgctacgaccattgctacacttcaccctagcacaaccaagacga

accgaatcgcgccacacaacttgagtataatgcaagcactcaccattaaaacaagaattt

gaatcgtagtcatagttttgtatctcatcgacccacatatcgacagcagctgttgcagta

aggtcaggactgccccaagctagattttctccatactggcctccagagtgcactaactgg

caatttcctttaagttggttcacatagttctttgcaaaagatgcaacgtttgcatcccat

gttataggaccaacacctacttcggaacgtgctcggttgtgagcgttgaggtagtcttgt

ggtgagttttgagcatatgtggtatgtgttagtatgaataacacagccatgagagggaca

aacattgagatcattggtataattgtggagaaaggttatttgaactattgatggttggtc

ccgcgg

>fava_dn_rep_c433

atttcattgcaattgagaaacggaactttttcattgaccttatcaactgaaacaagttat

agcaaattttcaattttgacaacaactttacagaatatatatatatgaacaaaatctaac

caacttaaccctatttaatgctatgcagattgaactacagatgaaaaatttaccataaga

aagaataaagagaatttaagataaggagcatctgggattaactgtttagtaaacagacat

ataaggaaacaagttgcgtacacatattacatacaaataagacagaaactaaatcaaaag

caaataaaatttaagagcttctcaatacatacttattttaagctcttttaacaggatcaa

agttggaaactccaataacagctccaaaaatagctgttagcacagctccaccagcaaaaa

tgcttagcagaaagttctggagagaaggtgagagatcagatccagctgcataagccacat

caggtataaccatggaagctgtcaaggatgcagctgtaagtgctgtcactgccttctctt

tcaaagatgcgttgattacaaccttagattttgactgtgtggttctctttggttgtgtta

atggtagtggcctgaaaagggattcggaggatggatttttcagcttgttttgggttgaag

aacaaagtggtagagctgttgcaacagatgcagaagccatttttttggtcttgtttgtgt

tgcttgtgatgttctctgctgaaaagttttgtggtttgatactctgataaggtaatggtt

tcacataaattttgataggtggaaattggagattttcagatgaggtggcagatgttggtt

atcctacgtggaacattctccacctgatatttggaagataaggtcatcatgtcataatct

tatacatttacatggtcgatacattggttgtcgtt

>fava_dn_rep_c434

ccgcggggaaataagtaagagaagaaagaaacaaaaaaaagcagaatgctagcgataatc

tgttccagttttgtgtttctttttcttgtaataaaattgtattcctattcttcagaaacc

aaaaactcacctccaagattacctttaataggaaatcttcatcaacttggttcattcccc

caccgttctttttacgctctagccaaaaaatatggtcctttcatgcaaatgtattttggt

aaagttcctattcttgtcatctcatcagctgaagcagcacgtgagataaccaaaactcat

gatcatgtctttgcaaacagaccccccaagattaattacgacattcttttgtataatttc

agagatgtttcatctgctccatatggagagtattggagacagttaagaagcatttgtatg

ttgcatcttctcagtgccaaaagggttaaatctcttcgcactgtgagagaggaagaactt

gtttttatgatggataaaataagagactattcttctaaatcactgcctgtgaatttaagc

gaattgattgcgtcaaaaactaatgatgtagtttgtagggctactttgggaaataagtat

agtggtgaaagtggaacaggatttgctaagttgatgttggatttcaccgagttgcttggt

acttttatggttggggattatgttcctagccttgattggatgacccatctttctggatat

tactccagagcaaagaaagtcgccaaacaatttgacgatcttttggagggtgtagtcgag

gaacgtttcaataatccgaaaggtgatgatgaagaacagactgatttggttgatgttttg

ctttggatccaaaggactgaatccctcggctttcctatcgatagaacaaccataaaggct

ttgttactggacatgtttgttgcgggtacagacaccatatcaactttgctagagtgggaa

atgtcagaactgttgaagaatccacacatgatgaagagattaaaagaagaagcaaggaca

gtggctaacggaagagcatacataacggaagacgatttgagtaacatgaaatacttaaag

gcgcttgttaaagaagcactacggatgtatcctccgatcccactactagtccctcgagaa

tgcagacaagatgtgaaagtagacggttacaacattaaagccgggacaagagtttttatc

aacgcttggggaattgcaagagatccacgatattgggatcaacctgatgagttcaggcca

gagagattcttggatacttcggtggatgttaaaggaattgattaccagttgattccgttt

ggatcggggagaagaggttgtcctggactagtgtatgctatggctgctaatgatcttgtg

ttggccaaccttgtgcatcagtttgactgggagttacctggtggtgctggggccaaggtc

gatatgtctgaagcatttggttttactgtccacaggaagtttcctcttatggcatatgca

gccagcacttcctaatcacaactcaatcagggagtaggaaccaataatccagttttttca

ataatttatttatggcatatagacttagtttgttttaaatatttcataatatatagttaa

acatggatatgtgactatatacatgtatgtgtctatttatgtctgtttatttaaattgaa

ataattacatatgagacttgaaattaatgtatacaatagttataaatcctgagatgattg

gatgaatttagtttaaggggactcaaggggct

>fava_dn_rep_c435

tcaccaaataacacaggttaaagagtaaaagtgaagccatacttcaaaaatataataata

taaaagaagcaatatcagccttatatttctgctcgcccttatcttcctgccgtttccttc

caactcttcatcttctatacgcaggagactccacaggggaagccaacccaagccatgcca

atcatggcttccttttcactaccatccttcattcaaatctcaaccttatctcaaaaactc

accacccctcaaaacaactttcactttgctataacacaaagaaactcagaactttcttgc

tctttatcttcttcttcttcttttccttcacacaatattcttcaaatatcacttccttta

gctgcttcacttgcaattcttctctggtcaactcctgcacatgctggattcatgtcagga

atctcaggactagaagctgttcctggtcctcagctgcctcagattgatttcctcaacaga

ataaatgaagaaaaccagaagagatatgctgaaaatgatgcgagaatcaaagagtctccc

ttggtgaagaagttgcttgaacaatctaagttgaataaagagaagaatagtaaagaaata

gagaacaagtactgcttacgtggggcagaatggggagttggtgattgctcagcagaagga

atgacaatagaggagagggacaaattcatagcaatgttgaaagagaaggttggagaaaag

taatataatgcagctagatcatagaaaagataaaattttaggcctcttgcagagtttcaa

aattttgtttttgaatcctatttactatacattggactattattatgtatgattgtaaca

gaaaacacttgctataaattttgtcactagttttgacagaagctcaactttgtagcttca

ccatttccatgttatatttgctcttcaaaagcaaatccaaacacatacatgttatggctg

gtacgtgaaagatattttgacttgtgattttccgcaactccaaatatgctagtgttggaa

catgtccatgttccatattgaattatgattaactttt

>fava_dn_rep_c436

tttttttttttaaaaaactgagaattttattaaggggaaaacacttagtcacacacaaga

catgtttcctcaaataaaataagaaaagtacatatggtatgattatatgctaacacagtt

gaaatataaatagttgtaggcttttattgaaaggaacacaacttttcaagcatggtactt

caatggaacacttgggatagatttttttttccactcaaactgattaatgaatcggcttaa

caattggagtcagctttggaatacccttgtaagtgggaacataaacaggtttcacaatgg

gaacataaacaggtttaacaatgggaacataaacaggtttcacaatgggaacaataggct

ttttgaagaaataatgcttatgcttaagaaatgtctcaggctcaacaacagaggggatag

gttttttcactatgattggtttttggaatggcttataataaattggtttcttgtgaaaat

atcctttgagtttgtgttttagatgaaaaaatcctttgaatttttcatcttcatttggtc

cttctaggcttggctttgtcatcccttcatttgcaagcttcccttccgagttcacttgag

tccctgacggatcatttcttgctaaaacagaagtagcataaaagaatgttacgagaagca

acaaagaaacaaagaaagcctttctcatgattgaaaaattgaacaaaagttgtttagaag

tggaaatgtttgaagacttagcttcccgcggccaatttgcgctgggttcaactccggtgg

cagaaagaagggatttatatctttgcacaa

>fava_dn_rep_c437

ttaaaatataggcttacatttataaaattttgagtatgactcttataaaattatctacca

taaatgcatgtattcccctcaagtttggggccaatggtagagggtgatgctatcaataat

tgtgggtctagagagagatttagattgaagaacaagttgatgaaaggctatcaatactct

gatagacgcagaagcaaagaatttacagtacaggtttatcaatatttgaacagagggagg

atatgttgaggtgaattttacacaagcaagaaatcaacagcacacaaaaggaacagatac

atttttacacaaatgaatcaagccttactgaactcctgtttcatagcatactataataac

tatcttcctcactcatccccaggtaacacaatatggctaacacaaacacaaagaaagaag

agagttccattgcaaaggctgcccaaccgatcacaccagcatgctttaaaatgctcggaa

tggcaatgcctccgagagttgaggctcctgtcaagaacttcgcaatattcacccagttat

tatcagattcagaaaacatagaagaaccatcagacccagcaaagaacagcaaaggcattg

gaagaagcacataagttatcgcactaagcatcggccaccaattattatacaaagcacaag

ccagaacttgcaacacgattcctcctgagactaaaattgccaaaaagaccagcttccctg

tgttcaagcagtaacgcatatatccaggcatgtcatccattttcaagtcacctcatgttc

cagcaagcaagcaatattactcaaaagccaacaaaaattgaaattgaaacgctttttctg

atgtttatttatgatgataatatattaactacacgtcgataacatcggtgcgtatatggc

actgaagaatgggataaagagcagcagcgtagcggtccgatcttggaaggaaactctgtc

cttacctcaccacacggtccacgtcttattttctgaatggattt

>fava_dn_rep_c438

aagcatctaagtagtaagcccaccccaagatgagtgctctcctatgggttctgcccctac

ggggacggagcaaaagccctacggggacggagcaaaagccctacggggacggagcaaaag

ccctacggggacggagcaaaagaagtttatagaatagagggtcacggcgagacgagccgt

ttttcattaaccataggtgtcaagtggaagtgcagtaatgtatgcagctgaggcatccta

acagacccatagacttgaaccttgttcctacatgacctggtcaattcgatcaggcactcg

ccatctatttttattgttcaactctttgacaacatgaaaaaaccaaaacctctgtccccc

atcccttgaatagatagggatggaagggcagaggcctttggtgtcctctccagtcaagaa

ttaggacctcacactcacaatgaaaagtcaatatgcttttctctcatgtctttctttcgt

ttatggttcaatattctggtgtcctaggcgtagaggaaccacaccaaaccatcccgaact

tggtggttaaactctactgcggtgacaatactgtaggggaggcccttcggaaaaatagct

cggcgccaggatgatacacttattcattcttattctctttgtcaatagcaaaagagaata

agaatgaataaggtcgtcttattctaaaccccaattatcaaatctctcacacttcacacc

tcgggacgcacaatccttattcttataaggagataggcgctttcacatcttcttaaccaa

aaatagctgggaagaggaaaggtttattatagggtactccggggaacagatctagtggag

acagggtggggcctgtagctcagaggattagagtacgtggctacgaaccacggtgtcggg

ggttcgaatccctcctcgcccacaagcggctaaaaggggaaggaccttggggataagaaa

agaaactatgattgaggttgaaatcaaatagtcttacattttttctttcttaaagatatg

aaaatatcttatatttaataggatagcccgagactatcgaacaaaggaatatccctaatc

tacacataggtattgtattgaccgggagtccccaatatagcagaacagaatagtatacga

tgagatagaatggaatagaaacaaactttacactttcttttgattccaaattattgaatt

cgaaatgaagaaaatctccccaagtaggatttgaacctacgaccaatcggttaacagccg

accgctctaccactgagctactgaggaacaacggcaaattctatcccatctatattatca

tataataagatatctaataatatattatactaagacatagaaagaataatatcacataat

aaaacatagaatatccttaacaaacttatatgcttaacatatttatattatatgcttaac

aaacataggaaatatcagatactaacacagaaaagaatatccttaacaaacttatatgct

taacatatttatattatatgcttaacatatttatattatatgcttaacaaacataggaaa

tatcagatactaacacagaaaagaatatccttaacaaacttatatgcttaacatatttat

attatatgcttaacatatttatattatatgcttaacaaacataggaaatatcagatacta

acacagaaaagaataacatagaagaacaaagaaaaagaacacagaaaagaataacataga

agaacgaagcaaagaaaaaaaagaacacagaaaagaataaataaagaaattagttattta

tttagaaatgatgaagtattgaagtttatatcattaattattttcaaattaacaagaaga

agatgtttcttttgttctttagatcagaagaattgattttaatataatcaataacattaa

catagaagatataataaataacatagaaaatataatcaataacattaacatagaagatat

aataaataacatagaaaatataatcaataacattaacatagaagatataataaataacat

agaaaatataatcaataacattaacatagaagatataataaataacatagaaaatataat

caataacattaacatagaagatataataaataacatagaaaaacaaaacaaagtatatca

aagaaaaacaaatcgaacactcatagaggtattatgatttatagcttagtttggggaaat

tatctcgttggtttct

>fava_dn_rep_c439

ttttaaattccacctcatatattttatgtgttccaaactttttcttagaataaatgtccc

ttatcatattaatcctttatccatcataaactcatttcttcttcaaaattttctttaact

tagaaagagagagagagagagagaaaaagaaatctctcagaaacaatggcagcttgcact

gtctactcaacacaatctctaagaacaaacatctcaatcccaacatcatcaaaaacaaac

gttggattccatcaaaaacaagtacttttcttcacaacaaacaagagccacaacaacaga

agaacaaacagcaacaaaagatacttgataacatgtggagcaggtgactcacagacaatt

gtgataggtctagcagctgattcaggttgtggaaaaagtactttcatgagaagactcaca

agtgtttttggaggagcagcagagccaccaaaaggtggtaatcctgactcaaacacactt

ataagtgacacaacaactgtgatatgtttggatgattatcattctttggatagaactggt

agaaaagagaaaggtgttactgcacttgatccaagagctaatgactttgatcttatgtat

gaacaagttaaagctattaaagatggaaaatctgttcaaaaacctatttataatcatgtt

actggtcttttagatgctcctgagcttattaaacctcccaaaatcttagtcattgaaggt

cttcatccaatgtatgattctagagttagagaactcttggacttcagtatttacttagac

attagtaatgaggtgaagtttgcttggaaaattcagagagacatggcagaacgtggacac

agtcttgaaagtatcaaggctagcattgaagctagaaagcctgattttgaagcttatatt

gatccacaaaagcaattcgcagatgcagtgatagaagtattaccaacccaactcatcccc

gatgataacgagggaaagattttaagagtaaggttgatacagaaagagggtgtgaaatac

tttagcccagtttacttgttcgacgaaggttcaaccatttcatggataccatgtggaaga

aaactcacatgctcttaccccggaatcaaatttttctatggaccagaaacttacaaagga

aacgaggtgtcagttgtggaaatggatggacaatttgacagactagatgaactaatatat

gttgagagccatttaagcaacttatcatccaaattctatggagaagttactcaacaaatg

ttgaagcatgctgattttcctggtagcaacaatggaacaggtctcttccaaaccatagtt

ggtttgaagataagagatttgtttgagcagatagtagcaagcagggctgagactcccgta

ggagctgctaaagcttaggaaaaatttgccttatatacataatattggaaaaggcggtaa

ttgattttattataaaaaaataatttcttacttgtgtttttcacaattcctgtattgtat

tgtatgttgttttgtgtatcaaaaaactttgcttgtatttatttatgtaatgtaatatgt

tgttacttgtaatatagaggaggagaaacaggactgagatttcagttatgttcgtgtcca

tttgcatctaaaaaacctatcatgtagtagtagtaattatttagaaaataaataaaaatt

acaaatatcatgatataaaaataaaataaaaagtgaaacaatgaaaccaaatgactctga

tcacatatcacttcaacaaagagacaagagaagaggcaaatggcatcaaacagtagagta

ataatgaaacaattcttttaccctcttgaatcaaactcattttctcccaatcaagtaaac

tagagtttcacaatctcaggaatatgaacaggataacgatcaatgcattctgtccaaggt

ccatggaccggtgttttaatacttgtgttacatttccaaacagcactgttacatttaaat

ccactgccaaatgctatctgccaaaccctatctcctttcttcatccttcctttggattca

atgtagttaagctcataccaaagcgacgaagaagatgtgttaccgaatctatgtagcgtc

atcctcgacgcttccacgtgttcggttgatagttgaagattcttctgtaactcatcgatg

acagcacggccaccagcgtggatacagaaatgctcgaaagcttgtttgaaatccggtatg

tagggtttccatttagggttgaagatttttcgaccaatgagtgatatcagaaagagtaac

tgttctgaagcaggtaaaacaagaggacccattgttgttatatttgattttagagcttca

cctgcaattgccatgagatctttctgaagtgaaattccgactttgccctctttgtcttct

tcttcaaacacacatttaaatgctttatcatcacctcctttatgtgttctaacaacatgg

actaatctgtatttagccctccgtttctcagacgatcgattcgaaatcagaatcgctgca

ccgcccattctgaacaaacagttcggcaaaagcatggctctttcattcccttggtaatag

tttggtgttataatctcggtactaacaacgaccgcgtttgaattcggatgaacttgaaga

agatcgcgagctaaatcgatcgaaatgagtcccgcactacaacccattccagaaagattg

aagctcttgatgttacttctgagtttatatttgttaatcaccatagcagacaaagaaggt

gttggcgaaaaaagactacaattgactatgagaatgtctatatctttcggtttaagaccg

gttttctcgaacaaagaatccatagctgagaaaattacaagctcagcttcacctcttgct

gcttccatggttggtttcggcggaatataatgaatggaaggcggtaaacaagtttcttcg

ccaagaccagaccgctcaagaatcctcatttgaaactcgacacttttcggattatttttg

aggataagtctcgaatgttccatgaaagtagcgaaaggaacacgacaagtaaacgggggt

ttgaaacaagcatagtcgacaaggtagattgtacgaggcttcgacatgaaataaaccgtg

aatatgaaaatgacaaggaaagaagaacataggatttgaacgaggttaaagtgaagtgaa

ttccaaagtttcagaagttcatttggacctaaacgtataatttcaatggaaacaccaatc

atgataggtataagagtgagagtgatgatatggttaacaagatattggtaaccaagtttg

acatatttgagcttaactgagctagaaaaatcaggcaagattggaggcatgtttaaaagt

gcttttttttttaagtgattgagaagaagaagatggtttgatccccgcgntcccttcaat

ttgattattattgattgt

>fava_dn_rep_c440

tgttagagcaacttgtacaacgagagaaagctaaaaaaaaaaaacagtatttttatttca

aaaagttgtgacaattatttcaaaaaattgatggagcaccattgttgaaaagtcgcgcaa

aacgcgtccttcccaattcaagtacaaggggttattgaggcttatcaaagttaaccacta

gttactcaaccatgttttctcaaagtaaataaaaatcagaataacacattgccatgcgga

atgcccatatttgagtgtatagggactgcaaaaatctacacttaggtgactattttcaga

catagtaaccagtgaaagtgaatccccttccaacaatctcaccagcacaagaccatgcat

agagttccaaaccaaacaaagcagcaatgcctacatcttcaacctttaagtcttgtttgt

tcttccataattgcttgacataatcgagttccttcttgaaggcctcacaacgaccaggaa

tgctagcaagacgggtgtaaaacaattgcttcgacaaaaggttgcatttctcaattgtag

caggctcctgaatatactctttgttctgctccaacaattgcttgtagtaaacattcccat

gcctggatataaattgagacgcttgacatgctttcgattgcaaagatttcaacttcgacg

ccatcaaatcttgtgaagtgaacgagtgcagtaatcggagattgttcgccgccgctactg

caaagaacaaaggatgcggctctatgtgaaaactcaactctataggctaaccttgtgtgc

caattttcgttttcttttttttgtttttgattaatattttcttgagcaatttggtttggg

ttgcttaggccttgtgaactgtgaagggggtatgtaatgtaaagttactattagcaccct

ggtgtttttagagttgagattctctccattttt

>fava_dn_rep_c441

ttaaacaatatttgtgccaccgttattttctcacaaattgagataccgcttgaacacctt

agtcaaaaaaaactttcaataccaaaagttttggtgagacttaactttgattgaacaaat

tttttggttgagagagagtaatgagatcattatagacattacattttggcctagaacctt

aaagtatagggtgattcttgtcacttattatactaaagttttccaacattcacttttcag

ttcaatttatcactcctttttatgttgagatgagtaatacaaataatattgcaaacaagg

aaatcatttaaaatcggtaactatttcacataagttaattacacattacacatcacacaa

aacaaataaaacaaaccacacatcaaagtgtttcccaaccacctcaaaagcaaaactttc

aattcctatagagtttaatttctgcctactgattagcagcatattgattaatgagtgaaa

gagcattactagtaacttgagcaacatcaaccattctagatctaatagagcttttgattc

ttccatcaagtgcttttccaccaaacccatcactacaagtgcttccatcagtcaaagctg

agctaacccatgtctcaacattacttatatgccaattgaaatcttgactcttgatcttgc

aaagtttaagctctttcaatgaactgctaagcctatcaacaccatcacctatgacttcaa

cacaatcatgaattgcagcatatactcttggcttaagattcttgaaatacttgcactttc

taacaaaggcttttgtggtttgagttctgttgagactaagtgataaagctgtttggacta

attggtgtggatcttgttggatttttgatgcatagacagagagagattcaacacaaagtg

ttggatatagtgttgtgctacatgaggatttgatgaagtttgttggtgttgatgttgcta

tggtgacaagagttattagaaggaaaagataaatggaaaacatggttgccatgtttggtt

tggctgaggagagagaaaaacaagtttttgttttgtggttggtttgtggtttgtggtttg

tcccgcggccgtaa

>fava_dn_rep_c442

tgcagtttcttcattttttttttttttttaggaatgaagatgcaaatagatagtgaaaat

gaattactaatatatagtagatacattgtattgtaggttagaggacacaaacgcctctaa

tctaagtttgaaataaacatgtaatagaaataattaattgataaaaaggaattactcgag

taattcgaattcatcatccctgaggtgagcaacgaacttgagaggaccacgaggaccttg

aatatcttcagcaataaactcaaccttataaggaaaatttgcagaaattctcttatcttt

ccaaatagacacgttctgtttgatgttaccttccctaccactgagatcaacctctggaac

tttaggaacatgatacactttcagaggaaccttcaccctaacacgtgctccaatcttcag

cgatgaatcttctcccacgtcacaccttatcatcgtcgcgaatctcctgcttcgatgaat

cttggagttggtgaacggaaacggagctgccattgttgcaattgtagtggtactcgtcgt

gctcatcatgctcgatttgttgtgtttggttctttctgcgaggaaatgaaaatatcaagt

agggattgcttgcttaattgtgccgctctctatctctctctctttctctgctactcattc

atggatatggataataaaatacaaacgaaaacagaagtatccaatattattcaaggacta

ggatacataagattttactcatttgtccatccaaatcacgcatcgaagacactctccatt

tctcaataaatcaaaagctttgtttatgtccttaaactccacttcatgagagacaaactc

atccaactgcagttccttgtccatgtattgcttgagtaaaatggaaatatgtgacttagg

tttgagtcctccaaataagtttcccttaagggtcttcccatcatgaaggacctcactaga

actgaggctcaacttggatccaggtttatcaaatcccaacacaattgtttttccccaacc

ctttctgcaagaagcatacgcttcatgcaccaatgacgccataccaacacattcaaagca

ataatctgctcctccatcagtcatctctatgatgacctggctcacagatttgttcatacg

ttctccagcatggacaaagtgtgtgaccccaaactttttaccaacttcaaatttttctgg

gttgacatctacacctataatcttagttgctccacaaagtctagcgccctcagcgaccgc

tagtccaatagatcccatcccaaaaatagctacagtagatcctggctccacacctgcact

cctccaagcagcgcctattcctgctccgataccacaactaagaaggcacgccctttcagg

aggaacttctcggtcaatctttgttacattggcaatgtcaacaacagtgtactcgctaaa

actcgaaacgttcatgaaatgatgtatggtttctccatttgagtcggtgaatctagaagt

accgtgtctaggcatccaaggagacacctcaaaagggaaattagtacagaggttgctctt

ggttgatttgcaatctatacactctccacaatctggtaggaaaattggaataacaacatc

tccttttgtaacttcagttacatccttccctacactttccacaaccccagctgcctcatg

accaagaattctcgggaaaaagccaggagggtccttcatgttggaaaaagcaagatcgat

atggcagatagatgaacatagaacacgaatccgaacctcacgagccactggtggcgccac

gacgatctcttcaatcaccaatggctcaccgggtcttcttgcaaccgccgctttacatct

tataggttggccttctctttcaactgttactgtaattggtagtttatgttgttccatggt

gaagttgttttgtttctctcactggctctgcaatgcggggagtggt

>fava_dn_rep_c443

ctgtagcaaattaccccgcgggcaacaagcaaagaagaaaaaaagaacagtccctactaa

gatcagcgtctccttcattcatcacactgtctagttttttcaacaaacacaaatcattaa

ttgaataatttttttgatattggaagaaaaaaaaaccataaagagttttttacatcgttt

tttatgataaccgatatgaaaagtggtggtgcagatgcaggtgccttgggagccaagaca

gcgagagcttgtgatagttgcatgagaagaagagctcgttggttttgtgctgcagatgat

gcttttctttgtcatggttgtgacaatttggttcattcggctaatcagttagcgagtaga

cacgaaagggttcgccttcaaaccgcgtctgctaaggtaacaacgacaacagcaacagca

aaagcatggcatagtggattcactagaaaagcaagaacgccgcgtcataataagaattcg

tcgattcagcagcaacaacaacggcttaaggagaaggttctttttaatactagttttctt

ccgctcgttcctgagctcggaggcgaagaagaagaagaagaacgggaacgggaattgcta

gttgatattaatgagattgcggaggaggaggagcagttgctatgtcgcgtcccggtttat

gatgtcgacccttttgatttagagaattgtaatgtgaaaaatgatgctgctgattttgag

gacatgtgtgatttggatagtttctgtgagtttgatgttgatctagctgaatttgcagct

aatgttgaaagtttacttggtgttgggagtagtgagattcatgagaattcatcggaacaa

gtgtttgattataaacaagagaatgaaatggatccatccaaaaatgaaatattaaaggtt

aaagatgaagaacttgatgatttggaatcagtttttgatatgacaagtgatgaagttttt

cattggaatatagatcatgatgaatcaatggctcatcaagagaaagagtttatgcctttg

agtattaatagtagtgttggtgttagtgagagtgttattactaaagaaactaaaagagag

agatttttgagactaaactatgaagatgttattagtgcttggactaggcaaggctctcct

tctccatggactaccgcaaaccctcctaagttcaactccgacgatgattcgtggcaaaac

ttcttgggttcaagtggtgtagatggagaaataagaagcataagagggaacttaatagga

agtaatggagatggaggaagagaagcaagagtatcaaggtatagagagaaaagaagaacg

cgtttgtttgcaaagaagataagatatgaagtaaggaaattgaatgcagaaaagagacca

agaatgaaaggccgttttgttaagagaacagcatgttttgctggaggagcagcttcattt

ccaactaattatcgctgacatgaataatgaatatgatatttagttatattaattgagttt

ctatgtttgctttttctgcaaacatggattgattaatatttggagcatgtggatgaatgt

gtatagtatgtattataataatgttatttactttgttagtagaattgttacttgggatgt

ttcaatgtatctctctagcttcatgtttaattagtaatatttgttaatgcgtgagacatt

tttagtgagaagagaaagagataataataaaggaaataaaaatttgtattattcaattgt

ataagtaatgatacacactatgactatttatatacaa

>fava_dn_rep_c444

ttcctacaaattgttttttagaggatagttactttagagcttcctggacaaggttacgaa

aatatttactaggtttaacatgttttgttgttgatgacacgaagcatccaacacaacaaa

attacagcttttcttcaaataggataaccaaatgatcattatatttttttgaaaaaagaa

ttttaacctaatctgttttgacttattgataacatcagacaaataaactgtcgcaaattg

tcttcaccaaactgaagaaggcagataatgaaacaatacaatacaattcctatctgaatg

acatgtatgctagcactaacattattatactcagccccttatcggtggctcgcagagttc

agtattgccgtttcctgacataataatttattttttcccgaaaactcaatcatgtagaag

gaggaacacaaccaattcagttgtcaccataaacccacttctctgaattgctagtgaaac

taaccaattcctctggcttaaaccacaacttaatctcatccttggcagtctctggaccat

cgcttccgtggatgatattccttccaacaacaacagccagatcacccctaatggttccag

gcgctgatttctgtggatctgtggctccgattagtttacggccgtaggtaataactccct

ctccttcccataccattgcaataacagggccagaactcaggaagtcgcacaatccattga

agaagggtctttctttcagatcgtgataatgctgttgggcaaattcctttgtagggatca

atactttgattcccacaagcttgaaccctttcctctcaaaacgagaaataatctctgaaa

tcagccctctctgcactccgtcaggcttaatggcaatgaaagtgcgctccagctctgcag

catgcacctcctgatcttggagcatgtaagctgcagcaggaagagtgagtgctcctgcaa

tccaggagctggatccatttccagaaccagttctcccataatttgaagcataaaccggaa

cctttctaatggatgcaacagctgcagccgccgctacggcgcgtccttgagagtgaaatg

aagcggagagaagagacctggcggctctggaagcggatttgcagagatgtgaggccatgt

tcgtcgtcgactacagatcgatgagtcgatggcagaatttgtgaatgagtgatgttcccg

cggtcttctgttctcactcgccttttattctttttctt

>fava_dn_rep_c445

ctttttttttttgagcaagggaaaacaatttattccacaacgaggagcaagagaaaattg

aaggttctatttctcaatacatgtttagaaagaaaaaataagcaagtgtacaaaattaca

aatggcacatgataggtacatatattatatataattaacaaaccttattttgctctcact

tccagttattggcaacaatgtcagccaaatcaacaaccctttgtgagtaaccccactcat

tatcataccaagcaatcaccttaaccatgtcatcacccatgaccattgtcagtgaggaat

ccacggtagatgacacatcagtgcacctaaaatctacggacacgagtggctcgtcacaaa

ctgagagaataccagtcagctccttggctgcactctctctaaaagcctcattcacttctt

cagcaaatgtcttctttgaaacttgaacggcgaggtccaccaccgaaacgtttggtgttg

gcacacgaagtgcaataccgttgagcttgcctttgagtgttgggaggacaagggccactg

ctttagctgctcctgttgatgttggcactatgttaagtgctgctgctctcgcacgcctta

ggtcacggtgactcgcgtcaagaagacgttggtcaccggtgtaggagtgagtggtagtca

tggtacccttgataataccgaatttctgatcaaggaccttgacaaagggagcaaggcagt

tagtggtgcaagaagcattgctgataatgtcgtcgtcatgggtgtaagcatcagcattga

caccaaccacataagtagggatgtctccttttccaggggcagtgatgagaaccttcttag

cccctgctgtaatgtgcctccccgcaccttctctgtccacaaacactccagttccttcaa

tcaccaagtctatccccaactccttccaaggaaggttggcagggttgcggtctgagacaa

ctttgataacctttccatcaactgagatgccatcagtaccaacaggcttaacatcagcat

caaagattccaagtgtcgaatcatacttgagaaggtgagaagcttgctttacacctccgg

tgtcattgatggcaatgacatcaagaggcgagtccttgcggccgtgccaacatctcaaga

agttccttccaattcttccaaatccgtttatggctaccttcagttgttttgcttccacta

caagacctttcttgtatcttccactacttccaactgcattggtttggaaggcaacaacag

aatgaaaatcacagttgcgtagactagagagttcagagaagcctttcccatttgccttaa

tagctggtttggctacagaaaaagtagccgaagccatgattgagcctacaaggtgaagtt

tgtagtatacaagttttgcagggaagcaaattcttagttagatgttgttattattatgta

gtactatgaagtatagtaaggaaataag

>fava_dn_rep_c446

aaccgcgggggtgtgtgcctcaacatccgaaaaaaaaccctaatttcccttcatcttcat

ccttcagccactgcacacacattcactcaccgtcgcaaccatgggtcgtgtcagaaccaa

gactgttaagaaatcctcccgtcaagtcattgaacgttactactcccgcatgaccctgga

ttttcacaccaacaagaaaatcctggaggaagtcgcactcattcccaccaagagactccg

caacaagatcgctggattttctactcatctcatgaagaggattcagaagggacccgttcg

tggaatctcgttgaagctgcaggaggaggagcgtgagaggcgtatggatttcgttcctga

tgtttctgctatcaggactgatcatattgaagtggacaaggaaaccctagacatgcttgc

tgctcttgggatgtccgagattcccggtgttgtgcaggtggatccggttcctgttcggcc

gattccgttcggtcgtggtggtgcagctgctggtggacctggtggaaggaggttttgaga

ttgaggtttaatggtttttgttttagagttatttggattttgataatgaatgtgttattg

agttttggattttgaatcttatgtttattttggtgttatggaattcctatgtttattttg

ttttatgataattaactctctctgttatgaattcatattactgcataatatttggataat

gttttaaccaaattgttcattgttatggcttattggttaattttcttgaatgtctttata

aatttaatcttatttgctgtaagattaacatggattgttctgtaatatgtttgcaaaatg

aaaaactggttagagtcca

>fava_dn_rep_c447

tttttttttttcaaaaaaaattaccggattcttaaaaaaatccgatagctcatattaggg

aaaaaaattccattaattcataggaaattttgtttgacgtgcttttctcatttaaactat

tccacattacacatggctctgtccattcctacgataaatttgatacaatctgaaaatttg

acacaacttcagagcatagagcagcacttgtgtatacataaaactggcgacagttttgaa

tataaatcaggtccgcacaattgattcaaactaagcagacaagtataggaaactcaaaag

atatccatgagttactacatcaaggtccagctgcctcaaccatttcttgctccacggtgc

taggggtaaccgggttgtaatggtagtgaagttcaccaacatcatcaccagacaatttct

tccatccagttggtcccacgtagtaaacgctagcaactccaccactagctccatcacgaa

atgttgcatgataaattgctcttcgagccagttcagaagcttcctcaactgacatgtcat

atttgtacccgctgtccaatacaccataagcatatggtgaaccagatccaactgagaatc

tagtgcctttaagtcttccaccttcactatcaacataatatagaccaggacccgtttcat

cccatccagcaatcatggtaccgacagataatcccattccacgataagagtataaaatat

ttgcaagtaacttagaggctcctgttacagaaattcttcttttatttgccaactcatgca

agcggcacttgatgccaagattcctatgccaaaattggcaatcagcagcacctccagcca

ttgtcccaagcatgtatggattaatttcaataattttcttcacagactgtgatgatatat

atcctcccatactagcccgagaatcagcagctaccataacaccctccttaaaaataaaag

caagagtcgtagtacccttcgcaggcttcaccatctgaatagcttctttaaggaaaccat

caaagtcgttggaattgggaagctcgaaggatggaggtgcggagatttctccggtgacgt

tgcttccgtgtgaaatcagtgaaggaaacgattcgagaccagatgtatcgagcttcatga

ttggaggttagtgactgtgaccgattgtggtagaagaagggaattgcagtgtgagatctt

gaaaatgttgagttttgaagcaatgtgagagaaaggaactgatttcagtatgtgttggct

tgggggaa

>fava_dn_rep_c448

atggtcaaaggagagacaaagcgttttgtttgtgttctcgagcgaaaccagcagggttgt

tattgtgatttgaaagagagtgagggatgctggaaaaccctccggcaccggaatccgcca

ctgcggtgaagcgctatgctccacccaaccagagaaaccgttctacccacagacgcaaat

catctgatcggcttgatcgatcaaacagtgttgggagcgatttagaaaagaatcaagttg

cttcttcaagaaactttcaagttccagatcacggagatgctagcagccctaacattctta

atgagaatcattattcaagattcattgctttagaaggatgtagtagcagtgcagcttccc

agcttctcaatgaccgttggacagctgcaatacagtcctacaataatccgaaggattcat

ctgaaaaaccagttatgtattctggtgggacatccgtttggactcagttcaggcttcctc

atcagattatgtcaccagctgcgagtgttctcccttctgtgtcacagcgggacttcctgg

gagaacttcgtcgccaaatgcagagcgccaattctagtttcaagacatgatggatgactt

ggattttgtaatctagttattgcctgtttgtgattttataatcaagttcttgtcacagct

gaatgctttaagaacactttacttgactacctgtttgtgactttgtaacatttgatatgt

ccaaatctccacatgatatatgtaagacagaatcagaatatatgtgacgcctgtgtaggt

ataaattgggcaaggattaagctccatgcctttggtgattttttctctatctttgggata

tcttacattaagtttactctataggcttgctaatt

>fava_dn_rep_c449

cttttttttttttgaaagcaacacaaaggaaactaaaattgcttgataagagagggaaca

attgttaggtgccaaaataagcaaataataatatcaagcattatagggtatctttataaa

tactaaactgtttatcaaaaatcattctactatgaagaacataacaacggattccagcac

aagtcagactcattactgctgagcacattgaactctctgagcaccaccaggcatatcatc

atcctcgtcataagactcctgcggagattgcttcttcctccttgactcttcatccatgtt

gacatcatgaagtgttgtctcctcacactcatctatctccatgtctgtcaactgtgatac

aggtcgtgctggcagtattgtctccaaagccttgacctgatcaagagtcagagactctgg

aaattccacagtgaagtgaatgtaaagcttgcccttcatgaatggtctctggtacattgg

cattccttcatcattaattgccttataagaatcaggcttgacaacttctccaggatttga

tttaatcaagagttgacgaccatccaaatgagtcagggcaaactggaaaccacataaagc

ttcagtgagggacagactgtgttctacaaacagatcttcaccatttcttttgaacttggg

attctccttctgttgaagcacaaatactatgtctccagtaactgtatccggcgcttcatc

agcttcaccagggaaggtaatcttctgcccattctgcattcccttttcaacatgaacttc

aagtactttcttatgttgaacaaccttctctcccttgcactgtgggcatcgatctttgtc

agaaattgtctctccagtacccttacagtcattacaaggatgttgcatttgttgaatcat

attagcaccgagatgcctcatagaaaccttcataccacttccttgacaaccagaacattg

catggaagccccagacttagaccctttcccattgcacttcgaacacagaacatttctcga

gagagaaagcttcttagatgtcccaaggtaaagatcctccaaagagaccttcaaggggtg

aacaacatcttcaccacgtctctgccttctaactctactaccaccacctggaaatcctcc

accaccaaagaaagacgaaaagatatcaaatggatcatggcctccaccaccactcattcc

ttccttcagcgcatcttcaccatattgatcatatatctcacgcttctctggatcactcaa

aacctcataagcttgtgcaagctctttgaacttttcaggatcaccacccttatcaggatg

attcttgatagcagctttcttgtacgccttcttcaaatcatcaggagaagcgctcttcga

aacgccgagaatctcataatactttgtgttatcacttttcttcggtgcacctcttccaaa

catcttaatcttctgtgaatttcacacccaaattcaatcgaaacctaaccctaattttgg

aagatctcaacggaattgtagagttgaagttgtacaatgcccgcgcgg

>fava_dn_rep_c450

tgtttgactaccttacttataacttttgaaaataagcttataagttattttaagagctca

aataagataacccaaatacattacatacatacaacattgacataaattcgcataaaagta

tgtaatcccataagaaacacaaattgtttcttacaaacatcaaattgaaacagtatgtta

ctaatagcaattagcatacattattcatataaaagttcagcattcatgatcatgctcaaa

gttgcatattgaattacatatattgtcctctgcatcacttccatcgcttgccaaactttc

catgcttaaacttgccaccatacctgccatgcttcccatgtttgccaaattttccatgct

tatacttcccatgtccataaccatgatgataacctccatgtccatgtgacatatggtgag

caccatatgcagtcgccagacctgcgaacaatcctataccagctccgcgtcctgaaggat

acgcggacggataggcgtgtggagcgtgataagatggttgatgataacctgaatgaggat

aaccagaaggtggataccctgctgctgcatgtggtggatattgtggaggatagtaacttc

cttgtggtggatatgctggttgtgggggataatagcctccttgaggtgggtaggaggaat

aggaaccatgtggtggaggataggatggtgctgttggatagcctgttatatgtgaaaaca

gacctctctcagtagactcttgaatgtttctaccgtcgctcatcttgattcgctcaatgg

aaaactgcaaacgttctttatgaaatcattcaaaggtgacaagtgaattcttgttggtgc

cacaaaaaattaaaggaagtgaaacttcacctattcttatacttataatttctctatttt

gaaaaagcacacgttgtttctacgcaatatggaatatatgtctgtctatatatatttttt

gcaagcataattgctagtagtatgttttttcttcctttgaaaacttagcctattatat

>fava_dn_rep_c451

accaccagcatcaacatatttgcatgtgttttacttttttttttggaagcaaacaaatca

tattaatattacatgtcacaagtgacgccccattaaagaaaaatacaataataaatgaac

gtgttacataacaatatatgaatgataaataatcattaataataaaaattcatatttcaa

aagaaacctaatcatgtcatcattttcacttcctcacagaccgtaatgaagtgaaaacaa

aaacacaaaatcctatgcacaaaccaaactcaacatagttaaatcctcaaaagagaaacc

caaatgcaagagctacgagtgaagcaaccgcagttggaacaaaaagaacactagcatcag

aagcaggactaggagctggggaatcagcagcagaaacaccacttgtagctatcaccaaca

aagcaaccataactgccacaaaaacattcatcttcattgttttaaagagagagaatattt

tgaaacagaaaaagattagtggttgtagtggcagtgggtccccgcggccataa

>fava_dn_rep_c452

tcaaaactctacacattcatgtaatgtaaacattcagttgaatgcaatattgagaatcag

ttattagcataccagagttttgttacaaaagtaaaatttcattatcacatgccacagtcc

caagaaaaataaatttaaaactggaaaattcggcaaactatcatttatctcaaccaaaag

gtaacaaaagtttgtcgtaaccgtcaatcaagtactcaaacaagaacacagctttaagta

aaaaagcgcatgacagactgaaagcacatacgatcactggtaaactgatacgatcgcata

attcaaatgtcgatctctcaagatacaaataaatgttcatcaaactcaagaacaatgact

ttaggtttcagtctcctttattaactttctaacggacataagctgtaaataaaattaaca

gctagtaacactagcttgatgctagagatcttcaatcctcgtcgtcatcgtcgtcatcac

ctccagcacccctatctttcttaacatgagtagtaccgcctactttgctgatcggcaatt

tcattgcaccaaagggagtatcaacatcaatgtgacctttcatggtgtaaccgagtcctt

tacctctaatcatatcccacagcgcagaaccaaaatccttaggcctaaaggtaatcggaa

catcaatgtaagttattccgtttttctcaagattcgcagatttagcgagttctgcacctc

caatgttatcatccccaagccaaacctcataatcaagtgcattgagtcctagatcaaaat

cattcatgttttccaatttcaaatgaagattcgcaaccgtctcttccatcgaaaactttt

tgaactgaatcttctcaatatcaatgtcaggcttatacggaatcggaatttttcctgttt

tatccaaaggtaacgtaaaccttcccaagacaggaacatcgaagataagatcaaccttaa

tcaaataaggaataatactcccgggtttaatatccgcataggttttcttaatatcatcat

aaatcaaagtaagaggaattttcacagtttcctctccatgagcatggatagtacccgcat

ccggtatcaaaccggaaacaagcttccttccatcactctcaatcaaataattgatatcaa

tcaaaggaattggaaccggattagggttcttaataagaacatcaacaacaagatcaatct

tatgaagattaactgtcggaatgtgaatccctttcacatccgctgtcggtttcccgaatc

caataactccctcgatcttctcaccaaggtcatgaataaaatccttcactttctcaacaa

atccgtctttcttttcctctttatcatcttccttgtccttctcagacttgaacaaacccc

tttccgacacttccgggacatcgtccgatgtcgacatatcgaaacagtaagcaatcaatc

aaccctgaaaattgagatgagatgagtgatgaaagagaaattgtgactgatattcaacta

tagtgagatgagaattttgtgggaagaaagtatattcgataacggaatgaacggtcagat

attagccacgaag

>fava_dn_rep_c453

tatactagaatcaaagaaataaaataagattaacaatatgagaaatcaaacaagtgacga

tatttgttggaagagaatcgtgagtggaagttagagttcagaaaccgaacaaagaaacat

gtctctcagttggtgcgttaagccaatttctttgcagctacaattccacccaacaacatc

atgcctccactcccacgtttctggaacatcaacaacaacaacaacctcgtcactgtctct

actacctcgttcatctcacgttctctctcaaaacaccgttttccactcccaacccaaacc

acacctcaaacgctcttccaccacaattcgctgttctgcgttgactcctgaattgaagag

cacgttggataaagttgttacttcaaacaaaatagttttgtttatgaagggtactaagga

ttttccacagtgtggattctcaaatacagtggtgcaaattttgaagtctttgaatgcttc

ttttgaaactgtaaacatattggataatgatatggtgcgccagggacttaaggagtatag

cagttggcctacctttcctcagctctatattgatggagagtttttcggtggatgtgatat

cactattgaggcatatcagaatggagaattacaggaactgatagagaaggcaatgtgctc

ttgaagcttcatttgctcatgagcagatggaatgaatgagttccatattattatgagtac

ttacattgtaataatcttgacatttggatacaaaatagaatatctcattgaatggacaat

tatagtgctagttgctgctgcaaactttgttaaattaatcatttttctgtcttttgtaat

actttcatttctgaataactactacaagatcattggttcaatttgataaataagtcttct

tctgtgtacattgtcatggaatgagctttgttgccttaaaaaaaaaa

>fava_dn_rep_c454

ggccgcgggactcttactaaatcttataacaattctagtatctaatctaatatcatatca

tatcctatcaccaaaaatgactatgaaaaaagagcaaaagaatgaaactgagaagaaagt

tgacgaaggagctaagaaagacgacagtctccctcctgtcgtttaccaattcgacttgca

ttgtgaaggatgcatcaagaagattaaacgatccgctcgtcattttgctggtgtggaaac

tgttaaagcagatctgtcttcaaacaaagtgaccgttaccggtaaatttgacgctgttaa

actacaagataaactggctgagaaaaccaagaagaaagtcgaaatcatctcagcgccgcc

aaaaaatgacgccgccgccgagaaaccgtcggagaagaaacctgacgagaaaaagcctga

agagaagaaggccgaagagaagaaaccagaagagaaaaaaccagaagaaaataagcctaa

acagagtacagtggttttgaagatcaaattacactgtgacggttgcattactaaaattag

aaggatagttatgaaatttaaaggggtagaaacggtaaaccttgacggagataaggattt

ggtaacggtgaaaggaacaatggaaccgaaggagttggtagagtatcttacagagaagct

aaagagaaacgttgaaatggttccggcaaagaaagatgaggagaagaaagagaaagacgg

tggtggagagaagaaagatggtggcggcggtggtggtgagaagaaaatcgacggtggaga

tcaagcgaaagtggaggttaataaaatggggcatcagtacccgatgcaatctccaatgta

ttggtacgatggaggtgcggggcagaatagcaattacggtatggatcagtttcatcaagg

tggttattgtcattatgatcaacgttatgtggggcctgggtatatgatggatcaaggtgg

tggctatcctatgcaggcatcacaggttacgttttacatgaaccctaatcatcctccacc

gcagatgttcagtgatgagaatcccaatgcttgttctgttatgtgatggattgtttttga

tcgagtgtatacatgcttacacggacgctatacgcatgatgtaatttgtataaaaaaaaa

tattaagtatatataaatattaattccagggaaattttacgataaatggtagagttaatt

ttgattttaaatcaataatatttgatggagttattactattatataaaggataactggct

ggagaatcctgctcagatttggaagagagaaaaaaagtttattttatggaaaatgcttaa

tattaaaaataaatttaaaattctgctcattactcttattttatttatatggttattgaa

aagaaatt

>fava_dn_rep_c455

aggttaggtttcaaggcccaaaatcatataggttaaactaacaaagtttttggccaaaaa

acgctagaaaagtttgactttttccttcttgtttaacctagcacgctgctcgttcatccc

ccttcttctgcttcaccgttctccaccttcgactgtgatttttcagctgcaaccatggtg

agagtcagtgtgttgaacgatgctcttaaaagcatgtacaatgctgagaaaagagggaag

cgtcaagtcatgattcgtccatcttccaaagtcatcatcaaattcctcatcgttatgcag

aagcacggatatattggagagtttgaatatgttgatgaccacagatctgggaaaatcgtt

gttgaattgaacggtaggttgaataaatgtggtgttattagtccccgttttgatgttggt

gtcaaggagattgaaggttggactgccaggcttcttccttctagacagtttgggtatatt

gttttgactacctctgctggcattatggaccacgaagaggctaggagaaagaatgttggt

ggtaaggtccttggcttcttctattaggccagttttgggagtctttataaattcagattt

tgattaccctctgatgtaatggaggatgttttattcccttagtaatatcaagaattgcag

gagctgtttgtacttttttttaaattttgttttgcaaactcaagtttgtcactataattt

atgtgatgtcttctttattatctttcttataccaaaacatgtccttgtggggattgtttg

aatgggagatatgagaattagagatgttgaaattggtgctgttggttagtagtgttgatt

ttgcatttactagatttaattctgaaattattgtcatgtggctttgagaaaaatagaatc

atttgttgtgaaggtga

>fava_dn_rep_c456

ccctaagccttaagaaccctaagcatccccgcgggaaccctaattttcacagaagcaggt

gaagcaccgcatcgagagagaagaaccctaagaagatgacgaccaggttcaagaagaaca

ggaagaagagaggccacgtcagcgccggtcacggtcgtatcggaaagcaccgaaagcatc

ccggaggacgcggtaacgctggaggcatgcaccaccaccggatcctgttcgacaagtacc

atccaggttacttcggtaaggtcggtatgcgttactttcacaagcttcgtaacaaattct

attcccccattgtgaacatcgacaggctcgcttcgttgattcctcaagaggttaaggata

aggcgattaaggagaagaaagcgccggttattgatgttactcagcatgggtactttaagc

ttttggggaaaggggttttgcctcagaatcagccttttgtggtgaagacaaagctgattt

ccaagattgctgagaagaagattaaggaggctggtggagctgttattcttactgcttagt

ttttttttatttggtattgttttcgaacaaattagggtttttttgcccttttttaatttt

tgctgttttttattgttggtttagactgttttgatattgagatctatgctatatttttgg

gatttaaatttgttgtttttatttatttgcactttcctttaatttattcacttcagtgtt

atttggatctatcaattttattgcgggaattgttaggatattagggattcctatgttact

tatggattgttaggatatttgggttttcaaccatctgatttcagttacacattggtgtga

aatcaaatattaggttgccgaaatgaactttgatgaaattttgtcatgttaaacaccatt

tggctgatttattgtatgaggcactgttgttcgtctctaattattctgacttaaaatttt

ttttatttgtattgatggagatttc

>fava_dn_rep_c457

tttttttccaaaagctaagaaacaaaatttatacatgaaacttttctagttagatcatcc

aattgaggtaagaatcatctcatgataagcaactcataatgtgaacatttgtaaagagct

tctaaagaacagaaggaaaaacaaatatgagaaaatatgatttaaactaataaggatctc

aatgttttgtagcagcagtactagtagtagcggtcaagatgaacattacaatagagatca

aaatgaaataaggaatttatacacaacttcagtacacaagcttcggttcaaactcggcaa

aatctacagatcctgattcaataggtggcatcagatatgcagccaagagttcagacacca

gggcagcaatcagtggaaccctcttcaagttctttatcactgaattgtcatcactctcac

caatagcaagaatcttctggtttatcaccaccatccggtccaacttacgcttaaactctg

ggttctcgacatcaagaacagctgggaaaatccgtgcagttgttcggttcgtctcaatga

tgacatgcatgtcaaattctttagtgttaagtccgattccttcatagaaatccgtgcgtt

ggcaatcattcagatacattgtgacataaaccgataaacagaagaatcgtgaccacagct

tggccttccagtcattaagaaattgcggctgtgctttcatcaaggcagagaagaagtctc

catgcctgttctcatcttggcaccagttctcaaagtacttaaaaatgggataacattggt

actcggggttttccttgagatgtctgtatatggttatgtatctccagtatccgattttct

cagacaaatatgtagcatagaagataaatttgggcttgaaaaaagtgtacttcctagctt

ttgtcaaaaatcccaagtccagtgccaaattgaaatcagataaacccttgttcaaaaacc

ctgcatgcctagcttcatccctagacatcagagagaaaatctcagccaccacagggttgg

ttttcttaagcctccttccaagctctttgtaaagaagaaacccagagaattcagcagtac

aagacctctcaagaaactccacaaagatctgtctaagaggaccatcaagtttatcagcag

cttccttaaactccttgttcctcacaaaatgagtctgattgtaatcagttttgaactcct

gaagcaaagctagaaactcatcctcattaaggttcttgttaatctcagtattgaaaagcg

tctccatttcatcaaaatcagtggtgtagaaccttggtgtcaaaagggtctctttgattg

cagttttgggtttcttgcttggttttgttgaagcaggtgatgatgttggggtagcagaca

ttcttatggagtatgaaactgttctacgtaaagggttgaattttggtgtgatgttgctga

aatttgaaatgggtttcaccagagccatttcagctgccataattggaggaaaataaatta

tgggtttgtggagatagtgaagatcagaactaaaaatcaaaactttgaagtttttttgtc

ctgtgaggactaagcaaatgagtaatttagatgagtttgtgtgatagaatctcatccaat

gaaatctttgaaagttaattgaaacatgacatgtgtcatttgttgtatgtttgttnnnnn

nnnnnnnntggttgtggtggttacatgtagtagatgagaacttgt

>fava_dn_rep_c458

attctcattctattttctaattccaactccatcccatccttctcagatctaaccttctct

tcacaaataaaacaaatattcacatatttatattcattttctctctctctctaaatgcac

gcactatgcccaatctccacaaatttaagcatcactcacacaccaaaccaaagtgactaa

gagtattagtaaccaagtcatattccaagttcaaaatggcttccaaggttgctataatcc

ttgccctcaacatcctctttttcactgctgtaacctccaactatgttccatgtcctccac

cacccccaaaagataacagtcaccaccactcacatccaaagaacccaacatgtcctagag

acacaattaagcttggtgtgtgtgctgatatattaggtttgattaatgttgaacttggta

agccaccaaagacaccatgctgttctctccttgatggtcttgctaatcttgaagctgctg

tctgcctttgcactgctcttaaggctaacgtcttaggcattaaccttaatcttcccatta

atttgagcttggtacttaattactgtggaaagggtgttccaaacggattcgtttgcgctt

aattaatatgcttgtcttgtgtcactgtattcgttattcttcatatggtgtgtggatttg

tttttgccaatgaacgcttcgttcatacatgatttatttgttgttattactatataatta

ttgttgtgatggttggaaattgaagcaaatttgtcttctttttcagcttcactaatgatt

agtcttttgtattccatgtaagtgaaatttcttgtattgataaaattcttcgtttaagat

cggggtattttgacatgatatgttctgtgtttgcttttcatgaaaaacaaacttacttta

ggttagttaaactgcagaagaaataattacccctagtcttaaaataagagttacatgtgg

accaagannnnnnnnnnnnnnngttctataaaaaaataacatttaaaattta

>fava_dn_rep_c459

caagaaaaaaaactaaagagggtcttatataactatgttgttcgtgcaaacaagagaaaa

gaattagaaaagtaaaatcctctagtgttttgaatcattgcttcattacaagagcactaa

aaacttctttcattttttctgtcgacttcaattttctatgaaccatagaatactaatgtc

aagacatagatagatcaaccaaggtttcatattttaaagtaaataatgaattagatttca

aaactcaggaaatctaggattgagtcatacatagagaacaaacacaaagggtcaaaaaca

agtaatcctcaaatgcaacaaaactgttctggatttggtttaacttaagcgtgtctattc

ctcttcttaaggccagatttggcaactttgaggcttctgtgaactgcactcaaccttgca

agagctgccttctttaaatcaggcctatagtaattgtctcccacctgattttgaacggcc

tttgccattctgcggaactccttcttcatcactgatctctgagacaaaacagaaggctta

ttctgtttcctcggcttcgttgtagctaacaacacagattgatctttaccagccgcctga

atagcaacagttttcttgtttgccaatccagagaacttgtaagagttgagattgtagagg

ttgttgctctctctactgaattgaacgctctggttgccatttccgaactctttgaccaag

aaggagttgttcttcttcacgatctcccatacgagtggccccggaacggtcgacattttc

tcctctcgccgccgagaacaacgaattagggtttcgtttggtttgggtttatatatattt

gcagtt

>fava_dn_rep_c460

gaaatacactccatttcattccataattttttttaccctccaatttgggcaaaataacaa

aattactttattccatcatggtgtacccaaacaatgtaatatcatcttcattccattttg

ctctgttccatcccattccactccattttttttacgtatccaaggcttaaggtcttgcag

gtctctctgcaccccaattacttaaaaaaatcttaatattttttgaattttcattcaaat

gtttattgtgttcacaataaactccatgtatagatagatgaaaagttaaaaagaagcaaa

tagaaatatatttcacctatatataggctgctaaatcattacactgcacacaagtcataa

gaaaatactgaaagttgaaaacatagaacagaatgataagtgtgccatagagcaaagttt

ggcacataactatcgcatcttcatacaagtaagactcatctaaacatctctcaaaagctc

tctcttacactaaccacggcaacatagattgtgaaaataagcttcttctacttagacttg

attctcttctcaacttcctccatactttggatatctttctgaaacttgcaaatccggaaa

atagaccagtagagaaggcatgctgaaatacacagtatcacgtttctctgagctttgtat

gtagttttctcatatcgatctctctcagcagctgtacaaacatcggaggtacacgacaac

ctatgctcattcttccagtatatatcaaggagctgaaacccagcaaagggaacaatgaaa

agcgcaggttgaagaatgagtgaaatcagggaagtcaaacggttcttcaagggctttggt

gatggaagggttagaagaatcgctatagcagcttcaatagccaccacataggtgagtatc

atccactgcaatgccatctctcaatcaaaatcggtgctttctga

>fava_dn_rep_c461

gtttcccttttctccgagtttagggtttttagaagtaagcataacaaaacaactacggtt

acacactttcttccaatttttctggcggcgattcaagagcttcgaaatggcaagaatcaa

ggtgtatgaactgaggcaaaaaacgaaagcagatcttctgaatcaattgaaggatctcaa

agcagaacttgctcttctccgcgttgctaaggtcactggtggagccccaaacaaactctc

caaaataaaagttgttcggctttcaatagctcaggttttgactgtgatttcacagaagca

aaagtccgcgttgagggaagcttataagaagaagaaatacttgcctttggatttgcgtcc

taagaagacaagagctattagaagacgtcttaccaagaatcagcaatctttgaagacaga

gcgtgagaagaagaaagaaatctatttcccattgaggaaatatgcaattaaggtgtaatg

ttacaattgttgcatgacttctgatttgtttttgacttttatttgaagtgtttgttgttg

ggttcagtttttctttcctcttgagtttaactaatcatatcaagaaatgaaattattaca

ctagttctctttgttttccccctcattccagtttccgattctttattgcttgaacaggtt

gtttgctttataaactgttttttgattctgtgtaattcgttatgagaatgtatacaaata

ggccataatatatacaatatctctcacctgcattgtgtgaagcttatcattctcaatgtc

t

>fava_dn_rep_c462

aaatttgttgattggcaggtaatggtcaacctgtcccttaattttgcaggaaatatagca

acaaatcttctataaattcagcacacattaatccaaccttgaatccaaaaaaaatcaaag

aaatataacagagaaaaaaaattatgggttctgtgtctgaaactgtttgtgtcaccgggg

ccgcgggtttcatcggatcgtggctcattaagagactcattgagcacggttatacagttc

gtgccacggttcgcgatccagataacattcagaaggtgaagcatttgttagaactgccgg

gtgcaaatagcaaattgacactttggaaagctgatcttgctgtagagggtagttttgatg

aagcaattaaaggttgtacaggagtttttcatgtggctacacctatggattttgagtcca

acgaccctgagaatgaagtgatcaaacctgccataaatggattactagacatcatgaaat

catgtctcaaggcgaaaactgttcgcagattggttttcacatcatcggccggatccgtca

acgtcattgaaaatcaaaaactcttgttcgacgagagctgctggagtgacgttgagtttt

gtcggagagttaaaatgactggatggatgtattttgtttctaagacacttgctgaacaag

aagcatggaaatttgctaaagaaaacaagatggatttcatcacaatcattccacctcttg

ttgttggtccttttcttagtccatctatgccacctagcttaatcactgccctttctccta

ttacaggaaatgaggctcattattcaattataaagcaaggtcaatttgttcatttggatg

atctttgtgaagctcacatattcttgtttgagcatatggaagttgaagggaggtacatat

gtaatgcatgtgaagctaatattcatgacattgcaaaattaattaatgcaaaatttccag

agtacaatgtccctacaaagttcaagaatattcctgatgagttggagcatattaaatttt

cttcaaaaaagatcaaagatttgggattcgagtttaagtacaatttggaggatatgttca

ctgaagcaattgacacatgcagagaaaaagggcttcttccaaaaactattgaaggtccaa

aaaattaatggagtcacaaagaaataatcttacgttatgtgtcgccatgtgtgtgttatg

gtttaaagataatgtttttctttatgtgttgaatgagttttcttttgtagcctgaataat

tgtgcaagtccaagatctcattttatattctaagttattgttaagcatgttgtgtttcaa

gtttgaaaagtttcatccaagttttgatagaacaaaataaagtggactttaaggcaatga

aacacaaaattgacatcatgagaaaaatctatgatgcagaaaaaaagagacaataacatt

tgtatttattttatcgtatagacaataatatcttagaccaaaaaaatatttttttttgca

gtatttactcaatccaaatttttttgttgc

>fava_dn_rep_c463

gggctcttgatttgattgttttaattttacactgtttcagactctaacaggaccgagtct

cttatttagctgaccatccaccacaccaccaccacacactcaccgcgctacaaaacataa

catggccaccgaaaccagcaacgacccaaaatccgtctacgatttcactctcaaggatgc

taagggaaatgatctagatcttgctacttacaagggaaaagttcttctcattgttaatgt

tgcttccaaatgtggcatgaccaattcaaattatgtggagttgaatcaactatatgacaa

gtacaaactaaaaggctttgagattctggcatttccaagtaatcaatttggtgaggaaga

acctggaaccaatgatcaaatattggatttcgtctgcacccgcttcaaatcagaattccc

catctttgataagattgaagtgaatggagagaactctgctcccttgtataaatttttaaa

gtcaggtaaatggggaatttttggagatgatattcagtggaactttgcaaagtttctagt

tgacaaggatggccaagtagtggaccgatactatcccacaacctctcctcttagccttga

gagagacatctctaaactacttggtgttgaatgaatgcatccatgaactcatcttggaat

ggaagagaggattactgtcttcttgaatgttaaatcatcacttgttgactttatataatg

tttcctttttgaattcgtaatgttttgtacaatggattggtttctcacaaactgaacaag

acttgaatatccttgcattcttccatccctttaactaagatttaagtgctttttacaaag

taacttttgttaattctacactcactatattgagtcctgaac

>fava_dn_rep_c464

atctcgatttaataatacagcaaccgaaagcggtgtcgtttcacgcctttcatcataaca

caacattggcttctccgtgaataaagttactatattatagcaataatcatcttcactctg

gatctcactaactctttattctcttcaaactctatcactgtttttcctttctctgatcta

aaaaattcaaatggctaaagatccagttcgtgttctcgtcaccggtgctgcaggacaaat

tgggtatgctcttgtccctatgattgctaggggagttatgctgggccctgatcagcctgt

gatcctccacctgctcgatatcgcgccagcagccgaggcactgaatggtgttaaaatgga

gttggtggatgctgctttccctcttcttaaaggagttgttgctacaactgatgtggttga

ggcatgcactggagtcagtattgctgtgatggttggtgggttccctaggaaagaaggtat

ggagaggaaagatgtgatgactaaaaatgtctctatctataagtcccaggcttctgctct

tgaaaaacatgctgctgctaactgcaaggttcttgttgttgccaacccagcaaacaccaa

tgcattgatcttgaaggaatatgctccatccattcctgagaaaaacattacttgtttgac

aagattggaccataacagggcactgggtcaaatttctgaaagactgaacgttgaagtttc

tgatgtgaaaaatgttataatctggggtaatcactcatcatctcagtaccctgatgtcaa

ccacgcaactgttaaagtctcttctgtcgaaaagcctgtccgtgaactcgtagctgatga

tgcatggttgaatacagaattcataaccaccgtccaacaacggggtgctgctattattaa

agctagaaagctttcaagtgcactatctgccgctagctctgcttgtgatcacattagaga

ttgggttcttggaactcctgagggaacctgggtatcaatgggagtttattccgatggatc

ttacaatgtaccagctgggctgatctattcattccctgtcacaactcgcaatggcgaatg

ggaaatagttcaaggtctctcaattgatgagttttcaaggaaaaaactggacttgacagc

agaagagctttctgaggagaaggctttggcttactcatgtctctcttagaatgcttgttt

ctttgagtagtgtcaatggaggttccattgtttttcaagtctcaatttttgaataatgta

tctctaagatattgagaaactattgatggatatggttttgccttttatgcaaatcatcac

ttgtattgtaatctcttattaagctccttctataggatgtttgactttgagcatatgcca

tttgaatcaggattaatgatatcatttgaaactttttgttatgtgcatcaaataacttat

tctacattaaagacttttgtattgcatatgtgaataacaaatgaaaagtgacatcttttt

gcgatatttatt

>fava_dn_rep_c465

agatagggtgtcaagtggcaaattcttcaaaagctaccaaaacaaaccaattccaactgt

ttctcaatcaaaaaatggccaccctattttcatctccatctcttcactcagttcgtcttt

cttcttctatattccctgtttcttcctcttcttctcaatctcttcaattttgcacttttc

catgccaccctcgcttgaaggcactgtcaattaaaaagagccgctgtttacttgtggcta

aggcagtggaagatgaaacccaacaacaacaagaaccagttgttgctgagattgaacaac

aacaacagccagcatcaacctcggagcaacaaaccgttgttgttcccatttcaccatcag

acactctcaccatgctctttcaggcagagggaacattgaatgaaacagctgttccatctt

tgaccaaagttttagagaagattgagggtgttaaaaatttgaaggttaatgtatacgagg

gactcacaactttagagttggaaaagcagacaaccatccaagcaacaggagtggcttcta

gtcttgtagaaaccatacaaggtttaggcttcaaattacagacattaaacctgagttttg

atgatgaagaagttgttgcagttgcctagacaaaatctacttgtgtaacaatgattagac

tccacaaaatttcttgattaaaacctactgcacaaaatcttttctcttttgtatctgaag

ggtgttaactaattttgtgtattattagtttttctctgcattcatacagtccaggtatcg

attatcagctgcattctttttgatattggtttagtgggggttcaatgtggtagtagagtt

cctattcggatagttttaatctcttttaaaaaaaagttatggcatctttccatgacgaat

gggagcatcttgtttgaactgatcttaaattctcgcctcataatttagttgacacccatt

ctcttttggcaga

>fava_dn_rep_c466

cgcggccgtaatggccactctgggctctaccaaaaatctgagccagaaggttttcgactc

cgcgcggcttctccaagaacctcctccgcaattccatcatggctgaacaaactgagaagg

cttatctcaagcaacccaaggtgtttttatgctcaaagaaatctggcaagggtaagaggc

ccggtaaaggtggaaatcgcttctggaaatccgttggtcttggattcaagacccccaaag

aagccattgaaggagcctacattgacaagaagtgtccattcactggcaatgtttccatcc

gtggtcgtatcatagctggaacttgtcatagtgctaaaatgaatcggactattattgtca

ggaggaattatcttcacttcattaagaagtatcagaggtatgagaagagacattcaaaca

ttccagctcatgtgtcacctgctttccgtgttaaggaaggtgatcatgtcattattggtc

aatgcaggccactctccaagacagtgaggttcaatgtgttgaaagtgatcccagctggat

catccagtggtgcaaagaaggcattttctggcatatgagatttggttggtcctcgtagta

tagagatcttatcatgtatgttgaaagtaattccgatttctgtatggtgaagttcttaac

aattttgataattttggtttgcttttgaagttctgtactctgatttgataagatgtttta

aattatgtttatttttccagacatatgttattatggttgttcaatactagttgcaattat

tttgaaaaaaaaaa

>fava_dn_rep_c467

atcacatgttgtctctcacatgcgtataatatatagctataaataaatgattcagtagtc

atgcgaggggattcatccaaaaactaaataaaatattactttactttgattcttaaagat

gagaactacttttgccttatgtcctctcttgcttctcttgtttgtagcagcgatagaatc

aagaaaagacctaggagaacattggaagctggtcatgaaagacaaagacatgccagaata

tattctaggtatgcttgatgcaaacactgacaagaacctcaacattatcaaacaatcttt

taaagattctaaagaaaattttgaaccaagacctaatgtttcatcctatgaggatgatga

cattggtgttattgaaaagaagaaatttgtcaaagattttgaaccaagacctaatgtttc

atcctatgaggatgatgacattggtgttattgaaaagaagaaatttgtcaaagattttga

accaagacctaatctttcatcctatgaagacaacaatgccaataatggtaaggaaaataa

aaaagccatcaaagattttgaaccaagacccaatgtttcagcttatggagtataagagac

atattgtagcatcatttgtacgtgtactgtagcatgttttcacttgaatctgtagcttca

attgtgttttgtagtatgctatcatctgtatctgtaggctatcatccgtacttgtttgta

gcctatcatgtgtatctgtgtatctgtgtgtcttattgtaaaataaagaattaactcatt

gagttagttaattagtgagtgagaattgttagtagctggcatttataacctgttaaagca

ggtcatttatcaacaaatatgtgtgcaccactctaattgaatttgaagtaataataaaaa

atagtttaacagaattgtaaatcaaaaaaaactctagcacctatagataatatgtgaaga

gagagaatgaaaactcaaaagtgtatattgtgaattgtgattgtgatcaagtggtgtata

tctttcatgaagatgtggtgaaagttgaaaaatgttcaacggtttcaaagggaattaaag

agagtggtggtgggtgacagaacatcatagagagagagagagagagaagaagaaggtgag

aacaagaacattaactagagaatgtgaaaaaaggaaaaacaaaaaacatgagtattgaat

agtgtttgaactatgcaatgtttgtgtctgattttctctttcttagtgtgtgtgagtcag

ttgcataagtcattttcagattgcataagtattttttttctaggggtgaggtatttctct

ctcctagtttgattagttatgaatgatgattgtgat

>fava_dn_rep_c468

aaactaaaacatacagatttcgaagacattccttcccatcaatttgaaaacaaacatcca

aagtacaaagtgagannnnnnnnaaatttgtgcaagttttatatcacattcccagtataa

cataatttcatttgtgcctcaacttaaataactccaaggtcttgtaaatctcttggaaac

ctaagccttactaaaccattcatcatgagaaggctattactacattcatacacaggataa

taattattagaaaacatggatatcataaggaacaaaaaacttaataattgtaaagccata

aaaatctaatgatacacaaataaactcaagattatactaagttcaatagcaaaactgagt

aacacatatgtctcggttaaataaagaacatttcttctaaccattaaaaacttgagaaaa

ttgaagtaaatgaaaacaagatagatagtcccccatcatgtgaaaccataataaaagtaa

acaagacctatttcttcttatctccagcaccaccagccctcatctttcttagaacattgg

acatctcttctctcttcttcttggcacgcttgtgggttccgagcttcctctttgcaactt

tcaatgccctcttgtcttttccaaccttaagcaactcagtgatacgtttctcataaggtg

caaatccagctacctcccttatgagatttctcacaaagtgaactctcttgcttgttttcc

ccttgcgagctgagggacgtggtgccaagtccttcttggtgacgatgtgacccttgttca

atccaacgaaaagacccgtactgggttgcttaggagccatctctcctctcttgccggtga

tttccaaaaccctaacaccagagtgtgcggttgaagagaagaatctaaactagccccgcg

gcc

>fava_dn_rep_c469

aaaaaagcgcccgtctcataaagaacaaaataactgcattctaaattgcgccgtgtgcgc

acggttagccttatctgattctatctatcattcattctttcagagtaacaacattcatct

tcttcaaagcaatgacaccaatggcatcatcctcttcactcaattcaattcctatatccg

ctccttccatttcttccccacttcgtgcattctccactcctctctttcctcaatcaaatt

cccactttcaccaaaccaggaaccaccatttcaacttcaccgttaaagtacaacccctag

atttcaccggttcgttcttcgaaggcggattcggttcaggcgatgatccgatttctcccg

gaaccggttttacaaccgccgttgaagagaaaccggaaccgcaatgcccgcccggactta

gacagtatgaaactatggttgttttgagacctgatatgtctgaggatgaacgtcttgaac

tgactcagaaatatgaagagcttcttgttgctggaggtggtatgtatgtagaggttttta

acaggggtattattccactagcatataccatccagacgaaaaacaaggacggagaaacta

acacgtatttggacggcatctacctcttattcacctacttcacaaagcccgagtccataa

ccgcccttgaggagatgctaatgagggatgataacgttatccgatcaagtagcttcaaga

tcaggatcaggaaaaggaaatattagtttatcttggcgtctcgagtagctctgataaaag

gatggatagaaaaaaaatgtagcattcatcatttgttgctgtggtatgtttttttcattt

tcatatggattcaagtatttttgtaacatggttgtatcaaattaaaagagggatgtatgt

gctattacttaaaagccaatgagtctacagaattttgttaaaggggttcgtattagacaa

acagtagaaactgtcacttgaaaaaataacatgtttctaattccttacatataatcacag

gatccagtaatgtgaaaaaaacactaatacaaaatctgcttgagcttgaattccacttag

ggtttgaatggagcaaagacaactactgaattgtgtccaccaaatccaaatgaattggag

atagcaacattaacttcatgctttttcttaaaattagggacagtatcaattgtaacatcc

tcttccaaattatcttggttaatagttggatgcaaccatccagtagttattgttttgata

gttgctatagcttccaaaccaccagcagccccaagacaatggccaatcattgacttagtc

ccattcatttttagttctgatgtatccttaaaaacctttttgattgcattaacctcagcc

aagtcaccagctagtgttgatgtagcatgagcatttacatagttcacctcttcaggggaa

acaccggcgtcttgtaaactcttgcttatgcaagatgaaactccaaatccatccgatctt

ggatcagtcatgtgatgagcatcacatgttatggcaccacccaaatattcag

>fava_dn_rep_c470

cacctcgtcaacctagcaagcaaagccttagagcctaaaaagaaaatatttaaagagaga

ttcttcaagtgaaaatgaaagtattgaaaaagtatgttcttaaaaaggatgattgttatt

ttgtgaaaaggaaactcgagaagcaagatacaccatagctctaacttttataatctaatg

tgatatagagagatttctcgtagtagcctctttgtgccacaactaactcgtccataaata

taactatatactatcctacaactcttttctaagttcgtttaatatttcatatgatctaca

tgagagagtttgaaaatgactctagtttgagatttgtcactagtgtgtgcaaggccacaa

ttgggttcataaatgtaccaaaacagaaaacaagtttttaagaataagaaaactaatgct

caagaagatcaagagtcacatattatgatattacacacaacatatagttcatttctgaat

acattatacagataaatacgacaatgagagaaatactaaaataccccttagctgggtctg

aaaatcttctcagcctcgcttgacaggatcgaaattggaaacaccgataacagcaccaag

aatggcagtaaccacaactccaccagaaacaatactgagcaagaaattcttcaaagaagg

tgaaacggtagcagcttcagccacatcaggaatcaccatttgagctgtcaatgcagctgc

tgtcagccctgtcacaactttctccttcagcgatgctttcacttgaaaccttccgtttga

ctttgatgatggcctcagagtcagtggtgttttgaagagtgcatgaggggttggtggttc

tatcttcttggtggcggcacaggttactggcacagccattgagattattgaagttgaggc

cattgaattggttgttcggagtcaaagggagtgggtgtggtgagtgtgttatgagagaga

ccatggtggagactatggagaatttgaggtggagattttttggaatatttggttatgtct

gacgcggcagcttctcagtgtgaaatgaatggataaggttatctcttttgtgtaatcatc

atcaact

>fava_dn_rep_c471

agtaaagtgactttttttttattgtaactattggcgtcatattgtatctcaatagttatc

ataaggtttccgggaaatcccgaactttacaaaatcaagaattttgcatacaacttatgc

aacaagttttgtttattagaaactcccaacatgcactaacatgtaacaatgacagaggcc

acaaaaaagtaacagaggcttaacacattcaatgttccaggagccaactggttcagaagc

tattaaatatcttcgacatcatcactcagtcggtatcaactattcaaattatatcacata

caaataaatgaaggtgatttaatccacaatctagtaatacagaacaatttgattgcaaat

ttttatttattgcaaggggtgaaaaaaaagcttcagttgttgtcttcacggccataatca

taaattatctcaatcccttcaggtggaggtgggttgcgagtaaacttggaaccaccaggt

gcccatggaacaatataaggatcaggatggcgccatttgttgtagctagcttcagcatcc

actattaacctatcaatggtatcaggatcttccacgtgcttgttttgctcgaaccgatcg

cggaggttcgcagcgtcgtcgtaaaataggtggcgatgtacggcccagttgagagtgtct

ttaagggcgcggcggtatagagtccgaaccttctccttctgcgccgcgcgccgagcaacg

tacgctgccgatgttaagctcatttctcgatttcttttcaatctcttctctttctctttt

ttcacttccactttgtaacaatcaccagaatatcaaataaatcattttgatctttggtaa

attt

>fava_dn_rep_c472

aaagacgatgatcgttatcaagacgagtctctgaaaagaaaagctataattttccatctg

tttgtggagtaaataattttgaatttgttgttccaaatggatccagaacagaccttcata

cgtgttcaggagagattctcgcagatgctaactccgaaagtgagagtgactttggagtat

ttatatctattcatagccatcactttgttctgtattcttgttgttatgcacgccaattac

gttcaacagcctggttgttcaggtgagctatctcaagtactgaaattagaagcacagctt

attcagattaagataaccagtgctggtttgtggtcacacaacgattccgaatccaacata

atagatcttcctgaagtagaagctgtaaaagatagcacagaggtttctgaagtcaatcaa

gatgattcgacatttttaggttccaagtttttgtggaactggattggttcaggtgctagg

aagggaaatttggcattcaagttttggaagactgaaagtgaatttcttgaacatcaagct

gagacctctacaagcaaccaaaaccccaggccagtggttgaagatgccgtcatcaaaatt

gataaagaggagccacgaagtagttttaccttatcagccaaagagacactaaaaggggca

attatacattttggtaaaaagtggtataggcgaatctcattcatttggagacacacaaag

cagataattggaagtttccagaagctgtgggatattactggtgtacacctcaatcttgat

attcccaaatggatgcgtattcttcatttggacaaggttaactctaatgcagtgcaatgg

cttaaaaagaaaacaaagttgtctgaacccacatatctatacaccatggaaaaggggtat

ttcttgttacccgaaagtgccaaggctcaacataatatccgtactgtgaatgtaagcata

tcagcctggcactcttgcttcggaaacaggtggcaacaactactcataaacagatttgta

ggatatgatacaattttaatcaacagtttattgagttctcctggtcaaggttatctttat

aattatcaatcaaaggaattctttaaccttagttttgctctagaaattcctgaagttcca

gataagtttggagactaccttgtaacgaagtgtggcgtgcttatgatgtctttatttgtt

ttttttactaccacaatgtccgtgtcatttacattgagagaaacacagactcgcatgctg

aagtttacagtacagcttcaacaccatgctcgacatcaccttcctacatttcagttgatc

tttgtacatgtcattgaatcactggtctttgttcctattatgattggcattctgtttttc

ctttttgagttttatgatgatcagcttcttgcttttatggtcttgattcttgtctggttg

tgtgagctatttactcttattagtgtccgaacaccaatatcaatgaagttcttcccccgc

ttctttctgctgtattttttggtctttcacatatatttcttctcttatgcttatgggttt

tcatatttggctctctctacaactgcagcttttatgcaacaccttatcctctatttttgg

aatcggtttgaggtaccagcattgcaaaggtttgtgcaaaatcgacggtcacaacttcaa

caacacccagacttccatattacctcatctactattcttgcatcaacactacacatcaca

agactgaacacaaggaaccagggagtaaatgccacaaatatgccttctgggacagggttc

agacctggttttgatcaatcaatgcctcagaacggaacaggagttactgagcctcaagga

cagtcagaaagtaaccctgcacatattcctgggcaagctgaaaggggtcccaaccctgga

tccatgaatcttagttctatgctattatggatattaggaggtgcttcatctgaaggcctc

agctcatttctttccatgttcagagatgcgagggaccaaggacaagtttataccgaagct

cctaggcatgataacagtgagaatcaggagagtgacagacaatgatgaaaattttctatt

gaatggagaacttttaatacaggaaggaatggtgtatattattttttggttttatgcttg

ataatatataggtggcccgaatgctttctcgttgttccaaatgaaaataaaaccgaaatc

aggtccagatctttaccatataaggtgtagtagtgaaagtgtgtagagactcacaaaact

atcctattatcaatggctgtttcatcttatcaatctatcatgttaagctctatggccaac

atttctagcaggtctagagttaaccaaattagcaacatcccttctgtctacattccaagc

ttgagaagaaatgttagcctcaaagttcgatctatggctgaggaagagcagaaagagcca

attgcaccaccatcgacaccaacaccacaaccagcctctactcgtataccaaaaatgagc

acaaagttttcagatttgatggcttttggtgggccagcacctgagaggatcaatggaagg

ttggctatgattggttttgtagcagcaatgggggtggagatagcaaaagggcagggtttg

tttgaacaattgtctggtggtggagttccgtggttcttggggacaagtgtattgttgtca

cttgcttcattgattccattttttcaaggtgttagtgttgagtctaaatctaaagatgtc

atgtcctcagatgcagagttgtggaatggaagaattgctatgttgggtttgattgctctg

gctttcacagaatatgttaagggtacatcccttgtataatagacttctttggaaacacat

ttaatgtctataggatttgtggttagaagccaatctaggaatcgtttttgtatattctta

tgtagttactcctttgaagtacttcatattcccatgttactagagaaatgtaattgaaat

ttctttctacttgaaagagcttctctaatttctgaatgatagaaaaataaaaatttgata

tattgaaaattttcattaaaatgctgagannnnnnnnnnnnnnnnnnnnnnaaaataaat

ctaatagactttgat

>fava_dn_rep_c473

tgatttaaaaatgaaaaataattgtccagctccaaaaatacaccacacaaacgttaccca

acggaaaactatcttaattcttaaactgctagtaacattttactgttactatattgccaa

ttgaagaggaaaattgtggtacaatttcaaacaaaacttcaaaatatttaataggaaaca

tttcttaatcacgaaattcttctggtattcaaccttaaaacaaaagataaaagaagtcta

gctcctaacataaataatcaacttcaaaatttttaacatcatacatttttcctagccatg

aggctaaattacagataaaaaaaactagaatgcattgaacaaaaaaacattaatgccgaa

atcaagcatcggcgatggtaacctcaacttcaacaccaggttcaatggtgatggaggtaa

tctgcttgaccacatcaggtgaactgtagaggtcaatgactctcttgtgcacacgcagct

caaacctgtcccatgtgtttgtaccttctccacagggtgatttcctggtggtaatgtgaa

gaacctttgttggcatcctcactggtcccttcaccctaaggcgcttatccttagcaccac

gaaccaaatcactgcaaaccttttcaagattttgaacatgttttgacgacagagtgatcc

tgatcttatggatctgctcttgtgattcttccaatccaggcttagtgggttttggtgcag

cgtatgccatgttttcccctgttacttaaaaaatgcggctggttatgctctgttcttagc

tgcttggggaagatggaagcaattcacggccccgcgggtttaggggatttctggtttatg

ttctctttcttagggttttctttgggct

>fava_dn_rep_c474

aaattaaatatttttttatgtcattttatatttcaaagtaactttatcaattagtgttac

taaacactataaatttatttagttagtttatttgtttcagctaactcactagccatccat

aaatttgtaattagaaaaaacttaacctttatacttgatcgtaaatttaataacatggga

catggcttttaccatattgtcatagcaagtagcaacctgtagtaactgatatcttaatat

atattttttgaaattctgcttaactcaatggaaaacaatttactaccgcagtgaaacaat

ctagttttgaccattatcccttccatcacctcattcacatcaaatgttttatgttcatca

attctaatctcgcatttctaaattatgcaacatgcattacgtgaggggaaaccaaacgaa

taaatttaattttcagtcttagcctccaggaaccagttgaatcagcttctctgcttgatc

aggaaagaaggtgatgaatagatacgcacaaataagaacagttcctaacacagctgaaat

tttcccttggatttgcaccaaaccagctttgccagcattctccaactcggtcgaaccaac

cgattcaaatgtccctggtttcacagtttgaagtccagatgatatgcatgctatattttg

gtaaccagcttcatctaacttatttgctgctgcagcagacctcattccttcctgacatac

aaccaacagtttactttcaggtgatatctgactcttgacagattttaagaaatcaggatt

tggtctagtgaatggtatcccaaagaacaacccagaaaaattattgtggacagtcctcaa

caagattgtaccgggatcattgtctgtattttcaacaaaaagaggaacatgataacatgt

tttaatatgagctctctcaaattgagatttgtcccgaacatcaagaacattaaacccatt

tactgctacaagttccttagcttcatcagctgttacaaattccacttctgcttttattct

aaagttccttctcatttgacattgaggtagttgtttccttgctagggtccttgtattatg

agtttttaaaactaaattgcaggaggtgtagaaagcagtccctgccattgtgaggatgtg

tattttcattcttcccgcggtcgtaa

>fava_dn_rep_c475

tagtagtgaatcaatgaaagggaaaataaattggaagagcatatgcaagaaaatcattca

ctttgtattatattttgaacaatacacaggttataatctctatgtgcataactatctccc

ttaaatttttctttcacttaaaaagatcgaaaagggtaaaatggaaatgaaacaaaaatg

tttgctgtacagattgccacgagttaaataatgtatcataccacaaaactcctcaatatg

ggcagcacaaatgtattgtacatgcatctttttaagcatctttttaagcagctcacaagg

taacacatcaaccacaaaatcataattcaagatagctcaaattagaataataccacaatc

aaacaattcaaataattgaaatgttgacaatgcaaattcacagttcaaggttgaactatc

taattcggctgagtttttcaatagataatacgttatctcattttttgaatagatgcatat

tactttgtcacggtaaagcagaaaaattcttaagttacttgccttgacattctaatagtt

tctttcttaagcaggaattataacatctatgatattccagcttatagatgtctcataaga

ataacaaaatcaaccactccgggcttctctccttgtggcatcgtgcaatagttcaatgtc

cacaccttctggaggcaattgcacatatctcaccaccgatcctctgatgaagcagttcct

tacagaaagcatgtgagggtacttgtcttgatcaacaacacgagtattttcgagcttgat

attaaggtattgatcaacagaatgcaatgtccctctgatggccaaatcgttcttgagttc

taccgtcacttctcttcccaccaaatctttgaagtacgagaaaaacaacattttcgctac

tatgaagaactatagagagtgaaagcgaaaaagccagtggtagtggtagaaaacaaaggg

ctcgaccccgcgg

>fava_dn_rep_c476

tttttttttcaaacagataccatgttaatttcggaataagctcattgaattattatgctt

ttgaaatgtacaagacagtactatatatactgttaagtaacaaatcaccaaaactaacta

aactgaattatttgcatcatgtaataagcttgttaatatcattcttaaaacggcctgatt

tcacaaatatctccaacttatttagattaggaccctccttagaatatgtagatgcagttt

cgacagcctaacttatattgcataatgcaaccccttggacagattcaccacatgcaacac

tttgccaaaaacatcataatagacaactttggcattcattttgtcaagttcaacagacat

aaatccttggccatcgtagtaaaatttgactccatatcccccatttttatcaaagtcccc

tttccatgcctttgaacctccaccactagttaagaactctatttggctgctggtgctact

tatgtgttccaagcaatggtcgtgcccattgatgtacatatcaacactgttggcctcaag

gatcggtaggaggtgtgttaagagctctttggtgtcgccatggtgccctatgcttcgtac

aggatgatgtcccacaacaatcttccacttggcactcgaatcccttaatgctgtttccag

atccttcaagaggtttgataaatattttttcctaggaagcacacctctccaatcatactt

gtgatcctctggcttcaaaaagtacttgtccacaaaaggagttgtatccacaaagaagaa

ctcggcgatttcattgtgaacgataaaagatctttggcaaaaccatctatggtcaatagt

ctgaagaattggatttaattgagcttcaacatcccccctatagtcatggttgcccaagac

attataccattgcttttgaaggctgttggcagtgtagatatctgagaaggaatattgaaa

tgcagggtcatgtataccagtcaatccatcgtcatagaagttatctccggttgataccac

aaagtcaatgtttaatttgtcagcaactcttcccatctgaaaagcaacttgagactgatt

gtaagttcctctccttccccagtctccaatgacaaggaagctgagagatccatctgcttt

gacagaagggtggtcaaagtgttggagctcagctgatgctacacttacagcaacaataac

actgatgaatgcaggaatccgtagccagaagaggcttaggcttgttgttcctcctaaacc

acccatgttatgttgtgtttgtgtgcagtactcctaacagacagaacactaattatatat

atatagagagagagctaaggaaaaggatagtaatagtgtccatataatgtgatggatacg

ctacctagctgcttcaaggaacttatgat

>fava_dn_rep_c477

gacaactaaatttactatattacctctcctcttaaaagcaagatgaaacttcaaacaatc

taactaaatttcttggtcgatcatcaaactaacaataacatagacaaggcaaatttgata

gtttcatgaacaacttagtacatagattcacaaaggataagaatatgaaaccaaaacatt

acatcccaatgttcatttcattacaatacaagtacataaaacactaaaatacactcagtt

gcggcaaatcctagctgttggcatggtaacacaaacaggtgcatatgctttcttagcttc

tggagaaccacgtttaggttcatcagccaaagccatcccagcaacagaacaaacagctgc

tgctgcagctgcaaacatgatgttcctccttccattggtttcttccttttcattctcata

actcactttcatcttttctacttcaactcctctagcagcatttgccaccaccagcttcct

ttgagttaccactggtgatcgggtggcgaccgctgagctgccggcgaagaatgatgctgt

catggttagtgatgccattgatgtggtgtgtagtggcgtaatcttgattgaattctgaaa

actagtgcttgtatgtgtttgttgtgtgtgagaagttaagttcccgcggccgtaatggcc

gcggaacttaacttctcacacacaacaaacacatacaagcactagttttcagaattcaat

caagattacgccactacacaccacatcaatggcatcactaaccatgacagcatcattctt

cgccggcagctcagcggtcgccacccgatcaccagtggtaactcaaaggaagctggtggt

ggcaaatgctgctagaggagttgaagtagaaaagatgaaagtgagttatgagaatgaaaa

ggaagaaaccaatggaaggaggaacatcatgtttgcagctgcagcagcagctgtttgttc

tgttgttgggatgttatttataaaacttctgaagttgttaaaaagagtttaactttagga

gataggtttttggttgatccatttcaaagaagtggaatttgtaatgaatctgaaagtgaa

agtgtttacaagggtttaactgtgagggaagttgttaggaagcgtagaagtgcggttgat

atggatggagttactggaatggagagggaaacattttatcagattttgtcacattgtctt

ccatctggttcagaaggtggaaagaagcaggggaaacaactctctttgccgttccgagcg

cttccttgggatgccgaggttcatgctgctttgtttgttcatagagtgacggggttacct

cagggattgtatttccttgtgaggaatgaagatcattttgctgagttgaagaaagctatg

aatcctgactttgtctggaccaaaccggagggttgtcctgatgagcttcctctatatgaa

ctccttaggtcggattgtcggcagcttgctaagcagctttcgtgtcatcaggacattgca

agtgatggttgttttagccttggtatgttggcccgtatggaacctactttgcgtgagaag

aacgtttggatgtatccgcgcttgttttgggaaactggagttcttggacaggtgttgtac

cttgaagctcatgcagttggaatctcagcgactggaattggctgcttttttgatgaccct

gtgcatcagttacttgggttaaaagggtcaaccttccaaagcctatatcattttactgtt

ggaagtcctgtcgtggacaagcgtataatgagcttaccggcctatccaggccccgaagat

gttgcgtgacttcacattgctattcaacgttaaggagaagtgatcttgctccctccctaa

acacttagattgtaataacacacctttgtatatgctattcctctgcatagatagaatttt

gcgctcacacacatccacgcatttgatattttttttaccgaaattgtagggcatgagctg

agaatcccatctagtagtaattttggtcatgaaaactctaatttagtccatgttttacct

attggtgtgacataattacttgttaatatagtttgtttttcttccgtactcc

>fava_dn_rep_c478

tttttttcaccttagaagctcccttatttaataacagatagaaaaatgagtaaaaccata

taagttctcatttatttatttcataatattgcattgacgagaaattatttgtaattaatt

ccaagtctaaattaagcaatactgacaatattacattttgttttattggcggtagaaaga

aacataaaagagtggtggaggtatttatatatttcaagggcagaagacaattctatagtt

ggtaccacctgggcatgaaactgtttttgtatcatcagtgggatactgataagcatcggg

gcaagaagattgttggcaagttcttacattgttgcacccattagaggttggagcaaattg

cattggaatgttgaaaccatcaatcacagataaatcaaagtaatcctggttgtttccacc

gttaagggtaaattcggcaagtgtagttggaggctgaccagataagctgcaacttagggc

accaccacagtcaccggtttgacaactaccacggcctgagccgtcaaaattgcaaccagt

tcggccccaaactcggccagatgcggttccagctgggatgtctatactccatgtttcgcc

agagtttaactgcctaccgccgccgctggggatggcagctggccagacagtgtatgagca

ttggttaacgatgtcaaatctcgctgcctgtgcagatatcaagcacagactaaatagaga

aaatactgatatttttattacagacatttttctgggagtgaattaaacaaataataataa

ggatgagaagagagtattttgtgagtcgcggtgggaattgaggc

>fava_dn_rep_c479

tgggttatttgcaaatgtgtgtttatggcatgacttatgtgggaagctacaatcatgaac

aagattacaataacaatcaagtttaaatgagagctaaaggaagaatagaacaatggctaa

tttcaaagatgatggatgttacatcaaatggagaggctataaatgatgatggtacactaa

atcaatttgaactgaagtcaaaaatacaaaaatacaagatttgagcagcgagagaaatgt

gtctgaagttctcagtttagtagataaaaacaaaataacaaggattgtttcaggcacttt

gttgaggaacaggagtataccaaccatgatgaatttctattgctctctttcttctggcaa

gccgagacttgcgatgtgctccagtgttcctatgcaatgtgcccactttcactgctttgt

caattacagagtatgcctctccaatcatcttctcaattgacaggatttcttccgcttgtg

catcagatttctttttgagcagagctagtgcttccaaaacctttttggtacgtgttttta

tctcagatttacgagctttgttataaacgcggcgtttctcggcttgtcgtgctctcttaa

ctgcggaatcaaccttttgaggagccgcttcgcaaacaacaacgccgcgcctctgtacgg

aactcaacgataccctaccttgtgaaaagaccaagtgagaaatgttgcgggagaaagata

aagaagcggaatttgaagttagggtaaggtttctcatttgggaaggaaggttcagcaatg

agcatgaaactgacgccatatttctctcttatcccttcacttcaggttgaatctcaattg

gattgctcaactcatatatcccgcggcnnnnnnnnnnggctaacgggtgcttttagcaag

g

>fava_dn_rep_c480

cccaagaatgaaatggaattctcataaataaaatcagaccaaagaatttaacacataagt

ttgctttcaactttgtccaccactaatttttccctttgggatgtacacatataacaaacg

tacgttcatctgtgcgtagttccagtatcatttcagttgtacaaacaacaaaacacagac

agaggaaatgatacataagagataacaaggaaacagattaaatataagaaagaaaaagga

atggtgactatatgaaatcatcctaagaataacttattttccccaacggggaacgatgat

ggaagaattgaaaaatggcgacaaacaaaacacattaaaatagaaatataacttatcagt

ctcataatgataaccaaattcaaaatttgcaaaacaatgtatataagacaaatttgctat

taccaaaatttcagatgcgcaaacaatcttgatctaaaaccgacaaatattgattaccac

taataaataatcaaacactgcaacagtctaattcattctcctgatgagctcattgatgta

attctcacggtttccagcatcacctccttcaacatagtggttcctcttcttcttcaagcc

acccaaaggagccttgagcttgaatggccataggaagttgtttgcctccttgaaatgggg

tccaacagtcaaaatctcatggataagatcttcaatgcatatgattccatgctttcccag

accctgctcaatgattgagttgtcagtgagaggagttctctgcctgtctagctttccata

acctctcttgtaaatgagttccctaacactcttcaaattagggtatccataagtcacata

tggctcaaccctgtgcaacatgttcaatgtagctttgttaactttgaggaaaaccccatt

aaaaacctgacgcaatcgaaatagttgcaagatcttccttgtttttgggtccatggcatt

aataccacgaatacgaataatgaacaagagcttagcctctggatcaacataaaatccgcc

tttaagcttagcctcacgcttcaaactaatcaattccttttcctgctcctcgtattcctg

agaatactgcttagccctgctgtaaatcagcttacggttctcagcccttttcttcttggc

ggcttcaaattcttgatttttagccaaagcccattcttcagctctcttctgtttcttcag

aaaagactccggaacatttgtcttcacctctttactcatctcgagggagcaaatgaatct

ctgtgaaaatgcgaattagggttgttcggcgagtggagtgatgagttagctacccc

>fava_dn_rep_c481

ttttttaagatgcaaatcatcagtaaataattatccatgctgttgtgtctataatatata

taatcatttgtannnnnntttttttttttcgagacttcaacatctgcagataaatgagag

taagttacaagaaatatcctgtgaatacgaagtcaaacagctcattttatttcccttttg

acaaatttgataactataaatcacaagttctacttttacaactttaaccaagcattgaat

aactaagattcagttgatgaggttgcatcgtccttgtgcttgatcataccagaatccaca

agaatgggaatctccgctgcaagccacggtgcaagacccaagcaaagtgcatgtgcaata

ccaaatctagggcaatttttcgcccagagaggcttgaaatgcacagtcaaacaaatcttc

cctcctctatacatcttctgagtttttccatccaattgcggcagttcaagctccggcgcg

gtggaagggtaggtaaccggaatatcaaactgaagatcaaattcatacttgagaagattg

taaacataccaacatttaccggtccagcgagtaccttcgggattggaagcggagatccga

aaccaatcgttgtcgttggatttgttcatctgagtgtatgcgatgagggatttgtactct

tccttcagacgcgccgtccatgccgctccgtcgcgtggaccggactttgtggttaacagt

gggatctgtgttagcgtcgatttcgtgttcgggtcccaaccttccattgttctctgtttt

cttttctcttgtgttcttttcgctttcaacacacgcgcttacttatgctgcataacttgt

ctacactctctctctggttttagaattcaaccggtttagtggttcaattanntggaccca

caataggtctatttctaagttg

>fava_dn_rep_c482

aaaaaatcaaataaatgaccattcttgacaactaaatgaaaagagaaataccacgataaa

atctacatgtccattcttgacaactaaatcaaatgaaaagaaaaatacccagataaaaat

tgaagacagcaaaataattaaagacagtataataacatggtattggtcttatatttaaaa

agacaataaaaataatgtaccagacagaaatccaaaactcccaaaccagttctagaatag

aaaaaactacaaaaaggtggacacaattcatgaaaatgtaacatgcatttaaactctctg

ctcaatctaaaatcaatcaagcatgtctgttcctcttctttgcaccagactttgcaactt

tgaggcttctgttaacaacgctcaaccttgcaagagcagccttcttgagatcaggtctgt

agtagttatctgcaacctggtttaggacagcctttgccatcctcctgaactccttcttca

tcacagatttgtgaagcaaagcagaaggcttgttctgcttccttggcttggtggtggcca

acaacacagactgatctttaccagctggctgaatagtaacagtcttcttattggccaaac

cagagtacttgtaggagttgaggttgtagagattgttcggctctctgctaaactgaacac

tctgagtgttgttaccaaactccttaaccaagaaagagttgttcctcttcacgatctccc

aaacaagaggtcctggaactgttgccatcttctccttctacgtgaaaacctgttgttgag

agaaatgagattcggaattcagaaggagagaaagccgcaaaccagtatatgtcgagagaa

agatccccgttgcataatcatacttatacgttccattggtttgcgagagggatgttagta

ttccctgagaactggtgagaacggtgagaagcgtagcgactaagtaccactgcatcttct

tctacttcgctagatccacactcgctcagtaaggaagacgagtctaaactaaacgcaccg

ttttactaccaccttcgatgtctatattgttttaagatt

>fava_dn_rep_c483

ttataaaggattaaatccattctttttttataaatattaagtaatcattcaattctacat

ctatgcgattgtgcatatgcattgttttaagctcaggtaagaacaaataaactcattatg

ccgctatgatgttttaggttttcatttaatttttaccagagagtaattgacagtacagag

aaattgattattaccacagatatacggaaaatccatatggaaaattaaacatgaacagag

tcaaagaaataccatatctaggatccccaaatccaccaaattattattgcaaatgaagta

tgggatgaatcaaccagatgctgtcttcttttgctggaaaacaactgcaaagacaggaac

tgcaacaccaatgctgaaagcagtgaacactgcaagagacattttcaacccctggtgctt

catctggtccaagttgtacatgtgatatgaatgcatatagtctggctcgctgtggccatg

tccacctcccatcctcttcacaccagttgaatgaaaacctctactcactgatttggagat

ggaggattcagaagaagcgataagcttcgcagcagatctgagtccgttgttgagtgccat

cgcaaacgctaaatcgattccgtcgccttctcgaaaatgaaaacccttcttttctttcgt

ttttgatgctgcccaatttttgataaaatataaacaaccccccggtcatatcttccaaat

cgtctacactaacaatccaaccttctcccccaaaaggcgattttagtaaataaccaaata

taatgcttggactaagggttaagttggtaatttttcttacatctcc

>fava_dn_rep_c484

ttttttttttttgaaaataagatatttgtttataatatagtgaatagaggaaggagtatg

cgttttttcagtctcttttaccctcaaaattcatgcctaatgaacatttaggaatattaa

cacttctcatcaagcagaaaaaaccatgcatgcttaattaactgctccaatccacgttag

gaatttgaaaagtactaatcaaactaagcacaatgtttacaaccaatgccaaaagacatt

tatatattcatataacgaccaatacataagttacaagaactaagaggaagaaaaggtatc

aatgatggtagtgtgaagaggatcactcaaatgtgtagcccaattgttaagaggtccttt

accagtagcagcagcttgaacagcaaaaccaaggaaagcaaccatagcaagacgagcatg

tttgatttcagccaattgaagtgttgctttcttctctgggtcagctgctaaaccgagtgg

gtcgaagaattttccacctgggtatagtctcttttcggagtcgagctctgcgtttctctg

gaattcaatgtagccaattactagaacttctacccaaatcaatgttgtgattgagaatgg

aagtggttgtccaagatatgttgatccctccactagttcaacctttccggcgtcttgcca

tgtaactccggtaagccattcaacggtaagagcaccaagagtagcaagcatagcccatct

cccatgaatcagctcacattctctaaacctctgaagtccaaagacttcagtataaggctg

aaacggcgtcgatttaacatcatcaaactccgtcctagttccgatcacatctccggcaag

attcttcgccagattctgatcaagagaatcaagatcaaattgcaaatactcagctggttt

ccccaaaccaaatggatcgaaaccgtaatcgccgaccaagcttccgtctagatactccgg

cgactttgctccgggaaaccataagggacgatcggtggttggagccgagcgggacggttt

ctttggtgcggctttcttctttccgaatccgaagccgccgaatttggcttgtactcggcc

gatattggattggacttcacggagacgagttcccatgaaggatgaagtggcagcggcggt

ggcggtggcggttgcggttgccatttggtttgtgctaatgtgcgcctagggggggtggga

atagaaaattgagtgttgttgttgtgagtttttgaaatgaggatgagtgatagtgttgta

tttnnnnnnnnnnnnnnnnnnnntgattatggataatctttggttgggtatttttggccg

ttggatttcggagagtatccatttgacggtgaagattgggtatggcttatcctcttgctt

atctggtgttgat

>fava_dn_rep_c485

ttaacttttgaaaaataccaaaaaataaataaaataaaaagaaaagaaatacacgcaaca

aagcataaatcatcacacctcaatatactcaacatcattcagatcgcaggactatcacct

tccatgctcaaaaacttaaaggagcatataaatgaggttcagtacttgttgaataactac

aaattaaaaaattgtcatgggtttaatgattttgcaccctgaaccaagacatgtagaaat

taagagctccaaaatgaaggacaaaaggttcattggaaaagtgacaccctaccacttatt

gcctccaccaccagataaacaacctatcaatcacaaaataatcaggtctaaggcttcaaa

gccacagcaagaccgaccttcgcactcttgtcaatggctgcagtatcaacttccccaact

agagaaaaccgtgctttgggactccagtcatgctggatcagagcacttgccctgccatag

ttgttcacccgagctttcaacaaggttatggggtcaagagcatgttgtgtgccaatggtg

agtatattttcattgcttgaaaagctatgtgaaaactccgccccaacagcagtgttagtc

agtgggctcacaacgtggtaatacgaggcattaagggtgtcgcctctgtcattgagagtt

agagatgcaataaggtcagcgtgagtaacattcagccctgcattgtacttgataaagtcg

ccggtggcagtatcaaaagaaacatctgacccaacagaaaccaaattgtttccaagcaca

ccagagatgttaacaacaggacttgctgttaacccaatactagtattgatcccggcataa

tcatgctggtactgtaattcaaccttgccagatttttgatctggtagagtgaagctaaaa

attgtcttgagaccaggtgcagcttcatccacagttatggttgtgagtagattagagtta

gtgtcaactttaacatcagtagtgatgttcttgttcttcaactttgtactaacatcagcc

aaaaaaatctcacctttcctgactccacttgaagtaacttccaatccagtaaaagtcttg

gtggtgattgtgaacttgtggtcattctgatagtctttgaacaaaagatctctggctctt

ttgccgatgtcggagtaaagtccgggaccgttcaccatgattttttgccgagaaagtgaa

gcggaaaatgcgagaaaatgtgagaaaatggtcccgcggtgaaaaagacaaaccctaata

atgaggagggagagggtggaatgtggccgttagatgggaaagttgaatcgtggc

>fava_dn_rep_c486

agaggagaagcactcaacaacgaagtttattttcttgaataccattattatttatacatg

acttctcattacaaaaagattgtattattatgaatgatacatggtttgttctgtcaatta

ttcgacgaatttatttagacaaatcagttttatgaatatataaatttaaaaagagacaaa

gtactaataaaacgatgatatttgatatattacaaatcataacacacattcaccacgttg

attcaaatcaaactcttcaacacgtatagaaaaatacataagcacaagagatttaagatt

atctatacctcttttatttagcacaataattattgattcatgaaacttattactttgaat

gtgcatcatgtaccatggtattatcagttaccattgtcggaagaaacaccgtgtccacca

tgataaccgccgtgcccatgaccatgacggtgaccgtgacggtgacggtgatggtgtcga

tgaaaaccaagaaatttggcatcattaagttcatttgacttttcaacaacctcttcagta

aactccctagctgagacctctgaggaaataagaagaaccattgccaagaggccaagaatg

agaattgttcttttggaatccattgtactcagatcaattgaaatgtgatatttggtagtg

aaggatcccgcggccgtaatggccactctgcgttcccgcgg

>fava_dn_rep_c487

cggggacacttcaaagctaacatctaaaaaagtttaggtgagtgaagtgttgaaagagag

aagatatcaacatggagatagctggcagctgtgaaacaccactaccacttctctcactga

atcatgtctctatattgtgcagatctgtgctggactctatgaggttttatgaggaagttt

tgggttttggtctcatcaaacgcccttcctctttcaaattcaatggagcctggttgtaca

actacggttttggaatacacctgctagagaatccaaactatgatgcatttgataacccta

tgtatgaaccacgacccattaatcccaaggacaatcacatatcattccagtgtacagatg

ttggacttgtgaaaatgaggttggaagaaatggggatgaagtatgttacagctttggtag

aagatgaaggaatcaaggtggagcaggtgttcttccatgacccagatggatacatgatag

aactttgcaactgtgagaatatcccaatagtgcctatttcttcttcttcgtgctctttca

aggcaagaagccagagcattaagaaggctcccagcaaatgtggtttcatggaaaatgtta

tgatgcgaagcttgagcatggatatgatgaactttgcgttttaagtttccatatggtcaa

aataaggtggtgaaaataatttacttttatttgtctgtaagatcagcaaaggtttttatt

atcatcaaaacagggaaaaaggtgcagagcgttgttaatttatgtattcaaatatccaat

actatgttgtaggatgcttgaaataataataaaattgtcaagtaaaataataaaaaaagg

cacgcagagacatcatattgaataaaacagtataaatgaaaaggtatgggttgtttggga

gatacattttcaaaagtacaaaaccatacttaataagttattggaatcaaacaaaacatc

tgtctaagtatatagatctctgatctattaactgagcatattcaaaactaatgcaaggag

aaagctttattcttggtcctataaaagaagtacataaataaatagaataacttgaataat

gaaaaccagcaaccagtgtactaacattaccaaaaccaactccacagagacattcacaat

ctgttgctctgcaaaccatagccaatgggaaacatgaaattactcggccctggattggcc

agcgcgggaagagaggatgatgccaacaatgagtccatacaacgccagtgcctcagcgaa

aatgaggataagaatcattccaacgaaaagctttggctgttgagcatttgctctaacacc

ggcatcaccgacgataccgatggccataccagcggaaagcccggccaaaccacaagcaag

accagaagaaagatgcgcgtaaccatcaaacagataataagatttcgccttaggattaat

accagtactgatgataaccgcgataatcaaaccgtagatacctaacacaccagccataac

aaccggaacgatcgatttcataacaagttcaggtctcatcactcccatcgatgcaactcc

gacgccactcttcgccgtaccgtacgccgctcccatacaggagaaaacgagagccgcggc

ggcgccgaggaagccgaagaagggagcagtttcgtcgccactgaatggagccattttgga

tcgatctgttgcgttctgattctcggatctctgatcggtaattcgttcttgctttctcgt

ctctcctcctttgatattttgcctcgtgtataatgtcagtcacatccttatgtgtagtat

tagcttctgat

>fava_dn_rep_c488

acgcaaggtttagactcgagcgccgagagtcaccgcgggacttcacagttcacacaacaa

ctacgcaaggtttagactcgagcgccgagagtcacacagagagatacgcaccgtcggtaa

ttgcagcgtcgtgaggtagcggcggcgaacaatttccgatcactgcatcattcactcgac

aagatttgatggcggcgaagttgcagcagttgacatccaaggcgtgtcaggcatcccaat

ttatagcaaaacatggaacttcttaccacaaacagctgttggagcagaacaagcaataca

ttcaggatcctcctacaatagagaaatgcaaccttctggctaagcaattattttacactc

gcctcgctagtattcctggtcgttatgagtcctttcataaggaacttgattatgccaaac

aattctggaagaactggaaagagataaaggtcgaagatgcaggtattgctgttttgtttg

gcctggaatgctttgcatggttttgtgctggcgagattgttggaagaggatttactttca

caggttactatgtctaacagtgaccactttataacgtaatgatttagcggggctctatac

tctcctaaatccaggagtaatgtcatttataacattatgttgatgttgattttgggcatt

ctacaagcaatttgtcattctttttgaggaaaaaaggttgcaaaactagtggttaataag

cctcggcaacctcttgtatttgaagcgggatagtctatgtgctgtttgttcttttatcat

gttagaaagctagcgacataaatcgaccataattattttcctgtgatttttagtcatttt

taatatccatgtttttagttttgcatgaaatacactgagaataaattgttgaatttttgt

accgactcttcttgatgttgaatttaagctagcctgagtacatattactgcaatatatag

caacagggctattataaatgaattatc

>fava_dn_rep_c489

tttttgtaaacatctccaaaccactcttaatttaccataagcaaatttatcaacaacaat

taacaaataatctaatcacccccacacttgttgttcaatcaaagcacagaacaatcagta

cgaatcttgccattcctcgcagttttaacaccaacacgacccaacttagtcatagcacta

ataaaattggagttgaaaacgttaccactagaagcaaaagagttaaccgttgccctagat

cttgtgtcagtgaagaggatttggtctgaagtgaataaaccttttccctgttgaagattc

tggtaataaatattgtcgaatgttcttggagttgttgggtccatgttgatggctattctt

ggatcaacgtttctaggacactgttgttgtaactggattgcgtattgtttgttcaatgtt

gggtcgattggattttggatcctattggagaatctgttgcagtgagagaatccaagggtg

tgtgcacctgatagggcaatcatgtctgtttgagtgagtccattgctagcaaacagagca

ttcagctggttcaaattgaaccctggctgaggcaatctcccatttacatctgaagatctt

gaaaccaacccatcaaatcttcccaattcaacagtataagacggtccaccagccagatta

acaacatctctagcagccaaagcaagaatatcagcacaagaaactttgtttctacactgt

ggaacagcatctaacgccgctttcgctttaataactgtatcaaatccatcaccagccaat

gacaaattatcagcgttatccttctccgcttggttacctccactcgacgccaccaaaacc

gaaccatcacaaccctgaacaaaacaatcgtgaaagaagagacggagagtagcaggaatg

gttacaaaagtctgttgaaatttcttttgaacagctgatctgacaatggactgaacattt

ggacaaatgttagcgtaatggttcggactaagctgagcaaaactagtataaggaacaaga

cacaatgctaacgcaaatgacaagacgagtataacactaaacctatccattttcgataga

agaggtgtttgcttccccgcggccgatgaagtcaggggtatatatatagaagagagatat

atgaagttgttgtggtcacgactcacgagccagttgtattcaaaatgactttaacagcag

agagaaaaaaagtattattctatgaataattgaataatggtttcaaaagggacaattgta

tatggctgccttaaggtttgtggaatcaatggtgactgggaaggattaagagagttactt

ggtttgaaacttctttgtataaagctttttaggcaagttggggaagatttggattttgag

gtaaaaacagggagagggagccaggtaggggctgcttggaattcagcgcgttaagaattt

gggttggtgttca

>fava_dn_rep_c490

agtatgcactttgcgcacgggtgcgtactctagcggtgaaagtaagtttcactaaaataa

acacgtaatctgttatcgttgttccctcctccctcttgcagaacgaaacgaaatcgttcc

atttcccggcgacggtgaacctaacatgctcctcgattagggttttgaaactcttctcta

gttcacttttaatttcaataaacaacgctcaacatggtgtctctttcactttcatctccc

aatttcgccaccgctttcctccccaagaaactgcccattcgggaaattacgcggaaacct

acaacgaattctgatgtttcgtttcgtactagatgcgcggtggatatgccctatggaggt

aatgtcccaaaattcccccgagtcagtgtttgggatcctttccgacgccttggagtaagc

cgtgatgcttctgaagaagagatttgggggtcaagaaattttctgttgcaacaatatgct

ggacatgagaggagtgtagaatcaatagaagctgcttttgaaaaaatattgatggctagt

ttcattcagagaaggaaaacaaaaattaatttgaaaagcaagttgaaaaagaaagtagag

gagtccccaccatggattaagaacgcgctaaacattgttgaatttccaccaactgaaatt

atcctcagaagattatttctctttgccttcatgggtggctggagcattgtgaattctgct

gaaactggacctgcttttcaggttgcaatctctttggctgcttgcatatacttccttaac

gaaaaaacgaagagtttggctagagcgttcatcatcgggtttggagctcttgtggctgga

tgggtctctggctcattgctagtacccaatattccaacaattttgttgcgcccaacttgg

acccttgaactcttaacatctttagtagtttattttttcttgtttgttggttgtactttt

ttcaagtgagatgtggcaactattcttccctagtttcattcagtgtactgcactcttgat

gaattcctaggtaatcaaactacatatttttggctttaaaaaaaaaaagtacatact

>fava_dn_rep_c491

ttttttttttgtgaaaccgagaaaataaagcaaattgtgacttaattggaagtatttaaa

caagttaacacatgggcttaatcatgaaatagcagaaaattcaaaatatcataacaatcc

atcaaaaatttcaacagaatttaagccagtatcccaaagacaacaaactatagatgccaa

aaatagtaacctaacaagaaataactcagatctgctgagatttgttaaggatcactccct

catacttcacttggaaccatttcattgcatcatccttggtaacacggtgttggattccaa

cacgggctttgcacctacggcgacgaccgacacgataaccagggcgctccaaaacaacaa

aaaagtccataccatagataccagtagatggatcatacttgattccaagatcaatatgct

cctgaattccgaagccaaagcatccagtgtcactgaagttccttcttagaagctcatact

ctttcaccttcagtccactctccaaaagttgcatggccttgtcacctctaacagttacat

agcatgcaatcttctcatttcttctaataccaaaggaacggacagtgtaccttgctttgg

agaacactggtgtttgtccactgagttgctcgagcaccttggcagctctggtaagacgat

ctccactctcaccaacggagagttgccattacggctatagggagggttaacgtatacaag

ctgttgtctttggggtttcattcgttcatcgcttcatctctccagccgccgcaacacttc

aaccagatcaaatggcttccgaggcgaaattggcgaaccctatgagggatatcaaggttc

agaagcttgtcctcaacatctccgttggtgagagtggagatcgtcttaccagagctgcca

aggtgctcgagcaactcagtggacaaacaccagtgttctccaaagcaaggtacactgtcc

gttcctttggtattagaagaaatgagaagattgcatgctatgtaactgttagaggtgaca

aggccatgcaacttttggagagtggactgaaggtgaaggagtatgagcttctaagaagga

acttcagtgatactggatgctttggcttcggaattcaggagcatattgatcttggaatca

agtatgatccatctactggtatctatggtatggacttttttgttgttttggagcgccctg

gttatcgtgttggtcgtcgccgtaggtgcaaagcccgtgttggaatccaacaccgtgtta

ccaaggatgatgcaatgaaatggttccaagtgaagtatgagggagtgatccttaacaaat

ctcagcagatctgagttatttcttgttaggttactatttttggcatttatagtttattgt

ctttgggatactggcttaaattctgttgaaatttttgatggattgttatgatattttgaa

ttttctgctatttcatgatgaagcccatgtgttaacttgtttaaatactttccaagtaag

tcacaatttgctttattttctcggtttctcatatgggcgtgtttatgaaccttgttaaaa

taaggtcacaagttgttttattctcttgtcttctc

>fava_dn_rep_c492

cgggggtctttcttgtagggtttacgaatcaatcgtcgttgctgcaacgtttccacatct

acaatgggtaagggaactggaagtttcggaaagaggaggaacaagacccacacgctctgt

gtgaggtgcggccgccgcagtttccaccttcagaaaagtcggtgctcggcgtgtgcttac

cctgctgctcgcgtcaggaaatataattggagtgttaaggcaatccgtagaaagaccact

ggaactggtaggatgaggtacttgcgcaacgttcctcgccgattcaagagtggcttcaga

gagggaactgaagctgcacctaggaagaagggagctgtcactgcttaagctttttgagga

ttgttgttttgttgggtactgagttttatagaattagtttgatatttgtgattggggcaa

gtaattttgaactactgtttttacagcctcattgtttcggttttattttctatttgttct

gtactttcttgatatttcgatatgattataaaactatttcagtgttcagtttgtttggtt

gtgtggctttattagttttgagtaagctatatttaattattttaatgtagttcgagtatt

gtggtatgtttctgtcatatagttgcttatttaggcaatgagtaatgagtaattataaga

gcagattcttttcaatcttt

>fava_dn_rep_c493

gtcatcaccaaaatatttaaagcattttttctaggtgtttaataatgggaccctcttttt

attcaatgagttggaaaatttaatcacacacacaaatgggccctaatttaaaggaaacat

acaatacattatctccaatctaaagttcttaaataattgagattgacccatatatatcaa

tcaatgcattcttttattaaaatgttgacatcttgatatgattaatcatatttcaaatta

tgaagaagtaggaagctaaaacaaatagtgaaattccataaagcagctttccctgtttgc

taccactcttgtcttgagctactcctgtgtttgttccttttccattggcattactagctg

gagaaactggggtagtactatttttctccaaagttttctcaaagtcctcaaatatcttag

cctcatgagatttaggtgacaaatgcaaaagatcaatacactgagttatattagtaggtg

agttacaatcagcaggtaacttaagagcaagtgtgacattaatactaaaaccaagattag

ggtcatcatgatccttaataagaatacacaaacatctcttactcttattaacaacatctt

taacaccagtgcaacaatctatagttggtgtctttgattgaccactcacaaaaggaagac

aattagccaaacctatgagtttgtctgtgcattctgctttgtcttggttcacatctgaag

tcacaagaccaaacataaggactaggaaaatcaaggaagagaagagtgataaattttgtg

cacttttgtaacccattttgatgatgaaagagtaactcaagacaaaacaaacaagatgtt

tgtgatatagaatagatacacctacacctctcgttttgatgtatgtttttatttatggta

taaggacttgtttgcttgattgtgagttgtgact

>fava_dn_rep_c494

aaaaacacttcaaatcacatataaataacatgaaaccctagtccattttctgatcagacg

gcaacaatgacaacccgattcaagaagaacaggaagaagcggggccgcgccggcgccgga

cacggtcgtatcggaaagcacagaaagcatcccggaggtcgtggtaacgccggaggtatg

catcaccaccgtatcctcttcgacaagtaccatccaggttacttcgggaaagtcggtatg

cgttacttccacaagcttaggaacaaattctactccccgatcgtgaatatcgacaggctt

tggtcgctggttccggaggatgtgaaggagaaggctgcgaagtcgaagggcactgcgccg

gttattgatgttacacagcatggttttttcaaggttcttgggaagggtgttttgccgagt

aatcagccgattgttgttaaggcgaagctgatttcgaagattgcggagaagaagattaag

gaggctggtggtgctgttgttcttactgcctaggtttttgtttagttggttttttgaatt

tcagtaatttgattctatacgtttgaacttgatcttatgatcttatgttggttatgttaa

ttttgtttttgtttagatttttgaaaatagtgtttctggtgataattatattatatgctt

ctatacctgtttgtttatcattctgattttggtattaaatgttgttcttgttttgttatt

aggtttatcaaggatagtgtggactatattgaaatgaaatggagtaggctagaaaggggt

tatttggaaatgaaattgggatatttgtctttgatgctggtgttttttgcaggcatgttc

tttgaaaggattttcacaattatattgttttaattctgaacagagctcatttccgaatct

tttaattcaactgtcaatgtatttgttgggttttgttttctttctcacctttaaaaggtc

ctgtttgatctatgcattttgatctgtgcatgattacttgttcttgctgcattctagcaa

tggtaaagagactcacgtttggttgtgt

>fava_dn_rep_c495

cgtgtttaatctagttcaattaaggcactaaatcatgaaaagcacatggaataaatcact

tttagcagactattaaggacaagcccctgaatctgctatccatcttgcaaagatgatggc

ctcgtaataactaaaattcataaaaatcaataaaagttcacaatagcaaaaatgtaggaa

ggaattgctccagaagaactccatttcccatcagaattagaatcttaaatttatgaaaaa

gcaactatctgaaccaaattccattaaaaatatcataacaaagcataacatcactatctc

atccagcctagaagactagctcatgtggcttcacgccggccttgagagaaaacctggcac

caagataagtcttcaaatccggaacgctctcaatcgctttaatcaatgcggcatcaacag

ttttctgttcatctttcttctcctgaggtaaaacttttttctcctctttgtcagattcaa

agaattcaccttcacctttgctcttctttttcttggcatcctttgagaaatacttatcat

caaagttgctcacattgacaccagagatatctactttggtggatgtgccaatcacatatg

actgattcacacgtctcaagggaactccattaattttgaaaggaccggttacaagaagca

atccagaaggaagctgcttcaaaaacacaaccctctttcccttgaatcttccagcaagaa

gaatcagcactgttccaggggtaatactagccctgagcttggtaggtttaggtttgtgct

tgttaagaaggggtttcttgacatcatcagctgggtaaaacttcggaggtttctcagcag

gagattcaggtttaggtttgggatcatgacgaggaaaaacgccgccgtttttggccttga

tagcccagatacctctcttgtggtacatctgagactttgagaattttccaattcctcgga

ttaaatcggggtttctgctaaccctaggtgctctctccttcttcggcgccattgctgcta

cgaaatgcaaaccctagtttctgtcactgagcaaatgagagagcggaagattctgctagt

gttgtatggagttaaattt

>fava_dn_rep_c496

gagaaacaatatttcatagttgaatgttttatatggagaatgaaacctttttttttcaaa

agaaaaagaaattgtatgagataaggcaagcacatagaaagagtaacaattatctattat

ttgtgtttttaaatgaaagcactaagtttcaaccaagttcataattccccaaagatagta

gtagtagaaaaaacactaagacaaaagcataagataccgaaattaaagaaaggaaaacaa

aaatagataataaccatagctgaaacatgatcatgcatgtttatgttttataagttcaga

gttggtcacaatctgcgataaccaccggcttcgaggccttcccggagctagacccgactt

tctcaatctccttcgccacctccattccctcaaccacctgaccaaaaaccacatgcttcc

catcaagccactccgtcttggaagtacagataaaaaactgagatccattcgtccctggac

ccgcattcgccatggaaagaattccaggaccggtgtgtgtcttgatgaaattctcatctg

cgaatttagctccataaatggactctcctcctgttccgtttccagcagtgaagtcaccac

cctgacacatgaaattaggaatgacgcgatggaaggacgagcctttgtaatggagaggct

tcccgcttttgccaacacctttctcgccggtgcagagtgcacggaagttatcggcggtga

gaggagtagtgtcggcgtaaagttccatgacaatacggccggcggggttgcctccgacgg

tcatgtcgaagaaaactttagggttggacatttttttgggaggtgagaactgaaaatttc

ctatcggagaagaagaagtgttggtgtcccgcggcc

>fava_dn_rep_c497

acgtgaagatttaaaattcaataaacataactaaaaaaaaaaaagtccggcccataaaca

gacagcatataagaaagcccaaaatccaacattagggttcttattctcagtgtgagcgtg

agtgaagggagagagaggcagcaacagaaaccctagaatcagaaaaaaatgcctgccggt

catggtttacggtcccgaaccagagactccttctcccgtcccttccgcaagaaggggaca

atagctcttgcaacctacctccggacataccatatcggcgactatgttgacatcagagtt

aacggcgccgttcacaaaggtatgcctcacaaattctaccatggacgcaccggtcgtgtc

tggaacgtcaccaaacgtgccatcggtgttgaagtcaacaaacaggttaggaacaagatc

ttgaggaagaggattcatgttcgtgtggagcatgtcatgccgtctaggtgcactgaggaa

ttccgattgaggaagatccagaatgataaattgaaggctgaggccaaggcaaagggagag

gtgatcagcaccaagcgcaagccggagggtcccaaacctggttttaaggtggagggtgct

acattggagactgttactccaattccttatgatgttgttaatgatcttaaaggagggtat

tagaactttatttctcttttgtttttctttttaaggagggtattagaactttatttctgt

tttgtttttctttcttggactgcctgagttggttttctttatctcaactttccttcaatt

ttatgataaatcaaactatgtttttttttttgggttcattttcatactgctatattgttc

tttccaagaacttgaggatctattggtatttaaggttagagttctttgctgacagtagaa

tttagctgctaattttatt

>fava_dn_rep_c498

gtgggaatttcccaatttggaaatttatagtatcagtattaaatgatagtatttttatga

ttacatatatgtttcattagatgatttagggttttatatgcctccgtgatccataaccgg

tattttggttgaaactatggctaatcccactcaaacagtgcccttgaaacttgtggtgaa

caaagagacagagagagtcttatttgctgaagttgggaaggattttgtggacattctatg

cagcttcttaacaataccattaggagccattgcaagatttatccaaaattgcaaaaacat

agaaactattacaacactaaagtgtactcatgaagatggctgtccacaaaatgtaatagc

ttgaaaattaacatagcccaatggaatgtgaatctgatgttaccactatttaaacactaa

acacaatcctgcaagtatatcatcaattacaaaatactcttggacaaagcgtctctatat

tctcgatgttgtcttttttcttcttcacattttcaccaaaataagcgagattctacatat

tacataagcctattacttctccaatggaagtgtttattcctattaagtataacttccatt

caaaacagtgatttcaaatactgtagacatctctataatataaaaagtacaatcacagtg

ataaacagaaaattgacaagattattacttattttattgtttaatgtttagacgtttgga

tcaaactaaacaaacatgtcagaaacttttggaagcttgatctcacccttttgagaaaga

ggaatagtgaaattgccaataacaggaagatcaataaccaaaccaatatccaattgataa

tcaatgtcccaatcagcaccaatatccttagccaaactaatcaatatgctatgaggcact

ttcaccaacacttcaagcagtgttgtgtcacttgctttcaatgatcctggatctggtact

ctccctgatgctatctcccttgcagcacttttgaaggagtaggtgatctcacagattgga

attgaagttgaataaggatttgaaacattgaccttagccaaatactcgacactctcgcgg

gtcacgcgcttgaaatcaacgtccgttatactcgccgttggtttcggcatttcaccgatt

ttctcagcaacgtaggttttggctttgtctagtagctgcgacattttcgacatcgtaaat

atcagaatcacacaagacaccttcaagtttcagcccactctcataatttatacgcaaaaa

cgaaaaagacaaaaaagtagctggcacgtggggaaacgcgtcgcacgtgtctccaacgcg

ttgccagatccggaatc

>fava_dn_rep_c499

aattgattgaaaatggttgaagttttgtggtaggttttagttatcggagaaaatgaagag

aatagaatagtagtaggagtgaccatgaagcaactgaaataacaataatattttcgaata

taaatatccaaaaataccattgaatatttaaatccaacatttaaataataataataacaa

tgtaaaaataaaaatttgaaagatacatttccgaaataccttaaaaattaatttaaagtg

cattcgatattcaaaagtttatgaagttccctttaaattcctttgtacaggcagaagccc

aagcccatacgaatgggctttgaaaagtgaagaaaaactcaggagtatgtgtaagaatcg

cgcgaagcaagagaagagccatggccaaggcgaaaccccacattatcttcgcatacggca

cgttgaagcgaggatttcccaaccatggtttaatggaagatctgaaagccaaagacgacg

ccgttttcatggatacatcccacacggaagatccccatcctctggtcgttggaccccacg

ggataccgtacctgctcaacttacccgggtcgggtcatcgagtgaacggcgaggtttacg

ctgtgtcggacgatgcggtggttaggctggacgagttcgaaggtttgaaagatgggtatt

acgaacggattccggtcgtggttgtgagggatggtggggagaaggtgaaggcggaagggt

attttggtcatagaagctttggggagattttgtggaagagtaagggtgaggttgggttga

tagaatatggggagaatgaagcgaaggagtatgtgaggaaagaggttagacctggtcgca

aaaacagtattcttgacatcttaattagttagtttatgttttctcttctcattttctggc

aattttgtaaaacattgtgtttttatgctgttttcatcttgttcaagcattacattacaa

gatgagttcatcaaacacacaggtaccaaacaatcttcatataagatcacacgtattttt

ttttttgcttataatccctttatttagtacaaaaccaaattgcagaataggggccaatag

gtcatcaacagttacaagcacagttgtactgttgagtgcattcagcaaagcccttgcctg

agtcagcatctggaccttcagttctaaaaatagaacaactccatccttcaccacacttgc

agaaaacttcactgccactgtcactacttatctcaattttaggtgaaacagctccaccat

caagtgtacaggtgcaattttcaccgcatttaacgaaaactttgccagcatcaggcccat

cagtcctagttataacacatgtccaaccttttcctgtcttgcacaacacattgcccaagc

ctaggcctgagtttttatcttcagctgggactttcccttggtcttgagtgaagctcgagg

actccatattctcagaatcccgcggcctgtgagatttctttattaaaatannnnnnnnnn

nnnnnnnnnnnnnnaaaaatttgttacaaattt

>fava_dn_rep_c500

atcctgttatagcagctactaatataatagcaaaaagaggaaacagaacaaaagttcaat

gaaacttttgcagatctatcaaatctgcaagagaagtaaatcacaaagaaatatgtaaga

cttccactgcactcttagtatatctaaataaactcgggtagaatttttggcaaactcaag

aaatacaattaagtagcaaaactgaatttaagctaaatttaaaaatgccagaatacaaca

atagtcttgattaaaagaaagcactgtaaccaaaagtaaacacaaaaagaagttgcatcc

ataaccatagtaatctatagccttctaccccttctaccactctttctgcgtgtgctgtct

gaaggaattggggtcacatcctctatgcgaccaattttcattcctgaacgagctaaggca

cgaagagcagcctgagcaccagggcctggtgttttggtcttgtttcctccggttgcacga

agcttgatatgaagagcagttattcccaactccttgcatctggtagctacatcttgtgca

gcaagcatagcagcatatggagatgactcatctctgtcagctttaactttcattccacca

gtaatgcggacaagggtttctcttccagacaaatcagtaacatgaatgaaggtatcgtta

aacgaagcaaatatacgagcaacaccaaaaacatgttccccgtctttaacagcaggacca

agagtaacagtctcttcctttggctctctaacctttctcttggacgacattgttgaaggc

ctggctttgtttggaggattcctggttcaatcctgcggcgtgcagcttcttcgttttcgt

ttgaaagggagaagaagaatttattgtcgattagggttttgttaaatttggtgttggatt

gggcctttttgttacccgctgggctcatctata

>fava_dn_rep_c501

tatcaaagtttataatgttataccataaacagagcagaatatgatgcatttgattgatac

cagtattctctttctccnnnnnnnnnnnnnnnnnnnnnnnnagtttcaaatttgaaggga

tgaggctagtttgcactaaaagacaagatattcgcattgaaatgcatgaaattaaactta

aaacaaactagataattattatttacacatggatcattgttaaattacattacaaactat

atgagcaaatatgcctctcttttttttggttaactgcaacagttgcatattaaccaattc

cataccctttattttttttggtctaatttgtcatcactttaaatagaagatcttgtggaa

gaatcactctctggcatcatcagcttattggtttctttcaacaaatcaagtaatgtgaat

gaatgtggtggaactatcacattcatgtccttcccaacattttgaagtagactctggatt

ggaaccaccttttttggctgcgagaaggaattttcatccattacatttgcagatgtaagc

acactttttgttgacccggaagactgcaatgaatttgggtccaatccattgatagaaatc

ttgaggttcacggcactggcaccgtagttcacagcctttattcgtatgtaagttttctta

tcaacagaattttgccaagtaattgctgatgcaattagtgttttcgaggaattcgtttga

agtgatgaattcagaagtgttgctccatttgactctgcgaaaaataactgcatccagtag

ctaggagttccgtagagctgaaaagagttgaatacaattgcatcggggttccacctccta

tcgttggcatttacaaaaaggggagcatagctagccatatcgataatatcactattcttt

tccagtccaatgaggaatccagcttcggctagggctgccaaaagacttccttgcccggca

tcctttccggtcaccgcatactcacttacaaatgcctttggaccacttcgtgttactcga

ttaaatgtcgtagatctagaaaacatgtcatcagcgccagtatagatatgataatcataa

agatcggcagggtgatctagaggagtcgaagaagcgtcgcagtttgagatgattttaata

tctggataagcatgtcttattgcactatagaacctcaggtagtttccacgatagttcttt

ttcccacaatcctcattgccaacagcaacatattttaggttaaatggctccggatgtccc

atagccgctctgagggaaccccattttgaagtcgggtcacctctcgcaaactcaagtcca

tcaagggcttcttgcacaaaaggtaaaacggcggatgtatcaacttcatcggtatggctt

acaccattattgaatacccatattggtaaagcgtccaagtcttctgatagttggagaaac

tcaaaatagccaagtccatcatcagtccagtacttccatacatctccgaagtgccctggt

ctctcctcccaaggtccaacggaagctttccatcgaaatgcatttcttaaagattcgcct

tcgacaaaacaaccacccggaaatctgataaaacttggtttcatatctaccagcatttga

aaaagatcatttcggaagccatgtcccttatatgtgtccactggcatagcagacacttgg

tccaaccatatcacaccctttgcagttgtcgttaattgaagtctcgagtttcgactcgtt

gccttggcctccaatatagtctcaaccttggtccaatttgaaaaataagatgcagaacca

gtgatcacggttgaagcgaggtttcccacaccgttcggtccagtgaacgaaactactaga

ttaagaggtccagttgaacgagcatagaagacgattttatatttcttcccttgctcaata

ttcatgccccagaagccagggttataaacgccaacaccatctgcggggcaggtattgtcg

cataacacctccaatcgaagtgcaaccttgttacgttcaaagatagatgtacgatccgtt

tcaacattaatattcgtttcatttccaataattgaccacggatcaatatttgaaggagtg

ttaggccccccggcttcaaatcctcggttgctgacaagctcggcccacaatccaccagct

ccagcatgattgatttcctcaaagaataatccaaatagagtatctggtattggcctagca

gatgcttgggaagcatctacaacgagtgttgcattaagatcagcatgtactttgacatca

aagcattggaccaaaagacagacaagtatgaagagttgaagcataaaaacacagcatgag

tccttggataaacccatttttttatatgttcacaacattcaatatgatatgtgtgttgag

taatattgggtgaaagggtgagtggtttttatagagaaagaaaagtcgaatgatgaaaga

gaataagaagagtgatgttggattttgatttggttttggttgaaaggtttgttggaaatg

caaagtgaattgtgagtgaatg

>fava_dn_rep_c502

tccaaagagtataaatttacaaactttttatactcttaaggaaaccactcttgtaggtag

aatcagaatacaacaaatagaaagtaaaggatttgagtgtcaaacatgttacataatata

tatctgaacaatatcatcatttcaacaggtattattcttatttacaatgcacggttatca

taaatcatttcaaacccaactcagatgaaaagcaacacagacgcaacagtgataccgatg

acggctaaggtggcaacaactgaaccttggtcaaatgatttctccaagcctgttcttgag

aaatgctggaaagccaaatacgctgcaatcaataaagaaattgcagcaccttcttttgtg

tcccttaatcttatagcctcataaggagctacaatcaccaagagtaacgctataaatggc

aactcaagttcaccaggaatatggaagaagagacgaacgagaagtgccaaaaatgctgac

cacacgccatatctaccttgtatccaggcaaagacagctgcaggtgcttgcaaagcaaat

aatggaacaagaaatgacttgtgtacagtggtgccctttgccagcactaaaaccagagca

gaaacagtagagatccactggacattaggagtagtgaaaggataaggagcagcatatgaa

cggacacgaaacgctgtgctggttctcgacttgtcggcgattcggattccgtgatggcga

gtgaaggatctgagacataggggattgaaggtgcggttggagaaagaaaacgatggtgaa

gagaaggatggatctcttgttgcgcagttgaggggagagaaggcagttcgtgcatttgca

gttgaagttgaagacagcacgcaacctgatgacatcattttctttctctttgttatgcta

cctgaattttgagaccaaagatttaactatttgagtctacccagacacaccgacgacccc

acttccagcttcttaacactctaacacatacgttcgcttcccttcttgttgcacacacac

ctcactcaagttaatgctaccagtgtat

>fava_dn_rep_c503

ttactgccgcggggtatttgccaaatcaaatatcatggtaaaattgaacaatgacccatt

cggattaattgataatattagttgatggatcatttgtaaaaaggttttcttaactcctaa

gttatgtcgagtagaccttgttcttgctataattcttaattcatgcgttgtagggggggg

atttatgtcaccacaaacagaaactaaagcaaaggttgggttccaagctggtgttaaaga

ttataaattgacttattatactcctgaatatcaaaccaaagatactgatatcttggcagc

attccgagtaactcctcaaccaggagttccggctgaagaagcaggtgcagcggtagctgc

agaatcttccactggtacatggacaactgtgtggaccgatggacttaccagccttgatcg

ttataaaggacgctgctacgagatagagcctgttcctggagaagataatcaatttattgc

ttatgtagcttatcccttagacctttttgaagaaggttctgttactaacatgtttacctc

cattgtaggtaatgtatttgggttcaaggctttacgcgctctacgtctggaagatttgcg

aatccctaatgcttatgttaaaactttccaaggtcctcctcacggaatccaagttgagag

agaaatattttgaagcaaaataatgggttactggaaaacaaaagttcttcctaaactcaa

gaaagtttttgagaagaatggtactaagaaagctgctgaggcctctaagtcctttgatga

atcaaaggagggaatcaatacagagtttgaggaaaagaaaagtgaacttgaaacaaaagt

tcttgcaatatatgaggcttcttcaactgaaatcaagggtgtgatcaaaaagcgagatga

agcagggctaaagaagaattcaacagaagttcagaaattcatagatgaacttgttaaaat

tgattttcctggatcaagagcagtatcagaagcatcttcaaaatttggaccagccttagt

ttcaggtccagttctctttgtttttgagaaagtatcaactttcatagtgacagaagaaaa

aactgaagaagcaactaaaacagaagaggaaacaagtggtgtaaaagatagggaaattgt

ggttgaagaaggaaaaaaagaagaaacaagtgaatctgcagcagagtggccattacggcc

gcgtgactcatcacattccttcctctctttccctatcatcaatggctccaatctcaaaac

tcctctctttcattttcctaacaacccttttattttctctccaaattcatgctagagaaa

gccaattcttcagcaaagtcacccatgttgtcaacaacaatgtcaatgaaagagaagttc

ccaatcccaacaatgaaggatcattaaacaaaccagatcaacaacaacaacaacaagaac

caggtttcatcccagaaactgaaaacagctatggcttatacggtcatgaatctggtctat

accctccatcaaccacctcaaacaatgttgttcccaacacttaccaatcatatgaaaaaa

catcatcgtcacaagatgaaaacaaaaatgcttacaacaattacttcaccaccaaccaga

tggattcttatagcaacaacaacaagtacccaaatgaaatcagtggcacaaaactcacag

caacatcttacaacaacaacaacaacaacaattacgacaagtattactacaacaacaaca

agtatgcttctaatgatcctagcaacacaaagttcacagaagaagcatacaactccatgg

aaaaccaaaacaacaacaagtattactacaacaacaacaagtatgcttctaatgacctta

gcaacaaaaagttcccagaagaagggtacaaatcattggaaaatcagaacaacaataaca

agtattactataacaacaacgttgctgctactgacaaatacaacttcaaaaccaatggtg

ctagtaacaacaattacaacaatggtgagagacaagggatgagtgacacaagagttatgg

aaggtggaaaatacttttatgatgttaactctgagaaatacaacccaacattattcaatg

gagactcatctaatggggttgtgaactctgagaattggtacaacaaaaagggttactttg

gtagtaacaacaatgtcaacacttatgagaataacaagaattctatgaatggttatcaga

atcaagaggagtttgagagtgaccaagaagaatttgagccttgattgattgaaacaaacc

gaagagagttcattattatccctttgaattttggatgtggttgttcaatgtttttatgta

ttttagattaaaaattacaaacaaa

>fava_dn_rep_c504

cccacaccttcaccttgtggttctcaattcctttagacctttcttctcctttctttcaat

ctctctactctcaaaatcatcactcatatcaatatacttttactccacaacttcaaaaaa

ctaataagctgcggttctgttccatagaatagagatatttcaaatgccatgaaagctagg

tgtatctcattctttctttgatcttctgatggcacttacaacgaacaaggtttccagcgg

tcctatcttgactaatagaacgacactttgtaaatcaaatggagaacattattttccctc

atcagccaggatcaacagagtacagttttcaaaagttagactggaacatagacatcagag

caatggatttatgctcaatgagagatcaaccttattcaatgactggtttgttaatggaaa

accagtaggtttaacctccaagacatctcctgtctcatgtaaatcaacaggagcaaataa

taccgaagagaaagaatgtgttacaacttctgaacatgccgaagatgggaaaaatgaccg

cgctcattctgttcgtggcttggctgaagcttatagatttgcctgtaacgatgcaaggtt

tctctcgcggggcataatgaggatggatgcgcgtgctcgccaagatgttgcttttctagg

gacagaatttctcaaacttgatgctcgggcgagaaaggatactgagaaaattgaccgtgg

tgtgaaggaaaaagctaagcgccttaatcgtattgctactatcttgaaagatatagctga

gtccagattgaaaagtgctgccgataagcactggagtgatggggctttagaggccgattt

acgccttgctgacttccgcgccaagcaacgtgccatggaagatgcccttatgtccttgga

gttgatcaaaaacatccatgacatgatggtgagcaagacgtatgactttcctattttcag

agataagggctctctttcagaaaataacgtcggaggccgaatcatgcttgaaaagaacgg

aaaaacgacaaattcctttttcggggatgtgacagcagaacggattactgctcttcagga

agcttactggagtatggcatcagcactttctgaagcagatggaattgattacactgatcc

tgaagagcttgagctgttgatcacaactcttatagacctcgatgcaatggatggcaaaca

aagtgtatctttgttggcagagtgttctagctcccctgatgttgggactagacgagcttt

agccaaggcgctggcatccgcaccatctatgtggactcttggaaatgcagggatgggagc

attacagaggctggcagaggatagtaacccagccattgcagctgcagcatcaaaagctat

ttatgaactgaaaaagcaatgggaaatagaagaaggggatagctggaggtttatgatgga

tgaaagcagcaaagaagaaaaatgaaagctgagaatctaataattaagataccaattaaa

aattgtatagcatgaatgcctccctaggattctaactagtgtagttttagtccggatttg

gattccatgcagtgggaaaaacacattttccctagtaaacaatctcggacgttaatatga

gattgaatggtttagattacacaataacaaaatcaatttagatgatcacgactattaatt

taagatcggacattgagattgaaaactgcattatgcagtactgcaccaaatccaaatcca

aatccgtattaaaccatatgatcaccatggaaaatgtgcttgtgtaagatgccaatatgc

tagttatatgctacttattgtatgatgaactttcctgcaaatggtttgttgagacttttt

ttgccatctttgccataaaaatgcataaagtagccacaaaaatgtaagacctaatctctt

agaccaaaattgtgtagctaac

>fava_dn_rep_c505

gcaaacaaagacaaacataataaaattaagttatttttttaatatcatggatatacatat

catagtgcttatacatacattgagtggtagtaactagtaatacatattttaatcaatagt

actcaagctgaatactatagagtttacaatatttgtcacaaacttgatagataatattac

agcttttaagggcatttgggtcctcctctttgggtcttccaattgttgtagcaaggacaa

acctgcttattgccataagttccaggaggcacacacaaacatttagcacaacacttttga

cagaagaacaaacatggtttcttgtattgtgtacttgagcatctatcactgcaacgtggt

ccacattcttgtggttgaagacttccttcagtgtttccttgatgtggaaattgagtagtg

ttactgtgtttgctaggttgtggagctggaacctcaacagtggatacaactatctctgca

tggttttgtagaggaagaagtagcatttgaactaggaagagtaaaacaatgcacattttt

cttgccattgctcaaaagatagaagatatcacaagtgtcccgcggcaagttacaagattt

atgtctttttatagatgaa

>fava_dn_rep_c506

aaaaaagtcgggcagttctatagctattttgtatttgtttcatacacatgtactacataa

ttatataatttttatcaggtggtattgacaaacacaattcaaccactattattgcaaaat

gtgataaagttgtgtaaacattataatcagaattcatggccaatagtacttgtcataaac

aagactaagcaaacttcataaccatttatcgcacatttacattatcactatcactatgga

ttaattacaagaagatcttactaacttcaaacatagatagccacaactaggctcaaattg

tctcaactctgaaaacaaagtaacttaaaagcattttgtaaaagacaaattgaaactcaa

acagaacaagattattctactttcttttccactgaaacctctctatctcctttcatgtta

gctttcccctgcttaaatgccttggatatttcatcaacaggaatcaaaggcaaatagcca

acaaaaccataaccctttccagccatggccttgattacatggttgtatttctcagaccag

tgagcagcggttggaaccgccatttctgcaactgcatggaaaggtggaaattggttgagt

ccaacccacagcttcactgaattgattagcaacaaactcttggattctgtagcaacatat

tttacagcagctcgtggtccttcagattgagccacattcactgccttccctgcttcatgt

gaccctttctgaatcaaagttttggttttatctgcaagctgcttagcaatagtaggagca

tgttcgttgaacttgtgagtagcttcatccaccttttggtcaacaaaaaccaggacatca

tcagggacatccttgaatttgttgtagacaggaccaataacagtggttactgttccctcc

actgtttcaacagcggatctcaaagacccagaattcttctttgcggaattatacagatat

gagacaaacacgaatgtgtgaatagcagcgatcttcacaaaccctaggcgctttagttct

ttgtccttgttctcagccttcacttttacctcgtaggtttccattgctttgtctgtaatg

gaacttgcttgttactaaagttgaaatatgtttataatgaaacagtaataacttatactg

caaatcgaaaggcacacagaatggagtgaaacaacggtgacgaattttgagagaaaaatt

tcaacttgcttttgggattgagactcgcagcatggggcggcttatatcatcatctaaaat

ctcatccttttatcacaacaacaaattaaaaacaaaacaccattctttgtacgtcaaggt

gggccccacatttacaccgactcctatggatctttctagaccacactaggtatttccaca

tgccccgtggcggtttctctaaatttgtgaacag

>fava_dn_rep_c507

acgaagctgatcaaccgatagagccgtgagatctctgcttcaacacttcagaggaatccg

ttttctctgatcggaatctggaatcgacgaatcggaatgggtttcttgataacgacacta

atcttcgtggtggttggattcatcgcgtgcctctgcaccagaatttgctgcaatagagga

ccttctgctaatctatttcacttgactctggttactactgcaacagtatgctgctggatg

atgtgggcgattgtatacctggcacaaatgaaaccgctcatagtacctatactaagtgaa

ggcgaataagtttatgaacaaatgtagcttcttgtatgttagataatatggatctcagat

tggttggtggagagaatttgatatcaattctcatgagtcattgattatttcctaaggttg

gatatgtatgtatttttttgtgttggataatggcatatctgtttttgcctgatgttgctg

tgttgtagttgttgtattcaaccctcattcatttcagatgttgtttcaatttcaagtcat

aaatgaaaaataaaaatattacacattagtcatcaatacaaaaagaaaatgacttatttt

aatcaatactataagtatgcattatttgata

>fava_dn_rep_c508

aattgtataaatatttaatacattaggcgtgttaaattatgcgtttttctttaatacagt

ttgtttatttttagcaattgtggcggttgtgttgaatatatcatcggtattgtgaagtcg

tggtgagagtgtgtatcttcatcttcacctaccgacgctgtatcaatggagaaactcaaa

ttttctcggcaggtagagtggtggcagaagaacatcatctatcctgttcgaagtctttgg

atttccatcgccacacgttttggaattcgtaaaactgggctgaagaagctaaaaaatgat

gtgagggcttgtgaatatgaggatattcatgtgatgtgggagatgttgaacggaaatgaa

tcattatcagagtttggtggcaattctccaaacaaaatcaagaagatgcatcacttaaaa

ttgttcaaatgggcaagatgtgttgctccatatactagtcacacctaatcaatcatcatg

ctcttgtgtgtcatcatgtgcgtgagtttatttctcttcacaaaaaggctgctatacttt

gtttcgtgtggtttttttcttcaaggaataaagagattagggtttcatgtagaacatggc

ctcttctctcatgtgaatcagctcaatcttattattgttggtgcacagtgtgtgtgagtg

ttgtaaattgcaaactaccgaatttaattcctactaataaaaaattcaaatcattttgta

gatatcaattgtatccaccaacctgatgtagccactatggtatgaaattaacatagcgag

tcatcagtttagtcgttgaagttataagatttgaaaatttcgatccttgaaattaacaaa

gttctaaaatgttttctgaaaaattgaaaatcatcgatcacaataactcttaaagaatca

atttcaagggaactaaattgtccgacccaaaattactagtgtgatcaaggattttcgttt

acaaggatcattttagaattttgtctatttcaaggactaaaatttccaacatctacaatt

tcaggactatgttggcgattcacttgaaaataatgtaaagttttgttgtgtgatttatca

ttttgttactttggagattcatgattgtgtcgcagtttttacattgtatcccttggtttt

atatcagaaaccatctgcattatgcagaagattttggaatggttctctaaatcatagcta

ttatgtgtgtaattttagatgtgtttttatgataaaagtcgaacatttctaatgatctga

tggttatgtttaattcagt

>fava_dn_rep_c509

atttttttacatttagtagaatctattgatctattagaaattttttaatcttgctttgat

atataacgaaatatgtattcttatagaccatttttgctatccgtatctaactagctaata

tttttggatataaggttctaacgaatcagtatttttctttttttattattatcatattgg

attggcaaaaatttatcaattccaaaaaatccacatttttgcgggcgaatatttactctc

tcattataaattgattaaattgaagatgtaatgtagggttagtccacaactcctcaaaaa

agaatgaattcttagttccttccgccatcccatctaatgaatcagtaagatttcaaattt

taatataatcttttgtattcacaggttccgtcgttcccatcgcttttcaatttatggtta

ggtctaaattctacaatggagcctctttaattaagaagattccatttttttttattttct

tattttcttcttttttgttgaatcaaccttctcagttttcttgattcgtggctcttgatt

cgtttattctaggaatatattcatccatatatggatgaattttgaagattgatgctttat

tacactactttttatgagatgacccatagacctttacataggagaattctatatcattga

tatcctttttctatctttctttcaccccttcatttatctgtatattcttattgctttaca

actaataatcttatttttttttttatttcagttgctataattatatcatcacatagatcc

ttattttgattttagattttcggcggttctttatatccagataacgggaagcgatgagta

ggttatttattctttagtaattaattcgacgaatttttgtagaaaattaaccactctaaa

aaaaaaccaacgagtcacacatgatggttatttctcaattccatctctattacagaaccg

tacatgaaattttcacctcatacggctcctcgcgaataaatcgattgaaaaatattgaat

cgagaaaataataataattactacaataagatacaacagaatataatcgcgggatttttt

tacatttaatagaatctattgatctattagaaattttttaatcttgctttgatatataac

gaaatatgtattcttatagaccatttttgctatccgtatctaactagctaatatttttgg

atataaggttctaacgaatcagtatttttctttttttattattatcatattggattggca

aaaatttatcaattccaaaaaaatccacattttcgcgggcgaatatttactctctcatta

taaattgattaaattgaagatgtaatgtagggttagtccacaactcctcaaaaaagaatg

aattcttagttccttccgccatcccatctaatgaatcagtaagatttcaaattttaatat

aatcttttgtattcacaggttccgtcgttcccatcgcttttcaatttatggttaggtcta

aattctacaatggagcctctttaattaagaagattccattttttttattttcttattttc

ttcttttttgttgaatcaaccttctcagttttcttgattcgtggctcttgattcgtttat

tctaggaatatattcatccatatatggatgaattttgaagattgatgctttattacacta

ctttttatgagatgacccatagacctttacataggagaattctatatcattgatatcctt

tttctatctttctttcaccccttcatttatctgtatattcttattgctttacaactaata

atcttatttttttttttatttcagttgctataattatatcatcacatagatccttatttt

gattttagattttcggcggttctttatatccagataatgggaagcgatgagtaggttatt

tattctttagtaattaattcgacgaatttttgtagaaaattaaacactctaaaaaaaaac

caacgagtcacacactaagcatagcaactccttcattataaaatgattaattaatttttt

tttatttaatcttttaaaatttttcatttcttctttgagaataagaacgtagtaagaatt

cgtcattttttgttttctcaaaagatggaacgttataaggaaaaggttattagtcgttgt

ttataaatatccaaactttgattcctaagaccccataaatagttcgaactggataggaac

aataatcaattttagctcgaatggtttgtagaggaatcctaccttctctgatccattcga

cacgtgcaatttcgtttccgtcgatgcgtcccgcaatttgtatttgaactccttttgtac

ctgcacctgcctcttcagcgaatttttttatagttttttttgcattgctttacgaaacgg

aattctattctttaattgtctggctagaaattctgcaagaatattcgggtctctataaac

atttggaattcttgtaatttcaatgttgatttgtcggttcacacaatttatttttttttc

acattagtatgtaattcttccattctttcagggttatcctctattactaagtttgggttt

ggaagtcccatattgattatcacgtgaatcagatcgcgtgtttttttaatctttattcgt

ccaattccctcgccatcgacagcggttgttgttttttgtattttttgtatataattcttg

atacaatctcgtattattttatcttcttttacattctcggaataaattcttggttgtgca

aaccaaatagaatcatgactctggtttttctttaggcagccagagaaaacaagacaattt

atttggacacagacattaaacttgctattcaatatataaacaagaaagatattcaactct

ttagtgagagcaagaagcaaaaatagttgcaatgaagacaaactccaaaatttaaaataa

agtacaagcttgaactctagaatttgtcctcatcgtgtcatgtcctcttttatctacaag

cttgtaatcgccctgacaaacttcaaagaccaagcttgtagatgattgacttagtaaagt

ggcaaattttcatccattggaaaatttcaaaggacctcagatttgaggatcattagaaaa

tagctatgagatcagtgatctgattgatataattttgctagctaagatccataacattca

tgtaataacagaaaacaactgttcaggtcaagaaattacgattgttctctcgctaactca

tttaaccatcattcaaaaagttcaaaaacatgtgttctaactaggtgaaaactgaccgca

gacagaatatcacgctctagcctccgatgtctgattgtaaaggtactaatttctcttctt

cttgcctcctttgcctgattttgatggggtagctaccaggtacacaaacataacaattat

gctgatcaccgagattaaagatccatacttagccagcaacctcttagcccattcaaactt

cttctccggaggcctgtcagcaagaacatctaacggcaatatgggagttgaaagtgccac

ctgtaaaacagcgtttgtggggacacggaacttgatgacagctggttcaccagaaaatac

tccctttgattttgcctccagctcaaatgtgtgagagaggacaccaccagcatcaagctt

ttcccatgtctttgaagtggtaccactgattatactgaaaaggtctgttggccaactctc

atctgagagactcacatcatatgccgtcgaggttccttggttgtagatatcgatggtgac

agagactctttcagcgccagtcttgagccggttgagagaagccttcttgtgagcgacgat

gaagggaacgtcggaggaagcgtgtgagaatagcaagagagaagctatgagcagaaaagc

aatcagagatttggagattggatccgccattggcacccacagagagagaaatgaagagag

agagagatgaagagtcaatgagatcgtgtgagtgttatttttatttgtggttcattat

>fava_dn_rep_c510

caattttgcnnnnnnnnnnnnnnnnnnnnnnnaaaaaaaaattagaacttattacatatg

ttgatcatagtacatgcgacttgagcctctaaacactccaaaatagtttcgattaacagt

tttacatcacattcacaaatcatcataattaaacatatccaacaccaaacatatacataa

acatcatatcagttcataactcaatacaacaagttcaaactttacaatttcaacatcatc

aatatatcaatatcatcattacaacatcataataccatcatgttcaattttttgttttat

tattaaaaaaaaagataaaaataattactaagctctgtatttctgaatcttgtttttgag

ttcatctttcttcgcaccaacaatcttatcaacctctttcccattcttcaacaacaaaaa

cgtcggcatagcctgcaccttaaactcctgcgcaacatcagataattcgtcaacgtcgat

cttgatgaattcaacatccgtgaattcgttagcgaaagcatgaacaatcggttccatcat

tttacacggtccacaccacgtagcagagaaatcaatcacaacgagtttagacgagtcttt

gatttcgttgaagtggagttgccagcgtgcggatgagtggaatgttttgacggaatgatc

ggaggattctgatgattgtgagggggcggcggcggcgtcgccggcgggagggaagagaga

agtgagcccattggtttgtgcgttgacttgcttgataactacgcttttttgtgaaagaga

ttcccgcggttcaaatgtttagaagaaactgtaatgtgaacttgatatattagcc

>fava_dn_rep_c511

gttgttctctgtgtattcctaaaattataacttgtatattatattaagagtgatggattt

tattaacttagagtgaaagtgatacaaatatgaggagtactcttcatcatcatgactctg

atctcaattaacataacataacattaagtaggggggaggaaccagattatttttacaaca

ttattattattatttttctgattcccctagtgatctcatatcatatcttttcatcagctt

tgttctcaacagcaacaacaaccttgctggtttcctcgttgctggttggcttctcatcag

aatcaacatgaccaaccacaacaatattcacctgtgccttctctttacccatcgcaacta

gcaagacacaacaacacctaatataattatatggacttgtgaaggaaactagaataagtg

atcccgcggccgtcacttattctagtcaatgacnnnnnnnnnnnnnnnnnnnnnnnngtc

tactgaattgggtacaaaactaggggatgaatctatagaaattc

>fava_dn_rep_c512

ctcacgcgtttcggtcttcccgaaagctctgttctgaacttcaccagcatcattttcaca

tcaacaaaccatggcattcttcttgaacaaatccaccctagcttctcattttcgatcctc

atctcagaagatcgaagatcccgtttctctctcgcgccgtcagttccatgttgaacccgg

aactcgtgaaaaagctctcttggcagaagattcagctctcaagccattcaaatcatataa

gcagagtgtaaaaaagctcaggaaaattggggatgttttaactattgttgttgttgcagg

atgctgttatgaaatatatgtcaaggcttcaacgagagaagaggctcgcaagcagtagtg

ttctgaaagtgcagaatgagatgaacatttggatgctagttttctttacattgtattttt

atggcaacaatttggatgttatgatagccatgttagaataaactgaaagcatttgaaccc

taattgcttgtgcatgagtagttttttcaccattcaagatttgtgcattctgataatact

gattgccagatcatagttgagagagttgttatggagtgtgttgtttattgtaattttgta

gtcaagctttcactgttctatgaatctggtgatcatttagtaacttactgccaatcaaag

atcttttgccagtttcttttaaaaaaaaaa

>fava_dn_rep_c513

gcacataacnnnnnnnnnnnnnnnnnnnnnnnnnnnnnnnnnnnnnnatcacgaccctat

tttaatatccaaatcaaccttctagtttatcatctgaattgtcccgcgtattgtcaaagt

tccaataatataataccttaacactagggttcttacttttcgataactgaacggagaatt

gagcaacactgcatctagcctcgtgattgtttgtcatatgtgtatacttttcaaatacac

atttgtgacgagctcatacaaatgaagtgaatctatggaccaatgtttgtccatacataa

aacttacaaatacaaaacaactaaacataggaaaaaactgtagaatcaattgttgaacat

tggaagcattacaaatctcaatattgcagctcggatgctaataattgcagcccattaaca

catccactaataattacacaagttcaatgtatgccataggtgcattatcaccccgccttg

gttgggttcttaaaattcttgtataaccaccatttctttcgccgtaacgttcagaaacct

ctgcaaataaagcatgaacaacctgcttctcatagatgaagccaagggcttgtctccttt

tatgaagagaaccatcttttgcaagggtgatcattttatcgacatactttctaattgcac

tggctcttgctctggtggtcttgatacgaccatgtttaaggagttgagttgttaggcctc

gaatgagtgccttacgctgatcagggggcctgttgagttttggcactttccttccatgtc

tcatagcatagactcgactaccgttgctaataacggtgaaaccttgatcgaagccaataa

attctgatgaagcaggaccgaagagagggagagtacgaagtccggtgaatggaggaagta

ggggtttggatttgactttggaaatccggagttttggcggagaaggaggaggaaatgcgg

tgaagcggactaatgaagccgaaggagaagtaatagaaggtaaggcggagcggagagagg

acgtgttccagcggctggtgggtgttgtgttgttcgtgggggtagccatagctgccacgt

tcgccattcttggttgaggaagaattgcaacttcagaaccagtgctttgattttacagga

taaaagaaaagtccccgcgg

>fava_dn_rep_c514

acccaccaagtcaacaaaagtaactggttgttagattaaaagccttaaatctaatacaca

cctaacagagttaggtagaaatagaggctcagatttgtcctcacttatttacaaaaagtc

caactttgtgatgttaaaataaacagagaaacaaaaatggttggaggaggagaaaatgaa

aagaagatgaaaatactgtgcctccatggattcagaaccagtggcagtttcatcaaaaaa

caaatcagcaaatgggatccttctatattctctcaatttcatatggaattcccagatgga

aaatttcctgctggagggaaatctgacatagatggcatttttcctccaccttactttgaa

tggtttcaattcgataaggatttcactgagtacactaacttggaagaatgcatcacatac

ttgactgaatacatcatagccaatggtccctttgatggttttcttgggttctctcaaggt

gcaacactttcagcacttttaataggttatcaagcacagggaaagttgttaaaggagcat

ccacctataaagttttttgtatcaatatcaggatctaagtttagagatcctagtatatgt

gatgttgcatacaaggatgtgatcaaagctaaatctgttcattttattggtgaaaaagat

tggttgaaaattccttctgaggaacttgcttctgcttttgagaaacctgttataataagg

catcctcaaggtcacactgtcccaagattagatgaagtttctactgatttgttgaaaaat

tgggttgccgaaatcgtaaccggagaaaaggttggtgtctcagtttgtgagcatgaaaag

aaagaagaagttgatagtaccaacgggatggaaataaacatgggaaatgctgaaaccgta

gtggtggcccaagcttgaaaactttagattataaggcagctgaatttgattgaaattgtg

tttttagtagtgattcttcatttagtttgctgaattatcgagtactattataataaagtt

agatgaatttgctatcaaaaaaaaaagttagatgaatttacctcgaatcatgaatatgtt

ttgcttgaattaatcgaattgaatatgatcaacttctttgaatttaaa

>fava_dn_rep_c515

actttttaactagatattgaaagtatttatataagccttcaaaaggagagattaaatatt

aagataagatagtaaatatgtaaacattagaagtttcattattatttgatatttagttca

caggaatacagttcacaagatacacataaacataattaaaacactttatttaattaaaat

tattacccttaaacacacatatacatattagggaagccccattcaatttccaatcctggc

tccagtgtcgaacttggcctttccgaatttctgagttcctccaaatgagttgttgaatgc

atttttggttccgttgttacggtgggtagtgtaacctccattggtaatttttgcaccagt

actgaattgagcctcatcgtaatcttgtgtgccacacttgttgttaaaggcatcaacagt

tccattgtttccaccatcgttcccaccaaagatattcccgaataaaccagccatcttata

gtatatgatatgatctctttcaatatatacttgaaatgctttgttttgtgttgatcccgc

ggcc

>fava_dn_rep_c516

caaatgggccaaaactggatcagcccaagaactaccattgtcactaccttttaaaccctc

aaaccctaattccaatggatttgcggcgctgcagaactctctacactttctcgtcgtctc

cgttcacgcttccttccatttccgtgtgaaaatgtcgcttggagaaactgcttgctcata

cgctcttctcatccttgaagatgataaaattgcagtcactgctgacaaaatcaccacttt

gttgaaaagtgcaaaggttgaagttgaatcatattggcccagcctatttgttaagcttgc

tgagaagaaaaatattagggatttgattgccagtgctgctggtgctggagctcctggtgc

tgttgctgccactccagttgctgctaccggtggggcagctgctgctgctccagttgtcga

ggagaaaaaggaggaaccaaaagaagagagtgatgatgacatgggatttagcctgtttga

ttaggttctcttttaatataattcagtcaatttttgttgtggtacctttagctatctgaa

tatgaatattaagttttgttatcacttgtgtccttttaaggattcaaccaattaaatgtt

acattattctgtcttttcattgttttgtttgaatgtttcactgatattaaatcatagttt

acttgtgagttagatcaaaactaagttatgcctttatctaacctctttgattggttaata

attagtattgaacatagtaatgatgtttaatggtt

>fava_dn_rep_c517

gaaatttcacattatttagattctcaagactactaaatatataaatgtgtgatggtatct

tcactaaggactacaatgtgagtatgtgacccaaactcacattaaacttagtatgagagt

gggtgttgaagttatatgaaacttacacaagctctctctatgagctaacttttggggaca

aggaaggtaattgaaatataatctaaatacaaatcactaaaaacttatagtaggcagata

aaataaaaacactgaaaatgcctgttttattaagagtgtacacaagtgagccgctatagc

tctcatccacactacaaacaacatagttacattacaacacaactatatataagttgtagc

taccagcaacattgcatatcccacttatctttgctttcagccataacctcaactctttca

aggaagtgtataaggtccacacttgtaaccgacaggacgatcagcaatattgcaacgttt

agggatagtaacagcaactttagggtcagcaccagacattttagcaacgtttgataacat

aacagcacaaagacaacctggattctgaccaagtttcttaacctgagcacaacagctttg

agacacatttgcatcttcatcagatgctgcactagcacaaggagctagcttaaaagcttc

attatctggtgttgtaccccttccacattcaccagcactttcaactccatatatccctgc

aacaaggactacaaaaccaactagacacaacaacttcataagaccctccattttttcttt

tcttaatgtatgtctaatatttcttgttctatgtccccgcggccgtaatggccactc

>fava_dn_rep_c518

ttacccgcgggcacgactccattaatctaaaatggctccaacaattctgcccctggcaac

tttatttttagctttctccgcttcttttctccggatcggtgaagcggaaacagatcaact

cactgcactcaagcttaattctcatattcttcaggagtcaatcgctagagaagttaacga

acacccaggggcaggatggaaggctgctattaatcctcgtttctccaattctacggttgg

acaatttaaacgccttcttggagtcaaacaaacacctaagaatctactgaatagtatacc

tgttgtaactcatccaaaatcatcaaacttgccaaaggagtttgatgcaaggacagcttg

gcctcagtgtagcactattggaagaattctagatcagggtcactgtggttcttgttgggc

atttggtgctgttgaatcattatctgaccgtttttgcattcattttggattggatgtacc

cctttctgttaatgaccttcttgcatgttgtggatttctttgtgggtctggctgtgatgg

tgggtatcccattgctgcatggcgatacttagcacaccatggtgttgtcactgaagagtg

tgacccatactttgatcaaattggatgttctcatcctggttgtgagccaggatatcaaac

tcccaagtgcgttagaaagtgtgtaaagggaaaccagctttggaagaagtcaaagcacta

tagtgtcaaagcatataaggtcagctctgatccccaaaatatcatggaagaagtttataa

gaacgggccggttgaagttgcattcagtgtttatgaggattttgctcactacaaatcagg

agtttacaaacacatcacaggatctgcaataggcggtcacgcagtaaagctgattgggtg

gggaacaagtgatgaaggggaggactattggcttcttgcaaatcagtggaatacaaattg

gggagatgatggttacttcaagatcaggagagggacaaatgaatgtgggattgaagagga

ggttacagccggtttgccttctcccaaaaatgttattagagaagtgaccgacacggatgt

tgacgctgacgtttccttctgaatgtaaatgtaatatttactataacggttaaattttat

gttggtattgaaaatctgcaccaaatgttgtctgttatatatatgtatatttaaggaaaa

cgatcttaattatgaaaaagatgtatggagacagtaagcttttgtggtttatacaatgag

atcacaccaaataagaggctaattgacatatttatagtacagtctacttgcaannnnnnn

nnnnnnnnnnnnnaggatcgcacgaacctttcaaactcagttgaacttgctacaatgctg

gtgaaatcagaccaagtgggaatagaggaacttgctaaaattgtttagctgaattgttca

tgtttcttctcctgttctcctcttgccgttcctctggagatagtctatgggatacggcgc

atttcctctttttttgcttcccataatttcgcatggttcctctgcttaatattttgtctg

tgttattatatcgcagagttcttaaaaatgcccctaaaaataggtttgtcctgacatgga

agcattgctttgtggtttgtgcttccattgctctttggtttgtgaaagctcaagaagggt

gaagtaaaatattgtgtggaagaatttagcggaagaagagtttataaactgaaatatcct

ttgatccgaacttgtaagaacttaactttgttgagttgtttccaatgatgttctgaaaga

aactcttattgttgttgtggaagtgcaagctattcctttccttttcaaatgagagtcgtt

taatgaagaaatgtaataaaatggtaaaataaacaacagaaaaattgatcagtttcatc

>fava_dn_rep_c519

ggccgcgggatcctataattcaacaccaaaaaaaaacattagtgtttatttcataacaat

ggcttccaacaagtttagtgcactcattataatcctttctcttctttcctattccacatt

ttcccatgcttgtggttcatgcaacccaaaacctaagcctagtcctccaccaacacctaa

accttgtcctcctcctaaggttagtcctcctactccttcaactacaccacctacggctag

tcctcctactccttcaactacaccacctacggctagtcctcctactccttcaacatcaca

aaaatgtccaaaggacacattaaagctaggtgtttgttctgatcttcttggacttgttaa

tattgtggttggaagtcctgcttcaagcaagtgttgtgcattgattcagggcttggctga

tttggatgcagcgatttgcctttgtactgctattaaggctaatgttcttggtatcaactt

gaatgttcctatcacactcagtctcctactcagtgcttgtgaaaaatctgttccttctgg

tttccaatgctcatagactttcatgttataggttcaagccatacaagtacttatgtttct

actatgatgtttttattttagttattgtttacgtaaaagaattaaggtgacatcatgatt

ttgctaaattatcatgaatccaagtatggattataagtaaaatgtgtacgtcctttcaat

aatggggtaagtttgtttgccttatttaccaactttgttttttcaattctaataaatttg

tagaatctttgtctcaaaaaaaaaaaaggctaggttgacttccattgactattgagattt

ataattcatctctccacaatctgtatacatgtaatttggattcataagataagttctgaa

gtcataagcaaaacaatgactagtaaatctctgagcgagatagtgagttgtgcaacatct

tcatggaagaatacacatctagaatctgaaatataacctaatgttacaagaatcacactg

ctgttc

>fava_dn_rep_c520

ttaattaaatcacagagcaacacaactgcttaaagtttaattataaatgaaagtattatt

catacacatgcatgatatacagataatacattaattcataattcactccaaaatcaaggt

acgcaaacatgcttcaataaccacactttcgcacctcataaattttttttattgtgatta

cacgggcctaagcccatagacctcatagtttaaaatggatagaattgcataaaacaaaat

ccttttacatcccaaatgatacagatgagtagctgatactcatagcaatttactacacag

ttactacacagcagcaaagtactctttgctaagtttggggtctggtttcatggacttctc

accaggcttccacccagcagggcaaacttcgtctgggttctcctgcacatactgcaaggc

ctggagtgttctctttgtctcgtcaacacttctaccaattccgaggttgttgatggtaga

atgttggataaccccttccttgtcaattatgaacaatcctctcaatgctatcccctgatc

aggaatgagaacattgtaagattttgatatggatttggtgacatcagaaaccaaaggata

gttcaagtcaccaagaccaccagactttctatctgtttgaacccatgcaaggtgcgagaa

cacactgtcaactgaaacacccaatatctctgtattaagttctgcaaattctgcatggcg

gtcactgaaagcggtgatttcagtgggacaaacaaatgtgaaatccaatgggtagaaaaa

gaggataacatatttcttcccaatatactcagatagtttgaccttgataaactcctgatc

gaacacagcttcagcttcaaaatccggcgctgagttcccaactaatggcaattcactaga

agccctaacgacgaaactgcggcgggaggaagaggggcgggtgagggaagtgcgaaggtt

gggaagggaattggaggcattggggatggagagagaagagagacgaggagaagagaattt

gggggagaagagtgtgttagggtttggagataggagagaagcagatggagctgagcaggc

catgaatgagaggcggagtgtgaggtgggaggaaatggaagatagagatagggagatagc

aataaagtgtatatgtatatacttcacctc

>fava_dn_rep_c521

tgaaggccataatataatataaacaggctctttgccgaatgcgatcaaacgccatattac

tataatgccctcattaaaaccacgaagaataaagatataaccacagggttagctaatctt

taaattgaaaagcatcaaaagaaactattgagacgattgaagatcatgaaaggaaacaac

aaaggtatttttgctccacaagaaagagttgcacaaacaattaaaattagaactatctca

gataaaactcaaaataaagggtttaattggagtacaaaattctcagaaattcaactggac

ttaaattaaagaaactagagcatcaagcaaaagcaaaatgcttaactttcagtttgttcc

ctcaacctcctgattgtgctccggacagtaacggcactagcagtgttcaaagtgtaagct

cctcctgctttcactttgccacaatccttgcatccccatattcctacagcttttctcttc

acagcatactttccacaaaattcacaaaaaaatttgctgtgctgactaacttccatcttc

ttaatctgcttacgcagactagcaccataacgggtaccatatttgccaacaattccagcc

ttctttgtccttttagtcatttttacagagatcgaagaagctgcaagcttggtgtaagga

aatggcagcgattgaacggaagaagcagagcccgcggcc

>fava_dn_rep_c522

aaaaccaagagagagagagaggatggataaatggaacaaagtcggactcataaaaccaag

aatacatacaacctatataagatgaacctgaaaaaaatgcaacccactgtacactgggtc

acacatttcaaacagatctaattaccctgaatttgttgttgtcatacaaatatttgtcat

tacatgtcgatcaataaatatgccatagcaatactatacagcaaagctgggatgttatat

tacatagtctatcagagaaaacataaaaatactccagcttccaacgaaatagccatggat

gaagatgaacatgtccaggacatgccatacacatctttttcatcattcacataccctttt

cggttttccctttccttatcccttttctaacttgattggctagggctgctatgggaaaac

ataactcggcctatcagtaatagattaaaagatttattgacaagaaaaacagacaatgac

tatgctttaaataaatcaagtacatacatcatagaaaaggtaaccaaccacattatatag

aaagagcctctggtaacgactctggagtaccgctgctaccgttgagaacaatcacattac

catcagaatcaacatccactatgacagaatcaccctctttgatctctctagcaagcatct

tctcagccatgctatcctccaaaagtcgcatgatggctcttcttagaggcctagctccgt

aactaggattatagccttcatcaaccaccctgtccctaaatctttctgttacttgaagtt

caatttctttgaccttcagtctctgaaacacctccttgagcattatgtctgctatctcct

tcacttccaattttgtgagttgtctgaaaacaatcatttcatccaacctattcaaaaact

ctggcctgaagtattgttttagctcctcagttaccaagctcttgattctattataactac

tatctttctcatcgtaatccaaatcaaatccaattcggcggcctcctttctcaatcacac

tgcttccaacatttgatgtcattatgagaagtgtgttcttgaaatccacagttctgccct

tgctgtctgttagtcttccatcttcaaggatttgaagcatcatgttgaatacatcaggat

gggctttctcaatctcatcaaagagtacaacagtgtaaggacgacgccgaactgcttcag

tcaattgaccgccttcggtgtaaccaacatatcctggaggtgaaccaataagtttggaga

cggtgtgcctttccataaattcactcatgtcaagacgaatcatggcttcttcagagccaa

agtagtatgcagccaatgctttggccaattcagacttccccacaccagttggaccagaaa

agatgaagctggcaattggacgattagggttcttcaatccaactcgggctcgacggatag

cccgacaaatggctttcactgcttcatcctgaccaatgattcgcttgtgtaaagtatctt

ccatcttgaggagacggtcagattcatctgctgagactttatcaacaggaatgcctgtcc

aggaggacacaatatgttgaatgtcaacttcagtcacaataggaccttcatctgcggtct

cagtctctgctttgctcatctccttgcctttctctataagtgctgaaatctgcgccttga

gatccatttctttatcacgcagttctccagccttttcaaagtcttgattgcgaacagatt

cctctttctccttaacaatcttccttacctccttgtcaagttcttttgcctcttctggca

actgtgcatgttgaagacggactcgtgaaccagcttcatcaatcaaatctatagctttgt

caggcaaaaatcgatcactgatatactggtacgaaagttgcgcagcagctacaagagcct

catcagtatagcggagcttgtggtgaatctcataacgttctctaagtcctttcagtattt

gtatggtttcatctacagttggttctggtactttaactggctggaatcgtctctccaaag

ctgggtctttttcaatgtgcttcctgtattcatccaatgttgtggctccaatacactgta

gttcacctcttgcaagagctggctttagtatgttagcagcatcaatagcaccttcagctg

ctcctgctccaattagagtgtgcacttcatcgataaaaagtataatctcatcactttgtt

tgatttcttccatcagtttcttcaacctctcctcaaactcaccacggtatttggttccag

ctacaagcagacccatgtcgagggttatgaccttttttccttcgattgtttcaggtacat

caccattagcgatccgctgagcaagaccttcagcaattgctgtcttcccaacaccgggct

ctccaataagacaaggattatttttggtgcggcggcctaaaatttgggtgacacgttcaa

tttgtggctgccttcccacaacaggatccaactttccctcttctgctagcttggtcaaat

tggttccatactcctccaaagttggcattttattgttactacttcctgagccaacagtag

cagtaacactgtcagcactctcacccaccatgcgaataacctgtgtgcgaatattagtag

gatcagcgcccaggttttcaagaacacgtgctgctacaccctcaccctctcgaagaagac

ccagaagcaagtgctctgatccaatataattgtgaccaagttggcgagcttcctccagtg

aaagttccaatacacgctttgcacgaggagtaaatggaatctcgacagcaacaaatccac

taccccttccaattatcttctccacttcaacacgtgcatctttaaggttgattcccatag

acttcaaaaccttggcagcgataccagttccttcaccaataagccccaaaagaatctgct

ctgttccaacaaaattatgaccaagacgtctcgcttcctcctgagccaacattatcactt

tgattgctttctccgtgaaacgctcaaacatggctctaggtatgaatctcttagccctcg

ccctccgtgaagaagttgccaatgataccttggagcgaaaatctagtccaggtctcagca

tagtattgaaattacaaagtcccgagaaacctgacattcttgatccgcttgtttgcaaag

cacacatcatcttgactgatcgcttagactttcccgatcccttatgttgactctgcttgt

gcccagccactaggccaggaacactaagtgactgagccaaaactctagccatgattccta

aatatctgccggaagcagttccccggataaacggataagataaagcgaatcaagcaagaa

aaattgatatttaagaaattagtaaaaagagaccagaagaaactgataagggagaagaag

ttgttttgtatataatgatagaggaagcaaagttgaggaaggaataaaattgtggtggca

aatcg

>fava_dn_rep_c523

gagcagaagttttccgatgatttcaaagccgcgggatccctttctatatattcttctcta

acacattgtttcaaattaatcatgccttttttctcctcaaaaacacttgtcctgacctgc

accttatgcttatttctacctctagctttcggccacgaattctcgatcgtgggctactca

tccgaggacttacagtccatggataaactcattgaactcttcgagtcatggatgtcgaga

cacggtaagatttacgagagcatcgaggaaaaactactccggttcgaagtgtttaaggat

aatctaaagcacattgatgatagaaacaaggctgttagcaactattggcttggtttgaat

gagtttgctgatttgagtcaccaagaattcaaggataggtatcttggactaaaggttgac

ttgtctcaaagaaaatcctcaaatgaagaggatttcatctatagagatgtccattttcct

aaatcagttgattggagaaagaaaggcgctgtcaccgccgtcaagaatcaaggccaatgc

ggtagttgttgggcattttccacggtcgcggccgtggaaggaataaaccaaattgtgaca

ggaaatttgacatctttgtctgagcaagaattaattgattgtgacacaacatataacaat

ggttgcaatgggggtctaatggactatgcattctccttcatagtacaaaatggtggactc

cataaagaagatgattatccttacattatggaagaaggaacttgtgagatgaaaaaggaa

gaaactgaaactgtcacaattagtggataccatgatgtgccacaaaataatgaacaaagc

ttattgaaggcacttgccaaccaacccctcagtgttgccatagaagcttcaggaagagat

ttccaattttatagcggaggtgtgttcgatggacattgtggaagtgagctagatcatggt

gtttcagcagttggatatggaacagcaaaaggtttggattatatcacagtgaagaattca

tggggggcaaaatggggagagaaagggtttatcaggatgaagagaaacactggaaaagct

gaaggaatttgtggactctacaagatggcttcttatcctactaagaagaaataagattta

aaatttcctatattgttgatgaaagaatttcataatttctgtcttatttctgtgctatga

atttctaattttcatgttattttctattcaagcaatgtaatatcttgaattcaataatac

tcacaatttggcaatgcagaaaaaaaaagaaacaaacagatgggtgcagaactactctaa

accatgtacccaaattaccactacccttagaactccattcccaaatcctccttttctctc

tactccaatcaccagactcagtagtgaaccacaaaacacacaaactcacacagagggaag

aaaaaacctccaaaatcaccaccgcaatggccacaatgatcatcgcttcatccaaaccag

taatccgaattcccacaagctccctccccttcttgttggcacccccctcattcgccttcg

aaaaagccgccctgttcgacttcaacctcactctccccatcatcgtcgtcgagtttctct

tcctcatggtcgccctcgacaaactctacttcacacctctcggaaacttcatggacgaac

gtgacgcctccatccgaggcaaactgaacagcgtgaaggacacttccgaggaagtgaagc

agctggaagatcaggccaatgctgttttgagagcggcacgtgctgagatcgcagtggcgc

tgaatcagatgaagaaagagacgcaggctgaagtggaagagaagattgcggaagggagga

agaaagtggacgcggaattgcaagaggcgctggccaatttggaaaagcagaaggaagaaa

cggttaaggctcttgattctcagatttctgctcttagccaggatattgttaagaaggttc

ttccc

>fava_dn_rep_c524

agtgaagttattaaacgaaatcagtgagaagtaatgattttgcacatgtaaaccatcgca

aaataagagctacttaaactttataagcaagtgattttttcattatccaatatctgagat

aagtcacttgaaaagagaaaaagaaagttgcagcagttcaatattttgttcaatattttg

tattttcgacagtaaatgaaaccatgcacgacttaagcccggcagagacaaacatatgca

tttattacaaaatggaaaatacttccagctcacttcatttttccaagaatatattgaaaa

catcaaaatggctaacaagatggttggagaattcactcgtcaactggcctttgccctagc

acagaaatgtcttcataccttttcccaacattattcctttcccataacttgtgaactccg

taatcaacagcccgttctccgaggaaagcgccggcgatgacgaaggtaacgtagacagag

ttgcggcgcatgaggaccttgtaaaggccttcgaaaacgcctcctcctcttcttctagcg

gcggattccatggtgagatacggtggtttgatttgatttgaatgttgtacgcaatctgat

ttgggttctagggtttgtttgttaccacgcgctcttgttctctctgatccttctgcgttt

caaatgtaaacccccgg

>fava_dn_rep_c525

aataaatctattgcggcctcctattgctacaatctttcaattatatctatccccggcagg

tcggttactgctgctccttgcctgcggaaagcaatggcgtcagtcatctctctctcaaac

gcaatcggattctgtagcagcaaaaaacacaataccctctctttccgttcagaaggagtt

gacatgaagattctttcgcggcggcacggttgttcaaaagttggaaatggccaaagatgt

gttcgattgtatggcctatttggagggagaaaagatagtgatgacaaaagtgatgacgaa

ccttctaaggcaggaattcttggaaacatgcaaaacctctttgagactgtgaagaaggca

caaatggttgtacaagttgaagcagtgagggtgcagaaagaacttgcagtggctgagttt

gatggttattgtgagggtgagctagtaaaggtaactctgacaggaaaccagcaaccctta

aggacagaaatcactgaggcagctatggaattgggatcagaaaaactttcgcttttaatc

actgaagcatacaaggaagcacaccagaagagtgtccaggccatgaaggaaaggatgaat

gatcttgcgcagagcttagggatgccgcctggacttggtgaagggttgaaatgatatttt

taacagagattgtaatatgactcgggcctaaatttgtatgcaaaaaatgctagatgggta

gaattatattttaatttattttccatcttgatatttaagcctggagttatttgcaatttt

gaaggattctcttttctcacctttttatagacctttttaattggaaaaatgttatgaaat

ggaagggaatagaatgagatgtaacaagggtagaatgaaatgatgtattcgagtgaagaa

atcaaactttgttagacatgatcttatccatgacgagtcacgctaatcttctaaattctt

tttgg

>fava_dn_rep_c526

tttttttgtatcacctcattatgaatattataagtctcaatttgtaatataaaaatcatt

acaatccaagcttttatcacactatttatcaccatatttgttacaatatttggaaaaata

ctaacaaacattaattttaaattaagaaaaaaaacttatcgaagaaggaaaaacagaagc

tagaaaaaccgattaaatgcatatctggataccaaaactcaaatacaatctaattaaagc

atactaaattaaatttattatataactattttacattcagatttagatgcttatatatat

aaaaaaaaacacttcaacagacgattgagatcccgacacgtggcagaaaaatgaacaaaa

cagaacactatttacatgcaaaactcaagagagttgtatccatgtgatcactgatcaacg

gtgcttgctccttgtatccccactgtacatccaatcatagacagtgggggtgttgggaag

tggtttgtcgaaaacatgagcaccaattgtcttagtagcagaattactcccgggattgaa

cacgctcctccaaacgttgtcaactttacgcgccgatgaaggtgttgtcggtgtcaccgg

tgtcgtcggtgttgtcggaatcgacgtgctcctcattaattgactacttcctccttcatc

tttgacgttggtggtgagttttctaagcttgtcaaggccacgttctggttgagggccagc

aacaatatcatcccaaagtttatctaacattttgtttgatagatgagtttgatgatcccg

cggcc

>fava_dn_rep_c527

atgccaacatcaaccacacttatatgaaaaattaaacacattcattatttttaagagatt

attaggtagaagaagtttaggttgtttgtgaccaacaaaaacatttttaaacttgaaagt

cacaaactatgtcaactcgatctacctacctaccaccaccttaattttatttctctaaca

aacaaacaaccttataaaccctctcatcaccctctcatcacaattgtgctttctctgtct

ttaacaaagataataactatggctactgaaactgttttggttgatgaaatctcttaccct

tccaagatcactactaccaagcctctttctcttcttggtcatgggatcactgacatggag

atccattttctccaagtgaaattctatgcaattggggtttatttggaacctgaagttgtt

aaccatttgcaacaatggaaaggtaaatctgctaaggagttggaagacagtgatgacttc

tttgatgctctcatatctgctcctgtggagaaggtaattaggcttgtggtgattaaggag

attaagggtgcacaatatggtgttcagattgagactgctgtgagggaccgtttggcagct

gatgacaaatatgaggatgaagaagaagaagctttggaaaaagctattgaatttttccag

tctaagtacttcaagaaaaattcagtcatcacatatcatttccctgcaaattcttcaact

gctgagattgtggtatctttggaggggaaagaagattcaaagtatgtagtagagaatgcc

aatgttgcagagactattaagaaatggtatcttgctggctcaagggcaatatctccttca

accattacatctttggctaacaatctctctgaggaattgtctaagtgaagaaaactttgt

gttgtgtaatcttgccaatctgtgttctttgttgttattttgtactaattagaacttttt

ccactttgtttgcggaattctactttttaatttatggaataatgttaatcttgtgtgaca

tgagtgctctataaattgttattctttaaggaagttagaaattgataaatttcactctgc

atataaatataataaatagtttttaaagtatgttaatttattatatgttatagaaattac

taaggt

>fava_dn_rep_c528

ccatcaaagcaaaaatagaagaaatcaaatctcgtttgaattgatggattttggtttgtc

aacattgatcattatggtatttccaacacatatcattttcaaatcaacaacatacctcca

tttctttacatattgcctatcaccatctctttcaataattccttttgcaatatccttgtt

ttccatgctctgcttgaagtagtcttaaatgaatagttttccctcatgtattgcatgata

tcgaaaattctcacattttcacttgtttgcattttttttaccactgcttagatttggcag

atccattgttcaaaactgtagtatatgtgtgagttttcaccaaagtctttgattgataag

tgtatcttccacctactttagaacatagaactacaaacccacatttgcccttactaccct

tactccataatttacattttttgcaaatttaatttttcgactatttaaaacaagtcactc

aataattgcttctttaaattgaaataaagaattaaattccacacaacttgaattcataat

ccttacttagcaacccattccttcatactaagaatatttttcttcttcagattcatttgg

atcagagttatccagttcatcactcaagtattgcttctcctctacatctttgcttttaga

agtgacaaaaacaggtgcaagcactgttatcttctttttggtgatagtttctttttaagg

actttgaagcataaactttgattttatatgacttctctttgatttcaactctattggaac

cattttgtggtagattcaatttaaccccttcaaaaccatcattgaatactgtggttctct

catcttcaatatcatcaaatctcactcctttagcatcatcatggccttccaaacttcctt

cttcatcaatttttccatccttattaatttcatataaactataacctttgtaccaactca

agtaaatataaaattataaccatggcaagcagaagctaaagatttcttataacatacatg

agcaagtaaacaagttttaattgttgtaggcacttgttagcctaacaattggataagact

tgcacaaagtagtgatgaaaaacagaaggaagcttttgaagttccttttatttaccctct

tctgcagcatacagagctttgatttcttcaatatcgtcatagctacccagaaatatcgga

gatcgttcatgcaattttgttggaattaaatcaagtgccctttccttgccggtgaaagac

tgtcctcctgcctgttccaacaagaaggacattgggaagacctcatacaatacacgaagc

tttccacttggactctttttgtcagcagggtacaaaaagacacctccataaagcaatgtg

cgatgaacatcagctaccatgcttccgatatatcttagagactttgctgatgaaccatct

gtgggaaacttgcatttctccacataagcggcagtagggccatcccagttcttggcattc

ccttcattcacagaataaatcttgcctttctttgggatcttaatgtcagggtgagttagg

atgaattcaccaagagaagggtcaagggtgaaaccattgacaccacttccggtgcttatt

acaagcgtgcaagagcttccatacatgcaataaccagctgccaacatgttcttcccaggt

tgcaggacatcttctatgactggttcatggttatcttgcatcatataaatcccaaaaata

gttccaatggaaacaccacaatcaatgttggaggagccatccagtggatcaaaaacaaca

cagtactttccacgtagcgaaggctccacaaatattgcctcctcatcttcttcagaaacc

aagatggatgttctgccactgcttttcaaagccttgatgaacacatcattggaaagaaca

tccagcttcttctgttcttcaccttgtacattagtctctccagcaagcccaatgagctta

gcaagaccggccttgttaacagcggagcaaacgaatttgcatccaagaacaatgtgactg

agcaagatggtgaaatcgccgcgagattcagggtgctttgattgttcgttgagaacgaaa

cgggttatggtcatcaaatcggtgcgttgtgcatcggcgatgtggtccatttcggattcg

gattcagttcaatactgtaaccgtttcaatggatgaatgaaatgaatgaatcgtaacaga

aagtgatcacatatatatgcgtgcgcgcgtgcgtcctcgtttatattgctatttaatttt

ccatcttgaccacaataaataaataaataaattatcaagaataggtgatgggagaagaag

gagaaaaggatggggctatcagcttgatgaaatgacacatggcattaacagaatcaaata

accattcgtggctgttgcgtcggataacgatttcgatacaacaattttcgtctttctctt

tggactctgtgtgt

>fava_dn_rep_c529

accaaaataaatggaataccttttggctcccaagattagtttcttttctctcatgatatc

agacacattgcattatattatcatttcatggtcactcttgtccaccgaaaggtcttatac

tctcatgattacattaatgagaattaaaggaaaacaaaattgtactacaaaaacatgtga

agcttgattcttcacaccccgggactcatgtttcccatttcagaaaataatgaactcaat

tttgtgttagaagccaatttttaatgcacatgtaaatagaaacaaggggacgtatgataa

aaataacccagtaattttttaaggacaattcaataccaagcttcacaagcaaaataaaaa

taaaactaattagtaacaaaaataaaaataaaagcaagactgataaattatacattttta

acacctcccttggccatctcaacaccattgtcatcatgttcaacaaatctagaccaatat

gggtgtgatttccatactctgtccatctcttcaattggtattcccttagtttcaggcagc

atagtgtatatataaattgtcatcaccaccacaaagaaggcaaagaagaggaacaatcca

aacttcatgcgacaaagcatggtcaagaaaatctgtgcaactaagaaggtgaagagcatg

ttgaccgagacgttgacactctgcgcagcagaacggatctccaatggaaaaatctcacta

ggcaccaaccaaccaagaggaccccatgaccaagcaaatccagcaacgtaaatgcatatg

aaaagcacaactactatagcataccactttggtaaatcaccaggttctccacttgttcca

aacttggccgcaattgaaactgcaactgcaacctgacaaataagcatttgaacaccacct

tcgagaaaaagagctcttctcccccacttatcaactccataaattgagacacaagtagca

acaacgttaacaacaccggtgataacagctgacataagtgaagcatcatccttaaaccca

atcgagttaaacaacacaggcgcgtaaaacataataacgttaattccagtaaactgttgg

aagaacggaatcaacacagccatagtaagctgaggtctatattttctctgcaacaaattc

ctccaaggattttcaacctgcatcgacgtttcactagcagccacaagatcattaaactct

tcatcaacatcttcaactccgcgaattctcttaagctgtgccttggcaccatctcgatcc

ccacgctcgatcatggaatttggcgtgtcgggtaggattagcgaaccaatggttattata

agtgcagggaccatagcaccacctaaactcaatctccatccccatccacctttgattttg

gcaaaaaagtagttcaaaatattggccacaagtattccaattgtaattgacaattgaaat

ccaatattcaacgctcctctgtacttgtatggagccatctcagacaagtataatggcaca

gactgattagcaaacccgataccgaatccgagcaagatccgaccgacgatcaacatcgca

acgttttgagcaaggccattaatgagagcaccgaccagaaaaagcaagcctccgaaaagc

atggagagtttccgaccaaaccttctagtgatcgttgaagcaaccaccgacgacaaaagc

gcagccaggtacaacgaggatgtgaacaaggtcagtgtctcactgtcgtattgacagtac

tgattcttcgaatgttgcgcgtttttcttccggtacaccgccggaaaaaatttctcaaga

aacggattcattgaagtaacaccacctgaaattcctatatcgtagccaaaaatcaaacca

cccatggctgcaacaacacatgttatggtgacgaaaggagttaagtttccggggtactcc

ttgttccccgctccgatggggattccggctgcaggcatttttttctccgatgaaggatga

agaaaacaagaatagtgaaagaaataagaataagagtcccgaagagaaagtaggtttttg

atttatatagagggatgagtagaaaactttggctt

>fava_dn_rep_c530

ccgcgggaacgcagagtggccattacgccgcgggacattgtcatagcttgttgaatcatg

gctcttcgtgggaagctagaggtcgacattgaactcaagtcaaatgcagacaagtactgg

caaaccataagggattcaacaactatattccctaaggccttcccacatgactacaaaagc

atcgatgtcctggaaggagatggtaaagctcctggttctgtccgccatttccattacgcc

gaaggctcactactcgccaagtcttcgaaagagaggattgaggctgctgatgatgaaaag

aagacagcaacctattctattgttgaaggggatcttctacagtactacaccaaattcttg

ggacacatagctgttataccaattggagaaggatgtgagctcaaatggacagctgaatat

gtcaaaactaacactgatattccagatccaactattgtcagagattttgcagtcaagaat

ttccttgaggtggatgactatattcaacaacttgcatagaacttcaatgtgtttcatgaa

ccacgagctagtttattttagtaaaataatgtggttgaatgattgaaccattttctaatt

aatatgttgtatgtgtgtagtaatgtgtctctccttcattgttgttggcttaataaattg

ttgccttaattattttgttaagcaattaattggaaggactaaagattgtctcccatgcat

attacattgaaatgaattaaagaaaacgccgttaaaaatt

>fava_dn_rep_c531

gctaagcagtggtatcaacgcagagtgccattacggccgcggggtccacacctcattttc

ttcaaagtaacacagattacagctgagttccaaattctcaaagacccgaaaatggcttca

acaatcatgtcattcactccatcaaccactagactgtttgcaacaacttcagcaaaggga

acaggtggtggtagtagtagcaaagaagagaaaggttttctggattggattcttggagga

atgcagaaggaagaccagttgctagagactgatcctattctcaagaaggtggaggagaag

agtggaggaaccactagcaatgggcgcaagaacactgtggctgtgccacaaaagaaaaag

ggtggtggctttggaggcttgtttgccaagaactgaaacaattcatttgtttcaattttc

ttttacatgtttcaaatgtatgcaatattgttgtataagtatttaccacttagctatcta

tatcttgacattatgaatatatatattttatgttattaattaattatgcttgatcttgtg

tcatattctattttcattgatagaatacacatgtttggattgaccgtaaatttgacagaa

tcatagtggagtaatgcgattttggttaaaactagtttttggagattcaacaaaattgcg

aagtcgctgtgattttgtcaaactctgcgaattcgaacatgcactaattggactcaaggt

atcttctttagcgaaattgtcagtttgaaaccattcaaatgtgatgcttgttaagaatcc

ttttatgattaagggaagatgatttactcaatttgcttaggatatgaaactatgaattca

ttgcagcagtgtgtaatgtagggttaggttgcttgtttattacttgtaaatagttgagtc

tcaaatcttatattccaaataaaataagtatgtgtttgttt

>fava_dn_rep_c532

ttttcgtgaagcaaacactcctttcttggagttccgtttaataacagataacagcgttga

ttttcttctctatttttcgatccccgaggttcatttcaccctaattatatgcttcatttt

ttcaactcttacgttttaccttttcaaacttcttaatcgatcgccaaattattacaggaa

aaatggcaaagagttacttcaaacaagagcatgatcttgagaagagaagtgctgaggctg

ctaggattagggagaaatacccggaccgaattccggtgattgtggagaaggcagaaagaa

gtgatatcccaagtattgacaagaagaagtaccttgtccctgctgatctcacagttggac

agtttgtctatgttatccgtaaaagaattaaactaagtgcagaaaaggcaatttttattt

ttgtggacaatgtccttccacctacaggagcaataatgtcagccatatatgatgagaaga

aggatgaagatggttttctttatgttacttacagtggggagaacacctttggggatatga

tttaccagtagcatttcatagatttacaatattatatgctctgttacttcttcattggct

tgttcataaattcccaatgctactcaataacaaccaacattgttctcattgccatgtaca

gttgtatgtaaattctaagaaattgtttaatgttgaaaaaacatctttcctcgttcaaaa

aaaaaaagnnnncattgcttgttcatcgttcaaatggatgggaacatttggaaaaaaaca

tggttctggataaaatattatggtaaaagttgatctatgtagt

>fava_dn_rep_c533

agtaaacactagttacaactcagcgcacgttagaaactataagcttgaatacactaacgg

agaaactaatgaaagtggcggaaattacaaagaaacacttcaattaatttatcttctttt

ccattcaacccaatttcattctccaactaatctcttctcttcaacttcctcttccaattc

cattgtctgcaatccctctcgtgtcctctcccatggattccactgccgccattcgatcct

tccactatcccataggcactatgtcccatctgcgatcttcaattgatagggcagctgtag

ttccctgccataaagtcagatggaactctagtaagtgcgtctactctcacaccaggggta

agaacatactggtttcatgtgcaaaaacagttgaagctatcaacataaccaattctgatg

acagcactctgcaaagctcattagagaaaaagccattgcaaactgctacttttcctaatg

gatttgaggctttggtattagaggtatgtgatgagactgaaattgctgaactgaaattaa

aggtcggtgagtttgagatgcatcttaagcgaaacattggagcaacaaaagctccattgt

ctaacatttcaccaactataccaccacctattccaagtgaacctatggacgaaacagcac

gagctactccacagccattgccaccaaaatcatctccagaaaaaaccaatccatttgcaa

atgttacaccacaaaagtcatcgaagttgacagcattggaggcgtctggtaccaatacat

acgtgttaatatcatctcctacggttggtttattccgaagaggtagaacgattaaaggga

agaagcttcctcctatctgtgaagagggtgatctaatcaaagaagggcaagtcatagggt

atgtggatcagttttctacttcacttcccgttaaggcagatgtagctggagaagtgttaa

agatacttgttgaggaaggagattccgttggttatggagatcacattcttgccgtcttgc

catcgtttcatgacataaaggatccagcattttccagctattgaagatttaattatgcgg

catttatattggcatattggtaaacgttttgaatagattgagaaataagtgtttgtgctt

acatattaggggaaggggttcctttattgaagatcatacactgtataccaatttctcaaa

attgtatttttattagctaattcctagatcaccttactatgtgtcttagtgatgtacaca

aacttgagactttgcacactatgaaccgatcctagtgcctggagtttgattgcttggttg

atttatttatttttctctaaaagaagttgtagaacttgttttgagatataatactcggaa

cataatggataaattatgcattgctagaatgtatagaaatgttgtctttttagtagtct

>fava_dn_rep_c534

aacatattttaaccgtattttttttgtatctggtgttaatataggtgtctaaatatcatt

tcagtaaaacaaatacttttccatacaataagatgttgaacaacgagacaactacatcta

agaaatctataaatgtgagtataaaaaatgaaatgctaaatttctgataccagaatcaca

cttacatgaatatacctcaagccttggtaagctcatcatcatcatcatcatcttcaatat

tccaactcaagtcttcttcttccacggccgaactcaaccgcttccgtatatcaattttgc

ttggactaccactccccactgccttcttttcatcaaatctatcaagttcctcaatctcat

cccactcaagatctttctcctccacttccttcacaccattaaaaaaattataaataatta

agtagtaacttgataaatctcttaaataattatgtaaagacataaatttgaacttgaatg

gtcacttatttatcttaaattcttacccaattgattatttgataaaaaagtaatctttaa

ttttaactaacaaatataaatatttcaaactcattttatgttttactcctattttgcaag

gggaaaaaaaaatcaaatcccataaaccacaaacataaacaaaatttgataacctcagag

atatcattttccttttgtagcagaaaatgaattcaccggcaatggcttcccaaacttcca

ccttcgccgtctccgtttcttctatcgcaacccctctccggcgccgccgcaacttccccg

tcgtacgcgctcaagtggaaccatctgataaatcaattgaaattatgaggaagttctcag

agcaatatgcccgtaaatcagaaacatacttttgtgttgacaaaggagtaacttctgttg

ttatcaagggtttggctgaccataaagattcattgggtgcaccactctgtccttgccggc

attacgacgataaagctgctgaggctgcacatggattttggaattgcccgtgtgttccca

tgagagaaaggaaggagtgccattgcatgctttttctcactcccgataacgattttgccg

gcaatgaacagactatcaccttggatgaaattaaagaatcaacatcaagtatgtaacaca

gattattgtgtattaatgtactcgtgttagttgtttcaatgtgtaacatttgaaaaacaa

tggaagagaaatatgtaacattatatgtaggtaaagtatatttctaccaaaaatcacatg

taatttttttcatttgtgctgttcataagtggcctaacccatactttgtcacaaaatctc

catttcacaacttgctttctttggctgctgatgccaaaattaagctctattttaatcaat

ttcatatctttaccgaaaaaaaaannntcttgtgattgtttggtc

>fava_dn_rep_c535

cacaagcgacgacgactgcttcagtagaaacaggttcgagagagagagagtctgtgaagt

gaaggttgagaagagaatggcgagaacatcttccaccacgaaggatgcacaagacctatt

ccgcgcgatttggtctgcatattccgccacacctacaaatctcaagatcatcgatctcta

tgttgcattcgctgttttcaccgctctccttcaggtagtttacatggctctggttggaac

atttccatttaactccttcctttctggagtactctcttgtgtaggaactgcagttttggc

tgtttgtctccggatccaagtcaacaaagagaacaaggaattcaaggatcttgcgcccga

gcgtgcttttgctgattttgttctctgcaatttggtgcttcatttggtgatcatgaactt

ccttggttaaattggtttggtgcggttttgttgtttccaagagaaccctcggatgataaa

gatcaatagtagtattacatacatgagttttgtcaaacagtatttaaagacagtcgattt

cacaaattactgtatttcatgttgaggagctagatattgttctcttttaacctattgata

ttgttgatgaattacagaaagtttttcgcatggatatctaagtctcaatacttctccaaa

aactaattacatgtttggatagatggtgaatttggctgaaattttagtgacatgattgtg

tttaaatt

>fava_dn_rep_c536

ttgttttatttatagaatttagtgattagtgattaaattaaagagataatgataatttaa

agactaaattaaaaaaacattgtaaaaagaaaatcttaataattttcttgtttattttgc

tttggttaggttgaagaaaacgcatcaacaagaagacgatttctgattcgaggtatcgct

gcgctgataagaaaagaacagaaaaaatgggtttcataatggaattcgctgagaatttgg

ttttgaaattgatggaagatccaaaggaaagggatcgtagattcagggaacacgtctata

aggtgaaagaccgatgcgcaaagaccaaggagatgtggagttaccctatgcggccatatg

ggttttggacattcgaacgccacaattctcagcttgcttgggatgctcagattagtcaag

ttgctggtcgaagagacccttatgatgatatccttcgtcactacctcgcctcttctaaat

aagtggtgttgaactttgtctcaactcacaagctttctgggtgaaaacgtgggaatttgt

cgtgggaatttgtcatatttcctttttgaatattctgtcccgttgcgtgttgcactgact

acaccgggtttgtaatgcactaaattggtgtttcaaataaagtttccgtagaactattgt

gttatattatatatttttttccattgagaatttaaattattcgccaattgctcttcctgc

ctattggtttttattatctccttatttacatagtcgggagattgtttgtttcaaccttgt

ttaacatatcttttttcccgctttttatagctcttttataaaacgtaatgatttgttatt

gtctcc

>fava_dn_rep_c537

aacttgcgaaaatcttaacacactacttaagattcaacctgaaccgtaagattcaactcg

aacgaacgatccagatatatgcagaaacctcgataacctcgatcacgattcactatacag

aaaagattggtgagttaactcagtgacggccaacatagattccgatcggaagagaaagaa

agcgtcgattacggcggcggaggaagaaagccggaacaagaaggaagcgaaaatgtcttc

gtccagacacggcagcggtgaggttagggcttcacacatactgatcaaatacgaaggttc

tagaaggaaatcttcgtggaaagatccagaaggttacgtcatcaagaacaccaccagaga

gagtgccgttgctcagcttaaggcgatgcaagccgacattgtttccggtaaggtgacgtt

tgaggacattgcttctcgctactccgattgcaattctgctaaacgcggcggagatctcgg

ttcctttggtcatggccggatgcagaagccatttgaagatgcaactttcgctcttaatgt

tggtgaaatgagtgacattgtggaaactgaaagtggagtccacataatcaagagaaccaa

atgattattattacatggagtaatctttccatttagagaaaacagtagcatttatagatt

atttctggaatcttattcagctcagaatctgtcaatatattggtgatttggacatggcag

agctgttaaaaacctatatatgtatgcttgtaattttgaaaatcatttatgggatttgta

ctgtattgtttttggtagaatcctgtcatgaattcattccaccactaccaagtttaaacc

tggtttcattgtcacctatatgcttatagtttctgattgttttcctcctagaaccaccat

cagcatcgcacctccatgtagcttgataaacggaatgtgttgttgagttttaaacaccca

acattgcatgatttatgtaattatgatgaatcttgtgagtcgtgaggtatatgctacgag

ttatttt

>fava_dn_rep_c538

ttttttcggaactccaattatattaatgatgtgatcaggtattgattgaacaaattcttg

aaatttttgcctcaacaggatttcgaaactcttatttaagatgagatacatcatatatag

tacataatacaacataacacaagttcatagattctcatatatataaaatatcatatgatg

aaaatagataggcttcttcattgcagatacacgacatttaacctcaagattatagcttcc

aatgtagaatcgtggccacatctttgacattttagggagaagaatactcagtaatcaaga

tgtggtcattgatatatggttgatgttcatacggagaaagtggatgaaaggccaaattca

agtccccaaagctcaaaggatcctttcacaaagtcatagtatcctcccttgagtgccaat

gtcttgttcaccaatccctctctcacaaatgggtaggtgagaaggtttccaagggaaaca

ttcacagattccttctcacagtgtgtgcatagctctgcaaaaggtgcatctccatgttgt

gatttcacctttgcctttgcaggtaaaccaattttgacccactcctcaatgaaatcagtg

gagtaggttccatcaaatggaaaggacaaaagtcccttgataccaccacaagcactgtgt

ccaatgacaacaatgttggaaaccttgagatgcagaactgcatactcaattgcagcacca

cttccagaatattttgcctggtcatatggtggaaccatgttagcaacatttctgaccaca

aaggcttctcctggctggaaatctaggacgtgagatgggcagactcttgagtctgaacat

gcaaacaccataaacggagggctttggcctttggcaagttcaccatacaaagctggattc

ttgtcatatttctctttcttgaaatgaaggaaaccagttttgatcctttctgaggcttca

gattttggaatgccttcagatgaggatgttcccaattgagctgtgatttgctcaaccttc

tcagcagctgtggctttcagatcagtcttctccctcaacaatttttggagttcttcaata

gcttcttcatagccctttcccatttcttctctcaaaactggggtgatgataggagatgat

gaagcgaaaacaggtttgtcttgaataagagagggaaagaagaggaagaagaagatgaag

aagaattgagagatgcaaaaataaatggtcttagtgtagttcttttgatagaagttttgg

caggggacaaagaagaaagactaaagccgtttatggaagaggtagacattgatgcaatga

ataatcaaatgacaatgctatatagtaaactatgatgtgtcttccccgg

>fava_dn_rep_c539

tatcaaccagagtggccattacggccgccgggattccaaataatcaaaaaagaattacaa

tggcttcttcaattatggcttctgtgagtcccaaaccaactcctttcactgttcagaaat

cttcagtgaaaggacttccttcaatttcaaggcctttcagagttgttgctagtggtatca

agaagattaagactgacacaccatatggaactggtggtggcatggatttgcctaatggac

ttgatgcttctggcaggaaacaaaagggaaagggtgtttaccagtttgtagacaaatatg

gtgctaatgttgatggatacagccctatctacgagcccaaggaatggtctgccaccggtg

atgtctacgccggaggtacgactggactggcaatctgggcagtaactctagctggtcttc

tagctggaggtgcactccttgtctacaacacaagtgctttgtcacaatagactctgttaa

gttcatatttaagttgtaacaatatttcatgtagctgaaggatatacttatcaaattatt

gtggacatagttccttctgttctgttctataaatgttgtgtggatttcaagaaaaataat

ctgtttatataaccatgcatcactatgttaatcttattgtgcacatagttccttcaaaaa

aaaaaagtctcccttatagtaaaacaaaatgatagtgttattaatta

>fava_dn_rep_c540

gatgaacttattggaaatggaaataattcggactaacaaacaagttgacagccaacaaca

gtctcgctcgcagaagtgtcgagagcgaatgaacaacaagcacgaagaagaaaaatcaaa

atcaaaataaaatacaacacacccactcattcacacaccacaaagattcctcctgttccc

gctttctctctcgatctgctactgctaggactcggagcagtatggattgcaccgccggca

cgtgtttgtttccgttacatcgctgcaaaaccattcacctcgtgagacacgctcagggga

tccacaatgtggagggagataagaactacaaagcgtacttgaatcctgaatatttcgatg

ctcatctcactcctctcggatgggaacaggttgataatttgcggaagcatgttcgatcct

ctggattgataaacaagattgatctcgttatagcttcgcctttgatgaggactttgcaaa

cagctgttggggtatttggtggggagggatacagtggtgataaaacggatgtggtgcctc

ttatggtggcaaatgcaggaaatagctttcgtgctgcaatttcaagtcttaattgcccac

caattgttgctggcgaactttgtcgtgaacatttgggagttcatccctgtgacagaagaa

gaagtgttagcgagtaccagtttctttttcctgctgttgatttttcactgatagacagtg

atgaggatgtttggtggaaggaagatgtgagagagacaaaggaggaacttgcagctaggg

ggctgaagttcatgaactggttgtggacacgaaaagagaaggagatagcgattgtaacac

acagtggattcttgtttcacactctaactgcatttggaaatgattgccacccctcggtga

agaaagaaatatccaagcactttgccaactgtgagcttcgctccatggtcattgtggata

gaagcatgatagggttggaagcatcaacaactaactatccaggcaagataccttcagggc

tggacaaacctagtgatgctgtggatgagaacgttgagaagcagggcgtttagttttgct

aaatgtatgtctttagttccatttaaacattgaactgctgggcatataatcattactatt

tgaagctgttgactcatacaaaatctaacatgattgtttcagactttacttggtgcgaaa

aaaaaaaactgaaaaaaaattctgaaagagccatgctattgagattcatccatatggtca

tgatggttacaaagattgagatcttaaatcctgataatcttgtctctggattcctcggac

taggaaaaagaatcttcaagagcttcgagaaaaaacaaaaccgagaacttgtatttgctt

ggatcaatttcaatctttcaaactgagacaaatctatttctcctttgaaaagtccctgta

gcaacaaataaatatataaacaccaagtattatcaatgaagtggcatatgttttatcctc

gccagatttactttacacattgaatctaaacttgagcaatgattattccgttttcattga

acattggacctagatgagatttcatcctcaaccatatctacaacaacttgaggtggaagg

gatccatcagattcttcttcggattcttcgtcagattcttctttgctgggcaactctgat

ggtctgataatgcagtcatttacagagctacattggcgtgagtcattgggctctttgagt

ggctcaaaatcatcaacaattgaattaattctaaaacttatagcaatctccttttcaatg

taaactagcatgctgtctgcttgaaactcatcttccatcatgtttctcaacatagtcttg

aaatttttcaatgctgaaaaagatctttcggttgtaggcgtataaaccggaagagtcatg

ataatacgaagtagtctgtcaatcaagtagtaaactnnnnnnnnnnnnnnnnnnnnnnnn

nnngtacacaattcttggatagttgataaattcttcaactttgaatcttgacgagcatca

ataa

>fava_dn_rep_c541

cttgattaaggacttccattcttttttatcaaaatcatagacaatctattgcaatctaaa

gtagcaaatacataaacaaaacgaaacaaaacaaaaatcaagtgccttctatgatttttc

agctgagttcccagtgtctctaaagcttaataaaatgattatcatccggaaacatttgag

acaaacacaacgaaatgcaaataactttgtagtaaaaaacaatctattgcaaacaggtca

aagagtgtaaatgaaatattaatggacatcatgaaaaagaagaatacagtggacgatcct

aactctagaacacaactgtgtcgagcatcattcccaaagtctgaacaatgtttgacgaca

ttaaattagaaacatgctcttcaagatgcttcttagcatcctgcctccccacattggcaa

ttgcaagcttttctgggggaagctcatcttcctcctgcactgccttgttgtacttggttg

ctaaactaagcatctcctgaacagtctgttcattagttttagaatgtgaatcgaatcgtc

tgagtgtcaaaccatcagtccatttcttcttatgaaggttaagcaacatcttctcttcaa

gctcgttcttcctgtaattgattgctattgagtaataatgtcggttcaagccatggatca

gagcttggatagatggcttgttcagatggccaaggttagaggttgtctgtcgtggttctt

gacccagcatcatagtttgtggattaatcagccggaatgcatcaatcaccactttgcctt

taacactctgaataggatccaccacaactgccactgcccgttgatttaaagcctcaaaac

tctgttgtgtattaatgtccactccagaaagccagcagccaaatccaggatgtgaatgat

accaacctacaaccatctctggtcttccagtttgtttcaacatatcaagcatattagttt

gaaaaacatgatcaacagcttcaacactaacaccagtcccactctgcggcatagcaaaca

catcaacaacgcgcacagtatactcatcaacaaactcccccaacatcaaacccataactt

ccataggaacaccagcccttccgtgtttgagcattttgagaagagcgagagaggagatgt

aaacttgttccgatgaatctagggtgggggaatccggtggtggatggccgcctagtgctc

cacctgcacccgcgaacattctttgaagtctctccatacctgacatgattgttcaggatt

tacaactgatgaatgaatgaatgaagtaaaataaaatctttttaggttgttttcttttat

aacaagttaagaattattctgtttttt

>fava_dn_rep_c542

atattctaacacaccataaaaaaaaacaccaaaaagaatccataaaaaacccatttgagg

ttgagacataaaccttaaccagaactagcatggcttggttaagaagcctctcaaaatttt

ccaatattaaatcatctcaaagagctaataaaactgacccgtttcaccttttaccatcct

tcacatttttctcccatttcagctcccaacctattgaggaaaagccttatgttaagcctg

ttgagcattcttcaggtttggaaccaacaaagcctcatgagaaaccaagggtggttgtgt

tgggttcaggctgggctgggtgcagactcatgaaagggttggaccctcatatttatgaca

ttgtttgtgtctcacctaggaaccacatggtgtttacccctctactggcttccacttgtg

ttggaactcttgagtttagatcagttgctgaacccattggaaggatccaaccagctattt

ctagagagcctggttcttatttctttcttgccaattgtacttccattgatgcacataatc

atacggtggaatgtgagactgtaactgatggagagcagacaatagatccttggaaattta

cagtctcttatgataagctagtaattgcattaggatcacatccttctacttttggaattc

aaggagtcaacgaacatgccatttttcttcgtgaagttcaccatgcacaggaaattcgta

ggaaattgctcctaaacttgatgctatctgatgttccaggaataccagaagaggaaaaac

aaaggcttttgcactgtgttgttgttgggggtggtcctactggagttgaattcagtggtg

aacttagtgattttatcatgaaggatgtacgccaaagatatacacatgtgaaggattaca

tccgtgttactttaattgaggcaaatgagatattatcttcttttgatgaccgactcaggc

tctatgctaccaagcagttgacaaagtcaggagttcgtcttgttcgtggcattgtgaaag

atgttaaagctcagaagattatcctaaatgatggtactgaggttccatatggtttgttgg

tatggtctactggtgttggtccatcacctattattcaatctctagatctcccaaaagctc

ctggtggaaggattggtgttgatgagtggcttcgtgttccttcagtacaagatgtgttct

caataggtgactgcagtggatttgttgaaagtacgggaagaccaacacttccagcattgg

ctcaagtggcagagagacaaggtaagtatttagcaaatatgttgaacaaaattggtaaag

caggtgcaggccatgcaaacagtggaaaagaaatagaacttggggatccatttgtttaca

agcatcttggaagtatggcaactattggcagatacaaggctcttgttgaccttagacaaa

gcaaggagggaaaagggatagcactggcaggagttctaagtttttttatttggcgctcgg

cgtatattacacgcgttgtcagctggaggaacagattctatgtatttatcaactggatta

caactcttgtgttcggccgtgatataagcagactatgataaactaacagtggcagaagaa

agaggtaattgactaccggacttgaaccttgaaggacaaatcaattgaaacttgcatctt

gaaaccaattaagattcatttttctattgtagattttcagaaatcagaagtatgaccatc

ccaagtcctattgtatatattgaaaatcacgaaatcatttttcatgctcatagataaact

attcatgaaatgtgtgatcataaaatgaaatcaatttatttatttacatgtacatagatc

atcattggattgaaaaatagttgtaaccagttactcttgcaaatgatattctgtttgaat

gtataattaggaacttaggatagagtaagtacaaattattatttagatagtatacaagta

ataatgtgatcacactgctcacactttattattcttcacaaatcaaaatagcttgaagag

ggatgatgatcttgaagttttctgacttcccaaccagtgctgcatatggactccgatagc

atccaaatacatgctactcctcccatgaaaacctaccacctttccctctgttgtggttga

agtaaaaaatttccctacttcatcaccaaatggaccatattttcctctactagtatagaa

agtcagtgacttaatcactatatcttgttcatcggtagtaattgaaccacaatagccgga

tatgcaagacagaacctcatttggatactccaattttatcctatgtgtggtgcttcctcc

attaccgccatgcttcacagaccatacagattgcttgtttcgatcatactcaatttgaat

cgagcaaattccttcaggggattttgtcaaataaatctgcttaatatcagaaaaaactcc

atcatcccagagtctacctccatccccaccccatggacctggtccaaattgagccggttc

tttaaccacaccacgagaaacctcttcaagtggtgctaatttagaaggcacaatttttgc

tggccatttagcactgtttatttctgtaatacttggttctcttgagatgatttccatgga

gggagatgttgctactggcactattacttttccttctatcacatgtacaccaagagcatc

tagaaataaaccttttctaccatgaattccaactacctttccttcattcagccttgttgt

gaaataagttccatattcatctccaaatggtccataaacccttttggtagtgtggaatgt

aagtgaccttatagtagaaggtcccatatacatcaaagatccatagtagcctgacatatg

tgttaggacttcatatggaaaatcaaagatcatcttttcatgtttgaatcctccagctcc

accatgtttgtatccccatactgcctctccatctagatcatacaaaactctaatccacac

aattccaacgttgcgcgacaaatcgatttgtctgattccagtgtaaggtccatcatcaaa

gacatatccaccattaccaccccaaggcccatatgtgaccacaccttcaactattgacgg

tgccttcaccatcgaaactatcttttcttttggttcaatgctgttaggttctttatagga

gtaaatcttcttggttggtgaagctttgacagaactatctttctgttttacggcaacaac

aatatcaaagccttggctgacacttccttgtattacagaatagccaacactctcagtagt

gttagtcatgtggttctgtgagcttgacaaagctttggatggttttggctgctgatagga

cctcaaatggactccaatggcatctatgtgccaaccatacctgccatggaatccaacaat

cttggctccagtcactggcaaggcaaaatatgttccttgtacaactccaaatggtccata

gattttcctgttactctcaaaactcagtgatctgatgaagattggtccccattgatttaa

gctaccatagtatccatcaatggatgttaggaattcatctgggtaatcaagcttgatctt

gtcaattttatggcctccacttccaccatgcttaagtgaccaaacagaattctcgtcctt

gtcatattcaatttggatggaatcaattccttcatcatgaactatcaccaattgccttat

tgttgaataaacaccatcatcccaactgtatcctccactgcctccccatggtccaactga

tacaggtttctcctctaagctcatcttctttttcctttgttattagactaagagaagttg

aacacagaaggctgcaagtgttaagatccctcggttggagaaggcttaaga

>fava_dn_rep_c543

gtggccattacggccgcggggattagggtttgcgacttcttctgctaaaatgggtaaggg

aacaggtagttttggtaagagaaggaacaagacccacacactctgtgttaggtgtggccg

tcgcagtttccatctccagaagagtcgttgctcagcatgtgcttacccagctgcacgtgt

caggaaatataattggagtgttaaggctattaggagaaagactaccgggactggtaggat

gaggtacttgcgtaacgttcctcgcagattcaagagtggattcagagaaggtactgaagc

tgcacccaggaagaagggagcagctgcttctgcctaaggctttaattatagtagtggagc

tatttttgtgttggatcattgatctttggaaacatcactctatttttagttttctactcc

ctccctcttttaatatataactgttttatgttttaaaatgagattgtttgacattttgtt

gttcaattggaatttttgactcaagcaattgtgatttactgtttctgtatgatgactctt

agaaaattgtactttgcctgtttgtcaacttttagaagataattgtactacactgtttat

taattgatgctagttttatgcgttttaggcttttg

>fava_dn_rep_c544

aaaacttcatcttttcgcctaaacttgacacaaatttaattgtttacatgtaaaaataat

taaactatcatttgaagtttcctggccaaaatttagtcatgcacagttaacatgaaccaa

cttaatttatctcaagtttactagattaagaggaagactattagattaatgttcggatac

ctcgttttcatcacttcagcatcgtcaataggtctcgggattcaaatcagaggccatttt

tattaaacaaactcaacagatttcatcaggcaatcataattcactaggttcaccaaacaa

acaagaaagaacaaacaacaccctcaactcactagaaactgattaatatccgcagttgca

caccgcacatacaggaaccaaacacgattaaataaacgagaaaaggaaataagcttgcgg

tgaataatgaatactagtactgcctatatgacttccagtgcctgcacaatatcagccttc

aaatcctcaaaatcttcaattccaaaactgaagcgaaccaaattgtcataaattttccac

ttcgccctttcagatgcaggaagatcccagtaagacataatggccggttggtccacaatg

ctctcacagccaccaaaagagggagcaatatatgggattttcagtgaatcaacaaatttt

attgtggtttctatatctccatcgatctcaaaacttactacgcccccaaaaccagtcatc

tgcctcatggcaagctcatgttcaggatgacttggcaagcctggatagtagacacgcttc

accttgggatgtgcctctaaaaatttggccatccccattccagttgaattctgctgctgt

acacgaagatgcagtgttttcatgcctctgatgaataggtatgcggcattcgggttaaga

acaccgcccaaaatatgatgcaaaatacgaatttctgaaatcagcttaccagagccactt

atgcaaccaccaatgacatcatgatgtccaccaatgtactttgtacaagagtgcataacc

aaatcagcaccaagggcaattgccttctggttcaaaggtgttgcaaatgtaccatcaatg

cagagcaaagccccatgcttgtggcagagctctgaaaccagcttaatgtcaacacatctc

aggaatggattggtaggagactcagtgaagaatagagacaccttgttctgctccaatgca

gattccaaggctccaacatcagcaggatcaatcacagaggccgtgatccccatcttagga

agcacagtctctataaacaccctagtcttcctgtaacaatcagttgtagtcacaagatgt

ccaccggctggaatcagcgccatgagcataacaatgctagcacacattccagatgccaat

aagacagttgactcagccccttccagttcactcaatttctcctctaaaacagtactggtc

ggatttccatagcgcccatattcataactagccagacgtttctccttataatcaataaga

tcggcagtcttcttgaaaaagtaggcagaagtgttcaccacaggagtggtaattgcatca

gtcacaatagccctaccaagtctttcagcggcatgaatcgttttgcttgcatcggttttc

aaaaacgaagaataagatctacggttagtctgtacagccccatttcccgcggcatcgaca

acattctcgtcatcggcgtcgaggtccaaggggatcggcgccgtcgcagcatcaacaggt

gacgcaacggccggcaccggattcgccccagaggcggggctgccggcaccttcattagac

cacgaagcagcgacgatttgcgcgacaccgatgttgctgcaattacggcgagctttggta

cttagctgacgctgaaagtttgggggaaaacggaggatgggagatgagacgccgtagaag

gcggaggagagagaggcggcggggaaatggcgaggggtgctgcggaggttttcggtggag

gggagggaggcgaagtcgggatcggagcggcactcgaaggtgaagacgcggtggaggctt

gaaacggccatttttgtcgtcgttttggtgaggttaggttttttttttttcctacaacta

ctcactctagcgacgaaatgaatgtgaatctcgaaaatctaagtttt

>fava_dn_rep_c545

ggtattgaaagggttttgaatgagtgatctgtcttttttttttgacaaatgtaaaagtaa

gtgatatgtatatcattaatgagagtcaattaagcacatcatgaatgaaggataccaggt

tgaggatgctctaagatcctcatgactaaaaaatgtaaacgaaacaatcatattcagtag

aaaatatagtgatacaacaacattgttatcaaccatttgatttgcaggaaacattggaag

ggtaaagaatataaaattattcagatgtaggaattgatgtaactacaggaggaggtggct

ctttattaatgttaacagcctttcctttctctctaagcctggcaacttcttctttcaggg

cttcttttctcttcttgctagccattactttcgcctgaacagcactgacattgggatctt

ttttctcctttggtggagaaggtggaggccttctagccgctgctcttgcactttgctcat

tgctccaactcaaacccaacactgctgcaccacttaatcccatcaatgccctccggtgca

gagtgtggtcatcacggagaggagggacttgctcatgattagagacctgatgttgatatt

ggttgccaatgcagcacactcttggagtggtaaaacacccatttttcccagaacacaggg

aggagctattccaaggtctcgcagagagaggtgttacggctgtggtaggaaccggtgaca

gcaaatgagccatgattttgactggcttcagctttgaacttttgagaaaacagagaaacg

agaagagagatatgtagttactgattaaaaagaaattattaaaatggagtttgggtggtt

atcttgtgactcgtgtcaccaacaacaaaaaggaaaacattccgttggcagtgcggacta

gaacttggggtggatattgattagggatgaagtgtgcaatttgattgcggaagtt

>fava_dn_rep_c546

ttctattgatgtgtaaactccaacattttcaacatagttgcaacatgacataacaaatat

actactatttttaactaacgacggaaattgttagtccagtcgatttaattgttatgatct

ctggaaaggaaaaggactttggttttcctatagctctgtacatagtacaataattaccgt

aaagttgaatcatgtatcataatatataggagtagtacatatttaaacatgtcactttgc

aaaatattactggcagtaggtaaacattaaataaatgtacacacaaagcaataaataaat

aaaccgtaatcacccatccttaatgcttctatccaataccatcattgcagttccaaccca

gaagctaccttttcgatattaataccaataaaaaaacaactttattttaatcatacataa

taatcaacatcaaagaaaaaacccaaaacgcgtgaaaagtcgcgtagatcttgtaacgat

caatgatcaatgaccactgatcagacggtggaggaaaccttaacgagcgacttgatagta

atacttggacccaaaaccgacggtttaaattcaaacgtaaaagtatcataacgcaagaag

aattccaccaaatacaacctacacaagagcaccaccagatccttagccggacacatttta

ttatcaggtgtagcctcatcagtctcacgtccattggaccagaacacgtgcttcaacatc

ttctctccatctcccacaaacctatccgcaacaaaatcctcaggattctcgaaaatctta

gcatccttagtagcgaacggttgatatccaaatatcatctcacctttcttgatctcaaaa

gacgcatcgtgactctgcaccaccagatcctccctcgctttcgcgtattgatacggtaca

gcaggttcaattctcaacgtttcgtagaccactgatttagtcaaagtcaacttatccaag

gcactgaaactgacaccgttttcttccttaacgacggttctgatttcgtcggaaagtttt

ttgtgtaaactctcaccacctaacccgacccattttaacagaatcggaaactggtttgtt

aaacctccaaaagcgttgaaacctagagtgaaaacaaggttgtgacatgcttcgtctctt

ttgattcccattttttcagcttcgtttaagacagaagttcctgcttcccataaaccttcg

taaagcttgtcgtagctagatttcactgtccaggacgggatcggtatggttctgatgaga

gcatcttcgatgtagttgaatatcttaggaagaccagctgtggctaatggggcaagctga

gctgcaagccagacttggacaagagttgggccggagtcgccgatttttgtttcggttggg

tttttatcagtgatgaatttgaagaggaagttgaaggtggcggttccgatggaggagttg

aagtttgctttgccgtgtttggaaacatctttctcgagatccgtgaagtgttcagagagg

ttggtgatgaagagaggaataaaggtgttgtgttttgaggataagataaagaagatcaac

cgtttgaggaggctatgggaagattctgcggtgtcttgaaaggcgcaggtgcggtaaccg

ccgaagaagttggtggaaggcatgaaagtgccgtcgaggacgtcgcgtttttcgactttg

gcgttgtcgaagaggatggggaaggaaacaccgtcgaggagagcgatgactttagtgtca

ggtgcgatgaaaccaccgggtggcatgttgactctgatgacagtggagttgtatttctcg

actcttgacgcgaagtatttgtcgcgtccttggttgtagaagtagtcgtgtctgtcgtgc

atgggtccgatgatggggagaccgtagcttcctgggattggtttcaaagggaggttttgc

caagaggttgaggatgatgaggtcattgttgtaagtgtttttgttgtgtttggtctgttg

aggggccccgcggcc

>fava_dn_rep_c547

ttggagcacatgattgggaaccactttaagatgacattnnnnnnnnnnnnnnnnnnnnnn

nnnnnnnnnnnnnnnnnnnnaacatagtttctcttgaacccgcgataacaaattgaggct

gcgtggccatatttcccagaaactacattaaacagaagtaccacgaccaccatgagtggg

atatgagatgaaatattcgttatcaaaaggctgaggaggttattcatacatgagagagtc

aacattatgatattaacacacaaagataatagaataattctgagctactaaagctcatac

atacaatacaatataaattcatcaacagaaaccaacataattgcacagaaggccaatgaa

tcagcttagttcattccactaaaccactccaacactgtcaaaggttcacttgcacaagaa

aaaactccattggactaaccataacgcaacaataacatctactttacactgatagctttg

aatgaaaaacttttttttttactgtcacaagaggtaaattatttactacttggaggagga

ccgccaaagagatagtctaaggaagatccgccacccggggcagcgtgaactttggtcgaa

ggtcgatcagtgaggaagttgccacagttttggccttcggcgcgatggtaattgttcttg

agacacccaggaattccagctggagtttccttattgataggtggtgaagcaaccctaact

ggcgaagcaatgccggctagtgaagcaatagtagctggtggagcaacggtagcattttgc

aaacgcccgccacttgaaggttcaacttgattgtttacacgttgagcattgtcggtaggc

tctccggtaccgaagagataacccaaggaactctgaccaccaccactactgacaccacga

cccattttcaaaccttgtgtggcaaaaccttgatgagaaacgaagtcggtgagttgaaaa

gtgaagtgaaccacactatgccccagctaagttaaggatatatagatatatatttattaa

ggagccaatttttcttttgaaagtatattagatggtccaattttttttatagaatatttt

taatatactcttatgcatttttagatgttgttgacgaaattttcgtaatactcataaatt

atattaatattcgaactcactacgttatagttttttatcgaat

>fava_dn_rep_c548

cactacgggtctaaattgacatgtctaattacattcatgtaactcacttgacgcataaca

attgaattactcctccgacccttctattctcccatataaaccaaatatatagtattcaat

taaaatttctcattcccctcccctaaactcatacaatctccaaggcacatcagaagaata

actaacaaaacttggaaatagagagcacatgaaacacttgacatgtaaatttcacttcat

tgttgaacccacaaggtctacaaatataaaactattaatgcagtagttctataggaattc

ataaaagatctaccagtttcttcaagagcaatcaaactgaaatacctatgataaaattaa

accctcctcacacaaaaatacaaaaactcccaaatgcatatatgtccttgttcttggttt

aaacttaagcagtcttgctttcaagaagcttctcaatcatatctgctgcttcttggacag

tggtaatggcttgggcactttcttcttcaacactgataccaaactcctcctcaagtccca

tcacaatctcaaccgtgtcgagagaatcagctccaagggcagaaaatttggactctccag

tgacctcggtcccatctgccaatgccaattgttttttgacaatgtcactgaccttggtta

cagtttctggtttagcggcacatgtaacttgaaagcgatgtaccctagattgcaatgaaa

gtgatggaaaggctttccctttaattgaaatggaaacagaattcgcgttagagaatctgg

caccaggtaccagtgattggttgaatgcggtgagggaaaatgaagttccggtgatggaag

ccattgtagagagagatctggagtttgaaagaagagaaaaaaggacggaggcgaccaaga

gtgtgagttgttgttgtaaagagagagagtcgagtgtgggttttataggagtaatgtggc

ttcgttattttcgaagacctttttttttttcttcttatgtatactatcgtcgttttctct

ccgtttcgtttttatcgtagctcgtaacgtccgttaccccccggcccaa

>fava_dn_rep_c549

tcactaagtctcagattaatctttactttttcaagttaacagaaaaagaccacattatgt

aacttcaatcaaattcagagagaaaaatcatgagttatgaatgttatctgtttagagtga

tcaccgacaattcaaaggccaaaaacatgtgaaataacatttcaaaagatatcataatgt

aatcaactatgtaaacaactatgtagctcaactgtcataaaatgggaacaaactaatcat

gctctcttgcactgaaaaatcaagaatcaaatctttaacttcaaactaatcatgcttttt

gcactgaaaaattaagaatgaaatctttaacttcagcattagccctatccaacgccgttc

ttccatctgcatctttcacgtcatatttcgctcccgctttcaagagttcctcaattttcg

gcaggtcgccttctgttgaagctaacaacagtaagatgtccacagggtgaatgttttggc

ctagcagagcctccattttatcgtatgttccctctacggcgagcatccccatatagttga

agtactgttcaacatcatctctatcaaaatcatcacgagactgactaccataaatcccag

caccttcagcatcaacaccattcttaccaaatctgaaccagcatgtgaatcttgaaccat

tggagggtccaattttagaggtgatgcttgagcttgaccatccatgtttcttaactctga

cgccaagaaaccgagaagaaactgagattttcgaagtgattatagatttggaatttgaaa

tgatgggttggtgagtgatgcatgtgattgaagccattgttgcgttgtgttgagttcaac

aacaaaagttgtttcggtttttatgtatgtatgtttgttttatcggagggaatcccgttt

catgttctatccat

>fava_dn_rep_c550

taaaaagtgaaatagatttggagtattctgatttggaaaagtgtgttgagtgttgagtaa

ctttaggacgaagaataagaaattgggttttcttaaaccctaatctgaaacgaaaacaga

atacgcaagcctcatccgaaaatggggaagaatccaaagaacgagctaggtagggctctt

gtgaagcaacacaatcaaatgattcaacaaaccaaggagaaaggtcgagtctacaagaag

aagtttctcgaatcgttcaccgaagtttctgatatcgacgccatccttgaacaaggggat

caagatgaggagcagcttcttgatcttcctgttcctcctcccactgccctcatcaatctg

gatcctgtgtctgttactggttttaatggtttgactcctgaagaaatgaagaaggagcag

aagattgaagaggcacttcatgctagcagtcttcgggttccgcgcaggccatcatggagt

gctgaaatgtctgctgacgagcttcatgccaatgaaacacaacatttcttaacttggcgt

cgcagcctagccagacttgaggaaaataagaagcttgtccttactccatttgagaaaaat

cttgatatctggagacagctctggcgggttgttgagcgcagtgatttgcttgttatggtt

gttgattcacgagatccactgttttatcgctgtcccgaccttgaggcttatgtaaaggag

gttgatgtgcataaacgcacattgcttttggttaataaggcagatcttttacctgctcct

gtcagagagaaatgggcagaatactttcgtgctcatgatattctctttatcttctggtca

gccaaagctgctactgcagttttggaagggaaaaaacttggttcatcacagactgataac

atggtaagtgcaaataacccagacacgaagatatatggaagggatgagcttttagcccgc

ctgcaatcagaggcagaagagatagtacacaggagaagaaattcaggctcctctgacaca

gggtcatccactattaaatcctcaggtgaaaatactgctagttcatcgtcaagtcatgta

gttgtgggatttgttggatatcctaatgtagggaaaagttcaactatcaatgctctagtt

ggacaaaagaaaaccggtgtcacctctaccccaggaaaaacaaagcactttcagacatta

attatttctgaaaagttgatcctttgtgactgtccggggttagttttcccatctttctca

agctcaagatatgagatgattacttgtggtgtgttgccaattgataggatgaccgaacac

agggagtgtgtccaagttgtcgccaatagagtccctagacacatcatagaagagatctat

aacatcagtcttcctaaacctaagtcatatgagtcccaatcccgcccccctctagcatct

gaacttttgagaacatattgtgcttctcgtgggcaatctacttctagcggacttcctgat

gaaaccagagcttctcgacagatactgaaagattacattgatgggaagctgcctcactat

gcaatgccgcctaacgtgtcaaatcaagaattagcttccgaagattccactggacatgac

caagataactcacatgtgtcagattcatctgatattgaagattcttcagatgttgaaact

atacttacaccaaaacttgagcacgtgctggatgacatcagttcattcgacattgctaat

gggcttgcttcaaacaaagttgcggttaagaagtccaaggaatcacacaagcaccacaaa

aagccgcagagggcaaaagacagcgatggatggatcgatctgggagaagaagaatgggag

caggaaagaacacggcagagttcttcaggagaagagacgagtggaggaagcacccgatgc

ttgggaatcagtttcgtcacgctactcctggcctcggcatcgctctcgtcgctttcggtg

tctacctcgtcgctgagcaggtctacaacaagaccaatgcttcttcctcttctcaccatg

tgaaggctggggaacactgaggaggaaattcatcgattcatcgttttccttttgtgtttg

tttgtttaaattgttggttaaataagcttactttgcaggggttttcctacaaaatgtaat

ttgctttggtcaaattttagactctccataatgaatgaatctatgtatgataacatttta

tgtctcactcatttttttgagtgagtgacttgcaattcagatatgctctactccgctagc

taaattctttatgcttgtatgaaatcacttgtgcttgaccgttctatgttatacatacct

ttttcatttcat

>fava_dn_rep_c551

ccgcgggatatactcattcacttaaacacacacacctcacactataatactcacattata

atcttttgtttcttttttgcttcaatgccatccctaaaactcttcactatgactttattt

gtattagtgttggttttaggcttgtggtgtactagtgaggttcaagctgatcctcaagta

ccttgttacttcatatttggtgactctttggtagatgatggaaacaataataacctcaat

tctttggcaaaagctaattatcttccttatgggattgactttgaaggtggtccaacagga

aggttctacaatggataactaccttaaccacacaaactaaccagttccacaaaccatagc

cataaccatggctactctaaccttcaactccacaacactcaaaaccccatctttcaacta

ccccaactccatcatcaccaaactctcctcatcaaaacccaccatcaaattcccattttt

caccaagaaaaaactcttccttcaacttcaaacacgttctgcatcagaaacttcagtaac

aacaacaacaacccagaaagacattgaagaagacaaagaagacccaactgctgaaacatg

ttatcttgaccctgaaactgatcctgatgagatattaagctgggagttggatttctgttc

taggccaattcttgatgctagaggaaagaaagtttgggagcttgttgtttgtgataaatc

tctttcacttcagtatactaagtattttcctaataatgttattaatagtatcactcttaa

ggattctattgttgggatttgtgatgagttggatctacctgtgcctagaaacattcgctt

ctttagggcacagatgcagacgattattacgaaagcgtgtaaggagcttggtataagagc

tcttccgagtaaacggtgtttgtcattacttttatggcttgaagaacgttacgagactgt

atatacgaaacatcctggatttcaaaagggatccaagcctcttttgccattagacaatcc

gtttcccacgaaacttcccgaagatctttttggtgaaagatgggcgtttgttcagttacc

ttactcagctgtccgagaagaggcctcggcctcagaagaaagatttggctatggttctgg

actagatcttgatttattgggcattgaaatcgacgagaagacgttgatcccaggactcgc

tgttgcatcttctcgtgctaaaatattatcagcttttatgaatggattggagctctgcgc

cattgaagcggacactgctcgcgctaacctgactctttcggttgcgatttcgactcgata

tgtatatgcgacgtataaaaaaagtcctacatcgactaaagaagctgaagcatgggaagc

agctaagaaagcttcaggaggtttgcatttccttgcaatccaagatgagttagattctga

aaactgtgttggcttctggcttttgctagacttgcctcctccacctgtataatcatgcaa

attattgcttcacaatattcacagaacaaaaaatataagatttatactaagagtcttatt

actcaaatattgataagcacttattgattgagtaaaaataaacacacattcattaaacat

ctaaaaaaaatcactttttattc

>fava_dn_rep_c552

attaatttcttttgggattctgcggatttatattatataataaaaaacaaaaataaaaca

agtcgtagcattagtaaaaagagtcacagtcttcacaccgagcaccaccaaacacaaaaa

gctgatccagatttgcactttaccttacctcacctcacctcacctgcttcccacacacac

ttccaacaacaacatccatggcggtaacttcaacatcactcgcttctcaactctccattg

gcctccgccgccaaccttcccccaagttcgattcccttacttcccaatctctctctttct

tcgatcccaatattcgcctttccgtttcttcccccaaaccctcccgttccgttatcgcca

tggctggttctggaaagttctttgttggtggcaattggaagtgtaatggaaccaaagact

ccatcagtaagcttatcgctgacttgaacaatgcaaaattggagcctgatgttgatgttg

ttgttgcacctccctttgtatacatcgatcaggtgaaaacctcacttactgataggattg

aagtatctggccagaattcttgggtgggaaaaggtggtgctttcaccggagaaatcagtg

tggaacaactgaaagaccttggatgcaagtgggttattcttggacattctgagcgaaggc

atgttattggagaaaaggatgagtttataggaaagaaagctgcttatgctttgagcgagg

gtcttggagtgattgcatgcatcggggaattgttggaagaaagagaagctgggaaaactt

ttgatgtctgtttccaacaattgaaggcttatgcagatgcggtgcccagttgggataata

ttgttattgcatatgaacctgtatgggccattggaactggcaaagtggcctctcccgagc

aagctcaagaagtgcatatagctcttcgtgattggcttaaaaataatgtctcagctgaag

ttgcatctaaaacacgaattatttatggaggatctgtaaatggagggaacagtgctgagc

ttgcaaagaaagaagatattgatggatttcttgttggtggtgcttcattaaagggtcctg

agtttgccaccattgtcaattcagtgacatccaagaaagtcgcggcttgattacttattg

tggttttggctatatgagagcattaaggaagagcactaaggaaggtggcaataatattat

ttggactattagagtttggtcttgattcatcctcttttatcattttctttgcaatcaata

ggataatcatcctctcagttttctaaagaacctgtgttttatggtaatagcatttagaga

tattaaagacaataaatgtttcttaataattgttgtttttcataattcacgtttatatca

agaaatcttggtctaaaaaaaaaa

>fava_dn_rep_c553

atagtgagaaagaaacacatattatatatatatttgaaggagagaaaaggctttggttca

ccaacccccgcaatataaaaaagggtgatcattcggtctaagcggccactcacgctgata

ttcactcacgctgacagatggcttgcaggcagttatgggcttcaagagctgcttcttatc

tcaggatctccgtctttcacagaaccttctctaatgttttgaacaatctcaagtatgctg

attctcatgaatgggtgaaagttgatggaaactctgcaaccgtcggcataactgatcatg

cccaagatcatttgggcgatgttgtgtacgttgaattgccagaagttggagcagcagtga

caaaaggagatgcttttggtgcagttgaaagtgtgaaggcaactagtgacgttaactctc

cggtttctggaaaagttgtccaagttaatgaagagctcagcagctctcctggtttggtca

actcaagcccgtatgaaaatggatggataattaaagttgagttgagtgacagtggtgagc

ttaacaaattgatggattcagaacagtattccaagttctgtgaagaagaagactccaatc

attaatagtttttgcaatatctttcagtactccccatggtgcagatgagatgatatcaat

ttggaagctaagaattgcttcttgttttgtatttctgctctttttatgattttagcccga

gcttctgatgcagtggggagtttgataataaaagaaccatgagtagtttttattgctgac

atagtgtcaattttatcatatgattcctattttaatttgatgtccttgttttcaactaaa

cagactcatttcgagatatatgcatatgcaatggttgcagagtgattcgtgcagaaagaa

ttaataccgacatttatatatggtgttaaagtagtaaccctgctatcctatcctgtctta

ttttcttttccgtttctttgctaaagtgattgggttacgtggttgatgaaagaatatgga

gttgtcccttatgacaagttcaacaaccaaccttgttttcccattttcatttcagaaaat

ggcgttgtccttctaatgaacaaacatactacccaacttgtcctatataacagaaatact

ggtcagttgattaatttattggaaagtaccaacactctaaggttcaatctacatatttgc

agtgagagtcttgtatcactgtcatagtaaatcacatttgtgaagccttttaagttttgg

ttaaaataggtttacttttgcttcatatctacttatgttgggatgttgctttttattatg

aacgattgtcttacaagagttataacctttatataataaatagcagaacttgttatgttt

cttctgttgctgttaagatgnnnnnnnnnnnnnngttgtttatagtttgtcatttctcag

aagggggtcttcgcataaacctgttggttattttgttttgcaacttcaaacagaaatatt

attgaaaaaatcagagtttg

>fava_dn_rep_c554

ataagactcttaattagaattaaacacaacaaattcacaccaatcatcaaatttgtgact

aatataaatgacataatttataaaaatatcattcacataatctaaaatagttaaaatcaa

attttatcttcactgatttagaagacaacattgtacaataaaatatcctgtgtgatctat

taccgcgtagacttccttacacttagatggaaaatcggaaaaaaaatcattaaaaaagtt

gcttctgtttgacttcaagaataggaggatcaacatcaattcccatatttgctgcagtgc

ctgctataattctcatagctgattcgatagtcgaacaattcaagtccggcaacttctcag

aagcaattgtacgcaactgatcaattgtaacttttcctactttttgtgccttggggtctt

tcgaacccttttccactccagcagccttaagcagtaaaacagaagcaggtggagttttca

atatgaaagtaaaacttttgtcatcaaagacagtgatttcaactggaataacataaccag

gtttatcagcagttctggcattataatccttacaaaaagccataatattaacaccctttg

aaccaagagccggtccaaccggtggagcgggcgtcgcctttccggcttcaagagccaatt

ttatcactccaataactttctttgccttgccaccagcaggctttgggggagccatggcag

tgattttgaagcgtcttgtagttcgagaagtgggaagagaaaaggggtttttggtttcaa

tggaaacgccattgggctttagggacaatttaagcagcgaggaaaacatcgaagaagagg

aagaagaacatagtggcggagtggtggagaagagagtggtcgccattgaaatgggacaag

tttttttggataaaatatgaagtatctactgtcctacacactgtatttcctcttcgttca

caatatctatctatctatctaattgctttttagtttttacttatcataaatattt

>fava_dn_rep_c555

tggccgcgggggtcttcaatcccctcagttttgcgccgataggtttccggctgctcaatt

cctaactcacaacatggcgagaggtttgaagaagcatttgaagaggatcaatgcccccaa

gcattggatgcttgacaaattgggcggtgcttttgctcctaaaccctcatctggacccca

caagtcgagggagtgtcttccacttattctcatcttgcgtaacaggttgaagtatgcttt

gacataccgtgaggttatcgccattctcatgcaacgccatgtccttgttgatggcaaggt

caggactgacaagacctatccagctggattcatggatgttgtatctattcctaaaaccaa

tgagaacttccgtctgctctatgacactaaaggccgtttcagacttcactctgttaggga

tgatgaggcgaagttcaagctttgcaaggttagatcagttcaatttggtcagaaaggtat

tccatacctgaacacctatgatggtcgcaccatccgctacccagaccctgtcatccgggc

caacgacaccatcaagctggatctagaggaaaacaagattgtcgatttcattaagtttga

tgttggcaatgttgttatggttactggtggaagaaacagagggcgtgtcggtgtgatcaa

gagcagggagaagcataagggaacctttgagaccatccacgttcaggatgctactggtca

tgagtttgcaactcgtttgggcaatgtgtttactatcggtaagggtactaagccatgggt

atctcttcccaagggtaagggtattaagctaaccgttatcgaagaagctaggaaaagggc

tgctgcagctcaacaagcagttgctgcttaaatctcttttgaatgaatttataatagact

tgagtgtttggtagttttattgacgctattggtttcttttgcattttgtctgagatttta

tcttattgaattttgatttgttgctctatgcaacatttgttgtgtctgctgctttattta

ttcgtgaaggaatattttcactcggttgttttgaatcattttgatagtacataatctcta

tagccaatttgctagtgcaaaccaaatatacttgtcatactgaatgattatggtaaactt

ggtagtatcataataatattcagttgggtccagacccttctattgatagaannnnnnnnn

nnnnnnnnnnnnnnnnnnnnnnnnnnnnnnnnnnnnnnnnnntttatgaaagtatataga

catgtttgtccaaaacaatattatgtagttgaaaaattgaaaagttgttttg

>fava_dn_rep_c556

ataaagaatttaacctgaaaaaaattctgataaaggaaaaaagaaaaagaaagtataaat

ttaataaaaaatttgaatatttattaaaattctacaaaaacgaagattgtactccgattt

tgtacaactgggctagggttcgttcctatccttcccagaaacggtgcctccttcaaattc

aaacacaacaaggtgttcgtttcattcttctataaaacgctgcgtttcgcaaccggtaaa

cggcgcttataatctcgtcgtttactcgaatcataaccgtttctggttcgagggtttgag

atggacgatacggtgcttgacgatatcatacggaagcttgtatcggcgaagaacgggagg

acgacgaagcaggtgcatcttacggaggcggatatccgacagctttgtacgacggcgaag

gagatttttctcagtcagcctaatcttcttgagcttgaagctcctatcaaaatctgtgga

gacgtccatggccagttttcagaccttttaagactgtttgaatatgggggatacccacct

gaagccaattatttatttcttggagattatgttgatcgtggtaagcagagcatagagaca

atatgcttactccttgcttacaaaatcaaatacaaggagaacttctttcttctcaggggt

aaccacgaatgtgcctccatcaatcgcatatatggtttctatgatgagtgcaaaaggagg

ttcaatgttcgcctctggaagacatttactgattgctttaactgtttaccggtcgctgct

ctagttgatgagaagatcctctgtatgcatggtggtctctctcccgaacttaaaaactta

gatcagatacggaatattgctcgccctattgatgtgccagatcacggccttctctgtgac

cttctgtgggctgatcccgataaagatctcgagggatggggagaaaatgatcgaggtgtg

tcatttacatttggggctgataaggttgctgagtttctcgagcaccatgatcttgatctg

atttgtagagctcaccaggttgtagaagatggatatgagttttttgccaaacgtaagctg

gtgactatattctctgcaccaaattactgtggtgagtttgataacgccggtgctatgatg

agtgtggatgacacattgacatgctctttccagatacttaaatcttccgataagaagggg

aaaggtggttttggcaataactcatcaagacccgggactccacctcataagggtgggaag

acttaattaacagtctcgagctatgtaactacgaagtcagtgagctggatgatggctttc

attctcaacataggttaaaggcttatctttctgctgcctgtcatgattgatgattcaaaa

gatataattgcattttgcctgctttctttggcaacgataactaaagacagtaaatttggt

taatttggttaatttgattctttattgtatctgcaaaaattgaatctctttgtatgaaga

aggggaaccaccttcttcaattttcgacatcttaggatccgacttctatcattgatacag

gaaatgaatcagcatggcaaagaaaagtgtaattgagagggagaagaagaggcaaaaatt

ggaacaaaaatatcatttgattcgacgatctccaaaaaaagaaatgagcaaagctcaatc

gttaagtgagaaatgggaaattcagggaaagttggaagtactaccgcgtaatagtgcacc

tacacgtcttcatcgacgttgttttgcgaccggaagaccacgaggtaattatcgacactt

tggattatctggacacgtacttcgtgaaatggttcacgaatgtttgtggccgcgtgacaa

caaaatcaaaaacaatacaaaccaaaccaaagaaacgcagacaaatagaaaatggcagga

atcttgaacaagattggtgatgcccttcatataggaggagacaagaaagaaggagaacaa

cacaaaggagaacaacatggtgttgtaggaggacatgggcatggtactgagtaccaagga

gaacaacatggtgttgtaggaggatatggacatggtgctgagcaccaaggagaacaacat

ggtgttgtaggaggatatggacatggtactgagcaccaaggagaacaacatggtgttgta

ggaggatatggacatggtactgagcacaagggagaacaacatggtgttgtaggaggatat

ggacatggtactgagcacaaaccagatcaataccaagggggagagcacaaggaaggaatt

gttgacaagatcaaggacaagatccatggtggaggtggagcaggtgagaagaaaaagaag

gagaggaagaaacgtgaggagggccatgaacatggccacgatagcagcagcagtgatagt

gattagatctcaactactcatcttgaggtgtatggaaccatatgtgacgactatgtcctg

tgttttgatttcttgttataattatatctatgtatgtgagtgagattgtagttgctaatt

cgagttgttgtaaaaataattgcggttagaatggtttgtgaaacccatactgctttgtta

atgtttggtatgtatattatcatcaagattttaaggagctctattattagtaactttttt

tggtgtcaaaatatcagtaaaaattaatcaaatataattgattccaatttggacaaaat

>fava_dn_rep_c557

acaacacaatacacataaaatatcatacatattctacgcgtctgtttagagagagaaaga

gagaaagagagagagagagtggacttttgttccttattttgtgttttattcttttgtttt

ggtcaaataataaaacactttcactctttcttattgcttccatccaaacataacaaaaac

tcatcaacataataaaacacctttcacttcacttctctttcttttgttagctcaattatc

gttcctcttcagtttcatcttcaatttcttcatggtttgttcaagttcaagttcgtcttt

tctataagatatctgttaacatggtttcttcgcaaggaaatgctgatcaaaatggttcgt

ctgataaagaacagcagcaagtgagccctcagacggaggttgcactgtcacaaggggttg

acgacgtagctaacaatgaatcatcggattccgatgtcaatgccatgccgaatgaattgc

ctccatcgccggacgcttcaccgctcgagacacattcccagagccccggtacaccttcta

atggattgcctctgtcgcaatctactcagcgcacttccgctataatacgcgaaaaggtga

catcagatggctttaactggcgaaagtatggccagaagagcctcaaggcagaaggattta

gaagatgctattacaaatgttcgcattcaaactgcatggcgaaaaagaaatttgtgatgt

cacccgaaggaaacttcgaaaattacacttacgccggtcagcataatcatcctaaacctc

aattgaacaccgtaccatcggttgaacgtgttcttcccgttgttgaacaaggaccgcaac

aatcctctttggctgacatggaaggtcaagacaagtcatcggttgagtatgaatctacgc

ctcaacagatcagtcctttacgttgctgcccaccttcaaatgcttctgaaactcatgact

caaagcgactgaagaaagacgataacaacactcataccgttggagttgacgtgtcaactg

gtgaatctcgcttggttgttcaaactccaagtgcatccggtatcgtagaagatgggtatc

gctggcgcaaatacgggcaaaaaaacgttaaagggaatgaaaatccaagaaactattatc

gatgcacgtctcctggatgccatgtcaaaaagcatgttgaaaaatcccctgagaatccaa

taaatatcgtaactacctatgagggagaacatgatcacgaaccccctactgcaagacgcg

tgcattccttgtaatacaagttccgtgcacatcaagtgaacatgatactgttaaggtttc

tggtgatgatactcctagtactacaagtgaactcaataaggttcctgaagatataaatag

ttggaattgacataggttatatatattcctattctaaccttctattttcgctttttctag

ctcatctttatttgtctggcccgattgattgcatattcaaatcacagtgatttattacat

tgaatgtacattttggtacatatctatgtaaaattccaatatattgtgattcgtctcgaa

ttcatgttttgagcatggtgatgctttaagttgtctttcttactttgattgagtttaaaa

aatgttatgcggagtttctgggtcgggtttatgtccctaaattgttgttcttttctaggt

cgggttaatgtcccttaggatatttgtgcttttttaggtcaagtttaagtcttttaggtc

ttttgttcttcaataagtcgggcttatgtttttcccgaaatcgggtttatgttttcccaa

attatttttttttttttgcactcaaatatttgctatttgtatatattgtatgttgtcact

atcaacactaaaaattaacaagtaagctagaagcttgtcattcacaaattggaagcatgc

tgtagagggtaattccatattctgagatattacataataaatattacagaaactacatac

aggtcattcaagcagcaactggcaatggagtagcaggagtaacccgattttcagctggaa

tttcaacaaactcagacagtagagcacggaattcatctccactcaatgtctccttttcta

gaaggacttcaacaatcttatcaattgcctcgcggttgctccttatctgcgacaaggcaa

tttcatatgcttcatcggatagtctcttgacagcggcatcgatgccctctgtaagctttt

ctgacgtcgggttcctcgtcatcattctcatgataacatcgccactttgcgctgatgagt

ccataagagaccaaggaccaatatcagacattccaaatgtcactaccatctgtcttgcta

tgccggtgatttgctgcaaatcaccacctgcgccagtcgtaacctcgggctcaccaaaaa

taacttcttctgcagccctaccacctaaccctccaacaattcttgcgaagagttgctgtt

ttgagatcagggttgggtcatcggaaggaatgaaccatgtaagaccccgagcttgaccac

gaggaattaatgttaccttttgtacagcatcatgaccaggagtcaaagttccgcaaatgg

catgcccgacttcatgatatgccactaaacttttgctctttccatcagtcattaatgttc

cttccattccagccacaatcctatcgatagaatcatcaatctctttagatgaaatccctg

accttcctcgccgaccagctaatatagcagcttcattcaagagatttgcaagatcagctc

cactgaaaccaggagttctcattgcaatcacttcaagagaaacatcattttcaaactttt

tattgttagcatgaacctttaggatatcagtccttccctttatatctggaacatctacag

atacctgtctatcgaaccgtccaggtctcaataatgcagagtcaagaatgtcggccctat

tagttgctgcaataacaatgacaccggtgttaccctcaaatccatccatttctgtcaaaa

gctggttgagagtctgttctctttcatcattccctccaccaattcccgtccctctttgtc

ttccaacagcatcaatttcatcaacaaagacaatgcaaggagcattctctttagccttct

tgaacaaatcacggacacgagaagcaccgataccaacaaacatctcaacaaactcagaac

ccgatatagaaaaaaacggaacgccggcttcaccagcaatagccttggctaacaacgtct

ttccagttcctggaggaccgacaagaagaactccttttggtatacgagcaccaacagaag

tgaacctctcaggcttcttgagaaactccaccacttccataaaatcctgcttggcttcat

ccaccccagcaacatcatcaaacgtcactccagtgttcggctccatttgaaacttggctt

tagactgaccaaactgaagaggaaatccaggcccgccgggacctcccattccaccagatc

gcctcgagagaaagaacaatacactaatgacagcaagagggaaagccaaattcccaatca

agttaaacaaaacagaacccgattcctcttggttattatgagctgcaaaatcaatgttct

tttccctcaatttctgaagaagctcttgactaagtccaggaagttgaaccctaacacgct

gcagcctgtttcctaactcaggagaaacagcctcaacaatagctatagttccattctcat

acaaatccactttggtaactctatccttgtccaaatactcaagaaaccttgaataagaca

tcctcgacgacgaaacaccttgttcatcagcagcataagcttttccatttcccaataatg

caggcaaaccaacaccaacatttccaagcaacaatttcagcatcccccttctaccctcct

gctgaccttgatctaaagtagcctttacagagaatgattttgatgccctattactcatcc

aagatgagaataggtatcttccactcaagtccttaccaagagtaattctattactatgag

tagataaagcatttccaacaagacatgctgacgatgctgccatcttgaatgtgaaagaga

aacgagagagtgtgagtggaagttcagagcctcttggagaagaggaaggtgatgaatgcc

gnnnnnnnnnnnnnnncaagaataaaccggtaggagaagatgac

>fava_dn_rep_c558

tgaggtgataaaggtacaatgtctatttcagtccatagaaaaggaatgcaaccatatatg

ccgcataaaatttgacacattcatagaatttgcattattatcaacacgtacatacataat

cttggatacaattattttatactcaattcatattaagtagcaaaatgtaataccatataa

atggttgtacaaatacaataataatcaaaattattattccaaaaataatatgaatttctg

tcttgaatatcattcgttttaagtcttcttaggaacaagtttggtcttaatttcatcaac

ctgtttagtatcaatctgaaaagcctgagcaagaacatcaccaggaacagttggtgttga

accaaacagagctgaagcaattgaaaaagtaccaggaagttgactgttaaaagccgaaat

cacagaagcagctttatctccattgttcttctgataatgcaccaaaccttttggaaaaac

aaagacttcacccttttttatagacttagaaatgagtttgtctgatgtagtgataaaacc

aacatctaattcaccatctaacacaaaaacaatctcagtggcacgagggtgtgtgtgtgg

tgggttaagtcctttaggtttgtagtctattcttgataaagacactcctaatgtgttaag

tccaggaattttgtcaacattggctggagtaaccaaagatcccactgtgttgttgatagc

tttaggattcgctaaaccatcgaaaaagaaatcagctgttgttacatttgcttctgcttt

gcatgcaaatccattcaccttaacacctgaagagggaagagctacacaaaggtcttgaag

tgtgtcaggatcagaagccaaggtaaatgaaatggcacagcataaaatcaccaaaattga

ccaatttgttggcgacatggctattaattatgtttataaaaaagtgcttattatatgacc

agagagaagagagaaaagtttgagcaagatggtcccgg

>fava_dn_rep_c559

cgatttcaaaatgtactctccattcattagatagataagatcaccaaaacttcgtgatct

gctggcgaacttattccaattcaatttcaataagagatctcaatattatgccttgaagag

gactcgaacctccacgctgtttaacacgagattttgagtctcgcgtgtctaccatttcac

caccaaggcatccttaaagggaatcatattctctgaatatgatatctatcttatgtcatg

gttcaatattattatactccaattctaatccaattgcaacatagaatacaacatctcaaa

tagaatacaacatctcaaatagaatacggtacgatataatacagtatctactatataata

cagcgagaatagaattctttctcgactaaataaatatagcttctagatgaacgaaaagct

agtcaaagggtcgattcaaaattggtagacgctacggacttaattgtattgagctttggt

atggaaacatagtaatgttaagtgaaaacataacataatataatacagcatctactatat

aatacaacatgtactatataatacagcgagaagagaattctttctcgactaaataaatat

agcttctagatgaacgaaaagcaagtcaaacgggggatatggcgaaattggtagacgcta

cggacttaattgtattgagccttggtatggaaacatattaagtgaaaactttcaaattca

gagaaaccctggaattttaaatgggcaatcctgagccaaatccttctttccgaaaacaaa

aaactaaaagttcagaaaaaaaggataggtgcagagactcaatggaagctattctaacaa

atggagttgacaacattcaattgattaatgaagatttctaacttctatttgtaaattgta

actattttttttagttttgattcgatcacaattgaaacgaaacattagaatcaaatcaat

tccaactggaagaaaaaatggtgaatattcattgatcaaatcagtcactccaccaaaata

tgatggatcttttgaataactgattaatcagacgagaataaagatagagtcccattctac

atgtcaataccgacatcaatgaaagttttagtaatcggaaaatccgtcgactttaaaaat

cgtgagggttcaagtccctctatccccaaaagccgattgaatttgctcatttttatggcc

ctttcccttttttttattgagttgacatagactcaagtaatttccctcaaattaggatgg

tgcgtcaagattttaatggtcgggatagctcagccggtagagcaaaggactgaaaatcct

tgtgtcaccagttcaaatctggttcccggcgactcattatgtatgagtatctattcccat

atttcttttcaaaaatcaaatattgggaggaatagatacatattgattagtggtcttaat

aattatacataatttatctaggcgtacagagatatgctctttctagatgaaaaaggaatc

gatgtatattctaggaagtatgaaaatgaatgattctagtttgtttcgattttttctgcg

atgcaataacaaatgttcatatttcaacaatattcaaatggttcaattagttggttgaac

gaccaaaaagttgaatctagcggagttcataggtaagaatagtcaaaatttatctgagat

acattatcaaaaaatatggtccatttctttgtattttctttgatcgtacttttttatttt

tcgtagccagatatctactactcggaatagaaataggaaattgacttcattataggactt

attaatcttgtaaaatttttaaaatatcgggtaggagtccatgccgctactcggactcga

accgagatgctctagcactgcttcctaagagcagcgtgtctaccaattccaccatagcgg

ctgactcgaaatcataataaccaattatttagtcaaagtcaatagtcaatcgtcgagatt

gaataaagtgaagtccaatatttattgagtacatttctttactatttactaaacatttat

tctcaattgataataaaactaaattgataataaaaagtaatttaattttttctaatatat

aatagaaatgaatatagaaatatatgatatatgcaatcattttcaatttaagtaaaagaa

gttttgatttctatattcatttctttttttttcccatgcagtgttcgttttcaaatttaa

caacgacggggaatgggttttttgtagtttatactttttcaaataaaaaaaagcactgtt

gaaactagaactatctagttctgacttcaatccagtaatgaaaaagaaattttttttgta

gttgcttatgaatcattcaaaaaaatgagattatatatatatctagaatatatgtttata

tatgtttcatattctataatagtattagtataaaatgactattaaagaatactctttgaa

tcgagctaattcagattattctcgtccaacatttgtatttcttacaataacacaactttt

tttttgattacgtggcaaaaaagggcgaggttttgtatttattacaatacgataactttt

tgaggtacctgtcaaaatagagtcagctaatgaaaaggctttcaatgctgccttatatcc

tttttttttccaaaaattcttacgaatttttttttttttgatatagaagtgcgttttttt

ggaactggcatacaattagtatagttttttcgttgctatagtttgatgtgaaagacattt

gttctgtaaatgaaaacgaattattctgtaattagccgtatgtcttatctatctaaatta

actctttctctttttttccatcgaaaggtaaagtaaaaacattgaatattataaaccttt

tccaaatttttcatagataatagatatatatggatctcccgcggcc

>fava_dn_rep_c560

gtgcttgtaggtctttatcatcattttttttcgtgaaaatctgcaccaatgggcagaaaa

tagataaccaacgaattccacgcatagtctgattccttggacatcaagcaatgaactcca

aactgaaattatacatctgcaggatccaaaaccccccaaggaatgttgttatttataaca

taataatgacatgaagataaaattttaacaccgatgattgaaaatagttcactctactcc

taatacattcgccattaatatattatatcaaaaagatcattcctctgtctcgttcgcgtg

gccaaccctactcaaccacggccagcaagtcgcttgatttacaaaagcagtgcttcgctt

cagtccccaaatccacctcataagcactcatatcagtgaatagtacctttgctccagcct

ttacattttcagcctcagcaccaacacttaggacttccccagtaagatattgttcaaatt

taacagctgacccgggcaacaaaattccaccagtggttgtttttgatggctcctggagac

gaataagaaccctatcagcctgaggcacgaccttagttggttcccattgcttggaaattg

cgttaatcttgaaagagtccctcttgaaaagtgggagtttcgggttggagaaagaagcgg

cggttgatttgtgaaggaaaggagttggtaaggggagaaatgtggaagccatgggaatgg

ataagagaggtaaacacagaagagtggattgaagagttaaacccttgttaggtttattat

ccacaaagtcacaagttacaaaatgtcaaagagtcccgcacctgtgttttttctagtctc

atttgtgactgtaccaccggccgccctcaaatgttcaatgaaagctgtactagctgtttt

tgatactttagattttgtctggaaacatggcaattttttgttgtttggattgggatactt

tagattttgatactgacatcattcttctctttatttggataatcaatgtatgacttctag

cactttcactttaaggtaattattgactaaaggatcagaaacaggtttttataattaagt

tttcattgtcgtgcgaataatggtttcattatcatatatagcaaaattcaaatgatgaac

cattaatacatttgtttgtcatatgctaagactgacttaggcatcaaatgaatatttggt

tagaatatttgtcttgaaaaaattgtttaattaataaa

>fava_dn_rep_c561

ttacaaaaataagcaacaaaaaatatcatttgaaattatcaagaactcgaaaattggcat

aggaataccccagttctacatgcttatgaaagttctacatgctcacaaaacagcaaacct

tgtattgtgttcattcaagcatttacattatttacttttacggagatagaacagaatgat

gaaacctcataaaacagaagtttgttcaccatttttataataaattgtaataaactattg

attatacagctgcagcttttagggaaagaaaatctgaaattttgttggtgtaaaagtctg

gttgaatggcgttgtttggattctgaaacatagccgttgtggtgactcctgagaggacaa

gaagagttttgcagccaccattttgaccaaataatatgtcggtgtctaatctgtccccaa

ccatgcatatttgtgactttgaaatgccaaatttgttagctaagtagtccatcatgaaag

tagagggttttccaacaactagtggttcgcgttgagtggatccaacaagagcaccaacca

tagagcccccacctgcccattcttgagcatctgtgagatgagtaacagcatctctatttg

tagcaataaaaagacatccagggttttcccgtatacagagagttccatactggattttat

agtagttgaaatagcgatcaaaccccacaacaactgctccaacatcttcatcgtgctcca

tcaagaaccctggcttaagctcgatctttttcccgccatcttccggcccaccaacatact

tgtatccagcaagctcaagctctttcaagatgccatcttctccaatgacataaacctttt

tatcttttggaaaatcaatggatttcaaatacgcaggaactgcaaaagacgatgcaaaaa

tctcctcctcgttaacattcaggccaagtgtttcaaacttctttccatattgtttcctag

acttagttgagttgttggtgacaaaaaccaatctttttccctttgcccgaagcatgtcca

acgtttcaggcactccttcaattaaactgtcacctttccatataactccgtcacagtcga

aaataaaagtctctacggaatcaatgagctcatcagcattctgaagcggttgagccaatg

ctttggttgctgccattctgaatcgattgttattggaactagcaatgtttgttttccatt

tgagaaacgcagaattggagagtaaagggttacgaggagagaaaaatgatgtataatttg

tgggaagtgaatgtgagcataatctttgagtgtgagaaacaacagcaacgctagtgctgc

tcctaagcatccttgtttgtaatttcacaagtttatgtttatgtttatgtttttgtttta

ttttaggttgtttatttttgtggtgtgtgcatgggtaggtgcacttgttcaatagaatct

tcttggtgggagacgagggcaaatgtttgatatgcaaggagttggatcggagggaactac

actactcagatataagattccaactcgtggtctacttggattgcgtaatgcaattttaac

tgcttcccgagggacagctattctcaacactgtatttgattgttatgggccttgggctgg

tgacatgaatactcgggatttaggctcgctggttgcttttgagggtggaacaagtacttc

ttatgcaatcgctagttcacaggagagggggcatatgttcattggtcctggagcagaagt

ttataaaggtcaaattgttggcatccatcaacgctctggggacttgtccttgaatgtttg

caagaaaaaagctgcaacgaatattcgttccaacaaggaacaatcagtgattcttgatac

accattggattatagtttggatgactgcattgaatacattcaagaagatgaactagtaga

agtcaccccccaaagtataagaatgtgcaagaatcccaaacttgcaaagaaaggaaagta

gacttccaccgtgattctgtgaagcagtaacttgcagatgttttataaggcaactctgct

gaaactacataacttcactaccgtttgattcaatgttgcatatatttgtctgaaactgca

gaatgatataggagttggcaaaataccttattcatcatagcatttgttcacagttgattt

agaggataaaattatactgaatttggtccaggtagccgattctattcagtgggataagac

ttgcttgttattgttggaagtccatatttattgacagcaaggaacatcaaaatacaaatt

gttaatgatatgattatgcaatgttcaataactttactagtcttcatatattgtcgatac

tgtgtatttttgccattaatcatccgatatccagaggaaaaaaactctagtaatttggag

tcaattgcctgtctctcatgtaacagaccatggttgtgttacttgtgtatagacttcata

atgcaaggcgactggatttttctattaaatcattaaataataggcttataaacttaaccc

at

>fava_dn_rep_c562

cacctaatgcgttatttgatatctgctcttcaccatggtcgaaaattgataatgcaggga

ccatatatggttttgcatacttacaactgataggtaactttacttcatctttttcatagt

taaaatgttttcccataagaagtggtattggttctctctcaagtttccccagctctttga

aatggtccaatgaaaaggtttcatgctttnnnnnntaatatacaagtccacgagttacat

cattctgttctgaacattggtcgaaacttgtgaggacagattggaaattcgatgcgagga

gtaatcaactcattctctgaattaagggatttaattttgtcaatttggtggaaattaatt

tttatatattgataatagaaaagagggaataatattgttcctttctaaaattatggttcc

tcttaccaaaggggaaagggcagggggcagatgcttttcttagcccccatttaattaatt

aaaatatatgatagatctgcagcagacttttttttgttcctttcttcccagaacaacaat

ggttgcgaattccacttccgataatgccgcactctcaggtgccgcgaataatgagaagca

gaattgggccacatccccttccaccgttttcaatcacttcgccacaagtggactctccgt

cgcaattgcaacatcaattactcatcctctagatgtattgaaagtgaggctacaaatgca

acttgttggccagaaaggtcctttgagtggaatgcattttttcagggacaattgctttta

actgcggtgaaaaatgaaggacctaagtctttgtatctgggtttgacgcctgctttaaca

aggtcagtagtttatggaggactacggttgggtttgtatgaaccctctaagaatgcatgt

gatcatgcctttggctcctccaatgttttagttaaaattgcttcgggaatgtttgccggt

gcttttgcaactggactgacaaatccaatggaagttctcaaggtacgtttgcaaatgagt

tcagacatgggaaagcgtggcccaattttggagcttcaaaggactgtatctgaagaggga

atcaaagctctgtggaagggggtgggtcctgccatggccagagccgctgccttgactgca

tcccagttggctacctatgatgaaaccaagcagattctagtcaagtggacatctctcaga

gaagggtttcatctgcatctaatctcaagcacagttgcaggaatactgagcaccctaata

actgcaccagtggatatggttaaaacacgtctcatgttgcaacgacaatccaagggtggt

gtaatatataaaaatggatttaactgtgcataccaggtcctgcttacggagggtccaagg

gcgctatacaaagggggctttttttttttccccaatttccgcatttgatttctccaataa

attaaatcaaacaagtttgtcacatatttctcctcataacacaacaaacagaaacacact

actacattctgaaaatcaaacaccaaacaccaaacacttcaacctcatttgaggtcaaca

acaatgacccttttaaaaccttaaaaaaaagttcaacacaacataatcaacaagtaaaac

accatttggtatataaaaaaattaccataaataaatgaatgaatccaaagctgctccaat

agcaaaaacaataggcatgagaaggagactatggcctctaagagttggtgcaaaagattt

tataggtgatgcttttggtggtggaggtagattttcaacatgaacagctattttcattcc

accaaaacaaaatcctttgccacttatgaaatagtaatgtcttgtcacattcaatggaac

aacatctcttccagctccagttgtccagttgtgaagtggatgatctgagttgcatgtttc

ataatctgttttgtttacttcaagtacattcatttggttcctatcatacacaaaaaaaaa

agccaatcaccattgtagaaatgcttgtccttagcccaagtagtgtagttgaaatttgtg

ttccaacctttgcttccaccgaccaaaatccttgaggctgatgccattggtagcatgaga

agaatagcaaaacctagagagagaaacaccattgttggtgaaattgatgaactaaagagt

cttttcttgatgccctccattgatgtttctgaagatgctcaattctatggtattgtttaa

actccggaacactctgttctgtctatgatgttctgttcttttggcccttttaaggaacct

tttgttttcttttaggttttctctttaaat

>fava_dn_rep_c563

aaaagtgatcgtagcaattaagttatatcgaataattttccttcgcattgataatatgca

gaattcatcacaacatctctcaatatgctatcaaacattgataacgtttagctggaaatt

atttcaccaaacaatcacaagattttatttctttctaacaaacaaataattttgcatctt

tctgttacacattcaaattaagaactagaaagtaactatgaaaagttaaacatattcaaa

catttcccactttcccaattctcagataaatcggaaacttagcagttttagggccatgat

cttccccccaagcaaccttaaaattttcaacgacatcctccccaagaagctcaactcctt

tctcctttgccgtctgataagccgaccacgatcttatataagttaacaaaccatcaaagc

tcatcaaagtctccgtcacaaactcaaacggccccgtatgatcaactccgtccaccgcct

caaaaggaaaatcaatgcttctataattgtcttcaaccaatttacgcgccgaatcccaat

aaggttttgaatccctataatagaattgatcaaacacaatgtctacttcttcattaattc

ttggcaaaaagtaacaccaagcagcaaggactccgtgaggtttgttgagtacatatttga

cctgttcgtagaaatttgggaggttgaaccaatgaagtccttgagctatagtgacaaggt

ctatggttccttgtggtgctacaatttgttcaacctcgggaatggacatggttggagggg

tgagttggtattgcacattagggagtttggttgcaaattcaagctgtttttcactaacat

ctgtggctatgacattcttgtacaaatcagctaaagatttggaggcttggccacttccgg

tggcgacatcccaaacaaggttgtgagaaggagtttttgaagctataaattgaaagagtt

gtggtggataacttggccttgcatttgcatattcctttccctgtttcacaaaaagttctg

ccatctctctctaataatgatcccgcggcgagtcagtatgagtcaactcggtctcatatt

a

>fava_dn_rep_c564

aggcctaattgaattgaacccttgaaaccggcttgcaaggtgactaggtgaggcatcctc

acctataaacttatattgcattcgatttgggttggacctaactcaaccttataaaatcgg

cttgtaagatataatggttcctctggacaattacatttcaaggggaactgttcatttcgt

tacttgtaaagaacctgactatcaacagagtttgtggaatatgatttcttctcttatggc

agataaaaatatggaggacggtgatattgagccagccccaaagcttattgaagttgtctt

cctaaactgcagaggactggtggaccactgggttgagccgtatctcagaattacagttga

gcgattgcatcgaactggaaaatcaaatttaaaatgtcttctcatgcaagtgattgcaga

tggtctatactacaatgctgcattgactctcagcatattgcaaaagttaggtgttgcatc

tgaaatcttcaatctttggtttcatatgctacaagaagtcaagaagagtggtgcaagggc

taattttaaaagggaacatggtaagaaagtttgctgccttggtttaacatccttgctggt

gcttccagctgatcaattgccaggagaggctttaggccgggtattccgtgccactcttga

tcttctagttgcatacaaagatcaagttgcagaagcggcaaaagaggaggaagaagaaga

agaagatgacgatgatatggatggctttcaaactgatgaagaagttgaggacgacagtgg

ttctgacagggaaatgggagctgatgctgaagatggggatgaagctgccagtagtaatct

tagaaagttggctgatcaggcaaagtcattcaggcccaatgatgatgatgatgactcgga

tgatgacttcagtgatgacgaagatttgcagtccccgattgatgaggtggatccttttat

tttctttgtggatacaatgaaagttatgcaatcatcagatccaataaagtttcaaaactt

gacccagacacttgagttctcttatcaagctcttgcaagtggtgttgcccagcatgctga

gatgagaagaggtgagattgagaaagaaaaatcagagaagtcgtcagctatcagagattc

ttaagcttttgggtgtacattcttgcaagtggtgttttgcccagcatgctgagatgagaa

gaggtgagattgagaaagaaaaatcagaaaagtcgccgcgggggcacagttcaacctcaa

acgaacaaattcaattcatttctcttattcgtgatctaacttttcctcaagatgcagata

ttcgttaaaaccctgaccggaaagaccatcactctcgaggtcgagagttctgacaccatc

gacaacgtcaaggctaagatccaggacaaggaaggaattccccctgaccagcaacgtctg

atcttcgccggaaaacagcttgaggacggtcgtactcttgctgactacaacatccagaag

gaatctacccttcatcttgtcctccgtctccgtggaggtatgcagatctttgttaagacg

ctcaccgggaaaaccatcaccctcgaggttgagagttctgataccatcgacaacgtgaag

gctaagatccaggataaggaaggaatccctccggatcagcagaggttgatctttgccgga

aagcagctcgaggatggaaggactttggctgactacaacatccagaaggaatcaactctt

caccttgtgttgcgtcttcgtggtggtatgcagatctttgtgaagactcttaccggtaaa

actattaccctcgaggtcgagagctcagacaccattgacaacgttaaggctaagatccag

gataaggagggtattcctccggatcagcagaggttgatctttgcgggtaagcagctggaa

gacggaaggactcttgctgattacaatatccagaaggagtcgacccttcaccttgtgctg

cgtctgaggggtggcttttgagctgctgttggtgttgtttcgtgtttgtgatgttggttg

ttttgaaatggaacttgggaactgatgatgatataggaaaacaaaatacttaactaaaat

atatgatcctttcttgaatggacgcgttcgtggagaaatccaaaaaggcttttcaccctt

cattccaaatgaaactgacacaacaaactacattttgataaataagattcatggtctcct

tctttctattaatagtaatgatccagaatttgaacagaaaatagatcaatttgatagaaa

attattattaaatgaaattggttttttttttaacttaatcagtaaattttcggaaaaatc

agtatcaagtttgaattttgacggactttatttatttccagaacatgaacaagtaaaaat

atattccgaagaaaaaaaaacaaaaagaaaattattattggacgcaattcgaactgatcc

tactaataatacaatttttaatagaaaactatgtagtgaaataaacgaaataagtaaaga

agttcctcgatggtcatacgacttagtcgacgaattggcggtattgatggacgcgagtcc

aaaggagtctcagattcgttccccaaaagctgaccgtatagttctttttaatagtaaaac

agattctaatgacaatcagaatagtgctgaaccggacagaaatactgaatttgctgtagt

tcattattcacgcgaaccagattattgccgagacctaatcagaggatctatgcgccctca

aaggcgtaaaacagtaacttataaactctttcaaggaactgcacactcccctcttttttt

ggagataatgggcgactacgcgttcttgtttggtgatcttgtctatgatatctcccaata

tttgaaagaatatttcaggaaaccgggtactgataattcggaatttttggcgtttgagaa

aacgatagaacagaaagagatagaggaaaacaaagacgagtctgaaataagacttagaca

aatcgaagaagcctgggagagcattctctatggtctaataatcagaagttttgtattact

attccaatcaatatttagaaaatatattctattaccttcattgataatcacaaaaaatat

cattcgtatactattatttcaaaatcctgaatggtcagaagatttgagggattggagaaa

agaagtccatatttactgcacctatcagggcgttccagtatctcacaccgcgttgccaac

aaactggttctgggagggtattcagataaggatcttattcccttttgttctgaaaccgtg

gcacaataaggtacgatctactgaaaaaaaaaaaaatctactgaaaaaaaaaaagatcca

ctgaaagaaaaaaacttatggtttttaacaggttatggaacacaagtcgaatcgtacctt

gatgatcatgtcccaaacccttttttatttttgggtcccattttcttaaaaaataaaaaa

acaattgaaaaaagatttgaaaaatcgtttttttctagttctaaaagtttttaatgaaag

aaaaaaggggtctcgaatcgtgttagaagaaatcgaaaataggttcaaaaaaagagatga

attgagtgaaagcaaaaaaattcaacaatcagtaagagtaatccaatgattgaggaatcg

cccgttctaattcaatctatgaattggacaaatttcacagaaaaaaggataaaagatctt

aatgttaaaacaaagacaatcttaaaacaactagaaaaaatgacagaagaaaaaaaagag

ggggttataactttagagattaatttgaattctaccaaaactacttatgatgctaaaaga

ttagaattactaaaaaatagtttgaaaatattaaaaagaagaaccgttcgattaatccgt

aaatcctatttttttttttcaaattttaatgggaaggatatacatagagatcgttttctc

tatcactcgtattattatgatgtctggacaacgttttcttaatattattgaattaacaga

caaaattgtaaatgtatttatttccatttacactaaaaaaaaaatgaacaaagagttcat

aaaacaaatgaaagtataattccatttatgtcgattatagacaaatcttggaatattaca

gatatgaattcacagaattcttgtgatgtatcttctttgtcacaagcctatgtatttttt

aaattatcacaagttagtaatggatataaatataagttaagatctatttttgaatctcat

ggaagatctttgtttcttaagaatgaaataaaagatttttttttagaatacaaggaggaa

gattaaattccaaattaagacataagaaccgtcccgattctataatgaatcaatggataa

attggttaaaggttcattatcaatatgctttacctcagagcggatggtcaaaattagtac

cacaaaagtggaggaagagaatccatgaacatcgcgtggctcaaaagaaaaatttagttg

aatatgattcatctggaaaaaaccaattcattttttacaaaaaacaaaaaaaacaacaag

tagatttattagtatcctggcccacaggagccccagccggtctcatgtcattgctgaata

ccggagcaaccccttgatactgcggcgcatgaaccatccctccaggcgcagtatagtaaa

tatgtctcccacttgcactatcatacgccacttgcgcatacccagcagctcccgcattat

ccgctactccagctggcctaactaagccatatccttccgcataaccagaaggtttgacct

gctgagccggtgctaaattcccctgacccgcagacgaaaacgcgacattctgatgttgaa

cgccaccatacaccggctgttcccggtatccatcagaagacattcgctgcactgcgtagt

acccctgagtcacctgcgccgccggtggacgcatcacctgaggcgcgtgatagaatgttc

ccggtgtcggcatcatgtaaacatgctggtctcctccaccggaagtgttcatccctgtca

tcgggaaaccgtcgctggaaaattgtttttccggccagtaacccgggggttgatgaacag

cagttggcgaattcgaaggagccactttctccagcatcttgtgcgtgtaaaaatcgcctc

cggaagtagcagcagcgggatatccaccggtgaatccatctacacttctccgatacaagg

actgctcactctcagaaacctgcaaacgctgtaattgcctctgaatctcaacactatcat

ccgcaagtccacgtggcggatactcaggttgttgcgcaacaagatctggcacaggctcgt

gatatttcacagcagcataggaaggaggttgaaccggtggagctagggttttgttatcaa

gaccaaagaggaaatcagcgttggattgaggtttaagtagatcgggttgagaaatcgaaa

tcgaaccagaatttggagtgatgaatatgaagagcctcattcgaactggttttgaggaag

gacgatagaggcgatcgtactcatgcatgaggtgttcgaggtcgtcttctgttgtgacag

agataagagcgtcaagttcttcaccgggaagctggtacttgaaacttatctcttgtggaa

tagcgtcgcagagagtggagagcttggagaggaaggcttggaacttgatgtttcggtcga

cggctaatattttggtgtcgccgccgatataagaaagctggttgtcgtggctgcgtggtt

ggatctttccgccgtagctgcacatgaacttggctttgtagttttgctggtcttcccatg

acgatggattgtcgaaatcgatatctcttgaacgtggtgacgagtggccggaatctggat

atgacggaggatattgataggtctccattgtaataagtgtttcacaagagttcgctttat

gcttctttaatgaagaccccgcgaaaaagaggaggcgttttctgcttgattaatttttaa

atgtttttatattttattattcagatttttctaaggtgttgtttcgggtttgataaatga

gggtgacgttgtgattgctatggcttcttgtttgaacacgtggtt

>fava_dn_rep_c565

tctttcaaccttgatcaagcaatcactaacacattgagtggaaagtatatgaattccccc

tagacttgctccaacgaagagacactataagtgaagttgtttattttaagggactactca

aagtactctttctcattcctttgagaagaacaacacctattttattcaacaatagtttca

tattttaaaaaggaaaaaagagaaaagaatatacaatgagtgaatgtagcagagtagcca

cacaacacaagtataacataatccatccatacagagaaagcatgtagttttacaaatata

taaaaaccttaaatgagttctccttaaaggcaaaacataagataattagaacaagttttc

atcattgtatcaagatcaaatgatgaggataaagcttttcataatcttggtaatgcacgt

ggatgagtgcgaggagtgtcccaatctgcaacaatgtgttcacgggtgatttctttgagt

gaacggcatccacttaaagccattgttatctcaaactcatcgcgtaacatctgaagaact

tttcttactccaacttctccttcagcagccaatgaaaacaccacaggacgtccaataaat

atgccagaggcaccaagtgccaatgccttgaagacatcagttccacggcgaacaccacca

tccaaaaatacaggaatgcggccttgagcagctttcacaacctcttctaaagcacttatg

gtggctggaacataatcaagttgtcttgctccatgattggacacaattattccggctgca

ccactttgtactgctagccttgcatcctcagcagtgagtacacccttcacaagaattggc

aggctggtgattgtctgaagccacttcacatccttccagcttagagtgcgatcaatttga

ccagcaacatatgaagcaagtccagagtcattagcttggtccatctttccgaggtccaag

ccttcaaagttcttcaatgtcaaaaacggtggcaaaacaaatctgtttttgatatcagct

tctctgcgaccgagtcttggagtatcaacagtaagggcaatagccttgaatccggctttt

tcagctcttctcacaagctgagcaaccacattcctatccttataaacatatagctggaaa

aagcgaattccaggtcctgttgaagccacttcttcaacacttgaagttgcccatgatgac

aaagtcataattgttccagcagctgatgcagctcttgctgttgcatactctccttcagga

tgagccattttctgcatggcagttggagcaatcatgattggcatggatattttgaagccg

aggacagtagtagtcatgtctatcttgctcacatcgataagaattcgcggcctaaacaaa

attctggagaaggcgtttctgttttcttggagagtccactggtcttcagcaccagatgca

tagtaatcaaacaccatcttcggcaacttctgcttcgcgacattctcatactcactgacg

ttggttatctccattttcaaaggtgtttgtagaaaactaagcaagagagtgcatgcagag

gaaggagtagagaggtgagaaattatagaaactccccgcggccgtaatggccactctcgt

tgataccactg

>fava_dn_rep_c566

gttttggtcaaataacttagatgtcgccctgcatttgatatttcatcatagctacaaaca

actgctgagattgattgaccaagataaattacagtatgtcaaacttactgtattatttat

taatagttgttcaaacttacaatgctgtatgtcaaaatgaaccagcaaacataacaagat

tgaacttcaagttctcacaaagcaaagaaaaaaactaaaaccacactcaaagagaaaggt

aaaactcaatcttcttggcccaaaagagtgagaaggagcttcttgtaatcccctgaagtt

tccttagctacaacatgctcaaaaggaacactgtttctcttgtgataaacatctgagatg

gatttcagatcatgttgagccctgctcacaatcacacgggtgagcgcatcctcatcggtt

ccaatcttcttcaccgcatctcgcaccaccttttcataatacttaacatgatcggtgaag

gaacgaatggtggtgtacaaagccttctgaaaatcatctgatccttcatccaacagcttc

ttagtaatggaagtaccgtgagtctctctgtaacggttgaaagttgcaatcagctgagcc

ttactccttgtagtcaagatcctaacagcttcttcctggcttcctttcttctctttcaca

gcctcatgaagaatatcagcttcactttgtgctaatcttgcattcacatcatcaccaaca

tacctaaatgcagtcaccaaccctaccaaaagctggcgaagatggcctgaggtatgagta

gccaaatcctcttccaaggagtgcttatagcgcttaatataagcgcgtctcacattaaaa

agctcttccggggacaaaatcgaagcaatctcaacaaccacattgtagttcttaccactc

ttgatagcaacattggccaaaacagcatcacgatcagccggttccaagatccaacggtac

acagctttctcaaagtctcctttcagttcagattccaaacgtttgataagatcctcttgg

taaatctcttcataagctcttctgatttgttgaatctgatgagagtttctatgaccaaga

attgttatcactgttctctcatcagtaccccaaccttcaaaagcctttcttagagattca

gcatcttcttgtggagaatggttgctaggagcaataagagtagccatagttgaatgtgta

ttttctataatactaagtgctaactaagatgttgtgtccccgcgg

>fava_dn_rep_c567

tttttttgttaactaattgatattatactcgtagcttatcaatttgtctcaaatttccct

ctctttaaaacttcagaggatagtaaaaaaaaataatgaagttgtgcatcatcaaaacaa

tatagcaaatgagtgaaactcttcccctctctcaaatattggttttagtttcaacttaat

gagaggatatggttctacctcaccagaatctcaatatttgagaaaaacaaaaaagagaaa

aacataagtcaaaacttgaaattcaaggtctgtcctatggaaggaaatgaaaatgacatg

gtacactattcttacaacttgagtttagtaatgtcgatcttcttacgaggatacgtgtat

gcttcatagttaatattttggcccaaggtactcaattttgtgaaagttgttataagatca

acctcataatcggttcgcgagacagtttcgcggctccgttggtattttgttacccatgga

gcaacgtctggatcagatgctaataaggcttctgttgcatcctggtccggacctatgaat

gcagataaaacagcaagctggcgagaaccaaggcctacagcagaaaacttatctttcatt

tcctgaacacttgctttctcccaaattggaattctcccctctggatccggctcttgagta

tcagccctaccaaattgtcggtcgaacaaaccccactgtccatttgatccatatgctgtg

tacaacaagttccctttttccacatttccaccgcatttgcgaatcgcagaagctaaaaat

gtagcctttgttgcactttgtgctgcatactggattagatctgcataggaaatcggtcca

cctttcgaatatgaatctatttctttttttgcttcctctatgaaattcaaggctgcagaa

aaccccttattctcaggtctacttatttctgagctgaaccgtatagagccatttgggcca

cccgttttcgttgccttatcataagtcaaagcatcatttatcgccagtgttagtatggat

ggaacaatatcgggatttcccttaatggccttataaagagttcctttaatttcagattga

aactccgaacgctgtctgcgttcaatcaggtcagcagcattagccgttccaacaagcaat

ccagagtttgttatcgattccaaaccaatagtgaccccgaagcatttaagaatgtctctt

cgtttccaaaggaactgttgttcttcatcagtaacatctgttttgattttagaacaacaa

atggaaaccgaacgaggttggtgtgtggttgtagttgttttaggataatgagaacgaagt

agtggatggagagaaggaagagttgaggttgagaggaaagagacacccattgttgttgca

actacaaccaaattgtttgaggtgtgtgtttggaagtggagaaggggaacaaaggtgatg

caatagtgtaagatcttttgtggtttgtttgaggtcattttaagattagataatatatat

tatccacttccatccattgctattggattctcttatttctaccccaattc

>fava_dn_rep_c568

actatcaaacagtgccgacatgaaagtattgtgtacaaaaaatggaaatccaagcatgcc

aatagtgagaggtggatatcaaatcttctatctttgccttgggccctcttccttaacagg

tggacttcacaaaagctatcaccgattattaaccgtccatgaggttactattttgggacg

gagggagtactaactatgtactatatgatatatattacttttctctactttcaagaataa

tactataatagtactcatcttttttagaagatcctaacactcaaacaaagctaggaagta

agaaacaagtgtgagtgtgaaagatgcctcgtagcagtttttcaggatcactgtcttctc

ccaagttggatgtggttgtagacatgggcaatccattccttaacctcactgttgatggtt

tcttgaagattggagctgttgcagctacaagatcagttgcagaggacacctttcatataa

ttcgaaagggaagtgtctcaagtaacgattttgagaaaactctgaagaagatgtgtaaag

aaggtgcatactggggagctatagccggcgtatatgttggaacggaatatggtgcagaga

ggatccgtggtaccagggactggaagaatgccatgttaggaggtgcagtgacaggggctc

tggtatctgcagccagcaacaacaaaaaaagacaagattgcagtagatgccattacaggg

gcagcaattgcgacagctgcagaattcataaattacctcacctgaaagacaagactggat

gtgttattacacttggtcttctttgtcaatgttactggctatgtaatgattttgatgcat

ttactaataagatgtgctattcagctctctgtcacttaacatgttttgtcaatgacatgc

tcactgttcagctcaattttcagtctttggataaatggattatggtataaggaggactga

ggttaaacattcgccgaatcaagtatcggtttgcataaatataattgaggttataagcat

atatatgcgggcacgcgcatgtgtgcgcgcgatgaaaccatgtgtttcaaccgctgagta

gtctattattataaactcatggtagggtatagaaaataatattccttgaactacagtcca

aatatgtctcattagagcatatacatatactccaaaggtcacctattatgttggactctt

gttggactctgagttcataaatggaaaaactttaccgacatcagatttctactcacactt

gtaagttcctttccttcttgccaaaaccttctcatctgaaacattgatcccttcctttcc

ttggtgcggggaccatgttccatgctaaaaacttgcacctgtaattaattgcaagataca

taaattagaagacttttgtctaatggttgtaagaagaacagttaaagttggctagctagt

tttgtgcaataaaccacttatctatataatgttgtattctcatcatctcaatacactttg

aattctttcattccacttttaaatctttgcaaatgagttttttctttgcatacagattgt

aaactctaannnnnnnnnnnnnnnnnnnnnngtaattcttacg

>fava_dn_rep_c569

tttttttttttaaaaaagttgaaaagtcatgccaagcttttatcagcataaaaagtctaa

ccttaaaggctttttttttttcagcaagtaaacagatgtaattatcatataaccctaaag

ggagacacaatagagctacaatgcagtaacttattgcgtttacatcatcataacatgctt

tgatttagaactaacatcagcattaaaacaggtcacacaaaataagagaagttttcatga

tcataatcgtaattacagagctcaataatgaaaatatgaaatcatgataaaagagcccct

tctatttctttccacttttcttgaggccagatcctccaaaagatcccttctgtgaggcct

tagctttcaactcctttagtgccttctcctcatcctttttcttctggatgttagccatgt

caacctcatcataatccttcttatcagactttggtttcttcaaaggcttagcttttccac

cttgcttggtcgacatggttacggtgcagagttagggtttcggtctgaaaacgaaaacga

gagagaaaataacaacacgcgacaaagaaagaccttacccgaactcccgcggccgtaatg

gccaccggagatgcaccagcagatgttgaatcaactgaactacctgagtttgttaagagt

ctgcaagcaacttgggacaaagttgatgataaatatgcagtgagtacattggttgtagct

agtgtgattgcattatggagctcaactggtttgatttcagcaattgataagcttttcaat

tccctgaatacagaaagcaagacccaatatattatttgccagccaatgcttcttcaaagc

ataccaggcgcaaaaaaaagtgccagggactgcagcaaagagctgtgaccttgtaaactc

aatctcgatagagcggaaatatggaaaacgccagactatgaggttctcattccaatgatt

tggtagaaagcgtttaatagatggcaacaatgtggcggatagtgcaacaatcccaagtac

aaagaagtaggctgtcaacacagtgttcaccaagtccttagatagaaacttaaacagtaa

gaaaagcgataatagcatcgcactcccaacaaatggaaaacgcatagcatgctcatttga

cattgtctcagtaggtggagttggtttgacggatcggtaacatcccacaaacacagtgat

acaagcagtcaaaacaacatttaaattcggatctactttcacaacaaggggtgctaacgt

taaacctaacaaagccaaattcgcaattcgctcggtgttcttcattcttcctaatgcgat

acaaaaactatagatctactgaaaataaccgttcggagaattgcctccacgagagagtca

aaggtcaaagaggaaggtaaagtgaaaccgttgtttcgtcctggtgtgttatgagaagag

agaaccgtttgtgattacgtggcgaggtgtgaagaagtgtgtgacgaaaaagatcggtta

gttactgtgatagtaaacat

>fava_dn_rep_c570

caagcagtggtaccaacgcagagtggccattacggccgcggggtctgcttccatcaccca

ggtttcagttcagattcaaactagggttttttcgccatcaatggctcctaaaggtgatgt

ttcgaagaagactgatccaaaggcacaggccttgaaagctgctaaggcagtgaaatcagg

gaccgccattaagaagaaggcaaagaagatcaggacaactgttacatttcacaggccaaa

gactttgtcgaaagaccggaaccctaaatatcctcgcatcagtgctacgccaaggaataa

gcttgatcattatcagattttgaagtttccactcactactgagtctgctatgaagaagat

tgaagacaacaacacattggttttcattgttgacttgcgtgctgacaagaagaaaatcaa

ggatgccgtgaagaaaatgtatgacatccaggccaagaaagttaacacattgatcaggcc

cgatggcaccaagaaagcttatgttaggttgacacctgattacgatgctttggatgtagc

aaacaagattggtattatctaaggtaatggagggttgtatcaagacaatttttatttaga

ttttagacttattttggtaattgttggagacattttgaatcaaagttaaattctggattt

tatgttttgttatttgtgtatcttagttcaattgcaattaaattttgttatttacaattt

taaatttgacagtaagtttgatctgaagtttgttcttagatttggtggcaatattttcaa

tgaaagaatgtacttaacatgttaaaagaaatatcacggttgtagcatcaaaacc

>fava_dn_rep_c571

gtgttaaatttactcctcttcattccacaacaatagtttaaatacatctccaaagtaaaa

ccctaatggatctaaaaatgggtcatgtcaaaaagagagaaaaagaaagaaacatattaa

attaaacattaattgagaataaagtctttaaaatgctcgtgcctgaatgagattcttgtg

ggccttctatcagtgacactgtaaatttatagactactaaactaagaccatgctattgaa

tatggttgatgatactggttttatttctcagctttttgttcattatcagtatgatctaac

acaaacatgtaaattcatagctggattgcaatatcacagccatcaaacatgtaaattaaa

aggcaaaaaaataataatccaagtttcaatctccaaggataaggattgtattttgactgc

catggaattgtaactcatgctttgctaacaaactccacatcaaataagagtacagaatca

ggaggaatgacacatgaacctcctttacaaccagccccccttgaaccatatccaagttct

ggaggaagtttcagtgtacgtttacctcctgcaaacataggaggaattccatcatctcct

aaaatgcctacatcccatcctttgataacctctccaacaccaatgcgaaatgtaagtggc

ttgccgcgattgtaactgctgtcgaagacttttccattctccaatctccctacataatgt

gccttaataagctgtcccttaacagcctgaggaccagtgcctacaactttatcacaaaag

gaaaggcctgaaggagctactgttaattcacatggagctacttcagcagctgctgttgca

attggtattattggttgtaataaagaatgaagaaggccaaaactgaaggagaagcctatt

gcgtctcttctgctcaattgattcgctttttgtataagaactggtggtggttgtgggact

tggatagaacatgttactgttactgaaattgaatggttacgtttttggggttgcaaagag

gtgaggcaagtcccaactgaatatggtgccagtgagttcatctctactcagttcaggata

tggccatgggtcagtttgtagtagtatggatattgtggctcatattgtttcatgtt

>fava_dn_rep_c572

cgtacaaatgatatttctcatctaaacatattgggctcaatattaaacatgcaaataccg

cacttaaaagtacggacaattttttcatgatacctagcaaaggatataaatataaacaaa

ttacccatcaattatgaaaggtatatgaccaaaataaatctataaatcagaaaatgagtc

acatttaattgtatctccattacaggtatggacattgcatatagaaacccaatagaaagc

tcaaaagtaatacaaagcccatgaaacaaattcctactacaattttaggcaccaagatat

cctaaacatgaaacacaaattcaagttgatttggaagatgacagagaaataaaagctaga

taatattttcctctgcatccagaattactgctccacaagactcaaaaaggaaagctagat

aatattcacccctgcatccagaaccaccaatccacaacactcccgataaagaattaagcc

ttattggctatgttgttggaaagccagtccaaaccttcataaagaccctccccagaggtt

gcacaggtgctctggatgtaccagtggcgctgtctcaaagagtgaagacccaacttgtca

gtaatttcagcagcattcattgcattgggaagatcttgtttgttggcaaagacaagcaat

actgcatctctcaattcatcctcattcaacattctatgtaactcatctctggcctcaaca

actctatccctgtcattgctgtctacaacaaatataagaccctgagtgttctggaagtaa

tgcctccacaagggacggatcttgtcttgacctccgacatcccaaacggtgaagctaatg

ttcttgtactccacggtctccacattgaacccaatggtaggaatagtagtgacgatctct

cccagcttgagcttgtaaagaatagtggtcttaccagccgcatcgagaccaaccatcaaa

atcctcatctccttcttagcaaataaccgattgaacagctttgtgaatgtcagccccatt

tctccttcccttattcgatcctccctttggcgatgaaagcttcgtgtgtgaaggaagctt

tggagtaacgcgagatatgagagttgtaagagaattgacttttatgagaggagagaaagg

gtatcactggaattaattttattt

>fava_dn_rep_c573

aatgttgtgtttatttgaagaatgattctttcctatgtaaattacacataagacaatcta

tttattggccatttaaaaattttgaaaattacatttttttacttatgtatttttacaatt

gagggtttaattaaattgaaaaattgatttaactttttacaacctagtacaagccgatct

gtcggtgtagaaaagatcttgcaaattttattttacctcttcaaaagctaaaggtattga

aaacatacaagttttacattcaaaagtgataagattcgagcgacacattaagtgttattt

tgaagcataattcctactttgcttcttttgcagccttgtttcttcctagataattcttag

caactagttcaatgtttgccaatagatcgatggaaacaagtgaagccttagctgcttcct

caacttcatactctactgtagatttgattatgctcgaatcttcacctttctctataactt

caaaacgaactctatatagtgtaaatccaaaatccaggtatccaccttcaaccacctcta

cttctttgatacgcctttcattatcaatcttcgtgaacttttcattgtaactagctggac

caggtatacctggagtgaatgtgactttgagaatagttccaactcctccatctccttcaa

ctatttccaccttctgaaacaattgtggcatctcttcacaaacgagttttccaataccaa

gtgtgccaaagagttcccatgcttcacttgctggcacatcaagctccaactcatgctcta

gttgaccagacatcttttctctttatcacaacaatcacaaaagcttactctagttagttt

taaaagtgtagagactttgttacttatttatagatatcaatatatgttatatgaaatacg

taattaagaacaaaatatttaggaatattacgttacattcaatgaaaccatttagttaag

ggctatcttttatcttttcttctatgtttttcggtgaatgta

>fava_dn_rep_c574

ttaataataaaacaaaacatatatgcaataaaatgaagatcaaatgcttctaattcacaa

acgaaatacattccataataatcttttgacacaaattttgtttttgcaatctaaaaaaag

ataaaataaacccaaacaactttcttttatgtgtatttaaaaagtacccacaaagacaaa

tgaagaaccaaacacatatcatcaagaaagaaatcaccacacaaggccttaatgatagta

attaattaaacaaaacacccccaataccacaaaacaacacaatcattccatcagaatctt

caatgagatgtagtctcatccttatgatgatctttgtgttcaccatcagtagcagtctta

gcatgataaccaggaagcttctccttaatcttttccaaaatacctttcttctctccatca

tgaacagctgttgttgttgctgctgtctcagtgtgaacaggagcagcaacaacaggtgga

ggagttgttacatcctctgctttcttgtgtcctcccggaagcttctccttaattttgtcg

aggaaacccttcttttcctcggtacctgttgttccttcaacaacctcaactttctcaact

ggcacagatgtgtcctctttcttcttcttgtccttcttcttctttcttttctcaccatct

tctccttcctcttcacttgacgagcttgatgagctgtcagatcggtgaagtttctccaag

agactggtgtgtttcttttcaccttcctcttcaactttggtttctgatggtgcttcatac

aaagtcaccttatgaccaaactcgtgggaaatcgcctcatcttgaggaggtttatgttct

tcatcctttttcttaccaccgagaaaatcaaaaagacctctgtccttgatctctgtctca

gagttggttgctgcggatgtggtctcctcgtacttgttctgattctcctcagccatgatg

atgatcactactaacaagatggatcgaactggaaatcaattaacactaagtaaaatggat

tatgttgctgaaaggcaaagtatgagatgatgatgatagtgttgttgttttgatgatccc

cgcggccgtaatggccaatctgcgttgatagcagctagaagaccgtctgagtgctatgga

a

>fava_dn_rep_c575

agtaattttattaataataataataattttctcataattctagaaaacatttgacataag

gaatatagtaataacaataattctgaaaatttgaaattcataaaattaaccacagcaatt

aatttcttataaaactcatacattaaaatggaaaatcatcattatcctcatactacccca

aatgtgaaagcatctcaggtgacatgcgaagcaagcgaagacttctctccattttaagtt

ctgtaatacgtttaacagttgcattatacttgttcagattatcaagactaataatgctct

ggtagaactgatattgcagcgttgcaagttgctccaactttccaatctgataatctagat

caccaacatatactcgatttagtttttcaattacatcttttgcaattttctcgagaagtt

cagattccatcctgttgacggagcaatcccaaccagagagattggctgcttctctcaaag

cctccctccactttatcacctttcccatgttcccttgaaatcttttctcatggttagcga

aagcctccccataacttcccgtttggttgcgtacatgagaagcttctatatcataaaaaa

taggcaaaacaatttgtccattcttcttcttacattcaagtattttcacaagctcgtcca

aacaccacttagagttcccgtagttcttcgaaaaaacaacgacggaaagcttggcctctt

caatggccctaagaagcgccgacgatatctcatctcctcgttgaagattgtaatctatat

atgttccgacatcaagtctggttagagtagcatgaaggtggctagtgaaggtgtagcgtg

tgtcctcgcctctaaagctaaggaacacttcatgcttttgttgaggagtattggaagagg

ttgaccatgccatgtttattggctatagttagcttcgtgtgcccgcggcctatgtgaacc

t

>fava_dn_rep_c576

tttttttttgattgaaagaggataagcgctatccattgagtaaagggaggctttcctaca

ctacagtgattcgggcggttggtggccccttcgagagttgcgggtccttgccgctgcatg

agtggtagctcacgctcaaaactcctccgacacgagtcctagtcgttgcgctgcggtccg

tttcccggcttcgcttgcgcgattttttgcacttattttattattggtttaatccatccc

cgaccctaatgaatcgagtatgtatgaaggggtcagtaaagcggttcgggttctcggtcg

gttcccgttcgcctgccccccctcatagtccattagtgggtagggtggtcaacttagagg

gcgcccgcctcctcttcagacgtgtcttcgacaacctggcggttgttcggtctccgcttt

tggaggcgggagtgcttggtcgctggggtttccttttcctcaaccaattcggttgtgtca

gccctgagattggttggtctaacatttatgagcaggctggctggacaaagatggatggaa

ggtaagatagatttgagttcaagtggcacaggaccttcgatcgaccatggggcgtattct

acccttaccgaattcattctattgaagcgtaacgtaaaacttctcatgttgcatttcttt

ggtttggaagttccagaatgttgtctgtggatttctaacaaaggaaagccccccttttct

ttatgccatttgattaatgaaagttccgtgctgctcgccactccccgaggccaaggacgg

ggtcgaaccgtcattccaggatttgcagtccgatacatttccattatgttacccagccaa

acccgccacctcgtgtgccaaagtcaaacggttgttcttccttccccgctccctattttt

ctacatatgcggtggcgggcggagcactcgatatgaccggcggcgggttaccagagaaga

ggggacaacttatttatcccttgccttgctccattttccttttcttattttaagtagcaa

gccactccactactggtacagaggccagaagtttgcttcctccatatgttcaggcaaaga

acatctagaagctagcttttgtcttgtaagctaccatgcctaagaataataagataatta

aatttggcttaccaaagcaaagtggtattactaaaaaaaagtaaataagcaaccagttcc

gttccaagtgacatggcttttcactattccttattcctttccagagaagcccatccagcc

cagcggatccattgtttatgcggcagtgcgatgctgcttaaaaacgtaagcccttaggta

taggttggggaaaactccaaacaaaaaagggccttattcttccttatattaaatataagt

tttagaaaggggcatccctaccccctcaattacaagctagcgcgcaaaggcttttcagcc

gttgttgctttttttttttgcaggctcgaaaatctctctttcgcctctcctccctgtctc

cattctggttgattcgcttcttccttcgcgcaagcgcttggttcgccccttttgtgttgc

attttttttgtgatcccccgcttcagtttgcgtttttcggatagccgggatccgacgttg

atttcctatttctttaaatgtcagctccatgttttgggcggcccatctggttagttcagc

tatcactgctggaagcccctgcggttgttcggctgcgtactccccgtttgcgtatctaac

actcccaaagcatctttttttttcatatttcttccggtcggcttcgtgcagatagatgtt

tgccgggggagattggtgctcctgaggtatgcccttccggtgggttgttatattttctgt

ctattccatccagtgcccgcactgcgtcataaaatcttcgtcttcgatctctcttcgcaa

cgcaataaagaagtctaacagtttctctcggcatctgtcttttaagtcattgatctcata

tctataagcaggggggtctgcgttcactattaagcctacgcccgtccattcctttaaagc

tttatcccgcccttagactcgttacgcttaacgcccacttactgaaagactcgttggctc

cgcgctctattcggtcggaacccggtttgagaaccttctctcatagattcctttcaccaa

gtcatcgccagcaatcagctcttatcccttggtgtggtgtcggcgagttcctttcttgtc

ttgcccaaaaacttgcttgattctcattggagaaagactctcccgcggcaaggttttaca

catagggccagctgctttctttatctcgcttgccgttagtctgtcaagcgcctttctttc

ggttggggtccgtcccggggcttagaccgcttgggttctatttttatcgaaggcagtcaa

cgtgtcagccattcctctcccattagacttgtaaatcctttgctctttttctttctctcc

cccgcgggaacaccacaacattttattacataaaattttcatatactaatggctcttcaa

actgcttctttgcttcctgcttctttctccattccaaaagaggggaagattggtgcatct

ctcaaggactcaactctttttggtgtttcattgtctgattctttgaaaggtgatttcacc

tcttctgcattgaggtgtaagagggaattgcgacaaaaagttggtgctgtgaaggcggag

acagcggctccggctactccagcagttaacaagtcatcatctgaagggaagaagacattg

aggaaaggaaatgttgtaatcactggtgcttcatctggtttaggcctagctactgctaag

gctttggctgaatcaggaaaatggcatgtaattatggcttgcagggactatctcaaagct

gcaagagctgcgaaatctgctggattggctaaggaaaactatacaatcatgcatttagac

cttgcgtctctagacagtgttcgtcagtttgttgagaatttcaggcgttctgaaatgcca

ttggatgtgcttgttaacaatgctgctgtttactttccaactgctaaggaacctagtttc

acggcggatggctttgaaattagtgttgggacgaaccatctcgggcattttcttctttcg

cgccttttgcttgaggatctaagcaaatctgattacccttcaaagcgcttgatcattgtt

ggctcaatcacaggtaacacaaacacgttggctggaaatgtaccgccgaaggctaacctc

ggtgacttgaggggacttgctggtggcttgactgggctaaacagttcggccatgatagac

ggtggagattttgatggtgccaaggcatacaaggacagcaaagtctgtaatatgctcaca

atgcaagagttccatagaagatatcatgaggaaactggaatcacatttgcttccctttac

cctggttgcattgccacaacaggcttgtttagagagcatattcccttgttcagaactcta

ttccctccattccaaaagtacataaccaaaggttatgtctcagaagaagaatcaggaaag

agacttgctcaggttgtaagtgatccaagcctaacaaaatctggtgtttactggagctgg

aataatgcttctgcttcatttgaaaaccagctatcacaagaagccagtgatgccgagaag

gctcgcaaggtgtgggaggttagtgagaagcttgttggtttggcctaaagttagaggctc

caacatctatgctgagttagaggcagaggcattgccatggaaggaaaatgatgcccacaa

gagtgaaaatcatgatatcatgttgagtgcattaattgttgtgttagataggaacttttt

tttgtctttatgtaggagttgatgaatcaataaaatcattcaaccatttggcaaagtgct

tataaacttttctgaaataactgtgcatagtgaaatttctcctaaatcaaagcaaaaaca

ctttcattttgacaaaaactacactttcaagattctaaacacttcaagatagacgcatac

attgggttctaaagcacaaaatcaaatacatctcctcagccacccgaaatattacggttt

tggtgacgggaatttacatctgtagtgatatacatatgttgctttatctatgaagtactc

aaagttcatagccaggaaatcttgccacctgtggatcaatcatgtctagaaaggtatatg

ccattcctccaagaccttcgaataaggaataaggacgatccccaccgtgcatttttcctt

cggtgatcaatttttgtgctctatcaagtagaaagcaagaaaatcctttagccttgtaca

aatactccttgtttcctgtcaatctgtataaagaaagaaagacataggtgttcccactga

cgccgtgacaaattccaactcgcctaagaagaccactcttccatactacttctcctgcat

cttcagctgcttgcgaaaattccttatccccaaaaacctcggctgcttttacgagcgtaa

gggttactccaggagcaccatgacaccaatgcacaagacggtcagattcgtttccttcac

tggagggataattgccactcgaaaaacgattcttaatcatgtagcgtagtgtacccttta

catcttctacctcatctggcttgagttccatatccattaaaacattcatgattcctgcga

gtccatgagcagcaccccaatatttcttcccatgccattcatacatcaaaggacaccttc

cctttggagccaattgtctaccagctgttataacttcttccacaattggtctcatgtgag

tagtctgtattgtgtctttatcgatatgcttgtttaagaatgaacaggcccataagtaac

ctgctcttccatacagtacctcatagggcaaatcacggggaagctcaatctttttgaatt

gtctaaggtaatagtcaagtaatctttcatcaccagcatgcttagctatgacagcaccaa

gagcacaaacaccggcacgcccacagataaatgtcacacggcttgagttttgtgaagcag

aatcacaagcttcgacaatcttcaagcacaagttaagatcatccaagttcttggttactt

ggtaggccttgaaaaccaggtatgccgttccaagaacccctgtataaagagtatagtcct

tcacacagttcccactcgaaccccatgtctcactcacgaccgattgtttcaaatgaagtg

ctgcagcttggagttttgaggcgagggattggtaaggtagagaaaggagtgaggtgagtg

agtgtggtgttggcgtatgggaaggagtctctgccacgaatggtggcaaatcattcggaa

agaagcggtcggccatctccgaccaaagctgagagcttgagaa

>fava_dn_rep_c577

taacacaattgcaaaattcgtttgttcgtgtgtttttgttgtatctctaatatccccttc

tctctgaaatctatagatccttccaagaaagggaacaactatctttgtctttgtgctgtg

tgttgtctttgtgttttcgagcactcagaagcaaacccttttcgccatgagacctcagaa

aacatcgatccacgccatctccacatgggtccggcgacaaccaccaaaggtcaaggcttt

tctcgccgtcgtttccggcatggccgctcttgttctcctccgattcatcgttcacgatca

cgataatctctttgtcgctgcagaggctgttcactccctcggtatctccgttcttatcta

taaactcatgaaagagaaaacctgtgctggattatcactcaaatcccaggaattaacagc

tatgtttttagctgttaggctgtattgtagttttgtgatggaatatgacattcacaccat

acttgatttggctactgttgtcactacggtgtgggttatatacatgattcgatttaagct

gaaggctagttacatggaagagaaggataactttgcgatatactatgtggtgttgccctg

tgctgtgttagccttggttattcatccatcaacttctcatcatatattcaacaggatttt

ctgggctttctgtgtatatctggaagctgtttcagttctaccccaacttcgggtcatgca

gaacaccaaaatcgttgagccattcactgctcattatgtatttgcactgggtgtagcaag

attcttgagctgtgctcattgggttctccaggtgttagatacccgtggacatttgctggt

tgccttaggatatggtttatggccgtcaatggttcttatctcagaaattgtccagacatt

catcttagccgatttctgttactattatgtcaaaagcgttttcggaggacagcttgttct

acgtcttccctctggagtggtgtgattaaagatttatgccctcgcattttcttttaactt

aatcaagcatcatgtttggttactcatgttagacttagagtacactttgtgttgacatgc

ttattctgttttggtggaaagtgtaaagttggggatgaaagtaaactaaatatattttat

gttatgcggtatgtttatttattatggttctaaatgttctatactacataaagtctctat

ttgattagagtttttgttaaaaaaaaaaa

>fava_dn_rep_c578

tggataataaaatatgacattgcttctttcaataattataaaacttctcaccacgactag

aggcaatgtgcaaccaatacatattcggttctctctgcgaagttcaacaatacatgttta

aagaaatgtgatgacaacgaaagtagcaacaaatcacacacacaaaaatcgacctaagag

gatttaatagtatgaaccaaattttttttttttaaataaaaacaaagaactttaatttaa

tcaattccaaaattttcaatacacaaagtaaccaccagatctcattacttcacttacaag

ttaaaatacagaaaaagctactcttaaaatgcagatccagtaaacaagtacactgttaaa

accctaaccgcttgtgaacttggtcacagctttagtaccctcagacacagcatgcttagc

caactctccaggaagaacaagcctcaccgcagtctgaatttccctcgaagttatcgtcgg

cttcttgttatacctcgcaagcctcgaagattcagctgcgagcttctcgaagatatcgtt

gatgaaactgttcattatgcccatggccttgcttgagataccaatgtcagggtgaacttg

cttcagaaccttgaagatgtagatcttgtaggtttccacgctcttcttgtttctcttctt

cttcttgtctccagcggcggcaccatctttggatggtagcttctttcctgccttgggttt

cttctcggcgggagttttctcggctatagtggatttcttctcttccacgggcttcttctc

ggcgggtttcttctctcctttttgtgccatttctctttggaaaatgaagaaaaagtaaga

tgcagattgaagaactggaagtgtgaatccccgcggcc

>fava_dn_rep_c579

aagttttaagtgcatgctagattggattgctgagaaagaaacaaagtacaaaaacccaac

tctctaataaacaaagctgcgccacactccatctttctccctgcacggtgaagttgttgt

ttgtgtgatttcgaacatttgatttggtaagagatgtctttgcaaggacaattggatagg

ttcaagaaacagcaagagaagtgtcaatcgacgctttcgagcattgcggggagccaggtt

ggtggtagaaaacctagtaatccagttgctgcacatgcgtcgatcaatgggagaaatcag

actactggtgtcaaattctccaaagatacggagaggcttcagcagattaatagtatacgt

aaagctcctgttggtgctcagatgaagcgtgttattgatcttttgcttgagacgcggaag

gcgtatactccagagcaaattaatgaagtatgctatgttgacatgcgggctaacaaagat

gtttttgacaatctgaggaaaaacccaaaagtatactatgacgggcaacggttctcttac

aaggcaaagtatggccttaaggataaagctgagcttcttcaactggtacgtaagtatcca

gagggcattgctgttattgacctaaaggatgcttatccctctgtgatggaagacttacag

gctttgaaagctgcagggcagatttggctgcttgccaacttcgattcacaggaagatgtt

gcgtacccaaatgaccccaaagcaaacattaaggtggatgatgacctaaaacagctattc

cggagtattgagttgcctcgtgacatgattgatatagagaaggatcttcaaaagaatgga

atgaagcctgctaccaacactgcaaagaggaggagtgcagcacaaattgatggcccttct

tccaagcccaagcctaagaagaagaagaatgaaatcaccaagagaaccaagctgaccaat

tcccatcttccagagctttttcggaacctaaacaattcgtgatcaactcactgcaagttt

gcaagtgttgcattaggtcaagtagataatttgttagcccaagttagatcagcaggttga

aaaatattcaggagttcttattgctctcttccctactcttgatatatgtttttaatcttt

gtacatcaaggctgtagcttatggactggactagtcctacccgatattttaaaaatttta

caagattaataagtcctataatgaagtcaatttcctatttctattccgagtagtagatat

ctggctacgatagatcacaaaagcaaaatcaaagaaagtcacagaaaaagaaaatggcag

gaatcatcaacaagatcggagagactcttcacatcggaggaggacacaagaaagaagatg

aacacaaaggagagaagagtcacgacgagaagcatgaaggtaaacacaaagaaggtttgg

ttgagaagatcaaggacaagatccaaggtgatgaacacaaaggagagaagagtcatgatg

ataagcacaaaggtgaacacaaggaaggtgtagttgagaagattaaggacaagatccatg

gtggaagtgatgaacacaaaggtgagaagaaggacaagaagaagaaggataagaagaaga

aagaacacggtcatggacatgatcatgatagtagcagcagcagtgacagtgattagatta

gatctaatgttgtttctctctttctatgttcattaagtatggtgtacaataccgtatggt

ggggagtcactggaaattctttctaactatgatggagtgatgagtctagaataataattt

agagagagagaaatttatgtgtatttctgttttttattttgttattgacttattatgtgc

taatttgtgaaaagctcacaacttttagtttggcttggatgtagattggcatatatataa

gaataaatatgttaattattaataatttctttctttatatatccata

>fava_dn_rep_c580

aacaaaataaagggcagcaaaaactattgagctgtgattggtgttattaaaagtattgat

ggaaatggacaattttattgatcacaagtttcttatatactagtacataaaacaagatcc

tgcaacaaaaaattgagacctataattaactacaaaatatacatggcgaaatcaaatgtg

atggagtcatattgctcaatcaattcctttgagctaaaccgattgttttctcgtgtctat

gcgtaggggttatttcttattaaatattccaaaaatcgatttgatggcattgatggcgtt

ctcgttgataaatgattcatcaaatcttttcagtaatgggtcgaattccggggatgccct

tgcatttgcaaggtcaggatcagttctgattctcttaaagtcttcaaaaccagaatttag

agcttcttcaagtgaagaaagtgcagcttgagtctggtttagcttagagtaacaacaagc

aacattataacttgccactgctgcttcatcaggctctggctttgttcccaaaactgactc

aaatttctctaatgcttcctcgtactttgcattcttgtatagtcgtaagccttctctcaa

atcactttcccgacgctctttcaattcttttttccttaggtagttttgcatttgaatttc

cctgagtttattactaactacaccagagttcctctcagctctgatgatctccttatcagt

gaactcaccaccagtttctatctttccatatcttttctgcattctcatgagtaacgggcc

tattctttggcgaattgtgtacattgttcttccatattccgcagctggccaaatttcggt

cccaaaaacagcactggtggcaagtactttatcaccaacagtgaagactccggctttatc

agctgacccaccaggagcaatagcatcaatgtaagttccaccatctcttcctttcacaaa

ctttattccataaggctggtcaatctcaacctcatactcttcatacggttcctcttcttc

tccttctttgggtgcttctgacccaacatcagatgaagctttaaccacaaaaaggaaggg

ttttgagaaatgggtatttgagaaactacaagaaaaatggtttcttgaagaaaacaactt

ggtggtaggaagaaaacaagttgggttttgcttagagttgatttgggtggttcttgttgg

caacgaaagtgcagagtagtgaccactggcggcggcggtggcggatatggataatgacat

ggttgttaagctcaaaactttgatttttcttttaagttctgtgaattgaattgaatagtt

gagtagtagaatgaagggtgaatgagagcagaaagaagaagaagaatatgagaggttgca

agtagatttgctgaagtggctctcaaggcacatccattcaatt

>fava_dn_rep_c581

aggaccacagtagaaaagaagacaaaaataatgacacacctataaccaccatacatatat

gaagattctcttactatnnnnnnnnnnnnnnnnnnnnnnnnnnnntcaattcgttgtatg

tatataagatagtgagagtnnnnnggccggggtaacccaaaacatcaaacattgataact

cattttgtgtaatgatgatatctcagaagacaaagttgtttctgtctttcttcatcttca

tttccatgacctctctctcatttgccttatctagtgaatactcactttcaagcaatacca

acttggacaagttttcctcttctgatgaacaagtgcttgaactcttccaactatggaaga

aagaacacgggaaagagtatgaaaactcagaggaaaaagcaaagagatttgagacattca

aaagtaatcttaagcatatcaatgagatgaatgcaaagagaaaatcaccaactcaacatc

gtttgagtctcaacaagtttgctgatatgagtcctgaagagttcagtaaaacctacttac

aagaaataccctccaactccaactatggtgatagaaagcttaaagacgaagacgacgact

gtgataaccttccagcttctgtagattggagagaaaaaggagctgtgaccgaagttaagg

accaaggaaactgtcagagccattgggctttctctgtcgctgggaccattgaaggattaa

acaaaatagtgactgggaatcttatcgacttatctgcacaagaaattatggactgtgacc

ctgctagccgcggttgtgctggtggtttttactttaatgcatttggttgggttattaaca

atggtggaattgacacagaagctaattatccttatacagctaaaaatggtacttgcaagg

aaaatgcaaacaaggttgttagtgttgacaacctttatgttttggatggatcagaagaag

ccttgttgtgtcgcactagtaaacaacctgttatagtgagcctcgatgcaactggccttc

aattctatgctggaggagtatatggtggagagaactgcaaaaaaaattcaaggaatgcat

ctctcgtgggtataatagtgggctatgattctgtggatggtgaagattattggattgtga

aaaacacatggggaaaagattggggagtgaaaggttacttgtttatcaagaggaatgtta

ttaaggaatggccttatggagtatgtgcaatcaatgctgcggttggttacccagtcaaag

aagctttatcttcagccatgtagtggttagaggaaagaataataccctaatccttttgag

acctataggtttctctaagcaacttatcttgtaatgctattatggattacttgtgttgtg

tggtaatttaataatacaaggatttctatatgcgaaaaaaaaaaggttttagtacagttt

ttttttaaatgaaaaaaattagttt

>fava_dn_rep_c582

tttcttcaattgtttatttttcttgatcatggatcccttaatcagaatttgctaccagca

cctcctccacctccaggattgtgttcagatccagtcttcacaacatcaatgatatggcca

ccactcagttcttgacccttcaatggtgcctgtagcactggtgttgcatttctatatatc

aaatatatcaccatctgaattattccaaacacaaatccaagtgtatttggtagagcaaca

tagtagtccttgaggagaaggccatagaagaaccacataacagcattgatggttaaaaag

aaggacaagaaaaatggcatgtattcgacgctcctcgtccttatgactttgctgatgacg

aagaggggtgcagcgaagacactgatgttgaacacaagacaaatccagccgataatagcg

agacgtttcgctcccttggcaaggtaaagagttgatagaagcatggctccaaatccaaac

acattcagcaagagaagtagttttatggtagaaagcctagatttctttgaggcataaatc

aggaaaattatgagataagctgattcaacaataattccaaaagtgttaatggtgatgaga

agaagagcagcttctcttttgacaaatgcatagtatatccaaagcattgcactgaaaagt

gcaacaacataaggaagtgactgaaatccttcagcagatttcttcttgaagattatgtaa

aaagttggtactggtgaaagaaacactgcaaaggaaatgatgttgcctagaaggccaaag

acaaaagcccaagtttcacgatgcatggccatttcttgaatctaagtaaaaaactattga

atagtttggaatattaaaaaataattgtcactctctttctttctcaaagtgaaaaccctt

agttgaaggtagctctttgtttctctctctaaattgcaccccgcgtcatcctcctcaaaa

agaagaacccttcatatagtttcagctaaaaagttttcttctcgttctagaaggaagcag

ccaacaaccaccacgaccgctgatcaggagtcgcaaccaacgaatgaggttgaaataccc

ttttactccgatgaagattggccgtttccgtatagacctccaggtacctcgaaggagcct

gatttctttgaaggtcctcagtgggacactgttgggttcattggagagtggttgtggatt

cttggtggtgttttcgcggtatttggtggtgggtatgctgccataaattacaatctagga

gcatcagatttcaagaacactcctgcatacaaggaatctgttcagtctcaagaactttta

gaacaacccgaaacatctgaatcagatatcttcgagtcaaatcctacagaggtagctcct

agtttgaactagtccattcatagataccagcaaacatactttgtttagcatatatgaatc

cgaatccaggtgaatgttccttaatttgtacggcaatactttgttatatttttgaactct

tagaagttgcgaccgtttgatcaaatcattgtcgatgatcttatcttatatatagataaa

aacattgaaatttagagatgatatttggttcaaatgtggcacatggatagtttggtttgt

atattgtatggactaaatgtgtcttttgatcttctttgtttggtttagcattttgttaca

aatcttgccaaaaagatgctctaggaatcacttggggcatgtttgtattgacttgtgaaa

atgtttgaaaggacctatgaaaacaacttacaatatgttcttaagctattttcagcttat

tttcacaagtnnnnnnagatagttt

>fava_dn_rep_c583

gtgtattgttgtttttttgtcaaatatctttttttttttgaaacaacacaatttcctttt

atcatcttaatgcaaactaaagatacaaagacaaattcatacaactagagtcattgttct

accatcttcagtacagagtaataaaaattttcttcgtatcaggagtgcttgtctatttat

cctctgcatcatcaaccttcttgtcttcggttactttatcgagtccaaaatatttggtaa

taatatccggcacgacgaaatttgatatgaagtatactgatataagcagggcacttactg

gaagaaacgtagacgggtcaccgggtttgggcggagaagtagcagcaacaacaatgaaat

ctggaattgccttagtccttaatgaattactagctgaagaaaagttcttgcaatgtggat

tagtcctgcttggttcttcaccccagagtagttttgtagtatgctccattcctatatgat

gttgttgctgcaatttgagttttgcatgcatgagaaacaatggagaatggaggttgaggc

atgtcatggtgaagcttagttcttcactgtggaaaacttctcttcagaaagatacagttt

tggttcactgtgttcaactagtttgcttgggataatgtttgagaggaggatatttttagt

aaagatgatgttatccttacattacagctacagataaggtcctattttatctttgtatgt

ttgttataggcacttctatgctattattgttatcaagcaatggt

>fava_dn_rep_c584

tgaattagtnnnnnnntgaatttaatggtcaaaatgaaaaacatgcgacaattggtagag

agtggaggggacggtgattagaccacacctcttgtaaattgaaaaacgaacatttcctga

gccgcccttgctttccattcgatctcacctttccctttcacatctcatcaattcatcaca

cactaacaaacggctggtagcgatgtcaactccggctaggaagagactgatgagagattt

caagaggctgcaactagatccacctgcaggaatcagtggtgctcctcaggataacaacat

catgctatggaatgctgttatatttggtcctgatgatactccatgggatggaggcacgtt

taagttgactcttcaattttctgaggattatccaaacaagccacccactgtacggtttgt

ctcccgaatgtttcatccaaacatttatgctgatggaagtatttgcctggatatattgca

aaatcagtggagtcctatatatgatgtagctgcaattctcacttctatccagtcattgct

ttgtgatcccaatccaaattctcctgcaaactctgaagcagcacggatgtatagtgagaa

caagcgcgagtacaatagaagagttcgggagactgtggagcagagttggacagccgacta

aggtcatcttgatatacttgttggcccgtgatgtcaattagattgcattgctacgtagga

gggtcatgcaaactccaaagtcttttgtatttcttcttctgaatgtcagtttatcttgtc

ttttcctcatggtagtttatgcattgtccatgtcagtctatgtaatataaaattctgtca

acttgcatctgattgttgtcaacatagttcaatgtacaagttgtagtcttctgaattggt

tgtgatcaaaaggattaattaaaccgggtccaattctgttcacattgtataatttcccgc

atttaatgtttgattgatatctgttgttgatgtaaaaaaaaaaa

>fava_dn_rep_c585

ttttttttttactagaaataatttattcattaattttcataaatataaaactaacatttc

agtacaaagggcaaattggagaattattacataatacaataaagaagttacaatgagaag

ttcagaatatatgaagccaagagatgaaaaaatccagagctttggtggaaaaaacaatcc

ttcccaaaagaaaaggactgtcatgacagaactattatagtttgctgccatgaccaaaac

cctagtgaaaacaaatggcctcatataactcttaactgccaatagtaaccaagataacaa

taacacggaaactcgatcctcaacgtgccccttgactaaaacctcaacacaaacatcgtg

tatagaacgtagttcaaatacgcgaaaaatatggtaaacaccaaccactctggacgaact

ttggtacagactgctgtgatgaaattgaaacgacaaaacatggtggcaaaaccgatacca

agcagagctaaaaatatcaggtgccaacatcagaaagaaacctaaaatccagaacccggt

agctatgttctcagagataaacagctgttctaaaagcggcgtcctaccagtaggatgcca

aaaaactttaaacagatttccttctaattataaggaaacctgtaaaaacaaatattgaac

aactgcttgctctggaaaaagagaaccgagtactgcggtgaccaagacccaccgataatt

aatggacccaaaaacactaccatgcaacactgattagaagtatagatagatattaaggct

acctcctcagaagaggatgtcacatatccagagactttcaataagcttgtcaacggtgca

gatgctgatcgacttgcaattttttatatttaatccctcgagggaatcgcccaatttttt

caaagcaggcatgctcttgtttgtaaccaaggagcagcctgccaaagaaagtacatccag

attgggcaggactgcatgggcaagggcagcaattccatcatcggtgatatcgcacctgga

aacatcaagatcgctgagtgctggacagttgcctgcaatttccatcaaggtagaatcacc

gattcttttacaaccgttgaggtttaacactttgagagtctttctgtgcaacttgaccat

ggtcgaaactcctacatctgtcagattaatacaacggcttagattaacaatagccaaact

ggacttagacctctcaagcagagaaagaaaccctgtgtctgttattccctcaagtccaat

caactcaagacgctgaagccgagtacatagctttccaaggatggcaagagtagaattacc

aactccggggcagttacggattgataatgacgaaatagattcacagtgagatactgcagt

caattccagtttcagatctttcatcccatagcagctcacaagagtaagagccttcaattt

tgaaccacagttataaaggaggccaaaaagcccaaattgggtaaccctatggcactcttc

caaatgtaagctcacaattgatggggcagccttggagaatgataccagcccattgtccga

cagaaaagcacacttatgaagctgaaagtttttcacattaggacaaccctttcctagagc

ttccagcccaatatctattactccagagaacgacccaattgtgagcgttgttagtttctg

cagtgcatggccattacccatcacccacaatcccttcccggtgacatatgggagggaatt

aagggcaagatcggtaattgcaaatccatattgtccaataactgctagtgagtaatcaga

aacagctagcgattcaagtgtcatctttgttagaacaaaggatgctgaagataccatgcc

aacaattcccagatcaccaactctaggacagttcttgatagatatagaccttagattggg

acaccacttcccaatagctggtagaccttcattaccgatactaggacatgactctatcga

taactcggtcagcttaggacagttctttgcaatttcaattaaagccttgtcagaaatatt

ctgtaacttgcaaaggtcaagcttctctaactggtgacaaccattagcaatctcaatcaa

gccttcgtcgctaatagtatcaacatcccaaagagtgaaaatctttagagaagggcagcc

atgagcaacggccttaagacctgcattagtcaacgcagaatctgaattgcacccattaat

cgaaagctttcctaaccctcctcgagatgctgtgccaacagcaacggcagctagtctaac

atctgacgcctttttcccctccaatcgtcgagaaagacacccttcatcaccaaatcctag

atccccaccatctttgtttccatttccgctaccaacatccttgcaaatttcatccttgca

aatattgcttagaagcataagccagcgcttggataccgaagcacacaaactcctatcttg

gcctgcaggcaaccttctaaggatctcaaacaagcattcatccggcaatgtttcaataga

tgactccggcttcggctcaaaccacttatctccactgaaatcaaatggaacaccaatgcg

agatctcttctgacgaggcttgtaagcatatttttgacagccaagggacaagaagagacc

agtttccttaggattaaggtatactgacctcccagtacaaaaatcatcacttccagtaaa

accaaaaaccttagacatgataagtaaaatctgaactgttctccaaagagaaggacaaac

aagaataagatgattataagagagaataaaaactcttgagagcaaaaaagcatgagagaa

acccctatgtgatagggtaaaaaaaaagagagagaaaagaaaaaaatgaatgtcagaaat

tgtgaaagagt

>fava_dn_rep_c586

ctattttaatgtcataatgttattaatctctaactcctcaagaccaatctctcattgtga

tgcattttgaaatctttctctctcttaatactcatcttttttggttatgcaacactgact

gcagtaacgggagttgtatattcattttctaccatatcttaaaactctcatgatttatca

tattaaattaagactaaggatgaaagatgaaaaacgccattagaaatgacagagagaaag

accaaggaaactaaaccttatacatagaatatcatacattatttatgctataaaataaac

acaacaaaactactacacatataatataatagaaccttatcccttattctacaaattcat

tacgcaacaagggctagccataactcttcatttctcacctcctcatgactgtttcctcca

aatcaactatgctgctggtgcaatatcgatatcgccatcatcgtcatctccttcactctt

tctttggttgcaaaagcttatgaattcagttgcagagtgattatccatgagatacatggg

aggagtagattctagggaacttatgatcaccattgcagctcaaaaatgttctagagaaga

gatagagtgtgttttggttgtaatgtgagcttaatatgttgactcaagatccccggccgt

aatggccactctgcgttgtttatacactaaaaattttactcaaatttactgcagtaaaag

atgtacctgtattgtacaaatttaatgtagaaaaaatgtat

>fava_dn_rep_c587

tttcctaactgagctgctcaagcttgacaggttacttgttcatgtcaataaggaagaagt

aaactttcttcaatacacatcatttaattaaattgtgtaacaagaaatcatacaattttg

tctgatcattttacattttggacatatatggatcagacagaaactggaactttcaaggcc

tctaatatcttatcatctagctcaaattttatggccttcttgtctctcccaatctttatg

tactcctcaaaccgttcttttatgtcttctggtaagtttgtggttgaactccatcccaat

ttacttttggctgctctaggctcagcatagaagtgcacattacggaaagggaaagctttc

tttgcatcaattccaatagcttttggatcataatgaacaatgttaacagcacgccctgct

gcctgagcacatagtttggctattccatttagagtcactgcacggtcgctcacaatgttg

aaaatgccatgactcgcagcatctggattctcaacagctaaagttagcattgaagataag

tctctaacatgtgatatgttagtgagttgcaatcctgagccaggaattagcacaggcctg

tccctaacaatcctatcaaagaaccattcctcgcagtctttgttgtttccagatcctaac

atatactgtggcctgaatactgcccaactgtcaaaagtttcttcaatgtatttctcaact

ccaacatgaccagcatctgctttgacagcatccccttcaacatgaggaggttcatccgtt

ggtttgtagattccggcactactgatgaacaagaattgcttggcacctgcactcttggcc

caatctatcaccggcctcgaagcatcaagatccttgccattgttgtccaaaaccacatca

aatgcttcccctcctacagcacttcctacatctgcaggactcccccatactgtactgcct

ccagcactcacaatttctgagaatctgttaaaaggaggtttcttcatcttatctgagctt

tcatcaccaacagtgagaatggtgacagagtgtccagcaccaagaagctctttcgcaaag

tagaatccaatcacagcatgtccaccgctgttggtatttacgatcaacaccttcttcttc

tctgctgcactggcactgatgctgaaggtagcatggttagcaaaacgtttagaagttaaa

gaaggatgtgatgaaaaggttgaggagattgagaaagaggaggattgtagtgaagaagag

aaagaaaggcgcaaagtggaagaatgtgaaattgggaggagattggaagaggttgcagag

aagagcaaggtaggtgaagaagaagcaagcatggccattggttcttgagagtgtgagaac

cagcagttccagataaagctataaagccaactacttactttcttcctttcgg

>fava_dn_rep_c588

ttttttttttgggataaactaacaaccatagttaaatattgataaattgaaaagtataac

aaagattcacacaagtgatgcaaatacagcttaattttaaccaataaatttattgaataa

cataaataaatcccaacctatcggttatagttctgactaagtcgatatgaaaagtaatgc

aacataagtatccccacaatataaagtttatggcgtacaagtgacaggtttattacttta

cttatggcacgccataagtattctcacgcatggtttttttattttctgaagaaatgagct

agtgagcatcagttctcccgggttccatcatcgcaaatatgaaaacttacatgaaacaac

acaagtggccaacgatcctggcaacagactctgcccttgaaattcattagacccgctgaa

acttcgactgctccaattatatataaataataacaaagaatgaaatatgatagtaatgta

caaaatcttcaaatcctgtctggctttggcattatagctgcccaagataacctttcgcgg

atctaaattaagagacagggcttgtttccagtagcactcagagaaggttgattgtgaagg

aaacgtatgggtgtgtatccgcctcacctgctaggttagccattccagcccctacctctt

catcagttttccagtatgcactgtttgagctatcactcaacccttcaaggattgatcgta

ctttcttctcagcttttacagatggagctaggacgcatgccaagaaatttggcggcaatc

catatctcagaatgctttcagaaaataagcggacagcacagaagtgcatccaagagctga

aaacctctccataactggtatagcaccattgcaacagagaacccctcaaactttcttgat

cttgcatcaatttatccaactcctgctttcggccctcatgtgtttctggactgtattcaa

aatcacgaatttggaaccctttttcacgtgcactagttctaaaattgtctgccacacgat

tgaagagggttacggtataaagagcatattcgttatcctcatgcaacttcttagaagacc

ttgggaccacatagctagtcaatgtttcgtagcttgcgagccaatccttctgtgaatact

tggaaacaacggcaaggagggtagttaaatgttctgaatttataatgtcctcaggtttta

ccaagttagaaagatcacggactgctaagcttccagtttgctttcggtttatagcattga

gctgacttcggatattattatactcggaaacacgaaccttgagatcatcctcaatctttg

caacttgagcatgaatgccatccacgatctccttcagaggcgacatagtcgggtacttag

catcatcccaaacaaatctagtcaaataggaatcaacaggaactccatcgacggtaagcc

cagcagtattaacgccagagactctctcgagttcctcaatctgacgcctgatcttgatag

aaacaccctccatgaaagcattagacttgttgagatcatcgctgagggagaggagtgaat

ctagggttccgacgcggagattgggaatgttgaatctgtaaagaggagtgtcgaaggaat

gcttggagatattttgctgcaattggttccatattgtggaggaagagctgttgttctgga

cgggaagagacaccacccagtacctgtttgccattttacttttacgagcttctctgtttt

cccgtctattatctatgtacctcaacgcccaacttcggatattcctaggcctgattggaa

gtttggcttgtgtaaaatacctgctgccgagactggcatcatgcttttcccaaataatta

agacatttcagtttttgtttcatttgttccatgctactctcttgtttgttgctttgggtt

tattagataagaaaaattatgattcttcattaaatttaagtgtttactgacaattatcaa

taatcatgaaattaaagctttggctttctttgtatacttaaatttggaacttttacatta

cgaagaataattgttaagtgtgaaatttgtac

>fava_dn_rep_c589

gcacttttaactcaaatttattcataaactatttagaaattatgaaaataagaaattatg

aaaatatgggatgacacgaataatttaggtgggaagttaaattatccaaacaatgatatt

gtgatctagataaataaaacttgtatggttaagaatgattggaggaattcatttagccaa

aacggaatactatactttaatttttcaatcttgtattgactggacaatgagaaaagtctc

ttctccactacaaattacaataccacatcatgatcctaaagaaaacaatctcgtaaagaa

atttccagttttggccttctctgcacatgctacatgaacatatcttccaagcttatccac

ttgccttgcttcacccttagtgtcctttttgcacactttgcatgttttgtctacccaacc

ctcttcaacataagccccatccacttcaacataagatttaaaaaaatctttatatgttcc

accaattttccttctaagagcaccatactgttctttgctcatttgttctccggactttgt

ggcttctctagcatctgctataagctgagcatcttgttcggatctcggttgagcgaaatc

aagagcatcagttacacttgtaaagaaagattgcttgttcttatcttcttctttcttctc

ttgtttggctgaaactatgatttttttggttctattgtagttattggtattattatgtac

tagttttgattttgaagtgaaagtggaataataaggagaggtcaatggcaaagtcccaag

acccatttaagattcttgagtaatcaccaccttgattccttgaatgaatttgaattatgc

ttcttgtattgtgtgtaatatatgtgcgtttgcttgaatttttttgaaatttttggggca

tgaaattgcagccacaagttggtgttaattttgtccacagcactgttgccactaccaaaa

cttagtggtta

>fava_dn_rep_c590

cggaaatcttgtcgtaaaatnnnnnnnnnnnnaatctcttttgattgctaagcattaaaa

agttacaaaatatacgaatacttacataagttgtgtcttcaatagtgggcataacaataa

aataaaaaaaaagacaaattaacctcaaataattgcttattttaaaataattaaaatttg

agattatgctagaattataaacaagagtaataggatgagtagtttacttgtaaagatagt

atatatgaattattcaagctttacttcaataaaaattaatacacaacagaaattataata

aaatggagtttttatttgaatggatagtatcaatcagttatcataggtgcggcactcatc

agtctccgggttgtccttgcagtattcctcaagagggtccgaccccttattcttatccct

ggcgcggctcgccaccgcattcatttcctccacctcatcccacgccgccacacactcacc

gctagtggcgtctcctgcacatgtctcctctgcgttctttatgctctcttccacctttcc

agttatgctatccggtgatgcacgtacgggtctaatgcttaacatcctgcccgaactcac

tgtgccggttctcttttggttgagcatgaaaactggtgccaacctgatgggttgacactt

ttgaagtgactctggtctgttcaaaattactcggggagttgaaagactcatactagccat

tgttgctgccattgttgcagctattgagtttttttttttgttgggttaacaaagatgagg

ttttttccgtgctcggcaactctgtcttccaaccatctcttttagacttaccatctgact

gcatcttatccacaaccttctgtttttccgcatctatataactcttcacaccaggaatca

atttgatgaagttcatgaagaaccctaggagagtagctttgactcctttatcacaaaaaa

cttggaaaaccgaacgaactacgttcgcgacaaagagagtgaaaagcggaacgagaatca

gagcgagaggttcgtattgagatagaaacgaattcgcggtggttcggaagtggttaaact

gagaggtcaacacagaagaatccataacgcgaaaagaacaatgaagaagaaggaagaaaa

tgaatggatagaaaacggaaaaagtgattataagaaaagttgttttaaaggatttactta

agaatgattaatagtaatagtaatcgtaatcgcttttgctagagttgcgtcgcgtgtcgt

tgtcacgtgcgggt

>fava_dn_rep_c591

atttcaataaccactatcattgctagtatcattgttctatctcacaacaaaccgatcgaa

tcgacgactaaaccaccactctctccgccgcaccatgtctgaagcaccgtctcagccacc

actcccttcctccgagacacctcctccttactggtgctaccactgcgacaaacacgtctc

cgttgaaacggtcgctaatgtccctgacgtcatctgcggcgattgcaaaaacggtttcgt

cgaatccatctccatctccacaccttctcgagctcgctcaccgtcctcttcctccgatga

tccttactttggttcgcaatttcttcaggttcttcgcctaatcgctgaatcgtcgcgtga

agacgacgcgtctcctccccctcctccgagtcgttcacctgacgacggtattctcagaat

cgaactcggtggttggaatcaccacaacgacgaagaggatgatgatgcgtttcacacgga

cggtgaagatgatgaaggtagcgtcgaggaggtggaagatcgacctggtaacgaagatcc

gaatatggacgatgaagatatgaggcgaaggagacgcgatctgttacggcatcgaattag

ggatttggcgacgcgaacacgaagtatgcagaatcggatcttggattgggcggagatttt

gatgggattggaagataattcaattgaatttcgtcttcagggaccggaatcggagcggta

tgtcggaaatcctgaggattacgttgacgcggcggattatgaagcgttgctgcagacgct

ggcggagagtgacggaagcgggaggagaggggctccgccggcgtcaaagtctgctgtgga

aggtttaccgacggttaagattgcattggagagtgaagtggttgcttgtgctgtgtgtaa

ggatatgcttggtgttggagatatggcgaagagattgccgtgtgggcatgagtatcatgg

agattgcattgttccatggttgactagtagaaattcttgtcctgtttgcaggtttgagct

tccaacggatgataaagagtatgaagaagagagagcttcgggttcggggcgtggcggagc

ttccaccgtttgagtgagtatgttttgttgaatcattgtaatatattccatgctttctat

ttatcttctatggacttcccttttttaatttttgttattaaatgccataacattgaattg

acatggttgataatcagattcgtaacatggttgataatcagattcgtaacatttaaagaa

cttcattaaaagtaccaaatataaattatcaaaaatataggttcccctcgagatcaaagt

aagcatgagaacaatatactttgtctacacgaatacaaaactttttaaaacaagaaaagg

aaattgagtttgtgacataacactaatccaattcgaccaaaactcccaaaatgcttttaa

ctgcggacaggagctcctccagctacggcagggcgaggcgcttgaccaggctgaccaggc

cttggtgcatcatccctgcgcctgatgaaacgctgaggagggtcacgcttcctcctatca

gtgcgagatctgactctcacaacatgagaatgaattgcacacgacacacagtactgcatc

ttcacatacagcttaggaagagtgtattggtcataaacacaagcttcctgaacatctcta

acagcagcctgttcaacaatgttcctcacaagaaatctcttgattgccttatccttagga

cagcattttccgcagttggagcaacggatgaacttgacatggccacggccatgcttgttg

cgtcctccattcctacgcttgaaagtcattgttgaagctcaagctttctgtgattatcct

tcgcagacaaagggttcttcaatgccgctagggtttgtagaataacaagcc

>fava_dn_rep_c592

ctttttaacatttttctaaattcttnatcaaatccattaaaacttcacttgaaaaaaatt

gagagaatgagacggggagagaatcctctcccgtatatgaatatgaggcaggtagcaatg

cagtcacacatgcatattcactgtcatctgatgatcgcacggtctagatcttaacgcaga

ttttaactttcgggaccatcttgatcagacagtatccctactgtgattgcacacgatcca

gacccgcccactggccacttgtcatgtctaatgcaattatgcaccacgatcaacctaaag

ctttactaatttggcagtgtcaaaaataaatatgcacgtcatcggataactgttgcgtac

ctatcagtaactttatacttatcacgaaagcacaaactggttgagtgataagtaactcca

aattatacaaacaaactaccctaacatcagtcttacatctcatagacagagtataaaaag

cacacattcctcaagataatttcttaattgaccatttgattgtgctctaaactatatata

ctatgcatctgaatgagaatccaacataggcactgaatgaagagatccaacataggtatc

tgaatgagaatccaacataggtttaagcttctagttgttatatgagcatgagaaagcctt

gataaggagtagcaatcctctttgttttggaatcactctcaacaaaattgaaatccacct

gaaggctcatgcagaggatgaatctgaggtggctgcctcttgtcctggcttttgacgccc

agcagcaggagggccatccctccgtctttcataccttctaccctcactctttgatccacc

actacgttttggttgataagtagggtacttgcaaggaatgatttccccgtttatgtactt

gtcacctccatagtctttatttttaacatctatgtatgaatctggcaggacccagagaac

accaggtaaacccttgaattcctcggaggttgcttcatcaacagtgcattgaaatccagt

gtaagttgtggtgctaaaggcatacatgttcttctttgcttcttccatgcttcccaagac

agatgcaagagtttgaagataagtatcaatcatttgctcccgagagggtttagggtcttt

aggaaactccattacaatgagccaatgattgtaatcacagccaggaagcataatcgtctc

tttcggctcattgcttccgcttctcttggaagagtaatcttcatcaaccgccgcacgaat

ctggatagcgtttctggtacgggttttggtacggatagagaggttgttgaaattgaggga

tgaggttttggggtggttaggtagggttagggttagggttttggaggagacggtgaagga

agaaactgttgccattggaaatgggttagccgatttgagaaatggagaacaagtttggga

tactgccattgtgaacaacgataaggctcaacgttcaacccgtttgtggagatgatgtat

tccccgcg

>fava_dn_rep_c593

agtctatcgtgatggaggctatttccatgctactggtactattagcctattaggtagcaa

cagcactcagttatacgacaggcagtattaggtggcaacaacacttagttatacgaaaga

cgatagatctgccgttcaaatttgagcacacaactcatattaaaaaactaaaacgacgta

gtagcataatccaaaaaaatcaaaattagaagaaaggagagaaaaaaaacgcacacacaa

atattcgcatctccttgtagatctactgctaacccttcggttcctcccttcatccattca

atcctcttcgatctccagctccgatccgtcgtcgcagattgtgaatcgaatggaaggaac

tggaggcagcagtgcctctgctgctgctgtgcaacctgtgaatcaacggttgcaggaatt

ttcgaagctatttcaatattatttggataaatctacgccacactctacgtatagatggat

tgggacttttgtaattgcatctatataccttttgagggttttttatattcaaggatttta

cattgtgtcttatggactggggatctatatgctgaatctgttgattgggtttctgtcacc

tttggttgatcctgagatggagccttctgatggacctttgctacctactaaagggtctga

tgagttcaaaccgttcattcgccgccttcctgagtttaagttctggtattctttcacaaa

agctttctgcattgcatttttgatgactttcttttctgtctttgatgttcctgtcttctg

gccaatattactctgttactgggttgttctgtttgtccttacaatgaggcgccaaattgc

acacatgatcaaatacagatatattccattcagcttgggaaaacagaaatacggtgggaa

gaaatcttctgcaagtagcagtggctctcgggcagactgaagctttgccgtgtcatgatt

tgaagaacacagatctcaaagttaggttttgtgggcattcagggtacttgtttttccctt

tttatttgaaggaatttgtagtcagagtattatggatgcctatatgcagttacagaaagt

acttgttttatggttttatgataccagaagacatgctaatgctggtagtgaacttcattg

ttgatatagttttagatttactgcaattttagtcttgtattgccattggactatcatgat

ctctggacagatttcacacaaccttgatttttctccttctcaggatatttctattgcctt

ctgcatctttacttgttaatcttagtattttagattgactattgactctgtcaaattacg

catattttttctcctaaa

>fava_dn_rep_c594

catatggtttgtacaattgattacatgtttttaacaacaagttttaaataacgaaagaag

tttaaaatagtaccattcaacattattcatattttcactcgaattaaaataataaaaacc

acacgtaaaaaagtgtccttaaattttgtccttattatcatgggacccatgataacaaac

atggtgtactaatacctgacatttcacacccatctatccacttatttaatattaagaact

caacacaacaggaggtatgaaatttgcaaaacacacatataaaagatgaacactacatta

tcattagtatgatagtaataaagtatataatctaaaatttaggcagcattgacggcaatt

ttcatcccagattggcagtgaccagcataattgcagatgaagtagttttgtcccctagca

agcctgatctgatcttttcctgatcgataaactttggctcctcgtggggtactgcagttg

tcatatgctgctttgttcacagccaccacattgtgagcacctgggctatagttgaagaca

agtgtgtcaccggccctaaagtgttttcctttgggccaagcaacgatgttaaaggtccaa

ccaccgttacctccaactgtgtaggtaacagcatgggccaactcggagtgaagaacaaaa

aagcagactagtagaaccaatgcactacctcttcccaaagccatgttttctagtttggtg

gaaaagtgtgaaagagattgatttggaggtgagtgaaattggaatgatgtggtaagggtt

ttataattgaggagtgagtgaaattggaatgatgtggtaagggttttataattgaggagt

gagtgaaattggaatgatgtggtaagggttttataattgaggagtgagtgaagaggcatg

cct

>fava_dn_rep_c595

tccatggtgtcagtagcattgcaaaagaattcaccaaacaaaagttatatttaaactcct

ttaagcacagaatgtcacaacatcatcgttgtattgtattaattgtgtttcactcataca

ggcataaacacaaatttatttaatagaagattagattattaattttgtattgtcaaattc

atggcaattacatttgcatatattccttaatggccgcaggtgcagttagtgcaggtgcag

ttagaaccacacttgcaatccccctcatgctcaactgtctcaacatccatgacaaagctc

ttttcagtctctacaatggtcacaccgaagttgtttcccttcccgcactggctcttgtca

gcacaatcgcagttggtgcatgagcttgacatgtttggtagtttgaaagaaagaaaagct

ctgaaaaagaaattagttgtagaattgaatgatggtgctgtgccgcggccataa

>fava_dn_rep_c596

ttttctttttttttttggccataccaacataaattatatatatatagttctagtcataaa

taggaaatctatgactatattgtacacaattgtctcaaagatagcattatatatttttgc

aaaaagcatataaaaattgaaaatgaattgtagtttaacattttaagcacttgctccaac

aggtacaccttccttgagccttgatgaaccatacaatgtttcttcaaacctaatgatctc

attttttgatccataagcaacttgagttctatcatcaatgttttcaacaaccttgtctag

cacagatttataaccatcactgctgtaacctccagcattttcaatcaatagtcctaatgg

agctacctcaaacaacagtctcagcttcgctttcgcagttggcgatgtcacatttgtaaa

tattcccttttcttttacaataatctggttgacatctggaaccattcctccagtgtatct

caatgtgtatttttctctgacatagtaatcaaccaacttagcataattggtgttgtcaaa

tgtagctctcaaatttccaggggagaatagttttccttcaccaatttcatgagtctcttt

gacatgctgccattttccttcatcaagcaggagaaactcatgagtaccagggaaatcttt

aagagcaagaacatatgtagttctaggacctaaaactcccatagctgcagcaacttgttc

tcttcctgtgactccaatcaacttatcaccaggccaaacaccaaagatggtgccaactgt

gaaatttgtgtcaactatgcttgaaccatcaagagggtcaaatgcaacactaaatccacc

ttcagttggacctcccatgtcttgaagctcaggaatttcttctgagcaagcatacttgca

gaagtgagagtgttttaaggcctcaaaaagaaggttgttagcaagcatatccactgcaag

ctgttcatccccaaaagaattaacacattgtgttccaccacaagaagctgtcttcacttt

gaaagaaattgttcttaatgcttctcccatgcacaccatcaacctaatcagacccttatc

cggtgtcgccttcgacaagaattcttccaaactgtcaccaatttcacatttggttaggaa

tgaagtagtctttgttcttgaaaacttgagtgttgacttggatgacactctaagagattc

tccaaataaagagctagatttcaaactcctataaccagaagagggagtgatagagggtag

ggagtgtttggaagaaacagtaggaaagaaagctccacgggagtaacatgctataccagt

ttccatggctgcaaactgaaatctatgttgaaaagttttgaggatgttgtgtatgttatt

gcattggaaaaagagagtgagaaggggatatagcaagttatagatatgtggctaattatg

catgtagttccacgtggcatgtgatgagtagatatgctaatgagactgcattttgaggtg

taaatatttagcatcg

>fava_dn_rep_c597

aatgaatgcacagtttaaaacatgcacaaattntaacggagacaaaaacaacaaaactgc

atacttgaattgcaatgattttttttatacaaagagattattgttccacgttcttttaat

gactttgatttgcatgcatacatacaacagccgctaatttagacagaagacatcaattgt

gctataagtgaagcagagaagcaaattcttatccctagtgtataacatatctcatcttaa

caattttatttaaacttatgtaaaaaactctttcaaataatgaggtccaggtttgaaggg

atcagactcggaatacgcctctggacggtaaaatccagcatcattcactcgattactcaa

agcatgaagaatggctttcttctcacctccatactcatccgtgttgctctcaacataagc

actggcctcagctgctatctcattcacaaaatcatacctattatcttctgataacagtaa

cttctctatgtccaattgcaagagctttccctcatcctttatcttcctcacctgttcctc

cagaaaaggaatgcgctccattctgaagggcaaagattcgtccttttttgtcattttcct

tcgcctccagatttttcttccaccaccctcctttcgcatttggattatcatctgcgagtt

aacactgaagcttgggctccgggaatttccagctcttaactctatcttaggtaaagcgga

agaaagagtgaaggaattgatgcggagagccatgggcgctgtccaccgcagtttggttca

ccttatctgaaatgaaaattttaaatcaaattactccaaatagttcgccaaatcttagct

tttaccttgtgaaagtttatgcaaata

>fava_dn_rep_c598

gaagaaaaaaactcaaaaaagaactcaaatataaaggggttccatcaataatgacgcatc

ttgaatttatttcgaaacaaataattgagaattgagacgacacaatttatttaatttatt

atttaatttaataagagaggaagcaaagacgattctaaacactattttcctccaccagtt

ccccaagtccacacggatacgcacctgtctttgtcttccttctagctttcttcccaactg

ttgatcttttttttataatagaactcattttagaagagattttccatttaaaatatgcac

gggaataacagaacacataacaaaatagagtacagaaaaaacataaaacgacataaaatc

aatttcaaattaagtctcacattcataattaacaccattcaaaagtcaaaactcttcaac

acaaatcaaaaaagaaatttctaacttgtttaacatccattaataacataacttaattct

tagctgcttgcttttctttggtcttctgtatcttcaaaactttcttcacaattttttgct

cttcaacaaggaaagcacgaataatcctttccctaacagctcctccagataaaacaccac

catatgctctgttaactgttcggcggttgcgagacaatctggaccccttgtattcagtag

gtctcaaatgtggaatcccttggatcctctttccagtaaccggacacttgggtccactcg

ccctcttcttagtcgtctgataaaccaactttccaccaggagtcttaacaactcgatgct

ggttggatttggtggcatagctatgcctccttcggtacgtgagtctctgaaccatggtga

actttgttctgtctgaaaccctagaaaatgaagaaaatgagagaggcgtgaaaatgtctg

tgagagagagtataaatcgtgtgggtggatattctcaaaaccct

>fava_dn_rep_c599

ttttttttttgagaattttgttgtatggtacatcctctttgaaggaatgaaccatagatt

tgctgataaagttaaaactgcagactacaaagtttggtaaacaactcattatgaataaat

acataatctttttggtacatatccgcgactcacaaaacaacacttctcttgtgctctaat

cttgtgaattaattgtttatttcacactaagaaaatgacactaaaaaatataacattgtt

gtctatattggctgtctgtttgatatctgcacaggcagcaaggtttgacatcgtcaaccg

atgcccctataccgtctggccagctgccactccctacggtggtggtgtgcagttaaatcc

aggccaagcatggaacttatacatcccagctggtacctcatcaggcagaatttggggccg

aactggttgcaactttgacggttctggccgaggcaactgtcagaccggtggctgcggtgg

tgaccgcagttgcagcttatccggtcaacctccactcacacttgctgaattcacccttaa

cggtggaaacaatttggattactatgatatatctgtgattgatggtttcaatgttccaat

ccaatttgccccaacctctaatggatgcaacaaagtgcgaacttgcacagaacgttcttg

ccctgatgcttatcagtatccgagtgataatactaaaacggttgcatgcccaggtggtat

caacgcagagtcgtcttctgcccttgaaacttatatcaaacctataaaatatatagctcc

accacccttttatgtttgtttctacagcaaagaaacaaaatatagaataatatattgggt

gtctgcattatgtgtaatatgtataacaatattgcttaagttactcgtgaaattataaat

atatattttctcgagaatgcaatatggaataaattctcttatttgcttttgaattctgat

gattataacttctactactatttgcaccaaagtattgtcgagttgtcttggtctgtatgt

tttcaatgcttgacatatgcctccttctcaagcaaaatttgaattgtatttgaaaaataa

tatttaataagactgttaacttttaaaaaaaaaaagaaattccagaagttcaaaagaaaa

aaatgccaaatcatcaataaacggaaagagagggattcgaaccctcggtacaaaaattcg

tacaacggattagcaatccgacgcttt

>fava_dn_rep_c600

cgccgcggggacatagaactaattaacaagagacaatagacataacttaagaagaagaag

aaaaatggaggctcttatgaaattgttgtgtcttgttggttttgtagtccttgttttagg

gatatatggagttgaaagtgcaggtgaatgcggaaagggtacaacaccagataatgaagc

ttttaagctagctccttgtgctaatgcagcatctgatgaagatgcaaatgtgtctcaaag

ctgttgtgctcaggttaagaagcttggtcagaatccaggttgtctttgtgcagttatgtt

atcaaacgttgctaaaatgtctggtgctgaccctaaaattgctgttactatccctaaacg

ttgcaacatcgctactcgtcccatcggttacaagtgtggaccttatactcttccttgaaa

gagttgaggttattgctgaaagcaaagagtgggatatgcaatgttgctggtagctacaac

ttatatatagttgtgttgtaatgtaactttgttgtttgtagtgtggaaaatgagctagca

gctcacttgtgtacactctgaataaatcatgcattttcagtgcttttatttcatctacct

actaaaatggttgtttttaagttatattttgatttatgtttatttgttatcacttagatc

ctacaaaaagctcaatattaaataaagaaaagttgaaatattatacaaataaaataatat

ttccaattttc

>fava_dn_rep_c601

cgaaacaatataaggaaaaatgttgtttccctagagatagaaatgcttttatttcaaaat

acacaacataactcattttacatggaagggaaaccattcattttagcttaagaggtattg

atgtcacagttatcccgataataaaccaaagaatttgtccaagcgattcataatgttaat

gaaactataagcccataacattatctaacagtttaactcctgctgaagcttgttgacaaa

ataagctgataagcaggaaatagcacaacatctttacaaacataacaacatgaaaccata

aaacaaaggtaacacgtacaacgcattgtattattattttcaccaattgctgcaatgtaa

tccttgaagacatgcactaatcgtcatctccatcaatgtagcgtctttcattagcggcct

tagctttctcaagcatcttatcgagatcttgttcaacctgagcctcccatttcacaagct

ggttcacgaacacaacgcccaatcccattcccaatacgtgctcccacggatggcgcatgt

aggggagtttgcggagagcgttggagtacatctgggtacccaaacccaatagggctccga

tcgtcgtcgcgctcagtactaccatttttttttgttctatcgcagactcactccgttcta

gactatgatgttctcaacatctataaaaccttttttttctctattata

>fava_dn_rep_c602

aacatacgaaaataagaagaaaaaaacatgtttcattgtcacatggccacaagcttctaa

ccatatcacatggtctaaattctacgtactatccatcccatatttcattccatctcaaaa

ctcaacctctaaaccactcttcactatggtactttcttcctgctctttaacatggacttc

accctctttatcccataagctaaacttgccacattcaaatcgtttgcttcgaaatacttc

aacttcatcttccaacaatgtctcttgtgcaatggaaacatcttcgagttcagaaagtaa

gagcctgagaagacctctattgttgggtataggagcattagcagcaaatttgcagccaac

aaaattactctttgctgatgaaataccggacagatatagagcttttgtcgactttacaga

cggatattcgtacgtttatccctcggattggaaagaatttgacttcacagctcatgattc

tgcattcaaagataggtatttgcagttacaaaatgtaagagtgagattcataccaactga

gaagaaagatatcagagaattgggtccaatggaagaggttgtaactaatttggtgaaaca

tagatattctgcaccaaaccaaagaccaacaatatatgacatgcaggagcggacgataga

tggaaaacattactatacaattgaatatgtaattgcatcgcgaaattacgctagcgcctc

ctttacaacacttgccataggaaatggaagatcctacacattgattgttggagccaatga

aaggcgatggaaaaaagtacgaaatcagcttaaagtggtagcagactcatttaggctcat

agatatttgatatgttacagaaccatgcaagacatgtaccaatttggctacatataaagt

ttgaaatatatgtaaatttttgcaagtgaatgatatcatctgtcatacaaatacaaaatt

tcttctagttgataatacattgcatatttatataataaactttatcaatcaccgag

>fava_dn_rep_c603

attcaaatgaaaaatccgaatggcaatttcgnnnnnnnnnaaaataaccgattaaaataa

aaataaagttctttttgtaaaacttcaaaatttaaacggttctcctcaatttcatttcac

ttcatcgcattctgaaaccgttgtggagctgagcaattatcactacatccatcgtcgtca

tcatcaacctctcatggatagccacgattctcaagacgggaaaagccccgctgatatgac

agcatttgtgcaaaatcttcttcagcagatgcaaggaaggttccagaccatgtcggactc

cattattacaaagattgatgatatgggaaatcgtataaatgagttggagcagagcatcag

tgagctgagagctgagataggagtggagatctctcaagcacaagcagcctctgagaaggc

aaaggaagaagagtcgaacaaagaagagggttcgtcttaaagtgacattttagtttcaat

ttctcattgttcttgtcttgcattgtacgatgattggtatggtgcatcaactatctctgt

ctatgttgttcagaaattaaagtgttgtgtggctgaatactgctgatcatttgtagctaa

atcatcttacaattttacgatttagcaatattcctacaatttaatgttacaaattacttt

gtttcccgcctaccctctgttattttcccagcatgctgtaagtcgatgaatggatccatg

gccccaaatccttgggctgatgtactaatacccatttatttgtgctttattccattattt

tgatacggggttcaacannnnnnnnnnnnnnagttttttttccctaattcacaaaagaag

agttggat

>fava_dn_rep_c604

ttttaaataaataatttatttggaccaataaatgattacatcttcttttgagtgtttata

tctactataaagtaattaaagtagtacataaacttgtttaacttataatagtaggtaatt

tcttagttcactgtgacagaccaataaatatacttcaccatattaaccttccatcgtgca

ccagttcagtggtgaatctaagaaaattacacattagcgtggaaaggctatctcttgtat

tctactgcagaagtttagactttttataagaaagacatacaattttaattacaagcattc

caaaaggagggactaaggtgctgcatatattttacatatgatgatatgacaggtaggacc

atctgcttacttattttgaccgaccaaccttcaaatatatttttaagttttattgataag

ggtcacacagcaggtttatactttctctactgtagcaaacaacacttcctttgctatctt

ggctccacgttctttggccatatacaagcctacatgatgcataaactcctggggatgggc

taaagctcctttgttatttgtggctacgtcaagagggacattgaaggtggcacatgcgtt

tgaatacaccaccacattttctagaggcttcagaaaaccacggtttttagcagacattgt

agagcatacaaaatccagaacacatatatctgtgcatacacctaccaccaccactgtttt

gatcttattcttcttgacccaatctacaaaaacatttgaaccatcttgttccattgagcc

aacataaccatcaaaacaatccttacgtcttattgttacattggtttcgttctctagcca

tctcaatgctggaactagatttgattcatcagtcccagcaatacagtgagggggataagg

ctcctctggcttgttaggctggtgagagtcaaggaaagccataactggcaatttcttctc

acagaactctctcgctagccttgctgattcactgatcatttctgaaatttgtttattgga

ctctcttggtgccagatttccagcaccaactgtgcagaagccatttatgatatcaacaag

aacaagaccatttacagtgtcctcagctaaaaccaccgactcttgttccagaggaatctc

gttcttcagaagttcaactgtgtgtgacaccatcttggtttccacttcaatgtcacacac

actattgtttatcttatcttttataattataatacaccaaaactatatagtattgcgtaa

gtgggggaaaataacct

>fava_dn_rep_c605

gaatttcatcaagacacacaccggtcctggaattctttccatggcgaatgcgggtccagg

gacgaatggatctcagttttttatctgtacttccaagacggagtggcttgatgggaagca

tgtggtttttggtcaggtggttgagggaatggaggtggtgaaggagattgagaaagtcgg

gtctagctccgggaagacctcgaagccggtggttatcgcagattgtggccattacggccg

cgggagaaacaaatggcgagcactttgttgtccgtacactgctctcttccctcccaaagg

aaggaaaatattggatgcttgagtggtttcaggacttcattgtgtggacaagttgtgctc

aatccaaagttctctgggagacaagtagcaggaagccatggaaaccgttttgttgttaac

tcactgtttggatttggaattggaaagaaggtgaagagtagagtgatcagagagactgtg

attcctgaaccagattaccggattccaattgtactacttggtatagccggcgggttggtt

tacacagataatctagtacctgctgtacctgtgggtctccttggattgcttctgttattc

cagacaactagagtgagatttgtctttgatgatgaggctttggaggtaaagataggcgat

cagctagacgaatcgggcgagaatgcttttgttggaggaaaaaaccgctggaaatactca

acctttgttaattgggaattctggtggccaaacttccctatcttggtttacttcaaggaa

acacaaacaaagcctgaaggacaaattcatttcttcccaataatttttaatgggaagcag

ctttatgatgttatggttgaaagagctggctcttcaaaaactagtgctccaaaagaatcc

taaagaattagttgcgtggcttgtgcctcaggaatcggtgtctctagacagtaattgttt

tggtttttacagaattacagatttgaaaccattgatatcttgggaaccctgcaaggaggt

ttctgaagtttgggttgttgcttgtggctaggggaagatgatgcagtgctctcagatgtt

tcaagttgcatacaagttgacgcttaataacaattcatttattattatttagtcttgcta

gcacataagcatatcacattctatttatttgagctgggcagttatttactttgtactgga

acatatacaatttcacagtacccatttattcgaaacatggttttttccaaaaaaaaaaag

aaacttcatattggttatgacaaatcaaacatgagaacctgttgtattctgtatgtctga

taaacaaatgtaactggaaacttacgattatgacaaatcaaacatg

>fava_dn_rep_c606

gtaaataataattcagtctaataaatttattaaaaaacacataaaactcaaatccaaaac

ccatcttttactaatcattttgagttgatcattatacatattatatttgatctctttatt

tatacattcatgtagcatcatagaaacataacatagactacataacacaattttagtggt

taattgtgattttagcaaactaactaggatacctaatgtagagatacaatattaaacagt

ttcacacacttacataaatatgataatttagaagtgaaattcaaagggatgcatatctat

tccttttacttctttccaaaacctcgttcaagaagataatcaactagattgtcaaattcc

tcaccactaatttggccatctttgtcagcatcaagtttcttgaggcaagcatttgctctc

catccagggatgtaggaacctaaatcttttagagctttcttgatctcatcctttgtataa

caaccatctttattcacatctgcatctatcaatgtttgcattattttgtttccagcatcg

atttgatattgaggcatggccttgtgatgactggcgtgatgaaacacgggcatgttaaaa

tgataactattgctctttcagttatcaccctaagtgtattgagtaggtatgaaatgaatg

agtatcccgcggctaatggccannnnncgttgcaccttcttctgaacatcctgcacctgt

ttcttcaatt

>fava_dn_rep_c607

agaaataaaataaaaggcttcatttaacatcagcggcaaatggcaattacggccgcgggg

tatacaaagtcaacagatctcaataaaaaaagatatataggatttatggctacacaaacc

gttgagggtagttctagatctggtccaagacgaactgttgtaggagatttattgaaacca

ttgaattcagaatatggtaaagtagctccggggtggggaactacccctttgatggggatt

gcaatggccctatttgcggtatttctctctattattttagagatttataattcgtccctt

ttactggaccaaatttctatgaattagagaattagattcacaagaaccaactaactagct

tttcaatcgaaaagtcaagtttagcatttaaatcgttaatttttagcccattccattttg

atttggtagttcgaccgcgaaacttctttgtttctgtatttccggaatatgagtgtgtga

cttgttataatggatcctattgatagtacagaacagaaaatggatacgtcatcttatctt

gatagatggctttcccccgtcagatatttattctagtatctggagcacggaatatagaaa

ataaatagattctaaagtgttagaactatgattcatacttcatatttatattgatattta

gacctcgcaaccggacgaaaaaaaaaaaaagaattataagaattcatttagaaaaagaaa

tggaattttattctttaaaactgccaccgcttatcatttctttgtctttttttttttttc

attctatctattaattgaataaatgatgatccagcagttcttactcagggaatttatgga

cttcgattaaaggttttttggggaaatcatcgtggttctggtatgaatctcaggttttta

aattgattctatagggtcttaacaagaaaattcttattaatcataaaaaaacagcagtaa

aatcttattacatacacaatacaaaaaaatgaagtaaataatagaatattcaagaggccg

gtaaggatcaagagaaaagacgagtgagccgacttgaaattttgacattataaacactaa

gaatagcttttggatttctatttttttttatcttcaaaaaagactggaataataggatta

agtgaaaaagtttaagactttctattacacgtatccgttttccaaataaccagtatttga

gtatttttgtttgagccgtacgagatgaaattctcatatacggttctcaggggggtccct

tggtttaccttgtgtgacttgttataatggatcctattgatagtacagaacagaaaatgg

atacgtcatcttatcttgatagatggctttcccccgtcagatatttattctagtatctgg

agcacggaatatagaaaataaatagattctaaagtgttagaactatgattcatacttcat

atttatattgatatttagacctcgcaaccggacgaaaaaaaatgaaagaattataagaat

tcatttagaaaaagaaatggaattttattctttaaaactgccaccgcttatcatttcttt

gtcttttttttttcattctatctattaattgaataaatgatgatccagcagttcttactc

agggaatttatggacttcgattaaaggttttttggggaaatcatcgtggttctggtatga

atctcaggtttttaaattgattctatagggtcttaacaagaaaattcttattaatcataa

aaaa

>fava_dn_rep_c608

ctttttttttttaaggttttcaaccccaaaatttcattgatctttaacaatttttccttc

aaagttttaaacacaattctaaacaggagcaacaattaagaaataaaatgccaatacaaa

ccaaaagagccaattttaaatttcaaatgtctgaagcaaaacagctgtgggcaacttata

cattcatcgatctatatgttcttcatgcatagttcaaccggaatacgtcattcaaaacat

aatagcttccttgcggtgttggtatcaaatggaacatctggctgaacttaagagcatgct

gttcgccggcgagctggagattcccgctaacgaaaacaagcataccaccgttagccccag

acggctgacaatcaacggttgtgatggagtgatgacactgctggaaagggagagaagtga

gtttggcaacgatgttctgagagccctgaatcttctgaccttcgaaggtgagcatggagc

tttcctggtagagagaggcgagaccgggacggttggtgtcgaaggtggtgtagtagtgct

cgacgaatgcctttgccaacgcgtctggatccatcgatgctgattcaatcgccgccgctg

gtgaactcttttttggttttacgctttactctcacgcagccacgcggcttattctagtat

ctggagcacggaatatagaaaataaatagattctaaagtgttagaactatgattcatact

tcatatttatattgatatttagacctcgcaaccggacgaaaaaaaaatgaaagaattata

agaattcatttagaaaaagaaatggaattttattctttaaaactgccaccgcttatcatt

tctttgtcttttttttttttcattctatctattaattgaataaatgatgatccagcagtt

cttactcagggaatttatggacttcgattaaaggttttttggggaaatcatcgtggttct

ggtatgaatctcaggtttttaaattgattctatagggtcttaacaagaaaattcttatta

atcataaaaaaaaacagcagtaaaatcttattacatacacaatacaaaaaaaaaatgaag

taaataatagaatattcaagaggccggtaaggatcaagagaaaagacgagtgagccgact

tgaaattttgacattataaacactaagaatagcttttggatttctatttttttttatctt

caaaaaagactggaataataggattaagtgaaaaagtttaagactttctattacacgtat

ccgttttccaaataaccagtatttgagtatttttgtttgagccgtacgagatgaaattct

catatacggttctcaggggggtcccttggtttacctatctcaataaagtctatgattggt

tcgaagaacgtcttgagattcaggcgattgccgatgatataactagtaaatatgtacctc

ctcatgtcaatatattttattgtttaggaggaattacacttacttgttttttagtccaag

tagcgacgggtttcgctatgactttttattatcgtccaaccgttacggaggcttttgctt

ctgttcaatatataatgactgaggctaactttggttggttaatccgatcagttcatcgat

ggtcagcaagtatgatggtcttaatgatgatcttgcacgtatttcgcgtgtatctcactg

gtggttttaaaaaacctcgcgaattgacttgggttacaggtgtagttttgggagtattga

ctgcatcctttggtgtaacaggttatcccttaccttgggaccaaatcggttattgggcag

tcaaaattgtaacgggtgtacccgacgctattcctgtaataggatcctcggtggtagagt

tattgcgcggcagtgctagtgtgggacaatctaccttgactcgtttttatagtttacata

cttttgtattacctcttcttactgccgtattcatgttaatctagcttcagctcttaaagt

gcagaggaaaattataaaaaccttaaatgagagtatggatgagctcagcaaaaacaggaa

gagcatttggtggaggagatggatcccaagagaggaatgaaagttgtaaatacataatac

cctttttttaaattcttgtctatagtttgtatattgaaatcaattatcttgttcagatag

agcaatgttttatacgaaattatgattagaaatgaaactgacttgaatgttatataatat

tcagctgaaacaaagtagagaattttattttgc

>fava_dn_rep_c609

caaatttccctttctttaaaacttcagaggatagtaaaaaaaaataatgaagttgtgcat

catcaaaacactatagcaaaaagacacataaatcacaccacatacttgcacatgtgaaat

cataatgaagaatcattataatacaagtctataagaccaaacagtaattaagaagtctag

attaaagtcttggaaatgattgcagtttataagtcttacaattaaacaaaatttaataca

tagaaactaaaacatgtaaagatatagattcattcatcttaaaatcctaacaaccacagc

tctccaaccttagacagagtaaaaaaatgacacaccatttaatctcccttcttcctaaag

gagcaaccttccgttagcctcgcacggccacccgttggctggcacaaaacagtctgacag

ttaccacacaccacgacggtctgagagtggctaaagacagtagtaatgttgaaacaaccc

tggcatttaacatccatgaagaaagagtttggtgattgaacaagacgcttgagtttgtgc

tttctcttctcaagctcagcaggagggtttaacaaatcaatatcgttttgaagaaccatt

tttgcagaggatattggctcggacctaagagaaactgctcacagaaacaaaaaccctaaa

atcttcagagggcgcgcggccgtgtgatgaaaaagggttaaatataatggttttcggacc

cgacccgacccaac

>fava_dn_rep_c610

gaacatagataaggaaaagatgatagataaagaggagcaacatgagtggtggaaaatgaa

gcagcaaaatctcccttaaccaaacaaaaaaagaagaagtttatgtcactactttactcc

gtcgattatccgacgtggcagctgagattgtgattcaagcgcgcgcatcaccattcaatc

atatatccatggcggtgcctgctccaacctcccctctctgcctccgtgatcttcacacaa

acaaccgtatcttccgtcaccaaacctcattcctcactccctcccgtaacaataaactac

atctaaccgtcgtggccatggctcccaaaaacaaggtgaataagtacaatgataactgga

agaaagaatggtttggagcggggatattctacgagggaagcgaagaagtagaagtggacg

tattcaagaaaattgagaagaggaaagttttgagcaacgtggagaaagcagggttgttat

ccaaagctgaggagttaggcgtaacgctatcttctattgagaagttaggtttgttttcta

aggctgaagagcttggtttactaagcttattggagaaactcgctactgtctctccctctg

ttttggcttctctgtcgcttcctactcttgttgcggctgttgcgacggttgttctgattc

cggatgattctactgctctggttgttgttcagaccgtcgttgcggcggctcttggtgtcg

gcgcggttggtttgttcgctgggtcggttgttctaggtgggttgcaagaagctgattgag

tggttcagtacacacgtttttttactgtggttgtttttgttgtaactacattgtagtaac

taatactttacttcattgtttaatctttaatcaaaaaacaaaaatttgttttctgttttc

aaaaaaaaaattactaatttcatgttgaccgttaaaaaaaaa

>fava_dn_rep_c611

ctttttttttttcaacaaaacactcggaaattcaaaattttcattgaatgataaatacga

ttaatagagtattgtgacatgccgattaaacaaccactgtgaggagaatgtataataaac

ttcaactacaagaaaattgtgttaaaaaaaactaaatcgaaaacttgaggagaaatcatt

ataatttaagcatcatttgaacttgttggtgctccaaaaccaccagatccacgaccaaag

ccaggccttccaccaggacccccaaatgagggtctgtaatcagcaggagcacctccctta

tctccaccaaattcaccaccaggtcccctcggacctccacggtacccatcacggtcacct

ccaaatctccgttcaccttcaaaccttggtggacccctagggcgatcaccgggtggacca

ccgaaaggcctcccagcaggcttagcctgtttcttcaaagtagcaggcacaatctcagaa

ggaagattaaggtaagtcctaagaaactcaattccatcattcgtcaggaaccagtaataa

tgcatccacgcaaacgtttccctaacatactcccttgatttgaagctctgcataagctta

atcacctgcaaattaggaacatcgatttctgggtgctttggcaaattgaaatctttcttg

gcatagcaaacaccctcttgaaagaggtatttgcagatttctttgcggtttttctctgat

atgatcatggtgacgagggagtggagagacaacgaaagagtgttctgccgcaatataaac

cctaaaaccaactaggttgtgttttttcttgttataaat

>fava_dn_rep_c612

ttctagttttttttttcgagaaagatataatagttcatagtagccaaattagtgcagaga

tagaaaagtggtagagtgagaatcactgaattcaactacatcacagtgttgtaattcaca

agccaaaatagtttttacaaattcatacaacttccttaattcatctcatcatttgatcta

tgttgctcttttgagaatcatatgcgagagcaaccaattcgttcttattgcggttaaaat

gatatctcaagaaacaactctcgtagtagtaatacaaatcttaatatctcgagaaacaga

acatcaattgatctatgaaacttccttggcggcagaaataacggcttccttggttatacc

gaactccttgtatatttttcctgctggagcacttgctccgaaccgatcaatacctatggt

ttttcccttgcttccgacaatcttgtgccaaccgaaagttgatcctgcctcaatgctgac

tcttgcagtaacagaagcagggagaacactctccttgtattcatctgactgatcatcaaa

aagttcccaactaacaaaagaaacaactcttacagtttttccttctttccttagatcgtc

ggcggcagccgcagcaatttccaactcagaaccagttccaatcaagataacatcaggttt

gttacctgatgagttgtctgaaatggtgtaacctcccttttctactccttcaatagaagt

tcccgcaaggttgggcaacttttgccgagaaagggcaagaattgagggtctcttcgggtt

aagcacggcgactctgtatgatccagcagtttcattaccatcagctggacgaagcatcaa

aacattcggcattgcccggaagcttgccaggtgctctattggctgatgagttggtccgtc

ctctccgagtccaattgaatcatgggtcataacataaataactccagcttcacacaaggc

ggaaatccttatggcagctctcatgtagtcagtgaagacaaagaaagttgcacagtatgg

aataaatccagggctatgaagagcaataccattgcagattgctcccattgcgtgttccct

aacaccaaaccgaacattgcgctcttctggagtagccttttggaaatctccaaacgattt

caacaaggtcatgttggaagaggcaagatctgcactaccaccaataagaccaggaagaac

cttagccagggcatttagatttgcttgcgacaaatttcttgtagcatcggcaggggtttc

tggagtgtacgttggaagtgctttctcccaatcagcaggtaattcacgattaataatagc

ctttagctctgcagcttcctctttgtatttcttctcatactcagcaaacttagcattcca

ctcagcttcaagtgcagcaccctcaggggtatggcgactccaatgctttttaacatcttc

tgggacatggaaaggctcatatggccatccaaggttctgccttgtagcgtccacttcttt

agaacccaatgcggctccgtgaacactgtaggaattagccttgtttggagaaccaaatcc

aatggtagtggttaccttaatcaaagtaggtttgtctgtgacagcctttgcttccttaat

ggctgcacgaatttcatcatacccggtatttccgttctttacccaaatcacatgccatcc

aagtgcctcgaaacgttgatcaacattctcagtgaatgcgatttcagtgtcaccgtcaat

tgaaatgtggttatcatcataaagtgcgataagtttcccaagaccccaatgtccagcaag

cgagcatgcttcatttgaaattccttccatttgacaaccatcacccaatataacatacgt

gtagtggtcaacaatctcactgtcgggcttgttaaaccgtgcagccaaatgtttctcagc

aagtgctaaaccaacaccgttggcaataccctgacccagaggacctgtggtgacttcaat

tccataagtctcaaaattctcaggatgtccaggagttttacttccccattgtcgaaactg

cttcaagtcttcttccttgacactgtgatagccggcaagatgaagaagagcatactgaag

catacaaccatgtccagcagacaaaacaaagcgatcacggttaaaccaagaaggattgtt

gggattatatctcataatttcatcatacagaatatgccccattggagcacaacccatggg

caaacccgggtgacccgaattggctttttcaacagcatcaatagcgagaaagcgaatagt

gttaaccgatttctccaccagggaaacttcagcggttttgtcgagtgtttcaacagcggt

tgcacggacggttgttctgcgacgcgaagcagttgaagaggaggtgagtggtttgggttt

gagagaggggaaggagagggaggaagtggaggagagggaggtgcggtcggagttgagaaa

aacggcacgtgcgaggagtgcttgagagagatgcagagaagaagaagaagccatgataga

gagaatgaagtgttttttgagtgatggatagatggatggagagaatatagaaaagaaaaa

ggaatggttggtggtgtgaggtgtagaggctgtggaaaatctcagcctatgcttcttgct

atctatcttctttcc

>fava_dn_rep_c613

aaaaaaaaacagctgaattgatccctaacagataaaaaacctagtactctttcatagcaa

cttatgaacctctcaaaacagatagagtgcaaccctcatcctagtagaaaaaacccaaca

ctttaccaccggcattctttctcctagcttcttcgtgatccatgataccagcagaggtag

tcaagacaatatatccaaactgtcttgaaggaagaagcctagcagtccatccctcaatct

ctttaacaccaacatcaaaacgaggactaataacaccgcacttgttcaatcttccattca

attccacaacaattttgccagaccggtgatcatcaacgtactcaaactccccaatataac

catgcttttgcatgaccataaggaactttataatcactttcgaagacggcctaatcataa

cttgacgcttcccacgtttttcagcattgtacatgctcttgagagcatcattcaagacac

taaccctcaccatatttgcagagaaactgagtggtgctgcagctacacaaactctctctg

cgttgaggtgaaatcggtggcagggttagggtttctgtcccgcggccgtaatgg

>fava_dn_rep_c614

atacgtgatacatttccaaaacctacctacaccacaaactaaataaataaaaacgcccat

ttcattgctaattctctaaaatcaaaaagatttgatttttctatctgggtcttttgtttt

gttggtgaatctgatcagagaaagtaaagggccatgtcgtgttcgtcttcatcgggatcg

gatgatgataatgaagggtttgagtcttatcggaaaggtggttaccacgcagagtagcca

ttacggccgcgtgattcagaagcttgtgttgttgtttgtgtcttaaatcatcttttgttg

tttgtgtttcgtcgatttcatcctctgaaaatgtcttgctgtggtggaaactgtggttgc

ggaagtagctgcaagtgcggctctggctgcggcgggtgcaagatgtatgctgatttgagc

tacacagaatcaacaacttctgagacacttatcatgggagttggatctgagaaggctcaa

tatgagagtgctgagatgggagctgagaatgatgggtgcaaatgtggagcaaactgcacc

tgcaacccatgtacctgcaaatgaagtggtgacagtgaaagttaccccgtttagtagcag

gaaaaataataataataataagtttatgattatgtagtagtatgttttatgtgaactctg

gtgtttggtttaaatatcaaatacccttttggagttatataataaaataaagttgtttta

tgtttaaatctttgagcaaaatctctatggttttggatttgtgatgggtgtattttaacc

gttgtttctttgtttgattatgtttggttttttctctttagtgggatagaagcttttagt

aatgactgaattatttggatattgctcacaaaactagattcatttaatcattatgtgtga

tt

>fava_dn_rep_c615

ccgcggggatatcaatctcaccataaattcatttttcaatatatatccatttacaactac

accatggctgaagaggagcaccgtagccgcggcctcttccaccacaaaaaggatgaagat

aagagacctacaggtgatgactacgactccggtaattacagaaaaccatcacacaatgat

gagttttctagtggtgacaatgaatctagttacgaaaaatcatcctatggtaatgaaaat

tctggtggtgactatgaaactggctataataaaacaacaactaattatgaaacatctgat

aactatggtggtactggtagtggatactctgacacccaagcaaccaccggtggtggctat

ggtggctctgacaccagaaaaactaccggtggtggttacggtggtggtgactatggtgac

tctgataccagaaaaactactggtggtggctacggtggtggtggctatgatgactctgac

gccagaaaaactaccggcggcggctacggtggaggtggctatggtgactctgacatcaac

acaactactggtggtggttatggcggtggttatggtgactctgacaccaaaacaaccacc

ggtggtggctatggtggtggttatggcgaggatacaaataatcgtgaagatgaagttgat

tacaagaaggaggagaaacatcacaagaaacttgagcatcttggtgagtttggtgctgta

gctgctggaggctatgctttgtatgagaagcataagtcagagaaggatcctgagaacgct

cacaggcacaagatagaagaagaggttgcagcagcagctgcagtgggatctggtggattt

gcctttcatgaacatcatgagaaaaaagaaacaaaggaggaagaggaagaagctcatgga

aagaagaaacaccatttctttggctgaacataggtgtatataatataaatacaatataat

aataagcatacaagcattatggagatattcattttgcttagctttatcctatgcttttgg

gttatttatgtgtatttattgctattcagttcatctaattgcactttaatttgagtggtt

tggagaatgtaatttccagtttggtatcatatatggatctgtatgttgtgtcggtacaca

atatttatagtgttgtaaaaggatgtgagatgatttcaataaaattattagttctaccat

gaaaaaaaaaacaaaggcaaatcaaacccactgatcattctcaccgcagaaaaccgcaac

acatataaaccctaacatttatcaacatcttccataactaaacccttcagagattcgaga

aagaataagaagaagtttttgaatcatagataagagacaaacaaagccagaatcagaaag

atctcactcaaaaacaacgaaggaagtcaaatatgagcgctagggtttctgaaaccccct

tcaaggtctaggcgaaatcgttcagtttctccgccgtcgctgccgctttcagacgatttc

tggcatggcagatgcttcttctccggcaggctctattttagaaaacattgagggaaactg

agagatctgaagaaaggagagtgagacaagagtctcttctaatcataatctctcttactc

tcttaattttttaaaatcaattttnnnnnnnnnnnnaatctggcggtgacatttgtcacc

nnnnnnnnnnnnnctgtttggttctacgtaggtatctcttcctaagcaaccatgctccct

cgcttcatcaccatcagat

>fava_dn_rep_c616

ggccgcgggcatggactacacacacttttgcaccaccactaaagtcatggccaccaccac

agcagccctctcaggaacaatggcgagtgcctctttcatccgaaggcagccgattaacac

aagcttaacagcattccctaacacgggtcaggctctattcggtttgaaagccggacgcgg

aagtcgtatcactgccatggccacttacaaaatcaagctcatcacccctgaaggaactaa

agaaatttcctgcccagataatgagtatattcttgacgcggccgaggaacaaggccttga

tcttccatactcgtgcagggccggtgcttgttcttcgtgtgctggaaaagttacacaagg

ggaagtggataattcagataatagcttccttgatgatgatcagttagcagctgggtttgt

tctcacatgtgtcgcttaccctcgttcggatattgttattgagacacacaaggaggaaga

acttgtttcttaaacatgatatttgtgtgtttttttcttctataaattattgtttttgtg

gattgttactatgttaactttatctagctgttctgtcatctgtttttgtttccgttgctt

tgtgaagaatgggttagttacactgatgaatattaagtgagttgctttaaatctctttgt

ttttctttctcttcttccctcttcgtattgatcaaggcattgaatacgtattgattttga

cagtaatgttggagttttttatgcatagattttggcagtggttcaaccattcaaagctcg

tacgtagtttatttgtgtaactagctctgaaataaacatgttaatattcactaattataa

gactttttttc

>fava_dn_rep_c617

ccgctatagctttaagttcaccaaacatcactttgcattccgacccaaatccctcacctg

tgttgcagccgcctgcctaacaactctttcttgcatcaatggcggaaactgccgacaaac

ctcaaactctcgcagagcaatactatgtgaaggacaaacaagacaaagtagaggtagttg

aaaaacctgttgctgtcgaggaagttgaaaagccaaaggaggctacttccgaggaagctg

ttgttgagaaagctgaagaaaacactctagagccaagcagtgaaactgttgtcggagaaa

acactctagagccaagcagtgaaactgttgtcgatcaaagcactctagagccaagcagtg

aaactgttgtcgaacaaagcaatctagagccaagcagtgaaactgttgtcgaacaaagtt

ctggtgatcaagaggaagttgaagagaaacctgaaattaagctagagactgctccagtag

atttccgttttccaactactaatcaaacaaggcattgctttactcgatacattgaatatc

atcgatgtgtagctgcaaagggagatgatgcaccagaatgtgataagtttgccaagtatt

atcgtgctctttgccctggcgaatgggttgacagatggaatgagcaacgcgagaatggaa

cattcccaggcccactgtagtgtggcatatgtctgagataaatgcacaattgacttgcag

tattacatttaaaattgcaagagacaaacttccaaacacttcagggaatgttcaatgtta

cgcattgagttaataagaaattttgcttttccatttttaaaccagctttagttgtaaaat

tgcaggaaatgcttctggctaaatgtccagatatctgtcttgttcattactggattcgta

actgagggattgatgtctgaggtttttattcccaaaataataatttttttttaagatcaa

tgttttatcacccaaaataatattgtcttgactcg

>fava_dn_rep_c618

ggccgcgggatctaactgctctcatctccgttcgcatccgttcgcagtcttcgccgccgg

ttgaacgagatttacaaagggaaaatgagtcgggctagtgcagctggtgccaagggaaag

aagaagggtgcaaccttcaccattgactgtgctaagccagttgaagacaagatcatggac

attgcttcccttgagaagtttcttcaagagaggattaaggtcggtggcaaagccggtgct

cttggtgattctgttactgtttccagggagaaatcaaagatcactgttacctctgacagc

aacttctccaagcgttacttgaagtatttgacaaagaaatacttgaagaaacataacgta

agagattggctaagggtcattgcttcaaacaaagataggaatatctatgagttgaggtat

ttcaacattgcagaaaacgaaggagaggaagaggattaattgtatgttattttcttttgg

caagtgcaatagtattgtttcagatatgatgcattgcttagagtttgtgtaatttaatga

ttttgtcactctttaagaattcataattcagttttctgctttaatacagttcttgtggta

ggattcattatgtttattttttaattatgctgcacacttgaggacccttgtttgttaggg

ttgtccagttggaaagaccaaccaagccaggttcggttgacacgggtcactcatccattg

tcattattagtatgcaatatttttaactctttttgatattattccaatttttttcttctt

attatgctttctgaaatcagaatttaaagaacattgataaagattaatgaatattgaaac

gaatatatttttgc

>fava_dn_rep_c619

aacttaataaccaccactttaaccacaactatactaatttatataaaccaaatgtctttt

tcaaagaaaaaccaagatcaactgcatagaagttgattatataaaccgaatatctttttg

aagaaaaacctagatcaactgcataaaagtggtttactgaagaaaggaaatctggttcct

tgtgtagttgagaactttcgattgcgaaattagtcctgctggtgaaaataacaaaccttc

attgagtaaacaaacacataaaatattgcaataacatgcataaaccaatttgttgagaag

taaacgggataagagatgctcggatcccatatctcagatcagaaatataatttgggattt

aaatctcccaaaccaaataaatatttcgacaatttatccttgaagtttttttttgataaa

tcacaaccgaaatttctaaacacaaacaagtacaattaagcatcaaacacaaccaaccaa

caaaatgaactcattttcaatctgaaacagttataaagcaaacaatttcaatggcctttc

aagtcatatatttgatggtctatcttcaagcatagttcaaacggaatatgtcattcagca

cataatagcttccttgctgtgttggtatcaaatggaacatctgactgaatttgagagcgt

gttgttcaccgccgagttgaaggttaccgctgacgaagacaagcatgccggtgttgacag

tggatggttggcaatcgacggtggtgatggagtgatggcactgctggaaagggagagaag

tgagcttggcgacgatgttgggggaacctagaatcttctgaccttcgaaggtaagcatgg

atccatcttggtaaagagtggcaagtccggcacggttgttgtcgaaggtggtgtagtagt

gctccacaaatgcctttgccaatgcgtctggatccatttctctctctcctctatattact

ctctactctctctacgctctctacgctgccgtaatggccactctgcgtttcttcaagcat

agttcaaacggaatatgtcattcagtacataatagcttccttgctgtgttggtatcaaat

ggaacatctgactg

>fava_dn_rep_c620

aatttcaaaaactccgtattgaggaataatcatttttacttagaaaaatcaaactccatt

tacannnnnnnnnnnnnnnnnnnnnnnnnnnatactaatccaaacatacactaaactagg

gggcagatacgataagataagtgatagtgttgtctattttttttaggaagcaaagtccgg

tcatgttgatttttttcccaactatgtttagaaatctataagcaaatcggttgtaaaaca

acctgcttggctgctgtattgaaaattttgcagaccaaaccagggccacataaagataga

ccagactaacaaaataagaaaattgcccaacaataaagcaaaaggaaaacaactgaataa

gcactcctttaaaaatgaacattatccagtagcatagatcaagcaccatggatgccgcaa

aaagattcttattttcgaaaactatcctatattaagccttctaaaagggcagctctttag

taacaacctcaatcaaaccaacattggttgaaaccccaaccattataataaaacacagaa

tacaacatgtttaagcatcatcaagagcaaggactccaggtagtggcttcccctcaagaa

gctctaagctggcacctccgccagtagagatgtgactcatcttgtctgctagtccagcct

tctccacagcagctacagagtcacctcctccaatgatggttgttactcctttgccactta

tttctgccagtttcttggctatggcctctgttcctgctgcaaacttatcaaactcaaaaa

cacccattggtccattccaaatgacggtttgactcttgtccaatgcttcgttaaatgtta

cgatggaatcaggtccaatatccaatcccatccatccgtcagggatgcttgatgctggta

caatcttggagttagcatcagcggaaaacttgtcggcaattaccacatcagttggaagca

aaagggaaactccgttagccttggctgtctcaataagtgaagttgcaagttcaagcttgt

cttcctccacaagagaggacccaactgaataaccttgagctttgtaaaaggtaaaaacca

ttcctccaccaagcaaaagaatgttaaccttcctcaacaaggattcaataactccaatct

tggaagacacctttgaaccaccaacaatggcagtaaacggtttcttgggatttgacacag

ctccaactagataatcaagctccttctgcataaggaatcctgcaacagagggcttcaaat

atttagcaacaccttccgtagaagcatgagcacgatgggcagtgccaaatgcatcattca

cataaagatcagcaagggaagccaactgctttgcaaactcaggatcatttttctcctctt

ccttgtggaacctcacattctctaaaagcaaaacaccaccttcaggaatttgtgcaacca

acttctcaacctcctcaccaatactgtcctcagcaatcttaacctcggttccaagaagtt

cagacaaccttggtacaagaggtttcaaactgtatttaggtgttacaccctttggacgtc

ccagatgggaagagaggatgactttagcgccatagccagtcaggtacttgattgtaggaa

cagcagcacggattctggtatcatcagtgatgttcaggttatcatccaaaggaacgttca

gatcaaccctaacgaaaaccctctttcccttcaaatcggcttccttcaatgttccaacgc

ttctctttgtcgccattttgttctacttttcacaaacgattgatggttgagatcggagtt

cgaagacggagtgtgagaaggagggttctggaagatttatagagagaaatgagactcgtg

cgaatcgcacgtgtgagatggtgtttgtgttttccttgtttgggaagcgctttttcttcc

cgcg

>fava_dn_rep_c621

actcaccacgctccgcggctaatcgcagttacttctgcgcagtctcattttcgttcgatt

agggttttttctcaacaagttctctcccgttttcaaccgcaatgagggccaagtggaaga

agaagcgtatgaggaggttgaagaggaagcgccgaaagatgagacagagatccaagtaga

ttgcgtcttcagccatcgtcgtctgctcttagttttgtttttccctttcgtgttgttttt

attttgttatttaataaaactaccattttctatattttatttattgtattttcattattt

gtatgaaatattgaaatgcaacaaatagtatgcaacatagcaataaaatgtgttgcaatt

gcgtgtgacaatgtgccactcaaaaactggattcctcagttgaaggccgtgtcaagaagt

atctcaagctgttaacaagaacctgttgaagaggcttgataatggctttacccttatagc

tgacatgaaactttgccttagccaacagaccctcagtgtcgaattctccggattcaacag

caatgttaatgtcagtaataccctttacaagatcaaccaataatccaggccgatcggctg

tctccacatagaacaaacttctctctggaccgtcatcagtgattattaagtgagttgcta

tgtccacatctatctgttcctttggaggtacaagtccaaaagccgctcctagagctaatt

gggcacttgattccgggtgatacgtaatcatgttatttaaaattgttaaacggattgctt

ctaacaactctggatcttccactttcctacctgtatcagcttttgtgattgaaaacttgt

tgtgcttgccagaggaatcaagatacacatttgccttaacaacattcagtcccaagtttt

tcaatgcactcattgtgtcaagaagagcgccaaggcgatcaccaaaggtaatctcaacga

cagttgcgtccggatcagaatcttgatcaattataacaacaggagttgggacaatatcag

ccacactttgatttccatcctcgacattagaaacagccgtggctcgacggatgattgtaa

tagctgaagataataatcttgttttgtgaagagtgagacatggtttattagtattatcat

acaacgaaccggtcttcaagataggggcaaagcaactactattattatcagaagtgaggt

gaagtccaacactacaagcagccatagccacagccattattgaaagagttgaaggataaa

gagatttttgagagcgaagaatgaatgaatgaatgaatgaatttatctataaaatggaaa

taaatctgtgacgttcacttctaatcttgtacaactaacaaaagagt

>fava_dn_rep_c622

aaagaaggcnnnnnnnnnnnnnnnnnnnnnnnnnnnnnnnnnnnnnaatgaaattaccaa

gaatgcatttaagctaaagatactattatatagaatataagaaaccaataaaccataaaa

aagacaaacaaaattgtatcacaatggaatagttgacaatgttttaacaattgctcagta

atgagttcaaagaagcaaaacacaattaatattaaaaaaaactaataaaaatgtgattta

ctataatcacacgaaactaagattcctccaaaaagatctttatctcatcatgtggtccaa

aatcatgcctttgaggcctgcttctcttttgtcttttgaattttaaggaccttcttcacg

attttttgctcttcaaccaagaaagctctaatgatcctttccctaacagcacctcccgac

aaaactccaccatatgcacggttcacagtcctacggtttctagacaaccttgatctcttg

tactcagtgggcctcaagtgtggaattccttgaattctcttgccagtaacagggcactta

ggtccactagccctcttcttcgtggtctgataaacaagcttaccaccgggggttttgaca

accctgtgctggttagattttgttgcatagctgtgacgacggcggtaggtgagacgctgc

accatcttcgatcaagctctgcctcaccgtttggtttactagggttttcagaggaagtga

atgatgcgccgtaatgaaatataagagagcgattcgattt

>fava_dn_rep_c623

ttaatggcttctaggattctgctgaattccattttgtcttcattcttgacagtggtcaag

cacaaaacagctgcagttttcttgtgaactattgttccaaggcgtgctttgcccttgaca

atgcagtagggaatttccatcttcctgcacaatgctgggagccatacaacaagctcaatt

gggtcgacatcgtgagcaatcacgacaagttgtgccttgttctgctcaataagataggta

acatggttgagaccgtatttcacattaataggcttctttgtctcgactgttttgccctca

gattcagcttgagcccttttcaatagacgctctttcttctcagctttgtcctcaggccta

tacttaaggagcatcttaaataggtttgtggcaaggtttttatcgagtgtcttggtgaac

tggttgagtgcaggtggaaccttcaacctctgcttgaggatcctcttcttcctctggatt

tgaacagtcttaggccatttcacaaacctcgtcaaatctctctttggaggcaaggcacca

ccgatcccaaattgcttaggcctcttctcgaaaagcgggttcaccactttctctggctgc

ttcttcttagcagctgcaactggtgcttttccaccgcgctttggtcccattgctgctgtt

gttgatggaagtggctgattacgaagaaagagtaccgaaaggagaa

>fava_dn_rep_c624

attatggtcatttcaaaaatctatacaaacgaacacattttcacttctcctttttcgtta

tccttttttccccaaaatctccggaaaatggcagcgctatcaagccaccacctattctcc

actttcactctctttaactcgcccaaaacgaccaaaacaaactcaatttctcgaactagc

aacacaagttcaattcgatgcgctgattcgagcacaagcacgagcacaatcgagaatcag

cgagtcacagtgagtaacgggaacgattcattggaaatatgtagagttcttaacggaatg

tggcagacgagcggtggttggggaagaatcgacagagatgacgccgttgaggctatgctc

cgctacgctgacgccggcttatctaccttcgacatggccgatcattatgggccagctgaa

gatctatatgggattttcatcaatcgagttcggcgtgagcgtccaccagaatatttggaa

aaggtcagaggtctcactaagtgggtgccacctccagttaagatgacaagtagtttcgta

agagacaacattaatgtgtcaaggaagagaatggatgtggcttccttggacatgcttcag

ttccattggtgggattattcaaattcaggatatcttgatgcactaaaacacctgacagac

ttgaaaggagaaggtaaaatcgagactttggctctaaccaattttgataccgagagatta

caaattattcttgaaaatgatattcccattgtgagcaatcaggttcaacattcacttgtt

gatatgcgtccccaacagaaaatggcagagctttgtcagcttacaggagtcaaactaata

acgtatgggacagtaatgggtggtctattgtctgagaagttccttgatgccaacatcaac

attccttttgctggacctgcaataaacactccttccctccaaaaatacaaaaggatggta

gatgcttggggagggtggggtttattccaaggacttcttcgaactcttaaacaagtagct

actaagcatggcgtctctattgcaaccgttgctgttaagtatatactggaccagcctgct

gtggcaggatctatggttggtgtaagacttggcttgtcagaacatattcaagactgcaat

gctatattttcgcttgttcttgatgaagacgatgtgagcagcataagagaagcatcaggg

aaaggaaagaatcttcttaaagtgattggtgattgtggagatgagtacaggggtgcatga

gtcttgctcattgcaatgtcaacttgatgattgttttcacttatataggacaagggaggg

tgaaaatataaaacaagggaaattattcactcccaattttgtctcagtagaagacttgta

tgtaatactttcagatgttgctttgtactggtcaaaacacaaaagaaaggaaaacattgt

tttaactaattaaattaataacttttatttctgataaattaatttttctatggtattctt

gtttgtt

>fava_dn_rep_c625

aatttgggggactttgtagcaaatataatgattttaggttagagcaataacttgttatac

gacccttgttcattctttcaacacaaacaacgacttgaattattcattcatcagaacaaa

ccctcacagatcgattgagtgagaaagaagaaaacaacaacaatggagtgcgttttcgga

ttagtcggtaacggcttcgctattgtggtggctgatacatccgcagttcacagcatcctc

gttcacaagtccaacgaagacaagatcatgctcctcgattcacacaaacttgtcgccgct

agtggcgaacccggtgacagggttcaatttactgagtatattcagaagaacgttgctttg

tatcagttccgtaatgggatccctctcaccaccgccgctgccgctaatttcacccgtggt

gagctcgctacagctctccgcaagaacccctactctgtgaacatccttcttgctggttat

gacaaagagacagggccatcactatactacattgactacattgcaacacttcacaagctt

gaaaagggagcttttggttatggttcctatttttcactatcaatgatggacagacacttc

catagtggaatgtctgtggaagaagcaattgatctcgttgataagtgcattgtggaaatc

agatcaaggttggttgtcgcacctccaaactttgttatcaaaattgttgataaggacggg

gcaagagagtatgcatggcgccaatcagtccaggacacacctgttccttcagcttgagtt

aaatcttgttccttgtcttgattgaacttattttcttatgcatgtgcttttgatatgtca

ctttctctaaatgattttgagatatctctgaaaatcacggtctctaaatagtgaaaagta

agacattttgttgtttgaatgactagttctacttagatttgtggcaatgcatgagattag

cttaaaatttaatttgaaaagtgttaatacgatccttaaaatttaatttgaaatgtgtta

atgaaccaattct

>fava_dn_rep_c626

taataacttttttttttgccaacaactgcccttgaaacattgttccaagacaattttctt

ttattaaggaatgcccttgaagcacttgttagcacccatacgaaagtaatcttcactttg

aattagacaaagtatgaccaactgacagaaccaactagagaaacagaattacaaaatcta

gacatgacatcaaccagatcagtctaacagctgaatctaactatgatattaccaatcagg

tcagtctaacagctgaatctagctatcatattattttttaacaaatattaaggtggaagc

ccaaaactaaaaagactacaaagaaataaaacgagttgacacttcccttacatgtatatt

ctagtttataacgacaatagcaatagtagcttatatttttacatatcccagcaaagcaac

agtttcttctgcattacctttgggaagtgaatgtgctcaggtatgtaggtaatttgcagc

catcattaactccagagttaattccggttcaataggaaactctctgtcctttccactggc

gaattcaagatgccaataaaaatactgacaaatcttctcaagaatagtggtactgatctc

aggaaaagtcacttctccgtgctggctttcagcaaaactccctggagaagtaagcatgtt

gcgaatggtttgagaaaccatggcagcttctttgtcaaccacgaattcgaaaccctcgga

actgatcagtttcactgtttcttctttcttcatcttttcctttttcgtttcgtcgatcgc

ttctggtgactcgaaaatctcaaatcccgattacaaacggttgttgttgaatcggaaaac

ggctgggtcccacttataacccgtttatctatatgtatat

>fava_dn_rep_c627

atcccacccaaaagaatctcctttgaatgataaccatctctttccacacattgaaactca

aaacaacactaacttaaaagataaggcaaataagtgtttccatgtacgtggcatgacaaa

aacccaataacccgattctcatccgacgtggcatcatgtaaccaataaacaatgactcca

ttatagaaagaagttattcataatttgaggttttgcgcgaccgccatggctacagcttcc

gcttctgcgtttcgcttactaccttcttccttccaatccaaaagccacagtaaccatcac

ccaacccccaaatcattcaacctccgcttccctcgccgtcacaacaccctatgcgtttcc

atctccaacaccaatgtccgaacaaaccctaacgatctagtagcttcccttctttcaaag

gtggtgcaaacagatggaggagttttactgcaaaaggaacaacataaagaggtggcggaa

gctgttgaggaattgcagaagtattgtgtgaaagagccagtgaaatgtcctttaatattt

ggagattgggatgttgtctactgttcccaacctacatctcctggaggtgggtataggagt

gcaataggcagacttttcttcaaaaccaaggaaatgatccaggttgttgaagctcctgat

attgtgagaaacaagatctcatttactgctttaggattttttgatggacaggtttctcta

aaaggaaaactcaagcctctggatactgaatggattcaagttgtatttgaagcgcctgaa

ttgaagttaggatcatggaaagcacaatatggaggtcagagtgaagttaaactccgaatt

acttatgtagatgaaaaaatcaggttaggattgggatcaagaggttccctctttgttttt

caaagaatatgaacttgaatttaaatcaaagttcccttcaagaaaattctgccaagtttg

taccttgccactctgcacaagttcatgtataatatgattacttaaaaagaaaaaagaaac

tttccaagacagtattatctcttggctacagagtttgtctcaaaatcactaaggtgttat

attgaagttacagtgtagagaaaccggctttccgtagagacaatctttgaagtatgctgt

gttttcttatggttcaagtaatcagaatattttgaccaacaagtttagagtatgtacaat

ggtttacatattcaacactcttctttttcattttttatattccacatcatattctgtttt

ttccacctaataattcaacattcattcaacttttattcactacaatgattttgtttaata

aaattcagcactctacccatt

>fava_dn_rep_c628

aatttaaaactccacatcactctcttcactcaaagtaagaaaaaaatctagatctgaaat

aatacaaattcgcatctctaattcccgttattctattattccgatttggtgaatcgcatg

aaacccgacccgattcaatttctctgaattccgatcaaatcctccagatccgtcgttttc

ctccgattagggttagggttcaccagaaaaaatggcggtctccgatggcgtcgtcaagaa

agtgatcctctcgtacacctacgtagcgatatggatcttcctcagcttcactgttatcgt

gtacaacaagtacatcttggataagaagatgtacaattggtcttatccgatctcgttaac

catgattcacatggcgttttgttcgtcactggcgtatcttcttgttcgcgtgtttaagct

ggttgaaccggtttcgatgtcacgcgatctgtatctgaaatcggttgttccgatcggtgc

gctgtattctctttcgctttggttttcgaattccgcttatatttatctctctgtttcgtt

tattcagatgctgaaagcgcttatgcctgttgctgtttattcgattggagttttgcttaa

gaaggagggttttaagaatgagactatggcgaatatgatttcgatttcgctcggggttgc

tgttgcggcttatggtgaagctaagtttgatacttggggtgttacgttgcagcttatggc

tgttgcttttgaggctacgcgtttggttttgattcagattttgcttaattctaaggggat

ttcgttgaatcccattacgtcactttattacattgctccttgttgtttggtgtttttgtc

tgttccttggttgattgttgagtatccttctttgagggataactcgagttttcatttgga

tttcgcgatttttgggaccaattcgctttgtgcttttgctttgaatcttgctgtgttttt

gctggttgggaagacttctgctttgactatgaatgttgctggggttgttaaggattggct

gttgattgcgttttcgtggtctgtgattaaggatactgttactccggttaatttgattgg

ttatgggttggcgtttttgggggttgcgtattataatcattccaagttgcaggcgcttaa

ggcttcggagacgcagaagaaggctcttcagaatgatgaagaggctggaaggttgttgga

acagagagacggggaagggactggaaggaagatcgacagccagaactgattcagaactga

ttcagcttgtctttctcatttttggatgcaaaattctgtctgttggatgaggcgcgtttt

gcttttgggtatatgcggtggtgtaatttgctacttttaggtaattatttaattttactc

ggattatcatcagtattattatgtcattttatttcatttcagcttatttcattcatattt

tagttgtgtatcaggtctgagattggcgtctgtgtcttatatatgcttattcagtgtttc

gtattaatgatcagttaatgaatttagtttccttgatgctctagaaagcttgttatcaaa

ttgctgtttttctttctcaag

>fava_dn_rep_c629

tgatgatttagtttttattaggcaatttgatgttatagtnnnnnnnnnnnnnnnnnnnnn

nnnnnnnnnnnnnnnnnnnnnnnnnnnnnnnnnnnnnnnnnnnnnnnnnnnnnnnnnnnn

nnnntttacacataatgttaattgcatttttctcagtttaatttttaaccagtaacacaa

aagaaatacttattccaagccatactctcacttttcttttactacaaaagaatatgctat

tgtgtagagaaaaaatatgaagtacatggaatataaatttagccattttttgttgaagtt

tgaaagacaaataatcagattcatcagaaagttcaattgatggaagcttattctcttgtt

attataaacaagggagtagcattttttgagccacattttgcacaaattgcaagaaaagtg

ccacagataacctacctataacccacacatttgaaaatggaaaaattgagcagcaatata

gtaaggacccctcaaattaaagtattaaaattggaagtcggaatcatcccattgaagaac

agcagaatcaatattctgttcccttttgagcctgtcttttaggaaccaatcacatacctt

tccagcattgagctcctctattggacaaccttcttgttctgataccacttttagtaaatt

caatgaagatatcgttaaccggcatagaaaacgctcatgagtgctcaacccagacatctc

aaccaaaccagcttcttcaagtgtcaaaaatgttctcctctcacttttcttttctcctgc

actgcatttgatttctcttctatgtactttaatcttcctctgtgctatcaggcttgtttg

tgtttgtctatgttgatttataccaacgtttgaaggcttgaacaaaattgttgtttgaag

acaaaatttataaggttgtagagaaatcataatttccttgctgagaagaatatatcaatg

caattgggacttcccgcggcc

>fava_dn_rep_c630

ggccgcggggagcatattctcattcacttttcaattctattctttcgccacacgccgctt

gtttgttcccacagctttctccgatggcctccttcgatgaagctcctcccggaaacccca

ccgccggcgacaagatcttcagaactaagtgcgctcagtgccacacagtcgacaaaggcg

ccggccacaaacaaggacccaatctgaatggtttgtttggaagacaatctgggacaactt

ctggatattcctattccgcggctaacaagaacaaggctgtaaactgggatgagaagaccc

tgtatgattacttgcttaaccccaagaagtacatcccagggacaaagatggtgtttcctg

gtcttaagaagcctcaggatcgtgctgatcttattgcgtatctgaagcaggccacttcat

cagaatagaattttcgtatgattttcaaacatggagttttaattttcgcacatttgattg

tgtggtgcaaaccacttctcttggcaaaaataaagcagacttgattagtttgcagtgcta

ttttgttccttttgcattgcttctttagattcacaaagattggctggactaattttaaga

cattggctgttgaaagcccaaacattcttatgaaattttacttaaattgaaagcatattt

ttgatttcacttgtttcacaagtagaagcaatactcttgtgttttctttttttactttag

tttgcttaattgaatgcaacataatgcaatttctttccgaaaaaa

>fava_dn_rep_c631

gccgcaggaggacaacacaaaaaagtggtggaatcctaggatttcaaccacacatctctc

atcacacaattccacttcactgctctatcaccaaacaaccaaccactagcaactaacaat

ggcaatggagatcactgctatggcttcaccaacaacaagaccctctcttccaactccttc

caccttcaccaagcctcaacatagacgcctccacaacatatcgttaccaacatcaaccac

catttcactactcgctcttttcacccctccaaatgaagctagagctgctgttacaatctc

caaggaccaaattgtttcttctatcacccaagtggagcagactattgatcgggttcaggt

ggtaggttctggttttttggacacagcacagcgtgttgctggagctattgggaatgtttt

gaagccggcgttgccaattgtgcagcaggctggtcaagaggctctgaaaattgcttcacc

ggctttctctgaggcttccaggaaggctcaagaagcacttcaaagttctggtgttgatac

ccaacctgttctcactgctgctaagacagtggcagatgctgcacaacaaacaaccaaagt

gattgaaggtgccaagccaatagcctcatcaaccttggacactataacttcttcagatcc

cactgtgattgctggaacagctggagcactatttgttgcatatctcttgtttcctcctat

ctggtctgtcatctcctccaaccttcgcggttacaaaggagacttgacgccagctcaaac

actagatatgttatgtacacaaaactacatcttggttgatgtaaggacggagaaagataa

ggacaagaccggtattccccgccttccgtctagtgctaaaaacaagatggttgccattcc

attggaagacatgccaagcaagataaaaggactagtgaggaatgtgaagagagtggaagc

tgaaatagcagcattaaagatttcatatctaaagaaaatcaacaaaggctccaacattgt

gatcttggactcgtactcagactcagcaaagatagttgcaagaacgctaacgaggcttgg

atttaagaactcatggaccgttggtgatggattttctggaggcaaagggtggttacagag

tagattaggtacagattcgtataaattttcgtttgcagaggtgttgtcaccatccagaat

catccctgcaggtgttaaaagttttggaacaaccagcaggcaatctagtcagaaacttct

tccaggagctgactgagattgtttttcctcactcaatgctgaactctcaaatatatatgt

gccttgattaatgaagtccttcaaattatattcatttgattaaatctctcttttaacctc

ttcctcttttaacctctttcaatttagattttgtgtgggagaacaatgacaactagataa

gtggttttaagtaataaaataataataataatagactagaatacaagaaatttgtgtatc

cagtttgagattacatcaagaggagagaacaatttttaaatttattgtatttcataagtt

gttataagatattacaatatgagttacaagc

>fava_dn_rep_c632

acatgacccccttaatgaaaaggttggtttgagggtgagaattaggccacgctagaaagc

caatcctgccttgttggcttctctaagcatacagatttcatttcatccacccacacaaac

aacacaaccaaagtaactccaatctcacatcacattctctagtatctctatctcatatct

cccacccaaaagcaaaaacaacaaacaacaacatgttaccactttgttcttcctcttcca

tggctgctaacccttccctcactctcccaaccaattctttcctcacattactccaccctc

ctccatcttctcgcaacacatcatcctcatcatcatcatcactttcttcaacctcattca

actctcagtttttaggcctcaagttatctcattcttccatttccataaccccttctccac

tttcatcttcttcactcaaaggagcctttgtttttgccaaggttagtaaaggctcgaagc

caccgggttttacgctgaaagatcaggatggtaagactgtgagtctctctaagtacaaag

ggaagcctgttgttgtttatttctaccctgctgatgagacccctggttgtaccaaacagg

cctgtgcttttagggattcatatgagaagttcaagaaagcaggagcggaggttgttggga

taagtggcgacgatactgcgtctcacaaggcatttgccaagaagtacaaacttccatata

cactgttgagtgatgagggtaacaaggtaaggaaagaatggggagttccaggcgatttct

ttggttcattgccagagagagagacttacgttattgacaaaaatggcgcggttcagctcg

tttacaacaaccagttccaacccgaaaaacgcatcgatgagaccttgaaactacttcaaa

gcctttaaagcttctgtatcccttctgtatgaaaccgttgtaagtaagtagctgattttg

aacactttggttacatgataatgttaaaaatactactctttcttctatagagagcaaaca

attgtaacgtatcgcgatgaaaatgttaccttcgattgtcacaaacactgtcacatatca

gtatcttttacatatcgtaagttcgagtttgctttgtgaaataacaaagttcagcaattt

atatgatttatttaattaacatggctataaaccatgaaagaaaaatg

>fava_dn_rep_c633

acacattttcttcaccttatctccgttcttcttttctccaatttttccttttttttcttt

ttcttaacactccaaattaaacaaccataactgaaatttcctctattacactcaccacca

caatgctcaccgcaaccaaagtagctttcacattaacaccccctcgtccatgttctacac

ctcccccaacacctttcgttcctatctcttcttctctttctcgtcctcacctcattcact

tcaattcccgtcgattatgccttcgccggagactttttctactttctcccaaagccaccg

cagatcaacaagggaaggttgaagaatttgaaggagaagctgttgatagtaatgtccttc

cgtattgtagcatagataaaaaagaaaagaaatccgtcggtgaactcgagcaagagtttc

ttcaagcgctccaatcattctattatgagggaaaggcgattatgtcgaatgaagaatttg

ataatctcaaggaagaactcatgtgggaaggaagcagtgttgttatgctaagttcggatg

aacagaaatttcttgaagcttctatggcgtatgtgtctggaaatccaattttgaatgaca

aagagtatgatgacttgaaaatgaggctaaagagggaagggagtgaaattgttgttgagg

gtccaaggtgcagtcttcgaagtaaaaaggtttacagtgacctttctgttgactatttaa

agatgtttctgctgaatgtcccagccactgtgattgcattaggattgttcttcttccttg

atgatctgactggatttgaaatcacttacttgctagagcttccggagccttttagtttca

ttttcacttggtttgcggctgttcctctcattgtatatctagctctatcactaacaaggg

ccattataaaagattttgtaattctaaagggtccctgtccgaactgcggaactgaaaata

cctccttctttgggactatactgtcaatttcaagtggtggttccaacaacaaagtcaaat

gctctgaatgtgcaactgaattggtgtatgattcaactacaagattgattacattgccag

aaggaagtaaataagtaaaagcctaagtggaggtatcctggaactatatccattgttcaa

gcaatgggagctcatcaataggtgttactaagtgacaaatttatccaagagaaacatgcc

ttattatgtgtcatgttatcactgtcatacataatgtgtgtgatctcaatagtttgaatg

tgttactacttgtaagtaatatggactctgtacagcatacactgcattgttactggaaga

gttatgagggtgcttaacaccaccaatgagtctaacctaaataatgtgttaggagttttg

ttacacgcttttggagaattttccttcctctctgccacggtacatttgcaggaacgtcaa

taacaaataaggaaaattgagtatattcaaattagagaaaagatattttgacacaacaaa

cttacacctacacccagtattacattcacaaatacatgaacagtgtttttactattttaa

aaaaagaacacttactctgaactctttcccggagtttcaaccggaacataaagcaggcaa

gggactcgcacgtggtgacctgagagggaattcgcttacctgcttccctttactatcgta

gtaaatcacgccggagaaatccaacggctcgcacttatctcccatcatgatcgctttctg

ccacacaccgccgtctccccaatcatccttccctttcttcttcttcatcttctcgataaa

cggcatcgctttgttgcttatgttcttcagaagcttcttcggtgactttatctcgattct

ccgattctcgttcgccggcgccgccttcagcttcaacggtaaacgacttgctcgcttcac

aaccatcatcatcagcgttgttaggttgatgataacacggttcgctttgcgcttcggcga

ttctcgggtgattatgtcgaccatggtttcgttaaaacttagtgaggctttcaccccgcg

cgcggnnnnnttgttgtgatagggtgctgcttaattatt

>fava_dn_rep_c634

ttcacattgtataactcatatagttgtcatttgcattctggtatagttgtcatttgcatt

ctggtctggtttattgaaattttaaataacagtggcaaaattttctcatcttttatttta

aagtgttcctggttcactcgagagttttaaataaactacaagcacatgataatgatacaa

acaagatagtattcatagtttatattcccactgccaccatcactttaaacaaacacaagc

tttgaaggttcttgcaggataaaaaccacacctgtatatgcctatacagctacactacag

cttcgaaactggtaaactcagggcttttgaattaacaatgcttgggcatgcagctggagg

ttgagcattttcattgaggaatggttccaccctgttggcatcaaaccataccgggtagcc

tttgatcttccccagcacattaagagcggctagtgtcgccattccctcacgggtccactt

ggaagcagatgcaatgtgcggtaccacaatggcgttcttcaactctgcaagcccgggttt

catgtacggttcttcctcaaacacatcaaggcctacacgaaacaacgggttctgtttcaa

gtgctccactagtgcagcttcatcaataacaggtcctctactgcagtttataagaattgc

ttccttcttcatcttggcgagtctttccttgttgactagatgataagtagttttatccaa

gataggatgaagactaattatatcagcctcctggagaacctcgtccatggacgtcgccct

tttccaagtcaccggtgtttcaccgtttgcctttaagaaatcagcataagctgtgacaaa

tttttcgagtcgcgtagcttggtagagatcaaagtatatcaggttcatcttgaacccttc

aaccatcattcttgcataggctgatccaatacggccagccccgataacaccaactgtttg

tcccttgagcaagtttccaacaaatagatgaggtagccatccatcatatagtcctgccct

catgaactcatctgcctcaactatccttctagcagctgacaacgacagagaagctgcaag

ctctgccgtagtctctgttagcactccaggagtatttccaacagcaacaccgtacttgtt

tgcagcattgacatcaacattgttataaccaacagccatattactgaaagccttgcctcc

agctttgctcaaagcagagaacaactcttctccccagtcttcagtcaactgtccaataac

tccatcacacttatcaccaatcaaggcaatgatatcttcaacagacagaatagttttctt

ctcagtacatatttcaagacgaacgtcttgctgaatgagaagattgatccaacgagttcc

aggcattgatttagtgctaacaactctgtattttccgtttggattccaaacttctattga

aactggtttcgccattgaactagttgaaggagtgagggtggtgaatgatgatgaattttg

agtagatatttgcttttgtggttattgttgtagtaatatataagagaatagattaattta

gatttgaatatccctgttaataattttctaaccaatgaaattgatgtgttgaggttgcac

tgtaggggtgatttggataaagcatgtgatctgggcccttttcatgtaacgtgaggctgt

taggtgtttgaaaggaaaagagatgagtt

>fava_dn_rep_c635

gaataatatacttacataaatcatttccactcttcaatattaattaaatatcacaattaa

ataaattatattatatttccacactcgttatgtgacaaatagaataatataatttttaaa

ccgaaaaactctaaacattgaaaatatgtgttggtagaaaattggaatacaggataaact

ccaagaaagcaagtgtgcaatcatatattaattataaaattaatacaaactgctccactt

attcattaaaccataactaaacctaacgcatgttactaatttcagcaggtgcaacagatt

taacagcagcatcaacaaacacaacagaataaggatcagttacagttaaaatcaaccttg

atccaccttcttcaccaccaaacactttcagtccaagataaccacaatcaccgtcattat

ccaaacaaaacccaagcctataaacacctcgagatccatgtttcgctatcaaaaactttc

cactaacagtttcaacaccaagatagttttcaggaccaccaataccaacacagttcagtt

gagtggtgttatcaacaaacagcaaccattttgatgattcagcgcagttaggtttctgag

tgaactcgatttcaagatcagtaccggttaagatggtaccagtggtgctttctggtatgg

tgaatttgactgggatgccaaggttagcgttgttttggaggacggttagtggacattcag

agtcagagactttgtttagggttagtcctccggtattagggttgtcacttgctggaaaaa

tgaagtattggttgccatgggtaagggggttaccgttagtgtctacaacttgagaaggag

cagcggtgtttgatggaagattggtgatgaaagtcaagagaaggaaggaaaggatgagag

atagaaggggattcattgttatgtgttttggtgccgcggcggggtggagttttcgatagc

ggattctctttttaagcatcggtggataagaagatgcggtgttggtggtggtggtgtcgt

cgttgttgttgttgttgagaaggatggttcttctggtgttgaaggttgttcgtttgggaa

gtggtgttgatggtgggaatggtaaccaccatggttgtaggtgagtgggttttgctgtta

tcaacattgcgatgctattgttcttaattgatgatagtggatattgcttgtgtttacgtc

acgggattttttttttttttccttcttctttcagtttctgtagtgttctagtgggaaatg

ttt

>fava_dn_rep_c636

cgcgggatccacaaactcacaagtcacaaaccaatacctccattgtgagagtttgtgagt

gtgtcttctcttttcttgtgagtaaaacctattccaacaaagacacactaagatatggca

gaaacatgtgataaaatgatcaaagggatacgcaaattgaagcaacacaaagtgagggac

aggagttgccaataaaggatggaacaagagcacagtcagacatttttgaaagtattgagg

ttaatgttcctaaggttggaggaaagaacagtggcaaaccataaacaagatcatggatag

ttcctttattagaattaacatcaccaattttcctacttaaatgaagtctagaaagatttt

gtgcacttgtacaaatctttctcagtgcccttacggctccattttcatatatattttgct

ttttttttggttaaataaggttcaaactttttatcttgtaagattatactcggttccata

aagagtggagaaattgtgcagactaaattgcaaactaaccactgccatcacaaacatgag

aatgatcaaaagaatattaaaccagattcggctcttccattcctgtatttggtttcagca

agtctttctgatccatcattattttcgaccacacaagataagataggagtacaaatgatg

agaacaaatacataaaagaagtaatcatcccattatttccttggatctacctatggctca

ccaccaagctccactcgcagattgtcatatggatatactttgggtgcaaacggtgtcttg

gaggctttatcattccacacagccaataatcccagagccccaaaacagctcaatcctcca

catagccaagccaaagcctcatactttccaacagtatcagcaatgcgatctatacaaggc

tcaggatatgcagtaccgttgtcccatgtaagttcttcattcacaggaaggggtttgttt

gggacatagtgtttgccgacggggagacccatgccggagcggaggcggagcgaggaagct

atcgatctgtgaacgacgccgtttccgcctaagatttgcgatgctacgttggttaatcga

cctgccatgattgttacttcgattcttcttcttcgttcttgttcgatttctggttgatta

ttgcctaatgtttatgttggtgccacacacagtccaaaacggcttcgtttggctttgctt

tttatttt

>fava_dn_rep_c637

acgggcaaaagaggagaagacatccattgattagacccaaaaaaaagtttatcatcgcca

ttcaannnnccccatacttatcatttacattttccacaacaatattccaatccaaaaaaa

aaagtgaaaagaaaaaatgctgagaatcgcagcgtgcgcaaagagactcgtgggaacggc

gtcgtttgaagcgccgattcccgccgctagggttcttccacgattctaccacgagagggt

ggtggatcattacgataatccccggaatgttggatccttcgataagaacgacccgacggt

aggtactggtcttgtcggagcacccgcgtgcggtgatgttatgaagctccaaattaaggt

tgatgaggtcaccggaaaaatcgtcgatgctcgctttaaaacctttggatgtggatccgc

cattgcttcctcatccgtcgctactgaatgggttaagggaaagcaaatggaagaggtttt

gaccattaagaacactgagattgcaaagcatctttcactcccaccagttaagcttcactg

cagcatgcttgctgaggatgctataaaagcagctgttaaagattatgaagctaaaaaaag

cctccgctgctgctgccactgcgtgatttcttccatttcttgaataatgatgaaaaaaag

cattagtatggtcgattgcacgtcattagaacatctttagtcagacttttcttggttggt

tggaagtacactgacatattgtattattatgaagtcaaataaattacttgtattgctggc

tatagtttgttatttgcattgtttgtttgtagaaatcatataatatatttttgtttaaat

atgtacacaatacaatctgagtatgtacatgtaatatttggaagtccacggctttatcct

tacttgggtgcacagcaccatattatcattacccgatttagtttatcaaccatatttttg

tg

>fava_dn_rep_c638

taggattcgaaccggagatcttaggagggtgattcttaatacatattcttagaacatagt

tggttaaatgtcaacggttnnnnnnnnnnnnnnnnnnnnnnntgcaactggtctgtaaca

tgatgtgaaattggcatgcagccacaaacacaagtattattgaggaggctataatacttt

ctctctcttctctacttcttctatatagccaaaccaagttacattataacaaaaaggaca

tacacacatacacacccacacacacccttaaggagaaagtaacaagcattttcagtttca

gagagacgggaaagtaaagagtgtttgttaaggtttagatttcattgagaagccaaagat

ggctagtttgattggaaaggagaatggtttgaacatgaaggagactgagctttgtcttgg

tttgcctggcggcggtggcggtggcagtggctgcgaggttgaaactcctagagctgctgg

aaagagaggtttctctgagacagttgatctgaagctcaatcttcaaaccaaggaagatct

gaatgagaatctgaagaatgtttcaaaggagaagagcatgcttaaggaccctgccaaacc

accagctaagactcaagtagttggttggccaccagtgagatcatacaggaagaacatgat

ggcacagaaagttaacaatactgaggagaacgagaagacaacaagtagcactactgctgg

tgcatttgttaaggtttccatggatggagcaccttaccttcgtaaggtcgacttgacaat

gtacaaaacctacaaagatttatctgatgccttagccaaaatgttcagctccttcaccac

tggtaactatggggcccatggaatgatagacttcatgaatgaaagcaagttgatggatct

tcttaacagttctgagtatgtgccaacctatgaagataaggatggcgactggatgctcgt

gggagatgtcccatgggagatgtttgttgaatcatgcaagcgtctccgcataatgaaagg

atcagaagcaattgggcttgcaccaagagcaatggagaaatgcaaaaacagaagctaaat

acatatgttatatcaaagtccagtaccaaatggacctataagaaaagatatatgaagcta

gcactacctgtggattattggagtgttgttagtttggaatgtgtcttcagtgttttttgt

tttgttcgtatatagattcaactttaattttcaagtatttgtaatcgacaagaagcaata

aacttaacaccaaaccacctatatactatatagtttgccttgtttaattattattattat

tatttactattatgtgattgcttccgtgctgtcatgtttgttgtgtgtgctatataatat

atattatatactttgaattttgatgtatattttatattctctcaaagagattcttcttct

tatatattttatttatttagtaatgttttaattaatttgtgtact

>fava_dn_rep_c639

atcacaaaaatccaaaatttactgcataaatacgcaatatttcaatttcaatctgttgat

tctttttcatttaggggatttcaatcctttaacctaaagcagattagcaaaagaaaagaa

ccaaattcactcagcaagttcagtgtctaatttgcaatcgcaagttgacctctgaattat

tgcacagggaaaagaagcccgcccactagagttataaaaataagggctcacacaacaatg

caggaataccgatccaatcaaaattacaaacgttttatttaagtattgttcagaagctta

aatagacagttaatcacgaatgctaatgatgttcactcacaaccccatcctcctgacctt

tcctggcagcagctcttattttgcgttgctcctccttgtaaaccctgtttcttcgttgga

actctttggaatggtggagacattcgaggtaatcttcacggaggagagtacagtctttgg

gttctctgcaagtagacatacactcgctgaaatcgatccagaaatcgtagcatcggcctt

tgtttccggtgattccccatcctgacgccatttctacagcttcacctagtttcttctgct

tcgtcgatctcgctcaattttgcttctcaattgcaacttcgatactctctcgttcttcat

cttcttacaacgttaaattaccaaaaggccatcactatttcattttctttg

>fava_dn_rep_c640

tacatgagtgatgatgtctttcagagtaaaccaaaccaaaccatctcatggttatggcct

tatcttctacttgttctctctttctcaacaccactgattattcacctcacaaatcactcc

attgtctaccatcttcctcttcttcttctcactttccgttcaaacctctcatccaccaca

ctcacaacacccttcccctctctcaaaaatggacaactaatgtatcattctttacttctt

tctttataaaacccaaagatgttagcatcattaaggaagaccttcttcaagccattgctt

cgcttgatcgaggagctgatgccactcctgaagaccaacaaagtgttgatcagattgcac

gcaaacttgaagcacttaatccaactaaggagcctctcaaatctaatttacttgatggaa

aatgggagcttatttacactacctctcagtcaatcttgcaaattaagagaccaaagtttt

tgagatcagttacaaattatcaagcaatcaatgctgatactctccgagcccaaaacatgg

aatctcgaccattcttcaaccaggtgacagcagatttaacacctctgaatacaaggaaag

tggctgtgaaattcgataccttcaaaattggaggcttcatacccgttaaggctcctgaca

cggctcgtggcgaactggaaataacatatttggatgaagaacttagggtatcaagagggg

acaaaggaaacttgttcatcttgaaaatggtggatccatcttatcggattcctgtatgat

gtgtcgtcactatagcattttatcgccatcgcgttttcttcctctcaatggattcaaact

accaatagagctttctgtacagaagtggttgtaaaatcgtttgtccccttgagtgtggaa

atctcggattcaaaataagcggttgtaaaatcttttacgtattgatatgaatatattatc

ttttacatgaaagacaagaaaaacatttctcaatctttttttattagtgttcttgctaaa

caccaaatatccggccttcttg

>fava_dn_rep_c641

ctagaacattctttaaagtgattgccttggtttgtctagattctagaaaggacaaaagat

gagaaatgagtgaaccaaatgcaaagataaacttaacataacatttatcatgtttcaact

ccaaatatcaactggaccaaattattcaatggcatcatcatgtgtaggaactctatcatc

acccaactcctctctacttttctcacaaaccaaacatttggcttcaacaaagagaagagt

tgttgtaagagcatctgtcacagaaaaacttggcatcaagattgagaagaaccctcctga

gtccaagctcactcaacttggtgttagacagtggcccaaatggggttgtcctccaagcaa

ttttccatggacatatgatgcaacagagacatgctatcttcttgaaggaaaagtgaaggt

cactcctagtggagctaatgaggctgttgaaattgctgctggtgacttggttgtgtttcc

taaaggaatgagttgcacttgggatgtttctgttggtgttgataagcactataaatttga

ataatatgaagatggaataggactttgattattattgatcatgaataaataaattagatt

ttatttgtactaatgttttgtaaaattaaacaaagttcatgtcatgttagttatattata

taactatgattttgtaattcttttacacatttttttgtgaccataaaaatagatttccta

tataaatggatggacaattgttattggataaaaaaaaaaggtggt

>fava_dn_rep_c642

caacaaagtataattgaaaatataattgatgtgtatacattggattttgtttgtattttt

aaatgtgtgaattatagaagatgataaaaattgagatataaccaacacatacaatgttgc

atacctttgatatttaaataacacaacttattatacatatatctacaatcaatactacaa

ttacacatagacaaggaagtatgaaatatccatggcaataaaagtggtttttcatttctt

ctagtaaacacccttctgataatcaggttcacagaaagttcctccaagatcatcacaagc

ctctgcttggaaaaccgtcttagggtcattgtattcaagatagtctctttcaatgttctc

tgaagctacagctttttttgtgtcattacttgcctctggctgaagcttcacttcaggata

catgtctgccaaacaagcttcccctccaaccgtttcacatgcctcagctggaaacacaga

gaatgctgagttgtagttgactgttgaagggtcagagttcccggaagcaactctgattgc

agtgtaacgaggtggagaagcacgtgaggcagtgaatttggtagggaaagtggtaagatg

tgagacactctttggtgttgtcagaggcacaacggtggtggtggctgcaaaggttaaaga

tgtctgcattttggagtgtgttttgtgaaatttggagtttaatcactagtaacgttttga

ggatgtttggatccaatttatagaataggaatatgacatctgtaactctaactataatgg

ataa

>fava_dn_rep_c643

attaaaatgtcactcaagacataggaacaaaacaagagctttatgataaggaacctcaaa

taataaggtaacaattatagaagtcatcagaagtgtcaatagtaaatttccctctccatt

cctttataattgtcacttttaaattttttccacaaatcaaaaaagataatgattgttatt

actttttaaacaataaatttcattttatctataatacccttaattatttatcacatgtat

ttgactttttctctctacaataaatgattaaggataattttaacaaaattacaattaatt

aatactatcttgaactttgtaagtgacaattaaaaatgaacaaaaaattattctaaaagt

gacacttaaaaagaaaaggaggtagtagtaaatactatagaaacagagagcttccgtaaa

tacatatatgaaaaaattgaaaatatcaagacaatagtttacaaatgtaaaagccaataa

agtacataatttaacctaataccatccagatcggatcaaacaagattataatttcaacat

gatgtatgtatggaacttttaccctttgggtgctcaaggtgccagcctcatcaagtatga

tttcttaccaaaaagtcaagaattttaggagaatgatttaaggtggaaaacttggcaagc

tggtgcatatgccttcttggctgaagcttgttgaaacttctttccagtaatttcattttc

ttctaaacttgtttgaacgaaaaaccttatccaccaagatgaactccgcaatttggctgg

acgaccaaatttggccaattgttcacctctaactctctgttgaccaggataacctgaaaa

gataaccacgctgtcagaagataccctcactaactctggcaaggttttattcaggtattt

tggagataagtagtccaatgcatccgacacaatgacaagagaaaatgactttgggcgata

aggtaaaggaaactttatgtcagccacgcgcacaatgcccttgcgtacaagacttttgca

ctttgcgcctgcatcatctaattcgtaaggttctattccccaagcctcggtatcctcttc

tgctaacaaactagataccacagtacaagtgtccgggccaacatgcaaaaccttatgcat

gctgtctccatatgctttcttcaaaacaggcaatgcttgtagtacttctaatgtacatga

tgaaccaccttcaagtttattcgcatccttaatatcaatactagctccacctgaactgct

gtaaaaataaccaatcagaagaaacgcccccacaataacaagcccaatagatatcaaagg

agagttttgagatttagactggatagaggcaacaaatggtatacttccaccatcacctaa

ccgacgtgaaggatttactggcctccttgacatgataaccgcaatgagtcttcagacctg

ataaggaaatcgatagattccttcagatctgatcttacagtgaatgagcggtgactgaaa

aagactatgaaagccgcgtatttaaattcaagcacagagattttttttattttttatttg

tgtttttatatgctatgcttttttattttagaaaaaagctacgtacatgttctatgcgtt

tttatagtgagttgaaataagattttagtttgttcagtttcctcgttnnnnnnnnnnnnn

nnnnnnnnggctattggatttctcattttcatggtttcgtttgttaaggatcgcgatttt

caacacttgtttgctaaggggtgtgttttgttgcatgtgtttatggcgttgtggaggatc

tactttgaaaggaaggtggaggatcttgcttgggattggcttaggcaaactgnnnnnnnn

nnnnnnnnnnaggtgtacaaagatatgtattgtattttatatatttatagttaaagttca

tcatccttttgagtgtgggactttttgcaagatatgttggttcagtggggaacatggttt

cgttggacccttctatactcttgactcaaatcaagtgtttgcatgagtgtttagcttaat

aaggattcatgttatggttcg

>fava_dn_rep_c644

agcagcataaacaaagaggaatttgtccgcgggaaacacaagacactctcaaaaatctct

tcaatccccacccaagtagagatcaatctagtgagaatgatgaagatgaaagccatgtct

gcaatgatgcttgctctagtgcttatggttgaggctgtttccattgctcaggctgcaaac

tgtgatccaatgcaattgagcccatgtctaggggcaatcatgtcaaacacatcaccttct

cctggttgttgcagcagattgaatgatcaaaagccatgcctatgtgaatacgctagaaac

cctaactttaaacagtatgttaactctcctggttctagaaatgttgctagatcttgtggt

gttaccattccaaactgctaagaaccacccttaactatatactagtatatctatgaggta

ctctattcttaaatactaatgatgtcatttaagtttgtaagctatactattatctttgga

ttattaagttattattagtataagataacaaagctatatcaaatgtattgtagcttcaaa

cttgtgatatgtatttaagaatcataaataaaaaagtggtggtgttattattatgtatat

tcagagttacattaatttgctagaacaaattaattataattctgttatttataatatatg

tggtttgagtacaagtttgagtttaagaattttttaatc

>fava_dn_rep_c645

tatatcaacatggattcaactgttacaaatgccattccagacttcagagttgtaagaccc

gtacggactgcgaatcactgaaagcaaaggagcaagtcctaagtgtcaaaggtttactta

gtgagaactaaccctgaaaattcattttatatgaaagttttgatcattagtagttcagat

ccctagtaacccatttcagcattcactatttggcgaagtaaacaaactcgcttgtgataa

aatgaccgattgaattttagttaaatgcacatctttcaaatcataccattataccattgt

atttgtaaaatgaagtgtcagataaatcataaaacgttcatcttataagttgtgatacta

gggttgtaactagggaaaattttctatatctgccctgacagtaagcatacaaaatgatta

agctctcccactttgctttttagatctggagtcagttgttgaaaccgctgatccagagag

cttgttaacctcatagagcaatacagccgagaacaggaaacatgtagtggtgcatactcg

gtgaaccgtgcttgcagcaatcaaagcaactaattctgctactatgacagcaccccagcc

agtttttgcaccggctgattcctgaaaatttccaatgaaagttaggagcacaagaaatag

cagagagctggtgaaccctccaagtattgtgtacagttccgacgacgcaagtttcccacg

gtacatttcttgaagcgagagaataacaatgaataaaagaaaggaatacgtcatggaact

cccagaccctgccatggcctcactaagatcaagaagatgcagttgaactttcagctcaga

tctaactgccacacgcgatcgcagtgttttaatatatacgagggttaatctcggtattta

atataatcaaaggacaaaaccgtctaatcactgaaatttcttatgccagtgaaacgacgc

cgggtaaaccgttgtggtctctatcggctgcgtttggatggaggagttgccggagctgcc

ggagtgtccggtttgtcttcaaaactacgacggcgaaagcgcgattcctcgagtactatc

atgcggccacaccgtttgtgaagcttgtcttgtggagcttcaggagcggtttccgaacac

tattcgttgccctgcgtgtactcaactcgttaaatactctcacaatcaaggtccgtcttc

tcttcctaaaaatattgaccttcttagaatctgcctcgaacaacactcttcatctaaaca

tcagaaatcgaatcagaaatcaaatcatgaacggtatttgatcaattatggatattcctc

tcggttttggtctgatgagttttatgccgcttggaaggattggattcttccgaacgatgt

cgtttcggttgacgagaatggttttggtaggtttagttcaggttctaaaggtagggtttg

cttcggagttaatagaactgtgagtctcgttcctgttgttagtttggttctggttagtga

tttgaagtttaagtttagttatgttgcttgggttattaagtgcttggaggggatgaatga

ggttgtgagggaggggttgggtttgattctagaaggttctgtgaggcagggtaggctttg

tagggtttatgggttatggagtgaggttgttggtggtagtttgtatctggtgtgtgagag

gcacagtggtaggtttttggattgaaggtgtcattgctttgaatttggaaggtttggttg

ctggatttttgggacttgaatgcttctcttttgatgagcttggtggtgtttgtattgatt

tgaatgaggtgtcgaaagacgggccaggagcacctggaggacaatcctggactgcacaat

ggttaaagtttgacaattcttacttcaaggacatcaaagaaaaaagggatgaagatctat

tggtattgccaactgatgcagctctttttgaagatccttccttcaaggtatttgctgaga

aatatgctgaagatcaggaagcattctttaaagattatgctgaagcacatgccaaactca

gcaaccttggagccaaatttgatcctccagagggaattgtgatagatggttctccaattg

taaagggagagaagtttgtagcagccaagtactcctctggaaaggagtaaaaacaagtgt

cttgttttcttgtccaagtatcttttaataaatacaagacgaagcttttttcaaatcgtg

tgattatcacatgtaattttggaagtctttaaatgaagaagaaaggaataaaattcaata

gtgtgcctatttgaattgcagagagagctgtccgatgcaatgaggaagaagatacgagct

gaatatgaggcagttggcggaagcccagataaggctctaaagtcaaactatttcctaaac

atcataattgttgttgcagttttggctattttgacatccctgcttggaaactaattggat

tttgttttactaaaattcactataattgaatctgcatttgtgcacatgttagtttcttcc

tataccatttacatattaatggattgatgaatcatataaccatattatcaagataaataa

atactctagggtcagggaagtagaacaaaatatgttgcttatgtttcccctctctttccc

tccaaactccccaaacaaagcataagtctaagaaaaacaaagcaaacaaagcttatcgaa

t

>fava_dn_rep_c646

acaccatcactgaccatacattttcaagtgtcagctaaggttacatgtgatagcatattc

ctgaaagaaaaatatgtgcctctaacacaagcctttcattcccattcacattatttctct

ttatatcctcacattgagaagtactcatttttcagcttctctatctttctacttcactta

ttttgcatccattcaaaagaaccaaactgtgttgtgcctatgatgtctggtgttcttctt

tgggtgaactgtggaaccaaagagaatgccattcctttgaggggtctaggaggaagaggt

gtgagaattcagaagagatttagacactgttctggaattagctttgctagtttttcaagt

gcagttgttgaacctacaaggtcttcagaggagagggtgtatgaagtggtgctgaaacaa

gcagcactagtgaaggaacaaagaagaattgttaagaagagaggtttgaatttggatact

aagcctattgaaggtgatttcactaatggtgatcttttgagttctgcttatgataggtgt

ggtgatgtttgtgctgagtatgccaagacattttatctaggcacacagttgatgacagaa

gagaggagaaaagctatttgggccatttatgtgtggtgcagaagaacggatgagttagtg

gatggtcctaatgcttcacacatcacaccaaaggcattggacagatgggaacaaagatta

acagatgtttttgaaggtcgtccttatgatatgtatgatgctgcactttcagatactgtc

acaaagtaccctgttgatatacagccctttaaggacatgattgaagggatgagactggac

ctaagaaagtcaagatacaataactttgatgaactatacctttattgctactatgttgct

gggacagttggtcttatgagtgttcctgtaatggggatagccccacaatcaaaagcttct

acagagagcatctacaatgctgcattagcacttggcattgctaatcaacttaccaacata

ctcagagacgtcggagaagatgctagaagaggaagggtgtatcttccacaagatgaatta

gcaaaagcaggcctatcagatgacgacatttttcgaggccgagtaaccgacaagtggcgc

aatttcatgaagggacaaataaagagagcaagaatgttttttgatgaggcagagaaggga

gtttcagagcttagttctgctagtagatggcctgtttgggcatctttgttgttgtatagg

cagattttagattcaattgaagctaatgactacaataacttcactaaaagggcttatgta

ggaaaagctaaaaagcttttatcactacctgttgcttttggaatagcattttttggccca

caaaagttaaccaaaatgactacaagatgaatattatattgtgctacaaaagtatggaat

agttctgttttgatcttgtaaatgtagtcccctagatcagaatggtaaaaattataatgt

gattttttttagtttaaaatttaaatgtatacatctcaatctcaagctaagcaaatattg

aagaagtcattcattatcttgttaacttgttttgtaagtaaatattgatttcattcttac

ttgttccctctttccatgggacccacatgccacatttg

>fava_dn_rep_c647

gccgcggaagtagctctcactgattcctttcgatctaacagaatatccgttcgtgcaaca

atggcgattagggctcgtcaccctaacgttctctctctcgttcttatctcctttctctcc

atcgcttctgctaaggttttcttcgaagaacgcttcgaagatggatgggaaagtcgttgg

gtaaaatctgagtggaagaaggatgagaatttggccggagaatggaactacacctctggg

aaatggaacggagatgctaatgataaaggtattcaaacaagtgaagattaccgattctat

gctatctctgctgaattccctgaattcaccaacaaggataacaccttagtcttccaattt

tctgtcaagcatgaacagaagcttgactgtggtggtggatacatgaagttgcttagcggc

gacgttgatcagaagaaatttggaggtgacactccttacagtatcatgtttggtccagat

atctgtggttacagtaccaagaaagtacatgctattttgacctacaatgacacaaaccag

ttgatcaagaaggatgttccatgtgagactgaccaacttacacatgtttacacctttatc

atccgtccagatgcaacctacagcatcttgatcgataatgttgagaagcaaactggtagc

ctctacactgattggaatctccttcctgcaaagaaaattaaggatcctgaggccaagaag

ccagaagattgggatgacaaagagtttattcctgatcccgaggacaagaaaccagaggga

tatgatgacatccctaaagaagttgcagactctgatgccaaaaagcctgaagactgggat

gatgaggaagatggtgagtggactccacctaccattgccaacccagagtacaagggacca

tggaagccaaaaaaaattaagaaccctaactacagtggaaagtggaaggcaccattgatt

gacaacccagacttcaaggatgaccctgacctatatgttttcaagttgaagtatgttggt

attgaattgtggcaggtgaaatctggtacattgtttgacaatgttgttattactgatgat

cccgagtatgccaagcaactagctgaagaaacatggggcaaacaaaaggacgctgagaag

gcagcatttgatgaggctgagaagaagaaagaagaggaggaatcaaaggatgatcctgtt

gactctgatgccgaagaggatgatgaagatactgatgatgctagccacgactctgatgct

gaatcaaagacagaggctggtgaagacagtaatgaagccaataaagaggatgtacatgat

gagctctagggtgtttgcaaaagttagcaaaagttttattcctgagtggcagttatcttt

ttttagctgtgtagtatttttctctagagataaagcaaaccgttaaaattgtaagcaaaa

ggttgagctttttgcttcatcccattagactattcagctgattagaacaaacaacaatgt

ttcgtttgaatttatcgtttattatgttttgatcgagtttgccttttatggatgcctcaa

attctaattttccccaaggatttccataaaaggagaatactatgcattggataatccatt

aaactaatttattagaaaaaaataagcacac

>fava_dn_rep_c648

ttttttcttacaattttttttttttttttttaaattagaattatgtagctttatttaata

aataatgtcactatgaaaaaatagtggtatgcattattttttcttgttgccgtcaaatct

ccaattcaacttttaaaacacactgagatgtgaagaaaggttttttaatcttttatatca

ttcaccacacaaacatagcgcagcattacaagagaagaaaaggataacagcttggaaata

aatttccatgcgtgatgttaatttatgatcatggagtgcacaacattgtcaattgctata

actgagtaaaagatgcatcaatagtcacaagaagaatgttcagcttcactgtccaggaac

caggatccattaaaagcatctattcagaatcctgtgtgagaatctgagtaggagcttctg

agcccttaggttgagctagctttggtggagatccatcaaagagacgagaacattctgtgt

aagcagatcctattccaactccagcaccaaaagcaatagatgcccagcgagtctgaggac

tcctgaataagaggagaccgccaaatgcaccggcaaacgaagagtagacgaagcggcgga

cggtcagatcaagggaggcatcccatttcgcatccaggtcatactctccaccaattattt

ctctcttctcacccatcgattccaattattacgtcttctgctttctctttctctttctct

ttctctaatctccccgcggnnnttttctgtatcattgtacaatttttgtaatgtaatttg

aaattcagt

>fava_dn_rep_c649

gaatataaannnnnnnnnnnnnnnnnnttgattatatatgcattatgctggttgtaattg

ggaataacttggagctttagttataagaaacaagcaataggttgacataacatggtaaag

gaaacttgaaaatccaaaaattccagtgaataagataaaatgtgacaagaccaactaaaa

gactaaagagaaaacatagcattgaatgagtctaatacataagacacccagtaactaaaa

tcaaaatttcacataatcttttatcacatctctcaaaaacataaataaaaaactagaagc

agattatcaaggtgcaataacaatgcgtccggcaacttggtccaaatccctccttccatt

ggaagtgatcttcctgccacccttggtgtccatttcaatgatgttcatgttctgcaactg

ttgaaggatgttacgggcaatggaaccactgctcttgcagaaatgaggtgggcgactgcc

attcctctggctccctccgtaaatcctctggaaagcaccaacacctagtccacccctcaa

gtagatcttccttgccatggaagcagctctgatatagtaccagtcaggatcataaggagc

caactccttaaacttggctgtcttcacaatatctgtccactccggaagctccatctttcc

ggatcttttgagatgggaagaataagctttgacgaactcgtgaggagaaacgtctttcac

agtccttgcggtagccatggatgagcagcagatgattctcctgagcttctctctcactgc

actgccagaaactagggtttcacaacgctttttcccgcgggaatacttaccactatacta

caacgact

>fava_dn_rep_c650

ttctctaaaggatagtaatgataataatatcaaaaatttttttttacaaaagaaaacggc

caattatattaaatggtaatcaattacattaaaatggcgaattaatttattttcttgaac

aaaatcatatcaagggataaattgattaaaacaaacaataaaacaaacattttaacattt

tagaaacattacaaaataaattattatcattttggcacaggcagagtttgagcaaacaaa

ctacataccttgtccaagtagcttttattctaaacattcactctcctagtgaaaaacaaa

gcctacacaactttgtctaacatcacagttattaaaactagcttatctcagtaatctcac

cgagcctcaagctatagtagttgagttacatggttcatagcaaaagtcagtgatatcaag

acaatcacacttcaaaggtaatgactcaaaacacctacaagacttgcaagctgagtgaca

agttggtttaacatcagtacaacgacactgaggaggtattgattttgtgcaaccacaatt

atcacaacaaggtccacatgttgtcgatttgacaccattggataggacttgagtgatgag

ggagtttggttcaaaacgagcttcaataattgtggccaaactcaagaataataataacaa

agcaatcttcatcttcaactccatctctctaatctctttcttcctttatgaggatgcccg

cggcc

>fava_dn_rep_c651

atctcatccacatatctttggtaatgctcaaaaccactggatttttcaatttttccaaca

ccttcatttttgtgattttttcaaatctgagtgtgactatgacttaatcaaaataattca

atttataaactatagtgactgtgattgaatttgacataagacaatggtgaatgaccaagt

ggaaattctaacgaccacacattcatcaaattttaacctatagtttctccgattaattcg

attctcttctttttctctctctgatcttaactttagaaatggcattcccaatcccacacc

ttatctcttcaccagcatcttcatttctaccgtacaatcctctgttaaacctcactccac

tttccttaaaccctaaaatcccaccgccgccgccgtcgcatctaaatgttctccaccact

ctcgccgccgtaagactgattctattctccgatgctcggcgtcgtcttacccggagaata

acaacgcgaactctcctaatcaagatgacgtaacggaactccctctgtttcctcttcctt

tggttctcttccccggagctatacttcctctccagatcttcgagtttcgctaccgcgtca

tgatgcacacactcctccacactgatctccggttcggcgtcatctacaccgacgcagtta

ccggaacggcggaagtcggttgcgtcggagaagttataaaacacgaaagactcgttgacg

atagattcttcttgatctgcaaaggccaggagcgttttcgtgtgaagaaagtggttcgga

cgaaaccctaccttgtcgcgagtgtggcgtggttggaggatcggccttctccgtctgctg

acgtggatgttgacggtttggcgagcgaggtggagacgtttatgaaggatgttattcggt

tatcgaatcggttaggtggaaaggcggagaaagaggttggtgatttgaggaggaacttgt

ttccgacgccgttttcgttttttgttggaagcacgtttgaaggcgcgccgagagagcaac

aggcgctgttggaattggaagatactgcggcgagattggtgagggagaaggagactttga

agaatacgcttaattacttgtctgctgcttctgctgtcaaagatgtttttccatcttctt

catcttcttcttcgtctccctcttgatgcacgggaaaggtatgatttgtattgtattcta

tcaacaataggcaatagcaccaatgtactatactgggaaaaagttgtaaattctctttta

agtgtaatttttcatatgatgatcgtgcctgtttattaaattttggatggcattttatat

cataatatagatgatactagatagaaattggtgttgtttaatcagtgaatcacatttgat

agaaatttatgacaatgttttaaaaaactgtcctgcgaaaacggtcgcacaaaacgatat

tggaaggtgccgatatcgatactgttatttaccatttttatgatgaaaaaacaattgtgg

ttctnnnnntgatccaaacagtgacttgtttgtccc

>fava_dn_rep_c652

atcaaacggttcacattcactaaaaataaataaacaataataaataaataaaataaacat

ttctccaaaaatgggtgctgaaaattagggtttcctccgactaatacatatacttttttc

tcgaaacccttcatttcaccgttcagctcaaaacccttctttttcttcatcttccatttt

ccgattttctctgaagaagcaatggcgaagggtccaggtctctactctgacatcggcaag

aaagccagagatcttttgttcaaggattacaacactgaccagaagtttactctcactact

tattcacctaatggagttgctataaccacctcaagtaccaaaaagggtgaggtttttgtg

gctgatgttaacacgcagttgaagcacaagaatgtaactactgatgtcaaagtggacaca

gcttctaatctcttcacaacaatcactgttaccgaggcagctcctggcctcaaggctatt

cttagcttcagagttcctgaacaaaggtctggaaaggtggaagttcagtacttgcatgac

tatgcaggagtttccgcaagcgttggtttgacagcaaacccaattgtcaacctctctggt

gttattggaactaatgtaattgctctcggccatgatctttcttatgatacaaaaactggc

gagttaacaaagtttaatgttggtttgaacttcaccaaagacgatttggttgcttcagtg

gttctgaatgagaaagctaatgtcctgaatgcttcatactaccatgtagtcaaccccttt

accaaaacagctgttggtgctgaggtagctcaccgattctcaaccaaagagaacacactc

acacttggaactcagcatgcattagatccattgaccactgtgaaagctcgattcaacaac

ttcggaaaggcaagcgctcttatccagcacgagtggcgtccaaaatcattgttgacgatt

tctgctgaggtggacaccaaggcaattgaaaagagtgcaaaggttggattatctttgact

cccaaaccttgaaaggaatttgagtccttttaattgaggtaggcattgttagcaaaaagc

acataattttctcccgagactgaatttgttgttttctgaagttgggacacaattgttaat

tcatgttgttttgatgattgcattttaagcaatacagcggttgtataaataagtaacata

gtctttctataaacaaaaatgttactcttaaatcttcataatcttttggcatataatctt

tggttgcactgtgttatcgtgctccttattgattatttttcagcttgatttataagctat

ttgcatgtaatgatgcacgtttttgcttttgcatgtgatgatgcttgtatttttttatag

tttcttttagagctgtgttatttatacacgatttcaaacatgcatttgcatgtgtttcta

atgcagtgaatttttcctgtaaaaaaa

>fava_dn_rep_c653

tattttaattctaaaaaagaagctagattttaaccaaataatgaagattagctcgttgga

ctaatctaaaactgaagtacacgcgcgcacacacaacaacccaagcaacactttgatttc

atgaaaaattgaactcacaacacttgaattgattcaattccctttcaatggctacttctg

tttctgcttcatcaatcgctaattgttccatcccttcatcatcttcatcatcttcttctt

catggtgcatagtctcatcctcacgcgtcacgtggcggaggcgtgactcttgtagagcta

tggttcaagttcaaactggtgcacctgctgcgtatgccaaggaaatggaacgcctttccg

ctaaagaatcgcttctcctcgcgtttaaagatgcaggagaatttgagggcttagtctctg

ggaagacaactgaatatcaaagaattgatgtgaatgagaggataactggccttgagcggc

tcaatccaacacctcgacccacgacgtcaccttttctagaaggtcgatggaaatttgagt

ggtttggtcctggatctcccgggttatttgcagctagggttatatttgagaattttcctt

caagtttagctaatttttcgaaaatggatgtctttatcaaggacggaaatgcaaagatta

ctgcaaacacaacatttttaaactcgatagaaagcagtgttattctctcaaccaagttat

ctgtggaggggcctcttagaatgaaagaggagtatgtggaagggattcttgtgtcaccaa

cagttctggaagacagagtacccgaacagctcaaaggtgcacttggacaggcagttaatg

ttttacaacagctaccggtacctttacgagatgctctttccaatggactaaaggttcctc

taagtggaagctttcagagactcttcatgatttcttatcttgatgaggaaatacttataa

taaggaacacatccgggattccagaagttttaacaaaaatcgaaacatcaccatcgcctc

taggagaatcaaatctagagtatgaaagctagtaattctgtatggaaagtgaactatttt

tccttggttttcgtcgaggtgtaaagtcaaactcttgttccttctaactatctatgtgaa

tatagaagtttcagtttgacactgataccatttcctacaaaaccgtatttcatgtttctg

tatcacaacaaaagtggttctatgatctagaacttgaataatgctaatcattagaccaat

tgtgaaaatttaaaaggaaaatttgtctgtatttttttgtttagttccaatttatatagc

tgaaaaatatattgcgtctgtgcaatgattttaggcatactcgcgcaattttcaaatgac

atttttttggtttcacttaaa

>fava_dn_rep_c654

atatgttgaaagttcatattcttcagtcattgatgaacannnnnnnggacaatactcaat

acagttaccacaaaaaatacagattccgaaatcaatactgtaatttagcaatcgtttctt

tctaatatccgtttccgttttccaatcaacaacgggtaaatctataggacatacacgaac

acatacttcacaagcaaaaatttatcaaaattcagatacagatacagaatcttgcaaaat

tcagatacagaatcttacaaacatccagaaattcggatttccaaaatcctataaacaaga

aatccataatctccaagcaccggttaaaggttggattagaaaggctactatcacatcatt

tgtggtagctactcctttcacactttaatttcatagaacaagaaatgatcaataatgtcc

aactactgaaatataaaaacaacttaaaatataggaatattatctaatgacaaaaaatgc

aacatttcaaaatgtcaaattgacaagataatagtagctaaaaccggaatttaggtatac

atcaaaacttctgtccgagcaacatcatttcacaaattaaggtgcgattacaattcgtcc

agcaacttggtcgagatcccgtcggccactggatgtaattttcctgccacccttggtgtc

catctcaatgaggttcatgttctgcaactgctgaagaatgttacgcgcaatagaaccgct

gctcttgcaaaaatggggagggcgacttccattcctctgactaccaccataaatcctctg

gaatgcaccaacaccaagaccccctctcaagtagatcttccttgccatagaagcagctct

gatgtaataccaatcaggatcatagggagccaattctttgaattttgcagttttcacaat

atcagtccactcaggaagctccatcttgccagatcgtttgaggtgagcagagtaagcttt

gacgaattcgtggggtgaaacatccttcacagttctcgaagaagccatggctaatgctga

ttatcgtttggtaatcgcaaatgctttcgccgctgagagagagtgaagaacgaacccagt

gcctattagggtttcagtttcatatattattcataatt

>fava_dn_rep_c655

acctgcatgcaaatcagccataactttaccaaatcttctttaaaatagaactcaaaacac

aacttcatccgcgagtttgacaaaatcactaataccgtaatttagttcaaacttcaagta

tagtggcacattgtgattctgtcaaatagagtctcaattcaaagatatactaaatctcta

tcaacacaatcatgacatcgaaataaaattatctcaccacattcaaaataaaattgaaaa

attcattgaggcaatttaacaaacagcatacaagcaattacaatcatagtcactacttct

tcttgtctccaccaccacttttaatccctctacgtctagaaggaataacaatctcacctt

tgagcacaggaatctccataatctccgctaactcaccgtacttcttacggaacttctcca

cccgatcatcactcatcaggttggatgaacggttacccaagtaaaattgatggttacctg

accaaacatcaacaacattctctttctgagttccacctgttgtcataacatgctcgttgt

tgcaataaaccttagcatcttcgtagaattttgggtgtatgtcttttttcttacaccata

ctccaaaattacccacttttttggcttgttgatgctgaaagctttttgtgggaagtggaa

ggaatggttttccttgtacaaaggtattggttatgcactgcgccatttttttgagatatg

gatttgaagaaaaggatgagaaatggagattgtgtggatatatgttgttcttgttgtg

>fava_dn_rep_c656

tattcaacgcagagtggccattacgccgcggcattccatctaaaaatttcagcttcgtct

gagtcatggctttagccatttcatccacttcattatcaagttttccaaacagagaaatat

gtcaaaaggttttatatcaaggaactacaacaagttcatggttaagtagaagaagaacac

ttacgattagaaatgcgaaaaaaggtgtttccgatttgtgtgtaccacttcctccagata

ggccattgtggtttcctggtagttcacctcctgaatggcttgatggcagtcttcctggtg

attttggttttgaccctcttggattaggctctgatccagagttattaaaatggtttgcac

aagcagaactagtacatggaagatgggcaatgttaggagtatcaggaatcctaatccccg

agatactcgaaggaatgggctacgtaaaaaacttctcatggtacaatgcaggttcagtac

actatttcgcagacccaataacactattcattgttcagctagccttaatgggctgggttg

aaggtagaagatgggccgatatcgtcaacccgggaagcgttgatatcgagcccaagtttc

caaataggcctaacccaaaaccagatgttggttatccgggtggactttggttcgatccta

tgcagtggggaagagggtcacctgagcctgttatggttttgaggactaaagagattaaga

atggaagacttgctatgcttgcttttgttgggttttgttttcaagctgtttatgttggag

atagtcctattcagaatttgatggctcatcttgctgatcccggtcactgcaacattttct

cggctttcacttcaaggtagtgctatgatttgagaagaattagaatgaacctgcacaatg

ttgtcctagaaatattctactgttatttacatgtaatgtgtttcacaatctgagtcttat

tattgatacgagatttacattcttttaaacaaagtgaaagtagtattgttttttgtattg

ttttatgttttccttgtaatctataatttggaaagtttggtacggtagaataagatttta

gtacggttgagtaactttctgatacgatgagagaagactaacctacaatgatagacggag

ggatttttcttatatagtgagagcagctaaaaccattcgcgtgatatgaggcgcattgtg

tgggtagtcgttggatataatcaaccggtgatagaaatatttatgatacaaaattgtctg

atcttagatgaatgaggaattcgatgcgctttatcgtttgatggtgcttttgggtcttgc

aaacagcccataaacatgtttgtcaagcatattcctctcaagatcgtctttaaagatgtg

caaatctatctaaaatataagagtttcataatttttcgtaaaaaaaaaaa

>fava_dn_rep_c657

ttttttaaataaaataaaaaacttaaggcagataacaagatagatgagcgcacgtgacaa

tatcccacaacatattgtaacttggaagttggaatggcttcaccaaagtagttttatatg

cagaacactaaaaattatcatagtaagtatcacaaacaaaggtaaatgagaatgttattt

tgccaacacacagatctttggccaatatttcgttcccgtatgcaatacatataaccggga

aaatcacagaatttcacaaaagacaatggagagtgttatagacatacgaagctattttca

ccctatccaacagcatcaacgtcagaacaggattcagacgagctcacatgatactgatgg

tcttgaaggcttacaatattgtacagcactggttggttttcttcgcttgcttgtagaaca

tctgcgacgctgcactcagatcagaactcttctcaactaaactatccagcttctcccctc

gtgcaagaacactatcaatggtcttgtgaaggatgatttttgtttcatccaactccctct

ggattctcatcaacttgtcagcctctgcaggatcctggaatttcacgagagcatcattca

aatagggccatgtttgagtactatcttcctgaacagttctccatgattcgccaaaacttt

tttgatattcatccagcacctggttgagaagagaaaatgcactccgaactggatagtgat

catccataaatcccactgcacagagaccatttctgttgtacgcatgcaccttgtactctt

cgtgctgaacggattgacgctgtccttgaggggtacgattggctacggtgcgccctacga

aaacgatgaactctttgacgctggagcgttgaaagtaaccgaagtggctcacatctgaag

cgtttgccagaaacaccgattcagaaccgtccccggtggatttaagcaccagaagagccg

tgatcttcatgttttctgtgaattttctttgtcttattctctctctttctttctggaaac

tttttcgcggctttgttttgttcttgttttgattcgtacgcagtcggtagaaggagctct

gatatcccgtgaatggtaattgacgttacgccttttatacggtgcgaaagcgtgtcnnnn

nnnnccgtaatggccactctgcgttgatacca

>fava_dn_rep_c658

aaaatatgggccccaaaagttaatactttgaatcgactgtcttcccattgttgaacatat

tataaggattaccatgaaacttaaaccgactcaattggaagaaattaatagacaggcaaa

caaatgaaaattctcaggaaagctaaaaataccaaataaatgtatttaagttacagggta

caacagcaaaatctttctatactagccacactcaaaggttacacaacgaaaccttggtgt

tgaaagccattattaagaaacaaaagtaagctatcaaagattgccaatagtctagttgat

ttgtctacagttcggacgaggttgatttttcttcgcttaagcttttgtcgttcactccca

caagctcagtgtcaaatataagtgttgcaccacctggaatagttggtggggaaccctgct

ctccatagccaagttttgctgggattttcagttttcgcttctcaccaagacacattccca

ataatccttggtcccatcctttgatcacttgaccaccaccaagctcaaaatcaatcggac

tatttctttcaaagctagaatcgaatacagttccatcagtgagttttccccggtagtgta

ctttcactttatcgcctttgtgtgcctgaacttcacatgatgctggtttatacttaacgc

caatctgcaactccgttacatcagcagcattcttcgcagcaactaatgctgatgcaatga

tgaagatggtgaagataaggaaaattgaaaatagcttcatcgtgtagatgagatgttttt

tttctttcttacaaagtcactgagtccccgcggccaccaccgattaccaccaccgatta

>fava_dn_rep_c659

tttttttttattcaacagatagttctttatggaagctcttaatataaaaaatcatgtcat

gcatagagtacaatgatatttcttgttatttcnnnnnnnnnnnnnnnnnnnnnnnnnnna

gtggccattacggccgcgggattcatccaaactataatcatatacaatttagtttgattc

ttaaaagatgagacctgctcttgctttactgcctctctttcctttcttgatcgtagcagt

tgcagaatcaagaaaagatcaaggagaatattggaagctaataatgaaagatcatgacat

gcttgaagaaattcaaggactccttgacacaaacactaaaaataatttcaagactttgaa

gcagtcatttgatgataaggaaacgaagaacactgttaaagatttcgaaccaagacctaa

tatttcagcatatgaaaacaaagacattgatgataaagaaaataagaaaggagtggaaga

tttcgaaccaagacctaatatttcagcatatgaagacaaagacattgatgataaagaaaa

taagaaaggagtggaagatttcaaaccaagacctaatatttcagcatatgaaaacaaaga

cattgatgataaagaaaataacaaaggagtgaaagattttcaaccaagacctaatatttc

agcatatgcaaacaaatacgttgattctgaagaaaataagaaaggagtaaaagattttga

acctaggcccaatatttcagcatatgcaaacaaagacagtgatgttggggaaaagaagaa

aggagtagaagaatttgaacctaggcctaatatttcagcatatggagacaatgaaattga

tgctggaaaaaagaagaaaggagtagaagattttaaaccaagaccaaatatttctgctta

tgggaacaatgaaataaacaataagaaaaaggagaaggtcgtggaagattgtggaccaaa

accaaatatttcagcttatggaaacaatgaaattgatgctgaaatctaagaggatcttga

accaagaccaagtctcactaaatataatgcataaatgtaaaagagtgcaactttaatctg

tgatggagtatatttaaataagtggttgatgtttcatgtttaatgattaataagtatgta

tgatgtatttatgtatttaagctttttgaataattattatatatttgtgaggttaaagat

tgctagtggaactttcacttgtttaccaaactttattcttgttttcttaaaccaacatgt

aagaacttaactatcaatagtattgttgtggttattagagaa

>fava_dn_rep_c660

agatctcataataacgccgttttggtaaacgaaaaaagtgaaaagtggaatcaatggaag

aagatttagattttgggaagaaggtttcgataaacgacccaatggaagtgatttatccat

ccaaaaccgttcttcttcttcgcgattttctcgatattcaacaacgcagagcccaggcat

attccaaactcaaaagtggcttttctgactacatgacttctggaggggagttggcttacc

agcagctttgcagtggaatcactaaagagtttaacgaatgctcaaaacaagtcctcgaaa

tggagtcactgcttcagagtcctgactatagccgcgttgatctagcacaaatccttagag

ctgttcaagatcaggaaaagcagaaactgcatctgacagctaccattcagctgttaaaga

aagctggccggccatctgaacgactggtgagccatgacaattgcaaatttacaaagcata

cagagcatgagtgtgttcatgtcaaggaaatcactgaggcttctgggactgaagaagcag

aagcagatgctgagtttgataatgctctcaacgaagctattagaggtgtacaggatgctg

taatggtcatcaatgaacatctagaagaagtcagatatgagattgcagcacttgaacttg

aagcagagtgatactttgctgattcattactgaaatgcttagtgctaacatatattggct

tgtgtctacggccaatggaatgtacataggcaccctaaaatgcagttcatgaattttgtt

tgtcacaagcaatttatattagagtagacaaaagaatatcaagaaaatttatttatatgt

gcttttattactttgttactttatggaagtgcatggttatttggatgagtactctgattc

aaacctctgaagttttggttgactctactttgtttttatgaaaaacgaggaaactcatat

tcataagagagataaagaccatactgattactaatttggtatcattgtatattaattgaa

aaagatggtaaactagacaacacaagtctcatatttataggtctatatgacacaaatttg

tatctaactctagctgaattatgactaaatcataacctttgtca

>fava_dn_rep_c661

ctcatgcatacagttcaacatatcaacacaatagttaagttcttgagtttaagtttaaga

aaacaagaataaagtttagcaaacaaccgcaaattccactagcaatctcttaattaacct

cacaagaatacaacaattattcaaaaagcttaaatacataaatacatcaaacatacttac

taatcattgaacatgaagtagcaatgacttatttaaatatactccatcatcatatataga

ttgaagttgcactctattacatttatgcatcatatttagtgagacttggtcttggctaaa

aatcctaagtgaattcagcaccaatgtcattgtttccataagcagaagcatttggtcttg

gttcaaaattttcaagggctttcttatcaccaaatgactgcttagtgttgaaattgtttt

tagtgttggtgttgacaagtccttgaatttcttcaggcatggcttggtctttcattacca

gcttccagtattctcctagatcttttcttgattcaaccattgctacagtcaagagaagaa

agagaggcaataatgcaagaacagttctcatctttagtttttggatgaatcccgcggcca

a

>fava_dn_rep_c662

catcacattcacacagcacaactactcaaaaccaaacatttccgtgactacaagtggctt

ctatctaaagtaatggagacgaccccagccacacaaacacaagacatgttagtctttaaa

tactcaaattacaggccatggaagctaattatcaccataaaaacaaatcgaaaataccaa

aaatagattagataacacgggatcgataaataacaccatttttttcaccaaacaagcaag

ccattgaatacatttgtcgttgacgattatacaagcacaagcacaatagtaaaaatcagc

atccacattgtcgtcgcaaaattgagttgaggtgaccatcaatcatcgagcaaatttact

taaactaataaaacattaaaatacagactacaaaatcagtttaaaataaaaactatttaa

atctagagcactcattcatgtttccagagagtatcatctcttcttcggaaagaagctttg

ttaggtactgctacttgtgtgtagatacagttaccgtgtatgaagtgaattctttcgcaa

acagttgtaaaagattgatgaattgccgccacataactcttccaacataatcctctcttg

atcaagtgaaagacaaaatcctcaaaaatcacgctttggtctactttcggcaggtgagac

ccggatagctctgccattcaagtccgcgccatctaagaattcaatagcaccgttgacctc

ttcagcactaccgtaagtcacaaatccaaagcctttagatcttccactttccctatcata

gactacccttgcctctataacctttccttgttcttggaacaatgactcaagtgccaagtt

gtcaacaccccaagaaagattacccacgtgaacgcggtgttcgctatatgaaccacctct

tggaggaccaccaaaacgagaattctcacgaggtggaggagctggtccagaattcactct

caattcccttccctccactagataaccattgaactgctgaacagcagcttgaacttcagc

agcagaagacatagtaacaaacccaaatcccctgcttctacctgtagtcttatcataaat

cacctcaaccatctcaacgttcccggcactttcaaaaatctctgcaagttgagcactatc

aacactgaacggaaggttcccaacaaacagccgctggttggcagagtaattccttgtagc

tccatcatcaaaagtatccacctcctggtcaaactccgacgaaaccgcaacacgggaaac

gaaccggtaactcaaagacagcgttttacgaagggagacagatgaaaacggtttgaaatc

cttgttgagtgagaacgaagggagtgaagagaaacattgtggagttttggttcggagaga

gggaagggctagggaagttgcagaggtagacattgttggggaaatggataaggttgtagg

gaggggaattagggtttattgtgtgagggtttagcgaaatggtgtgagaagtgtgatatt

tatgggagggaagggaacggtgggaatcaaag

>fava_dn_rep_c663

ggccgcgggaattctcacaagcaaataataacttacataataacaagaaccattaaagag

tagtagtgagttgcaaaatgaaagttctttacttccttgttagcatcttggctttggcat

cttctgtttcctttgcttatgacccaagccctctccaagacttttgtgttgcaatccaag

atcccaaagatggtgtatttgtgaatggaaagttttgtaaagatccaacgctagtcaaag

ctgaagatttcttcaaacatgttgaacctgggaatacttcaaatgcattgggctctcaag

tgactgcagtcacagtagaccaactattcggattaaacacactcggcatttctttggctc

gcattgattttgcacccaagggtttaaacccaccccacactcaccctcgaggcacagaga

tccttatagttctagaaggtactctttatgttggatttgtcacttccaatcaagataata

accgtctcttcaccaaagtactcaacaagggtgatgtgtttgtgtttccaattggtctca

ttcactttcaacaaaatgtgggatatggcaatgcagttgctattgctggacttagcagtc

aaaatccaggagttatcactattgcaaatgctttgtttaaatctaacccacctatttctg

atgaagttcttaccaaagcttttcaagtggataagagtataattgattatcttcaaaaac

agtcttggtatgacaacaactagtttgagagcatatgattagtcatttcatttccaatga

attatctacatactttcatatttattgtaataaataaaaaccatgtgaggaatatttaat

tccatatctcctttaatttaaattcattctagaacaataaatatttgtctaccttacttt

ggaattacaagtatttttgtgagtactaacatatatttcaattaataatttgataaaaaa

aagttgtaagtgaaagaaagtaacttcaaaagaaacttaa

>fava_dn_rep_c664

tgaataacaggtcaaagttactatattaggagatgatcacaaagaaaagagatatacata

atttattggagagacaatttcactgaaaattcaaatgagattttaatagctctcgctagt

aaaattacattgacgtatttttccggtacacttttataattttattttttcacaatgatt

cattttcttaaacaccaagatatataattgagaaatctgtcaaccacaattttatttgca

ctaaacctttaatacaatagataaagcaaacaaagaaggtttgtattcttcatataatag

aagaacaataatgaaggagtaagaaatcaagaatattgcaacaacaaaaaaaacacacac

aaagtacacaaatatgacaaagaaacacatgaaattgtaaatatggtgccaatgaataat

taatattctaagggcactgaaaaccagagttgttcaatccacaattgttgagaatgacac

tcaaagcaataggaacatcaaggtgaagaccaaggacattagctttaagggcagtgcaaa

gacatgcagcaacttcaagattagcaagacccttaattagggtgcaacaagggagcgttg

gtggagaccccactttaacatttaccaacttcaacacatttgcacacacacctaatttaa

gtgcatttataggacatgtagcttttggataagggtcaactattgggtttgggattggga

ctgggatttcagggatgatatagttggagttcaccatagtgatgaagaaaaaattgagac

ataggaaaaatgcaacactaataatcttagaagccattttacaaaactaattagttttgc

tataggatgcttgtggattagtattaatttgagtgatcccgcggccgtaatggccactcg

gcgttgat

>fava_dn_rep_c665

tttgctctaataaaattatttatgtattaaattttagcctttttacgataattaaaacta

cttatataaataattttgttgggattaaaacgcatatgagaatccatgttgttgtaataa

ttgaatacatgtgacaatagttacatagaaagaaaattgattaatcagagaggaatatca

aggccttttccaatctcaactccaatcacttcaagtattcccttgtttattataagctct

acaatgaaagttccaatgagtccaatcatgcatgctctggagttccatatttctgctgtc

tttgtaaagcctagaaatggtggtttaaacttaggctgtacttttggaaattcaacacca

gaaggagttttgagtgcttggacagtgaaggaaagtgtcttgttgtgaagtgatggtgat

aaattcaagctatggttttgaaggaataaaacttggtggtttgtagagtgaattggttgg

gttcctaagatgaaggatgctggtaatttggttgatgagatagccatggcaatggcaatg

acaatagatgctaactgcaaccaattctacactgtggagttatctactttttccattttt

ttcacttgtttttgtgtggttcatattggaagaagattgggttgacatacctcatacgtg

t

>fava_dn_rep_c666

aagactaacatcaacatacaagtgtttaaagagcagggacgcttgtggatgttttcttaa

aatcaatcttgtcaaccttaacctctccatcaataagttcatatacatagaccacaacac

gcagagcgtcgatgtccattagcacaaaacttgggttcacgtcataggtgatgctgctgt

aggcaccagatgcagaacctggatttataaccacgccgccctcgtgtttgtatgcggtaa

actgatgggtgtgacctgtgacaaggatgtctacgtcaagctgtctctgtaacatcgcca

gtgagtccaggtctccccatggaataacctgatgaccatggcatagtcccaacttgaatt

ggccaatggttagtgttttggtctctggatatcttgaatcttcatcatactcaccacgag

ttatatgcaagtcgggacaaagagtctttaggtagtcatggacttctttaatacacaagt

ttccagtacatatgatgtgctggatctttccagggacaagcatggacttaaactttgcag

gaatatcaggcgccctgtggggtatgtgcatatcccccagggccaataccagcaccatcc

tcaataatcaaaaactgaaagttgagttcgagaaccttgtcttgtttgataatgacggaa

atgagagagagagagacactccgatcagaagctggtcggtagaaacaaaacaccggcaga

ggcaccaattccacgtcaaatcaatttcaccccgcgg

>fava_dn_rep_c667

cgaaacaaaatttatttattaaaaccatagccatattttaaaatagaaggagtaaggtaa

tactgggagtacctacatttatttatttttaagcaatctaactactattaactcattcat

caattcatagtactagtacatttttattaatgatcataatcaacaataattattttactc

ataaccattacattattatagtgacacttaagtaaaacaagaaaaagaaatacatgaagc

aaatcataacaagaacacaaagccatgtcctagaaaaaccaataaccaaattctaacgag

gaaccaacaatgagtttcaaaggttacaatacaatgtataagttttttgtggcagaaggg

gtagcttatggtttcccgaggatggtagtttctgcaatcatggaagtaacaacatatggg

tccatgttagatgctggcctcctgtcctcaaaataacccttcccttctgcttctgtgtcc

cttccaactctaaccgaagctccgcggttcgcaacaccccatgagaaggtatgaatgtcg

gctgtttcgtgtttccctgtcaatcggcgctcgttgccttctccataagcagcgatgtgc

tcggcgtgcctcttgccgagcttctcgattgctttcttgatgacttcgtatccgccgtct

tctctcatagacttggtgctgtagtttgtgtgagcaccagcaccattccaatcaccccca

actggtttagggtcaaagctaagaaccacaccagcaatctcggtgatcctctccaaaatg

taacgagcaacccatatctcatcaccagcagagataccaactgagggaccaacttggaat

tcccattgaccaggcatcacttctccattgactccactgatgttgatgccggcaaaaaga

caggctttgtaatgagcctcaacaatgtcgcggccaaaagccttgtcagcaccagcactg

caataatatggtccctgaggtccaggataaccaccaactggccaaccaagaggccacttg

atgtctctctgcaacaaggtgtattcctgctcaataccataccatgtttcttcaacaaca

acatcaggatggctaaaaaccttggcagctgcgtgtcttttgttagtaggaataggttct

ccagcaggagtgtatgcatcacacataaccaagatatggttaccccttctgaatggatcc

ttaaaaatggcttgtggatataaaataacttcactatcttctccaggagcttgtcctgtg

ctggaaccatcatagttccacttggggagctgtgaagggtcagtaactggtccaggaaga

gtccttgctttgcacctcaagtccaaaccagaaccaccaatccatatgtattcagcgatg

attttctcggtggtgcctgagagatcaaggttgatgagatctgaaagaagagacatggtt

ttgaaaaagtgaagaaaaaaaatcctcgaaatcgaatctgtattgatcgttggatgaatc

ctcagattcgatgcgaaaaggataaaatatggggttttatttatagctggttttcttcct

attcctggtcttct

>fava_dn_rep_c668

ttagacctgtagcaaattctaacannnnnnnnnnnnnnnnnnnnnnnnnnnnnnnnnnnn

nnnnnnnnnnnnnnnnnnnnnnnnnnnnnnnnnctttttttttttaatcacaattataat

atattagaaaacttgggaagtacacaatccacttaatgtttgtttgaaggtcatgaaaat

gtaacttcagtaaaaataaaataaaaggcttcatttaacatcagcggcaaatggcaatta

catgataaagaagggaatgcatttgatattctcccgaatgaaatgtgaattagaacagat

tgacaatcagatagtgattatctgatgcaattcgaattgtagcaaatactttgtcatact

gaaatgagatcaatccgctatgaacagttacagtaatctaaatgaagccaaactgttatg

gttgattgtcattatgtgtgaaaagaattgatatcagtacatttggaattttgcctttgt

tttctcaaaaacctcttccgcaatcatggcctcgttaccatcagctcttttaatttctaa

agctgctttctgatagaatccagtacctcgcaatgcatctgcaataaaatcatcaaaccc

aatagcgattgctgcctctgcctttgctccgtaaaccagtctcttaattcgtgagaggtg

gattgcaccaaagcacatgggacagggttcacaagaagcatatatttcacaatctgaaag

ttctatttgcttaagcttcttacaagcctctctgattgctgtgacctctgcatgagcagt

tggatcagtgtaattcagaaccatgttgtgacaactagcaactacttcgtcgttatgaac

gataacagcaccaaaagggcctccatctccacaatctactcctttatatgcttcttcaac

tgcttttcttaaaaatgtgtggtctctatcctgcacagcttcctgatgaccagcaaacgc

agtagctactgaaacggttccgtccttagtttcaacaacgttaacttcttccatggtagc

gttcgaaatcggagaattgtttggttagaaagaagaacgatgaagaaaaacaaaaatggg

atggtcaagaaaatagagtccacgaaaattaataaatagtataataataactgaa

>fava_dn_rep_c669

aaaaacaagtatcttatactgtctcactagatccacagagaaactcagagtcaccactat

atctcatttaatcactcaaccaccctatttataatttccattcacccactcacaaacaca

ctataaactcactctcatacacctcactttccattacaattattctctcctccttcacca

tgagaggaaaaccagcattgctagctcttaaaccaatcttgaccacagtttttgttctac

tattttcagtaaccagtttctcaaaactagcaacaaaacaccaaacaaaccatcttcatt

tccaaaaccatctccaagtagccaatttagcatgcgaaggaacactctatccaaaccttt

gtgtctcaactctatccactttcccagatcttacctcaaaaacaatcccacaattgattt

cttccatcctcaactacaccataaaagaagttaaacaatcctcctccaatgtgaccggtc

ttcgagtccgaaccagaagtctcaaaaaaacactcgaacaaagagctcttgaagattgca

ccaatctcttcgacaccacaattgaagaactcaaaaccagcatttctgatctctctcaaa

ctccaaccacaatagctcttcacaataaacgtgactgtcaaacgctactgagcggcgcaa

tgacgaatatgtacacatgtttggatggttttgcttatagtaaaagtaacataagacaca

aaattgagaatcggttgtatgatatttccaaacacgttagtaatacacttgccatgttga

agaaagttccggcagtgaaatccaaaacttccaactccgaggctttccctgaatatggtg

agatgaaagacgggtttccgtcgtggctaaccaccaaagatcggaagctactacaagttg

cagcgaatcaaaccaaattcaatctcgtggttgctaaagatggaacaggaaattttacca

ccataggagatgcattgtctgccgctccaaattctagcacaactaggtttgtgatatata

taaaatctggggcttatttggagaatgtggaagtgataaggaaaaagactaatttgatgt

tggtaggagatggaattggaaagacagttgtgaaggcaagtagaaatgtggtagatgggt

ggaccacttttcaatcagccacttttgctgttgtgggagacagattcatagcaaagggta

taacctttgagaactcagctggagctagcaaacaccaagccgtagccgtgagaagtggcg

ccgatttctcagccttctaccaatgcagctttgtggctttccaagacacactctatgtcc

actccctccgtcaattctaccgcgaatgtgatgtttatggcacggtagacttcatctttg

gcaacgcggccgtagttttccaaaactgcaacctatatgcgcgtaaacccgatccaaacc

aaaagaatctattcactgcacagggaagagaagatcctaaccaaaacacaggaatttcaa

tcctgaactgcaaggttgcggccgctgcggatttgattccggtaaaatcaacgttcaaga

gttacctcggtcgtccttgaaaaaaatattcaagaacagtttatttaaattctgttatag

aggatttgatagatccagcaggatggttagagtggaatggaacgtttgcgttggacacat

tgtactatggcgagtatcagaatcggggtccgggttcgaacacaagtgctagggttaatt

ggccaggttacaaagttattactaatgcaactgaggcaagtcaattcactgttaggcaat

ttcttcaaggcactgagtggttgaactctactggtattccatttttccttgatttgagtt

gaaaggtgtaatggtaatatggtgtcaccattatgtactatgttttcaaatgtgtaataa

taaaagtctttgatgtttttttggattctatgaaatgtgtattttgaatttatataggct

tcttagcatgcttgttggaataaattgattgcatggttgcatatgatacaatgtttttat

aggcattacaatagtaatgatctgctacagttatgtaggcgtgttatagacaagccagtt

agtacatattttcaattagttcatacaaggaagtgactaaaatttgaactcc

>fava_dn_rep_c670

tcacgcgtattgaccgaaccttttatctattaactgaccgctgtttttccttatttatga

ctcacattcacaccataataactcttgggcggtgagcgaacgatcgaaccaactaacgat

cgttacgttctccaccgtcgcattccttccgcctctttggttttcccagcttaattaaca

caccaaacggaacgtaactctaggcttcaagtgcgcagaagaaggttattacagaagcca

tggttcttgacaacattctgtcttcatcttctccacgtttaaagtctccatctatccgga

gacctaaggatggacttggtagctggtcaacacttttccaaaggcaccgcttcctcttat

ctgctctcgtgctccttactattctatgtactgtttatctttacttcgctatcaccatag

gaagtggctcctgttctggattgagtggagcacagaaagcttcatgccatatggagcttg

tcaaggattctatttccaacggcaaactgaaatttctttaatatttcaaatattattatc

ggagtttttgttgcatccctccaccccagtataggtgtgacatgaagtccaaatacagtc

ttctgttacaatatctgtgatttgttgatgtgtaagatacaggcaacaagccatttctta

gatacattatgttaaggttgatgactggttactattgtaaaagtttgattttacctattg

tttatgctagtctgaaacccttttttgagcattgtcagcaataacggtctcttgaatttt

gggtcaaagttcttttcacctaaaagtaaaaatagttgtgttgattttcttgttcg

>fava_dn_rep_c671

aaaagaatccgtgatcttaatgtcaccccttaattttccaggaaacataaaacgcaacca

aattatcaggagatgcttgataagtctgtagctacagaaacttcaagatactactagttc

aaaaataattccacaaaaaaaaaaatacaaatagaacacagtctaattaaacaaaaccca

attaagtagtgagctccataatggccccaacaatgtctccattgtgagtcttgagagcct

tgacggccttggtccgggaaactcctgcttgtgtcatgaccaaatcaatgtcatggggct

ccacaccagtttcatcaacctcctcttcttcctcaggctgtgcaccagctattgcacctt

gatcttgttttgccattacagatcccatatctggcatcctaaactgctgagcagcctgcg

actgcaactgagagctcaggtcttctatttttgcttccccgaatataatgtaggtctcgg

aatttggactcttgaatacatcgggttttgagatgaaaaaatgaatgtttttggttctct

tgatggtgacccgactaacgccagtaacaggtttcagtccaagcttcagcattgcttttc

gactcttcttctcgcttctgctttgctttgaaccctcggtaccacctagagcgccatctt

ccttgtcatcatcgtcctcgtcatcatcgtcatcatcttcatcgtcatcttcatctttgt

catcctccttcacgtcctcaataattggagcctcgtcttcctcgggttggggtttcttct

tgagggtagtttcgtcaacggagggaatttgttggtcgagttcgggctcggtgactgcat

caataactggacctggagccatggttagagaattgagagggttgggatttgactcggcgg

aaaaggtgatcactgtgaagtgtgtagagattaattggattagatagagagcaagggata

acaacactagctactacttattcggcttcaattcaagggttttcattttttattttgggc

cattcatttt

>fava_dn_rep_c672

aaccactccaaaactctttgaagtcttcttttcccaatttaatttttttattaacatatc

tactattaacgttgtacgagtctaatttcaatgcaattgtaaataaatccaaatttacat

gcatgaactcactttttcaatttgaaaaagcatctatttaacagaaaaaacttgtctcta

taacaaataaacatatgcatcaacgatcaaacttagcaaagtcgacacaaaatttgatct

taagatacaactcgaacacaattacatttttgtgtcaatggatcaaacggtcagcaactc

cttttccttctgtttgtagatgacatcaaccctctttatataatcatctgtcagcttctg

caagtcgctagacaagtccttcacattatcttcagagagctttttttccttttcaagttt

ttcataagctttcaaagcatctcttcttatgttcctcaaagccaccttcccttcttcaac

ttgtttagccactatctttgttagttcctttcgtctatcagatgtcaactgagggatact

caaccttagcacttctccatcgttatttggagtcataccaacgtcagagttaactatggc

tttctcaatagacttcaagcttgatttgtcatatggttgtaccaacagagaactagcatc

aggagtgcttatttgagcaatgctcttcaagctaactggagatccatagtattccacctc

aattttgtcaagcattgccgggtttgctctcccggttctaatggaactgaaattcaaccg

aacattatcaagagtcttttccattctgtttttaacatctttctcaataaatgctttttc

agcttcaatttcttcaatagtagcagccctcacaattccctttcttctatgcaaggtcaa

gaatcctttagagaggagaggtttcccattcctagggagtgtgaagttgcgagggaggtt

tagtgtggcataggttgaagagcgtttgacgctgtaagaggactctgcagaaagaagagg

tttgggacggttttggaggatgtaacggagtggtgttgtgggagagaaagaggttgccat

gagatgagagagacagccactctacgccttgtgttgtttgttgtctttctttgtggataa

caataacaacgtttatgttcgttcgtgccacaaccgcaccgtgtcacaatgttttttccc

acagtat

>fava_dn_rep_c673

ggccgcgggatctcttacaccggaatctggtcattgggatcgcagatacgccgcatctgc

taaaatacgccttatccaattatgggccttctcgaccagttgtgggacgataccgtcgcc

ggccctcggcgggagaacggtctcggtaaacttcgcaagcataacacgttcgctgcccga

tctagctccggcaaggaattggaggctggaagtgtgagatcgttcggtgaagaaccgtta

gtgccggcgacgagagtaacgcgtagtatcatgatcgtgaaaccgccaggctatcaaagt

ggttcgcctccggcttctcccgccggttcaactactccggtgtctccgttttccggaact

cgagaatcatttcggtttcgacgaaggtcggtatcggatgcgttcgagaagaaaaaccaa

gacagatcaagctcttcttctcctttcgatgtgtgagaaatgagcccgccacaatttcta

agtctccggaagatgttaccttttggtcttagatgtaataatttttgtttatttgtgtgt

atgggttgagttgtattgaagttttgtgctgcagtttagtaatatagctcttttaagttt

gaaaggaagtccaaagggtagtaatatgtatctatgtgtagtgtatatagagtttttaat

ttactatgttttgttgttttttcatgtatgtaagttttctcctccggaataataacctat

attcaatttcttgttttgttatggattttcattattttgataaaaaa

>fava_dn_rep_c674

aagttcgagaagcactatttataacgtacatacatacccattacgaccgcgagcgctaac

cactctaacaagccccctggcgcattttccagatctgcttcttccacagccacccacacc

aaccaaagaaactagattttacttttcaaaaagctgttgaaagatatgggtcgtggaaat

agcagtggtggtggccaaagttcactgggttacctttttggaagtggagaggccccaaaa

ccagcaacaactaatgcccaacctcctgcagtgcatgtagataatgcacccccttcaaaa

ccagccgctgctcctacagcaatagacccaaacaagcctgctggtatcaatagtaattct

attgatggcctgaacaccggcaacttcatcacggaccgaccctcaaccaaggttcatgct

gcccctggtggtgactcttctctgggttatctttttggtggtgcgcctggggatggaaaa

tgatactgcttttgagaaccttttcttgagaataatctcttttattcaagttgtgtgaac

aaaatgggtggtgtattataggttatggttgttgggataagttgatcaagagtgttatat

tttagatgtgcttaacatctattacagagctgtgtgtcaaacaagttttattatcaattc

aacatttgctttcagttctgtattttatgcattcttgttatttattttaatactgaaaca

ttcactgaatttaattgatagtgggagatgaaatgcatgcataactatcagtttttatat

tcataatacaccttcgatcaaaccttcaacttattctaggcttagccctcactctcacca

aacaagacacaccaccatccaacaacaaccttacaaaaccttattcatcaagcaacaaca

acaactatgaagctgaaccatctctaacgttgggtttatctagttactacgaagaacctc

ttgatttttccacacaaacaaactcacctctccatagcgttgtttcatctttctctagtg

gtagagtcaaaagagaaagagacgttagcagtgaagaaattgaagtaacagagatagaaa

gagtttcttcaagaattagcgacgaagaagaagacggcgccaccgcaactaggaagaaac

ttagacttaccaaagatcaatctgccatgcttgaagaaagcttcaaacaacacagcacgc

tgaatcctaaacagaagcaagccttagccagagagttaaatctaacagctcgacaagttg

aggtctggttccagaaccgcagagccagaacaaagctgaaacaaaccgaggtggattgtg

agttcctaaagaaatgttgtgaaacattaacagatgaaaacagaaggttacaaaaagagg

ttcaagaattaaaggcactgaaacaagcacaacctttgtatatgcctatgcctgctgcaa

cccttactatgtgcccttcttgtgagaggcttggtggtggtgttaatggtggttcttcca

ataagagcaatttttcagtggctcctaaacctcacttttacaatcccttcacaaattctt

ctgcagcatgttgatttgttactatgaaaagggttaaaagaaaaaagaaaagaagaaaac

aacaagagtactttcctacacaacctttgtattttttggttgaggggaaatgtttgtgaa

ctacagctagcttctgaaatggttaaaacgtgtaattaattaattaatactgtagaaatt

agtaattagtaattagtaattgaatatcaatattacttcttttttttattactttttttt

tattttcctttattatatatgtttgtaaatttttatattagacatcatctatatatgaag

gtataactattttttatcaagaaaaaaa

>fava_dn_rep_c675

atcaacaatatttaaaaaatgtgtttcccatattgacaagttatcatccttttataaata

tgggcaatacttggttaaattgaaagatgaatcatactcaactctttttttttacagcaa

aaactccaacatttgcattagtgaatgtataaaattaatacaaaagacacggaatcgaac

acacacatactttaacaacactaaacacaatgaaggtgagaaagatgcaacataatcatg

gagaaagtagtgagatctcaaaatcattctggactacagggggtcctgaggtgcactaag

cactatgttactctcatgattctcctgggaactctatcagttgaaaatgattaggtttgt

gctgtaaaatagaaagcaatttgcttgttttttacttcaccaaagtataggacacaatat

caaacatgctatgctacattgctaattctctacgccttattcacccggatcttttgacct

tctaacaaagcgttgttgaaagaagagatggcagcctctacatcctcatctgatgaaaat

gtaacaaaaccaaaaccactggattttgaggttccaggaacccgtgaaatcttggcacta

agagcttttcctttttcagagaagaaatttttaaggatatcagatgttacacttttagcc

agatttccaacatataccttgtatggactgtcaacaaattgggattcctcggcctgaagt

gacaaccctcctgttgttgttaaaggtttttcggttatattcactttaatctcacgtcct

ccaatttgagtgccattgagtttctcaactgctgcatttgcatcctcaacggttttcatt

gtaacaaacgcaaaacgacggctccttttagagtatttgtcatacataacctcagctttc

tcaacagcgccatgttcttgaacaatctgagcaagctcatcattcccaacagtccgagga

atgttaccaacgtaaagctttctaaccgccggagccgtcgcggttgcttgttcagaaacc

gttacacatctcctaaaccggcgttgcaagaacagtgatgtcttgggtttcaagttatcg

aagtttggtggttgtgttgtgagcgaaaggaaattgggttgggagagaagagaaagagaa

gacattgttgtggatagatggaagtaactggaaattgtggttttgtttttgtttgcaaat

tttagaggtttatcttgttatcttggcgt

>fava_dn_rep_c676

tttttttgttaacttggtaaaggcatgtcctatggtttcatatattgaagagtgcaaata

gcgtgaaaacacatactcctaccaaattataaatagacatgtttcatacaatagtaacta

agaaatgtacaaatacatcccttttcactcaaataaaaaaaaacagctttatgacacaaa

acaaaaccccgcacgaagtgcaaacgtaatgcttgatagtatatgattacaatatatgac

cctcccctccatggtattatatgatattctgaacttttatactatacctctcccttgtcc

ttcccattaatttcatgacctccaacttcagcttctttaatctgtctttctatatcagcc

ttcttttccagaagtgcttctagttttccattcactttttcaagcttttgtttcagtgtg

ctaacatcattggtctttgaaggttttttcttcttctctcccttgtcttcttttttcttt

ttcttctccttaacttctttcttgtcatcttctttttctgattccttcattgtttcttct

atatcaatatcccttacagatacttcaccctcttcttcctcatttttatccttctttttc

tctttcttgtcctttttcttgtccttcttttcttcagcatccttgtctccatcctctttt

cccttcttttcctttttcttctccttcttcttctcttcaccgtccttgtcttcatcttct

tttcccttctcgttcttttcctttttcttttcctccttcttgtccttcttttcatcacca

ccttctttattcttaacattctctacatcaattttctcatctttttccttcttttccttt

ttcttctccttatccttctttttctcttcatcattatgtttcttctctttctctactgat

ttagtctccacatctttgtctatctcaaccttctcttcttttacaccgagtgttttatcc

tcgatcttgatttgaataggatcctccatatctatagtggttgatgtttggggtttgtag

aaagtaatgaagagcttgagaaatgtgttggacctccagttgaaaatatttgaccccttg

tatatggtgaacttgacaattcttctgaaccacacctctcttttcgttcatccatcatta

tttgactcgaacgacactctgtgctacaaaatcctttctcacctctatacatgtatatat

cttttccgtgtagactctttccgcataagtgacatgaattaagaaaatttgatgtcggaa

ataatggttccggttcagatcttcttacaacaccaacaacgttgttgttgtaacgttgtt

gcctccgaacttcaccatcatcataataaaccttagtgaaggttttattaggcacgtgac

atgtgacatatgtgtattcctcatcttcttccaaactttccatgtgaatttcattcatat

cataatccatttgaaacccgtttgtgtttttggcagattgaatttggattggtcttgatc

gattcaagtttgatgtgcaaacagcatgtttaggaagaacatcacatgttttgttgttgt

tgttgctgttgttgttgttatcgagagctgcaacgatcttgagaccaacgctactgaggt

cgtagttttttaaacctcttggtgattgcatctttgtgtcgaatgggcctcttggacttc

cggtggtgtccatgagacggtgaccggagactaatagctccgacaattttccgatcattg

gagagggtcttttcctcaacatgatcggagaagagaatggagaaggtgggttggtgtgtt

gaagaagggaatgggagaatagaaagatggagagagtagtgggt

>fava_dn_rep_c677

aatatagtaattaatctcttaaattaattacatatgaaagttcaagaaatatgaaaaaat

taattccccttgaattattctcatgtacttcatctgtgtttgatacagcaatttggatct

attgcaccaaagtagataaaatggtgctaaggataattcccagcttgttctgaatttttt

ttcttcataacaagaaaattagatatagttcctcacatagacactgattttgaccaaact

tcctaatatacacacaaattaaaatgtagtttacatcttttccattgtattcaaataaaa

tatcctttcagttggtttgtatgtaggactcagcaaaataacacaatcttatggcaatta

agcctaataattattacagtatatattacacttgcatgttttattaatcaaactttcctt

gaagagtcttgtgagttatgatcactgaagtttcacaacaatgcacgagatatcgtcatt

gcttttcctgttaaccgcttcttcagtgagacgctttgctgaagtccgagcatcctttat

atctttgatgcaattaaccgcttcttgattcgacattaccttccataatccatcactggc

taatataataaactctgcatcatcatttatcttctcaactgtcacatatggctctgaact

caagtgtttcttcaagcttttgtcaccaaatgccctcgacactgccaaccgtccatcaac

tcgtggaacgtcaccgggaaaccttgatacgaaaccacctctatttttgatatcctcgtg

ttctgtggttggttcatgatcgaccgagagttgtttggccacacctttctttgataagac

agcccgagagtctccaatattggctacaattaacttctgaccgttaaccaatattgcagt

aaccgcggttgaaccccctctacccaattcacctgacttctctaaaatactagaatcggt

tatactatacgctctcttgacagcatcttcaggttctctccagaagtcaggctcttttaa

gatattatcgaacaaatgagactgtaggtagttaggtacattgtgaccagcatgaccatc

aaatattgcaaacaagcctaattcatggttatcaatttgcttaaatctggcaacaagata

atcctccatttcatgatatgattttcctttaaccaagtgaaagccgtgagttatgtgttt

cgacatcttgctcttgcctttccctgaatcagggtctgataaaccaaacccaactttttc

cttcatcttatgaaggatatctctggcagccatgagtgaatggtgttgaggaagaaggaa

gaaaaatagttgagttgacttgacttgagagttgagagtgttgagtggtgaatgtatcaa

tgatgaagatggtggacaataattatttatggcctccacagattgaaaaagacaa

>fava_dn_rep_c678

ggccgcgggactcaaaaacccttctctcatctcaatcttcaattctcctttttccaactt

ttaacgttagagaaacacacagagagtgttatccatggctagcagtagcgtggcatcagc

agcttgtggatttgtcttgcccctcaatgttgctgccaacaacacaacctcctcactttc

ttccaggactaacatgttgatgtttcctataaaatccaacaatattgctagttctagact

tgttgtcagggcaaccgaagataccgccgatgctgctacccctaccgccagttctcccgc

cgctgatacagctactaagcccaagccaccaccaattggccccaagaggggtgctaaggt

gaagattcttaggcaagaatcctactggtacaaaggatatggttctgttgttactgttga

tcaagaccccaacactcgctaccctgtcgtggttcgattcaacaaagtgaactacgccaa

tgtatcgacaaataactatgctttggatgaggttgaagaagttgaatgagaccggtttta

ttgtattttcgactattgttatgctattgaagttactagcttaaggtttttttcatgtca

tgttaaaaaatttgtatcatattctcttatatttatctacctcttggtttctataaacta

cattcattgtgcagttgctcataatcttaaagtttacttgttagataattactactaatt

aggtatgttagagccttatgtttaggccaatgcaactactgaaaatgacctagtagtgaa

ggtgaaatagtctttactcatttaagtatatgtaaagtagaaatgtgtgttttcttaagt

atccttatgatatgtaagaagcatgtgagtgaaattagtttttcttaagatcatggcttg

gcccttagcatagtttccttcttttaagtaaaaggatattctcaaatgatatcaataaag

taaatttatcctcaaagtacaaaatgtatcactatgaaggggtaacaacaattgggagat

gaaaacatcgtatggaccattttgtaattgatatctagattttttaatattcatgactag

aatatttttttgttcaactttatctcctttaacttatcttagttcnnnnnnnnnnnnnnn

nnnnnnnnnnnnnnnnnnnnnnnnnnnnnnnnnnnnnnnnttttcatccacaccattaaa

atcgcatatttttatattttatgtatgtttgagtattaatcatggggctgtgt

>fava_dn_rep_c679

ggaccccaaacacactaactcaaataaaactatccaatccatggatcaattaaataacca

aacattactagtttttctctcttgcatgtttctaatatttaacattattgttccacatgt

tgaagcaagagcattttttgttttcggtgattcacttgttgacaatggcaacaacaacta

cttagccaccactgctcgcgccgactcttatccatatggcatcgattatccgactcaccg

tgccaccggacgtttctctaatggccttaacatgcctgaccttatcagtgagaaaattgg

ctcagaacccacattaccatatctgagtcctgaactcaatggagaggcactactaaatgg

tgctaactttgcttctgctggaattggaattctcaatgatactggagttcaattttttaa

cataatcagaattacaagacaattgcagtattttgagcagtatcaacaaagagtgagtgc

attgattggagaagagggaacagcgagattggtgaatgaagcattgtttttgatgacttt

aggtggaaatgattttgtgaacaattattttgttgttcctttttctgcaagatcacgaca

atttcgactaccggattatgttgtatacctcatttctgagtatcgtaaaattcttgtgag

cctgtataatttaggagcaagaagagtgttagtaactggtactggtcctataggctgtgc

accagcagaactagctcagcatagcagaaatggtgaatgttatggagaacttcaggaagc

tgctaatctgttcaatcctcaacttgctcggttgttagctcagctcaactctgaaattgg

tgctgatgtctttatttctgcaaatgccttcgcaatgaatatggatttcgttggcaatcc

tcaagcattcggatttgctacatcaaaagttgcatgttgtggtcaaggaccctacaatgg

gattggactatgtactccagcatcaaacctatgtccaaacagagatgcaaatgtattttg

ggatccattccatccaagtgacagagcaaacagacttattgtagacaggttcatggtagg

atccagtgaatacatgcatccaatgaatctcagtaccattatgcttttagattcaaagac

ctaattaatacagtaatattgtaccttattccaattaatatttccagcacaatatatata

ttgattgtttagactacattgttattgttgctaagtgtgcaaattatgagtaaaatattt

cgaagtttgggaaatcatgagaattgtggcgatgagtgttctaatttagtgagtaaaatg

ttggtaatttttttgtcaagaggtggagggttataacctctaagctttgtatctcatatg

gttgaatattaagtgttgtaacatcatgtttggttgataacattatcttatccgctttgt

cg

>fava_dn_rep_c680

ggccgcgggatccttcactaccaaatatcacatttcaatcaatcttgagttgagtacaat

ggattccaaaaaggcaatcctcatccttggcctattggccatggttcttcttatctcttc

agaggtttcagctagggacttaactaatgccaaagaggaagttgttgaaaagtcaaatga

agtgaatgatgctaaatatggaggctacggtcgtggaggtggttaccataatggaggtgg

ttacaacaatggtggtggttaccacaatggtggaggtggttacaacaatggtggtggtta

ccgcaatggtggaggtggttacaacaatggtggtggttaccacaatggtggaggtggtta

caacaatggtggcggttaccataatggtggaggaggctacaatggtggaggtggttaccg

tggtggaggaggacatggtggacatggcggtgcttccaacaacggtaactaataatatca

acattctcagtaataagtatcatgaagtaataatgttgggtgttgaaaaaaagaggtata

gatagagagacatgaatcttttatggttatgtatcatttatcttgcaactcgatagtata

tatatttcacaactctataaatcactatgtatatgtgatgatgagttttatttccaatca

aagtggtgaatgtgtacttatcatgtgtaaactaatatatcaaatttcatccttgttagt

aaaaaaaaaagaaacgaatattgaaaggaggcggagggttttatagaggggggaggagat

caagaacagaggggcgataatacagaaacaaggtgaggaattacagaaagggagaagaga

ggacgaaactgatttacagagaaatgggacgattggggtttcactatcggaaaccacaat

cgtcgttgcaaagctagccgccatctccatcacaatcgtctccaccgcgccactgaaatc

tcatccatcgtttaacacgatgcatcctccgcctcggatcttcacacaacagtcgtcgta

atcccagccacaacggaccatctaagagccaacaacggtgaacgacgtcgtgaatccttc

cacgagcacacacctacaaccaccgcccagggtttttttcttccctattttttattgcac

ttttattttatttgagtcatgattattgatctttgagggatttgaaagtagtgataagga

taacgatttggagagggtttttgcttcttatgcttttgtgtgttttgggaatgagagaag

agaaagtgatttcaagaaagacaaagagaaagggaaatgcttcagtctctctctttcacg

aacaatatgtagttttttttgtgtgtttctatcaccgccacatgttagaattctaatggt

ttttaggtatgttattc

>fava_dn_rep_c681

ttataaaaaattgtcaaagcaaaagcaaccattccaagataacatcataaagtagttgaa

gaaccgcggcggttgtgggaggagaataatgcttccgccaagggtagttgcagcagtgtt

gctgttgataataacagcaagtttgattggagcagagggaaaacctcgtcgaattgttgt

tgatacagatgttgatactgatgatttgtttgctatgctttacctcttgaaagtcaacac

ttcacagtttcaattcgaggctgtgaccattagtgcaaatagctggactaatggtgggca

tgctgtgaaccaagtttatgacttgctttacatgatgggaagagatgatgtttctgttgg

tgttggaggtgagggtggaatgctccctaatgggaccatactcccaaatgttggtggata

tctttcaattatagatcagggaatgacaacagctggaagttgcagatacagacaagccat

tcctgtcggactcggaggacgcttagatatcgattcaaattacggtatcagaaaagcttt

cctaccacagggaaaaaggaaatatacaccattgcagcaaccaactgctcaacaagtgtt

gattgaaaaagtatcagcaggtccaacaactctgcttatgatgggagcacatacaaatat

cggaattttcctaatgaataatccacacttgaagaaaaatgtggaacatatttatataat

gggtggaggtgtaaggtcaagtaatccaaccggttgttgcccgaagaacgcttcgtcttc

gtgcgtgcctcgacagtgtggtgatcatggaaatatgtttacagattataatactaaccc

ttatgcagaattcaatatctttggagacccttttgcagcttaccatgtgattcattctgg

aattcctatcactcttgttcctcttgatgcatcaaacacaatcccaatcactgaacaatt

ctttgacgcgtttgaaaagagtcaagacacgtacgaggcacagtactgtttcaaatcctt

gaaaatatctcgtgatacttggttcgacgaccagttctatacaagttattttatgtggga

ctctttcatggctggagtggccatctcaatcatgagtaaacccaataactataatggaga

aaatgaatttgctgaaatggaatatatgaacataacagtagtcacttcaaataaaccata

tggtatatctgatggatccaatccactcattgatggcctaaaagttcccaagttcaatat

tcaaaaagacggggttcatagtggtcatattcaacaaggactcagagatcctctttgctt

tgtgaagaatggcaaaggaaaatgtcaggatggatatacaaaagaggaaaatggtccaga

ctcagtaagagtacttgttgctacaaaagcaaagcctaaccgagatgttaagagttcact

tgacagagaatatttcataaacttcttaaatgttctgaagcagccaaaacaagcagggag

atacaacttcactacacaatttccctactacaaagaagtaacttacaagccagattttag

taacaaaacattgggaaaaccagttgtgtttgacatggacatgagtgcaggagattttct

agctcttttttatctccttaaagttcctgttcaagtaatcgaccttaaggcaatcattgt

gagtcctactggatgggcaaatgcagcaactatagacatagtttatgacatacttcacat

gatgggtcgtgatgatataccggttggtcgaggagatgtttttgcactgaatcagacaga

tccggtgatctcagccgttggagaatgcaagtatgttaaagccattcctcatgggagtgg

cgggtttttggattctgacactctttatggtctcgctcgttatcttccacacagcccaag

aagatatacaggagaaaactcggtgaagtttggagccccacgtgatacggatcaccctga

acttagacaaccattagctatggaagtttgggagtctgtcttgcaaacattgaaacaagg

gaccaatgttacagtattaaccaatggacccttgactaatttggcaaaggttgtttcaat

gaaaaacataagctctagaattcaggaggtctatgtaatggggggacacataagcagtaa

cgcagaagacaaaggaaatgtgttttcagttcctttaaaccaatatgccgaattcaacat

gttcttagatccttatgcggccaaaacagtgttcgaatcagaagttgacatcacactcat

tccactcagtactcagcgcagagtgagttcgtttgcaacagtgagaaaaaaagttgcgtg

ggactagaagaacaccagaggctgtgtttacaaagcatctattgtcaagcctacaccgtt

tgaagaaaatcaatcacagataccaccatatggacacattcctgggagaaattctaggtg

cagttgtcttggctgataaggcttcaactccgaagacaaaatttgaggtgaagtccatta

aagtcttagctagtggcaatgaatccacagatggaaaaatagtggtggatgagaaaaaag

gaaagttagtcaaaatattaagcaatgtggaggaaaatgcttattatgatttgtatgcaa

acaagcttggtgatcagtatcagtcagctaaggtggcaagctttaaagcacagaagagac

agtggagtcatcctcatgatgataagcctagcaaagaaaacggtgctcaactacatgttt

aagagtggttacaaccagcacaagactctgctatatgtaattttatcaacaataatacaa

tatgaatagctattctattcgaacaataatagtaacattgttgagtaatttatgaaaaat

attatattgaattacaccattcatacaaatcttcgatacagagggtagaatccagagagc

actcggtaaaaattctagcatgcgttacagctcttcctacagtatccaggaaggtctgaa

gatccaaccatatacacaggattgttagtgcattctccaagggaagcccatctcacacag

ctatcatgctgatcggagcatccccctccagcgcctacaatcttatcaaacgagtctaca

tgaatccactttgtcgctgaccatttctccccttcaagtactgggcatccggcatgaaga

ctgttagtatccgggctagcatgtgtgtcaagactgaagaaaagaagtgcatcccctcgg

cgtggtttcactgctattccttttttggcacactcagagaggtcactacttttttcagaa

cccctgcgacgtcgaggttcctcagcttctgggaatacagtttcaccgcctttggtcaca

ttagtgagatacatgagaacagtcgcgagacgatgtcctccctgaactatattaacttta

tcagtaaagtaatcataatgcgggtcatatttctgtccatgctcgtatctcaatacttgt

atgtcttccccattttctttaggaagaaaggtccaagcagaaattttgtcctcaatacca

gaaataataggatccttgtttttggaaatgaacatgccagagcttgtccgaacatcactt

aactgactatctccagaaagattatccgcaacagcggatctcttgagctccgatttcgct

aaagaaatcaagtgatcgcattccaagtccgtgagaaaaccttcataaacaaaggctctt

ggattccatgaaatctgtttgactttggaagggttgatgatggaactagctgaaccagcg

taggagctttgaacttgatcggtttgagagatcagtaacaagaagagcgaaaaccaaatg

accctactcatttcagatttagattctaaacttcaattttttctcaggaatttatctgtt

cggcctggaacttgactttacctcaactaatctttgtttagtctcactagtctgctgttt

ttattttacatcataattataataatgagaaaaaaaatagctgcaaaatttatcattttt

taagacatatttagcgtgaaagttcaaaannncaaaaatagtaagtattttgtttgatat

>fava_dn_rep_c682

ggcccaataagaaaacagacaggagcagaagcgcatttacgtgaatttgggtttacaaca

aatcatatacaggtgcatttcgggatttagggtttatctcagccgccgtagccgccacca

acgaacaatggttctcaagactgaactctgccgattcagtggtgcaaagatataccccgg

aagaggcatcagatttattcgaagtgattcacaggtttttctatttgtgaactcgaaatg

taagaggtatttccacaaccgtttgaagccttcgaagctcacatggactgccatgtacag

aaagcagcacaagaaggacattgcacaagaagctgcgaagaagagacgccgtgctaccaa

gaagccatactctaggtctattgttggtgctaccctggaagtcattcagaagaaaagagc

cgagaagcctgaagttcgagatgcagctagggaagctgctctgcgtgaaatcaaagagag

gattaagaaaacaaaggatgagaagaaagccaagaaagcagaagtagcatctaaggccca

aaaatcaggcaaaagcaatgttcagaaaggaggtttgcccaagggtcctaaattgggcgg

cggtggcggtaaacgctgattagttccagttttgggtgcaaattaaatacattttatttg

aaacttctgtactagtaatattttatttgcttgatttgattactttgatacctaattatc

acatgtttttggagcttcagatagtatctggttacttcatgcattttgctagtggttcta

tatttttttctgttaatgaatgatcttggaagttttattttgattggcagtttgactttg

aaaatcaaatgctcaaacattaattatatatgaagagacttgaattgcatcttcaaaaaa

a

>fava_dn_rep_c683

taatccaaagatcatatcatttagatatagatttgctataaaagagaatcagttaatcat

atcatatagatagaacctctagaatatgacacaattgatcaatgaatcaaaagtgaatat

atataacaactcttgattttgcaacaatcagaaaatccctaaaaggaaccctaattttca

atcaaatttgcttaacttcaaatagcacagaaattaaactatgatcaacaagtaatcgtt

tcgttttcattcaaacagagaatttatagatctacataacaaacagaaaagcggatagaa

atagtaaaataacaacaaaaaacaacacggaacacaaatccactagacttatctcaacca

gtgcttcaatcggtcctcttaagacgctccagacgctggttaagcagatcaatcttaacc

acaagatgcgtcaccgaatacaaaagccagtagaacaaaagcgcagccgcgataagaagc

gcgttcctctgactcttcataatcgatttctgatgacggagatgctccgtcggagtgcaa

gaatccgcttcacagcttggcctcgtctcgtacttccagtaaatgtccatgagaagaaag

aggcaaaacggaaccaccgagagaaacggtttcaggagattcttcgtgacggcgaccaga

cctttgcggagagcgtcgaggccggggatggtgaggagaagcaccattatcgcttcggct

gcggcggcgtaaccgagtaccacccattccaacgccattttttcagatcttgaattgctt

ggttttcttcgtttgggattcggagggtgagagtcccgcgg

>fava_dn_rep_c684

tggaagtgtgtataagaaagggataactattcccaagcaacattaaacggatagcaagtg

tccctatgttaagggcatctctagtgacacttgaagaagatatacttaacctttcttcta

cggttattacttcttcttattaggttccagtagttctagtaataatatccaattcttttt

cttcgctttgcttaattcacgtttcatcaatggatggaaaaagcgaagaagaagaactcg

aagtcaccgaatgggaactcatcaacaacgatgaatcaccaatcgttgaattcgatccca

attcttcatccgttatcagatccgatcatttctcgatcaatccggttcatcaaaacccta

attcggttagttcggtttccagttccgaaatcgatcgaaacttcgatgattcctacgtcg

caaatcagcttttccctaatccaattgttccggcatcatcttccgacaataattatcggt

ggaatgattccgataattccgttatttccgatgatatttctgatatttccgataattctg

atagggttgttgcagatacagccagtttgaccggtgatattggttttgattctgaggaaa

aagtggaagagaaagtggtgaaaggggagaatgagagattcgtgtggtggaaggttcctt

ttgaggtgttgaagcattggatgagtcctcttttgattcctgttccgttatctgttgctg

ctgctgctgcgtttttggggcttttgatattggggaggaggctttataaaatgaagagga

agactcagactttgaagcttaaccttgctcttgatgataagaaggtagctcagctcatgg

gtcgtgtagcacggctcaatgaagccttttcagttgtgaaacgtgttcccgtggtacgac

catctctgccagctcctgctataactctgaggcctgtgatgagtctgagatgaagaaaga

atttgacaaattacttggtttgaagtagtgtgaaattgcaataatgtttgtaatgtcata

aaaaccctttagtgtgaaaacatgtatctataaatgtattgttatgtatgtatgtatcta

tgtgtgttgggttcgaattcacgaagctactcaatggcttcttgtatatgacattatatc

ttgtttgttgatttctatatcttgtcaacatatttgttatggggtaatattggaatttta

aaatgtaatttatttaattatgaatatttattggaaaatttccaaaaaagtaatatattg

taacactaacttagttttatcaagttgtaacataataaaagtggttaaatcaa

>fava_dn_rep_c685

tttttttttttagagaataaaataattcacataataccatggcaaaaataaataaatata

ttacaacttgatatgatacatgaagaacatgttcaatcaattcttccattccacatccct

tagaaattaattaaattttcgagtgcataatgtctaattcttatcaacaattacaaaaca

tcattatgcaccaacaccaaaacatcgatctcgattctaatagatctctccatttctttt

taatggtggtggcggacacattccgcggggataacgggaaacctagcggaatcggcacaa

taatcatacaccatatgattagccctaacccaaataagttgatgactttgatgcaaagtg

agttcagacaacataggttcatcccaccaatatttcttatcctcactactactacatttc

ttggcattatcaattgatgtaactggtgttggacattcacaagcattgatttcgaaatct

ttataagtagcaacaaaaggtgcatgagaccaatctgttttaacacgaccaccttgtgtg

gcccaatcatcagcattccatattgaactgtaaacacccattgcttggtcttttgggaaa

gggattcctctgtgttccaaatttgtgtgaactctaattggtgtctcatccactaaaaac

actacttgacgttggttccaaaagattgagtaggtgtgaaaatccttagatggatcaaac

caaaggttaagtctttgttctctattaccaacaccattcacatacacatttgtttgaact

gagtaaggttcaccagttgtgttacccaaaaactcaaaatcaaattcattatgagttggg

ccttctgatgacatatagaaagcagtaacagttccagcagagtcaccttccacaagctta

agctgaacagtcacttttccaaacatatatttgcttttagatccaaatccagcaccggaa

taattgtcaagtttgagtttgagtaaatctccttcatgaacaaaatggtccatagcccaa

ccaggttgaaaaagctcctcaaattttgaagaagcaactaaagcaaaaaacaaaaaacac

atagaaagtcctaagagacaagacatttttgtcatgttgaagaaaatgcagagacaaaaa

acagatgaacaagaacaaagagaaagaaagtgtgagtgagtgaagtgaagcttctactct

gaaattgtgtgcccgcggnnnnnnnccactctgcatttttttcaacattccattcaccac

caccaccacctcctccactgccaccaaaacctccacttccacctccaatggcagcaagtc

catccgtcgtaaacccctcacacgaaataagcaccaaaaccttaaaattcttaaacgact

tagcaataagctccaagctctcatccgtaataaccatcctcttgagccgaatctcttcca

gcaacggcaaaccatacgccatagccgcgatccacggactaacataacctccccatcctt

ccggcaccaaattgaagtccgcaaagtgcggtttccctttcagcgaaacggatctcagct

cgggaaaccgtttcgtcaccgttgaaggactcacggcgtagcagtttccaacgaaaatcc

gccgcctgcaccaccgttcgatctcgtaccatgatttacacactaaagagatcgagtttc

ggtctttgtcacattgaatgaaggagaacacgtactcgagaacttcctcagggaaacgaa

tcgtcattttctgcatgtgaatgaacgagtgttgagtgtggagtgtggagtgtggtgcag

ttttgagagttagatctgagacaccgttaacgagcttaaagtgtttgtgaaaatgcgtga

gagagagagaagataagagtggtttatgttgagtttgagtgtggttagaaactaactatg

gttaaataagtgatggattattgatcattgatggtc

>fava_dn_rep_c686

aaattattaaaagttactaggtaaatagttctattcaacaatagaaagttacacaatgta

ttaaaaaaaaaaactaagaggaaccagccaaaatagcaaaagaaatgaacagtaagtagt

gaacaataacaatgtttatttttcaagacataaacttttatttatataaataatcacaat

aacaatacattacattacatcataggggaatgatctataagatcagcgcaagttttaaca

aattaagcatgcgcaggttcaaaggaagtaaggtttcggaagtgaacccatatcttctca

taaacaacagcttcatagatcttcgaaacggatccttcgccggcggagagtgtgaggtgg

tagttagtccctgcaacaacctgagattcacctttgatgattttgtggaatttcaatttg

gcaccactttgcttgtcgtactgagtgacggcgaaattggcgatctcgatgacgtgagga

tcgttgatgtcggagattggggaataaccaccgaccagcggcgaagatagtttccttgca

gccgcagaggctaatagaagaagtgtgagaagaattagggtttgaaatttcatgatttct

ttttttttttctttcagtctttagagagagaaagagagagatggtgttgtgttcccgcgg

ctgagactttagacggttatttat

>fava_dn_rep_c687

caagattgcaaatttcacacctttcctcaataggtaaattcatgaataatttcagctatc

agaatatatatatgctgccttcaattatgtatttgagaactacaagtatacaatacatat

gccactgaacaagagaaaaaatacatgtaagaaaagaatacatacgaatgtgttgaacgg

gtgaaataattgtgcttaaactgaaacaacaatttacctgaatccccatcacaaaagata

caataaatacagtatgcaaaaccaagatgataaaagagccccgttctgatatccaaagcc

gcgaacaaacaaaaggtaagtcaaggaaaccaaatttttatcagattaacgtgtaagaag

tttggtcttgtccaaatcattttgtaatggcctcattagtcttctgtggttcagtccttt

cttctcttttcttcttttccctgtcaatatattccttcagcagctctttagcttgaacca

gcttagaaagcagattgcgatacacgtccaagtcccgatccacacttcttttgttgatat

tcaaagccgcacaaagcttttccaaaattgtcggttccattgcatttgccaactcaagaa

ggcgaaaaaggcctactgcaaagaatcgactataactgaaatccccctttccacctgctc

tctctgcaatatcctttaagatcccctcaacttctccttccctggatgaaaactcaataa

gggaacttgaattttgagcacgagcccactcttccaacttctgtgcatctactctgtatt

gttctggatcttccttcaatgcattaatgtatgcttggaagatggcatctctgtcctcat

cacttggatacccgtccatgagctgatcatatacagtaacaaagccaagtgcaaatacag

gatcgtagcggtatgacttcttgtatctcatcagatgttgctgcacaattagctcttgaa

gcacggagttgtaaatactaggtattggccgcttatatgccttgagaaaatttaactttg

tctcagaaacagttggaagagggtctgaaacagaagaggaacaccgaacagccagttttg

aaacggaattggaagttcgaactccaacacagtgagacgaaaagctgaaacgaaagcgaa

cggcatctgaatcggaactaaggaagcgagtggaagaaactgtgagtttcctttgagaag

gagattgagtgggtgttgagaaagagaaggaagaagttacggcggccatcggagcagaga

gagtggtggccggattcagaaatttacaggcatagaatgaaaatttgggagaagaatgtt

cagagggagagttgcatcttggttgcttagagtgtgagtgttgttgtcattctgtggatt

gcggggaaaatcaaaatccnnnnnnnntccgttcggtgtgatattgaaattctt

>fava_dn_rep_c688

gagtattttgttttgtggccacaaattaatatctgggaaatttataggttctcttcatta

tctatcatttcctttcctagttggattgctccaaccaaagcatcacaatggacacaatca

cctccacttcagcatcactaacactctctctccctgcggttccgcttcgtacactttctt

cttccatctccgtatcctcatctcttctcttcccaccctctcacttatcgtccacgctga

gactctactctgaaaaaacaaggttctccaattctccctctgcggttcccaagtttacag

tgacttccggtatcacggaaatcaacgagactcagttcaaggaaactgtattggaatctg

agcgtccggttcttgttgagtttgttgctacttggtgcggtccttgccgtttgatcactc

ccgctatggaagccctagctaaggaatacgaagacagattaacagttgtaaagattgatc

atgatgctaaccctcagctaattaaagagtataaagtttatggactgccaagtctgatac

tcttcaagaacgggcaggaagttccaggaagcaggaaggaaggtgcgattacaaaggcga

aacttaaagatcatgtcgatgttttgttagaatcaatatcagttacatagtggttctata

tgtattccaaattgtgacttttttttggaaaactagtttacatggaagcagacctatttc

tgttccatgtctcgtatgagggtttagtaggcttttacgtttccaatctcttgtaatctg

tatcatttctaaagctgcatatacagcatatattatggtaaagaaagcttgttgaaactc

aagttccagcccccttcctatcaaatccagcgccaatcaacactagactaactaacgact

cctctttgatatataataaatgtctttccacattttactgttttaaaggtattgtacata

tttttcattttataaattagtttgagatagtatttgtgaatattcattcattcatgatta

tatagatcccaattaatgttggaat

>fava_dn_rep_c689

cttttttttttactgaaccaacaaccaagctctattcataaatgacaacttctgaaaatg

atccatgaagtacagaactgatacaaaataatcataccacgtctcaacgaaaacacaaca

actaaatattattgcaaattgcaatcaatacaaacaaaatgtttcttacacagatcacta

atctagtttactcaacataaataaatacatggaaatagtttgataggctttattccatag

taacgtcaattttataatgaatatataagcgctataattaaggaaaaacataacaaaact

ccacccttactctgcccaactctctaatgaagttcacaatacacgaatctgtgctatgtg

cgctacaagcaataataaaattgatattactcggctctggattggccagcacgggaagag

aggatgatgccaacaatgagaccatacaatgccaatgcctcagcaaagatgagaatgaga

atcataccaacaaaaagcttcggctgttgagcattagctctaacaccagcatcaccaaca

atcccaatagccatgccagcagacaaaccagcaagcccacaagaaagcccggaagaaaga

tgagcataaccatcaaacaaataatacgatttcgccttagggttaatcccggtactaata

ataacagcgataatcaaaccataaatacccaaaacaccagccataacaacaggcacaatc

gatttcatcacaagctcaggtctcatcacacccatcgacgccacaccaacaccgctcttt

gcagttccataagccgctcccatacatgagaaaattagggcagcggcggcgccgaggaag

ccgaagaaaggagcagtttcatcgccgctgaaaccagccattgttgttttgcgaaaatgt

gaagaggatgaagaagagaacgaagaaggtgatagaaagattatagatctattattattg

aatatactcttttttttttgtgtggttttatttgtcgattttaatttgttttggattgga

ccaaaggaagtttcttgagtggtcacccgctacgtttgttatcacgcacaccgcgtgttg

ttgttatattct

>fava_dn_rep_c690

aaagactagtttttataccacatttgctcaataatcgtgctagattagaggcacttgaaa

atatacaatatcatatcagggcaggaacaaaattacgtaccaaacaaattgctaaacata

atttaaaactttcgagatagtataccaacaaaattaactcaaaaatcttctcaacataga

gaacaaacacattgacacaagacaaccaattccattgagcaatctaaacacagactactt

aataaataaagacatcataaaattgcaacaacaccaaaatttcttaatcaataaatagga

aacaacctctcaaaaagactaaagagctttatcatcatcaccataactaatctcccttct

tcctgaaggagcatccctctgtgagtcttgctttgccacctgttggctggcacaaaactg

tttggcagttaccgcaaaccacaactgtttgagagtgactaaacacagtagttatgttga

agcatccttggcatttgacatccatgaagaacgaattaggagattgaacgagacgcttga

gtttgtgcttcctcttctcgagttccgcaggtggattcaacagatcaatgtcgttttgga

gaaccatggtgagaggttgaagagtgagacagatcgcaaaccctagaagatgagattgaa

cggggctgcgactgtgaacaaaagctgaaagcc

>fava_dn_rep_c691

aaaaagtgatttaatagaaggaaaaaaaaaaagacaaaaaaagcgatttaatgataggaa

ttacccaattcttggtgcgatcccaacattatatacaacaacactagacccaagtcttca

ctctcagtaccaaaatcccaacgttttctaaaccctagcggcacgcaattcaaatacaca

agatggcagatcctgaacaccgtgaagaggaggaggcacctgccgtcggcgatgaagaag

ataccggagcacaagtggctcccatcatcaaacttgaggaggttaccgtcacaaccggcg

aagaaaacgaagacgccattctagatctcaaatcgaagctttaccggtttgataaggatg

gaaatcagtggaaggagagaggggctggtactgtcaagtttttgaaacataaggttacag

ggaaagttaggcttctcatgaggcaatccaaaaccctcaagatctgcgccaatcatttca

ttatacctactatgtctgtgcaagaacacgccgggaatgagaaatcttgcgtttggcatg

ctagggattttgctgatggtgaactcaaggatgagcttttctgcattcgttttccatcca

ttgaaaattgtaaaagctttatggaaacattccaagaagttgccgagtcccaaaaacaag

ttgatgaccaggaatcatctgcagcagctacccttattgagaaattaagtgttgatgaca

aggctgctgatgcagagaagaaagatgaagagcaatctgcggaaaaaactgagaagtctg

cggaaaaaactgagaagcaagcatcaggtgagaccagtaaggcagatgcagataaaaaag

ttgaagagcctactgcttgagtattatttattaatatttcatactatgcaatctgattag

gttgtggttgagttttttctgtccatgtctttggatactgtcatacttaatggttgccag

attgtcattgtcgttcaattgaactgtttgtgtccttgggttttttcagcaagtgagatt

agggttgggtgtgaattttgagacttgaacttgttgagcttttggggtctgtgagtttat

tgttattattttcattctacatatgtatttgttatgggtaatgagtcgtgttggttggga

ggggtagtcgggaatggacaaagtcataaacaaaggttttgtttacaattcgattgagtt

ccatattttggttatagacatttagtcatttattttatttttaatcagagttttcttttt

gtttcggtgaaattcatcaacttttaaaagttacaactgagtctcttataaattggtgat

tggtttattgtgcaataataagttcggtgaatggttgttcagttgtttggatgagaatat

ttatatg

>fava_dn_rep_c692

ttttaatgtagtttaatatgaaacgtatatagtaaataattcccccaatattttaaaatt

aaaaaaacaatgcggcgcttttttgagcattttaagtttaacgtttctccacacttctct

ccttttctactctctctcttgtttttgcgagtgtagcaaaagagaaagagcacacagagg

aaaaaccagattcgaagaaacaaaaaatggcgggttacagagccgacgacgactacgact

atctcttcaaggtagttctgatcggcgactccggcgtcggcaaatcgaatcttctttctc

ggttcacgaagaacgagttcaacctcgagtccaaatccaccattggcgtcgaattcgcta

cgcgtactttgaatgttgattccaaagtcattaaggctcagatctgggatactgcgggtc

aagagaggtatcgtgcaatcaccagtgcttactatcgcggggcagttggtgcacttcttg

tatacgatgtcacacgccatgcaacatttgaaaacgtcgacaggtggctgaaagaactgc

gaaaccacacagattccaacatagttgtgatgcttgtgggcaacaaagcagatcttcgcc

accttgttgctgtatcaactgaagatgggaagtcttatgctgagaaagaatcactttact

ttatggaaacctcagctctcgaggctactaatgtagagaatgcatttgctgaagttctga

ctcaaatctaccatattgttagcaagaaaacggtagagggtgctgaaaatggaaacgcat

ctgtgcctgccaaaggagagaaaatagatcttaaaaatgatgtctctgccttaaagagag

ttggctgctgctcaagctaagatcggaaaacataattcgaattggtttctatcggctctg

ttgtatgtgtaatattctaagagagatacaaactagaagaaaaaacataaaatgtaattt

aattattcatagcatgttagactctatgtgtaatcttaaaaaacaagtttgatgtttcaa

taggagttgctggggaaacaagtttcttgctaagctacatagtctgtacttgtagtgttg

tagttatttttgaaatgttttcatcaaacaatcaaataattgattcatttgtttagagag

aaaatgggtgatgcacttaattttacagcgtnnnnnnnnnnnnnnnnnnnnnnnnnnnnn

nnnnnnnnnnnnnnnnnnnnnnnnnnnnnnnnnnnnnnnnnnngttggttag

>fava_dn_rep_c693

ggcagaaagatcataatcaaatcatatagaattcaaataacaaatgtgacaacattaatg

gccacacaacagaacgaaatcactaaacatttacataaacagtggcgcatctgtaaccat

tttttaaaacaaaactaaaagcagtggccatgacattgcttagaatagcaatttcatttt

tgaaattaactgaaaggaaaaaaaatcatgatggaggaaatataacacaaaatgattata

ccatagtaaactccaacaacattcaacattgagtaaacaaaatctttagtgaggagagaa

ttaacaaccaaaataacaaaaagtatctcagaacaaacaacaaaaaagaaacaatatcaa

ccagaatctacttgatcttcttctttggcctcaattgattgctgtgtccacacttcttct

tgcggcagttaacagccctaggatgaagacgagcatagcatttgcggcaaatggttttgt

cttgattgtatttcctcgccaaagccatcaaagaaggttcaatgattccaccacgaagcc

tgagaacaagatgaagagtagattccttctggatattgtaatcggccaaggtgcgtccat

cttcaagctgcttaccggcgaaaatcaaacgctgctgatccggagggataccttctttgt

cttgaatcttggtcttgacattgtcgatggtgtcgctgctctcgacctcgagggtgatgg

ttttcccggttagggttttcacgaaaatctgcattttgcggcttcttcaattctctctgg

atttacaactatggtg

>fava_dn_rep_c694

ggctataaaatcatgtagttcttttgattacttaaaatataaaatgtacaattgttttac

aaacatccttacatatcccaatatcttctatgaaatcttttatggaaaataggataatat

gcaaaactattttgcaggagcaatgcacctttatgagccttacggcataaaacataatat

aagtttctttaaaaacaagctcatcagagaatgttatacattacccccctttattttttt

caaaataataaagaggtattgtgtgttgattgatggagaacaaaattgaaaagacaatta

aaaccaaaagagttgaacaaaatttagtctattacaaattcattccatagaaaatttaag

cagcaactgcttgttgggctgcagcagctctcttcctagcttcctcaatgacagtaagct

tgatacccttacccttgggaagagatacccatggctttgtacccttgccaatggtgaaca

cattgcccagacgggtagcaaactcgtgtccagtggcatcctgaacgtggattgtctcga

aggttcccttatgcttctccctgttcttgatgactccaacacgtcccctattccttccac

cagtaaccataactacattcccaacatcaaatttgatgaagtcaacaatcttgttctcct

caagatccagcttaatggtgtcatttgccctgatgacagggtctgggtagcggattgtgc

gaccatcataggtgttcaggtatgggatgcctttctgcccaaattgcactgaacggacct

tgcagagcttgaactttgactcgtcatctctgacagaatggagacggaatttgcctttgg

tatcataaagcaggcgaaagttctcatttgttttggggatggacacaacatccatgaatc

cagcaggataagtcttgtcagtcctaactttgccatcaacaaggacatgcctttgcatca

gaatagcaatgacctcacggtatgtcaaagcatacttgagtctgttacgcaatatcagaa

taagtggcaagcattcccttgacttgtgtggtccagatgagggcttaggagcaaaagcac

caccgagcttgtcgagcatccaatgcttgggggcattgatcctcttcaaatgcttcttca

atcctctagccattttggatgaacaaaaacaagacaatagctacaatgccagagaaacaa

actgaagagtggaagctgccgctttaggttaagagaaaaaccctaattgta

>fava_dn_rep_c695

ccacattgagggccgcggcaccactccaatctcatcaaacaacaatgggttcaacaacaa

caacaagcgaaacacaaataacaccaacccatgtctcagacgaagaagcaaacctcttcg

ccatgcaactagcaagcgcttctgttcttccaatgatcctaaaaaccgctctcgaacttg

atctcttagaaatcattgctaaatctggtcctggatctcatctttcaccgcttgatattg

cttctcaccttcccacaaacaaccctgatgcacctgttatgttggaccgtatcttgcgtt

tgttggcttgttacaatattcttacttgtagtttaaggactctagaagatggaaaggttc

agagactctacggtttggctactgttgctaagtacttggttaagaatgaagatggtgttt

ctatttctgctcttaatctcatgaatcaggataaagtactcatggaaagctggtaccacc

ttaaagatgcggttcttgatgggggcattccattcaacaaagcatatggaatgacagcct

ttgaataccatggaacagatccaaggtttaacaaagttttcaacaagggaatgtctgatc

actctaccatcacaatgaagaaaattcttgaaacctacacaggttttcaaggccttaaat

ctcttgtggatgtaggtggtggtaccggagctgtaatcaacaccattgtctcaaagtatc

ccactattaaagggattaactttgatttgcctcatgtcatcgaagacgctccttcttatc

caggagtggagcatgttggtggagacatgttcgtcagtgttccaaaggctgacgctattt

ttatgaagtggatttgtcatgattggagtgatgaacattgtttgaaatttttaaagaact

gctatgaagcacttccggaaaatggaaaagtgattgtggcagagtgcattcttccagtgg

caccagacacaagcttggcgacaaaaggtgtggctcacattgatgtgatcatgttggctc

ataatccaggggggaaagaaaggacacagaaagagtttggggatcttgccaaaggtgctg

gatttcaaggtttcaaggtcaattgtagtgccttcaacacatacatcatggaatttctta

aggtttaaggtttttttggcatgttgatatgagttttgagattgcgattgttgtggttgt

gtttctatttaccaaagctttcacaaagaataaataaataaacgatttgtgtttctatac

tggagaaaaattataatgagaaacttcattgtaatattacctatataaaacaacattgac

tcattatagttttatgtataatgagcttaaataaaattgactcgttaatcaaaaaaaaaa

nnnnnnntaaatgatttctaaaaatacttgga

>fava_dn_rep_c696

acacgcattttcactatctgaattcttcacaacaccgtgaagtgtagaaccgaaccatgg

aggttgcaaccgccaacgcagcgtttaccgtcgcagtcaacaagccgatgatgttgttcc

cttcttcatcaagaaccataacccctctcaaagccattaaatcctcttccaggaactcta

tctccaccgatttcatcgctcctacaactgcaatgaccggttcctctagccatttctccg

gcctcaagctccgacctccttgcctcaatccggcctcctttcaaagttccggcgctaaaa

gaagcgtcgttactatggttattcctttctcaagagggactgcgtgggagcaacctccac

cagacttggcttcttacttgtacaaaaatcgaattatttacttgggcatgtgtctcgttc

cagctgtcacagagttgatggttgctgaatttctttaccttcaatatgaggatgaaaaaa

agcccatttatctgtacgtaaactccactgggacaaccaagggaggtgagaagttgggtt

atgagactgaagcacttgcagtttatgatttaatgagatatatcaaatctcctattttta

ctctctgtgttggcaatgcttggggagaggcagccttacttttggctgctggtgcaaagg

gtaaccgttctgctttgccatcatcaactataatgataaaagagccaattgcacggtttc

aaggtcaagcatcagatgtcaacattgcaagaagagaaatcacgagagtgaagacagaat

tgattaatttgttagccaaacacactgaaaaaacacctgagcagattgaagctgatgttc

ggcgtccaaagtattttagtcctactgaagctgttgagtatggaatcatagacaaggtat

tgtacaatgagaggggctctaaggatcgtagcgttgtttctgaccttaaaaaggcacagc

ttatttgacttccaggagattaaaccctattttcttgaagtttagaaatttgcttggaaa

tgtagatcagcgaaaatgaaggacataggagatcagcagccaaacgatacaactgtgcca

agtcctggtacagttaacctgaaaccttgtaatctcatgtttataacaccatagactgca

tttgcgtcccctaagaaaaggtagtaggaacaaatatctacgtgtttgtaaaatgctgta

tacatgattaaattctggtttattcaaatttgaaattttatgtaaaaaaaaaaa

>fava_dn_rep_c697

accaataatcgaaaaccaaaccttcctcttcaactctcttaacacttcttcatacactct

cctctctccactccgccaccgattcatcccaagctcaatggccgttactttctcagatct

acacaccgaagagggtctcaaatccctcaacgatttcctctccggaaaaacctatatttc

aggggatcaattgacaaaggatgatatcaaggtttatggagctgttactgagaagccaag

tgggtcttttcccaatgctgttaattggtatgatgctgtatctacaagcctttctgctag

ttttcctggaaaagctcaaggtgtgaagtttacaggaagtggtgctgctcctgctgcaga

agcacctgccaaagcagatgcgcccgctgctgaagatgatgatgatcttgacctctttgg

tgatgaaacagaagatgagaagaaggcagcagaggaaagggaggcagctaaaaagcctgc

caagaagaaagaaagtggcaagtcttccattctgcttgatgttaagccttgggatgatga

gactgacatgaagaagctggaagaggctgtccgcagtgttgatattcccggtttaacctg

gggagcatccaagcttgttgctgtcggatacgggatcaagaagctacaaatcatgttgac

aatcgtcgacgaccttgtctccgtggacgacctcattgaggagagactcacagttgagcc

aattaatgagtatgtccagagttgtgacattgttgcattcaacaaaatctaagttaagtt

tggccattttcaagcggctggacagaatgttcttccccctctcctctactccgttccttt

cagaatttcatgtttggtttgtttctgtggtggttttgttagaagttatttatggagtaa

aagagttagagtcatgttatgaacttatggtgtaacttaaatgttataattcattttaag

caccaagcacatttacatttgtgggattaacattacataaccgtatgcttctattttatt

tttgataagttttactagaagtaattgagtgaaataatgatcttattcaatttcatgaca

ctatatattttcatgttatctatcacaacattcttccctatttcgaa

>fava_dn_rep_c698

aaaaattatgcattttatttatttttatttttcatattctttcaacacaaacttgnnnnn

nnnnnnnnnnnnnnnagcattactaaaacgacaacacatctcatcacttcactgagaaat

gctaagcactttcgcttttcaatggctctgaaactcccaatcactttccccatcttctcc

tcacccattgacccgaacccaaactcgaactcgaacccgaacccgaacccacgtttatcc

accaaactcgttccaccctcaaaagcaaccagcgagcctaaatcccgagtattcatgtca

ccagcccaaggcccataacaatcaaatacagtgttgagaatagctgtccctcgggaagca

gttaaaattgcattacgcaatccaagtagaccacgagttggaatcttatatctgagtagt

gtagttccctccgatccaactccttgcatatcaaacatttgccctcgtctcccaccaaga

agttcaacaacagctcccatgtacacttccggtacctccacagttgcaatctcatatggc

tccaacactttgtcattaaccttcttgttgataactttgggaggcccaaccatgaattca

aatccttcccttcgcatattttcaatgagtatggtgatatgcagagttccacgcccgctg

acaatgaatatatcagctgtttccccatcttcaaccttcatagccagatttctctcaagc

tcacgataaagtctgtcccttaaattcctactggtaacatactttccctcacggccaaca

aaaggtgaagtgtttatggagaaagacattttcactgttggttcttccactttgatggac

ggtagtgccttcccagtcaatttatccgcaattgtctccccaatctgaatatcagaaatt

ccacatactgcacatatatccccagcttccaccctttctgcagggaccctaaagaatttt

tcgtacacataaagttcgctaactcttccatatctacatgaatcttctgaagtacaaacc

ttgacttccattcctttttctaaaaccccagcttccaagcgcccaattgctatacgccct

ttatgtccttcgaactcagtacttgtaacaagcatttggagtgagccatctttatcaata

cgtggtccagggatacatctcatgatggattcaaacaatggcccaaggtcgtctgccaga

ttttcaggagacagccctgctttccctttaataccacttgcatatatcacctgaaagtca

cactgttcatctgttgcatttagctcaatgaagagttcaaaggtagaattgacgacaaaa

tcagggcgagcagaagtcctatctattttattcacaaccacaacaaccgcatgaccaaac

tctaaagctttctttaagacaaaccttgtctgcggcattggcccttcgacagaatctacc

actagaaggattccttcgaccatattcaagatgcgttcaacttcacccccaaaatcagag

tgacctggagtatcaatgatattaatctttgcatctttataagtaatagatgtattttta

ctgagtatagtgattcctctttcacgctccaaatcattagagtccattatcctttcttgc

acagtttgattatcacgaaacaccttagtttgtttgagcatggcatcaacgagagttgtt

tttccatgatcaacatgagcaacaattgcgatatttctaatatctgatcttcgcatcaat

ttcttcttctcctcagtttttggctcggtgacttcggaaattgaacatttgattgagcta

ttgttccttgaagcaattcgaagagatggttttgaagaagaagataaaggaagtccaaag

agtttgttacgtttagataagaagagtggagaagaagaattcttaggaagtgttgtggag

gagaataatagagtgttttgaaagttgagtgccattttctccattctcctcagtcctcac

tcactctctctgtgttcaagttacagagaaaagagagagatgaaggatttggggaaaggg

tgtgtgaggctatgaatgaatttttagtatattatcgttgagttgagttggaaatgtaat

attagtggcacaggaatggatatgatacttgactgtgtgggaattagccaactaggtttc

ccttcctatggattactttcttccacatcgtctctcgtaac

>fava_dn_rep_c699

ccaatcctcaacctgtgctcgagagatagctgtccatatactgataaggatgtatgaatt

ctcgagaggagaggcgccatgatggtccccattgcattttttattcttgcgaaacacagt

aatataaataccatatagtaattgatattaatattatatataatgtcttacaacccctgc

aaaagaagcaggctgaagactacacatcatcacatttgcatgtactatagcagaacagtg

ctgctttacaactgtcaaatattaaaagcgattttcatgaaaatgaactaagcaaaaaca

cattagaaaaaacccaacagcaatcctatcttagaaacagaaaataaattaacacttttt

catgaaggcaaaacgcggaaactttgcttccttttcttctctttttcttcatcgctttct

ttattttcaaatattcttcgttcatatatatcatagagagctatggcagaccactatcta

agagtggtatggagggggaggagaaacatagaggtaaggcttcttggttggagttggtgg

aggagagtggtgaactgggtgaggaatgtatggaggataagtgtgaacaggtggaggagg

aggagaagcgtacttgtatggcttctttggtggtgagtaaactgggtgaggaacgtaggg

agtggggaccggtggtggaggagatgagtatttatatggctttttgggtggtgagtaaac

tgggtgtggaatgtagggtgagggaaccggtggtggaggagatgagtatttatatggctt

ttttggtggtgagtaaactgggtgaggaacgtagggagtggggactggaggtgggggaga

tgagtatttgtaaggtttctttggtggtgagtacactggatgtggaatgtatggagaggg

aaccggtggtggtggagatgagtatttgtaaggcttcttaggtggtgagtaaactgggtg

gggaacgtagggagtggggactggtggtggaggagatgagtatttatatggtttctttgg

tggggagtaaactgggtgtgggacgtagggagggtggattggtggtggagaagagtattt

gtatggtttcttcggtggtgagtacactggatgtggaacgtagggagtggggactggtgg

tgggggagatgagtatttatatggtttctttggtggtggtggtgagtggtagattggttt

agggtaaacatggtgggttggaggtggtggagatgagtatttatatggctttttatgtgg

aagaggaggtgagtggtaaactgggtgtggatgagggtagtatggaggtggtggagaatg

gtagataggtttagggtagacatggtgcactggtggaggaggagatgaatatttgtacga

tgtctttggtggtggtggagagtggtagactggcttagggtaaacatgatgaaccggtgg

cggaggagaagcatatttgtacggcttatggtgtggtggcggtggagatttgtaatggta

aggatgcttaggaggagagtacaccggtggtggaggagaagcatacttgtacggcttatg

gtgcggtggtggtggagatttatagtggtaaggatgttttggaggagagtacaccggagg

tggtggtgaagagtaatggtaaggtggtgaaacaggagggtacacggtggctggtggtgg

aggagaagaatacaagtagctgttggctgaaatttgggatggtaaggttacaaacagcat

ggctaaaagaagggtcaaagtggcagaggccattgaagacctcatttttttggttctttg

ccttcaacctcacttgcttagtgggtttttgatgtcccgcgaaataaaaacaaaaagaat

acaagaaaaattagt

>fava_dn_rep_c700

gaatttataaatactttttaaatctatgattaactaagtcaatattttttaaaatgatct

gctctcaacatacacaagccaagtgtaaaattttaacataacaataaatcaggcttgaca

ggatcaaagtttattaagtattattatcaacataggataacaacatattatgatcagtga

tcacaaactggtacacaatcattattacaacaccaaacaaacaagcttgaaaatgaaaca

taaccacggcattaatccatatagcaccaatattcggctgaaaatgcgtcgcactaattc

aaacgatgctttattgaagtatatcagatgaaggctgctgcaattcaattttcactgttg

aggcagaaaacgtttcttgtaagtattaacagtttcttgaggatccacttttgtctgtgt

ataagcatcaatcttgttcagctcctcgatccatgttgcaagtttaggtcttccttctgt

gatgttaaacttgaacaattcaggaaggataatgtggaatctttcaacgaatggaacata

agcaatatccaccaaactgaattgaccaagaaggaatgggccatcatcaaatttaccaag

ggcgttctccaagtactcaagagcagtactggctagttgcacagtatcccctttgaatga

gccgtacaagtctttgacaaaggtatcaacatgggataacaactgctcagcaaactcttt

cttggcaggatcattgggagagagaggtgttccttcaaagttggcatccacatatttgac

taaatcaagactttctcccaataccttgccattgtgctccaaagatggcaccttattttc

agggtaaactttttccttataccatgcaggcctgttttgaaggtcaataggaaccaattc

aatcttgtcttctagtcccttgtagtttctagtgatccatacacgttgtgcaaaggggca

agtataactgatgtacaaccttgtggttccgtcgaaaaggggaggtggctccgaagttga

agttagcggaggaggacgaacggtgacagaagtcattgatttgattgaattatatgaata

agaagaatgcagtgttgcttgtttacattgaatcgagtgaattgttcttatacttatagc

atcgaatattgtggtgtgtgtcagcgatgacggttgcttgtttatgatgaagaagttttg

agaatgaaaagtttgacagcgtgatgtttacgtgtctggatagtaatctgattagaaata

tgtctacattcattc

>fava_dn_rep_c701

tttttttttttaagccaaatattttatgctattcatttttcaacacagtatacttaaata

gagttaagaaatggctcggataacattcagtgcagatatgaagtaaaaaagcacagtcta

gtagttacaatgtcagcagactagaaacggttcaaattggaaggcaaatctgaatttttg

gctgggaaaaaccctgaccacattcagaatttaggagcactccaaacattcaaatacaag

tctatcatgaacataaatgtctgtgtaaagacttcaatcttttcttagctccatgaattc

acgatgaataccactggcgtttaggatcacaatttcaactctatccccagtgtatatatc

tctctcagatgcagatgcaaaaacagttttgaccagatcaactgcttctgattcagacag

tggagtaacagcatcctgggcaggcaaaaggagtgggctgggggacttcagttggttatc

aagaaaaggcatgatgagagtggatccagaaccctgggagctatatcctaccctctcata

ggaaccaacagcatcatatgtgaagacacatccctttccttcactgtcaaggccgcccaa

cacattaaaagaataataaggaaagaagcgtttgtaataaagtgtgtttgaaagcaactg

agccattgcagggcagctcatctgcttgttgtgctgatgctgatatgtcaaatgtctagc

agacaaaaccttctgcaaagccttcacatcagcttgaaaacccgaagaagccataacaca

cttctcagctaggtgagagattttggagtaatcacgagtgaggatgttgtaaccagagga

catcctcgtatctgctgcaatcacacagtagtctgatccagcaacagcgacacaggatcc

tccattgttgtcatatggagaccaattagcgtgttctttagtcatggtgaatgggacgtg

acggagtaggtctgatgcggtggtggtcggaaagagactagggtttcagatcggagattt

gaggcactagagatagggattggtttttatgcagttccaattctattctattattgtgtc

tttcccgcg

>fava_dn_rep_c702

cataatttaatgttatataaaacacaaggatatatctctaatcaattcacgtgtaacact

gatgaagtacatataacgaggggccgcagctatttaacatattaatggagtgtaacacaa

cattcataggatgtgcttgtaaagggggcattgggacagtctttctctcaagaggggcgg

aaattttcacaatgataaaagttaaacaatcaaataaaaccagacacgatattctcaaat

taatacaggcatcaatatcaaattaatacaggcatcatagaaatacattgatagtcttcg

cgtatcgtatcatttaagcacattgtataagtaggcagcaatacccagaattataatcaa

caaaacaatatcaatacagaagtttcgactggatcgaagctgattcaccgtgtctttaag

tctaacattagtattcttaagatcagatgatgccctgtccaccttggtgtcgatctcatc

catcaagggaacttgtctatccagttcctcattcatatcatgtgccatgtctttcaaagt

atccaatccttctgcaatcatatccaaaccttgatcctgttttattctccgcatttcata

ttcctgtctgaattgacttgactgctcagttgatttgaagtattcatcatcaaatcggcc

atccgaatcaaatttaatctcagcgcgtgaggcagaagaagctgcccagccaccagtttg

ttttggtgcaacaggagtcccgtccgggatagcttggattctttccggtaatgcaagaac

cagatcattccgggcagcaaattcttgcgacgaaagcccttttacctttttgacagccaa

cctctgcaacttaggaacttcctccaacaaccgagacttcgtccgacgaatctctgcatt

gatcgctacagcagaagccttccctttctccttggaagcggtttctgctttctgaagtag

gttatcgatgtcggagtcaacggaggcgtagagtctgacgaaagcgtcgtcgccggtgac

attggcatccttgttcttgtcaaggtcgtatttgtcgtactttttgcaaatggaatcgac

tcgtgttagaaggtcgatgacgctcatggtggttgtggtggtgttttgggatcaaaattg

gatcttgactagagtcaacaagaagaagtggtgaattgggagttcgatctgcttgtttca

gttctcaactcaaaagaggacttcagatgagagagagagagagagagaatgagagaccgt

gctttaattttaattttttaaataaa

>fava_dn_rep_c703

tatttgaaacattgatgtgtcaggttaaaaattaagaccagagttgatgcttcatgaact

ggtatttatgagactccaaaaaaatttctggtacatatgtcatgaagagtgcaactctta

acctcataattatttgtgtacaatatcatggtttactggaaatgttgtgctaattagaga

atgcaaaagacatttgattgttatgattgttatggaacctaacaagctttgacatgctaa

tattacattaacattaacagagatttgaaggttcagattcaatcttttattagatggagt

atattatattttatatacgtgcacggtgtgcacctactactttacatatcaacaacaagc

aagtgcatcgacagttcagagatcctcattgggaaaagcttccagaaactctattatttt

gtttcgttcatcctctgtgagattcggatcatcgccagtcactctcccccgaagagaggc

acgatcaccgtccgcatcatcaacgacatcaataaaattgttgacatggccatctttgtt

gggagaggttataagctcatccttattgcatgtccactcaccatcaacaatgtacttgta

ctcgtagtgcccttcaaacatttccttcttgagaaaccatgaaccttgtttgtcatcaaa

agctaagggcactctctgcccccatccaatatcaagtcctgaaatttccacattagagca

attgctgtgtccccatgacaaagtgactggcttcttactgagtcctgtaagaatatcggc

cgttgcactttttatggcatccagttttggaaagcacgaccttttgctcagaagtagtgt

agtagcttcattgagtttataacccagaatccaaaacatatatgttaacgcaacagctgg

agctcttcccagtccagcagtgcaatgtatatatgtcacacctccattagatttaactgc

cttgtataatttactaattacagcaggaagtcgcttccgtaaatcaaatgaatcaaagtc

ccttatctcagcgcgcaagtgttgaatgtcattgaaagttttggcatactctcgtatggc

attgatatcaactccaaaatattctaggtctgggtcttgttgcaagcaaaatatagtttt

gactccgattttacgaagcttatcaacatcttcaggagtctgcaagcatgatccaacaat

caagtctggtaggatgaagttgtagttcattcctaattcatgcctataagtcaaaacagc

acccatggcttctgtcatgttgttgctgtatgtctcagacttgaccacctcctcaacatg

agagctacttgtctctgcacttgatatagaaccagaagcagccttaagtgacatggttgg

agagttagggatgaaggatagagaagaagagtggtgggaagaaggagaagtaacaagtgt

gtggaagggtaaaagagagaatcttgatagattgtgaatgtgaagacagttcatgctgat

tctgataactctttcctcttctgcttcttcagttgagtgagcaaaattcgtttaagatcc

attttctttcattttcttcccttccttgcctttcctcttctagcttctgttttatatata

ctccagctagtgtatcttattgatattattattattattgttgttcttgttggcgttttg

tgtttttgtatgattcaacttcgaaccaataaaa

>fava_dn_rep_c704

tttcggccgcggcattcttcaccttcttcactcttctcttccagatccttcgattccacc

gattcaaccagccatggtgaagggtcctggtctctacactgacatcggcaagaaagccag

agatctgttgtacaaggattatcacagtgacaagaagttcactatcagcacttactcacc

tactggagttgctataacatcctcaggaacaaagaaaggtgaactgtttttgggtgatgt

taatacccagttgaagaacaagaacatcactactgatatcaaagtagacacaagttctaa

tcttttcacaaccatcactgtcaacgaacctgctcctggtgtgaaggctatttttagctt

caaagttcccgagcaaacgtctggaaaggtggaactacaatacctgcatgaatatgctgg

aattagcagcagtgttgggttgaaagcaaacccaattgtcaacttttctagcgttattgg

aactaatgcacttgcttttggtgctgatctttctttcgataccaaactcggggagctaac

caaatctaatgctgctgtgaacttcgtcaaggatgacttgatcgggtcattgactctaaa

tgaaaagggtgatgtgctatctgcttcatactatcatgccattaacccactgtccaacac

tgctgttggtgttgacatcagtcacagattctcaactaaagagaacaccttcacactcgg

cacccagcatgcattggatccattgaccacagtgaagggtcgcgtcaccaattcaggcaa

ggcaagtgctcttattcagcatgaatggcgtcccaaatcactcatcaccatttcaagtga

ggtagacaccaaggccattgaaaagagtgctaagattggattgagtttggctctcaaacc

ttgatttagttttgatccttcctggcaagaaactgaggtgtaggcaggctttaggaagca

tatagtttgtcagattgatgttggttctcttcttgggtaagaagtgttcaaggtgttttc

tttgcagttgggcaattcaaccttgaagaaataaataggttttttatgaaccaaatgtta

ctcttaaatgcccccacaggcaaaacatgcctcttttattgaattgtgaattggtactct

ttgagttttgacagcaaaattttaaaatttctggtatcatggtaaatcttttgcagcatt

tcttatatatcttgtggtatgatgatcgtttatttcctacc

>fava_dn_rep_c705

tctcttcacttctctttttgtggtgtacactctttttggatttgaagcttttgttttcac

tcacaaactcactcatcctttccaccacaagaactaacccaagaacattgaaagtgtaga

atggcttccatgattgcaaccttgccaccaccattgctacttcatcatggtagaaattca

ttttcagggaatttccagagtttctctgtttctcttcttgcagggagacggaacagcgtt

gcatttgttgtgaaggcttctggagaaagttccgaatcctcaacttcactcactgttctc

aaatctgttcagaatgcctgggataaacccgaggaccggttggggctaatcggtttgggc

ttttcagctgtagtagcattttgggcctcgacaaatttgatagcggctattgacaaattg

ccagttctcccgttttcgtttgaactaattggaatacttttctcaacgtggttcacttat

cgctatctcttattcaagcctgaccgggaagaacttttccaaatcttgaacaagtcagct

tcagatatcttgggccagtaagatgtgctgctgaaatattgctgcagattatgattttaa

gcgcggattatcattaatcagcggcgataaagtttactcctatgtcaaattacagtgcaa

gtttgaaatcttaacttatgcattaatgtgattttgttggatgtaactcaataccaaaca

catactaaatgtatagtttcttaagtttcatattaaaaacactacagtttcacattaaag

tcgttccaaacatatattattaatagcattttcagtcatatcatcacaattaagtttcac

ttccccctattttaaagaataaaacacagtaccatata

>fava_dn_rep_c706

ctctcagatttcatactagaataacgtcagcgataaacgcacagagtatttttcgtcgtt

ttctaacagttttttgaattatggctttgaaagagagattttctgaagaacgttacgtaa

gctgggaggaggtagtggtgtcgatggataagggcaggagggaggttcgttattttctga

aaaagaaaagcggtggtggttcggatctcgctcttataggtaaagagaagactcccaggc

atatgtcttatcactatgctataagagactcttctttgggcccttttttaaaactcaaat

cgcgtagagaagttcttgattggttggattcaatcgtttcagattcttcttccagggaag

cgactatggttgttggaaaatatgcttatgaacctgagattggaacctttaaggataata

acatgcagaaactgcgcaattgtacacaagagttctcatgggtagggttaccacagacat

gtaggaaaagaagaaatcactatcaatcatttgagcggaacggttttcaaatatctgtga

atgattttgtgtttgttttagcagaagagaacaaacgtctggttgcctacttggaagact

tgtacgaggattccagaggcaacaggatggttgtagttcgctggtttcacagaattgatg

aggttggtattgttttacctcacagttttagtgacagagaagttttgttttctctttttc

ttcaagatctgagtattgaatgcatagatggtttgacttccatcctcagtccccagcact

atgcaatgtatcggaatgaagttcgtcacgcctttcccgaaccatttgtgtgcagcaaga

agtttgataatgatgatgtcaaaccctttgatataactcaaattaatggttactggaaac

aggaaatactgaggcacatgtgtgccaactctgactcaaagtctaatgggagtgcaggga

aatccgaagatggcccagaactggaagaaaatctcccatcttcttctagggtcagaccta

tgaagagacagcggtgtacaaaagttgacagaacagatgcggctgagttagttgcacttg

aatcggaacatttgagtaatggtaagattgatgccaaaaccagcagtggaaacaattctt

taaaacttgccgggtccaccaagatgggcaccatcaaagagacaaatgccacaaccagcg

gtggaaacaattccttaaaacttgctgggtccaccaagatggccaccatcaaagagacag

atgaacttgcttcacattatttagctgtaggttccaatgttgaggtcctctctcaagaca

gtggaattagaggatgttggtttagagcttcagttatcaaaaagagcaaagataaagtga

aggtgcaatatttcgaccttctggatgctgaggatgaagctatgaggcttgaggaatgga

ttttagcttctagaattgtggtacctgatgatctgggtctccgactacctgagaggacca

agatccgaccacagacaaacaaacgtggaatgtcatttgtggctgatgttggttacattg

ttgatgcctggtggcatgatggatggtgggaaggcattgtgattcaaaaagagcttgatg

ccagatatcatgccaaatatcacgtttatttcccaggtgaaaaggcggtctccatttttg

gtcctagttacttacggcattctcgagattggacagggaaagggtgggtgaaattaaggg

aaaggccagatattgttgcttctaaattatcattatcaaacttgaaaacaaaacaaaatt

tatccaaatctgattacgaaaaatcaaatgcagcacctttgggggatcaatctaagcaga

ctaatattcactcaggtttttctgaaagggatatacttattagaaagcccgaggttgttc

cagatcttttgaaggatgttttattatcccagttaaggtggaagtcatcccgaaaaagaa

atcaaggtggtacctctcaacagaaggaaaagtatagtgatagacacaggaaattgtccc

catcgattctagagtcggatgattcagatagttttgcgatttcagcttcattgaaagttg

accaagatgactacaaataccagggagatccttccattttcagctcctcagtcgtcccat

cgttaaccaacatggttatgtgtaggtgactccatgttgttttctttagcatggtgtctg

cttttagggttacctacctcatcataccagtatttatttatttatttatttaacccttaa

aacctttcttcatccattcctaacttaggaattagtcttttgtgttaccttttttttttt

gggaaaataacatctcaacaatattaaatatcaaatacataatattaaaacaacagtcca

aataaacacttgtaactttgaaataagaaaaatttgaaccaaactcaaaaggatattttt

aacttttattttatatatttaaatagattacacaaccaaacacacccatataatcacaca

gcctaacacacccatatcattcaacgatagttgtggcaaattggggcagatctgtcctct

tgatcacttctgctgctacatggaggtatccgtcttcgtttcacaaggactctcttttca

tctgaaaatgaataaagttgagaaacatcgttaatgttccttgctaatgccatggatgaa

aaattcaacatacagaaaaagagaagtacaaaaattagtgaacatttcttagaagccatt

atttttcctagcttatttcaccccgcggcgtaatg

>fava_dn_rep_c707

ctttttttttttcgaggcgaaaagaaaaggtattgatatgattattataagttcagagct

tttgacaaaaacaatcataggaatctcaggttcgaaaaattgtgttgcctagaactatga

tagctgaagttacaacagaaacctatttaaataagtaaacaggcagaggtacaaatgaaa

acattatgatattctagtttgcagaagcagaggtctttgacccgattccttgaagccgtc

tcatccatttgttctgtgaagaagtaacatcagtaggctgtaatcttgtttcaagacctt

gtttagattgttctgtgattggaagctcagcagggagtgctgcagcagtgctagtgaagg

cattcaggacaaggaatgctaggatgggagagagatccaatcctccaataggaggaataa

gtccccggaatacgttcagatacgggtcacatacggagctgagaggagcaacaatagcag

gaggagcattgggaaaccaggtaagaacaagcctacaaacaatcaaagtattgtaaaggt

tcaaaaaattctgaagcccattagccaccactaacccagccacagaatctccaggcaaaa

cagcagcaaaattgtgctgaatattaaccaacctcctgttcctatagttactgagccgaa

tctggaagcatgtatcgtggaagtgagaggaaagtgagagtaatttgtttaagatagggt

tttgagaggcgagggagaggagaagtttggaacaatgatcaactgctttggaaatggatg

tgcggatttcaggtggtggtgaaattgggagtttgaagaatgggagtgctattagaaatg

gtgattgggggtttcttgaatgaaacgaaatgttgttatgagtttctgagtcggtaccat

tcacagccattgtatgggtcatgcgataagataacgcagatatgaggagtattgtagtat

attcttccaacgcccatccacgacacacctatgagtttttaattatatatattaaacaaa

tat

>fava_dn_rep_c708

gccgcgggagaagcacaagtttcaatagttgaaaactaaagaaacaacaatgaacttctc

tctccatctaacagtcacagctttgtgttgtctagttgttgtgtttggagggttaccttt

ctcttcagatgcacagcttgatccatccttctacaaaacctcttgtccaaatgttcattc

cattgtacgcgaagttctaaggaacgtctccaagacggatcctcgtattcttgctagtct

catcaggcttcattttcatgattgctttgttcaaggttgtgatgcatcgatcttgttgaa

tgatacttccacaattgtgagtgaacaaggagcttttccgaataacaactcaataagagg

tttagatgttgtgaatcgaatcaaaactgcggtggaaaatgcttgtcctaatacagtttc

ttgtgctgatattcttgccctcagtgctgaaatttcatctgttctggctcaaggtcctga

ttggaaagttccactaggaagaagagatagtttaacagcaaaccaatcacttgctaatgc

aaatcttccaggccccgcttccaacattactacactaaaatccttcttttcccgtcaagg

cctcaacactactgatcttgttgcactctcaggtgctcatacgatcggtagaggtcaatg

caaattcttcgtcaatcgattatacaatttcagtagtacaggaaatcccgatccaactct

caacacaacatacttgcaaacattgcaatcattatgtcccaatggtggaccgggtaccac

cctcgccgatttggacttaaccactcccgatacattcgactcgaagtactactctaatct

tcaagttcaaaagggtttgtttcagagtgaccaagagttgttttcgacaacgggctcaga

cactattgccattgtcaatagcttcaacaacaatcaaactcttttctttcaaaacttcaa

ggcctcaatgattaaaatgggtaatattggggtgttgactggatctcaaggtgaaattag

aaaacaatgtaactttgttaatggggcttctgctggacttgattcagtggtcactagaca

atcgtctcaagagggtgttagctcatactaagtaagttgcataaacttaaattatctaat

aaattaaagtgtgtgttttagaggcacatacatgttgtgcttttgtgatggatggtgata

gttttgtactttctacaatatggccattgatgcagtgtttgtaatatctattttcctata

ttatgataataattatgtgttatacaaaaacgagatatggtttggagactgaagtattga

ctactagtttttaactatcaatgatgatatcaaatgtggtttaatttaa

>fava_dn_rep_c709

catgctttaagaatacaatgcaattttgatacagaagagatgtaactttaaaatcaaggc

agccagaatcatagggataaagccctacatttaaatttcttattcaacatgtcggaagac

tcatgctacagccatacattctatagagatttgacacaatttgaaagtaaactaaagcta

atcacaacaacttaattccaaatgatgaatatctcaaatctcaaaagcctctgaacatga

taaaaatttatgtccttaaaataacaaagaaataaataaacacatattaagcacctttat

tcatgatacagaacaatgtctctcacctaaacaaattgtgcagaagaaccagcatcaaca

ccgagacatatccagccacctccttgaaggagcaagaacacatatctctataaatattta

tatttagaaaatgatatcctcgtctttatcagctgtaccagacaacaagttctgagagga

atcataagaaatccaagtcgctcctttctgcaattgcaacttctcaacagcttgttgctc

cctagcaagcaccatcttcctttcaatttccctcctcttataaccctgaatcttcttcaa

cctaaagaaatcctctctctcaagctcatccaactcacccttaatgtaactaatagtatt

ctccaaccgcggcttcacaacattctccaaagcattaaccctacggttagtagtcttgat

agcatcatcaagggtaaggaaagatgtctgaagcgacgcaagctcaacaagaacctcaat

tgccttaatataagcgacacggcactgctgcacctgctgaccacctctagccaatccagt

aaggtcattcttcgaggcctcgccgtcagcagtataatcaaatttcggaagcttcactcc

agcaacgttctcagtacgagaacgcaccctgagtgaagcttctttaacattctcaagcac

gacgtgcttgatgttgtcgccagctacatacttagcctcagttaatgcaaaggatgaggt

tttcattatgtcgcccatcgattcctttgtcgaaacaatctttttcaggatctgacgaaa

ctgaacagttaaagcatcacttttcttcttcagaagagcatggcctcttgttgctccaac

caaacgtgctttcaccactccaagcattgtcactgtggggaccacattcaaacgctgtga

ttgccccgacatctcttcttttttccttttctctcttctactgaagttgctgatcggaga

tcgagaacggagaatgatcgagagagaagtgaaagtgcaaaggaaattgttggattcggt

tgttattattatttttgt

>fava_dn_rep_c710

ataaatagtaagtaataactagtgttctagagacacctttaaaaaattttaaataatttt

aatacatttaagatttatgcatattaaaaatgcgatactgtttattaagcttacatactt

tatagttgaagtgggaggattatttttttctagcctccaaaaacaaagacaggtacacta

gttactctacacaatattaatagaaaagctctacggacccaatatattcattgcatgctt

aatattatggtggcaaagagaaagatattaaatttcaatacttttgttagtgagggctca

caaagccagaaaatataatgtaaagaacaaagcacttattcagcgtttgtgtcttcaaca

gatttcttatattcgggcttcagctcgtaagttccttggttggcacctcttttattgtat

acgcacagttcattcaagatctctttcaagaattgcgcgggttgatcagtttcttggaca

agttgcttcaaagcccagttaggctgtctttcgaatagtttgaacataatatcttccagc

tctccccgatctcttcttgttctctttgtatcagtttgtttgactggctgtgtcctcttc

ttttccttaaaattagatgaaactaagccaaccattccaggcattggcctcatgtgcact

cctcgaatatcctcaataacctgtacttgtcgatttttaatcatagatttctttgtcctc

tccctgcacaattttccataatcatctatgttttcatgacgtggcttcatatcaaactta

tgctctactttcccttccattgaaaccttgtcaccttcacttgtctcagagaagatgcac

ataggaacgaagtctttaaacatattcaaagaataagattttgccatattcacagaatca

gtgccagacatctccattgtaaactgaagatgagcaggatcgtcttcaggaagaagagga

tcaatagagaaaacgactttagaaagcggttgagaaggcggatgattctgccatgactta

gcaacagccaccggacacttcatcagccacaccgatctctctgcctttgaagtctccaga

gttgaaccgctacttccgctgtaaccgttttcttcttccatcttgactctctctttctct

tccttcatctacggttgtttcgctgattcgattcttcttcttctagtcaattat

>fava_dn_rep_c711

ttgacgtggttaggcagtgaagaaaagtaaaataagtagaataacgtcgtctccggcaat

tcacgtcatttgcaatcaatcttcgtctcttacaattcatccttcattcgactccaaaat

ctcaaccaaatgatccgattcattcttttgcagaaccgccagggcaaaacccgtttagcc

aaatactatgttcctctcgaagattccgagaaacataaggtcgaatacgaggttcatcgt

ctcgttgtgaatagagatcccaaatacactaatttcgttgagtttcgtacacacaaggtt

atatacaggagatatgctggattgtttttctcactttgtgttgatattactgataatgaa

ttggcctatttagagtgtatccatttgtttgttgaaattttggatcacttcttcagcaat

gtctgtgaacttgatttggtttttaacttccacaaggtataccttatacttgatgaattc

attctagcgggtgaactgcaagaaacaagcaagaaggctatcattgaaagaatgggggaa

cttgaaaaactagagtgaaacattacagttcagcagagtcaccagggctaggagattctg

atgatgatggtgtacttgggcatttggagtgattcttgtattttaattgtgatatgtgat

acacaactttgatatttcccccccttgagccatgtattatgtacactaccaaggcttctt

ttggcttgtgttaatatattgtagacattcatgaagtcagtttttagtaaacttttttca

tatagaactaatggtatacaccaaagatatacagttggataatggtaatgtagaaataaa

caacttcaaaataatatttttagataatatagaactaataagtgttggaagtattgtaaa

tgtttcattttcactatggagactgagaaaattctatccttcctttctagtttctatcac

ttcacatatatgcaacaagtgtgctgacaggttcaacacttgaagcaacattgtaccaaa

acttaagaacctgtttctaatttcgttaaatatagtattgagatgaagaagaccttgtcc

gtttgtcttggatgggggaaatgagctgctgctgttgtcgtccagcgattttcgatccga

atatttcttgtgatgcggaggtatgagggaaaatggcttgggcacaaggaaccattgcag

aagagtcatttcttccactttgccaattaggattttgggtcctcataacaatattgcaat

tgatagaagacctaccctgagctacttgttgctgcattgcccaaaagttttgctgctctt

gtttactatttgaggctgttgaactatattgagttccacaataataatgaggttgttgca

accgaggctgtggtcctacaacagacaactgatcaaggtaaactgttgaggagacgtaac

gactctgttgcgtaggatttggcacgaccctttgttgtcgtgctggtacttgcaaatttg

acaatggttccaacctacttacaactttattataataattgttgttgtcgacttttaatc

cataacttccggttgacaaagacaagaagttgaaactttgcttttgaggatcatatactc

caggtgttggtgtagtctgtgatatcgcaccatagtgggattgctgcaaaaaaatactac

cattcttactttcattagtattcctcacagtagcataagtagctctatttctcattccgc

tcacgttgagtacttctgaaagaacttcacgcttaggcccatcgggcactctcgtttcat

tacattcgtgaagcttggactctttgatagctccttgttttgccacctctgaacgccgaa

ttgtatgactgatgtaaacatgagtcgcacaccttttccgtaatcttttatgactaacag

cagatgacactgaagacaagcctggccacaatgcaagactttcattttttatatgttcct

tgatcaagtgctgtttttgcctgttatcaacatttctgaccgagtctaattcgacatctg

atttcttttcgtgttgttgtttctgtgattgtctggacatatttaatcccattgctaaac

ataactcagagccgtgcaatgcaactttgtcgcaatattcacgcttaaccatctcaggca

tgctctgaagatttaatgatggagtactgctatgaggtgccaccaataaatttgccttct

cagaatgttcttgaccaatagtttctttcaaagaagtaatttttgaagcttctctaggta

aacaactttcaggacaaagacttgcatcctgaccggttactgaggcttcaagagcagcat

tgacattctccttcttgtcctgcaaaactgaggagttctctatcaaagactctccatata

gttcctttctagcatgattggaatcactgtgagggaacattgcagccaaagcatatagag

tctcagctacctcttcctcgtctttcgtgatggaaccaaaaactccttctcttcgggaca

ggttttggctaatttcctgtttttttgacttctttacattatccttttctgaagattcta

ttccattgttctcatggttcaccttttctgcatcaagtaagatgaattcgcgaccgcgct

tcttcatggcagaacgtagcttccttggaacagaagcaccatagcatccattcaaaaaat

tattaggaatcttaaatctctttgggtcttgttgttgtttttcacaacccatttcattga

cagcactcaagcttctaactttttcttctctgttggatttcttcaaaaatttgaaagcag

cagcaccgaaaactatatagataagaaaaggagagaaagtgagaagaagcgttatctgca

aataa

>fava_dn_rep_c712

tccttttttgaaactattaagattgattcattcaagaataagcgatgagttcaaagcaaa

catgtccttccacacagaatcaatcacatgaaattcctttaaggaaccgaaattcaacca

aaaagtgttctgttgtgctagaattagcattagcatgctcataatctgcttctgtagagt

ctcttaaaaagtaccttacaatttgctttttctctatggcttcttttctttccttatctc

ttcccaaactaaacttgataaaagcctcttcagctgctacaactacaacacctctttcaa

ctcccgaggcactcaatgagaaatttggaagaaaaggcatcaaattcttggagactaata

gcattcccattgttgagttgacggttagaaatggaagctcattgagcctaagattacctg

atgctcatgtaacatcttacaaaccaaaagtgttttggaaagacgatggattcgaggaat

ttctttacacaattccagctagtgaaacaggtctttataaagctaaaggtggaattggtt

tggtcttgaatgaagtgttacaacctggcgcgaaagagttgcttccttctacgctagaat

ggactgtaaaggatgttgattatgatgccattgatgctctccaggttgaattggtaagca

ctaatagatttttcgacatgacttatattgtgacgctctatcctgtgagtatggcaacag

cggttatagttaagaacaaagctccgaagcctgttactctaacgaatgcgatactaagcc

attttaggtttaagagaagaggtggtgctgctattaaaggtcttcaaacatgctcatact

gttcacaccctcctcttgcttctccttttcaaatcttgagtccgtcggaagcaattaaat

ctgagcctagtagattgatttcctttggtgctgagcctgaattgaagcctggttcatgga

ctcaacagggagttcctattactctccttgagaataagatgagtagagtttttgctgcgc

ctccagaagaaagatcaaaggcgttttataacacaccgccttcgaaatatgaaatcattg

accagggacgtgagattttctacagggtgataagaatgggatttgaggatacttatgtgt

caagtcctggttccatgtctgagaaatatggaaaggattactttgtatgcactggtcctg

cttcaatgttggtgcctgttactgtgaatcctggtgaagaatggagaggagcacaagtta

ttgagcatgataatctctcataatcatgttaattaacaaatattaagtttacatgattat

ttgaaacctttcctttcgtttctccttttctttctctctgcactgttcttcctatgtata

tttgtttagctgatttatgtccctatagtacactactatcaataaaatagattacataat

gagataagcctgctgtttgaaaaagaagagcaagattgagattttcccagcaaggaaagg

actatctgctatcaaatgattttattaataatagttgaaacaacctaaacagagaaaatg

gcatatgtatgtgaaatttccacagatcatttttctgtgtctatattgaaatgctgagac

atatttatatacaaagccgnnnnnnnnnacagaggagaggaaagtggttatcccgcgg

>fava_dn_rep_c713

gaaggggagtgttggaataatatagcaaatgtgagtaagtggggaaatagtcctacattg

gatagaaatatggagacttaaacatatataagtgggagaatccattcacctatcacctca

aagttttaggtggataaatgatgtgtctctcacaaaagtgcttattcatacactatatat

taatcctcctaatgtaacttcctaccacagtatgtaactcatatcatggagaatgtacat

attgttataagattcaggcttccaattacatagtcaattctatcactgcctccttgaaaa

ggatttaagctgcataaatcatcataacgatattttatttataaggccaagctgacaagg

agttacatggtacacagatggggtaaaagaagcaactggtacaaacagactatagtacta

ctaataaaattcacgaaacatgcaccataacagtatccttatggattcttcaaataaata

tacgtccgacaactattttttcaagtcatctaaacttcacttgatatcttctgcttactg

ctgctagccttcttcttaggctgaggtttctggtcagttttctttggtgctgctgctgct

ttggaggctctagttctgggagtggttccactagtttttgcagcaggctctttcttaacc

tgccctgatggctgagagatgccagatttatcactaggtttctcagctttgagtgatgcc

attttacacactactcttttgtttggtagcttcaggttcccgcggccatattttatgata

ttatgttatatttgcttcaataatatgtatca

>fava_dn_rep_c714

atgagtttctcatttatactttagttttatttaactattattaggttttgttataataat

atggatttgggtttatgatatatctacttttgttagcttcctacttaaatatagttttgt

gattatttttggttagatagataggaacatggcatgtgaatgtggaattaatggtgttct

attcaaatgtttggttggttttaatgttttttaaagaaatcaaaactgtgtttttttaga

catttttttgttggtgtaccttctctcttagaggtggaaatagtttttttttttaattaa

aactaacatagtatattaaagcattgattcagtcatgacaataacaaaaattgttcaccc

gaaatatccaaaatgataaacaagaattctcaaaactaaaacatgaaaattagaacaata

aaccaaaatcaagcatcagctttcttataaacataggtcaaaccacacttgccacagtaa

tgacgatcaaagtgattagccatgaaagtccccgcaccgcactctgcattggggcactcc

ttcctcaacctctgcaccttcccagaatcatcaaccttataaaactgcagaacagcaagc

ttcaccttcctatgcttgtgcttaatcttcttcggcttggtgtacgtcttcttcttgcgc

ttcttggcgccaccacggagacggaggacaaggtgaagagtggattccttctggatgttg

tagtcggcgagagtgcggccgtcttcgagctgtttgccggcgaagatgagacgttgctgg

tccggtggtatcccttctttgtcctggatcttggctttgacattgtcgattgtgtcggag

gattcaacctcgagggtgatggtttttcccgttagggttttgacgaagatctgcatcttc

gaagggattagggtttgcgccggagaggggaattgggtgatacgagagtttcagtgtttg

tttgacgaatttatattggggaattatttttgttattgtcatgactgaatcaatgcttta

atatactatgttagttttaagaaaaaa

>fava_dn_rep_c715

tacaatttgggaaagcaaattacgtgtttactattttgccacggtttccgttctcttctt

ctccaccccttcgttacttggcaccaaacgcaggtctccatcacattttgccctaaccct

ccttccccaaaaaattatggagggcaaaatggggattttgtttctgattgtgttgggtgc

tacttgggcttgtgatgcaagagaattagtaaaccttgaattaaacaaaaaaacggaccc

gtgttcattttgtgaggaatataccactggggtacttgattatctaaaggataacaacac

tcaggtagagatcattgattcacttcacaatacatgccatcaacttcggtctttaaacca

gcagtgcactaaattggtggattattatgctccacttttcttctcagaaattgctccaat

acagcctgatgaactctgtgagaagctcaacctttgtgagtctgtgaagatttcttcgaa

agttcaaagaaataggtgtgacctttgcaaagaaactattgaagccttattggttgagtt

gaatgatcctgacaccaagctagagatattagagaaactactgaaggcttgcgattctgt

ggagaagtacaagaaggagtgcaagaaggcggtttttgagtatggacctttgattctagc

gaatgcagagaagttcctgaagacaacagatatatgcactactgtattacatgcctgccc

tgcttccactattgttagtcaggaagacacaaacatggaagaattaccattgctttctga

ctcttaatcattcttatggaagagatactatatctacattaaagatcaatgtgcttgttc

ctaaatctgtccttttgctgaaaaggattaactctcattgattatctaagattgtaatta

tggaatgcaaactttatattatgtatttcttccattgtgtaagcagtgaaagtgtttctg

tatgaaactgaatttctaaatgctgaatggaaactgttaatcttctttttaaaaaaaaaa

a

>fava_dn_rep_c716

actctatgtataatattgttttagttaataccctatataatgtctccacaattagataga

ttgatagatcacgctttcaagagggagaccttcctaccttacaacacgtccccttattct

tatgacctatgaccccaattatatttatgcaaagctgaaacttgacatgttttaaaattt

gaaccatgtttatccgccgaacagtgaacatgtcattgccaaaagatgttaagagtagtt

tattattacataaccagcaatacaaagacaaaggagaccaagtgtaacaacagatccagt

ctcgttcattcaggtaaggtaatttatgaattctgcagctgtcgcaattgcagcccctgt

aatggcatctattgcaatcttgtctgctttgttgttgctgaccgccgatgccaaagcccc

tgtcaatgcacctccaagtgtggcattcttccagtccctggtgccaaggatcctctctga

accatattccattccaacatacacgccagctatagctccccagtatgcaccttccttaca

catcttcttcagagttttctcaaaatcattgcttgagacacttccctttcgaagaatgtt

ataggtatcctctgcaactgatcttgtaactgcaacagctccaatcttcaagaaaccatc

aacagtgaggttaaggaatggattgcctgtgtctataatcacttcaaccttgggagaaga

cagtgaccctgaaacgctgccactagccatgtttcacactcacacttctttgttacttcc

ttgtttttcttagtttccaactttgagtgctattatggatgtgattgaaagtatacacta

gcacatttttaaatagtactattatggatggttaataaaccgtgatagtttttgtgaagt

nnnnnnnnnnnnnnnnnggcccatggcaaagataaaggatttcatatcatctctcatatt

ggtgtgcttg

>fava_dn_rep_c717

aggttttaaaagtttgcatggattaatcttaatcaccctttattcgattgtctcatacta

tcttaatcacccaagacagcagacacgtttcaaagcaaatgagtaaaagaaaattaagaa

aactaccagtatcatcatcatcatcatcatctcactaaaaatgtagactagatatgagca

gaaacatagatttatcattaacacaagtactatacttagcacaaacataccattcaagtg

tatgtaaagaatgggataatctaagccaattctgaaaccacacgatggattatgtgacat

tggtccactatttagtgacagaacaaatcttacatgaaaagggcagcaagcagcagacca

ataactgaaacaggatccggttaggaagagcagcacccacctttttgcccaactggttgt

cctctaatctgcactgttggaggccttgcattgttcgcggatggttggctcgccattcta

tccttgatggcagcagccatggccatgaatgccccttccacatttgtagcatcttttgca

cttgtttccataaaaggtatgccaatttgatctgcaaattctttggctgtatcatatgat

acaactctatttgctgtcagatcactcttgtttccaaccaaaagcttgttaacattatca

ctggcatagcggtcaatttcactgagccactgcttcacattattgaagctctcttcatct

gtcacatcataaacaataatgattccatgtgccccacgatagtagctactggttattgtc

ctaaatcgttcttgtccggcggtatcccaaatctgtagtttaattgtcttcccatcctgc

tcaacagtccgtattttaaaatcaactccaatagtgcttatgtagctctcaatgtatgaa

tcatcagcaaatcttagaagaaggcacgatttaccaacaccagagtctccaataagaagg

agcttgaacagataatcatattcgggattcatgacggggaacgtgaagtggccggagagg

gagagtgatcggatcggtgctccacgtggagatggaatggcaccaaacggagagagaaga

gaaggagaagaagaaggaatgaagaggaaaatgaaaagggatttttttttctgttttgaa

ttataaattgtgtggacctt

>fava_dn_rep_c718

cttggaattgatttgcagacatctcttgcggtacatgagttgtcttcagnnnnnnnnnnn

ngatgatgtggttaaatgcaatttgatatacacgtgcacagtcacagtcacacgcgcaca

tttatatgtcaaccgtccaaaaaccttaacaatggagcacttgaatactactccaaactc

cccaacaaagaaagaggtaatgaacagatgcttttgtctcattcctccaaattgggattg

gataatgttacattgctcataaatcagatgattgataatagtagatagcatggaaaatga

gatctcaagtgtgcactaaaatacattacagaaccacatgaaagaaaacataatgaaaac

caatcaattactgcagagagtactatttattcataaaaaaagcattagcctagtgccact

acctaggactgttacttattatttctagttagctctctctttcactcctgctccagctcc

ctcagtaaatcctcattgtcaaccaacacagttttccatccatctgggtcaagaaatgaa

acaattttcgtgttaaggccgggaagcggtcctgcttgccgagtgatcttccctccaagt

tcttgcgtgactagggttaatacctcagcgcttttgtatacatcatcggtaccaattgca

atctgcgcataagcatttcccttagagtactcggtgacaccgtagttatatgtcaactcc

aacacagttgactcttcctcccctttatacccaagtacagcaacagtgtacttgctctca

ggattatcattcttcctcaccaccttcaagcccaaagccttttcataaaactttatggcg

cgttctaaatcaccaacgcggagcgtgatatgagcaaaaggatcaacaattacagatctt

tggatgagtgcaaaagtgtagccatcaggatccttcacaaaggcgataacagttgttccg

ccctgaactggaccagcctccctagtgatgtttccaccaaaagcccgaatgtgctcaacc

aatttgtaaacatcttgtgttgcaattccaaaatgtccaaagccatctccaacatcataa

gaactcactccataattatatgttaattcaatagaaaaatgtgatttttcatcgccaaat

ccaacaaaagcgttagcatatttctcatctggaacatctctttgtctcaaaagcttcatt

ccaagagcttgagtataaaacttgatggcgcgatcaaggtcaccgacacggtaaacaaca

tgaagaaatcgacggttatcattctttggccagtcaatgtcagccattgatcaagtgagt

gaggaaagtctcccgcggccgtaa

>fava_dn_rep_c719

ccggtccacatcacaaaagatttcaagggtttaggatttaaagtagctttgcatcttgtt

ctcgtggctgcgtcttgtttttcaaaatcgaaatacgtttctaggaaatttcacaacaaa

cctcaagatgcagaacgatgaaggacaaatcaccgagctctacatccctaggaagtgctc

tgccactaacagattgattactgctaaggatcacgcttcggttcagattaacgtaggcca

cctcgatgaaagcggtgtctacaatggtcacttctccacttttgccctatgtggcttcac

tcgcgcacagggagacgcggacagtgggattgatcgtttgtggcagaaaaagaaaactga

acttaaacagtagatgacatgatcacatcttcgttgtgtttctttttgttaggttttatg

tgttgagttccaccattcctagagagatattgaattattttgatcaatgtattgcaaaat

ttgaatttcctatgataccttcaatgcattcaaagttgctttttaaaattatttagtttt

ttttcgcatttgcagtcatatggaatacttgctgtattatatacttgccctattataata

agtagggatggaaatagaccggccgggttaggtttaccaagtttgagtctgacttgcatg

acgtgtagtctgcgtatcaaatgtttgtttttaggaaaaaagtctgattgacctattaac

ctatataaaaaattaattagattaaatgtttatttgtattgatttatttaagtttattta

ttaagatatgttttgcg

>fava_dn_rep_c720

ttttttttttttgatcaactttttcatctcacatttaaaatataataaaaaaccaaagaa

atcaattttaaggggcacggtggatttacaatatcagtatcccaaagaacagatgtcaca

ttagagtaaatactgagcatttaaatattacaatgaaataaaagatattaaggatataag

tacattaacattgttgatacatattgaactctatatttatgattacaagaaaattcaagc

aagattaaaatattagaatgaaataaaagaagcaagcatcctcagatagcgacactatgg

agaacaacagtttcaatggttagtccaggtccgaacccgaacagcacaccccagtcaagc

ccttctccagtagtcttaagtccatcttgagctgatttcttacgcatctcatctaagatg

aacaatacacatgcacttgacatgtttccatattcacttagaatttctcgtgtggccctc

atcttttcaggtttcaagcctaacttttgctcaacttggtctagaattgctggtccgcca

ggatgagctatccaaaagatagagttgtaatcagatatgtttaatggctggaatgcctca

accagtgctttgttgatgttctttgaaactatcccgggaacatctttaagaagatgaaat

gttagtccagcttcgcgaagatgaccatcgatggctccttcactgtctggagcaattgtt

tgtgcagtccaaaccatctcaaatataggtttctcaatttctggaaccggatcagaacca

acaataagtgcagcagctccatctccgaataatgcttgtccaacaagactgtccaaatga

gtatcactagggccacgaaatgtaactgctgtgacttcagaacaaacaaccaacacacga

gcacctttgttgttctcagccaagtccttagccaaacgaaggaccgtgccacctgcaaag

caaccttgttggtacatcatgaatcttttcacatatggacgtagacctaagagtttggtg

agttggtaatcggctccaggcatgtctacaccacttgtggtgcaaaagatcaagtgagta

atcttggattttggttgaccccattctttgatagccttcactgcagcctccttccctagt

ctaggcacctctaccacgaccatgtcttgcctaacatccaaggaaggtgccatatattca

caaatactaggattctctttcagaatctcttcggttagatacatgtatctcctcttaatc

atagatttatcacacatgcgttggaatttctccttgagtacagtcttgtgttcactgttt

gtgatcttaaagtaaaaatcaggataagtgctttgttcaacacagttagctgggtttgcg

gtgccaatggccaagatagttgcaggaccttctgccctctgagccttgcggatttcagac

acactaaccatcttggtttctttggtagcagaactgagtctgtttacagaacttgttgta

gaaaaggaaagggttggaagagagttgtggtcccgatgtatggggtcagatggttttata

tgcagaagagaagagtagtgaatattgaagggtaggtagctgaaaa

>fava_dn_rep_c721

gtgaaaaagagcttttcaatgggtagctctacggctatgtaaagcacatgctaaagtcat

tacatcggactgtttcaagtatcctgatatgctataacccacaaccccactaatgatgac

cgataatggagattccttttctaaatgaaatcatctacgatagagacaattacggtaaag

gagaaattgttcgcataaacacttcgtgctattcttagcacataaaatttcaaaataata

atatgcatcaaccaattgaagattgcctcatgttttatttagacaaaaaaacattcacat

ggtaaaacccccccaaacactaattaaattgcaagagtttcatctcaagagtatctatca

tttatggcaccatatttcatgacaaaggtgaaccaattaattcaaaatcaatgaacaact

aaattcaagactcacgggacttcacaatgaaattctggcgactaataggattcatcacca

ctggtggaccagcagtgcgaggccatgcctgaaacttcttgtcggtctcttcaaaccact

tctcgtttgaagctgtcgcacacacagaacctccaaaaatctttttgtctgaaacataat

aatcacagacccatgcaactccaaatgttgcaactgtagcacctaagatgtacttagctg

acatgatgaaaaatcaaactgaagcttgagatctatttgatttgtgcaatgatgcaaaat

gaagaaatggcagagaaacgcgacttctactactccgaaagagcccc

>fava_dn_rep_c722

gtaggagtattaattatacttctagattttcaacaacaagatatacttccagattttcaa

caacaagatatacttccataactccatgttaatagtactaccatttttaatttcaaacat

aaatttctggaccacaaagatcacatacataacatactagtaataattaacttgcataat

gtaatgctggtcactaaattatataataaaccagcaattttaaaaccttaatcactatta

catttttttcaaactcataactaggtgtgtaaatttgtgtatctatggacacttatatct

gttgccacgagtcaacatatcagtgtagcatttgccacatttttctctgttgccataagt

tcctggagggacacatttgcaacggttacaacatgttacacatgctctcaagcataagtt

cttccttgaatgtaattggcacctataaccacatagtggaacacaatccttcaatgactt

gacgactggagtaggtggtgcaagtggagttttagcaataggtggctgagaagttggtgg

cggagttggtgttgtgaccggtggtggtgaaggtggtttcacaataggtggtgattgata

aggtggtgtttttgccaatggaggagaaggaggtgatggtggagttgttggcggtgtctt

caccggagaaggaggtgttggagccgtcactacaggtgttggtgatggttttactggtgg

agctagaacagaaggagctgaagggtttgcatagttcaccactattttgagatcttcctc

ataagaataaaccttagtggccaccacaagaaaaatgccaagaagaagaatggattttga

agccattttttggagcatggacaagaaagaaagaagagtaatctagtgagtgttcccgcg

gccgtaa

>fava_dn_rep_c723

aactgtaattttgaagtggaaaaaacagaaagaaagaagattgaagataagagataccaa

ctagaactaaacatgagccaaagttgaaccgtgaaaggtgtgaaccaacgccatgtctgg

cattaccacctccaccgtccccgttaaactcaaactctccgataccagaatctctcctct

attctcttcttctgttcctcattccctcagtttccactattctccctcctacaccaccgt

tcgttacggtgttaccctccgcacttccgcctacggtaacaacttcagaagaccaccacc

taagagagatgtcgacgaagaagagaaagctcttgacctctccaaaattaggtcgaatac

ggttaggcttattgatcagagtcagaatatgattggaattgtgactcttgatcaggcaat

tcaaatggctgaagatgccgaactcgacttggtaatagtatccgctgaggcagatcctcc

tgttgttagaataatgaattacagtaaatatagatatgacttacagaagaagaaaagaga

tcagcagaagaaaagtgctgctagtcggatggatttgaaggagctcaaaatgggatacag

catagatcaacacgattattctgtacgtttgaaagctgcacggaaatttttggccgatgg

tgacaaggttaaggtcatagtaaaccttaaaggtcgtgaaaaagaattcaggaataatgc

tattgagcttattaaacgttttcggaacgatattgggaagcttggaacagaggtggctaa

aaacttccgtgagaggaacatttatataaccctgattccaaataaagctgaagtgcagaa

acctcaagaaacaccaaaaaaagctgcagcagttgaagtctctgtcagtgccgaagcata

gttgaaaatttccacgtgggattctaattgtagaaggcatggattgtgagaaactgatat

tgctctgttttgaaatttttgaatgttgtatgtatagcatgcatataattcatgtgataa

tagtatatagttcattagcaatttaactatagaggcaaccttgtccattaacagatttct

tcagtaataatcacaagatcacccgggtcgaaaaaaaaaaag

>fava_dn_rep_c724

tatagaacaacttcaaatcaacggtccagatattattttcaaacccatgacgtgctgaaa

cttttgttaaatatctatctaaattcccataccactgggtgtctgcgcttcccatattct

ctcatgagccttcttgatcacttgtgggatgacaccgtcgccggacctttgccggagaac

ggtctcggcaagctccgaaaacaaccttccttcctttcccgatctatctccgacaaggaa

tcagagggtggaaacgtgagatcctacggtggggattcggtagagtgtgcggtgaaagtg

tcacgtagtatcatgatagtgaaaccagctgggtaccagagtccgggtggatcagctccg

gcgccccccgccggttctactcctccactttctccgttttccggagctagagagccgttt

cgttttcgaagaaggtcaatatcagatgcgtacgagagcgaggtcaagacaggccaaaac

agatcaagctcttcttctcctttcgatgtgtgataaatgaggatgagttagtgatagtca

ctggatccatgagtttcttttccttttggctctagctgtatccaatgcagttttgctttg

tgtgtgcttgtgtgtgtttgatgtatatatagaggttgcgtagttcttgcaagtaattag

catgttgtttatttagtctcgagagatgctaatagtaatgcttcgtttgtttaaatatat

aagaagttataaactgtagagctgtatctaacctaaccggttaatgctagtatatgtatg

tatatcagtatatatgtgtatgcaaaaaccttgttattgtgctttgctttctttctaaaa

attctctcacgataaaatctcgttatagcctcaaaaagcatttgacttaggggt

>fava_dn_rep_c725

ttgccactacctcacaagacaaaaatgatttgtctccattagtcacaatttgtgtggaca

gtatcatattagatattgtattaccatatttcaatgatcttttactaaaaaaagaagcat

agtaacacaaattcaatggctctttcttgttacaccttcatccctccttctttaccctct

ctttgtctacacaatcatcgttcatcacatcttcctcttagagtttctcatataaatgct

tccttaaactcttctgattcaaaagcaccatcatcaacatcatcacaacaaagtgtgcca

tcttcaaccccttatgttgaatctaggccacatgagcctgcttttaactatgcctttgcc

aacactaatggcaacccacttcttaggatggttcaaaatacagagtcttccattgaaagg

gtgattttcgacttccgtttcttggcactctttgctgttgcaggttccttggctggctca

ctcctttgctttctaaatggttgtgtttatattgttggtgcatacaaagtgtattggtca

agctgtgtcaaaggagttcacaccggaaagatggttctgcttctggttgaagcaattgat

gtttatcttgctggtactgttatgttgattttcggtatgggcttatatggattgttcatc

agcaacacacctcctgatgtgtcaccttctgtcgaccgtgcccttaaaggatcctctctc

tttggaatgtttgctttgaaggagaggccgaagtggatgaaaataagttcacttgatgaa

ttaaaaacgaaagtggggcatgtaattgtaatgattcttcttgtaaaaatgtttgagcgg

agcaaaatggttgtaattgccacaggattagacttacttagttactcagtatgtattttc

ttgtcatcagcatctttgtatattcttcataatctacataagcaagattagaatgtagta

tgcactgtaatattctgaattcatatatatgtaaatggagaatcatatggttcaaaattg

ttatggttgaaaacttctgatcacatcagctgtgacctgacatataatgtttgaacatct

ctacattatatgcattcagtacctcattgtatgctatataaaattttcaaataaagaatt

tttgtaaaat

>fava_dn_rep_c726

aaaaaaaacaatgaagtcccattattatcaatattattattttatttccacattgtagaa

ataaaattgtgaatgctactaactcattttctttccaaatgtaaaagtacaagacatgaa

aagctcaacccaattgccacattgaaaacactaaccatttatttcaaaatgagatgaaat

aaagatgatcaatctcatagaaattccaagatttaccctgttcacaatttaaagtctcac

ctaccattaaatatacaagacaatcaacaagaaaaatacacaagacactactacatcttc

tatgtacacaagttatttcaaaagagattacatattctaatccgaagaaacttgcggtac

aagcaagttatcattactatgagattcgaagtttcttctaacgaggttggtgaattcttt

gaggtgcacacttgaggttaacatattgtggtggcttctcaaagtgctttgatccaacag

gtgacttgagacccctttcatttggggacaagatcttcagaagagccgaagcgtccttgt

taacaccatgaagaggcttcttttcctcaacaggttcatcgataaccggacactcttgag

cttcgtcggacatcaccagtttctcaaactcattctctagaaaattgaagtcttcttcta

tgccaatatcaaaagtttctgccagcagattttctatatcatcatttgtagtatcaatac

cttcacttgcttcaaaatcttcctcgttctggctcaaccaatagttatgaaaccatggtg

aagatttcacaagctcccaccattgaggtgaaaaatcctccacttgacgaaatgcagcag

gtacgaagagaggagcattcggattcagagccgaacttcttttagataccaatgccatta

gaaattgtcagccaaatacaataacttcagcacaatcctattaagagtgttttgcagagt

gatgttatgattgtttacaaaaatcacaatcacagagtgagttcccgcggcc

>fava_dn_rep_c727

attctagctataaatccctttcgttttcataaaccctcgcttcgtactctcttcactttc

ataaaccctttgtcgccgctaactcccatctcactgcatcgaagatggttgttccccgag

gtcgtggtggttctggtggagggttcagaggaggtagaggaggtgacagaggaggaagag

gtggtggtggtaggggttttggtggaggaagaggaagtgattttaagccgcgtggtggtg

gtagaggacgaggccgtggaagaggtggtcgtgatggtggtagaggtggaatgaaaggag

gaagcaaggttgttgttgagcctcatagacatgacggtattttcattgctaagggtaagg

aagatgctcttgttacaaggaatcttgttcctggggaggctgtgtacaacgagaagaggg

tttctgtgcagaatgaggatggtacaaaagttgagtatagaatttggaaccctttccgtt

ccaagttggcagctgcagtgcttggtggtgttgacaacatttggattaaacccggttcta

aagtcctttacctaggagctgcttctggaacaactgtctctcatgtgtctgatattgttg

gtccaactggagttgtgtatgcagtggagttctcccaccgaagtgggcgtgatttggtta

acatggctaaaaagcgtactaatgttattcccattattgaagatgctagacatccagcca

agtacagaatgttagttggcatggttgatgtcatattttctgatgttgctcagcccgatc

aggcaagaattttagctctgaatgcttcatactatcttaaagctggaggcaattttgtta

tatccatcaaggccaattgcattgattctacagttcctgcagagacagtgttcatgagtg

aagtgaacaagttgaaggcagatcaatttaagccatctgagcaagtgacccttgaaccat

ttgagagggaccatgcttgtgttgttggtggatacagaatgcctaagaagaagaaagatg

cttaaaattttaaccttttctgcatttgtcatcatttaaatctgtcatttcaccaactta

ggatttcaattttgttttgaagaatactcattatctgggatatttattagagtttgtttg

cctaaatattaattatgttgtttttaatttcaaaaaaaaaa

>fava_dn_rep_c728

atcattgaagatgacaaccagttcagaagatagaatgaaaacaaaataaactggttgtaa

cattaacattatagaaattggattacaaattaatacaagcaaattcatatcaaatcaatc

tctcccaatagctgcaacatttcaacctcataatatacatagacgacaaccaacaacaaa

aaatcgaaagagaaaccctaaccctaacccccaaatccatagagagttctgccgcgggga

atccgaagcgaaaacaagaaagcgcttacagtaacagagaaagtgagtgtttattccgat

tcttcatcggtgataaaacgcgattctaaccatgccgatcagaaacatcgccgtcggaac

tcctcaagaggcctatcacccagacaccttgaaagccggtttggctgagttcatctccac

cttcatcttcgtcttcgccggttcagggtccggtatcgcatacaacaagcttaccgacaa

cggtgccgccactcccgccggtctcatctctgcttccatagctcacgcattcgctctctt

tgttgctgtctccgtcggtgctaacatctccggtggacacgtcaaccccgccgtcacctt

cggtgctttcgttggtggaaacatcaccttactccgtggtatcgtttacatcatcgctca

actcctcggatccatcgtcgcctccgcactcctcgtgttcgtcaccgcatcgtctgttcc

agcattcggtctatccgcaggagtaggagtgggtcccgctttggtgttggagattgtgat

gactttcggtttggtgtacactgtgtatgctacagccgttgacccaaagaagggtaatat

tggaattattgcacctattgctattggtttcatcgttggtgctaacattttggttggagg

agcctttaccggagcatccatgaaccccgccgtgtcattcgggcctgccgttgtgagctg

gagctgggccaaccactggatctactgggccggcccacttatcggtggtgggattgctgg

gcttgtctatgaggttcttttcattaacggcacccatgagcagcttccaaccactgatta

ctagatgggaaatggaaaaatggaaatcgaaatttgaaattgtgtattattgaattttct

tctgtgtgtttttgttttcttgtgtactttaatttatcactatttggagggatctttctt

ttgtgttgaaaaatggtggccgttgatgtttggttgtacggttcgtgatgatgaatggtt

aaattattttgtacatgtatggcctagtaggttctttgtagggtccaccactaaatcaat

gttatcatttcaggtctggttggttctatcattcaacttctttgttttctttcccttcaa

ttttttcttaccatagttaatatcattctcattttgtttgaatgatattgttggatttat

gtttcaatattttgaatttaaaattttttcaaaaaaannnnnnnnnnnnnnnnnnnnnnn

taaacattggcaatgaaaaaaaagtttttctt

>fava_dn_rep_c729

atcagcgttcagaatcacacatatagcaattgtcggcggcgagggagaatctgaagaaga

tgtcgtccggcgtggccaacaacgaggaagacaagaaacccactgatcagggcggcgcac

atatcaaccttaaagtcaagggtcaggatgggaatgaagtgttcttcaggatcaagagaa

gcactcaacttaaaaagcttatgaatgcttactgtgaccgtcaatctgtggacttcaact

ccattgcttttctctttgatgggcgtcgcctccgtgcagagcagactcccgatgagcttg

aaatggaggacggggatgaaattgatgccatgcttcatcagacaggaggttctgttgtct

gaaagaaacaatgatgtttcggctatattatgattagaattagtttttaacttagagcta

ggttatgcatataatgcatcttttattatctactatcatatgatatagctgttgtagctg

aactttgtaagtgctgcaatgcttcctttgttaatatgattatgagtggattataatgtt

tctacctgtgaaagcttatacttgtttctcttgagcttgttcaataaattcgggtgttga

tatatctctactctgcgtgtattggctttaatcatgtgaattggtgtgtcattcataata

ttgatcatgaatcaacctacatatttatgt

>fava_dn_rep_c730

gagtctccaaaaacttgagacggttccgttgaggtctgataaactgattttttatgttaa

aaaggactagtaaacctttccatttcataacaaaacaaacaaagattattactttacact

ctttccagaaagaaacaagacatgaatcacaatttctgttccatattacaatccaaatta

acagtagtaaacataaaaacttagcaataaaaaaacaagacaaacatagatataaatata

gtgaaacaccctaattgaaatttccattcagatagtaaccgatcacctaaattaggttga

atttcaatttcaataaagcatcagaaaatatacactaccaaccatgaacaactctgttgc

acctatctttaatccatttaaaactcttcctaatggagcctttgagtttaccttcaaccg

aataaactttataactagcaactctttttttcctctgcatctcaggatcactaaagctcc

agcttttcgaagccgaaccgttcgtcgattttcctttcttgaacttaccatcgttgctaa

tactcatctggttctgtgtctgtgctggatgaaccgaggaagcataagaagcactgtaac

aacgcagatcttgcatacggccgcgggatccttcactaccaaatatcacatttcaattga

tattaaattgagtacaatggatctcagaaatgcaatcctcatcctaggcctattggccat

ggttcttctcatttcctctcaggtgtcagctagggacttagctgagacttcccctaatac

cgaagaggtaaatgatgccaaatatggtcactatggtggtggtagtcatggccatggaca

cggtggtcactatggtggaggtggtcatcacggtggacatggagttgtatccgacaacga

agtggttgaaaagtcaaatgaagtaaatgatgccaaatatggacactacggaggtggaca

ctacggaggtggacactatggcggtggtcactatggaggtggacactatggtggtggaca

ttatggtggtggtggtggacatggtgcttccgacaatggaaactgattatatcatcaaac

atgatgcacattcaaagtaataaatttcatgaatgagtaacgtttggtgttgaataaaag

aggtatagatagtcttaaatctcttgtggttatgtattatctttgtatatgtagtgtgag

tttgattcgaatctatgtgatgaatgtgttatcatttgtaatatatcaaatatcacgtct

tttaggtttatatatccatactaatgaaatttctctaagaaaattggtcgattaatcgac

agaacaagccatatataatt

>fava_dn_rep_c731

gaaaataataattaatttatcatttctagagcgactttacaagtgatactaaaaaaacaa

tttcagtaacatttataaggcaaagccaatatctatatcgcaatgactttaaaaccaact

tttattttctaaacaattgtgtctaacattgtcttaataagcaaaaccaaataaacacgt

gtttgcttgaaaagtgaaaaaaatcatcactgcattggagaattatgttgctggatatac

cgtttttagaaaaaaaattgcactagagtgaccaaaatactcaaatatcctgatactttc

cgaagtctacataaatttgtttctaacggtgtcttaataagcaaaaacaagcaaacagaa

aacaacatgtttgtttgaaagtgacaaaaatcaattgcgtaatacaacaactctattgat

ctccaggaagtgacttgatgatatcagaatcaccaggatccactatgctgagacagcaga

ctctgtagtactttccacaagcagttccgagatcaacattgtttccattatagtgatgaa

ctccaaccttcgccaacatggcgtagtactcaatttcagatttcctcaatggtgggcaat

tgttagcaatgataatcaatttaccttttgagctcctgagagatttgagaacagttttat

aacccaaagtgtatttcccactcttcatcacaagagcgagtctgttgttgatagactcat

gggtcttcttggttttcttagcagccaccattgttgggtagaatattacgctaaaattca

accagaaaccctagactacgcaaaaccagaacttggtgaagcgcgcgattagggttttgc

cgattt

>fava_dn_rep_c732

atttaaaagcaaaataataataaattttcaaaattacaatattcaacaagaaattatgat

ttaatgattaggtagtagtacaaaagaagtgaatacagtagagaagaattgaaagcgatc

acaaacaagattattattattttaaattaacattcgaaattcaacatgtattgaaaacaa

aacacttaaggattttattttccacttgttttggatatttatgataaaagatatgagtcc

aaattccaattaagggcacttgcgtttgccaccacgagtagtcaagctggcataacaagg

acacttttcttggtttccagcagtaccaggtggcacgcagttacatcttctacaacatgt

cccacatgctctttcacacaatcttggtcgagatgataaacggcaccttgcaacacatgc

tccattacaatctatttgctgaagaagagaaccttgtgtttgtgtatattcagattgatg

tgcagcatcaacaaaatgaataaagagaagtgatgcaagaagggaagcagctagaagctt

agagaaagccatgattgagaatgttagttaagtgtcccgcggcc

>fava_dn_rep_c733

cgggatcacagttcaacctcaaacgaacaaattcaattcatttctcttattcgtgatcta

acttttcctcaagatgcagattttcgttaaaaccctcaccggaaagaccatcactctcga

ggtcgagagttctgataccatcgacaacgtgaaggctaagatccaggataaggagggtat

tcctccggatcagcagaggttgatctttgcgggtaagcagctggaagacggaaggactct

tgctgattacaatatccagaaggagtcgacccttcaccttgtgctgcgtctgaggggtgg

cttttgggctgctgttggggttgtttcgtgtttgtgatgttggctgttttgaaatggaac

ttgggaaatgatggtgatttagggccttatattgaggcccgttagaataaatttggtttt

atgtatgtgatcttcttatggaatgtgtgaataattagagtactttaccttggtctaatg

ggtgttctttttattttatggtattatgttatgattatgattgagtctgtatggttgtta

atggggaaacagatatactttcagtaatattaactacttatattttgttttatgtatgct

gggtcatcccttgctagatgaaagttcaacaatggtatgttcatgtgttatctatgaaag

caaatatgaacttggtgactagaccctgtttgtttgcttggcaaataagcttctactcgc

taagatgtgtgtaaatgactgatatttatcactaactagcaatccgtgctaaattagtgg

taataggtgaagctaaaaatcgttatcactccaaacttaattttccaatccatcaaacca

gctcaactagttg

>fava_dn_rep_c734

ttgcaaatctctttatatattcacacaattattatataatccatcatcaacagaacgcgc

ttttctttccaagcaccttcgttactcacctacctaccaaacatgtcgctgccggaatgt

tcggcgacgcaactagcgcagctcatcggtcccaacgctacaaacgcggcagccgccgca

gatttcatctgcagccagttcacagccgtgggaaataagttcgtcgacactcaatatgcg

gtggataacacgtaccttctattctcagcttatcttgttttctctatgcaacttggtttc

gctatgctctgtgccggttcagttcgagccaaaaacaccatgaacatcatgctcacaaac

gttctagacgccgccgccggtgggctgttttactatctcttcggtttcgccttcgctttc

ggtggaccttcaaacgggttcatcggaaaacatttcttcggcctcaaagatgttccgtct

gaagctttcgattacagctacttcctctatcagtgggctttcgccatagcagccgctggt

atcaccagcggctctatagcagaacgaacacagttcgttgcttatcttatctattcctct

ttcctcaccggatttgtttaccctgttgtttctcattggttttggtccagtgatggttgg

gctagcgccaccaacactggaaacctcttgtttagcaccggagttatcgatttcgctggc

tccggtgtcgttcatatggttggtgggattgctggtctatggggagcgcttattgaaggt

ccacgaattggacgattcgatcataacggtcgcgctgtcactcttcgcggacacagtgct

tctctggttgttcttggaactttcatgctttggtttggttggtatggtttcaaccctggt

tccttcaccaagattctaagctcttatgaatcaggtacttattatggtcaatggagtgcg

gtgggaagaaccgcggtaacgacgacactggctggatgtacagcggcgttgactacactt

ttcgggaaaagaattctctccggtcactggaacgttaccgatgtctgtaacggattacta

ggcggattcgccgccataaccgccggttgttccgtcgtggagccatgggcagccatcgtg

tgtggatttatagcagcggtggttctgatctcgtgtaacgtactcgcggagaaagtgcgt

tacgatgatccattagaagcagcgcagcttcacggaggatgtggcgcgtgggggataata

ttcaccgctttgtttgctaagaaagagtatgtgaaccaggtttacccgggtaaaccgggt

cgaccgtatgggttatttatgggtggtggagggaagttattaggggcacatggccgcggg

actcacaatctcatttctcattacaaaatttatatctctctcagattttttttagctcaa

ttttcaagcttttttccctcactcttttgcaagcttcaatggcaacaagagaggttaata

agcctcagtcacttcctccttaccctgagatgatactgaaggctcttgatgcgttgaatg

aagcaaacgggtcaaacaaatcagcgatagcaaacaacatagagtcgatttacggtgaac

taccagaaggccatgcagttcttcttagctatcatctgaaccagatgaaagagagtggtg

acctagtttttgcgaagaacaactactcaaggcctgatccaagtgctccacccaagagag

gacgtggtaggcctccaaaggcgaaggatccactggcctcaccaccttcagctgttgtgt

ccccaccgaggccaaggggtcgtccacctaaggatcctaatgcaggaccaaagactccca

agacttctggaagtggtaggccaaggggcaggccgaagaagattgcgaggactgaggatg

ctgatgtttcaactccaagtcctgttagtgttgctgctgtgaatgttgatgttgttgtgc

catgtgttgctgctgttcctacttcgagtgggaggccaaggggtaggcctcctaaggtga

agcatcagttggcacgagtgagtgcgtcgtagatgatgtgggaagctgaaagatggagga

atgatggtgtgtagaactagaagagggtgtggttttatattagtttattgttactgttat

gtgttgtgtcgttaatataattgctttgtaaaatttatctagtggtagtttagtttatga

gatggtttgttggtgtttctaatgtcaatgaagaaagttactttggtctgaaaaaaaaaa

aggaggaaatgaggggacgataatgaattgagtggttgtggaaggaagcggaggaacgcc

atgagttagtttcacggtggtgatggtggtggaaatggaatgagatctatactcggagaa

gctgttgttgattattatattctttaaagat

>fava_dn_rep_c735

taactccattaaactcagtcactaaaatcatgatctatatatggaacatatattacatca

aaatctctgcatacatagtcaagaattcaaaaactactgcacattatttcaacaaaacca

gatatcaaaatcattcatatgatttaaatcaatcccaccaaacaaaagggaaaaaattgt

cataatgaaaaacaaccaagtttacaattatatcatccataaacataacataatataatc

cttataaacatcaaactgaaaaatcctcaaaaggttcttgacaagacaaaaggaaaacta

ataagaaaactataactaacttattcgccacaagagaataaagctccacgcttgcttaag

cttgtaacttgttcttgatcttgatgccatcaacaatataaataacccaacaatcttgta

gctttatttacataatcggcgaggcggggcagatgttgttcagaaaactccgccggagat

ggcggacatgtcatcaaagctccagagattcattgtgtttccacaatcctgaactgaatc

agtaccgagcagagacgccaatgaagcatcactccagttatcatcaaacgagttctcgaa

gaacttcagctgggattcgatatcagcaagctcctcagagagtgtctttgcagactcatc

ttgcataggcagcatgtcctgagcattgttagacagcagattgatctgagattcgccttc

ccgaggagcaggagcggaaaacacagacgaaatctcaggggttttcggggcttgttcgcc

ccaacctagatcagaataatcaaacgagttgctcgaatgctctgagctgaagtaggcatt

aacatcagcagcagctgcaagctgaaatccgtttcccgcgaacgctcccacgttagcaaa

ctggttgttaaccatcggcttctgttcaacctgatccataggggaatagtagctctccat

attctcgccgaaattgaacattgtgttcccattcggtttgaacttattcatcggctccgt

ctcagcatttggcttaacacgttttgtgcaagcatttggagtctcttcagggaagttcac

cttggctttcgcgccgcggattctcctagcttcagcatcataagctctcgcagcttcctc

agcagtgttgaatgttccgagccaaacacgaactcctttcctcgggtcacgaatctcagc

tgcccattttccccacggacgttgtcggatacccctatactgattcttcctctttctctt

cgaatccttttcagccggctcattcgatttcgataccacaggttttacagcactcgatcc

acgagagaaggtccttgaagatttgttattggcagaagagaaagagaaacctttcacacc

gagcatcaacccttcatcttcctcttcatcctcgtcctcatcaaaatcagaatcatcctt

aaactctctgaacccagcctcgaaatcatcgtcaagcaaaaacggtttcttcaacccgtt

tttcctcaaactcggccacaagatatcagcagtcacacggcgggaaccacccaccgccgc

cgtaggtgggatgaagtcggagataatagcaccaccacacatgttttctctctcttttca

ctactctcagatctttggtttttctctataaaaaaacagtgaaactttcttttctaccaa

ttgaagaagattgtttgctcaatggttgagatttttacaaagtttttgttttgaatgttt

tggttgtgtttggtatgaggtaggacttgtgtcttatatatat

>fava_dn_rep_c736

ccgctaaagaaaaaggagcaccacaacaaaataaaacaaatccagccgtgaattgttaca

cgatagaacccatcacattcttatctcctcgcgaaataatagataaaactaggcaatgtc

ttcctttcaccaccacactccgtttcccatggcttcatgtacaaccaccccaccttcatc

atctctattcttatcaacacacacaaccaattcacaagacctctctccttccaaccgttc

tttccctctctccagaaccttcttcctctccccactcgttctcaaacctcttcgtctaac

ctaccaatctagggtttccaattcctttgtttctgcacttgccgctggcgtcgccgaaac

gcttgatgaagacgaagacgaggatgctgttcctattaacactgttactgtcgcaattaa

gcctaagactgggaaagctgcattgccactcaaggctgatagggtgagatctaagagatt

cttggaaattcaaaaattgagggagcttaagaaagagtatgacttgaagacagcaatttc

tttggtaaaagaaacagctaaaaccaagtttgtagaaacagttgaagcccatttccgcct

taacattgaccctaaatataatgatcaacagttaagagctacagtaagtctgccaaaggg

aaccggaaagcctattaaagtggctgttcttactcaaggtgaaaaattcgatgaagcaaa

aaaagcaggagctgatttggtaggtggagaggacctgatagaacagataaaaggaggatt

catggaatttgataagttaattgcttctccagatatgatgcctaaggttgctagcctagg

aaagattctggggccaagaggactcatgccaaatcctaaagctggcactgtaacgtctaa

tatttctcaggctatatcagaattcaaacagggaaaagttgaatacagggcagataaaac

aggaatcgtgcacttgccttttggaaaagctgattttccggaagaagatcttcttactaa

cttgctctctgctataaaatcagtagactcgaacaaaccttcgggcgcgaaaggtgtgta

ctggaaaagtgcacacatatgctcagcaatggggccttctgttaggttaaatatcaagga

gatgcttgattataggcctcaatcatcagaataaagcacttgaatatgcttgttttgttt

tagtcagctttctgggtcgtatcttgttgaagatgttacaaggttcttgtcacaaaaaaa

aaagttaccaaggttagatagagggaagaacttgggatatactttggatggttatatctt

ttgttaaaaaattctactaaaagtgtgcacacaacagtctttgacccaggatgaggattt

tccttgtattattattactttgtaactaacaccttagatctcattatattatctttctat

ggtaataaattttcatttttgctcttgaagtgttgataatgagatctttttcacttgcta

tgcatcaacttgtgttagttgtactcatttcaattggtaattggtaattgttattaccta

tgtttgtattttgattctataatcaatacatatctgtttgtgtatatgtgcatataatag

tttcatatttctccattataaatttgtagcattaatcaaggaagatttgtatgagatgtt

gacttttttcttgaaagcatgtgagtgatacattaaatgcaaaataagtagaaaataaac

atgtatatctactttggaatataaaaacatgtatctacaattatgaattactaacattaa

agagtttnnnggagtatttcacaatgctttcagcttggttgttttgttggaatttgatgg

gaagtgaggcaagtaggagaaagtgagggagagaaggaggagggtttgatgaaaggggac

ctttggcaaaggcaaagagaatgagcttgaaagtggaaagccatgcaaggaaaaatgaag

aaatgactcctagatgcatagaggtaagcttcaatgggagaagtagaaagatgagtatag

gtggaaaaagagctataagtcttccttttcctttgggaatcactctccctattatgtaac

tataccaaaacaatgttattgctatgctccataccatgatgaagttactcatttctcttt

ctttcatatcctactcactccccgcggcc

>fava_dn_rep_c737

aatcaaagtcaatcactttcattttctcttccacattgttatacttcatcatcatcaagc

actaagaacaagatacaaataaaaactaagaagaaagcagtgaagcattcaatgaagaaa

ctgaaagaggtgataggaaaaatgaaggacaaagcatcacaaagcaaagctgcaatttta

tcgaaacaaaaaactctttctctcttacgcgccacaacccacgactcttttgcccctccg

acacacaaacaccttagcacccttctctcctctggcgacggttcacgcgccaccgcatct

accgccgtcgagcttctcatggaccgcctccaaacgactcacaactcagctgtggctctt

aaatgcttaatcgccgttcaccatattatcaaacgtggcacgttcattctccgtgaccag

ctttctgtttacccttacaccggtggtagaaactatctcaacctctctaacttccgtgat

aaaacaagttcgatctcatgggaactctcttcttgggttcgatggtacgcggaatatata

gaacaccttctgtgtgcttctagaacactcggttactttctccgggaaacgacaacggtt

aaggagacgcatgaggaaagagtttcgagtcttgtgagtggtgatctgttgagagaaata

gattctttggtggcgatgatggaagggattgggaaaagaccaaatattccaacaatggaa

cagaagaacaaggtggttgttgagataatggatttggtggaagatgatgaagttgtggtt

gtgaatgaagttttggttagagttaaggaatttggggagagggagagattgggttctctt

gggtttggagaagttgtggagttggtttgtgttttgaagagattggagatgtgtagagag

agaatggtgatggtgatggaggagaagaggttttggaatttggtgatagaattgaaggaa

aaggttgggaagatgaaggtgtatagggatgaagggaaggtttacagaacggtgggaaaa

gacagaggaactgagtctgttcggttttgatttcattctccttctttcttttatgttgga

attgttgtgtatagatagtagaaataggttctaatgttttgtttgctgaacttctttctt

ttaagtagatattttagatagtagaaattgatgtaggaattcacaatgttgcatgataga

aggttttggttgaagttgaagctgaagaatgtgcacttgatatttgttaaattctgaagg

ttgggaagattaaacaacttaacaaccttttctttcttaagcaataataagcttgtaaat

ttcgtgtaattgttctgtttgcgaaattgaatcctctaacaaattataattaatgactat

ctaatatataactccacatacatcttattataggttcataatgagttacagataacattg

atcatacatatgaagcaccatttattatacgtacgggccatgatgttattacaacaagaa

aaaagaaaataaagcacaccatttattatagtactggttggattcagaggtggtaatatc

agtaaccaagcatattaaactccttttagtgtccagttggcttttttgttccatgaccac

gaggattattcatgttattgttgtatccatacggattggcctgataaacatctcctccat

atgttccacgcctctcatatctcacttcatccctcttcgtcgtgccagaaccgacatgct

cctccttgtaaacctcatactcatgttctgacacataatcattgtggccaccaccatgtt

tgaatggctttacactgctaccatgatgaggtgagtgattagtggtagcaccaaaaggga

acatttggccattgccatgaccatgcttattatcatagtcatgaccatggccatgaggct

tgctgtcctgataatacatattgtggttagagtcatggccgtgatgttttggcatcgagt

aatcagaatcattgtcataaccaggtttcatgcctggatattgttgtgtctgtggatatc

catggctctgtagctgttgagcctttgtttccatccacgagctatcatcaccatagtatt

gctgttgtgggtatccgtggatctttctgctttgcatcttgttggagaaagggaaagtaa

caattcacacaagttttggtgatagctgcttgaatgatcccgcggcc

>fava_dn_rep_c738

gaaaaaaagggctactcaaactcctcacgatttagatcactaatgaatccctcccccgaa

tcaacagtgacaatatacagtttatgaaaacatgtaaacagagatactcatttttcatag

taagatatattggcaacccattagagtaagcccactttacccaacatcttttatcctgaa

tgtcagaattgtatcacagagaatattgaaaatactgtgtttaagttcatcaaatgtagt

gcagctactacaatgttcacaatggatggatttcaaagacaaacaagattaagcaaagaa

aaatccatcaccgagatgtctccttccgtaacacgttcaacaagaaacctattcattttt

taaataacagtttttaacaactcaagtactggatcttagatcatcaaatgtccattggag

aaccaccttggggttcggcaccaccttggttttcagcgccaccttgatcagcaccaggag

cctgatccggggcagcatcctgctcagttgctacctcaggttcagcaggtgcactttctt

cctccctcacaatctcaaaagtatcaatcaactgttgaaccgtttcctgatccaaaatgt

ggaatggctcaccaactccaaccacagcaatggtacatacggagcttctgagcttctcac

cttgcaaggactccctagttgcaattagtgcatctttgattagatcttcccgggaggagg

ctgtgaagttgttaaacctgcgttccaaatatgtctttgcagcttgtgagcgagaaccaa

ttgcaaaagcttggtactcaaagtagtttccgcttgggcagttgtaatagaggtgagccc

ctgattcatctaatccagctaccagaagaccaactccataggggcgcttccatgatctct

gagtgcaaacctgagccttatcggcgagctgaacaactaaccgaccaacgggaagcggag

actcataagtgtaattatagttaatacattcagatcgcatgtaacgggaaagaacacggc

cgtcagccgttaaacccgcgatagcgactccgatatgatcgtcaaccttgaaaatcttct

tctggtgagaagacaattcagaattggctttgttaacgcaggcgagaaccacgtgcgtct

tggatcggagacctattgcggctgaaccttgcttcacagcctccatggcgtactccactt

ggaatagtcgtcctgctggactccatgtagtgacgtcggtgtcgtactggtttctgaaca

tggttgattttgattggatttgtgttcgccgtagaaatgggattcagtgagtgtgtcttg

gaggtcaccggagaagatcggagttgagaagcctgggatcgatctagggatttcttcttt

tccgcgg

>fava_dn_rep_c739

caattctttatgaaatctcgtcttaatcattacacagtctaagaactttcaaacgtaggc

cttttcgaagtctaccttaaacacaatcacctcttttttcttatgtttgacttcacctac

aattttctttattattaggataccatcaagaatttgtcttcttaaaataaaagatggtta

tgtgtatgagatgatagacccaattacatttctaagtctgtttttcaaaacctttgctat

aaaccttataaatatagacaatctggtgatggagatttgatttccaccaaacaattcaaa

caaagccattgcatttattgttcattacttaacttcatgctaagtttgagaatacaacat

gtctaaaagctgaaatccaattgaaaaaacattagtactatatttcagattgacaactca

aatttggatacaatgacaccatcattatctgcataaagccattcaccatccctaataaaa

cttcctccaacataaacaccaacatgtttttccccaaaaccttttttattcgacctaagt

ggatgagaacccaaagctctaacaccaacttgacacaaattaatctcatccacatctcta

acacaaccattcacaacaattcctgaccaacccatgctttgtgccaactgcaccaaattc

cctccaaccaaagcacacctcatgcttccacctccatcaacaaccaaaactcttccttct

cctttcgtttcaagaagttctctaaccaaaacattgtcttcaaacacttttacagtaact

attgtgcctgagaatgcttgtgactgaccatatatctggaacactggatggagtatcctt

aaatcaccagttgatagatgtgatatgtttgtgtcacatatctctgctgttgctagagaa

gacatgttacaatactccaaaaggcagagaaggaagtggaacgagtttgtgatgatgatt

ttgactttgtcacaaagtggatgaaactcatccaaagcatgtgtggttctcaaactacaa

actccaatatatgatgattgcgttacccttgatatccgtgtcacgttatctagtcgataa

gaat

>fava_dn_rep_c740

atgaagcgaaatcacccgcgaaaccgttattgtgattcaggataaggcagatagatacac

acacacaaatacataggaacacaaagcgagttcattccaatggcgactctgcaattcaca

ttcaccgattcaactactccatacagaccgttgttatctcaattaccttctaaatctatt

cattctcgaactcgaagtttcgtcgtcaaagcttcttccgaactctccagaaccagaaga

cagttcatcgcggaaaccacggctatatcggtgttagtaccttctcagttggtgaaagct

gaagaaactctttctgagtgggaaagagtttatctacctatcgatcctggtgttgtgctt

ctggatattgcttttgttccggaagaccctaaccatggttttcttctggggacgaggcaa

acccttttggagactaaagatggaggaaacacttgggctccgcgatctataccatctgca

gaagatgaagattttaactataggtttaattctatcagcttcaaagggaaagaaggatgg

atagtgggaaaacctgcaattctgctctacacttctgatgctggagaaagttgggaaagg

ataccactcagtgctgagcttccaggcgatatggtatacataaaggcaaccgctgacaag

ggtgcggagatggtgactgatgaaggtgcaatatatgttaccgcaaatagaggatacaat

tggaaggctgctgttcaggaatcagtgtcggctacgcttaatagaacagtttctagtggt

attagtggtgcaagttattacactggaactttcaatactgtcaatcgatctcccgatgga

aggtatgttgcagtctcaagtcgtggcaacttctacttgacctgggagcctggacaggcg

tactggcagccacataacagagcagttgctagaagaatccaaaacatgggatggagagct

gatggaggtctgtggcttcttgtccgtggaggtggtctttatctcagcaagggcacaggg

ataaccgaagaatttgaggaagttcctgttcaaagccggggatttggcattcttgatgtt

gggtatcgttcaacggatgaggcttgggcagcaggtgggagcggtgttctcttgagaact

aataacggtggcaagtcatggatccgtgacaaagctgctgacaacattgctgcaaactta

tacgcagtaaagttcattgatgacaagaagggatttgtattgggaaatgatggagtcttg

cttcgctaccttggatagagctttaattcaacatggtttgtgaactcaattgcaattttg

tagcagcagattaaatatccatgcttagtactattcattacggtgaagagagctattgga

acaaaccaattccttgatgtcatgctgaaaaatgtgaggagatgtggaaacattcttcac

cactatgaatatattatgtcaatttaacagtgtaattgccatactactattacatttaaa

tgcaaagtgttatatatgttgtaatgttgaagccgagtgcataatttcaacaaccagtaa

ctcataaataacagagaaagctaagatgatttcaaattaacttcaagactctaagtaaca

ggtggcttatggtagaacttgcgtcatctcaaacgtcatacacagacaatgaaaagggag

tgatcaaattaaaatgaaggcttaaatgtattttttgtcctcctagtttggagtttttaa

agaagtagttttcctattaaaaaattagcgaatttat

>fava_dn_rep_c741

aaaccacttaatcatatcacttttatagctgccgcatcttatctgttactttccttccct

tgtatagacaaacaacaacaacaacaacacgcagaacttcttctcaatccaatctttctt

cttgctttctaccacgcatatctgccattaatacacaaacatgcactccttaacatgctt

accactttcttctcctccatctatatcatctctaaagccaaaccactacaacaatccttt

attcaatgcaacttcaagaaaaacctgttttaccaccaaagcattactctcatcaactaa

agaatctatcctcaaggattttcgcgaaagaacagctcttaagattatcacaggcttgca

gaattttgataaagataatgttgcttctgttgttactgcagcagaaaagggaggagcaac

tcatgtcgatatagcatgcgacccggaattggtgaagctagctatcagcctaacttcttg

tccggtttgtgtttcttctgtaaatcctgcatcatttccagctgcagttgaagcaggagc

actaatggttgagattggaaattatgattcattctatgacaagggaatgatttttactgc

agagcagatattgagtctaacaaaagagacgaggagaatacttccatcggtagttttgtc

ggtcaccgttcctcacacactaagcctccctgatcaggtcaaacttgcagagttactaga

actagaaggtgtagatattattcaaactgagggaggaaaatgttccaatcctacaaagtc

cggtgttctaggtttgattgagaaggcaacaccaactttagcggcagcgtattctatatc

acgagcagttaagatacctgtcatgtgttcatctgggctaagtgctgtcacagcacctat

ggctatcactgctggagcttctggtgtgggtgtgggctctgcagttaaccgactaaatga

cgtggttgccatgattgctgaggtcagaagcattgctgattcattgaagacatcatttca

gacgcgtgaagtggaaacttatagacagtagctcaatatatgtagaaaggatcgagttaa

gttgttgtaggcagtgaataaaacatgggtctttttctattttagacttttgtcgtagtt

ctcgtttgtgtgaggaaaatattataagcaaaaaaattataactattcacaaaagcaaac

aagagttgtgactacaactgaatatacaactttaagtcctcaagtagtttcaattaatac

aggtgaagccttataagcaagacaacgaatacatagttttactcgtcatactctgactcc

tcatcagtttcttcataatcaccgctttccattttcttgttatactctgttgttgctcta

tcaaattctgcacgtttttccgttgctatatcatagtattgaaccttctcctcataagtc

attgttttccacttatctccacatgccttgccaacatcacgcattgactttacatctggg

ttctgctcttgaaagtctttccgaaaatcctccaagaagtagaagaaagcagtggggggt

ttcttgggcttcttagcatcaatcttatgtttgttctttctttgctttgctttggttatc

tctcttgactcagtctgccgcacggatctcttaattcccctcaacactagtttgccggag

gaagtggaggcggaagcagagggtgaagaagctttagcctttttcgccatgtgagacgtt

ggtgcctctcaagtctcaactctgcaactatgatgtggtcacggtttaacattcctc

>fava_dn_rep_c742

ttggaaaatggctgtctgctgtcaattgtcatacaagattgcaagaatttacaaaggcac

atatcaaggtcaccatgaaaccctggtgtttctattatgcaagctatagtgaaaggttac

caaataccacatttatttgtcaaattcttacaattttacttgttagttcaagatttcaac

agagaaaaaaatcacaattgaatacatgccagatatggaacaaaagtccaccaaaaaaat

aaaatttgacgaacaaccattagtccatacaaatcttaaggaccatttcactgatcattt

tatttatcagtgcgtaataaaacagaataagcagcagccaaggatcaaaaaaggattatc

agcgataaaggctttcagatctcaggttctctgcaatttctgtttcccatctatccccat

tttcaagcagtttctctttgtcatatagaagctcctcatgagtctcggtagaaaattcaa

agtttggcccttcaacaatggcatcaactgggcatgcctcttgacaaaatccacaataga

tgcacttggtcatgtcaatatcataccgagttgtcctgcggctgccatcttcacgctcct

cagcctcaattgttattgcttgtgcgggacagatagcttcacaaagtttgcaagcaatgc

aacgttcctctcctgttggatatcgtctaagtgcatgctcaccacggaaacgagggctaa

gagggcctttctcaaatggataattgattgtaacatttttatcaaagaagtacttcagcg

tcagcattaaacctcgaacaagctcagtgagaaaaagtgtgttgatgctacgttcaaaaa

cagcactccaatcctttgacatttccttggaaagtttttccctttcttcgtcctctaatt

tggtagagtagaggtgtgaactcaatcgcatgccataattttggggggtgtgcagtcctt

gacctgccatagccatatggcgggtgcgaagggcaagaagtgatttccgagctaagaacg

cggccattttagcagcggtggaatgcgaaaacgaagcctactgttcagaatgggaatgga

aattcgatgaacaccgatttgttgaagggaaaaaaacaatggggggcactgttttgtgtt

tctagcgacagcaacgtgtacaattgtgggccttacttaaaa

>fava_dn_rep_c743

acaagagaagggagtaataactgtgtaaaggacattaaagtcatttactgaaaacaatta

gattcgaaatctaaagcaggaaagaaagagtagaacaaacgcctgaaaaaaaaaaacgag

gaagaaagaaagggcttccactcaacgcgctttctctcttcctcttcctcttcctctcac

ccaagccatgagcctcttcggtattggcagaaaccagagaacattccgcccaaaaaaaag

tactccttctggaagcaagggagctcagcttagaaaacacattgatgcgacattaggtag

tggaaatctgagggaagcagtaaagctacctcccggagaggatttgaatgagtggctagc

tgtcaacactgttgattttttcaaccaggtgaatctgctctatggcaccctcacagagtt

ctgtactcccgagaattgtcgcacaatgtctgcaggacccaagtatgaatatcgatgggc

agatggtgtgcaaattaagaaacctattgaggtatctgctccaaagtatgtagaatatct

gatggactgggttgaaacacagcttgatgatgaatctatattccctcagaagcttgacct

gtgggatcatgatgcaggttctccatttcctcccaactttaaggatgtcgtgaagacaat

attcaagcgtctgttccgtgtatatgctcatatttatcattctcactttcagaaaattgt

gagcctcaaagaagaggctcatctaaacacctgcttcaagcattttatactcttcacttg

tgagtttgggctgattgaaaaaaaaggaactggctccacttcaagagcttatagacacca

ttatatattaaagttatgaattgtgaaatatcttatttaggcatcataaactatgtatgt

gtattactctagtcagagactggtttttaatatctgacttgagacattctccttgtatgt

tctgtgttgaatgagtttgggaactgggtctaagttatcctccatcacagaaaactcaca

taaagtataaaaaaatatgcaatgtgtgactttaaagtattggctattgtttgcaactga

aaaccgtacttcatgttcgatgttttggtgatgttttggtgtgaatttggaaggcttttt

ctaatattttcattgc

>fava_dn_rep_c744

cgcggggcgcgagtgataaacacacgcacgaacaagatgacggccgcgggatcaatattc

atacaaaacttgcaaaaacattcaattcatacagaattaacaaaaacattcatacagaag

taacaaagtgtgataatggcagaaggaaaaggaagagtttgtgtaacaggaggtacaggt

tttattggttcatggattatcaagacacttcttgaagatggttacactgttaataccact

gttagagctgatccagagaaaaagagagatcttagctttctcacaaatcttccgggtgca

tctgaaaaactaaaatttttcaacgctgatcttgacgatcctgaaagtttcaatgaagca

attgaagggtgcgttggaatattccacactgctgcttcagttgattttgaagagaaagaa

ccagaagaaatagtgacaaaaagaagcattgatggcgctttaggaattctaaaagcatgc

aaaaactctaagacattgaagagagttgtttacacttcaagtgcttcagcggtttattgt

caagacaaagaaaaagaaaaagatgtaatggacgaaagttattggagtgatatagacatt

cttagaaatcataaaccatttggttgggcttattcagtttctaagacacaagcggagaaa

gctgttcttgagtatggacaacaaaatgggttggatattgtgactgttataccaactatt

gttgttggacccttcatttgccctaagcttcctacctctgtttatggtgtactaccttac

ttatttggtaacaccgataacagtccaatatctcgtttccacatggtgcatgttgatgat

attgcacgagctcatatattcttacttgaacatcctaatccagaagggagatataattgc

tcaccattccttgcaactattgaggaagtagttgacatcgtttcttcaaagcatcctgaa

tttcaaattccaaagtccaaattgcttgggggacctaaaggtcttgagcttccacattta

acatcaaagaaactcacagatgctggatttgaattcaagcatactatagaggaaacgttt

gaggatgcgattcaaagttgcaaggaaaaggggcttctttaaaccattgaattaacaatg

tgtttgatcataattaataaataatatgtcactagacatggcttcttaatatttgagtta

ttttgtattcgagctttagttggggcttctgatggacggtgttttgtgactgagtgtttt

ttatagagttttctccccttttctaaacatgacttcttaatatttgagttatttactatt

tgaacacataagtttataaatttatcattaaaaatagtgataaattgatttggagtgaat

tgattatataaaattgattttgaataaaagtgagttgaaagtaaagagatttatgtttgg

ataaatttatgtaaaagtaagttgaataataaatttccgtgtaaaataaatcacgaaaaa

aaaa

>fava_dn_rep_c745

aaatttgttggtttgtaatattttcctttcaacaagtttcgcattaacacttcagtccgt

ctctccttttcaatattagagataacttcttcttcctctttcattcacccaacaaataac

aatgtctcattcttctcttctcaccccttcctcttctcttctttctcctatcccctcttc

ttcttcttctcctcactctcacagttcttctcttctctccgggaaattgaggaaatctgt

ggaatttagaaagactactctaaaatctcctgtgaaagctgtttataaggatgaattttg

gggacctcagaggagtttgcgtaagggtatttggtccataagggaggatttggatgtccc

gtcttcgcctgtctatgctcaaggacaagggcctcctccaatggttcaggagcgtttcca

gagtgttataagccagcttttccaatatagaataatccgttgtggtggagcggttgatga

tgatatggcgaatatcatagtttctcagctcctttatcttgatgctgttaatccaaataa

ggatattgtcatgtatataaactctccgggaggatcggttacagctggtatggcaatatt

tgatataattaggcatatcagacctgatgtgtccacagtttgtgttggactggcggctag

tatgggagcttttctgcttagtgctgggaccaaaggaaaaagattcagcttgccaaattc

aaggataatgattcatcaaccgcttggtggtattcaaggagggcaaattgacgtaaatat

tcaggcaaacgaaatgctgcatcacaaggcaaacctgaatggatatctggcctatcacac

tggtcaaagtctagacaaaattaaccaggatactgaccgtgattatttcatgagtgcaaa

agaagctaaagagtatggactcattgatggtgtcattatgaatcctctcaaagctctcca

gcctttggcagctgcagaagaaggtaacgatcgggctattgtttgatcttgatccaaggt

atgagaaatgttgtttttggttggctgcgcttagtatagctgaaattttcacagatgttg

atatctgtataatggtgtagtaagaaatgaggccaaaatgatacttagtaatgtattgcc

tcaaagtttatactgttaaaatcttccttaagggatcctggattcacatttttccttcac

aagcattctattttcttgtgataactgatgtctgatgattatatgttgttatattttatc

tcctttagacttgtgattgacttcatannnnnnnnnnggcatggaatgtccagttatacc

acaagatgctcatggtacccaatg

>fava_dn_rep_c746

aagggtgtagaaaggaagaacaaatcagccacactttacttcttcttcttctacttccac

aaacccacactctcacctcaccgctcaacacaatctgaagagaagagagtaaagaaagac

ggcggcaatggcgacacgggtggctgcgagatatgcgtcgagaaggctatttagcagtgg

gactgggaaagttcttggtgaggaggaaaaagctgtagaaaatgcttacttcaagaaagc

tgagcaagagaagttggagaaacttgctcgcaagggtcctcaaccagaagcaaagccagc

tgcaggctcaagtggtacagtaaccgatgccaaaccaagtagctccggacatgccgaaac

atcaagtgccaaagtttcaactgacaaataccggaattatggtgttgtagctggcactat

aacaattcttgcagctgtaggatggtaccttaagggcactgcaaagaagccagaagtgca

ggattgaagatttcagttgtggtgcttgttttttatttcaatttggttgcaacaattgca

caaaaataaataactacacaccactctgcttttagcatggattacttctttttgttcaca

ttgttttacatgtgacctatcacctatatatccctaatgtctcttagcatgatgagcatc

aacagtgaaactttaacatgtgaaatgagaagaatattttgtaagaaaaatcacatttgg

tgacatttcaaccgagctgctgcatcgattgatgaaaaggtttattttcacaagtgtttt

gtgctaagattgatgaatttcttgacttctcttttatatatacatacatagtgaggaatt

ataacagcctcttttatatattcactttaaaccaaattctagatgaaaatagctatgaaa

ctaaaccctatcttgatatatcagtaaatcagtgatttttgttaaaatacaatgagccat

attcaataaaacg

>fava_dn_rep_c747

ggtagaaatttctatcttagactcttataaaatgcattcatcaatcagttttaaaagcat

ataacaaacaaggcagtaaaatnnnnnntttttttttcgaatttgaattctccccaacaa

tttttctctgccaacattttatttcttggttgtaggatacatgcaggataatcacgttcc

acaaagatcagagtgtataggttacaaagttttactagattaaatttgtgtatttgaata

gcttctaacagcttacaaagtgggcggagggcgtcgaggaaaaaatgacactcttctaac

agcaattcaaagttcaaaaacaaaatcacaaaatgctgacaaccaggggagtttgagact

aaacaactttatcaatatgaaataaaacaatcagtttgtcatatgaaatagaaatagact

gatgcaactttcacagtttgtcaccaccagtcaaatagaaatttattgaattaatatctt

tcaccagctgtaaacatccaaaacagagtacacaagaatacttcaaaacatgccaagggg

agttacatgctcattgaggatccagagttctttctgcattctgatagcatatcaaggtaa

aactggcacttgctaatatcacttccgtaattgttcaggcaatcctggaaagcctttgaa

tgaatgttgcatgcatcaccaccaaaagaattggcagttggtgcaggggcagctgcagca

gcctcattaactacagcttcatgttgaactgtacgaggacccataacagcatccacagcc

ctgtgtgccacagcacttccagtgccaaaagccataccttgagctatggttgaaccaaca

ccactaagaataccaccaccaccactctgagtgttagctggaggaggagcacggttgacg

ggggcggggcgtggtgcagcacgtggagctggacgagcagatcttccaccggagctacga

cgagccatggatgaagttttagggcaaagcggcgaaagagggcagagggtgattttgaga

ggcggaaggaaacgagagcttataattgaagattcccgcggccat

>fava_dn_rep_c748

atgcaagaannnnnnnnnnnnnnnnnnttggtttagaagagagtcagaagagaaaaagaa

atatgtaggacccaccatttcttttcaatatctttaaaatagagaagaaagagaggagaa

gtgttaatttgatactaagttgtcatttatacccctattttcttctaattcaaaattatt

attaaggacagtttggtatttttaataaccatcaacatttctctcttctctatttctctt

atacttgtcttataacaaacaatacatcaaatctttatttctcacatctttctctcttac

ttatttctgtcttctaactttctcttcacaaccaatcacaacagacataattggatatat

gaattaggaggtcattatacatgtttacaattgaatgacacaaatacaatattggccaaa

ttacttatacagagaacctgcaaggccagaagagagaggtatcctcagttcacaagtttc

tatttttgcttatatctcagcaaattttgtgatagtgtacaattgtagagcattgcatat

aacaaaacttggaaagatgaaactaggttgtcaaagctgagatacagacttcccattaat

aaaatgtatgacctgttcgacagcgagatcaagtgcggtagttacagatgctaaattctg

caggaattcttcagcagttggtttgtcaccgtcaatgatatcagtcactgctttcacaaa

tattgcaggaactttcagtagatcagcaacataagcaacagctgctccctccatgtcttt

aacagttgcatcatttgcagtgattgatgattcatcctgctgtgtcatatctaaagagtc

accagtagacaacttaccaacctttagattaagttccttcacaaggttgggtgtttcgaa

ggctttacgtgaaccaactccatacagatcaaaaacaggaataggtattcttctgtcatg

gaaagcacagtcagatataatgaaaacatcaccaacagtagctcctctgtccttgaagcc

accagccgtgcctgcatttatgatcaagtcaggcttcaatgcttgaatagcagcatatgt

tacaagggcagatgatattgtgcccacactatcaaccccagaattaggatcttttccggg

ccaaatcaagtttatattgagatctttgtatttgccatgataacgaagccaaggaacacc

ttgtggaaacggagagtgaggatcttggatgaggtgaaatttattgacaacaggttgagc

ctctgtctgcatagcaatgacgatgacgatgttggaaattgctttgttgccggaaacctc

cctcgtaactgattgcggaccagccattgctttatcacttttccttcttctatttctatc

actcactacttttttatatatacattttttgcctctcttaaaatattcatttcccactgt

aaatgtaaataccctatnnnnnnnnnnnnnnnttttggattaatagttattttgctcctg

acgatgcattgaatcaaattttaaagttgattg

>fava_dn_rep_c749

ttaaaccattataaattaaatatatttgtggataagttaacatgcaccatccaaaccgaa

cttaacaagaattagattcaccgaacttaacaagaattagattcggattcaaattcaaat

tcttcattataatgtcatctactcggtgaatatttcaaaataataacaatttttcaatta

caaaataacaacataacacttagaattaacttcattcaaatttcatctggaaaataaaat

agcactaataagctttgaaatttcaacagcaatacatcacatgaatcaatcaagatctaa

aactaaacctctttaatactagttgatttctcatcaacttcaaaagcagaaacaggaaaa

gttctagaaagtccagcaggagtttggaaagtgattttaccagtaggtggatcattaaca

taaatatcacttagtgtaacccaaatgaaaagctcttttgtttttacaccattcagtttc

ttgatcttacctttttcaacatgagctgtgacttcagtagcataagatgttactttccca

atcttttcaaatttgtgtgttgtgcttttcttctgtttcagccaaacaaaaccggtttct

cggttgtaaccaacttcaatgatgtctttcaatggtaatagaccgttggggagagcgatt

tctcgaagaagctctttggatttcagttgacagatttggtctccatggtatacctctgat

ttggacttgatttcttcggttaccgacgacatcgttttgagttgctgttttttggtttgc

tttgttgttccccgcggccgtaannnnnntcatgcttgtgctccaacctgaaatataagt

aaaaatattccg

>fava_dn_rep_c750

aaagaaaatcacaatgtagaactcctatataaatatgtgacaaatttatagatcgaggga

caaatatagaatcatatagattcttctcatcaacacaaccatggctaaaataaattttcc

aattgtgaaaatttgttttataattttgagttttattgggatagtttcatgccaattgtc

aactgatttttactcaacaacatgtcctggtgctcttgtaaccattaagaatgaagtaga

ttctgctgtgagtactgaggctcgcatgggtgcatcattgcttcgtcttcaatttcatga

ttgttttgttcagggctgtgatgcatcagtactattggatgatacatcaagtttcacagg

tgaaaaaacagcaggtccaaatgctggttccttgagaggttttaatgtaattgacaccat

aaaatctaaagtcgaagctttgtgtcctggtgttgtctcttgcgctgatatcatcgctat

agctgcaagggattctgttgttgctctgggtggacctaattggaatgtacaattggggag

aagagactcaaccacagcaagtttgagttctgctaactcagatctacctggtcctgattc

agaactgagtgatcttattaatgctttttctaacaaaggtttcactaccaaagaaatggt

tgctctatcaggatctcacacaattggtcaaggaagctgtagattcttcagaaacaggat

ttacaacgaagataacatagactccacctttgcaacatcattgcaagcaaattgtcctac

tacaggtggtgacgacaatttatcaccaatagataccaccacaccaaacacatttgataa

ctcttattacaagaatttgcaagaccaaaagggtctctttcactcggatcaagtgctttt

caatggcgattcaacggactcagatgttgatcaatatagtagcgacgcttcgagttttgc

aactgattttggtaatgcaatggtcaaaatggggagcctcaatccacttactggggcaag

tggtcagattaggactgattgcaagtttgtcaattcatgaggttttgggccttttgggaa

tataatgtatgttaatttgggtgcttttattgtgtgtttgaactttaatatgtatcaatt

tcaatgtttgtaattgtttaaactattgtgtaagctcgtataatgcataaataataatca

agttgaaaattgtggaggtaaaaaaaaaaagccttattaaacatcaccttattacacata

cacacaaaaacttt

>fava_dn_rep_c751

gggcatttaaagcaaaaggttatatgaacaaaatcgataaaagcatcaattactatataa

tgaacagtaatgccagtttgaacaagctctccacagaatactccacagaatccgggcatg

agcagcttatttaaaattacttcagttttcaaatgaaagtatttgtagccaaaaaagaaa

aggatttctattacaatgatcctagacccaagttttgcataacattaccagcagcaactc

tttaatgttgttcataacaaggaaattttttttgacaagattagaacacccacgccagcg

atgtaatcgatagtcccagtccccaattattcgtattctttatagtctttggttccgaag

ttcttgcaccacatttgggaagggatagggcgaaccgggtgatgtggtcgttgctcgagt

ttagcggaaagtttgaagattgggatgcagacaaggaagacgccgaacatggcgatggcg

gtgttgcgtttccagtgtttagggcgacagtgagggccaccggtcatactccaaaccttg

tatctgaaatctccattgccgccgttaccgtgatctgctgcaccacctcccattttttgg

ttgttgttccgaaatggaaaaatcgaaaccctcattcgatcgatgaatcggttctaattt

tacagagttgggatgggattgggaggaagtgaagaaagagaatgaggtccagtgtgtgac

tgatca

>fava_dn_rep_c752

taatataattaattgatttagaatgaaataaaccttacaaatccattaatttttcagaag

cttatatatatagtaattgccttaccacactacaacttcaatactctctatttgttcatt

gattcaggcagtgatattatttcagaacctacctactggtttgtgcaacaattgaaaagt

tttgacaaccaagcaagaaccggttttaaaattggttagtaacacaagcaatccagttga

ggaattaaaaccgcattgcacttatacagagaagcaagcaagaataacaatcaaaactga

atagaattatttaaaggttgcactttaaaccgaacgcaagaataacaacagcaagtttca

gggtacttagttcatctactcttatttgagtatttttattacactacataactggaattt

gaactcctctttaagaagtcgacccggaaagagctccagcagatgtcagcagaggtacca

gcttaccctggctgtgcaagttggttgttgtgtcacagccgccaatgtggtttccgccaa

taaaaacatttggcacagtcctctgaccagtccactgagctaatgctgcttgaatctcac

ttccatcagattcagtatcgagttcaatggctttcacactgacactcaaatcagcgaaga

gcttcttgatttgaacacagaaagggcaatagcttttgctgaaaacgacgacggggttgg

aagctacgatctccttcgccttggctagtgccattttggtatatgctatctttgcttttt

gtttttgcagtcgaaaaccctacacgtcccgcggccataatggccactctgcgt

>fava_dn_rep_c753

ttttttttttagagaatgtaaaacacataatttcattccactaaataaaaaacaacttaa

cagccacaaaatacgcaattgttccattcaaaaccaataacaaaatcaaaactagttata

acaataacgataacaacaaaataacaaaaccctaatcaatggtttgcatgatatcttcag

gagcaaacttagtccccttctccttatccgccgcagcgcgccccttcgccttcctatcaa

tcaaactcttccgatccttatccaaacggagtttcgtgataaccaccttagaaggatcca

acccaacgttaacagttgaaccgttaactttctcacgagttatgcgctcgatgtgaatca

cccactttttgcgataaacctgaacaatctttccttcacggcctttgaatgttcctctaa

ccacctgaacttcatcgtccttgcgaaccggtaaggagcggacgttgtatttggatcgaa

ggtcgccggagagtggagcactcattaggacacggcggacgcttgatggggcggtgaagt

gggccttgcggctcttgcggcggctgctggaaacccttggattgaacttcatcttctcct

ctttctctctctcacactctctctctctctctctgtgaatgaaggattaggtttagggtt

tcagttttcttacactt

>fava_dn_rep_c754

ctcactattttttaactttaataataataattgttttgtgtagaagaaggacaaccaggc

actttcttcttctccatcgttcagattttctctgaaggaggtgcttctctctttctctcc

aaaattctcgatccatttcctcaacccgacccgaatccagatccatttcaccatgtacgg

tttcgaagctctcaccttcaacatccacggcggttaccttgaggcaatcgtccgcggtca

ccgctccggtctactcaccacctccgattacaacaatctctgccaatgcgaaacgcttga

tgatatcaagatgcatctctccgctaccgagtacggcccttatctccaaaacgaaccttc

tccgttgcatactacaacaattgtggagaaatgtactcttaagctggttgatgattacaa

gcacatgctatgccaagctacagagcccttgtcgacctttttagaatatatcacatatgg

tcacatgatagacaatgttgttttgattgttactggcaccttgcatgagagagatgtaca

ggaacttctagaaaaatgccatccactgggcatgtttgacagtattgccactctggcagt

tgctcagaatatgcgggagctttacaggttggttctcgttgacactcctctggctcccta

cttctctgagtgcattacatcagaggacttggatgacatgaatattgaaataatgaggaa

cactctttacaaggcataccttgaagatttttacagattctgtcagaaacttggtggtgc

tactgctgagataatgtctgaccttcttgcttttgaggctgatagaagggctgtgaatat

taccattaacagcattggtactgagcttacaagagatgaccgtcggaaactgtactctaa

cttcggtttgttctacccatatggtcatgaggaactcgccgtctgtgaggatattgacca

ggttcgtgctgttatggaaaaataccctccttatcaatctatttttgccaagctgtcata

tggagagagccagatgcttgacaaagcattctacgaggaagaggtcaagaggcattgctt

agcatttgagcaacagtttcactatgctgtgttcttcgcatatatgaggttaagggagca

ggagatcaggaatcttatgtggatttcagaatgtgtggctcagaatcaaaagtccagagt

tcatgacagtgttgtcttcatattttagtctgattattctttgtgatagcttgtatgttg

gtttggacaaactattatgtacaatttctgcattctgcgtccaagttgcccatcgataga

gactagacaatcactatttagaagattgtgatacaaataagtaaattcttatgctgaaga

aatcttctgtaatatagaagggagttctttttgtcaactttgattaaaatatgttaagtt

ttaaaaacgctacattgaatttgtatgttgttaattgtgatatttataataaatttctta

tggaataaacttgataacttggtccaaaaaaaaaaa

>fava_dn_rep_c755

caaaaacaaaggaaacactctcaagttagaaacatctgtgtgctgtatccatctacatct

ctcttcctctttatacttccatttcacctttatataccctcctccccattctcattcttc

ctacaccatcaccacattcatcttctcattctctctctctctctctcaattccttctttt

caggaatttggagagagtagtgatttgatttttgaagaaaacaatcaaatatgaagtttt

caatgatttttcttgttagcaacattctacttcttctaagttcaccagcatttgccaaaa

ctgtaagtccaccggcaccgatcgcgccaacaccgactccaactccggccccagcacctg

caccagattatgtaaaccttactgaactactcaatgttgctggtccatttcacacttttc

tcggatacctcgaatccacgaaagtgttggacacattccaaaaccaagcaaacaacactg

aagaaggaatcactatctttgtaccacaagacagtgccttcaattcattgaagaaacctt

ctctatccaaactcaaagatgatcaaatcaaacaagtgatccttttccatgcattgccac

atttctattctctttctgatttcaaaaaccttagccaaactagctttactcccacattcg

caggaggcgattacacgctgaatttcaccgatgattcaggaaccgttcgcattaactcag

gatggtctattactaaagtaacaagtgctgttcatgcaactaaccctgttgcaatttatc

aagttggtaaggtcttacttcccgaggctattttcggaactgatattcctcctgcaccgg

ctccagcgcctacgcctgatatcgcgcctgctgctgattctccaactgaaaaaagtgctg

acagtaaatcttcgtcgccttcgtctttgcctgatggctcttctgcttacaaggtactga

gctatggaatctggagcaacattgtgttggcaacttgtggtgtattggtgatgttgttgt

gaagatgaagatgaatttgttcagtatttaatttagatttattgtgttgatgtatctact

tttgatgatgatatttgctttgatttgaatccatttttttgtgaacataatgtgctattc

aatatatgtatgagaatgtgatttctattgaacacttttttttaaaaaaataaataaata

acttgtagcatgttattgtcatgttatattatgatgttattgccaatgccatttctacat

tta

>fava_dn_rep_c756

gtagcaaggcctcaaaaatttgtattnnnnnnnnnnnnnnnnnnnnnnnnnnnnnnnnnn

nnnnnnnnnnnnnnnnnnnnnnnnnctttttttttgaatcaatacctcatcacatatacc

acaaagtcattaagatacacatctatcgtagtgagaaaatcaataacagataggtaacct

acaaatgtcgctacaacaatgcaaaattaagctattgtggtcacaagcttaagatttgtt

ccattactagtgagaagcacacatcatatagttcatagagttgtattcaaagggagtaat

atatttaaagacataacataaatctactaacttgttaaccaaattgatacacagaatccc

tcaaaaattctcacaagaacaacaatatagaacactgaaggaaaatctgattattcaatg

gctcataaaaagggttttttacccaatatgaaggaaaaaaaatcatttcttgttataacg

agcaacctcaccgtcattgggaaagaaacccataattctcccagcagaattctggtaagc

atacatgaatccacccatgacaccaatcaatccaccggtcaccattgaaggtcctctgat

tccaggcttaatcccagagagatacccgacggtgacggaaacaccggtgatggtgacgaa

acggagataatcgagggtgttgaagtttccgacgacggtggtgaatggagggttgcgatc

gatgactgggtattcgggttttgtggaggctgtgatgtctgtgttcatctttgaaagttc

tctatggatcgatttggctatgacagtcgattgctacaagttcaacctcgagaagaagaa

taactgtgttggtttatgcttgtgctggtgaaagtgtttctctatctgtgtgtaatttgt

ttgg

>fava_dn_rep_c757

tttatttataaaatcaaaattatatatttatactgaagttaaacaagtacatataagttt

attcaaccaacaataataatagaactagaaaattggaagttcaatactgattgaggaatg

aggatcaattgcataccaatagtgataacaagggtcaatccctggtagatttgcttccag

tagtccctgttttctccctttacaattccattgaacttgatactattactatagctcctt

agactaacactctgggatttgattttaacagtgaagttttaacaaaacggtaaacatgtc

tttcttcattttgctcaagataatttgttgcattggctgccttacaccttgcttacaaaa

tgaaccattgcaaacacaaaggaaactaatacatgtctttttttttacggcagcaagtaa

tggatgatttatttgaatttatggctctcggcctaggtttacagttacaagcaatttatg

tgcgaagagaataactcaggcatgataaaatttacactaacaaacccggaagggtcctac

aacataagctcttatacaaggttgcaagaacacttgacaccgagggtggggaaaaaatta

caaactgaaaactgtcttacacggcaactggtgtggcttcgtcgagagcgaggacacctg

gaagctctttgccttccaatagctccaaacttgcaccaccaccggtggatatgtggctca

tgacatcagcaactcctactttctccacagctgcaacagagtctcctcctccaataatag

ttgtcaccccttttccacttagctcagccaacttctttgcaatagcctctgtcccagtag

caaacttgtcaaactcgaacactcccattggtccattccatatgatggtttgggtggtat

ccaatgcttcattgaatgttttaatagaatctggaccaatatccaatcccatccaaccat

caggaatggcagatgctggcacaatctggctgtttgcatcaggagcgaatttgtctgcta

tcacgacatcactcggtaacaagagtgacaccccctttgccttggctttcgcaagtagtg

ttgtagcaagctctaacttatcttcctctacaagggatgaaccaaccgaaagaccttgtg

ccttgtaaaatgtgaagatcatccctccaccgagaataagtttgtcaactttttctagaa

gtgactcaatcactccaattttagatgagaccttggaaccacccacaattgcagcaaatg

gcctttttggggtcgatactgccccaacaaggtagtcaagttccttttgcaaaagaaaac

cagcaacagatggcttcaagtatttagtgactccctctgttgatgcatgtgccctatgag

cagtaccaaaagcatcattcacataaagatctgctagtgcagcgagctttttggcatgct

cgggatcgttcttttcttcctccttgtaaaacctcacattttctagaagaagaactcctc

cctctggaagggaagccaccaacttttctacttctggaccaatactgtcatcggccttaa

caacctcgattccaatgagttctgagagccggggaacaagaggtgccaagctgtatttgg

gagtaacaccctttggccgtcccaagtgactggaaagaatgactttggcaccattctgaa

taagatatttgatagttggcacagcggcacggatcctagtatcatcagtaatattctgat

tatcatcaagaggaacattcaagtccgccctaacaaaaaccttctttcctttcaactccg

ccgcactcaaatcccccacactcttcttggccatggacaccacacctctcacccctctcc

cgctcactgacctcaccttggacgccacctgcactgccagcaagggatcggcagctgcga

agccgagtcggcggagtgggcggccgccgaggaatgaggagaggtggacgcgggaggaag

aaggagtggcggatgaggtggaggaggatttcgtggtggggaggagggagagagttgtgg

gggctgtagctgaagccattgtagagagagtgtgagagagtgaagtgaatgagagagagt

ttttgaggttatggttgtgaatgtgatgatgtgaggtggaaatgaatggaagaaagatgt

ggacgtcaagaacaaagggagaatgattgggtgtagtttcggggttttg

>fava_dn_rep_c758

aaaatagttnnnaaacttctttaaaatagtaaataaaaaaacttccttagatcaacacaa

tcagaaacattttaaagaagttttcaagaaacctgtagtttctgttaaactaaattgagg

tcacacacatacattaacaaagtggatgataatgcgcaattaagcattcagttcactttc

agaattcaaaacaagtaaattatggcattatactcaaaacacagaaaataaatattcatg

ataaaaacttcaacagcctttaacaaaaatgctaacaaagtagtgaaatttgtctaaatt

aaaaaaacaatatcaaagtctagattagacatagtctgggatgaaaatacaaaagcttta

ctctgcatcagacgcaacttctccattcttcttgttgtgcttccttgcatacctctggtt

cctcaaaaactttggatccatccctttggtggaagtgtggcgatgtctctttggcttctt

gatgccgttcttgtgggccttgtaagactggttgtgagcggtgtgattcttcgacttagc

catttcctaattcggcgattcagaagatcgatttgcgctagggtctcttggtaagaacag

aaggagg

>fava_dn_rep_c759

attatatatagaggcaatatagtgaaagaaaagcatctagccaaaattaacaaatatagt

atatgcataatgcaatctttatccaactgtcactaatgtggtatgtgaaagtggtacttt

ggcaactacaggtgatccgggtaaagtgtcttgtatctgaaacaattgataagtggcaca

tcaatgaatacaaatgttcagaacaaatcaattgtagaaaataagactgaaaaaacaaaa

ttaaggccatatctttgagtgggatttaatccaatctgttaattcctttgcttaataatt

acatggttcataaaatgaaatttcctttcttgcacaagtaatatgtttttgtgcaatttt

acattttcctcttatagtcattcttgcaacctgacttgtgcttcgtctaaacccacccaa

agctctagctgaggaaagaatcgggatataaacaaatcaaccaagttaccagtaatacat

ttgaaagaagaaaagcaaaaagagcttggaatgtggacaatgggcttctcaaattttcag

gaggacgatctctgtttatcaacccttgtaactgatcgcctcgtcttccctccattgcat

cccttaaaggcccccaacattccattctgttgtatatttttgagctagaatggaaatata

ctcgaacgatagaatccagcaattctggatgctcgtggaaaagtgcagaatctccgcatt

gaatagctttgagtaccaaaggcatttccttggagaaaatgtgatatctaaattcagcag

cgagatctaggaaggaattaggaccactaacaaagcaatgaacatgtaggcacatttcat

ttttcactttcttccattcagctacaacatcatccttctggtaccaccctctcaactgct

cgagtttgatgacattggaaaccgctaaagtgagattagctgttaaatcacagtgagata

gaatgtaggttcttggaataacacttgcatatctgtttatctgatcttccaatagaacaa

ccttcagctttgaagcttcaaactttgttggaggacccaatagcctaaccgtctcaaaaa

caatggaattgtaggaaggtgtggaattagtagtaatggaggagcatctgaaagatggct

ttaacatgatagggaaaagctttgtaggtgaaaatgaaaaggcattatgacataatgatg

ccattgaagatgcataataatagagtcctcttcatctttcaaatgccaccaccattatct

tctttccttgttctccgttgttgtcaagagttaagatagagagagagagaaagaggagtg

atgtgggaaattagtgttctcagtttggtttgttcatgtggactgtgtgattccgttcag

aatccacaatactccaaaatctctatcaatccatttcttccacacattgtctttcttt

>fava_dn_rep_c760

tcagttatttcgaagcattagattagtctttgagtttacgtttttttatttatttttaaa

tgtacaaaattatgtgagtgatttttttttggttaaatagaggtactcttatgtgagttt

gaaatcttttttattgcaacgacaatctctaggcataatttcatataaaacataaccact

cgtttatattcgacagtgtatctatctattatagtagtaaacgacgacgatgaaaacagt

gacagcaacgtcgtttgatgtctccgccgcttgcttccatccacgccctcttaatctacg

ccataagaccaacatgactagtttttcgtttgtaagatcctccaactttaataagggaca

tagaagcgttgtggttcaacaaaagtttttgaacttccgcatgatgtgtagtttaaaggg

gaatttggaggaggttgttggagttccatcttcagtgcctgtgcgtgtagctcacgacct

ttttctagctggccataagtatcttgatgtaaggaccactgaagagtttaatgccggaca

tgctcctgaggcaattaacataccctatatgtatagagttggatcagggatgacaaagaa

tagcaattttgttaaagaagtttcttcacattttagaaaagaagctgaaattattgttgg

gtgtcaacttggtaaaaggtctatgatggctgctactgacttgttagcttctgggttcac

tggagtcacagacatagctggtggttatgctgcttggacccaaaatggacttcctaccga

aagatgaagcattctagttggacttgaagctgtatatagaataatgagtagtattctggt

tggatcatgtaaatgcataaaaactaagggggtgggtgaaatggttttgtatacttggta

caattaaaattaatcttaattctcacaagtaaacaacaatgttaagctctgtctctgcaa

tttcaattacttcactacaaagttacaaccaagcactgaagttcatttaacctttgtctc

tgcaatgtcaattgctggttgaaagctgttttcaaatcccctttgagctttggcttaaac

ctaagtctcagaatttttggaaggactcttttaggggctaaagtcctacaatttcagtta

aataattttatccttttatcttattctcttaaatttgaa

>fava_dn_rep_c761

aataagttcaaaatgaactacaaagttttaacttcatttaaaagggttctgatatcaaac

caaataatcacgttggctctccattaaaaaaaaacttaaacatatcaataacaggaatac

ctcctattaataggttttgaaattcttcaatgagccattactctacaaaatgagatgaac

aatgtataatggaaatttcaagcaaatattgcctagggattgaaagtaatataaattagt

ccaattctactcaaaaagattcggataatatccaacagaaacaaaaatattcaaaaccaa

aaacaatggtttaaatatcctcatagcaaaatctggcaatagattaacaaaagtactgtt

taaacaaaccctagtagagacttccatcactcagccacattacataaacaagttgggctt

cttggccttgtatgtggtcttgagcttcctggttggtggtctgattttcttgaaaaccaa

tgggaacttgatcttggagttgtggaactgtttggtgctctccctcttgcagagcttagc

cgggatggtggctgttttgataatctgaatgcaggggaaacgaaccctgtggcgggaagc

catctcattgtacattagatcaacagcaccattaagagtggtgtcacggaactccttgta

catgttgtggtagccagttctactctggtacctcaaccatataccatagttcttaatctt

ggttggattcttctcataaatctcgttgatagcaaggatttgaccattgctcttcttcac

cttcttcagcttcctcaggaaataccaaaacttggacttggcgcgaacttcattggtagc

ccaaagcttcatgcgataaatcttcgggtgttcatctgattccgtgggcagagctctccc

aaccacctggtactgatggaacctgaaagaaaccatcgtgagagaatgggaaatggtagg

gttgatctctgagtgtcgccggtgcctcagtatagtgaaggggaaatggtaggtttttga

ttgtataaatatgatagaaaccctaattttatttt

>fava_dn_rep_c762

ttttttttttatacaatgaccttttttttcttcttcttttcataaagtgcaaacactgtc

aacaattaatttggcgtcttcgataatttgacacattgtcatggcgaaaatttgatacat

tatcatatcacatcttttacaaatgttatacactatccttagaactagacacctcagtgt

cttgcttctcctgttcgctcccagcaatagatttctcagatggctcccctcctgtggaat

caggttcctttttaagctcagactcgaactcctttgctgcctgttggaagcttttgacgg

ttttaccgatgctgcgaccaacttcaggcaatttcttgggtccaaaaacgatggcagcaa

ctccggcgataacgacgagttcgggtacacctaatccgaaaagcgcgttacatgtgaaac

cattgtttcttctgatttgcttgcgtggagtaatagaaacacttgaaattgaagatagag

aggaagaggaagagagagcgaggtgtttatagggtttttgtgttgaaattggggatggag

ttggaattgaaattgaagaagaaaacaaaacgcagttcattatcgtcatctcttaggtct

tgcttcacttcgcactcccgttgagattttagatttttatttt

>fava_dn_rep_c763

tagtttaaaaaatttagctcccattcatgaagaaaatgatgtaaaaagagtgttaaatga

agcgaagcaaaaacaccgcacatgatggaataatatgcattttggcaaataagccaatta

aacaatacatatcacaaattatggaataggccaatacaaaatagttgacaggttttgggt

ccatgataagggcagattctgggtctaacttggcataaacaaaggggtcaccttcctttg

aaccatctcttccatctgctcagctgacaatatggatgtctcactttcataattgccatt

ttgcatctgctggtgtaacttctcaaggattgcagattgatcatccaagttaaacttgcg

catgtgccgcagcatctcaagttgctgttgctgtaagttttgttgatagctctttttttc

tgcaaattcttcttctccaagctctgcttcatcgcaggccatctcttctttggcaaggaa

tttataaaactcaatcatcaaatctcggtcattctcatattcagtactgatttttcccag

gcatgaaataccaaatcttgggtcaatatccatctccaaaaatatttcctcctgtatagc

agttgtcacagcaacagcctcctgcttatccagaacagcatctacaatccttttcttcac

atctggctgggtagtaagaatagtgaatcgctcaaaaagatggaaaagttgttctctgga

taacattccactgctctgcgttccagaagccattaaagatccacagaacaatcccgcggc

caat

>fava_dn_rep_c764

aattcataagattacgagaaacatatacattgtaataaataacctctacaattatctaaa

aataaaaaaattagcatatatgctgctttttttttttttttacgcaaacaaactaaatag

attttattttgatctactgtcatgttctttccactatgatgtaatcaagcattcacacat

tattacatatagaatgagcttgactataaaaaacaacaagaacttcccactaagtaggat

ttattatatggatcaagttgtaaaagaacatcacttgatggtagtgttaaaaactataca

ctgaattatcaaaattgaactcaaactagaatctccactgtctttgaggacatggttgag

attgattcacctaagagaggacattaaaattccccgctggcgaaaaacactatcaggcgt

ggtggcttcaaaaaacaaaagcagcagcacagccagcatggctactcttgcattccaagt

ttcagcactttttgtcaaaccccattgccacactatcaccggcgatggcaactccctgtg

ttgtgagtcgtatgttgccaataactcctctacgctgccaagcggaaccaaagactgtcg

agcattgatgtttgaaactgccatggcaccaacatatgggagactttcaatcacagcatc

tgccaaatcagaaatgaaggaaggttcgcatcctagagcaggaacacggccccatttttc

tattccagattctagagccaattctttgtattcaacatcaatttcttctagagtttcaat

atgttcactgacaaagctaattggaacagccaacagacttttcactcccttttttccaag

ttcaactatcgtcacgtctgtatatggttttaaccattccacaggtccaactctactctg

ataggcaagggtgtaagcatttgttatctttcttgtctcaagctcttccatgatcaaatc

cacacattcctccatctctgctttgtatggatcaccggcctcttccacataagcaagagg

caccccgtgcgcactaaagaatatcatgacctccttaggggagtcaaaactcttcagctc

tttttcgattaaacttgacatggcctttatgtatccttcacgttgataccatgaaggtaa

taccgtgtgctccatgttgactagatactcatcctctcggaatatgctttccagtagacg

taagctcgagccactggttgatattgaaaattgtggataaagtgggagaacgaccagctt

tgtaattccatcttttttaatctgttcaatagcttcctcagtgaatggatgccagtaacg

cataccaacatacacttcggcagggacattctttgcaaaaagagatttcttcaaatcttc

agcctgtgcatcagttatgcgtcgaagtggagaaccgccgccaattgaagcatagccttc

tctgctctttggagcccttagaacagatacaaactgagccagtggcttttgaagaaaact

aaatattcttggcaatcgtataatatccgggtcagcaaaaaggttaaataaaaaaggttg

cacatcttctagagtctctggacctccaaggtttagcaacaacactccaatcttgtcatc

accattaagagttgtttcagaaacatcttgagctgttcgagttactaaagcttccaccga

gaacgcgtgtttgttgagtgattggttgaaaaccaaggattgtgcttcagatcgctgggg

agaaaaaccggaagttatgtaacttttcacatgattagagttaacataaggctccacgaa

ggctccagagcataatgactttttttgtgaggtacagattgcctgtggcaataacatagg

acgcttgaaattaggagaagcatgggtgagtgtatatgaggaagaagtagaaggtgatga

cgtggcatgggttggatttggaaagttcatagcttaataattgataaatcaacagcagca

gcagcagcagcaatgtatggttgaggccttgtttggcataaaaaatggagtgtgagaagt

gaatttaggagaatggtgataagtatacccaagtcgggtgggtgcaaatagtatggagga

gaatgagagttggcacgtgtctggtgagtgctatgagctgttgcttttctttttccctat

ggttttggatttatggtatagagagaggaaacgttagccgttggtagctgccgtactctc

tcctttctgataaaatgtactacaataaaccaat

>fava_dn_rep_c765

atgaaacacttgaagattttaacaacttccaaaacagtttttaacaagattttgaacact

agttcttatgaggttttcagcattgcaggaatatatgtgacatccatcattcttaaacaa

aaccataaataaccagggttggcggaacagagaatggccggccgtgacacagtggaggaa

ggtgccggagtagatcgaaccgacagggggaagagaggcagagttgcattttgtaagatc

aacacagcattttgccaaaatgatacataaattccacactacatatactaatataataaa

tcattcagaaataacatgaaaatcaattacatcgaacttcagaaatcacaaggcagaaaa

caattgtccgcttattaaccaaagactgagactaaaaacaaacagtttagaacttctagt

gcagtaaaccacatcaagcaccaacaagtgctttaaaaaaggtgttatgaaatacactga

gcatcgaagattccgattccgattccgatctaataacctgcgaacggcgaataaccatga

ccacagacctttattaagctacaatatgaagccagctcttttcctgaaacattaacttga

ctgctactgccatgaattaactactttctttgccatctgctgcacgaagcgagcctaatt

gcacaggagcttgggtttgcaccactttcgatgacccgtatcctgaagtatcgatacctt

tagccgtttccttctcaagctgttccttaatccagatgtaagtaatcctcagtccatcct

tcaacttcattgttggagcccagccaagtttctctttgataagtgtattgtctgagttac

gaccacgaacaccctctgggcctggaatgtgatgtataggagttttcttgtcatcaaaac

caagaacgatctcagccatctcattcatgctgaccatttcatcacttccaatatttaccg

gctcacggaagtcggatttagtcagtcgaagtacaccttcaacacactcatcaatgaatg

tgaatgatcgagtttgcaaaccatctccccacatctcaaatttgtctgtagaagtgatcg

ctttccgacaaaaagcagcaggagccttctcccttccacctttccatgtaccaaaaggac

catatatgttatggaaccttccaatccggcactcaattccaaaatctttgttatagtgct

tgcatatctcctctgtcgcaagcttctctagcccatacgcatcttgtggctcagccggcc

atgcatcagactccttcaagctcacattagtagtttccaactgtttaaattccgggtaga

tacaagcactagaggcataaaagaacctcttaatgccgttaatcctagcagcttcaatca

tgttaaagctaatcatagtattgttatacataataaccgaatgattcgactgaataaaac

ccatcccacccatatcggccgcaagattgaaaacatggtcaacccctttagtaaccgtaa

ggcagttatccatgaccctaagatcaacaagatggaattcatcacagaacatatcctcag

tcatgtgctcatttttcttccaatcagaagcaataatgtaatgcccctcagtcttgagac

gccgcgctaagtgcgatgcaataaaaccgccagcaccagtgatggaaattttcagcttct

ctgatggccagtaaggctctctctcaagattctcataggtgtattctccataattggttt

gtccagtacttcccattctgaggagagggaaatgagagagaagagatagtgataatgtat

ggagagagaatagtgtcccgcggccgtaatggccactatgcgtt

>fava_dn_rep_c766

ccacaaccattccatccattaataacttaacannnnnnnnncgaactataacaaaatcag

tccactcatattgatcatccactttggcatcactatagcagcacaaccagatacattagt

tttcaggtctacatttaagaccgaaagctatagcagtaaaaccagatacattaaagacaa

atcccatacacttcaacaacacttctaataagaaaactcttgcttgattttatacttatc

acttgtacttctcttagcaaccagtattcacctaatcaaatctaccaaacacagaataaa

atttgctacacagacaaaggacggagtgaaaaatgatttaaaattttactatgcattctg

gaaaagcaataaccacagagcacatgacattgttagaggaaaatatgtatcatggaagtt

atgtgcaacacactctcaatggtgcaccgtctcactttacaatcacaggttacccaagtg

tacattattgtcatactttgaaacttcttttgcacttgaaacaccagagtctctatttaa

actttcctatgcaacttctgtctccaaagaaattgtgattatcatccgttaatcatgtac

aagctcttgtgttcttacaaaatgagtttctcgtggaaggcctttcaatcttgcacaaag

ggttctttagctggttcaagcactataggctccaatttatctgcttcatcatctcctttg

ggtataatgagtccataacccccttcttttcttttgtatataatattaatctccccagtt

tcttcatttcgaaaagcatagaagtcatgatgaacattttccaactggtcaattgcttca

aacacggttaagggaggcatgtcaaagtattttgttctaacaacctcttgaataagttgt

tcttcctcctccttctgaggggaaatttcaacttcatcctcttcatcctctgacggtaat

ggaattatgggctccctatatttcaacctattggatcccttcatgtgtcgaccatgatcg

gtttctttttccttgatctttctcaactttctctgaatgatcgaggacaccaagtctata

ctcccataggtagtttcagcgtgttcctcagcccgcacaaccccgtgcctcttagtaaac

aaagtcacctcacatcttcgcgtcctgggccctcggccaaattctcctcctccgcggata

gatagcctgacatcaacttcccttacaacgtagctatgcttctgaactgctctccccact

ttgtcctcgatgtgctgcttcaccgcatcattaagctccatgtttttaccctggatgatt

aatttgacggaagagagaggaccgtcccacgacatgcgaacaacgcggtgtttagtggtt

gatgttgttgttacaacatggttaaatttgagcaattgttttgtggattctcctttcaag

aatgttgtggagggtagtaacttggttttggaggagatttgaggagatggagagagtaca

gaaaggcagcgagtgggagaaggggaagatgggagaaagttagtctggaagttgaagtga

gtcgccattgccatttgagtgaagatggttgtactactgcagtgtcaataaacgaaggaa

gaaagaggaagtggtggtggtcacagcgtttgatgacggttgcttgtctgtttccttttt

ggatgacgatgaatattcacacgcgggccattctattctatccatacct

>fava_dn_rep_c767

tttttttttttagtaaagtaattacttgattgagcatgaaactggtgacaccaaagagat

aaggcaagtttaaggttttatgaacaaattataatagtacagtaataattaaatgaaata

taaacaccattgtacattaccaatcaagaaaagtaaatcaagaccctcaattgcgacata

tcctagctgttgggttagtgacacaaacaggggcatactttttcttggcttctggagtac

ctcttttaggttcatcttcatctgccattgcaacaccagcaacagaacaaacagcagctg

ctgctgcagcaaacatcaactctcttcttccattgcttccctccttgttgatgtcatatc

tgaccatcttctccccttcaacagccctgacagcgttgataacgaatcttctctgtgttg

ctacggctgaccggttggttacggccggaaaaccatggattgaggctgtcatggtgatag

atgccatttctacaatttgatcctctatggtactcgctaccttctatggtcccgcggcca

taa

>fava_dn_rep_c768

ctagtttaaccatgaccaccataaagatttgttactggtaaaaaaggataaagaaacttt

ttttttttgaagaagaaaataagtttcccttcattggttaaattaaatcaagagtcacga

tcactatgagtatatacagatgtattaattaacaacagttggacctttaacagcaagcta

cattaacttttatcctattgatcagtactgagtactgacacactaaaacactactacctt

gggacattcgggaggaggcacaactcaattaaagacaaaatgatacctcgaatccacgta

gccctcaacatcccagagaaaacaaccaaaagtcacagcttctaaaacaagtctccttaa

acccccaaagctaattttgatgttttcatcctttgaataatcaactgctccctcaggtgt

tataaccatttcaggtcttccaaacaatgcctctgtgtgcttctcaatgatgctcacagc

ctccttagatcttattgtcgcatatctctggagagtatcagcgtcaaaggacatgacata

attacgcaacctggtgggctttataccatagccaaatccaccaggactggcatcgccgac

ttgagtagcaggtcttacatcatccatgatagtttgatgatcagcactctgttgtggtac

atttggaagaacattgatggacttctcgagttggaacctttcgtcaactcgcttaagaaa

gtaaccatacatgactgatgctgcatagacctgacccaccctgagtttactgatttgagc

aacagatgataaatctccagctctgttacccaaaattagagccaagtggttttggatcat

ctcatatgcttcttgagagtgaaggttctcaagtttcccaacttcatttgcccatgaatc

tagtgtagcagatagatcttgcggtatcaaagaaatattagcatccatgaacttctgcac

aaccaatgagtacaagatctcttccaaagctgtcctcctttcattagccttcacctcagc

aattctcctgtacaaaacaagatcggtggaggtagtatgagcaggatcggcgtcgcggtc

agattggagttggttaagctgctgatcgacggcagcaggcataagatgggggtgattgat

caagatctgagaaagaaactgtccgattggggactggaagtgaagaggtgcaattttgtt

gttgttgttgtggtcggatgttgttgctctaactacaaatcctcttgttctccttctact

agaaattccaagagagagtctcccatgggtatgaacttgcttggaagtgcataatagctt

agaagtaggaaagtgaagaaggcgagatccgttagtaggtcttggaacaaagattgatac

tccggtggctgccacgtccatatgtgtggattggattagcttcgtgaactcaacaggact

gccccc

>fava_dn_rep_c769

attggtaatatgacattatttggagtgggggaatagttgaaaatctacaccattaactaa

cgcaaaacattcacaaacattttgtttttatttagccatcaactcaaaacaagcaaatta

aaaacaaaggcaccaacatttatgtgcattgcttagcatagttaaagcctgaatcacatg

tactcactaactcttttattaatcctacaaataaaacttaactaaatcccagcagcagca

gaagaaccacatgattcatcatttagacatctttgcagccaattctctgaccaacttagc

agttacaagtgctgtcatgttattagaaacatcacgttgtggattgtattcaacaacatc

tccaccaacaatattacctttgagattgtgaagaatgtttaaaacatctctgaaagaaag

acctccagattcaatgtgagacacacctggagcaaagcctggatcaagagaatcaacatc

tattgatatatatacaccttttacaccttcacccgtaccaagtgtcaaattctctaaata

ttcgcggtcttttgcaaaatttctcatttcatgtatctctactccatatttttcgccttg

ttgtcttccatcaatatttattgatcttataccaacctgcactagtcgtttcgcatattt

tccctccataattcgagcaaagggtgaagcatgtgaaaaataattgtcttcaaagttttc

atagagatccggatgtgcatcaaaatgaagaatatcaactggtcctccaagcttctcaga

cacagctctaacaataggatatgatattgagtgatcacctcccacaattaagggacgcaa

tggagcatgattcatcactatcttgacagagtcgccgataaaattcattattctctcttc

actgactcctaaatttcgtagatcttggatagggatatcacccacatcagcaaaaacatg

cggctcgattaaattcttgccttcttcagttgttgagtttgtgctgtcatcccaaatggc

gtgcctaacaagaggaggagcaaatgtggcgccttcatgatatgatgcattgtgtcccaa

aggcactccaagaagagttgaagttgctatagcaccacccgcaatttctcccttaagctt

tgctttttctttaagatatgtaagtgctgcttgaatcacactatcttggccatcttttgt

caatttagctgctctttcttgaaattgggagaaactggagcctaatggtgctgaacttga

gatcattttataatttgcttttccttgaagaagcgaaggtgaagcatctatcacacgagt

tgctctgtctagtgtctgcttgaagcctttgcgtgctaaaattgacatcattttcaattt

ctctaataaagaaaaatggctgtaacttcaatgaccagtttatgaaactcactatgtccg

cggccgtaa

>fava_dn_rep_c770

gcggtaaaattgcaatcagacaacaaaatcacaaaggtatcgtaatttcgtcaaactcat

gcatctcaaatgattataacattctgcttccaaaatattattggtataaaagaaaactta

acttaataacatcataaatgcaaaacatatattctgagaaaaacatgaaaatatcaatga

accctcaaaagaaaatcgagacaaacaaaactaaatcatagcggattagtcgacaaagct

aacagtcccactagaacaagtttaaagaacacaaaggagcacaaaattaacaaacgtagc

agctctaataaattttgtgcttgtactataatcgctgaaacctaaaattcctccaaaaag

atgtttatctcagtttcatgaaacttaagcctttgaagcttgtttttccttggtcttctg

gatcttcaagacctttttgacaatcttttgctcctcaaccaagaaagctcgaataattct

ttctctaacagcacctccagataaaactccaccataagcacggttaacagtcctcctgtt

tctagataaccttgatcttttgtactccgtgggcctcaaatgtggaatcccttgaatcct

ctttccagtaacagggcattttggtccactagctctcttcttagtagtctgataaacaag

tttaccaccgggggttttgacaacccgatgttggttcgacttcgtggcgtagctatggcg

ccggcggtaggtaagcctctgaaccatcttctctgaccttgattttcctcacaaaacgga

caagaatgtagatgatgcggtgaacaagtacccaaaaccctagaaaatccgaagatgcgg

attacgataattaaaacgggcctgacccggtttaggaactatagggctgcttctttttgt

caattgaccccaaaatgacttatacttctaagactggggtgtggatagaaaattaaaaat

tgcaacaaaaaaaaacttaatattattga

>fava_dn_rep_c771

gataagcaaatatttttattttatgtttttacaaaatattttcctttttctgaaaaataa

aatcgattctcgaaaaaacaaacaaaactagtgaatagtgaaagagaaagctagcgtttt

ccatatctcatttatcccttccgcagctgcctgttccgcccgaagatcttccgagttccc

acttccgtcttcatcgtcctaacagaatcgcatctggctgaaatggcatggagaggaaat

ctttcaaagaacataaaagagcttcgacttttgatgtgccagtcatcacctgcaagcgca

tctgcaagggcatttgttgaaaagaattataaggaactaaagacattgaacccaaaattg

cccatattgatccgtgaatgcagtggtgtcgaaccccaattatgggcaagatatgatctg

ggtgttgagagaggcattaaattggaaggcatgacagagccacagattttgaaggcactt

gaagatctggtaaaagcagggcaatctttgaaagcttgatattactgcttattcactttc

tgaaatggaataaatgttgtaatgttgcacttcttattacaatatgtttctagagtcatt

ttaagaatgaacctgttgattctatgttgaatgaccgggttatgcattttgaatacattt

attgcagattgaacctatttatttcattcagctagcatgtataagattataattagatgt

tcttgtaacaaatccatgaatcaaaaggggaaatgttacatgattctatgaggattctac

gcaaaattatgttttctcaaatgtcaaaatttcattagattgttttgctggttc

>fava_dn_rep_c772

aattattaagtactagcaagatggaaaacaaagagagaatgtgcaatcagatcataaaag

ggaaaataaattagaatggtcattaaaagtgattttctcatcatatattcgaacaagcta

ggtttgtatatagtaattgtgatggctttggagaaagtttgttggccacactacaagagt

agctcacaaatttgccagtgccatcacaagtcttcattatcaactcatctttgaaattca

attaaactcaactatgaaacaaacaccaactttgggaacataaggatgcattcttcattg

caagatcattctacttcacccagataatcatcaatttcagctggtgtcaataccctgaac

tttttgtccgcgccaattatgccaatttcaatgttttttgctgagatctgtccctcaaac

ccttccttcagtgtcaatattgctgtgtggactgcatcatcgagttccatatcatcagtg

tacctcttttcaagaaatgtcttagcattagaaacatttttccccatagcagaagctttc

caagaaaaatatgaaccagatggatcgacctgatatagttgtggtccattatcatcaaat

ccagcaactagcaaggacactccaaaaggcctaacaccaccagactgagtaaattcctgc

ataaccgcggcaacttcccttactagttgagtgacagggattggttctttatataatcgg

tgatactgctcagcttgtttcctgctttttcgtaccaaaactcggaaatcaggacccatg

ccactgtatacaacacctatatttggtgttaataactgaattttctggactgatgcttca

tcgaccaaaatagacggcagtttcttctctgttgcaatgacaactccatttgcagcttta

attccaagagaagtttgtccagagccaacagcagtcaaagcatgctcaatctgaacaagc

tttccagaaggactaaaagttgtaagagaaaacgagtattgactatctcccatgataaat

cactgagaagtggagagagagaagagagagagtgttcggagaggtttggaggtttggttc

tgtcccgcggccgtaacgataacggtcctaaggtagcgaaattccttgtcgggtaagttc

cgacccgcacgaaaggcgtaacgatctgggcactgtctcggagagagactcggtgaaata

gacatgtctgtgaagatgcggactacctgcacctggacagaaagaccctatgaagcttca

ctgttccctgggattggctttgggcttttcctgcgcagcttaggtggaaggcaaagaagg

cccccttccgggggggctcgagccatcagtgagataccactctggaagagctagaattct

aaccttgtcccaagacagaccaagggacagtctcaggtagacagtttctatggggcgtag

gcctcccaaaaggtaacggaggcgtgcaaaggtttcctcgggccagacggagattggccc

tcgagtgcaaaggcagaagggagcttgactgcaagacccacccgtcgagcagggacgaaa

gtcggccttagtgatccgacggtgccgagtggaagggccgtcgctcaacggataaaaaaa

aaaagnnnnnnnncggaggcgtgcaaaggtttcctcgggccagacgg

>fava_dn_rep_c773

acagtacattcctctttccgaaatccgccatggcctcagcctcagaacaacgcatttgtt

tctgcttgtgcctctttttcttccttcgtctctcaacctcaaccacagagaccccaactc

cagcgccgtggccggaacagttccactcagagctattcgtcaagggattcggcactcttc

gaaaaaccgacttatggtacgattggcccaacggacgcaacttcaacatcaaccagcatc

agcttggtattctcaaatacgacgctgaatggaacaacggcacctcttttgtctacactc

tagaccctttcaaccgcacttgccaaacgcttcatttcgatgttggaattctccgaccca

attggcttgaaggtgctaactatttaggtcaagaatatgctgataattttctgtgtaatg

tttgggagaaagttgattttattcactactacgaggatgttgttactcgaagacccgtca

agtggatcttctatgatggaatggttgctcatgtgatgacatttgaggttggtgcagtgc

tggaggatgagcattggcaggctcctgtttattgttttagtgagtcagagcctgaacgtc

ggttacacaacataatgagcagctccttattcgatttagaggctgctgttggtggtggca

gtggttttcatcgaacattaatgagtgagatgagatcggccgcccgaaaatagtacatgg

tttataattgtgaggttagcatatgtagtgtgtttaacaatctacctatatgcactttat

tttattcaaaacatcatcatttgttaccggtaaagaatggaagaaaatattttagcgaca

caccaagctttacatcatcaaatcccataggcatcaagcattggcttatttgtatgcact

gccacttacattctagctttctttcccaagcattcaatcacattttcaaagcttcttttg

aaagaatccagcacctcgttttcaagttgtgatccaacacgactccaatcaatccccaat

ctctcgattgcattgtaaatgctttcggcctctgatacatttgcatcaatggttcttgaa

agaataccatggtccatgaatgcttttagagcagggtctggtggtatggttgaaatctgc

atatgcaccaaagaatgaccacttattttgttaactttataatgtccattcaatgtaatt

aatcacatgtttgaactgcgttaatcagttaaattattgtcgaaattaccgtatctggtc

caataagggaattaacataaaaagtatctgggtaagatggatttttcacattggttgatg

cccacatcaatctttgcttcttggcacctctcttctccaagagctcccatcttggacccg

aaaacattttctggtaaagctgataagctaacactgcttgagcaactgcggcctttcctg

tgagatcaagagcctcagcagtgccaatctgctcaagtttcttgtcaattgcaacatcca

ctctactgatgtagaaagctgctgcacttgaaaccttagacagatcattcatactagaag

cctcaagaccatccaagtaagcattaataacttcttcatatctagggagagagaatatga

gagtgacattaacacttatcccaagagaaataacttcctttattgaaggaatagattcat

caattgcaggaattttaatataaacatttggacatgcaaccgttttatgaagccattttg

ctgcttcaattgtccctttggtatcatttgcaagcttgggagaaactgcaacagatacat

agccatctactccctctgattcaatgtaaattggttccaagagtttgcaagtatcatgta

tgtctttcacaaccaattcccagtaggcactttctatgtctttgcctgcatctaccaaat

ccctcagctgttgattgtaagcatttgatgatgatatagctttttcaaatatcgcggggt

tggtagtaacacctctgattccagatgcaatgaacggaaccaaatctgagacgggtcgac

agagattatcatagtaaggagtctgtctttgtttctcataaagatcatgaagaatagtct

ctgtctttgtcattttcacatcaccattgttcatttttgcacacaaacacttcaagctca

agggtctattataatattgctataaacttggtctagtttgaaaatgaaaatcaagtgtta

ttttgatggggttttgttggtttaacagaacatgtggtgtttgagattgaagccatatgt

tttttcatggaatggacaatgggaacacaact

>fava_dn_rep_c774

aaattattctcggtaatataaaaaaataaaaaataaattctgggcagcatcgtgagatct

caaccgagggtgaggtttgcaatctggaatagttttgtatcgttcattcaaattcaatca

tataaaatcaagctctgtaaaaggagcgaacgaacgaacgatgaatgtgccgctacttgc

tttgatcgttgcacttttcattccaaatggagttttcgcaagtccttccacagttcccgc

gttcctttggtcgtctcattataatctggtttcagacgatggatcgaaggggtctgttaa

ttatcaggtcatttctccaaaagacctagcaaaatctgttttatcggaagcaggctggtc

aaattttctgtgcaaagggaagacatttgaggatcctctcgatctggcacttctatttgt

tggtggagagttacaatcttcagatttaagtttgaacgcagattcatcgctttcaaactt

gcttaaggactcttttttcagatccaacacttccatggcatttccgtatgtttcagcatc

gcaggatgtagatttggaacactcattggtttcaggatttgctgaagcctgcggagacga

tttaggaattggcaacgttgctttccttggatcctgctccatgggcaatggaaatcctga

agaaactgcagctttgcactcagttcaagactatttgacccagaggacagaaaagaatca

caaagggaaaacagatttggttgtgttctgcaatgaacagtctcaagcttcgaaaaatgt

tgacagaacacaatctgaaggtgaagttttatctgagcttatcagctctgtagagaaatc

tggggcaaagtatgcagttctttacgtgtcagatctctcgaagtcaatccagtatccttc

ttacagagagctgcaaaggtttctagcagaaggtacaatagggaatggatcagtcaattc

tacagtttgtgacggtgtttgccagcttaaatcatcgcttttggaagggctttttgtggg

aatagttttgttaataattttgatatcgggactttgctgtatgatggggattgactcccc

aacaagatttgaggcgccacaagagtgatgatccatttgttaaagctaataattgttctg

gtgccgggtctacttcattaaactattactgctacattatttgtcatttttatggaggct

cggttgaatgtcctgtaattttctacttttttcagggacttttcatgttgaatattaaaa

taatttgtaccagtatataaaagcaaaacgtctatgtaggttttaattttgatcccttgg

tgttgtaaaattttaagaagcaatttcagaatatgttgagtgttctgttttctcatacca

ttcattacggattggatgctgttggtctctttctgcatgctctagtttgtcactcttttc

acccttaattaagttaatttt

>fava_dn_rep_c775

tttttttttttagaattgtattttattaaattataaaaaattgtctttttttatttgatt

tgaattgttcgaattgtataatcatcaattattgacattggtaaacggcccaaagcaatt

tgattataattcaattcatgacgatcataagttgaaagttcatattcnnnagatttataa

actcgaacacaacatgctacctctatatatatatatatgaagatgaatacacagaggaaa

cctataatattagtccatacaaaatgactaattattctaaacatcagcactttttacaat

ggaagtgctgtattaacattgttacacgcatatatatttatacacacacatcgacacgca

gaaaaccatactgataaacatcaaatagtggtgagactacaatacataaaattccacaag

taataatactatttattctaaaagctgagctaaaacatgctccaccacatacttcgcaac

gatactattcgttttctccgtgggatgaaaagagtcccaaaacacaaatttggaagcatc

agaacaactgaacatgctgcccctgctgcatgcataacccatctcgaacattccagtagc

acaacatgccactgaggctgattcaaacccgtagagttcaggtttctttatgatatgcaa

cataatgtgatatggatttgaaaagaacagcttcaatccagggagctcttgattgagctt

agtagttatattctggagtttaccattgaattcaagtgctacgttattgtagcttgcaac

acaaccattctgctccataaaatttgtagttctctccaatggcaagcatcccatgggagg

tacccctccaagggaaatcttcctagcaccaagaccatatagactccttatgaaattttc

ggcaattccagcgagaaaagtttggtactgttgaattgtgtattgtgatgccctaccagg

catggtatagtaattctctaggaaatcatttgttccaatactcataaggtgtaatgattc

agaaatgctctcctttgctttggtttctccaagatatgcagttagattcttttggtattg

tttgtagtactctaattgcttccataatggtatcacaccaagtacagctgaagttgcatt

atcataaccagtagcagcagaggcaaaactgacaccagttgcaaaatctgaaatattatt

cttaggatccaagtaagcaggcacatattcttttataccaaaagcttctgatatgaaatc

ggtcggtattctcccattcgagaatcttccggtcgcttttccgcccaggaagtcacgtcc

atacggttgaaaattgctccgtgcaaccgtgggaataaagttgttgttcccggcgtcaac

ggaggagtcgccgaacacgataattgccggaacctttgcatttgtcttgatcaaaatgag

acagaaaatgtgaagtgaagataataatgctagtaggtttgattgcaattccattttgtg

ttggtaaatttttttgagtacgtgagtatggagaagtgtgtgtgggttcccgaggtgtga

cttgtgaccaaataaatattggttaggaatggaagtttgatcttttgaaaattgggtgaa

ta

>fava_dn_rep_c776

aaataaattcgttttaaatggtagaggggaatttaatattaaccccattttacatgatag

gaaacacaattgagaaattaaactttcccgaccttacattaaacttttttttaatggctt

tacatgcatatttttttacatgtaataaagagtctcatttttatatttccatgttcgaaa

actctaatctcaatccaattataaaagagtttgagcattaattacaaaacatgactagtc

tatgcataagcaacaaattaatgcaccaaattcatctcatagttatcttggcaaaaggaa

ttgtaagcatgttcttccatatcaaacccatcatcacaaaccattaaatggtcaaccatc

acactcatctcatctccacaagactcccaactcccatcatcaaattggaaggatggtatc

aaatccatatcaacccattcaacccctaagtcatgtccctcatccacttgtcctatgttc

catgattcattgtaattttcttcaaaattgctcttcatttgtaactctatgcttgttgaa

taatcattatcacccatcttggtttcactaatttcagcttcaaaagatcttatcaagcta

tctaatctttcatcatcattttcatcacaaggtaactcttccattagtgacatgagaaga

ttatcacccttgatttctatatcataagccaaactttcacaaacttgtgaagccatttgt

taaatattattaaattggtttaatttgttataaccttgaagaagagaaaaaggatgaaca

ataatagagagaatagagaatgagagaaaaagcttaagatgaattccccgcggccgtccc

tttcacatcctaacatgagattgtccaccaaatacaatggtggaacaaacttgttaaaag

ggttaaaacat

>fava_dn_rep_c777

aacacccataatatattttttcttttcttaattaatctagagtggtgttttataaggtat

cgtgggttgagacagggtagcgttagaatcgtcgttggtgaatagttaggatggcgaacg

gtggcggaaaggtatcgttcaaggtgaccctaacttccgatccaaagctacccttcaaag

tgttcagcgttcctgaggctgcaccttttaccgccgttctcaaatttgcggccgaggaat

tcaaggttcctccacaaaccagcgctattatcaccaatgatggtgttgggataaatcctc

agcaaagtgcaggaaatgtcttccttaagcatggttcagaactacggcttattccgcgtg

atagagttggtgcatctgagatgttacaggatgtctgagttgaacactttctttatctat

tttgttggctgttgtagtttaataaacactagtgctttatttatatatgtaattggtata

aacagtgtccttttgacaaagtttagtccaataatgaatagcatccacctccttccatag

tgaatgtttgtaatgcaggattttgcaaaaaataatcagcaagtgagtttgtaatgcagg

attttgcaaaaaataatcagcaagtgagtttgtaatggaaataggggaatgtatatcttc

ttgctatcgacaaagattgtcttctgatctgcaaccttgtcattttcaaatttttatttt

tgaaatgttctattcttgtaaatctagaagcactttctaatggctagttaaatccaaaaa

aaaannnnnnnnnnnnnnnnnnnnnnnnnnnnnnnnnnnnnnnnnnnnnnnnnnnnnnat

tattgttat

>fava_dn_rep_c778

tgagtttcatgcaaactaatattatcaataagacaataacaatgtatcttcttatttaac

aacaaaattgatagaaattgggcaataacatttcatacaatcccaattgcaacttgtagc

ccaatcattttcatttctaacaaaataatagttgcaatctaacacacaacactttctagc

aactaaaataacaaacactcctacatctaaaaataacaacctagccgaaacatttattca

cctaatccaacaatgcaaaaaacagcaactagcttcataataaaccaaatgattcagatt

cttaatcttcaagaagagcttgatcaaagtgccacacaccatgaccctttccaactaatc

tcagctgtgtcacatactcagaaatcttcttaattgattgaacctgttcatccaaaaact

cgctctcaatgaaatcagccaattgaggatcattgttactatcagccacagcatgaagat

ccagaagcttctcattcactaacttctccaaagacagtgccaattccatagcatacaatg

catcacccttttcgtgatcgaactccgacggaggactcacaataggatgcaacaccactc

ttccaccacgaagattctgatacttcataagcttctcagcatgctctctttcctcttcac

tagattccttaaagaacttagcaagtcccttcaaagctatgttatctctgtcaaaatacg

caaacagagagtggtacacataggaaacattgtattcaacattaatctgttcattgatgg

cagcttcagaatcattgttgaaattctggcgggccagagaaacgttgggtgcaatgggag

tagcaaggtagtctttcttgacttcttcgaacggttcaaagatgacaccagtgagtgttg

cgggtgagtttgttgcagcataaaccttcacgttcttgttttttgaagaagatgaaagaa

caaagctcagagaggaagaagagggattcttcttctgaacgtggtgagggagaagaggtg

aaggagaaagggttgagagtttggaagaaagagccattggagaagattgaagaaatttgc

ggtggtgaatggttgtgtcccgcggcgggaatcgactgaagaggcccctttgcattcctc

ttctcctatttgactagtgacttcactgcatcatataattggaattttgtgattggattt

tttttggattgtgttttgataatgggcaaggttcctgtatagggttagattatttccact

ttcaataaagtccttaggtacactgattatgttggtaaagttggtgcttgtatatgatga

gtatgtgatttagttgctttccatcatctttcattatccttatttggattaatgaatacg

tataatgtatgtgtatacatgaataatggcaaatagatagatcattattcttcctttatg

gatgactgttttctaaagcgaacttatttgtgagatgctagacatcacaactacattttt

tgaaaaagggtgctctggtgagtaatgactacatgtaaaatatagatgattctacaagtg

gctactcagaaagcttattgaaatccatggcttcttattgaactacataaccatgggaaa

ctgaaaacttgagaatgaagccaaatttagagtactaaagagtgaagttgagagagtgca

aagtgaagcatggttttgctttctttacacctaacttgaaacactcagattcctgctgac

acgcggcagccactgattccttcatgctctcaggtccacaaaccaaaactccaacattag

atccacgtgtttcattcttgaagttgcctaatatatctttaaagttaggccttcctccaa

aatgaacttcatgttcttcaagatcacttttaatttcagctgaactttgatcaaagggtt

tgatttcattctgcgacatcggcgggatccctttcttcaaccttctccatctcaacacaa

ttgctatcaatgcgcagcaagataaagctacgacaaaagcagctatcagaagtaaatcca

caatccaagagggagttttttctttcaacaatttggacttttttctggatggaattacag

tatggttgaaacagattagaaaaataaggaaagtgagggaacaaaatccagtgatagcag

ccatccaaactggactttcaagtccatatgctgcataatattttgagctcacactgtcca

atttcatggttttcactttcacgaactcgtttagcagctccgtaattcccaggtctagct

gcgtttcttgtgtaacaaacaatttcagtttcaaatgaaacttttggcttgcttggttga

gtagcaaatgcgtgatcgaatgtaacagacaaacatcttgtgccctctttatgacatgta

caagttggatctttgaaggaaaatcatttttgttggtggttgaatcaatctcggccaaaa

cgctcagaaatggtgttattccacttcctccggcaaccataagtacactgtcatgtttta

aaaagtctaatgaagcaggtccataaggtccttcaactgcaacaggtatgccctttcttt

tatcagcacctttgtttagttcagtttgtatcaaatcatgaagagtattagtccaccatc

cttgacatttaatcatcacagacataatgttttcatcggctttggaactcgatattatac

tgaacgaatgccattgaagatgtgatatagttggtatcttcatatatacaacacttgtag

ggttatactttagccttggatctttggggagaattaactccagagcttcacagggaaaaa

atctagctgaaactatgcaagttttcgggcttgattgaattatacggagcagtttgtcga

ggctaaaaagaaaaactcctggaaacaccatatagaagtgcttatctccagcatgaaaca

agaaaaatactagaaagagtatatacaaatgatgtgtgtagtagaaaatctcaaactttc

ttcttctaatttgtggaagtgaagtaacgaagataactagcccgataacaagtgcaatct

ccccagcaaggtatattcgtcccgtcttttgccacttccaaatctcatgttggatatggt

ggctgacaccccaaacaaacaaagtgctgccaccatgtattgtagcaaaaagtatcattg

cagttccgatccatgtatgatacctaactgaagcttcgaattgaataccaaatatgcgaa

atagagacaatcctcttaaaatcggaagaagaagcaaagccaaacaagcttctgctaaca

aaccaaaccttgttgctattctgagatacttaagttgccataagttcagtttgagtgatt

tgtcttgcatcaacttattgaaatcgttagaaattcgagcataataagtccatgccaaga

agactataacaaggaaaactaccagtatttcaacactggttaagattccaaatatactat

tcaccacaagtggatttgagaacactgttgatgaactccttgcacttctggttctttgag

atccagctttcaaatccaagaaaagaagtccaattactgcaatagcaattataggaaatg

tataaacaccaaagtttagaccatagtatccaaaaattgtattattggcactttgttctg

catgtttccatactcttgtccaaacttgagttggcttcagaatccaaagggaaatccaac

ctgcgcatatgaaaataatcaagagtttgagaattgcaagtaaagtagagcttgtcatct

ttgactttactactatcccagacttgggtcttttctctctacttcatctataatatgctt

agattttatgat

>fava_dn_rep_c779

tttttttttttgccatggtaatctcaatattcatttcattcacgatgatgattaattaat

gtacagtacagaaaggcataaatgtgaagtaataaacaagccaagagattcgaggcgttt

tatgaaacagatattttaaactgaagccactcggacaggttaatacaaaaattggttgag

catctaataatttgacaatgttccaattaaggcaagctcgaatacgtgcaagcctcgcat

tattgaaggcaaaatgtatcacacacttttcagcaggcatccaggtcacggcatggagga

ggtttgggctcacttaacaatgtagtattccccccttcaggtccactcctaatccgttgt

ggattttgtgtgccgtgcggtggcttctcgccctttctcagcttttcaactatctcaact

actttctctggggttacatcttcaaaataattataagtatatccttctgaaccattagag

taatcagccactgtaatcattggagcattcacacagcatcccatgcattccatttctcca

acagaaaagaaaccatcttgtgttacttcatttcgtttcactcctaagtgtttcagtaat

gcttcttcaatacctcgtgaaccacgtatcatacaaggtgtagttccacagaccaataga

tgatatttgccaaccttagatcgattgaacattgaataaaatgtggcaacctcatatacc

cgaataggagcaacctctataaccttagccaccgcatccattgcagaaacaggaagccag

cctccatgctgttgctgtgcaagatccagcagagggatgacagcagattgcttatagttg

gatggatagtgagataaaatctccttcaccttttctttgttggcattgttgaattcccaa

ggaagattggggttattgtccggagagtcaagatgatagttgagagcggttgagaaaacc

ctcgctggctgacggaaaatctgacggatctcgttgagacgattcgcggctaagcgtgcc

agcattttgggagaaacgagaaggaaccaaaccacggatcggagagaagagtatgagaga

gaaagagttgtgaggtttgtagtgt

>fava_dn_rep_c780

tgcatcagtattattgtttccactcaataaacacctcactactaataccaacacacgacg

cgaaaccctctcatttcctctctctctctctctctctctctctccataaccaccttttcc

tctccctcttgtttctttcaaaactttcaaatctagatcgagaaaaaaaatgagagaaat

catcagcattcacatcggtcaagccggtatccaagtcggaaactcctgctgggagcttta

ctgcctcgaacatggcattcagcccgacggcatgatgccaagtgattcctccgtcggagt

ggcgcacgatgcattcaacactttcttcagcgaaaccggttccggcaagcacgttcctcg

tgccgtttttgtagatctagaacctaccgtcatcgatgaggtcaggtctggcacctaccg

acaactcttccaccccgagcaactcatctccggcaaggaagatgctgctaacaatttcgc

cagaggacattacactgttggtaaggaaattgtagatctgtgcttagatcgtgtcaggaa

gttggccgataactgtaccggtcttcagggatttttggtcttcaacgctgttggtggtgg

aaccggttctggtttgggttcactcttgttggaacgtctctctgttgattatggaaagaa

gtccaaacttggtttcaccatctacccttcccctcaggtttcaactgctgttgttgaacc

atacaacagtgttctctccacacattctctccttgagcacacagatgtggctgtgctctt

ggacaatgaggcgatctatgacatttgcaggagatcccttgatattgaaaggcctactta

caccaacttgaacaggttgatatcacagattatatcatccctcactacctcgttgaggtt

tgatggtgctattaatgttgatattaccgagttccagaccaatcttgtgccttatcctcg

tatccacttcatgctttcatcatatgcccctgttatctctgctgctaaggcttaccatga

gcagctctctgtgcctgagattaccaatgctgtgtttgagcctgctagtatgatggccaa

gtgtgaccctaggcatggaaaatacatggcttgttgtttgatgtaccgtggagatgttgt

tcccaaggacgtcaatgctgctgttggcaccatcaaaaccaaaaggactgttcaatttgt

tgattggtgcccaactggtttcaaatgtggtatcaactatcagcctccttctgtagtgcc

aggtggtgaccttgctaaggtgcaaagagctgtctgtatgatcagtaacaacactgctgt

tgcagaggtcttctcccgcattgaccataagtttgatcttatgtatgcaaagagggcctt

tgttcattggtacgttggtgagggtatggaagaaggagagttctctgaagcccgtgaaga

tttggctgctcttgagaaagattacgaagaggttggtgcagagggagcagaagatgatga

tgaaccagaagattattgatgtcctattatataagtgcaagaagtttttttctcttctat

ttcagttgattatttcaagcaatagagggtaatgctttgttacattgccctccactctta

ttgttcttttatttcctgtgtgatatagttccatcattcaaacttgtgaaatggtctaat

tttgttgtaggtactgttgtttgcagcaactatgcatttatcattgctattatcaaatgt

tcattatctcttgttcatgcaatagtttctacaacattttctttgatattcataacattt

gacgaacatgtctgtaatctttcttttttcagttgttcaggttttgtttcttagtatttg

gttctggagagataggatcatgacatctgttgcttagctcaagtgaaatagaaatgaatg

aaaatgcattgcaaaaat

>fava_dn_rep_c781

aatcataatcattttcaaatagtagagagaaaaattaaaacaaaggaaaaaaataataag

attatgatgtaacaatcaattggctgattatcaaaatacttgttctaaaagacgattact

tttacattaaatattcaaatctaagatgaaaccagctgacaaaaaggaacatttgaaaat

taaataaaaaattgaaatagtatccttagccgttaaaaaagagaaatcaggcaacaacac

tgactttaaaagtgaaaatttcttgcactcagaagttacgcctaggcctttcctcagcaa

cattcactctgatcgcccttccgttcaagctctgaccgtcaagagcagcaatggcctcat

tcatttcagcctcatcggtcattgtaacaaagccaaaaccgcgtgaccggcctgtctctc

tgtcatagactatacgagcgctctcaaccttaccgtgttcgctgaagagttgctccaagc

gaccattgtcaaactcccatggcaagttaccaacatagattcgtagagaggaatcaaagg

cacgtggtgggcgctcaggtcttggtgctcctcttggagcggctttattaactgtcaacg

atcttccatctagctcatagccactgaacttgttcacagccctctcaagttcttcagccg

tactcatcgtcacaaatccaaatccccgacttctgtcagtatccctgttataaataacct

cagcagtctcaacagtccccggagtctcttcaaaaagcccagccaatctctgactatcaa

catcaaacggcaaattcccaacaaaaatcttcaattcctccggtggttcctcctcgtaac

cctcaacctcaccatcaccatcagcatcagcatcagcatcagcactctcagtctcacttc

cccacgtaggctccccttgattctcccatgtagctccaccctccttctcatcctgctgag

cccagtccgaagtctgggccacagatgtcagcaacgaagagtaacgggttcttctcttac

cagtactgagggaaagagagtgcgaaaccctagatgaattatacgtagggttttggattt

tgaaaggcttggcggagtttgagatgaatggggatttggttttggagtagaagagggagg

gttgagagagtaaacaggatttggccattgtgagggatttgaaggaagtgatagacatat

ctgaaaattcggagagaaaggaagtgtagaaagctctgtggttgtttgtggataagggta

gatatgatatgagaaatgaatgtttgaagaagtgtgagggttttatgttgcctttgggga

tagattgtaaatatataaggaaaaatattgcagatattatcgaaatactttctattcccg

cggccgtaatggccactctgcgnnnnnaccaatgcagagtaaggaagtttggaggataag

gtggcgtttggaagtggggttgttgttnnnnnnnnnnnnnnnnnnnnnnnnnnnagagat

gaaggatttggtattggcagtgctaagttttggaagtg

>fava_dn_rep_c782

tataaaatagaattaaaaaaaaaatctaaaatttgtattaaaaacaaaacaaaacaaaac

aagcccaaaaccccacacaccagtcaccggtcaccacccaataccaataattgatcattc

tttgcccaatcccttctgtattcaagctgtcacctctctctcttcagcaatcccaaaacc

catttcactcttcattccaatgttctaaaacatcataacatcaaaactctcaaaacatgt

ttcaatttctcctcctcctcgttctctcaatctcaaccatttctgcatcaaaaaatgcaa

cttccatctacgaggttctccacaaccacggtcttcccatgggtattttcccaaagggtg

tcacccaattcaacgttggtgaagatggaaaattctgggtgcatttagatcaagcttgta

acgctaaattcgagaacgagttgcattatgatcgaaacgtatctgggtcgttgagttacg

ggaagattgatgggttgacagggttggaagctcaagatctttttctttggttggctgtta

agagtattcgtgttgatgttcctagctctggtttgatttactttgatgttggtgcggctt

ataagcaattctcgttgtctttgtttgaaaccccacctgaatgtgttgctgttcgtgatc

attccgagggtcgattaggaaggctccgctataaacttgatcaaggaactttgggaagag

atgttctatagacagagcaggaaagggtgaatgaagtttttgtgtagcgaaattgtcatg

gatgtattcattcggagagggtataaattaataggtccaacgaattcggtcctaatgtcg

ttgttgtttgtgttatctcgcgtatagtgcagaatttagtgtagtagaggcagccaatgt

gtatgggaaagggaagcaacaagatgaatttcaagaatctgtttcgcaacgaaaagatgg

gctagaatggagcattttgttgaaatggatttaggtgacaagtatgcaaagattgtatta

taagaatgtattgggggggtattttattctgttgtatcaaagtttttatatcagtttata

atagacagaagaattatctgcacttttgatttgaaaattcatgattatcatttacattat

gtttactcttttccttgaataaaatttatgtagctctttaaggcttaaaaaaaaagtt

>fava_dn_rep_c783

cttttttttttgcacacacaacacaaccatttatagtacatatataaatatgtttaactt

agaaaaagtaaccataaggtcactatagtaatttacacacattatttcagtacaaattca

aaatacttccaaaatcacaataacacataggaaaaacaaccatgctgaaaacacagctta

taaaagcacataatatctctctcaagaggaaaaacacacagacacacacatactaacatg

tatctcaagcatcattatgtcctaaaagtgccaaaagcattttctcatagtcaccagttg

tgtccttcacaatggcacggtcaagagggacactatccctcctttgatactcatcagcca

caattttcaagtcaatttcagctcttgttgccacaactcttgtaagagccccttcatccg

ttccgtgcttattgatcgacagacgaactatcttagcaaagtacttctcaggacgagtca

agcacttgacagttgatctcagcaatgacagaaactcgtcttttggatcttccttcaggt

ccttgtttatatcttttccaaatgcatctttgtagtgattcaaagtagcattaacttgtg

ctttgctccttgtggccagaatcctaatgagatcgtcgtcgttataagcctttttcgaaa

tcttttcgtgaagtatctttgcctcagcttttgctatagtcaagttcacttcatctcctt

catagcggtgagaactaaccagaggcaataggagcttccggtagtctccagttgtgtgat

gagcaacatcttcctcaatagacttcttataaagaacatgataagccttctttgcagaaa

acaattgatcagaagatctagtgcaagctatttccaccagaacttgattgctcgatgtcc

attttttcgttgcttcgttcgccaaaaaggcatcgcgttccgcggattcaagcgcccaaa

gatgaactagtctctcaaagtcactggtaagctctttgtccaaggccttgaggagatctt

ctccgtaagtctgagcataagtttctctgataaccttcctctgactagcattcctatgag

ccaagatcgatataatcaaatcttcattggttccccaacctgaaaaggctttgcgaagct

gctcaacgtcatcggagacagggggaggagaatgaggaactgttagggtcgacattgtca

attattgttacttccccgcccccttccaatatctcagtgctacactaatgcagcctatat

atatttggtcaaattttacctttcaaagcttgtgttccagctatatcttccaatgtaaga

tttagagtatcaactgaccctcttctaacttcttctccccgagtagccaggaagtcctca

atgcttcccggttcgccaaaccatccaagatgatccgcaatcaagtgaagattattacat

ccaccgcatcttgctactactacacctttctcatatgattcccgacaaactgtcttaatg

gacctcgtttcacagaccttgcaggtgaaaatcatagcaagatcgtgccttgaagatgtt

ttcaaattggaattagccgaaaactttatggcagagttctcggtaatgctaacagatgta

gtgacattatcggagttcacactagacttcaaggtgtcggcttcattattttctacatct

tcacaagcgtgtttgcttggatttgtctgtgtcgagaccccccttttaagaagtccgtat

ctgtttaatattgtattgatagaagaatgccaagaggaagcttgaacgatggggtagcga

tcatagtggagcgattgtgttggaacgaatcgcctctgcaaccgcaaaatcctcgccagc

gccgccatactatgctgtggtgagctccacaacttttctaagaatttgggctttgagttc

tttcatgttctgccggttcatgggctaaagaatggttctctccgatgtgcattacttct

>fava_dn_rep_c784

aaaatttataatttttaaatgatgagatttattttgtgaggagaacccctgacacgcagg

cagggtgatacactctatcctattctcttttcttcttttcttttccccttccctctactc

cttgcatctaattccttcattttacacttaatttcattttccctcaattctaacttatta

ttatccttgcaaattctcttcttacctaataaacctaaatccttctttttttttttaaaa

tcataaactatttatttctaaatttagatcacatataacaagattcaatcttgttatttg

cattgatactacatataacatgttcataacttggccatacacgcggtacacattatacaa

acacaaagcattggtaacaaggacatcttatttagtagatattgatatgcatctgtgcat

tcatgctttagaaatcgagttaatcttccgcgggtgggtgatgattaccataaggtggac

cacgaccataaaacggtaaacacgggcgagtcgggtcggttgtgcaagaagttgatgttt

tctcctcaattggtttcttgttggatggaggaagcttgtaaccgtcagtgaaaccttgag

gagcaagcataagagcagcaaggaagaacactaataaggaaaagaaaaaggaattagcca

ttttaaatgctggtttaagtatcggttcataccacctagggaatattgaggttttatagt

agtattatgctcaatgagagtgtaaagtgtcaaagaaagtct

>fava_dn_rep_c785

cacaattttttttatcacacgatttaaaattaaatagaagatatcatttataatttcctg

aacactcgcaggaaaaaaaaaaggaaatgacgggtatcgtattgcataaaaaaagttatc

cttgtatctataaaaatacaccgtatagagtaagttatccccattaaacacaaccactca

tttctggatcggtttccactgcagttactacttgatgcagtcactgctgtcattcataca

tagaccattattagatgggtaaaaacccatttttattcaagaataatacaaattacaagg

gttccagatgaggaacccggtgactttgcgaccaaaacaagtcacaccgacactactagc

gacgcaaataaaacaacgacctcattattaagtagaaacatagaaaaccatctagattta

tcaatctaaacctacacgcgcacggtcgaacccaagatagagaaaccagatccagatctg

acggcatcaaccgtcttcagtttcgcctcggcctggtccaccttatacgggcagttgtcc

ttaacagaataaagctcatacggcgacgtgtcggtagccagccactgttccacggtgaag

agagtctgagcacatggagagtggaccccagttgtagtaacctcaacataattaacatac

caaccgtgatgactacccgccccgtctgaagtcaagttcatcttgcaaacgggtgcgtcc

aaacaaggtcctctgccgctgaatatgtcaaggttgctccgctcgaagtagttgtaaccc

ggacccattaacccgccccatgcttcaaggttgttgattcttatgccgtagttttttgca

tcgtataaggtgaggattatgtttgagtctgttcctgccttgaggattgacccggttctg

acgtacaccgtgtagacacagtcctcctcagatgtgacgattccggcgaagaaaaatgag

aatagaaagaggagagcgaagatatgagttgggtttgccatgagataactgtttagtgat

ggagatttggtaatggatgaggttcttaaatagaggagtgatatctggtggatggagaag

atgagatttagtatctagctaatataaagatgtgaaaatttagtctctggtagtataatt

ttagcggctccataacagggaccattatacgatatttgtttgtgaaatgaccatataaat

tagaagcacgttctccacgcgacaagaatatgcttaaaatcatatttatacttgttt

>fava_dn_rep_c786

gtggtatcaacgcagagtgaacaaatcacattgtggctggcgtgcgtgcggttgatgctc

ctccgctggttcttgttccttccattctatccatttccctttactctgcaaatcacaatg

gccaacacaatcacaacgacaagttttctaacaccgttccacttccattcccctcgttgt

tccattcccacacccaattcggctaaagcttttacagttccgaggagaaaagcaatatcc

ttaatcttcacaacttacattctctccgacattggtacaacattagctcaacaacaacaa

caacaacctcgtaggttccgagaatatgtagattcatttgatggctattcgtttaactac

ccatctaattggattcaagttcgaggtgctggtgctgatattttcttcagggatccttat

attcttgatgagaatatttcggttgaagtttcttcaccttcgtcttcgaaattcaagact

gtggaagatttgggtccgccggaagaagctgggaagaaagttcttggtcaatatcttact

gagtttatgtctactagaattggtgttaaacgtgagtctagtattcttaacactacttct

agagttgctgatgatggtaaattgtactatcaagttgaggcgaatataaagtcctatgct

agtaacaatgagcttgctgttatgccacaagacagggtggttcggttggaatgggatagg

aggtacttgtctgttctgggagttgagaacaatcaactgtatgagttgaggttacaggtg

ccagaaaatgtgtttttggaggaagagaatgagcttcgtcgagttatggagtcgtttcga

gcgaataagattcttcgttagaactggaatgtcgaggaatcaagatgttgtatgatcttg

tatgtgtttttgatgagttggttgagctggtaatgagaaagattcattcatttcattctt

gatctgttttgttataagaacttataattcaaattattattgaatctaaccttataaagc

atacccttatcaaggtaaacatgaggcgactttcttgagaaagctgtcttttagaaatct

ttgaatgcaaatcaccaattgttattataacagaatttttattgtgattatagcagaatc

tgtatagcagaactgttatttgtgattataacaaaaaatttatagc

>fava_dn_rep_c787

ggcaaggcaatgacatcaaaattgttaacaaaaaaaatatttagaatgtcaattaacgac

tgatttactatgatgataactgaacattacaatatcggtgctacaatggttttagaatct

ggacattaatagaggcaataagaaggcactaagaggaacaaccaggtatttgtctagtcc

ataaaaattgcatattgccaacattactattttcacacagcatattcaacatgttgacag

gagaaatagtcattaaactgaaaagggtaaagataaaatcagcattcttgttggatggac

atgtgctcatcatttccccgtgttcatcattaaagttatgttgtctcctttaagaagaat

cctccctagtgtctttctgctcttcttcttgacgtttacttcttcggcgtcatccaaaac

caaattcatgtactcatcaaaaccaatgattcttccttcaatcctcaaatccttttgctc

aaagagccaaatctgaatacgagctttgctttgaagaaacctgaagatcaagttaatggg

ttgggtcataaccctctgaactttggtactcgccatggcagctaggtttggagatgcgat

gagccgttgtgatttgcagagacgcgatgagtgaaccctaaaaattgagcaaaccgctat

gttcctcactcagtgaagggttttagaaatatatattccgttagtgttttgagtgattaa

tatatg

>fava_dn_rep_c788

cacaacatannnnnnttttgaaatgcaagatcatttttaaataagtctaatacagtttgg

tgagaaacaaacttagaactaatctctttgaggaaacttgaagccagttagatctgggct

cactgagcttcaagatttcttatagtaacccttgaaagaatatggagaaacacaatcaat

tttcttcgttaatcaattgaatgttgtacatataattacaaggatggcaatcagaaatta

aaaaatgaaaacctagtcccaactaaagttaataagcctcattctcaccgattctgggct

atgaactccaagcctaagacaaactgtactgaactccaagcctaagacaaactgtactga

actccaagcctaagacaaactgtactgaataccccttgaatacctgtgattgtgggaggc

aacggctactgctgcagcaccaactacgccaagatgagattgtgtctgcagcagttttgc

atccatagttgttacatgatcatttaggttattgttacttcctcgccggcggtatgggta

attgttcaaatcagtggttggtgcagcttgtaagttgtaatgctgggactggaattcatt

cttgtcttgtgaaaagtccttgtgtgtgtttgatgtagtctttgggttcgagggatgggt

actaaagacattgtgagtcgagactgatggagtagtatttgggtaaaccatccttgggtc

gttagaagattttgtgattctctcatattcgtagtgtagaactgggcctaccactctccc

tggcttagctggcacccgtggtgggggctgtacactccttatctgatttccagaattttc

tccaactcctctcaaactcttattagatgtctcttctgccatttgcttgttctcatacaa

agcaagggttgactgcttactgggaagaatcttactagggtgaacagtggaccttggaac

agatacatgctttctttcaggagcaggcacaggtccacttttaccatcattttcctcaaa

ataagcaaattgctttcttaattgatctattgcactaggatagaggaaattggcgctttc

ggttccattcaagtaatctttgagcagttgaggatgatattccagtatttcccgatatac

tagtaccctaacatcgtcctttgttatacgtctcctctcaaactcaaattccaattttga

gattggcttacaagaaggttctctttccacttttgccaagcccttaaagtaaggatcagc

tagtgcctgttcggcagttggtcgatcttttggatcaaaggctagaagcctctgcaaaag

gcgaagtgccaatggatcagcatctgagaatttgtgttcaaatggcacaggaggtttctt

ccgcattcccgacaagtacttccttgcattatcatttctaactcctgtaatagtttcagg

caatggtgtcccaagaaggtcggtaatcaaatctaactgatgcacgacacttttgccagg

aaacagtggctttcccaacagcacctcggcaaaaatgcatccaatgctccatatatcaat

tgctggtgtgtacttagatgagaatgatccacacagctctggggctctataccatcttgt

agcaacatagtccgtccaaaatattgatgttggagcatcattaaatgaaactcttgccag

tccaaaatcacagattttgagcttgcaattggcgttagccaatatattcttaggcttaag

atctctgtggtacacattagctgaatgcatatacttcaaagcacgtagcatctgataaag

aaaaaactgatggtgttcccgagtcaggtcatcattagctttgatgacttgatggagatc

agactccatgagctcaaaaaccacataaatatctctaaactctctctttgatggcggcaa

catgatcctcttaatatcaacaacatcagggtgtcttaaaagcctcagcaacttgacttc

acggaggatcctaaccgcgtcagaaatatgctcaaacgcatcatgaatcttctttatggc

aaccttttccccagtgtgtgtgtcaatcgctgcacaaacaactccatagctcccctttcc

aataatctcaagaatctggtatcggttcgcttccccatactcagtgaaaaactccaactc

tttcgaatccttcttggactgaattttctgcgtcattgttctctttaaacggagaaatag

caattgggaaaaggattggtgttatcgaagcttcaaattgttcaatagcgagaagaattc

gaagctccgatgaagatggaaaccctagatattgcagcagaagcgcagaaatttaagaga

agccaaaccgacgagagagatggaaatagaaacgaaagggatttgtgaacagaagaag

>fava_dn_rep_c789

agtaagaaagagaaacaaagcaaaattggactccattactctcttaaagcttctaaaaga

ttccaatgacatgcatccacaaaatatcactacttatcttgttatctacatgcattatgc

catccataaaggtatctaactaataatcaatataaaatctatcaaaatagataactgaat

tttattaagatccaaacatggcagaagttaagaacaagcaagtgattttgaaggattatg

tttctggctttcctaaagaatcagacctgaacatcatcgacagtaccataactctcaaga

ttccacaaggttccaacgatgttcttcttctcaaaaatctatacttgtcttgtgatcctt

acatgcggaatctcatgaacaaaccacaagactctctatcttctcgtgcctttactcctc

agtctccgttagctggttatggtgtgtctaaagttctggaatctggacataaagattata

aggaaggtgatttagtatatggtgttactaaatgggaagagtacagcttgatccctgcag

ctcaagtaattttcaaaattgagcacactgatgttcctctttcctactatactggaattc

ttggtacaccagggcttactgcctatgcaggtttctttgaattaggaactccgaagaaag

gagagaatgtttttgtttcagctgcctcgggtgctgttggtcaacttgtcggccaatttg

ccaaattgcatggttgctatgtcgttggaagtgctggaactaaagaaaaggtggatctgt

tgaagaataaatttggatatgatgaagcctttaactacaaagaagaaccagacctcaatg

cagctttgaaaagatactttcccgaaggcattgacatttactttgagaatgtcggaggaa

agacacttgatgctgtactcttgaatatgagaatccatggtcgcataccggtgtgtggaa

tgatctcacagtacaatcttacgcaacacgaaggcgtaacaaatttggcacatatcatat

ataagcggattcgtatccagggttttatcgtagctgatttccatcacctgtatgccaaat

tctgggagtttatcctgcctcatgtaagagaagggaaggctgtgtatgtggaagacatag

ctgagggacttgagaaaggtcctgccgccttggtgggaatattcagtggtcagaatgttg

gtaaacaagtgcttgttgttgctcgtgaataaattacgatgctttcatttgatttttttg

tgtgttgaataagaagctctctttgtatgatggccttttgcagtaacgtttctcttcttt

agagttgattgtaattgcttgcttctgtgatatcttgtgataaataaaacctctgttata

tgtaattttaggcataaggcaatgccatgacagaaaaaaaattacaagaacacaatacaa

aagtactatctttaagcccaaccctaattaagatgttatctcaaaaattaactaattaat

taatagtagtaagattattacatgataatatatgaattgaaactgcgaattgagaaaacg

tagaaccaatagagaaaaacaacggagaaagattgaactaagtgagttctgcgtctgaaa

gaggaatttatgatgaatccagaaagaagaacttcactgagaacatgagacatgaatgtc

aaaaaattaacataaaggtattgaaattttcaatcagaagcatcaagattcctcagagga

agaacacaagagatagagagaaaagagacaacagtatacatcatcatgagcagaagcttc

tttcccaatagaagcggaaatgccctccctgtatccaccaaagaagacgacgatgcagcc

gcagcaggaggaggcattcggagacgtctatcaagttcctctctgaacagcatccctgca

gcagcagctaattggtcatttcccagatccaagtcgctcccgttctcatccgcaggtgat

tctatcagaaattggtggctctggattttcgcatggatcctctccaggaaacctatcttt

gcaacagatcttgaaatgaacgagcaggaaacaaagcacctggcatccgccgacagagga

agttggaaacatgttatctacaggacgcgttctgagatcaaaagaatcaccaattctgat

cactctcttcttcctcaaacctacacgccaaatccgcaaacaatgatataacattcatca

ccaatctataatactggcgagtgtttaattattttttgtgaacgcgtcttcaaataaatt

agtcctgcatcaattcttcaaattgcttctgctagcttctgcaataggctacctcataaa

attccttcttccttctgctatagtatttttgtttgaataatcgtgtaatattcattaata

ttgttgccttgtccgtggtcataaacgtgacaaaaaaggttgtacgtaattactagttgt

attttacatattattcttgttattctctctctgtgtataataatactataactaggatct

tttgtggattgcaatatcatcaaatcatatatttaccaaaaaaaaaa

>fava_dn_rep_c790

aataatctaccatcaaggccagaagttatagatttatacaaatcaaaaggtattggtgga

atgcgtatatactatccagacgaagaagccctacaagcacttagaggttccaacattgaa

ttgattcttgatgtgcctagggataccctttcttctctcactgatgccaatcaagccaca

aattgggtaaccaaatatgttacaccctactcacaagatgttaaaatcaagtacatcact

gttggaaatgaaatccaccctgattacaatgaggcccaatacgttctcactgcattgcaa

aacattcaaaatgcaatttcagcagccaatttacaaggccaaatcaaggtctctatagca

atagacatgactctgattggtaactcctatccacccaaggatggtgttttcactgatcaa

gcaaagtcatatatacaaccaataatcaacttcctagtgaagaatggatcaccacttctt

gcaaatgtgtatccgtactttgcttatatcggaaatgaacaagccattcatcttgattat

gctctttttaatcaacaaggaaacaacgacgctggttaccaaaatctgtttgatgcgcag

ttagattcagtatatgctgctcttgggcaagttgggggatccaatttgcagatagttgtg

tctgagagtgggtggccatctgctggtggagatggagccacaactgcaggcaatgctgcc

acatattataataatttgatcaatcacgtcaagagtgggaatgggactccgttgaaacct

ggtgtggctattgagacttacttgttcgccatgtttgacgaaaatcagaagactggtgca

tcgactgagcaacattttggtctctttaaccctgacaaatcacctaagtatcaaataagt

ttcaattaattaaacagataattcaaagaaatatgtaactgaataaacgaataattgtgc

caatgtgcacatgatgttgtgtttgcatgttcctatttatataaattgaattaaataaat

gaaagatcatatcttgtttaattccaaaaaaaaa

>fava_dn_rep_c791

gccatttagtccaatgaatgcccttttttaataaaatagcattattccagaataacaaat

aaaaaagacaggcacaaaaagcaattaagaataagataagagagcttaaatgactttgta

gtgagacacaataatcaaaactcaggctgtcaatattaaactctagattcaaaaactgga

atccatcagacaaaaatgctgaggatatacaacaaacaataatagcaaataagtttgaaa

tatattggaaaagcaatcattcgtcctcttggctgcgcaacctggccagtttgtttgtga

caacaacatccaattgtgctgcacgttcaacgatttcctttcttttcctggtcgacacat

tgtgtgctatctcagcacagtatgttctgttatgcatcatgaggagttctagatccttga

cattgtggacaacaaactttttgaatccgttaggcagatagtggcgggtcttcttgtctg

aaccataaccaatgtttggcatcaaagtaactcctttgaattttctcctaacacgtgagt

caataccctttggcctgcgccaactttccttgacacatattctcctgtcactttgaggcc

taatgaacctcttgacacgcttcttgataatctttttggttagcaatggaaccgccatct

tgtatctttgtgattgatgatgagaaatcccaacagctagaaatacctacaaaaccaaac

cctagtcttttcttaaaatgggttgggtttgttggcccaatacaatgtacaagttc

>fava_dn_rep_c792

ggccgcggggggaagttgaatcgaatttgaaagtgttgattcaactaattatttcatatg

atcatggttttgaatttggcctccccagttcaagtaaaaccctcatttttctctgataca

attaacacctccataaaacttccagttaactttgcgttgaagaaaaaagagaatgacaca

aaaataataaagagaagagttcattgctcagcagcaaattcaatacaaccagcttggcct

ggaacagctattcctgaacctagtgatttcaaaacatgggatgaaccaaaacccatctct

gtcttaggttcaactggttcaattggaactcagacacttaatatagttgctgagtttcca

gaaagatttagagttgtaggacttgcagctggttcaaatgttactcttcttgccgatcag

gtaaagacatttaagcctcaagtagtagctcttagagatgagtctaaaattgatgaactc

aaagaggctttggctgatattcaacacaaacctgaaatcatccctggagaacaaggtgtc

attgaggtcgctcgtcaccccgatgcagccacggtagttaccggaatagttggttgtgcc

ggattaaagccaacagttgcggcgattgaagcagggaaagacatagcattggcaaacaaa

gagacaatgattgcaggaggagcttttgttcttcctcttgctcacaaaaacaaagtcaaa

attcttcccgccgattcagaacattctgcaatttttcagtgcattcaagggttgccaaag

ggtgctcttaggaaaatccagttaactggttccgggggcactttcagagaatggcctatt

gaaaagatgaaagatattaaagttgctgatgcattaaaacatcccatttggactttagga

agaaaaataactatagattctgctaccctcttcaataagggtttagaagtaattgaagca

cattacttgtttggagcagactatgatgatattgagattgttatccatccacaatctatc

atacattctttggttgaaacacaagattcttctgttattgcgcagttgggtttacctgat

atgcggttgccactcctttatactttgtcatggccagaaagagtctattgttctgaaata

acttggcctcgccttgatcttaccaagtatggcactctaacatttcaagcaccagacacc

aagaagtttccatctgtgaatctttgctacgctgccggacgtgccggaggcaccatgacc

ggagttatcagcgctgcaaacgagatagctgtagaactgtttgtagcagaaaagattagc

tatctagatattttcaaggttgtggagctaacatgcaatgaacatcagaaagaattggta

acatctccttcacttgaagaaattcttcattatgatcaatgggcaagacaatatgctgct

ggtttgcaaaaggctttgagtttttaaatgtggctaattgttgtcaacactagcatattt

tgtttatgtattttgttgatgttcctaagttgtataaaatgtaaagggtaacaatatgtg

ttaaaaagggtttcagtggaaactcttaaagcatgctgtaatattttgagatgataatgt

catccagtatatagtgctaatttctttttgctaattatttaaatatcatgttttttgcca

attagcaaagtaaacttttaatccaaaaaaaaaaa

>fava_dn_rep_c793

ggctatcagttcgagaagcttccggaggattggagatgtcctacttgcggcgcggcgcag

agtttctttgagagtaaaagtgttgagattgctggctttgctcagaatcaacagtatgga

cttggtggaaattctcttacttctggtcagaaaactgtgcttatttatggtactttgttg

tttttctttgcactttttcttagtggttacttcatacaatagttgttattatcattcttt

tgagtttttttttttaatttctgttggatttgagttgatgatttgttggtcacaagtgta

tgtaatgttgtttgtcaatactcaatagtaagagccaagtatgaggattatcaatcaatg

atcttacacactacaaaccatttcttctctacaaatttctctatggaggaaaactaggct

agcccaagtttttgttattttggtaaattatctatttattgaatgtgactcttgatgatg

atgaaggaattgaagggttgttgttgtattaa

>fava_dn_rep_c794

gtgtggtcacgcgctaaatggcgtgttaccataccataacagcaatttcagtcttagcgg

taagaggaagagtagtggctatccatggttcccatttcaaacacaatcctatcaaatggt

tttatctctaaaccctatcatcaccttcgcctcaacaacgaagttggaaacgaagaatca

tgattcatccaggtattctgttggagcatccttacccttttttgtaacgtctagaccgag

aatgtttgtcaaggagaaaagattcgggcgctgtttttcagttacaaatagtgatcaact

ttcagcgaatgcgagtaataaggatattggcagtgctgcgaatgatcagttaccgtccac

gaatcctccggttgagtcagaatcacagacaccagagggttccaatgggtctgcagcgtc

tcctaattcacaatctgtaaccacacgatcgtctcagaaagtgagagagaggataaaggc

ggctcgggttctcaaccagtccaaggaaccgaaagcgtctaagtcggatatgggcagtag

tgttttggctgcatttagagagagcgataaagggaagaagaagagaagatctggacttcc

agaagcccctggtaatttgtttgacgatagcaaacgagggatgccgaagccaggtttgac

ttttgattttcccggcggttctgatctgtttctgattatcttttcgtttgtttttatcag

cacagtgatgtttggaacaactttcattgtgtggaaacttggtgcaatccattttaatga

ctactaagatgagatttcaaagattcacacttcaccagatatatagaatagcttatttat

aatattttgacaagcttcagcttctttaaacttcatgaactgctgttgatattcagttta

ctgtgtttttgatgctcagctttttctattttctctattatttttgttattatttcatat

ggtggagaaatttaattcagaggggatgcccaagtgaatatgatatataatgctaatatt

tatacatat

>fava_dn_rep_c795

aagtaaaaaagttttctaccattagcttaaaaattaatctctttgaaataaaggcaactt

ccctcctctttacctaggctctgccaaatggttgctcctaagaatctaacagacataaac

atagacaaacacacagacatgtggctttaaataattcaattgacttcacaatcaaaatgg

tgaacattaaacaatactatttgattatacatgggtaaaaaaatcaaattattcttcagt

aatagaccgaagttctttttttaagacaaataatacatgcttgagcaatgaaaatctacg

catgctggaaatcatgatcgcaaaagttaactcagcttcttgcacctttaactaataaat

ctctatgccttgtttgcaatgttgtttgagagccagtcaagtccttcataaagtccttca

ccagatgtggcacatgtgctctggatgtaccagtgacgctgacgaagagaatgaagacca

agcttatcagttatctcagcagcattcatagcatttggaagatcttgcttgtttgcaaaa

actagaagcacggcatctctcaattcatcctcattcaacatcctatgcagctcatctcta

gcttcaacaacacgatccctgtcgttgctgtcaaccacaaaaataaggccttgtgtattt

tggaagtaatgtctccacagaggacggatcttgtcctgacccccaacatcccagacagtg

aagctgatattcttatattcaactgtttccacattgaacccgattgtaggaatggtggtg

acgatctctccaagcttgagcttataaagaatggtggtcttaccagccgcatcgagacct

accataagtatacgcatctctttcttggcaaacagccggctgaacagcttcgtgaaagac

aatcccattttcaactattcttgatgaaaagagcgatctgggaagaagaagatgaaagcg

gatctcttctccttgtaacaaagagaaaagaaatggaggttctctcaccgttaggcctac

gcggatttatagaatgagaaacacagatccaaatttcgtaacgtgtttattcggtccgtc

>fava_dn_rep_c796

ccaagggagcgaaacgtctcgttattatcggctggatttgtcttgtgttcaacatcagtg

tcttcgctgcacccctcttcgtcatcagcaaagtcataaggacgaggagcgtcgaataca

tgccatttttcttgtccttctttttaaccatcaatgctgttatgtggttcttctatggcc

ttctcctcaaggactactatgttgctctaccaaatacacttggatttgtgtttggaataa

ttcagatggtgatatatttgatatatagaaatgcaacaccagtgctacaggcaccattga

agggtcaagaactgagtggtggccatatcattgatgttgtgaagactggatctgaacaca

atcctggaggtggaggaggtgctggtagcaaattctgattaagggatccatgatcaagaa

aaataaacaattgaagaaaaaaatacttaacctaaactttcaatttcatataatattgta

aaatgcaagaagggtgtctagtttcttgtataagtgtttagttttcattattattattaa

ttaatgtaaaggtgtggttttaatttggtgtaattggttatggccatgaaagcaattata

taacaatattcctaagctttggccatatattataactctaataaataatttcaagttcac

ttttccttgtatgatcaatgttccaatattaatgaaaatgaattgttttagtaaaaaaaa

aag

>fava_dn_rep_c797

atttatgggctttagtattcaacccgcttatttttattcttattctcgttgttgggggaa

ggtggaagcattcaacttttcaaaacctcatttgcaagatatcaaacattcaacttttga

aaacctaaatcaaagccatggatacaaaattgacagaagtttctcagctcttggatcgat

tcaaggctgcgtttctccgaaacgacttcgattccagctccaatctcctttctcagctta

aggttttactaacagggttcagaagccttccacccttgtttgcagatacacctaatgcag

ttcaggagttaactatagcaagggatatatatgagcacgctgttgtccttagtgtaaaaa

ttgaggatcaagatgcctttgaaagggatttcttccagttgaaaccttattatacagatg

cccgtaatcgtcttccacaatctcctcaggagtacccaatacttggtctcaacctgttaa

gactacttgtgcagaataggattgctgaattccataccgagttggaattactttcatcca

ctgctctagagaatccctgtattaagcatgccgtggagttggaacaatcttttatggaag

gggcttacaaccgtgttttgagtgctaggcagacagtgccacatgaaacatatgtttatt

tcatggatcttttggcaaagaccatcagagatgagatagcaggatgcagtgagaaggctt

atgattatctttcaatcaatgatgctaagcaaatgttgttgttctccaaagaccaggaac

tcttggagtatattaatgaggagcaccctgagtgggaaattaagaacggctctgtcttct

ttcaaaaggcaaaagaatctgcaccttgcaaagaaataccatctctgcaactcatcaacc

aaacacttagttatgctagggagttggagaggatcgtctgaaaagaaatgcatgcaaatg

ggtctgagttttcagaaattgctaatgctgtcttttaaacttgtttacttcttattgtgg

aactataaaaatgatttaatctctgtattgtttcttggtgtaccttttcctttcttgtgc

taatattataacatgactataactccaattgttcgggaaacgcaatgaaacaacaaaagt

gtatcactggaaatgataatgaaagcttttaccaatgatttaatataaccaacgataata

gactcgtttctgc

>fava_dn_rep_c798

cttgaacttaaacttgtagtaatttcttataagttgactttactattctcaagctagatc

ccaaaacacatgagttaacactaatttactaatcacctaaaataaccaatacaaaacagc

accatgacataattaatgaattaatatacattcaaatatttgacatactatatagtttta

tataacaataagtaaatgagaatgatataatacaaaaagcaaataaagcactattaattt

ataatgtacaacaacagcatttgcaatacactactaacccactttcggcttcatagtgtg

tctgccaatcactaatagaataattaacattaatcatcactccaagactcttcaacttct

tcttatcatttttaaattacagattagtaacactaaaacgcgcgtccacaccatatctac

ctgggtctacccgcattagatccagcattcggataacctgccccaccaggaacctgatgg

taaccaccagaaccgtacacgtgtccctgatgctgattctgctgatgatgatgctgatgc

tgcatgatcatttgaggagcaccgtattcgaaaaccgaagatgaagaagaagaaggagca

acgtctttgtcacgtgccgcaacggttctctcaccttcgatctctctgaaacgttgaaga

taaatcttcaacggttcaacgtactcttcaaaccctaacgtagtcatcgcccagagtaga

tcgtctccgttgatcgtcttccgtttctctctctgacacttatcggaggcttcgccggta

atgaagcttatgaactccgagacgcattcttgaacggtttctttcgcgtctttcgatatc

ttcgcgttcgccggtaacgctttcttcatgattctactcacgttcgctatcggtaggaat

cggtcttgttctcgaggtgatagctcgctattacctgcgttcggtgctcctcctgactcg

ttgtctgaatcggccatcaactcggtgtcactcactgcgacttggaagcattgacggtgg

tcggagaagtgtggtcgcggctccggtaaggagatgagagtggagggaaatggaatgagc

ctcgcggcgg

>fava_dn_rep_c799

gaaacatctatctagcgggccgcggggacaaaacaatagggatcgatcgagagcataggc

acagatctagcactagcaccaccatttccgtgcctctcgctcctcccactttctctctcc

acaaatcatgggattcccatccaacggctcagaggaaaagtccctcaagtttctaatata

cggtcgcaccggctggattggcggactcttgggcaagctctgcacagcccaagggatcca

attcgagtacgggtcgggtcgtcttgagaaccgatcctccttggagtctgacatagcggt

ggttaagccaacacatgtgttcaacgctgccggtgttactggtcgacccaacgtggactg

gtgtgagacgcataaggtggagactatcagggctaatgtggtggggactcttactcttgc

tgatgtgtgccgtgaaagaggactggttgttattaattttgctactggctgtatttttga

gtatgatgagggccatcctcttggatccggtcttggatttaaggaagaggatactcctaa

ttttattggctccttttactccaagaccaaggctatggtagaggatctactcaggaacta

tgaaaatgtatgtacattgagagttaggatgcctatttcatccgatttatccaaccctcg

caatttcatcacgaagatatctcgatacgagaaggttgtcgatattccaaactcgatgac

tatattggatgaacttcttcccatttccatagagatggcaaagagaaatctcacaggaat

ctggaacttcacaaacccaggagttgttagccataatgaaatcttacagatgtacaaaga

ctatatcgattctgacttcacatggaagaatttcactcttgaggaacaagccaaggtgat

agttgctccgagaagtaacaatgagctagatgcaagcaaattgaagaaggaatttccaga

actgttgtctattaaggagtcactcattgagaatgtgttcaagccaaatcagaaggttaa

agcttgaggagcaaggttcacaattttatcgaatattatcattgttttaattatttgttt

cagtttgtgtggatgctgcaaagatatgttgtaatttttatttgattcaatgtctgtaat

ttttattatgttgtatttttattgactccatgaataaattagctccaagttactcaagaa

gaatggaaattttgtatgcttttgcttgtatgattattaattgtatttgactttgagcat

caagctctaaggggattataagcatggtggtttttgaggaaaaaaaaaag

>fava_dn_rep_c800

agataagaataatttctctccctacaaatccttttnnnnnnnnnnnnnnnnnnnnnnnng

taccattcagcctgcaatttgtttttgccactgacttcaaccaaccaaatactatgttaa

atttatttttatccgcaaatcttaactcaacgtggtaagaatatattccaataattataa

ctttttttatatgcaatagaagaatagacataaagcatatattattctcttgtgaattga

atagttttcactctaaaaagccactttactttgtttcttcaacatattgttgcaagaaag

tgttgttaaacttgatggcaccttcttcttcttcctttcctcttcgttgggagagcacag

gagatcaatggtggtatgcaacacctattgatttagcagctgcaaatggactatatgatt

tagtccttgagcttcttcatcttgatacaaatcttctcattaaactcacttctcttcgta

gaattcgccgactcgagactgtgtgggatgatgaatctcagtttgaagatgttgccaagt

gcagggcttatgttgcaaggaagcttatggttgagtgtgagaaaacaggaagaggagaga

attctcttgtgagtagtggatatggtggttggcttttgtatactgctgcttctgctggtg

atttggagtttgttcatgagttgttgacaagagattctttgttggtttttggtgaaggag

agtatggtgttactgatatgctttatgctgctgctaggagtaagagttctgaggtgttta

acatgattcttgattttgcactactagggaaagattgtggtagtgttgaattggatgaag

gtggtggtggtggagttttcaagaaggagatagtgaatagggctattcatgctgctgcta

gaggagggaattgggaaatactgaagaaggagcttattttaggaagtgcttttcagattt

tagcttatagagatgctcaaggatgtactgtcttgcattctgctgctggaagaggccagg

ttgaggtggtaagaaatctaattgtattatcacctgatatcatcaactcaactgattcac

aaggcaatacagcattacatgtagcttcttacagaggttaccttcctgtggtagagattc

taattcaagcatctccctcgttaacaaagttaaccaatcaaaatggagacacttttcttc

atatggcagtgtatggtttcacaagtactgatttctgcagactagacaaacacaccgagc

tcatgaatcggttattatcaaccaaaaagatcgcaaacatgaaggacataatcaatgtca

agaacaatgctggaagaacagcaattcatgtagctgtgattcacgatgttcaatgtgatg

cagtggaatcaatgatgtctgtggcatcgatcaatcttaatatccgcgacgcaaatggaa

tgacccctttggattatttaaaacaaagaccaagatcgaaatcttctgaagttttaatca

aacaattgatttcagctggaggaatctctaattgtaaggattatattacaagaaatgcga

tcgcgactcatttgaaaactcgtcatggtattcaaggtagtcctggaacatcctttagaa

tatcagattctaaaatattcttatacacacaaattgagaacgaaaattcatctgatattg

ctaataccgatcaagcaagcgtagaatcaaatgcatactcaatcgaaatagaggactatt

acgagtcagctgcagattcctcgtgtaacagcaagactagttctgccgattctgttgcaa

aacggttgaagaatcttctcaagttcaaggaattcaaagacgacgattctgttaatgatt

cttcaagttcaaggaaaaacaattcggaagagtttccgatctcgttaagacaaagatact

cgaaacagtgttctcttccaaacaacaaaagaacactatctataaggacattatttccat

ctccatctgcaaaaaaatacttcaccacaggcctaacacaaggtgtaatcaaggtaaagc

cacaaaaagaagggtctaatttatctcattcttcaagcaataagcaagaacacgtcgaca

caatggacccggcttcttgttccgacggacaaatagatgatgggggtaggggtaccctac

agttgaactataataaggggagaaactttaataagaaattgatgaacaaatatttttctt

ttggagcacaagatcagagtttggaagatgctaacagttgtacaatgtcaaaacacagtt

ctaagcgtttcagtttagttgcataaatgttaccatctatgaatgagcttactattttat

caattaatttttaactttcaatttctaacaagccactttaactatatttttcaaagatat

tatttttataatttagctttaaaataatatttcagctcaaaaagggaatgaatgaggtaa

ctttgtggcaaagagaaaggagagaaaacatgaattcatgaaatggttttctatgcaaaa

ttgatgttccattcttcaatgtcatcatttcaacatgtcaccgagacccggtggcattcc

caagctctgagtaagatctctcatcctttccttcatggccttcacacttttctggtgcgc

atccttgtaagcctccgtgactaaaagtgcaactttttcagatcccaactccatagcagc

gtcggtgatttgtgtttgtactggttgttgattgcctgatagtgtcacctttattaactc

accctcacagtaaccatcaaactcttctgctgcaagttctttctgtattcggctagattc

agtttgaacaaccactttcgccttcttcactgcctcaaacagattttgcatgtttccgag

cattcctgcttgaccctcattatcttcttttcctcctccgaaaagggcttggactcggaa

acatctatgaccagatccaactttttgtagtccacgccgggatactagtctcatgtcaac

tacattgctactggagcttagtttacgtagagatgatgaaggtgggaggttgtttttgca

ttgagtgactccatggatgtggcctgcgagagaaaccgtaaacgccattggttagggtat

tgtttagacgtggtggggaagaagagaagagaaaatgggtaggataggagtgagaaggaa

atgcaagcggtgtatcagttaaaactcaaaccgaaccaaaacacaacgctccctgaatag

gattgtgttacattaactttaacgaaactcgattcagattactgttac

>fava_dn_rep_c801

ggccgcggggacttgagaaagattttgtagcagactaacatatagccatgtcagtatact

ctacttcaaagcttaatttctttttatgcttattatcaacactttttctactttcatctc

ttcatgctcaagctgaatctcagaatcttaaatactgtgaaaagaatgtagagtatgcag

tgaaggttagcagtgtcgaaatattacctgaccctgtggtgagaggagagccattcacct

tcaaaatagaagcttatactgatgaaccaatacatagtggtgatttaatatatgaaattt

catatgctgggatagaagcacaacctgcaatattttaccatgctctcagcgaggaaacac

cgcttcccgtccgtccaggccgctttttgctcactcatactgaattattgcctccagtca

ccccgccgggtacctataatgtgaagctgaccttcgatgatgagggcggtgatcagttaa

cttgcatcgtatttcccttcaccatcggtgctaaatcttcagtatctgtttcttaattgt

ctaggactgctctgaaatttaagcattgtttatggtttacttttgaactcaaatgttaac

tagtcaagtgtcaagtttcgggctatgactgcaatgttcttctactcttaataataaata

agatgtctgaccctaatttaactgtcaaaaaaaaaaag

>fava_dn_rep_c802

taactgaatccgaatcctagatctaaacatcctttcaactcgttaagatcctcgtcggtt

aagcttttcgtccttggaagatcttcactgacgaagccttctaacaacactcccttctcc

ttcttctttttcatctttcttttccattcacagcttccacttctttcaatacttttcttt

tttttttttaagagaaatgaaaacaataatatttgcttcttcttggtaactcagtacata

gtacagatagttcatgacacatacaaaagaagcaattcaagtggtacaacaaagacataa

taattaaagaaagaaacacataagcattaaaaagagcacacaatgatgagaggtcttatt

tagattatgtatgtatgaccctgctattaattatagtatgtatgacccgtgaaataaaca

caaagtactagctgggtaggaagctagtaagtagtcactctttagtgtagagcttttgaa

gctgtccctcaagtgaatatgtgggggctgaggcattagcattccaaacaaaaactcctg

caagtaatgcacgttcaatgaggattctgcatccatcaatacaggcttgttgactcgttg

gagttggagtacttgtatcactgttgaatcctgccaagattttaagcgctgtaccataat

cattaactagattattaaagaagtttataaattcatattctgagcgaaaatactgattgt

agaacttgtagttaatccaatcaatattgtctggcttgttagtgtacaattcgtggtatt

ggaggttgacaagctctgtgggagaaatggacaccacctttatggagttggaaacatcgg

gatcatccttaaggcgttgtatgacttgccctatgcaagtgctaaattgattcatcttac

cgatattgatattgatgtattcataattaatatcaataccatcaattaggttatcgtcgc

tgacacgtggagtatagttctcgtagtcttggatgagctctttgattgatgttacggcat

tatcaatccaactactgtcatcctgaggattgaatggaaattcagcacctttgcctccta

tgcttattaccacattcacgttcttatgctctttcttgagattcgccacttttgttgagc

taaagtcatcaaagttccaagcgcgcttgaagaatcctgtacctgctccatcatcataag

tgtcattggcaaatcccagaatgaaatggaattttgggatgtcgccaatgatctcagatg

gaaaaccatttatattttctccaacaatgtactctcggaaaatggtaggcttcacaacgg

ctttgatatccggttctgtcattgtgactttagttggttggtggtggttcccgcggc

>fava_dn_rep_c803

atacagtcaatctgattaagtgtgaaatgaaatctaagggaagcattcaatatataaaca

atagattataaatcttattacaatttgcagtgtattgaatacacattatctccatgtata

catttgcatacaatgaatacataaaaacacataaaagaacggaaataaaaaatttcagga

tcttaaccttgtgataaacctcaacctttcttctacttagcagctacaatatcttgaaat

agtaactccaaatctttcaagcttatgttactcaagtcagggtagattgctacggcctct

ttgaaatcctgatcagccgtggaagacgtgtaggtaaccacgcaggacattcctgctttt

gttgctgcctgtaaaccaataacactatcctctaccaccaagcagtttttctctgataca

ccaagcttcttagaagctgtcaaatatatggatgggtcgggtttcttttccttcacgtca

tcaccagcaaggaagcaatcaaggctttgaaagcgctcgattccaataaggttttcaaga

caaagaataactgaacttttagttgctgcagagcaaacagctagcaatttcccggcgtct

ctggcctcgtccatcaatctcaaaactcctggcctaggttttacagagccagacttgatt

atatctttgtatctctctgtcttccaatcctgaagagtgtcaattaacttggcttgttcc

tcatcattagctggaggagtttggaacaacgttgacgaaggccacccatgttccttaaag

taccatcgcattttgggtttgccgccaccgatttggttctggagttgatcatagaattga

atatcccagttgagtggttgagaagaagaagaaggaggagaacgaacattgaagtgaaga

aaagcatcgttgtaagcttgacgatgtaagtgttcggattccaagatgacaccgtcgcag

tcaaatatgagagcttgaaacgaagatgaagatgaagatgaagctgagagtttgaagttg

aagaagagacggtggttattatggttaatgttgaaagaaggtaagttaatagtagttgga

catcgaaaactgtgtttggtttgagagagataagaatgggaagtggagggagtgaaggag

agagtgtgagtgagagtagaagccatggaaatcttcattaaccatcaaccattcatcttc

atcatcactttattcatcactctttcttttgtgtggctctgcattgcaattaacttttaa

attctatttttttct

>fava_dn_rep_c804

aaaacaaagcatgcctctcttaatttcaaaacttatttcacatgtagattgacatttaga

agctgacaagaactgcaagataagccacattttatttctaacattgaaatattactaaag

gtgtgtttgtttcaagtttagaaattacattcttgggaatacaaaggtaaacagtgagga

aactcatgtttgttacaaggttgaaaaaaattattcctatgaataatgattccaaagaac

aaaataacttcttataacttcttttattcccatagaattattctcatgttaaagaattga

aaagtggattcccattaaattttatatacttttttttaaataaattaataaaaatttatt

catggaaatagtaaaaattttgccaaacaatttttgagaagaataccttggaataaattt

cctacatttattttccttggatagtaattccaaagaataaattttgacaccttgaaacaa

acgccccctacagtccgatacataaacaacctctgaaaatttaatttctttaaaataaca

caagtaaaaatccttaattttattttttcagaagaagaggcccaacattatctccagttg

ctttgttgtgctttacatggcttccatcacatagaggaaaagttcctgatctccaacatc

tgcaataagcggtaacaggcttagagacttcattaagcacaacagaatcaacaacttttt

cttcattcttcctcacatctggatttatactactcacacccccagcttccgctttcacca

ccaccacactcctaactctcctaccaaaggaacaagtgttgttgaaattggtgcctaaga

atccatttttggtctcaccaaaacgtggcttttggcaaaatactacaccaacttggctta

gaacagattccattgaatagggtgtgaatttatgtttgtgttcaaaagttcttgggtttg

aaatgggttggtggtggatgagataagaagagagaagtgaagtgtttgttacacgtgttg

atggacttgtggatagcacactccactttctatggtgtgtggctttgtggtaaaaggctc

aactcttaagagt

>fava_dn_rep_c805

agagttataagcatatgtaactactcaacacgttctcatttaatggctatgcctaacttg

agtaacttccaccatccttcttcaatacggcgctattttcggtttcatatataaacagaa

gaaagttttaggtctgcaaaagcagaaaaatggtgaaaatgagtatcaaagaaacacttg

tagtactctttgcatctttcttgttcctatcttgtgtctatgcacaagatactattgtac

cagctatcatcacatttggtgattctgcggtcgatgtagggaataatgactaccttccta

ctctttttaaggctaactatcctccttatgggagagattttgttaacaaacaggctactg

gaaggttctgcaatggtaaattagctactgatataacagctgaaacacttggttttaaga

gtcctgcacctgcatatctcagtccacaggcatcagggaagaacctccttactggagtaa

actttgcttcagctgcatcaggttatgatgaaaaggctgctgcgttgaatcatgcacttc

cattgtcgcaacagttaagttatttcaaggaatatcaaggcaagttggcacaggttgctg

gcagtaaaaaagctgcatcaatcatcaagggttcattgtacttattgagtgctggaagca

gtgactttgtacaaaattattatgtcaatccttggatcaacaaagcaatcactgtggatc

agtattcatcttacctactagactcattcaacaccttcattaagggcttgcatggattgg

gagcaaggaaaattggagtgacttcacttccaccattgggatgcctaccagctgcaagga

ctttattcggtcaacatgagaatggttgtgtctcaagaatcaacgccgatgcacagggat

ttaacaagaaaatcaactcagctgcatcaaaccttcaaaagcaacttccaggtcttaagg

ttgtgattttcgatatttataagcctctctacgacctcgttcaaaacccttcaaatttcg

gttttgctgaggcagggagaggttgctgcggtacaggaacggtagagttaacatcgttat

tgtgcaatcaacattcaataggaacttgttctaatgcaactcagtatgtgttttgggaca

gtgttcatccttcagaagctgctaatcaagttctcgctgattctttactcgtacaaggca

tatctctcatcacataaggaacgactcgtgacacgattgtttatctgtgctaaatattat

tgtgtacaagtacaactacacattattattaaggtctttcaaaagtttcctgtttttcag

gattatgctacaagaatctctattctatgtatcttatatgacgcaaatttcagattaagt

tgtttgtaaaacatattataatacacaagtttctgcttactatttataaacacattgtaa

tatctttcgttattgaacgacaaatacctcttggactgcacaaacgatttctcttctccc

tcgcgcaatatttgtcccaaattgtggaaaaccaggtacctgccacgtcagtaacataac

agaatgtgtgaaaa

>fava_dn_rep_c806

atgaagatttagcaggttatagcagggacagtctattatccccctcctgcattagttacc

attagatgttatctcagacctttatatcatccatgcaaaaatctaattttgttacttgat

ggatttacattcatggcatctaaacttataattaaataggtatttatacattgaaccatc

aacttgtgcatgatattgaattaggtaaccattttacaagctttcaccaacatatacact

ccctaaaaaaacattgtaagccttacaacttgctaaaacatgttcaccatttgtttcttc

aaggtttacagtatcatagttgtcttagcccttctcacttgggagtgaaagcagcaaaaa

tagtagcatgaccaggatcagcaaggtgagcaaagaggttatcgataggaccggtgccgg

tgtaaatgtgctggaaccaagctcccataacagccaacatagccaatcttccattcttaa

tttccttagttctcaactccttaagcttctgaggagaagcacttccccaaccaagtggat

caaaccaaagtccaccagggtaccctacatcagttcctgtgagcttgttgtttggaaaga

tggggtcagtgttgacacaacctggattgaggatgtcggcccaccttcttccctcggccc

aaccgatgaaaagaagctcgactatgaagagtgtggttgtgtctgtgaaatactcttgct

caccagcagtgtaccatgaaggtgtgttcaagataccaatctttgttaggaattctggaa

tgaaaattccagctgcacctaacattgcccatctgcagtgtacaagctcggcttgaacat

tccatctcagactctctggatcagatccaagaccaagagggtcaaatccaaaatctcctg

ggagacttccatcaagccatggaggaggggtgcttccagggaaccacagaggtctatcag

gctcagcaactgtacacacagttgtagtagatcttactctaaaagatgatgcagtcttat

tgttcaccttcagtttcctcccaagaaaagcttttgagcttcccaagggtgatccgttct

tctgggaacttggagtagagatggcaacagctgcaatggcagaagaagcacaagcagaag

ccatgacacaggacttgtcctcaagagataaaagttattaagtttagtgagtttgtggtt

gatgtgaatccgcggcagaaa

>fava_dn_rep_c807

aagaatatagcccctagtacgacaaacaggcagacagctagacttttgattagaggttaa

agggagaactgcgaggagaatcgagattggcgcttagaacgcaatgttagggctatttgt

acagacagacataggggtagattgcttgtacgaggataaataaaggaatataattgttat

ggcgttaggaacgagtccacacttggttccgaacaaatgactagaattcgttgagccagt

ggcggaaggataagggagaagagcaataagctgcttgaatcttagaaagaggagatccct

ggtgaggacacaattaagaccagacagccggatcagagagatatgcaccttgaatttgga

aaccctcgaaaaagtggttagaacagatgctttccccacctaggacttagaccgggcatt

tcagtgcttggtagtagcgcataccaggacgagaggttatggccccttttggatagtgga

aaaattgtaccgaacgagacctgctgacgttgtttattttgggtgaacttaaacttaact

tttaattagctcaatcaaaatgcctatctacgcagagagcatgccccccttcaagactta

agacaagggaaaaaagagaagacagatgccgacacacttttttgagatgagctaaggatg

cctaacgagcagacgaggaaatgcagtagcggaaaggaatggaccactaatggacaatgg

tcccgcgaacgagacccgaatactgactgcgcaggcgaaaagaaccccgtgactaagact

gacttgacgaagctagactttggcccattgacaagaacggaaataaccaataaactgtaa

cagattgcagcatggggaaggaagtagaaaatagaagcgactgtttgaagtttgccggct

taaggctgaagataaagtatatatccatttcatgacttttgattagagtaggtttaggag

aggactaggagactgagatgcctttgaggagcgtttagggcgatgagaaggccgcgagga

cgtcaggcgaaaggtaatttttctaagtgaggtagcctgccccatatcctcgctgcgtgg

gcgtccttgtagaaaggaactcaactcaaaacgaaccgaaaacaactgcttaatgctttg

gccgctgaagaggaagaacaagagtctggttgcgctttggggctacttcccctaccactg

ttaaatagcatcctcctcccttaatagtaagcaggctggcttctcccaatagaatgcctt

aactgatgagtgacgaaggtagctaacctccatcgcattatccaagaagccctcttttcc

gagtctggaaagcagctaattgaaaaggatgagctagttaatttaattagccaagaaaga

ctaagaagaatgacggattttgaattctcggactaaaccccgtaatcttcctgcctctgg

tcgagctaatcgacggtagcgagcagaagaagaagcgaagctacagcagtcccattcctt

cttatctacgtaagacatagaagagagttcagctagctttccgtaacgggctactaacta

cgactttgcttccgcagcacgtcgaaggtcttcgtcaggtgtcggaatcacattttgatt

gtggtctatgaactcaataccagataaagaagaagcagctaacaactgaaagaatagagg

gaaggtagcatttcacacttctctcttcgtcattctaaagcaaggaatgaagtagcccgt

atagcaagaaggaagggtataggaaatgcaatgaacttcatcgtaatagatagatcataa

tttaatgcgtcgaactcgtatatttagagtgacccctctatgcctactctactaatgacg

gcatctaattaataatatcgtcataaatagtaggcacgatgccaattccactccattatg

agctttcctaattctcccagttgtgcctcattctcctaggaattctaatgacggctacga

tacaaaggaagccgaagaagagctagcaactgtcatactataataaaggcagtcgcagct

agatccaggttaatctaactcatgtgctattaactcaattcttaaatcataggtagctac

ttccttccaacttaaggcatctgacgctcaattattattgttgttgtcaagtcttggact

ctgttaaaataggaatctgtaaagtgttccatcaactaaataccctgctgcttctcaatt

cctgattaaagaagtgccagaggaattcggattaaagaaagtctcgtaggaaagggagcc

aatgatagatgctttgaaatagcgtaatagaaagaaagattgtctcaatatcaaggcaag

tcggaggcgagtaattgaataaatcccttccatggattttccggaagagtcactcgccag

aacaggggaagggaattcggaactcaatcaccaagtggaccttcccattgtctatgcctg

caacaaaggaagaggtgagctaccaactcgtcacctcccctctcgttccacttctgtctg

ttctttcattgtaaagtgtcggactctggtctttttggaacatgaaagtgttccgtgagt

gagatttcccctctcttctttaatcttttcttcccaaagctcactaatttagtgatagag

gctctaatgctgctagcgacgggtaggtactaggaagagttgacggtattaaatttatcg

acctatagcgaaaaaataataagagaagaaagctgctagcagagggtggagaagtcgtct

ggttatcaaaattatgtatagaaagaagattcgtcttgccggtaaatgctttagactgaa

ccttgttctctatccgctagcacgcagacaataactaggacaaatttatggcaaagtaag

tgtgcatcgaaggtaaataaactcgagtcataacatttctactagtgcttaggtgggctc

attccgaattaaaaaaaaagtgcttagtttcgttggaaaaaccaacgcaaatattgactt

tcaagtctctctttgggggggagcagagcacgttgacagaggttcatatgaggcgtgaag

gtgcgttggctctctatgggaatactatatcaagtgatcttaaatttatgctttccgaaa

cggaaatgtctttagatagtctgtcaagtacctcttccactaggtcagacgctggaagcg

ccccgactgacccggtggagatacttgatagaatatctactgaatcttcgaagtgggtcg

atcttagtggtcggcaattacctcctgaatggagtatgcctgaccttgttcgggcggtaa

tagccgacgatcgtatttacaatgagggctttcttacatttttgtattacgatatgatgt

tacagggacaggatgcttggttatgcgaagaaattttaacatttttagacctcattaatt

atgtcttctagaaatgttaaattttctttttccatgctttttctttggtcaacaaccaag

cacagctctctatagttattcactactccgtgcaggaaagactatctttctgtctggagg

gaaggctttttggactaagggtaccatgatactttctgttttgtcgagccttgctttggt

ctctggtttgatggttgtacgtgcaaaaaatccggtacattccgttttgtttttcatcct

agtcttttgcgacacttcaggtttacttattttgttaggtctcgacttcttcgctatgat

cttcctagtagtttatataggagctatagccgttttattcctattcgttgttatgatgtt

ccatattcaaatagcggagattcacgaagaagtattgcgctatttaccagtgagtggtat

tattggactgatcttttggtgggaaatgttcttcattttagataatgaaaccattccatt

actaccaacccaaagaaatacgacctctcttagatatacggtttatgccggaaaggtacg

aagttggactaatttggaaacattgggcaatttactttatacctactatttcgtctggtt

tttggtttcgagtctcattttattagttgccatgattggggctatagtacttactatgca

taggactactcaggtaaaaagacaggatgttttccgacgaaatgctattgattttaggag

gactataatgaggaggacgaccgccgagagacctcaggtcaggcggggctacccgctgaa

tttaagcatatcaataagcgaaaaaaaaaaaaaagctagggttttacgcttgcttctgca

tctgcaaaaatgaccaagttcaggaagctgggtcgacccaccggtcaccgaatgtccatg

ctcagaaccatggtctcgcagttggtgaagcatgagcgtatcgaaactactgttgccaag

gctaaggaggttagaaggcttgcagataatatggtacagcttggaaaagagggatctgta

tgtgctgcaaggcgtgctgctagttttgtgcgaggagacgatgtcattcacaagctgttt

acagaactggcttatcggtacaaggatagagctggtgggtacaccaggttgcttcggact

cgtattcgagttggtgatgctgctccaatggcctatattgagttcattgatcgagataat

gagcttagacaatcaaagccaccaactcctcaacctcctcagagagcagcccttgatcct

tggacacgatcccgtctcaccaggcagtttgctcctcccaaagtggaaaaatctgattct

gatctctgattcattcgaatgtattgcagaaactatatgtttttgtccccatagcttgag

atatctgaattttagctttgcttattttataaatcttctcattgcttgcaataataaaaa

taattatcttcctactacagagaaatgttctagaataaatcatttcactgaaaccgcaaa

cgatcagggaaatgaaaaatctaaaagctccatgtgtacatgattacatgtttaccgaca

caaactgacaattatcgatgtaatttcgtaactgttacaatggtgaatgtgtaaacaaga

caggttaggtctgtttacttgccatatgaatatactatttcaatagaaaaa

>fava_dn_rep_c808

tcaatatttcttttatttcatctaannnnnnnnnnnnnnnnnnnnnnnnnnnnnnnnnnn

aaaagttagagaattgtgtccatcccgttgggtttgaatcctctacctttcatttcctta

atttcacctaaaatgtgagaaattgaagccaatttttcttcataaatggaaaatgaaatt

gtaacttaacaactcacactacaaagtttgagttggcacatatacatatacttgtctcta

tgtactcacactacaaagtttgagttggcacatatacatatacttgtctctatgtatgaa

atagcaactcgcacttcaaagtatgagtcgagaagagagtcatcacatcataaaagccaa

accgaatactataatcctgttttctgataacatacatgttgatattaaatcataaagaca

gacaacttattttcataacatagcatcaagactcttattattcgtgaaaagccagaactc

tacactcaagcaagctccttcagaaaatcttgattgtcaaccaacactgttttccaacca

tcgggatccaagaaagaagtgatctttgtgttaaggccaggaattggccctggttgcctg

gtaatcttccctccaagctcttgagttactatgttgattacctcagcactcttgtataca

tcatcagtgccaatagccacctgtgcataaccatttcccttggtgtattcagtgacacca

tagttatatgtcagctccaacacaactgtctcgtgctcctctgcatacccaagcatagct

aaagtgtacttgtattgaggtctatcaaccttcctcaccatcttcaaacccaaagtcctt

tcataaaaggcaattgagcggtctaagtcaccaacacggagcattacttggcataatggc

tcaggggttgaagctctttcgagaatttcaaaagcataaccatcaggatccttcacaaag

gccataacagttgacccaccactaactggaccaggttccctagtgacatttccaccctta

gcccgggcgtgttcaacaaatttgtagacatctggagttgcgatagcaaaatgtccaaag

ccagttccaatatcataggaggtcactccataattataagttaattccacaacaaaatta

gtttgttcagagccaaatcctagaaaagcattggcatatttctcttccggaacatctctt

ttcctcaaaagcttcattccaaaagcttcagtataaaacttgatggtgcgatcaagatca

ccaacacggtaaacagcatgcaggaagcggcgcttatctttctttggccattccaacagc

tcagcattagattgtgcagcctcagccatattcaaatgggttcaaatactcagtgagaga

gagagagagagagtcacggaggattgtaagagaagaagaacgatctcaaaaaacaaagga

agatgcattgctttaataacatttaataattgatagtaatagtaatgtaactatctacgt

ggcaaacgattatgagttggtgcatca

>fava_dn_rep_c809

taaggatgcaagcatcaactaattcaaaatcaaaactattacaatcaaattaaaactaaa

taaaattattacaaaacaaattaaaataaactacttaattctcttgttctctcttcttca

tgttgttgattagtcctcaccaacaagagagtctccaataaaaggcattagatgcaacaa

tgaaagaaacagtgtattttattaaaaataaaaatagcaaattttttcactttcaaatgg

gtacaaaagaggataataataataatatattattttgtattatacagatatctctaaaga

gcataaatggaagtaattacaaaaatgagcatgtaacccaaatccctgaagggatgagag

gaatattgttctgtaagtatgcactggcagcagcaactccacttcctaacttcactggat

aacccacatctttcagtatcatctccacaccagcaagacaccccaatagttgcaactcat

tcatgttgccaaggtgtccaattctgaaaaccttcccagcaactttgtttagtcccaaac

ctaagctcaaattgtatcttttccatccccttcttacaatttcagcaccgtcaatgtttg

gaggaacaacaacagcagtcacagtgtcactgaaccactcttccttttgtgtgcaattct

tcaaaccccatgcctccacagcaagtctggttgcggtacctaaacgattgtgccttgcga

aaatattttcaagtccttcctcaaaaatgagatcaagagcagccctgagaccatagagaa

gctgaatggaaggagtgtatggccaataggttcctaacttgtagaatttgaggtagtcac

cccaatcaaagaaaactctaagtgatttagcagttttcgaagcttcaatagctttagggc

ttgcaaccacaaatcctatcccagtaggaagggatagggctttctgagaaccagttatag

ccacatcaactccccattcgtccatacggaaatcgagagcacaaatggatgacactccat

caacaagcaggagagctggatggctgtatgcatcaagaagttgtctgactttggctaagt

tattggtgactccagttgctgtctcattatgaacaatgcaaatagcctttatggtgtgtg

cagaatctgaagcaagttttgactccaaaatgtcgagatcggcaccacggccccattcac

tttccacaacatcaacattaaatttaaggcgttgctgctgatcaatccaaagcaaactga

attggcctatcaggaaagatacagtacgatctccaggcgacaatgtgtttgtcagagcac

tctcccaagcaccagtgccagttgtagggaaaagaaagggtgttccggtggtagtcttga

aaatcttcttgacatcctcaagtagctcttttgtgagcgctggaattgcaggagaacgat

agtcctcgttgttccggctcatagcccgaattacctggtcaggtatgttaaccggccctg

gaacaaagagatggtttcttcctggtccataaaaattgtccatctttcttagcttgaaat

tggtccctctttgaactcaagcaactcttgcctttttttagcaccaaagaatcaccaggg

agaatttcatatagtttggtaaatatggcagaactaagagctgccactgctctatgctct

tagtggaactttattatataaaactataaatatgtaataaaagaatattgtaaatctgag

gtggggccaaatgatgttcaagtagtagataagagggagaaaaataaatagcaccttgag

gctaagaatggatgaaatggaactcataagttctggcttatatttttgcactca

>fava_dn_rep_c810

cttttttttttgatatgcttaaattnnnnnnnnnnnnnnnnnnnnnnnnnnnnnnnnnnn

nnnnnnnnnnnnnnnnnnnnnnnnnnnnnnnnnnnnnnnnnnnnnnnnnnnnnnnnnnnn

nnnnnnnnnnnnnnnnnnnnnnnnnnnnnnnnnnttgttacatacaccaacctatttata

ccatttacatggcgtaaaccatttctgtcagtgaactaatggaaaatacattccgttgca

acatgttgaactaaacccacgcacgagcatattttaatttcattttgtggtgaataaggc

aaaaatgtactaggttcataaatacattcaaataagagaatatagtaatgaactaggttc

aaacagccaattcatatatatatggtttacttcatgctgttgttactcataacagtagtt

caatcaattcagaatcatgctgacgatgaatgatcaagagccttcatttcaaagtctcgt

ggacagctgaaaaatcaaggtctcctaatcccaagcttctggctttcttgaatgcctcat

ttgctgcagctgcgactggcataggtacagcattttcatctccaagtgcaagagccaatc

tcatgtccttctgctgatgtttcagtgggaaagccggggcataactcttcttgagcattg

acggtcctttcagtttaaacatgccgttactgatagcaccaagatccagcacatcaagaa

gtgtgccagggttcaagccgcttttttcagcaagtgttagtccctcagagaaagcattca

tcatacttcccattaccatgttgacaacaagtttcatttttgcaccatctccaacctcac

cgagaaagaaagattttttcccgattacatcaaatgctggaagtgcttcctcatataatg

ccttgtgaccagcagcaagtattactagttggccatcttctgcaggcttcttgctacctg

aaacaggagcttcaaggaaatcaccgccttttgctttgattgcctcagatattttgatag

atgtctcagcatcaacggttgacatgtcaatataaccttttcctttaatttgctcaagaa

caccatccttatcaaacacaaccgacaaagctgccgaaggatcagacaacattgcaattg

tatacttgcatttcttgacaacagcagcaggtgtttctccaactgaagccccgtgttcaa

caatttcatcacacttggagagggttctgttccagacagtgactttgaagccatgtttta

gaaggttgattgacatggctttgcccattatccccaaacccaaaaacccaatctccattt

ctgcagttcagcgtggatcactctcttcggtctcagtgttctcttgttcttcttctcaac

ggagactctttatagcgtgaatgattgatatcgttaatatgtcagagttattaatagaat

gcttatcattctcttatttttttcttcctcaaaacc

>fava_dn_rep_c811

ttttttgatccgtataaagaaatattgcataactattgagagccattgactaattctctt

acccaatccgtatcattgcataagaacaacttaagaagacaagcaaaacataagcctgaa

tgctgaatctcaataattaagtcataacaaaacattgtttatactaacaagcactcaaaa

taaaatgcactaaacataacaaacaaaatcatctcaggttctttccttcaaaataaactg

caaaatacaagtgtagtctccgaaaaagaacaattcacttgagaggaatgaaccgggaag

agtgagtagcaccaataccaggtctaccgtgcttaacaggcttgtatgaaatagagaact

cggcaaggtaatgcccaatcatctcaggtttgatctcaacctggttaaatgtcttcccat

tgtacactccaataatgcttccgatcatctcgggtacaataatcatgttacgaagatgag

tcctaacaggttctggcttctcaccctgtggtgcctcccttttcgctttgcgaagctttt

gatgagagccatgggctttctggtgagacctctctggaaccttctacgagcacgagcact

gaagagcttgacgagctcatcagtggacatgtcgagaagtgcatccaaatcaacgcctct

gaaactgaacttcttaaacgttctcttctttggaatcccggccgcggcggctgctgcatc

gacttcaacgtccgccattgttgagatggttctgctccttcggccgcttctgggttttca

actgggagaacaaactcgttagggttttcgtttcgttttttataattagggttttgtaat

gca

>fava_dn_rep_c812

ccgcgggatgttacattatattccacccacccacctctctgtctttttcctttcctttat

ttttattattatcactacataaaaaaatatcacataatcaacataatcaatctctttcat

ttttctaccaatttcccatcataataacttgtttagcaagaaagacaagatatgaagaac

taattattacttcactttttacataatttattcttaccaaaaattcttttgaccaattga

gttagtttcaagatttcaatatggggaaaggaagtggttgcataccaagcaagaaaaaaa

caccagttaatgatgatccagaagagaatgaaccacttagtgttgttgaaactgaggttc

caaaaaatgaaacaactagcagaagcatagatgttgtagcaacaacatcagaaagattga

aaaagctgaaagtttatattgttttttattcaatgtatggtcatgttgagtcattagcaa

gatcgttgaaaaaaggggttgattctgttgatggtgttgaaggggttttgtaccgtgtgg

tagagacacttcctaaggatgtgttggaactcatgaaagcacctgagaaggttgatgatg

ggattcctttgatatcttctgagaatttggtggaggctgatggcttgttgttcgggtttc

cgacgaggtttgggagtatggcggggcagatgaaggcgtttttcgattccaccggccagt

tgtggagggagcagaagcttgctggtttgcctgctgggttttttgttagtactggtactc

aaggtggtggccaagaaaccacagcgtggacagcaataacacaactagttcatcatggaa

tgctgtatgttccaattggttatacttttggtgctggaatgtttgaaatggattcagtta

gaggtggatctccgtatggtgctggagtttttgctggagatggttcaagacaagcaactg

aagctgagcttgcacttgctgagtatcaaggtagatacatggctaatattgttaagaagt

tgggacagaaaagctagattcttttgaagagatcaaaattaagtttcattcaatgttgtg

attgaattcaaactcaaatgttgtgtttacttgttgttggtataagattggtaggatcgt

gaaccttgtcattatctcttggggggaaaaatgtacactttgtaaagttcatacttttgt

gattatgtgatgtttataataaaatttatgactgaggtgtatttcatggggattaattgg

tgtcttcataatggattcatagaaaaaagttcggtatccaagtagctcatcacttcgtgg

atccagaatttgtttgtcacagggttttaaattggagaatttctttctcatcttgttatt

gttatgttgagttaaacttttgaaattcaaatgtgtatatctaaatacattttgtaaatt

tatgacattgagttatttcaggg

>fava_dn_rep_c813

aacataaaaggaagtgcaaatatatttatgttggaattacaaatctagtgaaccaaaata

aaaaacaaatctagtgaaccaaaataaaaattcaaaattgatgtatagaaactaagatat

tcagaccatttctttttattgatctatatcattttcagcactaacaaagttcataacacc

aaaagcatatgataacttaagttgattcaactgcaagaaaatatttcagaaacaattcat

gtaaattgaaacaagtcatactaggataatccaaattacccaaaaatgttgatctaaaat

aaaaggtacaagcaacaaaaaatttaaggaaataaaaatgaaaattcaagttaacaattg

attccactcccaactcaaattcactcatcgtcgtcctcgtcttcatccactgcagaattt

agtgcctccaagcgagcaatcaactttgtccttctctcatcataagtgagcttcttcagg

ttgtacctcttgtgttcctttggtggctgctttccagactttttgattgaggggtctgca

cgaatagcagcatgaaccttcttgtaaagttcctcgattccttcagcttctatccctttc

ttgatatattgactgaagtgagtttggtacttttcaggctcatcttcaatcaatgtcttc

atataggcagtaacatgtccaccaaagatatacttcctgtgaatctcagcatcaagttcc

tttttctccttgtcaaaaccagcaaaccttttgtcactgtgaggaatatccaaacctcca

tccaaagctcccttaagtgccccaaacacccggttaccggttgtggtcttgaccaaacca

acatcaaggagagcacggaaaggcctcctggaatcagccggttcgactgaataatcttct

ccggaagcctcgacattgccttcatattcctcatccatttcaagagttttaagaactcgg

cgagccaaaaggagaccagtgcaataggctgcagcatagtttgtaagacctacttcaaga

ccataatgaggtagctcatgtgcataagctgctgcaagaacaatatcaccggcaatgcta

gcagagacgatttgcgcaacaatatccttgttggtaaatcgaacaacgaaacgatatttg

ggagtgttatatttgttcttgtcctgattaatcaaacgaatcctggctctgtaatcagtc

ttaccctctcttcttctcttgaacttgacttgatacctcttgaagtaggcctttgatttc

tgagacttgacgaaaaccattctgaaggaatttggccgcacctcgaacagcggctggatg

agaagagagagagaggtcgagagccgaagccttcaaagttctcgattccaatt

>fava_dn_rep_c814

aaactatgcatctttaactatgaaactgcatcgatctccaccaattaataatccctcacg

attgaaaccagcaatccaaatttgcctagcacctaatctcgtatgatcctgcttttgtaa

tatatcgaacccactcaacagtgttgtagccactcttttcccacaccttaagaaatcgga

ctcgtaaaccagatgctgtgaacataggaacctgaaactccatctgaattggtggtctag

tccatgatttcttttcagtcattgtggagataagttcaatttctgcactcagagttggct

cagtttgcccaggaaattttcttattttccaaaccaaacaatcaatagctgcattatatt

ttgctcgaccagatgtgactgtgaaactcgttttagctgtttgttttggcactggaattt

tgataacgactcctaatgcaaacatctttgcaccaaagacactctttaccttaacattta

cttccatccgtgttctaccaagttccttgatggttggcaatactttgaagggaagattaa

caccctcagtgatacgatacttcattagttcaaattcaccatctggaggcacaaaactaa

cagtcttttccgaattgaacctcgtcaaatttacacattgatggaaagtgacatcatcaa

gctcgatagtttttccacttttagtaggacgtgacttaagttgcgactctttctcaaggc

caatcttgtcattcaaacccaacttcaaatcaggcattccagaaagaaagcacttcataa

gaatcttccccgttacatcacagcgggaaaacaagacgagacacattcaccttcttccag

agtagtagaacatggctggaaagcaaggtgatagatattattccattgaagcaccacttt

tgaatgatgagaaagtgaatgattcaaatactactaatactaatactaaggatccagaga

gcttcataatccatcatgtgggtagcgcttctgtattcaagacatgcttcaatctactca

atgttatctcaggagttggtattgtttcaatgccgtatgccctagcatcaggaggatggt

taagcatatttcttctgtttacaatagccatagcatgttgttatacaggcacattggtta

aaaagtgcatggacatggatcctgatatcagaacctttccagacataggtgaacatgcat

ttggatcgaaaggaaggctaatcgtatcgataatcatgaatactgagctctacttagctg

tatcaggttttcttattttagaaggagacaatctaaacaaactgataccaaatgtggaaa

ttcacataggagagtttaccattggtgggacaacattgtttactatgattacttgtcttg

ttattttgcctactgttttgtttgagaatatgagtttgttgtcttatgtttctgctggtg

gtgctttggcttcttcgattttcattgtttcgttgttgtggaatggtgcaattgatggaa

ctgggtttcatggtgaagtaagggctttgaattggactggaattccttctgcggttagtt

tgtatgctttttgttatagtgctcatccggttcttcctactctctacaactctatgaaga

ccaaaagtcagttctctagggtcctaattataagcttttcaacatgcacgtttggttatg

cagcagcggcatttctaggttacttaatgttcggacaagaagtcgaatcacaagtaactc

taaatctccaaacaggaaagttaagttcaagaatagcaatatacacaaacttagtaaatc

caatagcaaaatatgcattaatgcttacaccagtagtaaatgcagtaaaagcaaaggttt

caagcaacaaaagggtcacacatatgattattagtacatctttactagtaacctctcttg

ttatagcagtggcaattcctttatttggataccttatgtcacttgttggagcattgttaa

gtatctcagcttcaactttagtgccatccatttgttacttgaagatttctggagattaca

agagatttggaagatcaatgattattaattattctataattataatgggagttgctattg

ctgtttttggtacctatacatctcttgtggatattattcaaaacttgtaagttgcataag

tttcattggttagagcatacatattggtggattatcaccgttgtgaaacaaatgtcaaag

ccattgtatgacgagataataaatgtttgaatttttgagagttttcaaatttaactacgt

tcttcttgcaaaaaaaaaa

>fava_dn_rep_c815

aataacgtatatattttttggttaatacaataataaaattgataatgacaaattttttat

ttatattacaaaaattttaaaagcgcttttactactattcacataaacattttaacatag

tacattagtaaaaatctactagtcacatatatgcatctcttgtcatttaaaataaacata

tgaaggcatgcattcaaatacacactttgccatgcatgtaattttacactatctccaaat

tttccatacttataagaaaattagctgtttttgaagcttctttatctttctcctctttgc

tctggtgtcaaccctccctttccacatgtagggcaaccaattagcccaaatccttggcac

ccaaagcacttccatcgttcaaatatatttccattctttttattctttcctgttcccttg

catcctggacattgaatagctcctttgtcttggcaatttttacaactattaaatatcgaa

ttgagtgataatcctcctccagattttgaagcagaagctatgactgtggttcttttcagt

gtgaatggttgaagtggagcttgaagaatagaactagacagtggagctaccgaaccgttg

caacaacaaacagatatgaaggacattttttgtgtgatggttggtgtgtgtatgttgcaa

acaaatgtgaatgaagggataataagatgtgtttcatgtcatgtgtggttcttgtttttc

cttttt

>fava_dn_rep_c816

agttgttttgtcacaatgacacattatatccaatcagaaaacagaaccattaaaaaatac

taattagaaacactctaaataaataaaaaaataaaaaagtttttggtagaaattggcctt

ggtttggtctttggttttctcgaattgttaactgaagaaagtggtgaagtttggtagaga

aagaaagaagaaagaagcaatgggtatgatcggtgagactgtggattctatcaaatctct

tcagatccgtcaggtcctcactcaggctgttagtcttggcatgattgttacatctgcact

gataatatggaaggcattgatgtgcatcactggcagtgaatctcctgttgttgttgttct

ttctggaagtatggaaccgggattcaagcggggggacatcttgttcttacacatgagtaa

ggaaccaattcgagcaggggagattgttgtatttaatgttgatggacgagagattccgat

cgttcatcgtgtaattaaggtacacgaaaggggagataatggggaggttgatgttctcac

aaaaggagataacaattacggtgatgacaggcttctgtacgctcatggtcagctgtggct

ccaaaggcaccacattatgggtagagctgtcgggttcttaccttatgttggctgggtgac

aattatcatgacggagaaacctatcatcaagtatattctcattggtgccttggggttgct

cgtgataacttcgaaagagtaatgatgaatcttaaccaaattacggtctgacctccaaga

tactatgagcaaatgtcatgttttttttgaacttgaaggtgtcattttgtaccattaaac

agcataatgttaagaattaatacagagtagttgaattttataatctttttattgtttgtt

ggttgattttatagtttcttggtgcagtatgactgtatttatatgtttagttcctcaaaa

atagtttactttttgagaaggcaaaatggattctagaaattgtcacacatgtttcagtgt

tttcaattagaattgattttgcctctgaaatttattacaaattgaagttagaatttggat

attttatctctggaatttattcttagcttgaaaa

>fava_dn_rep_c817

ttttttttgcttgtaaaatttccacttttataccggttttttccaacaacgcctaagata

catcttgataggaggatactatagatttcataggaaagaagtccaaaaataaaagaaata

caacaatctaaaacaagtgaaccaagaaaaagcttttgcaaaaaaaagtgaacagaaaaa

ttctgaacagcattctatcagctcaaggggaagcataagtacattttattagacactttg

tcattgatcaaaacaagtttattaaagacttgttagattcctataaatcaacagaaactt

caaatttgtcccaaaatggtctctgctccagaaactttgacatcagatggagattcttca

acattaagagccttcacttttccatctactacatatgctgaccatctttcagatcgaatt

ccaagcaaaccagcagatagatcagtagttaattccaaacttttgtgaaagctcccatca

aagtctccatagaactcaatagcatctttggcttgaatgttctcagcccaagcattgaca

gtatatggatcattaacagccacacaaataacagaatcaatccctttagctttaaacttg

tcaatattatccttataaggaggaacatgtttgcttgaacaaactccagtgtatgcacct

gggagaccaaagatcacaacttttttatccttgaagatttccttgacaggggtagtggag

aatttggattcaacaccttggtcccaagtgcgagctttctgtagagaaacattggatgcg

gcagaaagtatatcagttccagttgcaacttttgcgtaggacctgatcccgtgaagaact

gaagacagtgattttgtggttggtgaagaacgaagtcggttgattacagaggttgccata

tcggtaaattgtcagagattgtggtgagtgtgcgatgtgatgtgatggatccaaccagat

ctgttacatttcaaaacattcggttatgttttattattatcattgttactgctagtggag

cacggagattaattatcaaaatataataaaggtacaaaattgatgtggttgaacgtgagt

taacactcgttagaagcaatattttagatttttaagctattaaattcttcaaacttaaaa

tatgttaaaa

>fava_dn_rep_c818

aacatgatataagtaataattcattaatcactgtaccacaactttaaacatcaaaaagaa

tgtaacacgttcctgctttatatatttgattcgaaaagctgatgactaagcgtgcattta

tatacttgtgcaaaattaaattactttagtaactttatgcaaaagtaaataaaaagaaaa

gccaaggaataatcttatgcttgtgggaggtggttttgaaagatcaacgtaatcttaaaa

tttcatcagnnnnnnnnnnnnnnnagtgagcagaaatccaagaatcataggtcatacatc

ggttgtgtaatgtgttattaaattctgacaacaaaccagtacagaaatagaatttacagt

gtcacaactcatataaatactatgatccaaattacagaaataccttatggcaagcatata

gtgttataaccttataacagataaccttatcctgcaaataccagatggagaatgttccac

gtaggatagccttattgtgtccattagttttcagctgagtctaccaagacactctccact

aagctcaaattccacacaacaacatccttaacaacattagttcactacaacaaccttaat

ttgaagttgaacaagatatattcacacatgtcactcctccaacttaaactagcccctaat

gcattcacacctctctctcatccatctcatagattcagacccaacagaacaatcaaaatt

agagcagttggcacggtccctgatcagaagaattcagaaaccactcaatctaacatcgaa

ccaccttctgtaggctttgcatttgtcagttcagtgttgctaccagatggaacaccagat

gtgcattatcgtactgctaccggtggacagaagcttaggaacataatgctggattctaat

attgaactctatggcccttatggtagagtgttgttaaactgtggaggaggtggaacttgt

gctacatgtatggttgaggttcttgaagggaaggagcttctgaatcctcgcaccgagaaa

gagattgaaaaattaaaaaggaaaccaaagaattggagattagcatgtcaaacagttgtt

ggcgaagcagattcgacaggggtggttgtgattcaacaattaccagaatggaaaggacat

gaatggaaatatgaaaagggtgatgacgaatcagtagagtcttcctgagaaaatctttta

tttgaaatcttgtaactagaagttgtaaccttgaagataatatatctctttctctttcag

aatttatatatcttcaagaaatactgaataaattcattggttgatttgtgaatgatacaa

aagttctttatacagaaaaggttacagcaggaatatacaactaacttaactaattagttt

atcctttttataatgccaaacttatatatcaagtacaaaccagaggacctatgtgaatga

gccggttttatgctttagttcaaactgaaatctaaatacaactgccatgaaaagatgtct

taaattttcaatgactaaatggatccacgccacgatcaaatgaaatgttcaacagtcatg

catgatgctgaaggacagatttnnntaggccgaaaaaattgcgtgcatttaatttgtgaa

ctgattttacatagtttagtacaaaaatgttattattctttttttatcttaatgaaacaa

tggaggtctatgaaggtagacagaagcggtaacgaattgggttcagtgggtttcgattct

gcaatcaatcgctcctatacaaactttatgttatttgacctgagattcttgggcaaaata

tatgagacaaattgatttcagatactccaccccctcctaggcatctgaatcatcctgctt

aaacttgcctgaacaaagagaagaagaaaaatcagccttctcgttatatcagttattaag

gaaaatctatagaaattccaaaatgaaggagaaattttttattatgagcagtctggtgcg

ctaaatggtgggagccccttgtgtacaccttgcaatgatcgcagctgaaaataaaccaag

atcagagcaatcccttttcctcctcaaatgccatcagacgtggaggttgatgtccataga

taatgtcatgagtttttatgggatctagagacacacagccatttcctttgaatatccgga

tcatcgctagatacggttggcacatgccaacttccattggctgattcctgaataactatt

gaatatcgaggaggtaaaattagggaaactccacgacttgaggctgatactcgaacctgg

tgcttttctttccaccttgggaaaaaattagagcccagtagttggaacaagtggtctctc

atttcatcatttgtcaaaagtaagcagttacaactaacagcagcatataaccagtaccag

tcgtcattggaaccgtgaggagtagcataaagaacaccgctatttttccaattttctata

agtcttttgttagttgcattccgagcaggaccactagttactcgatttacatgcaaaatt

ataagtggcaacctcttggtagggctcagttgacgcaattgtcgaacaacataattaagc

tgagaaaagctgaaatcagcaatgttggcaagtcctacatttgcaccatcaacaaccgca

tcgaatggaccattcttctcaagccatttctgaaaatgattaaagttggccttaggttct

ttttcgcaagccaatttagtcaacgaagcagcaaagttttcggtttcttttgtatcaata

tcaatgctcacgagcttctcattgcacgaaagacacactccatcctcatccacctgagta

tgaacaaccttccattgcccacttccaagccatccctgaccatgccatcccccacctccc

ctcacaaacccttcttttattttctcatcatcccattcactctttccaatcttctccgca

tactccgagttaaaccaatcctcaacaaccttcaaagtcgactcagacagctgtctaacc

acagcacgcaaccgatgcaatatctcatacaccttatcttgattcctcgtttcaacgcca

acttccaaaagcgcacaaagctcaggctcctccgccataacacccgactcaatcatatca

gcatcaacttcataagctttcgcggcatcccctcttttacaaaacccataaagagcagga

ccataagatctcaacttaggtgcaatgtcaacactcttcatttgtttcaacaactcaaaa

gccatttcaggatcttccttagcagcagcaaccctagcagcattagtgaaagtagcttca

ttcggttgaatgttatcattcaacatttgctgaaaaatctcaaaccctctttgaagaccc

aaatgagaagcacttgaagcatcatcaccaccagattgaacagtacaaaggtataataat

ttattgtaatggttgagatcgaggagaacacctgatttacgagattcgtcgtatagagaa

agtgcttgaagaacatcaccggatttagagcaatggctgagctttttgtgtaaaattcct

tcgggtgattcttgagcagcttttcgtctggctttggtggaaagcttaatagttcctcca

ttgggtgatgaatcaggtgttcgaggtaaagtgttggtggcggaggtagtgaagggaaga

atacggtggtttatggtgtttttgagagagggagaatgagaatcggaagaagagagaaat

gggcgatgggtgagaatggagaaaaggggggtgagttttgccatggcagcgcttccacgc

aacatcgcagttcattaattaatcaaattttggagtttgaagtactacgttgttgttggt

gtgttcaaaaccctatccttctcccgctttacttatcacttccacttatgttttgttnat

tacatttcataataacatcatgaatattgatttaaacttggtcaccaccatctcctccac

catgaccacctccaaaaccacctcctgcacccccaccatgaccacctccaaagcctccgc

ctgcacctccacctgatccacctccaaatcctcctccaacacctccacctgctccagaac

cacctccaatgcctcctccagaacctcctcctccacctaaaccaccgccaccaccactac

cacctccgacgc

>fava_dn_rep_c819

acatgccttgaaaattttccattaaaactgccgagcagagaacatttaagttgtttttgg

aaacactctgagttgtttccaaaagatatcttagtgtatgtttggctagcagtacaaaat

agagaatcacagcaaagaaaccacggtagcctatctttttcacagtaggattgtccacaa

acgtgcggcaacagccaaaataagtagtttcaaacacacttagctcacttacgtaaattt

cttcaaatttttaaaacttgggccaactcaagactatcaacaaatggtataaataatgac

tagaaaaaggtgcgagatgggtagcatgaactaaaacaaaactaacacctacgcaaaagt

gacacagccatagtgagggttggcataaaagtaagacatctcaatcacaccacgaataaa

tcaaaaaccccaaatctatataaaaaatgttaacagttcaacctcaaacacataatttta

tagattacacaaaagtgccagttcaagacaaaagccatcaatcccaacggtccttcttct

actcaaatgcatcatcatcgtcatcaggaaggggttgattagcagcttcgagaatttcat

tttcatgcctttgttgtaaggcaatatcaatttgaacttctggtggagccagtgcaggag

attcgacaaagtgcaaattagcatcacctgcaagtttcctggcaagatacaggaaaggct

tctcaaagttgtaattgctcttagctgaaatctcatagtactgcaaattcttcttcctgt

ggaaagtaacctgcttggccttcacttgcctgttcttcacatcaaccttgtttccacaaa

gaacaattgggatgttttcacaaacccgacaaagatctcgatgccaggtaggaacatttt

tgtatgtcagtcgggcagtaacatcaaacattataattgcacactgcccatggatatagt

atccatctctaagaccaccaaacttctcttgaccagcagtatcccagcagtagaaacgga

tctttccacagtttgtgaagaaatccaatggatgcacttccacaccaatagttggttcat

atttcttctcaaattctccagtaagatgcctcttcacaaacgtcgtctttcctgtgccac

cgtctccgacgataacaagcttgaagctagggtaatcaacggtttgctgattcggcaacg

ccatgaaatgcagtcgttggtttcagagggttgaggagagaaagaaaaagagagagcgag

ggtttatatttcacaagggttttcgaatttgagtgtggcctcttctagcttcaggttgac

gcagctttctccacccttggattaaat

>fava_dn_rep_c820

acagaggctattcagacccattttccccctttttcgagtctcaacaatcgtctcattttg

tgtacaacacaagcgcagggaaaagcgaagagaaccaacgaagaagatgttgagggttgc

atccaagaggctttcatctctctcatggagagccaaccacgccgcatccgctttcgtctc

taagaaccccatcgcaccgccttcttccgatgaacgcagatctgatcccttttcgatcca

ttccgaattctttcttcccttcagaggttttgctagtgaatcactaatccatgcaaaaga

aaacagcattcttcctgaaatccccgcaactgttaccgctgttaaaaatccatcttctaa

gattgtatatgatgagcacaaccatgaacgatttcctcctggtgacccaagcaagagagc

atttgcatactttgtcctaactggtggaaggtttgcttatgcttctgtgatccgtcttct

tgtcctcaagcttgtcctcagcatgtctgcaagtaaggatgttcttgctatggcttcact

tgaagttgatctctccagcattgagcccggcactactgtcactgttaagtggcgtggaaa

gccagttttcatcaggcgcagaaccgatgacgatgtgcagttggcaaacagtgttgatat

tggatctcttcgcgaccctcagcaagatgccgacagagtcaagaacccagaatggcttgt

tgtgattggtgtttgcacacatcttggttgcattcccttgccaaatgccggagactatgg

tgggtggttttgcccatgccatggatcacattatgatatttctggcagaattaggaaggg

acctgcaccatacaatctggaggtaccaacttattcattcttggaggaaaataagttgct

gatcggttaaagaccttgagctgctcaatagtggaaggagttcaaaatatttttgattta

ttggataatgtcgaaatgtgccatggacatacttggccggggaatctttattatttgttg

ttttagcaagtgtttatgtatctccaaattttaagtattactgtttgacgatacttgtat

caataattgcctggctttttgtttacaatttacaaatgaattgtttaaaagctgatgact

tcaggcataacgcattgtattttatcatatcataaattacatttcgatatgtgcttaaca

aataggatgtgtcgttggtttttgcatacaatttgtgtatatgattacatctttaaaatg

aagaaacattacaatcttaaatgaactaggtgcttatggcaaatatcaaaactgtcagct

tgctgctataacataattgttcactaacatttctgtgggctgtttttcacatgcatactc

atctgtgtacattatgtttcaaaaagacccttaacataaaccatgcccttttcactaaac

ttgagttcctttctcatttgctccacttgttggtcatcaccttgaacaacgatctctgat

aaggttccatcttccttaactattagtttcaccttaatttgctcctttctggatgctcta

gtccagtaataagcacccgccaatggacttaagatggttaaccaaaaccaattattgcca

acatctggcacagttatggtaagcacaagggaaacacttgcaagactgatacaggtgcag

aaagttaacagtgctgcttgccccctgctaggaaccatcactccttcaaacgtgattgtt

tctcctctatcaaccactgaaaaattattacgagtaaagaatgacagcacctcccctgca

accagatttggtgccttatttccaacttgatctacaaatgtcttcttcacgattttggat

ttaacagaacgtttgatgagggaccaaagaccaggaacagagataacaaagagaccaaga

gaagtatagtaactagccaaagaataaccagttgtctctgtaagcaaaaaaagagaattg

gggtttagtagttgttgttgttgggtgatgaattcggcactctcgtgaagggatactcga

agtttctttggttttggagttggaagatggtggtggtgccattgttgtagtttgtttgtg

tgaaatgggagtggaggtggggatagcaacatcatcttggcagccattgattttgcaggg

aatggatagggaaagaagatgggtttagcagctgtgtttaatggtctagattgatgggag

tgatctaacgggctagattgagtgagtttatttacacaataaatttttcaaaaagcgatc

aaagttgccaagagacacatagacattgaacatgtggctctaatgc

>fava_dn_rep_c821

accttatcaactcattacgtctttcacgttgactaagaaatacactaccagtgtcggcaa

gtcttcaaggcgtgttgcagaaaaaccaaatctccaaaacctgaaaaaaatggccatcta

aaagatctgaatcataatgttgaaaatagcatttctcagctatatatgaacgctgcaact

cacaaaaagaattggcattttgcagtttacattttaggtgaattcacaactccattatgg

ttctttcttttgtttacagtttgatttctcgtttgatagttgcgatcctggctgtttttc

aattccaaactaaggagcaaacactgtctttgaagcttctggtaaacacagatactaaca

aagttgtatttgctgaagcagataacgattttgtagacattctatgtagcttcttaacat

ttccattaggtaccattgcaaaacttgttcagaaagattcagaaatagggcccattacaa

ttggatgtctgaactctctcagtcaaagtgtgcaaaatcttggcttattgacaaactata

actcatcggaagatttttaccgctttctcaaaattaacattgatgatactgtgcccacaa

attacttcatgtgttccaggtttgatgaatttcattgtgaatgttcttatttgagcttag

gcaccaaaaaacattattgtgaacatggccatcctttgtcccgtttagtttctctgtcat

tacccatgtttcagttttgctttggatttgtcaaacctgttacaaaatttgttattagcg

atgatttaattgttttgccgcactccatggatcacacaatctttgatctcaccaagaact

ttggaattaaagacacaagctcggttaaagaaatgactgtcaatgtcactaaggagaagg

tattggatctgttaaagtgttcattgttttccaagacacctctaaccgatgtgtttttag

gaatgaaacctcttattgagagatcaaggattttctcttgtgatgttaaagaccttatca

gtggtgatatgaatatcactgtgaagctagtcataagaaaatcagatgatgttatattgt

atgctcaagggggacaggattttgccgagttgattatacgatttctaacttttccgttgg

gcggagttttacgcaagttagaggggaattattctttgggtagtattgatggattttgca

aaagcatagacgatttaaatgaagacgagtatttcgttttcaaagaaccaaagaaaaggc

ttctcgggctaaatgttatgcattattattgttattttcaacgcgatactactcccaatc

aaagaaaaactgaggtcacacttttcaaaagtaatgaagtttgtaatgacgggcacaatt

tgaaaaaaatgaatttggttaataccattaccataccgcctacagaacgtcctggaagtt

atatcaagacaccaaaaatgtatatggttacagatgatctcgtcatagagccgttgttgt

ctccaatttcatctgtatttttacttgaccgtttcaaaatttctcttaatgatttggagg

aaaaagttgtcaccattggcatcaaagagagtctaagcatatttaaggaagctttaaact

caacatctgcactcacaaatggtctccgtcacctgtttacccaagttaagaaggagaaat

aagaaagatgaacattgtgttgttgtttaagagtatgtcatgggaaagttagttatgggc

aatgttatcaaggatgaacattattgctagaatttctttattgttgtggttattttattt

ttagcactattgttttaagacctttaaatattggagtacctaattctgagtttagaatga

aatatatgcaagatcagtttcgttttttaaaaaatagtcattaactgtatttcattgcta

agcacaagtcgtgctaaagataagatttacagtacaccaagaaagataattgggctacaa

gctttt

>fava_dn_rep_c822

caggaggtaatgagaaatactaaataatgtgcaagtaaaatacagcaacagccaacacaa

acagctttagaaactgctaccaaacagaaaatctaaaaaattgtttatgcattagtgaat

ctagtaaatatgagacttttagcatttctataataataacttaaactgcaaaacagcaaa

gagcataaaattggagtacatattaatacagaatatactctataacaatacatgcattaa

ttcattgagctatacacaatacacaaaccaaagaacaaatgcattaactactccatagaa

tcaaaactgggagaaaaccatcccaattggccattaatttgtttgagtcttgcttcttga

ccctttgaatctccaaatgcagtagtgaaagggtatacatatgttacttttgctgccata

cacctataagcaaccacatcacctttcactagaaactcaagtacatctctatcaaatttt

ccatcaacttcagcttgcagatatttcccatctggagtatcttccactggctggaatttg

acatttttctgagtctctataatagcttcacgtaatctctgaatagcattatccactgag

ttttctggaatagtccaaggaaaatcaaaattggatgactttggatttgttgacacacaa

cccggattagtagagggacaaggcctaatttttccgttttctagtccaagttgcaagttc

tttgattgagaaaagggagttgtaagagagattggagggggtttagaagaattgagggaa

gggattgcatgagaaggtagaggagaggttaatgttatggcaatggctagagagaagaga

tttggtatgaatttgggtgttaggtttggtagtttgggaatggtgttagtgtgtgtttga

gaagaaacagtaagaggaagatgaccaagaaagaaggtcatcatttttattccactcttc

ttctcatccactcactacatcatctt

>fava_dn_rep_c823

agaaagttggatgttgaatattcagatatcgaaactattaaaccacattaaatagagcgc

tccgcaaacactttctaactccctcactcatttctcacctaacatctcgttctccgcatc

ttcatcctcatctcttcttccgatcacgatcaccaccatgggatcccagaaaatcaaagt

cgccaatcccatcgtcgagatggacggagatgaaatgactcgagttatctggaagtcaat

caaggataagctcattttgcctttcttggagttggacatcaagtactttgacttaggcct

tccttatcgtgatcagactgatgataaagttacagttgaaagtgctgaagcaactctcaa

gtataatgttgcaatcaagtgtgcaactatcactccagatgaagcccgtgtcgaggagtt

cggcctgaagagcatgtggaggagtccaaatgggacaattaggaatattttgaatggaac

tgtcttcagagaaccaattatttgcaaaaatgtccctcgccttattcctggttggacaaa

gccaatatgtattggaaggcatgcttttggtgatcaatatcgggccactgatgcagttat

taagggacctggaaagctgaagctggtgtttgttccggaaggccaaggtgagaccactga

tttggaagtttataacttcaccggtgaaggaggtgttgcattggctatgtacaacactga

tgagtctattcgttcttttgctgaggcctctatggcaactgctcttgagaaaaaatggcc

actttatcttagcaccaaaaatacaattctgaagaaatatgatggaagattcaaggacat

atttcaagaagtttatgaggcaagctggaaatccaagtatgaggctgctggtatatggta

tgagcatcgactcattgatgatatggtggcatatgcactcaagagtgaaggaggttatgt

atgggcatgcaagaactatgatggagatgtccagagtgatttcttagctcaaggttttgg

atcattgggtttgatgacatctgtattggtttgccctgatggaaagactattgaagcaga

agcagcccatggcacagttacccgtcatttcagggttcatcaaaagggcggagaaaccag

cacaaacagcatagcatccatctttgcttggacaagaggtctcgcccatagggcaaagct

agatgacaatgctaaactcttggaattcactgaaaagctagaggcagcttgtattggagt

agtggagtctgggaagatgactaaggatttggcacttattcttcatggatccaagcttaa

aagggagcattatttgaacacagaggaattcatcgatgctgtggcagccgagcttaaatc

taagatctctgcatagatttgtcattttagatattggaaaagcgacgagattgaagagga

tcggatgcactttcaaaagaaataagaaagaggatagaagcattggtcttggctattttt

tcttccttcatttgtgttttggatgggaacgcatgggctgtgtgcttcctttagtttgaa

ttgtgtaactttgaattagaaatttaaatggattttgttttgctatctttctgctgattt

tgctttgcatgttttatttctacaaatattacacctttaaaaagaaaggaaatatttctt

tcaactactgcaaactaagtaacattgaatctggatcaaaggtgctgtactcacctaaac

ttcaactggcgtaag

>fava_dn_rep_c824

tttttttttttcaaatcataccattataccattctttttttttttgaagataatgaattg

atgcattgatagtagacatgagtttttctcatggattacatgactgaggaagtctataat

ttgctttagatacaaactttttctaaattgccaaaaacacatgtgaatttgctttagata

tagtaatagatttattgttcaatccagaatgtaaataagaaggtttttaagataatgaca

acgaacgacatttgaattcaaaaacgagtagtaatctgggaaaacaaagccttaaagtcc

tttgttggtgaaccttttccctcgggtttagatgccaagtcaaatgccttaaatttagcc

tcctcaaaatttagtgcctggatgcagacttctgcaacatcaggtctagctatagttctg

gtttcagtctttagaagctcgtcatccttccctacgaggagttcccggatacctccctct

ttgtcttgcaagccaccagcccttataatcgtatatggaacaccagaatcggccagatat

tgctcagcctttcttttccaaaccaatatgttcccattacccaaactgttcaaaggatta

ttaaggtctgttccgcccatagatcctactaacacaatctgctttactcctgcagcttta

gcagcatcgatttgatttttctgcccaagccagtcaacctgttcggggtatgcaccatca

tcgaaataaaactcgggtctattcccttgtgttggatcaaatccaggtttcatcaatggt

actccacttgtcagtataatgagagcatcaataccttgaatcgcaggaacaagagtttca

acatctcttatatcaccaacaaaaacatcagcagcagcaccaattttctgcttgctttct

tctgtcctaacaaaccctctagcaacatattggtctgacctctctttcaatatcttataa

accaattgccctgtgcgaccaccagcaccagtaacaagcacagtagtctttgaagattga

gccatagcaacaacagaacttgttctaactccaactctcttcacacccttaagagttctt

gatgttggaagtgaaagagatgaggtagaataacaaggataagaagaagatgaaatggtt

aatgaggaagaagaaactggtaaaggaattgacggtgcaagtagggagtatttatggcat

tggttgggaaaacgtgtggtagcggaaacaaagggaacacgtgtagtagttgccatacct

atcagtgtagttccattactgtttttgaagagagtgaagaatggaaggtttcacttcatt

gtacatatagcgtcttctgttggtatctattgatattaagacattgttgtttctgattaa

tgttgttgtagtactagcactttcgatg

>fava_dn_rep_c825

atcaccggtttcataatcgactcaattttataaataagaaaaaatgaaagatggataagt

tttgtagaagaaaaagaaagaaagatagtgtgtattgaaaccaaaaatggcatgcttggc

agcagcctcatctactgcttcacttgtcttttcttcctccttctgctcgtctcgtctctt

catcagaacactcgctctaaggtctgcttcatcaccaccgcgtcttttttctaccaccgc

agtacctctccgcagcctcgtggctcgcgccactctcggcttcactcacccctccatcaa

ggaaacccccaagctctctttcacggcgaaggatactgaagcggcggaatggaaagggga

cctcttagccgttgccgtgacggaaaaggacgtagctagggacgacgagtccagattcca

gaatccaattttgagcgcgctggactccaaattgggcgggttattagctgaagcctcgtt

cgaagaggacttcgccggaaaagttggacagtcaacggttctccgaattgcagctggaat

tggatccaagagagtcgccttgcttggcctcggagcttctgcttctggtccggccgcctt

taaaggccttggtgaggctgttgctgcagctgctaagtccgctcaggctgtccacgtcgc

tgtcgttttagcttcttcttctcaaggactctcttcccttaacactgcctctgcaatcgc

cactgggaccgttctgggaacatttgaggataccaggtacaagacggagtcgaagaaatc

gttccttaaatcagttgacattattggtctcggaactgggcctgaagtggagaagaaact

taagtatgcaggagacgtttcttctggagtccttcttggaagggagcttgtaaattctcc

acctaatgttctcacaccaggagtattggcagaagaagcatctacgattgcttcaacata

tagtgatgtttttactgccaaaatattggatgctgaacagtgtaaggaactgaaaatggg

gtcctatctggctgttgctgcagcctccgcaaatcctcctcattttatccatctgcatta

taaacctccaactggacctgtcaatgtcaagttggcgttagttggaaaaggtttgacttt

tgatagtggtggctacaacataaagaccggacctggctgtatgattgagctcatgaaatt

tgatatgggtggatctgctgcagttcttggagcagcaaaagctcttggacaaatcaaacc

tcttggagtggaggttcattttatagttgcagcttgtgagaatatgattagtggaacagg

tatgaggcctggagacgttctcacagcttcaaatggaaaaactatagaggttaacaacac

agatgctgagggtaggcttactctagcagatgctctggtgtatgcttgtaaccaaggtgt

tgataagataatagacttggcaacattaactggggcatgtataattgctttaggaccctc

aattgcaggtgcatttacatccagcgaggagctggctaaggaagtttttgatgcttctga

agtgagtggggagaaactatggaggttgccaatagaagaaagttattgggaatcaatgaa

atctggagttgctgatatggtgaacactggtggtcgacaaggtggtgctatcactgccgc

tcttttcttaaagcagtttgttgacgaaaaggttcaatggatgcatattgatttagctgg

tcctgtgtggaatgaaaagcagcgttgtgcaacaggatttggtgtatcaactttagtgga

atgggtattgaagaatgcatcttaaatgcattcaaatcttgtccacgacctcaattttat

catcgattctgtctgttattagttttaacttttcaaattttaaacaaaatcataattggt

tttcggtttttcgtttcagttgtgtttttcatttttaaaaaattcttgtaagtcgccaaa

atttttccttcaatttttattacaatacattttaataacaacggtccttccgtaaaaaaa

aaaa

>fava_dn_rep_c826

agaaataatttccatattcggacttgtaatgtaactataaattctgaagtgtcagattta

gtcctagttaatgaaacaaggctagaaccactgccaaacaacttgacaaactttagaaaa

atctttctaatttggtgtgaaataagatgaaactgaagcattataagaggcccatttttc

caaatcgagacacaaacctggctagaaacaaatcaccgccattcatgcattcaataaatc

tcaattatacaatatcctaatgttaaaagtcgacacagcaaagaggaaacaaacgtctcc

cacttaaactaccactgaatggtacaaaggagggaaaattcattcggacaatccttataa

ggaattttcaaaggtttctaattgcttggagtagcttccttactctctgtagttgcctca

ccggcactcttgccagcagcatcatgctcactaccgatctttccagcatcttgttcacca

ggctttgacacttcttgaccttcatcatcactctcatcaatatcatccattgcgtcgcca

ccacccattccaccaaatttagagaagtccattcctcccagtcctcccattcctcccatt

cctcccagtccacccattcctcccaatccttctagtccccccattcctcctaatcctccc

attccgcccaatcctcccattccgcccattccaccttcaccgccaaatttcgagaaatcc

attcctcccaagtccacatcaccaccttcatcctcatcttcatcaacccatttatcccaa

tccactttcacataatgtggagcctttccttctccacgcagcaacctcttccaccatcca

ctctcagctttctgcactacagcaattatgcttcgcactcctacactgattttgctctcc

tctacattaaccttgtcaaaaagctccaatttcagctcatacttattgtctccagcccca

gcactaccagaaaagataaactcaccctcaggggtaagatcaactttagcatctttggaa

tcagccaattgcactgtgatgtagaccttgtctactctctgagcccacttaacctctgga

tgacgactcatattggatttggttgaagaactcgaagaggaaaaaggtaggtaccgtaaa

accctagttccttctttcgttggctacagagaaaaccccaaaaaaatgacacttattgag

ctatcgc

>fava_dn_rep_c827

aaaaagttgtaaaacccgaggtgagtcaggtttgtgactgtgaatttgtgatcctctctc

ggcggccgtcactgatctgcgatttcaccatggctccaaagaaggaaaaggctcctccac

cgtcctccaagcctgcaaagtctggtggtggcaagcagaagaagaagaagtggagcaagg

gtaagcagaaggagaaggtgaataacatggtgttgtttgatcagggaacttatgataagc

ttctcactgaggctcctaaatacaagctcattactccttcggttctatctgatcgtttga

gggttaatggatcacttgctaggagagcaataagagatttgatggctaaaggtttgatca

ggttggtatcatcccactcgagccaacagatttacaccagggctacaaacacctaaattt

atattctcatgttgtattggtattttgacgagtcatgattaagtatttttctctatccat

gggcgtatgtacctcattctagtttacctgttaagttaaatggatgttttttctccttat

catcaatgaaacatacctgacttggatcttattgtttctgtttattgagctactatttgg

ttcactatccttgctaaaccgtattttgtagcgtaagagaatattgaaactgatatgggg

aaagttttaattggttatattcccagaaatttacatatataatttccttagacaagtgta

tttatcaatgtcggtacaaattcttgatcacaatataa

>fava_dn_rep_c828

tttccatccacaaaagaaaagatagcacatttcacatggatagttgatgatataatatct

catcttaatctttcttggattctttctcatctcaccacaaacttctcttccctcatcaac

tcctcacaatatatatccactccctcaaaaacacacttcctctaacatgggattatcaca

caaaaaaagtcccataaacatggcaacagacaaagaaacaacaacaccaacatcatcaac

acctccaacaaaccaaggcacatggaaccaactcctaggaagcaacaactgggaaacact

actaaaccccttagacctcaacctccgcaacctcatactccgttgcggtgacttcataca

aacaacctacgatgctttcaacaacgaccaacactccatctactgcggtgcaagccgtta

cggaaaatcctcgttctttaacaaagtcatgcttgaaaacccaacacactacactgtcgt

ttctttcctttacgctactgcacgtgtatctgttcctgaagccttcttgcttcactcact

gtcacgtgagagttgggaccgtgagtctaactggatcggctacatagctgttacttctga

tgaacggagtagagaattgggtcgccgggaaatatatattgtgtggcgtggtacgacgag

ggaccttgaatggattaacgtgtttggtgctgcgcctgagtctgcatctggcttgttgag

tgctaaaagtctcacccaactcaacctcccaaacaagaataaagatgatggtagcagcag

cagtgacaatgaggatgacaaaagagtgccgaaaataatgaagggttggctgacaatata

cacctcagacgacccaaaatctcctttcacaaaaacaagcgtgagaacacagattctaaa

caagataaaaagtttgttgaacatctacaagaatgaaaatccaagtgtggtattagtagg

acacagtctaggagcaagtttatcaattgtaagtgctttcgatttggtcgagaacggcgt

aaccaacgttcccattgctgcatttgtatttggttccccacaagttgggaacaaagaatt

caacgacaggttcaaaaagtttccgaatttgaaggtgttacacgtgaggaatgttattga

tgttataccacattatccagggaagttgttggggtatgagtatacaggtgtggagttggt

gattgatacgaggaaatcgacgagtttgagtgattcgaagaatccgagtgattggcataa

tttgcaggctatgttgcatgtggtggcgggttggaatgggagtgagggtgagtttgagtt

gaaggtgaagaggagtgtggctttggtgaataagtcttgtgattacttgaaggaagagtg

tcatgtgacggcttcgtggtgggtggagaagaataaaggcttggtgagaagggaaggtgg

ggaatgggttgatgcaccagataaggaggatctacctgtgcctgaagaatattgattatg

tttaatgtctatatgggaataattcatgttaccatgctctactatcatgttgtataatat

tgaatttgtgttgtcatttgtactgtttgttcatcgatttcagcgattaatcaatgatat

ttatttccctccttatggctaaaaaaaaaaaaannaaccattct

>fava_dn_rep_c829

ttctgagtaacttggatatcatataacaatacctatgttgctttatatcccatgattatt

tacttcagattaacaagttcttccattcattggaaaaataatatcaggtgtacagaagaa

ggatcagggaagatccttcaaagaaaaaaatggtacagtaactcaataactagaattcat

gaaagcaatatcatatcttagtaaaaataccaaataaaatggaggaataaaataaaacaa

ctctcattaacagaaaaacttaactactagtaaaatataagcagcgcatttaagtacatc

atcatgagagcatcatgttagaagagttcttgtgaaagtgtctctggctgtagcccatgc

ctgagttagtgtctgctctgataagggggcatcagcatccatgttatgggaggtcacttt

ttcagctataagaggtccatttgtttcaagaataatccctccgtcaactatctcgtcaag

acataaaagaatgagatccaagttctcaagtgcctcacctttgtcaacattgctcctcag

caaaagggtgactgcatcgaagaaaccctgaagaactgatgccaaaatgagctcattttc

atcatcaccgccagtgacaaagaaatgcaggtcttgcacaaatttgtaaacaatgatatt

gttctcaagcagtgttatctcagcttcagtccgcgcatttgtcttaacagtcttagtgaa

cacaaacttctcaaaagctaattttgaactgtttgttggccagtcatctgagtaatactt

gactgccacacgcttcccttcagaatctaaaagaagaatgtttttaatcgaaggacacaa

gccgttggatgccatgtccagggagaggagaaggaagatgcagtgtgtgtttgttgtttc

aagtttggagatctgcgatatcaaataaagggtcggtaggaataacgaggggcaatttta

atatagcctt

>fava_dn_rep_c830

tgtattggggagacattcaccgtattttccaaatattaggtataagtgtattggggagac

attctttttattttccaaatattaggtataagtgtattcactgttaaatagtttattatt

gaacattattgaacatacgcatcccaaaatcttaaatttagcaataaaacgttctgctaa

ttataagatacacccaagcctaatgttctgcttatattatcaaataacagaaaggaacaa

tgataatcatacttaacaacacttcataaactctcttgacagttccattttttatttgct

tggaaaatatcaacctttgatagcatgctgcaagaactctgattttatgtcaacttgtga

attgtggtcatgtcaatgacctccccaagggtcccttgctcaagatggtcccaccatttg

aataagaaattgtccacaactagtctgaaccaaggggacagcttcaaacctccctcccct

gcgtcggctttccttaacagctccttcaactggtcacggttcacatatttgatatcagcc

acttcatcaggatttggattaacgttaacatcccggatgataaacagcaaataatccagt

tcatgctctccccatttgccatccgaaggtgccttatagtgtatgcggcccagtggggtg

aactggtcaactgggacatcttgagcaggaataccgagctcatccaaaagcttcctctga

gctgcatttcttactccaagggcattctcttcaataagctcagattctcggtacaatgga

tggctgcagcaggtgtttgtccacacaagagggaatgttacctttgttgcagatcgttgc

tgaagaagcaactcatactttgagttgaacaggaatacactgaaagctctatgcagccaa

ttttcagattcaatcttttcccacaagtggcagttgtatttagattcatgaccaaccgca

tggtcactctcatccactaaaatgcattcgtcatcgaacatgagacggcgctgaacggcg

tccatgccagcatcaacggcagcaggagtttcacccatggaggtagcagtagagagaaac

gcggagagaggggtcttagggttgaagaaaagcgaagaagatgaacgagatctggaaaag

gtagaagaaggaggagggaaagaatgagggaagagtgcggttgtaaggttgattttaaac

acttgccggttaagcgtcgcgacaatcaaggattgtgccattctcatttcctcattaatt

cctacggttttattaacccaatctagctggaagtaacaacaccttt

>fava_dn_rep_c831

actctagtattattatacttttcttcttttattctctcactctttctatctctaaagtct

aaaaatttctttcaatattctccaacatatcattatttggtttgcttctaaattgcaaaa

catagagaacaagaatgaagaaatttgtactcaagttagaattgcatgatgacaaagaga

agaagaaggctctcaaaacagtctcaactctttcagggattgatgccatttcaatggaca

tgaaagacaagaaactgacagtgataggaacagtagatcctgtaaatgtggtaagcaaat

tgagaaaattctggcacacagaaataatagcagtaggaccagccaaagagccagagaaga

aagaagaagcaaagaaggaaggagagaagaaagaagaaggaaagaaagaagatggaaagt

ggtaggtgacgtttgggggttttgaaaggctaatatttgagtccaaacattctctgaatg

gtcgtgtcttttccagttaatcttgcgactctgtgatgtcagtcaaatgtagtatagatt

agagagtgagttttgagatgaacaatcaactactcaaaatgtcatatgtaattgcttgcg

gtggtactaccagatttatgataccgttatcattaccaccatctcttaacagcttcgtca

ttacacagaagaacaagaagacgaagaagaagggtttggtgttagattattgggggttta

gtaatagtcgaaggaatgtgattattaaggcatcaagcgacgtcgcttcaccatccatct

gggagaattggaaacctcccaaatcatcttccactccttctttcagcgatattctatggc

cttctgcaggggcatttgtagccatggccatattgggaaaactggatcagctattgacac

ctaaaggactctcaatcacagttgcaccgttaggagctgtttctgcactcttatttgcct

caccctcagccccttctgcacggaagtacagtatgttgatggctcaaataggttgtgcag

ccataggtgttttggcgtttacaatatttggacctggatggcttgccaagagtgctagtg

ttgcagcttgtgttgcctacatgatttacaccgattctgttcatcccccggccgtaagca

tgccattgcttttcattgatggtgttaagttgcaaaccttgagcttctggtatgttttgt

atcccggagtagctggatgcattcttctctgtttcattcaagaggtggttctatacatga

agaaaaatttcaaattctgatcctgctttgctttgcacaaccgtgattgattgctacatc

tctccattggtagccagggaccattgttctgctataccttccaaattccgatttgattta

acaagggtctcaggagaaacattcttagatcagaattcagaactgttagtgagccttctc

caactaaaaataaaaatattgttctagatgtctcttctagaagtaaggctcaagtggaaa

ttatgtggatggttcaagtttgcagcatgcaagttggaacgttgtatatttgaatgagtt

cattgtgtgtattgtgtattgtctgaaaattatttgacagtaaaacttctaattctaaac

attgcttacaaatggttcctcctctggtaacaatgctgagcaaaataatgacatataggt

aaaggcaccaatgattctacaatgagaacaatggagtctctcctactttagtccatttta

attagtttccacccgatctagacaagagatggaaaagtaatacaggacaaagagaggcaa

ctttcccacccctgaatcatattggatgtaagtatgtgtttttctaaataaacatgcttg

ttttttcccctccagatatgcatttttgaaataatgtgattattatttagaaacacgtta

tgaaaaaa

>fava_dn_rep_c832

atgtctataaataataatgtactcctctactttaatcaaatgagaaatgttcagtttttt

catcattcaatgttctaatactagcaacgtataaatcaaatgactgagaggtagatactt

atttctattcttatagaagtagtaatttacaaatcaaagaactaataattgtaaaaatat

atgtaacctgttcatcatacacaaaaacatcaatgattagctcacttttcttgacaaatg

atttctcaagtggaatcttcaggttggatatcatcgttattgaacatgattcttggcttc

cccatgctgaaattcaaaacccttgcattcatattatacttgatcactccatcatataac

aactttcttatatagtgctctatatctgaaccaatcagtcgaagtgtgtattcattcaat

ggagcaccctcgtagtgatcgacaagctcctgaaagtaagaataaaccaagttacaactc

tcaggtttccacacaaactcatcagtaggatcaagaataagggtaagcttatccatctgc

gtttgatcaaaagcactagtttccggatcaatatacgcagcatagattcgaacatgtcgt

gtggcgcagctcaaccactgtcctccatattccctactcatacctttgttttttacattc

acctgaggttcaactggtcttggttctgttccctttggattcatttgttggttttctggt

atttctttctcttctattaccttcacaccttctgggtctttaacatcggattgttgttga

catgatatgaaaaatggtgttctggtttttggttctcttcttcttcctcctcctgtccag

cctagtatggataatttggttgaaaccgagtaagaacaacttgtggccatggttgatttt

tgatgcttttttgttgcttttaatggcttacaagtgggtcttaatattagtttgcatatt

tcttagcttccccgc

>fava_dn_rep_c833

gccaaaatgaatctcaactgaccaccaggatattaacttattgttttgaaacattttctt

agtagatgcatttatttttcttataagaggcacacacaaaatagaagaagttgaatatga

aggtttttgcaatacttgaaagaagaactagtagcaaaacaagttttattttgtcctaaa

cagggagaatgtaaaatacataacttacactactattaagaaaagtaactaattctttct

attttgctactaaacaaaaagcataaatgactccattaatagtcttttttccaacaaaag

tatttacaaataactgtaataacagaacaccatccacaaaatccaagaagaatttaacaa

gtaaaaaaacaactttcccattcccaaacccacttgtgcttatgaaatctagattctcat

acaaaaactatagttgagatcaccgagaggatcctaacttcggttttcgaaacaagaata

cacagctaaaaaagttgtagggaaatggtttggggagaatgtgtccatgataagagaaga

aaaataaaacatccaaaacttctgaggaacacagaaaggaaaatttcatatttccaacca

tactccaaacttggactcgggatcagaaatcaaaggatcataaattttgtatggcgaaac

tcgacttctttctcccttcatacattcttattgattgatattgataggatcggttcgacc

ctgcacggccatcctctccgagacatcagctaaagaactcaagttgcacctgatcaacgc

ctctacaaagtaacacgtttcatctctcgtgttcccttcaggcacgtccacgacaaatga

ttcaatcaccattgtacctggtcttccatcaataacctccggatgcacagtgattatgga

agaatagttcctgagcctatgatcaccaccaacaatcctgataccgagaatatgttcttc

atcatcaagctgttccaacctctcagtactagttgtagctggaagaccagatttaacatt

cacttctctaacacttccaatgccaagatcaccttgcataatacacctgctaacaaatgg

tttatatttctgtggctgatcaaatcttctaaccagagaccaaacaagatgaacaggggc

tttgatgtgtttaacaagagcagaggaacactgattgtctcttaaatcatgtttgtgatg

tcttcttatgtactgtgtttcaatagcagtgtgttgttcaccaccgttgttcatgttgtt

ggatgattattgttttggtggctagaacaaaagatgaagaagaaaaagatagaacaagtg

gtggagagggataagcttgagagagagaaaagaaagtagctggtgtaaaaaagtgtcaac

cttacttcccgcg

>fava_dn_rep_c834

tttttttttagtacgaaagcgtagtttattaatttcactccaccacataataattataaa

atatatgacttctctttttctacagacaagaaatgtccaaaaacaaggaggatacattat

ctatttaatggcctaagaaacaaccacctagtattatatcaacaaactcattaattctac

aacatttgctcatattttagaatatgtggttgcaatctatggcttgttttcatctctctt

ctccacttatcataaattcaatctcatctgctatatgttgttccaatttgtttgttaaac

acagatgaaaaatacaggtctcattcattcattcacattttccttattcattatttagtt

caaatcacatgcaggatctaaaacttaactgaagatccaaacaactaaacactaaatcca

cataattataaactttaagcatccacaatatcctctgattcaacagaagccaaaataagc

tctggttcttccctaccagattcacatgttccatcaattgcattgttcttccccaatgcc

ttcaatgccaaatgagcagctttctgtccagatatcatcattgccccaaatgttggaccc

attcttggggcaccatcaatttctgcaacttccataccagtaacaatcataccaggaaca

acctcccttgtaagacgaacaatagcatcttcagcagtattcatatcaagtgctttcatc

cccggaacaaaatcaatcattccaatactcttcaacctcttgacaccagtagcaccaaaa

ggcccatcatgtccacaagaactcacaacaatcttcgcctccataacattaggatccata

cacgactgtgtatcatggttcatcgaaaccaaagcccagttcgtcacaacaccagcaact

ctaccttccttcacaatcaaatcctcagcagccacagcattgaagagcttcacgttcggt

ttcgcgagaagcttactcatgatggttgaagtgaacaacgcagcgtgtttgatgacaacg

tagtcctcttgctcgtcgtaggcgacaccgatttcgtcgaggaagaggtgagctggtttg

cggacaaccatggcggagaagagttgtcctcctagccatgcaccgccgccggggctgacg

gactgttcgatgatagcgatactgatgttgggatttttgcttagttcgtatgcgcatgag

agaccggcggaaccggcgccgacgatgacgacgtcggtgtcggcgtatgtgatcatgtct

gtcatgtaacggcgggtcatttcacgggagacgatggattctttgatagggttgaatttg

aatgagtctagatcgtaagggggtgtggttagtgacatggagattgaatttttctgggag

gtggatttcaggagggtgggggtgcgagtaagaattggtcttccattgaagaatgaggat

ttgggagaagtgatgagagatgatgcagtggccatggctgccattggagggtattttggg

aagagaaaatgtttgatgaaaaatggtatatttgtgagggtagaattgaatgccgcggcc

gtaatggccactctgcgttgattccactgctnnnnnnnnnnnattaaggggctaaattga

ttgatgcttcaaaatctttgataaatacatatgtttgtattttataatatccaaaactaa

tttataagaagaaaaattattccgaaaataatataaggagagaagaatttataaaa

>fava_dn_rep_c835

ggtggtttccgtctttctcttcttcctccttttctatttcactctcacaatctcaatcgc

tttggcgctctaaaaccagatctcgctttctctctctaggtttcatcaatggcgaccgct

catactcctcgggaagagaacgtatacatggcgaaactcgcggaacaagccgagcgctac

gaagaaatggtggagttcatggaaaaggtctccgccaacgccgatagcgaggaactcacg

gtggaggagaggaatcttctctccgtggcgtacaaaaacgtgatcggagccagacgtgcc

tcgtggcgaataatttcgtcgatcgagcagaaggaagagagccgcggtaacgaggaccac

gtcgctgttatccgtgattaccgatctaagatcgagtcggagctttcgaacatctgtgac

ggtattctcaagcttctcgattctcgtctcattccttccgcttcctctggagattctaag

gttttctaccttaagatgaagggagattatcacagatacctcgctgagtttaagaccgga

gccgagcgtaaggaggctgcagagagcactctcgccgcttacaaatctgctcaggacatt

gcaaatgcggaactaccaccaactcatccaattaggctcggccttgctctgaatttctct

gttttttactatgaaatccttaactctcccgatcgtgcctgcaatcttgcaaaacaggct

tttgatgaagctattgctgaattggatacccttggagaggaatcatacaaggatagcact

ttgatcatgcaacttctccgtgataatctcaccctatggacctctgacatgcaggatgat

ggtgccgatgaaattaaagaagcagccccaaaagcagatgagcaacagtaaacaaattca

attgcctattagaatcaaacttctcctctataagttaatgaatgaaggaagaaggtgctt

gttgccattctctttgggtttgagattttaggcattcatgtctgttgagttctggagtcc

tgggctgttttagtttgtccttccatttttaagtgttttacaaattattgcctattttat

tgaggacactgaagtgttattatatggaattggaaatttaagattcccttctttctagta

atcaatatcactttagctttatttgtatcggtcgtgaactcatttggtggtgcatcaata

tcacctgttataatctagggatgcccactcaaaaaaaaatcgtgacaacacatcatgagt

aacctttttaattttatatttaattatttgtgaacaaaaaa

>fava_dn_rep_c836

tttttcatattaaaattataatatatcatttttgtaacataatcatgtgatgaaaataat

gtcaatcataacagatgtagctgcatttctatcagaaataatggagataaaaatgtatat

tgatctttctccaaatttttaggcatacaagtttatatatcactttagaaagagcataaa

cctatttaaaataaaaggaacataaagtaaatggaaaaaaaagtttacacttactctgcc

tcaactcaaagcacaaagcttcaattaagaacttatttaataagaccatatcaaataagt

ttttatgtcttcattgcttttacaatccaagagaaaatcaagataatctatacaaaagag

aaatgaactttattttccatttcttttgcgacaaattattttgaaagcttaatgcaaata

accttgtctgagaaagtcacagttctgatgtaatctttctcgagtttattgagtataagc

aactgaaaatcgttgtgcggtgcatgaagacactgatctttttagagagtaggagggatc

catatataaccgtgtgtttgaacatatccagctttttcgagcaactcatcggcttctgca

ggacctctgcttccagatttgtaagggattgacttgagttcacccttatcaattctgtgc

aaaagtggtgtaaagatctcccaagatgcctttaactcgtctcggcgaacaaaatgttgt

tgatcacctctaattgtgtcgagaattagacgctcataggcctctggtatggtcaccccc

tggtagcgttgtctgtatgacaaatcaagttcactttgagctgttgacatctccagacca

ggttgcttgaccgtaagcttcatgtacatagcttcggaaggttgcaggcgtataacaaac

tcatttctaccctgcttctgacacttgaaaatatcgccaggaacatccttgaactgaatc

cgtatgtcagcttttcttgatcctagggctttccctgcttttagtatgaaaggaacaccc

tcccatctttcgttgtgcacacgcagaacaacagttgcaaaagttggagtgttggaattg

tcaggtacagttggatcatctctatagccctcatactgtccaagaacaacatcattgtct

ttaataggaagtacggattgaagaaccttcactttctcatcccggatatgctcaggcctg

agagaaacaggcttttccatagcaaccaagcaaaaaatctgtagcagatggttctgaatt

atatcccggataattccgtattggtcgaaatatcctccccggccttcagttccaaaatct

tctctgaaaactatctgcacattagcaatattgtcgcgattccatagaggcaagaacaag

cgatttgcaaaacgaagtaccaacatgttctgtactaattcctttcccaagtagtgatca

atacgataaatttggggttcttcaaacaaccccccaatttggttgctgagttgctctgca

gattccagatccttaccaaaaggcttctcaacaacgacacgtgtccacccaccaagatca

gatttattcatgcaagtagtcttgatcatcttggaaaccgatggatacaccgaaggagga

agtgcaagatagaaaaggcgcctagaagagccctctgtagtgtttgtttcagattcatgt

ttggaaatctctttatccaacaaacggaaatcatcctcagaatcatatgagccacttaca

tatttgaccagatgcaaaaacttcgatacagcctccaagtgttcagaggaagcatctttg

tccttaataagatagccacgtaagcggtttctcaattcttcatcagatatttttgttctt

gcatagccaaaaataagaacgtcatcttctagtaggaatccctgcttgtacagattgaaa

agtgctggaaatgtcttcttcttagcaagatcaccagaagcaccaagcacaacaattgag

agtgacccagtctcaggtccattttcattatctaccgatagaggagaatcattcgttaaa

gcgagtctttgctcacaatgccaatcagtcgttcccattctctagcagtaccaaaaacta

gtaatatagtataatccacagaaccttgtatgctgataacgttgaagatgaaaaatcgaa

atccaaagctttggaaattgtaaggttccccgatggaagagttagttgaaaagtactttt

aacaacttaacattttgagtccaaccaacgccactgatacaagttgcaagtcttttcaaa

tttgca

>fava_dn_rep_c837

acagtattaaatctctagagaaaaaactgatccgcctatatggttatacaagactcgaga

gattaatctctgcaattatgcgcaaaggatatcccagactaataccaaaaaaaaaggata

actgtaaatcttgtgtactcatgcacaaatattaaagactcatgatgatgaactcattga

gtttaaccaagcaaaatatcaattggggaaaagaatttatactgaagattcaatttataa

cctccgaaggaactgaaggaaaactttcacaaattgccaattccataacagttttacact

acttgctttgaaatgcgagaatcatttaagtatccttgttgtctttttccctccattcag

ttcctcctccgcaactcaaaaaagtatgttgagccgaccagtccttcagttggaaactaa

gtttgtgcagaatacgaaacatcaaaagcagatcagatcaggatttcttaggcctcttag

aagttccttctcttttatcgatctctgcagacttcttcttgtccttcctttctctcttct

tcagagcagtcatatgggcctttaatatagttgcataagaagcttgaaagcgttgatgat

cctttggtccaaccgaagcagaaatcgtctttttcccatcagtggcccgtataagacatc

tatactcaattggtttaccatcagtagccagtttattcctctgaagtttagatttcaagg

atgatcgtttgagagtaacccaaacagaacccttctcggtactgcgctcgaacaaactgg

tgagttcattgagaaacggatctaattgtagcaaaaccatggttgagagagagaagtttt

tgatgctgaaacgcagtcgtcttggagagaacttaaagcaaatgaactataaaagagtga

gcacggaaact

>fava_dn_rep_c838

tccacctcagattttcaaaatctctatcttaatttaatgtttttctccctcaccctctca

tctacctttctctataactacacaaagagagaaaaaattcatatcaattagtatatttat

atattacactcttctgttgccagcaaaatttctagaaatgggtgaggagaacaaggaaga

aggaaagaaagaagaaagcaaagaggaaaagaaggaagaagagaaaaagggagaagaaaa

gaaagatgaagaacagcctcctcaagagatagtactcaaagttgatatgcattgtgaagc

ttgtgcaaggaaagttgcaaaagcattgaaaggatttgaaggtgtggaggaggtgagtgc

aaatagtaagagcagcaaagtggtagtgaaaggaaaggcagcagaccccataaaagtact

caaaagactacaaaagaaaagtggtaagaaagtggaacttatttcacctttgcctaaacc

tcaagaagagaaaaaagaagaagaagaaatcaaaaaaacaccaaaggcagaagagaagaa

agatgagggtcctactgtggtaacaatagttcttaaaataagaatgcattgtgatgcatg

tgctcaagttatacagaagagaatccgtaagataaaaggagtagaatcagtggaaacaga

tttaggaaatgatgaagccatagtaaaaggtgtaatcgatccagcaaagcttgttgatga

agtatacaagagaacgaaaaagcaagcttcaatagtgaaagaggaggaagaaaagaagga

agaagagaagaaagaagaggagaaaaaggaagagaaaaaagaaggagaagaagagaaaaa

agagagtgaagaagaaaacaaggaagaagaagatgataataaaagtgaaatcaagcgtag

tgaatattggccatcaaaagattatattgattatgcttatgcacctgagattttcagtga

tgaaaatccaaatgcttgttctattatgtagacttgttattgttatttataatatttctg

tattttagtagtagcttattagattaaattaattttctattgctatgtaatatagtataa

taatgaatattttagctttttgatatgtttatgaactttcaatgtattttgctgcaaact

catttatgttggactgtttgtgttaaggtgttattgagttttgccctgcaatttctgtat

acctataatgtttgacaaaaataatatcaagtctttaatgttgacactt

>fava_dn_rep_c839

ttttttctaaaggattgctcaacaaacacttaccaaagaaacaacatcaattaatacttt

attctttttagtaacttggtgtgccctctcatttattaggcaacatatttggactatgag

tcaatgactcacatgaaaacacttagcttccctcccaccaagaaattggaactccgaata

aattaatcttagtaggtattgacttccactccggcgcaaagcactaaatcaatcttagta

gttatttcaaataagaagttgctttaataatccataacaaatgagccctttaacaaccaa

ccaacagcaatcaaagtcagtaaaaactactttgaaacttacttttaacctaaattcaac

agattaaacacattcaataacatatcatgtatcaaattattagttacataatccattgtt

gaaacacagcacaaccaccggagtattattgccctacgaattcccgacggctcaccgcat

atcgtttcctacattttagccaggaggccagtttaactgacgaccaccaataacgtgcac

atgaatgtggtaaaccgattggcatccttgtggcccgtcattaattacaacccggtagcc

ttcggcaagaccttcttgcttcgcaaccagctttgcagtgtagagaagtcggccaagaat

atcgaagtgcctctcctcagccttggagataccagtcaagccatctctgactttaggaat

gattaaaatgtgcacaggagcttgaggatttatgtccctaaaggcgagcaccttatcatc

ctcataaacaacagtagaaggaatctctttgttgatgatcttgtcaaatatggtgggaga

atcggagtcgggaggaatggcagcaagggcagcttctttctcagaagccattgaagttga

atgagtgaagtgtgatgtcaaaacagaaattctttcacctgtgttgttgttgttcttcat

atagattgaaaacgtgcttattcagataagctgtaatgacttaaatgtccatgcttccaa

acgcaccgtatggttatagatcgcttttgcaatcgaaaagtttttagtttttatattatt

ttgcttgaaaaaa

>fava_dn_rep_c840

gtcatagtcctacatgagacccgagcacctgcataaagcactaagcagcactagccatgg

actcaataaggatctggtaaccttctggtacagcactctgagtcagtacacattctgtca

atgcaggaacatcagcattcacgtccaaaacccttgatagtatgagaagaagatgaaaat

ctgaaattcgcttcacaaatggaagacttttagtccgttcaagatgattcttcagcgcat

ttgcagtcacttgagtactccgattctcaacgggaaatgttgatgatagaggaccctgat

gatcagcaattttgacaaccaccaagaagaaatcattatcaacctctttagtatccttaa

ccccaacaaccacatccttcttcatgacagacaacttaggatcatgatcctccttaacct

cagtgtcataccaaccttccttaaacaacctaatacacacatcactcatctgaaaagcct

caaaatgcacatcagcaccacccgcttcctcattcacctccagcttgacgacggcagtaa

cccactccttcaaaccactctcagcatgatactcagcagcctgcaagacttcccgataag

acaaagtataatcctttttggcctgactcacactctgagtaaaaataaacccaaccctcc

tcatccccaaccccgcggcaatagcttccacatatttctcctcctcggaatccctaaaaa

acataagattctcctccaacccttgctgcggcggctcatatataaaattgacctcaacct

tcccctcctcagaaacagtcccatacataaacccacctctcttaacagcaaaagccagcg

tctcattgacatagtgctggaacgcgttagcgcaatcacgatcaaacgaaacaatttcgc

aatgcggattctcttgcctaccaacacgcatctgtttggcgatgagatcatccatggtca

tcttcttaccgaaagaaccggcgggactgaaagcagctggaccttgaaccctacgttcgc

cttcgtaggctaaaaacacgattgagccgtgggaaaggtttaaggcagtaagaggagtgt

tagggtttgacatgtcggtgaatcggagaagatcttcgcgagatttggcgagtaagaggt

tttgattagtggagagggtttggttgtgagtggggattcctagttgtttctcgattatgg

atttgaggtcggagatttttgtggcgtttgggttttcgagttttactcgttcaaggccat

cgcggcttcgaattcggagcatcatgattatcgacaagatggagacggaattttggattt

ttgatgttggaatggaataactgacctgagaggaaaggattgcttggaacggaataatgg

aaaattggtggggaagaaaagcagttacttcagctttgagggagaaagggaaagttacta

tactgcagcaccgtttgactttatgttatctaagtgtgtgggccccacaacgactctcaa

ttatcatgggctccgtgcccgcgtttcttcgtcaggcctt

>fava_dn_rep_c841

caaccgaaatagatccaataaaatcggaccaaaataacaaagaagaaaaagaaaagaaaa

cctgagtattacactccatttctgcgaagaaaagtgtgcgtgaaagagaaaaccagaaac

cgatcgaagatgtttctccgtgcggcggcgcggccattgatggcgaggataaagcaaaca

acagggatcgtcggactagacgttgttccgaacgcgagagaggttctaataggtctttac

agtaagacactcaacgagatccagaaggttccaaaagatgaaggctatcgtaaagctgtt

gaaagctttaccaatcacaggcttaaggtttgtcaggaagaagaagattgggagaagatt

gagagtcgtcttggttgtggtcaggttgaagaactcattgaagaggctcaggatgaactc

aaactcatcggctatatgattgaatgggatccttggggtatccccgatgatcacgaaatt

gagatggttgaaaatgatgcaccaattccgaagcacgttcctcaacaccgacctcttcct

ctccctaaggaatttcgagatacgttagaggcacttatgtctcaaccaggaatgacagaa

aagctagaggctcttgtgtctaaatcagtaaaagatggtgctgctgccagctctagtgaa

gaaccctcaaagccaaagccatgaactcagctatgctttgaattctccatttcaacgtgt

tttcccttttattgcattattaaactaaactggttgtgttaagtagtttcagggacaaat

cctctgctccaaaacaacttggaaggtttgaaataacaagtaatatcattgtactctatg

catgacatgattttttatattaagagcttccataaagaactatctgttgaataaatcagc

attttgaatatcatatgctttatttatttatttatttattaaaacttcaattggcatttt

gtttatgtttttcatctagaggtgatctgtgtaagttgtaagattgattgatccttttaa

atgtttgcactttatatctcatcttatgtaaattgaatgtataaaaagtgaatgacctat

cattagacattattcatcaaagttcaaaa

>fava_dn_rep_c842

cgtcaaaaggagcagacaaagtaacacagtccagttttttaaacaacaatgttcgaatcc

aacttgatattatcccccaaaataaccacaaaaccaagcattcatctgtgtagtccacat

ccaacgttatcagcaatgaacgaacgtagattactttgttattttcggttacatcaacta

ctacaaacataaacggccaaaataaacaagaatctaaagtcatgacattttaacataaac

aaaacaataacataactactgtacaaggaacacaagcatcaaatctagtctcctaagctt

gctttctctgaatccatcgacctttctcttctcacatcaaaaagacaggagttaggagac

tatagatcataaatatatctgtaatctttgaatccaagatgctcgagatatagactataa

tcatcattatacgccattttttaagcaacgcttatgatgttatctccgttgttgaaacat

ggcattgcgtgtaaagctgagtgtgttgatgatgtcttcctttgatcagatgagaaggaa

agcccaagtgagagtgttggaggttcaatagatgaacttgaactagaggtgatatcagag

acagtggaagctttaggatctgaaacaacaaaaggaattggatggaatagattggttgtg

aaattttgtttcgcatttccttgtcctagagtgagttcttccattggactctcaccaaca

cccaaatgatatactgacataaaaggaaattcattgctttttctagtttcaggggctgca

ggacacatgggctgtacttgtgaaacactgtcttgattcttaacctggtcttcctccatt

ggaattgcagacaccgagtcggtggtgatatcaaagagactagatctacggcgacggcga

ttgaggttacttcgccggagaaagtatttctgagcatgacttgcaacctgcgtcggcgtt

cttgttttcacatagtttcttgagattcctctccaatcacctttccctactttctgcaat

ccaaccaagaacagcttgtgctcttcctccgtccatggaattcctcgtttacgctcgcgg

tcgcggttcctgacggagttaatcggaacggcgtcgtcagcggaggcataaccggcggcg

atagcgtctttattattatcgtccttgatattgttgttgttgttgtcgtgagattgttcg

tactgtgagagattgttcatgctaacactcttcctcatggaatcaaccacaactctaact

ccgaagagcatgaattcgccggagacggcggcgggggaggcggaagacataatcggaacg

agcgatgattctagaagaaaactgactcggtgagaatcggtggaaaacgagtgagtgagt

gagtgactcggggaaaggaatagaaatagatagagaaagagtgagtgagagaaagaagag

atggatagagagatgagactcagtgcgtggtggtgaatataagagtttcagacggcaatg

aggt

>fava_dn_rep_c843

atctcataaactatttcctttcattcatgcaattcaccttcatatgtatgtttgcttata

aaaaaaacagtacactatacgtataatgtataaatatgtaagacaatacacaccaaatat

atatctctatatgtatatcaacgcatacattacaacaaaactagagctatatatagtgaa

attaattaaaacttaaaacacacgtacacatacatgttcttcaaacatataactaagcat

ttatatttatacatgtctctataatagtatatatatgaaagtcttatattacactaagat

gattacttattggattatttatattatatagtacttatatttcaggtaccaacttccaag

gattcatccactgaacgtgataattcaatttggtatgagcttataaggaggcttagaaaa

agttggtgttggatcatatcgtccataatcttttgtattagccctaagaagcctctcatg

gataatgcttacttcttcctcatctattgccttcattgttgatacctcatttactgcatt

catatcttcatctgtgattctcatgaactttgatgctcgacctgcaaaagaagttgataa

aagtgatccagaaatgaggagaaagaggagtgtaaacttcttgagaaacatgaaatccat

gatgagtgtggtccaagttggtatttttctacatgtacctttcacaaaacaaatgtttcc

tatgtatattgcaaggacaaattgttcgcaacaataactgaagttgacagttttcttaat

ttgaaataataagaattgaatatttctatacaaataggccccccattgttaaagaatact

tgatgagaaaaggctattattaatctggagaataaagttgtctgctttttcaaaaatgag

aaaaggctagacatttgatcatgaaagaataatgataatcttgtttgcatacatatgtgt

acaaaagaaataataacatctagcatgtacaaaagaaataagaacatctagcatgacaga

caaacgaaacttcaaaaaatattatcttgaatgtgatgatttgtttgcttttaaagtaag

atatatttgaaattaaagtgattcatgaaactgaagtgataataggatagaaatcataac

attatctgttaggttaatttaattatttgttttttaatatgtttaaaagacttagtcttt

ataggtgttgttagtgttgttagtttgtttagtgggtagctagttttgagggattttaag

tttgtggctttgttgaattgttctgttgtactgcctatttataaggttatctattttcaa

taaaagcaaactcttgaattc

>fava_dn_rep_c844

ttttatacagtaaaaaatccattaaattgtatcagtaacatatcaaatctatttggattc

acataaaccaaaaagaaaaacaatatgcaatggaaaccagaattagaaaacaatatgcaa

accaaatgctaataaggtcttatttctaagggttttattttttggttcagtacacacagg

gtaaagcatatcccacacaatcatatataaaacatagaaacacaatagaagttccctaat

atatgttctgtgcaatcaccaagcaatttatccttcccaaaaacatcattaaattttact

ggcaaaagaaatgccttcctttgcaagttgattcacatcctccactgcaggcttgactct

tgcaactttggcaatagctttatactcatcaggagttccaccctccaaaaaggccccaac

aactttcccctctttaatccagtatgtcccaaaattaggctttgatgatgcaggatcgtt

gtctccaaatagcactgtctcaccaacattgtcaccgtagaattgccaagacaggtcaaa

tgaacgggaatagaagtatggaaggtagtcatactcttcaactgattgtcctacatctgc

tgcaatgatggcctttgcagcctgctcagctgatttgcgagcatgatcaacgtgttcaac

tcttctcacgtcattgtacaatttcaaagggaacgtagcaacatcaccaacggcatatac

atcataagcacttgttttgaagaaggcatcagtcttgattccaccattttgctcttcaac

ctgccctttgaataaggatatctgaggccttcctccaacaccaacaacaacaatatctgc

ttccaggaccctaccatcctttagttcgacttcttttacctctccatcagagttggtagt

gaatccaacagcaactgtccctttaatgatattgatccctttattggaatagtatccctc

atagaaagcagcaatttctttagtaaaaagtcttggcatacaccaaggttctgggtagac

catggtaacatcaagattattgagcttcaaaactgcacttaactccagaccaatgtatcc

tcctccgacaactacagctttcccattcttctttgccttgattgcctcgtacaatttgtc

agcatcatcaacctccctcaggtaaaatatgtttttggcaccagctccttgtacaccgaa

atctgacaactttataacagctgagcctgttgcgataaccaaagtctggaaatcgaagtg

ttctccttttgcacttttcagaaactttgcagcaaggtctgcacttacaatttctgtatt

gagatacaaatctatccctttctcactgtaccactctggaagcaatctttctcccccact

tccaacacaggtatggaacccaggaagcctagcaggagactctggaaaaaggtaagcctt

gctcagagcaggacgctcataaggtgcaaccgcttctttagatatgattgcaagctcccc

atgcttaactccttgtttcacaaactcccttgctgcataaccagctgaaactcctcctcc

aacgatgatatacttgtacgaatgcaccatttttttgattgatgtgaaaatcgctgcaaa

ttctgctgcgtatatattcagtagaaatgaaaatgaaaatggaaatgaaatggaacttga

ccctttttattggtcagatccattttat

>fava_dn_rep_c845

tgcggacaaaataagtgccattcgattaaactaaccagcaaaagatgcaaggtttatgaa

gatgaaatcgtaatgcctatgatccaaaaatatatatcaagcttttgtttctgatttcaa

tgtcttgctcaacttcctggatctagatggaagctgttgaaatatattattttcatcaat

tcagttttcaacacaaaatacatgctgaaaatgttgttcagcaactttgattacaccgag

caaaaaaccggggaaataaacaattgaaaatgtaaacttcaactacagtacttagcctgt

aagagctgagttgagagcgctatgcacgtcaactccaatctgagcttcagctttctccaa

gtccgttgttgcggagttgagtttctgagtgaactcttgaagacccttttgcactaggtc

tgcatcaagttggttcactggtacagcctctacagcaataatatcagcaacagagtttgc

atggatgaaagcgaaaccactgcttacaaaatacttggttgtatcagttccttcttgtac

agtaaggaccccaggtttcaactctgcgattgttgcgacgtgtcctgggagaacgcccat

ctcaccggttgtagctgggattataacactgtcaacctctttagcggccaattgagaatg

atagggaagaacaaaattaacagtgagtttggtagggatggtagaaggaacaggtggacg

agttttgaggaattcgagaggcgtcttaggtggatcgatattggggctaactttcctcca

cgcttcaacgaaggaggaattggtggtgggggtagccacgtcggtggagaagcggcgagt

ggcggcgcgggagaggaaagtggatgtagcgcggcggaacatggtaacggtagggttgga

tcgctcgggctcgcaagtcggagagaaaatgaagagagagaaatgagtacgattgcgtga

gtgaaacaagagagaccccgagagagagagagaagaataata

>fava_dn_rep_c846

ttattatctgttcatttctggtgtatttatctatattatcatgtttggagttcataaaag

aaatagtgatcggaagaagatgaagtttctaagtgttctcatgcttgtaatattgggaag

tagcatcaaggcctatagtgaaatttgtcaaacttctttgaaagaaatcagtaatatcaa

tagccatggaccatatattggtatagctgtgccaaatgcctatgagttgaagcctcttct

tcaatcacccagctttgtgcctcataacaagtttccttacttcgactttgccggaaaaaa

atttcgcattggtgaattggaaaataagaaagttattgttgttatgagtggagagggtat

gctaaatgcaggtcttgccacccaattgttgcttactttgtttaatgtagaaggagttct

tcattatggaattgctggtaatgtaaattctcaattccaagttggggatgtgactattcc

acaatattgggctcacacaggactttggcattggcagagatttggtgaagacaatggaga

tttcagcagagaatttgattatctaaagttttcaaattacaataactatacaaaacattc

gaaatcaatggaaaatcttttgaataagatttggtatcaaccagagcaaatttttcctgt

ggatgggactcccgaagtaaagcagcgtgctttttgggttccagttgacaaaaattactt

tgaaatcgcaagaaagctcaagaatgttaagttgaatagctgtgttaacaaaacttgctt

gccaagaaagccaattatggtgagagtgaagaagggggtgagtgccaatgtgtttgttga

taacaaagcttatagaaatcatttgaactccaaatttgatgctactccaactgacatgga

aagtgctgctgttgctttagtttgtttccaacacaaaatacctttcattgccattagagc

attatctgatttagctggaggtggttctgcattgagtaatgaagcatctatctttttatc

actagcctctcaaaatgcatttgatgttcttgtcaaattcatctctttgatgatttggta

gcgttaatgtgtttcttgaagagtaagcaagggatatatttttagggagaaagaaatcat

atgtttcaaattctctcttatgttatgtactaaaaaataaaagcatagtggaatcttttg

tattttctctgctttcactttattttatatttatctttatgattaagattacgtacgtgt

ttacctttaacatgtgcgggtagttttatcatagacttaggatcatgaggatcgtctaaa

cgacataccttatgtaggctcttaatttggtatcttttatgagaaatcaggcattcacta

tggcaaaaaaa

>fava_dn_rep_c847

aagtgaagattattgaatggatttcaactttccatacggcaagttgtcttgaaaatctaa

agtttctccttaacttcagaattttcaataaaatttataatacaaaccaaaagataaatt

tatgaaacaaactaaaaggtagattggacatgtactcttcacattgcacactaacatcaa

aaagtactgaaacgaatagcaaccttttcaatggcaccgacagagtaaagatatatggcg

gataaaagtaaaaatctcaatcacacgaagattaaattgaaattcataaatctataccaa

aaaatgtcaacggtacaaccacaaaacgcataacgttacagaatataacggcacaaactc

aacacaaaggccaacactctcaagggtccttcctattcaaatgcatcatcgtcatcatca

ggaaggggttgattagcagcctcaagaatctcattctcgtgcatttgttggacagctaaa

tcaatttgaacttctggtggagccagtgcaggagattctacaaagtgcaagttggtatca

cctgcaagtttcctggcaagatacaggaaaggcttctcaaagttatagttgctctttgct

gaaatctcatagtactgcaaattctttttcctgtggaaggtaacctgctttgccttcact

tgcctgttcttcacatcaaccttgtttccacaaagaacaattgggatgttttcacagacc

cggcaaagatcacggtgccaagtagggacattcttgtatgtcagccgagcagtaacatca

aacattataatcgcacactgcccatggatatagtatccatctctgagaccaccaaacttc

tcttgaccagcagtatcccagcagtagaaacgaatcttgccacagtttgtgaagaaatcc

aatggatgcacttccacaccaatagttggttcatatttcttttcaaattctccagtaaga

tgcctcttgacgaaagttgtttttcctgtgccaccgtccccgacgatcacaagcttgaag

ctaggataatcaactgtttgctgattaggcaacgccattgaattgaatggaatcgattat

ggagaagacgctaggttgcgttgtgtttcagtgtgaggaatgaatgaagaaaggaatggg

agggtttttatatacgaaaaagggtttcaattatgtttt

>fava_dn_rep_c848

cacaaaccttttcttttttctcactctttctctctctctagaaaagaaaagagaaagaaa

aggttggagagaatatagcaaactccaaaaccatcacaataaaccgttgttcgatagatc

tgtggagaagaagatgggacataatcaacgatctctgatctacgcgttcgtttctcgagg

aagcgttcttctctccgagtacaccgagttcagcggaaacttcaattccatcgcgtttca

gtgtcttcagaagcttccagcttctaacaacaaattcacctacaactgtgatggccacac

cttcaattacctcgtcgataatggctacacgtattgtgttgttgcggatgaatcggttgg

aagacaagtgcctgtggcttttctggagcgtgtgaaggatgattttgttgcgaaatatgg

cggtgggaaggcttctacagctgcgcctaacagtcttaataaggaatttgggccgaaatt

gaaggaacatatgcagtactgtgttgatcatcctgaggaggtgagcaagcttgctaaggt

gaaagctcaggtttctgaagtcaaaggtgtcatgatggagaatattgaaaaggttctgga

caggggggaaaagatagagcttttggttgacaagactgagaaccttcatcaccaggcaca

agacttcaggaactctgggaccaaaatccgtagaaaaatgtggctgcaaaatatgaagat

aaagctgattgtattggcaattttgatagccttgatcctcgtaattgtcctcccaatcgt

tcttcggaataaataaatgccactctcccagacatttatcttgttatttcgactttcttg

tgattatatatgatatgatgaggaaagataccaggtttatggcatgattttgtatgtagt

caagatattaacctgtataccgtttacctttaagcttttacagtatgaagatgatgatga

tatatgaatgattttatatgcttttttggttgtttatcgtagttcaatttcaattcacct

gatccatcttgttattattattcgtgcaatttaagttcctttgtgtatgaatattcgttt

tcccgagggaaccaataaaagacttcaaatat

>fava_dn_rep_c849

gcgggaacgactcaatcaaactccgcgaatccaaaggcaaaaaacactcgtgtctgaaac

taacttcaattcaattcaaacatgttatgcggcgtcgtcagcacttccttttcttccatc

ttacccagaaataaacaaccctctctctcctttcattccaaatcaatctcatgttctctc

ccaggagctcttgtagaatcaaaagcaacaaatgttgctgaaccgcttttgcttaatgcg

gttcgaggactagatgttgaaagacccccggtttggcttatgagacaagccggcaggtac

atgaagagttaccaagctatctgtgagaagcatccttcattccgcgaaaggtctgaaaat

gttgatcttgtggtggaaatttctttgcaaccttggaaggttttccagccggacggggta

attttattttctgatattcttacccctctttctggaatgaatattccgttcgatattgtg

aaagggaagggtcctgtcatttttgatcctgtttactcagccggccaagttgatcaagtg

agggagtttgttcctgaagaatcagttccatatgtcggtgaagcactatcaattttgagg

aaagaggtggataataaagctgcggtcctgggttttgtgggagctccattcaccttggca

tcatacgtggttgagggcggttcatcgaagcacttctcgaaaataaagagattggctttc

tctgaacccaagattcttcactcattactacagaaattcacaacatccatggctcgatac

atccaatatcaagcggacaatggagctcaagctgttcagatctttgattcatgggcaaca

gagcttagtccagtagattttgaacagttcagtttgccttacttgaagcagatcatcaat

accgtgaagaaaagccatccagaactccctcttattctctatgcgagtggatcaggaggc

ttacttgaacgaatagccgcgactggtgtggatgtagttagcttggattggacagttgat

atggccgatggtagaaggcgattgggaccaaatgtagccatccaaggtaacgttgatccc

ggtgttctttttggtgcgaaagagcttatcactgataggatttatgatacagtgagaaaa

gctggtgtagggaaacatatattgaatctaggtcatggtattgtagtaggtacacctgag

gagaatgtagcacatttttttgaggttgccaagggagtgagatactaagttaattatgat

tttttttatattagcatttttgttcatttaatttaagttaatataagattttggtttgtt

aataattttattttgcagcaataagttactgtagttagttacatgactaaacaggtttta

catgatgacactttgccaagtttacattttgcataatccacttgacttttattt

>fava_dn_rep_c850

ggtcttcttcaacacaacgcactctttctctctctaaaaccattctctctctatataact

ctctctttctctgttttcttttttttctttttcagattctgtttcttcaaaatatttcat

ctctctcgcattgatgtcggaaaagggtcgcccactacccaaattcggtgaatgggatgt

caatgatccagcctcagctgaggggttcacagtcatttttaataaggcaagagatgagaa

aaagacaggtggcaatacagagtcacctggaaagactgccactgaacctcaaagcaaacc

tgcatcagatcccggcaaacctcagagtaaaaaatggttttgctgcatacaaaaccctcc

tgcagaatcataacaatgcggttcaggtgaaagagaacatcgatgtttatgtgtaactag

ctccagagtattttctaaaagataggagctgtaaaagtgagctgcaatgggggaaaggaa

agaagagaaagtattatttatatatgtgtatcagtgttgtggtttatgattaggattcaa

ggttcttgctttgccttgtattatgagctagagcgttgcttgtcacactgtactccttgt

attatcaaactagtcctattttattgatgcattttattcctttgaataaaaaaaa

>fava_dn_rep_c851

caagaaataaacagaagtgagatcgagagagtatataaatggaagaagtgtaaaaacgag

atcttcaacaagaagattaacctcagtgttagaagctactagaaactgtgaaatctcact

acaatgaggaagaggactatcccttccgcgcttcttcttctctgtattctctttctcttt

gttgcagatcaaggtcaaaagtttcaggcgaatgcggaggataattccgatgagcttgta

gatccacctaaggtggaagacaagatcggtgctgttccgcaaggactttcaaccgattca

gatgtggcaaagagagaagcggagtcgatctcgaagagatcgctacgaagcaacgcggag

aagtttgagtttcaagcggaagtttcacggcttatggatattctcatcaattcgctttac

agtaacaaagatatcttccttagggagcttatttcaaatgcttctgatgcattggacaag

attaggttcctttctctgactgataaggaggttttgggtgaaggtgacaacgccaagctt

gaaatccagattaagttagataaggagaagaaaattctatctattagagacagaggtatt

ggtatgacaaaggaggatttgataaagaatttggggaccatagctaaatctggaacttct

gcgtttgttgaaaaattgcagacaagtggtgatctcaatctaattgggcaatttggagtt

ggtttctactctgtgtatcttgttgctgactatgttgaagttattagcaagcataatgat

gacaaacagtatgtatgggaatccaaagctgatggagcatttgcaatttcagaagataca

tggaatgaacctctaggacgtggaacagagattagattgcacctcaaagaagaggctggg

gagtacgcagaagagtttaagctcaaagaattggtgaagagatactctgaatttattaac

ttccctatcaatatttgggcaagcaaagaggttgacgtggaggttcccgctgatgaagat

caagatgaggactcaactgatagctcctccaaggaagaaaatgaagatgaagatgctgat

aaaggcgaagacgaagaggaaaagcctaaaacaaagacagtgaaggaaacaacttacgag

tgggaacttttaaatgatgtgaaagctatatggctgaggagtccaaaggaggttacagag

gaagagtacaccaaattctacaactctcttgccaaggattttagcggtgacaaaccttta

tcatggagccactttactgctgaaggtgatgtcgagttcaaggcagtcctttatgtgcca

cctaaagcacctcaggacttatacgagagttattacaattcaaacaaatcaaacttgaag

ttgtatgttagacgagtgttcatttcagatgaatttgatgaattgttgcccaagtatctg

agctttttgattggtcttgttgattctgacactttgccactgaatgtatcacgagaaatg

cttcaacaacacagtagtctgaagacaataaaaaagaaacttattaggaaagcccttgat

atgatccgtaagcttgctgaagaggatcctgacgagtccactgacaaaaaaaaaagaaag

gaaaaaacaaaagg

>fava_dn_rep_c852

accataaggcttcacattttcttctcattttcatgtacctgtgctaaccttcagttaaca

actaaaacactatttttaatgtaataacaaaacagtaaaattgaaagagcaagacaacaa

cacaatactgaaactgaaagtaccagcccttattattgttctacattgcaccataacagt

attagtattaaatcccataacaatttaactcaaatttcaaaaacaccaattgaacagaaa

caaccttcatcaccaattcaaagactcgccgattgcggtggcggaggcggcggcaccaca

taatcaaccaccaccacactccccacacgatccctaactttctccttctgatactccacc

agctccttatccgaatcaacttcctccaactccctccccccgggaaacacagcaagcgaa

gcaaccgaaaaacccttagccctcgccggcgagaaattctctccacaagtaagttgacta

atctgtttaaaactctccggtattcctctaataacctccacaacctcactgctaccaacg

ccctccttcaacttcaacaacgtcacacgaatcgccgaccccgttgccggaaccaaatcg

gcatcgtcagcaacccaatccaccgccattagatcatcaataatcgggagaacgcttccc

ctgacgacgcctagatggcttggatgcgccgtgtaggcttcgagatcttctttggacttg

taccgactgtggagcatgtgagtgaacgttaacgcggtggagcgattgcgtaggagaggg

cctacggtaagatgaaggacttcatctagggagatgagggaactgagtccgttaagcatg

gaggttacttttgatggttcggtgttttctttgactttgaagaggacgatgtgttcgatg

gcttgggttttggttgatgacgtcatcttgatcgtggatctgaacggcgaggaacggagg

tggtgtgtgaggtgttttggagatgctgatgatgggatgcggaatggaaaagagaggtgt

gttttcaaacacagcattgtttgtttgcaatgggaaatggtgtagtgaaagtgattggat

gctgctgatgagtcacttgattcgtcaatcgattttttttagaaaatagattcattgcat

gttgttctta

>fava_dn_rep_c853

aaacttgaaaataatctagcatacaatttcctccaagtagattcagcagtccaaacaaat

agcagatggttataggttatatccgactatgtaagctaatgaccaaaactagcttctgcg

tgttaatagcatttcaaccttcaactccttgagaattatcttgcaagtcaaacatataat

gttgtttcaatcattactttcttctaaacaatatgtaatttatataattttgcatttaac

acgcacaacatctcagaaatccgatggcttcatgaggacactagacagagcacaaaaaaa

cacaaccttataaccgtttaaatgcacattcaaaatgccatagtcgatagtacaacaagg

aggggcgggggacatcaatattctccaggtttgcaaccaaagtaactattaatacaatgt

caacaaggggagagaaaaggaagagatatcaacctaagtgaagtaaataggccaattgaa

gtagtatgaatcccggtgtgccttgtcccatgtcttgttccacatgaactcatatggctg

gttcttcttcatcgctcttttccaatgatattgctcaaatagcattaccctatcatgcca

gttgaaggtggtggaaccggtgtgccaaaagtagggattgatgcagttcatggcggtgaa

ggcaagggatccgatccaaatgttgacgaaacggtgggggtcggcatggggaacgcctgg

gtcgccgcgataagcagagctgaaatacttgtccattctttccgccttctgtttctcaca

caactgcctcctcttctctcttatccacttctatgtgaacgactatgctgattagattat

gt

>fava_dn_rep_c854

ttttttttttatagttcatgaaaacaataatatttgattcttattgataactcagtacat

agtacatatagttcatgacacatacaaaagaagcaattcaagtctcacaacaaagacata

ataattaaagaaagaaaacagtgagaaaagtgtaataaagaaacacataagcattaaaag

aagcacacaatgatcagaggtcttatttagattatgtgacatactatacatagcatgact

cataaggccctgctattaattatagtatgtatgacccgtgaaataaacacaaagtactag

ctgggtaggaagctagtaagtagtcactctttagtgtagagcttttgaagctctgcctct

agagaatatgtgggggctgaggcattagcattccaaacaaaaactcctgcaagtaatgca

cgttcaatcaggattctgcatccatcaatacaggcttgttgactcgttggagttggagta

cttgtatcactgttgaatcctgccaagattttaagcgccgtaccataatcattaactaga

ttattaaagaagtttataaattcattttctgagcgaaaatactgattgtagaacttgtag

ttaatccaatcaatattgtctggcttgttagtgtacaattcgtggtattggaggttgaca

agctctgtgggagaaatggacaccaccttcatggagttggaaacatcgagatcatcctta

aggcgttgtatgacttgccctatgcaagtgctaaattgattcatcttaccgatattgata

ttgatgtattcataattaatatcaataccatcaattacgttatcgtagtcttggatgagc

tctttgattgatgttacggcattatcaatccaactactgtcatcctgaggattgaatgga

aattcagcacctttgcctcctatgcttattaccacattcacgttcttatgctctttcttg

agattcgccacttttgttgggctaaagtcatcaaagttccaagcgcgcttgaagaatcct

gtacctgctccatcatcataagtgtcattggcaaatcccagaatgaaatggaattttggg

atgtcgccaatgatctcagatggaaaaccatttatattttctccaacaatgtactctcgg

aaaatggtaggcttcacaacggctttgatatccggttctgtcattgtgactttagttggt

tggtggtcccgcggcc

>fava_dn_rep_c855

gggagaaacttttagtaccattaccaggtactagtacattgcagtaatatgaatagacta

aacactagactgaagtgttttttacttttcagaaagtagtttgcacttgtcacattgtta

aacttaaaccaattactacacactatattggatatttatcttcaagtaataaacatgaaa

catttgtccataaatatttaatgagtacaaaggatgacagaagagaataacttgaagaaa

aagatgacacactaaagagaatacagaggaaagtgtaaaaaggctccctctgtaacgaaa

gagactttttaacctaacacttatccacaaatcttgattacacaattgagagaagttgca

aacatgcaaatacaaaggaaatagtatcagttagcattgctactcctgaatgatccaaga

gctttagcagcacctgctcttaagatgaattggtggtagaaggccgcgattgccgcccca

acaaacggtccaacccaaaatatccaatggtcattccagatcttttcttcgttgaggata

acagcagaaccaaggcttctagcgggattaatgccggttccggtgaccgggatggttgcc

aaatgaaccatgaatacagcaaacccaatgggaagtggagccaaaaccggcacatgagaa

tctctagcacttctcttaggatcagtggcagagaaaacagtgtaaaccaaaacaaaagtc

ccaacaatctcagcacctattccaacactagtactatacccatcatgaagaaaattagct

ccaccaccatacctatcaaagtaagcactttgaaaagccttaaccaacccaactccacaa

atagcccccaaactctgagacaccatgtacataactgctctcaccaaagacaccttacgc

gccaatagtagcccaaatgtcactgctgggttaatgtgaccccctgaaattccagcagta

cagtaaacaaggacaaagatcatgccaccaaaagcccaagcaataccaagaataccaaca

ccaccacactcatcaccaccagctttaacatcagtttggatactgtaaccaatcacagtc

aaaacagtaacgtaaaggaaaagcaaagttgcaatgaactcggcaataagggccctgtaa

aaggaccattttgtgagttcttcagcatcaatcagtggtgctggaggagggtcatggtag

tctttgttagagaaagagccacgttcagcaacttcaacgtcttttgccattgggattacc

cttttgaaacaacacagtaaaacaagaatggaagttgttagagaagagagaaaggaagaa

acttggatgatggggaagtgtgccgcggc

>fava_dn_rep_c856

gattgaaaactgtgtctggtagttactgcaaaatttcatttcgagtgagacacctgcaac

caatgccttgcaggtcttataaatcatggataaccctaaattaggagtttggtttccaaa

tattaagattacaacaacctcattatacagcaacgcttgctctccaatcttgtgttttca

catattacaacatagatactcatgataatgaatcagaaacaataatacaaaaggaataat

attaggtaaattaacaagtaaagttcatatttgcttaaatcagatttcactgtccaactt

gagtgtttaaccatgtaatgtaaggaatccttactagataaacctcaacgtccttttgga

ttcatgatctgtgaaaacacagcactaggagttaacccatcagaatcttcactagccaag

cttctcccaccaatgctcatagacatgccactgcttttggagtcagtcacattaccatca

taacccggcatcgcatccatgtccttctttccttttgaatctgtaaacaatggctcgtct

tcaccgcatattccgccaaaaccgctgccactctcctccgcgctttcttgcaactgcaaa

gcaaattccaggttccatagcacatcgcccatggatggcctttcaatgccttggtcgttc

acacacttcatcgcggtctcggcgaacttcttgaaacattctggagctatcttgcccttt

agataaggatctgttatttgatcaaggatgccttttttgtagcaatgatatgcccactca

gctagactgacttgctccttagcaagtgttgggttcaaagccgggcgagcacagagtatc

tcaaatagaaccacaccaaatgagtacacgtcggatttgtcggtaagttgttgcctcctg

aagtattctggatccaagtaaccaaaactaccttttaccacagtacttacatgagtatta

tccaatgtaggacctgttttcgacaaaccgaaatcagaaaccttggccacccatttctca

tctagtaaaatgtttgttgtcttcacatcacggtgaatgatggtatgttttgcaccggtg

tgaagatagtgtaaaccgcgagcagctccgatgcatatctcaagcctttgcttccatgga

agtggaggtttctgggtcttgtatagatgctccctgagtgttccataggccatgtgatca

tagacaaggatcatttcggtgttctcttcacagtatccgatcagtgaaacaaggtgacgg

tgacgaagtttggagagcatttcaatttcagtttggaattcgtgcacaccctgctcggaa

agtggattcccacgtttaattgcaactttcgttgacccaccgtcaatttctcctttgtaa

accttgccaaaccctcccacaccaagaatcaaagcctcgtcgaagttgtttgtagcagcc

ttgatttcagcaaatgagaaatggcgacaaagattagccggaagagaggatgcataactt

cctgtcgtgttggtcttggcagaagctgctgagtgtgaattaccgtaaagagaaagtgga

agccagccagacggcccttcagttgtactagaatcttttccactcctgcgtcttcgggat

ttgctaagagcaaatagacctatcacaagtaacaaaacaaatcctccaacaacacctcca

gcaatgattcctatatgattcttagatttaccgtgactagattgcctcgccaatgaggcg

tcaattttttcttgcaccacaagaggaggagggtttgtccctgccagattacctttacta

tcactgattttgaatatctctattccattcaagattgcgtcgtagtaatttggcttatca

gatttatctggatgcagtgcaagccaaaggtcctgtcgtggcggcccattgggaacaaat

acaacataatctttatgcaccggaaccccatttgaatgtggaaggctataactatttgcc

cagccaataacatcagccccatcctcagcagtttgattagcaaggaatatattaaatacc

ctttgattaatcttggttatattcgtcgtgccttcacaaaaatgaagtctcacaagatag

gaaaacccagaatcaattgagaaaatccaagtcaagttgtaattcaaattgatattggga

tccggacccatcgatctggccgtcgagtagacatcagtcggagcaatataactcggtgtg

cccggaggatactcaatcttcactgctgaatccgcaatctcggtaactccaagtccggcc

ccatagatgtaaggcacatcatcgttccacgacctaaacatgccggtatcgtgggagggt

gagatatcattcccacccacgtttaacctataaacattctcaagtgcagtgctgttgtca

attgggaagctagtacctgtaccaaccatcagtgttgttccatcaggagaagtataaata

tcaggcatggacacaacctcaatcccattaacaaatgcataggctttagatgcatttgca

gatggagtaaaagtcacattcaaggttccttcatcaacatgaatcgcatattccttcaca

atatattcaaaattcaaacccaaaatagactcaaaagcactgaaattcctaagcacggta

tacgattgcgctgtcacgccaaaccgtgcatcagatatattgaggctgttataagaagcc

ggataaaagtacaaccggaggaacttccaaccagaagccaccggaaaactgtaggtataa

gccgattggaaaacacgcgccgtcatgaagggaatctgaggaacagcaggatcttgagtt

gcagcctgtgctgggttcgatttaccagttgaagaaccaaacttggaaccattatcagtg

gtccattcacggccatcaggatcagtactaacagcaggacctccacaactcagcaggatt

ttatccgtaggcttaaaatccgctgctagaacaagttcaatagctaacaccaagcacaca

aacagtggaacacaaacatagtatccattcatgttcctcttcatggtacacaacaaaccc

ttgttttatttttttcctgcttacaacttatgtcatgggaacaaaatgagctgaaaaggg

aagaaactcggtgagtcaaagcggaacgatcccgtgaatttagggttgattagatttgct

agttgaagtttccagttgagagagagagagagagagagattgttgaagaaggagatggag

tgagaaaattgcagat

>fava_dn_rep_c857

ggagctggtaaagaataaaaaataaatgcaaatatgaagagggaacccacatgtattgtt

aggggtacgcacttatctgattcccaagaatacatcacacagtcacactcactcccagaa

taacaagaacatcatttgttgaatgcaattaataacccagtcatggccaacactttttat

gcttcttctccccctcgtcttcaaattcaacacctaagtcaacatcaacagcacaaatat

ctaacttcttccgcgggcgtgtttcctatccgctttctcagtcatggctagcgtatcatc

atcactccttctagcatccccctctccactctccatccaattccaatcctcctcctctca

cctttccttctctcgctcccaaaatcttcactcaccgctcaccacttcatcattgccgtc

gctctcagcttcttcaatctcatctctttcaccaaccactccttctgtgtattgtggcag

aggcgataagaaaactgctaagggaaaacgcttcaagcattcctttggaaattcaaggcc

aaggaacaagaacaagggtagaggaccacctcggatttatgctccaccggattctaccaa

aaaagataagcttgaagatgatgaagtgcttaatattgacattatcgaatgaatccctct

tttttatggttgattcttgtgttgcattgtaacattttgagtctttcatgattttgtagt

ttttgatgtatggatttcatttggcatagaattaaatgttttcataaaaagttgcattga

attatgaagaactctagcacaacctgaatgtaaccaaacttctacaaatatattattgac

actataaattctctctccattctacttttattacaacaagaacataatgtaccaccatac

cagaaaatataaattacaattgattgaagcaatttatttttaaaattattacaacacaca

taaaacaccccataccataagggtctaacttattgagacaaaacatgacatggcatagta

aataaccagacccaaaaaacataaatcaacaagacaagattcttattctagctatttaca

agtgcacaatgtttcctgatctcaccctgtgaccctgtcttaactttaatttgacccatt

ttctcaattgatttagcaaattcagcatagaaattttcaagtgacccttgaagaaattga

gtcaccaaagactttgtaactgaatttgtcaacaaagcagaatcagattcaaacaaacct

ctccttttgacaacttgactataataactaagatcaaaagtgtttcgacttccggggtcc

atttcgactattgtagtattgtcattgatgttcttgcatttgaatgtcttaagatttgtt

gcatattcactgtcaagtgatggatcttcatcaccctttccagtaaaattgtacaaacgg

ttcgaaaatgaagtgcaaagagagataccaattgtgtgagcacctgagaggaggactaag

tcttttagatcaagtccttgattggcaaagagagtttggagggtggtgaagttgttaaat

ggagcaggaatgttttgattggattccaacaagtttgatattactccatctcttctacct

gttggaactttccaatagggaccacccgtgactacaatagagtctctagcagataaggct

atgatatcagcacaagaaacaacaccaggacattcggcttcaacaaggctcttgattctg

tcaataaaatcaaatcctctaactgtaagatttggaggagcatttttttcagcttggttg

gttgttgagttcagaagaactgatccatcacatcccctgacaaaacaatcatgaaaatgc

attcttattaaagcagccgcaagcgatggcgcattgcggatatgttcatgaacaaatttt

tgaatgatttgttcggcttttggacagctcttagaataaaatcctagctccagctgagca

tgagtcgatgaggccaaaagacagataatcaaaaccttgaagtacctactcagattttcc

attttcacaaatgtatatagctggaagctataagagtatgagatgcttgtttgaatccat

aacagtggtgagtgatttatatgtagtagagacagaagaaacaaagaaaataatagagat

ggtcaatgcagctagctaacatgtgagataaggtgacacacacatgctactttgctatcc

atttgcagttaataatagtttaaaattggtttg

>fava_dn_rep_c858

aattagttgactaccacttcaccaattccattttcaacctcaaactcaaatttcatcata

cgaacacaaccacatataaacacacgaatcttttgttcttgtctttgttgtttcaacaca

aactcaacatgaaaaacaacatcatcaacaacaactacacaaatccattttgaattcttt

ctctattgaatggagctttctcttacactaacaccaaacccttgttcaacttcacttgct

ttctctaacaagatatgtgagtttcatcaatggaagaaaagggtttctttctttgacgct

tcaagagttgtttcggttcgttgtgttaaagcttctgcagagagaacaggtgaaactatt

gatgacagaggagagactagaaccgggtttactacaccggctgctatggaggtcaccacc

acattcaaccaaggtttcaatgatgctgctgctgctgactttcctgtttgggagaaaatt

ggtgctgttgtgagactcagctatggcatcgggatttatggcgcgatggctgttgcgggg

agttttatatgttcgattactgggattgattctcttggaggttttcatctttcaatagat

gctgttcttgaaggacttggatatgcagttcctccaattatggctcttctttttatactt

gatgatgaagttgtgaagctatcgccccatgcccgtgccattagagacgtagaagatgaa

gagctatggagttttttctatggcatgtcccctttgcagtttatactgatggttgctgca

agttcagtgggagaggaactcttctatagagctgcagttcagggagcactggctgatata

ttttttcgaggcaataatcttatgcaagatgttcgaggaatggcgtctttgacaggagtt

cttccgccatttgttccatttgctcaagcacttgcagctgtacttaccgctatccttact

ggttctctctactacgtcgctgcctctcccaaagatcctacttatattgttgcgcccgtt

ttacaatctcccgccagtcgtcaagatttgaaaaagctctttgaagcatggtacgagaaa

aggcaaatgaggaaaatctattcaccacttcttgaagggctattggctctctacctcggt

ttcgagtggatccaaacaaataatattctttctcccattatcacacatggaatatactct

actgttatattgggacatggcctttggaagatacacgatcaccggcgaaaactaagacaa

agaatccaacagctgaaatcagaagaaagaaattccaactagattttgaaatttcctcca

tgactgatttttgtggaaaatctgtatatgtattagaggtgttaataatgtaaacaaaag

atgtgtatacttatatgttaggtgagttctcgtacctacagaatatgcaaagtaagtttt

gattataggattctctaattttaaatcattagtgtttgtaataaaaattggagtaatcca

aaagttgtgttaaatttaataaaatagcatatcaattttctcaactcaaaaaaaaaaaca

ttactgaatcagtattggt

>fava_dn_rep_c859

cgcgatacgacatcgttttagattgcgagtaaccactatctctctctactctagggtttg

ataattaaatcgagaacagttgtgaagaaaacgagtgttcatctcttcgttccgaatcct

ctcctttgtgcttaccttcctcgctcaaccagaatcgatctcatggcacctccaccagga

ccttattctggcaccagcaccctagctttggtcgctagagcttctgcgttctctttcggt

gttgtttatggatccattaagctcaagttcctaaagtcagaagccaagtctcaacaggaa

gctcgggccaaggctcatcactaaaagtaatgagatggatggagctgaatacccaaataa

ttcatgacgtggaacagtaccatgttttctctcaaattttttcttctgatttaaatcttt

agttaatcataagcattgagtttagtgcattgtgaaaatgttcaaattaagatgattttt

gccttgttttaacaactaccttgtctctgtttgagtttcaagtttatattgcgagggcct

gtatttttttgttactcttctaatttacttttgtcttcctttaggtaattactattttgt

tatgctttttcaaattttcagatttgttcttaattgagttagtcgatcactgttttcttg

tggtaaacaactggattttttttccagaagccatcaatccaccaatcccatttcttgcaa

tacaaaatccaatttatttctaggtcacagtagttcctctattagctatgattttttggg

gctgtttctggtgtgatatcgacattgagctagctatgattttttggggctgtttctggt

gtgatattgacattgagctagctatgattttttggggctgtttctggggtgatatcgaca

ttgagcttgttcaagaattaaagacacctctctattggacaatatgtggttatcttctcc

tctcaccatttctggttggaattttggaaatgttaatcccatttcgtctgttgtctcaac

atcttagttactcttttttcaacctatcgataagaaaaaaaaagtcccatttcttttgtc

gtctcaacatcatagttactatttttccaatgtattaatattgataagacaaatgcaaat

gctatttttttaacttttgaatgctat

>fava_dn_rep_c860

ggccgcgggaccaaaccacactctaatctcattctattttctccacaacttttcaactca

gtatcatggcatctgacaaaaaaaatcaagatcggtatcaacggatttggacgaatcggt

cgtttggttgcgagagttgctcttcaaagaaacgatgttgaactcgttgctatcaacgat

cctttcatcactactgattacatgacatatatgttcaagtatgactctgttcatggccag

tggaagcattttgagctcaaggtcaaggactctaaaacccttcttttcggtgagaaagaa

gttgctgttttcggaactaggaaccctgaggagatcccatggggtgaagttggagctgac

tttgttgttgagtctactggtgttttcactgacaaggataaagctgctgctcacttgaag

ggtggtgctaagaaggttgttatttctgccccaagcaaagatgcaccaatgtttgttgtt

ggtgttaacgagaaggaatacaaatcagaccttaacattgtttccaatgctagctgcact

accaactgccttgctccccttgccaaggttatcaatgaccgatttggaattgttgagggt

cttatgaccactgtccactccatcacagctactcagaagactgttgatggtccatcaagc

aaggactggagaggtggaagagctgcttccttcaacatcattcccagcagcactggagct

gccaaggctgttggaaaggttcttccagaactaaacggtaaattgaccggaatgtcattc

cgtgttcctaccgttgatgtttcagttgttgacctcactgtcaggcttgcgaagaacgca

acctatgagcagatcaaagctgctatcaaggaggagtcagagggtaaactcaagggtatt

ttgggttacactgaagatgatgttgtatctactgactttgttggtgatagcaggtctagc

atatttgatgccaaggcaggaattgctttgaatgaaaactttgtgaaacttgtttcttgg

tatgacaacgaatggggatacagtacccgtgttattgatctgattgtccacattgcttct

gtggcttgatatgagtctgagcttggattgcatcgtgtgacagtgttctaaatttttgtc

tcactgagaatttatcagcatactgtgaatttcaattctagaataaatttgtgttgttgt

gagtgatgtcactgagacacaaagtgtctgcgtttttttgtttgtatttcaaattaatag

acggccttaggtttttcctcctcttgttttagagatggatggcttttttcattttgggta

atcttcttaatataaggaatattttgtttgatatttctgtttaggtcggctgtttatatt

taagttgattgagttttgttggctgataaatccaatgatataaatttgtttttaagaaag

tttgattcatttcatatcataaattatttgatagtatttaaacatctaaaattggacttt

ttttt

>fava_dn_rep_c861

ataaaatacaaatcatataaaatcaaatctaatctaatctcctagagctgtacgcacgat

acgcttcaagatatgatctcaaaaattaatcatgattaattaataaccgaacgagaatca

attataaacataaaataactagataagaacaacagaatcaaagagagagaaaaagaaaga

atttcaatcttgtgtttacagtctctcaacagtcatcaatcaccatcttcttgatttcac

aacaatcttcttcttcttcttcttcagtttctctctgttcggaatggggatttatcttag

tactcctaaaactgagaaattttctgaagatggagaaagtgataatcttagatatggttt

atcgtcaatgcaaggatggcgtgcaacaatggaagatgctcatgctgcatatactgatct

ggatgagtctacttcatttttcggtgtttatgatggtcatggaggtaaggtggttgcaaa

gttttgtgctaagtttcttcatcaacagatgctcaagagtgaagcatacttagctgggga

tattggaacctctcttcaagaatcatttttaagaatggatgagatgatgcgtggtcaaag

aggatggagggagctatcaatcttgggagataagataaacaaatttactggaatgataga

agggttgatttggtctccacgaagcagcaatggtattagcagagttgatgattgggcttt

tgaggaggggcctcattctgattttgttggaccaacttcaggaagcacagcctgtgttgc

agttattagaaacaaccaacttcttgtggcaaatgctggtgattcacgttgtgtaatatc

tcggaagggacaggcatacaatttatctagagaccacaagcctgaccttgagattgagaa

ggaaagaatttcaaaagctggtggttttattcatgcaggaagagtaaatggtagtttaaa

ccttacaagagctataggtgacatggaatttaaacaaaacaaatttcttcctgctgaaaa

acaagttgtaactgccaatccagatattaacactgttgagctttgtgatgaagatgaatt

tatggttctagcttgtgatggcatatgggactgcttgtcaagtcaacaattagtagattt

tgttcatgaacaactgtgttcggaaacaaaactttccactgtgtgtgaaagagtatttga

ccgatgtttggcaccatcaactgctggtggtgaaggatgtgataacatgacaatgatctt

ggtccagttcaaaaaaccttctcagtccagtgcaccagcagaagagcagtcctcatcaaa

tgaacaagctgaatcacaagttgaaccaaaacttgaaagaagtgaagtttagtactgtat

tctatagcaactgttcaaatgtgttctatataataccgaatagacacagttcaaatctgt

attctacatatataataggcacacacactcactcctatgtatagtactagtgtacacaag

tgcatattctgttttctgaatgtccattcccctttcatctgacattctatgtactagtta

tcttgatcactataataatatcagagttttctagtgtctatatatcattccataaatctg

ttctgcgtgatctaccatgttgnnnnnnnnnnnnnnnnnattgattgt

>fava_dn_rep_c862

aatctcaaactagggttttgcactcattcacctctatcatcatcgtctctcaaaccagtt

tacaccattgagtactccacagactcatcaccatcaccaccttgcaccaatggtaggagt

agcattcgatgtcggcgccgttcctttcaacgccgacggatggggcccaatggattccgc

cgccaacaacaacaacaacaacagcctccctctcaacgtccccttcgcccctttctctcg

ctccgacaaggtaggccgaatcgccgattggactcgcaacttcaacaaccagacccgatc

caaaaaccccgccgattccgccttcgatttcaccctcgacgattcctttccaggcaacac

cgatgacgacgccacattccgcttagtcgatggtaaaccacctccccgtcccaaattcgg

cccgaaatggagataccagcagcaaaagcagcttcctcagcgtcgcgatgaagaagtcga

agccaagaaacgtgaggcggagaaggagagagctcgtagggatcgtctttaccatcagaa

tcgttcgaaccctaataatcctcgccgtgaagctgcggtttgtaagtcatcggtagatat

tcagccggagtggaatatgcatgatcagatcccgttttcgactttcaccaaactatccta

caatgttcctgagcctgaggatcttctcctatgtggtgctcttgagaattatgatcgaac

cttcgatagaatcaaccctaagaatgagcgtcgtctcgagaggtttaagaatcggaactt

cttcaaggttacaaccactgatgaccctgttatcaggaggcttgctaatgaggataaagc

gacagtttttgcaactgatactattctgtcgactctcatgtgtgctcctaggtctgttta

ctcttgggatattgttgttcagcgtgttgggaataagttgttctttgataaacgtgatgg

gtcgcagttggatttgctttcggttcatgagacgtcacaggagcctttgcctgaagctaa

ggatgatattaactctgctcattctttgagtgttgaagctgcttatatcaatcagaattt

ctctcagcaggtgttgattagggatgggaacaaggtttttttgattttcaagctactgct

ggtaagagaaatgctgttgcttttcctgtttttagtagtgctagtcccggtcttcagatt

cttgattttgcactttttgcaagcaatttctctactcctttgattgcaaagaccactttc

cttgatcctgttcttatcaagaaactcaagagtgttcttggtggaagcggttaagcctta

tttataatagttgctatttcattcaacctattttctaagtaatgcttatgcttataatag

ttgttatttcattcaacctattttcttctttttttatttggcaaaccaaacttatatata

ttattcagaaatacttaatttgcattatacaataataatactagagcctgatcatggacc

aatacatttattccatacaaagcaattataacgatgcatgcttatatatcttacatcatg

ccattacaaacaccacactaattatacaatataaatataatgccttaatgacttatagag

caaccttttctcccttgataagatgagcatcaatatctctagtgcatttattaaggtagt

ccatgtaaccatttggagggtcaatatcctcgttgattttctcatattcaataacccatt

taacaacaccagtaccatcagccttatcacttacttcaagggtcagcttaaagaccttat

agtgcttatcaatatcaccaccaaacagcttccacgtgttccttttgttctgtttatcaa

cttcttcaatactctcgtagcatgtgtgtacctcaccatctatgacatatgtccagtgtt

taactgtatcagtgtcatgccagtcttctccctcatgcaacttagcatggtgaactcttt

cacaatgaacttgcacttcgtgaaattcttccgcaaatagtttgtaaaacctttcagcgg

gtgttttgataccaagttcagtaataagcctaccagagagcaccatttttgagaagaaac

taaatatatataaataagagagaaaagaaaagagttgctatgatgtcccgcggcct

>fava_dn_rep_c863

accttcatttcgcaaacatccgcgtcgcctcgcccccctctatccctcctccctcttcat

cacaacgattctgcggcggcgcttcaacaacctaatcttctgtttgcactttcaggttgc

tgaagatgggagctggtggccgaacctctgttcctccttcgagcaggaaatcagagaatg

acactgtgaagcgggtgccatttgaaaagcctccatttagtctcggccagatcaagaagg

ccattccacctcactgttttaagcgctctgttatccattcattctcctatgttgtttacg

acctcaccgtagccttctgcctctattacattgccactcattatttccagaaccttcctc

accctctctctgtcttggcctggcctgcctactgggctgtccaaggttgcgtccttacag

gtgtttgggttgttgcacatgagtgtggccatcatgcattcagtgactatcaatggcttg

atgatacagttggccttattcttcactctgctcttttagtcccttatttttcatggaaat

acagccatcgtcgccaccattcaaacactggttcccttgagaaggatgaagtctttgtgc

ccaaacaaaagtccagtatctattggtactccaaatacctcagcaacaacccactaggaa

gaatcctcacaattactatcacactcactcttggctggcccctttacttggcgctcaatg

tttcaggcaggccttataaggggtttgcttgccactttaacccacatggtcccatttact

ctgatcgtgaacgacttcaaatatatgtatcagatgcagcaatacttgcagtatgctttg

ggctttaccatcttgcattggccaaaggtcttgcctgggttgtttgtgtttacggagttc

cactgctcgtagtcaatggattcttggtattgatcacattcttgcagcatactcaccctg

cattgccacattacgattcctcagaatgggactggttgagaggagctttagcaactgtgg

acagggattatggaatcctaaacaaggtcttccataatattactgacactcatgtggctc

atcacttgttctccacaatgccacattatcatgcaatggaggctaccaaggcaataaagc

caattttgggagactattatcggttggatgaaacaccattttaccaggcattatggaggg

aagcaagagagtgtatctatgttgagccagatcaaggtactgagaaaaaaggcgtgttct

ggtacaacaataagtggtgatttttagagctgctaaagcttgttatctatcaggtgaaaa

tgtttgaacttctctttggatgttagcacttggagaataattttgttgaatcctgatatt

tgtagtagtaaacagtcctaaagtagtttgcttgaattctgaataagatatagcatttct

gaacagtattattgtatttattgcaccatgatttgaaatcagtgcattatctatctgttg

agaaacttattgttattcttgttcttgatttgtctgtaagaattatgaatttcgcatatt

ccgtgttagactaagaattatgaatttgtctgtaactttatgtggtcaagtctgtttgat

aggagaataagatgtgtaactctcgaataattcattccaaatttgggtggaggaaggggc

agatcattaggaatgttagtgttataaatctgctttctgaacactaagaggcatgtaaaa

taaatgaatcgtgtatttttttctgcttttaccttgttttaatatgtttcttatccagat

gatcagattttatgtcctactattgtgaatattgcccttcattctcaacactattgttga

ggaaggagtataagctactacataatggagcaaaatatt

>fava_dn_rep_c864

ggccgcggaacaagtaaaagattagaaaatttcatgcaaaactagagagttttttttaat

tattattatggaggttgctattgaattggatgatgatttgttttttgcaaatttgagcaa

ggaaatcgctctcttaattatggatgaagatgaagatcctcttgtttctcaacctcaaaa

ctctcttcaggctttttctagaacaactcatcctcctcgacagtttgatatcttctatgg

tcaagctttggtgacaagagaaagcaaaggaacaggagtgtttattccacaagcaacaca

accaagaaggaagaacaagaaaacaagatctaattcatactcaaaatatccgaaacaatc

tcaagaaacaagaatggattctcaagttcataacaagaattcttagaaacagtgaaataa

tatggatgaagttattatatagatccttctagttaattaaagctgcacgattttatattt

tctagtgattaatttgattattatattgtttaataatttatgtgtttaattaattcttta

tagcctttaggcaataattgtatggctaattatgaagttttattattttatttatataaa

ggtgaatatatacatgggatgggaaaaccatttttagttatatatacacctattataaat

attagtcatcttataggagggttcaaccaaaggttttgtaatcttccactgtgtgtacaa

gcaagcattttgtggctatctagttttctatgatagataagtttgtccttttttcttgca

atgatgttaatgatagtgtttttttagtaaaaa

>fava_dn_rep_c865

catggaattggttactctcaataattatnnagaataaagttgttcttttgcaatgtgata

cctttaatagtttagactaagacacgtacaaataatgtttgtttgagcaacattatttat

taccttgttaataaaacttacacattggcaagtaatggacagaatacaaacatctaatat

aaattgctactttgttggttcaaaatgtctgaatctaatgcacaaattttcaaacaaaat

gtaaacaaagaaaacaatataaacttctttctcaaagcatcaccatcatacacatgcaca

gaaacaggtttttcactaacctagggtgagagcacccatcaaagcatggagaatatctaa

tctagttttctcatcgacctagaaaaaaggtatggttgaaggttgaacccaagttgcaga

cagaaaccaaaccagtagagacatttatgataacatgtgattagtagtgtcttttggttt

tcatgcggatccgaagaagagttccaaggttggagcactgaacacattagctgcagttcg

ttctgttttatatggtgatatgtggagctgatacttgataaacctagctaactgagtgac

gatttcggagtttgtatcaatatgccaatctggttccaattgccgaacaaaggacgagcg

tccgttctcagtggtgctgaagagaaccttgtccttgaccaaaccacctgaagtgaatac

ccctgcttcttctaacgcgacaagcactcttttctcgctttcgtcatccagaacccgctc

catgagatataaatcgcagaatttagttatttccaacaatacttctaacactgaggatct

cacagttgcttgtttctgaagctcctctggactgctttcctcgagaatcactccaagaag

acggcatgttacctttcttccttcactcaacttctgcctcactatctgccccaaggttgg

cttgacaggttgaaagaactcatccacgacattttgtgcccttgattcatctgaaggtaa

acaagcatcagaaggaacaaccgatgcatttgaatgtgtgctgctgactccaggattact

agatgcagcaccctgccgttttcgttgcctcccttgattatcactcggagatctcaacag

cctccatgtgaaaacaatagcaacagcaagaccagcaaccgctccaatagtgcgtatatc

gaggttatggaaagagaggttgaagagatcggagatcttgaaagaaacataagctccgaa

acgtttgatgaggtgaaagaggtcttgtttggaggcatcatcctcaccagacgcggccat

gtttcaccgttggcctcctcaccggaatttgcagaaatgaatgaatgattttgaagacaa

aaggaaaagagaaaaagaagaaagatagtgttcttttgttggtgttgtgttgtgtgttgt

gtctccgtttgccgtcaaccactggtatgagctgtcgtcaaagcttcaatgttttttttt

attgggaattccaattacatcgatttaccatgttcatattttggtaatatcttatcttat

gtagacatgtcctaggttaaaagatacgaaggtaagtgattgtgtttttaactttaaaat

>fava_dn_rep_c866

ttttttttttggactgttataaattttatctcaatttattttgcatttggaacacatatg

aaattattaatttccacacgtaagatcgaaagatctataacaagatacaaaggaacaaag

gaaaaaagtcagaaaaaaacccataacaaaatgggtagctaggtagctttattcaaaaca

cacaatagtagaaaaaggtgatggatccatcatctttgtttcatcaacaatgtttagtgc

aaaaacatctatgacggaatccacggcagtgtccaccggggaaacgttctgtttggcaca

cagaagcacagttggtgtcactcaaacatgttcctttgaatttgtgacttttagattcac

aatctcttgcctctgccaccatatttggcccagtggccacaagaatgagaaggaagacaa

agatggtggaaacaagagaaattgaacgagccatgaataattctaagagaaaaaaataga

gctagtttagaatttgaagtccccgcggcc

>fava_dn_rep_c867

taaaggcgtttaattctaaagcttgggagtggttgatcaaaatacctccaaaaatttagt

tatttattttttctgataagtataaataaataaatatatagtggggtttatcagcgcatc

tcaaatcgagtctcccccatttctctctcgactttcagaagcaatggatttactcacaac

ctctccttctcgctcactctcacaccttcccacccttttcccaaaacagcccatttcttc

cttcaaccctaaaacaccgttttcccaatttccgctggtgcgagtgagttgcgtgagtct

tcatcgttgattgttcttcctggatttcccaagtctccaacttttctattgatttctgct

ctaaggtgaaaaaccttgttcttttattcagagttgttagaacgatgggaaaagacgcaa

aagcgggagggaagggtaaaggaaagcaggctgcaagtggaagtgatgagaatgcttcca

agggaaaaggaaaaggtgggaaagctgcagatggacttggtacttgcacttatgtcaaag

caagacatgtcttatgtgagaaacaaggtaaggttaacgaagcctacaagaagctgcagg

atggttggcttagcaatggagataaggttcctccggcagagtttgcaaaggtagctcaag

aatattctgaatgcccatcgggaaagaaaggtggagaccttggatggtttccacgaggaa

agatggctggcccatttcaggaagttgccttcaacacgcctgttggtgctactagtgctc

ctttcaaatcaacgcgtggctaccatattatcttatctgaaggaagaaagaactgattac

taaatcagatagaagctgagtttcaatggaattggagcacttaagtgtttttgggtttaa

attatcggatgttgcttgtttttcttctgtgattgtggttgataatgtaaaaccaataat

gtaaaccaacactttaccatatgtatactgatattattcttgctttaattgcatctctcg

taacgcatattattcttgctttaaaaaaaaaaag

>fava_dn_rep_c868

tttttttttttttgaaaacataaatctaagatcatcatattatggccattttatcatcat

catcctatataaacatgaaaaaaagcacagtagttaatcgacaaataaaactgaaataat

acaacagtatgttataagagtacaattcagaatccaataaaagataacaacaacacaaca

cacaaacattaacaaaaagtaccgagtcaatcagaaggagtgggaggcggaggaacaaca

gcaaccgccgatgcatcagccggaatcacaaccgtaaaatcaggaggcagtggaggcaac

ggagcatacacagtcggcttccgattcctatcggacagtcgtgttttcttcggcctcgtc

ttcatgtgatgagcagtcgatttcttctgtggcctcgatgaacactctatcaacaatcct

ccacacacatttcttctttcagttcgaattgctttgtttctcaatgaatttggtgccatc

gctacaccacacccaaatatgcttgacactgacgccattttttctatctcttttctctgc

tccgctctgttgcggtttctcgtttctctcactcttatcccttatcgttttatctctccc

cgcggtaatggacactctgcgttgatacnnnnncttcagaatcagacaactcggaactcc

cttcttctt

>fava_dn_rep_c869

actacctacgtttgtgtctttctactatctaaagatcactgtcatcaaacacaacacaaa

agtttttcttttttaatagaaattccattaatttttttgtgaatgacccttttgtcctca

ttcacccactagcaaaaaaaatcagctttttagttttataaaccaaaacaagttttcatc

tttcaattccaaaaccaccaaacaccatggcttccatggcaacccttttctccaaagcct

ctttcccttctcactctttaaccaaatccttcgacacccatttcgcaccttccataaggg

tcaacgtagctgtaaaaaacaaggcgaaaaaccatcagaaactccgtgtttcgggtggtt

tgattgaaccggatggtgggaagttggtggagcttgtggtggaagatgggaagagggatt

taaaaaagggtgaggctttgttgcttccgagggtgaagctttcgaggattgatgttgaat

gggtacatgttttgagtgaaggatgggccaccccgttgggggggttcatgagagaaagag

agttcctccaaacgcttcatttcaattcactaagacttgatgatgggtcttttgtgaata

tgtctgttccgattgttttggctattgatgatgagcagaaacatcggatcggtgattcca

aaaaagttgctctttttgattcaaagggtaaccctattgctattctgaatgatatcgaaa

tttacaagcatcctaaagaagaaaggatagcccggacttggggaacaattgcccctggac

taccttatgttgaagaaactataactaatgctggagattggctgattggtggtgatctag

aggttatagaaccaatcaagtatcatgatgggcttgatcattttcggctgtcaccttcag

aactccgtgacgagttcacaaggcgcaatgcagatgctgtgtttgccttccagctccgga

atcctgtccacaatggccatgctttgctaatgactgatacccgcaagcggcttcttgaca

tgggttacaagaatccagtcctcttgcttcatcctcttggaggctataccaaagctgatg

atgttccacttgattggcgaatgaaacaacacgagaaggtacttgaggatggtgttcttg

atccagagacaacagtggtatccatattcccatctcccatgcactatgctggaccaactg

aggtgcagtggcatgcaaaggctagaatcaatgcaggggcaaatttctacattgttggcc

gcgatcccgcaggcatgggccatccagttgaaaaaagagatctatatgatgctgaccatg

gaaagaaagtgttgagcatggcacctggactcgagcgcctaaacattcttccttttaggg

ttgctgcatatgataagactcaaggtaaaatggcattctttgatccatcaaggcctcagg

atttcatcttcatatcaggcacaaagatgcgcacacttgctaggaacaaagaaagtcctc

cagatggatttatgtgccctggtgggtggaaagtgttagtcgattattacgatagtctag

tactttcaagcaatggaaaagtcccggaagctgttccggtctagtgttgtactttaatgt

atacccgattgggagaaacctttaagtttatgtattctcctctgctaagacactgatttc

agaaagatctttgttgcctaatccctaataaaatgaaaagtttgggatggcatttgcata

gagacacattgcctctctctgtaagtatggctttgtagtgttgcctcttgcagttatagt

gccacctctttgtaatatattattgtgattgtgcaaaggatttggtttggtttgagtgtt

tacattcaccgtcattcatactattactgttttcagtgtgaattgctattgcttcaaatc

ttgaactcgtgttagtattaaactatatgagatcagagaatatttgttaaaaacttagat

tcaacctg

>fava_dn_rep_c870

ggacaaacaattaagaatcatcaaaattctattgtttggatctaaaacatcaaataaggt

tttggtgataactaggtagcattacaaaaatgtggtcatcctcacaacagagcttcttca

tccccacaccaacccttgttaactcaaatttcagaaacagcaggaaaaccctagtcatcc

actgcagttgcccacaaccacaacacaacaaccaagaagaacaacaatctccaactccat

taagtaataggaagaagaatgagaacaagttagggaaattagcaatgattgcagtggctg

ctggtgtgttaacgtttggatcagttcatgatgcatcagctgctaaaactggtggtagaa

ttggtggccagtccttcaaatcagccgctcctcgcaactccgcgccaagaatcaacaata

ataattccaggaccaacatctatgtaaatccacgggtggcgcctccactagttggtggat

atgggtacggtggtatgccatactatggtggtgggtacggatggtctccattttctttct

ttgcaccaggtccaagtgtagcagtaggcgttggaggtggatttgattctatacttctgt

ttatgcttctcggtgctggtgctgctgttgtgaggagattcttcggatcaagaagaaatg

ttgatgatgatgacgacgaatactagctcgctagctagaagtacaagaaggtttacaata

cttgattgaagaggagagtgtttgtgttaaatgtagatgtttaaaaaaatggaagatcaa

acatatatcatatatgtgaccaaatcattttgtattttgagctatgcatatattatactt

gaaagagaagcattgatctgagatatgaaatctggtttatagtaaaatcaatatgaaata

gtgtctcaaatgatttaacatgtcacatgtctacctcagtgaaaattatacaaacatttc

tcctgctagctaaacaagcaaaaacaagatactacattaaatttattataaaaaaatccc

atggtaccaaagaaaagactccaacaattttctacaacgatgaagaagaagaacgtacgt

ctttttcaatctgtgtcatgaaagacgcaaactgtttaacaagagccacagatttctcct

tcctaggatcaaacaaatgaaacacatgtccttcaccttctgtctccaccacctccacag

aaccactccacccactcttcttcactgcctcataatagctctttcctctttccaccagaa

aatccttctccgcgacaagcactagcaatctcccacatccaagactcgaaagcttgggat

ctttaggagcatgtatcatcgggtcatcaaatcctccataactagggtacagaaactcta

taagctcgtctttatcatcgtttccgaagtaaggatgaatcaaaaccattcctctaagct

tcaaatctccaaagccttgagttgagcctctcatggccatgttatgtgcgatattcgatc

cagcgctgtctcccgcgaagaaaacaatgttataatcggcgtggtctttcagccagggct

cggaggattgtgagacccattgaatggcttcccatgtatcatcataacagatgggaacag

ggtgttctggagctagtctgtaatgaacggagaaaacaacgacgttggcatgtaaagcga

cgttgttgaggtgatggtggtagccagggttgaagggagtgcagatgcagaaagcgccgc

cgtggatgtagataagaagagggagtttttgagaaggagtggcatttggtgggaggtaga

gacgagctccgatgccggtgtttgttgtgaatagcaatgtcttaccctttggtgattctg

tccttgacattaccatcattgctcagattggtgaagggtacaatttgagcataaaggagc

ttgtgaagaagcttcgcatggaagcattgcagtttatgtcatcataataacttgagcagg

gcacttgttcattccgtgtggaaatggtagtagaagtatgtatggcttatttagtactag

taggtgctttaatcaattcagattgggagttatgtggaaatggtagtagaagtatgcgtg

gctaaggtggcttatttagtactagctagtacttgctttaagcaattcagattaggagtt

atgtggacttttgtattatttgattttacgaaaataactttaagtaggacatgacaactt

tttcttcctccacactaaaaaggtggaatctatcttttgaattaaaaaagagacaatttt

ataataatttacaaactttgtaatttggtttaaagataaacaagataattttgtgtgtgc

g

>fava_dn_rep_c871

tttctaagtaccagaaacggcatcgttcgatcagagattgcactctcacttccaactttc

aacccagcgcagaagaagaatcataaccaaaaaaaaaaaacacgaagctccaatctgaaa

acttcaaaaccagaaactgaatcatcaacatgattcattttcaacggttgcctgaaccct

aattttcctcaacttcttctcaacactgatccaatcccgatccatttccgatccaatccg

acccgatccaggctcggaatcactctccaatgtcaggtgccgcctctgctctcttccttc

tcgacatcaaaggccgcgttctcatttggcgcgattaccgcggcgatgtcaccgccgttg

aagccgaacgcttcttcaccaagctcatcgagaaagagggtgatccacagtctcaagatc

cagttgtatatgataatggtgtgagctatatgtttgtacagcatagcaatgtttacctta

tgactgcaactagacaaaactgcaatgccgcaagcctcattttcttccttcatcgcatag

ttgatgtgttcaagcattattttgaggaattggaagaggaatctcttagggataactttg

tggttgtgtatgaactacttgatgaaataatggactttggttacccgcaatatactgagg

cgaaaattcttagtgaatttattaagacagatgcttatagaatggaagttaatcaaagac

ctcccatggctgtaacaaatgctgtgtcgtggcgaagtgaagggataagctacaagaaaa

atgaggttttcttggatgtggtggagagtgttaacatacttgtcaatagcaatggacaaa

taattagatctgatgttgttggtgcattgaagatgagaacatatttgagtggtatgccag

agtgtaaacttggcttaaatgatagagtgttattagaggcacaaggtagagcgactaagg

gaaaagcgattgacttggaggacatcaagtttcatcagtgtgtgcgattagcccgatttg

agaatgatcgaacaatatcctttatcccgcccgatggatcatttgatttgatgacatata

ggctcagcacacaggttaagcctttattttgggtggaagcacaagttgaaaagcattcta

aaagtcggattgagattacggtaaaagctaggagtcaatttaaggaacgtagcactgcta

caaatgttgagattgagttacctgtgcctgctgatgccaccactccaaatgtgcggacat

caatgggatctgcagcatatgcaccagagaaagatgcattaatttggaaaataagatctt

ttcctggaggcaaggagtacatgttaagggcagagtttcgtcttcccagcataacagacg

aggaagcagctccagaaagaaaagctcctatacgtgtaaagtttgagataccatatttta

ctgtgtcaggaatacaggtaagatatttgaagattattgagaaaagtgggtatcaggctc

ttccatgggtgagatacataacaacgggtggagagtatgaactaaggcttatttgagatt

tgtaccctcgattggtgtttgaatttcaccaaataactgtatcattcaatgaacaacgac

tgaagctggtggacagcggagggagttttaagcgaagttatttagaatatatttggccaa

ccacaacagaattgatgtcttgaatgttaatatgaattgagtgttggccgaaactgttac

gattcccgttggtcaattgtgtgttgatttatatattttagacccctttctttttgtaat

cagaacgtccatcattatgtcgaattgtaatttggtttacatttttaagagaactactgg

ttttgtatttggaaggagaccaattatagggagatagttgattgtgatacaacccaattg

tgaaggattaaatgtttaattgtggatttgcaaagtttaacgcatcaatggcagaaaagc

atagttgtggcatcagttacttggatcgatttatgctataatattcttttaaagactgta

aaaaaaaaaatgaaaaaacaaaaggccccct

>fava_dn_rep_c872

aaaaaaaagtgtgcagcccacgtaacgagggagtacaaaacacaaaccctaatttcaacc

tctctcactaacacggcaaccttttctcggtcgccgccgtattctccaccgtccccatct

gaacgccgcatcagatctttcatttgagtacttgagttcaagatttccatcagtcatggc

aactagacttcagtttgagaataattgtgatattggagctttctctaaacttacaaatgc

ttattgtatggttgccattggaggttctgaaggcttctacagtgtatttgaatctgagct

atctggtgttatccctgtggtgaagacatctgttgctggcacccgcattgttggtcgtct

ttgtgctggaaacaagaatgggcttctcttgccccatactaccacagaccaagaacttca

acatttgagaaacagtctacctgatcaagttgttgttcaaaggatagaagaaaaactatc

tgctctggggaattgtattgcatgtaatgaccacgtggccctcactcacaccgatctcga

caaggaaaccgaggagattattgcagatgttcttggagtagaagtcttcaggcaaaccat

tgctggtaacattcttgttggtagtttctgtgctctctcaaatataggtggtttggtcca

ccctcacacttccgtagaagacttggatgaactttctacactacttcaagttcctttggt

tgccggaactgtgaaccgaggtagtgaagtgataggtgctggaatgatagttaatgattg

gacagcattttgtggttcagatactacagcaacagagctttctgtaattgaggatgtttt

caagctgagagactcggaccctagtgtcgacaagaggaaatccctcattgacagctatgt

ttgaaaattggtaatagtaatgacttttggtgtaagatgttttaaattttggttatagtt

ttgtctaatacaatgttccaaaaaggatttgtttgggggattggttgttgattgccggga

cctataaatcaaaggtatatttggtttagtatggattaatagaaaaatgatgcttaaact

tgtgtcgcccgttttgaaatagatcaaatcatgatgtaatttttgtctgtggttatccaa

tgcccttgattttgttaagttcaaaaaaaaaaa

>fava_dn_rep_c873

tttttttttggataacatagtttaataaaaaaatgaaaatagtagaagtctaaatatttt

ttacatgggattcttcaaaagacagaaaggcaaatggactattaaccataaacataacga

aacaaagttgttactgcaacatacacatacatgttaaacacttaatgaaaaggaattaga

agaaatactagagtttaaccaattcaacatttgaggcatcctatgggcaattgacacgtg

gcatatttaattaaaaataaaagcaacaaaacaacacaatacacacctagagagagaatg

aaaacaaccgcaacacattgaatggttcttcttatttctcaactgatagcttccaatttc

ttcattctgcatctcttttgcaagaaactctttgagaagaacagtagctgaaccatgaag

tttctttgtgctttgagtgccggtagttgtggtggtgtggttgcagttctggccggagca

gatgaagggaggcgcttgtgggtatgatggatccagcgtgaatgcggaggcggggggtgg

tgacttcacgctttcgattccgttcttcttctttctctctgagtcgcggcaggtatggct

tgttttggcatcaatgacgaaactgtcgagatcgcatatgtgtggggcttgcggagctag

tggctgcgattaagagcggtggaaggagaagaagttgagactgatttctattggcgacag

ttccgaggttattgttcttgttatttctcttctgcacaagaaacaaacaaaagacaaatt

ttccagattttttatttgaatcttttcttcatgtttttttgagtgaaactatgttgtggt

ttgaatggtgttacgtttttacaggttgaaattaatggtttgtatttgtagtgaaaggga

gaagatgacgataagaattgtcactgaacatgtaatgtaaaaaggtgcgaaacattgtta

gagcgtgaatgttttaatggaaatctgtgagaatggtgaagggaattggaattgggaagt

ggaattcaactacgccatagccataggtgtgaaggttctgttttggggtttaggaattgt

ggaagggtttaatggtgataacggaataaggaggcgtaggctggtaaggtggattggtaa

tgggtttgtatgctgtgcatttttttagagggtaaagggtaatgatgattgtgatgaaaa

tgaaagggaattggattgatgagttatggtttgggtagtaaaatgaaaaaggttgaccgt

ccgtgatgaagatgtgaagcagcttgcagaatttcctaaggagggtgaggggaaattcag

agattagaagcagctcttttggaattttagtggtgattcttcaaatgatgaagaatctga

gatgatgaagatgatttgttgaagaatcaagattgattgcacagagagaaagttgaaaga

ttatgcgtgagacgttgaacagagacgataagagtgcagcatatgcgtttgcagcattgt

agaaacgatgattgttgaagatgaaaaaaggagaaatgatattgatgataggaattgcat

agaatctttgagacaaagatgatgaagattccgggaggaaatgccgagagagatgaagat

ttatggagatccttcagagagttttaaggaaccgtgacggtgatgttgcgaagtagttga

aaaacacagagtgaatgaatgtagatgatgaagcttgatatgccggttaaggattccgtc

ggagaattgatgccaatgggagattttcgaagttgaagactgaaagcgattttggcgatc

gatcaagattcttggagaatgatgtggccggttgaagaagaaagagattgccggttttct

tgagctgaactggtttctttggtaggaagtgcgaaaaaggttgtaagaatgggaagcatt

agagaagcagaagaaactacaaaaacaatctgcatgtagctctctacacggaatgatgaa

cgagccatcaacttagatgcaagtgagcttcccaacatcaaaattctgtactgaggagaa

tgtttggtgatgcagctcgcaatgtaagttaagaagtaagtcacacaagccctcttccga

ccctgtttatcagcccaagatcccacaattattccgaacaacattgaagacccaaagcca

gcaataacgagttgtccaatatccccctttccatatccatgagtactatgagagaataaa

tgagaggataattatttttgaaggaattgaaagcggaggaagtattgattcgatccttat

tgtttttgctaagctcgagaactgcaacaacagcggctaatccaccgaacacggcgtaga

agaacaactccattttcgcgatgcagagaaattccagtaccaataaaattttggatcaca

gtctctttttttgcagagtgtgtgtgaaggattagttgtgtgaaagatagatttagcgta

gctttgtgtaaatttttttttgtattttgatcaatgatgtaatgtaagacggtgtagtga

gttataatctggtattgaatttttgtaatagatcttgcaatgtgacttgaaggaagtgaa

attataacagactagtgctttggaaatgtaatgttctctttctcaaacatagatttgaat

ttttccttcatttgctttaatttgaatgaatcttcaagcatct

>fava_dn_rep_c874

gggatccattcatgagtattttgatagtgaaataaaaaataaaatattataaataatata

gtttatcaagttggtggaagaagccgaatccaaatccaattcaatttcaatcggagaaga

gaataaggatttgtttcatggcgacttcaaagcttcaagccttttggaatcacccagccg

gacccaaaaccattcacttctgggcccctacatttaagtggggtatcagcattgccaata

ttgctgatttttcaaaaccacctgagaagatttcatatcctcagcaaatagctgtcatgg

ctactggagttatctggtcgcgttacagcactgtaatcactccaaaaaaattggaacctt

tttagcgtaaatgttgcaatggcaggaaccggcatataccaactctcacgcaaattgcgg

caagattatttcccaaaggaggaggctgtagcaaaagaatgatgacgaagaaagtgaaat

gacagaatttgaaaataagttattggagtttgatgccctaattgcaattttcacgttgaa

cgtttatctgaattataatttttcagaagtgtggctcattttgtagttgccaaaggcatg

aactgttaaatgttttcaattctgtgtgtatttttcatatggtacttgaatcttgtagaa

ttagggactcaagataagtaggaaatctcaacaccacatgctcctattttatccatcttt

atatttgttatgttcacagattgtattatgcactatattcagttgatctataacaactct

cttatcactttgaagggctccaccttagtgacagctcacacgctcttccaatcaacccac

atttcacgtacccatcaatcattgaattataagaaacagaatccctctcagccattctat

caaacatttgctccgcaaaatcaacacacccacatctcataaacaacccaatcaaacaat

tctgcaaaaacaaaccacacccaatatccgtcttacacaacaacccataaacctgcattc

cttctttcatcaaacccatattcaaacaagccttcaaaaccagtgaaaaactatacttat

ccaaacaaaccccattttcaatcatcaaacacagcaaaaaaagagctccttttggatcac

taccgtgagaataagacctgataacagcattccagagaaaagggtcgtcatttccatccc

tttcaacacgtaaagcatggttctttaagaagatgtaacgagcaaattcaatgagaggtt

tatggggtgaggaaacgaaagtgaggataagtttggtagtgagggacgtgtttttgatga

atcctgttgttattaaacgtgcgtggagttgattcatgtgttctttggttgtgaattttg

ggagcaaaatcaatgtggggattgtggaagaccatggttggtatgatgaagaattagcac

acacaagcatcaatgttattcaaacaaaatcaaaaagcttttttttttttcaagtaataa

aagttgtgtagtgcaaacaaaggttgcattgtgtcatgttgattcatgggttgtttgtta

gagggaaaagggataaggttatctttgttttaggggaaaaagggatggtt

>fava_dn_rep_c875

ttattaaaaagtctaaaatacataaagtgggggagggaactcataacacagtattttctc

tcatttgtttctcatctctaacttatcatcatcacttatggctaatgataattcagcaag

agttgaaaccaatgatattgaaaacaatgatactgttttagatgtaaacaaagattcctc

tcaagactcttatgttcctttcttgcaaaagttaatagcagaggttgtgggaacatattt

cttgttatttgcaggatgtgcttcaattatagtgaacaagaaccacgacaacgttgtcac

acttcctgggatcgcaattgtttggggacttactttgttggtgttgatttactctctcgg

tcatatctctggtgctcatttcaatcctgctgttaccattgcttttgctactacaaggag

gtttcctctcatacaggtaccagcctatatactggctcaactcctggggggtacactagc

aagtggaactctaaaattaatattcagtggcacacatgaccagttctcaggaacacttgc

aactggctctaactttcaagcttttgcccttgaatttatagtcacatttcaccttatgtt

tacaatttccgctgttgcaaccgataatagagcgattggtgagttggctggaattgcagt

tggatctacattattgcttaatgtgttggttgcaggaccgataacaggagcatcaatgaa

tccggttagaagtttaggacctgcttttgtacacaacgaatatagaggaatatggatatt

tataatttcgccgattttgggagcggtggccggagcatgggtctacaaccttgttaggca

cactaacaagccattgcgtgagatcaccaagagtgcatccttcctcaaagaagcaaagcg

tgggggaaacaattgatttcaggagttggaaaaatttgccataaagtatcaggagcagaa

tcaataatgtttatacctgtgcaaatgtctcttagataagatgagaaaataaaaaaagtt

gtttgtgtaatagtgactaaggtactatggattttgatcatgtgcagttttaaaaggggt

tgcatcactgcatgcacttcctaattaaggggctaaattgattgatgcttcgaaatcttt

gatacatacatatgtttgtattttataatatccaaaactaatttataagaagaaaaatta

ttccgaaaataatataaggagagaagaatttataaaatttctttcaaagtgcataaaacc

ttgattaatactattatgaataaaaagtctgaatgacttcattaacttgt

>fava_dn_rep_c876

ctttttttttttcggtgtaaaaatgggtgtaagaatagcacacctgttataactatatta

ctataacattatttagcaaacaacgcggacaggtaatttaattgttgctttcctgaacga

gagtcaagaatcacccactgaaacttgggtgtgaatgtgaagcacgaattctaaaacttg

actattgcaggaacaataaatattacaaaaatttggctatccaatgacctaataaaatac

aacaatttgatcctgataaggagttcacttgaaaaaacggggtgcggcaactttaaaaaa

gctctgataatacgacctaccatacatgtgttaccgatttagggtcgaaacttataatct

aggatgttaacttttaaaaaagtcaggtacagttcgggaaagaatctccccgccggcatc

cttaccaatctccatcatatcttcataagtataaggactaattctagatgtttcaagcac

acgggttccatcaggggaagcaactaatcctctaaacaagcaattgccatccttgtctct

gctagcataccctgcaataggggttcggcacgacccatccaaagttgtaaggaaggctct

ttcacaggaaattgctagtcgtgtttcttcatgattcagcgaagcaagatattctgccat

tttatcatcgttactcctacaggctattccaattgcaccttgggcaacagctggaagcat

atcatcaattgatagggttgaagttacattttctgtcatatttaatcgtttcagtccagc

caaagccaataaggtagctttgacaaccccatcactgagttttctaagccttgtttggac

attgccacggaaattatcttgcacggttagagatggatatctgtgaaggatttgtgactt

tcgtctaagtgaagcagtaccaataacacttccagccggtagatcagctagtgaagccgc

actcaatgatataaatgcatctctaacatcctctcgtggaaggttacacggtaaaatagt

cttctctggtaagtatgtaggaacatctttcattgaatgaacagcaatgtcaatgtcccc

atttataagcgcttcatctatttcttttgtgaacaatcccttcccacctatgtctgcaag

tggttgcgatagtattttgtcaccagttgttttaataatcacaatttgaatagctccttc

ttcagctaactccgtatgcgatgccatgagtttgtctcttgtctcgtgcgcttgcgccaa

agctagcggacttcctctggtaccaattctgataagagcggtcttattctgctgggtttg

ttgctcaacggcaagggaagccctgatgcgacatttggagaaaggtgaggttttgaacga

tgacaaagggaacgaggaacgaggattggaaggtgtagaaggaagagagaacgcggaaga

ggaagaaagggtcatctccatttgggtgtgaagaagtgaaattgggattttaaatgtgat

atgatcttaagatagagctttattatttgcaagatcttcaagatagaacctgtggtcccc

accttacccaattacacattacattcttgttattttgtcttcaaattttgatcattggga

aattttgatttt

>fava_dn_rep_c877

gtgtccttgaaaaactttgttccccattctgtcctcacgaaagaaaattccatgcttttt

agaatcccaaagggaaaattctcctaaaaaatttgcactagtcgatgcaagttttacatt

tacatccctatatgaatatcacgtgaaaccaggtgaacggtatatgcatcaaccaatttt

aattgtttttcgccggattatatgtaatgaaaattaaagagaaaagatttctgaacaaga

ttacatagagttcaattcaatataattcacaagcttcacagtgtcatttccacacaagac

aaaaactgaagttaaaaacagagacaaaaaaaagaataaattttatccatcatcactcaa

caactttcaccccagaaaacttcatgatccaccacaaaaggtaaattgaagggttacata

gaagaagaagatgaagaatcacttagtgatgatgtaaatagcatagagaattccaggaag

atagccaaaaagagtgagcaccaaacagatccagaactccacttcacagccaaatttgag

gaaaacaccaagaggtgggagaatgatggcaagaatgatatcaatgaaggtagctgtact

cattttgtcttgagttcaaatttgaaagagagaaagaaagtggtagaaaagaagaagaac

agtttgttgtttttgttgttacttgttgggtaggatgagtttaaagacaatgaataatgt

tatgtgatcccgcggccattaacggccaagatgatccgcatgttgtggtcatcatctcag

cagtagatgatcgttctcagttggttacactggctggggtggtcacacggtgatctgaca

gactctcaa

>fava_dn_rep_c878

cgaatagaaaggtgccaggtgtcatgcgtattgaataaaacactaaacttaatcttagcc

gcgagaaactgtgtttcttcaccttcacccatggcttcaactacactaggcacttcttca

attgcgtttcttccttcacgctacctttcttccccttcctccaacccttccattcacaca

ctctctctaacctcagggcaaagctgtggtaggaagttttgtggaggaattggaattaat

ggcacaatgggaaagtctcggtttcctgttgtcaatgttgctactgaagttaactctgta

gaacagggtctgaatactattgctaaagaaagccagaggccagtgtacccttttgctgct

atagttggacaggatgagatgaagctttgccttcttcttaacgtgatcgatcctaagatc

ggaggtgtaatgataatgggggatagaggaacaggaaagtccacaacggttaggtcattg

gtcgatttacttcccgaaatcaaagttgtttttggtgatccatataactcagacccagaa

gatccagaagtcatgggaatcgaagtgcgagaccgagtaataaaaggggagcaactttcc

attgtgttgagcaaaattaacatggttgatttgccattaggagctacagaagatagggtt

tgtggaacaatagatatcgaaaaagctcttactgagggtgttaaggcatttgagcctgga

ctactagctaaagctaatagaggaatcttatatgtggatgaagttaatcttttggatgat

catttagttgatgtgttgttggattctgctgcatcaggttggaacacagtggagagagag

ggtatttctatctcacatcctgctcggtttatcctgatcggttcgggaaatcctgaagaa

ggggagctcaggccacaactgcttgataggtttggaatgcatgctcaagtggggactgtt

agagatgctgaactgagagtaaagattgtggaagagagagctaggttcgacaagaacccg

aaggaatttcgagagacttacaaagccgagcaagagaagctcacggaacaaatcacctca

gcaaggagtttactttcttcagttcaaatcgatcaagatctgaaggtgaaaatctcgagg

gtgtgtgcagagttgaatgtggatggattgagaggagacattgtatcaaatagagctgct

aaagctttggctgctttgaagggaagagacgaagtgagtgtagaggatattgctactgtc

attcctaactgcttgaggaaccgtcttcgaaagttcattctggggtcgatagactcgggt

ttactcgtcaccgagaaattttatgaaatattcagctgaaggtgatagatagatagaata

ttctctcagctctggtatttgtttagattgtaacctttcctatggcggatttttttcgtc

tttaaaaaagagtaacattcataaattccttgcatacagaatattctaagcctttacttc

gaagaatgtgttgcataatgatttgaagtgagaactggaactagagtgtaaaaaccttga

ggatatcgaatattgcttcaacttcagctgcatctaaaactggcgaaaatgcatccttgt

tggacagtattccaaccaatggaacaattgtgatgccataattaaaataactctgtaagg

gaactaagttgtgatgcaattctacagaagatccaattaaactagatctagcagtgaagt

gatgctaaaagtagacaagatgtacataaaaaagaacatctaaatttcatcctggaaaag

ccttcaattttgaggatttgagatcctctccatttctaaattaattaatttaaaatccat

ttacaagggacattgacag

>fava_dn_rep_c879

agtggaacaagtgcgatgacccataagaatttaataataacggaattgatactcaagtat

ttttcttcctattaagcagaaacaccgaaagaaagaaaggaagaacggagaaagaagaag

aagaaaatggagaagtttcagatctggagaagtatagctttcgcagcgttcgcgttcgca

cttctctcccaatcggtgtccgcagacgacgtcgttgtgctgtctgaagagaatttcgag

aaggaagttggtcaagacaaaggagctctcgttgagttctacgccccatggtgtggacac

tgcaaaaagctagctccagaatatgaaaagcttggtaacagcttcaagaaatctaactct

gttttgattgccaaggtggactgtgatgagcataagagtgtgtgcaccaaatatggagtc

tccgggtacccaacgattcagtggtttccaaaaggatctctggaacccaaaaagtttgaa

gggccacgcactgcagaatcacttgccgagtttataaatacagaagcaggaactaatgta

aagattgctgcagctccatctagtgtagtggtgctaacacctgaaaccttcaacgaagtt

gtcttggatgaaaccaaagatgtcttggttgagttttatgcaccatggtgtggacattgc

aagagtcttgctcctatttatgaaaaagttgctgcagcttttaaaacggaggacgaagta

gtgattgcaaatttggatgccgacaaatatagggacctggctgaaaagtatgaagtgact

ggatttcctaccttgaagtttttcccaaagggcaataaagccggtgaagattatggaggt

ggaagagacttagatgactttgtggcttttatcaatgagaaatctggaacaagtcgagat

tcaaaaggacaacttacttctgaggctggtaaagtagagaacttggacttgttggtaaag

gagtttgtagctgctaacgatgaagagaagaagactgtatttgccaaaatcgaagaggaa

gttgggaagcttcagggttctgcttctagatacgggaagatttacttgaaaactgccaag

aatcacctggagaaaggttctgattatgctaagaaagaaattcagcgcctagagcgcata

cttgaaaagtctatcagccctgctaaggccgatgaattcactctaaagaaaaacatcttg

tcgacatatgatgcctgaatttaaggttcaatttctaccatctaccctttaagctagcct

cccacagcaactacttctgatgattgtttgacattgacgtctacattgctgggaggtttt

caacatcgagacttgtagtacttaatctgagtttgttttttaacgatcctattatatatt

agaacaattatggggtgatgaagattattctgtgcctcattgtatgtagtttacctgaga

tcgattttgaggaggacgagaacttttacaagctgtttttttcttggtttatggtataaa

cttgtcacgaagatggtcggtcaaatacatggcaactacaatggtttagtatagaacagc

gacggtagtaaatgcatgacaactacatgaatactattttacccgaagagttaaacaatt

aaggaaaactggatatggatttacgttgtgagtaaattattttagttcttaaatgtgtaa

gatgaaaaatatattttagaatttaattaactatttaaaaaaggatttaatgattaaaat

ggcataaaagaaaactttagtagactaatttgtaattttgatatttttagaaactatacg

aaccactttttatgtaaatttagctgattactcaaatatnntttctattcggttctg

>fava_dn_rep_c880

ataaggtggggactagcccttcattgcaaccaacaacttgtaagatatctttttttccct

tttgcaccaaaaccaaatttattatcattattatcctaaacatggctactatctttggtt

ccccaccaattttctcactccctcttagcagaaccactcatctcatcgcatcttcatcac

aaacacctccaccacaacaacctcagccctcaggttcatcctctccacagccaacaacaa

attcgaaagaagatcagacagtgcaagtctctattaactcacagcaacagaagcctatta

aaccagtagtttcatcaactaaggttgattccacagattggatagccacttccttgacta

gacggtttggccttggagctggtcttgcatgggttggttttcttgcttttggtgttgtct

cggaacaaatcaaaactcgcctcgaggtttctcagcaagaggctaatacaaggaatgtcg

aggaggcggaagaagtgattctgcctaatggcataaggtactatgagttgaagattggtg

ggggagatacgccgaggcgaggcgatttggttgtgattgatataacgggaaaagttgaaa

gcacaggtgaagtgtttgtgaacacatttgaaggggagaaaaaatcattggcactagtaa

tggggtcaaggccatatagtaaaggagtgtgtgaagggatagaatatgtactgaaaacta

tgaaggcaggtgggaagaggaaggtgatagtgccacctgaattgggatttagagagaatg

gtgctgatttaggttctggtgtgcaaattcctccacttgcaacacttgaatatattgttc

aagttgataaagtttccattgcaccagcttgatcattttgattaaatgtaactcttctac

taatctctttaagccaaattgtagggtgctttggatttgtcatatgataaatgttcattt

ttttatgttatcattttctcagtgaaaatatactatcaaacacagacatggacgtcatac

acgacacagacaccgacaattgaacataaatacaaatattggtattggtgtccagctgag

acccatacaaacttcttttatatttgtgttaggttacttgcttgatacactttaaatttt

accagattttgtgatatgtccaaaatgtttatgatga

>fava_dn_rep_c881

acgtcggaagaagttgatcgcactgggagattcagatctatgcaggaataacgaaagcaa

cttgtcttccttttcattgactgatacgatacgagtcatttttgggtcccactttgaagc

ttcttttccactgattcccagatctcatccaacttatataaacctctttgaagatccatc

ttcttcaatctcaacctgcttcacccaggtaactctctccaattttgcaggtttggggtg

cttgattaacaacaagttataatggctgccctgaaaccaggtgtgattgctttgtttgat

gttgatggcactcttacagctcctaggaaggtggctcctccagagatgttgaagttcata

caagaacttcgaaaggttgtaacagttggagtggttgggggttctgatctagtaaagata

tctgagcaacttggccagacagttaacgatgactatgattatgtattttctgagaatggt

cttgtggctcataaacaaggaaagctcattggaactcagagcttgaagacattcgttgga

gatgaaagtctcaaggaatttattaattttacgcttcattacattgctgacttggatatc

cctattaagaggggaacatttatagagttccgtagtgggatgctgaatgtgtcaccgatt

gggcgaaactgcagccaagaagaaagagatgagtttgagaagtatgacaaggttcagaac

atacgctcaaaaatggtttctatacttcgggagaagtttgctcatcttaacttgacattt

tccattggaggacagataagctttgatgttttccctcaaggctgggataaaacatactgc

ttgagataccttgatgatttcaatgaaatccacttttttggtgacaaaacttacaagggt

ggaaatgatcatgaaatctatgaatctgaacgcactatcgggcacacagttactagccct

gaagatacaataaagcagtgtaaatctctgttccttgaaaattgagatttttgcagcagc

atatcattaccgatactgaaaacaagatggagcaatggttttcagaggagaggatcacag

gatttagatgtataatttattctcattgtaatttatattcgcgctttaaattttggatat

aataagacgcttatctttcgataaatttgacctctcgataatttgagaatactcaagcac

gtggtctactttaaaaacaaatattgccataatggtatcatttcattaggttattcaaaa

ttgtgcttcctctgcaagtgtattttgcagaagcaatgcaatggtcattgaactccttta

ggaactgtggtaacaaacatctattgcttcttcatcacaaacatcagttttataacaact

agatcgattttgattctgttaattttcaaacactcaaacatttttttgttaagtggcttt

attgaaattcag

>fava_dn_rep_c882

cacagaacatgacccccaacaacctaaacctacatagcactaaaactttgaattaaagac

gtgtcacgtctccgatatcaacatatgtggttacatcaaatcgcctctatgcctacttgt

tggtgtctgtttcgtgtatgtgctttatatacttaaacacttcaaaatgggactaattaa

aatatccaaacacatcaatcatgacatggattccaaacaatacaccattgacacgtcgac

accagaaatattttgaaaaaattaaatagttgaatgcattttcatatgttggtgctacgt

aagtgtgtgacactaacacatgtcaaacaccaaacaccgtgaatgtatttccataggtgg

gtgctttgtaagtgtccgacactaacacgtgtgtctgaaaaccaaataccttaaatgtat

ttccatatgtaggtgctttgtaagtgtcagatactaacacgtgccgaacatctttttcaa

ccttaaccaaacagaaggcgtctacaatacatgaactaaaattcttattgaaaatgaaca

aaactgtcaaaatacaccagaaaaaaatgttgacaacaagggcataatcagaacaataga

aaatcaggattaaacctgcaaacagatattaaaccccttccaatttcttcatcttggaag

tttgtaaattaccctccttcctcaacctcctcttttgaacaagcccctttctcctcaacc

ttatcttcttggcaagcttcatcttcaccgtcttctcttccctctctttcaatttcgatt

ctaatggaagcttgtctacagaaacaacaccactctcttccttgccctcacttccttcta

ctacatctgcaacattagaatcaccaccagcagcagcggatgcaataaaaatcgcatttt

ttctcggtttcacaacaatgggtgtcgagattcgaagttccttcgaacagttggtgggca

aaccgatttgtactctagaaatagggcttgctgtgggatgaggggaaatggaggaagaag

atgaaagaggggtgagtgtgaaggaattgaagcagagaagtgccatagtgtttttttttt

tcttttctttttcaaatgaagaattggtttatatgatgttatgttaaggataagaaaggc

acatcacatgtgtgtcttcttccatctccaccacttaccaccttcgctaaggaaataata

ttaatgataattttttgaaatttatttgttagggttaaatatttagattattagaacatt

gagaccg

>fava_dn_rep_c883

aaacacacaacaaaagaatcaaatttcacgattcagaactgtgtgagagagactcctcaa

gagtgaaacgaaaaaatgggaaggaagaaagggtatgtcgagttcgaggaatctccaccg

gacgattttgatccggcgaatccttacaaggatccggtggctatgttggagatgagggaa

cacatcgtcagagaaaaatggatccaaatcgaaaaggctaagattattagggagaagctt

cgatggtgttatcgcattgaaggtattaatcatcttcagaaatgtcgtcatcttgttcaa

cagtatcttgagtctacacgcggtattggttggggcaaggatggtcgccacccttctctc

catggaccaaaagttgaggcagttgaggccgaatgattttcccatggataaataaaaggg

aagttttattacccaaatgatgacatggtggaggaatgcatattgttcaagctgttgtgt

tttgttagttttccatagtcagatttcctgtgaactacaattgtattttggcttggcttg

gcttggcttataagcttttattgcttgatcttaccatttaaatgtaaacaagtatggaaa

accttatgagcaaaagtacatctgattgtgagtgaaataagtttcttttgttctttgtta

tatgactgttgtgtcctctagtccatttctaatatttagcatgctttgttaattttaaaa

cctcatcatttgcagttttgatcatgtggatgtgattcgtgtattgatttgatacaaaac

tgaattcaattgataatccctagtgtagtagtgtatttgttttgg

>fava_dn_rep_c884

tgccgcgggagaagatccaatttacaccttctcccctcaaacttcaatacaagattttgc

aaaatgaatcaagcagctgcaagacttgatgttcccctccagatcagcacctcaacagtt

gtcaaagataggaaatcgattgaggatttacgtagtttgcgacttatcacagcggttaag

actccgtatttacctgaaggaaaaattgatctggaagcatatgataacttggtgaatata

caaattgtgaatggcgttgaaggtattcttgttgctggctcaactggtgaaggccactta

atgacatgggatgaacagataatgctgattgctcatacagtcaactgtttcggcgacaaa

gttaaagttatcggtaatgcaggaagcaactgtacaaatgaagcgattgaggcgaccgag

cgaggttttgctgttggaatggatgcagcacttcatataaacccttactatggaagaacc

tcaatgaaaggtttggttgcacattacaatagtttgctttctataggacctgtcattata

tacaatgtacctgcaaggagtggtcaagatgtccctcctagtgtggttgaaattttggct

caaaaccctaatttggttggtgtgaaagagactgtgggaggtgagagggtgaaaatgtat

actgataaagggattgttgtgtggggagttgatagtttgagccatgaagctagatgggat

tgtggtgctgttggagttcaatctgttgcaagtaacttggttcctggtttaatgaaagaa

ctcatgtttgagggtaagaatcctttattgaattcaaagcttacccctttgtttgattgg

cttttttcagagccaagccctattgctttgaacactgctcttgctcaacttggggttatc

aagcctgtttttaggttgccatatgtacctctaaatatggaacaaagggtagagtttgtg

aatttggtgaaggaaatgggtagagagaattttgttggagaaaaagatgttcaagttctc

gatgatgatgactttattgttgttgctcgatattagtttaaattgtcattttcatgtctt

attttaagttactttatgtattggcttttatattgggaagtacttatgtatacatgaaca

ctagattcatgcatcgaccaatggaaggttgctcctattttacaagttgtattgtgtgtg

aaatgaaacttctcattcacttcatagcagatatatatgactgaaaaagattccctaata

gtgactattttccagcattttctattgttactaagtttaaactacctttggagtttgtga

tattttcaaatctcatttnnnnnnnnnnnnntatgcttaatagttattctgaaggcaaca

aaatatccattaaaaagtgaatggcaacaacagaaccaagacaacaagagtacttaaatc

taggaaactcaagtaacttttgattgatttttctatttaaggtttcatg

>fava_dn_rep_c885

ttaaagggtaaaaaaaccaaattagctgggcattaccacttaacaatgttatttaaccga

ggatcaacaatagagaaaacaatcaaacttccaaattacaaccaaaatgatacccaacta

cgatgaatccacattgaaaataagagcaatgtctatgaaatttatgtaaagattcagggc

actgcgcctctctaaacaaacaccaaacaactatcacttaggcttcggcaaatccaagct

cggagagcttaagatgtgcttcggcataatcagcaaagaaggcatcttcatccgcagcat

atttctcaacaagagggcggaatacagagtcagacaaaagtgccttatcacttggcaact

gaagaaggccttccttctcaccagtcaacaactcagtgaagtatgagttgtcaaaaatga

gaggattagaagtccatggtccctcaaatccagaacgctccttgtgtgcagctccaatgg

tgtgaccaccagatagagcaacaatgtcctgatcgctaagccccatagcttttccaaaca

catccctcaaatggtcagaacccttggtggcatcaggcaaacgaccctctggtggtgact

cgggcttgtcctcccgtccggggtggaaaggaacttcaggtccaccagtgatctcaacag

caacaacaccacccaactgatagaaatcagcatagctcacaatagggaattgctccttaa

gaggctccaaaagcctgacagcaatatcaagaccgttgttagcaccatgagcaagctcag

ctggatgcttaatggttccgaaaggaccaccagtctttgtcttcgaatcaaaagtaccag

cagagtgccatgccaaacggagaattagaggagcgcatttcttctcagcaatgaaacctc

taagcttcctcttggccttttcaatggccttctggtaatcaggactaacggtagggtaag

attttcccatagctaacgattctcgaaatctaagacgaagaaatgccaaagttaaaccct

agtgacacgaggagagcacgagccaagagtgaagagcgtccccgcggcaa

>fava_dn_rep_c886

ccgccgggacatcgccgttgggtagcatttcatctccagaacgcgatcgaactcggagga

agcagccgccgccaaaatgccttcacacaagacattcatcatcaagaagaagctcgccaa

gaagcagaggcagaacaggcccattccttactggatccgcatgagaaccgacaacaccat

caggtataacgctaagcgtcgtcactggcgtcgtaccaagcttggattctgaggttctat

ggtcacttggtttagttttctttgttttcgtttgaaagagacaacaacttttgttttgga

gtttttaatttttttgattattactctttattgagatcagtgtttgttatgtaaggaagt

gattgtggtttttaatcacaatttaacttattaagtgattatgatgatttgactcatggt

tttagttttgtttaactcaatggttgctctttaattctagttctgtttatgctgttcttc

tgattgagcttttgattgagattatgctcttatttttcgt

>fava_dn_rep_c887

attaacatacagtataactgttttatttataaattgccttagccttagttgaagcaacgg

gaaatcgaataatggcgctgacaacctctatccgatttggttctctcgttcttcccccgt

caactcccacttctagttctcactctcacgccccttctttcacttcaccctcttcacgct

tctcgtggaaattgggagcaagtagaaaaattactcaaaaatctgctttgaaggtgaagg

ctgtttacgctaacgagaacagtttttcaagtcatgaaatctggtccatcagggaagatt

tgcaagttccgtcttccccttatatccctgttcacgcacaaggacagggaccccagccaa

tggtgcaagagcgtttccaaagtgttattagtcaactttttcaatatagaattattcgtt

gtggagggccggttgatgatgacatggccaacatcatagtttctcagcttctttaccttg

atgctgtggatcctaacaaggatattgtcatgtacataaattctccgggaggatcagtta

cagcgggcatggcaatatttgatactatgagacacattcgacctgatgtgtccactgttt

gtgttggattagcagcaagtatgggtgcttttctcctatgctcagggaccagaggaaaga

gatacagtttgccaaattcaaggataatgattcaccaaccgattggtggaattcaaggaa

cgcatgctgagatggctattcaggtaaatgaaatgctgcatcataaggcaatcttgaatg

gctatatctcctaccaaactggccaaagttatgagaagatcaaccaggatactgactggg

actttttcatgggtgcaaaagaagccaaagagtatggactcatagatggtgttatcacga

atcctctgatagctctccagccattagcggctgcagccgaggttaatgacagggctatca

tctgaaattaagagagattgttgcttcattgctgagtaaaccatagtgttaagtaggagt

gttcatcttcccatgttgatacccaggtgaaaatatggccaaagttgaggtaaaaatgaa

ctcatgatgttttctttttcccttcaaatcttcaatattttttattatgttgggatcatg

tatcatttagtttataaccggttttgaataaaaccaaaatttcaatcaaacaatatcatg

tgatgatgaacattattctgtttaactcttttcacgtcatcatttttgtgaatatcatta

gttatgtagacactgccacggagtgccgatgtgtttgaatataattggttct

>fava_dn_rep_c888

gtggggtgttcatagaaaagagggcgtcgcccaacgagtggcagattagatttttctgga

gtctcgctcatagatagaggaagagtacgcgcgatagcgcgctnnnnnnnnnnnnnnnnn

nnnnnncagatagcccttcgggcaaccaaccgcacatcaaattccgatcaacaacttgga

ggtatggctgagtggcttaaggcattggtttgctaaatcgacatacaagaagattgtatc

atgggttcgaatcccattccctccggcacggaagtggaacgggcgggcgaaattacgtga

gagaaagaacctcttggtgttggtggagtcccccggaggacagaatagcactacttagtg

agcggagagcccgttgcgcgttgttttgaccggcctatcttatttttataagcaagctcc

ctccggcagtccctgggcggctctcggttcttgagcatgttgggagaggagattaggcgg

cagttgaaagagctgctcgaaagcttgacgaacgaaaaaaaccctttctttcttgttatt

agtaaagggcttttccctgactagtaaagtggtaagtaaggtagggcgctattcgatgaa

gaaaacatactttaggaaagtggttcaggtagctcagctggttagagcaaaggactgaaa

atccttgtgtcagtggttcgaatccacttctaagcgggctttgggtccaaaggacaggta

gccgagccgggttttattaaattaaagttaattaaagaggaagccaaaatcaaatgtgag

cgctcctgcactatacggctttttggcgtcgccctatcttgtcttgctggagtggaggca

tattttataaaaaaagcggttgattaggttctcgcgtccctatgccccaaaaaaggatgg

gcgagttcggttcgagtgttcatcgatcgggtggatctatatgctgagggggtgagacga

ggtgtagcgcagtctggtcagcgcatctgttttgggtacagagggccataggttcgaatc

ctgtcaccttgaggtgatgggctgaaagtgcacagcccggcacagcctctttaggctggc

gaaatttaggcacaatttattacaaaaagcaaatgaactatattcttcgatccatgcttt

ttgtaacttccacgggttttgcgcatcaatgggttctttattctgatcacccattcttga

agaagctttctctctcttttggttggtttatctgataaaattttgatcttctgagtcaat

acaatcatatctttagcttctgtggatgctgtttctcggggaatttttgctgctgatcca

aggttgatgcactagagatggagaggatgctcgtggtggggcttctttgtgtgcacccag

actctgagaagaggccaagagttagagatgctgctaggattattaattgtttgtgtatgt

gcagtccttctgtgtgtagaacattggctcatgcgtccagtccaatgggtggcacctgac

tgttcccagttcagttggaacagaagtcaccagctttcattggcgggaagctgactgatg

tcttcctagttgacgacaatgctaaacctgtcacgaccgtagtgggtcaaatagaatgat

tcctatattaccttgcataaagaaagcagctcggtctatgagtccaagatatccgtacct

gtcaagtggaagagtcagagtggcagagtcttggagagcgcagagtctaatctggtaaat

atcttctagaataaggcggagaacggataataactcggcaaatgtccgatttcggagata

agtgtgcctttcttttgatcaatagaacaggaagaggagtcagccaaaaagaaaacaaaa

agtaatgaccaccaagatatgcatagtgattcgatcttttgatcacccttttttggaaaa

ccattttggggggcttccgccttacacacggaagattggattgcctgaatcacgagtctt

atatactgtgttacgatcacctcatattgataaaaagtccagagaacaatttcaaatgga

aatcaagaaaaaatatctggtcataaaaacagaaaagcatgaattgcgaaataagttttt

ttggttaaaacgccagcgtttatttggagctcaatatgaaatcctatttttttgcaagac

ccgtttggataagggaaaactccagagattgctttgaagtaagatccttgcgttgaccct

ttcctgaaccataaccgggggggagaggaaagaaaaggggatcaaaatttcccatcaata

aagtgagggctttccggaccttccccacccctcttttaattctcccttcccctctcactc

cccctttttcaagaaggaagccccgctccctaagaaggggcccctctctgataaggaagg

aaacgaaagaatctccattttatgacaaatctggtccgatggctgttctccactaaccac

aaggatatagggactctatatttcatcttcggtgccattgctggagtgatgggcacatgc

ttctcagtattgattcgtatggaattagcacgacccggcgatcaaattcttggtgggaat

catcaactttataatgttttaataacggctcacgcttttttaatgatattttttatggtt

atgccggcgatgataggtggatttggtaattggtttgttccgattcttataggtgcacct

gacatggcatttccacgattaaataatatttcattctggttgttgccgccaagtctcttg

ctcctattaagctcagccttagtagaggtgggtagcggcactgggtggacggtctatccg

cccttaagtggtattaccagccattctggaggagcagttgatttagcaatttttagtctt

catctatctggtgtttcatccattttaggttctatcaattttataacaactatcttcaac

atgcgtggacctggaatgactatgcatagattacccctatttgtgtggtccgttctagta

acagcattcctacttttattatcacttccggtactggcaggggcaattacaatgttatta

accgatcgaaactttaatacaaccttttttgatcccgcaggagggggagaccccatatta

taccagcatctcttttggttcttcggtcatccagaggtgtatattctaattctgcctgga

ttcggtatcataagtcatatcgtttcgactttttcgggaaaaccggtcttcgggtatcta

ggcatggtttatgccatgatcagtataggtgttcttggatttcttgtttgggctcatcat

atgtttactgtgggcttagacgttgatacccgtgcctacttcaccgctgctaccatgatc

atagctgtccccactggaattaaaatctttagttggatcgctaccatgtgggggggttcg

atacaatacaaaacacccatgttatttgctgtagggttcatctttttgttcaccatagga

ggactcactggaatagtcctggcaaattcagggctagacattgctctacatgatacttat

tatgtggttgcacatctccattatgtactttctatgggagccgtttttgctttatttgca

ggattttactattgggtgggtaaaatctttggtcggacataccctgaaactttaggtcaa

atccatttttggatcacttttttcggggttaatctgaccttctttcccatgcatttctta

gggctttcgggtatgccacgtcgcattccagattatccagatgcttacgctggatggaat

gcccttagcagttttggctcttatatatccgtagttgggatttgttgtttcttcgtggtc

gtaacaatcactttaagcagtggaaataacataacaagggcgaacattccttgggctgtt

gaacaaaattcaaccacactggaatggctggtacaaagtcctccagcttttcatactttt

ggagaacttccagctatcaaggagacgaaaagctataaaaaaaaa

>fava_dn_rep_c889

gagaataaatataatagcaacattgaatcgacctggtaacagaataaatataatagcaac

actgaatagacctggtaacagccactgaattacaaataatagcaacacaagaaacacaac

ttttaacgccaaaaatataatccagatgaaaattaataagagaaaatttgatctcaatct

agcattgcatatgcgtccatgcttatgcacaaacaatcaaataaatgttgtattcccgtt

tgacagaagcgtgcagtaatcctaacacgcaaaattatatcattcctacctaaagttttc

agcaactggaagggatgcgagcaaaatcgtcctaccaaaaattaaatgaccaaatgcaag

aaaaatgtaaacaatgtctgaacttggacaacctcagcagccctctgtttatttatggtc

cctatttctcctttactgtgttactcaagcaaaatataaattgtcaccataggttgcttt

atgcctttacgatctcagcaggaggagctgcaccaccagttgttggcagccccacaaaga

ctttgaaagcatcatatattccccactgggctcctgtaagtgttccaatcataacgatac

ggagagggagacctcgggtgaaaagacccacaacaccaaacttcttaacagcatcaccaa

ctgtggcacctttagcgttgttcagaaaagatacaaggttatcagcaggatgagacacaa

ttgcacaaagtacaccagcaacatatccaccagcaaaactgacaccaagttgcagagatt

tgctgcactcgctctttggcctggggatggcatgtttatagatttgctcaacaatggttt

caaaagaagcaaatttcatcattgtgtatggaatttgtcgtccccatagaggcactaatc

ccttgtacaaccccaaaactccttcagatttgacaaattttgggagcccatcaccaagac

ctctagcaaaaccaggctgagtttgaacacgtactttcacagcttcaaatgggcaaagtg

caacatcggcaatgacctcagcagaagctgaaccagcaaggtagatcaaggtcttgtact

tgtttgcatactctggaccagcaatatcagaatagtacttcttaaagaactcatagaatc

caaatttgcaagcaccctgtgcactgtaaccaagcaaggtaggcacccagccacggaaaa

agcccttaactccctgctccttgagcaaaactccaaaccctgatgagatgctcttgtact

tggtaggatcaatctgcatattacacttgactaagtcaagaggagtgacggtcatgtgag

taagaccacagctcagaattccaccagcggtacaggcagcatagaaagccggcgagtaca

tctcgatcttaccggattccttaggagaaggaatcatgaaagatccagttccggtcaaag

aaggcgacgcggcggcggaaggagcggggccggagttgatgatctggtgaataaccggta

gggttttaggggaggaaccgtagagaaatgaaggaatcatgttcttgcggacggaagttt

cagatgacggagccatttggagaatcgaaatgaacgaaatacgcaaagaagtgcgagaga

ggaggtaagaaaatggttggatttctcgctcctcagtctctcccaattcttcgccgtggt

cgcactctctctgtaatgtgtaggcttttatatgcaccaatcactgctttggttctttac

tttgtatcgttatgccagtttctacaaagaccgaaatgaccctcattctttgttggaatt

attgcgttgacaccatcgagaagaagagttgatgtggagatccccgcg

>fava_dn_rep_c890

ttaggaacacctcatctgccattgctgagataggcgacttgaacaaaataagattgaagt

tggatgcagaaatgaataaatgaatggctatgtatactgtttcctttgggactaattgtt

tattgtaaaaactcacataaacaaatagaaaggagattgcatgaagacttgttgccagag

ctgtgatataaaacaaaatttagaacagcttcttgaaatgcataacaatgcaccatgcat

aaacactcaacactcaattcaaagaacaaacagatcaaagatcaatcacaactcactagt

ttttccaaacctttcctactaaaacgacaaagcttggcacatcaaagtgcctttttatta

cataactattctatcagaaagcttaaaacaactgaaaccatctagtgcatgttcttattc

ctactagaagcacaaagattggcagatcatgaatttgccaaactcactgtcaatatccaa

cctactcaatgcctatcttcttcctgatctccacaatgaactgatagaccttatcctgat

caggaatagactcagaaacactctcaatcttgatacatctcttagcccaaccaatgaact

tgggacactccttctctatatcgaggttaccaaaagtctcatagcctttaaaccaagtgt

agaatggaataagtgctacatccacataaccaagcttctctcctccaaaataactcttgt

ctcccaactcttgctccaacagtttgagggcttctatgaatcccttctttgcagcttctt

tctcttctccatttttggtccaaaggttccttccaatctcatagatcttcttgtcaacaa

aatcagcccagaatctagcttgtgatctctgataaggatcagaaggcaacaatggagatt

tatgattccaaacctcatcaatatactgaacagcaatgagagattcacaaatgggtttac

tattgtgaatgagaacagggattttcttgtggatagggttcatctgtaacaacaaagggc

ttttgttcctcaagtcttcatctttgtactcatacttgatacccttttcagcaagggcta

ttctgagcctcattccgaacgggcttggccagtaatcgagaagagtcactgcatcggcca

tggatgtagttagcgaataagaaagagagaagattagacgttattgtggaagaagaaatg

agtggctgtgataaaacaaacaagggttagaagtgtatttatagagtgacatttggaatg

aaaaagaatgtagatgtgaagtttgtggaatgtaga

>fava_dn_rep_c891

cttacagatgttgcgtaggactttcggccgcgggagttcggaaattgagagaaaaaaatg

gcgttgcattggttagaagctgtactccctctcggaatcattgccggaatgctctgtgtt

gccggaaacgctcagtactacatccacagagctgctcacggacgaccaaagcacatcggt

aacgacttgtgggatgtcgccatggaacgaagggacaagaagctcgtcgatcaggcttct

aattaatcacaggttgtttaatcagcgtgtttgtgaagagaataaattgtttaaacgatg

ctctccagctgttccaaggaagcagagtagatctcaacaattaaatttccatattttctt

gtatgaaatatgacgtaatcattatgatatacatatgctctaatcaattgtttcttttag

ttgaatatggtgaagttgacatgtgatctctatgatgatatataatttctgttgcttttt

aaaaaaaaaaagaataatgacgaatcaagatgagaggggaagagtgaattttggtgtgat

tattctactgctgaagaatcctggattgagagggctgttaactagttttttggcatgagg

tttgaacacatggtgcaacaagttttatgagcttttgtgaactggatatggcaggactgg

tttttatatatggtgcagctttcagcattagcaaggcattgccacaaagctgcaagttag

caagcattttatttattttttgttagatacgggctcagttattatagttttctggtttca

atgcaggtttggaaatgttttatggatgcaggttgcctttgtttgaatagggtagatctg

atacatggctttggcagggcatgatgttgtttgaagcttgcagaacgttttgaagaggat

atggagaagatttgtaaaggcctttagcatagcctgaaggttaacaagaggggcttggtc

gtgaagattggacatgggttgcgactcaagtgtgtacctttgtaaagtttctatgtaatg

atgtaatatagatttgtaatgatttcttttgtggattattggtgtaattgtttgaatttg

taatggttttgatggtaattgaaacggttttggattaaaaaatgtgtgaatggaattatg

attggaacttgg

>fava_dn_rep_c892

acaaaaacaaggtcaacaataggaaccacctccaataatcattcaaatgactatatgagt

gaaactacataatacttctttcaaaatcactacaaaaacaaaattagagtaaggccaatg

atatataattccacaatcactttacatatagtgataaacccaagggcaagttgtacaaga

tgtataaaaagaaataaaaaccagactgcacgataactaatttttcattcaaaacgaagt

tatatacaatcatcacattgctagccttttctacagcttttattcactaagatgaagcag

ccatactcacatcattatgatcctcccctaaaaatctagccacaatagtatccacatcct

ccaaaccaactccaatacaaagagggttactcggaaccttaacagaatcatcaagtccct

gaaccatgccatgatccaccgagttctgaattctcacattaggaccacttttacacttcc

ccatacacttacatccaacaacagaaccaacaccttcaacacccaccaccttctcaaact

cctgcaacaatgcagcagcccctgatttcttgcatttgttacccatgcaaacctcaatcc

tcttttgagcagcagggataaccacattagtctctttcttaaacccaacaacaacagacg

catcatttgttgaacacaaatccatgacatgatgacctggagtggaagcttcaacatcaa

taatggtctgcaattgcaattcatccaccggctttgaagcaacattaactccaacacctc

ttaaggtgttcatgtcaaccactttatcacaatcactatcactcgattcagaagacgaag

atgacgatgaagattcagatttatcacaagttttcatcttggagggtttaagtttagcct

ttttcttctcttgtttcatttttttcttcaattccttcttctctgctctaattttctcaa

gctctctcagtaattcctcaccaccatccgtgatgaggttttgttgaagatgatcgaaga

gattaggttgatttgaatcagaagcaaaccctaattgagggaaagaagaaacgttttcag

agaaacgtttgagcaatttcatcttcttcttattgcttttaacaggtcctctgttcttca

attcctggtaatactgcaagtgaccctcgtcacgaaaatcaaaccccgttgacattctca

ctctaacaacgtctcttcgcggaacaaagctacgacgattggcgttagttccggcgccgg

agacgtatgttaactgacggagaacagtgccggaaacctccatgtctgacagtgacggtg

agagtcgttgaagatgaaaattgaaagaggtttttttttctgtttctttttgttatgggt

ggttgttgttatttgtgttatcgaaatcgtgttttt

>fava_dn_rep_c893

aaaatataaactaattgtctaacaatataataacactattaagtagcagacatgattttt

atattttaaaattagaatctaatattttcatggaataacccattatcccagaattcgtcc

ataggaaatgggaaaaagtactatataacgatcttgttccaactcataaatcaacactaa

gagatgtattgtactatatatctcataatcctaaggtttagcatagggcacactattttc

agttctaactctgaagttccccagacctaagaccgcaaacaaataatgcacagcatgaac

accaaaacaagtacagaaagctgaatatgtgtattatctcaaaaatagctagacacaaaa

aaggaaaagtacaagttaattatctatgaatgaaaattttgggggaatttctctcccatg

atcataaaaaatttaacctactacattccgccggttttactacccaaatgtgagcaaaca

caacatctctagagctcgtcgcgagagtcttcatcttcaccctctcccgatgcaccacct

tctggagctccacctgccctctggtatacagcagtgataattgggttgcaaacggcttca

acttctttaagcttctcttcaaattcttctttctcaacagtttggttctcatcaagccat

tcaagtgcttctttcactgcagtttcgatcttctccttttcatctgactccaacttgtca

gccagtttgtccttgtcattaacttggtttttcatgttgtagacataggtttcaagagag

ttccgagcatcaattctttctttgacttttttgtcttcctcggcaaactcctcggcttca

cgaaccatccgctcaatttcctcctggctcaggcgtcccttttcgtttgtaatggtgatc

ttctctgatttgccagtacccttgtcttctgccttaacatttagaataccgtttgcatct

acttcaaaggtcacttcaatttgaggggtacctcttggagctggtggaattccagacaga

tcaaagttcccgagaagacgacaatcctttgtgagactcctctcaccttcaaacacctga

atggaaacagttgtctgctgatcctggtaggtggtaaaaacctgagattttttggttggg

ataactgtatttctaggaatcaactttgtcatcactccgccaacagtttcaattccaagg

gtaagtggtgcaacatccaacagaaggatatctttggtttcttctccaccctctccactc

aaaatacttccttgcacagcggcaccaaaggcaactgcttcatcagggttaacacccttg

tttggttcctttccgtcaaagaaatccttcaaaagttgttgtacctttggaatcctagta

cttccaccaacaagaacaatctcatcaatttgattcttctgtagtccagcgtcgtccatt

gccttcttcacaggccccatggtcttcctgaacaaatcattgttcaactcttcaaatcga

gctcgggtgagtggctcggagaaatcaactccatcaaaaagtgattcaatttccacacgg

atctggtgctggctactaagagctctcttggcacgctcgctctccctcctaagcttgcca

agtgctctgttgtccttgctaatatcctttccatgctttttcttgatcaatttaatgaag

tactccattactctctgatcaaagtcctcacctccaagatgtgtatctccatttgtagca

agaacctcaaaaacaccattgtcaattgtcaagacactaacatcaaaagttccacctcca

agatcaaagacaagaatgttcttctctccacctttcttgtccaatccataagcaatggca

gcagctgtgggttcattgatgattctagcaacattgagaccagcaatgacaccagcatcc

ttggtggcctgcctctgagcatcattgaaataagctgggacagtgacgacagcgtcacga

attgtcttgccgaggaaagcttcagcggtttccttcatcttagtgagaatcatagcactg

acttcctcagggctgaagaccttggtctcaccatccttaactctgacctgaatgtatggc

tttccatccttgttgacaatcttgtaaggaacaagcttcatgtctctttgaacttcctta

tcttcaaattttcttccgataagtctctttacatcaaagatggttctttcagggttaaca

gctgccaaattctttgcagcctccccgatcaacctctcgtcatcagtgaaagaaacccag

gacggtgtgatacggttaccttggtcattggctatgatctcaacatggccattcttgtaa

acaccaacacacgaatatgttgttccaagatctatcccaataactgtccccaacttggtg

gtcgcttcctcttttgcaagcgatgatgcaaacagacatccgatggaaactacggcgaga

agaagcaaagaccggcgtgaagacgacggagccatagtggtttgttgtgagttcgtcacc

gttcaatgatggagtttgtattgatcccgcggcc

>fava_dn_rep_c894

gcagtaacaaccaatcagttaaacccttcaaatttgttttggatgcgtttccaatataca

tatatataatgaagctgcaattttccacacatcaaacataacaccattacggtataatag

acttggatagtaataaaaaaccacagtagaaacaatgatttttaaatgtatatttttata

gatcgtgattccaatatccacacaagaaacttaaaacaagttttctttctaataactaga

aagttgaagtaaaaccaaacatagagattatggaaacgaacaatctaaagcaaacgaaag

cgaagcaacttccatttatgcagcaaagaccttagcaatcttctcagttggaaccaacgg

aagataggcagaaactctgtaacctttctccgcaccaaaaaccacagcttcattatacct

ctcactacaataagccgctttcggcaaaaccacatttgccacctgaggaaatagcggaag

ctgattcaacctcttccacgccgaaacagcgcactcctccgcctttggctcatacttcga

gtaaacagttttggccaatccagaagccgattcaaccactccggcacggcggacttcggt

cacaacagatctagcttgagtcgaaaccttcttgacgttggtaggaacatgacgatccag

ttcagaaacggattcaccgaccttgcgatcgacgtatttgagaagctccacggggacttg

gtggaatttgtcgtagactggaccgatgacggttttcacggcttcttcaacggtgtcgac

gccggttttgagtggaccggatcgttctttcgcgtaggaatagaggatcgcgcatcgcat

caaggcgtggatagttgcgaattgcacgaattgaagatacttgagttgttgcacactttg

attattgttaacgttctcttcagccagttgttgttgcggttgagactcggattgagcttc

ggcggtcatgatcgaaaaattgatcggtggtgatttagtagtttaggtttcagggaaatt

ttgggaatcccgcggccgtaatggagagtatgggggaagatagaaggaagagattttttg

ggtatgaaatctctccc

>fava_dn_rep_c895

tttgtggtgaagacgaagctgatttccaagattgctgagaagaagattaaggaggctggt

ggagctgttattcttactgcttagttttttttatttggtattgttttcgaacaaattagg

gtttttttgccctttttttaatttctttttttttttaagcaatcgcaatatattgattta

aatttaataatcaaaattgaaaattgcatattacacaataacaaagtcagtttaaacaat

cacaatctttgatttagatttaacacagaaattgcaaattgcatggcgcagcagttattg

cacaggaatctatttccatttcccctaaaaaaaccgcatacatggtacatagaaagaaat

aaaaaactcaacctagaattctgagaatctcaacttataatgtatcaccaatgatcccat

gaagaacattacagcagcgcacatgtctcttattaatcctcaaatggcatcatacattac

agagcaattataaccataaaagttcacacttcacttattgaccaacttaagagattttgc

cttctcctgaagtgtatccagaaggctatcaaaggctttcttaaatgaatcaactccttc

aagttcaagctggttaccgacattgctccaatcaatacccaacttctggatagcattgta

tatgccttcagcttcagatgcatttgagtctattgtcctggctactgcaccgtgatcgat

aaatgctagaagagcttggtctggcatggtagatacggtgtcgggtccaataagaggagc

tacatataaggtgtcagagtaagcaggattcttaacactggtagaagcccaaaggagtct

ttgcttcttggctccctttttaactagagcttcccaccttggaccagaaaatttcctttg

gtaaagctggtaagccagtgctgcttgggctactgctgcctttccacgtagattaagggc

ctcaggagtgccaattttctcaaggcttttgtcaatgagagtgtccactcgactgacaaa

gaaagaggcaacacttgtgactctagagaggtcatttaaaccagatgcctcaagaccgtc

caagtaagcatcaattacagcttcatatctttcaagagagaatatcaaagtcacattcac

acttattccattggcaatcacttcctttattgaagggacacaagcagctgtagctggaat

cttaatataaacattagggcgagaaacaaatttatgaagccactttgcagcttctatggt

tccttgagtatcatcagcaagactaggagatacttcaacagaaacatagccatcaccacc

atctgtttgatcgtaaatttgttcaaatagtttggaagcatcttgaatgtccttaactac

aagttcccaataagcactttcaatgtctttcccagcttgcacaagttccctgaactgatc

attgtaagcatttgaagatgagatagctttctggaagatcgctgggttacttgtgacgcc

tctgacaccactttcaataagaggaatcagatccgtaactggtcggcagaggttatcata

ccatggactctgcccttgtttctcataaagatcatgaagcacagtcctcttagcaggaac

tccatttccatctgtctgtgagcatctaaccctaaaagacgctttgaaagggctttcgtg

atgaattcgaagagaattagttctgaatttcgtcgttgatgaaggatcgaaagcgaggaa

gtttgttttgatggatctggaccgaagcgattctggtgaataagcgcttttttgtgcgtt

tggcgttataaccttcgacagtgaaagtgaagaagccattgaaaattgtttgcaactatt

gtgaaatgaataaaagctgcttttattttaagttatggtttggttgatttcatttataat

>fava_dn_rep_c896

tccagcaaaacaatattctagcatctaagattcaagtgcgcaatagatttttttactgca

ccatatagctaaggtatccattaaatcaaaatcctcattagctctagaaaaaaatgaaaa

catgccttttttaagacatgggtattcaaaataacatgtaatgcttaaatacttgcaact

gagtataataatttcttaaaatagtctgaaactacaaaatttgggaggtgggtcttttga

caacattgaatgcaacaagagtcaaaccatatgtttagctaattaaaaacttcaaagcaa

tcaaacatttttaagcaacaggaactgggacagggacctcaatatcatttgccacgacag

cagcaggtcgaacatactcttcctcttcctttggagtgtgaatggtgacaatatcaggga

ggggagtcttgggaccttgtttacctttaggatcccaatcaagcatgatctttaccttga

taccgagaacaccctgtctaaggagcacatgtctcactgcagagtcaatgtaatccttga

ctggctgtccagaagaaatcatgtacccatccttgaacttcatggatttggctctttggg

ctcttaattttccactgacaatgacctcgcaacccttggcaccactctccatgacgaacc

tcaaaacaccatagcaagccctgcggacagcaagaccaccaaggagcttgtaacgaagag

actcggcctgagcaatggcacaaagtcctctgttgttaaccttctcagcataaagctcca

cactgttctccggaaacttaaacctcttctgtaccactgaggttagttctctaatcctcc

ttcccttctcaccgagaacagcttgggtgcgagtagctctgatgatgatttcagtgcgca

tcggggtaaccctaacctcaacacctgaataaccatcctcggccaactctctggtcaaaa

cttcattcaattcagcgaaaaaaactccgtctgcgacgaattttcgctttttgctcattt

gtgttgccatgattgagaaacggaagaaaactgcaatccgcagctgtggaaaggggggag

gggcacaaatgaagaactaccgcaaaaaccctagtgtttgatgcccgcggcc

>fava_dn_rep_c897

tacgggggaaagtagcggctgagtattgcgatctcatcttcttccgaaaaaacccatact

caaaacccttgacggcagcgttcgatccaaaatggacccaaacatgattgctgatatggc

ttctcttccagcggaagatcaacaaagaatgaccgccatgattgaccagctccaagtccg

tgatagcttgagaatgtacaataacctagtggagagatgctttcaagattgtgttgatac

gtttaagcataaatccctgcaaaagcaggaggaaacctgtgttcgaagatgtgcagaaaa

atttctaaagcactctatgcgtgttggcatgaggtttgctgagctcaaccaaggagctgc

aactcaagattgaagtctgctaacattgggaaacttttattgaatacaattttgtttagt

tatatagttttgctttttaaatacaagacactgtcgaacatatgtatttcttaattgcta

tgatcgatcaaaattgatgctaaaatcttaataagtttatgaagttgtattcattatgcc

ctagaacactggtgcttgattgggaattattcaatattagtagtactcaatttctgtctg

ttcatgagatcctttcattgttatgatcgaacaaaattgatgctaaaatctaaataagtt

catgaagatatattcattatgctttagaacgctgatgcatgattggcattattcagtatc

agtactcagtttttgtgtggtaatgagatgatggttgtaattacatgctgtattttatta

atctcagtttatcattataaaaatgttctaatgttttattgaactacatattaatggaaa

ttgatcattg

>fava_dn_rep_c898

cgccaaaaagtacattttatacaacagttattcaccttaaacattgaacacaatccactc

caataggtcctgcaaatcaaagatcttatttgtcttgaattctcaatagagagtcaaaac

caaccttgtttaatatttccggcgcattgttctcatgttcgactttcagagcaatttatt

attattatgttttaagaaagaagcaattgaggtcatgtcggcaaagatagaggtttgaac

ttcccttatctctcatatgttccattgaaaagataacgacaatcagtttggccataatcc

agtttgcaaagctaaatctcatgtgatgagttaatagaatacagttcaaaatgaatctta

atacaagaaaagagttactgaaacaagtgaactcagcaacataactacttcttcccacta

ggctccaaatttaaatcctggaggttcagcaagagatcatctgaattcaagaaaaccttg

ttggcagaatttgaaattgtttgagcaatttccctagcagcttcaattttccttaaggtt

ataaatgcagggttattggaaatagcttgaccaatcaactgagcactcttggcttctccc

tgtgctctaataacagcacttcttttgtcttgttcagctttttcgacaacaaatttagct

ctctcggcttcttgagcagcaacctgcttggcttcaattgcagctgtgaattccctccca

aatgtcaaacttgtgattgacacatcgtccaaagcaatgttaaattgtgatgccctttca

gtcaatatcttccttatttcacgactaacagcctctctctgagtaatgagctgactggca

ttgtactgtgcaaccacagatttgagagtttcgtgtataatagaaggcaagaccctttca

ttgtaattctcgcccagagttcgataaactgtgggtaattggtctggcacaggacgagta

agaactctaagtccaattttcaccatttggagatcccgacttcctgaagtgctctccact

aagtgaggtcgtgcacgaacatcatagatcacaggcctctcaaaccacgggatcataata

tgtgttccttcaggataaaccttatctttgacaccaacaagacggttaaagacaatagct

cgatgaccaccctcaacattatacaaggtgtttgtagccccatacacagcaagtccacca

ataatgctaactttaagcaaagcagcgattccgctaccaggaaccttcgggatcttcacg

ttgttgaaattcatcttgcggagagtggttgtggagattggggttcggtggaatgagggt

tcagtggttgtagggttttagattttggagcaagacactgcttgcgactacttcgggtta

tgtgttttagaatttctgggtcgggtacccgataact

>fava_dn_rep_c899

ctttttcatgaagagagaaagaaaactaactctatgcttccacactcttatcnnnnnaga

gtggccattacggccgcgggacacaaaacaaaacacatccaaagagaaagaaattagtta

tggcaactttaggtggcactaatgatgtggtggaatatacacaaaacagcgttgagatca

ataaccttgctctctttgctgttcaagaacacaacataaaacagaatggagttttggaat

ttgtgagagttttaagtgcaaaaaaacaagtggtttctggaaccttgtacgacatcgttt

tggagacaaaggatggtgggaaacaaaaggtgtatgaagctaagattttggaaaagccat

ggttgaactttaaggaagtgcaggaatttaaacttgttagtgaaaatgatgatgctcctt

cttcgtctaccgtttaattgggttattgaagatcatgcttcgctcccaagtcaagaatgg

gatatataaaaggaaatatatattgtctatacaacgaataagtgaatcatatatattttg

catctatttaataattcatgtcccttgtatttcttttcgtttagatattgccacctatta

aagttggatatttgtctagtagattaatgtttcttagtaatttgtgaaataacgaagatt

gtatttaaatttgagatttactattgttcttttaaaaatcaaagtaaaatatattattaa

ttaaaacaagtcaatctaacacgagatgtattacgagatgtattgataatgataagaaag

aatttaaaattacaatttaaaattctgaagaaaaaaaa

>fava_dn_rep_c900

ggccgcgggattcagtatttcattcttgttcttgttataagaaaagaaaaagagccatct

atatccgactagagttatgttgggtgtattcagcagctccgtggtgtccccaccggacga

gctcgttgcagccggcagccgaacaccgtcgccgaaaacaacatcggcggctctgcttaa

gcgtttcactgagagcaacgcctcaacagtgtcagtagaagtcggagatcaagtccggtt

tgcttatacccaccagaacgagtcgtcgttgcaaccaagactgtttggtgtaaaggatga

aatattctgtatctttgagggagctcttgacaatttggggagcttaagacaacaatatgg

actagccaagtctgctaatgaagtagttttggtgattgaagcctataaagctttacgcga

cagagcaccttatcctcccaaccatgttgttggtcatctcagtggcgactttgctttcat

acttttcgacaaatccacttccaccctctttgttgcatctgatcaatttggtaaggtgcc

actgttttggggtataactgctgatggatatgtagtgtttgcggatgatgctgagttgct

caaaggtgcttgtggcaagtcacttgcttcttttcctcaagggtgtttctattcgacggc

cgttggaggattgatgtgctatgagaatcctaaaaataagattactgctgtgcctgcaaa

agaagaagaaatctggggtgcaaccttcaaggtggaaggtgcaacagttgtagcagccag

agagtgagttgagttgagaatgagatcacaatgaggggcaatgtaattaaattgcttaat

gagtgaagcaaagttgtacaagtgtaaccttgttcatttgatgtataatttaatgtttca

atcaatgtatagttttcaatctaataataataaatgataattctggttgtggttctaaaa

aaaaaaaagtctctgttcagtttcacaaacaaacaaacatatccactgttttggtgcctt

taccatcaacatcaac

>fava_dn_rep_c901

catttaccaggcaaggccatagcatttgcagagttgattccaaataaaggcagttcagac

ttgtaatcgcaaaggcaagtaagattgcaacggtggacaacatcgcagcattttgcatcc

gggtttggcggnnnnnnnnnnnnnnnnncgcagagtggccattatggccgcgggataaca

tagtgagtaagcacctggttaatcaattaggagagtatggcacattctagtgggaaaact

ttgatgcagtggcaggtagcagcattgctctttgccatgttgggtggaagtctagcagtt

ttattatgtaacatagaatcaagtgagctaaaattatgtcgtgacgctgttaccggtgaa

aacccgccaaacccggatgcaaaatgctgcgatgttgtccaccgttgcaatcttacttgc

ctttgcgattacaagtctgaactgcctttatttggaatcaaccctgcaaatgctatggcc

ttgcctggtaaatgtggtttgaaaaagccgtccaattgttaagtgaggtgaaggtctcct

ccattaagagcttttcttcatgtgttttgctaacactagagaagctttaagcagcatttg

atgtcctccaaataaagacgtattttattttgatcagtgtgttttgtccatttatttgca

agcagatatgtagtgtatcagtgtctggtgttgtagatgttgttaaggtgtttgatgtac

aagaatacatgtcttaagaatatcaagaaaacatgtatcattgcctcccacagcaacctg

tagtctggttaataaaacgtttgttccttacttccttggattcttaattcaatgttgtag

attgtgtgaaatgtgtgtcccaagaagcaacaactagctatgattaaatatgataatctg

aaagagataatgtttttattattattgattagagcagcctgtgctcttaaatagaataca

atatatttaggaaaggaaattcctaataaatacttatcattgccctcaatcaattctata

agtaaggaccaataatttctataaatacatacacagctaatcaaatcctatttatgggat

tttaatcctatttaactataaaatataccactaattaacacatgtacagctcgttctgac

aggatgattgtttcagggtatattgagcaatagaatggcaattgcagttaagtaactctc

aaagtattctggacaaggcatacaacacaattctgagagcacggtttctcaaggctttca

ctgtctcgggcaacttctcactttccggtgttcccacaacatcataatgcttctcaatac

aaatcaaccaccaataggcatcttcgctgccgtcaaattatggcatgtctattttcctct

attatgattttcttgcttcagatcatatctgctctgataccagttgataggaattagaga

gcctagtctctcccaaaccaaacaacatacaagatccctaatctctcacaccctatattt

ataattctgacttcctatagcagttacaaaacagttacatagcagttactgcacacacaa

acatttcataataatagaataaactgcctaatatgcctattactaacaaattcttgcgag

gatcttatgttaatctctgactattttcctaacaaaagcttaggctctttcatttttggt

atgcacctttgtta

>fava_dn_rep_c902

cgggacaaacccatctaactcacaatctcatacaattaataccactccttgcaatgccta

cttctttagtcctttgtttttgtctaattataagcttagttgcttctgtttctgctcaac

aaccacgagctttcttcgtttttggagattctcttgttgacagtggtaacaatgattttc

ttgctaccacggcccgagctgacgcttatccatatggcattgattatccaactcacagac

ctaccggacgtttctctaacggcttaaacatcccagacctaatcagtttggagcttggct

tagaaccaacacttccgtatttgagtcctttgctagttggagagaagcttctaattggtg

ccaattttgcatctgctggaattggaattctcaatgatacaggattccagtttttgcata

taataagaatctacaaacaactagagctatttcaagtgtatcagaaaagagtaagtgcac

acattggttcagaaggagcaagaagcttagtcaacagaggacttgtactaatcactcttg

gtggcaatgattttgtaaacaattactatttagtcccttattcagcaagatctcgtcaat

tttctctcccggattatgttagatatctgatttctgagtaccgcaaagttcttcggaaac

tttatgatttgggagcacgtagggttcttgtgacaggaacgggaccaatggggtgtgcac

cagcagagttagcaatgagaagtagaaatggtgattgtgatgtggaattagaaagagctg

catctctatataatcctcaacttgttgatatgatcggaagtcttaaccaagaaatagggt

ctgatatttttatcgctgctgatgcatatagaatgcatatggattacatatccaaccctc

aagcatatggatttgtaacatcaaagatagcatgttgtggacaagggccatacaatggag

ttgggctttgtacacctgtgtcaaacttgtgtccaaacagagaccttaatgcattttggg

atgcatttcatccatctgagaaggctaatagaatcattgtccaacagattctaaggggaa

gcaatcagtacatgtaccctatgaatctcaccaccatcatggctctagattccaggactt

aatcatccatctatccagaaaattaagttaatgtttttttgttagttatgacttgtgtgt

aatggtgtttcaattatgtgtgtttctttgatttatcacatcatttgtatcccatagcta

gtactattagctagtggtttgtgatgcttggtgcttgagtaatggtttgtggactcaaat

tgtccttcgtcttttgccagtaataaaatttcttcttttgatcgaaaaaaaaaaa

>fava_dn_rep_c903

actttctcttatcatcacatcacaattttcaaaccctctctcttcatttcttcttctcta

aaatttcttcatctctctctttcttaagaattgcttaattttttttcttccacttttttt

ttttaaacaaaatcatttatattaaactcaaaattttcttcacattgtaacaacagaagt

acaagagatcttcatcatcaataaactcaaacccagctcaaggaaagaaaataatagaaa

ataacataaaaattcaccatcccaatacttacatccacaaataagaaacaacaacatttg

cagcaaacaatgtaacacctgcatagccattcaaaccaacagcactagaatcatcagccg

gactatccttcgatcctttcggagcatcagaagcccgaacacttttctccggcttagtcg

gtgcaggcgcctccgcaggcgcagccgtcggtgcttgtccaaaaagtgccataggaagaa

gcacttgatcaacctgataaactgcaagctgactatcactataaatagtattagaaacag

ttgtatcaaccacaccagtagtcacgttaacttgatttccagaagtagtaacattaagag

gatactctccatcatcactgtttccggcttgcgtgtgtaaggggttactagcagtttgaa

actgcgagattgtgtaaagtgttggtagaatatgaaactgcaaaagttgaagctggtttt

gtgtgcttatcgagtttaaagttcctgattttaggtttgagaatgcattatcggttggtg

cgaaaattgttaagccttggtttgaattgttgagctgcgaattgattcgatcggatactt

gtgttgctttaagtagcttgatgaatgttgtgaattgtcctgctttttctaggacttttg

ttatgttcaatggtcctgatggggctggagatatagctgtttgagctgaaactacaatga

agaataggttgaggcagaaaagaggaaggaaatggaaaagctttgccattgctatgaaaa

tttggacaactagaagaaaagattgtgtttagttagaagtggaatccccgcgg

>fava_dn_rep_c904

tgcagtttcatatggtaacaaatacaggactacaaacttgacactgcgatgcagcagcgt

ttcatcgacgaggccttcttctcagcctagattcatccaacacaagaaagaagcattttg

gttttacaggtttttgtctattgtgtatgatcatataataaatccaggtcattggactga

agacatgagagatgaggcacttgagcctgctgatcttaacgaccggagaatgcgagttgt

ggatgttgggggaggaactggattcactactcttggtatagtgaaacatgttgatgctaa

aaatgttacaattcttgatcagtctcctcatcaattggctaaggctaagcagaaggaagc

attgaaagaatgtaagatagttgaaggtgatgcagaagaacttccgtttccaactgatta

tgccgaccgatatgtttctgctggaagtattgaatactggcctgatccgcagcgaggcat

taaggaagcatacagagtgctgaaaattggaggcaaagcatgcattataggaccagtata

cccaactttctggttgtctcaattctttgcagacatgtggatgctcttccctaaggagga

agagtatattgaatggtttaaaaaggctggtttcaaagatatcaagcttaaaaggatagg

tccaaaatggtatcgtggtgtacggcggcacggcctaatcatgggctgttctgtcactgg

tgtcaagcgtttatcaggtgactctccccttgagcttggtcctaaggtagaggatgtaga

gaagcctgtaaatccattggtgtttctctcaaggctcattattggtgcaattgcagctac

ttactatgttctagttccaatttacatgtggatcaaggaccaaattgtcccaaagggtgt

gcctatttaatcaaagattggagaatcaggtgaaattgggtaggaaatggtggtcaagtt

ttgatggttgaaattctcaaggcttaatttgttgaaatattctgatattttaatagtaaa

ctaggtctagtgatattttaatacctgaacaaagagatacttattattctttctttgaat

aaaaattgttgttttccacaaaaaaaaaaag

>fava_dn_rep_c905

gaagaagataactttgtaccctgatgaatcttgagtaaggcaacaaccttaatctatagg

gaaaactctaatgtcaagagccctttgatcccatatcattgaattaatgcaatgcaaatg

ataaaatactggtggaaacgtgctgccgccactgcagatttaccaccgcaaaaccgcaaa

tttcttctcaatttggagagaactaaaaaaagttgtgggtctcacaatattttattctcc

cttcacaatttcactggctcttccaatcaagagaagttactcatttctcttcaacctctc

tcgccttaaattctctcttaccaatttctatcattccaatcagagtgttaaggtctgaat

ttatatcttttttatatgaatggaacaaagagagtcacaggcatcaactctttaccacca

gaaaaatccaatcaggaccattagagtatattatgtggtgcatatcctcttatatattgc

aacataatccaaaccatccatgtcttatcatttctatgatacaaacatcaaagtgttaat

ttccttggaggaagaaccttagtattaacttgtacaatttttcttatttcattgttgttg

tcaccactctatggcctccttattgcttagtggtgctgaaaatacaaagctgattagtac

catagttccatctggattaggttctgttagtctaagctttcacaaaaagtgcttcttcaa

a

>fava_dn_rep_c906

tcttatcatttctatgatacaaacatcaaagtgttaatttccttagaggaagaaccttag

tattaacttgtacaatttttcttatttcattgttgttgtcaccactctatggcctcctta

ttgcttagtggtgctgaaaatacaaagctgattagtaccatagttccatctggattaggt

tctgttagtctaagctttcacaaaaagtgcttcttcaaaaataatgcagtttcatatggt

aacaaatacaggactacaaacttgacactgcgatgcagcagcgtttcatcgacgaggcct

tcttctcagcctagattcatccaacacaagaaagaagcattttggttttacaggtttttg

tctattgtgtatgatcatataataaatccaggtcattggactgaagacatgagagatgag

gcacttgagcctgctgatcttaacgaccggagaatgcgagttgtggatgtt

>fava_dn_rep_c907

ccgcgggagctagcacagtcatagagatcaacaaacaaagcaaccacaaaatttatgcag

ctttagatcaacaatctctcggtgtctctcctctatggaatggtagatttgagttgaaga

gtttccacattggtaagatcttgatctcacaatgggtaaatcaccaggaaaatggatcaa

aactgttttgtttgggaagaagtcatccaaatcaaatataccaagaggaagagagaagct

tgttaataaaaaagaaggagttgttacttccaaggtttcagaaactggtttggctttgga

gccaaccaccgataccattaccattcccagacacgaggaagagctggagctggagagtag

agaagcagaaaatgttttgcctgataatcaagaaattgacacagtgggacctgttcatca

agatgcaccacttgatccagaaatattgaggcgggaggaagcagttacgaaagcacaggc

tgctttcaggggttatttggctcggagagcattcagagcccttaaaggcataataaggtt

acaagcacttattcgtgggcacttggttagaagacaagctgttgttacattatgctgtat

gtatggaattgtcaaattacaaggacttgttcagggacaaagagttagacagtctgatgt

tggatttgaaattcatgagaagtgcaatctattaaagcttcaggatgacaaacctgtcaa

gccaattgctatatctgataaaattctgaagctgtcagccaataatttcatccgaaagct

tattgcttcctctactacaataatggccctgcgtttgcaatatgtccgcggtgatccaaa

ttcagtcctaagttggttggagcgctggtcagcatctcggttttggaggccaattcccca

acccaagaaaatccgggatactaagtctcagaaaaagcagggtaatattgcaactggaga

tgctcaaacaagcaagtcaaaacgtacccacaggaagctttctactgcaaattttgacct

agccccagcacaagcaaatcctgaatttgaaaaaccaaaacgaaatgtaaggaaatttcc

aagccaaccctctgatcctgtgctggaaaatccccaaattgagcttgagaagattaagcg

aaacttgagaaaggttcataacccagttgttgagacttcagttctgtcagaagttgaatc

tgagactcagaagccacatctggaaaaagaaacagttgcctcaagtgtcggtgtttcaga

acaagcagtcatcagttctaatgaaagaatcaagaaggaagcaatgaaaatcatttccag

tgagctggatattggaattactgctggaggtttagttagtaaggtgtttgacactccaag

cagttatcaggtgaatgtggaatcaaagcccttgacagatattaccagtaaagatgagaa

catttctgatgatgaaatgaaaaatgagtccatagatttagttgagactagcaaagatga

aaattctcacttaaccaatggaggtttgagtcacaaggaagatcaaacaggtagtgaaaa

tcagaaaccaatccgaaaagcctcaattgtagctaagcaggaacgtgtcgagaacggtgc

acataatagcccgacagtacctagttatatggcagcaactgaatctgccaaagcaaaatt

aagggctcaaggatccccgaaaattggacaagatggaagtgagaaaaacaacaatcctcg

gcgacattctctgccatctttaactaacagcaaaatcagttctcattcacctaggacaca

gaggccagttcattcaggtggtaaaggggcccacaaaagtgacaaagctgtatcttctgt

ggttggaaacggcaaggtagttcaagcagaatggaagaggtaatttgaggaagactggtt

tttttgaagttgacatgaagaagttgaaacttgacatgttaatgtgttatcattgatgga

aattgctaaattgactgaacttataggtgtgtgtcatcaggtttttcttcacttgctgca

ttttggcatttgagagtgatttgtaaatcataggtttttagtttttgcatgaagcaacta

tacccaaatttatgtcctgtttgttttagaacattatttctgggctgtttatattgtttg

ttttcgctcccctgaagtgtgtgtatggtctaaacacgtaccatcccaggtgttacaacg

aactctgtgcttggaatatggacctaaaaatcttatggtaacatgaatttgtccctgcca

aaaaaaaaaa

>fava_dn_rep_c908

ttttttttttttaataatgaatatgtttctttgattgatataaatgagatgaatttcatg

aaaatacaagtagaaacacgaagaagctaaaagcttcggacaaaataggatacttatagc

aacacaacaagaagtgatactctgaaattcatttgaaaggagtactacatagtgggataa

gaagcatgcgcagctatgccacactgaccgctagggtcaccactttccctcaacaccctc

atgtaccctccttcaccccaatctttaccccattgattcttaatcaaccaatactttgtt

ccatcttccgttactccataaccaaccaccgtaactgcatgcttgaaggttgttccacat

gttcctgaaaataccccttcctggtataactgaaattcactgccgacatcgatcccaact

gatattggttgttgtgctacagcttgtagtagttgctgttcatcattggcaggtacatct

acaaaactagttatttgagctgcggttgtcatctgatcatttatttggcaggtttgttga

acttgttggtatggataatcagattcactaacgataccgttggtttgtattatagatttg

aaggcattatccatattacctcctccacaccctgagctctgttgatcacagtcaagcaat

tgttgctccgacaatgagatcaagttaccggttttgattttcgtaataccttctacagcc

gccacagctgaaaatgcccagcagcatccacatgtgccttggttcttaacatcagtgaca

gctccttgttctctccagtcaaaatttgttggaacatcatcattaacatccaacactact

gcatttgattccaacttggaggaagaaagttggtttggaatcttaattccagtgtgtgaa

gcaataaactcttcactagttaaatcagaatatggatttaggccaagcttgtaactcttg

ttgccagcattgtcaaatttctctatgtattccaaattctctttgaatatttgtaagcgt

ttctccatttcgaaactatcagtgtaggtgcgcccatgtttcatcatccattgctgatgt

ttttcagcaacagatgattcatacagagttcgagacatggctgtgtaagcacatgcccac

aagatcatgatacaaacaacaaggaatttcatgctgttttttgggtagctagctagacag

tgaagagaatgttttgtgaaaaaaggatatccccgcggcc

>fava_dn_rep_c909

aataacataaactatagcaaattcaactaacatagttcatgcaaagacacaccacctaag

ccataactctattaagtgaacacattatcaacacttgtttctttctttcttccattgctt

tcattgattctttttgcaccctgcatttcatcatgaacctcaccactctctttctctttt

ttcttcttcttatctcgctaaattcccattttgccctcgccggcaaacgcaaggtccaca

tcaccgacgagttagacgacgtgttcgacgatgaggaagatgatgattggaaacaatggg

gcaagaaacctgctccctctttcgctccctctgatatatccaagatggatacttcgcaga

tccaagaggagatgaagaaacgccacaccggacccgtcatcggattcgtcaagctccgat

taggtgttcgccggactccggacatggtagctgaacttgccatgaaatggacacatgttc

tgagaactggagctgttggaataaggtttacaggtgttgatctgagtacaatcatgttca

acatggacagcatcaaagacatagaggagttgaaggaatttgtcttggatcaatcagaag

catatgaaattaaaatgggggatcaattttttagaagacctggagatctacccctagatg

aactcattcagaagcaacatagtgagaagggtgaagcagataatgctggtccagaggaag

ctgatgggaatgtgaaaacggaattgtagttaatcggtacagataaaatcctctagaaat

ctgaggaaaaacttgaactttttgttttgtgttgattatgacggttgcagctatgaaaag

agtttttgaatctcataccatttgcagttgtaataatagaaattcacaaggaagtgatta

ttgtttatacaagaataaaagatggaagaaaatattcgactaaaccattgacgattcaat

tgaaactattatttcagcttggtgctgaaatcagagagctcaagaagctgaacccttggg

tcggctgctgcctcgtccctctctttctgagtgttgaacccccaatccactagatacaaa

ttccaattatctaactctggctctttgatgacatttttaagggttgcaaacctatcttcc

acaaagtgtagtgtcagtccttggtgttctggcattttttgaagtttcttcagcacttct

accttaggtccggttcctagaccatatattctttctggtggtatagttattccagcaagt

tctctaagtaacgcatcggcaaaccggctctgttttgtggtgacaatgtacactcttgag

cttgcaaaccttaatgcatcagtaacaccaggatagaatctgttggcttgaatccaacca

gtgaaatccttctccaaccaatcatctctaacctttccaaataaatggatcagatcatct

ctattttctttccactcttccatgacaataggcttcaattcgaaccagctctctaatatg

ccctcaactgtgagaccctctgccaccaaagattttctgatggaaggaactcttgtctcg

agcaacaacctcacgagtaaaagagtttcatatccagtttccaccactggtctcactcta

atcatctgttcaacaatccaatcttctgtggcagaatccacactttcaaacagggtcggc

catctcaacttggcagccttgagagcagagatagctgtctctccacatgtatcacaaaga

actccatcaaagtctaatgcatagagatggcccatttgcttcctttcctttcaatgttcc

caaaaccaatccaaatatcccgcggcctaggaagaagataagattttgcat

>fava_dn_rep_c910

gggaggtttttgtgttgtacaacactgcggttcagccccttcaaatcatcgtctaccttc

ttcgtcctctcatctccgctcttcaccatgagcagatcagggcagccaccggatttgaaa

aagtacatggacaaacagcttcaaatcaagctgaatgcaaatcgcatgattgttggtact

ctccgaggctttgaccagtttatgaatttggttgttgacaacactgtggaggtgaatggc

aatgagaaaaatgacataggaatggtggtaattagaggaaatagtgtggtaactgtagag

gcacttgaaccagtgaacaggagcatttgatatatgtttcttgtaaacatttataagtaa

attgtaaggtttaaagtttcaacagataccggttactgctgggtgttaaatgtaattggg

aatcttggtattgtcatgctagcggctaatatgaggttatgtttctggttgattttcaat

ccaggatacctacatagtggatggtattggtttaactaaaatgatgtatatgtctttttc

agttgaattgatagttttctattgacatttcaattctctctttctattctctttaatcta

aaatattagtagttgatagtgacaattacaattttcacttcccttttattttgtaggaat

tacggtttacacttaatgttttgttttaaggaattac

>fava_dn_rep_c911

agttatatttatgatgagataatattttggaactaatattgataaaaaaagataaagaaa

agtattaatgccattttacatgatcacatgttttcatcgacaaacaattactgagaaaat

aataaaacaatcaaatgagttcaatgaacgtgtgtaatcaggatgcaaaacaaattaaaa

acaaactacagccattttacacatcaatttgtcaatatttgtcagcactcagcaataagc

caatacctcaagacttgctcaaaggatccagtacatagaaatagcaaaacaagaaggaca

aaagaaaagaaagaaaggggcctattatgataagcatacatcatacaaagtatggtttat

ttatatagtagcgtgcataaagccactgtgtttagaaccataattgcatatacattcctt

acttccacttcttgaatccatgcttgcctccaccatgcttgccaaactttccatgcttgc

catgctttccccccttgaacttgccatgttgcttgaattttccgtggggcatgtgagcac

caccatgaccatagccaccatgtgcatagcctccttgtggatagtgcccaccatgagagc

catgagagccatgggcgagatgttgagcaccataagcagcggcagctgcggctgcacccc

cagcaagcattcctcctataccgccatgtccaccatgtccatgagaccctggaacactat

gaccagaagaaccaggataaccagcaggtggatacccttgttgtggaggatacccagctg

gaggatatcctccttgttgcggaggatacccagctggtggatacccttgttgaggagggt

acccatgttgcggaggatacccttgttgaggaggatacccatgttgtggagggtaacctc

caccatgtgcagcaccagctataccatgagcaagatgtgaaaacacccctttgtcagatt

catcatgatgtccatccttaccacctcccatctgtttcagaccaaacaacaaacaccttg

aaaatagaaatgaaattgtgaaattggaatagaagttgaagaagagagaaatagggatct

tgtgaatatatatagaaatagaattgaaagagtattgaatggagacataaagaggaagag

gat

>fava_dn_rep_c912

ccgccttgagcaagaaaacaaaagtaagaaaaacaaaagccattgctcttcattcccaaa

cctcagtgtgaagaccgaagagcacagacaacccaaaacgtcttttagtgatctaatcaa

atcgaatccaaaatgtttctcacaaggaccgagtatgaccgaggagtcaacacattttct

cctgaaggccgtttgtttcaggttgaatatgcaatcgaagccatcaagcttggatcaacc

gcaattggtttgaagacgaaagagggcgtggtccttgctgttgaaaagcgtattacttcc

cctctactggagcctagcagtgttgagaaaattatggaaattgatgaccatattggttgt

gcaatgagtggattgattgctgatgctcgtacacttgttgagcatgcacgagttgaaact

cagaatcataggttctcttacggtgaaccgatgactgtagagtccacaactcaagctctt

tgtgaccttgctttgcgcttcggtgaaggcgacgaagaatcaatgtctcggccatttgga

gtgtctctcctcattgctggccatgacgagaatggacccagtctatattatactgatcct

tctggcacattctggcaatgcaatgcaaaagctattggttcaggatcagaaggtgctgac

agttctctgcaagaacaatacaacaaggacttatctcttcaagaagctgagactattgct

ttatccattctcaagcaagttatggaagagaaggtcactcccaacaacgttgacattgcc

agggtagctccagcatatcatctttataccccttctgaggtggaagcggtgataagtcgc

ctatgatttccatccatttgtgtgatttatttctcctgtacgactatttaattagctgtg

tgccgaaactttttttggcacacaatgttgcttgtaattctgcctttatggccaaatttg

tacgccacactagactctcatgtcatatttgctgcaattcagtctagtgcaaattgagga

caaatatgatgtttcaatgttagggaaaatacaatatattaactagagctgttcacaaga

tccctgctatcatatatttttactgtgagatgagataccatgggtgatggtggttgcgac

taatgattaaagcagtgtcttcatgcgttgatcttccaa

>fava_dn_rep_c913

tccattgcatagtttttgtcccacacaatgtactaggctaccacaagttcattatccatt

caagataagttatggttgtaacaaaacaaaagaaaacaaactaaagtgaagatagataga

gtagtatctgcaatgagttccattggacaaaccatgttaatggcaataacagttactgta

aacaagtatgcatcttccaatatacaagcagttcataacagaaaacaaccaaaccaaacc

ttaaccagcaccaacataggatttggaaggagaggacttgtcttatcaactgttgttgcg

gcaactcaaatccctgagtcaagaactcagcttcttcaaaaatatttgaagaaatcagag

gaaaacaaggagaaaaatgacaaagagagagtgaatagttattacaaacgtaactacaag

gattattttgaattcatagaagggagtttgaaaggaaaagatggggaaatttctgaagca

caaaagggtattcttgattggcttgaagctaacaagtgataataacatattacttttagc

tacactctttacaatttttgtacattatatccaaatagttttgtagtcaattgtagattt

tgattttaattgtatcttgtttttggtttagactggtttcatgtctctttgaaatacatc

tagtacccaattttgttattgttagaaattgaatttaattaaatggggatattttagctc

gaaaaaaaaaag

>fava_dn_rep_c914

agggcttttaaaagcacagccaacataggattagagagccacttccagtcagtacaatca

atcaaaaccctcttctcttcttccctctaggttttgcttttttcctctcttcttctccgc

tttttcacttctcaatggctaacaacaaagaagctttcaacatctcagatctcagttccg

ctctcaatgaagaggatcgggctgacctagtcaacgctctcaagagtaagatacagagtc

ttgctgggcagcactctgatgttctggagagtctgtcccctgttgtcaggaagcgagttg

aggttctcagagagattcagggtgaacatgatgaattggaggcaaaatttttggaagaga

gagctgctcttgaagccaaataccaagttttgtatcaaccattgtacactaagcgttacg

acattgtgaatggtgttgctgaagtggaaggggtagcagttgaaactactgctgcagaag

aggacaaagagaaaggagtgccttcattttggctcaatgctatgaaaaacaatgatgtct

taggtgaagagattacagagcgcgatgagggtgctctcaagtttctcaaagatatcaagt

ggacaaggattgaagaacctaaaggattcaaacttgagtttttctttgattccaatccct

atttttcaaactcggtgttgaccaaaatctatcatatggttgatgaggacgagcctatac

tggagaaggctattggaactgaaattcagtggcttccaggaaaatgcctgacccaaaagg

ttttgaagaaaaagcccaagaagggtgcaaagaatgctaaaccaattaccaaaactgaaa

cctgtgaaagtttcttcaactttttcaatccaccagaagtccctgaagatgatgaagaca

ttgatgaagatatggctgaggaacttcagaatcagatggaacaagactatgacattgggt

caacaataagagataagatcatcccccatgctgtatcatggtttactggggaggctgctc

agggagaggagtttggagatctggatgatgaagatgaggatgaggacgatgatggtgagg

aggatgatgaagatgaggatgaggacgaagatgatgatgatgaagacgaggaagagacta

agactaaaaagaagtcatctgctagtaagaagagtggaatcgtgcagcttggcgatggtc

agcagggtgaaagaccaccagagtgcaagcagcagtaattaatttattcagagtgtgatg

ctgctgctgaaagttgttttggggtcctagtgcctggtgaaaaagggttttcaatttcat

gtggtttttcatggtccttaattcattttcctccccttagttttatttatagtctgaatt

tattgagtctcttgctcctttttgtcccctccttttggagagatggtgttatatagtcag

ggtatgttgtgatttattggtacttcaattttaggtctccttcctagatacttaaagttt

gtgcttgattgttaccaatatattaaactttgattagcaaaacgttgctacaagttttcg

tgtgagaagaaaaattgtaatgattttttttaagtgcattcaacatgttacagttttatt

gattgtttgtttgtattcagttttacttcagaaatattatatatatatttttacaaaaaa

aaa

>fava_dn_rep_c915

ataatttaatacgtttcttgttctctcaatatttctgtatttaaagaaaaatttccataa

gctgagttgtcacactatagattccaccgttcaaaacgtaccggtgatttcgaatattga

cgaatcaagataacgcaaaaagtcacaaacccctttcgtcaattctcctcctcttcctct

gctctaagtgattcgccatggctgttactatgaaacaaatggctctttttgtttcgttat

ttggtgtcatctccttcatacttggagttatagccgaaaacaagaagcctgcttctggta

caccggtagctgtgaaggatggtgtgagttgcaagtttccgtctgatccgaccattgcgt

tgggttatctttctatcgtgtttctcattgcgtcaaccgtggttggatatctttctctgt

tttacccttacaaaggaaaatctgttccacaaggggttctctttaaacacactactttct

ctgttttcttcaatgttgctttgtttacatctggactggctgcaactatgctgttatggc

ctgtaatacaggagcagattcaccgatcacgcaaagtgcattttgatgctgcctatgctt

gccctacagcgaaaactggtctcattggtggtggtgcctttttatcccttgattcgtgcc

tcttttggttggttgccctcatgttagctgacaatgttcgggaggatcattttgacgatg

gaaagggtgagcgtaatggtgagggtaatgctgcttttgacgcttatgatgcaagtttag

tcatataggagcagaaatgaattaaagttgttaagattgttaataaagccaaagtggaaa

catcagacatcactgttatgttgttagttattgtttcaattattatatgatggattttat

tatggatttaaatgttttatgtggttgttctcttgtattttcattgtggttcaagcaaac

acattcctgtttatgatcgagttggtctcttttgaatgtttacacggtagaccaaccgcg

aggtagtcaatttttacttaacaattatttgtgtttgttttgagtttccattttaaaatg

acaggtttttgcac

>fava_dn_rep_c916

ttttttttttttaataacaagcagagatttatgattgatattagctataattagctttta

cattacacaagacaaccaaagacaaagcaactatagccactttattttatttaaaaaaag

caaatacatatcgttacataaattggttccagtatctctgcttcaagtgttgatgtgttg

cacttcatttgcagttgcaagggtcacaagtgcagttatcaccacacttgcacccacctt

cctttgaagcaacgctcatttcagcaccttcaaactggatcttcgccggacccacgccaa

gaatcaccgtttctttggtttccacttccgagtagctcaatccactagaccttttgttgc

atttgcagctatcaccacagttgcaactacttccacaattacagccagacatttttttca

gcgactaagattacaaagcaaagaacaattgatattgcttggttgctgttttcttcacta

caattgatgttttcggccgtaa

>fava_dn_rep_c917

ttttttttttaaaacaaaaatacttgatctatctattattgtttgttgacaaaaacatta

ttattgttgaatgcatgtttctttattggtggtttaccaattacaaccgacaaaatactc

ttttgagagactagttcaatgatatatactgtaactttagtttcttattggagagacctt

aaacggccagagagggcaaaacattgtagttcaaatcccacagctcaaaagtgccattaa

caaaattgtaatgtgctcctttcaaagcaagtgttttcttcagaacagcttctctcacaa

atgaataggtcaataagtttccaagagatacattcacagcttccttttcacagtttgtac

actgctctgcgaagcttaagctgcttgtatctgttttaatcttggactttgctggattac

atatttggacccaattttctatgaagtcacttgcgatggtgccatcatctgggatagaca

tgagacccttgatacctccacagcagctgtgtccaattactacaatattctccaccttta

aatgtagtacagcatattcaatggccgcccctgctcccgaatacttggtcttgtcatatg

gtggaaccatgttggcgatgtttctgaccacaaaagcttcacctggttggaaatccaaaa

tgtgggaaggacaaacccttgaatctgagcaagcaaacaccataaactttggactctgac

ctttggcaagttcactgtacagatcaggatttttttcgaatttctcagttttgaattgaa

tgaagccggatcggatcctctcatccgggttaaacggttttgcagcggttgcctctaact

ccgccgtgagctccctgatcctaacggccgcgatgctgcctagctcagctttctcctgga

gaagcttggtcagcgatgcgatggtttcgtcgtatgattctgcagccatgtcttccttcg

agacaatcttgctagtacaacacagcatcatgcactttttgaatgccccagccatcactc

tttttgctcctcttcgccctaagttatacgtaagaggtttctttctttaacatattaacc

actaatatttcgtaataaaaaatcatagataaattgaagagtgagaagaaaagatagtac

caatgtaactaaatatgattagagagattggacttatgcttgtcttgccctatgaaatga

gaggaaaattactaatgaagtttgaaatgtttgtgttacatttggactaaaaggtaatga

aaatgaatttgaggaaatcatgtatatgaaaaggataaagaaagcgaaatgtgagttaat

atcagctatgggtttgtagttgacactacttccttatctctattctctcactaccaatct

>fava_dn_rep_c918

atttgatcatactatatgaaatcttcnnnnnnnnnncttgctatacgtctttcttgtaag

tatactgtttaaatcaatatcaatagaaaacctttgcttctgtatgaatcaatattatta

cattccaattcctttccaatattatgtaaacaaattttaaaagtaatatgtacaatatga

ttgaatacttgtgtaaaatttatttatatgtttgtaattttgagctcaaaaaggataaca

agcacctatatcgaaaaaccaaatattatcagcaaccaagcaagagatgtaacaagatgc

taaaggtttcttttactcaaagatgttataggtgtgacaacgatttatacaaaattccac

actttcgaactatgttcaaaagtaaaaatgagatagttttcaagtgcaacccatctatga

ggcaccaatgttggaatgcaaaagtgtgtccagtacagtataactgatagttgcgtcaag

aaacatcaatccttaaactgcctagaaaagttggttgaatttaagacaatgttcttctat

tgaaagactccactgttatgttggcagtaaaagcaggaattgaattcattttcctaatga

atatatcaacctgacgagatacatcaggctgagatgtgtaatgattcaccaaaatattgg

tattccctgattcatattcaaattcaagtttttcatcatcactttgtgcataaattgatt

gcaacgtaaatcgtgtcactgggattccatttggtcgttggtgttcatccatcacaatct

tgtaaccaaaaatctcacagcatgccctccgaaaaactgaaattctgtctgcaaacacag

tcttgtatctctcttctcgcttctccagtgttgcaatttgttctttcaatttcaatatct

tatctgaaatgtagctatctaccagctttccagtttctcctgattgactcttcaattctt

cgacagcttttaatttctcttttgttttctgcagttcattttgcagagcttcgatagttt

gtttggcttcgttatctacagtaagtgtatttaccatccgcagaacttttgtactagcag

aagaaaaatctccatgacccaactttgcctctaataatgatatctctgagcggagtcggt

cactctccctctccaatgactttattcttcttgcttcattgtgcaatttatcgttgagta

tctttatttcctcatgttggcgattattaaccactctcagctcgtttagggtgctttcta

gttctttgatatactcatctttcttcataagagatgattcaagttcctgaatgggattag

cactacttgattcatctccagcttcaccattcttcaatttggcaagaattcttaatttgt

ttctttcctctgtaacaactgcaagcgttaattcaatctgtttaatctctgattttaata

cttctgctttctctttggccaacacagcctcagtttcggcattttgtttaccaatctcag

cagcatccaaagccacctccagttgtttcaagcgactactgccttcaccctccttctgtg

tgctataaattgcctctttctgtaaagctgcaaatttagcaggcatgtcctcaaaacatg

agatgccaggtatgtctttaatcatcgatctccaagatgatatttgatcctctaacttct

tcatatttgactggacatcctgtaatttagataactctgattgtgccctttcacggcggc

tcttttcctccagcagtttctccttcaaaagctcaacgccctcatgatttgattttagtt

ttcttgcttcccttacttcagtttcatagtgttgaagctcttgttgcaattgcttaacca

aaacactactctctgaagaagaaccttgttgagatgaagaaattgctagtttcttttcaa

cttcaatcttctggtgcaaacactcgttgaattggtcctgaagatggtccagttgctctt

ttagcagctttgcttctctctccgcattattagctctgcactccatttgttcaagatttg

cagttattctagaaatagaaaactttgagtcagtttccagttgtaaatgatcatcctgaa

ctctccttttatctctctctacgctgtctgaaagacggcccaacctcccttctaaagatg

ttgctttctcttcagctgaagcagcacgagactcagctttcatgcgaaggttagtctcat

tttggagcttaacctggagttcagtgttcaagtgtatatgttttctgaatgtttcttgag

aatcggtaacctctttcaaaagttgatcctgtagagccttttcacgggcttttgcggcag

cgagttcctgttcagtgtataagaactgctccataaatttcttcttgtcagtatcaactt

ttttgaaattatcatcgagtgtctccaattgagattgataatcacgaacttgattttcag

ctttagttaatgcatctagaaaatccgatttgaccatttgacgacactgataggtgcata

gcatgtgctcggaaacggagggttcctccggtggatcctcgtagatcacgacctggtggt

taacctcggccacggcatctgtatcagcgtcggcacgtcgcctctttggtggaggagttc

tcagcatcattcttcttccttcacgttgagtttcccacaatacaatgcccaatttgaaat

ggaaaaacccaaacacaaggcgcaactttattcaaggtatcaaaacgcacggacacatta

cggctcgacccgaacataacatcagttggtttcgtgg

>fava_dn_rep_c919

cagaataatataagatttagattgaacagtattatataagtatattagaaacagaatacc

gtgaatccgctatagtctagttggtcagtattataatataagatggaatttttctttttg

aatttttgacagctgtatcatgtgtatttggattgaattgaaagtttgataaatgaccag

catggataaaagaagagaaaaagaggcaaaatcctgcaattttatttgatggatcaaatc

agggtaacaacatgctctacaaattttattgagtttccaatatattttgtgcaattctaa

tgttgaacaaaattcttaaccctaaccctaaatgctccacttagtttgtgcacagttttc

gcaatcccaaacatttcttcttttccaatctctctctctgatgaaaccaacacaacttga

gttccgcacaattcatcatatattctctggaactcttccaacacttccccaactattccc

atcttcttcttcttcgtcaacatcttcaccaacccaaccacatgccgctcaaaattcccc

tgctccaccaccttcctcaccgctgaaccgtcgataataccaccatctgatttaagtttc

aatagtcgctgaacatccctttggactgagtgaagtgtattggtggtttgagctacatct

atgattgcagcagcatatccagtagcaggcttgtcatggaatattggagagggttgtttg

agtttgagagatgtagtgtttggagaagagaattgcagagaatgattgttgtgagagaaa

tttatgattttttggggttttggttgtggaagacgattagggattggatgaggagttctt

gtgaagtgatggaattcatgatttgttgttggtgctggtggagggagtgatgagattttg

agatttgaaactgataaagtttccatggaacaagaagaagctcttaggtgaagaacctaa

ccagaagataaggttaaggccacttcccgcggcc

>fava_dn_rep_c920

ttctttttcggctatctaatactagaacttggaaagaattatggggtcttgttcttattc

cacttagtcaaatttggtgactagtatgttatcttctttttattctattcagtataactt

gcttgtgtaaagttcgtctattatgggataagcttttttagatcacggaaaagaataaaa

gaacaatgatattttccccttgatacattacattactcccatggactcaatgaacactct

tcaggcaaagtcggaaaacgttccgaatcattgcaataatcataaatcactaggtttctt

tgaacccaagcataatccattttctgatcacctgatagatgccatgaaccatattgatcc

caccagttttttgttgtggttgaaacacatttaggatatggatcttcccattgacaagca

tccacactgaagtctttgtaggatgacacaaacggtgctaatttccaattcgttttttca

agtccacctctcgtcgcccattcgtccgcattccatacacttgaaaacaagtacattggt

ttctcattcgggaagaaatcgttcggtttctcgttgttcttgaatactcttatcggaact

ctgtccacaaaaaacactagctggtgattattccacaggattgaatatgtgtgataatct

tcagttggatcaaaccatagcatatgtctcatctcgcgattaccggttccattcttgtac

acatttgtctgaaccaaatatggttgtcctgttctgtttcccagaaactcaaaatcaatt

tcatctcttgttggccctgcaccattttctgaacacatataataagctgtgacaacacca

gcagaatcacctcctaccaatttcagcttcatactaaaccacccaaatctatatctttgt

tttgtttgaaatccacatcctgtgttattgtctaaggagagattccagatctgtccatca

tcagaggtagtgaaatgatcttcagaccacattatgctgaaattatcttcaaaagatccc

ttagtcatagctgcttcagctggatcataataagcagcagctatgatagctgcaatgaaa

agaagaagggaaaatgaagatcttgcatccattttgtgtgttggatctctgaactcgagg

tttggaagttcattaaataatactagattttgaaaatggtttctgcatatacagttgtaa

catcatatgcaattgttctactttgtttaacttattttcatgtgatgaatgtaccaattt

gcccattcacnnnnnnnnnngaattaaactgagtcattttcacattgtgaaccttgaatt

gtgatgttgaagcagtgaattccgtcttctt

>fava_dn_rep_c921

aagagggtcccaccttctatcctctcaactcctcccgtatcctgcgaaacacctgattac

accccaaacagacagttaacaagaaccacaagtatctcaacaaaacaaaaacatcctcag

tgcagcgacattgtccatatatcactctctacttgtttatcgtctttcctcattttgatc

ctcatggagtcacgagtgctgtcacgcgccaccactctctcctccctccccactctcaac

aaactgcaccgccttcccctcgccaatgcttccctcctcgccgtgaagacgattggatca

ctctccgatggcgggaacctcgtctggggaagacaacttcgtcccgagctctgttctcca

cttctcaagaaaggtccttcactcctccgtccttgcctcgccactgctgaaggaaacgac

tccgccggtgaagagaaggtagctccggttgggttcttcagccggtatccagctcttact

accggttttttctttttcacttggtacttcttgaatgtgattttcaacatcctcaacaag

aagatctataactatttcccatatccttactttgtgtcggtaattcatttagctgtggga

gtggtgtattgtttggtgagctggaccgtgggccttcctaagcgcgctcctattgatggt

aacctgctgaagttgctgattcctgtggctgtgtgtcatgcattaggccatgtaactagc

aatgtctcatttgcagcagttgctgtttctttcacacataccgttaaagctcttgagcca

ttctttaatgcggctgcttcacaatttatacttggacaatcaattcccatcactctatgg

ctctcattggctcctgttgttatcggtgtgtcaatggcatcattgactgaactctcattc

aactggcttggattcataagtgctatgatttcaaacatttcctttacatacaggagtatc

tattcaaagaaagccatgactgatatggatagtaccaatatctatgcctacatttcaatc

attgctctaattgtctgcataccaccggccttaattattgaagggcctacactgttgaag

accggtttcaatgatgcaattgctcaagtaggtttggttaagtttgtatcagatctcttc

tgggttggtatgttttaccatctctacaaccaggtggccacaaacacattggagagagtg

gctcctcttactcatgcagttggaaacgtactcaaacgtgtattcgtcatcggattttct

atcactatcttcggcaacaagatttccatccaaactggcataggaaccggcattgcaatt

gctggagtagcactctactcattcatcaaagccaagatcgaagaagagaagcgacaagca

aaagcagcttaaggtgtcaacttgaggatgtcctgcactgtaaatcatcacaaatttctt

tctaaataattgactttgtttcagtatattgtactatctttctgatagcgcgcatttcat

tttcgttctttatttttgtcaaatctttcccaccttgttcattcagaaaacaatgacttg

ctgatttgcagtcttcttttgatgactgaaaaatgatgatgtatccattattgccataaa

atagctcttgaatctataataaaagctgtattcacgataaaaaaaaaaaggaaatgtata

gaaatgctcttgaagtgataaaatgagaagtaatgatcttgtaaatgatctattttctaa

taaccaagtctataagaaacatgtatactaaggttgagagacacatattagatgcaaaca

tattttaactttagtct

>fava_dn_rep_c922

tttttttttttagaaacatgcaagaatttgtttatttattttctgtaactcatataacac

aacacaaccaccagcaacatgctgcatccaatggatgaaccataatacatattaacagta

gcataataaagttctcatgatgatgatatacattagtgttaatgtttatatatatgcaca

tatttattcacaaacatatttaaagtatacacaagggaagaagcaaaagatatgtcagct

atctgctcttccaattcccatccacgacatcgacattctatgctggatttatatatatac

aatttagagtgtccaggatctgttctttgctttcttttctgccctgaattgattaagtct

gtccctccacaaagcagcgtcttgatagcgctcttctacaaccgctttcaacatgttgtg

caaaagattgaactccttggtttcaacacaaggcttaccatttggtcggtctagttcagt

aaataatgggccatctgaaccaggggactgtggtgacagtttgcctgattcaacaactct

cattccatcactgtatgccaaatacttgttgacttgtataggcaccttgcatctgacggc

aatgtttatagcatctgaaggtcgaagatcaaagctcatacattccgactcatttccaac

cttggaaagatatagctgagcaaaatatgcctcctgaaccctcttagtaactctcacagc

tctgacttcataacccatcttgtcgatcatctcgttcactacttgatataaagtaggtct

agctatttgaacattgcgtatagctgccattaacaagacacttggcatctccaaaacaat

tatagggagaagtagaccagtcccatcttccatcttcaacacaattgctggatgtggcgc

ataattcggaagaagtcctccatgagggctgttatgaacgcatctcaagtgtctaccatc

cctcattttgactatgaacccatcagctccactcttaacctcaactgcttcaagaatagt

agagttaacataatcttcatgattttgattaaaactctcagccatactaccactaccatt

agaagatgagttgaaactacaattcacagctttgcacagctttctcatattaatatggca

agaaagtgaattaggtttagctttggtgctaccagaaaatccccaaaattcagttctact

acaaccaccaccgttgattggtccaatcaacgacatgggaacactgcacacagttggtcc

tttcacacaaaccattttatccatcaaaattcaaaaccttgaaagatgaatcaccgaatt

agggtttaattagatttaattattgaaagaagggaaattaagatctgaaaatgaggaaga

ggataagagatttatatttttcttgtttgtgtaaggtattggagtgtgatcaagatattt

taatttagaaaaaagtatgaaccgttcttccaccgcaagcatcatcatcaattttggata

gaccataaattgaaattactactttacccttctggtttttgctttctcaccttctggt

>fava_dn_rep_c923

aagagggtattcaattgatgaaaattcaataaaattgaacaacatgtcactctctcagag

ccagatgatatctcgtcctctcaccaaatcacttttctccattcccataacctatgcatc

aatccacccccaaaggttttgctgccattctttaccaaattccaccaaagatggtgtgaa

ccatgtttttgatgaacctaaagttcaaatattgaagcacaaaatggaaccacttggtat

taccttagataactcttgtttgcctgggaactaccataacctattttgtccaaagtgtaa

aggtggacagttaatggagaggagtttatcttttcacattatcactgattgtgaatttgc

aatgtggaggtgttttagacctacatgtggttgggctggtcaggtttttgtagatgacaa

ggaattggacaataagccttttgaaaaaatggatgagaagagcttaggattggaaccact

tggtcctaagcttgttgcttactttaaggagagactgatatcagagaaaaccttaagcag

aaatgctgtaaggcaattgtcgcacgataagaatgttattgcttttacctataaacaaaa

tgggttgcttgttggttgtaagtacagaacaacggagaaaagattttggcagggaaaagg

cacaagcaaggtactttatggacttgacgacattactcacacaactgaaatcatcattgt

cgagggggaaattgataagctttcacttgaagaggctggctatcaaaattgtgttagtgt

tcccgtcggtgcacctagaaaggtttcttcaaaagatttaccaccaatagaaaaggatac

tgcatatcaatacctgtggaactgcaaagcatacttggataaggcagttcgcattattct

ggcaaccgataatgatccacctggtcaagctttagcggaagaactgacacggcgcctagg

acgagaaaggtgttggcaagtacgatggccaaagaaagacgaatccaccttcttcaaaga

tgcaaatgaggttctcaaatacatgggagctgatgcattgaagagagcagttgaaaacgc

agagtcatattgtagtacacacaaggtaactccatgatttccacctaggaatgtgaagat

ggttgcggttgtcttgctacggctccggacaccattctaaaacttgtgaatgtttaaaag

aacactaatctgttaaaacctaagctcttgtaagttttgtatctctatgttccaaatcaa

gatgtcaaagaaaactgtagagattttgtagatagtgttgtcattcaatttcctttcggt

gtctgatgaagagaagggagggagaacatttatggcttgtatgaagttttttaaatcaga

aaaaaaaaactttggtaaaaaaaaaaagg

>fava_dn_rep_c924

caattatttttatataaataaattggtggtgtnnnnnnnnnnnnnnnntccaccgtcttg

atagtgaaagcatcatatctttgtaaatcccatttttttctttcattgaagacacttagt

caacaagtgacagcttcaaaattactaaactactcttccaaactctataaaagaccacac

aaccttaagacaaaaacacaaagggacaaaaacaaactcactttgatagttactaaattc

aaagataatattgaagtgcagcaatggcaacaaatcaggaagaggtgaaacttttgggag

ctcttggaagcccatttgtgtgtagagtaactcttgctctaaaactgaagggaattgaat

acgaatttgttgaagaagacttgaagaacaagagtgagcttcttctcaaatataatccag

tttataaacaggttccagtttttgttcataatgaaaagcccatatcagaatcccttgtta

ttgttgaatacattgatgaaacttggaaacaaaatccaatcttgccttctgatccttaca

aaagagctttagctcgtttctgggcaaagttcatagatgacaaggttatgggcccttcat

tcaaatctgcattcacaattgatgagaaagagcgtgagaagaatattgaagaatcatctg

aggctcttcgggttcttgagaatgagttgaaggacaagttctttggaggagaagagattg

atattgtggacattgctgctgtgttcatagctttttggatccctttgattcaagatatat

cagggttgcagttgtttaccgctgaaaagtttccaaagctttaccaatggagccaagaat

ttcttaaccatcctgtggtgaagaaaagtctgcctcctagagaacctctttttgccatat

tcaaaggtcgctatgagagcgtttttaatgcttcaaaataaatcatatgaaattcagaat

ttcatgtgtcattgagtttttgttgttaggatttcatttcgaaataatgttttgtaatgt

gagatgagatttctttatgtgaaatctaaggatattgttgtgatttattcatatttggat

ttaaagaaaaagtcatctttctatcaaaaaaaaaaanntcaagaaacatgaagtatctac

aaccttacattgattccattttaatgatttttttaaaaggcaaaatgataataatgaaaa

agtc

>fava_dn_rep_c925

ggttttagtgtgcatttagtaaaatcctcccttcttcctgtaagaataatagtcacgaaa

ggaggcacaagttttttcattcgattcattttcctagaatcaaaacggcgtaaaaaccaa

atcaaatcacaccgaacctactgatctgagagagagagaacaacttcgcgatgagtttcc

aacgcgcaaccaaactgttctcatccatcgcatcgcgccgccaacacctccgaaaccttt

cctcaacgccgtcgtcaccggcggtgttcgtcgacaagagcacacgcgtgatctgtcaag

gaatcaccgggaagaatggaacgtttcacactgagcaagcaatcgagtacggaacgaata

tggttggtggtgttactccgaagaaaggcggaaccgaacatttagggcttccggttttca

attccgttgcggaagcgaaagctgagactaaagctaatgcttctgttatttatgttcctc

ctccttttgctgctgctgcgattttggaagcgttggaagctgagttggatcttgttgttt

gcatcacggaagggattcctcagcatgatatggtacgagttaaagcagcaattaacagac

agtccaaaactcgtttgattggaccaaactgccctggtatcatcaagcctggggaatgta

aaattggaatcatgcctggttacatccacaaacctgggcgcattggaattgtttctcgtt

ctggcactttgacatatgaagcagttttccaaacaactgctgttggccttggacaatcca

cttgtgtgggtataggaggagatcccttcaatggaacaaactttgttgattgcctaacaa

agttcctagcggatcctcagacagaaggtatcgttctcataggagagataggaggaactg

cagaagaggatgctgctgcactgattaaggaaagcgggactgagaagcccatcgttgcat

ttattgctggattaactgctccccctggccgtcgtatgggtcatgctggagctattgtgt

cgggtggcaagggtactgctcaagacaaaatcagcactcttagggatgctggagtcacag

ttgtggagtcccctgctaaaattggaactgccatgtttgaaattttcaagcagaaggggt

tggtgcaatagtttggcttaatttattttcattaagtcactttgattttgaattgactac

atggtcaaaatacttgattcccattgataataatgggataatttttcctattttattttt

caaaaatacgaggaggcgccttagaataaaataattcaagatgctttttatattttcgga

aatttgatacatttcaaattgagttccagcttttgtggcgctggtttgtaaacacatgta

gttagttttcaatgaatttccatctacttcagtttgtagtgtctgctaagttggatagtc

agttatgtgatcctaaatgtgtcgtcctatatggtttctggcaataattaggcaatttct

tctttatactgtgggacacatctctnnnnntctcaaaataccattgattttttcagagat

acatcctcgaacgtacaaaatctggtgttttgccaaaatttagtttag

>fava_dn_rep_c926

gccgcgggatccttcagccgctcaaaacaagctagggtttcgtttctgctgtgagatccg

tagcttgttagcctttgaatctatatcaaaatgagtaagcttcaaagtgaagcagttaga

gaagccatcacaggaatcatgggtgattccaaggacaaaggaaggaagtttgtggagact

attgagcttcaaattggcctcaagaattacgatccacaaaaggataaacgtttcagcggc

tccgtcaagttgccacacatcccccgccccaagatgaagatttgtatgcttggagatgcc

caacacgttgaagaggcagagaaaattggtttggagtcgatggatgttgaggctttgaag

aagctcaacaagaataagaagttggtgaagaaacttgccaagaagtaccatgccttctta

gcttctgaggcagtcattaagcaaattcctcgtctcttgggtcctggtctcaacaaggca

ggcaagtttccaacacttgtctcccaccaagaatctcttgaggcaaaggttaatgagacc

aaagctatggtcaaatttcagctcaaaaaggtgctctgcatgggagtagccgtgggaaat

gttagcatggatgagaagcaaatctttcaaaatgttcaactcagtgttaactttcttgtc

tctttattgaaaaagaactggcagaatgttcgttgcttgtacctcaagagttccatggag

aagtattaccgtgtcttttaaggagtttattttatccaattttcaaatattctgcggctt

gttatccctctctccctctcaaaacattagttatcctaatagttggatataatatttcat

cttgtcacaattttgctggattatttatggttgatttttttttctgttcaaatacttgac

tattttaagcgtgctgtttttggtgctctaagcatttctgatgtaaacaaatcttatgtt

gcgacatgaaaacctgtgtttcacactttcactggtgtgtacaggataagcttgtgttaa

aggcttaaaaataaccctaatgattttttnnnnnnaacaacatacatatatctatgctca

acataaaaaacttatttgtgtaatgtaagttttagcttcatttaa

>fava_dn_rep_c927

gccgcggggaagtccttgaaattgtgttgtctagaaacaaatataatatggctgttaact

tccttttgtttgtgttcttttttgctagtcggtttatggctggatttaacactgagctac

cactttctaactatgaaaatactatcaccattctgagtattgatggtggtggtattagag

gagttattccagcagtaattcttaaccacttggaaaaggctcttcaggttaaggataaag

aagcatcacttgcaaaatattttgatgtgatatcagggactagtactggaggacttgtaa

ctgccatgctcgctgctcctcacccagatgaccctactcgtcctctattcactgctgaac

aagttatcaacttctactatcaatatggtccttcaatattcaacgaaactagtggttggg

atgctacataccctggtacaaagtatgatggaaagttcttacataaaatagcaagtgaat

tattgaaagatacaagactcagtgagactttaaccaatgttgttattccaacttttgaca

tcaagaagtttcatcctgttacattctcaagttttaagttgaaggcagtttcaagcttag

atgcaaagttgtcagatatatgcattggaacttcagctgcgccaacttatctacctccat

attactttgaaaatgatggtgctgaattcaatttgattgatggaggtgtggctgcatgta

atccggctatggctgctgtgagtgaagtgatgaaacagaagcgtgcaaaaatcttgttgt

tatctataggatgtggaactaaagagcttacaggttttgatgctgatgttgcaactcatt

tctcagctgcattttgggcaacatctggtcttgctgttggtacttatgatattgcttcta

aagatataactgaatattaccttgataatgtcctcccaaacatccgctcttctcgcaact

accttagaattcaggaatataagttggatccatcgatggatgccctggataatgctacaa

tagctaacattaaaaaccttgaaaaggtaggagagagccttctgaaaaaaccagttttga

agatgaatgtggatacctatgagccagaggtaaaaacaaaccaaggtacaaatgctgagg

ctcttgagaaattggctgagactttgtacaaaataaagcagtttcgtcaaaagaagaaca

tgattgaaaagcttatgggacgaccaattcttaaaaatatcccaaacccttttgctactg

caaattaggcatgaggactgtgtaatggaataaatgcaaagttcatatgcttgtttgata

aattattagacaagcttataggcttgtttgataaaaaaaatatgtgtgcatgagcatgat

gtagcttaagcatatcaataaatgagtaatgtgtggatcgtgtgtttcgatgtttaattg

cttgttttgtgttttatttttactaaatcaaagttaagtcattccattgttcaatataga

ctaagacgatacatgtcaaaacatttgcgaagaaatgtgaacaagttaaagatgtaatta

cgggcatcataatagccaaggaaattaaactttacaa

>fava_dn_rep_c928

ctttttttttttgcatcttaaactctttaactagtcaataaattttaaaagtatatcaaa

attattcaatagaagcctggtttctggcaagtgaaatccaaaaaacaaaggggttgaaca

gtagcctgatacaagtcataaaaataaaaaacaggttacagctagtttgatatttatatc

gctccataattcaaacggtatgttccactgtccatagaaacatataaccaattttcaacg

ttttttcactacttcaaggtatcgttgacaacagtatcaaactggaagttcttcacacag

taaattcagcttcaggatcactttctttaggggcagcagcaggcactttcttcttgttgc

ctctcagacgtgaagatatattttcaatggcagagtttagttggcctattttttcattca

aggtctccttggtcccctctagcccttcgtcataatatattggttttctggccctccgaa

acccatattcattttcatctagcaaagatcttctgatctggggagcaaaaacataagcta

gggttccaaaaatagcaccacccagaagaaagccagcaatcaattccccgcctccacctt

ctctgttatcacggtattctgcacgaagtggtgtagctaggttccttctgctcacaatgt

ttgatttcatttgaacagcaaaattaggtccaaccagagaggaattacccttggctaaaa

acagctcagaggacttgagatgagatccaccaactgaaatggataatggtgcgaaagaag

tcgccatggaagagtgagtttgtttgaagcgagaataatgaaagaactggagagaaagag

aggtgtgttgtaatttgtatggagttatcgggcgagtattacgaggagagactgtgcaaa

ataatctccaccaaaaatattgttaaaaatctccgccaaatgaaagtatctaaatctaaa

taattgaaaacaaaaaatgtgtcatat

>fava_dn_rep_c929

agcgcgggtgttaactcgctcttgttatcccgtaacatgtgcttttcagcactttgaaga

aacagcaaggaaaaaaaaatggcgctaccccttcttcgaattcctggtacttcttccatt

ccatttgtcaccacaaagcctaagtcttttccttcgagaaccttccgcaagacgacggtg

gcagcagcggagcttcaaacaccgctggtagcgttgggcagaaccgatttccggtatccg

aatgttgaagctcctagccataaggtcaaagtccatgacagagaacgcggaattgttcac

gaatttctcgttcctgaggaccaatatattttgcatactgctgagtctcaggatattacc

cttccatttgcctgcaggcatggttgttgtacaagctgtgctgttcgggtaaagagtggg

aagattagacagccagaggcacttgggatatctgccgaattgagagagcagggttatgca

cttctttgtgtgagtttcccatgctctgaccttgaagtagaaactcaagatgaagatgag

gtatattggcttcaatttggacgttattttgcaagaggaccagtggaaagagatgactat

gccttggagttggccatgggtgacgagtaacctgggtacaaaaactaaattcaaagaatg

caagttttgtttccttcattcttttcatatgtaccactgaaatttcaagttaaagacatg

ttcgagtgtccaatatgtgttagttttcgacactcgtactacactaacatatactcatgt

gattatattctattattttcattttctcaaattatattttcattttggtaagttacggtt

agtgtcttcatgttagtcttagtgagtgtcgtattcattgtttgtgtctttatgtgtccg

tgcttcaaaactcttatccccctatgtttgtgtattctctgctgtcattttgttatcata

tactacattattattgtttgcataatatagaagatttgacatcttttggatcaaaaaaaa

a

>fava_dn_rep_c930

agagtaaaagcttgaaaaaataagtggacatnagatagagtttcttaattatcttatccc

tactagtacctacccaacactaattacatggattttgaaatgatatttatctcgaagcta

gagtgtgccgtgggatgtttctacaattgatttgagatctaaaagtaaaacaaagagaag

aatcaatggcaacaagaagcaatcaacttcacattgccatgtttccatggtttgctacag

gtcacatgactccattccttcacctctcaaacgaattagccaaaagagaccacaaaatca

cttttctactaccccataaagctcattttcaacttcgccaccttaatcaccacccatctc

ttatcacattccacaccatcactgttcctcacgttgaaggccttcctctcggcacagaaa

cagcctcagatattcccatttttctcaaccacttactcgccattgccatggacaaaaccc

gtgatcaagttcaacaaacactgaccataataaaacccgactttgttttctatgacaatg

cttactggatgcctgaaatagctagagaaatcggtatcaaaaccgtttgttacaatgttg

tttccgcttctagtttggctattgcacttgtccctgcaagaaaagtgaatgattccatta

ctgaggaagaactcagggttccaccagaagggtacccttcttccaaagttgttcttagtg

gtaatgaagcaaaggctttgtctttcatttcactaccatttggagaaggaaacatcacgt

tctatgacagaatcgtaatggctatgaaaggaagtgatgctataggaattagaacaacga

gagaattagaaggtaacttttgtgactatattgcttcacaatatgaaaagcaagtgcttt

taaccggaccagtcttgtcattggatgattccgcgtttgaggagaagctggaaccttatt

gggctgattggcttgatggatttgaacgtgcttcagttgtgttctgtgcatttgggtcac

agatcaatttagagaaagctcaattccaagaaattctgttgggttttgagctttcagggt

tgccttttattgtggctcttaaagctcctattgggtgtgaaacagtagaagaagctctgc

cacaaggttttgaagaaagagtaaaaggaagaggaaaagtttcaaggggttgggttcaac

aaccattgatgttgaaacattcatctgttgggtgttttgtgaaccattgtggctttggtt

ctatgtgggagtcattgatgagtgataaacagatagttttggtgcctcatcttgctgatc

aggttttgaataccaagttgcttgttggtgaattggaagttgctgttgaagttgaaagag

gtgaaactgatggttggatctcaaaagagagtttgagtaaagctattaggttggtgatgg

atgaaggaagtgaggtgggtgtgagagtgaagattaaccatgctaagtggaaggaaaatg

gaggaacttcggtgttaatcaatgcttacattgataagtttctccaaaatcttcaacatt

tctcaaattagtacgacttaatcagtctttaaattgaataagtggtacttgatcactctc

cttaattcggtactagaactatcttatccaccactcattttataataaataaacgtgttc

ttaatatttcaaaaaaaaaaag

>fava_dn_rep_c931

cgcctctgtaccaactaccaaggcaaaaactcatttatggcaatcactgaatgaagtttt

attatactacaataaaaatactacgaaacatgttgcctctttccaaaggcagatatttca

tttatggcatcacttaaatgaaggtttattagtatactacataggttacagatttgatca

caattgcttttttcttcttcttgattcattgtcaatcttctcaactacatacacaatcaa

taaaatgaaaagaaaacattccaatttggacacatactgaaaacacaacacattttttat

caagcattgaaaaaatttccatgtttgcttcaagtgtaagtcatctacataagaattaca

gatacacagttgccaacatgtgaaatggaaaacagtctcggcattttgaacacatcgacc

acgcaaaagccctatgatggccagttcggaatattcgctgtagcttcattaaccattttt

acaagggtcaccacactttctggtacaccgggtggtggcaatgtcagattttttggagga

gcaacactatcataagatcttttatcaaacctccattctccaatcttcccataggtcagt

tcgcccttcatattatagtcacaaccataagtgtagtgaattatgtacttctcaccaagt

tctttgtcccatggaggctgaatcataaagtctttgtataatatgttatgaacaccatga

agagcagacgcaacggcataagcatacatttcaagcacccatccaaaagctttatcagtt

tcaggatcttttttcatcgccaacgaaacattcatccaagtaggagcaatcttcttaagc

gattccttcccaacaatcactggcgaattcccaattggatctatattcgtcacaggtcca

ttttcctcggagtagtacttccttaacacattctcatatttcttcggctcaatataaaag

aaagggaacgccgctcccattccatttcttgctaaattcggtataggtttcacaattata

tgatccggctccgacatcaaaatgtaatcttctttgatatctgcttgttgaagccattgt

acaaatgcccatggtctattaagcactatgtaaccctgatccattccagaaggcaaaggc

tgagcaacaaaagtagggatctcatccataagctgatcagctttcccagaatgcaaaatc

cttgtaaatccacccatatcagaatctgaatcttccttaaacttcttgaaccaataatac

ataaccctacactgccaagtattgtaaacactatctgaagctgttacagcagtatgaaac

aatctcctagaagatttttgcttctccaatggcatcttgataattgggtcaatggtaatt

gaaggacgacgtgaaggaccaccaggaaaatcttgtttcaaaggagcgtttcctgaaatg

attatgttgtatgttatgagtgttactgaaaatgttatgaggattgtgaagaacatgttt

ccacaacccatttttttgtttgtttctttttcttgttctagttttgattcatgagaagaa

gaagaaagaagaagatgaggcggctatgtgttttctaactgctatattgtctgtatccag

atgttgaaagtggaaactcttaaaaagtcttctacactttctttttctattaatgtttca

ttaagattaattaataaaaaacatgcaatttgtgtttttttttttagaatttttcaatgc

atcccttgattctcttagtcatttggcgaaattttatttatgcctatgcttctgtagata

tatatccaaaagtattttaaaaagaaaaaaattgttttcttc

>fava_dn_rep_c932

agatattggttctgcctcttggtgttccaaactgatcataaacatttcatattttttata

ttaatttatagtataagatcataatcaccaaatttgtggattggagaagagtgaaagtga

agccatttgtgagtaactcgagaacattcaggttgcatcaactaatagataattcaatac

aaatgcctttgaatgtgtttcaagataaagatggattgccgaggattgtattgactgatc

caaatggttcttcagctgaggtgtttctctatggaggccagattgtttcttggaagaatc

aaaggaaagaagaactgctttttgtgagcagcaaggctaaatggaaacagcataaagcaa

ttagaggaggtatatcagcttgctttgcaatgtttggtgaagttggttcgcttgagcaac

atggaatgacaagaaacagaatgtggtcattggatagagatccttcaccattacctccaa

tagacaatcattcatctgtggatttgatattaaagtcaacaggagttgacttaaagaaac

cacatagttttgagttgaggcttcgcatatctctgagtgctggcaagctcattctgattc

ctagagtcagaaacacagacaacaaaccattttcttttgcattttcactatgcaactatt

tatctgtatcagatataagtgaagttcgtattgagggattggagacacttgattattttg

ataatctggtgaacagatcaaggttcactgaacaggctgatgcaattacatttgatggag

agacggacagggtatacttgagcagcccgaataaaatcgccataatagatcatgagaaga

aaagaaccattgtactccaaaagaatgcacttccagatgctgtggtatggaatccatgga

ataaaaaggccaaggctctacccgatttgggagatgaagattacaagatgatgttatgtg

tgaactcagcagctattgatgttcctattttcttgaagccatgtgaagagtggaagggct

atcaagaactctctactgtttcatcaagctattgcagtggacaattggatcctcgcagag

ttctttatggctttcattgatgcagtcaaatcaatcaaggtttatatataatatatatat

tacattgcttttgagaaaacaaaaaggtgtgcatggaagtcatgctgatagatatatagc

ataacataaagcccttttccaccttacaataatttgagagaagcttagcttgatggcatt

atgccatgacaattactaagcaattttttctgtgtatttgttaggttccagtcaaattat

tcagagattggaaactggtgattttgtgtatacatacttactatttgtaaattaggaaaa

ataacatgaacttttttctttctttctttctttctacgccttttgttaggagaacctgca

aatattgtggccctatgatcattattaacatgtttctatccgcacctataccttctctaa

ttgcaggataattaatgcacactaacaacataatcctcatctaaagagattaaacagaat

acaaatgcttgctcgaaacaggtttttcttgcacatttgattggtgttaacatgataaag

gagaaagataagaaaacgaaacatcttaatcacgccggaatattgtgaaccgtaaccggc

aagaaacaggtcctaaggaaatcataatttaaaatccaacatggaatgtatacattctcc

atagattgtgctagctgtacaatttaacacaaaatgtataaattggagacaaatgaaaat

atatcactcgtcgtcatcttcatcttcgaaaccttcagcaggcccggcttcatctccaat

gccttcaagtagttggtcaacatcctctcccaactccagcaacctagcactaagtttatc

aaccttgctttgctcttgcccgaggcataccagcagatcattcagttcaacctcactctc

cttcatagcttcttcccttgcttcagcttttattgcctctacatctgggagtgttgagag

ccctccacccctcagtgctttcacctcattttccagttgaaggttagattgctcaaggct

attgtatgcatcggatagactcaatagatcagcttccatctttccagctaaattctgata

catatttgcctctgattcaatttttgctttttcttccttaagcgtctccaaccttttaga

tgcttcttggaaatctctacggagtgtctctgtttggactctgtcgacgcctccactcac

tctttgttcagactggaaactactgccagtcttagccaggtcctcggccaatgtaccatt

tcgaagaacaagatcctgtatctcagagtgctgattctcaacaaaagctttcaaccgctt

tatatattcaccatcactttcaccactcttctgctctatttctgacggtactaccacaac

tttagtttttggacgactataaacctccacaatctgttctctaatatttgcttccagcct

tttgacaaaactaacaaagtaagaatccaagattgaagcaagaatcggatgatccgcatt

tttcttttctgacaaatcattctcatctacatcttgaatatctgccatactagcagctga

agatctagagaatgatctatgtgtgagtgatgactccgcgttagcgaaaacaaaactctt

atgcatctcctcaaattttaagaagtacgaactgagccctattttctgacttattaaatc

aacaatggcaaatgcatcttttccgctatcaggggaattgttatagatcacacattcacc

caacacaactgctgcaaatccccttatacacactgtttctgacgaatttgatactagctc

aagaagataggttaggtggggccgggcatctaggaagcagtgcactgcattgtggcaatc

tgctagccaggtaaccagtaatttcaaaataacagcttgaacatatgaatttcctgatgt

attattagactttccatctttggatttcattgaagaggcaagggctaaatatttcaccat

tcgatgcatcagtggttcgggagctcctaaagattgcatgggtgcctcaatttcaattcg

caaaaccctttccttgcatgggagattgtccttcaatatatgtgatagaacactagcagc

tctactacaagtctcaagatcaccattgttttcacctaaagtaagaccgtgtaatagcat

gcttccaaaagacatattaacatcctcctcatggaatgaatgattcacggaatatggttg

tggaattagagtagacgctagcattgcttggccatcagcatttttctcacaaaagttttt

gaaaacataatcagctgcaatgaactcttgcatgctagaagacctcaaaattattcgaag

tatagaattcaaagcaggttcgacttgtggctcctctccaagaactttgctagcaagaag

atcgagattcttcgaatctccaacaatcaagtccccaatacatttcaaggccgcacagcg

aactgcaacgggtacccattggctttcaacacctaatatcaacaaaatgtccagtacttt

tttctgaaccagagctgtcttatttttctgtttattagcatctttcccaggatcggcatc

agaacctcctttaagtaacaatttaatggtttctaatgcactgagtagattgattgtctt

ttgttgagtaaaagagtaagaacttgccctcagcttcaaaatcaatatcaatggatcaag

tcctatagtctctctaagtagtacctgattggatgcattgctacgaatcaaattgtttaa

caattcaaggcagtcctgcacgactacaccaccatcagaatttccctcctcccttatgat

actgaatatcttatcgtaagcaccttcaaagacaataatcttttggatctcctcagcttc

acgggtcaagtgagtaagaagcaataatgcctcattccttatcacctcgcgatccataag

catgtccattagccgagttatgccacgggggatagtcaatatagtttcctgtaacctctg

tcgagaattagtgagaagggcggtcaatatttgcagagtataatatcgtacataaaaatc

atcctcttccaacaaacttagaagaagagggatactttcttcttctctggaaagtaaatc

agtattcatcaaatctggctgaacttcattacctgatacttttgcatgattaataggagt

caatgcactcacaagagtttccaaggcaccgcgaaccatttcaacatcatgacgttcttc

cttcaagacacctaacattataggaaggcccattgccccaaatgctaactggaaagcttt

attttccgagacaacagcctgaagctcagtaatcgcatttctcctatcatcgggtagttt

accgttgtttattcgatcaagcaaacgctcaacatacctatcttcatttgaagaattctc

gttaccaaaaacaagcccaacaacacccttgtaaccagacattaaatccattttcatagc

tcagagatttgttgaaggagaaaaaatcgaagcgaatctgaatggaattggtgataattg

agatgtgttgttgattgaggaattgggagaaccctagaaaaagtgagagatgaatagagg

gaaagagatgattgggattttgttttgagggcaaaattcgtgttttgaggttggaatcag

aagaaaaagaagaagaaggtttctgtgttgtttattttcattgttgaagatttgtaagtt

>fava_dn_rep_c933

aaatatgtatgtacatgtagaagtaagttcccttgttttaggtgaaccaaagataacata

cttctgaagccttggaagaatcaaggacattccaatacaattctacaagaacatatatag

aacatggttacaacaagtgggataagctataataatgaacgagaagaaccaaaaacaact

ttaaataccaattcttacagttttataaaaaatacattaatctatcagcagaaactgatt

ctcaaatgaagagtaacttcctatttattaaaactaaaggaatatttagttattctgctc

aatggtgcgtttagcgccctccaaggtattcttcagcaacattgtgactgtcattggacc

cacaccaccaggaacaggagtaatccaaccagcaactttagatacttcctcaaaatctac

atctccaacaagtcgataacctgatttcctagttgggtcatcaacagaatttgtgccaac

atctatcactgcagctccaggttttatccagcttcccttgatcatctttgcctgtcctgc

tgctgcaataacaatatctgcttcacgtatgatattttctggttgacttgtgtgtgaatg

aacaatggtaactgtagcatctgctttcaagagaagcaatgaagctggtaatccaactat

gttgcttctaccaaccacaacagcatttttcccctttatacttacaccacttcgtgataa

tagttcaagacatcccttgggagtgcagggaagaaacaaagggtctcttcctttcattgc

aagcttgccaatgttcaaaggatggaaaccatctacatccttcgtaaggctgatttcagt

caaaacattctcttcatttacgtgcttaggcaatggaagttgaaccaatataccatgtac

atcggggttagcgttcaattcgtgaacatttttgataatttcagcttcagaagcttcctc

tggaagatcgatatcgaaggatttgattcccaattcagcgcacgcttttctcttcattcc

aacgtagctttgagagtcctttcggtttcctactatcaccactgcaagtcccggaacctt

gccgtacttttgggagaggagacgaacctcgtcggcgatttcagatcggatagtttgtgc

aacggctttgccgtcgattacggtggccatttgatcggtgaatctgagatcgtgcggtgt

ggttttctgttacaatgcaagttcacggcttgttctttagttgcgattgcgaattgcgat

gatgttaaaaggtttgaaaatgatatgccctcgtttttacggattaatttgtctattcaa

gggataaacaagtggataatatgcttttgttggtgatggtgatttggaagatgagagtga

agtaaagggtaaacttgcttctttatgagtaattttgtattgttatttgtttctagttag

tacttgatttggtgtttaaggtgtaatgaactatgaatgttgtcttttcttaacaacatt

aggaaaattttatatagcacgaggtattattttcgtaatgattcgaaatatagtgtctta

gaatcatgaaatggcaggtataagatgagaaactattggtgtaaaaaaaaaaa

>fava_dn_rep_c934

tttttttttctaccaaaaatttcaatattttattgagaatatgaatagaagttgggacaa

tttcaaatgaatagtagtaatccagacacaaacaatatttatacatgagagtgaaaatcc

aaaaaccaaaacaaaaagggagaacaaacactttacgacataataaaattcccaaaaaat

taggaattaatatagactcaaaatagattatacataaagacaaacctatatagagaaaat

ataaaaataaggtgagtcttgattactggatcaacgtttgatatgcttgactctgttcca

tcctttcttgaatgaatttccaacagcattaattcccttattgaaaaatctctttacctc

ctttgctctgaacttcactctatgttcaaatccacccatgatgagacagagctgcttttt

ggttaagaaagaaaagtatacaacaaaacttagtacttcccgcggc

>fava_dn_rep_c935

atttttttcaacttaatacaaccaacttaaatcttgaattacttgaggttttcatacaat

aataaacttataatgttgctaaaacatatttttattgacaatgtgtgaatgtgtcttacc

atttataacaagatttggttcacaactaaaaaaacttccaattcatggcagttttgccct

gtgttccttaagaacaggccaggataatagattgtttccttgcaagtttagaaatcacaa

tataaagataccatactgttgccctcattgtagcaaatataccaaaatggtctcaagaca

aaaaatcacacaacataaaccatccaattttgcatggagggtgtgagcatctttaggtta

taaacaatgatcacaaaccatgcaaaaattcatacccttaaaagtatgattcgattcaaa

gcgcctaaaatgtggtgtactacacacgactagttaacatactttttagtacttggtatc

tttagagaagtatgcatataaactcatacatctatagatggtcatgttctttatccgaga

tcaggcacagtgaaatgctcatcatcatcgtcttcacggtggacttcaatcctaggtttt

ttgtaagacatataagatttaggagctttatatgaggcaacattgctagcatgtgttaat

ggaagttgaggaacttcagccgctgctaaaccttcatgaggaaactgttcgttgaaaaga

cctgcgtctgataaccactcaagctctccaaattccatagattgctttttgtctgaagaa

tcaaagtctgtaaattccagtaagtcatcaacagcccaagaggaatcaaaactaggcact

agctgtgcaggaacttcaataggaatttgttttgtatcttgattagatggttcaatttgg

cttttatcattacttttagtgcaactagaacccaaagcgacacggatacctgtcgcgaga

aagcgctggtggttcgcagaaagactaccagctacatgaattggttcatcacagtcctta

cagaacagtgctctatcttcaacacaaaatatgaaagctggcttatcttggcagatatca

catttgggaagcttattagaaagagattgaaggagaagcctttgatgcttgctagcaagt

ttgttagcagcatgaacttcaacatcacatttagcacacaaagcagcttcatcagcacaa

caaatcacagtagcaggagctttctcacagacatcacactgaattttcatcttctctgat

ccctcacaccttcaaagatccaacaacaactctactaattcaagattattaactttttga

aagccaaaaaatgggaaaaacgttgatgggtgggtgaagaggtgaatgatgtgacagtgt

ggtagctagatgaatgtgttttggaccgtggaaaaggaaaatgaagatagggatttgaat

ttacgtgtgaaaaacagctataaagtggggtaggtttggggtttgtgtggctgt

>fava_dn_rep_c936

aaatatttgggaaaagaaaaacagtgaaagcgtggggtagtgggtagtataaagtttaga

aaccgtcgtccacgctcacgggtgggtgcttattcatattcattctctctcattcagatc

atcatctctttacgcgtaataatcatgactagttcttcagctgcttctcgcaagattctc

agtaagattgcctgcaataggctccagaaagaactcgtcgagtggcaggttaatccacct

tccggttttaaacacaaagtcaccgacaatctccaaaggtgggttattgaagtggctggt

gctcctgggacactttataccaatgagacctaccagcttcaggttgattttcctgagaat

taccccatggaagccccacaggttatattcttgcatccggctccgttgcatcctcatatc

tacagcaatggacatatttgtttagatatattatatgattcatggtctcctgccatgact

gttagttctatctgcattagcattctgtctatgctttcaagctcaactgcaaagcaacgc

cctgaagataatgatcgctatgtaaagaactgcagaaatggccgatcacccaaggagaca

aggtggtggttccatgatgacaaagtatgatgaccagttttcgcgaataagttcacaaaa

gtaaagtagaggatgttgtgggaaaaaacataatgagaaaccaggatcactttaattaaa

tggaaaattattctcctgtaaattaaaattgcctggtaatttgtaaaggaagaggaaatc

aggaaattatgtgagcaactttgatgatcaaagaattcagaacgatgttgttaatctcag

tgatatttgaatacggatattgatggctgattaaccttcatttggtttatatatgtcata

attggcaatatacgtgcgttatatttgtggtttcgaacaaaagtcatgtttggcagctaa

attgtagactagtttccatttagtaatcatactctttggaatggttcaacttacgagctc

tagaaagatgaaatcttgcttttgattttcttttacagttg

>fava_dn_rep_c937

gtttgtttatcatgataaaataacatttgaaccatcaaaagcatttttaaagaaaattac

taaaaatacatttgatcttaaagaaattacaaaataaaaactactgatgtaattattttt

aagagttgactgtaaaaacagacccttatagtgtgttttgcacctgaaaaagtgttcaaa

gtagccaaaacacaacaaagcataaacgtattattaattgtgtagcatagctctactcag

aatcctaactaatctatctttcatgttcaaagtttaaaacatcaaattgtctaaattgac

agatctccagtcttctcaaaagcataattaataatgcgaactaaaaaagccatacaagaa

agaaaacataaacactgaattattctaaatttccacatgtgctcagctttgcttgccacc

gggtctaggaggtccaaagtagtttgttatcagtcccattaacgaaaacatcatagccat

cattacttgtttgagatcattttcaacattcctcatgttagcaatggattgcgcacagaa

tatgagagccaaccacgaactcagcttatacctaaacataacaccagcgacgccgaatat

gacggcgatgaaaccggcgtaatcgatcggaagatcttgcggagcaatcaccggagctac

gtatggcttcgccgccgatggctgacgtggatcgtttccgtgagaagacatattctgatc

cgctttctcgcgtcttcctccctcgacgatttctctaacgggagtttactcttactttcg

ttctttaatttattttgttttaattttt

>fava_dn_rep_c938

cgcgacacaccacaacgcaacgcagccctgtgatttcgatcttttccctttattatctct

ctcgaatttctttttcttgagatcgttcttttctagggtttcgttctttctctctcgttt

tctccgcgctttcccttccaagttcccactcgtatctattttgttcgattcgatcttggc

ggtgggtagaaggttttgaaagctgcggcattttaatcataccttatcaaaacaggactg

aatgagctaatggagtctaaaggaggtaaaaagaagtctagtagtagtagtaattcctca

tcattattctacgaagcccccctcggatacagcatcgaagacattcgtccaaacggtgga

atcaagaagttcagatcagctgcttactctaactgcgctcgcaagccatcctgatatacc

gtcctgattctccttcccgtgcatgcaagcactttactattagctttttttgtttttctt

tagctttttttactgctctccttcaaaaaacatctttccaattcctctccttgctattat

acttttttttctctcctcgtttcttttgtatttctgtctgtttgaactgtggccatggct

gtttctgcaattggtttcgaaggtttcgaaaagaggttggaaatatccttttccgatccc

ggacttttctctgaccctcaaggaaggggtttaagatctctcacaaaatcccaattggat

gagattcttgcaccagctgagtgcaccattgtttcttcccttgcaaatgaggatgttgat

tcctatgttctatctgagtccagcctttttgtttatgcctacaagatcataatcaaaacc

tgtggtactaccaagttactgcttgcaatcccaccgatattgaaattggccgagtccatt

tctctagatgttagagctgtgaggtacacccgtggcagcttcattttccctggtgctcag

tctttccctcatcgccacttttccgaagaagtggctgtcctcgatggcttctttggcaaa

cttggatcaggcagcaaggcttatattatgggtggatccgatgaagcccagaattggcat

gtctactgtgcctctgctgattccgtgagcccagcagattctgtctacacactcgagatg

tgtatgaccggcttagacagagagaaagcatccgttttcttcaaagaacaaacaggttca

gctgccgagatgaccgtcaactctggcattagaaaaattcttccaaattctgaaatttgc

gactttgattttgaaccttgtggttattcaatgaactctgttgaggggcctgctgtatct

acaattcatattactcctgaagatggtttcagctatgcaagctttgagactgcagggtac

gatttgaaggcaatgaatctgaacgaaatggtgatgagggtgctagcttgtttccaacca

actgagttttctgtagctgttcatgtggacaatgcaagcaagtcatttgagcaggggtgt

ttgctggatgtcaagggatactgttgtgatgagaagagtcaccaaggtcttggaatgagt

ggatctgttatctaccaaaaatttatcaaggcttccgactgtggctcgcctagatcaaca

ctcaagtgctggaaagatgaagatgaagaagagtagcttttgtgtctttattatgtttgt

ctgttttaaataagtgtagccttgatggcttaaacacattgtctagaaggttataacctt

cattgttattttctattatttgctgtttgtgtctagaaggtaataaatagccttcattgt

tattttcgtttaaactatgatattgaaataaagtgcatgttatgggaaaaaaaaaaagta

agggttctggctttaacattgccaatttgcttaaaccttatcttgcaatgggtctatttc

agtgtatnnnnnnnnnnnnnnnnnnnnnnnnnnnnnnnnnnnnnnnnnnnnnnnnnnnnn

nnnnnnnnnnntttcaacctgattggatcaatgagccaag

>fava_dn_rep_c939

agtcaatactaattttccaataactgaccaactccatttaaactctctgctccttcttcc

accgtccacaaattttccttaacttcttctcctcgatcggaatccatggcgcagaacaga

ttcgtcattgaagtcgaaaaagcaaaggaagccagtgatggcagaccttctagaggacct

gcgtaccgtagcatcttcgctaaggatggatttccggctcctgttccaggtcttgatagt

tgctgggacgttttccgaatgtctgttgagaaatatccaaataatcctatgcttggtaac

agagagattgtcgatgggaaggcgggcagatacaagtggcaaacttacaaggaagtatat

gatatggtgatcaaagtcggaaactccatccgtagctgtggttatggagaaggtgtaaaa

tgtggtatttatggtgctaattctgcagagtggattatgagcatggaggcttgcaatgct

catggacttctttgcgttcctttatatgataccttaggttctggagccatagaatttatt

atatcccatgcagaagtctcaattgcatttgcagaaggaaagaaatatcaaacatatatg

agaaaagagattagcatgaaactataggtagaataattggtttcatgccaaagtagacaa

aattgtgaaggctatagtaagattatagatagatatatggaggttgccgtggaacttgat

gatgatctattttttgcagacttaagcaagcaaatttctttactaattatggatgaagat

gaagaagaccctttcacttcttgtcatcaacaatctcttgagtctttctctggagcaatc

tatcatcctcctccacagtcgaatatcatgtacgagcagatggttttgagaagacaagaa

agcaaagggacaggtgtgttcatcccgcagtcagcaacacagccaagaaggaagcaaagg

aaaggaagatcttcttcttcatatgcaaaatatcaaaagcagtctcaagatacaacaaaa

acagtttcttcaaaataatttcaaaccaatataaataatggatgattagatccttgtgca

aaactgaaacttctacttatctttaataatgtattagttgatcgtagttttagattcttc

tgttccaatttgttttctctttttgtgatatatttcatttataatttgtacaactatgtg

ttatttaaaacaatttacctcgtttatttttggtaatattctagttagtgtcttttttat

ttataatattgttctgttgtgctaaagaagttgaagtgaaaccagcagtgacgccaaatt

ccccctcatatgtatctaatgtcacttgttggctttgtctcccaaacgtcgcgctgttac

tcatactctgatcccttccgctccccgccacatgtgccattcttccacgctttgcccctg

caaacatcccaccttcctcctgcgctgtagccccgacggcgctacaagaccccacgggag

tggggccaccgaccatgcatgatccaattcctcttataggaattggatctcgtgcatgaa

gcctatgctctttcttaagattggagcagggaaccaacgcgtccacaaccatgccactgg

ttgtggttgcagctgtggctgctgcaatggctgcggcacgttgagggtcaagccatggga

ctaacatgttcccatagacaccaccactcgtagcaagaaacggtggtttactgtgattcg

gaaagctagtagcttggttcactatggactccaaggtgccactaccacggggcttttccc

atgtttccttggacggtacggttgctaatggtttaaccggaactcttggtaaccctaagc

catgcatagaaatttgtccattttcccatgtcagttctgcaacatcatagtcaaacatgg

gaacatcatgaggggaagttgagttagagttggagcgaagagaggctgatcttggatggg

gttgtggattttcgtcaacttcccagctaggaacacactggctcattttgagcaagaatt

taaactagttctccttaattgtgtttttgctctttttcacatgttgggaaaggaatattg

attgtagaaagagagtggaa

>fava_dn_rep_c940

ctttagcattcaccaccaaaaaactcaattcgcgagattttcactccttttatttcgtag

ttcaatctcctctcagtttcacgttaactccaatttagggtttaccactcctccttctcc

gatggctcccgatgctaaccgtgccggtaccggtgccagcaccagcggaaccgccaaaga

cgaagcagcggcaaagaagaagatcgaaagtgaagatctgtcagatgaggatttggcttt

gaagcaacaactcgagttgtatgttgagagggttcaggattccgagccggggttacagaa

agttgcccttgagagcatgagacaagagattcgtacttcaactagctccatgacctcagt

tcccaagccactgaagtttttacgcccccattatggtactcttaaggcatattatgaaac

tatggcggaatccgagttgaagaagtacttggcagacatattatcagttttggcccttac

aatgtctgcagaaggagaacgggaaagtctcaaatacagattactaggttcagagggcga

tattggttcatggggacatgaatatgtcagaaatttggctggagaaattgcacaagaata

tgctaaacggcagagtgaagaggccccgattgatgatctcatggaacttgtactacagat

agttgcttttcacatgaagcataatgctgaaccagaagctgttgatcttttgatggaggt

tgaagacctagatatgtcgattgagcatgttgacaaaacaaattttagaaggacttgcct

gtatctcacaagttcagctaggtatcttcccggtcctgatgatatgctagttctggacat

tgcatatttaatctatctaaaatttgaagaatatccaaatgcactccaaatcgcattgtt

cctggataacctgcagtatgtgaggcaggtgttcacctcttgtgatgatgtgttgcgaaa

gaagcaattttgttacatacttgcacgccatggaatcacttttgagcttgacgaggaaat

ggttaatgatgacgaagaccgagaagtgttgcaggatatcataaacaattcaaagttaag

tgaaggatatctgactcttgctcgtgatattgaagtcatggagcccaagtcccctgaaga

tatatacaaggcacatttgcttgatggccgtgcaagtgctggtgcgagtgttgactcagc

tagacagaaccttgctgctacttttgttaatgcatttgttaatgctgggtttggtcagga

taagttaatgactgttgtatctgattcttcaagtggcggttcttctggaaattggctttt

caaaaacaaggaacatgggaagactagtgctgcagcaagtctgggtatgattttactctg

ggatgttgactctggacttgcacaactagacaaatacttccatagtaatgataatcatgt

cattgctggtgcattactgggagttggaattgtcaattgtagtatcaagaatgactgtga

tcctgcaatggcactccttggtgattatatagataaagaagatagttcgatcagaattgg

tgcaattatgggattaggaattgcatatgcaggttcccagaatgagcaattacgatacaa

gttggcacctattctgagtgatcctaaagcttcacttgatgtgattgctttcacggcaat

tgcattgggcttgatctacgtgggttcttgcaatgaggaagttgctcaagcaatcatatt

tacactgatggaccggagtgagtcagagttgggagagccccttacacgactactaccact

cggtcttggtcttctttatcttgggaagcaggaaagtgtagaggcaactgctgaagtttc

aaagacttttaatgaaaaaattaggaagtattgtgacatgacactactgtcatgcgccta

tgctggaactgggaatgttctcaaggtgcaaaatcttttgggtcattgttcacaacatct

tgacaaaggtgaaatgcaccagggtccagcagttcttggaattgctatggtagcaatggc

tgaagaattaggtgttgagatggctattcgatcgttagaacatcttctacagtatgggga

gcagaatattcggcgagctgttcctctggcacttggtctgctgtgcatatcaaatccaaa

ggtcaatgttatggataccttaagcagacttagccatgatactgattcagaagtagcaat

ggcagctgttatctccttgggtctaataggtgctggaacaaacaatgcacgaattgctgg

catgctacgaaatctttcaagctattactacaaggataccagccttctattttgtgtgcg

cattgcccaaggtcttgtacatttgggaaagggacttcttacacttaatccttatcattc

tgatcgggccttactgtcaccgacagcgcttgccggactaattactatgttgcatgcttg

ccttgatatgaaagctattgtgttgggaaaatatcactatgttctctatttcctcgtttt

ggcaatgcagccgaggatgttgttgaccgtggatgagaacttaaagcctctatcagtgcc

tgttcgggttggtcaagcagtggatgttgttggacaggcaggtcgtcctaagacgatcac

tggatttcaaacacattccacccctgttctcctggctgctggggataacgcagagttagc

cactgaaaaatatattcccatatcgccaattctggagggttttgtaatattgaaggacaa

tccagattatagggaagagtgagggccttaatgatgttttttaacttctttcggtgtgtg

aaaagtgcttagttttcttttgaaatagttaaaatgtaaaaggaattatatgttaaatta

tttctttttaatctgccaatttatagacatttttctaaagtgcatttgatgatgcattat

tgcggcgccgattaatttgaaagatagtttattaagtatgcaggcaattaaatttcattc

gtaaaaaaaaa

>fava_dn_rep_c941

tggtgtttaattgaagttggagttgagtgtgtaaaataagtagtgtttgttttttgttgt

taccgatacttgttgaattaatggcttctacaactgctgcttctgctttccttcaattcc

actctctttcttctaatcctcaacttttcctcaccaaattccataattaccctatctccc

tttcttcttcttccaaacggacttccaattataagcgcgtttgcgcaaaagctgtttcta

ccgactcacaaaaacctctcacacaaagtaatcaaaacactccctcatcatcatcatcca

aactggttcttgttcttggtgcttcaggaggtgtagggcagcttgttctcgcttcactgc

ttcagcggaatattaagtcacgggtgatacttagggatcttgagaaagccaaggcattgt

ttggtgaacaagatgaggagagactgcaggtattcaaaggagacacaaggaagcatgaag

atttggatccatctatgtttgagggagtcactcatgtgatttgctgcacaggaacaacag

cttttccttcgaggcggtgggatgatgagaatacaccagaaagggttgattgggtgggag

tgaagaatctaatatctgcattaccttcctcggtgaagagagttgttcttgtgtcgtcaa

ttggtgtaaccaagtgtaaagaattaccatggagcattatgaacctatttggcgtcttga

agtataagaagatgggggaggattttcttcagagttctggctttccatttaccataatta

gacctggaagattgactgatggaccatacacatcttatgatctcaatactttactcaaag

caacagctggtcaacggcgagcagtccttataggtcaaggagataagctagtgggagagg

ctagcagaattgtggttgctgaagcttgcatacaggcactagatcttgaagtcactgaaa

accaaatatttgaagttaactctgttgagggggaaggtcctggaaatgatgctcagaaat

ggcgagaattattcgaggcggcgcgttctagctaactagacttattgtcaaaacttgtat

atcattaagaaccaagtatatgcatcatagttaggggcttcaaatttacaattattgtat

agatatacacacttctttattcattattgaatacacacaaaaacacttgcttcattaaca

atattgccaattatacacacagaagcacttgtttcataatgtaaatcatgtgcaagttta

ttattcatcaatcacaggttacacaagacaacattttgtgaaagaaatttttggaatttg

gtggtgtactacatgaattggcacaatcaacaatttcagatccatcagctccatttttct

ttgctttttcacactcagtcaagaaagtattgaatgcgtcctcaacatatttaactgcat

cagaaacacacatgttctcgactaacttgtccgggtatatactctttctatgatctccat

ttagagttgctgccattagtaccaattcttcctgaaattcactcaacttggctcgttctt

tagcagccctctctcgtagtttaattgggtcggccaatttttctggacaatcagttcgcg

ggctggttagagttaaaaggctctcaaatgttccagcccatgcat

>fava_dn_rep_c942

ccgcgggtcaacgcagagtggccattacggccgcggggtcttcatccagttgaattaata

caaccaccgttctcgctgtgtctgggaatcgatcattttgttcaatggcaatgagatcgg

cactgagtcgaggaagcgtgactcgttcaatggactcaacatctcgtggagctttcactc

gcttcttcagcaacaaaggtaaggttctcagcgaggaagaacaggccaaagaaaacgtct

acatccagaaatgggagagggagaggttggagaaacagaagcaagcggctgagaaggcta

aggcggagagagccaaagagagtgctgatgacaagaaatctgatgggactcaaaagagct

gattttatagatcatgctagccatgacatgaatgtcggatgtgggaagtaatcatggtga

ataaaacacattgactcttagtgaactattgtctctgtatagattgtgtgtttcttattt

gtagtttctttaatggtgttgatgagaccatgttgcatccaggtttttttgatgtaatcc

ttctttatgaagtttggtacattatgtttcgtgatcaaaaagtgaagcacaagctagttt

gtcgttagaaaatttcgggggatctttttacggtcccttgtttttcatgcatgcattaga

atgaatgcttgaaaacacataagctctttcatacctacgttggagtgaaaacgcacaaga

ttgtgcatgttagaaataaattcttctatctttcatatggaaac

>fava_dn_rep_c943

cgcttaaacatgtgatatgagcatcttatcttctcattatccattcattgcagcaacatc

ctcttcattgactattacctatggcagataccaagagcaatcccaatgctcagattcttg

aagagctcgaggctctgagtgataccctttacaaatcacacacgtctgccacggctcgta

gaacagcttcgcttgtcttgccgcgaaatactcctgttccatctattgaagatgatgaca

atgactatcacacggctgaagcttatggtgaaagtagtaacaaaccgcaatcccgtcgca

tgtccttgtccccgtggcgatcaaaatcaaagcttgaggatggaatttcaaagacagaaa

ccaaagaggtggtggctaagactaagacatcaacgactaatttgggtgagaatgagaaga

aagggatttggaagtggaaacctatgcgggcactttcgcatattggaatgcagaaactaa

gctgtttgttttccgttgaagtggttgctgctcaagaccttccttcttccatgaatggac

taaggttagctgtttgcgttaggaagcatgaaacaaaggatggtgctgttaagacaatgc

catcacgcgtttcacaaggagctgctgatttcgaggagaccctttttatcaagtgccatg

cttattacaccatcaacaatcatgagaagaggcgtaagtttgagccacgtccctttttga

tatacctttttgctgttgatgctcaagagcttgattttggaagaagctatgtggatttga

gcgagttgattcgagagtctgtggagaagagccaacaaggatcgcgagttaggcaatggg

atacgagcttcgagttatctggaaaggcaaaaggaggcgaacttgttgtgaaacttggtt

ttcagattgtggagaaagatggaggagttgatatatataatatgaatagcaataccaata

gtccaatggggaattcaaagtccagcaagttgagtagtttctcatcttctttcgcacgca

aacaatccaagtcatccttcagcgtgcctagtcccagaatgacgagcaggagcgacgcgt

ggagcccttcacaagcacagcaaggtgccagcattcaaggaatggacgatttgaatctcg

atgacccgaatccagttcatgattcctcttcttctgcccagaaagtcgatcatgaccaca

tagaacaggtggaggattttgatcttccagattttgaggttgttgataaagggattgagg

ttcaagagaaagaagaagatgaaggagaggaatcagataaatctgtagaagagaaaccgg

ttgcagacgaggttgtgaaggaagtagtgcacgatcatgtgcaccatgctagattgtctg

agcttgattcaattgctcaacaaataaaagctctggagtctatgatgggagatgatgata

tgaataacatgatgaaaatagatgaagagacagacgcactggaagcagatgaagaaactg

tgactagggagtttcttcagatgcttgaggaggatcaagacaacaaaggatacttattca

accaatctgaaattccatctttacaacttgaagaaagacgtgacaattctccggcagatg

gaggagaatccaaagtatatctttcagaacttggtaagggcttgggttgtgtggttaaaa

cacgggacggtggctacttggcttccatgaaccctttagatgttgcagtggctagaaaag

atactccaaagctggcaatgcagatatcaaagccttatgtgttggcttcgcatgaatcga

tgagcgggtttgatttgtttcagaaattagctggcattggtcttgatgaactcaactccg

aagtcttatcctccttgatgccaatagatgaactgataggtaaaactgcagagcagattg

cttttgaaggcattgcttcaaccgtcgtacaaggtaggaacaaggagggagcaagttcca

gcgctgcccgcatagtttctgctttgaaaagcatgtcaaacattattagttcaggaagga

aagaacggatatcaacgggactttggaatgttgatgaagatccagttactttcgagaagc

ttcttcctatttcaatgcagaagatagagtcaatgacagtcgaggcactgaaaattcaag

ctgacgtggctgaggaagaagctccatttgatttttctgcactcggctcaaagaaagggg

ctagtgggaaagatcttttggcttctgctattccacttgaggattggattagagaccaaa

gcttaggcaacaacaagggctctgcaactgcaagttctgatggcgaacctgagaaagtca

cactgatattggttgtccaactgcgggatccaatgagacgctatgaaacagttggaggcc

ctatgatggtgcttattcatgcaactcgcgttagcacggacggggctggggagggtaaga

ggtttaaagtaataagcatccatgttggaggtttcaaggttaggagttctacaaagaaga

atgtatgggacaatgagaagcagaggctaaccgcaatgcaatggttagttgcatatgggt

tgggaaaggcagggaagaaagggaagcaggcagtggcaaagggacaagacctgctatata

gtatttcatcacgggtagtggctgatatgtggctcaaaaccattaggaacccggatgtta

agcttgtaaagtgattagttatgccgaggaaacagtgttgcattcatgtatcataaaaag

cttcattcatgtatcatagaaagttctataaaatagggtctgagactgagtaaccctatt

gattattttcattttgatatttctttttttttctcacaaaacatccataatgtagttata

taccagatacagatttctcacaaagtataatgatattacaaatgacttgacttctacaca

acaacagagaaaagctattaaagaattcgcacacttccacacagttcaaaattattgcat

tgttgccaataacttttaagaacttattagctttaccattttcaaaggtataggcacaac

aacaatgtagccaatttaagatgatgcagttttggtgtttgggttgaaaggacgctgctc

agcacaggtaagaacatcatgggtcccaccttttccacggccgacaccaagaagatcaag

ataatataggaaatgggaagcaatattgttcatggagtcaacatcaccctgtccacaaac

cccttcaccatagagaatgttcattgtagcgccaaaaccaggtacacggtgctccattgt

gtcgttcttggtaggcttccagttaccgacgaaggcgtcgtgggcagagggttgtgcctt

tttgacgggcgtcatccatttccaaattgcagcttggaatgcaagggttgcattctgctc

tatgtattctggatggtcaagtagattcaccttcagagcttctccagcagctccataatt

gtagttccagtaaataggaatggctccacgtccgtagtattcagctccaggagtgcaggg

gtatgttaatttgtaatagtcatcacagtatgtctggctaggactcatttcatgattgta

acatagaccccaggccgtaggtcctccagttgccactccataaccacaagaggttttgga

tccaacgtgtccaagaaaagccgcaatctccatcatctgcatagttttattcccggtagt

cccaaacccctgcggctcaaaaagcgcagccgcattaataaacgaatgataatcccaaaa

cccaacggcatgagccaccggagtgttcctcttcgaaaacagattctcgaaatggtacgt

ttgaaaataatcgcttatcgtcaaattacaacagtaaatcgaccaatccttacattccca

accttgcgtacagtactttttgccgtgcttgtattttacttgtaccttcttagtgttcga

atcctcgccgttcacggtggttgttgcgtcggcgagaagaagaacgaataagagtaacaa

aacgactgcgttttgtttctccatgtgaagaaatgagaaaatgaatgaaacgaaaatgga

gtgatgataacaataataataataaagtgtttagtgatagtggttgaatgtgtttatatg

tgattgtggatcgaacggtggaggattgattgaacggtggaggattgatcggacggtgga

aaaagtgttggt

>fava_dn_rep_c944

ggccgcgggatagaacaagctaaacattactccttcatcttttcatttctcgctctagaa

ctttcttcagtcactttcactctctaaaactcgttcttccaactcagaactcagtactca

agatgcgtgagattcttcacatccagggaggacaatgcgggaaccaaatcggagccaagt

tttgggaggttgtgtgcgcggagcacgggatcgactccaccggaaggtaccaagggaaca

ctgaaatgcaactcgagcgagttaacgtgtactacaatgaagcgagttgtggaagattcg

tacctcgagctgtgctcatggatctggagcctgggactatggacagtgtcagatctggtc

cgtacggacagattttccggccggataatttcgtttttggacagtccggtgccggaaata

actgggcgaaaggtcactatactgagggagctgagttgattgactcggtccttgatgttg

tgaggaaggaggcagaaaattgtgactgtcttcaagggtttcaggtttgccactcccttg

gtggtggaactggttctggaatgggtacacttttgatttccaagatcagagaagaatacc

ctgatcgaatgatgcttactttctccgttttcccatctcctaaggtatctgatacagttg

ttgaaccttataacgctacactttcagtccaccaacttgttgagaacgcagatgaatgta

tggttttggataatgaagctctctatgacatttgcttccgcacccttaaactcaccactc

caagctttggagatttaaaccatttaatttcagccacaatgagtggtgttacatgttgtc

ttcgatttcctggtcaattgaactcagatcttcgcaaactggcagtgaacctaattcctt

tccctcgtttgcatttctttatggtgggttttgccccactcacttcccgtggctcacagc

agtacagagctctcactgtacctgagctgacccaacagatgtgggattccaagaacatga

tgtgcgctgctgatcctcgccatggtcgttatttgacagcatctgccatgttccgtggta

agatgagcacgaaggaggttgatgagcagatgatcaatgtccagaacaagaattcatcat

actttgttgaatggattcccaacaatgtgaaatcaaccgtctgtgatatccctccaactg

gtcttaaaatggcttctacgttcattggtaactcaacatctattcaagagatgtttagga

gggtgagtgagcaattcacggctatgttccgtagaaaagctttcttgcattggtacactg

gagagggtatggatgagatggagtttactgaagcagagagtaacatgaatgaccttgttt

ctgagtacaagcagtaccaagacgctactgcaggtgaaggggggtatgactatgaagatg

aggaagaggttcaggaggagggagaggaagtttgaaaactagtattttaagaactagtat

ctttgaccgtgtgttttgtttgtgtgtgtggtactctctatgaatgacctttttcgcatg

ttttacttgttaaagctgacgcttattaatttctgaaagattgaagcatataatccatat

tagtcatgttaagggtttggattctttaaatttcttgtgtaccaaaattctcctaatcaa

gtcgaattgtaatgtttcgactaaagtgaattatcaaacaattattaaaacaccgagaac

cattttttttacaaacactgaacagtttggtacattatttcagggatccaaaaatattac

catctaaactgaagctgaatgcttgacatgtagtacacaaatatcgtgttgataccaaaa

caatctttccacaatttaagaaatatgaatgtataaagaatgaaatattatataaaagtt

ttaagtcgnnnnnnnnnnnnnnnnnnnnnnnnnnnnnnnnnnnnnnnnnnnnnnnnnnnn

nnnnnnnacctttgtggtgctgaagaaatggtgcctttgt

>fava_dn_rep_c945

catgtatttatttatttaaacctacttcactccaaaaccaccatcattcattcaacaaca

acaacacctctctcgcctctcttttttctctgctcagttttttcgcatccatccatcagt

aatggcgttggaaggtgttctcctcggtatgggtaatccccttctcgacatttccgctgt

cgtcgacgaagatttcttgaaaaagtttgatattcagttgaataatgccattctcgctga

agataagcataaatctatgtacgatgaaatgtctgccaaatttaacgtggagtatattgc

tggaggtgctactcagaattcaattaaggttgctcagtggttgcttcaagtacctggagc

cactagttacattggttgcataggaaaggacaagtatggcgaggagatgaccaagaacag

tagacttgctggtgtaaatgttcaatattatgaagatgaaaatacgcctacaggaacttg

tgctgtttgtgtggttggtggcgagaggtcacttattgccaacttagcagctgcaaattg

ctacaaggttgatcatttgaagaaacccgaaaattgggcattagttgaaaaggccaagta

tttctatattgctggctttttcctcactgtatcccccgaatccatacaattagttggtga

acatgcagctgcaaagaacaaggttttcacaatgaatctttctgcaccctttatctgcga

gttcttcaaggatgtacaggataaagctcttgcgtatgttgactatgtttttggaaatga

gactgaagcaagaacatattccaaagtgcatggctgggagactgacaatgttgaggagat

agctataaagctatcccagttgccaaaagcaaatgaagctcgtaaaaggattactgttat

cacacagggcgctgatcccgtgtgtgttgctaaagacgggaaagtgacattgtaccctgt

aattctattacctaaagagaaattagttgatacaaatggagcaggagatgcttttgttgg

aggattccttgcacaattggttcaggagaagcctattgaagaatgtgtgagagctgggtc

ttatgcagcaaatgttgtcattcaaaggtccggctgcacttacccagagaagcctgattt

tcattgagtgattctcctgagttcatcattattattattcttattattatgaagttttga

ttattgggaatgattttagttgcattttaatagttacactcttaactggtttgggatttt

tattaagtcctggctaaggagaaggggtgaaaaagttgcagagggaatcattactgaaat

aagttcatgtatttgaagtcctatctggcagcatttctgagtttttattgtgcctcattt

gaagtgtttttccttttcttttttggttactatttagctatatgatgtcctatcaggtca

tcgtttataattttgctgtttgtgatcttagctttctttacctatctatttttgttgtga

aaactttttcttgcatctttctttagtatcatgtagggctggaaatgagttgagtcgaac

tcgtctcagttcagttcgactcattaagaattagtcgagtttgagtttatcacgaatttt

tttttagcnnnnnnnnnnnnnnnnnnnnnnngatttgagttagtttatcatg

>fava_dn_rep_c946

aatctatcaaaatcactatatataaaaccccaaaaccctncgcggggatcactctcatac

ctaaatcttaacctaacctctccaaaaaatactcttcttcttcatcgatcatgtcgcact

tcaacagcaagtaccatgatgagcttattgccaatgctgcctacattggcacacctggaa

agggtattcttgctgctgatgagtcaactggaacaatcggaaagcgtctagcgagcatca

atgttgagaatgttgaatccaacaggcaggctcttagggagcttcttttcactgctcctg

gtgttcttcagtaccttagtggagttattctctttgaggaaaccctttaccaaagcactg

ctgcaggcaagccttttgttgatgtcttgaacgaagctggtgttcttcctggtatcaagg

ttgacaagggtaccgttgaactagctggaactgatggagaaaccaccactcagggtcttg

acggtcttggtgcacgctgtgctaagtactacgaagccggtgcacgttttgctaaatggc

gtgctgtccttaaaatcggtgccaacgaaccatcagagcactctatccatgagaacgctt

acggattggcccgttatgccgttatctgccaagagaacggacttgtccccattgttgaac

ccgagatccttgttgacggttcacatgatattctcaaatgtgctgccattaccgagcgtg

tccttgccgcaacttacaaggccttgagtgaccaccatgtcatccttgaaggaactctct

tgaagccaaacatggtgaccccaggatccgacgcaccaaaggttgcacccgaggttatcg

ccgagcacactgttagagctttgcagagaaccgtccctgccgcagttccagctgttgttt

tcttgtccggtggacagagtgaagaagaggccagtgttaacctcaatgccatcaaccaaa

tcaaaggtaagaagccatggactctttcattctcttttggaagggcacttcaacagagta

cccttaaggcatggggtggaaagacagaaaatgtgaaggcagctcaagatgctttgttga

caagggctaaggctaattctgaggccactcttggaacttacaagggtgcctctaaccttg

gtgctggtgcctcagagagtcttcatgttaaggactacaagtattgatcaatgttggttt

cttctcccttaaagactagtagagggctgaatttcttgtttttgagtatagtcggttcac

tcacgacaaaataatcggttggagtttttgatttttctttgtaccttttgataatttctg

tttccatctgtttggtcaattatctggcggatttgtctagaaaagtttggtttcacgaga

cactattataagtgaagatctttgttttctttgcaattatctatctagctttgttttctt

tgcaattatctatctagctttgttttcttcacattttaataaaattatgcttttctattg

tgtaaaaaaaaaa

>fava_dn_rep_c947

agaaaatataacgttatatatttatctttgatgtttgacctcttatcaatcatcaatata

cagctggttctgaatcaagcgaagctaatgtattctcttccagagaaaaaccatataggt

ttattgcaacaactttttttattaaatatatggttacacgtacaaagataaaatcatagt

catcatcatctacatatacgagcgagcaatcacacaaaacaataatgagaaataaataaa

taaaactttgtaaccgactaaagtaagaattcaatagaattaaaaatatgtatagagaaa

gaaagacatctaagctaacagcacggagacgaagattccagctaatgaagtaacggcaac

gaaggttttggtaatagagaatgcagcagagggtgcctcggctcctcctgggcctgatgc

tgggccatttgtggttggtgccggcggagaagaagtgtttggggttggtgatgaaggaga

gggtgctggagaaggagttgttgtaggagaaggtgttggtgatgtgtttgtggaggaggg

tgatggagcaggtgctgtattaggtgataatgatcgggtaggtgcgggtgcaggagcttg

ttgtgctatgcatgaggtagctaacaaccccagtatcacaaacactttaattgcaacaga

acccatcttttatatgtgttttttgatacagaagaatgttgagtcaaatatgattcttcc

aaatatatggtttgtatgataattgatttgtgtccccgggagatgtaggcagattagaga

aaaattgaacgaaaataggtttggattcagactaaggatgataagatgatgaaaatggag

gtgagaagtggaagaacggtgaggtctcaccgtgcccgatacggcgaggttcgtttctgg

cgaaatgatttggggatgaaggttgagtgaacttgagagagacgaagagtcattattttt

ctctctttttcatctgaagcaatgcga

>fava_dn_rep_c948

ccgccccccttccttcatactactcctatatctaataaaaaaatatctaccaagcaaagt

ctaaaaatatactatagagcaagataaccattaaatggttttaggagaaccttttcctat

aaaataagcgatgaaaaatgggaagtaattgtgttctagagttactgaacgcttaaattg

ctcaatttcgttctaaatattgaaaatgtatagaaaatgtaaaccacatcgaaatacata

tgtccttagaaaagctctttgcataaccaagttcatgtctccatatcacttgctcttctt

tggttctttagtatattgagctatcagagtttcaacatccttccaaaagaggccttcaac

taagactccatccttagtgaaccactcagtaacacttttggtaagatttacgggttcatt

tgcagaaattgattttggatctgcactttctaccgtaagtgtgtaaatgtcagaaaatct

gggtaatttggaggaaactaccaaaccagtgctggtaaaggaaccagccagaggataagt

aaagagaatggcgttcttctccttagtatatatgatcagctgcaacaacccgttgaagat

tacatacaaggcgatgcaggcaagaagaaaatccctattctcaggaaacttcttcttgta

aaactgagcaaacagagcaatgacaataataacagacccaatcaacagcctcacattgct

caatctcacgtcttcagaatacccacgtcccttaaccacctcagaaacagactcatcgag

aatgtgcttgatggagtgatgatctaagagattggctttcttctgtgatttggatgccat

tgaaatgttcgaaaagacttcacttgactcaaatgagaattgcgtatttggtggtgaact

tgcactgtgaataaagcttcgattttatgtgtgcgtattgcgcactcttcttcttcttgt

tgttacttttttttattacagaaaatt

>fava_dn_rep_c949

gagaataagtgcgagaaatctaatatcataaaaacaaaaaagactaaaccgatgtatata

ctattttacaataaaatgcaataataatttcattacacaacaactcaaacacttgttatg

aaaacaatcatattaaagcaaagcagtctaaaaccctaatcactttaatttccataaaac

aaaagcaaaacaaattagctcactcactcaacttgaaaataaagtaaaaacattgatgca

cccttaaatcaactaatacttatgctcctcaatggttcttctggtattaattctgaattt

ggattgtcctttgttaatcccgtaggagtcgtttctgctgctatttctgctctcggtttg

tataccgatctaagaaagttataagcaagtgctccagctacagcgccaataattggacca

actacatatatccataaacctttgtaaatatgtatcacaattgcaggtccaatacttctc

gctggattcatcgatgcacccgaaacaggccctgctataaatagattcaacgttatcgtc

attccaactgcaatactcgcggaatcatccactgctcgatcatccgtagtaacagctgaa

ataacaaacattaacagaaaactgataatgaactccatagctaaagattgaccactcgat

ccaactggcacagttccgaaaaatgattttggcgtaatatcaaacattaaagacagtgtt

aaactcgcaagtgttgaaccgagtaactcagcagcaatgtaaagtggagcttctatgatt

ctgattcggcgaaagatggcccaagttatggtaaccgcagggttgaagagtcctccggag

atatgaccaactgagtaacacatgactgttacaaccaaaccccaagtgatgcatatacct

ggaaatgttacggagccatagattttgtccactgctacaacaccacaacctgcaaataca

agaaaatatgttccaataacttctgctataaccttttgtattaaggttattgcacggttt

gatgaacaacaaagtttcaagatggaatggtttccttgatgtatgattgccattgatatt

atgcttgtttcttaactcacatgtaatttagtgagataggtgtgttgtatatatatcagt

gggaagttacaacaagtgtg

>fava_dn_rep_c950

cacataacaaaaaagagaggtcgatagtgtatgactggttgtttgtgtgtaactaactag

aatggctctgactctgagagttgcgaatgtaaacctagggtttgagtcatgttcccgttc

atcttttgtttctctcaattcctccacatcccgtagcttcgtgtttcggtgctctcaagt

tcagcaattgtcaattactcaagaatctgagaattcactcatggaagctctcgttggcat

ccaaggacgaggacgttcttcttcttctccaaatcaactcaatgctattgatcgtgctat

tcaagttcttgaaagtttagggggtgtatctgatcctacaaactcaagtttgattgaggg

tcgctggcagctcatttttactacaagacctggaacagcatcgcctattcaggcagcagc

atccatcaaagatggaaaaagaatcctttttaggtttgacagagctgccttttcttttaa

atttcttccatttaaagttccatatccagttccatttaagctacttggagatgaagcaaa

gggctggttagacaccacatacttgtcgcattccggaaacctacgcatctcaagaggaaa

taagggaaccacatttgtgctgcagaagcaaactcaacctagacaaaagttattaaccgc

catttcgtcgggggtgcgtgttagagaggcaatagatgaactcatttccttaaataagaa

atctgaggataaagagccagaactagaagagggtgagtggcaaatgatatggaattcaca

gactgtgacggatagttggttagagaatgctgccaatggtctgatgggaaagcagattgt

tgggaagaacggacgaataaagtatgtggttgatatcttgcttggactgaagttctccat

ggctggaaattttgtaaaaagtggcccaaaagtgtacgaggttacaatggatgatgcagc

cataattggtggcccatttggatatcccctagagtttggcaagaagttcatcttggagat

tttatccagtgatgagaaggtcagaattagccgtggagacaatggaattatttttgtaca

tattcgcacggacgcatcgcggtgatcgaattagtaatgattttatcatgggagtcctcg

aagaagattggaccattcctgttaaccacatttgattgctatctatatatacaaatgata

ttattaatggtaatggccataaagaaattgaattcttcaatatctcgttgttcgtaaaaa

caaatgaatgccaaggcaccgtctcaactcaagtagttggcccaaaaacattcccat

>fava_dn_rep_c951

tttttttgcaaaacaagttttcttttgatttggtaactttaaatgccaaaaactttgaga

tccttacatcatgactattggaatatagctatcttttcatttacacgcaaaggtttacca

aaaggcaataacatgatttgatttattcaagtcggtgaatgtttcaaaaacaagagtatt

taaaagaaagaacaaacatatataggagtaaatttcatttcatattaaataaataggaat

aacatctagataaaagtaaagtaaacttaataatcgaaatttcaacaacaccaaccaata

acatgcataaaactagacctctttaacaccaccaacagttccattgactttcttaatctc

tttcacatcacttgctttctcttcaacttcaaaagcagaaactgggaaggttctagaaag

tccagcaggtgtttggaaagtgattttaccagtaggtggatcatcaacatagatatcact

aagtgtaatccaaatgaaaagctcttttgttttaacaccattcagtttcttaatcttacc

tttttcaacatgagctgtaacttcagtagcataagaaacaagttttccaatcttttcaaa

cttgtgtgttatgctattcttctgtttcaaccaaacaaaacctgtttcttcgttgtaacc

aacttcagtgatgtctttcaatggtaagagaccattggggagagtaacttctcgaagaag

ctctttggttttctgttggcagatttggtctccatggtatacctctgatttcgatttgat

ttcttctgttaccaacgacattgatttgagttactgttattcgaaggaaggtgatgttac

tttttgagttgttttggtgtgtgcccgcggccat

>fava_dn_rep_c952

tgtgttgaatatatgagcccataaaacaataccacgtgtcaagagaatagtgaaggcaca

gaacctgggtttaccaaccacccacccattcactttcagttatccatttccaattccata

tacacaatcccagtcctgcaacaactactttttagttttctccgccaatctaaatccctt

attcccctgaaaatggcgttgccggtggtggttgacgcagaatacctcaagcagatcgaa

aaagctcgccgcgatctccgagcactcatcgccaacagaaactgcgctcctctcatgctc

cgcttagcttggcacgatgcgggaacttacgacgccatctcgaagaccggtggacctaac

ggctctatccggaacgaggaagagtattctcatggcgctaacaatggtttgaagaaagct

attgatttctgtgaggaagtgaaggcaaagcatcctagaatctcatatgcagacctttat

cagcttgccggtgttgttgcagttgaggttactgggggtcccacagtcaactttgttccc

ggaagaagggattcaaaaatatgtaccagagacgggcggcttcccgatgctaaaaaaggt

gagtcgcatctccgtgatatcttttatcgtatgggcttgactgacaaggatattgttgcg

ctgtctggggcacatacactgggaagagcacatccggagagatcagggtttgatggcgct

tggacagaggaccctctgaaatttgataactcatactttgagatacttttggaagaagat

tctgcagggctgcttaaacttccaacggacagggctttagtggatgatcctgaatttcgc

cgttatgttgagctctatgcgaaggacgaggatgcattttttcgagattatgctgaatca

cataagaaactttcagagcttggctttgtgccaagctcaaaggcgaatgctcctaaggat

gcgaccattctggcacaaagtgctgttggagttgtagttgctgctgcagcggtcatcctc

agttacttgtatgaacattccagaagagggaagtagacttgatttgctaagctctgttgg

atgtcacgtttgtgtggcccataatgagtagtattgggttcatttttgcaatttcatcct

atgttttcttagtaccaataagaaaaatgctaagctctacagctattgggtatatgaata

gactataaattcaactgaatactccatttaagagcagcatcagtattactttgtacatat

aaatttcagtagtctcagttatgtgctccttttctcttgaagatcagaaaaggcgtgtag

ttaagtgtagttaaatcgttgttagtttatgttatttgcaagaaattcagaatttgaaaa

catggattatacactagaagaattactatacgcctgaggatcaatgatacttatatattc

gacgtgct

>fava_dn_rep_c953

tactacaacaattattctgaacaagcaatgttgcctcacacgcacaccctaactccaccc

acatcaacacctttcaccaacannnnnnnnnnnnnnttttttttttttagaaaaatacaa

acatagcatataaacaatttgtcaaatctatggattcacatatacaaatacatatagata

tattcatcataatgcttctaatacaataatcatccacctccaacccctttctttatcaac

tccctaaactcatcatcagtacttttaagcatcaatgacaaaggcaaattgaccccacta

ttaacaacctccatgtatctaactttgatcctattctccaaactaaaagagaaatactga

ggaaactctctcaactcctccattttccctcccatttccgcagaaaaatactcaaacttt

ggctgaaaattattctcaatactaaaagtcaacaatgtaggacacctcaaaaccatacac

ctaacctcttctttactaaatcccatactctccaaatgcttaagcttaggtattagtgtc

ctctcaacattggaaaccaaaagcacacaatcttgataagccaaagcctctaaatctctt

aaccctaatctccttaaataaaacaaagctggtttaagctgatccaccacactagaagta

agcaaccttggacacttgttcacaacttttctaaagctatgatcaggcactttgagatca

tgcacaagaaaatcaaaaactgggctgagatcagttttgatgctcgaagtgagaatcttt

ggacacatgccaaagattctaggaaggtctttatgttggattcctttggagacaaggaag

gagataatggagtgaatggattctaaggaagcagtgtgaagggtagggttttcggaaagt

gctttacctgaatcaatgcccattacttcaagacataggattttttctttgaattgaagg

gggagtttttcatgagttggtgtgtaaagagggtggttttggaggagacttttgggtttg

ggagttaaatgggttgggtttggtttgggaattgatgaaggtggtggtggtgatggtgat

gatagtttatctgatgagactgtgcatagtgaagatgagtgtagagctatggttctaaca

gatgggaacatggtggtgctgaggatgtatccccgcggtgctacacttgaaccagctgaa

tgccctactaaaattactttctcagcttttaataagtcaaagaagtgcaaggtagcaagc

actgaaaatgccatggagtaagcatttagaggtttagcatctcctgttgcagaagataag

ttgaccctagatgttaatccaaacgctggcctatcaaaagctaaaactttcgaacatgta

gcctcagccaaaggcttcataacttttttccaagagaaaaccgaggctccaaatccatgt

aacagaatcataggcaaacacatcttgttggtttgttccagggaattggattcagaatca

tatatcttgtggtgtatatgcactcctttaaactcacaaaaacaactgtctggatcagcc

aaaagcttcggatccactaattcatcttggtctattccagctatctctctccttttcttc

ttcaatcccacatccaacaactgctctgaaccagagacggaagaagcgaaagcagtgaag

gtgctgttgttgtgatgagtagcataataatttggaagccatgaagaagcgaaaagcttt

agacgagaagaacggttattatatgaatgtgtagaagtaggagtaggaggtgtgacacta

caattcaccgcgattctcttcataatctcaaaatcgcttctcagcttctcttcgcttctc

agcttaagcttaatgctctgttaacgcattcagacagtgccttcttctgt

>fava_dn_rep_c954

gcaaaagactaatttgacttgatttaaattaaaagttgaaaagaatgtaaattccaacat

ttcaaggcatctactgttaaacaagtgagaaaacacttgtctaggctaattatttttatg

gaatttccatttcaatagaaagaatttgtaacgctaatgcatagctgcaatggtatcaat

caaatcatggattacatttcagaggcaataatacacataactagcacagggcaataaatt

tatgcaaaactcagcacaccaaagctcgtgactaaatggaaccaaacaaaactaatacat

taaataaattataaacaaaaacaatgaaaatttcttttataaaggaaccccaattgagtc

cataacaaacaaattcaaattcaaaagaagaaaacaccacattactattgaagatattaa

taagtaataataactgtaaataaaatttcccaaaccccatacatgaaacaaaaataattt

acaacccaaatgaacaaaataaccctatataaaagaatataagtaacccacccaataact

ttcacaacagtgttataatccttataatgaatacaaaacaacacccttagtattcttctt

caccttggtatcatcattctctttgttaatgaagaattcagacttttttctttgaagccg

tttttactcagagatgggtatggtcttggacctgtaaagaagcttctttttctttgatgt

ttggttatcaatggtgagtacaactttgccaggttcaccgattttgaaggtgttgttgat

tactgtttcatcagctggcgagattttcctgtttttctgtactatcactgtgtatccatc

ttcagcactaggcacaaattctgctccatagctcacattccagtccaccactctgatttc

ccataccaaggtgcttttctcagaaattgggaactcaacagcatgttttgttgctggttt

gataataacctctgtagcagggtcagcagtggtgaattcctgttcaccctctctgctaag

tcctccatattgaactggcacttgctgaggagctatatatttgaaaagggtatcagcaga

tttggaggagccagcaaatacaaatttgctcttggtcctctgtgtcaggaaaggactaat

catcctagaaaaggcaaggtaccaccaaggaacattgataaaaatctgtttggcaacaaa

ttcgggatagttatcctgaagcaattgaagtgcctgattagtagcttgtctaagttcctt

cttaacgaatccaggagaatttttaagatcattaatttgaacaatagtagcgataccaga

tggagcaaagtcgagctttctaacacttttctccaaagactgaatcctccaacggataaa

cttgtttctcttctcatcatcagagaaactcttttgatacaattccttgttctcaaactc

tccaaaaacattgtagtaaaccgggtgaccttctttgtcataaccatccgtgaaaacaac

tttgtcccaatcagttccaagatcttcttgaagaagtgcttcaattccaaattcctttct

ccaaagaacagtttgtttgatcattgtgaaagcttccttcaccttgaaatccctggctcg

aaggaacttgagaagaatcacatcacttctttcatcagctagcaagggaattccccatat

ttcaacttcttctggtgtagaaggaggaacctccacatcaacagcaacaggctgttcacc

atcaccgttcgtaaccgcaacagatactatgctttcctgaatagcttcaaccgttttcgc

accgtcttcttcagtgctcgaagctattttctcaacaacttccaccacagtctcctcgac

tttcttggcttcaggtgcttcaggttcggttttaggttcctcggaagcagaagctttttc

ttctccaactactactgtttcttccttcttctcttctgttacttgttcctttttctcttc

ggttttctcctcttcttcttctggttttttctcttcttgtgcgattacttcaggtgttac

cggcttagcaggaggagcggtgaactcgtgtttgttaagtgcttcctgaataagctgttt

gagttcatcgagtgcttttttctgaagatcaggaagttcagaaaccacgttagtttcttc

cttgaaagaaaccgattgagaaattttgttatcagcgacagtttcatcagcaggcttagc

agtctccgccacagcttcagccggcttctcgggatcttcagcaaccggagtaacgtcctt

gtcagtggtggcagcggtggcagcaacaacttcttcggcgggtggtggtggttgtttgac

gggagcttcagaggtggttgtgagcacttgatcagctgggttttggggttcttgagccat

tagagtgaagattgagatgaattagggtattggaatgaaagagagatataaaagggagag

agtggggttggtttgtactctgcttatcttgttgctgagaaggtgattgtgaccaccaag

cataatgatgatgaacagtatgtgtgggagtctcaagctggtgggtctttcactgttacc

agggatacatctggtgaggttcttggaaggggtactaagatcaccctttaccttaaggag

gatcagcttgagtatcttgaggagcgccgattgaaggatttggttaagaagcattctgag

tttatcagttacccaatttctctatggattgagaagactattgagaaggagatttctgac

gatgaggatgaggaggataagaaggatgaggagggtaaagtagaggaagttgatgaagag

aaggagaaagaggagaaaaagaagaagaccattaaggaggtgtctcatgaatgggatttg

gtgaacaagcaaaagcctatttggatgaggaagccagaagagattggaaaggaagaatac

tctgctttctacaagagtcttacaaatgattgggaagagcatttggctgtcaagcacttc

tctgttgagggtcagcttgagtttaaggctgttctctttgtacccaagagggcacccttt

gatctttttgacaccaagaagaagcccaacaacatcaagctttatgtccgtagggtcttt

atcatggacaactgtgaagagttgattcctgagtatcttgcctttgttaagggtattgtt

gattctgaggatcttcctcttaacatttcaagagaaacattgcagcagaacaagatcttg

aaggttatccgtaagaacttggttaaaaagtgccttgaacttttctttgaaattgctgag

aacaaggaggattacaacaagttttatgaagctttctctaaaaatttgaagcttggtatc

cacgaggattcccagaacaagggaaagattgctgaacttttaagataccactccaccaag

agcggtgatgagcagaccagtctcaaggactacgtcacaaggatgaaggaaggccagagt

gacatctactacattactggtgaaagtaagaaggctgttgagaactctcctttcttggag

aagttgaagaagaagggatatgaagttctgtatatggttgatgctattgatgagtatgct

gttggccaactcaaggaatttgagggaaagaagttggtatctgccaccaaggaaggtttg

aaacttgaagaaactgaagatgagaagaagaaacaggaagaatcaaaggagaagtttgac

aatctctgcaaggtgattaaggaggttttgggtgacaaggttgagaaggttattgtttct

gaccgtgttgttgattcaccatgctgtcttgtgactggtgaatatggctggactgctaac

atggaaaggatcatgaaagctcaggctttgagagacagcagcatggctggttacatgtca

agcaagaaaacaatggagatcaacccagagaactccatcatggaggagctgaggaaacgt

gctgatgctgaccgcaacgacaagtctgtgaaggaccttgttctcttgctctttgagact

gctctcctcacttctgggttcagccttgacgagccaaacacctttggaaacaggatccac

agaatgttgaagcttggattgagcattgatgatgatgcagctgaagctgatgctgatatg

ccaccgttggaggaagctgatgctgatgctgagggaagcaagatggaggaggttgattaa

gttgctgttttaattttttagtatttttttgagggaattaagcttttttttgttgatgac

tcttagatttttttacctttttggttatgtttttgctgttttccgtgcactattggcgtt

attaattaaatgcaagatgggcatacttttatggattatattagtttgttgcacgatcat

gtttcgttgaatcttgattaaatttttcctatcttgaatgcctgaatatatgatatactt

tggcgtgcaagtgatatgtgaagttttataatatttattggaaaggaaatattaactaaa

aaaa

>fava_dn_rep_c955

ctacttttgaaaactacatgtgagcaaatctatgtcatgtaacattcaaaaaatgtgagc

aaatataccttcagacaattaagtcaatcatctaagaaacattaaacacaactgtgaaat

ccaaattacacaacagaagcccaaattaaactaaaagggattttagtatttttgctccgc

ttctagaatttttaaaagtaaccaaaaataaaaaaattgaaataactaactccctcccat

ccaacagaaacaaccaaaacactgtataccatttcataacatctgatatacaacactcat

atagtcctacccaaacacagctataccatttgtaagataatcaaacagaacaaccaaaac

ctctacaatgatgtaaacctaaacccttcatagaaatatccaagttaatttgcaactggc

aaattaatttaaaacagattatgaacattcctatgcataacacaaatttcaaatggtata

accaacaaatctgctaatggccatattctatagtcataaaataaaataaatcataaactg

tgaaacaccatatccaaatctcatagcaactcaaaaacaatcatatagcggcatcaaata

caaaaacaaaaccaagatcacaaaccctagagttcaaaaatttcaaaaacgtaacaaaga

actaaacgatcaaacttgatctaaagatcttgaacagtcaaaccgggaggaagagattgc

ttcaacttatcagctttgtcagtatcaaacacacaaagtgtgtacaaatacttcgagcaa

cgaaccttgaacttcaccacatccttactcctcttgattttcactgatctcgcatccttt

ctacgcgccgtcaacagaaaatccttgatctcgtggatctgcttcgccattgattggttg

tagtcctccaaaagggttcttcctcgcctcctaagaaatccccctcttttttttttcctc

cgcttattgatatgcttaaattcagcgggtagccccgcctgacctgaggtctccccgcgg

ccgtaatggcaactctgcgttgataccttttttttaaccggaatcattctgcgtatatta

aatgaggtagataaatataatcatttattcattaaaaaaacataaaacttaagacttgga

attaatcaaccatttctatatggggattaaggacgaggcagccccgtccttgtccttccc

aaattcttcatctgcaagctctccatgtttctccttgcagtaaatgtacaacaccgtaag

aaccacaatattacaaagcgaaaccgtagcaattattggagaaatcactaaaattagtac

caagctataacgagttatccacaccaacaccaattgcaatgacccaaacaagaaagagat

tgacaagatcaacctcttcattcctctcaccaatttccaacttcttctcaatggttcaaa

accccaaactgattccaccaccacgatagctttcactaagctcaagttaattaacaaata

caccacaagagggagtaaaacaagcattaaaaccatgtaaataaacatagggtgagttgc

gaggttgatatctccaagataagttatgagaatcaagacaactccaatcagaagtgttag

gaggaaaaagatgaagaagatgatgacgaatgtaacgatgtcggtggcgagtaacgggaa

gaaggaagtggagatagatttgatggcttctttgagtttaatgggttgattaaaatagaa

gtggtagacgctgtaagtgatggagataaaggctccgtgtgacaagattgaagagaaaag

gaggaagaggagagacaaggaggtgatgttggtagggtttgtggggggttgttgttgttg

aaggtgtttgagaacgaggttgtagacgagggaaaagaaagagagaggtaggaggaagat

gagggagagagtgaggtagtgacgaggttgagtatatattatgcgtttggattcggagag

aacatcccagatttttggagccatggtcatcatggtagtaaaagctaggtgaagaagaga

ttattgatgcagcccgaagtgggattgatgtggatctatatata

>fava_dn_rep_c956

tttttttcgaaaaggcaaatgaacattttttattcacagggtcacacattcacatttcaa

acgctacaacaaaagtgtcgcaattttttatttccatttcataacaaatatcttatcatt

attaatattattattattatgacaacgataacttataacatttcattcaagtttgtacac

acgcattttcagaagcatcatcatgcttggcaacacagaacaaccacatgagttgggtgt

tactaacatattgtagcaagtacctctaataaggaagcagcaaattgcatttgggtttct

tcatttatcgtaaagaaaagcggcgtgtgcacgaaaagggatttgataccgttctgctcc

gcgaaacgaagggaatgatagtaaacataattgcacacaaaccttcctgcatcatcagat

gtcattacatcataacctttatttgccaaggctttagtgatctcctccacaggaagggta

gactcccgtatatgtgaaactgtgccatctgaaggaacaattggaactttctgtggcttc

catcctagctcatcagggcaacgaaatgtagcctcgttaacagcttgacgctctatagcg

aaccttgttgcaccgctgttaaccccaaaatgcagccagataattttattggaactcgaa

gattcagagtccttggcagcaatggcagtttgcaatgtctggtacagtggaatgaggcct

ccttgtccagcagtatcaagaatgctgcagctcccaataactaaacctttcggcaaaccc

ttcgttttcacatattccaccaaattattcacaaccgtctccgttggattatgtgaaact

ccgtggaatttcttgaaacctgttatataaactgttgttacagccgcagaaggaccttca

gatcccatttgctgatgatggtctattaatattcaaagcttcaaccagaaagtattcgat

aaaataccagaaaacaggaagaagaaaacgttcaagttgaataaaagagaagatgatgac

acaccacaccgttgttgagtcttatctactcgaagcaaattaactggtgaagattccgat

tagatttagaaagaaaaaaactatataaataaagaaatataccaaaggaaagtgcgtgtc

gttttcattcataaaataaagaaagcactgttatggtaattcgttcgtgttcttgttctt

ccaaatgtgcgttacctctgtaaatattctttctagtaactgtttatca

>fava_dn_rep_c957

aaatggaagagaaaagaagaagtgtggagggaaactgaagggtttcaagtaaagtagtga

attgatctttagtatacgtgacaatctgacattgaattagcgctggaaatattagggaga

ttatgtttttacgggtcccaaacataaatctttattttaattccagattccaaaatagtg

ggactaaaacccaaaaaaaatgataataggaggaccaaaattacaattagaaagacaaaa

tgggaaaaatgggaagagttgtttatagatagatcatcaatacttgcacctaatttctaa

ttttcaaaatcagatagatcatcaatacttgcacctaatttctaattttcaaaatcagat

agatcatcaatacttgcacctaattttcaaaattttctagttttcattgactttgaagaa

gcttggagatgcatacaatagagtacctagaagataacctaccatagaatacctagaaaa

aaggtgttcgactttatttatcttaaaaagaaatgcaaaaagtcccaataatgtgaatca

tgtttgaggcaggtttctaagaactgaatccatcaacaactcatttcatgaaataaaagt

tttacacttcaatatcaaacttgaagctctgatttattccacaattcattgccggatgga

tcaattaaaggctgaatgtgaacaattccatgaagatattctcaattttctttagaagca

attgccctcttggcaaaagatggcaaacagaaagctgctgaatgaatctcacggttgtaa

aatttcaatggtcttgccacctgctggcaatcattctcatcaataggattcaccggatgc

ttgaaatcaacggaaggcccctcggttgagcaaagcataaaaccaatcatcccacttggg

tatgtaggaacagtggtccaagcatagttgacagaccctttgaatacctggcgacagttc

accacaatgtcctcaatgatgtgcatatgaagccatatactttccgcttgagtacacaca

actcctcctggacgaagagcccttgcaaccgactcaaaaaagggcttttcaaaaagctct

tgagcaggaccaataggatcagatgaatccacgataactgcatcataagttccttctgga

gctgccttcaaaaatgcaactccatcaccaatgttaagtgtcacacgtggatccgcaaaa

cctactgctatatcagggaaaaattctttggagacatcaacaaccatcttgtctatttcg

caaatgtctatcttttcaactgaagagtggcgtgcaacttccctcaggactcccccgtct

cctccaccaataaccaaaacctttttggggtttggaatagagcaaagaggaagatgagtg

atcatctcttggtaggcacactcatctctttctgtgagctgaatgactccatccaaaata

agaactttgccatatgttgctgactggaagaccataacatcctgataatcagactttcct

tgaaacaaaattttttccactttcaaggagtgtgcctcccctggccacattgggctgatt

tcggagaaccagccagggataacagatgataaaccattaggttgaggctctttggaaaga

gtatcggagacaccgtttacttcgtcttctcttggccgctttaagggagaatctgtggag

gggagtgtggtttctggtgccgccatgtttgcaatgagtaggtaagttactcgccccgcg

gcgta

>fava_dn_rep_c958

atcacttcacatgaaggataaatacacaacatacattagaaagcggtgaggcaagagaaa

atacacaacatagaagagaggaataacctcaaactctgtctcagttttttctgagagaga

taactcctgcaaagtacatagagcaaagtttgcaaatcacaaacattttctgaacaaagt

ttgcaaatctaacaacaaacaagaagatctgtctttgttaacacatacataactttcttc

tctaaatccaaatttgtttcatcataagtttctgcgaaagattgtctttcttttgctgca

tagatcttaaatttggaagcaaccctttaagactttggaatggagacagaagagaggaac

cagagagcgtgcaaggcaacagaaccagagttcttcttgcagtggggaagcaggaagcgt

ctgagatgcgtgagggtgaaagatccgcggatttcaactaggctcaacggtggtgtcaga

cggaaacttaactccggagcggataataatcgctccggtgttaccgagaaagaaacttca

cataatcatcaccaacaacaacatcacaaccgtctcacaaggaattccgacgcggcgatt

ctgcgaccggcggccggagatagtaggaaatcggcatcgccggagaaggaagatcggtac

tacacgacgagggggtcggcggaggaaaacggtaaagttactggcgatgttaacaacggt

gaagagagagctcttgtttggcctaagctttacatcgctctctctagcaaagagaaggaa

gaagattttcttgctatgaaaggttgtaagcttcctcagagacctaaaaagagggccaaa

atcatccaaagaagcttattgttggtgagtcctggagcatggttgactgatatgtgtcaa

gaaagatatgaagttagggagaagaaaagtaacaagaagcggccaagaggattgaaggct

atgggaagtgtggaaagtgattctgaatgatgcaaacatttaagaggaagatggatctgt

aacaacaacacacaaaatctaaaatattttggaagatggaagaagatagtattttgagtt

gatgatgatgattcaagtttttttaaatttctttctcacaatgtttttgtaaacaagtaa

gatctgatctgatccatttcaatcaatgaagaaaattattttctcaaaaaaaaaa

>fava_dn_rep_c959

cgcggggataacaaaaagccagagattcctctgtagctatctatcaacaaaaccgtgttt

ttcttcttcttcttcttccttcaaagcttttcattatcatgtcttcactttcagatctca

tcaatttcaatctttcagattccactgaaaaaatcattgctgaatacatatgggttggtg

gatctggcatagacattagaagcaaagctaggactcttcctggaccagttagtgaccctg

caaaactacctaagtggaactatgatgggtctagcacagaccaagcaccaggaaaagata

gtgaagttattttatatccacaagctattttcaaggacccattcaggagagggaataata

ttcttgtgatttgtgatgtttacactccagctggagaaccattaccaacaaacaagagat

acaatgctgccaaaattttcagtcaccctgatgttgctgctgaagaaccatggtatggta

ttgaacaagagtatactttattgcaaaaagatatcaattggccattaggttggcctattg

gtggatttccaggaaaacagggtccatattactgtggaactggtgctgataaagcttatg

gacgtgacattgttgatgcacattacaaagcatgtatttttgctggaattaacattagtg

gcatcaatgcagagtggccattacggccgcgggaccattcaaacagctatcatcaaaact

tgtgtgaattgatactttcccttcctccaacaagatgcaaaacaggaagatcaatggata

tccacaacagcaatactatgatgatgatagcacatggatggaaactaaggctcaacagct

acagagccatggatatccacagacacagcaatatccaggcataaaaccaggttatggcaa

tgattctgattactctatgccaaaccatcatggctatgagtctaacaacaatatgtgtca

tggtcaggacagcaagcctcacggccttgactctaaccatgatgtgtatcatcgtcagga

cagcaagcctcatggccttgactctaaccatgatgtgtatcaccgtcaggacagcaagcc

tcatggccttgactctaaccatgatgtgtatcatcgtcaggataaggttcctcatggaca

gggaaatggttatggctatgataacaatcatgggcatggcaatggcaatggccagatatt

tccgtttgctgctaccgctaatcactcacctcatcatggtaatggtgtaaggccattcaa

tcatggtggccgtggccaaaataactacgtgtcagagaatgagtatgaagtttacaagga

ggagcgtgttggtcctggcgtgacgaggagggatgaagtaagatatgatgagaggcgtgg

aacatatggaggagatgttcatcaggccagcccatatggatacaacaataacaggaataa

tcctcttggtcatggaacaaacaaagccaattggacactgaaaggagtttaatatgcttc

attactgttattactacatatacctaccgtactaataaattgtgtgctttgttttctgtt

ttcttgttttcttgttgtaataagttgccatgagtatgcatggcgcttcatatatgtgat

cgatgttatctgtaactcattatgaagttataataagagttaaattagactaaaaaaaaa

aaaatacaagaaaataggttgaatgaaataacaactattataagcataagcattacttag

aaaataggttgaatgaaatagcaactattataaataaggcttaaccgcttccaccaagga

cactcttgagtttcttgataagaacaggatcaaggaaagtggtctttgcaatcaaaggag

tagagaaattgcttgcaaaaagtgcaaaatcaagaatctgaagaccaggactagcactac

taaaaacaggaaaagcaacagcatttctcttaccagcagtagcttgaaaatgcaacaaac

cttgtggaaaaaccatgagctcacctttcttcaatgttttaatataaacagtgttagctg

aagaaacaaatccagcagtgatgtgaccttgtgtcacaataagaagttcacttgcacccg

ggtgactgtgtagagggataacacctgctggaccaaggtctaacctagctgctgagagac

caagaccattaaggcctggaaattgtgcgacgaatgcaggcgtaacggcagcgttgatta

tgtttgttacgtttgctggctcaattaggcctgaatatgcaaagtcttccacggttactg

ttgtggctgacatgcaagggaagcctgaaggggtgtctgagcctttgagatctgctacac

agaagtcttggactgaggcattggaaatggagaggaaaagtgagaggaggaaaatgattt

gaaacatgtttggttgtgtttgatgaattgttggtttgagtgtcaaagagatagaggctg

tttattttgcttcagaatctggtttaactaaaagatttcaaccaatcaagctactcgagt

cttatgttcggaactacaaaagtaatgcagctagcatattggcgaatggaaataacagtc

acgctgccacagatgagtcaactactttggagttgaattcaattaaggatgtaatcaaat

gtgtggaagatcacaaacttgaatcagaatttaggctcgatgaagtgaaaaagcgggcct

cccagttggagaaaagcagggctgagcgaaggaagacttcaatatttggaagttcaagat

catttgtaagtggaagtggagttggaagtggaagtaaatctcgaaagcgtggcagtcgag

ggagtggatcaagctctgctcgtccagccaaatcagtcagaactagtgtatacccgtcat

cttacagtcgcaggaacctggctcctatacatcctagtcctagtccaagatattcagcac

cattacattacccaagccaaactatgttggaaaattcaactgctgctaatcattatgcag

caacctatggaaactccatctctcaaagcccagctggaataggaatagcacaacaacaac

attactcataccctgttgacaacaacttaggtccttctaattacctatccagtagttctt

atgcaggacagactagctatggggtatatgattatggaagtgctgctgctccaccaactt

atccatacacagtagatcaaaccagctacaggggttgagttttctttttctccctcagcc

atttatttcctgtgttgtcttcttttgatgctactgtcatctaattcatttctttctgtg

catgtcaatattggaaatagaacagtttcctaggaacatatttttatcaaaaacatatat

atctatggtgccaattcaaagtggattgattactgatgaacttttatgtaatagttaaca

ttataattataattgtattagttgatcatttgataaatgaactgtaacaattttttaaaa

ttgtttt

>fava_dn_rep_c960

aaaggagagaaaattaaaaaggaagacaaatcaaacacacttgatgtatagtataatgtg

tcacaactcaaaccgcaccatatataaaacaaacaagcagttgataattgtatgacttag

ttgtaatcaggattagccaaaatatatccctcgagagccttgaaaagaccatcaccctta

gctttgtccttcttgagttgctcttcactaggagcatcatcaccttttgtgaagtatgtc

acactcaactttccaacagatcctccatttaggccttcagacaatttggcctcgaatgag

atcttctcaattgtgtctggaagcccatcaccaccaactatactgtagttgtgaacccag

ttagtaacatctactaactctattttgtgcaacacatgcttggtttcaccatcttcaaca

taagttacttttttaatagatccagcaccgccgtttccttcaacgatttcaatacttttg

atgacatcaacgacctttggagtaagagtgtcaacatcaatcacaagagctttgtagagt

attgcgggtgctacagtagaagtggtttcatcctcaaatttgaaaacacccatgatagtg

atgaattataatgctaacaaattaatattaacttagaagatgctttagtagctgtgtgcc

gcggc

>fava_dn_rep_c961

cacatcgactaaatttacacgaagacagacccacacaacagtttgatcatactcaatcaa

atacataatcaaatatccaaattcacaacacatggttcttcaagaccaattcagaacaga

taaataagatttaatatccaaatcaacaaagaaaagtaacttcaatactaaatgctttct

tctattaccattgaatttcacaaatactggtacaccagaaagaaaataaactcagaatat

aaccaaagacctgaaacctaaaactaaaacaactaactttattcttcaatacgatctgaa

aatcaaacaccttcacttcacaaaccctaattcaaacactatttagtctatattcacgta

tatcagacttataggaaattacaatagatccattttcctttcatcatcaacatatgaact

aaccccagaacctaactgtaactaagtccccatcaggaactaaacactgaaaaattaaac

ctccgttactaacacctttgcttcgctttcaacgctttttcatacgttcacaccctacgt

gtgatcctcttgaaaaaataaaggaaacacgctagggaccctgcgaacaaacataaccat

ccacgaatatcatctactagtgaagatgaaagtaaacaaacgaaaatcaactaatcattc

caaatcaagccgtaacaaccgcctcaaatcactggtggcaacaccgttcttcctcaaaaa

aaacggaaccggaacagaactagggggaggtgaagtaaaaaacgcggatccagcataaat

tccgtctttcaaattcgattccgaaatcataatctgcttctgcatggcaaccggatcagg

tccaaaccgatccgttgatcccaacaccaaatcacgtttgtctaaatccagatccagatc

cagatccagatccagatccagatccagatccagatccatggccttcagaacgagtcctcc

atcattaatctccggactcaacttctcacctcttttgagaatcttaacttgtcccataac

aagattcaccggagcatcagtacgatctccggtttgtctccgatcgttttgtttcgccgg

cggagtggccaccggactccgtttccggcgacgattctgattgacataggatttgcagtt

ggagttagggttagggctacagtttttgcgagatggaatcggtgaggaagggactgacag

gacgtcgttgggaagaaagcgacgttgaaggcaatcgtgtgaacggagaatcgcagttcc

catttctagagagagaaagaaaacgtgtgttaacggaggaagagagagaaagcattacag

ttgtattttatggatcccgcggcc

>fava_dn_rep_c962

tctttttttttttgcagaataaaatagttaattttttattagatctcaatgtagaaaact

tatttataatcacaaaaattccatttaaatccttcaaaatatgtaagacaagcattacat

atagatgtagaaagaaaaaaaaacataaattaaatacaaagaataatcaaacccatataa

aaaagtacataaaactgaaaataaaaataaaaaataaaaacttgctcatcaagtaagaat

agccatgcaaatcaaaccaaacccaacaacaaatccaaacccatcattccttccggccgc

actatcatccttgttgctagacgccggagccggagcatcaccttcaacggcggcactgct

cggagttggagttttagaaggagttttcgacgacttaggagcgggagaagaagccggagc

cttaggaccaaaaagctcggtaggtaacaaaaccttgtccaattgataaacagccaaagg

aaacttctgcctcaaagcgttgttgataggttcagtaacaactccagaggaaacattgac

ttggttaccttgtgaagtgaagtaaagaccccatggtccacttttgtcagaagcttgtgt

tctaactgggttactcacggtttgaaaatcggaaagagagtagtattttggtgtcacatg

gtagagaatgagttgcacttttttatcgtccgagaggttgttgatagttcctgatggaag

attttggaaggcgttgtcggttggggcaaagatggtgaagccctgtgaagttgagttgag

ttgggattggatttgagtgagttgttgggtttcattgaggagacgaatgaggaaggtgta

ttggctaccttgatcaaagattccggtgatgtttattgggccggaaggtgctggtgctgg

tgctttttgggctagagttaggaagggaatgagggtgagaagaatgagaacaagaggtgt

agtggtagccatgttgatgagttagtgaagatgaggtttcatttgtgtggagaagtgaag

atgtggttttatttgggtaaaggaataatattattaataagttaaaattttggtgaggat

ttgttggcatgagctggctcaaggcttgcattgatattcccttagttagttgacaaatga

ataagaaaatacaccaaaagtaatca

>fava_dn_rep_c963

ttttttttttgcagagagagaggcaaattgcataaatccattaagaaatgtcaaaacaaa

aatgtcaaacaacaagtattactgcaaaattaaaaagacaaagattggaaaaatacaaaa

acacagttagactgtaaaattctaattctccacctaaaatagagatgttccctttccctt

cttgtcaccaccgatttcaaaatgcttgcatctcttaatggcatgttgggaaacatgttt

gcaactttgacactgaagcctcaagacaattttcttagtggtcttagcctttttgtgaaa

aacaggcttggtctgtccaccataaccagattgtttgcgatcataacgcctctttccttg

agcagcaatgctatctttaccctttttgtattgggtaactttgtgtaaggtgtgcttctt

gcactccttgtttttgcagtatgttttctttgtctttggaacgttcaccattgttgcgca

agggtttgagatcggctgaacaaacgaagggtggagaaggaagcagagtctccccgc

>fava_dn_rep_c964

tgttattattgttgcaaatttattaattttattttggttttttttgttgttttgttttaa

tctctaaaatatcacaaatgatgaaaatgaatattacacatatgattagaaattaattat

tggtttatcatatggtttatatgactccacagcattcactttttttgtttgttcacatta

gatctgaattgagtaaccaaaaatggaagctcttgtttctcaattcaccttcctctcaga

gcaatctctccaggacaaagccttcgatccatccacaatcgaagatctgatgaaactctt

cgagatcgagtcctacaaagcctgggcagccgcagagctcgaacaagaaaccgaactcga

agaaaccgaacacgctatgcaagaagccgaggagcatctcgattcggttatggaaagcgc

catggatgaattcacaagatttgaacaagagatggagaggatttcaagagatgaagttca

gagtttagttgaaactggtgagagagcaaggaagatggggaattttatggagaaaactgc

tgctattgcttctaacaagtatatggaagctgctttgaattctgctacttcttctttcaa

atcagcttggaaaggaatttcttctggaaaggttcatccttcttcatagatgtgtcttta

tgtatctatatgtggcaaatattgcactcaatctaatatttctttttattttgtttaata

tatctatggatttgttttcttgttttcactgatttcaattcaattcatctaaactttttt

ctcatgaaaacaagaaaaggaaagtgagatgctgaatgaacatgaataagattaactaaa

actgaaaacgaaactagattcattgtttttgatagaggtataagatgaatcactttttgt

agtcagaattttgttgtatcctaaacgagatatcctaaattcacgcctttcttgtatcct

aaatcaaattaaatattttattttaattttaatttgtgatt

>fava_dn_rep_c965

actcccatttccatctcttgcaattctctcacacacaacgttacacagccaccacaacaa

ataataccgttaaaataacaaccgttcttaaacctcaccaccaccaccaacaacaacaac

aacaacttcaccccctttttaacaacaacccactttttactactcattcatttttcattc

cctcactttacactccatacattctttatttcagatctgcaaaacaccaaaattcccttt

accttcaacccaaataatattaagcttattgcagaagtgaaaaaacatgaagtttcttca

tgtttcatgttttgttgttatatgcttgttgtctatgtggaaaggaaattgtgaacctgt

tgaagataaagaggttttgcttgattttgtgaacaagtttccaccttcaaggacactgaa

ttggaatgagagtttctcagtttgtgataattggactggtgtaacttgcaatgaggatag

gtctagagttatagcgattcggttacccggtgttgggtttcatggaaatgttccacctca

tactattagcaatctttcagagcttcagattttgagtcttagatccaatttcattactgg

gttttttccttctgatttttctaatttgaaaaatttgtcctttctttatatgcagtttaa

caatttgtctgggtctttgcctgatttttcagcttggaagaatcttactgttgttaattt

gtctagtaataggttcaatgggagtattcctgtttcgctttcgaatttgactcagctttc

gggtttgaaccttgcgagtaatgatctttctggtgatattcctgatctgaatttgtcgag

gttgcagacgctgaatttgtctaacaatgatttacatggcactgtgccgagatcgttaag

gaggtttcctgattcagcgtttgttgggaataatgtaactcttggaaattatactgttgt

tccgcccgtgccttcaacggtttatgatgcgtcttcgacctcggggaaacgtgggaggct

aagtgaagcggcggtgcttggaattgttgttgttgggagttttcttgggcttgtagcttt

tggttttctgatgtttgtgtgctgttgtgctagaaaaaaggatgaagatggttttgaggt

tgctgcgtggagaaggaaaggctccacattatgtgaaagtcgattgggataaatgggttg

atgaagatgaggatgaaggtggtgatgtggacttgggaggaatggatttctcgaaatttg

gcggtgaaggtggaatgggcggaatgggaggattgggcggaatgggaggattaggaggaa

tggggggactagaaggattgggaggaatgggaggaatgggtggactgggaggaatgggag

gaatgggaggactgggaggaatggacttctctaaatttggtggaatgggtggtggcgacg

caatggatgatattgatgagagtgatgatgaaggtcaagaagtgtcaaagcctggtgaac

aagatgctggaaagatcggtagtgagcatgatgctgctggcaagagtgccggtgaggcaa

ctacagagagtaaagaagctactccaagcaattagaaacatttgaaaattccttataagg

attgtccgaatgaattttccctcctttgtaccattcagtggtagtttaagtgggagacgt

ttgtttcctctttgctgtgtcgacttttaacattaggatattgtataattgagatttatt

gaatgcatgaatggcggtgatttgtttctagccaggtttgtgtctcgatttggaaaaatg

ggcctcttataatgcttcagtttcatcttatttcaagctgtttggcagaggttctagcct

tgtttcattaactaggactaaatctgacacttcagaatttatagttacattacaagttcg

aatatggaaattatttctatattttccaggtt

>fava_dn_rep_c966

cttttttttttttgcaaataagagattcattcattttcatacagtgcttattctccaggc

accacttattcaatcacaaccaacaatattttcttaaaacatctaacaagtacaagcatt

tgtctctcaatgtagtatttcacatatatagaagtaatactgttaccgtagatgataact

taggcgatatagtacattggatttggaagtttgaatggtcttataccgctagggttcccc

tcaaagctactgaattggtcacctaagattccccaaatgcgataaccattactagttaaa

tgttttctcacttgagacttgtattctgccactgacgccaattcattagcaggatctctc

aaaataagacttgtccacccataataaccaacattgacaaggttatcgattgttgctgat

ctgagatactcccttctcccagaaatcaaaatgatctgaattcctcttgatttaatctca

ttgaagagcctcaatgagtaatcaagagccggggctttacccttgctcatccactcctcc

aaagccgtcacatttattttctttcccagattattcttatagaaaggaactgtagaaaga

agagtatcatcaatgtcaaaaatccaagcatcttgaccatctttcttcagattgcagcta

gtactaagataaaccaaacattcttcagttgtcctttccgagtccacactgtattgagta

gatctaacatatttaccaatgtattcagtacattcttcaggcacaacttggaagtctcga

atattatgcagctcaacgttgattctccaactttcacaataattcttcaaactgatcttc

actccattgtgtgtcttctgttttaatatgttccagtctgcaactgttgcaggaaccaaa

aaacatatgaaaactaagcagaaaacaaatgatctctccattcttgacggaaaaaaacat

gtaaagagatatggagagtactgggagataaatggacccaaatatt

>fava_dn_rep_c967

gaaattttttttttgcgaattgtcattgttatttttatttttagtactgtttttgttgat

ttgaacaaagtttcaaattcacaatgagtactaaattattttactgaaatagtatggaag

ttattttatttagcaagattacatgtaggatcattcattgtgatcctttattactatact

ttatacttgatagcattaacatgggtaattttcttaagcaaatggcctttggttgttgca

atccaagttaccaccagtgtccactcccaatatttgacaataccttttataaaatccgac

ccgatcatcggccctaggattttggccttgcccgcattcaagcccgccattgattatgtt

ggtgatcacaccatatccggggacccgtcctgctgacctatcagcagcagatggagtcca

tcttccagtaatcacatcatggcttgatggcttgtttgcctgtggggtcatccaaaacca

catggctgtcttgaatgatatagttggatttgtggacactaagtccggattgtttattaa

attttcattaattgctttgcccgcttgaccataattgtagttgtgagtgagttgaattgg

tcctcttccatagtatcttttaccaggagcacatggccaatctttagatgtacaaaagtc

tccttgaacattttgttcggacacaaagcaatatccccacgcatatggaccatctggtgc

agttggccaccctcctgtggtttcatgagaagtttgagccaagaaagcagcgagttcctt

cttctttgtgttatcatcaccagttgtaccaaatccattgaaagatctagcggcagcaat

aaacgcatcataggtataaaatccatgaccagcacatcgtccatcgtttctatatttaag

cattcgatcaaacagagaagaaggaataagccttccaacatctccaccaccagtactagg

tgtcggtgttgtcggtgttgttggtgttggagaagatttgcattggctttggcaaccatc

tttacaataagcatcagtgccaccacaaaaaccaaacttgctgcaacataacccatttgg

gcatacagctccaccagcttggctaccacattgttctgctttggatccaagaaacaaagg

tagtaaacataggataaagatagatagttttggtgttcttttcatttttgaagactagtg

agtcggttttgggcgtgaaatgaggggaggtgtttatttatagatgaggtagaataggat

tacttgttttcttatgtacttgttttcaaaattggctgctttttgtggccttacttt

>fava_dn_rep_c968

cgattataagcaaaagacaataattccattcttattaaaaagtaattaataaaataaaaa

caaggcccaggccacagcaacaatcttcagaaattttacaaataattgagttattgagaa

acaatgtttcatccatttcagtacacctaaaccttcaacgttgtaaaaaggtgtatttgg

gttggctagttacctacatttcccaattacatatagtcaaaagggcaggattccaaatac

aagacatgggggaaacctacaatcagaagtctcgaaaagtaaaagaaaaggacagaccac

atcaacgtttggaatactgaggggctttacgagctttcttgagaccagctttcttcctct

caacgattctagtatctctggtgagaagcccttgttgtttcaaaggtactctatgacttt

cgctaaccttcagcaatgcacgagcgatgccaagggtgatcgcctgagcttgaccagaga

gaccaccaccctcagctttaacaaacacatcataactagtttcatatcccaaagttacta

ggggcaccttaatgtattgaagccacaacggatttccttgaaaatattccttagcgtcac

ggtaattaatgacgaatttgccggttccttccaggagaacaacacgagcgatggcagttt

tccggcgaccggtgccgatgatagtttgagcggcgaaaccaccgggaagtctggatttaa

cgtacttcttgaggtcagcgatttcgggttcggcgggaggagcgggtgatattgcggata

cggttagggaggggagtttggaagagcgagagagggagaaggttctggggagagaaagag

aggttggtttgtgagagatattggaagaaaaagagagtgaagagagggaagatgtgagag

aagataaggaaaccgccatggttttcacttcaggagctaggtatgggtaacgtaaccaag

tgaaggatgtagccagagaaagaggataatggtttttatttttcaaaaataaaatggaaa

aaagtatactatgttgctgagattgttatccaggagaagcacaacatcgatggaaggatg

gttgaggcgaagaaagctgttcctagagacgaccaaaacgtattgagtagaaccagtggc

agcatccacggttctcccggtcaaggtcgcacgagaaagatatttgtcgggggtttagct

tctacagtgacagagagtgatttcaagaagtactttgatcagtttgggactataacagat

gttgtggtaatgtatgatcacaacactcagaggccgagaggatttggatttatcacatat

gattctgaagaagctgttgaccaggtcttacttaagacgtttcatgaattgaacggtaaa

atggttgaggtcaagcgagcagttcctaaggagttatcgccaggacctacccgctcacct

ctcagcggatacaattatggtctgagtagggtcaatagtttcttgaatggcttcaatcag

gggtatagtccaagtgccgtaggaggctatggacttcagatggacggtaggttcagtccg

gttgctagtgctagaaatgcatttgcaccatttggatctggttatggaatgggtatgaat

tttgagccgggtttgagtcctggatttagagggaatgcaaatttcaatggcaatcttagc

tatggacggggcttgaatccgtactttatcggaagctccaataggtttggcaatcctgtt

ggatatgaaagtggtaacggaggaaacaattctttcttcagttccgtgactcgaaatttg

tgggggaatggaagccttaactatgggacaaattctgcgaattccaatgcatacattgga

tcgggaagtggaaatgttggaggaaatgcatttggcaatactggactcaactggagttct

tcgccaatatctggacacgggggagggaacaatgtgtcaccgggtagtggaaacctcgga

tacggaggtggtaacaatggttatggattggggactgaagggtatggaagaagcagcggt

tcctctcttgcaccaacatcttcatactctacatcaaatggtggaggtggtgttgatggt

tcttttggtgatttttacagcaatagttcggtctatggggactctacttggcgatctgca

aattctgagcgagatggatctggtcccttcggttacggactcggtggtgcagcttcagat

gtttctgtcaagacttctcctggttatgttggtggttatactgttaataaaaggcagcca

aataggggaattactacataggatgacacgaacttgtaggcgaagataatccagtgaagc

aggtgaattgcgaaattttcacaccactgttgttactatatttcggtttttaagacataa

ggtgtttggtttatctagagctaactgaagtgcctgttttgaggattcagatatagagat

agaggttacaagggaatgttacggcttgagttttttgagttattattattatttaggttt

tgggtttttgggatttgagtgagatgtaaaatcttattgcgggatgatatagagaaagtg

tatcatgcatgagtaagcaatataaaaaacatgaaaaacttttcttttcttttcgggtag

ctttttgcatgtaatgtcctgcaaagttgttccttctactcagaaattgtttgttccttc

aaccgttttggtttgattttagcatattgtatctgaggattttgttggtttactagtgtg

atacattgacgggatacccttattggtccccgtgaataagttaccaatatattctgatta

attttttgaatgccaatattttaggctacatattcaaaaaaaaa

>fava_dn_rep_c969

gtaccaattaatcttttccaaatctcattgcatcattgaatagaaataaaacaatcagaa

caattcaagtatatataaaagtgctaatcaaaatatctattttattttcaaaataaatga

aatcaaatttacatatatactcaattgagtttgaatcactttcaaagtcagggtcatctc

tactagtatcagcaaacattcaagggtattacttcctttgttttcataatgtctaatgga

gatggacaacagtgaattaggcagagaggaagactaaagtattttattccgagtatgaat

acagaattttgtagcacacaacagtcgccgagggtgcagtaacactatccttgagcttga

agttagctcaaaatatcttctacttcaagggatgagtacttgaggatggcagcaataatc

ctttctttgtctctggttgggaatgcttccagcagtttcccatctttgtagaagtggaag

agaggcactgccctgattctaagtcgatcggcaacctcagattcttcatcatactcatca

attacattatgctttagaaacataacaggagcatcatggctaccagattttttgcacaac

tttgcaaaaccttgttctatatacctgcaacttccacacgaggttctgaagaaatccacc

acaatcagagtttcagtccctttagacttttctagaattttgcagaactcttgatcagtc

ttgaactctctcacacattcaaccggacagacatcatcatcttcatcaggcaaatcttgt

tgatactcgtcgacagcaactcgtattttaccagggaagaaatcatgtctgattttcata

acaggttcaaatcttatcccaccaagacatagtttttcatgactgtggtggttaggaact

gagccgaaaactaaatgagaaattccagacagaagcggtttgtgtaagtacagatctaat

ctcacactgagaaatgacttggtatgcagaatattctgcgggtgaatgacactggcaacc

agtggcatgttttctcaaacaaaatctcaaaacatgttattgaatggacattacaaagta

gttatggcagtggaggcaaccattttaatcctcagtagcgtttgcaggatggtggaatcc

aaaaccaggttttgcctttgccatatctgttcagcatacatttacgaatcaggacaagac

acgtgtactctaccatatccacccactg

>fava_dn_rep_c970

cgcggggatctcaagcaatcttttccctttataccatctaagtcccaattacagtttttc

ttcttccatggctgcctcagatgccgagtaccggtgcttcgttggtggtctagcatgggc

cactgacaacgaagccctagagaaagctttctctcaatacggtgacatcctcgactcgaa

gattattaacgatcgtgagactggaaggtcaagaggttttggatttgtcacattcgctga

tgagaagtcaatgaaagatgcgatcgaagggatgaacggtcaggacatggacggacgtaa

catcactgtaaaccaagctcagagccgtggtagtggtgggggaggccgtggtggcggtgg

atatggaggaggccgtggtggtggaggatatggtggcggcggaggctatggtggccgtcg

tgaaggtggaggaggatacggtaatggtggaggaggatatggtggtggaggtggttacgg

aggaagcagagaccgtggatatggaggaagtggtggtggaggtggatattctcgtggcgg

aggtggtgatggtggaaactggagggagtagtgatttagacgcttatcgtttttatgatt

atttaagtttaattctagatgttaagatctacggattgatctagaattgggtattgggta

cttgttctgtaacggctctgtttttccgtattttgtgtgtctctgaatctggtgtatctc

tgaatctggttgtgtataacgatcaattaaaacaatagtgagatattagtggcgactgat

tttgagtatggctttgttttcttatttaccttcaatttatgatgaaagtgacgatttaat

agttttgtataagttgaatacgttcaactaaagctgcaatataatcttaagagagcaaaa

gccttttacatgcttatatatccatgttttgaaaaaaaatacaaaccagtaacatttgca

gctttgctaggtctgcaaatatatttgttgcctagtgaataagcataatggtccatatca

tttattaactcttatccatccatttgcattttacaatccaaaagcaaaagccgataaaca

cagggaattacaagcaaaacaatgccataatacttaaaacttagtaagtttcaggtgtgt

gtgcaatgaaactgatgcattgaacttgacgaacgttgtcgaaaccgatgatacgaacga

aagcttgagggtaagccgcaacaacttcatcaagctccttcaagacttgagcagcatcag

tggtaccaaacataggaagcttccacattgtccagtatcttccatcatagtatcctggtg

acttgttgtgctcacggtacacaaatcctttctccaactcaaattccaagcaaggaaccc

atcccttcctgagaaggtattcaacttctttcaacaattgatctctggtcaatggtggca

aatatgaaagagtctcaaacttcttctttccaattggaggccacacctgcatgcacttta

ctcttccaccattgcttgtaatggaggtaatgtcagtgttgaccttcttaactgggaatc

ctggcatagatttgaggctaccaaatagagcccctgcggtggattgcaccgtagaggcac

ggttgactgtagtcacagctgaagaagatatcatagaagccattgttgttataattgagt

tcccgcggccataatg

>fava_dn_rep_c971

taaaagaaaaagagtgtatatcacagcatagttccatcattcccaaatgtgtggccacag

tttggtgtttgttaaccctcaaatctgtgagccaatcaacagtatataagacaaaccaga

tagatgagttattgaatgcttggtgtgccatggcgtccgttttcaaagtatcttcattta

tctctcctctacaccaaaagttgaattatgttaggttttcgcccaaatttcagcaggtta

gtgtaagaaatgggagatcgaatgttcccgctacgagagtaaaagctcagactgcggttg

aaggggatgtacttgaaaatgaaccagattatggagttgttaatgttcaccatgttggag

ttttatgtgaaaacctagaaagatctcttgactactatcaaaacattctaggtcttaaga

taaacgaagcaaggccacatgataaactttcataccgaggtgcttggttgtgggtagggt

ctgaaatgattcacttgatggaacttccaaatccagaccctttaaccggacgacctcaac

acggtggtcgagatcgtcacacttgtattgcaattcgagatgtttctaagctcaaagcaa

tccttgataaagctggtataccctacacgcttagccattctggaagaccagcaatcttta

ctcgtgatcctgatgccaatgcactcgagttcacacaaatagatgattaatgtttctgga

tttttttaccactcttgttgtccacgaaacaaatgactagaaactagaaatatatgaatg

tatgtaaatgattgtatatatgtaaaaggctgaacagagttgctgagattcttcacacca

tatatagaagccatggttctcaaaataaacttcatttcattgctttatatttatatatag

caaaaatgttaacaatttatcttaaaggtgtattctaaaaattgtctcaaaaattaagtt

tgaaagatagttcatcctcttcctctttaaaaaaaaa

>fava_dn_rep_c972

tttttttttttaaaaaaactaataaaaaatagttgtaatatgagagtgattgtaacatta

aataaaatcatcacatactcaatttattttggcacacatcataatcttattgacagtata

tagattatctttggaacacgaagataagacgaagaaaactatcaataaaatccattatag

ttgaaacagcaaaatctcaaatgtctcaaccaaagggcaggagctagtttcaccaatgtc

acattattgacaagctgaacaaaagtaagaacaacaaacttgaatactatccaatttatc

ctcctcgcatgagctcagtacctgtggtgcagcttctcttgttccacaaagtgcatagca

tacgagtaggtgccgacgacgggagtaaccaagagaagagcgctaatccaattttcggag

accttgtgatgaattttggttggcaaatccttccacaaaccgctcatgattttctgctgg

aaaggagaaagagcgtacacaacggatttcaccggcacgatgtttttccccatcttcttc

ttcttcgattggattcaacaaaaaaaaacgctaaggaatgacgaaccagaatgataatga

gaatgcgtaatggaaataataccctaccgttttttgttgggtttgttattcgtagctatt

ttcgattggaccgggtttgg

>fava_dn_rep_c973

gggaatcatcggcatgtccttgtatgtagtataaatgattgagctcgcatttccttttca

gttttcaacgccacattctacacacagatccatcctgaaaacaacacattgacaatggcg

acattgaagatcccatctcacgttcctcatccatcagaagacagtgaacaattgcgcagt

gcttttcaaggatggggaacgaatgaaggcttgataatatctatcctgactcatagaaat

gcagcccagcgtaaggcgattcgtcaaacttatgctcaaacacatggagaagatcttctt

aaagatttagacaaagaactttcaagtgattttgagaaagctgtgttactgtggaccttg

gatcctgctgagcgtgatgcctttttagctaacctagcaactaaaatgttgactgcaaac

aacgctgtcatcatggaaattgcttccacgagatctccactcgaactccttaaggcaaag

caagcataccaagcccgtttcaagaagtctcttgaagaagatgttgcctatcatacttcc

ggtgacatccgcaagcttttggttcctcttgtgggcacattccgttatgacggggatgag

gtgaacatgacattggcaaaatctgaagctaaattgcttcatgagaagattgcagataag

tcctacaatcatgaggacctgatcaggattgtaacaacaagaagcaaagcgcagttaaat

gcaactttgaaccactataacaatgcgtttggcaatgtaatagacaaggatctggaaact

ggttctgaagatgaatatctgaagttattgaagtcagtaattaagtgcttgacctaccct

gagaaatattttgaggaactcttaaggctggctataaacaagatgggaactgatgaatta

gccctaactagagtggtgacaactagagctgaggttgatctgcagcgaattgcagaggaa

taccaaagaagaaacagtgttcctctggatcgtgcaattgacaatgacacttctggagac

tatcagaaaattctcctggcgttgttgggacgtgaggactagagttcttactctgtctag

tagtcatgagatggcagtgtactactagacttgctggtaacctgattgtgagatgtatga

gatatcctggtggctttaaataatatgttttgaatatgtactgaaattcaaaatagaact

ttgcctccttcaataaagataacatccttgtctaaaaaaaaaaaagngaaaataagtgtg

gccaaaatgtaaattaaa

>fava_dn_rep_c974

gcaggatgaatattttattattataaataattagtgacgtgataatcacattgcaactca

tttttttactacacggttattcgatcacaaaataacttctgtgaaaagctttcttcatgt

agtgttaatacttgcacgaataagtaattattgaagtctcatcaattcaatcaacatgaa

taaaaaggacatagctagctagaggtacatatacccgtgacacacacttacaccattaca

aaactacaaatataactcaactttagaaattaaatccacacaatagagtacttagctcaa

aatctttcaccgtgtggttagattcaaaccttaccatatgctaaaattgaatccatcaat

ataaatagagtaccaatattaccattacccaaacaaatcttgtaatgtcatattgacata

agattttatgtaatgaacattttattaagtacatagctcacgagcaactccaaccctaga

attagggacgtcaaagaggagacggtggttttgttgctgcatgttggctatgacattcaa

aaccgagttcacattatcgggtgcagacgccatggccaagcacgttgtacttccggcggt

actatgtataagtatgttgtcttcaggaagtgtcacgttcatgcctgtgaacataaaggt

tattgtaggtacaactattggcacattgtagcatgtgtcgaaccctcctaaggttgtcac

agttaggtttggcccaactcttcgacgaaactcatttcgaaccgcctcatatgctggtgc

aactagtcgggtgaacacggtaccggaatcaaagatggtaccagctccggtggacggatt

gaatgccaatgcggtcggaggaatatcgacgattttccgaccaaccctaatagcttccaa

gttaacataatagagtgatgatcttctaggattcttgagaagaggggtaaacttaatcct

cttgggctgagcactaggcccaagtcttaaagacccagagtaattaagagacttgaagct

tggcaagcagtaagagaatatggactgatagaggttttgggtttgagataaaagtgataa

tgggcctcggcccaagcccaacagaccttgtggtggagtagaagttccagtggtttttga

aacacaaccaaaggtgtaactaggaattgggtcagtggctaaggtaacagtgtcttggac

caagttagatgatatagtggaacttccataggttaggttaaagttgcatgaattcacacc

acaactagggtttggtacctgtttgcattgaggagcatcacaagtaacattctgaaaagt

agttgatttttcaggagcaaacaaagtggaagaacaaccatcacaagcagtacaaggaat

ccaagcagcatcattactagtatccatagccaaaagcaatgtttgaggtggagtaccaat

tttagccctaacaatataagttggactttgtatgatctgtctaccagaagcaattggtac

aattgatctcttagctactaaactatcaaggaattgaagtctagtttggtcttttgattg

catttggaggatggtttcctcaaatgaaagtggttttgatggcttaaatggggaacatgg

actgaatacatggatcacttggagtgttgagctatggtcttggatgtcacatttagggtt

tagtccttggactagggagaggaataggaacaagagtgaaagtgtgagtttcataattgt

tgtggatgaggttttggttccccgcggcctaa

>fava_dn_rep_c975

aagcactagcaagcacttcaccggggatcactcactcaacaatgtctccaagcaaaactt

tcatcttcttagctctcttatccttttcaccacaactcttcctaacaatttcttctgcag

tggaagataatggccttctcatgaactactacaaagaatcatgtccacaagccgaagaaa

tcatcaaagaacaagtcaaactcctctacaaacgccacaagaacaccgctttctcatggc

tcagaaacatcttccatgactgtgctgttcagagttgtgatgcttctttgttgctgacat

ccacaaaaagaagcttgtctgagcaagaacatgacagaagctttggtttgagaaacttta

ggtacattgataccatcaaagaagctgttgaaagagagtgtcctggtgttgtttcttgtt

ctgatatccttgttctttcagccagagatggagttgtttcgctaggaggtccttatattc

cattgaaaactggaagaagagatggtagaaagagtagagtggatctgttggaggagtacc

ttcctgaccacaatgaatcaatttcttctgttcttgacaagtttggtgccatgggaattg

acactcctggagttgtttctttgcttggagcacacagtgttggtagaacacattgtacaa

aactagtgcaccgtttatatccagaagttgatccagctttgaatccagatcacattccac

acatgctaaagaagtgtcctgattcaatccctgaccctaaggcagttcaatacgtgagaa

atgaccgtggtacccccatgattcttgacaacaattactatagaaatattcttgacaaca

aggggcttttgatagtggatcatcaactagcacatgacaaaagaacaaggccttatgtga

agaaaatggccaagagtcaagagtatttcttcaaggagttttctaaagctattacattgc

tttctgagaataatcctctaaccggttcaaaaggtgagattagaaaagagtgcagtgttg

ctaacaaacaacaccatgatgagccttgaatgaaagtttcatggtggaagaagaagatga

agtgatgtgcaagatattatgcagaaataaaaatgttcatggagattggagaacttaatt

gcttttaattactattaatctttctttgagatatgtacctttgttttttatttatttagt

attagtactatggtagctcaatgttgagcatgttgaagatgatgtgaatgtatggtattt

aatatggtccatgaatggcatgtagtgacaatgtttatgatatttgtgcacatcaagttt

gtgtcttttatatgcactaataataattaacaccgtgtgcattg

>fava_dn_rep_c976

ctcacgtactatttgacaatttaaacttgcatacaaattttctccagtttatataaaagg

tagaggcagcttttggctaaaggcaaactgttcgacccaacaaagctcaaaccgccaaat

atgaaactcgtaaaaagatctcaactccacaactcgaacattaaaaatttaagccccagt

aaaacgacaatagtgagaaaagcatagttcgtaaagccagacaccaacaaattttataaa

aacacataacaaaactgaaaaacaaaaacaagtacgcaataacttctaagcaggtgtttc

acagaaataacatatgaatccgtttccttcctaacacagcatagtttaagttctttttgt

aggaaagaaaacacctcaagtacctaagttgtaactcattaaacatgttatatcaaacaa

taatagccagcaggtggtttaattcttctcagtagtgctctccagctcccttctcaactg

aagagagaaatcatcactaacatcgtcatcatcccaatcatcttcccactgttgagctat

ttctttaccttcctccttgtcatcccactcttcattgatttcgaactcttcgaactcatc

atcgtcttcgaagagatcgatcttaacatcctcggtaacagcctttgtttcggtcgccat

aggtgaatctgaatcaggtattaggagaatgaaaactgcaatgatcctttgagtattcgc

tattgatgagattataagaatttttttctgcgtgtaaaggaagtaaaaagggtttggaac

actgtcaaaaacggtgtcgttatatgcttcattcag

>fava_dn_rep_c977

gactaatataaactatcactaaattttttattaatctttaatcgtttgatcatgaaaagt

gttattatcattccaaagtcgcacatgaatagttgtaatttgtcttctacacctttctaa

aactacataggttccataatggaaaggataaatctgatgatacaatatatccaggtctaa

tgatacaagaatttacattgcctgctgtaagtgtatcaagatacaatgtgcaaaagtagg

tgacaatatacccaccaactacaatcctctctataaagtgatcagaaggtagaaatgaaa

gtttaagaatcacggtaaattgattgtggcatccacttgagatgcaaattcaaatagcca

gattttgcgccatctagctcaaaacgttctttgtattctccttccagtatcactctggta

agagtcaatatcactctgcccatgtagtcctttccaaaggtgtcatggtcataaacttca

acaagtagcatatcatgtaatccatcctctacaacaaagtcgaatgtttgattccaaaca

ggattcaagctgttgttcacaaccctggttttgttctttgtttctgctttcttcaatgta

agaacaacaaaaggatcagatttccccatgaaatccacagcaggcaagtcttcggctgat

atcacagtaacagaaaggactcctctaataataacctcttttttcttctgtgcagcttca

ttttcatttccattagactctactccattcgttgaacttttaataaccttttccaaggat

gtcattgagtagctgggagaaaatgggttggtaaagctattctctgtgccatatggacaa

tacaaaagctccaaatgtacctgtcctctattcttgttatctctttggatctccaaatcc

ttgagcaacttcaaccacacatcttttactttaccaggttggagttcactaagctttaag

tcagcacatccaattaattccgacgcctgtaagccctcattatcgtacactttaacatat

aagtgctgagtggacgcatcttcaactataaattcaaagtgttcattccatattggattc

aaatcattgttaattgtcttgcttttcttcgttctgttgcgtaaagggcgtatgtacaat

acagcatatggatctgattttccaatgatatccttgtttgttaattcctttgcttgcaca

agcttgacttctaatatccccacaggcttcaactccagatcactataatctccaggcaag

atgggtacaatttttctcacgggccaagtaatagaatcttcaacggcatcccgaattgcc

ccctcaattgcatcgtataatccaggtattgttgatatgtcaccaccaatgactttaagt

gtaaaatccagctttttcttttgtcttagtgaatagcaaacagctccaaagcccggaaat

tcttcaaccagtggcttaaaaatcaacctgaagacgcctgtaaatcctatatttttaacc

tgcacgggcagagcaacaccaatgagagttttaatatcaagtataatacttggattacca

tcccactgcatttctaactccattgtaataccatctccatcatcttcaattatagacacc

cctgtaaactgtggtgcaacagaaccaagggtaaacttggaaaatttgagagcagccaaa

accgtaggtctgtattcttcaagaatcggttccaccgatgtcttaataagctcagaagca

gcctcattgacatagggccagatcttggtaagatgcgagttaagccaatctaacttttgt

ctggaagagaaaaccacccaagaagggtagaactgagcaggtaagagttttctagaatca

tccaccgacattctcgcaaaagccgcgatggtggttgcgagctgggatcgtcgggcggag

cgggaattctcggcgcggacgaaggcgacgatgatggcgaggccgactaggattccgagc

accacgccgaaaatgaaacccattctgcgagaatcgcgtttcccctttcccggagaaatc

gaaggattgggagttgaaagtgtgagtgtgataaagtggagtatggtaaaaattagttac

agtttaggagaagagtgaaatgaaaaatgattagtaataatgatggaagttgaatcagtt

gagagaaagagagagtattggtgggaatgtttgcggcttttt

>fava_dn_rep_c978

acccttctttttctctgtccctttttatataaacctaaaaaccctattaacccctctctc

tctatcactttctttctctttctctctacaatttcaatcaacatgtccaccagcaccgat

caccacgaagaggaagatgtccccgccggagacgacgaagataccggagctcaggtcgct

ccgatcgtcagactcgaagaggttgctgtctccaccggagaagaagaggaagaagcgatt

ctcgacctcaaagcgaaactgtaccgttttgacaaagaagggaatcagtggaaagaacga

ggtgctggaactgtgaagtttctgaagcataaagttactggaaaagtgaggcttctcatg

agacaatccaagattctcaagatctgcgcaaatcacctcattttgcctaatatgactgtg

caagagcatgctgggaatgaaaaatcctgtgtttggcatgcaagggactatgctgatggt

gaattgaaagatgagcttttctgcattcgatttgcatccattgagaattgcaaaaagttc

atagaaacattccaagaaattgctgaaaccctgaagaaagaggaaaacgaggaagtaact

gctgctgctgaactccttgtgaaattgagtgttgatctgaaatcagatgcaggaaagaaa

gatgaagagaaatctgaggacaaaactaaggagtctgaggacaaaattaaggagcaagaa

tctgcaccagaaaaagaaagcaaaggagattcagagaaggaagttaaagagtctggttca

tctgcttgattatgatctcttcatatttccaacacaaggcaatatgataaggttaggtgg

ttgacccatgtgccactcttatcatgctctgtcatagtcctagtcttcggattgttttgt

tgctttgctacgttattgagtggtccatatcctttgttgagttattgtcagcaaagtagg

ttttgagccaggtttgaatccccaaatataatgattcgcgctttcagggtctggagttat

ttacattctattcatgtatttgtcattgaattttgagtgagtcatgttgctatcggggat

gggggaagggtgcagtcgggaaatagtagtacattactataaacaaaggctttgaatgca

tattgctgagtataactttttatagactcgtattactggtacataacttgcttttgctac

attttctcttttcatctaaaaaccaagctttctagttgt

>fava_dn_rep_c979

tgctttatagatttatagaagagtttgtattagttactacaaaactaattccagttttta

tgctgctacatacatacataccaataattgtaccactgaaaattttgatccctactttcc

gaccattagttcctttggactttgcttaatcctaaactaatttattttattaaatttgag

atccttaattggcaaaagttcagtttcttaattgatttatcatataatatcctcagctaa

tacctcattaagatccctaaaacctatcatatacacttagatactctatacatttttaat

ggtacaacacttgtagcataggaataactaatactgattcagcattctgttcatttttcc

ttgtatttaagcagcaagcaccagcactttgatggtgccttgactatttacagttagtat

ggtggggcgatcgctcttccagcataccgcactgatgaagtaagatcccgcctcatcctc

cgcatcttccatgtctagggtaccaaatctatgccacgtcagaggcttcgaaatttcctt

gtggtagacaaatacttcgtttgtttcactgccacatgcaatgtattcactgcttactgt

aaggcctacaaagtttttctcgtttgcatggcctctgaaagtacgaactggtaagttttg

cttcacgtcccataaccgcagtgtactatctgttgatgctgacgcaagttcatcgttgga

caaaaatttcacatatgaaacagccttcttgtgcccagtgaaaacatggacaggccggct

aatatttctcaaatcataataatggatatgatggtctgctgacccaactgcaatgtaatt

cccggatccaggattatacttcacgcagcatatgtttgctttcatgtctatatttataac

actggcttcctgattcgtgcaccaaactttgaccttacaatcatcactaccggatacaag

catagagggatccgttcttgaaaaatcaacactccatgcacgcttttcatgctcttcata

ttccattaaactctttcgagtggtcacatcccaaacagttacaatgccttcataatcact

actagctatttggttcttagcatatttgttccaactcaagcaactaagttttgaacgtgt

agtcatctccacaacaggacaatgagcatctgtgggttcattcacgaccgcagaaaaatc

aaaaactttgatacgtcgggaaactccggcagtagcaaacaaatcatcatcacggtcaaa

ctctatgcttgaaacaatgttggctgaatgaaatatatccccgtgtcttatttctgcaat

gactctcaatcgactgtagcgtgtgaaagttgttaagactgactgaaaatcatcaagacc

gcaactataaccttctcgacttatgaaactcgtatccctttcctgttggccttgtggttt

atctgctgcttgcctccgtttttgtagataacattcttgtaggtcattgaactgtgtatg

caccctcttctttctaactagagcaagacccgattgatttatatactgtgaatctgatcc

actgatgggatctcttctctggattccatgcccatgagagctgatttgtgattttccatc

atttttcttagtatggctccctgaagacaaccctcctcgaagatttaaaggactagatgc

gaggccactgctattcaagtccaatgatgaatgccgtgattttcttccaccagaatcatc

aagcatccgcaatttcacagagtacctgtcccgagcacgatacaagtccattctatgttt

ctccacagcaccaatgtcctcctttatgaactggagatcagtttgcacctccttcaactc

atcaactttttgcttgcgtaagcaatgcaagaaatctaacaatatttgcatatttctctc

agcttcttcttgttccatttttctcttcttctcagtaaggagtaacaaaagggtgtcgag

ctccttcattgtcacttcacatcccttttgtactgcctgccggaaatgctccacaggaga

agcagtctttgatatttgacgatctgatgtcttttttagtagcttatcgagcaagaagtt

cgggaacaaattactgttagtgaggtaatgaccacagcaaggacaatcgcttttgttccg

gagatgagtgatgatacacatgtagcagaagctatgaccgcaagcggtgagaaacgcgtc

tttgatgatctgcatgcaaatcggacataggaagtccttgtctaactccgacatggtggg

aaccggaaaagcgtcgccggcggcggtggtgtcggtggagaagtttttggaagggtctag

tttcactactgcagggactagaggtccaactgagtgttcttccatggtagtggatatggt

tttgttgcgagagaaacggtttcgacgcg

>fava_dn_rep_c980

ccgcgggagattagggttcttatcatacgacggagcagtcgagcatctcagcgaaacgaa

aatgccgtcacataagaccttcatgatcaagaagaaattggcgaagaagatgaggcagaa

caggcccatcccttactggatccggatgaggactgacaacaccatcaggtacaacgctaa

gcgtcgtcactggcgccgtaccaagcttggattctgaggttcttgttttcctgagtttaa

tttcattagggtttttgttgatttttgtttaatttaagtaatacaatggatattattgaa

ttgtaatgccatatttttgagctatgagaatttattgttgttaattacttgcttaacatc

tttttttaaaatcaattgggattctttgcactctaatttgaattattatacatttcgatg

attattttattagttttgatgttttaagtaatgtctagctgatctatttcattgtaattt

ttttcctttagagcaattttgtgtaacaagacataccaattaccactagtatgccattaa

atagtttnnnntctttgttt

>fava_dn_rep_c981

gtcaaagttagttatattcaaagtaagttatgagttttatgaaagaatacatacggttgg

gatggtgtatgttacaaaacaggttgatatgaagaaaagtatcatactattttcaaagtt

aatgttatatcagactaagcacactttatgacatagaacaataactttggttggtgtatg

ttcttgtacataattcatttgaagacaaccgaacatactcttttatcaagtactataata

catttgtatctaatccagtgttgcttattatgaatatataagaacaaccgaacaactgga

aattattacatatacaatgaccaatataatttctttagtttgtcaaaagagaactacctt

ctttgataagaacaaacacactgccaccatcacctcttgagatacgcagctcttcatcaa

ggtatgtagtaagcaaccaagattgagctttgctgttggaaattggaatcttcagtggag

gctgattggaaattgtttgcacaactgatgaagcagtgctttggacagaagtaagtatgc

ctttgaatggtgatagatcaattttttgccccaagaattccacattttctggtatctcta

atgagtctgtcagctgtggagtgcctatgataccttcctcaaacttgatctgcaaacgat

ttggacttcgaatttcgaatttggcgttggtactaatggaggtggttgtcaatggaccgg

caaactgaacagagttttggacagttaaactttcagagtcaatagtctgagatatttctt

caaccttgagcaatggtagtagtccactcgacaacaacggaaataaacctgcaaaggatg

tatacgcgagaatccattttccattaagcagagtcaaagcatcattagaagcaggagtcg

gattcttagcctcaagctgagtaataagctcaacaatctctgctctcgtttcactggtcg

ccttcaacccacgatctgtcccataaaacgaaccaaccaaagccttcttcaccttctccg

tctcactatccgccaatttcacccccccaaccgccacagccggagaaggcactttcttct

cttccccccactcatcgtcgctcgtgttagaccgaacataaaccatccgcctcgaaacat

ctgccaacacaacaggtcttccgaactgtttgcttcggaatccgacggtagtggttggcg

gagagatcgaaggtgtgacggggagggtggtgatgtggagaagctggttgaggtgagaga

gagacgccatttttttttgttgtgagagtgaagagagagaaaacggggattttatggagg

agacttgagagagaggaacagagaagcggtgggtttgtaagattccaacaagacgcgatt

acctacggttgagatttgtttgtcgtaaagaaaaccaaatgagatatcttttgaggattc

agaggcgtacgaaaacgttgttgcgaaacgaaaggagtgtgttcttagaatcaaagacaa

acttgtcctttcttttaattcgttaatctacat

>fava_dn_rep_c982

aaagagtcaaaagtggtcgtttgtgctcattattacgttgggatagtcatcattatgtca

ttaaccatccaccgatgaaaagtatcttctcatcatgaatgccattggatataaactatc

attcaacaataaataaatgacacacttatgttactttacatttattattcagaaaacaga

aacctccatgaatcaccaaactcaaatgaaaaagtacatgtttattcctttatatactaa

caataaggacaagaaaaaagaatgacaatgtaaaaagaaacaataaataacaccccagta

attctcttcttttccatgaggttattgctgttcttctttccctgatccacaatgaatttt

ctttctttaatttattctaaagatagtactgatttttttccttttcaagcagctggtgac

tgtgtaattgaagctatcatgccgcgggaagcaccgctcaaccattaacaaaaaatctct

tacactttctcacagtttaatagagaaaagagaaagaagaatctcctttttctttcttct

tctctaaatcagcataatcttttattcttcatttcatttttgttaatcaattaattacgc

tgtttcagaaatgtttatcgagaatttcaaggtagagagtccaaatgtgaagtacacaga

aactgagattcaatctgtgtataactacgaaaccactgaagtcgttcatgaaaaccgaaa

tggcacttatcaatgggttgttaaacctaaaactgttaaatatgaatttaaaaccgaaac

tcatgttcctaaattgggggtaatgcttgtgggatggggtggaaacaacggttctaccct

taccggtggtgttattgctaatcgagagggtatttcatgggcaacgaaggataatattca

acaagccaattactttggttctcttactcaagcttctgctattcgtgttggatcttttca

aggagaggaaattcatgctcccttcaagagtttgcttccaatggtcaaccctgataacat

tgtttttggtggatgggatatcagtaacatgaaccttgctgatgccatggctagggccag

ggttttcgacatagatttgcaaaagcaattgaggccttatatggaatccatggttccact

ccctggtatctatgacccggatttcattgctgccaatcaaggtgaacgtgcaaataatgt

tattaagggtacaaagagagaacaaattaaccaaattatcaaagatattcgggaatttaa

ggaagtaaacaaagtggacaaggttgttgttctctggactgccaacacagagaggtacag

taatttagttgtgggactcaatgacacaacagagaacctttttgctgctgtggacagaaa

tgagtctgagatttctccttccaccctgtttggaattgcttgtgttatggaaaatgttcc

tttcatcaatggaagccctcagaacacttttgttccagggcttattgatcttgccatcaa

gaacaacagtttgattggtggtgatgacttcaaaagtggtcagaccaaaatgaaatctgt

tttggttgatttccttgtaggagctggtatcaagccaacatcaatagtgagttacaatca

tcttggaaacaacgatggtatgaacctctcagcaccacaaaccttccgctccaaggaaat

ctccaagagcaacgttgttgacgatatggtcaacagcaacgctatcctatatgcgcctgg

tgaacatcctgaccatgttgtagtcattaagtatgtgccatacgtcggagacagcaagag

agccatggatgagtatacttcagaaatattcatgggcggaaagaacactattgtgttgca

caacacatgtgaggattcccttttggctgcccctattatcttggacttggttcttcttgc

tgagctcagtactagaattcagttcaaatctgaagctgagaacaagtttcacacattcca

ccctgttgctaccatcctcagttatctgacgaaggctcctctggttccaccaggtacacc

agtggtgaatgcgttgtccaagcagcgagcgatgctggaaaacatcatgagagcttgtgt

tggattggctccagaaaacaacatgatccttgagtacaagtgaagcaggggatagaacat

agttggaattttcaagctgaatcataaaacttgctcttttctttttttcttcttttgatg

tttgctgatgttttctcaagtgtagtatttgatttgatagtctatgctctcaagtgcttt

agagcttgaatgttttgcgtacatggaaatatataaatatttgtgctcagttggtgttta

tccctctattctatattttcttatagtatttctaat

>fava_dn_rep_c983

ggtatcaaattcaattaacatataataaatgaaaattgttctagtatttattttgtttgt

aatacatgaaacaagggtaatgaaaaagtaatgaaacaatttgcaagactaaaggaaaat

aaaagctgtgaaatgtatactataccaaacctatggtcgtataaatttacaactaaaaaa

ccaagcttccaagaaaacaaatccaacaaatcaatggaatgaagtttttttttcttattt

gtaaaaataaataaaattctaactttttttttttcggagctcgatagataattagatacg

ccaatgatttttttccgtgtttcataatatggttcaccaacatgttaactactcgattac

gataaattggatcggattttgcgattttttttttctgcagtacctcgtcgtgacatgagc

gtaataatccatttttttataacttataggggctaaaatattttattttggctttttggt

tccatattttgaacgcccttgtcgacgatcctttactgcggcagcatctagggttcctcg

aacaatgtgatatctcacaccaggtaaatccttaacccttccccctcttactaaaactac

agaatgtatttgtaaattatggccaataccgggtatataagcagtgatttcaaatccaga

ggttaagcgtactctggcaactttacgtaacgcagagtttggttttttaggggtaatatt

atacaccttaatacatgttcctcggcgttgagggcatccccgaagccgcggaaaacaagt

gtaggagagagacatcaaaatctatttttattttattttattttaaatttattatggatc

accataaaactcattctctattctatttttctctctttcttctcttcactttcacaattt

cagctttcgaccttgcaaccatacccttcaatgatggctattctcctctcttcggaaatg

gcaacgtcgttcgttccctcgacggtaacgccgttcaactcctccttgatcgttacaccg

gttctggttttatttcatccaatatgtatcaatatggattcttcagtgctaatataaagc

ttccatcgaataacacagctggtatttgtgttgcgttttatacatcaaacggcgatgtgt

tcgagaaatcccatgatgaattggactttgaattcctaggtaacgtagctggaaaaccat

ggcggtttcaaacaaacttgtatggaaatggaagcactcatcgtggtcgtgaagaacgtt

atcgtctttggtttgatccaagtaaaggataccatagatacagtattctatggacacaaa

aaaacgtaatattttacattgatgaagttccaattagagaagtgttaagaagtgaagaaa

tgagaggtgaatatccatctaagccaatgtcgctctatgcaaccatatgggatgcatcaa

attgggctacatcgggtggaagatacaaagtgaactataaatatgctccttttggagctg

aatttaaggacctagtcctcaagggttgcactactgatccagttgaagaattctccgaca

tcgagggttgcgccgatcaaaacgcccatcttgaagcccaagattacgcccaggtgactc

ctatacgtagtatgaaaatgcggaggtttcgccaacgtttcatgtactactcttattgtt

atgatacgattagatatcctattccaccgtcagagtgtcattttaatctggcggagaaac

aaaggtttagggagactggaaggttgaaattcggcgggagtcatcgccgactgtctaggc

ggagaggccgaaattcaactcctgtagaggacacagatcagagtgatatgtgatgtttga

ttatatgtaaataatagtgtgattttaaatggaactttttgggttttggcattgtctatt

gtttgataccttaataagttagtccttaaaaagttgtgttttttggtgttatgcttttgt

tacttggtgtgtgttgtttcagtaggtggagtataaaatcggtatataaatttgtatgag

tgctctaaagtatatgtcaaaattcatattgtgggacggttcttgattatctatggttga

tatttgtttcagaaagaaattgagatgggaaattatgaaaaatcaacgttgactattgtt

aggatgatgtatagcccatgaaagaaagagaaagaacttcatcaa

>fava_dn_rep_c984

ccgcgggattagaaaagtgatcatgtagtaaagtgaggcaagagagaatgcaatcaagta

ggcttttgttgtgttcctctcaatccttgtttgggccagccgtcactactactctccccc

cttgtcttagattcccaccacactccatagcaatcgcaagttcaacttcatctccgattc

gcttacgacccctgcgctccactacttcatttccagtaatggcttccaaggattctctag

ttcaagacccgcgtatagctggaatctcatccgcgatccgtatcatccccgactttccca

agccagggattatgtttcaggatataaccacactgcttctcgatccaaaggctttcaaag

ataccattgatttgtttgttgaaagatacagagatcaaaacatttctgtagtagcaggcg

ttgaagcaaggggctttatttttggcccacccatcgcgttggcgatcggagcaaaatttg

ttccaatgaggaaacccaaaaaattgccaggagaggttatctcagaagagtattctttgg

agtatggaactgacaaaatagaaatgcacgtaggggccgtacaacctggagaaagagcct

tagtcatagatgatcttatagccactggtggaaccttaaatgccgcgattaagctactag

aacgtgttggggtgaatgttgttgagtgtgcttgtgtgattgaactaccggagttaaagg

ggagagaaaagctgggcggcagatcattgttcgtgttagttgaaggagaaggagcttgat

atttgtggatgtgatcatgttctaattatttaccgtttgagctggctcttgttaggagca

ttattgatgactttagatgagatttattgcagaaaactcaattgtttgtgagctcagttc

acattaagggttatttttaaaataaaaacgttgtgtttttttagtaaagaaccaaaagag

tggtcaggctcgagcttttctgtatgcaacattatcttattttgatatcgaagacttgtt

ttccaaaaaaaaaaagaatacaaaccaaaccaaagaaacgcagacaaatagaaaatggca

ggaatcttgaacaagattggtgatgcccttcatataggaggagacaagaaagaagttggg

cttgctagaaggaattccaacaatgttgaagggcgaagtggcaatgttgaagatgaaacc

tcagctgcattatggtgaggatgattgtcctatttctgctcctgatgggtttcctaagga

tgatgatctccattttgaaatagagctgatagagttttttaaggccaaggttgtcactga

tgatttggaagtgattaaaaaggttgtacacgaagggcagggttgggaatctccaagaga

accttatgaagtgaaggcttggatttcagcaaagacagctactgggaaattgatcacgtc

acagaaagaaggagaaccatacttctttacatttggaaaatcagaggtacctaaaggtct

tgaaatggggatcggaacaatggttcgggaagaaaaagctgttatatatgttacaagtca

gtatttaaccgagtctcctctgatgcctgttgtagaagattctgaagtccagtttgaagt

ggagcttgttcactttattcaagtgagggacgtgcttggagatgggcgtctgataaaacg

tcggattcgtgatggaaaaggtgattttccaatggattgcccccttcatgacagtctact

tcatgttcattacaagggcactgttcttggtgaagaaaagagggtgttctatgacacaag

agttgataacgatggtcaaccattggatttctgctctggagaagggcttgtaccagaggg

atttgaattgtgtgttcgtctgatgctaccaggagagatggctctagtcacatgtccacc

agactatgcttatgataagtttccaaggccttcaaatgttcctgagggtgctcatattca

atgggaaattgaacttctatcttttgaaatgccaaaggactggacaggaatggactttaa

aagtataatgtacgaagctgaaaatatcagaaacaagggtaacaggttatttaaagaagg

aaaatatgaacttgcaaaggcaaagtatgaaaaggtgcttcgagaatttaatcatgttaa

tccacaagatgacgaggaaggaaaagttttttctgatacacgtaatttgctacacctgaa

tgttgctgcatgctatctaaagctgggagaatgcagaaagtccattgagacttgtaacaa

ggttttagaagcaaatcctgcgcacgtcaagggtctttatcgtcgtggaatggcctacat

ggctaatggagattttgacgaagcaagggctgatttcaaaatgatgatgaaagtcgataa

gtcaactgcatcagacgcaacagcagcccttataaaacttaagcagaaagagcaggaagt

tgagaagaaagctcggaaacaatttaaagggctatttgacaagaagcctggagaaattgc

agaagttaacgctaatgaagatgaagatcaggtcacccgtgaaagccagaaagatggtga

ggtacataaagacgcatcagatggaacgaactcggaggattcacatgaagctgtgcctga

tgcagatcaaaggggttggttctctcaattttggccaaccggtagtagaatattttcatc

tcttggacttcaaagatgtaccatactataatttatcataaagaatgtagcaaatggcaa

taaatcttcaaatggaggcattcaaaatcactaccacaggttattagagagttcaggcat

gttaccaggaatgttttttttgttcagttttatattgtaatcttaaacgcttgtaaccgt

atatttaataaaagagagctgtatgcataataggaatacagaggattgatgtagagaatt

gtagtctaaacaaactgaaatcatagtagaaaatttgaaacttaaatttatgct

>fava_dn_rep_c985

gagtaaacgaaacggagaagaaaatcgatctcttcgtttcactctcaaaaccaaattatt

gtcttctatttttcactattcactcttcatttttctcattttgatttcaatttttgttgt

tgttgttgtaacatatctatttgatttcactagtcatttgtttcttgttttttttgttgt

tgttgttgtatctttcccttcacgtgaaagtgaagaacttatctagagtttgctcatata

ttttccgtggaacttttttttcataacaaccaattcagaatagaatttggttgttggaac

gtgtgagaaagaacaagaaaatatatggttggtacgaagatgagatccatattatgggta

ttattgttgttgtttggttgttttgtgattgtttctggtgaaagacagcttgtgaaaatt

caagtaccaagttttaatgttacttctatgattcaagaacatactagttttctcacgaaa

gctgttaatttgttatggaaatctgatggatcgggatatacacacgtgtggccggaaatg

gagtttgggtggcaaatagtgttgggttcttttattggattttgtggggctgcgtttggg

agtgttggtggagttggtggtggtggcatatttgttcctatgcttagtcttattattggt

tttgatcctaaaacatctacggctatttctaaatgtatgatcatgggagcagctctgtct

actgtttactacaaccttaggctaagacatcctaccttaaacatgccaataattgactat

gatttggcacttctgattcaacctatgctcatgcttggtatcagcattggagtggtcttc

aatgttgtttttcctgattggatggttactatattgctcattattcttttcttaggaaca

tcaacaaaagcattctttaaaggaatggagacatggaataaggaaaccataatgaaaaag

gaggctgctaggagacaagaatcagttggttcaggagaatacaaagctcttccaactgga

ccagatgttgtcactgaaaaggaagacaccaaggtgtctgtgtttgaaaatgtttactgg

aaggagttcgggctactggtatttgtttgggtttcattccttgcactacagattgctaag

caaaactacacaactacatgctccacggcatattgggtactgaacttgttgcagattcca

accgcggttggggtaactgcatacgaggcaactatgttgttcaccggaaaaagagtaata

gcttctgttggtgatcaagggaaagaatttacagttttccagctagcgatctattgtgtc

ttcggcctgttggccggtatagttggtggaatgctgggactaggaggcggattcattatg

ggtccactttttcttgagctgggtgttcctcctcaagtgtcgagcgcgacagccacgttc

gcgatgacattctcctcgtctatgtctgtcgtggaatactacttgctcaaacgatttcca

gttccttatgctctttacttaagcatggtggctactattgcagccttggttggacaacat

attgtgagaaggcttatcatattatttggaagagcatctcttatcatctttattttggcc

ggcacaatattcatcagcgcggtctcactaggtggagttggcatttcaaatatggttcac

aagattgcaatgcatgaatatatgggatttgagaatatctgcaagtatgggtcatagcac

ttttaaggagattatttttttagtgtgtttaaattacatccaagttaggtcaagagctaa

ggtggacaagatagattatttttatcctgttagaaggtgtgatcttttttattgccaagt

catggagtgattctgtaggaagctcagaaacttatatgtattcaccctgttcaagtcatt

tagatttttaagacttatgtgatgtgaatctgtgtcgttcgatgaaaatttgacaataat

ccggtttgtgaaatgctagtaatatattcaatagctcactattgattggcaaaacgaaac

cctcacatatttattgtctatttttttcttgggactaacttaagagtttaagtctacatc

ttgtaatgtgtccgttttattttgtttttc

>fava_dn_rep_c986

ctcgtgagtggtggctgcgttgtctctttcctgaactcattcgacgatttcgtcttctcc

cgtgcctctgtgttcacaacttaaagacagaagaaaatgtctggtcctaggattgctcat

gctaccttgaagggtccaagtgttgtaaaggagataataattggaataacacttggtata

gctgctggtggtctctggaagatgcatcactggaatgaacagaaaaagaccaggaccttt

tacgatttattggaaaagggtgagattagtgttattgcagaggaagagtaaaaatgacgt

gtgtctattctttccatctacaggctattttgtttactgttatattcaattggctcttta

agtgtatctgcgccgagctatttacacattgaaaaaataagttgttgccctgaattgttg

actgactatgttggcttgtagaaattttatgaaacaatgtttcattgtgtttgtgttgtc

ttgttgatgttcctccaaaagtgtagttttttacatatttttcccggctgtattattaga

attaacaggtgtataagaccagcattttatgtcaccacatggcaagtattcagtgttttg

ttggtaaattataatatttgttaacctttccatctaatcctaaattttcaaactgtacaa

ctttacaagtacagacttcagtattaggaggcaaaagtgtatcctagaatattacctaga

atatcctggtattcaaatttaaactgcatccaaatgtggtgtatctcgttacttgaattt

aacctggatccaaatgtggtgtatcttgttacttgaatttaaactgtaaatttctctgt

>fava_dn_rep_c987

catttttagttggcgaagggattgaagaaaaaccgtctatacaaacgtatgtaaaatata

tgaacgcttctgcattttttccatatttgttagattaatgaaattgaatatctaattcga

tttctttttttttatttattgaatttttttttttttttggaaaaattcatgataacatca

aattcaagcaatacatgtttgaaatgaaactgagttattaatcaagatgaagccttgtac

ttgacatgagaaacataaaaatggcattaacatttatgtaacactcagtttatgggaaaa

ttagtcaagaaaggagaaatgctgcagcagactatgaaggcttatagtattttgtgggta

caaacggcattttcgtgacaactccttcattctgtttacctcgaataatgatctttgctt

tggtacctgctttgtgcaatccagatttgacatatccaatagctatgttcttcttgagac

aaggactgaatccaccactggtaacttcgccaatgttgttgcctccttcatcttgaatct

cgctgtggcttcttggaggtggaccagaagaaatgaaaccgacacgccttatggaaggac

catctgcgagctgtttcaggataacatcggcacctagaaaaccaccttctgctctccttc

tcttacctatagcccatgtcagtcctgcttcaataggtgtaatgtgctgttccaagtcat

ttccgtataagcacaatccagcttcgagacgtagactatctctagcaccaagtcctgtca

atcttattttcccttcggatttttccagcagtgccttggcaagttccactccatgttctg

aaggaactgagatctcaaatccatcttcaccagtgtaccctgtccgtgtgagaaagcact

gcgagccattgatgtccaacacgcggaactctccaaagtacagcttgctcaaatcctctt

ttgtcagatgttgaagaacaggagcagcaagaggaccctgcagagcaagtagagatctct

catcgtgaatgtgccatgacacatcaccacctttagccttgaatgccttcatatgctcct

caatatgagccaaatctttatctctgcacccagcattgacaaccaagtaaaggtgatcat

cagtgaccttagtaattacagaatcatcgatagcccctcccttttcattagtgaaaacag

tcaatgtcccggtcccattggcaagagcagcaacatcagcaatgacaagcttctcaagga

atgtaacaacatcctttcccttgagactaagaccacacatatgcgaaacatcaaaaagac

taccgttctgcctacagttcaaagttgagtcgatgattgagtctttgtactgaataggca

tactccaaccagcaaacggaaccatctttccgccatgagcaacatggaagtcatacaaaa

ctgtcttcttcagctcagattctgtggcgaaacatcttcgtgcaacagccttcttatctc

ctccattggcaagacggcgagtgatggattggccaagttgccacaagccccctctcattt

tagagattggattggttctgttctgttctgttctcaaacgaaatacactagagaagaaaa

aggctcgtttatgtgttggttttggatacaaatacaattaatatatttattttgtttatt

taatttaaagttaaatttaataaataaaatagaagaaaaa

>fava_dn_rep_c988

accattttccctttttagcttaatattttgtctcataaaaggtatcaccactatatatcc

attcttcttgagttgagatattcttactagagtgaaaaaagtaatatgaaaattcagtgt

gatgtttgtgagaaagctccagcaacaatgatttgttgtgcagatgaagcagctttgtgt

gctaaatgtgatattgaagttcatgctgctaacaagcttgcaagcaaacaccagaggctt

cttcttcaatccctctctaacaagcttcctagatgtgacatatgccaagataaggcagct

ttcatattctgtgtagaagatagagcactcttctgtcagaattgtgatgaagctattcac

cccgtcggcagtctttctgccaaccatcagcgcttcctcgcgaccggtatccgagtggcc

gccgcgagtactaactgcgccaaagacgacgaaaagcctcactttgaaccaccaagcagg

aatcctcaacaagtttctgttgaacctccttctcaacaagtccccaacttctcaccctct

tggggtgttgatgacttactggagttagcagattatgattcacataacaaaaaggaatcc

atgcaatttggagaaatggaatggttcactgaggagggactgtttggtgaagagtttaat

caagaagctatttctgcagctgaagttcctcagcttccagtgactcatcatgctagcaac

cattattcgtcacacagaaactcaaagtctaacaaaaagccgagaatcgaactcatacgt

gattatgattatgatgatgaagatgagtacttcacagtgcctgatcttggatgaagaaaa

tgattatgtgggatggaataattatggaaatttaattatttttctgagattcttttaaaa

gaaattgtaaagaaaagttattcttccaaattaagattttgaatttgtatattcatactt

ctctcagagtacaatagtttaagagttgaagtatgttttttaagttgtatggagtttatc

atataaactttcatgtgtttggttgtagaatacccatgtttgtttcttgttacttcccac

ttttataaaaaaaattgtttatagctaaaaaaaaaa

>fava_dn_rep_c989

cgactaaagtactaaggaatttatgtaaaatcagataaaatcccattttatgtgttgaat

ccttgattacacttgctgtggttagaatacaaaccattatcgatagtacattatggaaat

agttaccattttctgtttgaaaaacttagtgctgtatgtcatcaaaaataactcatgata

actcaaaactgatggctgacaaaacatattaaggaaaatacctgacaacataaataacaa

gtttttaaatgtctaatacaaatattaaaacttcaaatctgagtttgataaacaaccaaa

aacaagggcaaaaaacaattgtaagactgttatgcagtctagaacactaactcatgaggc

ttcacacctgccttcaaagaaaacctagcaccaaggtaagtctttagatcaggaacactt

tcgatggcttttaacaaacctgcatcaagagtcttttgatcatctttctttgactggggg

agcactttcttatcctccttgtctgcttcaaagaattcaccctctcccttctttgttttc

ttttgggattcttttgagaagtacttgtcatcaaacttttcaacgttaacagaagaaaca

tctactttggttgatgtgccaataacatatgattggttgacacgcctcaatggaactccg

ttaatcttgaaagggccagtaacaaggaggagtccagaaggaagctgcttgagaaaaact

actcgctttcccttgaatcttccggcaagaagaatcaaaactgttcctggagtaatgcta

gccctaagctttgtgggctttggtttgtgtttgttgcgtagtggcttcttaacatcatca

gcaggatagaatttaggagccttctcgacaggagcttcaggcttaggagctggatcgtgg

cgggggaaagcaccaccgtttttggccttaatagcccagagtcccctcttgtggtacatc

gtggacctggagtattttccgatacccctgatcaattcagggttacggctgactctgggt

gttctttcggttttctgtgttggtgccattgctgctgctgctcttcttcttccaacaaaa

aaaccctaattctgtccccgggnttacccacactttcacaccgacagcaaaa

>fava_dn_rep_c990

tttttttctcaaactcctttgcaaaatttgaatactcttgtttggttcaaattgaatagc

gtacattaaaaaatggtgaactcttggtcaaaagttattttgcttgtgcttacattgatt

tcctgattcctagtatactgaaataacctttttgttttctcttgaataataaagaaaaag

tagaggaacaacatacatgtaaaaagaacaaatatatcaggacctgaaataacctaaatt

catatactgctccaaatgtttggctctcttcctgttcttaagcttactaaaaccacataa

ctcaatttgtctaactctctccctactcacgcctatcatttccccaatctcttgcagtgc

cttcatccttccatcgtccataccaaatctccatctaatcacctgtttctctctcggatt

caggcagtctagtaccttttctaggtcctttttcattaactgcttaatcagctgttcttc

agctgtttcagcttccggtgcagaaagtacttctgaaggtttaagactctggttgatccc

tattttccgctctagagatctgggagattttggggccaaataaacagcagtaagcgtctt

cattgacagcccagttgcttcagcaatttctttatcgtctggttgtcttccattttcact

gtacagttgttttcttgcctcccttactttgtaagttgctctcaccaagttgaatggcaa

gcgaataattcttgactgatcagaaagggctttcccaactgcctgtttaatccaccaatg

agcataggttgagaacttaaagcccttagaagcatcatacttctctgcgccttttacaag

gccgcggcatccttcctggactagatcttgcagactcatccctgatttctggtaattctt

agcaactgatatgacaagtcgtatattgcttttaatcattttttctttgcaacgctcacc

ataatttaatctctttcttagggtcttctgatctacccctgccattgtagcccattgaga

aaatgtgggctggctaccacatttttctgtaagctcctcttgaagtttttcgagctttat

cagatcctgtattcctgcagacaagttaatttcttcagatacagtaagaagctttgaaga

tctggttgcagttctcaagtaacgaagtggatcagaatagtcaacctgttgtgtccgaac

acgcttttttctactggtggatccagacctaaaggacacaatattagtggcagccttccc

agatgctctgagtcttctagcttttctttccgtttggcgtccagatcttgcatctatact

atccaacaattctttgtcgatgtcttcaagttcctcaaaggtaggctcctcctcttgggt

tgaagtattgtcaacatgttccattatttccagtccagtagcaacggatccttctgctac

agcatcttgtgcttccatatgttgaacccatttgagaagtaaattttcaggcgcggaagg

aagttgtggcctaaacaccggctgttgtggagggttgtcttttactaatagagttgcatc

ctttgcaagttttgcagcagctttagcaagagcaagggcttcagaagcagcataggctat

tgccgcgtcttcactagtaagcagtgattctgcagatgaggctgctttaaagttttccgc

tattgccgcgtcttcactagtaagcagtgattctgcagatgaggctgctttaaagttttc

cgctattaccgcgtcttgactagtaagcagtgattctgcagatgaggctgctttaaagtt

ttcctgagaagtgacataggaccaaggagtgtttgcagcatcgaattcaaggagtgcggt

tgatggtgccgaaggagacagaatacagtgtgggcgtcttaatttaatttgaagaatgat

atgatgatggcggtttgtagggataggaataggagttgtgaaagtgttaaagtgtggcaa

gaaacaagacattatcttcccgtttttaaggatgagaaatgtaattcggaggtttcatag

ggcggaacacaacagaacttaaattcaacggataaagggccgctctttcttcttctcttc

ttcccgtttcattcggataagaaactaacaacaagagggtgagctcgcttcaactttctt

tttttat

>fava_dn_rep_c991

aacaagcattagtccataattggtttaaaaccattcagtcctttcaatgtcatcacaatt

agtgttagtaccaaggttcatccattttccttgggaaacccccaaaaacactgcactctt

gaaacaccgttcaacacaattctccacttgcaatttttctccatctttctcttccatggc

taccgatttcaatcttccacaattggattcttcttcttctctttcagttggacaaactga

tctcttgatcgtcggtcctggtgttcttggtcgtttagtggctcatcaatggcgtcagga

atatgaaggttgtcaagtttttggacaaacgataaccactgatcatcatgaggagttgat

tcaattggatattagtccatctttgaattggactaaatttcaacacaagtttccctatgt

gattttttgtgctcctccttatcaatcttcagattaccatggtgatctcaagcaggctgc

atcatgctgggatggtgaaggttcttttctgtatacatcaagttctgctccttatgattg

taaagataatggtttatgtgatgaggattctccagttgtgccgattgggaggagcctaag

gactgatgtccttttaaacgctgaaagtatcgtgctcgagtatggaggttcagttgttag

actatctggactttataaagaaggtaaaggtgcgcatgcttattatttagagaaggggat

tgttgaatcgcgacctgaccacatcctgaatctaatacattatgaggatgcagcttcact

ctcagttgcgatcttgaagaaacattttcgtggaaagattttcttaggctgtgataatca

tcctttatccaggcaagaattgatggatctagttgaccaaagtggaaaatttagtaaaaa

gttcgagaaattcacaggaaccgatgatcctcttggtaagagattaaacaactcaaaaac

gcggcaggaagtagggtgggagcccaagtattctagctttgctcatttcctcgagacctt

atgatcaatgttgacattgtatataaaacattatcattcatttcttgaatatgtgttttc

tctactcctctacctggagttagagttagatttactttgaaggtgaacactgagcattga

taagtcataatactacagaaggacatgtgtgaatcattttgattaagcataatcatgaaa

tattatcattacattttccatatgaattttttcctggatatcatttaaaa

>fava_dn_rep_c992

aaacttttctacttaaattattaataacaaaaatgaaattaatttgattctcattctcac

aactgagttacatataaaaaatgttacattaaattgcaacaagtttcacaatccttgagc

caatacaagtaaaaagcaataaaaataaaaaaatacaaaaaaagactttcaattcaacat

tctactactaataacatctatgatcttcttctccattgtaaagaataataatctgttaga

tctaacctgaaagcatcaaaattctaaactagatttattcaatctctatgttcacaaaac

aaatgtgttagtatgttgtgaagaagaaaaaaaaatctaaagttttactagttagcaaca

acattgtctaatgaaaacaatcatagaacagaacaaaacataaccagaaaaagcataaaa

ccagaagcagcattcatcgtttccatgcataaacaagcaaagaaaacccaacaggaacaa

ccaattcattctcctcaacactagaccattccaaaaccgatgaacttccaccaaaagaac

cacctgaagacgccatcgaaaccgacgaagaagaccaactcttcctcattttccccatct

cataacctcccaaattcacattctgctgtgtccacaaactcatcaaactcttatctttac

ctctttgatgttgatgttgttcctcttcatcttcttcctcgaatttgaacatgaaaatcg

cgtgtccgtcgctgttatctttcacaaacaaccaattataaacatcccacgagatctgaa

cgggtacaccatatatttcaactctttcgtttcctctgaacttccatttcagtctcttaa

tctgcaacactttcgttccatcaacagtgaaacaaagcttagagtgatctcttccaccgc

aatctatctgaatatcttttttttttttccgcttattgatatgcttaaattcagcgggta

gccccgcctgacctgaggtctccccgcggccgtaatggactctgcgttgataccactaat

attaacaggtgacttgaggcattatcacaactcacttaaaactccatacttatttgttaa

agcacaaagatatcaacacttaaaaaagatctgcaattacaaatcatagactttcaagac

cttaataaattccatgtcattggcaaagcaccaagaacaaacaaagaaaccaggttatat

atgatatgtttcatggcacacacacagaaacataaacaaaaggaaaacagctcacgagtt

gagtattttcataactacaaatatttgaccagaagccatatgataacaaccaagaccact

ataccaatcacagataatagcatgcacaagcatgagcaaccacccttagtgcgtttgttt

aaaattgccaagttcttctgcactctcctcagccgagaatccgtgacatctacatgttcg

tccaagtcatcaatgagtctagtgtgcagagtcagctcttcattcactgccaacgcaata

tgtttcgtacttaatacagtttcctccaatttctcaaggccctcatcttgctctttcata

acttgccgttgcagaccaacaagtttattgttatccaagccaacggttctgctcattgca

tctggttttatttctggaccaagcaaactatccctatttgcaaagtttgacatgttcaac

gttgaagccatttgattaacctttgacctcaaatttgcaaggttgtccttgcgacgattc

atttccttctcagactttgcagggacccttgacaaaagggattgcaagctatccagtctg

gtccccaatattgttatcttccttcttatagcagatgcatgacgctgtgtttctggtcca

gatgaaggaaatgaattatgttcagaaatcatgccactgatatcatcggcaagtttcatt

gcttcattatattccttcatccatgagtctgaagaagatgccattggccaggaaatttag

gagaaaatagcttcgtctgaattagggtttcgcaattgagatgaaagaggatcgatcttt

gatacgatgccgtttaaggaaggtaagaaatagaagcgtaacgacggcaccaggaagcgg

caccgttagtctgacggtgcgtcgaataaggaatctgaaaggaacgaaataggtcgctga

acgacgcgtcgtttatggtgtagtgtatactacctttgcgctacgcctacgcgggttatt

ttgtttgttgtgaatctctgacatgtaaaaagtgcgag

>fava_dn_rep_c993

caaaacacaaaactaaatttttattaaatttttcactaaataacaacattaatatattat

taactcacaataaaggattttcataattgatttcaagtacactcatacaatataaattgg

atactaaaacttaaaatcaagaataaaaatcttcatgaataacagttattcatattaatt

tataattaattttcttgttatatttctaaaaagtaaatgtagagtagaataataacagta

ctagtagtacccaattgcacggttcacggagccagcaccaccatagtgggtcccactctc

cccactcttcgtgctcacccacgtggtccccactcttttctatccactcgcgtgcccacg

tgttcttctcccttctcaaacgacgtcgttgttgttctcttctctctttcctcgtcaatg

cttggaagaagatcgcgacaataactgaagctctcgcctatcataaatctccaatgaaaa

gtctctcaacgttcgcggggtggttgttgcggcggtgctcggcgccatgacggtggctct

cgccggagattttgacgacggtgacgttgaacatgggatggataaggtggaggcttgttg

gagttgaactacgggagttatcatggatgaaacgaaaccggagattggtctagctaatgt

gaaaaactgaggctgattcgctgggaccacaaagaacgcgtttccggttggaacaacggt

gtttgatggtatggcccacacagggaccatgttttgcggtaacggaattgcagtttgttg

cgaaattatcgcggtgttattcatgtttgtgtttacgttggaggttgttactaaacctgc

tgaaactgaaacggcgctctcgtttatgtccacgtaagcactgttcgctggtcgtttgcg

tttctttttttcaggcgagtcttcggattgctgttgttgctgttcctggtcggagttctt

gttgggggaagtggttgggattttgagagttccgttgacggacatcgcgatggcggggac

ggtgccggttccggtggcggcaatgatggcgggttcggcgtgctcgaggagccatcggat

tgtttcgccgtctgatttatgtccgagttctcgggtgagttggaagatccgagccgcaca

cgtggcaggcattcgaatcctccggccacgaccttccacttttgtgtgacggtctttagt

ggatgctcttttttgaaccgatagttgggcttgggcttgggcttgggcctgatattgagc

ctgagtttgagtttggaataacgtcttgttgacttgcatggacataggtaaacccgtctg

caccggcatagccttcggtatcacctttattgcggagccatcttcagattccggttcctc

tttgggttctccgagagagagatcaatgtttctagaatcttcttcagccatcatacttga

tcagaaagaccaaaacaacgccaacaaattaggaggatagtgactatgaaggtgttttgt

taagagtgtttaactt

>fava_dn_rep_c994

aaagaagcaattatctttttaatgtaataaggaagaagcaattatctttttaatctatat

aagcatgaacatactgtactcattttttttgtctcatctagatatgttacattaaaaatc

ggttacatagcattatttccttaatatcatctttgcttaaaatgtttgtgcatgccataa

caaaattaaacaatgagaatgtacatatacatatatatcactcatggttgtgagagatgt

aactatttacagtgaacttccttaatccttatatcttcaagcttgaagagtgaagacaga

tggaaatgtttctactttgttgttgatggttggaaaagcagcattaaataagcagcaatg

catcattatcaagcttcttgactagtggttagtccagcaatttcacttagtggcttccac

ataaactttctccaatttccacattcagcaatgaccctcacattaggccttagtgtaggc

agcagagcaaggaattgcaattctgccttggagagaaccttggcttcaatgatccacacc

accaatcctttggcatttggtggtaatgcttccaatctgtaatcaacttccggcggaaaa

taccaccttgctatcggttgctttcctaaatgagcaggacaaacaggatgaatcttggtg

agagtggtttcaggatcacctagaccacgagctgtaaggtcaagaaaatagtaaccttga

cgttgaagacgtgacaagtaacgtccttcatatccaccttcatgaggagtatatattgca

agtgctttatgttcttcaacatcagaaagccaacgtccaatatctaatttaaccaaatct

cctccaatgaaatctcctaacccaactccaccactgcatttcactaccctattacttttc

ttactgttacattgttttcctctccaatccaccatcatcaatatctcattgttgttctgt

gttttagccatgaaaccgtttcttgtttgttgatgaacattgcaacatgttggtcttcta

caacctaagctagctgaggccaacattttggttgtttttgtttatttattgatgaatgtg

gtacatgtttttttcttcatatgttgaaagataaagaaatattggtttggttggaaatta

gacaagggatgaatggtgtgagatgtatggtggagaatgagataaagtt

>fava_dn_rep_c995

taagactagctggaagaccttcaactgaaggagnnnnnnnnnnnnnnnnnnnnnnnnnnn

nnnnnnnnnnnnnnnnnnnncaggcaaagaatgcaactaatannnnnnngttgattagtt

ctactcgaagttccagcgacactggattactagatatgattgaagggaagcttgctgttc

ttcggtttcagataaaaataaaagaggaactagaggctatggcctccagttcagaggtct

tacagagcacatccaattctgttgaaaatggtattgtccctgaggccagttcaactatcg

atggcaatgtaacacgggagaaagccaaggagttatcatcagatttgaagagcataactc

agttatataatgaatacgctgttccctttaagctctgggaggcatgcctggagatgctgt

actttgccaattattcgggtgatagtgacagcagtattgtcagagagacatgggctagac

ttatagatcaagctatttcaagaggcggcattgctgaagcttgctcagtgcttaagagag

ttgggcctcgactatatcccggtgatggaactgttttacagcttgacattatttgtcttc

acctggagaaggctggactggagagattaaattcaggagtcgaaaccgttggagatgaag

atgttgcgagagcgcttgtttctgcttgtaagggtgcagcagaacctgtcttgaacgcat

atgatcaattgttatcaaatggggcaattttgccatccccaaatcttagattacggatgc

tgagatcagttctagtggtacttcgagagtgggctatgtctatttattcacacaggatgg

gtacaggtgccactggttcttccataatattaggtggtggattctcattggaacgaacag

ttgctagccaaggaattcgggacaagatcacaagtgtggcaaacaggtatatgactgaag

tacggaggttagcccttccgcagagccaaacagaagtggtttaccgcggctttaaagaac

tcgaagagtcactcataagtcctaattcttacggtcgattttgatctttgttgcaatata

gtatgttgtatcaattgctcttgtgaaacactattatttgaaataagtttcaaatgttcc

atattggaagtgatttctttttctttcatttttaacccagtcttccaaaatcagtcttgt

gtaatcaattaagaatatcatcatatcatatcttggggtatataatattctgcctcccta

cctccatggacgtgtcaaccaagcaatcatttgagcctaattctgtttgatgttttcta

>fava_dn_rep_c996

aaagtcttcctaccaatcacatgtgcaccaaaacaaagcacttcacttcacttcaactta

cttggattgccaagcaaagttcttgcaagagctgtttgatgaaaacattgaaatgatatg

gcaaaaactgttgtgtcatttgttctggaacaacttggccaatttgtattaaaacatgct

ttccgatcagtagtagaagaaaaaactttggtgaaaggagttgaaagtgacttcaatgaa

atcaaagatgaacttgagagcatccatgccttcctcaaggatgcagacacaaggtatggt

gatgatggtggaggaggaggatccaacggagttaaaacttgggtgaagcagctcagagaa

gtatcttttcgcatacaagatgtcattgattactacatcatgtatgaggcagaaaaagtc

aaccatcctaaagcttctgagattgcttctgagattcaagacatcaagttatcacttggt

aaaatcaaagaaagaagtacaaggtttgagtttcggtctgaaggtggatcaggaagctat

agagggcttaaagctcctagaattggcgaccctcgaatggatccttttttcattgaagag

actcaagttgtagggtttgagttcccaagagatgaattggttagtcgcttggtcggggga

aacgatgagctcatgttggtttcggtggttggtatgggaggactcgggaaaaccactctc

gccaagcatgtttttgataacgagctagttagaggggactttccttgtagatgtttcatc

acggtttctcaatcatacactatcagagacttgttgatagaaatgataaagaagttttgc

aagatctgcaatgagcctaatccagagggtctgcataacatggatgatgaaagtttgatt

actaatgtcagaaaataccttgagcctaaaaggtacttggttatatttgatgatgtttgg

acagataatttttctgatgagattaaacatgccttaattaataataaaaggggaagtaga

atccttgtgaccactagaagcatgcatgtagcagtgaattctaagaagtcatttcttgtt

catgttcatgagctacaatctttgcctctaaacaaagcatgggaactgttttgcaacaag

gcatttagagggcagtgtccaacagaacttgaggatgaatccaaagaaattgttcaaaaa

tgtggagggctaccgctagcaattgtggccattggtggtcttttgtcaacaaaagccaaa

accatatttgagtgggaaaaggtgagtcaaagtctgagaatagagttacatcgcaatgtg

catttgactgacttagtaaagattttatctcttagttatgatgacttgcctttccatttg

aaatcatgcatgctttattttggtatatatcccgaggactacataatcaacaggaagaga

cttactagacaatggatagctgaagggtttgttacgcaagaggagggaagaactttggaa

gatcttgctgaagagtacctcatagagttcatacatagaagtttagttagtgtttccaaa

gttggatttgatggaaaagtcaaaagttgccaagtccatgatttgatgcgtgaagtgatc

attagaaaaatgaaagatttaagtttttgtcatttatgccataaagatgacgaccaaatc

actcttggaataactagacgcttttctatagcagctatctccaacaatgacttgacaaac

actagcaactcgggaattcgtgctatatttgtcttcgacaaaggtgagtttcctaatcac

ttggtagatggattaattgctaagttcaagcttttgaaagtgctggattttgagaattct

ttgttgaattctattcctgatgacttggggaatcttttccatctaaggtacttgaactta

agtcatacaaaagttacgattcttcccagatccatcggtaagctagtcaacttagaaacc

ttggatctaaggcaaactcgagtgcatgagttaccagaagagataaacaacctcacaaag

ctaagacttcttccggtttattatagaaaatatgaaaggcattattctatgttgaacttt

acaataggtgtgcaaatgcaagagggtattgggtgcttgatatctttacaaaaactttac

tttttggaggcagatcatggtggaatagacctcatccaagagctcaaaaagttgagacag

ttaaggaagttaggcataaggcgcgttcggggagaatatggaaatgcattatgtgctgca

atacaagagatgaagcaccttgaatctctaaatattactgctatagctgaggaggaaatc

cttgacttggattttgtatcaactccacctaaacttagagtgctcaacttgaaaggtaga

ttagcaaacttgcctaattggattccaaacctcaagtatcttgtgaagctaaggcttggt

ttatctaacttccaaaatgatccactagactctttaaagaatttgccaaatctgttgagg

ttgaatttgtgggatgatgcatttgctggtgatagtttgtgctttaaagttggagggttt

cctaagctgaaggaacttgatctcactagattgaataaactaagttctatcactattgaa

aagggggctttgctcagtcttcatcatttcagatttaacaacaaccctcaattgaaggag

gttccgcaagaccttcgacacttggaaaaccttcaattccttggatttgctgatatgcca

cctgaattggttgaaagcattgatccgcgcagagggggagaatgtcattggataatcgag

catattcctctagtactcattcggcagaaagtaggatcaagattccatgactatgagttg

tatcctattcctactatatctaatgtctgaactatcaaggttcatattcttcatgttgca

gctgagatgaagatgcactttgtctcttcattccttctatggtgtgcttaataaataatt

tacttgaattgaaccatctgctatatgatcaaggaatctgtactctattaatagaccaat

gcttatgttttctgtcatttgtgtgtggtgttgtttattaatggttgttgtgtggatgct

tttctgtagacagtagaaaggatgtttcaatttgctatgaatgaaagaagtatgttattt

acaggatgtgttacattgatgaattagttgcagcatagtattataatggatgcatatttg

tctagtagagtactcaatagtaagcaattaagacaacactgattcattattt

>fava_dn_rep_c997

atcactttagcatctacccgttaaaaataaaatttgtgttgaaagggtnnnnnnnnnnac

agtatcccagctttcagctttgtgattcctggataaaggagtaatgctgttagtttatgc

aatttattacaataataactggatacattttatgcaaatattgtttgtggtaaatacaaa

gctcataaaaacatgttcatttcggtataatacaaaaccagatggatgagtaaacacata

agccaaaagcccaaacaagtttggtctagcagatgcatagacacaacaatcaattgaagc

tccattgaggagacctctactgtttcaactatgcaactaaatatacaaccattttaaatc

ctaaaatggtgagggtgttgtttcctcttattctaatgaaagagaaataaatgtgtgtcg

tcaaagaaattggtgttggatccgtctcacttctggttttgttccacctttgctcgaacc

ttttgctccaaaacagatatttcagtctcaaggctgaacattctgttcaaagcatgcatg

ttctcaagggtctcagatagagtgcggtcgtcactcccatcacaccttaaatgttgcatt

ggaccatgaagcattctattcacaataccccgactcagatcatccacagctttccgcgtc

ttctttgagatatcatcgcccatcttacccaagcatttttcaagctcagcaaccctgatc

ctttcagcataggccctcaattttttaatggtgggaacagtttccagtgagtccctccaa

gcttcaaattggtttaactcttcattaatgatcacctgtgcttccattgcttttcttagc

cgatcctctttgtttgcagccacaacctctttaaggtcgtcaacattgtaaagtcgcaca

gattcaatctccgagacacacgaaccaacattccgaggaacagaaatgtcaatgaaaagg

cgatggcctccaacattttgacttgtttgaggaaggtccttgacatcgtctttcaagaat

aatggggtttctgatgctgtactggtgaatacaacatctgcttcaccagcacaggagagc

atttctgaaagaggtttgtaaataatttcaatatccttcagatcttcgcgtattgctgca

actctttcctctgttctgttgacaacaaccacccttgcgcaaccttttgaagccaaatgt

ttgatcactagctttcccatcttcccagcaccgataaccagcattctggcattatcatga

gaggctccaggtaacttcatataggccaactcaacggcggctgagctcacagaaactgcc

ccagcagaaatattagtctcggctctcaccctttttccgacagttattgcatgcttgaat

agaccactaatgtttcttccaaatccattaactccttgtccaactttcacaacttgctta

acctgagcaaggatttgaccttctcccaatacaagagactcaagacccgctgacacttga

aaaatatgttgtgtggcatctttgttgtaaagtaaaaaccggtggtcacaaagctctgaa

acaggaacggaacttgtttttgacatccattccatgacctctttgacaccacgatgttgg

gatagagcaacaacatatatctccatccggttacaggtactcagaacagctgcttcttca

atatgattgagactgcacagctctgcaatagctctaggccattctgcttccggtatggca

agcttttcacgcatttccaccggagcagtgtgcacactgagcccaatgacaacaatgctg

ctcctttcctttgtatacctatccgctgaagaagttttgagcagctcaagaggagagagg

gagctactgctgccattattctgaagagaattttgagcatcacaacgaaaaatccctttc

tgaatgaagaatgttctggatttggtgagaagtgaaaaatgggaatgagtggacggtggt

gatggccttgaagaagaattgaggaagagagagtcgaatttgggagcagagaaagtggtt

gaaacggccattgtgagagaagaaccctaaccctagaaatggatttgaaagataggagga

ggaggaggaagaggaagggggagaaaaaaacggtgtaataaaattgacccttccacacac

tcggtgtggttgtgaagatgatgggtttttagacagaaaattgtttgggatgtgtttttg

tccg

>fava_dn_rep_c998

acatacacatactaggacacactctctttaacctcaaaactagttctcattccaatgact

acatgttcgccacgtggctcatgctttcaccattaaccatacaaaccgttttcattggaa

gacacactttttctctctttctatctcatactcctcacaaatcaaataaaaactcatcaa

cacagaagaagaaaaacacagtttcaactaagttgagttgagttgagttgagaaattcac

cacacaactcaactttggttaagagtgtaaaaatttctcataccccatcttttcttttaa

ccaaaatttgctctttattgtaaaaagattacatttttagtttgtttttcttagtctaga

atttgggttggtgatcaatggcttctttggtatcaacaccatttactctaccaaattcta

aagctgttcaactttcttcactttctcaaaagcacctctttcttcattctttccttccaa

agaaaaccaattactatggtgcctcaaaagcctccttaagagtgaaatgcaatgcagttg

gtaacgggttgttcactcaaacaacccaagaagttcgtagaatagttccagagaataagc

aaaatttacctactgtgaaaattgtgtatgttgttcttgaagctcagtaccaatcatcag

tctctgcagctgttagagcactcaacaccaaccagaaagatgcttcttttgaagttgtcg

gttacttagtcgaagagcttcgcgacgtatccacatacaaaagttttgaaaaggatttag

aagatgctaatatcttcattggttctttgatctttgttgaagagcttgcactcaaagtga

agactgctgttgagaaggaaagggacagacttgatgcagttttggtgtttccatcaatgc

ctgaagtgatgagattgaacaaattgggatctttcagcatgtcacagcttggacaatcaa

agagtcctttttttcagcttttcaagaagaagaatacttcttctgcaggatttgctgata

gcatgttgaagcttgtgagaactttacctaaggttttgaagtatttgccaagtgataaag

ctcaagatgctaggctgtatatactgagtcttcagttttggcttggtggatcacctgata

atttgcagaatttcttcaagatgatttctggttcttatgttcctgcattgaaaggtgcaa

agatggagtattcagagccagttttgtttttagataatggaatttggcaccctttagcac

cttgtatgtatgatgatgtgaaagagtatttgaattggtatggaactagaagggatgcta

atgagaagctaaagagctcaagtgcacctattgttggtttgattttgcaaaggagtcata

ttgttactggtgatgatggtcattatgttgctgtgattatggaacttgaggctaaaggtg

ctaaggttattccgattttcgctggagggttggacttttcggggccggttgagaagttct

tgattgatcctattactaagaaaccttttgtgaattctgtgatatctctcactggcttcg

ctcttgttggaggacctgctaggcaagatcatccgagggctattgaagctttgatgaagc

ttgatgttccttatattgtcgcattgccgctggttttccagacaacggaagagtggttga

atagtactttgggtttgcatccaattcaggttgctcttcaagttgcattgcctgagctag

atggaggaatggaaccaatagttttctccggtcgagatcccaaaacaggaaaatctcatg

ctcttcacaagagagtggagcagctttgcaccagggcaatcagatgggctgaattgaaaa

gaaaaacaaaggaagagaagagactagcgatcaccgtcttcagtttcccaccagacaaag

gaaatgtaggaactgctgcctacctgaacgttttctcctccattttctcggttctaaaag

aacttgaaagtgatggttacaatgttgagggtcttccagagacttcagaagcattgattg

aagatatattgcacgacaaagaagcgcaattcagcagcccaaacctcaacattgcttaca

aaatgaatgtccgcgaataccaaagtctcactccctattctgcagcattagaagaaaatt

ggggaaaggctcctgggaatttgaatgcagacggagagaatctattggtatatggaaaac

aatatggtaatgtattcattggtgttcaacctacatttgggtatgaaggtgatcctatgc

ggttgcttttctccaaatccgccagccctcatcacggttttgcagcatattactctttca

ttgagaaaattttcaaagctgatgctgttcttcactttggcacacacggttcccttgaat

tcatgcctggaaagcaggtaggaatgagtgatgtatgctaccccgacagtcttattggga

atattcccaacatctattactatgctgcaaacaatccttctgaagctaccatagccaaac

gccggagctacgccaatactattagctatctgactcctcctgcagaaaatgcaggactgt

acaaaggtcttaagcagttaagtgagctcatcgcctcgtaccagtccctcaaagacaccg

gccgcggacaacaaattgtgagttcgattatcagcacagctaaacaatgtaatcttgaca

aggatgtggatttgccggaagagggcgtggagctcccaaccaaagagcgtgatcttgttg

tcgggaaagtgtatgcgaagatcatggagattgagtcccgccttttgccttgtggactcc

atgtcattggtgagccacccacagctatggaagcaattgctacattggtgaacatagctg

cacttgatcgtgccgaagaaggtatttctgccctcccttctatactagctgagagtgtag

gaagaaacatagaggaaatttatagatcgagtgacaaaggaatattaaaggatgtagagc

ttcttaagcaaataactgaagcatcacgcggagcagttacttcctttgtggagcgcacta

ctaataagaaaggtcaagttgttgatgtatctaacaaacttacctcaattcttggatttg

gcataaatgaaccatggattcagtacttgtcggacaccaaattttaccgagctgataggg

aaaagcttagaactttgtttgggtttatgggtgaatgtttgaggttgattgtagctgata

atgaagtaggaagtttaaaacaagccttggaaggtaaatatgtagagccaggacctggag

gtgaccccatcagaaaccctaaagttttaccaacaggaaagaatattcatgctcttgatc

cacagtctattcctactacagcagcgatgcagagtgccaaaatagttgtagagagattgc

tcgaaaggcaaaaaattgataatgggggaaagtatcctgagacggttgcacttgtactgt

ggggaacagataatattaagacatatggagaatcactagctcaggttctgtggatgattg

gtgtgaatccaatatccgatggactcggaagggtcaaccgtgtagaacctgtaagtcttg

aagagcttggaaggcctagggttgatgtcgtcgttaattgctctggagtattcagagacc

ttttcatcaatcagatgaatcttctggatagagcagtgaagatggttgcagaattagacg

aaccagcagagcagaactatgtaaggaagcatgcattagaacaagctgaagcccttgggg

ttgaagttcgagaagcagcaacaaggatcttctccaatgcatcaggatcctactcctcaa

acataaacctggctgtggagaattcttcatggaatgatgagaaacagctccaggacatgt

accttagcagaaagtcttttgcttttgattgtgatgcccctggtgtcggcatgactgaaa

aaagacaagtctttgagatggctctcagcacagcagacgccacgttccaaaacctcgact

cgtcggaaatctccctcaccgatgtcagtcattactttgactcagacccaactaaccttg

ttcagaatctaaggaaagatgggaagaagccaagtgcatacattgctgatactaccacgg

ctaatgcccaggtgcgtaccctatccgagacagttcgacttgatgcaagaaccaagctat

tgaacccaaagtggtatgaaggcatgttatcaagtggttacgaaggtgttcgtgagatcg

agaagaggcttaccaacacagttggatggagtgctacgtctggacaagttgacaactggg

tgtatgaagaagccaacacaacattcattcaagatgaggaaatgttgaagaagctgatga

gcacaaatcccaactcctttaggaagcttgtgcagacgttcttggaagccaatggacgcg

gttactgggaaactgaagaagaaaacattgagaagctgagacagttgtaccaagaagtgg

aagataaaatcgaaggcattgaccgttgaagtatgcacttataattgagatcaatatata

gtaccttcggtatctctgaaacactaccgccttgaattcatagttattgccaatggcatt

gttaatgtaattttatctcctctcctgttgtttccctgttgtaatttttgttgactttca

gggatcatgttcctttgtgcagctgcaaattccaaataaacttagccaatgtatcttcca

cttttaaatataaagataggtttctgtaatatatagattgctcatgcagctgcaaattcc

aaataaacttagccaatgtatcttccacttttaaatataaagataggtttctgtaatata

tagattgctcatgcagctgcaaattccaaataaacttagccaatgtatcttccactttta

aatataaagataggtttctgtaatatatagattgctcgtgttacaacatagtatgatttt

tttgtcttgaa

>fava_dn_rep_c999

atccatcatgactttgtctagtcaaactttacttgccgatataaaataattttaccccct

cnnnnnnnnnnnnnnnnnnnnnnnnnnnnnnnnnnnnnnnnnnnnnnnnnnnnnnnnnna

ttttaagtctattgatttgtttgccaagctagcaacgaatcttccaagtccatataattt

ctccactttgtcaaaattttcatcattcgctgcattctccttcgttaatcagggtttttc

atcgacttcatctcgtttcgctcttcaagaatcgatcaacaaccatgactcgcggtaacc

agagagaaaaagatcgtgaaagggctcaatctagagttggtggttctaagggaaaaccag

gaaagggtgatgggttgacccctgagcagcgtagagaaagggatgcaaaggctctgcagg

agaagatggctaagaaagcagctcaggatgctggagggaataatgctggtggaagcaaaa

aataggcaatggtttggttattgttactgatttggagcttgatgaggtgttgttgatttt

gtacgattatggaaaattgaaaatatttggattttgtattctgataatcttttttttggt

agtttagaattaaatgtgtgtattattgtgttatgggttaactgaataaataattggtgt

tttcttgaaatgggttgatgttggatgctgagccaatggcttgtattgtgtttctggttg

gatttggaatattaccatttttgtgatgattttgatcttttgctctctgccctgtc

>fava_dn_rep_c1000

aatcgaaaaaaaattctcgttgatcaaaactcaaattaaggtagatatgaaaagcattgg

aaagagacctaataaaataacactgaattagcagacaaaacatagcaattgagtatagta

cacaaaataagtaagcaaacacattcataatttaaaattggcatgttcaaaagaaaacta

atttcgaacaaaattgtactgaaacaactattatatgaatggattgtgaactctgacttc

tcaaggtcatcacttcttgttgtcctccttttctgcctcagcagctctcttgagcctagc

accaaggcgacgcttgtttgtgcgttcaaggcggagcttgtagtaagctttgaatgcctt

catttcatcagtaatcttaacaagttcaactgctggcttctccctcacaatgggcaagta

cgatccttggacttgagtggcattagcaagttcctcaggtgtagagtccccagccttggt

cttgcgtccacgtcttgggaaaacaaccaacttagccttgtatgtcttcagcctctgcac

attagcttgaagaccctccaaagaacggttcctgcggcgatgatcaatagcaatgccaat

ggttggagcaagcttctttgggataccagcagccttcaactcttcaagagaaaacccttt

accagctctgagtttcatgttgtatttggctgtttggccatgaacaattggcctaagagg

tccagcagtaggcctggggaaaatcttaactgccttcttttggcgagccaagcgtcttct

tgtcttccttgctggttggttaaaccatgtcttcacatagttttgccagtgcttgcgaaa

atgttcatttgggataacattgttgtgcttaaccatggtgccttacctgcaaggttgctt

ctgactcgagctgtagggagtggaagcccgcggccnnnnntttgtcctccatcttttgtg

tgtatgggtttgggagggttttggaacagtattcaaagagagacacattagtgtttgtgt

agt

>fava_dn_rep_c1001

aaaacagttttaccaacagttctattttattttaaagtaaatacagtgagtcatccaata

aaaacatatccttttattatgtcatatacatttttaaaaatgatagtctaatttaattgg

ataataagtttatcatgtaattattatgtaaatgagaaggttccctccttatctgattac

tgtcgtggttggtaaccaataacaatggcgacgcaatcttatcttgtcaatctcaactct

ttccatacaggaatcgcaggaccaaattcaatattcaaacctgccaattttggctttggc

atctggaaaagtttgaagcatccaattagaagaaatgtcaatgaaagttcccgaatcatg

caatgttgttcatgttacacacaagttggagcagcttctgcacagaaatgtcttgatttg

aatccgaatttgaatagcctcaacctcaacataccagaaacactacacacttcagctgat

ttcgtgaaaaggttgatgattttcggtgatcttgatcctgctactgcgaaattcgctatt

ggatttctaggtccgttcctctcagcgtttggttttctctttatcgcgagaatagtcatg

tcttggtatccaaagcttcctgtgggaaagtttccttatgtgatagcttatgctcctacg

gagccgcttctcgttcctactcggaaagtgattccacctctagctggtgtggatgttact

cctgtggtttggtttggattgcttagttttcttaacgaaatattagttggtccacaagga

ttactagttcttctttcccaacaggttaattagtgtgcacatttggtgtgtttgtgaatc

acatagttttctatcttatcattctttttggatttcagcttaatatccataccttgaaat

gcaatgttacattttcttacaattcatgcactatttagaatttggtttccaagaggatat

cattgaaacggtggaccacatcaatagtctatacgttctttaatcataatcatatgcctc

atagtttgttaggttctataactaattattgttaccttaggtatacttatgttgtttcta

ataacaactttgatgtatga

>fava_dn_rep_c1002

attagtttgtcaaaaactatcgtttagttnnnnnnnnnnnnnnnnnnnnnnnnnnnnnnn

nnnnnnnnnnnnnnnnnnnnnnnnnnnnnnnnnnnnnnnnnnnnnnnnnnnnnnnnnnnn

nnnnnnnnnnnnnnnnnnnnnnnnnnnnnnnnnnnnnnnnnnnnnnnnnnnnnnnnatga

agtgaaacttggaattttttcaatactgagaaaagaatacaatatttttccgtcactttt

tacttctaaagctgcattcatagaggaatggaaagtacaatagtttctcaagttgataat

atcattgtgatgtacccccattaatcatgagtttggactcaaaagaagttacttaatgga

taaacatatacacaattgtcctagctaaaaaatttagggacaaacaacaaaaatgaactc

tacatagtatgtgaaaactactttttgttttattaaaagcaataaaaaagcaagtttgaa

atctgaaaccctatatttcatcccaccgagttttagaatcaacgcttctcccacttctct

gacggcttccaccacctcgttcattcgaaaccctaatggcaccgaagaaggagaaggcac

caccgccgtcgtcaaagccggcgaagtccggcggaggaaaacagaagaagaagaaatgga

gcaagggtaagcaaaaggaaaaggtcaacaacatggtcttgtttgaccaaggtacctacg

ataagcttctcactgaagctcccaagtacaaactcatcactccgtcggttctttccgatc

gtctcagggttaatggatctttggcacgtcgtgcaatcaaagatttgatggcgaggggtt

tgattaggttggtttctgctcatgctagccaacagatttacaccagagccactaatacat

aagcggtttcgaggtgtttctttatttttcaggtttaggggaaaattatgagtttggttt

acatgtttttgttgccgcagtttgttttgtttaagatattgttaaattttgttttaaatt

aatgatgattagtttaatctgggcaataatttctctagcaattttgatgaatgcagtatt

gtttatatgcctccgcgatatattgttaaatgatgggtttgtttctgagaaattgattca

tgtaactgagaaagtttttatgattggtaaattgtttaagatgatttgttgatattatgg

ttatgctatattagaattgagctcttatatgttt

>fava_dn_rep_c1003

cgcaactgcatactcaaatcagcaatcttgggtcaaataatgtgtttttaacatgaataa

tacaaccttagtcatatcaactactgaattatatgataaaacattactaaattttctgac

tccaaattactggtgggacatgaaacataactcataacacaaacaaagaagcatacattc

atgtgcggaaaaaaaaggacaacacagaagaaaagaaacaggaattaatgataacagtta

cctaccttacatgcagaaactgaaacaagaaacagactctaggatcagctgcaaattgag

tgaattttgaagtacatcacaaagttggaaacttctcaaaggaagacaacatttattatt

acattctactacaagaagaactataataatttatctacgaagcacaaacaccaaacaccg

acacatctgtagaacaccagacacgccttcaatctgaagtgtcagtatagtactgtactt

catagatgataataccggataaagtgattgatacatagccattaagactccaaaaaggaa

tctattgcctttttgtgacttgccggtttgccatactataaccaaagcttctgacccttt

agagaaaaatgcttaattatttgcttctgctattcagaaaaaagcacatccctttttgat

atgagaagccaatagctaaagcagaaagcaaactgttttggaaaaatggagggagagtgt

aaaggatgatgtaattaatactaatcataattagtactagtaactaattaaccctctcac

acttctctccatgtttgattttaagaaccatggattgatttttgccaacaaagaaccttc

attgctaataacaaagattcaaagtgtttgataatggattggtttagaggatggttaaag

ggctccacaaaagaaaagtagtggggaagttggtgtctctgatcattgttttaaatactt

gtgatggttgagatgtagtataagaaagtggtgtgtggagtgtatgtatc

>fava_dn_rep_c1004

caataccgcaactcaccatcagcaccaacaccacaatattcctccgagtttctttccgtt

ttcgaacactagagtaagcatttctccacctaatgactactccgacgaacatctcaacaa

ttggtcatgtgattctccgccattaactccaccgtttgttcatctctgctaattttttta

actaaccaactccctcatctgttattttcatattatgggaaaaatatacaagtcctacaa

atttcaccagcatttacagcttatgggaaatgacattgaaaatgaaggaaaccattccca

gctgacaaagttaaaagctattatttccttgcgattattacgctcgacaacaaaaaaaaa

gtcaggagaagacaaatcgaagggtctccatgaatcagggacattacatcagaaaaaaac

aaattacattacatgcactgtttctaaaatatttcttctatgctctttgctcaacatatg

aacccgcaagtccagaagttccaagaaccgtaacctttccccactcagaaccaaggccac

tgcatctcatttttaagtgtatcatctttgtacccaattccgattccaccttcttccgca

gatagttctcatcaggaccaaaagcattcaaataggtggtgtagcgatcaaaaattgtat

gcagagctttgttcacatgatcaggcaaaggctttacatcctcgccgctaatgccaatgc

tgtcatcaatttccattttcaacgtaacaagaatcccaccaaactcttcaacagcctggg

cagcacgaaacacattagtcacaacctccttaccagcctcatcatcagtactttttgcca

acgcatgcttagcatcagaaacaacagcatccgaaatatcactccaattttcagccaaca

aatccttgaaaacactgtaaagctcagcatccttaatctcaggcacatgattcacatctt

cactataaaatcgcaaacttccaactccattcgagaacggagacctgcaatctgaggaag

gagagagaggattgagaaagcgaggggaagagagggtttgaagtttgttggtagcggcgg

tggcggcggcggtgaaggcgcgggaggaataggatctggaagagttgagtgatcggaata

aggatctcaatcccaacatcgtcttcatttttcgcagttttggtttggaaaagcttgata

gtaatttttgttacttcgttttcgagtttttttgt

>fava_dn_rep_c1005

gggtcccaatttttgttcgttccaacaaggaagagtctgtctggcggtgacagacaacca

tggtcgaccagtccgacgacctcaaccaactccttgacagtaagcgctctctctctcttc

accgccctctttctctctctaactccttctattcaatccatttcaggtgctttggatgat

ttccaaaccctcaacctaaattcttcccttccaagaaccggagaacaaagtaggaacaca

aaagagtctccttctttaccttcaggggttcaaggattgggattagggttacctgatttg

agaagcaagagaaaagggaaacagaaggtttctaaagatgctcatgtttctgaggctctt

aataagttaagggagcaaactaaagaggctgttaagggtcttggatttgcaaccccacct

gctggtgctgatgatttgggtaaagatggtatgatggaggattgggttaaacagtttgaa

gaacttgctggctctcaggatatggaatctattgtggagaccatgatgcaacagcttctg

tccaaggagatacttgctgaaccaatgaaggaaatcggagaaaggtatcctaagtggttg

gaagatcataaagccagcttaagccaagaagaatatgaccggtattcacagcaatatggg

ctgattcggaatcttaacgaagtttatgaaaaagattcgggaaacttcaccaagattgtt

gagctcatgcagaaaatgcaagactgtggacaacctccaaatgatattgtccaggagctt

gctccggattttgacttagccagtcttggccagttatctccagaaatgtctgatggttct

cagccaaattgttctataatgtgaagttgtttcctccctgttatcatgcggcagctgctg

gtttgcatattctcaggaacatttatttttcacttcagaataatatagtttcatatgttt

tcttttcctgcttactgtaaggttacaggatctcactttttatagcctcctttcaagatt

gtatatgatccaaggtttcttccattcacattttatgagaccaatgagaatgcgtcaagc

tgctagtttcaaacaaaatttacttcatgatagtttgaaaatactttctcatttaatctc

atgatccttgaatttgatgaccattatgaatcataatgaactatatgcaagtcacaacct

aaacttgtatctaaacaaaaacttcaccagtttctggtgttgaacatcaaagatgctctt

ataaaaagacaagaaaatttctaaagggggtcctatattgagggacaaaggtagtactta

tcagcaagtgtttcattacatgaaagaaaatgcaagtgctgattaaggtgttttttattt

tgtgattgtggacacattatatgtagtagacattatcatcctcttcttttgggcttacgc

ctgcaacttaataggaatattgtgcaccttgaaaggtgcatcttttacaccataaacaca

catgcacctggcattgaatcctccatgtatattatttctctactctcatgggctagcttt

ctggatatttctgggcacccataaaatggattacatggaaatgtgattttgtattattgg

agtagtgcgaagttggctattttaagactataattctgtctttaaatatagaacgttttg

gttatggtgttggcagttgacccatgataatcccgctagttttg

>fava_dn_rep_c1006

tcaatcagaaccatctaatcaacaccttgccgggcgagacagcaggaacttcctgactga

ggcataatgcagtcccaacatactcagctcaaaagattgaaatttgacaaataagctgtg

tacattaaagacacatttataagaatattgcaactaacaccagacctgcatgttgttact

ttataaccatgccatgtctcaaataaattaataccatgccgacattgccgttttttctta

ctctccaaccaaaaatttcttactctccaaccaaaaactaggtacataacaaaccataat

gcaactaaagatctctacatttgcttccctactaaattgtgattgataaatcaaatgaaa

tattaaatgaaaaagcagtaatcaatagataagaaaggcaaaggacaccacttcagaatc

ccgaatatataagcttcagagaaaatagccaatacaaaagagaataccatttggttctat

aatagtctaaaacaaaagagagattctcaaaaactaatgcagagggcaacaaactctatc

gaaatgaaacaaaaagctttgtgacgacaaatcagctgacaactctgctgatgaacacat

aaatctctcatatgttacttggatacatgaaaactctcacacgtgcacccattgatttag

gtggaaggttggacttgaacttagcacgaacaataccactgttaccatgaggtctagtaa

ccttgccccataaacaacggtaatgagtaccattcttcttcaccttagccttgtagatgt

aagccatcttctttccagcataccacgctacctcttccttggtattcactccctctattt

gaatcagtgatgtgtttgggtactggtttgatttggatctcttgtatccaaggatggtac

ccctgacgtaaagtctgatgcgttctccttggcgacccttcaccatttttgctgaaagaa

cgatgctgcttaacctaatttctgaatgacgaaatttttagggttttacagtcccgcggc

cgtaa

>fava_dn_rep_c1007

ctccacaacatatagatatactagccgagtatataacaacacataggcatatcaaacatg

catccaaatcaacaaacagattatcccattatcaataaatctaaaacatatgaacttctt

aaactcaatgtaaaacaggtagctcttctaaaaattcacaattttaacattcctgagcta

caaatacaaacaataaacattaatttgactgtaatagtaatggaagtcttcttccactgt

gtgaaagcattatattgaatctactatacatcacatgtaatgtaatactttattcccatt

agtgttcatgaaagattttacaagaatgccatgtaatttatcttctcgctttgttttgta

ccaaaatatcatcataacagctgaactgctgctatagcaggtattttttctcgcaatttt

tgtgttacagcaacacgcacagttatgaccccagcagctggacctgtgattcgaaccttt

actattggctcctcaatggctactagttcaagagatccaccagctgccccaaccaggtat

ggccttatttcctctaataccttttcaacattttcatcatttaactcaagaccggtctct

tcatcagatatcggttcaactgcaactatttcagggatcttttccattaatcgacgttca

atacccaatttcattgtcgtaacagaactcggacacgatccacaagcaccttgtaacttc

aaccgtacaacattgccatcgatttcatgtagagcaacatttccaccatcagaaatgaga

tacggtcgaatttcatcaagcacagtttctacattttcagcggttaatggcaattccaca

gctggatttggagtggcaacagcctttatagcaaatgggcgtgtagagtgcggtgaatga

aacgaaacgcaaggacgcagagagttattgttcgtcctcctaaaaggaacacgcttaccg

aaaaaccctgtaccctgtttggtggtggaggaaggttgaggagttctgcagtaagattgt

gaattgagcaccacaccttgcatcggtgtgttgatattttgacctaacaaacctaatcga

tcaatgttccaaattgttcttctttctttgtattgttcttctgtcaccacccaaacacca

tcgcaattcgcagcccgcggcc

>fava_dn_rep_c1008

tcgagtcgagcccctaggcccattagaaactagggtttcacggttttatatttaacctaa

ttttccatctcattcgtttgtttcgtgtcagcctcactccgaaccttcaccgtatctgga

tcgctccctgcgtcgtaagtgaaaatggtggagaagaccggcgccggtaggaaggaagag

gtggttaccagagagtacaccattaacctccataaacgcctccatggatgcacattcaag

aagaaagctcctaaagccatcaaggagataaggaagtttgcccaaaaagcaatgggaacc

aatgatgtccgtgttgacgtcaagttgaacaaggctatatggagccagggaattagaagt

gtcccaagaagagtcagggtacgaattgctcgcaagagaaatgatgatgaggatgctaag

gaagagctctactctcttgttactgttgttgaaatccccaaggaggagctcaaaggtttg

ggtactaaggttattgacgacgaagattgatgtgttttagctttgttgcaattttgaagt

ttaattttgttcagacattgaggatatgttgtatcacaacattaattttactttctctcc

gttttgtttgtgttttgtttgcctctcttaaaaagattagacatcagaaaatgcttatta

ccctcttttcttctcttcacttggatatactgagaaaatgtgtttattaggatttgctgt

gaactgttatgtgcaaggttgtcagaatcaagagtttactttgaatcaaaagatgatagt

aagatcggaaaattgtaaatcacaaccaaaatcggtggattctactcaaattgttttgtt

gccctattttttcaattaatttgttttgaatgagantttccatgt

>fava_dn_rep_c1009

aagaaaaaagtattaacattgataattatttgattctggaaagtactacaaactcagtaa

ctagacaagacagaaagcaaacagaagccatcgggggcaaacaacaattgtagcatcaaa

agagttaataatggaagctccaagatttgtcttcttgtccttgcttcttcttctccttac

agcttcaacaagctcagctcaactcactcgtggcttctataacaatgtatgtcccaatgt

tgaacaagttgttcgtgctgccgtgactcagaagtttcaacaaacttttgtgactgctcc

cgccactcttagactcttctttcatgattgctttgtcaggggttgtgatgcttcggtttt

gattcaatctccaattaataaagcagagaaggatcatcctgatgatgtgtcactagccgg

ggacggctttgacacggtgatcaaggccaaggcggctgttgacagggatcctaagtgtcg

aaacaaagtctcttgtgctgatattctcgctcttgctactagagatgtcgtcaatttggt

agggggtccattttataatgtcgatttaggaaggcgcgatggaagaatatccactttggc

cagtgttcaacacaatcttcctggccctagtttcaaattggatcaactcaacaccatgtt

taatcgacacggactctcccaaaccgatatgattgcattatcaggtgcacatacaattgg

attctctcattgcaaccgcttctcaaatcgaatctacagattcagtccaacaactcgaat

cgatccatcattgaatttacaatatgctttacagcttaggcaaatgtgtccattgaatgt

tgatcctagaattgccattaacatggatcctgtctcacctagaaagtttgacaatcaata

tttcaagaatctacaacaaggaaaaggactattcacttctgatcaagtgttgtttacaga

ttcaaggtcaagggctacagttaatcttttcgcatccaatgaaaaagcttttcaaactgc

ttttattgatgctattactaaattgggaaaggttggtgttaaaactggtaatcaaggtga

aattaggattgattgctccaaggccaactagaaaaactactcatatgatatgacatcatt

gttggatttcgattctttgcaataataaaagcgcgcagttattcgtaatagtcaatctca

aacattggtttgagattgtacaagcttattcgatttattttaaatgatctcgagtctcga

ctattggtttggatcagatggtcgggatcagtaactagggagtaaatttccatcatcatt

tcttttgagggtttatgatttttttatattttgtaattcattgttctattaataatcgat

gattgagttcttcaaattaaaaaagaagaaacacgacagatccaatgacaattccagcaa

tggctcctccagacaagttattctttttcttactagtgttacctccgtcggtagtgaaag

gagatgaatcgtttgtgtcggttccagggcaaagagaaagtggtggaccgcatagagaat

ttcccaaaaaggaatcttgagggaaagtctgaagcttaacaggaacggaaccgtttaaaa

cattgttagagacattgaactggtcgagggtcaaacgagtgagttccggtatagaaccag

aaagacggttgttttcaaggtagagggttttgagtctggtgaagttgttgaaggaagttg

aaatgggacctgagaagttgttaaagcccatgttgagtcgaaccaaatcgggcaagttga

agagaaagagagggatttggccggagagaaggtttcgttgaagataaaggttgcgaaggt

tgacacaggaagcgagatctgaagggagtgaaccagtgagagcattgaaacgaagactga

gagtacggaggtgagttagattggagaatatgccggtaggtatttgaccggagagagcga

cgccggggagatgaagctcgacaacatggtggtcttggcattggacgccggcccagttgc

agggagtctggttggttgcgttccagaaaagggttcggccggcgacggaggagcggagag

ctagtaatgcagctcgttcggaatcgagatccgctagaggtagtgatggagcagtgagta

gaattagtaagaggatgaagaacggatccatggatctgtgtttaactatttccatgactc

tgtcagatatacatggagtgaatgtgttagttgtatgaaaagaagaaatacagagagaag

gttcgtttgatggttgttgttgggtttagaggataaagaaagaatataaaaaatcaagta

tgagttggagatccaggctgtgagataatcaagacaacataacataacat

>fava_dn_rep_c1010

tttggccgcgggaacccaaaaaatcaaaacataagatatcaatgaatctatcaatctctt

cttttcttctttccacctcttccgttccaatttctctaaccaatttcttataactgaaat

caccaatttcaagaaatggccatggctatcactgcttccagtattcgatttcacttcact

gctgctactgaaaaacctattttctctgttccttttttcaagctatcttctaggtttact

catactatgccatcttactttcttcataaaacaaggttgttatcttctggaactacaatt

atccctcgagcagcaccacctgttactcatctgcaggatgctaatcaaggtgtgactgat

accgttccgactccgatagttataattgaccaagactctgatccaaatgcaactgtggtg

aagataacttttggtcatcggcttggagctctccttgacacgatgagtgcattaaaaagc

ttaggtctcaatgttgttaaggcaaatgtctttttggattcttctggcaaacacaacaag

tttttcatcacaaaagctgatactggcagaaaagtcgaagatccagagttgttagaagca

attcgtttgacaattataaataatctgattcagtatcacccggaatcaagttctcagtta

gcaatgggagcggcttttggacttctacctccgaaagagcaggttgatgtggacattgca

acacgcataaatatttctgatgatggtccagatcgaagtttgttctatgtggagacagct

gatcaccctggattactcgttgaacttgtaaaaagtattactgatattgacattgctgtt

gaatctggagaattcgacactgaggggctcttggccaaggcgaagtttcatgttagctac

aaaggcaaagcaatcagcaagcagctgcagcaggttcttgctaacagtttgagatatttc

ctgaggcgtccgacaaccgaggaatccagtttttgaagggcatctctttcaatttaaaca

atttgcatgacaatatattttcttttgaagtataaccaccatcatggattggtgaatgct

tgccttcattgtttcattcggaagagaaatgaagaataactggaatgcatttacatcatt

aaactgaattaacaaggatatgattgtaaagttattttttgctaatttcttacataaaat

tatttgcaacttcaaaaaaaaaaag

>fava_dn_rep_c1011

agattttttgatttgacatcatttttttatgtgtaaatctcttgtttttctcaacaacct

tttatttaaataaaacattttacaaacaaaattagtttacaagaaagcattcggttgaca

ctcatccattacattaaagtttatttgataagcaaacacactcaaaccaaacacacttca

tcctcatctttgttgctattctttgacaagatgagcatcaatatcttttgtgaccttttc

agccaaggccaagtatgaatctggagatgcacctgtaatatcctcatttaacttctcata

ttcaaaggtccatttcacaatcccaccaccatgttccttatcaatcacttgtattgttga

cttaaaactcttgtaattctcactcacttccccatcaaataagctatatgttagtgtttt

attgacatcatctatagcctcaattttttctttagcacttactttttttccttctattgt

atattcccagtgtttgacagaaccaacactttcccaatcaccttcatgcactctatttcc

atgtatttctggagatatattaggaacatgttcaagttgctttctaagaacatgaaagaa

cttagcagcagatgcttcaatatctgtttcactctcaactttcccacttaaagccatgtc

tatgctctctattaatgtttctattcatccccgctttctccctttctctcccaaatagat

atccctctctttcttctccttcttagacaaaatctctctataaaaccaaatccccaaaat

caaaatcaacaaaaaccctaaaatccccaatccaatagccaaccctctaacagttccact

cttatcatcatcactaccaccaccattagaattctgactcggtgccggagaaggaacagc

aacagtactaggttgtttcaaaacaggtaatttatcaaccggaacaaaaatagtatcata

atcatacaatttatcaccattaacatcaactatagacttctctgtagctccaaacatcaa

agcaatagatgaaacgttatcagaaggttgaacaacataagaaatcatgaaacgtgaact

gtttctggttttttcaggacacttgcagaagataggaaaaacagcatcaccacctatagg

gagattagtagcaacgagattcgggttaacaacttcaacagaagggtaagtagtgaggtt

ttgaaagttaaaggttgagacaatgtaaaaagtgtcttttggtttgatggtgtaggtgat

gttggagtaagagatggaaccgaaggtggtgttgatgaagttgcaggaacaggttatggg

gatgaggaggggttggttggggagtaaaggggtggaaggtgaagagatgttggatggttt

tgagattgctaggcggctgagggagaagaggtcggagatggtagctaagtcgaggtagtt

tggtgagtagctctgtagaatgcgtaggattgacatgggtagttgttggtttggtttttg

gtggtggtgcaggtgtagcctgtgttgctttcttgtcttgcagtttgggagttgaggaag

gggaggaagagtaggaagaagaacagggtggagaattggtggctgtgggctttgaaagtt

ttcatttttctcttgctctttgtttggttgttttggtttggttttatgctttggttcttt

tagacactgtttgtaattttgtggaatttgacgtccgttttgggatatgactattctatc

ttcgagtgaagtttcttata

>fava_dn_rep_c1012

acaataaccccttattaatttatttgattcgaaagtgttaacacaagaagaagaaaaaaa

atccttgcccttattctagtagcttttggattcgtgaatgacatgtttttaaaaattata

tacagtacatgaaaagtgttgacacaagaaaaaaaaatccttgccctcatcatacacaca

gacacagtacatgctttcatcatttaagttctcaagcactcacaatttcacacattttgg

catcattcaaggtcatcttaaaactccaaacattttggcatcattcaaggccatcctaaa

actccatataacagtaatccttattggttaacatataattcatcaagatctggcaagaga

aaacacattgctactcttacaaaaatgaccccaaatatgtccctagcactgctgctaatg

atttaatttttcaatgttgtatccatcaagaacacacaatacatcattataatctacgaa

ttcagaacagatacagctactcaagctgcttaatgaacattattaatcaaaacaatttct

agaaacaagaacgaaaactgccaacactttcgtctttttcttacgagactattaagcatc

ctgcattagagaataaaaacaatatcaataaacttcaacatgccattgtttgttcttctt

gagcaatgaaagcttttcttcccaactttctcctctcttctcagcaacccttttcagtgt

ttcttggatcccaggcatcattcctctcagaccacagaaatagatgtgtgctccattatc

caagagtttgaagatttcatcgctatattcctcaatcttgtcctgaacatacattttgcc

gccgttcttgttcttttcctctctgctgagtgctcggtcgtaccggaagttgtccgggta

atccttaaggtatttggtgaactcgtcgtcgtagaggagactatcggcatttgcgacacc

gaggaagagccaagctagtccaccaaacttgaatgttgggactgattccatgaacattcg

acgaagatagcctctatatggagcaacaccagtgccggtggcaatcattatatgggtagc

atttgggtcgtcctccggcaaaagcattatcttccctgaggggcctgcgatcttaatttt

gtctcccggcttagagtcacacaaaaaattactacaaacaccgttctttgaaggatcttc

cttccctgttacgggatcataataaacagcacggcgcacacacaagctggctgttttgcc

atcaaaattgtcaccatacctcgtggaagcaattgaatatagtcgaacattatggggagt

cccaggtttttttggattttcacctggtggaataacaccataactttgtccctcccagta

aggaacatttccatcatgattgatcacaatatgacaagtctccccaggagcctttggtcc

taccaatctttcaacagaaacaattgttgctgtataaggctctttaggcttgtgtaagtt

caatggaggctctgtaggattttccaattctaaaggggaaacagcaactttcggcacact

tgcttgttgcacagacatgcaaatcacattgtggtttctccaaccacggttcttagcttt

catatccaaagtaaatactggtgcccatgatttctccctaaggtttaagttcgatgcctt

aaacgcagatcttctgacggagaaatcactgctgacaggaacagttacagccatctgaga

cactgtcaaatgagacatggtgaaaaggagtgtgatctgaagtgattgatgaatatatag

taggaataaacaaaacaagtgaaagaggaatttgtgtttgatgatcctttggagagaggg

aaaggtcgtgcttttccctttggctcctcggagtgttgttcctcagtcttcacccaccat

taccaaccctataccaaaacttggaattgccaattctttttttccaaaacccnngcggcc

gtaatggccactctgcgtt

>fava_dn_rep_c1013

attaagattagttaatgaggaaatattgtttgaatcatagctgtgagggcaatagaatat

tccgtaacatttgagatagatagagaaagcggctgcttagaaagttagagagagagagag

agggagaaatagagttgaagacggtgtgaaagagagagagagagtcgcagttgaagtaaa

aaccataaaaaacagagcaagcaacagtttcttcttcttcttcctccgatctctacgatg

ccttctcgcagaagaactctcttaaaggtcatcattctcggtgacagcggtgtggggaag

acgtctttgatgaaccaatatgtgaataagaagtttagtaatcagtacaaggcaaccatt

ggagcggatttcttaaccaaagaagtgcaatttgaagataggcttttcaccttacagatt

tgggatacagctggccaggaaagattccaaagcctaggagttgctttctatcgtggtgct

gattgctgtgttcttgtatatgatgttaattcagtgaagtcgtttgacaaccttaataac

tggagggaagagtttctcattcaagcaaatccttccgatccagagaattttccctttgtc

gttataggaaacaagatagacattgatggtgggaacagtagagtggtttctgaaaagaag

gctcgggcatggtgtgcagcaaaaggaaatatcccgtattttgagacatctgctaaagaa

ggtattaatgttgaagaagcattccaaaccatagcaaaggatgccctgaaaagtggggaa

gaggaagaattatatctgccagacactattgatgttggaaacagcaatcagccaaggtca

accggatgcgagtgctgaacatatagatgttgttctcaatacaaaataaggtatatttat

ttgaaaatcattttggcatgtctagccattgctgtctatgaggtttaattgtacattggt

gtttgatcaagaaacaaattgttctagtatcatctgttggatgcttgtttggcttgtgtt

aacggatcacgttgtctccttttatgctagcaaaggctcattctgaagttatttcattat

ttatttttctttctcatactaaaaatattttgtaatcaatggtgattggtggttgttgtg

ataatgctgagttataaaacctgtttaaaacagttcagagtttctactcggtaatgtttt

attaggttatagttgatattgatctggtttaagtaatgctgaagggaagactgtgatttt

catggaaagaattgaaaataaataaaaggtgtcaagtcaagccttcgatgggatgactgt

tgatcgccgtggaaatctggggactgtcatgtaatgtatcgttgagtccattttatcttc

ataatgatagtaaatggataaattgaaacagtccattttatctgcataatgatagtaatt

ggatannnnnnnnnnnnnnnnnnnnnnnnnnnnnnnnnnnnnnnnnttttaccggggatg

caattnnnnnnnnnnnnnnnnnnnnnnnngtttggtgg

>fava_dn_rep_c1014

gaactataannnnnttatcattattgattctaccgtgaggaaaattgattttaaataaat

tgattctattttgattgctcctaaactacctgaaacaattttgattgaatcaattttgaa

tgccccaaacgtgaaaccaaatatacaagaaatggtgttgattacgcatctacacattaa

atatactgaattttaaccgatgtatgtatcttaaaaattgcgcactggataaaaccagaa

atccacttcaaaggacaatcgattacggccagatgaaaaaccactataactggcccttgt

ctttcatcacccctacaaacatgctcctataataaccttaaaataaaaaaagaccaggta

atgtcatgacaatgcattttacactatttattcatcctcatcgtcgtcatcatcatcatc

gtcttcagagtcgtcatcctttcccgcatcaccagcttcatcatcatcctcctctgcttc

atcagggtcctcgctgttgaaataattaagtggatttggccataaatcatccttgattag

ttctgcaacctcatcattaacgagctcatccaaatcttctttcccatcggtgtcactaaa

ccaggtaaagaaactgatatcagaagcagctcgcttgtttcctttcttctcttgactaac

tccatttggaatgtccttgccctctttccatttaatgggagtagcagtaacctttgttgt

tccttcttcaaggaaggtaaaagtctttacaagctttgtatcctcaaaatatggattagg

attaaagttaaaggtgattgaatagcctgatttgacatctttatgatcttcaacctcaag

agaacttaaatgcttaaatatcttctgatcttcctcattcaaaagttcgccaagagcagg

atggctcaaaaaagcagtcaaccagaaatcaggaatagacttgataagatcattgcgctg

atcatacagcggcttccttatctcattatacttctgctctatttccagaactttatcact

agcctcctcattgatcttttcgagctcatcttgcatttcctgtaacttttcaatggagag

gacaagttctccgtcgatttcctcggcgttctcgcccttttcagaaaccttaagcttctt

gctcttgtcggccaccatggttgttgttattgggaatgagagacagagggtctgttgtgt

ggaaaccctaagtagagacagagctcgttctagtggagcgtgtctaagtgaaggtgttag

ggtttttggggtttataacggtgaatgagtgttaacatat

>fava_dn_rep_c1015

gataacccagatctcggcccattcctactcaaactcgcccgcgacaccatcgcctccggc

gacggaccttccaaagccctcgactacgccatccgcgcctccaaatccttcgaacgctgc

gccgtcgataacatccccagtctcgacttcgccatgagcctccacgtcctagccgcgatt

tactgtagcttaagccgcttcgaagaagccgtacccgtcctcgaacaagcgatccttgtt

cccgatatcaaaataggcgccgatcacgctctcgccgctttctccggttatatgcagtta

ggtgatactttctctatgctaggtcaagttgataaatcgatttcttgttatgatcaaggt

cttcagattcagatgcaaaccctaggtgaaaccgatccgcgcgtgggggaaacgtgtcgg

tacttggcggaggcgaatgttcaggcgatgcagttcgataaagcggaggagctttgtaat

aaaactttagagatccatcgtgcgcatagtgagcctgcttcgctcgaggaggctgcagat

cggaggcttatggcgttgatttgtgaggcgaagggagattacgagcccgcgttggagcat

cttgttttggccagcatggccatgattgcgaatggacaagatactgaagtcgcttccatt

gatgtgagcattgggaatatttatatggcgctgtgtcgatttgatgaagccatattttct

tatcagaaagcgcttactgtgtttaaatcagcgaaaggagagaatcatccgtcggttgcg

tctgtgtttgttcggttggctgatttgtatcataggacagggaagcttcgtgagtcgaag

tcttattgtgaaaatgcgcttaggatatactcgaaaccagttcctgggacgaccgcggag

gagattgctggtggtttgacggaggtttcggctatatttgaatctgttgatgatccggag

gaggcgttgaagctgttgcagaaggcgatgaaattgttggaggataaacctggacagcag

agcacgattgcgggtatagaagcgcggatgggagtgatgtattacatgattgggaagtat

gatgaagctagaagcgtgtttgagagtgctgtgttgaaattgagaaccagtggggaaagg

aaatctgcattttttggggttgttttgaaccaaatggggttggcttgcgtgcaattgttt

aagattgatgaagctgctgagttgtttgaggaagctagaggaattcttgaacaagagtgt

ggacattgtcatcaagatactcttggagtttatagcaatcttgcagcaacttatgatgca

atgggaagggttggagatgcaattgaaatcttagaatatgttttaaagttgagggaagag

aagcttggaatagcaaatcctgattttgaagatgagaagcggcgtttggctgagttgctg

aaagaagctggtaagacacgagacagaaaagcaaaatctttggaaaacctgattgatcca

aattcaaagaggacaaagaaggagagtaccaagaggtggcctggtttgggatttagaatt

taattaaccacggataattttttactcaagtattattatatatattgatttgaatgcaca

ttctgtaacatagggtatctttgtatagctttgaatatgtaatttgggagcatccctgtt

ttcttgatgtttgtttcctgcatctgcttttcttgttcttattcctattgtgttgtgacc

ttctagatgtaccccaaagttcctgttgtccttttcttgaaaatattgaaagcaattttt

tttattctgagtgaataaagtttgatgtgtttaattcatcttgtgtgtaagcggcgagct

cctacaattttgttgcctatttatggtaattatgaaatgcatttaaac

>fava_dn_rep_c1016

tacggccgaaacatcaattgtagtgaagaaaacagcaaccaagcaatatcaattgttctt

tgctttgtaatcttagtcgctgaaaaaaatgtcgggctgtaattgtggaagtagttgcaa

ctgtggtgatagctgcaaatgcaacaaaaggtctagtggattgagctactcggaagtgga

aagcaaagaaacagtgattcttggcgtgggtccggcgaagatccagtttgaaggtgctga

aatgagctttgcttctaaggaaggtgggtgcaagtgtggtgataactgcacttgtgaccc

ttgcaactgcaaatgaagtgcaacacatcaacacttgaagcagagatattcgaaccaatt

tatgtaacgatacgtatttgcttttttaaaaataaaataaagtggctatagttgctttgt

ctttggttgtcttgtgtaatgtaaaagctaattatagctaatatcaatcataaatctctg

cttgttattataaatggtttatttggacatatttaatgcatattatgttattattata

>fava_dn_rep_c1017

cctcattctttctccttctctctcattcgatttcgtctctccctctttctcctttcaaac

gccaaccctaacctacacctttacccgacggcggtggtggcctccacttcccctctaaac

ccaaacagacaaacgagaaaaatagatgagatatgcaagctaagttggttaacccattac

ccctgttcttacttctcagcgaatccgttcattctcctttaaaatttacgatgttgatct

attttgttcttttttgttgacaggaaggtgacggcggatgataagtgacgtcgatgatgg

agccgctagggtttcagatattgaaaccattggctagctttgtgattggatcgatgatga

agattgagcatgagactcctcccttccgattttgtaaaactattcatttctggcactggt

ttctgtttaagcgaattggaactggttaatggagctggttaattggtgtagtagtttgta

tagatgttttgtaaaggtctgaaatgcaagtgttacctgatgctggtctatgcagagctc

ccaggtgtaacgacatgacatgaagtttgtcatggaggaggcctaagctaggagaagatt

aaagaagcaatagcataaagaaagattggagattggaagtttggccatggggaggttgaa

tatgttttgaagctgttaagaaatggagatgcactaatgatgagcttaggtttaagatat

gtagattttggcatttccatgtaaactctctttgtgatcttctttgtaatggtgaccacg

atgatgttgtaatcatatggtttaactttgaatgtattttgtaattattcatggggtatt

ggttttgtgaatttacattaaatctcaatattttataaggaaaaaaaaagcaaggaaaaa

aagagagtgattggcgatagtgtctgttggcaaagcgaagaagaagaggaagaaaagcag

aaaagaaagtggataagaggtgaacaccacacgtgtgaaccatgtccacttcaccatgct

tctcttcacctcctccaccttctttcaccaaaaccaaaccatctccaatgtgtagcatga

gtaaggatccagttcgagagtggattctctctgaaggaaaagcttcaaagataaccaaaa

ttagtcctgttggtggtggttgtataaattttgcaaaccgctatgataccgatgctggtt

ctttctttgttaaatcaaacaggagtattggaccatcaatgtttgaggcagaagctcttg

gtttaggagctatgtatgaaaccgggaccatccgcgtgcctaagccatacaaggttgggc

cgctaccaactggtggctctttcgtcattatggaattcatagaatttgggggatctagag

gcgatcagtctgtactagggagaaagctggctgaaatgcataagtctggaaaatctagta

aaggctatggtttcgacgttgagaacacgataggcagcactccgcaaataaatacctggt

catcagattggattcagttttatggagagcatagattaggttaccagttgcagctcgcat

tagaccaatatagtgacagaaccatttttgaaaaagggcaaagactggtggaaaacatcg

caccgttgtttgagaatgtggtgatagaaccatgcttgctacatggagatctctggagtg

gaaacataagctctgacaaaaatggagagcctgtcatattggatccagcatgttattatg

gacacagtgaggcagaattcggtatgtcttggtgtgctggctttggaggctcattctata

attcttattttgaggtaattcctaaacagccaggctttgagaagagaagagacctatata

tgctttatcactatttaaatcattataatctctttggctctggatacagatcatctgcca

tgtctatcattgatgattatctagcttatttaaaagcttaggattataacaattattgaa

caagcttattcaaatgaggctcttgtacatatcagtaacattttctcctaagttctcttc

attttgatgatcaaagataaattgagatgggtaggttgaattaagatgctaacatatata

gtgacgagcagtaccgatcctctccttcgtaatagaaatggannnnnnnnnnnnnnnnnn

nnnnagtctatagaagcttaatcactagatggataacaatta

>fava_dn_rep_c1018

agtagcgagtaaaatgaggaaaagggagagtgagagaagaactccttatcatctagttga

agcctactcttttcacttttcactccatatcttcatttcatttcacttcacagccagaat

agaattgtggtaactggtaacagattccatgtcgttgctgctattgccacctcaacctcc

cccaacactccctcccttcaccgtcgctacttccactatcacccttaaacccttaccctc

cttcaaacccccacgctgcactccaaactccacactttccatttccctctctccaccaac

aacaacacacacagatgcaacaactccaaagaaggttctgctccccatcggtttcggtac

cgaggaaatggaagccgttatattaattcacgtcctccgccgtgctggtgctcatgtcac

cgtagcttccgtcgagtctcagcttcaaattgaagctgctggaggcaccaaattagtcgc

cgacgcttccatttccgattgctccgatcaaattttcgatctcatcgctttaccgggagg

aatgcctggttcagcaagattaagggattgtgatgtgctgagaagaatcacgtgcaaaca

agctgaggaaaataggctctatggtgctattaatgctgctcctgctgtcacacttttgcc

atggggtcttttgaagagaaggaagataacctgtcaccctgcatttttccacaagcttcc

gacgttttgggctgttaaaacgaacattcaggtttcgaaagggcttacaacgagccgtgg

ccctggaactgcttacatgtttgctttaaccttggctgagcagctatttggggagtttgt

tgcaagggaggttgctgaattcttgttgatgagaaccagtgatgataatgtatctaagaa

ggagttcaatgaaattgagtggtctgttggtcaccatcctcccagtgttctcatcccaat

tgcacatggttctgaagagattgaagtagtgacactgatagatattctaaggcgagcaaa

agctaatgtcatagttgcttctgttgagaaaactcttggagttatggcttctcaaggaac

caaaattgttgctgataagttacttagtgatgttcaagagtcagcacatgatttgattat

tctcccgggaggaactgctggcactcaaaggctaagcaaatctagaattctgaagaagct

tctaaaagaacaaaattcagctggaagaatatacggggccgtctgctcctcacctgctat

tctacacaaacatggtttactaaagaacaagaaggccacagctcatccctctgtcttaga

cgagcttaaagacggagctgtaaatgatgctgtagtagttattgatggcaaactgatcac

tagtgaaggacttgccagtataactgattttgctttggctattgtgagcaagttatttgg

gaatggaagagcgagaagtgtagcagaaggccttgtttttgagtatccgaagaaatagag

catgtagtttcaagtacttgtaggtgggatagaaaaattataggcagtgtactctagccc

aggcttaactttgctccacattctgaatagttgaatgactgatactactaattaccatct

cctctgagtctcatttttcgagggatggttgtatatgaaaaaaaatacctgcaggttaca

cgaaatttgaacgaagtggtgaaaaatccaaatggttctccattttacacttcattgaac

ttcttccaccaaaaacgaccaaccacccatgtacaatgtttgtgaaactttcacattcat

tttagttttaacattgagattatataaccataactcgaatatcgtaatcataactaacaa

tgatttcctagatttacaaccccttatgctcggtttgtcccaattgaattagaaaaaatt

atatacacttgaataacaaaccaataattcatcaatgcacacactacaaacatcatttat

atagactataacatatcaacactacaaattaaagcttgcaccagcgacaaccgctgttac

tcattatttcagatttcacctgtcccaagaggttgtcttgaggtatttccagaggcccag

gctttataatctttcacaagctgtgacataaaacggggaagcataatcttgagtatctgt

tcaaggacttgagtcccagcagactcaattgcttgctttggtattgctttgaacgcgaaa

ggaatatcaatgcttacctcaattacagtgtctgatgcgagttgctgcaccagtgactca

tttgagtcgctctcacacgatatccggttcaccattatagcgtcaaacttatcgttctgc

gcggcgaccatgggcgagccctcgagcttgcaggataagagcttaatgcaacagccatga

ggttgctgttcaaccttgacgagcaaaacgggacaaacctcaaaagtgaaaaacttaatt

gtgtaaacataacacctgaatgtatcctcgctcacacgcacgatcctctccgcgtccaac

actgaatattgactcgccggcaacttcatgtattcaataaggggacgttggagttgttga

accgagatggactccttacggcgagcaacaaagcgggctttctgggagggaggaagagga

gttgcggaagcacgaagcggttgaaaacaggaaatgggaggaatggtacaggaagccatc

cgattccgaagtgtcgtgggaaggaatggatagcgagcccgtctcttgtggatgtggtca

gtctggtcatgcacacgtcactgctaacttgtttaaaacaatgttgaaaattgaaagtga

gatttttt

>fava_dn_rep_c1019

gtaaataattgttggaataatatagtaagtgtgagtaaatggaaaataatctcacattga

ataagaatatggagatttccatatatataagtgagagaactcactcactcacctatcatc

ttaatgttttagctgagtaagtggtgtttctctcacaaagatttaatatttttgacaaat

tcacgaaaatatcttagatatgaaaatgaccaccaataaactagtagtattccatattaa

tgggaaacagaaagtaagaagataacaataaaggaaaacgaagataaagtgcaggtttcc

acaattcgagaaaccattacatagcagagagatgcaagccaattaaatgtcctacagaaa

atagataagcagtaaacgaaagaacccaccaaagcagacttcactttgggttaggtgaag

catttaaagttccaactcatcacaatttaatggcgattaatccggtaggtactgcggcaa

gaacaaaggctagagtcaccatcatcacgtcgctgatcatgactggtctaagcttatgtg

agccacttggggtcggtggggtcggagttccggtggatgatgcggctgatgaaggcgggt

tgatcttggatgagtttttgaatattgctgcgtccgaagaggttggagatagacccagaa

gcttaggacaatcagagatgttggctttaacaccacaaacagcaggaagttgaagaagct

tatcctcttgaatccccatgctcttactttcagggctccccttatgagtctgttgaatga

tatagcatagacactctggatccgtatcctttatactcttcgttgcatcacagcattcct

tcttcggcgccggtgctttccccgtcgcgaaatccaaacatggaatcacctttgaaacca

cttgaccacatgttttggccaagtcttctgctccgtttgctgtgttggaacaaactacga

tggtgagagccagaacacataggcatgtcaatgtttggtgtttcatctctgttctttgtg

tgtgaattgtgatcgagtgagctttgagtttgggaagagaagagaagagaagaggcgttt

taaattggtgtgtagaggatgaagagtaaatgaatcgtcccgcggccgtaatggccactc

tgcgttgatacccctgattattagagatataattacgttgtactccatggtagataaatg

t

>fava_dn_rep_c1020

tatcataaaattttggagcaagagaaaaaaaaagtgtgaagcaaatcgcaagttggagta

gtatcggcaaaaacgcaacggtgcgaagtgtggaggcagttgagacagaattcggagtag

cttcttaatcccaactcaattcctcttctcaaattctctcagagataataatggacccag

aagaacgtaggtacttggaggatgaggattcatcactcatgaaaacttttaagggtgcaa

ctactggtttagttgctggcactatctggggcactgttgttgccacctggtatgatgttc

ctcgtgtcgagagaagtgttgcccttccggggttgttaagaactttcaagatgatgggtg

gctatggagccacttttgctgccattggaggagtctacattggtgtcgaacagttggtgc

agaactacagggggaagagggatcttgtaaacggtgctgttggtggatttgttgctggtg

catctattctcggttacagaggaaggagcatctcaaccgcaatttctgccggaacagcat

tggcattcacatctgcagtccttgattttgggggacagacattaaaacatgatgagggga

gggagtatgctgcattaactacaaagaaaagacccagtaacgatgcatgattttttattc

atgcacctttatgaccaaagacatttctagttgattggagagagtgaatcactctttgtt

gagccagttggaattacataatcggatgtcattccatattttcttttataagttgtttgc

tgaactggttgaattccaataaatgtactatgtattttgtttctaagagataagctataa

cctcactgaaattcctttggtcttatatactaaatctatttttgttaccataagatattt

gattcagattgaattgttgttgagctatggtggttcagccatgttgtttttaatagatat

tagcattagtcatctggtttatttgtgtttt

>fava_dn_rep_c1021

gtggatatggtggtggggaaggagcaggtggtggatatggtggtggagaagcaggtggtg

gtggggtaggtggtggttctggtggaggtggaggtggtggaggtgcccatggtggaggat

acggtggtggtgctggaggtggggaaggtggtggccacggtggatattatccttgaattt

acatgaaagtcagtgttattatgaatgaaaacctagctcctcctaaaaataagatgtggt

taagaggatgcttttaaactactgccataatttatcttttacgtgaaatgttgaatgaaa

gcaaattaatgttgtgtactcgtaaaaaaattattacatatatatcatgatatgccattg

ctttgcatggtatattaagaacataaatctaattgctcaaactctaaatgagtgaaattt

ctaattagaaactgccg

>fava_dn_rep_c1022

atttaatttagatcatgaaaaatctctaattatatcgtaattattatttcaatttcaaat

tacannnnnnnnnnnnnnnnnnnnnnctttttttttttttaacattagaacctggtccac

ataaaaacatagggtaaagtgttaacaagatatcatagtagtttcattcaaactcaagct

caagcaacctcgataaaatcatttcttgaattcagtccggccgattcaattgagaaacca

aagcaagtacactctataaaatacaagtgtaggacggaacaacagaacagacaagcatat

aacaaagaaaatcatcaatattatgtccacccaacagaatgctggattacagtttatgta

agatcatcctcttcaagttctttggctttcagcttttccttcaccctcaaaagaagcgta

gtcttccaagaatccagaggggtcatgctatcaaattccttgacaacatcagtaaacttt

gcaacatcttcttcatcaactgccgcagcaacatccgccaataatctatattcacgtgtt

cctgaaaatgttggatctaattcctggtatttctctaatgcattgtttatggcaacaaca

tcccctttgcagagttggcaaaggccagcattaagaagatggcctttcactccatatttc

agcaaattattatttagagactggcgtgcgatctcttcataaatctcaatcgacttctga

tattgttccagctgagcagcgaattgggccactttctgcttgcactggtttgcagaagtt

gtcacctcttcactttgataaaaatcagcggacttttcatagtaaacaacagcctgctca

atattttgttcggattcatacaactctgcaatttccttcaaatatctagcagccatggag

agccggccaatatcacacaaaagattcacagcatgatctaagcatgatacagcctcactt

atattagtctttttatagcaatgcgcagcatcaacataagcctgtgaagcctcgtgcttg

ctttccaacttcaaatgacaattcgccaacttaacataagtcgatcccgctttgtcccat

gatttagcgagtttgaacgaattggcggctttatcgaagagatcggcggcatcttcgaat

ttcgagccgaataagccccaaccgctgagcttcttctcagctttcttctcgaaatcctct

gcccttgctaactgatcggccattttactccgatcggaagcagaaaatggatcgattcac

tgagttgttgaaaaaattaggtgaagaagaatcataagagaaaggggggggatagagaga

tattagagataggactctgaagccctgaaaactgcaaatgaaaaagtgggaaataggaac

aagacgaaacgaaaaggtctaagccagcgaa

>fava_dn_rep_c1023

aggttataacttatggaccaggagtcccaaaattataagcaatagccacaaatttaacca

actgatgtaaatataataataccaaaactgtaataaaaccttacattgtatacctgaaaa

acgtctttgttatctatcaagaaaaaaataaataaatcaagctgtttaaatttatttttg

aacacacataatgtatgtttgatggttcacaatgttcaaagaactaaaggaataacaaaa

cataaccccccaactatataatatgcatagaaacaagatgtcgcaattataatttttaaa

acaagtttctaaccatatctcaactcaataaacaaagagtagacatcaaagatcagattc

taaggttccacagttgtcacctgttgccactctccatctactgcttccttccataatgta

acgttattgcccccatcagccactgccaatatatttccagtcaatgaccacgaaactctc

caaacaggggtattgaaatcgttcaaaatcttgccatcccattgatccccctccttggcc

acagtccatataatcaccttcccatcctgagaagcacttgcaatagtagacttcggaagc

cccaaatttggtgcccaagcaacatctctaacccaatcagtatgcttatgaagagcaggg

aagcagtccatcttccaatgcccgttgttaagcttccacaccttcacagtactatcacaa

ccaccggagcaaagcttctgaacagggtcaagcaagccagaaccaacaagagcgccaggt

gccatggaaggagcccacgacacagaagtgacaccaacgggatgagcttggtcaatcctc

gaactgtcccaaccaccatccgctcgtgcagtgaaaaccgatatattcccatccgacgaa

ccacaagccaaacaaagacccaactcatgcggcgcccaagcaaccgagttcacagacgac

ttatgctcatcaaacacatgagcttgaatccattcgttttgattaccttccttccacaca

atcacacgcccatcaaacgagcacgacgcaagtagagaaccaaatttcgggtgagcccac

gtcacctgccaaacaggtccttgatgaccagtaagagtcgctaaatgctgtgaggccgag

ttactcactccaattatcttaatcgtatgatctgaagaagccgtagccaaccgtttacca

tagtgatccatcgcaacatcatgaaccgtatcttgatgacccgtttcaaccttctgaccc

ggcattatttttctagaaatcgagagagcagtgcgtagtgtatttgctttgatcttattt

tttactattt

>fava_dn_rep_c1024

ttttttttttttagcataatctgaaaccagtttttttttcaattaaatttaaacaaaaga

accataacaaatcttaatcctaagaacttttcacatctatcacctacatttctaatcttc

cttttttcctcctcctaaatttaacccaacactttctcagatttacatttcccaccccaa

aatctcaaacgtcaaatcttacatagcttgtaacgactcagacggccacaaaacatccgc

cgccggttcagtaaaatcaaacaacccatccccagtcacaaaatccccggcaccggtgtt

tccaccgtcaagctccggcaacaacccgacaccagaaccaaacccgtcgtagccagtaaa

gtcgtcatcaaaccccaaaaacccagtcaggtccaccttctccggttcagtcctacccgg

ttcatcacctgaaaccactgttggaggcaacccaagttcatcatcagaagcctcaaacag

gtaacccaaatcattctgcatctcgcctagaccggtcagctgaaccgggtcactatgaac

cgggtcgatttgaaccgggttggtctgaaccggttcaaaagcaggagtgagaatctcttc

ctcaaagctcttgataaccgagtcaagacccaccaccgagtcacactcagtcttgtcagt

aacattctcaacatcattttcagcatcatcaagtatatgaaaaagatcatcttgatgaag

ctgaacacccgagtcaaaaaccgagtcaagacacgactcaaacgagttaactcgtgtcaa

ctcggactcagactcggacaagttaacattattaacatcagaatcagagttaaccttccg

aatcttagcctcaaccgagtaaccaacagagtgagtctgagaatcaaatgactcgttatg

aactcgctttctcttgtcggtggaaatctccattggagagaaagaaaatggaaaaagaga

gttttagagagagaaaaagagaagtgttttggtgggcgcggccgtaa

>fava_dn_rep_c1025

caagctttcacttgcaaaacatttttcactagatgctgaacaatatatataatatatgac

actcaattaggtgctttccgggttaagattatcaacactaagataaaatcctcagataga

gccaaaagggaaatctgagaggaaaaaactaactgaaaacataaaatcatatcacaaacg

aaatataatcttacataaaattgggctcattaaatcatcaccgaaggaagaaaattcaga

atacacagtaaaatacaggaacagaatatggttatacaatacaagcaatatttgtcccta

agcatggtcagacctgctgtcgtcctcgctggtttcatcagcagcgtgtcgccaagatga

tgttagattcctagcttttctgtccagttgaagattttccaaagcatttgatgcttgaat

tacatcttcgggaagttcttcattgtcatatcgccaattgtactcatccttcctgtatcc

agacccagaggatcctcctcttctatcggatgcttgagatgaaggaacagcccttcctag

tggaggtaagccaccagcaacagatgatgaagaatgccttcgcgtgttccgtgattctga

gtttggcaaaggctcagtttctgaagtagtaaacctcctaccactagcccttgcttggga

taagttctcaaatttcaagaatccttcaagcacatccaagagcacggggctgtctccatt

tctattaagatcatatccatttttgctactaaactctttcaactcagacttccaaaaatc

tttctccaaattacattctggaagataaacttttagcgagtgagtaagctgtgcccagtc

aaggtattcacataccagtgcagtaagaagtctaccagaaggagaagcgtgaagctgttt

agcacgatcgttacagctacctaacaatgcaggaggcagagcttgatccttttcgatgac

acgatcttcttcttcgatagcttcaaagacactagctcttaattcagctcggatcctagc

gagaacgcctttcttctcgagagtgcgagtcacgagggttttgaggtccatcatctctct

cgtgtaatcgtccattcttttcgattttcactttccaaacttcttcttctttttctcttc

tcttctgtgcgccaaatccccaatttttatttattccattttttttttaatatatactnn

nnnnnnnnnnnnnnnnnnnnnnnnnnnnnnnnnnnnnnnnnnnnnnnnnnnnnntaataa

atagtactatattggtattcttct

>fava_dn_rep_c1026

tcaagttaaaaataatatatgaatgagtattnnnnnnnnnnnnnnnnnnnngatgcggat

tatgatgtgaattcattgcaactattacagatactgcgatattacagccgcatcacgtga

tatggactacaatttaaaaccatacttcttggatacatttataaaagagaaagacacaat

gaaaatacaatttgaattcgtatccatacttcaaaaacaaaagaaacaactaatctatgt

cttccaaaaccatgtgagattttctagaaaattactggtataaactatcgttcaaatatt

gatgacaacaatatatgattattgtttcactcatgcaccccaatacataattgaaaacac

taggaaacacaaaacgggttattgaaactaaaatccgaatccaatcaaataacaaacaaa

caaaaccgattctcacacggcagcattcaaaatagaaaactcagatccaattccatcgtg

agcagatgaaccggttctgggccgggcctgatccaatttataactacaaagattcctgac

ggcccacagctgataaggactagtatccgtcgcgagccattggtcaacctcgaactgctc

ctgcgaacacgatctgtggggcccagtactagtaacctccacgtagttacagtaccaacc

gtgatgtgctccgtcaccgtcagacgttacgttaacggcacaaacgggtccgtctaagca

aggtcctcttccgctgaagatatcaaggttaccacgttcgaagtaattgtaacccgaacc

cattaacccgccccatggaaccagattcttgatgtagatgtagtaaccgtatttgtcgta

tagtttgattccgatctttgagtcggttcctccttttaaaattgaaccggttcgaacgta

gaccgtgtagacgcaatcttcgtcggagtcggatctaacggtggcggtgaaggagagggt

gaagagaaagaaaatagctatgaagaacgtcgtggttgccatttgtgtgaaaatggtggt

gaggtgcccgcggcatatgtggttagatcgaacggtgatatcgacacaactgaggatgaa

cttaaaaaattcgttgctaaacaggtggtgttttacaaaagaataaatcgagtattcttt

attgatgctattcccaagtcaccttctggcaaaatattacgaaaggatttaagggctagg

cttgcagcagggtttgcaaattgaagtacaatcaataatttccaatgtattttataaaca

tgttggtccaaatacaacaactcaatttcataccttatagtttgttatattaaaaacttg

tatcttatcatcacatatcatatgatgcttgtttatggttcaactaatgattcaaatgtt

cctttattttggaacaatgtatttagttagaacaaaaaagtgaagattcaattcaaagtg

ttttatttggaatgaaaaagaggatttgttgcttaaaaaaaaaattattg

>fava_dn_rep_c1027

atggacatagatttcataagatatctaccattcatttttatcatcagagccacatataaa

taacactcgtaactcgtcacattcaatcacaatcttcaacataacattgtcaataaacat

attcatttgttaactcagtagtaagcactacaatactacaatggccgcttcgacaatgtc

actctcttcttcatcactggttggaaaggcaatcaagcttaccccgtccactcaagacct

tggggtgggaagagtcacaatgaggaaaacagccactaagaaagtttcctctggaagtcc

atggtatggaccagaccgtgtcaaatacttgggtccattctccggtgaagctccatccta

cctcactggtgagttccccggagactacggttgggacactgctggactttctgctgatcc

agagacattttccaaaaatcgtgaactagaggtcatccacagcaggtgggctatgttagg

agccttaggatgtgtcttccccgagctcttggctcgcaacggagtcaaattcggcgaggc

tgtgtggttcaaggcaggatcacaaatcttcagtgagggtggacttgactacttgggtaa

cccaagccttgtccatgcacagagcattcttgcaatatgggcaactcaagttatcttaat

gggagctgttgaaggttaccgcattgcaggtggacctcttggagaggtgattgacccatt

atacccaggtggcagctttgatccattaggccttgcagaagatccagaagcattcgcaga

gttgaaggtgaaagaactgaagaatggtaggttggccatgttctcaatgtttggattctt

tgttcaagctattgtgacaggaaaaggacctatagaaaatctcgctgaccaccttgcaga

cccagttaacaataacgcttgggcctatgccacaaactttgctcctggaaagtgagaaaa

aacaatcataagtgactaacttgattatcacaatttggatttcttattctaataaactgt

gtaataatatatggtgcagcatcaagagactgaaataaatgtgaatgttatcatatctag

tgaaacgacaactcactgtgtaactgattcttacaaaatatcatatcatgtcacttgtgg

taaaaaaaaaaag

>fava_dn_rep_c1028

agcatacatgcaagcagctaaatcgtagccgtaccatatatcatcatataacaaaacaca

catcaacacaccaacgttaaagtttggttgaaactgaaacagaaacaaacttacacctga

taaggcattaaccaaaccatgtcactctccaccacaccttctcacctcaccatccttcct

tcaatttcaccactttctcgcagattttccttcactttccggcaaaacataaaccaaaaa

acttatcctacgactctcttgtcttcttcttcaacaaaatccactagattcttggtgaag

tctcagtctactacttcacctgcttcttccgaggctgtgaaagtgatatctgtcccttct

gaaatgaaggcttgggtgtatggagaatatggaggtgttgatgttttgaaatttgattcc

aatgttgctgttcctgatgttaaggatgaccaggttcttgttaaagtttttgctgctgct

cttaaccctgttgatggtaaaagaaggcagggaaagtttaaggctactgattctcctctt

ccgactgttcctggctatgatgtggctggggttgtagtgaaagttggtagtgaagtaaag

gaattcaaagttggagatgaagtatatggtgatgtaaatgagaaagcactagaagggcct

aagcagtttgggtctctagctgagtacacagcagttgaggagaagttgttggcactaaaa

cctaagaatttggactttgctcaagctgcttctcttcctcttgcaattgaaactgctcat

gaaggtttggagaggactggattttcttctggtaaatcaattattgttctcaatggttca

ggtggagttggaagccttgtaattcagctagctaaacaagtttttggagcttcaagagtt

gctgccactgcaagcactagaaatttggagttgttgaaaagccttggagctgatttggct

attgactacacaaaggagagctttgaagacttaccagaaaaatttgatgtagtttatgat

gccatagggcaatgtgagagggcagtgaaggctataaaagaaggtggaagcgtagtagca

ttaactggtgctgttacaccacctggattcagatttgtagtaacttccaatggagctgtt

ctgaagaaattaaacccttatttagagagtggaaaagtgaagccaattgtagatccaaaa

ggtccattcacctttgatcaggttgctgaagctttctcttacattgaaacaaacaaagct

actggaaaggttgttatattccccattccatgaatcaaaaagttataggcaattttgtta

tgtatctaattatgttcacattatatgagtaaataacagttcatatatcagcaaaaaata

aaggtacattcattgcttcaaaaaaaaaaagattaagttgttttatgaaagatgattatc

gaaaaaaaaaaa

>fava_dn_rep_c1029

gatttccggttcaaccaaccagtccgatccaatttataaaagattgatttgaaccttata

tacttttagaaccaaaataaatgtggaaaatgcagtgtctattacaacattctaaatata

agatggtgtcaagtaccattagtttccaatatcactttaggaaataaatttcctttaaac

ttcaagaccatcatttccaacagctacaaacttaaacaacttattcttataacatcttat

tacatagaattccttcaaacaaacacaacaatactaccaaaagctcaaattttgagtcag

aaggatatagtgactctgccaatggtttcacaaataaaacatggctaaagaaataaaaga

tcaacaaattgcagattcaattttcaaatgtccaacaccacaatgatagcacttcatcac

ttgttaatctttctgcagtgtttcctgatttcaccccttgaacctgttaaaggagtaatg

tttcccaacttaaccatagacttggcaaattgttcaaagaaaatatcatttcttgctgca

taagttttcactaaatctgcagatacttgattctttgtcaacagaacttcatcagaactt

aacagacccttgttgaccaataagttcttgtagtagttgttgtcgaattttaccggggtg

acaaagtctaggacaaataaattttggtctccaccagatcttggacaccgagtgcgcaat

tgagcagcatacttttggtcaagagtgaaatctgacttgccattcccagtttggttgtag

agtctctgtctaaagctagtgcatcttgaatcacctatagtgtgactaccagatagagca

actagatcaacaatgttaagtcccttaagcttgaatttagttaagatggtttggaatgtg

ttgttaggagcaggaatgttgttattcgagccactgatgcttgctccaagagaatccctt

cttcccaaaggcacgtcccagtttggtccaccagtaagaacagttgaatctctagctgct

agagtcaaaatatcagcacaagagactgtatgaggacactctttctctactgcagattta

atttcttcaatgacttcaaatcctctagctgagtttcggtttggattcgacctcttttca

ctgatgattgttccactgctatctagcaacaccgatgcatcgcaacccttgacaaaacag

tcatgaaaatgcagtctcagcaacgaagcagccatgcgaggttctttagcaacagccttg

gaaactacagacttaacaatctcttcaacttgtggacacgaaccgtcgtagaattgaggg

tacaggaaatctcctactttattttcacagtgacagaaaggagcaaagactagaagagaa

aaaagcaagaaaaagtttatagaattgaccattatgtctctttcaagatcacttcaagga

acagaagttattatgatagagtagttaaacaagtgctttcaactggattgaatcccgcgg

cc

>fava_dn_rep_c1030

tcagagtttcttggatgcaccaagcagaaaaagtaagttgtctnnnnnnnnnnnnnnnnn

nnnnnnaatcaaaccctaagagaaagggcaataagtgtttagcagaacaaccaattttta

aaaatgtcatttcatgttatcaacaccaccagtaactagacagaaaaaagattctcaagt

gttgtactatagtttataaaactgctcaggatttgtctgaatactgaaatccagattcac

cccttcattacaaaatgcacaccaaacaatacttagagtacaatttatttaaggtagact

cacaacacacacataacaaagatactgtaatataataccaacgatactttttcttccaat

taatcagtctcaacacctaatccatacaacacaccagaatacttctccgtttcaccgttg

aagaaagtatccctcaattttctaaatgcacaagcagtgagtaaactatcggatccagct

tgatggtcaacaccaagcctttcaacttccaacaattccgcaagcttgttcaaatcacca

tgtaaactgttacggaatttcatcagatattttatatcacaaagcattggaaaataaagg

ttgatcaaatcaaagaaccccgattgcgtatcgggcaaagttctgcaagtcaaaagcttc

agcagataaccaaaaccatatccaccgtaaaaagtaatccattttatgttatcattcaac

acaattcccgaagacagaagaagcgtgccgaactggtccctgtcgatcccagggacgtcg

tcgatcccagcgatgtctttgcggaaattgaactgccagatacaaggattcccagtgtca

caagtaggtaggtttccgttctcatcagagaaagtgagacctaactgaatcaggttcgac

atatcaacattatccttcaaagtctggtagttataatcggtgatgtgcttgaaaacccct

ataggacggagaacaacaccaggaaactctgtatccatagctacatagttgaaataatca

acaacttcacggatcaactcgaattccttttcgagattattgttccaaacctctctgatt

tgaaccgaatcgcctttggatgataaatccgacatttcaagagaattgataaccgatcga

atcaaatctgaagaggaatcgaatagagtgatttgagctacgaatcgagagcttcaggta

gaggatctgaaacgaaaacaagcgtaaattgaatcgatttagaagaactagggtttgagg

tttggactagacttaaaagtttgaacagagtgagagcaaaaagtgagaagtggggtagtt

ttgttgagaaatatggacacggaatgtcgtttannnnnnatatgcgaccatctataatct

atttatannnnnnnnnnnnnnnnnnnnnnnnnnaaaaagacattaataatttttgctctc

attttgccacatcttcaaattgagagaattttggttcacaaattacttttttt

>fava_dn_rep_c1031

agcttccgggaggcagacgctgttgatgtccggnnnnnnggggatccaagaacaagagaa

cgacgcaaacagatagagagagaaagtcagaagatttttagagagagagagagaggtgaa

caccgaccggacgttcggtgctccgctcatcacggttgtggccccaccgctccgtatttt

aagctctaaaggatcacatgtcaaacatagaggatagcaaagaacttggacagatggacg

tggatgcatctggtgggtcagttaacatgccatcgccccagaagcaggaggaaactttaa

agaaaaaatatggaggaatgattcccaaaaagccgccacttatttctaaggaccatgagc

gtgcttacttcgattctgcggattgggcattgggaaagcaaggtggtggtaagcctaagg

gaccacttgaggctcttcggcctaaactacagccaacacaacagcaaacacgttaccgga

aatctccatacgctccttcgggtgaagaaggtggaagtgttccatctgaggaggatgcac

cttccaatgagtaaaagcagcaatcaggcttttgatgatttgctgattattgttctggta

tacctcagtaggtataactttttgttgttttcaacaataaaataagtttattattatgta

ttcatattaatattaattagcacaagacatgttatttggcctgcagctatgctgtagaca

cttcctgtgattgtgatttgtgttttaatcttcttccccctcccttcatgttttgtagtt

cttagttttgaaaaggaaaaaaaaagtaataagaaatttatgatggaaagtggtcatgat

gtaaaaatggagtaggcaaatattgaacaagattaaaacaaaaatgttgttcatgtgttt

ggttttggatcataattgttcctttcacctttaattttaaaaaatgtgttgtaaataaat

tatatttaatattgtt

>fava_dn_rep_c1032

ttttacgaaagaagtctattgttccctggtgctaacaaattcttcctcccctaacaattt

aatcactaatatttttctacataaaatgcatttaaaagaaatcaaaagtttagatgattg

gagattctggtatgccattttcctttacatcagatttgcagatgagatatagaaatatat

aatgtgcaaatactataatactaaatacaaatagaaatgttattcagagcacttttagtc

ttactataacggtctaatattggtgactgcacgaattcgtaacatttatcataattaaga

aacaagtcatgcctcatgaggtcctttgagaccgttacaacattctaacagcattgaatc

cttaatttcctcaagtgaaaccaccaaaaaaagtaagaactagagcatcaaccaaaattt

aaaccaccacctgagtattccacctcctgagcaggtgctagagaagacacccaatcggat

gtgtgagttggaagcagtcccaatgaattgagcttccaaacacctagtgccatgcctccc

aaattaagagcaatgaacaacaacttaggcccaagaaggtccactttggcgtctttgtaa

ggttcaaacatctttccaacagtttgtaaggcacttattggctgccaaagagccgagaag

gtaatcccaatgctaaacaagtgaacagtgttacccgccatccacatcataaaccccatc

atcaacaaattcttaaaaggagcttgtgcaacttcccaagctttctgagatttccaactg

gattcagcatccttcttctggcgactaagagttgaatcgtcttgatcgagggaagcgcgt

gagaaaccgggcggatcgagaatatcgcgagaagtggaattatcggagaattcaacggcc

catcttcttccggaggatccaatcgcaccctttcctttctccattgaattgaattgaaga

aatctctgattttgattttctctttctgcgtttgtcgctgctactagtggagagaatccc

aaatcccaaatcccaacgagggtttttttgtatcgtgaaatccacggttgcaaaactact

atatactaatgtcgt

>fava_dn_rep_c1033

atggatactgatccatcccaattcccaactatagagttatcacttgtaagttaattataa

agtattcaaaatcaaaacatatcttatctgtgtgtgtgcatgtgtgtattttgtttttct

tttgttccataaagattcaaaatgttttagaccacgtatataattttaaaaaaatgtact

tgacttcaataatatctaaataacaaaaacaagtagatgtgtggtgtgaacaccattgca

caagacacccaaatgaaaatcccaagactgtgttctggtttcacctcccaacttgcaaga

ttctcctacagaaactacacttccttcctcacacatggtatccatgtctttcaatgccct

gatgaaattggaattgtagcaaaactatcggattgtattgcttctaggggtggtaacatt

cttgctgctgatgtttttgtacctcaaaacaaaggactcttctactctagaactgacttt

gtttttgatcatgttaaatggcctcggttacaaatggatgaggatttcttaaagctttct

aaaacatttaatgctgtgagatctattttaagagttccatctcttgatcctaaatataag

gttgctgttcttgcatcaaaccaggatcactgtctgattgattcattacatggatggcaa

gatggaagattaccagtggatataacttgtgtaattagtaaccacgatcgaggtccagaa

agcgaagtgattcgctttctggcaaggcacgatattccttatcattatttaagaacaaca

gcagagaataaaagagaagaggagatcttaaagctggttcaagatactgattttttagta

cttgcaaggtatatgcagatactatcaagcacatttttaagaagttatgggaaggatata

attaacattcaccattgtttgttgccatcatttagaggcggaaatccatgtaaacaggct

tttgatgcaggtgtgaaattaattggtgcaacaagtcattttgtgacagaaggatgtgat

gctggacctattattgaacaaatggttgaaagagtttcacatagagatgacttgcagagc

tttgtgcagaaatcagaaaacctggagaaacaatgtctgtcaatggcaatcagatcttat

tgcgagcttcgagtattaccatacgaagaaaagcgtactgttgtattttgaggacaaaca

tcaactatatatattgtaataatgtaacgaagtctgtaattcttcaatcttattactcat

tttgtgtcttcatttttggtataatataacattcttaagagtttcattgtttggcaaaat

tttacatgtcctactctatcgaggcatatacaaaatccaaacaaatcaagtgctgctact

aatatttaataaattcatagatctttctgatgcttcggccgagttggcgtgaccgagtag

aaattctatcccatcaacccatttttgcttgtcaagtttgctctcggacttaaattctag

aagaccttgtgcagttttgaggccgaaatagatctcttcagaaacttccctttcttttcg

atatggccatgcagagtcttcattacaaactccataaaccacacatttattctttttgga

gaaagttcctccaatgtgcttgcttttgatcttaattttgacctgatatttcttgttgat

gtaaacggacacatgtttccatcgtaacgaacctttttctgtaacctgcaacaaatctcc

ctcacatggagaatggttttccggcatttgaccctcgaaaataggagaacaatgtgtttc

cgctactcctctatcatagggacttatcgatgcattcttctttgcttcttttggaaatcg

ggctcttaatgccgcttctcctctcaaagctgttgcagctgcagcagtgagagtgattag

atcaccgggtgtctgaatatcaaccgcagatttaacagtagaagcgacacgatcatgatc

agcaccagcaaggtcagccatttcaacacaataagatgctaagagctgtgtggctgaagc

catagcaacatccaatttagagtgactgtcactactagcagctacagaagccatcgcgga

agctagtccagcaatcgaaagagcagaatgcacacgcgcattctctacacgtgctcgatc

tttcctcttaacatttgtgttggcacccccgtgttgcttctgatgcaaccattttccaat

tgtaccctttcgacaattcgtagaagctatgatcttgcctgacgttaattgcggtacaga

aatagtttctggcaatgtttgaggagtgttatcaagaaaagtgtgtttgtgttgtttttc

taaaagtgcttttgaaatctcagaagcagataaactccaagatcttgataagaactccat

aggctcaagaggtgttggtgggtcaggaatttcaggcaacgataacattaacttgagttg

atgattctctttcatatcctcaaactctaaaccttgccattgccatgactcgtttctcca

atgttgaaacggaaacacatccctttcacttttcggttccatcaatttctaaccgctcaa

aatatctcaacacaaaaataaatcatctactttatgatccgaattctgtttaaaagagat

tgtaatttcaatgaagagaagaaactgaggttcaagtttataatagaattaaagaatgaa

aaaggacaaggtatactgttttaaaaaaattaaaataaaaaaaaaaagatcggttgtgaa

attaagaaaaacaaaaccctgtataagtgggtatgggttgttgtcgatgaagtaatggaa

gaagaatattgagatgtgtgtgtgagagagagtgatgaatgtttgtttagtttatagcgc

gtttttgtaggtaactacttttttcgatggttctattacggagaaccaatccatatcact

ttgcttggtagaccatagttttcgagttt

>fava_dn_rep_c1034

acatttttcaattatttttttattattcacctgacaaataaataaaattaaaataaataa

ttggagtgggttttcttccttgactgtcaccagcgcattatagcacaccacaaaaaaaag

cacaagaactgtcgctcacacaaaataccattacacatgctctgctttccttcgtagttg

attagtattagagtggttacaaccggaagggtttcagtggagatggcgtcggttggcgtg

gcaccaacttcgggttttagagaaaccagtggtcatggtgaaattggtgttgatgtattg

ccagaggaaatgaatgatatgaaaattagggatgatagggaaatggaagctaccgttgtt

gacagcggcaatggaacagagactggacatatcattgtgactactattggtggtagaaat

ggtcagccaaagcagactataagctatatggcagagcgtgttgtaggacatggatcattt

ggagttgtcttccaggctaagtgcttggaaacaggtgaaaccgtggctatcaaaaaggtt

cttcaagacaagaggtataagaaccgggaattgcaaacaatgcgccttcttgatcaccca

aatgttgtctctttaaagcattgtttcttttcaaccactgaaaaggatgaactatacctt

aatttggtacttgagtatgttcctgaaacagttcatcgtgtcattaagcattacaacaag

ttgaaccaaaggatgccaatggtttatgtgaagctctatacgtaccagatctttagagca

ttatcttatattcatcgctgtattggagtctgccatcgagatatcaaacctcaaaatcta

ttggtcaatccacacacccaccaggttaaattatgcgactttggaagtgcaaaagtcttg

gttaaaggcgaaccaaatatttcgtatatatgttcaagatattatagagcacctgagctt

atttttggagcaactgaatatactacagctattgatgtctggtctgttggttgtgttttg

gctgaactgctgcttggacagccgttgtttcctggtgagagtggagttgatcagcttgtt

gagattatcaaggttcttggaactccgactagagaagagattaaatgcatgaatcctaat

tatacagaatttaaattccctcaaatcaaagcacatccatggcacaagatcttccataag

cgcatgcctccagaagctgttgatttggtatcaagattattacaatactccccaaaccta

cggtgccaagctttagatgccttaacccatcctttctttgatgagctccgtgaacctaac

gctcgtttgccaactggtcgtttccttccaccactatttaacttcaaacctcacgaactg

aaaggagtcccacttgagaccttggtgaaattggttccggagcatgcgaggaagcaatgc

ccgtttcttggcttgtaatatgttgtgaaatgtaacaatactgcaagtgttgttttgtct

atttgatgatatgatatttattagtatctttgttgtaattggttgccctgtgaaagaaaa

tttagagatatatgctaccccatattacccaacaatgatgggtattcactattcagaata

ccctgtttcgtgtatcgcggcagaatgtaacatgcaatagaagacaagtgtttgcaatta

tctaaatgttctatcagtatttgtatttgtggagctaatgatgagatgttgttaaattta

gttatgcttagcatggttattacaatttggaagttgtggcgatggccttatccgagtttt

tttttt

>fava_dn_rep_c1035

ttaactccaagaaagcatttgtgcaatcatatattaattataaaattaatacaaactgct

ccacttattcattaaaccataactaaacctaacgcatgttactaatttcagcaggtgcaa

cagatttaacaaaacaaaaatgatttcattccaagttgataattgaagccttacaatgct

attaaacagtactgtattagcgcttagactttaatggagaaatgaaaagttaaatccagt

cacaatatgaatgaaatcaaatcatgcgtcaatgctattttggaagtaaaataatttgca

atgtaaaaggaaaaattacaggcttgtgttctggtatggctacaaaactgcttagatata

atttctcactctaacaatgaggaatgttactagatttctacaactacaagatcttttcag

ctgaaggggataatgtattccagtttgatctttagtaatcttgtcagcttgcaacctttg

ccacttttcgttgttccacttgaccagatagcggacactgcatctcagtccagcttattg

gaaccatagttgcaccagcactgctttctgcagaaacaacaccaagctcattctttgccg

ttgtcagaaagtacgcccgtgcatctccaagtgaaagcacaagagctctaacaatgtcac

caggacgaaaagacaagtgcatgtctactttatcaatctcagtagccctaacatcttgct

gcctaatgataccggtaaatttttcgcgaacagacttttgagcaacacacataatatcag

caaaagcggttttcgccatcactttggtaaccctagcaatgacaatagatccaggctcag

gaacaggtccatgagctttatgaccagttacttcaatagtaggtctctggtcaggagagt

ccgaagcgggaggtatggtgtggcggaagccagttaaggaggcgtaaacggtgttgttgt

gaaccgccgcgtatgttccttttccggctttaacgtcggaggttttgcctactacttctc

ccggtgttaccatctcttctactgcccccattaacgcttttttctctccgtcgccgctat

aaagctttcctttgttttttggggctgaccaactcactcctagtcaatttcattgtgaat

tcgagaaccctttcaaat

>fava_dn_rep_c1036

cgcggggataattgaagtaacaaatatcagtaccaaatattgaaagatttaacgaaccct

agcgcagtcaccaccgcccggacgttcagccatcgtctcccgtctaagtgttgtgaaaag

tgagatggggaccaaggcaaagaaagctatgaagaagaacatgaaaagggttaccgctaa

aattaagccatctgagggtgctgattttttgccattggcgggtggtccgggtcgcaagct

tcctgaacttaaacctctggagaatacttcacctgttttgtacattggtagaattccaca

tggcttctatgagaaggagatggaagcttattttgcacaatttggaaccatcaagagatt

gagaatagccagaaataaaaagaccggaaaatccagacattttgggttcatagaatttga

atctcccgaggttgcaaaaattgttgctgatactatgcacaattatttgcttttcgaaca

ccttcttcaggtttatgtgatacctttagaaaatgttcatccaagattatggaaaggatt

caattaccgttacaagcccttggactctctccaaattgaacgagagcgacatgacaagga

aaggacactggaagaacacaaaaagttggttgacagggttatgaaacgtgacaaaaaacg

ccggaaaaggatagaggctgctggcattgattatgaatgtcccgagattgtgggtaacct

ccagcctgctccaaagaagataaagttcgaagactgagtagatgaaggctgatgatggtt

tagcatgtcatgaaggatgatgaccagaaagtgtgttttgtaggatttttaaatttgcct

aatgaactatcctctttgtaggggaaagagcctctagttttgagaatctctgaacattat

attagggaaatatttgtagaagtgttatacatttcactattcattagtttttgttactgt

taatcacctttattaagccaagctgaaccagtttattggttttggatactgaagaacatt

tatcaccttcatttagactttgttgatagtaaaaaattatacatcttgaaatatcgtaag

aaaatgcatttaaatgatcttatgcttaggtcaagttgacgata

>fava_dn_rep_c1037

gcttgaaaaagcctttaattccttgtaaatatatttattttgatttgaaaattttatttt

ataagtattaataaattgagcctccaacattgtgtacatggaatagtaaaaacaaaatat

gagcaaattgaaaagaaattatagttgtatattggtatctaccacaccatttgttcatga

ataaaacacacaacagaaaacaatgcttatttcaaaatcaaacaagacaaatacatataa

ctctgctgaagaaatagatccaacgacagtgtcatccttcaacaggacttctaatgcatg

attgttgcctatgttgtattggcagtgttttccaccacttcacaaaagtagaaccctcca

aaaacacatctttaccaacttcacctttcttataccactcttctatctgatcctccaatg

ctgaaactctttccttaacatcttcaaaagatttcttatcattactaagatcctccactt

cagcccaaaaacaagactcagaataaccaccttctttcctcctctcacgatgctccaacc

atctctgcgtatacctgtatctctttggccttcctcctttatccatgtaagcgcgtccat

cacggttcctcgaatgtcgataataattagcaatgtccaaaggctcaactagtcttctaa

actttgttcctgcctcaatccaatcttctaccccttcaaattcatccggaagttcatagt

ttagtagcttttcgattatctcgtcccatactcctgctagatctagtctcttcacatttg

ctttaaaatcttctggatcttcttgatccttgaatccgtcgtagtaaccctttttctgat

gcccccacatttctctgtacttttccacttccttcatcttttcttccataaatcctttct

tatcttctatggctttttcgttgtttctttttcgttcctctaatgctgctgctgcttgaa

tgcataaccttgctcgtgtgctcaggccaagatcatttaaggctgtgttgatggttgcaa

tgtcacggcccgtagtagtattctcggacaaaggaatcttctctagttggtccaagtaca

cagcattctgaattcctaggttttttggcaattccgcgccataagtgaaatgttgtcgca

ggcttttaagagaaacttcatcagtttctgcttcagagcttaattgagcagagaaaaaca

ggacctgcagaattgcatcagggtttttgatgacaatctgttttgcttcatttccacttg

tagtgcagaaaatgtaagttccgaagggtctataagggctcaaaggaacaaaatttgcaa

tagtttcaagtgttgcttctgttgttcccattagcttgcaagcagcatgtcttgtgacag

ttgcagcacttgatatcaactcaaagtagaaatctgaagttgaagagattcttccgagag

acgattcgctcatgaatgatctggttttaggatctaagagttgtgaaactgtgtcaaatc

tctggtcaaaagaagatagaggagcgaggaggatccttggcactatgtcatatctcagca

caaaatggaaaaagtagttagaccacttttctctccttgtagcatgagaaaaaatgtgat

ttccaactaatggagaaccaaatgtaacacataggggagggataccaccatgagattttg

ggttttggtagttttctaaggcccaaagagttgccagaattgccagtggtccaccagatg

aatgtcctgcaaaaattatttgcttctgcttttttattgccgtgtccacctgatctttaa

gtaaagacttagccaaaatctcttggaatctttggagaaagaattgattaaccaaagcag

gatcattgttaccaatacttctaagtgaaggaaatagagtgagatctattttgatttgac

caaaattagttttggaataccaatctttgaaaccacctgatgctggaaaggttatgataa

cttctttagggttgcttctactgttcttttcaagaaggtaaggtgtcttgtaactgtgag

ccttcaaggatgcagaaagtgatttttcaattctttctggactcagttggttggtgttga

ttccagttccaccacctcttgatgcagccatgtgtctgtctcagttcttgttctatgctt

ggttctcagttccttgtttctgtgtggtgttggggaagttttgttaatgttaaggtgctg

tgcttacaaaggtgaacacagatgtttttttatgttgttatgaagactttgagtagtagg

tt

>fava_dn_rep_c1038

tttaagggacacaccttcaactcaatctcaccatcaacacatttctcagtttattttcct

tcttgactgataattttacgaaacaaaacattccccaaaagatggcaaccagaagaggaa

gatgcagtcagtgtggaagggctatacaagtagaagcctacggctatgcgaattccatgg

ccatgatgtgttctagttgccactctcaaggtttctccatgcaacctaataacccttttg

tcaataacaacaacaacaactcacctcctcctccggccgcctatggacgacgtccgtctt

ggccacatacactgatgggcccacaagctccaatgacaccaccttctccatatggtaaca

agagggctgtgttgtttggtattagctatagtaatagtgctaacagccttaaaggctctc

ttaatgatgttcacagcatgaagtactttctcacacaaaagttaggttttcctacagatt

gcgttcgcatgctcacagatgaacctggagagagaaacccgacgagaattccaacaaaat

ataacatgcgaatggcaatgaagtggttggttgagggctgccaagcaggcgactcattgg

tgcttcacttctcaggacatggatcaaggcaagtggaccatggcatggatgaggttgatg

gctatgacgaagctatatgccctgttgattatgagcgcgaagggaagattcttgacgatg

aaatcaatgctacaattgttaggcctctaccttatggtgccaaacttcatgctgtcactg

atacatgctttagtggcacagttcttgatttaccattcatgtgcaggatgaacagaaaag

gttattatggatgggaagatcatagaagccacagagctggttacaaaggcacaagaggtg

ggctagcagtttgcatttcagcttgtgacgatgatggaagtgcagcagacacatcggcct

ttactggcatggaaagttctggtgcattgacttacagcttcatccaagccatgcaagttg

aacgaaaactgacttacggtcaattgcttaatgccatgcgttcttcgattcgtggcgcta

gagaaggaatgtttggcccgaatcaacaacagcatgtcatggacaatcggcagcagtatg

ctcatgatccacagctgtcatcttctgagaaattcgatatttattcgaagactattgcaa

tatgatgtgacacataactcggagtggcattgaataataaaatgaataagaataacagac

gagcggtggcagaaatttcgatcgataaaacctcaacccgaagctcaaacaaacgcatct

tatcatggtttcttcggatccccgagaggaagtccttcaggcatggtatatggatgatag

cgatgaagatcaacggcttccccatcacaaagaacccaaggagttcgtctcattggatca

acttgctgaacttggagtccttagctggaaattagacgccgataaccctgatacagaccc

ggagctgaagaagatccgtgaagaacgcggttactcttacttggatgtttgtgagatctc

cccagaaaagttgccaaattatgatgagaagatcaagagcttctttgaagagcatctgca

cattgacgaggagatccgttacgctgttgctggaagtggttactttgatgtaagggatcg

caatgaggcttggattcgcgtgtgggtgaagaaagggggaatgatcattttacctgctgg

aatttatcaccgtttcacattggatgagggcaattatattaaggctttgcggttttttgt

tggcgaaccagtttggaccccatacaatcgccctcatgatcatctccctgcaagagggga

atatatcaagacttttgtggagaaggatgttgccaaccaaactgttgacgccactgcata

atttggttctgcttgattgtagcatgtgataaaaaggactaaggctttgattctaaaatg

atgtttgaaaataataataatgatctctttcagaaagaggctcatcctcctatgtttggt

atgtaaccgttaagcctggatgttagttatatatttcatgctatcctgtttgttacctgt

gtgttgtataattaaaaattgcatatacaagtattattatcattattattatcaatactg

ggacagaataaacagacactaatagaatgttaatgtattataatgtgctgttttggttgc

aaaaaaaaaaag

>fava_dn_rep_c1039

tgaacaagagaataacctaaaaataccgtttgtaaaatcagcaacaatcaaaattcaaaa

tcaatcgcacatacaaaacaaattaaacagaacacatgtgaaatcgcaatattctaaaca

tgagattgagttgattaatcacaaaacagtcatcaccgtcatatcaaattaacacaaaaa

tccaagaaacaaaccacagcaatatctgtcatgaagatgaaaacaggaaacagagatgta

ctaatcatcaataagaagaacaggcactagttgatatcagcgtcatcgaaatcgtcatgg

aaggagttgaagaaatcggaatcggcgactttggaatcgttgccgccaatgccatcggcc

aagacctcgaaattgtcgacgtcgtcgacgtccatcgccatcgtcgttatctggtcctcc

atctctccgaacaagtccattgctctcatgatcgattcgctttcttgcgttcttttcccc

gcggctaannnnntggtctttggtcctctttcatgacaat

>fava_dn_rep_c1040

gccaataggaaattgataaaaagttttatcnnnnnnnnnnnnnnnnnnnnnnnnnnnnnn

nnnnnnnnnnnnnnnnnnnnnnnnngcagccctccaaattcgcttaatattattaacatc

ctcaaagttaggcaaaatcgattgtatgtacttttattttatgaaaaaacaattgaaaaa

aaaaaactttcgacgtatccattttggcctagtagcataaaaatcaaaagaaatatgtga

agatttcacggcaaaagaaaatactaatagcaaccatatttagtcaaacgcatggagatt

tattatagcgacactagcctttattttgaacctcctatacatcaacatgcagcgaacaga

ccattcaaatacaaacacactaaggtttcatagcatttttcaagtaaggttttaagaatg

tcatctcgagtgatgaaaaacctcctcgcaatgcttgaggctgaagaagccgaagcatca

aaatacagcaacaaccaaggtactgctgatgcatttaacaatcatggtggcgggaaccaa

aatttctctggtgccagaatcaacagcggtgcaaattccggggaccgaaagaaataccgc

accaccaacaatgttggtggccgcactgtaaataacagtggtcatttcattggtaatggc

aatggagggtatactgagggtaactctgacgcatccacaacgaactttaccaaatagtta

catgtatctgtgattataaatatgctatatctaagtaataataaagttgaaggtatatat

aggttcaatggattatggttgtatgcttatgtatcggaggttataaataaagacactata

gtgttgctataacaaatctccaaatgcttgactatagtctatatatagttgctagtagta

tttatatttgggttgaaatctctatatatttcatcggatatttattttatgggaccaaaa

aaatctaatttacttgtagctaactgtactaactactctctactactccgttcaagttgg

agggtgaagattatgagtccaacttagacataagatcatggaaagcctttgttcataatg

actttataaggatgtcaatttgttgttgcttggttggcaagtaagttgtttgtatagccc

atattgtatgtggtctcgtgtggcaagacaatctacttcaataagtttagtcctctcatg

gaaaaagtgatttacttccaaatgaaaaattacttgattgttataatataaagttgttga

tcctagacattgaacttgaagactagatagtaaacttcttagtcaaatgatttttaacag

tagcatgtatcataactctatattctacaccactagaagatctagaagtggttaccttgt

ttatttgatttcctgatattggagcattgctaattttcttgtcttgatcttgaagtggtt

aaatgtttatttgttttcttgt

>fava_dn_rep_c1041

aaaatcttaattttacattttaaaaaagtatcaatttaatcacttcctaagttcatattc

atagtaacttaggtaggtatctaatattgtatttttttttatgaaacaactcgatgttta

ttactttagccaccctttcttttgcatctttcatatccaaaatatagaattacacttaca

aacacacatcatacacaatagtgtctaactcattacaagttctatttgcagtttttcagg

aattattacaagagaatagaaacaaacagattctaagtcacacatagcattgacacattt

acgttccaactagaaagaagttcatctgacttgtccatgaggcaatggtcgaccttattt

tattccatgttgtcatcactctttccatatcgcaaaatgaatgtttacccttcgcttcca

cttctcgaacagtactaagatatgatgcttgggtgcagattttgggtgcatcagtgcctt

gataagctttgcagatataacttatgaagttttcaaaatcctcgtagagtggttttccat

ccacaactacccatggaacatattcgtgaggaggctgtagagcatttgtttcatttgcta

actttaggtccaactcttttccgcgttcactacgataacaatcattaacaagtgttgaat

ccagacccagtttctcataacaagattcccattctttgcgcttaccctgaagcgcaagat

cctcaatacaataaatgaaagagaaatgcttgtccagtttcggccagatatcaatagcac

acgcctcaaccgtattcagcaagcactcatatttaccatgctggcaatcaaaggttttgt

tacctttgagtttagcattaccccaaggaacgagtttaaggtcaacaatggatagaagat

cgtgctcgaaaacttgaggaagataattgacgatgaaatcggcgctgtaaggacacaaac

tttcgtagtataattcaagagaaaccttgttgtgagattgtgagggagagaagaaggaag

agaaagaaaggattaagatcagagaaaacgaaaaggtgttggaacaaaccatggttgctg

aatctcacaggac

>fava_dn_rep_c1042

attaaacaagtaaaagaaaagtgtagaggcagtgctcaacccgtcaagacaaagccagca

ctgtttgcaattgcatttctcagagagagaaaccgtttctagagagagaagctgttttct

agagagagaaaactccgcagctatgtcatcttccttcgacgcattcaccgatgacttgaa

cgtcaactcttcttcaactcaccacttcgctacctccgatcaagacgacacctactccgg

ttttggcggctactccaacttctccgccgacggcgatgtcaccgtcgatcatgccgccga

ggcttcaccggacatcttcggattcagtgatccgaatcccggttactctccgtctccatt

tgaatcggtttcagttgagaacgggaacgataacggaaacggttacggtgagagtgacgg

tgtttttgtctctgatggtccgattcttcctccgcctggtgagatggagtcagaggaagg

ttacgctcttcgcgagtggcgtcgtcaaaatgccattgagctagaggagaaggagaaaaa

agaaaaagaaacgagattgaagattctggaggaagctgaggagtataaggtggctttcta

tgagaaaaggaagcttaatgttgagactaacaaggttcaaaatagagaaagggagaagtt

atttgtggctaatcaagagaagtttcacaaagaggcagacaaagcttattggaagacaat

tgcagagctcattcctcgtgaggttcctagcattgagaagaagagaggcaaaaaagatca

ggataagaagccatcaatcactgttattcaaggccctaagcctggcaaacccacagatct

ttccaggatgcggcaaatactgttaaaactgaagcatacaccaccacctcacatgattcc

tccaccaccactacctgctcctgctaaagatgccaaagtcggtaaggatggaaaagaaac

atctgcgaaagcaactcctacagctgaaggtgcatcaggatcccaaccaaaggatgtcac

ttccaatggccctgcagaggaacccaaggaagaagcctctgcaacggaggagcaacctac

tgcataattttgccttgtttccttctctcaatctttgtctgatatccctttttgcacctg

ctgtgtttaatgaatcttttggtcctgtcattagttcgtaatacttattgattgcatttt

agtacctatattaaggtttttaatggattaaatatactatgaacaaatatgacatgtaga

ttcaatggtcatggttataaatagatgcgtattgactttataatgaaaaaaaaaaag

>fava_dn_rep_c1043

tttttttagctatattaaaaatcttcaattttattaatttaatagaaaataataacactt

atctctacaaaattcaataaatatggaaatggtagtttataagaaatattaacaatacac

aaataatatttataatctatctaattatgaatatatttgacgcctctctctcttagactt

aacaatctccatcttcaacatttcaagtaagaaatcattccatgtatcaatcggtcctgg

caaacaccaatgtacacaatcattatacaatgtcacattctctcccggcaaatgtccata

cctacttggatgtccgtctggtcgcaataacattgattgtgtagtatcaagcaatctgat

tttcaaaccatttctttttgcccatttttgagaaattttatactcctccaattgaatcat

atataactccatattagtcccctctaacctagcctcattactcttaaatggcttagtcct

cacacaattcccaccttgattccatataccattctcaaaatgagatggtgcaaatgtcct

aagaaatgtcacacccttaaaattttctaaactattcagggctctaaatgctgtcctaaa

agcctttctatagccataatacattgttaaatcagtcacattttctaagagacaataatg

gcatccaacaatttttcccttctcatagaaaaccattggcttctgaaaccaatgtcctcc

attgattatgacatagtcaaaatcttcaatttctgatgtccatttttcatcaaactcatc

aagataaatattgtagagaccagtgtgtgttggcccatctgaactttcttgttttgtttt

gacaagaaatggtgtccaataaactgccatggtgaaattgtaacttggatatttccatct

catgaaatagtcatcttttgtgtatgatacatcaatgggccattccacccttgatagtag

acaaatcaaagactgcatgtgatttctcccaacagaatctccaacaaaagccatagattt

tcctctaacgatttcaagaaactgaaaaggattgaaaattggcaattcacactcatttgg

tttccatctccacttcatgaaatcagaatctggccttccatatttcatacaattttgatg

ttcatgaatagcccaacatgttttgtttgtgtaatatggtgcttttggatttggaaccca

ttcaccactaaatatgtcacaagttttcaatgatgttgaaggtaaagtttcttgttcaat

gtttggagaagaggatgatgatggtagttgtttagacttgttcatcattacgagtaatga

tgaaccaaagaaaggataagataaaggggttacaagaacaagaattgtgacaaagattgc

tgttaagattagttttgggatcatttgttttttgttgttgttgttgtttccatagagatg

gtctttctcttggaacttcatttttgaaggagactgagagaaaacaagagaaagggtccc

gcggctgtaatggccact

>fava_dn_rep_c1044

tgattcaagaatgacaaggtaccatcttcctgaagaaaacaaagattctgaaattgcttt

ggtgccccacactgacagaggcaccatggccataatattccacaatgaagtccaaggttt

gcaggtgttaacaaaatcgggcaattgggctaatgtaaatattcctccaaatggttttat

tgtacttgttggtggtatgttgaaggcatggagcaatgggagaatccaagcaccaaaaca

cagagtggtgacaagaggagacaaagagagattgtcatttattctttttggtgtgccaaa

tcaagaaacacttattaaggtgccatctgaattggtaaatgaagatcaccctcttcgtta

caagcccttcaagtatgagcctaggagttaataggtttttagtacttgaacaccttgttg

gaatttgatgtcatattatatgattttctttatgtcataataatgcagagtatttagact

ctcacttttttattgctcgcaacaatagtgaaaattgaaagagggtttcaatctcacttt

tgcaaaattaaataaaatgtatttctttctttctttcctataatgcaaaattatctaagt

tgctatgagtttcactctaatcttatttaagtttctatgagcaaacgagtactatttttc

ttttcaa

>fava_dn_rep_c1045

gaagaatgaagttgtttaatatatattnnnnnnnnttactgccaataaggtttagatgaa

aatgtttgcgagacatgagagtcatattgctgataatatagacactacaaagtataagat

caataaagccagaaccaattttattcaacacatgcacatattacatatattcgatttttg

gtaggtttcaaattcagatttgcatcataaccaaagtacactggcttagtttcatacatc

aaattacattcatttttattttaaagttacttgtaattaattaggttttttttttttgct

acatttttaatatttgctatgtagctaatttattcatacttgcacacaatcaccacacat

attaatttgtactttgagctgcaaatttcgattcccaaactccaatttattattattggt

acatcttctcctcagttgcagttcttcctgtggcgacggttctcattgtcactgtcacac

tcatcactgctactgccactgtcactgctgtgacctgatatgccatccttgatcttctgg

aacatgtttctcttctctttcctgtttctcccttggtgttttgccgcagtggtagcagta

acaacagtggtgccagtggcagcgtgattgtggcgtgttccgttgcatgtgttgctttga

tttttggcaacatatcctggttgataaatcacatctgtctcgctatagtattcggttttg

gtttgtgttccatcattgtggtgtcctttgaacaagccggtcaccttctgtccaattgag

ctatgttgctggtggtggtggtgggttttctgttggcagctttgttcagaggttttgtag

cactgagtagaagccattgcaacaaagtgagtgttttttttgtgatatttgtttgcttgt

tgcttatgctttttcccgcggcc

>fava_dn_rep_c1046

tttgaaagttgttcagaattcataatgtattttcaataactagaaagaaacatcgaggat

gaacaaatatttaagaatggaatgtagaaatcaaccttaaaaccgcaaaatccaaatatt

tttgaagtatttagttataaaagcaatttagttgagaatgcattccaaaggaaattcaaa

ataagtattgcaatacatagatcaaaataattcaatatctaagaaaggtggaactcaaca

cacaaaatttgtcaaaaggaaacacaacaaagatgtaatcatgatctattgcttaagttc

ggttttctttttctgccacagtcggtcaattccaccgtcagcatctccctgtgcgcgagt

gaagccacatagagcgaaggtagagaagtgaccattgtagacaccactctcatccaaatg

acctacgttaatctgaacagaagcatgatccttagcagtaatcaatctgtttgtagcaga

acacttcctaggaatgtagagctccgtgatttgcccttcatcgttctgcatcttgagttt

gagcttgcgaacgagaacagacctaacaacagaatcggcgacacccccgcggcc

>fava_dn_rep_c1047

attaactgcaacagttcttcttatgaaaggaatttactcgttcaatccataactctaaaa

aatataataggtctatccttcggacagacagaacatttcaataagtaaaattgatagtta

tcaaggtaactaataagtctagaaagaggacaattccttctaacttggatagagtactag

aaacaatataacgcacaagttaaaaccaaatggtcaagaaaatcaaattatatcaagcat

tttaatccaataaatcacattgtcaaaattgaaatacaaaaccgacaaaacctaatacaa

tgaacaaaatcttgtgtttcaactcttctagtcaaacaaactaaagcccatatcatcatc

agactcttctttttcttcaaccttcttttcttcctttgcttcagccgcaggcgcagcagc

agccgcagcaccaccaccagatgcagcggaaacagcaatagctcctccaccagaaggaac

tgaagccaatttctctcttccactggcaattagttccgcaaaatctttgcccttgacttc

cctcaacagcaactctatctgctcatcctcaccctcaactccaactgagcctaggatgcg

tttgacatcaactgcggaaggggctgtgttacctcccaaaacggccaacaaatatgccgc

gatgatcttcatatttggctgggttgggatttcgcagcgaccgcttgtacgaactgtgag

gtccccgcggccat

>fava_dn_rep_c1048

ttgacgtgaattcgtaatcaaccattattaccttctcaatctcatccagcagagtagtca

gaattacaataaacactcctaactaattgaattgacgcaatggattcaaaagcttttaat

taagctttgtgtacttaaaaaaggtctaaacacaaggtgtagaatggatatgtaccatat

aatgcctttatttgataagtcgtaaagtttgtttctacagtacctaatgaaacgtacaaa

ttttacattttaacaaaacaaactattccatcgctctttaagggcttcataggcctgagg

acaaggtatagccaacaatccatgttttccattaatacaggtttcagtagctaggttcag

ccttaataatgtattgagtgccttgtcaattggcatttcaaccttcactttaaaaacttc

atacatgaatctctcacattcctctcctacactttgccgagaagtcacctggcccttatc

tgctttgagcagcactgcataagccaaaatagcttccttgtattgctgctgttcagaagc

atctaaaagaaagtgcactgagccaaagccacttgctaatgtcttttcataaagtgtctt

gttcactaatagctgataccgatcccatgtctgtttgtagcctagagccacacgactccc

atatattattagagcacttacggcaactacatcaagtaatacagctgatggggaagataa

gacattctcaaatttgtagtttataaaatatgccaggagtcccaaaattgtggcaatatc

caagcgcacagtgtctatgatgcggaatgatagctttttatgaggaaaaaccaccgctaa

atctggaaaaggaattctctcatagatctttaactgcaatgatggaacctcagctttatc

tttaccattggtttctccaacttcctctgtatacaatagaattaattcctcaaaggctgg

ttcctgcaggactgattttgagaaaagtatggaaatggaagtttttaagattttccaagg

atcatttccgcagtacttccttgttgcaggtctccatatatcactaagtgatatccttga

taaaaaagtaggccttagataaggttcaggagagtcgccttcattttcaacctgaaaagg

tgtttctgatttaaggggaatgagtccaatccaagaaagcaatctcctcaagagcctttc

acggggaccaacaggtgacaaaatcccttcatatcgagctactgccctctgagctgccag

ccaaatagggagttcttcatttggtacttggtttacacctatttgctcatccaaagcatt

gtcattatggagcagtgacttttgaaatactgacagttcctttagccaaagcctcaatct

ttctgtccaactgtgcactacatgtttctgactgaaattttcataaagctcgctgatcct

ggtgccaagttttgctagtggttttgatataagagagaagattgtttctagaagcttgga

ctgtaagtaatccagtttttcaacaattaataggccattctgcttctcagttgcataacc

ccgcctaaatattatggcattagactcatatgctttctcccaatcaacatatattggcaa

ggtaaggagatagtctgtattcaatgcagatgtcaacattagatcccgagctgagagttc

ttcgaattgtgcatcagaaagaagttgcatgaaagcacgttggaaacgagtagcaatggt

aactctggagctcctgtcataattgttgttgggagttgggtcgagagaacgcaaaagatt

gcggaggttgagagcatagtcggaaaggacagagttttgtggttgaagctgctgcatgtg

gtcgtggtagacaagagggtggtcatcctgagttaggagtgaattggagaggtgataatc

tgaacgcatctcctctaagatggttttgtgttcagcatgaagtatagagtcgagacatga

ggtgagaaggcgaaagtgatgagcagcatcttgatgaagcatgatagagaggatggcatc

tagaagttgggacttggaaacggggatgtgtttttgtctgggcacttgtattttagaagt

ggttttgacatccttggagttgtgttcatgtgaggcaacagatatttcctgaagggttga

acaggaaagcctgtgtgtgagacgtagtagtgatcgtgagtggtggaagaggtttggtgg

tgggaaatggataataatatgagtatgatgacatgttgaaggaagcagcagcaccatatc

aaccaacggcttttgctaatgatgtgaaacaaaaaataatttatttatttggtttaaaga

taagtgggtccacgtggaggcagggaagagtaaaaatctttaaaacctaatcttgttgtt

tgtggaggcgtccgtccgtggtcatgacgatgtatcagcaggcaatcccaaatcaattta

tgtttattaggaagaataggaatacaagttgtatgtttataagaaatgaaaagcagcagc

agcagcagcgcaagtcgtgggcgagagacagagtaatagatttcggtaaatataagggga

agatgctgggaagccttccatcaagctatctgaagtgggtttcaaaaaacttacgtgcac

gggattttgaggaatgggcaattcttgcagatcaagtccttgtagatcccgtctacagag

accgcatcgagtgggagtttgctctcaacgtgttagatggaaactctgctagatccacta

gatccactgaacccgcagattctcaactccaggagataagcgagaggtttggttgggata

agattggttggagcaaagttgatttcgaccttcttggcacttctaaaggaggcagaattc

ccagacttggtaatccaactgaacccaaactcccacctgctgctactgctgctgttccta

ctactactactactactagtatcagtatcactaggaggatggagagaagagacagactca

aaatgagatcgacgcagcagcagcagcaacagcaacaacaacaagttgtgcctcctgctg

ctgatattaaacaagttgccaacccattcccaggacgtcaggctcttctccgcaaagcca

tcaaccaccgcgcagattcctaaaccacgtctcatatcgtctctttctttcttcttttct

agaaccatctactgtcttttttttgttattatttataagcaccatctattgtaatgaatt

ccattattcaaatttcatcgtctttcctcgcctcacttatcaatgttgctttttacccat

ttatcatttcaataaacacctactttatcatttcaataagtgaggattggatataagttt

gatgttcaaattcaaacaccatacccacaatttcaatggaatttgtttacgatagaatga

aattgatatttgtttacataatgaggctgatttttgcaaggttggcaagcaattagcaac

acgactcggatgcaataaattaacatatgtaccgtgttcgggaggagttnnnnnnnnnnn

nnnnnnnnnnnnnnnnnnnnnnnnnnnnnnnnnnnnnnnnnnnnnggatttctt

>fava_dn_rep_c1049

tattccaaatgaattacaataacannnnnnnnnnnnnnntacaatgaacaaagtacaaac

aaactataaatcaannntgcaaaatgacaaacaatcaaaagaacaatcaaataatgcatt

atacatgcattatacactacaatttcaataattcacttgttttatctattatggatagat

ctcaataatagaccttaagcctaaaattggagttatcttatagtcactaaaaccagctga

agaaatcaacttaatccattctctcttgtttctctcttttccggtgactaataccatcat

caacatatcaaacaagagttgtgtttcaactgattcattaatattttctttctcattgtc

taacaccatctctatgataatcactttcccttctttacctttcttctctaacgattcctt

gcatttcttcaatatctttacacattcttcgtcattccaatcatgcaatatccacttcaa

caaaatggcatgtgcctgaggaatttcttgaaacatatctccaccaacatagtttaggtt

atgaattccttctaagccatcaacaacatgtggaagatcaaacacaatgcactccaattc

tggaaataacttggcaatagcctttgccgtggtccctgtgcctcctccaacatcaaccaa

tgactccaatttatcgaacacttccctacccttctccagcaacaaattagtcactaattg

agaatcacttgccatggaatcattaaacaaattgttaaatatggggttatgtgcagcata

atcccacaaaaccctcccatgtgttgtttcaaatgccgtaggatcctcgttttttaacca

agtggacatttgatgccacggacttgtcaatactggatcaagcaccgcctgcacaaacgg

tgccacactcattggagtgtccttaagtaataatctagatacatcggttaacataaactc

gatttctagctcattatccgtaacattttgttgcgagaaaaaaccagaataagtcatgat

tcgcatcaaacgatacatgtaagggatttttgaaggatgaagtggtagtgaagaaatgag

ttttgagagtgacatgggtttgccatagttatgtaagatatcaggtatgcctaaatcaac

aacacatttaagtgacatggaattgatgaagttgaaaatgtgattccatatgtggctttg

agctttgagcaaattggaagcaacatcttctccattttggaattccatagcttgctaata

ctcccgcgg

>fava_dn_rep_c1050

taacgatctaaaccgctttactcgggacagacagaataatcttcttcttcattcttcact

ttccttcaacgatccgcacaaaatgcttgcgattttcgacaaatcggttgctaaaagccc

agagggtctccaaacccttgagtcaaactctgtttcagccttaaaagacggctttctccc

acaacatttctcatctgtttaccctggttccgtcaccgttaaccttggtccttccggtgt

cttggcctattctcttaatcaacagaactttctcctcccaaggctgtttgcggttgtgga

tgacattttctgcttgtttcaaggccaccttgataatgttgctaatcttaagcaacagta

tggattgaataaaactgcaaatgaagtgattattgtcatagaggcgtatagaactctaag

agatcgtggtccatattctgcagctcaggtcgtgagagatttccagggaaagtttacatt

tatcctgtttgacagtggcgctaaaaccacatttatttctgcggatgcggatgggagtgt

tcccttcttctgggggactgatgctgatggaaatcttgttctttccgatgagacagagac

ggtaactaagagctgtgggatatcttctgcaccattcccaaaaggatgtttctttacaac

atcgggaggtttgagcagttttgagcatccgcttaatgaattgaagcctgtaccaagggt

tgacagttccggccaagtatgtggtgcaactttcaaggtggatgcagatgttaagaagga

accaactggcatgccaagggtcgggagtgctgctaactggtctaatgatatctaattgca

attctatttgtttgaactaaggcttttatcttagctgtactcattgaattttcaagttct

ggttttctattctcatcctttgataatgaggagtctgttgccatcatatgatagcttgag

gttctaatctattgagacgtggcttatcatattagtttgctggactgagtactcaattgg

cacatatcatgtaataatattgacttgtcactctgattcatcataatgtgatgattattt

actgtcttcgcctgatcttatttcattcaggttcatatgcatcctacttaaactgtaatg

tgatggatattattatgtaaaaagccaactcaccccagaaagtcaaagacaataagattt

tgaaatattcagttacattgtaattgccagatatcagctgcttactctataaaaattaca

ttgtaattccagatatcagctgcttactctataaagaaaacatatacaaatggtactaac

atgatatctatggattatactaagtgttcgagtaacaacacttttaatacaagctataag

catatatcaagaagattaaatttactaaagtctctgttgtggaatattctcaaaagtcat

aagcagctgcttcagtcctttgcatcagcaatgccccctggtgttatctggaacactgct

tcagcctctggcaggtttggtgcgtcaaacactttgcacacacgctgttcacctttccct

ttcctgaacatcagccttattgtggctgcatgagctagcacatggcctcctgctggtttc

tttggatcagttatgaacataccacctcctggnnnnnctatgacttgatttgtcatataa

actgcaacattgaattcctcagcaatcttaatcatgcgggaaagcatctgtgccaatttt

tgctggcgttcagcaagttctcctcttccagtgaagtccacccgaaaaagagcaatcaca

gaatccacaatcaaaagtctgaaaggttcttcagccatttttgcagccaaaccaaggagg

aggttatactgatgctcataagtgtaggcacgagcatagatgatattgtccaatacagcc

cctggatccatgccaaatctctcagctatggcaacaattctgtcaggtcgaaaagtccct

tcggtgtctatgtaagcaacttttccgttgcctcccttcatattagttggcagctgagtg

gaaacacagagagtatgagctagttgtgt

>fava_dn_rep_c1051

ctttttttttttgaccaaaacgcaaatcaaatgaataacagttgaaaaggttacatataa

aagcatgcatgtttgctttgcctagctaggattaaactaaacatacatgcaacaatctga

attaaaaacaaatggttttacaaataattaatatcaaacaaacaattacaacagtaatta

aatccaatcctgagagtgaagaagagatagagattttctcgacatcctctcatgtcgatt

tcaaccattcactggtttagagggaacaagtgtaaccgggaggtggagtttttccacaag

acaaaagaagctgaagagcaagtggaacatagatattaaggttaagaagcttaatcttaa

gagttgtgcaaagacatgcagcagcttggatttcagcaagtccttgaagaagaggacaac

acttgttgacagcagggtcaccaagtccaatgtgaaccaaaccaccaagtaaatcaacac

aagcacctagtttcaatgtgtcaattggacaagtttcttgtgatggtgtaggcgtaggtg

cagctgatggtggtggacatgaactccctcctttgctcggtggattaagcaccggaggaa

tggtgactggagggattggaacatgtggaagaggtacaactggagggactgtgactggtg

ggattggaacatgcggaagaggtacaactggaggaacggtgactggagggattggaacat

gcggaagaggtacaactggaggaacggtgacgggagggattggaacatgaggaagaggta

caaccggaggaacagtgacgggagggactggaagaggtggaagaggtacaactggaggga

gtgtgataggtggtattgtgacaggtggtttaacagttggtggtttaacggttggtggtt

taacgataggtggagtgacgattggttttttacctggtttgtgttttttatgtggttttc

cacaatagccacaaccaagaataggtgtagctgaagagatgaaaagcatgcaaatgatga

gataagcatagaattttgaggaggactccattttgagagagaaaaagggaaactagagtg

aagtggagtccccgcggccgtaatggccactctgcgttgattttaggttttgttgatgct

aattgagtgataaacgaatctatgcattttggtgtttcgagttcagatccatctttttgt

tcagaagatttaagcattggtaatgctaatgatgaagatgaagattccaatgttagggtt

tagatatagaaagtggtagttgaagttggattagagtttctgttaagaatttaacagttc

ccataactggggaagatttagatttgaatggaacgctaaaaattggaacggaatcggaat

gtgagttattgttaggggtttttttagggttatgcgatgaaataacgaagaagagtacat

aagaaaaggatgaagaatttgctgtaggagcagaaactaaacattgagcatggggttttg

ggtatggttgttttcttcttttcttttttgttgggttggtttggttaggaaccaaaacca

aaccctaattgatacgaatgaatgattgtagatgtagataatgataaaatattggttttg

tgagagagtgagagggagagagagagagagggggattaattaattagggtgtggtttgtg

tgaaggaaataatagctgaaaaatgagcagaaaagaagttaatcgataatcgatcaaaca

aacctggggagggagagagagagagagagagagagagagtttggagagaaaagtaatcag

agagagagagagtatgataatgaaaagaaacagtgc

>fava_dn_rep_c1052

ataataaataaatgaaaattatttatttatttatttttgacatatgaagggtgagatctg

gtgtgggtacatcggaaccagcagcattttgtgttaagagtgaaatcgaacatcggcaaa

gatgggaggaggacacggccacggcggtacaacctacaaaggtgtaaccattcatcaacc

taagcgctggcacaccgtcaccggcaaaggcttgtgcgctgtcatgtggttttgggtgat

gtacagggcaaagcaagatgctcctgtagtactgggctggaggcatccctgggagggtca

tgatgaccacggaaccggccattaattttcttaggctccagatacaaaaacaggaagatc

ggaacttgaagaatctatgttcctgaatgcgtgttaatcaaatggttactttgtttcatt

caataaattggctatataagatttttttacatgattccatgaacatagcaatgtttgagc

accttatttgttaacttggatgaaggtttatggtagctgttgtctagactatttatccat

gttttgtatgagaagtttatgttaatatactgaatttgtgttaactatgacaaggttgtg

tatgtgagttaataacaatttacttgtgggttaatgaactgatctaaaaattgtgagctg

atattttgcagctccagtcaatgggaactgggttgacataggcagcgagcaatttttgca

gttgttcatttcctctatttcaaagaggttcctaagcaaatggagatctaagtgtgatct

acatatgtattgattaccttttcttcttttggcaaaataaatgtcatttgtgtgggtaga

tttctggagggggttcaaattgatgaaatgttggatttaagttcataaaaattattgaat

cccg

>fava_dn_rep_c1053

aataataaaagataattttataaaaatgttacttttattaactcaatcatgacttttctt

aatctccctgtaataaacttaaacaactattattttgagacaaagggagtagaattatta

ggtaattgctcagaaacagatagactcacttcagttatgcagaatttccattggttaaat

aggaattatcctttttaattaataaatgtgtaaagtttttggcgcacattccgattcaga

caattgtctcaaataatctaaccaacatgtaaagggaatgggtgaaaaagcagctaaacc

tagcagaagtgggaaactataagattaaaaaaatttgattgatggttagcagggtccaga

ggaaatcagtttaggtaaggaactcagcatcaacttttgtaatctccagcttctcagcta

tccaacgcaataggctctggcaatggattcaatggaagggttgaagttggtacatgtggc

tggtgttgaagttgtgctggtccaaccaatttcttgtcgttcggcactacattctccact

ccaagtgctttcctcacttcattagcaagttctatcacatagtcctcctcatgtccaaga

ttatgcatagccttttcaagaagaaaaagatagtccctgttgtttccacaagggccatga

gcagttgctatttgcctagccatgtcctccagcggagcaggtcccagatagtatttgttg

tttactttgtcaggagtagacgtgaatacaataactccagtaagagcaggatgcagtgaa

tctccttccttatagaagtttacaagagtcttttgatcatactcacattctcgccgctcc

aaatactgcataacaagtttctccttttcagggcctcctcgaacacaataagcaactccc

cagcaaagtgccccttctttctcctccaaagtgcaagttcttgcagggttttcaggtgtt

cctctatgatcaatacatgctaaatcaaacacccttctgtagtcctttatgtaaccaaca

attttttcatcatattcaaatcctggattccatgccagtgaaccatatccaaaaacccag

aaaaccatagctattctatttatttggatctggaaggtttgggagaaaagatagaaagga

tttgaaaagagagggaagctgaaacgattagggtttatgaatgaaattagggttacagag

attcccctctttctcaattgaatttctttctatttctctcaggcggccgtaatggccacc

tctttctcaattgaatttctttctatttctct

>fava_dn_rep_c1054

gagtaaaaaaacatggtataatcttttggagaacctattactcacttcaagtctgtacaa

gtgtaaaataaacttagaatttccgacacaaaatgaaacccaaaaactaatccagccaac

ataaactggttataacatcaaataagtactcaaaagactatcatgtaattcaaaaacaaa

gatctgaccatgttttattgggcaagtgtagaacaaatacaaaggatgtctaatttgcaa

agtggagtaagcaacaaagacaattaatcacatataatctttgcataacccatccacaac

atggaaagaaaggttgaaccacaaaaagaccatgcaatgccaaacttgttctggaagcat

ttgcaaaagagccactactcaagctttacgggctctctctttggaggatccaaatccaaa

taggtaaatggctcagttggttcctcgccaggtttagatcgcatgcactgccagaagagc

ttgaaaggtcctggaacagtcttgaatgggtttccctcaaagcaagcctggataacatct

tcaacgcgatcattgcatcgatgagctttaggttcaggatgattctctcctgttattccc

catggactctctctccgaggaactggccttgtttccgtcatcttcttcttcttccttcgc

aactcgtttctcaactcaactcgttactcactttgtttggtgaagaaaaag

>fava_dn_rep_c1055

tgaatgtgaaagtgagagcaaaacgttagtagcacacaattccagaccaattggtttcct

tgggatatagtagtattatttattaatttctctgtcgaatgcgaaacaaatagctatttt

tttatttttctgattatccaatccattctctccctccttcacattttgacttcaccatgg

ccgtttcaatttcaacctcctccataaccctatcttctttgaactcctgcactcgtgtgg

gtgcttcttcttcttcgccgcaacttcaatttcctgttcgaactcgctttcttccgattg

gtactcgccgtcgtcccgctttctcacgccctagattcctggttcaagcaaagaaacaaa

cgttttcctcttttgatgatttgcttgctaactctgacaagcccgtatttgttgacttct

atgcaacctggtgtggtccttgtcaatttatggttcccgtactcgaggaagtaagcacgc

ggcttaatgatctgatacaagtagttaagattgatactgagaagtatcccagcattgcca

acaaatacaaaattgaggcattgccaactttcatcatctttaaggatggagaaccttttg

atcgctttgagggagcattaacagcggataagctaattgaacgcatagaaaccagtctca

aagttaagcaataacccggttaaaatgaagacatggatgtttgtctattggagtgttgga

tcatatacatgattggataggaggatcaaactgctgcttcaacttaattcagacattttt

ctgtatttatcacattttttatgccaagatccagaccgatgaaaaattagattgtttaat

tagtagtagttttaaaaaactcaaaagtaaactcagttagtttgtgtgctcttcaatatc

tttgttttccgtcatcgaattcatcaatatcttaattttctcgtacccaatgtataattt

tgttgtcttttgggggttcaaaaacaaattaaacctgtacaaaagttggagttagtttct

gtttgcaaagcacatattgaacctttgccattcaaaaaaaaaatcttct

>fava_dn_rep_c1056

accagtttcaaagttggcatataaaaaagtggacatgactttacgagttgagaggtgaga

cagtctttgtgggagactgtaattgtacttgatggaagaaattgaacacaagttggtgaa

cgtgggagctctcaaactccacatagctgaaattggaacaggtccaaacgtcgtcgtatt

cctccacggtttccctgagatatggtactcctggcgccaccagatgattgccctcgcctc

tgccggattcagagctatcgcccccgatttcagaggctacgggttgtccgattcaccacc

tcaacccgaaaagactaacttctctcatcttctccacgatctcctagcaattctcgatgc

tctttctctttccaaggtgttcattgttggaaaagattttggaagtcgtcctgcatattt

gttttccattctgcacccagaaagggtcttgggagttatcactttgggtgttccatatgt

tccaccacgccctgcactgtatgataaagtcctccctgaaggtttctacatttttagatg

ggcggtaccaggaagagcagaggctgattttggacgctttgacgcaaaaactgttgttcg

aaatgtttacatccttttctcaaagagtgagataccaatagctgatgaaaaccaggagat

aatggatttggttgcacctgatacccctcttccgtcttggttcactgaggaggatcttgc

aacatacggagctttgtatgaaaaatcaggattccgaactgcattgcaggttccttacag

atcatttggtgaagactttaacttgtcggatcctgtagtcaaagttccagcactcctgat

aatgggcgggaaggattatgttttcaagttcccagggattgaggatttaacaaaaggtga

aaaggcgaaagagtttgttcctaatctggagataacatttattccagagggaacccattt

tgtgcaagaacaatttcccgaacaggtgaatcagctcatccttgccttccttgccaagca

catttgatttgtggtggaagtttagcagtattttgtgatttatggtgaaataaatgtgca

atagccatctataatgatgcgattaactttatggatcacccttgacattttggagtttgc

ttgatagaatggaataatataatatttgtgattcacttaagttattaagctgtgattttt

gcactatggaattatttaaattcaagataggcagcaatgtcgtgtttttcgttgtgcaaa

acaattatagctaatgctacttatttaaggcacttctcatccnnnnnnnnaatcatgaaa

caaagt

>fava_dn_rep_c1057

taattcaaacctaatttttcatttattgttatataaaatcccaacatgcatggcgattga

gctgaaacatgagaaatgaaattcggaccaataaaactgatgttgagggtagggaaagtt

acctctttatttaagaccaaggtcaatggcacatcatatcccattcccaaccgtacaatt

atttcttacatagtgcagcactagtttaaggaatgcaattctagtttctatttgcagaat

gcaactattttgctctacaggcagtcttcaagggctctccctaacatgttacagtctgtg

gccaactataaaacaacaattatgctttagttcatggcgaataatcaaggctaatacata

gcgatattggtattcaactctaatggaatgcttctgaatatatactcaactagagttgga

tgtaacataaagagtctcgggcaagtgaaagaaaagagtagcatgagaggatattatcaa

ccctccactttcggacgccaaccttcaatgacatcctctggctgtgcccgcgtgttgatg

aatgattccctcgagaaaatatccttcaagtaagatttcaaaaagttaagtgaatcaggt

actgcccatttcttataatgccctaaagcaatctccaaatggtatacctttggtccaagt

gacaggtcagctgcagatatgtctttcccgttgataaaaggaccattttccttgaggtaa

tcgttgaagatgcttagttcatttagtaatgcttgttctgttccatcattaggatctttg

ctcttaagaaatccaataaatgtagaaaagatctttgatccaactgtagccttttctggt

ggagtcgccaatggcgggctaggatacttctcttccaatatttgtgtgataacatctgaa

tcaggaacccacttcccatcaatgtttatcacaggaactttaccttcagggctgatttca

aggagccattctggtttgttccttaaatccaccaatttcgtctcataaggtaaatgtttt

tcctccagtgtcagcaataccctttgagtaaaagggcagtctccaagcttgttgggtgtg

gtgagagaagctttgacagcaatttccaagggctcggaaggaggaacggaagacattgaa

actctgaggggtctgaaggtgaaacggtcgttgaagcggcttgttgaaacgacgtagttt

ggacggtaacgaaggtggttgacggtggcggaaagagcacacgctgaaacttgaattcgg

acggtggacatttggaaacggtgacgtttgtttgtagcttcgaatagaagatgatgaggt

tgtgtttagttctagcaagtgtttatgtgtcatcgtatccgc

>fava_dn_rep_c1058

ggccgcgggactcattatttgtgaacaagagagatcaacaaagcagaaggaagaaaaaaa

aaaccttttgtgaattcttatcatggataacaagacatcatcgtcaacaagaccaaaaac

tgctgatttggatgcacctcttgataccataggatttgagtttgaagatatatcagcaga

gaaagtttctggcaatcttcatcttacccaaaagtgttgtcagccatttaaggtgcttca

tggaggtgtatcagcattgatagcagaatcactagcaagtattggagcacatattgcttg

tggttataaaagagttgcaggtattcaacttagcattaatcatttgaaagcggcttttat

tggtgattttgtccatgctgaagccacacctttgactgttggcaaatccattcaggtgtg

ggatgtgcgaatctggaaggttgatccttcaaactcacaaaacagatcattgatagcatc

ttctagagttactcttaaaagtaacatgccagttccagaaaatgcaaaagatgccggcga

cttgctaaagaagcacgcaaaattgtagatgtttatttaaataagtacgatttaagttaa

actcaattatgcatggaattacaaatattcatggagtcataaatatataaatattcatga

tagacgttgttggttttttggttactagggaggcaatgagattttaaactagctcctcag

gtaaggttaaaattgtggtgattgtcatgaaaaaaacctgatgtaaaagttagttagata

tcgcaaattgtttgaaaagattgtttcaatagataaaaattgagtactttataaatgagg

aaattcataaattcaatgtttatgatttttgatggatatatgtatagtgtctcagttgta

tagttactctatgatttatcagtgatatcaaagtcgataattgaacttagcgccaa

>fava_dn_rep_c1059

aaatgcaaagaactataagagaacatatggataagatgatgagtttagttttagtggtgg

aaatgcattagatagatagatggatagttcttagttttggtcatctttggatggcatttt

gtatatcaaactctctatcattccaagggaggaagacaagcccaaattgcttatttggtt

ggaacataggcagaaagagagttgatgacaaacctcaaattaaataccatgatattgatc

tcactttctcaacttctctggttgccaaaacatttttgaaaggtaaggaactaaaatgct

gctatagggcatcaattgatggatttagtgcaacaaatttccatcaatgttgtgacttta

aaggaccatgtgtaataataggctacacaaacaactctttcaagtttggtgcatttaacc

ctgagggatatagaagcacagatgattactatgacacatttgatgcatttctattttatt

ggatagaaaatcaaaatgaacccatcattttgcctaaggttggtggaagtggtgcagctt

tgtttgattatgctcgtggcgggccgcagttcggggctgacgggcttctcattgggcctc

cgttagcacctgttatgggtggttttgctgggcctgatactaattctggtgttgggggct

tgacacaagccaagtctagattgggattgtcttatgctaaaagggaagatggcaaggagt

ctatctttggggatgagtcaagagcaactattcaagaagttgaagtgttttgtagcccta

aaattgcaagcttatattagaacttcattgcaagggaaagaatactataccatactaaat

ttgtttttctttcaacagaaatgttgatgttacagagagattgtactttaaaaggaagta

ctcattatttatcattataagttataatgtcacgccgagtaagatttcaactaattagtc

ccttcaatgatttatcattaatttgatcataagcctcataag

>fava_dn_rep_c1060

cgtagcaagggagagaacaatgatattcctctttcatttcattaaaggtgagaagagttg

gacaacttgcaatgggtgagtcagataaaaaagtttggggcaaacaaaaaaaatcaaccc

aaatgtcatgatgtgtttgttttaggttattgctgcaggcttattttgaaatgtccggta

caccacttgttcgcatattataagttgcctaaactaacatcattcactattacaggtaca

tcaccatttacattttacaatacattacatacattaatgtttatgatctttactgaattc

ttcaaactatgtaactatgtttcgtgtttgattgcgagagcctcagtttacactgatgtc

aaacactccacaaatctgtgatctgtgattccaaatacttcccaagtaatgcacgaggta

acagaatccaacagctcaaatcaaacaagcattgtccaatttattccaccataacagcct

tggagctgtcaaaatctgatgaatatgaaggaggtagatttacaaccccaagaattatga

gttaactttgttaacttttcctcccaatgctttcagattcgtagcttttcctcccaatgc

tttcaaattcgtaggttttcctcccaatgctttaagattcgtagcttttcctcccaatgc

tttcaaattcgtaggttttcctcccaatgctttcagattcgtagcatttcctcccaatgc

tttcaaattcgtaggttttagctcaatgtacttccctagcccaaggtctggtggaagtct

aaaaccagctatgcgaattcgctttaatgaattaatctcaagtccagcatttttaacaag

ttcgcgaacttcatgcttccttccatcatgaaccacaatacgaagtcgagatctttgcgt

atctggcatacgtggaagtaactccacactatcgggtacacaacggacaccctcaattgt

tgttccctcacttatggccgttaaatgcctcttatgaactagaccattaactgttgcaat

gtattcttttgaaaaattagatgacggatgtgaaagtttttgagcaaaatctccatcatt

agtcacaataagtaacccagtagtagcaacatcaaggcgccctacagtaaatagtcgtgg

tgggggtactcctgaatgcttcttatcccaagtgctcaaaaaatcatcaaatagactagc

aaatataagctgaggcatgaaattatattaatcagagtgaagagctatacatcctccaac

gccagtcctaactaatctcaaatacgaacaattaccaggaaatagcataaaacaaaaaca

ctacacgagaacctaaaattcctgactaacagaaccaatcagatctttcccttttccagc

aactttcttaggcaacaaattctgatgaatattaggcaaaacacctccattagcaatagt

aacagatgccatcaacttgctcaactcctcatcattcctaacagcaagttgaatgtgtct

tggtacaatcctcgtcttcttgttatccctagctgcgttaccagcaagttccaaaacctc

agcgcaaaggtattcaagaacagcagagaggtaaactggtgaaccagatccaacacgttg

agcgtaacgacctgctttaaggaaccgagcaatacggccgacggggaactggagaccggc

tttggttgatcttgagacagatttggtttttggtttgcctcttccacctttctttgttgt

tgccggtgtacctgttgccattgcgccttttttatgaattcttgtaagagattgtgttgt

tgttaattgtgatgttgtcccgcggccnnnnnngttgtagcattactaccacttattgat

ttttacatacaactaaatatcgatcgatattgtttt

>fava_dn_rep_c1061

ggggaagagaagttgaaactttttcagataattaaaatgaaataaaagcacaagcaaaaa

acaaacaagactcattgatgagacatcatacccatagaaaaatgaaaacagagtattctg

tttggacccctaatcaagaacttgactttcttaaacttagaatatagtgaatagagagac

atgcataacataaagttggagatataaaaagcacaaaatccattcaaagataataataat

ctcactatatcccaaaattaataaaaagtaaaatcaaaatgaaattcttaacacaatttc

aaacctaaggaattacatattcttctcctcaaccttacttctcagatgagttaggtcctc

tcttcttgccaaatcccaccacagcagtgacgaatctgcgattgtactgcatacgcttgt

gagcacgaccacggggcttcttcttcttgtcctgtttggccacctttggtgtttggcctc

tcaccttaccggcacgagcgagtgatccgtgaaccttacccatggttgcgaagatgaatt

ctctgctgaatcctctcttcaatcgcagtagctggaggcgcaaaagcagtacaggaacaa

tgacgatgagtttatgctattagtattgaaact

>fava_dn_rep_c1062

gggggcaaaactaggttagagccgttttcactctccaccctttccccaaccattttccag

aaacctcaccatgggacgtgtgattagagcccaacgtaagggagcaggctccgtcttcaa

atcccacacccaccaccgcaagggacctgctcgtttccgcagtctcgacttcggcgagcg

taacggttacctcaagggcgtcgtcaccgacgtcatccacgatccaggtcgcggtgctcc

tctcgctaaggtcaccttccgtcatcccttccgttacaagaagcagaacgagctttttgt

ggcggcggaaggtttgtataccggtcaatttatctactgtggtaagaaggcgactttagt

tgttggtaacgtgcttcctctcagatctatccctgagggagctgttatctgcaacgtgga

aggacatgttggtgaccgtggtgtttttgccagagcctccggtgattatgctattgttat

cagtcataaccctgataacgacacctctaggatcaagcttccttctggtgccaagaagat

tgtgccaagtgattgtagggctatgattggacaagttgctggtggtggtagaactgagaa

gcctatgttgaaggctggtaatgcttaccacaaattcagagtgaagaggaactgctggcc

taaggttcgtggtgttgctatgaatccagttgagcatccccatggaggaggtaatcacca

gcatattggtcatgcaagtacggtcagacgtgatgctcctcctggacagaaggttggtct

cattgctgccaagaggactgggcgtcttaggggacaagctgctgcatctgctgctaaggc

agataaataagaagtttaagtttagattgtttgaatcatatattattgcctgtgttccaa

ttttgtttgttaaactatttctgttttgttttgagaaccattttccccaaaaattattga

gcttttgttagacagcatttttatggtttcatatttcttctctacttacagattaactct

gcgttgttaatggctaaattgtttattcttttgcaattagctatccactacaacttcttg

gttttaggttgaaattgatcagtcttatatatgtctgtttctgctgacttattttaaatt

tcatcaatattttttagcttcaaatgtatactgaaatacatttttattttttcagtacat

attataataagatgccttttacaatagcttgtactaaattgtcaatataatttctttagc

attggaagaaataataaaatggttggttatttctatccaactatcattc

>fava_dn_rep_c1063

caagagaaannnnttttaagtggtttcaatattgcacaccacaacaagtgaaacagaata

tttatacaactagcataacagaacattcatcaacatcattgaatttaacaatgtagcagc

aatacaaaattcaattgattaaaaagtttacaaattcataaaactttattattgtactct

tcttcacaatcctaatcaaagtaatcttacctttttcttattcaaacaaacaacacaatg

ctattgggactttccttctctctctagattaatcaacccaaaaccaaaaccagaagatag

aaaatgaaagagaaagttcttaaatgagcaaaaactattagttctaaagaacctcttttg

ctgcagcaataacagcctccttagtgataccaaactccttgtatatttttcctgctggag

cactagctccgaaacggtcgataccgatcgcttttcctttgcttccaactattttctccc

acccaaacgttgttccggcttcaatgcttaccctggccgtgacagcagcagggaggacgc

tctccttgtaagcatcggattgctcgtcgaaaagttcccaagaaacaaaggaaacaactc

tgactgcctttccttcctttcttagatcctcgccagctttgtaagcaatttccaattcgg

aaccggttccgatcaaaatgagatcaggtttgtttcctgtcgagttgtctgagacaacgt

atccgcccttttcgactccttcgatggaagttccaagaaggttaggcaatttttgtctag

agagagcgagaatagatggtctcttccggttgagcacggcgactctgtatgctccagctg

tttcgttaccgtcagcgggacgaagcatcaagatatttggcattgcccggaaacttgcca

gatgctcgataggctgatgggttgggccatcctctccgagacctattgagtcatgagtca

tgacgtatataacaccggcttcagatagggcagaaagccttatggcaccacgcatgtagt

cggtgaaaacaaagaatgttgcacaatatggaatcagtccagggctgtgaagagcaacgc

cgttacagattgcgcccattccgtgttctctaacaccgaatctaatgttacgctctgctg

gagaatcactttggaagtttccagatgctttcagcaaggtcatattggaagaagcaagat

ctgcactgccaccgagcagaccgggaagaacctttgctagggcattaaggttttgctggg

atagatttcgggtcgcatcggctgggatctctggagtgtatgtcggaagtgctttctccc

aaccagaaggtaaatcaccggtgatgatagatttcaatacttcagcttcctccttgtact

tcttttcgtattcagcaaacttggcattccacttagattcaactgcagcaccctcgggaa

tgtggtgactccagtgttttttgacatcctcgggcacgtggaaaggctcatatggccatc

caagattgtttcttgtggcgtcaacttctttggcaccaagtgcacttccatgcacactat

aggagttggatttattcggagatccataacctatggttgtcgtgaacttaatcaatgtgg

gtctgtctgtgacagcttttgcttccttaatggcagcgcggatttcatcaaagccggtgt

ttccattcttaacccaaataacatgccacccgagtgcttcaaaacgtttgtcaacactct

cggtgaacgcgatttccgtgtcaccgtcaatagaaatgtgattgtcatcataaaacgcta

tcaacttccctaatccccagtgtcctgcaagagagcaagcttcatttgcaattccctcca

tttgacaaccatcacccaatatacaatacgtgtaatgatcaataatctcattgtctggct

tgttaaatctggcagctaagtgcttctctgcaagggctaaaccaacagcattggcaatac

cctgaccaaggggacctgttgtaacttcaattccaggagtctcgaaattctcagggtggc

cgggggttctgctcccccattgacgaaactccttcaaatcctcttccttaacactgtcgt

atccagcaagatgaagcaaagcgtactgaagcatacacccatgcccagccgacaaaacaa

accgatcacggttgaaccaaaacgggttcttggggttatacctcataacctcatcgtaaa

gcacatgacccaccggagcacagcccatgggcaaacccgggtgacccgaattcgcctttt

caacggaatcaacggctagaaaccggatcgtgttgactgatttctcaacgagtgatgatt

ccgcggttggggtattggtagtaagggttgaggcggaggaggctttgatgatggtgcggc

ggtggnnnnnnnngaggaagtggaagatgaagccatgattattgttgttgttggaagaga

gagaagatg

>fava_dn_rep_c1064

aacggttcaaagcaaaagctatacacattactgctatgttttttcagaaaccaaaattca

ttgaacaagagagtgtgagcttggaaatctcaactatatcctccatccatggttcccact

cttacatccaattcttctcttataccaacacattccaagataacatttcgaaatagtcca

tcaaggttcaatgtctacgctaagaaatctggaggatcatttccttcgtttggattggga

aaagaaaaagaccaatcaaatggttcggtgaattctaataataataatagtaataataat

aatagtaataatccttttggtttcgactttgggaaattacctgatgtaacttctttgatt

cctgttgggaatagcaattcttctgcgccaggcttttcgtttgggagtccaaggcggaag

gattcttcgactgtttttgttgctggtgctactggtcaagctggtattcgcattgcacag

actttgcttagggaagggtttagtgttagagctggtgttcctcaacttgcttctgctcaa

gaattggctcgtcttgctgctcaatataagattatatcaaatgaagaggcaaagcgtctc

aatgctgtgcagtcaagctttgatgatgcagactcaatagcaaaagcgataggcaatgca

agcaaagtggtggtcacaattggtcccacagaggatggaccaacgactgaggtctccacg

gcagacgccttgcaagtgattcaggctgctcagttggcaggtgtaggccatgttgcagtc

atctatgacgaaagcgtcagtgcctcaaccaacaacgtacttgatggtatctcctcattc

ttcaacaatctcttctctcggtctcaatctttgtcgattcaagagttcttgcagaaagtg

attgaaacagacgtcaaatataccttaatcaagacaagtttgactgatgatttcgcgcca

gagagttcttataatttggtcgtctcaggcgaagaaagcacccgttcgaacgactataaa

gtatcaaagtctaagatagcatccttggtggctgatgtcttctccaacacacaggttgca

gaaaataaggttgtgcaagtgtacactgacccaaatgctcccttgaagcgtgtagacgaa

cttttcagcactattccggaagatggaagaaggaaagcctatgctgaagtattagagaaa

gccaaagcagaagaaggggccagagcggcagctgaaacagcaaagacgccttccaaacca

gaagctccagagaaggcagaagtagctggaaatggagcagcagtggaagatatcttgaac

aaggcaaaagattttggtgcagggttttcatggcaaaagttcagctcacaggttgctacc

tcaattcaaaagcctgatgagaatgagaaaccaaaggtgcaagttgccacagtgagggga

caggcaaaggctcggagtctcattccgaaattagcagtgactaaacaaacaagtccaaaa

aacagtgtgagtaaaccaaaggaggaaaaaagaaaagcagaagcaccaaaggagcttagg

aaggtatttggtggcctgtttaagcaagagactatctatattgatgatgactagtggaga

tggaacatgtcttgcacggcttaaaactgcgttgttcgagtccgtgttaatgttggtaat

ttggttgaacgtgtatggttttgtaaggatgtcttttgaggcaagatatttatgtagctg

ttcttgttctgaaccatgtaatcagattatcaatcaggtggttggagtggcttcttatat

atttcatcagtttataaagctagatatctcactctttaacaaaaagttgttcaaatcatg

aaactaattgtatggttgattcacttggttgtttgatatttataattggcaaaatttatc

atgggatccttcaagtcactagtttgataatcgataagaaatttcttacaatctcttttn

nnnnnnnnnnnntactccctcg

>fava_dn_rep_c1065

agggtttctaatatccgctataaaacgttgctagcttcactttcccgcgggagctgccat

cacctagggttccgacagatcgaaacaccgttcacgatgagagccaagtggaagaagaag

cgtatgaggagattgaagaggaagcgtcgaaagatgagacagagatctaaatagtgcgct

attttcaatcaattgtagttgtttctggtttttttcatgtatgtttctgttgtttagatg

gaacttttcaataattgtttactttgttcttgttttggttttacaccctaactctgcgta

tcttaaaattttgctattcgcgtttttattctcgacttgaatctgtggtgttcatttgtt

tagtattgctgaatttcctttggatttactacttgtgttgaaaacgttttggtttaaagt

tttgttctaagaaattcattagtaggttatattgctgttgccatttcattactcgggttc

tcaatggtgtaattcctcacttataaaaaactgtgtatcttgattttgatgcag

>fava_dn_rep_c1066

aataatgaaaagcgaagttgcttcattggtcccacaaagcctctcaagtccttccttatt

ccttatctactgcaacctccaccctccaataaggttctaatacttggaaccgaaagagat

aacaaaaatgtgggtatcaaccatgtcttactcatctcttcacaaaccatattattatta

ccataatattaataatttccatcaaagatgcctcaacaccaacctccctaaattcacaac

aagagcttcctctgctgcacctggtgttgacctcaacacccttgattctgcaattgcaaa

gaaagatagcaatgctgttaaagaggcacttgatcagctgagtgaaattggttgggccaa

gaaatggagttcccagccatatgtttcacgtcgtacgacatcaattcgagagctgacaac

tttagggattaaaaatgcggagaaccttgcaattcctagtgttagaaatgatgcagcttt

tctctttactgtggttggaacaactggattcttgggtattctggcaggccagcttcccgg

ggattggggcttctttgtgccatacttgattgggagtatttccttagtggttttgggcat

tggtagcacatctcctgggcttctccaagcagctattagcagcttttcagcagtttttcc

ggattatcaagaaaggatcgctagacacgaagcagcccactttttgattgcttatctgct

tggcttaccgatttttggatattcgttggatattgggaaagagcatgtcaatctcattga

tgagaggcttgaaaagcttctgtatagtggccagcttgatactaaggagctagacagatt

ggctgttgtatcaatggctggactagcagcagaaggtttaacatatgacaaagtgattgg

tcaatcagctgatcttttcacgctccagaggtttatcaacagaaccaagccacaactcag

caaagatcagcaacaaaatctcaccagatgggcagttttgttcgctgcttctctcttgaa

aaacaacaagggaatccatgaagcattaatggcgtcaatggcaaagaaggcgagtgtact

ggagtgcattcaaacaatcgaaagcgcatcatagcgcatcacagttcaacgttaaactct

tataagctgttacatacaaattacagtctcatgtataaacaaagtttacaaacttatttt

tctttcacttttttatataaaactgcaaatcattactaaattggtaattgaacatcacaa

cacacaaacgtgattcaatattaaattagtgtatcattcaatttatccaagatacaatct

aaatatacaatatacatatacactctgtatactagtactaaatactaatacatgagatgg

atggatcttccttatactatatgattattatgccgcgtgtatcagatttcatcgtagaat

aaaccgtcaacgatttgatcaattagcgaatcgatgaaggagttgtagctaacggtgaag

attcgatccactttgttacgcgcacttgaaaaccaagcaaacaaagccgcgaaagcaacc

gcgcctaaacaaactccaccggcctccgttatccctgcaatatccattttcctgaacacg

gagtttccggtgaccagctccaccgtaaccgtcgccgcaaacaccatcatggcgagacga

ccggagatgatttcgaaatcttggcttttcttagagctctcaatgtattctgaaagcgtg

gcgaagaaagatgacacgtcatcggtaacttgagtatcttgtttacgagatttgattacg

gcggcgattggatcggaaccgtacgatctgagggtgcatagccgcggttgtagaacgatg

tttgtggcggcgccttctggttcggttagtttgggttttgggatggaaagctgaaccgga

gcgggtttatcgaggagaggacgtgagggcggtttgggcgggcggaggtcgcagtgagtg

acgcgcgtggtagatgccatgaaagaatgagattggttgggagtgagagttgggaagagg

aagatgaatgttatggtgttaagtgaagtgagtataagtcgtaatgaaaacgtgtaagtg

aatgcggggaagaagttggatacacgtaactcacatgactatcagacgaaatttt

>fava_dn_rep_c1067

atcattattgtgactattatggtcttttggtctggcaagaattctggattactggggatg

ttgatggacgtggtgtcccggtatcaaatccacagggtccactagaccatgaccttttct

tgttttgtgcaagagacacagtcaagcttcttagaaatcatccaagtcttgccctctggg

ttggtggaaatgaacaagttccaccagctgatataaatgatgctttgaaaaatgatctga

aacttcatccttatttcaaacatgaagacggaaaaggaaaacctacagaattaggggatc

ctagtcagtatcttgatggcacacgtatttatatagaaggatcgttgtggaatggatttg

cagatgggaagggaggcttcactgatggaccttatgaaattcaaaaccctgaagatttct

tcaaggatagtttttacaaatatggattcaacccagaagttggttctgtgggaataccag

ttgctgctaccataagagcgacaatgccttcagaaggatggcagatacctctgtttaaga

aactttccaatggttatgtagaagaagttccaaatcccatatggaaatatcataaataca

ttccatattcaaagccagacaaggttcatgatcagattcagctttatggtgttgtaaaag

atcttgatgatttttgtttgaaggcacagcttgttaacttcatacaatatagagccctat

tggaaggatggacttctcgcatgtggagcaaatatacgggtgtgttgatttggaagacac

aaaatccttggactggtctgagaggtcaattttatgatcatctacatgaccaaacagcgg

gcttctatggctgtcgatgtgctgctgagccaattcatatacagcttaacttggctacat

attttatagaggttatcaatactacatcagaagaattgtctaacgtagctattgaagttt

cggtatgggatcttgaaggaacatctccatattacaaaacacatgaaaaactatctttct

tgcctaaaaaggtaacaccttttgttgagatgaagtatccgaagtcaaaaaatcctaagc

cggtctactttcttcttctcaaactttacgacatgtcagacaatagaattttatcgagaa

acttttattggttgcatcttcctggtggagattacaagttattggagccttatagaaaga

agaaaatacctctcaagataacatctgaggttttcattgaaggatccgcatacaaactcc

aaatgcatgtgcataacacatctaaaaaaccagactctaatagtttaacatttgagcgcg

gttctacagctacagtaagagatagttgcttagttgcagactcggtagaaagtgtacata

acggggctggaaaaggacaagaagtcggctggtttaagaggttacatagacactttgctg

ggaaaagtgatggtttgaaggtttctgaaattaatggacatgacataggtgttgctttct

ttctacatttttctattcatgcttcaagtaaggaccgcaaagagggagaagacacaagaa

ttctcccggttcattactcagataactttttttccctggtgcccggagaggccatgacta

ttgatatatcgtttgagtctcctctgggtgtcgctcctcgagtgactctgaatggctgga

attaccatggacaaaccatacacgaggctctttagagatgtaacagacaaccttgattac

tttaaaggcatcgaggagagcttcttgagttttgttatttaagttttcatttgcctactt

caacgtcttcaaagaacatagaagaattcatatttatgagtatgtcgcagataaataaga

ccatgttattttaatgttttcattgttttgtttttcaattggtggaataaaaaggaaggg

tgagattatcataccaaataacgttcttttttttttaaagtatgctaaaagcaagtctct

ttcttgtttccttttattcagtgtaacacatccccagatgaatacatgcgatgcaatata

gcatattataaaagatgtaaggtttttaaatttaatatttaaaaacaacaaatctacacg

gatcacaaataagaacaagtaagtctgaaaaagcatcatcaatcaagaatcatcacacgg

acttcatgaaacggtttatcgaagagaagacgcctgccgctttgtcaagcagactctgcc

cttgctctgactccgcatcttcattgttgctgaataaaatctcaatatcttcggtatcag

gacgaccggcatattcctttgcaagcagatatctatttggtctatccttgtcatcactga

acactttccacaatccgggataacaccgaaagactgcaccaaatggcattggtcgcatat

gatagacagttgtaaaggtgcttaaaaagtagcgccttaactgccgtacgttaaatccaa

ctccaacatccgcgctatacaaacgtggattccacatgataaggggccttggtggatcat

cggagagggtggatgcaattttctctacatattctaacatctgataatcaggaacaatca

tgaccactatctcatcaccactgtctacaggcttccgatcgcttaaacttgcaaaactaa

ataaagcatctttccaccgatattttagaagagctgcagcaccagcatccggaaatacgg

ctttaactttctgacactgtgtttcttcaataagtgtatccaagaagacccttgacaact

cccagagctccaactgtgcgccttcatcatctaaaaattgcagttgaggaattaaaagtt

caactatcgctctcattcctcccgatgatacaaatgatgaagcagcctgacttgattgta

tcactgcaccctccaaatccggaggtaagcaactatcatcttcctcgttactattaacag

gagtatctgtagtgatagtttctagtgaggatggagattcggcctcttgagaaggttcgc

cttggaatttctcaaacttggcgcgaactttggaggtggaggttgcagagtgaaagggga

taccattgtggttgtagaaagatatggaaaagggagtgggtgtggttgggacaagtgttg

tggagtggaactggaagttgcagcaggttaagctccgacaacccattttctgttgctccc

aaagcccgtggataattctaattattgcattatcaccgtgagttaacaatggcatgtttt

gtaaaacaagtgtctcatcttaattgtttcaaattcctggatacaaaccgtaaggatgat

ttattcttgtgctctactccatccatctcaaaataagggttg

>fava_dn_rep_c1068

acggtcagaataacaaaatcttaaattagtacttttagtttacaccgatagaagcttaat

gtttaccctgcccaaaaaaaactcacaattagatggatttatgaaggggaaaactgatta

tttattaacagcttatttgaatgcgataatggcaatgataaatctcaataaagaaaccat

aaatacatgaaatgataacagcattcaagttgtagttaaaaacttgaaatgataaatctc

aataaagaaaccataaataccaactgcttactaccttatgtaagagaattctctcaaaga

aacactgtcatttgaaaattatttgagtgagatattgaatgaacttggtttcatctgatt

gctacgattatactgataattaaatcataatactaaaagaaaaaaacttcagttggtcag

tttacctttttaaaaccaaaatacagtattgatcaattttgcaaaaacataaaaggatag

ataaatggtgcttagagacaaacggttgatgtcagcattcactatgccttcgtctttcca

cccctcggccaaatacaaggcaccgaatttttcttggccgcaacagaacattgatcgctc

ctaccacctttactgctgagctctgtttggagcttacgccgtctcattctgttctctaat

cgcactcctcttgatgcacagactctgcgttcaatttccaaagcttctgtgaaattccaa

atcctgacaacaccttcttcacctccacacacaattcgatcagctcctatatctacagaa

taaagattattcttatatccatgcaacatcctctgtggaggctctacattaactttgccc

gaatgatttgcctttgaagctctcttccgcagaacacgcttgcttgtccgcagtagcttc

ctcacatcaattagagccagtttgccatcacttgatgcagacacaagccaaggaaactca

aaggcaagggaattcacaggaccagagtgagggtcccatgtagcaacatgcaacgcttcg

gtgatatagctatcacgaacaatctcgtacatatgtatagaaccatcttcacctccggta

aaaacaaaatcccctgaatggcttcttgccaaagcataagtattaccaacatgagcattt

ggaacaatgctcaccaaagtaccactattagtatcccaaacatacaaatccgagcccgat

gtggtgacaactgtagtatcatgagggacaagtccccaaacccagtcactatgcctcaac

accacaatgcatttcgtcgtgagacggtcccacacgcgaacagtcgtgtcccaagagcca

ctataaatcctcgtcaaatctaaagcaagtgatgtaactggaccatcatgaccccaaagt

cgaacctcggtattcttgttttgcgagcctttaagatcaaacagatggggcaattcttca

acagccctccaacaatgaatgaacccaccggtaccgccagcaaccagtagtctcgtatct

gctgcaactgcacgaatggtgcagccaagaggtttacacgaagcgattgacaacccatct

tccatgttccacatcctcacaacagtgtcatacccagaggtaaaaatgagctttgcagaa

gccaacaagaaaacagttctaaccgcctcggtatggccatacaacacatccatactatac

cttcccataaaagtcttactcctaaactccctttccacaaaaatctccttccatgacttt

tcatccccaacaccaagaggttctggaagcccccacctttcataatagaaccccttccaa

gcatggtgctcagaagcaagactgcggagaatcgacgaaacacacgaaacaattccaaga

tctttcggatcaaggcaattgagaatctcagatatcaatgcaggaggaaggtcggtaatc

gaccgacgaaccgcatcaaccacatcaccagaatccgatgattgattcaacccagtggca

gaacctttgattcctttcttcaacttcaacttcaattttcccttaaaattctcacctttt

acagattcagatttggtatttgaaatttccttggaattggattttggatcaactatatct

atatctaaggctgaattttctatcaatttttgaggaactttgttactctgcagacaatca

aattccataaaaataaaaggatcctaagctatgaatgaaaagggttgaaaattgttcaaa

ccctagaaaatgaattgggaaataattgaaaccctacttttgagggaaaatgaggaaaaa

aaataagaatacttcaaaaaagttcaacgaaatacctgagaattgaaatgggtggtgaag

aaaatgggaatgaatgggaaaggagagtgaaagggtttgtgtggtt

>fava_dn_rep_c1069

accaattagaatcgaagctcttgttaataacacttaattgacaaactttattttcctact

tataactactttttattgtcaagacacatattaactaaactataaattctattaaccaaa

aatgttttggagaaagggttgaatatctctcgtactccttctcgtttcgatattatcttt

gattataaaaaaaataaagtgcaagagtcaagttaaagatacatcaacaccaattatttt

ttacaagagcatgcaagttacactgttcttcttctatctcccatccttaatcactaacat

tggttccaatgatacacttcatagaatagaatggatgattaggcactgattctgatacta

tgagatgtcacaagctgtttactcttcaagtggatgattttcaactaagtcatatagttc

atatccaattccaaatccaagcattcttgccatatcatttgcctttgattctggcttggg

aatgttgtgatgcttcaaccatgaatacacaataaaaactttcttcataacaaacaattc

caatgcttctggattaaaaacaactcccattttcatattcatctggtgtggaactaacat

agcagcataccgagctagctcaccagctctccagtccaacaaaactggcaaccaaggata

agcagcatcaagtctcacaaaccacagtctaatatccggatactccgataactcacgagg

atcgcgaggatcttgtctcgtatagttgatgctaaagccaatagaacgctcaagaaactc

ttgcggttttacagataatggatttttcatgccggttgcagcttgaaaaggggacaagtc

aagactactaacatcatcattatcaattacaacattgaatcttccttcagttggaggcat

aggaggttcaactttagattcatcatcatcatcaaataaagagacatcgaagtttccttg

tgggttgaaggcaacagaaactgcaacatatctacgtggatgagagaaaggacgagaaat

tggaagggaaagccatgaagtttgaggagaattggtttgtctaggcaaaggagatagtgg

aaacaatggtttgaatctgatagttgtagccattaccagaaacagttaatacagtttcat

tattttatctatctacaaatttctcatgccttatgttttttcaacaatgttatcctcgcg

g

>fava_dn_rep_c1070

ctacttatattaggacatatctaacaaaattttacttggaatcaacacatgcattgccat

ctgatttgaaccaaaggcgaaccacactaacctaattcaacaacattcaaggcttatcct

attaagtaaactagattacttttatcaaacaatgccacaatacttcataaatcatacatc

aaacttgaaatgaaaataacaggatattataatttataccaattcgagacacagatggtg

attgataatgtaattctgcccgattttgtgcctatttttataccattattgcgaagagta

gctgctatctatcaacaatacataccataccacaaccctaaacaaataacacagtcacca

tccactgtcagatatcaactacactgcccgaaaactgtctgctttatccagtctcgctgt

tcctgagctggatgactcgcccattgtctcaggagatgcggcggcatgagctgtttccct

atttagagaagttggcatacctgaaatctccttcattaaatgtaacctcaaatcacatgg

tcgagtcctacttccaaccctcgcaatcacaaattttgaataattgtccaaagtgttggc

agcagcttcctttttgaaatctagctcttgatcttctttcatggctactgtgtcggaaac

caccgcaaaattgatttgatctcctttcttatccttctgatgagtttggttgaagaagac

cctaatgatgatggtgacgagacagaggacaaacaagaaccggtgtagttttatttttcg

gggaaatct

>fava_dn_rep_c1071

attgctttaaactctctctctctttctctctctctctgtgtctcttctctcttctcttct

ctccctttgtattcctacgcgtcttcatttccattcacaacactctctctctctaaacat

tgccatagatttggaccagagcaagacggtgttgttgccttcttaaggaacaatccaact

acaattcttcaacttctctaaaatatttatttattttctttcaaattttttcgatccgtt

tctgggtttttgtctgatgaaggttttgtgttgtttaagaatgcaaaattggatctttat

gtgatttaagttactggggtttttcgattgaaacaagggttggtgactactgttgtacta

cctagttcgtgagttgtgttgtgttgtgagtgtcagtgatgcataccggtggtagactca

ttgctggttctcacaacagaaacgagtttgtgcttatcaatgccgaagaaaatggaagga

ttaagtcagttcgcgaactgagtgggcagatatgtcagatttgtggagatgagattgagg

ttactgtcgatggggagccctttgttgcttgcaatgagtgtgctttccctgtttgccggc

cttgctacgagtatgagaggaaagaagggaataaggcctgccctcagtgcaagaccagat

acaaacgcctcaaaggtagtccaagggtcgaaggtgatgaagaagaggatggtattgacg

acatagacaatgaatttgattatgaacttgatgaatttggccagcaaccacattcagatt

cattgttttccggtcgccttaacactgggcgtggttctcatactaatatatctggtgcaa

attcagaacatggatcgcctcctctcaatcctgaaatccccctactgacttatggggaag

aggatcctgagatatcttctgatagacatgctctaattgttccaccatacatgaatcatg

gaaaccgagtccatcccatgccttatacggacccatctgttccactgcaaccaagaccga

tggttcccaagaaagatattgctgtatatggatatggaagtgtggcttggaaagatcgaa

tggaggaatggaagaagaggcagagtgacaaactccaagtggtgaagcatgaaggcgatg

gcaaagatggaactttcagtgatgacctcgacgaccctgatatgcccatgatggacgaag

gcaggcagccactttctaggaaactaccaattccttctagcaagataaatccatacagga

ttattattgtgcttcgtctcgtaatcctcgggcttttttttccattatagaattctccac

ccagttaatgatgcatatggcttgtggctgacttcagtcatctgtgagatatggtttgct

gtatcttggataatggatcagtttccaaaatggtatccgataacgcgagaaacatacctt

gatcgtctatcactgaggtatgaaaaagaagggaaaccatctgaattgtccagtgtagat

gtctttgtcagtactgtggatcccatgaaagaacctccactgattacggcgaacactgtt

ctatctatccttgctgttgattatccggttgataaagttgcatgctatgtctcagacgat

ggtgctgctatgctcacttttgaagcactttctgagacatctgaatttgctaggaaatgg

gttccatttgtcaagaaatataatattgagccccgtgccccagaatggtattttggtcag

aagatggactatctcaaaaataaagtgcatccggcatttgttagggaaagaagagcaatg

aagagagattatgaagaatttaaggtgaggattaacagtcttgtggcaacagcacagaag

gttcctgaggatggatggaccatgcaagatgggactccttggcctggaaatgatgtgagg

gatcatcccggcatgattcaggtcttccttggtcatgatggtgtccgcgatgttgaggga

aatgagttacctcgcttagtttatgtttctcgtgaaaaaaggccagggtttgatcaccac

aaaaaggctggtgcaatgaattctcttgtacgggcggcagcaatcatcacaaatgcacct

tatattctgaatgttgattgtgatcactacattaacaatagcaaggcacttagagaagcc

atgtgctttatgatggaccctcaacttgggaagaagatctgctacgtacaatttcctcaa

agatttgatggaattgatagacatgatagatactccaacagaaatgttgtatttttcgat

atcaatatgaaaggattggatggattacaaggtccgatttacgtcggaactggatgtgtt

ttcagaaggtatgcactttatggatacgatgcaccagccaagaagaaggcgccaagtaaa

acctgcaactgtttgccaaaatggtgctgctggtgctgtggctctcgaaagaagaagaat

ctcaacaataagaaagataaaaagaagaaggtaaagcatagcgaagcatcaaagcagata

cacgcacttgaaaatatagaggcagggaatgaaggagccatcgttgagaaaccatccaat

ttgactcaattgaaattggagaagaggtttggacaatctcccgtgtttgtagcctctaca

cttttggataatggtggagttccacatggagtgagtcctgcatcacttttaaaagaagcc

attcaagttatcagttgtggttatgaagacaaaaccgattggggaaaagaggttggatgg

atttatggttctgtgacagaggatatcttgactggtttcaaaatgcattgccacggttgg

cggtctgtatattgcatccctaagcgtcctgcattcaaggggtcagcacctatcaatctt

tcagatcgtctacatcaagttcttcgttgggctcttggttctgttgagatctttttcagc

aaacattgtccaatttggtatggctatggtggtgggttgaaattgttggaacgattttcc

tacataaattcagtcgtatatccctggacttccctcccattgatcgtctattgtacttta

ccagccatatgtcttctcaccggaaaatttattgtgcctgagataagcaactatgcaagt

attgtgttcatggccctcttcatatccattgcagcaacgggcatcctcgagatgcaatgg

ggtggtgttggaatagatgattggtggagaaatgaacagttttgggtgattggaggtgct

tcatcacatcttttcgcacttttccaaggtttactcaaggttttagctggtgtcgacaca

aacttcaccgttacatcaaaagcagccgacgacggagaattctcagagctctacatcttc

aaatggacctcgctcttaatccctccgatgacattactgataatgaatattgttggtgtg

attgtcggtgtctcagatgccatcaataatggttatgactcatggggacctctgtttggt

agattattctttgccctttgggtcatcatacatctttatccattcctcaagggtttgctt

gggaaacaagatagaatgccaaccattgtgttggtttggtccatcttgttagcttccatc

ttgaccctcttgtgggttagagttaacccttttgtttcaagagacggtcctgtgttagaa

atttgtggattgaattgtgatgatacatgaataaatgaaggtgtttatgaagaagcattt

gaatacgacacgcttcacatcacaaaaaacgcgttgcacaaatttttggatggaagttgt

attttttcggtggtgtgtgtagataaagaggaagaggaaagataatggaagggaaagaaa

agggggagtttgacacattgttgcctgcaatagaggttttcttttcatttattctttgat

tatattttctgtggtttttaagtgttgttgttattctctttttatctttctagttgtttc

actttcatgtattcttatataagaaagaggcattgaattatatatgataagttattccct

cttcaaaacagagggataagcagtctcaagaattagagttatgtcatattttaaggtatg

gttttttgttgaaacctttgtgacatgagagatttcttcctcatttgtaataccatcagc

tatggatattcttgttcctcctctcacatgttgtttatgttttcattcttcacactgtat

gaaacttttggttagatacaatcaatgtttgatgtcactagaaacttgtgatatgctagt

agtactattgtataacattctcctacatatatgttgcatgaattaaatatttatttaaac

ttgtgatgtgcttgatgtccactcatttggtgtgtggagttaaaaaa

>fava_dn_rep_c1072

tcgaattttactgcaaaacgatttttattgatgattgtttatgaataataatcatcagtg

tttcaatctagtgggggtaaaaacttaatctactttatttaaaaaatactattcaaattc

aaaatattatacagggacagggtcagataagaaaacatatgggtggtcgatataacaaat

ctctccaaaaattcaataataaaaaaagccctcctcaaattgatggaacgaaaaatcttt

gggagctaatcaacctctccaagagcagcaaccagacacaacctaaggcaggcaggcagg

caatacagcagtatcatctccagtaatcaacaaaatttgacactatgtttacatccgaag

catctaagcagatcctgcagcaaccttcttccgcacaatcctttttcgacgagctttagg

agctgatggttccccagacccgtctgcattgctttgcttgatgcccaagagcccaagccc

agaaacaactgcattctttgatataaaccatttagatgatgacggctttcctacattttc

agaagtcctgttgtaaaccttcaagagctcatctgactgagccagtggatctgcctcaac

tccaagccaggcacgcttggaagtctctcctaattgaacgttcaagaccctgagagctgt

gccgaagaacaatgcgactccaataacatcaaaatgactgaccattgctctatcaaatcc

gcagagcagttgatatctcaggttagcatagattccaagaaaagcaccataaccaagtgc

attgctgctcgcagttgggacagtcacagataatcttccttccttcttcttagccaaagt

gcttgacaatgcaccttgtactgcgcccgtgcctaaaccaaccatgcacaactcagcagc

cttgaacaaaaaggcttgaattcttttttgtaggtcgaattccctaagaggatagctcat

ttcaaatatgttgtttggaagcttctgcaatgtgttttgcaaatcaaattgaaatgtgtt

tccatatgaacgacaaggagcaagggaccaactcaacaaaaactcatttcatttcatttt

cgtcctctcttctctctcactccatcctttcacttcaacacaatccattcccattcaaca

acacctcttttttttcatcttctcccactcagttcaactttattatacctgttgggtatc

tgtaatgcgatcagttgatgaatgagaagaggaattcaaagcgtcaaaaatcattcaaaa

tgcctggaactgacttcaacgatgcacagaatctcgacgacgaaattcttttccctgtcg

aagagattgcgcaatacccgttgcctggatatgtgtcgccgacttcgataagttttagtc

cggacgatagtttgatttcgtatctgtttagtccggataatacgttgaacaggaatattt

tcacttatgatttgaaaagtaataaacaagaattgttgttcagtcccccggatggggggc

ttgatgagagtaatatttctccggatgagaagttgaggagggagaggttgagggagcggg

gtttaggagtgacgcgatatgaatgggtgaagacgaactcgaagaggaaagcagtcatgg

tgccattacctgctgggatttatttccacgatatttccctttcgaaaacagagcttaagc

ttccaagtataccagcgtcgcccactattgatcctcatctgtctcccgatggatctaagc

ttgcctatgtgagagactttgagttgcatgttatgaatctcttgtctaatgaatcaaagc

agttgacccatggcgcaaaggaaaacggttttattcacgggcttgcggaatatatagcac

aggaggagatggatagaaaaactggatattggtggtcactggatagtaaatatattgcat

ttactgaagttaattattctgaaataccgctttttagaattatgcatcaaggtaagagct

cagctggcccagatgcacaggaagaccatccttatccttttgcaggagcttcaaatgtta

aagtacgccttggggttgtttcggtagctggaggctccaccacttggatggatcttgact

gcgggggagtgaaagaactggacaacgaggatgaatatttggcaagagttaattggatgc

atggaaacattctcactgctcagatcataaatagacaccagactaaaataaagatcgtta

agtttgatattagaacagggctgaggagagatatattggtagaagaaaacaaaacctgga

tcaacattcacgactgtttcacacctcttgataaaggagttaccaagttttcaggtggat

ttatctgggctagtgagaaatcaggatttagacatctctatcttcatgatgcaaatggga

cttgtttaggacccatcactgaaggtgaatggatggttgagcaaattgccggtgtgaatg

aggcaacaagtctaatatatttcactggaaccttagatagtcctcttgaatccaacttat

actgtactaaacttttcgtcgatggaactcaaccacttcaggcccctaccagacttacac

atggcaagggaaagcacattgtagtccttgatcatcatatgcaaacctttgttgatatcc

acgactctcttagttgtccgcctagggtattactttgctcattagaggatggaagcataa

tcatgcctttgtatgagcagcaaataccagttccaaaatttaaaaagcttcaacttgagc

ctccagagattgttgaaatagagaccagtgatggtactatcttgtatggagctctttata

agcccgacgtttcaagatttggacctccgccttacaaaaccatgatcaatgtttacggtg

gtcctagtgtacagcttgtttctaactcttggcaaaatactgttgacctgagagcgcaat

acttgagaaataaaggcatcttagtttggaagttagacaatagaggaacttctagacggg

ggttgaagttcgaaggctatttaaagcacaaacttggacaaatcgatgccgatgatcagt

tttgtggagcagagtggcttatcaaagaagggcttgcagaatttggacacattggattgt

acgggtggagctatggcgggtatctgtctgctatgacactgtcaaggtatcccgacatct

tcaagtgtgctatagctggtgcacccactacatcgtggggcgggtatgacacattctaca

cagagaagtacatgggattgccatctgaaaataaatcagcgtatacaagaggttctgtga

tgaaccatgtgcacaagttgaaaggaaggttgttgcttgtgcatggcatgattgacgaga

atgtacattttaggcacactgcaagacttatcaatgcacttgtggcagctggaaaaattt

atgagctgatcattttcccagatgagcgccacatgcctcggcgatatagcgaccgagttt

atatggaagaaaggatgtgggaattcatagaccggaacctttgacatcatatattttttc

aagcatctttcttttctttgttatattgaatgacagtgtagtatctgaagttcttttaca

catgtaaaattgtttcttaacttgttagcactttcacccaagacgattggtgatgcattt

ctatacaggaaaaataaaaacttctcttggatttgatcaaaaaaaaannnnnnnnnnnnt

gaatataaacttgcctttgccaa

>fava_dn_rep_c1073

ataatataatattatcttttcaagactttttttgcaaactttgaaatactaaaatagatt

atagctcaatgattagccaaattacaggctctccatacacaacaatgcaaggtcccccgt

caatagtatcagatggtaacataacaattttgtcatattcttatttagcaaattgtaaca

gacaagtacatttgttacataggatttagtcagaacattgtaaccaaaaacacataaatg

atgttcagcagcaattctttaaatacacaacaatgacttgtagtcaacatgccctaatgg

tggtgatcctgaacatatcgtaggccgcaatatttgcagacagctggcttggccaagtca

aggcatatatattcaattgggtgtccaagggcgggatcagtatctccttcacaagcaact

atcctgccttcaaccttaataggtggtacttcattgatcagctccattggagatttcttg

cttgtatcctgcatccatttggccgtatggttgctgatttgagtggttacgaggctgaaa

cttctggtggcggtggtcgttgccggcgatgatgacaattttacgaggttcttcaaaata

ctcgaagccatctcacaaaccctaagttcactcacaaatcaagaaacaattttgattaag

atcaatgaatgaatcaaaagggaacttgattgaaactcaccctgaggatacaaaacgacg

ttagattgggaggttttggtttgtttccacagttttgataatgggccgacccatgttttg

gt

>fava_dn_rep_c1074

tttttttttaaggatcaaaaatatctttcatgttagtgttgaagctgagactcttgttgt

tgacttctgtaaaaaatgtataccaattattttaactaataaagtcaataattggagtta

aggatcctcataacccatctctcccaatgaaatattctatgtatgctatacaaatatcac

ataattatagtgttcattgcttccatatgtaattaggtagcttctctaaggaattaagca

accttcacttatgcattacttccttctgccaatgttatcaaccttgtagttgaatcatat

accatcacagttgcacagttctcacatttgactttgttggttgaatcaccacttgaaatt

gacaatattgtcccaaagaatgaggtgttttcagtaccacaattaggacatggacccttc

agaatcaagaaatcttttatgattgcatttgtaattgactgtgctaaccacagaatgaaa

ggaacagcagcaaaccaagtcaaaatgaaactgaaaggttctggaatcgctatcagataa

ttaatctcaaatccagttacatcatcaagaaagaagaacaatcctaatgcaaccacagtt

gctggaactttcagtaacaatgccttgaaatagtcaacagtaaggtcactataaaccttt

ctactacgaagactgcaccggggaccttcagccacaatttcactcccttccatctttagc

ttgagttttaattcatcaaactctttatcagtcaagattggatttccagacacataagca

atagacgcttcaagaaatttctgctcagaagaacttagcattacaacgctgcttccttcc

cacatgagttcttctttgagattatcgaattcttcattcgacataattgcctttccctcg

tagtagaaagcttgaagtgcttgaagaaattcttgttcaagttcaccgattgatttcttt

tctttttggtctatgctgcagtactgtaagattttactatcaacaacagtatcttcctgt

tgttcggaggtggccttgggggaaaactcgaataggcggcggcgaaggcaagagtggcgg

ccggcattgaatcgaataggatgatggagtctagaagcagaagtggatgataagaatgtt

ggagaggcagtgaatggacatggacatgtcaatgtgaaggctaataatttgctagccatg

gtgatgataaactagtgatgaatgaaggttattattactacgtgtatttgagggtgaaga

ttgtttggagggaaacaaaagagagctcaacgataaggcaacggctaacgtgtggaggga

gttggtggcatattcttcaccacttt

>fava_dn_rep_c1075

acattcagttagaggtggtagcaaacttcaaagagaaaccgatccaaaaaactctctcac

actctcatcttcaagctagagattctctctcatctctcgaggattcacgaagattccttc

atcttcttcatcctcacattgaagagaagttacagtgtgtattggttctgctctgagatc

cacgaatcctcagctacatcaccatccccttcaattaccgtaaaccatctctgtccgatt

caccataataagcataacctccatctccatctattctctacccaccaaaaaacgagtctc

cgcccttcaaccccaatttcaccacctcgataccaccccaaacgacgttgttatccatcc

actcatagacctcaacgtcgaatacaatcccgatctccactcacccatcccagccatcat

ttgcttcacaactacagcaccataaattatgtcaacaaccaaaacaaccaaaagtaggag

caaccacatcgtagctttcagagaaagttatgctacttcgcactgtcggagcaaatcgtc

ttctctgttcattttcacattctcgcttcaaaccttccttctctctccgacccatttcat

ccatggctgaagaattcgtcaaaggaaccgtcaattctaacggcgtcgccgttctcaccc

tcgatcgccccaaagctctcaacgccatgaacctagatatggatgtaaagtataaaagtt

atcttgatgaatgggaatcagatccaaatgtcaaatgtgttttgattgatagcagctcca

ctcgcgcattttgtgccggtatggatattaaaggggttgttgctgaaatacagaaggaca

aaagcacacctttagtacagaaggtcttcacagctgagtattctttgatatgcaaaattt

ctgagtacaagaaaccatatattagtttcatggatggaataacaatgggttttggaattg

gcttatctggtcatggtcgctatcgtatcgttacagagagaactgttcttgccatgccag

aaaatggaattggcttgttcccagatgtagggtttgctcatatagcagctcaaagtccag

gagacggagctgttggtgcttatcttggattgactggaaagaggatttctactccatctg

atgcaatatatgttggccttggaacacattacgtgccttcgggaaagttaggttcattca

aggaggcccttttggcaacacacttttctcaggatccccatcaggacatcaaagtacttt

tagcaaggtatgagtcaaacccggattctgaggcacagttgaagttgcttttacctcaga

taatttcttcatttggaagaaataagtctgttactgagattattaaggatctaaaacaac

accagtcaagcactgactccaatgtttctgagtgggctaatgaagctctccaaggccttg

gaaaaggtgctcccttctccctttttttgaccaataagtacttctctgcagttgcctctg

cagtaggaaaaggcaacggtgaattatccacgcttattggcgtaatgaaaactgagtatc

gcattgccttgaggtcttcactgcgacatgattttgcagaaggtgttcgagcagtattgg

tcgataaagatcagaacccaaaatggaaaccctcgagtttggaggaagttgataaaagtg

aagttgaagctgtctttaaaccattaggagcagaggcgagggagctgtctgtgtaatttt

caaaggaaagctatcattttcattgaatgcattaatgaattgtatgaaactgcatcaaat

aaacagacaggtgttaccattaggtcatgtgtctggtgttgcttgtatcgttgttcatca

ttgatactgtaacataaaatctatgatcttggaaatcaaaggacaatgttgcttctgaaa

aaaaaaagagaaaaacaacaaagtaccataaataagcacagttttctgaccagaagtaag

agaatttccaccaagtccatactgttgattctgagcaaagccagcaatctcaacaccttt

actctc

>fava_dn_rep_c1076

tttgagaaaaattaattaaaaactatatacagcaaaacacttgccattgaattattttcc

ctgccttgttataaaaaatctgtaatacaatgttcttatcacattattctaacacaggat

gaataaaagttcagtcttaactataatttcatagcaaagtgcacgcacacgcgacatttg

tgtatcacaataaagaaacccgtcgaataacttatactatcacataatgtaatcttttca

gcaaaaagaaacagtttgcatcgaacaagtcggtctaaactacctctctcttttcaaatt

ccatcgccttagttgtgcttttactgcttggacacaatatctttaagcttttccttcaat

tcaccagtctggtgcatattaagaataatatctgatccaccaacaaactctcccttaatg

aaaacttgtggaaatgttggccagtgactgaaggattttacagcatttttcagttcagga

tcctccaaaatgtttctagcacttagaggaacatcatattgttttaaaactttaactgca

agagaactaaatccacactgtggaaaatcaggcacgcctttcatgtatagcataacagga

ttatccttgacatcctgctcaataatgtttgacagagaagcgccagaactgtcaagctta

ttagcaggcttgaaatcatcatgtgtgtctggatcattagacaccttggttgagtatttc

attccataataacatgaactagttagacgtgtggtgagagagcgagctgcgattcctttg

aaaagaatggccgccatggcagttttgtatcaattctctctgatgcgacgaaggaatgag

tcgcttctctaaatgcatacaacttgcaaattgaaaactaaataggttgaaaaaa

>fava_dn_rep_c1077

aaacgacgccattcatagcaaagccacctcttcaagaccacgacgactagagtgttgccg

ttttgttgcgttgcacttgcagtaacgagaaaggatcaaaaccttctcaaaatccaatct

ttaagatctgcaaaactcaacaatcgcatcaattttatcattccagaaaaaatcccaaaa

aaaaaacccaaattgaataaataaaaaagggggaaatttgatggagcgaacacagggtaa

gagaagcgctaagatgtggatggttttgatgtgtttggcgttcatctttgttgatcggat

tgaatcgctttctgtgacagtgaatgaagttgaatgtctctatgagtatgtgctttatga

aggggatactgtttccgggaattttgttgttgttgaccatgatattttctggagctctga

tcatccaggcatcgattttaaggtaacttctcctggaggtaatgttgtccaagatatcaa

gggaacctctggagacaagtttcaatttaaggccccagtacatggaatgtataaattttg

tttccataatccatactcgacacccgagactgtttctttctatatccatgttggtcatat

tccctctgagcatgaccttgccaaagacgagcatttggacccaattaatgttaaaattgc

cgagctgagagaggcattggactctgttacagctgaacaaaagtacttgagagcccgtga

tgcaagacatcgccacacaaacgaaagtacacataagcgcgtaatcttctacactgtggg

agagtatcttctgttggctgctgttagtgcattgcaggtcatatacatcaggcgcctttt

cagcaagtctgtggcttacaatcgtgtttgagttccctccctcattaatcttattatgct

ttctatcttttagttaaatttaattaaagggtttaggggcttataatataaacatgtttt

ctctgtgaaacattatgattattattcttggtggtgaacattttgttgttctctttacga

cttatagaggtcacttgtactttgttacaaagtttttctattccaaagtttttaaatcgt

gtgagactaatgttatgcaaaatggtaccctatactttgatgtt

>fava_dn_rep_c1078

aagaaaaatattatcactttttataataattccactctcttccctttatactctctcatt

ctctcttaatcttaacaaacagaaacaacgaaagccactggcacacgatctggcacacga

tctggcacagtggttccttgagaactaggcagcagcgatcaagaacttcacggaggcgcc

atggggcctcacggcggggatgaaagatctggcctagatcttgaaaggggttttcgaaac

cctaacttaaagcgaacaaagtcaacggaaataaggagatttgagggaggattcacaatc

tgagatgaaagataaaccttctggactgggacaattgaagtttgggagcgattttagagt

tcttcggtggtgttcagtgagttctcttcctctttctctctgattttgtaacgataacgg

ttagcatctctttctctctcagccttatgaccgtatctctcttgatttctgaagggtttg

tgtaggaaaatgttgtggttgtggacgttgtaaacatccttccattcaaccttcatctct

ctctctatatctatctctcacacacataaacaatgtctgtaattacaatacaatctccaa

aacattgtgcaacaaaacaagtactaaaaatagaaagaaactacaagaaaatcttgtttg

ctttctcaacatttttcaccacaatcctcttactcatactcctcatctatttcatcctca

aaccatcaaaacctcaattctccctccaagagcttgacatctatcaactaaacctcaccg

gaccaatcctcaactcatcaatccaactcactctcctctcaaaaaatccaaaccagaaag

ttagcatttactacgacgagtttcaagtctatgccacttacaagaatcaacaaataacta

ctgactcatttctgccacccttttaccaaggacaacaagagactaatcttctctcatctt

ctttgataggaaatgctttacctgttgctccttccattggttatcaaatgggccatgatc

aagcttctggaagattggttttgaaccttaaagttaatggaaaacttcgttggaaaattg

gaacatgggtttctggtcgttacaggtttaacgttaactgtgtttctgttacggcttttg

gaattggaacctctatgaatgtgccactgcctcctatgacttctcagtgttctaccataa

tataaatatattgttaattagagagttatgtaagttcttcatgttttttacttttatttt

ttttttctcaaaaagcaaatacttatgatttttaagagcaaattccatataacatgtaaa

gaaagcgatgtaagttctacaaaattaagattaaaactagcattattgtaatcctaatat

ggtccagaaaagatctaggcagttgcatgaagtttaaattaaccgatacacccaagatca

tataacaaaaaaacactccaaaatagatatataagtcctcatcttccctagtttaagcct

tggtctcgagaagtttgtcaatcatgtcagcagcttcttggacagtggtgatgctctgag

cactttcttcttccacgctaatgccaaattcttcctcaagtcccatcacaatctcaaccg

tgtcgagagaatcagctccaagagcagcaaatttagactctccggtaacagctgtaccgt

ctgcaagggccaattgtttcttgacaatgtcacacaccttctgcacagtctctggtttag

ccgcacatgtaacttgaaaccgaagccctcttggttgaaaggtaatggacggaaactttg

cacccttaattgagaatgaaactgaattaggaccagagatcctggtacaagaaaccatag

atttgttggagaggagggacatggaggttccggtaatggaagccattggagatctgagat

ctgaagagggacggagagtacaaagtgttgaaaacgaaatgaaacaaaaatgagttgtga

aatagtgaggaatgtatttataggaagaacaatgtagtacagtgtttattggtttggttt

ggtttcttgttggggttttgatgtttattcaacttt

>fava_dn_rep_c1079

aaggcaacaacttcacgtatataatagtattcattaaactagtattgatctgttatatac

atatttttcaggtgtaaccaagagactactgctgctgcataatgatgacacaacacaagc

caccacaaaatattcaaaaatactggcttatgtctcatatagctaacttctatggatgtt

aagattaacaagttacaccaacttctgctctacaaaatgtacacaaaatatataactaaa

caaaaagtatactttgataaatgccctgtcatcggtcatgcttcaacctctttgttagtg

gtatgagtctcatcattttcaccatccccttcttcctcttcatcagctagttttgttagc

tctttctgaatcataatctttagagatgcctttcttggggtgagatccttattgaattgc

ttaccgagtagcttcaaaatgtcggtaaatgtcgccgtattgaagtcaacttgtttgaaa

atatcacatattgcattgcgtaactgatcatcactggggtttgacttctctttgtttttg

ccctttcttttagtaaccttctcagtattgtctttggaagaagacttggtgaaagttgag

gttttctgtttttcatttttcttcttccttgaaaaaacctttgggcttccctcactaata

tcatcatcggactcggagtgtgtggagaatgatttcttagaagttctctttcgtggcgtg

cgaggttttttctgaaatgtcgtttcctttgctttgcctttggcagcgaattctttctcc

ctggaaaatattttatttattttactcattttcttcacatcttcagattcattatcggat

tcggaatcatttctatcttcactttcagatttctgaggcatttcatcctcagatctgtca

ggaacacctttttcatcttctttctcagaatcactttcgatgtcagttgtagtatttctc

tcttcatctgaagaatcttcatttttcttccgactcttagcagagcgtcttgacgttgat

gttccagatctcgatgaaccttgctttgttatgtgcttgcgcttttttcctttaactggc

ttttcttgttcttcaagcaacacatcccttctcacatgaggggcaaccaaaaagtttatt

agctttgcaataatatcttccttccttgtatttgcctttgcaacttgtatgtcaagcaca

tcgcagatatccagcaacttctctttattacacttgtcaaatttttcttttactttaatc

atttgcttttcctcattttcataccacacaaaaccagaaaaccttgatatgtttttctta

acctcagctgcctttcctctcctacaaaagaggatagtatggagaaattttaaagtatca

tcaaccttccttctagataatttaaatgccacattgggtatatcttttaaatgcatacca

ctaccctcatcgatgaaaaattctttggatatatctctctcatatgattctaccaacctc

tcaactgatttcctatcacgtacaggacgattggttgcacttgcacttgcaggagtcctt

gcctctgtctctttcacctgcttctcatggatgtttcgtctcctgcgcctttttaatcct

ttctcaactcccatgtcttcagaatcgtcttcatttgacttatagtcaaccttatcatta

gcatcctcctcctcttcttgactttccatcttttcactgatttcatctttgtcttcaggg

atccccttctgttcctttactttctttttcaccttctccatattgccctttcctctccca

cgcttcttcgatgctttctcaactataatatcttcttcagttgacttatcgccaacctta

accttatcattctcctccttttcttgacttttttcctttttaccactctcacctttgtat

tcagggacatcctcaaccttttcttgacctttatccttttcaccggtctcatgtttgtct

tcagggacatcctctttttccatgacatctaatgctagtttcttagcttcaatgctttct

tttccttctttactatccatagtttccttaacatcggtttcttcccccaatttgtcatcc

tcaaactcattatcatctttcttatttatttttgtttcattcacaaaatcatctttctca

tcatcttctgattctttcaagccaacatcttttttatcatcttttatttcctccccagca

ttaagccccttttcctcttttaattcttccccttcatcggctttcttatcctcttttaat

tgccccccttcatcaactttcttatcctccttaaagtcttccctttcatttactttctta

tcgtcttttaattcttcctcttcattgactttcttatcatcttttatttcttccgcttcg

ttgactttcttatcctcttttaaattttccccttcgactttctcatcctcttttatttcc

tccccatcaccaactttgttatcatcaattacaacatcacagtcatcagctttcttatcc

tcttccacttctagttcttcctccccatcagctttcttatcaacttccttttttttttta

gtcaaacattttggagatttattatagcaacactattgcctttattttaaacctcctata

catcagcacataacctatattataaaatatatgttcaacttcattattccttgaatatag

catatttataatcacaaatacataaaactatttgcgatagttaattgtggatgcattaaa

gtcaccctcggtataccctccattgccattaccaatgaaatgaccactgttatttacagt

gcggccgccacgattgttggtggtgtggtatttcattcggtccccggaattcgcaccgct

gttgattctggcacgggagaaatcttggttcccgctaccatggttgctaaatgcgtctgc

cgtaccatggttgttggatttcgcagcttcggcttcttcagcctcaagcatggcgatcag

gtttttgagcacacgagatgacattcttatactcttacttgaaaaatgctatgaaaactt

ggtgtttttttttgtatttggatcccgcggcc

>fava_dn_rep_c1080

ttttttttttttgagacaaattcgcaataaacaattaatactagatcatcattcatcaca

gatcattgtacaaaagtacaaacaattcatttttacgccatatagttaaaatggcttcca

ttgtttacaaacatcaatcaagaacaaactgcatacattcaaaagaataaagaagaaaac

aatcaaattttacactactgtctaaagcatggcctaaggccatctatatacactgcactc

tgcacaccttttgctttcaaaccaattcaagagaaaataaaatgtatagaaaccaaaata

tatgctataatgctatgcatcacaacaacggaaaaaaagaaagcaaacgttttaattgag

caaacttaattcattctgtcgcattatttgactttaatgtcccccttttgatccgaggac

aggtttttggaggatggagatttaccagtgatagctttcttcgctttcacaagtgactgt

tttacccttgatataagattatttgattgcttctgtgctggcttggctggagtttctttt

ggaaggtccttggatactgtcgctgtatcattttccttagcggcttcaacctcagttttc

actggctcttcgatttgttctttctccgaattattctctcctgtttgaatagttttttcc

tcctcatctttttcttcgaatttagacgccagtgtttctctaacagattcacttaccgct

ttagagatttcatccacctttgtgttagcttccactgcttcaggtgtagctgtctttgcc

tcattctgttcggcagccttcttgatctcgatctccgcctctcttgaggatacttggaca

gtatcaggtgcttctagttccttctttacattttctgcagcaacatcaaccttttcctcg

atactggtagcgttctccgtaacaccgaccacttctttcttgacatcttctacaacaatc

ttttcttcaacttcaggagaaggttccaccggatttgcagagtcatttagtgaaacatgt

tcagtattcaccgtggtgttagtttcactttccccttctttgacagtggagtttttgatt

gtgccgtctatagattcttcacttttttctgtgtctttctcaggccccacaacttcctcg

cctatttgtaattgttctcttggcttttcctccactccattagacagagggtctatgttc

tcttcttttacaggaattgcttctgattccgagttgccagcctctttcacttcctcaata

tccgagtctttaggagggtgaacctccactgtatcacctattggttcttgactttcagat

gcttcctttggcttctcttcttcctctttctcgggtgattgaatttctgtttctgtaggc

cctgcattgtcgttgtttgtcgctttcagtatcgcggcctcaaactctaattctcttgtt

ttttcttctacatcaatggcactattagtttccgtagactgttcagggattgaagatgtg

tttggttgcacctctccctttggttctgttgattgttgtactggttctttctgattttcc

tcaatttcagtaaccgcgggctcttcttttactgcttctgtgggctcaacttcagcaacc

acgagttcttcttttactgtttctgtaggctcaacttgagtaaccacaggctctgctttc

tctgtttccgtgggccctgcaatgtcgttgtttgtatctttcaatatcgctgcttcaaac

tctaattctcttgttttttcttctacaacaacgacatcattagtttctgcagatttttca

ggaattgaagccgtgtttgattgctcctcttccttaggttctgttgattgctgttctggt

tctttttgattttcctcaacttcagtaaccacaggctcgtcttttactgtacctgtgggc

tctacttcagcaaccgcaagttctttttttgtttccgtaggctcaacttcagtaaccaca

gattctgcttttactttttctgtaggctctactttgttgttgttggtctcttccgctatc

tctgtcgatgtcttttcctcattggcttcaccagagggttcaataattgcaactgtgctt

ggttcctctgcctcttctgatttttcttgcgattctttttctggttcttgggttacttcc

ctctcttctatggctacaggctcggacaccttttcgacacttgtttctgaaatttcagcc

tgtttcacctcatcaacttcatagtgattagtctcagtcgcatcacttgaactttcagtg

gtgtcgactgatttttcgatgatttcagcttctggaacatcttctgttttggattggctc

tcttcggcagtttctacaggttccttttcaatagtttgtagtgtttccttcacatctggt

tcagtcaatggtggctcgtcttgctggcttactgctgtgtcaagcactggttctgaaaga

ctgataggatgtgattccggaactagatccttctcctcttccacaccgtctgcaactgca

acaggttctgatggtacagaattgtcagttttttcttcagcaacaggaagcttatcagcc

ttctcttcatctactttctctaccgccgaatttggcgcatcatcaactatgtcaggtttc

ggactatcatcaacaggtaccttcaatgctgaagactcgtcacccttttcgatctctgct

acagcaagctcagccttctcttcatccacttgctctacatctggatttggtgcatcatcg

attatgtcaggtttcaaactatcttcaacagaaaccatcaatgctgaagaatcgtcaacc

ttttcaatctctgctgccgccagctcagccttcacctcatctccttctgtcgtggacgaa

ggtgtagcttggccttgtgagtcattgggcttgcctccttcctcattggctaaggaattg

atcttttctgcaatagcttcgtccttttccagcttcactaattccttcaatgctgaagaa

ttgtcacccttttcgattgcttctgatgcaagctcagccttcacctcatcccgttctgcc

atggacgaaggtgtatcttgactttgtgcatcatcggacttgtcaccatcttcattggct

aaggaatcgatcttttctgcaatagcttcatccttttccggcttcacaaattccttcaac

gctgaagaatcatcccccttttcgatctttgctgctgcaagctcagccttcacctcatcc

ccttctgccatggacgaaggtgtagcttgaccttgtgcatctttgggattttcaccatct

tcattggctaaggaatcaatcttttctgcaatagcttcatccttttccggcttcacaaat

tccttcactgctgaagaatcaccccccttttcgatctctgctgctgcaagctcagccttc

acctcttctccttctgtcgtcgacgaaggagtagattgactttgtgcgtcatcgggcttg

tcaccatcttcattggctaaggaattaatcttttctgaaatagcttcatccttttccggc

tttacaaattccttcaatgctgaagaatcatcaccctcttcgatcttcgctgctgtaagc

tccgccttcacctcatctcctgcctcggatgtaggagttgcttgaccttgtgcatcgtca

ggattgtctccatcttcattggctaaggaattgatcttttcttcaatagcttcatccttt

ttcaacttcacaaatttcttcaatgttgaagaatcgtcatcctttactatctctgcttct

gctgcaagctcagccttcacctcatcgccttctgtcgcagacgaaggagtagcttgacct

tgtgcatcgtcgggcttgtcttcaacttcattggctaaggaattgatcttttctgcaata

accttgtccttttcctgcttcacaaattccttaaatactgaagaatcatcacccttttcg

attgcttctaatgcaagctcagccttcacctcatccccgcctgtcgtggatgaaggagta

gcttgacctggtgtatcgtcaagctggtctccatcttcattggctaacaaatcaatcttc

tctacaatagcttcatctttttccggcttcaatgctgaagaatcgtcaccctttacaatt

tctgctgctgcaagctcagccttcacctcatctccttctgttgtggaaggtgtagcttga

ccttgtgcatcttcgggcttgtcaccatcttcattggctaacgaattgatcttttctgca

ataccttcatccttttccggcttcacaaattccttcaacgctgtagaatcgtcccccttt

ttgatctccgctactacaagctcagccttcacctcatctcctatcgtggacgaaggagta

gattgaccttgtgcatcctcgggcttatctccatcttcatttgccaacgaactgatcttt

tctgcaatagcttcgtccttttcctgcttcacaaattcctgatgagtttcagaagccatg

atgagaaaaccaagagtgagtgaagttacaagtgaagttgttgagaacttaaggtgtaga

aattaaccaataaagaaagttttcaagtgaggagaataatatggttggcaggaaaaaaga

agtttgtttttgcaatgaagaaaaaaaaggcagaaacagagatgtttgnnnnnnnnggaa

gaggggagaagataggcccatgcgggaacaacaaccaacccaatcccaatcccaaagag

>fava_dn_rep_c1081

gttatgaattgtcctatacttcgaccaatatttaaaaacaaatggtaactatagtcatgc

aattggatgcagattgggcgattcattgtttacataagcacagaaaattgatgtttaaga

aagaaattctatatttaaaatcatcccctggatacagtttgtgactcttctatttcttaa

cacaatgacagattaagcacatagtttggttacatgggaggttgttgagtgtgaagcttc

ctccgcaatttattaatagtaacaactacattgacatagatcttatcttccatgttgctt

aggaaacagaaataagcatgtctccaagatgcaaagagtaatatggagccccaaaatacc

caaaatccagtgaaaaatcctacaatcagggttgtgtagaatggatttattaccaagccc

cttcttttgtctccatagttgtcattttcatgttcttcaatgtgactacaattcgcatat

ttgaacgaactatttttaaggcatgcttctgtgagtggagcaccacaaaggtgcggatta

ccaatgtagcagtcattgtcaaatgtttcaaactgtccatcggatggaattttcccagac

aaagtgttgtatgataagttcaaaaaagtgagaaaactcaaagtaaccatggtagtggga

atgacacatgataactgattcctcgacaaatccaatgcttccaagtttttcaattcacca

atattggaaggaatggaacccaccaactggttccttgacaaatttaaagctttcaaataa

actagctttgtaatttcaataggaatgccttgtgttaaatgatttgatgacaagtcaatc

acatatatgtatttatataaactcccactaaaaaacaactccattcctttaaatcgaaga

ccaaataaatattttctagatagatatatcgaatgactttccttaattgtaagaaacacc

atataggattgatcatttatactttcatcggtagccatagcaggaaatacacatcttgga

atccccccctcaattgattctctgaaaggtctaatatccgtaaagactttagttggcata

acgatgtggggatattttcatcaaaggaattcctactcaatatgagtgtctctaggatca

acatttctgtaccaatccaatatggtatgggccccctcaattgattcgaccttagatcca

agaaagtcaaaaaatgacaatttcttatagtgtttggaatatttccagaaagattgttgt

tgtacattaacaatacgtggagatacacgaggctaccaaaggaatcgggaattaatccaa

taaaattgttttcagcaagattaagaacaatcatgtttgtcccatatttccaacaatttg

gaatgactcctgacaaattattaaaagataagtcaagagtttccaatgagttattgacac

ccaacatttcacacacttgtgaaattgttccataaaataaattgttggagagatccaaat

cccttgagttaggaggcagtcgaggcaagggacatgagaaattattgtagctcaaatcaa

gttttttaagtttgattttttgtgagaaatcatgtccacatctactcagctcattgtaag

agagattcatatactcaacattaggtgacaaatcccaaaaccaattaggcacagtatcag

agatgcttgtatttgatacatctatatgggagaaattgttttgtgtttgaatccattttg

gaaattgaggacccaaaagacactttgccagacctaaagtttcaagttggaatgggggga

cccaatttttgctcatgttcaaagttacatagttaaaagataggtccaacactttgaggt

tgctaaagttagctagatgaacatcactgatctttcccatcaaattgtttccggttaagt

ccaacacaaccagttttgatagttgaggaaggattttctccaatgaatcatttaggtgat

tataagaaagacgcagttctttcaaactactttggccannnnnnnnnnnnnnnnntgatg

gtattgagccagtgagcatattgtgt

>fava_dn_rep_c1082

tcattagcaataatatagtgtaataaaattagtactatgactgaaacttttccttttaag

aaaaaaaactgaaataaaataaaacattagacaaacagatccaaattctgaagtctgtga

atcttgaatactcgttcgagcgtgtgcatacacaaaaagggggttatcatggagaacaca

aaattgttacttctctttcttttcttcttctcgatcaatcttctaccttctacactcgct

atctggctcacgctaccttcctctggtaccaaatgcgtttccgaagaaatccagaacaac

gtcgtcgttttggccgattatgtcgtcgttcccggtgatcataccctatcccccactctc

gccgtcaaggtaacatcaccatatggaaacaatcttcatcataaagagaatacaacacat

gggaatttcgcatttactactcaagagactgggaactacctggcatgcttttgggtggat

ggtggtaatccaaaaggtggtgacgttagtgtgaaccttgactggaaaatcgggattgca

gccaaggattgggattcagttgctaagaaggagaagattgagggagttgagcttgagctg

aaaaagctagaaggagcagtagaatctatccatgagaatttgctctacctgaagggaagg

gaagcagaaatgaggatcgtgagtgaaagaacaaatggcagagtggcatggtttagctta

atgtcattaggtatctgcattgcagtttcagttttacaattgtggcatttgaagagattt

ttccagaagaagaagcttatatagattatgctactatgctagtgttaatttaattttact

agtgaaacatctaccataaactaaagcttttatttaatgagtcctacttgcatcttacaa

gttgtag

>fava_dn_rep_c1083

catttcaacctgcacatgattcatatattcatagagacatcatattatacacaatttttt

acatcatatgatacaatatacatgttaggtaaacaactttagttacttcaatgatataac

aaacttagaaaattgttcaatttaagtagacaaactttatcagtaaaaagaagaacaaac

tttgtagtcacaataataataaagcacgcacgaagttacatcgatatacttaaaggaatt

gcattttttttcttccattttgaagctgctgaaacctaaaactaatacatttcttccatt

cacatgctacaaatattatcaagttgttcttgaaaaatgtcaatgaaattagccattcta

gacctttcaagagccaaaccaacctcaattccacctccaccatctctacaatccgaaaga

gaaatcgacccaaagccatcaatatgaactgcatcagatttcacaggcttcccccaacca

aaatcagtctcataaatagcaagttttggtgaacctgcaaccactaccaaagacagacct

ggttttgataattccctataatcaggcatcaacacttcatctactaacaaagcatcactc

ttgaattctcttatcttcctttcaatagcatttgcagccacaacaataccatcttttcca

accaactcgcccctctttacaaccacagtataagaggcaagacaatttccaaaataggtt

tttggtaatgaatattcaggacgatcgcgacaatctgctaaaaacaagaagttgcataga

tcaccaacagcaccatcaccacctgtgtttttctctgatcttaccatacaaaaccaaatc

aaggaacatgtcacaacaaaagttgacatatgttttgttgtgtctttacatttatcaaca

aaccatttcttcagtttctgaacttgttcatgactcaatattaaactagttcgtaccttg

tcagcataactatcactataagcatcttcatgttggtaaatgagcttaggagccttaggg

tgtttaagaactttgtctctttcatgggaaggaagatcaagaggtagagatgataaacta

ttgtcgttgtttgctatggcttttgaaagataggcccaatatttcatgaaatgatgaagt

gattttccgtctgcagcgacgtgtttgaaagtgaggcagatagagaagccggagtttggt

agaagggtgagttgaatagccattaaagggatggcaattgtacggttaggttcggtacga

ggtggaggtaggttagcaacaagagggtgccagttttgagcgtcttgtgaatcagatatg

aggaggttgaagtctgcggaggattcagagacggtgaaggagagagagtcgccgttgagg

tagcgtatgtaaggggggttatcggaaataggagggatgatgaggttggaggagaatggg

aagaagtgttggagggtgagagaaagagagtgtttgagaatcggagagaaattcttcctg

tgagagaggttgtgagtggtcggggagaaattgttctggccatgagagagttgaaggttt

tgggagagagtggcatggtttagtttaatgtcattaggtatctgcattgcagtttcagtt

ttacaattgtggcatttgaagagatttttccagaagaagaagcttatatagattatgcta

ctatgctagtgttaatttaattttactagtgaaacatctaccataaactaaagcttttat

ttaatgagtcctacttgcatcttacaagttgtagcaagagttttttttgttatgaattgt

cctatacttcgaccaatatttaaaaacaaatggtaactatagtcatgcaattggatgcag

attgggcgattcattgtttacataagcacagaaaattgatgtttaagaaagaaattctat

atttaaaatcatcccctggatacagtttgtgactcttctattt

>fava_dn_rep_c1084

ccgcggggacctttgcaaaggaaaagatctgaaaatgcgaataatgcattgaaccaaaaa

cacatacccaaatcactcccaaatcatctctgcgggtttgcgcctgggatttttgccact

ctcaaatcatcgctgttgttcattcttcaactttcaaagcacaaaaatggaagggactgt

tttcgctccggcattggaaggaataaagaatgttaagtctgaacagggagagattctgtc

acagcctttcttggaagtatgcaagcagatacttcctgttatagataagtttggagctgc

tatgacccttgttaaatctgatataggtggcaatatatcgagattggaatctaaatattc

atctagtccaaccaaattcaactacctgtatagtttagtacaagtagaggttgaaactaa

aactgccaagtcatcatccagttgtaccaatgggcttctttggcttacgagagcaatgga

tttcttggtggcacttttccggaacttaattgagcatacagactggtcaatgtcacaagc

atgtacagattcctataacaagactctaaagaagtggcatggatggctcgctagttcaag

cttcgctgtagcgatgaagcttgctcctgataggaagaagttcatggacgtgatacaggg

aggtggcgatgttaatgctgacatagagaaattttgtaccaccttttctcctttcctcga

agagaatcacaagtttctggctcgatttggcttggatgaactgaaggcttcatgaagcat

ggattcagtgtctattgatgaatggctcttgcatgctcaccctcttttttggttgtgtgt

ttatgtttatgcaccatgtttctcttttggcatccaaaataataaacatgataacaaaaa

aattcaatgacttatagtgttgtttaccttacaaatgacttgttttctgcttggtggtat

gcaatgttgtgacatatgcaactttgtttggcatgaagtaaatttattgaaatatagttt

tgcatgtggttaattgattatatttaaatttgacgtgataaaaacttcaaagtttgaat

>fava_dn_rep_c1085

gcaatgccagcctgaattttatatgtgcaaattaccaaagagatatactacatgtagtag

ttgttcaaagattacatatttatgcataaatacacaaatagacccgacggtgaagattta

ataatttctcaattggtcgctgtgcaaaagaaagaatagaaatatgtcattttcctccga

gagtgcagtttcaagccttttttttttctttggcgaaagaaaactttgatccttgccaca

aacctctcatattacctcaaaatgctcctatgcttgcggccagcactccccaaaagaatg

tataatcaccaaaaatctggcaatgacatgtttagagccataggccacaactattctatt

ttacttgcacatgtcataattgctgacttcatcgtttctttatctaaaacagtttgaacc

atcacctcttgtattttgtttcctgatcgggcaagcaacttgtactgatcctcaaagatg

gatgccatataaacctgaaatgtgggagctaaaccagggctcctgaaaaaacggcctttt

gaaactgactggactccctcaatttttgcaattcttttataagagattcaacttcagacc

actctgccttaggttgagcagcttccaatctcccatcaaaggattcatgatcccatacta

cagactctaaggattccatcaatggatcaggcgtgacctcctttaatgataaaaatatgc

gcccaagagcgaagtttaattggataacttttgcctctacaacctgatcaatcttaaaaa

aattagatgggtccagcgcgggatcccaagatatttctgactgaggaattagtgaagaaa

ctccttcaacctcaacaaaaactccaaagtaagttatcttctggacgcgacatttcacta

tatcaccaacttgaagtctagccataagttttctctttttctcaactagttcttcattct

cttttggtttgagtgaaaatatgagctttttcaattttctgtctgccaataacacatatg

ctttgattttctgtccaataaatgatgataagaaattgactttctctttgtcataaatcc

ttaacaggtcttccaatttcatatctggagaaattttatcttcaaatttactagcattcg

tatgagaaggagaatcggccgacaaattattaatttcagcatcattatttgtcgtagtga

ccaggttttgcttgtacattgatggatccaagcccttcttccttaaccatgactcgaaag

ctaagaatttccatttggacaaaagattacggtatggcagaaaccctaccaaggtaccaa

aagaaacaacgaaacccttggcgctgcagcttattagttccacatcatgtctatctccat

tcttaaacaaatcttctgccttggtccaataagcgtctttgccttcctgaatatctgaca

catcaacaaaattgcctgattcttcactgtttccgcgacttccgggatcaagggaaacag

tcttttgtccaacaagtattgaggtttgtttcacatattgatctaatctttttggctttc

cttgtattgcagcttcaacggacaactccgtcttagaaccaacggaatttaaatcagacg

gttgatttaaatcgatcggctcctgacggacctccgactgtttctgctcatcttgctccg

attcatcatttgggactacgactcttgcatctccaaactgctcttcgtctttgtcacttg

gtctgtgtggctgttccaacaacatcaccttcttgacttcatcacttggatcttcagact

gctcttgttcttttttggcggttggtctgtttggctgttccaacaagttcaatgtaacga

cttcatcacttgaatcttcaaattcctcatgttctattttactgcttggtttgggcggct

gttccaacaagctcaattcagcgacttcatcacttgtctcttcaaacttctcttgttcta

tttttccgattgatctgtgtggctgttccaacaagctcaaattggtgacttcatcacttt

gctctacaaacttctcttgttcttttttgccgcctgatctgtgcggttgttccaacaagc

tcaaattagcgacttcatcacttggctcgtcaaacttctcttgttctatttttccgcttg

gtctatgtggatgttccaacaagctcaatctaccgacttcatcactcgtctctacaaact

tctctatttctttattactacttggtctgtgcggttgttccaacaacatcaagttagcaa

cttcatccctcggctccacaaatttctctagtcctttattactacctggtctatgcggtt

gttccaacaacgtcaacttagcaacttcataacttgtcacttctttcggcatcttcaacc

caccatctctattctcgccaattgacaatcccggcgtcctcaacaatgtcacgtcactaa

acttctccttcacttgtccactcagactcaaatttggttttaacatcaacctcgaagaca

catcctcctcttcatccttgtccgttttaaacaacgacggcttcctcaaaataaccttag

gaacactacttttcctaacattccccggctcactcggcgtcaagctaggtttcttaatct

ccattgcaacattcttataatcatcatttggttggagttgtggttttggaggtgaattgt

tagcccaaggtctttcaacttcaaatggaagttcctcaatttccacaactttacctttct

ttcttttataaaatgatttctcaatatcaagataagaagcatcagggtttgcctttcttc

ccattatctttgcaagagtgagtttaggatcctcgccaagtaaacgaccgaacttgaggt

ccatgaggtcttgattatctaaattaggtatattctggtcaagttcatcgtgtgagcgtg

atgatgaggcgaaaaccttgttgatttttctctgatttagataaacagttcttcttgtta

ttgttgttgttctgttggaggatggaaaccgtgggagaagagtagtggcggtgaggaatg

aggttttgctggtgagaggaagagactccatttttgtgttaccttaaacaaacttcatct

caacggttttctttcagtctcgctgttctgttctcaggagggttatccagcagctcgtag

tgacgtggtttctcgg

>fava_dn_rep_c1086

aaccaaagtagaccggggcaacaagagaaccaatcatagagatgaataaggaaagaataa

accatgtacctcattgtttgatcttaccatatccagcacaaggtcacatgaatcctatga

ttcaattctcaaaacggttaatacaaaaacaagttaaagtaacacttataaccgtcactt

ctctttggaaaactatcagccacaaaaacctttcatcaattgaagttgaaagcatctcag

atggatatgatgaaggaggcttatcagcagcagagagcttagagatttacaaagaaacct

tttggagagttggatcagaatcactttctgagcttcttcaaaaactttctacctcagaaa

atccaccaaattgtgtcatctttgatgcttttttgccttgggctcttgatgttggtaaaa

gttttgggctacttggtgtggcttttttcactcaatcttgttctgttaatagtatatatt

ttcaaactcatgagaagtttattgagttaccactttcaaaatctgagtatttgttgcctg

gattgcctttacttgcacaacaggatttgccttcttttttgtataagtatggatcttatc

ctggttattttgacatagttgtgaatcaatttaacaatattggtaaagctgattggattc

ttgcaaattcaatctatgaactcgaaaatgaggtggtggattggttagtgaagatttggc

cattaaagacaataggaccctcaataccatctatggtattggataaaagacttagagaag

acaaagaatatggcattaacctctcagatccaaacacagaattttgcatcaaatggctta

atgacaaaccaaaaggttcagttgtttatgtctcatttggaagcatggctggtctaagtg

aagaccaaacacatgaactagcttatggattaaaagatagtgaaagtttctacttatggg

ttgttagagattgtgatcaaactaaacttccaaaagggtttgaaaaaacatcacaaaaag

gcttaatagtaacatggtgtccacaactactagtgttgacacatgaagctgttggatgtt

tcgttacacattgcggttggaattcgactttggaagctttgagcattggtgttccattga

ttgctatgccactttggactgatcaagtcaccaatgcaaaacttattgccgatgtttgga

aaattggtgttagagcttttgccgatgagaaagagattgttagaagtgaaactattaagg

attgtataaaggaaattattgagtctgaaaaaggaagtcaaataaagaaaagtgctttga

aatggaagaatttggctaagagttcttttgatgaaggtggaagatctgataaaaatattg

aagaatttgtgaatgctttagctcaattttgagtataacattattgtattggtgttcttt

tgataagttgtttgtttttctttcttttatgtcatgtacttaagttgtgttttattttga

atataataaatttcatttgatatggagtttgacaatgatgtccactccaagccaaaaaaa

catgaggtgcactgtgcggttctctgtaaccaatccttgtcaaacaagttcaataatttt

agtgtaattaaacacaaataattggtatccaaccaaataatgtaaaagcgacaaatatcc

aatcacgtgcattaaacg

>fava_dn_rep_c1087

accattttccgtcggcctctttggtctgaaaaactcaacactaacacggaaaggaaaaaa

aaagaagcgaattagggtttaagaaaccgccatggatttcacatcagggaagaacgcatc

agaaaacaacaacaacaactcagaaaactctcaccaaatctccgattcttcttcccaatt

accttccgattccttcaatatcaaagaaaccgaagaacgacaagcacgtgaactcaaagc

tggtcttcaccctctaaagagcaagtttgtattttggtacactcgccgggtacctggaat

tcgaaatcaatcatatgaggacaacataaagaagattgtcgaattcagtacggttgaagg

attttgggtctgttactgccatatttctcgtcctgcttctttgcctagtccaacagattt

gcaccttttcaaagaaggaattcgtcctttatgggaggactctgctaactgcagtggtgg

aaaatggattatacgattcaagaaggctgtttcaggtcgtttttgggaggacctggtgct

tgctttagtgggtgaccagttggattatggtgataacatatgcggtgcagtactaagcat

tcgatttaacgaggatatagtgagtgtctggaaccgcaatgcttctgataatcaggctgt

aatggctttgagagattcgattaagcgtcacttaaagcttcctcacagctacgttatgga

gtacaagccccacgatgcttctctgcgagacaattcatcttacaggaatacttggttgag

aggctagagatacaactgtgtttgtccaagcccccttgactgatgcttttgtatacttgt

gcagcttaccgccgccttgttatcattggctaaagtctaatgatgatgttgggtgtttca

gaactggttctgacttttgaatttaatgtacgaagcaacattttgaatgctttattgtag

tttcatttggttacttgaggattcactctatcaaaattgtgttctgtcttaacctgttgt

tgtaaataactttaatccttttgcttttcacatcttgctgaaggaaagaagcatgacttt

gttcatccagatgacaaattactcagtattttaatatgaatgcattgatctttgaatgca

aaaaaaaaga

>fava_dn_rep_c1088

cttttttttttttaagacacaagaaaaatccagtctcgtttatttatttgacagtattat

agaaaatcatgcaactggagtagaagacgtctctactagaaggaaaaggtttgatgtcac

tgaagagagtagacatttgaatagtgctctcaaagttcataaatcagacaacaggtgaac

aatgcggatataaatttaatgtatgttaaaagacttacttacgccctgccaaattataaa

agttcgatttctggcccaaaagttccggatcagaccaaaagcaatcactcttttatagtg

tagagttctttataatccttgtaaaaataaatactcggaaaatcttgaatcttttaccgt

ttccacgagccctttcatgcaatcaattattcatatatcgttaagttcaaacataatctt

ttcaaatcactgtcttcactctgatgaagcttctttctccttcttccccgagagataccc

ttccgcctttgcccatcgggtatgttgatagagctgaacagtctcatttgaagcatgaca

tgcaagaagtagataattgcgtggctgaaccatccatgcaaatctcatgaacaacgctga

ataaacacacattgctcctgtcatgttgccagagatcatttctggaggtttattgatgtc

agccaaaccagctgcgacgaatccccagttggcaacaggtccccaaaagtgagatgtttt

aggaccaactggactgttccaaaatgctttgaaggcagccatatgatatcacttctactg

gagacagatgacaaggatcggcgccgttgattttagggaggagaacggggaacgaacaag

gattggaatccaaatctctgataactgttaacgtcccacggaatggtccaacaacactct

tctgttaaacgacattgcatttt

>fava_dn_rep_c1089

taagagtttcaaccataatatttccgccttttatncttctttttttttttaaaatcataa

actatttatttctaaatttagatcacatataacaagattcaatcttgttatttgcattga

tactacatataacatgttcataacttggccatacacgcggtacacattatacgaacacaa

agcattggtaacaaggacatcttatttagtagatattgacttgcatctatgcattcatgg

tttagaaatggagttaatcttccgtcggtgggtgatgattaccataaggtggaccacgac

cataaaacggcgaacacgggcgagtagggtcggttgtgcaagaagttgatgttttctcgt

caattggtttcttgttggatggagaaagcttgtaaccgtcagtgaaaccttgaggagcaa

gcataagagcagcaaggaagaacactagtaaggaaaagaaaaaggaattaggcattttaa

atgctggtttaagtattggttcataccacctagggaatattgaggttttatagtagtatt

atgctcaatgagagtgtaaagtgtcaaagaaagt

>fava_dn_rep_c1090

ggcgcgcggccacataacaacaacaccacaaaccttcatttctttctctcttttccctca

tagtcaaaaccgtttcatcactcttctctcaaggttattagaaaagcaaaatggtgaaga

tttgctgtattggtgctggatatgttggtggtccaaccatggcagtcattgcacttaaat

gtccatccattgaagtggctgttgtcgacatatctgtatcccgcatcacagcctggaaca

gcgatcagcttccaatctatgaacccggtcttgacgatgttgtaaagcagtgtcgtggca

agaatctcttcttcagcactgatgttgaaaagcatgtctttgaggctgacatagtgtttg

tttctgttaacaccccgactaaaactcagggtctcggtgctggcaaagcagcagatttga

catattgggagagcgcggctcgtatgattgctgatgtctcaaagtctgacaaaattgttg

ttgagaaatcaactgttcctgtcaaaactgctgaggctattgagaaaatcttgactcaca

atagcaagggaatcaaattccagattctatcaaacccggaattccttgctgagggaaccg

caatcaaagatctttttaatccagaccgtgttcttattggaggcagggaaacccccgaag

gctttacagctgttcaaactttgaagagtgtttatgctcattgggttcccgaggagagaa

tactaaccacaaatctctggtctgccgagctatctaagcttgctgctaatgcctttttgg

ctcagaggatttcatcagttaatgcaatgtcagcactttgcgaagccaccggtgcaaaca

ttcaacaggtggcctattccgtcggcactgactcaaggatcggacccaagttccttaacg

ctagtgtcggtttcggtggttcctgcttccagaaggatatcttgaaccttgtttacatct

gcgagtgcaacggtcttccagaggttgcagagtactggaaacaagttatcaagattaacg

attatcagaagagtcgttttgtgaaccgcgtagtcgcgtctatgttcaacactgtttcaa

acaaaaagattgccattctcggattcgcattcaagaaagataccggtgacacaagggaaa

ctcccgccattgatgtgtgccagggactactcggcgacaaagccaaccttagcatatttg

acccgcaagttaccgaagaccaaatccagagggatctatcaatgaacaaattcgattggg

accatccaatccacttgcagccaacaagtccaacaactgtgaagaaagtgagtgtggttt

gggatgcgtacgaagcgacaaaagatgcacatggtatctgcattctgactgaatgggatg

agttcaagacacttgattatcagaagatatatgataacatgcaaaaaccagcatttgttt

ttgatggaaggaacattgtggatgctgacaagttacgtgagattggtttcattgtttact

caattggaaaacaattggatgcatggctcaaggatatgccagctgtggcataaaatatat

tggtcatttgattccagaatgaaaagtcaagaagtattgagttgaggcactcagttttat

tctttaatattttttgttaagagtttaatttttgttttgacttgttcactttcccatagt

agttgatataaatggagtatacatcatgggaatgatggaaactttatgtttaattactct

gtttttgtactcatccattactgtatggctgttatgaatgaacttttgtttgattaatct

gctcttgttatcaaaatattggttcatgttttcaaattttatggaaaatatttttggcct

aaaacactcttgtgttatgtagctaaccttgggtgtgctttttggaaacgttttgttatt

caaattggctacttgtttgggttgatttgtatcatgagatctgattccaaggctgtcaat

atatgctgtaattgaaattgagtcactgttgggaatatatgtgtatctaaacctttgttt

cgctctctatcaagtttgtgtttccaattttgaaaaaaaaaaag

>fava_dn_rep_c1091

aactgaattcagttcagagttagttacacttaactaactcaactctctatctctctcttc

ttcttgtttttcacaatattcatctttcttgattcctttgttccaacactaattagtagg

agaagtttgtacgtatttgatatttgatattcgtacatgaaaatctagtaaatacgataa

actagatcttatatttcatagaagcaaaaacataatacaaagcataaatctactcatctg

aaaaacagtaaatcatcctcctcagaatgactgctacaattacttgtgaggaagatcacc

taaaactttacagagatacaaactgtgaaccttagttttgctgccaaaactgccattctc

acattccagtgagctgccaaaatcgctgattaaatttattgccagtgccatgtgacagaa

ccttgagcaaaatactcaatccaccatatcacccatcaccttaaggcattcacaaatgcc

gttaatgattcaacgtctctcttttctgaaggatacttaattggccttgaagagtgttta

gggaagaagaggattgtggggaagcttcctagcattagttcactctttgcatattctttt

tcatcaccatctgctctaaattttccaaccttcactcctgaccctgctaaattctcggcc

aaatccacatatgattcttccatagcctgacagaagcggcaccatggtgcataaagcaca

acgatccagggctcttttcggtcctgcaatttggccagattctcaatcccagccctgctc

aagctgaccacattctcagagttgaaaatgtcagcaactgtggcagagccatttgcatgg

gccacaccatttccattgccattattctgggaagcatcatcctgtttcaaattacccttg

tgaagaccacactcctttgctttggcatcctcccaccaccatcttccctctctttcatgc

tgtccaggcaaaactggccttgtgcatggctcacaaccaatggaaacatatccttgagaa

tgcaatgaattaacaggcacatccattgttcttaagaaggtccatatgtcaatgccattc

acattagcaaccgggttccactttaccaggctgccactcccaccatccaatccctcaaaa

actggatcaacctgaacaacaggtatttcagacctagtaccaggtgactggtcttttctc

tgtccagtaatccatgctctaagacccttaagagctcttcttaaaggtctcaccttcctg

attctgcagcactcttgatgaccatcctcataaaatgaaaagagccccttggttcgcact

aatccctgaacctcaacagcatcagggaacatgtattcaatgcgaattccatagcgcttc

tcaactgcatcaaacaatctgtatgtttctggattcagtctcccggtgtcaagactgaat

accctgtatggcctacccgtcaaatgtgcatactcaattaaagcaacatcttcagcacca

ctgaaagcaatagcaatatcatttccgaatttctcaagcgccttgtccattatttcaaga

ggcgaagcattttcaaggttcttcgctaattgttcaaaatcttcctcttccttctcaaga

atctcaggagcaacgatagttgctgcaagaggaacaacaccagaaccgtttctttgagat

tcaacattaagagtcctcaccaaagaacgtctcgacggtaaattcaaaccaccagacgaa

ccatttgttctctccaaaatccttaccgaaccaatttgcaaacctttcgtctctgaggaa

gatccaatccgcgagaacaacgaactcgaatttaccgctgatgatgatgaagtaacggcg

agagccattggagaatgaacgaagtattgatctaagttcgttccgagattgaggtgtaga

ttctgttgatttttttgtgctgtccccgcggccgtaatggccactcttctttgataccac

gatgctgagaaagtgcaacaacataaatctccattctgttacacgtactaagaacagctg

cttcttcaatatgattcaaaccacaaagctcaccaatggctcttggccattcagcttctg

gtatagcaagtttctcacgcaactccaccggtgtagtatgcacactcagcccaatcacca

caaggctgctattctccttcgtatatctatcagcagcagaagttttaagctgttgtagag

gatccacagatgaagaagcgtcacagcgaatagttgaacttgaacctttctggctctgaa

caaggctctttttgtttctaccatgaaatgataaatgagttgtaacataagagaaagaag

aagaagatgatgaacaatgtttaaggaagaatggttctgatttcccaccagagaaaaaac

tggttgaaacagccattgtttgttattgttaatgttagagaaaaataaggttgtttgtta

gtactactattgttaattttgtgttggtgtttctgaaaatgaatgatgatgttggaaaat

aaagatgtggaaggatttgaa

>fava_dn_rep_c1092

aaaccagataacatgcagaggtgtaattagctccttaccactcaaatagcaaatttgagc

atttgcaagttcaacttcccggagtgaaacttaaatcaccaagatggtctaaatgttaac

agcaagtgagtatagcaacaacggccggcagcatcagccggtaacaaggcctgccgagct

gaacttgccaagagtgctgtggatcctgtcttccatcttagagaagctagtggctcgaaa

tgaaaagcttgtggatgagctgaaccagcaactaaataaactgagctctgactcagtaag

agtagggaagagcttgaatgcctttcatggcgtacgagcaccgggcataagtatacctaa

gtacttggaaaggatatacaaatacactaattgtagcccatcatgttttgttgttggcta

tgtgtatatagacatgctgactcataagcaccctgattctctagtcttatccttgaatgt

tcacagactgctagttacctgtgtcatggttgcttccaagatgctagatgatgagcatta

taataacgcagtatacgctcgagtaggaggggtcagcaacgcagaattgaacaagcttga

attagagttactctttctgttggattttaaggtcgtggttagctctcgagttttcgagag

ttactgtctgcacttagaaaaagagatgattgtaaacggcgcaggcttgaagattgaaag

aacattgtcgccaaaatcttttgagaatgaagtttcagttgaagataagaaaagctcttc

cccacctccaattgtggattatggattgacataacataagtcctttttctttctgttgtc

aattttggggtcccttgccctgtttagtcctttatctttttcttcatggctaagcaatta

ttggattgttcattggccagtttgttatttatatgaatataattcctatgaaactgtgtc

taatttgatttagttgtgacaagacataatcatatccattgttctcttttcttttcattt

tttttatttcttaaaacattgttactcttgtagttttgagttctcatttgaatttgctta

ctcatgggtttggatttatttatgtttcgatgttttaattttaaaagcaagtttcttgca

aaaatcagtgtaaaatttccaaccaaatacaaaagctacttgtatacaaataataataag

taggtgnnnnnnnnttgtattgttactctaaattttaaaaagattaaaaaatattttgat

tatttttnnnnnnnnnnnnnnnnnnnnnnntgagatgcg

>fava_dn_rep_c1093

aacgcagagtggccattatggccgcgggatctccacatcaactcttcttctcttaagtaa

gctctaattatacttagtatctacaagtgtaaatttaattaagagatggcttccaaaact

tgttccttccttgcaattttcctcacaataaacctcctctttttctcacttgtttcagct

tgtggctcatactcatgcaaccctactccaaaaccaactccaaaaccaaagccaaaccca

aatcctacccctaccccttcaactggaacatgtccacatgatgcacttaaacttggtgtc

tgtgccaatgtcctaaaagaattattggatctaacattaggccagccaccggtgaaacca

tgttgctccctccttgatggccttgttgatcttgaggctgcagcgtgtctttgcactgcc

cttaaagctaacgttttgggcattcatcttaacctacccatctcactcaccttgcttctc

aatgtttgctcaaggaaagttccacgtgatttccaatgtgcctaaataaaatcatatact

atcaatgtatcgttgtgttatttaaggtttttgttggtttttggaattcgatttttgagt

ttttgattattgtatacgtgcatggatgattattaaccactcgttatgatctttccattc

actattttgttttgtattgtgaaagtattacgataaataaattccatatgtttgcttata

tgtaaatctattggaataaaatatattgtgaagtggtgtaatttatttgattgagaaatt

aattatatatacatagttgtttgttaaaaaaaaaaa

>fava_dn_rep_c1094

atacaataagatattatgattttgacttggagcaaacttgtgtgctgcattcacctgaga

aaagactgagcaaatttcttctgatgcaaaagttcatatggctttacctccactccaaca

tgaaatagacgggctttcaattacgcgattcaaaactcaccacacctaaatcttgataca

ccacacacacagaaaaaccatgtctagcttcactcatgctactacactactccatgctca

cataaaaaccaaacacaaaatcacttcaagcaacaaccttaacaacaacaacaacaataa

cctttcatcttctaaacaaaatgcaaccaatagaagaaacttaatttcaacatttctatc

aacatcaatggttgttggagtttccattggcacaacaccattagcattagcacagaattg

gggcactcgttcttttctcagagagcatttcttcgagcccgggctttcgccggaagacgc

ggtattaaggataaaacaaacagctgaagggttacataatcttagggaaatgttggaaac

tttgtcttggaggtatgttatgttttatataagattgaaacagaattatcttgaacagga

tttgaaaaatgctatgactactttgccggaaaatcgccgcaaggagtatgtcaaagttgc

taatgaattggttgattacttgactgagatggatcggcatgttcgatcgccaaaggtata

tgaatcgtacttatactatgagaagacattgaaatcaatagatgagcttgtagctatgtt

agtatagtgcaaagatgaagcagcagggtattggtattttgaatttagttgtataaaaca

cattttggatttttaattgaatatatatacaaggtttacttggatgtaaaactttaagta

tattatttatgtacatgtttgtcattagaatatgctaacttacactctttgaatcggtgt

ttgatactcgtatgacattcatatatcattaaaaaaatatttaaattgctcataaaaagt

atccaacacaaggctaaagagagtaacacagtttgatacaaatgtaaacacactatggag

gaatacacacaacacaatttcataaatggagaaatccatgaccaaatggcgctagcatat

aacatcagtttcgagagattgcaacttgtgacagaattattatgataacgaacatagcaa

cagcaatgtaagaaccaataagagagccagaaatacgttcacagaaactgttgtattgtt

ggcaaataggaaaccaattggcttgtgtgtttccataatgtgctatgtacacaatcgatg

ctgctgctgaggctgctgctgttagtacacccatcattaccatgtcaagaacaatgagta

gaattctagtttttactgcagtgctcctcactatatggaatattgacaggattagtgaaa

gcacaagatatccacaaacaactgagtttcccatcacaaaaaacacaaatgagggaagat

catcatattgagctctgaattgtataaaagcagtagcaaatggaagcctctcatcagttg

ttgccatagcaatagcactagctagtgttgcaacacctgcaattattctcaaaataaaat

ccattattgacaaccctcttttcactcctttgcttgtagttgcatttctaggcacttcac

caagttcaattgaacctcccttcatattttttctaacttaattaaaaacaaactcttgct

tagaaactttatatatgtatataagtacttgtgcctcttaactatctaaaacagacactt

atatagttgtagattttttgttgtcaaatttagtatttcctagaat

>fava_dn_rep_c1095

tttttttttgattagaagattaatagattgaataaatgatgataaaactgcattaagaca

acaaaacagagttggatcattcataatgagaatcatagggcaggtaatcaacaacaaaac

aaaaggacaaaaatatatctagaaagcactattccatagcagaacacattaagctggata

gaattacaggtccagcacattcaaacctggaggaagtgattgtttcaacttatcagcttt

ctcagtatcgaacacacaaagggtgtaaagatacttggagcatcgaaccttgaatttgac

aacatctttgcttctcttgattttcacggagcgcgcatccttcctccgggccgtgaggag

gaagtccttaatctcattgatttgcttaggcattgttggtttggctgcgatttcctcccc

aataatcacagtcggcacgctgagacgaaacttgagaagttaggaaccataaact

>fava_dn_rep_c1096

acaaaagaggggcccaaaacacaaaaaccgcggcgccaaaacatttccagaattctccaa

aaccagaacgttcgtcaatggcggttttaggcctttcgacttccacactcccctccttct

atcttcgctctacaaccattcaccgatccaacccttccaccatcccccgctccttcctct

ctcatcctcctcctttccctaacttcactctttccaatccacactcccaaatcgccgttg

tctcgtccgattccaagacctcttcatttgacctaagtgccccagatttggacaatagcg

gcggcggcggaagtattaaaggtaacggagacgattttagcggcggaggaggcggtggtg

aaggaggaggcggagacgatagtaataagggagaggaaggatcgagcggtgataaaagga

agatggctttgtcaatgtcgcagaaattgactctaggttatgcgattctcgtcggagcgg

gtggtgtaatgggctatctgaagagtggtagccagaagtcacttttagctgggggtttgt

cttctgcactgctgttttatgttttcaccgagctacccggaagacctgttcttgcatcat

ctgtgggccttggtatatctgctgcacttcttgcggtgatgggttctcgtttcaagaaat

cagggaaggtctttccagcaggtgttgtgtcccttgtgtcattcataatgactggtggtt

acttgcatggaattatgcgtagtacacactagaatgtaaataaaataaaaacagtgagaa

atcctataaactgcgctgaatacaattcccactttgttgtgaaacttgtggtttgtactg

tagacccgaggtttatgtttgtcatacacaaacatttgttgtgaaataaagatgatgaat

gggaactttcctttggccttgaaaaaaaaaaaga

>fava_dn_rep_c1097

cgcggggctctcaaaacttgttctcgatcgctcagtctctctctgcggctgaagattctg

aacgagtaacatccttcaattccgtttcttctaagttaagacatcatgggtaaagaaaag

gttcacattaacattgtcgtcattggacatgtcgactctggaaaatcaaccaccactggt

cacttgatctacaagctaggaggtattgacaagcgtgtgattgagaggtttgagaaggaa

gctgctgaaatgaacaaacgttcatttaagtatgcatgggttcttgacaagcttaaggct

gagcgtgaaagaggtattaccattgatattgccttgtggaaatttgaaaccaccaagtac

tactgcacagtgattgatgctcctggacatcgtgattttatcaagaacatgattactgga

acctcccaggctgattgtgctgtcctcattattgattccaccactggaggttttgaggct

ggtatctctaaggatgggcagactcgtgaacacgctcttcttgcttttactcttggagtg

aaacagatgatctgttgttgtaacaagatggatgctaccacccctaagtactccaagggc

aggtatgaggaaattgtgaaggaagtttcttcctacttgaagaaggttggatacaaccca

gacaaaattccattcgttcccatttctggttttgagggtgacaacatgattgagaggtcc

accaaccttgactggtacaagggaccaactctccttgatgctcttgacaacatcaatgag

ccgaagagaccctcagacaagccactcaggcttccattgcaagatgtttacaagattggt

ggtattggaactgtgccagtgggacgtgttgaaaccggtgttgtgaagcccggtatgctt

gtgacttttgctcctaccggtttgacaactgaggttaagtctgttgagatgcaccacgag

gctctcactgaggctcttccaggagacaatgtcggattcaatgttaagaatgttgcagtc

aaggatctcaagcgtggttttgttgcatccaactccaaggatgaccctgccaaggaagct

gccaacttcacctcccaagtcatcatcatgaaccatcctggacagatcggaaacggttat

gcaccagtgctagattgccacacctctcacattgctgtgaagtttgctgaacttatcacc

aagattgacaggcgatctggtaaggagattgagaaggagcccaagtttttgaagaatggt

gatgcaggtatggttaagatgattcccaccaagcccatggttgtggaaacttttgccgag

tatcctccacttggtcgttttgctgttagggacatgagacaaactgttgctgtcggagtc

atcaagagtgtggagaagaaggacccaactggagccaaggtcaccaaagctgcagccaag

aagaagtgatttcacaccatagttaatcttttccgagtcttagtctgtttttcctgttta

attttaaagtttttgccggtttcattaagctaaacttacaaaactgggttcttgatcgac

ggtggtcagttttccagtttgtttgattttcctggtactgttttttggtgttaatttatg

tttcagaactacattttgttgctgagggatgtttaaattgcattaatttagacatttttg

tctaccctttgttaagttatgttacgattgtggtttaacatttttatctatgattttgtt

gtttgactgagcaggttattgaacttgcatgatagtgcctgcaaaattaatactagtacc

tatgtcttaacaattaaaaaa

>fava_dn_rep_c1098

cgagaaaaattatatgcataaaanntaaagcgaatttaaatcaacaaaacgagcttagat

atagaagaaatccattattatgtaccagaaacaatgataacaaccataatactcctcaaa

tcatataggaaaaaaataagcaacaagatccagaaatcaacatctgtaatctctacacga

aagaaaaaacgagagatagagagagttttatatactactaaactagtcaaatctcatcct

ctaataaaaaaccacacaaataaaagtaaactcatcgcaatagagtataatacgctcaga

tccggtgagcagatccaaaagcaaaagcaacaacggcggcaataccacctgcaacgagcg

atggtgaaacagcggatgctggagaagcaggacttggagctggagcttcggcagcagcgg

cggcggaaatgatggcggcaagtgtcgccaatgttgcgagaacggtgattttagtggaag

ccattggtggttgtgttgaggttttgatttttcttcgctagggttttgatttgaaggaat

cgaaatgcccgnnnnccgtaatggccactctgcgttgttgcggaagagnnnnnngaggat

gagaaagcaggaatagtggaag

>fava_dn_rep_c1099

ctttttttttaagaaagaacacatttaaagaaatggaatttaaggtccaaaaaaacatag

gcaggcatgcaagattacattcattctggacacagtctacttagaaatttcggcgtcctg

catattcaaagtaaacaattacaaggtataaaaccagggataatatctcatcatctgaag

atcccacttctagttaaaccaagaacaaatattggtaaagaaaaacagaaaactgcaatt

aaatctaggaagcatttatttgtattttgagttgtaagctgtcttagccagatccatcga

agacccattttctcttcaacaaccgtcgtgctttatccgtcgttcaaagatgtcggaagt

ggaaccaccctttagaccacgcgtgaagattattgagaagcaaaagtatttccagaatat

cccaaaacacacatacctgaaagggccattggataaggttacatctgtagcaataccaat

tgctctcgcaggaacttcattgtttctgattggtcgagggatctacaatatgtcacttgg

aataggaaagaaagaatgacttgtgagttcctgatcgaagaggtgggtgttttgtggctc

aaacttaagagagcttcctcaatatgtactgtctgttttccttgctttaatgttaagtgt

cttaataaatatgcttgattaccgagtttgatcaccaatggaggtttagtatgtgaatga

atacggttctctgtatgtttgatgatgtaattggttgatgagttagtacttgtgtgttat

cattgattgttctagttcatgtttgtctctgtctagcaccttctatagaagcattttttc

gttggtggacattttgattggccttttagcaaaccactgaaattgcatgcgaaaacaaag

tacccgagtttgaaacaaaggtcaaattgacatcttannnnnnnnnnnnnnnnnnnnnnn

naagatgagtgaaacttaaaatgcagtttttttttagtcaaactannnnnnnnnntagga

tgtgttaatgttctatgcttacatttattgagttgattgcatagtcggannnnnnnnnnn

nnnnnnnnnnnnnnnnnngtctggtttagctgtgaattagtctatggccaattttctcaa

gggcacatagactgagaatcatatttaagtttctggttga

>fava_dn_rep_c1100

aataaaaacctgcggtttggttggttgggttaatctaaatctaaactctcaagtctcagc

accaaccaaacaaaaagataggattatagaaaaactgaaactcaagtgtttgtgttttat

gtagttgaaaatcaatcaacaaacaaataaaacaatggcatccttatccctctcatctcc

tccatctctctcctcttccttccacaatgcctccacgcgccttaacctacgcacacctac

ctctcttaaactccgcacccgttcctctcctctctccgtcaaagccataaccctaaccca

agacgatctcaaaaaactcgccgccgacaaagccgtcgagtacgtcaaaagcggcatggt

cctcggcctcggcaccggctccaccgccgccttcgtcgtctccaaactcggcgagcttct

caaatcaggcgaactcaccaacatcatcggtgttccaacctccaaacgcacagaagaaca

ggcacgatctctcaatattcctttatccgtcctcgaccataatccacgcctcgatctcgc

tatcgacggcgcggacgaagttgatccttatctcaacctcgtcaaaggccgcggcggtgc

tctcctccgtgaaaaaatggtggaagccgcttccgaaaacttcgttgtcgtggtcgacga

cacgaagctcgtctccggtttaggtggaagcggtttggctatgccggtggaggttgttca

gttttgttggaaatacaatcttattaggcttcaggaattgttcaaagaggaaggtgttga

cgcgaaactgagagtggatgagagtgggaaaccttatgttactgataattctaactatat

tgttgatttgtatttcaagactccgattagggatgcgaatgctgtcggaacggagatttc

tgcgcttggaggcgtggaggggcgtgggttgttattgaacatggctacatctgttatcat

tgctggaaaaactggggttgaagttaaagacaagtgattttgatttcttgattgtaattt

ggattcggtaaattatgttttgagttttattttggttttttaacatgtctatgcgttgtt

ataattttatattttcgtgaaacaaataatagaagagagaagcaacaactttttatgctg

ttaactctgttactgatgtgtaagtgtaagtgtctactgtcttagtactccctccattca

aaattgtttgatttgcagaacatagttactttctcaaaataatattttcaatgtaatgtt

aattattttttatttgtattctctaattatatgaaaaggatattgtaaagatatggcgtg

aagacttgcactgcactatcaaggtcttgggtttgctgggagtttgttcttgaattgtgt

tgaggactatttgaatatttatagtttttgttacttaggtggctatttgttttggtacaa

tattttcaagtgttgaattaacttttgttatttgttgtgtggttattgattactataatg

gactaccctatgttaatta

>fava_dn_rep_c1101

ataatcaacgactagagtgaaacgacataaaccctttaaaccctaactccgagttggtgt

agttgcgttaaccctttctcttttcatcatcttccgcagcaaacatcacccagaggcgaa

gatggtaggagacaggtcaaagaagttgaaactttctgagaatcccgaagatgctgacgg

agacctcatcctctccatcgagaagttgcaagagatccaagacagcctcgaaaagatcaa

tgatgaagctagtgataaggtccttgaaattgagcagaagtacaatgagataaggaaacc

ggtttatgataagcggaatgagatcatcaaatctattcctgacttctggttgaatgcttt

tttgagtcatcctacccttagcgaacttttgaacgaggaagatcaaaagatattcaagta

tttgagttctcttgaggttgaagataataaagatgtaaaatctggatacacaatcaccct

caatttcaatcagaacccctattttgaagattcaaaactttcaaagacttttaccttcct

tgaagaaggaacaacaaatatcactgctaccccgataagatggaaagagggcaagggaat

acccaatggtgtcgatcatgagaagaatgggaacaaacgggctcctgttgatgtcagttt

ccttagttggttttgtgactgtgagcagaaagatgatgatctgcttgacattcatgatga

ggttgcagaattgatcaaggatgatttatggtcaaatccactcatttatttcaacaatga

ggagcttgatgaagaggatggcaatgatgaagctgatgatgaggagaaagatgaggatga

ctcagaagatgacgacgatgagggtgacgatgaggaagacaattaaaaatacattatgtt

tgtgtgacctatctatttattttttctggtttgagtatgtattaggcttatgggcgtgca

gaccatgatggcctttctttcagggcattaactcatgtctgcaagtgaagctacacaggc

tggtagttattggttctctgaccataatttattgattaccctgtcaagttatttcttcta

gtttcatccatcgtgcacttttagtgatatacttatatactcatggtatattagctttct

ctcatggatttttgttataatcttcatagaaaaatttacacatttactatatatgctatc

tcagctattttcctcctgcacgataactattgtaggaaagaatctttcttccgttcaatc

tttacatgcataactatattgttgcaactggaacataaatgcaccatcagttgaatttat

attgtggattcatagaca

>fava_dn_rep_c1102

tttggcggtgtataacagcgaagttctataatcattttttctccaacgcagttttaaact

tactctactctctctctatattcaacgccgccgttttggaacttctcttcgctctttctg

tttccaaaaaggggcaaattattaatcacgcggaaataaaataatcgattttgccaccaa

tttttagctcccgaaatcggatctcaggagggaatttcttttgagaataaagaaacagaa

cgcagtccttgtctatactgctcggaattagttgaatggcggataaatcatgcatcaagc

gtcttcagaaggagtatagagccctttgtaaagagccagtttcccatattgtggcccgtc

cttcaccaagtgatattcttgagtggcactacgtattggaaggaagtgagggaacacctt

tctcaggtggtttttactatggaaaaatcaagtttcctccagaatatccatataaacctc

ctggaatcagcatgacaacacctaatgggcggttcatgacacagaaaaagatctgtttat

caatgagtgattttcatcctgaaagttggaatccaatgtggtctgcatcaagcatactta

ccgggcttctttcatttatgatggacaccagtccaaccaccggcagtgtaaacaccacta

ctgccgagaagcagcgtctagcaaaatcttcactcactttcaattgcaagaatgctacat

tcaggaaaatgttccccgagtacgtggagaagtacgaacagaagcagctttctgagcaaa

ctgcttctgagcagcgggtatcatcagacgcacgtcatgataaaagttcaaattctattt

ccgaaaggaattcaaactccacaggagaagacacgaaaggggtagagggattaaaggaca

tgaggagaaacagaaaacaggcatttccaacatgggtgatgttattactcttctccatct

ttggggttgttatggcattacctctacttcagctgtgaaacttttatgggaataagttat

atcgataaaaggttttcatggagctgcttgttcttatgttctgcttaggttatgtcatat

ctaacaggatttgtataggaaatattggacatggagggtgtgttaaagatatgattttac

tccttgtgtctatgttttgccagtttcttggtattgatacttgtcatgttatgtgtactc

ttcaaatgttgaagcttgtcttctagtcttggttggtaacacgctgctttgctacggact

gcattctcatatgtcatgttaattcaagttgatgttgcgaccatctacttgatttttttt

tcatttcataaaaatagattctttaacgttgtatacatcccaacatggatcattacaaag

gttgccaccacattggcctcttttcaaaacattttcaaacccccacacaaagcaccatta

caagcaatggaaaggacaaggcaagggatctaggcaagtaacgaaggacccatgggtaac

cagggatagaaagggtgataataccttccctttctataatctaccccccgaacccaa

>fava_dn_rep_c1103

gccgcgggacatcagagctgcatcttggtttataactataagcaatacgttctttgaaag

aaacttctcgatcatgtctatcatttttctgcttgttgctatactggccattggactaga

atttacaggtgtgcaatccgtaggagtttgctatggcgtgaatggcaataatctaccatc

aaggccagaagttatagatttatacaaatcaaaaggtattggtggaatgcgtatatacta

tccagacgaagaagccctacaagcacttagaggttccaacattgaattgattcttgatgt

gcctagggataccctttcttctctcactgatgccaatcaagccacaaattgggtaaccaa

atatgttacaccctactcacaagatgttaaaatcaagtacatcactgttggaaatgaaat

ccaccctgattacaatgaggcccaatacgttctcactgcattgcaaaacattcaaaatgc

aatttcagcagccaatttacaaggccaaatcaaggtctctatagcaatagacatgactct

gattggtaactcctatccacccaaggatggtgttttcactgatcaagcaaagtcatatat

acaaccaataatcaacttcctagtgaaaaatggatcaccacttcttgcgaatgtgtatcc

gtactttgcttatattggaaatgaacaagccattcatcttgattatgctctttttaatca

acaaggaaacaacgacgctggttaccaaaatctgtttgatgcgcagttagattcagtata

tgctgctcttgggcaagttgggggatccaatttgcagatagttgtgtctgagagtgggtg

gccatctgctggtggagatggagccacaactgcaggcaatgctgccacatattataataa

tttgatcaatcacgtcaagagtgggaacgggactccgttgaaacctggtgtggctattga

gacttacttgttcgccatgtttgacgaaaatcagaagactggtgcatcgactgagcaaca

ttttggtctctttaaccctgacaaatcacctaagtatcaaataagtttcaattaattaaa

cagacaattcaaagaaatatgtaactgaataaacgaataattgtgccaatgtgcacatga

tgttgtgtttgcatgttcctatttatataaattgaattaaataaatgaaagatcatatct

tgtttaaaaaaaaaaagttgtttgtgtaatagtgactaaggtactatggattttgatcat

gtgcagttttaaaaggggttgc

>fava_dn_rep_c1104

ataaatactagacacatctccactcataaatcacacagctactaaagcatcttctaagtt

aatattaatttgctagcattataattcatcactatcatgggtgttttcaaatttgaggat

gaaaccacttctactgtagcacccgcaatactctacaaagctcttgtgattgatgttgac

actcttactccaaaggtcgttgatgtcatcaaaagtattgaaatcgttgaaggaaacggc

ggtgctggatctataaaaaaagtaacttatgttgaagatggtgaaaccaagcatgtgttg

cacaaaatagagttagtagatgttactaactgggttcacaactacagtatagttggtggt

gatgggcttccagacacaattgagaagatctcattcgaggccaaattatctgaaggccta

aatggaggatctgttggaaagttgagtgtgacatacttcacaaaaggtgatgatgctcct

agtgaagagcaactcaagaaggacaaagctaagggtgatggtcttttcaaggctctcgag

ggatatattttggctaatcctgattacaactaagtcatacaattatcaactgcttgtttg

ttttatatatggtgcggtttgagttgtgacacattatactatacatcaagtgtgtttgat

ttgtcttcctttttaattttctctccttttttttttttcaagttccaaattcaagtcata

ataataaatatcagcaatcaaacaagccttcaacccaaggaaggagatccaaaaactgaa

aatacctagtctagtaaataaaacaactcaatctgaaattcggataaagccagtgccgga

aagataaatagtcattcaccagggaagtaaatcccagcagcataaataacttaagaaaca

aggctgtcattaagtgacaatgaagacaactgatcagcagcaccgccagcctgctgctgt

gcaacattcctaaggacatccattgcctctgccacctttgctttcagggcttctggtgac

tcaagtaagtgcaaaacttcagtctggtccatctccagaagcatgccagtaactttggca

gcattgtcaggctccaactgttccacaagcgggtaaagattctcacccagcatcgtcctc

tgctgctctggagatgcattagccagatgagaagccaaagcaccaatgggaatttgctgt

gaaagtgatgcatcacgaaggggcatcccacccacatcataaggaacagaatacatgcct

ccagccactccagccatgggaccatcaggcatgcccctaccagaaggatagcgatacata

cgccccctaggaagcatctgctgtggcataagaggaactggctgctgggactgctggact

gcacctccacgtcttccaccaggacgctgcccttgctgaaccattggcacaaagtaattt

ggcactggacccccaccaggcctcattccaggcacaagttgttgttgatacccaaatcca

ggctgggaaggaatgatggcaggagggccttggccataaaatatttgttgacccatacct

ggaccaccagggggatacataggcatgcgaggagcaacagattggggcatcgcaacagga

cgcatttgagcaaattgagcctgcagcctagctcttctatcttctttccgctgggccaga

gtcacatacagaggtttgcttaccaccattttgccattcatctccaagagtgctctagat

gcctcctcaggagtagagaatgcaacaaacccagatcctcgacttatgccattggggtct

ctcattaccttgcaagaggtaattgtaccataagaggagaacagctccttaagtttgtca

tcaacaatgctatcatccaaatttttgacatacaggtttgccccttgatatttatcagcg

gcttccttcatgctctgctcaaatttttgtttcagttcatgctccctttcagatttcttc

tgagcttttccaacataccattccttgtcatcaattttctttccattaagagcctcaaca

gccctggcagcatcatcggtactctcaaagtttacaaatccaaaacactttgatttccca

tccccatccctcattactacagcactggtaattgttccaaattctccaaaaatctgcttc

aactcatcatcggtggttgtttccgatagattcttaacaaaaacattgttgaattttgcc

ctgtcagctgtagtctctctctcttgcttgcgaaggaagggtcccacataaacttgctta

tcattcaacagcataccattaagcttttcaatagctttttgagcagcctcctcactgtca

aattgaacaaagccgtaaccttttgattggccagatccatcaacagctaccttgcaagaa

aggatactcccaaatgtagaaaaggtatcatgcaatgccttatggtcaattgccttgtcc

aaattcttaataaatatatttccttgaccacttttgcggatgctgggatcacggtgtgaa

tacataatacgaatgggcctgttgttcaaaggagtgaagttcagaacatccaatgctctg

gcagcatcttgagggttgctgtagttcacataaccataaccaagtgacctcctagtggtc

aaatccctgcaaaccctaacagacacaacctgacccaattgattaaaaaggtcataaagc

tgagaatccgtaacattgacatcaagatctccaacgtaaagcgaagtcgtcacaaactga

ttcccaccagccgcagccgcagccgctgctgccgccgcagcgccaccgttaggacccgtc

atcgcattctgaggctgaacctgaacctgagccatctcacttcctcaacaacaaaaaaac

gatcttttcccaaaaaaaa

>fava_dn_rep_c1105

taagatggtgaaataatattaagccacaatcactaattcaccaacaaacaacagccacaa

aaaatacaagataaaaacattatgggtactgaaaatgtacaagatagaaaacattatgtt

cttgttcatggtgctggccatggagcttggtgttggtacaaggtaaagccaatgatagaa

tcttctggtcacttggtcactgtaattgaccttgcagcttctggaatcaacttgaagaaa

atcgaagatgttaatacaatttcagagtattctgaacctttgttggagttaatggccgca

attcctcctaatgaaaaggtgattcttgtgggtcatagccttggaggatttaacatagca

cttgccatggaacgatttccagaaaaaattgctgttgctgttttcttaactgcttttgct

cctgatgttcaacataaaccatcatatgtcatggaaaagtatcttgagagcatccctacg

gctgattggttggacactgaattcttccagagtggaaacaaaagagcaatattgtttggt

tccaaatttttatctgatagactctatccacgctcttccccggaggatcttgaattggcc

aagagtttagtaaggtcatcgtccctctttatggaagacttgactaagcaaaacaagtta

ttctccaaacaaggttatgggtcagttccaactacttttattgtttgcagtggagacggt

gcaattccattgaaatttcaactctggatgatccaaaatgctggcattaatgatgtattc

gagatcaatggctcagatcatatggctatgatttctaagccacaacaactctgtgattct

ctctatcagattgctactaaatacgcatgaagtcatggaatagtctttgacattatttat

tggatttgaattttatttgagttcgaataaagggaatgtggagaagtggctcgcgttgca

acttcagcttatttctccatgcaccatgtatgttatatacaacttacttcatatgtttat

ggataatacaaggttatgtcgatgtatataaacttacgaaatatttaaatgttcctttgt

taaaatattttatgctccttttgtttttatgaaataaaattgttcatttttgttactaaa

aaaaaattc

>fava_dn_rep_c1106

ttaggccgcgggatccattttttttctctccattctaccctttctttgtttttctattca

ttgcaatttctgaaggagaacatgaccattctcgttgagcaaattccctttgggtcacaa

tttgatgataccaaaaatgtgcattctgaaaacaataccaatgaacttgttcttgatggt

ggatttcatcaacctcaaaatgattctcagaattcattgtttctcgctcctgaaatcaat

tcatttggccataccttcagaaattatgatgaagaaagtgaaaggcaaaaaggggttgag

gaattttatagattgcaacatatcaatcaaacatatgatttcgtgaagaaaatgagggag

gaatacaagaaactagacaaagctgaaatgagtatatgggaatgttgtgagttgttgaat

gatgttgtggatgaaagtgatcctgatttggatgaacctcaaattcagcatttgctgcaa

tctgctgaagctattaggaaagattatcctaatgaagattggttacatttgacagctctc

attcatgatcttggaaaaattcttctgctgccgaaattcggtgaactacctcaatgggct

gttgttggagacacatttcctgttggttgtgcttttgatgaatcaaatgttcatcacaag

tatttcaaggacaaccctgatgtcaaaaattctgcttataacactaaaaatggggtctac

accgaaggatgtggactagacaatgtgatgatgtcatggggacatgatgattatatgtgt

ttggttgctgaggaaaatggcgcgactttgccaaaagcaggattgttcatcatcagatac

cattccttttaccctttgcataaggaaaatgcttacactcacttgatgaatgaacaagat

tttgagaatttgaagtggctccatgtatttaacaaatatgatctctacagcaaaagcaaa

gttcttgttgacgttgaagaagtgaagccgtattatctctcgcttatcgagaagtatttt

cctgcaaagctgaggtggtgatgagtgaacattaaagatactacttaagatcaagggttt

gaaggaaacatatactcatttgttctggctagtgattttcaaaataattaagggattaaa

gggttaatcatttttttttcatgtagtttcaataagaaatacatatgtttgtattgtaat

gcagtttgtattatgttgcaatcaaataaacaattgatttcataaatcaataaaaagaat

acattatttcttattatttttatggtcgtatttatttgtatgcatataattgtgtaacca

accactatcatagaaactattccgttaatttacaaaaataaaaaaaatcgtggttaagca

cccattatgtaggagtgatattatgatgtaaatgtaagcttgcagcattgtttatttaag

gcaccgtatttgtggttctacacaaaaaaaagaaagtgactagagttgtgttgaataaga

gagctcgacacagagaacagttgagaaaagaagctgaagcaaagaggttgaagaatttct

cgaaagaaattgatagcatcccaaaaatctttgcggaaattaagaatgaagacgaggaaa

aaaagcgaagacttttgaggcgacaaactgctaaacaagagaagttaaaagtatgtccac

ctcgcataggaaagcacaagtttcagcctgctcctactcaagttttattaagtgaagaga

taactggatccatccgtaggctcaagggttgttgcacccttataaaggaccggtataaaa

gcctagagaagagagggttgattgttcccaaacccagaaggaacgggtagagtcctcttc

gatgttggcttacgtaacttaagtcggcccccaaattcagcatggcttgcttctatgtgg

caagaagatatgtgatggataacaggataaaacagtttttttaactgaaatttttgtcta

tgacatggttgttgtctcaattttgtacgaattttcagttgccagaaaatcaatatctaa

tgtattttgtatttgatatttcgcatactattatactggaagttaatggttagagcttca

acatgtagaagattagcaatctttcttttagttcaagtgtttttggagtttattcatcat

cacattttagaaaaatccatctttctattgattttgct

>fava_dn_rep_c1107

agagtgaattaaaaattgtatcgggagttgggaaaatgggtataaataaggaagtgcgtg

tcggtgccgcgggagtcgataatgagcagcagcgtaggaaaggttgtgtgcgtgaccggt

gcttccggttacatcgcttcatggatcgtcaaatttcttctccgcggtggttacaccgtt

aaaggcaccgtgagagacccaaatgatccgaaaaagatcgaccacttgcttaaactggac

ggcgctaaggagagactgcaactgttcaaagcaaatctactcgaagaaggttctttcgat

tctgttgttgaaggctgttatggtgtttttcataccgcatctcccttctatcacgatgtg

aaagatcctgaggctgagttgattgatcctgcgttgaaggggactctgaatgttctgaaa

tcatgtgctaaatcaccatctctcaagcgtgttgtcttaacatcttctatggctgctgtt

gcatacaacggaaagccacgtactcctgacgttgttgttgatgacacttggtttaccgat

cctcatctcgctagggaatctaacctctggtatgtgctttcaaagacattggctgaagat

gctgcttggaaatttgttaaacaaaacaacattgacatggttactattaacccagccatg

gtcataggccctctcttgcaaccactcctaaacacaagtgctgctgcaattctaaacctc

ataaatggtgcagagacatatccaaatgctgcttttggatgggtcaatgtgaaagatgtt

gcaaatgcccatattctggcgtatgagaatgcttctgctagtggaagacattgtctggtt

gagagagtcgtcaaccactcagaaattgtcaagattctaagtcaactgtacccgtcatta

caactcccagagaagtgtgcagatgataagccgaatgtgcctatatatcaggtttccaaa

gaaaaagcgaagagcttgggcatcgaatacattcctttggaagtgagcatcaaagagact

gtggaaagtttgaaagaaaagaagtttgcgaagctttaaattgtggaatcatgaatgttt

agtttatgtttttaatgcctatatatcatgaatgagttatatgaaggtggtaggtaatcc

aatattgaacagaccttgcttagtatcttgcttatttttttccacccattttatcaaaac

ctatgttttttatttttaactttctgctatggtacttggtagtaattattgttttatcat

taagtttatggatgtaattgatagttaacttttattatgttttccttgcttagttgctta

gatagtaataggaaccaagtccttttgtattaattgttggcgcaatggaaagtttatgtt

atttatgcaatgaatcaaagcaagacaagattagaaac

>fava_dn_rep_c1108

tttttttttttaaaataaactctagcacgtttaaatagtcttttcaacaaagactcgaac

caacttcgtatcatgattaggtttcaaataaattgacaacagatggaaaatacaaccaat

gtcaaggtaaacataattcatagcaatacacaaactaagattagttcttcctcttcttct

gcttaatgctttagaaagcatttgttggggatatgcaaggcatctggattcttccagata

acaaataagaaaggacgcttcatatgatcagagttcatcatgataatcgtccatgtaatc

acggcgatgctttctatatctgtctctatgtctgttccggtgtcgatcataacctctatc

ttttctcctcctttcaccttcttctcttgctctttgagcctcctcctcttcttgggcttt

ctttattttctctgcttcctcaaaaccctccttttcaatctctctgttggcaaaaacttc

atcaacaacttgttttgcataatcaggatcatcacaaatcaaaatgttgtcaaataccga

accacctttaacctgccaaacttctatgccgacatatttgataggcttaagcacataaag

atcggggtcatcttcgaactctggattatctaaccatggagttttccatttgcctttgta

attaggattcttgattttctttcgtttccatggtcctttgtatgctggatttgggatctt

cggccgtttccatattccatcttcatcttcatcccaactatcaggctctttagccttcgg

gtcaggaatttctgctgggattgaatcatatccctcaggcttgaccgaattagggtcttc

aatgtattctctgtcatcccagtctgcaggcttttttgccttgacgtctttgatttttcg

tggagggagaatatcccaatctgtatacatgctaccggattccctctctctattatcaac

caggacgctgtatgtagcatccggcctgaggatgaatgtgtagaaatgagtcaacttgtc

tgtttcacactgcaggtccttctttatcgggtaattttgaccctggtacgagactatgac

atggagcttcttcgtgtccgtgccgcataaatctggtccaaacattacactataaggtgt

atccccaccaaacttcttttgattaacaaatccagagaggagtttcatgtaacctcctcc

acactcaatctcttgttcgaattttatagagtactgaaaaaccagtgttcggttcttgtt

agtgaattccggtatcttcgcagagatggcaaagtgctttgcgtcattagatgtttggat

acctttatcatcgggatctccagcccatttcccagccgtgtgcttgaacgaaccggcttt

gccttcgcttcttttccagtccgattttacccaccgactttgccatccatcttcgaatcg

ctcttcaaaaatgatttctgaaaaggaaatttgaactagcaacaaacagaataacataaa

catcttgagatcagttgaaacattttccgccatttgcggaataagctatttttgctctat

cactcccgcggcgtaa

>fava_dn_rep_c1109

cgcactcacaaaatactcacttctcttctcatccttattattatttgtttaattcactcc

cagaaaaatgtctgtaataaaaatatcactattgtctgtctttactctctgcttgatatc

tgcacaggcagcaagatttgacatcgttaaccaatgctcttacactgtctggccagctgc

cactcccagcggcggtggtaggcagttaaactccggccaaacatggagtatagacatccc

agctggaacctcatcaggccgagtctggggccgaactggttgcaactttgacggttcagg

ccgtggtaattgtcagaccggtggctgcggtggggtcatcagttgcagcttatccggtca

acctccactcacacttgctgaatttacccttaacggtggaaacaacctagattactttga

tttatctgtgattgatggtttcaacattccaatgcaatttgctccaacctctaatgggtg

caacaatgtaagaacttgcagacaatcttcttgccctgatgcttatcagtatccgagtga

taatactaaaacagtttcatgcccaggtggtaccaactatagagtcgtcttctgcccttg

aaacttatatcaaacctatcaatggataaataaataaataaacatttctatatatatagc

tcaacctcccttttatgtttctttctaccgcacgtaaacgaaatgtaatattttgtctac

actatgtgtagtatattcacaacaatattgcttaattatgacttgaaattatttataaat

aatttctcgccaatgcaatattgtgaaataaataaatgagagcttatatggatccagtca

ctttctttgtcgaacttgtttctttttatgtttaattatattttttgaatattttatttt

ttgagatttgtat

>fava_dn_rep_c1110

ttacggccgcgggagaaaatcagataatgtgatatggcctctcttggattctcactctac

ccaccgctcaaattagggtttccttccaaacctcaaatccttcccatctccactctcaac

ttctcttccaccttcagatgcattggggctagaagattgcaaataaatcaaaagaaattg

actgtatctgctacaaactccaactctagaggtggaaaagcatctaatgaagggtcagat

gtaaaggaaactagcaatgcttcaaagggacctcctcttcttactattttgtttggtttg

ttggtatttttcattgctgttcggtgcattgggttcattgtaatgtggttggtaggtttg

attgttaatgtgccacctccaaaataaaaacatgtttcttgtaatgtcttgttgtaatgt

tttcttgtttatatgttaaactattaggatcctggttcaccatgtgttagtgtcactgca

gtgacacttcagatgcttttagggactctaataattgagttacttgctccatttgtgcag

aaatgattgttacaatggttggtatgaaaattaccttttcgatgcttaaatctatcactg

gctgattttatctaaaaaaaaaa

>fava_dn_rep_c1111

gtagaggagcttccaagtaacatcttcaaagataaagcaaaatgaaaannnnnnnnnnnn

nnnntggtatcaagacagagtaacaagagtgagtggtgaggtcagagaagaagaataaga

gagagaaagaagaagaagaagagagagagagagagagagagagtgagagagagagagatc

aaggaagaagaagaagaaagatgggtggaggaatggaagcaaacaagaacaaatacatag

aagaatggggagctgttagagagaatctggaacacaatttccgttggactcgccgcaatc

ttctcctcgttggtatcttcggtctcgctgttcccgtactcgtctacaaaggcatcgtca

aggaattccatatgcaagatgaagataacggcagaccatacaggaagttcaagccttaaa

gtatgtaagatgttttgtaataatcaggggttatgaaagttagaccttaatgaggctaca

acatgtgttttttcttaactgtccaatggccgtttgtttatgtagtttggttctatcaaa

ccattccttgtttggctgtcttcattgatattgctgtgatgttaaattgctactgttcaa

aatcaataatatatttggaaaacaaagcatgagtttgagaaaatattacattgacaacta

taagattgtatttgctaggttaaaagctaatggtgttactcaaactctattatattcctt

gcagtaactttcattagatgtttttgttgtattactcatattagcaatgaaatgctttga

cttttattctgtaaaaaacaagtaaatgttacaagaagtcatgtacatggaaacaattgt

aaccatgaaatgaatgataaatcaaagagtttcattaaaatgttaggacaagcctgtcac

aaatttttaa

>fava_dn_rep_c1112

aactagaagaataaagaaaaacttcaccggaaaccctttataaaagcaagacaaagccgt

gtctttgtggaaccaccgttaacacaaacgcaagagaaacaagaagagaaaaaaaccatt

gagaacatattcgtctaaactcacatatattatacattagagtagaataagagaaagtga

gaaacttccactcacaataagcattgaattctaaattctaaattccgaaaccaacatggc

aaacagttactttgcaaaaagaaaccacctctttctttcggtttcaagtgacagagactc

ttctctatcattaacaacggattcagattccatttttgagttccaagaatcagacattta

caattccgaccatgcttactcggcggagttttgcaaatctctccatggctctcgctcggt

aaagaaaccgtcttcatcgaagacaaaggacgccggaggaactgcagcgtccgtgccagt

aaacatccccgattggtcgaagatcctcggggacgagtacacgaagagaattaagatgga

aatggagggagaggatgaagacgagagtgggtggttaccaccgcatgagtttttggcgag

gaagagagtggcttctttgtcagtacaggagggttttggaaggactcttaagggaagaga

tcttagtaggctcaggaatgctatttgggcaaaaactgggttccaataatttaactgtct

tttgtttcttttcttttgttctactgtattacatgtgttttttgttttgttttgattttg

gtaagtgtaacttcgcatgagatatatgtgttgtgattttggtgaaagatatatctactc

tctaggtttagtagagtaaaatacacgtgtgttataaggtttcagttattaattcgaaac

ctggtgtttgtaatgtcctcgagaatttcttcaacattattaatgtcagtatttgttttg

gaagaaatgattagatcataacaaattaacaatgaccaggcaatgctctcttattgtgtt

tttaattaataatttacacggcaagtggatcagctgataaggttaaggagctgtatttcg

agtttcttcggtaggagatcgcagcggttcactcgcctctttctctcttcaacgacgtcg

tttctcttcgccggaaaaaatgtcagggaaaggagcaaaaggtctgataacagcaaacag

agacaaggacaaagataaagataagaagaaatcaatttctcgttcttctcgtgctggttt

gcagtttccggttgggcggatccaccgtcttttgaagacgaggacaactgctcatggaag

agtcggggcaacggctgcggtttactcggccgcgatcttggagtatctgactgctgaagt

gttggaattggctgggaatgctagcaaggatctcaaggttaaaaggattactccgagaca

tttgcagcttgcgatccgaggggatgaagaactggatactctgataaaggggactattgc

tggggaggagtaattcctcatattcataaatctcttatcaacaagtctaccaaggagtag

attcttataactgatggaattgctattgtggttgcaggatagaaaatttatgttactact

gattatggatttgttgttgggtacaatttggtttaatatgtagaacctctaatggttaag

aatgtcagttgattgtgtataaacttgatattgtttttcagcttgattaatatggtatta

atttcaattatgctaaaaaaaaaaag

>fava_dn_rep_c1113

aatacatggatgactgagattggagctgcatgtaaaacatgagagggagcaggagaggtt

ctgtatccatgatacnnnnnnnnnnnnnnnnntaaattcggagggaccaatttgcaattt

agataatatatatcgagaccatgttcatctcaaatcaaccatctaactaaatataactat

atcaaatggagaacgctggcggttcacactacaccactcatatttccgattcaaacaagt

ctcaacccaaaatgcattagaccgttgtttacagtccataggaatatatttattagtaca

cgtgcatcatagatgaaaccattacagtcataacaatttactacataaggtagaacacag

aattgtggcatatctcagctagtaatgaattacctagctggacaagtactttgtcatcca

tgattggaagcgagaccacgccagctgaactgaagcttcatcttcgtcaggcaatcccat

gttcttcctcctagttattccttctggagacctgttcatgaatgcatgagtgttgccagg

gtatatgtatacctcgtttggaactccggatgcctttagcttctcctccaaagcctttgc

agccgtaacatctgaaaagccaacaaaattgtccagctctccaaagtgagcctgaacagg

agccttggccttagcagggtctgcaagttcagaagaaggaactccatagaacgccacaac

agcatcaacatgtggaaccagcacagagcttgcaattgcgagagcaccccccatacaaaa

tccagttacaccaaccttctttgatccatttgatttaagccagtcgactgaagcatggat

atccttcacagcacctggccaatcaagaccgtcaaacaaatgctgtgcttctgcaacatc

cagaccaacctttcctctgtacagatctgggataagagctttaaatccaagactaagttg

agaaatcaaggcagcatggtttttaatttcaaaatccacaccccaccactcttggagcac

aacaattccaggagcatcttgtttgccaatgacatatgcatcaaatgttgtgttgtcatc

tctttgaatctggattttgttaaaaggggcagagtcagccatggaacgaactgggaagcg

agaagcaaagggtgaaatggtccgaagagggtttgtggcaaagaatttgggcgcagatga

tgttacaaacctcaacatttctcacttaaaacactctgtgatcttatatgcaaactggta

caggttctcgagtgaattaattaactcaatggtgttaataattaatttattttctaatta

tatattttttttttcactgtcggtatagtctggggttaattctgacaatcaagtgttttc

aacttc

>fava_dn_rep_c1114

atgatagaatgacagatagagtgatgtgaatcaatcataaccaaaaaagaaagcaagaaa

atagtcagtaactactttttcttcatcttctccttagatccaacaaaagactttgtaatc

aaatggcaaaaaaagctgtgttgattgggatcaactatcctggaacaaaagctgagttaa

gaggatgtgtcaacgatgtctggaggatgcacaaatgcttgattgaacgatacggatttt

ccgaagacgacatcaccgttttgatcgacaccgattcctcgtacaccgagcccacgggaa

agaacatacgccgtgtgctgtcggagttgatccgatccgcggaggatggagatgtgctgt

tcgttcattatagtggacatggcacgcgtcttccggctgaaactggagaggatgatgata

ctggttttgatgaatgtatcgttccttctgatatgaatctcatcactgatgatgatttca

aggaatttgtggatgggattcctagaggatgtaggctgacgattgtatctgattcttgtc

atagtggtggcttgattgaagaagctaaggagcagataggagagagcacaaaaggagaag

gagaagaggaaaaatctggatctggctttggattctcaagctttctgcataggaaagttg

aggatgccattgagtccaggggatttcacgtcccttcgggattgcgccatcataaagaca

ggggcgaagaagatgaagaagctgaagatgggaacagcgaactcccgcatgggaactatg

gttatgtaaagaataagtctctgccactctcaaccctcattgagatactcaagcagaaaa

ctggaaaagatgatatagatgttgggaaactgagacctaccctgtttgatgtttttgggg

aagatgctagccctaaagtgaagaagtttatgaaggttatcctcaacaaactccaacaag

gaggtggtgaaggtgaacatggtgggatactgggaatggtgggcagtcttgcccaacagt

ttctcaagcaaaagctggatgagaatgatgagggatatgcaaaacctgccatggaaacaa

aagtgggaagtaagcatgaagtatatgctggatcatcaaagcgcggtatgccaagtggtg

ggattctgatgagcggctgtcagacagaccagacttcagcagatgcttgtcctgccggaa

atgcagctaatgcttatggagctttcagtaatgcaatacaagctataattgaggagactg

atggttcagttacatattcagagcttgttcacaaggctagacagaagctacagaaggccg

gtttcactcaaaagcctggactctactgcagtgatcaccatgttaatgaaccattcgtgt

gctgataagttctgatgcagacaaagagaataatgagtgttcacgtcccgtggctgcaaa

atttgcattgtattttttttaacaaacttggcaattgagattcgtgtgtttctatgtgtg

tttttatgatacagttgtattactgtgattggataatcagtgttgctctaaaggaatatt

tacttagataaaaaaaaatataaaaagagaatatgtatttcaactattatattaataatc

attgatgaatttgatacaaattggagtcagactgactggattatgtgatatagcagccta

ttcagactggactatttaatacagcctagttatctgctgtttcagtgaattatctttaca

aatatacaaaattgaattatatgctaaaatgggagataaaaattgataactaaatgagac

tgcacaattgtgttgaatggcacagaggatgagtctttcctcccaaaaaaatcgggcatc

aaggttgagccttttcctaaaacattaattgcttggtgacttgcagaggacactgaataa

tggaaacgtcaagcaactatcaaaaggcttcaaactttgttataaagaaaatgtgaggct

tcaaactttgttaaaacaaaaatgtgagcaaagtctattatcacacgtcaatcatcaatt

tctttaacccatgctaaaccttgaagaacagtttcaacagctgcattaggaacaagaagc

ccaacaattcgttttttatccacggtagtagtttctatgcgaacaactcggagcctccta

tggcttaggcgggcctgcttagaaagggttttttctattgttccccaaacaggaagaatg

aggccacccaagacatttacttcctgtagtcttcttccgactgtacagaagctgccaatc

ttacaattgggaccgtgcatgcactgtttagatgaaacttcatattcctcttcccaacca

gtttgagccttttctattgacgaaacttttctgtatttacttttcaattcagatagaggc

atctcccggtttgattccccaacgggaggacggactatcttatacatacccgaagcagaa

cattcaaacgctaaaatgaaatgacgttttcccaaccattccctcttggactcatagaag

ccatcattggctgagccaagcccatctttctgtttttcattcagcatgttacttgctgac

tcccacgtgatcccccgatccaagatgaatgtaaacagaacggttgaagccgcagtcaat

tgatcaatatgaactgtctttggagtcccttgtagttctatgacattagctttcaagtca

actattcctgtgtcgaggttaccttcaatgcgtgcattccgaacgagaagatccaagata

ctcacaaataactcaaatagcccgttctgaatatcaggaggtagccccaaaagccggttg

aggaatcgtccaacttcgtgcatatctgaatcaataattcgaccagataaccttccagga

tctttaccgtttccaagaactgtgtccctaactatcccaacagatacaagagcagctttt

gcctgcataatgaaatcttgaatagtatctggtctctcagatgaacatcccggaggaaca

acaggtagagaatcctgctccattattcctttgtacataatcaccagagccctctttcca

taagcactatcataattataagcacttaaagaaggcccagccctgcgg

>fava_dn_rep_c1115

tttttttttaatgaaaaataactatgattttattaatattaatacatgaaatcaaaattc

caaaacatggacacgagtttatctgtgacaattcatagtatgatgaatccaaacattaat

tgaaaatcaagaaagttaaataaaaacacaaacacaaacacaaacaaaaaaccatgacat

gaatccttgaaaccaagaactagtacttaggacttaggagcaaagttagacttgattttg

tgaacctctttggtaccaatttggaaagcttgagtcaagacattatctggaacaggtggt

gtagcattaaacaaagtaagaggaatattcactgtccctggcaattgactattaaaccca

gcaataacagtagcaggttcccaaccattgttcttctgaaaatgaaccaaacctttaggg

aacacaaatgtttcacctttagcaatggtttttgcaatcaattgattcgttgttgttatg

aacccaacgtctagttgaccttcaagaacaaacaccatttctgttgcacgagggtgcgtg

tgaggcgggttgagtccaccgggcgcgtagtcgattctcgccattgaaacaccaagagtg

tttagtcctgggattttctgaacgttggctcctgttactaatgaaccgaatgtgttgttt

gttgcaccttgtttgactagtaggtttgagaagaaatcttctgcgcttactttggatgct

ggtttgcatgtgaatccgttcactaaaatcgcagaagcatagtctgcaacacatagatct

tgaagagcatcggcatctgctgcggtggcggtggaggcggtggctaacaccacaagcaac

aacgctccgatgagtttcatcatagatttaatttgaaggagaaattttgatgctaagttc

ccccgcctacctttgtaacatttactcttatgttttgccgtgttttgaaattctnnnnnt

tatattgc

>fava_dn_rep_c1116

aatataaaattgaagtttgcaaatttaaaacgtaaaaggatagtttggtagttgaagagg

gatgcagataggatatcaataagataaccgttcgagggagtaaggatagtgttattttag

agagtttatttttcatttcccattcaattatctcaacttatccatttttctaactgtaca

tggcagtttcaatggcggctggactattctctgcttccactctaactctccgcccacaac

tccaacacaagtttccgttttccgcacctcctccttctgtacggatggttccgctccgaa

cagtcacttatgccaccgtctctcaaccacctgccgacggaaaaattcctggtttaatga

aacccaaaaaatctcccccgagatgcaagacctcatcggacttcccgaggtttctcgaac

ccaagcgctcaagcatatttgggcctacatcaaggaaaacaatcttcagaaccctggaaa

caagaggcttattcggtgtgatgagaagctgaagaagatatttgcagggcgtgatgaagt

tggtatgcttgagattgctggtttgattagtcctcacttccttaaatgatggccatgttt

tggtgtatggaattatgattttaagcttttacaatgagtccttcaacatagtagccttag

tttttagttttggttagtatttggaagaacaagcttatgttgaagattttgttttgaatc

atgtaagttcttcagcacaaacccgtagtagctatgtgttaggtatttattggacatgtt

attgctatctatgttctccttataactaaaattctgcgcattgtgcttatgaaaccagta

gtttggactttgattctacaataaagcttctgcagggtagagcaggcaacatttccagag

cctgataaatttccatggttgagggaccgttaactccaattcattagttttggcagaaac

ttgatagtaatttgaaaagacctagaaacttgctttaaaatgtagggatggtgaaggaac

ttcctcatatgaagcttatctcattacaaactagcgaataaagtgacatttaaagactaa

tcactgaattgttgtgttgggcttcaagggagagtacaaatgaataannnnnnnnnnnnn

nnaagcatgttcattcaactttaaaaatggtttagattttagaatctgtatataccattc

gaattttggaatttggaaacatcagaaacatcatgca

>fava_dn_rep_c1117

tattgtataatctttaatcaaaagatgaaaatagtttacaatgtactagtcgatgatatt

attattgtaataaaggaaacaatttacgtagatgatatccaaaaaaaaatagaaagagtt

tattagcaagacgtttaagcattcattaattgaaaagtgaaacattacaaagtgtggtat

tggaatttgactttatgtaggccaatttctataccaaccagcccatgcagacaccaatcc

actaataccaagagaaagagtgtgattacagaaaggatttggttcaatttcagccaatcc

tatgatcaaatctgcaacattagcagccacagccataagcctcattattctatcaccttt

tattttctgaatctctttctcagtttcttctgatttttcttttccctctgaacttttaag

ctttctctcagctttaaacccttctttcaacataatgaaatcagctataatgaagaaaac

ataacccaaagactcaccaaaagctgatatgaaactcatcttctttgctaactttgcatc

caaagttccaattcttgacagccaaagaaaatggtcaaaaaagaaatacaccatttctcc

tgaattagaaagcactgccagtagcctcaaagtgggagtagaaccaggatttcttctaag

agtgttgaatccagtgagaaatcttccagttctgaaggcttttctgctgaggccagcagc

tacttcccaatttttgaacctcttggatatgtcagggtgagtggcttccacgcgccagtt

tgcaagcttggagacatattgaaatgtcttcacaagcttgtctatgccatctctttttgc

taagaagattaccaatttgtccactgtatcattcatggtgtggaaggattagtttaggaa

tatggctttggttcatgtaggatatggtctcgttgtgtttatttcaaacttgtaagtacc

aaacaatgttttcatgtgttaaactagagtcacaagaactgggttttggat

>fava_dn_rep_c1118

gcttataagttgttgcatattattaatttatatttataatcatgtgaatgttaacaaatt

gaaaaataaattcaattttataaatgataatttagtagacgtgtaatgtgaacccaaaca

gatcccgtgcgatagcacgggtttcttactagttgttataaaaataaaagaacttaatta

gaagaaagaaaatataatggaaaaatccaaactatagtaacaaaaaagtgcaattaaaca

tgaaatgatcatgatttggtacataacttagtcaacataagcaagacgagcaccagcgtg

gctgtaaagaaagaccaataaggtgtgttttccattaatctgcataggacaatgtgcacc

acaaacataaaatacaaatcacttccataacttcctcaaagacatgtttttctctgtctc

cttcttcatgaccttggccacagccatctcaaaatcctcctgggtcacatgtaccctcct

ctccctcaaagcaaacattccagcttctgtgcatacagcctttaactcggcaccagatgc

tccattcatcttctcagcaatcttcttcaagtcaatcccacgcattaaattcattcttct

tgaatggattttcaagatatcccggcgagactcttcattagggtttggaaattcaatctt

tctgtcaatccgtccaggtctcaagagtgcttgatctaggatatcaatccgattggtggc

catcaaaaccttgatcttattcgaagcttcaaatccatccaactggtttagaagttccaa

catagtacgctgcacctcactatcaccattgccacttccagattccattcgggctgatcc

aatactgtcaatttcgtccatgaagataattgatggagcatgctccctggccataacgaa

aagttccctgaccattcttgaaccttctccaatgtatttctgaactagttcagaaccaga

caccctgatgaatgtacaatcagtgtgatgtgctactgccctagccaacaatgtttttcc

ggtaccaggcggcccatagagaaggacaccctttggttgagctattccaagactttcaaa

cagctcaggatgtttaattggaagctcaatgacctcttttatttctttgatttgctggtc

taaaccaccaatcatgtcatatgtagaatcaggaactttctcaactttcatcagattgac

caatggatcaactttgcttggaagaactaaatgaagcacataactgtcattgcggagagc

aactctagtagatggcgtgatcttcgtgatgtcaatatttttgtcgatatcaacaacata

tttcccttccggatggaccttgaccaacactttgttcttgcccataactttgacaacttc

accaacatatgagccaggctcttgaagaagctgcagctcttcacgaagcatcctcaccct

agaattgagctcgttacgttgagcctcgagacgattaagattatgagtcttttgacggag

aaggagttgtagctcatggatgtgctgagtatagtactgtcgaagaccctcgccctgctt

ggttagcttggcagagcaatgctcctctggtgcaccatccgcacgcttcacttctactcc

aacagcagccatagatcaagcacttccctctttagaaattcagcgtagattcttgttgtt

gttctcttcttcggtagcgatgagattgtgttgtgagcgtgaaaattgagtcttctcttc

gaatcttgtaaagagaggggtgtgcgtgatgagagggttaagtatcc

>fava_dn_rep_c1119

aatctcaccatctcaattttacatgacaccttaactcatgaacaatttttccacagacca

tttttatatacatatatattacaataaaacactgaattaagattagcttcctttgacacc

aaaaatcttgacttgctctttgaacttgtttttctttgttggaaatgaattatgcaacag

ctgaaatacttcggttgcgcctatctctattgagacaatcaaaaccttcaatcctcactg

cagcttgtgcgtgaccaaacttcattggaacacagcttctagctgaggaagatggtgaag

agggtgccatggaaaatggactaaaatttccatgaccattgctattgccaaattgctcat

cttggatcacagggtttgaagccctgctaggtggagatccacaaaaaaatgggggtggtg

atgatgccatttggtccccaaatctttccgtatagctatttccctgggggagaatgatat

caagaagatcttccctgacacctgaatcttcaagctctgattgatagctgataatttgcg

gcttatagggtctgatatggttgttgttgttaatgaatgagtgattcatcaaaccaaatc

tacgaggcttagggcaaaccacagattccatcctcatctcatcacagcttctcatggcgt

tcttttgctggtaaccacagtaattcatctttctctcaattttcacacctgaaaaccaaa

gatcttgataaccaagaacacctgaggtaagctgaatcagagactcctctaagaggagaa

gaggaagaaaaaaggaccaaagaaaattttgggtctcagaaaacaaagagatggatagag

agaagacagagagagagagagagttcaaagggtattcatagagagaaagtggtaataaaa

aaggggaaaaataaatagtgagggtagttttattctatattaaggtgcatagatggtaat

gtatgaccgcaattatgnnnnnnnnnnttaactattaaaacattggattctata

>fava_dn_rep_c1120

atgtaacaacaggtatcgaacggttaataaccgacgcattgagcattgaaccgtttatca

catcggaagtggttgctgccggataacccacccctgttgctgttgttgcccaaattagga

acgaagatgaagatgagtgtttccaccttcactcatctatcttactcttctccctccatt

tcctctatcttccattctcttccctctacccttatctccaatcctttcccttccaaactc

gccacttcatcaccataccctctcaaaaagatcaaagccttgggaattcaccaaactagt

tccacttcacatggaagcaaacttgatgcagtcaagagtaggatactgctccagatgttt

caaagggcaatgccaatggaaccccttacaaccattttcagacaacaatcccagatgaat

gactttacattggaaaatcttcaagataggaaaaagtcatccaaatgggttgcagcattc

ttgtttggactaactatatcaataatttctcccgatgtttcatatgcaagcagctcagta

aagataaatgagatgtatcaggttggggagttgtttgacttaggaatccaattaatatat

ttgctattgttgttgggcttgctcggggctggaacttactatgtgattcgccaagttctt

gttcgcagagagcttgacctttctgccaaagagttgcaagagcaagtcagaagtggtgat

gcaaatgcgactgagctgtttgaacttggtgcagtgatgctgcggaggaagttttaccca

gctgctaccaagtttttacttcaagcaattgataaatgggatggagaaaatcaggatctt

gcccaggtttacaatgcacttggtgtcagttatgtccgtgacgggaagctagagaaagga

gttgcgcagtttgagacagctgtgaagattcaaccaggctatgtcacggcatggaacaat

cttggtgatgctctcgaaagtaaaaaagaatatgtgtcggctctaaaggcatttgaagaa

gttcttctatttgatcctaacaacaagattgcgcggcccaggagagatgctttgaaggga

cttgttggaatgtccgaaggagttactatcagatatagagagaagaaatgattgacattc

ttctaccaacttgccctataaatatgaatttatctaaaactacacagttttcaatcaata

tcttgatcatgtttgaaggaattttggctatagtgatgtatttgaggtagaatttttctt

cttattttggtagaattatgataacatggtataaactcatctactcagatcatttactca

ttgctaatggcatagttgaggaaaattattgctgggaatgattgaaagacgagtcggtgc

tcaattctaaaatcggctacaaaagtcgttggtctagtgcatcatggtgtcagacttcct

tgttttctttctcttataattggtcattttcatgtgttgtaaaatgacattagcataaac

ttgtcatctgtatcttcatcgatcaaatcatgtttatagaaatcattgatgttcatgttc

tagataagtgaaaggaagttataatgagtgttgctggttcttttttttgttgaaattttt

acttggaagagttatgttggtttgaaatttggataccgaacatggtaaatgaatctactg

agatatagctgtgggattttcatgttcnnnnnnnnnnnnnnnnnnnnnnnnnnnnnnnnn

nnnnnnnnnnnnnnnnnnnnnnnnnnnnnnnnnnnnnnnnnnnnnnnnnnnnnnnnnaaa

ctcttt

>fava_dn_rep_c1121

atctagtagttaaaaatttgccagcgtgtttatttattcgaacatcggttccaagccctt

gttacatctcacgaacacctcaaggcgcactttagcattgtcttcataaaaaagaacaaa

gcgcgtgatctcacgcgctccatcgttcttctcttgcaaccatagaaaaacacacacaca

gactctatatctatctcctctccgattcaccaccgtcgccgtcccttgttcccgatccaa

tttccttggacggaatcctgtgaaaatgatgagacgggatccagtgtttgtgaggaagaa

gcaatcatcggttccatcattggttgatctttgtgttcagaaagctatagataatgtaag

ataccttggaaatgttggtcctgttgatcatcatctgcttgagcaaattttgccgcattg

cacgcttgatcagttgatgcatatcgagaaggccagcgaaggggatttgagccctgtaac

tgataagttgtggaaaaagttcttcgagaagcagtttggttcaaattgcacaaaagaagt

aattaagagaatggcagctaaaagagtgtcattcagatggttgcagttgtacgaggctaa

agtaaaggaaatggccgaggcagagaatgaagcactcgaccgaatcaggcaacgctacaa

gtcagaagatgcaagaaaacagagtaggcaagtaaagatatgcgccaaagttccaccttc

aagtaaacgaaaattttggggagataatggacctggatacaatgtgtcaaatgtgaagag

caacataatgaagaagtcaaagatagaatttcttaagtgccgcgaggtaaaaaacattgc

agcaatgaataaaaattctatccagagacctgccagttcatcgagcatgatgaggagtgg

aagtatgtctggagttggttcaacttcgaaagatcccaaatcttcaaagaggttatttta

ggagcaaaataagaaagatagcaatgctatgtatcattatctggttttaaattgtgatga

cagttacatttatcttgatgctactaaaaattgtattatcaattatcattatttgcttgt

aaaatttgcttggtttttatgtgctgttgtattatttactgtgtagatagcatcaagata

tgcctaattgttgtaatatgttgatttccatatataaatgcaattacagatcattttcgc

atcgaaaaaacatgaatatgatgtagctctagtctgc

>fava_dn_rep_c1122

atacaattattgtaatataataaaatcacatatgaatgacaaattccatttcaacctttg

acaattacacacgcaatacccttcaccttccacgaaccttcattcaactttaccaatctt

tatttaacttgtattgtttttccacaattttatcacctacttaattttctcaaacaattt

cttagagtaaccatgaacatcaacaacaacaataacagtgaacaatccttttggcaattc

agtgatcaactaagacaccaagcatcaaacttagcaaatctgtcactaaatgattcaatt

tggagcaacaacaaaagacctgatcaaagaagaaacttcgatgtcaaaaccaatggagtt

tctgatatcaacaactccttcactaatttcaactcaaaacctagtgatttcaatgatgga

tggaagatgatgaataattctaatggatcatcacttttttccatgccacataataattcc

tttgctggatttaataagggggtttattcttctccttatgctaacaacaacaataacctt

aaccacaacaacgttaacaatatcaatcttagtggttacaaatcaggttttaaggttgct

gatgagtttcaactacctaacaaaggtgtgaagaagaatctgaacaacaatagtagcaac

aagaagcatggagataatactgatgttgtcaaaacttctggtgacaagaaattcaaaact

ctgccaccgtctgagtcactgcctaggaatgaaactattggtgggtatatctttgtttgc

aacaatgatactatggctgagaatctcaagagacagatctttggtctgcctccaagatac

agagattcagtgagaacaataactccagggttacccctttttctgtacaactattccacc

caccaactccatggtatttttgaggctgcaagttttggtggaagtaatattgatccaaca

gcttgggaggataagaaatgccccggcgaatctcgtttccctgctcaggtacaagtgatc

aatagaaaagtttgtgagccactagaagaagattcctttagaccaattcttcaccactat

gatggtcctaagtttcgtctcgaattgagtgtgtctgaggcgctgtctcttctggatatt

tttgcagatcagaatagtttcaatgatattttcaaggctataccagcataaaatggtaga

agtaaaagagaatacaagaaccttaagttatgaagaaaaatagaaaatgaatattgtggg

cttgtaaagaaggtcctaatatatgacaatgaatatggtggaggaaagattgaatattca

gcctatatatgtataagtatatgagattcaatgttatgtgtatatacaggctgttatgcc

taatttccccccttttattgtgagtgtatatgaacatacataaaagaatacaataagaat

gaactaatttatcttatggcctattatgtactctaatttatcttctttgcatgttgatta

acctaagccataaagcatatattcacatgtgttttgcccaacgttatactcatgatagtt

atgaatgtttgatttc

>fava_dn_rep_c1123

aattggcaagctttcttcgctggtatgtggctgttgaactttatgaccctgcatatgcaa

aacgattttactgnnnnnatgaaattccggaggagaatatgatgaagatgggagctggta

tgaacggaggggaagatgggtttaaacggtggcagagtttggtacgacagacagaattaa

ctgctcaattatgttcaatcactagagatgttgggaatgtccgaggtaacacacagaaga

agattgaaaagcttagacagctactttctggtcttctaagtgaacttacctattttgatg

agccaatacgatcgcctctgacaccagacgtgctcatcactgggattgtgccctctgagt

cgtcaatatttaaaagtgcattgcatcctttgcgtcttacttttaaaacagcaaatggcg

gaacctggaaaatcatatttaagaagggggatgatcttcgccaggaccagttggttgtcc

aaatggtatcactaatggatcgattgcttaaattagaaaatcttgacctgcatttaacgc

catacaaggttctagcaactgggcaagatgaaggcatgatggaattcattccatcccgtt

ccttggcgcagattttatccgagcatcgcagcatcataagctatttgcagaattgtcatc

ctgatgatcatggaccttttggagttacagccaactgtcttgaaacgtttataaaaagtt

gtgctggctactctgttatcacatatatacttggtattggagacaggcacttagacaatc

tcctcctaagagacgatggaagtcttttccatgttgattttggttttatacttgggcgag

atcctaagccatttccaccaccaatgaagctctgtaaggaaatggttgaggctatgggtg

gagctgaaagccaatattatacgaggttcaagtcctactgttgtgaagcatacaacattc

ttcggaagtccagtaacctaattttaaatctattttacttaatggcgggttccaatattc

ctgacattgcttctgatcctgaaaaagggattcttaaacttcaggaaaagtttcggctgg

acttggatgatgaagcctgtatacatttcttccaggatcttatcaacgagagtgtgagtg

cattgttccctcagatggttgagaccattcatcgttgggctcaatattggcgataacctc

aatatctgttgggtttcatcacaccattgtaattaaccagcatcttatgctgtctagagg

cctctgaagcagttttaaatgtgtgcagctttatattctctctgaaattggaagctggaa

ctccataagcagagtggcatgtcagacacgactgcagctgtgctgtcagctatttgctgc

tctagtttctgaactaattatcctggtcctagtgaggttgtccgcaaatgggaatgaatt

attacatttccattcttttgatatcgctgtgttaagatgcggtataacttttgctgtttg

tatagaaacccacgtagctaatcactagccaacatacgttatgtaaatagcttcagctat

gatcccatactttttgcttaattggcaagagataaattgccaaaatcgcatctgtttagg

ttgtgaacactgaagaattcgactttatttatttacttattagggtatattgtgtatagt

tcgtatatcaggtacttgaattgaacaatacaaaatttaggaatatatattatgggctct

aagatcgaaaaaaaaaaa

>fava_dn_rep_c1124

atccaaaatagagacaaactctttggaattgtggctcacattaatattcgataacacaag

gtcacagcttttcttatgcatttaccccaaaaaacatgactctattatatgaacttgtgt

tatcgatactacataggtaaaccgtgccagaccttgttgatctataggatgcatttaaaa

tctcaattactatgaatgtgtccgtgtcttgtttacaaataatgtaaagtactaataata

gatcaaaatttcaatgtaaacttcaaaatattcaacaaaggatttaggtgacagcgttgt

gtcattccttttatgcatttacagtatttcttaattgcacaataaacagagagatgtaaa

cttgatctcttatttgacttgaaaatgttagcacaccaagcatattgactatatgaagtt

gtgacgtgtaccgcaatataaatgacagatctttactttgtttgctgtgctttctctaga

gaattcctcagtaaggatataacttgagctggctcaggggctccaaacactgaacttcca

gcaacaatgcaattcgctcctgctgatgcagctgtgtctatggttgaaggccctaatcca

ccatcaacctctatgtcaagtaatggatacttcttcctcagtatttgaaccttttccatc

atctctggcatgaatttttgtcctccaaatccaggctctacagtcatgacaagaaccatt

tccacaggattttcagattcaaccagaggaaaaacctcttcaaccaacgtcccaggcttt

atcgctacaccaggcttcatgccttgtgacttaatacttcggataagttctttccagttg

tcttttgatgtctctatatgaaatgtaaagccagaagcaccagcttttcccaaaggttca

acataatcaagaggatttgtaaccatcaggtgacaatccaaatatgcctctgtgtgcttt

ctcaaactttcaatgattggagcgcccatagttaaattcgggacaaagtgcccatccatg

atgtccatatgaagccaatcagcgccgtaattgaccatccgatgagcttcggaagccaaa

tttgcgaagtcggatgagagcattgaaggagcaatttttggtgtcactgccatttttttc

gcgtcgacgagttctggtggttggttaccgaacacaaagcaaaccaagcttcttaattgc

gactcccgttgttgggtaattaatcgcttttaaataagtcaaaaccggtaggtaaattaa

tcacttttaaatacttttctgctggggcaagtttggacccgtaaaccccaaaaggcg

>fava_dn_rep_c1125

caaactaacacacctaaataaaataaatatcaagaatgagtaacaacttttaaacaactt

ttaatcaactttaaaccaaacaataacatcagagcatacaaaaacttttactgtagcata

gatattttttaaaaattacagcagcagttaataaattaagaactgatgtccgaacagtgc

agccaataaaatccatatgcaaaattttaaactacatcttctcgagtatcaatacccgcc

tataaaaaaactccaaaacaaggctctgcataatggcattcaagaggaacttcttttttc

ttctatcaggaagatagatacttaagcactaagctaacttgcctttcaatgaacttattg

ggatcttactcaagaccttactcaagaccttctcatcaaacactgcatactgctttttaa

tctcgatgaacgccttttcagccagagggtctatcttgtcctcaaacttatcataaaaga

aaggaactgtgtgcaacaaaacaaaggttaggtagaacaaggtcaagaaattgacacagt

ttcccaattttgatagaatccacaagctaacaacaacaccaaggagtttcttcaaatctc

ttccagaaccaatatcacgcaaggcagtaaatcctctgttaatttcaattctcaatgcag

ctgcaaactgcagaattggttcttcaggaagatgaacaactgggatgtgaggagcagtct

tgtgaataaatgtatgagcattggaccacaagaacaatagtgcaagaaccagaatcgaac

tgtgacaaatgagggtaagaaagtggtactcaagcagctcaaagaaaacccaaatagcag

ttgcaacaccgagtgcaccaccagatatcttcttgttcttccataataagacatcagcag

gctttccgccgccgagaacggagtggacgggcttctcccttccaaaaaggcgaaaaacct

tgtccttaactgatgaaatctccggtttaacagattcggaatgagaatcggagtcggaat

cggaggacgattcgtcaaagtgaagcttctctgggatcttttcaaacaacgaatcgccgc

cagtcgctgccgtcacggtgcttccggattcgccacccatttttcttcgaaaccctaaat

attaggaaaaggaataaaattggagaaaaagttctttcggttaggggagcctggagaatc

gagtgtagtatgggtttttatagtgttgtgtttaacgaggtttggattccgaagctgagg

ttcggg

>fava_dn_rep_c1126

aaatcatctcttattaattactcttgaataaaaaacattcatttagttatttcattaggc

ggaggcgtcttctaccaacattaactcctgtatatattcattttacgattatgtagatga

ttacaactacgatggataatcataaatatgagcctgaaaactacaaatttcagcgagaag

tcagnnnnttaagttccaaaaggtggagttatgatagcatggccaccattgtcaccctta

atcacatacttctaatcaagcaaaacaattattttcgaacctaaacaatcaacagcaatt

ttaaggccgatgacaatttaaatctcttcacaacctcttcaaccaataaaaaatcgttaa

aaagcttagcttccccgtatatacaaacactcataagtgactgtacacactattcgacac

aaacatactacacctattgtataaaactgttgtattggaatactatagctatgccctagt

ggactgactatacactagttacccccataccacaaaaatatgaaaagtaagaatggaaaa

attttacagccaatctacaagcataaaaaattaatcttcagggcgttggatctcacccat

catgatccggttactggataccttgaaaccaagaagagagggatctatctgtttcccttt

ctttcccttctttttgccacctcgccctgcctgaccaccatcaaccgactctgccccagc

tccactggttggatgcacatctgcctcaggaagaacaggctttttaagcatatctataaa

tgacgcctctgacatagcaccttcactgaaggatgaagatctaaaccgagcctctttctt

ccctgcagcttgagcatcagtcaagctagtagttgacaaatttccagacggctctcgtct

cccttcactggatgtaaggtttaataattgattctgattgacatgagttgtagatggttg

gtctgactgcacgtccggggaggacaaaacccgagatacatgaggccgcttattgaaagc

atgatcaaacccttttgttgaaggaggaatcctgtcactagaaacatcatctcctcgtga

actattcaaacccatctcacgaccaaaagagccaccatcacctcctgcactgcttagtga

gctatgtctactgtgagcaatagcaggcatttccattgaaggcattgtgctttccacttg

ttctgacaagtctgacatcccaggaacaaacaagtctttttccatagatgacttaccgat

ctgaaaaggaattctataattgggatgcaaagcatccttattcgtagataacagtgattg

ctcttcaagtaatgaaccagatctggaccggagaggcactctttcagcattccctagatt

gttgaaatgatcatttatatgtatgttagttaaattatcgtgcattagcccactggactt

cgcactctgagtcctttctagaaagggatcattcagatgagattgctgatccagaggaag

ctcgaaaggatgacttgatgtgttggcctcagggacatgccaagatttatcgtgacttct

tgatgaaaggggatgccacttatcaacacttgatgattgtgcagattgaagacccagttt

ttgatgaagaaggtccacaaaacctcgtcctgaactctcttcatgacctccagcagacgc

agatatgctcagatctgctgatgtaatgctatcccctaactctcttctctgcctcataga

ttcaagttgttgaagatgcaaccgtggatcaatccagttattatccacatggccattgtt

ccctgagagggaatgatggccaaaaagatcatcagatgattgtaggttgtgatgagaaga

caaagaacccagttgatcagttgggtgcatgtagcgacggcgttcaagtaattctcttcc

ttgcacagaaacaggtgaagacctctcaaacatcatagaatgatcatggaatcctctttg

gttctgctcaagatgattccttcccataaaatttaattgttcatcttgtgcgacaagcct

ctgttgttgtttgtggatttctgagacattaaatccagctgaatgacccagttggtggtt

tgatggatttctaactaactgtccagtttcattaattggccacgacctaccgaaatgtct

ttctccatccagacctaactgctgtctaagagccatagataactgctgagcttgcacctg

ctcttgctgaaaacgaacatgttctgatggcaatatatttccatgccttgcttgcaacaa

gagctctgacaaatcagcctgccttccttgcccagcattaagggccatattagcttgaat

atactgctctgccggtggatcaaggtgccctgaagaatgtgaattctgctgcaagtcgta

cnnnnnnnnnnnnnnnnnnnnnnnnnnnnnnnnnnnnnccctagaaagatcgtgttttga

ttgt

>fava_dn_rep_c1127

tatggttagaaaccttatctaaaatattgaaagtccaagcttctcttggtgctctttaag

agaccaaagacaaacttcatccactcaataactcacccctatcgttattgcaatagccaa

acaaacttctttccttaaccaccaccaacccttattttacaccactctctctctctctca

ctctatatgcttcattgacacaaggctcatttccacttgaacaccccatttccaaccttt

gcagtttttgccatttttcaactctttattgaaaatggcgcagattttggcaccttcgac

gcaatggcagatgagaatcacgaaaacctctccgtgtgcaactccgattacatcaaagat

gtggagttctttagttgtgaaacaaagtaagaaagttgcgcgttctgctaaatttagagt

gatggcagtcaactctgaatatggcaccatcaatagggtggaggatttgcttaacttgga

catcactccattcaccgacagcataattgctgagtacatatggattggtggaacaggaat

cgacgtgcgcagcaaatcaaggaccatatcaaagcctgtttcgcatccctcggagcttcc

taagtggaactatgatggatctagcactggacaagcccctggtgaagatagtgaagtgat

cctatatcctcaagcaattttcaaagatcctttccgtggcggaaacaatattttggtcgt

ttgcgatgcgtatacaccagcaggtgagcctatacctacaaataagagacacaaagctgc

tgaaatcttcagtaacccaaaggttgaggctgaaattccatggtatggaatagaacaaga

gtacactttacttcaaacaaatgtgaaatggccattaggttggcctgtaggtggctatcc

tggtcctcagggtccttattactgtgctgccggagcagataagtcatttggacgtgatat

atctgatgctcattacaaggcttgtatatatgctggaattaacatcagtggcaccaatgg

agaagttatgcctggacagtgggagtatcaagttggtcctagtgtaggtattgaagctgg

tgatcatatctgggcctcaaggtacatccttgagagaattactgaacaagctggagttgt

gctctctcttgatccaaaaccaatagagggtgattggaatggtgcaggatgtcacaccaa

ttacagtacaaagagcatgagggaagatggagggtttgaggtgataaagaaggccatttt

gaacctttcccttcgccacaaggtccacattgaagcatacggagaaggaaacgaaagaag

gttgacaggaaagcatgagacagccagcattgacaatttttcttggggagtagctaaccg

gggatgctcaatccgtgtgggaagagacactgagaagaatggcaaaggttacttggaaga

caggcgtccggcttcaaacatggacccatatgtggtaacagcactactggcagaaagtac

attattgtgggaaccaactctagaggctgaagctcttgcagctcagaagcttgcattaaa

ggtctaaatctattgactttccaggtggagactgtgaggagctgagaagctcttttagat

gatttatatttatattatgaaattcttgtttcctggacaaatgaggctgccatacctagt

tactggttatgagtgaatgcacattgtatttgcatatggtatggacagaatttgcattgg

tgatttgcttgcttatatctttgaatttagttttctttttctacaaattaatcactcagg

tctcagctcatgtacaccttaaaatgattggttcatgtgtttctagaagcatgtcaccta

taatcctcttttctaacaact

>fava_dn_rep_c1128

ccgcgggacaaacaaccgagtgtccaccaagcttaacgtgcaccctcaattcaatcgtga

tacaaatcatcgaaaaatcgaaggcgattcattctctctccgacgacgcaacccttatct

ccggcaaactttcatttcctagcttcttgcacgatttcattttggtgccaatcgtggtgg

tgaattggtgccctacctcaaacgacaatgtcatctctacatagaggggattccaggagg

ttcaacaataaaccaaaagcacggcatcacttgacaactcagaagagacaagagattaag

gaagcttttgaactattcgacactgatggctcaggtactattgatgccaaggagctaaat

gttgccatgagagcccttggatttgagatgacagaagagcaaattgagcaaatgatagca

gatgtcgacaaggatgggagtggagcgatcgactatgatgaatttgagcacatgatgacg

gccaaaataggagagagggactctaaagaggagctcatgaaagctttccatattattgat

caagacaaaaatggtaagatatctgcatctgacatcaagcgcattgctaaagagcttggt

caaaattttactgatcgagagattcaggagatggttgatgaagcagatcaaaataatgat

caagaggttgatccagaggagttcgtctcgatgatgaagagaacaagctaccgtcactag

tcatatcatcatgtagactacatggagaagtagttacctttgtttatgtgcaaaaatcag

atgatgttgtacttctgctagtgtgcaaattgtaggaatgatgaatcaaatgttgtttat

tttgataagagatatgaatccattgctatgattttctgtttcctatgatcatatagatgt

attctggaatttaggtatgttgttcggaactaggtttgagtctaaactagtagcaatcta

tgatttcaaaataaatcagaattcaaaatataataaatcaaatatgaaccaattgcaata

taggataaacaatctgtatggtagagaatgttga

>fava_dn_rep_c1129

agtagaatacaaccctagagcaaagcattcttggtttgttttattcttcttcactgccaa

gtttgtgcgttttaactctcctttctcttccttcaccgccgcaccaaccgttttttctcg

ctgcacttcccaatcgattttcctctttccatctctccaattttcgtatccactgattct

atttcacccttctctttcgttcatcgaaacacgaatctcacgtttatcgatccaattatt

cgtgctttctttagacgcacgaggaaaccctaatctgacaaatggggaagaagaagccat

caattcgtgacgacgagaatcctccgcaacaaggcggcggtgggaaatcgaagaaaaagg

ctccaatcatcgacgacgacgaatactccatcggaactgaactttcagaggagccgtcgt

tgccacagccggaggaaaaacctgggaagaagaaaggtaaaaaagctaactctaaatctt

tacaagctaaagataacggtgatgattttgataacgaggacgatgatgatgttcctgaga

tcgtgtttgctgggatgaaaaaggggaaatcgaagaaaagtaggggtggaagtgtgttta

ctgctgctgctggttttggtttgcttggggatgaggaggatgttgataatgatgatgacg

atggcgacgaggatgaaccagttgttagttttactgggaagaagaagtcttcaaaaggaa

acaagaagggcggttctttgttttcagctgttagttttgatgagattgatggagaggatg

agagtaagaatgaaaaggtggaagatgatgatgaagatataggaccaatcactttcacag

gtaagaaaaagaagtcatccaagtctttgaagaagtcggttagcttattgagtactgata

ttccggatgaggatagtgtttctgtatccgagtctgctaagggtggcgatgataaagatg

aagacgatgtacctttagttgaatttactggtaaaaagaagtcctctaagaagaaaagtg

gcacggctgctgctaaagtgggtaatgaaattgagtttgggagtgaaggtgtagatgttg

ttgagactgagaaacctagtgctggagttagtagtactaatgttgacaatggcaatataa

gtaagagtgaagaggtcgttggaacttccaaaaataagaagaagaataagaagaagagtg

ggagaactgctcaagaggaggaggatttagatgcgattcttgcagagcttggtgaagccc

ctcctacagcaaaaccttctgctcctccaaagcaggatgataatgtccagcctgctctgg

aagttggttctgcccctggtgcttcaggggaaaaagagggagaagaggagactgtagaat

cagctgctgcaaaaaagaagaaaaagaagaaagaaaaggaaaaggagaagaaggctgcag

cagcagcagccggaagtgcaacggcaattgaaactgaagaagtaaaggctgagacaattg

aaccaaagaaaaatgatttgaaggccaaagcagctgctgacaagaaattgccaaagcatg

ttagggagatgcaagaattactagctcgaagaagagaggctgaaataaaaaagaaaagag

aagaggaggaaaagctaaggaaagaagaagaggagcgacaaaggctagaggaacttgaga

gacaggcagaagaggctaaacgtagaaagaaggaaaaggaaaaggaaaaacttttgaaaa

agaagcaagaaggtaaattgttgactggtaagcagaaagaagaagcccggcgattggagg

caatgaggaggcagattctcaatagcacagggggtgtgactctccctgctggggacactg

gtgcaccaaccaagaaacccatttatcagacaaagaaggggaaatcaactagtagaaatt

ataatggtgctgctgctgtcaagacagatgaaattattgaagcaaaggaaactaccgctg

atttgggttcagaggaaccaaagaaggttgaagaggtggagtcggtgcagaaggaggaca

aagttgaactttcggaagctgttgaagaggatagagtggaagaagatgatgttgaggatg

aatgggacgccagaagctgggatgatgtcaatctgaatgataaaggtgcatttgctgatg

aagaagttgactctgagcctgaaatgattgtcaaaaaagagattaaaactggcatacctg

ctaaaaatgctggtgctacaagtaaaactgtttcgaagcatgtcgctggagaaattgaag

atagaaaacaggccaaagttgtggttgaggacaaaaagaaaaagcccgacccacaacagt

ctgctgtgtcttctaagccgagtgatgcaagcctccgttcaccaatttgctgcatcatgg

gtcatgtggacactggcaaaaccaagcttctggattgtattcgaggtaccaatgttcaag

aaggcgaggctggcggtatcacccaacagattggcgcaacatactttcctgctgagaaca

tacgtgaaagaactaaggaattgaaagccgatgcaacactgaaagttccaggtctactgg

ttatcgataccccaggccacgaatcattcaataatctgaggtctcggggttcaggtctat

gtgatattgcaatcttggttgttgacattatgcatggcttagagccacaaactaaagagt

cactcgaactattaaaaatgaggaacacagaattcattgttgcattgaataaggttgata

ggctttatggctggaaaacatttcgcaatgctccaattcgtaaagcaatgttgcagcagt

ctaaggatgttcaaaatgagttcaatatgaggctcgaacagattgttaccgagttcaagg

aacaggggctaaatactgcattgtattataaaaacaaagaaatgggagaaacattcagca

ttgtgcctacaagtgcaataagcggtgaaggaatcccagatatgttattactattggttc

agtggacccaaaagaccatgattgagaaacttacatacagtgatgaagtgcagtgtactg

ttttggaagtcaaggttattgaaggccatggaactactattgatgttgttttagttaatg

gcgtccttcatgaaggagatcaaatagttgtacctggaatgcagggtccaattgttacca

caattcgagctttattgacacctcatccaatgaaggaacttcgtattaagggatcatata

tccatcacaaagaaatcaaagctgcgatgggcatcaagatcactgcccagggacttgaac

atgctattgctggcggaagtttatatgtggtgaagcctgatgatgatttagaatatatta

aaaccgcagccttagaagacgtggagtcggtcttgagcaggattgataggagtggtgagg

gcgtttgtgtccaggcatctacccttggctcattggaagcattactggagtttttgaaaa

ctccagaagtgaatattcctgttagttctataagcataggccctgtacataagaaggatg

tgatgaaagccagtgtaatgcttgaaaagaaacgagagtattctactatattggcattcg

atgtcaaagtcacgcccgaagccagggaacttgcagaggaattgggcgttaaaatattta

ttgccgatatcatttatcatctatttgatcaatttaaggcttacatggaaaacattaagg

aggagaaaaagaaagaatcggctgatgaggcagtcttcccatgcgttctgaagatcttac

caaattgtgttttcaacaagaaggatccaattgttttgggagttgacatccttgaaggca

ttttgaagattggaactccaatttgtattccttcccaagatttcattgacattggacgca

ttgcctccattgagaataaccacaaaccagttgattatgcaaagaaagggcagaaagtag

ccattaagattgttggcagcaattctgaggaacatcagaaaatgttcggcagacattttg

agattgatgatgaacttgtaagccatatttcacggagatctattgatattctcaaatctg

attatcgggatgaactaagtaatgaagaatggaagctggtggtgaggttgaagactcttt

tcaggatacaatgagcattcatttcttataaacaatgtttcaacaaaatgtttggtataa

tgcctaatttcaagggattttgcgaggattttgcggatgggttcaccttgtaggccaaac

ttctggggcaatgatgcagatagaagcctatttataggggttgactgacttgtacaagaa

actcctatgctcttgcagctgggaacttgtggccaaaagcgtaactgtcaaggacaaaag

gtccaaattttgtgatttactatacacatgtggtatacatggagcgaataaaagtttaat

tttaaatttgttaaatgaggaaaaggaaatattcacgggagtatttgtcgatgttacaaa

aaaaattgtccacttttggtttccatggtttacaagttttcaatttgttgttgatgcatg

gtatttaatgctcagttattttcatcttttggtgttccatctcatcaagtac

>fava_dn_rep_c1130

atatcaattaatttagttggaaaatgagaaaagaagaaaggaaataaattgggaaaggat

aaagcgaaattcttctgagctctatggcttcaaccattcttccattaaccaatcttcaat

tcgcttcgtttcattctcaccactctcttccatccatcgccaagtggggatggagaagag

agcaagatgcaggcatggcattacatagaactcaaagtcaagcatttcgagtccttgcta

atcccaatgtatcttcaggaaaggatggttcaaaaaaggatgtaatcatggttgatcctg

tggaagccaagcgattagcggccagacagatggaaagaattaaagcaaaagaaaaactca

agaaaagacgccaaattgaggcaattaatggagcttgggcgatgattggcctcacggtgg

gcctagttattgaaggtcaaaccggaaaaagtattacgactcagctgcaagactactttg

gagcaattattcacttgttcgtgaagtagacaatcccaaagatgcttggatacatactaa

gaaatgcagattctttctcacattctttgtgaatatatctgttaaaggtttaccgataag

gttttttcttaacacaccacaaaaccatacaattttcgctactcatttttccttgttacc

tgttaatgctacatatgtaacatgatttttacaaaagagttatgctgttagccaattatt

gttgctcgatatggatgtttacccgctcattgctttttcctggttctggtatttattgtt

aaaatcttaacatttttccccttactg

>fava_dn_rep_c1131

tgccaaaaaagtcacaatctcttaaaaagttgttttggttgattctctctgcagtacttt

ggttgtctttgaaaaaaatggcagaggaattgatattgttagatgaatggctcagtatgt

ttggtatgagagcaaggatagcattggctgaaaaagggataaagtatgggtacaaggaag

aagatcttgacaacaagagccaactgcttttgcaaatgaacccaattcacaagaagattc

cagttcttatccataagggaaaacccatttgtgagtctataaatattgttgagtatattg

atgaaggttggaatgatagagttccatttctaccctcagatccttatcacaaatctcaag

aaaaattttgggctgattttgtcaacaagaagattggtgatgttggggggaggatatggc

ctgggaagagagatgagattgaagtggcaaaaaaagagttgattgaagggttaaaggaat

tggagaatgtgcttggagacaaaccttattttggtggtcacacatttgggtttgttgata

ttgctttgatccctttctacagttggttttatacttatgaggagttatgcaacttcaaag

ttgaggcagagtgtgataagctgattgcttgggaaaagagatgcaaacaaagggagagtg

tttgcaaatctgttgctgatgagaaagaggtgcatgagtatgttgttaactataggaaga

gatttgacttggattgagaagacattctggaatctgtgagatattgaacatattagtttt

gtctttttgaattggttttttctatagtatttggtcatgtcttgttgatttcctttaaaa

tgttttctaataatgttttgggcaaaattttgacttgagaaatgatgatttcctactttg

ttgcttatggttccaatcagcctcaaattcaatatatgatataactttcaaatctctgca

gtcttttaatgtaaatttatgtactaaccatcatgtctatagattttgaagta

>fava_dn_rep_c1132

atcaaaagtgaggcaaccgtctcaactccactaaaaataccatttccactaaaatatatt

tatatttacattattttatttcatctcatgaaacccccaccaatttgtgctgtccatttc

aacaactccattctcaaacctcccaacaacaacaacctcaattttcgatccaatttcatg

ttttttccaatcctaaatcgaagttgagagtcgaaaaatacgatgatgttgagtttgggt

gtagttggtgggtccacaaagtgtcttcagctctatgattccagttacagaatcaacaac

aaaagattccttaatctaaacaacaacaacaacaacaacggtttcagtttcaatacgagg

tggcgtttcatggcacttgaatctgactcttcgtcttttgcatcttctgttgattctgac

accaccgataaaaacgctgctgctactgggttctgcattatagaagggcctgaaactgta

caagattttgctaaaatggaacttcaagaaattcaggataatattcgaagtcgaaggaat

aagattttcttacatatggaagaggttcgtaggcttagaatacagcaaaggattaaaaat

gcagaacttggaatttcgcaagaagagcaagagaatgaacttccgaacttcccatcgttc

attccattcttacctcctttgacttcagcaaacctcaagcagtattacgcaacctgtttt

tctcttatcggcgggataattctttttggtggtcttcttgctccgagtttggagcttaag

cttggaataggaggcacgtcttatgcagattttattcaaaatgtgcatcttcctatgcag

ttgagtcaggttgatcctattgtggcatcattctcgggtggagcagttggagtaatctct

gcattaatggttgttgagataaacaatgtaaaacaacaggagcagaaaagatgcaagtac

tgtcttggtaccggatatcttgcatgcgctcgttgttcaaacacgggtgcacttgttctg

attgaaccggtatccaccttcaatggtgcagatcagcctctatcaccaccgaaaaccgag

agatgttcaaattgctccggatctggcaaggtcatgtgtccgacatgtctttgcactgga

atggctatggcaagcgaacacgatcctcgaattgatccttttgattagaggtttaatatt

gtggtagatatcctaagtagggaatttgatattgtatgatgagtatcgtatatgtttccg

ttatgaaataatgaagtttctgtaaatatgtataaacacaataatatgcaaaagaaagtg

gttaaggatctaaagacatcctttattttatagaattggatatgttgcagctggaaaaaa

aatggttactttcttggaatggtcacaagaaattaggagcca

>fava_dn_rep_c1133

tatgggacccaaccatatgggggtaatgagaaacacacgaatttgttgggacattgaagt

tgaagaaaacctgatctcttctcctctccactcgtcccttcaaacttcaaacccagtttt

ttttctcttttgtttagccatgtcttatactaaatcttgcatttcttattctgctttacc

cttcttccaacggaggggtctgcaacctgcttcttcaccttctcatcacacaggaacatt

cattccacagtgtttaaatggaaacactagttttactttaacaagaaaaagattaagtgc

caagttttcaaagaatgacagattctctatccctagaaatactacttcttcttcttcttc

ttcaaactccactgatgatgacacttctaatcagactaagaagacaccatttggatattc

aaggaaagatgttatattaattggacttggtgttactttcctcggcattggcttaaaagc

cggattagagtatgttggagttgatccactacaagctggaaatgtggttcaacttgtgct

ggttttgggcctcactattggatggatctcaacatatatcttcagagtttcaaacaagga

aatgacatatgcgcagcagcttcgcgactacgaaagcaaagtcatggagaaacgattaga

gtccctgacagaagcagagctagaagtactgcttcaagaagttgaggaagagaagagtcg

ttagctatagctagtgtgacgaagaggtgaattgaagtagcttgtttgtatataaacaac

gaagttttacagcagtaattaaacagaaatgataatctctatatggactagcttagaaag

gtttctgctatgtttactacagttcactctctcaaacaaaatatagtgtttggtatctta

gcaaaatgtaatatgcaagattattcgcaaaacacatacagaagtgacacattttatgtg

tgaagatggaaatcacataattaggtgtttcttaatcattacactatgagaaaatgaagt

ttgccataattattggttatataccagaaccattcacgtgcagatgcgtctctattttgt

ataaacatgacagagtcatttcacgtgcatgtgctggtttgaggggcacttggtttgata

ctagctaataggggacctttcagaacaaattcaggttatttcttgtccatattgaaaggc

atac

>fava_dn_rep_c1134

tgttatttgttggatcagaactccactaacaggcaactctgaagcaattaccttattcca

ccaccactatgggttcacttgtttgagattgttgctcaaataaagtctgcattcgcatga

cagagcttccaacttcctctagtttctgcattagcagcagctccctagagcgagtagaat

cgtatctcaaagacttggatggagacatcatcaaatgtgaagagtcttgcgatgttgacg

tttgtcttgaaactatctcatttcttatgtttgataggcgcatcataacagccttcctga

aatcgttaggatttgcaacgccgaggattttaacatcgtcgctgggtggccttctaactc

caacattttcaattctaagcgcgggacaccaaaaaaagtgtcttagctttttcacatcta

ttgtaattgtggcaaattaatatttagatctttgccaattacttgaaaatgggaagccag

agttacttaacggttttgattatatgtcttgtagcaataattggatcaagtcatggtcaa

ttgcagttaggttactatggtaaaagctgcccaaaagctgaagaaatagttttgaagtat

gttcatcagcatattcctaatgcaccatcactagcagctgcactcataagacttcatttc

catgactgctttgttaggggatgtgatgcgtccgtgcttgtgaactcaactcaaaccaat

caagctgaaaaagttgctattccgaatcttacacttcgaggctttgatttcatcgacact

ataaagagtcttattgaagctgaatgtcccggcgttgtttcttgtgctgatatattgtct

ttgtctgctagagactcagttcatgccattggtggaccttattggaatgttccaacaggt

cgaagggacggaaccatctctaaatcagcagatgttttcatcagccttcctgctcctttt

aacaacctcaccactctcctaacactctttggaaatgttggacttgatgccaatgactta

gtcttgctttctggtgctcatacaattggtgtttctcattgcccagcgatttcaaacaga

ctatataattttacgggaaaaggtgatcaagacccggatctagacagtgaatatgctaca

agtttgaaaacatttaagtgtaagaatatcaatgaccaaaccacaattatcgaaatggac

cctggaagtcgcaatacatttgatcttggttatttcaagcaagtagtaaagagaaggggt

ttgtttcaatcagatgctgcattgcttaaaagttctacaacaagatcaattgttgcacaa

catcttcaatcaaatgaaaaattctttactgaatttgctaagtctatggagaaaatgggc

cgaattaatgttaagattgggactgaaggtgagatcaggaaacattgtgcatttgtaaat

taataattgtgtatctttgtttatttgcatttgctttgaaagtcttgttatttatttcat

gtcaatgttgtgatgacttcatattggaggtaccttatgatttggctctttttgtaataa

tatgggaataattggttaaatgattccaaagtttgatggcaaaaaaaaaaagcggaactc

agaaacgaagagaaatgggaaacataccgtaagttttctt

>fava_dn_rep_c1135

aacgcagagtggccattacggccggggggtacagcttcgcaccacaacaatttcaagttc

aagcgaagcaacaatttctctctaatcttcttctactaccttcaagatactaaagatggc

aacagagactttcctatacacttctgagtcagtgaacgagggacatcctgacaagctttg

tgatcagatctctgatgctattctagatgcttgtcttgaacaggattcagacagtaaggt

tgcatgtgaaacttgcaccaagactaacttggttatggtttttggagagattactaccaa

ggcggatgttgattacgagaagattgttcgtagcacttgcaggaatattgggtttgtttc

tgcagatgttggtcttgatgcggataactgtaaggttcttgtgaatattgagcagcagag

tcctgatattgctcagggtgtgcatggtcatttcactaagaagccggaggaaattggggc

tggtgatcaggggcatatgtttgggtatgctactgatgagactcctgagttgatgccttt

gagtcatgttcttgcgaccaagctcggtgctcgtcttactgaggttaggaagaatggtac

atgtgcttggttgaggcctgatgggaagactcaggttactgttgagtattacaacgacaa

gggtgccatggttcctattcgtgtccacactgttctcatctctactcaacatgatgagac

tgttaccaatgatgagattgctgctgatctcaaggagcatgttatcaagcctgtgattcc

agagaagtaccttgatgaaaagaccattttccacttgaacccttctggccgttttgttat

tggaggtcctcatggtgatgctggtctcactggtaggaagatcatcatcgacacttatgg

tggatggggtgcacacggtggtggtgccttctcaggaaaggacccaaccaaggttgatag

gagtggagcttacattgtgaggcaggctgctaagagcattgttgcaagtggactagcaag

gagggccattgtgcaagtctcttatgctattggtgtgcctgagcctttgtctgtctttgt

tgacacatatggaacagggaagatacctgacagggagatcctcaagatcgtgaaagaaaa

ctttgatttcaggcctggcatgatctctatcaaccttgatctcttgaggggtggaaatgg

caggtttttgaagactgctgcttatggtcattttggtagggaagaccctgacttcacatg

ggaagtggtgaagcctcttaaatgggagaaggcctaagtcatatctgtggtgggagtttt

cagcctttgcctctatctactatttttcaatacttttaatatcgtgtctattttttcttt

gtttatggatttttttgccgcagcaaatataaaaagtgctctctgtatgttgtatcttcc

gtgcatttcacatttatatcaatgtctaaaaaagagtatcctaccccttatattgatgcc

ctctgttgttgattaactcatgcaatttaccccaaacaaatgtttggttttggacagaaa

tgtgtatgaagatttggtatgatttgtttgtgaattatgttaacaattgcaaaatatcaa

tctctaatcaagaactttttgaaataaactactaaagtgcatgctttgatttttttggaa

taaccaactttttaa

>fava_dn_rep_c1136

aacaaaatagaagcaattgaagtgaaaccctaagatggaatcacacggtgccggaccact

ccgttcccggagttcccagtccgcttctccttcccactccgcctctgcctccgccacctc

ctccatccgtaaacgcaaactcgtatccgaagatcacgcgcctccgtttcctccttcttc

tttatccgccgacacacgcgacggcgcacttacatccaatgacgacctagaaagcatttc

cgcccgcggcgccgattctgattccgacgaggactcagaggatgcagttgttgacgacga

tgaagacgagtacgacaatgataacggttcctccatgcggaacttcaccgcttccaggct

caacaatgctcccgctgctccccgcaatagtaagctcaaaactgaaaactccactgttaa

gatcgaacagtccgatggaactaaagactccggaccttctgttaccggtgctgctcccgg

cgccggttctctccccggcattgtggttaaggaggatgcaacgaagatattcaccgaaaa

tttgcagaccagtggtgcttacagtgccagggaagagagtctcaagaaagaggaagaaac

tggaaaactcaaatttgcatgcctttcgaatgatggtgttgacgagcatatggtttggtt

gataggattgaagaatatatttgctagacagcttcccaatatgcccaaggagtacattgt

tcgacttgttatggataggagccataagtctgtaatggttataaggcgtaatcacgttgt

tggaggcattacatatcgtccatatgcaagtcagaggttcggtgagatagccttttgtgc

gattacagccgatgaacaagtaaaaggatatgggaccagattgatgaatcacttaaaaca

gtatgcacgtgatgtagatgggctgacacattttctcacatatgctgacaataatgctgt

tggctattttatcaaacagggatttacaaaagagattcacttggagaaagatcgatggca

aggttacataaaggattacgatgggggaattctcatggaatgtaagattgatccaaagct

tccttacactgatttatcaactatgatccgtcgtcaaagacaggcaatagatgagaagat

tagagaactgtcaaactgtcacattgtttatgctggaattgattttcagaagaaagaagc

tggtattcctaaaaaaattgttgatgacatccctggtttgagagaggctggatggactcc

tgatcagtggggtcattcgcggttcagaactttagggggttctactgacaatgctgcaaa

cctaaaacatttaactgcatttatgcgttcacttctcaagcagtcgttgcttgatcacgc

tgatgcttggccattcaaggaacctgttgacccacgggatgttcctgattactatgagat

catcaaagatccaatggatttgaagacaatgagtaagagggtggagtcagaacaatatta

cgtgacatttgaaatgtttgttgaggatgtgagaagaatgtttgggaatgcacgcaccta

taattctccagaaaccatatattacaaatgtgctacaaggctagaagcccactttcaaag

caaagtgacatccgtcctccagtctggcaccaagattcagtagcgacatttcaacagcgc

tgcacactgctgcatttagatatatttcttgataatcatgtcctatacaaatcattaagc

tgcgtaaatagtagtttcgacctgtaccaatatcttgttctaacgagtggcagttcttga

ctgaaacttttgagtgtgccatatttataataagtattacatttagtggtagtattgtgt

tagactcttcaattctgtaactgttagattattagagttagtggggaattattatattat

taatttagagtgtttaggtttagaataaataagggaaggcatcggggaaaaaagaaaaag

gaaaagaagagaagaaaagaaaagagagtggaagatacgaaattgaagagaaggagttga

aagttggaaatcgaaatcgaaatcgaaatcgaaaatcaaaatgcctgtgatggagaagct

gaagatgttcattgttcaagaaccagttgtcgctgcttcatgtgccatcgccggttttgg

cctttttcttcctgctgttgtgaggcctattctggactcatatcaaacaaccgagcaacc

tcctcaacctgctttgagtgatgtggttaaaggtatggctggtaaaaagtaggctgattt

tactggagatactagtggataattgccaaaatctctgcttgaatttttctttttccgttc

tgtgagtaataaaagtacctagttacaaaacatttgattgagcatcaatttgttacaatc

tatcagttccagattcatccagaggcttcatttcatgtttaggttaagtgtttggttgaa

tgtcaagtttattctgcccccttattgattttgtctctaatatcaaatgcactttctgaa

ttgttcctcttgtgacaacataaaggagttttgttaagctgacttgagacacaagtactt

tcttatgaatcttacatacaaaatcaaaattgagtaaacgattgctggtctctcttttca

ctactaaaataaaactttagcactaaaaagtgaagtacataagtggtgcagacgggctta

tatgtagttattgcatgaatgagcataaatatatttcacattgaataaaaaaacatttag

tatgttttcataattattgtattgaatagagataccttgtattttgagataaatgtattt

cacgttgaataaaaaagacatctagtatgttgtca

>fava_dn_rep_c1137

acatgattaaatttatatatagagatatatatagtttgaaggaaccaccaaaaaatactt

gtaatcaaaatagaatggaggaaaacattagactcaatttgccaaatgcaatagaataga

aactttgttagtttcctttattaatagaaacactcttagttcttcccaagttaaaaaaca

tagctaggcagtattacaaatacaacacaatacacaacataaagctaactactagctaat

aatttcatgtccaccaccacatcatgcccaacaacagagtagaactcattgataattata

tagaaaaatagaataaaaaagaaaaagaacaaaggtatcatttgttgagttattctccaa

agtttttcagagtagaccagtggaaagagtggaaagaggagaggtgagaggaggctgaga

tctgatcctactctttcttagcctttcagcaatgatgaaagcaagctcgagtgattgaga

agcattgagtcttggatcgcagtgtgtgtggtagcgtgagctaaggtcgtcgaatgtgac

agttctcgacccaccaatgcactctgtcacgttttgacccgtcatctctagatgaactcc

tcccgggaagcttccttcttgctcatggacatcgaagaatgccctcacttcagccctgat

ggcatcgaaaggacgagttttgagaccacatggagcctttatggtgtttccatgcatagg

gtcactgacccatgtgacaatttgtcctgctctacgcacggcccggatcagatgtggaag

cttcaccctcatattctcagctcccattctagtgattacagtaattctccctggtttgtt

ttggggagcacatctcaaatcgagtctcccccatttctctctcgactttcagaagcaatg

gatttactcacaacctctccttctcgctcactctcacaccttcccacccttttcccaaaa

cagcccatttcttccttcaaccctaaaacaccgttttcccaatttcgcagaaccccagtc

agttgcgtcctcacaacctcatcgccaaaaccctcaaacttcgattcccatcttcaaaaa

cccactatcacctttcaactatcttcccctcaaacgccggaaagggccatgcgaggcgct

gaatcggatactatgggcttgttgttgagggagaggattgtgttcttgggaaatgaaatt

gatgactttgttgctgatgctattatgagtcagttgttgctcttggatgcgcaggatcct

actagagatattaagctttttatcaattctcctggtggttctctaagtgctacaatggct

atctacgacgcggtacagcttgtgagggctgatgtatccacaattgcaatgggcattgca

gcatcaacagcctccattatcctcggtggtggcaccaaaggtaagcgtctagctatgcca

aatacgcgaatcatgattactcaaccactcggaggtgctagcggaaaagctatagatgta

gatattcaagctaaagaaatcatgatcaacaagaataacgtgtgcagaattatatcatcc

ttcactggtcgctcaattgaacaagttctggaagatattgaaagggatagatatttgtct

ccaattgaggcagttgaatatggaattattgacggggtaattgatggagacaagattatt

cctctttttccagtgccagaaaaagtggaaccaatcacactagacatcgatgttccggat

ttcctaatcccaaatatccctgatgatgagatatactagctttatatacaaatgtgatgt

tgtaacaaaatgaaagtttcaggattattgtttgtaggatgctgcgacaatatttgtctt

tcaactatgattcagttatggagaaatttgtttgattattgttaaaaaaaaaaaaag

>fava_dn_rep_c1138

gagccatctcaaaatagtccaaaaaaggcttattagaattcaagatgatatcgaataaaa

tgctagtgtttatacaataacgccttgttttgcaccctcacaaaagtttcaaaaaatggt

atctcaagcgctatcagttccagcatttcccaccttcactatctcttctcacagcaaaat

cccttcccaacttgccttttctcacagaaacccctgcacaatagcttcttccttgccaag

cccctatgttcattcatcaaaaatcgggttatcaagtaaaacccatgggtttcctttaaa

acttgatgagaaaaatacacatgatgttagtacaagctatggtgtaattgaagccaagaa

ggggaacccgccaataacacccgcagtgatgactcctggaggagctattgatctctcatc

tgttctgttgaggaatcgtataatattcttaggacagccagttaacgcacaagtggctca

gagagttatttcgcagcttgtgactttggctactattgatccagatgcagatatactgat

gtacatgaattgccctggtggaaacacatactcggtgcttgcgatttatgactgcatgtc

ttggataaagccaaaggttggtaccgtatgtttcggagtagctgccagccaaggaacact

tctccttgcaggtggggagaagggaatgcgctattcaatgccaaatgctcgcattatgat

gagtcaaccacaatgtggatctgggggtcatgttgaggatgtgagacgttcagtgaatga

agctgttcaatcccgccataaaatggataagatgttttgtgctttcactggacaaccatt

agaaaaggtgcaagagtacacagagagggagagttttctatctgcttctgaggctttgga

gtttggtcttatcgatggtatcctggaaacagagtattgaatctagactatgtgatgtat

atttgtatttctaaaaagttgataattgtaactcacgagtcatgaatatgcaactatttg

gaaattgactttcatagaatggttaatctagtttttctttttctgctattatggagttca

aagttgataacacaatgttaatgtgcaatatgtttgaaacatgcttatatgtatacattt

cttctcttttgaaatttctataaatattaatagataac

>fava_dn_rep_c1139

cccacttcattcattcattttccgatcttcaccgctccggttcttgattccggttcacgt

aaaaaaaccgaaaaagaaaaaccgaaccgtaaaatctcggtgttctgatggaagctcacg

gtggaggtctccgccgcgtcttggtgcttgccttctgcatcgccggaatctggtcggcgt

atatataccaaggcattcttcaggaaacagtgtcaacgaagcgattcggtccagacggtg

agaggtttgaacacctagcggttttgaacttggcgcagaacgtagtctgtttaatctggt

catttataatgataacgatttggggtggtggaatttctggtggtgctccttggtggtctt

attggagcgctggtattactaacaccattggtcctgctatgggaattgaggctttgaagt

atattagttaccctgctcaggtgctggcaaagtcctcaaaaatgattccagttatgctga

tgggttcactcgtatacggtataaaatacaccattccagaatacctttgcaccttccttg

ttgctggaggagtttcaacatttgcacttctaaagactagctcaaagactatcaacaagc

tagcacatccaaatgctccacttggatatgggctatgtttcttgaaccttgcttttgatg

gatttactaatgcaacccaggattccttaaaagctaggtacccgaaaacaagtgcttggg

gtattatgttgggcatgaatttatggggaaccatatacaatatgatttacatgtttgcat

ggcccagtgccagtggatatgaggcggttcacttttgcaaactgcatccagaggcagcat

gggatatttttctgtattgctgctgtggtgctgtgggacagaattttatcttcttgacca

ttagccggtttggctctttggctaacacgaccatcaccaccactcgcaaattcgttagca

ttgtcgtatcttcactattgagtgggaatcccctttcaacaaggcaatggggatgtgttt

ctatggtattctctggattatcataccagatctacctcaagtggcagaagttgaagagat

tgcagaagaaaaagaaagccacgtgaagccctgcatcaattttgaagttcaatatcgatt

ctatacccgaagattcaaagaatcagatttaggtttatatacaaagaagtattaatatta

gttgttgtaattgtaattgaatagatcccttttgagaagtgaaaaagtttaggtcccatt

ttcttattcattgtgttaatttactagttgcccgtgctttgtaagaactgctagttttga

tcttaatagcatacaaaaagctatattctatgtaaacatgattttcagatcatctcatct

tctgtagtttatttagattatcccaatattgtatgagttgcaaaagaagcttaaacaatg

ttatgagcattaaaagtacatttctaaaattgtttgctttcagattttgttttgttctat

caaattgatgctgtgttatgtttatattacttcaattaaatcttacaatcaaaatgcaat

aacttctgggcttaaaaatgtt

>fava_dn_rep_c1140

tgggaacatatttgcaatatcagaaaactaagaagcctcatatgcaatttgtgtaacttt

catttcactgactcagttaactcttgaacaagcaaaaccctctttatattttcttcttct

ccgagaaaaccaaaagtaacagaggctcatcgaagaagaagaaacaaccaaatcttcaat

accaaaatcaacaatcgttcaacaaaatgagtagcatcggaacaggctacgatctatccg

tcactactttctcacctgacggtcgcgttttccagatcgagtatgctgccaaagccgtcg

ataacagcgggactgttattgggatcaaatgcaaagatggaattgtgttgggtgttgaga

agcttattccttccaaaatgatgcttccgggttccaatagaagaatacacgccgttcatc

gccactctggaatggctgtggcaggattagctgcagatggcaggcagatcgttgccaggg

ctaaatctgaagcaaataactacgacagtgtgtacggtgacccaattcctgttaaggagc

ttgccgatcgcgtggctagctatgtgcatctttgtacattatattggtggctcagacctt

tcggctgtggtgtcatactaggtggttacgacagagatggaccacagttgtacatggttg

aaccttctggcgtctcttataggtacttcggtgctgcaattgggaagggcaggcaggctg

cgaaaacagagattgaaaagttgaagcttgcagatttgacctgccgacagggagttattg

aagtagctaagattatatatggagttcatgatgaggccaaggataaagatttcgaactcg

agatgagctgggtgtgtgaggaatcaaatcgtcaacatgaaaaggtcccagaggagcttc

tagaagaagctaagacagcagccaaagcagctcttgaagaaatggatgcagattaaatag

agactaaaattatgtatcccaaagatgtatttgattaacttgtaatagagcattgagaac

atctatagaaaattcataggctttaaattaaagattgctctgaaaatttggatttttgta

tgcttcttgatgatttctgttgctgcggagaactgtttcaaagtgatttaaagactgctc

tcatcaattaatttagtgttgaattttaatcttttccatgccattattggaacctttggt

ttgagataacgttgtatgaaaccaaacacattattttcctttttcttacataggattaga

agcatcaagttttagcc

>fava_dn_rep_c1141

cgggactggttcacccctccaaacacaccatcaccatcaccatcaccacccttccactct

tttcctctacacacttcttgtaacaccgtcttaaaacagtgatgaaagagcattgagatt

tgagtgtgttgtttcgtatgaattgtttgatggatgaagaaggagaagcggtgagaaaac

aagaacatgttgttgttgatgatgttaacttgtatagctagttcttctattttttaagat

ccattttcactcatgaagagactcaccacctcagattcttcatctcctctcatcacaatc

tctccctcaacagaagaacatagtccgaggaacaaacatgtgtatggtatggagtttcaa

tccatgatgttggatggatttgaagaggaagtttgtgttgaagaagcgggtcatcattca

gagaagaaacgacgactgagagtggatcaagtgaaggctttagagaaaaactttgaagtt

gaaaacaagcttgaacctgaaaggaaggagaagcttgccatagaacttgggttgcagccc

agacaagttgctgtttggtttcaaaaccggcgggcaagatggaagacaaaacagttggaa

agagattatggtgttcttaaagctaattacgatgctcttaagctcaagttagatgcaatt

acacaagacaacaaagcttttcataaggagattaaggaattaaaattgaagctggaagaa

gaagaaaaaagtgcaagcagtgttttggtgaaggaggagatgataacaatgcctgaatct

gatgaaaccaagcataacccgtcttcggaatcatcgattcctagtttagaatctaaggat

catttgaattatgacagcatgatcaacaacaacaatgagtttggtgttggagaagcttct

tcactctttccagcggacttaagagatggttcatctgacagtgattctagcacaatttct

tcatctggaggaattcttcaaagtcatcttttgttgtctcctgattcttcctccatgaat

tgcttccaataccagaaatcttatcatgtgaaaatggaggaacacaatttcttaagtgca

gatgaggcgtgcaattttttttcagacgaccaagcaccaacgttacaatggtactgtcca

gatcaatggagttgaacttaattgacacaaaatgatgaagagagaaaatggtacctcaat

ttctttgtaaaatagggactggtttggtagcaaaactatggctaggttttcttcatgtga

aggaaagaacagagaaatggttggggcaggctgcttgtcccattaaggatggtttgggat

taaggtttgcatatggctttgggatggggttgaataattagtaagtggtaatgatgaaaa

tccacggtgtaaattattttaattcaagcataaaaaattgctttatctttctattatttt

acttttaaaattaataaagttcaagttttgttatgtttt

>fava_dn_rep_c1142

attttcaaattgtattaattaatttttattaacatatcactatttttttaatcactattt

atgttacatattttttcactatctataataaacactcttatacaaacattaattcattct

ctggtaagcatcacagttaagcataggctaatacatgaagtaggaaaaacatccatccca

ttcttagcttcataaagacaggtgagattcgtcttaaattgaaacttcagctgttttaaa

ttgtatataatataacaacagttaatgttgtcaccaaggatactaataactgtaacaagt

aaaaaaacattgatgacagatggacatttaaattagacacatacggcatgagcaagaacc

atccatggtaataaacaagaagaatttctgaaaccaaaaataagtagcagcagaaataat

tgcaaaagacactttgtctttcacaataaaatgattcaatgatctacccatgtaaaaatg

aaaacaaaaatagaattctacaacagacagtcaacaatgtctcacattccaaaaccaaac

acagaccgaatcctctggaaaatcagatccctccatcccttttctgtattctccaccaca

gtcgcgttcccatatctgtcttcttcaggcacatccgtgtgtgttaacttcacaactgta

actccagattctggctcctcaaacacaattctcaccaatgaatgcttcccatcaggccag

ctcccgaatctccatttctgaacaatcaacttcccttcctccaattccacattctccccc

gtcacagacccatcaaagatactaaactcaccaccaacctccttactaatcctcgcatta

ctctgcgtaaatcctttccatctattctcatccatcaatatctcaaacaaatccttcgcc

cgacaattaaacttctcattcaaactaatcgttttacaccccttcttcaccttctccttc

tcattcttcgtcgcaaccgcaacaacctctgccggctctttcttcttggctgccgcaaca

accgaactagaattactcctctgcggaggagcaaccttcttagtctcaagctcatcttta

acaggtccgcctttggacatactctccacccaaaccctaaccttctccaaaatcaacggt

ttccctttcacaacaaccgcatccttcaaactcttcccaataatcccttcatctttcaca

ctaaccctaacctccggatcctcatcggcgttctcatcggaaatatacggaatctcaaaa

ctgccaacaagcttatgcaagatcttcccttcggaatccctcgcttcaccttcccacgaa

atcgacacactgatctcatatcccggaatgatcttccccttccgaacattaacgtaggct

tcgccgtcgagcttctcgaccttcttaacggtggcgtggaaattaccctcgccgtcgagg

attttgaggttgttgaagagcttgttgaagaaggttctagaccattctaggcaattggtt

tccgaccagtgccagttgtggacgtttgtgccgtcgggtctgtcttcgacgatccagcgt

ttgtcgccttcgccgtattttgccattgaaattgggaatgtgaaagcgaatcaaacgatg

gtagtaataatgtaataacgttggtgttggggtgaggatatatatgattgttgtttgatt

tggaaggatctagagaaggaagaaaagaaaccctggtttcaactccaacaagtgaggctt

aggaaagatctagaagacggcttaactaggagttcgtcgttgtggtctaaattttt

>fava_dn_rep_c1143

ctttttttttttcaagaaaagtcaaagcatttattaatatctcaaattgcagcagcagcc

acacatgaccttacatgacttcatagaacagtagaacatataaccaatctgaactaacca

tattagtcattgaatttcacataaaagtatatcaatttcttgtagcatcaaccaattaca

tagtatataatcaataaacttacacatatttatattaatatttcattataccatgtagta

acaatctaacataacatatagttcgatcaattccaactcaaacacgataattctccagct

gaacatctttagcagtcaatggcacatactccccaagataaacttcaaggtgatcagcca

aagcagacttatcaatattctcatgatgataactcttgcaaaccaaatacaacacagttt

gcaaaacaagcccaaacaagaacaaacaagacagtaacacgaaacacaatatcccataac

ctgttctgtctacataaccaaaacaccatgttccatccacaaccactaccttaaaaacca

accttatcaagaaaaacgaaacattcagcaccaaaaatataaatatcgacaaacccattt

tccctttaatcaactccctactcttcaccatagctttgaatccatacaaatcttccaaca

ctgtcacaacactagctagttgccatatcaaagtcaagtaaacaaatccaataaaataga

atattatcaataaaataagcaaaacagctccaccgttttttatattaagtgtaagcgcta

ccaagataataaccagaaaggttagaaaattgtaagcaaagaaagcagcataggcgcata

gaaaagtgagcatgagtctcttccaaaccttagggacaatcttgatgacttttttgaagg

agagatctttggatgtgaagatggaagctgttgtatagacaatcgccgcggtggagagga

gagagaagatgagaaggaaagtgaagtagaataatttgaagagtaaaagtgtgatgaact

gtgatgaaactatgtcggtgagtttgtcgtattttggtgtgccttgttgggtttggtcga

gttgttgttcggtgtgtttgattttgttgaagatgaggtcggagacttctatgtggaaga

ggaagatgaaggagagagggaggatgagagttaaggtgatttgtgagaagatttttcgcc

atgagaagatgattttgaaggtttgtctgtagcaaccgaagaatcctaggaattgcatgt

cttcttgttctctgtccatggtttttggtttggtttggtttgatttggagaagagattgt

gatgaagatgaagaggcggaaaagactatatgggagaagactaatgttgataagagaatg

agtcaatcccgcgg

>fava_dn_rep_c1144

caacaaattaaaaaagataaaacaaacctaacactaatttctttcgccggtactaacaat

agagtttaagaagtcgtcattgtttgagcagattggaaaacaaaatggagacgaccgccg

cactcactggcattcgactgacttcttcgatccgcccttcccgtctttcctccccttttc

tttcccccgcatttcctcccaatcgttctctctccctcaagcttcacaagcagtttcctt

caacttcccagaacgttctcgaagcttcctctcttctcagaccttccgcctctgtttctg

ctacctacagtgaggcgattcagttggctgatgtagattgggataatcttggatttggta

ttcaacctactgattatatgtatttcgcgaaatgtgatcaaggtggaacgttctcgaagg

gtgaactgaaacgatttgggaacattgaattgaatccatctgctggtgttttgaactacg

ggcaggggttatttgaaggtttaaaagcctaccgcaaagaagatggtaatatactcctct

ttcgtccggaagaaaatgctttacggatgaagatgggtgccgagcggatgtgcatgccat

cacctagtgtagaacagtttgtggaagctgtgaaagacactgttttagcaaacaaacgct

ggataccacctcagggtaaaggttccttgtatattagacctttgctaatgggaagtggag

ctgtacttgggcttgcacctgctccagagtacacctttttaatatatgtttcacctgttg

ggaactacttcaaggaaggtttgtctccaatcaatctgattgtggagaatgatctgcatc

gtgcaactccagggggcactggaggtgtgaagaccattggaaactatgctgcggttctta

aggcacagtctgcagccaaagctaaaggctactctgatgttttgtaccttgactgtgtgc

acaaaagatacttggaggaggtttcttcctgcaatatatttgttgttaagggtaacatta

tttccactccatctatcaaagggactatcctgcctggcattactcgaaagagtataatag

acgttgctcaaagccaaggattccaggttgaggaacggcttgtgtcggtggatgaattgg

tagatgctgacgaggtcttctgcacaggaacagctgtggttgtatcccctgttggcagtg

ttacttatcttggcaagaaggtatcatatggagatggcattggaaaggtttcacagcaac

tttataatgtccttaccagtctacagatgggtcttgcggaggataccttgaattggactg

ttgagctgagataagaacactgagagtagccttcactgcatcttttgctgaaatcttttt

cttaaattttggggttatgtagatatactgtggtgtttgtattactatttattgaataaa

ttatagcaagtagaacgtaaaaaagataattctgcatgtttattttctagccaactggga

tagtcatcatcagaggattagttaaccttttatttggttgaaaaatagacattctagcag

tggtaattgagtagccttgagatttgatgagattatatgaaatttaaaatttaatgttct

tggggttgattatccaaatttcaacttgcaatctatgtttaatggataaaaaagtcagac

ttttgaaaataaattgctaat

>fava_dn_rep_c1145

agaggaaaannnntctgttaacatgattaaaattcaaccaaacatctatgccggttttga

aatatgatatagaaacgttaaaatacacgtagtcaaaaacctgaaatttgtttcgaagtt

cttttacatatgatatactttattaaaattccaacaaaatgtcagtggcataaatttagc

agtgaaaagaaaacctcccaattggaaagatgcatggattccatcagagtgcatttcatt

agaagcagcaataaaagggtatacaatttctgctgcacgcgcaagcttcatcgacaatga

tctcggatccttgtcttttggaaaactggcagattttgtcatattatccactgattcatg

gaaagattttgctgaaacagcatctgcatctgtagaggcaacatatgtttctggagtgag

agcatatccttgaaactcttcatagtgccgaacattcgggcaaattcaagcccaaaagtc

caatccagcccaaaccgcaaattcaacattatttcggaccattcggatccgatttgtagg

ttgggcgggttactctgtcatgtttcaacaaggttgaacattaatggtataaaaatgttt

cattaaataagtagtttgaacttacaaattatcaagtgtcaattctaggtagaatgaagg

aggtttcaaccttaatcagaatagagttaaaacactacaagagtaattaaaaagtttttg

taatttatataaaaacattagtttgtccatgataaatgcatcattaaataaatatgaacc

tacgaaaaatcaagtttcaacaatgccatcacttcctggaaacatgcatgagtttgaaaa

ggaccattctgaatggcagtctatacattcttatcaatggaatttatgtgtacaaagatg

tttgattttcgttgttgttgttgaattaagatgattgagaagatgatgaaaatgaagtat

ctttcaggcttcttgctgctcagaattgatgctcacaattgtaatgtgtgtcatactttt

tgctgctgaaaaaacttacttatatatgtaacagaaaatggttagagacagaaattcgta

ttatgagttgatgcatgttagcatgagtcactcatgttagttatg

>fava_dn_rep_c1146

agaaagcctttttatacgctgatgaataaaaagtgtaaactagtacattttatagattca

catcaactatccagcccttacacaagctttatataatccaaatatcacctttgaaatcag

tttgttcaaaagacgcaatatctaacatgttacggggctttcaggcaaaaaaggacatca

attttacaacaatgtctatatttcactcatttgaggcataaaaacaatacctaaaactta

tattctaaggacaacacttcaacatcaagcaattacattgccttccaaaaataacaaaat

taaaagaaaatttaaccgacgtcaactacattcagttttacattagcgaaaacccttagt

gtaaaagatgtatgagtgcttactgaaatagaatggaaaaaaaagtcttacgacgatgtc

acctctgtctgtccagagggcaagaaactttcccaccatccagattcatatttacgatac

ggtgccacccattttgcaggcttctcaaattccagattagtaggattggaggctaatgct

tcttctaggctgtcagcttcaaaacttcctgataaaacgcggtccagtcttaacctcatg

aatgtgatccatggaccattggttgacacaagagcaacagcaggtctgcgcaatctcttg

gtgaagtcagggaaactgtccaagaatttaggctcaatgacaagccagtaatcttgttct

ttattacgctctccgtagtagcgacgtcgttcaaatagttgctcttggaaatgctcctcc

tcatccaacataaattttgcatttgcaataataaaatagtacttgttgaactccttggta

gcaggagcatcagaagagctaacgttatcagattgaagagacgcaacagcagtggtaaaa

ggtgtttgtttcttgaaggaagagagtaaggtgaaattttggttggtggaagagaccaaa

ctcattggcaaagagtgagttttgtaggaaagattgtggtttctgttggaagaggtgaga

gtggattgagctagcattgcagttgatgaactcaaagttagagaccccatactcagcatc

ctgatcttcttgttactgttgttgttgtggttgtagttgtgatgagataaccccgctaag

gtttttctgttacttaatatgtagagagagttaggagaagatgtgatgacagcaggaaaa

gaatagagaaagatggcaattggcatacagagaatgggtcaataggaagatatcaggaac

aaccttatctagttatctaaaatataacttatataagtccccgcg

>fava_dn_rep_c1147

tttttttttttaaaaaacttcatcttatctatattggaaatgatcagaaatactcattgc

tcaacattaaatgtacaaaaacaatgaaaccaaacaagatatcccaaaaggagtaataaa

tgcaaagaagctacccctggtgatactattaagttggtcaataaatttatgatgaaacta

gtagttatatctgtccccaagtagaccaattagcctcacttccaacacgcggcatcatct

gattccttgactgagaatcaacattgaagttggctccacacataacaccctcactgtcaa

tcctaggcattgctttcatcttctttgttggatgttcaaagttcaacagaccatgctcac

tatgaaacaaacacccatttggaaaaggtgcaaatgatttagcacaaccagacttaacaa

gctccacattttcagaaataaccactgacccatcagctgcaataccccaatacagtccaa

tttggccatcagagccagatgcagcaaaaactgtttgatttttatggtcatagatcacaa

atccaaaactcccttcaaattgtttaaggacttgatcagctgggtatggaccccggtcgc

gaagtgtcctatacgcctcgatgatgaagatggcctcattgcttccctttgatagtccat

attgcttgttcaattgactaaggttgtgtagatttcccatgaaagcacaatatatgttgt

ccaaaccactaaacaacctttgataaatggagggtgtgtttgaaggggaataagctaata

aagcatcatttccaaaagtcatgtagaaagcatcggaggaattgcatgagatgaaatctc

taagaatttcgtgacttggtttagctttcttagggtttgaattctgagaagctggactat

taagctcttttggtgcattcaccaacttttccttgaaaattcccaacatcttttcctctg

tttaaagtgacaattagcaagaaataatgagatcagatttggtgagatagattgaaaatg

gataatggatccccgcggcct

>fava_dn_rep_c1148

gggggccttttgttttttctttttttttttttgcataaaaagatagctaaattattgtca

taacttggtcttacaattacaagtacagtataacttatatcacatagatttagctcattt

aaccaacaatcatcacactctttttgttgtttcccttttattccagaacacaagtacatt

cacaaacaaaaacatacataaaagttcacatgccatattgccctcatatcacaacaatat

gcacctaaaatccatcacacataatgcaactatgctattacatcacaccggttaattatt

caccaactaactttgagcaagagcgagatagaaaatgtcatccagtctgtcattactagg

agagttcccaccatataccaataagccctcacgaccacctcggcgagcactggcgaatgc

gcaccatccacgcggcccaggatggccaccggattccaccttatcgtccaacctcgtcca

tgataatgtctcggtgtctaatgcataaacctcgccagaaaattgaccagcacccatatg

accttggtcactaggatctatttccccaccatatacaattatgtgtttccctgtcaaaca

agttgaaaacacactacgggccgttggtttgagcccacctgtttccacttgggcccatgt

tttatgagccagatcaaagcaatgaacgtcatcgacttccattcctgcgaaaccataaac

aacccatatttttccttgggaaactgttaggcccggcccgcctcttcccttgcaagtttc

accgggagatggaaattccacccacttaccatcaacaacatcaaatgcccataaatcatt

gagccttccagcgaccccgcagccgccaaaaacatacacatgtcggtcatcagccgtcat

ggaatggtagctccggtgaggaggcccaatatctccacttgaaaccaaggcccagttgtt

ggtcttagtatcaaaagaatagagttcattgagttcattgtgttcaacatctcttccacc

aaatacatagatggtttctccaactgcagccattgtgacaccaacgcgtggcggcggggt

ggtgccggatacatcggctactgaccatgtcaaggtatctaagtcgtacacgtgtaactt

gttatcaacggggacacgaggcacaaactcacccccgaacgcatagaccttttgccctac

tacagcaatagcatgagaacttcttgctccttgtgtgattcctctttgatcaagcttgac

ccaattgccatgaagtactgccattgttgggatacacttgctgtgtgtggctcaatgtgt

tcccggattgtatgta

>fava_dn_rep_c1149

cttttttttttttgagagaatatattttattgctctgcaaatgacgactacaaaaactaa

aggaagatttattttaaatgacagctactactaatctatttatacacacatacgagaacc

atatacacaacatactaaaatactaaattaccctccaataaattcacctttgacttctac

cttgtgatttggtaaagacataattggaaattgataaattttcaaagatgccaaggttga

tgaacacataacgttagaatagagggttgaattaagtccaatcttaccttttaagatgaa

gcaataaccgttataatctttaagacctaattacataacttcaatctcaacaactaatag

ctacatatttactatttttcacatacttaagtttaagtacaccaacttcatgccacataa

tttcttgagacttcaacttcaatgacattaatagtgctacaattttgaagttacgaattc

aaccatgtgaattagcaagtttgtttatcaaagccaaagcattgctagtcaattgagcca

cttgcacaattttccctctcactagtgtctgagtattgtcctgtgcattaatctcttgga

atccgtcggtacaagtgctctcatccgtcagagcagcactaacccaagtttgtacatcat

ttattgttagctcaaaatttgaattcctaagatgactcatctcacctatagaccttctga

gttcatcgacggagtcactcaacacttcgacacaatccttcatagctgcaacctctcttg

gcttcaaaccgcggcttctagagagcgttgacatgaaagctgaggttgattttgccgatg

caagtgtcacgtttaaggctgtgttcgttaaaagaacgcgatttgtttgaatgaaatcgg

catgcttcactaaggatgcatagcatagtcttggataagttgtggaactgcaagagtttc

taatgaataaggtgttggtttctcttgcttgtataaagtgtgtgatgaaggtaaatgcaa

aagttatgagaaggaacttggaagaaaatgcttccatttttttttcttgctattttattt

tatatcacaatttggactttggtggatcccgcggcag

>fava_dn_rep_c1150

aattaactataatttattatttaatttaaaaacgataataacagtaacagtattgacatt

cttcaaaactgtttcgtcgcatccgtttttacagtcgcgaaccgttttttaaaaccctcg

tgaatataaggttttgagaaacatataaggtatattgagtcacctctattgtatttccat

aataggtaccagccttattattgatatatattggattgagttttatgatattcatgatta

caaaatctagaaacacccgtaaccttgtattgtactatagttatacaatactataaggct

caaaaccttagttgatatttctacaattcttccttatctgaccactagacccagtaagtg

gactaaggtttcccattttgatcattgcattagcaaaatcagctccaaaacttgcagggt

tgttaacataagaattaacttgagaatctgtggaaccaccattgaaaagcacttgatcag

agtgaaagagaccctttttgttttgcaagttcctgtaataagcattgtcaaatgtgtttg

gtgtggtggtatctagtggggacaaatttgcatcaccaccactggtaggacaatttgctt

gcaatgattttgcaaaggtgggatctatgttattgtcgttgtaaatcctgtttctgaaaa

atctgcagctaccttgaccaattgagtgtgatcctgaaagagcaacgatttctttaactg

tgaaacctttggcagagaaagcattagtaaggtcacttaattcagaggagggacctggca

aatctgagttagctgaacttaaacttgctgttgttgaatcccttcttcccaattgaacat

tccaactaggtccaccgagaacaacaaccgagtctcttgctgcaacagcaagaatatcag

cacaagagacaatattaggacataacctctctactttggactttatggtgtcaattacat

taaaacccctcaaggaaccagcatttggagctgctgttttttcacctgtgaaacttgatg

tatcatccaatagtactgatgcatcacaaccttgaacaaagcaatcatggaaatgaagac

gaagtagtgatgcacccatgcgacgctcgttgataacagcagcaagtatttctaatttaa

tggttggaagcaaaagaggacatgttctgctataaaaattcgaggacaattgagcagaag

ttgtgccaataaggcaaaagagtaagaaaaaaacaactttggtgattggaaaagtcatgg

taattgttatttaaaggaaatattatataagaggatgcaaggaaatcatacgtttatggt

tagtgctttgtgcccgcggtccttttgaataaaataaaagtcggtg

>fava_dn_rep_c1151

gcggggtctccaaccaacaccaacccatcccatcccactctctctttcctctatccttcc

ctttatattaataatcctctcacacctttaaacttcagggctcaacctcaaaccagctct

cacacattcagaggcaacccttttttctgccataaaaaacaatacaatttcctcacccca

acgcaataaacactgacatctataatctatcatccccaatcccaaacaatcatattccat

ttttgcataaagcaaaatggcttctccaccagacacaagcaaaaccgtaaagcttgaacg

ttacaatagctacctcagaaaagttaacagcacaaaactccttaacgcatcgtccaaact

tctcttccgagccacacttttaatagcactcgtccttgtgttcttcttcactttcaatta

ccctccactctccgactccaccaaccaccacttccacacccattcccacttcctcgcctc

cgcattcggcggcggcggtgcatgggaacgacaaatccgtcactccgccacaccccgccg

tcctaacggtttcattgtacttgtaaccggtgcagccggtttcgtcggttcacactcttc

cctcgctcttaaaaaacgaggcgacggtgttataggtctagataacttcaacacctatta

cgacccgtcgttgaaacgcgcgagacaagctttattaactcagcatcaaatcttcatcgt

tgaaggtgacctaaacgacgcgccgctgttaacaaaactcttcgacgtagttccgatcac

tcacatccttcatctcgcagcacaagccggagttcgttacgccatgcaaaatccacaatc

ctacatcaaatcaaacattgccggtttcgtgaaccttctagaagtttcaaaggctgctaa

tcctcaaccagcaatagtttgggcttcatcgagttcagtttacggtctcaacactgagaa

tccattctcagagcttcaccggacagatcaaccggcgagtctctacgccgcaactaaaaa

agccggcgaggaaatcgctcatacttataaccacatctacggtctctccctcaccggact

tcgcttcttcacggtgtacggtccttggggaagaccagacatggcgtacttcttcttcac

aaaagatatcctccatgggaaaaccatcgatgtttaccaaacacaagaaggaaaagaagt

cgcgcgtgattttacctacatcgatgatatcgtgaaaggttgcataggggctttggacac

agcggagaagagcaccgggagcggcggaaagaagaaagcaccggcgcaattacgaatcta

caatctcggaaacacgtcgccggtaccagtggggaaactcgtatcgattcttgaaaactt

gctcagcacgaaggcgaagaaacacatcctcaagatgccacgaaacggcgacgttccgta

tacgcacgcgaatgtgacattggcttacagagatttcgggtacaaaccaacaacagatct

atcaacgggtttgagaaaattcgtgaagtggtacgttcgttattacgggattcagtcagg

gctaaaaaaggaaaatcatctcaataacgaaatacccgaggattccgcttgatttgcaat

ttgcatcatatcacatgcctctcattttcaaaaaatttaaaaaaaaaccttctctcattt

ttcttgttttttcttttttggcttttgtgaaataataattatatttttttaaaaatgttg

ttaattattattgtgtcggtgatgtaagaaatatagatatatggtatagaaaaaaagaat

gatataaatgtgaaagaaagatcaaagaaagacaaaggaaagaagaaggtgtagatctat

gagttggaggaagaaaagggaggtgtaataattatgagtggggtctactacaaaggttgt

agatctctctctttttttattcatatataaatttttaagaaaaaaaatgaaacaatgatg

atattattaaacaaattattattatgtgacatctaccaatcaggtgacacaaaaaaacac

tcatcaagagtagaaaagaagatgaggatacagtaggttaactcttgcatgtcctattat

tgttttgagttttaattacagtatgcatcaagtgccaactttgggtgatttgagaaatac

taatggatggtactctagtttcatcttattgattgtctcctatttttctgatactaattt

tgcatttattgttattcatataattgatacagaaatttaaattacatggatagtattcta

ttctattctattatatgtacttggtctaaaccatatatcaatacacatccgacctgaatt

ttgaacactagtttgcacttgacagtggaaatttcccaacaaaatcagtattaagactac

ctaggtaaagatggtcctcaaactccacagctgaagttaaaaaactaatcaccttccctt

catgatcaccaaagtttctgataatattgcctttagttccaacatttaccaccattgctg

acttggttactccattgatcagattaaatagccatggaaatgaagctaccaagtgcttgg

acaccttagaggtgtgcacaaaccccagtctttcagaagcaaattgtttaaccaaagcga

tccaaaatgagccatcaggagcaagattgatattgtcaggtccaccagggaggttttcaa

tgaagatatctgttttgcctttgttaattccttttagccaatgcctcacacacctatatt

tccaactttcgcaaaccacaacataatcttcatccttagacaatgcaacaccatttgcaa

aagccaactcatcaagaaccacaactgtctcattcaacatagggttatacttcaaaagtt

tcccatgaggtcttgcctcaagcacatcaagatgccaattatgtaaaccaaattttgtac

tcgcaacactaaaatatatatttccatctgatgcttctattacatcatcagcaaacagta

attgagaaccattaacatgtgaaagaataacactgaaaccatcttcttctgtaaccttaa

gcaaacctatggaagcatcacagacaatgagaccaccgtcttttgctgttgtgattccaa

gcaaagaaggactatctatatgcttccaattctcccagtttccattcttgatcattcttt

taatccaaccgtctcgcgtagctgtatagatgataccttctttatccacacaaacatctt

ctggttgcttcagaaaaccttctccgagtttaatcaaattctgtaattggttgtttttgg

atgacgatattacagaaggtgttggttcaaacaaaagtggatctatgggggagaagtaga

agacttgaactgtgagagaaactaaagctgctagaactaaggttgatgtcagtgttacac

aatggttttttgtggtgagaggaagattattcatattagaagccatgctcttggagttgt

gttgctctgctattctatatagcctataatggaggttatttataacaatatcttgaatcg

tttaatggtaaatttttggctatgcggtgtatat

>fava_dn_rep_c1152

tcggatcctgttacagaatgtgtgtcacacttaaaaannnngatttttaggttcgtgtgc

acaaactatgaataaactagtttttagatatataagaaatgtcattcatatgtccaatgt

cttaagatcttagatggagatttgatatctctttgtcttgtaatcgtgaaacattgtttc

attgtttcttactacctcttttgaactctttaacaaatagtatcaatattctggttcaac

ttgatgggggaaaagggagtgggatattgttattagatcctgttatagaatgtagaattt

gacttcttgttgctcctgcgcttcatactctccagcagagatttaaatagaaaagcatga

ttaatcctgtaccgaccaatgatagtagttccacgattgatcctaggatgatgctctcga

ataccaaataactccgcacagacatataacacttaaatcacactaaattatacaccattt

tttaacacagtagtactttgtacaaatttacaagttcagcacaaacaaatgacttttttc

actatgtctgatacaaaagcaaacatgttttgtaacaaaatggcaacaaggtcctgcaaa

ctatgttttctttacaagaaatggttcaagaacacaagacttaggattcttcataggagg

acctttttctcttctctctagttcatcaagcctgaaagtgatcaacttatcccaattccc

tccttcaaacagcactcctgctttaccatcagtgattctttgaacaatgccgcagtacat

gtaaaatggattattttgattcttcacaatagcaatcattcctggaagtaacataggaag

ttctggtggtttcgccttttctgacgatgaaagtttaagtcttttgtcatctgcatcagg

ttttggaacagggttttcttcaatgaattttctcaaccctttttcacctccaggaaaccc

tccggttaatcccattaactctttctcaaaaccgtcttctgcaacactttcaaaggactg

tgttgagttgttactagcttctgcaacactttcaaaggactgtgttgagttactagcttc

tgcaacactttcaaaggactgtgttgagttgttactagcttctgcaacactttcaaagga

ctgtgttgagttgttactagctgcttctgatgatggtggttgattctgatcatcggaacc

aatttctcttttgagctctttttgaagtccttcttctcctttacagagtcctctgcctcc

catgatttgcagcaggtcgaattcggcacatatccgtactgttttgtaggctggtttgct

gtgaaagatggaagctttattgttgtgaggataaagatgagtgagattttcttggcctag

gaatttggatcttagaagagagccatgaagggtattaagggaaacaaaggaagacatttt

taactaactgttacgaaaattggctaaagacagcactattgagagcagaaagtggaagag

agagaatagaatcataatctaatcctgtccatagaataattggataggcctatctttttc

ttgaaatgaaaaactagtagaaaatcttcatcccatttaatcttgaccacaaagttgatt

ctttgagacgagaattgct

>fava_dn_rep_c1153

aaaacatccttaaaatctgtttctctatactatatccactccataaaannnnnnnnnnnn

nncttcactttcattattcatcttcactactactcttctctttctcttcaccatatataa

ctagcattcattccattcatctttttcttttgcttagtgacaaagtaatcaattatggca

ccaagcttatctgggaaaagagagaagcttcttccggtgaaggatagtgatattgcgtat

tcacagataggaattggtttacaacaacaagaatcagaagaaactaagatgatgaggttt

aagaagaaggtgaaaagtgttgctaacatggcgtatgaaatgggtcggtctgatccgagg

aagattatctttgcggctaaaatggctcttgctttgattcttatatcacttttgattttt

cttaaagaaccgtttaaggatatgactcgtcactctgtttgggctattctaactgttgtc

gtagtctttgaattcagtataggggcgacattgagtaaggggtttaacagagggataggg

acattatcggcgggaggacttgcggtaggaatgggagagttatcggcattggcgggagat

tgggaagaagttatagttattattaatacgtttgttgttggattttgtgctacctatgca

aaactttacccgactttgaagccttatgagtatggtttccgcgtgttcttgattacttat

tgctacattaccgtatcgggttatcatacgggtgaatttcttcatacatcgataagtaga

ttcttgcttattgcacttggtgctactgtatctttgggagtgaatgtctgcatatatcct

atttgggctggtgaggatctgcataaccttgtcaccaagaatttcatgggtgttgcaaca

tctttggaaggggttgtaaatcactatcttaattgtgttgaatacgagaagataccatcc

aaaattctgacttatcaagctgctgctgaggatccagtttacagtggctatagatcagct

gttgaatctacgagtaacgaggactcattgctgggatttgctgtgtgggaaccacctcac

ggtcgatacaaaagtcttaaatatccatggaagaattatgtcaaagtaagtggagctcta

agacattgtgcatttatggtcatggccatgcatgggtgcatactttctgaaattcaggct

ccggctgagaagagacaggttttcaaaaatgagctgaagagggtaggttccgaaggagcg

aaagtgttgcgtgaacttggaagcaaagtcaagaagatggagaaactaggcaggggagat

ttgttatacgaagtgcacggagctgccgaagcattgcaacagaagatcgataaaaaatct

tacctcctagtgaactcggagctctgggaaatcggaagccgtcccagagaagaaaccgat

gataatcattccaagggtctctttgacatggaccaagatagaaaatttctagagtacaag

tctctaagtgaagctgtacttgatcttagatcgattgaagttcaaaacaattgggatgga

gaaactacccatagtattaacaactcagctgtgcctgcttctattgccgatgaaaacatg

tgtatgaaacaacaacaacaacaacaacagtcttggcctgctcgtgtttactacaaacca

gatgccaaggcaaaagtggaagaatcaaaaactttagaaagtgctagttcgttgtctcta

acgacgtttacatcacttctgattgagtttgtagcaaggcttcaaaacttggtggattca

tttgaagaattgggtgagaaagctaactttaaggacccccttgaaccagttgtgataacc

tctggcggattttggaccagattgttcaaccgcttcagatcctcgaatagtttgcagtaa

ctgcatcgttttgacaaatgcgacagaaatatttagagctagagcaaatacttaactttt

ttacattgtatttttaaaatgtatacagatcttcctatttatatcatgatatcataaatg

aatacaattttgaactaaaataaagtcgactcgtcatgttgcttccccgtgaagacgata

ttttggcaccgcaagcaagtgtttggctcttgggattgaaagaccaaacttttctgtcat

gtccaaatcatttggagtcatattcgagggaagatcccaagaaaaacaatgcaccagttg

agtgacaacaagtttcactgtaattagtcccatgtttattccaggacatcctcttcgacc

aaaaccaaatggtataaactggaaatcatttccgcggtaatctaaatttttatccatgaa

tctctctggataaaattcttccgcattgtcggaccatatcttagaatctcttcctatcgc

ccataaattcacaatgattcttgatttcttttctaggaaataaccatcaacggtagcatc

ctccgttgactcgcgtggcacgagtggccctgcgggatataatcttaacgtctccatgat

cacaatatctaagtaacttaactttgacaaatcattttcctccaccactttgttgacccc

aaccacattatctaactcttgttggagatttttcattactcttggatgtctcatgagctc

ggataaagcccactccacgacggtagcagaagtttcgaatgcagcagcaatcatgtctaa

gactatagcctttatatttgttcgatcaacgacgttgttttgctcatcagagggaccgat

cggctggtgcatctgtgacagtagaatgtctataaagtccttatgcttctgtcctccatt

ctgtacatctgaactttctttatgctccttaactatcttctccaagacttggtcaagttc

tttatgagttctcttgtatcttcttcttataccctgaagatcaaaaactcgaagaaaagg

aacataatcagcaagattaaaagcaccagaaaggttcgtcccttgttgaatcagcctctt

caaatcaaaaacctcatcaatactacaccccaacaccatcttacacacaatatccattat

aacatcatgcacaaactcactcacatcaacaacctcacccgaactcgcagctttcttcaa

caacttcaaagctaactccatttctctcttcctcaaagacgcaaacgtctcaactttaga

agcacttaaaagctgcagagtgcaaatcttcctcatgttacgccaatacgcgccatattc

agcaaaaattaaccccttagaaccgtaagataggtagtgcgtggcttcgagtttaggacg

gctggcgaaagcgaggtcgtttgctttgaggaattgttcggctgctgaagaggaagagac

tatgactgttgggacttggcctaaacggagtgacatgatgggaccatgttttttggataa

ggcttcgagggaacggtgtgggagggtgcctaacatgtggaggttaccaatgagaggata

gcctggtggacctggtgggtgttttctgtcatgcttgggctgttttgaacggaggatgaa

ggtggtggtgatgatgagaatgaaggtgagtgtgaaaaagaggaatagttgaatcattgt

tatggctgaaagttgaaaatgaaataatgttctttctgttaaaacccaaaaaaaatagaa

ataaaaaagaaaaaaagaaaggttccacaagtgtcgttaaattgcattgcatcgcatcat

atcatattcaccaccaccgaatagaagcaatctctgaaggcaaagtgtttgagttagact

gtttgacaatcaaatcaagatccatctttcttcttcttctgtctttttcttatttcgaaa

ccctaaacccccttttcattccgatccgagatccaagaaaatggatgaagagtacgacgt

catcgttttgggaaccggtctcaaggaatgcattctcagtggtcttctctccgtcgatgg

cctcaaggttcttcacatggatagaaatgactattacggtggagaatccacctcgctcaa

tcttattcagctttggaagaaattcagaggagatgataagcctcctccacatttgggttc

tagcaaagactataatattgacatgaaccctaagttcattatggctaatggtatgttagt

gagggttctcattcatactgatgttacgaagtatctctattttaaagctgtggatggaag

ttttgtctttaacaaagcaaaggttcacaaagtaccttcaaatgatatggaggccttgaa

atctccgcttatggggatatttgaaaaacgacgtgcgcgcaagtttttcatttatgttca

agattataatgagagtgatcctaaaactcatgaggggctggacttgacaagagtgactac

taaggacctgatagcaaaatttggccttgatgataacactgtggatttcattggtcatgc

tttggcgcttcatagagatgatcgctacttgagtgagccagctctggacactgtgaagag

aatgaaattatatgcagagtctcttgcacgatttcaagggggatcaccctacatataccc

tttgtatggtttaggagagcttccccaggcatttgcaaggcttagtgctgtttatggtgg

tacatatatgttgaacaaacctgaatgcaaggtagaatttgatgctgaaggaaaggttat

tggtgtcacatctgaaggggaaactgccaagtgcaagaaagttgtttgtgatccatcata

tttgcctggcaaggtgaggaaggttggaaaggttgcaagagcaatagccatcatgagcca

tccgatccccagcaccaatgactcccaatcagtgcaagttattctcccacagaagcagtt

gggtcgaaagtcagacatgtacctattctgttgctcgtattctcacaatgtttgcccaaa

gggaaaatatattgcatttgtatcaactgaggcagagacagatcagcctgctattgaact

taaacctgggattgaccttctaggacctgttgaggagatattttttgatatgtacgatag

atatgaaccagtcaatgaacccactctagacaactgctttgtatcaaccagttatgatgc

taccacacactttgagtcaacagtagtggatgtcctcaacatgtatacattgataaccgg

caaggttctcgacctctctgtggatcttagtgccgccagcgccgcagaagagtaaagaaa

aaaaatgatgatttcctttgtgtttgttatgtgttgccaatggaatccttacattggact

ttatgacaaatgttaacagtttataataagtttattatatagattgtttttgttttggaa

aaagaatactctgaatactgtagaacagatttgtttccttcccacacccagatgctccca

tgtgcgtaatacatttgttatcttaatgtatgatgtgtgcggcatttcttttagtttcct

aatagcctttattttcgtgatctagtatgtattttttgaggaagtctcgagtaaattgtt

atgttcgg

>fava_dn_rep_c1154

tttgcagggcatcccagcaagttcgttgcatttatttagttttccaatcttcaaaatatc

atttatttgtcaactaaattcattcaattagatgcaccaacataaacatcatactgtaaa

ccatccaatcttattttagataattctattttgtaaagcatgaaatgttacaactgggca

cagtagttaaagacctctctcactgtgcaatcctagcacatcatgaaatccatggttaca

accgcttcaaactagttattagcacaaccaactctaatatgcatcatcatttgcatctaa

attcagacaaggaaccatcactgtcaaaatgcaaaccagtagctactaatggcaactttg

atcttgagcagcatcactcttatcaccggcaacatcaccagtagcaacatcgtcagtaga

acttggatcctcaacgtcaacttcagaaccatctcctggagcataagctgagcgccttga

tcgagcctgctgatgagttggctgcagttttggacggagtgcttcaagtggtcctttagg

cttttgtgctccttgcttccccaaagcccaatcagcagaatcaaaataagcacgttcatg

atccttggatattagtggaggcttctttggcaatctccctccatactttttctttacagc

gtcttcctcttgttgagaagtgggcattgagttttcattatttgcatctttctcagaagc

tttgagatcatcctcagaattattattattattactattattatcacagtgaacttgatt

gttcacatcctcatcaatattattagcatctgacattgcttaatctccctctctttcgta

atgggagaaacagaggtttgaaattggcgatcttgggttgagtgaaaggagaagataaaa

gttagagattgctatgtgatgagcatctcgaactgcgttgcgacaaagtcacggtatata

taggaactagaacatactaaaacactacaatcatccattttattaaataaaatatatttg

gatcttgcaacatgttgcaatttgcaaatgaa

>fava_dn_rep_c1155

ctttttttttttcggatcaaaacaaagaaaacttcattcataccttaataatttacaaat

ttattacaagcagtggatagagaaaaaacgttacaaatttcaattttgaaagaaaaaaaa

taagccaagaggttcaagacatcatgcaaacagtgaacaaagtttaagcttcaaagtacc

aacaacaacacttgctcattttaagggtatgtttcagttgctggtgctggagcatatgaa

ccaataaagatgtcaccataaatcaacccagccaagcctcctccaatcaatggtccaacc

cagtagatccagttatcagcaaagtttccactaaccacagctggaccgaatgagcgagcc

gggttcattgaaccgccactgaatggaccggccactaagatgttggcacccacaatgaac

ccaattgcaataggtgcaatggtgcccaatgaaccctttttggggtcagctgctgtggca

tagacagtgtaaaccaacccaaatgtaataataacctcaaacactaatcctgcaagaggg

tttaatccagcagcaactccatgggttggaacactctttccggtgacgaagctgaggagg

agagatgcaaggatggcgccaagtaattgggcaatccaatagaagagaccagttaggatg

gtgatgttgcctccaatagctaatccaaaagtgacagcagggttcaaatgtccgcctgag

atgttggctgctatggacacaccaacaaatagcgcaaatgcatgagccacagcaactgcc

actagaccagctggatcaagagctgcatctgatgttagatcattgtaagcaatggctgat

ccaactccagcaaacacaaaaatcaaagtggcatgaaattctgatagataggccttaagt

gaggcaacgctaaaagagtcatcaaaggaaccaagagttatcttcaccattttggcaatt

cacttgaaaacaaagagctaactaattaaccaaagactaagatgttttttttttgaacct

atctaatcaatcaaatattaattgatgatgcattcccgcggcc

>fava_dn_rep_c1156

taataagaaattccaaaaaaaaaacgaaatttgaatcccggtgtcgaactccggtcgttc

attggcaccgccgtcgtcaatgtcattcgccaactgtgttctacgctccaggattttagg

ttccggttttttccgagcgtaagaaatctaagaagaatagaatgggtagtagtagcaaga

gtaggaaatcaagaagaaaaaggaaatcggatgattctgcatcggaatcaacttcttcgc

aatccgatgattctgatagaagccatagaggtggtgatcgacggtcacggcggcggaggc

gaagccgacatgactcgggatcggatagttcttctgatggcagtggtagtgataagggtg

ggaggaagaggaaatcctccaggaaaattactgaagaggaaatagcacagtacatggcta

aaaaagcgcagagaaaggcattgaaagttgctaaaaaattgaagactcgtactgtgtcgg

gttattcaaatgactcgaatccatttggtgactccaatcttaatgagaagtttgtttggc

ggaagaagattgagcgtgatgttgttcaaggtgtgcctattgatgaattttcagttaaag

ctgagaaaaagagacagagagaaaggatggcggaaattgaaaaggtgaaaaaaagaagag

aagaaagggcacttgaaaaagcacggcacgaagaagaaatggcattgttagctagagagc

gtgctcgagctgagttccaggactgggaaaaaagagaagaggagtttcatttcgatcaaa

gcaagtttaggtctgagattagattgcgtgaaggacgtgccaagccaattgatatcctta

ccaagcatcttgatggctctgatgatttggatattgaaataaatgaaccatacacagtct

tcaagggtttgacagtgaatgaaatggaagagcttcgtgaggatatcaaaatgcatttgg

accttgacagggccacaccaacccatgtagaatattgggaggcactcctactggtcagtg

attgggagctagctgaagctcgaaaaaaagatgcacttgatcgagctagggtgcgtggag

aagaacctccagctgaggtgcttgcagaacagagaggtctgcatggcagtgttgaaccag

atgtgaagaagcttttgcaggggaagacacgtacagaattggaggctttgcaggcgcata

ttgagtcagaaatgcgtactggtgcagcaaagcttgttgagtactgggagactgttttaa

agcaccttcgcatatacaaggccaaggcttgcttaaaggaaattcatgctaaaatgttac

gcaagcatttacatcaccttgagaaaccatcagaggatgaagaagataaattggaagatg

ctcatgtaatgaaatctgaggaagatattgaggactatgttgaatttcaaccttcaaata

gacctctttctccagaacccatcaaaggagaggagggtcaagaagttgaagacgaggctg

ggtccttttcacccgaactgtttcatggtgatgaaaatgaggaagctattgaccctgaag

aggaccgggttctactggaacaaaaacgtatggcagtaaaggaagagcagcagaggcgaa

ttcaggaagcaatggcatcaaagcctgctccatctgaagataattttgagatgaaagcta

tgaaagttatgggagctatggaagaaggagatgtaatgtttggctctggtgctgaagtga

atctggattcacaggtttattggtggcatgacaaatacaggcccaggaaaccaaagtatt

tcaaccgtgttcatactggatatgagtggaacaaatataatcaaactcactacgatcatg

acaatccacctccaaaggttgtacaagggtataaatttaatattttctacccagaccttg

tagacaagataaaggctccaacttacacgattgagaaggatggcagcaatggcgagactt

gcatcataagattccatgcagggccaccatatgaagacattgctttccgcattgtaaata

aagaatgggaatattctcacaagaaaggttttaagtgcacgtttgaacgtggaattctac

acgtatacttcaatttcaaacgccaccgctaccgcagataatttcaggcactggcttact

ttctggtgaatttctcttgcaagcttgaggaaactaaatggctacctttttgagtaaaac

atacggaaagtgaagtgtttcttttagatatttggaaaacttgtgttactcgcagcaatt

ttttttatcaaagttgaaggaatcagtaatggtgaacagccactgtcgcgaacaattatg

aatgaatggtctgcttggaaacaattgatgtatggtgcaagaacacaaagcatgaaaaca

actattattgtttgtatttatgtggtctcatttcatgaacttttgtactcctagcaaaga

caagtcctgtattgagatattaaatattgaattgaaaacaattaatcaatttttttagca

atgtttcaaccttttcaatgtttcttttatgccagtccagaaacaggtaaaattctatgt

tcattcacatcaaagactaaagcactttgaatattaaacccagcattagccttcaacaat

ctttacagcagtcccaaatgggctttgtttaatggcaagcaattcaacatccacctctat

tattgcatttggaggaatttcttgaactcccttgcttccataggcaagttcaggaggaac

aattaacagcctctggcctccaactcgcatgccttctactcctaaatccaaaccttttag

gacatttcctcgctcagattcgcctacatcaaatccatagggcgttcctcctccaacgcc

cattccttgtctactagtcataaatgtgattcccctccatttagcaacatagtgaattgc

gacacgcgatcctttcacagcttcagctccatttccaaccttcaaatcatagtacttcaa

accatttggcaaggttttgaaatcactctcaggaattttagctcctcttagcgctcttct

actagtgctaacagcttccgccacatcacatgcatagactccagcaaatgttgtgaggaa

acaagtgagtaatgctcttcttccttcagtaactggttctacagaagcactactggaaga

agaatgtgaacagctacatgggaataacctatttgaatttttgttgaacagttttttgtg

tgtttctttggagttgaaaagagagaagttacttgtgaagagattggaggcgaggaaagg

attgtgctgatgggtgagaagagagagttccatgatcttggtttgttcttttgttattct

atggtttattaattagatttttatgtttatattttctttttttagtttggtttgagagga

aaaagagaaagaaaatatagagagaggat

>fava_dn_rep_c1157

ttttaagattgaaactcaaataaaaaatttattttaaataaaaaagtacacaaataattt

agtaattatcttcatacaactattcaaagggaaaagcagtatcaattacattatcatttt

aaaacaaaatggaataccttaaaattttattcattctctacacattacttaatggctcaa

aacaacttaaaagaagatcaccaccaaattatatacaaggagaatttctctcttcttggc

ggcgatctcctttatagtgcatatttgatatcatagtgcatatttggtatcaggataagt

ttgtcgaggttatttcgacaaaccctgatacaaaagatgcagatataattcatcaaagag

aattgactccctcacgagttgggaaacttactcatatatctttccattcaacaaatcaca

caagcattgggattctcttccatactttgaacataataatatgttgtcatgtgaggatta

taagccctataagccttcaccatttctaaaacagggtcaggtgctggtgctggctctttc

ttcttctcctcttctttcttctcttcctccttctttccctcttctttcttctcttcttct

ttcttcggttcttccttcttttcctcttctttcttctctccctccttctttccctcttct

ttcttctcttcttctttctttggttcttctttctttggctcttctttcttctctggctcc

ttcgccggtcctaccgagattaaatccgcttgccaatattttcgaagtttgctcactatt

tttactggatccacaattcctaccactgttaatttcttctccttcatgtccatggtgatg

gcatcaatccctgaaagtgttgaaactgtcttcaaggctttctgttttgccttatcatct

tccaaatctaatttgagcacaaacttcttcattttagggttgctatatgggtgtatgaag

atgagaggaagagaaacttgaaaatgagagaagttagagagaaaaataaaaaaagggtaa

gtgataagagagaagagagagtggtatacgaaatcaaataatgttgaaacaatatggaaa

caatttaataccgataaatgagaatggaaacaaggttttgcagagtaggagagagaatga

taaaggtagagagagagagagagagaaaattaaacatgcaactctactatgtatagaaag

aat

>fava_dn_rep_c1158

ctaaaaaacacctctttatgaatggnnnnnnnnnnnnnnnnnnnnnnnnnnnacttttaa

cctcaatatgcatatgcagtcaatagcatctaaaacatctaaactaacatttttttcttt

taaagtataaagttaagtaatgactattttactgattttcgcttagtagagtgatctacg

aaggatcatgttatctttgaagtcaaatttgaaaaggcagatgattcattaacttggata

tttcttgactacataataattaggtttcagttctgtacaaaatggagagaatggataagg

tgtgtgtattctcaaggaatttgtctgtactggtcaactgtagtcatactgaaaaaatag

ataaacgtaggggctaaaacaaggggacatatcatggctcaatttctgttcattttggtt

tgagtgcttgggaacaaagtgaacttttattctctttagtacgacacaacacaagtagaa

aattttcaataaaaagtaattgtcttcagctcatgacttgtctacaatcaaaatggtttc

tcttcatttctaacaaaaaagcagccagaaggactcccatcagggaacaatgccaacctc

acaatcgattcagcaccttcatcaggtgtcaaataacctgtattgtagtttatatctgtt

ttcacaaagccaggacaaacagcattgatgcatattgatgaatactttctagccagaatt

cttgtgtaggcacatagagcaactttcgagataatgtatgtagacatattaataggccaa

cctttattttctactattgaaccctctttaaaatcctttagaaatttgttcataacttca

tcaatcttttcttcagtaaggtttccaacatcacttagtacttccttaggccatccattc

ggaattttctctaactttcccatggatgaggaaacattgacaattcttggtgaagttgaa

agctgtagaaggggaataagcgcttcggttaattccttagctccatagtagtttgtttta

aaacctgcttcagtttgttcatagctctcagttattattttactccaatcaatctggtca

ccattttccaaagctttagcagcaaccagggcctcaccgtccacgttaactccaggaatt

ccagcattgtttaccaagatatcaagttttccaaattgattctggataaaatctgcaaat

gatgttatacttgcaggatctgtgacatcaagttgatgaaaaaccacaaggtcaggtaga

taaagttgcttcaatttgtcaacagcttcaagacctctattttcatctcttgctgtaagc

acaacagtgacccctttagaagccaattgtttgcatactgcaaatcctattcctttgttt

gctcctgtaacaactgcatacctttttgttgtttctggttctgccattgatagcaaatat

gagtggactaaaataaagatggatttgtttgtcccgcgg

>fava_dn_rep_c1159

attcccaatcttctcttttccaatcacactctttgtcattttctcaactaaattgtggct

ctctcatattcaattaccctttttcggtacattttttttcagtgtctttgcattctacca

cgtcactataacccatccttaccagaaaaattcaacattctacgttggttaacgataatg

tttttgtttcctcgcaggttcttttaacgtggaaattcttgagttttcaactttcatttc

gtttgctaaagctcgatgggagacgtggctaaggatcttacagccggtactgttggaggg

gctgcacaactagtatgtggacatccctttgacaccatcaaggtcaagctccaaagccag

cctacaccactccctggtcagcttcccaagtatgctggtgcatttgatgccgtcaggcag

acaatagcagccgaaggtgcagggggtttatacaaaggaatgggcgcccccctcgctacg

gtagcagccttcaacgctgtcttgtttacagtaagggggcagatggaatcattattgagg

tcacatccaggtgcctccctcacagtgagtcagcagtttgtttgtggagctggagctggt

ttcgctgtttcctttcttgcttgcccaactgaattaatcaaatgcaggttgcaagcacag

agtgcactagctggaacaggagcagcagctgtggctgttaagtatggaggaccaatggat

gtggccaggcaggttcttcggtcagaggggggcgtgagaggtcttttcaaaggcttgatt

cccacaatgggacgcgagatacctggaaacgcgataatgtttggcgtatatgaagcatta

aagcaacgctttgcaggaggtaccgatacgtctggcctaagcagaggttctctgattgtt

gcgggaggtttggctggtggttccttctggttccttgtttacccgaccgatgttattaaa

agtgtgcttcaagtagatgatcacaagaatccaaaattctctggttcattggatgctttc

agaaagatcaaatcctctgagggattcaaagggctatataagggttttggtcctgcaatg

ggaagaagtattcctgcaaatgcagcatgcttcttggcatatgagatgacaagatcagct

ttaggatgattagttgttatggtaaagcatgatatcaaatgctcatgtttctttctttcc

ccttttgttcccaaaacttttctcattccatcaattaccatatggccagacattctcgtc

ttttgttactgcttcaataaaaagtagttattctttaacctcaagttatacttacatcct

tatgattgaatttgtttcctaaaaagaaactgaaattgtaagtgttgtcaatatttgtga

aacataatttagagcttatttgatatgatttattttttttgaagtgaannngttgttgaa

tacacgttggaatatcaataaaaatttgaaaactagtgagagat

>fava_dn_rep_c1160

aaagataatcgttttcgcgaaatcatctgaaatcgttagaaaatagtacatggcaatcgt

tagaaaatagtacatggcaatcgttagttctccttataatttcacaggttcattacatgc

cgcgtattttgacatttatccattcacaaaggaacctgcagcaaacttaggacctctcct

actattttctacatagacctacccaaccaaacttcaccccctaaggaataagtacagaaa

ggcagaagaagaacgaaactcagaccaagatatgtgactgtgaagaataccctttctcct

gacctttcattttaccaaaccatacaattaggaaacaaaaaatagattaaacaaaagtaa

actactaatctacgggtctgcaaataccaaacaacaatgtatgcattaaaaagcggtagc

attcgcccactagccttcccctcaaaaccctacttcagacacagcttcggttactcagga

acagataaatcatcatctccagaagtccctgaacttgtcccaactacctgattcttgcat

tcaatcttctcactcactaagcaatttgcactttcattcgttgcttcttgggttttcgat

tcattacttctatcgatcatttcattcttgccactctctttttgatcctcgccatcattt

tctttggcaaaagcttcatctggtacacactgttgtgtggctaattcaaccttaagctca

tccttctctcccttaacttcatcaaacacaaagccacctcccatttcaatacctttgaac

tcatccagattcgaaaccatctcgcttggaacagactcttccataaaaacatcttctaca

ccttcagcttctgatactccatcattctctgacttaacagcagcattgccctcaatgttt

tcatttctctcactttccatcattgaatcaaccatctcagcattgttctcaattcccata

acaggtgcatcagatcccccatcactctcattatcagacacattctccacaacttcctca

ttgacatcttccacaacctggttctgactctgcctctcaagaaacagcatcctccttcgc

aaatcacccaactccctctccattaagaaaagacgctccctcaaagtcaaattctcttcc

tccaactgtgcaatatgagaatcctgatcatcaaccctattctctaagacattattataa

tccccacctccataattcggatatatcatctcaaaccggctcaaatcatggtccagcctc

ctctcaacctcgacatgttcctccacatcactctcactcgacccatcacccccatcttca

tcctcatcatcatgaacatctcgaagcctaatcaaacccttcatcgacccatacccatca

acaccccactcttccttcgccatatctcccaacatctccctggaaggagacaatataggc

gtaggaagcacagccggagtcacaggacaaggcgacacaagcgatgcaattccaccagat

ttcttagcacgaataatataggaagtggtattcctgggcgcaaaaggagcaaatctagca

ttaaacttcttcttaggatagaacttacgagctttcaaacggttctgagattgcaactcc

tgaagagtaggtggttgataaccaagactctgattgggtttttccattctcctaacatct

ttcctcttatcaccaccaaccttcttccccttccagttgttgttgttgttattattatta

ctactacgacccggcttagcgaaattagggttctgaaacttgatgttaggatccaaagga

agctgttgagaccaaaccttatatccaccacgattcatcatcggcgccagattctgattc

atcatctgctgattcattccttgattatggttcatctgagagagaaactgttcctgagac

tgattaataatctgcggcggattcatcttcatcttcatcatctgagactgaggctgctga

ggcggattcatcttcatcatctggttcaaattcatcatcatcttcgattgatcgttcatc

gtaacaaacaaaccctaactacaaattccttgaaatttcaacgaaaatagttacgaagaa

aacaagcaaaaaaattatcaccgatcagaaattcttgatcggtggattcagattcaaaaa

accctaaatcgatgtttagtgaaattaagaaccgattaaaaaattaagcgaaggtgaaat

cgtgtaggggaatcgagatttcacaaaggtgagagttgagaaaccttgattttgattatt

atttttttgtgatttactattctgaaaagagtggttatcgtttcttgatgtcccgcgg

>fava_dn_rep_c1161

aatcatcttctaccatttgaagttgaggaaatctcaaaataatgaccaacctcaacaaat

atgttttgctttaggtaaagtttctcaattatatacctaacctgcccatgttccttgtaa

cagaaaaaggttaactcaactaccgcaaaagccatcaaaaattagttatttagagatgaa

atcagagactttttctatcttaattaatagaataacacttgttttaaatgcataacaata

acaccatggaacaattttattactaagaaatctctacttagcatatatcatctcatacat

tatcctttgtactagaaaccttaatgtcttgcttctcttgctcactcgcaacagagattt

cttctgtaggatttggttccttttttagctctgtctcaaattccttcgctgcctgttgaa

agcttttgacagtttggccgatgctgcgaccgacttcgggcagtttcttgggaccgaaaa

caagagcagcaactcctgcaataaccacaagctcaggcacacctagaccgaagaatgcat

tgcaagtgaaactctttgttgttctggttttgattttggctttcttcaagagaagtgagc

aagtgttagagttggaagctgagaaggataaattggaataggatgaagagattattggta

gcttagtcggaataactgaaggtgaagaaatggaaagtgttatctccatttttggtttag

attctttttactggtttctgctaaatgagtggatatcaatggttaagattgggttgagga

ttgggtgggactgtgttaagattttcaatggttcaccctatattttgtgattgcgtttgt

accattttttt

>fava_dn_rep_c1162

gatccaagagcgtgtttggctgtacaagaacatattcgcgttttagcgattccaaaagcg

cgtttttgagtcaaaaaacgcgtccccaaacagacacaaataggaaagtggatacagaac

attctagaagtagaaaaacaacgcaaccttggctgtcgctttttctctctctttctcacc

ttattatccctttcccctgacccaaatcaaatgcaattcttcttcgatcaaacccttttc

aatttcaactttctccctcaattctcaccttccgaatccttggtttcagcatggcttcga

agcgtatcctcaaggaactcaaggatttgcagaaggatccccctacctcatgcagcgcag

gtcctgttgctgaggatatgtttcactggcaagcaacaattatggggcctgctgatagcc

cttattcaggtggtgtgtttttggtttcgattcattttcctccggattatccattcaagc

caccgaaggttgcatttaggactaaggtattccacccaaacatcaacagcaatggaagca

tctgtcttgacattctaaaagaacaatggagcccagcattgaccatttctaaggtccttt

tgtcaatttgctcgttgctgacggatccaaatcccgacgaccctttggtgcctgagattg

cacatatgtacaagaccgacaggaccaagtacgaagctactgcacgcagctggacacaga

agtatgccatgggatgaccaaatcatcctctcagccgtttaatgcaaccatgcaccgtga

ttttatgtaaaaccaaagatattttgaatgattgtttttgttattgttgtcaatggaact

caaccattgcaagcgaaattaattgtcatttgtgaatattcacgagacatttggtgcaaa

ctttctggttgtttacatgctattctcaacgatgatgataaatgaaaattttaattacca

acaaaaggtaattgctgcctgattattcaaaaaaaaaaaaagactaatctaataaaaaaa

cagaaacagaacacatggagggcatgacaatgaggagaagcaaatgcttaaagtaaaaga

aaagctagacaaatgttttaaagagaagttactggaattctgtgatgtgtttgacataac

agcaatcaacaagtcaacaataaaaaaggaagatgttattactaagctgatagacttttt

gcttgcccctcatgcaaccacaactgtgctacttgcagaaaaagagtccagtaaaaagcg

caagcgtcctgtaaaacgaggtacatcaaggtctaggactagtacttcaagaggttctgc

aaagaggcagaagaaaactgaaagttcttcaggagtggagaaaaagagcacaactgacac

agaagatgaatcagagggagaggagaaaaatgaagaaaatgaggatgaaactgacaatga

cattcctgagaaatctgaagatgaaacggcagagaaatttgaaagggaagataaaagcga

ttctggtagtgaatctgatgatgtgaaaaaaaagaggaaacattctaaatcatcatctgc

gaagaaagagtcctctggaagaagcaaaatcgagaaaactatggtcacaaataaatctcg

ctcaccacctaagagagcacctaagaaatcatcaaatacccaaccaaagtctgatgatga

tattgatgaaagtccaaaggtattttctaggaagaagaaaaatgagcaagggggaaagca

gaagatatcaacttcaactccaactaagtcttcatcaaagtccaaagagaaaactgaaaa

ggttgccaagggaaaaggcaagaagaaagagaagtcctcaagtcccagtgataatcagtt

gcgcgatgcaatatgtgaaattcttaaagaagttgacttcaatacggccacatttactga

cattctgaagctactcgctaaacaatttgatgtggatctcaacccaaagaaggcagctat

caagctcatgattcaggaggagctgacaaaactagctgaagaagcagatgatgatgaaga

cgatgatgatgaagaagaggccgagaaagacgaaggccgatctaccggccaggagaccta

agtctgattgtggattagacattactgcttggtggttaggtatttagcaatgcatattgt

aattctcatgtacactttaagttgtgtagaataattggctgtatcttattcttaattctt

aacacccctccaatctgaagatatcaaaaacatgtatttgccataatctagtgcagcctt

ttgtttggctgccagcatgtagtttattgtgtacaccaacatgatttttaggtttttctt

ttctagtttaaacaaatgtctatttaataaatatctaacctttgaagttgacacctactg

tgcatattcatgttgggtgatcaagagtctctatctttctattcaannnnnnnnnnnnnn

nnnngagaaggacccctcaccccacttgtatc

>fava_dn_rep_c1163

ggacaaaacttgatttgatctaaatggcaaagacattaaatatgtttaaaaaaaaaaata

aagtttagaaaacgagatctgtgaagaaaatagtgtgtatccgattatagtggtttaagg

tttgagtttaataaccgaaagaaagataaattggtgatatctgtgtgtgacgtgcgcggc

gttccttgtttccttcctgagatggcattgaatgttatatcacccccactattctcactg

tcaaatgttgcagataccagaaccctaacaatcacagcattcaaccctaaccctaggact

atccccattcaccgacctctggggtgtaaccttaggactatccccattcaccgacctctg

ggattccgagtccgcgcggctcctgctgctagactgaaagcgagggcggccactggtaat

tcagagtcagaggaagtaatcatacaatcaaaagtgacccacaaagtattcttcgacatc

agtatcggtaatccagttggaaaactagtgggtaagattgtcatcggactctttggggac

gatgtcccccaaactaccgagaacttccgggccctttgcactggtgacaagggttttggc

tacaagaactccaccttccatcgtgtcatcagagacttcatgattcaaggaggagatttc

gacaaaggaaatggaactggaggcaaaagtatatatggtcgcactttcaaagatgagaat

tttaaactctctcatactggacccggaactcttagtatggctaatgcaggtcccaacacc

aatgggagtcagtttttcatatgcactgtcaagacgcaatggcttgatcagaaacatgtt

gtttttggccaagttttggaaggcatggacattgttagattgattgaatcgcaagagaca

gatcgtggtgatcgtccacgaaagaaggttgttatcagtgactgtggtgagctgccaatt

gcttgatatgttttgtttttttgcctgctcaccaacaaaccgagttcttgctctctgcaa

ctttttttgtttcactaccataattctctatgacttgagtacctttcttattttgatact

aggacctacaaactcttatcagctatttgtattctagtagagcaatatagttggagttgc

tatcatattggttgggagattttagatcttatttgattctgaatagtatccttaaaataa

cccaaagtgcacagattcagcatggaatcaaatgtgagggaactctggtgaaatttaata

agattgtgattgacttttctaagaaaaaaag

>fava_dn_rep_c1164

ccgcgggacttaactcgaccaaactgaaacattctacgtgttgtttcttccacactcttt

gtttctcgtttttcgtgtcttgttttctttatccattttctttgtttcttaaaggtttgt

agaaaatactttcataatgtgtggcatacttgctgtacttggttgctctgatgattcaca

agctaaacgagttcgcatacttgagctttctcgcagattgaagcaccgtgggccagactg

gagtgggctccaccaacatggtgataactatttggctcatcaaaggttggccattgttga

tcctgcttctggtgatcaacctctcttcaatgaagacaaatccattattgtcacggtgaa

tggagaaatctacaatcatgaagagctcagaaaacaattgcctaatcacacgtttcgtac

gcaatgcgactgtgatgttattgcgcacctgtatgaggagtatggagaaaactttgtgga

tatgttggacggtatattttcgtttgttctgctggatactcgcgacaacagtttcatagt

tgcaagggatgctataggtgttacttccttgtacattggttggggactagatggttctgt

ttggattgcatcagaattgaaaggactgaatgatgaatgtgaacattttgaagtttttcc

acctggtcacttatactcgagcaaagaaagagagtttcgtcgatggtacaatcctccatg

gttcaacgaggctattattccgtcaacaccttatgatcctctagttttgaggaccgcctt

tgaaaaggctgtgataaagaggttgatgactgatgtgcctttcggtgttttactatcggg

tggtttggattcttcgttggtcgcatctgtcactgctagataccttgctgatacaaaagc

tgctaagcagtggggagcaaagttacactctttctgtgtaggccttaagggtgcacctga

cctgaaggctggaaaagaggtagcagatttcttaggaactgtccatcatgaatttcagtt

tactattcaggatggtatagatgcaattgaagatgtcatctatcacacagaaacctacga

tgtcactacaataagggccgcaacgcctatgtttctgatgtctcgtaagatcaaatcatc

aggagtcaaaatggttatttctggagaaggatctgacgaaatctttggagggtatttgta

tttccataaagcaccaaacaaagaagagtttcaccaagaaacgtgccgcaagatcaaagc

tcttcataggtatgattgtttgagagctaataaatcaacttatgcatggggtctagaagc

tagagtgccatttttggacaaggactttatcaaggctgcaatggacattgatcctgagtt

taaaatgataaaacatgatgaaggaagaattgagaaatggattctaagaaaggcctttga

tgatgaagagaatccatatctgcctaagcacattttatatagacagaaggaacaattcag

tgatggagttggatatggctggattgatggcatcaaagaccatgcagcaaaacatgtcac

tgacaaaatgatgttcaatgcatctcacatctttcctttcaacactccaaataccaaaga

agcatattactatagaatgatctttgaaagatttttccctcagaactcggcaaggcttac

agttcctggaggacctagtgttgcatgcagcacagaaaaagctattgaatgggatgcttc

ttggtcaggcaaccttgatccatctggtagagcagcccttggagtgcatgtttcagctta

tgaaaaccaaatcaacccaattacaaaaggtgtagagccagagaagattataccaaagat

aggagtttctcctcttggagttgccattcaaacctagtatgagacatagcaaatattact

tgcctaagaaataaggatattattatactattagtattcaatagagaataagataaccga

aaaatttgcctgatgtgtattttatccaggcaaatttttatatgtataaggctttctact

tagctgtatttatgtgttttgatgttgtgtaatccacatcttgtctttgcttttaaatcg

atgtggtgatttgaacactttcatattgtaatttggctttttaagaagagttgtgtatta

tgttaagtttgaatgcaagttctactatttgaatactgcttagaaaaaaatgtctttaca

t

>fava_dn_rep_c1165

atataataaagtcaataaaaaataataatacaaatttcaaagaccgtaaaataaaataat

aatgtaaaataacttggagaaactgaaatttaaaattgattattgatcctcaatacaaaa

acactagtaattcagaacagtatgaaatatttaaatataaaagatagcttcttagataaa

ctgaaataaaagatagtttcttagataaactataaaaaaatttaacatctttttatccgg

ttaaatcaactcttttgacagttatgcagatgcatcacgattgcgagtttaaaccctgac

atcccacttattttttatatttcatcgtcccagaggtcgttgagactgggcttggttggt

ccagggatcaaacgattctgcttaatttgatcaattttctcatgatcctcctttgtcaat

gtccaatcaaagatactcaagttttgacccattctctccttatcatagctctttggaaca

aaagtgattccttgttcatataaccatctcagagaaatttgtgcaacagactttccatga

gtatcagcaatctctttaagcatatcattctccataacctcattcgctcctctgcttgca

cctttcctcaatggtgaaaatgctgtcaacactattccattttcattgcaaaactctctc

aacttcttttgttgccatgcaaggttcatctccacttgattaaccgctggaagaacagtg

gcaacagatagtagcttttgaagtttcttgacagagaagttactaactccaatagcttta

gtgagtccgagtttcaaagcttcttccaaggattcccacacacctttcacatcaaatggc

aacagatcagccacatcaattggaaatgaaaactttccaggctgagagctaagtggccaa

tgaatcaaatacaaatccaagtaatccaattgaagagtcttgagagatttttggagagca

ggaataacaagatgaggatgattttcagtaacccaaagtttggaagtaacaaaaagctct

tctctagtaacaagaccaagttgaatagcctcattcaaagcttcaccaagtgcttgttcg

gagccataagcagcagcagtatcaaagtgtctgtaaccttgtttgatggcttcaatgatt

gcttcttttgtgtctttcttgcatgtgaagtcaggtgctgatcccattccaacaacaggc

atcttaatatgagcagatgtgttggtaagaacctttgatggaatttcaacactgcccatg

ttgtttttgttgttcttgtttagttgaagctgttacaatttgaactatgtttaatgatat

gctggggtttatttgcaagatggtaatactgagaaggcgttggaggtttacggatcaatg

aagggtttaggttcggggtgtttgccggatgaattgacgtttacgatattgattagaagt

ctaatgaagaatggagaaaatgagcttgttgaaagtttgaaaaaggagagttttgattat

gttaatgcacctgataaatttgttcagaaagttcagcaaatacatgcaaaacgacatgtc

aatctggttgtgtaatgtgattctactgtactggttttattggagagatgctactttcat

tattccacacatttgaacctcaattcaacattttttgccaatttggtaataatgatggca

tatgcgaatgtgataggatagccgaactatatagtcctgttgttgcaatgtatttgtgct

tttgatttgaacagattatatatacttgtgttgttgcaatgttgatgagatcatttatac

ttttcgatttgaacaattgtaacaatgaggacgggatgttggagctctgttattctgttt

atgatatgctcttgcttgttgaaaaagtaatggtaggttttttagctggtagcattaaga

ataaaagttttgtatttataagtaatgtctgtgcatgcttgcagctttaaatcaagtaag

caaaacattattgacattgctagacatgaaaaaataatagcttctatctcaagttaattt

ttacctttcctttacaagatattacattgcaaaacaaatgtaaaattggttttagaaaaa

tattcatctataagaaacaagcatcacacaccaaaggggaaaagagtctaatggaatgag

gaaaagactgaaaatagaaaaactaaatacagactcataattgacatttaggtagcaaaa

tggctaggagagttgttgaacatgctgtgccacataaacgcgcgagagagtaccatctcc

agctcctgatcggtccagttctgagttggaaggtgttgaaggaacattactaactcttga

aaatcaagcttccgaatttcatctgaccacgtaagaagaaaactcgcaaatatatacaca

aggaagtctggtaaggcatctccttctgctagatatgtgtcccaaaggcgcgtgacaagt

tggaagggtatctcacggattagaagacagttgaaccagcggaaagcaaattgaagaaat

tccagtccctgattctccatatggcttgaaactggttcatcgatcctcctgaccaattcc

ttcaacttaaaaacaagcctttgaattcccggttgagcaaatgtgtaatggtcttgcata

ccatcaagcaattttgacaagcaccaatagcagtcagcctctacattagagattttatct

gaagataaatcggacattgtccaattctctatgcttccttctaaatattcagataggaaa

acaacaaaaaatggcgtgacaaggtcatttatcccctgaacatatccacttgctggatgc

cgaatggcccatgcgtaaagaatacgctccagtgatttctgaacttgaggttgttggaag

aatgaaacatcaggtacagttcttggacaatcaatgcaaatctggcgaagcatgctgatc

tcatcatctgagcgttctgtatcaggaatatcataatactgagaaacacaatcaagatac

tcgagccgcttccttctcaaaaccccctcccttctatctgaattagttggtgcatatccc

aagagaactctccatactgtaggacgcatataatctggaacaccactccaagctaattca

cgcaatttgtctaatataaccattgttccagaaagagcctttgtgaacttcataactctt

gcagaatcagtagctctagcacccatggaagttttccgtacatgtacttcctcagacgga

ttctctacatgcgaggttgatgtttttgatttactatcactagtaatacttccaagtgat

ttgcttccactttgaacttcctcctctactgtctcaattgcgcgattacttgtaccggta

tcactataactcctttggaaaatcggtgagtataaacttgaattatcgttcggatccctc

gggctcaataatattttaccaggaatgttacgacccttgagtaacccttgaacatttctg

agggtttggttgaaacgagaatcgaggattcctatggtagtgtctttaatgttgttgttg

atattgtcgttgttgttgttgttatcatcgttgttcttcatggttgatctgtggttaata

gtgagagtgttgttgaaatgagagcttagtgatgagtagtgaatgagtgcaacaaggaag

caacatcatcaacactcaaccaaccaaaataacatcctttttcatcaatgctaccaaagt

tc

>fava_dn_rep_c1166

cgatctcggacaaaggagaaaatcattttttttataaaaaatactgaatacgatataaat

attttattactcaaatgaatagattattgcatcctatacaactcacaaaattatgattct

caatatatttttctatacatttgtactagtacttccataataaaaacaatggaagttatg

gcatacgaaggaatgaaaatttcagccaataattgcaagcaagctttgtctgacaatagg

aactgaacatagtaaagatccaaatatgccattcagagcataagcaatggcacaaaatgg

gagagcttcaggttccttagcagataatgctgctgttccaagtccatgtgcactagaagc

agttgctattcctctggcaatcgggtcactaaatcggagtctatcaagagttgcttgcac

gaaattcgccccaactagaccagttactacaaccacggctgctgtgagagatggattggc

accgtcaaacaaggacacaatgctgagggccaacgctactgttatacatctgggtagaat

ggacacagttaaggatggttctaattgaacaagacgtccaacaagggcagttgagtacaa

tgagaatattgttgagatgatgaccgaggtgaaaatctcagctgcgtgccttttcacaag

ctttctttgcttgaacatagagaaggaaaatgatagaataacagaacccaaaaatcccat

taaaatgtcaccggctccgggattgttggatgcattggtaaggtaatatcctagaacagg

atcaagtcctaacttggaaaaatacccgaaagcaaatgctgtcaaaactgatgatagtgc

acagcaaattataggatggaagaccttcttcacactcgatggcagcccagtaccaactat

gtagcccaacactgtggatgaaagcaaaaacggaaggcatgttctggcacttgtccccaa

caaagttggataaaatagtgcactaacaaatgatgttaggagaatcccggtccacgtcca

cacttcaaaggtagaaaatggagaaggctttcccattggctcagcgtcaatcaatgttgt

tttgacggcttttcgtactgctatagctgtgaaaccagcaacacatagtgtagctagcca

tcctccaactacaataaggctgattttgatgccggaagcggcgggaatatctctaacaga

aaggggcaagacaaccaaagaaggaacatagaacaaagggagccatctctgaatgaacat

aaaagcaggctcaaagaagttcaccactgctgtagctgcagatggcacagttgagtcgag

aataatcaaaactgagaatatacaaaacattccaaacaatgcactagggaatttgattgc

agcagccacaaatgcttgcttcaaaaacttgtctgtagcaacaaaaagccccagagacac

aatcaaatgtaacaccccaaacacattttgagtgagtgatgaagtgtttccagaatcagt

ttgagctgatcgagttgaaagttgcctactttgagaacttccatgagtacccttttgcaa

caaactggaatccaacactgcaggagctattgggcaacacaaaaaggagcatttttgaat

atatgggagagaaagaggtgttgattttgcagagggggtggaacgaatgagaggtgttga

ttttgcagagggggtggaacgaataaacaaaaaaggatctggtcaatgatgaagagagga

aaattgtatttgttgagaaaactttggcttattcaaaaagagtgaaatataattttatag

agaatttgataactacggccggtttcgtgatgttatggttgtctgaatatgaacgcgttt

gaatagagaaatagagttggaaaaggtcatgctaatgtcattgtaatgtaaggactaagg

cgataggagcaagtaagtgatgttgagtacaaatgtatgtgcaagtggatataggtctaa

agttgacaagtttgacaatagttatgtggattattaattatctagaggacattaaattag

aatttgaatctatataaagaataactagaattttatat

>fava_dn_rep_c1167

agggtaaatagatgagttgtttgtgaaggagaacaaaagctgaagggataattccttctt

ccgattattatgtggtgttgatatggcgtcactcgtcaccccagcgcatgttagcaacat

caaaccttttcagtcttccaccggcaagactaaccgtgtgaggtgtaaactgtcgccacc

gccatcatggagggaaggccggcgaatggtgtccatctctttagttctctcccccttcct

tctcaatcctaaccgtgctaatgctgaaggaaattttatggacaaatatgttaagagaaa

aaagctagagcctctggagacttatgtaccggctgtaatactcacacagttccagatcga

ggatttggataaaacattagagggcgatgaaccccaatttgccctctgccggtctctact

tcgttctgggcctgcagcatctcttcgtataaatattcgagcagttgcacaatatgcttc

agacagtggcaatggtaaaactgcattcaacaatgttgatgaatgtttgaggtcaattga

ggaactcgactccttgcttctgcgtgcatcaagaaatgatcctggcgcctcagtcaaatc

aatgaagacaaacgtcaaatctgcccttattgctctagacagcctcttacaaacagtacc

atcggatgtgctaagtaagggaaagattatagctgattcatacttggaggatcgggaaga

cgttgaaacggagagcttggaccctggtttaaagcaattagaatcaatactatgatacct

tacaggcatacaaatcaaaacgaagagcttgaatcctgatttaatcatatctttttgaaa

atatcattgtttatatctatctctataccaacatgtattactttctcacatctatgttcc

caatcattgttatcaatacatgttcatattcggtccgttatactgagtttggtgcttcat

tggcttggttatttcacgtctcgaggtaatgtcaacgtgatattccagaagctaggattt

gtagcttcaaccctacatgtgttataagtgatttcattagtttccaagcgtataataaac

caatcatgttgatttattacaagtgcttaataactatgctcggacgtgtcctgtgtttta

ttttttgactgtataatcaagttggtttttagaaacttttggccccattgtttcaagagt

aacaactaaaaattgaataaaatttggaatttctgt

>fava_dn_rep_c1168

acacaatcaataacatcaaaaaatttgtacaatagtctcaattaaacaatcaatacgtga

gataaaaatactctacacaatggtccaataaaaagttataaatatgttaaattatttatt

ataaatagaagatgtcatcatgactttgagtcactaagctaatgtagaaaagcgagagaa

aaaaaaagaaaaagaaagaatcgatggcgaagaataatgttgaagggtttgaattggaac

agagtcacgggaaagagagagtaagagtagctcgcgtttggaagagcaaagatggtcaac

aacacttcgttgttgagtggcgtgttagcatcaatcttgtttcccattgcgtcaattcct

atattcgcgatgataattccgatatcgtcgctactgataccatgaagaacaccgtatatg

caaaagcaaaggaatgttcggaaatgctttctgtggaggagtttgccattctacttgcca

aacatttcacatcattttataagcaggttaccactgctattgtaaaaattgtggagaaac

cgtgggagcgagttagtgtcgaaggtcaacctcataaacacggtttcaaacttggatctg

agaagcacacaacagaggcagtagtaaacaagtctgatgcactgcaattgacttctggta

ttgaaggattgtctgtgttgaaaacaacccagtctggttttgagggttttgtaagagaca

agtatacagtgcttcctgacacacgcgaaaggatgctggcaacagaaataactgccctgt

ggaggtattcatatgaatcagtatatgatgtccctcaggagcccttttattttaccgaca

agtatctggctgtgaaaagagttctgctcgacactttttttggccctccaaaggaaggag

tctatagtccatctgttcaaagcactctctaccagatggcaaatgcgacactaaacaggt

ttcctgacatagcttctattcaactaaagatgccaaatattcatttcttaccagtcaatc

tctcaaacaaggacggtcagattgtaaagtttaatgacgatgtgtatttaccaacggacg

agccacatggatcaattgaagctagcttgaaccgcaataggtcaaggatgtagaaactag

tggcgcagcttacacactattgggcttgtaataatttaagatcataacaacaagtgaaca

tgtaaacaaagtgctgctttcaacaaacatgtcatactaatgtcagtgctggttttgttt

tctttgcatctgtgataaaaactgttgttgtgttgtaggttccttatgtccaataaagtc

aaatagtttccttatgtacttaaggggagtgaaataataaaaggttactactgtaaaatt

tatgatattgatactgtgctgtattttggtcattcaataatctcttgaaataatgatttt

tgcaaaaaaaaaaa

>fava_dn_rep_c1169

tttttttttttcgagaaaaaaaatggcaatagttattggaaataaaaatatcacgaaaaa

tcatgagttaaaaagatatgctccacctcaacatcaaaaccaattccagaaatatctggt

caaccgataaatccccaaagtgggttgacacgattattatagaaggctaaaacaaaacaa

aaaatgttgaaccaacaatttcaaaagaaatggaccgatatatcttaatatcgtcatcgt

caaatataatacagattgctgtcttgcaatttaaccagtcagtgctgatgctccagagat

aatctcaatcaattcggtggtgattgatgcttgacgagttcggttataagtgagggtcag

acgatcaagcatatccccggcattcctgctagaactgtccatggcagacatccttgctcc

ttgctcactgcaggcattctccaacaccgcattgaacatgacacaagaaaactggaactc

agacagattctgaagaacttcagattttgtctcacccccttcaatctcataggaatccag

ttcaccgagtgttcctccagcttcagcctctctttcaacaatctcaggggatagtacagt

tgacactgttggtataaattgcacgacagaatgaaatttattgaaaacaatcctaagtgc

atcatactccacattcttcaaaatgtcatcagccaagacagacacctgagtataattcag

aggattcttttgcagctcagtcaagctgagtgcaatgtctttctttgagtcacgaatcaa

ctgagccttagccttctctcccaatatgacgtacttggtctctttatcaggtccagaatt

caacttgctcatcgctctgcttatctttacagaggtggagttgattccaccgcaaagacc

tttgtctgaagaaacagtaataacaacattcttcttgacatcaacacctatagcatcgcc

gagaagagccgtaaatggctgccacagcccacgggaattttcagccctagtttgaacagc

tcgcaattttgatgcagcaaccatcttcattgccttagtgattttctggatattcttcac

actcttcatccgatttctcacaatttgcgtggaaatcgaacgagctcctccaattgaaga

ttgctctaacggagagatgagggtggatcggagagtgttgttgattggttgggaggacat

gagaggagccaaacgcttcccttcgcgtcggattgcagcagccatcgccatcgattgatt

ctttgatcgctgttttcggaaccctagctctttgatgcagttctgtcttaattgcttgtg

ttgctcagcggaatgaacgaataagaacgagagacactgcacatacctcacgcttctctt

ttttaatgactaaaaataataaattc

>fava_dn_rep_c1170

ggttatttgagtcatttcctaacaataaaaaagacagaaaactccctacgcgttcacgaa

gaccctaaatgaagtaaccattaaaaataaatacaaaaacacagcaactcttgatcacat

cacaaagaacaaaacgaaagcaaacagaacaaaaagatgagaacaagcaaccacttaatc

ggcgtgctaaacttcctaaccttcctcctctcaatcccaatcctcggcggtggaatctgg

ctaagcagccgagcaaacaacaccgactgtctcaaattcctccagtggccattaatcatt

atcggcataagcatcatggtcgtttcattagcaggtttcgccggcgcgtgttacagaagc

accttcctcctacgactttaccttgttgttatgttctgcgtcatcgccgtgctcatcggg

tttataatctttgcctatgttgtaacggataaagggtctggaagaagtctcgtgaataaa

ggctacttggattattatcttgaagattattctggctggttggaggaacgtgtggcaact

gatgggtattgggggaagattagttcttgtataagggattctaaagtttgtaggaaatta

gctaggaattttaatgatcagcctgagactgctgatatgttcttccaaagaaaactcact

cctgttcagtctggttgctgcaaacctccaacagattgtgccttcatgtatcaaaatgaa

acagtgtggatgccaggaacaggactaatagcaaacaaccctgactgcactaaatggaac

aatgaccaacaagagctttgttatagctgtgattcttgcaaagctggtgtgcttgctagt

ctcaagaaaagttggagaaaagtctcagtgatcaacattattgttatgatcattcttgta

attgtttacatagttgcttattatgcttatagaaacaaccgcaggatggataatgatgag

tcttatggtgaagcaaggatgacaaaatcacaaccaagtgcttttcatctttgattaatt

aactctctactatgtggcttttggacttttatgttaggattttgaggattggatttgttt

tgtatcaattatcaatcccttgtagtatgtatgtaagagtttggttttaatcttgcaagt

tgaaatgtttgatgtatttattatatttgtttgcagtttattagcctcttgtttttaagt

tgaaggtactcattttgtatttctgatggaattaagtattaatatagattatgtaaattt

ggagaaatattttatttttagtcatgagaaatggctctttgcttgcaaaaaa

>fava_dn_rep_c1171

ataaagttcataaaatattatactttttatgtagaaagaattatcttctacaatagtctc

acaaagcatgcataatatgaaattaagttaattttctttcaagttttgctctgtctctct

ctcctgaggtaaataaataaaaatcacacaatcttcagcagcattacaggttgtatagtc

tccaaataacataactattcaaacaaatgcctggaatttgctacagtataatagaagata

caccagaaaactgagtaaagaataatgggaggcatagacacacatcatcttccaattttg

agaagtgatcatttggttcattggttagattcggttgatttttgcttaattctcgggtaa

aatgacagggtccaagtacaaaataaaagaaatctcaagatgaaacacatgaatctaatc

attcaatactgcatagtatataaatttatcaggagcgtttaagtaaaaaccaaatctgga

aggaaaaaaaagggacatgaagagagtatgtaaaatatctcccttcagttatttcaatta

gaaaaaactaaagcaagcaaagttcttttgttacatctataacagcttgcaaagccaatg

ccattcttcccttcagagatcacctcttatgagcttgaacaaatttggagtttttcgcat

cctcctcagttagaccaagggaaaacttggtgattatagttccggtgatagcgaatccaa

cgacaaaaggccaattcttcttccattctcgcttgaagaaaactggccatggatcgaatt

ttctcatttttctctgtttctacgctttctctttcttcttcgtcccgcggcatcttcaac

gagtttgactctcctgggaacacttctaattccttggctccagatagccttgttcagctt

gacatcgaccttaacatcgt

>fava_dn_rep_c1172

atttcattatttaataaacctatgtattctagcaccctcaatggaaaactaattaaaaaa

aaaaagatgattcaatttccgtaatacagcctatatagtaacacttgtgtgaaccctgaa

gccacacacctcaaatcacaacaatcatggccaaaatctacttcatctccttcatcttcc

tcatcaccatctccaattccatacccaaaacccagtctttcgtccaaaccatctccccag

caacactcggcctccacaaaccccaaaaactaagccacctccgcttctacttccacgaca

tcatcggcggaaaaaaccaaaccgccttccgtgtcgcttcctctccatccaccaacaact

ctccgacgggattcggttcggtagtgataatggacgacccgttaacggtttcaccggagt

caaattccgaaattgtaggaagagcacaaggaatctacgcttcagcctcgcagagtgaat

tgggttttctcatggtgcttaactttgctttcattcgagggaattataatggaagtagtc

tgagtgttttgggaaggaatacggtggtgtccgccgtgagggagatgccggttgtcggtg

gtagggggctgtttagatttgcgactgggtatgctaaggcatctactcattcttcaactg

agttagaagctgttgtggaatataatgtttatgttttccattattaaaataagatttgta

ttattctgttttattttgatgcatagctatgtagtctgtgaagaaaataaataaatatga

aatgagatgcttgttagtacacgtagaatgaatgacctttcttttaatgataaattaata

aaaacgagttatgagtagaagaacatttttagaaactagaacaaaaagtttaaatgaaaa

acatggatgggagc

>fava_dn_rep_c1173

atgaaatttaagtcctctcattatcttacttcaatagtgacttcaatggtgaccattgct

ttggattagaattttctaggaccacacaaaaatggccctccaaacacataattttcctta

ttcattctaaacaataacaaagagacaaattctcaaacctttgattgaaaggccaagtaa

aggatcattagtaccataataaacaaccttatgtatgaaatatttacatgaatattttaa

agtaattataatatgaaaaaattgacagcaaatttacttctttttttttgtgtttcaaca

atgtttctttaatgttgcataccatgtttcttgtatgtgacagaaaagtaaataacatta

tttcatggcaaaggttcttgtgaatctcccaaacgagatagatgtagatatgcatcagtc

ccatatccttccatagagattatttgaagatcgccgccaaaataccgagcatacagccga

ctaataggaagaccgaatccataaccagccattgtcacattatcacctactccaagatct

gtatgctcattaaatggatttctagcagtactatacaggtacgtaaaaatcttccgcaga

ccacttctcggtattccacctccctcgtccgagatctttatggtaacatcttctaatcca

tcagcaactattattcttatgggcggtgaaactttgtcagaattcatgtaacgctcttcg

acagcacgcagtgagttcttaaccaactcgaataccataagatgcaagtgagctggaaca

tacggaaaactaaaatcaggatcgccgtaaatatgaacatcaggagcacttccatattca

cgacaacatatagcgcgtgcatcgtcactagcattcctcgctacctccacaggagacatt

cttgtgtctatataacctacgacatgaggattcggatttggattgtgcaattcgacgtgc

tgccctataagcattcggattccaattcgcgacatgtaaaaacgatcaaggaactgatga

atctcgacgatatcttcgttataaatctttggatttaaatctttcttcaattgctgaaca

cctaaggccattgtaggcaccacattgttgtgtctcacttttatggcctttatgacttca

gtgaactctttttcatcattcatattcttgatctcagaaaaggatcgaatatcgcgaaag

gaatcccaataccaatccctaaccttcaaaatagcaggtttgtatgacaaaccacaagga

agattttcaagttcaatagctcttctagcaatcctaatagcaagttccttatgaagaaac

tgtgcagaaatcagcaaattcttatcactgggctttgaaccaaactccatcatgtacctt

aaactcacacctgtctgtttgaaacaaccccatttgtgcacatcttcaaccaagcttttt

gagaatgtttcaatggctttctttgcagccatggctcaaaaaaagtgattttggaaaatt

aatcagaaatgggagatgaaaaagattgaaactttggattttccggaaagtgagaaattt

gaaggaagtgacttctttttcatggtgttaagttattttttgagaattgatttcaaggat

aagcttgggacaagt

>fava_dn_rep_c1174

atggtttctagtatgcgacgtcgtnnnnnnnnagtgtactgcggtaattagacagtgttc

tttcttcttctacactctcatcactgtcacgatctcatcacaaaaatggcaaacaaaacg

aagaaaaaaaccccaacaacctacgacgatgtcgaaaaagacaacatctggtcttcctta

atccacaaaggaacaccaacaacattcacaacaatcgctttattcgcattcctcacccgt

ttcttagtatccttgtacccttattcaggtttcagaaaccctccgaaattcggagatttc

gaagcgcagagacattggatggagataacaatcaatttaccgattcgtgaatggtataga

aatgggaccggtaatgatttgagttattggggtctcgattatcctcctctcactgcttat

cagagctggattcatggagttttgcttcggcgttttcatcctagttctgtttcgcttttt

agttcaagaggtcatgaatcatatcttggaaaacaactgatgaggtggacagtgatatct

tccgatgctctgacattctttccagcagttttgtactttattattgttcattataatcaa

ccttcccggagccgtaaaactgatatagcatggcatattaccatacttttgctaaaccca

tgtttgatcttaattgatcacggccactttcagtacaactgtatcagcttgggctttact

attgcagctgttgctgctattctctctgggaaagatcttgttggttctgtcctttatgtt

ctagccctaaatcataaacagatgagtgcatattttgcacctgcttttttcagccatcta

cttggcaaatgcctgagacgtgaacatccagtccttgaggtgtcaaaactaggatttgtt

gtcttaggaacctttgcagcggtgtggtggccttatctttactcaacaaattcctttttg

gaggtcctctcgcgtcttgctccctttgaaagaggaatatttgaggattatgtcgccaat

ttttggtgtgccacttcaattctcataaagtggaagagactgtttacaacagggccacta

aaacttatcagcttctctgcaacaatcctatcatgttttccttcaatggttcaacagata

aagtctccaagtaataaaggattcctttattgcctgctcaacagttctctctcattttac

ctgttttcttttcaagtgcatgaaaagtccatattgctgccacttcttcccgcaaccatg

ttggccatggaagatccctttattttcaaatggttcatgaaattcgccatgctctccatg

ttccctctaatctgtcgtgacggcttgattgttccatatttcgctctactcgctctcttc

atcctcgtactcaacacacccggtcaacgcagagttagagagaacaacaactatgtttat

aactacttaggcacaacgacgattcgccttattttattttgttcattagttcttcacata

gtttatttgacaacgcaagctcctaagaagtatcattttctttttgaagcaatgattatg

aatctgtgtttctctcaatttgtgattgtaactattggttgcaatataaagcagtggatg

ttaaacaaaaatgtcaaattactagaaaagaagcacatttgacattggatttaacttttg

aagttagatgagtttttgttttcagctatggtttataaattatattaattgaattaagag

attggcctgcatcgacatatattgtaatagttatttttttattttatttttagtcttgtg

agtttgttgttctcatcacatctctattattatttacattttaattgggtca

>fava_dn_rep_c1175

gggaattctttcttcttcactctttgatcacccaaatgtaaatcctttttaattattcaa

ttaattcccctcccacaattatcaaaaagcttttttattttagcaaaattttatatacca

aaatccggaaaaagttgaaaaaattccattattattattatggcttccgttcttccttat

ctttttcattttcactctcttctatctccgtttcccctttctcctctctcaaaccctttt

ttccatttctgaaattcagcccatgatatcacccaattcaatttctaaattgtaatcttt

tagtgtcctcactttacaaatttctccaacaacatgaaaagggatcgccaagaaacctct

gctggaggtgcgaatagcaacaacagcaacaactttgtaagtgtacgaggtgaatgttca

tcaatgcagaacaacaacaacacaaacgggaaacaaagtttgtggagagaagagaaagac

agcagtggtggaatggacgagttactagcggcattaggttacaaggttcgttcttcagac

atgcttgacgttgctcaaaagcttgaacaactcgagatggtgatgggtagtgctcaagaa

gaaggaattaaccatcttgcttctgatacagttcactacgaccctaccgatctatattca

tgggttcaaaccatgctcactgaactcaacccttccaccgattcacaaatcaatgaccct

ttagacgcttcgtcttccatcttcaacgataactctcagtatgatctcagtgttattcct

ggaatggccgcgtacccacctcagacccagaatcaaaacaacgaaagcgaaggctctgtc

agtaacaaaagattgaagacatgggagagtgaaacggaatcagatgtttttctgccggcg

ttatcaccgccggtggagactacaagacctgtggttctcgttgactcgcaggaaactggg

gttcgtcttattcacactatgatggcttgtgccgatgcgattcatcgtgatgatctcaag

attgctgatacactcgtgaagagtatcggaattttggctccgtcgcaaactggtgctatg

ggtaaagttgcttcgtatttcgcacaagctttctatcggagaatctacagggtttcacca

gatgaaaccattgattcgtctttatctgatgctcttcacatgcacttttatgaatcttgt

ccttatctcaaattcgctcacttcaccgctaatcaagctattctggaagctttcgccggt

gctgcaagtgttcatgtcattgattttggtctcaaacaagggatgcagtggccagcgctt

atgcaagcacttgcattacgtcccggtggtccaccaactttccggttaaccggaatcgga

ccaccgcagacgggtaacaccgatgctttacaacaggtgggttggaaattggctcagctt

gctcaaaccattggtgttcagttcaagtttcgcggattcgtttgcaccagtcttgcggat

cttgacccgaatatgcttgagatccgacccggtgaagctgttgctgttaactccgttttc

gaacttcacaccatgttggcccgacccggttccattgataaggttctcaacactgttaag

aagattaaccctaagattgtgactatcgtggagcaagaagcgaatcacaacggaccggtt

ttcatggaccggttcacggaggctttgcattattactcgagtttatttgattcgctggag

gggtctaattcgaacccgaccgggcccggttcttcgagtcaggatctgttgatgtcggag

ctttacttggggagacagatatgcaacgtggtggcttatgaaggtgtggaccgagtcgag

agacatgagacactgactcagtggaggtcgaggatgggttcagctgggttcgacccggtt

catttaggttcaaacgcgtttaagcaagcgagtactttgttggctttgttcgccggcggt

gatgggtatagggtggaggagaataatgggtgtcttatgcttgggtggcacacgaggtca

ctcattgctacctccgcgtggaagcttccactcagtgagtccaagtgagttgactcggcg

gagattaagagcgagttgaaattaattggactaattatgagaatatgaccaatcag

>fava_dn_rep_c1176

attcatggaccggttcacggaggctttgcattattactcgagtttatttgattcgctgga

ggggtctaattcgaacccgaccgggcccggttcttcgagtcaggatctgttgatgtcgga

gctttacttggggagacagatatgcaacgtggtggcttatgaaggtgtggaccgagtcga

gagacatgagacactgactcagtggaggtcgaggatgggttcagctgggttcgacccggt

tcatttaggttcaaacgcgtttaagcaagcgagtactttgttggctttgttcgccggcgg

tgatgggtatagggtggaggagaataatgggtgtcttatgcttgggtggcacacgaggtc

actcattgctacctccgcgtggaagcttccactcagtgagtccaagtgagttgactcggc

ggggattaagggcgggttgaaattaattgggctaattatgagaatatggccatcagtgtt

gtgggttggttgaaacttaaactttgatgttgattactgaaaagtgactcagtgagtgag

tgagcgagcgaggcagggaaaataaagagtaaaaaaaaaaaagtaaaaacaatgaaagtt

ctgtctgtaatttgtaattgtaatcttaatttcgatattattagtgttttttcttgctct

aaattaaataatttttttaattcatgagctagattagatgtatctgtttttcttaatatt

tctcttaatcatgcattaactttaattgaaactgactactataccatgtgataaaaaaga

taatatgtttatatttgattgtctcc

>fava_dn_rep_c1177

cgcgaccattgctagggtttctgatacacttcgcttcccctgcagcgtgtgatttggttg

cctcagtcgccgcaccattctcacacagcaaccatggctgaagttgttgttgaagaaccc

gttgctgtcgtttcagatccctctcagatcgaagtcaagctctttaaccgctggagcttt

gacgacgtccagttctctgatgtgtctttgagtgattacattggagttgcggcaaccaaa

catgccacatacgttcctcacactgctggtagatactctgttaagaggtttagaaaggct

cagtgtcctattgtggagaggctcacaaactctctcatgatgcacgggaggaacaatggt

aagaagctgaaggcggttggaatcatcaagcatgccatggagatcattcatttgctaact

gaccagaacccgattcaggtcattgttgatgccgtcgtcaacagtggtccccgggaagat

gctactcgaattggttctgctggagttgtgagaagacaggcagtggatatttcacccctt

cgccgtgttaatcaagcaatctatcttctcactactggtgcacgtgaagctgcgttcagg

aatattaagacaatagctgaatgtttggctgatgaacttattaatgcagcaaaaggctca

tcaaacagttatgcaatcaagaagaaagatgagattgagagagttgccaaggcaaatcgt

taagcagcttgtgacagtagtattggcatccccggattttgatttattgatgtatcttaa

ttaattgtgcgtttttgactttagtttcatagccttttgcttgcgtacataatgagtatt

tttgttccttcaacttaaattctgttttcgaagcccattatgtgtttgattactaattca

taaattataatatgataatttgaatgatggtacagtgtttgtaccacataatggctcatt

tatgcttaatcatgtgacatgcttaaatggcaaatatctgtgttgtacatcgcatagctt

tcaatcttagatactagtcccaatatgtaccttcaaaaaggttatcgtag

>fava_dn_rep_c1178

ctggatatgagtcgaagtagtgaaggcagatattaattgatagatttgagcactacataa

ccctctcatcgcctctcccttcctcatcctcaatctccaccgccttcaccatttccattt

catctctcacccaatcacaatggaaggatctggagtcgtcaccgtctacggcaacggcgc

cctcaccgaaaccaccaagaaatcctcccctttctccgtcaaagtcggcctcgcccaaat

gctccgcggcggcgtcattatggacgtcgtcaacgccgaacaagctcgcatcgccgaaga

agccggcgcttgcgccgtcatggctcttgagcgtgtccccgccgacatccgcgcccaggg

cggcgttgcacgtatgagcgaccctcagctcatcaaggaaatcaaacaggccgtcactat

ccccgtcatggccaaagcccgaattggccacttcgtcgaagcgcagatcctcgaagctat

cggaattgattacgtcgatgagagcgaggttctaaccctagctgatgaagacaaccacat

caacaaacataacttccgtatccctttcgtttgcggttgtcggaacctcggcgaagctct

ccgccgtatcagagaaggcgctgccatgattcgtaccaaaggggaagctggcacgggaaa

cattattgaagctgttagacatgtcaggtctgtgttgggtgatattagggttcttgccaa

catggatgatgacgaggttttcacattctccaagaaaatcgctgctccttatgatcttgt

tatgcaaactaaacaactcgggaggcttccggttgttcagtttgctgctggtggtgttgc

tacgcctgctgatgcggcgttgatgatgcagcttggttgtgatggtgtatttgttggttc

tggtgttttcaagagtggtgacccggccaaacgtgctagggctattgttcaggctgttac

tcattatagtgatcctgaggttttggctgaggttagttgtggattgggtgaagctatggt

tgggcttaatttgactgatcacaatgtcgagcggttcgccaatcggtctgattgaatgct

ttcatgaaagcttctctcttttgcatggaaatttctatctgaaagggttttatctttgtc

ttatcttttactagtgtttttttagttgtttcttatatttgattttaataaggttatatg

tttttgcttcttatgtttttgaactgtgttagatgaaagaaaaaagaaaaaaaagacgaa

gctagccttttgtggcttgtaatatgctgaatacatgttttagacatccaaggttattaa

ttatagtgtttaagctagtatgttgagtcagtgactagaaccattcttgtttgagtattg

aattctattgttatgttggatgtacttttttttttttgaataagttaatattggatgtta

tcttttgctcaattggattgtgtttcaagctatatcccttttgctgcccaaattattgaa

caattttatatggtactgttgcattccaatgctgcctttgaatgtatgtatagttcaagt

caagattataaaatgacacttat

>fava_dn_rep_c1179

ttttttgatgaagcaatatacgctcttttcttataaataaagaatattcagataaagcat

accaattgacattaccttaaatcaaaatcttataactccggatgcttaattatcaatgtt

gctaaatataccaagtcgaataatgagggcatttaacctttagacgaaactagcatccca

aaaacgaatatgaagccaataaataaaatccgtaaggtacaacagaacaaggtctaggaa

tataaaacatgttactgctctaatggtacaaggaacactgccaactctttcactaaaatt

actgacaatcagcatgtcctttgaaggtaatataaggaggaggtagtattagtgaacatt

cccatgacgatattatgatgtcaacccgcagcagatgaaacatcaatttccaatccagtg

gcagaaaaggcttccttcatttccgattccgggcttcatccttttccttctctcttttct

tgtacagcttttcaagaacacgctttgcctcacattgtggaaagttagataaatcatagt

aatgtggtgctatatctactaaccattcaccgcgtatatccgtcacagtacgtataaaat

tccgactggtaagaacatattcgttatagataacccactctggcttgtgatccaggcaat

ttgatggatgcaggtgtaccacctgattgtcttttacggttaaatagtgtcctgtccgct

caagatgagccacctgcatgaaatatcctgctagcattgcctttctaatgttgacgtaat

agtcacggctattgaagtcagtgctgcataacttcaggttaaaccgtgccatgatacgca

ctagctgttgtcgaacattatcagctgatttcagtgccctgttattgacaaagttgtcgt

agcaccaagatgcatcgtcattgttttgcttgtaggcatggtatacattcaagagtgtga

gatgatccccgtcaatgtgtccaaacctagcttttgcttcatccgcagctttctgtgcct

ccctaggccggataaagcaattgggtactgacaacatggcagaaattgaaagaatctcgt

ttgaacagttgaactcgggactaacaacaagcatctttgacatctgggggtccaatggaa

actcactcataatctcaccaagctttgttaagttaccctcatcatccaatgcacccaagt

aattcaacacttccaaagcccgcattaatgtttcaggggcaggagggtccatgaaatcaa

aatgcactaagtcgtctatacccaatttcttcagtgtaagaactgtgttggctagattag

atctcaaaatttcaggataagtttgcggctgaagatcattattgaaacttctctcggtat

aaagtctaaagcatttccctggctgagttcttccagcacgcccagatctctgatgtgcac

tcgcctttgatattggtgataccaacaaagactcgacacgaattcgaggattataaacct

tttgcttagcaaatccagggtcaataacatagactataccgtcaattgtcaaggaagttt

ctgctatgtttgttgaaaccacaatcttccttccaggagggcctccctccttaactggag

gaggagccgcatcaaaaatcttctgctgcatagctggcggaagagtagaatacaatggca

ccgctttcacagggcccacctgatcccccatatttgcaacttcttttgatattttgcgac

aagcgtcttctatttcttcctctccagtaagaaaaacaagtatgtctcctgcaggttcac

acatgtgaatctgcaccactgtcctaatagcagcctccaagtaatctctttctggttctt

gagtataaaaaatttcaactggatgtaaccttccaggaactttcattagaggagctccaa

aaaaatatccctgaaacttctcagcttcaagtgtagcactcataacaaccaacttcaagt

caggtctgtttttaagaacttccttaagaagcccaaacaacacatctgtagccaaagtcc

tttcatgtgcctcatcaagaataattactttgtatcgttccaaaagtgggtccgtcattg

cctctctcaaaagcataccatctgttaaatacttcaaaactgtccttgcactactacaat

cttcaaatcggatgctataaccaacctcttccccgatagtaacatccatctcttcagcca

cacggcgcgaaacagacatagcagccactctacgaggctgagtacatgcaatcatcatct

tcttccgtttatccggtgtctctaaatcaacagcttccaaaacaaactgaggtatctgtg

ttgttttaccactaccagtttcaccaacgagaatcaaagtctggttatccttcaaaacct

gcaagaaatcctctttctggtgccaaacgggaagtgttttcctcttctccaaaatctcat

agtaccgttgagagtaaggctttccgttccagtggttaacgaggctgttgccgattaaac

caccgttagttttcaccatcttagcggcggcttcgtcgacgacgtcgaacaaactcacct

ttctcttcctctctgtacccatcaaaaccctaataatggagcttcgattttttggtatcg

tttcaacgaaataggaatatacctttcagaaattcggtaattataaaatatagaggctag

aaaacacacgggcctcggcaatgctagggctaagttgttgtgaagacgaggaacgtcttt

ggcgttagggct

>fava_dn_rep_c1180

aaccataaatcacatttcttctttaattttttcattattattgtgtggtgataacaatct

tattcatcaaaattaatagaaattcatcgtagattttaatagatttccaacaatcataaa

acatggatcgacaaatttatttcttacaaaataaacatcaagaaaagcaaacatgcatgt

ttatatatcttacatgcatgccattacaaacattacactaattataacgatgcaataaat

atattgccttaatgacttatacaacaaccttttcttccttgataagatgagcatcaatat

ctctagtgtttttgccaaagtagtccatgtaaccatatggagggtcaatatcctcattaa

ttttcacatattcaatagtccatttaacaacagcagtaccatcagccttatcacttactt

cgaggatgagcctaaacgccttataatgcttatcaatatctcctccaaagatcttccaaa

ttatttttttgttttgttcatcaacttcttcaatactctcgtaacatgtgtgtacctcac

cacctatgacatatgaccagtgtttaactgtatcggtgtcatgccagtcttctccctcat

gcagcttggcatggtgaattctttcacaatggacttgcacttcgtgaaattctgttgcta

atagtttgtaaaacttttctgcggatgttttgataccaagttcattaataagcttaccag

agagcaccatttttgagaagaaactaaatatatataataatagaagaaagaaaagatttg

ctataatgtcccgcgggc

>fava_dn_rep_c1181

catgcctaataacacatccaaataaaaagcactcccctgattacaaattttttggtgtga

agtgctaaatgactcaaattttggaagccagttattagagatatatggatttatcaaaac

taactctcttgattttctcattcatcactacaccctgcagttcagacaagctgaatccta

aagattaatggctatatctctcgcacacttacacataaatacaagtgcatgcacattcag

ttttaagggagcatcttaagacagatattccaatcaacttaacagcataaaatccgaaaa

ataatatgcaacataatccaattaccactaaccataacacatccaaacaaaaagtactcc

caggattataatttttgtttcatactcaaattctaactcaaatctgagtcattggcacaa

aaggacaatgacaagctacatttaaagttctaacataatataagacatcattctcagtaa

gttttcagatttcacaaaggaatgcatagtttaacatacccaaaattctcaagtttccga

ggaaaagaaatcaaatagaccgcgccaaacctcaaaagttccagttagagggccaatatc

catgaatcccatatccagtcttccaattatccaaaaacacaacctgcttcacagccaaaa

agcaaaccaacaacgcaaaaatcataaccaccctctgcaccttaacactcttgaccttaa

caagcccatggctcataaacgccaaaagcaaacccacaagtgtatacaagaatccaacag

taaaaccctcagcaccaagctgcattccagatccctgatagaaaaacacaagcttcgaag

gatcgttacgatccaaaagaaacataggcattttccttataatattatgcattgcaccag

aaacactgaaaaagtaaacaaaaacagaacccgccaaccaaaccctaggatcatgcaaca

gagtttcaccggtaagaagcttcttaacgaaataagatagccagatcaagaatccaaccg

caatgagaatgattttgtttctcgagaggaggggaggtcgatgaattgtcccgacggata

acttggtttttgcttcgacgaattcggccattgattcagcgaggcgagagtaatctccct

gatccatgtgttcggaatccttgagtccgtgattaggacttacgagacggatgtgaggga

gggaattgacgccgaattgggagaaactgagttgcgactctttgaattcgatttcgcaga

agaagagttttgtaagagaggaatggttggcgttgttggtgatgaaggaggaagcgacga

tggagaattctttgtgaagctcggtgagacggagttcggatttgtcgcggagttgcgcgg

cgtcgaagaagatgaggagggagtagggtcttggggtattgacggaagtgatgtaacgag

cgatggactggtcgttgagatggatgattccggaagtggatcgggattgaagtgatagaa

gctcggtggttcggtcttcggttgaaccggtggtggagcagagggagaagatgatggtgg

ttagcgtcaagaatgcgaatcgtgataggttgagatgggaagccatggttcgattcgtga

agcagattactctgcgaagggttttgatttgggatggaggaagagactgaaatgaggaag

gaggtgtggaatttagggaagacttttgacaaatccacttcactcctctaacaccacgtc

ataattctcttttgtaaatattacatattt

>fava_dn_rep_c1182

aatattacttgtcatccatttataaagtccactcaactccatcagcaaattatcgaatcc

ttcactctagagttcttccagaatctgcattctcatttctctctctccatggccgctcga

accctactctgcagaaaaaccctatcctctgtcctccgcaatgacgcaaaaccaatcgga

gctgccgtagccgccgcatcaactcaatcccgcggtgtgcatgtcttcacgctaccggat

ctcgcttacgactatggagctttggaacccgtcatcagcggcgaaatcatgcaaatccac

catcagaaacaccaccagacctatattaccaactacaacaaagctctcgagcagcttcac

gatgccgttggtaaagctgatgcatctaccaccgttaagctccagaatgccatcaaattc

aacggcggaggtcatatcaaccattctattttctggaaaaatctggctcctgttcgtgaa

ggaggtggtgaaccaccaaaggaatccttgggctgggccattgacacaaattttggatct

cttgaagcattgatacaaaagattaatgctgaaggtgcagctcttcaggggtctggatgg

gtgtggcttggtctcgacaaagagttgaagaggcttgtggttgaaaccactgcgaaccag

gacccactggtcactaaaggagcaagtttggttccattgcttggaatagatgtttgggaa

catgcctactacttacagtacaagaatgttagaccagactatttgaagaacatttggaaa

gttattagctggaaatatgccagtgaagtatatgagaaagagagctcttaatctgaagtg

ctgcttggtgtgtggaacttgggatggcgggtttgcagcttgtttaggagatggaataaa

atgatgtgaagtgatagataagaccttcctttgatgtacttgcactttagaactttgagc

gatatatggccacaataacctttagagcctttttgcttctgggatgtactcgttttgctt

gaatgagagtctttttcaaattacggtcctctttccaagtttcttttgtgagatgaagaa

gttggagttatcaatatgcatgtttggttggaattgtttatatgaaaataattatttcta

gtttctcttttaagctttaagaacacttttcttttccaaaactaattaatgtttcgcata

gacaataagttagtaattttgcccctttccttgaatggtgttgagactttgtttcttatt

aagaggttgggttaggattggttgtccattttgtttaaaggccgtgatgactaccttttg

gatgtttgattaactaatcatgttttgtatgtcggtttgtagtttttgcttttttgggtt

tggtcttgttatccgtatcttttgtattggattgcaccccttgtgct

>fava_dn_rep_c1183

aaatcttaannnnnnnnnnnnnnnnnnnnnnnnnnnnntccaaacaaacaaaataccagg

catgagtggatttagcataatcagttgaaaagcatatttgagaagtgcaaacagattaag

catcataattccacaaaatgagattctgagacaaactgttgaaatcacacaaacttgata

taataatgataataacaaaatgaagaagtacaaatgaaaataaatcctaatcccatacaa

gaaaagcaaacaatgacaaacatcaagtccaataaacaaaagacagaacataaaaacaaa

aactagaaaagaaatcaaaacagaccaaaagaatgatgatcaagagtagatgggaacatc

agtccaagcaccagaaacatgaccattatcacaaacataagccctaacaacaggctcccc

atcatcatgaaagccaaaccctctcttcaaacaaaacgcagaataaagatcccaagtatg

attacaagaacaaaaagcacaaggcaaatcaatcttcttcctgttactacaatcattcac

cgtcttccaaacacgcgtcctcttgaaattcataaacacccctctgaaaagcctatactt

tctcttctcctccaaatgaacacaaccaggccaatcacaaacatataaacaatcgtcacg

gtactgtctcccaagtagcttactcccttgttctctactcagcgtccattttcctgtcgc

caagtgcgaactcaatgaaccagtaacctttcttttcttagattcaaccggaacatcaac

aactccagctcgaatctcttcatgggtaggttcttcttgatggaattcagaatctgcata

gagggtatcatgataaagatcgtcgtaaagtgaccaaatggaagaacattcattaggttc

attaggtcgagaagacgaagatgttgtcggtattggcaccgttgctgtggtggtggtggg

ttggtggtggtgttcgtcggggaaatcagccgtgttgttgaagaggataccggcgtgaag

aggaccgggacaacagaaagagagtttatgaagagagtgttcttgtgatgaacaagagag

aagctctgaagggatggtgagtgaggttttgttcttccagcagaaagcatggatgagaga

gttgagctttgaagagacgcaagagagacgagcccagtcgcgagggtcttcttcaacctt

ccatagtatgttgagaattatgtcgtcggctaattccgacaacatttcttttggtttcag

attgttgtttctgaaccacaagaattgtccaggaactggtgttggaaatgtgatgccgcg

>fava_dn_rep_c1184

aaaaactaatatgtaactaaaaggaacaaagccaaccaagctgaaattacagcgcatttt

tagagagatgcaaaattccatctataatttatctagatggataaagctatcaattgaaaa

accagttcaccaattagatctattattgatcttcatggaaacaacaaattcagaacggca

atcaaaacgtcatataatagaaaattatccatttctctagaaatgaaccagcaagtattg

ccaacaagtattattagttgactttcacattgtcacgtattaacatgttatccagatcag

agagtaaataaaaatagttaaaacaaaggagtctgcatgatatagcgccagcaatcacac

aattttagcaccgcgcaagaaatagaaaacaacatctaccatactagaatagttcctaaa

gtgagagcacatatagtactagacaagaggtggatattgctttgattgctgcttgaccct

tcttttatactccactgcatcctggatgaatagatgatagccttctgtttgggcagggtc

agaaggattaggctgatcaagcaggtcctgaatacccacgagaatttgcttcactgttat

agctggtctccacccactgtcctcattaagtatagacaagcaaacagttccagatggata

cacattagggtggaagaaactttgagggaatttacacttaggtggcttgctagggtagtc

ttcactgaaaatcattgtcagtgggtaatagccaccttcccaatcagtcccttgcttgcc

aggaatagtgcagtgccagaccatcaaattgatagttccatcagggagcgtttcaggctt

ggcaacgaaaccatgaggatgattctttctccatgactttcgttcctcggcaaggcgtcc

acgagcaataccagacatatttcgtcgctagatactttccgtcgcttctttttccaatcg

ataaaaccttctctctctgtctaactgtaactgcgccttcaaaatctctctctatgccta

acaaacttgcccttattctactacggtgacgcgtcttctagaatcttcgcttactccctc

tttatttcgtctaatcggatcaaatataaaactatggattagttccct

>fava_dn_rep_c1185

attttattgtatatttaacgattacggtcgagccagacccaaatggaaaaagagtactca

aacggaatcccgctacaaggctagttgcttcaaatacgcacccgcacacaccgtgtccgc

gtcactccaacgaaacacacagtctctctgcgtcaaaaaccacgagaagaggaacaacat

gcagatcttcgtcaagactttaaccggaaagaccatcactctcgaggttgagagcagcga

caccatcgacaacgtcaaggccaagatccaggacaaggaaggtatccctcctgaccagca

gaggttgatttttgccggtaagcagctggaagatggtcgcactcttgctgattataacat

tcaaaaggaatcaacgcttcacttggtcctgaggctcagaggaggaaccatgattaaagt

gaagactttaactggaaaggaaattgaaattgacattgagcctactgatacaattgaccg

tatcaaggaacgtgtagaagaaaaagagggaattccacctgtgcagcaaaggctcatata

tgctggtaagcagcttgctgatgacaaaacagctaaagagtacaacattgagggtggttc

tgtccttcacttggtgcttgcattgaggggtggtacttattaaggtagaatgcacaccca

aattcaagaaattagcttgttcaggcttggtcattttctagttcttggtaaatgtttgaa

ttgcttctgaacctaaagacatgaaaggaagctctatactgtccgtctctgaggatatgg

ttgttcttttatatgcttggtatttgtaattttggggattttattggtgctcggtacctc

ttggttatatgcttttaaacccaaaaattggcaacttaatgaaactctaaactaataatt

cataaaaacaaaaaatattatgagagtttttactctgattctgtgtacagggttgaaagt

ttgattttagttttaaagccaaaagcacaatgatggtggcctcggtggactcataaatgg

tctagataagatcttaacgtaattgtcagacagcccacaaatatgatgtatttattgtaa

gaggatttaacctatcaaccccggttgtacctagcatcccacatcttatatgcttttacc

gttaaatctttgcttaaagttatcggtttaataaaatatgagatactggaaatttaaaaa

attccggtaaaaatca

>fava_dn_rep_c1186

ctttttttttctaataaatctacttgttgttttttttgttttttgtaaaaaatgaatttt

tttttttttaaagaaaagggcaaccactcatatagttgttgagacatataaaacaagggc

aatattcaaaacataatagttatgtgagtcatacacgatacacatcccatatataagaag

tgcaagtttagggtccaagcaggcctataatgctaccgtgaccaagcttgatatcaatat

tactaggaattatctgcttccatgtgattaagttggaaagtcccttcgttattcttgtgc

accactaccttgaacttctcttgtttttctcctcgcttgagcttgagaagcaaatcaaac

ttagcaacatcatcgttgacctcagccttcgcatcagaaacctcatggagttcatagggc

actagtgaattggacctctgctggatggtcttaagagcatgatctgctgcatcctgaact

tgagggtcttctgttggcacagactgccaccccggcttggggccatcctttttgacacca

agatcagcagaagtaaatgaaggtccgtcaccatcaccagcatgcttgaactcatgaagt

tgtttaaaattcaaccagggtttaacccacacttgagcgttatagatcttcttctcaccc

gcatcaacaacctcaatagtaaggtgatgcattgtaccagcaacaatctgttcctgtgct

ttcaccactcttccaaactccagaagtgaattctgtttggcgttgtgttgatcgacggcg

aatcgagcaagggattcaacttcgagggagttttgagaacttggggaatcgtggatgcct

ccggtaatgggttccatttgggcgtgatcgaaatcggagcagtcgctggaagattgtaga

acggtgaagattgagaagaagaggaagaataaggaaagaattgaagaagaagttgctctc

attcggtttgaatgaagaaaacaattgggaacgctttgatttaacgcagatttggaaaca

gtctccgtgtttgcttaatgaggaagccacgcattccacacgct

>fava_dn_rep_c1187

gaacggccctcactcactctagacaaaaacaaaaacaaaaatatcccgccctccattatt

accatcttaacccccgcatttccctctcttcaccactcccacttccatccatataagtat

attctatttctccctcaaccgcttcatacaaaatcatcatcatcttcatttccacaaacc

ctaaaccatgagagagatcttgcacgttcaaggaggccaatgcgggaaccaaatcggatc

caaattctgggaggtcatctgcgatgaacacggcattgatcccaccggtaaatatgtgag

cgaaggcggtaacgatacgcaattggagaggatcaatgtgtattacaatgaagcttctgg

tggaagatatgttcccagagcagttcttatggatcttgaacctggtactatggaaagtat

cagatctggtccctttgggaagatctttaggcctgataattttgtttttggtcaatctgg

tgccggcaataattgggccaaaggtcattataccgaaggtgctgagttgattgattctgt

tcttgatgttgttcgtaaagaagccgagaattgtgattgcttgcaaggttttcaagtttg

ccactctcttggaggaggtacaggttctggcatgggaacactattaatatcaaagattag

ggaggaatacccggacagaatgatgcttactttctctgttttcccatctccaaaggtgtc

cgacacagttgttgagccatacaatgctaccttatctgtacaccaactggtagaaaatgc

agatgagtgcatggttcttgacaatgaagcgctttatgacatttgcttcaggacattaaa

actcagcacccctagctttggagatctgaaccatctaatttctgcaaccatgagcggggt

tacctgttgtctgaggtttcctggacagctgaactctgatctcaggaagctggctgtcaa

tctgattccattcccacgtcttcacttctttatggtgggttttgcacctctcacttctcg

tgggtcacaacaatatgtatcccttaccgtcccggaactgactcagcaaatgtgggatgc

gaaaaatatgatgtgtgctgctgatccccgacatggccgatacttgactgcatctgctat

gttcagaggaaagatgagcaccaaagaggtggatgagcaaataatcaatgtgcagaacaa

gaattcatcgtactttgttgaatggattcccaacaatgtgaagtctagtgtctgtgatat

tccacccaagaatttgaagatgtcgtccacttttattgggaattcaacttcaattcaaga

gatgtttaggagggttagtgaacagttcactgctatgttccggcgcaaagcctttttgca

ttggtacactggggagggaatggatgagatggagttcacagaggcagagagtaacatgaa

tgatttggtagcagagtaccaacaataccaggatgcaattgccgaggaggaagattatga

agaagaagaaggtgatgagcagccgtatgatgagcattagctgaatgatactctcgggta

atgcactaatggtaaaaatgtccaccatgcaactttgctgttatttgtgtatctacagcc

atatatgctctttctttattcttaattttacatgtttctctacattttgccaatttcatc

ttcaagtgtattattattaatatttctgagttgtttggaaatcggattgtgaatcgtcag

acaggattattctatacttctattccctaaatcatacgtatgtatttatgtatttgtgtg

gattagttttgtttcctaccaattcactttgttttaagagggctgggactttcccggcca

tgccatgaagccatccgctactccaagactaatgcttcatttggaagctaggaaaaaaac

aagaggaaaggaaatatatagaaaaaagtaaaaagagtaaatagatattgcacgctattt

ggatgggagagaaagagagtannnnnnnnnnnnnnnnnnnnnnnnnnnnnnnntaaaatt

gctagattacactcttgtaaaatcaaa

>fava_dn_rep_c1188

tgtttgtggaaatacatgcaccatgcactactcagtaaaatgtgtggactaatcacatca

tatataaagtaacttttannnnnnnnnnntataattggtgtatataattcgtgcaagcct

catgttcctaatgcttttcatatatgaacaaaccaagttaaataattgttcaattctgaa

gttaaaaaaattagaagttctcatgggattgtttatagcacactttgtgcaagggttagc

tctcacatttcttggcctatggcacataatcaacacaatcaaatcttaccttgtgaaagg

tccttcaaatttcactgtaaggttttggttccaaattaacacctttcaattcaaattcaa

acacttggaacttgttttaattttaatcttttctgttctagcatttctaaggcaagtcat

agatttcccttattttcactatgcttttaagcttgataactttgaacatgcaactatgtt

catacaccttgctttgtttgctggcttttcgctcgctgccgagttaacagattcgctcga

actcttttctggatttgtcgggattcttgtatcctcagttttcagtcaagagcttttctt

acttcattttcactccgctgatcatgttggacttgaaggtcattatcactggctgttaca

gcttatagtttttgtttcgcttgttgcagctctggctgcaactgtttttcccaacagttt

caacgctgcgattgtactttcgatttcggttatttttcaaggatgttggtttataaacat

gggatttatgctttggattcctgcatttgttcctgaaggatgtgtgatgaatatggctag

taagagtggcaatgaaatgcatggagctgtcacctgtgaaacgaaagaggcggattttcg

ggctagaggactagccaatttgcagttcagttggatactttctgctattatgatatttgc

aggagttgtatgcttgaaattggctagaaaatttaccattgtgaaccggctggagtacga

gagaattcaaagtaaagttgtagattctactgtggctaacgaaggtttcaagccgggaaa

gtaaattctcggacgcagtgtgtgtgaatgttgtaaacttcgagtatcgagttcgattcc

tactcataaaaaaaaaactgtccatgtgtttggactttttaaggtcttgaaggtatgtaa

gagactcattatgtgaaacttaataagatgtactatagtgtgttttatgttagttcatgt

ttatatagatttggtgtctagtaatcttaaagtacacttgagctttaaaataaccaaaat

tgaggagctgaacttcttaatatatatacagtttgtttataatagggaggggctccatgt

atagatttcatatagtgaaggaattcttgaaccac

>fava_dn_rep_c1189

atcgaatccaaaatttccttgaattttattatataattaggattaggcaactaaaataat

agaatatagaaaataaagaaatgaaaggtgtaataggcagaaagagaaaggcaccaaata

caaatttaacattgtacatataacaaaattacacagaaagatactccaaaaagggaaata

ttataaaatcatctcaaacttggaatcttttccttccacctaaatgatacattgagaatg

ttacaagcacgtgccatgacaacttcaagtagttggatcaaagtttgaggcatttgaatc

aagaacagaaacatggcatacctccaacaaggcctcttgtttagaaagctccccttttaa

tagcatcttgattagcatcaccaagagcagcctcattcaaatacttgtctgccaattgga

ctctcttgacattctcttgttcttggacaagcatgccaccatattccaacagcttttcta

gtgtcatctttggctgctcaaattctggtggtccttcctttgagttcacaagcctctttc

caattccttcaatacctatcccagaaatccacttcctcacttcgtcgtcatacactctag

ccctcaatgcaccaaagaaatcaatggattgaccagggaaggtgtcaacaagcctgacaa

tgtcctcattttcaatattgtcatgacggaatattccggtacagacaccaattcggtcct

ctcttgtaggagcccagtagaatttctccatacgtccatcacggatgagaggagcataca

atgttgagaaatcgttaccagtgacaatgatgggaacacgaggattctcttccttgttgt

acataccagggagttggacatttgtggggttatcagcaatgttcatgagtgtggcattca

ccatctggttgttgacagtgtattgggttgttccacccatacgacctgctcctgcatcaa

gatcgttgatgaacaaagcacacatctttcccttcttgattaagtcagcagcttcacggt

atcgctgtctgatcagttttgctggctctcctgcatttccactttcaagctctccggcac

tcatcatgattgggttgattcccatctttgcaaaaacaagctcagcttggaaagattttc

cctgtcctttgcctccccagacaccaagaatgagaggaaccttgatgtttggcaaggtca

tgaaatttttagtgatatgaacaacaagcttgtccataaaagcaggagcaatgtaaaaac

cactcatagtgttgtcaaagtcgtactgtttaagtccggtgctaacgtattcataagaac

tcatgactgcataatgagttccagtattttgtggagcttggaaaacagaatcaaccaaac

cctttcctctggtgatgtcttgttggtcatctgaaatatcataggccaaacccctccacc

tgtctccgtctgtctgcttttcagggtcaatctctttagcagcaaaaactttgaaacttc

cagaagaaactttagtgtttggatgccttgaagtaatcttcttcaagcttgtgccgaaga

aagatgaattggaaactgaaggtcctgctactactgagccattcaagctcataggagttt

tgacagctccaacagtggagactgaagcagccatggataagtggtttgattggtgtagca

caaaagaggaaggtagttgaagttgaagcaaattattctatgccgcgg

>fava_dn_rep_c1190

gtgtgaggttgaattcacatgcgttttgcaaagctgaactaactagccaagagggacagt

caaagactgagacacgtgatctctctctctgggatctgttgcggttccttcctcttcatt

ccttcgatccaccatctcagatttcaaacctacccattctttctcaagttgtaggtttag

atctatctctctaatcaccaattatgttcatctttgattggttctatggcattcttgctt

cccttggtctctggcaaaaagaagccaagattctcttccttggtcttgataatgccggca

aaactacccttcttcacatgctcaaagatgagagattagttcaacatcagccgacccaac

atcccacttcggaggaattgagtattgggaagatcaagtttaaagcttttgatttgggtg

gtcatcaaattgcccgtagagtttggaaagactattatgctaaggtggatgcggtagttt

acctagtcgatgcctacgacaaagagaggtttgccgaatctaagaaggagcttgatgcct

tgctctcagacgagtccctggcaaatgttccatttcttattctaggaaacaagattgaca

tcccatatgcagcttcagaagacgagctacggtatcatctcggcttaaccaatttcacca

ccggtaaaggaaaggtgagtcttatagactccaacgtccgacctcttgaagtattcatgt

gcagcattgtccgcaaaatgggatatggcgaaggcttccagtggctttcacaatatatca

aatagagtccgatctgtttgctggtcaactaggcactgttctgcagttaaatcacaagtt

acttaccttttgtttttgcttttgattggattatttgatacaactgaagtaatattacat

ggtaataacatttgtatctgtagaggttttttttgaagtacttgattatgttataggaag

gaatggtttggcagaaacttaaataattttgaagaatcttaatacagttttttcttctta

catgtgaatggaagcagtggtatcaacgcagagtgtcagattaagttaaaaagttacaga

ttttcttttggtgattttggagaattttcatgtgattagtagtaacttgcagtagtgatt

gttgataccctctccatggtccacaaacaagactactgcctctagcagtgtacagctttc

acattgcctttattaaaagctgtttttcaaagtagtgttcaggaaatgatccgaaaacgt

ttacgaccaatatttgtaatagaagtgttttctctgtttgtttactctaaaatttcagat

ggttttttcctcaccacccccctctggtacccgtggtgatgctccgagctgttctagtgt

cagttatagattgatatttccttttctttgaaagatttttttgtattctcaatagtcata

aagtattgattttattgccgaacaattgaatagtgtatttaatcgtcaatcatttaaatg

ctcgaagttggacatgtatgtgatataagatatagggaataa

>fava_dn_rep_c1191

aaattttttgatgtgtttcaataataaactgtgtctactaatgatggtttaaagctatac

tgtaacaagatggaattcactgcttcaacatcacaatcaaggttcacaatgtgaaaatga

ctcagtttaattcgtaaacatgggtgtggtcagaactacactggtccacagatgccaaaa

tatatcaagaggccgctcaatccatcaaagtaaagttaatttctatttaatgcattgaag

acaaaaaaagtattaaaacaacattgttgaattgcaaaataacataacaaaagcagaagg

caaaataatactaacaaaaactcaggcgaagtatgaacttcatttgagcaaaagaagcct

atgctgcaacaggcttggcggtagagagccttgatctcttcttagccaaactctcactac

ggcggtccctctgctccttcaatctggaggcgagaagtttttggtactcagctgcctcag

attttgctttagcaatccttttcttcttatctgcaattctagccctcttcctttgaagtg

tcaggggagtgaccaggcgctgaatctttggtgctttgctctgctttttcccagctttgg

ttgtgaaagttcgacggtatgtgttgacatacttcctgacatcatcctccttagaaagat

tgaagagcttgcggatcttggaagctctcttgggacccctcattctaggcttctcaacat

ctgtcaagcctggcaaatcattttcacccttcttcacaatcaccaagttcaaaacagata

gatctgggctgacaatgcacccacggacagacttcctccttcgctccccatttctccttc

cgtgtcctcggaaacaaggagttcctcggtgaagcaagaggcgaacgcgacccggagtta

acactccttgcttcattggaaaaccttgtttgtcacaccctccagtaattttgaaaacat

agcctttgaattcctctcccaaagcatcaccattaacctcctgagagatcctcttgtccc

aaaaagcacgaagcttctggtcatcatcaatttcaagcttcttctggcatccagtggtgg

gattcgcaacgttgaacttcatgttgaagtgtggatttggatctcagtgtcttcgttcgc

gttacggcaagtgagattaaagatgatggaaattagggttttttgaatacccggatgagg

tagtcacttaattggtctatgcttaagttgttaagaagttggacccaaatagcccatatg

agaatttttcctacctcctcatttatatctacaccccaacatattttcataatatctata

ttatccttgtaaaaattgtatcaaaaagtctcgaacaaaaaatatgatagtatatctaaa

aaaattcagtagtgtatttg

>fava_dn_rep_c1192

tttattttgtgaggagaacccctgacacgcaggcagggtgatacactctatcctattctc

ttttcttcttttcttttccccttccctctactccttgcatctaattccttcattttacac

ttaatttcattttccctcaattctaacctattattatccttgcaaattctcttcttacct

aataaacctaaatccttataacactatctcgattttctctcaacccaaaaccctttttct

cgttttttcaattctaacgatcaatgactctgtgaactctgtttccttgaagatggcgat

ggttccttggcgtggcatcagttgctgtttgagtgctgctgctctttactttctcggtag

agcaagtggcagggatgctgaaattcttaattctgttagcagggttaatcaattgaggga

gctagccaaactcttggatgaggaaatactccctttggttgttgcaatttctggaagagt

tggttctgaaaccccgattagttgtgagtttagcggtttaagaggtgtaatagttgaaga

aacggctgagcaacattttctgaagcacaatgaaaatggttcatggatacaagattctgc

gttaatgttatccatgagtaaagaggttccttggtatctgggtgatggaactgatcgtgt

gcgtgtagtcggagctcgcggtgccactggttttgtattgcctgttggaagtgaggcatt

tgaagagtcgggtcggtcaattgtacgtggaacattggattatctccaaggtctaaagat

gcttggagttaagagaattgaacgggtacttccagttggaacttccttgactgttgttgg

tgaggctgctgaagatctgaaccggaaccattatttccgacatcaaattttcttaattca

tgtcacttatgcggaaagagtctacacggaaaagatatatacatgtatagaggtgagaaa

ggattttgtagcacagagtgtcgttcgagtcaaataatgatggatgaacgaaaagagagg

tgtggttcagaagaattgtcaagttcaccatatacaaggggtcaaatattttcaactgga

attcttgcggtttaactattgattaattagtgtctaactctggattaagaaaatcaataa

ttatccagagattgatactgttaagtaaataataatatatacggctatactatggataac

tgtggaatatttgtatgagaattaagatagatagctcgatcgtaaccaaaacaaaaattt

tgatgagaaataatgagagagttagattatttttatgtaatttctttttatatgatgctc

aaggaatcatcaattatgaactcgaaatctcaattgcttagattgatttctgttgtttct

gaattctgtctcaaacatgaactaattgaaaataattatataaaaaaaacatcttttttg

tcatggaattaaaacattatcttg

>fava_dn_rep_c1193

tcagtgcaatttgacaaaacaaaagaaatagacaatactaatctatctattactttgcaa

tttgcaacattggaacaaaaaagcatgtttgagctatatgagaattgagccaaagaacca

tttaaacaatgaagaaataattaacaatgaggtcattattagcataaacagaatatagaa

ttgctagtaaaatgcactcagcaatcaggtacagtatgaagctccaaacacgagtggcaa

taaggtcagctaatcaagcaaataaaatatcatttagaagtttaaatgtatcaaaattac

agccaaaattgcaactacttatcgttctacttaacgtttgccgccaccaccacccaattt

tggacccttgggcaaacctcctttttggacattgcctttgcctgacttttgggcctttga

tgctacttctgctttcttggctttcttctcatcctttgttttcttaatcctctctttgat

ttcacgcagagcagcttccctggctgcatctctaacttcaggcttctcagctcttttctt

ctgtatgacttccagtgtagcaccaacaatggacctagagtatggcttcttgttagcacg

gcgtctcttctttacagcttcttgagcaatatccttcttgtgttgctttctgtacatggc

agtccatgtgagctttgaaggcttcaagcggttgtgaaaatacctcttacatttcgagtt

aacgaagaggaacacctgagaatcacttcgaataaatctgatgcctcttcccgggtagat

ctttgcaccgctgaatcggcagagttcagttttgagaaccattgttgcttcttttctggt

ggctgcttctacgctggtggtttctctaaaccctaatatcccaaatcgctaaaatctgcc

tcgggttatctatatac

>fava_dn_rep_c1194

atttgtatataggtccaaccttgctataattttgcattgtttatttaacctttgacactt

tatacataacatcttgaatcaatatcatgcatcacaatatgtgtacgtgaaaattcacat

gttcaagtattatgtatgttaattcctttgaaaaataacagtaagaaaaatccaaaaaaa

caagtgaaacaacaatcaatcaacaaaccaaacatcaaaatattcacaataactacaatt

ttcgggggaaaaacagtccctgatattactaacaacaaatttcagatctttgcatgaaaa

agaagaaacaaaacatcaagttgaaatattgattggaactcctgaacatgacaaaattgt

tgctattgtctttgttgttttggaggaaaagatgaaggaagactcaaattctgaatcaag

cctccttcgacgctacatccaacacctccgcaaactaccacaccacgatcccacttttcg

atgataccaccgcacacaccttcagatccacagccgggctgttcttccccttaactctca

agtactcatcaacttttattcttccatcaccatccaaataatttttcaaaagagtttcac

caagccaagacgaaccaagaggcttccctatacatacaaaatgtgtatctgcttctatac

tctctccaagtgaagtttgatatgttctattttcatttgtgaaagagtccaagtcaacag

attgtccaagtttgacatcaactttctttgatttaagccattttagagtctttcttgaag

cttttgtcccaatatattctagcaaccttgatcctttatgaactatagtcacctttttgt

caggaaaatcaacagcgatttccgctgctagctcgactccggttggacctcccccgataa

tcaaaaccgaacgcgctgattttatcttcgtattctctcctttgtattggtcgattcttt

cggtccttgttttcgggataagttcagtgtgaccggtggcgataacaagataatcatatg

aaattttacggccatctgaagtgaagacttcagattcagatatgttggcagcacttgata

taataaggtcaccttttttgaaatattctctatggtttatcactgtcctttctgcaaaag

atggctccaccattgcccttaaacttgcccatggaatctcaaaatactccttcggatcaa

taagagtgacattagcttgatcttgtaaagttttagcaaggactgcaccagcaactccac

ctcctatgatcacaactttcttttccaccatttttttaaactttctaacctttttttgat

caaagttgaaattaacttggtaatgattgtttttgtttatcgtttcaattaagatgaagc

aacaatataaatagatgaagttgcgtcattgcatggatcatcaaattgaaacgtcttggt

ttgaatttggacataaaaacgaccaaagccaattggtagctatagtcaactttacaagca

g

>fava_dn_rep_c1195

gagcacaaannnnnnnnnnnnnnnnnnntggtactagacccataacaagattacaaaact

acttcagatattgcaatacaaattttggataaacctcaccctaaatgaactcagagtctt

ctttaatcttaatggcatcatattcaagtttaaaatttgtaccctagaaacataaaatcc

agtttaggggcatgatgagtagggttgcaatttccatttcagatcattaaaaagacagtg

aacacagctgccaagatccaacttataattataacgaaaatgaaagcaattccacacaaa

actgtaattaaatataatctgagtcacaactcaagtttgataacagacataattaactaa

aaaccacaaaattacaagttcttcaaaaactaaaatcatcctagcatcattaacataaca

atgcaagagactgtaaatggactcccaatctttagcgatacttaatgaaaccaatttcct

tggcattgctatggaagcactgcctgcaacacatgagtccatacttcctgatcaatccat

gagagtttccacacacacggcatgtgcgagaaccaggtccgtaggttttggggtgagaat

tccagacattagagtgacccatggcttcgatttgcgattggggaaacgaagtagcagcac

cgatctcaaaaataaaaaccctagtcacgcaaccagaatggaaaccagtgtatttcttaa

cttatataggaggctcaaaact

>fava_dn_rep_c1196

accctaagctaaacaagaaaaagagaatagtactactgctattgctagtagctccttgtg

agagagaatcttagataaaaggacactcactccccttaatatctttacctttgtgctcct

caaaaacagagtgtgaaaatggcttggagctccacaagaatgaaatgggtcttcatgttt

gtgcctctattcttgtttcatctggctttcacaaccgccacagaagacggtttagtacca

aatggagattttgaagcaagtccatccaacggtttcccaaacgaagcaatagtagaggga

cccaccgaagtcccaaactggaaatcaaacggcaccgttgagctagtggaatcagggcaa

aaacaaggtggaatgatcctcatagtaccacagggtagacacgcagtaagaatcggtaac

gacgctgaaataagccaagaattaccggtagaaaaaggttcaagttactctataacgttc

tgtgcagctcgcacgtgtgcacaatttgaatctattaatgtgtcggtgtcgtccgcatca

cagacgatagacttgcagacactgtataatgttcagggttggaatccttatgcggtttct

tttaatgcggatgaggatagttttaagttggtgtttaagaatccgggcatggaggatgat

cctacttgtgggcccattattgataacattgctatcaagaagcttttcactcctgataag

cccaaagacaatgcagtaattaatggtgactttgaagaaggtccatggatgtttcgaaac

acgtcaatgggggtcctacttccaacaaacctagacgaagaaacttcgtcgctcccaggt

tggatagtcgaatcaaaccgagccgttcgctatattgattccgatcattacgcgattcct

caaggacggagagccattgagcttctctcaggaaaagaaggtatcatatctcaaatggtt

gaaaccaaacctgaaaagccatataccttaactttttcattaggccatgctgatgacaaa

tgcaaagagcctcttgctgttatggcctttgctggtgaccaaactcagaacattcattac

acgccgaattcgaattctacctttcaggtttctaacctcaatttcactgccaaagctgat

atgactagaattgctttctatagtgtttactacaatactagaagtgatgatatgacctct

ctttgtggccctgttgtggatgatgtgagggtttggttttctaggtctagcaggcttcgc

gggctgggctttgtaagacttgggcttgggattttgggtttagtgttggttcttgtttaa

aaggagttcattatgttatggtttatttaatgttattactactaagcttgataggttctt

ttgtatttggggtgtaggtgaagtcacattctccaaaattgttgtattgtgaactaatgt

taagtttatatagtttatgctatatttaaaattaaaaatgaaaatgtgttgctgcaaaaa

aaaaaag

>fava_dn_rep_c1197

ttcaaatctcactttccatccttttcacattaaaatcacacacacatactctctagaact

ctctttttaaatcacataaatatatataacttgcaaaagctacacaaataacaagattca

ttcaacaaagaagaacaaaaaatgaattccaaatggatatttttcctactaaccatttca

acaatgttagtaataggaaaaacacaatcaatatcatcagagaaacctttggtgaaaata

gtaaagggaaagaaactatgtgacaaagggtgggaatgtaaaggttggtctgtgtattgt

tgcaatgagactatctctgattatcttcaaacctatcagtttgagaatttgttctcaaag

aggaatgatccaacggcgcatgcggttggattttgggattatcgttcttttattactgct

gctgcactttatcagcctcttggatttggtacttctggtggaaaacatggtggacagaaa

gaagttgctgcttttcttggtcatgttggaagcaagacctcttgtggttatggtgtagct

actggagggcccttagcatggggcttatgctataataaggagttgagtcctgatcaattt

tattgtgatgactactacaaattaacctatccatgtagtcctggcgcagcatactatggc

cgcggtgccataccaatttactggaattacaactatgggaaaattggagaagccttaaag

gtggatctattaaaccatccagaatacatagaacaaaatgcaacattggcattccaagca

gcactatggaaatggatgacaccacctgagaagcacataccttcagcccacgatgttttt

gttggaaattggaagccaacaaataatgacactttgtccaaaagggtacctggatttggt

gccacaatcaatgttttatatggtgatcaagtttgtggccaaggtactgataatgatgga

atgaataacataatttctcattatctttattaccttgatttattgggagttggtagagaa

gaggcagggcctaatgaaattctctcttgtgctgaacaagctgcttttaaaccctctgga

ccaccttcttcttcaacaacttgaagagattctttacttgtttggttggttgttcttgaa

tcatgcaaaatctaatgtgtatagattactatgatgctatgtattttggtttctatgatc

actatatgattttaatatatttttggaacaatgtatgtatgaattgagtatttgatcaat

aagaattgtctcaacaattttgttgatatttgtgtgatttgtatttcactaattaaaatg

acttaaattggattcaaattatctaattgcagttatgtttgtct

>fava_dn_rep_c1198

tgaagaagacctattccctagtgcatttggtacgaattcgcatgtgaagttttgtacagt

aatccaattacatgtcttaaaaaaagtaacattttttatcattaaaataagaaatagaaa

tcattattttatcatagaagtaaaccagtcataagtcatatatcaaagaggatgctttat

ccatggcaaacaatattaaaaaccataccatctgtttttacaaactacaaccactcaaga

ttatttaactgcaaccaccatagaagaaaggaatagcaacccaagacatagcaaagactg

gaccagatcacctcattcatgcactgaaagcacacaacgaagacattgcccttcatgcaa

aagatcgaaagccttgttgatctccagaagtgtcaaattatgggtaatgtactcatcaac

cttgatttccttcttcaagtacttctctacaagccaaggcacttgtgacctactcttaaa

gccaccaaaagccgttcctttccacacacgacctgtcaccaactggaaaggacgggttga

tatttcctgccctgatgctgcaacgcccacaataactgatgttccccagcccttgtggca

gcattccaaagcagatctcatcacagagacatttccaatgcactcaaagctataatcaac

acctccatctgttagatcacagataacctgctgaattggtttctcatggtcttttggatt

aatgaattcggtgactccaaagtttttagctgtatcatacttgttgttatcaatatcaat

cccaataatccgtgatgcaccagcactttttgcaccctctgcaacagcaagcccaacagt

tccaaggccgaaaatagcaacaattgaccctggctcaacttttgcagtgttccagacagc

tccaaggccagttggaacaccgcatccaagaagacaaactttgtccaaaggagcatccgg

atgaatcttagcaacactaacatcatgaacaacagtgtactgactaaaagtggaagtccc

cataaaatgataaattggtttccccttaatagaaaacctcggcttccgatccgccatcat

aaccccaacaccagtagcagcacgaaccttcccacagagattggtcttgcccgatttgca

aaacttacattctccacactccgcctgataacacggaatcacatgatcaccaggcttaac

attagtcacaccttctccaacactttcaacaatccctgcagcttcgtggccgagaatgca

ggggaaaagaccttcaggatccttgccgcccaaagtgtatgcatcggtgtggcagagagc

ggtgaagaggatttggattcggacttcgttggcttgcggcggagcaacctcgacgtcttc

gattgataagggtttgttgggttcccaggccaccgcagctttgcatgtaatgacttgacc

ttgagttgccatggagatgagattgagagtcaagagtgagagagtgagagagctacttga

tgccacacacagcctcaacagatataaataaatctcccc

>fava_dn_rep_c1199

tagtaccatttgggtgtaaatttaatttaatcttgtaaatagatagatatcaacgtggtg

agcggagcgtaccctaaccatttccactccatttctcagtcccgatgtcctcttctctct

ccacccctttcctccaccattcttcaactcttcgccatggaaccaaccctttccctcacg

ccacttccactcccaaaccccgatgcttcaagcctttctccgcaaagtgctctctcgaca

acatccccaagcagttcagacaggaaaatctcaaagatggattgatggacaactacaaga

atgttcctcagtttctatatggtcttactccctcacaaatgaacatgttcatggctgcag

acaatcttacacacaagatgtctcaaagagttacagaggatagcatttcatctgccaaaa

gttatttggatcatagtggaatgggtagtgtatctagcatggacaataatgcctcttcaa

ggatcagcatgagtgtaagtatgtatagaggaggtggtaggggaatgggaagacctagga

ggtctccaccggatttgccttcttttctcttggatgctcgaatatgttatttgggtatgc

cgattgtgccagctgtgacagaacttattctggctcaacttatgtggttggattatgata

acccagagaaacctgttcacttatatattaactcgtctgggactcaggatgagaataatg

agactgtgggatcagaaacggatgcttattccatagctgatatgctttcttttgttaaat

gtgatatctatactgtcaatttggccatggcttttggccaagctgcaatgcttatgtctt

tgggaaaaaagggttatcgtgctgtcttgccacattcatcaacaaaattatttctcccaa

aaatccacaaatcaagtggttctgtcacagatatgtggattaaggctaaagagctggatg

caaattgtgagtattacattgagctgttggcaaaaggaacagggaaaccaaaggaagaaa

ttgtcaaagacatccagttgactagatatttccaagctcaagatgccatagactatggag

ttgctgacaaaatattgaactcgggagaagcttcatatgagaagcggaattataatgaat

tgcgtgctgcaagagcacttaatagaggtggccggaaccctcaagctgcttcttcgggac

tttaggtaatttcacacataaaaacattaggtcgcttgttcagttccctatccaatgccg

ttaaagggtgttagcgggtttggtttaagttgacagctagtatagaaaggtcaagcagct

atcttatatgaagccaaatggaatttgaaccagttaatgtctagagtctgtatctttgct

ctggtgttatctgtgatgtacttatattacttgaattaccagtgctttgtttcaactaaa

gaaaataagaaaattactaggcctaggggattattttctgaagttgtaaatatcaaaacc

gttatcaactaaagaaaattagaacattatatacactttgtttaattttttaatacataa

ttgggtggaggcctaatgcgtgcac

>fava_dn_rep_c1200

agtcaggcctcacgtaagtttgtgcatgtgagccgccatgtctgtgtctctacgcaatgc

tgctactactattctctctttgcatctccttctcttccttgttaatgctaactcatctat

ggattcatacaaaacactcaaaatgatcttcaacccaaacgccactgtcccacaatcacc

tccacgcacacccgctttcttcaaagacttcaccatcaaccaatcaaaacatacttgggc

tagaatctaccttccccgcaaaccaaactccataaagctacccctcattttcttcttcca

cggcggcggcttccttttctacagccccgctacagcttacgtccacagcttttgcaccga

cctcgccaacaaaactcaatccgtcgtcgtttcactcaactaccgcctcgcaccacagca

ccgtcttcccgcggcgtatgaagactccgtggagcttcttcgctggattaaaacctcaaa

cgactcatggtggaaacgtcatgctgattactcaacatgttatctcatgggtgagagtgc

tggaggtaatatcgcatacaacacgggtctacgcgcggctgcaatagttgatgagattaa

accggttatcatcaaagggttgatattgattcaaccgttttttggtgggacaaagaggac

accatctgagttaaggcttgagaagggtacgaatctacctttgactgttactgattcgat

gtgggaattgtctttacccgtaggtgttaatcgtgactacgtgtattctaatccaacggt

gaatgggggtgataaggtattggagaagattagtttgtttgggtggaaggttggggtttt

tgggtgtgatggggatcaacttgtcgaccgtcagaggggatttgtgaagttgcttgaggg

taaaagtatccatgttgttggacgattttatagtgggggtagacatggtatttttatgga

taataataccatgtctgaaaaagtgttcgatctagtcaagagtttcaattcatgaattat

gagtaatgcagtcaatactttgtcatgtattcctatgtgagaataaaccaaccctatcta

tcatcactaccctatctatcatcactatttgtaatggtaaacaatgaattttaacaagag

gcgtacacttctcttattttaatggtacatcttgtgtaatgaaatattttattttaatgg

tacatctaatatatgaaatatcttgcttccaaaaaaaaag

>fava_dn_rep_c1201

aacccaacccatttcaaaattccaaacccaaaccaagctgaaccacattcggttcctcac

aacacaaaaattccacaaaccagagataaaaatcgagagcagaatcaaacaacgttccag

aaagtgaaacagaagaaaaactagagagagaataatcaaagaaggtaaaaatgacggaag

cgatgataaggaagaagccaggaatggcgagcgtgaaggacatgccggttcttcaggacg

gtccacctcccggcggtttcgctccggtccggttcgctcgtcgaatccctaacactggtc

caagtgccattgcgatttttctcacgacgtttggtgctttctcgtggggtatgtaccagg

ttggacagggtaacaagatccgaagggcactaaaagaagagaaatatgctgcccgccgag

cgatactacctgtgctacaggctgaagaggatgagagatttgttaaagagtggcataagt

atcttgagtacgaggcagatgtcatgaaggatgtgcctggttggaaggttggtgaaagtg

tctataattctggaagatgggtgcccccagcaagcggtgagttgcgtcctgatgtctggt

gatgttattttagccatcactatcaagaattggtctgcttcctgaatatgttacattgtg

atactttcatccagcatctatgaaataaagttgtatctggaacatctgaaattgtgttgt

cgatttctaaatcaagaatgctttttcaatattttatttcacgttggctatcacccgtgt

catgtaaatcaagatgaatgctcatcattggcatagaacattgaatttctaatgcctttt

cactccctagggtaaaaggttaatttcagcctttttttttactccctgttgatcgtaagt

gatttagaaaataattccaacaattggttaatctgttgcttgtattaaccaacttttgca

agtcacctcagtagagtgaacttgaaatgttagaagattattggttaatacgttgtttgt

aggttttaatgctaacttttgacacacttgtccgtgttctcatccatttaaatctaatga

tggtgaccaacactttcttgtaatgaaaattttcaactttagtttatgtgaaa

>fava_dn_rep_c1202

atttattattcccaaaataaaatacttttatatttattaattatacttgttcctcgttct

tcactcagaaatgtacaccttatagctgcaaagaaacgccatagaatataagtcagatcc

agctacattctcttctcatctttgattcaaatccatggcttccaaattgcttctcattgg

tgtgtttgttcttgacttgattgcttttggacttgctgttgctgctgaacaaagaagaag

cactgctaagattgttccagacagggatgaaaactataactattgtgtctatgactcgga

tatatcaaccggctatggtgttggagcatttctattcctccttgttagtcaaatcgtcat

aatggttgccagtcgatgcttctgttgcgggaagcctctaaagcccggaggctcaagggc

atgtgcagtcattcttttcataatctgctgggtgtttttcttgatagccgagatatgcct

attggcgggatcagttgaaaatgcttatcacacaaagtacaggacccttttcgtgaatga

tcctccttcttgtgagacagtaaggaagggagtttttgctgctggtgccgccttcatttt

cttcacttccatcatttcgaaattctattacatcaactactcgagtgcaagggaaagttt

tcaaccatatcttggaggtggtgagaccggcgtcgggatgggaacctacaaatgagcttg

ttagtctcattattacatgaattgtgactatatctgaaactcaattccataatattttct

tgattatgtcgcatttttttatatttacagttttagaaggtatactagaatgtataagtt

gtatgttagcggttcttgttattgttgttgttattacctgttgtctattttaattgatga

tgattggattcattacctcgaacatttttcgggtgttctttgtcatatattttcttgatt

atgttgcattaattgatgaagattgtttctaaaatcagtacttgtcaagagcagaacatt

caagatcttgatcaatgttgtagaaattctgtgtgttgcaatacataagagttatgtaca

catgtaacttctaaatgacatggctaaaagaaaagaatgacccaatgattttgtttttgg

>fava_dn_rep_c1203

gactgtttctgggactgtgtggcgggctcatgctgtctcttctgttcgtaactggtttat

taactcaaggtagggctgggagacctatgttttgacaccctccgagaggagattaaaaaa

caaatagaggggttgctccaaatatattttcataatgctcttgttcttccccctgggaag

ataattcaagacattgcaacccaccttcatcacgattccgagtctttggagcaactcttg

accattttgaagaatcttactgaattaggtctacaaagtcaagagtttcaacaaattctc

ctctttctctcgcagtgaagtcccgatggcaaactatatataacatttgaatttctctta

cattccacgtttccgaaatggatcttatcaaatattttacattttctatgatcatcttta

ttttaggtatttggggaatcctccttaatagacgaaatattcttattatgttaatgtcaa

ttgaattaatgttattagctgttaatttgaactttttggtattttccgtttctttggatg

atatgatgggtcaattatttgctttattggttttaacggtggcagctgcggaatcagcta

ttgggttagccattttcgttataactttccgagtccgagggactattgctgtagaattta

ttaatagcattcaaggttaaacatgactcctagagaattaccaaaatacgaagttatctt

ttctcctttcgttctcttctttatttattttttatttttacttggttggcagggcagggt

cggggcctttatcgctgggcgagcgcatccgatttattctaaagtcctttcctaaaccac

ttcccgtttagttgctgaaagatagatagaacctttctaaatgagattgagttccacgca

tatgcaagctagaaagatgctatttgctgctattctatttatttgtgcattaagttcgaa

taagatattaatctataatgaagaaatgatagtagcttgttgttttataggcttcatcat

attcagtcggaagagtttaggtaatactttcaaagtaactctcgacgggagaatccaggc

tattcaggaagaattgcagcaattcctcaatcctaacgaagtagttcttctggaatctaa

tgaacaacaacgattacttaggatcagtttgcgaatttgtggcaccgtagtagaatcatt

accaatggcacgctgtgcgcctaagtgcgaaaagacagtgcaagctttgttatgccgaaa

cctaaatgttaagttagcaacacttctaaatgccatttcttcccgtcgcatccgtcttca

ggacgatctaggcacaaagtttcacttattagttaggaggagatttatcccccagtgtat

ctcgaaagcagaaaaaatagaactcattcgagagagcttggtggtcttaagaatggttcg

ggtggggggttctcttaagaataaataagacgaatagaatctaattcatgtttatgctaa

cagaagagcggatccaatacaaagacaacttttttctcatgaagcagctttgcaagaaag

gagttagttccctccgggggttctgatagccaatgcttcctacgctccagcccttagccc

tgaactccttagcacaagcactaagtaagcccgtgaaggaaaggccttcaatatcgcacc

tactaggacttcaatttagttttcgaataggactacttctttcttttctgtacgctaggg

ccgcccgcacgctttaagggttgttgaacgtattgcactaagaaagactcctgccgttgc

ggtcatattttatttgaaagccgctcaagcaatttctttaaagaagagcaagcaagacaa

gataaggctcgaaagctgcgaataaagtcgagctgctctaatcgagctgtgaaatcggtg

tggtgaggtattggaagtgttcagcgaatatagataaagaaactcaaaaagagcaccgtg

ctatactggcagagcttcagcaaaactaggtaggtagctttcgctaatgagagatggatt

gggggattatattaggtctcctaattaatggaattagttttatcttgattagtttatttt

taaagaagcattctccctttactcgatctttaattgaattatcgatagaacgttgatcaa

tattaaggaagagatcgaggatcacagcaggatcctatctgatctttgtcaacacaggac

tcgaagccccctgctcaaacaaaagggtaatccttcggaattttcaattcctttcggatt

tcttatattaattatagttgaggggagaagactcattgaaaattctatctatataggggg

gggagccattgtgaagcctagagaggcggaagcggcaaatcgcttctaccgagttccaca

acacttagcttagtagcttaactctttctactggcgtggcgctgctggatgcttttgatc

aatagaacaggaagaggaggaaaaaaaagcttatgatgagcatctatttgagtcgatcat

ttccaagatgtaattcaagtttattcttatgtagtggaaaggccttacaatctgaagttt

tacgcttaggggaagaaatgttcttggtggatgcaggacctgggacccccagaaattgta

tgcaagatgagcttacaggagtgccaatcaaccgagccaccaggtttgataataaggtgg

gattcctggatctagtggccggtgaatcactgatcaaaaagaagattttggagagattat

tcatcgatctagtggccggcgaatcactgatcaaagagcgagcagccgccaggtttaatg

atttggtgggatccacagatgtagtggccggtgaaccgcttcttcttcttccacgaagat

tcagacaaaaccgagcttggatgaaactgaacaagatttggcgaacgaatacaaaggtaa

aaggctttattattgataaagtcaaaggaggttattcagtagccatcgcgggtttcatta

cttttcttccattccgttctcacaacaaaaggaaaaaaaggaggatatcgaatgatcgat

tcaccattgagagcattaaccccaaaaggacgaatattgtggtgttctaacagcggcaga

tcaaacaataacttgatgagcgaaatctgctgacgaaaaaagtgcagtttttttaaattc

cgaagcgaaacctagcgtctcctgataacactctatcaccttaatccattcttttgttga

gggtctggcacttattacaggccgctctgtcattgtctgatttttggttgtctgatcaca

ctcgaaattatgtatctacttatcgtatttttgcccctgctcggtagttccgtagctggt

tttttcggacgttttctaggatcagaaggaaccgctataatgaccactacgtgcgtttca

ttctcttcgatcttatctttgattgctttttatgaagtcgcactgggagctagtgcttgc

tatctaagaattgctccatggatctcatcggaaatgtttgatgcttcttggggcttcttg

ttcgatagcctgaccgtagtgatgttaattgtggttacattcataagtagcttggtccat

ctttattccatttcatatatgtctgaggatccgcatagccctcgatttatgtgttattta

tccatttttactttttttatgctaatgttggtgactggagataactttcttcaattattc

ctgggatgggagggagtaggtcttgcttcatatttgttaattcatttctggtttacacga

cttcaggcagataaagcagctataaaagctatgcttgtcaatcgagtaggtgattttgga

ttagctcttgggattttgggttgttttactctctttcaaacagtagacttttcaaccatt

tttgcttgtgctagtgtccccagaaattcttggattttttgcaatatgagattgaatgcc

ataactctgatttgtattttactttttattggtgctgttgggaaatctgcacagatagga

ttgcatacttggttacccgatgctatggagggtcccactcctgtatccgctttgattcat

gcagctactatggtcacagctggcgttttcatgatagcaaggtgctcccctttatttgaa

tactcacctacggctttgattgttattacttttgcaggagctatgacgtcattccttgcg

gcaaccactggaatattacagaacgatctaaagagggtcatagcttattcaacttgcagt

caattaggctatatgatctttgcttgcggcatctctaactattcggttagcgtctttcac

ttaatgaatcacgcctttttcaaagcattactattcctgagtgcaggttcggtgattcat

gccatgtcggatgagcaagatatgcggaagatgggggggcttgcctcctcgttccctttt

acctatgccatgatgctcatgggcagcttatctctaattggatttccttttctaactgga

ttttattccaaagatgtgatattagagctcgcttacactaagtataccataagtggtaat

tttgctttctggttgggaagtgtctctgtccttttcacttcttattactcttttcgttta

ctttttctaacatttctagtaccaactaattcattcgggcgagacatcttacgatgtcat

gatgcgcccattcctatggccattcctttaatacttctggctctcgggagtctctttgta

ggatacttggccaaagatatgatgattggtttaggtaccaatttttgggccaattccctc

ttcgtactaccaaaaaatgagattcttgccgaatccgagtttgctgctccaaccattatc

aaacttatacctattctgtttagtactttaggtgcttttgttgcgtataatgtaaatctc

gtagcggatcaattccaacgagcctttcaaacaagtactttttgtaatcgactctatagc

ttcttcaataaacgctggttcttcgatcaagttttgaatgactttctagtcagatcgttc

ctgcgtttcggatatgaagtctcattcgaagctttagacaaaggtgctattgagatattg

ggcccttatggtatatcgtacacattccgacgattggccgagcggataagtcaacttcaa

agtggatttgtttatcattatgcctttgcaatgttacttggtttaactctatttgtgacc

tttttttgtatgtgggactctctatcttcttgggtagataatcgatcgtctttcattttg

atagttagtagtatttattataataagtcaataaataagaataaataataatagaactgg

>fava_dn_rep_c1204

ttttaccagtcgcgggaagattggttctttttgccgtgaaaaatcctcatcctctttttc

tatcactctaacaaaaaccattgaagcctctctctctctctctctctctcggggttgaag

atgagaatttgtggagcagcaaccagcagcgcttatagcaccgcgtctaattccctgcaa

gtgtttgtttcgtcttccttctctactccagccaaaaccaagttcctaccttcactttct

agattttctgtcaagcgttctatcttctcttctcaaacccgaccccactctactgtgatc

aagccctccatgaaccttctgaacaaacttggatttggcagcggacggtcatcagagaac

tacgattcagccattcctcaggggccggattgcgatataccagcaccaggtcagcagttt

gctcaatttggcgctggctgcttttggggcgttgaattggtcttccagagggtgcctgga

gtgagtaagacagaggttggttacacccaggggcttgtgcataatccttcgtatgaagat

gtatgtgcagggaccacaaaccactcggaggttataagggttcaatatgatcccaaacaa

tgttcctttgaaaatctgctagatacattctggactaaacatgatcctactacgccaaat

cgtcaggggaacgatgtaggaacgcagtacagatctgggatatactactacactcctgaa

caagagaaagcggctagagaatctttggaacaacaagagaagcaattgggcagaaagatt

gttaccgagatccttcctgctaaaacgttctacagagccgaggagtaccatcaacagtac

cttgagaaaggaggtagatttggtttcaagcaatctgctgctaaaggatgcaacgaccca

attcggtgctatggttaataatacttaataaaaaaatgtgagttcatcctaaaggatcaa

catgatatgacactgtattggagcttgaaaatgggcatagccttgaatcaaaattgtata

attttgttgtaaagtattgtgttggcaacatacaaaccaataaataaagaacaaaatgtg

ttttcagaatttgatcagattgtgaccaaaatatcaaagagacaccatatagtgtttcaa

atttgggtgttgagatattgcagtatctatacagtatctatacagtatctattacagtat

ctatacaagttacaaccaatattagtagtactataagatgattattaaattgtctgtaag

gaaatgaacatggtgtgtc

>fava_dn_rep_c1205

ggtatcaacgcaaagtggccattacggccgcggggaattcatttttcactttgtttcgcg

tgcagtagggtcagccaatgggcggtggaggatccatgaggacggcagccaaactcgccg

gcatcggcgtcgctaggtcaggcttcagaggaacaccggccgcgtgcccggtcgagcagt

cggttcgcaacgtatcgcgtacgtcatcgcccaccagactctcaacgcagggagcgaatg

tcggtgaggtgaagccgctgcatacggcggtgtcaggggatttgtctgactgggaattcg

ccgatgaaggcgatctgttcatgaccggcggagagcctacgccgagagtggtgttcggcg

atgtccctacctttaaggaagcgcaggaagctaccgcagaattgaaggaagctattgatc

aaatttacctttcatctggcaattctcaatgtgggggatcatctcctggtagtgaactct

ctgttgtgtctcctcttgctaaggaaacagggacaaaatcttgtcttgttgaggctattt

caagtccttcagtgcctaagcatgccattcatgcttttcaattgctcagcacaagtcctg

aggcccagactgttgtgcaatctattgcttgtgaccctaatatatggagtgctgtcatgc

aaaaccctgcagtcactagtttcttccaatcacagctagcagccgtagaatcatcaaatg

gggctgcttttgctggttctgaaactctagagattcctgaaaaagaggaggaaaaagctt

ttgattttatgagtattctgcagagtttgaagcttacagttactgaaatggtaagcagaa

tgtccaacttctttcagaacatattcccaacaattgagaaagacaaatcttcagctgatt

ctggtggagccaatttcatggattataagaatttgatgggaggaagtttcatggggttgg

cagttatggtaataatggttgtactaatgaagagagcctaaacattcactagaaagaatt

actactatctatcgagttttcttgtcgttttaaatgcttgtatatgtgaaatacatttga

agcaacaacttatgaaatttggagctagtactctgtatttctcttaaaaaaaaaagctag

atatagttttttttttaattgtttgagaatttttaaataggtttttaaactaaaaatgtt

tgtaaatacgtttctgaattaattttttttaaatgtttagatattttattac

>fava_dn_rep_c1206

aaacacttgcgcacacgttcacaagataannnnnnataagctcttctaagtaccacttta

gtgttgttacaaaaaagccattttaatattgttcaaaaagaccacaggtttcattatgaa

aatgaaatatcgcataaccaaaatacaacagaaacatcgaatataatcaacacaagatga

agattcttaagaaacaagattcctcaataccataaacgagaaatgcttacaaatatccaa

cacaaacatagctttcataacttgtaaatcaacttcatagctaaaacattgaaagacaaa

ttcatacttaataaaataaaaattcataattatgctatgattttaaatctccgaatcctt

gtgagccacagagtgatgacttcataatcctaagcggcggcttaggctcctacaggaacg

gaggcaatatcacccatcgacgaggatacctcggtctcccttcctttccgtcaaacagct

tcttcaagcgcctgtgctttgcgagagcccagttggtcatgatgaaagtagcaacggcga

caaatacataaccggcaatggtttgcgtagcaatgttgaagcccaaccactggtagatct

cggttgtataattagcacaagtaacaatattgaagagaaatccccgtgggatttggtatc

caccttctccagcaggtccgcgaagattccttagtataatatggcaatagaaattcgaaa

cttgacaaagtactccgaatccaaacccgatcttcatttgaaggtcacttactggtgtat

aaagtggatggttcacatagtaagcgatataacagccgaacgtccagtaataagcacagt

tgcggaaaacattggaaaggggcgatgttgcgtggctgaagcgatgcacgaaaaacgttt

ccagaattcgtttggcgtaatggaaacaccaatagtacatggcatatgtttgcacgggat

ggtggacgacaggttcgtctttgtagccgaaatactggtagacagcagggtagtaataga

agattggatagagaagcaacggccccaaatactcgaaaaagaaaagcgtacggtaagaaa

cttgtggacctaagtctttgaattgtacggttaaggtttctgagtttccattagtatagt

cgtttagactctttttatagtttagaacaatcggcttttcctttgatcctggttggacag

gaagtgtcaggcgctgcctcgatggatgcttcttgattcgcttgtgaatagcttcctgca

gatctgcaaccgtagcagaatcaccgagctcaatgccacctttgacaatttctcttccac

tcctagaaaccaaagtgaccttcatcttcaaaaccaaaacacacccacttcaccaaacag

tgtcagcaacacacttcactgaaagatcatatgcaagattcaatagaagtagtaaaacct

gaataggggaagattgtgcgcaatgtttgtgtgttttggaagtccctgagagtcacagag

caaattggatagaaataagatagaagaaaaaggagaatttaagatgaaggatggattaat

gtgaggtatggttagtagaagatgtatgcattaatgtcatatggttaggagt

>fava_dn_rep_c1207

acatcacaccaaaacaacacattcttacctttttacacctctctttctatcttctccatt

ttgccactcattccacaaagaaacaaaatcatcatcatcatcaagatatcaatacaccaa

ccctttgagcttctaaactctcatatgatagttcaataaaagttctaaactttgagagcc

tcacaaaatttctgtgaaatttaaattatgctatggaggccatcaattctactgttttca

cccccctttccgtgctatctgatagaacaaaaaaacccaccaaaatttcatgcaaagttt

caaacttttccacttcaaacaccagaaaaaacaacctaaaagagtttctatcaaagggtt

tacaaaagggtctaatccttgcagcaacttcagttgtgaatgtcaatgtaaaccatggtg

aatttgccagagctttaacatatgaagaagcattaggactatctccaagtgcttcaaact

atggtggtgacattgatgtcaatggttttgtagatagtgtggtaggctttgtaactgaaa

atcctgcaattcttgctggtggagttgctattttggcagttccattggttttgtctcaga

ttttgaagaaacctaagccttggggagttgaatcagctaaaaatgcttatgctaagttag

gggctgatgggaatgctcagttgcttgatataagaggtttggctgagattaggcaaattg

gtggcccaaatgttgggggtttgaagaagaaatcggtgtcgatagcttataaaggtgatg

ataagccagggtttttgaagaagctttctttgaaatttaaggaccctgagaatactacat

tgttcattcttgacaaatttgacgggaactctgaactggttgcagaattagtcaccctta

atggatttaaatctgcttatgcaattaaggatggcgcagaaggaccacggggatgggtga

atagcggtcttccttggattgaaccaaagaaagcattgagctttaattttggaagcttga

cagatactatcaatgaagcaattggagaatctgatggcttggttgtcactcttgggattg

ctgcagctacgggattgggtgcattagctttttctgaggtagaaacaattctccaacttg

taggatctgctgcaattgttcagtttgcaagcaagaagcttctttttgccgaggaccgga

agaaaacactaaaacaagttgacgagttcttgaacaccaaagttgctcctcaggagcttg

ttgatgaaataaaggacatcggtaaggctcttctaccaacctccaccaacagcgataagg

ctcttcctgcaccaacagaaactgttccagagctccctacagttactgccaccgcgcaga

aatcagaagttactcccgatattgtcgagcccgtacagaaagcagaagctactcctgata

ttgttgagaccgtacagaaacccgaagctactcctcctgacgttatctccgagaccaaag

tagaagcagcagcagaacctgctccagaaataaactcagtcccaaaaactgaaactgtgg

cagaatcaattcctgcgcaacctaaaccgctctcaccatacccatattatccagatttca

aacctccaacatctccatctcctacacagccctagagtgaagagaattctatagcaggca

aaatagttatccaaaatattcagcatgtattcttgttatgatcataatcataaaatttgt

gctactcagtaaccttgtttttgtaattgatctctgttgtggaggtagatgtactattag

ttgttatgttgtgatttagtatcatattacatcattcagttttgaataagcaaataaggt

ctcaaatatgcctttgtattattgattcatgaagtttcgattaagtaaagcattgagctt

ttggttgtgtaacactttgcaatttattgtcaagtatagaatttgtgttagc

>fava_dn_rep_c1208

aatcttcaatcctttgattaagaaatgcaaaagattcttcttcactgaaactctcttaca

aatacaaaaaaactggtaatgcttcaaaacgatgtcgtttcaccccttcctcaacaacaa

aatcccaaaccctaatttcccttctccgccattgaagacctggctcaactccaaaacctc

tgattccttcaacaacatggtccttgccatgcagcttaactctctcagtgaccccaattc

caaaaccctaatccccaatttcgacttcacacttcttctctccgacgaaattctcctcag

aatcatctccaaacttccgaattcgcaattgaagtcgaactcactcgtttcgaaacggtg

gttgaatctccaaggaagattaattcggtcgttgaaaatcttcgatttgaacttcgtttt

atcaggtaggttagtttatagattccctaatcttatacatgttgacttggttccttcgac

tttcgttgtttatcctcaaaaaggttccgttttggtgagtcacagtgttgtttcaatgcg

ggttgacactgggtggtgtttcgggttttccgaggaagggaaaaaattgcttccggttga

agcaattgataaggggctcaaggaagttgcgaaagggtggccgcgggacaagaagtggaa

gggtttcagttggatcgagtgtgtttgtgagggtttagggagcaatcttcttaacctagc

agagcagacttcaccttcctcaactctcttccattcacgatggccgacgatgactacgaa

gatatcgacatgggatacgaagaggagccagcagagcctgaaatcgaggaaggtgcagag

gaggaggatccggataacaaaaacgatgacattgatggtttaggagaaccgattgaaacc

gaggaaaaggaagatgatcaacccgttgagcgtcctcgaaggacatccaagtatatgact

aagtacgagcgtgctaggattctcggtacccgcgctcttcaaatcagtatgaatgcacct

gtcatggtcgagttggagggggagactgacccactcgagattgctatgaaagagctaaga

gagcggaaaattccttttaccatccgccgctacttgcctgatggaagctatgaagattgg

ggagttgatgaactgattgtggaagactcctggaaaaggcaagttggtggggattgatgg

cttgaactgacagtggtgtttatcctagatttgtgtgaagtgaacactgatctatgatcc

taccataaaactggcttatcctatttgttttgggtgtgtatctatgtatgtcttgttcct

tttactttaacccggtattatttttgtttttgcttgtctgtatattataaatctctaggc

ctttaacagtatatgctggtgtttgaactttgaagtgatatacttagatgggtttttgaa

ctttgaagtgatacttagatgggtgcttgaacatgttagtgatttgctgttacattttgg

aaataccagttagatgctgctgacattttagcgaaaaaaaaaaa

>fava_dn_rep_c1209

aaaacaaaatagtagattaatgacttcataatattgggaatcaatcacgggggaagactt

aaataatttatttttttggggagaagaagactttaatttagagaatattaaaaaatgcat

ttaatttagaaaagtaagtacttcatttaatcttttcttgattcaagtgggaggtaggca

tttgtgtgcattgaatgttgataacgtgactaatcactctcagaatctgttcgcactgtc

gacgccacgctctcttccctttccattccaattcacagatccattttcccatcctcatcg

gtttgttgaaataaatggccttagtcaccactgcagaagtgtgtgatgcaaacccacagt

taattttaagcggggagcttcgtgcccttcagccgattttccagatttacggtcgaagac

aggttttttccggaccaatagttaccttaaaggtgtttgaggacaatgtcttagttcgcg

agtttcttgaagagaaaggaaatggaagagtgcttgttgtggacggtggtgcgagtttgc

gctgtgcgatattgggtggcaaccctgtagtgcaagcacagaacaatggatgggcaggta

ttattgtgaatggatgtataagagatgtagatgagatcaacggttgtgatatcggtgtga

gagcaatcggttctcatcccatgaaagccaataagaaaggtatgggagaaaaacatgttc

ccataactattgccgggacaaggatctctgatggggaatggctttatgcagacaccgatg

gaattcttatctctcgaactgagctttctgtttgattctaattcatgtgtaagaactata

tttacaaatttggaagacatgtcattgtgtagtgctttctgtatttgtgtttttgaccct

tgtttcctagcttccacaaatttgcaatgctattagtctccatgataatcaaactagatt

ggattgtttgccccatatacaccaactttatacatgttttgatgagctgggggattcaaa

aatcacggcgagccatcatgattttgtgaagttacaacgtgttgtctgtcaaaattatag

tagtttatcaaagtgttgtctatgtctattttgaattgctgttatgagtacttgttaatg

gttgtatctgttacacgctaaatttggagagatccaaatgcaaaatatacacatgccttt

tttttttgggacaaaatacatgcccattttttgataaacaatagtattgtcattttttgt

ttgccatagaatgatgttgctgtctagaaaccaaatgtatgaggtaacgtgattctgcct

ctgattctaactactttggacatggaagaggaaaggtatcaaatgatgatgtgttgtatt

gtattctatgcatattaaatgtctgtctcattgtaaacatttctacgatgagtgactatg

ctttggttaatatgtcaaaagnnnnnnnnnnnnnnnnnnnnnnnnnnnnnnnnnnnnnnn

atttgacgacaattggtgtggtttgaggtttaagtttagaaatgcttcttgaaacctctt

gttcatatgcatgaaattgaaaagaaaaaaagtacaatgggacagcatgattt

>fava_dn_rep_c1210

aacaaacaaacaaaataacaaaatgaaaaacagaataacaaaaaaaacaaaataaaacaa

tagcaaatttaatattttcacttacctttggagtaattaaggaaagggaaagcagaggat

tcccaactccaaaatcttaggcgattcctttctctccttctctctcagcctcaatcatca

tctatggctatggctggtggggtttcaattttgccatatattcatcttcttctccttctt

gtaattgtttcatggtattgtagtgttgtaaatggttcaaattcaacaactatatacgat

gaattaagggcaaagggtcttcctgtagggcttctaccaaaaggaataactcggtatcag

atcaatggtaccagtggagaatttcaggtttggatggaagaaccgtgtaatgcaaagttt

gaaaacgaggttcactatgatccgaacattaagggtaccctaaattatggtaagatcggg

gaattatctggtatgactgctcaggaactcttcctatggttccctgttaagggcattcgt

gttgatctacctagctctggtctcattcacttcgatgttggtgttgctgataaacaattc

tctttgtccctctttgaagatcctcctgattgtaaccctcaggttaatgctgataatgat

gatgtgttgagggcagtttggtagaagataaggatgtcggcgcgtttcaaatagatcaat

cagtttagctgtgcttgtggttggtcttgtgatggtgtttgtgtacagtcacggtctttt

tctttttctgcttggattggaatcaatctcctctgtcaagggacaaaggatgacaatttt

ggtggtcaacaacaatggcctatatggttttggcaaaatttgtgctagtcagggatgttg

gataaggttggttttgctaataatgttctatatagtagtactcctaaggttttgtaattt

gtattctggtattgtcatttctacacacaagttttgtgtagattccactctattttaata

aaatttagggtaatgtgttggtgtttcttgtactgtaactaattttgattccctatattg

tgagtagtaagtaagtaccctacactttgttcttttatgtgagatagtccgctttgccta

tatttttcaatttttgcttgttggttcaattcagtggggcagttttctctaataatgtgt

taggtactgtttaaaatttgtttgttgttccctattgtattctatttatctgcattatat

tctgtcatggtcctacacagaacaagatttttgtgtagattgaacatggaaatttttgta

gttggcaaatacagtttgagaaattaaggatgaaaggacaaaggatgttttcact

>fava_dn_rep_c1211

gccgcgagtatatacttttgagtgagagaaatgagtaagtggtgcattttggtggttttg

gctcttgctctagttgcaacaagtgcaagaaatgtgcctgctggagaggctggtttgaag

gatgagaagacctttggtggatttggtggattttcaggaattggtaacaatggttttcca

tctggaggtgtaggtggtgacgctggtggtggtggacttggcggactaggcggtggtgga

ctcggaaatattggaggtggtggtggtggactcggtggaataggaagtgatagcggtgga

ctcggtggaattggaggtggtggccttggtggcctcgctggttctcctggactcggagga

tttaacgggctaggtggtggtggacttggaggttctggtggtggtgtctttcctcatcct

tgactgacaagttaattactatatatggaatttactctatttaataaaataaaacttaat

tatagtagaacttcgttatagcaggtttattgttattacagttgtttaatgttagtgttt

tatcaaagattatattattcacctaaagataaagtgttagcaagtactattttattttgt

tgtcgtgtattcttctttctgatgtaatgtaagggagtacatttcatgttgtacgagttt

attagttaaactgaaaatttaagtatatgaaatattatagattttaacatgtgcatgttt

ttctccaaaaaaaagtcattcataccnnnnnacctgtttt

>fava_dn_rep_c1212

acatatatgtacaaaaagcattgtccactccatccgggcttactaacaccactaaacttt

caagcaaccacatcaattaattaaacctccaaccaccatggcacacgacatctccaacga

ccaaccaccaccggaaacctcaacaacaacaacaccaccgctcctccgccgcaacttcct

ccgtctcccatcaaccctcaaactcaaaacctccctctggtcagaactctccggcgccgt

cggcgatttaggaacctacatcccaatcgtactagccttatcactagtcaacaatctcga

tctaacaacaactctcatcttcacttctctctacaacatcatcactggacttttcttcgg

ccttcccatgccagttcagcctatgaaatccatcgccgccgtcgccatctccgaatcgcc

acctctcacaattcctcaaatctccgccgccggtttatctgtcgccgccgtcctcctctt

cctcggaacaaccggtctcatgtcttttctctaccggtacctccctctcccagtagttcg

cggcgttcagctctctcagggacttcaattcgctttctccgccattaaatatattcgcta

ccagcaagatttatcatctggttcatcaaaaaccggtccagtccgtccttggtttagttt

ggatggactggctctagctctgattgctgttctgtttcttgttctcaccactggcgctgg

tgaggacaatcattcagaacaacaacaacacgagctagaagaaaatgattatgatagtga

ggagagagttgataatcttcgacgaaataaaattcgtcgaagattgaagattctatcgat

gattccatcggctttgattgtgtttgtatttggattattgttgtgttttattcgagatcc

ttcgatttttcatgatttgcgatttggtccttctaggattaagttgattagaatcacatg

ggatgattttaaagttggatttgtcagagctgcaataccgcaaattccgttatcgatttt

gaattcggttattgcggtttgtaagctttccggagacttgtttccggaaagagaagcttc

tgctatgaaggtgtcagtgagtgttggagtgatgaactttgttggatgttggtttggtgc

catgccatgttgttaaagcgaaagcggcgttagatgctgttccacagtgtagaaacaaag

tttcttgtgctgatattcttgctttggtggctagagatgttgttaatctggctggtggac

cgtcttatactgttgaattgggaagatttgatgggttggtttcaagatcttcagatgtaa

atgggagattgcctcagccagggttcaatttgaaccagctgaatgctctctttgctagca

atggactcacccaaacagacatgattgccctatcaggtgcacacacccttggattctctc

actgcgacagattctccaataggatccaaaatccaatcgacccaacattgaacaaacaat

acgcaatccagttacaacaacagtgtcctagaaacgttgatccaagaatagccatcaaca

tggacccaacaactccaagaacattcgacaatatttattaccagaatcttcaacatggaa

aaggtttattcacttcagaccaaatcctcttcactgacacaagatctagggcaacggtta

actcttttgcttctagtggtaacgttttcaactccaattttattagtgctatgactaagt

tgggtcgtgttggtgttaaaactgcgaggaatggcaagattcgtactgattgttctgtgc

tttgattgaacaacaagtgtgggggtgattagattatttgttaattgttgttgataaatt

tgcttatggtaaattaagagtggtttggagatgtttacaaaaaaggcattcatggggagg

gaataagttgtgttttttttcatgtaatgtttctaataattcatgtacacaagatacatg

aactttgagttgattattaaaaatgaaaataagtgtggccaaaatgtaaattaatttatt

ttaatttttgtattgaatacttttttactaaaaaaaaa

>fava_dn_rep_c1213

ttaaatatgagaaacgtataactgaagtttgtcaccttccaatcctaacttccactgtcc

ttctccttcaccacctcttccaatctctctctagctacccaatctccatcacctttaatc

cttctctcactatcaccattactcaattccacacctaaatgtgatgatatgatccaacac

tcaattccaccaccacaaccggagcttcttcaagttccggtctaaaacaatgcctccctt

tccccccaaatcattcttcaacgcacgtcaaaccatttcatctcgcagatcctccttcct

catcctcgcctctgtcctcttcttcggactcattctttttctcaaaccgtttcacaacga

tcgctgcctaaatagaaaccctagatctgttcgcgtcctatgggatcacggcaccacgat

caccggaaatgtcgcttctgaacataggcacaaggttatggcctttgtaggaattcagac

cgggtttggatctgttggtagacgtcaatctttgaggaagacttggtttccttccgatcc

tgatggacttcaacgcttggaagaagccactggtttggcttttaggtttgttattggtag

aacaaatgataggtggaagatgtctgcacttaagagagaaatagctgaatacgacgattt

cattcaattggatattgaagaggagtacagtaagctcccatacaaaacgttggctttctt

taaagctgcttatgctcttttcgaagccgatttttatgtcaaagctgatgatgacattta

tttaaggccagatcggctttcattactacttgcaaaagaaagatctcattctcagacata

cataggatgcatgaaaaaggggccggttttcactgatccaaaactcaaatggtatgagcc

gctatctaatttactcgggaaggagtattttcttcatgcttacggtcctatttatgctct

ttctgctgatgttgtatcaagcttggttgctcttaggaataacagttttcggatgttcag

caatgaggatgttacaattggagcttggatgcttgcaatgaatgtcaaatatgagaatat

tcacgaactttgtgctccagagtgtacattgacatcaatcgctgtgtgggatattccaaa

gtgttcaggcctgtgtaatccagaacagagaatgttggaacttcatcaaatggacagctg

cattcagagtccaactatgaaatctgatgattaggatatatctttctaattatatatctt

catgtcaacagaggacactttgatgtgagtttttttctgctgtaatttttctcttacctt

ctttagaaaggctttatgtaaagcatgagaaaatacgtataattagggatgaagatataa

ttttactatacttattttccaattttttatgacattcttgtttagatttcatatatgtac

aagaaactcatgtcattctttagaaagaaaaatgaaccattatgcctttgtttgaacaag

tgtgagttatgatggtgttaaggggccttttggattcatggtttcaatgtgggaattata

aaaatcaacttatctcttg

>fava_dn_rep_c1214

cttttttttttcagtctacacatacagaaattaataaataatttattaacttccatattc

tttagaattagcattctttatttcaacaaaatcaatttgtaaattttgagttttattatt

attatttttttgataaataaatgaatgttttcttattttctaatattttagaagattcgt

aagaacttagaagaaaagaataatgaaaaggtataaaaagttatgtaatttagacatagt

gtaatttagccatacttatagggcggatgtagccaagtggatcaaggcagtggattgtga

atccaccatgcgcgggttcaattcccgtcgttcgcccatgaatctcttacgggtggcaac

atgcaaaatacccgagaccggattcgggctttggacgacctcctgagggctctgctcctc

gagcggcaagaatcctaccaggaattccattcctcttccgcgtcacgccggagttcctgg

tcagcttctctcctccagcagtgacaacgacagtgacgccattaggacgccatcgttcag

cgcgttccatatttgccagccgtcgggaaggtttcacggcgtcatgaacatcggtgccat

ggtgttagagggttcaacaccgctcgtacatactaaagtcttagtagcctgatgcttgtg

cgtgacatcgaaaagtgctgcacgccccatataaatatttaatttataaaataaaaagaa

aataggcaaatcgaccgtttcacttagcgtacttaaatctctcgcgaattataaactggc

gctatcttgctgctgctggatatttttgatcaatagaacaggaagaggaggatttctagg

aaaagctatgactcagaaagaggataagatgagactttaccactgtggagcggctttttg

caaaaagatgagggagaaactcagtaagagatgtcggagaagcgaaatatacgagatcac

aaacgcagattgctcgcggcaaaatatgaattgagacgaaagctttataaagccttttgt

aaagattccgatcttcctagtgatatgcgggacaaacttcgttataagttgtccaagttg

ccaagaaatagttcctttgcacgagtaagaaaccgatgtattttcacgggtcgccctcgt

tccgtatatgagttatttcgaatttctcgtatcgtttttcgtggattagcatctcgaggt

tctttgatgggcataaagaaatcgtcttggtagcaaccgccaaacccataaaacaagggt

tagctccgcagctggtccacaagcaaggcttggtaagtaggtccattaccggccggctcc

ggaccgaaaagacctaacggagtaatcccttatctcggatcggaggggctaacggggcag

gaatcaaagtggggggcctatctaccgcctgtgtctatctcctgtcaagtatgctcccca

gacatagactacgtacagggtagtactcttggatataatatcaccgcgtgaacgtgaaca

taacattaagttacgaatgtcactcccgagggatcgaccactattctaaagaaagtaagg

cggagaactgttgttcattggagcgccggagtgcgggggttgttcccatcattgaagtca

gagtgtgggactgagccttccgaatggtaaaaaaaaaaaaagtgcttagtttcgttggaa

aaaccaacgcaaatatcatattgactttatctcgcccaacttctaaggatagatagaaaa

ggttggagagagttactttatgaaattgtctcctttctaaagctgcgcaaggcggcccca

cccctattttcccccctttaatgaaagaccctcgccccaaaaaaatgggattaattaaaa

aaaaagctttttgattgattcagggcgcagcgaaaccaatttatttcaaggcaaaagggg

gactgaggaaaagttttaagatgatatggaccctggacgagaacctccaattgcttaggg

gtcgcactctgtcccgcttggtggacgaaatattatctccttttagaatgatttctccca

cacacctttgccctctttcaccgaagaggaaagaaaaatcttccaagcggacagagacct

aaatttccattagattcattcctaagcttgctttgttgcagtaagatgatcagtccgaga

gtgctggggagaagataaagcggtaaaaacccctcagattcggtcaccgagcagtcgtcc

gactcttaagtaacccaggatgaccccttcgacgctcgcttctttcgctgtataccccct

ccatccttcggaggtggaagaaagaaaaggtactataaaaaatgttgtatttattctttt

taagtagggccccagaacgaaaaaaaggtgggggaacctgagttgtcacgataggaaaga

gcaaaaaatgactataaggaaccaacgattatctcttctaaaacaacctatattctccac

acttaatcaacatttgatagattatccaaccccgagcaatcttagttattggtggggctt

cggttcgttagctggtatttgtttagtaattcagatagtgactggcgtttttttagctat

gcattacacacctcatgtggatctagctttcaacagcgtagaacacgttatgagagatgt

tgaagggggctggttgctccgttatatgcatgctaatggggcaagtatgtttttcattgt

ggtttaccttcatatttttcgtggtctatattatgcgagttatagcagtcctagggaatt

tgtttggtgtctcggagttgtaatcttcctattaatgattgtgacagcttttataggata

cgtactaccttggggtcagatgagcttttggggagctacagtaattacaagcttagctag

cgccatacctgtagtaggagataccatagttacttggctttggggtggtttctccgtgga

caatgccaccttaaatcgtttttttagtcttcattatttactcccctttattttagtagg

cgccagtcttcttcatctggccgcattgcatcaatatggatcaaataatccattgggtgt

acattcagagatggatcaaatttctttttacccttatttttatgtaaaggatctagtagg

ttgggtagcttttgctatctttttttccatttggattttttatgctcctaatgttttggg

gcatcccgacaattatatacctgctaatccgatgtccaccccgcctcatattgtgccgga

atggtatttcctaccgatctatgccattcttcgtagtatacctgacaaatcgggaggtgt

agccgcaatagcacttgtttttatatgtctgttggctttacctttttttaaaagtatgta

cgtgcgtagttcaagttttcgccctatttaccaaggaatattttggttgcttttggcgga

ttgcttactactaggttggatcggatgtcaacctgtggaggcaccatttgttactattgg

acaaatttcttcttttgtgttcttcttgttctttgccataacgcccattctgggacgagt

tggaagaggaattcctaattcttacacggatgagactgatcagtgaaaaattatgacacc

aatcatttacgtattacaccaagaattaattgacaatgccaccttaaatcgtttttttag

tcttcattatttactcccctttattttagtaggcgccagtcttcttcatctggccgcatt

gcatcaatatggatcaaataatccattgggtgtacattcagagatggatcaaatttcttt

ttacccttatttttatgtaaaggatctagtaggttgggtagcttttgctatctttttttc

catttggattttttatgctcctaatgttttggggcatcccgacaattatatacctgctaa

tccgatgtccaccccgcctcatattgtgccggaatggtatttcctactgattgtttccgg

tttgtggcggagacggagggaatcggggaggtgaggactcatacggtggagatccatcct

ggtgtgacgaagatcgttgtgagagatggggagtctttggagttgtcgcttgatcagttg

gagattgatatgtggaggtttaggttgccggattccacgatgccggagcttgcgagtgcg

gtgtttgttgatggggagttgattgttacggtgccgaaggggcatggacatgatctgcag

aatgttgatggtggtgatagtggtagaggtatgggtggtagacttgtgcttgtacagtga

ttatgattgtggtgatggtgatgatgatgatgatgatgatgaagatgatgtttatgatga

tgatgatgatgatgatgatggtgaaaattcttttgtttgacacctttaaatgtgactttt

tagtatatgtgggttagatttttcaggtttaatgttgttgggtgtagctaatttcttttg

tatgtgaatttgattgttgcttcaacataaacgttcttccttcaataatggtctatgttt

atggcaatgtcaattttcgctattagtttttttccttgttttcaaattgtgcttttgggt

catgattgtgaattactattggtgtaaatatctgctttataggaattagatgcctttttg

cgtgatgcttttgaagtttgatgatgctttatttgagtattatgacaatactttatctat

ttaaatcaaagtggttattatgagcaaaaaaaa

>fava_dn_rep_c1215

cgttaactaaaattatgtaagactagttaacaacttaacattataccaagaggcaacact

caaaaaatgagaattgctttgaaattcagaagtaaaaacgtcccacaccctttgctattg

aatttagggtggaggaattatgagttcatagtcataattcatagctttcacaactaacta

ctaaaatcaatctgcagcaatacgtaaagttaaagagaagaggtacaacggttgtatatt

aaaatatccttaaaacttcatcaaaacatgaacaggaaccgggtattgttcaagtacatg

tagaaaagtatattcatattttctacaggactaagaaagaaaacatgatacagtcgaact

caaagctccattatgccatgcctccactggttgctgtcctgattatctgaaagagagatg

atccaatgacaacaaaaacgaagaaaccgagcaccaaggggccaacagggtagtcttttc

ctttcttaacagttgtttctggcacaaatcctctttttgtaatgtttttctcaaacttct

ccaccttcctaaccgacaggcgccttgatgtagtcatgtttaattggtaagaaattggaa

cggcggtggaaaacgaagatatcgccggagaatcgagagacggtgacggtgacggtaccg

gcagtggctctctctccgagtttttatttagagctttgtggatcgtgtgaaggatgatga

atgtttttagcggttatttattttccatttttattttcttattttcttatttatttactt

acccaatatacctaattccgttgaaacttgataatggatacccgagtataacgtcacggg

ttcttcgcgattggctgaagatgttaactgtgtacacgtggcaattggatacagggtgag

atttagtttaatacgtgaatgaatgagtct

>fava_dn_rep_c1216

caccatgtcaaaacaccattgggccattatacgtgtatttcctaacccaattttaatttt

cattttcccattttgttttttcatctctcgtcaacaataaatcaaccaaatcacaaactc

ttgacacaaaatcttcatccaaacgcgcaagagagaaaatggacgccattgattccgtct

tcgatccactcagagaattcgctaaggatagcgttaggctcgtcaagcgttgccacaaac

ccgatcgcaaagaattctctaaggtggctgttcgtaccgcgattggtttcgttgtgatgg

gattcgttggctttttcgttaagcttatctttatcccaattaacaacatcatcgtcggat

ctggttaggatgagggaaactgcaatcacctcaggatcaacttcaatagaacatcttagt

tatgttattttcagttttgcttttatccatagttcttatcttcggaagcattacagtttt

atcgaacatgtaaaattttattgtcccatgttagacttttgacctattgttcaaggagga

aaaaatttagatgaggaattgtacattggtttcattatttgctgtttggtttattatatt

gaaatatcaatttagtttggaaaaaaaaaaattttaattacattattttatttg

>fava_dn_rep_c1217

aatcaacacacgttacaatccttcgcatttgttcgtgtatatcaccctttaactctcaac

tttctttttaaaaagtctcatccaagtcgtctcctttatttcttccattcaatttaattt

cctctatcagacagacacacatcgtgtgtcacgacacaacacacactactactttcttct

ctctctctaattgacattatatattatacacaccccttaatccattcataccttactatg

gattttaagacacctctctttctcttgtttgttcttggctcaacttcccaagctctcaaa

ttcgatatcggcggaaccgatggatggtctctaaacccttccgagaattacaacgaatgg

tcagggagaaacaggttccaagtcaacgacgttctagttttcaagtacaagaagggttca

gactcggtgttggaagtgacaaaagaagcttatgataaatgcaacaaaacaaacccaatc

aaaaagttggaagatggtaacacagagtttactttggatcgatctggaccattctatttc

attagtggaactgatcaaaattgtgaaaagggtcagaaattaaaccttgttgtcatatcc

ccacgaggacacacaccatcttctgctgctccttcaccgtctaccaccactccatctccc

tctgtttctcctttaccatctgcaaccactccatctccttctgtttcaacttctcccact

gcaaattcaccatccgtcagtacgcctccagccggtgaacctacggctccacctcctacg

gcgggtggaccacctgctccatcaccgatgtctccttccatgggcgcttcaccgccatct

caatctccaactgtaccatcagacggtgctgcggcgccgggtgcagagtctccggcacct

ggggcaacagcggttccaccggactcatctgattcaatagcgccttcgagttctctggtg

tattccgttgcagttgttgtcggtgctgtgtttttgtgttactgaaacatgaattagcgg

tatgattatgttgtaataaatttttcggagatttatcattgctatgttcgattggttggg

tatgattaataaggcatcatttcgttattaccatttatttgggtgtttttgttgaagaac

gatttattgtagaatgattctatcatatttttcacttttgtgtatcattgttatgttatt

atgtgtaacaacaataaatttcatttatattcatactttaacatatagggttacaaatat

ctgtatgatcaactata

>fava_dn_rep_c1218

aaatcgttcacgcgataacagaaattgaccaacaatatcttaacgaggaaggtagaagaa

tacgcgtggttagttttattttacatctcaacacgatgaaaacagaaattgtaattaact

aacattacattgcattttccgtcaaaagcaacgtttgaacgcataaaaagagaaaaccga

cgcggagggactttagcttccaccgacatcttttttctcccaaatttctctccattgttg

ttctctgaatcatcatcatcatcatcatcaatggctgatgataacaagaaactcaatcac

gacgaagaggcaagtcctcttctccaccaccaacaacctaaatcagctgaacaacaacca

acacagcaggaactttttcatctatggaccgctgacggtgttcccatggcccatggaagc

gtaatgggccagcctattcctcgttctccttggaattccaacgtctgcgcttgtctcggt

caaagcgatcacttttgcagcagcgatctagaagtttgtcttcttgggagtgtggctcct

tgtgtgctgtatggaagcaatgttgagagacttgggtccaataatccggggacatttgca

aatcattgtttgcattattctggcctctatgtaattgggaattcctgctgtggttggaat

tgtcttgctccgtggttttcatatcctagccggactgccattcggcgcaggtttaatttg

gagggaagttgtgaagcacttaataggtcatgtgggtgctgcggaagctttttggaagat

gaggcgcagcgcgaacagtgcgagttggcatgtgactttgcaactcatttcttttgtcat

gcatgcgctctttgtcaagaaggccgtgagctccgccgtagaatgcctcatcctggcttc

aatgcccaaccagtattggttatgatcccccctgcagagcaggcaatgggtcgtggggct

tgaacaatattctttctcatcttggaagtgatattctggtgtatgctattacatttgttt

cgatatgatttcacacgttgttgaactgctgcctccttcttttccagattttgctgaaaa

gagtatcatggttgtttgaatttggtataaataaaaacataaagcaccattatattttca

gtctgtatcacctactattctgtattatatatgtatttgttgcaatcaacattttgtgta

tacgcttaaactgaatgaatttatatatgtattgagggcatggttcctctatggttattt

aagataagctgcaattcactgaaaaaaactctcccctctgttttaagtgt

>fava_dn_rep_c1219

ccagtaacccctgactagtattagtaagttgcaacaaacaactcccgcgtctctctctct

ctctctccttatagaccattctacttcctactacttttcagagtcaagtcaatgctaaca

ttacaaaccccttctacacagagcactgtagtgctttgtaaccaaaccaaataacctcta

atgtgaattgaatgaacccaacgcccccacttagttacaccctcccctataaaaaacaaa

catcaacaacataacacaagacaagataagaaaaaccaaaaacaagatggttcctgtatg

gctactcaccgtcacagtagcagccactttactgttaacaacaccaacagaatcccaacc

agaagcaaatttcaaatgcgccaccgaaagcgccacgtgtcggagcctaaccgactacac

ttccaccaacacaacaaccttgaacgaaatcgcaaccctgttcggcgtcaaacacttcct

cgacctcctcggagccaacaacctcccaaccaacaccacaaacacttacaaagtaatccc

caaccaagtcatcaaggttccgtttccgtgcaaatgctttaacggaaccggtaaatccaa

caatttaccgcggtacaaaatacaaccaggtgacacgttaagccatatagcggcgattcg

ttttgccggtttgatgaagtatcaacagatccaaaccgctaatattaaaaagataccgga

tgctaacaatataaccgctggtgatacgattttgattcctttgccttgtagctgtgataa

ggtgggtgggagcagtgtgatgcactatgcacatcttgcggaaaatggtactagcattga

tatcattgctcaaaaatatggtacaacatcacaaactctactctcaatcaacggtattga

ggatcctaacaaacttgaagccggtcaagttctagatgttcctcttccagtttgtaactc

taatgtgaggaatacttcggtggactttccgttgcttgtaccaaatgggacttatttcta

tactgccaatgattgtcttaaatgtaaatgtgactctgctggtagtagtaacaacagcaa

ttctatgttacaatgtgaggagtccggactaaaaccgataaatcattggtcggtttgtcc

gtctgcaaaatgctccgccagtgtgttgattgggaacactacatctactgattcgtgtag

tcggacgttttgtgattatgttggttatacttctcgcaatatctctgtagttcttgtaac

tcaaaacacttgtgcagtggcaccaagtggttcaggtggttcggattcaggggcatctag

gagcatcttgaatggctgggtttggatcaagcttctcattctcattcacttccttctgtt

ttttgtttatcatttgtagtacaaaccctgcatctttgttctttttctttccatgagttc

attgtttagtgtgtgtcttgtgagtgttgtcacttaatggaacatttttatgtttctttt

ccttctatttagtgcatgtttggattgatagtgagttaggcttaatcagagtgctaccat

gactttgtcacactcacattgatttcaaaggcacatttacactatttgtaatatagcatg

tttggaagaataagaatgttgtctgaactacaataatctgtatcttttttaatgtatgcc

ccttattgacatagccctctcaaacttttgagaggtccggtgtaagaagataaagttagc

tctgttgagtcatcaaatttttttaagggactctat

>fava_dn_rep_c1220

ttgaaagttgaaaccgtgagaaggtgaaaaaacatgaaaaaacattgacaataatgcaaa

acaacagatacaacaaaggtttatgcagattgtgcaaggaatagagcaacaatcaactat

atttcaagcctcatagctaaaaaagacaacttccctcgacacaaaaatgtaggctctaat

acaaaaaaaaaatgttaatcactaagagagaatggccatgacatctaaggctctcaaagc

aatatagtcagaaccatctttgcccttgaagtcattccccgcatatttggagtacagaac

tgtattccctggtgtaatggataatggtgttctgttgccttcatcatccacagatcctgg

accaactgctatcacagtgccaactgatggtttctccttggttgcttccgtgagcaacaa

accaccggcagtttttttctcggcttctgcaacctttatcaagactctatcgctcaaggg

tttaagatccttgacctcatcggtttcaagaataccaacaatgtcatcatctttaagtat

aagatgttttgaaccattgaactccacctcagtccctgcatactttgagtacacaacttg

tgcacctgcctttacactaatttccactttgttattcccaactgtcttcccttctccaac

agcaaccacctcacccccttgaggctttgattgagcagttgatggcagtataattccacc

cacagttttctcttctgcatccttaatttttaccagtactctatcacctagtggcttgag

taaggtatgctttggggcaacgacagtggcagctttaacaaccatagacgggaacaacct

ttgtgtgggagtaccgattctaacattcttagaacgaaattgagttgatgaagttcgaag

cccttcaaaggatgacaaattcctcgttgaaatcgatgaagcagtgagctgagtagtcgc

cattggaattttattttctttaacttatcaaaagtgtgccaggaaggttaggaaaatgtg

aatggataaaccctaagatttgattatattttgttgatagatagagggggcttctagaat

tttccgagtccaaatatagtgagttgcgggctgattcggtgaaacggtgagggggaggac

ttgcggcggaggaaagagcggcggcagtggcgactcggtgagtgggaataggtgccactc

c

>fava_dn_rep_c1221

agataaaaaaaagtttgaattttattataatgaaagataatccttatttccacgccaaaa

gtatttaaatacagaaaacaaagaaattctaatgggcatttaatccaaaacagaattaaa

tgaataaaacaaataaaatagaaattgttgaaattgtagaaaatagtaaaatgttgtttt

cgagcctgaaacacaatctaatggcacaaaaagtcatagacctctgcattgcaatttgtt

gaaaccgtgggccgattctctttctaaaaagatataatacgaatcattctgacattcgat

gctaaatttacgcaaattctatcgaaccgtttcgacacacttgcgcgcagtcaaccctcc

tagaaatcatgcagctttgtagacaaattcatcttgatatgcttcatttgacatcaatac

cttaaggtctgcattggttactctcttttttgtcccagccaatagcttgaatttcttgaa

cagaatctcaaattcatcagcatttagtatataaccatgctcttcaattttctttctcaa

agcttgacgtccactaagctttcctaatacaatactagtctcaactgatctttcaagacc

aatatcttcaggagatattatttcatatgtgcctttatgttttaacatcccatcctggtg

aatgccactttcgtgaagaaaagcattagcaccaacaagagccttgtgcggctggagaag

catcccggagtactcttcaaccattttgcttgtcctcaatatttgtcttgtaataattcc

tgtatgaagaccacctaagctatcattacccctgcattttagggccattacaacctcttc

taatgaagcattccctgccctctcaccaattccattgattgttacttctaattgtctagc

acctgcacgtgcaccctctatagtgttggcagtagcaagtccaagatcattgtgacaatg

agttgcaatgataacattttcaattccaggagtattcttttttatatcagcaatgaattt

tccatattcaaatggcattgttacgcctacagtgtcgggtatgactacagtggttgcccc

gactttaataacttcttccaaaacttgataaaggaattgcttctccgatctggtagcatc

ttccgcagcaaatgagatatcactgcaacctaaactctgtgcaaacttcaccgtatcacg

agccatattgagtacctcatctttcgtctttctaagtttgtactccatgtgaatgggact

agtggctataaaagcacatattcttggcttttttgcatgttttatggcctcccatgtaat

ggcaacctccttcttattgcacctacacacaccacatataacaggaacataaccatcatt

atcaacatcgttgccaacatcttcagctatcattttaaccgcatcgaagtcatcactgga

agcagaagggaaccctgcctctatgatatcaacgcgtagctttactagtaagcgtgcaat

ctcgagcttctctttgcaagacattgcggcaccaggggattgttcgccgtcacggagggt

ggtatccaatattcgaacgtagtttggtataggaatttggttttgaatgtgtaaaagatt

atcgtttggtgccataggagatttttgaggtgttgaaaactatactcggtgaaattgata

tgttttgcttctaattttatgtcactcatttatagaaatggatcagctacta

>fava_dn_rep_c1222

acaatgtccataaactttctctcctctatatttttttatttttataaaacagacttaaat

tttaaaccctcgtccattcacccaatttaagtcatacccatttaccaccccgatcaaaat

agtataaacgaggataaaatcctatatttctttttctatatagaaaacaagactacagaa

ccttacaagaataagaactcatattcatccaattcgacataaaaattcaaacaaaaacaa

gcatgacaaaccaatacctcacacaaatctccttaaattcctcaactatgtatccccata

atttattttgaagcagccaaactttcaaaacgagctttaaaaaaagataaaagtgtttct

cttggaggcaattgttccttaacaactgggtgattgttgaaatcttcactccatttataa

agcttaggatatttctcactactaaacaacttcaatcctgcagcttcttgtataacaggg

aaccaaaaagctataaaaacagcagtaatatcaacaatcccaatctcttctccaccaaaa

tatttccccttgagttcatcctcaagaaattgaagagccacttctacttccttgaaactc

tcttcacgttccttttcatcatgagaagaagtagctttccttaccgcacccaaaaactta

tcatctatgaaatttgaccagaaacgagccaaggctcttttgtaaggatctgaaggcaag

attggattttgcttccaagtttcatcgatgtattcaagaatcaaaattgattctgaaata

ggcttttcattatgaaccaatacaggaactttcttataaactggattatatttgatgagt

gcatcactaaagtttcccaatttttcaagcacatattcgtattcaattcccttcagtttt

agagctatatctaccctggctacaaatggacttcctctcaatccaaaaagtttcacttct

tcgttatttgatgccatctttgttcttcaattatttgcttaaacaaatgtttttgcttat

ttgatttgtacctaagatctagttctatatatagagccaggttctattctccacaaatgt

>fava_dn_rep_c1223

cagaaaccctagccaaactctagcatcagcggcaccttggtttctcatagagaaggcgaa

aatgaagttcaatccacgagtatcaagcagccgccgcaagagccgcaaggctcatttcac

ggccccatccagcctccgccgtgttctcatgagcgcacctctctccgccgacctccgtgc

gaaatacaacgtccgttccatgccggtgaggaaagacgacgaggtccaggtcgttcgtgg

taccttcaaaggccgtgaaggtaaagttacgcaggtttacaggcgcaagtgggtgattca

catcgaacgcatcacaagggagaaggtgaacggatctaccgtgaatgttggtgtgaatcc

ttcgaaggttgttatcacgaagctgagacttgataaggataggaagtcgttgcttgatag

gaaggcgaagggtcgtgctgctgctgataaggagaagggaaccaagtttgctcctgagga

tattatgcagactattgattagtctctcttttgagtaattctgttatttttattttggta

gtggaaaattattatatcaaattctgagaatttgttcttgttgacaatttgttctgctta

attttgtcttgttaatcaaactttttgatattttggttatcatatgttttttttctgtta

cgattcaatatgaatgtactgtttaattgtgtatttactgtttaattgttttgtataatt

taattttgataatactatacatgttttccttataaatattggcaattgctattgtgat

>fava_dn_rep_c1224

gccgaggcggccttctgttttttccnnnnnnnnnnnnnnnnttacaactattttataaaa

aaaaaggtaattataacacataaatagtggggttttattgcacaaagttacctttcaaat

atgttggttttacaattgatatacaaactaaatggtaattagtttatgaagagataccaa

catgcatatattacacacaatcacgcaaaatagaatgatcaatgatcctccccgtttgta

tcagttaacagcagcaggggaaggattaagagtgattaccacattatcatggactccctg

caacagtagctcgccatcgtaggtaactactttacgctttttggcagctcgtagtgtgcc

aacaagcgcttcaaatatatttgcgcaccgatcatcgtgaaagagtattccaaatgtaac

cttgtaagaaccatctgtttgaacctctccaagcctcttgatttcttccctgagcttttc

aacctcttcttctacattcatcattacaatattctttttctcggaccaacaaaaaaagga

aagtatctttctttatgtgtgtcccgcggcctgcctccctcattgcaatctcagcctgca

agcgccactggtagacacaacttggatccttaatcttaatgactatccacgactttatat

acttgtcccacgcatcataataagcttcgaggttattatttattatttccagctgaggat

caacggcttttacaacttcaaccggaacaggcttgaaaccaagcatccagccttcaaata

acacaacttcaagtggtccttcaatttcaggccatgttgaaggatcagcgcggtcacctc

tcccaccgaaagcagatttatcatatcttggtaacttcatcttgataccttctttcgaca

ttttggttaaagcagacagagtttcaacagaaataggaagatcatggcttcccgcatttc

cacgaaactcaagaagtgcatttcctggattagcttctcttagtttattctgaccttcag

ccgtcaaataaaaatcatctatcgatattgttgcagacttcctgccaaatgtttggaaaa

ggtaatcgagagcaaagacgagggtcgtctttccacagccttgaggagcactaaatccaa

tgactaaaggaggtatatcttctccgtctttgaatttggatcgatgctgagcaatctcac

tttcgcaccagaggaaaactggtatatagtaatgataaagcctaactttttgaggctcgt

tcaagaacaattcattaagctgaaacagtctgcatagttgtcgcccgtaggttaaccact

tatctatagactcggccacattctctggcgtcaaaccaattttgtcaagcaaaggtcctg

aggaaataaatgtgtacaaatcttgcacagaggaaacttgagcaggcgatgacggaaata

ctgaatgtacaggaccgttcttttgctcgttgctgacagacgtcgaactactttgcagcc

atgaacttccacttcctgactttgtgaaaagggtcttagaagagttagaaagggtagggg

cagaaagggaagaaagaagagaattggaaaagaaatgaaacttagaagtgttagaatttg

aacagacataagcatagttagaagaagttgaagaagaataagaagaacaacagaaagaag

atgaaattgtagggtgcaatgtttgagagaaaacattcaaagtagccattgttgtgttgt

tgtttttctctatctatctagttttggaacaaaaagtcagcaacaaatcttagccgccac

gtaatctttggcttgtgtaatttgcccaaaccagtgatttacaccagaaaaaataaaata

atatatagtacacctatattctattctattctattattcaatctatctaaacaatgtgtt

tgtgtatcgc

>fava_dn_rep_c1225

cgttttctttttctccacatccaccaccccatccgccaagcttgaattttccccaatcac

agtccctttattcttcgaaccactccgcgggatgcacttcaaattttccttctagctttc

tcatattcactttcatcaactatgtcaaatatcaatgaaaacaaacacattgcagtgttt

tctctcccttttagcagccaccctttaccactcctaaaccttaccatcaaactagctcat

caacttccaaactgttacttctcattcattggcactgcaaaaaacaaccaaacactcttc

tcaaaaaccaaaacaccaatcaacatcaaacactacaatgtgaaccatggaatcccagag

aatcaccaactacttcatggtagtgagctcaatctctatctccaaactggacaagagaat

tttcaaaagggtatagacttagccgtcttagaaactaaaacaccaatcacttgcattata

gcagatgcttttgtcacaccaacttttgatcttgctaaaacattgaatgttccatggatt

cctgtttggattccaatgtcatgctcactatctgttcattttcatggtaaagaaatacgt

caacattgtactgtgaacgatggaaacaaaacattggattttcttcctggtttatcagtg

ctacgtgttgaagatttaccacatgatcctctcatgactgatactgagaaagaaactgcg

cttacaaaagcacttgcgtcactttctacaattttacctcaagctaaagcagtaattgtg

agtttctttgaagaattggacttacctctttttgttcaagacatgagaagtaagttacag

ttaatgctttacgttccactttttaatttgaaaccaaaaacacaagtaaatgatgagata

gatgaaagtgggtgcatttcctttttggaggtgcaaaaagcaaaatcgttgaaagttgtg

tatattggttttggtaacactgtgatagaaccaccgaaacatgagattgtggcgattgca

gaggcattagaagaaagtgggtatccattcatttggtcattgaaagagaatctgaggagt

catttaccaaatgggtttcttgaaaggacaaagacacgtagcaaagttttgagttgggtg

cctcaaggtagaattctagggcatggttgtgtcggtgcatttgtgagtcaaggtggttgt

aactctatgcttgagggaatgtcaaatggtgtgcctgtgatatgtaggccttattttgca

gatcaagggataaatgcaagattggctgtggatgtttgggaggtcggtgtcattattgag

ggtaaggtttttagcaaaaatggtttgcttaaaggtttgaatttgcttcttgtggaggaa

gaggggaagaggtttaaagaaaatgctttgaagatgaagaagattattgaagaagctaat

ggaccaaaagggcttgcaacaaaggatttcaaaaaactcgtcgagttggtttcaagctct

tgacttatgcatgcaaccattattattattattgttattaaataaaaaacaatctactac

ttcttttaagtattttatggttggtgtatatgttttgatgttgttgttgttctttcaata

atgtatctttcatgtcttaaattcttcatttttcatgtccttcaataatgtgtatctcaa

gagatatttattgaattttatcttatgtctctatgattaatcttcagtttcattagttta

cttctttttctatctcaaactaaatcatcaatagtttctttcttatcgtttaattcaagt

acaatatcatttgtaaa

>fava_dn_rep_c1226

acaccgttgcggcgcaactactaatccatgagtgtcaacattgatcatattccaccgcct

tcaacggaaaagtaaagtacgctcacgtctccttttccttcaactaattttctgttttca

tttatatataaatacactattctctctttctagtttattatattttttattcattattcc

accaccgccatcatcttcattgtactttcctaattcaattatttgattcatcttcttcct

attcacgcttcaaccgaaaatagaataatccaatttcaactttcaaattttaatttaaac

tttagacttagatcatttttctataaaaaaaatgaattcgattcatttgattaacactct

cgcacttagtgctccgcatcggttctctttacctcacatcaattattcttctcctccttc

ttccgccgtctcgaaacggcagcgtttctcgccgtccgctaggttccgtctcgtctcagc

gcaaaccgtcaattcagtaccgcgcgcgaatgggagttatacagtttctgatttcatgac

gaaaaagcataatttacatgttgttaaaacttccacatctgttgatgaagctctggaggc

tctggttaggaacagaatcagtggtcttcctgtagtcgatgacgactggaatctggttgg

agttgtttcagattatgacttgttagcaattgatacaatatcagggggttctcaatgtgg

tgcaggcttatttcctgatgtcgacagtacttggaaaacattcaacgagatacaaaagct

tcttagcaagactaatggcaaagttgttggtgatttaatgaccccaagtccacttgttat

tcatgagtccactagtctggaggatgctgccaggttgttgcttgaaactaaataccgtcg

acttcccgtggttgacaaagatggaaagctggttgggcttattacaaggggaaatattgt

taaggcggctctgctgtcaaagcgctctagtgagaggctcacatagtttgacaaatacag

atttcaagtgctccttatctttagctatgtcctgtaatcatagtcatattttcattatta

aagatgcatttatcagcctaaaagggaagctgaatgtaactcttgatgaccttctgagac

ctttaaagtaggtggttaatacttaatactattgagaagaaagtgaattatcaatctcct

tactcaacaatgtttttgtgcattggattctttaggcttaaagacagtatatccagttaa

tctttgtatagatctgttaaatctttataggtattgggattggaatggatcattttgaac

actgtattaaacttatactgtaaattggttagcgttttgatttttatgttatatgatgtc

ctcgttcatattctatcactcacgaacttcttaattcctttatgtatttattatgacatt

attgaattgaataactagctaatataggcatgcttgtgacatttttctttgccaatataa

ccaacacattatttactaaatttaacattattttatacaaagttttgaattgataatgtg

aatagaaacattactattgttgttactaagaagtgaacaacacttgtcttgcattgttta

acaatctaaatatccttgtcagtctgcaagattttaagttcctgaatcaaagcatcaata

tcaccatatgaacttccaccttcttcaacagctccttttgcatccacagaaatacttttg

actctccttctcatttcctctacctcttcactctccaccatcaacttcttcactgcaaac

tccaccttctcccttcccaccaactctttcctctctacatcccaaaacccccactctctg

ctacccacctgaaccccaatcctcaacacatcagttaccagcttctcattgacaaattgc

tcagccgaaagtggccatgttatcataggcacaccagcacacacacattctaaagttgag

ttccatccacaatgagtcataaacccaccaactgcatcatgctccaatatcaatagctga

ggagcccaacctctaaaaattaaacctttatccatctccttcattctcttttcaaattca

tcaagagcccaaatttcactatcacttttagatgagttgagaatcttcccaaccacccaa

atgaatggttgatcggaagcctcgagaccataagcaatttccttgagctgtttcataggt

aagcaagctgcgcttccaaaactaatgtaaagaactgaattgggtttcttagaattcaac

caattcaagcaactttgttcatcaatagtgggttgtttccctctctctttcttatcttct

acgcttctgttgcagagagaaactggtcctacaagccatgttttctttcccaattcattt

ctgatgtaatcagcataaactggttccaaatcatagaagctattgataacaatacctaag

cttttttcctctatttgctttatcctatcaacaaactcagatttgtttctcctgaaactc

ggtacttgagaccttgtcatctcaactctatcaggaagaccagggacaaggaaaggttct

gagtcagaactcagattctcaaacacaacatgttttctcatattatcgaagacgcagcga

gggaaacacccattcccgttgaagacaatcattgggactttaagttcgtcaatgatgtca

ccagcccaacgatgaaacatgtccaccactatacaatcaggtctttgttggaggagtaac

agtttgagaggttcgaggagaaccgaggtatcgatcatgggaccagcagacatgtcagtg

tcaggtaattgggtgttttcaggaagggtgagagtgtagatggtgataggaagattcgat

ttttggtcgcgggtgattgagttttggaattgtgcagcgcaggaaggtgtggttaggatg

gttgatgtggctccatgtttggcaaacactcttgctgtgtctatcattgggatttgatga

cctccacctaggaaggggaagaagtatattttaactgtgccagtttcatggttcatgttg

aaatggagatgatggagatgatgatgatgatgatattcactttacttctaagtgagaaac

tgagttattcataaagacgcgggtccagttatttggaagatgggtgaattataatagatt

catgtcggtaggatgaattagaaaaggttgacatgcaaacgtcatgaatgacatattaca

gtctgatggatgtggatagagaatactcaaactataggaccactcgatgta

>fava_dn_rep_c1227

ggccgcgggatcaattcaccctcatactcaatcaatcttacccattcttcaatccaccgg

aaaactctcaaatccgatccatggctcccgcaaaggcagagaagaagcccgcagagaaga

agcccgccgagaaagcgcccgccgagaaaaagccaaaggccgagaagaagatctcgaagg

aaggagggagcgacaagaagaagaagcgcgtgaagaagagcgttgagacctacaagattt

acatcttcaaggttctgaaacaggttcatcctgatatcggtatttcaagcaaagctatgg

ggattatgaacagtttcatcaatgatatctttgagaaactcgctcaggaagcttccagat

tggctcggtacaacaagaaacctaccattacttcacgggagattcaaacggcggtgcgtt

tggttcttcctggtgaattggctaagcacgctgtttctgaaggaactaaggccgttacca

aatttaccagttcttagatttgatttttaggtttgttttttcacagatctagatttttct

tgagtttcggatgtagatttggtgttctgaatagctataattaagcgttttaattgaata

aatcatcttttatttcttagtttacataaaaatgatcgcatcttttataattttcagaaa

ttcacttaaatttctttaattatatttagatatgtatatccatttgagatcggctaagca

tttcacctaaatatcaatttcaacatctcctgcagaaatttg

>fava_dn_rep_c1228

gaaaatgtaattttataaaaataattaatgttgtaagagatatatcacttattttaaaaa

caatttatttttgcattattttagattggagagagtattattttacagtattttgtgcag

tcttgccacagctaaggaattagatggacttaaagaagtaccgatgagtcaaaatagtga

gtcagcatcatcttagcaaaggcaaccatctttttttgttaaaataaagaaaaaataccc

atctttttaacatataactttccaggagacccttaaatgtcccatctcttcttaacccga

aacattaatactgaagattttatgtcatcttgtggcacttgaagcatgaattgaactttc

atcttcatcggagctgtttttgctttcccgccttcatctccaacccaaagggatgcccag

ccaaggcgaacctctgaatcataaccagctttaaatttgaaatcttcaccataggtatgc

ttgtagacagcactccaatcgttggaatcacaattgtaccagtaacttaacttgtgagaa

ggagcaaaccttcgcttgaaggcaaatgagaaatcctttgaagggagagaaagtattgga

agaaatgacatttcatcgtccttgtagcgatatcttaacttgaattcttcatccttgtag

tgagcagtgcagagacccttgaaaaattgagattgaagagttccagtgactgacaaggaa

tattggacttgttgctcctccttctccttctccttctcttgtaaagaaatttcaccttga

ggaaatttgaaggttgctttgggcaatcgagtggagggaagaagtgttgatagttgaagt

gcatatccagggttatcaaggtttgcaaccattgtaacctctccttgttgagccttgaca

tcgtgagtaccaatgagtttcaattttggacccacgtcaatggaactgttaacgatagca

ctgtgatcttcaaggtcgtagtggagactgagatacttggatacgaaactgatctgaggc

tcagcgatctctcctttactgttattatgaaaagctaactttaacttggcaaggctgtcc

aaaaacttgcaagaaattttgtggaagaaaatggtgctttcactatcaaactcagtcgta

attcttagggcaggcctcttcggaaacggcagaggctccggagagtaattagggttttcc

ggaccagaatccatctctaccgtccactcactatattctccttatctcactctcccgcgc

taaactcacccgccgactctatatacattgtggtccacca

>fava_dn_rep_c1229

actgataacgaaatgtctcgtggcggtgacgcactttcttcaattacactaacctcatat

tactacaaaaacaaattcaaaaagctttaattgattttgcaaaaaaagcttaaaactaat

taaaagaaaaagctcaagataatcgattttgggtgccactgggaaatgaaatgttgaatg

tgtttagggtttatcagtgtatgctcgctttcgtcctcgtaatctccatcttcatttccg

tttttcaaggtagttcgtgatttgcttgcagttcagactttggtttctggtatagtagaa

ataaagatggcagctgagctagtcaatgctgcgacaagtgagaaactggcagaagttgat

tggacgaaaaatattgaaatctctgaattagttgctcgtgatcaaaggaaaactaaagat

gttgttaaagctataaaaaagagattgggtaacaagaaccccaaaactcaactttatgct

gtcatgttgctggaaatgttgatgaacaacataggaaatcacattcatgagcaggtggtt

gatgcagaaattattccaattcttgtgaaaattgtaaagaaaaagtcagatttgcctgtc

agggagcgaatatttctcctactagatgcaacacaaacatcccttggtggtgcttctgga

aagtttccgcagtattataaagcatattatgatttggtgagtgccggggtgcaatttcct

caaagagctcaacatgttcgatcaaatcgtcctagttcagaaccaaataggtctaataat

gtacccaaaaaggagcaggtcccacttagacatggaggggttcctcaaaaagcaaaacct

aacactgttcctgaatctagcactatccaaaaggctagtaatgcattggaagttctaaaa

gaagtccttgatgctattgatgccaaacatcctcagggagcaagagatgaactcaccctt

gaccttgttgagcaatgttcatttcaaaagcagagagtaatgcatcttgtgatgacttct

caagatgagaggatagtttcccgagcaatccaagtgaatgagcagcttcaaaatgttcta

gataaacatgatgaccttctttctagcaaggttacaacaaccgtgaatcactttgattgt

gatgaatcagaggagcaagaagaacccgaacagttattcagaagattacgcaagggaaaa

gcttgtgtaaagcccgaagatgaagaaattgaacctcagttccctcgattgagtttgctt

gaagagagactcaaccgtccactaatacgaccactagaaccttctcttgcggaagctcat

gcttctcctgtacctgttgtagttccacctccaagtgcaaagcttcctcctgttacgata

ccaccgccaccttcaaagcacacagagagagaaagatattttcagcataataaggacagt

agcactttggctggccacatcagaggcctctctttacatagtcgcaacggcagcagctcg

cagagtggaagctttgattttagtgattgaattcctaatagaagaatctccctttttaac

cttattttatcctaggttgtcttcaaaatttggtatttggaattgaaatttttgggggct

ggtgggggcgtgagttatatctatgctacagtatgtaaaatgtatgtaataatgagctct

gagataattgatcatgacttaaaagtttttggatgttccaggtgctattgtaataatgcc

ttttctatattcttttactagtgagagaacttcactttctccactccaa

>fava_dn_rep_c1230

tgaataattatgtagactttttagaaacattgaaaatgttaaaaacttgttttcattatt

tccaaaaatagatcttcacaaactagcatttcatcttctctatcacaactgatatatcat

cacattaaacaatggtttttcttcttccagtgatcatcaacaatggtgatatcatcacat

tcaggcaacatcttacaacagagatcacttgcaaaaaatgagagctaccataccttcggc

aatcacaagactggggatgtgggatttatgcagctttcggtgcttatagttataccaaca

ttttgtgttcgtagttttttttcagtataaaccagatatcttctgcgcgcaactgcaaag

cctaattcctcgagacagagaggaccacagagaatttaacactctacgtgcccagatctg

aattcaaacccaaaacttgtggttaagagagaagagattcattatcatttcatctcatcg

aaatgctcttgaatttgtacggatggttatagcatcattttcacatatacaatagaatga

aggtggaatcatacttcttgctttgagtgcttataccaacattttcatagataccaacta

caataactgtgattatttattcaactgatataaaagactttattacaagattcacatatg

ttgattcactctgtaagctaaacaaacaaagttctggatcaaagtgggagaaggaagaag

attactaacaagttaaatcatccaccacttattggaggtataatggccaactcatccttg

tctttaacaatggttgaatctaatgtatactcctcattcagagccagaaccatgcatcct

tgtatttcttccaagcgcggaaattgaaccagaagctttttcaaacaatcttgagtagta

ctcccagatgacacttccaatggtacctcattcaagccagtaagatcacgcgcacgtgca

aaaaacagaacttttatcttcaccaaagaactgtcttttacgatttctgtcatgtttaag

cagctatgcccaatctggttgatgtaatgtaacacttttcaacgaatcttttgcaaaatt

ctttacgaatcgcgccggtttagcgtgtaatgaaagtgagaaaaatactctgtattttca

ttcatacatttcttcccgcggccgtaatggccactctgcgttgatctcccctgcttctgc

tacttggttccagcaacagacatcatgaacagaagaaactagtcccagtactttatcaac

ccatcccagccacaagttgctactttgctctgttccagaggatgccactcacaaccaatg

cagacaccttcatgacacttgagagttctaaagactttgcaactcttccaatcccagaac

caacacttaccctcaccatctcctgacatgacaaagcgtccatccggcgaaaaattgacc

tgacaggcatatccagcaacaatatgtccagcaaacctcttctttttgttaagttgaaac

ttctcccttgtgctataaataagtatctgattatccaaactctgtgtagcaagccaatta

gcgttggggtgaagcgaaatggaaggcatagaatgcatgtgtggctcactaatatacttt

ataaccacaggaataccatattcccatactctgagggacttgtcatcacttgaagtaaca

aatctcctattgttatcaacaaaagtaatggtattcacagcccctaaatgctgatcatac

tcttgagttatctgccccgtattcatatcccactgaacaatcttcttatcactcatacca

gccaacaaaacattctgcttatcctcatccgggttaagcttcacaacataagggatcttc

ccagtagaaaaagtggaaatgacttggcccgtttcggtatcccaatacttaatattcttg

tcataaccagcactcaaaaacttggttccgtcattggtgaaacaaatatccctaacagct

ttagagtgtcccatgtaagttctcatacattttccagtattgaaaacatcccaaatctta

acctttgtatccatcccagcagagagaatcaaatgaccagccttggggaaaaaccgaata

gcagaaacccctttcgtgtgtccactccaagtatgaaccaatctcttgggtatataacaa

tgatcattactagctttcgcatctttaggaggtgcaatccaagacctcccttgataatcc

ctttcctctttcccatgaaaagtactcttatctttaacagcctcaaccttctcaccacca

aaaccactcttctcttcacctttcttcttagcatactcctcagcgtacttcttctgatcc

tccgttaactctccttgtaaaccttccttctttccagcccaaggactcttcttattcttc

aacaaccacgcctcggaagcagggttttcaatctcaacgccttcttcgttgttgttttca

ttctcgttatcgtcttcttggtcatcattttcctccttcttcaaggcttcgattctcctc

ttcttctgctcgcggtgaggcatattgtaaacagaagcagcattattcttctgcaaagca

tcgaaatcgccaatgtaattcgaggcggaggggtcagcggcgtaaccgaatttgagaaaa

gtgttatgctgttcatcgaagagaaagggttcaatgttggcgtcttcgacaaaacctagc

ttgtggttacgcataccttgtgctaaaccgtctttggcgtaagggtgagagggaccttgg

atggggggccagagctggtcgtaagtagggttgaaagcgacggcgtgttgagttggatcg

atgggttttgatagggttttagggtcggcgacggtgagtgcgagcatggtgtcgtcgact

ttgggtgcggcggaacgggaagggaggaggcggggtggggaggagttttccggtgagtta

gggttttgaattggggagtcggggtcgttgttgtcggagtattggttgagtagatccatt

gtgaagaattctattagctacgaagaaacacggtatgatgagaatgttgaacggtggtga

gagagagggagaggggaaaggagcactgcagcggcggaggaagaagcttggtgaa

>fava_dn_rep_c1231

tttttttttaaccaaaaaaaattccaataaatacttaatgttttcttaatcaataccaca

agatacttggtgacaacgtcacaaataatattaaataatagtaataataatacaagcaat

agaaaaatgtaaatgaatattttgttcacatttttggtaatgcaaactaggcagcattgc

cctcaagtcccagttttgatgttagccaagcacaaacatgatccgtctcttctggaattg

tgtagtggccaagcccattataagctttaaaagttacatcctgaaatccattagaactta

agcatttcgacgatttctcaccaaacttatatggaaccacatcatcaccttttccatgac

acatcaaaatgggaaaagactgagcacgccttgtagcttcatctacaccttgtaacttgt

cactcaaagtctttgaacatggaagccagccacttaaaccaacaacagcacttatgttgg

ctggataagcattgccattcccatattttccagcagtaaagcaggatgcagagtaaaggg

cagtcgctgctcccatgctaaaccctccaacaccaagcttaatgtctgcgggttctgtag

acaacaaattggcaacatgtgcggctgaagcatccaaaccttcaagatcatcgggagcgt

cttcagaaagctcttccacatcaaaccaggctgtagaaggaaagccaccaaatacagaca

ttggtcgagtaggagcagttggacatatccatttaatattgggaagaggaagggtctcta

ataactgggaccaactggagccattatccccgaggccatgcaaccaaactatggttgctt

ggtgtttacctttaggcctgactacataggttcttccaaactcaaatgctctagcagcag

atctaccaccaacagaaccaagtgatggtgcagcaaagttcatatttaccactcaacaat

agacctatggagcagtctcaaaactagaaagagaaggggttttacaaagaaagacgagga

tctcaattctacagacacccgctaaccttagtggccactctgcgttgata

>fava_dn_rep_c1232

acatcaaatctatgatttattagtagttttataatattaataaaaatataatgtgtaatt

tgggtatccaaaaaccgacccaaattaaaccgcataaaattggatcggattggattggat

ttttttaatatatcatacaaaaccgaaccgcaagtaaatttatctttggatcggatgatt

ttttattttaaaaccgatccaaaccgaaccgcgaacacccctaattacatgcactaaatg

tcggaatacgtttcacagttgcattgcgtccatagcaaagtactttgttacattgtaaaa

agataaatagtaaattgcacaagtatccaactctaagctccgaaatgaaggtgaagcata

gcaaatggatcctaacaccttatattccattaagagtgacaacaactcttcgtgaggtag

gatgatcccggtgctcacacagccatataccctgccaagttcccatgttaagttttccat

ttgtaatcggtatcgtgagtgcacaaccaaacattgatgatttaatatgagccggcatgt

catcgtggccctctagagtgtgcttccaaggtgcagatgaaccctcgggaacaattcgat

tgaggaacgtctcagtatcatcgcgaacatcagtatcgtaattctcattgattgtaagtg

aagcactcgtgtgctgcaggaagagatgagcgagaccgcacttgaatcccgaaaggtcat

gctcaatttctttcgcaatctttggagtgacgagatgacagccgcgtttgtaaggaggaa

gagcgatggttttttgggcccacttgggtgcggtggcttctgccatggattcctttgtct

tcacggtggggagttgcgatttcacgctgaatgggaactttgctacgcacagtgatgaca

cctgcatgattcgttgattcgtagcaaactataactccagaaagagaaaaaaataaagat

taaaaaattaaattagttctcattgattgcttaagtaccaaacttgtggacaagacaaga

ggtccccgcggccgtaatggccactctgcgttgata

>fava_dn_rep_c1233

gaagccgaattcctctttttccttttctgcgnnnnnttcctgcgatgtttcaaacaannn

atgtaaaaaatatccgcggggaagaattggcaaggaaagtcgtacgccaggttcaccagg

ttctaccactgcagggctcaatcatgctagttaggctatatgcttaacacatgcaattct

aacgaagtgctcttggacgactctttgatattccgtcagtaagtcagtgcatcaaaaaaa

gagtcatgctcttcctctttgaaaggaaaggggctttagttaagtcatcgattccaatcc

tatatttatttttttttgttggtcaacaaccacatatcgtttcttttccttcaagacttc

ggacagaaagagagtccggagggaatcattgagaaacaatcaatcatgcctcaactggat

aaattcacttatttggtcatgtcagaatttgcacctatttgtctctcattcgtgatcagt

ctgctagtttctttgatcttactcggtcatatttttgttaatccaacacgaaagttgccc

ccaggcgaggtttttcctatggctctcggtcttttatcttgctttggcactcctcttgcc

tttagtgccgacaccgtaccttccccttcggatttgtttacgtatacttccgacatgctg

gaagactcggcgagttccgggcgtagtagtagtacctcagcggtcaatcaaccgcttccg

ggggaacaagctatgcctcccgctcttcctgttatgcaggaagctgctctgccctacccc

tatcaaaataatgagattatagggggggacagtgttgaatccatccaacggaggcttttg

gggagattcccctctccttctgcccatgagattcaaatggcccggattgaagccgaagac

ctattcgaggtcaaggtcgatatctgtcaggtcatggcgggccttcacccaagcggggat

tggatgggacggggggctagagctctggataatcttcgtaccgccacaggggaggagtcc

ttaagtcagctccttcgaatgcgtgaggacctggagactgcaggtttgcagtccgcaacc

ttccggcaattggccgacagagtgccattccgggcggatgcggatcaacactcagccacc

taggacaggatgagatcgcgtgcaagacgcgacgctccctatgatatctgaggtcttttt

cccatgcatcggtcaacaaccaagcacagctctctatagttattcactactccgtgcagg

aaagactatctttctgtctggaggtaatttttttgtctcaatcaatcatgcctcaactgg

ataaattcacttatttcacacaattcttctggttatgccttttcttctttactttctata

ttttcatatgcaatgatggagatggagtacttgggatcagcagaattctcaaactacgga

accaactggtttcaaaccgggggaacaaaatccggagcgaggaccccaacagtttggaag

atatctttagaaaaggttttagcaccggtgtatcctatatgtactctagtttattcgaag

tatcccaatggtgtaacgccgtcgacttattgggaaataggaggaaaatcacttttatct

cttgtttcggagaaataagtggctcacgaggaatggaaagaaacatcttatatttgatct

cgaagtcctcatatggcgcttcttcttcaaatcctggatgggtgatcacttgtaggaatg

acataatgctaatccatgttctacacggccaaggaagaatcaaaaaataagaaaggtatt

gctttttttataatagttgctcgttcgttcataaattcactcgaccacttgttagtcttg

ctacacgacacgagtctagggtgaagttgaagcagacacgggcaagtaaagtcgacttca

caagtgattcaacataccatatatatggatggaaatcaaaaaggagataagggtctcgcc

caggtggctcccgcaaggtaacgacttcaaactcaaacaaagaacgttcttctccaaggc

atgagtcaaagaaaatgctgtatgtattgcaagacccgtcgataagctaaggctacgaca

tgaaggaaggccagaagaactacagaaccacgcccaccagagctaaaggagaccgaaggg

acttgcgggaggtgaaggtcgctggcttgcccgtgggccgtggtatctgacgggacattg

gcctccctcccgctacggctcgctgtacgttcctttagctataggcgtgtccaatccgag

aatagtccgttccacatagtgcaaggaagctcagtggggtttcatacgagggtcctgcgt

actgtgccatcgtccaaccagtacagtacggccatttcaaatcgccccgcgaagctttcg

ggaagaggggtagtcttgcgttcagaaagaaaggttatcgagattataaagagctctttt

gagtggggaggaatagtgctggaagggagcgagaccaagccgagccactaggagagtaag

cccttcccacgtgtaaagtttaataaaagaatgcagcctcaaccagagagagaaacctgc

gccgccggcggcgcagaacgcactaatccgcgactagcaagttttcctaaaccaaactta

ataaattgaattgttacttttaaaaaatgcttgctgaaaataaaagaaagaaaaaggatc

gagacaggaacctgactctcttagcataggcttgaagcccttagttgcaaggtctttgac

tgtgcccaagaaagggatgtgggacaggcagctaagcaaaaaaccaaccccgccgtacaa

gtacaactacaagagggggccctaccagtcatagtcttcactctcccccataagaactca

attcctggggttattgct

>fava_dn_rep_c1234

ctttttttttttaaacacaaaaaggtcacttgctttattgacttgatagtgaacttggta

gattcactatatacaactatattttaaacaagattacaacattactttgacttagaaaaa

agctgtcatcaattctaataccatatttactatagccaatgtttacatgataatagttgt

agcttctaatttcacagtagcccaaagaaacatacaacaaacccagagaagcctaaaggc

caaaagaccactggccaacttgaatcagatgcatcttttttgacagttgaatttgatgca

tcagttttggcatttgaatctgatttatcattttggacaccatatatttgttgaatcttc

tgcaaatcatcatcattcaccaattcaatcttctgtttcggcaacacaatgggatacata

atagcctcttccacagaagaatgttccaaccctaataaatgaccaatttgatgcatcaca

actgattcaaaatcaacttcatcctgtttaatatcactcttactcgaccattgttcgttt

atatcaaaatacataatcccaacatttttagctttgtttagataagtgcctccaaccatt

ttaccctttccgtctaagtctaaaactagtattttgatattcgaatttttaaacgacatc

gtctctgtaaagttcaacccagttacttttgaccaacggttaaacgcgttttgaaacaaa

gacttaatgttgtttgggacgttatctttggggtaagcatatgttaattggttccccgtc

caccatggtttaaaggacttggcgttgagattgtcaggaacaccacaacgtggctttgta

agtgtatcagttgtatttctatcaagcatacctgtatttttgagattaaaaaatttctga

aatttgattattgaagattcgtccaccagatctgaaggcgaagtggaagagtttaagaat

ccgtatttttctaagtagtccatcgcctccaacatttcctcggctttttctttagtcttt

tgtccaatttctccatatttcttgctcaattcttcatctggaagattgcgtaaataacgg

ggagggatattgattttggtctttgattcaatgagttttgtcaatccatcgaacaagccg

ccggagacatctgcaaatagaaaagatagtatgaagaggaatgtaaaggtgtttagtgct

ctgaatttcatattgtgttttggttcgggtttgagaaaatgtgagttaagcttttggatt

ttattgtagaattttgtcacgggatttggaaattattcatataccatggacattgcaggt

ttggatatgggggctgtatcttctccacttggataggataggacaagaatcaactaacat

taaaatatcttatttttcttattcaaatagacccctgagtagaacaccggacactcaaat

gaatgcattatagtactactgtctaaaccaagacagctgctaggaa

>fava_dn_rep_c1235

tgagatcttgctacaatgttttaataactatcgtcttcttgatagaaagatgtttgacct

ctgtttgaaaaataatacaacactatgatcaagccaaaaattatatattagcttcgaaaa

ttcggcgacaatcgaagccatattagttttaaacccaataaatagataggaactaacaaa

cattactattgtgtcacatattttatgagtgaccaagttgttttcttttctagtatttta

catcaatagatagagatactcaagacatcaacttcggtggaagatcataatactgttaca

aatgccaatgttcttcttcggtatgtaaattttcatatacactacaccccattacactat

gacataatacactccaattttagaatacacttaactcaacatagattgagatcctacagc

atcgtgttattcacgagcagtttgtgtcgtaacttacaagtccaagcaactctggacctt

ccaatcgagggttattctcaaagttattcaatggaatatgctcgaatggcccagaggtag

gaattgtaccacataagttattggtggagacatccactactttaaggcttgaaatagcaa

tgagttccctagggattgggccagttagtaggttatcattaagtcgcaaaaagacgagat

tcttcaatttccccaatgaaggtgggatggttcccgacatgttgttgttatacaagtcca

agctaacaagactcttcaggtttccaagctctggaggaatagttccttgaatgttatttt

tgtatagctcaaggtactgcagatgctctagcttcccgagttcaggtaccaactgtccag

ataggttagaattaccaagatccactcgagtgacccgattgtcttggttacaggtgacat

ggaaccaagtacaaggactgactagggtcggatcccagctctggaggatgttatcagggt

cggtgaggcttcgtttgagtgtgtaaagagcgtctccttcagaattagcatgaattaggg

ttagggttagggttaggattaggggaattccgaaaagggaaagtgaagtgaagatttgtg

ccatcttcttcttcactgatctgaggtttgttgatcgaattgaagagaaaacgcgtgtta

gggttcagagagagagagagagagagagagggaaatgggaaatgggaaatggaaaatgga

tgcttttcaaagagagtatcggttttgggaataacagagcagtaaaacatgataa

>fava_dn_rep_c1236

tcaagttgaatagcacatatgctagtttttttatggcttccaacgtataaataacaactt

gagcgtaacactgtacttttttcatttttagtcctgagggctaatgaatacactttcaaa

tattgtactaaacattatacaatggattgtgaaactagaatgtaaaatatttggtggaag

tagacaaaaatactaaacaaaaatagggttcacctaagaaaaaccagtgcatttgaatct

caacgatacaggactagcactatccaatcataatagtaaaccatttactaaataatttta

caataaattcagtcaaataccaaattataagaaatacggaacacctgtccttggccctct

catctgattgttctcctctgtttcagtgaaaaacttaacttctctctccatattttcatt

gaaactaagaatagaagctacatttccacaacggtagcagtaattaggtgcagaccatac

agttacaaggcctttatcttggaacatgtatttaaggccttcttgtacaagttgatgtgc

tcgacaaacaagatcaaggttgtttatgtgattgaactcagatgtaacccgagatccaaa

aagccaaccagctccacgcggactgactgcccatgtttcaatatcctcaggatcactcca

cattagatcacaaaatggcccctcatgaggaatttcacagttccgctctatgactcttat

ctggtcaattgttcggatgtctggagaaaggccgccatgaacacaaagtacagttccatc

tataattgcagaaagtgtaagatagtcaaagacatcagtacagtatctccaagcattagc

atttccatactttctctggcattcatcataaaatccatagacctgggtgagttgtctact

ttcatgatttccacgtagaagagtaatattagctgggtatctagcttttagaagcaaaag

gatggtgaatacttcaagactattgtaaccccgatcaacaaaatcccccataaaaatgta

atttgtctcgggaacatgaccgccggtctgaaaaagtttcattagatcatgaaactgacc

atgaatatcaccacaaacagttactggactattgacaggttgcacattggactcctcaat

aagaatctctttaacgtattcgcagagaagttgaagttcgtcttcgagaaggtgttggcc

gtctttaaccttcgagatccactggtccaaatccatatctcagtcactctctctctctct

ctctcttctccctcggcggtgctaaaatacgaaatgtaccgaaacaagaggagagaagga

ggaaatcagcgtctctcttctctactaacaattactttccctccagtcaacaaaaattat

taacatctacctaatat

>fava_dn_rep_c1237

acaattgatcacgagcatgcaaacttttccatatctaaatgtaactactacaattccatc

tcaaaggatgttattgtaatttaacaacaatctatcatcactctcgtctcaactatatat

aagagcctcaagccattccatgcatgcaaactagtttagctaataactaccttaaatcaa

caaccatgaaccataaattggttattaccttaacactttgctatgtaatttacatatgca

agagcaatgctattgatccatgttcatctcaaccagataactccgatctcaacgtgattc

ctatgtatggaaaatgctcaccattcaacccaccaaaaccagattcatgggacaacagaa

tcataaacatggcctccaaagacccagcccgaatgagctacttatcaacattagtagccc

agaaaaccgcctcttcatctcccatcgcttcgggccaggcttttaacatcggcaactacg

ttgtccgtgtcaaaatcggaacaccaggtcaacttctgttcatggttcttgacacaagta

ccgatgaggcttttgtcccttcctccggctgcaccggttgttccgcttccgccacaattt

tctccccgaactcctccactagttatgtcccgttggattgttccgttccacaatgtggtc

aggtccgtgggctttcatgcccagccacaagctccggcgcgtgtttattcaaccaatctt

acgccggttcttctttctctgccatactcgttcaagattcacttagattagctaaggatg

ttattgctaactactcttttggaagcattaacgctatctcgggtgcttcgattccggccc

aagggcttttgggcttgggccgtgggccgttatctttattatctcaatccggatcaattt

actcaggtgttttctcttactgccttccaagttttaaatctagctatttttccggttcac

ttaaactcgggcccgtgggccaacccaaaagtattcgaaccacaccacttcttcgcaatc

ctcaccgtccatctctctactatgtcaatttcaccggaatcagcgtgggtcgagtaaatg

ttcctttacccaacgaacatttatccttcgacccaaacactggttccggaaccataattg

attccggtacagtcataacccgtttcgtcgaaccaatttacaatgcagttagagacgagt

ttaggaaacaagtgacaggacaattttcaagcttaggagcattcgacacgtgtttcgtga

aaacttatgaaacattagcaccagctgttacgttgcatttcacggatttggatctgaaat

tgccaatggaaaatagtttgattcatagcagttctggttcgttggcttgtcttgcaatgg

cagctgcaccgagtaatgtgaactctgtgttgaatgtgattgctaattttcaacaacaga

atttgaggattttgtttgataccgtgaatagtaaggttggtattgcccgtgaggtttgta

actaggtagctagttaagataatcatttgtaatttagtttggatgtttttgtagttgtgg

ttaccatgaataagtttcacatgttgtgtgtttattagtgtaagtgtgattattgttact

ctcaaaggagaatagttattttgattacacactttagtattttctattttaaaaagctta

gcgtggaattaattaatatatcaatttggttttgatggaaaaaaaaactatagtta

>fava_dn_rep_c1238

ctttctgtgaagaaaaaaaatctagaaaatgcacaaacattatttacttgtcttcttcca

tatatgtggtcctggtacaaagtcatacaaataattgttaatcagtgtacataatatcaa

ttccttcctccttctcatctttcaagtgaagaattaaaccatgattttaaatcagaacac

aaaattgctttgttagttatatgcttacagataaaatctacaagcaaatgactagactaa

atatccactgttctctaattcctaatacaaggcaaaccaaactaggcagcaactatggtt

cgttcatcatccaaaatcccagagcttcaaaattttcgcaacggatttcgatcaagctta

aggccataaaaggtgagatgaatggagaaacaagtggatcttcgggaggtagctgggatc

ctgggttggaaattgaagtcccttttgaacaaagaccggtaaatgagtactcatctctga

aggatggaatcctgtattcgtggggcgaattgggtcccgggtcgtttttccttcgccttg

gaggtctctggttggcggtgttcacagtgcttggagcgccaattgcagctgcaagcttta

acccttcaagagagcctctgagatttatactagctgctggaacaggaacactcttcattg

tgtctttgattatcttgaggatttatctggggtggagttatgttggtgatagacttctgt

ctgcagtcattccatatgaagaaagtggatggtatgatggacagatgtgggttaagccgc

ctgagatcctagctcgagatagattgttgggctcttacaaggttaaaccagtagttaaac

tgctgaagcaaactctagttgggactggagcattacttgttaccggagttatgctattta

tctttgctacaccagtggagaatttttttcaatccaccttcaccacagaagaaaacaaat

caactgtccaaattcccaagcttaacaagttcaacctaagaaaggaggagttgcttaaat

tgcctgcagatgtgaaagcagacgacaatcttgcagctgctgctgccgaggctgctgatg

gaagaccagtctactgtagagataggttttatcgagcattagcaggaggccagtattgca

aatgggaggatctgctcaagtaaaggacaaagaggagacacgcatatgatttcatatagt

aaaagcagtttatcatttgttaaaatttgcaactctatggccaaagctttattctactat

taaagaaatccattaaaagcagtcaaagtaaaagcaaacaatgcctaaaatagataacta

aagatcacatggagcagtaacaaaatcgtatgacgtgaaaggaaaataaaggttccaata

tatatgcaagtagacatgcaatccaaattccataagcatgttagcgtttagggtctacgc

aagcaggacaagattatatttaatatatcatgagttaatataacctaaccaagaaggcaa

gaaaactgcagaaaatttgtcagctctgagacccttctggtaattggatgaaataatgtg

tgcagtgaaacaactagctttcactatacttgccaacgtaaagaatcttattttgggggg

atttatgcttgtgtaaagatatggtacacatagaaatagatagatgtatatccttaaaga

tgcagtcttctcttgcataacaattctgatgcaagtacctaaagaaagcattttttacaa

caatgttgtgcagctatgtttaggcaaggcacagcagctggttcttgaccgattaagttc

agtatttggccaacagatcacagcaccaggaagaacaaaagatgaattttctccaaccaa

gtaggcaagactaattgaggtctctaatgagttttcttttcagtactagcagaacatctc

aaccaagagtcattgcgtttttttttaaacggcccaagagtcattgtacacttgtaaatg

gatgggaaaatcttcctattgagcaagttttctgcaccagtggagaattcggaacatatg

tgctccaacagcattcctaaatgttttcttgcttgttttggcatattcaagatcaagtgt

tcgatatgcaagagcaaacagatacattgctggatgaagtcttctttcagcttgctcgct

ataatccaagccatcccagaatctttgtgatggcatagtgcgacccttcttatctttgcc

atatgtttcgacataccaaccccctatgtcatggaagccacatcgaggctcaaatagcca

aaatttgcttggtggtaaacgtgctctggtgcatctacactcacaaactgaaacgttatc

acccacatgataccagtgcatatgcttcacctgaaatttttatcataaatgacaatatag

ttacaagcatctagctttttaagttatccataatactgaagttctagcaagatcctgaaa

ctcaatccatgtttcttttaacatataaaatacaaaacagaaaagagttggtactggtgc

aaaagtaaaacccatttcatgttccaagtattttttatatgaagggttccacgtattaaa

ccaacaggactgtgggatagcaactttgcataccaacaatgtcctatgtttcataatcac

atgagtcttttcacgactgctgttgtagacatcacaacatccctcatctggactgctgcc

aagtccagatatggttcccaaatgcattctgcttccaactgtgctatttgcaaataaatt

tcttgaaaagccactgctgatatgtctgggatcttcatccaaattaaggtacacatggat

gtgatcttcagcatcttccttctgcatcgcaccagaacaacatccaggtaggattctaga

tgccgaagcaactaattttccagagacgtgcaattctaaatctctatatgcatctgatac

attacttgaaattttagagctgagaccttgttcagttccaaaatcatctggcctttccac

attcctgtgggtgttcacatccgttatagattgacaaaggcctgaatggaaggatctata

agaagttaacttttcatgttcagtagatgatatctccggaatcatgttgactaggcaaac

catcccaaaaagatcacggccagaaaccaagtgcaatacctcgggagtttcgtaagagga

cctctcctcagcctcaaaacagtcaggaagaagctccatggggatagaatcaattgcagg

accataatatgctgcatgaatagccgaataaaaatctctctcgagtacatcaaaatatcc

tgttccaaccaccaccttcttctccaacaatatatcatttatagctaatctgtaccactt

taaccattcaacaacttccatctccttgaatgtcgtagactgtgattggtaaatgcgcaa

ttttgaaccctgaacagaatgcttctgcataagctttttctgagataacaagtacatgtc

ataatgggctctcttcctagaatctgaaagaatctcatatgcggcgaggatttgaacaaa

tcgtctcgacgcagtggaatcgttccttgactcagcgaggtctgggtgagtttctttagc

caatttgcgaaacgacgctttgatctcagcaaatgagcttgtttccgatacttctaaaag

ctcgtacgcgttctctgcggcataatctgtccaaactgattcgaaggaactactgaagcc

atgttttctgcgactctgaaacttcaaaacgacgacgtagcgcgacgaaacggtgatgtt

cattctcaactatacttctgttgtgttcaccgatcaacacgcccatctttgtttgcatcg

caagcattcaacaaatccgtagcatatttatactcagaaggaatctcaagagctgataaa

ccagcttcaatgtgtttaaattccaaataaccaaatctctccttatcgaaaaaatcaaaa

aggcttcgaattctcttatccctttcctcctttgtctcctgtgatgctaacagaacatga

tccattgtcactggacccggtttcttcaccgggttgtatcgctcctccttcggcttcaac

cccattgatgataacactaaacgaatccagattcaattcaaattcaaatcccacacgttt

ttgagagaaggggttgtggttgtggttgtggttgtggcggttttatgggggggggggggg

gagggggggggggggnnnnnnnnnnnnnnnnnnggggggggggggggggggaaaaaaaa

>fava_dn_rep_c1239

taagagtgttgcaatagaccaaatcgagccaatgtattcaaaacgtgaaaaaactttatg

ttagtagaaaaagttaaatatcagaattatttaataaaaaccacttgactctagtgataa

gtgtgaagaattcagcgttgaaattgtagaaatatggcaataaattttgtatcttggatc

atcctttttcatctcctcctaatcctattgtgttgtcatggaaaccaagcaagggaattg

gttgaaacaaaaaccaaatcattggaaattcagcacaaccatcatcatcaacataatcat

atgactcataatattgatccttcagtgatggtgtttttcactttgaaagatttgaaagtt

ggaaaaataatgcaaatttattttcctaagagagacccttcaacttctccgaagttatgg

ccaaaagaagaagctgagtcacttcctttctcttcaaaccaactctcatatcttctcaaa

ttcttctctttttctccaaacactccacaagccatggctatggaaaacaccttacaagaa

tgtgaaagcaaacacatcaaaggagaagtaaaattttgtgctacttcattacaatctatg

cttgagttcacacaaaagactcttggttcaaattctgaaatccaagtttatgcaactttg

cataaaacaaaatcaagtgttacttttcaaaattacactatagtagaaattttgatggaa

attatagctccaaaaatggtggcttgtcacactgtgccatacccttttgctgttttttat

tgtcatagtcaagaaagcgagaatagggtgtataaagtattattggagggtgagaatgga

gataaagttgaagctatggttgtttgtcacatggatacatcacaatgggcacctagtcat

gtttcatttcaagttcttggtgttacaccaggaagttcttctgtgtgtcacttttttcca

gctgataattatatttgggttcctaagaggaaatcacaaggttcatctagcatgtgattt

gtaatagtactcgtgtgcttataatttgtgcttatgttgctaaaaaataattgtgttgta

ttcaaagcataagttttttatgagaatatgtaaaagctctactttaataattttgttgtc

attttgaatcatgatgttcttgattttattgtgtgcatttttttcttctacacaacttaa

aatcgtcgtcgcaagaaaaaaaattct

>fava_dn_rep_c1240

caactagccaaaaagttggatattttcaagtaatttttctgcagttctcaagaccgtcat

aatacagaaaacatcaagtgataattccttttcaaggacaacattcattcaatccttagc

ttagatcaatatgaagagtagctaaaattgcccaaaatcaattgatgtcaaacgatcatt

tgatacataactatcgataaattatcaattacagttaagaaatgtacaactagaggtaca

ttcaatcacaacttaggtttcaagtaaccttcagccaaatgcctgactgtaaagcaaaat

gatagaagggatataataagcatccctatgaatatttatgactcatttgatcatgttgag

accacagaattataatgctcaccaagcccaaaagcatgcttatggtaagaaagcttgata

ctggaactagacgaacaaataccatcgaccggtttatactccttccccatctccacatca

gggaaggaccctgaaaatatcacaatatgcttcttcagacagtgagtcaaagcaccgagc

tctagttgtcctccccatattgctgtggattctacttctttgcaataattttcaaacttc

tgagcaattgattcatcagagtcaccttcgattaaattctctgacaggcaaaatggaagg

aaatccgatgtatgcttcctcatgtaagcagctgccagttttcgaagttcttcgtatgta

tatggagatttacccccagagaggtggcccaactgattttcaacagctctgtaaaggcaa

tgcccatcaggctttatttcacacacagtcaaaccgagaggtttcaacttcctttccagt

ttctcattttcaaccatgcgatcacttataatgtcattctgctcggcttggattctcagt

tccctctctgcttcttgttgagctcttttgtcacgcctctgttttgctttgctgaccttt

gtattctcaggttgactactaacagttactccagctatggccttcaccaaattgtctaaa

ttgctcttttcatttccattaccactgctatagcctaatgtagacagttctttggtatgt

ttctctttgagcttagtagaaagttgagaaacctcttcctccacttgctttttcttagtt

tttttgctcagccttgcttcctctagctgcttgctttttcatttcaacttcttttttctg

caattgtgatatctctttcctgtggcgtgcgagaacctcatcacgagtctcttcctcctt

ttcagatgttttgtcagaaatcacttcttgcacctcatcaatttcttgggtatcctccat

tgcaaaaaccctggagagcttcctcgctgtttctggccaccgacaacaaagtcgctgcga

ctccaatttgacggagatgatgagttttgttgatttagggttccattgtcaaaatgtgtt

ttttctgtacaaattcttgatgggcctatt

>fava_dn_rep_c1241

cgttttggactaatcatttttaagggaaaaaacatcacatttcgccgctagtgcagtgtc

tgcaaattagggtttccaaatctccgatcattatcggcgccggcgcaagttcctattaca

atcttcaccatggccactcaaatgagtaagaagagaaagtttgttgccgatggagttttc

tttgctgaattgaacgaggttttgacccgtgagcttgctgaagatggttactctggtgtt

gaggttagggttaccccaatgcgcactgagatcattatcagagctactcgtacccaagct

gttctaggtgagaaagggaggaggattagagaactgacctcagttgttcagaagaggttc

aagtttcctgagaacagtgttgaactttatgctgaaaaggtgaacaatagaggactttgt

gctattgctcaggctgaatctctacgttacaagctccttggtggtcttgctgtgcgcagg

gcttgctatggtgttttgagatttgttatggaaagtggtgccaagggatgcgaggtcatt

gtcagtggaaagttgagagcccaaagagccaagtccatgaaattcaaggatggatacatg

atttcttctgggcaaccagtcaaagattacatcgactctgcagttagacatgtcctcctc

agacagggtgttcttggtatcaaagtaaagattatgcttgattgggatcctaaagggaaa

caaggtcctaaaacccccctccctgatattgtcaccatccacactccaaaggaggaggaa

tacaacccacctcaggaatacagccagcctcctcctgctgttttagcctctaccgatatt

gaggtgcctgttgcttgaagatcttttactttttctgaaccgtttcatggtagcttgtat

tttattttttcattaaggaagtttttatactctgttacaagtgtttaatcatcattttat

ttttattaaaactggttatggttcctgtaatataggactaatatgcttttggttgcatgt

ttttagttacatttctcccaccttttaaaattattctacttgttcctttgttggattggt

ctaaaccaaaacattttgtgtccaagtaattgccaaattgacttatttacttaccttacc

attttgaattaaaaattaaaaaacata

>fava_dn_rep_c1242

aattctgactccacaaattttcgttcggttaccgaacccactagacccaaacttcactgc

atcacaattcacaaccatagtgttccaccattccatcccaatatctcaaatcaaaattct

cattttcctaatcaacggttattctttaaactactactctagtctccgctgcacacaaac

acatacccctaactttctcattctatctttattcctttcagacacacttcacactctctc

tctaacttcatctttctttcccaaagcatttcctcttctgttaccagtcggtgttcgatc

gaagatgggagggtctggatccggatctggtgccgctgattttcttatggtgcttatcag

aaacattgatgttcttgctgggcctgtggttagtcttgtttatcctctatatgcttcggt

tagggcaattgagagtaagtctcccgtggatgatcagcaatggctaacttattgggttct

ctattccatgatcactctttttgaacttacttttgccaaaattcttgaatggattcctat

atggccatatgcaaagctgattctaacctgctggttggtccttccttactttactggtgc

tgcttatgtttatgagcactatgtaagagccttcttagccaatcctcagaccattaacat

ctggtatgttccaaggaaaaaggacgtcttctctaagccagatgacattataactgctgc

agagaagtacatcaaagagaatggaacagaggcatttgagaatctgatccatagggctga

taaatcacaatggggaggtagtcaccatacaaaatcacagtgggatgatagtcaccatac

aatgtatgacgagcattattgaatgcaatcttgtagcttctgcctcttgtggataagggg

gtgcgcgtgtgtacatgtttaacgtgttactgatttcgtgtctgtttttccgaggagata

cttaaggttcttgtccccctttatctgttgtcgtgtaactatcataggagtagtcatcta

tgttatgtttaatggttataaaacggatgctatatagattatgttggtacaacggtggaa

acttcttgattttctctggtttagtgtaataagagtatgggtgtgttgggaataatttaa

ttgaaagagtgaggctaataattaatacaacgtgtagtcaagcttagcttgatataatct

caaaaaaaatgacctctccacaaaaacacaacacgac

>fava_dn_rep_c1243

gatatttttgcacaaaataaaaacgaccaaaactataattcaaagaaccacacacacaaa

cacttgtctctctatctttctctctctaacatggcaatggcaatggcgcttcgtaagctt

tcatcttccgtcaacaaatcttcacgtcctctcttttccgcatcatccgtttactacaag

tcttctttgcctgatgaagctgtttacgacaaagagaatccacgcgttacgtggccaaag

caattgaattctccacttgaggttattgatcctgaaattgctgatataattgagcatgaa

aaagctagacagtggaaggggctggaactgataccatcagaaaatttcacttctttatct

gttatgcaagctgttgggtctatcatgacaaacaaatacagtgaagggtatcctggggca

agatattatggtggaaatgagtatattgatatggcggaaacactatgccagaagcgtgcc

ttggaagcgtttcggttggatcctgctaaatggggagtgaatgtgcagcctctgtctggt

tcgccttcaaattttcaagtttacactgcattgttgaaacctcatgatagaatcatggca

cttgatctccctcatggagggcatctttctcatggataccagaccgacaccaaaaagata

tctgcagtctctatattttttgagacaatgccatataggttggatgaaagcacaggatac

atcgactatgatcagctagagaaatcggctacactcttcaggccaaaattaatagttgct

ggtgctagtgcctatgcacgtctatatgattatgcacgcatacgcaaggtgtgcgataaa

cagaaagctgtgctgttagcagatatggcacatatcagtggattggttgcagctggtgtt

atcccttcaccttttgactatgcagatgtagtgacaaccacaactcacaagtcactccgt

ggcccacgtggtgctatgatatttttcaggaagggattaaaagaagtcaacaaaaaagga

caagaggtgttctatgactttgaggacaaaatcaatcaagctgtcttccccggactgcaa

ggtggtcctcacaaccataccattaccggtttagctgttgcactgaaacaggctacaact

ccagagtatagagcatatcaagagcaggttctcagcaatagctcaaaatttgcacagact

ctgactgagaaaggctatgaacttgtttccggtgggactgagaatcatctagttttggtg

aatctgaagaaaaagggtattgatggatccagagttgagaaagtgttggagttagtacat

attgctgctaataaaaacacagttccaggagatgtgtctgctatggttcctggtggcatc

aggatgggaactcctgctcttacttcaaggggatttgttgaggaggatttcgttaaggta

gcagagtattttgacgcagctgtgaatttggctttgaaggttaaggaagagagcaaagga

acaaaattaaaggacttcgtggaagtattgcagacgtcctcttacgtgcaatcagagatt

tccaagcttaagcatgatgttgaggagtttgcaaaacaatttccaaccattggttttgaa

aaagcaaccatgaagtacaacaaataagtaaaagaaagtttccatcaggacttcaagggg

atcagttttactgattaatggaagaagaggattatatatagcagatgcaaattattaggc

tggaactttcttatgtatggaacttgcaaaacttgatcagataccttcttgcaatgtata

gatatatctatttataatctattaaatgtgaattgcgacctgcgagcttttaagttgttg

gcatgtcaatgttttcaacattcatttgggtcctatttagataagcaactttattaaatg

tttatagcaagcacttatctgtgaaatcttaaatcaacacataaaatgatgatttttctt

ttcgac

>fava_dn_rep_c1244

caaatattttcctttatttaaaaaaataatcgaaaccataagaaaaaaacaacaaataat

catttcacattaattaattatttatttcaaacatcatgtcttgtacttcgtgattaaaac

attgcaaaaaacaagacaatatatatagaaatacaactaaagtaaaaaagaaaaaaaact

ttcatatataattaattaacttataataaattctttaatcacctttgtgtttttttaatc

gaaattagcacaggaaaacatagaaaaaacacacacagacaacaaacacttcaaaagtaa

aatgaattcttcactttgctaatgataccaaaccaaattctcatggctcaaactcatttt

gttcatcttcaaactgattcacaacttggttcttataaccattgtagcttccatggttat

tctggtagtatccattgttgtaaccattgttgttaccactgttgtcagtgttatagctgt

taccattgttgtggtaccaattatttgtgttaactcctctggaatatgaatcaccgtacg

ttgttgggttgtatttctcattgttaatatcatggaaatattttccaccttccaaaaacc

ttgtgtcactcatcccttgtttctcaacattgtaatagttattgttgttgtggaatggtc

tttcattgttgttttgtttttccatcacggagttgtaattgtaaccttctccattgtact

ttgtgtcactcaactcattttgactatttccaaaaggatccttgttgtaatagtttgtgt

tataggcatcgttgttgtagtacttgttgttgttttcgaactcggagttcgttgcggtgg

tggttggaaggtgatcggattcatggccatataaaccatagctgttttcagtttgaggga

tgaaagatggttgttggtcttctggtttgtttgtgagggttgttgattcttctttgttgg

ggatttgtgtttctttgttgaaatgagtgactttgctgaagaattcactgtctctggcta

tggtttgaagaggtagcaagagggttgtgaagagaaggagagaaatgaagtttgatgatg

gagacatagtggaagagggaagatgggtttggtttttttggagtaggtttagtgatgccc

cgcggttcactggcaagagtcatttcttcatggatattctcgccgctagtgggagctgct

gtctcgtttcttgtatacaaatgcatccgaaggtttgtgtatagtgcaagtaacccggga

caagcggcagctgcagcagcaccaattgctgtatttcttggtgtaaccggaatttccttt

tccgcctttcctcttagcaagacttttcctatagctctaagtcaagctttagcctgtgga

actgtcggggctttcctggttgacagaacaatcagaaaacaactcggacatcttctagac

aaggcaaacgctccacaacttgagccaaaagacgatacgtcgcaccaaaacataggcttc

ctatctgatattgcaggtccaaagggcactcagcttgaaatagtttacggagttttcggt

tacatgcaggttttatcagcatgtttcatgtcatttgctcatggtggaaatgatgtttcc

aacgcgataggtccgttagccggtgcactagctattctgcaaggcgctgcaaaaggagct

gagatagttattccaatcgatgttcttgcttggggtggatttggaatagttgcagggcta

atgatgtggggttatagagttatagctacgattggaaagaaaataacagaacttacgccg

acgagaggatttgcagccgaatttgctgctgcgtcggttgttttgtgtgcttcaaagcta

gggctaccaatatcggcaacacatacattagttggtgcagtaatgggagttggattcgca

agaggacttaacagtgtgagatcagaaacagtgaaggagatttgcgcatcatgggcggtt

actattccggttggcgctaccttatcagtgatttacacatggatttttactaagcttgtg

tctaatgttttatgagtgtgcattttcaagatataaagaacaggttatatgtatttggtt

acttaattcaattcactatctttgacattattgttcatttgttgagaaattcggagaaga

ttgctgtgccttgcagcacaagtatctcgggatgaaattttctatatttggtttctatgt

gaagtgaagacatgttgcgtttttgtggctaagttgaaataacttagcaatctttaagct

ttgaagaatgaaaattcagaacaaatggatgtacttaataaattcattttttaacctttt

tgtttttcctactcatattcttctctctaaaatatgtacaaaaaggaaannnnnnnnnnn

nnnnnnngaagatgtttggattttatnnnnnnnnnnnnnnnnnnnnnnnnnnnnnnnnnn

nnatacatgaagaggaattaggaattaaattacatggtc

>fava_dn_rep_c1245

tcgacttctcaatttcggaatcagaagaacaaagagaaaacaaagcagaagcttctatgg

attcctccataacactacagtggtagctttgcttcgaccatttcagagataattaatttt

attcgagtgtttgaattttcacgatttcgctattttcattttccaacactcaaaaaatcc

atctagggtttgcgaatttggaatccatggcacgagctcgcaatttagttgttgctgctg

gtttagtcgcttttgcttcagcaggcatggcttttcctttctacatggcttcctcgaaga

aaccggttattgatccaacaaaaccactttcgccacaagctacttttcgaggaccttata

taaacactggttcgcgcgatgttggtcctgatcatcagacttaccaaaagaagtgaacga

agttgagaatttgtttggccccaattgagattaagatcatgttatattatatgattagtg

taactggtttgagttattgtaaaattagtttcaaaatgtatgtcatttttaataaaaaga

tgaatgttgttagttaattatgacacattttgtagatgaatgttgtattgcttgttcaaa

tattttttttttttgccaaaagagaatatcattatttttataatttaaagttcacaatta

caaccatacatcacacatggggtgggccacattccaacttaaaagtaatgaaaaaaatgg

cccattcacccaacgcactcaccacctactaataataatcattgcaacaacacaacacat

tacacttgcaacaacatcctttacatacaaaaagggtaaataattaaaaccagataagat

caaatttcaaattaaaaacaaaaccacaaagctagcatggtaatggttttggcatcaact

accacaaaccgttggatgtaatcaacttgacttgaaaggaatggctctgatcacaactgt

gtggtaaagtgctgcaagtgctgcaccaatgaatggtccaacccagaaaatccattgatc

attccagccacgatcttggttgaagataattgcagcaccaagactcctagcagggttgat

accagttccagtgattggaatagtggccaaatgaaccaaaaacacagcaaacccaattgg

caaaggtgccaaaataggaacgtgagagtctctggcactacgtttggcatcagtggctga

gaaaacggtgtaaacaagaacaaaggtaccaataatctcagcaccaagtccatctccttt

tgtgtaaccaggagcaacaaaattagcaccaccgccttttgcaccgaagaaggcttttcc

ttcaaaacccttaaccacaccagcaccacagatagcaccgagaacctgcatcacgatgta

gaacactgctcttgttagagacagtttcctcgccaagaacaaaccgaatgtcactgctgg

gtttatgtgacccccagagattccagcggtgcagtaaacaagggcaaagatcatgccacc

gaaagaccaagcgattccttgaataccaacagttgcacacttggactgagatttgttgac

acccatgacagtcaagatggtgatgtagagaaacagaaaagttgcgacgaactcggctat

cccagctctgtagaaagaccatgaagtgagttcagaaggctcgaacaacggtgctggagg

tggttccttgtagtcctttccgtcgtcatggctctgagctgcaataccgatgggttgtct

ctctgggaatttgttggctccaagtgaaacatcttgttcctttgcttccatttctcactc

tcttgatgaatggaggaggaatatgaatgctatgaaaaaatgagtagtggtagtggttga

ggttcccgcggcc

>fava_dn_rep_c1246

atttattagcagccagcactctcggtgtnnnnnnnnnnnnnngtgtcgtggtcgaagaca

gtgccataggcctagcagctgctaaaggggatgtatctatagctcaacattgcttaaact

ctcatatttctttcctatggatgtgttattgttgttactgcctctattggttgctttgcc

aggcttgaatgtagtcgagagccgaaaaacactggctgtggagcactatgttgagtactg

tgctgtgagctgcagagcttacagtgcctcagtgacggagtttggagcggttggcgatgg

acaaacttcgaacacgcgggcctttcagacggctgtagatcatctgagtcagtactcgtc

gaacggcgggtctcaactttatgttccaccaggaagatggttgactggtagtttcaatct

caccagtcatttcactttgttcctacacaaagatgcagtcattcttgcctctcaggacga

aagtgagtggccggtgatcgagcccttaccgtcttatggtcgagggagggacactcaagg

cggacgatatagcagtctaatttttggaaccaacctcactgatatagtcataacagggga

caatggaacattagatggacaaggtgaagtgtggtggcaaaagttccataagggggagct

tagctataccaggccttacctgattgaaatcatgcactcagatgatattcagatatcgaa

tctcactttggttaattctccatcctggaatgttcatcctgtttatagcagcaacattgt

tgttcgaggcatcacaattctcgctcctgtgaattcgccaaatacagatgggatcaaccc

tgactcttgcacgaacacgcggattgaggactgttatatagtgtccggggatgactgtgt

ggctgtgaaaagtggttgggatgagtatggtatagcttatggaatgccaactaagcagct

ggtggttaaaagactcacttgcatttctccaaccagtgctgtgattgctttagggagcga

aatgtcgggtggtattgaagacgtacgggctgaggacattctagcgatcgattccgaatc

aggagttaggatcaaaactgctgttggaagaggaggatatgtgagagacatatatgttag

aaggatgaccatgaaaacaatgaaatgggcattttggatgacaggagattatggctctca

tgctgataacaactacgatcctaacgcgattcctgtggttcagaatattaattaccacga

tatggttgctgaaaacgtgactatggctgcaaagttggagggtatatctaatgcaccgtt

taccggaatctgcatatccaatgtaacaattggactagcaaagaaggcgaaaaaactgcc

ttggaattgcacgaatatagccgggatttcaagtggtgtcactcctgtgccatgtggcct

attgccagaccaaggagtagaggatattgcggcatgcaccttcccagaaggaggtttgcc

tattgaagatgatgtcgaggttcaaacttgtacttatagaataaatttgtgatgatagta

tagaagcaacggctcgatttcaagggaacaatccaaacaatgtaatgcttccaaaaactg

tttgtttgagacttattacagattttctgttagtccttttctataatgcaaaacaaatta

cttattcaaactcaaaaaaaaaaagcaccaaatttagtaacaaaatcaggaaaatcacca

gaaaatttattaccacccaaatcaagaaactgcaaaaccttacaagatgaatcaggtaat

gcaggttctggaacagacccagataaggaatttccatgaaccttaagagaaacaagctta

tcacacaagttccatacagaaggtggaaggtcaccaaccaaaagattgtcactcaaatca

atatcagtaagtgaagagccataaccaagctcaagaggaatggttccactcaatgaattg

atgttaaggtaaagactttgcagcataggaaactgcccgagctctctggggagagaacca

gagagatttgcagaggggagttgaagagacagcaagtgcaaaaaagggtcttttgagaag

gaaagattgttccattgaggtgaagagaggtcagtacatgagagagatgaaccgttggtg

aagacccatttgaggcctctccattgacagagaggagttgaggtgttccatgaggagagg

acgaggttttcattggtgccttggagtgaaggtttgattttgaataagatgagttgaaca

tcagaggaagtggttggaggaatggattttgttgaggctttgttgaagaaacagaagatg

aaaaggaagggaatgtagagagagagaagggttttgagaaatgccatgagagagagagag

agagagagggagagtgaagtggtgttt

>fava_dn_rep_c1247

tttcgcatcagaagagttgattggatttaggtcaaatgatatatttagtcattttcaaac

atatgcacttgattgacttcaaatattagtccagcaacaaagcaagaatacaagggaatt

taatcgaaaaacatagcagtaggaagtgaaaacaaagatgatataatagtgattactcga

cacataacacaagcataggaaatgattatctgataagagaaaagaatgatatgaacgaaa

agcaataagcaatcctgagttgtgatagtgaaacatagaaacaaaatagtagtatatgta

taaaaatcaagatgtacagcagggcttgtttgtagtggcggtttcagatcctccaatggc

gatggtcttgccttctttaatgttggcagctgcagcagaatcattggaagacgagagtga

cttcttgctgattatacggtatatctccccaagaatagtctggaaagccttctcgacatt

ggtggcttcaagcgcagaagtctcgataaaagaaagaccttccctctcagcataattctg

agcatcttccgtgttaacagcccgaagatgcctgagatcggttttattgccaatcaacat

gatcacaatgttagcatcagcatgatcccgaagctccttgagccatctggtaacattgtc

aaatgtagtaggtttggtgacgtcatagacgagaagagctccgagtgcaccgcgatagta

ggcactcgtaatcgccctgtaacgttcctgtccggcagtatcccatatctgagatttcac

agttcttccctcaacctcaagtgtgcgtgtggcgaattccacgccgatggtggatttgga

ttccaggcaaaattcattgcgagtgaatcgggaaagaatattggatttaccgacaccgga

atcgccgatgagtaccactttgaacagataatcgtattcctcatctcctctccgactcat

cctcgccttttttcaactattccgcgtctttctttttcttttcttctaattaaacgggca

cacacatcacacaactaattaacaagctccgtctaatttaatccaatcttcaactcgccc

actcaatccacgctatttagtctaaacgactcaaccattaaattcaacaaacatttttac

caaaaaaa

>fava_dn_rep_c1248

ggccgcgagggatctcaccttatccatcttctatcccataaaataaaaaaacctaaacag

agaacgtatctgttgaagaaacaaaccatagagatattagcatggccatggcgtctgtgg

ctcagcaactcacgggtgtaaaatgcacccctcttctatggaaaccaactctagcccaaa

agcaaagacgcagtggtgttcttcctattgtatgttccattgcaatttcaagtgcaaata

acagagaaagaataaagttgaaacaactctttgatgatgcttatgagaggtgccgcactg

ctccaatggagggtgtttccttttcttttgatcagtttactgaagctctcgagaaatatg

actatgagtctgaaattgggactaaggttactggcagtgtgtttggcactgatactaatg

gagcttatgttgacattgcttcaaaatccacagcttacttgccggtgcgagaggcatgca

tccgtaaaattaagcatgtagaagatgtgggcatgtttaatggcgtgagagaggaatttg

tagtcattggtaaaaatgaaattgatgatggcatgatcttgagtttaagatctatccagt

atgaccttgcttgggaacgttgtagacagcttcaagcggaagatgctgttgtgaagggta

agattattgatgcaaataaaggtggtgttgtggctgaagttgaaggccttaaaggatttg

ttccgttctcgcagatgtcaacgaaaataccgggagaagagattattgagatggacattc

ctctgaagtttgtcgaggtggatgaggaacagtccagacttgtccttagccaccgcaaag

ctgtagccggcattcagggacaattaggaattggatcagtagttactggcactgtgcaaa

gtctaaagccatatggtgctttcgttgacattggtggaatcagtggcctccttcatgtca

gtcaaatcagtcatgatcgtatatcggatattgcaactgttcttcaatctggtgatattt

tgaaggtgatgatcttaagtcatgatcgagagagaggccgagtaagtctttcaacaaaga

agttggaacctacacctggagacatgattcgtaatccaacgcttgtgtttgagaaggcgg

aggagatggctcagacattcagacagagaatagcacaagcagaagccatggctcgtgctg

atatgcttaggttccagccagagagtggattaactctaagcagcgacggaatcttaggac

cacttggttcagaattgcctgcagagggactggatctaagtgaaatacccccagctgaag

attaatgattgagggatttcaaagttcttaaccttttttgcgtttgttacacaatttgat

ctatgtattgttgtagaatgttaaacctaatgttatttttttcttctgttttgatgtatt

aatctttcctcgtaattgtaaaattgaaaaatatatatataatctattactatgtatcta

tttgaatcgaattcttgtctcgacaataatagcttcttgatttgacatgttgatgtcaaa

gtcttacatgttgtagttgaagtacgttcaagcatgcatcaatcgaaatatgttatgtta

ct

>fava_dn_rep_c1249

agaaaaagtagtttttatttcattactcttatacataaccagattcagagccgcgggaga

gaaccaaaacacacttgtaagaaaaacaatgaggtctctaatggcttctgcttctctcat

tcttgcagtagctatactctttctaggtttcccatctgagatttcagcaaaccactattc

ttattcctcaccgccaccacctgtctactcaccaccacctccaaaggatccatatcatta

ctcatctcctccacctccaccagttcacacttaccctcaccctcacccagtctaccactc

tccaccaccaccaattcacacctaccctcacccacacccagtttaccactctccaccacc

accagttcacacctacccacctcatgttccccatccagtttaccattctcctcctcctcc

agtctcttccccacctccaccagcctattattacaaatcacctcctccaccttaccacca

ctagatagcttattgttgccatcacactatatggatctaagcagaaagtgtgaaagcaaa

gaaagccatgcacaaagaagattgaggggattcttcacatatacatttcgtcagtgtttg

attaatattatttagagggttgcttctatttatttattttgagtttttgttgtataagcc

acagctgtagggtactgcctcaatatgcatgtgtctgccgttgtttgcatgaattgtaat

tttcaaagtttggttttgtctcaagctgactgatcagccaaataaatatatcccctatta

tagtaaaaaaaaaaag

>fava_dn_rep_c1250

actccataagtactccaccctccctctcccactctccaatcaactctcacctctcaatta

atctctagagtaatctgtttcttactcaaatctctctcattcttgaagaaaaccaaaaac

ctagaaacaaccatgttggccaaaatcctctacattgccttcatcatctacacaatgtcg

gttattgaagccaaaatccccggaatttactctggtggtcaatggcaaactgctcatgca

actttctatggcggatctgatgcttccggcaccatgggtggagcatgtggttatggaaac

ttgtacagtcaaggttacggagtgaacacggcggcattgagtactgctttgttcaacagt

ggtctgagctgtggtgcttgttttgagataaagtgtgataacgacagagaatggtgccat

tcgggaagtccttccattctgataactgcaactaacttttgtcctcctaattttgctctt

cctaatgataatggcggttggtgtaaccctcctcgtcctcactttgatctcgccatgcct

atgtttcttaagattgctgagtatcgcgctgggatcgtccctgttgcttatcgcagggtg

ccatgtagaaagcgcgggggaataagattcacggtgaatggattccgttactttaacctg

gtgctgattagcaacgtggcgggtgctggtgatatcgtgcgcgcgtacgtgaaaggaacc

cgaaccgggtggatgccactgagccgaaactggggtcagaactggcaatctaacgcggtt

ttggtgggccaggcattgtctttccgtgtctcgagcagtgaccgtagatcttcaacttct

tggaatattgcacctcctaattggcaatttggtcaaactttcactggaaagaattttaga

gtctaatgatattgagaaaactgagtttttttttttttactatttctagttggtgttttt

ttttactgtatgcatggtgagaatgagatggagatggagatggagatggagagagaatga

caatttaattgtgactaggtgttgatttgtctttcttgttattggtagggtttgatatga

taatcatattaatataatgaaggtgcaagagtaagactgtactttgaatttgtatgctat

tgctagagaggttaactggaagtgaaaagggaaagggaaagcgaaagctaaaagggtgaa

aggaagtgtaggaaaggctgatgtggttaataaaaatagtaacccgcagctaatttgtac

ttatgatgatgctgtaatatactactaatatattaattaattaccatttgcttataactt

aaaaaaaaaaagattttctttgttgaaaaaacacaaatggcacacaataacactaactat

ccttagcctcctcagcagctgtctcaaaagtctctcctgggacaagctctggaacatcat

catcattctcttcttgcgctgtggctgcgccagcacctgcttcaggaacctgcttattga

actgctcagctatcttcttcaggttttccaaattatcaggccctaattggtggataatgc

taggaagaatgtcttgcaacttcttggtttgaggagcaccactaacaacccatgtattag

cagcaatggatgcttgaactttggggtttgagaactggatgacaacgtcatccttaaaga

tattgacctcctcaatttgagggatggcattcaccccaattctcttcagggtgctctgga

gccttttatcatctgtagttgttgtcttgtggacagccttcttctttcttctgacagtac

cctttccaccagttcgaactgaaccggccatcttcatcaacttttcacgattcatctttg

tgaaatcagtgattttgaacacagggtaagcctcagcgtgaagaagaacgaaacaacgca

acgagataaggatccttcttataccacct

>fava_dn_rep_c1251

tgaaaacaaagaaattatatctttattgatggattgcaaccataaactgggtctaacagc

cattcaaagtatgttatcacattatatatgattttattaaaatactcttgcaataatctt

cagagacaaaatcagtaaaacaaaataacttcagagacaaaattagtaaaacaaacactt

gaaccctatagtttggctaaaaccaaactacatatatgggcaagcggtaatttttattcc

atttattatttgcatagacatgattaaaaacagagcctagtggtctttgtttcatcagat

gcatggtgacactaacatagttagttacattttttataacagaagttagtgatatcagtg

caacgacactgtggtggaaaagaccttgtgcaaatgcaacttttgcaagctgagtgacat

gtttctccaacatcagtacaacgacactgaggagggattgattttgtgcaagggcagaaa

ttacagcatgctgtagttgttgatttgatagtgtaacctgcatcaccattggagagcagt

tgagtgatgaaggaagttgaatcaaaacgagcatcagcaacagttgcggtgaagcccaag

aggaaaagcaaaaaagcaaacttcataattgtcttcttgttgataaactccattgttact

ctctttctctcacttgctctttttctattttgatttgtgcccctcaatagactaagttaa

ttattgtgctctcagttatatacaagggtagtttttgtatattgtgctcatcaaaatgta

gtggagctattgttctcacttatatacaagagtcgtgtaactaccttgaagttagttata

aaactacagctatnnnnnnnnnnnnttaattttcataacctcagtatacattttactgtt

ggaaatccacctatacactatgatgaatttctaccaccacttgatgataagttatgaaac

aaacaaataaaagaacaatggagatatttctg

>fava_dn_rep_c1252

cgcgggtatccatccactcgtatccatttccaccatttcttctctctctaccccctttct

tcactatagaaactactagcaaagctgtcactttttgccattgaacaagtatattgtaat

acattatatacaaataactttgttgtcagtgtcttaaattcttcatcctttctagcctcc

attcctccaacttcatgcatggttttttatttcaaaaaggaggaagcttttcctttgact

ttcactttatctggtttctgatttgattttctgatacccttttgcttcacaggagttcaa

aaaaatgggagaaagtaccttgttctctttgtagaatctggtagagaaagttaaatactt

ttgagttagtcttttagaatgccagttccacttgccccctacccaactccaccaccagct

ccatatacagcaccaacaacaaatggttcacagagtcaacttgtgtgttctggttgtaga

aaccttctggtctatccagttggagctacttctgtatgctgtgctgtttgcaatgctgtt

acagcagtgccacctcctggcacagaaatggctcagttagtttgtggaggatgccatact

ttactcatgtacatccgtggagcgacgagcgttcaatgctcttgttgtcacacagtcaat

ctagctttggaagcaaatcaggtggcacatgtcaattgtggtaactgcaggatgcttctt

atgtaccaatatggagcaagatccgtgaaatgtgcagtttgcaattttgttacctcagtt

ggggcttcagcaagcaccactgagcagaagttcagcacctaagaaaatcacatactatat

aatagcaaggtcattacagactctacaaccttggtttttgctacttactacatatcaatc

ttaaggaataataacaaagagtgcaaatctgattgaaaactttcttcacatgtatagagt

tatttttctcttgcgtgacgacggtgattgtattgaatatttttgatagaatgtatgaat

aagtgaataactatctgttctaagattcttgattttgagtgctataaaaattccatctct

ttggataaggtaaaacttattctcaacttttgttaaattgtagaacaagtggttgagaaa

gttactatgtgtgtatgtggagcaccagacaccatttatttctttcacgtacttttttgc

ccttttggaaatagaaaaggtccaagtatagcaaaagggtaaagcagtcaaatgagttga

ttcaacaagataacatctaggtccaagtcctagatatcattagatttttcagaatgaaat

tgagatgagaattcgtttccaaccagggaagcctttagttattctatggtttccttttgt

tgattttgacatgcatgactaaaaccatattaatcagaaattggatatacattgaag

>fava_dn_rep_c1253

gggactcgctctctcttttcagtttcttcatcttttcaaaacacaagaaacaaccatggc

tttccgcaccaaaccttcttctcccttcgactgcatcatcttcgatttggatgacacctt

gtacccatccagcaccggaatcggagcttccgtcaagaaaaacatcgatctcttcctaat

tgaaaaatgcggtttctcacaaaccaaagcctccacactccgagtcgaactttttaaatc

ccacggtagcaccctcgccggtttacgagctctaggctatgatatcaccgcagaagatta

ccacagtttcgtgcacggaagactaccttacgaattaatcaaaccagaccttcagttacg

aaacctcttgcgaaccatcaagcaaaggaaaattatttttacgaattcggacagaattca

tgcgttgaaggcgttggatcggcttggaataacggactgtttcgagcagataatatgctt

tgagactttaaacccgaatttaccgaactcgacccgacccgatgagtttcctgtcctctt

gaaaccttcgctggatgctttcaaaatcgcgattcagactgctaacgtggatcctcatcg

taccttgtttctggatgacagcgtaagaaacatcgttgcggctaaagaaatgggtctaca

tactgttttggttggagaaacagtgaaaagcaaagttggggattatgtagtggaatgtgt

gaataaagttgcacttgcggaagtgattcctggaatatgggggaacagagtggaagatgg

tgatagaaagatttcacgcactaacagcgaattggatgctttgctaagcgcatatactgc

tgttggagcttgaaaaactgaccttgtttgggagagtgggttaaggcatgatgttgtaca

taccagacaactagtcattcacccaacctaggcacaaagactgtgaacaaatattggaac

cttgtgtatgtatgttgttaatgatcttgtgaatgaataaataattgtatgatagatgtc

aaatctagtgttaatgtcaaatgaataaataattgttggtagtttttcctatatgaaaaa

ttagttgtgcgaatgagtattgagtattatttaatttgaaaaaataaatgattaatttta

ttaaaatctnnaaataattt

>fava_dn_rep_c1254

tgaattattgatattgaggtgtgaaggaatgtggaattatgcgcatagtggaccccacat

ttcacaccatccacatgctaatgaagttcatatttcaaaaaagatagccctcactaacca

ataaagtagagttggaacaagtgtaactcactccaattcatcaaaaccataataaaatat

tcctaaacccaaaaggcccttttacctatatacatactttacattttccatatgcaccta

acatcataaaaaactcatctaatacttagaatcctcacatcaaaccataaccaaacatca

ttattcccatcaaaaccatacatgaatcctcaaagtaaactacaactaaggatcctttat

tggatcttattacttgtttgtgttactaacactcttccaatcaaagctttggaatcaagg

aagctagttgagaagtgcacaccatgtggtgacaatccaatatcatcaccacctccaata

atatacaattcaccaccaccaccaattgtgtattatccatcaccaccacctccaatagta

tactactctccaccaccaccttctccgaagaaatcgccctcctcgaattgtcctccacca

ccatcttcgccatacatagtactcaccggaccgccggggaacttgtatccagttgatgtg

aatttcagtggatcaggagcaactccagtcggccgtggaagctttgccggattcttgcca

ctgctggtttgtttgttgagctttctagccttttggtgattagtttgagattgaaatact

caacaaaatgagtagaagatattacatatatatgtaaggtaagaggggagagaaattata

ttagtgcttaatatgttccatatgtgatcccaatttgctgatcatatatatatttatttc

catgtctactttttgtttctctgtagtttcgttgattcaataaattttctgtatcataga

taaagtaataaacctatgaagtcttttcttaagcttgaatgcaacttttaatttagatta

tgcagcagaaattctaactaaatttctatagtcagattaataactaagagatggtaataa

taagatattttttcgatttaaaagttttgggttcgaattcagttatattaaattctctta

aagaaaaacattgtaattcattctacagttgcacagacatatccggctggaaaaatctgt

ttagagttgaaaattattagtcaattataaaannnnnnnnnnnnngaatgaatt

>fava_dn_rep_c1255

aaatctccagccaaacaacaaacaaactaaaatcagttcttacacctccgatggtgattt

tctcgagaaaattctaacaacacaattttctctctttatccaatcattagatccgatcca

attcctctccactcttctttcagatcaagcatcgcattacagatttcaagaataatttca

ttttgattttcgatttgcgttttgaaatcgagcgaatcttggattcacatcgacatggct

atggtggatcaacctctatatccgattgctgttctaatcgacgagttgaagaacgaggat

attcaactcaggctgaactcgatccgtaggctttcaacgattgcgcgtgctttgggtgag

gagaggacaaggaaagagctgattcctttcctcagcgagaataatgatgatgatgatgag

gtgcttcttgcaacggctgaggaattgggggtttttgttccgtatgttggtggtgtggag

cacgctagcgttttgcttccacctttggaaactctttgtactgttgaggagacctgtgtt

agggataagtcggttgagtcgctttgtagaattggtgctcagatgagggagcaagatttg

gttgatcactttattcctttagttaagaggctggcttctggtgagtggttcactgcccgg

gtttcttcatgtggcttgtttcatattgcctaccctagtgcaccagatgcgttgaagact

gaacttagaaccatatatggtcagttgtgtcaagatgatatgcctatggttaggagatct

gctgctaccaatctgggaaaatttgctgctactgttgaagcttctcacttgaaaaccgac

atcatgtctgtgttcgatgatctcacacaggatgatcaagattctgttcggcttcttgct

gttgagggttgtgcagctcttggaaaattgttggagcctcaagattgtgtggcacatatt

cttcctgttatagtcaatttttctcaggataagtcatggcgcgttcgttacatggttgcc

aatcaactctatgagctttgtgaagctgtcggtcctgattccaccaagacggaattggtg

cctgcgtatgttcggctgctacgcgataatgaggcggaagtacgtattgccgctgctggg

aaagtgaccaagttctcccgcatattgagtcccgaactagccattcagcatattctacca

tgtgtaaaggagttatcgacagattcatctcaacatgttcgctctgcattggcttcggtt

ataatgggaatggcaccggtcttggggaaggatgctacaattgagcaacttctccctatt

ttcctctctcttttgaaagacgagtttcctgatgtcaggctaaatattatcagcaagctt

gatcaagtgaaccaggttattggtattgatctcttatctcagtcgcttttaccagctatt

gttgagcttgctgaggatcggcactggagagttagacttgcaatcatagaatatatacca

ttgttagcaagtcagttgggtgtaggcttttttgatgataagcttggagctctttgcatg

caatggctaaaagacaaggtatactcaatccgtgatgcagctgccaataatgtcaaacgc

ttagcagaggaatttggaccagagtgggcaatgcagcacattattccccaagttttggac

atgattaatgatccacattacctgtatcgaatgacaattctacatgcaatttctctatta

gcccccgttctgggctcagaaattacttccacaaacctgcttcctctagtcgttaatgca

tcaaaagatagggtacccaatatcaaattcaatgttgctaaagtattgcagtctctcatt

ccaattgttgatgaatctgttgtggagagtaccatccgtccatgtcttaatgagcttagc

gaggatccagatgttgatgtgaggtttttcgcttctcaagcgctacaatctagtgatcaa

gtgaagatgtccagctagatcaatctacataccgtctttagttattttttacttgtaata

ttcaatttgctcaatcctttgtattaagtcattgttaagatcctgctttgctttctttct

atcggatacctatgctaccaggttttctgtagttataaaaacattgtccggaagtgggat

gatttttgacgtacaatcactaattttatgattaaccattgtggttattttgttaaaaaa

aaaa

>fava_dn_rep_c1256

gtcgaatattgccaacaaattggagaagggaccatattcccaatcacatttcaaatcatg

caaaagaaaaagacattgacattttctttatccaaagcttaatcaaaaatcaacaaattc

atctctctgtcaataatagctctctcggttatctctaaagagctatcatcggagcttgcc

gaagaagagaaatccaccatgctgagaatcgccccatggcggaggaagcggcggcggggg

gctatcttgctccgatttcactttttttttttaaaacccagagaagctagtaattctcat

aaatcatagtcaaatgatagagacaataaaattcagagcttacacttttcccagtcatgc

ttaacaaagataaacactaatgataaaagagaaatagaaatccaaaaaatgcaacaccta

cagcacacatagaagttattgtaacaaagttttctctgccccaaaattcaacacatgcct

caaattccataaagtgacaagaggtactgcatgattttcaaaaaagtcttggcaaaaatc

ttctacgatagaaagaaaatacaggtttcagaatctggttctttataattaataaaagtt

acacgtttaaaagatgcatttgaatagggcctggtacttcagaagaatcagcagagtcca

aaactaaatgtttgatgcatctatgaaggtatctagtgacccatgtaaggagcgccaccc

gggccaggatgaggtctagcaccaccactctgccccaactgaggattctggtacagttgt

tgcaaccccggctggtttccatatcccgtaggagcctgattaccatacgcagcaggaggt

ggcccggcttggttcacagaagcaccaccaagacctccaagcaagtttccaagccccaat

ccagctccttgatttgcaagcaaagctgacaacgcctgaccaagtgctggattcaatcct

tgagctgggactccaggattataacccccaacaggccccggaggcgccatcaagtgacca

ccacctccaccatgggatggcccaccaccagaggaatacttgttcttctctttcctcgaa

ttgtaatgaggctggtgatggtgatggtgagaatgatgctgttgatgatggtgttgaggc

tgctgataatgacccttgtatcccttaggaccatcaacagccttctgacaatacaaagta

tgaccctcaaaagtcttattaggctcttccaaagccttcttagaactctcaacagacttg

taaacaaacagagcaaacccctttggccttccagtattcttatcaagacccaaaggacca

tcttcaacttccccaaactgtctaaaaaactcaagcaatttctgaggatcaatatcagaa

ctcacattgcttacaaaaatcttcctttgagtatactccgagactggcggaccattgggc

ggaggtgccggaacaggtccagcagaagccaactgacaagacgttgttctgttaccaatc

tgcttctgaggctgcttcagcgcccgtcgacaaccactcctatgcttaaacaaaataaaa

gcataccccttcgactttccagaagccttatcagtaaccgccttacagtcttcaatttca

ccgtatttctgaaacacgctggttagggtttcggcagttgcatcccatccaagtccatga

acaaaaattttcctgtgagcagggtccacatcagccagttgacgaacattctctgacaga

tctggatacttctcaatagcttgcttcacgagaacatgtaattgctccttcgtgaaaggc

tctagaagcttctcaacaggttcgtcttccaaatcgagatcttcctcttctccatcgttg

ttagtttccgtttcctcagcaccgttagcttcgaccgtctcagtcgaaacagtcgaagcc

gcggttggatttgaaacttccgcagcgtcttgttgttggttttggttttctagggtttcg

tcttctccttgttgctcttcgtcttcttcttcttgttcttctgctacttgctcttgttct

tcttcttccggatcctcttcaccttcttgttcttcgttttgaggttgatcttcttgtacc

ggagtgatttcttcatcgacggccatgttgtcatggcttggttcttgatttgttgctggt

tcgggttgtgcttcggctacttctggttttggttcggtttcgaggggtttggtgggttcg

gggtcggaagatcgaagctttcgttttttggccatggcggtgatggaatcggaattaggg

ttagggtttgggaagagagtgagaatgaggaaaattagtggctgatgtgatgagagaaga

aagtgattgtaaggagaaatgctgtgaagagctattttactaacacctgtcgggttatca

atgtatgtcgttgggctgggcgcctgggccagcgcataattgggc

>fava_dn_rep_c1257

cacggatcaactcgaattccttttcgagattnnnnntccaaaacttctctgatttgaacc

gaatcgcctttggatgataaatccgacatttcaagagaattgataaccgatcgaatcaaa

tctgaagaggagnnnnnnnnnnnnntttgagctacgaatcgagagcttcaggtagaggat

ctgaaacgaaaacaagcgtaaattgaatcgatttagaagaactagggtttgaggtttgga

ctagacttaaaagtttgaacagagtaaccatcctcaacattgtcaataaacctattcatt

tgttaactcagtagtaagcactacaatactacaatggccgcttcgacaatgtctctctct

tcttcatcactggttggaaaggcaatcaagctttccccgtccactcaagaccttggtgtg

ggaagagtcacaatgaggaaaacagccactaagaaagtttcttctggaagtccatggtat

ggaccagatcgtgtcaaatacttgggtccattctccggtgaagctccatcctacctcact

ggtgagtttcccggagactacggttgggacactgctggactttctgctgatccagagacc

ttttccaaaaatcgtgaactagaggtcatccacagcaggtgggctatgttaggagcctta

ggatgtgtctttcccgagctcttggctcgcaacggagtcaaattcggcgaggctgtgtgg

ttcaaggcaggatcacaaatcttcagtgagggaggacttgactacttgggtaacccaagc

cttgtccatgcacagagcattcttgcaatatgggcaacacaagttatcttaatgggagct

gttgaaggttaccgcattgctgggggacctcttggcgaggtggttgacccattgtatcca

ggtggcagctttgatccattaggccttgcagaagatccagaagcattcgcagagttgaag

gtgaaagaactgaagaatggtaggttggccatgttctcaatgtttggattctttgttcaa

gctattgtaacaggaaaaggacctatagagaatcttgctgaccaccttgcagacccagtt

aacaacaacgcttgggcctatgccacaaactttgctcctggaaagtgagaaaatacaatg

ataagtgactaacttgattatcacaatgtgagtttctgattctaatattctgtgtaataa

tatatggagcagcattaagagactgaaataaatttgaatgttatcatatctagtgaaacg

acaactcactgtgtaactgattcttacaaaatatcatatcatgtcacttgtggtaattgc

atttgttcattgcatatttgtctgataggtataacatgcatagaagtgtgaaggtatgaa

tctacatgttccatgtttcatcacaagcggaagtgcaaaaacgaatgcctaaaaatatga

atccacaatctaatatttatgtaattatcttgtacaattgtactaacttgtatatgtcat

tagtggaacagaaacatgtctgtgagaatgagaagccaatttttcttttggtgagccacc

aaattaatccggtagaacactcttaagttgatttatacccccaaaactacgggttgagtt

cgattgactttgagacttggtaactttggtaacttacctccatctctcgttaaatcaatt

tattttattcaccc

>fava_dn_rep_c1258

aagtaaaatnnngataaaattggaatattttaaaaaatgtaagggtatggaaataatgac

agtatggggaaaattttccctaccaaacctatggacaaaacccagtccagcccatttaat

aatttggatttaggggctagaagggcaaagcccagaacccacccgtaacaaatataataa

ggatcatatttttttataataacagaaataatataatattaataacatcaatatcaataa

attgttctggaatctaacaaaatcaacaagaattataatataatgctaacaaatatgatg

acaatgataaaattaaatctgcttgaaaactactctcctcctcatcaactataagttgtt

tcacttggtagcagcaacctcagaagctttggttgaagttgtctcttcagagacggtgtt

attaatctcatcccaagcttcaacccaagtgaccatggcgtcagcaagtttgacgaaata

gggatcaatagttccaagtttctccttgacttgcttggagagttcaatgtagcatttctg

aacagaggtgcaatcttttgaaagggttgcagcttggaaaaatggaatgatctcttcttg

ccagaagatacctttgtactcctttttcagattaacaaatgggttgcttgctttgctgtg

ccatatgtatggcagtccagttttcactccatatcccaagtggtcacagataacctttat

gcaccagccagcccacatatcgtcataacgtccaataggctgaccatcgcccataagtcc

aaagtacattgcaggtccaatcagttcccggttgaatgccagattcataccgcacatggg

aaacagagttcctttgggaatggtcataacagcatcaacaaacctagtgttcctctcatg

gggcttgacaagctgtgttggagcatcataatcaggtatgttgagccaaaggccgtgaga

aacggcagtggggacaccttcacgaagactgaaagggtatccacggacgaaatcagtacc

ttctctgtatggatcataaagggtgttgaagaaaaatggagtggatggactaaggagatt

cttaatgtgctgctcaagtgcattgatttcatggccagttgggtctttggcaacaaagca

atcatcgtcaatggtgtagatatacttcttcttagaaaccatatacccaaagcaacgaca

agcagaatccttgaaggagatgcaactcgccttaggacccaagatcctattaatatcatt

ccgattatacagctcataatcaaaaccttcaggaaccttgataaccttagacgggtcccc

atcttgaacaatgatgagatgatactgttcaaaaaagggtctccacatctccagaaaatc

aaggttccggatcgtggggatgacgatgtcgagttcgtctttcaacagaggggttggctt

ggaggatgaagccattgtagggaagagattgaatgagatgcggaaggtgtttgtgtttat

gtctgcttcggctctgtgactggtaacgttactatatatatactcgaagattaagcgtga

tttgagaaattaatggtaatggaaatggaaaaatgaaaatctcgtgggttttgttttggc

ccgtttgaacttaaggagcgggttttgtttggtgtactacact

>fava_dn_rep_c1259

ccaagctagttgtgtctaaaacagcaagagtaaaaacagaacactatcactctaatctac

tctcccaaccatatcactattcaaccacaccaaactattctccttctctccctacaactt

caattcaaatcccattcattcttccctcgcacagggctatattcggaattccaaatcttt

ccatggatccagcgccaataatcaaccatcttcccaacacgctgtcgtttcacccaccgg

agatctggcagtttccaccgtccacaacaccgttgttcgagcaaggcttcggagctttcg

caacggaacttcccggtagagaactggtcggttctgagctcagacttccgaaccgtggcc

ggaaaagatgcgacgcggaggaggattctgttaaaggcgtttccaccagcaacgacgtga

atgatgatgatggtggtgatgggaaaaggattaagacatcagggaagaataaagatgaag

aagttgtaggagaagaaaacagttcaggaaagcatgcggaaagaactacggatgagaggc

atcctaagcaagattatattcatgttcgagctagaaggggtcaagctaccgatagccaca

gtcttgctgaaagagctagaagagaaaagattagtgaaaggatgaaaactcttcaggatt

tagtccctggttgcaacaaggttattgggaaagcattggtccttgatgagataatcaatt

atatccaatcacttcatcaccaagtagagtttttgtcgatgaagctggaagcagtaaatt

caagaatgacacctggtatggaagtgtttcctcctaaagtttttgatcagcaaacatatg

atacaacccctataccatttgcttcacaagctacaagagagtacagccggggttcatcgc

cagagtggttacatatgcaggtgggaggtggttttgaaagatcaacgtaatcttaaaatt

ccatcagttggtatgcaaatagcaattttagtctctacttcagagctcccacaaatcata

cactcaaggtattaaagtttacatgaattcttgattccgttttctaaaccacattgtcgc

ttcggttagtatttatgtaagctactccatttgtaagtgtgttggatatgaattcattta

cataagatgtagcttagtggactataggctttatatgctggcattcttcatactttttaa

attatcattggtgtcaaagaatgatgcttgttctaaatacatcttattggttatatattg

aatattggtctcaacattctttttgtagactcgacatggtgttttgtttatgaagctatg

ttgaataaattgaaacccatgtaattttgttggtttggtttagtctattcagccatataa

agatcttgaactagcagagtttgcaaataatatagattattctattgcttgtttggtata

aagggacatgtatgtttaccttttctcgaacaaaatttnnnnnnnnnnnnnngcatttgc

ctgtgctgatctccgcaaggattgtcctgtttggagcctcaaggacacagaagttttttt

ttgtcttttattgtttttttataaaaaaatatatttgtattgtttgagaatagaga

>fava_dn_rep_c1260

aagtgatgagtttggttaaaccccctgttgtgttgttgtagaatctgcttcttatttcat

tcaccatttccgattcgacatggcggctttcttaaggaaggtagcaagggccactccgat

ctcattcggcgcccattccaaatcctcccgtactaatttccacctaccattcaccgctat

tgccgccgtttccggcggattctcctacctttactactcttcctctcccaatctggttca

ctcagatcaaattggagatgaggaaattaagactaaaaatatcgcactgaatcctgataa

gtgggttgaatttaagctgcaagacactgccagggtcagtcaaaatactcacttatacag

gttttcgttcgatcccactaagaagttgggtttggatgttgcttcttgcattcttacgag

ggctccgttgggacaggatgctgaagaaaaaccaaaatatgtcgttcgtccgtatactcc

tatatcagatccagaatctacaggatattttgatttgttaatcaaggtgtatcctgaagg

aaaaatgagccagcattttgcaagcttaaaaccaggtgatgtcgttgaagtgaaagggcc

cattgaaaagtttagatatacccctaatatgaagaagaatataggcatgattgccggtgg

ctcaggaattactcccatgcttcaagtgatcgaggctatattgaagaattcggatgacaa

aactcagatatcgttgctttatgctaatgtctccccagatgacatactgcttaatcagaa

gcttgacattctagcagcaacccacccaaatttaaaggtattctacacagtagataatcc

aacaaaaaattggaaaggaggtgtgggttacatatcgaaggatatggctgtgaaaggctt

acctagtcctagtgacgatactcttatacttgtgtgtggtcctcctggaatgatgaaaca

tatatccggtgaaaaggcgaaggactggacacaaggagagctatctggcatactcaaaga

ggctgggtacaccgaacaaatggtttacaaattttgagaaattttggttccatgcttgca

tctaattgattgctacaaaaattgtggttcaaatttagagagattttgtttctagcatgt

taagcgtgcatgtctgccgcaaaatttgcctcctaatgttatttctgaaagacgcagatg

aaaattcttacatgaaataagtggatagagaatcctaatgttatttctgaaagatgtaga

tgaaaattctaacatgaaacatgaaataaatctagaatttttagtggttagagaattatg

attgtgtaaatgcctcgtttgtttatgggggagaacttctcccatcgtcttcaaaagttt

aagaagtttagggtctgattat

>fava_dn_rep_c1261

atataaagtgatacagattattttataaatcaaaatatatatttacacgaaatttctatt

ttgatttaattgtaccagctttcaacagtataaaaaaatgaactatatatactcaaacaa

caaaaaactaatttataattttgtatgtccattcccatgtggatgtcaaaagttagttca

catacaactttatttaccaaacagcatcattaagtttgaacaggtaaaagcttcaagcat
[truncated: 42,951,224 more chars]
