# Supplementary material for: Development of new genetic resources for faba bean (Vicia faba L.) breeding through the discovery of gene-based SNP markers and the construction of a high-density consensus map
Source: Sci Rep. 2020 Apr 22;10:6790. doi: 10.1038/s41598-020-63664-7 (PMC7176738; doi:10.1038/s41598-020-63664-7)
Supplement: Supplementary file 3 — Supplementary Information 2. [file 41598_2020_63664_MOESM3_ESM.doc]

Development of new genetic resources for faba bean (Vicia faba L.) breeding through the discovery of gene-based SNP markers and the construction of a high-density consensus map

Carrillo-Perdomo E.1*, Vidal A.2, Kreplak J.1, Duborjal H.3, Leveugle M.3, Duarte J.3, Desmetz C.1, Deulvot C.1, Raffiot B.4, Marget P.1, Tayeh N.1, Pichon J. P.3, Falque M.2, Martin O.C.2, Burstin J.1, Aubert G.1

1Agroécologie, AgroSup Dijon, INRAE, Univ. Bourgogne, Univ. Bourgogne Franche-Comté, F-21000 Dijon, France ; 2Université Paris-Saclay, INRAE, CNRS, AgroParisTech, GQE - Le Moulon, 91190 Gif-sur-Yvette, France; 3Biogemma, Chappes, France; 4Terres Inovia, Thiverval-Grignon, France.

File S2. Faba bean gene-based SNPs.

fava_c13602_1045	fava_c13602	1045	A/G	tcttcttctttttccgcttggtttgacctgtcatttgatgctgcaagctttccacctccaagaagcttactaaatttgaaatctatcccagcttccctca[A/G]cccattttctgcggctaaaaacagctcacttttcttagaagacggatcagatgtcatcctctttgacaaagcactcttaagcaaagaaccaatttgatcc

fava_c13602_1280	fava_c13602	1280	G/A	aactcttgatctcactattcttctcctcaaccacatccctaactttttgaacaacaaccctagtcaactcatgtatggattccattccagctaaagaggc[G/A]cgtatattYtctccaatctctgtttcttgcggtacaaacaaagattcagacagttccgaataaccggcaYcatcaatgatcttaaaaaYactgcaaatct

fava_c13602_1289	fava_c13602	1289	C/T	tctcactattcttctcctcaaccacatccctaactttttgaacaacaaccctagtcaactcatgtatggattccattccagctaaagaggcRcgtatatt[C/T]tctccaatctctgtttcttgcggtacaaacaaagattcagacagttccgaataaccggcaYcatcaatgatcttaaaaaYactgcaaatctggttgtgaa

fava_c13602_1350	fava_c13602	1350	T/C	atgtatggattccattccagctaaagaggcRcgtatattYtctccaatctctgtttcttgcggtacaaacaaagattcagacagttccgaataaccggca[T/C]catcaatgatcttaaaaaYactgcaaatctggttgtgaatcttggacacaaagctcaactgatcaatcaacaaaggcctcaaYgattccatactggattc

fava_c13602_1369	fava_c13602	1369	C/T	gctaaagaggcRcgtatattYtctccaatctctgtttcttgcggtacaaacaaagattcagacagttccgaataaccggcaYcatcaatgatcttaaaaa[C/T]actgcaaatctggttgtgaatcttggacacaaagctcaactgatcaatcaacaaaggcctcaaYgattccatactggattccaacttactcaacttctcc

fava_c13602_1433	fava_c13602	1433	C/T	gttccgaataaccggcaYcatcaatgatcttaaaaaYactgcaaatctggttgtgaatcttggacacaaagctcaactgatcaatcaacaaaggcctcaa[C/T]gattccatactggattccaacttactcaacttctcctcacactcactaacaaactccttcatctgcaaaccctcactctcttccttccttaaactctcag

fava_c13602_1835	fava_c13602	1835	G/A	acaactcttcaacaatctcattcgccctctttataacaccataagtcacagcagccatgccagtatatttctgcgaccgcggtaacgcacttccacttcc[G/A]ccgaaaccgttcactttcgcggaaatcttctcaattccagtaaccatcatatgactcgacttctcaatctccgatcgcatcgattgccgctccatcgctg

fava_c13602_2072	fava_c13602	2072	C/G	gaacagcctcatcccgaagccggccaaactcatcccgtttccgtatagcctcttgagccagcaccttcaatcggttgaaagaagtctgcaactccgactt[C/G]gctttctccgcagcaagccgtccctgcttttcccgatcaagctcggcgagaatctctcggagtttttcaacggacacgtcatccgaggatttagaattat

fava_c13602_2186	fava_c13602	2186	C/T	caagccgtccctgcttttcccgatcaagctcggcgagaatctctcggagtttttcaacggacacgtcatccgaggatttagaattattagtaatcggaat[C/T]ggatcaccaccgtcgtcctcgacgtcgctgagcaccgcatcggcatctacgtcgccggcgccagccatttaattgaacagcatgcaatatttgataaacc

fava_c13602_2344	fava_c13602	2344	T/C	gcgccagccatttaattgaacagcatgcaatatttgataaaccctaaaaacaaccagtctctcaagaattttggattgagaaattctcgattttaatttt[T/C]gtttcccaattcggaaaattaaaaccagtggcgcaatgaaattgaatagttaatgtaaaaaatatagagaagatgaaaaagaaggtgtgtttttggggtg

fava_c14285_302	fava_c14285	302	G/T	cttacaatacatctaaacataataagtgtcttacaagttggcaagaataaaggaaaattcaacattccatggacatcacaaggtgttgattctttttttt[G/T]Kactttctaattttcttccaaagctttaaggtttcttttcttcgtcaaagattcttcaagattccattgctagtcacaatttcttgtaaatcaacaatca

fava_c14285_303	fava_c14285	303	G/T	ttacaatacatctaaacataataagtgtcttacaagttggcaagaataaaggaaaattcaacattccatggacatcacaaggtgttgattcttttttttK[G/T]actttctaattttcttccaaagctttaaggtttcttttcttcgtcaaagattcttcaagattccattgctagtcacaatttcttgtaaatcaacaatcat

fava_c14285_561	fava_c14285	561	T/A	agagaaacaataaaaggttcaattgaaggttcaagttttaacattccaaaatagcatcacaagtcccataatcttcaacaattcttgccaaatccattca[T/A]tataattcacaagagtgttcaataaaccaaagaaagMtcatacaatttcaggcccattttagtattcatattaatRtcaaaacaaacaaaaatagtggaa

fava_c14285_598	fava_c14285	598	A/C	ttaacattccaaaatagcatcacaagtcccataatcttcaacaattcttgccaaatccattcaWtataattcacaagagtgttcaataaaccaaagaaag[A/C]tcatacaatttcaggcccattttagtattcatattaatRtcaaaacaaacaaaaatagtggaatttatgggctaaacaaggcattttcgtctgggctttg

fava_c14285_637	fava_c14285	637	G/A	aacaattcttgccaaatccattcaWtataattcacaagagtgttcaataaaccaaagaaagMtcatacaatttcaggcccattttagtattcatattaat[G/A]tcaaaacaaacaaaaatagtggaatttatgggctaaacaaggcattttcgtctgggctttgggcaagaaatgaacatgtcagcttgagtaatctcaacct

fava_c14388_1175	fava_c14388	1175	T/A	gggcttaggaatagaaagtttttgatccaatgggtttggagccctattttgaatgcgtgtgcgggtcccaatgctattgatttgaattgagttgaaactt[T/A]aactattcattcttttcacgtcatactctaccttcctactccttccctatggttttctactagctacccgccactactgtatcatgtatagtatgtaatt

fava_c14388_264	fava_c14388	264	C/T	tactcttttcacttgcagtaagtgaacaaaatggagattttcaaacgctaaacacaaaattcaatcctaaaatcccacttccaccactcagaaccctatc[C/T]tcctcaaaacgttttgagggatcttcagatcttgttaaactcaagtaccacatgggaccggttctctcttctccgatcaacatctacctaatctggtacg

fava_c14388_378	fava_c14388	378	A/G	ttgagggatcttcagatcttgttaaactcaagtaccacatgggaccggttctctcttctccgatcaacatctacctaatctggtacggaaactgggctag[A/G]cctcataaacagcttatcaaagactttctactttcaatctccgacaccaccgcacctcacccctctgtttccgactggtggcgcaccgtttcactttaca

fava_c14674_1320	fava_c14674	1320	G/A	aatcaccgcattagggtttcttgattgaaaatcttgaagctggcgtttccagtcgtcgccgtagagtttgtggaggatgcaatcgaagggaccttgatct[G/A]ctagaggacggtcagagacgactcggacgagttcgattccgcgggatttggcgagcgttaggagtgagtcgcggatgaagctgttctgtttcttgggcgc

fava_c14674_365	fava_c14674	365	T/C	aaatctttgaactaaatctttttcttgataacagctgcaaattcagataaaagggtccatcggagagaaactctgactctgactctggttgtgcaaaaac[T/C]agcaacaaccactatgtttcacacaatcatttttgtactgtaacagacttagacttatctatttcatcagttaaaaccacagcattgcccttgtcaccat

fava_c14674_745	fava_c14674	745	G/T	ttgaacagattcagcttcatagccttcctcaacccactagcaatatcaacaataaaagcctgtggtggcatctcagtagtatcatccaaactcatcatct[G/T]ataaaacttctcatcatcattcacactatcccggttcgccagattcgaaatctgcgaaaaggacaacaaatcttccgagacccccaaaaccttctcctct

fava_c14785_167	fava_c14785	167	C/T	ctatcgccatggctatgcatccccacttagacagaatcctctggaccgaagaccaaatctcccaccgaatctccgaactcgccgcccaaatcgtccacga[C/T]tttcccccgccctcacctcctcccgtatttatcggcgtcgccaccggcgcattcatattcctcgctgacctagttcgcaaaatcgacctccctttcaccg

fava_c14785_281	fava_c14785	281	C/A	cacctcctcccgtatttatcggcgtcgccaccggcgcattcatattcctcgctgacctagttcgcaaaatcgacctccctttcaccgttgatctcgttcg[C/A]gctcaatcctacggctccggaactgtctccaatggcgcacctaccatttcttctgacttgaaggtggaagttcacgggcaacacgtcattttggttgaag

fava_c14785_395	fava_c14785	395	A/G	gctccggaactgtctccaatggcgcacctaccatttcttctgacttgaaggtggaagttcacgggcaacacgtcattttggttgaagacattgtagacac[A/G]ggacatactttatctagactcatttcacaYttgaaattgaagggagcatcctctgtttctgtatgcactcttcttgacaaaccagcaaggagaaaagtta

fava_c14785_425	fava_c14785	425	T/C	ccatttcttctgacttgaaggtggaagttcacgggcaacacgtcattttggttgaagacattgtagacacRggacatactttatctagactcatttcaca[T/C]ttgaaattgaagggagcatcctctgtttctgtatgcactcttcttgacaaaccagcaaggagaaaagttaatgtacagctKgtgggtgaaggcaaattct

fava_c14785_506	fava_c14785	506	G/T	tatctagactcatttcacaYttgaaattgaagggagcatcctctgtttctgtatgcactcttcttgacaaaccagcaaggagaaaagttaatgtacagct[G/T]gtgggtgaaggcaaattctaccggggatttgagtgtccagacaattttgttgttggttatggaatggattttgatgaacggtacagaaatttgcctaaca

fava_c14785_665	fava_c14785	665	C/G	atggaatggattttgatgaacggtacagaaatttgcctaacattggtgtcttgaagcctgaacattacagttgatgctgtatttgggagatggcacttgc[C/G]RcatttggaaccatgaagtattgattttgtctgRcacgcttcSacttgcccgcagagtgaactRatagaatagctatagaaaatatgaatttgttgtacc

fava_c14785_666	fava_c14785	666	G/A	tggaatggattttgatgaacggtacagaaatttgcctaacattggtgtcttgaagcctgaacattacagttgatgctgtatttgggagatggcacttgcS[G/A]catttggaaccatgaagtattgattttgtctgRcacgcttcSacttgcccgcagagtgaactRatagaatagctatagaaaatatgaatttgttgtacca

fava_c14785_699	fava_c14785	699	G/A	gcctaacattggtgtcttgaagcctgaacattacagttgatgctgtatttgggagatggcacttgcSRcatttggaaccatgaagtattgattttgtctg[G/A]cacgcttcSacttgcccgcagagtgaactRatagaatagctatagaaaatatgaatttgttgtaccatttaacgttgtcatgaaattattagttctgcgt

fava_c14785_708	fava_c14785	708	C/G	tggtgtcttgaagcctgaacattacagttgatgctgtatttgggagatggcacttgcSRcatttggaaccatgaagtattgattttgtctgRcacgcttc[C/G]acttgcccgcagagtgaactRatagaatagctatagaaaatatgaatttgttgtaccatttaacgttgtcatgaaattattagttctgcgttggatattt

fava_c14785_729	fava_c14785	729	G/A	ttacagttgatgctgtatttgggagatggcacttgcSRcatttggaaccatgaagtattgattttgtctgRcacgcttcSacttgcccgcagagtgaact[G/A]atagaatagctatagaaaatatgaatttgttgtaccatttaacgttgtcatgaaattattagttctgcgttggatatttgcagattttacaattatcttt

fava_c14870_1171	fava_c14870	1171	T/C	tgaagagttggaagcgagtaaagggaatgcggagaaactgaacatgaagttgaaatccgtggaagaagaaaaggagttgttagaatccgaaatgaagaag[T/C]taagagtgcagacagaacaatggagaaaagcagcagatgctgcagctgcagtgcttgcaggaggttttgatatgagtgctgctgcaagggctccggaaag

fava_c14870_1386	fava_c14870	1386	T/C	ataagaacttcgttggtggaacattcgagacgcctggtgttggaaggtatcatggatatgtaggttctcctggtatgaatgatgatttggacgacggttt[T/C]ggaggtggaaaaaagaagggttctggtataagaatgtttggtgacttatggaagaagaaaggccaaaagtgagatttctgatttctgcaactcttggatt

fava_c14870_1550	fava_c14870	1550	A/G	caaaagtgagatttctgatttctgcaactcttggattcacgacacaatcattgcgtgtcaccgtgattctgacaagttttgtcagaatcacggtgtcttc[A/G]cggtgagattctgtctagtattttaactatggtcatgtttagaggtgcaaaaggaaacagttgcttactgatatgtgtatgcattttttctgctatcttg

fava_c14870_468	fava_c14870	468	A/G	gatcaaggggatctgatttgcctcagaggcagtcaccgagaggtactactcatcagcttcgatcatcgagttctgactcggatccattgcatcatcgacc[A/G]gttactgatcgaagtccaaagctaggagagcggcgttctccgagaggtagtactcaatcggagacggtgaatcagaagaagctcggtactcgcattgcgg

fava_c14870_624	fava_c14870	624	G/A	aatcggagacggtgaatcagaagaagctcggtactcgcattgcggatttggaatcgcagcttggtcaggcacaacaagagttgaaaaacttgaaggatca[G/A]ctgtcttctgcagaagctgaaaagaaaaaagctgaagatgaacttgtcaagaaaagtgtttcaaagaaagctcaagaatgtgataataacaaaggtgaaa

fava_c14923_406	fava_c14923	406	A/G	caacctgctccttacacacaggacagaaatttttgtgtgcaacccattgttgtatacattcaaagtgatacaaatgatcacacttcagcctcccaacctc[A/G]tcatcttcttcaaactcctcctgacaaatgctgcaYtttttgtctacctgatgctttgatgcatcatcgtaaaatcgaagtttggtcttccttatgtgag

fava_c14923_442	fava_c14923	442	T/C	gtgcaacccattgttgtatacattcaaagtgatacaaatgatcacacttcagcctcccaacctcRtcatcttcttcaaactcctcctgacaaatgctgca[T/C]tttttgtctacctgatgctttgatgcatcatcgtaaaatcgaagtttggtcttccttatgtgaggttccatttcat

fava_c15078_310	fava_c15078	310	T/C	ctagaggaagtttataagacctaatgcaaaagtcaaatatttttgttaacattctattacagatgcattacattcatgcatcactagatggtggtctgcg[T/C]ccacgaccccgaacaacccgaccacggggagctcttccacgagctcttgttgtggtggaaaactgtgcagcttgtcKcaattgagccctctctcgagctc

fava_c15078_387	fava_c15078	387	G/T	gcatcactagatggtggtctgcgYccacgaccccgaacaacccgaccacggggagctcttccacgagctcttgttgtggtggaaaactgtgcagcttgtc[G/T]caattgagccctctctcgagctcgctcttgtaaattaatggtggttgtagttgacgtaccactaactggtgatgttggctttacctcttctgtttccgag

fava_c15078_661	fava_c15078	661	C/A	tactctcacttgcaacagaagcaggctcagctacctgatcactctcaacagatatttgttcattggcatcaactaatttatcagaaccatcggcggcatc[C/A]tctactaaatcaccttcatcattcttatcatcaccagaaacttcaggcgaattaatctcaccggcctctaaagcatcatcatcacccctagctggggaaa

fava_c15080_1023	fava_c15080	1023	C/A	ccgttttcgttttcgcctcacaagaacactaataacaatggtaaattagtaaccattgcRaaaggtggaacggaacgaaggactttgaatgataatggtg[C/A]tgctgctgagtttttgaatcatcatcagcatatttatgctgatgggaataattggggttattttcctggtttgccatcgcgtttatcattcaatcacttc

fava_c15080_1754	fava_c15080	1754	C/T	cagtttgagatggaatggagctgatctattgacaagagttgcacgaaagtctgtaggagaagagaacaaaactattaaacggatggagttgaatgaggaa[C/T]cctctcatatcttcttccccatcaattctcaggacattacaagatacttccttgcaccagcaacagaggcgcagaaagcacagcaagatgttttRcttga

fava_c15080_1849	fava_c15080	1849	A/G	aggaaYcctctcatatcttcttccccatcaattctcaggacattacaagatacttccttgcaccagcaacagaggcgcagaaagcacagcaagatgtttt[A/G]cttgaaaaaatactgcacgagtcattgacatttcacttttggaacagcttgacttctgctctcatcccagaacctgatagccttgtggccaggcttatga

fava_c15080_2163	fava_c15080	2163	G/T	ttttaatgccgctgtacaattgtgttgtatatttatagttaagtaatgggttgaactcctatgttaaaagagctaaaaaatatagtaaaacctttgtaaa[G/T]aatgRccagttatggaatgttcatacgttacaactaacaatgtagatgtcttagagatgcagtgactcttcacggaacggttttgatggaactgagtgta

fava_c15080_2168	fava_c15080	2168	A/G	atgccgctgtacaattgtgttgtatatttatagttaagtaatgggttgaactcctatgttaaaagagctaaaaaatatagtaaaacctttgtaaaKaatg[A/G]ccagttatggaatgttcatacgttacaactaacaatgtagatgtcttagagatgcagtgactcttcacggaacggttttgatggaactgagtgtagctga

fava_c15080_890	fava_c15080	890	T/C	gaaatccagtttcgaagctttcaatccaatgctcacttctctgcttcaagatcctgactcagtcggcgtctctacactcaccagaggtgacaaaatcctc[T/C]ataaatggtggatcaatgagttcaagagagttccgttttcgttttcgcctcacaagaacactaataacaatggtaaattagtaaccattgcRaaaggtgg

fava_c15080_982	fava_c15080	982	G/A	aaatcctcYataaatggtggatcaatgagttcaagagagttccgttttcgttttcgcctcacaagaacactaataacaatggtaaattagtaaccattgc[G/A]aaaggtggaacggaacgaaggactttgaatgataatggtgMtgctgctgagtttttgaatcatcatcagcatatttatgctgatgggaataattggggtt

fava_c15096_1213	fava_c15096	1213	T/C	atgctgtgactcctttgcttgaggatgctcttatggacagggatttggttcataggcagacagctgcatctgccgtgaagcacatggcactgggagtggc[T/C]ggtttgggttgtgaggatgcgctggtccatctgctgaactatgtttggccaaacatatttgaaacttctccacatgtgataaatgctgtgatggaagcca

fava_c15096_1620	fava_c15096	1620	T/A	tttatttagattgcaatttacacattttgacgtatttgttgacgatagaagggttgatggatgaaaatttgagttggactctgcagttatactatttctg[T/A]taWaaatatttttagaatttcgatgttgctcttgtgtgtttgttaacaatagttctcttatgtgatttatcctttagaaYaagtacaactatatcaaatt

fava_c15096_1623	fava_c15096	1623	A/T	atttagattgcaatttacacattttgacgtatttgttgacgatagaagggttgatggatgaaaatttgagttggactctgcagttatactatttctgWta[A/T]aaatatttttagaatttcgatgttgctcttgtgtgtttgttaacaatagttctcttatgtgatttatcctttagaaYaagtacaactatatcaaattttc

fava_c15096_1700	fava_c15096	1700	T/C	ctgcagttatactatttctgWtaWaaatatttttagaatttcgatgttgctcttgtgtgtttgttaacaatagttctcttatgtgatttatcctttagaa[T/C]aagtacaactatatcaaattttctaatttcgtgttttaatttgtgcagttgacgatgaaacacattgcagttataattttgtgtacgacgattgttgtcg

fava_c15103_394	fava_c15103	394	A/G	gagtccaactgtttcatccctttttgctgaagaagatgaaggtgtgtctaaaagaaaattttctcaaaaccaaatgagttcaccggttttgaagtccttc[A/G]attgcctttttccatatagatgctctttgcttagcatcctcaaaagacataacctttttaggtgtgcaaatcttgaaatgtgaaggactagtcacttgaa

fava_c15108_1024	fava_c15108	1024	C/T	cgcaRtatcaaaatgctcaccaccttcgacagatccaatctcaaatccRcaccacacatatagaaacatagaagaaatcaaagaagaagagaaagagtgt[C/T]agaagaagaagatttagcgaaaagaaagggtaccttttttattctttctcgccattttcgctttatatgttgcaatccatgtaattYctgagcatattga

fava_c15108_1111	fava_c15108	1111	T/C	agagaaagagtgtYagaagaagaagatttagcgaaaagaaagggtaccttttttattctttctcgccattttcgctttatatgttgcaatccatgtaatt[T/C]ctgagcatattgaaattggtcaagggtttcgattggggtatggggtttgttggggattaggttttattatgtttgcttctgtttaatcttgttgttgcgt

fava_c15108_128	fava_c15108	128	G/A	tgatttttccaagaggaaaattacaccaacaaatcaacaattctaactattcttttgcaaaaccttgttcctggtacatatttttccaaccttgtacatt[G/A]ccaactatcctcacttgttgtagatccacacattgcataaaacctaaaacccacttgtacatagttttaacccactcagtacattctcttcaaacaattt

fava_c15108_503	fava_c15108	503	C/T	acctgcattctaaaaaagtcatcaacctgtaccattaaccatgtcttattaacaacaatgacattagcgaattcaaatttcaccataaagaaaccatatt[C/T]gaaaaacaaatgcatacaactaacaaattgcagagtttatgtagcttagatttctgttcRcaacacaaatccattataggccctatgttgcgataacatt

fava_c15108_563	fava_c15108	563	A/G	acattagcgaattcaaatttcaccataaagaaaccatattYgaaaaacaaatgcatacaactaacaaattgcagagtttatgtagcttagatttctgttc[A/G]caacacaaatccattataggccctatgttgcgataacattaagcttgcatcaaacaatctctaaattcacataactagacatgtgcagatttaaagtgta

fava_c15108_724	fava_c15108	724	G/T	ctaaattcacataactagacatgtgcagatttaaagtgtattcaccataatttccagatgttcttctagttgttgatgtttttgttgcagaaatagtgga[G/T]caatcggaaccagagtctacaagcaccgaaaccgcgactttgaagctYcgaagaatctYacctcYaacaagcacRccaagccaccRatctgcaaattgat

fava_c15108_772	fava_c15108	772	C/T	aatttccagatgttcttctagttgttgatgtttttgttgcagaaatagtggaKcaatcggaaccagagtctacaagcaccgaaaccgcgactttgaagct[C/T]cgaagaatctYacctcYaacaagcacRccaagccaccRatctgcaaattgatggctWcYcaaccccaaYccccgagtcactgtatcaYaacaacaacctc

fava_c15108_783	fava_c15108	783	C/T	gttcttctagttgttgatgtttttgttgcagaaatagtggaKcaatcggaaccagagtctacaagcaccgaaaccgcgactttgaagctYcgaagaatct[C/T]acctcYaacaagcacRccaagccaccRatctgcaaattgatggctWcYcaaccccaaYccccgagtcactgtatcaYaacaacaacctctgcaagaccat

fava_c15108_789	fava_c15108	789	C/T	ctagttgttgatgtttttgttgcagaaatagtggaKcaatcggaaccagagtctacaagcaccgaaaccgcgactttgaagctYcgaagaatctYacctc[C/T]aacaagcacRccaagccaccRatctgcaaattgatggctWcYcaaccccaaYccccgagtcactgtatcaYaacaacaacctctgcaagaccatcacttc

fava_c15108_799	fava_c15108	799	G/A	atgtttttgttgcagaaatagtggaKcaatcggaaccagagtctacaagcaccgaaaccgcgactttgaagctYcgaagaatctYacctcYaacaagcac[G/A]ccaagccaccRatctgcaaattgatggctWcYcaaccccaaYccccgagtcactgtatcaYaacaacaacctctgcaagaccatcacttccactataatc

fava_c15108_810	fava_c15108	810	G/A	gcagaaatagtggaKcaatcggaaccagagtctacaagcaccgaaaccgcgactttgaagctYcgaagaatctYacctcYaacaagcacRccaagccacc[G/A]atctgcaaattgatggctWcYcaaccccaaYccccgagtcactgtatcaYaacaacaacctctgcaagaccatcacttccactataatcaacaccttgtt

fava_c15108_829	fava_c15108	829	T/A	cggaaccagagtctacaagcaccgaaaccgcgactttgaagctYcgaagaatctYacctcYaacaagcacRccaagccaccRatctgcaaattgatggct[T/A]cYcaaccccaaYccccgagtcactgtatcaYaacaacaacctctgcaagaccatcacttccactataatcaacaccttgttcaccatcatcaatcgcaRt

fava_c15108_831	fava_c15108	831	C/T	gaaccagagtctacaagcaccgaaaccgcgactttgaagctYcgaagaatctYacctcYaacaagcacRccaagccaccRatctgcaaattgatggctWc[C/T]caaccccaaYccccgagtcactgtatcaYaacaacaacctctgcaagaccatcacttccactataatcaacaccttgttcaccatcatcaatcgcaRtat

fava_c15108_841	fava_c15108	841	T/C	ctacaagcaccgaaaccgcgactttgaagctYcgaagaatctYacctcYaacaagcacRccaagccaccRatctgcaaattgatggctWcYcaaccccaa[T/C]ccccgagtcactgtatcaYaacaacaacctctgcaagaccatcacttccactataatcaacaccttgttcaccatcatcaatcgcaRtatcaaaatgctc

fava_c15108_860	fava_c15108	860	C/T	gactttgaagctYcgaagaatctYacctcYaacaagcacRccaagccaccRatctgcaaattgatggctWcYcaaccccaaYccccgagtcactgtatca[C/T]aacaacaacctctgcaagaccatcacttccactataatcaacaccttgttcaccatcatcaatcgcaRtatcaaaatgctcaccaccttcgacagatcca

fava_c15108_928	fava_c15108	928	G/A	tWcYcaaccccaaYccccgagtcactgtatcaYaacaacaacctctgcaagaccatcacttccactataatcaacaccttgttcaccatcatcaatcgca[G/A]tatcaaaatgctcaccaccttcgacagatccaatctcaaatccRcaccacacatatagaaacatagaagaaatcaaagaagaagagaaagagtgtYagaa

fava_c15108_972	fava_c15108	972	G/A	ctgcaagaccatcacttccactataatcaacaccttgttcaccatcatcaatcgcaRtatcaaaatgctcaccaccttcgacagatccaatctcaaatcc[G/A]caccacacatatagaaacatagaagaaatcaaagaagaagagaaagagtgtYagaagaagaagatttagcgaaaagaaagggtaccttttttattctttc

fava_c15112_1252	fava_c15112	1252	C/T	gtcctgaatcagtagccaaacttactaatgcttctttaaagagacctgaattaggctcgtttaaagctagctcagtcactttatcaaaatagagctgaag[C/T]tctctagataaaacatgcttaacaggtaatttgatgtcaaccggaaggttgtcatccttttgttcatgttttttgacatcggaaggagctgaaattacat

fava_c15112_1717	fava_c15112	1717	A/G	tcctagagtgcagcatgcacttgattgcttcctgcatgatctggcgcattcggtattcaacatcgggagcaagagaaagagcaacatcaggagataaatt[A/G]gtaatcccaatactctgtgcaataacttcgattgtttctttaggaacaatgctcatattcacctacactcactttgccttatgagatgtgcgatgtgagt

fava_c15112_327	fava_c15112	327	T/C	cctgaattcagctcttccttccaaacctgggctagggctgcagagatgtttaagccctgagtattgcctacgctacttttaacttcatctctagtgatcg[T/C]atttttcatctgtccagaagatgaYgatgaaccaatttttgaatcagccRaagaagcttgagttcccatctcctcttgcctatgagattgtgaagaattt

fava_c15112_352	fava_c15112	352	C/T	cctgggctagggctgcagagatgtttaagccctgagtattgcctacgctacttttaacttcatctctagtgatcgYatttttcatctgtccagaagatga[C/T]gatgaaccaatttttgaatcagccRaagaagcttgagttcccatctcctcttgcctatgagattgtgaagaattttccagaaccacactagcctctccat

fava_c15112_377	fava_c15112	377	A/G	taagccctgagtattgcctacgctacttttaacttcatctctagtgatcgYatttttcatctgtccagaagatgaYgatgaaccaatttttgaatcagcc[A/G]aagaagcttgagttcccatctcctcttgcctatgagattgtgaagaattttccagaaccacactagcctctccatcagtagctgttttctttgaagggRa

fava_c15112_476	fava_c15112	476	G/A	cRaagaagcttgagttcccatctcctcttgcctatgagattgtgaagaattttccagaaccacactagcctctccatcagtagctgttttctttgaaggg[G/A]attgatgttccaattccataggacttgccttacgtttattaggtggagaagtaagaacacttgcactggttttccagaSagtatgagggagaggagatgg

fava_c15112_555	fava_c15112	555	G/C	gtagctgttttctttgaagggRattgatgttccaattccataggacttgccttacgtttattaggtggagaagtaagaacacttgcactggttttccaga[G/C]agtatgagggagaggagatggaaaagctgggaacatctttagtggaatgtatatacattgacccgctgcacgcagcaaggctccataaacacgccaagct

fava_c15112_730	fava_c15112	730	A/G	gcaaggctccataaacacgccaagcttcatgccttttcatttcatttttctgtgtctcaagaagcatctccggttcaagaagtcgtatatatgtttcaag[A/G]tttggcaacaaaagaaggcgaaccacattgggtcccagtgctcctaacccctgaatagcaccataatgttgtgtgattgccttctttggatccaagaatg

fava_c15121_1065	fava_c15121	1065	G/A	tgtctgcatggaatttgacactgttgcatgaagagaaaaagaccatcactttttttgattggtgcctcttcaagaaagaatagagaacagtaaatctctc[G/A]gcacagggaacaacaacataaccctgtaRcaatccttcatttgtgaccttctttcttccatcatctacatcaatatagacaggagtagtttgaaatgaca

fava_c15121_1094	fava_c15121	1094	G/A	gaagagaaaaagaccatcactttttttgattggtgcctcttcaagaaagaatagagaacagtaaatctctcRgcacagggaacaacaacataaccctgta[G/A]caatccttcatttgtgaccttctttcttccatcatctacatcaatatagacaggagtagtttgaaatgacaggcgagcaagatcttcaaccttctttgtt

fava_c15121_142	fava_c15121	142	G/A	cagttttgtattccattaacattgtttttgagaagcaagtataaaagttaatgatagaaattgacaataaaataattacaaactggaaacatgatgaggc[G/A]ctattattcaggtacaaggtWaaaatgcagagacatttccagttgttgaaaattctgatggcttaaataatctttaYgttgactactttaaagttataaa

fava_c15121_1452	fava_c15121	1452	A/T	gatccagaagacggccaggagtagctaccaatatatttatccctttagcaaggtgttcagcttcagttcttctagctgaacctccaataatcaatccaag[A/T]gtttgtgaatgatactttagaagcttcttggcaacatcatgggtctgaatagcaagctctctagtgggacatatgacaacaacaccagctccacttcgag

fava_c15121_163	fava_c15121	163	T/A	ttgtttttgagaagcaagtataaaagttaatgatagaaattgacaataaaataattacaaactggaaacatgatgaggcRctattattcaggtacaaggt[T/A]aaaatgcagagacatttccagttgttgaaaattctgatggcttaaataatctttaYgttgactactttaaagttataaaatctaattttataaaattcta

fava_c15121_1857	fava_c15121	1857	T/C	attcggtactcataattccagaaccgctattatccttaacttttttctcttccactacttcagtttctacttccacctcctctttcatcttcttcttctt[T/C]ttcgaggattctccattgctacgRgtatcgttcaaatcSttttcttctgctacgtgttgctcctcaccaatatcacggagtctctttttgtcgcgcttct

fava_c15121_1881	fava_c15121	1881	A/G	cgctattatccttaacttttttctcttccactacttcagtttctacttccacctcctctttcatcttcttcttcttYttcgaggattctccattgctacg[A/G]gtatcgttcaaatcSttttcttctgctacgtgttgctcctcaccaatatcacggagtctctttttgtcgcgcttcttcttcttatgtgaagattgaaagg

fava_c15121_1896	fava_c15121	1896	C/G	cttttttctcttccactacttcagtttctacttccacctcctctttcatcttcttcttcttYttcgaggattctccattgctacgRgtatcgttcaaatc[C/G]ttttcttctgctacgtgttgctcctcaccaatatcacggagtctctttttgtcgcgcttcttcttcttatgtgaagattgaaaggtgttttctggttcgg

fava_c15121_219	fava_c15121	219	T/C	acaaactggaaacatgatgaggcRctattattcaggtacaaggtWaaaatgcagagacatttccagttgttgaaaattctgatggcttaaataatcttta[T/C]gttgactactttaaagttataaaatctaattttataaaattctaccaaaactttttgaactgtatctcgttgtggaRcgcttaatgcctcacaaactgcc

fava_c15121_296	fava_c15121	296	G/A	tctgatggcttaaataatctttaYgttgactactttaaagttataaaatctaattttataaaattctaccaaaactttttgaactgtatctcgttgtgga[G/A]cgcttaatgcctcacaaactgccgcatatcatcagtatttcgctttccataaggattgtttgggttgaatccatgtctgcctccctcaactctccgtgtt

fava_c15121_483	fava_c15121	483	G/A	caactctccgtgttttcttcctttgcttactgctgcttatgtttacactcacatttggaggatttgaaaaacagaacgaagctgcaacagcctgcagatc[G/A]aggcgttgaacattgaatatatccttggaggaatgtgaatcatatgctaatatgtatgatctatatgcttccttagccattttgttcaaaaaataatttt

fava_c15144_283	fava_c15144	283	A/G	ccccacttggaatgttagtgaaagtattaaatgggtgaaaaagctgaaaaaagatcatccagaagtgagggtgtttatcagcattggaggtgttggctct[A/G]aatttccattcgatcctgcagaaaaaaatcaatggatacacaacgccctgcaaaccatcWcaaaaatcttacttctctatgatgtcatcaacataagtgt

fava_c15144_343	fava_c15144	343	A/T	agaagtgagggtgtttatcagcattggaggtgttggctctRaatttccattcgatcctgcagaaaaaaatcaatggatacacaacgccctgcaaaccatc[A/T]caaaaatcttacttctctatgatgtcatcaacataagtgtagatgaccttggtagtcacataaacacaattgacggtattgatattcattatgatgtcat

fava_c15149_1334	fava_c15149	1334	C/G	agagagtcctcctgtgtctgaatttgagtcaccaaagttgtatattgctggaaattcacacttctttgattcacctgcaactgctactgttgaatgcttt[C/G]taataattgaacaatagaatagcgatatccaaagaatgctaatcatatgcctcattattgtaacaaaactaatgaaaagggctaagaaagttgagatagg

fava_c15149_640	fava_c15149	640	A/C	ttatggcagctgagtgaagttcttttctgagttgaacaagagccgatttcagttcacgattgaagaacttagcaacttcattatacgggattgcacatcc[A/C]gctttgtcgaattcacSgggttttactttgtgaagttctatgatatatggcaaacaaccaaccggaccagtattgtgaatccaaaatgatcttcctcctt

fava_c15149_657	fava_c15149	657	C/G	agttcttttctgagttgaacaagagccgatttcagttcacgattgaagaacttagcaacttcattatacgggattgcacatccMgctttgtcgaattcac[C/G]gggttttactttgtgaagttctatgatatatggcaaacaaccaaccggaccagtattgtgaatccaaaatgatcttcctcctttatcatatatattcttt

fava_c15162_219	fava_c15162	219	A/G	taaactatctattttcctctattaagctatataacaacaaacacataatttacaacaaatttacatattatgatataaaagttttcacaacaagctagta[A/G]taatatatacatacacacataacaacatagacaagtgatttactaatttatttacatttacatgagagaacaagcatgaacattatcatcattaaacatg

fava_c15162_673	fava_c15162	673	T/A	ctaagtccactttcaccgaatctatgcctttgagtttgtagaccgcgtttttaacaactctttcgcaacctgtacagcacatccttactttcaactcaac[T/A]gtctgtaaggaaattggacgaccctttggcatgttgaacttcttattgtgtttgtgattattatgatgagttggatgWgcaaacaaaccgcttataaagg

fava_c15162_751	fava_c15162	751	A/T	acatccttactttcaactcaacWgtctgtaaggaaattggacgaccctttggcatgttgaacttcttattgtgtttgtgattattatgatgagttggatg[A/T]gcaaacaaaccgcttataaaggatgcaacttcatagccaaaagttctatataataaggaagccatggtttagttgaagtaaagaagcaagaaaggaagaa

fava_c15198_3818	fava_c15198	3818	T/C	ctaagagcaagagtgaaaagtttcctccgccgcatgaagacggacaactcggatttcctttgggagcttcaaatcatattgatccagatatagttcctca[T/C]gatgtttctttggattcaatgtcatataccttttcaaaggaaccatttcaagcttggtcaggccccataggtaatactaccggtaatggtttcacaaagc

fava_c15198_3944	fava_c15198	3944	G/C	ataccttttcaaaggaaccatttcaagcttggtcaggccccataggtaatactaccggtaatggtttcacaaagcgaaagaaaaacactgcgaatactgc[G/C]aacgatgcattggatttatcaaaaccatataaaggaaccctcaaggataagagtaaaggaaagaaaatcatagtttaaatatactcattatcgctttcaa

fava_c15198_4102	fava_c15198	4102	A/T	ggaaagaaaatcatagtttaaatatactcattatcgctttcaaagagtcaaattataaatcaatctcattttttggttttggaatccaaatgctgtttga[A/T]gcacttctgaaaacatgtcatccttgcttcctaagtgaagttaattccttatcaaggtgtaaaccatttcctcccatttttacaccttcattttttttYc

fava_c15198_4201	fava_c15198	4201	C/T	aWgcacttctgaaaacatgtcatccttgcttcctaagtgaagttaattccttatcaaggtgtaaaccatttcctcccatttttacaccttcatttttttt[C/T]catagctttcattagaaaagatttttttttttgagaggtgttgagtctgttggtatgatagataattgctgtgatgtacattgtgattttttgattttgt

fava_c15198_567	fava_c15198	567	A/G	gtatccctatgagccctacaattctgatttgattaatcttgttcccgaggatgtctctaaaaggatcgatagtcttccaaaatacagtaaatatgatctg[A/G]tttcagctgttaagaggcagatcccattcttttaccaggtatcaagaccctacattaaaagtgacctatttgtcaaggaagctgaggccagatataaagg

fava_c15227_218	fava_c15227	218	C/A	agtaaaaataccagccctttaattaaattcattataaccacttatactatatagcaaatatataaattttaacccataagataaacatgattaccccaaa[C/A]aagtgacaagtcttccaatattcagtgcatcataaatgccataaaagaagcaagcatagaacaaaacatgatMaccacagaaattggcaaagcaaatcca

fava_c15227_291	fava_c15227	291	A/C	ccataagataaacatgattaccccaaaMaagtgacaagtcttccaatattcagtgcatcataaatgccataaaagaagcaagcatagaacaaaacatgat[A/C]accacagaaattggcaaagcaaatccagttccagtttccaattgtgaYgtgggcgctatctctgaatcttgtgccagaatttKtgtcatgcaaaacaagc

fava_c15227_339	fava_c15227	339	T/C	ttcagtgcatcataaatgccataaaagaagcaagcatagaacaaaacatgatMaccacagaaattggcaaagcaaatccagttccagtttccaattgtga[T/C]gtgggcgctatctctgaatcttgtgccagaatttKtgtcatgcaaaacaagcctatagccattaccattatcaaaacctgaaatggagagccattaaatg

fava_c15227_374	fava_c15227	374	G/T	atagaacaaaacatgatMaccacagaaattggcaaagcaaatccagttccagtttccaattgtgaYgtgggcgctatctctgaatcttgtgccagaattt[G/T]tgtcatgcaaaacaagcctatagccattaccattatcaaaacctgaaatggagagccattaaatgttgtcatttctagaaaatatcttatgtcactctga

fava_c15247_1009	fava_c15247	1009	C/T	tttatgaatgtgctagtgcactttcagaaggaaatgaaggaaaagcctcggctatgatagatgatctcaggcagatggtttcKattaatggagatccttc[C/T]gatagaattgcggcctacatggtggaaggacttgcggctcgtttggcttcgtctggaaattgtatttataaagctttgaaatgcaaggaacctccttctt

fava_c15247_1174	fava_c15247	1174	T/C	tttataaagctttgaaatgcaaggaacctccttcttcagatcgtctagcgtcaatgcagatcctattcgaggtgtgcccgtgttttaaatttggatttat[T/C]gcagcaaatggtgctattgctgaggctattaaagatgataagaaggttcacataatagattttgatatcaaccaaggaagccaatatataaccctaatac

fava_c15247_2069	fava_c15247	2069	A/G	atgggaggacaaaaacttgattgttgcttcagcttggaagtaaacatgaaaaccgagtaaatagacattttaatcctttaccatgtgagccggtgtcact[A/G]tagttcataaattttcgaaattagaaaaacatacttgaatgtgtctttccttaRgccgtttggtctcgtatgtgtcatctgttaattagtttgatcctct

fava_c15247_2123	fava_c15247	2123	A/G	gagtaaatagacattttaatcctttaccatgtgagccggtgtcactRtagttcataaattttcgaaattagaaaaacatacttgaatgtgtctttcctta[A/G]gccgtttggtctcgtatgtgtcatctgttaattagtttgatcctctgatgtgttttttgttagtcaatttagttcataaaattactgacaaaaacaRaat

fava_c15247_220	fava_c15247	220	G/A	aactaaattgcttcaattcttcaattttggttgcatcagcaaaggcatcctagaagatttcgactatatttcatagactgaaattttatgtgagctgatt[G/A]acttgaagtaaaactatgtcattgattatatcgtcagagttggctgatgcgtcttacaggaatgccaaactatactcgctaaagggaactgatgttgcac

fava_c15247_2220	fava_c15247	2220	G/A	ttaRgccgtttggtctcgtatgtgtcatctgttaattagtttgatcctctgatgtgttttttgttagtcaatttagttcataaaattactgacaaaaaca[G/A]aatacatactcagtgacatgggctcacatttagggactaaagtgactgttcactcatttttgttatatggttgcttgtttagtttatYtatgtRtttagt

fava_c15247_2308	fava_c15247	2308	C/T	ctgacaaaaacaRaatacatactcagtgacatgggctcacatttagggactaaagtgactgttcactcatttttgttatatggttgcttgtttagtttat[C/T]tatgtRtttagtttgtggttcatgatgYtctgatcagatgtatactatgtgcaaacttgatgtaatttaagtgtttaacatggctgaacgacattStagt

fava_c15247_2314	fava_c15247	2314	A/G	aaaacaRaatacatactcagtgacatgggctcacatttagggactaaagtgactgttcactcatttttgttatatggttgcttgtttagtttatYtatgt[A/G]tttagtttgtggttcatgatgYtctgatcagatgtatactatgtgcaaacttgatgtaatttaagtgtttaacatggctgaacgacattStagtagtata

fava_c15247_2336	fava_c15247	2336	C/T	acatgggctcacatttagggactaaagtgactgttcactcatttttgttatatggttgcttgtttagtttatYtatgtRtttagtttgtggttcatgatg[C/T]tctgatcagatgtatactatgtgcaaacttgatgtaatttaagtgtttaacatggctgaacgacattStagtagtatattacaacatataacagttatgc

fava_c15247_2404	fava_c15247	2404	G/C	ttatYtatgtRtttagtttgtggttcatgatgYtctgatcagatgtatactatgtgcaaacttgatgtaatttaagtgtttaacatggctgaacgacatt[G/C]tagtagtatattacaacatataacagttatgcttaggattgtaactacattgttgtttgcttgttaccataattttacatttcttcttaggaactcgttt

fava_c15247_691	fava_c15247	691	T/C	actttgagtcagatttattggaaaacgagagcccggattgctttgagtttgatggtgaaatgcgattgaaactgcaagaattggagagggcgttgcttag[T/C]gacaataatgatgatgacgacgatgatgaggaaaacatgtttagaactgtacaaagcatggacattgatcctgactttgctgagtgtgccaatctcgttc

fava_c15247_991	fava_c15247	991	T/G	ggacgccgaagcagttactttatgaatgtgctagtgcactttcagaaggaaatgaaggaaaagcctcggctatgatagatgatctcaggcagatggtttc[T/G]attaatggagatccttcYgatagaattgcggcctacatggtggaaggacttgcggctcgtttggcttcgtctggaaattgtatttataaagctttgaaat

fava_c15266_1437	fava_c15266	1437	A/G	gtttcattggatcaaattgaaacccagaattcagctcagagagtttcgcaacatgagacagttgacctggtaacccccaatcgtaattcttcacagaaca[A/G]tgaagcttctgtagtgaatgatgatttgaatcttccattgttttttggattagaatgtggaaatgggtttgttatagaatgaatgaaaatgaaaactgaa

fava_c15266_257	fava_c15266	257	C/T	atcttattactgtcattatttgacttataaaacaattaacaataaacttaagattcttcaaaaaacttgctattaatccctgttctgtacagatgcaact[C/T]RgaYtcactcgcgacgcWaatctcggtgtaagccggagcRaaaagaacatcaccttcggtgatttcgtaaacttcagatgatcctgtgttcatcgttcct

fava_c15266_258	fava_c15266	258	G/A	tcttattactgtcattatttgacttataaaacaattaacaataaacttaagattcttcaaaaaacttgctattaatccctgttctgtacagatgcaactY[G/A]gaYtcactcgcgacgcWaatctcggtgtaagccggagcRaaaagaacatcaccttcggtgatttcgtaaacttcagatgatcctgtgttcatcgttcctt

fava_c15266_261	fava_c15266	261	T/C	tattactgtcattatttgacttataaaacaattaacaataaacttaagattcttcaaaaaacttgctattaatccctgttctgtacagatgcaactYRga[T/C]tcactcgcgacgcWaatctcggtgtaagccggagcRaaaagaacatcaccttcggtgatttcgtaaacttcagatgatcctgtgttcatcgttccttccc

fava_c15266_275	fava_c15266	275	A/T	tttgacttataaaacaattaacaataaacttaagattcttcaaaaaacttgctattaatccctgttctgtacagatgcaactYRgaYtcactcgcgacgc[A/T]aatctcggtgtaagccggagcRaaaagaacatcaccttcggtgatttcgtaaacttcagatgatcctgtgttcatcgttccttccccggccgtgaccaaa

fava_c15266_297	fava_c15266	297	G/A	aataaacttaagattcttcaaaaaacttgctattaatccctgttctgtacagatgcaactYRgaYtcactcgcgacgcWaatctcggtgtaagccggagc[G/A]aaaagaacatcaccttcggtgatttcgtaaacttcagatgatcctgtgttcatcgttccttccccggccgtgaccaaaaagatcgaaggacctggaatgg

fava_c15266_447	fava_c15266	447	A/G	tcatcgttccttccccggccgtgaccaaaaagatcgaaggacctggaatggccgggaacaccactctttctcctttcgggagagtacaatgatcgatctc[A/G]aattcctcgaacggtgggatatatttgtttacatatggattcataggaacaccttgtaaaatctcaggaataccctgtttgtatgtgagcatggaacaaa

fava_c15266_711	fava_c15266	711	C/A	tgcattcaatacactccccagataaatacgcatgtggttcgtttgctccgaggaacaatgcttccccgggattgagttttacgtggtttagaaagaaagc[C/A]gctaacacaccaatatcagacgggtattgattctccaaccgcagcacRagcagctccttttctgtcaRtggcctcacctcactttccagttgaagccgat

fava_c15266_759	fava_c15266	759	A/G	cgaggaacaatgcttccccgggattgagttttacgtggtttagaaagaaagcMgctaacacaccaatatcagacgggtattgattctccaaccgcagcac[A/G]agcagctccttttctgtcaRtggcctcacctcactttccagttgaagccgatttatcaatctgttcactgcttcggttacgatttctttactagctgaca

fava_c15266_779	fava_c15266	779	G/A	ggattgagttttacgtggtttagaaagaaagcMgctaacacaccaatatcagacgggtattgattctccaaccgcagcacRagcagctccttttctgtca[G/A]tggcctcacctcactttccagttgaagccgatttatcaatctgttcactgcttcggttacgatttctttactagctgacataagatgagtgaacactgcc

fava_c15282_1158	fava_c15282	1158	T/C	gctctggcttagctccatttgactgttggctttgtttgcgaggaatcaagactatgtcccttcgagttgaaaagcaacaggagaatgcacaaaagattgc[T/C]gagtttcttgcttcccatccRcgagtgaagaaagtaaactatgctggtYtgcctggttttcctggacgagatttacactattctcaggcaaaaggtgcag

fava_c15282_1179	fava_c15282	1179	G/A	actgttggctttgtttgcgaggaatcaagactatgtcccttcgagttgaaaagcaacaggagaatgcacaaaagattgcYgagtttcttgcttcccatcc[G/A]cgagtgaagaaagtaaactatgctggtYtgcctggttttcctggacgagatttacactattctcaggcaaaaggtgcaggatctgtgtttagctttttga

fava_c15282_1207	fava_c15282	1207	T/C	gactatgtcccttcgagttgaaaagcaacaggagaatgcacaaaagattgcYgagtttcttgcttcccatccRcgagtgaagaaagtaaactatgctggt[T/C]tgcctggttttcctggacgagatttacactattctcaggcaaaaggtgcaggatctgtgtttagctttttgacaggttcacttgccctctcaaagcatat

fava_c15282_1321	fava_c15282	1321	A/G	tggacgagatttacactattctcaggcaaaaggtgcaggatctgtgtttagctttttgacaggttcacttgccctctcaaagcatattgttgaaaccacc[A/G]aatactttagcataaccgttagttttgggagtgtaaaatcactcatcagcttgccttgctttatgtcacatgctagcatacctgctgcagtccgYgaggc

fava_c15282_1416	fava_c15282	1416	C/T	ccaccRaatactttagcataaccgttagttttgggagtgtaaaatcactcatcagcttgccttgctttatgtcacatgctagcatacctgctgcagtccg[C/T]gaggccagaggtttaaccgaagatcttgttcgtatatcagttggtattgaggatgtcgacgatctgattgctgatctagacaatgcatttagaaccgggc

fava_c15282_1613	fava_c15282	1613	A/C	gggcctctgtaacttcatacaagtaatatgtccacctttgtgcaattggattatctatgtgcttgttttgaataaagaaaaaaaatctgttatgatgaaa[A/C]tgttgtgaagatttggaaacatggttWtcctaactgtgtagcctttaWggggactgaaaacctcaatgtatttgtagtgtattgagatgtagttgtattt

fava_c15282_1640	fava_c15282	1640	T/A	atgtccacctttgtgcaattggattatctatgtgcttgttttgaataaagaaaaaaaatctgttatgatgaaaMtgttgtgaagatttggaaacatggtt[T/A]tcctaactgtgtagcctttaWggggactgaaaacctcaatgtatttgtagtgtattgagatgtagttgtatttttcttaatctaagttcagaacatttat

fava_c15282_1661	fava_c15282	1661	A/T	gattatctatgtgcttgttttgaataaagaaaaaaaatctgttatgatgaaaMtgttgtgaagatttggaaacatggttWtcctaactgtgtagccttta[A/T]ggggactgaaaacctcaatgtatttgtagtgtattgagatgtagttgtatttttcttaatctaagttcagaacatttatttaaattataagataaagatt

fava_c15282_251	fava_c15282	251	A/G	ttcgccaattcggttcccatttctcgctccactcagagcacaatttctggaaagagctgggatagcttggtgttttgcaagtcggataatttcagaggaa[A/G]tatacggttgtgtgcagacgggttgaagctacggtgctcggttgatagagagatggatgtgagcacttctgctttggtggatgatggtgttgtYgctgct

fava_c15282_345	fava_c15282	345	C/T	gaggaaRtatacggttgtgtgcagacgggttgaagctacggtgctcggttgatagagagatggatgtgagcacttctgctttggtggatgatggtgttgt[C/T]gctgctSagtgcttaactgaagaggagataagggagcctagcatttcgacagtgctgatgaatttcaagagtaagttcgatccttacggtgcagttagtg

fava_c15282_352	fava_c15282	352	C/G	tatacggttgtgtgcagacgggttgaagctacggtgctcggttgatagagagatggatgtgagcacttctgctttggtggatgatggtgttgtYgctgct[C/G]agtgcttaactgaagaggagataagggagcctagcatttcgacagtgctgatgaatttcaagagtaagttcgatccttacggtgcagttagtgcgccgcR

fava_c15282_729	fava_c15282	729	A/C	ttcttaaaagcggtgacgaaattcttgccgctgatgatctgtatggcggagctgataggttgctgtcccaagtagttcccaggagtggagttttggtgaa[A/C]cgggtaaatacaagtgatttggatgaggtcgYatctgcttttggacccaagactaagcttgtatggctggagagtccaaccaatccccggatacaaattt

fava_c15282_761	fava_c15282	761	C/T	gatgatctgtatggcggagctgataggttgctgtcccaagtagttcccaggagtggagttttggtgaaMcgggtaaatacaagtgatttggatgaggtcg[C/T]atctgcttttggacccaagactaagcttgtatggctggagagtccaaccaatccccggatacaaatttctgatattcgaagaatagcagaaatggctcat

fava_c15289_1178	fava_c15289	1178	C/T	agaacccaattttcaatggatacttcttgaaataggcctcacatatgcttcgagcaacaggacgccttataagtacccttggtgcatacttcttctgctt[C/T]ttaagtctatatttctcctgtgagaatgatgttttcttatcaaatgttgcactattggcaattagagcttcaattatttcatcacccttagcaccctgtc

fava_c15289_1372	fava_c15289	1372	A/C	ccctgtcttcgcatgtcttctatatcttcaccagtgaggctttgtgcagtattattgtcgatcaacgaccggttgtccttcagctcactgttgggttgat[A/C]gtcttttaactcagtttggaaaKgaccatcttttgtttcaacggtgttatttacatcacctttgggttggaatggcgatagaaaagcaccgtcggaagaa

fava_c15289_1395	fava_c15289	1395	T/G	atcttcaccagtgaggctttgtgcagtattattgtcgatcaacgaccggttgtccttcagctcactgttgggttgatMgtcttttaactcagtttggaaa[T/G]gaccatcttttgtttcaacggtgttatttacatcacctttgggttggaatggcgatagaaaagcaccgtcggaagaagtgtcaagttgaaaaacagtacc

fava_c15289_1686	fava_c15289	1686	C/A	gtcgccgtcgttgatatcaagcaaaacgctgcaaccttcccatgtgattctggaactcgatttgctattgttttccgttgaagacatttttctgaatata[C/A]gatgatagatagaagatccttccactgtactgaaaatgatgtttttattttattttattatgtttgggtgcagttaacttgtttcagttatgctgatgat

fava_c15289_686	fava_c15289	686	A/G	aaaaaccattttccttccacgagtcaaggatctcttgagacgccttttctccagcctttgtagctttgtgagcttttagaacaggaaatgtggtattttc[A/G]gcaccaagatcagaaattccattttctggtgaatgagatgcttcctccatgctgaccgatacactcatttgatcctgcaaaattgactgRctttccacga

fava_c15289_776	fava_c15289	776	G/A	tggtattttcRgcaccaagatcagaaattccattttctggtgaatgagatgcttcctccatgctgaccgatacactcatttgatcctgcaaaattgactg[G/A]ctttccacgataccgacatcatgttccgggttttgttgaggtgattcctttggtgacaacaagtctgatatggaagaccttaaaattctcttccagattt

fava_c15293_1517	fava_c15293	1517	C/G	ttaccttggatttcttcccatacatcgctcaacggaggtggtggtggtgataattcttcttctctgatgtcgtcttcttctccatcttcttcttcttctt[C/G]tagtttgtttagcatggaagaggaagaagagcatttgagaagcaatattggtggattattaggttttcttgagagattgaggaatttggatttgtggttg

fava_c15293_1705	fava_c15293	1705	G/A	ggatttgtggttggtggatcttgagaaaagaggttgagaatgggagagagatgggaggtttgagttttggttgaggagactatggagaatgggtacaact[G/A]aagccatgcgagcaatagcgatccactatctttttctaatggaaatgaagaaagttacaaatggaaaagaagtaatagttatcaaaatgttaatggagca

fava_c15293_258	fava_c15293	258	A/C	atgatgattgaaacttagcattaacgtgaatatgataattaagcactaacttaagaagatgttcagtgtctggtggatgagcttccaattgaggccgttc[A/C]ggcaaaacccaacgtccatcaccacttctcaccatccctttattttcatcttgcctccaatgaggtggtacaccatactcactgagtagaaaatcacagc

fava_c15293_395	fava_c15293	395	A/G	cctttattttcatcttgcctccaatgaggtggtacaccatactcactgagtagaaaatcacagctcttattcactagtgcaatatctctttttgaaacca[A/G]ctcaaattttttcttcttcccatgatacccatctattaaatgcaaaagtacctctaaattatgtgcacaacctagatcaccatttYccttcaaaaaagga

fava_c15293_481	fava_c15293	481	T/C	tctttttgaaaccaRctcaaattttttcttcttcccatgatacccatctattaaatgcaaaagtacctctaaattatgtgcacaacctagatcaccattt[T/C]ccttcaaaaaaggactttctctatgatccaaagcaatctcagttccaacatgtgcataactccaaggaaaagacaaagcatcttctatatacttttgaaa

fava_c15297_1079	fava_c15297	1079	T/C	agtccttgaatcttcctctgaaaggtgagagtatcacaatttttcaaagtgagttaaagcatcaaaaaaagcttgtgaggaacttgaaaaagaagtctct[T/C]tgggccagaaatttggaggagatagttgagaaacttgtagatattgttacctatatacatcaagcaatttgcgagctccttggaaatcaYggtactggtg

fava_c15297_1169	fava_c15297	1169	T/C	agaagtctctYtgggccagaaatttggaggagatagttgagaaacttgtagatattgttacctatatacatcaagcaatttgcgagctccttggaaatca[T/C]ggtactggtgctgtcaagtatggtaaaggtcctcaaagactaggtgaatccggtcttgcactacactatgctaacataatcaatcagatatatatgattg

fava_c15297_185	fava_c15297	185	T/A	tctcttcattcttttctttgcttctcgcttctctgtacatagaggcttctgatttctgtgttgcattgcaattttgagttttgattgtggagtgaagagt[T/A]aaaatgggggcggtttgttcggctgggatggctggggaaaatgccgagtttggagggaagactttggggttttctgggaagattcttaagaagggagata

fava_c15297_1853	fava_c15297	1853	T/C	acaaggcattgggaactcagctttctcaagaggatagaatattattagaggaagtaattgcaaggaggaggagcccaggagttagcaaaagccaggaact[T/C]ggtgtttccaagaaaactcaagccaggcaccctctcagRaccaaaagtgctgggagttcacctgttagggagtccttcggcaccacactcatcaccaacc

fava_c15297_1892	fava_c15297	1892	G/A	tattattagaggaagtaattgcaaggaggaggagcccaggagttagcaaaagccaggaactYggtgtttccaagaaaactcaagccaggcaccctctcag[G/A]accaaaagtgctgggagttcacctgttagggagtccttcggcaccacactcatcaccaaccgccgaagctataacgttttggatataatggatggcttag

fava_c15304_837	fava_c15304	837	A/T	gagtactcgcattggcaaagttgggttacaaaacctgtgtagtaccaaagcaagccgtaaaagctttaggaacagaaggtctagaaaatattaaaatagt[A/T]ggttgcaagaatctgaaggagtttattaatgtagtattcagcagataaagRcaattaaattaaaacaagYtgctaatgttgtgctgatctctctttgttt

fava_c15304_888	fava_c15304	888	A/G	aagccgtaaaagctttaggaacagaaggtctagaaaatattaaaatagtWggttgcaagaatctgaaggagtttattaatgtagtattcagcagataaag[A/G]caattaaattaaaacaagYtgctaatgttgtgctgatctctctttgttttgtaagatgtacaatttgacactcagaagtttgtaaatatagatatactat

fava_c15304_907	fava_c15304	907	C/T	aacagaaggtctagaaaatattaaaatagtWggttgcaagaatctgaaggagtttattaatgtagtattcagcagataaagRcaattaaattaaaacaag[C/T]tgctaatgttgtgctgatctctctttgttttgtaagatgtacaatttgacactcagaagtttgtaaatatagatatactatagttttcgcctgtcttggc

fava_c15337_606	fava_c15337	606	G/A	ccaaggcttgcttaatgtttccaagggcaggatgatttgcttcactgtcactcaaactcatcctattcaattggctccacagactttcttctggaatttt[G/A]ccgccagcaagatgtacaatactaattatgacaaacgtgaaaccagacatgtacgctgtattcgcatccacaacatatttttcatataaatcagaaggaa

fava_c15337_708	fava_c15337	708	T/A	cgccagcaagatgtacaatactaattatgacaaacgtgaaaccagacatgtacgctgtattcgcatccacaacatatttttcatataaatcagaaggaag[T/A]tggctaatgagtatatatgattttgcatctgcgccactttgYgaagcgcgtgtctgcgctttagacgacgggacagacctMgaaagcaatctcatttcat

fava_c15337_750	fava_c15337	750	T/C	cagacatgtacgctgtattcgcatccacaacatatttttcatataaatcagaaggaagWtggctaatgagtatatatgattttgcatctgcgccactttg[T/C]gaagcgcgtgtctgcgctttagacgacgggacagacctMgaaagcaatctcatttcataaccaaacacggcggaaagcttgtccttagcctcattgatga

fava_c15337_789	fava_c15337	789	C/A	catataaatcagaaggaagWtggctaatgagtatatatgattttgcatctgcgccactttgYgaagcgcgtgtctgcgctttagacgacgggacagacct[C/A]gaaagcaatctcatttcataaccaaacacggcggaaagcttgtccttagcctcattgatgacaaaagtaggaagattcctctgatgataattcttggtaa

fava_c15371_191	fava_c15371	191	A/T	caacatctacgcaacagaattcttaataacatctttatcatcattacaacaacttcaaatcaacaactacttaataataatcaacttaaagttcaataac[A/T]gaataatcaataatcaaaagcaacaagcgttcatccYcctgagtgttacatatcagaacaatgacaccgactcgaactattgaaggtatacaacattcac

fava_c15371_228	fava_c15371	228	C/T	tcatcattacaacaacttcaaatcaacaactacttaataataatcaacttaaagttcaataacWgaataatcaataatcaaaagcaacaagcgttcatcc[C/T]cctgagtgttacatatcagaacaatgacaccgactcgaactattgaaggtatacaacattcactctcgattacagtggtaagtgtatgtatgcaaacgct

fava_c15371_369	fava_c15371	369	C/T	tattgaaggtatacaacattcactctcgattacagtggtaagtgtatgtatgcaaacgctttagcttctattctgcgcgaacggtttgatccgctggccg[C/T]gcaacagattgccatccagtgcagtgttgaagaacttgctttagatcttctgttggaggttccactgtccatgtgagatagtgttcactcggatgaaaca

fava_c15422_1370	fava_c15422	1370	A/T	aaccattcgagttcccgcctcccagattcgggccaatctgtggcctaacttgagaatttccaggaacaacaccacgaaactgagaatttccagaaaacat[A/T]ggattctgaggaaacccatacataggttgttgttgttgttgttgttgaccaggatgcataccatatggaacagcttgagaagggtttggtctgtgcattt

fava_c15422_1646	fava_c15422	1646	A/G	tcatgttcatattaggttgctgaagcaaattcacaatattttgagccaaaatttgtccttgaattggagacaaattcatttgaggtaattgtccttgtcc[A/G]tgaagaggaaacatttgaggagcaaaacctaaagcaSccatatgMggaggcatatgtaactgatttggggcattSatatattggggtggctgttgttgct

fava_c15422_1683	fava_c15422	1683	C/G	attttgagccaaaatttgtccttgaattggagacaaattcatttgaggtaattgtccttgtccRtgaagaggaaacatttgaggagcaaaacctaaagca[C/G]ccatatgMggaggcatatgtaactgatttggggcattSatatattggggtggctgttgttgctgatttggggcattgatatattggggtggttgttgttg

fava_c15422_1691	fava_c15422	1691	C/A	ccaaaatttgtccttgaattggagacaaattcatttgaggtaattgtccttgtccRtgaagaggaaacatttgaggagcaaaacctaaagcaSccatatg[C/A]ggaggcatatgtaactgatttggggcattSatatattggggtggctgttgttgctgatttggggcattgatatattggggtggttgttgttgcatgtttg

fava_c15422_1721	fava_c15422	1721	C/G	tcatttgaggtaattgtccttgtccRtgaagaggaaacatttgaggagcaaaacctaaagcaSccatatgMggaggcatatgtaactgatttggggcatt[C/G]atatattggggtggctgttgttgctgatttggggcattgatatattggggtggttgttgttgcatgtttggcataaagggttgcatcaagttttgatttt

fava_c15422_457	fava_c15422	457	T/A	ccatcttcaacaacatcttttcctccatcataaacttcaccccccatttctttcaccgaaaccgatggatataccagaggcttatcaggataatctttca[T/A]gaacgaattcgctttcatgaacctgaatacctgaaacaggtagctcYgatccctctttatatctgcaatcaagagtttctgtaataaacttggcttcttt

fava_c15422_504	fava_c15422	504	T/C	ttctttcaccgaaaccgatggatataccagaggcttatcaggataatctttcaWgaacgaattcgctttcatgaacctgaatacctgaaacaggtagctc[T/C]gatccctctttatatctgcaatcaagagtttctgtaataaacttggcttctttttgttttctgagaaattcttgtcagccagcttctgcttcttgccgtt

fava_c15422_944	fava_c15422	944	T/C	ctctggaagggtgattctttctccgtgcttcacgccatcgtagaacttcttgctcagaataattcgtagaaaatgatctttttggttgctgctgatgttc[T/C]tcggaatttaaaccaaaagctcccgctctctcattgttggggcccttacgattttttgaaaaccctgctctcctgtttcctttgttgctatcgcgaaatt

fava_c15427_1212	fava_c15427	1212	A/G	ttccggggacacggacaattccaaggctaggtcccaaacctagtaaccattttaacgaaactttgttgtgtagatcgtatttgtacttcctccggatacc[A/G]taatgccatacatacataacgaccaaaaagaagaacgacaggacaagaggaacccatcctccttggtgYacttttaggaacgccgccgatagatataccg

fava_c15427_1281	fava_c15427	1281	C/T	gtagatcgtatttgtacttcctccggataccRtaatgccatacatacataacgaccaaaaagaagaacgacaggacaagaggaacccatcctccttggtg[C/T]acttttaggaacgccgccgatagatataccgcttctaccacccaaaagaacaaaaggaatagtgtagcaatcacaatgcttttctgccacacgaagacta

fava_c15427_1560	fava_c15427	1560	T/A	tgtcctgaaatccaatggttacagcgagagtcagaaccataagtatccagtttatttctgggatgtagatctgtccaaacatgtgtttcgaggtgtgaac[T/A]actttgacgcggggaaaacaatcgagtgcatgacactgYttgatgatggagaaagtcgcagtgatggaagcttgacttccaacaattgctgccaatgtag

fava_c15427_1599	fava_c15427	1599	C/T	taagtatccagtttatttctgggatgtagatctgtccaaacatgtgtttcgaggtgtgaacWactttgacgcggggaaaacaatcgagtgcatgacactg[C/T]ttgatgatggagaaagtcgcagtgatggaagcttgacttccaacaattgctgccaatgtagcaataacaaagaccggccaaaatacaggttcaggtattg

fava_c15427_1727	fava_c15427	1727	T/C	gaagcttgacttccaacaattgctgccaatgtagcaataacaaagaccggccaaaatacaggttcaggtattgagtcgtaaaaactattttgtatagagt[T/C]gaggtttttagacaaaaaagctgcttggcccatgtattgcattaccaaacaSgggtatatcacaaccgcaaatgcaagccttattgacgtagcagtaaaa

fava_c15427_1779	fava_c15427	1779	C/G	aaaatacaggttcaggtattgagtcgtaaaaactattttgtatagagtYgaggtttttagacaaaaaagctgcttggcccatgtattgcattaccaaaca[C/G]gggtatatcacaaccgcaaatgcaagccttattgacgtagcagtaaaatggccgagatctgcaaacatagcttcggctccagtgatacagagaagtatcc

fava_c15427_2028	fava_c15427	2028	T/G	tgatataatgcggcgagatagcaaagactatttttggattccagagtatcgtattatagatcccaataataaaaatcgatagaagccagatgattacgat[T/G]ggtgcaaacataaatgcaattctgtgtgttccacaatgttgaagagcaaacatgccaactaatatgacacatgctagaagaacaagttcaccRttattga

fava_c15427_2121	fava_c15427	2121	G/A	ttacgatKggtgcaaacataaatgcaattctgtgtgttccacaatgttgaagagcaaacatgccaactaatatgacacatgctagaagaacaagttcacc[G/A]ttattgaatttcgttatcgtaacttttaRccctgataccgatgccagaactgaaatagctggtgaaagcacaccatcgcctatgaccatacaggcaccaa

fava_c15427_2150	fava_c15427	2150	G/A	ctgtgtgttccacaatgttgaagagcaaacatgccaactaatatgacacatgctagaagaacaagttcaccRttattgaatttcgttatcgtaactttta[G/A]ccctgataccgatgccagaactgaaatagctggtgaaagcacaccatcgcctatgaccatacaggcaccaaacagtacgacaacaagcaggactgttctt

fava_c15427_408	fava_c15427	408	G/A	ttactaagagtctctaacccaaatggtaaatttctacaaactacacataatatatcattccaacttcaataagactgatgtgaggaatgttaagagccac[G/A]gccggacccctgcaattcttccgcagaaacgagtatcccatgtcgatcacaatctttttcaagaacgacgatgatttccttgccttcacatacgagtgtc

fava_c15427_597	fava_c15427	597	C/G	catacgagtgtcccattatatacgcgattcccgcttccttggcttggattaaatctagaatctcttctttaacagtgttgtccagcccgggattgttagg[C/G]acttgaaattttactcgacgctttctgacttgcgggttctcgtcgtcgatggtggattgcaagcgtcgtagtgttatagatctgctactagggatgcttt

fava_c15451_1387	fava_c15451	1387	T/C	tcattcatggtattgtttccgaggccaagacggatgaagaatgcaagatgttagcaaatttcacgacctccatgattgagctgatgaggagatacacaga[T/C]gtagatccacattttaaatttgaataggattcttcttctaaatgatgccttgttatagctcagaMtttgttttaagtctgattatgttggcaaatttgca

fava_c15451_1452	fava_c15451	1452	C/A	acctccatgattgagctgatgaggagatacacagaYgtagatccacattttaaatttgaataggattcttcttctaaatgatgccttgttatagctcaga[C/A]tttgttttaagtctgattatgttggcaaatttgcaggtaatctacatatatcaagaatttatgaacaattttttactctcttattggtatgaatccctat

fava_c15451_523	fava_c15451	523	C/T	cttctgctgttattgcttggcttatgtacaccagggagagtttgaagcagttgttttataatgtgaagactatgcccaatgttaacattcttgagatatg[C/T]ggtaggcataagctggagatacttgatttgtcgcataattctattgttggggttgagccgaattatcagaggtttccttgtttgaagtcgctttcgttga

fava_c15451_754	fava_c15451	754	A/G	atcttttggtgtctgcttgtccgaggattgaagcgttggagcttgtgaatccggagattgcgatgtctgatggtcaagtgaccgttgagttgagtagctc[A/G]acgctgaagagtgtttacgttgaagcaataagtttggataagtttgtgttggaggcggatgggattgagtgtttgtatttgaaggattgtgctcttgagg

fava_c15486_1067	fava_c15486	1067	A/G	aaaccggagatttaccacggaatactggtgaatcgcggttatctggacctgatagaacagcgaggatgccggagtcttgagatagatcgaaactccggtg[A/G]cgagttaactcagtcggttgaggcgctggacgttgattttgattttgattttcagattcSgagctatcagatgaccagaaaagttcagcttcgttgagct

fava_c15486_1127	fava_c15486	1127	G/C	cgaggatgccggagtcttgagatagatcgaaactccggtgRcgagttaactcagtcggttgaggcgctggacgttgattttgattttgattttcagattc[G/C]gagctatcagatgaccagaaaagttcagcttcgttgagctcgtcaccggcggaggtggtgatactggaaggcggagagaaattgaaagcaccgagaaggc

fava_c15489_133	fava_c15489	133	C/G	caaatcaagttcttgttattaaatgcgtttatcaacactactttagaatataaaaatatgagaggaactattttgagttagaactaaaaagttgtgtaaa[C/G]accaaattgagcttattgcataatccagatacacaaacgtataaagaatttttcaagccataaattaactagggataagtagtacaagcttttagtaaag

fava_c15489_661	fava_c15489	661	T/A	ctggttcctgtgcaagaaactggttgcatggaaatgccaatatttcaaacccttgattcttatgctccgcgtacaactcattcaactctttgtaatttat[T/A]tccgtaaacccacaWtRtgaagcaacattgacaaccagtagaaccttcccacggtaaacactaagactcacatcatttttacggatatccttgacaatga

fava_c15489_676	fava_c15489	676	T/A	gaaactggttgcatggaaatgccaatatttcaaacccttgattcttatgctccgcgtacaactcattcaactctttgtaatttatWtccgtaaacccaca[T/A]tRtgaagcaacattgacaaccagtagaaccttcccacggtaaacactaagactcacatcatttttacggatatccttgacaatgaaatcatagatggatY

fava_c15489_678	fava_c15489	678	G/A	aactggttgcatggaaatgccaatatttcaaacccttgattcttatgctccgcgtacaactcattcaactctttgtaatttatWtccgtaaacccacaWt[G/A]tgaagcaacattgacaaccagtagaaccttcccacggtaaacactaagactcacatcatttttacggatatccttgacaatgaaatcatagatggatYtg

fava_c15489_776	fava_c15489	776	C/T	WtRtgaagcaacattgacaaccagtagaaccttcccacggtaaacactaagactcacatcatttttacggatatccttgacaatgaaatcatagatggat[C/T]tggaggatctgaagcatggagcaaagggctttgtaaagtccaaacctcgaagtaagaaatccatttaacctcRacttggtaggaggattgttgtgtaaca

fava_c15489_849	fava_c15489	849	A/G	tccttgacaatgaaatcatagatggatYtggaggatctgaagcatggagcaaagggctttgtaaagtccaaacctcgaagtaagaaatccatttaacctc[A/G]acttggtaggaggattgttgtgtaacaacagcgcaagaaaaaacatctccaaattgacaggaaatgagacgatccaacgttgataggggtgaaaatcgcg

fava_c15501_1099	fava_c15501	1099	C/T	atattcggtcaacaattcgataggtaagtgcatggttaccatatctgatcagattctctttgtctatcaagaaatcatgatctgtgtccagctcccaaaa[C/T]ttgcagtatataacataaaaatgctcataagagaagtacctcaaaaccttgttgatgtcttcctcttcatcagcatgttgcattgcatctattatgtttc

fava_c15501_2126	fava_c15501	2126	C/A	ttcgacgtccaatgacgccacatcagtgaccacctccatactcatctaaaccaattccgttatcgtatctctcattcaaaaaccaaatcccaaaaaaagc[C/A]caaaactcaaaacgattcctgaattcgatttgtttcctttttccaacaccggaacgagtgaatttcaatgcattcgaatcgaaaccgcgtaatcgaaacg

fava_c15501_445	fava_c15501	445	A/C	attagaagcaaagctggtacatagaacccaaaagctgatcttggggcactaagctttgaaaggctagtccatagattttggggcactaatcttttttcat[A/C]tattaattatttcaaaagggagcctctagtgattcatcccaaacttctgcacttccattagagacatcttctacgtcttcttccattgaaagcctaatat

fava_c15501_988	fava_c15501	988	C/T	caagacttggttcagatgatttgtcctcctctgacagtatgaagtaaacaaagtcttcatatcccatctttccctcaaccttactggtaaactttcttgg[C/T]acctgagaaaatattcggtcaacaattcgataggtaagtgcatggttaccatatctgatcagattctctttgtctatcaagaaatcatgatctgtgtcca

fava_c15506_1152	fava_c15506	1152	C/T	aagatgtccctgaaggctactgacatggctcaactgcatactatgtacagcttgtggatatcatgataagaaattcttccaatggcattgtggatattat[C/T]gtaaaaaatttatcacgtcatttttctcttgtttgcataagttctaagtaatttgtttgccacctctttctgattatcttgtattgatatattaggatta

fava_c15506_883	fava_c15506	883	C/T	cattaagcatttcaagccacttacactccaatgtgatgctggagtcagctttggctcatatgtttgaaggacggcatgttacaaattcaaatgtggacac[C/T]ttcagtatcagtaatcccgaatttcatgcaaaatattggagtcagttgcaagtgcttttgaagaaaatgttggccacggtgctttcgtcgRgtgcaaata

fava_c15506_974	fava_c15506	974	A/G	tgtggacacYttcagtatcagtaatcccgaatttcatgcaaaatattggagtcagttgcaagtgcttttgaagaaaatgttggccacggtgctttcgtcg[A/G]gtgcaaataaatctctcagccaaccatcttcaacgtctagcagatttggtgatagtcaaaagatcaaggaactgtacaagatgtccctgaaggctactga

fava_c15512_530	fava_c15512	530	G/A	tctgtttttccaaactaagaatattcccaaggctgatactccaagaagcaatgtgattaaacccattaaagccacgacccaataatattgctgccaccaa[G/A]tccgttttgatgaaggctcggcattccaatcaagcaacttataacttccgaatgtgtctgggagttgcatgcgatgttcagaaagccgggacaccatact

fava_c15512_783	fava_c15512	783	C/T	catctggagagggacccatttccaagtgaagaaagattcattaagtgaacctgcgatgtattaacatctaactcatgagctatgagtccagccaactccg[C/T]aatgtgaggcttcatatccRtgtagcttatgttaaatgaaatggaaattgttatttgagcaatttggagttcaccttgatcaatattatcctgcgatgga

fava_c15512_803	fava_c15512	803	A/G	tccaagtgaagaaagattcattaagtgaacctgcgatgtattaacatctaactcatgagctatgagtccagccaactccgYaatgtgaggcttcatatcc[A/G]tgtagcttatgttaaatgaaatggaaattgttatttgagcaatttggagttcaccttgatcaatattatcctgcgatggagctggagctggagctggaga

fava_c15556_1005	fava_c15556	1005	C/T	ttgaggatgatttaaggttgaagagattcgattttgcagagaaattcagagaatttgaagcagtgagaagtgaagttgcagagagaagaatgagagagag[C/T]gaagaggagattggaggtgtatagtgattctttaagaataaaatgtcgaKtgaatcattcYggtcagagtttctgttatatgtgttgtgaaaatttgtta

fava_c15556_1055	fava_c15556	1055	G/T	gaatttgaagcagtgagaagtgaagttgcagagagaagaatgagagagagYgaagaggagattggaggtgtatagtgattctttaagaataaaatgtcga[G/T]tgaatcattcYggtcagagtttctgttatatgtgttgtgaaaatttgttacaggKaagtaattYaaagcccttggatctgtgacagttagttagaagatg

fava_c15556_1066	fava_c15556	1066	T/C	agtgagaagtgaagttgcagagagaagaatgagagagagYgaagaggagattggaggtgtatagtgattctttaagaataaaatgtcgaKtgaatcattc[T/C]ggtcagagtttctgttatatgtgttgtgaaaatttgttacaggKaagtaattYaaagcccttggatctgtgacagttagttagaagatggagggcagaga

fava_c15556_1110	fava_c15556	1110	T/G	aggagattggaggtgtatagtgattctttaagaataaaatgtcgaKtgaatcattcYggtcagagtttctgttatatgtgttgtgaaaatttgttacagg[T/G]aagtaattYaaagcccttggatctgtgacagttagttagaagatggagggcagagatgaattcagttaagagttgttatgagggtttgaaatccagttat

fava_c15556_1119	fava_c15556	1119	C/T	gaggtgtatagtgattctttaagaataaaatgtcgaKtgaatcattcYggtcagagtttctgttatatgtgttgtgaaaatttgttacaggKaagtaatt[C/T]aaagcccttggatctgtgacagttagttagaagatggagggcagagatgaattcagttaagagttgttatgagggtttgaaatccagttattgctaagtt

fava_c15556_1244	fava_c15556	1244	G/A	agttagaagatggagggcagagatgaattcagttaagagttgttatgagggtttgaaatccagttattgctaagttggagttagttatagttgtgagaaa[G/A]ttacggttagtttgagatttttggttaSagaagttttctgttatgctgagctgtcgaaagcttatctaaaRagtacMtgattcccttcttcttttaaaac

fava_c15556_1272	fava_c15556	1272	G/C	tcagttaagagttgttatgagggtttgaaatccagttattgctaagttggagttagttatagttgtgagaaaRttacggttagtttgagatttttggtta[G/C]agaagttttctgttatgctgagctgtcgaaagcttatctaaaRagtacMtgattcccttcttcttttaaaactcaatScttctaattttattttatctga

fava_c15556_1315	fava_c15556	1315	A/G	aagttggagttagttatagttgtgagaaaRttacggttagtttgagatttttggttaSagaagttttctgttatgctgagctgtcgaaagcttatctaaa[A/G]agtacMtgattcccttcttcttttaaaactcaatScttctaattttattttatctgatttctgttttatgagatggcataaggactaattatatcatgtt

fava_c15556_1321	fava_c15556	1321	C/A	gagttagttatagttgtgagaaaRttacggttagtttgagatttttggttaSagaagttttctgttatgctgagctgtcgaaagcttatctaaaRagtac[C/A]tgattcccttcttcttttaaaactcaatScttctaattttattttatctgatttctgttttatgagatggcataaggactaattatatcatgtttccccc

fava_c15556_1350	fava_c15556	1350	C/G	gttagtttgagatttttggttaSagaagttttctgttatgctgagctgtcgaaagcttatctaaaRagtacMtgattcccttcttcttttaaaactcaat[C/G]cttctaattttattttatctgatttctgttttatgagatggcataaggactaattatatcatgtttcccccctttctttttcttagtagcagggcacaat

fava_c15556_1610	fava_c15556	1610	C/T	tattttcagttagaagtttggactttgtgttaaacctggctgttggttattagagataggattcaataagaagttatgttagtcattgtttctgttaggg[C/T]ggttaatgaacgaaaaacttgtcaaaggctacttcctgctaKtgacttgaatgcttaaaaattgtttagtgaaagttaggttagttcgatagttgaattc

fava_c15556_1652	fava_c15556	1652	G/T	ttggttattagagataggattcaataagaagttatgttagtcattgtttctgttagggYggttaatgaacgaaaaacttgtcaaaggctacttcctgcta[G/T]tgacttgaatgcttaaaaattgtttagtgaaagttaggttagttcgatagttgaattcataattagttaaggttagtgttagctacaatgggattttgaa

fava_c15556_1802	fava_c15556	1802	G/T	gttgaattcataattagttaaggttagtgttagctacaatgggattttgaaggaaataagtgtttgaaagagtggagtgtgatttatagttaacagggtc[G/T]caaattggtgtatgtgYatttRtgctaatttgtatggttttaaagtaattgaatactgaactggtttataatgtcgaacctgtcttgaactgacacatga

fava_c15556_1819	fava_c15556	1819	C/T	ttaaggttagtgttagctacaatgggattttgaaggaaataagtgtttgaaagagtggagtgtgatttatagttaacagggtcKcaaattggtgtatgtg[C/T]atttRtgctaatttgtatggttttaaagtaattgaatactgaactggtttataatgtcgaacctgtcttgaactgacacatgacttgtcttgttaaactt

fava_c15556_1824	fava_c15556	1824	A/G	gttagtgttagctacaatgggattttgaaggaaataagtgtttgaaagagtggagtgtgatttatagttaacagggtcKcaaattggtgtatgtgYattt[A/G]tgctaatttgtatggttttaaagtaattgaatactgaactggtttataatgtcgaacctgtcttgaactgacacatgacttgtcttgttaaacttggttg

fava_c15556_670	fava_c15556	670	G/A	cctaatttctcaaccttgtcccatctttcaacatctaacaaaaccctaggttcacctcgcaattaatcggcgatatctaagaattgaagctagagacttc[G/A]ggatttcatttcagtacgccttaatctacacattggtttgcgacttcataacaagaagaagatgaaaagatgaagaaggacgatcgtgtttacctaccgg

fava_c15556_779	fava_c15556	779	C/T	tttcagtacgccttaatctacacattggtttgcgacttcataacaagaagaagatgaaaagatgaagaaggacgatcgtgtttacctaccggagagagtc[C/T]ggtgctccttcagatcggagaagatgaccataaatttaRagaggaaacaatgcgatggagtgattgttgatgtgttggtggagattggaaatacgttgat

fava_c15556_818	fava_c15556	818	G/A	ataacaagaagaagatgaaaagatgaagaaggacgatcgtgtttacctaccggagagagtcYggtgctccttcagatcggagaagatgaccataaattta[G/A]agaggaaacaatgcgatggagtgattgttgatgtgttggtggagattggaaatacgttgataatgatgtgaataagaggtatgcgattgaggatgattta

fava_c15573_1203	fava_c15573	1203	C/A	gaccaccatccaatttcttaatccccaacgacttactacactccgccaaattcgcctgtttccccaaccaactcaaaaccttaaatccatcctcaaaagc[C/A]gccggataccgactctccggcgcaagccgatacccaaccgcaacaaccaccgcatcacatagcttcgcaatcctcctacaaaaataatcattMgccaccg

fava_c15573_1296	fava_c15573	1296	A/C	caaaagcMgccggataccgactctccggcgcaagccgatacccaaccgcaacaaccaccgcatcacatagcttcgcaatcctcctacaaaaataatcatt[A/C]gccaccgaatcattactcccactcacccaaccacctccatgaaactgcaccatcaccggtaacttcctcttccgatcatcaatcgtcggcgaataaccac

fava_c15573_158	fava_c15573	158	T/C	catctctggtacatttggagactccaaagtcaagaataatgcacaaaattttatattgttttcatgagaaatattacaacataatacacacaagaaagaa[T/C]cgaacaagtgttaccaaatgtggtgtgcatgaggtgcatcaatctcactggttccacctatccgactttcaaataactattttgaaccagtcaacaacat

fava_c15573_708	fava_c15573	708	C/T	catgaactgcgtctttatattcaagaacaggcgcatcaacattcaccttcctaagctcctctgagtaagctatggcacgatctctcatccagtcatgttc[C/T]gccactactgttaatgttggaggcatctgctttaaaggaggaccatggcctgggactagaggattggcggctggatggtctaggctaaattctttctcgg

fava_c15574_1094	fava_c15574	1094	C/A	tttgctatattctacacctctcatgggaactgtctttcgcttttcatcttgcttctttggattcataggaatatcaacattagaagacagcttctttgag[C/A]ttttcgcYgtgtttccttctcttRcctttctagttatctcatcatcatcatcatYgtcatcatcttgcttctttggatttataggaatatcaagattagc

fava_c15574_1102	fava_c15574	1102	C/T	attctacacctctcatgggaactgtctttcgcttttcatcttgcttctttggattcataggaatatcaacattagaagacagcttctttgagMttttcgc[C/T]gtgtttccttctcttRcctttctagttatctcatcatcatcatcatYgtcatcatcttgcttctttggatttataggaatatcaagattagcagataact

fava_c15574_1118	fava_c15574	1118	G/A	gggaactgtctttcgcttttcatcttgcttctttggattcataggaatatcaacattagaagacagcttctttgagMttttcgcYgtgtttccttctctt[G/A]cctttctagttatctcatcatcatcatcatYgtcatcatcttgcttctttggatttataggaatatcaagattagcagataacttStttgagcttttcgY

fava_c15574_1149	fava_c15574	1149	C/T	tttggattcataggaatatcaacattagaagacagcttctttgagMttttcgcYgtgtttccttctcttRcctttctagttatctcatcatcatcatcat[C/T]gtcatcatcttgcttctttggatttataggaatatcaagattagcagataacttStttgagcttttcgYcgtcttttcttctcttgcctttctagttatc

fava_c15574_1204	fava_c15574	1204	C/G	tgtttccttctcttRcctttctagttatctcatcatcatcatcatYgtcatcatcttgcttctttggatttataggaatatcaagattagcagataactt[C/G]tttgagcttttcgYcgtcttttcttctcttgcctttctagttatcacatcaacatctgatttcttattacttttaccgaacagagaatgtaatgatttgg

fava_c15574_1218	fava_c15574	1218	C/T	RcctttctagttatctcatcatcatcatcatYgtcatcatcttgcttctttggatttataggaatatcaagattagcagataacttStttgagcttttcg[C/T]cgtcttttcttctcttgcctttctagttatcacatcaacatctgatttcttattacttttaccgaacagagaatgtaatgatttggaatcatgtttctga

fava_c15574_1420	fava_c15574	1420	A/G	tttcatttgttgcttcggtttttccataaaagactctctcacttgtagaacgagtatttgattcttctttggactcttttgtgataggactgacaaaaac[A/G]tctttgataagcgtttgacggctgctcaatgaatctgattgagtttttgcagaaactataggttctaccgtcttttgaatggttgctgaagtagtttttg

fava_c15574_1867	fava_c15574	1867	T/C	tatcaggagaaacttcagtaacagtaacagatctgtcctgtttcatattgtttcctttgtcgacttcatcagataggcctaagttccaaagctcctccga[T/C]aacagactttgcctgttagatggtttcaaatcctttcgcacctccttatgttgagctgaaaatcgagatagattagaatctgagaatgctaatccatttg

fava_c15574_196	fava_c15574	196	A/G	aaagactaatatattattatttgtatacataccagagtgaagtaattcatttatatttttttacaattcaattagatacagaaatatgaagaccacttac[A/G]gttccaacaaatacaacatctattcccttgtagacagactgcagaagatagtttctaatgttggtattgagtattgactcgcattagagtggaccaacca

fava_c15574_2278	fava_c15574	2278	C/T	ctttagcttttttgtttgtataacgtcttctaactccactatcatcaccaaacaccgatttccctctctcaccctccaccggccacctacagcttcccga[C/T]tcaccttcttctccactgaaccccgacgtccgctgcggtggtccaccaaacacatcattaaaatcaacttccgattccctctgcgaSgtactcggattac

fava_c15574_2365	fava_c15574	2365	G/C	tacagcttcccgaYtcaccttcttctccactgaaccccgacgtccgctgcggtggtccaccaaacacatcattaaaatcaacttccgattccctctgcga[G/C]gtactcggattactattgctatcgccgtcacgcttttcaRaagatttataaaatcgctccattaataagaaattcaaaatctggcgggaaactgttagag

fava_c15574_2405	fava_c15574	2405	G/A	cgtccgctgcggtggtccaccaaacacatcattaaaatcaacttccgattccctctgcgaSgtactcggattactattgctatcgccgtcacgcttttca[G/A]aagatttataaaatcgctccattaataagaaattcaaaatctggcgggaaactgttagagatgaaaagagaagagtagaaaggtttatgagaaagagcaa

fava_c15574_440	fava_c15574	440	G/C	ttctgcaatgtatttttggtccgaagtcgcacccttttgctgcaacttatctggatgcagactgagtaaagctctttgataagatcttttcaccgcattc[G/C]cttcaattatatcaacaagaggcacaggcttccagccacagtttggccaaagtacgtattgtaatgttgacagcagcgagcgtatgtttccttccttccc

fava_c15574_575	fava_c15574	575	T/C	gccacagtttggccaaagtacgtattgtaatgttgacagcagcgagcgtatgtttccttccttccctttagaccactgttgtatcttcttgtcaataact[T/C]ggatttcttgaatttcttggttttgtgaaacctcctcctcatcttgggataatacttgaatcgtgaaRttctcatggaaggactcgtctttatctcctat

fava_c15574_643	fava_c15574	643	G/A	tagaccactgttgtatcttcttgtcaataactYggatttcttgaatttcttggttttgtgaaacctcctcctcatcttgggataatacttgaatcgtgaa[G/A]ttctcatggaaggactcgtctttatctcctatatcttccggaaatgaaatgYcatgaactgctgctgatgctgagatatcatcctgctgtgaaaaactat

fava_c15574_695	fava_c15574	695	C/T	gttttgtgaaacctcctcctcatcttgggataatacttgaatcgtgaaRttctcatggaaggactcgtctttatctcctatatcttccggaaatgaaatg[C/T]catgaactgctgctgatgctgagatatcatcctgctgtgaaaaactattggtaggcatatctgtgatctttatgttagagacctccttccggRactgttc

fava_c15574_788	fava_c15574	788	G/A	tgaaatgYcatgaactgctgctgatgctgagatatcatcctgctgtgaaaaactattggtaggcatatctgtgatctttatgttagagacctccttccgg[G/A]actgttcaScttcaccatccacatcattattttcccttacagctcctttctgcttgtatgcataaccctgaagtcgagattttgagtcaactttgggttt

fava_c15574_797	fava_c15574	797	G/C	atgaactgctgctgatgctgagatatcatcctgctgtgaaaaactattggtaggcatatctgtgatctttatgttagagacctccttccggRactgttca[G/C]cttcaccatccacatcattattttcccttacagctcctttctgcttgtatgcataaccctgaagtcgagattttgagtcaactttgggttttgtttcagc

fava_c15589_1023	fava_c15589	1023	A/G	ggccttcattactcgcattattgaagatagcactcaactcaagcataattttaggtggttcacttcaatgattggtagaaaatcaaacctcaagtgtctt[A/G]catcaaaactttgggaagttggagtcactgtagtgaagacaactgaRtttgtccagggcaggacatcccgatgggggcttgcttggtctttcttgcctat

fava_c15589_1070	fava_c15589	1070	G/A	attttaggtggttcacttcaatgattggtagaaaatcaaacctcaagtgtcttRcatcaaaactttgggaagttggagtcactgtagtgaagacaactga[G/A]tttgtccagggcaggacatcccgatgggggcttgcttggtctttcttgcctattgtgcaacagaagtcatcaatttccttgccgaataagaataacatgt

fava_c15589_1295	fava_c15589	1295	T/C	gcaaggatggggccattaatgtgttggaaggtgtcaagtcttacttttccttgcatggtctctcatgcacaatgaacacctcctctttcacggtggatgt[T/C]gtggcaacaaaagatgactgcaattcaattttgaagaatgaaatgcccataatcaatccatccactgattatcaacctacagagaagaagacatctgata

fava_c15589_1396	fava_c15589	1396	G/A	gtggcaacaaaagatgactgcaattcaattttgaagaatgaaatgcccataatcaatccatccactgattatcaacctacagagaagaagacatctgata[G/A]atcaagtgttaatctctcttgtgatagatcaggcctttgtatatcggtttttcagcaaatccctgggacactgttggtgaaaggttcagtacaagataaY

fava_c15589_1621	fava_c15589	1621	C/T	tcagtgattttccgcaaactggaagaagtgttacagaataaattttgtacccgatcagtatagggttcttgattatttgatttatcatttggagtggtga[C/T]tgaatatgttacatgattctgagttgttcacaagtgtgctaWtttccggggtcagtatttatagaaatcaagtgaagRtgttggtgtaaaaaaaggaaat

fava_c15589_1663	fava_c15589	1663	A/T	ttttgtacccgatcagtatagggttcttgattatttgatttatcatttggagtggtgaYtgaatatgttacatgattctgagttgttcacaagtgtgcta[A/T]tttccggggtcagtatttatagaaatcaagtgaagRtgttggtgtaaaaaaaggaaatagaaggcaacaataatttctggagcactgttgctccagaaat

fava_c15589_1699	fava_c15589	1699	G/A	gatttatcatttggagtggtgaYtgaatatgttacatgattctgagttgttcacaagtgtgctaWtttccggggtcagtatttatagaaatcaagtgaag[G/A]tgttggtgtaaaaaaaggaaatagaaggcaacaataatttctggagcactgttgctccagaaattttgtgtggtactaactactaaaccttacgtgaaca

fava_c15589_228	fava_c15589	228	C/A	gagaaagagagaagaaaagcgaggcacgattcaccccaacaacaagtactctgaaaacccacctgattttcctcatttagcttctctctacccatccttc[C/A]aaccttttctccaatactctcataacggacaccctagaatcgattggaccgatttcaacgccactcgcgaactcacccgtatcctactcctccatgatca

fava_c15589_350	fava_c15589	350	T/A	ataacggacaccctagaatcgattggaccgatttcaacgccactcgcgaactcacccgtatcctactcctccatgatcactctctcaactggtggattcc[T/A]gatggccaactctgtcccactgtaccaaataggtctaattacattcactggcttcaagatcttctctcatctaacatcattgttccagacaccatttcat

fava_c15589_584	fava_c15589	584	C/G	taggaactggagccaattgtatttatccacttcttggttcatcccttttcggatggacttttgtagcctcagatgtaactgatgttgcaattcagtgggc[C/G]gagataaatgttaatagtaatccacatgtttctgagttgattgaaattaggagggtacaatgcaatgcaactacttccgggttgttgcaggatgaagagc

fava_c15589_722	fava_c15589	722	G/A	tgattgaaattaggagggtacaatgcaatgcaactacttccgggttgttgcaggatgaagagcccgtacttagtgccacggaggtagagaatttacccaa[G/A]ttgcctcttgatttgaatgcgtgtgagcataacagttatcgtggacctcccatacttgttggtgtagtcagggaggatgagaagtttgatttttgtatgt

fava_c15589_910	fava_c15589	910	T/C	gatttttgtatgtgtaatcctccgttttttgaaagcttggaggaagctggacttaatcccaaaactgcttgtggtggcacttctcaagaaatggtttgct[T/C]tggtggtgagaaggccttcattactcgcattattgaagatagcactcaactcaagcataattttaggtggttcacttcaatgattggtagaaaatcaaac

fava_c15656_1218	fava_c15656	1218	A/G	gagattccatctggctcatctcctccccactggtggccacttcccagtcttcagcctcaattgaattataacccactgcctgcaaaatttttactttctt[A/G]cgcaaatcatcaactattttttctgtcggccttgtctgaagctcttttttcattgccacaatagaagcttccttttcattgagcattgcagtaaatttct

fava_c15656_1755	fava_c15656	1755	T/A	atttcttcatatttgaaacactttctttagcactctgcagctggtcttgtagtagttgctcccgttcttttaaaatttccattgttttctggttctcctc[T/A]gctagattgcgatgcttaatctccacaatttcttttactttctcttccatctgctgctctaactgcctgcttcgctcctcaagtcttcgtattgtggcct

fava_c15656_1893	fava_c15656	1893	C/T	ctttctcttccatctgctgctctaactgcctgcttcgctcctcaagtcttcgtattgtggcctgctgattcttcaagtgagttgcctctgttctaaattc[C/T]tcaagctcaactttcattttccggttctcagattctagctcagacagggtgacatcttgctcagcaactgaggcaagagctggataaggatcgggggctt

fava_c15656_2286	fava_c15656	2286	G/A	tttctgcaattcgaagcccttgttcatctaacacagatttctccttctctaagtcaaaatctttccagaaggcggagacgacggagatcggagaatttgt[G/A]ttgtccctStccgatccacttggcgaagactccatttttgtcaccgatgaatatcgatatcgactttgtgagagaaattgaagtggtgatcgctgcgagc

fava_c15656_2295	fava_c15656	2295	C/G	ttcgaagcccttgttcatctaacacagatttctccttctctaagtcaaaatctttccagaaggcggagacgacggagatcggagaatttgtRttgtccct[C/G]tccgatccacttggcgaagactccatttttgtcaccgatgaatatcgatatcgactttgtgagagaaattgaagtggtgatcgctgcgagcttttctttc

fava_c15656_291	fava_c15656	291	A/G	gtacaacatattagaatattgacaacttgttttcattacattaaatgaccactggaagtaagttgcatagccactacagtcctcgtggaagtggaagatc[A/G]gtggttctttctccaataagaggttcatctgagccatgactaagataactcaaagctgacattcgatacagacaggtaaagaccaagatatgcaacccga

fava_c15656_756	fava_c15656	756	T/A	aacggatttttccgtataatttaacattatctcctttggtcttttccaattccgctgttaataccccaatcttctccttcaactgccttatttcctcttc[T/A]gtctctctcaagcgtgtcctaaatcgatctctctgactacatattactttaagcatagagctctgatcttgatctaaggcttgcctttgatccatattct

fava_c15656_879	fava_c15656	879	A/G	atcgatctctctgactacatattactttaagcatagagctctgatcttgatctaaggcttgcctttgatccatattctgatgctcagacacctcactccg[A/G]ttagcctctgatagatcccagtcgtccaggaaagttcctttatgatctttggaactggaactatatcccttggatatatcatcttccaacttttgtatca

fava_c15660_1429	fava_c15660	1429	C/T	agctcgtactgcttacaacaagtgcaggcctgtttggcttcaaacatatgtttgaaccaacctcagttgtaaatacactctcattttggatttggctact[C/T]gtcagcgaacttccgttactaacacaatctctaacaccaagaggatgataaaagtgttcatYcagtctgcaaaacaacttgtgcctttcttccagcgaag

fava_c15660_1491	fava_c15660	1491	T/C	tcagttgtaaatacactctcattttggatttggctactYgtcagcgaacttccgttactaacacaatctctaacaccaagaggatgataaaagtgttcat[T/C]cagtctgcaaaacaacttgtgcctttcttccagcgaagttggatgaaagaaatcaggaggttggtcgtacaatactgtagcattaatYccccagttttga

fava_c15660_1579	fava_c15660	1579	C/T	aaaagtgttcatYcagtctgcaaaacaacttgtgcctttcttccagcgaagttggatgaaagaaatcaggaggttggtcgtacaatactgtagcattaat[C/T]ccccagttttgagccaattcatgctgcattgcttttgttacgcatagagaagcgtctgccattttcccataccgattctcaaaccatttgtataaagaga

fava_c15660_325	fava_c15660	325	T/A	tgttgcctgtgaactgcaccttgaaatctgcttgaaggatttgcaggctgattttatgaatggagctgagatttcaaattttgatcctatcgtctctttc[T/A]gcgagactctgcttaagtttagccataccgtgatgagcRagtcaccaaacaaacaaaaccgtttatacgtgaaagcaaagccaatggaggaagcgcttgc

fava_c15660_364	fava_c15660	364	A/G	tttgcaggctgattttatgaatggagctgagatttcaaattttgatcctatcgtctctttcWgcgagactctgcttaagtttagccataccgtgatgagc[A/G]agtcaccaaacaaacaaaaccgtttatacgtgaaagcaaagccaatggaggaagcgcttgcagaggccattgttgatggaaagattgaaccaaggcaaga

fava_c15660_562	fava_c15660	562	T/C	agactccaaaaaccgttcgaaaagtactgtttaatgagtttggttggcataaagaactttccaaaaaaaaaaatcagacttttatttacaacaaaagcac[T/C]aagtcaaaaatagacagatgttgaagatcttgctgcataaaaatcgacttatggtgctaaataatcaaccatctataggatcaatcaaaccaacaaatct

fava_c15660_689	fava_c15660	689	A/T	atcttgctgcataaaaatcgacttatggtgctaaataatcaaccatctataggatcaatcaaaccaacaaatctatgaaattttttgttacaagtcaaag[A/T]cgtcttctaagaaRacagaaaatcaaaatcttgatataacctcagttatcagtggctttgcatgttcttcccattcagtaacccaccttgatgaagaacc

fava_c15660_703	fava_c15660	703	G/A	aaatcgacttatggtgctaaataatcaaccatctataggatcaatcaaaccaacaaatctatgaaattttttgttacaagtcaaagWcgtcttctaagaa[G/A]acagaaaatcaaaatcttgatataacctcagttatcagtggctttgcatgttcttcccattcagtaacccaccttgatgaagaaccggtatcgagtgcac

fava_c15662_1558	fava_c15662	1558	C/T	aagatgaagtgagaaaagccttggacttcattgattatgtctatcaaatatttggtttcacatatgaactgaaactttcaacgaggccagagaataagct[C/T]ggagatgatgaaacgtgggatagggctgaaagtgctctcaaggaggctttggatgagtttggcaagccatggcagttgaatgaaggggatggtgcattct

fava_c15662_1811	fava_c15662	1811	A/C	gtgtgcaactctacagcttgatttccagcttcctgatcgttttaagttggaattctcagctgaggatgaagccaaaattgagaggcctgttatgatacac[A/C]gagccattctaggatcagttgaacgcatgtttgccatacttttagagcactacaagggtaaatggcccttctggctcagtcctcgtcaagcaattttgtg

fava_c15662_2143	fava_c15662	2143	G/A	agtacaactacatcttggttgttggggaagaggaagctaatacaggacaggtgagtgtaagagtcagggataaggcagaccatagcgttatgaccattga[G/A]aacctgctcaaacacttcaatgacgaggttgcagctttccactgaWaaagcaaatttatacccccaataactttttgcaggtgtacRcatgtttgcatta

fava_c15662_215	fava_c15662	215	A/G	ccgtttctattcatcttccaccgtattcgccgccgcatccgccgccatggttgttcacgctaaggacgaatcctacctaagcgcaaccatcccaaagcgc[A/G]tcagtttattcgaggcgattcaRgcggagcaacagactcgccgcctatcaYtgtcaccggatccgatcaaggtaacactccccgacggaaaagtgaagga

fava_c15662_2189	fava_c15662	2189	T/A	acaggtgagtgtaagagtcagggataaggcagaccatagcgttatgaccattgaRaacctgctcaaacacttcaatgacgaggttgcagctttccactga[T/A]aaagcaaatttatacccccaataactttttgcaggtgtacRcatgtttgcattatttacatttcaactgacaatgtagtctaatttttagacgattttga

fava_c15662_2230	fava_c15662	2230	G/A	ttatgaccattgaRaacctgctcaaacacttcaatgacgaggttgcagctttccactgaWaaagcaaatttatacccccaataactttttgcaggtgtac[G/A]catgtttgcattatttacatttcaactgacaatgtagtctaatttttagacgattttgaagtttaatgtattttcattacagtcatgattgatgtgatgt

fava_c15662_238	fava_c15662	238	A/G	tattcgccgccgcatccgccgccatggttgttcacgctaaggacgaatcctacctaagcgcaaccatcccaaagcgcRtcagtttattcgaggcgattca[A/G]gcggagcaacagactcgccgcctatcaYtgtcaccggatccgatcaaggtaacactccccgacggaaaagtgaaggaaggaaagaaatggcagacaactc

fava_c15662_2402	fava_c15662	2402	T/G	tttcattacagtcatgattgatgtgatgtgaatcttacctggagtgtactcatgtagattcatatattgtttgttgtagacagaatgccatccttattcc[T/G]agaactaaagaggaatgttggcaatatttgtgataatactctttttgaggcattttttagcac

fava_c15662_266	fava_c15662	266	T/C	tgttcacgctaaggacgaatcctacctaagcgcaaccatcccaaagcgcRtcagtttattcgaggcgattcaRgcggagcaacagactcgccgcctatca[T/C]tgtcaccggatccgatcaaggtaacactccccgacggaaaagtgaaggaaggaaagaaatggcagacaactccgtttgatgtggctcgtgaaatctctaa

fava_c15662_661	fava_c15662	661	G/A	ggccttgtactaccagaggagagggtttctattatgacgctttttacggtgaattgggtttgaatgatgatcactttaaacaaattgaagctggagcatt[G/A]aaggcggttgcggaaaaacaaccctttgagcgcattgaagtgacacgtgaccaggcgcttgagatattttctgataataagtttaaggttgaaattatca

fava_c15664_1037	fava_c15664	1037	A/G	accgatcaaccgccacaagactattatatYgttgtctcttcgcggtttacctctcaagtgttgacagccacctccatacttcattacagtaactcggcga[A/G]aactgtttctggtcctcccccagatggcctcacggttcaaatcgattggtctctcgaacaagctcgctccctcaggaggaatctttcRgctagtggaccg

fava_c15664_1125	fava_c15664	1125	G/A	gtaactcggcgaRaactgtttctggtcctcccccagatggcctcacggttcaaatcgattggtctctcgaacaagctcgctccctcaggaggaatctttc[G/A]gctagtggaccgagacctaatccgcaaggatcttaccattatggtttgataaacacgacgcgtacaattagacttcaaaactcagcacctattatcaatg

fava_c15664_1433	fava_c15664	1433	C/G	tcggacaatcctacgggcggtggtggttacctccagacttctgtcatggctgctgattttcgaggctacgtggaaatcgtgtttgaaaatcctgaagata[C/G]Ygtgcagtcttggcacattgatggtcatcacttctttgttgtaggaatggatggaggacaatggtcagcttcaagtagatcaaattacaatttgggagac

fava_c15664_1434	fava_c15664	1434	C/T	cggacaatcctacgggcggtggtggttacctccagacttctgtcatggctgctgattttcgaggctacgtggaaatcgtgtttgaaaatcctgaagataS[C/T]gtgcagtcttggcacattgatggtcatcacttctttgttgtaggaatggatggaggacaatggtcagcttcaagtagatcaaattacaatttgggagaca

fava_c15664_1545	fava_c15664	1545	T/C	ggcacattgatggtcatcacttctttgttgtaggaatggatggaggacaatggtcagcttcaagtagatcaaattacaatttgggagacacgatttctcg[T/C]tgcacagttcaggtatatcccaagtcatggagtgcagtgtacatgcctttggacaatgtgggaatgtggaatgtgagatcagagaattgggctcgtcgtt

fava_c15664_1764	fava_c15664	1764	C/G	atcttcgcgtgtattctccggcaaattcgtggagagatgagtatccaataccaagtaatgctttgcggtgtggtcgagctataggtcattgaactaagtc[C/G]ttaagtttcattcattcKatgtgaaacactgacatagacacggacaccagacacgattatgaatRgtcgcattcaatgttgaagacatggctacataatt

fava_c15664_1782	fava_c15664	1782	T/G	cggcaaattcgtggagagatgagtatccaataccaagtaatgctttgcggtgtggtcgagctataggtcattgaactaagtcSttaagtttcattcattc[T/G]atgtgaaacactgacatagacacggacaccagacacgattatgaatRgtcgcattcaatgttgaagacatggctacataattcgttttccttcaaaggct

fava_c15664_1829	fava_c15664	1829	G/A	cggtgtggtcgagctataggtcattgaactaagtcSttaagtttcattcattcKatgtgaaacactgacatagacacggacaccagacacgattatgaat[G/A]gtcgcattcaatgttgaagacatggctacataattcgttttccttcaaaggcttaactcgagccaattttcctttgtacYagtcaataatactttagaag

fava_c15664_183	fava_c15664	183	G/A	catttataaagctctaaagccaaaggaaaaaaagaaaagtgttagttgaagaaatggagtactcaatgagactaagttctctctcattattgttactagt[G/A]tttctYgttgtgacaaactcaaatggagaagatccttatagattctatacttggaatgttacctttggagatatctatcctcttggagttaaacaacagg

fava_c15664_189	fava_c15664	189	T/C	taaagctctaaagccaaaggaaaaaaagaaaagtgttagttgaagaaatggagtactcaatgagactaagttctctctcattattgttactagtRtttct[T/C]gttgtgacaaactcaaatggagaagatccttatagattctatacttggaatgttacctttggagatatctatcctcttggagttaaacaacagggtatat

fava_c15664_1909	fava_c15664	1909	T/C	caccagacacgattatgaatRgtcgcattcaatgttgaagacatggctacataattcgttttccttcaaaggcttaactcgagccaattttcctttgtac[T/C]agtcaataatactttagaagacaatatacagtccctagaatgctttgttcattctcttaaatctaaatttaaatcattggtacatttgccacatgacatt

fava_c15664_927	fava_c15664	927	A/G	ttcaagggcataagatgaaactagttgaagtagaaggaattcacacattacaaaacacttatgattcccttgacattcatttgggacaaagttactcggt[A/G]ttggttacaaccgatcaaccgccacaagactattatatYgttgtctcttcgcggtttacctctcaagtgttgacagccacctccatacttcattacagta

fava_c15664_966	fava_c15664	966	T/C	ttcacacattacaaaacacttatgattcccttgacattcatttgggacaaagttactcggtRttggttacaaccgatcaaccgccacaagactattatat[T/C]gttgtctcttcgcggtttacctctcaagtgttgacagccacctccatacttcattacagtaactcggcgaRaactgtttctggtcctcccccagatggcc

fava_c15665_1009	fava_c15665	1009	T/C	ggatgtcttctcYgcagcYtgattcagttgtcaaaaccttgtgcagttctagtccaaggtttgcctttcctgactttgaaagtgcatctttaataactcc[T/C]ggcacaggcatttcaagcaacacattttcRttgatagatttggcagccttgaatacttgatgaaccacccttccctgatgaaccagcctctttctttcgg

fava_c15665_1039	fava_c15665	1039	G/A	tcaaaaccttgtgcagttctagtccaaggtttgcctttcctgactttgaaagtgcatctttaataactccYggcacaggcatttcaagcaacacattttc[G/A]ttgatagatttggcagccttgaatacttgatgaaccacccttccctgatgaaccagcctctttctttcggtgtcagaaagaccagtcttaggcacttgag

fava_c15665_1177	fava_c15665	1177	C/A	cccttccctgatgaaccagcctctttctttcggtgtcagaaagaccagtcttaggcacttgaggagatggaagccaccatcttttactatgttgctcact[C/A]gtgtctttcccttcggtccggcttcctccctctgcataccaaaactctgttttcaccatcgaatcaagtgcctcgataagcatagagtccaatttctgga

fava_c15665_122	fava_c15665	122	A/C	ttctctatttcggctaagagctgagtaattaatcaatatatgattttgatataataatactcggtttgcttctctatttccatttgtaacttcattttct[A/C]ccaacattttttcttcaaaggcaatgaagaaattcatgtctaacatataatcagagcaaacggatcagtttgcttctatatattcaaaccYgcgatcaat

fava_c15665_1699	fava_c15665	1699	T/C	ctgaatgttgaattgcataaggtttctctttgacataaaactgttgaggactttcagagagttcccattcttcaagtccttgttcatcctcccttttcat[T/C]gttaaacatttcgaagagaatgatccaaaagcatctttgcttgaagaacagcttgaatcatcttcatcaaatgaRtcagttatgcatccatcacctctac

fava_c15665_1774	fava_c15665	1774	A/G	gtccttgttcatcctcccttttcatYgttaaacatttcgaagagaatgatccaaaagcatctttgcttgaagaacagcttgaatcatcttcatcaaatga[A/G]tcagttatgcatccatcacctctactagttctgctttcctcttcataagattggttatttagaatgcaactctcaagacccttatatgtcatgattcttt

fava_c15665_1911	fava_c15665	1911	T/A	tcctcttcataagattggttatttagaatgcaactctcaagacccttatatgtcatgattctttcttgatcatgatcaatgctaattttcctccccctgg[T/A]gcaacaacctagtcctttcttcatcttaaacatccctttcactttgacaattgctctgtggcttcaataactcttactctgaaccctcagatggcattct

fava_c15665_213	fava_c15665	213	C/T	tcattttctMccaacattttttcttcaaaggcaatgaagaaattcatgtctaacatataatcagagcaaacggatcagtttgcttctatatattcaaacc[C/T]gcgatcaataataacaataacatttacaaatctaatatcaaaaataaaataaaaaaacctctatggagaatttggagtagacaaacttgcacaagcctct

fava_c15665_337	fava_c15665	337	C/T	ttacaaatctaatatcaaaaataaaataaaaaaacctctatggagaatttggagtagacaaacttgcacaagcctctctactaatgcaccaaacactact[C/T]ctgcttggtgtagttgttattacatccttggatttggcatcaaaagattcatgttcaaaatctgaagtactgttgcgaKattgaccatctgctttgttca

fava_c15665_416	fava_c15665	416	T/G	actaatgcaccaaacactactYctgcttggtgtagttgttattacatccttggatttggcatcaaaagattcatgttcaaaatctgaagtactgttgcga[T/G]attgaccatctgctttgttcatcttttgaagtaaggattgcctaatatgtgaatccgtYatccaagtttcagagatatttgttccaggagaggaacttag

fava_c15665_475	fava_c15665	475	T/C	catcaaaagattcatgttcaaaatctgaagtactgttgcgaKattgaccatctgctttgttcatcttttgaagtaaggattgcctaatatgtgaatccgt[T/C]atccaagtttcagagatatttgttccaggagaggaacttagtgaaccagaattgcttagagaatcctcttgcaatatatctccaattctagttagtatgc

fava_c15665_921	fava_c15665	921	C/T	gtcctctctttcaaagagaatataacagcttccaatttattaatggtctcgagagccatatgttcagatttcaaattcagagatttaaggatgtcttctc[C/T]gcagcYtgattcagttgtcaaaaccttgtgcagttctagtccaaggtttgcctttcctgactttgaaagtgcatctttaataactccYggcacaggcatt

fava_c15665_927	fava_c15665	927	T/C	tctttcaaagagaatataacagcttccaatttattaatggtctcgagagccatatgttcagatttcaaattcagagatttaaggatgtcttctcYgcagc[T/C]tgattcagttgtcaaaaccttgtgcagttctagtccaaggtttgcctttcctgactttgaaagtgcatctttaataactccYggcacaggcatttcaagc

fava_c15666_1073	fava_c15666	1073	A/G	aagcttggcaagcgtgattctttgcttctgtgaatatcctcgccgagatgttccccacctgaggcataggagagccattatctgtattgagaagcattcc[A/G]gtctcaggcacaacttcaaataggttggcacgattttcctcgtgaaaaaatcctggctttgcactgccagtgataaccacatcaaagtattgaagccatt

fava_c15666_126	fava_c15666	126	A/T	aaatagtgaatatcaatgaaaacttatttacaagttgcaaagtaaccggtcatctgcaagtaatatcaaatgaaaacactcaatttctgtaataaaaaaa[A/T]tWaaaaaaaattgaatagcacccaatttctgtaattgctactgcacactatatcacccccacattaggggtggaccgaactatacaattttttatgatat

fava_c15666_128	fava_c15666	128	T/A	atagtgaatatcaatgaaaacttatttacaagttgcaaagtaaccggtcatctgcaagtaatatcaaatgaaaacactcaatttctgtaataaaaaaaWt[T/A]aaaaaaaattgaatagcacccaatttctgtaattgctactgcacactatatcacccccacattaggggtggaccgaactatacaattttttatgatatat

fava_c15666_1805	fava_c15666	1805	T/C	taaaagaccaatttagtaactcgctaggataccccaaatcgtaaaccagctttttaattgtgccttgataagcaagagattcaaaagtttcaggcttgta[T/C]tgtgccaaagtataatccatgtcaaatcccacagcaacaatgttcttcatattcagtgaccggttacaaaatatctgtttgcctatatcagttctagatc

fava_c15666_1932	fava_c15666	1932	T/C	tcccacagcaacaatgttcttcatattcagtgaccggttacaaaatatctgtttgcctatatcagttctagatccatgttcaggagaagatgaccataca[T/C]RtggYtgtttttccctatccctgccaattgattttgagtcatgcataccaacgtcgtggttctcgtccattgaatattgagaatgcaacttgctagcacc

fava_c15666_1933	fava_c15666	1933	G/A	cccacagcaacaatgttcttcatattcagtgaccggttacaaaatatctgtttgcctatatcagttctagatccatgttcaggagaagatgaccatacaY[G/A]tggYtgtttttccctatccctgccaattgattttgagtcatgcataccaacgtcgtggttctcgtccattgaatattgagaatgcaacttgctagcacct

fava_c15666_1937	fava_c15666	1937	C/T	cagcaacaatgttcttcatattcagtgaccggttacaaaatatctgtttgcctatatcagttctagatccatgttcaggagaagatgaccatacaYRtgg[C/T]tgtttttccctatccctgccaattgattttgagtcatgcataccaacgtcgtggttctcgtccattgaatattgagaatgcaacttgctagcacctggtg

fava_c15666_307	fava_c15666	307	G/A	tatacaattttttatgatatatctagcatcttttttccccttgtagaaaacatttaaaacatcatatggcgcatcaaattacgaaaatgagcttgagaag[G/A]gagatgggagccagcaacgcacaacagatcagaatacaactttccaacccaacgggatgcagccataaatacctaaaSttatattYctcgtggctcagaa

fava_c15666_385	fava_c15666	385	C/G	ttacgaaaatgagcttgagaagRgagatgggagccagcaacgcacaacagatcagaatacaactttccaacccaacgggatgcagccataaatacctaaa[C/G]ttatattYctcgtggctcagaaccgagaattccaaattcatgctgcataaaatcttcactgggtctgtaatacttgtctggagagtgYaacgccaggtta

fava_c15666_393	fava_c15666	393	T/C	atgagcttgagaagRgagatgggagccagcaacgcacaacagatcagaatacaactttccaacccaacgggatgcagccataaatacctaaaSttatatt[T/C]ctcgtggctcagaaccgagaattccaaattcatgctgcataaaatcttcactgggtctgtaatacttgtctggagagtgYaacgccaggttagatacttg

fava_c15666_473	fava_c15666	473	C/T	taaatacctaaaSttatattYctcgtggctcagaaccgagaattccaaattcatgctgcataaaatcttcactgggtctgtaatacttgtctggagagtg[C/T]aacgccaggttagatacttggctagtataaaggcaggcaaatctctcaacctgatgagcaaagcgagaattctgataaccagttttcataagctgtcccc

fava_c15671_1242	fava_c15671	1242	C/T	aaacgaatcacttaatttggaagcactactacgataaagaaaagaacatcaattataataatttgaacgctttacggcccagcagcctttccttgatgaa[C/T]caacttcgcatYtatatccctgagctttttccggatcttagaaagatagtattcttggatgatgatgttgtagtacaacgtgacatatcttctttatggg

fava_c15671_1254	fava_c15671	1254	T/C	taatttggaagcactactacgataaagaaaagaacatcaattataataatttgaacgctttacggcccagcagcctttccttgatgaaYcaacttcgcat[T/C]tatatccctgagctttttccggatcttagaaagatagtattcttggatgatgatgttgtagtacaacgtgacatatcttctttatgggacttagatctta

fava_c15671_1517	fava_c15671	1517	G/A	aaatacacaaactacttgaacttttcacatcctctcatatcatctaattttgatgatgaacagtgtggatggctttacggcatgaatgtctttgatcttg[G/A]tgcttggcgaagaacaaacataacggaRacctaccggaaatggttgaaactcaacctcgaatcagagacagcattgtggaatccaggagtgcttccacct

fava_c15671_1545	fava_c15671	1545	A/G	atcctctcatatcatctaattttgatgatgaacagtgtggatggctttacggcatgaatgtctttgatcttgRtgcttggcgaagaacaaacataacgga[A/G]acctaccggaaatggttgaaactcaacctcgaatcagagacagcattgtggaatccaggagtgcttccacctgcattgattgcatttgatggtcaagtgc

fava_c15671_1866	fava_c15671	1866	C/G	ggcttgaaattggtttcccagaatatagaagtttgtggagtagatatgtaaattcctctaataagtttataaggagatgtagaatcataaggtgaaggaa[C/G]aaagttccaccaccacaRgtatttattaagattccatcattgacactgcgttggattagagggaagaagcttctgccttcagtctccgattcatacgttc

fava_c15671_1884	fava_c15671	1884	G/A	cagaatatagaagtttgtggagtagatatgtaaattcctctaataagtttataaggagatgtagaatcataaggtgaaggaaSaaagttccaccaccaca[G/A]gtatttattaagattccatcattgacactgcgttggattagagggaagaagcttctgccttcagtctccgattcatacgttctgtatgatattgattacg

fava_c15671_2109	fava_c15671	2109	A/T	agggagggagtacatacatatatgaatgtactaagaaatatcctactggaagcagtatctgcagttaagaaagatataaatcatcattgatctagccgtc[A/T]tctgacaatactgtaaatttgttcttgaaatggagaaactgtttcgtatgttttgaaatttcatcgaggcggttctgaagtcttgttgcataaaacgcta

fava_c15671_2247	fava_c15671	2247	T/C	actgtttcgtatgttttgaaatttcatcgaggcggttctgaagtcttgttgcataaaacgctaggtaatcgcttacagacaggtttagacttcatccttt[T/C]ggtgttgaaagtaccattgttggacatcaaatttagtttgtatatcattgttgctttatYtacttattttgctattttgtgtatttggtaaacacRagaa

fava_c15671_2307	fava_c15671	2307	T/C	ctaggtaatcgcttacagacaggtttagacttcatcctttYggtgttgaaagtaccattgttggacatcaaatttagtttgtatatcattgttgctttat[T/C]tacttattttgctattttgtgtatttggtaaacacRagaaatagtgaaaaagtaacatgtatacagatgtaactgatataaaatcaaaagagatattaat

fava_c15671_2343	fava_c15671	2343	A/G	ctttYggtgttgaaagtaccattgttggacatcaaatttagtttgtatatcattgttgctttatYtacttattttgctattttgtgtatttggtaaacac[A/G]agaaatagtgaaaaagtaacatgtatacagatgtaactgatataaaatcaaaagagatattaattgtatttattttggaaatttatctcatgttttcttc

fava_c15671_323	fava_c15671	323	G/C	atttgcacagagagagagagattagttcatggaatcaaggctatgaagttttatatatctgcaaagggaattaagagattcacgatatcgaacggcggag[G/C]taagggatcggctaaggatacatcggcgacggcggcaggggttccSactStaactgcctcttccagtcgccggatttcactccggacggtgcttccggtg

fava_c15671_369	fava_c15671	369	C/G	agttttatatatctgcaaagggaattaagagattcacgatatcgaacggcggagStaagggatcggctaaggatacatcggcgacggcggcaggggttcc[C/G]actStaactgcctcttccagtcgccggatttcactccggacggtgcttccggtggtgttagttctcggactcgttctaccgtttatctttgttagagtcg

fava_c15671_373	fava_c15671	373	G/C	ttatatatctgcaaagggaattaagagattcacgatatcgaacggcggagStaagggatcggctaaggatacatcggcgacggcggcaggggttccSact[G/C]taactgcctcttccagtcgccggatttcactccggacggtgcttccggtggtgttagttctcggactcgttctaccgtttatctttgttagagtcgcgat

fava_c15671_873	fava_c15671	873	C/T	cacacggtgttcctcaaagtctacattgtctttgtctaaagttggctgaagaatatgctgtaaatgccatcgctcgatctagcctacctcctcccgaatt[C/T]gcttctcgccttgctgacccttatctgcatcatctagttctcctaactgataatgtccttgctgcctctgttgttgtaacttctacagttaaaaattcag

fava_c15672_1044	fava_c15672	1044	A/G	ctgtgtaatctttcatattgaaaaaaatgttaaaagtgaaatctgcattatcacaatcttccagcgtagaatgaacaagggtctcatctaaatcaagcac[A/G]agggttactgattttcttcttggagattgcttaggacatagtgtgggttggtcatttaattctacttctgatagttctggtaaactcttgataaatgact

fava_c15672_1274	fava_c15672	1274	T/C	tcatctttgacatcggactcctggttgcaggatcttatctgagctatagcagaatacaagctagcactttcacgaaccacgataccttctccatatgaca[T/C]gatatctggagtacagccaactttagcatcaYcttcacgagcaggcagggtcgtgtactgttcagaagcacaaaccacggaKggYtcggtggatccaaaa

fava_c15672_1306	fava_c15672	1306	T/C	tcttatctgagctatagcagaatacaagctagcactttcacgaaccacgataccttctccatatgacaYgatatctggagtacagccaactttagcatca[T/C]cttcacgagcaggcagggtcgtgtactgttcagaagcacaaaccacggaKggYtcggtggatccaaaatcagataggtacttggtttcaccaacatcatc

fava_c15672_1356	fava_c15672	1356	T/G	taccttctccatatgacaYgatatctggagtacagccaactttagcatcaYcttcacgagcaggcagggtcgtgtactgttcagaagcacaaaccacgga[T/G]ggYtcggtggatccaaaatcagataggtacttggtttcaccaacatcatcatccaatgaatttccacaaaagggtaaacttgtaatgatcatgtcagaaa

fava_c15672_1359	fava_c15672	1359	C/T	cttctccatatgacaYgatatctggagtacagccaactttagcatcaYcttcacgagcaggcagggtcgtgtactgttcagaagcacaaaccacggaKgg[C/T]tcggtggatccaaaatcagataggtacttggtttcaccaacatcatcatccaatgaatttccacaaaagggtaaacttgtaatgatcatgtcagaaatgt

fava_c15672_1881	fava_c15672	1881	G/A	tctctacctcttgagcagatatcacgacttcagaacagggatttttggaaattactttcgatttttgacagatttgaagatcatttctctctgctaaaca[G/A]ctcacacttactttgctcttcatatttagggatggcatttgacttgcacttataatcggtacaacaatcttgctttcggcacatttgtggggaaccaacc

fava_c15672_2020	fava_c15672	2020	T/G	ttgacttgcacttataatcggtacaacaatcttgctttcggcacatttgtggggaaccaacctccctcttgacactcaaagcaagcagcagaagagtctc[T/G]Kctaacaacggaatcaacaactttctctctctagtttctctctctagaaatccattgatttacagttgcgcgagtcagagagccccgcgg

fava_c15672_2021	fava_c15672	2021	G/T	tgacttgcacttataatcggtacaacaatcttgctttcggcacatttgtggggaaccaacctccctcttgacactcaaagcaagcagcagaagagtctcK[G/T]ctaacaacggaatcaacaactttctctctctagtttctctctctagaaatccattgatttacagttgcgcgagtcagagagccccgcgg

fava_c15672_511	fava_c15672	511	A/G	ttgtatatgttaatgcatctcaacttgaatggaaaggggcatttagcaactatttagaggctaaatttcctaaacctagagaaagtagaaaagatgctaa[A/G]tttgttccaaggaaaaaccttattctttgttaccgtatctctttgcaatgataggacgcacatcatcagcatcagccaatgtctctagaaagggaagtaa

fava_c15674_1303	fava_c15674	1303	G/A	gtggatggggtggaaaccattcaagattacttacacaagtgattatttccaagaactgtatgaattagctgtggagctcataagaaagggttgtgcttat[G/A]ttgatcatcagacacctgatgaaataaaggagtatagggagaagaagttgaacagtccctggagagacagaccagtttcagaatcgttgaaactttttga

fava_c15674_1893	fava_c15674	1893	G/A	gcctgatgacattagctggtttgcggcgtagaggcatgacttcaacagcaatcaattcttttgttcggggaattggaatcactagaagtgatggcacttt[G/A]atatccgtgggtcgccttgagcatcatgttagggaagaaatgaacaaaaccgctcctcggacaatggctgtcctacatccactcaaggttgttattacta

fava_c15674_2521	fava_c15674	2521	C/A	caaagtggtaattccaaatgcatttggattgtcctctctccgggatgcaaaagttggggacagtttccagtttgaaagattaggctattttacggtcgac[C/A]aggactctactccagaaaaacttgtttttaatagaaccgtcacattaaaggacagctatagtaagggtggaaaataggataatcatgaatggaaattaaa

fava_c15674_366	fava_c15674	366	C/A	taaagatcggtttagacgaacgcacagctcgaaacaccatcgccaataacaaagtcactaccaatctcactgctgttatccacgaggccggtgttaccga[C/A]ggatgcagccgatcggttggaaaccttatttatacggttgcaaccaagtaccctggaaatgccttgccgcatcgcccaacattgctgcaatatgtagttt

fava_c15674_891	fava_c15674	891	G/A	aaaaaccttctaaaaagaagaaggagaagcctgctaaagtagaggatacggcagcacctgttgctactcctgaaaaggcatctgaagaagatcttaatcc[G/A]tattcgatattccctaatcccgaggagaatttgaaggtgcatactgaagttccttttagtgatggttctattctgagatgttgcaatacaaaggcgcttc

fava_c15675_1330	fava_c15675	1330	A/C	aaattgcaatcatgtggttgccatctccaatgttgataccctagatcagttcttccatgcttattgcaagccaattgatcagacatataaggacaatctg[A/C]ctcatYgtacaatggatatgaagcattatcaaacacccatcgacccgagaaaacatcgcagcttttagcactcactctctggcgaacggaactcttSgaa

fava_c15675_1336	fava_c15675	1336	T/C	caatcatgtggttgccatctccaatgttgataccctagatcagttcttccatgcttattgcaagccaattgatcagacatataaggacaatctgMctcat[T/C]gtacaatggatatgaagcattatcaaacacccatcgacccgagaaaacatcgcagcttttagcactcactctctggcgaacggaactcttSgaatcacca

fava_c15675_1427	fava_c15675	1427	C/G	ctgMctcatYgtacaatggatatgaagcattatcaaacacccatcgacccgagaaaacatcgcagcttttagcactcactctctggcgaacggaactctt[C/G]gaatcaccacgcctagcaatttttctaccagtatactctgtagtagagttgcatctactgaatctatccaaaacctcttgagatttgttgaggtgactag

fava_c15675_162	fava_c15675	162	C/T	gatgcaactctattctcattatcagaattcgaatctcggactcatggttaaaagatccaaactactaacacacaaaagaccgtgtcgaaaatgcctatat[C/T]cttgattttgtgaagcaactctacacaWgcaagcattctattcaaaacctctaatacaaagagaMRtaaaaagtggaataaattacatttaaaagattgg

fava_c15675_190	fava_c15675	190	A/T	tcgaatctcggactcatggttaaaagatccaaactactaacacacaaaagaccgtgtcgaaaatgcctatatYcttgattttgtgaagcaactctacaca[A/T]gcaagcattctattcaaaacctctaatacaaagagaMRtaaaaagtggaataaattacatttaaaagattggtttYttatccttccatttttttcagtYg

fava_c15675_227	fava_c15675	227	C/A	taacacacaaaagaccgtgtcgaaaatgcctatatYcttgattttgtgaagcaactctacacaWgcaagcattctattcaaaacctctaatacaaagaga[C/A]RtaaaaagtggaataaattacatttaaaagattggtttYttatccttccatttttttcagtYgattctcttgatctcttcgcccccttgtatcaYactca

fava_c15675_228	fava_c15675	228	G/A	aacacacaaaagaccgtgtcgaaaatgcctatatYcttgattttgtgaagcaactctacacaWgcaagcattctattcaaaacctctaatacaaagagaM[G/A]taaaaagtggaataaattacatttaaaagattggtttYttatccttccatttttttcagtYgattctcttgatctcttcgcccccttgtatcaYactcat

fava_c15675_266	fava_c15675	266	C/T	gattttgtgaagcaactctacacaWgcaagcattctattcaaaacctctaatacaaagagaMRtaaaaagtggaataaattacatttaaaagattggttt[C/T]ttatccttccatttttttcagtYgattctcttgatctcttcgcccccttgtatcaYactcatatgatcatattcaatgaacaaaattgaaatgtgtaata

fava_c15675_289	fava_c15675	289	C/T	aWgcaagcattctattcaaaacctctaatacaaagagaMRtaaaaagtggaataaattacatttaaaagattggtttYttatccttccatttttttcagt[C/T]gattctcttgatctcttcgcccccttgtatcaYactcatatgatcatattcaatgaacaaaattgaaatgtgtaatagatttcataaaaagtggaaWagt

fava_c15675_322	fava_c15675	322	T/C	agagaMRtaaaaagtggaataaattacatttaaaagattggtttYttatccttccatttttttcagtYgattctcttgatctcttcgcccccttgtatca[T/C]actcatatgatcatattcaatgaacaaaattgaaatgtgtaatagatttcataaaaagtggaaWagtaactcgttccacgtatcaggcacccctggcaag

fava_c15675_386	fava_c15675	386	T/A	agtYgattctcttgatctcttcgcccccttgtatcaYactcatatgatcatattcaatgaacaaaattgaaatgtgtaatagatttcataaaaagtggaa[T/A]agtaactcgttccacgtatcaggcacccctggcaagcaccaatgtatgcaatcagagtaagatgaaggattcgacaactgttccggacgaagaggctccc

fava_c15675_623	fava_c15675	623	T/C	gatactcggaaagttgagtaacattgaggacagaaacttttgaatttaaactgctaaggatgttctctacactgctcatggtaggtaaatcagaaccact[T/C]ccccaatagccctcaaagtcaataggatccttctcgccgtaacagtttccttcatttcccggattccattctcggctccagaggtgtgttggggacatgg

fava_c15681_103	fava_c15681	103	C/A	ttctgatttagtttccaccacactaacgcccaaacccctctcgtagatctacttcacaatttccgccgccgtgcggcttcaccaacgccgtcgYgtYcct[C/A]ccttcgtttaaatcgctgctcttcctcctctgtttctccgtttaaaggtgttgttaggatatgatctatgccacttgcatcacaacattaaagtcactga

fava_c15681_1090	fava_c15681	1090	C/T	tatgccttataaattatgtttgccaagtgatttgaagatagattggagaaattttaatatgctcaacttggaaggaatgtaccgtttctgattcaacaaa[C/T]cttgaagccttatatacccaaattacatcaagacttgcatctRgattgcctcaagagtgttttatgtttgtgttaccactgaaaagatggtattatggga

fava_c15681_1133	fava_c15681	1133	A/G	tggagaaattttaatatgctcaacttggaaggaatgtaccgtttctgattcaacaaaYcttgaagccttatatacccaaattacatcaagacttgcatct[A/G]gattgcctcaagagtgttttatgtttgtgttaccactgaaaagatggtattatgggaaaaagccgaattgStcSgaaggttatttattatttgatcaaat

fava_c15681_1204	fava_c15681	1204	G/C	atacccaaattacatcaagacttgcatctRgattgcctcaagagtgttttatgtttgtgttaccactgaaaagatggtattatgggaaaaagccgaattg[G/C]tcSgaaggttatttattatttgatcaaatgttacctctgttcatttacctttagagccgcttgctttttaattaaattttctttatgttaacattatata

fava_c15681_1207	fava_c15681	1207	C/G	cccaaattacatcaagacttgcatctRgattgcctcaagagtgttttatgtttgtgttaccactgaaaagatggtattatgggaaaaagccgaattgStc[C/G]gaaggttatttattatttgatcaaatgttacctctgttcatttacctttagagccgcttgctttttaattaaattttctttatgttaacattatataatt

fava_c15681_211	fava_c15681	211	C/T	ttaaatcgctgctcttcctcctctgtttctccgtttaaaggtgttgttaggatatgatctatgccacttgcatcacaacattaaagtcactgagctccag[C/T]gcactagttttgcattcttcgttgaattttcgagtgaaatcatcctctaggtgttctcttaggcagataagaatggaaactaatcttagttgtagcacag

fava_c15681_96	fava_c15681	96	C/T	ggttctgatttagtttccaccacactaacgcccaaacccctctcgtagatctacttcacaatttccgccgccgtgcggcttcaccaacgccgtcg[C/T]gtYcctMccttcgtttaaatcgctgctcttcctcctctgtttctccgtttaaaggtgttgttaggatatgatctatgccacttgcatcacaacattaaag

fava_c15681_99	fava_c15681	99	T/C	ggttctgatttagtttccaccacactaacgcccaaacccctctcgtagatctacttcacaatttccgccgccgtgcggcttcaccaacgccgtcgYgt[T/C]cctMccttcgtttaaatcgctgctcttcctcctctgtttctccgtttaaaggtgttgttaggatatgatctatgccacttgcatcacaacattaaagtca

fava_c15682_1024	fava_c15682	1024	G/T	cacctctccattggccttgaatttgtagctttcttttgcctggaaccttcttttccgtgtcctgtgcaatatgtggcacttcagtttcctcggaattctg[G/T]cgttcacgtgtaagaggactaacttccaaatcttctgaattggatacattgtcaatcatattttcctctggaactgctttgacgacttcttcaattgtgc

fava_c15682_191	fava_c15682	191	G/A	atgacgagattcacaaaaatttatcaatgaacattcatgggtgaaggaaaagataatcatttctataaatgtatagcataaataaatatctgttacaaca[G/A]agtatcaRttaaggagtccttagtagctttcacatcatcatgatttttattgttcaccttctttagagggttcacctcccacaccattagaggatttgtc

fava_c15682_199	fava_c15682	199	G/A	attcacaaaaatttatcaatgaacattcatgggtgaaggaaaagataatcatttctataaatgtatagcataaataaatatctgttacaacaRagtatca[G/A]ttaaggagtccttagtagctttcacatcatcatgatttttattgttcaccttctttagagggttcacctcccacaccattagaggatttgtccacatgcc

fava_c15682_2533	fava_c15682	2533	G/A	tgattctaaacgatgcaggcaacggtgttctgagaattgtgatgaaattttcccattcttgtggatcgactattaactgttccttgtaatatgaatcgaa[G/A]gaagggttctgagtagcgaaaggagcccaatgagtagtttgagttttatcggaggataaggaagcatctggatcggatttggaacgcttccagacgtttt

fava_c15682_481	fava_c15682	481	G/A	ccaaccttgccctacccttccagcatccgatggctattgttgactcatcaacattgagggactctgtagcaactgtgttccctttacccataactataac[G/A]caacagcctggcaatagattttctgcctttttaccaaactcggcatcagcaaaatctgtaaattttacatctttgtcctgcagaagatgcttgaagtcYa

fava_c15682_580	fava_c15682	580	C/T	cRcaacagcctggcaatagattttctgcctttttaccaaactcggcatcagcaaaatctgtaaattttacatctttgtcctgcagaagatgcttgaagtc[C/T]acaggagatgcctgtataatttgctttgttatgtatgggagaataaggggcaatccttcagatgatatYcggaaagcacatggtgcatcagtaccttctc

fava_c15682_649	fava_c15682	649	C/T	catctttgtcctgcagaagatgcttgaagtcYacaggagatgcctgtataatttgctttgttatgtatgggagaataaggggcaatccttcagatgatat[C/T]cggaaagcacatggtgcatcagtaccttctcgtgctgtttgtctttcaaacatcttcatgccaactgaggttattttcagttgctgcccagctgagaagt

fava_c15682_811	fava_c15682	811	A/G	caactgaggttattttcagttgctgcccagctgagaagttcaactcaagaatatccttgacaaacttggagacataataaattcttttcacgtgacttgt[A/G]tcactattacgtgtaataaggtggccattgaatggaaaacgctcatcaatcccataaaagtcccttatactattaataaYtgtgtcatctttaaaaaaga

fava_c15682_89	fava_c15682	89	T/C	gtgtttctcacaaacaatttatgcaatattttccagcattgaattatgcatactgatacaaatatgaaccaaggtctgtcttagaaat[T/C]aatgacgagattcacaaaaatttatcaatgaacattcatgggtgaaggaaaagataatcatttctataaatgtatagcataaataaatatctgttacaac

fava_c15682_891	fava_c15682	891	T/C	attcttttcacgtgacttgtRtcactattacgtgtaataaggtggccattgaatggaaaacgctcatcaatcccataaaagtcccttatactattaataa[T/C]tgtgtcatctttaaaaaagacaacagggtcaacacctctccattggccttgaatttgtagctttcttttgcctggaaccttcttttccgtgtcctgtgca

fava_c15686_1193	fava_c15686	1193	A/G	gcaatgatatagatgctgcaaaagtggcagccatgagagctgctgaattagttaacaagaacttagtagggcctaattccttgactacggaccaaaagaa[A/G]aaactgttgtggggaagcaagaagagtacacccactgaagagtctggccatcgctgggatatggcaatgtttgatcgtgagcgacaggaaaaattcaaca

fava_c15686_1466	fava_c15686	1466	G/A	gagctgagaagcagaaggaactccagctggatctcgagaaacagtacactgcaggtctccggcgaagagatggccgtacagttggattaggtctttaaac[G/A]ttcaccagcagaattttgcatcctttgactttggcagttattYtgagtatacaaaataYtcactagtgttattcactggattaggtStttaaacttatca

fava_c15686_149	fava_c15686	149	C/T	tgaaaagtgtttttggaaaggggaagaagaatcgacagcatttgttcaaacgacgagagaagagggaagaatcaatcgagggtttcatttctcctcctcc[C/T]cctcctcacaattaccctatcaatcaatcatcctcatcataaaaatccctaatttcgcactcgctttgtaggatggattcaaactcacagtctccgccgc

fava_c15686_1509	fava_c15686	1509	C/T	gtacactgcaggtctccggcgaagagatggccgtacagttggattaggtctttaaacRttcaccagcagaattttgcatcctttgactttggcagttatt[C/T]tgagtatacaaaataYtcactagtgttattcactggattaggtStttaaacttatcatgtttgttatatatgtttgggagttcatcacgtgtatgatcta

fava_c15686_1525	fava_c15686	1525	T/C	cggcgaagagatggccgtacagttggattaggtctttaaacRttcaccagcagaattttgcatcctttgactttggcagttattYtgagtatacaaaata[T/C]tcactagtgttattcactggattaggtStttaaacttatcatgtttgttatatatgtttgggagttcatcacgtgtatgatctatgtatgatctgatatt

fava_c15686_1553	fava_c15686	1553	C/G	taggtctttaaacRttcaccagcagaattttgcatcctttgactttggcagttattYtgagtatacaaaataYtcactagtgttattcactggattaggt[C/G]tttaaacttatcatgtttgttatatatgtttgggagttcatcacgtgtatgatctatgtatgatctgatattttgaggagcttatcacagtcgtacaaaa

fava_c15686_1657	fava_c15686	1657	A/T	aaacttatcatgtttgttatatatgtttgggagttcatcacgtgtatgatctatgtatgatctgatattttgaggagcttatcacagtcgtacaaaattt[A/T]aatgcgacggtttgatgctagaaggatatataatggagctgggagaggtgtgagaaaacatagatagtgatgaggtttcatcgatatcaacgcaaacaaa

fava_c15686_260	fava_c15686	260	C/G	attaccctatcaatcaatcatcctcatcataaaaatccctaatttcgcactcgctttgtaggatggattcaaactcacagtctccgccgcttggcgattc[C/G]gacaccaaaaatgtatttcgcaagccttccggggaggcagctaataggaattatcggcgccgttcacctattgacggctcatcatcaccKgatgatggta

fava_c15686_350	fava_c15686	350	G/T	ttggcgattcSgacaccaaaaatgtatttcgcaagccttccggggaggcagctaataggaattatcggcgccgttcacctattgacggctcatcatcacc[G/T]gatgatggtagtcccaggcgagaacacagctcaggtccatatccatctagggaagattctgtgagagtctctcaccatcattcaaggaagcttgacggaa

fava_c15686_737	fava_c15686	737	A/G	gagaggagagtgatagcaggtcaaaagattatgggcgtagtatggataagtattcacgtgaaaaatatgaaaggtctggatctggcaagaggtgtgcgga[A/G]tatgatgaggtggagaaggaaagacgtatgagggacggggatggtcgagatgaaagaaaggattctcaaagaagttctggagaacacaaaagtgaccgtg

fava_c15687_1362	fava_c15687	1362	A/G	gttgtaagccgtgtggaagctatggagattatcctgttgagaggtgtcttagcagtatggagagggcttacaattttgcggataatcaggtgctcaaact[A/G]tatgggttcaggcatagtggtctgttgagtcctaagattaagagaatcaggaatgagtcggttactcctttggagtttgtcgaccagtttgatattcgaa

fava_c15687_999	fava_c15687	999	C/A	tcgaccaaaagtcgtggattgctatcaatactgggagttttctgtttaggaattgtcagtggtctttggatttgcttgatgagtgggctcccatgggtcc[C/A]aagggtccggttcgtgaagaggccgggaaggttttgactgctaatcttaagggaaggccggcttttgaggcggatgatcagtctgcattgatatatttgt

fava_c15695_1213	fava_c15695	1213	C/T	taattggtccacctcctcctcttccaccataaacaagcaaccgcttttcgaccattgtggcagtatggccgcatctaggttgtggcaatgttccagaaac[C/T]gagagctccgtccactcgagggatattgtgtccaaaacataaacatcggatagccacttctttccatcccatccaccatacataacaatcttccggttcc

fava_c15695_1507	fava_c15695	1507	G/A	aaaagtctcccaacctttgacctccagaacggccaccaaaaacaaacatatgacaatcaatggaaacagcaacatgaaaagctcgtggactgggacccac[G/A]tgtccatccgaatcacttccagtgcactctggcttataccatagcttagcctcaatatcatagacaaggatatcagtgagaaacttcttatcgacaagac

fava_c15695_1663	fava_c15695	1663	A/T	tatcatagacaaggatatcagtgagaaacttcttatcgacaagaccgccgaagacgacaaccttggatttgccgatgttaacagcagaatgaccactgcg[A/T]ggttggggagRagttccggcgtaatcagaagatgtagctccgacccagtaatacattgttttgWtgtctttcgatcttccgattccggtgacgatatcag

fava_c15695_1674	fava_c15695	1674	A/G	aggatatcagtgagaaacttcttatcgacaagaccgccgaagacgacaaccttggatttgccgatgttaacagcagaatgaccactgcgWggttggggag[A/G]agttccggcgtaatcagaagatgtagctccgacccagtaatacattgttttgWtgtctttcgatcttccgattccggtgacgatatcagatcaaaggtcg

fava_c15695_1727	fava_c15695	1727	A/T	ggatttgccgatgttaacagcagaatgaccactgcgWggttggggagRagttccggcgtaatcagaagatgtagctccgacccagtaatacattgttttg[A/T]tgtctttcgatcttccgattccggtgacgatatcagatcaaaggtcgtgtaatgtactcaccgtttcaaccttctttcgttgttgttttttctgggttta

fava_c15695_354	fava_c15695	354	C/T	aaaagttttggtatatcatccatacgcaactgagaaacgtttttgatgtgataaaatcgatgcatcgcgtgttcatcaaatccaggttgctcttcagatg[C/T]gttctttcctagatgaagccggtgaattagacgttggtaatcccgaagcaacggcccgagctctttgagtttgactatatgaggctcRgccttcttccag

fava_c15695_442	fava_c15695	442	G/A	gctcttcagatgYgttctttcctagatgaagccggtgaattagacgttggtaatcccgaagcaacggcccgagctctttgagtttgactatatgaggctc[G/A]gccttcttccagtgttcacggagcgcctcaatagccggtgaacttgcagaaacattttcacctgctaatattgaagctaattcaagaagctctttatctt

fava_c15695_547	fava_c15695	547	C/T	tcttccagtgttcacggagcgcctcaatagccggtgaacttgcagaaacattttcacctgctaatattgaagctaattcaagaagctctttatcttccaa[C/T]tcattcacaattggaagattagaattggactgtgaaactgatatttgcaatctctgttgcaattctgagagaggaaatttctctgtttggttttccttga

fava_c15695_654	fava_c15695	654	A/G	acaattggaagattagaattggactgtgaaactgatatttgcaatctctgttgcaattctgagagaggaaatttctctgtttggttttccttgaattgag[A/G]ttgaaaattaccattaattgacgctgcatcgttactttcaggaatatttctgggtggagatgcagttactctatttgcaatcgggtcatcttgagggact

fava_c15695_970	fava_c15695	970	G/A	tggacagccgcttccactgtgctgaaactctgtccaatacaatgcaatcgttatagtagatatcatagcgactcaaccatccaccggtcccatgccctcc[G/A]aacatcaacaaatagtgtcctgcatttgtaactgtctggccacaacggggggaaggagcttgacctggaagctttaactgagtccacccgggcgtttcat

fava_c15704_1493	fava_c15704	1493	G/A	tttgtgtagtagtttcaagttcttgtttgagcctctctgtggtacttataatgtcaccggtgctagcactacaactgtttaaagcctttgctatcctgtc[G/A]cagcattcagcaagcgaatcgacctcattctccacgctatccaaagccagttgcgcagcatgtgaagcgtcgaggaattcgtggttgatggacagggcgc

fava_c15704_1595	fava_c15704	1595	C/T	agcattcagcaagcgaatcgacctcattctccacgctatccaaagccagttgcgcagcatgtgaagcgtcgaggaattcgtggttgatggacagggcgcg[C/T]ttttcgatagttgatcggaggttgcggcgtgcttgaggagtgttctcRtcgtagaaagaagagagtgtgtttagYgaagacaagagatccggcgtgtcga

fava_c15704_161	fava_c15704	161	G/A	caagaactcgcaagtgattatctatcattgctccaagccttttcacatactcagtagcaacctcatatccagataaaggttgttgaatagcacacaagca[G/A]ttaatgagaaagatctttgagggtgtctcactagtcagtggtgaggaagcagagcggctgtttgacaagattgcatcaaccgatgatttagtaagttgac

fava_c15704_1643	fava_c15704	1643	G/A	gttgcgcagcatgtgaagcgtcgaggaattcgtggttgatggacagggcgcgYttttcgatagttgatcggaggttgcggcgtgcttgaggagtgttctc[G/A]tcgtagaaagaagagagtgtgtttagYgaagacaagagatccggcgtgtcgatccttgattctagaactttcttcagcttccgggagagtcccggcgcca

fava_c15704_1670	fava_c15704	1670	T/C	attcgtggttgatggacagggcgcgYttttcgatagttgatcggaggttgcggcgtgcttgaggagtgttctcRtcgtagaaagaagagagtgtgtttag[T/C]gaagacaagagatccggcgtgtcgatccttgattctagaactttcttcagcttccgggagagtcccggcgccaaacccgccaccgtcgttcccattctct

fava_c15707_182	fava_c15707	182	C/T	taactttttaaccgaaagttcataaactttttctctgcttttcactcattttagaaaccaccgttatcgccgccgcacacgacggcgattttctaccgaa[C/T]YattttttctttggtttcatgtgtttttctRcctctRttgccgttactScgcgttgcctcatctaacggatgggttgcgtgcttgggacggcggccggtg

fava_c15707_183	fava_c15707	183	T/C	aactttttaaccgaaagttcataaactttttctctgcttttcactcattttagaaaccaccgttatcgccgccgcacacgacggcgattttctaccgaaY[T/C]attttttctttggtttcatgtgtttttctRcctctRttgccgttactScgcgttgcctcatctaacggatgggttgcgtgcttgggacggcggccggtga

fava_c15707_213	fava_c15707	213	G/A	tctctgcttttcactcattttagaaaccaccgttatcgccgccgcacacgacggcgattttctaccgaaYYattttttctttggtttcatgtgtttttct[G/A]cctctRttgccgttactScgcgttgcctcatctaacggatgggttgcgtgcttgggacggcggccggtgacggagatcaWcgtcgtcgacggagagaaca

fava_c15707_219	fava_c15707	219	G/A	cttttcactcattttagaaaccaccgttatcgccgccgcacacgacggcgattttctaccgaaYYattttttctttggtttcatgtgtttttctRcctct[G/A]ttgccgttactScgcgttgcctcatctaacggatgggttgcgtgcttgggacggcggccggtgacggagatcaWcgtcgtcgacggagagaacagcgtaa

fava_c15707_231	fava_c15707	231	C/G	tttagaaaccaccgttatcgccgccgcacacgacggcgattttctaccgaaYYattttttctttggtttcatgtgtttttctRcctctRttgccgttact[C/G]cgcgttgcctcatctaacggatgggttgcgtgcttgggacggcggccggtgacggagatcaWcgtcgtcgacggagagaacagcgtaaggagtcttctga

fava_c15707_293	fava_c15707	293	A/T	ttggtttcatgtgtttttctRcctctRttgccgttactScgcgttgcctcatctaacggatgggttgcgtgcttgggacggcggccggtgacggagatca[A/T]cgtcgtcgacggagagaacagcgtaaggagtcttctgaaggaagaaataatgtcgttagggttaaagagaagcaaacgaatcgtcatactggagattttc

fava_c15710_1384	fava_c15710	1384	T/C	taggcatgtattcataaaccaaaagcctttgatctccatcagtacaatagccaatcaacttaacaagattagaatgatgcagcagactcagcataagaac[T/C]tcagtcacaaactcctgaaaaccttgacggccatcatgactcaattgYttaacagcaacaagctcgccggttgaaagacggcccttgaaaacttttccaa

fava_c15710_1432	fava_c15710	1432	T/C	agccaatcaacttaacaagattagaatgatgcagcagactcagcataagaacYtcagtcacaaactcctgaaaaccttgacggccatcatgactcaattg[T/C]ttaacagcaacaagctcgccggttgaaagacggcccttgaaaacttttccaaaaccaccttccccaatcaaattcgcttcattaaaacccctcgtcgccg

fava_c15710_1621	fava_c15710	1621	T/C	ccctcgtcgccgtcgcaagctctcgaaaaccaaaactagccgcagccgttctacttttcccactcatcacactcttcccctttccctctgaacattctct[T/C]ttccattcaccctctgatgcattWgttacaccattccctggagtatgagcagatcgagaaccattatcaacttcaaccctactaacatcttttttccgac

fava_c15710_1645	fava_c15710	1645	A/T	gaaaaccaaaactagccgcagccgttctacttttcccactcatcacactcttcccctttccctctgaacattctctYttccattcaccctctgatgcatt[A/T]gttacaccattccctggagtatgagcagatcgagaaccattatcaacttcaaccctactaacatcttttttccgacgagaaacaaagcaagaaaaacaac

fava_c15710_1807	fava_c15710	1807	A/T	catcttttttccgacgagaaacaaagcaagaaaaacaactcatctctcacacaaataaactcaaactaaaaaataaaaattaaaaaataaaaataaaaat[A/T]aaattaagatctgtctccaggtcacagagagaggatctctctagcatttatcctctctcaaaacctaggagaatatgatgaagagatcaattgaacactg

fava_c15710_201	fava_c15710	201	C/T	tggcattgtttaagaaactaaaaaaaaagtaacttagtctaagaaatataagtaaaacactcgaaatgatagatacatcaatttctatacagatgtttcc[C/T]ttttctatttcttaaataatgccctgataacacttgttagcattttcctttactttgtagcagagctttgactgtcactcaatgacactccatctccatc

fava_c15711_1638	fava_c15711	1638	C/T	ttttggttgaagatttttggcgacggatcaagcatacaaccataaaaatggtaaataagagcataattaaggcaaccaatgtgacaattaagagaatggt[C/T]ttgaaaggaaaatggtgagcatggctatggtaattgtttcctgaatgtaagtgatggtaatggcgaatgaaagtatggttctttgtagaaacaataggtg

fava_c15711_207	fava_c15711	207	G/T	agtgggtgtttcctgagaaaataaaatgaacatctagaacaaagtgcgtctataactaatgcaaatgggtctggccttattggccctttaggcttatttg[G/T]gctgtYtRttttgctctttttttctcctctactctcttctMctttcagtagaggtgaaggaaccatgcaaatctaatttgccaacatgacgatgtcctat

fava_c15711_213	fava_c15711	213	C/T	tgtttcctgagaaaataaaatgaacatctagaacaaagtgcgtctataactaatgcaaatgggtctggccttattggccctttaggcttatttgKgctgt[C/T]tRttttgctctttttttctcctctactctcttctMctttcagtagaggtgaaggaaccatgcaaatctaatttgccaacatgacgatgtcctatcaagta

fava_c15711_215	fava_c15711	215	G/A	tttcctgagaaaataaaatgaacatctagaacaaagtgcgtctataactaatgcaaatgggtctggccttattggccctttaggcttatttgKgctgtYt[G/A]ttttgctctttttttctcctctactctcttctMctttcagtagaggtgaaggaaccatgcaaatctaatttgccaacatgacgatgtcctatcaagtatS

fava_c15711_248	fava_c15711	248	C/A	aagtgcgtctataactaatgcaaatgggtctggccttattggccctttaggcttatttgKgctgtYtRttttgctctttttttctcctctactctcttct[C/A]ctttcagtagaggtgaaggaaccatgcaaatctaatttgccaacatgacgatgtcctatcaagtatSaaattacaaaattttcaccatatatatgtccaa

fava_c15711_315	fava_c15711	315	C/G	RttttgctctttttttctcctctactctcttctMctttcagtagaggtgaaggaaccatgcaaatctaatttgccaacatgacgatgtcctatcaagtat[C/G]aaattacaaaattttcaccatatatatgtccaaacgccatttttagtccaacaaggtagagcagttattKctaggctgctgatgctcttctcacacagaa

fava_c15711_385	fava_c15711	385	G/T	ttgccaacatgacgatgtcctatcaagtatSaaattacaaaattttcaccatatatatgtccaaacgccatttttagtccaacaaggtagagcagttatt[G/T]ctaggctgctgatgctcttctcacacagaattttgaagataatgcagacttaataaattggtatttgtttctgaaatcttagtgaactggaaagagattg

fava_c15711_738	fava_c15711	738	T/C	ctactttttctctgtttgtcaaccttggaagagcccaagagacaagaacatgttctccagtggggcgcttaatatcaaccggtacacgtcctgtcagcaa[T/C]tcgagaagaactaccccgtagctgtaaacatctgactttgtagtaagcttacccgtggaagcgtactctggtgccaaatatcctgtggtccccaacacac

fava_c15711_87	fava_c15711	87	A/G	ttggaactataatttagatattataaaattaaaaggagttaaattaaaacatattatacaataaaatatctatacaaccaaacaca[A/G]tagaaaaaacggatagacgagtgggtgtttcctgagaaaataaaatgaacatctagaacaaagtgcgtctataactaatgcaaatgggtctggccttatt

fava_c15713_1068	fava_c15713	1068	G/C	gcttacttctccgaaaggtggctactggaaggagggtagtatgctaaaaccaataggatcttcttctcaggataagagagtacaacaattcaaagatggt[G/C]ctgatttacatgatagaagggggaatgctgtccgtgtaagagaaaaccaggacgctgccactgctatgaagtctcctagtcatgcaggatttcattccaa

fava_c15713_114	fava_c15713	114	A/G	ctgttatttattattctttcgaagcaaaaactgaacatgccgaatctttcgttctgagcttctgagttgaatcgttcactgagtctaactaccgagtttg[A/G]cactgcaccgatcttccagtatgttcgacggcttgctcggtcgtggattcgccgcgaaatgtaaatcgttgatgaaattgacgaRgaatcggatcgatgt

fava_c15713_1209	fava_c15713	1209	C/G	agaaaaccaggacgctgccactgctatgaagtctcctagtcatgcaggatttcattccaagaacaatttgaatgagccatttgctgttaatcatggcggc[C/G]tacctgatcttgataactctcagagaaaagttcaaaaggatgaaactcYtagggtaaagccttactacagtaatgcaatcccaccaccttatgtaaagcc

fava_c15713_1258	fava_c15713	1258	C/T	tttcattccaagaacaatttgaatgagccatttgctgttaatcatggcggcStacctgatcttgataactctcagagaaaagttcaaaaggatgaaactc[C/T]tagggtaaagccttactacagtaatgcaatcccaccaccttatgtaaagcctaattctaaactcaagaccagcacacaaagaactgagYcagtatctcca

fava_c15713_1347	fava_c15713	1347	T/C	ggatgaaactcYtagggtaaagccttactacagtaatgcaatcccaccaccttatgtaaagcctaattctaaactcaagaccagcacacaaagaactgag[T/C]cagtatctccacattttgaaaatgatgccatctcagtaaatccttcaatacatcaaaaacctgatgctgcttctgcgacagagagaatccgatctggttt

fava_c15713_199	fava_c15713	199	A/G	taactaccgagtttgRcactgcaccgatcttccagtatgttcgacggcttgctcggtcgtggattcgccgcgaaatgtaaatcgttgatgaaattgacga[A/G]gaatcggatcgatgtgataaggaggaagaggaaagcgacggagaagtttctcaagaaagatatcgctgatctgttggcgaatggattggatgttaatgct

fava_c15713_659	fava_c15713	659	C/T	ttgctgcaaatttgaatccaatgtcttttaccttggagcagaaggttcgcttgatgcaagagatttcttcagaattttcaataaagtgggactccaaagc[C/T]tttgaactgaggatgtcaaaatcYtctgctttcgcacagggcYacaatacttataagcctgaYcattcagctggtcataataaacctttacaaggccagg

fava_c15713_683	fava_c15713	683	T/C	cttttaccttggagcagaaggttcgcttgatgcaagagatttcttcagaattttcaataaagtgggactccaaagcYtttgaactgaggatgtcaaaatc[T/C]tctgctttcgcacagggcYacaatacttataagcctgaYcattcagctggtcataataaacctttacaaggccaggatgaggttttgttagtgaaaagtc

fava_c15713_702	fava_c15713	702	C/T	ggttcgcttgatgcaagagatttcttcagaattttcaataaagtgggactccaaagcYtttgaactgaggatgtcaaaatcYtctgctttcgcacagggc[C/T]acaatacttataagcctgaYcattcagctggtcataataaacctttacaaggccaggatgaggttttgttagtgaaaagtcccgactattctaacaaggg

fava_c15713_722	fava_c15713	722	C/T	tttcttcagaattttcaataaagtgggactccaaagcYtttgaactgaggatgtcaaaatcYtctgctttcgcacagggcYacaatacttataagcctga[C/T]cattcagctggtcataataaacctttacaaggccaggatgaggttttgttagtgaaaagtcccgactattctaacaagggacacaaatttcgtaatgaca

fava_c15714_1376	fava_c15714	1376	T/C	tgacgtggacttcaccaatatcacccacgcctcttatagatgtaattacgttgttcatattaaaatcttctgcaaatgggatgaatgcaccatcagctcg[T/C]acaaatgtttctgggtttgttccagcaaaaatgccgtaccaaagatcatcggtaataaacgaYgtggctctctcaactgcaccaagatatacaccRggtc

fava_c15714_1439	fava_c15714	1439	T/C	aatcttctgcaaatgggatgaatgcaccatcagctcgYacaaatgtttctgggtttgttccagcaaaaatgccgtaccaaagatcatcggtaataaacga[T/C]gtggctctctcaactgcaccaagatatacaccRggtccaagactgggtggcaacggatgaaacatcttctggtttggatagtccaaatcaacagcaacat

fava_c15714_1472	fava_c15714	1472	A/G	ctcgYacaaatgtttctgggtttgttccagcaaaaatgccgtaccaaagatcatcggtaataaacgaYgtggctctctcaactgcaccaagatatacacc[A/G]ggtccaagactgggtggcaacggatgaaacatcttctggtttggatagtccaaatcaacagcaacatgtcgatttgtaagaaaaccaacttctcgacttc

fava_c15714_2267	fava_c15714	2267	T/C	gatgaccatagtagtttctttccaggtccagcgctgactcctccgattgagttgatccagaatggtaaacaactctcaagtccaaatgattccgattcat[T/C]gatcagctctcctacatcacactaaaggcaatgttgttggaggtagaaaaatggcgtggaagaagaggaagttgaaggttttcatgccctagtagtagta

fava_c15715_1960	fava_c15715	1960	A/G	ttcaagcaaacccataaaccagaatatggattctcttgaataatcttctgacttcaatctgctatctcagaagaaaattaatgttacagcacacagtagg[A/G]ccatttggctggatttttattaYaataatgttacacatgttaacacaatctcctaatataaactMatagctatgatacaattatgttggaaattaatRgt

fava_c15715_1983	fava_c15715	1983	T/C	atatggattctcttgaataatcttctgacttcaatctgctatctcagaagaaaattaatgttacagcacacagtaggRccatttggctggatttttatta[T/C]aataatgttacacatgttaacacaatctcctaatataaactMatagctatgatacaattatgttggaaattaatRgtggttaactgattggttggttaac

fava_c15715_2025	fava_c15715	2025	A/C	ctcagaagaaaattaatgttacagcacacagtaggRccatttggctggatttttattaYaataatgttacacatgttaacacaatctcctaatataaact[A/C]atagctatgatacaattatgttggaaattaatRgtggttaactgattggttggttaacttcttgaatatgaggacaaggcctttctggttctcctcttgt

fava_c15715_2058	fava_c15715	2058	G/A	ggRccatttggctggatttttattaYaataatgttacacatgttaacacaatctcctaatataaactMatagctatgatacaattatgttggaaattaat[G/A]gtggttaactgattggttggttaacttcttgaatatgaggacaaggcctttctggttctcctcttgtccacaaaatcttcagatcacctcttttctttcc

fava_c15715_2552	fava_c15715	2552	A/C	cactcttatcacctcgagctaccactatcatcacactctcatggaaccaccatttagagacaaagacccataagatgtcatataggaaagcacaacttag[A/C]agaacagtcccaaccttgagatttggaatgcgaacaatttgaagaactgtaattaYcaacgcgataccaagaatatcttgaccaatccacgcatacggcg

fava_c15715_2608	fava_c15715	2608	T/C	gagacaaagacccataagatgtcatataggaaagcacaacttagMagaacagtcccaaccttgagatttggaatgcgaacaatttgaagaactgtaatta[T/C]caacgcgataccaagaatatcttgaccaatccacgcatacggcgcatgacgtttagctgcccaaacaaccgcaaacactatgcagaagggagtaatagYa

fava_c15715_2707	fava_c15715	2707	C/T	aYcaacgcgataccaagaatatcttgaccaatccacgcatacggcgcatgacgtttagctgcccaaacaaccgcaaacactatgcagaagggagtaatag[C/T]aagtgtcagatatggaacagctccaaagaacggtatcttcacatatgtttgcgcaggatgtcgaaaccatctgaaacatgataatagagccaccgaacaa

fava_c15715_2918	fava_c15715	2918	G/A	cctctatcccgccaatgcaaaatagaactaccagaacttcaacaaaccataacgacattaatttgtacagcataaccaagaaacaagaagcaagcacaac[G/A]aataaaatcgctgctgtagtactgatttccacgtaaccacgagaaccaacactttctgcaacatattcttctgaagcatcctttaaaagcttgtcttgct

fava_c15715_3155	fava_c15715	3155	C/T	aagaagcacataaaatggtaccaacagccataagccataaaaacacttccgcaacatcaaccaatggacgcagtggagagtataactgtatggccactat[C/T]gatttattYtgtatgtgtctttccaagtttaagccagcatcttgtggaagcataacagcaggaattccaatatcaacatcagtttcatttacctcacaaa

fava_c15715_3164	fava_c15715	3164	T/C	ataaaatggtaccaacagccataagccataaaaacacttccgcaacatcaaccaatggacgcagtggagagtataactgtatggccactatYgatttatt[T/C]tgtatgtgtctttccaagtttaagccagcatcttgtggaagcataacagcaggaattccaatatcaacatcagtttcatttacctcacaaaccatcttga

fava_c15715_3536	fava_c15715	3536	T/C	tatgagtggcatgtttttctttggattctaatgtaggaccgaatcttgcaccgacaccaacatactcatcgttctccacaccatcgatccaagttggaac[T/C]ttcaccagaacaaagttgttctcacagccaggccttgtgggagcaacatcatcatgatgaactatatcaccagccaaaaccaaagaaacaccaaacaata

fava_c15715_3704	fava_c15715	3704	T/C	caccagccaaaaccaaagaaacaccaaacaataacacataagcaacactacgtatagcttccaatgaaaccatgctattaactgatataactgagataac[T/C]KagattctcgaatggaaaattRagaagaaatcaaaagggtatctgaaaaagcaaaaaagagtacttttttatttgttttgaggaagtgggaagtagtggc

fava_c15715_3705	fava_c15715	3705	T/G	accagccaaaaccaaagaaacaccaaacaataacacataagcaacactacgtatagcttccaatgaaaccatgctattaactgatataactgagataacY[T/G]agattctcgaatggaaaattRagaagaaatcaaaagggtatctgaaaaagcaaaaaagagtacttttttatttgttttgaggaagtgggaagtagtggct

fava_c15715_3726	fava_c15715	3726	G/A	accaaacaataacacataagcaacactacgtatagcttccaatgaaaccatgctattaactgatataactgagataacYKagattctcgaatggaaaatt[G/A]agaagaaatcaaaagggtatctgaaaaagcaaaaaagagtacttttttatttgttttgaggaagtgggaagtagtggctggcggtggagagagagagaat

fava_c15718_1135	fava_c15718	1135	G/A	aggaattatggggctttctcctgatcaaggcatgaatgctaatcttgtgcaaggtggagggattggtatggttggtttggcacctggggctgttcaaatt[G/A]gtgccgtgtcacctRctaaccaaatatctggtgataaacttggaaagtctaatggcgatacttcttctgtgtcgccggttccttatgtatttaatggcgg

fava_c15718_1150	fava_c15718	1150	G/A	ttctcctgatcaaggcatgaatgctaatcttgtgcaaggtggagggattggtatggttggtttggcacctggggctgttcaaattRgtgccgtgtcacct[G/A]ctaaccaaatatctggtgataaacttggaaagtctaatggcgatacttcttctgtgtcgccggttccttatgtatttaatggcggtatgcgaggaaggaa

fava_c15718_1266	fava_c15718	1266	G/C	gtgataaacttggaaagtctaatggcgatacttcttctgtgtcgccggttccttatgtatttaatggcggtatgcgaggaaggaagggtaatggagctgt[G/C]gagaaggtaattgagaggaggcagagaagaatgataaagaatagagagtcagccgctagatctcgcgctcgcaagcaggcttataccatggaattggaag

fava_c15718_1503	fava_c15718	1503	A/G	ttcagaaaaagcaggaagaaatcatggaaattcagaaaaatcaagttaaggaaatgatgaatttgcagcgagaagtcaagaggaaatgcttaagacgaac[A/G]caaaccggtccatggtagattgaagattggattattattattccgcagttaaaacattatgtaaatacataaatacatgttttttctgtagattgaggct

fava_c15718_764	fava_c15718	764	G/C	ggagtttttctcgatctagcgcgtgttgggaataatagtaatttgggtttggcttttcaggctcagcagatgaataaagttgctggttttatgggtaatg[G/C]tagaatgaacggtagtactaatgatgatccacttgttggtcttcagagtcctactaatttgccgttgaatgtgaatgggattagatcaacaaatcagcaa

fava_c15730_1066	fava_c15730	1066	G/A	agggtttcaaagagccttgcaattggatcccaataatgagactgttaaggaaaatataagggtagctgtgcagaagttgatggaagagcaacataatgcc[G/A]gtcacaatcagaattcgagatcatctccagaattccaaaaccattctgcaaggggttcgacaagccacRcagctccacctacatttgcctcaatgcaatt

fava_c15730_1135	fava_c15730	1135	A/G	gcagaagttgatggaagagcaacataatgccRgtcacaatcagaattcgagatcatctccagaattccaaaaccattctgcaaggggttcgacaagccac[A/G]cagctccacctacatttgcctcaatgcaatttaaccccagcaatttggcaagcatgttcatgtcggctacaaatgcacatcaggggtcRcattctcagga

fava_c15730_1224	fava_c15730	1224	G/A	cgacaagccacRcagctccacctacatttgcctcaatgcaatttaaccccagcaatttggcaagcatgttcatgtcggctacaaatgcacatcaggggtc[G/A]cattctcaggaaagtcaagaaaatgccaatagtagtggagctaatgaacccggaattcagtttggggctaatgttaatgtaaaccatgatcaaattcctc

fava_c15730_1395	fava_c15730	1395	T/C	atgttaatgtaaaccatgatcaaattcctcaggacctaaggggcgcctttcaatctgtgatggggatgttctcagggaatgtacctcctgggcaaccaca[T/C]gatcaaatgaatggaagtcaagaagatgccaatagtggtgaagctaacgaaccagaaattcaatttgggggacgYgttgatctaaactctgatcaactcc

fava_c15730_1470	fava_c15730	1470	T/C	ggaatgtacctcctgggcaaccacaYgatcaaatgaatggaagtcaagaagatgccaatagtggtgaagctaacgaaccagaaattcaatttgggggacg[T/C]gttgatctaaactctgatcaactccctcaggaactaagagtctttcaaaatgtgatagatatgttatcagggaatgtacctccagggcaaccacatgatc

fava_c15730_1622	fava_c15730	1622	A/G	gtgatagatatgttatcagggaatgtacctccagggcaaccacatgatcaaacgaatgaaagaacagcaccaaactaagctattgaagcattcttccgag[A/G]ctgtaaagactctcacaggttacagagctttgagttactattgtttcaccgaggcaaccatacttttccaggttacatatcctgttgtttggatgtgtaa

fava_c15730_1758	fava_c15730	1758	T/C	tactattgtttcaccgaggcaaccatacttttccaggttacatatcctgttgtttggatgtgtaatttactgaagcacattattgtgacttaagtttaga[T/C]tattattacttgcaattctttctgtatatgttgtgatgcaaatattcttgcggatctcatctcgatagattacaagttgaagaagcagggaggctgttag

fava_c15730_187	fava_c15730	187	C/T	cataaaaactccaaagaataaaaagatgaacactttaataatctagctttcttcttcaattcccaaagccatgtctcacaatcgcatcaccaccgattct[C/T]ctctcKcgcgtcgcatagttctctctttcctccatttcctcaacaacgttgaacctagccccggggttgatgctgagggaattgaggttgctagggagtg

fava_c15730_193	fava_c15730	193	G/T	aactccaaagaataaaaagatgaacactttaataatctagctttcttcttcaattcccaaagccatgtctcacaatcgcatcaccaccgattctYctctc[G/T]cgcgtcgcatagttctctctttcctccatttcctcaacaacgttgaacctagccccggggttgatgctgagggaattgaggttgctagggagtgtttggt

fava_c15730_483	fava_c15730	483	T/C	gtgaagttggtagggtggattctatgaaagcttcgagttcactctctgatcagaatgctgctggtgataaagaccatcctgaggcgtcggaatccatgga[T/C]gaagatttggcagaggaccctgacatttctgtgtctaaagatgaactctgtggacaattctttgctgctttagagaaaaagcgctatttcaggRccaata

fava_c15730_577	fava_c15730	577	A/G	catggaYgaagatttggcagaggaccctgacatttctgtgtctaaagatgaactctgtggacaattctttgctgctttagagaaaaagcgctatttcagg[A/G]ccaatattgatggaggtgatgacatagtgcaattggagaaagcatctcgcttatttgataatggttttacggagatggaaaaatctgggtatctgcagtt

fava_c15730_825	fava_c15730	825	G/T	aaatgcaatccaagcaatacaatgatgcagttgagttgtataattgtgcaattgcgataaatgaaaagaatgctgtttactactgcaacagggcagctgc[G/T]tatactcaaattaacaaatataccgaggcaatagaagattgtcttaaatctatcgagattgatccgtactacagtaaggcatacagtcgtctgggattag

fava_c15731_1181	fava_c15731	1181	G/A	agaatcagattctgccattgatacaaaagtataaagttgtccatttaaatcgaactgatgctcggttggcaaataatgggcaatctttagagattcagaa[G/A]ctgcgttgccgagtcaattttagtgctcttagatttactcctcagattgaggagttaggcagaaaggttatcaatcttctgaaacagaatggcccatttc

fava_c15731_1457	fava_c15731	1457	C/T	tagaagaattgacaaggatgagatatgcttatccttggtggaaagaaaaaataattaattctgatttgaaaagaaaagatggtttatgtcctctaacacc[C/T]gaagaaaccgctcttgcgcttagggcctttgacatagatcaaaacattcaaatctacatYgctgctggggaaatatatggtgggagtaggagaatggcca

fava_c15731_1517	fava_c15731	1517	C/T	ctgatttgaaaagaaaagatggtttatgtcctctaacaccYgaagaaaccgctcttgcgcttagggcctttgacatagatcaaaacattcaaatctacat[C/T]gctgctggggaaatatatggtgggagtaggagaatggccagtctagcaaagaactatccaaaattggtcagaaaggaaacactgttggaaccgtctgaac

fava_c15731_340	fava_c15731	340	A/G	gagagataccggcgattgtttcagatcagtcgccggcgatttcttcgcctccgtttatttatcctgaattcggagatctctctgtttcacgttccgattt[A/G]gatttagtgttgcaaactgtggtaatgcgttttggaatttgaggtttactcatttgaatttggatcaattgttagagttaattcgagattttagggtttg

fava_c15731_590	fava_c15731	590	C/T	ggggaagagaagacaagagttgggaagggttgatgaggatgagtaagaataaggttgagaagttgaagaatacggtgacggtgaggtcttccaggatgaa[C/T]ttgtggatgatacgggctaccacgtcggtgattttatggatttgcattgttcagttaactgcatttggtgatatgtggggtcctagagttttgaaaggtt

fava_c15732_1577	fava_c15732	1577	G/C	actcaaatatcgaagagctctatttgcttaatcctagagctcttcttccttgttttgttgtaatttgcaaaccatgaacctgaaaaagacccgtgtcttt[G/C]cacacgtctggattcacggtgaatttgacagaatcactgggtgctaccatatagtatttcaacaaaaYcacagtagcactatgttttctgttaactcgcR

fava_c15732_1645	fava_c15732	1645	T/C	aaaccatgaacctgaaaaagacccgtgtctttScacacgtctggattcacggtgaatttgacagaatcactgggtgctaccatatagtatttcaacaaaa[T/C]cacagtagcactatgttttctgttaactcgcRgcgatttccaaaatgcgcttacatattgtgtagttttatgaactcttaccacccttctattttggcct

fava_c15732_1677	fava_c15732	1677	G/A	ScacacgtctggattcacggtgaatttgacagaatcactgggtgctaccatatagtatttcaacaaaaYcacagtagcactatgttttctgttaactcgc[G/A]gcgatttccaaaatgcgcttacatattgtgtagttttatgaactcttaccacccttctattttggccttttttctatggaggttctttgctttacatatt

fava_c15732_200	fava_c15732	200	A/C	ttacctacggataatgcatatgcatggttgagttttttcatggaaagttgaaggaaaacaaccaaagttgttatcttttttgtttttgattttcaaagaa[A/C]gtgaagattttttttgcatagggaaaaatggaccgttttggaagatagaaaaagagaaagagaagggaatcgttgaagggtaatatgccaacagtgtggt

fava_c15732_598	fava_c15732	598	C/G	attggaagcagtgagtttctcaacccaataactcatgaggttattttgagtaactcaaggtgtgagcttaaaatcactggttttcaagaaggggttaata[C/G]tggtggaaacaatgctagtggaagtagtgctggtggttcttcaacttttgttggtactttgagacctggaacacctggWcctggagggcaccctacaatg

fava_c15732_677	fava_c15732	677	A/T	gttttcaagaaggggttaataStggtggaaacaatgctagtggaagtagtgctggtggttcttcaacttttgttggtactttgagacctggaacacctgg[A/T]cctggagggcaccctacaatgcattactttaacccttcttttaggacttcttctactcctccaaggaaatctccttttctRttatcagaagggtctggtc

fava_c15732_758	fava_c15732	758	G/A	tgagacctggaacacctggWcctggagggcaccctacaatgcattactttaacccttcttttaggacttcttctactcctccaaggaaatctccttttct[G/A]ttatcagaagggtctggtcttaatggtgctggtgttcattcaagtaataggatgtcacttgagactgatgctaatggctcttcaactgttacttgtcaca

fava_c15732_860	fava_c15732	860	A/G	tatcagaagggtctggtcttaatggtgctggtgttcattcaagtaataggatgtcacttgagactgatgctaatggctcttcaactgttacttgtcacaa[A/G]tgtggtgaacagtttaccaaatgggaagctgctgaaactcaccatctctccaagcatgctgttactgaacttgtggaaggagattcatcaaggaaaatag

fava_c15734_1362	fava_c15734	1362	C/T	ggtttattggcattgaattgtaccctttctcaagtgtgtagtagcatctttttagatcttctcgatatagaatttcgtcttttccgaaaattgatagaag[C/T]gcaacgttgaaRgtgaatgttttcatttcaaggtaagtggtgattagatttccgtcccatgatttgagacaagattgagcaatggattcaatgttgtgaa

fava_c15734_1374	fava_c15734	1374	A/G	ttgaattgtaccctttctcaagtgtgtagtagcatctttttagatcttctcgatatagaatttcgtcttttccgaaaattgatagaagYgcaacgttgaa[A/G]gtgaatgttttcatttcaaggtaagtggtgattagatttccgtcccatgatttgagacaagattgagcaatggattcaatgttgtgaacaatgcttttga

fava_c15734_1479	fava_c15734	1479	T/C	atgttttcatttcaaggtaagtggtgattagatttccgtcccatgatttgagacaagattgagcaatggattcaatgttgtgaacaatgcttttgatggc[T/C]tctggcatgaaggtgcgaagaacaagtcttctaaggttagtatggtaatttccttgatgaaagaaaattgcttgttttcctaacatcctttctttgcttg

fava_c15734_1602	fava_c15734	1602	T/C	caagtcttctaaggttagtatggtaatttccttgatgaaagaaaattgcttgttttcctaacatcctttctttgcttgctggaaaagttggtttgaaaag[T/C]tgtgctttggttaacacaaattttgctgcttctggacttgaaatcatcacacaaggacaacccaaaatatttgacttgaacatagcaccataccttttga

fava_c15734_1841	fava_c15734	1841	C/G	atttggaaggtttcacctatgtaagggtaacccatagagccaggagggagaggcaattgtatagcattagaagaaaaaaagaaggatttgaatagaagaa[C/G]Sagaatgaagagaagagaaagaaagtagaaaaagaaagtgatggtaaaatccatgaaaaaagaaaatattgaaatgaaagaaggtgagtgag

fava_c15734_1842	fava_c15734	1842	G/C	tttggaaggtttcacctatgtaagggtaacccatagagccaggagggagaggcaattgtatagcattagaagaaaaaaagaaggatttgaatagaagaaS[G/C]agaatgaagagaagagaaagaaagtagaaaaagaaagtgatggtaaaatccatgaaaaaagaaaatattgaaatgaaagaaggtgagtgag

fava_c15734_281	fava_c15734	281	C/A	tgacaaaatatttcaaaataaaaataaattgggagaataaaaaactataaaccaaaaaaaatttagcctattaacactgtttactcttcctatataacct[C/A]tgtacagttgacttcttgttagtgcaaaatttataaggactaaaatttaatcttccttttgtttatgcatttgttRtatgatttcttctcttcaccactt

fava_c15734_357	fava_c15734	357	G/A	ctgtttactcttcctatataacctMtgtacagttgacttcttgttagtgcaaaatttataaggactaaaatttaatcttccttttgtttatgcatttgtt[G/A]tatgatttcttctcttcaccactttttgttgtcaaaagttgaatcaaattcacatgcttttgaaaccattccaaatttagaaagttgcttccttgctata

fava_c15734_549	fava_c15734	549	A/T	cttgctatatttggctagcctccacttttttgtttggttcatatttttattgtttcttggaatataatttgattggtaagccattttgtggaagagcaaa[A/T]ggaccatattgaattccatttttttcaccaaccacagaccacctgtactttgtggttagatgatgtacaagaaccaaaatctctaactttgctaattcat

fava_c15734_836	fava_c15734	836	G/A	tgaggctccttgaaattttctggactgtgatgtatgtttctgaaaagtggcaatactttccatccttttggtataagatacccttgatattcgacatctt[G/A]ggttgcttctcgaaaagtaaaagacaaaattgatgctactcttagtgtttcttgtatgacccttgaagttattggcatattttttgtgtcctcccaagtt

fava_c15736_1410	fava_c15736	1410	A/G	gttctcatcttttccacaaagactgtgttgataagtggttgaagataaatgcattgtgccctctttgcaagagtgaggttggtgaagacccaacaggatt[A/G]gggtctggagaagatgccacccaaacaacaggatgagagcagagttgtaaatggagtcaccaatccctaggcttaatctagctatatacaatacatacat

fava_c15736_1601	fava_c15736	1601	A/G	atacatacattttggatgctctcattatccttaagacctgagaaatggataagctcatcttgcaggttaagagagagaaagaacaaatcacctatttata[A/G]aattggtggatgctacctaggtaatggtggtgggttcatacacaataagtttgcgtttttctctctcataacatgacagaagacacttatatcttctgaa

fava_c15736_173	fava_c15736	173	C/T	tctttacctccgtcactcacacgacgccgtttcatcttccctctgtcgtttccctcttcttcttctccctctcgatgccgtttatctatccaattcttct[C/T]tccctttaccgtccctagtcaacgccgtttcgctccttttctctgtcgtttcatcgattcgcatctctctcttaggttcaattgaagcttgatggagcga

fava_c15736_322	fava_c15736	322	T/G	tttcatcgattcgcatctctctcttaggttcaattgaagcttgatggagcgacctcacgaaagtcatcatattatagaaatagctgggagtgtcgagcct[T/G]cagcttctacatcttcctcgtctcgtgtagacgaStcgcatcgtgaagaaagaataagtggagcgcggttgcctgttggtcagccttcagtttctacctc

fava_c15736_357	fava_c15736	357	C/G	gaagcttgatggagcgacctcacgaaagtcatcatattatagaaatagctgggagtgtcgagcctKcagcttctacatcttcctcgtctcgtgtagacga[C/G]tcgcatcgtgaagaaagaataagtggagcgcggttgcctgttggtcagccttcagtttctacctcgggtgtatcgaatagtaggagttcttcgttagtaa

fava_c15737_1853	fava_c15737	1853	G/A	acaaattggacatggcgaaaaacctggtggcatcattcccggttatgcggccatccgcatctgaatcagcgtgattgaaccagtgttggtaaatgctctg[G/A]tgatctttggagcacgaaccgatcggaatcggatccaactccattgataggagaaaaRgaatactactctgttgcgtttcacaataacaagaagaagatt

fava_c15737_1911	fava_c15737	1911	A/G	atctgaatcagcgtgattgaaccagtgttggtaaatgctctgRtgatctttggagcacgaaccgatcggaatcggatccaactccattgataggagaaaa[A/G]gaatactactctgttgcgtttcacaataacaagaagaagattgaagtttaatcagctaagttgagtattaatttgtttttgcagtttgattgcacacgct

fava_c15737_290	fava_c15737	290	T/C	acttacattaattgcatcaataaacccgtaatttttgtatattacaaatgtcactctaactaaagtactggaaaatttaatcatagggattcttaaaatt[T/C]ttcaagaggttaggaatgtcataggcaagcatgtcatcaactactcgtatcatttttggtttcagtttttcaaacttgtcaatattataaccacgcaagc

fava_c15737_431	fava_c15737	431	T/C	ctactcgtatcatttttggtttcagtttttcaaacttgtcaatattataaccacgcaagctctctctgaaatgctcaacgtttggaaagtcaccaggagg[T/C]aaatggaattccctttgtacctttccaaattcacctgccaagttatcaattagtttttgctgagctttacctttgcctatcattgcaggcatctcYtttt

fava_c15737_527	fava_c15737	527	T/C	gaggYaaatggaattccctttgtacctttccaaattcacctgccaagttatcaattagtttttgctgagctttacctttgcctatcattgcaggcatctc[T/C]tttttaagatgactgataatgtaagcatgtatcttaactgctcgggctcgttttacaaactcgttgattcttcgatcacaagccRccttgggtatatctt

fava_c15737_612	fava_c15737	612	G/A	cattgcaggcatctcYtttttaagatgactgataatgtaagcatgtatcttaactgctcgggctcgttttacaaactcgttgattcttcgatcacaagcc[G/A]ccttgggtatatctttcagatctgaaagaaggtcttcctgctctttttcaaagagttcctttccgattggaccactgacagcatcatttacaggcttgtc

fava_c15737_767	fava_c15737	767	G/A	gttcctttccgattggaccactgacagcatcatttacaggcttgtcgttaaaagagccaatatacacacgaatgacttcaggtgtgtttagcaccttccc[G/A]agtgaccacattaaggctccataaaccctcatcagttgttgagtatcaacttgatctgccttgtttaaaaccactcgaattttgtcatcatgtcccctta

fava_c15740_1028	fava_c15740	1028	G/T	gtcaagaatacggaactgatgatgataatagtgactatgatgattattatgatgattatgaagaaaatcatcgagctgaagacgagtcacttgctgagaa[G/T]ctcaaactctcgcttagcaaccatgtgatggatacccaagaacttgatggtggtggcaagtacaaggttcccgcagaaggtgaggaggaagaggattccg

fava_c15740_1193	fava_c15740	1193	A/G	aggttcccgcagaaggtgaggaggaagaggattccgctgccgatgtgattcgccgtaccaaggaatatggtgagcagtatgaagttgaagatgatggaaa[A/G]gatgctgtgatttttgaagaaagtagYgatgagtcagaagtttgggattgtgagactattgtttcaacatatacaaatttagataaccaccctggaaaga

fava_c15740_1220	fava_c15740	1220	C/T	aggattccgctgccgatgtgattcgccgtaccaaggaatatggtgagcagtatgaagttgaagatgatggaaaRgatgctgtgatttttgaagaaagtag[C/T]gatgagtcagaagtttgggattgtgagactattgtttcaacatatacaaatttagataaccaccctggaaagattgaaacaccgggggcaactaggaaaa

fava_c15740_1544	fava_c15740	1544	A/G	cgaaagactcgatgattgaaaaacctgaacagtacaagagaaaacaacatggcctagagtcaaaggaggagaaaaaagagagaaaggctgctgtgaagga[A/G]gaacggcgtgaggcacgtcgcactaaaaaagaaatgaaagagctttataagtgtgaagctaatcgggcacaaagagctgcagctgtatctggcccttcat

fava_c15740_884	fava_c15740	884	C/A	tattgcaagtttctacttattccactcttgccggtgattgtggacctttggatgggagttcaagtggtgcaacagatgtgcactgtgtcgtcgagaagcc[C/A]agaggccgtcgtctgttagatgaacaattcgatttgYttgaacgtcaagaatacggaactgatgatgataatagtgactatgatgattattatgatgatt

fava_c15740_921	fava_c15740	921	C/T	ttgtggacctttggatgggagttcaagtggtgcaacagatgtgcactgtgtcgtcgagaagccMagaggccgtcgtctgttagatgaacaattcgatttg[C/T]ttgaacgtcaagaatacggaactgatgatgataatagtgactatgatgattattatgatgattatgaagaaaatcatcgagctgaagacgagtcacttgc

fava_c15742_1480	fava_c15742	1480	A/G	cgcgggcagccatatgtagcggcgttcttccaggaaatccagatccaccggttgaagcggaaacaagagccccgttgtctaaaagggaatgaagacattc[A/G]agccgtctatgacgagcagcaaggtgcagtggcgtagcaccgtttccatctcttatgttcacaaatctggcaaatccccaagaatctgcaacaggcgtcg

fava_c15742_459	fava_c15742	459	T/C	gcgaaaaccgagtactacatccaagagaattgaatgtctcatgtataattacatttcataattggtaaaataaaactgcaaccgtctgtcaaaagttcga[T/C]cgataatcgtattacaacgactcaaacagaccatataagaatatagctcatctattgtcttgcatgatcttagtgatcttttagacaatgWgaaaaaatt

fava_c15742_550	fava_c15742	550	T/A	aaagttcgaYcgataatcgtattacaacgactcaaacagaccatataagaatatagctcatctattgtcttgcatgatcttagtgatcttttagacaatg[T/A]gaaaaaattgaattcaaataacatgcttactacaataggtagctattccaccgcaagtgtaaaatcaccattcatatgtcaatggaaacaaagaatctgc

fava_c15742_703	fava_c15742	703	T/C	gcaagtgtaaaatcaccattcatatgtcaatggaaacaaagaatctgccggcaatcatcaagacttaagaaatccttcattgcttttcggtatcaatctt[T/C]cctgaattgtgaccagtgatccttccaaatgagctaatggctgacaaactcttgaagctgctgctcccttcgctgaaatttgattttcttgacctccttg

fava_c15744_2058	fava_c15744	2058	C/T	ctcatacaataacacttccaaatggcaaagagtcgactgtgagactggaattgcaagaatttcactcagctctggaagaagcaaaagctgatattgttgg[C/T]ctctgggcattgaggttcttaatctctcaggacatgctttccgaaagcttattgaagtccatgtatgtttcttttcttgctggatgctttcgctcagtac

fava_c15744_2541	fava_c15744	2541	C/T	ctaccttcccctttgctgacaaagtattgcaatgattgcaagataaccaaggtgcaaaatgttccatgcttgcaaaatctttgcgccaacattaaaaagt[C/T]acttgatccatatctcttcgtgcttcttgatgctactttaaataatggagtacattgtaccaaaaggaaaataaaataatggagtactgtatctctatgg

fava_c15750_1905	fava_c15750	1905	G/A	gaaccctgcaaatatagatgggtagcatgcaagactctagcagcctttactgcttagtggctggattcatacattagaaatttgtttgattagaaatatg[G/A]ttaatatttatgaatgctcttttatttttcttctttttcaaaccataatatccttgaactctgcactgtctctttcatacaaatacaaaccatagaggga

fava_c15750_191	fava_c15750	191	T/A	ccatcgtctgaactcaaccaggttcctaattacttcactccatgcattctccttccgattcacccgagaaattatgagagctttatagaatcacatgctc[T/A]ggttacggttaatactttattctttgatgtgatgaaatgattgagatttgatatgaagaatgtttttcgcctaactgttttgattctaatgagggagtta

fava_c15750_360	fava_c15750	360	T/A	gcctaactgttttgattctaatgagggagttatcgtggcgttgtttcggccacaagattgatcagagaaaattgcttattcttgccgccgtttttaccgt[T/A]actggatttctgtttcagttttttgtacatgcttatgtagccgattcatcRtcctcggatagcactYtacagtcgaatgactttctgaaaggggagattg

fava_c15750_411	fava_c15750	411	G/A	acaagattgatcagagaaaattgcttattcttgccgccgtttttaccgtWactggatttctgtttcagttttttgtacatgcttatgtagccgattcatc[G/A]tcctcggatagcactYtacagtcgaatgactttctgaaaggggagattgttcaacaggttcgcctcactctttctccMtctSttgtttcgcctatttcgt

fava_c15750_427	fava_c15750	427	T/C	aaaattgcttattcttgccgccgtttttaccgtWactggatttctgtttcagttttttgtacatgcttatgtagccgattcatcRtcctcggatagcact[T/C]tacagtcgaatgactttctgaaaggggagattgttcaacaggttcgcctcactctttctccMtctSttgtttcgcctatttcgtttaacaagtctgttca

fava_c15750_489	fava_c15750	489	C/A	atgcttatgtagccgattcatcRtcctcggatagcactYtacagtcgaatgactttctgaaaggggagattgttcaacaggttcgcctcactctttctcc[C/A]tctSttgtttcgcctatttcgtttaacaagtctgttcaaccagtatcagttcagcatgacactgttgtagatattcattcacaaccaaagaagaatataa

fava_c15750_493	fava_c15750	493	C/G	ttatgtagccgattcatcRtcctcggatagcactYtacagtcgaatgactttctgaaaggggagattgttcaacaggttcgcctcactctttctccMtct[C/G]ttgtttcgcctatttcgtttaacaagtctgttcaaccagtatcagttcagcatgacactgttgtagatattcattcacaaccaaagaagaatataaatct

fava_c15751_1538	fava_c15751	1538	G/A	caaacgacttcttgcaatagaaacgatgaaacagaacctgcccagtggccataactgcttgtggtaatctcagcaaaatgccgctttcttggatgagatc[G/A]cagccatagattctaagggcgatttcagtagcttcatctatgccatcttttctcgaaggagaatttgttaactgctcatccgtgaggtaaaatgtatcaa

fava_c15751_174	fava_c15751	174	T/G	aacgagatcataatgaaggccaaccactataatcttctttccaaaaagcagagagacaggcaccagaacaccgtcttgcatgcttagaaaaatataataa[T/G]gcctaaatattgtYgtagctactgttttatatgctttggagcaaataaaaatacaaactaataatacaaaggaaaactgggaactacataaaaaaaaaac

fava_c15751_188	fava_c15751	188	C/T	gaaggccaaccactataatcttctttccaaaaagcagagagacaggcaccagaacaccgtcttgcatgcttagaaaaatataataaKgcctaaatattgt[C/T]gtagctactgttttatatgctttggagcaaataaaaatacaaactaataatacaaaggaaaactgggaactacataaaaaaaaaacttRtattcatgcat

fava_c15751_277	fava_c15751	277	A/G	ctaaatattgtYgtagctactgttttatatgctttggagcaaataaaaatacaaactaataatacaaaggaaaactgggaactacataaaaaaaaaactt[A/G]tattcatgcatagaccaatagaggtaacaatagaacttaagaaaacaatgtgggcacatcttggttacttcaatgatgtctatgccggttctctcttgat

fava_c15751_419	fava_c15751	419	C/T	aaaacaatgtgggcacatcttggttacttcaatgatgtctatgccggttctctcttgatgagtatgacgagctgtagtagtcacggtcacggtcatgtga[C/T]gaatagcgctttgacttctctgaatgtcctgaatcctttgctctctccctagaacgatgaccatgatccttgagtttgaccctctcagattcttctcgtt

fava_c15751_666	fava_c15751	666	G/A	tattcgttccctctctttgtccctgtgtaactccccgctggcctccattttacggtctggtctggaccttagtgtgggttcatcttttacgtctctctca[G/A]cagcaatgcctttggattcatcatcagacttcctagcatcRttcaacttatcactggcttgctttaccaaagcacccttgctaccactcacgtcaatatt

fava_c15751_707	fava_c15751	707	G/A	cctccattttacggtctggtctggaccttagtgtgggttcatcttttacgtctctctcaRcagcaatgcctttggattcatcatcagacttcctagcatc[G/A]ttcaacttatcactggcttgctttaccaaagcacccttgctaccactcacgtcaatattagcttctccgggagctcccttcaacgctgacgtctcaggat

fava_c15751_833	fava_c15751	833	G/A	ccaaagcacccttgctaccactcacgtcaatattagcttctccgggagctcccttcaacgctgacgtctcaggattaattggtgagctacttcgtggagc[G/A]tcctttgtagctgaatgagactttgattccaatgatttattggagaatgtaaatgagtcccagtccttgcaaattggtaaatattgtgccttgggaagac

fava_c15756_1242	fava_c15756	1242	T/C	ctagccgtgacgctgagcgtgatagtactccagagctatcacctatagcaacaaatgatggtagtccagagggtgcagggtttttgtcaaaccggaataa[T/C]gatgaggctgatgaagatgatcctttctcaaagcgaagaaaaatggatcttgacatcactcctgtagttaagcctatccgggagccacgggttgttgtac

fava_c15756_1488	fava_c15756	1488	T/A	gtaaatatgggcagaaggtggtaagaggcaatcctaaccctaggagttattacaaatgcacaaatgctggttgcccggttagaaaacacgtggagagagc[T/A]tctcatgatccaaaagctgtaattaccacatacgagggtaaacacaatcatgatgtaccaacYgctaggaataatagtcatgacatggcaggaccatcag

fava_c15756_1551	fava_c15756	1551	C/T	atgctggttgcccggttagaaaacacgtggagagagcWtctcatgatccaaaagctgtaattaccacatacgagggtaaacacaatcatgatgtaccaac[C/T]gctaggaataatagtcatgacatggcaggaccatcagctgcaggtggtcatcaggcacgaattcggcaagaagaaagcgatactattagcctcgaccttg

fava_c15756_1678	fava_c15756	1678	C/A	aggaccatcagctgcaggtggtcatcaggcacgaattcggcaagaagaaagcgatactattagcctcgaccttggaatgggcattagcccaaacgctgaa[C/A]atagatcaaacaatcaagggaggatgatgatgcgtaatgaatacggcgacggccaaaMtcacgccggcaattcaagtttcaagtttgttcatactgcacc

fava_c15756_1736	fava_c15756	1736	C/A	attagcctcgaccttggaatgggcattagcccaaacgctgaaMatagatcaaacaatcaagggaggatgatgatgcgtaatgaatacggcgacggccaaa[C/A]tcacgccggcaattcaagtttcaagtttgttcatactgcaccgcctccgggatactatggtgtactaaataacaactcaaatccatatggttctagagaa

fava_c15756_2064	fava_c15756	2064	C/T	ggctggtaatgcttcaaaggcttgttttctctttgaaaatgctgatagttcttatatgatcctatcacagtatcctttggcagaactagcaaaagctgtg[C/T]tctttcaggttgggttttatatctgatgtttgtataattatatgtagaaattcttctgtacaaatatgaagctgatactaaaattgcttccacaagaatc

fava_c15756_267	fava_c15756	267	A/G	tcacactatctctcaccacaatggatgccgacgccggtgaagctcaattccggcaacaaactctcggcgagatggatgacaaacaccacgatccaaaacc[A/G]gattctgcttccgacggaSctgccacctccggaaacaccagcggagctagatacaagctgatgtccccggcgaagctcccgatctctaggtcaccgattt

fava_c15756_286	fava_c15756	286	C/G	aatggatgccgacgccggtgaagctcaattccggcaacaaactctcggcgagatggatgacaaacaccacgatccaaaaccRgattctgcttccgacgga[C/G]ctgccacctccggaaacaccagcggagctagatacaagctgatgtccccggcgaagctcccgatctctaggtcaccgattttaactattcctccaggact

fava_c15760_1031	fava_c15760	1031	G/A	cataccctttcctagcttatgtgggtaatccagcggatattgatataaattacgctctttttcaaccaacaaagggaatttatgatccaaagtttaaact[G/A]cattacgataacatgctcgatgcccagattgatgcggcttatgctgctttggaggatgctggattttataaaatggaagtcatagttacggagacagggt

fava_c15760_1370	fava_c15760	1370	T/C	ttgcaatcttcaacgaggattcgaagaatggtcctacctccgagaggaattatggattgttcaaggctgatgggagcatatcatacgatgtcgggtatca[T/C]gggcttaatgYtggatattcatctcatttgtcctttaaggatgtcaataatccaggtctgtcgcgctYctatatgatcaccacgatattStgtttatcag

fava_c15760_1381	fava_c15760	1381	C/T	aacgaggattcgaagaatggtcctacctccgagaggaattatggattgttcaaggctgatgggagcatatcatacgatgtcgggtatcaYgggcttaatg[C/T]tggatattcatctcatttgtcctttaaggatgtcaataatccaggtctgtcgcgctYctatatgatcaccacgatattStgtttatcagctctgatactg

fava_c15760_1438	fava_c15760	1438	C/T	gatgggagcatatcatacgatgtcgggtatcaYgggcttaatgYtggatattcatctcatttgtcctttaaggatgtcaataatccaggtctgtcgcgct[C/T]ctatatgatcaccacgatattStgtttatcagctctgatactgatgattttctagagttagtttccttggcaatgaagggatttttttgtcttggcttgt

fava_c15760_1460	fava_c15760	1460	G/C	tcgggtatcaYgggcttaatgYtggatattcatctcatttgtcctttaaggatgtcaataatccaggtctgtcgcgctYctatatgatcaccacgatatt[G/C]tgtttatcagctctgatactgatgattttctagagttagtttccttggcaatgaagggatttttttgtcttggcttgtcttgatctcttactatagatca

fava_c15760_404	fava_c15760	404	A/T	ctcaattccactagttcatcatcatgattttccggttctgtcactcttttctgcttcttcttatcttcactcaatttgcgttgctgcaagcgtttacggg[A/T]acgtatggtataaattatgggaggattgcagataacattccctcgcctgatcaagttgttaMtcttctcagagcctcgaagataaggaacattagaatct

fava_c15760_466	fava_c15760	466	A/C	atcttcactcaatttgcgttgctgcaagcgtttacgggWacgtatggtataaattatgggaggattgcagataacattccctcgcctgatcaagttgtta[A/C]tcttctcagagcctcgaagataaggaacattagaatctatgatgctgatcacagtgttctcaaggcttttagtggaaccggacttgaaattgttattgga

fava_c15767_125	fava_c15767	125	T/A	attaactagttactaacacaaccaaagaaaactatctagaaaaaacctgactaatgagatgacacataaagaagtccagatgagacaattcaggtatgtg[T/A]atgcttattagRtgacgtgtaaaactatactttaaacctatacaaatttacattatgtacgtacaatgactgaggataattttatcaagacgcttttcct

fava_c15767_137	fava_c15767	137	G/A	ctaacacaaccaaagaaaactatctagaaaaaacctgactaatgagatgacacataaagaagtccagatgagacaattcaggtatgtgWatgcttattag[G/A]tgacgtgtaaaactatactttaaacctatacaaatttacattatgtacgtacaatgactgaggataattttatcaagacgcttttcctcatctatgatct

fava_c15767_1740	fava_c15767	1740	G/A	gaacactcaatccgaacaaagaaaagtaagcacttggaggggcatatatctcttcacatgcacatgcaaggtaatattctttttcttgacatgtagggac[G/A]taagctacaacaaattttcctgattttttgaaattcaaaatgtgccttcgaatttcttccactttaccccacccacagtttaaactatcaatatggagat

fava_c15767_1968	fava_c15767	1968	A/G	ctgctttcaaaaaattctcacaaatttggggaagagaaagtcccggagagaacctactcttcacttgatcagatatctggccacgcagcttcattgtgag[A/G]acgctRcctttccgaacacgctcccatggatacgcaatcaacatcttaaccttaacaagaaaattcctccacccagtaataggYtcaaattcgaactcgc

fava_c15767_1974	fava_c15767	1974	A/G	tcaaaaaattctcacaaatttggggaagagaaagtcccggagagaacctactcttcacttgatcagatatctggccacgcagcttcattgtgagRacgct[A/G]cctttccgaacacgctcccatggatacgcaatcaacatcttaaccttaacaagaaaattcctccacccagtaataggYtcaaattcgaactcgccactcg

fava_c15767_2052	fava_c15767	2052	T/C	gcagcttcattgtgagRacgctRcctttccgaacacgctcccatggatacgcaatcaacatcttaaccttaacaagaaaattcctccacccagtaatagg[T/C]tcaaattcgaactcgccactcggataatcctcatcggcgattctaacgttttcaccatcaatagccccttgtttttcttcttcttgaactttggtggagt

fava_c15767_2271	fava_c15767	2271	T/C	cgcgaagggaaatgcgtgttctcgtcggagtttgaggaagataggggaaagaagaaaaagagaagcgatggtgaaattgaagcgaggaagtagaagacga[T/C]gatgatgatgatgatgatgaggtgctgcggtggtagaatgaggtgagaggaggaggtgtgggtagaagcagcgacgccatttttctgataggaatgaaat

fava_c15767_281	fava_c15767	281	G/A	tgtacgtacaatgactgaggataattttatcaagacgcttttcctcatctatgatctcttgaacaagctaaagagtacatggaaggaaaactatagaaac[G/A]aactgaattaaaatgtttgcaatgaattatgaaaaactatagagaactaagataatcttttataattgacaaaatggggttggagttaggatatccttcc

fava_c15767_519	fava_c15767	519	A/G	ctccatctgaaaatgtcaaaccctgtagcagttcctttaacgtcgtttcaactccagttagtgaactccctgcgcctaataaaatctcaggtagagaggg[A/G]cttgatctggatatctccacaacagtaacctggttttcttgaggtatattggccttcaattttgctatggcaatggctcgggaaagtccaccaatagcat

fava_c15767_631	fava_c15767	631	T/C	tatctccacaacagtaacctggttttcttgaggtatattggccttcaattttgctatggcaatggctcgggaaagtccaccaatagcatcaaccaaacca[T/C]gtgatgccgcatctttaccagtccaaacccttccctgtgctacctcctccatcttatcRacagtcattgatctggataaagctgccttgtctcgaaattg

fava_c15767_690	fava_c15767	690	A/G	caatggctcgggaaagtccaccaatagcatcaaccaaaccaYgtgatgccgcatctttaccagtccaaacccttccctgtgctacctcctccatcttatc[A/G]acagtcattgatctggataaagctgccttgtctcgaaattgtttataagcattctgggcagatttggcaaatagctctgcctcatctggtctaaaagaac

fava_c15771_1120	fava_c15771	1120	C/T	gtgctgctcttcctagcagtgtttttgaagctgaaagaccaggatgcaaattctctgcaatgcagcgcgttgccacgtacttctacaagggtgcgttgta[C/T]ggatctgttggctttggatgtggtattgttggtcaaggaattgcaaatatgatcatgaatgccaaaaggagcattagtaaatctgaagacgacataccga

fava_c15771_325	fava_c15771	325	A/G	cttctttctcacaaaacaacaaccactgttacctgcggcacaacaaaaaaaacatgacactctcttcactcagtttcaccatctccaacaccaccacaac[A/G]acatcatggaacacacaaaacacaccacaacccaacaRcactgttccatctcccaacactttctgttccatcaccctcaacaaaacacaccttttcagaa

fava_c15771_363	fava_c15771	363	G/A	cacaacaaaaaaaacatgacactctcttcactcagtttcaccatctccaacaccaccacaacRacatcatggaacacacaaaacacaccacaacccaaca[G/A]cactgttccatctcccaacactttctgttccatcaccctcaacaaaacacaccttttcagaatccactgcattaacccacaacctatctcacccttggaa

fava_c15771_482	fava_c15771	482	C/A	cactttctgttccatcaccctcaacaaaacacaccttttcagaatccactgcattaacccacaacctatctcacccttggaatatcccaaatccgatcaa[C/A]ctgatatcggtggcggaggtgatgRcggcgatRatttcggcggaggaagaggcggagatgatggagatcacggagaagMagaagatggagatgaagagtt

fava_c15771_507	fava_c15771	507	A/G	aaaacacaccttttcagaatccactgcattaacccacaacctatctcacccttggaatatcccaaatccgatcaaMctgatatcggtggcggaggtgatg[A/G]cggcgatRatttcggcggaggaagaggcggagatgatggagatcacggagaagMagaagatggagatgaagagtttggacctctgttgaatttcgaagcg

fava_c15771_515	fava_c15771	515	G/A	ccttttcagaatccactgcattaacccacaacctatctcacccttggaatatcccaaatccgatcaaMctgatatcggtggcggaggtgatgRcggcgat[G/A]atttcggcggaggaagaggcggagatgatggagatcacggagaagMagaagatggagatgaagagtttggacctctgttgaatttcgaagcggttatgaa

fava_c15771_561	fava_c15771	561	C/A	gaatatcccaaatccgatcaaMctgatatcggtggcggaggtgatgRcggcgatRatttcggcggaggaagaggcggagatgatggagatcacggagaag[C/A]agaagatggagatgaagagtttggacctctgttgaatttcgaagcggttatgaaagaagttgaagctcgtggtgttacgttgccttctgatatggaagaa

fava_c15772_1794	fava_c15772	1794	G/T	aagagtcacctgcactaggattcactcctcttgaaccatctgttctacgtgcatacatatcagctgctagaagattgtctcctactgtcccaagggaact[G/T]gaagagtatattgccagtgcttactccagcattcgccaagaagaagccaaatccaccactccacattcctatacgactgtcagaacattgctcagcattc

fava_c15772_231	fava_c15772	231	G/A	ccgaattcgcagacgctaatggtgaagcgaaatacatcaacatcctgcaagatgttgcgaaccacaaagctcgatctgttgaaattgatcttgacgactt[G/A]atgaactataaggatctagatgaggagtttctgactcgagttactgagaatacgagaagatatgtagggatttttgctgatgcgattgacgagcttatgc

fava_c15775_1091	fava_c15775	1091	G/A	taattgcgggacagactacaacagcagcggcaatgacgtggagtgtaaaatttctacatgacaacagagatgcacaggacatactcagggacgaacagtt[G/A]tctttaaccaaaatgaagccagaaggagcttcacttaatcatgaagatatcaacaacatgcgttacggtttgaaggtagttaaggaaacactaagaatgt

fava_c15776_700	fava_c15776	700	A/T	tgtgtttacgcgcttctaattatcactctcgctctctttattacctatgtaatcctttgttgggtgagcttcttcctgttcctcctgctttaagttctgc[A/T]gatgaaaatctccgtttttcggcttttgggtttgatcccaaaaacaagagtttcaaaattattcagcttgtttttgtttccaaatcggatcaaatcgtcg

fava_c15776_826	fava_c15776	826	G/T	ttgggtttgatcccaaaaacaagagtttcaaaattattcagcttgtttttgtttccaaatcggatcaaatcgtcgctgaaatgtaccaatctgattcaag[G/T]gatagcgMgtggactgtgattccaaacgctccctctgcaaaacccaagtccaaaaattgttcctttgatccctcattcaatggtgcaattcattgggtca

fava_c15776_834	fava_c15776	834	A/C	gatcccaaaaacaagagtttcaaaattattcagcttgtttttgtttccaaatcggatcaaatcgtcgctgaaatgtaccaatctgattcaagKgatagcg[A/C]gtggactgtgattccaaacgctccctctgcaaaacccaagtccaaaaattgttcctttgatccctcattcaatggtgcaattcattgggtcactgaggat

fava_c15780_1359	fava_c15780	1359	C/T	atacagcagcttcagtttcacgtttaagaagtcttcttactgaaatccaggtgtcctgtcctcctgcttcaaagagggactccacaggctcggccaatgc[C/T]ttggccagttgtttctcaaaattagttgttatctccaacaattttgcattgcgcacagataaagcatgtgaatcaatatcgcggagaagcttatctcgca

fava_c15780_1613	fava_c15780	1613	A/C	tcaaactggagcaaaatagaatgagtcaatgttttaacagatgaagcaaatccttctccattgtttagtgattgatccagcttagtcttaaaatcgtcta[A/C]agctttagagcgtagaagccctatcattgttgtataagcagggtgcactatatccaatgcatttgattccaattgcttccgttttgcttttctcacagat

fava_c15780_208	fava_c15780	208	A/C	aataggggttctttatattctcatttaatttacacattacagtaaataagataagcaaaatatagctgtaaaatacaaaactgatctcttaaagcaacta[A/C]gacgtgcatattataatatgatgccgacctttttcaatgtaaattaccatgatcataattcagcttcagaaacgtttgttctttgcctctctaacaagtt

fava_c15780_2653	fava_c15780	2653	G/C	catctccctgaaagaagtgttgaaaagatgattcatcaaagtactctttccgctactttgcggtcccatgatagcaacgacggcgtaagaaagtccacac[G/C]cggccatgtttgaagtcttgatgaaattatcgagaccggagacgttgaagactccgtcaccgtcaatgagttgcgtggcgcagcaatcaacggcggttgt

fava_c15780_390	fava_c15780	390	A/C	tttgcctctctaacaagtttgtggttgagaattcactgtcatcattagatgagccaacactggatagagccgaattggagattgagcttgatactgagtc[A/C]ggtgtttgcgcttgacttttgaatatctgagaatctgaacgatgttgttctgttgcttcaggtgctggatttccctgagcttcttcggcaagacgtttaa

fava_c15780_84	fava_c15780	84	A/G	ggcgaagaaatacttggatccacttatacgctgcaataatacaatcgggaagatgattaatcagcttcgggcgcacagtgaag[A/G]taagattatcagaacagaacccaaataggggttctttatattctcatttaatttacacattacagtaaataagataagcaaaatatagctgtaaaataca

fava_c15785_1020	fava_c15785	1020	C/T	gttgtctgtgtatgcaagtcagctccttgagaaagaacactctggatgtcatgctctacttagagatgataagtgtgaagacttgtcaaggatgttcagg[C/T]tattttctaaaataccccgtggcttggatcctgtatctagcatatttaagcagcatgttaccaccgaaggcatggcattggtgaagcacgccgaagatgc

fava_c15785_2015	fava_c15785	2015	T/C	attcctctgatagactgagttattctgagataatgactcaattaaacttattggatgaagatgtcattaggctgctccactccttatcatgtgcaaagta[T/C]aagattcttctcaaggaacccaacacaaaaacaatttcacccactgattattttgaatttaattcaaagttcactgacaaaatgaggaggatcaagattc

fava_c15785_2147	fava_c15785	2147	A/T	caatttcacccactgattattttgaatttaattcaaagttcactgacaaaatgaggaggatcaagattcctctacctcctgtggatgagaagaaaaaagt[A/T]attgaggatgttgaYaaagaccgaagataYgccattgatgcttcaattgtgcgtattatgaagagtcggaaagttttgggataccaaaatttagttatgg

fava_c15785_2162	fava_c15785	2162	C/T	attattttgaatttaattcaaagttcactgacaaaatgaggaggatcaagattcctctacctcctgtggatgagaagaaaaaagtWattgaggatgttga[C/T]aaagaccgaagataYgccattgatgcttcaattgtgcgtattatgaagagtcggaaagttttgggataccaaaatttagttatggagtgcgttgagcagt

fava_c15785_2177	fava_c15785	2177	T/C	attcaaagttcactgacaaaatgaggaggatcaagattcctctacctcctgtggatgagaagaaaaaagtWattgaggatgttgaYaaagaccgaagata[T/C]gccattgatgcttcaattgtgcgtattatgaagagtcggaaagttttgggataccaaaatttagttatggagtgcgttgagcagttgggtcgcatgttta

fava_c15785_593	fava_c15785	593	G/A	actatttggaccgctactttattgcccggaggtcacttccaccactaaatgaagttgggttggcttgcttccgtgatttggtttacaaggaactacatgg[G/A]aaaatgagagatgcaattatttcacttattgatcaagaacgtgagggagagcaaattgatcgagctctattaaagaatgtattagatatatttgttgaaa

fava_c15792_1236	fava_c15792	1236	C/G	acagcctggtatttgaatagcatctggcagatctcggtactcacatgatgtttccttatccagtttgagaaaatagaggtacgaggacaaagcagtggct[C/G]cagaaggaaagtaaacataggacaacaagtcgaattccttagccaaatctagtgcttctgtggcaaatgaatcaaccaccaaggcaacaaagcgtgtcgt

fava_c15792_318	fava_c15792	318	T/C	catattttcataaaacttatttttctataccaaatttctccacttgagtgctaactcagaaagagtctttgtagaagacccatcttctttaagtgcacta[T/C]tagcaccttctctcaattccttcattctattgcgcatttttctgccttcttctccttccatgagacacttgatcaacaYagcaatttctacttttWccac

fava_c15792_397	fava_c15792	397	T/C	ccatcttctttaagtgcactaYtagcaccttctctcaattccttcattctattgcgcatttttctgccttcttctccttccatgagacacttgatcaaca[T/C]agcaatttctacttttWccacaataccgttctcattaagtcttggcctcagtcctactttgaggccttcactcagtaaaaccgcattcgttctttgctca

fava_c15792_414	fava_c15792	414	T/A	actaYtagcaccttctctcaattccttcattctattgcgcatttttctgccttcttctccttccatgagacacttgatcaacaYagcaatttctactttt[T/A]ccacaataccgttctcattaagtcttggcctcagtcctactttgaggccttcactcagtaaaaccgcattcgttctttgctcagcaaaaagaggccatgt

fava_c15794_1444	fava_c15794	1444	A/G	cgtggcacaagctttttcacaagagaatgccatctgaagcagtggatctagtttcaaggttgcttcagtattctccacatctgcgttgcaccgcattggc[A/G]gcatgcgcacacccgttcttcgatgatctgcgagacccaaaagcaagcctgccaaatggacaacctctacctcctttgttcaacttcacacctcaagaac

fava_c15794_1599	fava_c15794	1599	C/G	aatggacaacctctacctcctttgttcaacttcacacctcaagaactggtaaatgtgcccgacgatttgcgtcaacgtctcattcctgagcatgcaagga[C/G]ttaaatttattcatccagtcattgtaaacctgtaaacatctataagggtatcgtaaatgcgctttgaatgactagaatcgttaccttcgatgtttcttgc

fava_c15794_1874	fava_c15794	1874	G/T	tctgacaagtgaagacaatgatgacatggataaagtgacactatcagttgttgtaagtgtttggattgtaaacccttttgtagcattagcttatgatttg[G/T]tgtatgcttaggttctgaaagatagtgagtttctaacaatcatgtaattactaagcaagtgagggaagtattaaatgaaatacatttttttaaagaacaa

fava_c15794_493	fava_c15794	493	G/T	ggagttctaatgtagctgcagtaacgaagactgagaaatccggttttgatgaacttccgaaagaattacatgagatgaaaattagagacgagaaaagtac[G/T]aacaacaacgagaaggaaatagaagcaactacagtgagtggcaatggaacagaaacaggtcaaattattaccacaactattagtggccgagatggacaac

fava_c15795_1121	fava_c15795	1121	C/A	attcgtcatagaaaccgtatacctgtgtgatctgacgactttcatggtttcccctaatgagtgttatccgatctggatacctcaccttgagagcgagcaa[C/A]agcagaaacgtttcgaccgaataaaatcctctatcaacaaaatccccaaggaacaaatagttagtcttagggcaatcacctcccactttaaaaagctctt

fava_c15795_1453	fava_c15795	1453	T/C	ggttcgcatcgtttcagctgctcaatttgtctgtctaggtcagacatctctcttcactttctctctctagaaacagtgatgatgatgatgatgatgacga[T/C]gatgatgatgatgacgatgatgatgatgatgattcagtcactgcctcagagcttcttccactgcttcttcttgttcagttttcgttttctcagtttttgt

fava_c15795_227	fava_c15795	227	C/T	ttttcctccacaggattatatggactcccaatattccctgatccaatccaactcttcctaatcaagttctcatatatgcaaatgtccttttctttctaaa[C/T]aaaccctctgtccatcacatcttctgagtgaacaaaggtaagaataacatatttccaaatcaatcatactaattcacaacactgctacaataataatgat

fava_c15795_713	fava_c15795	713	G/A	tgtagcaataatttggagctgaccagacagtaactatctggttattgaacatccatttataaccttccattaccaactgatgagctcggcaaatatagtc[G/A]atgttattggtgtggttaaaagaagagacaacgctaccgccaaacaagaaaccagcaccacggggactcagaccccatccatctacaatatcttcaggat

fava_c15798_1101	fava_c15798	1101	A/G	atctcaaaattagcacaccaaaatctcttggaactctaatgcaaagtgaagcatcttcaattaatggattgaaatgaatattcaagtgaatgaagaaaca[A/G]agttaatataagatacctttcattatttcatacaccatttgtttcccYcattccatgKtgctgcaaatctttgttaagataagatggtaatgccattcca

fava_c15798_1149	fava_c15798	1149	C/T	aagcatcttcaattaatggattgaaatgaatattcaagtgaatgaagaaacaRagttaatataagatacctttcattatttcatacaccatttgtttccc[C/T]cattccatgKtgctgcaaatctttgttaagataagatggtaatgccattccattatgtgcttctaaagaacttgaagaaaattttcccttccttggccac

fava_c15798_1159	fava_c15798	1159	G/T	aattaatggattgaaatgaatattcaagtgaatgaagaaacaRagttaatataagatacctttcattatttcatacaccatttgtttcccYcattccatg[G/T]tgctgcaaatctttgttaagataagatggtaatgccattccattatgtgcttctaaagaacttgaagaaaattttcccttccttggccacaaccaaatct

fava_c15798_1580	fava_c15798	1580	A/G	tttactactactcgtttcaccgacttcttcgttctctcgacccgcttccgcgttcatcttcttgaatttcccaagccgaacgggtaaaacccctttatcg[A/G]catcatcttcaacaacaatttccttttgtctattaccaaatctgttttctccgttacaaggacaaccgtcgtcttctctaagatcatcgaaatcaaacat

fava_c15798_2041	fava_c15798	2041	A/T	gaaaaagctgttgaagctgtctttgaagtgcatcagaagttgaaatttcattatggtgttgtctattagattgagcagaagatgatgaagatggatgctt[A/T]atgagaaatctaacaagtaaatgaagtaaaccagatatgaaaaaaagaacagcaagaacaactatgatgaaaagaacagctggactaatcttagtactag

fava_c15798_2339	fava_c15798	2339	T/A	gtattcatctttttgatgaattctcaaatgggtatgttcaaataacaatttaggatacatagtttctgacatcaatctagtaacaaaacacaacaaaata[T/A]tctcctccaaccaaacaagaacctagtcaaatcacatctatgttacaagaacaaaagtcctaaaaatctaaactttatacaaatttcagacaagtaaaac

fava_c15798_848	fava_c15798	848	T/C	cctcataatcaagttgtgattcaatataaggttcatgtccaaatagatacacacactaaagattcatattgactagattcaattatgtatcaaatcgata[T/C]tcgactagcatatatggtaagatcatatcaacatttacaaccacaaatccaatttcatcataccatgaagtcagaagatttttttccaagaaagaaagga

fava_c15800_1804	fava_c15800	1804	G/T	tgaatatgatttgtatgaatatggcttcccttggcaagtgttgagccacaacactggtagcacaggaaggcagaggaaacaaatacatgtttcttgcaaa[G/T]atgctttcacttgttttatggtttcagagcccaaatatggcttagtctttRaggttaaaaaccatagcccaaatatgttaatcatattttcagaaatgac

fava_c15800_1855	fava_c15800	1855	A/G	cactggtagcacaggaaggcagaggaaacaaatacatgtttcttgcaaaKatgctttcacttgttttatggtttcagagcccaaatatggcttagtcttt[A/G]aggttaaaaaccatagcccaaatatgttaatcatattttcagaaatgactcaagagtaaagtgaatttgaagattgtcatttgtcacagcaactgtgaat

fava_c15800_440	fava_c15800	440	G/A	ataggggtgtcgcatgttcaccatcatggagctttagatgggatactcgggggcgtgttgctggtgaaattcagaatccttcctatcactcatctcatgc[G/A]gtcagtagaaatgtcagcatggagtttaaaggctcgttaagttcttacagaagtaatttatcggatgggggaagcgtcctggacaattctgcaactcctg

fava_c15800_897	fava_c15800	897	G/T	gatttctgataatcagatcatgggtctaaaatcaccagacagctcaatttttgaagggagggtatcatttgtactctccgcttgcagcaatgaaatcgaa[G/T]ctatatcccaatgtggatcttctgatggctggtcgatgcataccttttctgagctggtggcctcctcacaaaggggaaggtggtcttttgacagtgagtg

fava_c15801_1485	fava_c15801	1485	C/A	agatataatacaatctatcaaatctccatctggactcttaattgtcttgacgggaggcttgttgatcttcttcaagtgagctctgattctgttcaagtta[C/A]gcaatgcgaaatctggtctaaaagtttggttaacattgttggtgtgagagattaattgtgatgaagttgagtttgagagaatggggtgtgacaaaatagg

fava_c15801_701	fava_c15801	701	A/G	tgtggtaccctcttgaccgcgaattcacgatttctcctccaaattgtaccatgcttgcatgatttctcaaatgactaaatatgtttgctggccaatatcc[A/G]actagtagacctgatccRaattcaagccaccaatgtccatgctttggatctttccaaaccattaagccaatatcaaactgtcttccattatatattgatc

fava_c15801_719	fava_c15801	719	A/G	gcgaattcacgatttctcctccaaattgtaccatgcttgcatgatttctcaaatgactaaatatgtttgctggccaatatccRactagtagacctgatcc[A/G]aattcaagccaccaatgtccatgctttggatctttccaaaccattaagccaatatcaaactgtcttccattatatattgatcttggagagattgcagctc

fava_c15804_123	fava_c15804	123	A/G	tcatcgttttctctcacaccgaaccattcacgcctccgtcgccaccgttccttccatccccgcctccctttccttgctctgtcccgcgcatctaggtcac[A/G]ggtttgcttgtgaaagatgagtttgatatctcaaaatgctcgttctaaaatagagaccaagagggaggaggacattaaagaaaaggagggtgaagatggg

fava_c15804_1519	fava_c15804	1519	C/A	gcataaacagcacggcgctttgtactaatccaggagggacacattttgcagctggctcagacagtggagttgtgaacatttacaacagagaggaatttct[C/A]gggggaaagaaaaagcctatcaaggccattgacaatttgagtacctcagtggatttcatgagatttaatcccgactctcaaatattagctatctgttccg

fava_c15804_1840	fava_c15804	1840	G/A	gttttatggctgtcggaaatgcttcaggtaaagtgttactgtataagttacaccattatcagcatgcttaaggtaacttagaaacaccggcatcgttttt[G/A]WtRtatttcatttttgttttatgttttatggtgggaatagtgaattttgagactttagttgtgttttatcattaattaatgaacatgatgcttcagttag

fava_c15804_1841	fava_c15804	1841	T/A	ttttatggctgtcggaaatgcttcaggtaaagtgttactgtataagttacaccattatcagcatgcttaaggtaacttagaaacaccggcatcgtttttR[T/A]tRtatttcatttttgttttatgttttatggtgggaatagtgaattttgagactttagttgtgttttatcattaattaatgaacatgatgcttcagttagc

fava_c15804_1843	fava_c15804	1843	G/A	ttatggctgtcggaaatgcttcaggtaaagtgttactgtataagttacaccattatcagcatgcttaaggtaacttagaaacaccggcatcgtttttRWt[G/A]tatttcatttttgttttatgttttatggtgggaatagtgaattttgagactttagttgtgttttatcattaattaatgaacatgatgcttcagttagcat

fava_c15804_262	fava_c15804	262	A/G	ctcgttctaaaatagagaccaagagggaggaggacattaaagaaaaggagggtgaagatgggcaagagtctgatgttgatgccgtggaagtgaagaagcg[A/G]aagcgagatggcaaggcagaagagttggaattagaacaagtgaaagaaatgaaaaagctggagaactttttgttcgggtctctctattcccctcttgaat

fava_c15804_385	fava_c15804	385	A/T	agttggaattagaacaagtgaaagaaatgaaaaagctggagaactttttgttcgggtctctctattcccctcttgaatttggaaagggtggtgatgatga[A/T]gaagtggaagctgggtctgctgcaaaggcttctgatttgttcttcaccgaccgttctgcagatagtgttgtctctgtttacaaggaggatgctgacttgt

fava_c15804_769	fava_c15804	769	C/T	accgaggaactgactgggcccaacttgattccggggagaaaatggatggatcttctgatgatgagttaacagatgatgataataaagctgctgttgtgag[C/T]cgaggttacaaagacatggatgatattctgagaacaaatgaagacctggttgtgaaaagcagctcaaaattgttgccaggtcatattgaatactcaagac

fava_c15804_955	fava_c15804	955	C/T	ttgaatactcaagacttgtagatgcaaatatacaagatccttctaacggtcctataaattcggttcagttccatcgaaatggtcagttgcttcttgctgc[C/T]ggattagaccggaagcttaggttttttcaaattgatggaaaacgaaacaccaaaattcaaagcatattccttgaagattgtcccattcgaaaagcatctt

fava_c15810_1207	fava_c15810	1207	C/G	gtgggtactgattgctcttgcctttgccagagctacatgatccaacagccttactaacagagttgaagcaacaaagagtatccattcagttcaactactt[C/G]ttcacctgtgaaaaatttcaaattctccacacccagatgatcaatacaatgaaaaactcacaaaccaatccaaaaacttcaactttttgcagaaaaaatg

fava_c15810_348	fava_c15810	348	C/T	acctacaattttacataggaaaaaaaccaaacctatgttgcataaaattccaaactcataattggtttcttgagatttccaaacatatatatgaagaact[C/T]gtatcatcacttgaaacgaactacaatgcatgaaatgtcatccctactctctcttttcaatgcctctgcagttaaatgtttagctgctttttgtggatct

fava_c15811_129	fava_c15811	129	A/G	tgctacaaatttgaatatacaaatctgaatacatattctttatgttgaaggataaaccgttcacatcaaaacaaacaagtaacataatcaactttgtaag[A/G]caaatagattgcacaccaaacttgcaaaaaaaaattaaatgaaatttcattttgggggttcacatWtaaaaatgtattcccttaatattttgcttaaggc

fava_c15811_1608	fava_c15811	1608	A/G	tctctctaactgatcgaattctggaatcaagttccttgtgattgaggagctttttgcgataatctttgacggcattgttacgacgtcgtatggcatcatc[A/G]gactcggttgacgacatggtgttctaacgttaggttaaagactgaaactgaacacagatgtctgtgttgttgttattaagaatgaaatgttatagctgct

fava_c15811_195	fava_c15811	195	T/A	caaaacaaacaagtaacataatcaactttgtaagRcaaatagattgcacaccaaacttgcaaaaaaaaattaaatgaaatttcattttgggggttcacat[T/A]taaaaatgtattcccttaatattttgcttaaggcctgcgaatgtgctaggccaaccttggatactgtaagtatgaaaccttaacctatagttgaactgca

fava_c15811_372	fava_c15811	372	A/T	accttaacctatagttgaactgcaaatccaacaaaaactattcaagcttacaaagaaaaagtatgtcagttggtggctggaagtgactacttgctccaaa[A/T]gttctttgggcggtgctagataaacagatgtactacaaaaaatgtttttaatcttttccgaaatctgcattgtaatgggcgcttgcttccagtttctttg

fava_c15811_597	fava_c15811	597	G/A	tcatgaaatcttcatgaattacataatcgcgttctgcacgaatggcagccatcccagcttcagtgcaaacgttacgaagatcggcaccattgaacccctc[G/A]gcaagcttaacaactgcttcatagtcaatStcaccatgctttgcaattccagcagcatggattttgaggacttccatcctggactgctcgtttggtaatg

fava_c15811_627	fava_c15811	627	G/C	gttctgcacgaatggcagccatcccagcttcagtgcaaacgttacgaagatcggcaccattgaacccctcRgcaagcttaacaactgcttcatagtcaat[G/C]tcaccatgctttgcaattccagcagcatggattttgaggacttccatcctggactgctcgtttggtaatggaatctcaattttccgatcaagtcgacccg

fava_c15815_1236	fava_c15815	1236	C/T	tcaatgacagtctaagcagctcaacctgctcccaatctacaccggtattatttaaaactagaattcggatgcttttcagtaatggaagttcagatttata[C/T]agtgacattaagttgtaggataagttgagagccttcagagaagKcaactgttcacaaatgatgccaacatccttccactccgaaagaaggttcccagaga

fava_c15815_1280	fava_c15815	1280	G/T	gtattatttaaaactagaattcggatgcttttcagtaatggaagttcagatttataYagtgacattaagttgtaggataagttgagagccttcagagaag[G/T]caactgttcacaaatgatgccaacatccttccactccgaaagaaggttcccagagagatcaagWtSttttatattcggcaYtgtagtactaatttgacct

fava_c15815_1344	fava_c15815	1344	A/T	ttaagttgtaggataagttgagagccttcagagaagKcaactgttcacaaatgatgccaacatccttccactccgaaagaaggttcccagagagatcaag[A/T]tSttttatattcggcaYtgtagtactaatttgacctggaactccagggaaaccaacacccataaaYgacaacgacacactcgataactcctcRagtcgac

fava_c15815_1346	fava_c15815	1346	C/G	aagttgtaggataagttgagagccttcagagaagKcaactgttcacaaatgatgccaacatccttccactccgaaagaaggttcccagagagatcaagWt[C/G]ttttatattcggcaYtgtagtactaatttgacctggaactccagggaaaccaacacccataaaYgacaacgacacactcgataactcctcRagtcgactt

fava_c15815_1361	fava_c15815	1361	C/T	ttgagagccttcagagaagKcaactgttcacaaatgatgccaacatccttccactccgaaagaaggttcccagagagatcaagWtSttttatattcggca[C/T]tgtagtactaatttgacctggaactccagggaaaccaacacccataaaYgacaacgacacactcgataactcctcRagtcgacttagcttatcattaagt

fava_c15815_1410	fava_c15815	1410	C/T	tccactccgaaagaaggttcccagagagatcaagWtSttttatattcggcaYtgtagtactaatttgacctggaactccagggaaaccaacacccataaa[C/T]gacaacgacacactcgataactcctcRagtcgacttagcttatcattaagttcactttcacccaataacttaatagaaactcgtttattactagacgaaa

fava_c15815_1437	fava_c15815	1437	A/G	gatcaagWtSttttatattcggcaYtgtagtactaatttgacctggaactccagggaaaccaacacccataaaYgacaacgacacactcgataactcctc[A/G]agtcgacttagcttatcattaagttcactttcacccaataacttaatagaaactcgtttattactagacgaaaacacatacatttcatcctcttcatctt

fava_c15815_1623	fava_c15815	1623	C/T	catcctcttcatctttagtagaggtacttctataccttttttcaatagcttcaagaaatgaaatccctttagacagattcttaggacgaacaaaagaacc[C/T]gatgtttcggatttagcattgaaRtagcgagctccgtttagggatccgtcgtgttttccttctgcattgtcccaatcgattccaatccatgtgtcggagt

fava_c15815_1647	fava_c15815	1647	A/G	tacttctataccttttttcaatagcttcaagaaatgaaatccctttagacagattcttaggacgaacaaaagaaccYgatgtttcggatttagcattgaa[A/G]tagcgagctccgtttagggatccgtcgtgttttccttctgcattgtcccaatcgattccaatccatgtgtcggagtagctctctacagttccgacgtact

fava_c15815_1865	fava_c15815	1865	A/T	gttggattgccggatgtgtgtactcgttgccctaagcggaattcgggtgttgaattgggaggatcctgcatcttttgttttgttgtatggagtaagagag[A/T]ggtggaacaaaacctatagtgacacgttgatggtgtgtaRtgttgtttcactgtgtYagttggttttttcgttcctttgtggaaatgtagatcacgacca

fava_c15815_1905	fava_c15815	1905	G/A	attcgggtgttgaattgggaggatcctgcatcttttgttttgttgtatggagtaagagagWggtggaacaaaacctatagtgacacgttgatggtgtgta[G/A]tgttgtttcactgtgtYagttggttttttcgttcctttgtggaaatgtagatcacgaccagcttgcggtggctggtgaaagtcgacgaccgcgttgcagg

fava_c15815_1922	fava_c15815	1922	T/C	ggaggatcctgcatcttttgttttgttgtatggagtaagagagWggtggaacaaaacctatagtgacacgttgatggtgtgtaRtgttgtttcactgtgt[T/C]agttggttttttcgttcctttgtggaaatgtagatcacgaccagcttgcggtggctggtgaaagtcgacgaccgcgttgcaggttgtgacaatctctttt

fava_c15815_263	fava_c15815	263	G/C	attataaagtaaatttttttaatatagattacaccaaaaggcttataaaaaaatttctgatatttttatcaactaacttcgtctacaaggataactgaat[G/C]attgtcaaccccaaggtccataagagatgacgagtcattgtcaagcagcaagggcaatggagatccctcctcYtgaaaaaataatttcagcttcatggac

fava_c15815_336	fava_c15815	336	C/T	taacttcgtctacaaggataactgaatSattgtcaaccccaaggtccataagagatgacgagtcattgtcaagcagcaagggcaatggagatccctcctc[C/T]tgaaaaaataatttcagcttcatggacttcagcttaaaaaagctttcacagagaaattttagtttgccaactgtagttgttgctggaagcttctttgtca

fava_c15815_607	fava_c15815	607	C/T	ccgtgggccatcagaagctccaactaaaggcttttgatcctcaattccgtaaaaattcttgagctcggagaacctggggtgctgtttgatttcttcggga[C/T]tgacatgcaaccttgagaccacaagccggacatacctaatctcagagtcctttctttcacgagaggtaatctcgctcccattcaaYatctgaatgttcgc

fava_c15815_693	fava_c15815	693	T/C	tgatttcttcgggaYtgacatgcaaccttgagaccacaagccggacatacctaatctcagagtcctttctttcacgagaggtaatctcgctcccattcaa[T/C]atctgaatgttcgctaaacgagcgattgaaacaaatcttggaaccccacctcttgcagtatcagttattgggttcccagaaagcctgatttccaccaagt

fava_c15815_822	fava_c15815	822	C/T	aaacaaatcttggaaccccacctcttgcagtatcagttattgggttcccagaaagcctgatttccaccaagttaggaaacaagtttaatgaatcaacaga[C/T]gctagatcactaatgttgttgtcgcccaacagaagacatcgcaaattttgaaagggcttgcagtccgtaacttttgaatcataatactgcgaaccattat

fava_c15815_923	fava_c15815	923	T/C	gctagatcactaatgttgttgtcgcccaacagaagacatcgcaaattttgaaagggcttgcagtccgtaacttttgaatcataatactgcgaaccattat[T/C]RggataaaagagagaactcaaacaattcttgttcaaataaagctgctccaaacatcttagctgagaaagcttcatgatttcactccattccgctatacaY

fava_c15815_924	fava_c15815	924	G/A	ctagatcactaatgttgttgtcgcccaacagaagacatcgcaaattttgaaagggcttgcagtccgtaacttttgaatcataatactgcgaaccattatY[G/A]ggataaaagagagaactcaaacaattcttgttcaaataaagctgctccaaacatcttagctgagaaagcttcatgatttcactccattccgctatacaat

fava_c15821_1424	fava_c15821	1424	G/C	aagtcctactttttgattatgaaaactcgtatccttggtccaagaacgagttagttgatgagcaccctttggtgaccaaagttaagaactggaaagaagt[G/C]gaacaacaattgatgtcactggtagcttcatagtctacaacaaatgctggactagttcatgtttttgaaatgtctaacatttattcttgaagaagtgaaa

fava_c15822_112	fava_c15822	112	A/C	attcataatttcttcaagagatatcatataataatttaaatacaaggcagcaacactagaaaaagaaaatcaaacaataaaaaactactatactagactc[A/C]cccaacaatacattaatctaaaactaatcaagttccaaaatttaccataatcaaaataaaaaagagtggtcacatatgaatatagcaagtaacatcatta

fava_c15822_642	fava_c15822	642	A/G	tctccatcttcatctgctccatcctcacaaacccatctctcaaaaccttaatagcattcaccatttcaccaatagggtctctttctctttctctctctac[A/G]tctctctccctctctctcttccccaacccaatcttctccttcacaagcctcgttccaccgttgatataaccgcgagaacccgattccatctggtgactgt

fava_c15826_1101	fava_c15826	1101	A/G	tgcctgatgatgtacggcttagattacaatcacttgaagggtaggcatctgatcttctccttttctcagtcaggcacatctcagccatgtcaccatcgtc[A/G]tccatgagatgttctatttcatcacgaaccttctgaacccgctgagtcaaagcaagaaggtgacctttgaatcttcgaacacgttccagaaggagagtac

fava_c15826_573	fava_c15826	573	G/A	ccagtcatgtcaccttgaccaagttggctttgaatttttattctggaagcacttttttgtatctaaaataggatagtaaggagaaatacaatgctacaca[G/A]ccaattccagtaattaccataacccagttRaatccagatgagtagtcaaaaactgaggttttaaagttcattccaaatactcctgtcacYgcggcaaata

fava_c15826_603	fava_c15826	603	A/G	tgaatttttattctggaagcacttttttgtatctaaaataggatagtaaggagaaatacaatgctacacaRccaattccagtaattaccataacccagtt[A/G]aatccagatgagtagtcaaaaactgaggttttaaagttcattccaaatactcctgtcacYgcggcaaatattgcagccaccattgtggctgctgtaagaa

fava_c15826_663	fava_c15826	663	T/C	atgctacacaRccaattccagtaattaccataacccagttRaatccagatgagtagtcaaaaactgaggttttaaagttcattccaaatactcctgtcac[T/C]gcggcaaatattgcagccaccattgtggctgctgtaagaagcatctcaaactttattaggaggttttgaatattccccagttttatgttgagaaaatctt

fava_c15826_771	fava_c15826	771	G/A	atattgcagccaccattgtggctgctgtaagaagcatctcaaactttattaggaggttttgaatattccccagttttatgttgagaaaatcttcagtgtc[G/A]tctatgtattctttgagcgacgataatgtattgagagtattatcaatgacgataaagtatgcttccagcaacatttccagtggctgaatcctttctccat

fava_c15828_1390	fava_c15828	1390	G/T	ataaaaaagatgttagtgagaaatcacatcctaagtatgttaggatcgtgagtaagcagatggtgggaatatatgtgtctgtttgggtacaaaggaagct[G/T]agaaggcatattcatcatttgaaagtttctcctgttggagttggtcttatgggctatatgggaaacaaggggtctgtttcggttagtatgtctgtttttc

fava_c15828_1675	fava_c15828	1675	C/T	aaattctacaacgcactcgtttctcttcagtcttcgactcagatcaaccacgcacgattccatcgcacgatcaaattttctggtttggagatttgaatta[C/T]cgtatcaatatgtcggatggcgaggttcggaagctagtagStctaaaaaattgggatgaactcatgaattatgaccagctaagcaatgaattatgtaatg

fava_c15828_1716	fava_c15828	1716	C/G	gatcaaccacgcacgattccatcgcacgatcaaattttctggtttggagatttgaattaYcgtatcaatatgtcggatggcgaggttcggaagctagtag[C/G]tctaaaaaattgggatgaactcatgaattatgaccagctaagcaatgaattatgtaatggacatgtatttgagggatggaaagaagggttggtaaatttt

fava_c15828_1897	fava_c15828	1897	A/G	aagaagggttggtaaattttccacctacttacaaatatgaattcaactccgataaatatgcgggcaagttcacacaagaaggcgaaaaaaagagatctcc[A/G]gcatggtgtgatcgcatattgtggataggaaaaggaataaagcaacttgagtatcaatgYgcagMaaataagctctcRgaYcatcgaccagttagttcaa

fava_c15828_1957	fava_c15828	1957	T/C	cgggcaagttcacacaagaaggcgaaaaaaagagatctccRgcatggtgtgatcgcatattgtggataggaaaaggaataaagcaacttgagtatcaatg[T/C]gcagMaaataagctctcRgaYcatcgaccagttagttcaatcttcttggttgatgttgaagtaattgaccgccgaaaactacaacgagctctaaatttca

fava_c15828_1962	fava_c15828	1962	C/A	aagttcacacaagaaggcgaaaaaaagagatctccRgcatggtgtgatcgcatattgtggataggaaaaggaataaagcaacttgagtatcaatgYgcag[C/A]aaataagctctcRgaYcatcgaccagttagttcaatcttcttggttgatgttgaagtaattgaccgccgaaaactacaacgagctctaaatttcacaagt

fava_c15828_1975	fava_c15828	1975	A/G	aaggcgaaaaaaagagatctccRgcatggtgtgatcgcatattgtggataggaaaaggaataaagcaacttgagtatcaatgYgcagMaaataagctctc[A/G]gaYcatcgaccagttagttcaatcttcttggttgatgttgaagtaattgaccgccgaaaactacaacgagctctaaatttcacaagtgcagttgtacatc

fava_c15828_1978	fava_c15828	1978	T/C	gcgaaaaaaagagatctccRgcatggtgtgatcgcatattgtggataggaaaaggaataaagcaacttgagtatcaatgYgcagMaaataagctctcRga[T/C]catcgaccagttagttcaatcttcttggttgatgttgaagtaattgaccgccgaaaactacaacgagctctaaatttcacaagtgcagttgtacatccag

fava_c15828_2117	fava_c15828	2117	T/A	agtaattgaccgccgaaaactacaacgagctctaaatttcacaagtgcagttgtacatccagagattttcgttagagaggatgatgaagacatgttctag[T/A]agataacatggcaaaagattatgtttatatggttttgtttattcttctgcagggtgttaacattttggttgagatttgaagcMtactttttgagagtaat

fava_c15828_2200	fava_c15828	2200	C/A	atgaagacatgttctagWagataacatggcaaaagattatgtttatatggttttgtttattcttctgcagggtgttaacattttggttgagatttgaagc[C/A]tactttttgagagtaataatgtgaatatgaattagttYcatggaagagatatatgtaaataaatatacatgtgataacatactgtttttaaaatgtgtta

fava_c15828_2238	fava_c15828	2238	C/T	atgtttatatggttttgtttattcttctgcagggtgttaacattttggttgagatttgaagcMtactttttgagagtaataatgtgaatatgaattagtt[C/T]catggaagagatatatgtaaataaatatacatgtgataacatactgtttttaaaatgtgttatgtattgtgagaagaggaatagttctactacactgtgt

fava_c15828_568	fava_c15828	568	G/A	tgaatataaagcaaaaagtgtatgattttagcgaagatgaagcggatactgaaaccgaaagcgatgatgatgatacatcttgcaaagactacaagacgag[G/A]cgacacaaaagaggaaaatcagaaactttgcgcgttcagtacataaatacaaaggaagtgagagtaacaattggtacttggaatgttgctggagtacctc

fava_c15828_704	fava_c15828	704	A/G	tcagtacataaatacaaaggaagtgagagtaacaattggtacttggaatgttgctggagtacctccatgtaatgatcttgatattgaagactggctttgt[A/G]ctaaaaaaccagcagatatttacattattggttttcaagaggtggttccattgaatgctgggaatgtgctaggagctgaagataataaacctatttcgaa

fava_c15834_1056	fava_c15834	1056	G/A	agRatgaagccgaatgggatccaggcaaaggtgatgatggcgtttagcggatctggacgctgcacaaagcgaccgtcgtgaaagatgagtcggctcttta[G/A]acgctctagaggaactggttttgctgatttgcttggaggcaccatgtaMccttccttgcaaatagacatgaaatcatgatcagtttttcgatctccaagt

fava_c15834_1105	fava_c15834	1105	C/A	gatctggacgctgcacaaagcgaccgtcgtgaaagatgagtcggctctttaRacgctctagaggaactggttttgctgatttgcttggaggcaccatgta[C/A]ccttccttgcaaatagacatgaaatcatgatcagtttttcgatctccaagtccaatatcaggcaattcatcaccaaactctttYacaaccgccaaacgct

fava_c15834_1189	fava_c15834	1189	C/T	ttggaggcaccatgtaMccttccttgcaaatagacatgaaatcatgatcagtttttcgatctccaagtccaatatcaggcaattcatcaccaaactcttt[C/T]acaaccgccaaacgcttcaaatctccaacaagcacaccagatttcttcacgaatccagtcgccttcttcgtcttcggattcacctcgatctccgttccca

fava_c15834_1546	fava_c15834	1546	C/A	cgagttcgatatcgcggattttgagaccggcaaaggagatgaaaatcagtatctgaataccgagagattcagatatgaagagataagaaacgatgactaa[C/A]ggaagagaaagcagtagaataaagccacggaggaggcttccagcttcaacagcgacgagcatgaagtacggaaacgagcttcgagaaatgagaagagtac

fava_c15834_1648	fava_c15834	1648	G/A	gaagagaaagcagtagaataaagccacggaggaggcttccagcttcaacagcgacgagcatgaagtacggaaacgagcttcgagaaatgagaagagtacc[G/A]tcaagatcagccgcaacggtttcacgcggcgaagaagacgacgttactccgttacattccgttatcggagggaagttacgtttcggtttcggcgtcgcca

fava_c15834_829	fava_c15834	829	G/T	tgctgacgctgtaggtgacacatgacactttgcggccgagggcaatggcaatgatgatggggtcgagggcggtgcggtggttgcaaacgtagaggtttcc[G/T]ggggttcctggagatggtggSggaggacggtggccgctgattacaagcttgataccgagcatttcataggtgtagcgaactatgcgttcagggagtggga

fava_c15834_850	fava_c15834	850	G/C	atgacactttgcggccgagggcaatggcaatgatgatggggtcgagggcggtgcggtggttgcaaacgtagaggtttccKggggttcctggagatggtgg[G/C]ggaggacggtggccgctgattacaagcttgataccgagcatttcataggtgtagcgaactatgcgttcagggagtgggaggttgaagtagactctgatga

fava_c15834_958	fava_c15834	958	A/G	ggtggccgctgattacaagcttgataccgagcatttcataggtgtagcgaactatgcgttcagggagtgggaggttgaagtagactctgatgatggagag[A/G]atgaagccgaatgggatccaggcaaaggtgatgatggcgtttagcggatctggacgctgcacaaagcgaccgtcgtgaaagatgagtcggctctttaRac

fava_c15837_146	fava_c15837	146	T/C	catcaatttcttccccttcctttatcggcgtcaaaatgtcatcgtcactgatatctgattttctctatcaagccggcggagctgccgtcatcgacggcgg[T/C]ttggcaacagagcttgaacgccacggtgccgacctcaacgatcccctatggagcgccaaatgcctcatctcttcccctcactctgacctcattcgccagg

fava_c15838_1253	fava_c15838	1253	C/T	tgaatcctgacacgttattgccgttggcttattccatcaagataacaaagcgattagaggaactgaaactagagtacgagaagaagaatgggaagataac[C/T]cgaWcgagagaaatttctgaaaatgaaaaaccaattcttYcaagcatYcagagcaactgaagccRaaatataacacgcagaaggaaccgattaaaaagcc

fava_c15838_1257	fava_c15838	1257	T/A	tcctgacacgttattgccgttggcttattccatcaagataacaaagcgattagaggaactgaaactagagtacgagaagaagaatgggaagataacYcga[T/A]cgagagaaatttctgaaaatgaaaaaccaattcttYcaagcatYcagagcaactgaagccRaaatataacacgcagaaggaaccgattaaaaagccattt

fava_c15838_1293	fava_c15838	1293	C/T	gataacaaagcgattagaggaactgaaactagagtacgagaagaagaatgggaagataacYcgaWcgagagaaatttctgaaaatgaaaaaccaattctt[C/T]caagcatYcagagcaactgaagccRaaatataacacgcagaaggaaccgattaaaaagccattttggtttctaaatgtgaggcgttgtcactacagttct

fava_c15838_1301	fava_c15838	1301	C/T	agcgattagaggaactgaaactagagtacgagaagaagaatgggaagataacYcgaWcgagagaaatttctgaaaatgaaaaaccaattcttYcaagcat[C/T]cagagcaactgaagccRaaatataacacgcagaaggaaccgattaaaaagccattttggtttctaaatgtgaggcgttgtcactacagttctaagttctt

fava_c15838_1318	fava_c15838	1318	G/A	aaactagagtacgagaagaagaatgggaagataacYcgaWcgagagaaatttctgaaaatgaaaaaccaattcttYcaagcatYcagagcaactgaagcc[G/A]aaatataacacgcagaaggaaccgattaaaaagccattttggtttctaaatgtgaggcgttgtcactacagttctaagttcttgaatgtatgaaattaca

fava_c15838_1451	fava_c15838	1451	T/C	gccattttggtttctaaatgtgaggcgttgtcactacagttctaagttcttgaatgtatgaaattacagaaacatcataaagtatgtttttgttattggt[T/C]tgatggatcacatttattaatggaagacgtgttcaatgaccaaactaacaagcaaaaWatgtatttaagtgtgtttctgtatttttgttRcattcaagaa

fava_c15838_1509	fava_c15838	1509	T/A	tgaaattacagaaacatcataaagtatgtttttgttattggtYtgatggatcacatttattaatggaagacgtgttcaatgaccaaactaacaagcaaaa[T/A]atgtatttaagtgtgtttctgtatttttgttRcattcaagaactattgcgacaacgccacaaacatttaaagaccagaatggacctgcagaactattcgt

fava_c15838_1541	fava_c15838	1541	A/G	tgttattggtYtgatggatcacatttattaatggaagacgtgttcaatgaccaaactaacaagcaaaaWatgtatttaagtgtgtttctgtatttttgtt[A/G]cattcaagaactattgcgacaacgccacaaacatttaaagaccagaatggacctgcagaactattcgtaaatSgataacctctaaatgtaggtctctttt

fava_c15838_155	fava_c15838	155	A/G	tccaatttcatgattattttggatcgttccttcttcttctaaagaacctccctttccaatttcaatttcaaggtttttgtcattgtaatgtttgaggcaa[A/G]ctatgtcatttttgttctggtttgagagagttaatttctgctttttgaatggaaaggaaaagaagtgatgactatgaatcaggtccacttccttcaccaa

fava_c15838_1614	fava_c15838	1614	C/G	atttaagtgtgtttctgtatttttgttRcattcaagaactattgcgacaacgccacaaacatttaaagaccagaatggacctgcagaactattcgtaaat[C/G]gataacctctaaatgtaggtctcttttgctgctagtgtgagaatcatacccKtagagtctttaactgtgaccatatttcacatcctYtgtgcagttgtat

fava_c15838_1666	fava_c15838	1666	T/G	ccacaaacatttaaagaccagaatggacctgcagaactattcgtaaatSgataacctctaaatgtaggtctcttttgctgctagtgtgagaatcataccc[T/G]tagagtctttaactgtgaccatatttcacatcctYtgtgcagttgtatcagttaatgactgctttcatctaagtgtgttcttaggcttcagtgaatgtaa

fava_c15838_1701	fava_c15838	1701	T/C	actattcgtaaatSgataacctctaaatgtaggtctcttttgctgctagtgtgagaatcatacccKtagagtctttaactgtgaccatatttcacatcct[T/C]tgtgcagttgtatcagttaatgactgctttcatctaagtgtgttcttaggcttcagtgaatgtaaaaatctgttgactcaattcttcctgtttcctacta

fava_c15838_1819	fava_c15838	1819	C/A	taatgactgctttcatctaagtgtgttcttaggcttcagtgaatgtaaaaatctgttgactcaattcttcctgtttcctactagtgctgtgtaaagatga[C/A]attgtcatagaaattattattattgtttttgtgatagtacatatagaaagttttaaacttattgttgagtcaattgtaatgtaactcatcaaaactcaag

fava_c15838_260	fava_c15838	260	G/A	gtcatttttgttctggtttgagagagttaatttctgctttttgaatggaaaggaaaagaagtgatgactatgaatcaggtccacttccttcaccaagagc[G/A]ttggatagattYgggtttgtaaaaccggatgctaacacttctgagggcgttgccaagacgaatagaYctgcatatgagtatgagaaaattaaggaacgga

fava_c15838_272	fava_c15838	272	C/T	ctggtttgagagagttaatttctgctttttgaatggaaaggaaaagaagtgatgactatgaatcaggtccacttccttcaccaagagcRttggatagatt[C/T]gggtttgtaaaaccggatgctaacacttctgagggcgttgccaagacgaatagaYctgcatatgagtatgagaaaattaaggaacggagaagggttagaa

fava_c15838_327	fava_c15838	327	C/T	ctatgaatcaggtccacttccttcaccaagagcRttggatagattYgggtttgtaaaaccggatgctaacacttctgagggcgttgccaagacgaataga[C/T]ctgcatatgagtatgagaaaattaaggaacggagaagggttagaaaatggaggaagatgattggggttggtgggagtgattggaaacattacctaaggaa

fava_c15842_1106	fava_c15842	1106	C/T	ggcgtcatcttaagaagatcatccaataggaaaaggagagttgtctttgattactcagatgaagatgaagacatcatcagtttagcgtcacctgatgtcc[C/T]tagtaaacagtcgcctcaagattccatgcaaaatgacaaaaagtcattagagaaaaccactttgaattttgatttgcaggtagaacataaaccgttggtt

fava_c15842_1274	fava_c15842	1274	C/T	tttgatttgcaggtagaacataaaccgttggttaaggaagagaaagcaactgagaaaaaaacaactgagaaaaaagttttcctaccaccaagagaagatt[C/T]atcaattatgagcaagtgcacaagtaatgggaaatcatctactgagaaKctgcagagtagtgctcctgaaatttgtgtaaataaggatagtataaaaaag

fava_c15842_1323	fava_c15842	1323	T/G	ctgagaaaaaaacaactgagaaaaaagttttcctaccaccaagagaagattYatcaattatgagcaagtgcacaagtaatgggaaatcatctactgagaa[T/G]ctgcagagtagtgctcctgaaatttgtgtaaataaggatagtataaaaaaggtctctccaggtgaacctaagaggagaaaagtgatgaagaccaggattg

fava_c15842_558	fava_c15842	558	G/A	agctcttcaagcagcctccctcggtcgataattgcttgagagacaataggttctgtgggatctctaattcttttgtaagaaggaacgtggaaggccaacc[G/A]gcagcttctgccactccacaaataaaatctttagcaggctYgggaccaacaaagagtaacattgttcaccaacctccaaagaacacagtgcccatttcta

fava_c15842_599	fava_c15842	599	C/T	gacaataggttctgtgggatctctaattcttttgtaagaaggaacgtggaaggccaaccRgcagcttctgccactccacaaataaaatctttagcaggct[C/T]gggaccaacaaagagtaacattgttcaccaacctccaaagaacacagtgcccatttctattgctaaagttgatcacacatcacagaatgtggtgaaacaa

fava_c15842_705	fava_c15842	705	T/G	caacaaagagtaacattgttcaccaacctccaaagaacacagtgcccatttctattgctaaagttgatcacacatcacagaatgtggtgaaacaagtcaa[T/G]agtgaaatcaacggtacaggaaatacaggagttcatgataacatcaataaacctactgcagagaaagaaaagWcccttccagtgcccactgggaaaaaga

fava_c15842_778	fava_c15842	778	A/T	atcacagaatgtggtgaaacaagtcaaKagtgaaatcaacggtacaggaaatacaggagttcatgataacatcaataaacctactgcagagaaagaaaag[A/T]cccttccagtgcccactgggaaaaagaaaggacagggtgataaaagtagctctggtactggaggttcgttggcaagcttctggggtcgtccatctacaaa

fava_c15846_2118	fava_c15846	2118	G/C	ccatcttctgaccaatctccgccatttcttccggccgagtatgcttcaactgttcagcagcaagcttaaaatcctcaggattcatattcttcatactctc[G/C]gaagccatcctcatcaaatctggattcgacatcatctgttgttggatccgagccatatcagcgggagacatgcgactcatttgttcctgagcgattctga

fava_c15846_290	fava_c15846	290	C/A	ttaaaattagggtatggtaatgactaaagttactgaattgtaggattcttctcagttctaaatgataaacaattcaaaatctaatatattacagataaac[C/A]aaaaaaaatgcaatcacattctcaatctgaacattatggcattgatttccaaataaataaccaatggggtataaactaggtttcaacctcttctctccta

fava_c15846_693	fava_c15846	693	G/A	ttgcacattcagcccctctttgaagcctgtccgcccaaagcatcattttctccaatttctctggtgatatggatgacatggcttgctgagctttttcagc[G/A]tcttctggtgaaagatcgaaaccaaattgttttcccatgtttgccatcatttctgggctcatatttttaaccaatgatgtgaacatctgtcgcatagctg

fava_c15849_116	fava_c15849	116	A/C	taaggtgagatcaatgtcaagaaaccctttctttctacacccccttaattttcttctaagggttggtagcaatgtcactcaaatcagaaagatataagaa[A/C]atttaagttttatatttttttctttgtttggtaaagtatgttaacagctatagatatatacaatgaagaactcatgaaagcaYtagagccttttatgaaa

fava_c15849_199	fava_c15849	199	C/T	tcagaaagatataagaaMatttaagttttatatttttttctttgtttggtaaagtatgttaacagctatagatatatacaatgaagaactcatgaaagca[C/T]tagagccttttatgaaaactggttcttcttcatcaatctctgaacaacaatcacatgatttgaatcccaattacgtaacccaaagaagctctattggtct

fava_c15849_300	fava_c15849	300	C/A	tagagccttttatgaaaactggttcttcttcatcaatctctgaacaacaatcacatgatttgaatcccaattacgtaacccaaagaagctctattggtct[C/A]aaccaactcaccccatcacaaatccttcaaatccaaacccagttccaaatctcccaacaacaacaaaaaagcactctcacacaaaagcccgttcccatgY

fava_c15849_498	fava_c15849	498	T/C	tgaagcactttggagcttcctcaaaacccacgaaactctatcgtggagttagacagcgtcattggggaaaatgggttgctgaaataagacttccaaagaa[T/C]cgcactcgtctctggttaggcacgttcgataccgcagaggaagcagcttttgcttatgacatcgcagcttataagcttagaggcgaattcgcacgcctga

fava_c15849_932	fava_c15849	932	C/T	tctatgtctgatgaatgttcacctggtttgtcttctccagaatccggtgtaactttcttggatttttcagattcttgtcggtgggatgaagtggagaatt[C/T]ggggttgaagaagtacccttctgttgaaattgattgggaagcattatgagtctgagaagtgatttagcaaaaatatgtttttgggtttactatgtttctg

fava_c15850_1000	fava_c15850	1000	C/A	aatctttcggggcactccaattcagtttagggaatactgcaccgccaagggactcgacggattccttaactttcaactcaagttctggaaaagaaggtgg[C/A]ggagaagattcttccgcKtcatcatcggatScctctgaaacctgaaaatcttcttcRtcgtcaggattgcgaattctattgggtaatgcatcctcRttta

fava_c15850_1018	fava_c15850	1018	T/G	aattcagtttagggaatactgcaccgccaagggactcgacggattccttaactttcaactcaagttctggaaaagaaggtggMggagaagattcttccgc[T/G]tcatcatcggatScctctgaaacctgaaaatcttcttcRtcgtcaggattgcgaattctattgggtaatgcatcctcRtttaagacggaagcaggcagta

fava_c15850_1031	fava_c15850	1031	C/G	gaatactgcaccgccaagggactcgacggattccttaactttcaactcaagttctggaaaagaaggtggMggagaagattcttccgcKtcatcatcggat[C/G]cctctgaaacctgaaaatcttcttcRtcgtcaggattgcgaattctattgggtaatgcatcctcRtttaagacggaagcaggcagtagaaagggcttaga

fava_c15850_1057	fava_c15850	1057	A/G	cggattccttaactttcaactcaagttctggaaaagaaggtggMggagaagattcttccgcKtcatcatcggatScctctgaaacctgaaaatcttcttc[A/G]tcgtcaggattgcgaattctattgggtaatgcatcctcRtttaagacggaagcaggcagtagaaagggcttagagtcatcaagaaggtattcaacgaaag

fava_c15850_1096	fava_c15850	1096	G/A	gtggMggagaagattcttccgcKtcatcatcggatScctctgaaacctgaaaatcttcttcRtcgtcaggattgcgaattctattgggtaatgcatcctc[G/A]tttaagacggaagcaggcagtagaaagggcttagagtcatcaagaaggtattcaacgaaagattcgggaagttggtgaattaacgtcttgattgatacag

fava_c15850_200	fava_c15850	200	C/A	aagtgttgtcaaataaaaagttatggtagcactatagcacacctgtagcataacaaaatttcaacaaattatactatttttgcaatctgtgattgaacaa[C/A]actgctgcaattccaaagttgaaaattgagagcaagaatctagttatcatgcaccagcttctggagacctgtcttgRtttctcaactcttgatcagcatt

fava_c15850_277	fava_c15850	277	A/G	ttttgcaatctgtgattgaacaaMactgctgcaattccaaagttgaaaattgagagcaagaatctagttatcatgcaccagcttctggagacctgtcttg[A/G]tttctcaactcttgatcagcattccttagaaattgatcccaaccactcccttgactggtatccaagtaatcatatggaactgctgtttttaggcctggtc

fava_c15850_721	fava_c15850	721	T/C	tttcgagcagaacaggataaaaagtagtaacctcccgctgagaaactccgactaatttcttattctgtacaaaacaacgaaactccatatctggcttaag[T/C]gaYggataccatttccggagagcaagaaagaaatYatgcggtcgagacataagtttatcatcgcaagaatcataagcatgacacaagtcatggactaacg

fava_c15850_724	fava_c15850	724	C/T	cgagcagaacaggataaaaagtagtaacctcccgctgagaaactccgactaatttcttattctgtacaaaacaacgaaactccatatctggcttaagYga[C/T]ggataccatttccggagagcaagaaagaaatYatgcggtcgagacataagtttatcatcgcaagaatcataagcatgacacaagtcatggactaacgagt

fava_c15850_756	fava_c15850	756	C/T	cgctgagaaactccgactaatttcttattctgtacaaaacaacgaaactccatatctggcttaagYgaYggataccatttccggagagcaagaaagaaat[C/T]atgcggtcgagacataagtttatcatcgcaagaatcataagcatgacacaagtcatggactaacgagtcagaacctcggaacagaagcgcaatttcactg

fava_c15853_1205	fava_c15853	1205	T/A	gagaaggtttcaaaacattttcagagattgttgattcttcttgtgaattgttaacttcaattggatcatgaatgttctcttgtgtaccaatttgagattg[T/A]gattgagWttgagatccaagttctttcaatgatggactacttgaaactctagagagttttccattttccatcaatggagtagatctttgactttgactca

fava_c15853_1213	fava_c15853	1213	T/A	ttcaaaacattttcagagattgttgattcttcttgtgaattgttaacttcaattggatcatgaatgttctcttgtgtaccaatttgagattgWgattgag[T/A]ttgagatccaagttctttcaatgatggactacttgaaactctagagagttttccattttccatcaatggagtagatctttgactttgactcaattgtgtt

fava_c15853_127	fava_c15853	127	T/C	ttaattagcactatatcttacaattggtgccataataataagaatttgcaccaagctcctaaagattttacagtaaatacttttacaaaagaaaaaagaa[T/C]caccccaaaattaaaaaaaaWattaaaatcataaccctaatagtaataataattataataataatgctcaataattgatgaggttgttccaattaaattt

fava_c15853_1406	fava_c15853	1406	A/G	attgtgttgttcttccattaaaactcctacttccaaagcaagagaattttgcagctctttcagcaaaaccaggatcagttgagaattcggcaactgttga[A/G]ttcaaactcatgttttgaatgctcaatttaggaggagaattcaaaggggtgttgtaacatgaagtgttgttattgctattgatgtaagagtttgcagcaa

fava_c15853_148	fava_c15853	148	A/T	aattggtgccataataataagaatttgcaccaagctcctaaagattttacagtaaatacttttacaaaagaaaaaagaaYcaccccaaaattaaaaaaaa[A/T]attaaaatcataaccctaatagtaataataattataataataatgctcaataattgatgaggttgttccaattaaatttgttgtggatgtgtacaacagc

fava_c15853_1791	fava_c15853	1791	G/T	attattcttttgttgttctgatgtaagtgaaagtgattgccatgtaagatgtgattgttcataatgaagattagtgtttgaaaagaattggttttccatc[G/T]aggtaaaagtgMaaaagaaaaattgaatcgaccttatttaaacaggatctgttctataaactcgtactagtKtRtctttgatttctaagaagacacgtat

fava_c15853_1803	fava_c15853	1803	A/C	ttgttctgatgtaagtgaaagtgattgccatgtaagatgtgattgttcataatgaagattagtgtttgaaaagaattggttttccatcKaggtaaaagtg[A/C]aaaagaaaaattgaatcgaccttatttaaacaggatctgttctataaactcgtactagtKtRtctttgatttctaagaagacacgtattaaWaaaaagtc

fava_c15853_1863	fava_c15853	1863	T/G	agtgtttgaaaagaattggttttccatcKaggtaaaagtgMaaaagaaaaattgaatcgaccttatttaaacaggatctgttctataaactcgtactagt[T/G]tRtctttgatttctaagaagacacgtattaaWaaaaagtcgaacaaataaagttgaaagatgaaattgRtattatgaatatgaatgaatcacttacattt

fava_c15853_1865	fava_c15853	1865	G/A	tgtttgaaaagaattggttttccatcKaggtaaaagtgMaaaagaaaaattgaatcgaccttatttaaacaggatctgttctataaactcgtactagtKt[G/A]tctttgatttctaagaagacacgtattaaWaaaaagtcgaacaaataaagttgaaagatgaaattgRtattatgaatatgaatgaatcacttacatttga

fava_c15853_1895	fava_c15853	1895	T/A	taaaagtgMaaaagaaaaattgaatcgaccttatttaaacaggatctgttctataaactcgtactagtKtRtctttgatttctaagaagacacgtattaa[T/A]aaaaagtcgaacaaataaagttgaaagatgaaattgRtattatgaatatgaatgaatcacttacatttgaaaaaaaaaaaaaggtgagtttggtgatgag

fava_c15853_1932	fava_c15853	1932	G/A	aacaggatctgttctataaactcgtactagtKtRtctttgatttctaagaagacacgtattaaWaaaaagtcgaacaaataaagttgaaagatgaaattg[G/A]tattatgaatatgaatgaatcacttacatttgaaaaaaaaaaaaaggtgagtttggtgatgaggttgaagtgatgaatgaaaagagtgaaataggtggga

fava_c15853_398	fava_c15853	398	A/G	ttcatagtggtagttgatttgtcccgttcaaattctgaaattgtattggtgtttttctattgtcagtttgtccaaatcccatttgaacaattgtgtggag[A/G]tcatcttcagagaatgtcaatggatactgagaagaagcttcatggaatccattaagtgaagataagtgcaYRccaaggttttggcataaagcattttgct

fava_c15853_469	fava_c15853	469	C/T	ccaaatcccatttgaacaattgtgtggagRtcatcttcagagaatgtcaatggatactgagaagaagcttcatggaatccattaagtgaagataagtgca[C/T]Rccaaggttttggcataaagcattttgctggtgttgttgcccataaaaggatggtgctgaggaatcttgagagaatattgggtgtgccaaagaattattt

fava_c15853_470	fava_c15853	470	A/G	caaatcccatttgaacaattgtgtggagRtcatcttcagagaatgtcaatggatactgagaagaagcttcatggaatccattaagtgaagataagtgcaY[A/G]ccaaggttttggcataaagcattttgctggtgttgttgcccataaaaggatggtgctgaggaatcttgagagaatattgggtgtgccaaagaattatttg

fava_c15853_860	fava_c15853	860	T/C	ttctttcgcttattttctccctcctaactctttctgcaagactatggctgtcagtggcctgacctcttcttgctctgacatgaatgtaatcctttggagc[T/C]tcaggtggttttgagttactcttattctgtttttcatcacctccattattactatttgtacccccttttgactcttcctctgcttttactttaccatttt

fava_c15854_1270	fava_c15854	1270	C/T	gacattcaaagtttgcagtggtctggttgtgaaggaactgaacgcggtcatctccactgactcttatccggccgaagtgtgaaagatccacaacaacaac[C/T]ccattgtctgcggcatcgagtgcttcgtcatcattatgaaatgtttcaattattccatctccagaaacctcggcgcctKcagttttgacagtgtccagga

fava_c15854_1349	fava_c15854	1349	G/T	tgaaagatccacaacaacaacYccattgtctgcggcatcgagtgcttcgtcatcattatgaaatgtttcaattattccatctccagaaacctcggcgcct[G/T]cagttttgacagtgtccaggaaatcatggtcgatggggggaggagaaaggtcgaaaggtgaagattgagcKgaaatagagaaagacgttagattgttctt

fava_c15854_1420	fava_c15854	1420	G/T	ttattccatctccagaaacctcggcgcctKcagttttgacagtgtccaggaaatcatggtcgatggggggaggagaaaggtcgaaaggtgaagattgagc[G/T]gaaatagagaaagacgttagattgttcttgttgcggWggtKWgagtagaatggaaggtttgggaaagaagaaggtttgaacRatagcgcagatggagacg

fava_c15854_1457	fava_c15854	1457	A/T	gacagtgtccaggaaatcatggtcgatggggggaggagaaaggtcgaaaggtgaagattgagcKgaaatagagaaagacgttagattgttcttgttgcgg[A/T]ggtKWgagtagaatggaaggtttgggaaagaagaaggtttgaacRatagcgcagatggagacgatggatgatttSgaaggttgaagcaggatggcaatga

fava_c15854_1461	fava_c15854	1461	G/T	gtgtccaggaaatcatggtcgatggggggaggagaaaggtcgaaaggtgaagattgagcKgaaatagagaaagacgttagattgttcttgttgcggWggt[G/T]WgagtagaatggaaggtttgggaaagaagaaggtttgaacRatagcgcagatggagacgatggatgatttSgaaggttgaagcaggatggcaatgacagc

fava_c15854_1462	fava_c15854	1462	A/T	tgtccaggaaatcatggtcgatggggggaggagaaaggtcgaaaggtgaagattgagcKgaaatagagaaagacgttagattgttcttgttgcggWggtK[A/T]gagtagaatggaaggtttgggaaagaagaaggtttgaacRatagcgcagatggagacgatggatgatttSgaaggttgaagcaggatggcaatgacagcg

fava_c15854_1502	fava_c15854	1502	G/A	gaaaggtgaagattgagcKgaaatagagaaagacgttagattgttcttgttgcggWggtKWgagtagaatggaaggtttgggaaagaagaaggtttgaac[G/A]atagcgcagatggagacgatggatgatttSgaaggttgaagcaggatggcaatgacagcgttgacatctcagagtaggttttattgataacagagacgga

fava_c15854_1532	fava_c15854	1532	G/C	agacgttagattgttcttgttgcggWggtKWgagtagaatggaaggtttgggaaagaagaaggtttgaacRatagcgcagatggagacgatggatgattt[G/C]gaaggttgaagcaggatggcaatgacagcgttgacatctcagagtaggttttattgataacagagacggatgcgcgtttcagaaggagaaaacatgaagg

fava_c15854_172	fava_c15854	172	A/G	agaatatacacaattattgttaataaccttcaaaatcatagataaagaagaccagagtattctgatgcacaaatttatcaccaacatgataaagaagacc[A/G]gaatagaattcaaaaacagttttaaaaagaaattcaagtgttatttatggacttgttcatttctactcacatcatcaaatcaccaccacacttgagcata

fava_c15859_664	fava_c15859	664	A/C	cagccagatgaagtggttcttgggagggtttagctgaggttgtgataattggtgtgtctactatccagtgaggtattctttgggaattcattgagttctt[A/C]aaacaaattctttgatcatctagggggggaattctgtatataatttagagttgaacttcattcaagggggagcagcatttatctattctcttaacaagca

fava_c15865_147	fava_c15865	147	T/C	atctctgtcaattatagttgtcttaaatcgtcatcataaaacaaaatacacattttaatgtttatgaacaagagaatgatgtattttaagatccatataa[T/C]aattactatttcttaatttattgcatagtatcattgaaaataacttacaaaaaatacacaaccatgaggaagtagctagcaaatccatttggtttccaca

fava_c15867_1146	fava_c15867	1146	C/T	gaaaccctaacgccgtggattctcagaactggaccccacttcatcacgcggcgtcgagatgccatttaaaagcggtggagtttttacttcaacactccga[C/T]gtgaaatacgcgagagacataaacgggaaaacgccgtttgatattgcggcggagagtgaacacacgcgcttgttcggtttactgcgttacggcgacgcgc

fava_c15868_1387	fava_c15868	1387	T/C	caatttaatggtttcttcctgaccaagatactttgtccatctccttactatccaaactggatgtgagtagagagttgccagagcacgtgcttgagaacga[T/C]catcaccctctactttgggcaaaggaagagtttcattttccttgagYagaactagcgttcggaggattccgttgaccaagttgcctgcaccaggcctgag

fava_c15868_1434	fava_c15868	1434	C/T	ctatccaaactggatgtgagtagagagttgccagagcacgtgcttgagaacgaYcatcaccctctactttgggcaaaggaagagtttcattttccttgag[C/T]agaactagcgttcggaggattccgttgaccaagttgcctgcaccaggcctgagagcagctttggcaagctgcacattctcatctacaacagcataaggtg

fava_c15872_180	fava_c15872	180	A/C	atctcaaaacccttcaaactcatctcaacttgtttcactatttttcttttcctgtaaaagaaatctaaatgggtagctgtatgagtagttataaagcctc[A/C]tcctcttcttctataccaatcatccaaccaccaaaaccttcacaaaacaatgaaacaacctttctagttgaggaagaaacagtgaaggaagtactcacag

fava_c15872_287	fava_c15872	287	C/A	tcttctataccaatcatccaaccaccaaaaccttcacaaaacaatgaaacaacctttctagttgaggaagaaacagtgaaggaagtactcacagaaaccc[C/A]Yaaaccagaaccaccaaagccacatccaaacattagggcctttcacaagttcataaaagaaaaacaagacatcaaggttcacgagaagcagccattgttc

fava_c15872_288	fava_c15872	288	C/T	cttctataccaatcatccaaccaccaaaaccttcacaaaacaatgaaacaacctttctagttgaggaagaaacagtgaaggaagtactcacagaaacccM[C/T]aaaccagaaccaccaaagccacatccaaacattagggcctttcacaagttcataaaagaaaaacaagacatcaaggttcacgagaagcagccattgttca

fava_c15872_391	fava_c15872	391	A/G	accagaaccaccaaagccacatccaaacattagggcctttcacaagttcataaaagaaaaacaagacatcaaggttcacgagaagcagccattgttcatc[A/G]acaaaggMtatgatgcttcagaaatttgcaggttaagaaaaaccattcccgcaagtacttccgaaaaagccggcaaaagggtcaacgaatctccgatcga

fava_c15872_399	fava_c15872	399	C/A	caccaaagccacatccaaacattagggcctttcacaagttcataaaagaaaaacaagacatcaaggttcacgagaagcagccattgttcatcRacaaagg[C/A]tatgatgcttcagaaatttgcaggttaagaaaaaccattcccgcaagtacttccgaaaaagccggcaaaagggtcaacgaatctccgatcgagttgctta

fava_c15872_522	fava_c15872	522	T/A	ggttaagaaaaaccattcccgcaagtacttccgaaaaagccggcaaaagggtcaacgaatctccgatcgagttgcttaaaaactgttcctttcccggtga[T/A]tttgacgacagacgagatagaatggttcatggttcgaggaaatttgggtctatgaagttgattcagtgcaRagaccaactgggtcagaaaaYggtcaaag

fava_c15872_593	fava_c15872	593	G/A	ttgcttaaaaactgttcctttcccggtgaWtttgacgacagacgagatagaatggttcatggttcgaggaaatttgggtctatgaagttgattcagtgca[G/A]agaccaactgggtcagaaaaYggtcaaagagggaatacgtcgccggcRagatcccggggagaattcttgccgacaaMagtcccggtcaccggtcacacgt

fava_c15872_614	fava_c15872	614	C/T	cccggtgaWtttgacgacagacgagatagaatggttcatggttcgaggaaatttgggtctatgaagttgattcagtgcaRagaccaactgggtcagaaaa[C/T]ggtcaaagagggaatacgtcgccggcRagatcccggggagaattcttgccgacaaMagtcccggtcaccggtcacacgtagtgacacaggtgatRctaga

fava_c15872_641	fava_c15872	641	G/A	agaatggttcatggttcgaggaaatttgggtctatgaagttgattcagtgcaRagaccaactgggtcagaaaaYggtcaaagagggaatacgtcgccggc[G/A]agatcccggggagaattcttgccgacaaMagtcccggtcaccggtcacacgtagtgacacaggtgatRctagatccttcgtgagtcggagcccatctgtg

fava_c15872_670	fava_c15872	670	C/A	gtctatgaagttgattcagtgcaRagaccaactgggtcagaaaaYggtcaaagagggaatacgtcgccggcRagatcccggggagaattcttgccgacaa[C/A]agtcccggtcaccggtcacacgtagtgacacaggtgatRctagatccttcgtgagtcggagcccatctgtgagaagaacgaatcgatctccagttgcaag

fava_c15872_709	fava_c15872	709	G/A	gaaaaYggtcaaagagggaatacgtcgccggcRagatcccggggagaattcttgccgacaaMagtcccggtcaccggtcacacgtagtgacacaggtgat[G/A]ctagatccttcgtgagtcggagcccatctgtgagaagaacgaatcgatctccagttgcaaggggtagaacatcggtgccggagagaggacgccggaaaac

fava_c15872_874	fava_c15872	874	T/A	tagaacatcggtgccggagagaggacgccggaaaacggagataccggcaacggagagtaaacggtcttgtgctaaagagtcactggaaaatccactcgtg[T/A]cattagaatgtttcatttttctgtaggaYtctgtaattgcMcagaatggtgatggtgtcggtggcgccaccattcagccacggttgaccgggtgagctaa

fava_c15872_903	fava_c15872	903	T/C	ggaaaacggagataccggcaacggagagtaaacggtcttgtgctaaagagtcactggaaaatccactcgtgWcattagaatgtttcatttttctgtagga[T/C]tctgtaattgcMcagaatggtgatggtgtcggtggcgccaccattcagccacggttgaccgggtgagctaagtagggtcgactcacagtgaaagtaaatg

fava_c15872_915	fava_c15872	915	C/A	taccggcaacggagagtaaacggtcttgtgctaaagagtcactggaaaatccactcgtgWcattagaatgtttcatttttctgtaggaYtctgtaattgc[C/A]cagaatggtgatggtgtcggtggcgccaccattcagccacggttgaccgggtgagctaagtagggtcgactcacagtgaaagtaaatgattgtgattatt

fava_c15873_1360	fava_c15873	1360	C/A	aacgtacatatgcagtgtttccaactcaaatttcaaacaaaagtcatatttgcatacttaagtttgaatccatttcaactgtgtgtgatatgaaggtaaa[C/A]atttagtttgcaactgttcccgagtgaaaaccaaaccagagctatattcatgttttgctttgcaagSataagttgRacagtttctgatggcaatccaaac

fava_c15873_1427	fava_c15873	1427	C/G	atccatttcaactgtgtgtgatatgaaggtaaaMatttagtttgcaactgttcccgagtgaaaaccaaaccagagctatattcatgttttgctttgcaag[C/G]ataagttgRacagtttctgatggcaatccaaacacacgatattgaaaagaaaatgaacctattcggtttttggttccattagtaScttgcttgtatttta

fava_c15873_1436	fava_c15873	1436	A/G	aactgtgtgtgatatgaaggtaaaMatttagtttgcaactgttcccgagtgaaaaccaaaccagagctatattcatgttttgctttgcaagSataagttg[A/G]acagtttctgatggcaatccaaacacacgatattgaaaagaaaatgaacctattcggtttttggttccattagtaScttgcttgtattttagtatattta

fava_c15873_1512	fava_c15873	1512	C/G	gttttgctttgcaagSataagttgRacagtttctgatggcaatccaaacacacgatattgaaaagaaaatgaacctattcggtttttggttccattagta[C/G]cttgcttgtattttagtatatttattatgatatttagcggtaggcttgtaaacacacaattactagaattctgtctttcttataatcttatcttgacttg

fava_c15873_197	fava_c15873	197	C/A	gaatttacatcacaaaaccctaattccagatctagtttctctcataatttctgcttacagtcacaccggtttgcgtacgtatctgaattatacacctatt[C/A]tatcttccttgcacactcacgctacctcatcacggttacaacttgtttcatttaccagaatcattaatttcKctgcaaattaggttttatattatataca

fava_c15873_269	fava_c15873	269	G/T	gcgtacgtatctgaattatacacctattMtatcttccttgcacactcacgctacctcatcacggttacaacttgtttcatttaccagaatcattaatttc[G/T]ctgcaaattaggttttatattatatacaatttctattgtttgatttccacagtaYatatgatggccggccaaaaggatcaaaacaaatctgaatggatag

fava_c15873_324	fava_c15873	324	T/C	tcatcacggttacaacttgtttcatttaccagaatcattaatttcKctgcaaattaggttttatattatatacaatttctattgtttgatttccacagta[T/C]atatgatggccggccaaaaggatcaaaacaaatctgaatggatagatagaataagatctgaaggagccattccgtttcttgatcctgctaagtgttcaaa

fava_c15873_551	fava_c15873	551	G/C	cattcatggttagaggtcctgagtattttaccacaaaagttaagattccagcaagtgattatcttctcaaacctcttggatttgattggataacaagttc[G/C]acgaaaattggtgagatattaaagcatccaaatagcagagttaggaaggtgattgRgaatgagtttcccgtKggtgataggccttttgtgtgggcattca

fava_c15873_607	fava_c15873	607	A/G	gattatcttctcaaacctcttggatttgattggataacaagttcSacgaaaattggtgagatattaaagcatccaaatagcagagttaggaaggtgattg[A/G]gaatgagtttcccgtKggtgataggccttttgtgtgggcattcaatcttcaacttccaaccaaagataactatagtgctgttgcRtattttacgaacaaa

fava_c15873_623	fava_c15873	623	T/G	ctcttggatttgattggataacaagttcSacgaaaattggtgagatattaaagcatccaaatagcagagttaggaaggtgattgRgaatgagtttcccgt[T/G]ggtgataggccttttgtgtgggcattcaatcttcaacttccaaccaaagataactatagtgctgttgcRtattttacgaacaaagagcctattgttgaYg

fava_c15873_692	fava_c15873	692	G/A	ttaggaaggtgattgRgaatgagtttcccgtKggtgataggccttttgtgtgggcattcaatcttcaacttccaaccaaagataactatagtgctgttgc[G/A]tattttacgaacaaagagcctattgttgaYggctcactaatggacaggttcttgaaaggggatgatgcttttcgaaactcRagactcaagttgattgcSa

fava_c15873_722	fava_c15873	722	C/T	tKggtgataggccttttgtgtgggcattcaatcttcaacttccaaccaaagataactatagtgctgttgcRtattttacgaacaaagagcctattgttga[C/T]ggctcactaatggacaggttcttgaaaggggatgatgcttttcgaaactcRagactcaagttgattgcSaatattgttaatggtccttggattgtgagaa

fava_c15873_773	fava_c15873	773	A/G	ataactatagtgctgttgcRtattttacgaacaaagagcctattgttgaYggctcactaatggacaggttcttgaaaggggatgatgcttttcgaaactc[A/G]agactcaagttgattgcSaatattgttaatggtccttggattgtgagaaaagcagttggggagcaagcaatttgcataattgggcgtgcactttcgtgta

fava_c15873_791	fava_c15873	791	G/C	cRtattttacgaacaaagagcctattgttgaYggctcactaatggacaggttcttgaaaggggatgatgcttttcgaaactcRagactcaagttgattgc[G/C]aatattgttaatggtccttggattgtgagaaaagcagttggggagcaagcaatttgcataattgggcgtgcactttcgtgtaaatacactgtgacRgaga

fava_c15873_887	fava_c15873	887	G/A	ttgcSaatattgttaatggtccttggattgtgagaaaagcagttggggagcaagcaatttgcataattgggcgtgcactttcgtgtaaatacactgtgac[G/A]gagaatttYatggaagtggatattgatattggatcttctatggttgcatctgcaatagttcatttggcatttggttatgtgacaactttgacggttgacc

fava_c15873_896	fava_c15873	896	C/T	ttgttaatggtccttggattgtgagaaaagcagttggggagcaagcaatttgcataattgggcgtgcactttcgtgtaaatacactgtgacRgagaattt[C/T]atggaagtggatattgatattggatcttctatggttgcatctgcaatagttcatttggcatttggttatgtgacaactttgacggttgacctggcttttc

fava_c15875_1357	fava_c15875	1357	T/C	tcgcagaatgctggcctattctacggttggtacgcctgattatattgctccagaagttctgttgaagagaggatatggtgtagaatgtgattggtggtct[T/C]taggagccataatgtatgaaatgcttgtggggtatccaccattttattcggatgaaccaatggcaacttgtagaaagattgtaaattggagaacaacttt

fava_c15875_1885	fava_c15875	1885	A/G	tgtgggcgaaagtgtaatacctggaattgctgaattgaagaagaaaacgaaaccgaaaaggccatctattaagtccttatttgatgatgaatcagccaag[A/G]cttcttctaatcaacctgctagagggagcttcttaaatctgttacctcctcagatggaagtgccagagaaaaatgaatgacaatgatctaaactaatact

fava_c15875_576	fava_c15875	576	T/A	ttgaagcggcaaagcattacatagaaaatcattataagaagcagatgcagagcctgcaggagaggaaggaaagacgtaatatgctagaaaagaaattggc[T/A]gatgctgaagtcaccgaggaagagcagaacaatttgcttaagtatttggagaaaaaggagacagagatcatgcgccttcagagacataagatgggggctg

fava_c15875_738	fava_c15875	738	G/A	cagagatcatgcgccttcagagacataagatgggggctgatgactttgagcccatgactatgattgggaagggtgcgtttggagaggttagagtctgccg[G/A]gagaaggcaactgggcatgtatatgctatgaagaagcttaagaaatcagagatgcttcgtagaggacaggttgaacatgtcaaagctgagaggaaccttc

fava_c15881_1613	fava_c15881	1613	A/G	aggcctctagggttgcttgcctttcctcagcagcattataatcacctttacttacaagcccctgtgaagttgatatctcaatgagactttttctaaaact[A/G]gaggcttcagcatctattcttgcaagatcggaattgattttcaacccagtgatggcacgttgcgcaaactgaagcaattcctcacgcttcaccaatactt

fava_c15881_491	fava_c15881	491	C/T	tggctaattcagcttcatggtccaatacctgatctgatttaactgaaggggattttggttgttcatctgtctctgtctttgaaaactcagtaccttgaac[C/T]gaagcagaaggtgaaggtgtaccatctgactttttctcagatggtaatgtttcagtctttacaggtgggctttctatttcgagaattggtctctctatag

fava_c15882_1312	fava_c15882	1312	G/A	tcaaatttggtcctttaacaaccactccacatctatgttctgtagcatacctgactctgacttcatattgagggaaagatggaagcttcattccatcaag[G/A]gcagcacatagtatggggccttcttcttcaaagtgcctgtcagctctcctactagtgacaattccagttttcWMatcaagagttgcaaaatttgacttaa

fava_c15882_1385	fava_c15882	1385	T/A	gaaagatggaagcttcattccatcaagRgcagcacatagtatggggccttcttcttcaaagtgcctgtcagctctcctactagtgacaattccagttttc[T/A]MatcaagagttgcaaaatttgacttaaaagcaatatcaccaggcgacatggccaatccagcYcccatggattcaaatgcccctcgaccacgataataaac

fava_c15882_1386	fava_c15882	1386	C/A	aaagatggaagcttcattccatcaagRgcagcacatagtatggggccttcttcttcaaagtgcctgtcagctctcctactagtgacaattccagttttcW[C/A]atcaagagttgcaaaatttgacttaaaagcaatatcaccaggcgacatggccaatccagcYcccatggattcaaatgcccctcgaccacgataataaact

fava_c15882_1447	fava_c15882	1447	C/T	gcctgtcagctctcctactagtgacaattccagttttcWMatcaagagttgcaaaatttgacttaaaagcaatatcaccaggcgacatggccaatccagc[C/T]cccatggattcaaatgcccctcgaccacgataataaactcgaggatcataacccaacagagaaagatgagcggtatcacttccacaagctaagccaactt

fava_c15882_1552	fava_c15882	1552	A/T	tggattcaaatgcccctcgaccacgataataaactcgaggatcataacccaacagagaaagatgagcggtatcacttccacaagctaagccaacttctac[A/T]gggtccatcaatccattaaccccagcagatgctatcccatccaaattgggaagttttgctgcctgaagaggYgtcttgtatcccagccttggcagcgaca

fava_c15882_1624	fava_c15882	1624	C/T	cacttccacaagctaagccaacttctacWgggtccatcaatccattaaccccagcagatgctatcccatccaaattgggaagttttgctgcctgaagagg[C/T]gtcttgtatcccagccttggcagcgacacatcacccaacccatcaatcaaaacaaaggcYactcttttcttYggcaRctgtggactacccatttatctct

fava_c15882_1684	fava_c15882	1684	T/C	ctatcccatccaaattgggaagttttgctgcctgaagaggYgtcttgtatcccagccttggcagcgacacatcacccaacccatcaatcaaaacaaaggc[T/C]actcttttcttYggcaRctgtggactacccatttatctctttaatttttttctcaggttataaatagYagcagggttaaacagcaaataactcagcaact

fava_c15882_1696	fava_c15882	1696	C/T	aattgggaagttttgctgcctgaagaggYgtcttgtatcccagccttggcagcgacacatcacccaacccatcaatcaaaacaaaggcYactcttttctt[C/T]ggcaRctgtggactacccatttatctctttaatttttttctcaggttataaatagYagcagggttaaacagcaaataactcagcaacttcactctYtcgg

fava_c15882_170	fava_c15882	170	C/T	taaaataaatagtaacagttacatttatttaagctgagtttacattgttatttgtagaaaagacttgcaatagtacaaaagctcaaaatttgattatttt[C/T]tcttttgcacccatatcacttgcttacagaacaaaacagaaaagctttaacttgRaaaagcttttgcatctaggttgaggaaattctttataattcccat

fava_c15882_1701	fava_c15882	1701	A/G	ggaagttttgctgcctgaagaggYgtcttgtatcccagccttggcagcgacacatcacccaacccatcaatcaaaacaaaggcYactcttttcttYggca[A/G]ctgtggactacccatttatctctttaatttttttctcaggttataaatagYagcagggttaaacagcaaataactcagcaacttcactctYtcggtgcct

fava_c15882_1752	fava_c15882	1752	C/T	acatcacccaacccatcaatcaaaacaaaggcYactcttttcttYggcaRctgtggactacccatttatctctttaatttttttctcaggttataaatag[C/T]agcagggttaaacagcaaataactcagcaacttcactctYtcggtgccttcactaatagcacaagcagcgtattaccaaattcgattgacgcaaaattcc

fava_c15882_1792	fava_c15882	1792	C/T	tcttYggcaRctgtggactacccatttatctctttaatttttttctcaggttataaatagYagcagggttaaacagcaaataactcagcaacttcactct[C/T]tcggtgccttcactaatagcacaagcagcgtattaccaaattcgattgacgcaaaattccgacggaaagaagattctcagatggagcgattgagcgttgg

fava_c15882_225	fava_c15882	225	G/A	agaaaagacttgcaatagtacaaaagctcaaaatttgattattttYtcttttgcacccatatcacttgcttacagaacaaaacagaaaagctttaacttg[G/A]aaaagcttttgcatctaggttgaggaaattctttataattcccatcatttctccgccagggaaacgccccaagcatcccctcgcagctgYaatttcattc

fava_c15882_315	fava_c15882	315	T/C	ctttaacttgRaaaagcttttgcatctaggttgaggaaattctttataattcccatcatttctccgccagggaaacgccccaagcatcccctcgcagctg[T/C]aatttcattcaaYtcatataccgaatcaccgctataagattgagagcatttacctcctcctctatcttctgtttccaagtcataagccaagtcttcgcca

fava_c15882_328	fava_c15882	328	C/T	aagcttttgcatctaggttgaggaaattctttataattcccatcatttctccgccagggaaacgccccaagcatcccctcgcagctgYaatttcattcaa[C/T]tcatataccgaatcaccgctataagattgagagcatttacctcctcctctatcttctgtttccaagtcataagccaagtcttcgccagacttgacacttg

fava_c15882_451	fava_c15882	451	A/T	aagattgagagcatttacctcctcctctatcttctgtttccaagtcataagccaagtcttcgccagacttgacacttggaagaggaaatggatcgagaga[A/T]gttttgcgaataacggattcgccaattgcWcctacaaagtccttcaaccgacatatagaaaacggaactggttcaaagctatgatctccgtattctactg

fava_c15882_481	fava_c15882	481	A/T	cttctgtttccaagtcataagccaagtcttcgccagacttgacacttggaagaggaaatggatcgagagaWgttttgcgaataacggattcgccaattgc[A/T]cctacaaagtccttcaaccgacatatagaaaacggaactggttcaaagctatgatctccgtattctactggtgtagaatggtctcctgtgacacaaaggt

fava_c15882_868	fava_c15882	868	G/A	cctctccaggtacaaaaactcgggggcaagactgcgaaggagctgagagcgccttagctattgctgttgcttttgaagttagtagagttcgataatctcc[G/A]gttgctccaggagcttctagaatgtcaataccaagtgataagcctaaaccagcaattatttttgttggagctaccatgcatggtctcaaaccatgtttat

fava_c15887_1017	fava_c15887	1017	A/T	agcaacccaagcgatctccagcaagtgaatctgctccacactggagaactattgcaccgggttgatatgtttcaactactttggaaataatagtcttgaa[A/T]agtctagtgaagctagagtcatcaattccatccttgagtgggacatttattgcataaaacttcccttctctttctcctatttccttagcatcaccagttc

fava_c15887_295	fava_c15887	295	G/C	tcactctacacaaaatatggcattaattcaaacaagacagactatcgatataactttagtctacagcaaatatacaaaataatgatatcaaaagaactaa[G/C]aaaaatatacacaactcttgggttatccctaatgctttcttgagaaacaactaggtatttctatctagttccatgttcattgtctcttaaaacgaaggct

fava_c15887_401	fava_c15887	401	C/T	tatacacaactcttgggttatccctaatgctttcttgagaaacaactaggtatttctatctagttccatgttcattgtctcttaaaacgaaggctctccc[C/T]cgacaaaaggatgcacatagctgggggctgacttcatcaaagatccgtttgatgatcattgtcgttgtcaccttcataatattcatcatcccgctggata

fava_c15888_376	fava_c15888	376	G/A	tccatattcaactatggaactcttagagctaagtttccattataactttaatggaatcttgaaaaaattgacaaaatcttgaatcatcatcaatatctat[G/A]aggtttgtaatgtgagtcattaccattagaccatatgtagtagtcctagtattcattctctacaccccttttcaagcgtattgatcttcatgttccgacc

fava_c15888_605	fava_c15888	605	A/G	tcatgagaaacataatagcagagaatactcctcccaaaataactacccatttgaacatgtagctatggtcatcattccaagtatatgggatgttcatgcc[A/G]aaaatagcggtcaccagagagtagaaagatagacatacagttccagagctaagaaagagctccaactgaataagctgattacgatgattgtcgagttgaa

fava_c15889_1421	fava_c15889	1421	C/T	cacttaaatatgtgacaggccgtagcttcaactttttcttcctaagatcattaatcatgtctcgtattccaagccaaattaacgagtcaactatttgata[C/T]gaaagcctataaccagcttgttgctttacacgagacaaRggatgaggaaattcagttgttggcgtccaaggcattgtgaaaaaaatatgaattggaattt

fava_c15889_1460	fava_c15889	1460	G/A	tcctaagatcattaatcatgtctcgtattccaagccaaattaacgagtcaactatttgataYgaaagcctataaccagcttgttgctttacacgagacaa[G/A]ggatgaggaaattcagttgttggcgtccaaggcattgtgaaaaaaatatgaattggaatttgtagggcctctgcaacatgggtatgcccatatgctgggg

fava_c15889_413	fava_c15889	413	A/G	tatctgtcaagtgcaaaagagaaaaagaacaaaaccgttacctaggcaccaagagaatcaataatattaaacacaacagaaaatttacatacatatcaaa[A/G]ctcacactgaatagcatgcccggaaataaRcaatcatgtgactgtgacaacttacaagtcgagcacatctcgttgatgcagtaacatatataattgggat

fava_c15889_443	fava_c15889	443	G/A	aaaaccgttacctaggcaccaagagaatcaataatattaaacacaacagaaaatttacatacatatcaaaRctcacactgaatagcatgcccggaaataa[G/A]caatcatgtgactgtgacaacttacaagtcgagcacatctcgttgatgcagtaacatatataattgggatgtatatatagagtgaaaaRgggtggggagg

fava_c15889_532	fava_c15889	532	A/G	cccggaaataaRcaatcatgtgactgtgacaacttacaagtcgagcacatctcgttgatgcagtaacatatataattgggatgtatatatagagtgaaaa[A/G]gggtggggaggtggggggtgtcaatcagcttgcattggatcaggaacaaccaaaacatctacttaaagagaaaaatttggatgaagtcggctctgtatca

fava_c15889_656	fava_c15889	656	T/C	atcagcttgcattggatcaggaacaaccaaaacatctacttaaagagaaaaatttggatgaagtcggctctgtatcagtctcgggtttcttttgaggaag[T/C]tgcttaaaaaatgctttcacagctccagttaccccatcctcattttccatggccttggcaagttcaattgcatgatcctttaccttgggatctagcataa

fava_c15892_1352	fava_c15892	1352	G/C	cagcttcttcagctaccaaaccatcttttggggctagaaactcgcttagtgaatggactgagaaagagacatttgttcttcttgatgcgtggggtgatcg[G/C]tttcttcaacatggaaggaagagtcttcgatctgatgaatggcaacaagttgcagagaaggtatcagaagtttcaaaagttgataggacagatactcagt

fava_c15892_1667	fava_c15892	1667	G/C	tgtcttctccaccgcaacaagccggtttctcttgtggtttggactcaagagaaagtgtttttatgaagcctagagtttttgttaatcatgcaaatgggtt[G/C]ggtgacacgagggataatcccgcRaataccgaatccattggtgtagaaggtttagaaaggcctcgcgcaaagaaaagaaggaaaagtaggggcagtgacg

fava_c15892_1691	fava_c15892	1691	G/A	gtttctcttgtggtttggactcaagagaaagtgtttttatgaagcctagagtttttgttaatcatgcaaatgggttSggtgacacgagggataatcccgc[G/A]aataccgaatccattggtgtagaaggtttagaaaggcctcgcgcaaagaaaagaaggaaaagtaggggcagtgacgaRtcttcttccttcaaattacttg

fava_c15892_1769	fava_c15892	1769	G/A	gtgacacgagggataatcccgcRaataccgaatccattggtgtagaaggtttagaaaggcctcgcgcaaagaaaagaaggaaaagtaggggcagtgacga[G/A]tcttcttccttcaaattacttgctgattccattcagaatttcggtaagatatatgagaaaattgaaaataataaaaggcaRcagataatggaRctggaaa

fava_c15892_1850	fava_c15892	1850	A/G	aaagtaggggcagtgacgaRtcttcttccttcaaattacttgctgattccattcagaatttcggtaagatatatgagaaaattgaaaataataaaaggca[A/G]cagataatggaRctggaaaagatgaggatggatttcctcactgaattggaaacacaRaagagacagatttttgaaaaattgcagagtgagattttaaaac

fava_c15892_1862	fava_c15892	1862	G/A	gtgacgaRtcttcttccttcaaattacttgctgattccattcagaatttcggtaagatatatgagaaaattgaaaataataaaaggcaRcagataatgga[G/A]ctggaaaagatgaggatggatttcctcactgaattggaaacacaRaagagacagatttttgaaaaattgcagagtgagattttaaaactcgagcaaagag

fava_c15892_1907	fava_c15892	1907	G/A	atttcggtaagatatatgagaaaattgaaaataataaaaggcaRcagataatggaRctggaaaagatgaggatggatttcctcactgaattggaaacaca[G/A]aagagacagatttttgaaaaattgcagagtgagattttaaaactcgagcaaagagatggttagaatgatgattctgttgagtYgggtctctgaagtctta

fava_c15892_1990	fava_c15892	1990	C/T	actgaattggaaacacaRaagagacagatttttgaaaaattgcagagtgagattttaaaactcgagcaaagagatggttagaatgatgattctgttgagt[C/T]gggtctctgaagtcttatgcattgYattgtagttctcattgttgtgtaacacagaatgYMgaaaccagttgttgtatcatacttgaggcatttcgcctat

fava_c15892_2015	fava_c15892	2015	T/C	agatttttgaaaaattgcagagtgagattttaaaactcgagcaaagagatggttagaatgatgattctgttgagtYgggtctctgaagtcttatgcattg[T/C]attgtagttctcattgttgtgtaacacagaatgYMgaaaccagttgttgtatcatacttgaggcatttcgcctattcttcaaatttctgaggcaataYgc

fava_c15892_2049	fava_c15892	2049	C/T	actcgagcaaagagatggttagaatgatgattctgttgagtYgggtctctgaagtcttatgcattgYattgtagttctcattgttgtgtaacacagaatg[C/T]MgaaaccagttgttgtatcatacttgaggcatttcgcctattcttcaaatttctgaggcaataYgcttgccagccaacacttttcattttactggaatca

fava_c15892_2050	fava_c15892	2050	A/C	ctcgagcaaagagatggttagaatgatgattctgttgagtYgggtctctgaagtcttatgcattgYattgtagttctcattgttgtgtaacacagaatgY[A/C]gaaaccagttgttgtatcatacttgaggcatttcgcctattcttcaaatttctgaggcaataYgcttgccagccaacacttttcattttactggaatcag

fava_c15892_2113	fava_c15892	2113	C/T	tgYattgtagttctcattgttgtgtaacacagaatgYMgaaaccagttgttgtatcatacttgaggcatttcgcctattcttcaaatttctgaggcaata[C/T]gcttgccagccaacacttttcattttactggaatcagtcagaactggatagtccagccatgttatcattaccttgttggcaaagagttcattgctttctt

fava_c15892_474	fava_c15892	474	A/G	agccacactttgggaattaggattggtgattgattggtaatcactgagaaccaattttctatagttatagtttccttgctagttgtatttgtgaattgtg[A/G]tttgattctaaatggatgacattgagaatgatgcaattgcaaggtatccccctaacccatatgctgttagtcaccctcataatcaaggctatggttatca

fava_c15892_848	fava_c15892	848	G/A	atcatgttgatgaagatgatgacatggatgggaatggcgatgagaatgatgataatgaccaacaacaaagtgaggagtatggtgatctgaaacatcctgt[G/A]aggaattYtccgtattccccgccatttgaaaatgaaggtaacaatgatggggttgaagaagatgaagaacggtcagtgggggaagatgtaggtgacggtg

fava_c15892_856	fava_c15892	856	C/T	gatgaagatgatgacatggatgggaatggcgatgagaatgatgataatgaccaacaacaaagtgaggagtatggtgatctgaaacatcctgtRaggaatt[C/T]tccgtattccccgccatttgaaaatgaaggtaacaatgatggggttgaagaagatgaagaacggtcagtgggggaagatgtaggtgacggtgatgatgat

fava_c15895_1302	fava_c15895	1302	G/A	agatgtccgtttatattcatccatgtagagcataatgcaacagcaagaccagcatttacatattggtgctgaccttcaagccctagttttaaaccattta[G/A]taatttgacatctaatggggctgtcacttgaagaggtacgttcaactgagaagccttatctataagtacatgcattgcttcatcaggttgagtcaccgta

fava_c15895_1612	fava_c15895	1612	A/G	caataggtgcttgcaccacatttgttgcgtcgtatttgccacctaacccaacctccattatgcaaacatccacctgctcttctgaaaatatcttgaaggc[A/G]agtaaagcaaggaagtgaaaaaaggtaggcattggaacaccgtcatcagttttttcctttagtctatcataacaccaccagaaatatgctaaaaatttct

fava_c15895_1762	fava_c15895	1762	T/C	ttttttcctttagtctatcataacaccaccagaaatatgctaaaaatttctcttcacagatttccacaccatctaaacgaaatctttctcgtatatcaat[T/C]agatgaggagaagtgaaaagcccagtgcgaaaaccacagttgcgtaatatagattctgtgaaggtgcatgtagatcccttccctttagtaccagcaacat

fava_c15895_233	fava_c15895	233	A/G	gcaaagtttacgattacatgcaaatgcgactttgtattcctccccttattaaccgcccctttcttctttaaatacaatgacgaagattgaaagctcccac[A/G]aagacagctaccaagctgatgcttgtataaaccaatgtttcaatgtccaaaacccaaSatgttYttgtgttgataacattcaattagcRgtttccgaacc

fava_c15895_291	fava_c15895	291	G/C	ctttcttctttaaatacaatgacgaagattgaaagctcccacRaagacagctaccaagctgatgcttgtataaaccaatgtttcaatgtccaaaacccaa[G/C]atgttYttgtgttgataacattcaattagcRgtttccgaacccttcacaatgagatgcgaaagacacaaatatctcattcgaccccgtcgagaattataa

fava_c15895_297	fava_c15895	297	C/T	tctttaaatacaatgacgaagattgaaagctcccacRaagacagctaccaagctgatgcttgtataaaccaatgtttcaatgtccaaaacccaaSatgtt[C/T]ttgtgttgataacattcaattagcRgtttccgaacccttcacaatgagatgcgaaagacacaaatatctcattcgaccccgtcgagaattataatggcac

fava_c15895_322	fava_c15895	322	A/G	aaagctcccacRaagacagctaccaagctgatgcttgtataaaccaatgtttcaatgtccaaaacccaaSatgttYttgtgttgataacattcaattagc[A/G]gtttccgaacccttcacaatgagatgcgaaagacacaaatatctcattcgaccccgtcgagaattataatggcacttcatYacttYgaaatcagaaaata

fava_c15895_403	fava_c15895	403	C/T	ttgataacattcaattagcRgtttccgaacccttcacaatgagatgcgaaagacacaaatatctcattcgaccccgtcgagaattataatggcacttcat[C/T]acttYgaaatcagaaaataaacaaatctttcaattaagtccaacaaatcacttcttgattaatttcaacacgtcaccaacaagatgtatggaaccagtta

fava_c15895_408	fava_c15895	408	T/C	aacattcaattagcRgtttccgaacccttcacaatgagatgcgaaagacacaaatatctcattcgaccccgtcgagaattataatggcacttcatYactt[T/C]gaaatcagaaaataaacaaatctttcaattaagtccaacaaatcacttcttgattaatttcaacacgtcaccaacaagatgtatggaaccagttacaagg

fava_c15895_712	fava_c15895	712	T/A	ttaattccataccagttaaatcttcagaagccacgccgttattcttgcctttgttcccttgcatgagattttcccatactttttgtagagtaaactgcca[T/A]gacaggtcaatgttagaatcagttggtgttaaagcatgggatccaactttgtggtacaatgataaaccaggtacaaatagcgccttcttgaagtacacac

fava_c15897_1428	fava_c15897	1428	T/C	aaaagtccagcccgtgtacccccaccagacccacttcctcccggagtcgtaacagttgaccatgacatgcccgtttgtattggctcatgtacatcatcat[T/C]atcatcatcatcatcatcagtactctgagaatgggttagattttcctgagggtgagaaggtaaagcggaggcattgagttcatcaccttcttggccggga

fava_c15897_181	fava_c15897	181	A/C	aaacatacatMttatttcttctgagttctgatatattatttctagtttgagccataaatggatttggtgcattcaacagccactacagtcaatgatctcg[A/C]catcgaagatcagatggttaatttcaaatttctgatttgctgaaatcacaatgcacattatctaacggtttagaacggcagactgtagtgactgccgaaa

fava_c15897_91	fava_c15897	91	C/A	agagaaaatttaagccaaatatagcttaacccatttttattatctgaaaatgattgatttaattaaacaacaactaaagaaaacatacat[C/A]ttatttcttctgagttctgatatattatttctagtttgagccataaatggatttggtgcattcaacagccactacagtcaatgatctcgMcatcgaagat

fava_c15901_1020	fava_c15901	1020	T/G	ggactggtggatgtttcagctcagatattctatcagctgttgatacagcagtagccgacggagtcaatgttctatcgatctctttaggtggtggagtttc[T/G]tcttactaccgcgatagtctatccgtagcttcattcggagctatggaaaagggtgttttcatttcatgttcagcRggaaactcaggacctgagcctgtga

fava_c15901_1095	fava_c15901	1095	G/A	cgatctctttaggtggtggagtttcKtcttactaccgcgatagtctatccgtagcttcattcggagctatggaaaagggtgttttcatttcatgttcagc[G/A]ggaaactcaggacctgagcctgtgagcttaacaaatgtttctccatggatcacaacagttggagctagcacaatggacagagattttccagcagatgtta

fava_c15901_1725	fava_c15901	1725	T/C	tagctgcattttcatcaagagggcctaatttgcttaccctcgaaattctcaaacccgacatagtagcacctggtgtgaacattcttgctgcatggagtgg[T/C]gttacaggtccatcaagcttacaaacagatcacagaagagtgaaattcaacatcctttccgggacttccatgtcgtgccctcatgtaagtggaatcgcgg

fava_c15901_1959	fava_c15901	1959	C/T	ctgctgctataaaatccgcaatcatgacaacagcttatgttcatgataatactattaaacctcttagagatgcttcttctgccgagttttcaactcctta[C/T]gatcacggtgcaggtcacattaaccctcgaaaagcacttgaaccaggtttagtatatgatattcaaccacaagactactttgagtttctttgcaaacaga

fava_c15901_2190	fava_c15901	2190	A/G	ccaaacattcaaacaaaacttgcaagcacactcttgctaacgtaggtgacttgaactatcctgctatatcagtagtgtttcctgagaaaacaactggttc[A/G]gcttcggcgattcacagaactgttaccaatgttggacctgctgtttcRaaatatcRtgtgattgttacgccattcaaaggcgctgaagttaaagttgagc

fava_c15901_2238	fava_c15901	2238	A/G	acttgaactatcctgctatatcagtagtgtttcctgagaaaacaactggttcRgcttcggcgattcacagaactgttaccaatgttggacctgctgtttc[A/G]aaatatcRtgtgattgttacgccattcaaaggcgctgaagttaaagttgagcctgatacattgaaatttacaaggaaatatcagaagttatcttataaga

fava_c15901_2246	fava_c15901	2246	G/A	tatcctgctatatcagtagtgtttcctgagaaaacaactggttcRgcttcggcgattcacagaactgttaccaatgttggacctgctgtttcRaaatatc[G/A]tgtgattgttacgccattcaaaggcgctgaagttaaagttgagcctgatacattgaaatttacaaggaaatatcagaagttatcttataagatYagtttc

fava_c15901_2340	fava_c15901	2340	C/T	aatatcRtgtgattgttacgccattcaaaggcgctgaagttaaagttgagcctgatacattgaaatttacaaggaaatatcagaagttatcttataagat[C/T]agtttcaaaattacaactaggcaaagtgaaccagaatttggaggtttggtatggaaagatgRggtgcataaagtaagaagtcctattgtgataacatatc

fava_c15901_2402	fava_c15901	2402	G/A	aaatttacaaggaaatatcagaagttatcttataagatYagtttcaaaattacaactaggcaaagtgaaccagaatttggaggtttggtatggaaagatg[G/A]ggtgcataaagtaagaagtcctattgtgataacatatcttccaccaatRtgaagtYctttcaactagggctagttactagttaagcagtttttctttttt

fava_c15901_2451	fava_c15901	2451	G/A	ttacaactaggcaaagtgaaccagaatttggaggtttggtatggaaagatgRggtgcataaagtaagaagtcctattgtgataacatatcttccaccaat[G/A]tgaagtYctttcaactagggctagttactagttaagcagtttttcttttttgtaataagcaaattttctgtgttatgaagtgtatgcatttttgttaaag

fava_c15901_2458	fava_c15901	2458	T/C	taggcaaagtgaaccagaatttggaggtttggtatggaaagatgRggtgcataaagtaagaagtcctattgtgataacatatcttccaccaatRtgaagt[T/C]ctttcaactagggctagttactagttaagcagtttttcttttttgtaataagcaaattttctgtgttatgaagtgtatgcatttttgttaaaggaaatgt

fava_c15901_330	fava_c15901	330	G/C	aaaactcggcgaaaccagaagtgttttccagtcatctagattggtattcatcaaaagtgaaatcagttttgtctaaatcagtagaagctgaaatagacaa[G/C]aatgatgaagagagaatcatttacagttacaRcactgcttttcatggaatggctgctaaattaagtgcagaagaagcaaaaagactagaatctgaagatg

fava_c15901_362	fava_c15901	362	G/A	catctagattggtattcatcaaaagtgaaatcagttttgtctaaatcagtagaagctgaaatagacaaSaatgatgaagagagaatcatttacagttaca[G/A]cactgcttttcatggaatggctgctaaattaagtgcagaagaagcaaaaagactagaatctgaagatggtgttgtagctattttccctgatacaaagtat

fava_c15901_659	fava_c15901	659	C/T	gtggttggagttttggacactggtatttggcctgagagtcagagtttcaatgacgccggaatgacgccggtaccttctcattggaaaggtgcatgtgaaa[C/T]tggtagagggtttcaaaaacatcattgtaataagaagattgtgggtgcaagaatcttctaccatggatatgaagcagcaactggtaagattaatgaacaa

fava_c15901_762	fava_c15901	762	A/T	gtagagggtttcaaaaacatcattgtaataagaagattgtgggtgcaagaatcttctaccatggatatgaagcagcaactggtaagattaatgaacaagc[A/T]gattacaaatctcctagagatcaagatggtcatggaactcacactgcagctactgttgctggatctccagttcatggtgctaatcttcttggttatgctt

fava_c15904_1001	fava_c15904	1001	T/C	ttgtctgcattccattcagttctccttcagacaggttcttctgcatgctttcctcgccttcRtttgaaccctgtgtgctacctgataccaggttcatatc[T/C]gagacagctggtgacaattccagtctgagtttgctcgagaaatcttgatggataacatttccaaattccagtctaaaattactatgattgtcaaccaagc

fava_c15904_123	fava_c15904	123	T/A	aaccattcaaaacttgaatacatacaagtttaaagtacttgcatatattcaaaaatcgcatagtggatgacttttaaatacacattacctgtaggaaccg[T/A]tcaaaactcgagtatatacaacactgccagcaagtaattgacaatgSatgtttcagtctaaaaagaccaaagatatgttcacatgaaatagtttttcagc

fava_c15904_1364	fava_c15904	1364	A/G	ctataccatccaatcgatggtgaaaatcttccatctgaagctttgttgttgattgatgatgtttgtagtttaagagattggattcaagagaatttttttc[A/G]cgcgagagtttatcgagttcctctgcaaacgctgccctttcaggatctacaacagaaaccggaggatgactatgactgtgtataggtttcctgcggtgta

fava_c15904_1514	fava_c15904	1514	T/C	caacagaaaccggaggatgactatgactgtgtataggtttcctgcggtgtatattcttcagaagatgcttttgaccttttataaattcttcattagcaaa[T/C]tcccaacgctcagaatgtacttttcggaatccataggtRttgagctgccggatgaagctggagaaattgctgtgcttgaaataggttgggaggagaaggc

fava_c15904_1553	fava_c15904	1553	A/G	tcctgcggtgtatattcttcagaagatgcttttgaccttttataaattcttcattagcaaaYtcccaacgctcagaatgtacttttcggaatccataggt[A/G]ttgagctgccggatgaagctggagaaattgctgtgcttgaaataggttgggaggagaaggcgagcgaattcggtattgttccaaacgatgaagctgcagt

fava_c15904_170	fava_c15904	170	C/G	ttcaaaaatcgcatagtggatgacttttaaatacacattacctgtaggaaccgWtcaaaactcgagtatatacaacactgccagcaagtaattgacaatg[C/G]atgtttcagtctaaaaagaccaaagatatgttcacatgaaatagtttttcagcccacttgcaaaacaaaacaacttatagacaagtttcatgtcattgat

fava_c15904_434	fava_c15904	434	T/A	agagtgtgagattatccatgttcttggagttactagagattctatgaactgaccgcccttcctcttgctcgtcatatggatttgctcgatagttggatat[T/A]gcctcttcattgtctgaacagcctggtctttcggtaaggaattgttcccaaaagacgtcattcactcgacgtggagcagctggtttgacttgtccgttac

fava_c15904_537	fava_c15904	537	G/A	ctcttcattgtctgaacagcctggtctttcggtaaggaattgttcccaaaagacgtcattcactcgacgtggagcagctggtttgacttgtccgttacct[G/A]aagcttcctgcggtgaagctaaattgataacctcgttggccacaactcggtttaaggaaactccRttttctgattctttaccattggcattaactattga

fava_c15904_602	fava_c15904	602	A/G	gacgtggagcagctggtttgacttgtccgttacctRaagcttcctgcggtgaagctaaattgataacctcgttggccacaactcggtttaaggaaactcc[A/G]ttttctgattctttaccattggcattaactattgattcggcaatttcttgacaatctatctggggtgatctggccgagtatgaattcctgttgaattcca

fava_c15904_710	fava_c15904	710	T/A	attctttaccattggcattaactattgattcggcaatttcttgacaatctatctggggtgatctggccgagtatgaattcctgttgaattccaatggaca[T/A]gatgccagagttaaatttagttggcaggatatatgactatcaccttcttcactacttggctccagggagtgaagatttgggctctcRgtagttgttgctc

fava_c15904_797	fava_c15904	797	G/A	attccaatggacaWgatgccagagttaaatttagttggcaggatatatgactatcaccttcttcactacttggctccagggagtgaagatttgggctctc[G/A]gtagttgttgctcttcgtgataaacacgaatccatattaaaagtaaaagatgtcccagtatctgcaagctctagcgttgcaggtgcaaaagcaaggccag

fava_c15904_962	fava_c15904	962	G/A	caagctctagcgttgcaggtgcaaaagcaaggccagttcttgtctgcattccattcagttctccttcagacaggttcttctgcatgctttcctcgccttc[G/A]tttgaaccctgtgtgctacctgataccaggttcatatcYgagacagctggtgacaattccagtctgagtttgctcgagaaatcttgatggataacatttc

fava_c15907_1136	fava_c15907	1136	C/A	gacatgtaatgtttctgataaacaagattggaatgctcgtttatattctcaggattggggacgagagtcgcaagaagggtacaagaaatcagacttggca[C/A]gtcaatgcactcatagatataaggtgtacattgaagggtcagcttggtcagtgagtgaaaaatacattctagcatgtgattctcccactttacttgtgaa

fava_c15907_1276	fava_c15907	1276	A/G	cagcttggtcagtgagtgaaaaatacattctagcatgtgattctcccactttacttgtgaaacctcattattatgatttcttcacaagagggttaattcc[A/G]gtgcatcactattggcccattaatgaagatgataaatgcaagtccattaaatWtgccgtggattggggcaatagtcacactgaaaaggcacaaaatattg

fava_c15907_1329	fava_c15907	1329	T/A	cttgtgaaacctcattattatgatttcttcacaagagggttaattccRgtgcatcactattggcccattaatgaagatgataaatgcaagtccattaaat[T/A]tgccgtggattggggcaatagtcacactgaaaaggcacaaaatattggaaaagcagcaagtgatttcattcaacaagagttgaagatggattatgtgtat

fava_c15907_1441	fava_c15907	1441	T/C	ggggcaatagtcacactgaaaaggcacaaaatattggaaaagcagcaagtgatttcattcaacaagagttgaagatggattatgtgtatgactatatgtt[T/C]caYctcctaaattcctatgctaagctctttagataccaaccctccataagtgataaagctgttgaactatgtgtggaatcaatggtgtgtaatgcacaag

fava_c15907_1444	fava_c15907	1444	T/C	gcaatagtcacactgaaaaggcacaaaatattggaaaagcagcaagtgatttcattcaacaagagttgaagatggattatgtgtatgactatatgttYca[T/C]ctcctaaattcctatgctaagctctttagataccaaccctccataagtgataaagctgttgaactatgtgtggaatcaatggtgtgtaatgcacaaggat

fava_c15907_1831	fava_c15907	1831	A/G	tatttttactacaagattctagtgcaactggttgatgcgcagaacatatgaatatgttataaaccctagcattacgagtttggtttttaatcatcggaaa[A/G]aRaaaaaaaacaagcgaatgtcatcattgttagagattaatgtttttctcttccaacagagtttttggggttgataaatatttagMatacattgtctctt

fava_c15907_1833	fava_c15907	1833	G/A	tttttactacaagattctagtgcaactggttgatgcgcagaacatatgaatatgttataaaccctagcattacgagtttggtttttaatcatcggaaaRa[G/A]aaaaaaaacaagcgaatgtcatcattgttagagattaatgtttttctcttccaacagagtttttggggttgataaatatttagMatacattgtctcttat

fava_c15907_1917	fava_c15907	1917	C/A	ttaatcatcggaaaRaRaaaaaaaacaagcgaatgtcatcattgttagagattaatgtttttctcttccaacagagtttttggggttgataaatatttag[C/A]atacattgtctcttatatagttggctaaataaaatgtatctacaagaaatagtactatgcttcatcattctttgaattgatcaactttctccaaattttg

fava_c15907_210	fava_c15907	210	A/G	aacctcaacagcgacccaattgaagaacaatatcaaagttatcatcttttggtattctgagtaacaagaagctcgaaacatcaaaaggtacgcaggggaa[A/G]gaaaacttcggatcggggcaaattggtaatttggaagagacaattcatgaaatcgttgttgagatccgccgtgatattggttttgccggtggtgttaatc

fava_c15907_391	fava_c15907	391	C/A	ttttgccggtggtgttaatcgtcggcgcgttgatttacgcgcggttcgtcgaaacacctgacattttctccaaagtttcatcaaccgaaccagttctaac[C/A]acgaaagcatacgaaataccaacaaagcccctaaatccaMtagattgcaatggatacaacctcaccaaaaaatgtSccactRacaatcccgaaatctcat

fava_c15907_431	fava_c15907	431	A/C	gcggttcgtcgaaacacctgacattttctccaaagtttcatcaaccgaaccagttctaacMacgaaagcatacgaaataccaacaaagcccctaaatcca[A/C]tagattgcaatggatacaacctcaccaaaaaatgtSccactRacaatcccgaaatctcattctccaatcaagaccgttcatctaactccacgtgtccaga

fava_c15907_467	fava_c15907	467	C/G	ttcatcaaccgaaccagttctaacMacgaaagcatacgaaataccaacaaagcccctaaatccaMtagattgcaatggatacaacctcaccaaaaaatgt[C/G]ccactRacaatcccgaaatctcattctccaatcaagaccgttcatctaactccacgtgtccagattacttccgttggatccacgaagatttaaggccatg

fava_c15907_473	fava_c15907	473	A/G	aaccgaaccagttctaacMacgaaagcatacgaaataccaacaaagcccctaaatccaMtagattgcaatggatacaacctcaccaaaaaatgtSccact[A/G]acaatcccgaaatctcattctccaatcaagaccgttcatctaactccacgtgtccagattacttccgttggatccacgaagatttaaggccatgggccca

fava_c15907_616	fava_c15907	616	T/G	actccacgtgtccagattacttccgttggatccacgaagatttaaggccatgggcccacacaggaataacacaggaaatggtagagaaagcaaaagcaac[T/G]gcaaatttcaagttggtgatattaaaaggaaaagtgtacttagaaacatatgagaaagcttttcaaacaagagatacttttactctatggggtatcctac

fava_c15907_756	fava_c15907	756	A/T	ttagaaacatatgagaaagcttttcaaacaagagatacttttactctatggggtatcctacagttgttaaggaggtaccctggaatgttgcctgatttgg[A/T]gcttatgtttgattgtgttgattggccagttgttttaattgatcaaaataatgtYgttgccccacctccactttttcgatattgcggtaatgatctaaca

fava_c15907_811	fava_c15907	811	T/C	tcctacagttgttaaggaggtaccctggaatgttgcctgatttggWgcttatgtttgattgtgttgattggccagttgttttaattgatcaaaataatgt[T/C]gttgccccacctccactttttcgatattgcggtaatgatctaacattggatatcgtctttcctgattggtctttctggggatgggccgaggttaatgtaa

fava_c15909_1172	fava_c15909	1172	A/G	cctattatggcatgaaccaggtacagcctggtgcggagaattttcaacaatatgcacctggacatggacctgctgcttggggtgcatatgacatgcagcg[A/G]gctcagggacatcgatagacttctatcctgttacaaaatcttataactggttacctggactgccactttctagattggttcaaggttctRctcatgggta

fava_c15909_1262	fava_c15909	1262	A/G	acatgcagcgRgctcagggacatcgatagacttctatcctgttacaaaatcttataactggttacctggactgccactttctagattggttcaaggttct[A/G]ctcatgggtatcatgagaagttgtttcttagatgctcctcaaaattgtagttatatcagaaggcctttattggcttgatcacgcagtagatggtgtttgt

fava_c15909_128	fava_c15909	128	C/T	catctccgagacagaatacttgagactgagtacggtagtttcctcgatcaacaaccatagttccacctccagagttcatttttcacattcttcaacaacc[C/T]agcttgttaatttcctttactcacattttcaaaacctaaaaccacgatttcacttctctattctcaccaacttttaaaaccatgtcaaatagaaaccgcg

fava_c15909_1367	fava_c15909	1367	G/A	tgggtatcatgagaagttgtttcttagatgctcctcaaaattgtagttatatcagaaggcctttattggcttgatcacgcagtagatggtgtttgtctac[G/A]gtgtcattaattgRatatcttgttatatagtaacagccaattttagtggtgtggcatttatgctaagcttacacctttgcctttagcttattatgttttt

fava_c15909_1381	fava_c15909	1381	A/G	agttgtttcttagatgctcctcaaaattgtagttatatcagaaggcctttattggcttgatcacgcagtagatggtgtttgtctacRgtgtcattaattg[A/G]atatcttgttatatagtaacagccaattttagtggtgtggcatttatgctaagcttacacctttgcctttagcttattatgtttttcttccactcctatc

fava_c15911_1257	fava_c15911	1257	C/T	gttatcagttgcacagctggttcacatattccatacttcttttatttctcagtactgctggattttggatgaccaggttgaatgagggattgtccttgtt[C/T]gatgcaattcttattgttcccatgtttcagatagcatggaccctcttctcaatttgtacgggatttatctattttcaggaatatcaggtatttgacacat

fava_c15911_1591	fava_c15911	1591	G/A	agaaatgaaaagattagtagcgtcttctgaagaagcagcacacaacaaagacccacgatcttttgtcaaaggaatcctgttaaaggttacagatattctg[G/A]tcaaggcaaagacttcttgtgcattaagtcttggttttggggaggatacgatcaatgcatcttcagtttttgtgatgccaatgatgtcgtcaagaatgaa

fava_c15911_1918	fava_c15911	1918	A/C	cccttgatagtgttttgatagctgtagaatttccaaggttttggatttatttttttcttctattttccgattttattttaatgattggagtagatgagat[A/C]taaaccattcataattcaattatttgctctattgagcattcatgtaatcttccccactaaggaaaaagaggatMaaaaagtgttgtatattgtatatata

fava_c15911_1992	fava_c15911	1992	C/A	attttaatgattggagtagatgagatMtaaaccattcataattcaattatttgctctattgagcattcatgtaatcttccccactaaggaaaaagaggat[C/A]aaaaagtgttgtatattgtatatatacttgtaaatttcttgcttggacaattgaaaagagaatgaacgaacacgttttcatctaaaaaaaaaaag

fava_c15911_383	fava_c15911	383	G/A	atttcaagcaatcgtttgtttctcaggattattatagtgttgttttgaaactggttcagttggtggttacgactacggctgcaaaatcgttgatatttca[G/A]ctttggtttgtatttgtgtgaagaatttgagaggtaacttgtgttgggaagtggaggcgaattttgtggtgcatgtggtgtatccttcgcggtgccacat

fava_c15911_484	fava_c15911	484	C/T	ctttggtttgtatttgtgtgaagaatttgagaggtaacttgtgttgggaagtggaggcgaattttgtggtgcatgtggtgtatccttcgcggtgccacat[C/T]gtttattcatgggggagtgggttgttggagctttcatcaacctttttggtagtcttgctataaactttgggaccaaccttctcaaattaggacataatgR

fava_c15911_903	fava_c15911	903	A/C	aagtagtgacagtcaaggtactggttgccacggctttcattgttcttggaaatgtttttctagttgcttttggcaatcaccaatcacctgtttatactcc[A/C]gagcagttgacagagaaatataccaatgccgcattccttctgtaccttctagctttgattgtaattgttgtcttgcatcactccgtttacaagagaggtg

fava_c15913_1528	fava_c15913	1528	T/C	catgtgggaactgcagaggaaagtaagacagtttcaccaatcagctgaaacattttggttgcataatggatcaattgtaaacagaaacttggtcgaattt[T/C]ctagttggaaactccctgtacctttgccttgataagcttggcatgtagttttacttgtataaagtattttgttttgttgctatttttttagtcttgattc

fava_c15913_1668	fava_c15913	1668	T/C	ggcatgtagttttacttgtataaagtattttgttttgttgctatttttttagtcttgattctttacaggcatgttagttcatcattgtcgtctagtaatc[T/C]gtctatatagtatatataaaacagaacaacttttcattgattaatactcatatcaacttaacaatgatttgtttgtttttcctctgctgaannnnnnnnn

fava_c15913_173	fava_c15913	173	T/C	ataaaagttttataaacataaaatcatgtttaataatgaaggaagaaagaatgagtttgcatctgatacaccttcctatcttagaaccagaatctctctc[T/C]cgcactcattcctcctcactacttcccacaccttctttcttcctccatcttcaatctaatgtggagtaacaacaaatcaagaacaatgcatcaKcagcag

fava_c15913_267	fava_c15913	267	T/G	tctctcYcgcactcattcctcctcactacttcccacaccttctttcttcctccatcttcaatctaatgtggagtaacaacaaatcaagaacaatgcatca[T/G]cagcagcatccactatgcacacgcacgcaccagatcgggtccttactcctagtcgccgccaccttcttcctcacaagactcttggacgctccttgcaacc

fava_c15913_903	fava_c15913	903	C/T	taaatcgctccattattcttacccctgagggggaccgcactgataagagagatactagtgcctttaatacatggaaagatataatcattccaggtaatgt[C/T]gaagatggtatgaccaaggctgggtctaccatagtccagcctttgcctctgtctaagcggaagtacttggcaaattatttaggacgtgctcaaggaaaag

fava_c15915_1418	fava_c15915	1418	A/G	agttctcataagagtgaatgaataaatataaaatacagaattttgcggctttttattccctcagctaaaccttctcttacctacctaataaagaagcctt[A/G]gcagcagcaaaaaggcgattttctatatgcatcatttatttgttcttatcggtgtttgaggtgtgacacttgtggtgacttgaagttttttggtacccag

fava_c15915_1535	fava_c15915	1535	G/A	gattttctatatgcatcatttatttgttcttatcggtgtttgaggtgtgacacttgtggtgacttgaagttttttggtacccagagatttgagtttatga[G/A]ctttatgtaacagcggtgtcgcacggtacagtactgggtatctgtattctacctgataacttccttgcctcgtatttttagttgggacttggaaattttg

fava_c15915_164	fava_c15915	164	G/A	cattctcgaacggaaggccaaattttcgttcctcaaattctcaattcttatccaatacatgaaaatcataatcaaattacttaattcaattaattcctat[G/A]cacagaaaacRaaaacagtttaattaaattgaattgaatcattacatttttttcttcaattaattctttggtatttcgttttcaatcggattagcgaaga

fava_c15915_1665	fava_c15915	1665	G/C	agtactgggtatctgtattctacctgataacttccttgcctcgtatttttagttgggacttggaaattttgatatatgttatgttctattttttctatat[G/C]tgttttcataattaattttgaggatgagctgaggcatttgtaatttcctttctgttatattgggaacttcggttaatgactctattggtcttgaaaaaat

fava_c15915_175	fava_c15915	175	A/G	ggaaggccaaattttcgttcctcaaattctcaattcttatccaatacatgaaaatcataatcaaattacttaattcaattaattcctatRcacagaaaac[A/G]aaaacagtttaattaaattgaattgaatcattacatttttttcttcaattaattctttggtatttcgttttcaatcggattagcgaagaattttgatttt

fava_c15915_401	fava_c15915	401	G/C	aagaaaaaaaaatgaggaaatggtgcggtgaaagcgacgtcgttgaggttgaagggattgatgttagtgtttgtgttgcggcggatggtgatttttgggt[G/C]agcgttggctctgccggtggtccgatcgggaaatttagccttgaaagcgaacatgatttggcgcatatggttaatgatttcatagagaatgcaggaagct

fava_c15916_1322	fava_c15916	1322	T/G	agtgattcatgttaatttctactcctttgggacactaccctatttgagacaaggttagagaaaacattgaatagaggtgctgcagaagctgcttctactt[T/G]tgttccctgtgtggcttagaaaagtttacaatgatgttgcctatgggaagtatattgtRtttggagtgtattttttaattcttttgtatggatgagatat

fava_c15916_1381	fava_c15916	1381	A/G	gaaaacattgaatagaggtgctgcagaagctgcttctacttKtgttccctgtgtggcttagaaaagtttacaatgatgttgcctatgggaagtatattgt[A/G]tttggagtgtattttttaattcttttgtatggatgagatatgatgtaatgtgtgatttgctcttgtaatatacttggctttaaaggtacaattatggctc

fava_c15916_1513	fava_c15916	1513	C/G	gatgagatatgatgtaatgtgtgatttgctcttgtaatatacttggctttaaaggtacaattatggctctgtttggcaataccatattttgagcttatag[C/G]ttgtcccttatctgtttggcaagacagcattttgagcttatagcttgtcctttgtctgcttggcaaaactgcattttgtagcttgtgccttatttgtttg

fava_c15916_372	fava_c15916	372	C/G	aaacaactcaatcaaagtcattttcttctgaagatagttcagtgacttcaactgggtcttacccttctaatcataggtatcagaattgcagggatagtga[C/G]gaggaagatgaagaacttaatgattgggttgatgatcttagcgatgatgatgatgatggaattagggaagaacgtgatgaattgggtgtggagtttgaag

fava_c15916_669	fava_c15916	669	T/C	atgtggagactattggactcaaccctaatgctagagacagaagtgtttatgttcatcctgtgttgaacccggttgaaaacctatctcagtggaaagctgt[T/C]aaggctagaagaagaccaacattaaggcctcagaaagagaataatgtttgtYccaatcctgaatcacagRttgcatttggtgttaaggaagtctcagaat

fava_c15916_721	fava_c15916	721	C/T	tcatcctgtgttgaacccggttgaaaacctatctcagtggaaagctgtYaaggctagaagaagaccaacattaaggcctcagaaagagaataatgtttgt[C/T]ccaatcctgaatcacagRttgcatttggtgttaaggaagtctcagaatccgacaacacaccaaagaagtcgaacaaggaaattgcagttgatgctagcct

fava_c15916_739	fava_c15916	739	G/A	ggttgaaaacctatctcagtggaaagctgtYaaggctagaagaagaccaacattaaggcctcagaaagagaataatgtttgtYccaatcctgaatcacag[G/A]ttgcatttggtgttaaggaagtctcagaatccgacaacacaccaaagaagtcgaacaaggaaattgcagttgatgctagcctttctaattggcttggttc

fava_c15917_698	fava_c15917	698	A/T	actcatatgaatctgattgggttaaccttgttggagaaatcgactcttcttcgttacaactacaaacccctttagtagaacaaaactactatgatttgat[A/T]gaaccagatccagcaagccattttcaagatggaacttggagacaactacaaacccctttagtagaacaaaactactatgatttgatagaaccagatccag

fava_c15932_1266	fava_c15932	1266	G/A	gttccaatgttttctcaactctaaaaacagtaacaacctcaggcacagaaaccaacttaatcttccaagaaacaataacaccaaaactcgctccaccacc[G/A]cctctaatagcccaaaaaagatcttcccccatagattccctatcaagtaatcttccattaacatccacaatttccgcatccaaaatattatcaacagaca

fava_c15932_1707	fava_c15932	1707	A/G	gaccatcatagtcatgaccaccgcttcttattctgatttctaggttaaatttcttgcagcacaagattgatgcttggatgtgggaaacatgagatggggt[A/G]aggatgaaaagtggtttaggagtagtRgatgtgttgaatctaaggtttctgatgtaggagtttaagatgagtgaatatgaagggttgttagggaaataga

fava_c15932_1734	fava_c15932	1734	G/A	ttattctgatttctaggttaaatttcttgcagcacaagattgatgcttggatgtgggaaacatgagatggggtRaggatgaaaagtggtttaggagtagt[G/A]gatgtgttgaatctaaggtttctgatgtaggagtttaagatgagtgaatatgaagggttgttagggaaatagatgagttcagagattggatgagaaggtt

fava_c15932_189	fava_c15932	189	G/C	tatgcagcaatcttctgcctaataacactagttacaagaccaaattcatattctaaacatacatcatcaatcatctccactcactagccaaatgaacaaa[G/C]aaagtcccttaatcagggaagatacaattggaaataatttcctagtaacaaacttcaatctagatctgaactagcttcctaWaatatatgcatgcctctg

fava_c15932_271	fava_c15932	271	T/A	actagccaaatgaacaaaSaaagtcccttaatcagggaagatacaattggaaataatttcctagtaacaaacttcaatctagatctgaactagcttccta[T/A]aatatatgcatgcctctgtgtacatattttattataatgatacaccccttcataaacatagctgcttctattctgccataatactagtctcagatgcaac

fava_c15932_577	fava_c15932	577	T/C	ccaaatacttgcttcttcataaccaacatccccatctccattgacacctaaatcaacatctctgtagtttaaatatgagcttctaggtgacttggacaca[T/C]atggagtcatgtaatcatagagctttcgaatccgatctaaatactgattcacaacatcattaccttcttctttccaacttaccgagtactgaattttata

fava_c15932_718	fava_c15932	718	G/A	atactgattcacaacatcattaccttcttctttccaacttaccgagtactgaattttatatatgttaccagctctatgtgggaacggtgtttccacctcc[G/A]aaatctcactcattttcccaccataaggattgaatgtaagtgaaacctttcctagttccatcatcttcttccatataccatcaagatcaatttcagaaat

fava_c15933_1129	fava_c15933	1129	T/C	taggagtggacaagccagaatccatgttgagttttaattgtaatggggtccttgttcaagggaagactctcatgggatccttatatggaggactcaaacc[T/C]aaatctgatgtacctattcttcttaaacgctacatggacaaggaactgatgttggacgagtttgttacacatgaggtggagtttaaagacatcRacaaag

fava_c15933_1223	fava_c15933	1223	A/G	caaaccYaaatctgatgtacctattcttcttaaacgctacatggacaaggaactgatgttggacgagtttgttacacatgaggtggagtttaaagacatc[A/G]acaaagcttttgatttattggttaatggagagtgtctYagatgtgtaatttggatggacaaatgagtatgtttttgtttgtttcaatgtaattSttgcat

fava_c15933_1261	fava_c15933	1261	C/T	acatggacaaggaactgatgttggacgagtttgttacacatgaggtggagtttaaagacatcRacaaagcttttgatttattggttaatggagagtgtct[C/T]agatgtgtaatttggatggacaaatgagtatgtttttgtttgtttcaatgtaattSttgcattWagcccttgaattatttcaaYatgtaatttattgaat

fava_c15933_1317	fava_c15933	1317	G/C	gacatcRacaaagcttttgatttattggttaatggagagtgtctYagatgtgtaatttggatggacaaatgagtatgtttttgtttgtttcaatgtaatt[G/C]ttgcattWagcccttgaattatttcaaYatgtaatttattgaatgtgccattttttataaattattgttttggatgaactggatcatgtaggagactcat

fava_c15933_1325	fava_c15933	1325	T/A	caaagcttttgatttattggttaatggagagtgtctYagatgtgtaatttggatggacaaatgagtatgtttttgtttgtttcaatgtaattSttgcatt[T/A]agcccttgaattatttcaaYatgtaatttattgaatgtgccattttttataaattattgttttggatgaactggatcatgtaggagactcatcattgaga

fava_c15933_1345	fava_c15933	1345	T/C	ttaatggagagtgtctYagatgtgtaatttggatggacaaatgagtatgtttttgtttgtttcaatgtaattSttgcattWagcccttgaattatttcaa[T/C]atgtaatttattgaatgtgccattttttataaattattgttttggatgaactggatcatgtaggagactcatcattgagaaattttttttaagggcctga

fava_c15933_676	fava_c15933	676	T/G	ttgtctctagttttagtgagtacactgtggttgacattgccaatctactcaagattgatccagcaattcctcctaatagggcttgcctcctcagttgcgg[T/G]gtatcaacWggggtaggagctgcttggaaaacagcaaatgtggagccaggttctacagtagctatttttggacttggatgtattggattagcagttgctg

fava_c15933_685	fava_c15933	685	A/T	gttttagtgagtacactgtggttgacattgccaatctactcaagattgatccagcaattcctcctaatagggcttgcctcctcagttgcggKgtatcaac[A/T]ggggtaggagctgcttggaaaacagcaaatgtggagccaggttctacagtagctatttttggacttggatgtattggattagcagttgctgagggagcta

fava_c15933_900	fava_c15933	900	C/T	actaggataataggtgtggatatcaagccagaaaaatttgacattggaaagaaatttggagtaacagacttcattaattctggagaatgtgaaaacaaat[C/T]tgtaagtcaggttatcatagagatgactggtggtggagctgattattgttttgaatgtgttggtatggcatcattggtgcaagaagcatatgcttcatgt

fava_c15934_301	fava_c15934	301	C/T	gatgcttttgaccgagttactaagaagcaaaagttatccagttctaagactcatgagttgcttgatcagatcagacaggaaattgagcgtgttttagaaa[C/T]gatgcagtcagtcagtaacactgatcaagtgcttgatcataaccctatcctgaatgagctgaaggccagtatgcttaaaattgctccacttggccaaatg

fava_c15938_1732	fava_c15938	1732	G/A	aacaaaaattgtgaagaacagaagaggatctgaggagaaaaagtgaaacccagatggagagaaaatgagatgataggaaaatgcaagggataaaattggg[G/A]aaaagggttttgtctctcttgtgtttctgggtactttctctctctgaattctctttggtttctcggtttggtctgactgcaatgcacgcgagttgaacac

fava_c15938_178	fava_c15938	178	C/T	acattggaacttctaatacaatatgaattggaaaaaatttctatggcaccttttgtaatggatgctactaaacaatgaaacaatgaaaatggaggaaggc[C/T]gcaacatgttaaaagaagcacaacccaattacaatatgcttaacaataataataataatatagaatcaatcaatcaatcagaggcttctactaatatttc

fava_c15938_542	fava_c15938	542	A/G	cttccagaatcttgccatttgcttgagttctctgtctccaatctgaagggtcaacagaattcccatttaacaacttgtgattctcttctttgtttctgcc[A/G]tctttgttgtctttggtgcttaatggtagcaacagtgttgcagctgtcctgtctggcatttcttgtaatttcttctcaataaaaaacaccaccaaattaa

fava_c15939_859	fava_c15939	859	C/T	caaactttacaccctcaacaaatctcagccctccaagcaatctatccactttcgtgtcaacatcggaagagcgagcggttttacgatagtgaagcgcgag[C/T]tctaagcaatagagaaccggtccggctgaagaaggaatacgaaccggattgaagatttggttcgaactcatcggaattgtgggccatccattacacgggt

fava_c15946_1278	fava_c15946	1278	C/T	cgtcacaccttaatttctcagttccgagagtaacagccacgagaaatgttgccactggcatcagggctttaagcatctggatgaaagccacagaaatgta[C/T]aagtaagcagtgttcccaaaccacagacttgctgcaaagaaggcgcttatagggaccacacaggttgcgtatatatgaaatgtcattttaataggcgcta

fava_c15946_1407	fava_c15946	1407	A/T	ttgctgcaaagaaggcgcttatagggaccacacaggttgcgtatatatgaaatgtcattttaataggcgctacaaccttcagaacacggataaggaaaaa[A/T]gcaactccaccagaaaaagccatatggatcatagtgagagttatYggaaacggaaaattaaaatacagtgtggagagaacccatttgttatacaaaataa

fava_c15946_1452	fava_c15946	1452	T/C	tatgaaatgtcattttaataggcgctacaaccttcagaacacggataaggaaaaaWgcaactccaccagaaaaagccatatggatcatagtgagagttat[T/C]ggaaacggaaaattaaaatacagtgtggagagaacccatttgttatacaaaataacaccYgaggagagtgaaatgtaaaccagaaggtagatgtaggtca

fava_c15946_1512	fava_c15946	1512	C/T	ctccaccagaaaaagccatatggatcatagtgagagttatYggaaacggaaaattaaaatacagtgtggagagaacccatttgttatacaaaataacacc[C/T]gaggagagtgaaatgtaaaccagaaggtagatgtaggtcagcaaatgctgcctattcattattgtcgccattactgccatatcgatccgtgtaatggcac

fava_c15946_271	fava_c15946	271	G/A	cattgcatggtccagccgaaaagcctatacaaaaattaatcaatttactgatcaatactcttaacattcattaaaaaaatcaactatgctgaaagaatat[G/A]tatgtttcctctgctctgttttctttcatcttttgtttttcctccctcttctaaaggatatRtctctgtacttctcttgggaagaaaggtttagacatat

fava_c15946_333	fava_c15946	333	A/G	aacattcattaaaaaaatcaactatgctgaaagaatatRtatgtttcctctgctctgttttctttcatcttttgtttttcctccctcttctaaaggatat[A/G]tctctgtacttctcttgggaagaaaggtttagacatatacttgttttggttcctttagattttgacttttaaaactcattgttgacaagctaggtttctt

fava_c15946_663	fava_c15946	663	C/T	attgactatcttcattggtgttcacatcatcatctgcctttttctccatttgcagctcctttgctgattcgtcgggaataatttgaagtagagatgtacg[C/T]acatccctgatcttcaggtagttgtaacaaacaaccccacttaaagcaatagcatagccaataacattcagtcctgtaatttttgattcaggaaataaga

fava_c15946_828	fava_c15946	828	A/G	cattcagtcctgtaatttttgattcaggaaataagacagttgaaagagagataagtagccagtctttcaagactccagccactcgaatagttacagcccc[A/G]gttctaccgatcactaagaatgttgagaagttcaatgccaaagcacagagagcatttgaaaaaaatatccagaagtcaaactgcatatgtggagcttcca

fava_c15948_1331	fava_c15948	1331	A/G	agattgaagagagaaagaaaaaaactttaaaacggactgactataagataacgggcctagaaaagcagcttagtgacctcatggtttgtcttgagggtgg[A/G]aaggtggtagaacagctgccattgctaaatgacacaactggtggaactgtctctgacatttcaaaagaatcctcatcaacttgtagttcttagctgggag

fava_c15948_1448	fava_c15948	1448	G/A	tgccattgctaaatgacacaactggtggaactgtctctgacatttcaaaagaatcctcatcaacttgtagttcttagctgggagttgaaagagaaagaag[G/A]gaaggggaaagaatgaacgaggtaagaaaacacacccctgcccgttggagggttttttaaattcacctcctaaacaagcattgtgtaggcttacctgcac

fava_c15948_1595	fava_c15948	1595	C/T	ggagggttttttaaattcacctcctaaacaagcattgtgtaggcttacctgcaccatacaaaactaaactgaaggacggaggcacaagttgagatgcatc[C/T]gcctgttcttttgttgttaaaactttaatgtgtcgtgtttgtaaataaatagatatatacatagttttcaagagaggattcttcaagtgcttgtgcttat

fava_c15948_404	fava_c15948	404	G/C	ttgtcagaatggattcaatggaagatcgatacagtgttttgattcgcttcgatgaacagcaatcgactgacgcgttctacactcattataatggtcgacg[G/C]ttctcgtctctcgaggttgaggtgtgccgagttgtttttacattagatgttcaatatacaggctcgattgaacacgcgcaaccttccaatgccccttcaa

fava_c15951_752	fava_c15951	752	C/T	caaataaacaagatattaaaggcgcccttgcacccgaagaaatagctaaggtgttgaacttagaaactatggataagtctcggcactggatgatagttgg[C/T]tgtagtgcatacaccggtgagggtttgcttgagggatttgattggctggtccaggacatagcatctaggatctacgtgctcgactaattatggtcatgtc

fava_c15951_911	fava_c15951	911	T/A	tagcatctaggatctacgtgctcgactaattatggtcatgtcaatatctttgaaagggttcaaagttcaaacttagaatttaagagcattcttaatctac[T/A]atgctatcatcggaagacattgttatgcatctctaagatgtttYgcatggaatttttccttttctattaacatttaatctagtcagtgtatagagcttgt

fava_c15951_955	fava_c15951	955	C/T	tatctttgaaagggttcaaagttcaaacttagaatttaagagcattcttaatctacWatgctatcatcggaagacattgttatgcatctctaagatgttt[C/T]gcatggaatttttccttttctattaacatttaatctagtcagtgtatagagcttgtctattgcaaggtttttgtacgactttacagtgagctctttatag

fava_c15952_316	fava_c15952	316	T/C	ttcttggaaaccctagaattgatcacatgcctgataattatgaagaccttcctttggatttcaaccctctcgtcttcacttcactcgagagacatcttcc[T/C]tctcatattcttaccctatctagagacctcaaagctcactacatgtccaacattctactccgttatttgcctcacagtgaacgcgttaggatgcaaaaac

fava_c15952_835	fava_c15952	835	G/T	ccatgcttgataaattgatgaatgatttcatacgtcctatagcccgagttttcttttctgaaattggtggatccacactggactctcatcatggttttgc[G/T]gttgaatatggagccaatagagatgtggaacttgggttccacgtggatgatgcagaagtcaccttaaatatttgtctaggaacacaattttcaggcggag

fava_c15956_1406	fava_c15956	1406	A/G	acatctctgagacacacatactattatagttctcatcatcatctgctgcaaaattaacaatatcctctaactccatttcaaatatgcatttagcatcctc[A/G]tgtatcttgccatccaatccaatctccacatccaactctatcaatttatccacaagtgcgggtagcatggtactaccaacaatttctccaattgaaccac

fava_c15956_1532	fava_c15956	1532	A/G	ccacatccaactctatcaatttatccacaagtgcgggtagcatggtactaccaacaatttctccaattgaaccactctcaagctttaacatattttcaac[A/G]tacatgacaatctcatgcactgtcacatcRtaacgttttggtatattctggacaactattggcgataatcgcatgggagcaaggggaaccaaatcagcta

fava_c15956_1562	fava_c15956	1562	G/A	gtgcgggtagcatggtactaccaacaatttctccaattgaaccactctcaagctttaacatattttcaacRtacatgacaatctcatgcactgtcacatc[G/A]taacgttttggtatattctggacaactattggcgataatcgcatgggagcaaggggaaccaaatcagctatttgtttcagagcatcatgcacccgagaca

fava_c15956_1716	fava_c15956	1716	T/C	gggaaccaaatcagctatttgtttcagagcatcatgcacccgagacagaaccttattctttctgtcaactccattttcatttttcagaaaatccatgaca[T/C]gtttaggagcagtaaaatgYctcacaagcctctccaaacaccaatcaatatacattcccttcgaagcagccaatgatacgagaagctcaatcaaagcatc

fava_c15956_1736	fava_c15956	1736	T/C	gtttcagagcatcatgcacccgagacagaaccttattctttctgtcaactccattttcatttttcagaaaatccatgacaYgtttaggagcagtaaaatg[T/C]ctcacaagcctctccaaacaccaatcaatatacattcccttcgaagcagccaatgatacgagaagctcaatcaaagcatccatgatatcagtcgcatcat

fava_c15956_2187	fava_c15956	2187	C/A	actgatttcagcatcctcgtccattgtacgtaattcttcgtttgaatttggaagcaactgtcccatgctcccaaaaacaaagaaaacgcagagatggaaa[C/A]ctaaccgaatatgaRgStgaggctagggttttagatcggatctaccggtYcaaaagcggacgagctacagaaccggtggcggttttcgtaaaccggctca

fava_c15956_2202	fava_c15956	2202	G/A	ctcgtccattgtacgtaattcttcgtttgaatttggaagcaactgtcccatgctcccaaaaacaaagaaaacgcagagatggaaaMctaaccgaatatga[G/A]gStgaggctagggttttagatcggatctaccggtYcaaaagcggacgagctacagaaccggtggcggttttcgtaaaccggctcagctttgttatgtgaa

fava_c15956_2204	fava_c15956	2204	C/G	cgtccattgtacgtaattcttcgtttgaatttggaagcaactgtcccatgctcccaaaaacaaagaaaacgcagagatggaaaMctaaccgaatatgaRg[C/G]tgaggctagggttttagatcggatctaccggtYcaaaagcggacgagctacagaaccggtggcggttttcgtaaaccggctcagctttgttatgtgaaaa

fava_c15956_2237	fava_c15956	2237	T/C	gaagcaactgtcccatgctcccaaaaacaaagaaaacgcagagatggaaaMctaaccgaatatgaRgStgaggctagggttttagatcggatctaccggt[T/C]caaaagcggacgagctacagaaccggtggcggttttcgtaaaccggctcagctttgttatgtgaaaagaaaaaggaattagagaaggatggagcagcagg

fava_c15956_253	fava_c15956	253	C/T	atgaatgcttagtttcagtttctacgacaccattgtgggagtgggctggcctaaaactttgcaaaattgactggctttgacgacaagcctaaaacaaaga[C/T]gttggactagtggatagtctaactttcgacagcattctcgggaccYcgctccatcYgttccttaaacccatatttgaacgatttgggtgtgatagacatt

fava_c15956_299	fava_c15956	299	T/C	tggcctaaaactttgcaaaattgactggctttgacgacaagcctaaaacaaagaYgttggactagtggatagtctaactttcgacagcattctcgggacc[T/C]cgctccatcYgttccttaaacccatatttgaacgatttgggtgtgatagacattttgttcaagtcggggtcaaaatcaagacctgctccaacactgccta

fava_c15956_309	fava_c15956	309	C/T	ctttgcaaaattgactggctttgacgacaagcctaaaacaaagaYgttggactagtggatagtctaactttcgacagcattctcgggaccYcgctccatc[C/T]gttccttaaacccatatttgaacgatttgggtgtgatagacattttgttcaagtcggggtcaaaatcaagacctgctccaacactgcctatcatatcatc

fava_c15956_452	fava_c15956	452	A/G	ttttgttcaagtcggggtcaaaatcaagacctgctccaacactgcctatcatatcatcatcaaccatcatatcctttgcatttaagtcaacaaattcgtc[A/G]tctgatgcttcacttccagaatcactgtcttcctcRtcgtcatatgtagttctgactcttgaccagcggacaaagtgtggtcttaggtagccttcacttt

fava_c15956_488	fava_c15956	488	G/A	caacactgcctatcatatcatcatcaaccatcatatcctttgcatttaagtcaacaaattcgtcRtctgatgcttcacttccagaatcactgtcttcctc[G/A]tcgtcatatgtagttctgactcttgaccagcggacaaagtgtggtcttaggtagccttcacttttcttcaacaaacatggatcaaaYgggaagaacatgt

fava_c15956_575	fava_c15956	575	T/C	cactgtcttcctcRtcgtcatatgtagttctgactcttgaccagcggacaaagtgtggtcttaggtagccttcacttttcttcaacaaacatggatcaaa[T/C]gggaagaacatgtcgagtctatccatcccaccaaaagccttggaaagatcagcctcgagcaaatcttcaaacacaaataactctgctgacatgaaaagcc

fava_c15960_1315	fava_c15960	1315	C/T	cttcctctaacttctctttaagaaacaaaatctcagagtctttaagccgtgtttgccattcaaacttcttccccattatcctataggtttttataacact[C/T]ttaagctctttcgattcagctgcaacaatcgccccctctggcgaaggatcaaaatgaaattgctttttgaaccaacattgcttgagttcagataaggttt

fava_c15960_1424	fava_c15960	1424	G/A	tttcgattcagctgcaacaatcgccccctctggcgaaggatcaaaatgaaattgctttttgaaccaacattgcttgagttcagataaggttttcaattct[G/A]agactagtactttatcagcgtcttgaattccatcagggtcataaggtgattgagcatattgtacctgagcatatgaagcttttatagttgaaatgctagc

fava_c15960_1551	fava_c15960	1551	C/T	attccatcagggtcataaggtgattgagcatattgtacctgagcatatgaagcttttatagttgaaatgctagcaaaaactctcgaaaccaaagcttcta[C/T]ggcYaattttttcttaagctcttcatcttcttcacYStgtgtcatgtttccttcatcaactttaacattctgcagcccctcaacttgtgcaattccactc

fava_c15960_1555	fava_c15960	1555	C/T	catcagggtcataaggtgattgagcatattgtacctgagcatatgaagcttttatagttgaaatgctagcaaaaactctcgaaaccaaagcttctaYggc[C/T]aattttttcttaagctcttcatcttcttcacYStgtgtcatgtttccttcatcaactttaacattctgcagcccctcaacttgtgcaattccactcgttg

fava_c15960_1587	fava_c15960	1587	T/C	acctgagcatatgaagcttttatagttgaaatgctagcaaaaactctcgaaaccaaagcttctaYggcYaattttttcttaagctcttcatcttcttcac[T/C]Stgtgtcatgtttccttcatcaactttaacattctgcagcccctcaacttgtgcaattccactcgttgattttgcacgaagaacctttgtaagagttcga

fava_c15960_1588	fava_c15960	1588	G/C	cctgagcatatgaagcttttatagttgaaatgctagcaaaaactctcgaaaccaaagcttctaYggcYaattttttcttaagctcttcatcttcttcacY[G/C]tgtgtcatgtttccttcatcaactttaacattctgcagcccctcaacttgtgcaattccactcgttgattttgcacgaagaacctttgtaagagttcgag

fava_c15960_274	fava_c15960	274	T/C	ctacctactagtttcaacattattttgtacataatgaaagcaagatcatgtaaaagataccattccctgccactagcctaatatcaccaaaactttaggt[T/C]atatcctttcattaaacttacatttaaataaaaaacattctgaaaagaaactggacatccttaccacaccagtccccacatcacagttgctgctgctgct

fava_c15960_538	fava_c15960	538	A/G	taaaacctggaactactgtgaaaccaacttgtggctcagattccactgctattgctacttcatcatcattcacattcacattcacaacactctccatgta[A/G]acatcagagaatctacatcctttccctacttgaaagatttcagcttcagcctcaaaagaaaaggctaagcaatgcaaaagccataccctcttggccattt

fava_c15960_660	fava_c15960	660	C/G	ttccctacttgaaagatttcagcttcagcctcaaaagaaaaggctaagcaatgcaaaagccataccctcttggccatttcagcaaatgatgtgaacaagt[C/G]gttatcatcgggaaattcYccaccgttcaccatgttgattctcagcttcgtgtcaccgaaaaacgatgcctccatttgagggtggactagtctcaagtat

fava_c15960_679	fava_c15960	679	C/T	cagcttcagcctcaaaagaaaaggctaagcaatgcaaaagccataccctcttggccatttcagcaaatgatgtgaacaagtSgttatcatcgggaaattc[C/T]ccaccgttcaccatgttgattctcagcttcgtgtcaccgaaaaacgatgcctccatttgagggtggactagtctcaagtatttcgtccggcagaactttg

fava_c15960_877	fava_c15960	877	G/C	ttgcgaaagctgattttggtttcgaaccaagatactcttttgctttcgaggattttaactcgttgaaattcaaaaagaagaattgtgttttgtttctatc[G/C]ggaagaaaatcggtaggacgaagattgaaattagggaagttgaaagaatcgaacatctccctgcaaacaaaagactcgattgcaaaacacttgtgatctt

fava_c15962_1200	fava_c15962	1200	T/C	ttcctcccgaggaatcttcgcctgaaatggagatggttagccgaggcctagcgaaacacttgaaactagaacaaagagaacaccttatgcttctctttgg[T/C]aaagtttgcaatgaagacagtcacgggatYgcaagagaagcccttggtttgatgcattctcaaaattacagcgctgttcaacttgaagacaRccttgaag

fava_c15962_1230	fava_c15962	1230	C/T	agatggttagccgaggcctagcgaaacacttgaaactagaacaaagagaacaccttatgcttctctttggYaaagtttgcaatgaagacagtcacgggat[C/T]gcaagagaagcccttggtttgatgcattctcaaaattacagcgctgttcaacttgaagacaRccttgaagctgaaagataatatctgcctgcctgcagac

fava_c15962_1292	fava_c15962	1292	A/G	ctctttggYaaagtttgcaatgaagacagtcacgggatYgcaagagaagcccttggtttgatgcattctcaaaattacagcgctgttcaacttgaagaca[A/G]ccttgaagctgaaagataatatctgcctgcctgcagactgtagaatgcattcttttagaatatttatgtttaatcctttagtttttttatctgcttatga

fava_c15962_1409	fava_c15962	1409	T/C	taatatctgcctgcctgcagactgtagaatgcattcttttagaatatttatgtttaatcctttagtttttttatctgcttatgaaataatatttagatgg[T/C]ttattttagtgatgtataataataataatgataacagtaataataatattcaccataatcatcattaaaaaggtgtcttttac

fava_c15962_867	fava_c15962	867	T/G	tatgccgaaattctggcgtgcaagtgtcggttgaaccagcgaatattcgggactccttgtatcgaacatctgttaattttgttttagacgcttgcagcag[T/G]gcaccaacttactctacttcagttagtatttatggtgaggattctcaacaattccttgctggatttgcggaaaacatcggccttgaaaatgttcgcgctg

fava_c15963_1379	fava_c15963	1379	A/G	ggatgatcttcgacctggtgccatataccgaggtgtgagttgagggctggtgcatggtatgatgatcaatcttcaactatttgcattataaatgtcctag[A/G]gagttggagggaactattttgatctttgatgatatatattgtgcagcactaacctgaagctgtatgtgtttagtttgacaaagctgattataataatata

fava_c15963_142	fava_c15963	142	G/C	tcacgctgacactaaccccaaaactcagcttcaacattttttcttcgctacttcaatttttcacctagctaaatcattcactacaaattcaacaattctt[G/C]aattcattccaaaacaattgcaatttccaagcaatgtatttatcatccgaaaagcctcgtccaatcgatttctacaaagaagaatcccgcgacatgatga

fava_c15963_1503	fava_c15963	1503	C/T	ctttgatgatatatattgtgcagcactaacctgaagctgtatgtgtttagtttgacaaagctgattataataatatattactacaataggagagaatgaa[C/T]gagtctctatgccctctgtaaatatggattgacagtgagaaaaaaatacagctgttttgcttctacttctatgtagttctctattgattttgcggcctaa

fava_c15963_274	fava_c15963	274	G/A	caatgtatttatcatccgaaaagcctcgtccaatcgatttctacaaagaagaatcccgcgacatgatgatggaagtagtaaccgccaccgatctccaagc[G/A]ccgcagctccacctccaccaacaacaaccgcaaatgattctcgccgcagagagcagcggcgaagacccggagatggagatcaaagcccccaagaaacggg

fava_c15963_823	fava_c15963	823	T/A	aaggtattgatgatgccaacatttcttttgggcctgttgaagcttctgggagaccaacactcaatcttgagagaagtttggatcatgatggacaccctct[T/A]gccattgctgcagctgatgttgcagcaagtggagtccctccttggaattggagagaaactcccggaaatggtggggaaagtcagtcttgttgtggaaggg

fava_c15965_1351	fava_c15965	1351	A/G	tccagaaagacctggtgaacctgagtgtagtttcttcttaaaaactggggactgcaaatttaagttccattgcaaatttcaccatccaaagaatcggatt[A/G]caagatcacctccttgcaacctaagtgacaagggtctgcctttgagacctggccagaatgcatgcacgcattacagccgatatggaatttgcaagtttgg

fava_c15965_1770	fava_c15965	1770	G/A	ccatgatagatttatttaatggcactggttattatgcctaatgcggcacaagctttttgcagaatggcaataggacatatacatcccagatagaagtttt[G/A]ttatcttttgtttttggtttatttttttatctcttggattagtatttgttagaatttagaatattgctccatgattgtcttggaattttgattagatttt

fava_c15965_427	fava_c15965	427	G/A	gttggatttgaaagatgatgacgaaattcatgagaaaggttcaaactttgaagttgttgctgtagaatctgaaaagggtcaagagtgtcataatgatgat[G/A]gttggaatggtgacgatgatgggtggaatggggatgatgaggtgaatgactgggtKgagaatgtgaatgagaatgttgttgttagtggagaagttgatca

fava_c15965_483	fava_c15965	483	G/T	ttgctgtagaatctgaaaagggtcaagagtgtcataatgatgatRgttggaatggtgacgatgatgggtggaatggggatgatgaggtgaatgactgggt[G/T]gagaatgtgaatgagaatgttgttgttagtggagaagttgatcaagtggagaacaaggatgaaagaagcaatggtaatggtagagctgaactgtacccac

fava_c15965_674	fava_c15965	674	G/A	ctgtacccactgaggcctgaagctgaagactgttccttttatctcaagactgggacttgcaaatttggatccaattgcaagtttaatcaccctcttagga[G/A]aaaaaactcggcttttaaagagagagcSggagaaagagatgaattggagaaaagagcaggccagacagaatgcaagtattattcaaggtcagggggttgt

fava_c15965_702	fava_c15965	702	G/C	actgttccttttatctcaagactgggacttgcaaatttggatccaattgcaagtttaatcaccctcttaggaRaaaaaactcggcttttaaagagagagc[G/C]ggagaaagagatgaattggagaaaagagcaggccagacagaatgcaagtattattcaaggtcagggggttgtatgtttggaaaggattgtaaatttgatc

fava_c15968_1710	fava_c15968	1710	C/G	catcaaggtttatggctaaaattagtcatattaaagctatttattaatggctatcctgaaatcctgtagccttgaaggccttgttttgaatttagagact[C/G]ccgttacaaccttcaatggaatggctgccattatacataKatatgtatattatgtacaaattaaaaaacttccatcttttgtacgagttagaatttatga

fava_c15968_1750	fava_c15968	1750	T/G	ttattaatggctatcctgaaatcctgtagccttgaaggccttgttttgaatttagagactSccgttacaaccttcaatggaatggctgccattatacata[T/G]atatgtatattatgtacaaattaaaaaacttccatcttttgtacgagttagaatttatgaactttcacaaatttttagtaagtttcttttacttgaggt

fava_c15968_360	fava_c15968	360	C/T	aagctgttagggctgaagagtgggtgttgattgatgacgctattcttcactacaggaatgcgcaaaggatccttctcgaagctaattcaactcctgttcc[C/T]tcgtttatcacttccagtgagaaacagaaggtacaatcatatcgtcaaaagatatcaaaatggcagggacaagtttctgagagattgcaggctcttaata

fava_c15968_534	fava_c15968	534	T/G	tttctgagagattgcaggctcttaatagacgggcaggtagctcagtttccaatcagagcacctcaaaccatgcacaaactgctgcaattccctctaaact[T/G]tcaaatactaccaaaaaagtgttacaaaaggatcctcaaagaaaagatgaagtgaaYaaagttcaaagcccaaaatctagtcaaacatctggagcaaatt

fava_c15968_591	fava_c15968	591	T/C	gcacctcaaaccatgcacaaactgctgcaattccctctaaactKtcaaatactaccaaaaaagtgttacaaaaggatcctcaaagaaaagatgaagtgaa[T/C]aaagttcaaagcccaaaatctagtcaaacatctggagcaaattatgacccaaaactgKttgaaatgattaacactgcaattgtagaccgaagtccttctg

fava_c15968_649	fava_c15968	649	T/G	aaaagtgttacaaaaggatcctcaaagaaaagatgaagtgaaYaaagttcaaagcccaaaatctagtcaaacatctggagcaaattatgacccaaaactg[T/G]ttgaaatgattaacactgcaattgtagaccgaagtccttctgttcgatgggatgatgttggtggccttgaaaaggcaaagcaagctttaatggagatggt

fava_c15968_765	fava_c15968	765	G/A	ctgcaattgtagaccgaagtccttctgttcgatgggatgatgttggtggccttgaaaaggcaaagcaagctttaatggagatggtgattttaccaactaa[G/A]agaagagacttgttcacaggtcttcgaaggccRgctagaggtttgcttctctttggtccgcccggtaatggtaagactatgcttgccaaagcagtggctt

fava_c15968_798	fava_c15968	798	A/G	gggatgatgttggtggccttgaaaaggcaaagcaagctttaatggagatggtgattttaccaactaaRagaagagacttgttcacaggtcttcgaaggcc[A/G]gctagaggtttgcttctctttggtccgcccggtaatggtaagactatgcttgccaaagcagtggcttcggaatcagaagcaactttttttaatgtcactg

fava_c15971_323	fava_c15971	323	T/C	tgaagagtgttcgtgctccgtcaatctcttcgccgccgtccacctcttccggcaccggtcccgtcattgaattggtcagtacgtcgcttctcaatccgaa[T/C]cgatcttatgctccgcttagtactgaggatcccggtagttcgagtaagggtctaaatccaattactgttgggctacctccggcatgggtggatgtatctg

fava_c15971_725	fava_c15971	725	C/T	attccaatgtcaggaaaaatgtgcagcgttctcttgctactgaccttcaaagcctctctgtagagcttcgcaagaaacaatcaacttatttgaagcgcct[C/T]agacagcaaaaagaggggcaagatggagttgacctagagatcaacataaatggaagtaaatctagatttgaagacgatgacttggacaatatgatattta

fava_c15971_905	fava_c15971	905	A/G	acttggacaatatgatatttaatgagcatcaaatggccaagctaaaaaagagtgaagctttcacagttgaaagagaaaaagagatccagcaggttgtgga[A/G]tctgttaatgagcttgctcagattatgaaggatttatcagtactagtcatagaccagggaaccattgttgatagaatagactacaaYattcagaacgtgg

fava_c15971_992	fava_c15971	992	C/T	agcaggttgtggaRtctgttaatgagcttgctcagattatgaaggatttatcagtactagtcatagaccagggaaccattgttgatagaatagactacaa[C/T]attcagaacgtggcaaccacagttgaggatggccttaaacaactgcagaaggcagagagaactcagaaaaaggggggcatggtaatgtgtgcgacagtcc

fava_c15974_1314	fava_c15974	1314	T/C	aactagcaggcggatacaactcacagcgcgagagctccaatcgagtcaatttctcacaaccaaaaaaacaggaaggcatcctaaagaaatctccttcgcc[T/C]aattcRagattgaaatccttaatatcatttctggaaaggaaaagaatccactgatttatggcaggacagctcgccaagcttgcattgacaatgtgaaact

fava_c15974_1320	fava_c15974	1320	G/A	caggcggatacaactcacagcgcgagagctccaatcgagtcaatttctcacaaccaaaaaaacaggaaggcatcctaaagaaatctccttcgccYaattc[G/A]agattgaaatccttaatatcatttctggaaaggaaaagaatccactgatttatggcaggacagctcgccaagcttgcattgacaatgtgaaacttatgaa

fava_c15974_396	fava_c15974	396	T/A	tcacaacagaaaatgcagtgaactgtggtatgcatggtaactatcagcatactccacaaaatcccaaggacaagcaaaagacctgcatttgacaacatag[T/A]ccaaagttttaatccccgcggatgaaataWacttctgctttactagaagctcttcgaaacttcatcaactcaaccaacattttcaattgacattccatat

fava_c15974_426	fava_c15974	426	T/A	tgcatggtaactatcagcatactccacaaaatcccaaggacaagcaaaagacctgcatttgacaacatagWccaaagttttaatccccgcggatgaaata[T/A]acttctgctttactagaagctcttcgaaacttcatcaactcaaccaacattttcaattgacattccatatcatacatagaaggaatgatactcatcgtct

fava_c15977_263	fava_c15977	263	C/G	acccaagtttgcatggatccatgataactgaaaatcaataaatttaacaagaagcaggaatagattaattccacagcaacaaatactggttgacaataca[C/G]gaaaaaagtcttcacttgtggggaagtttaacgactttttgaagaaccaccgtccttgcccttataatgtcacggctacatgtatcagcttgagctccta

fava_c15977_445	fava_c15977	445	C/T	gtatcagcttgagctcctaaatcttcgacattcttcataaaaactcccaacttcttcttgatttcctctattgcaaccttcaccccttcttcttcctcga[C/T]ggcaaaatccacattcgtttttaaggattcgatctcaacttcaagtcggttaactagaatccgtatattgtccaaatcctttatagctacataggatcca

fava_c15977_716	fava_c15977	716	C/T	tccactttcccattgaaccgactgggatagcagtaagagcagctagagcagccgcaacgtgcggcgaagcaacggctgccgctacaacggagcagatcaa[C/T]acagcagccaccgtggcaacaaatatgataagcgagaccttcttccaagtacgaatttgtttcagcttcttatcaagcttgccttttctgacttgcaact

fava_c15979_2282	fava_c15979	2282	C/T	tgattcctgataatggtccttggaattataacttcatgggagtgaggcacgcatcagggatgaagtatggagtgaagcttgggactcctagggagtatta[C/T]cacgaagatcacagaccaacccatttcttagagttcagcaatatggaggaaggggagacaattgcagagggtgatagagaggataccttctcttagatat

fava_c15979_2409	fava_c15979	2409	T/A	cttagagttcagcaatatggaggaaggggagacaattgcagagggtgatagagaggataccttctcttagatatataaatgttggtttagtttatcttac[T/A]gaSacacacaagtggcagatcctttaatttctgaaacttgYcgtaagatgttgtattgtctgttgtttgtacgtcctaatgattttgtatgaatatcatc

fava_c15979_2412	fava_c15979	2412	C/G	agagttcagcaatatggaggaaggggagacaattgcagagggtgatagagaggataccttctcttagatatataaatgttggtttagtttatcttacWga[C/G]acacacaagtggcagatcctttaatttctgaaacttgYcgtaagatgttgtattgtctgttgtttgtacgtcctaatgattttgtatgaatatcatcagt

fava_c15979_2450	fava_c15979	2450	T/C	agggtgatagagaggataccttctcttagatatataaatgttggtttagtttatcttacWgaSacacacaagtggcagatcctttaatttctgaaacttg[T/C]cgtaagatgttgtattgtctgttgtttgtacgtcctaatgattttgtatgaatatcatcagttttgtatataaacactccgaatccgttgcctgtttctg

fava_c15980_1054	fava_c15980	1054	C/G	aaatttcatccccttgctaacattctgtgagcaaagtcccagctgctccattgaaggaatacttccccaatcctcaacaggggcaggtggtccaagtgat[C/G]ctggcagtttgatggatgagcgcacggcacatttggcttcgacagtcttacgaaattgagtataaacatccggcaaacaagtgacatcaaacggaagatc

fava_c15980_301	fava_c15980	301	T/A	cccagttgttccaaacttgagaggcacaacttgacgaatgtataaactaccaggaaagttccttttttctcttgggattgttagtaactgtggcaaccag[T/A]atgcaacatattcgccttctgggtcatatgtttgtgcttgtttcggaaYgctgaaataacgatcttcccgaggatcgttcccaactcctgatccgtaagt

fava_c15980_350	fava_c15980	350	C/T	ccaggaaagttccttttttctcttgggattgttagtaactgtggcaaccagWatgcaacatattcgccttctgggtcatatgtttgtgcttgtttcggaa[C/T]gctgaaataacgatcttcccgaggatcgttcccaactcctgatccgtaagtccagttcccataattagaacaYggatcataatccaaaagacatgtttca

fava_c15980_423	fava_c15980	423	T/C	ggtcatatgtttgtgcttgtttcggaaYgctgaaataacgatcttcccgaggatcgttcccaactcctgatccgtaagtccagttcccataattagaaca[T/C]ggatcataatccaaaagacatgtttcaaaccattcggcacccatgcgccaatctatgcccatgtcacgaactaggaaggaacacacaatctgccgtccac

fava_c15981_1272	fava_c15981	1272	A/G	gtgttattctatattagtagtgacggtgttgctccactatactatttgctctgtgattcaaaccaaacttgctttgttctctcagtttatggtgtacgcg[A/G]agacgtgattaaaggaggagagcagctcacattgcttgatccttatttttgcgacgttgatgttacctggaaggagaagcattaccagttcaaatctata

fava_c15981_206	fava_c15981	206	A/G	agaaaaaaaaaatccaagatcgatcaaggagtgtttgagtttatttcagtttcttgggtttaaatcacaagaactgagctaaagctcgtagagaaagatg[A/G]acaattcaagcggcgaagaaaaagaagcaacagcattgtcaagagctgcaaaatccactgacgaactttaccttctccgagacacctatttccctcaaaa

fava_c15982_1150	fava_c15982	1150	T/C	cagagtctagcaagatgtttgaagctttgaaatctctataaataacaggttgctcggcttcatgaagaaatgctaatccttttgcagctccaacagcaat[T/C]ttcattcttgtagaccatggtaatgatgctgaaaatcttctaaataactgattttccaagcttcctcttggtaaatattcatacaccaaaagtctatgct

fava_c15982_448	fava_c15982	448	C/T	gagatggagaactcaacacacttcctctaccattttgatgcctgtcaatccgcacacgaggccgtggctgtgattgcggccgtgattgtgattgaggcat[C/T]ggcgacctaggagatttaagtggatgatgcctatgaccattcctatgatattgactgttttctcttttcctctccttYgtggattcatcttcctttgctt

fava_c15982_526	fava_c15982	526	C/T	gccgtgattgtgattgaggcatYggcgacctaggagatttaagtggatgatgcctatgaccattcctatgatattgactgttttctcttttcctctcctt[C/T]gtggattcatcttcctttgcttgatctttttctttaaccacctcattattctcaactggaacagtgtaaacaaagggtccaattggaatatcatcaaaat

fava_c15982_673	fava_c15982	673	G/A	tattctcaactggaacagtgtaaacaaagggtccaattggaatatcatcaaaatcttgtagaggctccaatatcttaaccacattgctcattgtaggcct[G/A]ctcttcggcctatggctaagacattgataagccaatgaagctgcttttttagtacccatttcagagtactgtccttcaagttttggatccattattttgc

fava_c15982_842	fava_c15982	842	G/T	ctgtccttcaagttttggatccattattttgctaagttttcttgaatcaattaacattggtcttgcccaatcaactaggttttgttcccttgtaggacgg[G/T]ttttgtccactgactttctccctgttagcaattccaataaaacaactccaaagctatacacatcactcttagctgtcaaatgacctgtcatgacatattc

fava_c15982_982	fava_c15982	982	G/A	aaacaactccaaagctatacacatcactcttagctgtcaaatgacctgtcatgacatattctggggcagcatagccttgagtgcccataactcttgttga[G/A]acgtgggtgtcatctccttcaggtccatcttttgccaacccaaagtcagagagttttgcattataatcagagtctagcaagatgtttgaagctttgaaat

fava_c15985_1266	fava_c15985	1266	C/T	gcagaagctttttcttcactttcctttaagttgttgagaatcaacttctcttcttctagttctgattgaagcaatgtcgaagcttctttcaagctgttga[C/T]ctcagctgttgctttttctatgttcatcttcacttcctcgagttccttctttcttaaaacatcggtttcatcttctagttttgattccatgtaYgctgct

fava_c15985_1360	fava_c15985	1360	C/T	tgttgaYctcagctgttgctttttctatgttcatcttcacttcctcgagttccttctttcttaaaacatcggtttcatcttctagttttgattccatgta[C/T]gctgctaactcggctttcaattcgagcaacaaagaggaagaagcttccaattttgatttgagaactcttgctgataaaacttgttggtttagtgtctcta

fava_c15985_1758	fava_c15985	1758	G/C	ttgatagctacatcccttccattcaccatagaatcatactctctccttaaagagtccaattcttttttcaaaaactccaattccgaagccgcggaacttc[G/C]cattgattgttcaacctcaagctgtgccttggcttcaaaactggcttcatctgMaatgtcttgctccagctcctcgactttgcgaatcacaagttcggct

fava_c15985_1812	fava_c15985	1812	C/A	tccaattcttttttcaaaaactccaattccgaagccgcggaacttcScattgattgttcaacctcaagctgtgccttggcttcaaaactggcttcatctg[C/A]aatgtcttgctccagctcctcgactttgcgaatcacaagttcggcttcttctttaacctgaacctcatctctttctacactttctaaattgagtctgagt

fava_c15985_2116	fava_c15985	2116	A/G	ttttccaatccactatccccccgaatttggagacggcatctttgacagattcaaaaggtgcggctgtgtcaatgacatgcttaagatctttagcggaatc[A/G]gaaactggtgaagattctccactagactgactaggggattctgaaaagttctccatagatgattattcgtttccttcacaaagtggcacttcttaaaaca

fava_c15985_889	fava_c15985	889	A/G	gtgtcacaagagatgaagtatcaacttcttttttgttgcttcctcctctagttgattcacttctctccaatgctttgattgaatctttagccaatttttc[A/G]gaaactttcgaagctcctatctccatttttgtagcatgtaatctgttttgcaatgttgctaaacttgctttggctttttcagcttcttcttgtgcttgaa

fava_c15987_107	fava_c15987	107	A/G	accaaaatcaccgaaacaaggaagcaggaagcacaagacaaatcaagcgagttcaaagtctctaactcttaaaccctttatcacagtttaaacctctata[A/G]tttagtttagcgagttgaacacaaaagagacaacacatagatgcagggcgttgtaagaaaactaaccaagacattttcacacagacaggttctaggtttt

fava_c15987_1727	fava_c15987	1727	T/C	gtgaatatgtgcaattacattgtcaaaactgtaggtaaattatttccatcccttattttttgccccctccatattcacttttagctcagcaaaatcttgg[T/C]ggggagggtgacaagagaattaagagagaaggtgaggtagggttttcttacttattttgtggactgatagactagtaggtttctacgactgattgttgca

fava_c15988_1670	fava_c15988	1670	C/T	gagcagtttgttctgcagagatatgaaaatgagttgaggtttgtctgtgaagagcttattccataaaccttgcctggaacttcacatataatgtaatgtg[C/T]ttcaaattgtatttatctgaacagttccttttttgttaattattatttcttggatattcttcaatatgtgagtgccaaaatgacatgggcataaaataac

fava_c15988_546	fava_c15988	546	C/T	gccaacctcatgtacctgatcaattactgcaaagaatggaagaggttggatatgttatgcctactgaggtccagaagcaagcattgccgcatcttttttc[C/T]ggccgtgactgcattcttcatgctcagacaggttctgggaagacactagcatacttgcttttgatatattccatcatcaacactaaaaaatcttccatgc

fava_c15991_1426	fava_c15991	1426	T/C	atcattttcttgaggatgagaagtgaatgttgttgagtggatttccgaagacacatcgtcacctaacgtctgagaagtgaagggagaatcttgatattgc[T/C]tatcatgctgtgtatcagaagaaaaggggaYgccgtcaggacattcagcagcgggcgaagccgaaaacgcctgtctatcacttctgatcggatttgatgg

fava_c15991_1457	fava_c15991	1457	C/T	gttgagtggatttccgaagacacatcgtcacctaacgtctgagaagtgaagggagaatcttgatattgcYtatcatgctgtgtatcagaagaaaagggga[C/T]gccgtcaggacattcagcagcgggcgaagccgaaaacgcctgtctatcacttctgatcggatttgatggYgtagcttgcagcaatgcaggatttgcagtc

fava_c15991_1527	fava_c15991	1527	T/C	tatcatgctgtgtatcagaagaaaaggggaYgccgtcaggacattcagcagcgggcgaagccgaaaacgcctgtctatcacttctgatcggatttgatgg[T/C]gtagcttgcagcaatgcaggatttgcagtcaaaccaccaacaRgcgaacgatgaaaagaatccgagggtttcatgtacgcgctttccataagagtgggaY

fava_c15991_1570	fava_c15991	1570	A/G	ttcagcagcgggcgaagccgaaaacgcctgtctatcacttctgatcggatttgatggYgtagcttgcagcaatgcaggatttgcagtcaaaccaccaaca[A/G]gcgaacgatgaaaagaatccgagggtttcatgtacgcgctttccataagagtgggaaaaacaggtgaagatgaagaatgcatagctcttgaggcttcaag

fava_c15991_354	fava_c15991	354	G/A	caaatctagggagtgaaattaatttcatcccaaaaaatgaacttagtcattgttagctactgttaaagttgagtatatcattatgataagcagcagaatc[G/A]gaaattcgagcataaWattctagaagctatcattgaatcttcgcccgttttgttgggggtacggcagaaaactgatcatctgtattctcatgttcatcgg

fava_c15991_370	fava_c15991	370	T/A	aattaatttcatcccaaaaaatgaacttagtcattgttagctactgttaaagttgagtatatcattatgataagcagcagaatcRgaaattcgagcataa[T/A]attctagaagctatcattgaatcttcgcccgttttgttgggggtacggcagaaaactgatcatctgtattctcatgttcatcggtaacttSggcttcatg

fava_c15991_461	fava_c15991	461	G/C	cgagcataaWattctagaagctatcattgaatcttcgcccgttttgttgggggtacggcagaaaactgatcatctgtattctcatgttcatcggtaactt[G/C]ggcttcatgtgttgcatcctcRgaactttctttggtgttgcattttaattttccagacgttttcaatttgtctacaggagaggttaccccagtgtccaag

fava_c15991_483	fava_c15991	483	A/G	atcattgaatcttcgcccgttttgttgggggtacggcagaaaactgatcatctgtattctcatgttcatcggtaacttSggcttcatgtgttgcatcctc[A/G]gaactttctttggtgttgcattttaattttccagacgttttcaatttgtctacaggagaggttaccccagtgtccaagagtgcagcagaaggcgcgttag

fava_c15991_621	fava_c15991	621	T/C	ttttcaatttgtctacaggagaggttaccccagtgtccaagagtgcagcagaaggcgcgttagaaggggacgagcccttgctgccacctatctgtttctg[T/C]ttctcaaacatcatctgaagatgtttcccttggttttcaatttgaatctgcaactttctttgaatctcaagctgttcgtgaagccgtttctggaggtcca

fava_c15991_897	fava_c15991	897	G/A	tctttttctcagaagtttcttctggcgactcaggtttgtatcgggcagttctatacttctgcaggtggctttttacatggtagatagtaaggccgtcaac[G/A]ttcatcagattcaagacacctttYggagtggccttgtcactaccaccaagctggctgactgcttccacaaaagcctcgtgaagttctggactccatcgca

fava_c15991_921	fava_c15991	921	C/T	gcgactcaggtttgtatcgggcagttctatacttctgcaggtggctttttacatggtagatagtaaggccgtcaacRttcatcagattcaagacaccttt[C/T]ggagtggccttgtcactaccaccaagctggctgactgcttccacaaaagcctcgtgaagttctggactccatcgcattctaggcttagtttgaggtgcag

fava_c15992_1116	fava_c15992	1116	T/C	ggatcccgaagaattttgtaatgagagtattttcataacttcctacatgatggtagtatttaggaagcatattgagcagaacctttagctcagacttttt[T/C]agtgtctttatcacaaatttatcatcatgagaaataaagaatatgctgccactttttcctggagaagttatatcctttagaccagagtcgccgcaaatag

fava_c15992_1575	fava_c15992	1575	A/T	ggttttgaaatttgcccataaatgatttcttttttacttgcttcacactaaacttattttgccttttcatcttattatgcggtatttctgaaaaatttcg[A/T]attctctccacaagtagaactccttgcatatattccctttcaaaaaccatattactcgtaccagatgcttcagattgaccttcattagaggcctgtgata

fava_c15992_1680	fava_c15992	1680	G/A	tctccacaagtagaactccttgcatatattccctttcaaaaaccatattactcgtaccagatgcttcagattgaccttcattagaggcctgtgataacgc[G/A]gaYgatgaatcacggcatatacaatcttcagaagaatttggatgttcccagttcgcatccaacaacgaagtccttagagataaatgacgagatattttct

fava_c15992_1683	fava_c15992	1683	C/T	ccacaagtagaactccttgcatatattccctttcaaaaaccatattactcgtaccagatgcttcagattgaccttcattagaggcctgtgataacgcRga[C/T]gatgaatcacggcatatacaatcttcagaagaatttggatgttcccagttcgcatccaacaacgaagtccttagagataaatgacgagatattttcttta

fava_c15992_1833	fava_c15992	1833	C/T	tcgcatccaacaacgaagtccttagagataaatgacgagatattttctttatacctctggctcgaggtttatcagagagactccggttaattcttggatt[C/T]ggaacttgaaaactgttatcWtcgctattaagaatgctacaccatttctttagagacggttgtttgctcccaacgggatagaatgttccttttccgtctt

fava_c15992_1854	fava_c15992	1854	A/T	ttagagataaatgacgagatattttctttatacctctggctcgaggtttatcagagagactccggttaattcttggattYggaacttgaaaactgttatc[A/T]tcgctattaagaatgctacaccatttctttagagacggttgtttgctcccaacgggatagaatgttccttttccgtcttttaggcccatactccatgttc

fava_c15992_291	fava_c15992	291	T/C	gtgtttcccggaaaactttcttatccatgaaattgataaaacgttcagcataaactttaggatctgttactgatatagttataggatcaaactttattga[T/C]ttgtagatatgctctattttctttttgatattatattcYtgcaatatatcaattattcccatgaagagtaccacatcataaatctcaaaaagctctactt

fava_c15992_330	fava_c15992	330	C/T	aacgttcagcataaactttaggatctgttactgatatagttataggatcaaactttattgaYttgtagatatgctctattttctttttgatattatattc[C/T]tgcaatatatcaattattcccatgaagagtaccacatcataaatctcaaaaagctctacttctgattcttccaccttatcatcttgaattaagcatgtag

fava_c15996_1315	fava_c15996	1315	T/C	cgagatcgatggagtagagactgttggatatccagaaggttagagccaaactcagtccgcatatcaagtttactgtaagggtttctgaaggtgagattgg[T/C]ggcggtgcttccaccgccgagggtttggacgccggcgttacctcgctgactttttcccggtggtccatgtggtgtaattagattgcgaatcgagtcgaag

fava_c15996_174	fava_c15996	174	A/T	tatagaacaatagacatatataactacaaaaataaaaaccgagaaaagggtatatttcaaacaaaataggttacaccaaaataatgttttgcctttatat[A/T]tatcctaaataaacaagtaaattaatatcacaagaRttgctcgtatcgtctttacatgatacatcccaaataaacaagttagatttttgataaaatggtt

fava_c15996_210	fava_c15996	210	G/A	aaccgagaaaagggtatatttcaaacaaaataggttacaccaaaataatgttttgcctttatatWtatcctaaataaacaagtaaattaatatcacaaga[G/A]ttgctcgtatcgtctttacatgatacatcccaaataaacaagttagatttttgataaaatggtttaggaccttcccctaactaatgctttcccctaaagt

fava_c15996_374	fava_c15996	374	T/G	ttaggaccttcccctaactaatgctttcccctaaagtggtcttcaagttgtacaaagtatcttattctcctcttgtatgttgtggttgcgcttaaactca[T/G]gacagatcaatgattatttttccttgtaaattaggtgccctatgaggatagccttttttgtactcgcttgaaataKttatactccacgaacttgaccctt

fava_c15996_450	fava_c15996	450	G/T	atgttgtggttgcgcttaaactcaKgacagatcaatgattatttttccttgtaaattaggtgccctatgaggatagccttttttgtactcgcttgaaata[G/T]ttatactccacgaacttgaccctttttagaatgaatagaattRcaagtagctgcattttaccatatgcaaatttcatatcatcacattaKcgaaatacta

fava_c15996_493	fava_c15996	493	A/G	tttccttgtaaattaggtgccctatgaggatagccttttttgtactcgcttgaaataKttatactccacgaacttgaccctttttagaatgaatagaatt[A/G]caagtagctgcattttaccatatgcaaatttcatatcatcacattaKcgaaatactattaagagtcttcccttatattttctctcttttctatctcttct

fava_c15996_540	fava_c15996	540	T/G	gcttgaaataKttatactccacgaacttgaccctttttagaatgaatagaattRcaagtagctgcattttaccatatgcaaatttcatatcatcacatta[T/G]cgaaatactattaagagtcttcccttatattttctctcttttctatctcttcttaatgatttcaacaaagtatcaaaRttggctgctgctagttaattaa

fava_c15996_618	fava_c15996	618	A/G	caaatttcatatcatcacattaKcgaaatactattaagagtcttcccttatattttctctcttttctatctcttcttaatgatttcaacaaagtatcaaa[A/G]ttggctgctgctagttaattaatttaccaagtaacagaatcagacatttgcacttcattcatttttgacatgttgcaacctactacgatgagcaagaaca

fava_c15996_762	fava_c15996	762	T/A	acatttgcacttcattcatttttgacatgttgcaacctactacgatgagcaagaacaaagcctaatgatgcaactgatgccactaggtaaccagccaatg[T/A]tccatagcttacagaaataggccattcctgccagggcctctcccagtcaagtggcattggccaggccccaaaccatcccccaataactgctccatgggct

fava_c15996_895	fava_c15996	895	C/T	agggcctctcccagtcaagtggcattggccaggccccaaaccatcccccaataactgctccatgggcttgtaaacaaatcaggtactcaatagatccatt[C/T]ggctttgtgtgcgcaaatatacgtttccagtcggcccatgatgaaccaagaacacatgatgctggaacagttgtgaacaatgacatcataagagaccaag

fava_c15997_1189	fava_c15997	1189	A/G	ccaagtaaagatttaaatcaagtttgtaccatgcatgcgaactgttgttatggtatggatagtaaggttcacgatctgagaattatgctccaggattgga[A/G]acattatttgactttgcctccaagtttaaagaggttgtcagttgtatcctggagggttcctcagaaatgcagccttgattccctcgagcccaatggttca

fava_c15997_1368	fava_c15997	1368	A/C	ttccctcgagcccaatggttcagctgagaagagtgctgaagaagattaaagttctcatttcacatgttattttctcatatgtcgattggatattcagctg[A/C]aatgcggaaaaggaaaggtttttattataatttttttcaggttagctttagtactgtaaacgataagaattggactataatgctgttaatgtatagtcag

fava_c15997_197	fava_c15997	197	A/G	tccttttccgaacatgttctcaccgtctccgtcactaaatgtccttctcacacccgccgcgaacgctcccctctcaccgccgacgatccctttcgtcaac[A/G]cacctcaacgccgccgctaactctcccccctccatgtcgccggaatccaccccgttccatctccggcatMtcctcgttgcagcgctgtttttcttcgttg

fava_c15997_267	fava_c15997	267	A/C	ctctcaccgccgacgatccctttcgtcaacRcacctcaacgccgccgctaactctcccccctccatgtcgccggaatccaccccgttccatctccggcat[A/C]tcctcgttgcagcgctgtttttcttcgttgtctctctctcctgcttactcctcttcagagacgccggttactcgcRcttcgttaactcttactccttccc

fava_c15997_343	fava_c15997	343	A/G	tccaccccgttccatctccggcatMtcctcgttgcagcgctgtttttcttcgttgtctctctctcctgcttactcctcttcagagacgccggttactcgc[A/G]cttcgttaactcttactccttccctcgttttctaaccgttttYccctcgccgtccaacaattccgttacggctaggaatgaatatccacttgaaaagatt

fava_c15997_386	fava_c15997	386	T/C	ttttcttcgttgtctctctctcctgcttactcctcttcagagacgccggttactcgcRcttcgttaactcttactccttccctcgttttctaaccgtttt[T/C]ccctcgccgtccaacaattccgttacggctaggaatgaatatccacttgaaaagattctgaagaaggctgccatggaagacagaactgttatactgacca

fava_c15997_596	fava_c15997	596	A/G	aagcatgggctgcaccaaattcagtcattgatctttttctggagagctttagaattggagttcatactcgtaggcttttgaatcatttgataattattgc[A/G]ttggaccagaaggcatttctacgctgtcaggctatacacacctattgctttttgcttgttagtgaaggtgatgactttcatgaagaggcatactttatga

fava_c16005_1112	fava_c16005	1112	C/A	gttctttcgcatcttgcgctgccgagaacaaagaatgataatctgcccgcacggaaggatctgtacaaggtaccggcgacaacattgcagcttctaatat[C/A]gcgaaaaaataatcactaacggMagacttaacacttcctgcctgaatcaactgaatggcaagctttgcagccttactgaatttggcagggttgttgatat

fava_c16005_1135	fava_c16005	1135	C/A	gagaacaaagaatgataatctgcccgcacggaaggatctgtacaaggtaccggcgacaacattgcagcttctaatatMgcgaaaaaataatcactaacgg[C/A]agacttaacacttcctgcctgaatcaactgaatggcaagctttgcagccttactgaatttggcagggttgttgatatgagaagatatcttcttcatagca

fava_c16005_1249	fava_c16005	1249	C/T	cctgcctgaatcaactgaatggcaagctttgcagccttactgaatttggcagggttgttgatatgagaagatatcttcttcatagcatcaataacttgcg[C/T]ttcggaagcatccgtagaggttttaaacttcaaactctttttaggtgctgcagcttgggtgttatcggattgagtagggttaggtcgtttgagggaactt

fava_c16005_259	fava_c16005	259	G/A	ccaaagagacacttattttgggacaacttacaacactagtgaagaaaacatttactcttcttgtcttgaccgtttttgaagtttcctttgatttaccatc[G/A]tcctctctttctcttacagaaaatgtcctctctttttcttacagaaaatgtcagaaagaaaacctaaccaagccattgtgtggcacgtcggtctccacta

fava_c16005_527	fava_c16005	527	A/C	ttacatcaagttttccattaactgactttccttgcctcctgcgagtctgttgctcccgtatggaagcccacaatttctctacagcatccctctgatgagc[A/C]gtaaatcttgccactttatcagaggcatgcttcactaaaatatcaattacggtttggcaccaaggtgttttataccgccgagcagcaatctctagacagg

fava_c16005_632	fava_c16005	632	G/C	atcttgccactttatcagaggcatgcttcactaaaatatcaattacggtttggcaccaaggtgttttataccgccgagcagcaatctctagacaggttat[G/C]agggcttttctttgtaacttaaggattcgtttagtttcttcctcctcttcctttcttatagcgtcattctttgcacttttctttgtggattcaggaatca

fava_c16005_805	fava_c16005	805	C/G	gcacttttctttgtggattcaggaatcagggcatcaagtccaaatgggtcagcttcctccacacctttattttcctcatttgtggcaagcgattgaacac[C/G]ttcatctactgtaactgtaatRtctttcaRagacattgcttcttctgtgtcatcctcctctgtcgcaacaggaaggttagatatagcttccttgatttgc

fava_c16005_827	fava_c16005	827	A/G	gaatcagggcatcaagtccaaatgggtcagcttcctccacacctttattttcctcatttgtggcaagcgattgaacacSttcatctactgtaactgtaat[A/G]tctttcaRagacattgcttcttctgtgtcatcctcctctgtcgcaacaggaaggttagatatagcttccttgatttgcatagctgctttcgagaacacaa

fava_c16005_835	fava_c16005	835	G/A	gcatcaagtccaaatgggtcagcttcctccacacctttattttcctcatttgtggcaagcgattgaacacSttcatctactgtaactgtaatRtctttca[G/A]agacattgcttcttctgtgtcatcctcctctgtcgcaacaggaaggttagatatagcttccttgatttgcatagctgctttcgagaacacaaagctatca

fava_c16010_1225	fava_c16010	1225	T/C	aacgagcaacgttgccacactgtttgcacttaggcttattctgaccgcgttgcgatttcggcggcattgaagacatcggcgatcccaaacctagggttcc[T/C]accttcgccgtcgaactgttactaggcgcYgtcgccataaccgcataaataacaccccttttcgaRttcaaagggtaataattataagagagatgattca

fava_c16010_1255	fava_c16010	1255	T/C	taggcttattctgaccgcgttgcgatttcggcggcattgaagacatcggcgatcccaaacctagggttccYaccttcgccgtcgaactgttactaggcgc[T/C]gtcgccataaccgcataaataacaccccttttcgaRttcaaagggtaataattataagagagatgattcaaaataagaggaaaatgataatcttcttcgt

fava_c16010_1291	fava_c16010	1291	G/A	ttgaagacatcggcgatcccaaacctagggttccYaccttcgccgtcgaactgttactaggcgcYgtcgccataaccgcataaataacaccccttttcga[G/A]ttcaaagggtaataattataagagagatgattcaaaataagaggaaaatgataatcttcttcgtYttttgaaattgtgagagtaatcttttggaagagag

fava_c16010_1356	fava_c16010	1356	T/C	gtcgccataaccgcataaataacaccccttttcgaRttcaaagggtaataattataagagagatgattcaaaataagaggaaaatgataatcttcttcgt[T/C]ttttgaaattgtgagagtaatcttttggaagagagtgaagtttgtttcggtaattttacgaactgttaacccccgtgcgtcgcgttatagctatagcagY

fava_c16010_589	fava_c16010	589	A/G	cagtcctctttcttttatttgaagattcattaacttctttgttttgttcattgctgtcaccatccacagcgcacttcttaagcttctttaatccctcgtc[A/G]acaattttttttattcttattctcacaccatcgcttcttgtcgaatttgaccttagctgcaattttaacccaggtatcataatctcactgttctccattg

fava_c16011_320	fava_c16011	320	C/T	ccttgtctgaacaatataaatgtcttttccaacaagacatccttccacatcctgtgggctgccaaactttctctccaggaaaaacccaactgcacaaagc[C/T]gttgaccaagttgtcgccggaaaactggatcaactgtcagtggtttcttgctgtaatccacagtcaaatggataacctctccatccgcaggccctgcact

fava_c16011_463	fava_c16011	463	T/C	gtttcttgctgtaatccacagtcaaatggataacctctccatccgcaggccctgcactatgtacaatcaattcctcgctgaaatttgcaaaagccagtgt[T/C]tgcacaagcccgttaaatttcccacatgatatacgccatggtgtaccccgggttccagaagccagagtttcaccaaggccagaagcaatctcagcctcca

fava_c16011_615	fava_c16011	615	A/G	gttccagaagccagagtttcaccaaggccagaagcaatctcagcctccacagaattgttatcctggtctgtcgggctcatagtatgtagtacaaatgata[A/G]atctggtgaaagcatttcttggatcagaattgccattgaagcttccttctgaggcacaccagcagcccgccggctcaacactgccctccgtgtgtacagt

fava_c16013_741	fava_c16013	741	G/A	ggcttctagtttatgagtttatgcagaaaggaagtttggaaaatcacttattcagaaaaggcgttcaaccgatttcctggatgacacgaatcagtattgc[G/A]atcggtgttgcaagaggactagcattcttacattcccttgatgcaaatgttatttatcgcgatttaaaagcttccaacatcctactcgattcggatttca

fava_c16013_918	fava_c16013	918	C/T	acatcctactcgattcggatttcaacgcaaatctttcggatttcggcttagcaagagacggtccaaccggagataacactcatgtttcaactcgaattat[C/T]ggaactcatggttacgcggccccagaatatgtagctacaggtcatttaacactgaggagcgaYgtatacagcttcggcgtcgtcctattagagctactaa

fava_c16013_981	fava_c16013	981	C/T	caaccggagataacactcatgtttcaactcgaattatYggaactcatggttacgcggccccagaatatgtagctacaggtcatttaacactgaggagcga[C/T]gtatacagcttcggcgtcgtcctattagagctactaacaggacggcgcgtagtcgatgacgatagaccggcatattcggaagaaacgctagtggattggg

fava_c16016_1209	fava_c16016	1209	T/C	tattgcttcatgggaaatcggttaatacctcccttggtccttctgtggaggctagtgtatatactgactcaactggagcatgcgctgccttcatttctaa[T/C]tccgatgataaaaatgataagacagtggaatttcgaaacgcatcgtatcacctgccagcatggtcagtYagcattcttcctgattgcaagaatgtagtgt

fava_c16016_1278	fava_c16016	1278	T/C	caactggagcatgcgctgccttcatttctaaYtccgatgataaaaatgataagacagtggaatttcgaaacgcatcgtatcacctgccagcatggtcagt[T/C]agcattcttcctgattgcaagaatgtagtgttcaacacagcaaaggtttcttccccgacaaacaaagttgcaatggttccagagaagttgcagcaatcag

fava_c16016_1431	fava_c16016	1431	C/A	ccccgacaaacaaagttgcaatggttccagagaagttgcagcaatcagataaaagtcaaaaaacttttaaatgggatgtgttgaaggagaacccagggat[C/A]tggggggaggctgactttgtcaaaaatggctttgtcgaccacatcaataccaccaaagataccactgattacctgtggcatacaacaagcatacttattg

fava_c16016_1551	fava_c16016	1551	A/G	tcaaaaatggctttgtcgaccacatcaataccaccaaagataccactgattacctgtggcatacaacaagcatacttattggcaaaaatgacgagttcct[A/G]aagaaaggaagcgaaccagttcttgtaatagaatccaagggtcatgctctccatgcttttgtgaatcagaaatatcaaggcactgcatatggaaatggtt

fava_c16016_1722	fava_c16016	1722	C/T	aatatcaaggcactgcatatggaaatggttcacactcaccattcactttcaagaatcccatctctctcaaggcagggaagaatgaaattgcgttattgag[C/T]ttgacagttggcttgcagacagctggaccattttacgagttcgtaggggcgggagtaacaagtgtaaagattaatggacttaacaacaagacgatagact

fava_c16016_1869	fava_c16016	1869	T/C	gggcgggagtaacaagtgtaaagattaatggacttaacaacaagacgatagacttgtcttctaatgcgtggacttacaagattggagtacaaggagaaca[T/C]caaaggttacacctggtagatgggttgaataatacgaaatggacatctacttcagaagcaccaaaaggccaggcactgacatggtacaaggctattgtgg

fava_c16016_1978	fava_c16016	1978	C/G	acacctggtagatgggttgaataatacgaaatggacatctacttcagaagcaccaaaaggccaggcactgacatggtacaaggctattgtggatgcccca[C/G]ctggggatgaaccagttgggttggatatgttgtatatgggaaaaggtttagcctggttaaacggagaagaaatcggaagatattggcctaggctaagtga

fava_c16016_2101	fava_c16016	2101	C/G	ggatatgttgtatatgggaaaaggtttagcctggttaaacggagaagaaatcggaagatattggcctaggctaagtgaattcaagaaggaagactgtgtt[C/G]aagaatgtgactacagaggcaaattcaatccagataaatgtgtcactggttgtggagaacctacMcaaagatggtatcatgttccacgatcttggttcaa

fava_c16016_2166	fava_c16016	2166	A/C	ctaggctaagtgaattcaagaaggaagactgtgttSaagaatgtgactacagaggcaaattcaatccagataaatgtgtcactggttgtggagaacctac[A/C]caaagatggtatcatgttccacgatcttggttcaagccatctggaaatgttcttgtcttctttgaagagaaaggtggtgatccaacaaagattacattcg

fava_c16016_684	fava_c16016	684	G/A	cacaaggcggtcccataatcttatcccagatagaaaatgaatatggtttctatgaaaactattataaagaggatggtaagaaatatgccttatgggctgc[G/A]aaaatggctgtttctcaaaatacaagtgtcccttggataatgtgccaacagtgggatgctccagaYcctgtgatcgatacttgcaactcattctactgtg

fava_c16016_750	fava_c16016	750	C/T	aagaggatggtaagaaatatgccttatgggctgcRaaaatggctgtttctcaaaatacaagtgtcccttggataatgtgccaacagtgggatgctccaga[C/T]cctgtgatcgatacttgcaactcattctactgtgatcagtttacgcctacttctccaaacaggcccaaaatttggactgagaactggcctggatggttta

fava_c16016_981	fava_c16016	981	A/G	cagtagaagatgttgctttttctgttgctcgattcttccagaagggtggaagtgtacataattactacatgtatcacggaggaacaaattttggccgtac[A/G]gcaggtggtccattcattactacaagttatgactatgatgccccaatagatgagtatgggttgcctagacttccaaaatggggacatcttaaggaacttc

fava_c16020_1170	fava_c16020	1170	G/A	cttttgactcatcgcaattcaattcaatttgtgttgtgtgccagcaatttcttgctggcacaaacacgtggttggagagtgctggatagtatactaccca[G/A]aaattctgatggagaattaggtaatgttgagaaggaagaagtggtttttgaatgaaatttttagaataaaaaacaaaaaagaggaaagggaattagaata

fava_c16020_978	fava_c16020	978	C/G	tctcaaacatgctggtctctgttgccatgcccgttgcattatcatcaactgattttctctggtagttgaagtaccacaagttgatggatgaagatcctca[C/G]tctgattagtttccgtaagagaaaacggtgaatgaggcaccacttggaccccatggacaccttcaagggctctactgtgattctttgtacacttttgact

fava_c16022_1070	fava_c16022	1070	C/T	gggtggacacaaatatagatcaaagaaaaaccaaagtgttctttcttggaatttctcctacacacaccaacccaaatgaatggatctatggatcaacaac[C/T]gcaacatcgaagaattgctatggtgaaactgcaccgatcagtggaactggcacatcataccctggtatatatccagaacaaatgaaggttgtagacatgg

fava_c16022_226	fava_c16022	226	T/C	aaaaaccaaacttttttacatataaatttcaaaacagtccaaatatccctccctcttcaacccttttctctatcaccttctctaataatgttaacccttt[T/C]atcttcattgtttcatcttctttttttcacaYtcacccttcaatgtcacatagcttcttcagctattctatacagcttgaaacaccaccacaRcaatatc

fava_c16022_258	fava_c16022	258	C/T	aacagtccaaatatccctccctcttcaacccttttctctatcaccttctctaataatgttaaccctttYatcttcattgtttcatcttctttttttcaca[C/T]tcacccttcaatgtcacatagcttcttcagctattctatacagcttgaaacaccaccacaRcaatatccaccatcaaagaccaatgattcatgccaacca

fava_c16022_319	fava_c16022	319	G/A	accctttYatcttcattgtttcatcttctttttttcacaYtcacccttcaatgtcacatagcttcttcagctattctatacagcttgaaacaccaccaca[G/A]caatatccaccatcaaagaccaatgattcatgccaaccaaacaaactgtgcattgtttgtaggaacatggattcctgatgacacttaccctttttaccaa

fava_c16025_181	fava_c16025	181	G/T	tctacaacacccattgtccccttattcccatccttagttaacctttttgaaccaacccggcccaacaaagacggaccacaactaaacccactccggtcca[G/T]ccggctctccacattgaaccgctcgtatcttaaaaaactcctccccacactactcgttctaaaaccccttctctcactctgtaatgccatttccaccccc

fava_c16025_374	fava_c16025	374	C/T	ccacccccaaacttttcgaccggttgaatatcctgtcattcacaagcctatcatgaacctcatccggcaatctcaatgtgaaccgctcgcaatcttcact[C/T]gaccgaacagttaactggcccatcgagttagaccgggagaacaatctcgcaaacattaaacccattgacttcgaccgaaccggtttattcatggttccat

fava_c16028_1143	fava_c16028	1143	A/T	gacacacagaccaagccagatcaaagtccgacctcctagaaagaccttcaattgtaaaataataagtgtccgcatccggtacacactgaaacccttcaaa[A/T]ttttcaaacacccctaatgcagccttcccttttcccaattttgagaatgaactcactaactcattcaaaatggtaacattgataaccccattttctttct

fava_c16028_969	fava_c16028	969	A/G	gacataaatgagaagtcaaaaagttaacagaagacaaaggtggatacctcttgttctctattgctgccatatacacttgatgagcttctttagccttttc[A/G]cccttacacaaccaagacaatattctaccaattttttcaccgtccggaattctccgagcatcaagcatcttctgacacacagaccaagccagatcaaagt

fava_c16034_1684	fava_c16034	1684	A/G	tatcttcattcagcggagcatcataataaaaatactttctctgattctgaataccagctaaagcctccatatcctcttcattcctttccaacgattcctc[A/G]ggtaattcaggatataatctagataagttcatattattctcctcagtgctaacagaagacaaagagtcacaattctctttagtaacatgatYatgatgat

fava_c16034_1776	fava_c16034	1776	T/C	gattcctcRggtaattcaggatataatctagataagttcatattattctcctcagtgctaacagaagacaaagagtcacaattctctttagtaacatgat[T/C]atgatgattcctcaaattRccatccacttcatcatccagaggagaatatgaattgaatatgtgatcacaMggggataaacgYgaaaccctatcagataaa

fava_c16034_1795	fava_c16034	1795	A/G	gatataatctagataagttcatattattctcctcagtgctaacagaagacaaagagtcacaattctctttagtaacatgatYatgatgattcctcaaatt[A/G]ccatccacttcatcatccagaggagaatatgaattgaatatgtgatcacaMggggataaacgYgaaaccctatcagataaatcttctacaacttgcacgt

fava_c16034_1846	fava_c16034	1846	A/C	aagagtcacaattctctttagtaacatgatYatgatgattcctcaaattRccatccacttcatcatccagaggagaatatgaattgaatatgtgatcaca[A/C]ggggataaacgYgaaaccctatcagataaatcttctacaacttgcacgtgattcttagaatcgaaatttttgtcctccattSgtaaacctaaaacaccaa

fava_c16034_1858	fava_c16034	1858	T/C	tctctttagtaacatgatYatgatgattcctcaaattRccatccacttcatcatccagaggagaatatgaattgaatatgtgatcacaMggggataaacg[T/C]gaaaccctatcagataaatcttctacaacttgcacgtgattcttagaatcgaaatttttgtcctccattSgtaaacctaaaacaccaaattagggtttat

fava_c16034_1928	fava_c16034	1928	C/G	attgaatatgtgatcacaMggggataaacgYgaaaccctatcagataaatcttctacaacttgcacgtgattcttagaatcgaaatttttgtcctccatt[C/G]gtaaacctaaaacaccaaattagggtttatacatgcatataggaacaattaagtttaataatgaatttaaattttgggataaagaacgaaactttaggaa

fava_c16034_212	fava_c16034	212	T/C	tgtacgattacaatatctacatcttcgtgtacttatttacatgacaaataatgacaccaggaaactttacaatccgaatggaacgtagcagctgtacaaa[T/C]taatcactgtccaatggttctttttctttatgctgatgtccaagaggagaaaagaatatgaaatttgatgctgaaaaacttaataaccatcaatcttcaa

fava_c16034_496	fava_c16034	496	T/A	aagactggctgccataaaaacgagaattttcttgtgtgcgagtggtgacaatacttccttcaagtgaacaaccaacaaatgcacctttactgcagctgta[T/A]gtataacaagcagcatagccgccatcaccagctcgaatatcagcttcagctgcccttccaacaaYgccaacagcagcacttaaaccagctccaagtgaaa

fava_c16034_561	fava_c16034	561	C/T	gaacaaccaacaaatgcacctttactgcagctgtaWgtataacaagcagcatagccgccatcaccagctcgaatatcagcttcagctgcccttccaacaa[C/T]gccaacagcagcacttaaaccagctccaagtgaaagatgtgcattaccactaaatgtcttgacagcatcatttgttctcagaacaataatgaagtctgtt

fava_c16035_724	fava_c16035	724	G/A	tcaagtcaaaaagtgcttgattaacctcttcaggccgctcatggctcactaaatgaccgccatgaagatctaccatccgagccattggatgaaatctctc[G/A]gcaagtcttcttgcataatatagctgagcaattatatcatccctgccatgaatgacggaaacaagaaaacctgcagatttgattacttcgatctctgttt

fava_c16036_716	fava_c16036	716	C/G	gggtaacatacatgggagaaaatacagtagttgtgagcatgacatgtagcaaaaggacagacacaatggttaaactgtgtgaagtgtttgaatctttgaa[C/G]cttaaaatYatcactgccaatatcacttgtttttctggcacacttttgaagacagtcttcattcaggccaatgaagaagataaagatctattgcagctac

fava_c16036_725	fava_c16036	725	T/C	acatgggagaaaatacagtagttgtgagcatgacatgtagcaaaaggacagacacaatggttaaactgtgtgaagtgtttgaatctttgaaScttaaaat[T/C]atcactgccaatatcacttgtttttctggcacacttttgaagacagtcttcattcaggccaatgaagaagataaagatctattgcagctacagatccaaa

fava_c16041_2219	fava_c16041	2219	G/A	aagaagtggtgaaaggaaaaatgagggctgcgaggctaatggttttccagaacgggatcttgagtattatgtgaaggtgattagatggttagagtgtgaa[G/A]gacacattgaaaagaacttcaggcagaaatttctgacttggtacagcttaagagcaacccctgaagaaataaggattgtgaaaatatatgtagatacatt

fava_c16041_2432	fava_c16041	2432	T/C	agcttctcttgcagagcaacttgtggacaccttctcagaatgcatgtcaaacaagagatcatccgtacctgcagggttctgcatgaagctttggcattga[T/C]tagatttgaagctctcctgggctgtgatgaaataattatttagattKatgacattcttcatttagagagtcatcaaatatatttatttcaacttatcatt

fava_c16041_2479	fava_c16041	2479	G/T	caaacaagagatcatccgtacctgcagggttctgcatgaagctttggcattgaYtagatttgaagctctcctgggctgtgatgaaataattatttagatt[G/T]atgacattcttcatttagagagtcatcaaatatatttatttcaacttatcattcatattgttgttttaagaattttagattttacttaaattatacagtt

fava_c16041_325	fava_c16041	325	G/A	gtgactgatttgttttcgttatggctgcgaattcttcgttcgaaggtattgctcttgatccttctaaatgtaataaattgagtatggaggaaaaaagagc[G/A]cttgtatatgaaatatctaatgacggtgcatccgagatgctgcagtcatggagtcgtcaagagattttgcaaatcttgtgtgttgagatgggaaaagaaa

fava_c16041_976	fava_c16041	976	C/T	gccaacgcaccaaacttgatggaaacttttactgcatctcttgtgggaaagtaaatgacttactcggatgctggagaaaacaacttatggtagcaaagga[C/T]gctagacgtgtggacatactctgttatcgtgtctccttaagccagaaacttttacaagggacagaaatgtatctagaactttataaaattgttgatgaag

fava_c16042_1034	fava_c16042	1034	T/C	ctatcacgtctctgataagaaaaacggtgatatacttgtttattggtcttcgtcRcggtgtcttcctcaaagaaatgaacttgccaagaaactcctcggc[T/C]tgctacctagccattcatttggcaagtgcctaaacaatgttggtggtctagacatggctctttcgttctttccgaagtgtgaaaacgaYgcaaatactaa

fava_c16042_1123	fava_c16042	1123	C/T	aactcctcggcYtgctacctagccattcatttggcaagtgcctaaacaatgttggtggtctagacatggctctttcgttctttccgaagtgtgaaaacga[C/T]gcaaatactaaaccRaaatggtgggatcatttacattgtgccatgtctcattacaagtttgttcttgcaatcgagaacactttcaccgagagttatgtga

fava_c16042_1138	fava_c16042	1138	G/A	tacctagccattcatttggcaagtgcctaaacaatgttggtggtctagacatggctctttcgttctttccgaagtgtgaaaacgaYgcaaatactaaacc[G/A]aaatggtgggatcatttacattgtgccatgtctcattacaagtttgttcttgcaatcgagaacactttcaccgagagttatgtgacagagaagttatatt

fava_c16042_1356	fava_c16042	1356	C/T	gctgttcctatatattttggtgcaccaaatgtcatggattttgttcctccacattcaataatcaatggtagagaattcaagtcattggaaaagctggctt[C/T]gtacgtaaaggctgtagctaatgacccggtagcctatgcagaataccatgcatggagaaggtgtggtgtaatgggtaactatgcgaaaacacgatctgtg

fava_c16042_1500	fava_c16042	1500	G/A	taccatgcatggagaaggtgtggtgtaatgggtaactatgcgaaaacacgatctgtgagccttgacacattgccatgtcggttatgtgaggctgttagca[G/A]aaaaggtggaagaagtgcaagaagctagctagggcRaatcgtgttattttgatggctctgcttgacagagatagaaaagattgagtttattacttgtata

fava_c16042_1536	fava_c16042	1536	G/A	tatgcgaaaacacgatctgtgagccttgacacattgccatgtcggttatgtgaggctgttagcaRaaaaggtggaagaagtgcaagaagctagctagggc[G/A]aatcgtgttattttgatggctctgcttgacagagatagaaaagattgagtttattacttgtatagacattttgatggctctgcttgacagagatagaaaa

fava_c16042_367	fava_c16042	367	C/A	cacctaaccttgttgttgttgcttcctcttccaccagcatgctcctagtttctcccaaacccatcaacacaatcacaatcaccatcatgcttgccttcac[C/A]ttcttcctcatcttcttctccggtttcctccatttcccttccgtgtcaccctctctcccacccatccacgaatccttcacgcttccctctaccaattcct

fava_c16042_475	fava_c16042	475	T/C	tcatcttcttctccggtttcctccatttcccttccgtgtcaccctctctcccacccatccacgaatccttcacgcttccctctaccaattcctcctccga[T/C]cccttcaccgatctgctttcttctttcaggaaatgggattcgcgagtgggttgtgctgaattcagagagaaaacaaatggggttttgttgaatcaatcaa

fava_c16042_988	fava_c16042	988	G/A	atgtacagtcaacctatgccggttccctttttcacaatgaacggaactatcacgtctctgataagaaaaacggtgatatacttgtttattggtcttcgtc[G/A]cggtgtcttcctcaaagaaatgaacttgccaagaaactcctcggcYtgctacctagccattcatttggcaagtgcctaaacaatgttggtggtctagaca

fava_c16044_1174	fava_c16044	1174	C/T	tttgggatgaggattatcgagaagtgaatccattctactacgaagagtgctccagaatcgtttatcagaagatggactgaattgaggagaagaagaagaa[C/T]taacaaYgtttgtatcatcctgtttcatgtgaagaagaagaggaggagRagaagaacgtgtgtcgattgaggtcgccatagacagtgtttgagagttgag

fava_c16044_1181	fava_c16044	1181	T/C	tgaggattatcgagaagtgaatccattctactacgaagagtgctccagaatcgtttatcagaagatggactgaattgaggagaagaagaagaaYtaacaa[T/C]gtttgtatcatcctgtttcatgtgaagaagaagaggaggagRagaagaacgtgtgtcgattgaggtcgccatagacagtgtttgagagttgagagtgttt

fava_c16044_1223	fava_c16044	1223	G/A	ctccagaatcgtttatcagaagatggactgaattgaggagaagaagaagaaYtaacaaYgtttgtatcatcctgtttcatgtgaagaagaagaggaggag[G/A]agaagaacgtgtgtcgattgaggtcgccatagacagtgtttgagagttgagagtgtttgagtgtggaaggtaagggttgaataacggctagttttgaaaa

fava_c16044_606	fava_c16044	606	T/C	catttgccagtcttgccttctcaccaagaagtgcttccagctgaagcctcaccagatcatcttcctcaggcctagctcctttcgcaaatccatcacgcag[T/C]cttctgttctcttcttcaagcaaaccacatctctcttgcataaaacataaatctgatttaagacacttcaactcccttgcaagtgagcttgctttgctag

fava_c16044_756	fava_c16044	756	T/C	aatctgatttaagacacttcaactcccttgcaagtgagcttgctttgctagccattgaaactgcaagattttttgctttcttgatatgcttatcatgcat[T/C]ttctgtccattttctgtttgtgactctgtaacattttcttcatagaaYtcatcaattttccttttcactcctttctttgactcatctttctcttctgaat

fava_c16044_804	fava_c16044	804	C/T	tagccattgaaactgcaagattttttgctttcttgatatgcttatcatgcatYttctgtccattttctgtttgtgactctgtaacattttcttcatagaa[C/T]tcatcaattttccttttcactcctttctttgactcatctttctcttctgaatcctctttcttttctaactcaacaattttgcttcSgaacatatcagtta

fava_c16044_890	fava_c16044	890	G/C	ttttcttcatagaaYtcatcaattttccttttcactcctttctttgactcatctttctcttctgaatcctctttcttttctaactcaacaattttgcttc[G/C]gaacatatcagttaatgtaggtggtttccccaattctctggtactctgtaatgcattatccaaattattagacaaaagagagagtgtatgagaaactgag

fava_c16048_149	fava_c16048	149	T/A	aaagtggagtggctttactttggaagcaacagtacattcttgatttcatccaaacccagaaaaagagaactccatgactcagatcttacagtagaagaaa[T/A]ccacactctcagagctcagaagaagaaaatgtcgcaaccgcaacaacatctcaacagcaacatccccgtaagcgagattttctggactctcgtcgacaaa

fava_c16048_1641	fava_c16048	1641	G/A	aagctgaaacactgctctcgtcatcattaaataactggggtatggcgttggcatcatcagacacacttgatccagtttggggacaggttttaggcgatcc[G/A]ttcataaggagacttcttttgagattcatattttgccagacagtgttgaccttgtatgcaccagtttacaatcaaaacgaatttcttccgacatgtgtcc

fava_c16048_1759	fava_c16048	1759	G/A	tttgagattcatattttgccagacagtgttgaccttgtatgcaccagtttacaatcaaaacgaatttcttccgacatgtgtcccttctcttccaacactt[G/A]tcctgcccccaagctacacgtatcaaagtgtaattctgcatctagcaagcatttttagtgcaactaagcacttcatattttcYgaggatgtttctcctga

fava_c16048_1842	fava_c16048	1842	C/T	cttctcttccaacacttRtcctgcccccaagctacacgtatcaaagtgtaattctgcatctagcaagcatttttagtgcaactaagcacttcatattttc[C/T]gaggatgtttctcctgaaactgtatctaccaacgtggatcagttgtgagttgtagtatttgaagcaacaagggatccaatttccaagataagcaagaact

fava_c16048_2051	fava_c16048	2051	G/A	gaagcacataatgtatataacaaccgagaactgatttctgtcaacatttgtcaaaatgctccaatttgtggactttctgagtaatgcttttcaatgtatg[G/A]agttttttcttgcattaaaatYagaggtggacaaaatatatggttaactgaaccataatatttgagctgtacaacacaacttttaaacaatgaataatca

fava_c16048_2073	fava_c16048	2073	C/T	accgagaactgatttctgtcaacatttgtcaaaatgctccaatttgtggactttctgagtaatgcttttcaatgtatgRagttttttcttgcattaaaat[C/T]agaggtggacaaaatatatggttaactgaaccataatatttgagctgtacaacacaacttttaaacaatgaataatcaaatttaatttcgaaaacaagtt

fava_c16048_402	fava_c16048	402	C/T	ggcacgatacatatttctccaacgtcttcaaagtctacacacagttatggaaattccagcaagagaatcgccagaagctaatcgaagccggcttgaaacg[C/T]tgggagatcggcgaaatcgcctcgcgaatcggccagctctatttcggacagtacatgaggacttctgattttaattatttatcagagtcttatattttct

fava_c16048_762	fava_c16048	762	C/T	tggttgaggagtgtaagagagtgtttcaggatagtgattttaaggaatggaaggttgttgtgttggaaattgggaggtttttgaaggcggatactgcttt[C/T]atgaatgttagacccttgagatatagtttggttttggattctcaYcctgataatttgccacatgttcctgttgctgttgcRaagcggaatttgaaattgc

fava_c16048_807	fava_c16048	807	C/T	aatggaaggttgttgtgttggaaattgggaggtttttgaaggcggatactgctttYatgaatgttagacccttgagatatagtttggttttggattctca[C/T]cctgataatttgccacatgttcctgttgctgttgcRaagcggaatttgaaattgcgagatgctgtgttgagtagctttcatcataacgaggttaagtttt

fava_c16048_843	fava_c16048	843	G/A	tgaaggcggatactgctttYatgaatgttagacccttgagatatagtttggttttggattctcaYcctgataatttgccacatgttcctgttgctgttgc[G/A]aagcggaatttgaaattgcgagatgctgtgttgagtagctttcatcataacgaggttaagttttcggagctcactattgacacttttaggatgcttcaat

fava_c16049_933	fava_c16049	933	A/C	tttaggttgacaggtttgtttgtggttggagatgcgattccgtttcttggatggcttgatttgggaggacatgtgaaggatatgaaaaaagctgagcgtg[A/C]gatggatagtgttgtctatggatggttggaagaacatcgcaagaaaaataatgattcgggagagattgctaagactgagcaggattttattgatgtcttg

fava_c16057_1058	fava_c16057	1058	C/G	ggttggcatcaattgcaccacttgtatcYgtcatactgtcaactctagttgtttttctaactcgagcagataaaaatggcgtaaaaattgtgaaacatgt[C/G]aaaggaggactcaatccaatctctatcRatcagttagactttaacRgtccacatgttgtagatgttgccaaaatcggacttgttgttgctgtggttgctc

fava_c16057_1086	fava_c16057	1086	A/G	YgtcatactgtcaactctagttgtttttctaactcgagcagataaaaatggcgtaaaaattgtgaaacatgtSaaaggaggactcaatccaatctctatc[A/G]atcagttagactttaacRgtccacatgttgtagatgttgccaaaatcggacttgttgttgctgtggttgctcttaccgaatctgttgctgttggacgatc

fava_c16057_1104	fava_c16057	1104	G/A	agttgtttttctaactcgagcagataaaaatggcgtaaaaattgtgaaacatgtSaaaggaggactcaatccaatctctatcRatcagttagactttaac[G/A]gtccacatgttgtagatgttgccaaaatcggacttgttgttgctgtggttgctcttaccgaatctgttgctgttggacgatcttttgcgtcgatcaaagg

fava_c16057_1229	fava_c16057	1229	G/A	aaatcggacttgttgttgctgtggttgctcttaccgaatctgttgctgttggacgatcttttgcgtcgatcaaaggataccagctcgatggaaacaaaga[G/A]atgatgtcaataggcttcacaaacatcattggatctctcacctcRtgttatgttgcaactggttcattctctcgaaccgcggttaactacgcagctggat

fava_c16057_1274	fava_c16057	1274	G/A	ctgttggacgatcttttgcgtcgatcaaaggataccagctcgatggaaacaaagaRatgatgtcaataggcttcacaaacatcattggatctctcacctc[G/A]tgttatgttgcaactggttcattctctcgaaccgcggttaactacgcagctggatgtgaaagtttaatatcaaacgttgtgatggcagttacagtgatga

fava_c16057_1828	fava_c16057	1828	G/A	tccgcattgctttgttttgcaaatgcgaatttcgttaaagaaagaatcatcaaatgggttactcaagaagaatcagaagatgacaagggaaactcaaaga[G/A]tacaattcaactcgtaattcttgaaacctcgaatttggtgaatattgacacttctggaatWgcttctttggaggaactgtacaagaatttgtcttcacaa

fava_c16057_1889	fava_c16057	1889	T/A	ctcaagaagaatcagaagatgacaagggaaactcaaagaRtacaattcaactcgtaattcttgaaacctcgaatttggtgaatattgacacttctggaat[T/A]gcttctttggaggaactgtacaagaatttgtcttcacaagggaaacagttggcgattgtgaatccgaggtggcaagtgatacataagctgaaggtgtcga

fava_c16057_190	fava_c16057	190	T/A	cccaaacacttcaatatgacatcttcaccagtcgatacctgcaaattcgaggatttgcaggttgacattgagaagaacgtgccacaggatgtaaggtctc[T/A]gtggattcttaatgctcctgagcctccaagtccttggcatgtggctgcagattctgtgagaaaaaatgtttctaactttagagacaaagtttcctctcta

fava_c16057_621	fava_c16057	621	G/T	gggaacttcgcgagagatagcaattggtcctgtcgcggtggtttcgttgttgttatcatcaatggtgcagaaactagtagacccttctactgatccagtt[G/T]gttacacaaagcttatttttcttgcaactctctttgctggtatctttcaaacttcatttggactattcaggttggggttccttgtggattttctgtcaca

fava_c16057_986	fava_c16057	986	T/C	tcttgggaggatctttcttggtgttcatcctaacaactagatttctgggtaaaaggaagaagaatctgttctggttggcatcaattgcaccacttgtatc[T/C]gtcatactgtcaactctagttgtttttctaactcgagcagataaaaatggcgtaaaaattgtgaaacatgtSaaaggaggactcaatccaatctctatcR

fava_c16058_1038	fava_c16058	1038	C/T	tttcatttcgaattcgttcgggtgacatatatgtaacagtgccaacgaatgtagcacacattgcaactgaattctctaagccagcactaataccgaaatc[C/T]gtaattttcggctcccctttgagattaataagtaaattggcaggctttatgtctctatgaactaaatatctgactccatgcagatagcttagaccatgta

fava_c16058_1184	fava_c16058	1184	T/G	tttatgtctctatgaactaaatatctgactccatgcagatagcttagaccatgtaagagcttctgaaacatggacgataaaataggttcaggaatcattc[T/G]atgMcttcgtaaaatatctgcaagtgaccctccattcatgtactccaaagctatgcttatttgKccagaatctggWgtgtaaaatgcaccatgaaattcc

fava_c16058_1188	fava_c16058	1188	C/A	tgtctctatgaactaaatatctgactccatgcagatagcttagaccatgtaagagcttctgaaacatggacgataaaataggttcaggaatcattcKatg[C/A]cttcgtaaaatatctgcaagtgaccctccattcatgtactccaaagctatgcttatttgKccagaatctggWgtgtaaaatgcaccatgaaattccacaa

fava_c16058_1248	fava_c16058	1248	T/G	gaaacatggacgataaaataggttcaggaatcattcKatgMcttcgtaaaatatctgcaagtgaccctccattcatgtactccaaagctatgcttatttg[T/G]ccagaatctggWgtgtaaaatgcaccatgaaattccacaagaccctcataacaaggtgcttcacatagtgtccttatctcagtaagtagctgctgccgtt

fava_c16058_1260	fava_c16058	1260	A/T	ataaaataggttcaggaatcattcKatgMcttcgtaaaatatctgcaagtgaccctccattcatgtactccaaagctatgcttatttgKccagaatctgg[A/T]gtgtaaaatgcaccatgaaattccacaagaccctcataacaaggtgcttcacatagtgtccttatctcagtaagtagctgctgccgtttctccttctcaa

fava_c16058_1602	fava_c16058	1602	A/G	caatctcatcatcaacagacctagaagttgagtacttctgtaaccctaattcattgatgttgtaaaccccatatgatctactcaacaagtttacagttcc[A/G]ccatctgaaaatgtgtaagaatcatttgggtctaaagatgaagttgatgatgaaaaccccttttcagcatcaaataaaggtgcaagcttttttcttaatt

fava_c16058_408	fava_c16058	408	A/G	catctccacatatcagaaactgattgccaattatgaaggatcctgagactctgattgcaacaccgtcttcaccgtgtgctcgacactgaagcttctcaac[A/G]acgtgcactaacttttctggtggccaatcgccgattaatgttgttcggatactcgatagagttgtgaagatattgttaggaccaaYatgttgtttcccag

fava_c16058_494	fava_c16058	494	C/T	tgaagcttctcaacRacgtgcactaacttttctggtggccaatcgccgattaatgttgttcggatactcgatagagttgtgaagatattgttaggaccaa[C/T]atgttgtttcccagagaaactgaaaattgagtttttgttatataagttttttgtatgttgccacaactcgtcgggtccatcgaatagtaagtaataatgt

fava_c16061_116	fava_c16061	116	A/G	aattaaattactaccatagatttatagatccaattttctttaaaaaaatcttactcctatttgatgttttaaccacaaaataccccaattatactagttg[A/G]acaacttctgtctacaagacataaaaaaRctatatgaaattaRattttaagtttaMatatgcaaatggtaaaaactaaccttatttaacttctctatcag

fava_c16061_145	fava_c16061	145	A/G	ccaattttctttaaaaaaatcttactcctatttgatgttttaaccacaaaataccccaattatactagttgRacaacttctgtctacaagacataaaaaa[A/G]ctatatgaaattaRattttaagtttaMatatgcaaatggtaaaaactaaccttatttaacttctctatcagcaaacccaaaatttactatgaaactactc

fava_c16061_159	fava_c16061	159	A/G	aaaaatcttactcctatttgatgttttaaccacaaaataccccaattatactagttgRacaacttctgtctacaagacataaaaaaRctatatgaaatta[A/G]attttaagtttaMatatgcaaatggtaaaaactaaccttatttaacttctctatcagcaaacccaaaatttactatgaaactactcaactacatttcttc

fava_c16061_172	fava_c16061	172	C/A	ctatttgatgttttaaccacaaaataccccaattatactagttgRacaacttctgtctacaagacataaaaaaRctatatgaaattaRattttaagttta[C/A]atatgcaaatggtaaaaactaaccttatttaacttctctatcagcaaacccaaaatttactatgaaactactcaactacatttcttctatccaagcagaa

fava_c16061_275	fava_c16061	275	T/A	atgcaaatggtaaaaactaaccttatttaacttctctatcagcaaacccaaaatttactatgaaactactcaactacatttcttctatccaagcagaaag[T/A]taaataaaggatttacaggaaaacagacactggtgtgattagtatatagcagccaggtaaaggcctRgcaatagtccatgctagtatggaagcMgccaca

fava_c16061_342	fava_c16061	342	G/A	actcaactacatttcttctatccaagcagaaagWtaaataaaggatttacaggaaaacagacactggtgtgattagtatatagcagccaggtaaaggcct[G/A]gcaatagtccatgctagtatggaagcMgccacaggacgagcaaacgatcattctccataatatcttaatagacatcttcatcgttcccagcctctacatc

fava_c16061_369	fava_c16061	369	C/A	agaaagWtaaataaaggatttacaggaaaacagacactggtgtgattagtatatagcagccaggtaaaggcctRgcaatagtccatgctagtatggaagc[C/A]gccacaggacgagcaaacgatcattctccataatatcttaatagacatcttcatcgttcccagcctctacatcattaaaatcatcgtcRtcgtcatcata

fava_c16061_458	fava_c16061	458	A/G	agtatggaagcMgccacaggacgagcaaacgatcattctccataatatcttaatagacatcttcatcgttcccagcctctacatcattaaaatcatcgtc[A/G]tcgtcatcataatctacattctgattataatcaccatcgttaaaatcgtcttccgactcttttccctcttcattttcatcttcatcttcactttcttctt

fava_c16061_575	fava_c16061	575	T/C	cattctgattataatcaccatcgttaaaatcgtcttccgactcttttccctcttcattttcatcttcatcttcactttcttcttttttcttctttttatc[T/C]ttaccctcttccgccaggaactttttctctagttcttcaaagaatgccagttttttcgcatcagcttcagggttccatcgaaactttttccggYtcagca

fava_c16061_669	fava_c16061	669	T/C	tttatcYttaccctcttccgccaggaactttttctctagttcttcaaagaatgccagttttttcgcatcagcttcagggttccatcgaaactttttccgg[T/C]tcagcatctgcttagatttacctggaaccaactccttaacaaattcgtcgtaatataaaacctgagacagcgaatcacgagtRaaMgcgatcttcttctt

fava_c16061_752	fava_c16061	752	G/A	atcgaaactttttccggYtcagcatctgcttagatttacctggaaccaactccttaacaaattcgtcgtaatataaaacctgagacagcgaatcacgagt[G/A]aaMgcgatcttcttcttgtcagaaaatctttctacgtgcatccttttgcgcccttttgattcggcgtcatccaagagataaggagccgctttaaaataac

fava_c16061_755	fava_c16061	755	C/A	gaaactttttccggYtcagcatctgcttagatttacctggaaccaactccttaacaaattcgtcgtaatataaaacctgagacagcgaatcacgagtRaa[C/A]gcgatcttcttcttgtcagaaaatctttctacgtgcatccttttgcgcccttttgattcggcgtcatccaagagataaggagccgctttaaaataactat

fava_c16065_284	fava_c16065	284	T/C	gtccttcaaaccacgacgttgagaaaatgtcttacggttcaagtcctataggttcaccacctcatcaaaacttccactactataccgcttctcccattca[T/C]cactctcgcgaatcttccacctctcgttactccgcttcgctgaaaaaccctcgcaacatttcagcttcctcctggaagaaactgaataacaggaacagtc

fava_c16065_541	fava_c16065	541	A/G	cgtaatgttcggttgtattttggattcttcttgcttttcgtggtgctttttacgctcttttcgttgattctttggggtgctagtaaaacctacaagcctc[A/G]tattgttcttaagagcatagtgtttgagaatctgaatgtacaatctggaaatgatggaacgggagtaccaacggatatgttatcattgaattcaacggtt

fava_c16065_959	fava_c16065	959	A/G	tgaagagtgttgtattaccaatgaatctaacatttgtagtgagatcaagagcttatgttttgggaagattggttaaatctacattccatcggagaatcag[A/G]tgttcaatcactatccatggcaacaaacttggaaaacatcttaatttgactgattcatgtgtctacaagtaatcatttattcatatcatatccattgggt

fava_c16066_1247	fava_c16066	1247	G/A	tctttgcttgtgctcttgatcaggttggcattgctgagcttaaaactgccgttgaaaggacagggggccttgttgtacttaccgaaagttttggccatcc[G/A]gtgttcaaggattcacttaagcgtgttttccaatcgggtgattctgatctgggcctagcatcaaatggaatatttgagataaactgctctaaggacatca

fava_c16066_1460	fava_c16066	1460	C/T	ttattggcccttgtgcttctcttgaaaagaaaagcccattatgctcagacgctgttattggtcaagggggtaccagtgcatggaagatgtgcggccttga[C/T]aaatcaacatctgtgtgtctgttttttgacattgtgaggaaggaaactcctgacgctacgacgcaatccacaagcaatcaattttacttccaattcttga

fava_c16066_272	fava_c16066	272	C/T	gcacaaaacaggaagcgttaaacgtcgtcgttccagttgcagcaatctacactccaatcaaacacttcccttccatgccggttctcccttattctccact[C/T]cgttgtcgaacgtgccgttcgattctcaatcctttctgtatcgttgatttcgccgccaagatctggatctgtccgttctgcttccaacgtaaccattttc

fava_c16067_1437	fava_c16067	1437	A/T	gaattttgcctgcgaagtttctgaaaattggtttgatacttttgatactctactttatatgattctattaacgcatttctctgagctaggagcttcctca[A/T]cgtcgtgttttccttgagaatcagttcaacagagacatcagttgcaggYtggtKctttggtagtagttgttggtccatgttggttatatcagtcagaatt

fava_c16067_1486	fava_c16067	1486	T/C	ctactttatatgattctattaacgcatttctctgagctaggagcttcctcaWcgtcgtgttttccttgagaatcagttcaacagagacatcagttgcagg[T/C]tggtKctttggtagtagttgttggtccatgttggttatatcagtcagaattttcttcggagcagctccgacaccaacagaaacagaatctcccttcaaaa

fava_c16067_1491	fava_c16067	1491	G/T	ttatatgattctattaacgcatttctctgagctaggagcttcctcaWcgtcgtgttttccttgagaatcagttcaacagagacatcagttgcaggYtggt[G/T]ctttggtagtagttgttggtccatgttggttatatcagtcagaattttcttcggagcagctccgacaccaacagaaacagaatctcccttcaaaaataat

fava_c16069_323	fava_c16069	323	G/A	tgtaaatttgtgaagaaaattgaggtagcggcgaatcgaaggatgagtggcgaggatgaagaaaaaaagttgaaattgaagcgaaattcttctcggttaa[G/A]caattttcaacgcgaccttatggcgggtgcgttgatgggtggtgcgattcatacgcttgtggccccgattgagagggcgaaattgttgttgcagacgcag

fava_c16069_514	fava_c16069	514	G/A	gcagacgcaggagagtaacttggctattgttgctagtggtcggagaaaattcaaagggatgtttgattgtataattcggactgttaaggaagaaggtgtt[G/A]tttctttgtggcgtgggaatgggagtagtgttcttcgatattatccttctgttgcactcaatttctctcttaaggatctttacagaagcatattaagggg

fava_c16070_1308	fava_c16070	1308	T/C	atctcctctttgttataattataattctccagtggctcccatttggcagcggccagacgcacatcaaaacagttgatttcctttcctgacgagacgtaga[T/C]attatcttcattccctgacttgaaacacaaggaaagaatcgactcctggctagcatccatagtaaggcgcggctcatcattgcaYcgtaaatcaaaccaa

fava_c16070_1393	fava_c16070	1393	T/C	ctgacgagacgtagaYattatcttcattccctgacttgaaacacaaggaaagaatcgactcctggctagcatccatagtaaggcgcggctcatcattgca[T/C]cgtaaatcaaaccaacaaacacgaccatcctcgccggaagtgacgatgagacgagggttttgagacgaggcaatgcagcagttggtgctgtctttgtgac

fava_c16070_1500	fava_c16070	1500	G/A	tcaaaccaacaaacacgaccatcctcgccggaagtgacgatgagacgagggttttgagacgaggcaatgcagcagttggtgctgtctttgtgacctttca[G/A]ccKtcgagccttcagtttagtctttggaggctggatattgagcgcacgctgagcttcctgcgccacMgatgtcatttccgcttctctgaaacctaaattc

fava_c16070_1503	fava_c16070	1503	G/T	aaccaacaaacacgaccatcctcgccggaagtgacgatgagacgagggttttgagacgaggcaatgcagcagttggtgctgtctttgtgacctttcaRcc[G/T]tcgagccttcagtttagtctttggaggctggatattgagcgcacgctgagcttcctgcgccacMgatgtcatttccgcttctctgaaacctaaattcgYt

fava_c16070_1567	fava_c16070	1567	C/A	tgcagcagttggtgctgtctttgtgacctttcaRccKtcgagccttcagtttagtctttggaggctggatattgagcgcacgctgagcttcctgcgccac[C/A]gatgtcatttccgcttctctgaaacctaaattcgYtgtatctatatgttatgtatatatatggtagttttctgaggaaacggaaagaacgaatgtggtgt

fava_c16070_1602	fava_c16070	1602	C/T	cKtcgagccttcagtttagtctttggaggctggatattgagcgcacgctgagcttcctgcgccacMgatgtcatttccgcttctctgaaacctaaattcg[C/T]tgtatctatatgttatgtatatatatggtagttttctgaggaaacggaaagaacgaatgtggtgtttggaatttggatgatggaatcaggcgctatcgaa

fava_c16070_317	fava_c16070	317	G/A	attacttcttggtgcattaaaacacacataaaacctaaaaaactatgatgctacataatttaagcaggaatatccagactaaaatcctttgtgcttctaa[G/A]tttttcatgtcagggagcatgatctttcacacgcaacataaattgttWtgattgctctccaggaatactcagcttgaaactatgttacagaatagacttt

fava_c16070_365	fava_c16070	365	T/A	tgctacataatttaagcaggaatatccagactaaaatcctttgtgcttctaaRtttttcatgtcagggagcatgatctttcacacgcaacataaattgtt[T/A]tgattgctctccaggaatactcagcttgaaactatgttacagaatagacttttacggttttagatgtgtcgcacacaacaaggttgtctgtatcagctga

fava_c16071_130	fava_c16071	130	A/G	ctgcaatagattaaacgaatttgaacgaatattagttataacatgtttgctttcttacagtgaaataattcaatatttcctaattttatttaggctgtag[A/G]tttgtacctgcaaagaaccttacacctcaagaccctatctttaacataaaaccctggcataaccatgatcttaactttgcttggaccctgtgaccttgaa

fava_c16071_1310	fava_c16071	1310	C/T	ttttggtttcggactgaaatgacgaatgggaatctgcaaccgaaacaacaacattggagctcttgctattccttacactctctaactcactcgttatgac[C/T]gcagggttacatttattacaggaggacaccgatttRtaatcctctaaactgtttcttctcgtaggtgaggtctgaagaacagttatgtgatttgcacaag

fava_c16071_1346	fava_c16071	1346	A/G	caaccgaaacaacaacattggagctcttgctattccttacactctctaactcactcgttatgacYgcagggttacatttattacaggaggacaccgattt[A/G]taatcctctaaactgtttcttctcgtaggtgaggtctgaagaacagttatgtgatttgcacaagaaccagttacagatttggtgtcatcagcaggacata

fava_c16071_293	fava_c16071	293	T/C	ccatgatcttaactttgcttggaccctgtgaccttgaacacatatcttccatgtagcgaggatcgaagtttctattctcttcaactcttaaaactcccaa[T/C]ggtgggttggaagaaaaggcaagcaaatgtagcagccacatgcattttgcagtcacaaagaaagYttgaaggagttgctcaggccaaggcctggtacaat

fava_c16071_358	fava_c16071	358	C/T	aagtttctattctcttcaactcttaaaactcccaaYggtgggttggaagaaaaggcaagcaaatgtagcagccacatgcattttgcagtcacaaagaaag[C/T]ttgaaggagttgctcaggccaaggcctggtacaatttagagtactggtaatacagctcattttctgatcacaaaaYttgctaaattcttcactgtaatac

fava_c16071_434	fava_c16071	434	T/C	tgcattttgcagtcacaaagaaagYttgaaggagttgctcaggccaaggcctggtacaatttagagtactggtaatacagctcattttctgatcacaaaa[T/C]ttgctaaattcttcactgtaatactttgttccctttctcagtacctcattccagctcaaatttctaagggcaacgaatgacgagaattttgcttggcgat

fava_c16071_539	fava_c16071	539	G/A	taaattcttcactgtaatactttgttccctttctcagtacctcattccagctcaaatttctaagggcaacgaatgacgagaattttgcttggcgatcttc[G/A]tgcgggtctatgaatttcggacaaccgttcttttgaaaagcacaattctcaaagtcttggtagagagactgatttatgaatgcttcaaaatggtatagaa

fava_c16073_328	fava_c16073	328	C/A	ttccgttctaaaactccttcatggccgaccaagataactccacgcgccgccctcaacgtgaagcgaagaaaagagcagtagctgcactgtgcgagcaacg[C/A]aaacgaaagcgcgttgcactcggtgatataaccaacgacgtcgtttcagaRaccgagaaactcgtttccgatactcatagtcacagtcaccggcagaaga

fava_c16073_379	fava_c16073	379	A/G	ctcaacgtgaagcgaagaaaagagcagtagctgcactgtgcgagcaacgMaaacgaaagcgcgttgcactcggtgatataaccaacgacgtcgtttcaga[A/G]accgagaaactcgtttccgatactcatagtcacagtcaccggcagaagaagaagaggaacaccgctaaatctccggtgccggagaaatcggaagatcctc

fava_c16079_154	fava_c16079	154	G/A	tagaattttcctcatttatggttgaatatgtatgaaggtaaacttggagatagatccttaatggagaagcttgttaggtcttgtgacagagagtatatga[G/A]aatggctatgttaaagcatgaagaaactttcaaacaacaggtatatgaacttcatcgattgtatcgaatacaaaagataatgatgcaaaatatggaggcg

fava_c16079_506	fava_c16079	506	A/C	cagatgacgacgaggggatagatctcatcgatgagatggagattgagttgacattaggcccgtcgcgatatacccgtagcaagaaaggtgagacaccaat[A/C]acttcagattcaggacatagcttatcttcatcttcatctggatcaagtgacataaacaagataagaagatacatgacacatcaaaaaagagaagaaccaa

fava_c16079_704	fava_c16079	704	C/T	caagtggaattactcgtaacacctttggtattgaagaacagttaagacaacaagagagattaaaacagtcaccttggttttttcaagtgatgaatctaaa[C/T]atgacataagaaaaagaaaacatgttaatgtcaatgtgatatatttaaatttttctagctaacttttgtgaatgatgttagtgttgtgtgatcaRttgtt

fava_c16079_799	fava_c16079	799	A/G	ctaaaYatgacataagaaaaagaaaacatgttaatgtcaatgtgatatatttaaatttttctagctaacttttgtgaatgatgttagtgttgtgtgatca[A/G]ttgttatcaagttatggtggggtttgatatgtcacgcattaagaccactattttactttgcacagatccattactattcacatgtcacatgtcacatgcc

fava_c16081_1184	fava_c16081	1184	A/G	ttttattaatactaggtttatccatacaatcaaacatcatatcgacatcaggaaccaacccagggtacttcctcaaaagctgcagcataccccaaacagt[A/G]aacatagccctactctgaacacaagcataataccaatcaacaaacatcttccctccaacaatcaccactctaaaagccgcaaacttttgcgcttccgcca

fava_c16081_136	fava_c16081	136	G/A	aggtaagtaaataaataaaaataaaaattcccgcattgtatgctctgttaaaaaatgacgacctattacaaacacaaaattttgtaaataattacctctc[G/A]aggcactataaatttacattcaacatYgcttcttttcttttgatWccgaaaatggcactaagtgtaagcatatgttcttgcatattagatgttccaaatc

fava_c16081_1373	fava_c16081	1373	C/G	gcgcttccgccaaatgccctttagaaattctcgttttcatccacggttccagatctctccggatcgccttgaaaaaatccgggcaatccggaccattagg[C/G]gcttttgcKtttccggtggcaRtactgtcaccggagactgcgttaatacaagtgagataggagcactggatgattttgtaggctctactctgacgagtct

fava_c16081_1382	fava_c16081	1382	G/T	ccaaatgccctttagaaattctcgttttcatccacggttccagatctctccggatcgccttgaaaaaatccgggcaatccggaccattaggSgcttttgc[G/T]tttccggtggcaRtactgtcaccggagactgcgttaatacaagtgagataggagcactggatgattttgtaggctctactctgacgagtctcttcRtcga

fava_c16081_1395	fava_c16081	1395	G/A	agaaattctcgttttcatccacggttccagatctctccggatcgccttgaaaaaatccgggcaatccggaccattaggSgcttttgcKtttccggtggca[G/A]tactgtcaccggagactgcgttaatacaagtgagataggagcactggatgattttgtaggctctactctgacgagtctcttcRtcgaagggtttggtWgg

fava_c16081_1478	fava_c16081	1478	G/A	ttgcKtttccggtggcaRtactgtcaccggagactgcgttaatacaagtgagataggagcactggatgattttgtaggctctactctgacgagtctcttc[G/A]tcgaagggtttggtWgggaaaacatgccatggcgttggttcgaggttgtgacctaccacggttccggttcggctgacaacgtcRtccaccttgtagagaa

fava_c16081_1493	fava_c16081	1493	A/T	caRtactgtcaccggagactgcgttaatacaagtgagataggagcactggatgattttgtaggctctactctgacgagtctcttcRtcgaagggtttggt[A/T]gggaaaacatgccatggcgttggttcgaggttgtgacctaccacggttccggttcggctgacaacgtcRtccaccttgtagagaagaagaccagtgagag

fava_c16081_1562	fava_c16081	1562	G/A	tctgacgagtctcttcRtcgaagggtttggtWgggaaaacatgccatggcgttggttcgaggttgtgacctaccacggttccggttcggctgacaacgtc[G/A]tccaccttgtagagaagaagaccagtgagagagaagaaagagagagcgatgacacaaggaaagagataagtaggggtacgaggagagtgtttgggagaga

fava_c16081_163	fava_c16081	163	C/T	ttcccgcattgtatgctctgttaaaaaatgacgacctattacaaacacaaaattttgtaaataattacctctcRaggcactataaatttacattcaacat[C/T]gcttcttttcttttgatWccgaaaatggcactaagtgtaagcatatgttcttgcatattagatgttccaaatcttttgtgcagcaaaatataggtaagat

fava_c16081_181	fava_c16081	181	T/A	tgttaaaaaatgacgacctattacaaacacaaaattttgtaaataattacctctcRaggcactataaatttacattcaacatYgcttcttttcttttgat[T/A]ccgaaaatggcactaagtgtaagcatatgttcttgcatattagatgttccaaatcttttgtgcagcaaaatataggtaagatcactcactctagacatat

fava_c16082_1033	fava_c16082	1033	C/G	tatgatctttgtaataattgattcccttgtataatttctcattctctgagccagttcccttgagactctctgacgcttgcttaagagctctttcacgaat[C/G]gcacgagcatctttcgaaaagtcagtctcagtctccaaagttgcagttgctttgctatcatgttgaacttgaatctctttggaagactcaaaatgaaagc

fava_c16082_1135	fava_c16082	1135	G/A	cacgagcatctttcgaaaagtcagtctcagtctccaaagttgcagttgctttgctatcatgttgaacttgaatctctttggaagactcaaaatgaaagcc[G/A]tgtttctcagattcatcgttcggttcagtagatgcagagctttttgaagagccggaggaaaagaacaacttattgtcgggcttcatggtctttttctgga

fava_c16082_1590	fava_c16082	1590	A/G	agtgatagggtttcttcgaccgtagaacgcaattccagatctacgctcacgaagacacgtggacagaacctatgctccgcacgacggcgaaagtgcctct[A/G]tgaacggcggcggccaagtcttctacacacggaggtaggtcgcaaatcgactacgacaacatcagcaaaaacctatgagcgcgtgaagaaattggacat

fava_c16082_204	fava_c16082	204	G/A	atgggttggcaaagtatcaagggcaaagaatatatgcattccttgagcatgaccaatacactacaacatttatcattaatggcctattttaaacaagttc[G/A]tttctctaactgttatatatattctggggtctattggtcagataSStgcaaaaatgataggacctatgcgcatacctaatacttgttttcaactcattac

fava_c16082_249	fava_c16082	249	G/C	agcatgaccaatacactacaacatttatcattaatggcctattttaaacaagttcRtttctctaactgttatatatattctggggtctattggtcagata[G/C]Stgcaaaaatgataggacctatgcgcatacctaatacttgttttcaactcattacatgacatcttcagtttgacaaagttaaaatgggaaaacaaaagag

fava_c16082_250	fava_c16082	250	G/C	gcatgaccaatacactacaacatttatcattaatggcctattttaaacaagttcRtttctctaactgttatatatattctggggtctattggtcagataS[G/C]tgcaaaaatgataggacctatgcgcatacctaatacttgttttcaactcattacatgacatcttcagtttgacaaagttaaaatgggaaaacaaaagaga

fava_c16082_595	fava_c16082	595	C/T	tcttattctttgcatgatgcttcaatgcacaatgctcacaaaagtagtgtttgcacttggttgaaacaggatccacaaatggatttctacaaatgaaaca[C/T]gcaaatggtaatgaatcatcgtcggagtcatcttcatcgttcaaattagcaccctcctcttccgcatcctcacctgcagccaatctcatcctccttgatt

fava_c16091_1467	fava_c16091	1467	T/C	aaccttctttaagaagagatgtaaatcatgtcgttggagttgaaccaacttttgattataaagttgatgaaaatgagaatcaaggaaccgagattgaaag[T/C]gagatgcacgaagacacggaggagatcaatgcattgctttattctgacagcgacggttattctactgaggatgatgaagttaccagcacaggacattcac

fava_c16091_1751	fava_c16091	1751	A/G	aagacaaagaaacgtaagcttttcgatgtggcttatgacgatgtgcagtttatggatactgcaaattctctaaaacggaaccagccctgtgacctgaatc[A/G]gaacaatggcgatgataatgatgcagaatcaagatgctctggtagcaattatggtcaagggtctggtgaaatgggttctttagcatgcaccaagaagatg

fava_c16094_1379	fava_c16094	1379	G/T	cagccgccaacgcctttctaaaaggcgaacatttttcagcccaactacattcatcgagagctagggaggaatggcataatgcagaaaaacttaattctga[G/T]gcagccactaaaattttgagtatcaggaatagtgataatgatatctctagattggatttgcatggtcttcatgcaacYgaagcagttcaagcattgcaag

fava_c16094_1457	fava_c16094	1457	C/T	atgcagaaaaacttaattctgaKgcagccactaaaattttgagtatcaggaatagtgataatgatatctctagattggatttgcatggtcttcatgcaac[C/T]gaagcagttcaagcattgcaagagcatctccgtagaattgaaagtcaaggcttctcgaaaagctcagccccttcgaatggtgtgaagaagaatggccgcg

fava_c16094_1775	fava_c16094	1775	G/A	ctgctcttcctacagctgtaagaagttttctcagtgaaaacagataccgatttgaggagatgagaccaggagtaatcacagtgtggcccaagtttcggca[G/A]agttgacaacaattatgttgtcataccaagttcttgtgggaatctgaaagtttttatgctgtgtaattttatcatgtacttcaWactggagttactatat

fava_c16094_1859	fava_c16094	1859	T/A	ggcccaagtttcggcaRagttgacaacaattatgttgtcataccaagttcttgtgggaatctgaaagtttttatgctgtgtaattttatcatgtacttca[T/A]actggagttactatatcacacWatgcagtgggggagatttaatcatactttggaaacYatgtgtaatttatccttttaaagggattatattcatggagta

fava_c16094_1881	fava_c16094	1881	T/A	acaacaattatgttgtcataccaagttcttgtgggaatctgaaagtttttatgctgtgtaattttatcatgtacttcaWactggagttactatatcacac[T/A]atgcagtgggggagatttaatcatactttggaaacYatgtgtaatttatccttttaaagggattatattcatggagtattatttatccttttttaagtga

fava_c16094_1917	fava_c16094	1917	T/C	atctgaaagtttttatgctgtgtaattttatcatgtacttcaWactggagttactatatcacacWatgcagtgggggagatttaatcatactttggaaac[T/C]atgtgtaatttatccttttaaagggattatattcatggagtattatttatccttttttaagtgaaatttacatgtccgctatgttggtatggc

fava_c16094_466	fava_c16094	466	C/T	tattattttctgaaaaaaggtttctagattgtggtttccttagttcttgttctgtgtatgctgcacaagtcttctgacagagagataagtcatttctgct[C/T]gaattatggtgtatggaccacgatgaccgtttcagccggatcatcccttaagatgtcgtggtccaggggaaaacaatcttctggttggacagcttttgat

fava_c16094_597	fava_c16094	597	A/T	ttcagccggatcatcccttaagatgtcgtggtccaggggaaaacaatcttctggttggacagcttttgatctcaagcagaaaaacaagaacaatatcgaa[A/T]cggaagttgacaaggatcctttcccacctataggctcatctggttctatgcggcacggtgataagttaggcaagaagaaacatgttcccctgaagccttt

fava_c16096_1512	fava_c16096	1512	T/A	tcgagcactaaattgtctcgagtatggtttgattaaacgagtttataaatcttttcacgtatagtgatatttacggattcttttggtagctagacatttt[T/A]aaaaattctatttcgaatgaatcgaaatgaaatacactttaKMccatacagcatggtacaatacaattctaatgttatatcacactttcatttaattgtc

fava_c16096_1554	fava_c16096	1554	G/T	ttataaatcttttcacgtatagtgatatttacggattcttttggtagctagacattttWaaaaattctatttcgaatgaatcgaaatgaaatacacttta[G/T]Mccatacagcatggtacaatacaattctaatgttatatcacactttcatttaattgtcttt

fava_c16096_1555	fava_c16096	1555	C/A	tataaatcttttcacgtatagtgatatttacggattcttttggtagctagacattttWaaaaattctatttcgaatgaatcgaaatgaaatacactttaK[C/A]ccatacagcatggtacaatacaattctaatgttatatcacactttcatttaattgtcttt

fava_c16096_244	fava_c16096	244	G/A	ccttccttcgaaatccacatctatcttgccaaaccagacaatgtcgttccaacgcattttccaccaaacaagaccttatcgcacttcaagaagaagaaga[G/A]gaRgaagaagaagaWgatgatgaagaacaaagagtttcatattcagacgatgatacatctttcttatccctcagcgagaaaccYgaccgcaatttgggtt

fava_c16096_247	fava_c16096	247	A/G	tccttcgaaatccacatctatcttgccaaaccagacaatgtcgttccaacgcattttccaccaaacaagaccttatcgcacttcaagaagaagaagaRga[A/G]gaagaagaagaWgatgatgaagaacaaagagtttcatattcagacgatgatacatctttcttatccctcagcgagaaaccYgaccgcaatttgggtttgc

fava_c16096_259	fava_c16096	259	T/A	cacatctatcttgccaaaccagacaatgtcgttccaacgcattttccaccaaacaagaccttatcgcacttcaagaagaagaagaRgaRgaagaagaaga[T/A]gatgatgaagaacaaagagtttcatattcagacgatgatacatctttcttatccctcagcgagaaaccYgaccgcaatttgggtttgctagatgattacg

fava_c16096_328	fava_c16096	328	T/C	ttcaagaagaagaagaRgaRgaagaagaagaWgatgatgaagaacaaagagtttcatattcagacgatgatacatctttcttatccctcagcgagaaacc[T/C]gaccgcaatttgggtttgctagatgattacgagactgaagaactcggttatgattgcggtcccaatcaccgaagcgggtatgtggctttacttggaaaac

fava_c16096_681	fava_c16096	681	T/C	caagagaaaaggcacaagctagactcaatgatgatgcaaaatgttcgcagcgctgcagttaatgctgactgtgtactgtttctcgttgatgcatctaaag[T/C]gcctgaaaaaatcgatgaagtgttagaacaaggcataggaaaccccaaggataaaccccccactctattgattatgaacaagaaggatcttatcaaacct

fava_c16098_687	fava_c16098	687	T/C	agataattacaaagtctgatggatcattgacattagatttactcagacaagattctatcagatctggcgactcacaaaaatgtgggaagctcaaagtgca[T/C]gctgaggaatgtgttggctcaaagactacagtagagatgatactcaggtgttcagatttggaatatagggaccttttctcaaagagtgatccctttttaa

fava_c16102_1359	fava_c16102	1359	A/G	tgccgcaacaatgaccacatgtcatgtttgtgtaacataattaatttcttattagttacattaagttatttatcatatgtacgcactaaattagatgtct[A/G]ttgtaattttatgagaatgtccaaagaatttaagtcttcaggatccagttcctgttatgactggtatttttaatttaattgacagtggatatttcattca

fava_c16102_861	fava_c16102	861	C/T	ttgacatcgagtggaaacccaccttcagaaaaggtgttccggctgggaaagtagcagtgatgcagatatgctgtgacactagtcagtgtcttgttcttca[C/T]ctaattcattctggaatccctcaaaatcttcagcttttacttcaagattccacagttttgaaggttggagctgggattggcggtgatgctactaagattt

fava_c16103_771	fava_c16103	771	C/T	cacttatgaattatgttcaggcccatcagaatgtttttcgccaattggttgatttgattggaatcacatccattatggaggttttggttcgattagtagg[C/T]gccgatgaccatgtgtatcccaattttattgatgtgatgcaatggttggctgagagcaacctgctcgaaatgattgttgataaattaagtccatcttgcc

fava_c16104_1041	fava_c16104	1041	C/T	gcggactgggctgatctgtggcggcctgatcttgcggggaggatttcaatggtagattctcctagagaagttgttggtgcagtattaaagtatatggggg[C/T]ttcctataatgcaaatgacatcaatttggaagttaatggtggaagagatgcagttaaacataatttagcattgcttgcaaaacaggttcgactctttgat

fava_c16104_1351	fava_c16104	1351	C/T	tcccaaagtctggagcgagtttatgggctgacctttgggcaattcctgccgcctctcggattcaaactagtctgattggtggacgcgtaaggggaccgtc[C/T]ccattaatccatcagtggatagagttttgtctgcaatctgcaagagcacttccctttaagcaggaggtgattccaggtgcctctccccctcttcttcaag

fava_c16104_172	fava_c16104	172	C/T	tgcaaacacattacacactctttcaaccttcaccgcctcacccttccctcttctcacatcgaaaccctaactctaacccctacaatttctcccctctaat[C/T]cctacgRaacctcccaaatcccaattccaatttccgcataaccgttccccggtctccactatctccgcctcccgccgggacacttcagaaacgccgcaac

fava_c16104_1827	fava_c16104	1827	G/C	gaaatacatgccacaaatctgtgattttaacttccagaaacatccaaggttggttgatgttgtttgaagacttgctgaggtcgattttcaagattgttgg[G/C]ttgtaataatgctggtggagctgtacgagttaactgcccattcgcttgccctcttttatattgatagacttcaaaattttctgtaaaattcataaaaaaa

fava_c16105_1152	fava_c16105	1152	T/G	atactgttacacacaatgttacagatgcgggtgaaggttctagcatgtcaggcatgcagtcacaggttcctccttcagtttcaagcgtgtcaactagacc[T/G]cccacatcacctccactccctggaatattaaaggcaagagttaagcaagaacattgatctgcaagtttatgatgtcctcttgcYttttaagtatgtctgt

fava_c16105_1236	fava_c16105	1236	T/C	gcgtgtcaactagaccKcccacatcacctccactccctggaatattaaaggcaagagttaagcaagaacattgatctgcaagtttatgatgtcctcttgc[T/C]ttttaagtatgtctgtatatcttgtaaagaaatttaccgaattaatataccttcttattggtcatcagtacagacaagtcatttggtggtaggtcagaag

fava_c16105_1425	fava_c16105	1425	T/C	gtaggtcagaagcagattggtctgtttgcttctctattgtttaggtttaactggcaaattcttttactatcatccaccatttaataccttagcagaattt[T/C]ctccatattttttgtggtaacttttccggagctttagttagagagatcatggaatggggtccaacatcatagattaacttcagagttcactgatttacag

fava_c16105_444	fava_c16105	444	T/A	ctctcaaattaaaccatcttgatatacaggatgatgatgctgcatcacagggagtagttgctagcaagaagaaaaagagaggtcaacaaagggctgttgg[T/A]ggtgataaaagtggaagagggcttcgccaatttagcatgaaagtttgtgagaaagtagaaagtaagggaagaacaacttacaatgaggtggcagatgaac

fava_c16111_1318	fava_c16111	1318	A/G	actgccaggatatggaggaatcttatgttgacttggcagagaagttagcagggtcaggtgtgaatgttggaaaatttagagctgatggagagcagaagga[A/G]tttgcaaaacatgaactgaaattgggaagcttccccacaatattatttttcccagaacactcctcgaggccaataaagtaYccctctgaaaagagagatg

fava_c16111_1399	fava_c16111	1399	T/C	ctgatggagagcagaaggaRtttgcaaaacatgaactgaaattgggaagcttccccacaatattatttttcccagaacactcctcgaggccaataaagta[T/C]ccctctgaaaagagagatgttgattccttgatggcatttgtaaatgcattaagatgagagttatctagaaattatattgtttggtttgcaatccaactat

fava_c16111_1554	fava_c16111	1554	C/T	tgagagttatctagaaattatattgtttggtttgcaatccaactataagcactcttctgggttctttcattacaacacatatatactagtatcattcatt[C/T]cactgtggagtttaggaaaataataaaaaatattgaaggttggagaataatgggggttMSgtacagtaagtagggttgtcgtattagtgtactagtacta

fava_c16111_1613	fava_c16111	1613	C/A	ggttctttcattacaacacatatatactagtatcattcattYcactgtggagtttaggaaaataataaaaaatattgaaggttggagaataatgggggtt[C/A]Sgtacagtaagtagggttgtcgtattagtgtactagtactatctttgtgtgttagttaattaactaaagtaattatagttaaactacatgagtattaatg

fava_c16111_1614	fava_c16111	1614	C/G	gttctttcattacaacacatatatactagtatcattcattYcactgtggagtttaggaaaataataaaaaatattgaaggttggagaataatgggggttM[C/G]gtacagtaagtagggttgtcgtattagtgtactagtactatctttgtgtgttagttaattaactaaagtaattatagttaaactacatgagtattaatgt

fava_c16111_736	fava_c16111	736	T/G	ggcatcaagagtgctgcagagtcaggaaggtgagacctttaagaagggcacttaagggtttaagggcatggataacagggcagagaaaagaccagtctcc[T/G]ggtactaggtctcaaataccagttgttcaggttgatcctgtttttgagggaattgacggtggaactgggagcttgataaaatggaaccctgttgcaaata

fava_c16111_862	fava_c16111	862	C/T	ttcaggttgatcctgtttttgagggaattgacggtggaactgggagcttgataaaatggaaccctgttgcaaatatgaagggaaatgacgtgtggaactt[C/T]cttaggaccatgaatgtgcctgtcaattccttgcattcacaaggatacatttccattggatgtgagccgtgcaccagggctgttttacccggacaacacg

fava_c16116_1255	fava_c16116	1255	T/C	aacatcactttctgccctttttgttttattaggatcttcagtcatttcaaatttcccagtctggtcagttttctcagaattggaaacattgggggaagaa[T/C]gttgcacactggctgagccagtgaaatcattccatgaatcaaacgaaacagtagcttctttagtaccaacattatcagttgccttgttatcattatcttg

fava_c16116_1431	fava_c16116	1431	T/C	cagttgccttgttatcattatcttgccattggttatctcccatccagtcaaattccttgttagaagaactattagccccctcttccggttttgcatcaat[T/C]aaatttgccgcagggaattcaaaattgctggtctgggcagttatcgttgagctggaaattataccagaagggtcttgagtgcttgttgtgccagtaaaat

fava_c16116_627	fava_c16116	627	T/C	gattttgcctcacagtatcctcatcagccaccctgtttaaagaagcaactgtatgctgagcattgattgcagcaggagtactcagtggattatcaaactg[T/C]cttaaaatatcatgttgtgaaaatccactaaaatccatatcatatgagctgtttgatgatccaaataaattcatttcaaaggtcttttcagaatgagttt

fava_c16116_812	fava_c16116	812	G/A	ttttcagaatgagtttgattcagagtttgtaaaggaacatttttgaaaggatcttcttgtctagaggccgtgttagtaaagtcactccaaacatcaaagg[G/A]gcccgcgttatctttagtggtcacagtgtcggttgttttgttattgctaacttgccattggtcatcttgcatccagtccaaacttttaactttagaagca

fava_c16117_548	fava_c16117	548	T/A	taacccttagtgactcatttaatattttactccattcattccttttgttacaatgcagatttgttttatcagactataaatacttacaaaaatatgtcaa[T/A]gaagttccgttgtccagtcaaactctttccactctttggaaaagtttgccactgcttgcttctgtacgtccaatgctgcatacctttgtcgcctgtacat

fava_c16119_1196	fava_c16119	1196	G/C	tggatcctgtttggttgctgtacacagagatgaaaaagtgttataaaatgtaagatatcatttatctgcttttaaagatattgcagactgtcttgtgttt[G/C]tgtactgctagtagagtgctagtggtctgaaaagttgaagctattgagtgagagtgtcaatgaatatacatctagtacaatatctttgtgattctcacaa

fava_c16119_191	fava_c16119	191	T/A	tgaaaaacaccaagaatgaagaataataatataaccactaacaaatgatgatgaggatgaaacaagtttgagtttgcttcacctttttaaggaaccctag[T/A]acaWYcatcaaagagataaattcWcaaagttttcaccaagttcaaagcaattaaccttggatctgcaaaatataaagtttaacaaaacttttatatacca

fava_c16119_195	fava_c16119	195	T/A	aaacaccaagaatgaagaataataatataaccactaacaaatgatgatgaggatgaaacaagtttgagtttgcttcacctttttaaggaaccctagWaca[T/A]YcatcaaagagataaattcWcaaagttttcaccaagttcaaagcaattaaccttggatctgcaaaatataaagtttaacaaaacttttatataccaatgg

fava_c16119_196	fava_c16119	196	C/T	aacaccaagaatgaagaataataatataaccactaacaaatgatgatgaggatgaaacaagtttgagtttgcttcacctttttaaggaaccctagWacaW[C/T]catcaaagagataaattcWcaaagttttcaccaagttcaaagcaattaaccttggatctgcaaaatataaagtttaacaaaacttttatataccaatgga

fava_c16119_215	fava_c16119	215	A/T	aataatataaccactaacaaatgatgatgaggatgaaacaagtttgagtttgcttcacctttttaaggaaccctagWacaWYcatcaaagagataaattc[A/T]caaagttttcaccaagttcaaagcaattaaccttggatctgcaaaatataaagtttaacaaaacttttatataccaatggattcaattcaacaatttata

fava_c16119_504	fava_c16119	504	T/C	gtggaagctcttcttcatctgcagcttcaaccataacatccaacacaccaaacagtagcagccggtatgagaatcagaaacgccgtgactggaacacttt[T/C]ggtcagtacctcaagaatcatcgtcctcctctttctctctctagatgcagcggtgcacatgtgcttgaatttctcaggtatttggatcaatttgggaaaa

fava_c16119_762	fava_c16119	762	T/A	gtccatgtcctctaaggcaagcatggggaagcctagatgcactgataggccgtcttcgagcggcctttgaagaaaacggaggaaaaccagaaacaaatcc[T/A]tttggtgctcgagccgtgaggctttaccttcgcgaagttcgcgatcttcaatccaaggcaagagggattagttatgagaaaaagaagagaaaacgtccac

fava_c16121_1235	fava_c16121	1235	G/A	ttggtctgtagttggtagaggcattgggtctgtaattcgtagaggcacttggtttgtagttagtagaggcatttggcctgtagttagtatgcgggaagac[G/A]tggcttcttgggttggcaaatgatggaaaatttcctgtttggttataggtccttggaggtgcggcagctgtaaaattagaatgcaaatgaggagcctttt

fava_c16121_1351	fava_c16121	1351	C/G	gcaaatgatggaaaatttcctgtttggttataggtccttggaggtgcggcagctgtaaaattagaatgcaaatgaggagcctttttttgaatttccattt[C/G]ttttctggtatcagtcaaaatcttgggtaacctgctagttaaactgccagttctatgtgcatactgtgagctgttttgattctctaaggtagaagatttt

fava_c16121_1572	fava_c16121	1572	C/G	agaaccattgataggcttatattgcatgccaacagaatgtgataccgaagaacgataaggcatggcttctgatttatgaacgcctggttgttgaacataa[C/G]tggtgtaggacaggtcaggttgttgatcagcataatccccatggacatggacagtagttccttctgcgataggtgaaccattggctgaagccatcaagag

fava_c16121_311	fava_c16121	311	G/T	cagatggattaagagagagattcttggttaatttgaccagattttcttgattactaccacttgaatgcatctctatttcctcctctctcgccttcgtagt[G/T]ttatagtaaccatcattgttgtacacttcctgttttRggcctgtatgctgctgagtaatcttttgggaatgtattaacttctctcggttttcatagaaat

fava_c16121_348	fava_c16121	348	G/A	cagattttcttgattactaccacttgaatgcatctctatttcctcctctctcgccttcgtagtKttatagtaaccatcattgttgtacacttcctgtttt[G/A]ggcctgtatgctgctgagtaatcttttgggaatgtattaacttctctcggttttcatagaaatcaaaatcatccaatagacaagtcttagcttgataact

fava_c16121_497	fava_c16121	497	T/C	ggttttcatagaaatcaaaatcatccaatagacaagtcttagcttgataacttttaaatattttcagcatttccagaccctctttcagtccaatctcttg[T/C]gtgtccctactgaaagttacacacttgttctcattattttgaaggatgatgtgcacaaaccgggggttgggaacatcttttataatatgccacttaattg

fava_c16121_916	fava_c16121	916	G/T	tcgtgtttaatacccttgtgaatgtcatcttcgttgaaagatttaatcatataaaatttagcattttcatatttagttttgaaatctggaaggttatatc[G/T]atctctggatactgtgaatgcaaactcatccttttcaactgatgattgtggttgagaactatttttatgaaaccgaggaccccgagtcatttcctttgac

fava_c16122_1168	fava_c16122	1168	A/G	aaacaggttgtgatggtgctcccttgcttccactgctttacgtaattaccaatctggcattcaatatatccctgcttagcgtactgaagcgttcttctgc[A/G]gttgttgcttctcttgtgttaatgttgtcagtgccaatttctgtttatactctttcccttccattaccatatcttcccgagggaggaacagtcttgagtc

fava_c16122_1370	fava_c16122	1370	T/C	attttttctgcttggctgtgcaattcttgtatgtgggctttacacatacaacacatcccgacctgcaaagaatagtgcagaagctgaatgaaacagaagc[T/C]gagaagaaaataccagttttgttgttagcactgtagccacggtgaaatttattcccgggttgcatccaaattatattattattattatcatttttttggg

fava_c16122_1526	fava_c16122	1526	T/A	cgggttgcatccaaattatattattattattatcatttttttgggggaaacaatttagtttccatgtttttaaagggtttcttacttgctttttttttgt[T/A]tatgtgttaattgttattactcattgtggaaacatgttctccccgattaataataKgtagaaacagactaacgtaattggctgaaatgtaaacaactact

fava_c16122_1582	fava_c16122	1582	G/T	agtttccatgtttttaaagggtttcttacttgctttttttttgtWtatgtgttaattgttattactcattgtggaaacatgttctccccgattaataata[G/T]gtagaaacagactaacgtaattggctgaaatgtaaacaactactttttgcttattaattggtaatgtaaccgcataacataatacttgaatagatcagat

fava_c16122_193	fava_c16122	193	T/A	catattcatattcaacaacctcgttaatcccttcattcccttctcattcttcattctctcgattccatttccaaaatcgcaaactcaacaacaacactct[T/A]aacctaatcccaatgtcttcttcRtcacttcctacaaaatcagccttcaatttaccttccatttccagattcaaccgcgcacgtcgaaccgcaatcaaat

fava_c16122_217	fava_c16122	217	G/A	taatcccttcattcccttctcattcttcattctctcgattccatttccaaaatcgcaaactcaacaacaacactctWaacctaatcccaatgtcttcttc[G/A]tcacttcctacaaaatcagccttcaatttaccttccatttccagattcaaccgcgcacgtcgaaccgcaatcaaattccgcggttcgtcttcatcggatt

fava_c16122_320	fava_c16122	320	A/G	acttcctacaaaatcagccttcaatttaccttccatttccagattcaaccgcgcacgtcgaaccgcaatcaaattccgcggttcgtcttcatcggattgg[A/G]tcaattcttcttctaatcgaaccgttttgttaacctccggtattactgttagtttagctgttgcgaatcgcgtgctttataaactcgctcttgttccgtt

fava_c16122_670	fava_c16122	670	G/A	tagaagctttgggattggttgctggaatgtcttctgcagcaatacttcctggaccagtcatacccatattgaatcagtcttatttagtttggcagttaac[G/A]ttttccactcttatcctgaggagaaggtactcaatcagccaaattgttgggtgtttactagtagctgctggtgtcgtggttgctatttcaagtggctcta

fava_c16128_1003	fava_c16128	1003	C/T	acataatcaagggtattgatttcttggaattcaggccttctgaaattgccgcaggggtagcaatttctgttttaaaggaactgtcgggccaagaagtcga[C/T]aaggccatagcctatttcttcatcgtagataaggagagggttctaaagtgtgttgaattgataaaagatctatccttgatcaaggttggtgcatctaagt

fava_c16128_1144	fava_c16128	1144	G/T	ttctaaagtgtgttgaattgataaaagatctatccttgatcaaggttggtgcatctaagtatacaccattagtgcctcaaagtcctataggggtgcttga[G/T]ggagggtgcatgagttataagagtgatgagttaacaaatgggtcatatcaaaattctttacataatagtctaaatactaaaaggagaaaatttgatggac

fava_c16128_1460	fava_c16128	1460	T/C	aaatactgcagagatagatgctgcggttggtagagaaaattttgatgaaacaatgttgttgaggcatagcatatgtgaatgaagtgacatatgtaatatt[T/C]gagaggttataattgtgttgtcacatgtgaatctcctaactttgaatatcataagaaaatcaaatatataaaatcatttttttttaataatttaaatgga

fava_c16128_288	fava_c16128	288	A/T	atttaagcttcttccatggctgaaagctttgacagtgctacatctaatcttctatgttctgaaaacaatagaacatgctttgatgaggatgtggaatgca[A/T]tggtgttgttgatggttctggaatctcaacttcatgggaccacatgatgaaccttaacttggacaacgttggatcggattcgtttgtgtgttttgttgca

fava_c16128_551	fava_c16128	551	T/C	ttatttgatgagattgcgtagtggtgacttggatttgagtgttagaagagaggctcttgattggatttggaaggctcatgcttattatggatttggaccc[T/C]tgagtctttgcttatcSgtgaactacttggatcggttcttatcggcttatcaattaccgagaggtgtgagttggactgtgcaattgttagccgtggcttg

fava_c16128_568	fava_c16128	568	G/C	gtagtggtgacttggatttgagtgttagaagagaggctcttgattggatttggaaggctcatgcttattatggatttggacccYtgagtctttgcttatc[G/C]gtgaactacttggatcggttcttatcggcttatcaattaccgagaggtgtgagttggactgtgcaattgttagccgtggcttgcttttcacttgcggcta

fava_c16128_799	fava_c16128	799	T/C	tagatttacaggtcggtgaaccgagattcgtgttccaagcgaaaacaattcaaagaatggaactaatgatactaagcacattaggatggaaaatgcatgc[T/C]ttaactccttgttccttcatagactacttcatatctaagatcagYtgcgaggagaatcatcccgaaaagtcgtcgattacaagatcagtgcaactcatcc

fava_c16128_844	fava_c16128	844	T/C	caattcaaagaatggaactaatgatactaagcacattaggatggaaaatgcatgcYttaactccttgttccttcatagactacttcatatctaagatcag[T/C]tgcgaggagaatcatcccgaaaagtcgtcgattacaagatcagtgcaactcatccttaacataatcaagggtattgatttcttggaattcaggccttctg

fava_c16129_801	fava_c16129	801	A/G	aaccattgattcctgcattattcaccaagatatcaagtcttccaaactgattctcaacaaactcaaccaatgatgaaatactaaaaggatcagtaacatc[A/G]agttgatgaaaaacaaccagattacaaaacccaaaatccttcttcaatttctcaaaagcttgaataccccttttctgatttctagcagtaagaacaactt

fava_c16130_142	fava_c16130	142	T/A	acatgcttttggtgtgcatagaaaaccatgtagtgtaaaacattgtaaatatgttggctaacatttaagattactcgaagttatattttttgtatcaaag[T/A]ctgaaaattaacacagatcacttcatagtccaccatctgatttttgagttggtagacatccttttttgttgggtatagaagagtgacaatatctatactt

fava_c16130_1683	fava_c16130	1683	C/T	atctccatttgatttgttgatcaggaggaagttttcctgacctctgaccttgaaaaaacagtaaagattttgccaaagcttctctgtaatttggtttgca[C/T]tcaacattttcaacaaccaacaaacatgtgatgatgataagtaacaagaaaagcagtgaatttattttcatcatgctcaatggttgtgttgtgtgtgacc

fava_c16130_663	fava_c16130	663	A/G	cataacctaccatataggacattcttagaggattttcacctaatatatagtctacctgtctttttgctatggatcgtaaggtattcggagtgacgaaaac[A/G]ttgccgcagttaaatgtatgttttgttgctgacatatactttgaataggttgtaagcaagaatgttatagctgtcacatattggagattactctcgggta

fava_c16130_939	fava_c16130	939	A/G	tatcggcttcttgtctatattgatcaaagttcttatcgcgattcagcaatgctctctttgaaagaagaacatgtgcaccagcatatttgttatcccaact[A/G]aagatatcaggttgatcatcagctcctaaagatttcaccaatttgtaatagtaaattgcatttgttgctctaaaaagccatgcagctccccacaacagtt

fava_c16131_204	fava_c16131	204	G/A	gtagacagctattatcatacaacaaatgcctgattgaaaacctcactcaaaccttctgctaatccaaacaatgcacaggcaaatggagttaatggcaagc[G/A]aattttggcatggcacggcgaggatgaaaccaaaattacatttctattgacattctacaaaactgttggtgccagcacaaaattttcattaaatgattaa

fava_c16132_1236	fava_c16132	1236	A/T	cgtcaaatacaccctcggctcgcattactatgtaaccagtttttttctgtatttcttcctttgagtcattttcatcaaagatttcaccaacaatctcttc[A/T]acgacgtcttcaagagttacaagcccaacagttccaccatactcgttaagaacgacagccatgtgaaccttcctgattcgaaattctctaagaagattcc

fava_c16132_1967	fava_c16132	1967	A/G	ggagtgatttcagtgagaagcaaaatggcaacagtcatcactcctgttgctgcactaacaccagcttcgccaaatgctgcagttgcagcttctgtgacca[A/G]agcagtcgctgcaatatttaccacagttgtgccgataagtatggtagttaggaatcgagtaacatcgcttcggagtagtctgaagacaccattttcagtc

fava_c16132_2124	fava_c16132	2124	C/T	gagtaacatcgcttcggagtagtctgaagacaccattttcagtctctttttcagccaactcacgaaccttccaaggccaaagtgtagtaattgatgtctc[C/T]gcgagagagaaaaacgcagaaagacctaaaagcaaagccaaYaacaacccttgctccttaaacaaWcgMagaacttgcaaaacagttggccaagtactct

fava_c16132_2166	fava_c16132	2166	C/T	tctctttttcagccaactcacgaaccttccaaggccaaagtgtagtaattgatgtctcYgcgagagagaaaaacgcagaaagacctaaaagcaaagccaa[C/T]aacaacccttgctccttaaacaaWcgMagaacttgcaaaacagttggccaagtactcttaagcaaaagtatactcttcccaataacaccatatccagctt

fava_c16132_2190	fava_c16132	2190	A/T	ccttccaaggccaaagtgtagtaattgatgtctcYgcgagagagaaaaacgcagaaagacctaaaagcaaagccaaYaacaacccttgctccttaaacaa[A/T]cgMagaacttgcaaaacagttggccaagtactcttaagcaaaagtatactcttcccaataacaccatatccagctttcaccacaccttccgWRgcRaaaa

fava_c16132_2193	fava_c16132	2193	A/C	tccaaggccaaagtgtagtaattgatgtctcYgcgagagagaaaaacgcagaaagacctaaaagcaaagccaaYaacaacccttgctccttaaacaaWcg[A/C]agaacttgcaaaacagttggccaagtactcttaagcaaaagtatactcttcccaataacaccatatccagctttcaccacaccttccgWRgcRaaaactt

fava_c16132_2282	fava_c16132	2282	A/T	ttaaacaaWcgMagaacttgcaaaacagttggccaagtactcttaagcaaaagtatactcttcccaataacaccatatccagctttcaccacaccttccg[A/T]RgcRaaaacttccggacagccaaacaccaacaccccacaaacagtagcagccaaaacaatccctctcttcaccaaaaactcatctatgaacttcaaatta

fava_c16132_2283	fava_c16132	2283	G/A	taaacaaWcgMagaacttgcaaaacagttggccaagtactcttaagcaaaagtatactcttcccaataacaccatatccagctttcaccacaccttccgW[G/A]gcRaaaacttccggacagccaaacaccaacaccccacaaacagtagcagccaaaacaatccctctcttcaccaaaaactcatctatgaacttcaaattag

fava_c16132_2286	fava_c16132	2286	G/A	acaaWcgMagaacttgcaaaacagttggccaagtactcttaagcaaaagtatactcttcccaataacaccatatccagctttcaccacaccttccgWRgc[G/A]aaaacttccggacagccaaacaccaacaccccacaaacagtagcagccaaaacaatccctctcttcaccaaaaactcatctatgaacttcaaattagcat

fava_c16132_2594	fava_c16132	2594	T/A	ccatttgaaccatgaaaggttgtagaactgaaaggacctacacaatttgacacaataggaaataagcatctactgtttgtacccaaaaccttcattggga[T/A]tctctttgtgcgaatctcatgcatagggtaaagagatttcaaacccgaactgaagacatggtgacccaaaaccgaagattcgaWagccattgctagaaca

fava_c16132_2678	fava_c16132	2678	A/T	aaaaccttcattgggaWtctctttgtgcgaatctcatgcatagggtaaagagatttcaaacccgaactgaagacatggtgacccaaaaccgaagattcga[A/T]agccattgctagaacagtaaaatcataacactccagaaactcaaacgagttgtaactttttttggtgatgaaattgaaattgattaggcgatgcaaccga

fava_c16132_529	fava_c16132	529	A/G	aaagcaaaaaaattcccgtctctttattttcttggtctaaacaggttgtcaaaataacatagccagccaaagaaaaacaactacaaataaagcaaaagaa[A/G]acaagctgacatacatgaataacattatcaggcatcagcagcatcttttgcaccaaagggatcatgagttggaccaacatcctgaccaatgtgacgctgc

fava_c16133_1017	fava_c16133	1017	A/G	gttaaaggaatgcacaagaaaaatcaaggaaaaggaagagaaaatgatgaatcgcagtccagatgttttatcaaagaaaatcaagttggtaagctctggc[A/G]gggttgtgtttgaagttgatattggtctggcactaatgtcaaaaagatttgaggaaattatcgagaccattcctgtKgctgttggtgacattgataccat

fava_c16133_1094	fava_c16133	1094	T/G	aaatcaagttggtaagctctggcRgggttgtgtttgaagttgatattggtctggcactaatgtcaaaaagatttgaggaaattatcgagaccattcctgt[T/G]gctgttggtgacattgataccatttcYgttcatgaagtgagtagcaaaatgttgacaacggttattgaatactgcaagaagcacaacatccgacagaagt

fava_c16133_1121	fava_c16133	1121	T/C	ttgtgtttgaagttgatattggtctggcactaatgtcaaaaagatttgaggaaattatcgagaccattcctgtKgctgttggtgacattgataccatttc[T/C]gttcatgaagtgagtagcaaaatgttgacaacggttattgaatactgcaagaagcacaacatccgacagaagtatgttaatctcaaggactatgttaatc

fava_c16133_1374	fava_c16133	1374	G/A	tcttcaaacatatgcaagctacttaaaaatcgatggtttgcagaggctgacatggggtaaagaatttgatttgataaaaggcatgacacgagaggaaatg[G/A]ccRagttctatgcagctcaagaagactcaaattccagttattagaagagaagaactaKtagtcttcagatttgttaacagtctaggtaaatgcaaacttc

fava_c16133_1377	fava_c16133	1377	G/A	tcaaacatatgcaagctacttaaaaatcgatggtttgcagaggctgacatggggtaaagaatttgatttgataaaaggcatgacacgagaggaaatgRcc[G/A]agttctatgcagctcaagaagactcaaattccagttattagaagagaagaactaKtagtcttcagatttgttaacagtctaggtaaatgcaaacttctta

fava_c16133_1432	fava_c16133	1432	T/G	aaagaatttgatttgataaaaggcatgacacgagaggaaatgRccRagttctatgcagctcaagaagactcaaattccagttattagaagagaagaacta[T/G]tagtcttcagatttgttaacagtctaggtaaatgcaaacttcttacttttcctatatgatcctgtYttggttgaactcgattttcgaaccaaagttaaac

fava_c16133_1498	fava_c16133	1498	T/C	gactcaaattccagttattagaagagaagaactaKtagtcttcagatttgttaacagtctaggtaaatgcaaacttcttacttttcctatatgatcctgt[T/C]ttggttgaactcgattttcgaaccaaagttaaactgatgcctattattagagatataagatgttagttactagttggttagtgcctataaaataggttta

fava_c16133_158	fava_c16133	158	G/T	cttcatctccaggaccaccaccattggtgacgaactaatctcgaaccttgaccatgacatcttcgataacatcgatataaacctggcttgtagttgaagc[G/T]gccatcaagaatcgagcgaaattagagcaattgaatctccattttcgtcaccgaaaggaaagttgagtaagaggattattgttttggaaacaaagagaac

fava_c16133_259	fava_c16133	259	G/A	gccatcaagaatcgagcgaaattagagcaattgaatctccattttcgtcaccgaaaggaaagttgagtaagaggattattgttttggaaacaaagagaac[G/A]aaatcaaatacccttggaaaaagcattgatagttagggttttctactctctccgattccacagtttctcactctggttcaactctccaaagcttcatcRK

fava_c16133_358	fava_c16133	358	G/A	cRaaatcaaatacccttggaaaaagcattgatagttagggttttctactctctccgattccacagtttctcactctggttcaactctccaaagcttcatc[G/A]taaccctatcaacRcgtcaatgagtagcccaaagttcatcagcttagaagaagggtgcgattatttgcacaagggtatcaccaagctcctcaacattctt

fava_c16133_372	fava_c16133	372	A/G	cttggaaaaagcattgatagttagggttttctactctctccgattccacagtttctcactctggttcaactctccaaagcttcatcRtaaccctatcaac[A/G]cgtcaatgagtagcccaaagttcatcagcttagaagaagggtgcgattatttgcacaagggtatcaccaagctcctcaacattcttgaagctttacctga

fava_c16133_770	fava_c16133	770	A/G	gatggccaaattacatagctatgaccaggcgtctctcgaaatttttttgcccacttgaaaaacttgatattccaagattaaaagttccttctcttgaaga[A/G]accagcttcttatccttttatcacctggtatacgRaggKatgaataaagaaataatggatgctgtatttgctatggttgatcgggaacgggcaggagagc

fava_c16133_805	fava_c16133	805	G/A	tcgaaatttttttgcccacttgaaaaacttgatattccaagattaaaagttccttctcttgaagaRaccagcttcttatccttttatcacctggtatacg[G/A]aggKatgaataaagaaataatggatgctgtatttgctatggttgatcgggaacgggcaggagagcatattgatcaaacatttgctcttaacacattggat

fava_c16133_809	fava_c16133	809	T/G	aatttttttgcccacttgaaaaacttgatattccaagattaaaagttccttctcttgaagaRaccagcttcttatccttttatcacctggtatacgRagg[T/G]atgaataaagaaataatggatgctgtatttgctatggttgatcgggaacgggcaggagagcatattgatcaaacatttgctcttaacacattggatttat

fava_c16134_1868	fava_c16134	1868	T/C	ctggcattgctagatggaaggactatccttgtgcaaaatcttagagacaacaagtttgtatgtatctgtaaagttttggtgctgtggcttatgattatac[T/C]atcattttacRccagtgctagacaaatactgtaaaattttcacagcaccattgaacaatgttaaatgtaaaacagctaaggtctaccatcatttaggtag

fava_c16134_1879	fava_c16134	1879	A/G	agatggaaggactatccttgtgcaaaatcttagagacaacaagtttgtatgtatctgtaaagttttggtgctgtggcttatgattatacYatcattttac[A/G]ccagtgctagacaaatactgtaaaattttcacagcaccattgaacaatgttaaatgtaaaacagctaaggtctaccatcatttaggtagtttacatttgg

fava_c16134_686	fava_c16134	686	G/C	tggctacacctaactcattgaaacccctttcgttgcctcgcaatgccagtagtagtggtaatagtgggaggattggaattgccatcaaaatgccgccgct[G/C]gcgtcgtattcagatgtttatggcgatttgattcgacggaaggaggtcaattgtgggaattgcgatgacaaatgtggttctggaaactaccgaagtataa

fava_c16138_1577	fava_c16138	1577	A/G	tactcccattatgttcaacatcaatagcctgaaaaatccggtaaagctcaagctccttatcatccatatacttcttaaactcgacaaaatcaacacgccc[A/G]tctttgtttgcatcgcaagcattcaacaaatccgtagcatatttatactcagaaggaatctcaagagctgataaaccagcttcaatgtgtttaaattcca

fava_c16138_353	fava_c16138	353	G/T	aattgtcacaataatcaattgtcgactttattcttgtcgtcattgtcttcattgcaaaagtattcccaacaattgcaaactgtcagcctcaataatacta[G/T]ttatcaaatgcagcatataaatttcttcactccaaatccaaactttttttcatagactcataaaccatgtaagtgatgcttgcggacgggacaactttga

fava_c16138_608	fava_c16138	608	C/T	tttttctgaatacatcacccattccgttataagagctttgggcctgcattcttgttctgacgacctgtaatgggtaaacacatgttgctccaagagctcc[C/T]gataccgtcccacatcctaattgtaccagagggccgggttctccgtcctgaataatatacttttttgacatctccttcaaggtttcataagcagctagat

fava_c16138_944	fava_c16138	944	G/A	tttgtagtcgtgtcttaacaagatccataggatatattgcagtttgagctacacctccagctataccaccagccagcagccgccccatacctccaatatc[G/A]gccttcccctctccacctttagcctccacaatgaaggttttcatcatctcataggtataaaatctaatggcactctcaggggccaccttaagaacattta

fava_c16140_1714	fava_c16140	1714	G/A	aactgtgcgtcgttggaagaaacgcggaggcggagaagggagcggaaagaggttgatccattgggatattggattctcttctgaaatcccgatcgtgtga[G/A]gttaagtttgggtgagtagaagttggcgactacgctacggtgttttttgggtaatgctggtggttttacggcggttactccgccgaaaactatggcggag

fava_c16140_794	fava_c16140	794	T/C	agccggcaaggtaagtcatgatagccatcacaacccatggaacggcactaaaccacgccgcttgcctgagatccacacgatatactgagttgaagtatat[T/C]ggcatccaagaaagaacaacaaaaaaaccccagctatgcatggagtttgcgacaattagagaccatgttggcatctttgaaagcaagcgcctgaaaggag

fava_c16142_350	fava_c16142	350	A/T	gaaaaatcttcacccatttttctattaaattaagaaagaaaattctctccttgtgtcgtttgttttttggtttgtgctgagaaaaaatggcgacgagtat[A/T]gggataatggatagcgcttattttgtcggtagaaatgagattctgacttggatcaataatcgtcttcaacttaatctctctcgcattgaggaggctgcat

fava_c16143_196	fava_c16143	196	T/G	taatcgctttcctaccaaaatggcttctcacttactcaaccttccattcatttcccttttccacattacgccaacttaattcatcaacaaacttttcaga[T/G]tctctcttagtgcttttWgaagaagttggaatagaaattttagctgggaaaatggaggatgataagaaaaggagaaaaaacaagaagaaaaagaataagc

fava_c16143_214	fava_c16143	214	T/A	aatggcttctcacttactcaaccttccattcatttcccttttccacattacgccaacttaattcatcaacaaacttttcagaKtctctcttagtgctttt[T/A]gaagaagttggaatagaaattttagctgggaaaatggaggatgataagaaaaggagaaaaaacaagaagaaaaagaataagcaaaacaagaatgtagatg

fava_c16145_1038	fava_c16145	1038	A/G	aaataagttagaccaaataccacgaaaattatgtggYgagatattaattgtgaaagattacacatcattacaaaacagcccgtgaagaaactttttcctt[A/G]aaaagttttgcacataccaaccaatgcggctcttaagcgatgatctgcagacaactaaRtttctgtttctaaagcatatgttagggaatcatgacttcgt

fava_c16145_1097	fava_c16145	1097	G/A	acacatcattacaaaacagcccgtgaagaaactttttccttRaaaagttttgcacataccaaccaatgcggctcttaagcgatgatctgcagacaactaa[G/A]tttctgtttctaaagcatatgttagggaatcatgacttcgtcgtcgccgtgagttttctgtttcggttSaggccaatttggtatcaagctagttaagaga

fava_c16145_1166	fava_c16145	1166	C/G	ggctcttaagcgatgatctgcagacaactaaRtttctgtttctaaagcatatgttagggaatcatgacttcgtcgtcgccgtgagttttctgtttcggtt[C/G]aggccaatttggtatcaagctagttaagagaatagcgcatcgaatgtaatgttacatgttaagctgtaagataagggaatttgcatcacaatcaattatt

fava_c16145_1368	fava_c16145	1368	T/C	atgtagcatgtactagagttgattaaagccaatagctgaaaattaactggtatacgattatgacaaatctgagttgatagattgtataataaaaacctca[T/C]gagacaaatgtgatgcacatgagtttgtagtctgcaaaacagacatacagtatataaggtgagatgtcaattttccaagttgtctgtttttc

fava_c16145_214	fava_c16145	214	G/A	ctccatccacctcctcatttctttccacgccataaacaccctcagttcagaggtttagggttgttacatagaaagggaattgttgagtctgtctggggaa[G/A]cagtgctgtgtctcgaagcaatcRattgaatgtatcctcatgggatgataaaccctttgaaattcttccaaatggtaaaaggtcttacttggatgaacaa

fava_c16145_238	fava_c16145	238	G/A	ccacgccataaacaccctcagttcagaggtttagggttgttacatagaaagggaattgttgagtctgtctggggaaRcagtgctgtgtctcgaagcaatc[G/A]attgaatgtatcctcatgggatgataaaccctttgaaattcttccaaatggtaaaaggtcttacttggatgaacaagatgtcgttgcgtttattgaccct

fava_c16145_602	fava_c16145	602	A/G	atcccaagttagtacgaaaaagtgaatctacattgttgatcaactcaaacaaagatgatttgatggttgaatattataaatgccgaacaagtggaggacc[A/G]atgccaatttcctggattaattcgttcaRaaaggttatattttcttgcaaggaYgggaagacctatgggcggctcattggtggatcagttttggcttcat

fava_c16145_631	fava_c16145	631	A/G	acattgttgatcaactcaaacaaagatgatttgatggttgaatattataaatgccgaacaagtggaggaccRatgccaatttcctggattaattcgttca[A/G]aaaggttatattttcttgcaaggaYgggaagacctatgggcggctcattggtggatcagttttggcttcatttgctgattcttttgctcctctgtatttt

fava_c16145_656	fava_c16145	656	T/C	atgatttgatggttgaatattataaatgccgaacaagtggaggaccRatgccaatttcctggattaattcgttcaRaaaggttatattttcttgcaagga[T/C]gggaagacctatgggcggctcattggtggatcagttttggcttcatttgctgattcttttgctcctctgtattttacaatgacacaaattaaggaagtgc

fava_c16145_780	fava_c16145	780	T/C	tggtggatcagttttggcttcatttgctgattcttttgctcctctgtattttacaatgacacaaattaaggaagtgctgtcaactgaggagccttgtgat[T/C]tatcatatgaatttggggatgggatgtatgatatcaaagaacttccactaggatttccaaaaccagttaaacatctttatcctttcaacgatcaaattgt

fava_c16145_902	fava_c16145	902	A/G	ggatgtatgatatcaaagaacttccactaggatttccaaaaccagttaaacatctttatcctttcaacgatcaaattgttatatacgttcgctatcttgg[A/G]ccgggtgtttccgtaggacaagcatggcaagagggaaataagttagaccaaataccacgaaaattatgtggYgagatattaattgtgaaagattacacat

fava_c16145_974	fava_c16145	974	C/T	aaattgttatatacgttcgctatcttggRccgggtgtttccgtaggacaagcatggcaagagggaaataagttagaccaaataccacgaaaattatgtgg[C/T]gagatattaattgtgaaagattacacatcattacaaaacagcccgtgaagaaactttttccttRaaaagttttgcacataccaaccaatgcggctcttaa

fava_c16147_1551	fava_c16147	1551	G/A	aaattttgctgtttttccatcattgcaaggtgggcctcacaacaaccacattgctgcactagctattgcgttaaaacaagtggccactcctgagtataag[G/A]catacatgcagcaggtgaagaagaatgctcaagctttagcatgtgcWttattgagaagaaaatgccgcctagtaactgggggtacagacaaccatttatt

fava_c16147_1598	fava_c16147	1598	T/A	acattgctgcactagctattgcgttaaaacaagtggccactcctgagtataagRcatacatgcagcaggtgaagaagaatgctcaagctttagcatgtgc[T/A]ttattgagaagaaaatgccgcctagtaactgggggtacagacaaccatttattactttgggatttaaggcccttgggattgacaggtaaattttacgaga

fava_c16147_183	fava_c16147	183	C/A	ttttcttcttcttcttcttcaccatctgcaaaatctcaatgtcctattcctctatttcccttacacaatctctactcaaccatcactgcaactgtaaaat[C/A]tattcctcttcttgttgttgtttatcgatttagttgcagagtttcttcgatttcaagaactgtttttgatggacctctctcatcatcaatccaatttatc

fava_c16147_2024	fava_c16147	2024	G/T	ttgagagcaatagggacattattgagctccgggcacgggtcgaagcttttgcaactcagtttgcgttgccgggttatgacatttgaacataatgcagctg[G/T]tgggttgcattattggtttttttgtaaagtctacactggggggttatcgggtctgcttgtcacaaaagcaagtgatcttgatgtgcagataatttgtcga

fava_c16147_2348	fava_c16147	2348	T/A	attcctgctgactacaggggttggaacagagattcttgatcaacctaccgagaacatattcttctgagtaatctgctgaaaatgaaaataattaatctga[T/A]caaggttagtYaccttgaattcttttgcctgctgtttcatggtgtaaagaatggtaatcctgtttattcttgctacatattaccccgttttttcatatga

fava_c16147_2359	fava_c16147	2359	C/T	ctacaggggttggaacagagattcttgatcaacctaccgagaacatattcttctgagtaatctgctgaaaatgaaaataattaatctgaWcaaggttagt[C/T]accttgaattcttttgcctgctgtttcatggtgtaaagaatggtaatcctgtttattcttgctacatattaccccgttttttcatatgatagttgtactc

fava_c16147_842	fava_c16147	842	G/A	tgatggaagcgttaggaagtcatttgacgaataaatactccgagggaatgcctggtgcacgttactatggaggaaatcagtatatagatgaaattgaaac[G/A]ctttgttgtgagcgtgcgttgaaagcgtttaatcttgattccaagtgttggggtgtgaacgtgcagccgtattcgtgtacctctgctaattttgctgtgt

fava_c16147_956	fava_c16147	956	G/A	gtgcgttgaaagcgtttaatcttgattccaagtgttggggtgtgaacgtgcagccgtattcgtgtacctctgctaattttgctgtgtatactggattgtt[G/A]tctcccggggatagaatcatggggttggatactccttgtggagggaatactagtcatggatactatactcctaatgggaaaaaagtttcgagtgcttcta

fava_c16148_1191	fava_c16148	1191	G/T	aaggactgcttgaggactcttctctcacttattgaagaagccggcaaagctaatagattagtagacaatgctcgaacttcatatgtgaaagagagactcc[G/T]agccaatccagctttcatggagaggctgcatgctaagtccctgagattgagagaatccaatcaagttcctgcttgaggttttcatgtttgacgcaggagt

fava_c16148_1359	fava_c16148	1359	T/C	cctgcttgaggttttcatgtttgacgcaggagttttagaacatttcaactgccaaaattcattattccttttgtgatttttatctttatctttttagttg[T/C]aatctggattctagtttcagaaacaactccaactctgcatactgaggtaaggctgcgaagatcctcacctcacctctaaaatcttaaggagactcataat

fava_c16148_184	fava_c16148	184	G/T	ctctctacactcgcaatcttctccaccaccactcttccgccaccgccgcactcgccgtttcatctccctcccgattccgtctcttctcctccgatgaaaa[G/T]ccctctccacctccctccatcaaagaccccgcccccgcccccgacgaaatcgaagacgtcgacaacaaagagttcaagtcgcggattgataaatacctaa

fava_c16148_310	fava_c16148	310	G/T	accccgcccccgcccccgacgaaatcgaagacgtcgacaacaaagagttcaagtcgcggattgataaatacctaaaaggcgatgaacagatgcttccttc[G/T]atcatgcaggcgattatgcagaggaggttatcagggaagcacgaggatactgatgatgagttgatgaatgaactcagcatggaacctattgatgatgtcg

fava_c16148_457	fava_c16148	457	C/T	atactgatgatgagttgatgaatgaactcagcatggaacctattgatgatgtcgatgatcaggattttgaatctgattttgaggatttacatgaaacgga[C/T]gaggagRtcgatgatttgtataatgctagagatattgtcatgaagaaaatggttaaagataagtactttaatatggatgacaagaaatatgatgatattg

fava_c16148_464	fava_c16148	464	A/G	tgatgagttgatgaatgaactcagcatggaacctattgatgatgtcgatgatcaggattttgaatctgattttgaggatttacatgaaacggaYgaggag[A/G]tcgatgatttgtataatgctagagatattgtcatgaagaaaatggttaaagataagtactttaatatggatgacaagaaatatgatgatattgttgaaga

fava_c16149_1122	fava_c16149	1122	C/T	taacactaccaccaccgaccgatccaagctcatccacaactaccattgtcactttcccagtaacaaacacattcttcactgtcccctccaatttcctaca[C/T]cctgttgcagctgtaaagtgttgtataatatactgcgccgaWgacgaaacctcattaaggggtaatttggcatgaacaggaacaggaaaaagtggacaac

fava_c16149_1164	fava_c16149	1164	A/T	ccattgtcactttcccagtaacaaacacattcttcactgtcccctccaatttcctacaYcctgttgcagctgtaaagtgttgtataatatactgcgccga[A/T]gacgaaacctcattaaggggtaatttggcatgaacaggaacaggaaaaagtggacaaccaagaacactaagaagcatcttaaggttagatttcttgttga

fava_c16149_137	fava_c16149	137	G/A	attttcctttattattctctctttctctcctcactaaaagtgtctcacgcactaaaaacaacaaactcaaaatcaagtttacatatactatgcccaaaag[G/A]tatgggttagtactgtattacaattacaaaaccaatccttataSatgttaagtaacaattagtattgttttcgcttataaatggagtatccttatgcact

fava_c16149_181	fava_c16149	181	C/G	tcacgcactaaaaacaacaaactcaaaatcaagtttacatatactatgcccaaaagRtatgggttagtactgtattacaattacaaaaccaatccttata[C/G]atgttaagtaacaattagtattgttttcgcttataaatggagtatccttatgcactgcgctaatccctSgagccgcatggaacacaaaaaacacaaScat

fava_c16149_250	fava_c16149	250	C/G	ctgtattacaattacaaaaccaatccttataSatgttaagtaacaattagtattgttttcgcttataaatggagtatccttatgcactgcgctaatccct[C/G]gagccgcatggaacacaaaaaacacaaScataacaattttccatatYaatatatatcatctatgcaaggatgatttccaatcaagatgttgttgtggaca

fava_c16149_278	fava_c16149	278	G/C	ataSatgttaagtaacaattagtattgttttcgcttataaatggagtatccttatgcactgcgctaatccctSgagccgcatggaacacaaaaaacacaa[G/C]cataacaattttccatatYaatatatatcatctatgcaaggatgatttccaatcaagatgttgttgtggacactctttgttaagttcttgaggtggcaag

fava_c16149_297	fava_c16149	297	T/C	tagtattgttttcgcttataaatggagtatccttatgcactgcgctaatccctSgagccgcatggaacacaaaaaacacaaScataacaattttccatat[T/C]aatatatatcatctatgcaaggatgatttccaatcaagatgttgttgtggacactctttgttaagttcttgaggtggcaagaaacaatcaatagacaacc

fava_c16149_786	fava_c16149	786	G/C	tcatctccgccgtgttgtcgcttcgctcgactaggtctttttgatccgcccagagttttaaaacgaaacaatccacgccgttcatttgtttctctcccat[G/C]tgttgtgctgcactgaatacagctgatactgctaaagggtctagtccctgaattgcgcgacgaagaggacgaacRccacctttagccgcgtgcacgccga

fava_c16149_861	fava_c16149	861	A/G	cgccgttcatttgtttctctcccatStgttgtgctgcactgaatacagctgatactgctaaagggtctagtccctgaattgcgcgacgaagaggacgaac[A/G]ccacctttagccgcgtgcacgccgagccaaggtgtgtgacgccaagcaataatgccattacttcctgcaagaaccttttgaccaccaacaacaatctcta

fava_c16151_1014	fava_c16151	1014	G/T	gattgtcaccacaattgaSactttctccgatttggtttgggttttggttttggtcattggaaggattattgagactgatttcgggttcggtttgagtttc[G/T]gtttcgggttgagaattttcctcacacatatggtcattgttgaagtcattgttgaaatgggttgggatgataattgggatagaaacctgacaagaatctt

fava_c16151_1336	fava_c16151	1336	C/G	atctgttaaacaaacagcacattccaatgggcctttatcagttttaacatccttgatagctgagtaaaagaggatagggaaagtgttgaggagttcttgg[C/G]tgataccatgagaacatgaRcaaggacaatcagtggtgtggtttgtYcttgtgatgatgatgtgtgattcaacgcagtttctgatgtaRaaaaagacgat

fava_c16151_1356	fava_c16151	1356	G/A	attccaatgggcctttatcagttttaacatccttgatagctgagtaaaagaggatagggaaagtgttgaggagttcttggStgataccatgagaacatga[G/A]caaggacaatcagtggtgtggtttgtYcttgtgatgatgatgtgtgattcaacgcagtttctgatgtaRaaaaagacgattcctaggaagagaagtagac

fava_c16151_1383	fava_c16151	1383	T/C	catccttgatagctgagtaaaagaggatagggaaagtgttgaggagttcttggStgataccatgagaacatgaRcaaggacaatcagtggtgtggtttgt[T/C]cttgtgatgatgatgtgtgattcaacgcagtttctgatgtaRaaaaagacgattcctaggaagagaagtagacaaagtatggcaccaactgtgattgcaa

fava_c16151_1425	fava_c16151	1425	G/A	ggagttcttggStgataccatgagaacatgaRcaaggacaatcagtggtgtggtttgtYcttgtgatgatgatgtgtgattcaacgcagtttctgatgta[G/A]aaaaagacgattcctaggaagagaagtagacaaagtatggcaccaactgtgattgcaaaggagggttcccatttgtgatgagagatgtctgtgggaagag

fava_c16151_411	fava_c16151	411	C/A	tgataattagtactagtggatgtgtgtctaaaaaaatcaataaaaataaagagagaaaaaaagcacttgctacactcacatcatctcatcttttgaactc[C/A]acaagRaaaWaaWtaaaWaaataaataaaaataacgagttaactcactcatacgcatctaattttcacacatctcaagtaaaggcaaacgagttaactca

fava_c16151_417	fava_c16151	417	A/G	ttagtactagtggatgtgtgtctaaaaaaatcaataaaaataaagagagaaaaaaagcacttgctacactcacatcatctcatcttttgaactcMacaag[A/G]aaaWaaWtaaaWaaataaataaaaataacgagttaactcactcatacgcatctaattttcacacatctcaagtaaaggcaaacgagttaactcaattaac

fava_c16151_421	fava_c16151	421	A/T	tactagtggatgtgtgtctaaaaaaatcaataaaaataaagagagaaaaaaagcacttgctacactcacatcatctcatcttttgaactcMacaagRaaa[A/T]aaWtaaaWaaataaataaaaataacgagttaactcactcatacgcatctaattttcacacatctcaagtaaaggcaaacgagttaactcaattaacaaca

fava_c16151_424	fava_c16151	424	A/T	tagtggatgtgtgtctaaaaaaatcaataaaaataaagagagaaaaaaagcacttgctacactcacatcatctcatcttttgaactcMacaagRaaaWaa[A/T]taaaWaaataaataaaaataacgagttaactcactcatacgcatctaattttcacacatctcaagtaaaggcaaacgagttaactcaattaacaacaact

fava_c16151_429	fava_c16151	429	T/A	gatgtgtgtctaaaaaaatcaataaaaataaagagagaaaaaaagcacttgctacactcacatcatctcatcttttgaactcMacaagRaaaWaaWtaaa[T/A]aaataaataaaaataacgagttaactcactcatacgcatctaattttcacacatctcaagtaaaggcaaacgagttaactcaattaacaacaactttact

fava_c16151_617	fava_c16151	617	G/A	caacaactttactcatcttccatttcaatcttcgttcttgcttcatcattctcacaccccattaaaattcaacaatgacccatttattgaatcgacctaa[G/A]catcatcattcttcattcaagaaccttttttttcccgttgaaatcgatgttttcagaaaatgcacagaaactgttcgatgaaattcctcaaccacgtcgt

fava_c16151_932	fava_c16151	932	G/C	gtgtacttctcctcacacttcacttgttcatccagagaatgaccagttgaatttgacctcaaaagcttcggttttgatgcatgattgtcaccacaattga[G/C]actttctccgatttggtttgggttttggttttggtcattggaaggattattgagactgatttcgggttcggtttgagtttcKgtttcgggttgagaattt

fava_c16153_545	fava_c16153	545	C/T	gagttcgctaatgcctcaatgctaactttttcatctatctaaatttgggaacggccaaatatatcaaacgaagaaaaatatatcagaatttagtattaaa[C/T]attttgtcaaaggtggcacggatcatagagctgctattgtgtcacaattgttacttcttgttcatatttttattcaccaattccatgtatcaattagggt

fava_c16156_1374	fava_c16156	1374	A/C	gggaagccatcaatgaatttgagaaaaagtggctcaatgcaacaggccgatttggaggaaaaataatctccaccaccatctcagtgatgtttggaaggac[A/C]cttgaggttcctgaaagctgcgaaggagttgcccgctttacatttgactatctatgtgggcgtccgttgggtgcagcagattatattgcagtagctgaca

fava_c16156_1784	fava_c16156	1784	A/T	gaaggcagtaaacttcgccgtgatgtcttagcagaaggcaacgtgggctcaggaggtacccctgttggcatcacatccatactatctggtcaggaagaga[A/T]gtttacttttcagagagctgtctcacgcctgattgaaatgcaaactccattatacttggatggagtcagtgattttcatccttattttcagaggcatcat

fava_c16156_189	fava_c16156	189	C/A	cactaaacaaacaaaacgcaaaaacaatgagaagaatctggaaccttctctcccataacaaccctaacaaactcttccaatccgcattctcgatcccaac[C/A]aacttccacgtcactccctttcgctcttactgcgatccaccgcgcattcccaatcaaaaccgtccaggaccgattgtgcagtacaagaatctcgttgatc

fava_c16156_305	fava_c16156	305	A/G	ccctttcgctcttactgcgatccaccgcgcattcccaatcaaaaccgtccaggaccgattgtgcagtacaagaatctcgttgatcaagggaagttgcagc[A/G]cgattcttatcaagagagtgttgctacggagcttcagaatttgttagctaggttggagaattatgagagggaaatggaagagtatcatgttaatttatct

fava_c16156_537	fava_c16156	537	G/A	aaaggattctcatagaggaagttgagaagcagcagaacgaggaggattggtggaagaggttgaataataaaatcactgaaagatggaccgctgggaaaag[G/A]cccggtaatgtggagtcaggagtaggaaaatgggtgtcatatcttaaacgtgagaagaagctggattcactagttggtcgccgcccaactgctcctccag

fava_c16156_720	fava_c16156	720	A/G	gcccaactgctcctccagctcccagaggactttacatatatggcaatgtgggcagtgggaagacaatgctgatggacatgttttatagtgccactgaagg[A/G]atagttaaacatcgaagaaggtatcactttcatgaggccatgcttagaataaatgaacatatgcacaagatatggaaaaaacaaatggaagagaagcctt

fava_c16157_158	fava_c16157	158	C/A	catttacatattcatacatcactaaatatttacaagtgcacattatgaaagtctcataatgaaacaagttttgcccttatttaaaatgttaaaaagaaaa[C/A]acttgaacaaaacaaaacaatgttcaaggtgcaaaacatcatcatcatcataatttcaagttatagtcataatatcaatgattttaaattctttgSaaaa

fava_c16157_254	fava_c16157	254	C/G	aaaaMacttgaacaaaacaaaacaatgttcaaggtgcaaaacatcatcatcatcataatttcaagttatagtcataatatcaatgattttaaattctttg[C/G]aaaagcaatgcaaccatattttatctcatctacaaccacaatcgcagcttcatcatcggccgttgccgccgcgtYtgctccattttcataatctcctaaa

fava_c16157_329	fava_c16157	329	C/T	aatatcaatgattttaaattctttgSaaaagcaatgcaaccatattttatctcatctacaaccacaatcgcagcttcatcatcggccgttgccgccgcgt[C/T]tgctccattttcataatctcctaaatcattcaataataagagcaacaaggacacctaatgagtccattctcattgcattccaagcatctcttcaacaaac

fava_c16157_674	fava_c16157	674	A/G	gagacttaatcacttcagcaccaccaatgtattttcctctaataaagacttgaggtagtgacacattttcctctcccattacacactgcaattccttcct[A/G]taagcacgatccatggaaacatctctttcatcaacccaaaccctaaaccctttcagaatcatcttcaccgcattacaatcctcaaacgttctccgaattc

fava_c16157_782	fava_c16157	782	A/G	gatccatggaaacatctctttcatcaacccaaaccctaaaccctttcagaatcatcttcaccgcattacaatcctcaaacgttctccgaattcctctcaa[A/G]ctcgtcaaatacaccacaatccgttcctcagcaccttccaatcggaacaacatccccgattcccaccccaatttctcaccagaacgcgcttccgaaccta

fava_c16157_884	fava_c16157	884	C/T	tcgtcaaatacaccacaatccgttcctcagcaccttccaatcggaacaacatccccgattcccaccccaatttctcaccagaacgcgcttccgaacctaa[C/T]ctcgaagccgcgcgcggctccgaacctaatctcgaagccgaacgcggctccgaacctaatttcgcagattctttcggcgatgattcgaacaaattgcaga

fava_c16158_1989	fava_c16158	1989	G/T	ttgttactgttgttgttgtcgtcgtcacaaattgatacacaaccaccaacggttgtcccattagactgaagaaaagcaagattgttacatcccacagcta[G/T]aaacgtgttttcagcctgtgaaaatatgtaaggactgtctctaagattaacggtttttctattgttgatgctaattttgtttttttgacaatttgaacgg

fava_c16158_2109	fava_c16158	2109	T/C	gaaaatatgtaaggactgtctctaagattaacggtttttctattgttgatgctaattttgtttttttgacaatttgaacggtaaattgggttcataatct[T/C]aactttgctcSagtacgggtMaaaatggattacttcaagatttaacgacttcaagtaaggtttaggaacatcagaagaagtattgttgtcaaatttgcat

fava_c16158_2120	fava_c16158	2120	G/C	aggactgtctctaagattaacggtttttctattgttgatgctaattttgtttttttgacaatttgaacggtaaattgggttcataatctYaactttgctc[G/C]agtacgggtMaaaatggattacttcaagatttaacgacttcaagtaaggtttaggaacatcagaagaagtattgttgtcaaatttgcattctatttcaaa

fava_c16158_2130	fava_c16158	2130	A/C	ctaagattaacggtttttctattgttgatgctaattttgtttttttgacaatttgaacggtaaattgggttcataatctYaactttgctcSagtacgggt[A/C]aaaatggattacttcaagatttaacgacttcaagtaaggtttaggaacatcagaagaagtattgttgtcaaatttgcattctatttcaaaccatttgtgt

fava_c16158_914	fava_c16158	914	G/C	atacttttcatccaatagtatatttgttgatttgacgtctctatgataaattggctgagatgcaactgagtgcaaataaaatatagcaccagcaacttca[G/C]aggcaattctcaaacgcatgtcccatgtcattggtatgtcctcattttggtcatgcagatactcaaaaaggttaccattgggaatgaattcataaacaag

fava_c16159_239	fava_c16159	239	A/G	catgggaatttcagttgccaaacacagactagaaattctcaagcttgtcaacaaacaaaatgacaaagtgccaaaaaacaccaataagttttctttggtt[A/G]tcaaaaagtatataaagaagtgtcttagcaaggccattctccataaagaagaaaagaaaaacatgaacRtgatttcattgcaacaagagcaaaattggaa

fava_c16159_308	fava_c16159	308	A/G	gccaaaaaacaccaataagttttctttggttRtcaaaaagtatataaagaagtgtcttagcaaggccattctccataaagaagaaaagaaaaacatgaac[A/G]tgatttcattgcaacaagagcaaaattggaaccaagggaaatggagaagagcattggtaagtgaggaactcaaaggagaaaagggtatgcatagaaatag

fava_c16159_499	fava_c16159	499	T/C	atagaaataggaaaatagcattttcagggcctttagatggaagaatgtatgatgagaaaatggtaaataccaacaaaaaaatgttgaaattttcaggtcc[T/C]cttgatggaaagatgaatgaaagaaaaatggtttacacaaatagaagtcctatgagaaataggcctattgatggaaggtttgtggacactacaacaagaa

fava_c16159_744	fava_c16159	744	T/A	ctctacaacaagacaaaagattattatgattttgaggattctgaaatgtggcatgcattgtttgaagatcttaaaccaacttgattttcttttttgggat[T/A]ttcttggtgtgacattaatgttttcttatgcaatgatggttcttgaatattgtaatattttctttttggtgtttcttttttgtttttctctgtcaacttg

fava_c16162_1086	fava_c16162	1086	C/T	caacagcttcaacatgatgaattgacacaaacggtgctattgggtgagaggatagaagaccgtgagcatcaccttttatgtcccactggtgaaaaccatg[C/T]tcccgggtcagtggaatgccaagttcagtgatgcaggcatgcagtctatcatcactaccataaagcccagggtacctctcaatgcattcatcaagaatct

fava_c16162_1251	fava_c16162	1251	G/A	gcccagggtacctctcaatgcattcatcaagaatctcatagagggcctttgcaagaggatgacttattgcgatcccggcaccaccgtaggccattgagtg[G/A]ctaaaataggtattagccgaatggctctctgaagggctacctatgtaaatcatttcagaggaattatacttgctgagaacatcaactaggttattcacat

fava_c16162_2178	fava_c16162	2178	C/T	gccaccataagagatgtacgaagcacagaggatcgaactatgaagagcagcagtggcaacacacactccaaccaaacttgtttttacctgaaagtaagaa[C/T]cSttgatgaaaattccgatgaagatgatgttgaagttaaaaagttattttccaatgaaaattgaaaactgcatagaacaacaaatatagcaacccgttga

fava_c16162_2180	fava_c16162	2180	C/G	caccataagagatgtacgaagcacagaggatcgaactatgaagagcagcagtggcaacacacactccaaccaaacttgtttttacctgaaagtaagaaYc[C/G]ttgatgaaaattccgatgaagatgatgttgaagttaaaaagttattttccaatgaaaattgaaaactgcatagaacaacaaatatagcaacccgttgaat

fava_c16162_364	fava_c16162	364	T/C	tctctacatagaaattataggttcaaaaaagatcaattcaaggtgaaaaaaatgctgcatggaactagctctccactcaaaaggccaatcatgtgaaaaa[T/C]gcgMgagaStaatcctaacatgctatttcataactgacctacacaatcagtaacagagctaaacggccccttcccagttcccacattgtccaactgagat

fava_c16162_368	fava_c16162	368	A/C	tacatagaaattataggttcaaaaaagatcaattcaaggtgaaaaaaatgctgcatggaactagctctccactcaaaaggccaatcatgtgaaaaaYgcg[A/C]gagaStaatcctaacatgctatttcataactgacctacacaatcagtaacagagctaaacggccccttcccagttcccacattgtccaactgagatttgg

fava_c16162_373	fava_c16162	373	G/C	agaaattataggttcaaaaaagatcaattcaaggtgaaaaaaatgctgcatggaactagctctccactcaaaaggccaatcatgtgaaaaaYgcgMgaga[G/C]taatcctaacatgctatttcataactgacctacacaatcagtaacagagctaaacggccccttcccagttcccacattgtccaactgagatttggagtaY

fava_c16162_473	fava_c16162	473	T/C	Staatcctaacatgctatttcataactgacctacacaatcagtaacagagctaaacggccccttcccagttcccacattgtccaactgagatttggagta[T/C]gtccttgccagaattgccttggttgcaacagagacgacgtggaaccaaatgccaattcttgctcaaaggctgtgccactacttcgatctttcgcacattg

fava_c16168_182	fava_c16168	182	A/T	aacaggaccctttcatcatattttgtatcctctgccagtggtcctctccttgggagtaaagaaaaactactaatttctaggtaaatttatttgtgtctta[A/T]cacttagaaaatagatctcacataaaatgaagagacctacatgagagtgccggttatcggttagaaagtgttccttattctctaaaataaaacaccccat

fava_c16168_320	fava_c16168	320	C/A	tacatgagagtgccggttatcggttagaaagtgttccttattctctaaaataaaacaccccattactacaaaatggcagctccattgcagcatccttcat[C/A]tagtaacagaacaaaagaaaatctaagcaactacacagcatcattctttggaaattgactacttacaactatgcatctgcgacagtatgggcagtctcca

fava_c16168_496	fava_c16168	496	T/A	tctgcgacagtatgggcagtctccacaactgcgaatccaaggatccaaacaagcagagtgaaatttatgtccacattgcaaatggattagtttatctcca[T/A]ccgtgaaagattccaagcatatcccacaatcttgcagtatcctggattctgattccgtattgctgctgctaaaaacttcatgatgcaaacagtccagggc

fava_c16168_737	fava_c16168	737	T/C	aagaactggaaccttgccatttgagaagtcaagtcagtaactaaagagtcctcagttggaatttcacttgataactcactttcttgatcctcctcatcag[T/C]tggatctctattatattgcctgtttctatctacaggctctcctctcaacctYtgaaggagtctttctcgcgcaagtctcacacctacaggtaatctgtta

fava_c16168_789	fava_c16168	789	C/T	cagttggaatttcacttgataactcactttcttgatcctcctcatcagYtggatctctattatattgcctgtttctatctacaggctctcctctcaacct[C/T]tgaaggagtctttctcgcgcaagtctcacacctacaggtaatctgttactgccattcacactcggagtacctctcacactttgtccagaatcaaagttat

fava_c16170_1239	fava_c16170	1239	G/A	caaccaactccttgagatagagttctggagcaattctcatgaataatctcatgttaaggtcgttatgatgagttacaaatggcttagcagcagcaccacc[G/A]gcaatcatattcatcataggtgtttcaacctctaagaaatcaagcttgtccaggaaggacctaatgtaggaaatgattttagacctagtcttgaatattt

fava_c16170_155	fava_c16170	155	C/G	ttcaaaacaatatggtatctttcaaaagtataacaaaaaagacaacaagttataatctattaatccaatcaccaaaagatactcccaaaaaagaaattca[C/G]caaaaaaaatactgtatataaatagcactcatccaataagattagatgaaccgatacaaaaactattcatcaaatagacagatcagaactgagctcccat

fava_c16170_318	fava_c16170	318	A/G	ctattcatcaaatagacagatcagaactgagctcccatattaagaatcttgcatttctcctatacctgtaaatgattagattggggcaagatttaacaca[A/G]ctttggaggaaagctcaatcttgaggtttcatagcagggaagagaagaacctccttaatattttgggaRtcagtcaaYaacatggtcagcctatcaatac

fava_c16170_387	fava_c16170	387	G/A	aaatgattagattggggcaagatttaacacaRctttggaggaaagctcaatcttgaggtttcatagcagggaagagaagaacctccttaatattttggga[G/A]tcagtcaaYaacatggtcagcctatcaatacccaaaccccaaccaccagttggtggcaaaccatactccaaagcggtacaaaatgtctcgtctaaggcca

fava_c16170_396	fava_c16170	396	T/C	gattggggcaagatttaacacaRctttggaggaaagctcaatcttgaggtttcatagcagggaagagaagaacctccttaatattttgggaRtcagtcaa[T/C]aacatggtcagcctatcaatacccaaaccccaaccaccagttggtggcaaaccatactccaaagcggtacaaaatgtctcgtctaaggccattgcttcat

fava_c16170_528	fava_c16170	528	A/G	aaccaccagttggtggcaaaccatactccaaagcggtacaaaatgtctcgtctaaggccattgcttcatcgtcacctgattgccgatccttgagttgctc[A/G]gcaaatctttgtcgttgtaccacagggtcattcaattcagtatatgcattgcaaatctcatgtttgttaacaaacaattcaaaacgttctgtcaggccag

fava_c16170_777	fava_c16170	777	G/A	ttatgatgaatgtgggattgacacatgtttcctccaaaaagtgcccaacaagcttatccaacaagcgtgtagttgtttcaggagggggacatttgatttc[G/A]tacttcaaacaagcattcttcagatattgattagtttcatcactggccaagtctttgggaatactgaggccagcaatctgctctaatccttcaatcatat

fava_c16171_1104	fava_c16171	1104	G/A	ctaaactggtttgcatctgattttgcgtatgatgattataccattatttcttttgatttggtgaacgagtcctatcgaaatattttgcctcctaagcacg[G/A]tggggaacttctgtttgaggtgagcttggatgttttgcgggattgtttgtgcatcttttctcatagtcgcacattttctcgtgtttggataatgaaggaa

fava_c16171_836	fava_c16171	836	G/A	tgttttgtggaacccttccattcaaaaattcaaaacattaccttctttaggtttaggtttatgccaatatcgtgcttatggttttgggtttgatacctta[G/A]ttgataactataaggtggtttctgttctttgcgagatggataatgaaggatttcttMaagctactgctaaaattaaggttcatacattaggtactgattc

fava_c16171_893	fava_c16171	893	A/C	tttatgccaatatcgtgcttatggttttgggtttgataccttaRttgataactataaggtggtttctgttctttgcgagatggataatgaaggatttctt[A/C]aagctactgctaaaattaaggttcatacattaggtactgattcttggagaatgattccaggtgactttcctgccccattaactaggtggctgaaattggc

fava_c16172_1250	fava_c16172	1250	G/A	ctgcgattgaggttcgacttttaatggacgtaccataagcgctttgctgaatccagaatctttggcactatcggtaccactaagatataccggatacatc[G/A]aatcttgagtaacaccgacgggttttggtcgcttggggcatatgactaaatgtcgcttcaagtgagaagttccacaattatcttgcaacagctttccaca

fava_c16172_1440	fava_c16172	1440	T/C	agctttccacagtatttacatagaactttactaccttcggttgtttgaatgcgctgcatttcttcccatactttagatgtcattttcggacgctttgaac[T/C]gttaaaatcttcgcaatcataatctatctcacagctagttgtaatctcatttgtcattctctcctgggttctgcaattgtacccagcgaggtgaagctgt

fava_c16172_212	fava_c16172	212	G/C	acaccacaaatatgagaactattttttagttcatcaaggagctgggatgaaagcatctaatatagttaaaattcagttttgttatatgcaattttctgat[G/C]tacaRgcttgaatatatgatttagatttaccatccatgtcttcagaatttaccccatttaagcttttgttaaatttttctccaagctttaaccattcaac

fava_c16172_217	fava_c16172	217	A/G	acaaatatgagaactattttttagttcatcaaggagctgggatgaaagcatctaatatagttaaaattcagttttgttatatgcaattttctgatStaca[A/G]gcttgaatatatgatttagatttaccatccatgtcttcagaatttaccccatttaagcttttgttaaatttttctccaagctttaaccattcaacttgaa

fava_c16172_319	fava_c16172	319	G/T	cttgaatatatgatttagatttaccatccatgtcttcagaatttaccccatttaagcttttgttaaatttttctccaagctttaaccattcaacttgaat[G/T]ttttcttggattttcttggccatttccctctcatctttcaactgtggaacttgctccttcaactcatctctttgggctttSagagcttttgcttcttcaa

fava_c16172_400	fava_c16172	400	C/G	ttaaccattcaacttgaatKttttcttggattttcttggccatttccctctcatctttcaactgtggaacttgctccttcaactcatctctttgggcttt[C/G]agagcttttgcttcttcaaaaacttctctttttctcttYgcagccgtgaacttagccgattgaaacacRgatatgttagcttttatcgcatttatctgat

fava_c16172_439	fava_c16172	439	T/C	ccatttccctctcatctttcaactgtggaacttgctccttcaactcatctctttgggctttSagagcttttgcttcttcaaaaacttctctttttctctt[T/C]gcagccgtgaacttagccgattgaaacacRgatatgttagcttttatcgcatttatctgatcttctagctctttctttctttgctccaaagttgctagct

fava_c16172_469	fava_c16172	469	G/A	cttgctccttcaactcatctctttgggctttSagagcttttgcttcttcaaaaacttctctttttctcttYgcagccgtgaacttagccgattgaaacac[G/A]gatatgttagcttttatcgcatttatctgatcttctagctctttctttctttgctccaaagttgctagctggttgcataactcattttccaaagacaaaa

fava_c16172_583	fava_c16172	583	G/A	ttatcgcatttatctgatcttctagctctttctttctttgctccaaagttgctagctggttgcataactcattttccaaagacaaaacttccttgaactc[G/A]ttcttgtttgcttcaagactttcttcgagtttatctgcttttgcgatgttggagcttgctgactctatcttcttactagcgtcgttgtagtcacaactcc

fava_c16172_775	fava_c16172	775	C/A	cacaactccaccgagtgaattctctagaaacttccaatatcaacaacttcatttcggacgagattcccttgacggatgacatattggaaaggtattctat[C/A]gtagatttcatggcacagcactgtttggcctgcagtagaacagagaactctttggagagaaggtcttgcagtgttttcaatgctttgcttgtctcttcac

fava_c16172_900	fava_c16172	900	T/C	ttggcctgcagtagaacagagaactctttggagagaaggtcttgcagtgttttcaatgctttgcttgtctcttcacaaggcgtagtagtgttggatgatc[T/C]ggtgatgRccgtggtatcttgggcaggtgacttaaatgaggtctcaacatcgagagatgcataaaatgctttcatttcggcgtcatcaagagatagttcc

fava_c16172_908	fava_c16172	908	A/G	cagtagaacagagaactctttggagagaaggtcttgcagtgttttcaatgctttgcttgtctcttcacaaggcgtagtagtgttggatgatcYggtgatg[A/G]ccgtggtatcttgggcaggtgacttaaatgaggtctcaacatcgagagatgcataaaatgctttcatttcggcgtcatcaagagatagttcctcttgatt

fava_c16174_1452	fava_c16174	1452	C/T	atgtctgactcattcaaaagaagtttaacaagctcgacgtcgtctgagtccaatgccttgtgtattctagtgattcgttttagagacaaagcgtccacta[C/T]gggagcatcgtcttcaccatccttctgaagatcacggcggaacaRtttaactttttcggagagctcgtgaggaagcgctttctcaattgagatctggtca

fava_c16174_1497	fava_c16174	1497	A/G	gagtccaatgccttgtgtattctagtgattcgttttagagacaaagcgtccactaYgggagcatcgtcttcaccatccttctgaagatcacggcggaaca[A/G]tttaactttttcggagagctcgtgaggaagcgctttctcaattgagatctggtcaaggtctgatcgtgccaccctgtcaacacattgagcgacgagttga

fava_c16174_1816	fava_c16174	1816	G/T	tggaagaggcgtacatcaactccacagcaaagttaattgcaggtccacatgcatcatgagcacacacattatcaacacatgtagaaacctccattggaga[G/T]ggtttgagtttaccggtatatacatagttaagtaatatgagaaaagcttcatgtccaactttgccaaaaggcaacaaatcactcaagcaatacttcaatt

fava_c16174_2061	fava_c16174	2061	G/A	agctcatggaaaaacttactcctagaacctaaaatacaccgatgaactctaaccaaaactccctcaacaatgatgtccgcatcaccataatcatattcag[G/A]atcaatcaacagctgttccaaattagaactaagcttactcaaactgataacctccaggttacgagcatgatcattgccattcgagttgtgactaattgat

fava_c16174_2598	fava_c16174	2598	A/T	tgtgtgcaaattccataagaaaaaaaatacatgtatgaagaagttctttgtttgaatttccagagccaccaaaacagcataatcaaacaaaacccaaaat[A/T]gaagaaaacaaaatcacaatgaaaaaaaaaaactcacttttgaaattcaattttcagcaccgacccattaaacaaaagtctcaattttcaaaagcaaatc

fava_c16174_844	fava_c16174	844	G/A	cttccatcctagaaataagtcttttgctttgcattatgggagtctcattgaggtcaacctcccttaagtttccatttgaaccttttcccgccgaaagacc[G/A]gcaaactcggatgttgtctctgcacgagcaatgtccatggctagtttggcttccaaggggaagaacagtctcgcaaatgccacgcggttctcaaggtaca

fava_c16177_1309	fava_c16177	1309	C/A	cattttcattttccgagattgtctaggaattttctatacattgaagttctaaattttagttcctttaagtttattcattcaagttgaaagcaaatggagt[C/A]tttttccatgatgagagttacctgtggaagtttttggcgtaaaccttatacKggagagttggtgtagtgtctcagacagtggagttgctgcaatgcaaag

fava_c16177_1361	fava_c16177	1361	T/G	attttagttcctttaagtttattcattcaagttgaaagcaaatggagtMtttttccatgatgagagttacctgtggaagtttttggcgtaaaccttatac[T/G]ggagagttggtgtagtgtctcagacagtggagttgctgcaatgcaaagtaaagtcaaatgcctttctaatttacgtgaattcacgttatacaattaagta

fava_c16177_158	fava_c16177	158	C/T	tgtttgaagaagaacctgaatgctattcagtccatattcacacacaccaacccaaaaaccccaattcaccccacccacattataactcttctcttctatt[C/T]tcttcatttcattcgtaatcaccctcacagtcacacagaaacgacgtcgacSaagagaagaaatgaaaaccgagaagaagtcattgttacaagatctaat

fava_c16177_210	fava_c16177	210	C/G	caaaaaccccaattcaccccacccacattataactcttctcttctattYtcttcatttcattcgtaatcaccctcacagtcacacagaaacgacgtcgac[C/G]aagagaagaaatgaaaaccgagaagaagtcattgttacaagatctaattgaaatttcaggtggttgtgttgttaccgacggaggatttgccacacagctt

fava_c16178_1007	fava_c16178	1007	C/T	gtgatgaaggtagaaggaatMatcaagttgaagagatttttaggagaacatcttttcttaggacaYcaaaacatcatcaacatcctctaacaatcttggg[C/T]catgatcgcatattttggttcggagatttgaactaYagactatacttgaaagaccatcttgctagaYatttaataagaaagcatgattggaagggtttgc

fava_c16178_1043	fava_c16178	1043	T/C	tttttaggagaacatcttttcttaggacaYcaaaacatcatcaacatcctctaacaatcttgggYcatgatcgcatattttggttcggagatttgaacta[T/C]agactatacttgaaagaccatcttgctagaYatttaataagaaagcatgattggaagggtttgcaagagtttgatcaactScaaaaggagttagaagaag

fava_c16178_1074	fava_c16178	1074	C/T	aaaacatcatcaacatcctctaacaatcttgggYcatgatcgcatattttggttcggagatttgaactaYagactatacttgaaagaccatcttgctaga[C/T]atttaataagaaagcatgattggaagggtttgcaagagtttgatcaactScaaaaggagttagaagaaggtggagtttttgagggttggaaagaaggtaa

fava_c16178_1124	fava_c16178	1124	G/C	ggttcggagatttgaactaYagactatacttgaaagaccatcttgctagaYatttaataagaaagcatgattggaagggtttgcaagagtttgatcaact[G/C]caaaaggagttagaagaaggtggagtttttgagggttggaaagaaggtaacattgaatttgctccaacatataaatatgcatcttcaactagcaatatct

fava_c16178_1250	fava_c16178	1250	G/A	tttttgagggttggaaagaaggtaacattgaatttgctccaacatataaatatgcatcttcaactagcaatatctattgtggtggtggccttccaactag[G/A]tcMggagaaaaacaaagaacRccagcatggtgtgatagaattctatggtatggtaaaggaattgagcaacttcattatattagaagtgaaagcaaattct

fava_c16178_1253	fava_c16178	1253	A/C	ttgagggttggaaagaaggtaacattgaatttgctccaacatataaatatgcatcttcaactagcaatatctattgtggtggtggccttccaactagRtc[A/C]ggagaaaaacaaagaacRccagcatggtgtgatagaattctatggtatggtaaaggaattgagcaacttcattatattagaagtgaaagcaaattctctg

fava_c16178_1271	fava_c16178	1271	A/G	gtaacattgaatttgctccaacatataaatatgcatcttcaactagcaatatctattgtggtggtggccttccaactagRtcMggagaaaaacaaagaac[A/G]ccagcatggtgtgatagaattctatggtatggtaaaggaattgagcaacttcattatattagaagtgaaagcaaattctctgaYcatcgtcctgtttctg

fava_c16178_1355	fava_c16178	1355	C/T	gagaaaaacaaagaacRccagcatggtgtgatagaattctatggtatggtaaaggaattgagcaacttcattatattagaagtgaaagcaaattctctga[C/T]catcgtcctgtttctgcacttttctcaacacaaattgaaatcaaatcatctaYYagaggacttStggaatttgatcatgacYcttcaacaattttgcatc

fava_c16178_1408	fava_c16178	1408	T/C	ggaattgagcaacttcattatattagaagtgaaagcaaattctctgaYcatcgtcctgtttctgcacttttctcaacacaaattgaaatcaaatcatcta[T/C]YagaggacttStggaatttgatcatgacYcttcaacaattttgcatcctaatcatgtggccagaaaaggagaWgaggatgaaaactcaacttcattgtca

fava_c16178_1409	fava_c16178	1409	T/C	gaattgagcaacttcattatattagaagtgaaagcaaattctctgaYcatcgtcctgtttctgcacttttctcaacacaaattgaaatcaaatcatctaY[T/C]agaggacttStggaatttgatcatgacYcttcaacaattttgcatcctaatcatgtggccagaaaaggagaWgaggatgaaaactcaacttcattgtcat

fava_c16178_1419	fava_c16178	1419	G/C	acttcattatattagaagtgaaagcaaattctctgaYcatcgtcctgtttctgcacttttctcaacacaaattgaaatcaaatcatctaYYagaggactt[G/C]tggaatttgatcatgacYcttcaacaattttgcatcctaatcatgtggccagaaaaggagaWgaggatgaaaactcaacttcattgtcattgttaatgaa

fava_c16178_1437	fava_c16178	1437	C/T	tgaaagcaaattctctgaYcatcgtcctgtttctgcacttttctcaacacaaattgaaatcaaatcatctaYYagaggacttStggaatttgatcatgac[C/T]cttcaacaattttgcatcctaatcatgtggccagaaaaggagaWgaggatgaaaactcaacttcattgtcattgttaatgaaRaatgtacaaggttgata

fava_c16178_1481	fava_c16178	1481	A/T	caacacaaattgaaatcaaatcatctaYYagaggacttStggaatttgatcatgacYcttcaacaattttgcatcctaatcatgtggccagaaaaggaga[A/T]gaggatgaaaactcaacttcattgtcattgttaatgaaRaatgtacaaggttgataatacaagcaataaatctttagattataaaaagatgataaaaagt

fava_c16178_1520	fava_c16178	1520	G/A	tggaatttgatcatgacYcttcaacaattttgcatcctaatcatgtggccagaaaaggagaWgaggatgaaaactcaacttcattgtcattgttaatgaa[G/A]aatgtacaaggttgataatacaagcaataaatctttagattataaaaagatgataaaaagtttcagtcaggttatggtaactaggaaagtgacaaataaS

fava_c16178_1648	fava_c16178	1648	A/T	taaatctttagattataaaaagatgataaaaagtttcagtcaggttatggtaactaggaaagtgacaaataattgcttttctaaaaagccaacgatatat[A/T]tttttttggagatcagaattgtagaaaggttaaagttagaccacaaaatatggtaaatataatattattgaaaatcaagattaatctaataatgtaagtg

fava_c16178_215	fava_c16178	215	C/A	catcttataactcaattatatttaaataattttgtagatgaattattacctttttccttcttgtcatagatattggtgttaatgtgtttgtgaccttcta[C/A]aaatctttataaactatggagttgaagaaccaaaattcaagatataagaRgctatgcaattggttcaccgctaagcaaaaggaaaataaaccatcacaga

fava_c16178_265	fava_c16178	265	G/A	tttttccttcttgtcatagatattggtgttaatgtgtttgtgaccttctaMaaatctttataaactatggagttgaagaaccaaaattcaagatataaga[G/A]gctatgcaattggttcaccgctaagcaaaaggaaaataaaccatcacagagtttatacaaaattgaagatggagaagaaaatgagagtgatgaatatgag

fava_c16178_482	fava_c16178	482	T/A	agtcattagaatttgattcatgtatttcaacaaacaacctaaggatctttgtaggtagttggaatgttgctggaagatctcctataggaagcttagctgt[T/A]gatttagatgagtggttgaatttaaagaattctgcagatatatatgttcttgggtttcaagagatagtacctttgaagacattaacRgtaataggagcag

fava_c16178_569	fava_c16178	569	G/A	gaagcttagctgtWgatttagatgagtggttgaatttaaagaattctgcagatatatatgttcttgggtttcaagagatagtacctttgaagacattaac[G/A]gtaataggagcagaggattcatcagtagcaacaaattggaacaatctcataggaagaacactatcaaatgatcaaaattattataaaaaaattggaggaa

fava_c16178_753	fava_c16178	753	G/A	taaaaaaattggaggaaacaataatagaaattacaagatggtggcaagtaaaaagatggttggagtattcataagtgtgtggttaaaagaacaagtgtta[G/A]aaaaatattgtgtttcaaatgtgagagtttgttcagttgcatgtggtgttatgggatgtttaggaaacaaaggttgtgtagcagttagtatgttgattga

fava_c16178_927	fava_c16178	927	C/A	ttgtgtagcagttagtatgttgattgaagggacgagtttttgttttgttgttgcacatttagcttctggagaaaagaaaggtgatgaaggtagaaggaat[C/A]atcaagttgaagagatttttaggagaacatcttttcttaggacaYcaaaacatcatcaacatcctctaacaatcttgggYcatgatcgcatattttggtt

fava_c16178_972	fava_c16178	972	T/C	tgttgttgcacatttagcttctggagaaaagaaaggtgatgaaggtagaaggaatMatcaagttgaagagatttttaggagaacatcttttcttaggaca[T/C]caaaacatcatcaacatcctctaacaatcttgggYcatgatcgcatattttggttcggagatttgaactaYagactatacttgaaagaccatcttgctag

fava_c16179_1495	fava_c16179	1495	C/A	gaccaactgttccactcacccccttttcaagaggtagttgataaagaacagctgtagtaggcattgggacggatctaactcctgtttcgtctccaaagct[C/A]gatgctacagcaacgggttcacttataatatctgcctccacatttgaatcgtcaaaaacagcagcccgagcaacaacttcgacagaagagatgttcttat

fava_c16179_1924	fava_c16179	1924	T/C	tcccatgcaaaggtggtgcgactgccaaaacaccaacctcatcgtccagattgtcgccaatatgatcctcctcagaacctataaaactatcagccgatat[T/C]gtagttatagcattcgatatcaactctgcatttgccatcaacttaagtctcgaaacatccatatgagtagctatagttgaattatcacaaactgaattac

fava_c16179_787	fava_c16179	787	T/A	cttttgcacgtgggaggaatgtaacttctggttcaggaataatccatggcttcaaatatctatcaccaatagaccttgacattgcaagaaccccaaatac[T/A]cgatgaccattccattgtatcactttgccaccagctgcctcgattcttgcatattcatcatctcgatttggtttatgatccacagataaaggcattggtt

fava_c16181_1064	fava_c16181	1064	T/C	atatgaaaagcatgattacaattaggtaacaatctaagtttatcttgttccaaaaactcacttagacaaacagcacaatcaaaaggctctttcaaaccta[T/C]aatctctttgtaaatgaaaacaggaagagcatcaatgaatgcttgatccaaacctgaatcatgaagattgaaaagctgctgcaactgtctttgatatgca

fava_c16181_1311	fava_c16181	1311	C/T	aagagctagaagatctatgctttatgagaaacctaacaagcaagtgaagaaaacctagaatgaaaaatataatagctaatataactatgataaagagaat[C/T]gcaggacttattttgtttccagatgaagaaattggagctggctctgatgctttttggctataaggagttgaagaagaatatggagaaagaataggaggat

fava_c16181_1422	fava_c16181	1422	A/G	ttttgtttccagatgaagaaattggagctggctctgatgctttttggctataaggagttgaagaagaatatggagaaagaataggaggatttgtgatagc[A/G]ccattgttaatatggatttgagagtgaaaaaaatgcattttttttagaatttgggatttcaaaattcaaattagagagagtataaattatgaagtgggtt

fava_c16181_1615	fava_c16181	1615	A/G	agtgggtttgaataaagtttgaatctttgcttgatttgggataaaacccagctcagacatttgaacaaacaggtaatgagcacattagaaaggttattag[A/G]aagaggattaagcaaagattataaaaaaaatcagattYttttgttgggtgtttaaagagWttgagtgtggtaacaaaaaaataattataggataataata

fava_c16181_1653	fava_c16181	1653	T/C	ggataaaacccagctcagacatttgaacaaacaggtaatgagcacattagaaaggttattagRaagaggattaagcaaagattataaaaaaaatcagatt[T/C]ttttgttgggtgtttaaagagWttgagtgtggtaacaaaaaaataattataggataataatagtaagaggaaagaacatgggtgagtgagaaatgtcaat

fava_c16181_1675	fava_c16181	1675	T/A	ttgaacaaacaggtaatgagcacattagaaaggttattagRaagaggattaagcaaagattataaaaaaaatcagattYttttgttgggtgtttaaagag[T/A]ttgagtgtggtaacaaaaaaataattataggataataatagtaagaggaaagaacatgggtgagtgagaaatgtcaatgtttgtagttgtagttgaccta

fava_c16181_262	fava_c16181	262	A/G	tgcaaaatacatacaataatcataaatgatatagttgaaagacaattgcttcattaagttaagtgctttatggcataacttacatgttttggtcctaaag[A/G]aaagtgtgagttgaagtaagagctaaacaaatggattcattgaatatatgaaaatagtaaacaatacaaaaagactaagtaacttaatgtatcatgtacc

fava_c16181_367	fava_c16181	367	G/A	tgtgagttgaagtaagagctaaacaaatggattcattgaatatatgaaaatagtaaacaatacaaaaagactaagtaacttaatgtatcatgtacctcta[G/A]ctttactcatccatggcaaattttcatYgacaacaacacaattatgaaaatgtgtatctgatgaacttggtatcttattatctttcttagaccattgcca

fava_c16181_395	fava_c16181	395	T/C	ggattcattgaatatatgaaaatagtaaacaatacaaaaagactaagtaacttaatgtatcatgtacctctaRctttactcatccatggcaaattttcat[T/C]gacaacaacacaattatgaaaatgtgtatctgatgaacttggtatcttattatctttcttagaccattgccaaatctttgaaacagaaaaactttcacct

fava_c16181_717	fava_c16181	717	T/C	caacttgcaaatctgaatcacaaacaacatactgaaaagatcccattgagtagcatctcctaacatcgaaattacagctactactctctccttcactttt[T/C]tcaaccccttctccattatttgaacttctaaactttccaagcctaacagaaaaaacccttttcctattcattatatggttttccatattcttgttaactg

fava_c16181_909	fava_c16181	909	T/C	tgttaactgaaccaatacctataacacaatcttcatccctttgatcctcaaactcaaaaacaggatttttctctaacgaaaacccttgagcataaagtgt[T/C]cctctacataaaggacaagttgaatttgacaataaccatgtatctatgcagttaatatgaaaagcatgattacaattaggtaacaatctaagtttatctt

fava_c16185_1367	fava_c16185	1367	C/G	ttgttttcctcttcatcaaagcttcaaggtcaatttcagccaataaatccatagcatctgtttttgatctaaaagaatcagtggatctaaacgattttgt[C/G]gcattaaatgttttaatcggttttagagaagttgctctctccaatacKgtaggagataacaaatgtaatggtggctctggccaactcaaaccaatccttt

fava_c16185_1415	fava_c16185	1415	G/T	ccatagcatctgtttttgatctaaaagaatcagtggatctaaacgattttgtSgcattaaatgttttaatcggttttagagaagttgctctctccaatac[G/T]gtaggagataacaaatgtaatggtggctctggccaactcaaaccaatcctttcagtcccactaaccattctagatagtttactaatctgttgaggaggcg

fava_c16185_1699	fava_c16185	1699	G/C	aaaccattatcaactatcactcgcgagttggcaatatcagcagcatttgaatccttcaaactagtattcaatgagtcatgtgtgattggtttatctatga[G/C]tccgtcgaatacttcatatccttcatccaaaggatggatttcctcaccaatatcaccatcactttctagtagcaKttcttcttcaatatctggagcatca

fava_c16185_1774	fava_c16185	1774	G/T	tcatgtgtgattggtttatctatgaStccgtcgaatacttcatatccttcatccaaaggatggatttcctcaccaatatcaccatcactttctagtagca[G/T]ttcttcttcaatatctggagcatcatctgatggtttaaacttcgacggtgatggaggatcactgaaggattttatctttgatggttttggttgaactcta

fava_c16185_202	fava_c16185	202	A/T	ggatacacaagaaataacattactttcttgagagaaatacttttcaccccacaatttgacgaacaagtaatacaccacaccatagagggatttcactttg[A/T]acttattctttaccaacatacccaaagattcctatgaaatctcttcaaatRSaKttcagagtttacatattcagccaatctaacaactagctttaaaaca

fava_c16185_253	fava_c16185	253	A/G	caatttgacgaacaagtaatacaccacaccatagagggatttcactttgWacttattctttaccaacatacccaaagattcctatgaaatctcttcaaat[A/G]SaKttcagagtttacatattcagccaatctaacaactagctttaaaacatgttcataagatactgatgaacaaatcatacaactattttttctataagat

fava_c16185_254	fava_c16185	254	C/G	aatttgacgaacaagtaatacaccacaccatagagggatttcactttgWacttattctttaccaacatacccaaagattcctatgaaatctcttcaaatR[C/G]aKttcagagtttacatattcagccaatctaacaactagctttaaaacatgttcataagatactgatgaacaaatcatacaactattttttctataagatt

fava_c16185_256	fava_c16185	256	G/T	tttgacgaacaagtaatacaccacaccatagagggatttcactttgWacttattctttaccaacatacccaaagattcctatgaaatctcttcaaatRSa[G/T]ttcagagtttacatattcagccaatctaacaactagctttaaaacatgttcataagatactgatgaacaaatcatacaactattttttctataagatttg

fava_c16185_821	fava_c16185	821	T/C	cctctccgatgtacttgtctgaattaacctcgtacttataggttggtgggaagtttaacattccttctgaccatcctttaaatacacctttttctagttc[T/C]ttcgcaagctgatctttctcaaccaatttcgaccactgctttttcaagataagttctcttgttttttcatatgataagttgatacggtaattcagatcac

fava_c16192_176	fava_c16192	176	T/C	tttgatggttaatatttaacaaccaagtataaaggcatcaagcctttgatatgtatgttttagtgtcatagtacatagtatgagagtataagctaccctt[T/C]aaaaattgtaaaacaaaagagtaaacaaagttacaatcaaatatatatatttttcctttacttatatatcctttccctcacctctaattttcagaagaag

fava_c16192_662	fava_c16192	662	C/T	ctttcatcaacgaaccacattgtcttggttgtgtttggtagaaaactttggaaactaccaattctccttctttctcctcttcattgtttccaagatggta[C/T]tgatgcataacccaatttgttttctctggctttctttgctttctgtagttagtgtaaagaacaaggattttcttatatccttttaatttgccaatcacgt

fava_c16192_841	fava_c16192	841	T/G	ccttttaatttgccaatcacgtaaaccggtctcgtcttacctgttttgtgccaccttgtttcacttccatctgaatctgtgtgcacttttcttctttttc[T/G]tgttcctgttgtgtatgcttttgatggcctgtgaaagaaatgtctgatcaagccatctttgcttacacctggcaacttctctgggtgagtgcagcagatt

fava_c16194_152	fava_c16194	152	C/T	ttgttgcgatattagagtaagctcccagggatgaattatccctaacaaactgacctcactattacattgatatgttgcattgcacaggtgtatctcatct[C/T]atatgattgcactccactaattaaatatacaatcacatagcgtatcaaatttatatgtgtagaattactctctataaataatcgcagccattcaacaatt

fava_c16194_440	fava_c16194	440	T/C	gagcaatgtgattgacattttgcaatgcgtagcctccacgaaaggttcctggtaatgatgccgacgcttgagctgcttccataaccttctctacaacaaa[T/C]tgacccaatataagcacttcaaggatcttcctccaatcccaggacatctttacaaggaSgctgatagtaaataacccaaaaacagctccagaagctccaa

fava_c16194_499	fava_c16194	499	C/G	gccgacgcttgagctgcttccataaccttctctacaacaaaYtgacccaatataagcacttcaaggatcttcctccaatcccaggacatctttacaagga[C/G]gctgatagtaaataacccaaaaacagctccagaagctccaactgagactgtgtttcttggtaaaataagccaggaaacaaggtttgctccaacaccagta

fava_c16195_831	fava_c16195	831	T/C	cagattttgttagaccagatgagaggcttaatgacattgttggaagtgcttattatgtggctcctgaagttcttcacagatcttacagcacagaagctga[T/C]gtgtggagtataggtgtgatagcatatattctattatgtggtagtcgtccattttgggctagaacagagtcgggtatctttagggcagttttgaaagctg

fava_c16196_1077	fava_c16196	1077	A/T	ggatcttgataagggtcgacttcctggaaaacccgtttcaaggtggttgtttcttcgaaaatgactgtatcttccttcatgactacatctgcatattgaa[A/T]tttttttggttgtgggcctgccctcacagtgtcaagatctgatggaactttctcatcaataactgtttggtcgtcttcatcatcactggcatagttgtca

fava_c16196_1311	fava_c16196	1311	T/C	gcaatgggctggcatgagtcaaaagattgagtcggagtcgcatgtgaaccggaaagctttgactgaacccctcggatttcaaccgacgacgaagactcgt[T/C]agcggaggaagttgaggggaaggaacgcatttctaacatcttaagggaccgaccatgggaggaagtgtctgccaKtaggttaatcttcttggtcgttgat

fava_c16196_1386	fava_c16196	1386	T/G	atttcaaccgacgacgaagactcgtYagcggaggaagttgaggggaaggaacgcatttctaacatcttaagggaccgaccatgggaggaagtgtctgcca[T/G]taggttaatcttcttggtcgttgatacaggagtaaaaacagtaccatagtcaccaggcaatctaacattgtaggctcgtataatctcctcataatcctct

fava_c16196_1516	fava_c16196	1516	G/A	gagtaaaaacagtaccatagtcaccaggcaatctaacattgtaggctcgtataatctcctcataatcctcttccttctttttttctgcatcattataggc[G/A]cgtttaatcttctcatactcctctttcatctttcccaccttagcaaagtaaggagccttctcagcatacgacatMgctttccattcctcagaaccagctt

fava_c16196_461	fava_c16196	461	T/A	gttgtggacacgcttcttctccatctcgatggagtcacctataccagcgtgagcttcagctagttccgcctcggccaaggctaattgcttcttgagtatc[T/A]caactttctcttcagccttgaccttaactgcctcttgctgctttctatcagctgcaatcttgtctttttcttctttgagagaaaggagcttcttcaccaa

fava_c16196_860	fava_c16196	860	G/C	aatatccaaaggcttcgacatcaatcccttcagttctttgaaaagttccattgctttggaggccctagaatcatgctcagcaacaggggaggacatacca[G/C]acatagaaactacttgacttgggtccgtcagcaaagaaagcaagtcatttgccgggtcagtatcaacagtgtcgctgttacaatctttctcagcctggtg

fava_c16197_1029	fava_c16197	1029	A/G	cttcattaagtttaacSgctgaagaaaattcttcaattactgatctcacagcattctcatcaccagaatttaagctaatcaagcgcttctctagctcacc[A/G]attttctcagaagagacgaaatgtgttgctagtccagctgcaactaattcctttccattcaatcgcgctcccgtaagtgccaaatattctcctaaatWcg

fava_c16197_1127	fava_c16197	1127	T/A	ccRattttctcagaagagacgaaatgtgttgctagtccagctgcaactaattcctttccattcaatcgcgctcccgtaagtgccaaatattctcctaaat[T/A]cgctggcaaacgagaatggtagtatgagaaaccacaatcagtatgaaatccaaaacttgcttcaggagtggcaaaaactgttttttctgttacRactgag

fava_c16197_1221	fava_c16197	1221	A/G	ctaaatWcgctggcaaacgagaatggtagtatgagaaaccacaatcagtatgaaatccaaaacttgcttcaggagtggcaaaaactgttttttctgttac[A/G]actgagaacttcaaWgggatcatcaatgctgcacctccacccattgaaattccattcacaagagcaacctgagttttcttataggtactaatgtgatggc

fava_c16197_1236	fava_c16197	1236	T/A	aacgagaatggtagtatgagaaaccacaatcagtatgaaatccaaaacttgcttcaggagtggcaaaaactgttttttctgttacRactgagaacttcaa[T/A]gggatcatcaatgctgcacctccacccattgaaattccattcacaagagcaacctgagttttcttataggtactaatgtgatggcaaagccagtaaaatc

fava_c16197_574	fava_c16197	574	T/C	agctgaattctcatatttaccatcccacctgcatttttcactttctggaatctgaagctctaattccttgtcaaaaggcataaaaattaagtccaatttc[T/C]catcatctactttgtcaagtgttgcaggatcccactttggagcattgtccttgtcaatggtgagagctctaataccctcgtacatatcctcagatatcgt

fava_c16197_830	fava_c16197	830	T/C	gattgattccttccttcacgaaccgatcttaatgctattttcaatgcagtaggtgatgttcttttcatttctttcaaaactggacctatccatccatttc[T/C]ttccttgcttgattcagcttccagcRatttaataatttcttcaacagaatcctttgaaaagcactcatcgattattgatttcttgtttaagatactgtct

fava_c16197_856	fava_c16197	856	G/A	tcttaatgctattttcaatgcagtaggtgatgttcttttcatttctttcaaaactggacctatccatccatttcYttccttgcttgattcagcttccagc[G/A]atttaataatttcttcaacagaatcctttgaaaagcactcatcgattattgatttcttgtttaagatactgtcttcattaagtttaacSgctgaagaaaa

fava_c16197_945	fava_c16197	945	C/G	cagcttccagcRatttaataatttcttcaacagaatcctttgaaaagcactcatcgattattgatttcttgtttaagatactgtcttcattaagtttaac[C/G]gctgaagaaaattcttcaattactgatctcacagcattctcatcaccagaatttaagctaatcaagcgcttctctagctcaccRattttctcagaagaga

fava_c16199_1624	fava_c16199	1624	T/C	caattcttgaaatggcaacatcaaagcctccttggagtcagtatgaaggggtagctgcaatatttaaaattggcaacagcaaagacatgcctgaaattcc[T/C]gatcatctctcaaatgatgcaaagaatttcatcaagctatgcttgcatagagatcctgcaactcgccctacagcccatatgttacttaatcaccctttta

fava_c16199_2140	fava_c16199	2140	T/C	atcatacccttgcttcatatgaatcatatccaatgagatcaaatgccaagtttactcttgatccttggcatgaaatacctatgcacaaagcccacacacc[T/C]ggtggatccccaaggagacttatttgagtttctaaatgtaaatacccctaaaatctttcttggttttccccagcttgccttgtgctYcgWagttgagctt

fava_c16199_2227	fava_c16199	2227	C/T	aagcccacacaccYggtggatccccaaggagacttatttgagtttctaaatgtaaatacccctaaaatctttcttggttttccccagcttgccttgtgct[C/T]cgWagttgagctttattcaatcttaactctgaaagctcaaactatattaaaaaaactctggtccattctttctttataaatgagaaatagaagacggtag

fava_c16199_2230	fava_c16199	2230	A/T	cccacacaccYggtggatccccaaggagacttatttgagtttctaaatgtaaatacccctaaaatctttcttggttttccccagcttgccttgtgctYcg[A/T]agttgagctttattcaatcttaactctgaaagctcaaactatattaaaaaaactctggtccattctttctttataaatgagaaatagaagacggtagaag

fava_c16199_697	fava_c16199	697	A/G	ttacttttggatctgcttctgtttctggctctagcgttagctccaatggctcttgtgatgatccttcagcttttaactctcaaattaatagcgttagggg[A/G]caatgtgaaacaaagttYaatgtgacgtctaaaagccccggacctgggtcaagaggacctaccagcccaacatcacctcttcatccaaggttgcatgttt

fava_c16199_715	fava_c16199	715	C/T	ctgtttctggctctagcgttagctccaatggctcttgtgatgatccttcagcttttaactctcaaattaatagcgttaggggRcaatgtgaaacaaagtt[C/T]aatgtgacgtctaaaagccccggacctgggtcaagaggacctaccagcccaacatcacctcttcatccaaggttgcatgttttgagtcttgactcgccta

fava_c16202_1024	fava_c16202	1024	A/C	gcaagtggctagcaatcatggaccacctgttgccataagtagtgtgcaactttacaatagtatcttcttcttcagcagaaatgttccctcttttgagatc[A/C]gttctcaaatagttaatccatcttagtctacaactcttcccacaccttaacaatcccgcatttttgggcaatgacctccatgacccttccccattggcct

fava_c16202_712	fava_c16202	712	A/G	attcatacattgttgttgttgtggttgtggttgttttttctttctcttgttcatgtgttgttttaacttccacgtcatgaactaatttccctttattatc[A/G]ggttgagtaatagtactactagtggtgatttgttttttgtagatcttgtttttcttcatggcccaacgacttgttctaccacatttgcgtttaggaggag

fava_c16203_121	fava_c16203	121	G/A	aaggggtgtatgtagtgaaccgcaacagcatatgaaatgtttcgccgacttcgacaatgacgatgtgaaacggaggaagcatcaaataagccatggctat[G/A]acggcaagagctgtgattaagaagacgaagaatattctgggtggtgttactcttcttctacgctctaattctgacYcatcccgttccttcgtttccgaaa

fava_c16203_1258	fava_c16203	1258	A/T	gacgtagacgaagctctaaatttattccttaatattttataaaattaaattcttaccataagcataagtatatctacaacatttcgtgtatcagttattt[A/T]aaaagcKgaagttgttagttggaagcttgtgaatggatttataccaaatgagtttgttgatcatgttattttccttgtataattgcactgttgtcccttc

fava_c16203_1265	fava_c16203	1265	G/T	acgaagctctaaatttattccttaatattttataaaattaaattcttaccataagcataagtatatctacaacatttcgtgtatcagttatttWaaaagc[G/T]gaagttgttagttggaagcttgtgaatggatttataccaaatgagtttgttgatcatgttattttccttgtataattgcactgttgtcccttcattttga

fava_c16203_197	fava_c16203	197	T/C	aagcatcaaataagccatggctatRacggcaagagctgtgattaagaagacgaagaatattctgggtggtgttactcttcttctacgctctaattctgac[T/C]catcccgttccttcgtttccgaaaaagtttcttctctctctccaccgaattcccactctctcccgttctcaactcctcaYacttcattctatcgccattt

fava_c16203_277	fava_c16203	277	C/T	ttctacgctctaattctgacYcatcccgttccttcgtttccgaaaaagtttcttctctctctccaccgaattcccactctctcccgttctcaactcctca[C/T]acttcattctatcgccatttttcactttcacattcaggtgagacaagtgatgatggtgcaagcacagatggatgggaagaagaagacgaggctgagccta

fava_c16203_478	fava_c16203	478	T/C	agattggtgacggtggaagtggtggtggagtttccttgcaaaatgttccttggggtcagcgagcgctatctattgctgaagaggtccttatgaaattcag[T/C]gaggacatcaaactctatgctttcaaaactagtcctcgtggWtatgtttacgtgagattggacaaattaacaagcgaatatggatgtccaagcatggaag

fava_c16203_520	fava_c16203	520	A/T	atgttccttggggtcagcgagcgctatctattgctgaagaggtccttatgaaattcagYgaggacatcaaactctatgctttcaaaactagtcctcgtgg[A/T]tatgtttacgtgagattggacaaattaacaagcgaatatggatgtccaagcatggaagagcttgagagttacaatcaggaattcaagaaaagattagatg

fava_c16203_772	fava_c16203	772	C/A	ccccgggtgctgataggatactgaaagtgccagatgatcttagtcgatttgaagacatgccgatgagagtttgctatacagaaaatatagagtccaactt[C/A]cgtgaagtggatggagtattcttattagattctatcgaaaaagactcggagatatgtgtttggaaattggcagRtgttaaagagaatcgagatcctacaa

fava_c16203_846	fava_c16203	846	A/G	tatacagaaaatatagagtccaacttMcgtgaagtggatggagtattcttattagattctatcgaaaaagactcggagatatgtgtttggaaattggcag[A/G]tgttaaagagaatcgagatcctacaaagaaaggcaagccgttgaatcgtaaacagaaggattggagattaagattgccattcgacttgcatagaatggta

fava_c16204_1279	fava_c16204	1279	G/C	attacctcaaactaaccattcctcatttcaagggaaaacctgagttgggattagtacaagcaagatggtcatacgtgaacaaagacgagaatcttctgac[G/C]aggcttcaaaacatcaacttgtgttttcactttgaagttgaacaacaagtgaatggtcatttcttgaatttctttggattcaatggaaccgccggtgttt

fava_c16204_1816	fava_c16204	1816	G/C	ccttaacaatgttcatacctgaagcagaactacctctctgggtaatatgttacgtccctatcgtcatgtcattcataaacatccttccatcaccaaaatc[G/C]gtccctttcctcgtcccatatcttcttttcgagaacacaatgtcggttacaaagttcaacgcaatggtttcYggattatttcaactcggaagtgcttacg

fava_c16204_1888	fava_c16204	1888	C/T	tcataaacatccttccatcaccaaaatcSgtccctttcctcgtcccatatcttcttttcgagaacacaatgtcggttacaaagttcaacgcaatggtttc[C/T]ggattatttcaactcggaagtgcttacgaatgggttgtgacaaagaaaacaggtagatcatcYgagtcWgatttgtttgcgttagccgaaagagaatcaa

fava_c16204_1951	fava_c16204	1951	T/C	acacaatgtcggttacaaagttcaacgcaatggtttcYggattatttcaactcggaagtgcttacgaatgggttgtgacaaagaaaacaggtagatcatc[T/C]gagtcWgatttgtttgcgttagccgaaagagaatcaaagtcttcgaatgaagacaagcttcatagaagacattcggaatctggtttggagttattaggaa

fava_c16204_1957	fava_c16204	1957	A/T	tgtcggttacaaagttcaacgcaatggtttcYggattatttcaactcggaagtgcttacgaatgggttgtgacaaagaaaacaggtagatcatcYgagtc[A/T]gatttgtttgcgttagccgaaagagaatcaaagtcttcgaatgaagacaagcttcatagaagacattcggaatctggtttggagttattaggaaaacttc

fava_c16204_2075	fava_c16204	2075	G/C	cgaaagagaatcaaagtcttcgaatgaagacaagcttcatagaagacattcggaatctggtttggagttattaggaaaacttcagaaatcagaagtgatt[G/C]taaagaagaagagaaacaaactctatagRaaagaattagcacttgcttttcttttactcactgcttctgctagaagtcttttatcagaacatggtgttca

fava_c16204_2104	fava_c16204	2104	A/G	acaagcttcatagaagacattcggaatctggtttggagttattaggaaaacttcagaaatcagaagtgattStaaagaagaagagaaacaaactctatag[A/G]aaagaattagcacttgcttttcttttactcactgcttctgctagaagtcttttatcagaacatggtgttcatttttactttttgctttttcaaggtttgt

fava_c16204_226	fava_c16204	226	G/A	aaatataaccatgaccctttagtgttcatacatcattggtcaccattaattatcaaacccatcaactcaaaattcatttcttttttttggttttgatacc[G/A]aaaaaaagtttttattttttattttttatagtgatggcaccaaattcagttacaattgcaactgacaaagctaatgatttcagtttgttacaagtccatg

fava_c16204_775	fava_c16204	775	T/C	tgtcttttagagaggactatgttggtcctttggttttgcttgtgtccaagttttgtattttgctgttcttgattcagtctttagatcgacttgtgctttg[T/C]gttggRtgcttYtggatcaagtataagaagttgaaacctacgtttgatgatgatgtttatgatgttgaggatcctttgagctttcctatggttcttgttc

fava_c16204_781	fava_c16204	781	A/G	ttagagaggactatgttggtcctttggttttgcttgtgtccaagttttgtattttgctgttcttgattcagtctttagatcgacttgtgctttgYgttgg[A/G]tgcttYtggatcaagtataagaagttgaaacctacgtttgatgatgatgtttatgatgttgaggatcctttgagctttcctatggttcttgttcagattc

fava_c16204_787	fava_c16204	787	C/T	aggactatgttggtcctttggttttgcttgtgtccaagttttgtattttgctgttcttgattcagtctttagatcgacttgtgctttgYgttggRtgctt[C/T]tggatcaagtataagaagttgaaacctacgtttgatgatgatgtttatgatgttgaggatcctttgagctttcctatggttcttgttcagattccaatgt

fava_c16204_910	fava_c16204	910	A/G	aacctacgtttgatgatgatgtttatgatgttgaggatcctttgagctttcctatggttcttgttcagattccaatgtgtaatgagagagaggtttattc[A/G]caatccattggcgctgcttgtgaacttgattggccaaaagacagaatactagtYcaagtcttggatgattcRgatgatcctaatttacagatgcttatca

fava_c16204_964	fava_c16204	964	T/C	tggttcttgttcagattccaatgtgtaatgagagagaggtttattcRcaatccattggcgctgcttgtgaacttgattggccaaaagacagaatactagt[T/C]caagtcttggatgattcRgatgatcctaatttacagatgcttatcaaagacgaggtttcatcttggaaagaaaaaggcgtcaacattgtttatcgacata

fava_c16204_982	fava_c16204	982	G/A	caatgtgtaatgagagagaggtttattcRcaatccattggcgctgcttgtgaacttgattggccaaaagacagaatactagtYcaagtcttggatgattc[G/A]gatgatcctaatttacagatgcttatcaaagacgaggtttcatcttggaaagaaaaaggcgtcaacattgtttatcgacatagattgattagaactggtt

fava_c16206_535	fava_c16206	535	A/C	ttgcaatcagaggatcaaataaagctgaacctggatcaagaaagagtagaatactcttctatctccatgttgcttttcagatgctttcagtggttcttgc[A/C]acagtaggagctgctatgtcattgaaaaaatttgaaaattcatttgataacaatcatcaaagRttaggtctagcactttatggtgctatRttgctacaag

fava_c16206_598	fava_c16206	598	A/G	tccatgttgcttttcagatgctttcagtggttcttgcMacagtaggagctgctatgtcattgaaaaaatttgaaaattcatttgataacaatcatcaaag[A/G]ttaggtctagcactttatggtgctatRttgctacaagctttcattggatttttcagacctcagagaggaaagaaagtaaggagttattggtatttagtac

fava_c16206_625	fava_c16206	625	G/A	tggttcttgcMacagtaggagctgctatgtcattgaaaaaatttgaaaattcatttgataacaatcatcaaagRttaggtctagcactttatggtgctat[G/A]ttgctacaagctttcattggatttttcagacctcagagaggaaagaaagtaaggagttattggtatttagtacattggatactaggaacaatagtgtcct

fava_c16209_1005	fava_c16209	1005	C/A	cgatggaagtagagcctgtgattatgaacgataagagtatcaggaaaatccttaaagcattggactctgatgatgttgagttactaaagcttcttttaga[C/A]gaatctagtgtgacgcttgacgatgcctatgcgctacactacgcttgtgcttattccgattctaaggttgttcaggaagttcttactctaggattggcgg

fava_c16209_150	fava_c16209	150	A/G	ttccaagtcatgtcttctttggtggaatggacttgaaagcatttttgtgtcaacctatccgttgactcaatttgaggcttaaaagagtcatggataattc[A/G]aatgaagcatcatcatcattgagttttgtatcatcacacatatcaaatggttcaagcaatactcacaatgaacatgttgcaaatattgaaattgtgagtt

fava_c16209_1629	fava_c16209	1629	A/C	tgtcggctcttaaaggaaatataaaagaagtagatctaaatgaaactccttcttttagaacaagaaaactccaattaagattacaatcccttgtaaaaac[A/C]gttgagaacggtaggcgtttctttccgcattgttcggaggtgcttgataagtacctagaagacgagatgcccgatgttcatgtcctagagaagggaactg

fava_c16209_1743	fava_c16209	1743	A/G	ggcgtttctttccgcattgttcggaggtgcttgataagtacctagaagacgagatgcccgatgttcatgtcctagagaagggaactgaggaagagcaaag[A/G]acRaagaaagcgcggttcatggaacttagagacgaggtgcagaaggcatttcacaaggatatggctgaaaataaccattcaaatttttcatcttctttga

fava_c16209_1746	fava_c16209	1746	G/A	gtttctttccgcattgttcggaggtgcttgataagtacctagaagacgagatgcccgatgttcatgtcctagagaagggaactgaggaagagcaaagRac[G/A]aagaaagcgcggttcatggaacttagagacgaggtgcagaaggcatttcacaaggatatggctgaaaataaccattcaaatttttcatcttctttgactt

fava_c16209_411	fava_c16209	411	C/A	gtgatgctgagattgttgttgaagagattccggtcggtattcatcggtgtatactggcttcgcggagtcagttttttcatgaactttttaagaaagggaa[C/A]gatggagatttgaaggatggaaagggaaccggaaagccgagttatcagatgaaagatttggtgKKttatggtagggttggatatgaagcttttgttgttt

fava_c16209_475	fava_c16209	475	T/G	gagtcagttttttcatgaactttttaagaaagggaaMgatggagatttgaaggatggaaagggaaccggaaagccgagttatcagatgaaagatttggtg[T/G]Kttatggtagggttggatatgaagcttttgttgtttttttgcattatttgtatactggaaagttgaaggctccaccaattgaagtgatgacttgtgttga

fava_c16209_476	fava_c16209	476	G/T	agtcagttttttcatgaactttttaagaaagggaaMgatggagatttgaaggatggaaagggaaccggaaagccgagttatcagatgaaagatttggtgK[G/T]ttatggtagggttggatatgaagcttttgttgtttttttgcattatttgtatactggaaagttgaaggctccaccaattgaagtgatgacttgtgttgat

fava_c16209_768	fava_c16209	768	A/G	ttttgctttttcagaggtatcttctaaattttgttgataaggctcttgtagaagatgtcattcctattcttgtagctgccaatcattgccaactagacca[A/G]cttctctctcattgcattcaaagagtagcaaggtctgacatggacatcatatctttggaaagagaacttccttatgaagttatgaccgaaatcaaatcgc

fava_c16210_235	fava_c16210	235	T/A	caattcccatctctttatttttctgtgattcacaaaaacaacaataaaagcaatcaaagtatgagacaacaagaaataaaccatgatcgaatgtcctgag[T/A]tggccgtggttgttgtttcgtgaYgaggctgaattccatggagactcggtggtggtcggctRggttctgcacaagtgaagggagcatggtaacgattttg

fava_c16210_259	fava_c16210	259	T/C	gtgattcacaaaaacaacaataaaagcaatcaaagtatgagacaacaagaaataaaccatgatcgaatgtcctgagWtggccgtggttgttgtttcgtga[T/C]gaggctgaattccatggagactcggtggtggtcggctRggttctgcacaagtgaagggagcatggtaacgattttgatcggagataggctgtttgcRYtg

fava_c16210_297	fava_c16210	297	G/A	gagacaacaagaaataaaccatgatcgaatgtcctgagWtggccgtggttgttgtttcgtgaYgaggctgaattccatggagactcggtggtggtcggct[G/A]ggttctgcacaagtgaagggagcatggtaacgattttgatcggagataggctgtttgcRYtgggtcgcagatgggagagRggaggtctatgtcgaaggtg

fava_c16210_356	fava_c16210	356	A/G	tgaYgaggctgaattccatggagactcggtggtggtcggctRggttctgcacaagtgaagggagcatggtaacgattttgatcggagataggctgtttgc[A/G]YtgggtcgcagatgggagagRggaggtctatgtcgaaggtggaacgtcgaggtcgtcggtgtaggttgcattcRcggattggctgaggttgtgtgggtcg

fava_c16210_357	fava_c16210	357	C/T	gaYgaggctgaattccatggagactcggtggtggtcggctRggttctgcacaagtgaagggagcatggtaacgattttgatcggagataggctgtttgcR[C/T]tgggtcgcagatgggagagRggaggtctatgtcgaaggtggaacgtcgaggtcgtcggtgtaggttgcattcRcggattggctgaggttgtgtgggtcgt

fava_c16210_377	fava_c16210	377	G/A	agactcggtggtggtcggctRggttctgcacaagtgaagggagcatggtaacgattttgatcggagataggctgtttgcRYtgggtcgcagatgggagag[G/A]ggaggtctatgtcgaaggtggaacgtcgaggtcgtcggtgtaggttgcattcRcggattggctgaggttgtgtgggtcgttctcggcattcgaaggttgc

fava_c16210_430	fava_c16210	430	G/A	attttgatcggagataggctgtttgcRYtgggtcgcagatgggagagRggaggtctatgtcgaaggtggaacgtcgaggtcgtcggtgtaggttgcattc[G/A]cggattggctgaggttgtgtgggtcgttctcggcattcgaaggttgctgttgcgaacagaggagggRaaattttgttggggatgatgctgtgagatgttg

fava_c16210_497	fava_c16210	497	A/G	ggaacgtcgaggtcgtcggtgtaggttgcattcRcggattggctgaggttgtgtgggtcgttctcggcattcgaaggttgctgttgcgaacagaggaggg[A/G]aaattttgttggggatgatgctgtgagatgttgataaagttgacggtgagaggagtatgaggtttgatggagtcacggtgatgagaagagtggtggctgc

fava_c16210_683	fava_c16210	683	C/T	aagagtggtggctgcacagatccgtgaagggacggtgtgtcgtcgacatgaatgggttacggctgcgcaattgttctcgtggtggctgttcacccttctt[C/T]tgatctaggctcccagtttaaattgatgtggcatgtttattgcttcacactgttgtttttcttttcgaacccRctttctttgattcgtacgttgttgaaa

fava_c16210_756	fava_c16210	756	G/A	ttctcgtggtggctgttcacccttcttYtgatctaggctcccagtttaaattgatgtggcatgtttattgcttcacactgttgtttttcttttcgaaccc[G/A]ctttctttgattcgtacgttgttgaaatcattccttatggttggtccggatgattcactagaactgtgatcttctgcttgtcagtctttcttccttatca

fava_c16212_1417	fava_c16212	1417	C/T	tggctccatcaagccagcgatagctttatcaggtatattggaaattaagggaactaatattaagcacaaggatgagattaaggaagatatacatggtaaa[C/T]tggtgtctgcaaacagtattggWatttatcatgaccatttctatatgttttatcttgattttgatattgatggtacacacaactcatttgagaaaacaag

fava_c16212_1440	fava_c16212	1440	A/T	ctttatcaggtatattggaaattaagggaactaatattaagcacaaggatgagattaaggaagatatacatggtaaaYtggtgtctgcaaacagtattgg[A/T]atttatcatgaccatttctatatgttttatcttgattttgatattgatggtacacacaactcatttgagaaaacaagtttgaagactgtaagaatcacgg

fava_c16212_630	fava_c16212	630	G/C	tgtgttctagtttcactatggggtggtttggagaagagaagagtattagaactctgagggtagattgtttcatgaaagaaagtactgtgaatatctacgt[G/C]agacctattactggaatcacaatagttgttgatcttgatcttatgaaaatagttgagtatcatgatagagatattgaagcagtgccaacagctgagaaca

fava_c16212_754	fava_c16212	754	C/A	agttgttgatcttgatcttatgaaaatagttgagtatcatgatagagatattgaagcagtgccaacagctgagaacactgaataccaagtttcaaaacaa[C/A]gcccaccatttggaccaaaacaacacagtctcaccagtcatcaaccacagggtcctggcttccagatcaacggccatagtgttagttgggcaaactggaa

fava_c16214_122	fava_c16214	122	A/T	aattatttataagaaaacaatttagtacaattcttattcaaaaggaaacatgattacaaaacaaaaacattttggccacttagtataacataacaatttt[A/T]YtcagagttacaactttaaatcataagaaaacaatggaaaccaccaaacttatcttctacaaactataccattaccactgctKgcaccaccagtgcaatc

fava_c16214_123	fava_c16214	123	T/C	attatttataagaaaacaatttagtacaattcttattcaaaaggaaacatgattacaaaacaaaaacattttggccacttagtataacataacaattttW[T/C]tcagagttacaactttaaatcataagaaaacaatggaaaccaccaaacttatcttctacaaactataccattaccactgctKgcaccaccagtgcaatcc

fava_c16214_205	fava_c16214	205	T/G	tataacataacaattttWYtcagagttacaactttaaatcataagaaaacaatggaaaccaccaaacttatcttctacaaactataccattaccactgct[T/G]gcaccaccagtgcaatccatcataccatattcaataatctccaatccttcctgctcagaatctttagcagaagagaatatacaagcattacagtcggctg

fava_c16214_638	fava_c16214	638	C/T	agatatttcagcaacttctgggattgtaaccatgacaccgttctcgttcgatgttctctcgactctaagttgatttccttcagattgccatgtagaaagg[C/T]gcccttctgggattacRagttcctttccgtcataagagagcttcaaatggtcaacttcatcatcccaaattgttgatggggtggcctcaacagacaagtt

fava_c16214_655	fava_c16214	655	A/G	ctgggattgtaaccatgacaccgttctcgttcgatgttctctcgactctaagttgatttccttcagattgccatgtagaaaggYgcccttctgggattac[A/G]agttcctttccgtcataagagagcttcaaatggtcaacttcatcatcccaaattgttgatggggtggcctcaacagacaagttgtgagaatcaaagagaa

fava_c16215_2999	fava_c16215	2999	T/C	atcggctttggaaagaacatctacaagcactaaaatttgttcagcaagctgtgggtttacgagggtatgcacaacgggatccacttattgaatataagct[T/C]gagggttataaccttttcttggagatgatggcacaaataagaagaaatgttatatattctatatatcagtttaaacctgtgctgctaaagcaagatcagg

fava_c16215_3297	fava_c16215	3297	A/C	tagttcctaaagcagcagttggtgcatataattgaattccaacttattaaacgagaaggaacaacttggaaggcttctctttatccactgttcggcaaat[A/C]atttatgtaaagtggaaatttgatgctccatgtgtaaaattttataaatgtaacaaaccacgcccttagaatatgagccaagaataggttataaatccta

fava_c16215_3437	fava_c16215	3437	A/G	ttttataaatgtaacaaaccacgcccttagaatatgagccaagaataggttataaatcctaaatttagtgattattatatttgtaacagcattttcctac[A/G]cgctgagatgtgatttacagtgaatacgatgcatacaaatgatttataatttttagtactttgttgtaaaaaagtgttcaaataaaattaatacaaacac

fava_c16215_620	fava_c16215	620	G/A	agaacggagagtccttggattctctcttgcctgaagcttttgctgttgtaagagaagcttctaagagagttctcggccttcgtccattcgatgttcagtt[G/A]atagggggaatggttcttcataagggggaaatagcggaaatgaggactggagaagggaagactttggttgctattttgccagcttatttgaatgcattag

fava_c16216_1408	fava_c16216	1408	A/G	tgctgctagtgttcaatagccctaaattttcactagatcctaccgtagaatctttagaagtcaccgattgtgccttagaatgccttctagctatctgtgc[A/G]aattcatcttcttcctcttcatcactttcgccccttgtctcagaatagactgcggcaggagggctagctataggtgttgaaacagactccgaagggctag

fava_c16219_244	fava_c16219	244	G/C	cgcctgtatcaccgccactaccaccagccgctcaagagttcctagactctcacaacaaagcaagagccgaagtcggagttgcgccactccaatggagcga[G/C]aagctagcgaaagacacaagcttactagtccgttaccagagaaacaaaatgggatgcgatttcgcgaacttaacgacaagcaaatacggcggtaaccagc

fava_c16219_363	fava_c16219	363	C/T	agcttactagtccgttaccagagaaacaaaatgggatgcgatttcgcgaacttaacgacaagcaaatacggcggtaaccagctctgggcgggttcggcgg[C/T]ggtgacgccgagcaaagcggtggaggagtgggtgaaggagaaagatttctacatacatgcaaacaactcttgcgttgcgaaccatgagtgtggtgtttat

fava_c16219_513	fava_c16219	513	G/C	tacatacatgcaaacaactcttgcgttgcgaaccatgagtgtggtgtttatacgcaggttgtgtggaagaattcggtgcaacttgggtgttcacaagcta[G/C]ttgtagtgttaaggagaaagctagtttgacgatttgtttctatgatccaccgggtaatgtcataggggagactccattttgattcagtttcaagtaatgt

fava_c16219_669	fava_c16219	669	T/C	aatgtcataggggagactccattttgattcagtttcaagtaatgtttattagttattagtatctgtttcttgaattagaatgtgtaactttctaagtgtg[T/C]gcacaatgtgctaagagaggttaatatttcattatgcttaattaRtttagcgttatctcttgttatggttgttaattgagatggatctYttgttatgtgc

fava_c16219_714	fava_c16219	714	A/G	ttattagttattagtatctgtttcttgaattagaatgtgtaactttctaagtgtgYgcacaatgtgctaagagaggttaatatttcattatgcttaatta[A/G]tttagcgttatctcttgttatggttgttaattgagatggatctYttgttatgtgcattttattaggtgtagtttatatttatttctaatgaaatatgata

fava_c16220_338	fava_c16220	338	G/C	ttaaaagtcatgaacaccttccttatttatcttaacaaaaatcgaacaacaaaatcatcacaacttcatcgctaattcattataaatttgacgatcgaaa[G/C]aattctcaacaacaaatttgatacactttgacataacaatgtcagcgatttgcttactcagattcaaaaccaaaaccaaagcttagcacaaacaacaagt

fava_c16220_766	fava_c16220	766	G/A	tgatgcgtcgcggatcagagagcaaaggttattgtaagtgaatctcgcgtgaatctgacgaacagaggtagtgagctgaagtaaattgttgtagaatctg[G/A]aagcaRaSgacggaggatgtatgttgaatcgggaacgacggaagctgatgtaggtgcggtggtgttttgtttgacggtaaggagaagggatcgatggtga

fava_c16220_772	fava_c16220	772	G/A	gtcgcggatcagagagcaaaggttattgtaagtgaatctcgcgtgaatctgacgaacagaggtagtgagctgaagtaaattgttgtagaatctgRaagca[G/A]aSgacggaggatgtatgttgaatcgggaacgacggaagctgatgtaggtgcggtggtgttttgtttgacggtaaggagaagggatcgatggtgagcggaa

fava_c16220_774	fava_c16220	774	C/G	cgcggatcagagagcaaaggttattgtaagtgaatctcgcgtgaatctgacgaacagaggtagtgagctgaagtaaattgttgtagaatctgRaagcaRa[C/G]gacggaggatgtatgttgaatcgggaacgacggaagctgatgtaggtgcggtggtgttttgtttgacggtaaggagaagggatcgatggtgagcggaatg

fava_c16220_876	fava_c16220	876	A/G	acggaggatgtatgttgaatcgggaacgacggaagctgatgtaggtgcggtggtgttttgtttgacggtaaggagaagggatcgatggtgagcggaatgg[A/G]cgaagggagtattgtattacggtggaggttaaacgatgctgttgatggacgattgatgaagaaggaaataaattttcgaaatagattcaattttcccttc

fava_c16221_688	fava_c16221	688	C/T	gtggacatcttttccataaagtttgtttggagaagtggttggactattggaacattacttgtccactttgtaggactcctttgattcctgaagatgatgc[C/T]gcatcttgcttttcatgagcaatgattgaagtatgcacagggttcttcatgtacagtgtatatatttgtgacaattttgatgttgYtatgtatctgactt

fava_c16221_774	fava_c16221	774	C/T	cctgaagatgatgcYgcatcttgcttttcatgagcaatgattgaagtatgcacagggttcttcatgtacagtgtatatatttgtgacaattttgatgttg[C/T]tatgtatctgactttctttttagccttcttagtgctttgccttttatgatgtagagttagtggagggttttgttttgtttactatgttttttccctttct

fava_c16223_1205	fava_c16223	1205	C/A	gagttaatgggattggagacacttatgctgtgcaattgatcagtaaatttggcacattggagaggttgttggaatctgttgatcaaatcaaagaggatcg[C/A]attMgaaagaccttaattgaaaatgccgagcaggctcgattaagcaaggaattggcattgttgcgttctgatctcccatcctacatggtaccatttgcYg

fava_c16223_1209	fava_c16223	1209	A/C	taatgggattggagacacttatgctgtgcaattgatcagtaaatttggcacattggagaggttgttggaatctgttgatcaaatcaaagaggatcgMatt[A/C]gaaagaccttaattgaaaatgccgagcaggctcgattaagcaaggaattggcattgttgcgttctgatctcccatcctacatggtaccatttgcYgtaaa

fava_c16223_1304	fava_c16223	1304	T/C	gMattMgaaagaccttaattgaaaatgccgagcaggctcgattaagcaaggaattggcattgttgcgttctgatctcccatcctacatggtaccatttgc[T/C]gtaaaagatctcttgtttcataaacccgaggataatggcagcaaattcaacagccttttaacagctatcagtgcttacgcagaagggttttcagctgatc

fava_c16223_197	fava_c16223	197	C/T	tctttatactataacaccaaaatattcaaatggcatgttgttgtaaatacaactatttctttcttcactcgcatttcttgtggagaagagtcccatttcc[C/T]cgccgtcgcgtaaccggaaccgtcaccgctagtagctactctcgcaattttcaatctccgaaattgctcagttcttcacgcgcaccactctcgaagggct

fava_c16223_407	fava_c16223	407	G/A	aaactttagattcgcgtggagatggagttgtttctgcaagttctcagagcaatgcaacattggctccagaaattagttatggagctgctccacctgtgca[G/A]cttggttctgttagaaatgcggaagatgcggcgaacagtgattctttcaatggcagagttatgctcattgacggcacatcggtaattcatagggcatact

fava_c16226_1145	fava_c16226	1145	C/T	tgaaagaaggactgatatgttttgagcttatgcaaaaagtatacaagctgcaacccaaagtgcaacattatggatgcatggtggatatgcttagccgagc[C/T]ggacttgtcgaagaagcaacaaaactcattcaaaaaatgcctattgaaccaaatgatgtgatttggaagaccttgcttagtgcttgtcaaattcatgaaa

fava_c16226_636	fava_c16226	636	A/G	tgatgtacttgctgcgaggagtttgtttgatgaaatgccaaggcgaaacgtcatttgttgtaattcaatgatggctggctatgttcagaacggttattgc[A/G]ttgaggcattgaagctRtttcatgatattagaagggcgacagatatgttaccggataatacgatgctgttgatggttcttacagcagtcgcRcaattagg

fava_c16226_653	fava_c16226	653	G/A	ggagtttgtttgatgaaatgccaaggcgaaacgtcatttgttgtaattcaatgatggctggctatgttcagaacggttattgcRttgaggcattgaagct[G/A]tttcatgatattagaagggcgacagatatgttaccggataatacgatgctgttgatggttcttacagcagtcgcRcaattaggttgcgtggaggatggag

fava_c16226_728	fava_c16226	728	A/G	gttattgcRttgaggcattgaagctRtttcatgatattagaagggcgacagatatgttaccggataatacgatgctgttgatggttcttacagcagtcgc[A/G]caattaggttgcgtggaggatggagttgcgatacatcgttatttaatggacaatggttactctctgagtgataatctcggcgttgctctaattgacatgt

fava_c16226_872	fava_c16226	872	T/G	taatggacaatggttactctctgagtgataatctcggcgttgctctaattgacatgtactcaaagtgtggtagcatcgagaatgctatatcagttttcaa[T/G]gatatYgataaaaaatgtgttgatcaYtggaatgctatgattggtggcttagcgactcatggaatgagtgaaatggcttttgattttctaatggagatgg

fava_c16226_878	fava_c16226	878	C/T	acaatggttactctctgagtgataatctcggcgttgctctaattgacatgtactcaaagtgtggtagcatcgagaatgctatatcagttttcaaKgatat[C/T]gataaaaaatgtgttgatcaYtggaatgctatgattggtggcttagcgactcatggaatgagtgaaatggcttttgattttctaatggagatggaaaggc

fava_c16226_899	fava_c16226	899	T/C	ataatctcggcgttgctctaattgacatgtactcaaagtgtggtagcatcgagaatgctatatcagttttcaaKgatatYgataaaaaatgtgttgatca[T/C]tggaatgctatgattggtggcttagcgactcatggaatgagtgaaatggcttttgattttctaatggagatggaaaggctttgtattacacctgacgata

fava_c16228_1523	fava_c16228	1523	T/C	gcagtctttcacctcagggttcactgtctcctaatggtaccgatgcgaatggaggaaatggtgggagttctggaaatggcggcaaaaagtcttcttctca[T/C]gtaggattgatagtgttYgctgtggttggtgcagtttttgtggcctctttgattggtttctttgttttctgcttgctaaggatgaagcaaaagaagttaa

fava_c16228_1541	fava_c16228	1541	T/C	gttcactgtctcctaatggtaccgatgcgaatggaggaaatggtgggagttctggaaatggcggcaaaaagtcttcttctcaYgtaggattgatagtgtt[T/C]gctgtggttggtgcagtttttgtggcctctttgattggtttctttgttttctgcttgctaaggatgaagcaaaagaagttaagtagggttcagagtccga

fava_c16228_2045	fava_c16228	2045	G/A	tagccgggaagggtgcgtcagaatttaagtctgaaattgctgttttgacaaaggttcgtcaccgacatcttgttgctcttctcggatactgcttggatgg[G/A]aacgagaagcttcttgtgtatgagtacatgcctcagggaaccctaagtaggtatatttttaattggccggatgaagggctcgaaccacttgcgtgggata

fava_c16228_2654	fava_c16228	2654	C/T	ttgatatcaatgaggaaacacttgcgagtattcacactgtagcagagttagctggtcactgcagtgcaagggagccatatcaaagaccagacatgggcca[C/T]gcagttaacgttctttcatctcttgttgaacaatggaaaccgtctgatttgaattccgaagatatatatggtatcgaccttgacttgtctttaccacaag

fava_c16228_2801	fava_c16228	2801	T/G	atttgaattccgaagatatatatggtatcgaccttgacttgtctttaccacaagcactcaagaaatggcaggcttatgaaggtgcaagtcaattggagtc[T/G]acttcttcttcttcgcttcttccaagtttggataacacgcaaacaagtatacctactcgtccatatggatttgccgattctttcacgtcggcagatggga

fava_c16229_1043	fava_c16229	1043	T/C	ttgcccagattgaaactcatgactacatggcaaagatttttcattcattcatccaattcaacaatatattaatcgactttgatagagatgtatggggcta[T/C]atatctttgggMtactttaagcagacaactaaggctggggagattgggtcRtccactatgcctcacaaagtgaatcccattgattttgagaacagcgaag

fava_c16229_1055	fava_c16229	1055	A/C	aaactcatgactacatggcaaagatttttcattcattcatccaattcaacaatatattaatcgactttgatagagatgtatggggctaYatatctttggg[A/C]tactttaagcagacaactaaggctggggagattgggtcRtccactatgcctcacaaagtgaatcccattgattttgagaacagcgaaggcaaYttaggcg

fava_c16229_1094	fava_c16229	1094	G/A	tccaattcaacaatatattaatcgactttgatagagatgtatggggctaYatatctttgggMtactttaagcagacaactaaggctggggagattgggtc[G/A]tccactatgcctcacaaagtgaatcccattgattttgagaacagcgaaggcaaYttaggcgtggctaatggaggtttttctcatctaagcatgaagttgc

fava_c16229_1148	fava_c16229	1148	T/C	ctttgggMtactttaagcagacaactaaggctggggagattgggtcRtccactatgcctcacaaagtgaatcccattgattttgagaacagcgaaggcaa[T/C]ttaggcgtggctaatggaggtttttctcatctaagcatgaagttgccgatttcacgttggcagagggatttgactgattcaactgtcttaaggaccatgg

fava_c16229_1550	fava_c16229	1550	T/C	ttaccaaagaaagcttaagagacttcattgaaggcttagatattcctcaaaatgcaaagacctatctcttaaagttaacgccgcatacttacgttggaac[T/C]gccgttgaactggccagaactgtagaacacgaagtgaataatattttgaatggagcaatctaggaaataggttgtttgaacctgaaacagttttaKtgag

fava_c16229_1646	fava_c16229	1646	T/G	gaacYgccgttgaactggccagaactgtagaacacgaagtgaataatattttgaatggagcaatctaggaaataggttgtttgaacctgaaacagtttta[T/G]tgaggttttgattagttagtgaggttacttcattgctgccggtacgatttttttgaccWgattctcaccattgataatcattataaggttagcaattgtg

fava_c16229_1705	fava_c16229	1705	A/T	gcaatctaggaaataggttgtttgaacctgaaacagttttaKtgaggttttgattagttagtgaggttacttcattgctgccggtacgatttttttgacc[A/T]gattctcaccattgataatcattataaggttagcaattgtgatcattaatatgagaaactagagtgatgctgaaagatttgtggatttaattttatctat

fava_c16229_191	fava_c16229	191	T/C	caacaaccttcaataccctccgaaccaatcctctcccttttccctcttcccatttttccttcctttctctctctcacaccaccgccgccaccaacatgtc[T/C]cgacttWattccgccgacttccaactatccaccataacagctttatctccattagacggccgttatagagataaagttcaagacttggctcctatcatgg

fava_c16229_198	fava_c16229	198	T/A	cttcaataccctccgaaccaatcctctcccttttccctcttcccatttttccttcctttctctctctcacaccaccgccgccaccaacatgtcYcgactt[T/A]attccgccgacttccaactatccaccataacagctttatctccattagacggccgttatagagataaagttcaagacttggctcctatcatgggtgaata

fava_c16229_305	fava_c16229	305	T/C	ccgacttccaactatccaccataacagctttatctccattagacggccgttatagagataaagttcaagacttggctcctatcatgggtgaatatggact[T/C]aattactatagggtcattgttgagattaaatggctcatgaagctttctgaaattagtgaaatcacYgaagttcctccttttagtgaagatgctaagtctt

fava_c16229_371	fava_c16229	371	T/C	aagacttggctcctatcatgggtgaatatggactYaattactatagggtcattgttgagattaaatggctcatgaagctttctgaaattagtgaaatcac[T/C]gaagttcctccttttagtgaagatgctaagtctttcttacaaggtttgattgaccactttggtgaagctgatgttaatgagattaaaagatttgagaaga

fava_c16229_689	fava_c16229	689	C/T	ataaccttgctcatgccttgatgttgaaagaagccatggattctgttatgtttcctgccatggataaaataatcaaagctttgtgtacaatggccaaaga[C/T]aacgccSatRtctcYatgctttcYcgcactcatggacagccagcttcaccaacaactttagggaaggaaatggcgatatttgctgtgaggctaagcagag

fava_c16229_696	fava_c16229	696	G/C	tgctcatgccttgatgttgaaagaagccatggattctgttatgtttcctgccatggataaaataatcaaagctttgtgtacaatggccaaagaYaacgcc[G/C]atRtctcYatgctttcYcgcactcatggacagccagcttcaccaacaactttagggaaggaaatggcgatatttgctgtgaggctaagcagagaaaggaa

fava_c16229_699	fava_c16229	699	A/G	tcatgccttgatgttgaaagaagccatggattctgttatgtttcctgccatggataaaataatcaaagctttgtgtacaatggccaaagaYaacgccSat[A/G]tctcYatgctttcYcgcactcatggacagccagcttcaccaacaactttagggaaggaaatggcgatatttgctgtgaggctaagcagagaaaggaagga

fava_c16229_704	fava_c16229	704	T/C	ccttgatgttgaaagaagccatggattctgttatgtttcctgccatggataaaataatcaaagctttgtgtacaatggccaaagaYaacgccSatRtctc[T/C]atgctttcYcgcactcatggacagccagcttcaccaacaactttagggaaggaaatggcgatatttgctgtgaggctaagcagagaaaggaaggaacttt

fava_c16229_713	fava_c16229	713	T/C	tgaaagaagccatggattctgttatgtttcctgccatggataaaataatcaaagctttgtgtacaatggccaaagaYaacgccSatRtctcYatgctttc[T/C]cgcactcatggacagccagcttcaccaacaactttagggaaggaaatggcgatatttgctgtgaggctaagcagagaaaggaaggaactttctcaagttg

fava_c16231_330	fava_c16231	330	A/G	gattgggccgaagagtatgtaatcttgaacaagtacatggttggggcataaagcccattaggtttcaactatcgacatccgatggtcagaattcgctctc[A/G]caRtgttatttgaatgggcctggagaatgggcctattaccatgctggagactttgtcattgagaagcctaatgggcccattaatcttaaattttctttgg

fava_c16231_333	fava_c16231	333	G/A	tgggccgaagagtatgtaatcttgaacaagtacatggttggggcataaagcccattaggtttcaactatcgacatccgatggtcagaattcgctctcRca[G/A]tgttatttgaatgggcctggagaatgggcctattaccatgctggagactttgtcattgagaagcctaatgggcccattaatcttaaattttctttggccc

fava_c16231_541	fava_c16231	541	T/C	ttgcactcacaccaaaggtggtctttgcatagatggggcagtgatttgtcctaaggagttagcacataagttgaaacaattttagcttattcacactcag[T/C]Rgtgtttttttaattaagcatttttgtaagaggaaacactataaaaaaacacgccttgattgtgggggtttttaacccatttaaatgtaaatttttgtgc

fava_c16231_542	fava_c16231	542	G/A	tgcactcacaccaaaggtggtctttgcatagatggggcagtgatttgtcctaaggagttagcacataagttgaaacaattttagcttattcacactcagY[G/A]gtgtttttttaattaagcatttttgtaagaggaaacactataaaaaaacacgccttgattgtgggggtttttaacccatttaaatgtaaatttttgtgca

fava_c16231_644	fava_c16231	644	T/A	tgtttttttaattaagcatttttgtaagaggaaacactataaaaaaacacgccttgattgtgggggtttttaacccatttaaatgtaaatttttgtgcaa[T/A]ttttgttttgttttcggattaaatatcattggggaaaagggttgaagaaaaaaatgttggattttgagatcaaacaaccttggatcttatgtgatgattg

fava_c16234_1408	fava_c16234	1408	G/T	gtaccacaggtagtttgattcctagtaaagtttcaggaaaaatagtgatatgtgacagaggaggaaaccctagagcagaaaagagtttggttgtcaagcg[G/T]gcaggggggattgggatgattttagcaaacaaccaagattatggggaagagctagttgctgactcctatcttttcccagcagcagctttgggagagaaag

fava_c16234_1604	fava_c16234	1604	A/G	gaaagcaagcagtgaagtaaagaagtatgtttcttctactcgcaacccaaccgctaaaattgcattcggcggtactcaattaggggttcagccatcccca[A/G]tggtggctgctttcagctctagaggaccaaatatactcacaccgaaaatacttaaaccagacctcatagctccaggagtcaacattctagctggatggac

fava_c16234_1963	fava_c16234	1963	G/A	tcatgaccacatcctatagaacctacaaaaatgggcaaaccataaaagatattgctactgggacacctgctacgccgtttgactatggttctgggcacgc[G/A]gatccagtagctgctcttgatcctggtcttgtgtatgatactacagcggatgactacttgagcttcctctgtgccttgaactacacttcatttcaactaa

fava_c16234_2568	fava_c16234	2568	T/C	ctgaattacctgttgcgatattatgcaagtatgagaataattgtatttcacttctttagtctgttgttggtccagcaaagaagcctataatagagctgaa[T/C]ttgtgtctctttgctgtggtttcatgtaaagaataattttatacagaaaaaagggcaataacacatgtgagtaatggcattgaatggcaattcatattac

fava_c16236_1703	fava_c16236	1703	C/T	aacttcagctttttgctcttgtcgcaaagcaaccgacctttcatcagcttcgatcagttgagcagctggggtacattactgtgctcatgcagaggaatga[C/T]tgtggtgtacgtggactacagtttatcattcagtccacagtaaaggctcctggaagcatccaagagagagttgaggcatttctcaagatgtttgagacca

fava_c16236_2238	fava_c16236	2238	C/T	ttttagaaaatcacagtctctttatggttcatttaaaggtcttactgggcagatgaaattgtagcaggtggatcttttaggtttgtaaacttctttaact[C/T]acccaaacacttgaataaaagagttctttgtaatatcagttagtttgagcgaaaggtttccatttgaacaaaacgccaaacgtttccttttaattttaag

fava_c16236_518	fava_c16236	518	T/C	cagcggtttgtgagactaagtttcactatcaggataaaattcctccaagtgattatgttgttaatattgcatcaaatatgcagtactatcctccaaaaga[T/C]tggttggttggatcatcattgccttctaagtttagccccagtgttatccaaatggttctagaccagctttctccaaataacgttcgaatcttttgggaat

fava_c16237_1460	fava_c16237	1460	C/T	aacacaattccctcctttgcctcagaatgttttggaccatgacttccaaagcttttatggaatgggatatgattccaacacagcacttgataatttggga[C/T]ccaatgggaggttgaaaccagagttatagtatgttgaatatatagccttgtttctacatcagaagactttggaattttcctgttgactgcacaatttaac

fava_c16237_1574	fava_c16237	1574	A/G	gaaaccagagttatagtatgttgaatatatagccttgtttctacatcagaagactttggaattttcctgttgactgcacaatttaactgaagttggaaaa[A/G]aaatagaaggaaaataaaggttttagttgtataggatcttatttaatacctaactattggctcttcgaatttttagtagaagatgactttgttagacaat

fava_c16237_207	fava_c16237	207	G/A	ctttaatcatttatatgcatttcttgagccaactgctgagtcagggttgttctaatcaattgatatataattgaaagagagagacacatagaaaggtgtt[G/A]ttttcttggttgaattgagatgggtggtgaagaaaaagccatggagtttcaacatggaaatgagaacattatgacttcagaaatggatattagttctgtg

fava_c16237_571	fava_c16237	571	C/A	attataaccctcttgtgttggagaatcatcaaggaatgagtagcacctctcaccttgttcaatactcaaatcttggtggcatgatccacaaggttccttc[C/A]tatggaagtggaagtttctcagaaatggttggttcctttggccaacatggtggtgatcaYgtagctaaYacatcaggatatccaattccaccacaacatt

fava_c16237_631	fava_c16237	631	T/C	aatactcaaatcttggtggcatgatccacaaggttccttcMtatggaagtggaagtttctcagaaatggttggttcctttggccaacatggtggtgatca[T/C]gtagctaaYacatcaggatatccaattccaccacaacattataaccacatcaaggatgctggaatccaaaggggtcaaattcatggtgaacaatctcaag

fava_c16237_640	fava_c16237	640	C/T	atcttggtggcatgatccacaaggttccttcMtatggaagtggaagtttctcagaaatggttggttcctttggccaacatggtggtgatcaYgtagctaa[C/T]acatcaggatatccaattccaccacaacattataaccacatcaaggatgctggaatccaaaggggtcaaattcatggtgaacaatctcaagttgaggact

fava_c16240_1201	fava_c16240	1201	G/T	taacagcagcgcgcatcttgtcggacatgaaagcgcggtctggattgtcaacaatcggcgaaagagtcccgtcataatcgagaaacaatgctattttctt[G/T]ccttttgcacagttagtaatttgctcaaaagaYgaaagtgcggatggaaactctagcagccaggttaaataagcagcatcggcttcagaagacggaacac

fava_c16240_1234	fava_c16240	1234	C/T	cgcggtctggattgtcaacaatcggcgaaagagtcccgtcataatcgagaaacaatgctattttcttKccttttgcacagttagtaatttgctcaaaaga[C/T]gaaagtgcggatggaaactctagcagccaggttaaataagcagcatcggcttcagaagacggaacaccgtgaccaacatccatagatattttcttgtgag

fava_c16240_1592	fava_c16240	1592	G/T	ccttgtccttgttaatgttgcagtttcagcaaggagagatgtatgatttgccttcagttccattggatccagatcttaatcacaggcttcaatttccagc[G/T]acttgaaaattttcagttcccaaataacaatgcaagttccaaataaatttcctactaatgaatgaggagaagaagaagaatgaatgaatgaaagaggaga

fava_c16240_320	fava_c16240	320	C/T	acaaaagctttgacaaaacaaaactgagtagtgaaaaagttattcctatcttgaaaggtcttcttcagagcttaattctgctccaataaacccttgaaaa[C/T]ttctcaaccaaacccaaaatcagaatctgtcattaatataatagtatagtagaaacataatctcctctatacattatagtaaagagtttgatttccatgc

fava_c16240_475	fava_c16240	475	C/T	acataatctcctctatacattatagtaaagagtttgatttccatgccacaagtgatttgagaaattccatcacctcagaaggatcacaaagagagtaaac[C/T]gcgttgctttctttaggggccgaagaaactaagattccgaaacctttattaacttcactcaaaaccttaaatgcatcttcgtctgtccgatcatcaccga

fava_c16240_829	fava_c16240	829	A/G	caaaaacgcgttgtgccaccaaatcccaattctcttcgtccacattacgataatgtacagagacacagaacttattattctcaattttcgctcctttgat[A/G]tctcgtatacagtcaacgagcaacatccgtacctcatcaatcataggcaggaaatcagcagcaggttggaataaattaacttccttacccttcttatcag

fava_c16241_1017	fava_c16241	1017	T/C	aaaaaccttatctttagggctttgagttttcactgtttagagcaaaattcttcaccaaattacaccattttactgacaggaagaaagttataattttatg[T/C]ttaatttcttcttcttttttgataaaaaWttatttaggatgtggagaccatctaagatttcaagggcatgacggctgaattcgaatctgaaacatcgagg

fava_c16241_1046	fava_c16241	1046	A/T	tcactgtttagagcaaaattcttcaccaaattacaccattttactgacaggaagaaagttataattttatgYttaatttcttcttcttttttgataaaaa[A/T]ttatttaggatgtggagaccatctaagatttcaagggcatgacggctgaattcgaatctgaaacatcgaggttgtctagatctaagatctgtatatgtac

fava_c16241_153	fava_c16241	153	G/A	tccatatacaggaaacaagggatagccttctcccatactacctataccattcatcaaatgagagttacctaaaaacttcatgttcttcgaggagacttcc[G/A]atttcatcgagattatttaagggtaaaggcggtgacagaagagagtgagcacacatggtgattttcttgcatgatgatgtttcatgcttgaagctatgcg

fava_c16241_258	fava_c16241	258	T/C	catcgagattatttaagggtaaaggcggtgacagaagagagtgagcacacatggtgattttcttgcatgatgatgtttcatgcttgaagctatgcgtgct[T/C]actctttatctgtcaccccaccaccatctttctttctctctttcctYctcatctSttYtcctactatgtttacctatatttttagtttttgagacacYag

fava_c16241_305	fava_c16241	305	C/T	cacatggtgattttcttgcatgatgatgtttcatgcttgaagctatgcgtgctYactctttatctgtcaccccaccaccatctttctttctctctttcct[C/T]ctcatctSttYtcctactatgtttacctatatttttagtttttgagacacYagtcttgatgttaagatctggtttacataaattcagtctttgtcgtttc

fava_c16241_313	fava_c16241	313	C/G	gattttcttgcatgatgatgtttcatgcttgaagctatgcgtgctYactctttatctgtcaccccaccaccatctttctttctctctttcctYctcatct[C/G]ttYtcctactatgtttacctatatttttagtttttgagacacYagtcttgatgttaagatctggtttacataaattcagtctttgtcgtttcgattcgat

fava_c16241_316	fava_c16241	316	T/C	tttcttgcatgatgatgtttcatgcttgaagctatgcgtgctYactctttatctgtcaccccaccaccatctttctttctctctttcctYctcatctStt[T/C]tcctactatgtttacctatatttttagtttttgagacacYagtcttgatgttaagatctggtttacataaattcagtctttgtcgtttcgattcgattca

fava_c16241_356	fava_c16241	356	T/C	ctYactctttatctgtcaccccaccaccatctttctttctctctttcctYctcatctSttYtcctactatgtttacctatatttttagtttttgagacac[T/C]agtcttgatgttaagatctggtttacataaattcagtctttgtcgtttcgattcgattcagtcttttttttgttttccctttttaatgaaaacactaacc

fava_c16241_686	fava_c16241	686	G/A	atgtttttcaagatacttgtttttacattattcttatagggattcttctttgttctttctcagcatggtatccaacagttacttcatcaaaaccatacta[G/A]tttatatttagtcaatgaattttgattttatatactcagtcaacttgcttgtactagttttagtactaYtaattaattaaaatcatctggaaacacctat

fava_c16241_755	fava_c16241	755	C/T	atccaacagttacttcatcaaaaccatactaRtttatatttagtcaatgaattttgattttatatactcagtcaacttgcttgtactagttttagtacta[C/T]taattaattaaaatcatctggaaacacctatagtttgtctttgattcatactgccaagaaacatggtttgttttattagtttattttattgtttggaagt

fava_c16242_1478	fava_c16242	1478	T/C	aaattctaaattacacctgtttagtgtttaccatagagtttcaacccttattatcttgtcttcagctgttattttaagaaagtcttgaataatttattta[T/C]aaggaacaaggtggttccttatttcattttcctctcttcaaattggtcatttaattctatacactagatcataattgcaaggatctaacatgttcttgta

fava_c16242_439	fava_c16242	439	G/T	tgcaggagctcaaggaagagggtttatggaagaatttcaattcacaagaaggtgtcaatgaggatgtaaagatatcggatgaagacgatgaggaagaatc[G/T]tattcccaacccaatggaagtcagagtgaatcattatcattatcccaaaccaatggaagtcagagtgacagtctgaaccctaattcttgttttcccagat

fava_c16243_315	fava_c16243	315	G/T	aatactactatctaaatttccactgttaaatcaacaacccattacacattcctttcacgcacaacatacccttattcccagcaacatttttccttcccat[G/T]aatactcactcctttttctttctcttgggYtttcatctcttcaaatcaaccctttcactcccctctttctaatggatgcaaaaccaaaaccttctgtaga

fava_c16243_345	fava_c16243	345	C/T	tcaacaacccattacacattcctttcacgcacaacatacccttattcccagcaacatttttccttcccatKaatactcactcctttttctttctcttggg[C/T]tttcatctcttcaaatcaaccctttcactcccctctttctaatggatgcaaaaccaaaaccttctgtagaacctttgaagtatcagacatggttcttgaa

fava_c16244_364	fava_c16244	364	A/G	gctgtgaaacatttttcaactctgcccaaaattgttttgcataaaaaataatcaatgttacatacttattccgatacacgcaaagagtcgatcaaccatg[A/G]cgatgctggctgtgatcctctaactgcataaattaaatatctatccaagactgaaatcatttacagtttgatccctcaagtgaaagtccattttcttagg

fava_c16244_589	fava_c16244	589	A/G	ttgataacattctatccaagggattcaattgcacaaataaaatgggtacaatactacacggtgatgcatctacgacacaaacacaatacagcatgaatgc[A/G]tgggcagattacaagagYttttatcgtctcctaacctagtaccttgcgaagttcatcagtcaacttattgcgacttgatgcttccaacttgcttaaaagt

fava_c16244_607	fava_c16244	607	C/T	agggattcaattgcacaaataaaatgggtacaatactacacggtgatgcatctacgacacaaacacaatacagcatgaatgcRtgggcagattacaagag[C/T]ttttatcgtctcctaacctagtaccttgcgaagttcatcagtcaacttattgcgacttgatgcttccaacttgcttaaaagtgcggcaagaccttgaggt

fava_c16244_926	fava_c16244	926	G/A	atgcttcgtcgtctaaccaggcttcttctctacctttggcaatatcagaattcagataccagtctttgagcaagtttatggcagatacatgtgacaattg[G/A]tcaccagcagcatctctcacactgttgatcagtgagtgctcaccgattcttctgtgcagtctccgatagaagacagaacgcgagttacgccaatcaagaa

fava_c16246_227	fava_c16246	227	C/T	gtcaaattttatgtttgtgtatcacaactcaaaaccaaaacccaaaatatacaagaaaaaacagaaattatacaaattacaacattagtgtcatctcttg[C/T]tcaacatgtttggaaaattgagtaattgcactaacaaaaccaaattgaaaaattgtgaaattgaatgagtgtgttttagatatgcctccaaggtctttct

fava_c16246_822	fava_c16246	822	T/C	ctctaagtggttcctttctttttataatttctgtccaattgtagtccatgagtagtccagacattgcaagcagctcaaacaatttgttgtcatcataagc[T/C]ggaacYccccaacactcatcatgaaattctatgtatagcatatcactattttttgtgatccaattgcatcttttcaattcccctggctcagtgatcactc

fava_c16246_828	fava_c16246	828	T/C	gtggttcctttctttttataatttctgtccaattgtagtccatgagtagtccagacattgcaagcagctcaaacaatttgttgtcatcataagcYggaac[T/C]ccccaacactcatcatgaaattctatgtatagcatatcactattttttgtgatccaattgcatcttttcaattcccctggctcagtgatcactcctggaY

fava_c16246_928	fava_c16246	928	T/C	Yccccaacactcatcatgaaattctatgtatagcatatcactattttttgtgatccaattgcatcttttcaattcccctggctcagtgatcactcctgga[T/C]ttggctgttgatgtatagttttaggtacggttgattctcttctttcatgtgatgaagcagaaatcaaacgaagtgccaatgaaatcttttcatccaatgg

fava_c16249_1172	fava_c16249	1172	C/T	acttgaggaagtacgaggcttttcaaacattaccaggccatgcaaatactttggagtttagtcaaaaaggcttgcttgcttgtggtaatggttcatatat[C/T]caggttcttagggatgtttctggtgcacagaactattcaagttacatgactcattcaatggtaaagggttatcagatagggaagttagctttccgaccat

fava_c16249_1295	fava_c16249	1295	G/A	gtgcacagaactattcaagttacatgactcattcaatggtaaagggttatcagatagggaagttagctttccgaccatacgaagatgttctaggcatagg[G/A]cattcaatgggttggtctagtattcttattccaggagcaggagaacccaactttgattcatgggttgcaaatccatttgagacacctaaacagagaaaag

fava_c16249_1418	fava_c16249	1418	T/G	ttcttattccaggagcaggagaacccaactttgattcatgggttgcaaatccatttgagacacctaaacagagaaaagaaaaggaagtacagtctcttcg[T/G]gataagcttccagtcgagacaatcatgctagatcccagcaagatcggtactgtgaaatttactaaaaaagagaagccaactgtggaaaagatggatgctg

fava_c16249_1547	fava_c16249	1547	G/A	tagatcccagcaagatcggtactgtgaaatttactaaaaaagagaagccaactgtggaaaagatggatgctgatatggaagctgctgttgaagctgtcaa[G/A]ggcaaaaaactgaaaaagaaaacaaaaggaagaagtaaggcaggtaaaataatgcctaaaaagcaagatgcaattgccacggcaaagaggccttatttgg

fava_c16249_196	fava_c16249	196	C/T	tctcaccaccgtccctcgcgtcagaaaggtattgcaactgtatcacggtgcgagatggagataactcacgacaaggaagctcttcctaacagaaagcctc[C/T]caccgaacaggacattattgatgaactaaaattgagatcaRagaagtatctcagaggaaaaggtgctaatttgaagggtttaaaggataaaaagcttagg

fava_c16249_237	fava_c16249	237	A/G	atcacggtgcgagatggagataactcacgacaaggaagctcttcctaacagaaagcctcYcaccgaacaggacattattgatgaactaaaattgagatca[A/G]agaagtatctcagaggaaaaggtgctaatttgaagggtttaaaggataaaaagcttaggagtcagttagttgctagRgagaaattatatggagaRtctgc

fava_c16249_314	fava_c16249	314	A/G	ttgatgaactaaaattgagatcaRagaagtatctcagaggaaaaggtgctaatttgaagggtttaaaggataaaaagcttaggagtcagttagttgctag[A/G]gagaaattatatggagaRtctgcaaaagctgctgcccaggctgagaaatggattatgcctagtgatcaaggWtttttggaggctgaaggtctagaaaaga

fava_c16249_332	fava_c16249	332	A/G	gatcaRagaagtatctcagaggaaaaggtgctaatttgaagggtttaaaggataaaaagcttaggagtcagttagttgctagRgagaaattatatggaga[A/G]tctgcaaaagctgctgcccaggctgagaaatggattatgcctagtgatcaaggWtttttggaggctgaaggtctagaaaagacgtataggatcaagcagg

fava_c16249_386	fava_c16249	386	T/A	aaaagcttaggagtcagttagttgctagRgagaaattatatggagaRtctgcaaaagctgctgcccaggctgagaaatggattatgcctagtgatcaagg[T/A]tttttggaggctgaaggtctagaaaagacgtataggatcaagcaggagacaatagcccgtgaagtcgatattttaagctcaaggaaccaatatgatatta

fava_c16249_536	fava_c16249	536	G/C	caatagcccgtgaagtcgatattttaagctcaaggaaccaatatgatattactttaccagagcttggtccatatactctagacttcactcctagtggccg[G/C]tatatggctgttggtggtcgcaaaggccacctMgcaattgtggacatgatgaatctgagccttatcagagagcttcaggtaagagaaactgtgcgtgatg

fava_c16249_569	fava_c16249	569	A/C	ggaaccaatatgatattactttaccagagcttggtccatatactctagacttcactcctagtggccgStatatggctgttggtggtcgcaaaggccacct[A/C]gcaattgtggacatgatgaatctgagccttatcagagagcttcaggtaagagaaactgtgcgtgatgtggcatttttacataatgagctcttctttgctg

fava_c16249_863	fava_c16249	863	A/G	taaaaaaccattttctcctggcttctaccaacaaatttgggatgcttctttatcaggatgtttcaacgggtgatgtagttgggaaatatcgaactggttt[A/G]ggacgtacaaatgtgatgcaagtaaacccttacaattcggttgtttctttaggtcactcagccggtacagttactatgtggaagccaactagttcttctc

fava_c16251_1105	fava_c16251	1105	C/T	gcttggatcattcttcgggtacatgtgtggtcaataaagaaatcaaattggagcgacctggcatctcaggcacctcaattcggcccgataacaaaattgc[C/T]gcaacagctggctgggaccacagagtaaggatatataattaccgcaaaggaaatgctttagctgtacttaagtaccatcatgccacgtgcaatgctgtaa

fava_c16251_253	fava_c16251	253	A/C	gtcacatggaccgacttgacaagtcattctagagaaacgaaagaatgagtaaacgaccaccacctgatcctgtggcagttcttcgaggccatcgcgcttc[A/C]gtcaccgatattgcttttcatccctataagcctatattattctccggttctgctgacggtgaagttcgaatttgggatacatttcgacgccaaactgttt

fava_c16251_388	fava_c16251	388	T/C	tattattctccggttctgctgacggtgaagttcgaatttgggatacatttcgacgccaaactgtttcatcagcatggttacacagtgccgcacatgggat[T/C]gttgctgttgccagtagttcttcacttggaaccaataaatttgttagccagggtagggatggaactgtgaaagtttgggattttgataactctggtttgt

fava_c16251_949	fava_c16251	949	A/C	atgaagatggttccatgctttggtgggatgtacggaatcctggagttccaataagttctgtgaaattccattctgagccagttctgagcatctgcattga[A/C]gggtcttgcaaagggggtatctcRggagctgcagatgagaggattgtgatgtatagcttggatcattcttcgggtacatgtgtggtcaataaagaaatca

fava_c16251_973	fava_c16251	973	G/A	gggatgtacggaatcctggagttccaataagttctgtgaaattccattctgagccagttctgagcatctgcattgaMgggtcttgcaaagggggtatctc[G/A]ggagctgcagatgagaggattgtgatgtatagcttggatcattcttcgggtacatgtgtggtcaataaagaaatcaaattggagcgacctggcatctcag

fava_c16253_469	fava_c16253	469	A/G	tcttgtactcttccagacagatcacacagcttccttcatcctggcattggtcagatgaacaatatattgtttctgtcaaacacttggaaagcacatcctc[A/G]gacaatcctgtgttcacctggccaatcctctccccaagtgcaagaagttcctcatagctcatgttatctacgtccattctcatgtctctgtgctgatcaa

fava_c16253_649	fava_c16253	649	G/A	tcatgtctctgtgctgatcaagtatattcctcgagccatacagtgaagcacgctcaacaaccatgaaaccctcagaggaaaatctagcatggagaccagc[G/A]tcatcggccaatgatctatatctttcactcgaaattctagaccttccatttctgtcactgttccgccagccaacagtggtcaRcggccttggatgtctag

fava_c16253_732	fava_c16253	732	G/A	ctagcatggagaccagcRtcatcggccaatgatctatatctttcactcgaaattctagaccttccatttctgtcactgttccgccagccaacagtggtca[G/A]cggccttggatgtctagaagagtaaccttcaggtaccataggcaatccattatctgaaggtgccgcatgtcccaagcgcaagtttgaagaagccctgaaa

fava_c16256_1093	fava_c16256	1093	A/G	gggaaatgattttcagaggaactgacacggtagcgattttactggaatggattctggcaaggatggttcttcatccagagatacaacaaaaagtacagga[A/G]gaaattgatggtgtagttggaaattccgacgttattagtgatgttgatgttcaaaacatgcgttatctgcagtgcattgtgaaagaggttttaagggttc

fava_c16256_148	fava_c16256	148	C/T	cagaatttcagcttcttctttttcctgaccttgttYacattcatcctctcactctcagttttcaaactgctttgtgttttgttttgttttttttgttttt[C/T]ctattctggttaactccaggtgggtttgcttgggttttttataacaaggcttcgagttcggctSggtctgttattcctggtccgtctttttccggtttgt

fava_c16256_1591	fava_c16256	1591	G/A	tcaaatgggttcaagctgatgattctcctgttgatttggatgaatgtcttaagctttctatggagatgaaatcgccacttgtgtgcaaggttgttcctag[G/A]acctagggctaggctaaacttagtatttgatgttacttaacttttatgtgttttcggaaaggagaaagttggtcatggatcttgtggtgtttggtttctt

fava_c16256_212	fava_c16256	212	C/G	aactgctttgtgttttgttttgttttttttgtttttYctattctggttaactccaggtgggtttgcttgggttttttataacaaggcttcgagttcggct[C/G]ggtctgttattcctggtccgtctttttccggtttgtttgggctYttgtcaggccaaactcctcaccgtgttttRgccaagctcgctcggagtcaccgagc

fava_c16256_256	fava_c16256	256	C/T	ggttaactccaggtgggtttgcttgggttttttataacaaggcttcgagttcggctSggtctgttattcctggtccgtctttttccggtttgtttgggct[C/T]ttgtcaggccaaactcctcaccgtgttttRgccaagctcgctcggagtcaccgagctgagtcgctgatggctttttctgttggtctgactcggtttgtta

fava_c16256_286	fava_c16256	286	G/A	tttataacaaggcttcgagttcggctSggtctgttattcctggtccgtctttttccggtttgtttgggctYttgtcaggccaaactcctcaccgtgtttt[G/A]gccaagctcgctcggagtcaccgagctgagtcgctgatggctttttctgttggtctgactcggtttgttatttccagtgaaccggaaactgcgaaggata

fava_c16256_501	fava_c16256	501	G/A	gcttttggtgataggccggttaaggaatcggcttacgagctgttgtttcatagggcaatgggttttgcaccgtatggtgagtactggaggaatctgagaa[G/A]gatttcttcSacccatttgttttgtcctagaaggataaacRggtttgaaggttttagRagtgaggtggggttaaaaatggttaagaggatagagtttttg

fava_c16256_511	fava_c16256	511	G/C	ataggccggttaaggaatcggcttacgagctgttgtttcatagggcaatgggttttgcaccgtatggtgagtactggaggaatctgagaaRgatttcttc[G/C]acccatttgttttgtcctagaaggataaacRggtttgaaggttttagRagtgaggtggggttaaaaatggttaagaggatagagtttttgatgggtgaaa

fava_c16256_542	fava_c16256	542	G/A	gttgtttcatagggcaatgggttttgcaccgtatggtgagtactggaggaatctgagaaRgatttcttcSacccatttgttttgtcctagaaggataaac[G/A]ggtttgaaggttttagRagtgaggtggggttaaaaatggttaagaggatagagtttttgatgggtgaaatgggtagtgttgaggtgaaaaaagttcttca

fava_c16256_559	fava_c16256	559	G/A	tgggttttgcaccgtatggtgagtactggaggaatctgagaaRgatttcttcSacccatttgttttgtcctagaaggataaacRggtttgaaggttttag[G/A]agtgaggtggggttaaaaatggttaagaggatagagtttttgatgggtgaaatgggtagtgttgaggtgaaaaaagttcttcattttgggtcacttaaca

fava_c16256_736	fava_c16256	736	T/C	ttcttcattttgggtcacttaacaatgtgatgatgactgtgtttgggaagtgttatgatttttttgatggggagggttttgatcttgaggaaatggtgag[T/C]gaagggtatgagttgttgggtgtttttaactggagtgaccattatcctctttttggttggttggatttgcaaggtgttaggaaaaggtgtagggttttgg

fava_c16256_83	fava_c16256	83	T/C	aacgttagttctaatttgtcttcaagaaaacaaagaaacaatgtctccagaatttcagcttcttctttttcctgaccttgtt[T/C]acattcatcctctcactctcagttttcaaactgctttgtgttttgttttgttttttttgtttttYctattctggttaactccaggtgggtttgcttgggt

fava_c16257_1725	fava_c16257	1725	T/C	atgatcaacaaaaaggtggtaagcaagaagttccaggaatgaaaacagtacagacagttttggagcgaagtgcaacacctgtggtctataatgatatgcc[T/C]gatgctctctgtcttaaggattctctaactgctgtccgcaatgctacacaagctgccgatcgcatacatcaagtattcagaatgcagtcctttcagagga

fava_c16260_1025	fava_c16260	1025	G/A	tcaactcagaatttcatctgctgaaaccactgctgctgaagcagctcaactcatcgaaaagcaaattaaggatgcaactgagggtacaatatcatccatt[G/A]tagagaagtcaaaatatgctatagaaagagctcttgcggtggcagaggaagcaggcgaaaatgcaaaaaagWccatggaaacatttacagatggtacttc

fava_c16260_1097	fava_c16260	1097	A/T	tgcaactgagggtacaatatcatccattRtagagaagtcaaaatatgctatagaaagagctcttgcggtggcagaggaagcaggcgaaaatgcaaaaaag[A/T]ccatggaaacatttacagatggtacttctccatttacggaaattgcctctctccaggtggaaaatatcaagttacaaggaatgctaaatgatatagaatc

fava_c16260_1207	fava_c16260	1207	G/A	catttacagatggtacttctccatttacggaaattgcctctctccaggtggaaaatatcaagttacaaggaatgctaaatgatatagaatctcagttgac[G/A]attgcaagaaatgaggttgctagactgaacatagaattggagcacaccagacaacagatgaaggcatttgaacaaagggccatYgatgcagagaaagctt

fava_c16260_1291	fava_c16260	1291	T/C	tagaatctcagttgacRattgcaagaaatgaggttgctagactgaacatagaattggagcacaccagacaacagatgaaggcatttgaacaaagggccat[T/C]gatgcagagaaagctttgcttgatcttcaagagttaagcaagaaaaccactctacagcaagaagaggagatgaagtcattattggagaaagtgagaaaag

fava_c16260_1408	fava_c16260	1408	G/A	tgcttgatcttcaagagttaagcaagaaaaccactctacagcaagaagaggagatgaagtcattattggagaaagtgagaaaagatgtggcagacaagac[G/A]aaggcaatttccaaggcttttaaaactgacttgaaaaacataaaggctactattgatgcttctaaagaagttgtccttagtaaagataatgcYtacctca

fava_c16260_1501	fava_c16260	1501	C/T	acaagacRaaggcaatttccaaggcttttaaaactgacttgaaaaacataaaggctactattgatgcttctaaagaagttgtccttagtaaagataatgc[C/T]tacctcagaagatgtgaagcactgcagaggtcattaatggcatcagaagatgctttgaagatgtggagacagagagctgaaatggctgaatcactgctta

fava_c16260_2377	fava_c16260	2377	T/C	ttgtactcaatcatcgatgtgcacataaacagagtccaaatggtgtttggaacattttcggtggtaagctagcatggggacctgaagcaattgtatgtga[T/C]gatccgaattttcaggggcgtggaaatccttcaagtggggatattttccatgctgcaccaaatgttgaccattctcaagactttgtgcggaaagatatca

fava_c16260_2554	fava_c16260	2554	A/C	aagactttgtgcggaaagatatcaaggaatggttgaactggcttcgcaatgatatagggtttgatggatggcggcttgactttgtaaagggcttctctgg[A/C]acttatgttaaagaatatattgaagcatcaaaacctgcattttccattggagaatattgggacagtttagcttatgaacaaggaagtttgtgctacaacc

fava_c16260_257	fava_c16260	257	A/G	gattccactctcaggccgttgaaacctctccgattccgcgccattgcagcaccgtccttgattctaacctcttcctgccatttcgaaattccaattcaac[A/G]ttgatctcaaatgttaacattgatctaagtaaaatcgagataagtagaaaaaacgcagctcgcatagttcaaacaagtgaattttacaggcataaatggg

fava_c16260_2836	fava_c16260	2836	G/A	gaatacttcattccgctctgcacaatgaatattggagaatgatagaccctcaagggaaaccaacaggagttatgggatggtggccttctcgggctgtcac[G/A]ttcctagagaaccatgatacagggtctacacaaggtcaYtggccgttcccacgagataagctcatgcagggatatgcatatatcttgactcatccYggaa

fava_c16260_2875	fava_c16260	2875	C/T	tgatagaccctcaagggaaaccaacaggagttatgggatggtggccttctcgggctgtcacRttcctagagaaccatgatacagggtctacacaaggtca[C/T]tggccgttcccacgagataagctcatgcagggatatgcatatatcttgactcatccYggaactcctgttatattttatgaccatttctatgattttggca

fava_c16260_2932	fava_c16260	2932	T/C	tcacRttcctagagaaccatgatacagggtctacacaaggtcaYtggccgttcccacgagataagctcatgcagggatatgcatatatcttgactcatcc[T/C]ggaactcctgttatattttatgaccatttctatgattttggcatccaYgatgtaataaccgagttgattgaggcgcgaagacgggcagggatccattgtc

fava_c16260_2980	fava_c16260	2980	C/T	cgttcccacgagataagctcatgcagggatatgcatatatcttgactcatccYggaactcctgttatattttatgaccatttctatgattttggcatcca[C/T]gatgtaataaccgagttgattgaggcgcgaagacgggcagggatccattgtcgaagYtctatcaagatataccatgcaaacaatgaaggttacgtagctc

fava_c16260_3037	fava_c16260	3037	T/C	ctcctgttatattttatgaccatttctatgattttggcatccaYgatgtaataaccgagttgattgaggcgcgaagacgggcagggatccattgtcgaag[T/C]tctatcaagatataccatgcaaacaatgaaggttacgtagctcaagttggcgatgcactagtaatgaagcttggtcagtttgattggaatccctctaagg

fava_c16260_3230	fava_c16260	3230	T/A	ctctaaggagaaccgtttggaagggagctggcagaagtttgttgacaaaggatcagactataaagtgtggttgagacaataatttaatctagttcagtag[T/A]catagtcaataaagcaaatgataataaaattgaaWttttcactagcattgttgatttatataagttatgtaagttaattagtcatattcactaataagtc

fava_c16260_3265	fava_c16260	3265	T/A	agtttgttgacaaaggatcagactataaagtgtggttgagacaataatttaatctagttcagtagWcatagtcaataaagcaaatgataataaaattgaa[T/A]ttttcactagcattgttgatttatataagttatgtaagttaattagtcatattcactaataagtcatgtaagttaattattgtaccttaacatttacaat

fava_c16260_478	fava_c16260	478	C/A	tagcagctttgggagttcttaaacggtatccctttcttccgaggcacccttttgtccgtgggataacttcaactttgtcaagaagaaacagaaaattttc[C/A]tttgctgacaagaggatcttcaaatcagttcacacagttttttcacacaataatgatgatcaaatgtttacagatgaagttgttgatcaagttttgggaa

fava_c16260_627	fava_c16260	627	G/A	aataatgatgatcaaatgtttacagatgaagttgttgatcaagttttgggaatagaagatgaattaatgacagccaagaaagctctttccgaggcacaag[G/A]cagacaagaggctatagagaaagagagagatcaattgcttgaagaacttgcacgttctgaggccaaaaagcaggaatacgtcgatgccattttacatgac

fava_c16263_1079	fava_c16263	1079	T/C	cgccgacggcgttgcggcctttgcaaggcctttggaaggggatttgtgcagacatgagtttagcattttaccttgtggcttatgatgacattggtggcat[T/C]gcctgccgtcggcttggagatcctcctcaacgtttctcaacctatgccccRgttttctggacgtccaatgctacatttatggaatctccctttcctctag

fava_c16263_1130	fava_c16263	1130	G/A	acatgagtttagcattttaccttgtggcttatgatgacattggtggcatYgcctgccgtcggcttggagatcctcctcaacgtttctcaacctatgcccc[G/A]gttttctggacgtccaatgctacatttatggaatctccctttcctctagaggaagaatctctgtatggcagccgcattcatctacaacctgctcaaccac

fava_c16263_1264	fava_c16263	1264	A/G	tctccctttcctctagaggaagaatctctgtatggcagccgcattcatctacaacctgctcaaccacacaatgaaaatcatgagcagttccctatgtcgg[A/G]cattgaaggggaaaatcacattcagcagttccatttgtcWgacaatgaagtggtgaaccgaattcttctcataaacgcaagttatgatttagttattccY

fava_c16263_1304	fava_c16263	1304	T/A	gcattcatctacaacctgctcaaccacacaatgaaaatcatgagcagttccctatgtcggRcattgaaggggaaaatcacattcagcagttccatttgtc[T/A]gacaatgaagtggtgaaccgaattcttctcataaacgcaagttatgatttagttattccYgaccttgctgctggaacaataaatccaaggagtgctgagg

fava_c16263_1364	fava_c16263	1364	T/C	RcattgaaggggaaaatcacattcagcagttccatttgtcWgacaatgaagtggtgaaccgaattcttctcataaacgcaagttatgatttagttattcc[T/C]gaccttgctgctggaacaataaatccaaggagtgctgagggaaggatttggcagtaccaggatggtacttttggatttgggtttgttcgagacaactttg

fava_c16263_1654	fava_c16263	1654	G/A	gctggatgtcacctgtacattgtgacatagtgtatctttagctgtgaaattatgacagttttcttagatggtatcctagataatcagtttcaaggaacat[G/A]cccctcacctgtgagtcaccttttaataYgtgacatcctttggtttggaatttggacttcctttttttgttattttatatgtaagcaaaataatgggaat

fava_c16263_1683	fava_c16263	1683	T/C	gtgtatctttagctgtgaaattatgacagttttcttagatggtatcctagataatcagtttcaaggaacatRcccctcacctgtgagtcaccttttaata[T/C]gtgacatcctttggtttggaatttggacttcctttttttgttattttatatgtaagcaaaataatgggaattgacatcttgtcggtccattcttttagat

fava_c16263_184	fava_c16263	184	C/T	ttccaaatccaatctatctattaatcccaattcttcaatcatccaatccatttcaattcaatggcgaacaatctcaatgttccatgttcaatttcattca[C/T]cgatttccccgaagatgttcagctttgYatcctctcgtttctcggccccaccgagatcgcttcgtttgcatgcacctcgaagcaattcggttcactctgt

fava_c16263_212	fava_c16263	212	C/T	aattcttcaatcatccaatccatttcaattcaatggcgaacaatctcaatgttccatgttcaatttcattcaYcgatttccccgaagatgttcagctttg[C/T]atcctctcgtttctcggccccaccgagatcgcttcgtttgcatgcacctcgaagcaattcggttcactctgttcaaacgatggtaaactRtggttcacgc

fava_c16263_302	fava_c16263	302	G/A	ttcagctttgYatcctctcgtttctcggccccaccgagatcgcttcgtttgcatgcacctcgaagcaattcggttcactctgttcaaacgatggtaaact[G/A]tggttcacgctttgcgatcgaaggtggggttcgaaaacccaaatYgtgaaatggggtgaaggtaaaattccgtataaacttctttacaatactcttcatg

fava_c16263_347	fava_c16263	347	C/T	cgtttgcatgcacctcgaagcaattcggttcactctgttcaaacgatggtaaactRtggttcacgctttgcgatcgaaggtggggttcgaaaacccaaat[C/T]gtgaaatggggtgaaggtaaaattccgtataaacttctttacaatactcttcatgaatgggagaatctcattggtttctggcggagaagcggtccgggaa

fava_c16263_539	fava_c16263	539	T/G	gtccgggaagtgcattgatttcgtcgccctcgttggttttcttcgagtgggggccgtcgttcattttggcttttagggtttctcctgttgaatcgggatc[T/G]gagatttttgcttatcaggttaaaaaggtgccttttttgaagatgggtatttctcaagagggacaaattctgaatcttttggatcctgatggtagagctg

fava_c16263_752	fava_c16263	752	G/C	attctggggatgggaatgaattgattcctgtgaatgtttgttttatggggaagactcattttgttttggaggagaatgtgtttgggagtaattcgagtcc[G/C]gtgcattttagtggtgaggattgtggtttctttggagaggatgggattggaattggggttgggagtggatctccgccggatctgattatgttggagattt

fava_c16264_450	fava_c16264	450	G/A	tccaaagctgtgcggcttgtggatttagaggtgaactaatgtttggctctccaagaagactttgaatggatagtagaattgttcttacatcatatgctga[G/A]gaccacttgtcctgaagaatgtccaaacatatattgccatgaagatcaacattgggatggaaacagtttgtttcaaacttaactttaggaggcttaaaag

fava_c16265_1203	fava_c16265	1203	C/T	aagttagagaactactcaaagagattgagttgagcagtgaagagagcaagaatcagactgcatttaagtatgcaaagttatttagtggtgccactattgc[C/T]tttgcactcaccatcgttgttctggctttcacactaacaactgatggataatcatctggcagacctgaaattttattgtaccaYgMggttttgaattttt

fava_c16265_1287	fava_c16265	1287	C/T	gtggtgccactattgcYtttgcactcaccatcgttgttctggctttcacactaacaactgatggataatcatctggcagacctgaaattttattgtacca[C/T]gMggttttgaattttttgtgcgtagtttttgtgttctagtatttctggtagtagtttttgagtcaactgcaaggctaaacataaatgtcccaaaggtgag

fava_c16265_1289	fava_c16265	1289	C/A	ggtgccactattgcYtttgcactcaccatcgttgttctggctttcacactaacaactgatggataatcatctggcagacctgaaattttattgtaccaYg[C/A]ggttttgaattttttgtgcgtagtttttgtgttctagtatttctggtagtagtttttgagtcaactgcaaggctaaacataaatgtcccaaaggtgaggR

fava_c16265_1389	fava_c16265	1389	A/G	Mggttttgaattttttgtgcgtagtttttgtgttctagtatttctggtagtagtttttgagtcaactgcaaggctaaacataaatgtcccaaaggtgagg[A/G]tttctaatagtgtttagttttatgacctgttgtttcattttgtatcactaggcatgtaagttctgttgtatcaaaggatttgtagctaaaaaaaattgaa

fava_c16265_345	fava_c16265	345	G/A	tcctcggcctcgactggcacctgaatacagctcctcttccagacactgctcagtggtttctcttggcaatgaatgagaaacttcctgctccaattgttgc[G/A]atattaagagctcgtattattggattgcaccattttttgatgttgtttatgatgctggcattctctgtgctatttgggtcggtgaaagctcctggccttg

fava_c16268_347	fava_c16268	347	G/A	tgtacatagatagataaagaaagcacaaccttctatactcatgaagaatatccttcaattattctgtaaaaaacttgcaaattgtctcaaccgaaaatgt[G/A]ctagttatatataaataaaattatttcaacaaccatagcctactYtccacacMcacttggacatcaggaatatccacgccagctaaactgccccaacaca

fava_c16268_392	fava_c16268	392	C/T	gaatatccttcaattattctgtaaaaaacttgcaaattgtctcaaccgaaaatgtRctagttatatataaataaaattatttcaacaaccatagcctact[C/T]tccacacMcacttggacatcaggaatatccacgccagctaaactgccccaacacaaaaattgcacctgtactgtagtattcactagagactaaagaagaa

fava_c16268_400	fava_c16268	400	C/A	ttcaattattctgtaaaaaacttgcaaattgtctcaaccgaaaatgtRctagttatatataaataaaattatttcaacaaccatagcctactYtccacac[C/A]cacttggacatcaggaatatccacgccagctaaactgccccaacacaaaaattgcacctgtactgtagtattcactagagactaaagaagaactatacag

fava_c16268_738	fava_c16268	738	G/A	cagctgtaacctgtcgcatctcaactttcacgcgagaaggtggtcggggcccatgacgaggtgctttaggtgtatttgggcttggacacaatatcccatc[G/A]cgcttgcgcaaaaaaggccaacgctgagaaggacggggagtcataatatttaacatctcttgccgagagcgatctggggtagtataattgaagtgaaagt

fava_c16268_872	fava_c16268	872	G/C	cggggagtcataatatttaacatctcttgccgagagcgatctggggtagtataattgaagtgaaagtgccttgcaaatatgaccgcttttctaactctct[G/C]tgaaacatggaaaacaggtctcagttgatcatggtgcaggtggcatagtattttaaccaacaactcagtggggagagactcaagcctagattccgactgc

fava_c16269_1058	fava_c16269	1058	C/T	ggtaattaagaagaaagatgaagaagttgaagaagttgaaggccaaggaaagtgagataaacatcatgcattatttttatttctctattttttcctcctt[C/T]aaggatatcatttgtcttaagttgttcaaaactaacaatagaatcaatgagcatacaagaaaatatagaatctctctctaattttctcttaaaKattatt

fava_c16269_1152	fava_c16269	1152	T/G	ctccttYaaggatatcatttgtcttaagttgttcaaaactaacaatagaatcaatgagcatacaagaaaatatagaatctctctctaattttctcttaaa[T/G]attattttaagatgttaaaattagagattgatttatatttttttgtctctaatatttcgacctaaaWYttattttctttagtagtgattattttactttg

fava_c16269_1219	fava_c16269	1219	T/A	aaatatagaatctctctctaattttctcttaaaKattattttaagatgttaaaattagagattgatttatatttttttgtctctaatatttcgacctaaa[T/A]Yttattttctttagtagtgattattttactttgttttgtatacaattaatgcctttggtattaatttgtgtaatgtgaggattgtttttatatatataga

fava_c16269_1220	fava_c16269	1220	T/C	aatatagaatctctctctaattttctcttaaaKattattttaagatgttaaaattagagattgatttatatttttttgtctctaatatttcgacctaaaW[T/C]ttattttctttagtagtgattattttactttgttttgtatacaattaatgcctttggtattaatttgtgtaatgtgaggattgtttttatatatatagag

fava_c16269_314	fava_c16269	314	C/T	tttgttattacaacaactatagtctcacacaaccaagatacttttgcaagacatgtagaaggtattggactgaaggaggatctctaagaaatgttcctgt[C/T]ggaggaggttcaaggaagaacaagaaagttccttcatcaYcatcattagccacacctaataataataactcaccaaaaattcctgacctaaatccaccaa

fava_c16269_354	fava_c16269	354	C/T	cttttgcaagacatgtagaaggtattggactgaaggaggatctctaagaaatgttcctgtYggaggaggttcaaggaagaacaagaaagttccttcatca[C/T]catcattagccacacctaataataataactcaccaaaaattcctgacctaaatccaccaacccttcaagtctcacatctttcatctcatcaaaaccctaa

fava_c16270_1169	fava_c16270	1169	C/T	atcaacatctgtgacaattgcagccttgtccaaccacttatattgatttgagtgtaaaagatctccttctaaagaaattgcagcatcaagttccctattt[C/T]gatataaaagctgaggatcatcYggcaatgtctcgttccttttcttgacagcagcgacaatcacttgcgccaaacgaactaaaggactaccttgtggttt

fava_c16270_1192	fava_c16270	1192	T/C	ccttgtccaaccacttatattgatttgagtgtaaaagatctccttctaaagaaattgcagcatcaagttccctatttYgatataaaagctgaggatcatc[T/C]ggcaatgtctcgttccttttcttgacagcagcgacaatcacttgcgccaaacgaactaaaggactaccttgtggtttSacattcttataaaacggggagc

fava_c16270_1270	fava_c16270	1270	G/C	gatataaaagctgaggatcatcYggcaatgtctcgttccttttcttgacagcagcgacaatcacttgcgccaaacgaactaaaggactaccttgtggttt[G/C]acattcttataaaacggggagcccaacacgaataagattattgatatgagcatcgcaatggtaggaatgccaagtcctaaactccatcccatattgtctY

fava_c16270_1696	fava_c16270	1696	G/A	tgagttgtgtaggacatggtggaggacgaaaatagggtagtgtagttgatatagtgatactaatcaatccgagttcgtatatgaggcatccaatggtaat[G/A]gtccaaaaScgtccaatcaaggaatctgctagKagagcaccRaggagaggagtgaagctWgatgttccaccaaaattagtgagtgtgtttgaagctKcta

fava_c16270_1705	fava_c16270	1705	C/G	taggacatggtggaggacgaaaatagggtagtgtagttgatatagtgatactaatcaatccgagttcgtatatgaggcatccaatggtaatRgtccaaaa[C/G]cgtccaatcaaggaatctgctagKagagcaccRaggagaggagtgaagctWgatgttccaccaaaattagtgagtgtgtttgaagctKctaccaatggca

fava_c16270_1729	fava_c16270	1729	T/G	agggtagtgtagttgatatagtgatactaatcaatccgagttcgtatatgaggcatccaatggtaatRgtccaaaaScgtccaatcaaggaatctgctag[T/G]agagcaccRaggagaggagtgaagctWgatgttccaccaaaattagtgagtgtgtttgaagctKctaccaatggcatgttcaattcttgtgttaagtaac

fava_c16270_1738	fava_c16270	1738	G/A	tagttgatatagtgatactaatcaatccgagttcgtatatgaggcatccaatggtaatRgtccaaaaScgtccaatcaaggaatctgctagKagagcacc[G/A]aggagaggagtgaagctWgatgttccaccaaaattagtgagtgtgtttgaagctKctaccaatggcatgttcaattcttgtgttaagtaacttattaagt

fava_c16270_1756	fava_c16270	1756	T/A	taatcaatccgagttcgtatatgaggcatccaatggtaatRgtccaaaaScgtccaatcaaggaatctgctagKagagcaccRaggagaggagtgaagct[T/A]gatgttccaccaaaattagtgagtgtgtttgaagctKctaccaatggcatgttcaattcttgtgttaagtaacttattaagttactgtgaaatccagcac

fava_c16270_1793	fava_c16270	1793	T/G	aatRgtccaaaaScgtccaatcaaggaatctgctagKagagcaccRaggagaggagtgaagctWgatgttccaccaaaattagtgagtgtgtttgaagct[T/G]ctaccaatggcatgttcaattcttgtgttaagtaacttattaagttactgtgaaatccagcacttgcgaatctatcacacgcttcatttgcaaaaatgaa

fava_c16270_255	fava_c16270	255	G/C	ccataatatcacccatctttagtaaactctcttttatcctcatgtttaccatcttttaaattatgaactgatatatttgtaatctcttgctccaaatatt[G/C]ttccttctctctttcacatatttcacccaagggcttatatgtgtaaaaccaagcacatattatgaaataaacgagatttataacttgaatcccagtaata

fava_c16270_3197	fava_c16270	3197	A/C	tcaaaatattctattgcactctggtacagacgtgaagttgagtgtctctcgtgatggcattcttcttccaatgagtcaatctcttatcaaatctaagaag[A/C]ggactgatgtacaaagagtaactgcactagatcgtgctaatgccgaggatgccttgcttccaattcctaaagtttttgattctcaYgagaaagataaatc

fava_c16270_3283	fava_c16270	3283	T/C	tcaaatctaagaagMggactgatgtacaaagagtaactgcactagatcgtgctaatgccgaggatgccttgcttccaattcctaaagtttttgattctca[T/C]gagaaagataaatcatttcaagtcacttcagacaaggatgtaaaggacgatttgcttRcaagtggtactgattccatggaaatagatgatggtattgtcc

fava_c16270_3341	fava_c16270	3341	A/G	cgaggatgccttgcttccaattcctaaagtttttgattctcaYgagaaagataaatcatttcaagtcacttcagacaaggatgtaaaggacgatttgctt[A/G]caagtggtactgattccatggaaatagatgatggtattgtccaaagtgagactgatgtaattagcatggaggaccggatgagatcattaggattgcttta

fava_c16270_3607	fava_c16270	3607	A/G	aagctactcttccaaagaaaaaggtaagagcggctgttttgtctatggaaccaagtgaagcattcaagctactagaagtattgctggttatatggcaatc[A/G]aggtcatctagcagggagtccgtgcttccatggatgtacagcatagttgttactcatggtcatagtgctgctgaagaatctgcaacacatatgtttgatt

fava_c16270_3738	fava_c16270	3738	C/A	tggatgtacagcatagttgttactcatggtcatagtgctgctgaagaatctgcaacacatatgtttgattcggtgcacaagattaccaattccagagcgg[C/A]agctcttcagtctttgttacaactatcaggtcgactacaattggttacatcgcagattgataaggcttctcagaaaataagccatccgatgaRtgatctc

fava_c16270_3831	fava_c16270	3831	A/G	agagcggMagctcttcagtctttgttacaactatcaggtcgactacaattggttacatcgcagattgataaggcttctcagaaaataagccatccgatga[A/G]tgatctccaatcagaagaaagtgaagacgaggatgaatattatcacgaggatgacgacaatgaagaagatgatgcatccgaaataagtactgatgatgaa

fava_c16270_705	fava_c16270	705	C/T	aaccaaaacacacttattggaattatcgcttttggatcatccaataagttgtattctgcagcaacattttttctttttatttccactagtgctgaaacta[C/T]agtggctacaatgttgatcacaaaccctactcccattctttgtaaacacgttattcctgccggattctttgtgaatctacgcgcaaacgggacgaaaagg

fava_c16270_910	fava_c16270	910	A/T	cgtatagaatgattcctgtcatcattgttaacacgctgaaaatcgacatattggctggtgcaatttggaatgagggagtgaggtggcgatccatcgaacg[A/T]gcttgttgaattacgaaactacctaaatgtgatgaggaggttatgagtaaaattcctgaagcccaaattggaagcattctaataatggcttttagttcct

fava_c16271_376	fava_c16271	376	G/T	tgatgttacacaacttttatacaaagtacaaaacattagtttagtctaaactaactgtttcagcaggttatattcgcctctgagaacatatcagaataca[G/T]aatagcttttaacggtgatgtttacttcttttctcctctgttttctttttcttatgcttcRatttttctttggaSctgtgtttcttagaatgtccttttt

fava_c16271_437	fava_c16271	437	G/A	agcaggttatattcgcctctgagaacatatcagaatacaKaatagcttttaacggtgatgtttacttcttttctcctctgttttctttttcttatgcttc[G/A]atttttctttggaSctgtgtttcttagaatgtcctttttccgagtgacttttatgcgactttttactcttttttcgcttttctYcaaRaagctcatcgtc

fava_c16271_451	fava_c16271	451	C/G	gcctctgagaacatatcagaatacaKaatagcttttaacggtgatgtttacttcttttctcctctgttttctttttcttatgcttcRatttttctttgga[C/G]ctgtgtttcttagaatgtcctttttccgagtgacttttatgcgactttttactcttttttcgcttttctYcaaRaagctcatcgtctgaagattcatatR

fava_c16271_521	fava_c16271	521	T/C	ctttttcttatgcttcRatttttctttggaSctgtgtttcttagaatgtcctttttccgagtgacttttatgcgactttttactcttttttcgcttttct[T/C]caaRaagctcatcgtctgaagattcatatRactgcaacgatgtaggcctctgcggaccataaacaacacgRtattcccttaaatctggactagtcgaagg

fava_c16271_525	fava_c16271	525	G/A	ttcttatgcttcRatttttctttggaSctgtgtttcttagaatgtcctttttccgagtgacttttatgcgactttttactcttttttcgcttttctYcaa[G/A]aagctcatcgtctgaagattcatatRactgcaacgatgtaggcctctgcggaccataaacaacacgRtattcccttaaatctggactagtcgaaggctct

fava_c16271_551	fava_c16271	551	G/A	SctgtgtttcttagaatgtcctttttccgagtgacttttatgcgactttttactcttttttcgcttttctYcaaRaagctcatcgtctgaagattcatat[G/A]actgcaacgatgtaggcctctgcggaccataaacaacacgRtattcccttaaatctggactagtcgaaggctctccatcRggattaggcRgaaggtaRgg

fava_c16271_592	fava_c16271	592	A/G	gcgactttttactcttttttcgcttttctYcaaRaagctcatcgtctgaagattcatatRactgcaacgatgtaggcctctgcggaccataaacaacacg[A/G]tattcccttaaatctggactagtcgaaggctctccatcRggattaggcRgaaggtaRggcccagtttcatccatccttggaccaatagcacctctgcctc

fava_c16271_631	fava_c16271	631	A/G	catcgtctgaagattcatatRactgcaacgatgtaggcctctgcggaccataaacaacacgRtattcccttaaatctggactagtcgaaggctctccatc[A/G]ggattaggcRgaaggtaRggcccagtttcatccatccttggaccaatagcacctctgcctcgcttcttccttgaatgtagaaactcttcaagctcttcat

fava_c16271_641	fava_c16271	641	G/A	agattcatatRactgcaacgatgtaggcctctgcggaccataaacaacacgRtattcccttaaatctggactagtcgaaggctctccatcRggattaggc[G/A]gaaggtaRggcccagtttcatccatccttggaccaatagcacctctgcctcgcttcttccttgaatgtagaaactcttcaagctcttcatcctttaaacc

fava_c16271_649	fava_c16271	649	A/G	atRactgcaacgatgtaggcctctgcggaccataaacaacacgRtattcccttaaatctggactagtcgaaggctctccatcRggattaggcRgaaggta[A/G]ggcccagtttcatccatccttggaccaatagcacctctgcctcgcttcttccttgaatgtagaaactcttcaagctcttcatcctttaaaccttcaggag

fava_c16271_830	fava_c16271	830	C/T	atcctttaaaccttcaggagtatgcccgccttcttttttagttaagcatgaagctgaggcactagtgcttttatcggccccatcatatctttctgcactt[C/T]ttgacgggttatgatccctatatcttttgtcatcgctacttttatcccttgacgatgtgcttgaagcccgtttatttagctccaRttctttctcccgcgc

fava_c16271_915	fava_c16271	915	G/A	tatctttctgcacttYttgacgggttatgatccctatatcttttgtcatcgctacttttatcccttgacgatgtgcttgaagcccgtttatttagctcca[G/A]ttctttctcccgcgctcgccacatttcatttacttcaacagctcgatttgcttgttgtaccccgcgtacggtagcagtgaggaaacgcgaattcggccga

fava_c16276_1017	fava_c16276	1017	C/T	gtgcgttctatctagttacttagtctttcactcaatttttWWtaaataaaatgggtaccctaaatctatcttcgWtaacaataatccataagtaaaatgg[C/T]taYRttttggtacaatggtttgttttgcaaaattttggtttaatatatagtggttgttgattgagatgattttgcaacttgctagatttttttcactatn

fava_c16276_1020	fava_c16276	1020	C/T	cgttctatctagttacttagtctttcactcaatttttWWtaaataaaatgggtaccctaaatctatcttcgWtaacaataatccataagtaaaatggYta[C/T]Rttttggtacaatggtttgttttgcaaaattttggtttaatatatagtggttgttgattgagatgattttgcaacttgctagatttttttcactatnnnn

fava_c16276_1021	fava_c16276	1021	A/G	gttctatctagttacttagtctttcactcaatttttWWtaaataaaatgggtaccctaaatctatcttcgWtaacaataatccataagtaaaatggYtaY[A/G]ttttggtacaatggtttgttttgcaaaattttggtttaatatatagtggttgttgattgagatgattttgcaacttgctagatttttttcactatnnnnn

fava_c16276_593	fava_c16276	593	G/T	tactttttcatcgagcaattcgtttggttagccaaaaccgggttgatcgatccgaagaggtcgagaatcttgacgaagataagcgcttatgcggagctcg[G/T]tggttatttcggtagtgtggcgatgaaatttagggatttgagggttattgttgaggaagaaggttgtttgaaatcgagcattgagattgagggtttgaga

fava_c16276_836	fava_c16276	836	G/C	agagagaagaaattgatcaagaggttctcaattcttcaggatttcgctgatgggttgatggctttggatgatattttggatgggaatgcaccgttttcca[G/C]gccaattgtgttgtcttctgcagggcttttatcagcactcattagtactcacaagaattgggtttcttgctgagtttctggtgcgttctatctagttact

fava_c16276_957	fava_c16276	957	T/A	cagggcttttatcagcactcattagtactcacaagaattgggtttcttgctgagtttctggtgcgttctatctagttacttagtctttcactcaattttt[T/A]WtaaataaaatgggtaccctaaatctatcttcgWtaacaataatccataagtaaaatggYtaYRttttggtacaatggtttgttttgcaaaattttggtt

fava_c16276_958	fava_c16276	958	T/A	agggcttttatcagcactcattagtactcacaagaattgggtttcttgctgagtttctggtgcgttctatctagttacttagtctttcactcaatttttW[T/A]taaataaaatgggtaccctaaatctatcttcgWtaacaataatccataagtaaaatggYtaYRttttggtacaatggtttgttttgcaaaattttggttt

fava_c16276_991	fava_c16276	991	T/A	gaattgggtttcttgctgagtttctggtgcgttctatctagttacttagtctttcactcaatttttWWtaaataaaatgggtaccctaaatctatcttcg[T/A]taacaataatccataagtaaaatggYtaYRttttggtacaatggtttgttttgcaaaattttggtttaatatatagtggttgttgattgagatgattttg

fava_c16279_152	fava_c16279	152	G/A	attaatacaatgtatgcaactgcagaaaaatcttgaaaatcacattttttttccataagtatctcaaacaattttgttttgaatacattaaacttttctc[G/A]gtagggaacattaatctattaatttgtctttctcaatagaaatttattgaatttttattttataacctgattaaaattaaggataaacttcaataagaga

fava_c16279_285	fava_c16279	285	G/T	tcaatagaaatttattgaatttttattttataacctgattaaaattaaggataaacttcaataagagacttgaagccacatataggagtaattttgaaac[G/T]gctgaaacctgcatcatatataagtttcttccattctttctttgttctttctttaccaagaaacattgtaagcattactaaatcatactccaattgcaac

fava_c16279_504	fava_c16279	504	A/G	tcactattttcatcaattgatatatcaatgataatcacttttccttcttttcctttgtgtgaaatagcttctttactgttcttcaatatcttcaaagata[A/G]YtcatcgttccaatcatgcagaacccacttgagtaaaacagcatcYgcagaagggacagatttgaacatatctccaccaagaaagttcaaattttcattt

fava_c16279_505	fava_c16279	505	C/T	cactattttcatcaattgatatatcaatgataatcacttttccttcttttcctttgtgtgaaatagcttctttactgttcttcaatatcttcaaagataR[C/T]tcatcgttccaatcatgcagaacccacttgagtaaaacagcatcYgcagaagggacagatttgaacatatctccaccaagaaagttcaaattttcatttc

fava_c16279_550	fava_c16279	550	T/C	cttttcctttgtgtgaaatagcttctttactgttcttcaatatcttcaaagataRYtcatcgttccaatcatgcagaacccacttgagtaaaacagcatc[T/C]gcagaagggacagatttgaacatatctccaccaagaaagttcaaattttcatttccagttaagttaccaacaacctgtggttgatcaaaaacagtgcatt

fava_c16279_733	fava_c16279	733	A/G	gatcaaaaacagtgcatttgatgttcggaaatgcttcatgaatgagttttgtaacaccgccagttccacctgccacatcaaccaaagactccaaaccctc[A/G]aacacatgcttattctcttgaatagcaagcttaaacaatctcgaatcagccgccatagcatcttgaaacatactcaaattatcagattcagaatctttgt

fava_c16282_1339	fava_c16282	1339	T/A	aaagtcgggaaaacccggcaataaaatatcatgtactttctaataatactgaatagtgatattgtttttatgagtttggtaggtagttacagcatgacag[T/A]attctagatgggcaggtagctgataatcatatggcgaagcagttccactgttacccatttttgtttttctggtaaatgtaagaagaatttatttggcatt

fava_c16282_797	fava_c16282	797	C/G	catgtagactttgaatatgatgatgatgatgatgagatgatgatgatgatgatgatgatgatgatgatgatgatgatgaagatgatgatgacgacaacga[C/G]atggcatttgctatagcgcaaagcatgcctgggttatgccatctccaacttcttggaaacaagatggctaatgatggtttgcttgctattcttgatggtt

fava_c16284_302	fava_c16284	302	T/G	tgcgtttacacaaggacggaggccgtagttggatgtgaatcgtagggccggtttggtgcttcttctttggcgtcgtacggttgcacggctggggaaggtg[T/G]ctgctgctactgcttttgcgttagaggctggttcggtcgatgggggttRtgtggatcaaaagcgtgatcgaagggatgttgatcggaattcgatggaggg

fava_c16284_351	fava_c16284	351	G/A	ggtttggtgcttcttctttggcgtcgtacggttgcacggctggggaaggtgKctgctgctactgcttttgcgttagaggctggttcggtcgatgggggtt[G/A]tgtggatcaaaagcgtgatcgaagggatgttgatcggaattcgatggagggMaaaataagtggtgcgRatctgaagttgtaggtgacgagtggagggcgt

fava_c16284_403	fava_c16284	403	A/C	ctgctgctactgcttttgcgttagaggctggttcggtcgatgggggttRtgtggatcaaaagcgtgatcgaagggatgttgatcggaattcgatggaggg[A/C]aaaataagtggtgcgRatctgaagttgtaggtgacgagtggagggcgtggttccatgttgtaagttgtgatgcattgctSgtgtcggtggtgaaagtcgK

fava_c16284_419	fava_c16284	419	G/A	tgcgttagaggctggttcggtcgatgggggttRtgtggatcaaaagcgtgatcgaagggatgttgatcggaattcgatggagggMaaaataagtggtgcg[G/A]atctgaagttgtaggtgacgagtggagggcgtggttccatgttgtaagttgtgatgcattgctSgtgtcggtggtgaaagtcgcggacggctgcagcata

fava_c16284_483	fava_c16284	483	G/C	gatcggaattcgatggagggMaaaataagtggtgcgRatctgaagttgtaggtgacgagtggagggcgtggttccatgttgtaagttgtgatgcattgct[G/C]gtgtcggtggtgaaagtcgcggacggctgcagcatattctaaatctttttcttttgaattcaattgttttgttgctttcaaattctgacttatgcttgtt

fava_c16285_134	fava_c16285	134	G/C	aatacataagggattacacatacagaaatttgtttgttaaaaaggaaatgaacacaattttcatttacatgtacatcaatcataatcaaggaaaagtgtt[G/C]agtaaacaaaataaatctgtcttcaaaacaagctgatcKtttctaaattttgaatcatacgacatatatgaccaacagaggcagcWaaatattgtggtct

fava_c16285_1371	fava_c16285	1371	C/T	tcaacaaatcctgaagaacaagatccaaaaccgcaagcttcccggagccacgtggaccgaggagaagaatggaattgttacacgcctccgctactgaact[C/T]gaaatcatgaatttcaacttgctgtagtttccgtctggagagtctttgaagatgaaaccggtgtcgcagattctgctccggaggagattcaacgcttcca

fava_c16285_173	fava_c16285	173	T/G	aaaaggaaatgaacacaattttcatttacatgtacatcaatcataatcaaggaaaagtgttSagtaaacaaaataaatctgtcttcaaaacaagctgatc[T/G]tttctaaattttgaatcatacgacatatatgaccaacagaggcagcWaaatattgtggtcttcaggtgtttagccgtcacgttctattaacttttgaagg

fava_c16285_220	fava_c16285	220	A/T	caaggaaaagtgttSagtaaacaaaataaatctgtcttcaaaacaagctgatcKtttctaaattttgaatcatacgacatatatgaccaacagaggcagc[A/T]aaatattgtggtcttcaggtgtttagccgtcacgttctattaacttttgaaggatagcggggcaagaatgataagcctttagtccttgatacagttcagc

fava_c16285_810	fava_c16285	810	A/G	tgacagaggagtcaaaattcaaatagttagtgaagatttctttgaactttttgtcttctataatattttgaacttttttattaaattgaacagcataatc[A/G]gaaggaaggcttgaatctattggcaatgtcagcatatgcaccaataatctttgtgaatcttctattgaaggtggtaagaacaacagctttctatgtgaaa

fava_c16287_1023	fava_c16287	1023	T/C	aatgggctctttcgaattcaggtacgcaaaagaacatactgtgccactaaaagaaagaataccagaaacatcttttttgctgtatgcttcaatagaacta[T/C]tattcaaaaatggtattagcaattcaacttYatgcacaccatccgatgagcatggctcatcatcaagaaccaatttaccatcgatcagggcttttgcttc

fava_c16287_1054	fava_c16287	1054	C/T	gaacatactgtgccactaaaagaaagaataccagaaacatcttttttgctgtatgcttcaatagaactaYtattcaaaaatggtattagcaattcaactt[C/T]atgcacaccatccgatgagcatggctcatcatcaagaaccaatttaccatcgatcagggcttttgcttctgcaagctcattggcatgaagagatattgca

fava_c16287_1352	fava_c16287	1352	G/A	aagaacttaagactttccccagtttaaattcacaagggcggagactactagatgtaatatttgaagatagtgagcagtttctacaattccaccttctctg[G/A]ccataacaaatgtgaagaagcagtctttcgccccagttagcctctgaaagcggtgctgcttcagcaactcccttcacagtctggcaaagcattatggttg

fava_c16287_294	fava_c16287	294	T/G	tgggcaagatcttcctcattccttcaaaaacaaaatccaaacctgctgctgtaaagttcgaaagtataaaaattatcaaagcctgcagttttcgaaacta[T/G]gtRaaaatgtgcattcatgttgtggagagatgagggaatttatgtagagataaagccatcaacccttaaggaaaaataacacaaaaccaacYaagacaga

fava_c16287_297	fava_c16287	297	G/A	gcaagatcttcctcattccttcaaaaacaaaatccaaacctgctgctgtaaagttcgaaagtataaaaattatcaaagcctgcagttttcgaaactaKgt[G/A]aaaatgtgcattcatgttgtggagagatgagggaatttatgtagagataaagccatcaacccttaaggaaaaataacacaaaaccaacYaagacagatag

fava_c16287_386	fava_c16287	386	T/C	cgaaactaKgtRaaaatgtgcattcatgttgtggagagatgagggaatttatgtagagataaagccatcaacccttaaggaaaaataacacaaaaccaac[T/C]aagacagatagtagaaggatgagagcagcagctacaacactaatttgacctgacttcRccgatKtattactagaactctctcctctgccatcgagtttca

fava_c16287_444	fava_c16287	444	G/A	ataaagccatcaacccttaaggaaaaataacacaaaaccaacYaagacagatagtagaaggatgagagcagcagctacaacactaatttgacctgacttc[G/A]ccgatKtattactagaactctctcctctgccatcgagtttcaacttgtcctYcatgaggtgagtttctgaataagccagcactgctacatcccagaaaga

fava_c16287_450	fava_c16287	450	T/G	ccatcaacccttaaggaaaaataacacaaaaccaacYaagacagatagtagaaggatgagagcagcagctacaacactaatttgacctgacttcRccgat[T/G]tattactagaactctctcctctgccatcgagtttcaacttgtcctYcatgaggtgagtttctgaataagccagcactgctacatcccagaaagatttagt

fava_c16287_496	fava_c16287	496	C/T	agtagaaggatgagagcagcagctacaacactaatttgacctgacttcRccgatKtattactagaactctctcctctgccatcgagtttcaacttgtcct[C/T]catgaggtgagtttctgaataagccagcactgctacatcccagaaagatttagttttgaaagatatggcttctttttctggttccaaaattgtMgagaYc

fava_c16287_590	fava_c16287	590	C/A	tgtcctYcatgaggtgagtttctgaataagccagcactgctacatcccagaaagatttagttttgaaagatatggcttctttttctggttccaaaattgt[C/A]gagaYctcRgttggagcttccatggataacaactccatacagtgatcttttaaaacctcaacagtttcagaaggctgcaaRtaatcacacacatacgtcc

fava_c16287_595	fava_c16287	595	C/T	tYcatgaggtgagtttctgaataagccagcactgctacatcccagaaagatttagttttgaaagatatggcttctttttctggttccaaaattgtMgaga[C/T]ctcRgttggagcttccatggataacaactccatacagtgatcttttaaaacctcaacagtttcagaaggctgcaaRtaatcacacacatacgtccctgct

fava_c16287_599	fava_c16287	599	A/G	tgaggtgagtttctgaataagccagcactgctacatcccagaaagatttagttttgaaagatatggcttctttttctggttccaaaattgtMgagaYctc[A/G]gttggagcttccatggataacaactccatacagtgatcttttaaaacctcaacagtttcagaaggctgcaaRtaatcacacacatacgtccctgctaacc

fava_c16287_671	fava_c16287	671	G/A	tttctggttccaaaattgtMgagaYctcRgttggagcttccatggataacaactccatacagtgatcttttaaaacctcaacagtttcagaaggctgcaa[G/A]taatcacacacatacgtccctgctaaccatggtgcgaaaactcttctaggaaaaggaaggctgcattcttttctcaataaatgaagcacaagttgtgaaa

fava_c16297_1613	fava_c16297	1613	C/A	acgtagcggtttcaggtttctgaacatcaaaaaacgaaaccgaacttcttctcagagcagcaaaatccaaagctttccaccacagctcttcaactataac[C/A]gcacaRtccgcaaggtttcttctcgtccgataactcttataaaccttttgaagcttagttgctgcagaatcaagctctgcaaccggcctaggagaacaaa

fava_c16297_1619	fava_c16297	1619	A/G	cggtttcaggtttctgaacatcaaaaaacgaaaccgaacttcttctcagagcagcaaaatccaaagctttccaccacagctcttcaactataacMgcaca[A/G]tccgcaaggtttcttctcgtccgataactcttataaaccttttgaagcttagttgctgcagaatcaagctctgcaaccggcctaggagaacaaaaaacca

fava_c16299_457	fava_c16299	457	C/T	agattgatttgggtcctcagttcactttgaaagaacaacttgaaaaggataaggatgatgagagcttgaggaaatggaaggaacagcttcttggaagcat[C/T]gacatgagttctgttggagaaactctggagccagaggtgaagtttctgagtctggcgataaaatcagaaggtagagatgatatgattctacctgtaccag

fava_c16300_137	fava_c16300	137	A/C	aaaaataaaaagatctcaaagaggagaaaagttttctacccaaagatttgtaatgtacacagaatggaacaaaaatacaggaaccaaatgagtgccacca[A/C]gaagtcacgtgcatacatacacatactttacaggcagaataaaagtggactctagccagaattaagcaaggagaataaccttgtaatgaaaactcacctg

fava_c16300_442	fava_c16300	442	G/A	ttgttccatgtctggcccagtaatccttaagatccaacggtatagataaatcatggccttccgatgcaaacctactgagaagtcttgtaaaaacactgcc[G/A]ctcattttcctacctccaatgcgagcatggcgcttgtttggaagctgcggtagcggatacatcgacagtgtagtagtacaacaagatgattgccacccac

fava_c16300_658	fava_c16300	658	A/G	agcactgtcgtggtactccggtacacgtgcaaactgggacaggcatgacagattcatcgaaagtaatcaaattcaagccaacatcctgtctatcccattc[A/G]gatttgatctttgttccttcagaatatgccttcctatttaaatcctcgcccactttctttacttttgtaggtgactttgatgccttggaattatcctttR

fava_c16300_758	fava_c16300	758	G/A	Rgatttgatctttgttccttcagaatatgccttcctatttaaatcctcgcccactttctttacttttgtaggtgactttgatgccttggaattatccttt[G/A]tcatatttgttagatgagacgcgatggcttctgaagttataacagttactggggaggcatctcttattattatatctgtcgagctatacgctgcttctgt

fava_c16302_195	fava_c16302	195	A/T	ttaactcaataacaatgtacttaataacttcnnnaaaaaaacaagtgtagtagtaaccaatctagcaatcttaacaaattttcatacatgtcttcttact[A/T]tacataaaactaaattcaaatccaagttttccaaaataaccccttcaaagtttcaatgtcaaatctggtgtagatttttctatatccacacatggattca

fava_c16302_962	fava_c16302	962	G/C	tgagttgatacattggaaccaacttggcttgtaacttcagattcatattctggataagcttccacttcaagatcaatgctttgtttcatgatttagcagt[G/C]gaaatcttgttttatcttgggataagtttactttgaagccatttcaagaggaagagtttattggtagcaaaagtaatgcaaaatgggtgcactcatttcc

fava_c16304_1143	fava_c16304	1143	T/C	gatagagatagttgtaaattcctccaccagatcttattagtactgggggcttaaatgtctcccttgtaggactatctttcttagaatggttcccatcacc[T/C]ataagtattgttgcaccataatgctgagcaggttcaaaatcaacttggtccatgaaaaggttagatttagtatggttttcaatacaagcctccaaaaagg

fava_c16304_1611	fava_c16304	1611	G/T	cttctctgacttcaatgttggaactattgttgatacttatatagctacagaaagtctctcccaagtaaattgctccgaaggactgagggagaacgaggag[G/T]ccagagaggcccatggaatcggagaagtgatgaaggaggaagcggtcgcgataactggggtcggaatcgggagcgatgagatttgcggcggaaggggcgg

fava_c16304_1857	fava_c16304	1857	C/T	cgatacgaatgggaggatcgacattgaaggatgggcggcaaagtctcataaccctaaacgcaactgaatgagaagatgaagatcctccagtttgactcat[C/T]ttgttagaaattcaaatgcSaaatgttgatgatttacagtcacataagaatcaatacctagatcgWtttcaatgtgtttctttcaaaataataatgggaa

fava_c16304_1877	fava_c16304	1877	G/C	acattgaaggatgggcggcaaagtctcataaccctaaacgcaactgaatgagaagatgaagatcctccagtttgactcatYttgttagaaattcaaatgc[G/C]aaatgttgatgatttacagtcacataagaatcaatacctagatcgWtttcaatgtgtttctttcaaaataataatgggaatttgattcggggtttttcta

fava_c16304_1923	fava_c16304	1923	T/A	aatgagaagatgaagatcctccagtttgactcatYttgttagaaattcaaatgcSaaatgttgatgatttacagtcacataagaatcaatacctagatcg[T/A]tttcaatgtgtttctttcaaaataataatgggaatttgattcggggtttttctatcctaaacatgaaattcttagtttcatgtattcattatcgcaaaga

fava_c16304_261	fava_c16304	261	G/A	acaaattacaaaatgagaaactcgtgtaaatctttctatagctgtaacaaaagattggtttttagctctttaatggtaatcaaaacaactatgtcccttg[G/A]ttcaataaaagcaactttgcttcttacagttaagtaatcaaccattcaatactccagttaagttacagatgattgtagatagtccaataaggctctgtga

fava_c16304_364	fava_c16304	364	A/G	caataaaagcaactttgcttcttacagttaagtaatcaaccattcaatactccagttaagttacagatgattgtagatagtccaataaggctctgtgatc[A/G]tgtaggattcattgaccctgagatggaagacaatctgagcctataagtatcaaaRctatRaataagggacttggtcgatctgattaggcacatcaatgat

fava_c16304_419	fava_c16304	419	G/A	ttaagttacagatgattgtagatagtccaataaggctctgtgatcRtgtaggattcattgaccctgagatggaagacaatctgagcctataagtatcaaa[G/A]ctatRaataagggacttggtcgatctgattaggcacatcaatgatacagaatccaacctgtgaaSttcaacagatcaaccttttgaaaagatgtcaaccg

fava_c16304_424	fava_c16304	424	A/G	ttacagatgattgtagatagtccaataaggctctgtgatcRtgtaggattcattgaccctgagatggaagacaatctgagcctataagtatcaaaRctat[A/G]aataagggacttggtcgatctgattaggcacatcaatgatacagaatccaacctgtgaaSttcaacagatcaaccttttgaaaagatgtcaaccgcttct

fava_c16304_484	fava_c16304	484	G/C	gagatggaagacaatctgagcctataagtatcaaaRctatRaataagggacttggtcgatctgattaggcacatcaatgatacagaatccaacctgtgaa[G/C]ttcaacagatcaaccttttgaaaagatgtcaaccgcttctgatcaaactattgttgtggccacacttttaatccaagtccacaaaaatctctaaatcagg

fava_c16304_912	fava_c16304	912	T/C	ggtttgtaagattcagttttagcgtgaatggtctttgaaggttaatgatagatggaacttctacaacttgcaactcaatctctttctttgttgttggagt[T/C]cccaatatttgctgggtctgcaggcggccaggttcacccaaatttgttcgccatgttatttggagtttaccaagaacgttacttccatcaactttagttt

fava_c16305_108	fava_c16305	108	T/C	atataaaatttaaataaatttattaatttacacaaaatgtgattgaatattcattctttaaagtatacatgcaattgctaaaataaaaattacacatatg[T/C]gcaaaatatataataatcaatagtaaaattacataatgacttaagtaaatctatatcaaagactattgtagagaatgaatttgtgattcagatatactat

fava_c16305_1302	fava_c16305	1302	A/G	acattttcatattcggggtcacaatttttccaccaactcttctctcatcaccatctctagaagaagtattgttttgtagcttcttccttttctgaatctt[A/G]ctgtcatgatctgaattcgccaaacagtttcccatctctgttaaaataataatattaaccagatgacgacgacgaagacgatgaagatgatgaaaggttc

fava_c16305_277	fava_c16305	277	C/T	tagagaatgaatttgtgattcagatatactattattgtgagaagtcagacatcggtcattagtaagatgttcttttgatctcttatttttctgttgcgca[C/T]ttctcgattttttcactYtgggcttRWatttaatagtttgagccttttctaaaatttgcaatacttgctccatagaaggtctatttttagggtcagcttc

fava_c16305_295	fava_c16305	295	T/C	ttcagatatactattattgtgagaagtcagacatcggtcattagtaagatgttcttttgatctcttatttttctgttgcgcaYttctcgattttttcact[T/C]tgggcttRWatttaatagtttgagccttttctaaaatttgcaatacttgctccatagaaggtctatttttagggtcagcttctaaacattggagtataag

fava_c16305_303	fava_c16305	303	G/A	tactattattgtgagaagtcagacatcggtcattagtaagatgttcttttgatctcttatttttctgttgcgcaYttctcgattttttcactYtgggctt[G/A]Watttaatagtttgagccttttctaaaatttgcaatacttgctccatagaaggtctatttttagggtcagcttctaaacattggagtataagttgtgcta

fava_c16305_304	fava_c16305	304	T/A	actattattgtgagaagtcagacatcggtcattagtaagatgttcttttgatctcttatttttctgttgcgcaYttctcgattttttcactYtgggcttR[T/A]atttaatagtttgagccttttctaaaatttgcaatacttgctccatagaaggtctatttttagggtcagcttctaaacattggagtataagttgtgctat

fava_c16305_453	fava_c16305	453	G/T	aaggtctatttttagggtcagcttctaaacattggagtataagttgtgctataagaaatgctgctgctagaggatattgttgatccaatctttgatccat[G/T]atctttttgagtttctttttgtcatgtagatatccctttgtgaaatccaccaaattcatctcatatgttggcctatctgtgtcaagtgctattaagccag

fava_c16305_887	fava_c16305	887	C/T	ttatctgaagtatgcaaaaattccaatcctctagcagctcctatagctatcttcatccttatttcccaagaaagtggttctggacttttgaataggtgac[C/T]ttccaaactgcccttttgcatgtattcatacacaagcaggaactgatcttcctcccagcaaaacccaagtagtttaacaagatttggatgcgagaatttc

fava_c16309_1061	fava_c16309	1061	T/C	agctgaagttcttggtctgcttgctttggcgacttctgggtccgcatctggggttcttgcaacgtactagaatggtttgtttcctgattttgtactaaaa[T/C]gtttacaggctttttcctgatatgtttacgtgtttcctgaatgatatctgaagtccgattctctacaattgtaaaaccttcctcgaaagatttaccaatg

fava_c16309_1182	fava_c16309	1182	T/C	tatgtttacgtgtttcctgaatgatatctgaagtccgattctctacaattgtaaaaccttcctcgaaagatttaccaatggtgccaccgatatgagtaag[T/C]gaggatgtgattgcacctaatccYttaataattgttggactgtcccttttgagatttccctcctctaagtgattcaaattttggcccttgcccctagatg

fava_c16309_1206	fava_c16309	1206	C/T	tatctgaagtccgattctctacaattgtaaaaccttcctcgaaagatttaccaatggtgccaccgatatgagtaagYgaggatgtgattgcacctaatcc[C/T]ttaataattgttggactgtcccttttgagatttccctcctctaagtgattcaaattttggcccttgcccctagatgttacgccaggaaattgtgaccttg

fava_c16309_1634	fava_c16309	1634	G/A	cgccgcgccgaagaagctctgatagctttagcagcaagcgaagacgaagaagtgtcaacaccttcatagttcgaagaagagttcgaattcgaggaagaag[G/A]tgtaataggattagggtttcgaattggttcRttttcgaaaatcgaaggagaagacttggaagttccgtaYtgttttcttctatacgccatcgRaatcgaa

fava_c16309_1665	fava_c16309	1665	G/A	cagcaagcgaagacgaagaagtgtcaacaccttcatagttcgaagaagagttcgaattcgaggaagaagRtgtaataggattagggtttcgaattggttc[G/A]ttttcgaaaatcgaaggagaagacttggaagttccgtaYtgttttcttctatacgccatcgRaatcgaatMaacctctcgattttctggatccaattgaa

fava_c16309_1704	fava_c16309	1704	T/C	tcgaagaagagttcgaattcgaggaagaagRtgtaataggattagggtttcgaattggttcRttttcgaaaatcgaaggagaagacttggaagttccgta[T/C]tgttttcttctatacgccatcgRaatcgaatMaacctctcgattttctggatccaattgaatctctgcacgaaatggtttgaactagagggaaaaaaaac

fava_c16309_1727	fava_c16309	1727	A/G	gaagaagRtgtaataggattagggtttcgaattggttcRttttcgaaaatcgaaggagaagacttggaagttccgtaYtgttttcttctatacgccatcg[A/G]aatcgaatMaacctctcgattttctggatccaattgaatctctgcacgaaatggtttgaactagagggaaaaaaaacgaaactagcacagtgaggaacta

fava_c16309_1736	fava_c16309	1736	C/A	gtaataggattagggtttcgaattggttcRttttcgaaaatcgaaggagaagacttggaagttccgtaYtgttttcttctatacgccatcgRaatcgaat[C/A]aacctctcgattttctggatccaattgaatctctgcacgaaatggtttgaactagagggaaaaaaaacgaaactagcacagtgaggaactaactaccgct

fava_c16309_214	fava_c16309	214	T/C	nnnnnnnnnnnnnnnnnntttttttttgagaaataacagtactttgatatacataaactatttattttggcttgtggcaaatatacagaaaatagctgat[T/C]tgggttatatatgctggtattataataaaaagaaaaattaaaaaacactaataaagttacacaatataaggtacaaaattacatcaagctaatgtcaccc

fava_c16309_669	fava_c16309	669	C/T	aatcaagggacttaataggcacttggggtaggttaagaggattaacttcagagacttcctcattgctctcgtctaagtagacaacatcctgcattgtaag[C/T]tgatgatattcaacaacctccctcagaaagcgattctcacgggcataaactgagttctcatgggccaagcgagctttctcagctagaagagtttcaagtt

fava_c16311_1156	fava_c16311	1156	C/T	attaactcatcaactttcaattccataaatgactccttgacaatctcatcaccagttgcagctgaagccacaacacggaagccgttatttaagacatcta[C/T]gttttgcttgttgtcatttaagataccaactgtaatccttggtccattgctcttctggaacattcctgtactttgaacatcctgaagcaacgacgacaaa

fava_c16311_902	fava_c16311	902	A/G	caattgatttacatatttcatcatttactgcagtggcctttaaagtgatgcatgctgaaattaggtcgggtgagccaagtcctgtgagcagagattccac[A/G]atggcttctggaattccaatcttagcgaattttcgtgcatatccataaacttgggaagctagaacacgattgtcatcaggcgtcaaaagtacacgaatag

fava_c16312_1104	fava_c16312	1104	A/G	atcatagaattacaattagagctagaaacaagaaatttcacagaagagtctatgaacgcaggggaagcacaaagtgttcagatgcaagtgaagggccgcc[A/G]tgcacagggtttgtggaaggtatggctccggttttctctcgatccgcatggtattgtacatggcatctcatacagaacgaccttgtccatggatggggaa

fava_c16312_1519	fava_c16312	1519	T/G	tgaagtcttcaaagaacgctggaatcaagctactgaaaaggataaaacttgggttgatccattccagagacgcataagaagacggcacgctgatcactaa[T/G]accgtgtttgtttctgctgtagattgccttcgcaccacacctcaacacagtgcatgaccggcgacaatccaaatccaaacacatgctaactcggttgctc

fava_c16312_1686	fava_c16312	1686	T/G	atccaaatccaaacacatgctaactcggttgctcggacatgttaaagcttttttcacggttacctttatttatagccatagttacgaatttgtagctgag[T/G]tcttaattcaattcatttttgaaaaataacacttttctttttatattctttttgttgctaagccccctttttttttcctttcttggcacctcttctttct

fava_c16312_888	fava_c16312	888	C/T	acaaggctatacacatcgtcgctgagaatcagacaaagtggtggtttgctaaaagatttctgcatccggatatagtttctatatatgattatatctttct[C/T]tgggatgaggatttaggcgtggagaatttttctccgtcaagatacgttaaaatYgtgaaggaagaaggtttggagatatcacaaccagctcttgacccaa

fava_c16312_942	fava_c16312	942	T/C	gatttctgcatccggatatagtttctatatatgattatatctttctYtgggatgaggatttaggcgtggagaatttttctccgtcaagatacgttaaaat[T/C]gtgaaggaagaaggtttggagatatcacaaccagctcttgacccaaattcaacagagatacatcatagaattacaattagagctagaaacaagaaatttc

fava_c16313_1160	fava_c16313	1160	A/G	aattccaagcagtcatgaaatcacagcttagcagcagaagagacctgctgtgtctcaaaagtatgctttcaagatggtcatgacgtaaacaaagtccaac[A/G]ttgtcagattgatttccacgcatcttatattgtttaaatgccgtcataaatgcatgtttggcagctttatacaacggtagaagaatatgagttgaaattc

fava_c16313_1315	fava_c16313	1315	C/G	tgtttggcagctttatacaacggtagaagaatatgagttgaaattcccagtttatgatctctgttccagaagtatagttcgtcagagcttgctatcctgt[C/G]ggctgatagacggacggattcagcagataaatttgtSgaaattggagattcttcatttaacaaggaaaactgggatggatgaataaaccccagctcatca

fava_c16313_1352	fava_c16313	1352	C/G	ttgaaattcccagtttatgatctctgttccagaagtatagttcgtcagagcttgctatcctgtSggctgatagacggacggattcagcagataaatttgt[C/G]gaaattggagattcttcatttaacaaggaaaactgggatggatgaataaaccccagctcatcacgaagaggagttacttgattggaagaagaaaacgaaa

fava_c16313_242	fava_c16313	242	T/C	gagctgtaaatatttgaccaatactcttgcttcttgacttgagatatgagagctactttctcacatagctctagctcataaagtaattgaatagagagga[T/C]ctctctgggcatgacttatccagtatggtcttcaaatctacatcttttatcttctcaaggagctcattttcaagaggcttgggcaattgcacttttaacc

fava_c16318_1011	fava_c16318	1011	A/G	caattagcatggtYttgtcatgttgtgcaaagtagcatctagatatagtgatatccgtgctttgtctcgtgatgtctataagcccgtcatcataatcgcg[A/G]aggctgcaacggtctatccatatattcctagaattcggctttatttgaatcccatctacatcatgaccccttcccccctcaaactcaaggttgcatataa

fava_c16318_1257	fava_c16318	1257	C/T	taatcctctggcctcttccatctattgtcttatgagatgacacactcaggtatgacgaaagattaattgtacctgaaacctcaaaaacaatccatagcgg[C/T]tcctttttacgacatccttcacgaagtgaaccaggaccatcatctgaaaSggaggtaaccaaatagagaggaccgtggaggccaccaatggcgaggcggc

fava_c16318_1307	fava_c16318	1307	C/G	tatgacgaaagattaattgtacctgaaacctcaaaaacaatccatagcggYtcctttttacgacatccttcacgaagtgaaccaggaccatcatctgaaa[C/G]ggaggtaaccaaatagagaggaccgtggaggccaccaatggcgaggcggccgaaaccttcagcacggccggccatggcccKcagagtggaatccaaatct

fava_c16318_1388	fava_c16318	1388	T/G	ccaggaccatcatctgaaaSggaggtaaccaaatagagaggaccgtggaggccaccaatggcgaggcggccgaaaccttcagcacggccggccatggccc[T/G]cagagtggaatccaaatctccgtatggttgaatcggcgtcgacgccattttcgctttctgttccgagctttttttggatacttgcacgcaatgataatga

fava_c16318_237	fava_c16318	237	T/A	ttaacttttagcttagctaaatacattttttggctaaattgtgtttttcttcctttagtattgactcaaaactcattttttgttttgttttcaccactat[T/A]atccWgtccactggaScgactaatctgattctgagatcagttgtgacatgtggtttcaaccccctcccRatcgcaccagtatccagtccactgRatcRac

fava_c16318_242	fava_c16318	242	A/T	ttttagcttagctaaatacattttttggctaaattgtgtttttcttcctttagtattgactcaaaactcattttttgttttgttttcaccactatWatcc[A/T]gtccactggaScgactaatctgattctgagatcagttgtgacatgtggtttcaaccccctcccRatcgcaccagtatccagtccactgRatcRactaatc

fava_c16318_253	fava_c16318	253	C/G	ctaaatacattttttggctaaattgtgtttttcttcctttagtattgactcaaaactcattttttgttttgttttcaccactatWatccWgtccactgga[C/G]cgactaatctgattctgagatcagttgtgacatgtggtttcaaccccctcccRatcgcaccagtatccagtccactgRatcRactaatctgattctgaga

fava_c16318_306	fava_c16318	306	G/A	aactcattttttgttttgttttcaccactatWatccWgtccactggaScgactaatctgattctgagatcagttgtgacatgtggtttcaaccccctccc[G/A]atcgcaccagtatccagtccactgRatcRactaatctgattctgagataagttgtgacatgtggtttcaaccccctcccgatcgcaattgYgagRgatcg

fava_c16318_331	fava_c16318	331	G/A	cactatWatccWgtccactggaScgactaatctgattctgagatcagttgtgacatgtggtttcaaccccctcccRatcgcaccagtatccagtccactg[G/A]atcRactaatctgattctgagataagttgtgacatgtggtttcaaccccctcccgatcgcaattgYgagRgatcgaactgtgttcctcccttttttacat

fava_c16318_335	fava_c16318	335	G/A	atWatccWgtccactggaScgactaatctgattctgagatcagttgtgacatgtggtttcaaccccctcccRatcgcaccagtatccagtccactgRatc[G/A]actaatctgattctgagataagttgtgacatgtggtttcaaccccctcccgatcgcaattgYgagRgatcgaactgtgttcctcccttttttacataggg

fava_c16318_397	fava_c16318	397	T/C	ccccctcccRatcgcaccagtatccagtccactgRatcRactaatctgattctgagataagttgtgacatgtggtttcaaccccctcccgatcgcaattg[T/C]gagRgatcgaactgtgttcctcccttttttacatagggcttcataacatattgtctacYggcctacaaatggactgccaacctgcacataactggagaac

fava_c16318_401	fava_c16318	401	A/G	ctcccRatcgcaccagtatccagtccactgRatcRactaatctgattctgagataagttgtgacatgtggtttcaaccccctcccgatcgcaattgYgag[A/G]gatcgaactgtgttcctcccttttttacatagggcttcataacatattgtctacYggcctacaaatggactgccaacctgcacataactggagaacctct

fava_c16318_456	fava_c16318	456	T/C	agttgtgacatgtggtttcaaccccctcccgatcgcaattgYgagRgatcgaactgtgttcctcccttttttacatagggcttcataacatattgtctac[T/C]ggcctacaaatggactgccaacctgcacataactggagaacctctctgagagaatctgtagctgcttccattgtccaagttgggtaatattcactaggat

fava_c16318_744	fava_c16318	744	C/T	actcctgatgttctctgtcttccgccttttcggtataaaattcaaaagcctttttcttagctcctgcctcatatatgttgcattgggagtatatctgggc[C/T]tcaacactagcacaaatcgcatagatttcccagtttctgatgtaattgttgtacagatgaacttttccaaatctcacacgagggtgtctctgccttgttc

fava_c16318_888	fava_c16318	888	A/C	aattgttgtacagatgaacttttccaaatctcacacgagggtgtctctgccttgttccatcaaagaaacaatgatgaatcgtcacccggatgcatctatc[A/C]gcgacgtgtgacgggttagctccaattagcatggtYttgtcatgttgtgcaaagtagcatctagatatagtgatatccgtgctttgtctcgtgatgtcta

fava_c16318_924	fava_c16318	924	C/T	gagggtgtctctgccttgttccatcaaagaaacaatgatgaatcgtcacccggatgcatctatcMgcgacgtgtgacgggttagctccaattagcatggt[C/T]ttgtcatgttgtgcaaagtagcatctagatatagtgatatccgtgctttgtctcgtgatgtctataagcccgtcatcataatcgcgRaggctgcaacggt

fava_c16324_1368	fava_c16324	1368	C/T	tgtttgccgaggttgttccggtcaaacacaggaccatgatttatgcatttgatagagcttttgaaggctcattctcttcggttgcagctcctttggttgg[C/T]attcttgcagagaaaatgtttggctacaattcgaaatctgttgatcccatcaaaggatcttcacctgaggctcttgccttgtcaaagggacttctttcaa

fava_c16324_1512	fava_c16324	1512	A/G	atcccatcaaaggatcttcacctgaggctcttgccttgtcaaagggacttctttcaatgatggcagttccttttggattgtgttgcttgtgctacacgcc[A/G]ttgtattttatatttaagaaggaccgcgaaaatgctagaatgcaagccatcaaagaagaggagatgatgtgagacgctcgctaactaggaatggtagttt

fava_c16324_158	fava_c16324	158	T/C	tttggattgacgtacgtgatgggactcattcgaacctcaaaacatagcaccaaatcttctcttcttccatccaaatcaaaatcatcattttcaactccca[T/C]ttattctgcttctcaaaccatgaaaacaacaattaagatttatggcgtgtctctttcgctcattctcatcaacctagctgctattatggagcgcgccgat

fava_c16324_1698	fava_c16324	1698	T/C	taggaatggtagtttgcatgaatggaattcttaactcagtctgcttgtgacatggctggcactcatggagttcaaatcttcggaggtaaacatgaaactt[T/C]tgcaacaattcttcaaaatggtctagtttcttttatttgaagagtctatggtttccatcacgagttgaggctgataactgaacttgttctgagcaactat

fava_c16324_1816	fava_c16324	1816	A/G	atggtctagtttcttttatttgaagagtctatggtttccatcacgagttgaggctgataactgaacttgttctgagcaactatttcacgtgggaaattac[A/G]ctcacctcccaaattttagattgacatttgaccatgacttcctttatttttgcatttgWKtgacacactcttctcttgtacatactctcacgttatgctc

fava_c16324_1875	fava_c16324	1875	A/T	actgaacttgttctgagcaactatttcacgtgggaaattacRctcacctcccaaattttagattgacatttgaccatgacttcctttatttttgcatttg[A/T]KtgacacactcttctcttgtacatactctcacgttatgctcagMttaatattttatggcattagttgctttgctgaattgacttgtatcaagataagagt

fava_c16324_1876	fava_c16324	1876	G/T	ctgaacttgttctgagcaactatttcacgtgggaaattacRctcacctcccaaattttagattgacatttgaccatgacttcctttatttttgcatttgW[G/T]tgacacactcttctcttgtacatactctcacgttatgctcagMttaatattttatggcattagttgctttgctgaattgacttgtatcaagataagagta

fava_c16324_1919	fava_c16324	1919	C/A	cacctcccaaattttagattgacatttgaccatgacttcctttatttttgcatttgWKtgacacactcttctcttgtacatactctcacgttatgctcag[C/A]ttaatattttatggcattagttgctttgctgaattgacttgtatcaagataagagtacccaaatttgaccagttgaacgttgcctatcatttggtttcta

fava_c16324_312	fava_c16324	312	A/G	tttcgctcattctcatcaacctagctgctattatggagcgcgccgatgagaatcttcttccgtctgtttacaaagaagttagtgaagcattcaatgcagg[A/G]ccgtctgatttaggttacctcacgttcatacggaactttgtgcaaggactKtcgtcgcctctagctggtatacttgttattaactatgatagaccaacaa

fava_c16324_363	fava_c16324	363	G/T	atcttcttccgtctgtttacaaagaagttagtgaagcattcaatgcaggRccgtctgatttaggttacctcacgttcatacggaactttgtgcaaggact[G/T]tcgtcgcctctagctggtatacttgttattaactatgatagaccaacaattcttgctatgggaactttctgttgggctttatcaactgctgctgtgagtg

fava_c16324_750	fava_c16324	750	T/C	tcatggctggtcagacgttttggggagtacagggatggagatgtgcctttgttttgatggcgagtttgagcgcgttgattggaatccttgttttactata[T/C]gtggatgatccgaaaaaaagattttccccgactcaagatgctagtgatagttcagaaaggggtgattctatYtataatggcaatgctagtgtgacttcag

fava_c16324_822	fava_c16324	822	C/T	cgttgattggaatccttgttttactataYgtggatgatccgaaaaaaagattttccccgactcaagatgctagtgatagttcagaaaggggtgattctat[C/T]tataatggcaatgctagtgtgacttcagtttggaggcactcttgggcagccactaaatctgtgatcaaagttcaaacatttcaaataattgtcttacaag

fava_c16325_125	fava_c16325	125	A/T	tgtaataaatgcaaatcctaccaaagaagttcaactctgtccttgtttatcatccattcatcacataaccatgtatgacaacagggagttcattatttac[A/T]gatcttgcctcctaatgcttaacattctcaaggaacattgcaatcacataaataaattgaggctaatactacaaatcaaacgggtttctattggagaaac

fava_c16325_567	fava_c16325	567	T/C	aggcttagttggcatcttcccacgcttcaatataaaaactttctcaacatccaatccatccggccacgccgagcatacaaattccaacacattcccatct[T/C]cccctttcgaaatatccacaatcaaactaaggtgaagcctcacattcaccccagaactatcatctccaaacacaggcacatgctgaaacccatcaaacat

fava_c16325_805	fava_c16325	805	A/G	ttccccctaatcgtaacaacctgctcaccaggtcggtcttcaacaacaaaagaattgaattcagtcacaggctggtgtggaggagcgtattcagctcgat[A/G]ctcgatctcgtttcgaagtattcgcaaaatgttggattcaaagggtgattttgttgctgattttgtagagtgagttctgttaccgaaacctaatgtttga

fava_c16326_278	fava_c16326	278	T/C	ttccactcttttgctgcatagccgattccacctcctccacctcttctgtcgaatgtaatatgccttcttcaaccaaaatcttcatcaaagggcttcccct[T/C]tcaacctctgaagtgtatttagccgaagtgttttcRgtgtttggtgaagttactaaagtcaagcttttgaaagataMagaatctggacagtctctaggat

fava_c16326_314	fava_c16326	314	A/G	ccacctcttctgtcgaatgtaatatgccttcttcaaccaaaatcttcatcaaagggcttcccctYtcaacctctgaagtgtatttagccgaagtgttttc[A/G]gtgtttggtgaagttactaaagtcaagcttttgaaagataMagaatctggacagtctctaggatttgcatacatttggtttttcgatgaggagtctgcac

fava_c16326_355	fava_c16326	355	C/A	atcttcatcaaagggcttcccctYtcaacctctgaagtgtatttagccgaagtgttttcRgtgtttggtgaagttactaaagtcaagcttttgaaagata[C/A]agaatctggacagtctctaggatttgcatacatttggtttttcgatgaggagtctgcacaatcggctgcaaatgagacgaatggaaagttttttgatggc

fava_c16332_1159	fava_c16332	1159	C/T	ctgcttcaattcctgctaactcgttaacgaaatctttacagtatgctttaacaacatcttttggtagcatttgttatggctcgttattcactgctgctat[C/T]aggacactgcgatgggagatacgtggacttcggtcaaagattggcaacaatgagtgtttactttgctgtgttgatttcctttttcatctcgtggaaactc

fava_c16332_1567	fava_c16332	1567	A/G	ttaaatggagcgacagagttattatgattggctccacatccatgctgatgggaatggttttggttggattggcaatggtcgtggtggagagtgctgttac[A/G]tcgatatatatttgctatgcagaagaccctttattgattcagagatgggatgctgaatttttcaaccagatgtctgagacactacatcaccggcttcagt

fava_c16332_1828	fava_c16332	1828	G/C	gagaaaatgctgctatttgaggaatcaaatgcagtgttctcttttgttctacgacagtttacttatccatttgtatcaatcatagctataaacatatttt[G/C]atattgatttggtgtttagttaaattaataggaataggagaaggtttctggtggtgtaatcagcaatgtagaattggatgtgagtgaagagtatatataa

fava_c16332_844	fava_c16332	844	G/A	tgtatgttatatccgtcatagacagacttccatttacgatgttggttttgcaaaaagctgtgaagatggtttggaatcttcccgaggttatgagagtatc[G/A]tatgcatttatgtttgttgtgcttttatggatggccttatggtcatttggtgcagcaggtgttgtggcttcgaacttgagtaacgggggacgctggtggc

fava_c16333_1103	fava_c16333	1103	C/G	cctcaggcataccaagagcttctttcaactcatgtgataacatgccaggtttcatctccctcaactttacctcaaattcttttccttcatgatacaactc[C/G]ccaagggatgtcagttttggctttgtctgatacttaaaaaatgcatcatgaagaacctggtagtctatatccatttttcccattttaggttgcatacgtt

fava_c16333_1645	fava_c16333	1645	C/A	tcttcattttcttcatcatcagaatcagaatcagagtcggccttctttttagtagaagcattttctgcggcctcatccttcttatcagtttcctccgaag[C/A]agtgacattattgacagaaaatttctctgctatttttctatattcttcatccatgtcttcatctaccttctctgttatatagtcaatttcaatttgttca

fava_c16333_350	fava_c16333	350	C/T	ccttttcttgcatctttctttttctcttcttctcattctctgcaaccatgtcactgaaatcttctcgttgactacgcaacttctcttcctcccttgcttc[C/T]tcgtacttggcaggtaaaacattttccatggcttccaactcttccggctgtaaagtgacatccactttatctgacttctgacctttaagcaaatcgaccc

fava_c16333_728	fava_c16333	728	T/C	ctccagtaggagtgcttgatgaactatcaacagattgggtaccgtcttcaagttcctcctcttccatttcttcttcctcctcctcttcttcctcctcctc[T/C]tcctcctcctcttcYtcctccaaatcaccccagtgtttcgtcttatcaaccggctcctcctcatagttaggctggtcttgttgttgaacaccaaaaacat

fava_c16333_743	fava_c16333	743	C/T	ttgatgaactatcaacagattgggtaccgtcttcaagttcctcctcttccatttcttcttcctcctcctcttcttcctcctcctcYtcctcctcctcttc[C/T]tcctccaaatcaccccagtgtttcgtcttatcaaccggctcctcctcatagttaggctggtcttgttgttgaacaccaaaaacatctccatacagtgggc

fava_c16333_890	fava_c16333	890	A/G	catagttaggctggtcttgttgttgaacaccaaaaacatctccatacagtgggcgcccatattcatcaacgggaggcttgccccaaccaccagggtgata[A/G]ccaaaactagctccagcgggtatgggtgcattcagtcctgggatcttcaaatgRgggtaYgaMggtggaggaccatatctctgcatattaataagccatg

fava_c16333_944	fava_c16333	944	A/G	gcccatattcatcaacgggaggcttgccccaaccaccagggtgataRccaaaactagctccagcgggtatgggtgcattcagtcctgggatcttcaaatg[A/G]gggtaYgaMggtggaggaccatatctctgcatattaataagccatggaggaggagccccctcaggcataccaagagcttctttcaactcatgtgataaca

fava_c16333_950	fava_c16333	950	C/T	attcatcaacgggaggcttgccccaaccaccagggtgataRccaaaactagctccagcgggtatgggtgcattcagtcctgggatcttcaaatgRgggta[C/T]gaMggtggaggaccatatctctgcatattaataagccatggaggaggagccccctcaggcataccaagagcttctttcaactcatgtgataacatgccag

fava_c16333_953	fava_c16333	953	A/C	catcaacgggaggcttgccccaaccaccagggtgataRccaaaactagctccagcgggtatgggtgcattcagtcctgggatcttcaaatgRgggtaYga[A/C]ggtggaggaccatatctctgcatattaataagccatggaggaggagccccctcaggcataccaagagcttctttcaactcatgtgataacatgccaggtt

fava_c16335_1101	fava_c16335	1101	C/T	cataagcaattgcaattgctatgatttggattgaaaatccaattccgagtctttgaagcaatgtgattcccctagggtggccggttctctggcgcatgaa[C/T]ggaacgaaaagtcggtcgtacattggcactgagactaacatggagagggttacaaagctaccaagagatgccgctggaattttgaaattaggaccaaggt

fava_c16335_1707	fava_c16335	1707	C/G	acaataacaaaccagcagtaggaataccatatccaagtccccatcctaaattctcttgaatgtatacaaggcctaaagttgcaatcaaggcacctaaaaa[C/G]gacgtaaacatccaccagttaaagaaagaagcctttaactctttctcacgaggattaaaatcatcaaactggtcagcaccaaaggttgatatatttggtt

fava_c16335_1848	fava_c16335	1848	G/A	ctttctcacgaggattaaaatcatcaaactggtcagcaccaaaggttgatatatttggttttgtcccacctgccccaattgccatagtgtaaagtgctgt[G/A]tagaaaaatgcaacttgtgatgtcgaggctttgttacaaactccatttgtgcatgttggtttcaaactcttgagtgatacagctatagtaagaagtgtca

fava_c16335_2103	fava_c16335	2103	T/C	gagaatcggctatgtaagcaccaagaattggtgtaatccaaacagatcctgaccaagtattcacattcctaaccgatgaaacagtgtcttcatgaagctg[T/C]gttgtaaggtaattcaccaagtttgatgctacaccatagaaagccattctYtcaaatgcttcataaccaacgagaaaagcgcaagctttccatttgccag

fava_c16335_2154	fava_c16335	2154	T/C	accaagtattcacattcctaaccgatgaaacagtgtcttcatgaagctgYgttgtaaggtaattcaccaagtttgatgctacaccatagaaagccattct[T/C]tcaaatgcttcataaccaacgagaaaagcgcaagctttccatttgccagttttggatggaacagcaggtttgccacggaaatcaacagtgccatcttggg

fava_c16335_909	fava_c16335	909	C/T	ttgttccaagactctgcatatcttcaggggattgatcatagaaaaattccaacaatccaattgcattgaaaacatctgctataccaataagaacatattg[C/T]ggtaacagccaaaatatactcatagggacaatatctttaggacccaaaatgtgattctcttttatgacatgaattcttctaacttcgactgcataagcaa

fava_c16337_541	fava_c16337	541	G/T	ttcctgttttttggaaagtggcaattacaaatccagggtcatctcttgcattccatgacattgccggaacaataatctcccgagttttatcgaaatgaac[G/T]tggtcagaaaatgtatacctgtgagcactaaagatgatccttggcctcagagcttgtaaaatgtactcagaaacattcagcgggagagtatgaagtaaat

fava_c16337_730	fava_c16337	730	A/T	tatgaagtaaattgtacaggccagtcccaacaagctccctgttttttggcaccacatttaaccgctctataaaagagctagaagatctttcaaaactttc[A/T]gtgccaccaaagcgcttggttctggttcggtccaacggtaagtgcagcaRaaggacagggcctgatccagatgacagggtgctttccctcaagaaaaagt

fava_c16337_780	fava_c16337	780	G/A	accacatttaaccgctctataaaagagctagaagatctttcaaaactttcWgtgccaccaaagcgcttggttctggttcggtccaacggtaagtgcagca[G/A]aaggacagggcctgatccagatgacagggtgctttccctcaagaaaaagttatagtttgcatctgtagagtgcgtggagtgatttcttgtttttgttgtt

fava_c16337_970	fava_c16337	970	G/A	tttttgttgttgctgcatcagttaactcacgaagttctagactttcgctctctattactttttcaacatcaaaacgtaaactactgttgtcgcaaagtaa[G/A]gccacagaattcagtgaaacaaaactgacgttaccaatcacaaatgcaccacaaccagaacgatccaatcccggtaacttgcgagaaatccaattgactt

fava_c16338_1182	fava_c16338	1182	A/G	ctgtggctgtgtggacaaatggctgcacattaatgcgacttgccccctctgcaagtatgacattttgaaaagtagcaactatggtcaagaggaagtatag[A/G]acccctKttggggaatttgtatgctcttggttggcagtataataggcccagcgtgttaatatacttcagtacagatcttagttgttgctgctgcagcttt

fava_c16338_1189	fava_c16338	1189	G/T	tgtgtggacaaatggctgcacattaatgcgacttgccccctctgcaagtatgacattttgaaaagtagcaactatggtcaagaggaagtatagRacccct[G/T]ttggggaatttgtatgctcttggttggcagtataataggcccagcgtgttaatatacttcagtacagatcttagttgttgctgctgcagctttgtctttg

fava_c16338_504	fava_c16338	504	G/A	gattttagggtatgcgctacagtgtgttgttcatactgtgtgtgtttgttttgagtatcggagaaggataaggatgcgggaacaggtgagtacgaacggt[G/A]gtaacgcggttgggagtggtggaagtggggatttgagctttggttcaatggatggttctggtcagtatggggatgatggttccaccagtttgccgaagca

fava_c16342_1238	fava_c16342	1238	G/T	tcgggcactccaaccactaggagcagcagcagacctgtaatgtgaccactcactcaatgtggctttccctaatatgatagccccagctttccttaacttg[G/T]taaccacacctgcatctcttggcaccactgagccaagaagtgcataagagcctgcagtagtgttcagcttatccttagttgcaatgttatccttaaccaa

fava_c16342_1376	fava_c16342	1376	T/G	aagtgcataagagcctgcagtagtgttcagcttatccttagttgcaatgttatccttaaccaaaataggtattccatgcaatcttgacagagaacctggt[T/G]ccttttcccttctctctttgtcagctgcatctgcttgtgctagtgcatcYggattcacctccagtacccctctgacaacaggattttKgatattgatttg

fava_c16342_1426	fava_c16342	1426	T/C	tatccttaaccaaaataggtattccatgcaatcttgacagagaacctggtKccttttcccttctctctttgtcagctgcatctgcttgtgctagtgcatc[T/C]ggattcacctccagtacccctctgacaacaggattttKgatattgatttggttgaggtagaactcaacaagatRtcttgatgttaattggtttYtttgRa

fava_c16342_1464	fava_c16342	1464	G/T	agagaacctggtKccttttcccttctctctttgtcagctgcatctgcttgtgctagtgcatcYggattcacctccagtacccctctgacaacaggatttt[G/T]gatattgatttggttgaggtagaactcaacaagatRtcttgatgttaattggtttYtttgRaaagctagttgaagatcttgcactgttgcttccttgatt

fava_c16342_1500	fava_c16342	1500	G/A	gctgcatctgcttgtgctagtgcatcYggattcacctccagtacccctctgacaacaggattttKgatattgatttggttgaggtagaactcaacaagat[G/A]tcttgatgttaattggtttYtttgRaaagctagttgaagatcttgcactgttgcttccttgattgtgaatccactggccaaggttgttgttgatgatagt

fava_c16342_1520	fava_c16342	1520	C/T	tgcatcYggattcacctccagtacccctctgacaacaggattttKgatattgatttggttgaggtagaactcaacaagatRtcttgatgttaattggttt[C/T]tttgRaaagctagttgaagatcttgcactgttgcttccttgattgtgaatccactggccaaggttgttgttgatgatagtagcatagaaaggaacgtaag

fava_c16342_1525	fava_c16342	1525	G/A	cYggattcacctccagtacccctctgacaacaggattttKgatattgatttggttgaggtagaactcaacaagatRtcttgatgttaattggtttYtttg[G/A]aaagctagttgaagatcttgcactgttgcttccttgattgtgaatccactggccaaggttgttgttgatgatagtagcatagaaaggaacgtaagtagta

fava_c16342_190	fava_c16342	190	G/A	taSctctaaatcattactccttcaaattttcaacttacaacttcctaaggggtggtggctttctgatcaaagttgcttgctcaaatgaataagcaatttc[G/A]atcaactttggctctgaaccttttaaacctccaaagcaaattccaaatggtataccMttttcatatcctgctggaacactcactccaggataacctccaa

fava_c16342_247	fava_c16342	247	C/A	gctttctgatcaaagttgcttgctcaaatgaataagcaatttcRatcaactttggctctgaaccttttaaacctccaaagcaaattccaaatggtatacc[C/A]ttttcatatcctgctggaacactcactccaggataacctccaatagcaagtaYgctactaaaRgatgaaaaaRgtgttaccacagcatcaagtttatttc

fava_c16342_300	fava_c16342	300	C/T	ggctctgaaccttttaaacctccaaagcaaattccaaatggtataccMttttcatatcctgctggaacactcactccaggataacctccaatagcaagta[C/T]gctactaaaRgatgaaaaaRgtgttaccacagcatcaagtttatttcttttcatcactttctcaaacccattttttgaccatcttgtcatgtttaataat

fava_c16342_310	fava_c16342	310	G/A	cttttaaacctccaaagcaaattccaaatggtataccMttttcatatcctgctggaacactcactccaggataacctccaatagcaagtaYgctactaaa[G/A]gatgaaaaaRgtgttaccacagcatcaagtttatttcttttcatcactttctcaaacccattttttgaccatcttgtcatgtttaataatgcttgtttct

fava_c16342_320	fava_c16342	320	A/G	tccaaagcaaattccaaatggtataccMttttcatatcctgctggaacactcactccaggataacctccaatagcaagtaYgctactaaaRgatgaaaaa[A/G]gtgttaccacagcatcaagtttatttcttttcatcactttctcaaacccattttttgaccatcttgtcatgtttaataatgcttgtttctctgctttctc

fava_c16342_647	fava_c16342	647	T/A	gtctttcaggtatgcattcaaagacaatttgaattcaaaattcaaagcgataatttcactttgagcaacgataatgtcatcaatgtcatcaatcttcaaa[T/A]tgtcgaccaaaactgcacctctttgccttaatgtttttaggcgtaacttgaaagtttcatgcataaaagtStcatttccgaaattgtagaatagtctcac

fava_c16342_718	fava_c16342	718	C/G	taatgtcatcaatgtcatcaatcttcaaaWtgtcgaccaaaactgcacctctttgccttaatgtttttaggcgtaacttgaaagtttcatgcataaaagt[C/G]tcatttccgaaattgtagaatagtctcactactcctaatctcttccctcgtagtccatctttcttaagaaattgagcatagccaccttttgggatatact

fava_c16342_848	fava_c16342	848	G/T	tactcctaatctcttccctcgtagtccatctttcttaagaaattgagcatagccaccttttgggatatactttgatgcttcaattgttgctttatcaaca[G/T]tatcaatgccagctatggtttcaagaacataagcagcatctgataYtgtcctgcaaattggcccRatagtgtcctgccttggactaaYtggaactacacc

fava_c16342_894	fava_c16342	894	C/T	gcatagccaccttttgggatatactttgatgcttcaattgttgctttatcaacaKtatcaatgccagctatggtttcaagaacataagcagcatctgata[C/T]tgtcctgcaaattggcccRatagtgtcctgccttggactaaYtggaactacacctgctctactagtgagaccaactgtgggtttgatgcctactactgag

fava_c16342_913	fava_c16342	913	A/G	tatactttgatgcttcaattgttgctttatcaacaKtatcaatgccagctatggtttcaagaacataagcagcatctgataYtgtcctgcaaattggccc[A/G]atagtgtcctgccttggactaaYtggaactacacctgctctactagtgagaccaactgtgggtttgatgcctactactgagttaaaatttgaaggacata

fava_c16342_92	fava_c16342	92	G/C	gttttagaaaatagaaacaaatttttatttcatttcatagagaaaaactcattacatttttagtcaaaatcccatgttttgatttatttta[G/C]ctctaaatcattactccttcaaattttcaacttacaacttcctaaggggtggtggctttctgatcaaagttgcttgctcaaatgaataagcaatttcRat

fava_c16342_936	fava_c16342	936	T/C	gctttatcaacaKtatcaatgccagctatggtttcaagaacataagcagcatctgataYtgtcctgcaaattggcccRatagtgtcctgccttggactaa[T/C]tggaactacacctgctctactagtgagaccaactgtgggtttgatgcctactactgagttaaaatttgaaggacataaaatggatccatcggtttcggta

fava_c16343_1155	fava_c16343	1155	T/C	aggagaaaggcaacggaaagttgactccaagtgacaacatagttgaaatgtgtgacaggtggagatccaaggaactggacaaccttactccagatgaact[T/C]tacgccatctcggaatcagactataagtgttggtgtttggatttgaagccaaaattggcaaatgcttaggttattatgatcattggatctctccataata

fava_c16343_1317	fava_c16343	1317	C/G	atgcttaggttattatgatcattggatctctccataataccaatcaatcagtgtacatttcttagcaagtgtttggattatcttctttttgtgtcagtgg[C/G]atgcttttagggtgaagctacacttggtagcagaaacttacacatacttgaaatctaaatgttgccgtgataccaaatatacacttcagaaactatctaa

fava_c16343_141	fava_c16343	141	C/T	aagggtatgcactgcgctttcttctcccagcattgcattagctgttagggtttgcagtttcagtgtgtgtgactgtaatctgaatggacacagaagaaga[C/T]cctcctcaaaactcagctaaaagacagaaatgYtcagcatgctataaacaataYaagaagaaagaacatcttattcagcacatgaaaacctcttaccact

fava_c16343_174	fava_c16343	174	C/T	tgcattagctgttagggtttgcagtttcagtgtgtgtgactgtaatctgaatggacacagaagaagaYcctcctcaaaactcagctaaaagacagaaatg[C/T]tcagcatgctataaacaataYaagaagaaagaacatcttattcagcacatgaaaacctcttaccactctatccatgagccgcaatgYgtgatctgtagMa

fava_c16343_195	fava_c16343	195	T/C	cagtttcagtgtgtgtgactgtaatctgaatggacacagaagaagaYcctcctcaaaactcagctaaaagacagaaatgYtcagcatgctataaacaata[T/C]aagaagaaagaacatcttattcagcacatgaaaacctcttaccactctatccatgagccgcaatgYgtgatctgtagMaagcactgcaaatcttttgaat

fava_c16343_261	fava_c16343	261	T/C	aaagacagaaatgYtcagcatgctataaacaataYaagaagaaagaacatcttattcagcacatgaaaacctcttaccactctatccatgagccgcaatg[T/C]gtgatctgtagMaagcactgcaaatcttttgaatctctgagggaacatcttatgggacctttggctaaaggactttgttcagggattttctcgcaagggg

fava_c16343_273	fava_c16343	273	C/A	gYtcagcatgctataaacaataYaagaagaaagaacatcttattcagcacatgaaaacctcttaccactctatccatgagccgcaatgYgtgatctgtag[C/A]aagcactgcaaatcttttgaatctctgagggaacatcttatgggacctttggctaaaggactttgttcagggattttctcgcaagggggttgcaaacttt

fava_c16343_506	fava_c16343	506	A/T	agtgagcatagagaaacttgttgcctatctgcccctgttcctcttggcacaagtgaattgccctatacagactccaatatcgattttctggattcttatg[A/T]tSaaaatgatgctgaccggcgtcctaaagcaattgcattggactgtgaaatggttggtggtggaagtgatggatctctggaagtttgtgctagagtatgc

fava_c16343_508	fava_c16343	508	G/C	tgagcatagagaaacttgttgcctatctgcccctgttcctcttggcacaagtgaattgccctatacagactccaatatcgattttctggattcttatgWt[G/C]aaaatgatgctgaccggcgtcctaaagcaattgcattggactgtgaaatggttggtggtggaagtgatggatctctggaagtttgtgctagagtatgctt

fava_c16343_719	fava_c16343	719	C/G	gatgagaacttaatcttgcacacatatgtaaaacctcgactccctgttacaaattataggtatgatataactgggctgaccgaagagcatcttcgagatg[C/G]ggtgccacttaagcaagttcgggaaaagatattgcaaattttatacaatggagagtccattgcaaaagttagaatggatggtggaaaggccaggcttctt

fava_c16343_936	fava_c16343	936	G/A	catacgatttggattgcttggaaatgagttaccctgatcatttgttgagagacactgcaaagtaccgtccattgatgaaaacaaacggttccagccactc[G/A]ctcaagtatcttaccagaacatatctaggttatgatatccaaactggcacacacgatccattcgaagattgcatttcttcgatgagactgtacaagagaa

fava_c16346_1428	fava_c16346	1428	C/A	gctttgaggaagcagggcatctaccggtggtaccgacctcaagtccagagaattatcaatagggtggaaccttggcaacattggagaagaagaaggaaca[C/A]tagaattcctgatcacattcccatttgagaatgtgggtagagggagagaaatttgttggcttgcggggtttaccctcaagcgaaaatttctgtggaagcc

fava_c16346_1649	fava_c16346	1649	A/G	ttgactcgggtattgtttcagaagatccgtctccgacactaaggccaagccttgcattcacctgttctgccggtgatgatcggttgaagttgttcatcgc[A/G]taatcctgagtaggaagagtattccaagcactaccatctgtatgctgactatagctagaacttccaccgtcagaagactgtccacctattccttcacctg

fava_c16346_176	fava_c16346	176	A/G	agagttaaagaaatattcatccatccaacaccaatcatctcatgtttcaccttaacggggagataatcggtttcccgaaaataaacaaaaaatgataata[A/G]agaaattttggctaaattcttaaatggccatttaataagtaacaatggcaccagaaacttcaacttcaattaagcagaattttcctgactaattgtgccS

fava_c16346_1913	fava_c16346	1913	C/T	ccccgacaaaaacgctgttacgtccaccagaaggaagcaaaaatgaaccagaaccaatagatggtggaatgtgctcattcagctgaccactcgatttggc[C/T]Rcgttagaatgatcgacttccgcacgatagctattattatcaccaacattatcctgtaaagcagcatttaagttgagattttgtggcaccaaatgaggat

fava_c16346_1914	fava_c16346	1914	A/G	cccgacaaaaacgctgttacgtccaccagaaggaagcaaaaatgaaccagaaccaatagatggtggaatgtgctcattcagctgaccactcgatttggcY[A/G]cgttagaatgatcgacttccgcacgatagctattattatcaccaacattatcctgtaaagcagcatttaagttgagattttgtggcaccaaatgaggatt

fava_c16346_276	fava_c16346	276	C/G	Ragaaattttggctaaattcttaaatggccatttaataagtaacaatggcaccagaaacttcaacttcaattaagcagaattttcctgactaattgtgcc[C/G]gttttcttgacccctatgtgttcatcatcttatcttataagataaaatagaatattcaagacacgaatatctctcatgttgctaagcctgttgtcttaca

fava_c16346_425	fava_c16346	425	G/A	agaatattcaagacacgaatatctctcatgttgctaagcctgttgtcttacaaattggacacagattcttgtgcattagccactgtttgatacagtcggt[G/A]tggtaatcgtgcccgcagtccaacgatccgatatcatcttcgtttttatactcctcctgacaaacagagcagggttctgcatcactctgagaacctgatt

fava_c16351_1239	fava_c16351	1239	T/C	atcaggtaccgggtattgctcttcacatttatagcgatctaatgaccagagacgaataaccttatctcccacaccagtaacaaccgtacccattttcatc[T/C]gacactgagaaaccgcggttgaatgacccttccactgatcaacatgaaatgttccacactgaagcgccaatctatgctgctccaaagctacttcccctaa

fava_c16351_1478	fava_c16351	1478	G/T	tcgaagaatccatcagcaacttcctctcgtaaaactctcgcagcgatcgagactcaaggattgcattccagaatttgcaaacgagagagcagcgaacgag[G/T]tcgaacatgttgaggaaagcgaagatggtacagaggatgtcgccgtcgagggaaagtatMgtggttggagtttcgcggcgtttttctctttggttggtgg

fava_c16351_1538	fava_c16351	1538	C/A	ttgcattccagaatttgcaaacgagagagcagcgaacgagKtcgaacatgttgaggaaagcgaagatggtacagaggatgtcgccgtcgagggaaagtat[C/A]gtggttggagtttcgcggcgtttttctctttggttggtggaagtaatggccggagtaggaggtgtgtttcctgtcgccgccgtgaagattggttgccgtt

fava_c16351_320	fava_c16351	320	T/A	ggctgtttttgttacaatatatataaccccaaattcccaaagagtaaacaaataaatagatcataaattcttgattttacaatgtacagatatttttgtc[T/A]ttcaatttccacaatctgatgtctYtggcattgtgtgtggtaacaatcttcttcattccRacggcaaggcacgtaatcgaaggtcgggaattcttMggaa

fava_c16351_345	fava_c16351	345	T/C	accccaaattcccaaagagtaaacaaataaatagatcataaattcttgattttacaatgtacagatatttttgtcWttcaatttccacaatctgatgtct[T/C]tggcattgtgtgtggtaacaatcttcttcattccRacggcaaggcacgtaatcgaaggtcgggaattcttMggaatgacatcaatatttatgtaggtatc

fava_c16351_380	fava_c16351	380	A/G	tcataaattcttgattttacaatgtacagatatttttgtcWttcaatttccacaatctgatgtctYtggcattgtgtgtggtaacaatcttcttcattcc[A/G]acggcaaggcacgtaatcgaaggtcgggaattcttMggaatgacatcaatatttatgtaggtatcctcaggcaatctcttcccctcccttctttggatgg

fava_c16351_416	fava_c16351	416	C/A	tgtcWttcaatttccacaatctgatgtctYtggcattgtgtgtggtaacaatcttcttcattccRacggcaaggcacgtaatcgaaggtcgggaattctt[C/A]ggaatgacatcaatatttatgtaggtatcctcaggcaatctcttcccctcccttctttggatggaRcctgaatgacttcgatgtgttgacaacaatttat

fava_c16351_482	fava_c16351	482	G/A	cggcaaggcacgtaatcgaaggtcgggaattcttMggaatgacatcaatatttatgtaggtatcctcaggcaatctcttcccctcccttctttggatgga[G/A]cctgaatgacttcgatgtgttgacaacaatttatcatctataatacaacttgaaacaaYattgccatcattYtgatttacaaatcttaagataccatcta

fava_c16351_541	fava_c16351	541	T/C	gtatcctcaggcaatctcttcccctcccttctttggatggaRcctgaatgacttcgatgtgttgacaacaatttatcatctataatacaacttgaaacaa[T/C]attgccatcattYtgatttacaaatcttaagataccatctattcctccaacagccaatgtcgatttgtcatcttgcatgtgctgtatagagtaaatgaca

fava_c16351_554	fava_c16351	554	T/C	atctcttcccctcccttctttggatggaRcctgaatgacttcgatgtgttgacaacaatttatcatctataatacaacttgaaacaaYattgccatcatt[T/C]tgatttacaaatcttaagataccatctattcctccaacagccaatgtcgatttgtcatcttgcatgtgctgtatagagtaaatgacattaggactcactc

fava_c16351_685	fava_c16351	685	T/A	cctccaacagccaatgtcgatttgtcatcttgcatgtgctgtatagagtaaatgacattaggactcactcgtttctcccataacagtctccttgtcctca[T/A]gtcccaacaatatgtatacccaatagctgatccggcaaacagtagctgagatgagggttttaaacacagcgtctttatgcctctaaaatcacttgatcta

fava_c16354_1141	fava_c16354	1141	G/A	ccaactgttgttcgaactccttctttcctaccacaacttccttcaaccttttaaccaccattgtcaccccctcctccaaaaccgccttgtatgctgtacc[G/A]tagctccctttaccRagaacttcagccgaagcctttagtagatcttcgagatcaaaggtataagaagaaccttcaaagaaaaataacttattcttttcag

fava_c16354_1156	fava_c16354	1156	G/A	actccttctttcctaccacaacttccttcaaccttttaaccaccattgtcaccccctcctccaaaaccgccttgtatgctgtaccRtagctccctttacc[G/A]agaacttcagccgaagcctttagtagatcttcgagatcaaaggtataagaagaaccttcaaagaaaaataacttattcttttcagcagcctgcactccgc

fava_c16354_1473	fava_c16354	1473	G/A	agtagagagagtaaggcgattcctccaatgactagagcaagtataactgctatgccaaagcttttcttatggacggttgtagctttttggtcttgtgtga[G/A]tgcaggggaaggagatggggaaggagagattgaagagcagttactgagaagaggtggaccacataagagagagtttccgRcaaaagaagtagacgggaat

fava_c16354_1553	fava_c16354	1553	A/G	agctttttggtcttgtgtgaRtgcaggggaaggagatggggaaggagagattgaagagcagttactgagaagaggtggaccacataagagagagtttccg[A/G]caaaagaagtagacgggaatgttttgatggaatttggaatagaaccgttcaagttattattgctcaaattcaagtatctgagactcggaaggttgaagtc

fava_c16354_1741	fava_c16354	1741	A/G	gaaggttgaagtcgggtataggtccagatatagaattgttttggagataaaaccacgtgagtcttctcaggttctgaaactcagaaggtatactaccaga[A/G]aaggcattaaaggaaatatcgaatacaactagtttaggagagacagaagaaggaattagacctgagaagttgtttttctgcaggtgtgcgaattggagtg

fava_c16354_1876	fava_c16354	1876	T/G	taggagagacagaagaaggaattagacctgagaagttgtttttctgcaggtgtgcgaattggagtgaaggaatggaaaggatgttagaaggaagattccc[T/G]ctaagaccattggaatgaaggcttagaactctaagaScatctagttttcctattgtgttctccggaatcRaaccagttaatccRattcctggaagatgga

fava_c16354_1913	fava_c16354	1913	G/C	gtttttctgcaggtgtgcgaattggagtgaaggaatggaaaggatgttagaaggaagattcccKctaagaccattggaatgaaggcttagaactctaaga[G/C]catctagttttcctattgtgttctccggaatcRaaccagttaatccRattcctggaagatggatgcctacgacacgagttcggtttgagttgcaRgtcac

fava_c16354_1946	fava_c16354	1946	G/A	aatggaaaggatgttagaaggaagattcccKctaagaccattggaatgaaggcttagaactctaagaScatctagttttcctattgtgttctccggaatc[G/A]aaccagttaatccRattcctggaagatggatgcctacgacacgagttcggtttgagttgcaRgtcacaccagtccatgaggtgcaaattgaagacgagtc

fava_c16354_1960	fava_c16354	1960	G/A	tagaaggaagattcccKctaagaccattggaatgaaggcttagaactctaagaScatctagttttcctattgtgttctccggaatcRaaccagttaatcc[G/A]attcctggaagatggatgcctacgacacgagttcggtttgagttgcaRgtcacaccagtccatgaggtgcaaattgaagacgagtcattccagttgagcc

fava_c16354_2008	fava_c16354	2008	G/A	taagaScatctagttttcctattgtgttctccggaatcRaaccagttaatccRattcctggaagatggatgcctacgacacgagttcggtttgagttgca[G/A]gtcacaccagtccatgaggtgcaaattgaagacgagtcattccagttgagccttggtgcgtgtggaacagaagaagcaaactccaatagagcttgtcggt

fava_c16354_2154	fava_c16354	2154	G/A	ttgagccttggtgcgtgtggaacagaagaagcaaactccaatagagcttgtcggtccgagttcaagtcagaagctgctataatcaagctaaagagagaca[G/A]cgatgcgRttaatagaaaaagagcagcattagagaatttcagcttcatgtcttgtttgatgagaaatcaaggctagtagtcactagtgattgttccaagt

fava_c16354_2162	fava_c16354	2162	A/G	tggtgcgtgtggaacagaagaagcaaactccaatagagcttgtcggtccgagttcaagtcagaagctgctataatcaagctaaagagagacaRcgatgcg[A/G]ttaatagaaaaagagcagcattagagaatttcagcttcatgtcttgtttgatgagaaatcaaggctagtagtcactagtgattgttccaagttctgagct

fava_c16354_236	fava_c16354	236	A/G	caatcagccaaaagtttcaaaaggtaacaaataagttgatacatgaaaacttttactaccaacttgaaaacaaagattagtagtactattttacaaacaa[A/G]aattgcttattattggttgagcatatatctgttgacagaagcaaaagagtgagttatcttatggtgtttgcagattagaatattcagactccgaggatgt

fava_c16354_337	fava_c16354	337	T/C	aattgcttattattggttgagcatatatctgttgacagaagcaaaagagtgagttatcttatggtgtttgcagattagaatattcagactccgaggatgt[T/C]ctgttcttaaactcaggatgtttaatttcctctatcattctaacagcttcatccattctcggcctcatatcYggcgtcttRccgacacacgcaagtccaR

fava_c16354_409	fava_c16354	409	T/C	gattagaatattcagactccgaggatgtYctgttcttaaactcaggatgtttaatttcctctatcattctaacagcttcatccattctcggcctcatatc[T/C]ggcgtcttRccgacacacgcaagtccaatctgaagcatctgaaccatttcctcttcaacatattgtcctcgaagaagttcttcatcaaatacttcagctg

fava_c16354_418	fava_c16354	418	A/G	attcagactccgaggatgtYctgttcttaaactcaggatgtttaatttcctctatcattctaacagcttcatccattctcggcctcatatcYggcgtctt[A/G]ccgacacacgcaagtccaatctgaagcatctgaaccatttcctcttcaacatattgtcctcgaagaagttcttcatcaaatacttcagctgtccattcct

fava_c16354_619	fava_c16354	619	G/C	ctctcacaacggacttaacccatcttggaagatcgacaacgtcttcatgaccgggatatctaagcggagtctttcctgttagcatttcaagtagcagcac[G/C]ccgaagctgtatacgtcggacttctgtgtgatcttcttggaatcggttacttcYggtgctctatatccattcgctctagacattgttgctggcgcgttca

fava_c16354_673	fava_c16354	673	T/C	gatatctaagcggagtctttcctgttagcatttcaagtagcagcacSccgaagctgtatacgtcggacttctgtgtgatcttcttggaatcggttacttc[T/C]ggtgctctatatccattcgctctagacattgttgctggcgcgttcatcagaggaggtagtccaacatcggaaatgcagctatcgaattcttgtgttatta

fava_c16356_140	fava_c16356	140	T/C	gagagtactaacctgtttgctgcatcaacctcgaagctagttcaaactagtgggaggttgtgtgcatagatttgcaccaaaaaatccaaatacttgttga[T/C]taattcaattcaaattcatcaaaaaatcgaaatggctcgtggaaaggttcagctgaagcgaattgagaaccaagtgcatagacaagtaaccttttgtaag

fava_c16356_342	fava_c16356	342	C/T	gccgagcggggcttctaaagaaggctaaggaactctctgtgctttgcgacgctgaaattggccttgtcattttctccgctcatggaaagctctatgaact[C/T]gccacaaaaggaaccatgcaaggagtaatagagaggtacttgaagttcacacatgggactcaatctgaagctgtcactgaaccacatcctttgctaatgg

fava_c16358_299	fava_c16358	299	C/T	tcaaggttgctgcaaacgttcccgatttcctctctgcagactggtttgaatctcgcaagaaaaaaccgtttggtccaagattagatttcactgcagaaga[C/T]gcggttcatcaccagcttgaagcgttgaagtataatgatcagcctcgtcaagattatggaattgaagtcatgtatcggtttgctggatttgatccttttg

fava_c16358_749	fava_c16358	749	A/T	cagagtcactgcttcacaacacggatacattttctggtgggttagcttattaaaatacattcaatgtagaagaaatcagtttcatatttgaaaaacatag[A/T]aatatattaaatataYtattactatcctaatcatatagtctgtaaaatttgtacatatYtgtaagaaattcagctgtaattagggagaagattctctccg

fava_c16358_765	fava_c16358	765	C/T	caacacggatacattttctggtgggttagcttattaaaatacattcaatgtagaagaaatcagtttcatatttgaaaaacatagWaatatattaaatata[C/T]tattactatcctaatcatatagtctgtaaaatttgtacatatYtgtaagaaattcagctgtaattagggagaagattctctccggaggagatttcactgc

fava_c16358_808	fava_c16358	808	T/C	ttcaatgtagaagaaatcagtttcatatttgaaaaacatagWaatatattaaatataYtattactatcctaatcatatagtctgtaaaatttgtacatat[T/C]tgtaagaaattcagctgtaattagggagaagattctctccggaggagatttcactgcagagggagcagtggaaatctccacccttcatttgtaaataaat

fava_c16361_1909	fava_c16361	1909	G/A	caacacctaattgggttaagcataaggagccaagagaagtgcacatgtttgttgatttttttcttcaagagcttgaagctatagtaaatgaagtgaagca[G/A]attttgcctcaaggtacacgtaagcaccgcaggactgatagtaatggaagtagtgtttcatcaaggagtaaccccttgcgggaggagaaattgggtaggt

fava_c16361_376	fava_c16361	376	G/A	ccaatttggaagggttgcttcagcgacatgttgaaatggcagctgagattaagaatctcgatactgatttgcaaatgcttgtttacgagaattataacaa[G/A]ttcattagcgctacagatactataaagaggatgaagagtaacatttcagggatggagactaatatggaacagcttcttgacaagataatgtctgtgcagt

fava_c16362_1188	fava_c16362	1188	T/C	tgaactctccggctggtctgaatggacacggattttctccatccggactacttcagttgggccaatcacaaagtttaaacaattcttccggtgatcaatt[T/C]aaattccagtcggccataactcccgttaatcagaatattcttcagggtatgccaatgtctatagggtacgatcacttgcaaaacagcaaaggtgctattt

fava_c16362_1320	fava_c16362	1320	A/G	agaatattcttcagggtatgccaatgtctatagggtacgatcacttgcaaaacagcaaaggtgctatttctcttcaaaacatgaatactgatgtaaaacc[A/G]acttttcccatcccaagtcaattcccggatcaaagacctagagtaactacctctagttttcatcctccgagtctcggtatctcaaacaatgccttgatgt

fava_c16362_2369	fava_c16362	2369	T/A	tttagagtgataatgtcccctcttatgtactattaaaccagttttgtcctactagttttctttctgtggagtttagcttctttttttttttcctttttct[T/A]tcaagattcaagctctctagaattctgcatcatgtgctttaaataagaacttaatgagaaatataaagtgtattattcaatttctcattagtttaatctt

fava_c16362_274	fava_c16362	274	C/A	catggtaatggatgattccactgatctctttcccatcggaatgcgtgttcttgccgtcgacgacgaccccacttgcctcaaagtcttggaaactctcctc[C/A]gaagatgtgaataccatgttaccacaactagtcaagctataatggcattgaatatgctgagagaaaacaaagacaagtttgacttggttatcagtgatgt

fava_c16363_1126	fava_c16363	1126	C/T	ttttccctaatttaatatctagaaactttacttttgctcttccaggtccgttgaaagggtaaaatatccacattgcaatatcagtgaatgttcctccaga[C/T]attggtttcacatgtacataagattcYgcgctttgtaaatttccttttttgactctttctttgtttgcattatcaRcaggtaggtctagccaataggcac

fava_c16363_1153	fava_c16363	1153	C/T	ttacttttgctcttccaggtccgttgaaagggtaaaatatccacattgcaatatcagtgaatgttcctccagaYattggtttcacatgtacataagattc[C/T]gcgctttgtaaatttccttttttgactctttctttgtttgcattatcaRcaggtaggtctagccaataggcaccatcaatgttaggatcctgaggaaggt

fava_c16363_1202	fava_c16363	1202	G/A	aatatcagtgaatgttcctccagaYattggtttcacatgtacataagattcYgcgctttgtaaatttccttttttgactctttctttgtttgcattatca[G/A]caggtaggtctagccaataggcaccatcaatgttaggatcctgaggaaggttagtaccattttgttctattggaacaggacttgactcttgacctttctt

fava_c16363_1410	fava_c16363	1410	T/C	aatgctccattggagaaaaaccaattgactgaagaaggtaagtattcttcatcagaatgtagatatagaaatggagagtaagcttgaagtattgcctcga[T/C]ttgtttctcattaggcatgatttttgaaatagaattgagattcttcaaacatgaaatagatggagggttagtattcccaccattttgtgtaatgaaggtt

fava_c16363_1630	fava_c16363	1630	G/A	cttgagttcctctattgattggcttaccatcatataagttgatgtcattggatccccatatccatgaagaagactcgcattggtttgtcaggtctgatct[G/A]acacacatgattttgtcgaggggaggcttgtctggtgttgtggtgacaacatggcctaatgctttgtagccatcaggtggtactggtagccaaatataca

fava_c16363_1814	fava_c16363	1814	G/A	tggtagccaaatatacaaagtggaatcttgagtagccttgatggatgcagtgtttgatacagttgtgtaatcaactggtggttttaaagtgctattagtg[G/A]tacttgaaaagacattcttWgcaacaagaacatatccaaaaagaggtttgttgttgggttggctgtagctacctaacatagagaatccttcaggtattcc

fava_c16363_1834	fava_c16363	1834	T/A	tggaatcttgagtagccttgatggatgcagtgtttgatacagttgtgtaatcaactggtggttttaaagtgctattagtgRtacttgaaaagacattctt[T/A]gcaacaagaacatatccaaaaagaggtttgttgttgggttggctgtagctacctaacatagagaatccttcaggtattccaattggttcgtatactgagg

fava_c16363_1979	fava_c16363	1979	C/A	gtagctacctaacatagagaatccttcaggtattccaattggttcgtatactgaggcgccttgattgtcaaggccaccatcgtaagcaccccaaactttg[C/A]gaaatgttgatgcttgagacacttgtagcccacctagatcaagaattccacctgcaaaaccattaccttctggccaagaggttggtgcagttggaagctt

fava_c16363_2158	fava_c16363	2158	T/A	aggttggtgcagttggaagcttgaatgtgtagtcgatggggcgagagcgggacgggggccgggatcgccttttcttgaagatattcttattctctaaaat[T/A]gaatccattggtgcagcattaggaacaacctctattgcttcaattgccatgaaaagcacaaaacaaacacaaagaatcctcacactcacacccattttta

fava_c16363_435	fava_c16363	435	A/C	agagaaaatgccaaggtcttgtactactagaatttgaagctattgctgctttcactaatttgattttcttttttcaaattaaattcaataacccattaca[A/C]aattgcaccaatagcattgttgtcaatcaatttttttgaactctaagaatcaatgtaactaaaagaatctcatacttcatcgccattccaattattcttt

fava_c16363_604	fava_c16363	604	C/A	ctcatacttcatcgccattccaattattctttgcctttggacctgttggtccttcctctgcaagcacttcttttggtaatcccctaataatactttcaaa[C/A]ccatcttttaacttccctggcaataacttctccacattcttcaactcatcatctatactataatcaacatgtggaccccaccctctaaaaaaattcaacc

fava_c16363_765	fava_c16363	765	C/T	taatcaacatgtggaccccaccctctaaaaaaattcaaccatgctggctctttaacagatcctaaataatctgcagaaaccaacacaaattttcccatat[C/T]cataacatttctacctttattagtatcatctcttgccccaacaccattttttcctagcagaacaagaccagcatgtggatatgttgcatggccatgcaaa

fava_c16364_1229	fava_c16364	1229	G/T	gaacacaatgagaaaaaggaaggggataatggaaagagagattagaaaattttgattgtttatgaggctattgtctcatgtcttgttgttgtaatgtatc[G/T]ttttgcttgataaatatttatatttacaggtgattaatgaatgttaactttttattaatgttaaatggaagccattacgtcgccatgatgtggtagattt

fava_c16364_558	fava_c16364	558	T/A	aagccaagcgtctatggacctacggcctctcattcgcgacgttcaccggtttcatcgtcgtcgttctaaacaacttccaggatcagttagtcttctacgt[T/A]actccaaccgacgcaatagagaaatttcgaactaaccctaccaaagccaagtttcgattaggcggtttggttctcgaaggaagcgtcgtctatccatctt

fava_c16368_315	fava_c16368	315	T/G	attctaatttcagttccaattccttaatggagagttccgatgaagaaaacgacgccgttatcggcaatcatgcacccaaggaactaactcggttatcatc[T/G]agtagtgcaaaattcgttgatgaagtacttaatggtccaaatcaacgctgtttggataatttccgaatggataaacaagtgttttacaaRttatgtgata

fava_c16368_405	fava_c16368	405	G/A	ggttatcatcKagtagtgcaaaattcgttgatgaagtacttaatggtccaaatcaacgctgtttggataatttccgaatggataaacaagtgttttacaa[G/A]ttatgtgatattctagaaaccaagggtttgttacgcgacaccaataggatcaaaattgaagagcaactagctatgtttatgttcattattggtcataatt

fava_c16368_649	fava_c16368	649	C/G	gacgattagtcggcattttaataatgttttgaatgcgattatgtccatttcgaaggagtattttcagccgccgagctcggatgttcattcagttatctct[C/G]aagattgtagattctttccttattttaaagattgtgtgggagcggttgatggtatatatgtacctgtgacagttggtgtagatgaacaagggcctttccg

fava_c16369_1872	fava_c16369	1872	G/A	gtggaaatgctcctatgaagaagttgggatttggtctggttggatctggaaaaagaacaaccgtcccttctgttttccatgaagatgaagatgatgatgc[G/A]cacaaggataaaaaattgaggcctttggttccgattgattactcaacagaggaattgcaggctgttgaacctactgcttctgggccaacaccacccaatt

fava_c16369_2640	fava_c16369	2640	C/A	ctaaatcatgaatggttggttgttgtattttcctcactttcccttttctgctggatttatgttcttacaaccatcactatgtatgcgtctgccttaaaat[C/A]taagatggggctgcattataatgtaccattttcagtctgtattgcttcaagattgaagtagctatctcgataagccccccattttctttacttatgagaa

fava_c16370_1188	fava_c16370	1188	A/G	gtaatctcattgctggtgattcccatgcaagttatgctgttcttgtacctggacttgaagttacagatactgccatacaagtcttgataaaatgtctgaa[A/G]agtgaacacagggtcttaaagaaggaagcagcttgggtSctgtctaatatagctgctggttctgttgagcacaagcaattgatatattctagtgaggcag

fava_c16370_1227	fava_c16370	1227	G/C	ttcttgtacctggacttgaagttacagatactgccatacaagtcttgataaaatgtctgaaRagtgaacacagggtcttaaagaaggaagcagcttgggt[G/C]ctgtctaatatagctgctggttctgttgagcacaagcaattgatatattctagtgaggcagtgtctttgctattgcaccttctttccgctgcaccgtttg

fava_c16370_1557	fava_c16370	1557	T/C	ttgatttggttagatctgctgatattgaagctgctaggctagggcttcagttcatagaactggtcctgagagggatgccaaatggaaagggctcaaagct[T/C]gtagaacaagaagatgggattgaagccatggaaagatttcagtttcacgaaaatgaagacttgagaactatggcaaacagtctagttgacaagtattttg

fava_c16370_231	fava_c16370	231	A/G	cttcaatggccgattctgtcttctctagcaacaaaagagatcctattaaatcatcagttgggagtgttgcgggtagtagaagacggcaacatgcagttac[A/G]attgggaaggaaaggagggaatcgttggtgcgtgcgaaacggctttgtagggttggagttagYgagggtgaagttgatgttgacatgatgcttgatcaag

fava_c16370_294	fava_c16370	294	T/C	gtgttgcgggtagtagaagacggcaacatgcagttacRattgggaaggaaaggagggaatcgttggtgcgtgcgaaacggctttgtagggttggagttag[T/C]gagggtgaagttgatgttgacatgatgcttgatcaagagcagtcttttttggagtctcaaactttgttggcWgttgacaatttgaaatctgctcttgctt

fava_c16370_366	fava_c16370	366	T/A	cgaaacggctttgtagggttggagttagYgagggtgaagttgatgttgacatgatgcttgatcaagagcagtcttttttggagtctcaaactttgttggc[T/A]gttgacaatttgaaatctgctcttgcttctcaggggaagggtgcagtgcagaaaagagtaggcgctcttcaagaattaaggcgtttactgtcaagatctg

fava_c16370_651	fava_c16370	651	C/T	agttgcttgaagctgcttggtgtcttacaaacattgctgcagggaatccagaagaaacaaaagctttgctgcctgcattgcctttgcttattgctcacct[C/T]ggggaaaagagctcctcacctgttgctgaacagtgtgcttgggcactaggaaatgtggctggtgaagatgaagagttgaggaatgttcttctaattcaag

fava_c16370_864	fava_c16370	864	C/T	ctcttgctagaatgatgcttccaaacaggcgttcaactgttagaaccgctgcctgggctttgtctaatctaatcaagggtctaaatcctaaagctgctaa[C/T]gagctcattcgcattgatggagtattggattcaattgttcgacacttgacgaaagcggatgatgaatcagcaactgaagtagcttgggtagttgtttacc

fava_c16372_1171	fava_c16372	1171	G/A	tcgcggtgtcattcttgaacgccacgatcacctgcgcccccgccattcctaccccggttgggtttattccccaggaaacccaaccggctggagttggtgg[G/A]gacgcgatgaaggcgaccgagagcgtggagttttgcgggtcgtgggtgtaatggaggaaggcattgagtacggggaggtcgaggcagttagggtaggtta

fava_c16372_136	fava_c16372	136	A/C	aacaatacctacaccagtatattattataacttaaaaaacgctatgaaaccgactattatacatataacaactaacaaaaaaaaaggtgggaaggatcta[A/C]aaatatccaaacagaacattgaaaaccagaatcttctatgagaagaacaaacctaaatctggtaaaatgattcattcatcaggattatgttcccttgttt

fava_c16372_628	fava_c16372	628	A/C	agagggtaattccaacattacgatgaatactgaaagtaatcccttctgattcacttccaagtttcatgccagttgcccaaccagcaactccaagaacata[A/C]gcacataattgacaactaacatgaaggtagaaccaagctggatcagctgatgggaaagttttcacatatcttgccatgattactccaaggggaaacaaaa

fava_c16373_1434	fava_c16373	1434	G/A	tcttggaatccctcgaatcaaaggaaaaccacaccttggttcagccggaggatttcgacttgacaaaccttgaatcgtctgtgcctcccagcgttgcaaa[G/A]tacatcaaacacctgcaaagtgagacacagaagcttggcgttgagaaggaaacaatgcggttcgagttgacaagcgctcaagccatgattaatattcttc

fava_c16373_1762	fava_c16373	1762	A/C	gatatttagttatttcttgtttttctttagtaagaactagttcgtttctttgcctaaacatgtttgttagtgacatgcatcgttttaaaaactcctcctc[A/C]tcatcatcatcatgtatgttattgtgactttcagtttccctttcattctgttgttaaacatgttcatcttagttctttgttcttaccctttctatgaatg

fava_c16373_278	fava_c16373	278	C/T	ccccagcacaacatgggtcacgaaactctcttgctcactgcccctcctactaccacactagtgaaccacccactctcagtctctgtaccatctgcttcag[C/T]aacagcaactgctgcaccacctacctcccttgctcctggatttaggtttcacccaactgatgaggaactcgttatttattacctcaagcgcaagtttgct

fava_c16373_657	fava_c16373	657	G/A	ggaaagctaccggaaatgatcgtccggttaaacatgacttaaggactgtaggattgaagaaaactttggtgtttcatagtggccgagctcctgatgggaa[G/A]aggactaattgggttatgcatgagtacagacttgttgaagaagagatggagagggatgggtctggaccctctcagccacaggatgcatatgtcttgtgta

fava_c16373_870	fava_c16373	870	T/C	aaaataacataggacctccaaatggacaacgatatgctccttttgttgaagaagagtgggaggaaggattgggaatggttcctggagctgagcctgtggg[T/C]ggtcctgtttatcaccctctgcgtattgaaggcaacaacggcgtttcatgcagcgaagaaagaagcattgttgtgaaggacactcattccaacaataaaa

fava_c16382_616	fava_c16382	616	C/T	gctcataaatgaaatgctaagagcatcctatgatcctggtgtgcctgtggtcctagaaagcatctttagctttaatctgtccatacctcttaactatctt[C/T]ttgaagatgtcaaggaaaaggttataattatacagggaatgaaagatccaatttctgactctaatgcgacggtggctatgcttaaagagcactgtgatag

fava_c16382_759	fava_c16382	759	C/T	aagatccaatttctgactctaatgcgacggtggctatgcttaaagagcactgtgatagtgttataatcaagaagatagatgccggccattgtccacatga[C/T]gaggtaccggaattagttaattctattatttRtgattggatacataaggtggaaagtaacattttagcagggttctcagtgtaaatgtttttgacatcca

fava_c16382_791	fava_c16382	791	A/G	gctatgcttaaagagcactgtgatagtgttataatcaagaagatagatgccggccattgtccacatgaYgaggtaccggaattagttaattctattattt[A/G]tgattggatacataaggtggaaagtaacattttagcagggttctcagtgtaaatgtttttgacatccaacaactgtgcataatgattatatcagcagggt

fava_c16384_1074	fava_c16384	1074	C/T	ctatcagaaagaaaacaccaaacgacgcttgggctattacattcgtcttcttcacgtgtgttcctcttcttcttcttcacggtttgcttgttttctataa[C/T]ccgtttgataatcaggttcctttgttgttgagacaaacWtcggtgcttcttgaaaacgacaccgttctctgtacttacccgtttttgaacagcctcgttt

fava_c16384_1113	fava_c16384	1113	T/A	cattcgtcttcttcacgtgtgttcctcttcttcttcttcacggtttgcttgttttctataaYccgtttgataatcaggttcctttgttgttgagacaaac[T/A]tcggtgcttcttgaaaacgacaccgttctctgtacttacccgtttttgaacagcctcgttttcgccgcgtttggtgctgcttattgtgcttggtttttat

fava_c16384_1620	fava_c16384	1620	T/G	cgccggaaagaaagatggagaagcgagactccgtcgtggatttagagggtgttttgttgcgagggtagtgtggtaattttttcagtttattgagaaggaa[T/G]ttttgggttttatctggtgcatgcacctgacaagcggattcatgtaaattcatttgtttatctacaaaacaagagaagaacaaaatttcaaaatttctgt

fava_c16384_396	fava_c16384	396	G/C	agagaaaaaaaaaacacaggaaagaatcaatccaaaatctcttatccccgctgtcccatccggcccaaaattaaacccaaaccaaacccatacgcctctt[G/C]aatctcatcacaacaataacattcatcttcttccatgcttaacctccttctctctccaccacctctcctaccaccaccaccaggatcaacactctctaac

fava_c16384_777	fava_c16384	777	T/C	tctccgttttatctctctgtttcattttccacctccgtttcaaatccaaatcactaccctatttacaaaacttcaattccctctggactgttcggtttct[T/C]ctcgttcttttcatttttctctggtccgtcgtcgagcttctccggttaccgttcttccgtagaagatatttatatcaatatataccttccttagcagctt

fava_c16385_333	fava_c16385	333	G/C	tcattcgattttcaccataaggataatctgtttgagctcttcttgcatttctgaaagcacaacaaccaatggaataaatccctatgagaagaacaagcat[G/C]gtgacaatgagaactgataatttatgccaattccttcttaagttttcgagtacaccagctttgcatgaatcacattcataacatagcactgttggtgcat

fava_c16389_250	fava_c16389	250	T/A	caatacagccctatgctaatagtaaaatgtgaacaaaatacattgatgaggatagtcatcatatattaatttaacataactgcctcatcagaaaaataac[T/A]gataccagaagacactgcatatagagtcaagaaaaatgtagttgtagagagccaaaatcatgactcacaaaataagccattacttagcagtcccgcaaat

fava_c16390_193	fava_c16390	193	A/T	catctgaggcacctgcgacagtgatacaccagcggacgctccccacacaccRccgtgatagcgccgccattaactacctggttgcagtctgttaaggttt[A/T]tctgcgRccctacaagtttctttactgatcacttcaatgcagaaaagtgcaggaggtattacacgccacttgcttggaggtttatactgttatctaagaa

fava_c16390_200	fava_c16390	200	G/A	ggcacctgcgacagtgatacaccagcggacgctccccacacaccRccgtgatagcgccgccattaactacctggttgcagtctgttaaggtttWtctgcg[G/A]ccctacaagtttctttactgatcacttcaatgcagaaaagtgcaggaggtattacacgccacttgcttggaggtttatactgttatctaagaaaatgcat

fava_c16390_604	fava_c16390	604	T/G	aaaggaagcctgaagaggttcaaacattcatggaattgatgcgagaaaagatcgaggagttgcttcaacaggaaagcattattaacgaatacggtgttcg[T/G]ttacatttcattggaaacatgcagctattaacggaacctgtcagagttgctgcggaaaaggcgatgagagttaccgcccacaaccaagagagagttcttt

fava_c16390_947	fava_c16390	947	G/T	aagtaatggtatcgatttgcattttcgtgattcttgtaaagaaaaagaaacaaaagcttgtagaagtctgcatgaaggagcagaaggcgcagaagagaaa[G/T]attacttattcgagcaaaatttagaaaaacatagtgataattacagtgaagatgaaatcacgccttgcaatgaagagaaaaagttcaagcaaggtgagtt

fava_c16394_1093	fava_c16394	1093	A/G	atacggaggaatgcaagattggaactatatacacgctggctgtttcgaattgacgttggaaattagtgacaataagtggcctgttgctgcagagcttcct[A/G]tYatttggaaatacaacaaaatgagtatgcttaatcttgttgcaagccttgtcaagacgggagtgcatggaaggatatattcttcaggagatggaaggcc

fava_c16394_1095	fava_c16394	1095	C/T	acggaggaatgcaagattggaactatatacacgctggctgtttcgaattgacgttggaaattagtgacaataagtggcctgttgctgcagagcttcctRt[C/T]atttggaaatacaacaaaatgagtatgcttaatcttgttgcaagccttgtcaagacgggagtgcatggaaggatatattcttcaggagatggaaggccat

fava_c16394_1207	fava_c16394	1207	A/C	caacaaaatgagtatgcttaatcttgttgcaagccttgtcaagacgggagtgcatggaaggatatattcttcaggagatggaaggccattgccagcctta[A/C]ttaccgtcRgcggaataaattacacagttagggccggaagaacttttgYtggctaccatcgSttacttgccccgagagataaatatgaagtagtggcaac

fava_c16394_1216	fava_c16394	1216	G/A	gagtatgcttaatcttgttgcaagccttgtcaagacgggagtgcatggaaggatatattcttcaggagatggaaggccattgccagccttaMttaccgtc[G/A]gcggaataaattacacagttagggccggaagaacttttgYtggctaccatcgSttacttgccccgagagataaatatgaagtagtggcaaccatgcctgg

fava_c16394_1256	fava_c16394	1256	T/C	gtgcatggaaggatatattcttcaggagatggaaggccattgccagccttaMttaccgtcRgcggaataaattacacagttagggccggaagaacttttg[T/C]tggctaccatcgSttacttgccccgagagataaatatgaagtagtggcaaccatgcctggttacaagtcaaggaatacaacaatttggttagatgaagga

fava_c16394_1269	fava_c16394	1269	G/C	tatattcttcaggagatggaaggccattgccagccttaMttaccgtcRgcggaataaattacacagttagggccggaagaacttttgYtggctaccatcg[G/C]ttacttgccccgagagataaatatgaagtagtggcaaccatgcctggttacaagtcaaggaatacaacaatttggttagatgaaggagctatgacattgg

fava_c16394_589	fava_c16394	589	T/C	tattggaaatgtgcatggagacgaacccgtgggccgtgagcttattatgtttttggccaattggttatgcgataaccatttaaaagatcctctggccaca[T/C]tgattgtggagaatgttcaccttcatttacttccatcaatgaatcctgatggcttcaccttaaggaagcgtggcaatgcaaataatattgatctgaatcg

fava_c16394_750	fava_c16394	750	G/A	taaggaagcgtggcaatgcaaataatattgatctgaatcgggattttcctgaccagttcatctttgtaaacaatgatgaggattcccggcaaccagaaac[G/A]aaagcaataatgaattggtcaagagatatacgatttacagcatctgcaacattgcacgggggtgcacttgttgcaaattatccttgggatggtacYgatg

fava_c16394_846	fava_c16394	846	T/C	aaacRaaagcaataatgaattggtcaagagatatacgatttacagcatctgcaacattgcacgggggtgcacttgttgcaaattatccttgggatggtac[T/C]gatgataaaaggacgttctactatggatgtccYgacgatgaagcatttcgattcatggcaagtatatatagccactcccactacaacatgtcttcaagca

fava_c16394_879	fava_c16394	879	C/T	tacgatttacagcatctgcaacattgcacgggggtgcacttgttgcaaattatccttgggatggtacYgatgataaaaggacgttctactatggatgtcc[C/T]gacgatgaagcatttcgattcatggcaagtatatatagccactcccactacaacatgtcttcaagcaaggaatttgtagatggaatcacaaatggagcag

fava_c16399_1240	fava_c16399	1240	A/C	aaaatgatgaaggtggatgggagatggaagatttagagctgggccctgaagccgacacccctaaagcttctattggcacacagtcttcagtttttgtcgc[A/C]ccaccacctggcatgccagtaagccatatatgggtgcagaaatcatcacttgcggctgagcatgcagctgctggaaatttcgatactgcaatgaggttgc

fava_c16399_1555	fava_c16399	1555	T/C	ttgcagttgaaagaggttggaccgactcatctagtacaaatgtgagaggccccccagctcttcctttcaaattatcccagctggatgagaagcttagagc[T/C]ggttataagttttttactgctggcaaattcaccgaggctcttaagacatttgttaatattcttcatacaattcctttgattgtggtggagtcaaggaggg

fava_c16399_2179	fava_c16399	2179	A/G	cccgttttgtgccaacccaggaggggcagctttgtgctgtttgtgatatttcagtgataggggctgatgcttctggactgctatgttcaccttcccaggt[A/G]cgctgatcgagctataagaccaggagcattttataaaattttgtttcaggtcaattttcagcactagatagatccctattgttcagtttatattttcatg

fava_c16399_2401	fava_c16399	2401	T/C	atagttaagaggaattacttttttgctcttcagcttactcttttgttcagtttaaagtgattttcaatgtaataaatccatttcgtatcttgagggttgt[T/C]agtttttcatacacaagaaagtgaaaggttgtacctggatcaaatttatggttataggctttatgcttatttttgtttggcaaagtacatatcacttgta

fava_c16403_1925	fava_c16403	1925	C/T	tatataggtagtagtttgcaaccctcacaccagcattacatgtgagtttttaacctccaagcattactgaaaagactctagacatgctagcgttcaaatt[C/T]aaacattactgattatctactagtttttcaaaatgttcccttttctttttgcttgagtttcttatgatgtcagaaagcagatgtactcttatatatttat

fava_c16403_267	fava_c16403	267	G/C	aatttctccagcacctgcttgtttcgtgtagcaacgatttccttgcatcagaacccaggaatatggatgacatggagcaagtcaatgaaaataacagaga[G/C]tctatgatagagcagcttgctcaggcgtttcttgaattggaaactcagaagggatcttctgaggataaggttcaatgggttgaaattaagcaacattttt

fava_c16403_693	fava_c16403	693	G/A	tgtatgatggggagaacaaagataataaggtaagtagttcccccgagaactctccgtataagtctggtgaaaagtctgaaggtgtggctaatgaggttgc[G/A]ccattactcgacataaaaatattttgtgagaaaatggatggggatggtcttctggattatgtcgtggcgcataagaaaaaagtgtctgtttttcgcgaag

fava_c16406_1317	fava_c16406	1317	C/T	acccaatatattcataacttgtcctttgtataatgtcatcaccatagccccaccaactgtgattacagttccaatcacttttacttggcatcttaatttt[C/T]ttatgtccaatttctccatcctgaaaataacagccattacaaaagtcattgcagggagaatattgctaatagcacaagagtatgttggagatgtgaaYtt

fava_c16406_1415	fava_c16406	1415	T/C	ttYttatgtccaatttctccatcctgaaaataacagccattacaaaagtcattgcagggagaatattgctaatagcacaagagtatgttggagatgtgaa[T/C]ttcaacccagcatagtatagattctgatcaatcacaggcccgaggagtcccaatacaaacatttgcatgaacataagaaacgtaatcctYggccttatct

fava_c16406_1505	fava_c16406	1505	T/C	gagatgtgaaYttcaacccagcatagtatagattctgatcaatcacaggcccgaggagtcccaatacaaacatttgcatgaacataagaaacgtaatcct[T/C]ggccttatcttcctctcaagaactatagcgaatggagcaatagctgctgtagcaaaagcatgtctataaacaacaagaacatagtgactcattcctcggt

fava_c16406_1712	fava_c16406	1712	T/G	atacttttgttattatattcataccagcaaaaccaaattgaagacagatcatagctatgtatggcttgcacctttgaaacaaacttgaacaacatttatc[T/G]ccttccattactagtactacgttattgtttgtattgtatacagaggaagagaagaacacttgaatgtgtttgtgttgtgtattttcttcttgagtccccn

fava_c16406_251	fava_c16406	251	G/A	tactaaatcaagagagcaatacaagtatttataattaggtaactatattgcaactaaaatgtgaaaaggattgagaactaataactaaataacagtattt[G/A]YgcattatgtctctacacaactatcgactgacacagRaaaagaaatgttaactaactctaccttaacctaagaactcagatWattatctgcacatgctgt

fava_c16406_252	fava_c16406	252	C/T	actaaatcaagagagcaatacaagtatttataattaggtaactatattgcaactaaaatgtgaaaaggattgagaactaataactaaataacagtatttR[C/T]gcattatgtctctacacaactatcgactgacacagRaaaagaaatgttaactaactctaccttaacctaagaactcagatWattatctgcacatgctgtc

fava_c16406_288	fava_c16406	288	A/G	ggtaactatattgcaactaaaatgtgaaaaggattgagaactaataactaaataacagtatttRYgcattatgtctctacacaactatcgactgacacag[A/G]aaaagaaatgttaactaactctaccttaacctaagaactcagatWattatctgcacatgctgtccacactaagtggtctttcttaatcagccaccaaaaa

fava_c16406_333	fava_c16406	333	A/T	aactaaataacagtatttRYgcattatgtctctacacaactatcgactgacacagRaaaagaaatgttaactaactctaccttaacctaagaactcagat[A/T]attatctgcacatgctgtccacactaagtggtctttcttaatcagccaccaaaaatgctttagctaagttttcccaaaaaagaaagtaaagtgataatga

fava_c16406_525	fava_c16406	525	C/T	tgataatgatgaggatgcacatcacaaaaacctttcccaatttcttaactctacgtgacccttttttcattgcacttttttgttattgttattcttctct[C/T]aatagYatctacacgtttaaacttttggagcactaataattgtcacccttaaaactttagcaggttcatccttttgcatctcaatgtcatttatttcaac

fava_c16406_531	fava_c16406	531	C/T	tgatgaggatgcacatcacaaaaacctttcccaatttcttaactctacgtgacccttttttcattgcacttttttgttattgttattcttctctYaatag[C/T]atctacacgtttaaacttttggagcactaataattgtcacccttaaaactttagcaggttcatccttttgcatctcaatgtcatttatttcaacaccttc

fava_c16406_632	fava_c16406	632	A/G	atctacacgtttaaacttttggagcactaataattgtcacccttaaaactttagcaggttcatccttttgcatctcaatgtcatttatttcaacaccttc[A/G]gttactgattccaacactccattttttacacaacactcYgttctttctgtttttgtttccgcttcaatctctttgttttctttgtttttgccccatagaY

fava_c16406_671	fava_c16406	671	C/T	cccttaaaactttagcaggttcatccttttgcatctcaatgtcatttatttcaacaccttcRgttactgattccaacactccattttttacacaacactc[C/T]gttctttctgtttttgtttccgcttcaatctctttgttttctttgtttttgccccatagaacggagtaaagtcctattacgatcaaaattgctccaataa

fava_c16412_1018	fava_c16412	1018	C/T	atcccccgaccaagctaaaatattttgtgaatcaatatttacttgtttaacagaagaaagtccacccccgcatattttcggaataatgtcaccggtaaat[C/T]taaaggtacacagatttggagtagaaagtttgatttggataatggcatacgaattactatgcaaagctaaattcacaagtgtctcatttgaaatgctgaa

fava_c16412_1466	fava_c16412	1466	T/A	attaaagaaatatcaccacgaagagagattcctaattcccggaggtgagtattatgggagcaaacatagtttacaatcattttaaggagttgaggctcaa[T/A]aataccacgatgttcaagatcaaggtcgaaagcgtgaagttcagtcgagctatcacgaagagtaaaaatgttggacacaaatgtgtcaaatttctttaaa

fava_c16412_1628	fava_c16412	1628	T/G	gtaaaaatgttggacacaaatgtgtcaaatttctttaaagtgttaaatcttgaagaatgcaagataagtgttggaatagatttccagagatgtttccatc[T/G]ggtggataaaatgcaagtttgaacaacatgtttRgtgttcaagaatgataaaatgtgaatgataatacagtcgggtaagtcactcagcctgtcttcattt

fava_c16412_1662	fava_c16412	1662	G/A	ttaaagtgttaaatcttgaagaatgcaagataagtgttggaatagatttccagagatgtttccatcKggtggataaaatgcaagtttgaacaacatgttt[G/A]gtgttcaagaatgataaaatgtgaatgataatacagtcgggtaagtcactcagcctgtcttcattttcatgcgtttcttcaatttcattctcactatgcc

fava_c16412_1888	fava_c16412	1888	T/C	cctggtttcttcaagataatctcgccgccgtcgagttgtgacttctccgatggttttgaacgccggaaaatctgattgaaacgtgtagattcaagctttc[T/C]agggttctttattttaattttgtgcgaaggaataaaaaatttgacgttggatgaaaaactggtgccaagtgctatttcatggtggtgcgaactgagaact

fava_c16412_303	fava_c16412	303	C/T	cggccaaactgatattttcagtcacttatttgatatttttttaaaaaggttcatagagaagaccttgtaacttgtaggcaatatgagaactcttcacagc[C/T]cattattaaaaagtggagagttggtgttaaggttggttctccgccatatctacacttgatgatttattacccttttcctgaagattggaMgatgcagcag

fava_c16412_393	fava_c16412	393	A/C	tcttcacagcYcattattaaaaagtggagagttggtgttaaggttggttctccgccatatctacacttgatgatttattacccttttcctgaagattgga[A/C]gatgcagcagtggcaggctcggcggcagaagcaggagcagcatcagaagcagacttggcagccgaagcaggtgtgacagaggaggaacRcacagagaatc

fava_c16412_482	fava_c16412	482	G/A	tgaagattggaMgatgcagcagtggcaggctcggcggcagaagcaggagcagcatcagaagcagacttggcagccgaagcaggtgtgacagaggaggaac[G/A]cacagagaatcgcaaacgataattgataatcttYgcaccctttaaagattcttcaacctgcttcagattaaaagaactcgggtaatttgttgtgatgtta

fava_c16412_516	fava_c16412	516	C/T	cggcagaagcaggagcagcatcagaagcagacttggcagccgaagcaggtgtgacagaggaggaacRcacagagaatcgcaaacgataattgataatctt[C/T]gcaccctttaaagattcttcaacctgcttcagattaaaagaactcgggtaatttgttgtgatgttaactacagccaacggcgagttttgtcgcaagaagt

fava_c16412_737	fava_c16412	737	G/A	gcaggtggttccgaacgtgctttaaatgcctttcttaagttaacaacttctttacttgacttggcagcagctttctttaacatttcttctttcattaata[G/A]tcttaaaagtccatcatcaagtggtatcagttctacttccaatgacttcaagttacacaaagaagggagcttaaccttcaatagatcaggaattaaggag

fava_c16413_1024	fava_c16413	1024	A/G	gataatcaataacgatgtcaccaaaagtttcagcaggattgattagctccaaccaggtataagatcgtgtgagctcatatgttgcaaggccgccattttc[A/G]ttctgtaatgagagaatgacatttacagcatcatataatctctttgcRtccaatggttcaccaacaacctcaggtgcaattttagatagcaataggacag

fava_c16413_1072	fava_c16413	1072	A/G	ccaaccaggtataagatcgtgtgagctcatatgttgcaaggccgccattttcRttctgtaatgagagaatgacatttacagcatcatataatctctttgc[A/G]tccaatggttcaccaacaacctcaggtgcaattttagatagcaataggacagctttcagtccttctgctgtgcagtcagatattggccatccatggtctg

fava_c16413_1379	fava_c16413	1379	C/T	tttgagagttggaccaaattcgtcaattaggctagttgaaataattgcttgtgcagcaaatgcagtatcccatagttgacttccattgtagccctgcatt[C/T]tcatgccatcttcagcaacccacagataatcattgatcctgggaagatgcaacttgaaagcctctgaatttggatcttccacccagcagcaaagcatatt

fava_c16413_1628	fava_c16413	1628	G/C	ttcataatgtatatgctcaattacagtcttgatagccttttctctcaactttttgccaggccaattcatgaaaataggctcaacaaacttgtgtagagtt[G/C]cccaaagaatatcttgcacgagcggatgaggataatataagtcttcctttgcacacaaattgcgagcctgattccagtctatatcatggtatggaacggt

fava_c16413_187	fava_c16413	187	T/C	taagaatccgattttacattcacacactaaacatattatgattatgcactattttcttagagatcgtgctgaaaaaaatggtatgaaaaaacacaattta[T/C]gagtatataaRttcaatttagttgctctMtgtttagcaggatgccaaactcataaRctataaatcttttcaagaataaaaccgtggatgatgataaaagg

fava_c16413_1978	fava_c16413	1978	A/C	tttcaggaggtaggggattatttccagaccactcaaacactccaagtactgatagccacattttcccccaggatgttatatgagtagcaccaccatgctc[A/C]agaatccagtcacgtcccttttccatatccccttctccatcattaggtccctcccctagcaatcttagagtaacataacacaagacagagccaaacattg

fava_c16413_198	fava_c16413	198	G/A	ttttacattcacacactaaacatattatgattatgcactattttcttagagatcgtgctgaaaaaaatggtatgaaaaaacacaatttaYgagtatataa[G/A]ttcaatttagttgctctMtgtttagcaggatgccaaactcataaRctataaatcttttcaagaataaaaccgtggatgatgataaaaggtcgaaataact

fava_c16413_216	fava_c16413	216	C/A	aacatattatgattatgcactattttcttagagatcgtgctgaaaaaaatggtatgaaaaaacacaatttaYgagtatataaRttcaatttagttgctct[C/A]tgtttagcaggatgccaaactcataaRctataaatcttttcaagaataaaaccgtggatgatgataaaaggtcgaaataacttgaatagtgaccaaaata

fava_c16413_2389	fava_c16413	2389	T/G	aattcaatgcccttcttaacgttgttgtaacagcctcttccgtcacatcctcgatatctttgaccttgactttaggtaaaacttcgttcatagggttctc[T/G]tttgcaaactgaaggcgcataagtagatcatcactgtgtttgtgagtgaagcgattatcgtggaaattgcggcgagcggtttcaacRtcgtggagatctt

fava_c16413_243	fava_c16413	243	G/A	ttagagatcgtgctgaaaaaaatggtatgaaaaaacacaatttaYgagtatataaRttcaatttagttgctctMtgtttagcaggatgccaaactcataa[G/A]ctataaatcttttcaagaataaaaccgtggatgatgataaaaggtcgaaataacttgaatagtgaccaaaataaagcgatactagcaagcctgcaataca

fava_c16413_2476	fava_c16413	2476	A/G	tcatagggttctcKtttgcaaactgaaggcgcataagtagatcatcactgtgtttgtgagtgaagcgattatcgtggaaattgcggcgagcggtttcaac[A/G]tcgtggagatcttgcggcgatccggaatgaggatcgaactcccatacctgtcttccgacgtgattgttcagtgtccgaagccatggactacctccttccg

fava_c16413_901	fava_c16413	901	G/A	ttttttcaatataggcaacagctttttctatacagcgttgtatttcttctcgacgatgcccgggatatagtttcccaaatgttgctaaagcttgaattgc[G/A]gccgaggtacattccacataaggataatcaataacgatgtcaccaaaagtttcagcaggattgattagctccaaccaggtataagatcgtgtgagctcat

fava_c16415_215	fava_c16415	215	T/C	ggaggttgttcaaggctttgatccttgatgcaccaaaccttattcccaagcttatgcccacgattcagaatattcaactcgttgaaggaaatggtggccc[T/C]ggaagcattcaagaaataaccattgttgaaggtacYggcatcaaacatgttaagcatagaattgatgcaatagacaatgagaatttgacatataattatt

fava_c16415_251	fava_c16415	251	C/T	accttattcccaagcttatgcccacgattcagaatattcaactcgttgaaggaaatggtggcccYggaagcattcaagaaataaccattgttgaaggtac[C/T]ggcatcaaacatgttaagcatagaattgatgcaatagacaatgagaatttgacatataattattcagtgatcgacggggacggtaagccagaaattcttg

fava_c16415_562	fava_c16415	562	G/A	ttgaaggctgttagggaggaggcattggttgttctcaaagtagttgatgcatatcttgttgcaaatccagaagcctatgcttgaagtgatcttatgagat[G/A]gctttgcataattacattatattattatgatgtattttaagtttaataWgctatagtttattttaagccaaatatgatatggctaaKaaataagacataa

fava_c16415_611	fava_c16415	611	A/T	catatcttgttgcaaatccagaagcctatgcttgaagtgatcttatgagatRgctttgcataattacattatattattatgatgtattttaagtttaata[A/T]gctatagtttattttaagccaaatatgatatggctaaKaaataagacataattatgtgtttgtgttattggagtttggtagtaaaggtacaataagattt

fava_c16415_649	fava_c16415	649	G/T	gatcttatgagatRgctttgcataattacattatattattatgatgtattttaagtttaataWgctatagtttattttaagccaaatatgatatggctaa[G/T]aaataagacataattatgtgtttgtgttattggagtttggtagtaaaggtacaataagatttattggtctaattttgtttgtttgaaataaagtgattta

fava_c16416_348	fava_c16416	348	T/C	tttgtcaataatcactgtgtttctttctcaaactcctagcaaataacaagaatataactagaaaagccactccagcaattcctcctagtattatagcagc[T/C]gttttccctggattttgccctgaagaaattgaagagtatgaagattgagatgatctagatggaactccattaggataatagctaaaactaacaaaacact

fava_c16416_612	fava_c16416	612	G/A	ttttcacacactgtccacaatcagagtcaccaacatcaccttcacattgtcccaaaacataaaaagccatatagctagttgtataaaaaccatgaccact[G/A]gccacaccattttccatcacagaaaaagcagtgtctctcctctctccaaacccacttccagcaacattagttgcaccacaagtcttgtacagcatctgca

fava_c16418_226	fava_c16418	226	G/C	gaaaagtcctcatcagtgtgagaatacaaaagttttgaatgaacttgcataagtgtgacaataccaaattttatattcataaattgcaatatttattgag[G/C]tYcaaatcaataaaaggccaaaaatgaaaacttctcatcagctagtaSagtagtagcttaacaacaatttatcctcatgttagaaaattgactagacaat

fava_c16418_228	fava_c16418	228	C/T	aaagtcctcatcagtgtgagaatacaaaagttttgaatgaacttgcataagtgtgacaataccaaattttatattcataaattgcaatatttattgagSt[C/T]caaatcaataaaaggccaaaaatgaaaacttctcatcagctagtaSagtagtagcttaacaacaatttatcctcatgttagaaaattgactagacaatcc

fava_c16418_274	fava_c16418	274	C/G	ataagtgtgacaataccaaattttatattcataaattgcaatatttattgagStYcaaatcaataaaaggccaaaaatgaaaacttctcatcagctagta[C/G]agtagtagcttaacaacaatttatcctcatgttagaaaattgactagacaatccagattcaccacccaaatcacttttctttaccctcttactatttcca

fava_c16418_783	fava_c16418	783	A/C	attgttctcatcttcaatcatgacttcaatatattcgtattcgccagcaggacatcccaaggaagtggaccaagaagtttgatagagagaagcattaaaa[A/C]aatcctttctcattcttttaacaatccatctactaagaactgttgttttctcagaaccatgtcttcctctcataagcttcatcacatacttcataacctt

fava_c16418_944	fava_c16418	944	G/A	gtcttcctctcataagcttcatcacatacttcataaccttagcttcttctaaacctgtatcactcctcaaaacgtcctgcaaaacaaaaagctgtgttcg[G/A]ttgtgtaaagggtggtggtgattagaggaagtgatgaaacttgtaggtgagttgggtgaatgatcagactctataaagtcatgtaccatttgtaccaact

fava_c16419_261	fava_c16419	261	T/C	atagcactctatacagaaacaagatatgacatatcaacctatggagtatggaaggagaagaaaggatggtcctgaaattgatcatgagtgtgaaatgagg[T/C]tactatcttctactacacccggaggagactccggcggtacggccgattcttgaaatacagaagattcaagatccagaatcctccttgcagttgcttgaga

fava_c16420_1191	fava_c16420	1191	A/G	acgctggagtagtttctacaattcgtgctgctgcttcacattttgaggaattaggatgctctgttaatgaggtgtcgctgccatccttttcgcttgggtt[A/G]ccagcttactatatcctcgctttgtctgaatcatcttccaacttgtctcgctatgatggtattagatatggaaaccaagtttatgctgatgagctagact

fava_c16420_1557	fava_c16420	1557	A/T	atgatatcttgatttcccctgcagctccttcagctgcttataaaattggtgaaaagaaaaatgatcctttggctatgtatgctggagacatcatgactgt[A/T]aatgtcaacctagctggactgcctgccttggttttaccttgtggatttattgagaatggaactgcaggccttccagttggcctgcaaatgattggtgccg

fava_c16420_2024	fava_c16420	2024	G/A	atgtaaattgagctggcttgaattttccactctttgaaattgagaatgttcacaataagtcagtaacctcttctagttgctttttgttttgctatttttc[G/A]attgccgcaagccattggctagcatatgtaacaatagttaatttgttttatttagtgaaaatcagctagattgatcaagtgtaaatgagcaattggtagt

fava_c16420_870	fava_c16420	870	T/C	gtgatactggtggaagtgtgaggcagccagcatctttttgcggcgttgtaggtttgaagccaacatatgggcgtgtctcaagatttggacttatggcgta[T/C]gcatcatcgcttgacgccattggctgctttggttcatcggtcgctgatactgggattctccttcatgcaattgctggtcatgatagatttgatgccacct

fava_c16426_424	fava_c16426	424	A/G	aaaaaggaaagagtcatggtatctgactttgattgcaacatgatgcttgccaatgacttaattatagggtgtgaaacagacaaaatgatttgtgggtttc[A/G]taagtcttctgtgagcctttgatggggtccatggattgtgcccctaggtctgtggaggtggattagtttcaggcttgactatactagaactgtccggtgg

fava_c16426_609	fava_c16426	609	G/A	tagaactgtccggtggctgtgactggtatatcgcagatgccagtgagatgggcatgatgcaaagcgcctttgattgaagcaattgcatggcagctccaac[G/A]ttttcttccataagcttagcgacctgtttttctgtgccatcatttgaccacttgtcccaagccggccgatttcttccaccctcactgccatcttcctcga

fava_c16427_1018	fava_c16427	1018	T/C	aataagggagctcttagggacctatcaagRgccgcaaccttgccagcacacaattctcYgttaaacaagaatgagagtcaaaatttggagagcaatcaga[T/C]agtgcttagagatgctgctgaatcatcatgacagacttatattttgatttcaatggagggcagttccatRaaacaagggacatagtagagagtgttttcc

fava_c16427_1088	fava_c16427	1088	G/A	atgagagtcaaaatttggagagcaatcagaYagtgcttagagatgctgctgaatcatcatgacagacttatattttgatttcaatggagggcagttccat[G/A]aaacaagggacatagtagagagtgttttccctttgatgagaaattatagRYggtctagttagcgctgtttaaatgagcattatgtatttgatttcataat

fava_c16427_1138	fava_c16427	1138	A/G	gaatcatcatgacagacttatattttgatttcaatggagggcagttccatRaaacaagggacatagtagagagtgttttccctttgatgagaaattatag[A/G]Yggtctagttagcgctgtttaaatgagcattatgtatttgatttcataatatcatacggctagttcctttgcagattttgattgagtagccaaacatgac

fava_c16427_1139	fava_c16427	1139	C/T	aatcatcatgacagacttatattttgatttcaatggagggcagttccatRaaacaagggacatagtagagagtgttttccctttgatgagaaattatagR[C/T]ggtctagttagcgctgtttaaatgagcattatgtatttgatttcataatatcatacggctagttcctttgcagattttgattgagtagccaaacatgacc

fava_c16427_565	fava_c16427	565	T/A	ccagaaaaggttcaatctgtacttctgttgctcggaggccgtgaactacctccaaccgtaccttccatgccaatttctcctgacgaaaataaccggggat[T/A]tactggcactccacaaaaattcagcgtctctcaaagattagcttctttgattagatttcgtgaaaagcggaaggagcgaaattttgacaaaaaaattcgt

fava_c16427_866	fava_c16427	866	T/C	caaatgaagggttgatggtagacaataatggatcccaacagcaagatactgtttgtaggcactgtggcatcagtgagaagagcacgccaatgatgcgacg[T/C]ggacctgaagggccaagaaccctctgcaatgcttgtggacttatgtgggcaaataagggagctcttagggacctatcaagRgccgcaaccttgccagcac

fava_c16427_947	fava_c16427	947	G/A	gcacgccaatgatgcgacgYggacctgaagggccaagaaccctctgcaatgcttgtggacttatgtgggcaaataagggagctcttagggacctatcaag[G/A]gccgcaaccttgccagcacacaattctcYgttaaacaagaatgagagtcaaaatttggagagcaatcagaYagtgcttagagatgctgctgaatcatcat

fava_c16427_976	fava_c16427	976	C/T	gggccaagaaccctctgcaatgcttgtggacttatgtgggcaaataagggagctcttagggacctatcaagRgccgcaaccttgccagcacacaattctc[C/T]gttaaacaagaatgagagtcaaaatttggagagcaatcagaYagtgcttagagatgctgctgaatcatcatgacagacttatattttgatttcaatggag

fava_c16430_235	fava_c16430	235	A/G	cagaaaacacccaagctcaaacacttctcataaaagaccaactgtagacagatacatgaaaagaaggttattctattcatgatcaagcagcatgtacccc[A/G]tgaagaagtctttgatcaaagtgccaaacaccatgaccctttccaacccttctcaattgagccacatactctgatatcttcttaattgcttcaacctgtt

fava_c16430_481	fava_c16430	481	C/T	tgttgcgttctgccacactatgcacattcagaagtttctcatttgttaacttctccaaagacaaagctaattccattgcatgcaatgcatctcctttttc[C/T]acatgctcaaattctgaaggcacatccttgatggggtgaagcacaactcgtccaccacgagtgttctgatatttcataagcttttcagcatgttctctct

fava_c16430_64	fava_c16430	64	T/A	ttttttttgaacatgaacatgatagcctcttttttattgaagacaaatattacagagcgtgtt[T/A]aacagattttactccacaRttacattactctattctacaaacttagtacataggaaaaactaagctacaacagaaaacacccaagctcaaacacttctca

fava_c16430_83	fava_c16430	83	A/G	ttttttttgaacatgaacatgatagcctcttttttattgaagacaaatattacagagcgtgttWaacagattttactccaca[A/G]ttacattactctattctacaaacttagtacataggaaaaactaagctacaacagaaaacacccaagctcaaacacttctcataaaagaccaactgtagac

fava_c16431_131	fava_c16431	131	A/C	agaaaattttcaaatttcttagttctgttacgcattccatcgagcacttgataggcgcttgttcttgtcgtgttcgattctactgacagtgtaagctttt[A/C]agattccgaataacaaacgatgaacagttcatcgccggctcattcatcgatttccaccacggcggtcgtcggcggcggtgtcggaggtattagcaacgcc

fava_c16431_433	fava_c16431	433	C/T	acttgacaaggaggttaagtccttggatgaagataactggaagtttgaaggacctcgttctcggattcatcgtgcatcacgtcgaggtggtggtcatctc[C/T]ataggcccatggaaatttcaaagaattggggtttaaacccaccRaaatagttagactctgattacctcgaagcaccaggcctctgtttaattatctagYR

fava_c16431_477	fava_c16431	477	A/G	ttgaaggacctcgttctcggattcatcgtgcatcacgtcgaggtggtggtcatctcYataggcccatggaaatttcaaagaattggggtttaaacccacc[A/G]aaatagttagactctgattacctcgaagcaccaggcctctgtttaattatctagYRatgacggttatgttctacagataagagttattaggctgcattga

fava_c16431_532	fava_c16431	532	C/T	cYataggcccatggaaatttcaaagaattggggtttaaacccaccRaaatagttagactctgattacctcgaagcaccaggcctctgtttaattatctag[C/T]Ratgacggttatgttctacagataagagttattaggctgcattgatagaaaccattgcagttatgctacaacttctggtgatatattgttctggaagaac

fava_c16431_533	fava_c16431	533	A/G	YataggcccatggaaatttcaaagaattggggtttaaacccaccRaaatagttagactctgattacctcgaagcaccaggcctctgtttaattatctagY[A/G]atgacggttatgttctacagataagagttattaggctgcattgatagaaaccattgcagttatgctacaacttctggtgatatattgttctggaagaaca

fava_c16431_767	fava_c16431	767	G/A	ggtgtcctttactgtttgctgtatctttgcttcagttttgagagatcagtgattagttacacttgattgtttcaaaatttatgtattttaatgtagcgag[G/A]atcaKtaaaaacccgagataagttagagtgtttgctggtttgtatttttatatttaatctgtttatgtttggacaggatattaacctgtttataaacatt

fava_c16431_772	fava_c16431	772	T/G	cctttactgtttgctgtatctttgcttcagttttgagagatcagtgattagttacacttgattgtttcaaaatttatgtattttaatgtagcgagRatca[T/G]taaaaacccgagataagttagagtgtttgctggtttgtatttttatatttaatctgtttatgtttggacaggatattaacctgtttataaacatttgagt

fava_c16433_1279	fava_c16433	1279	C/T	atatatcatctgaaaataattctattgatggtagtaaaaataatgcatctgctgccaagttccaagatgaatcactcaatcggccagactttgccactga[C/T]tacagggatgccaaattttttgtcatcaaatcttatagcgaagataacgttcataaaagcatYaaatatggtgtctgggcaagtacaccaaatggaaaca

fava_c16433_1342	fava_c16433	1342	C/T	aagatgaatcactcaatcggccagactttgccactgaYtacagggatgccaaattttttgtcatcaaatcttatagcgaagataacgttcataaaagcat[C/T]aaatatggtgtctgggcaagtacaccaaatggaaacagaaagttagatgctgcatattgccaagcaaaggagaaacaagatgcctgtccggtttttctct

fava_c16433_1695	fava_c16433	1695	T/C	cagtttcgtcacattgttcttgaaaataatgataacaagcctgttaccaacagtcgagatactcaggaggtgaaattgcaacaggggattgaaatgttga[T/C]cattttcaaaaaccatgaaactgatacatccatccttgatgattttaatttctaYgaagaacggcagaaggcaatgcaagaacgRaaggcaaggcagcaa

fava_c16433_1750	fava_c16433	1750	C/T	gagatactcaggaggtgaaattgcaacaggggattgaaatgttgaYcattttcaaaaaccatgaaactgatacatccatccttgatgattttaatttcta[C/T]gaagaacggcagaaggcaatgcaagaacgRaaggcaaggcagcaaagcagcgtgatgagtactggattagttggggggagtgagcataggagttcttcgg

fava_c16433_1780	fava_c16433	1780	G/A	ggattgaaatgttgaYcattttcaaaaaccatgaaactgatacatccatccttgatgattttaatttctaYgaagaacggcagaaggcaatgcaagaacg[G/A]aaggcaaggcagcaaagcagcgtgatgagtactggattagttggggggagtgagcataggagttcttcggacagtactggtgatttcatcaagcaggtgt

fava_c16433_2251	fava_c16433	2251	G/A	taagaggtggttttgcataaaagaatgggtttcatatatgatactaagttttattgagtgagttgacttattgtttactttgaactctcttacattacac[G/A]ttcacttatttgagtaggttaggttttcttccccgtccttYtacatttttgtttaataattttgggcttgttagggtctgattcattttcatatatattt

fava_c16433_2292	fava_c16433	2292	T/C	tactaagttttattgagtgagttgacttattgtttactttgaactctcttacattacacRttcacttatttgagtaggttaggttttcttccccgtcctt[T/C]tacatttttgtttaataattttgggcttgttagggtctgattcattttcatatatattttttgttttctgttcagctttaattgtaaaaatgtcaaaata

fava_c16433_529	fava_c16433	529	G/A	aatcccttgttttccattctggatatggcttcaacccacaaatgccatacggaccatattccccagttacctcacctttgccttctgtaggtggagatgc[G/A]cagttatactctcctcaccaatttccatacactccaccttattacaaccacctgcttcctcctaccttaccatatctcaattcaccgtctccagtttctc

fava_c16433_912	fava_c16433	912	C/G	tccgattcctccaaaccttcagaaaggcagaggtctttgatgcctctatccacctctgtttctcaacagccaattggctccgtcaggtcatttgggccca[C/G]cgctggaatggcttctcatcaacagcgatcattgtatgggtttggatccagttcaaacccctatggtagaggatatctctctaactccggttctagcttt

fava_c16435_1274	fava_c16435	1274	C/A	gtagcaagttgtttattcagtggattggtgaattggtacaaggctgctctatttgtgagcataacttgtctcaagataaagagaagagaatggcatgaaa[C/A]gcttgaagaatggaaatagacatggaaactaggcttttggaatttcagttgtcatgtacaaacacttatagcttccttttcaatcacttatgaatttgta

fava_c16435_241	fava_c16435	241	G/T	gacatcattacctgctccgattttcaattttcttatattttctacatcgtctctctcttgtgataattattctgtcatttctttttccatgagatgagcc[G/T]attggcgtagatcttgagagggggaatgaaaccttagtggcttcctatttgRcttctcgcgatgtttcttaggtgagatcattctgagatttatccttct

fava_c16435_293	fava_c16435	293	A/G	ctctcttgtgataattattctgtcatttctttttccatgagatgagccKattggcgtagatcttgagagggggaatgaaaccttagtggcttcctatttg[A/G]cttctcgcgatgtttcttaggtgagatcattctgagatttatccttctttctctgtatctcttacaaatgatatcatcccttccctttcattctcgattt

fava_c16435_457	fava_c16435	457	T/G	acaaatgatatcatcccttccctttcattctcgatttttgaagggcttatttaaataaaatgatgaacaaaggtttaggttttgtgagttctgctgctat[T/G]tcatatgaatagtttccctccccttgaaatttgtgcattgattgcttatatgctgtgaaattagggttttttgtttggattctMtgatttgtttaacaRg

fava_c16435_541	fava_c16435	541	C/A	tgagttctgctgctatKtcatatgaatagtttccctccccttgaaatttgtgcattgattgcttatatgctgtgaaattagggttttttgtttggattct[C/A]tgatttgtttaacaRgcctaattctctcctccaatttttattgggatttttttattttcttattattgttctttcttcttggWttgtgatatgttattac

fava_c16435_556	fava_c16435	556	G/A	tKtcatatgaatagtttccctccccttgaaatttgtgcattgattgcttatatgctgtgaaattagggttttttgtttggattctMtgatttgtttaaca[G/A]gcctaattctctcctccaatttttattgggatttttttattttcttattattgttctttcttcttggWttgtgatatgttattacagtgaaaatgttgtt

fava_c16435_624	fava_c16435	624	A/T	ttttttgtttggattctMtgatttgtttaacaRgcctaattctctcctccaatttttattgggatttttttattttcttattattgttctttcttcttgg[A/T]ttgtgatatgttattacagtgaaaatgttgtttgatttggctcagatgctaattttttactgtcctgtttttaYtactatgtaactgtgatgttgtgatc

fava_c16435_698	fava_c16435	698	C/T	ttcttattattgttctttcttcttggWttgtgatatgttattacagtgaaaatgttgtttgatttggctcagatgctaattttttactgtcctgttttta[C/T]tactatgtaactgtgatgttgtgatctgaacttgcagtaacttgtttcttttgattccaatatttactgtgaaccaaacactgttggtgatttgtagaaa

fava_c16435_904	fava_c16435	904	A/C	attgttggtccatgattatttacttgaaatcttactttgttgttgtgaatcaaaataatatgaggcttttgttaggcttggcacatgattgaaatgatcc[A/C]tatatctgattccttcaaWcaacccctcataatgaattgaataatactttcttggaagcttcttgttcttgtctcattgattatcttgctaaccatttct

fava_c16435_923	fava_c16435	923	A/T	ttacttgaaatcttactttgttgttgtgaatcaaaataatatgaggcttttgttaggcttggcacatgattgaaatgatccMtatatctgattccttcaa[A/T]caacccctcataatgaattgaataatactttcttggaagcttcttgttcttgtctcattgattatcttgctaaccatttcttctcgtttttcatgtgatt

fava_c16436_1949	fava_c16436	1949	T/A	ccaacgcgacgttccattgaaaacctacgccaatttcaacgcttaattccaaaattattcacgacaagaaccgtgttcttcgatcggattacaaaaaaaa[T/A]aataaggcgaaaaaaacgaagatggatttttggggtgtgagattaataggttaattattaggtgggaaaaaaaaggaaaatgatggttatcgaggaaata

fava_c16436_321	fava_c16436	321	T/C	aataagtgttaatgtagacctaagctttacatcaagtatctcttatttacttcaagttctttctgcttctaagctttatcgctgctaagcggagatgagc[T/C]gcaatagaagccggaatttctgcatcaaacccatctctcacagctcactagcaacataagcaacaattgcagtScaggttaacccgRattttatatgtac

fava_c16436_395	fava_c16436	395	C/G	tttatcgctgctaagcggagatgagcYgcaatagaagccggaatttctgcatcaaacccatctctcacagctcactagcaacataagcaacaattgcagt[C/G]caggttaacccgRattttatatgtacaagataaaatgggaagaaattattaatgggaatcgacattttttaatttcaaaccctccattcccttgactgcg

fava_c16436_408	fava_c16436	408	G/A	agcggagatgagcYgcaatagaagccggaatttctgcatcaaacccatctctcacagctcactagcaacataagcaacaattgcagtScaggttaacccg[G/A]attttatatgtacaagataaaatgggaagaaattattaatgggaatcgacattttttaatttcaaaccctccattcccttgactgcggtttcataattct

fava_c16436_567	fava_c16436	567	G/A	atttcaaaccctccattcccttgactgcggtttcataattcttgacatgagaaccgttattagaagatgttgctactctatttggatctgacgatccaac[G/A]tgcgaacctctttgtccacttgaacttatgtgtgcttttccacggatagcttcggtagcacgagcacgaagatccgaatcagcgccaccaaatggattgc

fava_c16436_774	fava_c16436	774	G/A	tactatcagcaactcgccttgatgacccgcttgattcgcccaagacattggaatttgataacataacctccttactaacagcagcgtcactaggggttgg[G/A]tttttctgctttgacatatttaMagaatttaaaatgggccccgacattcgtcgtcttgaagagtctcctgaaaccaaaccaggaggacgcccatcttccc

fava_c16436_797	fava_c16436	797	A/C	gacccgcttgattcgcccaagacattggaatttgataacataacctccttactaacagcagcgtcactaggggttggRtttttctgctttgacatattta[A/C]agaatttaaaatgggccccgacattcgtcgtcttgaagagtctcctgaaaccaaaccaggaggacgcccatcttcccttccagtgtgtcggttagcacta

fava_c16443_1024	fava_c16443	1024	T/C	ctcctaaagcaatgttcttccttgttccccaatccaagaccggtttacccttcaaacgagacgcaacacttccattgcacatgtaaggataaactaaaag[T/C]ctttcggacgatgtcatgcaaaatccataaagtcgaagcagatttctatgtaccgcaaggctaatcatttcaacttcagtctgaaattgaatctctcctc

fava_c16443_1522	fava_c16443	1522	C/A	tcgccattttatgactttttggtttgcttgatggtaaaacatcttgattagtattgctcaagttcatggatattggcatcaatgtcatcccatgacagtt[C/A]ggttcatttccagtagcacaaacaagaggattaccaacaatgctaaatgatttYgccaaaattctcggtacaggcccactgagattgttgWaagataaat

fava_c16443_1576	fava_c16443	1576	T/C	tgctcaagttcatggatattggcatcaatgtcatcccatgacagttMggttcatttccagtagcacaaacaagaggattaccaacaatgctaaatgattt[T/C]gccaaaattctcggtacaggcccactgagattgttgWaagataaatcaagaaaagaaagctgtgtcatgttagctagagattctggacattctccaacaa

fava_c16443_1613	fava_c16443	1613	A/T	atgacagttMggttcatttccagtagcacaaacaagaggattaccaacaatgctaaatgatttYgccaaaattctcggtacaggcccactgagattgttg[A/T]aagataaatcaagaaaagaaagctgtgtcatgttagctagagattctggacattctccaacaagactgttattattaaacctcaagtattgaaggcttct

fava_c16443_1798	fava_c16443	1798	T/A	agtattgaaggcttctcaagtgtccaagagaaggaggaatttcaccaatgaagaaattattagagagatcaagagtttgaagcttaggaagtttgcctaa[T/A]tctgaagggattgacccggttatgttgttattctgtaacagcacaatttgaaggttggttaaatttcctatgctYggagatagaKtacctgataaacKtt

fava_c16443_1873	fava_c16443	1873	T/C	tttgaagcttaggaagtttgcctaaWtctgaagggattgacccggttatgttgttattctgtaacagcacaatttgaaggttggttaaatttcctatgct[T/C]ggagatagaKtacctgataaacKttgactcggagtacccaatccaatgacaagattttcaccRgaacatgtaaccatagtccaactacatggatcaactg

fava_c16443_1883	fava_c16443	1883	G/T	aggaagtttgcctaaWtctgaagggattgacccggttatgttgttattctgtaacagcacaatttgaaggttggttaaatttcctatgctYggagataga[G/T]tacctgataaacKttgactcggagtacccaatccaatgacaagattttcaccRgaacatgtaaccatagtccaactacatggatcaactgcatcaccatc

fava_c16443_1896	fava_c16443	1896	T/G	aaWtctgaagggattgacccggttatgttgttattctgtaacagcacaatttgaaggttggttaaatttcctatgctYggagatagaKtacctgataaac[T/G]ttgactcggagtacccaatccaatgacaagattttcaccRgaacatgtaaccatagtccaactacatggatcaactgcatcaccatcccaattatcaaga

fava_c16443_1936	fava_c16443	1936	A/G	acagcacaatttgaaggttggttaaatttcctatgctYggagatagaKtacctgataaacKttgactcggagtacccaatccaatgacaagattttcacc[A/G]gaacatgtaaccatagtccaactacatggatcaactgcatcaccatcccaattatcaagaacaccgtgtggatccactatagtctcttttatatccatta

fava_c16443_2247	fava_c16443	2247	T/A	ggcttcatcattttcaattgaaaatatgaagaaaaaacaaacaaacaaatagtactagtttgaaactgataatgatgataataatgatgctgttgttgaa[T/A]ttttttactaactgttttcaaatggatgatgtttcttaagtttggtttgatgttcaaaagaagaaaagaaaaagaaaagaaagtgagtttgttctttcca

fava_c16443_237	fava_c16443	237	T/A	caacatagattaaaccaaaacaatatcatacttacaaaataaaaaaagaaaaatatatttaaacctactcttaatttcattttcattctttagaaaactt[T/A]aagataaaatttaataatttccaaaacatttcaccttggtcctgaaagctccatagcttgaactaataacacagaatcatcagtaagatcagaatatcta

fava_c16443_910	fava_c16443	910	C/T	aatcaccgactacagcttcacaatagttatcaagtaatatgtttgcagctttcacatccctatgaattatctttggatcacattgttcatgaaggtatag[C/T]agtcccctcgctgctcctaaagcaatgttcttccttgttccccaatccaagaccggtttacccttcaaacgagacgcaacacttccattgcacatgtaag

fava_c16444_1098	fava_c16444	1098	G/A	gacaattctggccctgatcgacgtgatcattatagtgggaatcgttcacgcccgtactagagatttgtcattgaacattttatgtatactgtggtctgag[G/A]gatttattatttcagttggctggtttcttgaaatggatgctaagaggtaggttgttcgcttactttgagcaagacctcttcgctccttctgcYtttttgt

fava_c16444_1191	fava_c16444	1191	T/C	gtctgagRgatttattatttcagttggctggtttcttgaaatggatgctaagaggtaggttgttcgcttactttgagcaagacctcttcgctccttctgc[T/C]tttttgtttgttagttgggggttgactttttagttggggattaatttgattatatgaatgtttttatagaggcatgtggatagtctccaaataatcgctg

fava_c16450_856	fava_c16450	856	T/G	tcaaattcttcaataactttcttgaatggtatggctgtatcatgccagactctatcaactccaatttttccgtactttccttcaatgtattcatttattc[T/G]ttgttcggtcatacttgaaggcctcacaaacctgtggaaaatgtgttcaacttgcaatgtttttgcactaaccattagtaccggaaactcaagaatctca

fava_c16452_1542	fava_c16452	1542	T/G	tcgcttcaaagaaaaaccagacagtcatctggcaaaaacctctcacaaacgattgctatatggaaagagaacccggtacgcgccctcctctctgccagtc[T/G]gacgaagaccctgatgcagtttggggagtgaacatggaaacttgcattacgccttattctgaacatgaccacaaagcccagggtagtgaattagctccat

fava_c16452_1845	fava_c16452	1845	C/A	attactggaatctcatgggatcaaaaatctcatcaaacacagtcaggaacgtgatggatatgaaagcaaacatgggttcatttgccgccgctcttaaaga[C/A]aaagatgtttgggtgatgaaYgtcgtacctcatgaaggaccaagcacacttagaattgtattcgacagaggactcatcgggactactcatgactggtgcg

fava_c16452_1866	fava_c16452	1866	C/T	caaaaatctcatcaaacacagtcaggaacgtgatggatatgaaagcaaacatgggttcatttgccgccgctcttaaagaMaaagatgtttgggtgatgaa[C/T]gtcgtacctcatgaaggaccaagcacacttagaattgtattcgacagaggactcatcgggactactcatgactggtgcgaagcattttcaacatatccgc

fava_c16452_223	fava_c16452	223	G/A	ccctctcttctctttctccactctccaaatctctctttctttctttatcccaacccagttaacccttgaatctgataggtttcttagagagttttattta[G/A]ttgtgctatttaattcgaaataccgtggttgcttgttgataaggtgttttggatggtgaattcggYgcttcatgtgtaacggaacaaagatcaatgctgt

fava_c16452_2279	fava_c16452	2279	C/A	tctgaacaagatggcaatgaaattgttttcatcatccaaaagaaattgtggctcactactgaatcccttaggggtgcagcagaataggacaaaataacaa[C/A]tacatggtttatttaatttttYccctccattttctattttctgctgttacaaggaattaacagcatagcatagctacaactacattttgtagcattacat

fava_c16452_2301	fava_c16452	2301	T/C	ttgttttcatcatccaaaagaaattgtggctcactactgaatcccttaggggtgcagcagaataggacaaaataacaaMtacatggtttatttaattttt[T/C]ccctccattttctattttctgctgttacaaggaattaacagcatagcatagctacaactacattttgtagcattacataatgttcacattgttaagtttc

fava_c16452_289	fava_c16452	289	C/T	tgaatctgataggtttcttagagagttttatttaRttgtgctatttaattcgaaataccgtggttgcttgttgataaggtgttttggatggtgaattcgg[C/T]gcttcatgtgtaacggaacaaagatcaatgctgtgatcagttgtagtgcatatctgaggagtgagagttatatttggatttgtcttactttcaacagata

fava_c16452_645	fava_c16452	645	T/C	gactcggggcatcgtatttgggcgcagatgatgatgcagatagcaagcaacaagatgaatctacaagtttagtgcagggagatgcgatggtggatgatat[T/C]gtaccaaagagtttccctgtttgcgatgatcgtcattcagagttgattccatgcttagacagacatcttatctatcaattgagactgaagttggatctta

fava_c16453_1064	fava_c16453	1064	C/T	ttgatatgagtgtccttgttgaagggcttattgctagtactggttgggatgaacttgcgtatgtttggcagcatggaactgatcctagagcatcttgaaa[C/T]ggaaggtactaaaaacttaggtttatgtttttgttctccctttatttatatgtggttagattgggagggatgttgggaatttccaatagcttttttgagt

fava_c16454_1105	fava_c16454	1105	C/T	aaggtatctattgttaaaccttggactactggaataagtggacagcttcaaaaagcacttgtgacaggtgtgccaaagctaaagagaatagatcttgaag[C/T]agcatgtgaagattttagtaatgtaatcggtaaatcaccgattggtacattgtacaaaggaactttatctagtggaattgaaattgctgtagcttctgtt

fava_c16454_1272	fava_c16454	1272	T/C	atctagtggaattgaaattgctgtagcttctgtttctctgacattatccaagacttggacaaagaatttagaagctcagtttaagaacaagatagacaca[T/C]tatcaaaagtgaaccataggaattttgtcaatcttattggatattgtgaagaagaagaaccttttaccagRatgttggtttttgaatatgcRccaaatgg

fava_c16454_1343	fava_c16454	1343	A/G	aagctcagtttaagaacaagatagacacaYtatcaaaagtgaaccataggaattttgtcaatcttattggatattgtgaagaagaagaaccttttaccag[A/G]atgttggtttttgaatatgcRccaaatggaacactattcgaRcatttacaYgtgaaagaagctgagcacttgaactggggaccgagacttagaattgtga

fava_c16454_1364	fava_c16454	1364	G/A	tagacacaYtatcaaaagtgaaccataggaattttgtcaatcttattggatattgtgaagaagaagaaccttttaccagRatgttggtttttgaatatgc[G/A]ccaaatggaacactattcgaRcatttacaYgtgaaagaagctgagcacttgaactggggaccgagacttagaattgtgacaggaatggcttactgcctac

fava_c16454_1385	fava_c16454	1385	G/A	accataggaattttgtcaatcttattggatattgtgaagaagaagaaccttttaccagRatgttggtttttgaatatgcRccaaatggaacactattcga[G/A]catttacaYgtgaaagaagctgagcacttgaactggggaccgagacttagaattgtgacaggaatggcttactgcctacaatacatgcatggattagacc

fava_c16454_1394	fava_c16454	1394	C/T	attttgtcaatcttattggatattgtgaagaagaagaaccttttaccagRatgttggtttttgaatatgcRccaaatggaacactattcgaRcatttaca[C/T]gtgaaagaagctgagcacttgaactggggaccgagacttagaattgtgacaggaatggcttactgcctacaatacatgcatggattagaccctcctgtgg

fava_c16454_155	fava_c16454	155	T/A	tgatcaagaacatgaatctttctgcgSctttttggcatgtagttgtgtggttcttgttttttaacttgagtttgtgctgctattctttgaatgaagaagg[T/A]attactcttttgaagctgaagaagagaRtaactagcgacccttttgatgctttgtcaaattgggttgatgatgaagcttctcttgatccctgtcattggt

fava_c16454_1633	fava_c16454	1633	C/T	gatgatcatgcagcaaaaatctcggatttgagtttctcccgcgaaacagattcatctgaaaagaaacctgatggtagaaaagacattgatattgatatga[C/T]gcaatcaacaagtccagcgagtaatgtttatagctttggtgttttgttattcgagatagtaaccggtcggattccttattcgctggataatagctcagct

fava_c16454_183	fava_c16454	183	A/G	tttttggcatgtagttgtgtggttcttgttttttaacttgagtttgtgctgctattctttgaatgaagaaggWattactcttttgaagctgaagaagaga[A/G]taactagcgacccttttgatgctttgtcaaattgggttgatgatgaagcttctcttgatccctgtcattggtttggagttgaatgctcagatagagaagt

fava_c16454_1856	fava_c16454	1856	G/A	taaaatgggacaaacctatgaaggaaatggtggatgcaactctagcatcttaccaagagaatcaggtggaacaagttgcggagttgataaaagattgtgt[G/A]gatcctgattcggagaagagacYgagaatgaatgaagtgagtgagagattgagagaaataacgaaaatgtcgtctgagtttgtagttcctaaactttctc

fava_c16454_1879	fava_c16454	1879	C/T	gaaatggtggatgcaactctagcatcttaccaagagaatcaggtggaacaagttgcggagttgataaaagattgtgtRgatcctgattcggagaagagac[C/T]gagaatgaatgaagtgagtgagagattgagagaaataacgaaaatgtcgtctgagtttgtagttcctaaactttctccactttggtgggctgagcttgag

fava_c16454_2020	fava_c16454	2020	T/A	aaaatgtcgtctgagtttgtagttcctaaactttctccactttggtgggctgagcttgagatttcttctgcataagcatgttgaagttgaaatgaatgaa[T/A]gaatcagattgtagtttgtaattaRgtatgagaggttttagcgttttcatgagttttagtttgtttaaggtacaaaaaaagttgttacttgttgtatggt

fava_c16454_2045	fava_c16454	2045	A/G	ctaaactttctccactttggtgggctgagcttgagatttcttctgcataagcatgttgaagttgaaatgaatgaaWgaatcagattgtagtttgtaatta[A/G]gtatgagaggttttagcgttttcatgagttttagtttgtttaaggtacaaaaaaagttgttacttgttgtatggtctattataggatggttgatctcaga

fava_c16454_2230	fava_c16454	2230	A/T	gatggttgatctcagaaaacattttatatgaatgagatcaataaatgtatagtaaagttctctggagtgcttagatttttctagcagaaaggcccgagtc[A/T]gagactaactcctcgaagtagtgagagccgttagcatgttgttgagtgtttagattgatttatttgaatttgataacaacttatcaatgaaagctttaca

fava_c16454_305	fava_c16454	305	C/T	ctttgtcaaattgggttgatgatgaagcttctcttgatccctgtcattggtttggagttgaatgctcagatagagaagttgttgtcttgaatttgaaaga[C/T]ctttgccttgaaggaagtttggcaccagagcttatgaaccttgttcacataaaatctattattttaaggaacaactctttccatggaaccatacctgaag

fava_c16454_431	fava_c16454	431	G/A	cagagcttatgaaccttgttcacataaaatctattattttaaggaacaactctttccatggaaccatacctgaagaaattgtaggcttaaaagaattgga[G/A]attttggatttgggatataacaacttcagtggacatcttgatgctaattttggtcataatatcttatcacttgcaattcttttgctagacaacaatgagc

fava_c16454_662	fava_c16454	662	C/T	aactgaaaatgctttctgagtttcaagtagatgaaaatcagttaatcaatgcagctaacaagccatcttgttcagaaagatccatcaaatggcgtgctcg[C/T]gaaaatgaaggtcctaggagaatRctggaataYcgcRaactacatcgtcgtccttttcattaccatcgtaatcatacttcacctctctataggccttctt

fava_c16454_686	fava_c16454	686	G/A	aagtagatgaaaatcagttaatcaatgcagctaacaagccatcttgttcagaaagatccatcaaatggcgtgctcgYgaaaatgaaggtcctaggagaat[G/A]ctggaataYcgcRaactacatcgtcgtccttttcattaccatcgtaatcatacttcacctctctataggccttcttcttcgcttcccccatcggtcactg

fava_c16454_695	fava_c16454	695	T/C	aaaatcagttaatcaatgcagctaacaagccatcttgttcagaaagatccatcaaatggcgtgctcgYgaaaatgaaggtcctaggagaatRctggaata[T/C]cgcRaactacatcgtcgtccttttcattaccatcgtaatcatacttcacctctctataggccttcttcttcgcttcccccatcggtcactgaagtaccac

fava_c16454_699	fava_c16454	699	G/A	tcagttaatcaatgcagctaacaagccatcttgttcagaaagatccatcaaatggcgtgctcgYgaaaatgaaggtcctaggagaatRctggaataYcgc[G/A]aactacatcgtcgtccttttcattaccatcgtaatcatacttcacctctctataggccttcttcttcgcttcccccatcggtcactgaagtaccactggt

fava_c16454_901	fava_c16454	901	T/C	ccgagtccagattctccaaatcaaaatgcttctgattctccaaatcaaaatgcttctgattctccggatcaaaacgcttcagattcgcctcctcaaagtg[T/C]acYatcaaagaagaatcaagtgcctatttttgccggagttattatcggcagtgctgtgtttcttgttatttcaaccattggtatatatctttgtaaaacc

fava_c16454_904	fava_c16454	904	T/C	agtccagattctccaaatcaaaatgcttctgattctccaaatcaaaatgcttctgattctccggatcaaaacgcttcagattcgcctcctcaaagtgYac[T/C]atcaaagaagaatcaagtgcctatttttgccggagttattatcggcagtgctgtgtttcttgttatttcaaccattggtatatatctttgtaaaaccaac

fava_c16458_1294	fava_c16458	1294	C/G	tatacaatgctgcacttgacgcacttgggaagttgaggagacctgtggaagcactgaatctatttcatgcaatgcagcaacaaatgtccacatatcctga[C/G]ttagtagcttatcatagtattgctgttactcttgggcaagcagggcacatgaaacagctctttgatgtgattgatattatgcgatctccaccaaagaaga

fava_c16458_2100	fava_c16458	2100	T/C	aaacatgggatgtttcaagaagctaaagagttatttgagcgaatgttggtaaacacgaataatctgagtaataaagatgattacaaaatgcgagtgatac[T/C]ggatatatacacattcaataccatgttagatgcatgtgctgcagagaaaaaatgggattattttgactatgtttaccagaggatgttgtaccatggttat

fava_c16458_579	fava_c16458	579	G/A	accaaaattgataagaaagatgatgaaggctcttccacaggtaatggtaatgctagaggaaaacctaataaagggtttagggatgagaggaatcagcacc[G/A]gagtcgtagtttagagccaagatctgatgactgcggtttgattaagagtcaaggggaaattataggcgacaacgtaattagggctaatttcaatggcgtc

fava_c16462_1121	fava_c16462	1121	T/C	gcttccaaaattcaaggctttgcaagatacggctgggcgggaagttgataaaatgatgcttacagatgcgccactcttatttactcctgggcagttggcc[T/C]tggccgctctacgtacctcaaatgcactccataaagttgtcgactttgatagttttctgagcagagttttttccctYaagagttctRcacacacaatgga

fava_c16462_1198	fava_c16462	1198	C/T	tatttactcctgggcagttggccYtggccgctctacgtacctcaaatgcactccataaagttgtcgactttgatagttttctgagcagagttttttccct[C/T]aagagttctRcacacacaatggacgaacttcttgaatcattggatRcaattgattcttgggttagaaaatacgtaagtccgtcagagaaggaattgaaRc

fava_c16462_1208	fava_c16462	1208	G/A	tgggcagttggccYtggccgctctacgtacctcaaatgcactccataaagttgtcgactttgatagttttctgagcagagttttttccctYaagagttct[G/A]cacacacaatggacgaacttcttgaatcattggatRcaattgattcttgggttagaaaatacgtaagtccgtcagagaaggaattgaaRcatatcgaccg

fava_c16462_1244	fava_c16462	1244	A/G	tgcactccataaagttgtcgactttgatagttttctgagcagagttttttccctYaagagttctRcacacacaatggacgaacttcttgaatcattggat[A/G]caattgattcttgggttagaaaatacgtaagtccgtcagagaaggaattgaaRcatatcgaccggaaactgaagtcttgttggggtcaYagctctcatga

fava_c16462_1297	fava_c16462	1297	G/A	tYaagagttctRcacacacaatggacgaacttcttgaatcattggatRcaattgattcttgggttagaaaatacgtaagtccgtcagagaaggaattgaa[G/A]catatcgaccggaaactgaagtcttgttggggtcaYagctctcatgacgagggaaagaaaagggagaagaaatcaaagcacaagtccagaaagagctcaa

fava_c16462_1333	fava_c16462	1333	T/C	aatcattggatRcaattgattcttgggttagaaaatacgtaagtccgtcagagaaggaattgaaRcatatcgaccggaaactgaagtcttgttggggtca[T/C]agctctcatgacgagggaaagaaaagggagaagaaatcaaagcacaagtccagaaagagctcaaaagaagctcaaaatgtgtagtctcttctctagtttt

fava_c16462_1469	fava_c16462	1469	T/C	atcaaagcacaagtccagaaagagctcaaaagaagctcaaaatgtgtagtctcttctctagttttatattccgttcaattgaggtactcatcttctgaat[T/C]ccggaagggagttttttaggtttttactgattattattgaattgaacgcgaaggatggagtgttgaagRgcttgaaaaacattgacataatgaacaaacc

fava_c16462_1538	fava_c16462	1538	G/A	tccgttcaattgaggtactcatcttctgaatYccggaagggagttttttaggtttttactgattattattgaattgaacgcgaaggatggagtgttgaag[G/A]gcttgaaaaacattgacataatgaacaaacctgggtccattttgtttagtttagaggaaaggagctatattattttaaacttttaattgtttggagcaaa

fava_c16462_268	fava_c16462	268	T/C	actttttcaaatttgtttcacaaatcttcttctcatcataaatcggtctataactatatattcctctgtctcagcttccttctcaacggaccttacattg[T/C]gagggagtgataaagatccgatttccctccagaYtttgcttgagcactgcccgtaattaaacacagagagcactatactgatttcacttgttctcggctt

fava_c16462_302	fava_c16462	302	C/T	atcataaatcggtctataactatatattcctctgtctcagcttccttctcaacggaccttacattgYgagggagtgataaagatccgatttccctccaga[C/T]tttgcttgagcactgcccgtaattaaacacagagagcactatactgatttcacttgttctcggcttgaagaatcaagcagtgtttgcccctatttaaatt

fava_c16462_790	fava_c16462	790	G/A	aagaagtgtgcaacaatttccacttccctcataagatccaggctacagccctcatattttttaaaaggttctatctgcaatggtcggtgatggagcatca[G/A]cccaaaaatataatgttgacctgcatatatgctgcttgtaagatagaagaaaatcatgtatcggctgaggagcttggtaaggggatttcgcaagatcatc

fava_c16463_1185	fava_c16463	1185	T/G	gtgatagaacatcacagaaagctctgtgagttataaaactgtcccttcttgagaaaagggttccacattcacacttatactctttagtgccacaaatttt[T/G]gaatgagctttccaatcagattgaactgcataacgtttggaacacttttcacatttccatttcttctcaccatgctttctgaaaaagtgcttcttgattc

fava_c16463_162	fava_c16463	162	A/G	attaaaaaaaaccttacacctataattttaacaagaatttgtactaacttaatttgattaatatatagaacttaaaagttacattacacaaccgaaaaac[A/G]agtactagagaaatgtctacttagatagatcatttttaatccttctacagacagaaaattaattaatatctccaaattttggaattatacaattagtata

fava_c16463_1741	fava_c16463	1741	A/C	tgacatattttcatctacggacaaacctttcatcatctcctttagattatgagaaaacaaactatgttgtctttgcaaatatatgtcagagataaacaaa[A/C]ttttgtgatagatcaacttgttttccactaaaggatgtctgaactagtcggaatctagggcatgtggttSgtgcgtagtagtctgtatgagtgtctaaat

fava_c16463_1811	fava_c16463	1811	G/C	ctttgcaaatatatgtcagagataaacaaaMttttgtgatagatcaacttgttttccactaaaggatgtctgaactagtcggaatctagggcatgtggtt[G/C]gtgcgtagtagtctgtatgagtgtctaaatctcaaggtatcgagttcgaatcWtaatcatataaaaaacatYaaaaggaagagcaagcYgagaaaataat

fava_c16463_1864	fava_c16463	1864	T/A	ttccactaaaggatgtctgaactagtcggaatctagggcatgtggttSgtgcgtagtagtctgtatgagtgtctaaatctcaaggtatcgagttcgaatc[T/A]taatcatataaaaaacatYaaaaggaagagcaagcYgagaaaataattaaggtgaatagatcccaagatggatttScttcattcaaagttatatatacac

fava_c16463_1883	fava_c16463	1883	T/C	aactagtcggaatctagggcatgtggttSgtgcgtagtagtctgtatgagtgtctaaatctcaaggtatcgagttcgaatcWtaatcatataaaaaacat[T/C]aaaaggaagagcaagcYgagaaaataattaaggtgaatagatcccaagatggatttScttcattcaaagttatatatacacatccaaatctagctatagt

fava_c16463_1900	fava_c16463	1900	T/C	ggcatgtggttSgtgcgtagtagtctgtatgagtgtctaaatctcaaggtatcgagttcgaatcWtaatcatataaaaaacatYaaaaggaagagcaagc[T/C]gagaaaataattaaggtgaatagatcccaagatggatttScttcattcaaagttatatatacacatccaaatctagctatagtaagctaaaatcaagaac

fava_c16463_1940	fava_c16463	1940	G/C	atctcaaggtatcgagttcgaatcWtaatcatataaaaaacatYaaaaggaagagcaagcYgagaaaataattaaggtgaatagatcccaagatggattt[G/C]cttcattcaaagttatatatacacatccaaatctagctatagtaagctaaaatcaagaacttggtaagcaaagtaagtttcctagtttgtatggaaattt

fava_c16463_563	fava_c16463	563	T/C	gaaacaaattcctcaaagttattattagcaacaacaccttcaaattgtgaactttcattaccatatatcacatcatgcaaaagagaagattcttctgtat[T/C]tgtagcagtaacagtgacaccttgttcaaagcattcagaggctattgcagctttattcccatatggagccaaacaatgagaaaaaccagtcgctatttct

fava_c16464_1365	fava_c16464	1365	T/C	ttcttccaaaactttaagctgttcgagtatcgataacctctcttcctcaaaacttgataatgattcctccaaatataagacagcttctaccggagtgttc[T/C]ggttgctgctgcattcttgatgattgttgtgaaaaccattttcttcctttgtttcttgattgaaatcaatggacactccatcactatcctcggcgttact

fava_c16464_1813	fava_c16464	1813	T/G	tcttgaagcctattaatcattgccattgtttgattggctgctacagccgatgcatttcgttcttcttctagttctgcgtataaagttttcaaagccttcc[T/G]ttctgattttaaggctgattttaacttatcaagcgtcacctcgccgcattctatgtcgcttattacacttccatccaatgaatcttctgttcctgactct

fava_c16464_2124	fava_c16464	2124	T/C	catatggttacttgatgttggcattctcacttcaagtgacatggtgttaaattctacaaactcttcttcacctttgtcatgaccactatcatgttgaaca[T/C]aaaatctgactgagctagtagatgaatcgtcttgcattctttgatttgtttcgaaaagtacgtcttggccttgatactcatccacatgttcttgatcagg

fava_c16464_2706	fava_c16464	2706	T/G	cgcttccgcatcggtattattcatatcgaagtcaagaataaaatcttcattatctctaaacccttcatccacccccttgttatatctactttgatttcca[T/G]tttctgtggcaccggaatcaaccaattcaacaggaatcaatctgcaatcatcaccatgaatgaaaaaatcaagatgtttSgtttgaacttcctcaatttt

fava_c16464_2786	fava_c16464	2786	G/C	tatatctactttgatttccaKtttctgtggcaccggaatcaaccaattcaacaggaatcaatctgcaatcatcaccatgaatgaaaaaatcaagatgttt[G/C]gtttgaacttcctcaattttctcaacctcaacactactcagatcacaaacttcatcacccccatcatccttagcctttggaacaatcaatttcaaScttt

fava_c16464_2882	fava_c16464	2882	G/C	gtttSgtttgaacttcctcaattttctcaacctcaacactactcagatcacaaacttcatcacccccatcatccttagcctttggaacaatcaatttcaa[G/C]ctttcctcttccaaggtttcttctttccctttctctaaaccaatttcaagcttgtacaaatcatcaaccatagtttccttgacaccatcacaaacagaac

fava_c16464_3081	fava_c16464	3081	C/G	acaagcacaattctgctcaacctcaaacaactcaagaattccacttttttcttcaatcttatcccgattttcatgatgaacgactgaaaaacctgatcta[C/G]tgtgatctgaacaatgatcaacttctgaaacaaacacaccttctttgatcaaattcaccttctctgaattcggttcatactccaaattttccaaaatgtt

fava_c16464_3294	fava_c16464	3294	T/C	aaccacaacacaaggaggataaaatctgttatcaaaattaacaccacagcaagaacaccttaaaccctcttcatctttctgaaccccaccttcaacccta[T/C]caatttccttcatccatggaaaaaaatcaacacttttcgacgaaaacgaaactgaaacMgaacaatcctcacacatatcatcaaattcagcaagtttatg

fava_c16464_3353	fava_c16464	3353	A/C	ttaaaccctcttcatctttctgaaccccaccttcaaccctaYcaatttccttcatccatggaaaaaaatcaacacttttcgacgaaaacgaaactgaaac[A/C]gaacaatcctcacacatatcatcaaattcagcaagtttatgatgatttaaacaaaaaccgagtttcgaaatctcaagagcatgagcttcacaaacaagat

fava_c16469_1267	fava_c16469	1267	T/C	aattatcagaaaaggcatcgataaagcaatttggtagttcaaatgagtttgtcaaatcagtaacacatgcttttgaagggagtgattattctaccagtaa[T/C]ggtataagtccttccagttttattgattccgcaatccaagtatctgattgcgaatacgaaaatggcacttcttggggtaatcagatgggctggttatatg

fava_c16469_1378	fava_c16469	1378	G/A	cttccagttttattgattccgcaatccaagtatctgattgcgaatacgaaaatggcacttcttggggtaatcagatgggctggttatatggatcaatatc[G/A]gaggatgtaccaaccgggctgaatatgcatcgaaaaggttggagatcagagtgttgtacgccagatcaaactgcattcacaggctgcgctcccggaggtt

fava_c16469_1618	fava_c16469	1618	T/C	gcctcactgttgtcttctttggcaagcattctcctgttatgggcatcctctttggtaaaatccaaatcagggcaggcttgtcttattgttggcttacgaa[T/C]tggggtctgcgttcggtctttgaagtttgttatgcagctttagtcgcttattgcgtaatcaccaacaccggtatctttcctaagggRYtYgggctatgga

fava_c16469_1705	fava_c16469	1705	A/G	gttggcttacgaaYtggggtctgcgttcggtctttgaagtttgttatgcagctttagtcgcttattgcgtaatcaccaacaccggtatctttcctaaggg[A/G]YtYgggctatggattcctttgactctttttgtgatctacactatacatacattacaagagtatcaatcaaaagggctatcattaaggtattggtggaaca

fava_c16469_1706	fava_c16469	1706	C/T	ttggcttacgaaYtggggtctgcgttcggtctttgaagtttgttatgcagctttagtcgcttattgcgtaatcaccaacaccggtatctttcctaagggR[C/T]tYgggctatggattcctttgactctttttgtgatctacactatacatacattacaagagtatcaatcaaaagggctatcattaaggtattggtggaacaa

fava_c16469_1708	fava_c16469	1708	C/T	ggcttacgaaYtggggtctgcgttcggtctttgaagtttgttatgcagctttagtcgcttattgcgtaatcaccaacaccggtatctttcctaagggRYt[C/T]gggctatggattcctttgactctttttgtgatctacactatacatacattacaagagtatcaatcaaaagggctatcattaaggtattggtggaacaacc

fava_c16469_638	fava_c16469	638	C/T	caaaagtgaagaaggttcatcagaattcaaacaagaatggttacaaatgaaagatatgtatgacaatcttagccgaagagttgaagatgtgacgcgaaat[C/T]cgttgtcattaaaatttgagggggagtttgcagtgttcttgaatactgaaaaaagRaatcatccaagtatagttaaggttatatcggagaataaggatga

fava_c16469_694	fava_c16469	694	A/G	tgtatgacaatcttagccgaagagttgaagatgtgacgcgaaatYcgttgtcattaaaatttgagggggagtttgcagtgttcttgaatactgaaaaaag[A/G]aatcatccaagtatagttaaggttatatcggagaataaggatgatcttcctgatggactgcctcacttaatctatgtatcgagagagaagaagccaaatt

fava_c16469_859	fava_c16469	859	C/T	acttaatctatgtatcgagagagaagaagccaaattatgaacacaattacaaagccggagctatgaatgtgttgacaagagtctctgggttgatgacgaa[C/T]gcgccctttatgttgaatgtagactgtgacatggttgttagcaatccSaagattattcaacatgctgtgtgcattttgatggattctaagaagggaaaag

fava_c16469_907	fava_c16469	907	G/C	acaaagccggagctatgaatgtgttgacaagagtctctgggttgatgacgaaYgcgccctttatgttgaatgtagactgtgacatggttgttagcaatcc[G/C]aagattattcaacatgctgtgtgcattttgatggattctaagaagggaaaagatgttgcttttgttcaatgtttccaacaattttatgatggaataaaag

fava_c16471_107	fava_c16471	107	C/G	ttcactcattcactcactttcttcatcttctgcattttcaaatcttcaccatctttcagcaacaatcaacaactaggagattgttgagagatattctgtt[C/G]tgagatttggagaaggatttcatctctacgcaatgcgagggagacagcgagtagcatcaagggaaaccatagcagtgcaggatagtaaagatgaaaattc

fava_c16471_1204	fava_c16471	1204	C/T	gcaatttatttggatcggttgtttcaatgattctgatggataaacttggaaggaaagtgcttctactgggtagctttttaggcatggcagtaaccatggg[C/T]ctacaagtcattgctgctagttcttatgcatcaggatttggagcaatgtatctatctgtcggtggaatgttgctgtatgtgctatcatttgctcttggtg

fava_c16471_1312	fava_c16471	1312	G/A	tcattgctgctagttcttatgcatcaggatttggagcaatgtatctatctgtcggtggaatgttgctgtatgtgctatcatttgctcttggtgctggtcc[G/A]gttccttgtctcctcatgtccgaaattttacctggcaagatcagagccaaggcgatggcaatttgYttggctgtgcattgggtgataaacttctttgtcg

fava_c16471_1378	fava_c16471	1378	C/T	tgtatgtgctatcatttgctcttggtgctggtccRgttccttgtctcctcatgtccgaaattttacctggcaagatcagagccaaggcgatggcaatttg[C/T]ttggctgtgcattgggtgataaacttctttgtcggtctattctttttgcgtttactggaacaaatgggggcacaactSttgtacagtattttcggttcat

fava_c16471_1456	fava_c16471	1456	G/C	gagccaaggcgatggcaatttgYttggctgtgcattgggtgataaacttctttgtcggtctattctttttgcgtttactggaacaaatgggggcacaact[G/C]ttgtacagtattttcggttcattttgtttgttggctgtagcttttgtgaagaaatatgttctagaaacaaaagggaagtcactgcaggaaattgaggttg

fava_c16471_1777	fava_c16471	1777	A/T	catgaaattcggccattgcattagcttgaatgttaagcttttgtatgatgacttgcaaaacaagcaagagactgtacaacatgagtaatttttcttctct[A/T]tcgctatgttttgtgtgtatatgatgagacatacatgattagatgtcagaatttaccacattgtttaactttttttgttaaatacaatttatgaagaggt

fava_c16471_394	fava_c16471	394	T/A	atggataccatgttggggtagtcaatgaaaccttagaaagcatttctatagatctcggttttaccggaaatacattggcagaaggtcttgtagtaagtat[T/A]tgtttaggaggtgcttttattggatctctattcagtggatggatcgcagatggggttgggcgtagaagaagtttccagttgtgtgctttgcctatgatca

fava_c16473_1011	fava_c16473	1011	A/G	tcttagWagaatgttgaggagtcggaggtttctggaggcgacgactccgaagctgaaggtaggaatcattagaaagagagagatcctgattctgagaaag[A/G]gctagggttttagcacgagttcgaacaccgtaataggtggtggaggtggtggtKgtggtgatagtggtggtggtggaatcaagtaaaggtaattccgatt

fava_c16473_1065	fava_c16473	1065	G/T	gaaggtaggaatcattagaaagagagagatcctgattctgagaaagRgctagggttttagcacgagttcgaacaccgtaataggtggtggaggtggtggt[G/T]gtggtgatagtggtggtggtggaatcaagtaaaggtaattccgatttaggtttcggtttcttcatgtacttccccatcgaagagattgaaaggagaagaa

fava_c16473_235	fava_c16473	235	C/T	ggaatctggtctttatttctaagagtttgttcaagttccatcagtacaagtatagacaaaagaagagtaaaatacaagaacaaaaggaaattcctgtaac[C/T]tcatacagacctaatttgatttttatcccctaaaccctacaactacagcccttttttccccctttgatgatctgttagacaaaggactttgtatagttgt

fava_c16473_917	fava_c16473	917	T/A	aaaacattttccccaaagtcagggtcttcagcttcaggagttttccccttggtaggaattggggatttagggttttgattttggggcttattcttcttag[T/A]agaatgttgaggagtcggaggtttctggaggcgacgactccgaagctgaaggtaggaatcattagaaagagagagatcctgattctgagaaagRgctagg

fava_c16476_1069	fava_c16476	1069	A/G	agttgttcatctatactaatcgaagagtattgtcgatccggatcgtattcggtttctccaaacattggaacatagctgcaaggattagtagacgcacgcg[A/G]aaaaagataagaaagctgtccaacacacaatataaggttaagaataagatataaaccgatcaggccaRtgaatttgataaagatcaatataatccaMttg

fava_c16476_1137	fava_c16476	1137	G/A	aacatagctgcaaggattagtagacgcacgcgRaaaaagataagaaagctgtccaacacacaatataaggttaagaataagatataaaccgatcaggcca[G/A]tgaatttgataaagatcaatataatccaMttgcatcctcaacagactattatctattgcttgaataatattggtggcatccaaagatttaggaccacctc

fava_c16476_1166	fava_c16476	1166	A/C	gcgRaaaaagataagaaagctgtccaacacacaatataaggttaagaataagatataaaccgatcaggccaRtgaatttgataaagatcaatataatcca[A/C]ttgcatcctcaacagactattatctattgcttgaataatattggtggcatccaaagatttaggaccacctctaatccaagtcatctgcccagatggtcca

fava_c16476_1395	fava_c16476	1395	G/A	ccctggggatatttcggtgtttaatccaccggccaagatactcctcactcaatccacaagtctcagcacgctgaggcactggatacatttcagcggagtc[G/A]aagaagttgattccggcatgaaaagcttcgtcgagaagatgaaatgactcagtcaacgtgttctgttccccaaatgtcatggttcccaaacacagccttg

fava_c16476_276	fava_c16476	276	T/G	cttatacgcaggctttaagcagtttggtatttggcatcgatctagcttaaaaataaaaaagaattttgttgaattaaaaaagctactttaacatgggata[T/G]gatttggaaaatctgtagagtacttttacaatgtagatgtgagttagtcattttatctccccatgtacactctctctttttagtgtaattatttgcaatt

fava_c16476_705	fava_c16476	705	C/G	agctgcatgtatagctttattggataagctgtatctggattccccttctaaatactttcctttaaagagatttaacctagcatctggtggaccattgcca[C/G]gggacaaatatttgcctgaaagaatacccattgccagaggactataggccaacaagccaatactctcatgRtggcagcactcggccattgcagaatcgaa

fava_c16476_776	fava_c16476	776	G/A	ttaacctagcatctggtggaccattgccaSgggacaaatatttgcctgaaagaatacccattgccagaggactataggccaacaagccaatactctcatg[G/A]tggcagcactcggccattgcagaatcgaaagttctacatagcaggctatacgaattctgcaatgaaatgatctttgggtaggaatcaaatttctgtgcaa

fava_c16479_1730	fava_c16479	1730	A/G	tgtagacctgccccttcttggttgtaatttgccgtagtattgtttcaggttttgtcttcccaacagtagtctcacccgtcttcctgtcaagaaaccgtac[A/G]gaaatattattgacctcagcttctgaaacttgtaatctcaggatacccccagatagaatctcaacagctgataccatggcaaaaagaccacgctccatat

fava_c16479_2072	fava_c16479	2072	A/G	ttggttctacctgaaacaccaatcgaataccatcacgcgtatcaactgcaaccggaacgcacgaacaaaagtatccactattgataatccgatgaacatc[A/G]tcctgcacctcgcgaaccgtgagagccgaattaggtcgacaggctttgagagcctgagccgcttctgcttccaggtccttcctctcgagttcttcKccgt

fava_c16479_2168	fava_c16479	2168	G/T	catcRtcctgcacctcgcgaaccgtgagagccgaattaggtcgacaggctttgagagcctgagccgcttctgcttccaggtccttcctctcgagttcttc[G/T]ccgtctttgtttcgaaccaacacttcactgattaaaactctctcctcRttctgtcgagttttcaactgaatctcctccgccggagaaagagacaagcctt

fava_c16479_2216	fava_c16479	2216	A/G	ctttgagagcctgagccgcttctgcttccaggtccttcctctcgagttcttcKccgtctttgtttcgaaccaacacttcactgattaaaactctctcctc[A/G]ttctgtcgagttttcaactgaatctcctccgccggagaaagagacaagccttttctctcgtcggtgagtgacaacgtcgccgaacatagagaatgtgatt

fava_c16479_234	fava_c16479	234	T/C	gcaagcatccaactactgaataattttttaaacaccctgtcgtaaatagtcttactcagaagtcagaaccttttttgatgcttcatcttttctcttcaca[T/C]gatcctataacgtaaaaaaacctacatgaccgatccactttccattaatcatgcaaatcctgtagattatctcaaatcaagaggcaaatcagaataagac

fava_c16479_345	fava_c16479	345	C/T	cgtaaaaaaacctacatgaccgatccactttccattaatcatgcaaatcctgtagattatctcaaatcaagaggcaaatcagaataagacattccatcta[C/T]gggtgacacttagagaagcattagctgaattgaagacatttgagaaaaatatttacagagctttctgaataaggaaaagcccatgcaattaaaccaaaaa

fava_c16479_797	fava_c16479	797	G/A	tgacaccttctactggccgtatcatagggaaagaaatttctccggagccaacaacatatgatcgaccagagcccacaccaccttcttcatagcctctcac[G/A]ctattagttccaccaatggcaaaagcttcatagggagagaaattgcctaccacatggcccccggcgaaacttaaattaaaacgagcaggaccgatctcaa

fava_c16481_1110	fava_c16481	1110	T/A	ctagtggcaaaacttctgctttaacccccatgtggattgaaagaaaagatggagctttcacataaactttctggaaattctcttcctcttttggaagtga[T/A]tctccttttaccaactttccaaatgtgtcccatctggtagcatttgatattatggttttggcaaagaactctcttccatctgaaagcctcactcctacgg

fava_c16481_1236	fava_c16481	1236	C/T	tgtcccatctggtagcatttgatattatggttttggcaaagaactctcttccatctgaaagcctcactcctacggctttgccctgctcggtgataatact[C/T]gtgacatttgccttgtaaagtatctgactaccctgRtctaccagacctttcgccaacgactttgcaatgccaccaacacccccaacagggtagttaatcc

fava_c16481_1272	fava_c16481	1272	A/G	caaagaactctcttccatctgaaagcctcactcctacggctttgccctgctcggtgataatactYgtgacatttgccttgtaaagtatctgactaccctg[A/G]tctaccagacctttcgccaacgactttgcaatgccaccaacacccccaacagggtagttaatcccaccaaagtgtctgtcacacagaaccatgctagcat

fava_c16481_140	fava_c16481	140	T/A	gctgataaaattcatagttacagcaaggatatcttgtataagtaaaaatctgttcggataaaagaaaactgaacatacagatatcatttacattttacac[T/A]Ycacaaccccccgctcctgaatttggagtcttttgcacttacagaagcgcgcaactgcacgcagctaagcaatctgggccactgaattgcgacgggacag

fava_c16481_141	fava_c16481	141	T/C	ctgataaaattcatagttacagcaaggatatcttgtataagtaaaaatctgttcggataaaagaaaactgaacatacagatatcatttacattttacacW[T/C]cacaaccccccgctcctgaatttggagtcttttgcacttacagaagcgcgcaactgcacgcagctaagcaatctgggccactgaattgcgacgggacagc

fava_c16481_1452	fava_c16481	1452	C/T	cacacagaaccatgctagcattgatcattggagtctgcaaagcgttgacagtgctcactataaaacactctgcatctatgaaagacaacaactgtggatc[C/T]tgaatatacttccgggctatcgctccagcattttgtggtaagttataggctaatgttaagcattcgagcggcttctgaaaaaactgtccgaaaaggtaga

fava_c16481_398	fava_c16481	398	G/C	ttgactggacaacctagttctataaccgtaaatgtgaggaatctggtttcctccctagacaaagaataaagaaactatataaaaaggtagtgttatacac[G/C]ataatacatagactcatgggtcaaacaaatttataacactcatgccagtgtcctgaaccaaccaagcaaccgaagcagcatggcatctaaaaccggtgac

fava_c16481_633	fava_c16481	633	T/C	gagcacacataactccagagaaagctacagcaataacaccttggccagggaagcaactgtctccaacacagtaaagaccatctatgcttgtcgtattaaa[T/C]ggcattcctaRtaaccccttcggaatgctgcgtggcattggtccataagtaccctcgtcacgggctagatatcgtcgatgtgtttttggtgtccctacct

fava_c16481_644	fava_c16481	644	A/G	actccagagaaagctacagcaataacaccttggccagggaagcaactgtctccaacacagtaaagaccatctatgcttgtcgtattaaaYggcattccta[A/G]taaccccttcggaatgctgcgtggcattggtccataagtaccctcgtcacgggctagatatcgtcgatgtgtttttggtgtccctacctctataaattcg

fava_c16481_909	fava_c16481	909	C/T	cttgcttctttgcctcatattcgattcttgagagaccctgccagtcatccatggaagaagttgtgaatatatgaaggatatgacgaccttcaggagctaa[C/T]gatgaatcaagaatagttggtatactcaaaaagatacttccatatggctcctccaaattKctccagttgttctcgagcacgaaatggtgacaatctgtat

fava_c16481_969	fava_c16481	969	G/T	ttgtgaatatatgaaggatatgacgaccttcaggagctaaYgatgaatcaagaatagttggtatactcaaaaagatacttccatatggctcctccaaatt[G/T]ctccagttgttctcgagcacgaaatggtgacaatctgtatctagtggcaaaacttctgctttaacccccatgtggattgaaagaaaagatggagctttca

fava_c16482_103	fava_c16482	103	G/A	ttttttgaagatttaattattttatttatataatattaagatctaaacaagcacaaagtgtgatcctttattgctacatactcagatattaaRatgcatt[G/A]ctaacaggttggtttgcaaacaaggtaatttcagattttctctatgaaagtccaKaaattgaggaaatgtgattctccagattatccaRctgtctcacat

fava_c16482_1162	fava_c16482	1162	C/T	taaacaagtgacatggggatcaaggttccgtccgaagcacgtgcccactgcctttcagtgacataatgcgttgcatcaaaacctccaagaaccgagtcaa[C/T]cttcttcaaaacagaaacaccagccttcatatcatagtcatacacagagggaggcgtcttcagtgagctatatgaaaatcgcaagatactYgacgaaaac

fava_c16482_1253	fava_c16482	1253	T/C	ccgagtcaaYcttcttcaaaacagaaacaccagccttcatatcatagtcatacacagagggaggcgtcttcagtgagctatatgaaaatcgcaagatact[T/C]gacgaaaactcagacRctgWagaatccactgagtatactggatcgataaaactaacaaccagaccattttcaagactccttagtggttcaccaatagatg

fava_c16482_1269	fava_c16482	1269	G/A	caaaacagaaacaccagccttcatatcatagtcatacacagagggaggcgtcttcagtgagctatatgaaaatcgcaagatactYgacgaaaactcagac[G/A]ctgWagaatccactgagtatactggatcgataaaactaacaaccagaccattttcaagactccttagtggttcaccaatagatggaaggtgataaactgt

fava_c16482_1273	fava_c16482	1273	T/A	acagaaacaccagccttcatatcatagtcatacacagagggaggcgtcttcagtgagctatatgaaaatcgcaagatactYgacgaaaactcagacRctg[T/A]agaatccactgagtatactggatcgataaaactaacaaccagaccattttcaagactccttagtggttcaccaatagatggaaggtgataaactgttatt

fava_c16482_1427	fava_c16482	1427	T/A	gactccttagtggttcaccaatagatggaaggtgataaactgttatttttggtagagcattttctctctcatatgcaacaagatgatcgctaaaaagttg[T/A]atctcctgaatcttcacactttctctgtggggaataagaactgtagttgaggaggtattgttgacagcacaagctacgacctctgaattgaaaaattgat

fava_c16482_1530	fava_c16482	1530	C/G	ctcctgaatcttcacactttctctgtggggaataagaactgtagttgaggaggtattgttgacagcacaagctacgacctctgaattgaaaaattgatca[C/G]ttctcctcttgataaagaaatgatccccRcgatggctgacagtagtgtcaataccatccacacgctgtgtcagaactttaagaccttcttcaggtttgga

fava_c16482_1559	fava_c16482	1559	A/G	gaataagaactgtagttgaggaggtattgttgacagcacaagctacgacctctgaattgaaaaattgatcaSttctcctcttgataaagaaatgatcccc[A/G]cgatggctgacagtagtgtcaataccatccacacgctgtgtcagaactttaagaccttcttcaggtttggaaacatcaagataaaaattgaaccttgtaa

fava_c16482_158	fava_c16482	158	G/T	aagtgtgatcctttattgctacatactcagatattaaRatgcattRctaacaggttggtttgcaaacaaggtaatttcagattttctctatgaaagtcca[G/T]aaattgaggaaatgtgattctccagattatccaRctgtctcacatcacaggcatagaaccagggtattatatgctgctggtctcacttaagccatagatt

fava_c16482_1717	fava_c16482	1717	C/G	tcttcaggtttggaaacatcaagataaaaattgaaccttgtaattttactttccgatccaacaaacagatattttttgctctcggaagcttcaaggtcaa[C/G]agaaaaagtatcatccttttcctcgtaaagacaagtatcttttgattgttctgttcccagcacatgaagccatgccttgtaaggcctaagaatattatcc

fava_c16482_1880	fava_c16482	1880	C/A	catgaagccatgccttgtaaggcctaagaatattatccattgttatataaactagagcatcgtcaccagcccattcgaggttcgatgttacactaacaag[C/A]ggctctccaattgtagcttgagtctcaatatccatgacataaacagtatatatttcatctcctttggtgtcttctgcatatgctaccaacttgctatttg

fava_c16482_192	fava_c16482	192	A/G	taaRatgcattRctaacaggttggtttgcaaacaaggtaatttcagattttctctatgaaagtccaKaaattgaggaaatgtgattctccagattatcca[A/G]ctgtctcacatcacaggcatagaaccagggtattatatgctgctggtctcacttaagccatagattaactaatcataatcactccatctcttcactgctt

fava_c16482_2201	fava_c16482	2201	C/T	gaaccttttggttactggggatgagRcgtctacaacgttgaacatactctttcccttccaaagtttttgtataatagtaataaggcccttggcgtacagg[C/T]gcagacacgtcctcctcctttatccttcYccttatctcagcaaaaagcttatcttcaatttccttagttccggacatgacggaatcggtgtaagcattct

fava_c16482_2230	fava_c16482	2230	T/C	ctacaacgttgaacatactctttcccttccaaagtttttgtataatagtaataaggcccttggcgtacaggYgcagacacgtcctcctcctttatccttc[T/C]ccttatctcagcaaaaagcttatcttcaatttccttagttccggacatgacggaatcggtgtaagcattctcctgacggagataatcaagtacatcggga

fava_c16482_298	fava_c16482	298	T/C	tcacatcacaggcatagaaccagggtattatatgctgctggtctcacttaagccatagattaactaatcataatcactccatctcttcactgcttctggg[T/C]gttRcgtatctgagactaaagctgattagtSatgtttagagactttaggaagaatgtataagtgaaggcgtcttcctgaagcttctcaaatcttcccgac

fava_c16482_302	fava_c16482	302	G/A	atcacaggcatagaaccagggtattatatgctgctggtctcacttaagccatagattaactaatcataatcactccatctcttcactgcttctgggYgtt[G/A]cgtatctgagactaaagctgattagtSatgtttagagactttaggaagaatgtataagtgaaggcgtcttcctgaagcttctcaaatcttcccgactttg

fava_c16482_329	fava_c16482	329	C/G	atgctgctggtctcacttaagccatagattaactaatcataatcactccatctcttcactgcttctgggYgttRcgtatctgagactaaagctgattagt[C/G]atgtttagagactttaggaagaatgtataagtgaaggcgtcttcctgaagcttctcaaatcttcccgactttgaaaaatgtccagcaccaagctcgcatt

fava_c16482_509	fava_c16482	509	T/C	gtccagcaccaagctcgcatttaaacaacagtatgttatcatcagttttcaaatcccttagttttgccacaaactttgcaggttcagaatacaaaacacg[T/C]ggatcatttaaaccagctgtaacaagaatgtgtgggtaattatgtgccttcacattatcaacgggggaatacgacttcatgtagaagtagaattcctcct

fava_c16482_95	fava_c16482	95	A/G	ttttttttgaagatttaattattttatttatataatattaagatctaaacaagcacaaagtgtgatcctttattgctacatactcagatattaa[A/G]atgcattRctaacaggttggtttgcaaacaaggtaatttcagattttctctatgaaagtccaKaaattgaggaaatgtgattctccagattatccaRctg

fava_c16484_1155	fava_c16484	1155	G/A	acgtgtctgcacttccagtggcaacaaaccttccatcaggactaaacttagcacatctggcaacactcttgtgttctgaaacgtgccgtgtctcatgctt[G/A]ggaaaactctttgacgaaccttttaaatctgccacagaactgaaatcaatggctgttgcagccggacggggcaagggcaaggatgcacccaaatcttgaa

fava_c16484_1356	fava_c16484	1356	C/T	agggagacgatgaagtccctcttgacacatcgtccttttccgctgcaagacccttagcaaccaagtcaagaagcttgttggatggggcttcaacattcaa[C/T]ggtgtcatagtagcagaagcaacagcagtagcagcctgagtgagattgcagtgacgaagatgagcgacgataagagaattgaggtgtctgaagagtttgc

fava_c16484_389	fava_c16484	389	T/A	acatgattagaaggccattttgctactatttccgttgtcatcgcatcccagataatgacctcgttattgagttcatctatcgatagaataaattcttccg[T/A]cgcgttaaaaatagcctggaatcttagttgtgtatgcRcggcaccgagatattgtttgactaatcttccagtgccaacctcccaaagctttatggtagag

fava_c16484_427	fava_c16484	427	A/G	catcgcatcccagataatgacctcgttattgagttcatctatcgatagaataaattcttccgWcgcgttaaaaatagcctggaatcttagttgtgtatgc[A/G]cggcaccgagatattgtttgactaatcttccagtgccaacctcccaaagctttatggtagagtccttcccacaggagaggacaaacctttgatctcttgt

fava_c16484_528	fava_c16484	528	G/A	cggcaccgagatattgtttgactaatcttccagtgccaacctcccaaagctttatggtagagtccttcccacaggagaggacaaacctttgatctcttgt[G/A]aaaattgcactggtagcttctgctgttgcatgtgctgcagttattgagcgcacacagttggcagtgatgccatcccacaaccgaatagcaccatctttaR

fava_c16484_708	fava_c16484	708	T/C	accgaatagcaccatctttagatgctgtaacatacatggaacctgtacaagaatatctaacctggtttatggctccattagggctattatctggaacatt[T/C]gctgacagataacactgaaaggtatttatatcatacaaatgtggaatcgcatgatcagtgcctgccagcagaaagtccccagaagggtgaaaagatacag

fava_c16485_1620	fava_c16485	1620	G/C	ttgaaattctcaacagggaagatgaatttaggaatgggaaggcctaaccttgttaaattctctaaagctttgaaaggtatgggatggttgcataatgtag[G/C]caagaatggaaagaagaataacaccactaaataattttttttttgtgttttgtaacttttgattagacttagaagttgtgcaattgtttgatgatgaatg

fava_c16485_276	fava_c16485	276	T/C	taaaacaaaaatcatcacaaaatccattcacagtgccacttttctactaaaccaaaccattgcttctctttctctttgctttgtcccaaacacagaaaaa[T/C]aggtaaaatgagtgagttatctttctcaaacacttccaacaagaccaatgaattctctagtaacatcttatccatcttgttccattcttgtttctctata

fava_c16485_379	fava_c16485	379	T/C	gtaaaatgagtgagttatctttctcaaacacttccaacaagaccaatgaattctctagtaacatcttatccatcttgttccattcttgtttctctatatt[T/C]tcacaccctttgtactttttttacttccttttcttctctccatatatccttaaacttctctcttttctttctcctttgttcatcaccactacacttctta

fava_c16485_863	fava_c16485	863	C/A	ttcagttgaagctagtgatgtttatgagttttcagttgaagcaagtgaagttttgagtataattgaagaagagaaaaaggtgaacttggatgaagaaaac[C/A]aagttgagaaagtagaaaaagttgttgttgaatccattaaggaagaaaaggtgttagatgtgaagagtttagtaacattgtttcaagaatatgctgagtt

fava_c16486_1097	fava_c16486	1097	A/G	aaatggaaatgaagaaaatggaggtgagagctgaaagaatgaaagctgtggcacaagaaaagttaacaaataaaatggctgcaacaaggagaatagctga[A/G]gagaagagagctaatgcacaagtaaaactgaatgacaatgctttgaagactactgagaaagtagagtatataagaaggacaggacatgtgccttcttcat

fava_c16486_809	fava_c16486	809	A/T	caattcattcgggtacttcgactccaatgaggaatcaaaatggattgttgcaaatggaagctgcatcaggtaatggtagcacaacaactagacaatgtgg[A/T]gaaggatcaagtccttgtagtatggttgaaaagaatatgaagattgatgatcaagctagaaaaatgggtcctctagaatgtagagcaatcgcttgggatg

fava_c16493_1151	fava_c16493	1151	G/C	tcactctccattgttcaagcttcagccatcaccattaatcgttcttcgcctcttctcttcaccctctcaatcgcaacaagccttcatctttctcatctct[G/C]acgcacaaactctctacatacaacagctcaacacactgaaKataatccctcaaactcgattcctccgccRcgccgagtcttcaatcaggtctgtttcacc

fava_c16493_1192	fava_c16493	1192	G/T	ttcttcgcctcttctcttcaccctctcaatcgcaacaagccttcatctttctcatctctSacgcacaaactctctacatacaacagctcaacacactgaa[G/T]ataatccctcaaactcgattcctccgccRcgccgagtcttcaatcaggtctgtttcacctttgttcttgtgcgttcacgtacgcttcgtcttctccgatt

fava_c16493_1221	fava_c16493	1221	A/G	tcgcaacaagccttcatctttctcatctctSacgcacaaactctctacatacaacagctcaacacactgaaKataatccctcaaactcgattcctccgcc[A/G]cgccgagtcttcaatcaggtctgtttcacctttgttcttgtgcgttcacgtacgcttcgtcttctccgatttatgctccctttRtttgatctaggatcca

fava_c16493_1305	fava_c16493	1305	A/G	actcgattcctccgccRcgccgagtcttcaatcaggtctgtttcacctttgttcttgtgcgttcacgtacgcttcgtcttctccgatttatgctcccttt[A/G]tttgatctaggatccatgagtttctttaggtttaggtttttcgacgtgttcctttaagtcgtgtttgagaactctRagcatgaggtgatgatagagtagt

fava_c16493_1381	fava_c16493	1381	G/A	tcttctccgatttatgctccctttRtttgatctaggatccatgagtttctttaggtttaggtttttcgacgtgttcctttaagtcgtgtttgagaactct[G/A]agcatgaggtgatgatagagtagtgtttgaaaggtgaagaaatggctgaaggttggtgattatccatggtatcgccgctgcaaccgatttcggcgagaaa

fava_c16493_159	fava_c16493	159	T/C	cacaatttacaaattgcattcgagtgctatcctatttcgacttcattcaagataacaaacttccattccatttacaccttgaaaaaaattacacaagaag[T/C]gaatattacatcaaaatacttttgaacctatgtttgctaatcttcaaattgcaaacatcaatacaacttcattgatctatgctttaagcttccaaatgcc

fava_c16493_269	fava_c16493	269	T/C	atcaaaatacttttgaacctatgtttgctaatcttcaaattgcaaacatcaatacaacttcattgatctatgctttaagcttccaaatgcctcagttttt[T/C]attcgaactctatactatcatgacaaaccaaacatgccattatatgagcagYcatgccttgcattaatcatcatgctacaacattgaagcttagccatcc

fava_c16493_321	fava_c16493	321	C/T	tacaacttcattgatctatgctttaagcttccaaatgcctcagtttttYattcgaactctatactatcatgacaaaccaaacatgccattatatgagcag[C/T]catgccttgcattaatcatcatgctacaacattgaagcttagccatccaacctacataaaaaacaatcaaaagttcaagacaaaatcaagtttcRagcca

fava_c16493_416	fava_c16493	416	G/A	agcagYcatgccttgcattaatcatcatgctacaacattgaagcttagccatccaacctacataaaaaacaatcaaaagttcaagacaaaatcaagtttc[G/A]agccatgttcagacctgcaaaactgagccatgccatgcttacaccttgttgatcactttcaggccatcaaatcttccaaaaYtaatccaccttcatgcct

fava_c16493_498	fava_c16493	498	C/T	aagacaaaatcaagtttcRagccatgttcagacctgcaaaactgagccatgccatgcttacaccttgttgatcactttcaggccatcaaatcttccaaaa[C/T]taatccaccttcatgccttcttaagcattacagccttgctgcagcatgaccaaaagcaacagccttgcaataaacatacctacataaaaccccatcacca

fava_c16493_957	fava_c16493	957	C/T	ctaactataacaactaactaaatcagtcactgaaactcccaaccaactctctaacagattgtaacataatatgtcagctcatgtaactgaattccaccct[C/T]taatcgatKcaatcatcacccttcatcttctaaccaaaaatcaccaccaaaaggctcttcaattttctctctctctgaactccatcttcaccttcactct

fava_c16493_966	fava_c16493	966	G/T	acaactaactaaatcagtcactgaaactcccaaccaactctctaacagattgtaacataatatgtcagctcatgtaactgaattccaccctYtaatcgat[G/T]caatcatcacccttcatcttctaaccaaaaatcaccaccaaaaggctcttcaattttctctctctctgaactccatcttcaccttcactctccattgttc

fava_c16496_1932	fava_c16496	1932	G/A	gaaccacgccccctgcattgaatcttaacatgcttgatgccctctttttttaaatccgtcactggatagtatcgagaggtgtttgtcaaatcaatcacca[G/A]accgagtttcctccctaaaactctctgttgatgtatcacttgtttaaatgagtatcttttaccaggaaaaatgcagtcattgaatgattcaccaagagga

fava_c16496_700	fava_c16496	700	A/G	ccttattcccatccatgagtttcttctttccccgctcattcacaaaaagtagctcccggtcaccttcgacctcaaacagaaagtcgactgaatttaaatt[A/G]gcgtatttccactttaagagtccttcatgagtacgtggtacataaggatcgtcccaaccctgaaatatgagaccatctgcctcatgtgagagtcttttga

fava_c16498_1343	fava_c16498	1343	T/C	ttatgaataatgggagaccttagctattttttgttttctataagtaaaacggagccatacattacagctgatgcctagaatctggtatcttttacaacaa[T/C]gaatgatggatcgagttcaaactagaaccagcaagaagctatagctacaatttggaaaattttagcattcaaaatcacctaacctagacaaatattaaWa

fava_c16498_1442	fava_c16498	1442	A/T	aYgaatgatggatcgagttcaaactagaaccagcaagaagctatagctacaatttggaaaattttagcattcaaaatcacctaacctagacaaatattaa[A/T]aaaaaaaatgggttcaacagtaacacgctataaaagcctaattgaagacatcggattaataacaaaattaattttaagagaccRatactttcaagtggta

fava_c16498_1526	fava_c16498	1526	G/A	cctagacaaatattaaWaaaaaaaatgggttcaacagtaacacgctataaaagcctaattgaagacatcggattaataacaaaattaattttaagagacc[G/A]atactttcaagtggtacattcatatttgtttggtagcagaaaagagtctcatgaactttcatgatttcagattgcttctattagcgccgagcagttctgg

fava_c16498_2312	fava_c16498	2312	G/A	ccacctttgcggccacgtgaacaaaaaagtcatgcacatttgtcatgactttgggaagagactgcaataacgaggatccattattagaagcatttttttc[G/A]gattctaggagaagcagctgctctaactcttgaatccactgatgacattcaccaagatatttctcaaatcgaagaattgtttgctgtaaaaagggtgatg

fava_c16498_2641	fava_c16498	2641	G/A	ctaggatgatggaaccttggacgtaggatcataaacgatcgaacagcaacctctgtattacgtaacatatccttaacaactgacatcaattcatgtagca[G/A]agttttctgccgttccatagcagtgctgattccaccaagttcctgaacaatatggcttgcatcaacctcaaacccatcattcgaaacggaagaatcataa

fava_c16500_1120	fava_c16500	1120	T/C	catacattttaactcttcaactctatggacagtcgctatattccataaaatcggtactgcattcggattgttcgcttcttcctttgttacaattgcagct[T/C]tgtctaaccatttgaattgattcgagtgtgaaagtcttccttccaacgYtataggagaRtcaagctcgatattttggtataaaagctcaRgatcatttgg

fava_c16500_1169	fava_c16500	1169	C/T	atcggtactgcattcggattgttcgcttcttcctttgttacaattgcagctYtgtctaaccatttgaattgattcgagtgtgaaagtcttccttccaacg[C/T]tataggagaRtcaagctcgatattttggtataaaagctcaRgatcatttggcaatacctcgttcctctttttRaacgcggccacaatcacttgcgYgact

fava_c16500_1179	fava_c16500	1179	G/A	cattcggattgttcgcttcttcctttgttacaattgcagctYtgtctaaccatttgaattgattcgagtgtgaaagtcttccttccaacgYtataggaga[G/A]tcaagctcgatattttggtataaaagctcaRgatcatttggcaatacctcgttcctctttttRaacgcggccacaatcacttgcgYgactcgaacaaacg

fava_c16500_1210	fava_c16500	1210	G/A	aattgcagctYtgtctaaccatttgaattgattcgagtgtgaaagtcttccttccaacgYtataggagaRtcaagctcgatattttggtataaaagctca[G/A]gatcatttggcaatacctcgttcctctttttRaacgcggccacaatcacttgcgYgactcgaacaaacgggcttccgtttggtttcacagtcttatagag

fava_c16500_1242	fava_c16500	1242	A/G	tcgagtgtgaaagtcttccttccaacgYtataggagaRtcaagctcgatattttggtataaaagctcaRgatcatttggcaatacctcgttcctcttttt[A/G]aacgcggccacaatcacttgcgYgactcgaacaaacgggcttccgtttggtttcacagtcttatagagtgatgtgcccaaaacaaagRcaatgatagata

fava_c16500_1265	fava_c16500	1265	C/T	aacgYtataggagaRtcaagctcgatattttggtataaaagctcaRgatcatttggcaatacctcgttcctctttttRaacgcggccacaatcacttgcg[C/T]gactcgaacaaacgggcttccgtttggtttcacagtcttatagagtgatgtgcccaaaacaaagRcaatgatagatataaacattgcaatggtgggaatg

fava_c16500_1330	fava_c16500	1330	A/G	gttcctctttttRaacgcggccacaatcacttgcgYgactcgaacaaacgggcttccgtttggtttcacagtcttatagagtgatgtgcccaaaacaaag[A/G]caatgatagatataaacattgcaatggtgggaatgccaaggccccaaccccaacccatgtgatcttgaatataaaccacaatggttaaagcacttaatgt

fava_c16500_139	fava_c16500	139	C/T	ggtctgatatggactatgattacaaacaataacatagttttatgatattatgatatggaaaacaaaaataaacatggcaggtacttacatttgatcaaaa[C/T]atgtatacattcattatggtacaatatttctaccaacaagatcataaattcattcatcctttgcaaattctcttttYtcatcctccttaccctcttttaa

fava_c16500_216	fava_c16500	216	T/C	caggtacttacatttgatcaaaaYatgtatacattcattatggtacaatatttctaccaacaagatcataaattcattcatcctttgcaaattctctttt[T/C]tcatcctccttaccctcttttaaactaaccaatgagacttctccRttcacctgttccaaatctccttgcttgtttatttcactaatttcctccacaggct

fava_c16500_261	fava_c16500	261	G/A	caatatttctaccaacaagatcataaattcattcatcctttgcaaattctcttttYtcatcctccttaccctcttttaaactaaccaatgagacttctcc[G/A]ttcacctgttccaaatctccttgcttgtttatttcactaatttcctccacaggcttgtaagtgtaaaaccaagcacatactacaaaatatatgaagttta

fava_c16500_617	fava_c16500	617	T/A	tacaaagccgtagcactcgtcctcatactttccggcgattgatcgtatagaaactctaaatgtccaacagacataaaaacttcagccgcaccatgcaaaa[T/A]atactgaggtaccaaccaaaacacacttattggaataatcgctttcggattatccaataaatgatattcggaagcaacttttttYctcttaatttccact

fava_c16500_702	fava_c16500	702	T/C	ccgcaccatgcaaaaWatactgaggtaccaaccaaaacacacttattggaataatcgctttcggattatccaataaatgatattcggaagcaactttttt[T/C]ctcttaatttccactagtgctgcaactatagtgcctgtaatgttaatcacatagcctattcccatYctctgcaaacatgtaaggccagtaggattctttg

fava_c16500_768	fava_c16500	768	T/C	tatccaataaatgatattcggaagcaacttttttYctcttaatttccactagtgctgcaactatagtgcctgtaatgttaatcacatagcctattcccat[T/C]ctctgcaaacatgtaaggccagtaggattctttgtgaatctacgagcataYggaacgaaaaggcgttcgtagagaacaacaccWatcatcatagttagta

fava_c16500_819	fava_c16500	819	C/T	gtgctgcaactatagtgcctgtaatgttaatcacatagcctattcccatYctctgcaaacatgtaaggccagtaggattctttgtgaatctacgagcata[C/T]ggaacgaaaaggcgttcgtagagaacaacaccWatcatcatagttagtacactgaagattgccatattagccggcgaaagctggaatgaatgcgagagat

fava_c16500_852	fava_c16500	852	A/T	catagcctattcccatYctctgcaaacatgtaaggccagtaggattctttgtgaatctacgagcataYggaacgaaaaggcgttcgtagagaacaacacc[A/T]atcatcatagttagtacactgaagattgccatattagccggcgaaagctggaatgaatgcgagagatgacgattcatcatgcgagcttgttggattacaa

fava_c16500_993	fava_c16500	993	T/C	gcgaaagctggaatgaatgcgagagatgacgattcatcatgcgagcttgttggattacaaagctactttgatgtgatgctgcagttatgagtaaaattcc[T/C]gatgcccaaattggaagcattctaatcatacattttaactcttcaactctatggacagtcgctatattccataaaatcggtactgcattcggattgttcg

fava_c16507_1031	fava_c16507	1031	A/T	accatgttcatgttgctccaaccattgctgtcagtgacagtgctttgaaatttactcatgttctctacaatctgtccccagctgaactgtatgagcaggc[A/T]atcaagtatgagaaaggatcctttgttacttcYaatggagcattagcaacactttctggtgctaagacaggtcgctctccgagagacaagcgtgtcgtta

fava_c16507_1064	fava_c16507	1064	C/T	gtgacagtgctttgaaatttactcatgttctctacaatctgtccccagctgaactgtatgagcaggcWatcaagtatgagaaaggatcctttgttacttc[C/T]aatggagcattagcaacactttctggtgctaagacaggtcgctctccgagagacaagcgtgtcgttaaggaYgatgttactgaaagtgaactttggtggg

fava_c16507_1136	fava_c16507	1136	T/C	agtatgagaaaggatcctttgttacttcYaatggagcattagcaacactttctggtgctaagacaggtcgctctccgagagacaagcgtgtcgttaagga[T/C]gatgttactgaaagtgaactttggtggggaaagggctctcctaacattgagatggacgaggagacttttttggtgaacagagaaagagctgtcgattact

fava_c16507_1658	fava_c16507	1658	T/A	tgcattatctcatgcctaagcgccaaattctctccttacactctggttgcaatatgggcaaagatggtgatgttgcactcttctttggactctcaggtac[T/A]ggaaagactactctttctaccgatcataataggtatttgatMggagatgatgaacattgctggagtgagaatggYgtctccaacattgaaggtggttgtt

fava_c16507_1700	fava_c16507	1700	A/C	ctggttgcaatatgggcaaagatggtgatgttgcactcttctttggactctcaggtacWggaaagactactctttctaccgatcataataggtatttgat[A/C]ggagatgatgaacattgctggagtgagaatggYgtctccaacattgaaggtggttgttatgctaaatgcattgatctStccMgggagaaagagcctgata

fava_c16507_1733	fava_c16507	1733	C/T	cactcttctttggactctcaggtacWggaaagactactctttctaccgatcataataggtatttgatMggagatgatgaacattgctggagtgagaatgg[C/T]gtctccaacattgaaggtggttgttatgctaaatgcattgatctStccMgggagaaagagcctgatatctggaacgcaattaggtttggtacagtgctgg

fava_c16507_1778	fava_c16507	1778	G/C	ccgatcataataggtatttgatMggagatgatgaacattgctggagtgagaatggYgtctccaacattgaaggtggttgttatgctaaatgcattgatct[G/C]tccMgggagaaagagcctgatatctggaacgcaattaggtttggtacagtgctggaaaacgtggtgtttgatgagcacactagagaagttgattactctg

fava_c16507_1782	fava_c16507	1782	C/A	tcataataggtatttgatMggagatgatgaacattgctggagtgagaatggYgtctccaacattgaaggtggttgttatgctaaatgcattgatctStcc[C/A]gggagaaagagcctgatatctggaacgcaattaggtttggtacagtgctggaaaacgtggtgtttgatgagcacactagagaagttgattactctgacaa

fava_c16507_2424	fava_c16507	2424	G/C	agagtacaagaagagtaagatttttggccttgagaccccaacagctgtagagggtgtcccttcagaaattctcgatcctgtgaatgcgtggactgacaaa[G/C]atgcctacaatgagacattgttgaagttggctggattgttcaggaagaattttgaaacattcaccaactacacaattggaaagggtggtgacaacctcac

fava_c16507_2603	fava_c16507	2603	A/G	gaaagggtggtgacaacctcacagaagagattcttgctgctggaccaatcttttgaagaagcaatatcttgtgttgttaggatgttggaaaatgaataaa[A/G]tgtggScttRgaataattatattggttgggttaggttcatatgttttaatcttgtaYgagtgtaatgcatttgtctaatgtgttggtctttacatttttt

fava_c16507_2609	fava_c16507	2609	C/G	gtggtgacaacctcacagaagagattcttgctgctggaccaatcttttgaagaagcaatatcttgtgttgttaggatgttggaaaatgaataaaRtgtgg[C/G]cttRgaataattatattggttgggttaggttcatatgttttaatcttgtaYgagtgtaatgcatttgtctaatgtgttggtctttacattttttaaccct

fava_c16507_2613	fava_c16507	2613	A/G	tgacaacctcacagaagagattcttgctgctggaccaatcttttgaagaagcaatatcttgtgttgttaggatgttggaaaatgaataaaRtgtggSctt[A/G]gaataattatattggttgggttaggttcatatgttttaatcttgtaYgagtgtaatgcatttgtctaatgtgttggtctttacattttttaacccttata

fava_c16507_2660	fava_c16507	2660	T/C	gaagcaatatcttgtgttgttaggatgttggaaaatgaataaaRtgtggScttRgaataattatattggttgggttaggttcatatgttttaatcttgta[T/C]gagtgtaatgcatttgtctaatgtgttggtctttacattttttaacccttatatgttaagaaaaatgccatctcaatgattcttttccctagttttctct

fava_c16507_557	fava_c16507	557	C/T	cggggatcaccaatttgttttttccattacattgttacaccaaaagttgcaccacattttttcaaccatttccttcgtttctctatagttttttcatttc[C/T]agWtttcctcatttcctcatcaataattttctgaagatggctgaaaacggaaatggaaacgttgcaactgaaaacgggctagSgaagattcRgacgcaga

fava_c16507_560	fava_c16507	560	T/A	ggatcaccaatttgttttttccattacattgttacaccaaaagttgcaccacattttttcaaccatttccttcgtttctctatagttttttcatttcYag[T/A]tttcctcatttcctcatcaataattttctgaagatggctgaaaacggaaatggaaacgttgcaactgaaaacgggctagSgaagattcRgacgcagaaag

fava_c16507_640	fava_c16507	640	G/C	tatagttttttcatttcYagWtttcctcatttcctcatcaataattttctgaagatggctgaaaacggaaatggaaacgttgcaactgaaaacgggctag[G/C]gaagattcRgacgcagaaagctctgaacgggatttgtcacgatgatagtggtcctacggtgaaagcgaaaacSattgatgaacttcactcgttgcagacg

fava_c16507_649	fava_c16507	649	G/A	ttcatttcYagWtttcctcatttcctcatcaataattttctgaagatggctgaaaacggaaatggaaacgttgcaactgaaaacgggctagSgaagattc[G/A]gacgcagaaagctctgaacgggatttgtcacgatgatagtggtcctacggtgaaagcgaaaacSattgatgaacttcactcgttgcagacgaagaaatct

fava_c16507_713	fava_c16507	713	C/G	gaaacgttgcaactgaaaacgggctagSgaagattcRgacgcagaaagctctgaacgggatttgtcacgatgatagtggtcctacggtgaaagcgaaaac[C/G]attgatgaacttcactcgttgcagacgaagaaatctgcaccatctacacctaatacagggatttcatctccgtttgcttctctctctgaacaagaacgcc

fava_c16508_111	fava_c16508	111	A/T	aatattaaaattgacaaatagcattacgaagcacaagctgcacaactatttcacaatttgttacttcacatccacaccaRacataatttacaaataataa[A/T]aaaatcaataccactcttggaatatacaatattttacacatgaaacaaatcatatttaaaattttaaattgaaaaagaaaaaacttgtaaatcttattta

fava_c16508_90	fava_c16508	90	G/A	cttctttttgaatattaaaattgacaaatagcattacgaagcacaagctgcacaactatttcacaatttgttacttcacatccacacca[G/A]acataatttacaaataataaWaaaatcaataccactcttggaatatacaatattttacacatgaaacaaatcatatttaaaattttaaattgaaaaagaa

fava_c16509_1064	fava_c16509	1064	G/A	attgctttacctaatagtaacaatttgttgcaaagaccaaatcacccttcatttcttggtttgttaaatacaatgccatttggttctagttacaattggg[G/A]agtttcttcaaRtgatcatgSttttgcaaatcaagaagttgttagtgcaattacatctttgccaccaatgttgtctttatcaactggWaattcttctcaa

fava_c16509_1076	fava_c16509	1076	A/G	aatagtaacaatttgttgcaaagaccaaatcacccttcatttcttggtttgttaaatacaatgccatttggttctagttacaattgggRagtttcttcaa[A/G]tgatcatgSttttgcaaatcaagaagttgttagtgcaattacatctttgccaccaatgttgtctttatcaactggWaattcttctcaaatgttgtttgga

fava_c16509_1085	fava_c16509	1085	C/G	aatttgttgcaaagaccaaatcacccttcatttcttggtttgttaaatacaatgccatttggttctagttacaattgggRagtttcttcaaRtgatcatg[C/G]ttttgcaaatcaagaagttgttagtgcaattacatctttgccaccaatgttgtctttatcaactggWaattcttctcaaatgttgtttggaacatcacaa

fava_c16509_1152	fava_c16509	1152	A/T	gttacaattgggRagtttcttcaaRtgatcatgSttttgcaaatcaagaagttgttagtgcaattacatctttgccaccaatgttgtctttatcaactgg[A/T]aattcttctcaaatgttgtttggaacatcacaatcatatttttcttcaaatgtWaatgcaatggagatggaacatcatcaacaacatcaacatcaacaKc

fava_c16509_1206	fava_c16509	1206	A/T	ttagtgcaattacatctttgccaccaatgttgtctttatcaactggWaattcttctcaaatgttgtttggaacatcacaatcatatttttcttcaaatgt[A/T]aatgcaatggagatggaacatcatcaacaacatcaacatcaacaKcatcaaagacaaatcaataatcattatcatcaaatgttgagttcgtcgagttcgc

fava_c16509_1251	fava_c16509	1251	G/T	gWaattcttctcaaatgttgtttggaacatcacaatcatatttttcttcaaatgtWaatgcaatggagatggaacatcatcaacaacatcaacatcaaca[G/T]catcaaagacaaatcaataatcattatcatcaaatgttgagttcgtcgagttcgcaaaatccgttgaataattctctcaacaatccatctttcagtttgg

fava_c16509_1412	fava_c16509	1412	C/T	ccgttgaataattctctcaacaatccatctttcagtttggccaaatttctagaaaactcatcaaataaagaacaaggcttctccccaaagtgatcttact[C/T]aaagacacaaatacaaaaggttatcttatttgaattctcctcttctcaaaatttgcatggttcttgaagattgtgtgatgWgaaaactagtttctttagt

fava_c16509_1493	fava_c16509	1493	A/T	tccccaaagtgatcttactYaaagacacaaatacaaaaggttatcttatttgaattctcctcttctcaaaatttgcatggttcttgaagattgtgtgatg[A/T]gaaaactagtttctttagttatagttatatattgagatttgaataatcatcacttattgtttttttttttgtgagtgcctgtttggattttctatatagt

fava_c16509_208	fava_c16509	208	T/C	tgaaggttcaaatctacttcttcttcttcttcaacattattttcatacctatcaaatccaaaaccaagcttctaaatttcaaacttttctctaccttcaa[T/C]cttcatcattcactcactacttataagaatgagcctactaaaagtggaaggaagagaatcttcaccaagcaaccaacaatgaaagaaattagaagatgag

fava_c16509_715	fava_c16509	715	G/T	ttggttacttaatattgcaaaacatgaaattgatcaacttccaccattacctaattttccacctctaggagcaaatttcaacttcggttttcctcttccg[G/T]ctaacgaatcgatcaccatcgccaacaccgacgccaacacttgtcaaactcatcaacaattgttgaatttcaataggagtaacaatattcattgggacgg

fava_c16509_932	fava_c16509	932	A/T	gaatcgaaagaggtttcgagagaaatggttatggaaaaagggaattattggatgaataatagaagtactactcaagaagaggtaaatgttaacacaaagc[A/T]aggtagtaacaatgttgaagttcctagtgcaattgctttacctaatagtaacaatttgttgcaaagaccaaatcacccttcatttcttggtttgttaaat

fava_c16511_1059	fava_c16511	1059	C/T	aggaaggatgttgatcaaactaggtttgatttggaagatgtggttgctgagtgcttggctcaagagaatggatatgagtatggggttgaaggattagttt[C/T]gcaaaaagattctcagtatctttcattgcaaggtaatgcggatagagaaggcatcagggatgttgatatgctgcagatttcacctgaatgtttatcattg

fava_c16511_1391	fava_c16511	1391	G/C	cagaaaggttatactgagaaagttactagagaaggatattggggcggaaatcatgccaccatcatttgttaatggtcaattgaaatgtaaaataaggcga[G/C]agaggataaaggctgcaagaaagcgaataaaaagaataactcatccattacacgctgatctcgatgagactgtaactcctggcattttagatagcacttg

fava_c16511_1699	fava_c16511	1699	G/A	agaaggggcattacaataccttactaggcctacacgtgtttaaccccggcgttgcataagagaggtttaagatatgtttcagatttactggtgttttagt[G/A]atctattggatactgatattttctgattgacaacaattttatgatagagcagatattttcatactgcaaattttgtcctcaaactttttatatattcatt

fava_c16511_237	fava_c16511	237	A/T	ggcgccgttcaacccttcgaccaatacgaatccttcttcaacggcatcaacggcccacaaggaaccttcgtccacatcggaacctcaggttcaagtaaca[A/T]ctactcttacaaagagagaaaactattcgccgcgcgtaattccattgaagagttcacaaataggttagggttatcttccaaaacaatggaaatYaaaacc

fava_c16511_331	fava_c16511	331	C/T	gtaacaWctactcttacaaagagagaaaactattcgccgcgcgtaattccattgaagagttcacaaataggttagggttatcttccaaaacaatggaaat[C/T]aaaaccatgatttccgatatcaccgaaggcgaattcggtcagggaaactggtttcaggttctgattggggcctgttgttatgtggttatgcggaagggtg

fava_c16511_955	fava_c16511	955	G/T	tgttgccttgggggaaagatataactaagaagaatattgttaagaatgctccgattgtgattcagtatatggagaagaagtctatgtcaaaacctgttga[G/T]aagaggaaggatgttgatcaaactaggtttgatttggaagatgtggttgctgagtgcttggctcaagagaatggatatgagtatggggttgaaggattag

fava_c16512_1111	fava_c16512	1111	T/C	atgcttattctgatagtcattgtgacgaggggataagagaaggtgagcgttgtgccaaatcgtttatcgatcctgcacataataatgcgaagaaacagtc[T/C]tccgataatggagggaaagctataaatagatccgactctgactctgttgacctgttttatgatgcatgcgagattaacaatggaRgtgatgaagttcatc

fava_c16512_1196	fava_c16512	1196	A/G	tgcgaagaaacagtcYtccgataatggagggaaagctataaatagatccgactctgactctgttgacctgttttatgatgcatgcgagattaacaatgga[A/G]gtgatgaagttcatcaagtcaaagttccttcttcagaggctatgaaagagaatttggataataataaaaataatattattgatgctccgacatcaatggc

fava_c16512_2164	fava_c16512	2164	T/G	ttgaaaacacaggtaggaaggtctttgaaccagaagaatctgatcatacaatagaaatggttaaggaatattgggggccgaagaatgttgaagaaaaggt[T/G]Ygtgcagttaacaaagcaagttctcatgaagaacctgtccatgaaactaaacataggtgtcaagaagttgctggtaaaacaaacctgattcaggagacac

fava_c16512_2165	fava_c16512	2165	C/T	tgaaaacacaggtaggaaggtctttgaaccagaagaatctgatcatacaatagaaatggttaaggaatattgggggccgaagaatgttgaagaaaaggtK[C/T]gtgcagttaacaaagcaagttctcatgaagaacctgtccatgaaactaaacataggtgtcaagaagttgctggtaaaacaaacctgattcaggagacact

fava_c16512_221	fava_c16512	221	A/G	tcgtgctgcttctcttattcaacgtgtctctgactctgcaaaaatcacttctcagagtagcaaaacacaacgaaacaaaacacctacgaagaagaatctc[A/G]ccgtcgccatggagttcggagccaccgcagcgacgctaacaaagaagctacctaacggccttggcgtcagcggaagatccgcttacgacggtgttttcgc

fava_c16512_3587	fava_c16512	3587	G/A	aacaactcccgagtctagagaaatatataaggatttgaaggaggttagagttgtgtcagatgaagagattgatgagaatagatcccactcttctagtgaa[G/A]catctcagatacctagtatgtctgaatggaaatcaagccccttcaaagaaaaggaagttaaatcaattaacagcaaaataaaggagagtcatcaaacatc

fava_c16512_4669	fava_c16512	4669	G/A	gcgttcagcaaaagtacacttgtgagaaggttttcgatcttctaaaggaagcttggaacagattcaacatggaagaaaggtgaagagtgcatggtaattg[G/A]gcttctaatgaccatgcgctcgctgatgacgattctttcaagtgaataattcaattaaaagtaagctcatattgtatgtagtaggcaagtttagatggcc

fava_c16513_1012	fava_c16513	1012	C/T	atatagcgaaaattttgttggtcaatggtgcagatagaatgatgaaaaataaggatggaaaaacagcattggatctaagtttatgttacggaaaagactt[C/T]atgtcttatgaccttgctagattactgaaggtagtaccagccaactgagttctataatgtgcgagatgtaattgagcagaaccaggttgaagtttcagtt

fava_c16513_1142	fava_c16513	1142	A/T	ggtagtaccagccaactgagttctataatgtgcgagatgtaattgagcagaaccaggttgaagtttcagttaaatgttatgtgcttctaattggtgcttc[A/T]agcttcttcaatcgtctcggagatcttagagatagatctttctcaaaatgatgaaaYctttgaYggaatcttcgacacgaRtaatcattggaatattttt

fava_c16513_1199	fava_c16513	1199	T/C	ttgaagtttcagttaaatgttatgtgcttctaattggtgcttcWagcttcttcaatcgtctcggagatcttagagatagatctttctcaaaatgatgaaa[T/C]ctttgaYggaatcttcgacacgaRtaatcattggaatatttttgcagcatagctttaactcacataacttttgtgaaaaaccaaattcatagtgcagatt

fava_c16513_1206	fava_c16513	1206	T/C	ttcagttaaatgttatgtgcttctaattggtgcttcWagcttcttcaatcgtctcggagatcttagagatagatctttctcaaaatgatgaaaYctttga[T/C]ggaatcttcgacacgaRtaatcattggaatatttttgcagcatagctttaactcacataacttttgtgaaaaaccaaattcatagtgcagattataagtg

fava_c16513_1223	fava_c16513	1223	A/G	tgcttctaattggtgcttcWagcttcttcaatcgtctcggagatcttagagatagatctttctcaaaatgatgaaaYctttgaYggaatcttcgacacga[A/G]taatcattggaatatttttgcagcatagctttaactcacataacttttgtgaaaaaccaaattcatagtgcagattataagtgctcagcaaatttctcaa

fava_c16513_1417	fava_c16513	1417	T/C	ttctcaattatcatttcaaatatgtaacaaagatgtatctaatgggtgtttgtgttttttataatgaattgtaacaaagtagatgcagttcataaatttc[T/C]gtttcattgaaaagttgtttataaatattgattgacagtgaaccatttactaattaacgtgttcatactactaaaattt

fava_c16513_266	fava_c16513	266	C/T	gtttactccattttctatcatcaatactaattctgcaattaactccaaattcagagccggtttccatagaatttacggatgcacagaagaaaagttatgt[C/T]tcgctttctccagggatgattRtacRgttgcaaggaatgttgcaatcaaatcaagcagagaaataccttatttggaaacccaacaagaacatggaacttg

fava_c16513_288	fava_c16513	288	A/G	aatactaattctgcaattaactccaaattcagagccggtttccatagaatttacggatgcacagaagaaaagttatgtYtcgctttctccagggatgatt[A/G]tacRgttgcaaggaatgttgcaatcaaatcaagcagagaaataccttatttggaaacccaacaagaacatggaacttgggaagattctgaYagtgaagac

fava_c16513_292	fava_c16513	292	A/G	ctaattctgcaattaactccaaattcagagccggtttccatagaatttacggatgcacagaagaaaagttatgtYtcgctttctccagggatgattRtac[A/G]gttgcaaggaatgttgcaatcaaatcaagcagagaaataccttatttggaaacccaacaagaacatggaacttgggaagattctgaYagtgaagacgaga

fava_c16513_379	fava_c16513	379	T/C	gggatgattRtacRgttgcaaggaatgttgcaatcaaatcaagcagagaaataccttatttggaaacccaacaagaacatggaacttgggaagattctga[T/C]agtgaagacgagagccttggatttgaaagtgatggggaagagacacagacttcacaaactgYcggtgttaatatcaMtgcacaggacgagtatgagcgta

fava_c16513_441	fava_c16513	441	C/T	gaaacccaacaagaacatggaacttgggaagattctgaYagtgaagacgagagccttggatttgaaagtgatggggaagagacacagacttcacaaactg[C/T]cggtgttaatatcaMtgcacaggacgagtatgagcgtattaagaaagaggttgaacagcttttggaacctgaagaaagaaaaatattgcaacaaaatata

fava_c16513_456	fava_c16513	456	A/C	catggaacttgggaagattctgaYagtgaagacgagagccttggatttgaaagtgatggggaagagacacagacttcacaaactgYcggtgttaatatca[A/C]tgcacaggacgagtatgagcgtattaagaaagaggttgaacagcttttggaacctgaagaaagaaaaatattgcaacaaaatataactcctgacttggaa

fava_c16513_657	fava_c16513	657	T/A	aaaatatcgtctgaaaaatggaacccactgcactctcttgcgttatcactgcaaatttatagcatggaccggcttcttgaaaatggttacgatattgatt[T/A]cctcaataaggaaggtctcactgcacttcataaggcaattataggtaaaaatgaagcggttataagccatcttttacggaaaggtgcaagtcctcatgtc

fava_c16513_846	fava_c16513	846	A/G	agtcctcatgtcaaggataaggatggagctactccacttcactatgcagttcaagttggtgcaaagcaaactgtgaaattattgattaaatacaacgttg[A/G]Ygtcaacgttgcagataaccaaggttggaccccgttgcatgttgccattcaaagccggaatagagatatagcgaaaattttgttggtcaatggtgcagat

fava_c16513_847	fava_c16513	847	C/T	gtcctcatgtcaaggataaggatggagctactccacttcactatgcagttcaagttggtgcaaagcaaactgtgaaattattgattaaatacaacgttgR[C/T]gtcaacgttgcagataaccaaggttggaccccgttgcatgttgccattcaaagccggaatagagatatagcgaaaattttgttggtcaatggtgcagata

fava_c16514_2223	fava_c16514	2223	G/T	agcagggaggacggtcacggtctttaggagagtgacgatcgtcgtcggaaagatcgttttcgggaattagcggggaagaatcgccggcgccagcgctgtc[G/T]cgcttcatcggagaagaagcggttcgggagccgattgaggaggagtgacggagctccgccatcgagatggagcaccgtttctgtttctctctgtataatt

fava_c16514_395	fava_c16514	395	A/G	agaggaagaaaagtgggacatgaggtattgctacatgttagatgaaaagaaaaagaaaaaaaaccaccaggtttctcggcaaggagaaccctcaatagta[A/G]gaaatctattctttaaagtgataaccatcagtcgaacctcatttgctgcaagccattacatcttctccaaatctctatttactgtccctcctacctcagg

fava_c16514_537	fava_c16514	537	G/A	tttgctgcaagccattacatcttctccaaatctctatttactgtccctcctacctcagggtgaattaaccacagaccatgttttcacactttaccatagg[G/A]atatatagaactcataacttagatggtataattcttggaaatgtgaaagtcccagYgtaaagcatcaataaaaatagctacggtcaggctttacacatgc

fava_c16514_593	fava_c16514	593	C/T	agggtgaattaaccacagaccatgttttcacactttaccataggRatatatagaactcataacttagatggtataattcttggaaatgtgaaagtcccag[C/T]gtaaagcatcaataaaaatagctacggtcaggctttacacatgcaaggRgtcagagggttctcccacaggtcgtagttagggaaagtctcttctggcaat

fava_c16514_642	fava_c16514	642	A/G	atagaactcataacttagatggtataattcttggaaatgtgaaagtcccagYgtaaagcatcaataaaaatagctacggtcaggctttacacatgcaagg[A/G]gtcagagggttctcccacaggtcgtagttagggaaagtctcttctggcaatcccgtccgtgtctgatgagtcacaataacatcttctacaaaaggagccc

fava_c16514_834	fava_c16514	834	A/G	aaggagcccaacccatgtacggatccccaaaagacttggacattagtcctttgtcaggcttaattgactttagacggtgccccatgtatcttcgggcccc[A/G]attatcagcttagcaaaattgcctccatgggtcatcacaaatacatctgacttcaagcacactaagaaatcaagagctgcaaggctcgtcacatgctttc

fava_c16515_296	fava_c16515	296	T/C	acaaggaaaattacagggaaaattttgtttctctacttttttccctctatcgaggattggacacatgaagccacgttttagttcaatcattatggtagag[T/C]tcatctaattgacacattcaaggttgaaccaacattctcttagaatcttccctctttagggctcactaccaactagccatgtcaagaatccacgcaccct

fava_c16516_1265	fava_c16516	1265	C/T	aacttctagttcaggttgatggtgtaagcaacagtggtacaaatgaagatggtagcaagaaaatagtaatggtcttggcagctactaactttccatggga[C/T]atagatgaagcactaaggagaaggctggaaaagagaatatatattcctcttccaaatttcgagagtcgtaaagagttgatccggataaatttgaagactg

fava_c16516_1562	fava_c16516	1562	C/T	taaatggaatgagacggaagatagcaggaaagacacgcgatgaaattaagagtatgtctaaggatgatatttcaaaggatcctgttgccatgtgtgattt[C/T]gaagaagctttggcaaaggtccaacgaagtgtttctccggcagatattgaacgccatgagaagtggtttcacgaatttggatcagcataaacactcacca

fava_c16516_263	fava_c16516	263	T/A	ctatcatcttctttgatggtgctgttgcacaaatcaacaggcatctaaacagtgttgacgaccctttgattcgtgcaaaatggatgaatgttaagaaagc[T/A]ctttgtgaggaaactgaagttgtaaagcaacttgatgcagataaaagggcKtttaaagataatcctattggaagacgtcctccgtcgcctccaatttcta

fava_c16516_314	fava_c16516	314	G/T	gtgttgacgaccctttgattcgtgcaaaatggatgaatgttaagaaagcWctttgtgaggaaactgaagttgtaaagcaacttgatgcagataaaagggc[G/T]tttaaagataatcctattggaagacgtcctccgtcgcctccaatttctactaaatcttcgtttgtttttcagccgttagatgagtatcctacttcatcga

fava_c16523_1128	fava_c16523	1128	A/C	tcttcacatagataacatgcctcctattgctatgactcggagctgcattaagagcctcgttgattgtacggtagtgaccgctcccgtccaacgcaacaac[A/C]gcatctgcaaYggtaccgcgaggcttagatttaagaagctcttgatcagcctcggtcatccattcaggaaactccaagttttcatcatgcagggtagtgt

fava_c16523_1139	fava_c16523	1139	T/C	ataacatgcctcctattgctatgactcggagctgcattaagagcctcgttgattgtacggtagtgaccgctcccgtccaacgcaacaacMgcatctgcaa[T/C]ggtaccgcgaggcttagatttaagaagctcttgatcagcctcggtcatccattcaggaaactccaagttttcatcatgcagggtagtgttcctcggaggc

fava_c16523_1473	fava_c16523	1473	C/A	cttggttgctaagtgccgcacttagccaagcttccaggtttccctcgtattgcgcggttctgtcgccagctcgaattctcctcatctcacctaacgacca[C/A]gcaagctctgatacggagaaatcaagaagctccttgcaatcttctattgcaagttgctcgcggttattaacagaaaaagtactgatcttcgtcatgttgt

fava_c16523_1773	fava_c16523	1773	T/C	ttgtgaggcacgagttttggttttcaatatccatgcaggcttgtgcaatcaaagcttgcatgtcttgggtttgcatttctgagggaaattcatcttggaa[T/C]gagagaacctgatcaaatgaaggaaaaaggatgagaacgaaaaggaaatttacaagaaatgccatctttccgttgtgacagaatcggaaagacagacaaa

fava_c16523_390	fava_c16523	390	C/G	ccgtaaagaaaccggcggtagaagcgtctttgatgacatgataaccaggccaattaaccctacctgcaagtgatgaaccaggaccgtaatttctgtattc[C/G]ccgtaccagagagtgtccaatgcaaagtttccaagccactcgagccagcctcgaggttgaaccatactactcatgtatgtgttaatgtaaactgttctag

fava_c16523_813	fava_c16523	813	G/A	ggtagaattgtctcagcgagtgtgcgtaaagcgtgtcttggtgcccttctacgctacacctaaagaatgcagattggtcggaatccacacgcagtgccac[G/A]gcttgatggtttactggtccagctgtgttccggaatgtcatgtccctcgcaatgaaacccttcccagatacagcaaacgtggcagttcgaaaagttgtcc

fava_c16526_2134	fava_c16526	2134	T/C	atctgaatctgaatggaggtggcaccatcagatatttatagaaactgttgatgttttttaattctgagtcattaagcagatttgcttgctatatattttt[T/C]tccccacttgagtcattatgcagatgtcctctttggtaataaacaaacaattggagtgtatcttcaacactccatttctttgtaattctttgagtcttaa

fava_c16526_393	fava_c16526	393	T/G	tctcttctttacctactgactctgacctagccgatgcttttctccacagaaaacttagggaaggatctgcgcgaggatttgggatacggaaaagtaattc[T/G]actgtaggatcggattcttgggtggatgatccgattactcggcatataataggtgtttacgttaacaaagaattggaggatgataagaatcttttgtggc

fava_c16526_756	fava_c16526	756	A/T	taacaggggcatttatgattgcacatgccattggtgacataattactggtgatatagtagaacctgaagtgatcatagctgcagctccttctgttggtgg[A/T]ctgtttgattcattaactggtagtatgggcatatcttcccgggccaaacctatatctccacaagttccttcttcctctcctatgggaacagctggacaag

fava_c16527_230	fava_c16527	230	T/G	caagagacttaatagcttgttgttataaatacattatggaacatggtaaaaaaaaacgattgtccatcaatgacaatagttatttacaaaccattttcta[T/G]aacttgtRgcagaaaacctatgcacggctaagaaccactctaaactcatctStcattctctgaatgaccatttccactagtattatattacatatctaaa

fava_c16527_238	fava_c16527	238	A/G	ttaatagcttgttgttataaatacattatggaacatggtaaaaaaaaacgattgtccatcaatgacaatagttatttacaaaccattttctaKaacttgt[A/G]gcagaaaacctatgcacggctaagaaccactctaaactcatctStcattctctgaatgaccatttccactagtattatattacatatctaaacatgttga

fava_c16527_282	fava_c16527	282	G/C	aaaacgattgtccatcaatgacaatagttatttacaaaccattttctaKaacttgtRgcagaaaacctatgcacggctaagaaccactctaaactcatct[G/C]tcattctctgaatgaccatttccactagtattatattacatatctaaacatgttgagtaactttgggaccaacttcaacactatcaccttctgaaacata

fava_c16528_1003	fava_c16528	1003	T/C	tatctgttccaagtgtcttggccaaacaaggactaacttcaaattctttttcaatgtgttttggggatgatggttatggaagaatcRcatttggggataa[T/C]aataatagccYggaccaaggaaaaacaccattcaacattaggccattgcattcaacttacaacatcactgtcacccaaattattgtcggaggaaatgttg

fava_c16528_1014	fava_c16528	1014	C/T	agtgtcttggccaaacaaggactaacttcaaattctttttcaatgtgttttggggatgatggttatggaagaatcRcatttggggataaYaataatagcc[C/T]ggaccaaggaaaaacaccattcaacattaggccattgcattcaacttacaacatcactgtcacccaaattattgtcggaggaaatgttgctgatctcgag

fava_c16528_1550	fava_c16528	1550	T/C	cggttaccgtatagtcttcgatcgcgagaacatgactctaggttggagagaatctaactgttatgatgatgaagtctcgagtttacctgttaaccgatcg[T/C]acgctcctgctRtttctcctgctacggctgtgaatcctgaaaaaacatctaaccagtctaatggtcctgaaaYaataccatctagtcactcattgaagat

fava_c16528_1562	fava_c16528	1562	G/A	agtcttcgatcgcgagaacatgactctaggttggagagaatctaactgttatgatgatgaagtctcgagtttacctgttaaccgatcgYacgctcctgct[G/A]tttctcctgctacggctgtgaatcctgaaaaaacatctaaccagtctaatggtcctgaaaYaataccatctagtcactcattgaagataaaacctgcttt

fava_c16528_1623	fava_c16528	1623	T/C	gtctcgagtttacctgttaaccgatcgYacgctcctgctRtttctcctgctacggctgtgaatcctgaaaaaacatctaaccagtctaatggtcctgaaa[T/C]aataccatctagtcactcattgaagataaaacctgcttttgcatttacagtgtcaatttttctggttttggccattttttgagcatacatttagctgtta

fava_c16528_373	fava_c16528	373	G/A	agggtattttcggtattgacaacctacccgacaaagggactcgtgaatattacgttgctatggcccacagagaccgcgtgtttcacgctcgccgccttgc[G/A]gacggcggtgctattgatcagaaacttcttacgttttatcccgataactccacctaccagatttcctcgtttggatatttgcattttgcgaatgtttcgg

fava_c16528_517	fava_c16528	517	T/A	ataactccacctaccagatttcctcgtttggatatttgcattttgcgaatgtttcggttggaacaccggcttcatggtttcttgttgcattggatactgg[T/A]agtgacttgttctggcttccttgcaattgtacgaaatgtgttcatggtggtctacagaaatcatcacagaaaatcgagtttaatatctatgataataaga

fava_c16528_635	fava_c16528	635	A/G	tccttgcaattgtacgaaatgtgttcatggtggtctacagaaatcatcacagaaaatcgagtttaatatctatgataataagagatcatccacaagtaaa[A/G]atgttgcttgcaatagcagcttatgtgcgcagccggcactttgttcttcatctggtggcacttgtccatatcaagttcaatatttatcagaaaatacttc

fava_c16528_826	fava_c16528	826	T/C	aaaatacttcaacttctggtttcttggtggaagatgtgttgcatctaattacagatcatggtgatcaaactaaacaggctaacccactagttacttttgg[T/C]tgtgggcaagttcagactggtgcttttctgactggggcagctccaaatggcttgtttggattaggaatgagtgatgtatctgttccaagtgtcttggcca

fava_c16528_989	fava_c16528	989	G/A	aggaatgagtgatgtatctgttccaagtgtcttggccaaacaaggactaacttcaaattctttttcaatgtgttttggggatgatggttatggaagaatc[G/A]catttggggataaYaataatagccYggaccaaggaaaaacaccattcaacattaggccattgcattcaacttacaacatcactgtcacccaaattattgt

fava_c16529_1251	fava_c16529	1251	T/A	gaaaggaaaccttggcatggaaaagattgaagacccatctctttaccactatgctatcttttctgataatgtactggctgcatctgtggttgttaattct[T/A]ctgtgcaaaatgcaaacgaccctgagaagcatgtattccatRtagtaactgacaaattaaattttccgtcaatgagaatgtggtttctcatcaaccctcc

fava_c16529_1293	fava_c16529	1293	A/G	ttaccactatgctatcttttctgataatgtactggctgcatctgtggttgttaattctWctgtgcaaaatgcaaacgaccctgagaagcatgtattccat[A/G]tagtaactgacaaattaaattttccgtcaatgagaatgtggtttctcatcaaccctccttctaaagcaaccattgaagttcagaatattgatgatttcaa

fava_c16529_147	fava_c16529	147	A/C	aaagttatgattctagaaataaagaaatgggaacacaactcacaaaacaatgatgatctgatctaaacccaaatagaccaacacagtgatgtaacatctc[A/C]ctcccatggaacttgttcWattcttcttcttctcggttctgttttttcttcatgttgcagattccacaaccatWggatcttgtgaccaatgttatcgcac

fava_c16529_166	fava_c16529	166	A/T	taaagaaatgggaacacaactcacaaaacaatgatgatctgatctaaacccaaatagaccaacacagtgatgtaacatctcMctcccatggaacttgttc[A/T]attcttcttcttctcggttctgttttttcttcatgttgcagattccacaaccatWggatcttgtgaccaatgttatcgcaccaaggaatttgatgttact

fava_c16529_1746	fava_c16529	1746	C/A	aattgatttgaaaggtatggtgatcggtgcagtagagacatgcaaggagagcttccataggtttgataaatacctaaattttagcaatccactgatctcc[C/A]ataatttcagtcccgatgcatgtggttgggcatttggcatgaatgtctttgacttgaaggagtggaagaagcgaaacatYaccggaatctatcatcggtg

fava_c16529_1826	fava_c16529	1826	C/T	ttagcaatccactgatctccMataatttcagtcccgatgcatgtggttgggcatttggcatgaatgtctttgacttgaaggagtggaagaagcgaaacat[C/T]accggaatctatcatcggtggcaagatttgaatgaggacagaaccctctggaagctcggaaccttacccccaggYttaatcaccttttataaactgaact

fava_c16529_1901	fava_c16529	1901	C/T	tgaaggagtggaagaagcgaaacatYaccggaatctatcatcggtggcaagatttgaatgaggacagaaccctctggaagctcggaaccttacccccagg[C/T]ttaatcaccttttataaactgaactatccactagatcggggttggcatgttttgggacttggctatgatccagctctgaacttaacggagatagagaatg

fava_c16529_221	fava_c16529	221	A/T	agaccaacacagtgatgtaacatctcMctcccatggaacttgttcWattcttcttcttctcggttctgttttttcttcatgttgcagattccacaaccat[A/T]ggatcttgtgaccaatgttatcgcaccaaggaatttgatgttactgcatctagaaaccgaaattatcgcgatgagaaggatattgatataattgcaacct

fava_c16529_334	fava_c16529	334	T/C	caatgttatcgcaccaaggaatttgatgttactgcatctagaaaccgaaattatcgcgatgagaaggatattgatataattgcaacctatagcgatgctt[T/C]cggtcatattcggctggctaggttgaaaatgagggacttatcggattcttgggtttgggaaaacccgagtaatgctaacaatgaacagcaaaattactcc

fava_c16529_557	fava_c16529	557	A/G	caagtgattcaagatttgaagacaatctcaagcactcagcagatgaacatgatgctgaagaaggctttgctcgatttccacattccttaccgatgactcc[A/G]gtgaagatcaagcgtcggatgatgcgtctggaaagaagaaaagcccgtgctgctgaacttattaagcaaaacaaagaaacggacWatcgcattgtatcag

fava_c16529_642	fava_c16529	642	T/A	cttaccgatgactccRgtgaagatcaagcgtcggatgatgcgtctggaaagaagaaaagcccgtgctgctgaacttattaagcaaaacaaagaaacggac[T/A]atcgcattgtatcagcagcaattgaacRctccaaaggatttgatactactatcactggaaagtatagtatatggaggaaggaatatgaaaacccgaattc

fava_c16529_670	fava_c16529	670	G/A	cgtcggatgatgcgtctggaaagaagaaaagcccgtgctgctgaacttattaagcaaaacaaagaaacggacWatcgcattgtatcagcagcaattgaac[G/A]ctccaaaggatttgatactactatcactggaaagtatagtatatggaggaaggaatatgaaaacccgaattctgattccactcttaaactcatgcgagac

fava_c16533_1073	fava_c16533	1073	C/T	gaacaactggtgtaaggtgttttgcagcagcagccatcactatccgtcccacttttccatttcctgtatagaaaattttgtcccacttttgttgcagcaa[C/T]gcagtagtttcatccactgccccttccacaactcttatagacgagttatccaagtactcccctaacaatttcgccagcagtgaagatgaagccggagcaa

fava_c16533_1261	fava_c16533	1261	C/T	gaagccggagcaatttctgatggctttagaaccacaacattaccagctgctatagctccgacgactggatcaagggacagcaaaaaaggatagttccatg[C/T]ggagataactaacacaacaccaagtggttcaggtatgatttcagccgaagacggaaaagttgtgattgaagttttagccttttctggtgtcatccactgt

fava_c16533_1385	fava_c16533	1385	G/A	gtggttcaggtatgatttcagccgaagacggaaaagttgtgattgaagttttagccttttctggtgtcatccactgtttcaattccttgagcacgagttc[G/A]catgagtttttaaacataccaatctcgtagacgacggtttcaagtggcggtttagcaaggtcagaRcgaagagcatcaacgatttgatcctcttgttcag

fava_c16533_1451	fava_c16533	1451	G/A	tcatccactgtttcaattccttgagcacgagttcRcatgagtttttaaacataccaatctcgtagacgacggtttcaagtggcggtttagcaaggtcaga[G/A]cgaagagcatcaacgatttgatcctcttgttcagctatcattttcaaaagagccttcacctgcgatattctccattcataacttcgcgttttaccagaac

fava_c16533_185	fava_c16533	185	C/T	aatgtgaaaagtaaaatacaataaaaccaaaacaaatgatacaacaccagaaaagcagccacatttgaaaacaaattaaagcttaggaccatccaattag[C/T]gcacgaactacaccaggaacatcaccggctactagagccttcatcattttttgcttcgtgtcagtgtaaggtgggtaccttatcgatgaatcgcctgtaa

fava_c16533_377	fava_c16533	377	A/G	cgcctgtaaagccgcgataaagaactgcctttctgtgagtaaaggcatcaaaggagaattttccatggtatgcacccattccactgtcaccaactccccc[A/G]aatggcaatgtatgaaccacaagatgtaaRacagtgtcattgataagcaaacccccagcagaaacattcttcacaaattgctccttgaacttgttgtcgt

fava_c16533_407	fava_c16533	407	G/A	ttctgtgagtaaaggcatcaaaggagaattttccatggtatgcacccattccactgtcaccaactcccccRaatggcaatgtatgaaccacaagatgtaa[G/A]acagtgtcattgataagcaaacccccagcagaaacattcttcacaaattgctccttgaacttgttgtcgtttgtaaataaatatgccgcaagtggctttg

fava_c16533_515	fava_c16533	515	G/A	cattgataagcaaacccccagcagaaacattcttcacaaattgctccttgaacttgttgtcgtttgtaaataaatatgccgcaagtggctttgttcccgc[G/A]ttgatcaaatcgaagctttcttccagcttattcaccgtgactattggaagcaatggaccaaagatttcctcactcattatcagggaatcttgcggaacgt

fava_c16533_692	fava_c16533	692	A/T	ttatcagggaatcttgcggaacgtctaataaaatagttggagcaatcctcaatttgctttcatccttttcacctccataaactatcttgccagaaacctt[A/T]tcctcgtccaagaactttgtcaagcgagcaaagtggttagtgtttacaatgcgcgacaagtctttcgattctattgggctctttccaaagcagttttcta

fava_c16533_875	fava_c16533	875	G/A	ttccaaagcagttttctaattcagtctttaaagcgtctaccaatttcggagcaaattctttcgttgttataagataatcaggagaaatgcaggcttgtcc[G/A]ttgttacagccccatttgccggaaattatccgtcttgcagcaacctctaaattgacatttgaatcaacaacagctggagattttcctccaagctccagaa

fava_c16534_1656	fava_c16534	1656	A/G	agggtatgactgatattgctcggttgcttgttattcgttacctaaggaataatagcgctgttgatacgaggacacaggaaatgtctaagtggaaatcatt[A/G]ttggtccaagatataatcgatgatactgatcaggcaggccagtttgtagctgggtacaaaggcacagctgatgattcagagatatcccagtggcttgcaa

fava_c16534_2030	fava_c16534	2030	A/G	gatccgagaatcggtcctttcctgcttttggcagccttaatttctctgggcaccatctggctgagaagaagccaacaagctcaacaagctcaaccatctc[A/G]gtctgaacaaccaagttccacggagcctccctcccgtgatgaaagaaaatcgagacctactgaccgggttcagagacggtctggtaaaaatgcgccccca

fava_c16534_207	fava_c16534	207	C/T	ctcccccctcaccgtcaacggcttccacaatcaaagcctacgcacttcctgttattctcttcgccgcagccatgttctaccagctctttgttatccccaa[C/T]gccttccctccttctcattatgacgttctgcaaattgataggtatagttcgacagacacggtgaaggaggcttacaacaatcttgaatctaaatggaatt

fava_c16534_2145	fava_c16534	2145	G/A	gttccacggagcctccctcccgtgatgaaagaaaatcgagacctactgaccgggttcagagacggtctggtaaaaatgcgcccccaagcatgacagattc[G/A]gaaccacccaatgctttccagatgccactttcagactctgaatctgattagttttaatttggtttctccttaaagaaaaggagcttttcatgacataatg

fava_c16534_495	fava_c16534	495	G/T	acgacttatttggcattgatgaacagcatcatatcattgaggaggccaacaggcgttatgcagggaaaaatatttcacagttggacttccctttactaaa[G/T]KccYctttctctgagtctattgatcattctactaaagtcatctctgcaagtgatttccagtctatatttccagattcaaagctatggttaatYcagttgt

fava_c16534_496	fava_c16534	496	T/G	cgacttatttggcattgatgaacagcatcatatcattgaggaggccaacaggcgttatgcagggaaaaatatttcacagttggacttccctttactaaaK[T/G]ccYctttctctgagtctattgatcattctactaaagtcatctctgcaagtgatttccagtctatatttccagattcaaagctatggttaatYcagttgta

fava_c16534_499	fava_c16534	499	C/T	cttatttggcattgatgaacagcatcatatcattgaggaggccaacaggcgttatgcagggaaaaatatttcacagttggacttccctttactaaaKKcc[C/T]ctttctctgagtctattgatcattctactaaagtcatctctgcaagtgatttccagtctatatttccagattcaaagctatggttaatYcagttgtattc

fava_c16534_588	fava_c16534	588	T/C	tactaaaKKccYctttctctgagtctattgatcattctactaaagtcatctctgcaagtgatttccagtctatatttccagattcaaagctatggttaat[T/C]cagttgtattcatctgggagcaatcgttgtgctgagttctctaagtcttggaataaaatagcttctttgttggatgcatttgcaaacactggtatggtgg

fava_c16535_1314	fava_c16535	1314	C/T	caggcgagagatgaaagggttggtgtcgaagatgctccatgaaaatgcttctcagaatgatcttcggaaggagtcattatactcggcatgtgatgattgt[C/T]tgcagttacttcttcaccattttttgcgggctgcggcttcagacctgcaggatgtgagtcaaattRcacgtcaagcggataatttgcattggattttgga

fava_c16535_1380	fava_c16535	1380	G/A	gaaggagtcattatactcggcatgtgatgattgtYtgcagttacttcttcaccattttttgcgggctgcggcttcagacctgcaggatgtgagtcaaatt[G/A]cacgtcaagcggataatttgcattggattttggatattttgattgatagacagattgccgaggatttcctgaaRacatgggcRtctcaattagaattatc

fava_c16535_1454	fava_c16535	1454	A/G	cagacctgcaggatgtgagtcaaattRcacgtcaagcggataatttgcattggattttggatattttgattgatagacagattgccgaggatttcctgaa[A/G]acatgggcRtctcaattagaattatcggaagctcattctaaggtgcctgcagttcacaggtttgaggttagcagagttactgccaggttgtttgttggga

fava_c16535_1463	fava_c16535	1463	G/A	aggatgtgagtcaaattRcacgtcaagcggataatttgcattggattttggatattttgattgatagacagattgccgaggatttcctgaaRacatgggc[G/A]tctcaattagaattatcggaagctcattctaaggtgcctgcagttcacaggtttgaggttagcagagttactgccaggttgtttgttgggattggaaagg

fava_c16535_1940	fava_c16535	1940	A/G	acggagagcaagacaggacaagacaattacgaattacatctgcatcaattgagcagagctgaagacgatatttttccacggcagggtaaatggctgcact[A/G]tataattggtttgtcatcttttcaccttctcatgatcatctctatctgattgctctactattatcagtatttaaatcttctttcagaccccagtagtttc

fava_c16535_2182	fava_c16535	2182	C/T	tctcattgtctcgatgcttttgcttggatgaagaaaacattgtttattaacacctctcattttgttggaaataaccagagggaacattggtttaatagta[C/T]gacagggtgcttagaactgttttccttttctcacttcatatttgttgtattcaccaaaaaaaaacttcatatctattgtgtgatttactcgtaggctgat

fava_c16535_703	fava_c16535	703	C/G	gaaacccttatgatgcagcgaatttccgatttgttagcttcccggagccctgggaaccacttcaacgattcatcttccagtgatattaagcttacgctta[C/G]ttccaaagacggcttcagtttttcgatgaacgtgcatcgtcagattcttgttgcgcatagtaggtttttctctgtcaagctttctgatcggtggacgcag

fava_c16537_1023	fava_c16537	1023	G/A	tactacMccatctaaaaatagacctttgccagcatccttgtgagatatttgagaaggtttaacattgagagtgtagccaagttcgtccttcaacttctgc[G/A]aaagctcagcttgacttatcRgtctttgtttcRggaaaccattggcggaactaagctcactatttcgaggtaaaacWtttgtttgttgagatagtaactc

fava_c16537_1044	fava_c16537	1044	G/A	acctttgccagcatccttgtgagatatttgagaaggtttaacattgagagtgtagccaagttcgtccttcaacttctgcRaaagctcagcttgacttatc[G/A]gtctttgtttcRggaaaccattggcggaactaagctcactatttcgaggtaaaacWtttgtttgttgagatagtaactcaaacgagccgtcattcctggt

fava_c16537_1056	fava_c16537	1056	G/A	atccttgtgagatatttgagaaggtttaacattgagagtgtagccaagttcgtccttcaacttctgcRaaagctcagcttgacttatcRgtctttgtttc[G/A]ggaaaccattggcggaactaagctcactatttcgaggtaaaacWtttgtttgttgagatagtaactcaaacgagccgtcattcctggtcttttcatttgt

fava_c16537_1100	fava_c16537	1100	A/T	caagttcgtccttcaacttctgcRaaagctcagcttgacttatcRgtctttgtttcRggaaaccattggcggaactaagctcactatttcgaggtaaaac[A/T]tttgtttgttgagatagtaactcaaacgagccgtcattcctggtcttttcatttgtaagaagaatttcatccattaacgtgcaaaatgttttaatttgag

fava_c16537_474	fava_c16537	474	C/T	caactgcttcgtgttgctcagcctgtagttcaggagaagttcttcgtcttgaagagccctagttgctaccaaaacaacagttttcagagtagtaggaaca[C/T]gtgattcagtattttttgaaactctagatttgaaccaagaacttccaaatctcttcacatttacttctgttgcatttccaaataatatattaggaatgta

fava_c16537_599	fava_c16537	599	T/C	tagatttgaaccaagaacttccaaatctcttcacatttacttctgttgcatttccaaataatatattaggaatgtaggctctcatgtcattttcaaccaa[T/C]ggaaagtcataagggcaaattgtgacatttggaagcatcccttttgaagggtgattagcaaaatgtgccaacgctagtggattcctacgctcgagtacat

fava_c16537_792	fava_c16537	792	G/C	gagtacatcgtcactattatctacttttgaccctttctcgtctaccatttttccaccattccacatttctttcctgttacctccacagccccaaggttgg[G/C]cgtcgatgacagtcccatcatgtcttgtaatcaaataggggttctgctcatcaagatatccaggaatatgatgataataagctggagagtatactacccc

fava_c16537_929	fava_c16537	929	A/C	aggggttctgctcatcaagatatccaggaatatgatgataataagctggagagtatactaccccgggataaaaggctatcacagcaccaacatctactac[A/C]ccatctaaaaatagacctttgccagcatccttgtgagatatttgagaaggtttaacattgagagtgtagccaagttcgtccttcaacttctgcRaaagct

fava_c16540_1467	fava_c16540	1467	G/A	taatcatccaaatcaatatttcagtgatagtcaaaagatattgcacccaaagaaggactcttcaacctaaaatattctattataagagagtgatgcaggg[G/A]cagtcattcacagcaattaatggacRgatgcagagatggggatatcttttaccagcttccaaagtgattctaaggtggaattaggcattccagttatata

fava_c16540_1493	fava_c16540	1493	A/G	atagtcaaaagatattgcacccaaagaaggactcttcaacctaaaatattctattataagagagtgatgcagggRcagtcattcacagcaattaatggac[A/G]gatgcagagatggggatatcttttaccagcttccaaagtgattctaaggtggaattaggcattccagttatatattatcctacaagttgagtagggaatc

fava_c16540_1625	fava_c16540	1625	A/C	tccaaagtgattctaaggtggaattaggcattccagttatatattatcctacaagttgagtagggaatctggcacgataatactgtatttctatattgtt[A/C]Rcatctctgtagagactggcctgcatgtaatctgattatagggaatgaatcttgatttgtgttaatctcgcggttcataaatgtcaataagttttgatag

fava_c16540_1626	fava_c16540	1626	G/A	ccaaagtgattctaaggtggaattaggcattccagttatatattatcctacaagttgagtagggaatctggcacgataatactgtatttctatattgttM[G/A]catctctgtagagactggcctgcatgtaatctgattatagggaatgaatcttgatttgtgttaatctcgcggttcataaatgtcaataagttttgatagg

fava_c16540_818	fava_c16540	818	T/A	agctgtcaaagacactcatccttacaaacagaaaatggacatcttcaatcagagaacaggagaagcataggttgactccgattgtggaagccttgtgctc[T/A]agttatctaggtcctgatttttctcagcctaaggaatatgctgagatatctttaaaagacattgatcaacttgccaagacctcatttcctctgtgcatgc

fava_c16545_1049	fava_c16545	1049	T/C	aacctgtttcctaaatggaaaaagaactttatgttgcaccagacaattgggaaaaaatgccacaggagggacctcaattatcttggcaacattcgaactt[T/C]ggttcagactattcaagtatgattttgagtgtaccactagaaggtcatcttgagaagcttcaagaggctcaacaatgcgaYtttgatccaaMagaccaaa

fava_c16545_1130	fava_c16545	1130	T/C	cttggcaacattcgaacttYggttcagactattcaagtatgattttgagtgtaccactagaaggtcatcttgagaagcttcaagaggctcaacaatgcga[T/C]tttgatccaaMagaccaaaagagacaaggaaattacaaattcgtccccatttggatgaatcaaaagggtgcagtttctctatgccgagaaatgatatgtc

fava_c16545_1141	fava_c16545	1141	A/C	tcgaacttYggttcagactattcaagtatgattttgagtgtaccactagaaggtcatcttgagaagcttcaagaggctcaacaatgcgaYtttgatccaa[A/C]agaccaaaagagacaaggaaattacaaattcgtccccatttggatgaatcaaaagggtgcagtttctctatgccgagaaatgatatgtcgtaagagttgg

fava_c16545_294	fava_c16545	294	A/G	tatatactttgaagtttccactggtatataaacattttacaacagatcatttttcagggtcagcaaatcacacaaaaaccttataaagtgtaagtgcagg[A/G]tcactgttatacagcctttcgacctccactcgtttctatcaagcatttgttctcgagattcacaattgagtcagcaatgactctagcactggacttcatg

fava_c16549_1230	fava_c16549	1230	C/T	tcttgaggaaactagatgcagaactgaagcatgaaatctgccactgggcagcatattatgagcataggatgcgccttgggcagaaggcaatttttcacat[C/T]gaagcatttgtggctaagttcatgagcgtctacaaatccttcctcattgcaacatttggctgaagaaaggatgatggttttcttttccatgatatatttt

fava_c16549_762	fava_c16549	762	C/T	aagcccaacattctctccgcagaacaatggagaaatacagtgcttattgcagattaattctatgttgcaatagttcttcaagagtaacagaagcaattcg[C/T]tctcgttgtcttaatgtacgaataaatgcaccaagtcaggaacagattgttgaagttttacagttcattggtaagaaagaaggacttcaacttcctccta

fava_c16549_868	fava_c16549	868	G/A	ttgtcttaatgtacgaataaatgcaccaagtcaggaacagattgttgaagttttacagttcattggtaagaaagaaggacttcaacttcctcctaacttt[G/A]ctgctcgcatagcagagaaatctaacaggaatttaaggagggccatattgtcatttgagacttgccgtgtccaacagtatccttttactgacaagcaaac

fava_c16553_457	fava_c16553	457	T/A	aacaccagcaaatgattgaagaacttgtggaggaaaatgagaagcttaggcagatactggtagaagagctgaaagtaccatcgagcaaacttgaatcggg[T/A]tctacaggtagaattagaactaagttgccatgtactgattgttttgagtgccgtagaaagcaaagaagaaagtaggttcgaaatgataattactaatata

fava_c16553_751	fava_c16553	751	C/T	agtggacatagtaaaagcaaatgttcatctggccaagaaaaattaagaaaatagtacagcaaaagactggagtagagtaaggttctgtttggcgggaggg[C/T]gaatggagaggagaagagaggaattacttcaaatcttagttgtttggttcaatgttttggagggaagagatggaaacaaaatatggattcataggagaga

fava_c16558_1011	fava_c16558	1011	A/G	gagctgtttggatattgcaccatggacaggattttRgttttgtttcttgattttggatctgcttggcaaggcatcgtgaaaccagtttgttaaaggcctg[A/G]acataacttRggtttggaagtcgcctWcagggcttggtaaagtttggaatgaagatgaatgaacaccaagattaggctttaggaccttagKaatttttKt

fava_c16558_1021	fava_c16558	1021	A/G	gatattgcaccatggacaggattttRgttttgtttcttgattttggatctgcttggcaaggcatcgtgaaaccagtttgttaaaggcctgRacataactt[A/G]ggtttggaagtcgcctWcagggcttggtaaagtttggaatgaagatgaatgaacaccaagattaggctttaggaccttagKaatttttKtcacWcttttt

fava_c16558_1038	fava_c16558	1038	A/T	aggattttRgttttgtttcttgattttggatctgcttggcaaggcatcgtgaaaccagtttgttaaaggcctgRacataacttRggtttggaagtcgcct[A/T]cagggcttggtaaagtttggaatgaagatgaatgaacaccaagattaggctttaggaccttagKaatttttKtcacWctttttatgtattttgtactgaa

fava_c16558_1102	fava_c16558	1102	G/T	aaaggcctgRacataacttRggtttggaagtcgcctWcagggcttggtaaagtttggaatgaagatgaatgaacaccaagattaggctttaggaccttag[G/T]aatttttKtcacWctttttatgtattttgtactgaattttgggatgggatttgtatggttattttgtttggatttggactgtagttttgtttagataaac

fava_c16558_1110	fava_c16558	1110	G/T	gRacataacttRggtttggaagtcgcctWcagggcttggtaaagtttggaatgaagatgaatgaacaccaagattaggctttaggaccttagKaattttt[G/T]tcacWctttttatgtattttgtactgaattttgggatgggatttgtatggttattttgtttggatttggactgtagttttgtttagataaactctgaata

fava_c16558_1115	fava_c16558	1115	T/A	taacttRggtttggaagtcgcctWcagggcttggtaaagtttggaatgaagatgaatgaacaccaagattaggctttaggaccttagKaatttttKtcac[T/A]ctttttatgtattttgtactgaattttgggatgggatttgtatggttattttgtttggatttggactgtagttttgtttagataaactctgaataatgtc

fava_c16558_465	fava_c16558	465	G/A	tttgaaggttgaaagttgatgcgttgattgttgaagtttgttgaagtaggggatgagaaagcttgtgcgatgaagatagatgtgaaacaacagatttgca[G/A]tgtagatgtgaagaagatgatgatgcggttgtgatgctgttgacataggagcgatgaagatgcgtgaagataattgatgcgaaggatgtagaattgaagt

fava_c16558_600	fava_c16558	600	G/A	tgctgttgacataggagcgatgaagatgcgtgaagataattgatgcgaaggatgtagaattgaagtgtgatgaagatgatggctgaagttgcgtgaagtt[G/A]MagatgctttgttgatggaaggtggagcaaaagagaggaagaacgagtttcagagaaaggagtgagagatgaagattRcagagagaaggttatgtgattc

fava_c16558_601	fava_c16558	601	C/A	gctgttgacataggagcgatgaagatgcgtgaagataattgatgcgaaggatgtagaattgaagtgtgatgaagatgatggctgaagttgcgtgaagttR[C/A]agatgctttgttgatggaaggtggagcaaaagagaggaagaacgagtttcagagaaaggagtgagagatgaagattRcagagagaaggttatgtgattct

fava_c16558_678	fava_c16558	678	G/A	atggctgaagttgcgtgaagttRMagatgctttgttgatggaaggtggagcaaaagagaggaagaacgagtttcagagaaaggagtgagagatgaagatt[G/A]cagagagaaggttatgtgattcttctatgaatattaaaggatgaagatgatgaaaagcagagagaaggttgaagattcttctgcagaatctttcagaatc

fava_c16558_946	fava_c16558	946	G/A	aattatatgcaaggggaggttttggtttttaatagatgcaagagctgagtttgaactgagtttgtgagctgtttggatattgcaccatggacaggatttt[G/A]gttttgtttcttgattttggatctgcttggcaaggcatcgtgaaaccagtttgttaaaggcctgRacataacttRggtttggaagtcgcctWcagggctt

fava_c16563_1441	fava_c16563	1441	C/A	ttttccactgcttctcactcttaaacttcttcccacacaacacacaataaaactccttctcgtctttcttctcctcaaactcatcatcctcaaacaaatc[C/A]tcaacttcttcatcatcaaccttagcccaatcaggctcctcataagccatagccctctcctttttctccatctccaacttcctcttYctctccttMtcct

fava_c16563_1528	fava_c16563	1528	T/C	cctcaaacaaatcMtcaacttcttcatcatcaaccttagcccaatcaggctcctcataagccatagccctctcctttttctccatctccaacttcctctt[T/C]ctctccttMtcctcctctttcttcttctccatctccaaattcttcttcaccttcatatcaatcactctcttatctctcttcttcacaaaatcagccaacc

fava_c16563_1537	fava_c16563	1537	C/A	aatcMtcaacttcttcatcatcaaccttagcccaatcaggctcctcataagccatagccctctcctttttctccatctccaacttcctcttYctctcctt[C/A]tcctcctctttcttcttctccatctccaaattcttcttcaccttcatatcaatcactctcttatctctcttcttcacaaaatcagccaacctcctcaccg

fava_c16569_1036	fava_c16569	1036	G/T	aagtgagtagtgttgaagaaatgaaggttgatggtagagatggtggtgggaacaagagtgaatcggtgatgttttcgttgggattgactattggtggtcc[G/T]gttgttttgaaagttcctggtagttatcctggtagcgttggtttcaactcaaatggttctgtttatcttgatggaatgaaacttgttttcgaatctgaga

fava_c16569_1515	fava_c16569	1515	A/G	ggttatgatgatagcagggaactgttttctatgggtaggattgattctcagtggcttaataggactacaaacaaaggaagccataacaataacaatgtga[A/G]caatagttcWRccatggattttgatcaagagtctgaggctgatttgtttgaaattgcaattgatggttctggaaagtctacaRttacaacatcatagctt

fava_c16569_1525	fava_c16569	1525	A/T	atagcagggaactgttttctatgggtaggattgattctcagtggcttaataggactacaaacaaaggaagccataacaataacaatgtgaRcaatagttc[A/T]RccatggattttgatcaagagtctgaggctgatttgtttgaaattgcaattgatggttctggaaagtctacaRttacaacatcatagcttacaagttaca

fava_c16569_1526	fava_c16569	1526	A/G	tagcagggaactgttttctatgggtaggattgattctcagtggcttaataggactacaaacaaaggaagccataacaataacaatgtgaRcaatagttcW[A/G]ccatggattttgatcaagagtctgaggctgatttgtttgaaattgcaattgatggttctggaaagtctacaRttacaacatcatagcttacaagttacaa

fava_c16569_1598	fava_c16569	1598	A/G	taacaataacaatgtgaRcaatagttcWRccatggattttgatcaagagtctgaggctgatttgtttgaaattgcaattgatggttctggaaagtctaca[A/G]ttacaacatcatagcttacaagttacaacaaattgtgcaagtgtacatttgtatttgtcttggttctgccatttaacaactactcggcttgtacaaatag

fava_c16569_1731	fava_c16569	1731	A/C	ttgtgcaagtgtacatttgtatttgtcttggttctgccatttaacaactactcggcttgtacaaatagtacatttctttgtacaacacaataatgtagga[A/C]atttttttgtgtatttagagtttcaaaggtagattacaggtcccaagatgagaaatacaaaaatatttgtatattagaatattaactttacctaaaaact

fava_c16569_244	fava_c16569	244	T/C	cccaaattcaacaaaagatgtggatacatattgcagttatagcctccaccgttggatgcgtttcaaccatactcttgattctaatctgtcgccgttggtg[T/C]tacagaagaaaccgtagagattcttcttctgaacaagSaaatttaaccagaatagaaagttctagaactagaattcatcatcacatgccaagtgttcatc

fava_c16569_282	fava_c16569	282	C/G	atagcctccaccgttggatgcgtttcaaccatactcttgattctaatctgtcgccgttggtgYtacagaagaaaccgtagagattcttcttctgaacaag[C/G]aaatttaaccagaatagaaagttctagaactagaattcatcatcacatgccaagtgttcatcatcaaattcaaatagatcaaggtaacaagaataaaagt

fava_c16569_409	fava_c16569	409	T/G	gaactagaattcatcatcacatgccaagtgttcatcatcaaattcaaatagatcaaggtaacaagaataaaagtagcaatcacaatcattctctgtttcg[T/G]tctggaaaaagaacagcaacaggtttgttcagttggattaataatccatctatggccgttgatgcagtggaaaacggatggtcgagattcgctttcacta

fava_c16569_556	fava_c16569	556	G/A	catctatggccgttgatgcagtggaaaacggatggtcgagattcgctttcactagctataaaagttacatgccttctccatctaaaaggtctacactgtt[G/A]ggctcatttgcggcacctgattataaatcatcagaagctgagattagttgggaggtttcaagtgaatcagatgagtttatgcagaaggttaggttaaatt

fava_c16569_664	fava_c16569	664	C/A	ttgcggcacctgattataaatcatcagaagctgagattagttgggaggtttcaagtgaatcagatgagtttatgcagaaggttaggttaaattctggaat[C/A]aaaaaattgaatcaaattattcaaaatcaaaacggttattctagttctgttgtRaattctgttattagaactgctttgccgttacctggccctgttttgg

fava_c16569_718	fava_c16569	718	A/G	gtgaatcagatgagtttatgcagaaggttaggttaaattctggaatMaaaaaattgaatcaaattattcaaaatcaaaacggttattctagttctgttgt[A/G]aattctgttattagaactgctttgccgttacctggccctgttttgggaaattgtgtttttccgcaagagtcttattttgagattacgattttgaattcgt

fava_c16569_916	fava_c16569	916	G/T	cgtcgattagcgatgagtttgaatcagttaggaagagtgtggaaggtgataaggctatgcttattgcgaaagggaactcggaggcgttgattcatgttac[G/T]agtggtggtaagaatagtaaagtgagtagtgttgaagaaatgaaggttgatggtagagatggtggtgggaacaagagtgaatcggtgatgttttcgttgg

fava_c16570_1106	fava_c16570	1106	A/T	tgaaaagcatgcttgcatttgggcaacaatctaagtgtctcttcgtcttcgaatttcgacaaacaaacagtgcattctagtccttgtttggatcccttta[A/T]ggaagaaaatttaaagaaaggaagtgtttcaatcactttcttttcaacgcccaWaactatagaccttgatggaatttgtccttggttaagagggtttggS

fava_c16570_1160	fava_c16570	1160	A/T	ttcgacaaacaaacagtgcattctagtccttgtttggatccctttaWggaagaaaatttaaagaaaggaagtgtttcaatcactttcttttcaacgccca[A/T]aactatagaccttgatggaatttgtccttggttaagagggtttggSgttataagatgaagaggagtaacacgacagaatttgacataaacaagtaagata

fava_c16570_1206	fava_c16570	1206	G/C	WggaagaaaatttaaagaaaggaagtgtttcaatcactttcttttcaacgcccaWaactatagaccttgatggaatttgtccttggttaagagggtttgg[G/C]gttataagatgaagaggagtaacacgacagaatttgacataaacaagtaagataaaagttatgagaaacataatggaaagaacaattataacaacacctt

fava_c16570_1404	fava_c16570	1404	G/T	ctttactaggagttacaccttgttgcatctctggcatgttctggttgctatttctttggctttgagtgtttaaaagtagtgaaagaatcataattagtat[G/T]agaaagtggtttgtattactagtaaacatgttattatggacttttgtgtaataagatcaagagttttctttacttaagcatatttgagattgtatctagt

fava_c16570_282	fava_c16570	282	T/C	aagggattgatattatctatctttaccaataaagactcataaaccacacttttgcatactatattataagatagcaatctttgactaaatatattaactt[T/C]tgttaatccacttaaaccaacaatcaacatatactaatcacagtRctaattactgttcatcaaacatttgaagctaaatgtttatgttgcaataactgaa

fava_c16570_327	fava_c16570	327	A/G	acacttttgcatactatattataagatagcaatctttgactaaatatattaacttYtgttaatccacttaaaccaacaatcaacatatactaatcacagt[A/G]ctaattactgttcatcaaacatttgaagctaaatgtttatgttgcaataactgaaccgagtttgtttcttgtcttacaaaccattgaactgttctccttg

fava_c16570_455	fava_c16570	455	T/C	gctaaatgtttatgttgcaataactgaaccgagtttgtttcttgtcttacaaaccattgaactgttctccttgcaataggcaaccaaatcctccacattg[T/C]ctcatcatttccattccttcttctgctgccaacttccacaaaccttggaacatttgYaatctcagacatacacctcttctcgccaggattcaatgctgta

fava_c16570_512	fava_c16570	512	T/C	tgaactgttctccttgcaataggcaaccaaatcctccacattgYctcatcatttccattccttcttctgctgccaacttccacaaaccttggaacatttg[T/C]aatctcagacatacacctcttctcgccaggattcaatgctgtaaaagaattttcatcctcatcaggagaaaatcttgcacttgacacatcatgaagcatc

fava_c16570_745	fava_c16570	745	A/G	aagatcactccatctactccttgtagcaacagcatcagagatcaatattctatgatttatcatatgcaaatgttgtttttgttgttgtttccattttgta[A/G]cattgctgctaccaccttcttcaagatcaagaagaactatttcttgttcttgttcttgttcatctattttactctttctttgagaaggttctctttgaat

fava_c16570_876	fava_c16570	876	A/G	gaagaactatttcttgttcttgttcttgttcatctattttactctttctttgagaaggttctctttgaataaaaatctcaagatttggatcttcacttag[A/G]tttgaagggactcttaagaatcttgaactgaatgaaaaagtgaagtttcttatatcattttcttcaaccatgtacctacaaagaggacaggtagagtgac

fava_c16573_1018	fava_c16573	1018	T/C	ctgagcatggtcaggttatcaagtcWtctgaacttgaattaccttccttcagacatttattgatYtcctttggtggattggctgggttagaagagagtat[T/C]gaggaagatgataactttaagggaaaaaatgcaaaggaagtattcagtttatatttaaatacatgtccaaatcaggggagccgaacaattcgaacagagg

fava_c16573_1237	fava_c16573	1237	T/C	ttcagtacttccaagaaccaataagccgagcaatgctgggttgagtattgttgtattcttaaaacttatgtagttcagacatcgattctccaagttatgg[T/C]ggaaacagaaacagaaacacgggcaatttttcaaaaccagaattagaaattggccctctaacttcatcccgaattgaccatgatgttatgttatgggatt

fava_c16573_1465	fava_c16573	1465	G/T	agtactctctggttgtaagactcgcaaacagtgttccatctatctatgtctgtatatgaaatatttcagaaatagaattgctagtcataattttgtctca[G/T]caaagaaggcctgatgaaactctaggtctctttagatcgacttgttaagtatttttgttataaagataactttcataaacacttgtaagatgaatacaat

fava_c16573_259	fava_c16573	259	G/A	agcagcgcctcacaacagacgcagacgccgcaccggagctagacccaatcaacggaaacaacgacgaatctcacagtaacaagaagctcaagaagcataa[G/A]aagaacaaaacaatcgagatacccactgtcagcatagccgttcccgcttccatcattgacaacgttccaaccctagaactcgccactcggttggctagtc

fava_c16573_376	fava_c16573	376	G/A	agatacccactgtcagcatagccgttcccgcttccatcattgacaacgttccaaccctagaactcgccactcggttggctagtcaaatcgctcgcgccgc[G/A]actatttttcgaatcaacgaggtggttgtgtttgataataKaagtaatRcagataaagattatactgtggYggagaattcagatgatgaaagtagttcgg

fava_c16573_417	fava_c16573	417	G/T	gacaacgttccaaccctagaactcgccactcggttggctagtcaaatcgctcgcgccgcRactatttttcgaatcaacgaggtggttgtgtttgataata[G/T]aagtaatRcagataaagattatactgtggYggagaattcagatgatgaaagtagttcggcttttctcgtgcgaattttgcagtatcttgagacacctcag

fava_c16573_425	fava_c16573	425	G/A	tccaaccctagaactcgccactcggttggctagtcaaatcgctcgcgccgcRactatttttcgaatcaacgaggtggttgtgtttgataataKaagtaat[G/A]cagataaagattatactgtggYggagaattcagatgatgaaagtagttcggcttttctcgtgcgaattttgcagtatcttgagacacctcagtatttgag

fava_c16573_447	fava_c16573	447	C/T	cggttggctagtcaaatcgctcgcgccgcRactatttttcgaatcaacgaggtggttgtgtttgataataKaagtaatRcagataaagattatactgtgg[C/T]ggagaattcagatgatgaaagtagttcggcttttctcgtgcgaattttgcagtatcttgagacacctcagtatttgaggaaggctctcttcccaatgcat

fava_c16573_580	fava_c16573	580	G/A	ttctcgtgcgaattttgcagtatcttgagacacctcagtatttgaggaaggctctcttcccaatgcatagcagcttaagatttgtgggaatgttgccacc[G/A]ctggatgctccacaccatttgcgcaaacacgagtggggtccgtatcgggaaggtgtcacaataaaaggacattcaaactctggcacaacactagttgatg

fava_c16573_760	fava_c16573	760	C/G	ctggcacaacactagttgatgttggcttatttaagcatgttgcagttgaccaaatacttgaaccaggaagaagagttactgtggccatgggtgccaaccg[C/G]aatttggattctgatttaccacgcgaRgttgtcYcatYatccaagcctagggaagaaggagcttattggggatatcaagtacgctatgctcacaatatta

fava_c16573_787	fava_c16573	787	G/A	tatttaagcatgttgcagttgaccaaatacttgaaccaggaagaagagttactgtggccatgggtgccaaccgSaatttggattctgatttaccacgcga[G/A]gttgtcYcatYatccaagcctagggaagaaggagcttattggggatatcaagtacgctatgctcacaatattagcgcagtatttaatgaatgtacataca

fava_c16573_794	fava_c16573	794	C/T	gcatgttgcagttgaccaaatacttgaaccaggaagaagagttactgtggccatgggtgccaaccgSaatttggattctgatttaccacgcgaRgttgtc[C/T]catYatccaagcctagggaagaaggagcttattggggatatcaagtacgctatgctcacaatattagcgcagtatttaatgaatgtacatacaagggtgg

fava_c16573_798	fava_c16573	798	C/T	gttgcagttgaccaaatacttgaaccaggaagaagagttactgtggccatgggtgccaaccgSaatttggattctgatttaccacgcgaRgttgtcYcat[C/T]atccaagcctagggaagaaggagcttattggggatatcaagtacgctatgctcacaatattagcgcagtatttaatgaatgtacatacaagggtggctac

fava_c16573_943	fava_c16573	943	A/T	gctatgctcacaatattagcgcagtatttaatgaatgtacatacaagggtggctacgattgtataattggtacctctgagcatggtcaggttatcaagtc[A/T]tctgaacttgaattaccttccttcagacatttattgatYtcctttggtggattggctgggttagaagagagtatYgaggaagatgataactttaagggaa

fava_c16573_982	fava_c16573	982	T/C	catacaagggtggctacgattgtataattggtacctctgagcatggtcaggttatcaagtcWtctgaacttgaattaccttccttcagacatttattgat[T/C]tcctttggtggattggctgggttagaagagagtatYgaggaagatgataactttaagggaaaaaatgcaaaggaagtattcagtttatatttaaatacat

fava_c16574_146	fava_c16574	146	G/T	ttcaacgaactcataacaacttactacaaaatgtaacgaaatcgaatcaaaatcaaatcacaacaaacaaaccctaaaaccgaaggagaacggaacgggt[G/T]tctcttcatcttccccacccgttctcctcttttgaaatcggcgatcgatcgatttttggttattgttgtgattgaaaaactcgttttcggtggtttgttg

fava_c16574_414	fava_c16574	414	A/C	cttccagagtggatagatttgaatcgaatttggacattttggaaaatggattcttctcaaggttccaagttggctggtttggtgcagaatgcctccatta[A/C]gagggatgaatctagtttggatcgtgttcctgtttatgttaaggagttgattgctggtggatttgctggtgcgcttgctaagagctctgttgcacctcta

fava_c16574_553	fava_c16574	553	A/G	ttaaggagttgattgctggtggatttgctggtgcgcttgctaagagctctgttgcacctctagaacgggtcaagatactctggcagactaggactggagg[A/G]ttctataatcttggtgtttaccactctatgaataagttgttaaagcatgaagggtttcttggattatacaaaggaaatggagctagtgtcatccggattg

fava_c16579_171	fava_c16579	171	T/C	tgcacacaaggaaatggcatcaattgtaaaagtagtagaaaatctactactaaaagcatttgaatgtagtgggattaatttcttttcttttccaactcca[T/C]aacYtYtctaaaggacaaaagagaagcaaaagaaaaaatgagcaaaaagggttcaagttatacataaactcttctaagaaactaagcataattgttcMta

fava_c16579_175	fava_c16579	175	T/C	cacaaggaaatggcatcaattgtaaaagtagtagaaaatctactactaaaagcatttgaatgtagtgggattaatttcttttcttttccaactccaYaac[T/C]tYtctaaaggacaaaagagaagcaaaagaaaaaatgagcaaaaagggttcaagttatacataaactcttctaagaaactaagcataattgttcMtaRaga

fava_c16579_177	fava_c16579	177	C/T	caaggaaatggcatcaattgtaaaagtagtagaaaatctactactaaaagcatttgaatgtagtgggattaatttcttttcttttccaactccaYaacYt[C/T]tctaaaggacaaaagagaagcaaaagaaaaaatgagcaaaaagggttcaagttatacataaactcttctaagaaactaagcataattgttcMtaRagaag

fava_c16579_269	fava_c16579	269	C/A	caYaacYtYtctaaaggacaaaagagaagcaaaagaaaaaatgagcaaaaagggttcaagttatacataaactcttctaagaaactaagcataattgttc[C/A]taRagaagcactcaaatagacctaaacaaagtaaggaatattttcactagagatagatccatgactcattgttgttttggatggagtgtaaacagaagaa

fava_c16579_272	fava_c16579	272	A/G	aacYtYtctaaaggacaaaagagaagcaaaagaaaaaatgagcaaaaagggttcaagttatacataaactcttctaagaaactaagcataattgttcMta[A/G]agaagcactcaaatagacctaaacaaagtaaggaatattttcactagagatagatccatgactcattgttgttttggatggagtgtaaacagaagaagcY

fava_c16579_765	fava_c16579	765	C/G	ttaacctcaaatttgcttcataaaccatactatttgctgcatctgctctttgtccttcaggtacttccaatagcaactttgagacattgctagcaccaaa[C/G]actttgtgaacagctgcaaatttttgaggctcatgaggtgaaaaataaggagagaaaggacattcctcagcacaccttcttctcaaaagcttacatgcag

fava_c16579_946	fava_c16579	946	C/G	tctcaaaagcttacatgcagcacaaggtgttaccgtgtttaacgttgtccctgaagttggaccatagaaattcttccttcccattcgagaagatgtttct[C/G]cttctgataagttgatctttgctatatcatctaatctgtctctggacatccaaacatgcttgttgattgtcttgatctaagctgtgatttagatgagaaa

fava_c16587_1258	fava_c16587	1258	A/C	ttggagaggaaagactgttggtttatgagtacatgaaatatggaagtctagaagatgtcctgcacgacccgaagaaagctgggatcaagatgaattggtc[A/C]gtgaggaggaagattgcaattggagctgcaagagggttggcttttctccaccataactgtattccacatatcattcacagagacatgaaatccagcaatg

fava_c16587_1693	fava_c16587	1693	G/A	ataacaatcttgtcggatgggttaaacaacatgccaaactcaaaataagtgatgtctttgataaagagcttatgaaagaagaccccaatttggagatcga[G/A]cttttgcagcatttaaaggtagcttgtgcttgtttggacgatagaccttggaggagacctacaatgattcaagtcatggcaatgttcaaggaaatacaag

fava_c16590_1047	fava_c16590	1047	C/T	tgttggtggcagctatctgtgctggagcacccctcgcccttaaYgtggaggttattaaatctccacttggagtttctatcttgcttcttgttgttgcttt[C/T]catttgtcagcttttatagctggttatgcactaggtggttctatcttccgtgattctcctgaagtgaagccactacaacgaacactttcgtttgagacRg

fava_c16590_113	fava_c16590	113	G/T	accaccggttgactactacactagtatagagtatagaggagtataaaaagagtcccgtgccgtgccgtgccgtttgtgtctgatattcaaaacatgaatt[G/T]gatttcttcaggcagagtcaagcctcttcctctatcatcacacttccgtctccgatgcgccaacWccaccaacttctccaattYcccccatactactact

fava_c16590_1146	fava_c16590	1146	A/G	tYcatttgtcagcttttatagctggttatgcactaggtggttctatcttccgtgattctcctgaagtgaagccactacaacgaacactttcgtttgagac[A/G]ggaatgcaaagtagcctccttgctctggctcttgctaataagttcttcaaagacccggttgtgggtatgcctccagcaatttcgactgcaattatgtctt

fava_c16590_1532	fava_c16590	1532	T/G	cttattatacattatattacattaaaatttaaaaatacttcacatcacactaccggttcgttctaatacagtttttgtgagggtagtgtagaaccggtgg[T/G]atgaaataattgacacttatctatttatgtataagtgactttttcaaattataggaacatcaaaagcaaRtRtgtgattagcaaaatcacacttcatcaa

fava_c16590_1602	fava_c16590	1602	G/A	gtttttgtgagggtagtgtagaaccggtggKatgaaataattgacacttatctatttatgtataagtgactttttcaaattataggaacatcaaaagcaa[G/A]tRtgtgattagcaaaatcacacttcatcaaataagagctagcccatgcaaaacatgggctgcataaaataattgtaacagtttatttagttaacactttc

fava_c16590_1604	fava_c16590	1604	G/A	ttttgtgagggtagtgtagaaccggtggKatgaaataattgacacttatctatttatgtataagtgactttttcaaattataggaacatcaaaagcaaRt[G/A]tgtgattagcaaaatcacacttcatcaaataagagctagcccatgcaaaacatgggctgcataaaataattgtaacagtttatttagttaacactttctg

fava_c16590_178	fava_c16590	178	A/T	cgtgccgtttgtgtctgatattcaaaacatgaattKgatttcttcaggcagagtcaagcctcttcctctatcatcacacttccgtctccgatgcgccaac[A/T]ccaccaacttctccaattYcccccatactactactattcgcttggcggtttcaactccccgccttcccctcaaatgtgcgtccacaaacttctccgattc

fava_c16590_197	fava_c16590	197	C/T	attcaaaacatgaattKgatttcttcaggcagagtcaagcctcttcctctatcatcacacttccgtctccgatgcgccaacWccaccaacttctccaatt[C/T]cccccatactactactattcgcttggcggtttcaactccccgccttcccctcaaatgtgcgtccacaaacttctccgattctcatcctcccgatccgatt

fava_c16590_329	fava_c16590	329	G/T	tcaactccccgccttcccctcaaatgtgcgtccacaaacttctccgattctcatcctcccgatccgatttcgaatcactcttctcacgataccacgacgc[G/T]gaaacaaactattttcgacaYcctcaacaactcaaactcacttctKcctcctgcagtccttgccagtactctgctagctcttatctacccaccttcttta

fava_c16590_350	fava_c16590	350	T/C	aaatgtgcgtccacaaacttctccgattctcatcctcccgatccgatttcgaatcactcttctcacgataccacgacgcKgaaacaaactattttcgaca[T/C]cctcaacaactcaaactcacttctKcctcctgcagtccttgccagtactctgctagctcttatctacccaccttctttaacctggtttactaccagatac

fava_c16590_375	fava_c16590	375	G/T	attctcatcctcccgatccgatttcgaatcactcttctcacgataccacgacgcKgaaacaaactattttcgacaYcctcaacaactcaaactcacttct[G/T]cctcctgcagtccttgccagtactctgctagctcttatctacccaccttctttaacctggtttactaccagatactatgcacctgcattaggatttttga

fava_c16590_990	fava_c16590	990	C/T	atcgcttcttccctcgtatttgcaatgctattcgaccatttttgcctccactgtccgtgttggtggcagctatctgtgctggagcacccctcgcccttaa[C/T]gtggaggttattaaatctccacttggagtttctatcttgcttcttgttgttgctttYcatttgtcagcttttatagctggttatgcactaggtggttcta

fava_c16591_3684	fava_c16591	3684	G/A	tcttgtaaattaactctgcgacatcggattggtgaccaggattatgctgagactcaggtcgaatctcaatatcagcagattcatctacgatttcctgcat[G/A]gtgttttggatgaactcgtcgaacgaatcgagttgctggcgaactaaacctttctcttcgaagtaagctgaaataacagcccaagcatcttcttgagtaa

fava_c16597_293	fava_c16597	293	C/T	catatggacaaacttactttcatccaccttcagggagatactccgatggccgtctcatcgttgattttcttatggatgcattcgatttaccgttcttaaa[C/T]gcttatttggattcgttgggtttgccgaattttagaaaaggttgtaactttgcagcagcaggttcaactatcttaccagccactgcatcatcaatctgtc

fava_c16597_722	fava_c16597	722	C/T	ccggaatcaagagactatatgacgaaggagctagatatttctggatacacaatacaggtcctctcggatgcttaacacaaaatgttgccaaattcggaac[C/T]gatccatcaaaactcgatgaactaggatgcgttagtgcacataacatagctgctaaaaccttcaatctacagcttcatgctttatgcagtaaattccagg

fava_c16597_986	fava_c16597	986	G/A	caaactattcaagatacgggtttgaacaacctataatggcatgttgtggatatggaggaccaccattgaactatgacagtagagtttcgtgtgggaacac[G/A]aagatattgaatggaacatcgataacggcaaaaggttgtaatgatagttctgagtatataagttgggatggaatacattacactgagactgcaaatcagt

fava_c16598_1441	fava_c16598	1441	G/C	ttatgttcaaagaggaacaggttagagaggctcaagcatggatagcccgtgtccgcgagatggatgttttccagtcaacaacaaaccagtcattacaagc[G/C]gaattgcgagagcgcaatgaacaatatagtcagctatggatgggttttcagagacagtatgcagagatggagagagtccatttgcatgctattcagcagc

fava_c16598_2238	fava_c16598	2238	C/T	caaactgtggtttcatcatccgctggggagacacagtcggttgataaagatcaacttattgcttcccaacaaagcttacaacagatttcttcacagttct[C/T]tgaggccttaagattgaactcttttaaaacaaatggtgaaattaagaatccagtgacactatcgaatgatggacctgcaagccaaatattattaactgag

fava_c16598_2717	fava_c16598	2717	G/A	gaagctggaagattttgttactagccatcctgaattattttcgattgagggagactttattcaactccgcgaaggggcgcataaaataatagctgcaacc[G/A]ctgcagttgcaaaagttgctgcagctgctgcagcatcttctccttattcttcgtatatgcccactgtagcagttacgccaatggctcaatctcatcgcct

fava_c16598_637	fava_c16598	637	C/T	tcaatttctagggttagggatttcttattttctatttctatttatttttccttttttctactcggtgttttcttcactgacgtgagggcaccgtcgactg[C/T]gatttttcaatggaggccacggccgccgcagccgctgctcgcggcgtttctcttcagttacagactccgcctcggaaagaatggcgcgctgttgctgaac

fava_c16606_101	fava_c16606	101	C/G	tagtgtgaacaagaaaaagtctaagaaagtaatgaagcgaacgtcaataaaaaaaaaggaagggagattccaccgatctggaagacctatttgcagagtt[C/G]gaagctctttttcagtctRattSaSaaatattaagtatggggggttttgccattggttctgcatttgattcaaaatctggccaaatcataatggttgcgt

fava_c16606_1032	fava_c16606	1032	T/C	cacaaaacaactaccatttcctacagggtcWtttgaaatgattcactgttcaagatgccgtatagactttcatgaaaatgatgggattttgttaaaggag[T/C]tggatcgccttcttcgctcgaatggttattttgtttattcagcaccaccagcttacagaaaagataaagattttcctgtcatttgggataagttggtgaa

fava_c16606_120	fava_c16606	120	A/G	tctaagaaagtaatgaagcgaacgtcaataaaaaaaaaggaagggagattccaccgatctggaagacctatttgcagagttSgaagctctttttcagtct[A/G]attSaSaaatattaagtatggggggttttgccattggttctgcatttgattcaaaatctggccaaatcataatggttgcgttacttttgatgattgtttc

fava_c16606_124	fava_c16606	124	C/G	agaaagtaatgaagcgaacgtcaataaaaaaaaaggaagggagattccaccgatctggaagacctatttgcagagttSgaagctctttttcagtctRatt[C/G]aSaaatattaagtatggggggttttgccattggttctgcatttgattcaaaatctggccaaatcataatggttgcgttacttttgatgattgtttcattt

fava_c16606_126	fava_c16606	126	C/G	aaagtaatgaagcgaacgtcaataaaaaaaaaggaagggagattccaccgatctggaagacctatttgcagagttSgaagctctttttcagtctRattSa[C/G]aaatattaagtatggggggttttgccattggttctgcatttgattcaaaatctggccaaatcataatggttgcgttacttttgatgattgtttcatttta

fava_c16606_1263	fava_c16606	1263	G/A	tgctcgtaaagttcagactgcaatatggattaaagaaaatagtcagccctcctgtcttctgcaaaatgcagaacaaaaggttatagatgtctgtgatgtg[G/A]atgacgaatctaaRccttcatggaataccccacttaagaactgtatacaagtcagaaattctaataYagaatcttacaagctacctccaagtcatgagcg

fava_c16606_1277	fava_c16606	1277	A/G	agactgcaatatggattaaagaaaatagtcagccctcctgtcttctgcaaaatgcagaacaaaaggttatagatgtctgtgatgtgRatgacgaatctaa[A/G]ccttcatggaataccccacttaagaactgtatacaagtcagaaattctaataYagaatcttacaagctacctccaagtcatgagcgcctttcagtgtttt

fava_c16606_1330	fava_c16606	1330	T/C	gcagaacaaaaggttatagatgtctgtgatgtgRatgacgaatctaaRccttcatggaataccccacttaagaactgtatacaagtcagaaattctaata[T/C]agaatcttacaagctacctccaagtcatgagcgcctttcagtgttttctgaaaggctgaacaagataggtataaatcgagatgaatttacatcagattct

fava_c16606_1518	fava_c16606	1518	T/G	tacatcagattctctcttctggcaagaccagattcgtcattactggaaactgatgaatgtcaaagaaacagaaatacgcaatgtgatggatatgaatgcc[T/G]tttatggtggatttgctgttgcattgaRcaactttccagtttgggtaatgaatgttgttcctgcaagcatgaagaacaccttatccggaatttatgaccg

fava_c16606_1546	fava_c16606	1546	G/A	cagattcgtcattactggaaactgatgaatgtcaaagaaacagaaatacgcaatgtgatggatatgaatgccKtttatggtggatttgctgttgcattga[G/A]caactttccagtttgggtaatgaatgttgttcctgcaagcatgaagaacaccttatccggaatttatgaccggggtttgattgggagttttcatgattgg

fava_c16606_2047	fava_c16606	2047	A/G	gaaggagattttttgcaaccacacgggtaacttgtaatgttatatagttctagtgtatctgctttgtttattggttaatgaccattttgtgtacatgtct[A/G]caaggcactcaatttgtacatcactttcagtttagttagtaggaatagaagttaaatgtcctatatgatggcatggcatggagaatgtatgaattgatta

fava_c16606_227	fava_c16606	227	T/C	aaatattaagtatggggggttttgccattggttctgcatttgattcaaaatctggccaaatcataatggttgcgttacttttgatgattgtttcatttta[T/C]gtYggaaccctttttggcaacaacgcccccctttacatctccaaccttccatcttcatccaacactcYctcttctaatgcaactttcagatttaccaacS

fava_c16606_230	fava_c16606	230	C/T	tattaagtatggggggttttgccattggttctgcatttgattcaaaatctggccaaatcataatggttgcgttacttttgatgattgtttcattttaYgt[C/T]ggaaccctttttggcaacaacgcccccctttacatctccaaccttccatcttcatccaacactcYctcttctaatgcaactttcagatttaccaacaaac

fava_c16606_295	fava_c16606	295	C/T	gttgcgttacttttgatgattgtttcattttaYgtYggaaccctttttggcaacaacgcccccctttacatctccaaccttccatcttcatccaacactc[C/T]ctcttctaatgcaactttcagatttaccaacaaacttgctctcacttaccgcaaaacacctctgctaattccYgaaactggcgttaccgtatgccccttg

fava_c16606_368	fava_c16606	368	C/T	ccaaccttccatcttcatccaacactcYctcttctaatgcaactttcagatttaccaacaaacttgctctcacttaccgcaaaacacctctgctaattcc[C/T]gaaactggcgttaccgtatgccccttgaggtttaatgagtatatYccctgccacgatgtttcctaYgtYgYKacattgatgcctaatcttgatgtttcta

fava_c16606_413	fava_c16606	413	C/T	tcagatttaccaacaaacttgctctcacttaccgcaaaacacctctgctaattccYgaaactggcgttaccgtatgccccttgaggtttaatgagtatat[C/T]ccctgccacgatgtttcctaYgtYgYKacattgatgcctaatcttgatgtttctagaaaacaagagctggagagacactgtcctccacttgaaaaMcggt

fava_c16606_434	fava_c16606	434	T/C	ctctcacttaccgcaaaacacctctgctaattccYgaaactggcgttaccgtatgccccttgaggtttaatgagtatatYccctgccacgatgtttccta[T/C]gtYgYKacattgatgcctaatcttgatgtttctagaaaacaagagctggagagacactgtcctccacttgaaaaMcggttgttttgcttgcttccaccgc

fava_c16606_437	fava_c16606	437	C/T	tcacttaccgcaaaacacctctgctaattccYgaaactggcgttaccgtatgccccttgaggtttaatgagtatatYccctgccacgatgtttcctaYgt[C/T]gYKacattgatgcctaatcttgatgtttctagaaaacaagagctggagagacactgtcctccacttgaaaaMcggttgttttgcttgcttccaccgccca

fava_c16606_439	fava_c16606	439	C/T	acttaccgcaaaacacctctgctaattccYgaaactggcgttaccgtatgccccttgaggtttaatgagtatatYccctgccacgatgtttcctaYgtYg[C/T]KacattgatgcctaatcttgatgtttctagaaaacaagagctggagagacactgtcctccacttgaaaaMcggttgttttgcttgcttccaccgcccagc

fava_c16606_440	fava_c16606	440	G/T	cttaccgcaaaacacctctgctaattccYgaaactggcgttaccgtatgccccttgaggtttaatgagtatatYccctgccacgatgtttcctaYgtYgY[G/T]acattgatgcctaatcttgatgtttctagaaaacaagagctggagagacactgtcctccacttgaaaaMcggttgttttgcttgcttccaccgcccagca

fava_c16606_509	fava_c16606	509	C/A	atatYccctgccacgatgtttcctaYgtYgYKacattgatgcctaatcttgatgtttctagaaaacaagagctggagagacactgtcctccacttgaaaa[C/A]cggttgttttgcttgcttccaccgcccagcaattataaaattcctatccaatggcctcttagcagggattaYgtttggcggagtaatgtcaatcatacac

fava_c16606_581	fava_c16606	581	T/C	tggagagacactgtcctccacttgaaaaMcggttgttttgcttgcttccaccgcccagcaattataaaattcctatccaatggcctcttagcagggatta[T/C]gtttggcggagtaatgtcaatcatacacatctcgccgacgtcaaaggaggccaRaattgggtccatccRaaggatcagctttggtggtttcctggcggtg

fava_c16606_635	fava_c16606	635	A/G	ccagcaattataaaattcctatccaatggcctcttagcagggattaYgtttggcggagtaatgtcaatcatacacatctcgccgacgtcaaaggaggcca[A/G]aattgggtccatccRaaggatcagctttggtggtttcctggcggtggtacacatttcaagaacggagcatccgaYtacatacaaMggataggacatatga

fava_c16606_650	fava_c16606	650	G/A	ttcctatccaatggcctcttagcagggattaYgtttggcggagtaatgtcaatcatacacatctcgccgacgtcaaaggaggccaRaattgggtccatcc[G/A]aaggatcagctttggtggtttcctggcggtggtacacatttcaagaacggagcatccgaYtacatacaaMggataggacatatgattacaaatgagactg

fava_c16606_710	fava_c16606	710	C/T	atctcgccgacgtcaaaggaggccaRaattgggtccatccRaaggatcagctttggtggtttcctggcggtggtacacatttcaagaacggagcatccga[C/T]tacatacaaMggataggacatatgattacaaatgagactggcgatctacgttctgcggggatagttcaagttctggatcttggctgtggtgtSgccagtt

fava_c16606_720	fava_c16606	720	A/C	cgtcaaaggaggccaRaattgggtccatccRaaggatcagctttggtggtttcctggcggtggtacacatttcaagaacggagcatccgaYtacatacaa[A/C]ggataggacatatgattacaaatgagactggcgatctacgttctgcggggatagttcaagttctggatcttggctgtggtgtSgccagtttttcagctta

fava_c16606_803	fava_c16606	803	G/C	catccgaYtacatacaaMggataggacatatgattacaaatgagactggcgatctacgttctgcggggatagttcaagttctggatcttggctgtggtgt[G/C]gccagtttttcagcttatcttcttcccttggatatacgaactatgtcctttgcccccaaggatggtcacgaaaatcagattcaatttgcattagagcgag

fava_c16606_962	fava_c16606	962	T/A	aggatggtcacgaaaatcagattcaatttgcattagagcgaggaattggtgcaatgatttctgccatgtccacaaaacaactaccatttcctacagggtc[T/A]tttgaaatgattcactgttcaagatgccgtatagactttcatgaaaatgatgggattttgttaaaggagYtggatcgccttcttcgctcgaatggttatt

fava_c16608_1067	fava_c16608	1067	T/G	tttctagacaacaccccaagagtttaaccacatttctgtgatttatttgagagacaacatccacctcattgatgaattgttcaatttggctttcatctat[T/G]gttttggactttttgatagcaacaattatattatcttctagttcccctttgaaaactgtaccataacctcccctacctatgattaagctctcatcaaagt

fava_c16608_1553	fava_c16608	1553	G/A	cgatttgcccctcaggacagaaacaagtgtaattcccaaaagtgttgttacaatttgctttccttttgcatccatgatattctgttgcgcattcgtcaat[G/A]tctgagcagcctccaggaaggtatggatttccttcaaaacctttcgaacagttgcatctataaccgtcataatccggatcgctatcaacacagtcactat

fava_c16608_2119	fava_c16608	2119	T/C	tatccataactgtcgcaaccgacggttatgaatttgttttctgtgcttgaaatggtcaaagaaggaactctaagaaaaggagaatttgcagtactattac[T/C]actactattagtacaaaaatRcgagacgaaaaaggaaaacaccatttggcctttaggatttatgtccaggacttgaatatttgtaccacggattaatttg

fava_c16608_2140	fava_c16608	2140	G/A	acggttatgaatttgttttctgtgcttgaaatggtcaaagaaggaactctaagaaaaggagaatttgcagtactattacYactactattagtacaaaaat[G/A]cgagacgaaaaaggaaaacaccatttggcctttaggatttatgtccaggacttgaatatttgtaccacggattaatttggagttgtttttacaagtgagt

fava_c16608_2252	fava_c16608	2252	A/G	aggaaaacaccatttggcctttaggatttatgtccaggacttgaatatttgtaccacggattaatttggagttgtttttacaagtgagtatgaatttgga[A/G]ttcatgtaacatggtgtctgatcttgagttgttgagtttcctatgccaaatggatatggaatttggatatctccacaagtggatttgcagcctggtaggg

fava_c16608_881	fava_c16608	881	A/G	tgacatctctgtggataatcggtattgaggcagctgaatgtagatatgacaaagctccagctacttctgctgctatcttaagacgagttttccacgatac[A/G]ttattcaccttacctatttccgtgtgtataaaatcagaaagggtaccattggaaacaaactcataaaccaacaaaggaacttgtgtttctagacaacacc

fava_c16613_543	fava_c16613	543	G/A	gcaatgaaacatactctggaggagtgaccactcgggaacgagaatttatcaacgggaacaacggcgttgtagtcgccgtgtatggcgtaggaagggcgag[G/A]tcttcgaactaggaacttSaggaaacctatgaagatgagatcgaKgaRagaacagaggatgagagggaggaggaggtggggacggagcggagagtgagag

fava_c16613_562	fava_c16613	562	C/G	aggagtgaccactcgggaacgagaatttatcaacgggaacaacggcgttgtagtcgccgtgtatggcgtaggaagggcgagRtcttcgaactaggaactt[C/G]aggaaacctatgaagatgagatcgaKgaRagaacagaggatgagagggaggaggaggtggggacggagcggagagtgagagggagtggcgaagaagagtg

fava_c16613_588	fava_c16613	588	T/G	ttatcaacgggaacaacggcgttgtagtcgccgtgtatggcgtaggaagggcgagRtcttcgaactaggaacttSaggaaacctatgaagatgagatcga[T/G]gaRagaacagaggatgagagggaggaggaggtggggacggagcggagagtgagagggagtggcgaagaagagtgagagtgttacggggaagaataagcgg

fava_c16613_591	fava_c16613	591	A/G	tcaacgggaacaacggcgttgtagtcgccgtgtatggcgtaggaagggcgagRtcttcgaactaggaacttSaggaaacctatgaagatgagatcgaKga[A/G]agaacagaggatgagagggaggaggaggtggggacggagcggagagtgagagggagtggcgaagaagagtgagagtgttacggggaagaataagcggaaa

fava_c16614_497	fava_c16614	497	A/G	tcatggagcaaggttgcctcattggctaaaatagaaacaaaaatgagagagaagatgagacaatgagaaggaatcatcatggtttgttttgtcacagaca[A/G]gatattgttagtcttaggtttgtgtttgaaaaggggttacttggtaatcttgtcttgtgYggatgagattgaattttggtgtaacataaatgagtatttg

fava_c16614_557	fava_c16614	557	T/C	caatgagaaggaatcatcatggtttgttttgtcacagacaRgatattgttagtcttaggtttgtgtttgaaaaggggttacttggtaatcttgtcttgtg[T/C]ggatgagattgaattttggtgtaacataaatgagtatttggtggctatttatgtttaaatttgtgagtttagtaaatcaaactagctagcttggaagatt

fava_c16615_811	fava_c16615	811	C/T	ggtataacttcaaatctgggggtgttaatatcaagtatgtagcagttggaaatgagccatttttgaaatcctacaacagttcatttttgaatgtaacctt[C/T]cctgccctRcaaaatatYcaaaatgccctaaacgaacttggtttaggagactcYataaaagccacagtcccttccaatgcagatatctatggatatccag

fava_c16615_820	fava_c16615	820	A/G	tcaaatctgggggtgttaatatcaagtatgtagcagttggaaatgagccatttttgaaatcctacaacagttcatttttgaatgtaaccttYcctgccct[A/G]caaaatatYcaaaatgccctaaacgaacttggtttaggagactcYataaaagccacagtcccttccaatgcagatatctatggatatccagatccaaaca

fava_c16615_829	fava_c16615	829	C/T	ggggtgttaatatcaagtatgtagcagttggaaatgagccatttttgaaatcctacaacagttcatttttgaatgtaaccttYcctgccctRcaaaatat[C/T]caaaatgccctaaacgaacttggtttaggagactcYataaaagccacagtcccttccaatgcagatatctatggatatccagatccaaacaatgcggttc

fava_c16615_865	fava_c16615	865	C/T	agccatttttgaaatcctacaacagttcatttttgaatgtaaccttYcctgccctRcaaaatatYcaaaatgccctaaacgaacttggtttaggagactc[C/T]ataaaagccacagtcccttccaatgcagatatctatggatatccagatccaaacaatgcggttccctcatccggaatattccgacccgatttatccaacc

fava_c16616_1190	fava_c16616	1190	T/C	cgtacaaagaaacgaatcataagccggaacattagccgaatcccaaccaatctcaataaccatttcagcaaaagtctctttagcaccaacaacattccca[T/C]cactctccaaaccctccatcaaaatSgcatacgtatccgaatccggcctaacaagtttcttcccaatttgcaaataatcacaagcttcaatagtcctacc

fava_c16616_1216	fava_c16616	1216	G/C	gaacattagccgaatcccaaccaatctcaataaccatttcagcaaaagtctctttagcaccaacaacattcccaYcactctccaaaccctccatcaaaat[G/C]gcatacgtatccgaatccggcctaacaagtttcttcccaatttgcaaataatcacaagcttcaatagtcctaccactactacaaacagcactcaacaacg

fava_c16616_1501	fava_c16616	1501	T/C	taacatagctagcgaaaatcgaagcaaaagtcgaccgcgaaagcaacccttctcgccgcatggatttaacagcgtcccacattgcatcaaataaacgatt[T/C]ttacctaaaatgtcaatcacgaggttccaagcgtagggagtgtggttgtggttgatgcggtgaccggcccaccggaagaatttgaccgcctgagcgggga

fava_c16616_1723	fava_c16616	1723	G/C	cttgttcgacgtcttgtggtgtgacatcgatgccggaatcttcgagggatttttcgacggaaagggaagggtttgtggagatgatttcgaagagggattt[G/C]gttttgcctaacggtaatggtaatggagagagtgttgtcttgttttgaagaattggggctgatggggaaggagatgaaattgaatgatagcttctctttc

fava_c16617_349	fava_c16617	349	T/C	aatcatacttcccataaacctcaagacaccttgcattccccttctcagattcatacaacaccaacctctcattgctatctttccataaaaacctattact[T/C]ccataaaacccaacaagcttataaatcacaggcacaggtccaaYactatacaccttaatccaagacccttcatcccaataatccttcatcatccaaacat

fava_c16617_393	fava_c16617	393	C/T	tcagattcatacaacaccaacctctcattgctatctttccataaaaacctattactYccataaaacccaacaagcttataaatcacaggcacaggtccaa[C/T]actatacaccttaatccaagacccttcatcccaataatccttcatcatccaaacatcaaaacatttctctattcctcttacaggatagacaaYaacacca

fava_c16617_486	fava_c16617	486	T/C	ggtccaaYactatacaccttaatccaagacccttcatcccaataatccttcatcatccaaacatcaaaacatttctctattcctcttacaggatagacaa[T/C]aacaccaatagtatcatattcatcaaaaggtgctagagttttaaaacagtctcctgaaaaacaagaatactctattttgggcacttttatcttcctaaac

fava_c16619_131	fava_c16619	131	T/C	aaaataagtgtccaatatcactgttcaataaaataatcaagttttaaactgcacctttaaccctttagcttgaaaactccattttaatctccctgtgaac[T/C]aactcgatacttgttcacagaacttgttgattacatagggaatgtgcagcatcRttcactRattcacaaaactaacaagatttaaaatctttaagttaca

fava_c16619_185	fava_c16619	185	G/A	ctttaaccctttagcttgaaaactccattttaatctccctgtgaacYaactcgatacttgttcacagaacttgttgattacatagggaatgtgcagcatc[G/A]ttcactRattcacaaaactaacaagatttaaaatctttaagttacattgtaaatttataggggtatcaagagccagattctcaagattgtctttagcaac

fava_c16619_192	fava_c16619	192	A/G	cctttagcttgaaaactccattttaatctccctgtgaacYaactcgatacttgttcacagaacttgttgattacatagggaatgtgcagcatcRttcact[A/G]attcacaaaactaacaagatttaaaatctttaagttacattgtaaatttataggggtatcaagagccagattctcaagattgtctttagcaacacaattt

fava_c16619_314	fava_c16619	314	A/T	taaaatctttaagttacattgtaaatttataggggtatcaagagccagattctcaagattgtctttagcaacacaattttctggcagacatttcattcct[A/T]tctttccaccaggattctctccctctgatgacaRaaaagccaactgtcagaactcttccataacaatctgcaaacaattacggttacagacctctggtgt

fava_c16619_348	fava_c16619	348	A/G	gtatcaagagccagattctcaagattgtctttagcaacacaattttctggcagacatttcattcctWtctttccaccaggattctctccctctgatgaca[A/G]aaaagccaactgtcagaactcttccataacaatctgcaaacaattacggttacagacctctggtgttagagcaagtgaaatgttatttggcgcgtcttct

fava_c16619_560	fava_c16619	560	C/T	ccctgcttctatccttatcataatcatccctctccctgcttccactcttctccctcagccggtctcttcctctatcactgtcaaaatcataatttttatc[C/T]ctttttctttctcgttccctttctaaatcacgttctctatctctgtctttgcttgatggtttctgtcgtggactaccacttggactatggctgcggtgtc

fava_c16619_728	fava_c16619	728	A/G	gtggactaccacttggactatggctgcggtgtcgcttccttgagacatcatgcctatccctcctctctgaagatgttctactactgccttgaccatcctc[A/G]ctagaatctgatataccgtactccacttgcagccttttacgatgcaacaaaacttttttctcaatttcctccaaatttttaattcccctttcttcaagag

fava_c16620_1387	fava_c16620	1387	G/A	gtgcacccgtgtcccgaggatgatgatatctacatgaagaaccaaatttacactccccggttttcatgtaatgatgacattcctgctgatcaggcctttc[G/A]ggtaatgactgttccttctgactactgcctgaaggaccagttgaggaaccagaaggttgaaaaagtccagtaaaggcagtagtcggtgaaggtagctgag

fava_c16620_1560	fava_c16620	1560	C/T	aaggcagtagtcggtgaaggtagctgagttatcccataaatctgtggcgaagcaatgctcaacggagtactcgtaggaagcacaggacttgcagcctgag[C/T]ctgatgataagggctccagccttgaaaaggtaccatggtgggggacactaccaaaggcccgtatggaccctggacaaacgagccatgcagcacaggtggc

fava_c16620_1906	fava_c16620	1906	C/T	gatgatggaatttacaagtaacaccaaacttgcattgtcctgttttcacatagtaggtacagtctttctcacccactcgcaacggatatccataataatt[C/T]agcgacacgggggcaataactcctgctgtttgtttaggatggtgatacttgcatgaagcaccaaatttgcacgatcccgtccttatgtaatactggcaca

fava_c16620_649	fava_c16620	649	G/C	aaaagtttgaagcataaataagcacaaaaagtagacatgtggatgggatgtgcataatggacttgcagattctcttcaagttattcaaagagcaaggtgc[G/C]tgtatgatggccatggtttattcaagatcaaagRgacggccatgaaaagattatatgaacacaaagacaattgtttggtgcaatgagtgatgagcctgaa

fava_c16620_683	fava_c16620	683	A/G	acatgtggatgggatgtgcataatggacttgcagattctcttcaagttattcaaagagcaaggtgcStgtatgatggccatggtttattcaagatcaaag[A/G]gacggccatgaaaagattatatgaacacaaagacaattgtttggtgcaatgagtgatgagcctgaactgggataactttaaaaKaaagactatttttgag

fava_c16620_767	fava_c16620	767	T/G	ttattcaagatcaaagRgacggccatgaaaagattatatgaacacaaagacaattgtttggtgcaatgagtgatgagcctgaactgggataactttaaaa[T/G]aaagactatttttgagaccggccgagtgaaaaattgagatagaatggagaaataatgaggagtagattcacattgcttagcttgaggtgtgaaagacagt

fava_c16621_1020	fava_c16621	1020	T/C	taagtggaggcattgggatgccaagactcttcccaatcattactgatgtagcttcggtttttgatctattcactgtaacccctgtggtgatgtcagccta[T/C]atctgtcactacaatgttcataacatagataatgaactggaggattcttcatggatgcctggggttgttcgtacttcccttacactatgctcttcagtct

fava_c16621_1327	fava_c16621	1327	T/A	caatgacattgttcgtattagctatgctgcgcatcttgtgcttgtatttcctgtagttttctatggattaagggtcaacattgctggtctgatatttccc[T/A]cgtcaagaaggcctttggttgccgataacttcagattttcatcaattacgattggtcttgttgtctctatcttcctgggagcaaatttcatacctagcat

fava_c16621_1593	fava_c16621	1593	A/C	caatcactcttagggataaatacaacatagcaactaaaacagacaggattctggctatacttatgataatccttgcagtcttgtcaaatgctgtggctat[A/C]tacagtgatgccattgccttgatcaagaagaataaaacttaaggtgaataatttcttgatttgaacataacttaccatggttatataacattggtttttt

fava_c16621_1745	fava_c16621	1745	C/T	tttcttgatttgaacataacttaccatggttatataacattggtttttttggagaaggtatataaagaaactaccatgtgtatggacatggaataaaata[C/T]acccaagacaactgtccacatgattgRaagagacagaagaaataaataaaaacRtaaacMcgtttatttatacggtgtttgacaacatatttgttgtcaa

fava_c16621_1772	fava_c16621	1772	A/G	ggttatataacattggtttttttggagaaggtatataaagaaactaccatgtgtatggacatggaataaaataYacccaagacaactgtccacatgattg[A/G]aagagacagaagaaataaataaaaacRtaaacMcgtttatttatacggtgtttgacaacatatttgttgtcaaaataccatttctcttgtaatatgctct

fava_c16621_1799	fava_c16621	1799	A/G	aaggtatataaagaaactaccatgtgtatggacatggaataaaataYacccaagacaactgtccacatgattgRaagagacagaagaaataaataaaaac[A/G]taaacMcgtttatttatacggtgtttgacaacatatttgttgtcaaaataccatttctcttgtaatatgctcttcgctgttataatttgaggaggttctt

fava_c16621_1805	fava_c16621	1805	C/A	tataaagaaactaccatgtgtatggacatggaataaaataYacccaagacaactgtccacatgattgRaagagacagaagaaataaataaaaacRtaaac[C/A]cgtttatttatacggtgtttgacaacatatttgttgtcaaaataccatttctcttgtaatatgctcttcgctgttataatttgaggaggttcttattgaa

fava_c16621_1952	fava_c16621	1952	T/G	atttctcttgtaatatgctcttcgctgttataatttgaggaggttcttattgaatgtgcctcaatatcccaggcttcatctccctcataaatgtaagaaa[T/G]gaatttgtaggcacttgtgtgtgctattaatggccaaaggactgaatggatgMcccccttgcttatgaaaaatggtggcttatttatagtctgctttcta

fava_c16621_2005	fava_c16621	2005	C/A	atgtgcctcaatatcccaggcttcatctccctcataaatgtaagaaaKgaatttgtaggcacttgtgtgtgctattaatggccaaaggactgaatggatg[C/A]cccccttgcttatgaaaaatggtggcttatttatagtctgctttctaaaattccttggaaatttgaactctgtgcacttcacagtattgcacaattctca

fava_c16621_360	fava_c16621	360	T/C	gaagtcttgcacctgaggcagagaatatgaaaataaggaagaagagagaagttggtgatgcgaatgcacctttgttacctaagtctcaagaaacagatgg[T/C]gaatttcatgaatttaaYggagcttcattttctggggctgttttcaatttgtccaccacagttattggtgctgggatcatgggcttgcctgcttgcgtca

fava_c16621_378	fava_c16621	378	T/C	cagagaatatgaaaataaggaagaagagagaagttggtgatgcgaatgcacctttgttacctaagtctcaagaaacagatggYgaatttcatgaatttaa[T/C]ggagcttcattttctggggctgttttcaatttgtccaccacagttattggtgctgggatcatgggcttgcctgcttgcgtcaaaaagttggggatggtgc

fava_c16621_480	fava_c16621	480	G/C	gagcttcattttctggggctgttttcaatttgtccaccacagttattggtgctgggatcatgggcttgcctgcttgcgtcaaaaagttggggatggtgcc[G/C]ggtcttcttgccataatcctaacagcattattgactgggaagtcaattgagttcWtgatcaggttttcaagggccggaaatctttcttcctatggaagtc

fava_c16621_535	fava_c16621	535	A/T	gatcatgggcttgcctgcttgcgtcaaaaagttggggatggtgccSggtcttcttgccataatcctaacagcattattgactgggaagtcaattgagttc[A/T]tgatcaggttttcaagggccggaaatctttcttcctatggaagtcttatgggggatgcctttggtaaatacggaaaagcactgatagagacatgtgttgt

fava_c16621_723	fava_c16621	723	T/C	agacatgtgttgtaatcgctaacgttggtattctaattgtttacatgattattattggtgatgtgatttctggaactgcttcaagtgaaattcaccattc[T/C]ggtatccttgaaggatggtttggtgttcattggtggactgggcgtacatttgttcttgttttcacaacacttgctgtgtttgcacctttggtcagcctta

fava_c16621_912	fava_c16621	912	T/C	tggtcagccttaagcgaattgattcattgagattcacatctgcattgtcaattgggttagcagttgttttccttatcattgctgtggggatctcaatttt[T/C]aagattataagtggaggcattgggatgccaagactcttcccaatcattactgatgtagcttcggtttttgatctattcactgtaacccctgtggtgatgt

fava_c16624_1322	fava_c16624	1322	A/G	atggcttgctacaccggaatcagctgtgcagtccactaaaaccgtgtttggtataaaatgatttccatgcacatgttgaacaaatttttccaaatcagtc[A/G]cttcacctttttcctcacgaatttcgctccactttactaagtcgattcccgaatcatcaaggagcatcgacttggaacccatgatccccattacacgtaa

fava_c16624_2038	fava_c16624	2038	T/A	cagccattccggttccttccacatttacaagtgccaagttgtctattgttgtaaagcctttgacataattctttaatatcttcttatcttcattttcaat[T/A]agagaaggRtggcagacctttgttccaggagcagaggtattgaaaatattccttattataactggtatgccgtatcgaataactggactaattgttcgtg

fava_c16624_2047	fava_c16624	2047	G/A	cggttccttccacatttacaagtgccaagttgtctattgttgtaaagcctttgacataattctttaatatcttcttatcttcattttcaatWagagaagg[G/A]tggcagacctttgttccaggagcagaggtattgaaaatattccttattataactggtatgccgtatcgaataactggactaattgttcgtggatgtaaga

fava_c16624_2286	fava_c16624	2286	A/G	taagacagtgtcttcaaaatcacggcatcactaacttttctaggatctgcactgtacacaccatccacatctgtccaaatggtaacctgacgagcccgga[A/G]taaagcagccataattgctgcagagaagtcacttccatctctYttgagagtagtKggaatattttgacgcgtgcttgctataaatccagtagcaataatt

fava_c16624_2329	fava_c16624	2329	T/C	gatctgcactgtacacaccatccacatctgtccaaatggtaacctgacgagcccggaRtaaagcagccataattgctgcagagaagtcacttccatctct[T/C]ttgagagtagtKggaatattttgacgcgtgcttgctataaatccagtagcaataattaccttagatgggttaagagagtaccatttattaagtcttcgtt

fava_c16624_2341	fava_c16624	2341	T/G	acacaccatccacatctgtccaaatggtaacctgacgagcccggaRtaaagcagccataattgctgcagagaagtcacttccatctctYttgagagtagt[T/G]ggaatattttgacgcgtgcttgctataaatccagtagcaataattaccttagatgggttaagagagtaccatttattaagtcttcgttcagattccaagt

fava_c16624_2463	fava_c16624	2463	T/C	gctataaatccagtagcaataattaccttagatgggttaagagagtaccatttattaagtcttcgttcagattccaagtaatcaggatcaacatgatcag[T/C]actagtagaattcacaatgaggacttcccttgtgtccatccatttgcattcagtccctttcttcttaacagcataagacaatatctgcgcagaccataac

fava_c16624_2710	fava_c16624	2710	T/C	cagctatgtctatagcatgaagcattgcctttaagtttctaatatcttgatgcaaattggctaagaaagtagcaagatcatcttcatcaaataattcatg[T/C]gcagttaaactgtgcttctcttcaaccacatccagtgcagatatataagacttatcctgagactgagccttgtatatgagatcatacatcatatctgtta

fava_c16624_2872	fava_c16624	2872	C/T	actgagccttgtatatgagatcatacatcatatctgttacttttgacatagcagacaccaccaccaacttcctctcagaatcatcattgataattacatc[C/T]gcaacattgtttatcctcgctgaacttcccacacaagtcccaccaaacttgtgaacagaccaagtttctcctttKggtagctttttctcctctacagcta

fava_c16624_2947	fava_c16624	2947	T/G	cagaatcatcattgataattacatcYgcaacattgtttatcctcgctgaacttcccacacaagtcccaccaaacttgtgaacagaccaagtttctccttt[T/G]ggtagctttttctcctctacagctacatccactgtaaaatctgtaagtgaagagtgtatcttgaaggttggtgRgtctgttccccatagtgaactaacac

fava_c16624_604	fava_c16624	604	G/A	tgacttgtgcaccagctcctggtccacgaactatcagaggctgatctctataccttcttgttgtgaatgcaataatgttatccgagccagataattgagc[G/A]aatggatgatccttcttgtatcgtcgaagctccaccgaccccttttgattagccacgtctaccactcccacatatctcaacacttcccctgcagtgtcag

fava_c16627_1112	fava_c16627	1112	T/C	gtgcgaagatctgaaacgattgaattctagcttgtgccattatcgtcgaacaaggattcctctgaatcgacaccacatcggctttcaatccttgcgtgct[T/C]aaaccgcaaacgaatctagttcggattaggttcatatcagcaccgtcaaccttccggacttgcctttccgaaacgtggccgccggaagcaacactctcgc

fava_c16627_1466	fava_c16627	1466	A/G	gataattgatatgagttcattgtttttattttgtaattttgtaatagtaaaaacttggagagaaagaaaaaaaacgaacacacacgagaattagagaata[A/G]gtacaagaaagtgtgtgtgaggaaaaaacaaactgaaaaaatagaggttgagagaaggtggcgttttgtttttaaaggaaacggaaaccgccatgaggaa

fava_c16628_269	fava_c16628	269	G/T	aaatatataaaccagagctctcaatttcgcaagaagctactcacatttgttttgaaatgccatgtagtatggtggagaacttagttgaatgtgtggctta[G/T]ctgttatgagtctagagacctacacttgcggtctggtctgaacaacatagacttttccatctttgatgacaccttctatgtcctgaggagtgccatacaa

fava_c16628_621	fava_c16628	621	T/C	ccttcaagatcttcgccattggaatcagatcgaaaaattattgaccttcttataaatagcccaattggtttgctaggataacccaagacctgaggagaat[T/C]caaatcactttttttgcagatgaaactcaaagcacggccgggataagctccaactagtgtttcacctagtccctttacaacctcggtatatatttctgaa

fava_c16629_152	fava_c16629	152	A/C	caccgtttgaaattaataaaattgctaatagtacattgtatcacttcctaaaattctagaaaattgaagtgaaaatttagtaaaaaatgaactacaatgg[A/C]cttgcaattttaaaaccgaacatcatgtgagtttccacatgtgtaaatttccacatttcacgggactcttcactcatggttaaagctgtaactgtactgt

fava_c16629_355	fava_c16629	355	A/T	gctaggaaaaccacaaggcatttatcttctgtaagagagtacagaactagcatagataaaattaacgttatgattataattagatatttaaactttgcga[A/T]tcatgggcaaagtgtgacatgactgaatacagatgaatcacgtgaggctgagaaggcttactgaggcccttgatgttggccaaatatttcagtgctccaa

fava_c16629_504	fava_c16629	504	T/A	tgagaaggcttactgaggcccttgatgttggccaaatatttcagtgctccaaaagttcaatctttgcaagatgattattcatacgatgagtatcgtcagc[T/A]gataatcKatcaccaaccataagtttgaactgttcaaattttgctttttcgcactttaaYgcagcagttttagtagcctgtaaattcccagataaatcaa

fava_c16629_512	fava_c16629	512	G/T	cttactgaggcccttgatgttggccaaatatttcagtgctccaaaagttcaatctttgcaagatgattattcatacgatgagtatcgtcagcWgataatc[G/T]atcaccaaccataagtttgaactgttcaaattttgctttttcgcactttaaYgcagcagttttagtagcctgtaaattcccagataaatcaagcaaatta

fava_c16629_564	fava_c16629	564	C/T	tctttgcaagatgattattcatacgatgagtatcgtcagcWgataatcKatcaccaaccataagtttgaactgttcaaattttgctttttcgcactttaa[C/T]gcagcagttttagtagcctgtaaattcccagataaatcaagcaaattaaacaccactgggaaatttccaatacttttgaccattgtaatggaagcccata

fava_c16629_849	fava_c16629	849	G/A	atgcagccaaaccacactccccaaaattcaccaaaatgctatcttttatagcattagcgacattaaattgggttattataatagaatcacctgatgcttg[G/A]tctctattaggattcataaaaacctccatcaccatgtacctgtttttgaacaccatcgctYtttttctaggacccaaaaactgtgagaatcgcacaaccg

fava_c16629_910	fava_c16629	910	C/T	attaaattgggttattataatagaatcacctgatgcttgRtctctattaggattcataaaaacctccatcaccatgtacctgtttttgaacaccatcgct[C/T]tttttctaggacccaaaaactgtgagaatcgcacaaccgagagagagagagagacaggagaattttagggtgcagatgatctactgtaagtctggtgctc

fava_c16630_1179	fava_c16630	1179	G/A	tagaaaagatgcttatctttgatccaaacaaacgcatcacagttgacgaggcactgtgtcacccatatctttcatcacttcacaacacgaacgaggagcc[G/A]gtgtgtccaaggccattcacttttgattttgatcagccaacWtgcactgaagatcatatcaaggagctcatatggaaggaatctgtgaagttcaatccag

fava_c16630_1221	fava_c16630	1221	A/T	ttgacgaggcactgtgtcacccatatctttcatcacttcacaacacgaacgaggagccRgtgtgtccaaggccattcacttttgattttgatcagccaac[A/T]tgcactgaagatcatatcaaggagctcatatggaaggaatctgtgaagttcaatccagatccaccttgtcagtaatccctttcttattttgtgtaaaatg

fava_c16630_1380	fava_c16630	1380	T/C	atccaccttgtcagtaatccctttcttattttgtgtaaaatgtaacctctattgcctgtccatcgtcgttcagatgattcgctaaagacagaagctctct[T/C]cctttgtaatctaatcctaaaaccRaactacgacatggaaagaaacttctcttgaaaatcggatgttacacgcatttatacgagtaataaaaagctgttg

fava_c16630_1405	fava_c16630	1405	A/G	ttattttgtgtaaaatgtaacctctattgcctgtccatcgtcgttcagatgattcgctaaagacagaagctctctYcctttgtaatctaatcctaaaacc[A/G]aactacgacatggaaagaaacttctcttgaaaatcggatgttacacgcatttatacgagtaataaaaagctgttgtattgttctcaagttttctttagga

fava_c16630_1970	fava_c16630	1970	C/G	tatattaaataaattagacatttttctagaacaagccatagtctgaatctctagcgagcgtgatagcaaacgaatttcaaaaggttcaacttgatcctaa[C/G]gaaagtaaccgtaattctcgacagcaaggttggtcaatcgcctctcacaaacacaacatcctcaatacaagtgattcctacatagaattgtcgtttcacg

fava_c16630_360	fava_c16630	360	C/T	gaaacttgtttgaggtgtcttgtaagtacgtgcctcccattcgtcctattggtagaggcgcgtatggtatcgtttgtgctgctgttaattccgatacgca[C/T]gaacaagttgccattaagaagattggtaatgcttttgacaatatagttgatgcgaaaaggactttgcgagaaatcaagctccttcgtcacatggatcatc

fava_c16631_324	fava_c16631	324	G/T	caaagctcaaacccgatcaatcatgttttgagattacgttgatctcaaacgccgacaaagagaatcttccgatggagatcgccgcctcagcgacgtctcc[G/T]gtatctccactggagacgtcgctcggggaggagctaagcgccatgaagaagaaacttgagagactgagaacagaYaaagagaaaacagagaaaatgctga

fava_c16631_399	fava_c16631	399	C/T	agatcgccgcctcagcgacgtctccKgtatctccactggagacgtcgctcggggaggagctaagcgccatgaagaagaaacttgagagactgagaacaga[C/T]aaagagaaaacagagaaaatgctgaaagagagagaagcgttgtttgacgcRaagatgaaggagatggaagagaaaagcgagattcagaagaatctcgaaa

fava_c16631_450	fava_c16631	450	A/G	gggaggagctaagcgccatgaagaagaaacttgagagactgagaacagaYaaagagaaaacagagaaaatgctgaaagagagagaagcgttgtttgacgc[A/G]aagatgaaggagatggaagagaaaagcgagattcagaagaatctcgaaatccaggtcgatcgattgttccgattaaaggaactcaagtatcgatgcatga

fava_c16633_1070	fava_c16633	1070	T/G	ggagattcttgttccactgctcgctcactgattttagcatccaccaatgttttcttgtcttccaatgtttcttgtttcacagtttccttagctttggttt[T/G]ttcattttctgtggaaaactttgcatcttcattctcagctgcattagttttttgattttgtttcaccactgtttcttttgctggttcaatgattaKagtt

fava_c16633_1166	fava_c16633	1166	T/G	gtttKttcattttctgtggaaaactttgcatcttcattctcagctgcattagttttttgattttgtttcaccactgtttcttttgctggttcaatgatta[T/G]agtttcttctgaagttttcttggctgcttctggtattcctgtcttcacactctctttagctggataatgtagtgaaaatacttcctcaacatcatcaaca

fava_c16633_312	fava_c16633	312	C/T	atcagtcttcaaataattataagtaaatagaaagaaaaaaaaagaataacacaaatatagctgagaaatgtcacataacatagcaaggtcgacttcatgt[C/T]gcagccgagagtagaccgttgaattaagagagagaagctttatcactgtcaacatcagaagcctttggcgaaaccacagcatcaaaatagcttttctctc

fava_c16633_434	fava_c16633	434	C/G	aattaagagagagaagctttatcactgtcaacatcagaagcctttggcgaaaccacagcatcaaaatagcttttctctcctatgctagatgttgtttcca[C/G]tgctttgatctccgcagattctgctgttggtaaaaccacttcaattactttcttcttaggcggagaaactgccacatctcccataggattagtctcggat

fava_c16633_670	fava_c16633	670	C/G	gctttgtgtttcctccgaaaccttctcagctttcggtgtttctgtctttgtcacagtcacagttacctcaggtgcaatagcattttgattttgtttcacc[C/G]ctgtttcttccaccactggttcattgattttagtttctggtgtttttgtcttcacaaccacatcagctgcagagtcagtgattttgttttgcaatgtagg

fava_c16633_808	fava_c16633	808	G/A	tggtgtttttgtcttcacaaccacatcagctgcagagtcagtgattttgttttgcaatgtaggtttttcctcaacaacaacaacagcagattcttgtttt[G/A]taactggctcattgattttggtttcctgagatagtttctcttctgtctttggagatacatgtttcactgttggcttgctgattttagcatccaccacagg

fava_c16634_537	fava_c16634	537	G/T	ctcaaattcaatctaatgtggaggataagtatcctactatcttgttgcctaatcaatctgatgatttgtctcatctagctcttgatattggaggatctct[G/T]ataaagttggtgtacttttcaagacacgaaggccaatcagatgatgataaaaggaagaaaagtttaaaggagcgattagggctttctaatggtaatagaa

fava_c16637_1073	fava_c16637	1073	G/A	atgggtctgaggatgatgatgaSgataacaatgaaagtgaggacgatgagatggatgatcagattaatattaatgcacatggggatggagggaggaggac[G/A]gaggagttttttcgtgaacgagataaattaagtgaggaSgatggccatggccacggccactcttggccgccatctattaggatgaagaaacttgaggttg

fava_c16637_1112	fava_c16637	1112	G/C	aggacgatgagatggatgatcagattaatattaatgcacatggggatggagggaggaggacRgaggagttttttcgtgaacgagataaattaagtgagga[G/C]gatggccatggccacggccactcttggccgccatctattaggatgaagaaacttgaggttgaaatggcaaaggtttttcaagaccctgcaaagtcaccgt

fava_c16637_125	fava_c16637	125	G/A	aggaaacgctcaacacattctctctctatgctactattgattaatcgtaatcacttttcaacaggtaaaatcttaaagcagatctacaagttttctcatt[G/A]attcattttctgcttttaatttaagtcgtagattttcacttatagtatttgagttttttataatcagcaccgggaatttaggaattagggtttcagaatt

fava_c16637_1577	fava_c16637	1577	A/T	gtatcaacaacgcgcaagggagagaacctatcaacttggctagacaacagtagctcccggtaaggaatgatcatcattgctgaaataaataatgtatgaa[A/T]tgaaacccttttgattatgatgctacataatttcttagaacagttagattaacttagcacgttaattatatatagaaattcttgcccttgaaaatgaatg

fava_c16637_995	fava_c16637	995	C/G	gacaaagggtaccgagtagccatgatcttgacttgcatggttattcgttggaacacgggaatgactcgagagataacgatgggtctgaggatgatgatga[C/G]gataacaatgaaagtgaggacgatgagatggatgatcagattaatattaatgcacatggggatggagggaggaggacRgaggagttttttcgtgaacgag

fava_c16638_153	fava_c16638	153	A/G	aatatttaaccagtaaataaatacaatgacttctcattttctgtgaaaagtgttttcttgagaacatcagataagatgcgaacaaacttttttgcataca[A/G]tgaaaaaggtRtgaacagtgaaaggggRaaaaaattgtaaaggtaaatttgaYaggcacaaagYcWttcatgaactcaatgattgataacctagtggacc

fava_c16638_164	fava_c16638	164	G/A	agtaaataaatacaatgacttctcattttctgtgaaaagtgttttcttgagaacatcagataagatgcgaacaaacttttttgcatacaRtgaaaaaggt[G/A]tgaacagtgaaaggggRaaaaaattgtaaaggtaaatttgaYaggcacaaagYcWttcatgaactcaatgattgataacctagtggaccttgtatgtcat

fava_c16638_1698	fava_c16638	1698	A/G	atccgaatacggaacaattttaggcggaggaatcgaagtaaacttgaaaaacagaaaacaaagtgtaaaaacaatcggaaacgaggtagaaaacgccacc[A/G]ttctaatattcttcataacaaacattccaacctYactcaaaacagattcaacagaagcccttttcattctcataaccaataaccttaacctcggccgcaa

fava_c16638_1732	fava_c16638	1732	T/C	gaagtaaacttgaaaaacagaaaacaaagtgtaaaaacaatcggaaacgaggtagaaaacgccaccRttctaatattcttcataacaaacattccaacct[T/C]actcaaaacagattcaacagaagcccttttcattctcataaccaataaccttaacctcggccgcaacctcaacgaaaatctcttcttcaacctcttactt

fava_c16638_181	fava_c16638	181	G/A	acttctcattttctgtgaaaagtgttttcttgagaacatcagataagatgcgaacaaacttttttgcatacaRtgaaaaaggtRtgaacagtgaaagggg[G/A]aaaaaattgtaaaggtaaatttgaYaggcacaaagYcWttcatgaactcaatgattgataacctagtggaccttgtatgtcatcttgtttcctatcactY

fava_c16638_1902	fava_c16638	1902	G/T	caacgaaaatctcttcttcaacctcttactttcctctttaccataaccactcttcctaccactatcacccctacttctactaacaagtggctcaatttca[G/T]tatgaacaccattctgttgagacttgcaacaataataccgaaacctcgacaccggaactctattttctctaccataacactYaaatttcttgttcaatac

fava_c16638_1984	fava_c16638	1984	C/T	acaagtggctcaatttcaKtatgaacaccattctgttgagacttgcaacaataataccgaaacctcgacaccggaactctattttctctaccataacact[C/T]aaatttcttgttcaatacaaactcatgttgaaaattaaacttcattgaaggcaaaacaaatgaactagtgcataaacctctatgaaccctaaaatttcta

fava_c16638_206	fava_c16638	206	C/T	tttcttgagaacatcagataagatgcgaacaaacttttttgcatacaRtgaaaaaggtRtgaacagtgaaaggggRaaaaaattgtaaaggtaaatttga[C/T]aggcacaaagYcWttcatgaactcaatgattgataacctagtggaccttgtatgtcatcttgtttcctatcactYcttcccatcaatgatggcatccaag

fava_c16638_2151	fava_c16638	2151	A/G	agtgcataaacctctatgaaccctaaaatttctaatttgtaacctatttggggtcaaaaatgacccagtacaacaaggtaaagaaatacatgccatttct[A/G]ttccaaaatcaaccctatatatccataaaatcttccaatgttYtataaactgaattacctaacaaaattataaaaaaaataaaaattgaagcttgaaaat

fava_c16638_217	fava_c16638	217	C/T	catcagataagatgcgaacaaacttttttgcatacaRtgaaaaaggtRtgaacagtgaaaggggRaaaaaattgtaaaggtaaatttgaYaggcacaaag[C/T]cWttcatgaactcaatgattgataacctagtggaccttgtatgtcatcttgtttcctatcactYcttcccatcaatgatggcatccaagggaatagctta

fava_c16638_219	fava_c16638	219	T/A	tcagataagatgcgaacaaacttttttgcatacaRtgaaaaaggtRtgaacagtgaaaggggRaaaaaattgtaaaggtaaatttgaYaggcacaaagYc[T/A]ttcatgaactcaatgattgataacctagtggaccttgtatgtcatcttgtttcctatcactYcttcccatcaatgatggcatccaagggaatagcttact

fava_c16638_2194	fava_c16638	2194	C/T	ctatttggggtcaaaaatgacccagtacaacaaggtaaagaaatacatgccatttctRttccaaaatcaaccctatatatccataaaatcttccaatgtt[C/T]tataaactgaattacctaacaaaattataaaaaaaataaaaattgaagcttgaaaatgaagggatagagaatttcagagaaataactataactaggaaaa

fava_c16638_281	fava_c16638	281	T/C	RaaaaaattgtaaaggtaaatttgaYaggcacaaagYcWttcatgaactcaatgattgataacctagtggaccttgtatgtcatcttgtttcctatcact[T/C]cttcccatcaatgatggcatccaagggaatagcttacttagctccttgcttattttactagaccttagttgcttatcattgattccaaactttgctcttt

fava_c16638_716	fava_c16638	716	G/T	agaaacgacccgggcgacatagggccggatccaagacttccggtctgttagttgctgcaatgacaaccactctcatctctgattcaaacccatccatttc[G/T]gtcagcaactggttcagcgtttggtcacgttcgtcattgaagcttcttccacgtttgcctccaactgcatcaagttcatcgatgaatatgatcgatggtg

fava_c16639_848	fava_c16639	848	T/C	ccactttcttcctcctcctcttctcctcctcaagctccttctccaacctcgccctcttctcaacctcctttttcccccatctcaactccctctcctccat[T/C]tccatctccaattccaattccctctcactacaacaacattcccttctcttcaactcaaactcaaattcatccctcttccactctagtttctccttcgaaa

fava_c16643_1751	fava_c16643	1751	G/A	gattgggttttcgtcagtggtattaattggtcacgatgatggaggattgcttgctcttatggctgcacaaagagttcaaacatcaatgaattatcgtaac[G/A]taactatcaaaggggttgtgttgctaaatgttagcttgtcaagggaagtggttccttcctttgcaagaatcctcttgcatacctcacttgggaagaagca

fava_c16643_1966	fava_c16643	1966	T/C	tactaaggatggaaatcactcaagtggtgaatcggcgtgcttggtatgatgctaccaaattgacttcagaagttttggatctctataaggctccactatc[T/C]gttgaaggatgggatgaagctgttcatgagattggtaaattgtcatctgaaaccatcctttcagcaaaaaatgcagaatcattgctacaagctgtagaag

fava_c16643_205	fava_c16643	205	G/A	agggaacgaagcattcaatatcaaaacaaacggcttttgttcccaccggaaccctaactgatctccgcttccggtcaccgggaatgtcaagtataccgcc[G/A]tccgagaatcttactccggtatcggaagcggtttcggtcggttatcggatggtaaacaattaagcattctgttgaagcgatcgtaactacccgtctgtca

fava_c16643_2101	fava_c16643	2101	C/T	gtaaattgtcatctgaaaccatcctttcagcaaaaaatgcagaatcattgctacaagctgtagaagacattccggtgttagttattgcaggagctgaaga[C/T]tctctcgtctctttgaaatcttgtcaagctatggcctcaaaacttgaaaattctagactagttgcaatatctggatgtggtcatttacctcatgaagaat

fava_c16643_391	fava_c16643	391	G/T	actacccgtctgtcagttaggttgttagggcggttatggcgaagggaggacaatgcttgttcgagaagctttggaggtgtatccggacggttttctttgt[G/T]tttgtattggtggtgtcgctggttgttacgtcgcttccggtggttgttgcggtggtggatgttcttgttccgtgcgttctgatctcgaattttacttgtg

fava_c16643_532	fava_c16643	532	T/A	tggttgttgcggtggtggatgttcttgttccgtgcgttctgatctcgaattttacttgtgttaattgctacggtttcaaggagctttttcgacgttactc[T/A]ttcaagagttcgttgacggatattcctctcgtttcactcatacgatccttcatcattatatgtgtttattctatttgtgatggacctgctctttcacacg

fava_c16645_1212	fava_c16645	1212	A/G	aataacttcgtgagagcttcctggtcaccgctcttcaaggaggaaaatccggtaatggtgttaggagaagagttggaggagggtagagggaagcaagtga[A/G]gtggtgccatttcgtaatgtccatgtttctcctcatgtcccttgagaccaatcccagtcttagggttttggattgtatggcttcggagcatgtcttgcac

fava_c16645_280	fava_c16645	280	C/G	gttatagttgatgaagcaattcaaccatagaatattagaactaaagcgcaacaaagaaaagtagaattgtgaatagtttttcaacaacgctagttaatta[C/G]tcttctacatctagacaaaaattggYaatgtcacttcgaaacaaagctaagcccttctatgaaccaatgaccaaattagaagctcatgcgtcaaagtact

fava_c16645_306	fava_c16645	306	C/T	atagaatattagaactaaagcgcaacaaagaaaagtagaattgtgaatagtttttcaacaacgctagttaattaStcttctacatctagacaaaaattgg[C/T]aatgtcacttcgaaacaaagctaagcccttctatgaaccaatgaccaaattagaagctcatgcgtcaaagtactcttcagggagataaaacttcaaacca

fava_c16645_603	fava_c16645	603	T/C	ccagagttgaagtgtttctccataaggtgccacattcctggttttggtttccgaaaaggatcagcttcctttgttccagccttgcctgatttaccacctg[T/C]cccacaagctataaaaacctgaattgggaccttcactttctcaataaactgatttagccgtccaatttttgagtccacggctttttgtctttgattcttc

fava_c16645_812	fava_c16645	812	G/A	aatattggattcattggtgaaaattaccagtttgtagccatcgttgtataaggtttgcagtttatcaggaattaagggatacatgagggaccaagcatta[G/A]gacctgttatattcacagcagttttcgcaagacacccatcaaaatcaaatgcagcgattttacttgaatcttgcaaaccatcatccccttcaagaaaaat

fava_c16648_131	fava_c16648	131	G/A	taaatagcccattaaattacaaaaataacaaacaaatggcttaaaagctcattagttactggttactagttagtactttttgagaaatagaaatggtctt[G/A]acccctatgctttataKctcccttgaaatacttcctaataataactacctatgcttccaaatcagttgcMcatggaaatccattctcatgatgggttctg

fava_c16648_1376	fava_c16648	1376	A/G	tttgaacctcaaattttttagaagctagtttattatcaacaataggtgaatcagtattctgactcaatgactctgctacagaactccttacacgcacata[A/G]gtggtgaaagaatatcgaatgttgttttccaccttaggaaaggatgagtaccatggccgaaatacagtgaaatcaatcggaggcataaaagtgtcacact

fava_c16648_148	fava_c16648	148	G/T	tacaaaaataacaaacaaatggcttaaaagctcattagttactggttactagttagtactttttgagaaatagaaatggtcttRacccctatgctttata[G/T]ctcccttgaaatacttcctaataataactacctatgcttccaaatcagttgcMcatggaaatccattctcatgatgggttctggtgtacacaatttctgt

fava_c16648_1676	fava_c16648	1676	T/G	caatcgacgtacaataaggagaagcagctaaaagttccaacgcagaagacatgcttccacatattacaacattttctgctgctttaacatcaaaactacc[T/G]gagcgagtcaaaacaacattaagccgaccaggWagaggcctgaactgaaaaggaatgctctcccatgtttttcttcccataacaacggcattcttcttcc

fava_c16648_1709	fava_c16648	1709	A/T	gttccaacgcagaagacatgcttccacatattacaacattttctgctgctttaacatcaaaactaccKgagcgagtcaaaacaacattaagccgaccagg[A/T]agaggcctgaactgaaaaggaatgctctcccatgtttttcttcccataacaacggcattcttcttcccggattcattagtcgtcatagttatctccttaa

fava_c16648_2009	fava_c16648	2009	C/A	ggttaggcagtgggttgatactactaccattgccattgccaatgccatttccattgaagattacagaggaatcagcagccatagcagaacctagaaaccg[C/A]RataaacgcaaagattggttctgatgaaaataagSactgcacaaccaattcttttggaagtgcagtgcaatcgaaatgaaattgaggcgcaaaattttga

fava_c16648_201	fava_c16648	201	A/C	tagtactttttgagaaatagaaatggtcttRacccctatgctttataKctcccttgaaatacttcctaataataactacctatgcttccaaatcagttgc[A/C]catggaaatccattctcatgatgggttctggtgtacacaatttctgttaaattattggcRtgacttgactgcattaacgtgcgttcaacaaactgatgtt

fava_c16648_2010	fava_c16648	2010	G/A	gttaggcagtgggttgatactactaccattgccattgccaatgccatttccattgaagattacagaggaatcagcagccatagcagaacctagaaaccgM[G/A]ataaacgcaaagattggttctgatgaaaataagSactgcacaaccaattcttttggaagtgcagtgcaatcgaaatgaaattgaggcgcaaaattttgaa

fava_c16648_2044	fava_c16648	2044	G/C	ttgccaatgccatttccattgaagattacagaggaatcagcagccatagcagaacctagaaaccgMRataaacgcaaagattggttctgatgaaaataag[G/C]actgcacaaccaattcttttggaagtgcagtgcaatcgaaatgaaattgaggcgcaaaattttgaaatagtgatgaggtgagagtgagtcRggatggaga

fava_c16648_2135	fava_c16648	2135	A/G	gaaaataagSactgcacaaccaattcttttggaagtgcagtgcaatcgaaatgaaattgaggcgcaaaattttgaaatagtgatgaggtgagagtgagtc[A/G]ggatggagatttgtgttctgatttgtaatagttgtgggcggagaggtgaaacggagatgatgagcagagatgacaatgccggcagagactgccgccggat

fava_c16648_261	fava_c16648	261	A/G	acttcctaataataactacctatgcttccaaatcagttgcMcatggaaatccattctcatgatgggttctggtgtacacaatttctgttaaattattggc[A/G]tgacttgactgcattaacgtgcgttcaacaaactgatgttacttccataaatctatttgttgtgcctcatttcaaacagcagaaacaacctgaagtagag

fava_c16648_374	fava_c16648	374	C/T	attaacgtgcgttcaacaaactgatgttacttccataaatctatttgttgtgcctcatttcaaacagcagaaacaacctgaagtagagactaagatctcc[C/T]ttattttaaacagccatcttcatctcaatcttctggtgaggatcatagtcaatgagcttgaaatcagtagccacaaaagaatccatatctttcttctttg

fava_c16649_1003	fava_c16649	1003	C/A	atcctctgaagttattgatccaaacacgggtttgctttacacaaacatgtttgacgcacagattgatgctatttactatgcactcatggccctaaatttc[C/A]gaacaattaatgtaatggtcaccgaaacaggYtggccttcaaaagggtcgccYaaggagaaggccgcaactcctgataatgcacaaacatataacactaa

fava_c16649_1035	fava_c16649	1035	T/C	tgctttacacaaacatgtttgacgcacagattgatgctatttactatgcactcatggccctaaatttcMgaacaattaatgtaatggtcaccgaaacagg[T/C]tggccttcaaaagggtcgccYaaggagaaggccgcaactcctgataatgcacaaacatataacactaatctcataaggcatgttatcaataRtactggta

fava_c16649_1056	fava_c16649	1056	T/C	acgcacagattgatgctatttactatgcactcatggccctaaatttcMgaacaattaatgtaatggtcaccgaaacaggYtggccttcaaaagggtcgcc[T/C]aaggagaaggccgcaactcctgataatgcacaaacatataacactaatctcataaggcatgttatcaataRtactggtactccagcaaagcctgggaagg

fava_c16649_1127	fava_c16649	1127	A/G	gaaacaggYtggccttcaaaagggtcgccYaaggagaaggccgcaactcctgataatgcacaaacatataacactaatctcataaggcatgttatcaata[A/G]tactggtactccagcaaagcctgggaaggagctagatatttatattttctcattgtttaacgaaaatagaaagcctggtttagactcagagaggaactgg

fava_c16649_1458	fava_c16649	1458	T/C	cctccgaaattgatttgcagaatgctttggattgggcctgtggtcctggcgatgtggactgtactgctgtccagcctagccagccttgtttcgagccgga[T/C]aatctagtctcacatgcatcttttgtttttaatagctattaccagcaaaacggggcctctgatgttgcttgtagttttggaggtacaggtgtaaaagttg

fava_c16649_1615	fava_c16649	1615	T/C	ctctgatgttgcttgtagttttggaggtacaggtgtaaaagttgacaaggatccaagttatgataactgcatctacatgagagatggcaataacaaaaca[T/C]cgggaggtaatacaagagcaatgtcgtcaacttcatcttctcaacagaaaggaagatattcttcaatttctatgtatcttcttgtaatttgtctcttcat

fava_c16649_1821	fava_c16649	1821	T/G	tgaatattgaacaatcctgaccaggcaagtagaaaatgcagcatgattttgctatgttttgaagtatgctgcgttttggcatgcgagggaggtaatggtt[T/G]tgttgtaagcttttcatacaatgtcatgcatcagataagtgtaaaaatattttgctcttgtaaaatctttagatcagatagcccaaaatttcttgMtgtt

fava_c16649_1917	fava_c16649	1917	C/A	ggttKtgttgtaagcttttcatacaatgtcatgcatcagataagtgtaaaaatattttgctcttgtaaaatctttagatcagatagcccaaaatttcttg[C/A]tgttgtaaacttttatgtgccattaagataaggccataaaagtaagcaagataaatgacatctctgtgattcatttgtgtcaatgaataatgacagaaac

fava_c16649_507	fava_c16649	507	A/G	ggatttatgattctaacattcaggtcctaagggcctttgccaacactggagttgaacttatgattggtgttccgaattcagatttgctctcattctccca[A/G]ttccaatctaacRccgatgcttggttgaaaaacagcgtYcttccttattatccggccacaaagatcacatacataactgttggggctgaaatcacagaaa

fava_c16649_520	fava_c16649	520	G/A	taacattcaggtcctaagggcctttgccaacactggagttgaacttatgattggtgttccgaattcagatttgctctcattctcccaRttccaatctaac[G/A]ccgatgcttggttgaaaaacagcgtYcttccttattatccggccacaaagatcacatacataactgttggggctgaaatcacagaaagtagctataatac

fava_c16649_546	fava_c16649	546	C/T	ccaacactggagttgaacttatgattggtgttccgaattcagatttgctctcattctcccaRttccaatctaacRccgatgcttggttgaaaaacagcgt[C/T]cttccttattatccggccacaaagatcacatacataactgttggggctgaaatcacagaaagtagctataatacatcttcattggtagtacctgccatga

fava_c16650_1229	fava_c16650	1229	C/T	ccgcgtctccaaggataacatggataaattcagactcttccaatccaaagtagtttttggccaccttcaaaacctccttatcactatcgacaccgatgac[C/T]tcaaaacccaattgaattgtcagaaaagttagcaaagccccacctccaaccccaaggcataaagctttcggtctcagcccatttcgaatccgcccatcta

fava_c16650_519	fava_c16650	519	C/A	ctacatagatcaaactcagccataatgttctatccaagaaaaaatactgcttttctactgtgtatggccaatttccttaaacaacttttctacagaagag[C/A]aatgatacatgagaaaaacattcttcgtctggcatcattgtaccataactcttaatacatcttcaattagttttgcaatggaaaaagtatatacgataga

fava_c16652_269	fava_c16652	269	T/C	tacaattttcagggtacaatataacaattaaaaacaaagaaagcctaccctgcatataaattttcaaaaaattatagcttgggtaaaatgtctgatagtt[T/C]ctaaccaattctgcatctaagtgtgcacWctattcttttacaaagtttgaagagtaatactacagcacaatgctcaaaccttgaacaaaccccaaacatg

fava_c16652_298	fava_c16652	298	T/A	aaaaacaaagaaagcctaccctgcatataaattttcaaaaaattatagcttgggtaaaatgtctgatagttYctaaccaattctgcatctaagtgtgcac[T/A]ctattcttttacaaagtttgaagagtaatactacagcacaatgctcaaaccttgaacaaaccccaaacatgtatMtWttgttgccgcgaaaaggagcatg

fava_c16652_373	fava_c16652	373	C/A	accaattctgcatctaagtgtgcacWctattcttttacaaagtttgaagagtaatactacagcacaatgctcaaaccttgaacaaaccccaaacatgtat[C/A]tWttgttgccgcgaaaaggagcatgctcctttatgttccccttataaacagMcacattcatggaaatcactgaagcttcatccaagtaccattagatgca

fava_c16652_375	fava_c16652	375	A/T	caattctgcatctaagtgtgcacWctattcttttacaaagtttgaagagtaatactacagcacaatgctcaaaccttgaacaaaccccaaacatgtatMt[A/T]ttgttgccgcgaaaaggagcatgctcctttatgttccccttataaacagMcacattcatggaaatcactgaagcttcatccaagtaccattagatgcagt

fava_c16652_425	fava_c16652	425	C/A	aatactacagcacaatgctcaaaccttgaacaaaccccaaacatgtatMtWttgttgccgcgaaaaggagcatgctcctttatgttccccttataaacag[C/A]cacattcatggaaatcactgaagcttcatccaagtaccattagatgcagttaaaggagcacttcattaaaatatcttcaattatcaagcagtccaaagct

fava_c16656_107	fava_c16656	107	T/C	tgtaaacaatatcttagtttcaaattacaaataatatctaataagcaagttacacatagtgaacaaacatggacaagggcaacgttgttttgttggattt[T/C]tggccaagcgtgtatgcaatgagagtaaaaatcgcattagaggagaagggagtctcgtatgaatgtagacaagaagactttcaagctaaaagctctctac

fava_c16656_258	fava_c16656	258	G/A	agtctcgtatgaatgtagacaagaagactttcaagctaaaagctctctacttttagagatgaacccggtttacaaaaagatcccggtcttgattcataat[G/A]gaaaacccatatgtgaatcactcaacattgttgagtatattgatgaggtttggaatcacaaaccttctcttttgccctctgatccttacaaccgatccca

fava_c16656_362	fava_c16656	362	T/C	aacccatatgtgaatcactcaacattgttgagtatattgatgaggtttggaatcacaaaccttctcttttgccctctgatccttacaaccgatcccaagc[T/C]aagttttggggagattacatcgacaaacatgtatacaacattagaaggaagatatggttaggaaagggtaaagaacaagaagaaagtaaaaagaagttta

fava_c16661_1117	fava_c16661	1117	C/T	gaaatcattctcaacctgattaagaacatgtttgaggctatcaagtaccacattcgaaaatgatttatctgatgaagattgatgcccgtgatcaagaata[C/T]RactatcatcMaaaccgacattgtcagaagcgttgatggaattcggttctgaattccaatctattgtatttagataagatacaatagagtcataaagagt

fava_c16661_1118	fava_c16661	1118	G/A	aaatcattctcaacctgattaagaacatgtttgaggctatcaagtaccacattcgaaaatgatttatctgatgaagattgatgcccgtgatcaagaataY[G/A]actatcatcMaaaccgacattgtcagaagcgttgatggaattcggttctgaattccaatctattgtatttagataagatacaatagagtcataaagagta

fava_c16661_1128	fava_c16661	1128	C/A	caacctgattaagaacatgtttgaggctatcaagtaccacattcgaaaatgatttatctgatgaagattgatgcccgtgatcaagaataYRactatcatc[C/A]aaaccgacattgtcagaagcgttgatggaattcggttctgaattccaatctattgtatttagataagatacaatagagtcataaagagtacaagcaagat

fava_c16661_215	fava_c16661	215	C/T	ttctacagcaaagtggaagcactgcataaaatatttgaatacaaaggaaccaatgattctttctatgttcaaaattgtcatatgtacaatcttccaatat[C/T]ccaactttctacaatgcaacaatgactatagtttccattgaaaagcttcatgtgcaaatttactRtggtcaatgtttagatgcaaattgacgcctttgaa

fava_c16661_280	fava_c16661	280	G/A	tgttcaaaattgtcatatgtacaatcttccaatatYccaactttctacaatgcaacaatgactatagtttccattgaaaagcttcatgtgcaaatttact[G/A]tggtcaatgtttagatgcaaattgacgcctttgaagtccgtttattcatcccaaggatgatcaccattacagtgacRtcatcRtgatatttcctYctact

fava_c16661_357	fava_c16661	357	G/A	aaagcttcatgtgcaaatttactRtggtcaatgtttagatgcaaattgacgcctttgaagtccgtttattcatcccaaggatgatcaccattacagtgac[G/A]tcatcRtgatatttcctYctacttccagcYggaacattcatcaactcctccgtgctgaaacctgcagaatcagctgcttttgttactagtttttcaatga

fava_c16661_363	fava_c16661	363	G/A	tcatgtgcaaatttactRtggtcaatgtttagatgcaaattgacgcctttgaagtccgtttattcatcccaaggatgatcaccattacagtgacRtcatc[G/A]tgatatttcctYctacttccagcYggaacattcatcaactcctccgtgctgaaacctgcagaatcagctgcttttgttactagtttttcaatgagatatt

fava_c16661_375	fava_c16661	375	C/T	ttactRtggtcaatgtttagatgcaaattgacgcctttgaagtccgtttattcatcccaaggatgatcaccattacagtgacRtcatcRtgatatttcct[C/T]ctacttccagcYggaacattcatcaactcctccgtgctgaaacctgcagaatcagctgcttttgttactagtttttcaatgagatatttggctggatcgc

fava_c16661_387	fava_c16661	387	C/T	atgtttagatgcaaattgacgcctttgaagtccgtttattcatcccaaggatgatcaccattacagtgacRtcatcRtgatatttcctYctacttccagc[C/T]ggaacattcatcaactcctccgtgctgaaacctgcagaatcagctgcttttgttactagtttttcaatgagatatttggctggatcgcagtgaggattgc

fava_c16664_1134	fava_c16664	1134	C/T	acctgtgctgcggctgtgctggttatgccgcttttggtaatgatacaccgggaaatcttttaacaggctttgcaacttacaagctttattggctagtaga[C/T]ttcgcMaatgcttgtatagttattcacctagttggtgcataccaggtgtatagccagccactttacgcgaatgttgagaactggttacgatttagattcc

fava_c16664_1140	fava_c16664	1140	C/A	gctgcggctgtgctggttatgccgcttttggtaatgatacaccgggaaatcttttaacaggctttgcaacttacaagctttattggctagtagaYttcgc[C/A]aatgcttgtatagttattcacctagttggtgcataccaggtgtatagccagccactttacgcgaatgttgagaactggttacgatttagattcccggata

fava_c16664_1258	fava_c16664	1258	A/G	tcacctagttggtgcataccaggtgtatagccagccactttacgcgaatgttgagaactggttacgatttagattcccggatagtggatttgtgaattac[A/G]cgcacgacttgaaactcccattgctaccaaattttcaactaagtccactaaggctatgtttccgaacattttatgtcgtgacgacgactgtgattgcgat

fava_c16664_1657	fava_c16664	1657	A/G	gataatatctgcaaaactaagctgagaaggtttgagagaagttgaaattaagttgacgaatcagattgtggtgttagtttttcattcttattactttttg[A/G]aaccaaacattaaacataggagaatggtttattggttgttccctttttgatagcaaacgtcttatataatcatggagcattggtgagttcgagattctgt

fava_c16664_215	fava_c16664	215	C/G	catctatatatatatccatttcactccatcaagtttcttacaatcgaattttacgtaacgaagatcatgagcgaggaacgtctttctggtggcagtggca[C/G]cagtactccgttactgcaatccaaatttgaggagcagtatcaYgttaaaagaacRgggacagtgtggacggcagtggcRcacatagtgacaggagtgata

fava_c16664_258	fava_c16664	258	T/C	tcgaattttacgtaacgaagatcatgagcgaggaacgtctttctggtggcagtggcaScagtactccgttactgcaatccaaatttgaggagcagtatca[T/C]gttaaaagaacRgggacagtgtggacggcagtggcRcacatagtgacaggagtgatagggtcaggagtgttgtcgttggcatggagtattgcacagattg

fava_c16664_270	fava_c16664	270	G/A	taacgaagatcatgagcgaggaacgtctttctggtggcagtggcaScagtactccgttactgcaatccaaatttgaggagcagtatcaYgttaaaagaac[G/A]gggacagtgtggacggcagtggcRcacatagtgacaggagtgatagggtcaggagtgttgtcgttggcatggagtattgcacagattggatggattgctg

fava_c16664_294	fava_c16664	294	G/A	gtctttctggtggcagtggcaScagtactccgttactgcaatccaaatttgaggagcagtatcaYgttaaaagaacRgggacagtgtggacggcagtggc[G/A]cacatagtgacaggagtgatagggtcaggagtgttgtcgttggcatggagtattgcacagattggatggattgctggaccattagcgattcttttgttcg

fava_c16664_852	fava_c16664	852	G/T	ttgcagcaataatgtcctttgcttatgctttcattggaatgggactcgccactgataaaatcaccgaaaacggacattttaagggtaacattagaggtgt[G/T]ccaactttgacYggaatggaaaaagtatggctggttgcgcaRgcacttggYgacacagccttcgcttatccattctcagtaattcttatagaaattcagg

fava_c16664_864	fava_c16664	864	C/T	tgtcctttgcttatgctttcattggaatgggactcgccactgataaaatcaccgaaaacggacattttaagggtaacattagaggtgtKccaactttgac[C/T]ggaatggaaaaagtatggctggttgcgcaRgcacttggYgacacagccttcgcttatccattctcagtaattcttatagaaattcaggacactttgaaat

fava_c16664_894	fava_c16664	894	A/G	gactcgccactgataaaatcaccgaaaacggacattttaagggtaacattagaggtgtKccaactttgacYggaatggaaaaagtatggctggttgcgca[A/G]gcacttggYgacacagccttcgcttatccattctcagtaattcttatagaaattcaggacactttgaaatcgcctccagcggaaaacgtaaccatgagga

fava_c16664_903	fava_c16664	903	T/C	ctgataaaatcaccgaaaacggacattttaagggtaacattagaggtgtKccaactttgacYggaatggaaaaagtatggctggttgcgcaRgcacttgg[T/C]gacacagccttcgcttatccattctcagtaattcttatagaaattcaggacactttgaaatcgcctccagcggaaaacgtaaccatgaggagggcctcaa

fava_c16666_1250	fava_c16666	1250	T/C	tgaaagatattgctctgaagattctgcaaaatggaatttcaacagttcctgttattgattcatcttctgaagacggttcatttccacaactactgcatct[T/C]gcttcactttcggggatccttagatgcatttgcaggtattttaggaattgttctagttcattgcctatacttcaacttccaatttgtgcaattcccgtgg

fava_c16666_252	fava_c16666	252	A/G	aacctctttccatagggtttctacttttcacccaggatttcttaaattcctcgacatacgattctcgatttgtgcattgaattggtgctgcatttggttg[A/G]tttttttgctgtgaattgaatttggatgatagcatgctgtaaagtttagttcttggaaggaattttgtgtagctttttgttttgaattttggaatagaat

fava_c16666_929	fava_c16666	929	T/C	acttgtcaatgcaaacagcttatgaattacttcctgagtcaggcaaggttgttaccttggatgttgatttgccagtgaagcaagcatttcacatactaca[T/C]gagcagggaattcccatggctcctctttgggatttcagcaagggacagtttgttggagttctcagcgttttagattttattttaattttaagagagctcg

fava_c16668_133	fava_c16668	133	A/G	tttcatagatcatgggaagagaaagcacatagagatcacattccattttcttaactaggcaaggaaaattctctgacaaatcaatccattccccagaaat[A/G]ccaatgtacatatagctacagtStacatattcaaaaacaagagtatacaaatttcacaaatattatttttacaatttaaataaacaagaaagaatatgaa

fava_c16668_156	fava_c16668	156	C/G	agcacatagagatcacattccattttcttaactaggcaaggaaaattctctgacaaatcaatccattccccagaaatRccaatgtacatatagctacagt[C/G]tacatattcaaaaacaagagtatacaaatttcacaaatattatttttacaatttaaataaacaagaaagaatatgaaatttttcaatgaattggtaacaa

fava_c16671_457	fava_c16671	457	C/A	aatcaccatctctttcttactgcgacaaacccaatttgcgtcagcctcatttctcatggagtttgagattgataactccagtaatggtgatgtagtagga[C/A]atgttactactggagaggttagcagtagcRtagctgacaaagacaaagttgaYtgcagttctttcgatccaactgatgaacaggatcaagatgataatat

fava_c16671_487	fava_c16671	487	A/G	ccaatttgcgtcagcctcatttctcatggagtttgagattgataactccagtaatggtgatgtagtaggaMatgttactactggagaggttagcagtagc[A/G]tagctgacaaagacaaagttgaYtgcagttctttcgatccaactgatgaacaggatcaagatgataatatgactcaagattcttctggagtggaccaaat

fava_c16671_510	fava_c16671	510	C/T	tcatggagtttgagattgataactccagtaatggtgatgtagtaggaMatgttactactggagaggttagcagtagcRtagctgacaaagacaaagttga[C/T]tgcagttctttcgatccaactgatgaacaggatcaagatgataatatgactcaagattcttctggagtggaccaaatcccgttagcgattcctgcattat

fava_c16676_1654	fava_c16676	1654	G/A	aacaattttgaatcttccattggaagaaaccttggtgtttgaaacctttgaaataataaccttctttaaactgcttccaaagaaagatgaacttgaaaat[G/A]aagctactcctccagaactattcaagctcaatggagttgtagtgataggtttaacagcagagattgatgcagccatgtttatgttgttttgctaagatag

fava_c16676_234	fava_c16676	234	T/C	atgcactcaatctgattaacattaacattgtgcacactaaatctcaactatagagcacaacaacaatccaatattttaacaagatgataaaattattcga[T/C]ttattatctaaagtctcaRcttctcctccttaaYttaacctaataggagaatccttagattattcaaatgtgtataagcatgttccatcatcacttctag

fava_c16676_253	fava_c16676	253	G/A	cattaacattgtgcacactaaatctcaactatagagcacaacaacaatccaatattttaacaagatgataaaattattcgaYttattatctaaagtctca[G/A]cttctcctccttaaYttaacctaataggagaatccttagattattcaaatgtgtataagcatgttccatcatcacttctagcagttgggtcgaagttttt

fava_c16676_268	fava_c16676	268	C/T	cactaaatctcaactatagagcacaacaacaatccaatattttaacaagatgataaaattattcgaYttattatctaaagtctcaRcttctcctccttaa[C/T]ttaacctaataggagaatccttagattattcaaatgtgtataagcatgttccatcatcacttctagcagttgggtcgaagtttttggcatttcgatctgt

fava_c16677_1214	fava_c16677	1214	T/C	ggccagttattggaatccatgcacaaagacctagcatgaaatattcaaacttatctgagtcaagaaagcaaggacttcacacaccatcacatatgagaac[T/C]ggtagtggaagagaagggacatcgcgcgaagtaaagaagaaaagatctgaagaatcattagaagcaaacttgaagaagYcaaaacaagattcatcaacWa

fava_c16677_1293	fava_c16677	1293	C/T	cacaccatcacatatgagaacYggtagtggaagagaagggacatcgcgcgaagtaaagaagaaaagatctgaagaatcattagaagcaaacttgaagaag[C/T]caaaacaagattcatcaacWactaattcatcttcttcaaagttgcaagcacctaaagtcaagctaggagaaaagatcacagctcttcaacaaattgtgtc

fava_c16677_1313	fava_c16677	1313	A/T	cYggtagtggaagagaagggacatcgcgcgaagtaaagaagaaaagatctgaagaatcattagaagcaaacttgaagaagYcaaaacaagattcatcaac[A/T]actaattcatcttcttcaaagttgcaagcacctaaagtcaagctaggagaaaagatcacagctcttcaacaaattgtgtctccttttggaaagacagata

fava_c16677_213	fava_c16677	213	G/C	gagtgaagaatgtgttggaaacactattgctacctctactacaccattaaactggtggtatcttcaagcaaccaactctctttcttctaattggaatgat[G/C]ttaataaacttgcatggagcaatcaaatgaataatccaaattcttcatcttcttgtgatgatcaagatatatctgtttcttcgaattctttcaccaatgc

fava_c16677_948	fava_c16677	948	G/C	tggaggagtaggtaataaaatgttctctaatatccatgttattcaagacatcaataaggttaagcaagaatatcatcatcctcatcatcatggaaatcat[G/C]aagtatttggaaaatcatttgtaaatcccaatggatatcttgatggatttaataatagtcttaattcaattggagaaaatggaaaattttatcaagggtt

fava_c16679_166	fava_c16679	166	A/G	ccagagaaaccctgtcaaaaccacgttgtttcaaatatttgatctttgcacattcttcacgacctccaaaacttcgattcaattgaattgcagatgcatt[A/G]atatttaaaatgcctcctcaaaaggatccagataagttcttcatgtctatgtYtatcagtaatggagagaataagcaatttgattatttacgcgctttat

fava_c16679_219	fava_c16679	219	C/T	tcttcacgacctccaaaacttcgattcaattgaattgcagatgcattRatatttaaaatgcctcctcaaaaggatccagataagttcttcatgtctatgt[C/T]tatcagtaatggagagaataagcaatttgattatttacgcgctttatatgagggttgcattgctggaggtcttgctggtgttgctgtagaagcagcttta

fava_c16679_565	fava_c16679	565	C/T	caaagcagaaattgctggagatcttccctgagaatctaaacgctctggctcatattgctgcaggtactattggaggagccgcttcttctcttgttcgtgt[C/T]ccaactgaggttattaagcaaaggatacaaaccggtcagttcaaatcaacctcatccgctctccgtcttatcattgctaatgagggttttaaaggtcttt

fava_c16680_130	fava_c16680	130	C/T	atatcctaaccccataagaaaaaccaaacctccatcttcattcttcttcatcctataatcatattgcaagcacataaattataggtatgagtaattacaa[C/T]gatgatcgtaaccatgataatgataatttttatccatttggaaattttgatcatggatcRagtcaaagcaacttcaacaaaaaaatattgttaaccgcaa

fava_c16680_190	fava_c16680	190	G/A	atattgcaagcacataaattataggtatgagtaattacaaYgatgatcgtaaccatgataatgataatttttatccatttggaaattttgatcatggatc[G/A]agtcaaagcaacttcaacaaaaaaatattgttaaccgcaatatgttctttaatactagtattggttttggtgttggctcttcatctttacgccaggtatg

fava_c16680_427	fava_c16680	427	C/T	atcaactcagcctcaccgttgctcatgctcacgttcaattcgccgatccgtcaaactccggactcgatcccacgctcattgccactcttcccactttcct[C/T]ttcaaacaaaaaggaaaactagatgaagaacaaaacaacaatgtttctaataacattgttgaatgcgcagtgtgtttgagtttgttagaagatggagaaa

fava_c16680_536	fava_c16680	536	C/T	aaaaggaaaactagatgaagaacaaaacaacaatgtttctaataacattgttgaatgcgcagtgtgtttgagtttgttagaagatggagaaatgatgagg[C/T]tgcttcctaattgtaagcatagtttccacgtgagttgtatcgatatgtggttagcttcacattccacctgtcctatttgtcgaaccaaggttgaacctcg

fava_c16680_666	fava_c16680	666	T/C	gtgagttgtatcgatatgtggttagcttcacattccacctgtcctatttgtcgaaccaaggttgaacctcgccttgaaccagagacgcgcgagggtccca[T/C]ttggtttaaaccatcatcggaaagtacttccgatgctgctgattctattaacgagtctcccaagaatatttctcgattaagttcttttcagaggattctt

fava_c16686_1421	fava_c16686	1421	T/A	gagctagcttacagtttgaggaggcaactgtgagtgaccttctttatccttcaaaatcatcttctgatcacaattattatgatatagaattagttctagc[T/A]gtcttggaaactttcctaaagctttggaaaagaatgtcccctggtgctgtagataacacctactttttgagatcaatcagaaatgtcggaaaactcatag

fava_c16686_2175	fava_c16686	2175	C/G	taacaatatgataacaagggcaagaagtaaatcagaacatagcatgaaaagggcagatagagataaataattactaatataatcttttcaagaattcaaa[C/G]ttcaaaccttcctatgtatgcaatttgtagcctcaattctttttttttttaccttatcataaatgtgataactaaataatctttgtactgtcaaattgta

fava_c16686_279	fava_c16686	279	G/A	aatatgttttgttagtagcttaattttgtctggcaacagttatttcaattatcattgtatgtgaccaggaaaatcagaagcttgatgttggtgaatgaga[G/A]tattctgcatcagttgaaattcacttcttcaccaacttaattcagctttaatgttagaagcaactagagagtagagacaggcaggYacctcgaagttttg

fava_c16686_365	fava_c16686	365	T/C	gttggtgaatgagaRtattctgcatcagttgaaattcacttcttcaccaacttaattcagctttaatgttagaagcaactagagagtagagacaggcagg[T/C]acctcgaagttttggtatagacaacgataaacgaaaatgaagttcatgaaacttgggacaaagcYRgatactttctacacggaacaagctacgaggacat

fava_c16686_430	fava_c16686	430	T/C	atgttagaagcaactagagagtagagacaggcaggYacctcgaagttttggtatagacaacgataaacgaaaatgaagttcatgaaacttgggacaaagc[T/C]Rgatactttctacacggaacaagctacgaggacattgacatcagaaatagcctcagatcttgtgatacaaatcaatgatgttacttatcttctacaccgg

fava_c16686_431	fava_c16686	431	A/G	tgttagaagcaactagagagtagagacaggcaggYacctcgaagttttggtatagacaacgataaacgaaaatgaagttcatgaaacttgggacaaagcY[A/G]gatactttctacacggaacaagctacgaggacattgacatcagaaatagcctcagatcttgtgatacaaatcaatgatgttacttatcttctacaccggt

fava_c16686_573	fava_c16686	573	T/C	agaaatagcctcagatcttgtgatacaaatcaatgatgttacttatcttctacaccggtttgcgcttgttcggaaatgtggactcctacagaggctttgc[T/C]acgaatcWagtgagKcggagagtgttagtgtggaggttcatgatatacctggaggggaagatgcttttgaagtttgtgctaagttttgttatggaatttc

fava_c16686_581	fava_c16686	581	A/T	cctcagatcttgtgatacaaatcaatgatgttacttatcttctacaccggtttgcgcttgttcggaaatgtggactcctacagaggctttgcYacgaatc[A/T]agtgagKcggagagtgttagtgtggaggttcatgatatacctggaggggaagatgcttttgaagtttgtgctaagttttgttatggaatttcgatcaaca

fava_c16686_588	fava_c16686	588	T/G	tcttgtgatacaaatcaatgatgttacttatcttctacaccggtttgcgcttgttcggaaatgtggactcctacagaggctttgcYacgaatcWagtgag[T/G]cggagagtgttagtgtggaggttcatgatatacctggaggggaagatgcttttgaagtttgtgctaagttttgttatggaatttcgatcaacattagtgc

fava_c16686_869	fava_c16686	869	G/T	aactagagtctttcttcaattcatgcattcttgaaagctggaaggatcccattgcgacccttgagagtactgctacgttaccagagtggtctgagaatct[G/T]ggtattgtMagaaagtgcatcgattccatcattgagaaaattctgacaccctcacaacaggtgaaatggtccttcacctacaccagacccggttacacga

fava_c16686_878	fava_c16686	878	C/A	ctttcttcaattcatgcattcttgaaagctggaaggatcccattgcgacccttgagagtactgctacgttaccagagtggtctgagaatctKggtattgt[C/A]agaaagtgcatcgattccatcattgagaaaattctgacaccctcacaacaggtgaaatggtccttcacctacaccagacccggttacacgaaaaaacagc

fava_c16688_1142	fava_c16688	1142	C/T	ttcgcactggaggttttgatcatactggaaaacattattcatctccagaggaaatgaagaaggtcgctcttgaaaggtacaactcaatgagaggcaatgc[C/T]cctgagagattggtMtctggatctgatgatttcacaatgttcctttgggaacccttcatcaacaagcaccccaaaactcgcatgacaggtcatcagcagc

fava_c16688_1157	fava_c16688	1157	C/A	ttgatcatactggaaaacattattcatctccagaggaaatgaagaaggtcgctcttgaaaggtacaactcaatgagaggcaatgcYcctgagagattggt[C/A]tctggatctgatgatttcacaatgttcctttgggaacccttcatcaacaagcaccccaaaactcgcatgacaggtcatcagcagcttgtgaaccatgtct

fava_c16688_1733	fava_c16688	1733	G/C	tgcatggaaatcaatgactggctattgtgtggatgcaagatcgttttcaactataggatatgaatgtctattccacatactatattgccattgagattag[G/C]cactggaagaacaagttgctcaaagtgagtctMaggtaaRaaagctgcctgaattgcttgggggtttattatacgcttgcgagaatatacttcgcgattg

fava_c16688_1766	fava_c16688	1766	A/C	tgcaagatcgttttcaactataggatatgaatgtctattccacatactatattgccattgagattagScactggaagaacaagttgctcaaagtgagtct[A/C]aggtaaRaaagctgcctgaattgcttgggggtttattatacgcttgcgagaatatacttcgcgattgtttttggtattttgtcctattgtttatgaatga

fava_c16688_1773	fava_c16688	1773	G/A	tcgttttcaactataggatatgaatgtctattccacatactatattgccattgagattagScactggaagaacaagttgctcaaagtgagtctMaggtaa[G/A]aaagctgcctgaattgcttgggggtttattatacgcttgcgagaatatacttcgcgattgtttttggtattttgtcctattgtttatgaatgacMactat

fava_c16688_1868	fava_c16688	1868	C/A	ggtaaRaaagctgcctgaattgcttgggggtttattatacgcttgcgagaatatacttcgcgattgtttttggtattttgtcctattgtttatgaatgac[C/A]actatggcgattttgtttttttgttagtaattgtaaactgagtatgcaagggctagccctaagatggctttttgtttgcgccttgacagttgacactaca

fava_c16688_208	fava_c16688	208	C/T	agagaagaatcagaggcggtgaaaaattgaaaaatggaaggtgaaacagaggcgataatggagcagaggactgtagtcaacaacgtgatgtgcctcttga[C/T]gcaccaagatggagctcctcttggtgctcctatgtaccttcctcagaacataggtccacagcagctccaactcattgtcaatcagcttctacaaaatgag

fava_c16688_464	fava_c16688	464	C/T	aaacatacttgcagaaacacaaagtgtcggtggagaaagcattgcctatagtttgtcaacctcaagctgttttccggattcgtcctgtgagtcgatgttc[C/T]gcaacaatatctggtcatggcgaagctgtattgagtgttgcctttagtcctgatgggcggcagctWgcaagtggttctggtgataccactgttcgatttt

fava_c16688_530	fava_c16688	530	A/T	ctgttttccggattcgtcctgtgagtcgatgttcYgcaacaatatctggtcatggcgaagctgtattgagtgttgcctttagtcctgatgggcggcagct[A/T]gcaagtggttctggtgataccactgttcgattttgggaccttggcactcagacaccaatgtacacatgcacaggacacaagaactgggtcctttgtattg

fava_c16688_92	fava_c16688	92	A/G	ataaaaccctacttcagtctccctgcgtttactttccattgccccgtacacacataacacaacaatcaatagaactgagcaaagaggaacg[A/G]aaattagcagagaatagagaagaatcagaggcggtgaaaaattgaaaaatggaaggtgaaacagaggcgataatggagcagaggactgtagtcaacaacg

fava_c16689_1285	fava_c16689	1285	G/A	cgcattgttctcgttgcttaattccttggggagcaaatggtggaatttatatgatggaagttgaccgagttctaagacctggtggttattgggtcctttc[G/A]ggacctccaatccattggaaggttcattacaaagcctggcaaagacctaaggaagatcttgaggaagaacaaagaaagattgaagaggttgctaagctcc

fava_c16689_1459	fava_c16689	1459	A/G	gaaagattgaagaggttgctaagctcctttgctgggagaagaaaaccgaggaaaatgaaattgctgtctggcagaaaatggttgactctgaaacatgtcg[A/G]agcagacaagaagattctggtgtaaaattctgcgaatcaacagatgYggatgatgcttggaataagaaaatggaggcatgtgttactcctaatcctaaag

fava_c16689_1506	fava_c16689	1506	C/T	gaggaaaatgaaattgctgtctggcagaaaatggttgactctgaaacatgtcgRagcagacaagaagattctggtgtaaaattctgcgaatcaacagatg[C/T]ggatgatgcttggaataagaaaatggaggcatgtgttactcctaatcctaaagtccacggaRatcttaagccttttccacagagactttatgccgtccct

fava_c16689_1568	fava_c16689	1568	A/G	agaagattctggtgtaaaattctgcgaatcaacagatgYggatgatgcttggaataagaaaatggaggcatgtgttactcctaatcctaaagtccacgga[A/G]atcttaagccttttccacagagactttatgccgtccctcctaaaattgccagtggttatgttcctggaatttctKYRgagacgtatcaagatgatatcaa

fava_c16689_1643	fava_c16689	1643	T/G	tactcctaatcctaaagtccacggaRatcttaagccttttccacagagactttatgccgtccctcctaaaattgccagtggttatgttcctggaatttct[T/G]YRgagacgtatcaagatgatatcaaaaagtggaaaaaacatgtaactKcttacaagaaaataaataaactcttggactctggaaggtatcgcaatatcat

fava_c16689_1644	fava_c16689	1644	C/T	actcctaatcctaaagtccacggaRatcttaagccttttccacagagactttatgccgtccctcctaaaattgccagtggttatgttcctggaatttctK[C/T]RgagacgtatcaagatgatatcaaaaagtggaaaaaacatgtaactKcttacaagaaaataaataaactcttggactctggaaggtatcgcaatatcatg

fava_c16689_1645	fava_c16689	1645	G/A	ctcctaatcctaaagtccacggaRatcttaagccttttccacagagactttatgccgtccctcctaaaattgccagtggttatgttcctggaatttctKY[G/A]gagacgtatcaagatgatatcaaaaagtggaaaaaacatgtaactKcttacaagaaaataaataaactcttggactctggaaggtatcgcaatatcatgg

fava_c16689_1691	fava_c16689	1691	G/T	actttatgccgtccctcctaaaattgccagtggttatgttcctggaatttctKYRgagacgtatcaagatgatatcaaaaagtggaaaaaacatgtaact[G/T]cttacaagaaaataaataaactcttggactctggaaggtatcgcaatatcatggatatgaatgctggtttgggtagtttcgctgctgcaattcaatctYc

fava_c16689_1790	fava_c16689	1790	C/T	tKcttacaagaaaataaataaactcttggactctggaaggtatcgcaatatcatggatatgaatgctggtttgggtagtttcgctgctgcaattcaatct[C/T]ctaagttatgggtcatgaacgttgttcctacaYtagctgacaaacaYactctgggtgttatatatcagcgaggacttataggcatttatcatgattggtg

fava_c16689_1823	fava_c16689	1823	T/C	tggaaggtatcgcaatatcatggatatgaatgctggtttgggtagtttcgctgctgcaattcaatctYctaagttatgggtcatgaacgttgttcctaca[T/C]tagctgacaaacaYactctgggtgttatatatcagcgaggacttataggcatttatcatgattggtgtgaagctttctctacatatccgaggacgtacga

fava_c16689_1837	fava_c16689	1837	C/T	atatcatggatatgaatgctggtttgggtagtttcgctgctgcaattcaatctYctaagttatgggtcatgaacgttgttcctacaYtagctgacaaaca[C/T]actctgggtgttatatatcagcgaggacttataggcatttatcatgattggtgtgaagctttctctacatatccgaggacgtacgacctcattcatagca

fava_c16689_1949	fava_c16689	1949	A/G	tatatatcagcgaggacttataggcatttatcatgattggtgtgaagctttctctacatatccgaggacgtacgacctcattcatagcaatggtcttttt[A/G]gtctgtacaaagacaaatgtaacatagaagacattcttctggagatggaccggattttacgaccagaaggtgctgtaatattccgcgatgaagtcgatat

fava_c16689_2388	fava_c16689	2388	G/A	gagcgcgttggcttgattatcaccacacctgttgtataaggacggctaacctacgtagcaccgacattttagattgaaaacatggttggtgttcaacgcc[G/A]caaatgcctttatattcaatggtgtttatgtgtcRgtatcagatcttatgtcagtgtgtctgtattagtgctttataggggctgataagttatgctcaat

fava_c16689_606	fava_c16689	606	A/T	gagattactaagaaaggcgttgagtgtgatatagttccgaaattaagttttgattctcaccatggtggagaagttagcaaaattgatggggttgattcaa[A/T]gcctaaggtgtttaaaccttgtaaagctcggtataccgattacacaccatgccatgatcaaaagcgagccatgactttttctagagataacatgatttat

fava_c16689_877	fava_c16689	877	C/T	caccatttccatggccgaagagccgcgattatgttccgtatgcgaacgcaccgtacaagagtcttacagttgagaaggctattcagaactggatccaata[C/T]gagggaaatgtgtttaggtttcctggtggtggaacccaattccctcaaggggctgacaaatatattgatcaaatagcttctgtgataccgatagagaatg

fava_c16690_1095	fava_c16690	1095	G/C	tgtttttatcctttcccttcttagtgtctccactatcctccttcagaaactcggatgctaacttttgttgatcacctaacctctggctgctaccttctgg[G/C]ccaagccttgaagttttctttgatgtttttaaatcctcagggtcatcacctttgctcttcaaacttttcttattccaaccaaaccagctcttcttctctt

fava_c16690_1290	fava_c16690	1290	G/A	tctctttaaccacgccattggcttcagaattctcaagggaagccaatgagccattttcttgaccatcaaagaccccgtgctcttcatcttcacaagctgc[G/A]tcggagttccccattcttagggcagaatctaattgcattctttcctcagcagtcagtacatcatcatattcatcattgttttccccatttaacatacttt

fava_c16690_130	fava_c16690	130	C/T	aaatcttaatgaaaggaatgtaactaaagagaacaacactcaagaaaatctaaaatcgatgatcatcgacataggtgtagtagatacagatatcggtatc[C/T]aacRaaaattagaatgaaggaaagcaattgatgagcaagactatagctcttcaattttacttcaYatgacaaatacaacaatttctagaagatgatagct

fava_c16690_134	fava_c16690	134	A/G	cttaatgaaaggaatgtaactaaagagaacaacactcaagaaaatctaaaatcgatgatcatcgacataggtgtagtagatacagatatcggtatcYaac[A/G]aaaattagaatgaaggaaagcaattgatgagcaagactatagctcttcaattttacttcaYatgacaaatacaacaatttctagaagatgatagcttcct

fava_c16690_1866	fava_c16690	1866	G/A	tttggtctgatcgctggatgcgtaaaccatcaaaaccagcaagggtcatatccgcacgtagattagaaccacgcttccatattcgataggtatcagaagg[G/A]gctatgcgtccaataaacggaatcataaagctttcaaaatgaaaagttatctccatataaaaatcacgaatacgactcgcSgaagcaacaatccgcggaa

fava_c16690_1947	fava_c16690	1947	G/C	ttcgataggtatcagaaggRgctatgcgtccaataaacggaatcataaagctttcaaaatgaaaagttatctccatataaaaatcacgaatacgactcgc[G/C]gaagcaacaatccgcggaagcctacgacaccatttagcccaagccaaaggctgataatgccgcgcaataatcatcgcaatagcttcctctctagtacaca

fava_c16690_195	fava_c16690	195	C/T	tcgacataggtgtagtagatacagatatcggtatcYaacRaaaattagaatgaaggaaagcaattgatgagcaagactatagctcttcaattttacttca[C/T]atgacaaatacaacaatttctagaagatgatagcttccttggcaagtggcaacaacctttttaatagctaataacaattggttgcacaagaagtttctag

fava_c16690_2564	fava_c16690	2564	G/C	taactaatctcttcccataaccttcccctcccctaaatttcacacacccgaatcaattaaccctcgaaaaacgaataattgttcaatttctagatcttca[G/C]aaccacaattacaacttacaatgataactgtttttgctcaaaaatcacttcagtataacagaatttctccgaaatttcgaacattaactcaaaaatccaa

fava_c16690_342	fava_c16690	342	T/A	tggcaacaacctttttaatagctaataacaattggttgcacaagaagtttctagtaaattcttagagaacccctccaatgttgaaatgtaaagtcagctg[T/A]gggactgggcataataaagactctttctgtcgaYgcataccgtaatattactaatattcttctatgtcctcggtgctgcgatgcaccccatctccaggtt

fava_c16690_376	fava_c16690	376	C/T	gttgcacaagaagtttctagtaaattcttagagaacccctccaatgttgaaatgtaaagtcagctgWgggactgggcataataaagactctttctgtcga[C/T]gcataccgtaatattactaatattcttctatgtcctcggtgctgcgatgcaccccatctccaggtttagctactggcggcttcttatgtttcttactttt

fava_c16690_534	fava_c16690	534	A/T	ctccaggtttagctactggcggcttcttatgtttcttacttttggctttctttgccttcattcggcgtttcttttcattggcgtctacccatttgtagtc[A/T]gcgggtatgctgaaaggatcagcttcatccttatatctgtgactgtccgagtcactggactgcccccctcgacctcctttcatccatgaaatccacgatg

fava_c16691_1412	fava_c16691	1412	A/G	agcgagcgtcaaaatcagcagaatgagaaccattgagtttgccttcaagttcgcattgaatcttgagatgtttagaagcagagagaggacggagagagtg[A/G]aaattgttaggaagaaagagaaaagaagaaggtggtttgagaatcaaagtgtagggttttggagcgaagaccaaatccattacaataagaataagaagaa

fava_c16691_581	fava_c16691	581	T/C	ctgcacaatctaaaatgcaacccagcttattaactccctctccaaatgagatatcaaattcagctactttgtaaggccttgacaccttgctcttttgcac[T/C]ctaacacgaactttaaggccaacttcttcatctcctttggcagactttatcttccctatagaacgaatttcaagccgaaccgatgcaaaaaacttcaatg

fava_c16691_695	fava_c16691	695	T/C	taaggccaacttcttcatctcctttggcagactttatcttccctatagaacgaatttcaagccgaaccgatgcaaaaaacttcaatgcaattccaccact[T/C]gtcacttcgggatttccatagtagacaccaatcttgtatcttatttggttcaaaaatataagcgtacaaccagctttagatgcatttccagacatcttac

fava_c16691_90	fava_c16691	90	C/T	cgtctttcatggaaaattgaatatcattcacactaattagaatttttgtttctcatactttaatatatcatcattttccacaacaatta[C/T]gaatggatcgggatgaaaaatataaacaacctaatcatcaaagacacctccagttccatttctgttgaagaaagaatatttcaaaatttccaattctata

fava_c16697_417	fava_c16697	417	A/G	gtggctccacaaggacataaagtggctttccaagaagtttgcaccgtcccttaacctcaggcacaccaataacacaagcacattccactacttcagcacc[A/G]gcacgctctaaaagtcttattccYgctgacaaagttccaccagttgcgaccaaatcatcgataactatagcgcgttcaccggtttggacagccccaacat

fava_c16697_441	fava_c16697	441	T/C	gctttccaagaagtttgcaccgtcccttaacctcaggcacaccaataacacaagcacattccactacttcagcaccRgcacgctctaaaagtcttattcc[T/C]gctgacaaagttccaccagttgcgaccaaatcatcgataactatagcgcgttcaccggtttggacagccccaacatgcatttccagacaatcagttccat

fava_c16705_1284	fava_c16705	1284	G/A	cggcaggaaactcaccaccaggaaccctattagcaaacccattagcattccgcaaaagctgctccctttgcctggcgaattcatccctaccatcctcccc[G/A]tacttcctcttggccaaggaagcttccaagggattctggtaatcaggacgaggagggaaccgcatgtcgaagcggtctagaggtcgaggtgggggtggga

fava_c16705_337	fava_c16705	337	T/C	cataaataattccatgtatgtatagtccagaaactacatggagggtttgtattctctcctgctctttataactaccttcttccttgtatcgaaatcaagt[T/C]tgtcaatatcaaatgcggttcctagatagccatacaagaccttccttttctctccttttttctcatcaacctgcacaaggtttggattgttttcatcctc

fava_c16706_1006	fava_c16706	1006	G/A	ggtttttgtctttaacacgtacccaatcaccagctgccaagccatcagtgacccgttcgagagttgacacatgaattcttatagggtcgtgaataccgtg[G/A]accctcacaagaacgaatccgtgatctgcagtacgttctagaccaacaacagttccttctgggacatccatgttttgagctttgcatgagttgggagact

fava_c16706_1212	fava_c16706	1212	G/A	gatcggaccatgtcgcccacctgcaggtgatcctttgagaggaaccattcggtgtactcagtgctgcctgattttgcaattactttcatatttccttgat[G/A]tctccatcctccgtcattKcccagtttactcagcgagcttttaaagacgYgcagaatatctaccatcaaagggcggtttcttaaatcatactcaaaacag

fava_c16706_1231	fava_c16706	1231	T/G	cctgcaggtgatcctttgagaggaaccattcggtgtactcagtgctgcctgattttgcaattactttcatatttccttgatRtctccatcctccgtcatt[T/G]cccagtttactcagcgagcttttaaagacgYgcagaatatctaccatcaaagggcggtttcttaaatcatactcaaaacagccactaaggatattctcga

fava_c16706_1262	fava_c16706	1262	C/T	ggtgtactcagtgctgcctgattttgcaattactttcatatttccttgatRtctccatcctccgtcattKcccagtttactcagcgagcttttaaagacg[C/T]gcagaatatctaccatcaaagggcggtttcttaaatcatactcaaaacagccactaaggatattctcgatgggagaaggaaggccacttgggataagcgg

fava_c16706_1566	fava_c16706	1566	T/A	catgaatccgtttcaattgatataggacctccaacttctggtttccattgttctggagccatgtaattaggagttccaagcctctgagcgatatctgagc[T/A]aacgaatgaggaaccaagcaaaagatttggaataccaacatctcctaaaatcgcttgaccattgtcattgagaagaacattgcaaggtttaaggttcaga

fava_c16706_1674	fava_c16706	1674	C/T	gaggaaccaagcaaaagatttggaataccaacatctcctaaaatcgcttgaccattgtcattgagaagaacattgcaaggtttaaggttcagaacaagga[C/T]ccccttagagtgaagctccagaataccttgagccagattgatcccatacctcaaaacatcatgtaatgaaatccatccttctcttaatctagccatcttg

fava_c16706_1975	fava_c16706	1975	C/G	taaagtacaaatcactaaacttctccagtacaatcttcacatgatcctctctaaccggatacaacatcttagcagcaacttcatgatgttcatcataatc[C/G]tcagttgactgatgaagagttgcaagccaaacatcaccaaagggtcctcgaccaatccgatgctgaagtttcaaagtttcaggctcaatccacggattcg

fava_c16706_780	fava_c16706	780	A/G	gaaatccgaccggtagctgaggcccctccccttttacgacgccattcgaatcgagggcttaagatattctcttttggcctaacgaactgtcctacacaga[A/G]tgattctgacatttcgaggtcagaagggtttccgttccagagagtttgcagcccgataaatccaacagcagctctaccatcatcacgattgatggtatgt

fava_c16708_1028	fava_c16708	1028	C/T	tttcgtgaaacataaccattgttagctttgattgacttctcaactgatgatgcaatgaatgatttccaaaattcatctgaatccgctgaatcaggtaaat[C/T]gccctcagctccacttgtagtggtgtctttctcaggtaatagtccttYaatatcaatgttatcatctcttatttgcgttgtggtggactggtttttcttt

fava_c16708_1076	fava_c16708	1076	T/C	gatgcaatgaatgatttccaaaattcatctgaatccgctgaatcaggtaaatYgccctcagctccacttgtagtggtgtctttctcaggtaatagtcctt[T/C]aatatcaatgttatcatctcttatttgcgttgtggtggactggtttttctttctacgttcatccactacttttattgatgttgatattccaggaccaaca

fava_c16709_1357	fava_c16709	1357	T/C	ttatactccatctgatgaacaaatattctaatgacacgtctcgagtacacgatgaattgtgatttctgacaagtgacaaagcatttgatcaagtgtctat[T/C]agttggcttccttccttccaacatgcatctgtatatatgatgtatatatgttaatgtatatagttgcaatttttgatcatccattggtaaaagcttctat

fava_c16709_574	fava_c16709	574	A/G	cctttgccatcaggaaaatgaagaagaattggacgatgaggataatgtcgagaataggtcgcagcaggccttgttggaaggtgtgatgagggaacaacac[A/G]gttcagctcccaaaaaattccaccatagatgctgctcaatccaaggaattcacatcggtagccgRcagaaatatgtcataatgattgtggttctgatctt

fava_c16709_639	fava_c16709	639	A/G	aggccttgttggaaggtgtgatgagggaacaacacRgttcagctcccaaaaaattccaccatagatgctgctcaatccaaggaattcacatcggtagccg[A/G]cagaaatatgtcataatgattgtggttctgatctttgctgtgatgatggcatttgctatacttatctgcgttggcgatgtcacgaatccaatagatccgt

fava_c16710_1021	fava_c16710	1021	C/A	taataggaaccaagtttgatgattttgttagacttcccccagatttacaatggacaattgtgacacaggcaagagcatatgcaagggcaatgaaggcaac[C/A]ctatttttctcaagtgcaacacacaatataaacgtcaacaaaattttcaagtttatcatggcaaagctctttaaYttgccttggacgatagaaagaaatt

fava_c16710_1096	fava_c16710	1096	C/T	catatgcaagggcaatgaaggcaacMctatttttctcaagtgcaacacacaatataaacgtcaacaaaattttcaagtttatcatggcaaagctctttaa[C/T]ttgccttggacgatagaaagaaatttgaaagttggggaacccattattgacttctaaattgttctttacgttcatttctttgtatgtatatatgaaaata

fava_c16710_1215	fava_c16710	1215	G/C	agaaatttgaaagttggggaacccattattgacttctaaattgttctttacgttcatttctttgtatgtatatatgaaaataagagaagaaaaaagagaa[G/C]aagaaacaaatttggaaaaagagagagaaagaaaactagtcttccttttcgtattaacttgaatcaatcaacatttagcacctagctaggaaggtagtca

fava_c16710_345	fava_c16710	345	A/G	aaagaacaatacatacatgtcaatgtctaacaagaaagccaaatctcaatggcaaaactgattaaacaaacaacaaaaaagatgacacaacaactctgcc[A/G]gaaaatcgttcaggtcgacgtccgttacggtgttctccaaaggatatcctttgttggccacttttttcgattcatttggaataaactcatggtttgctct

fava_c16710_542	fava_c16710	542	A/C	ctctcttggaagttcatcacctcaatacacaaggttacctctccctggttcatcttcttcttcgccgccgtctccggccaccgttgacgacggtttctct[A/C]aagaacaaccacaaatatcaaatgggtatgagtcggattctaatttggttaacctcaagattagtttattgggtgattgtcatatYggaaaaaccacttt

fava_c16710_628	fava_c16710	628	C/T	acgacggtttctctMaagaacaaccacaaatatcaaatgggtatgagtcggattctaatttggttaacctcaagattagtttattgggtgattgtcatat[C/T]ggaaaaaccacttttttgatcaaatatgtaggaaatgaagtagaaaagagaagtttacaaatggaaggattgaatttaatggataaaacWttatctgttc

fava_c16710_718	fava_c16710	718	T/A	attgtcatatYggaaaaaccacttttttgatcaaatatgtaggaaatgaagtagaaaagagaagtttacaaatggaaggattgaatttaatggataaaac[T/A]ttatctgttcaaggagctagaatttcattttgtatatgggatgttgcaggtgataaaaagtccttggatcaaattcctatggcttgtaaagatgccgttg

fava_c16710_859	fava_c16710	859	G/A	atgttgcaggtgataaaaagtccttggatcaaattcctatggcttgtaaagatgccgttgctgttttgattatgtttgatctcactagtcgatgtacatt[G/A]aacagtgttgttggatggtacagtgaagcaagaaaatggaRtcagactgcaatacctattttaataggaaccaagtttgatgattttgttagacttcccc

fava_c16710_900	fava_c16710	900	A/G	gcttgtaaagatgccgttgctgttttgattatgtttgatctcactagtcgatgtacattRaacagtgttgttggatggtacagtgaagcaagaaaatgga[A/G]tcagactgcaatacctattttaataggaaccaagtttgatgattttgttagacttcccccagatttacaatggacaattgtgacacaggcaagagcatat

fava_c16712_1321	fava_c16712	1321	T/C	aggggcgttgcccactgactccagaagagattggattgctcctagctgctttggggttcaacaacagaacacggctctatcttgcttcccacaaggttta[T/C]ggtggaaaagctaggttggaaactttaagcaagttatttccttttatggaagataagaagagtctagcctctgaagagaaacttgccaaggttaaaggaa

fava_c16712_1845	fava_c16712	1845	T/G	gcaaattgatctgatgcttaaaactacaattatggcttctatagttttgcaccgtgatgatagatagccaacgagtgggtctcaatcgcgaaataaacat[T/G]gtctaagaagttggcatggaatgtaactaaaacatggctcaattactaagcacaatatcaatcatatttgtgtcaaattgataactcataggtttgctgc

fava_c16712_277	fava_c16712	277	T/A	agcttcatcccttgagccaaatcaaccactctcaaaaaaccgcttttctcggcgttttcctctccgtcttgccatttctctttcccaatctcttgcaacc[T/A]ctcgctcgtgcttccccttccatgttctccgaatggattgctcctaaacctaggcacgtgacattgctacagggcgctctgcaacacgaaacttctcttc

fava_c16712_571	fava_c16712	571	G/A	agggttatattcaagttttccttgatggaggattgaaccagcagaagatggggatatgtgatgcagtagccgttgctaaaattttgaatgcaactttggt[G/A]gtcccacattttgaagtgaatcctgtttggcaagatacaagttcgtttgcggatatatttgatgtggatcacttYatYgatgttctccgtgatgaagtat

fava_c16712_646	fava_c16712	646	C/T	ctaaaattttgaatgcaactttggtRgtcccacattttgaagtgaatcctgtttggcaagatacaagttcgtttgcggatatatttgatgtggatcactt[C/T]atYgatgttctccgtgatgaagtatctatagtgaaagagcttcctagtgagcactcttggagcacaagagagtactatgccacgggcatcagagatacta

fava_c16712_649	fava_c16712	649	T/C	aaattttgaatgcaactttggtRgtcccacattttgaagtgaatcctgtttggcaagatacaagttcgtttgcggatatatttgatgtggatcacttYat[T/C]gatgttctccgtgatgaagtatctatagtgaaagagcttcctagtgagcactcttggagcacaagagagtactatgccacgggcatcagagatactagaa

fava_c16715_307	fava_c16715	307	C/T	cacatgatgagttcttcaatctttcttctatactgtcttctcacattctctttgtattccatctcccaacacacaagctacagttattctctcgacttta[C/T]gccagcgaggaagttaagcatgtcatcctctggccatgaccacaaatatcatgaaactcaggtagcagatgagagaatggaaagtgcccctgccaatgca

fava_c16718_1480	fava_c16718	1480	T/C	gtggtttgagtgcttgtttcatcactctccgagcttggagattagatggacgctgttctgaactttgtgttaaggctcattcgttgaaaggtcaacagtg[T/C]gtacattgcagattaattgtaggagatggttatgtcacaattacgaaaggggaaagtatccgaagattctttgagcatgctgaagaggctgaggaggaag

fava_c16720_1048	fava_c16720	1048	C/G	cagaScattatggtataacacctatctttgaacattatacatgcatagttgaactctatgttcgtgccaatgagttgaatgagatttgcacattagggaa[C/G]acaRtgtcattgtcaaWgaaagcaagtcataatgttgcagagttgcttcaagagaRcaaactcactagtattgatgacatagagaaactcatacacaagc

fava_c16720_1052	fava_c16720	1052	A/G	ScattatggtataacacctatctttgaacattatacatgcatagttgaactctatgttcgtgccaatgagttgaatgagatttgcacattagggaaSaca[A/G]tgtcattgtcaaWgaaagcaagtcataatgttgcagagttgcttcaagagaRcaaactcactagtattgatgacatagagaaactcatacacaagcatta

fava_c16720_1065	fava_c16720	1065	T/A	acacctatctttgaacattatacatgcatagttgaactctatgttcgtgccaatgagttgaatgagatttgcacattagggaaSacaRtgtcattgtcaa[T/A]gaaagcaagtcataatgttgcagagttgcttcaagagaRcaaactcactagtattgatgacatagagaaactcatacacaagcattacagggattcggRt

fava_c16720_1104	fava_c16720	1104	G/A	tatgttcgtgccaatgagttgaatgagatttgcacattagggaaSacaRtgtcattgtcaaWgaaagcaagtcataatgttgcagagttgcttcaagaga[G/A]caaactcactagtattgatgacatagagaaactcatacacaagcattacagggattcggRtttatcagaattagttttagatcattctgcaactcccacc

fava_c16720_1164	fava_c16720	1164	G/A	aWgaaagcaagtcataatgttgcagagttgcttcaagagaRcaaactcactagtattgatgacatagagaaactcatacacaagcattacagggattcgg[G/A]tttatcagaattagttttagatcattctgcaactcccacccagccatatttcaaccgtccatatttgacttcactagtttgaaagccgtgggggatccag

fava_c16720_1485	fava_c16720	1485	T/G	ttgcaatgcatacaatatagccaactggatacctcaaatcacgtgtataacatactaattgaatcataatcatctccaatgatttttagcattatagaac[T/G]atacaagttKttcagaacagcataatgacgttaagtcgatcttgaagtcatgtatttttattgtgaatgtattttgagctccaaattaataaattttaca

fava_c16720_1495	fava_c16720	1495	G/T	tacaatatagccaactggatacctcaaatcacgtgtataacatactaattgaatcataatcatctccaatgatttttagcattatagaacKatacaagtt[G/T]ttcagaacagcataatgacgttaagtcgatcttgaagtcatgtatttttattgtgaatgtattttgagctccaaattaataaattttacattttttggtc

fava_c16720_355	fava_c16720	355	T/C	tttggagtgatgtttatgtgcagaataatatgatgaatctttactttaagtgtgggaggtatgttgttgatgatgggtggaaggtgtttgagaaaatgcg[T/C]gtgcggaatgtggtttcttggacgactgtgattgctgggcttgttggttgtgggaaattggatactgcgagggaggtttttgagaagatgggttgtaaga

fava_c16720_475	fava_c16720	475	A/G	ggacgactgtgattgctgggcttgttggttgtgggaaattggatactgcgagggaggtttttgagaagatgggttgtaagaatgtggtttcgtggactgc[A/G]atgattgatgggtatgtgaaatatggtaacccRattatggcgtttgatttgtttgaaaggatgctgcttgataatgtgaggcctaatgagtttactttgg

fava_c16720_508	fava_c16720	508	A/G	ggaaattggatactgcgagggaggtttttgagaagatgggttgtaagaatgtggtttcgtggactgcRatgattgatgggtatgtgaaatatggtaaccc[A/G]attatggcgtttgatttgtttgaaaggatgctgcttgataatgtgaggcctaatgagtttactttggtSagcttgattaaagcttgtactgatttgggga

fava_c16720_577	fava_c16720	577	G/C	tgattgatgggtatgtgaaatatggtaacccRattatggcgtttgatttgtttgaaaggatgctgcttgataatgtgaggcctaatgagtttactttggt[G/C]agcttgattaaagcttgtactgatttggggagtctcaaattgggtcggcggatgcatgattttgctatgaagaatggttttgaggttggacctttcttgg

fava_c16720_952	fava_c16720	952	G/C	atgtagttcctgatgctatcacttttgtaggtgttttgagtgcttgtgttcaaatgaatgttctggaagaagggtataagtatttcagtctcatgacaga[G/C]cattatggtataacacctatctttgaacattatacatgcatagttgaactctatgttcgtgccaatgagttgaatgagatttgcacattagggaaSacaR

fava_c16722_1424	fava_c16722	1424	A/G	ttcttagtgaagttcagagatctttgctagatggaggaatgcatgttgaggatctaccaccaatacctcaatctggaacttctcatttgggccagcacac[A/G]cttccatYagattctcctgaaagatttaaggaagttaatcatgtcattgggggtcctgctggcttgtggcatttcatataccgcagtatatacctggatc

fava_c16722_1432	fava_c16722	1432	C/T	gaagttcagagatctttgctagatggaggaatgcatgttgaggatctaccaccaatacctcaatctggaacttctcatttgggccagcacacRcttccat[C/T]agattctcctgaaagatttaaggaagttaatcatgtcattgggggtcctgctggcttgtggcatttcatataccgcagtatatacctggatcaatacgtg

fava_c16724_1220	fava_c16724	1220	T/A	atgaaattgtctttagggaagcaaagaaagatccaacagggcagcaggtttataaactcctggtaaacattcataaaggtttcgaacaaatatctgagaa[T/A]attctggcaacgaatagaRttagaagagaagtatctgactatgaaatgaaactggcagccacaccttccacttcaagaagcttagaYgtgagcaagttac

fava_c16724_1239	fava_c16724	1239	A/G	agcaaagaaagatccaacagggcagcaggtttataaactcctggtaaacattcataaaggtttcgaacaaatatctgagaaWattctggcaacgaataga[A/G]ttagaagagaagtatctgactatgaaatgaaactggcagccacaccttccacttcaagaagcttagaYgtgagcaagttacaatctgatcttgatggcgt

fava_c16724_1307	fava_c16724	1307	T/C	aaatatctgagaaWattctggcaacgaatagaRttagaagagaagtatctgactatgaaatgaaactggcagccacaccttccacttcaagaagcttaga[T/C]gtgagcaagttacaatctgatcttgatggcgtgattattagaaacaatgaatacccgaaatagcaggtacagttgagtgaagctgtttaaagaactaatt

fava_c16724_1713	fava_c16724	1713	G/A	gaaacacattaagaaatttcatttttcctattggtttgaatggttgagtttcatgtttgttacttggatttgttcattattgtttttcactaggcctagt[G/A]tatccaagaggcctgcaattaattattgtaatgtcagtttacagaacattgaacttgcaa

fava_c16724_236	fava_c16724	236	A/C	cgtttctgaggacctatattgaagttgataagattcgtgtttgagaggatttcactgtcatctaagagtggaaaggttgttgatggtaagaatcctagtg[A/C]tggaatcWaagatttcataacttattaagctcttttgaggattcaaccgaaaaggaaaaYgaagaagaggtagctgatgattattctaaagttgaaagca

fava_c16724_244	fava_c16724	244	A/T	aggacctatattgaagttgataagattcgtgtttgagaggatttcactgtcatctaagagtggaaaggttgttgatggtaagaatcctagtgMtggaatc[A/T]aagatttcataacttattaagctcttttgaggattcaaccgaaaaggaaaaYgaagaagaggtagctgatgattattctaaagttgaaagcaaactaaat

fava_c16724_296	fava_c16724	296	C/T	tctaagagtggaaaggttgttgatggtaagaatcctagtgMtggaatcWaagatttcataacttattaagctcttttgaggattcaaccgaaaaggaaaa[C/T]gaagaagaggtagctgatgattattctaaagttgaaagcaaactaaatgatttcaaaatggagactcaaactcccggaacaggaagtaccagagacaaaa

fava_c16724_434	fava_c16724	434	T/A	gcaaactaaatgatttcaaaatggagactcaaactcccggaacaggaagtaccagagacaaaacctctatcactgaggctagcatagatggatccaactc[T/A]gtgaatgatgaattaaactgtttattaaatgaaaagaaattagctgtagaagaggttgatgcaggaatggggtcatttggaaatgaagaaaattctgctc

fava_c16727_1066	fava_c16727	1066	A/G	ataatagggatatattggataatttctcaaatgagtatctttatcttgctgggattacttttattatgaaggtgaagaagggtcattttgctgagcattc[A/G]cctatgttggatgatattagtggggtgcctaattggaagaaggttaatagtggtttgcttaagatgtataaagctgaagtcttggagaaggttcctatta

fava_c16727_1247	fava_c16727	1247	A/T	cttggagaaggttcctattatgcagcattttctttttggatcccttattaagtgggaataatggtttgagttacattgctgaggggaagacatggaccgg[A/T]cactattatatttgtatccgttatataatcgctttgttggattacttgttctctgtcaacttgcttaatcttctgttcatgagagtaaaagataatggtc

fava_c16728_1003	fava_c16728	1003	T/G	cttccaacccagtttcagatacggatgttaagaaaaatgatgtttcctatgctctttcggctaaaaggtcacttcccttctctgatgcagacaacaatga[T/G]aataagcttggactaatgcagtctccaactgaaactgatggcgacgaggttgctgcaaagagattgttgatttttcctgatgagcaagttcagcttgaaa

fava_c16728_1242	fava_c16728	1242	C/T	tcttccagcaaaccaagcccagacagtgttgctggaagacttaggtctaggacaaaaaagcaacgatgaggataacttcattaatctagcccagggtatg[C/T]tgaagaggacattcaacattgcattttgaaattatcaatcaaacttgatRatttggtaagaaaacctgttttggcatRgtagttgtattagtatgtgaag

fava_c16728_1292	fava_c16728	1292	A/G	gacaaaaaagcaacgatgaggataacttcattaatctagcccagggtatgYtgaagaggacattcaacattgcattttgaaattatcaatcaaacttgat[A/G]atttggtaagaaaacctgttttggcatRgtagttgtattagtatgtgaagMacgttaagtataacagataattttaattaaagggtttagtattatgtgc

fava_c16728_1320	fava_c16728	1320	A/G	cattaatctagcccagggtatgYtgaagaggacattcaacattgcattttgaaattatcaatcaaacttgatRatttggtaagaaaacctgttttggcat[A/G]gtagttgtattagtatgtgaagMacgttaagtataacagataattttaattaaagggtttagtattatgtgcattctcttggaaaaaattagtacttttg

fava_c16728_1343	fava_c16728	1343	C/A	tgaagaggacattcaacattgcattttgaaattatcaatcaaacttgatRatttggtaagaaaacctgttttggcatRgtagttgtattagtatgtgaag[C/A]acgttaagtataacagataattttaattaaagggtttagtattatgtgcattctcttggaaaaaattagtacttttgaatatttgactttttgcttgaaY

fava_c16728_328	fava_c16728	328	T/G	accgcgagagaatgggaaaattggggattttcgatatctcactttccctcaagctcaaatcaaccccaccttcgcgaagatctccatctaaccctaaatc[T/G]cctatttctcgtaacctatctKccccttctcgccgttcttccaggttgcagaatgttgctcctattagttactcagaagcgccactgaagcaagaagtgg

fava_c16728_350	fava_c16728	350	T/G	ggggattttcgatatctcactttccctcaagctcaaatcaaccccaccttcgcgaagatctccatctaaccctaaatcKcctatttctcgtaacctatct[T/G]ccccttctcgccgttcttccaggttgcagaatgttgctcctattagttactcagaagcgccactgaagcaagaagtgggtgagaaaaagactagggttgt

fava_c16728_640	fava_c16728	640	C/T	atggtgttggaaaagatgggaaaaggatctatgattctgttcaaggcaaaacatgtcatcagtgcaggcaaaaaactcttggtcttcggacacgctgttg[C/T]gaatgcaacttggtccaggggcagttttgtggagattgcttgtacatgagatatggggagcatgtactagaagccctggccaatccaacttggaagtgcc

fava_c16728_844	fava_c16728	844	G/A	cttgtcgtggaatttgcaactgtagcttatgccgtaatgctaaaggatgggcacctactggatatctatatagaaaggtagtgcagttgggttacaaatc[G/A]gttgcacactacctcatccaaacccgtcgcgcagaagatgttgagaaaaatgatgatacttccaacccagtttcagatacggatgttaagaaaaatgatg

fava_c16729_1238	fava_c16729	1238	C/G	ctccaaaaagcatagcaaaacagtgaacaagttgattgaaaacgcaattatggaggctgagttaaatggagcaaaggtggtaagtctaggacttcttaat[C/G]agaaacaacttaatgcacactatggMctttataataaaaggttccaagaactaaagataaaagttgtagatgggagtagcctagttgctgctattgtgct

fava_c16729_1264	fava_c16729	1264	A/C	acaagttgattgaaaacgcaattatggaggctgagttaaatggagcaaaggtggtaagtctaggacttcttaatSagaaacaacttaatgcacactatgg[A/C]ctttataataaaaggttccaagaactaaagataaaagttgtagatgggagtagcctagttgctgctattgtgctcaacaacattccaaaaggaacaaata

fava_c16729_1518	fava_c16729	1518	C/T	ctatgcagtaaaaatatagaggtagatgtattatataaggatgagcttgaagagcttgaacaaaaggtcaccatgtcaaaaggaaacttggctctttcat[C/T]aattaatacttcaaagatatggttagtaggagatgaatgggaggaagatgagcaaatgaaagcaccagaaggatcattgttcataccattttctcatttc

fava_c16729_1791	fava_c16729	1791	G/T	aactcacactcgtgtgagaattggcttccaagaagagttatgagtgcatggcgtattgctggaataattcatgctttagaaggatggaatgttgatgagt[G/T]tggtgacactatacttgacactaagaaagtatgggaagccactactcgccatggttttcaacctctaaagatatattctcacaaaccatgtgtgactaat

fava_c16729_547	fava_c16729	547	T/C	attggcttcatagagcacttcatcatcattatttatactctcgttatcattctcatcatcactcttccattgttactgaacctatcacttctgtgacaca[T/C]ccatttgctgaacacttggcatattttaccctctttgctattccactgtttacaccattgttcatttataaatcttctgtggttgcaatatatggttata

fava_c16729_745	fava_c16729	745	G/A	atatcttttacattgatttcatgaacaatatgggtcattgcaactttgagttctttcctaagaaactcctggccttctttccacttttgaagtatctctc[G/A]tacacaccatcgtttcattcactgcatcacacgaaattcaggacaaattactcRctcttcatgccgatttacgactatatttatggcacagtagatgcat

fava_c16729_799	fava_c16729	799	G/A	ttcctaagaaactcctggccttctttccacttttgaagtatctctcRtacacaccatcgtttcattcactgcatcacacgaaattcaggacaaattactc[G/A]ctcttcatgccgatttacgactatatttatggcacagtagatgcatccactgatactatgtatgaaaaatgtttgaagagagcaaaggagtcaccagatg

fava_c16729_976	fava_c16729	976	T/C	agagagcaaaggagtcaccagatgtggtgcacttaacacatttaacatcatttgattccatctatcaatcgcgtctgggattttcttccttggcctcaaa[T/C]cctcaaacatcgaaatggtatctacatttgatgtggccttttacaatgttctctatgcttatgacttggattttcgggcgtgccattgttttagaaagca

fava_c16732_220	fava_c16732	220	T/C	tcatcacagattcagaggtgtactcagttgttgttgtatgttccgcagtaagacgcggttgtttcatccatgagagagaagaaaggctcaaagccttcaa[T/C]ctctccctgcgcctctctcagagacgcataccacaattgcttccacaggtggtatacggagaaattcgtcaagggtcaatatgacaaagaagaatgcgtc

fava_c16732_473	fava_c16732	473	G/A	atatgattcgtatattggaaggtgaaaacgttcttcaagacagtgctgttgcttctcagtaacttggaagattagcatatcatgtgtttgatgtaatggc[G/A]cacagaaaattgttctataatcactgRccaattcattcgaattttccataatttgtaacattattttgtttaaattatagaatcatagaatagctttaaa

fava_c16732_500	fava_c16732	500	A/G	acgttcttcaagacagtgctgttgcttctcagtaacttggaagattagcatatcatgtgtttgatgtaatggcRcacagaaaattgttctataatcactg[A/G]ccaattcattcgaattttccataatttgtaacattattttgtttaaattatagaatcatagaatagctttaaaacaaattaatgtggtcaacagttcaca

fava_c16733_397	fava_c16733	397	A/C	ttgacaaaattgtgtatccggtgtctgacaccaatatacatgattacattcaatcatagttatttctttacgtgtgtgtatgtatgtgttagtgtcaatg[A/C]catgtttggtgtcaatgtaattgcttctaatgtagtgtttgcgctaattctcctaattctctacacttggttgattggttctgattccattcttctggtt

fava_c16736_171	fava_c16736	171	T/A	catgttagtctcgcatgcgacattctaacaaatacaaacaaaacgtttaaatttgctaatttgattcacaatatatgacagaaagaagccttcgttcctt[T/A]tgtagatgaagactcacatttaattttacattgaataaacctagttacagggtcatataatacacttaacctcttacaagtacattatatgaataatagg

fava_c16746_2152	fava_c16746	2152	T/C	caaaatgaagcccttaaagaaagagtaagaacaacgttcaagcgaattcaaagttctgtgaagccaattcctctttcaggaattcaaagttctgtgaagc[T/C]gattcctctttcaccgccataggttgtgacattataaccactaaggaatacatttttgctctaattttacttcattggcgttRgcccccaatgtcctttg

fava_c16746_2235	fava_c16746	2235	G/A	tcaaagttctgtgaagcYgattcctctttcaccgccataggttgtgacattataaccactaaggaatacatttttgctctaattttacttcattggcgtt[G/A]gcccccaatgtcctttgtatcattactttttagcatccagcttcttttaatttaaatatgattaagttttggtagatgcagatagatcgtttgttatcat

fava_c16746_317	fava_c16746	317	A/G	gcggtattcatccagcttactccggcatggcggcgctgccgtacttcgacgaaattgatccgtcaactgtcgacgtgcttctcatcactcattttcattt[A/G]gatcacgctgcgtcgcttccttactttctcgagaaaacaactttcaaaggtcgggttttcatgacgtacgccacgaaggcgatttataagcttttgctgt

fava_c16746_527	fava_c16746	527	T/C	ttaaagtcagtaaggtttctgttgaagatatgcttttcgatgaacaggatattaatcgctccatggataaaattgaggttattgatttccatcaaacact[T/C]gaagtgaatggtattagattctggtgttacactgcWggccatgtcctYggtgctgctatgtttatggttgacattgctggtgttcgagtgctgtatacag

fava_c16746_563	fava_c16746	563	A/T	tcgatgaacaggatattaatcgctccatggataaaattgaggttattgatttccatcaaacactYgaagtgaatggtattagattctggtgttacactgc[A/T]ggccatgtcctYggtgctgctatgtttatggttgacattgctggtgttcgagtgctgtatacaggagactattcacgtgaagaagaccgacatcttcgag

fava_c16746_575	fava_c16746	575	T/C	atattaatcgctccatggataaaattgaggttattgatttccatcaaacactYgaagtgaatggtattagattctggtgttacactgcWggccatgtcct[T/C]ggtgctgctatgtttatggttgacattgctggtgttcgagtgctgtatacaggagactattcacgtgaagaagaccgacatcttcgagctgcagagaccc

fava_c16751_1160	fava_c16751	1160	T/C	taatcgatttccaaattgctcaaggaagccaatggatttcgttgattcaagcttttgcagctaggcccggcgggcctccgcacatccgcattacaggtat[T/C]gatgattcaacatcagcttatgcacgtggaggtggactacatatagttgaaaagagattatcaaagcttgctcagcatttcaaggtgccatttcaatttc

fava_c16751_1316	fava_c16751	1316	G/C	gattatcaaagcttgctcagcatttcaaggtgccatttcaatttcacgcagcagctatctcaggttgtgatgttcaactgcataacctcggagttcggcc[G/C]ggagaagctcttgcagtaaattttgcattcatgctacatcacatgccggacgagagtgtcagcactcagaatcatagagataggctcttaagattggtta

fava_c16751_211	fava_c16751	211	C/T	attcctctaaaacaaaaaatgttgaatctgtgcagaagaatgaacttgtagagattaattatagtttagacgccggtaagattcttagttattgattcta[C/T]gatgcaggcatcggagcggcataggagtatgagtatgtactatcaaccattgcaacaaatcgaatcatattgcttgccgcaatatcgaaatctaaatcat

fava_c16759_1039	fava_c16759	1039	G/A	attgaatttctcaatctcaacctccaacagcctcagaaaatcgttaacctctttggaaacctcgccttcatcgtccaaacgccgccgtttggaggaagaa[G/A]aatcatctttaggaacaatgagtttgagctgcttcttgagatccttgtaggagagaaacttgtcacgccaatccggcaaagtttgctcgatctggttgtt

fava_c16759_125	fava_c16759	125	A/G	atatgcattacaaaattccaaaacacctgtttctaaccaacagcttggtataaatatctacataaagcccacatattgttagcataatgatacaagctag[A/G]tgccaatttgagagagtgcatgacacctataaccctggctaaaattttacattattattaaaaagaagaaaaataatcacgaactggtYcaaaaaggagg

fava_c16759_214	fava_c16759	214	T/C	atacaagctagRtgccaatttgagagagtgcatgacacctataaccctggctaaaattttacattattattaaaaagaagaaaaataatcacgaactggt[T/C]caaaaaggaggccaaacttcacataaagtgtgttctcaacatagaaaagaaaatgttggacaggtcctatctatttggctactttctcggatactttctc

fava_c16759_329	fava_c16759	329	C/G	aacttcacataaagtgtgttctcaacatagaaaagaaaatgttggacaggtcctatctatttggctactttctcggatactttctccacagaagagaaac[C/G]ggaMacacgcRaagatgaagagttttaatcctctaccagagtctcgctatgcatgggaggYaatgagtatatgcttacagttgagcttccccctctaatc

fava_c16759_333	fava_c16759	333	A/C	tcacataaagtgtgttctcaacatagaaaagaaaatgttggacaggtcctatctatttggctactttctcggatactttctccacagaagagaaacSgga[A/C]acacgcRaagatgaagagttttaatcctctaccagagtctcgctatgcatgggaggYaatgagtatatgcttacagttgagcttccccctctaatctctt

fava_c16759_340	fava_c16759	340	G/A	aagtgtgttctcaacatagaaaagaaaatgttggacaggtcctatctatttggctactttctcggatactttctccacagaagagaaacSggaMacacgc[G/A]aagatgaagagttttaatcctctaccagagtctcgctatgcatgggaggYaatgagtatatgcttacagttgagcttccccctctaatctctttcagggt

fava_c16759_390	fava_c16759	390	C/T	tggctactttctcggatactttctccacagaagagaaacSggaMacacgcRaagatgaagagttttaatcctctaccagagtctcgctatgcatgggagg[C/T]aatgagtatatgcttacagttgagcttccccctctaatctctttcagggtgtccagtgctgaagtagttagtttgatgaaaacattctccatgttttgga

fava_c16759_859	fava_c16759	859	G/T	tacaagacctgtgtagttaagtgcactatagttctctaacaaaaccatctctccatgaaaatccactatttctctccctactgtcatcaactctccatca[G/T]aactcttagcccaagcaactttgtcYtgcaattccttccatttgataacatattcctcttctttctcaacaaaaaacccattgaatttctcaatctcaac

fava_c16759_885	fava_c16759	885	T/C	tatagttctctaacaaaaccatctctccatgaaaatccactatttctctccctactgtcatcaactctccatcaKaactcttagcccaagcaactttgtc[T/C]tgcaattccttccatttgataacatattcctcttctttctcaacaaaaaacccattgaatttctcaatctcaacctccaacagcctcagaaaatcgttaa

fava_c16763_1019	fava_c16763	1019	G/T	tgggttatataattcctttgttggctgcaactggtgctatgccgcttgatcaagagagttgggttgatgggcattttgcaaatgtggcttatttgattgc[G/T]ggaaattggttgaaatattggatggaaatcggtgcggttttgtcgattattggattgtttgaagctcaattaagtagcgctgcgtatcagcttttgggta

fava_c16763_1329	fava_c16763	1329	A/G	tttgagttttacggagattatatctactgtgaatttcttgtatagtttagggatgcttttggagtttgcttctttcctgaagttgaggaaaaaattccct[A/G]caatgaaaagaccttataaggttccttttggattctttggtttgagtataatgtgtttYgttccatctgtgcttttggtgtatgtgatgatagttgctac

fava_c16763_1388	fava_c16763	1388	T/C	tggagtttgcttctttcctgaagttgaggaaaaaattccctRcaatgaaaagaccttataaggttccttttggattctttggtttgagtataatgtgttt[T/C]gttccatctgtgcttttggtgtatgtgatgatagttgctaccaaaactgtgttagtggctagttctttcttaacattgttagggatagtgttgtattatt

fava_c16765_640	fava_c16765	640	A/G	ggatacctccatcctctgttagggcaacccctgccccttgccagaattctgattcttccgatgagttgaaacaacttaaattggaaatggcccaaatgaa[A/G]aaagatatgcaggagcttgttgcYgagaagaaaaagaaaaatgaaagtaacatgatgaaggagactttcaaagaacttgtaaagtggaaagctataactg

fava_c16765_664	fava_c16765	664	T/C	caacccctgccccttgccagaattctgattcttccgatgagttgaaacaacttaaattggaaatggcccaaatgaaRaaagatatgcaggagcttgttgc[T/C]gagaagaaaaagaaaaatgaaagtaacatgatgaaggagactttcaaagaacttgtaaagtggaaagctataactgaccatcgcctaactgaaatcatgg

fava_c16766_360	fava_c16766	360	A/C	tagcataagcgattcctctagcatgttccctatgttgttcagcaatgatggtaagatcagtgatgataggtttcacattcgacgtgagttcacccaacaa[A/C]gcctcgtaaatctgcacaatttcttccgttgaaggttgcgaatttctcagccttgggtcatcttcacgttgcttcaacaaagccttgaatcgaccaacga

fava_c16767_1089	fava_c16767	1089	G/C	gtagtgaaagatgctggagtttttgtagattcatgaagcgtgtttgcagaaggatgtcagtatgtgacccataactgtttttaattattcagtaaaagct[G/C]taaaRcaaatggagtgtatatgacctcttggttggacttaaaattatggcgaaaatcggatccatgatattatcaagaaggtccagtttcatgaggttaa

fava_c16767_1094	fava_c16767	1094	G/A	gaaagatgctggagtttttgtagattcatgaagcgtgtttgcagaaggatgtcagtatgtgacccataactgtttttaattattcagtaaaagctStaaa[G/A]caaatggagtgtatatgacctcttggttggacttaaaattatggcgaaaatcggatccatgatattatcaagaaggtccagtttcatgaggttaaattac

fava_c16767_1244	fava_c16767	1244	T/C	atcggatccatgatattatcaagaaggtccagtttcatgaggttaaattactcttgtgtgctacaagagaaaaaggagtgttacaagttatatttcacat[T/C]gatattccagaaaggttggccatatgttttatcctatacagcgggataataacactggctgaggattatctcatgctaaggagaggagtattcaaagtgt

fava_c16767_287	fava_c16767	287	C/T	actcgtcggtccctctccttccgccaaaactcaaattctcattcaaaccgcaatcacttgcgttctccctaaacactggaatggaatacactatggtgga[C/T]ttgaccatttggtactctttattgaccttgattctcgatttgatatcactcgtttctctcacttgcttcttcaYcgcatcatggRaccttacggggaaga

fava_c16767_361	fava_c16767	361	C/T	actggaatggaatacactatggtggaYttgaccatttggtactctttattgaccttgattctcgatttgatatcactcgtttctctcacttgcttcttca[C/T]cgcatcatggRaccttacggggaagatagtggagattatgataaatcgttatttgatttgtgtatgactcgttttttgtatgttcgttgttccgatagtt

fava_c16767_372	fava_c16767	372	G/A	atacactatggtggaYttgaccatttggtactctttattgaccttgattctcgatttgatatcactcgtttctctcacttgcttcttcaYcgcatcatgg[G/A]accttacggggaagatagtggagattatgataaatcgttatttgatttgtgtatgactcgttttttgtatgttcgttgttccgatagttttcaatttctt

fava_c16771_687	fava_c16771	687	G/T	tggaagaaggtgatgaatggtacgtatgataatggttgagaagtggagattcaaggagaacgcaatgaatcgaaggttttgagaagatgaaggttgaaat[G/T]cagaaacaatggagattatgtagagacctcaagattgagagagtcttcagattttatggggctgaaactttaatgatattggtggcaaaatcWtggcttg

fava_c16771_780	fava_c16771	780	T/A	ttgaaatKcagaaacaatggagattatgtagagacctcaagattgagagagtcttcagattttatggggctgaaactttaatgatattggtggcaaaatc[T/A]tggcttgtgacattggtgatagggaaaccatggttatgagcacttcRaagtcgtcgagcatgtcatgaaactgtttggaaattgatggtgtatatgRttt

fava_c16771_827	fava_c16771	827	G/A	agagtcttcagattttatggggctgaaactttaatgatattggtggcaaaatcWtggcttgtgacattggtgatagggaaaccatggttatgagcacttc[G/A]aagtcgtcgagcatgtcatgaaactgtttggaaattgatggtgtatatgRtttcaaataagcataagtttggaggatgcttgaaacttgaatattaggag

fava_c16771_877	fava_c16771	877	G/A	atcWtggcttgtgacattggtgatagggaaaccatggttatgagcacttcRaagtcgtcgagcatgtcatgaaactgtttggaaattgatggtgtatatg[G/A]tttcaaataagcataagtttggaggatgcttgaaacttgaatattaggagtttaggaacaacatgtttaggataggtcttgatgataagttttcatgtaa

fava_c16772_138	fava_c16772	138	C/G	gcaataaataagtataacttcattatatcataactagtacacatacatatatcacagaccatcactagaaatgcaggtcaatacactagacgatggacag[C/G]atttataacataaatttgaaagacRgaRcattttctacaaggtagtttaagattatatgttacattttgtgaaggaattcattcagtgttgagtgagcca

fava_c16772_163	fava_c16772	163	A/G	tatcataactagtacacatacatatatcacagaccatcactagaaatgcaggtcaatacactagacgatggacagSatttataacataaatttgaaagac[A/G]gaRcattttctacaaggtagtttaagattatatgttacattttgtgaaggaattcattcagtgttgagtgagccatattacatcatggacagcagatgtg

fava_c16772_166	fava_c16772	166	G/A	cataactagtacacatacatatatcacagaccatcactagaaatgcaggtcaatacactagacgatggacagSatttataacataaatttgaaagacRga[G/A]cattttctacaaggtagtttaagattatatgttacattttgtgaaggaattcattcagtgttgagtgagccatattacatcatggacagcagatgtggca

fava_c16773_1567	fava_c16773	1567	A/C	aagcgtgaatcacttccagtacctcgcgcaccatctggtaaatctaccatgccaactcgaagaacatctcttccgctgcctacaagaggcagaaacaaaa[A/C]aaacttatacaccaatgctagttatgtggactctcctgatgtttctgttgatgcaccacaaatcgacaagatcgcagaattcccaatggcttcgtatgag

fava_c16773_530	fava_c16773	530	C/T	ctcaccaggagatggagcttatatcaaaagttcacaatccgtttattgtggagtataaagactcttgggtagaaaagggttgttttgtatgtattatcat[C/T]ggctattgtgaaggaggagatatggctgaagctataaaaaaggccaacagtgttaacttttctgaagagaaactctgcaagtggcttgtccagttactga

fava_c16776_1041	fava_c16776	1041	C/G	tctcaggcttcaagtcagtgtggatgaggcgcatatcatgtacaaatgctacactttccaacaattgtcttcctagctcacggacaagatcaaccggaaa[C/G]gggcgataattgttcttccggagaaaatcatataagcttggtccaagcatctcaaatacaatacaaatatggttccggtagtcaaaccaattgcgtattt

fava_c16776_1364	fava_c16776	1364	T/C	atttctctatcccaacattctaaaacctgaccaaaagtcccttcaccgattttcctgaggattttataccgagaagttaaattttctccaagcgcaaaca[T/C]gtaatgtccatctttatcatcctctcgccattgRggagaacctttttccgcaMatccttttactgatataccaccttggtcaggaagtaccctcaatgtt

fava_c16776_1398	fava_c16776	1398	G/A	aagtcccttcaccgattttcctgaggattttataccgagaagttaaattttctccaagcgcaaacaYgtaatgtccatctttatcatcctctcgccattg[G/A]ggagaacctttttccgcaMatccttttactgatataccaccttggtcaggaagtaccctcaatgttccatagcttgttgtcacattagcaaactcttgtc

fava_c16776_1417	fava_c16776	1417	C/A	cctgaggattttataccgagaagttaaattttctccaagcgcaaacaYgtaatgtccatctttatcatcctctcgccattgRggagaacctttttccgca[C/A]atccttttactgatataccaccttggtcaggaagtaccctcaatgttccatagcttgttgtcacattagcaaactcttgtccacaatacattcccgaatg

fava_c16776_308	fava_c16776	308	G/A	aaaaaagtaccactatgaaaagaaaacttacgtaacaaatgttgcagttatctatagccaaacacaacacaacaaccacttatctattgccggtgacgtg[G/A]tgaaccctgtatttaatacctctggtatcgctccccggtaaaaaagggatgcctgagtgcttcatgcgccttcatcctgctcatggggtcaaactgaagt

fava_c16776_435	fava_c16776	435	C/T	atcgctccccggtaaaaaagggatgcctgagtgcttcatgcgccttcatcctgctcatggggtcaaactgaagtagaccctgcaagagatcaagaagatc[C/T]ccagctgagtgatccacatgctgcatcaccagattaggaagtcgaggtagtttcaagacagccttgatgctttctcgggagactgctccttcgggccagt

fava_c16776_606	fava_c16776	606	G/A	tttctcgggagactgctccttcgggccagtttagtctaccccttctgacatacttctctgcagggcggtctgctcttttcaacatgtgctgaggcattgg[G/A]cctaaaaccctctccatcatggctagatgctctaaattctcatgcgtctgaaacaaagcttctcccgagcacaactcaaccaaaatgcaaccaatactcc

fava_c16777_1256	fava_c16777	1256	T/C	catcaattcacgccaaacaggtttgttctcatatttaccagaattggcaaaaccagcagcctgttgtatctctgctagcctctcttccacttctttctcg[T/C]cttcgttcgttttcaacaacaccgatcttgcttcttcaattcggttctgcattaccaaccacctcggtgactcgggaataatgaaaagagcaaagccaat

fava_c16777_317	fava_c16777	317	C/A	aacaaaaatctccatgcttacataatcctactatgaacttaaatgaaactaaaagaaacagaataattgtacaactagtaaaagtttgagtatacaataa[C/A]aatgcaatttgccttcattttttctcatgccccgtatcagatatcaaattctcatgtcgcagaatcatgagtggaggtcataaagccttggacataacaa

fava_c16777_705	fava_c16777	705	C/T	gaagaaatagcggaaaacagaaagaacgttccagcaaaagaaatttttccagatacagataggaaagacatggcaacaagtccactgcagaccctattag[C/T]cacagctccaagtgcggatgcttgagcacgcacacgtaaagggaagatttcagatgtcaaaacccagcacacagggccaagtccaattgaaaagaatgca

fava_c16783_1721	fava_c16783	1721	T/C	cactttcaaatagtatttcttcttctatatcagggccatcatctgatggctgaaattttgatggagatggtgggtcactaaagcatttggtcttcgttac[T/C]gaaggtttgactctattaagtgcttcacgaatWatattctcccattttggaacaggccgagtatcttcagcaccaaaaatattgccggggtttaagggta

fava_c16783_1754	fava_c16783	1754	A/T	ggccatcatctgatggctgaaattttgatggagatggtgggtcactaaagcatttggtcttcgttacYgaaggtttgactctattaagtgcttcacgaat[A/T]atattctcccattttggaacaggccgagtatcttcagcaccaaaaatattgccggggtttaagggtacaagctcttgaagaccgaggacatagatgtcag

fava_c16785_176	fava_c16785	176	A/G	ttcttatgaatttactcacttccatcactattactgccacaaacttctgtccacctaacttggcactccctaatgacaatggtggttggtgcaatccccc[A/G]agaccacactttgatatgtctcaacctgcctttgagactatagccaagtatagagctggaattgttcccatcttgtatagaagagttgggtgcagaagaa

fava_c16785_290	fava_c16785	290	G/A	atatgtctcaacctgcctttgagactatagccaagtatagagctggaattgttcccatcttgtatagaagagttgggtgcagaagaagtggaaacataag[G/A]tttaStataaatgggagagattattttgagctggtgcttataagcaacataggtggaggtggagagatatggaaagtttggataaaagggtcaaaaaaga

fava_c16785_295	fava_c16785	295	C/G	tctcaacctgcctttgagactatagccaagtatagagctggaattgttcccatcttgtatagaagagttgggtgcagaagaagtggaaacataagRttta[C/G]tataaatgggagagattattttgagctggtgcttataagcaacataggtggaggtggagagatatggaaagtttggataaaagggtcaaaaaagaataaa

fava_c16785_515	fava_c16785	515	T/C	attggggtgctaattggcagagtctaagttatctaaatggtcagagtttatcatttagagttcaactcaagaatggaaagactcgtacagctataaacgt[T/C]gcaccttccaattggaRatttggccagtctttcaaaagtaatgttcagttttgattctcaactaaatactatcagttatatgatWtgaaatatgagtcaa

fava_c16785_532	fava_c16785	532	G/A	cagagtctaagttatctaaatggtcagagtttatcatttagagttcaactcaagaatggaaagactcgtacagctataaacgtYgcaccttccaattgga[G/A]atttggccagtctttcaaaagtaatgttcagttttgattctcaactaaatactatcagttatatgatWtgaaatatgagtcaacaatagtacatccttgt

fava_c16785_600	fava_c16785	600	T/A	tacagctataaacgtYgcaccttccaattggaRatttggccagtctttcaaaagtaatgttcagttttgattctcaactaaatactatcagttatatgat[T/A]tgaaatatgagtcaacaatagtacatccttgtaacaattatatagtatattctggtgattttctgtaaggaattattccaagtggttgtggctcagcaat

fava_c16788_1042	fava_c16788	1042	C/T	gaagcatagtcatcatgtcaattcatcaaccaagttacagaatcttaggtcttctggatcgcatgatcttcttatcgcgtggacaaaccgtttacagcgg[C/T]tcaccttcgcagttgccttcatactttgcagaattcggtcatcctataccagaaggtgataaccgtaccgagtttgcattagacctaattcgcgaacttg

fava_c16788_1198	fava_c16788	1198	C/T	gtgataaccgtaccgagtttgcattagacctaattcgcgaacttgaaggttcacccggaggaacaaaaagtttagttgaattcaacaaatcgtggcaaag[C/T]atgacaaagcttcatcgtgttgaagatttaccacaaaactcaacctcttctttgtcattgaaggaagctatcagcgcaagtatttcaaaaggtaagttag

fava_c16788_1345	fava_c16788	1345	A/T	cttctttgtcattgaaggaagctatcagcgcaagtatttcaaaaggtaagttagtttctggagcaaccgcaacaaataactccaacacaagatcatcttc[A/T]tcagcagcgtctatggtgcctacttaYgcaaatccgttttggattgagttattaaccctttcaaagcggtccataaccaattcaagaagaatgcctgaac

fava_c16788_1372	fava_c16788	1372	T/C	gcgcaagtatttcaaaaggtaagttagtttctggagcaaccgcaacaaataactccaacacaagatcatcttcWtcagcagcgtctatggtgcctactta[T/C]gcaaatccgttttggattgagttattaaccctttcaaagcggtccataaccaattcaagaagaatgcctgaactattcggaataagattaggtgctgtta

fava_c16788_1834	fava_c16788	1834	G/C	ctctaacgttttgggctgttggcctagacggtggattctccggctttttgttttattttgttatcatctttgcttcgttttgggccgggaattccttcgt[G/C]tcgtttctttctggtgtagtgcctcatgttatgttaggttaYactatcgtggttgcSattctcgcttaYttcttgttgtttagcgggttctttattaatc

fava_c16788_1876	fava_c16788	1876	C/T	gctttttgttttattttgttatcatctttgcttcgttttgggccgggaattccttcgtStcgtttctttctggtgtagtgcctcatgttatgttaggtta[C/T]actatcgtggttgcSattctcgcttaYttcttgttgtttagcgggttctttattaatcgggataggattccSagttactggatatggttccattacatgt

fava_c16788_1891	fava_c16788	1891	C/G	ttgttatcatctttgcttcgttttgggccgggaattccttcgtStcgtttctttctggtgtagtgcctcatgttatgttaggttaYactatcgtggttgc[C/G]attctcgcttaYttcttgttgtttagcgggttctttattaatcgggataggattccSagttactggatatggttccattacatgtcattggttaaatatc

fava_c16788_1903	fava_c16788	1903	C/T	ttgcttcgttttgggccgggaattccttcgtStcgtttctttctggtgtagtgcctcatgttatgttaggttaYactatcgtggttgcSattctcgctta[C/T]ttcttgttgtttagcgggttctttattaatcgggataggattccSagttactggatatggttccattacatgtcattggttaaatatccttatgaagctg

fava_c16788_1948	fava_c16788	1948	G/C	gtgtagtgcctcatgttatgttaggttaYactatcgtggttgcSattctcgcttaYttcttgttgtttagcgggttctttattaatcgggataggattcc[G/C]agttactggatatggttccattacatgtcattggttaaatatccttatgaagctgttttgcagaacgaatttagtgatgcagttaagtgttttgtgagag

fava_c16788_2102	fava_c16788	2102	C/T	tgttttgcagaacgaatttagtgatgcagttaagtgttttgtgagaggggttcagattttcgataatacacctcttgcttctgttcctgatgcacttaag[C/T]tgaagctgttggataatatgagtgaaactttgggaatgaatataacagcttctacttgtttaacaactggtactgaYttgttgaagcaaaatggagtttt

fava_c16788_2179	fava_c16788	2179	T/C	cttctgttcctgatgcacttaagYtgaagctgttggataatatgagtgaaactttgggaatgaatataacagcttctacttgtttaacaactggtactga[T/C]ttgttgaagcaaaatggagttttggatttgagtaaatggaactgtYtgtggataactgtggcttggggtttcttctttaggattttgttctatgtggttt

fava_c16788_2225	fava_c16788	2225	T/C	tgaaactttgggaatgaatataacagcttctacttgtttaacaactggtactgaYttgttgaagcaaaatggagttttggatttgagtaaatggaactgt[T/C]tgtggataactgtggcttggggtttcttctttaggattttgttctatgtggttttgttgcttggtagcaaaaacaagaggagttRaaaaaaaaggaaaaa

fava_c16788_2310	fava_c16788	2310	G/A	agtaaatggaactgtYtgtggataactgtggcttggggtttcttctttaggattttgttctatgtggttttgttgcttggtagcaaaaacaagaggagtt[G/A]aaaaaaaaggaaaaaatgttggtttgagtttgtgtaacactttgttactaaagttttattttatttattttatttgttaatctgttttacagtttgtttt

fava_c16788_2451	fava_c16788	2451	C/T	tttgttactaaagttttattttatttattttatttgttaatctgttttacagtttgtttttaatgagcaagtgtttgataatctatatagttgattagta[C/T]tttagaagttacttatgataaagtgtagtcaatactcaatagaataaagtgtaaattttgtatttatattaaatgcatgttcttacttattagatattaa

fava_c16788_658	fava_c16788	658	C/T	tgaatggtgaatcagtggagtctcgtttacttaaggtgatttcagcttacgtgatgcaagatgatcttctctttccgatgcttactgtggaagaaactct[C/T]acctttgcggccgagtttagattacctcgatcactctcaaaatcgaagaaaaatgcgcgtgttcaagctttaatcgatcaactaggactccgcaatgctg

fava_c16790_474	fava_c16790	474	C/T	aatatttcattttcaccttatggaaattattggagaaaactcagaaaaatatgcacaatggagcttttaacgcaaaagcgtgtgagttcatttcagccca[C/T]aagagaagacgtgttcataaatctcatcaaaaggattgattcacagcaaggatcaggatccgccatcaacatcactcaacttgtggtttcatcaactttt

fava_c16790_655	fava_c16790	655	T/C	ttgtggtttcatcaacttttgctattattacaaaggctgcacttggcaagaactgcaaagtgcaagaagagcttgcgtgtttgggaaatggagaatctgt[T/C]gcgggaggtttYRacataggtgaactgtttYcatctgctaaatggcttcaagttgtttctggtttgaggccgaaactcgagaggctgcatagacaaatKg

fava_c16790_667	fava_c16790	667	C/T	caacttttgctattattacaaaggctgcacttggcaagaactgcaaagtgcaagaagagcttgcgtgtttgggaaatggagaatctgtYgcgggaggttt[C/T]RacataggtgaactgtttYcatctgctaaatggcttcaagttgtttctggtttgaggccgaaactcgagaggctgcatagacaaatKgatgagttgctag

fava_c16790_668	fava_c16790	668	G/A	aacttttgctattattacaaaggctgcacttggcaagaactgcaaagtgcaagaagagcttgcgtgtttgggaaatggagaatctgtYgcgggaggtttY[G/A]acataggtgaactgtttYcatctgctaaatggcttcaagttgtttctggtttgaggccgaaactcgagaggctgcatagacaaatKgatgagttgctagt

fava_c16790_686	fava_c16790	686	T/C	aaaggctgcacttggcaagaactgcaaagtgcaagaagagcttgcgtgtttgggaaatggagaatctgtYgcgggaggtttYRacataggtgaactgttt[T/C]catctgctaaatggcttcaagttgtttctggtttgaggccgaaactcgagaggctgcatagacaaatKgatgagttgctagtaaaRgttattattgaaca

fava_c16790_754	fava_c16790	754	T/G	tYgcgggaggtttYRacataggtgaactgtttYcatctgctaaatggcttcaagttgtttctggtttgaggccgaaactcgagaggctgcatagacaaat[T/G]gatgagttgctagtaaaRgttattattgaacataaggaagctaagttgaaagaaaatcaaggccaatgtggtgaagtagaagaagatctagtagatgttc

fava_c16790_772	fava_c16790	772	G/A	taggtgaactgtttYcatctgctaaatggcttcaagttgtttctggtttgaggccgaaactcgagaggctgcatagacaaatKgatgagttgctagtaaa[G/A]gttattattgaacataaggaagctaagttgaaagaaaatcaaggccaatgtggtgaagtagaagaagatctagtagatgttcttctRaattttcagtgtg

fava_c16790_859	fava_c16790	859	A/G	agttgctagtaaaRgttattattgaacataaggaagctaagttgaaagaaaatcaaggccaatgtggtgaagtagaagaagatctagtagatgttcttct[A/G]aattttcagtgtggtaatgatagtttgaaaaaggggtctagagggtatatagaatgattaatgtcataatcctctctaaaattttgtttccttctttctc

fava_c16791_121	fava_c16791	121	T/C	atattcatacatcactctgtttttcctagaacttaccttaacttatcatcacaagttcaaaattattaatattgtcatacaccataatgccatcagtgca[T/C]tctagtcctttctacccaatggaaaatccagccttagcatcacttctccggcacacatccggcgaaaagcggaacaaattctccgccggtgggctcttaa

fava_c16791_286	fava_c16791	286	G/A	aaaagcggaacaaattctccgccggtgggctcttaaagatgttcaaactcttccctatgttaacatcaggttgcaaaatggtagcactattaggaagacc[G/A]agaaagcctatgttaaaagacagtgccacaacgggaacaatctttggttacagaaaaggaagagtaatcatagcaatacaagaagacacacgtcaaatgc

fava_c16793_111	fava_c16793	111	T/C	atcatggatttacaaaaactatagaatgaaagtgaaattttacaaaggaggaatctaccctacatgaacaaacacaatgccctggttttattattacaac[T/C]ggtaataattattatattatcaataaataataattaataaaatgcaattccctaaactgctattctttcactacttgataaactagatgataRttggRca

fava_c16793_1235	fava_c16793	1235	T/C	tgccgattccgctcagaatggactgaggcgacccggccacgtcacgagtttttggcgaatgatgaactgttgcttggggtttgttccaagcgttgatagg[T/C]gcggtgttgagtttgttccgagtttcgttgagtgttgactgactgagtcgttgagttagccccgcgaagaagctttcttcttcgtcgctgctctcagttg

fava_c16793_204	fava_c16793	204	G/A	ttacaacYggtaataattattatattatcaataaataataattaataaaatgcaattccctaaactgctattctttcactacttgataaactagatgata[G/A]ttggRcaaaaaaactcaaaccatattctaaaagctaaaaaatttcttactcaagaactggactttgttattgaaactgaatttaacttaactaccacacc

fava_c16793_209	fava_c16793	209	A/G	acYggtaataattattatattatcaataaataataattaataaaatgcaattccctaaactgctattctttcactacttgataaactagatgataRttgg[A/G]caaaaaaactcaaaccatattctaaaagctaaaaaatttcttactcaagaactggactttgttattgaaactgaatttaacttaactaccacaccattga

fava_c16793_556	fava_c16793	556	T/C	cagctggctgcttcttcccggagagcaattaatctttgctgcttcaaaagtgcatttcttctagccaacaataaatcaaaatcgttggtgaaacaatttg[T/C]gaatcgattttgagcagcaacattcatgtcaccaatgttcaagttcaaaccgtttaccttcttaaccggttgcataactggtccacaaccagttctcttg

fava_c16793_884	fava_c16793	884	C/T	catattgttgtacccgttggttttgaggatgaagttgaaaagacatagggacgttacatcttccagtttcgtaaccaagttcacgccttctgttctgaag[C/T]agtgactgtcgttgatgaaccaaccaattaggttccgtttcccaaacggaaccacactcttgcttctgtttcacttcttgctgaaccaagttagcatggg

fava_c16794_2089	fava_c16794	2089	A/C	gatcaagcacctgagagtatgagaagtagtgctactgctatatattgtgtaattatagctattggaagctatgctggtacatttatagtgacacttgttc[A/C]taaatatagtggcaaagaaaggaattggttRcctgataggaaccYtaataggggtagattggagtattattattatcttgttagtKgtgttcaagttctt

fava_c16794_2120	fava_c16794	2120	G/A	ctactgctatatattgtgtaattatagctattggaagctatgctggtacatttatagtgacacttgttcMtaaatatagtggcaaagaaaggaattggtt[G/A]cctgataggaaccYtaataggggtagattggagtattattattatcttgttagtKgtgttcaagttcttaatctcatttaWtttggaatttgtctttggt

fava_c16794_2134	fava_c16794	2134	T/C	tgtgtaattatagctattggaagctatgctggtacatttatagtgacacttgttcMtaaatatagtggcaaagaaaggaattggttRcctgataggaacc[T/C]taataggggtagattggagtattattattatcttgttagtKgtgttcaagttcttaatctcatttaWtttggaatttgtctttggttttatacttacaag

fava_c16794_2175	fava_c16794	2175	T/G	agtgacacttgttcMtaaatatagtggcaaagaaaggaattggttRcctgataggaaccYtaataggggtagattggagtattattattatcttgttagt[T/G]gtgttcaagttcttaatctcatttaWtttggaatttgtctttggttttatacttacaagcctttggaagaaattgctgaaatcagtaaggaagacgattt

fava_c16794_2201	fava_c16794	2201	T/A	gcaaagaaaggaattggttRcctgataggaaccYtaataggggtagattggagtattattattatcttgttagtKgtgttcaagttcttaatctcattta[T/A]tttggaatttgtctttggttttatacttacaagcctttggaagaaattgctgaaatcagtaaggaagacgatttggaacaggttgatgcaaatatcttgg

fava_c16794_2311	fava_c16794	2311	T/G	tgtctttggttttatacttacaagcctttggaagaaattgctgaaatcagtaaggaagacgatttggaacaggttgatgcaaatatcttggcttccaatt[T/G]agaagagaaagtttagaaggaaaaaaagctagtgatgtattgttcataatggttgatttgtattRttggagactttgattgttcaatttctctacaagtg

fava_c16794_2376	fava_c16794	2376	A/G	ggaacaggttgatgcaaatatcttggcttccaattKagaagagaaagtttagaaggaaaaaaagctagtgatgtattgttcataatggttgatttgtatt[A/G]ttggagactttgattgttcaatttctctacaagtgacttatataataagaaggaaatgtagagtttagaggtatttccttttYcccccttttttccaaag

fava_c16794_2459	fava_c16794	2459	C/T	aatggttgatttgtattRttggagactttgattgttcaatttctctacaagtgacttatataataagaaggaaatgtagagtttagaggtatttcctttt[C/T]cccccttttttccaaaggtgcttttattagagtgatggtttcattttcttaaatagtaatgtaattgtaaactattttggttatagcaactatgatattg

fava_c16794_731	fava_c16794	731	T/A	tcatccttgctaatgaggtatgtgatagatttgctgtagctggtttcaatggaaacctgataagttacctaactcaacaactgaatatgccattagtatc[T/A]gctgcaaacacactcacaatcttcggcggaacagccagcttcacacctctcatcggtgcWctcatttccgagtcctttgccggccacttctggaccatta

fava_c16794_791	fava_c16794	791	T/A	taagttacctaactcaacaactgaatatgccattagtatcWgctgcaaacacactcacaatcttcggcggaacagccagcttcacacctctcatcggtgc[T/A]ctcatttccgagtcctttgccggccacttctggaccattaccattggttcaatcatctaccaacttggaatgatgagcataacactatcgactatacttc

fava_c16799_1061	fava_c16799	1061	T/C	ctgtcattattgaatacatgtactttgcatttggccaaatctttggaaccaaaccaaaccaatcaactttctctagctcctcacaatacccttgtaattt[T/C]gaagccaaatttgtatttgggccacttttcgaattaattatgtccaaaattgtttctcggagttttggcgatttgattcttgaacttagtgttccgtttt

fava_c16799_1942	fava_c16799	1942	G/C	gtattgttagatacatcttcaaaccaactaataatcttttcatagtcaaaatttccactgccattgctgcgcttgtttttgttgttgctcacaagtgttg[G/C]ctccattagagtatgtttaaacaaatttaggtagaaatgaaacaaaagagaacactattgatgaaaatggttttgaatattggagctgatatgtttctta

fava_c16800_1009	fava_c16800	1009	G/A	aagcagatgtaattagagcctttgttgttagcttYgcaagcctacactttccattaacatcacYagtgRattcatctcggtcttcttgtgttcttatgcc[G/A]gccaataRaattgaagaagttggacggaaaggaataaagtcatagagaccattgccagatgtatttacaggaaYacgaccatcaaatcgatatatagcct

fava_c16800_1017	fava_c16800	1017	G/A	gtaattagagcctttgttgttagcttYgcaagcctacactttccattaacatcacYagtgRattcatctcggtcttcttgtgttcttatgccRgccaata[G/A]aattgaagaagttggacggaaaggaataaagtcatagagaccattgccagatgtatttacaggaaYacgaccatcaaatcgatatatagccttcatttga

fava_c16800_1083	fava_c16800	1083	C/T	tctcggtcttcttgtgttcttatgccRgccaataRaattgaagaagttggacggaaaggaataaagtcatagagaccattgccagatgtatttacaggaa[C/T]acgaccatcaaatcgatatatagccttcatttgacttgggtaccatgttatatctgcaaactcatgttcatgtccaaaactaaccacttccattcctaaa

fava_c16800_1360	fava_c16800	1360	C/G	caccaagaacaccaagtgatactttaactgcattaaaatcttcatcattattttgattaagaatctcaactttagcataaccatcttcagaatcagaagc[C/G]ctaacaatcttaatctcaaccacataatcatgaacagcactWcctttattccacaatgtgcttccatgagcaccagttcccattaatccacctatagtca

fava_c16800_139	fava_c16800	139	A/T	atgagataaaacatattgtttgacgatgatacctaaataacaaattacacataaattattcggtttatattgtcatatttttataaaattattcatcaat[A/T]cttataaaatgaccatatttaaaatcttgacacccctaatttaatgagacatgagaacaaacccttgcctccttgtagacYtttcctagtctacaaaaat

fava_c16800_1402	fava_c16800	1402	T/A	catcattattttgattaagaatctcaactttagcataaccatcttcagaatcagaagcSctaacaatcttaatctcaaccacataatcatgaacagcact[T/A]cctttattccacaatgtgcttccatgagcaccagttcccattaatccacctatagtcaaaccataccaatatggtgtatagggcaaagccaaaccMtttt

fava_c16800_1498	fava_c16800	1498	C/A	cactWcctttattccacaatgtgcttccatgagcaccagttcccattaatccacctatagtcaaaccataccaatatggtgtatagggcaaagccaaacc[C/A]tttttcgaagcttcatcgattaaatcYttcaacgttacaccactttcaaccgttatcgttttttcatccacgtcgattttcactaccttgttSaggtatc

fava_c16800_1525	fava_c16800	1525	T/C	catgagcaccagttcccattaatccacctatagtcaaaccataccaatatggtgtatagggcaaagccaaaccMtttttcgaagcttcatcgattaaatc[T/C]ttcaacgttacaccactttcaaccgttatcgttttttcatccacgtcgattttcactaccttgttSaggtatcttgtgcttattaatatcccatttggat

fava_c16800_1591	fava_c16800	1591	C/G	ccaaaccMtttttcgaagcttcatcgattaaatcYttcaacgttacaccactttcaaccgttatcgttttttcatccacgtcgattttcactaccttgtt[C/G]aggtatcttgtgcttattaatatcccatttggatcttccggacataccaatttcggtatgctgtgtgagtaacgcgtcgcgactttgattttggttttcg

fava_c16800_220	fava_c16800	220	C/T	tataaaattattcatcaatWcttataaaatgaccatatttaaaatcttgacacccctaatttaatgagacatgagaacaaacccttgcctccttgtagac[C/T]tttcctagtctacaaaaataccctttacttggattgcaatgattatcttcMgaacaaatacacaaaccttctaaagcacatccatttttcaatattgtaa

fava_c16800_271	fava_c16800	271	A/C	acccctaatttaatgagacatgagaacaaacccttgcctccttgtagacYtttcctagtctacaaaaataccctttacttggattgcaatgattatcttc[A/C]gaacaaatacacaaaccttctaaagcacatccatttttcaatattgtaacaccttcttttaatccaagcatttgatctgtccaagtactagaaaaaagcc

fava_c16800_502	fava_c16800	502	T/C	aaaactttccaacatttttgtacttcttagaaacaccttcaaatgccaaatttctattcttaccccaatgaggcaaccctccatacttaaacatcccaat[T/C]tgttcaatctcYtcaataatgtcttcataaagcctaggcatcattggatctttgctacgataatatgtgaaatcaaagtctatagcatcttcttggttcc

fava_c16800_514	fava_c16800	514	C/T	catttttgtacttcttagaaacaccttcaaatgccaaatttctattcttaccccaatgaggcaaccctccatacttaaacatcccaatYtgttcaatctc[C/T]tcaataatgtcttcataaagcctaggcatcattggatctttgctacgataatatgtgaaatcaaagtctatagcatcttcttggttccctaaataagcac

fava_c16800_816	fava_c16800	816	C/G	tttttcactaaagctacacttattttaaatgtggtttggtgaaaaaactctccttttattcttgaatcccaagcacatactgttatctttgcattatgaa[C/G]actatccaaacatgaccccgatgattgaagacggttttgaaatccaattataggatatccagtaaagattatgccattattggttaggccataagcagat

fava_c16800_943	fava_c16800	943	T/C	gaagacggttttgaaatccaattataggatatccagtaaagattatgccattattggttaggccataagcagatgtaattagagcctttgttgttagctt[T/C]gcaagcctacactttccattaacatcacYagtgRattcatctcggtcttcttgtgttcttatgccRgccaataRaattgaagaagttggacggaaaggaa

fava_c16800_972	fava_c16800	972	C/T	tatccagtaaagattatgccattattggttaggccataagcagatgtaattagagcctttgttgttagcttYgcaagcctacactttccattaacatcac[C/T]agtgRattcatctcggtcttcttgtgttcttatgccRgccaataRaattgaagaagttggacggaaaggaataaagtcatagagaccattgccagatgta

fava_c16800_977	fava_c16800	977	G/A	agtaaagattatgccattattggttaggccataagcagatgtaattagagcctttgttgttagcttYgcaagcctacactttccattaacatcacYagtg[G/A]attcatctcggtcttcttgtgttcttatgccRgccaataRaattgaagaagttggacggaaaggaataaagtcatagagaccattgccagatgtatttac

fava_c16801_1420	fava_c16801	1420	C/T	gctttgttggaagggaagctattgtaacaacacaatgcctgaatggatggtatgtggtgaagacattggacaatgcagagagtgtaaaattgcagtatcg[C/T]tcccttgctaaggtcWctgaggatccttcaaaacttgtctccagcaacaagggacctaattggcttcagatcagccgcactccatcataaaacgtggttg

fava_c16801_1436	fava_c16801	1436	A/T	agctattgtaacaacacaatgcctgaatggatggtatgtggtgaagacattggacaatgcagagagtgtaaaattgcagtatcgYtcccttgctaaggtc[A/T]ctgaggatccttcaaaacttgtctccagcaacaagggacctaattggcttcagatcagccgcactccatcataaaacgtggttgataatacKtctKcaga

fava_c16801_1528	fava_c16801	1528	T/G	ctaaggtcWctgaggatccttcaaaacttgtctccagcaacaagggacctaattggcttcagatcagccgcactccatcataaaacgtggttgataatac[T/G]tctKcagaaatgtgtaagcaatttatgttaagcaaccattttacataaagaagttactgctttatgttaagcaaattattgttaacaccccaaacaaaat

fava_c16801_1532	fava_c16801	1532	G/T	ggtcWctgaggatccttcaaaacttgtctccagcaacaagggacctaattggcttcagatcagccgcactccatcataaaacgtggttgataatacKtct[G/T]cagaaatgtgtaagcaatttatgttaagcaaccattttacataaagaagttactgctttatgttaagcaaattattgttaacaccccaaacaaaatcttg

fava_c16801_2106	fava_c16801	2106	T/A	caatatcatcagaatccattaattcttgcttttgtttatctgtaacattacttgcagtattctcttcaaaaggatcttcttgctttataattatgcttgg[T/A]gtWggtgaagtaatgtggtggttaacctgttcactttcatcaggagtgatgttgcatgtatgaatgccaatgtatgtaatttgatacatatcaggtttgt

fava_c16801_2109	fava_c16801	2109	T/A	tatcatcagaatccattaattcttgcttttgtttatctgtaacattacttgcagtattctcttcaaaaggatcttcttgctttataattatgcttggWgt[T/A]ggtgaagtaatgtggtggttaacctgttcactttcatcaggagtgatgttgcatgtatgaatgccaatgtatgtaatttgatacatatcaggtttgtctt

fava_c16801_2385	fava_c16801	2385	C/T	attcagagttcatgattcccttctgtccatactttctccatgaatgattatcaacaatggtttgggaaactatgctccatgtctcaccacacttccttct[C/T]ttgtagcaacctcttgaactcttYttaacaacRttacctgcatctccagatggtggatcaatgccggcgcctaSatcatcacaacctggttgagagacaa

fava_c16801_2409	fava_c16801	2409	T/C	gtccatactttctccatgaatgattatcaacaatggtttgggaaactatgctccatgtctcaccacacttccttctYttgtagcaacctcttgaactctt[T/C]ttaacaacRttacctgcatctccagatggtggatcaatgccggcgcctaSatcatcacaacctggttgagagacaattgaaagagcttctgtgaaagatc

fava_c16801_2418	fava_c16801	2418	G/A	ttctccatgaatgattatcaacaatggtttgggaaactatgctccatgtctcaccacacttccttctYttgtagcaacctcttgaactcttYttaacaac[G/A]ttacctgcatctccagatggtggatcaatgccggcgcctaSatcatcacaacctggttgagagacaattgaaagagcttctgtgaaagatctgagcacat

fava_c16801_2459	fava_c16801	2459	G/C	ctccatgtctcaccacacttccttctYttgtagcaacctcttgaactcttYttaacaacRttacctgcatctccagatggtggatcaatgccggcgccta[G/C]atcatcacaacctggttgagagacaattgaaagagcttctgtgaaagatctgagcacatttgcagcaagtttgtatgataagagagagccatcaccaaag

fava_c16801_511	fava_c16801	511	A/T	agttccgccgtaacgtttttgctgatcgcgccgaccacagggacacttcagatgtactgaattggagcgccggaactcccagcagcgacgattcggacgg[A/T]gaagacgatgaagacgacgatgaaggcgacgaaggtgacactgtagtagacacactcgtcagtgttggcgatgggagcaaccggagttcaaatgcaatta

fava_c16801_850	fava_c16801	850	G/T	ttggtgaagatcatcgagaaagattagggaaaactcagaactctgcaactgttgctgaaactgattgtgaggagtattattcccattatcttcaaggtgc[G/T]gaagggtctgcttctgtacagaaagttatgttggatgacaatggttgtgggtttagtggaagaaaggatgctgtttattcaagtgagtctggggagtcat

fava_c16805_1070	fava_c16805	1070	G/A	gcgcaaagccgattctgctttccaaacttgcacgacagtaaaatcatttcttgaagaaactatggtagcaattccagactcggttttggactcttcgttt[G/A]cgaaatcaccgcctgggcgtgaagatctttttgctaacattgacagtttattgcctcttccatacagaacaagagatgaggattctgtttacatccatta

fava_c16805_1196	fava_c16805	1196	G/A	tctttttgctaacattgacagtttattgcctcttccatacagaacaagagatgaggattctgtttacatccattagttatctgttttatgcaaatgactt[G/A]aaaaatgtaattcattctatgtagaaaggttaaattaaaacaaattagataagatattggttccactattatcaactattcaatgtggcaacaacttatt

fava_c16805_388	fava_c16805	388	T/C	caactgaactcgctgatgcttacggctatgatgagatcaacttcaattgcggatgccctagtcctagagtagctggacgtgggtgctttggtgcgcgtct[T/C]atgcttgagccaaagtttgttgccgaggctgtttcagtaatagctgccaatacaaatgtacctgtcagtgtcaaatgtcgaatcggcgtagatgatcatg

fava_c16805_798	fava_c16805	798	C/G	attgaagagaccaatgcagctctaaagattggcgctcacggtgttatggttggacgtgctgcctaccacaacccttggcatattttgggaaacgttgata[C/G]tggaatttatggtgtaccaaatattggtcttacacgtcgtcaggtcttggagaaataccaagtttacggagactcagtgttactacaatatggatttcgg

fava_c16806_1172	fava_c16806	1172	A/G	gctgtccatctataatgcactgcacaccctttcctggaatagcagagaaatctgaggcatcataaagccaccctgattttaattcattggcatcatttcg[A/G]ggaccRttagtaagagaggactcatcaaagaagtgaaaatggcgtgcgtattgtaatattgcttttgccagcgggtgttcactgctagcctcagcagaag

fava_c16806_1178	fava_c16806	1178	A/G	catctataatgcactgcacaccctttcctggaatagcagagaaatctgaggcatcataaagccaccctgattttaattcattggcatcatttcgRggacc[A/G]ttagtaagagaggactcatcaaagaagtgaaaatggcgtgcgtattgtaatattgcttttgccagcgggtgttcactgctagcctcagcagaagccacca

fava_c16806_416	fava_c16806	416	C/A	tttttatttttctttaatttacaactatttcaagtattgtagtaagtctgggttttctatatcttctaagaagcaaagaagaacatacaacactcacaga[C/A]gagagMgccatgcatgcaccagcaacccatggtggaagcttgattcccagtgaaggaaataaaacacccgcagcaactggtacggctataatattgtagg

fava_c16806_422	fava_c16806	422	C/A	tttttctttaatttacaactatttcaagtattgtagtaagtctgggttttctatatcttctaagaagcaaagaagaacatacaacactcacagaMgagag[C/A]gccatgcatgcaccagcaacccatggtggaagcttgattcccagtgaaggaaataaaacacccgcagcaactggtacggctataatattgtaggccatcg

fava_c16806_524	fava_c16806	524	A/G	ccatgcatgcaccagcaacccatggtggaagcttgattcccagtgaaggaaataaaacacccgcagcaactggtacggctataatattgtaggccatcgc[A/G]aatacataattcaatcgaatacgagaaaaggtctttttagaaagatcaattgctgtgatcacgtcttccaaattatctctcatcaaaacatagttagcag

fava_c16806_958	fava_c16806	958	A/C	ggcgtgattcccattttcttgaggccctctataacaacagaagcttctcgtttaagtgagtctgcaacccccaaaactccaatcaatataccgtcacatg[A/C]taccaggatccctgtcttggcactttcttcaagctcaacaacaaaattttccgcttcggtagagatgtttataccactttccaccagcagcttcctgtta

fava_c16809_252	fava_c16809	252	A/G	ggtctttacaagatgaccattatcattcatcactaatataaatagtaatatatcaccctcaccacctatcagtcatatcagaaagaaaagttacatagtt[A/G]ttacctaagtcttatagaaaaagtcagcaagaaaatacactaaaaaagaaagaaatactaacatagaaagtaataatgaagctatttatcaaatcatata

fava_c16809_387	fava_c16809	387	A/G	atacactaaaaaagaaagaaatactaacatagaaagtaataatgaagctatttatcaaatcatatatttactcttctttcaacatatgtgaaggaacaac[A/G]tcttgaagaagaccatggtccctaagaaaggaagtagttgtccctggattacaaagctttttctcttgagtaaaaggattgtaattataagggttactca

fava_c16809_552	fava_c16809	552	T/A	cttgagtaaaaggattgtaattataagggttactcaaagaggacaatgtattaaccagtagttgttgatgaggatgatgttgttgctgctgatactgtaa[T/A]aagtaRttgttctcttgcagaactgaacYgaagttagtggccggaaatccaccggagagtgcaccagtaggtggtggtatctgaactccggcgaaggcgg

fava_c16809_558	fava_c16809	558	G/A	taaaaggattgtaattataagggttactcaaagaggacaatgtattaaccagtagttgttgatgaggatgatgttgttgctgctgatactgtaaWaagta[G/A]ttgttctcttgcagaactgaacYgaagttagtggccggaaatccaccggagagtgcaccagtaggtggtggtatctgaactccggcgaaggcggaacggg

fava_c16809_581	fava_c16809	581	C/T	ttactcaaagaggacaatgtattaaccagtagttgttgatgaggatgatgttgttgctgctgatactgtaaWaagtaRttgttctcttgcagaactgaac[C/T]gaagttagtggccggaaatccaccggagagtgcaccagtaggtggtggtatctgaactccggcgaaggcggaacgggacatagctggacttggatgaatg

fava_c16810_2033	fava_c16810	2033	T/C	attgctgtttgcttcaaagattgaggagaaaatacccatctccccaacaattcctgcctctactcctaatgtagaatctggcttgagcacagcagctgcc[T/C]tcaagacaattgatcatcagtctggaataatgaataagacttttcaaattcctacttcttcccaaccaaatatctttccttcaacccaaatcagtggttc

fava_c16810_2365	fava_c16810	2365	C/T	aaggaggcgccattagtatatcaagtgtgtactcacaagggttgttggatacacttacacacgcattgcagagttcgggagtggatttgtcacaagctag[C/T]atttcagtgcaaattgaacttggcaagcaagcaaaaatcacaccaaacggacaattatcaatgtgtgtttctaaggatgacgaagatccttcaaaaaatc

fava_c16810_606	fava_c16810	606	A/T	aactcgatcctgtaagctcgtgatttggcgattaaggacttcgtgcttgaaagtatagttaactgaacgaatgcgtctccgatcttcgacctattgagga[A/T]ataaagattaacaaagagaattgtggtttctattaaattcggatctgagaagtttatatataccgatcgaagcagcaaattcttcatctctattttgatt

fava_c16810_711	fava_c16810	711	A/G	agattaacaaagagaattgtggtttctattaaattcggatctgagaagtttatatataccgatcgaagcagcaaattcttcatctctattttgatttcag[A/G]gttcaaatataatttagttacacaaagcggtaatagaattgtgttctatgaaatttgaatttagggaagactgaatttcaaaattccgttgaattaagta

fava_c16812_155	fava_c16812	155	G/A	taactgttccagcaacataaaagagtgcaacaaatagctggaacttacagaaacaaatacatatagctatcttttgtagtatcaactaaagatgactaat[G/A]acaagggagttgcaaaagaagttgaatggagaatcaaagttgaagatggtccatggaaaagagcagtagtttcttgcagcRtgtggacatttataactag

fava_c16812_236	fava_c16812	236	A/G	tcaactaaagatgactaatRacaagggagttgcaaaagaagttgaatggagaatcaaagttgaagatggtccatggaaaagagcagtagtttcttgcagc[A/G]tgtggacatttataactagtctttatttcaagctgtgcaaattttttaagaaagcttgggatattggtgtgaatgatccaagaaaatttatccattgttt

fava_c16812_436	fava_c16812	436	C/G	tgaaagtaggaatagcactcacagctgtttcacttttctactatttaaagcctttgtatgatggagttggaagaaatgctatgtgggccgtcatgaccgt[C/G]atcgtcgtcttcgaatacactgccggtgctacaatatataaaagtataaacagaatttgtggaactacYcttgctgggcttctagcctttggtgtacatt

fava_c16812_505	fava_c16812	505	T/C	gaagaaatgctatgtgggccgtcatgaccgtSatcgtcgtcttcgaatacactgccggtgctacaatatataaaagtataaacagaatttgtggaactac[T/C]cttgctgggcttctagcctttggtgtacattgggttgctagtagagcaggagatcaatgggaacctataattgttggagtctcactctttctattagctt

fava_c16812_769	fava_c16812	769	T/A	tattcatcttaactttctgtttagtttcggtatctggttatcgaattgacgagttgtttaacatggcacaacaaagaatttctactattataatcggaac[T/A]tcgttgtgcataatcgttagcacgactgttcggcccgtttgggctggtatagaactctatgttttagtcaccggaaacctcgacaaactcgcaaactcct

fava_c16812_912	fava_c16812	912	C/G	gctggtatagaactctatgttttagtcaccggaaacctcgacaaactcgcaaactccttgcaaggttgtgtggctcagtactttgaagcccaaactgcaa[C/G]tgaagagtctaacaaaaaaatcatgggttataaatgtgtgctaaactcaaaagcaacagaagaatcattggccaatttagctagatgggagcctgcacat

fava_c16815_1047	fava_c16815	1047	G/C	agcaatcactttagatgaggctgttttcaactcggcatcaRccctgtattatgatgcaaaatttatgcattggattgtggaccaagtaccagatcaatct[G/C]tattgaacactgctggatggagatttattattcctcaactgtacaagaaatatccaaatcatcgaatgaacttgaatatatctttgtcttctcctccggc

fava_c16815_1330	fava_c16815	1330	C/T	ccagtagcatgtatctcattgatgattcaagcttcgggtttggtcaaaataaacggaaacaaccttgtaggtaccataagattggatgactttggaatgt[C/T]gttgaaatggagcaacataggcaatctacgRatgttccttattcagccagctatgtggacgattattgaaacggtctttttgccgtatgcgaattcacat

fava_c16815_1361	fava_c16815	1361	A/G	cttcgggtttggtcaaaataaacggaaacaaccttgtaggtaccataagattggatgactttggaatgtYgttgaaatggagcaacataggcaatctacg[A/G]atgttccttattcagccagctatgtggacgattattgaaacggtctttttgccgtatgcgaattcacatctaagtaaagggttacctctgccgattattc

fava_c16815_340	fava_c16815	340	C/T	aaggctatttcctctatagtttcactccgattgccgaatattgataagggtgcaaagattccgtttttgggtaatgtttacttgctgctttctgatgtga[C/T]tatttatcaaattgatgttgcttcatcggttgttaagccgggagagagtgggattttgattgttgcttctggggtYaggtgtaatttgagtatgaRttgg

fava_c16815_416	fava_c16815	416	T/C	tttacttgctgctttctgatgtgaYtatttatcaaattgatgttgcttcatcggttgttaagccgggagagagtgggattttgattgttgcttctggggt[T/C]aggtgtaatttgagtatgaRttggtattatgcttatagtacttggattggtcctgttgagatttctgatcgaggtttagcacatgttcaggtcgaaggaa

fava_c16815_436	fava_c16815	436	A/G	gtgaYtatttatcaaattgatgttgcttcatcggttgttaagccgggagagagtgggattttgattgttgcttctggggtYaggtgtaatttgagtatga[A/G]ttggtattatgcttatagtacttggattggtcctgttgagatttctgatcgaggtttagcacatgttcaggtcgaaggaatggaagttgaactttcgttg

fava_c16815_987	fava_c16815	987	G/A	taacctcttacacaagaactcgaaacttccaattctatgcacaaattcatcgaaaatgctagcaatcactttagatgaggctgttttcaactcggcatca[G/A]ccctgtattatgatgcaaaatttatgcattggattgtggaccaagtaccagatcaatctStattgaacactgctggatggagatttattattcctcaact

fava_c16816_1288	fava_c16816	1288	A/G	ttggggagacagaaatgaagcaagttgactttaggaaatccttgcactttaggttggggctaccttatgatcgtccccttctaagaattgccaatgcctt[A/G]gatttttcaaaattgaaaaatggtggttctgcctctctScagaaacgttcagctttgctcagagatgtacacattgggattccaagcagYggggttaatg

fava_c16816_1327	fava_c16816	1327	G/C	ccttgcactttaggttggggctaccttatgatcgtccccttctaagaattgccaatgccttRgatttttcaaaattgaaaaatggtggttctgcctctct[G/C]cagaaacgttcagctttgctcagagatgtacacattgggattccaagcagYggggttaatgggggaactctttcccttgttcaaggttcctatgaatacc

fava_c16816_1378	fava_c16816	1378	C/T	ccaatgccttRgatttttcaaaattgaaaaatggtggttctgcctctctScagaaacgttcagctttgctcagagatgtacacattgggattccaagcag[C/T]ggggttaatgggggaactctttcccttgttcaaggttcctatgaataccatcattatcttcaggatggtttcaatgattcgggatggggttgtgcttatc

fava_c16816_1825	fava_c16816	1825	C/T	cattgcactttgagacccaaagtacacctattatgattggtggtggtgtacttgcatatacactactgggagtggattacaatgatgcaactggagattg[C/T]gcatttttaatacttgatccacactatacaggtacggatgacctgaagaaagtaatcaatgggggRtggtgtgggtggaaaaaagctgttgatagcaagg

fava_c16816_1891	fava_c16816	1891	G/A	tgggagtggattacaatgatgcaactggagattgYgcatttttaatacttgatccacactatacaggtacggatgacctgaagaaagtaatcaatggggg[G/A]tggtgtgggtggaaaaaagctgttgatagcaagggaaagaatttcttcttgcacgataagttttacaatctactgctgccacagaggcctaacatggttt

fava_c16816_2059	fava_c16816	2059	G/A	atctactgctgccacagaggcctaacatggtttgatagactgggaatggacggaaccaattcccaatttcttgcaatggaagttttttttttagttacat[G/A]tgtgattggatgttttgtaattttggcttgctgtgactaaaactaaaattaccggccatacctagagctggcctagtttttaaggccccaaaaatataag

fava_c16816_206	fava_c16816	206	G/A	actcagcaaaggatccgacccgggtatccattactggttaatcggatccccattcttaccgccacttaccatcgtctccatcctccgatgcatcaacacc[G/A]tcccctccYccacctctcccgatttacacaaagaatcaaacgaacttctaaccttaattccgaaaggctttcaactaatcggagccctagcttccggaga

fava_c16816_215	fava_c16816	215	C/T	aggatccgacccgggtatccattactggttaatcggatccccattcttaccgccacttaccatcgtctccatcctccgatgcatcaacaccRtcccctcc[C/T]ccacctctcccgatttacacaaagaatcaaacgaacttctaaccttaattccgaaaggctttcaactaatcggagccctagcttccggagacgacaccga

fava_c16816_331	fava_c16816	331	C/T	tacacaaagaatcaaacgaacttctaaccttaattccgaaaggctttcaactaatcggagccctagcttccggagacgacaccgatgctcgtgccgcaat[C/T]gatgccgctagtcaattgaggaagtttctttacggcggaggaacagtggaagacctaccgctgatcggaggtgtttccgatttggattccggcgatttcc

fava_c16816_657	fava_c16816	657	A/G	attaagttgccagtttattaccctctcaaaaatcctactgatgttgagaaagcatatgtaaaggcaactggagctgttattgccaagttaagagaccctc[A/G]ggctgtgtacatgttRgaagcattgagcaaaacctgtccagatcaacctccacctgccatagttcgaggtgtacaattagactttcacacagacctctcc

fava_c16816_673	fava_c16816	673	A/G	attaccctctcaaaaatcctactgatgttgagaaagcatatgtaaaggcaactggagctgttattgccaagttaagagaccctcRggctgtgtacatgtt[A/G]gaagcattgagcaaaacctgtccagatcaacctccacctgccatagttcgaggtgtacaattagactttcacacagacctctccagaaccaagcctttgg

fava_c16816_780	fava_c16816	780	G/A	ttgagcaaaacctgtccagatcaacctccacctgccatagttcgaggtgtacaattagactttcacacagacctctccagaaccaagcctttggctaagg[G/A]tgacgagggctttgatgcaagctcactatcctgctcatactttaccatcagcaataaagcagctttttccatagagagtgcagatactatccaagtgagt

fava_c16821_1046	fava_c16821	1046	C/T	gaaaattcttaaccaaactcttatcatcttcttgtttatgaaatgaccaagattctgttgggtataagtcatcaaagttcacaagtaagtctccagcgcc[C/T]ttccggcatctcctagaaggatgatgagcggtcaaaagctggagaaactttaaaagtgaaccgcgagtttctggttttatcatcttgtgctccaagatga

fava_c16821_1187	fava_c16821	1187	A/G	ggagaaactttaaaagtgaaccgcgagtttctggttttatcatcttgtgctccaagatgatgtcaaaagctaatgaagttgcctcttccacatcatagat[A/G]cctcttgcaatctgttgggggtctgatacattagatgaaagatgctcgttttgaaacttctgatcatctagaccaaaYgaactggacatttgtttgttga

fava_c16821_1265	fava_c16821	1265	C/T	ttgcctcttccacatcatagatRcctcttgcaatctgttgggggtctgatacattagatgaaagatgctcgttttgaaacttctgatcatctagaccaaa[C/T]gaactggacatttgtttgttgatccaagtaagcaattgatcagctgtgcgtgcatcatcaatcacctgtatatcactattcttttgtttcggttcccaac

fava_c16821_1466	fava_c16821	1466	C/T	taccacctatgaatttaggaggagcaccccaaaagagcataggataatgaccaacagaaaacttatcacagagcttagtatttatcttttctgcacaatc[C/T]acccttgtcaataatatgattccaggatgcactgcatcaggtccattaaataaccttgcaaccttttcgtactgtggcttataattcctgcaagcaggac

fava_c16821_1574	fava_c16821	1574	T/A	tcaataatatgattccaggatgcactgcatcaggtccattaaataaccttgcaaccttttcgtactgtggcttataattcctgcaagcaggacaccaatg[T/A]gcaaagaattcaacaatggcgaaggtagcaggagtatccttcaaaacatcatcaaaactactcgcatttagctccaccgcataatccctatSaattccac

fava_c16821_1666	fava_c16821	1666	G/C	caccaatgWgcaaagaattcaacaatggcgaaggtagcaggagtatccttcaaaacatcatcaaaactactcgcatttagctccaccgcataatccctat[G/C]aattccacttcgaYcagtatcgctaacctcacgaaggatcgatcgatgacccgaaaaagccgaagtagcattacaacaaacagatagcgtgcatagaaac

fava_c16821_1680	fava_c16821	1680	T/C	gaattcaacaatggcgaaggtagcaggagtatccttcaaaacatcatcaaaactactcgcatttagctccaccgcataatccctatSaattccacttcga[T/C]cagtatcgctaacctcacgaaggatcgatcgatgacccgaaaaagccgaagtagcattacaacaaacagatagcgtgcatagaaacaaacaggagatcaa

fava_c16821_819	fava_c16821	819	C/G	cacacttgaacacatcttgtaaaaatgttgtcggcattcctcacagacaaagaagttgtgaacaaaatcacagattgcagtaaatgtaaactgactctct[C/G]catcctcaatccttacagagagtgaatgtagaagaacccataagccacagctaaagcctctggtttcattcttactaccacgacagaacatccagtatcc

fava_c16822_1766	fava_c16822	1766	T/G	caactctagcaaaatcagaactttatcaaaccttgagaacacttcaggactgcatcaagcagattgccatgattacccctagcgaaaaagagattccatc[T/G]tcaagttaccaaRccctgagagaagagcagaagaagctcaaatctcttgtgcgtMgattaggagaatttacagcagaagctgagttggagccaagctttt

fava_c16822_1779	fava_c16822	1779	A/G	atcagaactttatcaaaccttgagaacacttcaggactgcatcaagcagattgccatgattacccctagcgaaaaagagattccatcKtcaagttaccaa[A/G]ccctgagagaagagcagaagaagctcaaatctcttgtgcgtMgattaggagaatttacagcagaagctgagttggagccaagcttttggttccttccatt

fava_c16822_1821	fava_c16822	1821	A/C	caagcagattgccatgattacccctagcgaaaaagagattccatcKtcaagttaccaaRccctgagagaagagcagaagaagctcaaatctcttgtgcgt[A/C]gattaggagaatttacagcagaagctgagttggagccaagcttttggttccttccatttaataatgcctgctacaacaatatgctgaaatctctatcaag

fava_c16822_1968	fava_c16822	1968	A/G	gttccttccatttaataatgcctgctacaacaatatgctgaaatctctatcaaggatggtagatctcttgctttttgtggcttactcaatggagcatgtc[A/G]cgcaattggcacagaaagatggagtaatttggatggacattcaaggtcgagggaatgagaatgtgaagatttttagggacagagttgacccaatattgaa

fava_c16822_2179	fava_c16822	2179	T/C	gagataacaaggatgaagtctattaagaaaattgaaagtgacttgaagagccaaaatgttcctcacgatctcgagtcacaagaatatctcaatgcagacg[T/C]attaaggatcttgagcagagatgaagaggttgatagcattacaaactctttcctccaacatctagaggaaattgctgacaaaactcataccaacaaagat

fava_c16822_2388	fava_c16822	2388	T/A	acttaaagttcagatgctttttcattacagctgcttgggattctgcaccagtagcttgatgagggaaatagtgaagattgagagtgaaataagcgagctg[T/A]gtatgtgggagaacccttcaagtgaaacaaacttcaaatagatttactttaaatgctctgcattcatagKaaatctatcactgtggttccaaaaatgacc

fava_c16822_2458	fava_c16822	2458	T/G	gtgaagattgagagtgaaataagcgagctgWgtatgtgggagaacccttcaagtgaaacaaacttcaaatagatttactttaaatgctctgcattcatag[T/G]aaatctatcactgtggttccaaaaatgaccacaataagagacttagacttactagttagccaatgacatgtagatagtatcttgtataaaatcatagtat

fava_c16822_350	fava_c16822	350	A/G	ttctatgtgcaaccattcaggttatgattttctctctacttagccttcaagttataagacctggtcacttcagcgactgcatggctgcgctggctgtggc[A/G]gttggcacgtttgtggtggcgctaccggtatcgacgaacttgttgactaaaaggatagcttttggacagcttgtgattatttatgtgagcacagttatac

fava_c16822_509	fava_c16822	509	C/T	cttttggacagcttgtgattatttatgtgagcacagttatacatggtgcggaagaaggagtggctacacattcaattcatgtggcttgttctactgcact[C/T]ggtgctatagcttctgttctggccatgttgctaccttatccaagagttgcttactatgaggctaagaagttctaccaattatacactgagaatacatctg

fava_c16823_207	fava_c16823	207	C/T	tctccgacagggaaaatctcaaaacaaccttaattttcccaacaaacattcttcaaacctcgtttgttattctcttggctgttcagattcggttctatct[C/T]acRcacttgctcaccacttcaatccaaagattcccatagagaaagccgttacacctccatcatcatggtatactgatcactctttcttccaccttgaact

fava_c16823_210	fava_c16823	210	A/G	ccgacagggaaaatctcaaaacaaccttaattttcccaacaaacattcttcaaacctcgtttgttattctcttggctgttcagattcggttctatctYac[A/G]cacttgctcaccacttcaatccaaagattcccatagagaaagccgttacacctccatcatcatggtatactgatcactctttcttccaccttgaactcga

fava_c16823_551	fava_c16823	551	G/T	atgtttgtcgtcatcatgcctctatccttgcttctggaagtggacaaaagtcctgctttgtctgcccttaccacggctggacatatggattaaacggaac[G/T]cttcttaaggcaaataggatatcaggaatgcgagatttcaatgtaaatgattttggtcttataccgatcaaagtagctacctgggggccttttgtcctta

fava_c16830_118	fava_c16830	118	T/A	gcgaagcaaaatcgtggaagtcattcgcgacaccgagaatacgcagtagcatcttcccttatatctgaaaatcgccgccaaacgaaacacaatcccatag[T/A]ttctcgttttcaactccgcaagtaagaaactctatcacacgagtaattcaccaaaaccctaacaatggggaagaaacagcacagcaaagaccgaatgttc

fava_c16830_1427	fava_c16830	1427	T/C	ataacggagttgcttttcatagaagcattaggaattttatgatacaaggtggtgatcctactggtactggtagaggtggtgaatcaatttggggaaaacc[T/C]tttaaggatgaactcaactccaaattagtccactccggaaggggtgtggttagcatggcMaacagtggcccacatactaaYggttcccagtttttcatcc

fava_c16830_1487	fava_c16830	1487	A/C	ctggtactggtagaggtggtgaatcaatttggggaaaaccYtttaaggatgaactcaactccaaattagtccactccggaaggggtgtggttagcatggc[A/C]aacagtggcccacatactaaYggttcccagtttttcatcctgtacaagtctgcaaatcatttaaacttcaaacatacagttttcggaggagtagtcggtg

fava_c16830_1508	fava_c16830	1508	T/C	aatcaatttggggaaaaccYtttaaggatgaactcaactccaaattagtccactccggaaggggtgtggttagcatggcMaacagtggcccacatactaa[T/C]ggttcccagtttttcatcctgtacaagtctgcaaatcatttaaacttcaaacatacagttttcggaggagtagtcggtggcttgactacactagcagcaa

fava_c16830_1649	fava_c16830	1649	C/T	taaacttcaaacatacagttttcggaggagtagtcggtggcttgactacactagcagcaatggaaaaggttccagttgatgacggtgaccgacctctgga[C/T]gaaatcaagataacaggtgtcacaacttttgtcaatccttacaccgaaccagatgaggaagaagagcaagcaaatgccaaagagaagaatgcaaatgatg

fava_c16830_1876	fava_c16830	1876	C/T	tatagtaatcctggagctggaacatctgagtctggagctacgggggttggcagtggtggtgttggtaagtacttgaaggctaagagtgttccaaccgaag[C/T]tgcagctgctgccgctgacacaggtacagccgttgtaggaaagaaaagaaaagcagttgtcagtggtggattcaaagacttYtctgcatggtaattatcc

fava_c16830_1958	fava_c16830	1958	T/C	agagtgttccaaccgaagYtgcagctgctgccgctgacacaggtacagccgttgtaggaaagaaaagaaaagcagttgtcagtggtggattcaaagactt[T/C]tctgcatggtaattatcccatattttaagtacaatttgtaaatcagtgtagacttttcttgtaataatttatcatgttaaaaaatttgacagaatagtgg

fava_c16830_275	fava_c16830	275	C/T	ccctaacaatggggaagaaacagcacagcaaagaccgaatgttcataaccaaaactgagtgggcaacggaatggggaggttttaaatccaaagaaaatcg[C/T]accgctttcaaacgtctccctttttactgctgcgcacttacttttactccgtttgagtttccagtttgtacacctgatggaagcgttttcgatgttatgc

fava_c16830_572	fava_c16830	572	C/T	cagaaggagaatatcactgtccagtgctaaacaaagttttcaccgagtttacacacattgttgcagtcaagactactggaaatgtcttttgctatgaggc[C/T]gttaaggagttaaacatcaaaacaaaaaactggaaggagctactcaccgatgagacattcactagggatgaccttataactattcagaatcctaatgcac

fava_c16830_866	fava_c16830	866	A/T	ataacataaacatgtcgggtgatatcaagcaaatgcttaaggagcttggaactgaggaaggaaaggaaactgcattgcatgggggaggtggtggcaaggc[A/T]caaaaagaaagggccgctgcacttgctgccatcttagctgcaaggtcgcaagttaaagaagattccaaatcgaatcctgatgcagctcctccggcactca

fava_c16832_148	fava_c16832	148	C/A	agaaaaaggcgcgctctctagctggctgctctcgtgtttcagcaattctgcagatttcaaccgaccgaatcaatctgaatatggattcactaagccctgc[C/A]gtctttgtcaacacggaactgttacccttttgtgttggaagaagggttcgcactgtgattcaagttgtccgatctgacggtgtacttgtcattggaaaat

fava_c16832_280	fava_c16832	280	A/G	gtgttggaagaagggttcgcactgtgattcaagttgtccgatctgacggtgtacttgtcattggaaaatcacccgatgagaagcagattgttgtgaaaac[A/G]tcacctgattcacccctaccagctcctcatacaccttttgttgaagtcatcggtattgttagcagtgacaagtccatcaaagctgagttatggaccaact

fava_c16832_573	fava_c16832	573	T/C	caagtttatctcaatatacaaaaacctttctatatgatgcaaatggaggatttaaacacttgtttttgttttcatgctaaataatctgtgccttttgtaa[T/C]tgaaatctataaacacttgttttttggatgtttactacacttgtctgttatcttcaatattttacattacccaaggtttagatatatgagaaatagattt

fava_c16841_148	fava_c16841	148	G/A	ctttctctacatagcacaccaactttcgcaccaaattaatcatagtactaattaatattaaaatgttgatactgaaaaaaagtacatgggctagctagat[G/A]gtacatagctaataactgcctagatacactgatcagcaccaaccatactcaaaatattattcaaacataaacaagattcctttctagctagctcgatcaa

fava_c16841_303	fava_c16841	303	G/A	tattattcaaacataaacaagattcctttctagctagctcgatcaaacaaaatcaaaacaagcaaaaacaaaaaagaaaaatataaagaaagtaacattg[G/A]tagagtaactatctctagctagaaaacataatagtctctatcaattctctcttcttcSacaccaaaccatgatggagaaatgaagttcacgataaatagg

fava_c16841_361	fava_c16841	361	G/C	caagcaaaaacaaaaaagaaaaatataaagaaagtaacattgRtagagtaactatctctagctagaaaacataatagtctctatcaattctctcttcttc[G/C]acaccaaaccatgatggagaaatgaagttcacgataaataggaagaccttccagaactagagtctctcgggccattgcatcggtagcattccattctatt

fava_c16844_1001	fava_c16844	1001	A/T	agaattgtccttggtgttcttcaaaggattgattcggaatatcaStcatattgaagaacggcgatgaagaagacggaaccgagtattgtccaaatgatga[A/T]gttKggtgatttaacggcggtgatgagattaagtgttgctcaaactttggcgccgatgttccgaggcgcaaaatattgttgttagtcgatgatgtttggt

fava_c16844_1005	fava_c16844	1005	T/G	ttgtccttggtgttcttcaaaggattgattcggaatatcaStcatattgaagaacggcgatgaagaagacggaaccgagtattgtccaaatgatgaWgtt[T/G]ggtgatttaacggcggtgatgagattaagtgttgctcaaactttggcgccgatgttccgaggcgcaaaatattgttgttagtcgatgatgtttggttctg

fava_c16844_1817	fava_c16844	1817	A/G	tagcagaagtagttgtggattgaggatgaagatgatgtgttatctgactttgattttcttctctgattccaaagaatgatgtcgatgaagaagccgccat[A/G]ataatactttgcaatagattaagaggaatcaaaaaggtaggaagaagaaaggatctaaggagacaagagagtataatgtgaagacgaggagaagagaaca

fava_c16844_292	fava_c16844	292	C/T	caaataaaataagcactatgagacagtaaaaagcaatatttctttttattttttcctcattgcatcaaatttgagcttccaaaatgttgacttgagtgag[C/T]tgattttaggttatgattcaaagaagccatagcatgctgttgttgttgttgttgttgattttgggagaatccagcatcactcatgtttStgaccattcca

fava_c16844_381	fava_c16844	381	C/G	acttgagtgagYtgattttaggttatgattcaaagaagccatagcatgctgttgttgttgttgttgttgattttgggagaatccagcatcactcatgttt[C/G]tgaccattccaccaacaccaagaaaatctaatgttaacttatcagattcaccaaaccttatattgaggttactctcattaccaaataaggaggatgtgtt

fava_c16844_945	fava_c16844	945	C/G	gtttccttgaagatccggaagttgcattagaccatgtagctgttgtggcttgtttgagaattgtccttggtgttcttcaaaggattgattcggaatatca[C/G]tcatattgaagaacggcgatgaagaagacggaaccgagtattgtccaaatgatgaWgttKggtgatttaacggcggtgatgagattaagtgttgctcaaa

fava_c16846_492	fava_c16846	492	T/G	taccaggtatttcaagctaatacagggaatccagattctccggaagatgaaggttgtaatgaccgaaggcggatgtcatattttacactcctggccgctt[T/G]ggagctaggggatgattctgctccgtctgacacgagtggtaaggaggaagtgaaaccattRtatgcatttccaatatggttgccacattttcgacagaga

fava_c16846_553	fava_c16846	553	G/A	accgaaggcggatgtcatattttacactcctggccgcttKggagctaggggatgattctgctccgtctgacacgagtggtaaggaggaagtgaaaccatt[G/A]tatgcatttccaatatggttgccacattttcgacagagaagcctagttcttcgacggaataagccccatgaatgtttatgaaaatgaggaacacattgta

fava_c16846_718	fava_c16846	718	G/C	cccatgaatgtttatgaaaatgaggaacacattgtacctcatcaacctgggtaaatctgttatcatcaatattgaagaatgatatgatgcctcgctttat[G/C]gacttgccgtattttgatccgatggatgtRgtattccggtttgaggaggataggttcagctcataaccacaagagccacagctgtaacgaacatctctct

fava_c16846_748	fava_c16846	748	A/G	attgtacctcatcaacctgggtaaatctgttatcatcaatattgaagaatgatatgatgcctcgctttatSgacttgccgtattttgatccgatggatgt[A/G]gtattccggtttgaggaggataggttcagctcataaccacaagagccacagctgtaacgaacatctctctgtgaagcggaagcatacgaagagaatgttc

fava_c16851_1443	fava_c16851	1443	A/T	cacaggatcaaggatcaaagataattatattttgttctaccaagaaaatgtgtgaccaacttgctcgaaatctcacacgccagtttggagctgctgctat[A/T]catggggataaatcccaggctgatagggatcatgtgttgaatcagtttcgaagtggaaggactcctgtacttgtagcgacagatgttgctgctcgtggac

fava_c16851_2187	fava_c16851	2187	C/T	ccgtcaaggctgcaccatctcaatataatgcccccgtcagctttcatcaagcaatgatggaacgcaatggaggaggcagtgatcgcaacaagaactataa[C/T]cgggagcgaagccgcagtcatagtccttatagacaggacaagggaaaaagttcaccatctaccaacagtagtcctcagaaagggtggggaggatcttcag

fava_c16851_2421	fava_c16851	2421	C/A	atggagaattggaggaagggatgattcctgatgaagaaggaatgataggtcatgatgaatcttctatatatcaaagtgcaaattagaattttggctattt[C/A]tactttgacaatatgttaaggtaattttggtccactatcagtattttgaaagacattggtacatggacatttgcaagatataggatctggtagtggaatt

fava_c16851_2636	fava_c16851	2636	T/G	attttgagaagctaagtaaaatgctgataggtgaagaagaacctagtaacaagtatgtttatggttgcctgataggtgaagaacctagtaacaaatatgt[T/G]gatggttttatgtttattattttggtaaaggtttagtttatatcttctacacacacagccagactagaaattattttaagccactatgaattgttgcatc

fava_c16851_648	fava_c16851	648	C/T	cattaactccagaggcttatagacgtcgtcatgagatcactgttactggagataatgtgcctccacctgttacatcatttgcgtcttctggctttccatc[C/T]gagattcttagagaggtacaaaatgctgggttctcagccccaactccaattcaggctcaatcgtggcccattgcccttcaaagtaaagatatagttgcca

fava_c16853_330	fava_c16853	330	A/T	acaaacaagataaaaataataattcaataagttgcagggaaaataaatagtaagtctcaataatgctatctagctacgaaaagatctattttggaaaaat[A/T]gcttattctgatatcttggacataatatcctatgtagcactgaccgtgtctgagtcagacacctacactgccatttgtcattacgtttaatttattcttt

fava_c16853_602	fava_c16853	602	T/A	taaaacccttcatggtacgccggaggaaaatgactttctttgctgacggtgagttttgaagaaaaaagtcttctgttccatcgggttgcggtaattctcg[T/A]tgtgtttcaattttgagtaacttcaagttatgtaaataagggaaatgaatcttgaatgaatSagggataaagttgagaatctggagagtcgttgaagaga

fava_c16853_664	fava_c16853	664	G/C	aaaaagtcttctgttccatcgggttgcggtaattctcgWtgtgtttcaattttgagtaacttcaagttatgtaaataagggaaatgaatcttgaatgaat[G/C]agggataaagttgagaatctggagagtcgttgaagagattgtcaatgattcgacaagggcaagctgagcaagccaatcgaatagaattgaagaaaacttt

fava_c16856_280	fava_c16856	280	A/T	agtaaaaaagctaagttcaacaggtcttcaaaaccatataatagcatgtatgaagacaaagagttctcctggattcccaatttggtcattcaatgacatt[A/T]ataaccctcaagaaaattatcaacattgttccaataatcaacatattcactcatttcttatgtagcaattagtgatYcacaggttatacagtttttccat

fava_c16856_357	fava_c16856	357	C/T	caatttggtcattcaatgacattWataaccctcaagaaaattatcaacattgttccaataatcaacatattcactcatttcttatgtagcaattagtgat[C/T]cacaggttatacagtttttccataatcaacctacagagctacctccttatgttatctcttgctgcagtaagttccttaacagaacaaaacaaaaacttct

fava_c16856_929	fava_c16856	929	C/G	atccccggctcgcacattgtcattgtccacaatctcatatgacaaatcaatattaggaaaacgattgcaaaatcgagcaatatccaaaagctgagaatcg[C/G]tcatgttcaataattcccgtctctcgtcatcttccatctctaataaatcaaagacagtttctatactcctccctgggttctcttggcatttcttagccaa

fava_c16858_1186	fava_c16858	1186	T/C	cttggctatacgaagagaattcttgaaatgaaatagttttagcaccagctacttccagtaaagactttagctgatccccaaatttatacaaatgcacaga[T/C]tccaacacaaaagtgtatcctttcaaacagttttcgcgagctttgggatcagcaactctgatagatgctccttccactgacactgttggaatatgtgaat

fava_c16858_1660	fava_c16858	1660	T/C	cagtgccaaaagaaacaaggtcgccatcatgtaatgctgtttctttgtttggtagctcgtgaacttttttcttaagcccgacattcttgctgacaaatgt[T/C]ccgtacttggagcagtctcttaactgtacatttggtagacgatttgacatgttcattgcattaacaactatttctgcatgaacccgagaaacccctttat

fava_c16858_802	fava_c16858	802	A/T	tatcatctaaggttggttccactccctcatcttttctcttgagtgaagtgccggaatcatcatcctcgtcatctaattcttctgtttttacatattttac[A/T]ttatttccaccacataatgcctcacttgcaatgggtgatgtggctgtttcaacttcagtatctgaatctgctacaattgtctcgtctgttgagcatgacg

fava_c16859_1071	fava_c16859	1071	A/G	aaggccgaacaccactaggaaatatgaaggttggtatgttccggcgtttcacatgggagacgtggatatccattcccggtttccatagagtgtacgcgtt[A/G]acagaatgcttgaattcatctacagttagcctaatatcaaattgttcaccttcgtttaccggaactccttgtttacgctgcagacccatgaagtaacagt

fava_c16859_1191	fava_c16859	1191	G/A	ctacagttagcctaatatcaaattgttcaccttcgtttaccggaactccttgtttacgctgcagacccatgaagtaacagtggtgaaaggatctagattt[G/A]tctgaaaattcgccaggatatgggtgacactgaagcatgccataagtgtaccgctcaatcttcagtgtcaactggcggagacgagactcaacccatcctt

fava_c16859_138	fava_c16859	138	T/A	ccggtgcaagatattctcatttacacacaattaaaattgatgcctYaagtttagctcaggattatttccttcaccaaaagcgttacataaataaacaaag[T/A]gctacaaatattttacaatacatcttcaagatgcagcagcttaatctttcaagtttctaagaacttttatccactgctttccctaaagaagtgaagttca

fava_c16859_83	fava_c16859	83	T/C	tttttgaaataagtgatgataatctatttttattttgccggtgcaagatattctcatttacacacaattaaaattgatgcct[T/C]aagtttagctcaggattatttccttcaccaaaagcgttacataaataaacaaagWgctacaaatattttacaatacatcttcaagatgcagcagcttaat

fava_c16859_849	fava_c16859	849	C/T	taggagctacatcttcatgaacttccttgtttggaggggatgaagatacaaaggtctttgaaaccctcgagttgttattatcctccaatcgctttctctt[C/T]ctttcatcatcggctccaagtgcagctgttttacctccttgagacttttcagcttgactttggccactaacccttgatttcgaactttgtctattctcac

fava_c16866_424	fava_c16866	424	G/A	cttcttattgcctccttttggtatctgcctatcagctgccctcttgcccttacaagtcctactgttgtgtccagtttgtccacatttattgcaagtgaca[G/A]ttgaaaagcttcttgaaagcacatgggagttctttggctcatcacctgttttaattctcatcttttttggacgtccaattgctcttctcatgacaggagg

fava_c16867_1064	fava_c16867	1064	A/T	gaatgggcttaggtttaactggtcagggtatggttgatcccataccagttaaggtcctaccaccaaagcagtcattagatcatgcactcaagtctcataa[A/T]gtagaagggaacacagagaagcaaaggaagaaacggaccagaggtggtagRagaaaacgtgagaagaaatttgcagaagcaattcgtgccgcaaaagaaa

fava_c16867_1115	fava_c16867	1115	G/A	aggtcctaccaccaaagcagtcattagatcatgcactcaagtctcataaWgtagaagggaacacagagaagcaaaggaagaaacggaccagaggtggtag[G/A]agaaaacgtgagaagaaatttgcagaagcaattcgtgccgcaaaagaaaaagaagaaccggcaccagatgtctttgcgctaataaatactcagctagcta

fava_c16867_1569	fava_c16867	1569	C/A	tgcctcaaatgatgttgttagcaaagaaaaagagaagagatggcttaagttttagttggtacttaggactcagttctgtttgtagttgcataatgtaatg[C/A]cctcaatgatgatacctaaagaagaagaWaaggaaggaaagctcacaggtctaagtaactctacactacgatacaagtattttgcggtgtactgtagtat

fava_c16867_1598	fava_c16867	1598	A/T	aagagaagagatggcttaagttttagttggtacttaggactcagttctgtttgtagttgcataatgtaatgMcctcaatgatgatacctaaagaagaaga[A/T]aaggaaggaaagctcacaggtctaagtaactctacactacgatacaagtattttgcggtgtactgtagtatgtagcccatgtacatgccctgttctagtg

fava_c16867_1700	fava_c16867	1700	T/A	aggaaggaaagctcacaggtctaagtaactctacactacgatacaagtattttgcggtgtactgtagtatgtagcccatgtacatgccctgttctagtgc[T/A]ggtggaagatgtgtgctattttattttaataaaccatcaatttgatttttaaactatcattttctttaattttgtctctaaattttataaatagaataac

fava_c16867_322	fava_c16867	322	C/T	cgatcaagattattatctgaagtagacgcattgccgcatagtactaatatttttgcggaagaagagaaagtagagccacttgatcctgctgatgttgaat[C/T]agagccactaggagaaaaatgcttttctgttggatctaaatgtagatttcgttacaaagatggacgttggtaccatggtcaagtgctgcagttggataat

fava_c16867_705	fava_c16867	705	A/G	tatatgggcagtctcaaatacaaatgcaggcatttggagagaagctgagcttgaatcatgggatgaaaaagccggagttggacaagttgtttttcgagac[A/G]atggaagttctgtgaagcttggtccacaagatatggtattaactgagtatgcagatgtgagtgacatagatagtgatttcagctcagaacaatccgatta

fava_c16869_1051	fava_c16869	1051	A/G	atcgagcaggagcaatcgcatagcattttatcttgtttctcagtaaccccaatttctcgcgattgtgaagcgctatcattgtcaataacgccaccacccc[A/G]gccccaagcgaatgtccaacaaatgtcagcatataagtcgggtactctgctattagcttcctcaaaacctcccattctgcatcaaagacccactcggctg

fava_c16869_112	fava_c16869	112	A/G	attcttatatatcaaaaatactattcttatttctctttctacaaatacttccttagaagattaacccaaataggcttttcaagttataatattcacatgg[A/G]ggccaaactaaaatgaacatcttaacaaaacatgtactaaaataaaataagtgcttaatgtaaatcaatgaatttttattcttggaaccaaaatagaagc

fava_c16869_1434	fava_c16869	1434	C/T	taatccttccgcaagattatccaatccggattaatcccataacccccttccggagcccactgagggttacgaatatcatcttcgtaaacagctaagatca[C/T]gcgacaaaaacgcggtataggctcgaactcttccgcagtagccagaccccaattttcactctcatagccWgcagtatacagacattttttccaaacccac

fava_c16869_1504	fava_c16869	1504	A/T	gaatatcatcttcgtaaacagctaagatcaYgcgacaaaaacgcggtataggctcgaactcttccgcagtagccagaccccaattttcactctcatagcc[A/T]gcagtatacagacattttttccaaacccacctagtacaacccaaacaataaacacactcaagtataggaagcccacataaaaatgacatcaatgcctatg

fava_c16869_1705	fava_c16869	1705	G/A	cttcaattcaagcatgttgacaaaatgttacaaacaaaaataacagttcacacaaaataaacttgcttcatacataacctatgccactagggttgtcaaa[G/A]ttaaagcWtagaagaaattcagcaaaaactgtaaaaaaaaaaatcgaaactttaaagtgaattaggtaccctggagagttttgaaagagacccaattggt

fava_c16869_1713	fava_c16869	1713	T/A	caagcatgttgacaaaatgttacaaacaaaaataacagttcacacaaaataaacttgcttcatacataacctatgccactagggttgtcaaaRttaaagc[T/A]tagaagaaattcagcaaaaactgtaaaaaaaaaaatcgaaactttaaagtgaattaggtaccctggagagttttgaaagagacccaattggtgaaagtat

fava_c16869_255	fava_c16869	255	G/C	taaaataagtgcttaatgtaaatcaatgaatttttattcttggaaccaaaatagaagcaaagaaggctctaataagcttaatatagagaaatattttaag[G/C]agttgtcttcttgaacaccatttgacctgtgtcatccacttcaaacatgcgcYcgacaaattcattccaaccttccgattgtttctcgtgagacRaggag

fava_c16869_308	fava_c16869	308	C/T	gaagcaaagaaggctctaataagcttaatatagagaaatattttaagSagttgtcttcttgaacaccatttgacctgtgtcatccacttcaaacatgcgc[C/T]cgacaaattcattccaaccttccgattgtttctcgtgagacRaggagcttttgccactagatcttccggaatcttcctcaatgtcaatttcatgaaatgt

fava_c16869_350	fava_c16869	350	G/A	ttaagSagttgtcttcttgaacaccatttgacctgtgtcatccacttcaaacatgcgcYcgacaaattcattccaaccttccgattgtttctcgtgagac[G/A]aggagcttttgccactagatcttccggaatcttcctcaatgtcaatttcatgaaatgttccatatgaggaaggtgagtatgcttgaggaatatccaaagc

fava_c16869_487	fava_c16869	487	G/A	caatgtcaatttcatgaaatgttccatatgaggaaggtgagtatgcttgaggaatatccaaagcaactgctctctgaagggctgcgcgatactcttgact[G/A]tgttcacgagctagagatttttgcctcaccatcttctgctctgccggaatatccatatgccgatctttctctagcattaaatcgagcgcctgtcgggatt

fava_c16871_954	fava_c16871	954	T/G	tttatgttggaggactccctccgacagccaatgagcagtctgtcgctattttcttcagccaggtcatggctaagattggaggaaacactgctggtccagg[T/G]gatgcggtggtcaatgtttacattaaccatgacaaaaaRtttgcctttgtcgagatgaggtctgttgaggaagctagcaatgcaatggctttggatggga

fava_c16871_993	fava_c16871	993	G/A	ctgtcgctattttcttcagccaggtcatggctaagattggaggaaacactgctggtccaggKgatgcggtggtcaatgtttacattaaccatgacaaaaa[G/A]tttgcctttgtcgagatgaggtctgttgaggaagctagcaatgcaatggctttggatgggattattttcgagggggcacctgtcaaagttaggagaccta

fava_c16873_489	fava_c16873	489	G/T	gtagcaagagactgtcccagcaaagcctaacaagccctgcttgttacaattgtcatttaatccaaacaaatttcgcaatacacagaaatacaaccaagaa[G/T]atgcattgttaccatgttgggtcgtctatacaagaagctcgtcttcaagataattatactcaaaagtgaattccgtcttgattctttgacaccatccatg

fava_c16874_296	fava_c16874	296	T/C	tctccaaagacttctgaatcttctaatgaggagagcggcattctcgttagtgcaggcgatcctatctcggaaacagttcaaactctcgaatcatctttgg[T/C]taatgataacaaccaattctcMaaaataaacgatgcggatgatcattctagtagtcctcaaggaacggtgtccgagtacgattcagtttctaataaYgga

fava_c16874_318	fava_c16874	318	A/C	ctaatgaggagagcggcattctcgttagtgcaggcgatcctatctcggaaacagttcaaactctcgaatcatctttggYtaatgataacaaccaattctc[A/C]aaaataaacgatgcggatgatcattctagtagtcctcaaggaacggtgtccgagtacgattcagtttctaataaYggaagctgttcKggtgaatcggaag

fava_c16874_393	fava_c16874	393	C/T	tggYtaatgataacaaccaattctcMaaaataaacgatgcggatgatcattctagtagtcctcaaggaacggtgtccgagtacgattcagtttctaataa[C/T]ggaagctgttcKggtgaatcggaagatactaaagagaaagctgcaaactccactatgcgtttggatattatacctggttgtgataacgacagaagggata

fava_c16874_405	fava_c16874	405	T/G	acaaccaattctcMaaaataaacgatgcggatgatcattctagtagtcctcaaggaacggtgtccgagtacgattcagtttctaataaYggaagctgttc[T/G]ggtgaatcggaagatactaaagagaaagctgcaaactccactatgcgtttggatattatacctggttgtgataacgacagaagggataagatccgactga

fava_c16879_1048	fava_c16879	1048	G/A	agacatcaaatgaagacgtgggatttggtaaaagaaggtatcatcgttgtttggtggtaactgcacgcgcacgcgcttttctttaccatcaggttagact[G/A]cttgttggtgttctcaaagcagtcggcactggaaacttaacagttccagatgttgaaagaattttgaatgcKaagtctattaaagctgcaagtccaatgg

fava_c16879_1120	fava_c16879	1120	T/G	gcgcttttctttaccatcaggttagactRcttgttggtgttctcaaagcagtcggcactggaaacttaacagttccagatgttgaaagaattttgaatgc[T/G]aagtctattaaagctgcaagtccaatggccccggcatctggtctctacttggctgaggtgaagtacgatctacctagttagtagacgttctgtgttatga

fava_c16879_159	fava_c16879	159	T/C	agttagattgtagttgaaaccgattcaatgacacaaaagtgtaagcacacagttgaatttgaagaatgcagtgataacagaccgtcaaagatttcaaaaa[T/C]cgactatcccaatcctatcgacgacgacggcgaagaagaagaagaccaaaagcaagagatgaaccaacaccgtaagattcaacgctacttggtggcgatt

fava_c16879_460	fava_c16879	460	T/C	gagtttctgttatttgttccagtcgaactgatgctggagtgcatgctttgtcgaatgtttgtcatgtagatattgaacgaatcagcaaaaggaagcctgc[T/C]gaagtgttacaacctcatgaacctgctgtggttagaagggctgtgaaccatttcttacagaagcaaaacggtgatttaatggttattgatgttcgatgta

fava_c16879_937	fava_c16879	937	C/T	ctctaatggatagagaacgacaaaataaagttgctgatgatcttcacggctttcacagcaaatctgaattcgccgttccttttagctctagtgcaagaat[C/T]gatgaagtaaagacatcaaatgaagacgtgggatttggtaaaagaaggtatcatcgttgtttggtggtaactgcacgcgcacgcgcttttctttaccatc

fava_c16881_1053	fava_c16881	1053	G/T	tctcagctggaggggttattggttcaactttgatcgctatctgtgtggttgttgttgggacaagaaatgatattggattccatcagactgctccattagt[G/T]aagtggagtggcgttccatttgcttttggtatctatgggttttgctttgcaggacattcagtttttcccaatatatatcagtctatggctaacaaaagag

fava_c16881_111	fava_c16881	111	T/G	cttcttttttcacttatcacctcattgagagagtcacagctgtcaagttttggcaaagaactgtatacacaatcatgtcgaacgagaagaaagagaacga[T/G]ttttttctcgacgctaacgaagaagaaaccgatatcgaagctgttaactacgagagcgaaactagtcatgaaaatgatggtggcagttacgatgaactcg

fava_c16881_1656	fava_c16881	1656	G/A	gaattgtggggaaaaaggcaaccaatacacaggttgctctcagtattgtaattactgcatgggggatagtttgcgctggttttggatcatattcatctct[G/A]ttaaagattttgcagcagctctgacagcttttcaatttcagcaagtggacagttacttatctttgcagccattggtatatgcatgccccattgagttata

fava_c16881_690	fava_c16881	690	T/C	agcaagctgggtgggccagtttgttggtgatggttatttttgcagttgtgtgctgttatacagcagaactaatgagacattgctttgaaagcagagaagg[T/C]atccttagctacccagatataggagaggccgcatttggaaaatatggccgagttattatatcaatcgttttgtacaccgaattatattcatattgtgtgg

fava_c16885_146	fava_c16885	146	T/C	tctaataaagtagtaggtgaaacttcacacaatataaaaattttcatctttcacttatttttctttccctatagcatcaatcattaacatttttatgttg[T/C]ttttctcacttSttagctatacattatacaaattctagagagatgactacaaaaaagataacatggaaatccatgatgttgatcttaaattgttacaaaa

fava_c16885_158	fava_c16885	158	G/C	gtaggtgaaacttcacacaatataaaaattttcatctttcacttatttttctttccctatagcatcaatcattaacatttttatgttgYttttctcactt[G/C]ttagctatacattatacaaattctagagagatgactacaaaaaagataacatggaaatccatgatgttgatcttaaattgttacaaaagtaaataccctt

fava_c16885_608	fava_c16885	608	A/G	gtttaatggctcaaactgttgctgttaaatgtttggatttggatggtttgcaaggtcatagagagtggctggcagagattatatttcttggacaactaag[A/G]YatcaccatcttgtgaagttRattggatattgttgcgaagatgaacaccggcttttggtgtatgaatacatgccaagaggtagcttggagagtcaactat

fava_c16885_609	fava_c16885	609	C/T	tttaatggctcaaactgttgctgttaaatgtttggatttggatggtttgcaaggtcatagagagtggctggcagagattatatttcttggacaactaagR[C/T]atcaccatcttgtgaagttRattggatattgttgcgaagatgaacaccggcttttggtgtatgaatacatgccaagaggtagcttggagagtcaactatt

fava_c16885_629	fava_c16885	629	A/G	ctgttaaatgtttggatttggatggtttgcaaggtcatagagagtggctggcagagattatatttcttggacaactaagRYatcaccatcttgtgaagtt[A/G]attggatattgttgcgaagatgaacaccggcttttggtgtatgaatacatgccaagaggtagcttggagagtcaactattcagaagatattctgctgcta

fava_c16885_840	fava_c16885	840	T/C	aacaaggatgaaaattgcattaggtgctgctaagggtttggctttccttcatgaagcagataagcctgtaatatatagagatttcaaagcttcaaatatc[T/C]tactagactctgattatacagccaaattatcggattttggcctagctaaagatggccctgaaggggaagaaacacatgtcacgacgcgcgtaatgggaac

fava_c16886_134	fava_c16886	134	G/C	cctatttggtgtgtagctgacatgaaacaactccgtctcttgaacttctatcaatctctccagttttgtagtattcagaagcgtttcagttccaagagtt[G/C]tMtaMctgtgaaaccaatggggtctaaaagactgaaaacacccttttcaagttcacaacaacccatttctaaccatgttattgttaaggaaaatgatgtt

fava_c16886_136	fava_c16886	136	C/A	tatttggtgtgtagctgacatgaaacaactccgtctcttgaacttctatcaatctctccagttttgtagtattcagaagcgtttcagttccaagagttSt[C/A]taMctgtgaaaccaatggggtctaaaagactgaaaacacccttttcaagttcacaacaacccatttctaaccatgttattgttaaggaaaatgatgttga

fava_c16886_139	fava_c16886	139	A/C	ttggtgtgtagctgacatgaaacaactccgtctcttgaacttctatcaatctctccagttttgtagtattcagaagcgtttcagttccaagagttStMta[A/C]ctgtgaaaccaatggggtctaaaagactgaaaacacccttttcaagttcacaacaacccatttctaaccatgttattgttaaggaaaatgatgttgatag

fava_c16886_1637	fava_c16886	1637	A/G	tcagaatccatagcggacaaagttcacgattcctacattctccctgatgtttttggcagtgatcattgtccaatcggcctcgttatgaagctttgaatct[A/G]caattgaagcactgatcactctgagcttatagagaatgttgatttttgaggaagcctatttacctaaaaaaaggRKtttttttgctttttgtcttgtttg

fava_c16886_1712	fava_c16886	1712	G/A	ggcctcgttatgaagctttgaatctRcaattgaagcactgatcactctgagcttatagagaatgttgatttttgaggaagcctatttacctaaaaaaagg[G/A]Ktttttttgctttttgtcttgtttgcttcattttggaaattttgatatccttgtacaacatactatggtcattttccttaagagatcatacaaaagagat

fava_c16886_1713	fava_c16886	1713	G/T	gcctcgttatgaagctttgaatctRcaattgaagcactgatcactctgagcttatagagaatgttgatttttgaggaagcctatttacctaaaaaaaggR[G/T]tttttttgctttttgtcttgtttgcttcattttggaaattttgatatccttgtacaacatactatggtcattttccttaagagatcatacaaaagagatt

fava_c16886_316	fava_c16886	316	A/G	tgttaaggaaaatgatgttgatagctattccattgaggttgaaatgttaagaaatgatccgtctattgtagacaccatgactgttccccaactccgcaac[A/G]cRttaaagagtataggKgttccSgccaaaggtcgtaaagaggatcttttgtctgccttgaagactttcatggtgccttttttgtgttctgcagaacaaga

fava_c16886_318	fava_c16886	318	A/G	ttaaggaaaatgatgttgatagctattccattgaggttgaaatgttaagaaatgatccgtctattgtagacaccatgactgttccccaactccgcaacRc[A/G]ttaaagagtataggKgttccSgccaaaggtcgtaaagaggatcttttgtctgccttgaagactttcatggtgccttttttgtgttctgcagaacaagatt

fava_c16886_333	fava_c16886	333	G/T	ttgatagctattccattgaggttgaaatgttaagaaatgatccgtctattgtagacaccatgactgttccccaactccgcaacRcRttaaagagtatagg[G/T]gttccSgccaaaggtcgtaaagaggatcttttgtctgccttgaagactttcatggtgccttttttgtgttctgcagaacaagattctcaaattaaagacg

fava_c16886_339	fava_c16886	339	C/G	gctattccattgaggttgaaatgttaagaaatgatccgtctattgtagacaccatgactgttccccaactccgcaacRcRttaaagagtataggKgttcc[C/G]gccaaaggtcgtaaagaggatcttttgtctgccttgaagactttcatggtgccttttttgtgttctgcagaacaagattctcaaattaaagacgaacaag

fava_c16886_464	fava_c16886	464	C/A	ttgtctgccttgaagactttcatggtgccttttttgtgttctgcagaacaagattctcaaattaaagacgaacaagggctgtttatttcttctgaaaata[C/A]gtcttcRgaaatgaaagccaaaaaggtRtcgggtgaagagcccgtcgatgatgttaatgatactctagagacagttgaacttaaccagggtaagaaaagg

fava_c16886_471	fava_c16886	471	A/G	ccttgaagactttcatggtgccttttttgtgttctgcagaacaagattctcaaattaaagacgaacaagggctgtttatttcttctgaaaataMgtcttc[A/G]gaaatgaaagccaaaaaggtRtcgggtgaagagcccgtcgatgatgttaatgatactctagagacagttgaacttaaccagggtaagaaaaggttWaaac

fava_c16886_492	fava_c16886	492	G/A	cttttttgtgttctgcagaacaagattctcaaattaaagacgaacaagggctgtttatttcttctgaaaataMgtcttcRgaaatgaaagccaaaaaggt[G/A]tcgggtgaagagcccgtcgatgatgttaatgatactctagagacagttgaacttaaccagggtaagaaaaggttWaaacaatcagaacctgRaagaaaaa

fava_c16886_567	fava_c16886	567	T/A	cttcRgaaatgaaagccaaaaaggtRtcgggtgaagagcccgtcgatgatgttaatgatactctagagacagttgaacttaaccagggtaagaaaaggtt[T/A]aaacaatcagaacctgRaagaaaaattgtcaaagcgacaaccaaaaagaagctgttggttaaatcagacRaggtttcagatgttaagccttctagggcga

fava_c16886_584	fava_c16886	584	A/G	aaaaaggtRtcgggtgaagagcccgtcgatgatgttaatgatactctagagacagttgaacttaaccagggtaagaaaaggttWaaacaatcagaacctg[A/G]aagaaaaattgtcaaagcgacaaccaaaaagaagctgttggttaaatcagacRaggtttcagatgttaagccttctagggcgaagagaaaagtgtcttca

fava_c16886_637	fava_c16886	637	G/A	agttgaacttaaccagggtaagaaaaggttWaaacaatcagaacctgRaagaaaaattgtcaaagcgacaaccaaaaagaagctgttggttaaatcagac[G/A]aggtttcagatgttaagccttctagggcgaagagaaaagtgtcttcagatgttgttagcattgtagcacaatcagatgaaatcagtacaacaaccactat

fava_c16887_208	fava_c16887	208	T/C	gctgcaaacgcccttctcacatgctaaccaactattaaaatcataatagctccccatatccatttcattgtaccaaacctctacctattttccgccaatt[T/C]tccctggttatccttgaatctgactcaaattgaatccttagagatgttgagacgtgaaatttcatgaagatgctgatgattgtacatgtctcactcggtg

fava_c16888_1560	fava_c16888	1560	T/C	gcatcataggggtactttttcagaaactgtttagcagcatatttccaattctcagcactgccaatgagaccattaaccataacgatcaaatgagtagggt[T/C]agaactgtgaccagaagcatcaacaacttggatatcaaaaccatcacctgcatcatcgtaatcgattctaaaacaaYtaaatctaggaaaatacttgttc

fava_c16888_1637	fava_c16888	1637	T/C	cataacgatcaaatgagtagggtYagaactgtgaccagaagcatcaacaacttggatatcaaaaccatcacctgcatcatcgtaatcgattctaaaacaa[T/C]taaatctaggaaaatacttgttcttcttcctcttcttcttcgYgttggtgcttttggttttgttattgagcttctccgccttgttgtttgtatcttcatc

fava_c16888_1680	fava_c16888	1680	T/C	tcaacaacttggatatcaaaaccatcacctgcatcatcgtaatcgattctaaaacaaYtaaatctaggaaaatacttgttcttcttcctcttcttcttcg[T/C]gttggtgcttttggttttgttattgagcttctccgccttgttgtttgtatcttcatcgacgctgttttgaacttggggaacttgttcttctgattcaacg

fava_c16888_1794	fava_c16888	1794	A/G	gttttgttattgagcttctccgccttgttgtttgtatcttcatcgacgctgttttgaacttggggaacttgttcttctgattcaacggaagccataaggt[A/G]Ygagattgagaagaaacagtgaaatttgatttcggttgggggtgtgaaatggaatagcagcagagtatgggggcgtaggaagtaattattatacggtggc

fava_c16888_1795	fava_c16888	1795	T/C	ttttgttattgagcttctccgccttgttgtttgtatcttcatcgacgctgttttgaacttggggaacttgttcttctgattcaacggaagccataaggtR[T/C]gagattgagaagaaacagtgaaatttgatttcggttgggggtgtgaaatggaatagcagcagagtatgggggcgtaggaagtaattattatacggtggcg

fava_c16888_523	fava_c16888	523	G/A	catcactactcagtggcaaaataagttggtgggaatgttcacaaactttctcccggctcgtctcccccaagaaaaaaaatagagtggcattactctatca[G/A]atagaagggttctataatcgaaaatcatcaatcatgtgttggaccacatcagctccatcggaattgatgctgtaagtctgaacctgaattgcattatgtg

fava_c16888_664	fava_c16888	664	T/A	ggaccacatcagctccatcggaattgatgctgtaagtctgaacctgaattgcattatgtgcgagatatttctgcctgctaccactgaagctgacatcaac[T/A]cggtcccaactcacagttgttaagccttggatcatttcctcttccaagtcaattttacaggatccacaattaactttggatttacttggaacttcctcta

fava_c16888_777	fava_c16888	777	G/A	acagttgttaagccttggatcatttcctcttccaagtcaattttacaggatccacaattaactttggatttacttggaacttcctctaaaacagaagtgg[G/A]ttttgctgtctcttcgtttacaatatgtggatacttctcgtgtcttttaagatgtcgacgctttggaagctcgtttctacgccttatagacgatgtgctc

fava_c16888_892	fava_c16888	892	A/G	cgtttacaatatgtggatacttctcgtgtcttttaagatgtcgacgctttggaagctcgtttctacgccttatagacgatgtgctccatccaaccagctg[A/G]tcataacgaatatttgcataggcaacgcggcgtttgaaggaccgcaaagcagacatgaatttaatatcttctgagtcagacaccatctggagaagaagag

fava_c16892_1006	fava_c16892	1006	A/G	gactctctattttaagactcaggtcttcaaagtcaactccaaagcgctttagtatgtgaaaagcagcggacttgaaggtatcaaccgatgggtctttaga[A/G]tcatttgtaactcctYtgaggctaatggtgagaggctccttggcgaacaaacacaacacaacgagcggttcaagaaaataaccaatggaccgYgaaacgc

fava_c16892_1022	fava_c16892	1022	T/C	actcaggtcttcaaagtcaactccaaagcgctttagtatgtgaaaagcagcggacttgaaggtatcaaccgatgggtctttagaRtcatttgtaactcct[T/C]tgaggctaatggtgagaggctccttggcgaacaaacacaacacaacgagcggttcaagaaaataaccaatggaccgYgaaacgccacaatcRtgaggaYg

fava_c16892_1099	fava_c16892	1099	C/T	ctttagaRtcatttgtaactcctYtgaggctaatggtgagaggctccttggcgaacaaacacaacacaacgagcggttcaagaaaataaccaatggaccg[C/T]gaaacgccacaatcRtgaggaYgattgattctgccacccataatagttcccggttcatacttcaatttRgtaccggtttggttgattacgacacggcagt

fava_c16892_1114	fava_c16892	1114	G/A	taactcctYtgaggctaatggtgagaggctccttggcgaacaaacacaacacaacgagcggttcaagaaaataaccaatggaccgYgaaacgccacaatc[G/A]tgaggaYgattgattctgccacccataatagttcccggttcatacttcaatttRgtaccggtttggttgattacgacacggcagtcatcgcagacagttt

fava_c16892_1121	fava_c16892	1121	C/T	tYtgaggctaatggtgagaggctccttggcgaacaaacacaacacaacgagcggttcaagaaaataaccaatggaccgYgaaacgccacaatcRtgagga[C/T]gattgattctgccacccataatagttcccggttcatacttcaatttRgtaccggtttggttgattacgacacggcagtcatcgcagacagtttcgaataa

fava_c16892_1168	fava_c16892	1168	A/G	cgagcggttcaagaaaataaccaatggaccgYgaaacgccacaatcRtgaggaYgattgattctgccacccataatagttcccggttcatacttcaattt[A/G]gtaccggtttggttgattacgacacggcagtcatcgcagacagtttcgaataatcgcaggagagaaatctcgtggcttcgaagaccaggccatgtctcat

fava_c16892_1312	fava_c16892	1312	G/C	tttcgaataatcgcaggagagaaatctcgtggcttcgaagaccaggccatgtctcattggtgcgaatgtcttcgatgataatgggagtggaagagagtgt[G/C]gagagaagcagcctttgtctgaagcYatggcttcccttgagcctcttgtagtttttctccgttttcaccatttgggaggaaggaagtggctgagcagcac

fava_c16892_1338	fava_c16892	1338	T/C	tcgtggcttcgaagaccaggccatgtctcattggtgcgaatgtcttcgatgataatgggagtggaagagagtgtSgagagaagcagcctttgtctgaagc[T/C]atggcttcccttgagcctcttgtagtttttctccgttttcaccatttgggaggaaggaagtggctgagcagcacggggcggagttgatggaagagccgcc

fava_c16892_225	fava_c16892	225	A/G	aaatccatagaacagttaaacaaagaaaaatattgaaataatgcttagaattatacataatataactaacaaaattgacaaatgaaaactcgattcacta[A/G]aMttcgctgtcttcatgagatctttcgagaaaggttcttcatgccacagccaatacatttgaggataacWgaatgtgtacccggatctggtttgataata

fava_c16892_227	fava_c16892	227	C/A	atccatagaacagttaaacaaagaaaaatattgaaataatgcttagaattatacataatataactaacaaaattgacaaatgaaaactcgattcactaRa[C/A]ttcgctgtcttcatgagatctttcgagaaaggttcttcatgccacagccaatacatttgaggataacWgaatgtgtacccggatctggtttgataataaa

fava_c16892_295	fava_c16892	295	A/T	aaaattgacaaatgaaaactcgattcactaRaMttcgctgtcttcatgagatctttcgagaaaggttcttcatgccacagccaatacatttgaggataac[A/T]gaatgtgtacccggatctggtttgataataaacttcacatcaagaagatccattatRtttctaagagttttaaccccgtattgggtaagctttccaacac

fava_c16892_352	fava_c16892	352	G/A	gagaaaggttcttcatgccacagccaatacatttgaggataacWgaatgtgtacccggatctggtttgataataaacttcacatcaagaagatccattat[G/A]tttctaagagttttaaccccgtattgggtaagctttccaacacgtaccttggaaatatcttgaggacatagagcacaaagaagaaatagcagaccctgat

fava_c16896_180	fava_c16896	180	A/T	ttcagtgttacagtaatgcctcgaaagcctagaaggcatcaacaactcaaatcttggccgattacattctccccttttgctcgtgctcttgctcattcga[A/T]gccgaagcgtcgttccaaacccMtcgagaaccctaccccttcctctgatagtaatggatctaccaagcatcctttagaagagaggttggaaggatctgag

fava_c16896_203	fava_c16896	203	A/C	aaagcctagaaggcatcaacaactcaaatcttggccgattacattctccccttttgctcgtgctcttgctcattcgaWgccgaagcgtcgttccaaaccc[A/C]tcgagaaccctaccccttcctctgatagtaatggatctaccaagcatcctttagaagagaggttggaaggatctgagtcatctgatggggagtttgatgg

fava_c16898_1285	fava_c16898	1285	C/T	gtgaagaaaattgcatttgatgttgcccttgcatctgccccattccttgcattgcagattgcctgaactgttgctgctgctgagcaagaagttgctgttg[C/T]tgctgttgaggtaactgctgcatcagtttttgccgttgctgttgttgttgttgagctgttggctgttgctgctgcagttgtgaggtttgctgctgaggag

fava_c16898_1497	fava_c16898	1497	C/T	atcatagttgtagaagacctaggtgaattggcatatggaattggtgaagctgttgcattgtcaaaagaagtggcactcgttgcagcagaaggagatgcag[C/T]tgatcttcccataagttgtggtcctgttgtctgtaacatcgaattctggcctcccatattatttgatgacattggggtattcttcatatatacattattg

fava_c16898_621	fava_c16898	621	T/C	atcctatattttttcagttgagctctattttacaaaatcgtaacagtttggcaatccaaaagaacatagaaaacaacaaagcagtttgcttctgtttttg[T/C]gagtcgtgtttttagagcaacRtggtKtgataattcattctaaaccgaatccaaccatgcccagtcctatcactgttgattttgttgtctctgttgagag

fava_c16898_643	fava_c16898	643	A/G	ctctattttacaaaatcgtaacagtttggcaatccaaaagaacatagaaaacaacaaagcagtttgcttctgtttttgYgagtcgtgtttttagagcaac[A/G]tggtKtgataattcattctaaaccgaatccaaccatgcccagtcctatcactgttgattttgttgtctctgttgagagaaattgggatgattctgttgtg

fava_c16898_648	fava_c16898	648	T/G	ttttacaaaatcgtaacagtttggcaatccaaaagaacatagaaaacaacaaagcagtttgcttctgtttttgYgagtcgtgtttttagagcaacRtggt[T/G]tgataattcattctaaaccgaatccaaccatgcccagtcctatcactgttgattttgttgtctctgttgagagaaattgggatgattctgttgtgcgttc

fava_c16898_763	fava_c16898	763	A/G	aaaccgaatccaaccatgcccagtcctatcactgttgattttgttgtctctgttgagagaaattgggatgattctgttgtgcgttctgaagtgtcatcat[A/G]ccagattgtagattctgagcatttgatgccatgccaccatatgcaccttgtgaattgtgttgttgttgctgctgttgctgttgttgtaaggacatcatac

fava_c16898_991	fava_c16898	991	G/A	acatttgatcagtcaacatctgggatggatgacttcgctgaggattgtttccagataatccatactgcatccgtggcagatgagactgtgaagatatacc[G/A]gctgaaatgttgggaatcatctgggtatttggtgttgtttgagcagcactaaataatgcattgttagcatgaccagaaaactggcttaaacgagtcatct

fava_c16903_122	fava_c16903	122	T/A	catgactaagcttaatcattctttaattccatatcaatgaagttcaaagaaattactaatattaaatataaaacaacaacgagataagcatattacaaca[T/A]catgatcatagctaggaaacaRgcaaagaaataaacattaattaattaagaatattcactggttaattaaaaatgatcagcataatcttgttcatgatct

fava_c16903_144	fava_c16903	144	A/G	ttaattccatatcaatgaagttcaaagaaattactaatattaaatataaaacaacaacgagataagcatattacaacaWcatgatcatagctaggaaaca[A/G]gcaaagaaataaacattaattaattaagaatattcactggttaattaaaaatgatcagcataatcttgttcatgatcttKtagatttgaagagacctcaa

fava_c16903_224	fava_c16903	224	G/T	atgatcatagctaggaaacaRgcaaagaaataaacattaattaattaagaatattcactggttaattaaaaatgatcagcataatcttgttcatgatctt[G/T]tagatttgaagagacctcaatgttattgttacttgaacttgaacatgaagatgaagtagaagaacatgtcttgttgtttaccatcataaaatcaaacatt

fava_c16908_1029	fava_c16908	1029	T/C	gaaacccagttcagcaagcctctgtgaacaccaagagaagtggcgactctcatcatcagcaacatgagcaaaatccgcaaagaacccctccccgagaaga[T/C]cactatagggagaaaatcgaacaacagtatcccaagccaaatcaattgcattgagctccacatgtgctagattatgaagcaaataagcattcagaggcaa

fava_c16908_266	fava_c16908	266	T/C	atggtgtaatatatctacgcaaagtaaacaataattttgccattccctttgggtcaagcttctcagcaatagttgatgcatggacaaatggaatttggaa[T/C]ttRcatcagcgctagcagtcaaagttgaaaaYtttgcaWatgaaagaattgcatcaccaaactctttggcttgttggctgttgggaagacaggttcctat

fava_c16908_269	fava_c16908	269	A/G	gtgtaatatatctacgcaaagtaaacaataattttgccattccctttgggtcaagcttctcagcaatagttgatgcatggacaaatggaatttggaaYtt[A/G]catcagcgctagcagtcaaagttgaaaaYtttgcaWatgaaagaattgcatcaccaaactctttggcttgttggctgttgggaagacaggttcctatatc

fava_c16908_298	fava_c16908	298	T/C	aattttgccattccctttgggtcaagcttctcagcaatagttgatgcatggacaaatggaatttggaaYttRcatcagcgctagcagtcaaagttgaaaa[T/C]tttgcaWatgaaagaattgcatcaccaaactctttggcttgttggctgttgggaagacaggttcctatatcagccaataactttccagttgaagacaaaa

fava_c16908_305	fava_c16908	305	A/T	ccattccctttgggtcaagcttctcagcaatagttgatgcatggacaaatggaatttggaaYttRcatcagcgctagcagtcaaagttgaaaaYtttgca[A/T]atgaaagaattgcatcaccaaactctttggcttgttggctgttgggaagacaggttcctatatcagccaataactttccagttgaagacaaaatgtgctc

fava_c16908_500	fava_c16908	500	G/A	gtgctcattatctctgccatgatcattcaggtggccctgtcaaacttaaattctctctttccatagcaatgatggaagctaccctctcataaacttcaga[G/A]aggtgttttttatcgtcctctttgtctttcttttctggattgcttgttgatRRagcatcataccaatcacggggaatgccagcttcctctcgagccgcat

fava_c16908_552	fava_c16908	552	G/A	ctctctttccatagcaatgatggaagctaccctctcataaacttcagaRaggtgttttttatcgtcctctttgtctttcttttctggattgcttgttgat[G/A]Ragcatcataccaatcacggggaatgccagcttcctctcgagccgcataattgaaaggaccttttagttccacgttgtattccttgagcaaatctttaaa

fava_c16908_553	fava_c16908	553	G/A	tctctttccatagcaatgatggaagctaccctctcataaacttcagaRaggtgttttttatcgtcctctttgtctttcttttctggattgcttgttgatR[G/A]agcatcataccaatcacggggaatgccagcttcctctcgagccgcataattgaaaggaccttttagttccacgttgtattccttgagcaaatctttaaaa

fava_c16908_669	fava_c16908	669	C/T	acggggaatgccagcttcctctcgagccgcataattgaaaggaccttttagttccacgttgtattccttgagcaaatctttaaaagtggagtctggggtg[C/T]ggcccattttttgRcagacagaaagaaaccagcaaagccccactgctacatgtgcaacttcctcttcaRcaattctagccacaattttagatgtcctatt

fava_c16908_683	fava_c16908	683	A/G	cttcctctcgagccgcataattgaaaggaccttttagttccacgttgtattccttgagcaaatctttaaaagtggagtctggggtgYggcccattttttg[A/G]cagacagaaagaaaccagcaaagccccactgctacatgtgcaacttcctcttcaRcaattctagccacaattttagatgtcctattatctccaaagccaa

fava_c16908_738	fava_c16908	738	G/A	gagcaaatctttaaaagtggagtctggggtgYggcccattttttgRcagacagaaagaaaccagcaaagccccactgctacatgtgcaacttcctcttca[G/A]caattctagccacaattttagatgtcctattatctccaaagccaactaatttttgcaccaaacgtggtccagcatcaagtcctctagcctcctggactag

fava_c16908_867	fava_c16908	867	C/T	attatctccaaagccaactaatttttgcaccaaacgtggtccagcatcaagtcctctagcctcctggactagtgggatcactgccaaacgtgcagcaaca[C/T]tgtYcgatgatttctcacattccctccatagcaaattgtgagctggcatatctccatatttgaaacccagttcagcaagcctctgtgaacaccaagagaa

fava_c16908_871	fava_c16908	871	C/T	tctccaaagccaactaatttttgcaccaaacgtggtccagcatcaagtcctctagcctcctggactagtgggatcactgccaaacgtgcagcaacaYtgt[C/T]cgatgatttctcacattccctccatagcaaattgtgagctggcatatctccatatttgaaacccagttcagcaagcctctgtgaacaccaagagaagtgg

fava_c16909_1301	fava_c16909	1301	A/G	agcaggtttttgttcgaaaggtgattgagctgcatgacaagtacctggcatatgtgaacgactgtttccaaaatcatactctattccacaaggctcttaa[A/G]gaggcttttgagattttttgcaacaagggtgttgctggaagctcaagtgccgaactgcttgccactttttgtgataatattctcaagaaaggaggaagtg

fava_c16909_1856	fava_c16909	1856	T/C	ttgacctcaatctccctgcagagatggttaagtgtgtagaagttttcaaggaattttatcaaacaaaaactaagcacagaaagctaacttggatttattc[T/C]ctgggtacctgtaatgtaagtgggaaatttgaaccaaaaactatggagctggttgtgacgacataccaggcttcggcactgcttcttttcaattcctcag

fava_c16909_2000	fava_c16909	2000	C/T	tggagctggttgtgacgacataccaggcttcggcactgcttcttttcaattcctcagataggctgagctattctgagataatgactcagttgaacttgac[C/T]gacgatgatgttattagactgcttcactccttatcatgtgccaagtacaagattcttaacaaggagccaagcacgaaatcaatattacctactgatcatt

fava_c16909_2428	fava_c16909	2428	A/G	attaagaagcggattgaagacttgatctctagagactacctagaaagagacaaagataatgcaaatttgttcaagtacttggcatgatgtgatcttgatg[A/G]cttcacaactgaagtttttaatgcagtcgctgcaaatagaaaaacaatacttgaaattggagaaaatcagttcctcataRtttccttaagggaggaatag

fava_c16909_2508	fava_c16909	2508	A/G	ggcatgatgtgatcttgatgRcttcacaactgaagtttttaatgcagtcgctgcaaatagaaaaacaatacttgaaattggagaaaatcagttcctcata[A/G]tttccttaagggaggaatagtgcttgtaaatttttcctccatatctaggcatcaactggttactgtaaatgtgtgctcatgttggaaatattttaatcag

fava_c16910_691	fava_c16910	691	A/G	ctgtgagaacagcattgagtaagagaacgccctgcacagcccatttttcaagattaccctgagatggaattgaacagccaagatcttgcttaagctcctt[A/G]aatatatttaccaaactagatggaagtttgattccctcgggcaccgagaatgaaagccccattgcttgaccaggtccatgataaggatcctggccaagta

fava_c16910_823	fava_c16910	823	A/G	ttccctcgggcaccgagaatgaaagccccattgcttgaccaggtccatgataaggatcctggccaagtatcacagccttaacagaatgaaaaggagtagt[A/G]ttaagagcattgaaaatcaaatgctgaggaggatacacagaatcatctctagtagaacaaatctccgtttcaacaaactccgagagattaaccacataag

fava_c16917_1184	fava_c16917	1184	C/T	gcgtgacccttggtaaacaaacacacccaccagaaaggatgcaacccttggaaccaaacaacatgaaatctaacagtcacaacccttcagattaattgtg[C/T]caattaagaacaaatgttaaatgcagtataaatagaagtacttgaattgtatagagagaggttctcgaaaacctgtgttgtaatattccaaaattatcta

fava_c16917_752	fava_c16917	752	G/A	ccgaatcacgttgtatgctgttactggattctcttgaaatggccaatccaaggcggcttgaaccagagataagaagatttgtacaagacatttataaagc[G/A]ggggaSaggccggagactaagcatctgatatctcaaatcccattattagtgcccaaggtgccacaacaaaaaRacggtactgaatgcgggaactttgtcc

fava_c16917_758	fava_c16917	758	C/G	cacgttgtatgctgttactggattctcttgaaatggccaatccaaggcggcttgaaccagagataagaagatttgtacaagacatttataaagcRgggga[C/G]aggccggagactaagcatctgatatctcaaatcccattattagtgcccaaggtgccacaacaaaaaRacggtactgaatgcgggaactttgtcctttatt

fava_c16917_825	fava_c16917	825	G/A	aagatttgtacaagacatttataaagcRggggaSaggccggagactaagcatctgatatctcaaatcccattattagtgcccaaggtgccacaacaaaaa[G/A]acggtactgaatgcgggaactttgtcctttatttcattaaattgtttttggaRctcgctcctgataaatttagcatgSaggggtatccttactttatgaa

fava_c16917_878	fava_c16917	878	A/G	tgatatctcaaatcccattattagtgcccaaggtgccacaacaaaaaRacggtactgaatgcgggaactttgtcctttatttcattaaattgtttttgga[A/G]ctcgctcctgataaatttagcatgSaggggtatccttactttatgaaaaaagattggtttacatttgaagaattggatcggttttgtgagaacttgaccc

fava_c16917_903	fava_c16917	903	G/C	gcccaaggtgccacaacaaaaaRacggtactgaatgcgggaactttgtcctttatttcattaaattgtttttggaRctcgctcctgataaatttagcatg[G/C]aggggtatccttactttatgaaaaaagattggtttacatttgaagaattggatcggttttgtgagaacttgacccgttgaactgtatgtccttgataagc

fava_c16919_644	fava_c16919	644	T/A	gaacatagtagaattgaggttgtggccattaaggttgctcccattattgtgttccttagtgattgaacagctaggatgtttttcttttcattgtccttca[T/A]catagcattaacccaatttcttctaccactagcattgatgccaacaatggttgtatgtggttgagttctaaccttatgccaaagccataaatgataacca

fava_c16921_2097	fava_c16921	2097	C/T	gcgacggtcctacacttaccgtccaggttcttactacagggtcttggccaactcagtctagtgttacatgcaacctgccagccgaaatatctgcactttg[C/T]gagaagttccggtcatattaccttggcacgcataccggcaggagattgtcgtggcaaactaacatgggctttgcagatttaaaagcaacctttggaaagg

fava_c16921_2334	fava_c16921	2334	A/G	tgtgtgtcctcatgcttttcaacaatgctgataaactcagctacaaggagatcgagcaagcaaccgagattcctgcttccgatcttaagagatgcctgca[A/G]tcattggctttagttaagggaagaaatgtccttagaaaggagcccatgagtaaagatgttgttgaggacgacactttctttgtYaacgacaagttcagta

fava_c16921_2418	fava_c16921	2418	T/C	ttaagagatgcctgcaRtcattggctttagttaagggaagaaatgtccttagaaaggagcccatgagtaaagatgttgttgaggacgacactttctttgt[T/C]aacgacaagttcagtagtaaactatacaaggttaaaataggaacggtagttgcacaaaaggaatccgaaccggagaaacaggaaactcggcagcgagtag

fava_c16921_2745	fava_c16921	2745	G/A	caacggaggtcaagaaacggattgagtctctcattgaacgagactttttggagagggatgataacgacagaaaaatgtatcgatatcttgcttagaattg[G/A]taaaaacttgtttgtttcagtaatgaatcacagtgatgcaggtgttgtatcataatgaatgaaccaggtgaaaatttgccttttaagtagctccattgat

fava_c16921_760	fava_c16921	760	G/A	taatcataacgctagtggtctcagttttgaagaactttacagaaatgcatacaatatggtgcttcacaagtttggtgagaggctatactcaggattggtt[G/A]caaccatgactgcgcatctcaaagatatagctcaatctattgaagctgctcaaggaggttcttttctagaagaactgaacaggaaatggaatgatcataa

fava_c16921_987	fava_c16921	987	C/T	tactgatgtacatggacaggacttacattccaagcgcccgcaagactcctgttcatgaactcgggctgaatctttggagagagaatgtcatttattcaag[C/T]cagattaggactcggctactgaacacacttctggaattagtacatggtgaacgcactggtgaagttattgatagaggaataatgagaaatataacaaaaa

fava_c16926_141	fava_c16926	141	A/G	cacatgtgatttcttaacttatgtcctaatgaacttttacaaggtaaattttactgaaaacaattctaatgaacttttacacaacaagatgcatagcaga[A/G]Wataagttacatacaaatagacacataatagacatatacaaagtgtttagcagcaaactacataatcaactcttcaacattttcatcatcttccatttct

fava_c16926_142	fava_c16926	142	T/A	acatgtgatttcttaacttatgtcctaatgaacttttacaaggtaaattttactgaaaacaattctaatgaacttttacacaacaagatgcatagcagaR[T/A]ataagttacatacaaatagacacataatagacatatacaaagtgtttagcagcaaactacataatcaactcttcaacattttcatcatcttccatttctt

fava_c16926_1501	fava_c16926	1501	T/A	ttgtggcaaccatagcatccgttatatagttcaccgaaagacataattttagaattcccggaagtattgatggttgaataggattaggtggttgatgaga[T/A]cccgaatttgagctgaacagttcaatcactgttactgaagaaatgtatccaatctcaagagaaataagtttttcagacgcagaagcccaaactcgagcta

fava_c16926_337	fava_c16926	337	T/C	tttcttcatcatcttcattaacatcagcttcaagaagccactgttcaaattcatccacctcatcataatcatgtgtatataaaccatcagacatatcaca[T/C]gggtcaggcccaagctcctcaccgtcgcatgctacatacaagaaaggtctacttctagctaaagcatctaccatgtctctattaacacttccagtcactc

fava_c16926_832	fava_c16926	832	A/C	caaataaaattgttatgcatttatcggtgagtctcttacaccctcgaagagacaaagcatatagtgaattaataacagttggtcgtaagtatgaaagtcc[A/C]gcatcagttatatcagagccatctattagtaaaattttcagtttcagaagtacaccaatggctcggagagcttcatccccaaggctcctgcaatctctca

fava_c16926_952	fava_c16926	952	C/T	catctattagtaaaattttcagtttcagaagtacaccaatggctcggagagcttcatccccaaggctcctgcaatctctcaagtcgagaactttaagttc[C/T]ctgtttgatgtcaaactaagaactgcatgattggttaagagcttgcaccatcttaagctcacatgtgttaaggtaagggatgttgcagagatatcatgaa

fava_c16927_218	fava_c16927	218	C/T	attggaacaactgctattacggccgcgagcatagggacaacagttcctggtgtgccaactccagttgtgtcaccaagagaaatctcaaagcagcccatgt[C/T]gtataattctttcgcaacatatgcgactttcgagggaggtgttggtccgtccaccgggcattcaacgacacatgatatataccctcgcacaggaatagag

fava_c16927_852	fava_c16927	852	G/A	tgtttttgaacataggattatactggtattcagagaatgaatcatcaatctttccaaatttcttcgatttcctcgctttggtgctagccttttggtttaa[G/A]gattcatctcgggagaagtctcttgtttgtcgtttccatgggaatgtctccgctgtatactYatcatcatcatcattgcaactgttagatgtgctgcaat

fava_c16927_914	fava_c16927	914	T/C	ttcgatttcctcgctttggtgctagccttttggtttaaRgattcatctcgggagaagtctcttgtttgtcgtttccatgggaatgtctccgctgtatact[T/C]atcatcatcatcattgcaactgttagatgtgctgcaattccttccctcaagccagaagtctcccatgctgccaatctcatctactctcggacgacaagta

fava_c16929_1460	fava_c16929	1460	G/C	aaggctgaaaaaaatgaaatgcatcatattaacaacgagagcatctcaattcccattttgtgacgctatcaactacaacttttgttttaatcaattagca[G/C]atgatYgtggaagtgatttttagctagctactcttgtgtaaactgccatcatctccaccagttgYtggctgaaagcaagcagatttattatctatctgct

fava_c16929_1466	fava_c16929	1466	C/T	gaaaaaaatgaaatgcatcatattaacaacgagagcatctcaattcccattttgtgacgctatcaactacaacttttgttttaatcaattagcaSatgat[C/T]gtggaagtgatttttagctagctactcttgtgtaaactgccatcatctccaccagttgYtggctgaaagcaagcagatttattatctatctgctacgagt

fava_c16929_1525	fava_c16929	1525	C/T	ctatcaactacaacttttgttttaatcaattagcaSatgatYgtggaagtgatttttagctagctactcttgtgtaaactgccatcatctccaccagttg[C/T]tggctgaaagcaagcagatttattatctatctgctacgagtattgtatagaaggggggaaggtgattagtgacgatgttcattcatatggtaaaactatg

fava_c16929_323	fava_c16929	323	C/G	tcaccgattcatcatcatcacaaaattcacaacaaaatcaaaccctctaaatccttatttttcttcaatttcccccaattgtactccctctccctcgccg[C/G]actccgccgtctccgccaccgtctccgattYgttctcctcctttcgtttccctttttctactttcttctctcccgtcctactcactctttcttcttcaac

fava_c16929_354	fava_c16929	354	T/C	acaaaatcaaaccctctaaatccttatttttcttcaatttcccccaattgtactccctctccctcgccgSactccgccgtctccgccaccgtctccgatt[T/C]gttctcctcctttcgtttccctttttctactttcttctctcccgtcctactcactctttcttcttcaacttcctctccgccttcgctttctccgccgcgc

fava_c16929_468	fava_c16929	468	C/A	cgtttccctttttctactttcttctctcccgtcctactcactctttcttcttcaacttcctctccgccttcgctttctccgccgcgcttttctttgttct[C/A]tccctcaaattcaaatcctctcagtcgcttcctatagttctgactgtcaagcgctggaatcggtggcggcactggcgtctgtggcggcggaggtggaggt

fava_c16929_707	fava_c16929	707	A/G	acgctggttgtgtttgaggttagggtttattcaaacggtgacgtttatgaaggtgaaatgaagggtggaaagtgttctgggagtggggtttattactata[A/G]tatgagtgggagatatgaaggggattgggYggatgggaaatatgatggctttggggttgagacttggtctaaggggagtaggtataagggtcagtataga

fava_c16929_737	fava_c16929	737	T/C	tcaaacggtgacgtttatgaaggtgaaatgaagggtggaaagtgttctgggagtggggtttattactataRtatgagtgggagatatgaaggggattggg[T/C]ggatgggaaatatgatggctttggggttgagacttggtctaaggggagtaggtataagggtcagtatagagatggattcagacatgggtttggggtttat

fava_c16929_876	fava_c16929	876	G/C	ctaaggggagtaggtataagggtcagtatagagatggattcagacatgggtttggggtttatagattttatactggggatgtttatgctggggaatggtt[G/C]aatggacacagtcacggttctggtgttcatacttgtgatgatggtagtagatttgttggacagttcaagtggggtgttaagcatggtcttggccactatc

fava_c16930_1195	fava_c16930	1195	T/A	ccgactcccttgagttccacatatttacaagggtttctaatatcaagtctcctcctggaagttgatcacataaattaaacatgagaagcgaattattctc[T/A]atagggcctaaagaactaatatcttggattgcttgtaaacgcaaaattcctaaaacagctttccaaattgcttcgtctcgtgcgacccctttaagttcgt

fava_c16934_595	fava_c16934	595	T/C	tctcttccttctccttaaccttcttcacctgaacctctaccacattcccatcacctctactcgtcgacacttcttcaaccctcctattcgacccaactct[T/C]aaactattctcatccttcacctcagaaacaatacccgattctacttctcggctaattacacaacccattttacctcaaatccaagcttcatttcctcgac

fava_c16939_1077	fava_c16939	1077	A/G	tgcctctgatttccccctgccggatggcaatcacctttgatatagccattagaaaggcaccttcatcgagtagctgtctcgtgaggctcaaatccctctc[A/G]ggtaaatcagctaagaggccaacagcagctgcttgctcttcagtgattccgatattttctgatatgactttaactagactactaagctgtcctacagatc

fava_c16939_1212	fava_c16939	1212	G/T	gctcttcagtgattccgatattttctgatatgactttaactagactactaagctgtcctacagatccacgaagcgcatcggctaattcagggcccatgtg[G/T]ggagacaaattctgaagaagttttatggaagctaaacggagatctttctgtggtgcttcgatgaactgaactaaactgatagtggcaccagagcttttga

fava_c16939_2130	fava_c16939	2130	G/A	aggagacagcctcctctctttctttagaaagctcgtgagaaaggaactttactaccgtgcgcacagtgtccccttcagccagcagttccttattctcgtc[G/A]tctccctcgaccacgattctaagtgtttcaagagctctacaacgaactttacggctgctactcttcagcatatctacaatcatcggtatgagccctgcac

fava_c16939_2436	fava_c16939	2436	A/G	gagccatatccagctgtacagcttcatttctcgcagtccactcttcaatggtgttccgcaaagccaaactaggattcaactctgcgctttttaattctcg[A/G]agagtcaacgggcaaaccagtttccgtccactctccttgcattccttgaaccatttttcgattgcttcacgctcaaaagtctgtccgttttctgaagtaa

fava_c16939_2604	fava_c16939	2604	C/T	cacgctcaaaagtctgtccgttttctgaagtaacaggatcacgcataacttgcttcgtcaaaggacatacaaaagcatcatatataggctcaatgtgcaa[C/T]ctctcgaaaagtaagctatcatccgattggctaccagggtcattatttccatcccagcttccagacatcatatcttcagttatctaactgtgtgaacaaa

fava_c16939_702	fava_c16939	702	C/T	ctactaacttaaggacggcctgcccttcgtaaagacaaaatgtttcttttaacgaacacctcccacggtggatcctacataatccagaagtatctggtgg[C/T]ttactgaaacatgaaaagaatgaagcacagaRtgttggttctggaatctcgggcaRtttggttaaattctttgattctaatgataaattctccaaagcta

fava_c16939_734	fava_c16939	734	G/A	agacaaaatgtttcttttaacgaacacctcccacggtggatcctacataatccagaagtatctggtggYttactgaaacatgaaaagaatgaagcacaga[G/A]tgttggttctggaatctcgggcaRtttggttaaattctttgattctaatgataaattctccaaagctatagcagaaaccatctgcacggtatcaagtcca

fava_c16939_758	fava_c16939	758	G/A	cacctcccacggtggatcctacataatccagaagtatctggtggYttactgaaacatgaaaagaatgaagcacagaRtgttggttctggaatctcgggca[G/A]tttggttaaattctttgattctaatgataaattctccaaagctatagcagaaaccatctgcacggtatcaagtccattagtttgaagcagttctatgaag

fava_c16941_423	fava_c16941	423	G/A	tttctcctaaaattgcagcaatggttgaagcaatggatcatgagataaaggaatctgctgaaggcaattctgccaaaagcactaaattaaatgctagttt[G/A]cctgaagagcggaatgataaacctgaagtacagaagacgagtgatgacgaaaaagaattaagctcgcctactgcaataacaattccagtggataaagagc

fava_c16941_526	fava_c16941	526	A/T	tgaagagcggaatgataaacctgaagtacagaagacgagtgatgacgaaaaagaattaagctcgcctactgcaataacaattccagtggataaagagcat[A/T]caagcccccctgctccacagcagtctgatcaagctactgaaaagcatgtcactcatacacagactgttgatactgaagctgattcaaatggtcagaattt

fava_c16944_1217	fava_c16944	1217	G/T	acattataaatgttggggctattacagcactatgttcatcattgctcggtggaacgttgcctcagccgagacttttgatggctatgggaagggatggact[G/T]cttccaccatttttttctgacataagtaagcacagttatgttcctgtaaagagtaccgtagttactggccttgttgctgcagccttggcatttttcatgg

fava_c16944_1361	fava_c16944	1361	T/A	ctgtaaagagtaccgtagttactggccttgttgctgcagccttggcatttttcatggatgtctctcaacttgctgggatggttagtgtaggtactcttct[T/A]gcattcaccatatcagcaatatctatgttgatagtaagatatatcccaccatttgaRgttccattgcccccttctctacaagaaccaattgatgcagagt

fava_c16944_1418	fava_c16944	1418	G/A	atgtctctcaacttgctgggatggttagtgtaggtactcttctWgcattcaccatatcagcaatatctatgttgatagtaagatatatcccaccatttga[G/A]gttccattgcccccttctctacaagaaccaattgatgcagagtcgaatgtctggagccatttggaaacattMaaaaaagatgcaaaaaataaatgcttag

fava_c16944_1490	fava_c16944	1490	C/A	tgatagtaagatatatcccaccatttgaRgttccattgcccccttctctacaagaaccaattgatgcagagtcgaatgtctggagccatttggaaacatt[C/A]aaaaaagatgcaaaaaataaatgcttagttgttcaagaggatatatccgttgattatccccttattcccaaaagtcttgcMatagacacatttgtacata

fava_c16944_1571	fava_c16944	1571	A/C	ggagccatttggaaacattMaaaaaagatgcaaaaaataaatgcttagttgttcaagaggatatatccgttgattatccccttattcccaaaagtcttgc[A/C]atagacacatttgtacatattggcaataggaaacaaattgttggttgtgtaatagcattcatgtgcttaggtgtgtttatacttacatttgcagctgctt

fava_c16944_2017	fava_c16944	2017	G/A	ggagttattattcatgttttctatggtagaaaccatagctctctcaaacttgatgatgccaattatactccttcaagctctcttgcttagttatatacaa[G/A]cctaagaaatatttctactacattagtggttacatgtaatcattgtacagaaRaatgtaaaatattttRtRgttcataggtcttagYcatagtctctttt

fava_c16944_2070	fava_c16944	2070	G/A	tgatgccaattatactccttcaagctctcttgcttagttatatacaaRcctaagaaatatttctactacattagtggttacatgtaatcattgtacagaa[G/A]aatgtaaaatattttRtRgttcataggtcttagYcatagtctcttttttttctttctcttatagatgaaagaattgataaagtgtgtattttgtaagaaa

fava_c16944_2086	fava_c16944	2086	A/G	ccttcaagctctcttgcttagttatatacaaRcctaagaaatatttctactacattagtggttacatgtaatcattgtacagaaRaatgtaaaatatttt[A/G]tRgttcataggtcttagYcatagtctcttttttttctttctcttatagatgaaagaattgataaagtgtgtattttgtaagaaaattatatgtaatctgt

fava_c16944_2088	fava_c16944	2088	G/A	ttcaagctctcttgcttagttatatacaaRcctaagaaatatttctactacattagtggttacatgtaatcattgtacagaaRaatgtaaaatattttRt[G/A]gttcataggtcttagYcatagtctcttttttttctttctcttatagatgaaagaattgataaagtgtgtattttgtaagaaaattatatgtaatctgtct

fava_c16944_2104	fava_c16944	2104	T/C	tagttatatacaaRcctaagaaatatttctactacattagtggttacatgtaatcattgtacagaaRaatgtaaaatattttRtRgttcataggtcttag[T/C]catagtctcttttttttctttctcttatagatgaaagaattgataaagtgtgtattttgtaagaaaattatatgtaatctgtctcattaaattagtccag

fava_c16944_603	fava_c16944	603	G/T	tgcttggttgattggctggtccttattacttgaatacggaattggtggagctgctgttgctcgcggcataacccctaatttggctgcacttattggcggt[G/T]ttgacaacttgccagcatttttatcctatcaacatattcctgggaYtgatattgttgtagacccacttgcagcaactRtggtattcatcgttacttggat

fava_c16944_649	fava_c16944	649	C/T	ggagctgctgttgctcgcggcataacccctaatttggctgcacttattggcggtKttgacaacttgccagcatttttatcctatcaacatattcctggga[C/T]tgatattgttgtagacccacttgcagcaactRtggtattcatcgttacttggatcctgtgtactggcatcaaagagagtacaatggtacaaagtatagtc

fava_c16944_681	fava_c16944	681	G/A	tttggctgcacttattggcggtKttgacaacttgccagcatttttatcctatcaacatattcctgggaYtgatattgttgtagacccacttgcagcaact[G/A]tggtattcatcgttacttggatcctgtgtactggcatcaaagagagtacaatggtacaaagtatagtcacatcagtaaatatatgtgccttgatgtttgt

fava_c16944_868	fava_c16944	868	G/T	gccttgatgtttgtgatagtagttggcggttatttaggattcaaatctggatgggttggatatgaactctcaatagggtattttccattcgggatagatg[G/T]aatgattgctggttctgcaacagcWttttttgcatacataggttttgatgcagttgccagtactgctgaagaggtgaaaaatcctcaaagagatttacca

fava_c16944_893	fava_c16944	893	A/T	gcggttatttaggattcaaatctggatgggttggatatgaactctcaatagggtattttccattcgggatagatgKaatgattgctggttctgcaacagc[A/T]ttttttgcatacataggttttgatgcagttgccagtactgctgaagaggtgaaaaatcctcaaagagatttaccactaggaattgcYgcttcWttgtctg

fava_c16944_980	fava_c16944	980	T/C	gttctgcaacagcWttttttgcatacataggttttgatgcagttgccagtactgctgaagaggtgaaaaatcctcaaagagatttaccactaggaattgc[T/C]gcttcWttgtctgtgtgttgtggattgtatatgatggtatccattgttgttgttggtttagtaccttactatgctatcaatcctaacacccctatatcct

fava_c16944_986	fava_c16944	986	A/T	caacagcWttttttgcatacataggttttgatgcagttgccagtactgctgaagaggtgaaaaatcctcaaagagatttaccactaggaattgcYgcttc[A/T]ttgtctgtgtgttgtggattgtatatgatggtatccattgttgttgttggtttagtaccttactatgctatcaatcctaacacccctatatcctctgcat

fava_c16946_1143	fava_c16946	1143	A/T	cttgccaatctcagcattttacatattgcatcccagcaccactgaatgccccgcatcgacagggtctccagggctgtaagctttccaaaatcaatcatgt[A/T]aactctccctgcactattggaaattgaaagctttttcagatgcttggtttcagggaccctaatccagctacagccttgtacctcaagagattcaatttta

fava_c16946_1297	fava_c16946	1297	G/A	ggaccctaatccagctacagccttgtacctcaagagattcaattttaggggaaatgagcgaaagagaacagtttccaagaccataaaaatccagcctaca[G/A]tccaataaaaatggcaggtcaattgtaattgattggacaccttcacacccaagcagtaacaacttggtcagatttgggcatgaccgaatcactgcattca

fava_c16946_1474	fava_c16946	1474	C/T	ggcatgaccgaatcactgcattcactgcagcatcctccaatcttgccccaacaacttcaaggattttaagattatggaactcatcccatttagggatacg[C/T]tcaatcaagacaccccacagtttcagaatttccaaattcattgctgcaccaatgcagtccaactttgacgggctctcatgaacaatcgcattgtcgccaa

fava_c16946_1594	fava_c16946	1594	G/A	gtttcagaatttccaaattcattgctgcaccaatgcagtccaactttgacgggctctcatgaacaatcgcattgtcgccaaggttatccattcgaagctc[G/A]agataagaaagagatggacctgcaagtgctagccatgaagcaaggccagaaggggagaaagggcagaaaacaattagctcYtctaatcgctcaacagctg

fava_c16946_1675	fava_c16946	1675	T/C	ggttatccattcgaagctcRagataagaaagagatggacctgcaagtgctagccatgaagcaaggccagaaggggagaaagggcagaaaacaattagctc[T/C]tctaatcgctcaacagctgataccattcttctcacaatgatgtcagaatcttcatcatgaggagggttatcaaacgcattgcgagtaaaacagagagatc

fava_c16946_1789	fava_c16946	1789	C/T	cagctgataccattcttctcacaatgatgtcagaatcttcatcatgaggagggttatcaaacgcattgcgagtaaaacagagagatctgatataagccaa[C/T]gagttcttccatcgcttagacacacaattgcaatacgccacatcacgtccattactgatatgcgacaatatgtattgaagaatcacatcaggcagagact

fava_c16946_369	fava_c16946	369	G/A	acatacttaccaaacctttgactattctagtcctcctgaataagtttgattatctatcaagaatcagtacaacaacaacaaccaaatattaatttctgcc[G/A]taatgttctattcaagactatgcttctatccaaataatttccacaacattgttttactgtgctattgacagtcatgagagaggaggtaaactctttattc

fava_c16946_522	fava_c16946	522	C/T	tttactgtgctattgacagtcatgagagaggaggtaaactctttattcttaaaaattaaatgaaaagagaatgtcaaattttaaatggcatgcaaaacaa[C/T]aaattaactccacagaagtaaagtcataatcaattcagaagtccagctgaagctgaaaatcagcatgaaacgccctgcattaaaaaccgcatcaacagat

fava_c16946_733	fava_c16946	733	C/T	tttattctattcgaattatctcacgatgcatgtatcgaaaccggcaaatctcatcaaaaaaatcatcagcactgttatgtgaacccttcatctgaagaat[C/T]ttgataaccatggtccttaaatttttSccatacttcaacaaggattcaagagtactcattttctgctcagcatttaatggtgatctaacagtgattaaga

fava_c16946_760	fava_c16946	760	C/G	gcatgtatcgaaaccggcaaatctcatcaaaaaaatcatcagcactgttatgtgaacccttcatctgaagaatYttgataaccatggtccttaaattttt[C/G]ccatacttcaacaaggattcaagagtactcattttctgctcagcatttaatggtgatctaacagtgattaagacctcctccaaacatggaatcacaaatt

fava_c16946_919	fava_c16946	919	A/G	taacagtgattaagacctcctccaaacatggaatcacaaattcagaatcaacatgttttagactgttcttctggcatagtgctgcaaacatggctccatg[A/G]atatcaaacttgagcagcttgggatggctgttgaaaaagtcaacaaaatcaatctctggaaagggttgaagagcatcatagtccccagtgaattccacct

fava_c16947_1175	fava_c16947	1175	C/T	cagatgatgatgctctagctttagctaatgccttagctgcatcgttggaatctaattttgcaagtttccatgcgcttattcgcactggacgctggttcaa[C/T]ttttttcctttgtcaggtggttgtattgcatctgggtccatagtagatggtaggcgccctggctccaattgatgtatcatttcatcctgggcatccataa

fava_c16947_118	fava_c16947	118	T/C	accataaatttagcatatgaaaacaccctatgatcaaatacaatactcaaatgcaatgatagtggtcaatgaattagtacagagaaaagaagattgtgat[T/C]cattagcctgtacagatgaaatggcgaacatactagaaccacatgtggcaacaagaagacaaactaaaaaccgataacatgtttacggatttttttttcc

fava_c16947_2219	fava_c16947	2219	A/G	cagagagtgccagtacagaataaacaccaaatgcgacatattcatagatatcctttccaagaaaaggagcaaagaaggcataatatgcaatgcataataa[A/G]aaaaaaacYgttatagccacaacctgaaaagtgtgaaaagggagttcccaaccatgacgtctacccatgttgatgactctgaaaatgataacagactaag

fava_c16947_2228	fava_c16947	2228	C/T	ccagtacagaataaacaccaaatgcgacatattcatagatatcctttccaagaaaaggagcaaagaaggcataatatgcaatgcataataaRaaaaaaac[C/T]gttatagccacaacctgaaaagtgtgaaaagggagttcccaaccatgacgtctacccatgttgatgactctgaaaatgataacagactaagaaatcagaa

fava_c16950_1483	fava_c16950	1483	G/A	gaaataaaattttgattagcttctactttggttagggcaggttgagtgttagattgagatttttgctggctacaactaattatataccggtcttcttgat[G/A]aaggccgtggaagggtttgatagcactgtcattgttgtcactatgtcattttgcaatagcaatcatcaagcttggacaatcttcctttggttttgttttt

fava_c16950_1639	fava_c16950	1639	C/A	atagcaatcatcaagcttggacaatcttcctttggttttgtttttgacagaaaaaaacagttatttaatgggttttgggtatgggaatcattaattagcg[C/A]tgtctttatatatgtttaacattataaggaagtatcagttttggcaattgctagttttcttttctagttttgaacacagatcatggtgattttgtaatga

fava_c16954_278	fava_c16954	278	T/C	ttataatataacagcctaaacttcttcatgtataaactggcttcaacacaaacatacccaagaagccgaggcctaaatggattatctaatacaaaattta[T/C]ttcactttttatttattaactgaaagggagattacattatgtattttttYccttctaatttcKgtgtacWatactgtaaacaaaaactaaaagctgaacc

fava_c16954_328	fava_c16954	328	C/T	aacatacccaagaagccgaggcctaaatggattatctaatacaaaatttaYttcactttttatttattaactgaaagggagattacattatgtatttttt[C/T]ccttctaatttcKgtgtacWatactgtaaacaaaaactaaaagctgaaccaYaaWaaatgaagttgtgaaRgggaaggatgccgcatacttcatgRcatt

fava_c16954_341	fava_c16954	341	T/G	agccgaggcctaaatggattatctaatacaaaatttaYttcactttttatttattaactgaaagggagattacattatgtattttttYccttctaatttc[T/G]gtgtacWatactgtaaacaaaaactaaaagctgaaccaYaaWaaatgaagttgtgaaRgggaaggatgccgcatacttcatgRcattcgtttggcaagta

fava_c16954_348	fava_c16954	348	T/A	gcctaaatggattatctaatacaaaatttaYttcactttttatttattaactgaaagggagattacattatgtattttttYccttctaatttcKgtgtac[T/A]atactgtaaacaaaaactaaaagctgaaccaYaaWaaatgaagttgtgaaRgggaaggatgccgcatacttcatgRcattcgtttggcaagtaattagag

fava_c16954_380	fava_c16954	380	C/T	tcactttttatttattaactgaaagggagattacattatgtattttttYccttctaatttcKgtgtacWatactgtaaacaaaaactaaaagctgaacca[C/T]aaWaaatgaagttgtgaaRgggaaggatgccgcatacttcatgRcattcgtttggcaagtaattagagtagagaaRtcaattcttgtgttgcatcctccc

fava_c16954_383	fava_c16954	383	T/A	ctttttatttattaactgaaagggagattacattatgtattttttYccttctaatttcKgtgtacWatactgtaaacaaaaactaaaagctgaaccaYaa[T/A]aaatgaagttgtgaaRgggaaggatgccgcatacttcatgRcattcgtttggcaagtaattagagtagagaaRtcaattcttgtgttgcatcctcccact

fava_c16954_399	fava_c16954	399	G/A	tgaaagggagattacattatgtattttttYccttctaatttcKgtgtacWatactgtaaacaaaaactaaaagctgaaccaYaaWaaatgaagttgtgaa[G/A]gggaaggatgccgcatacttcatgRcattcgtttggcaagtaattagagtagagaaRtcaattcttgtgttgcatcctcccactctcagaaccggcatag

fava_c16954_424	fava_c16954	424	G/A	ttttYccttctaatttcKgtgtacWatactgtaaacaaaaactaaaagctgaaccaYaaWaaatgaagttgtgaaRgggaaggatgccgcatacttcatg[G/A]cattcgtttggcaagtaattagagtagagaaRtcaattcttgtgttgcatcctcccactctcagaaccggcatagggatcaatgacaacatttatgactg

fava_c16954_456	fava_c16954	456	A/G	aaacaaaaactaaaagctgaaccaYaaWaaatgaagttgtgaaRgggaaggatgccgcatacttcatgRcattcgtttggcaagtaattagagtagagaa[A/G]tcaattcttgtgttgcatcctcccactctcagaaccggcatagggatcaatgacaacatttatgactgttggtttcctagcagagaatgattctgagaga

fava_c16955_1409	fava_c16955	1409	C/T	gctcggcgttcggcttcttaggcatcgtgcgaatttgattagcaaaagtgttaaatacatagatccgaagagttttctgaatacaaggcggatttttcag[C/T]gcttcctggatatcaaccttctttctagaaagcgcggcatcaacacgagaYtcaaactcgagaagctgtgtataaagagcagattcagRcaggatagcag

fava_c16955_1460	fava_c16955	1460	T/C	taaatacatagatccgaagagttttctgaatacaaggcggatttttcagYgcttcctggatatcaaccttctttctagaaagcgcggcatcaacacgaga[T/C]tcaaactcgagaagctgtgtataaagagcagattcagRcaggatagcagccaccctttcctgcatctgtttctcaggaagcttctgcttcttcctccgYg

fava_c16955_1498	fava_c16955	1498	G/A	ggatttttcagYgcttcctggatatcaaccttctttctagaaagcgcggcatcaacacgagaYtcaaactcgagaagctgtgtataaagagcagattcag[G/A]caggatagcagccaccctttcctgcatctgtttctcaggaagcttctgcttcttcctccgYgcagcaggcgtgagttccatcggtctcaacggcgagaaa

fava_c16955_1559	fava_c16955	1559	C/T	aYtcaaactcgagaagctgtgtataaagagcagattcagRcaggatagcagccaccctttcctgcatctgtttctcaggaagcttctgcttcttcctccg[C/T]gcagcaggcgtgagttccatcggtctcaacggcgagaaattattctgaggYgaaaatccaacaggccgcataggtggtttcaaaggaccccgtttcgcag

fava_c16955_1610	fava_c16955	1610	C/T	ccaccctttcctgcatctgtttctcaggaagcttctgcttcttcctccgYgcagcaggcgtgagttccatcggtctcaacggcgagaaattattctgagg[C/T]gaaaatccaacaggccgcataggtggtttcaaaggaccccgtttcgcaggcgtagacatagacgacgacgaaacccctaaattacccaaaccaccaacct

fava_c16955_2054	fava_c16955	2054	A/G	ttccaaaggatgatgaagagccaccaatgcctttggaagggttattattgttcacagacatcactatccaaatctcgatctggaatcaggagatccactg[A/G]ttgaagaagaagaagccgagtaagaacacactgtgatctcagtttacatagatttttatttgtaaattagggtttagcgagagaagaattgaaattgaag

fava_c16955_363	fava_c16955	363	T/C	tcacaaatacatcatggaaaatggaaaactaccatttttcaaatgcataacataaaggtataaattatttttcaaatatggaggagcgagcactaggact[T/C]ttgatcgatgtcatgtgcttcccggagcatcacttcctgcagctggtttgcgattcaaatatcggatgacagcatcttcaacccatggttggttgaaaaa

fava_c16955_467	fava_c16955	467	T/G	atcgatgtcatgtgcttcccggagcatcacttcctgcagctggtttgcgattcaaatatcggatgacagcatcttcaacccatggttggttgaaaaaatc[T/G]gatttacgctctttttcagcgttgcgactaggttctccagctgcagttttcagatccctgctttgagattcaatcaaggcattaataaattctactggag

fava_c16955_677	fava_c16955	677	G/A	aaccgagaaagaacgcccgtcttcttcgatgctcatgaatttttctgatgattccacatatcgcttcatcacatgtttcgatctctttgttcttctcaac[G/A]ttagccaatagtgcagacaattctctctgaataggaaaaggaacatcaacagtcacatcataacaagcactcccggctggactatttcccgagagtttga

fava_c16956_1535	fava_c16956	1535	A/C	atactgctgtaatagctgaaactggagattcatggtttaactgtcagaagcttcgtcttcctgagaattgcggttatgaatttcagatgcagtatggatc[A/C]attggttggtcagttggtgctactcttggatacgcacaggctgcaacagataagcgtgtgattgcgtgcataggggatggaagttttcaggtgacagccc

fava_c16956_1685	fava_c16956	1685	G/C	ataagcgtgtgattgcgtgcataggggatggaagttttcaggtgacagcccaagatatttcaacaatgatccgttgtggacaaagaagcatcatctttct[G/C]atcaacaacggaggttatacaattgaagttgagattcatgatggcccatataacgtgatcaagaattgggactatacKggctttgtgagtgccatccata

fava_c16956_1763	fava_c16956	1763	G/T	gacaaagaagcatcatctttctSatcaacaacggaggttatacaattgaagttgagattcatgatggcccatataacgtgatcaagaattgggactatac[G/T]ggctttgtgagtgccatccataatggacaagggaaatgctggactgccaaggtacgtacagaggaagatttgacagaagcaattgcaacagctactggag

fava_c16956_1955	fava_c16956	1955	T/G	ctactggagcagagaaagattcactatgttttatagaagtttttgcacataaggatgataccagcaaagagttgctagaatggggatctcgtgttgctgc[T/G]gccaacggtcgtcctcctaatccccaatagaaactcaattcctaatgattgtattgttataaaaaacaattataatttgtatattttggtgtactagatt

fava_c16956_2059	fava_c16956	2059	G/C	aacggtcgtcctcctaatccccaatagaaactcaattcctaatgattgtattgttataaaaaacaattataatttgtatattttggtgtactagatttgt[G/C]gaggacatgtattggataagtgcgacaaaagtgaaagtaagttggaataattgctcgtttgttgaaatcacgaactggtgtagctttactcatagcattg

fava_c16956_926	fava_c16956	926	A/G	gggaccctgttccattttttcttgcaccaagggtaagcaatcaagcagggttagaagcggcagtggaagaagcagctgcttttctaaacaaagctgtgaa[A/G]ccagttattgtgggtgggccaaaactaagggttgcaaaagcacaaaaggcttttatggagtttgcagaagctagtggatatccaatagctgttatgccct

fava_c16961_1007	fava_c16961	1007	T/C	acccattaacgaaaccgtttgtggaaagatttgctgcggatgaagatagattctttaaggtttttgctagctcaatgcagaagcttagtcttttgggtgt[T/C]aaaactggtagaagaggtgaaattagaagaagatgtgatcagattaattgatttttgttgtttttttgKtgagggtttgttctggattctggtttttgtt

fava_c16961_1076	fava_c16961	1076	G/T	gctcaatgcagaagcttagtcttttgggtgtYaaaactggtagaagaggtgaaattagaagaagatgtgatcagattaattgatttttgttgtttttttg[G/T]tgagggtttgttctggattctggtttttgttattgggtttcataaaaatgtaaactttattttacttagtttttttatatatattttacttacaccttca

fava_c16961_680	fava_c16961	680	A/T	ccatgtcacaaatcatcgacatcttcaccaaacgcggcttcaccgtcgaagaaatggtagcactttccggtgctcacactgtcggattctctcattgttc[A/T]gaattctcatcagaaatatacaacagttcttcatctcccaattctccaagttcatacaatcctcgttttgctgaagcgttacgaactgcttgccgtgatt

fava_c16962_1101	fava_c16962	1101	C/T	aacctacatttgtgaattgagacttgttgttcctttgtgacctgtcttgggaatttgattcaatccggctgaagtagtcaatttcctgcattatgagtga[C/T]gcgaccgtgcctcgggtgcctatttccaccggaggaaactcagcactctcttttgctgtgtcgattgtgtcaaggagggtagtggatttaatcatacttt

fava_c16962_1245	fava_c16962	1245	A/T	cactctcttttgctgtgtcgattgtgtcaaggagggtagtggatttaatcatactttctgtagttggtgctgaggaaagaggtaaaccttctgttacaag[A/T]agtgctgcttctggttttgttctagtgggggaaatgcagatactttgaggcatttttttcttcatcttatgttgtatatctaaaatgcatggcataaagt

fava_c16962_465	fava_c16962	465	C/T	taatgacaagagaattggatcaaatctcatctgaaccacaaaaaaagaaggtattttcaagaactaaacctagaacacttcatgtttctgttccatgtag[C/T]agtagtagtaccagtacaagtgcaaaccctaaaagactacaaccaagaaaatcattagatgaagccaKtggcagtggaagtggaagcagtaaaaatacaa

fava_c16962_533	fava_c16962	533	T/G	cctagaacacttcatgtttctgttccatgtagYagtagtagtaccagtacaagtgcaaaccctaaaagactacaaccaagaaaatcattagatgaagcca[T/G]tggcagtggaagtggaagcagtaaaaatacaaatcatagaacaaaagctaatagtaatagtactagtaggaatactcatagaataccgatgttaggttct

fava_c16962_828	fava_c16962	828	T/C	ggaatgcagaaaagacttgggctactcctttaacgacccgtctcaagaagctcggtttctcaaagctctctgagagcattatctaaagctgaaacttttt[T/C]gtgtcagacctcaggtttctgtaaccaaaagccgaggttccgtttggcctaccgccgtctgatacatccaccatagagcacatactcggtaggagcttgc

fava_c16964_1180	fava_c16964	1180	G/T	caggagacacagcatcatcttgccgcctttttgatgcaattgtgagtcactgtgttcctgtcattgtgagtgataaaatcgaactcccatttgaggacga[G/T]attgattattcccagttctctctgttcttctcattcaaagaagcattacagcccggctacatggtcgaccagcttcgtaaatttccaaagcagaagtgga

fava_c16964_1476	fava_c16964	1476	T/C	gtcaaacacaagcttccaggggttactctctccatccatagaaaccgaagattaaaaatctctgactggtggcgaaagagatgatgattttttgaacttt[T/C]gatactgaccttgtacattcactctcttcagtttctatgaccttgtacgtcttcttatctcaacaacttaaaatagagtgctgtgtagaagttttaatct

fava_c16964_466	fava_c16964	466	C/T	tccaaatgatcgaccgccgcaacgcaaccgaatctcccgtcaccgctaacgactttccgcggtggcccggaaattgggggctcaagaagcaacacagcgt[C/T]gagtactggWtgatggggtcgcttattcatgaaggggctgatgctggggaagctStgagggttttcgatcctgaactcgctgacgctttttttgttccgt

fava_c16964_476	fava_c16964	476	A/T	cgaccgccgcaacgcaaccgaatctcccgtcaccgctaacgactttccgcggtggcccggaaattgggggctcaagaagcaacacagcgtYgagtactgg[A/T]tgatggggtcgcttattcatgaaggggctgatgctggggaagctStgagggttttcgatcctgaactcgctgacgctttttttgttccgtttttttcttc

fava_c16964_521	fava_c16964	521	C/G	tccgcggtggcccggaaattgggggctcaagaagcaacacagcgtYgagtactggWtgatggggtcgcttattcatgaaggggctgatgctggggaagct[C/G]tgagggttttcgatcctgaactcgctgacgctttttttgttccgtttttttcttccttgagttttaacactcatggtcatactatgacagatcctgctac

fava_c16964_755	fava_c16964	755	G/T	ggaattattgaacaagtccaaatattggcagagatctaaaggcagagaccacatatttgctatgacacaccccaatgcgtttaggtttctacgaaatcaa[G/T]tgaatgctactattcaaattgttgtggattttggccgttatcccaagagcatgtctaatttgaacaaagatgtggtctccccRtatgttcatgttgtgga

fava_c16964_838	fava_c16964	838	G/A	ggtttctacgaaatcaaKtgaatgctactattcaaattgttgtggattttggccgttatcccaagagcatgtctaatttgaacaaagatgtggtctcccc[G/A]tatgttcatgttgtggattcttttacagatgatgaatctcaagatccgtaYgaggctcgctccacactgcttttctttcgagggaggactttcagaaagg

fava_c16964_889	fava_c16964	889	T/C	gccgttatcccaagagcatgtctaatttgaacaaagatgtggtctccccRtatgttcatgttgtggattcttttacagatgatgaatctcaagatccgta[T/C]gaggctcgctccacactgcttttctttcgagggaggactttcagaaaggatgaaggcattgtccgtgctaaactggcaaagatattgactggttataRtg

fava_c16964_987	fava_c16964	987	G/A	taYgaggctcgctccacactgcttttctttcgagggaggactttcagaaaggatgaaggcattgtccgtgctaaactggcaaagatattgactggttata[G/A]tgatgttcattatgagcgaagtgttgcaactgaagaaaacataaaagcgtcatctaaagggatgcgttcatcaaagttctgtttgcatccagcaggagac

fava_c16971_1320	fava_c16971	1320	G/A	tgtcacaagagaagcttcaaaatatgattgaagtttggaataagattgatgctgaggaagagtgcatggatgatgatcaatatcttgatgaagacgaaga[G/A]gctgatgaaactagcaaccttgatggagaagaggatgtgaatactgRgacattaatggaaactgaagtagccaatgagaagtcactgtgtgactctgatg

fava_c16971_1367	fava_c16971	1367	A/G	gatgctgaggaagagtgcatggatgatgatcaatatcttgatgaagacgaagaRgctgatgaaactagcaaccttgatggagaagaggatgtgaatactg[A/G]gacattaatggaaactgaagtagccaatgagaagtcactgtgtgactctgatgtcattgaagaaaaaggagactattccgatggttggttgtacgaggac

fava_c16972_220	fava_c16972	220	C/T	ttcttctaatggagattgcaacaacaaaagtccttgtattcctctcatcgcagctagtacttgcagctttcatgtttggaaacacatgcaagaatatatt[C/T]gaatccatcatcttcgtgtttgtaatgcatccatttgacgttggcgatcgatgtgttatagatggYgtcgagctaatggttgaagaaatgaatatattga

fava_c16972_286	fava_c16972	286	C/T	ctttcatgtttggaaacacatgcaagaatatattYgaatccatcatcttcgtgtttgtaatgcatccatttgacgttggcgatcgatgtgttatagatgg[C/T]gtcgagctaatggttgaagaaatgaatatattgacaacagtattcttgaagcttaataatgaaaaagtgtattatccaaattcggtgctggccatgaaac

fava_c16972_412	fava_c16972	412	T/G	atatattgacaacagtattcttgaagcttaataatgaaaaagtgtattatccaaattcggtgctggccatgaaaccgattagcaattattacagaagccc[T/G]aatatgtctgaaggtgtWgagttttcggttgattttacgacaccagcagagaaaattggagcactgaaagaaaaagtaaagaggtatttggagaggaatc

fava_c16972_430	fava_c16972	430	T/A	tcttgaagcttaataatgaaaaagtgtattatccaaattcggtgctggccatgaaaccgattagcaattattacagaagcccKaatatgtctgaaggtgt[T/A]gagttttcggttgattttacgacaccagcagagaaaattggagcactgaaagaaaaagtaaagaggtatttggagaggaatccacaatactggcatccat

fava_c16972_544	fava_c16972	544	T/A	attttacgacaccagcagagaaaattggagcactgaaagaaaaagtaaagaggtatttggagaggaatccacaatactggcatccatattttagcttggt[T/A]gtgaaggagattgaaaatgtaaataagattaagatgggtctttatgttaatcacacaatgaattttcaagactttggggagaaatgcaaaaggactagtg

fava_c16972_776	fava_c16972	776	T/C	agagctcaatatcagatacaaccttcttcatcaaggtgttcatatcagacagatagaacatgataccagttaatctcccatgatgtttcttactcattgc[T/C]gtgcaatttcatgtgtgtgtttcttttggttctgcgtatagtgaaagtaaaggccttaatagacaataatatcaaaagttttgtatatgattatcaaata

fava_c16975_1129	fava_c16975	1129	A/G	tagcaatatattgtacagttataggaatgcttcaattcaagaggtctatttatatatgttgatcagtggctttaactgttgatctctatgtataacctgg[A/G]ctttgagttagaacttggtgatcttgcaggatcttcattctgacataatacttttgaatataaattttcctatagataattttgggacacatgttcctat

fava_c16975_771	fava_c16975	771	T/C	atggaagtcaatcagaaacctcctgcacacattgtggcataagctcaaaattgactcctatgatgcggcgagggccatctggtccaagatcgctttgcaa[T/C]gcttgtggtctattctgggcaaataagggtactttgagagaactttccaggagacatcaagaatcatctattgtatcagccgaacaggttgatcaacata

fava_c16975_890	fava_c16975	890	C/A	gcaaataagggtactttgagagaactttccaggagacatcaagaatcatctattgtatcagccgaacaggttgatcaacataatatctcagactatggaa[C/A]agcatctgtccccacacataacaatcttgccgttttctgtggtaatgataaccaagctttggcagctgatcgttgagttgatgaattttccggtcaSaga

fava_c16975_987	fava_c16975	987	G/C	gaaMagcatctgtccccacacataacaatcttgccgttttctgtggtaatgataaccaagctttggcagctgatcgttgagttgatgaattttccggtca[G/C]agaagttgctagagttgttaacttttgcatagttctgctcatagcaatatattgtacagttataggaatgcttcaattcaagaggtctatttatatatgt

fava_c16976_1548	fava_c16976	1548	G/A	aatgcaactcaacggctacatcatcgtctctatcttcagacatcaaataactgatggcattgaaaactagtttctcgtacaaggttgcatgttcaagatg[G/A]gacttagcggtgtgaagcaaatatgaagcacgagaagaatcagaggaggaaatgaaatgagctgctaaggtgttggccaacacacagtagttatcatggg

fava_c16976_1728	fava_c16976	1728	G/A	acacacagtagttatcatgggttactgattccagaatcacacgcctctctctaccgtagctgagaacctgttgatagtaggaattgatggttgaaatgca[G/A]agttgagaggaagttgtgacatcgtaaccacaaccgtctagtttgatccttccttccattgttcttgactcctgttatgattcctagagtaagtaacaat

fava_c16977_447	fava_c16977	447	C/T	gagaacggagcggttgaagaaggaagtggtgtagaggaaaatggaaatggtgagaaaattgtaacgcaaatcgaatcgaaagacgtgacgaattggaacg[C/T]gaaaacagtggatRaagttgaagaaaaaattagggttttatctgtgaaatctgggaatgggaaacagaattggaagatcaaaaaactgaacggtggtggt

fava_c16977_461	fava_c16977	461	A/G	tgaagaaggaagtggtgtagaggaaaatggaaatggtgagaaaattgtaacgcaaatcgaatcgaaagacgtgacgaattggaacgYgaaaacagtggat[A/G]aagttgaagaaaaaattagggttttatctgtgaaatctgggaatgggaaacagaattggaagatcaaaaaactgaacggtggtggttttcgtcaatctca

fava_c16977_576	fava_c16977	576	G/A	attagggttttatctgtgaaatctgggaatgggaaacagaattggaagatcaaaaaactgaacggtggtggttttcgtcaatctcaatctatgaggaatg[G/A]tcgcaatgctggtggttatcatggtcatggtcatggtcatggacatggacatggacatggggatttgaaggataaacgaagttccaaggctgagatgatt

fava_c16979_749	fava_c16979	749	A/G	aatcaaaggtctttgggaagaactcgcagagtttcgtccaattcatcagtgtgtctgtggtggcgtaaatcctttgattgatcacatcaactgtgagtat[A/G]tgcttatattcttgctgggattgaatgaaagtttcaatccaattcgtggacaaatcttgctcatggaccctttgccatctattagctgtgttttttcatt

fava_c16980_1222	fava_c16980	1222	A/G	acttccttgacttcctgtacaaagcatcaaccggaagaagacatctcattcagtgacttggaggaatacggcagttatagctctgataataaattatcaa[A/G]ccacggggaggtcttcaggggctcatcacctgaaggttcgagtggctgggttcagctacacgaaaacaagagagatggccggcagaaagctatccattta

fava_c16980_166	fava_c16980	166	A/T	ctctctctcaagcgactcttccgtctcccccaagttctttctccgcatatttcgaaaacccttcgattcattcagcacaaaaagtagcatataaacttca[A/T]attcaaataataaaaacaaatatagcaattttatttatttttaaaaaattaaaaaagagaaaatatgtcttggctcttcaagtccaaccaccctaatcca

fava_c16980_464	fava_c16980	464	T/C	gcggcgtcgccaacttcctcgctccacctccctcatcatcttcttcctccggtgagtccaccgcaccttccataactctcaccggaatccggaacgatct[T/C]gtggagatcggaggaagtttcagaagctcactctcacttctctccccgagaaaagccgtcaccggaatctccaagctagcttctcaattgttgcagtcgg

fava_c16980_927	fava_c16980	927	G/A	aatacttccaagacttaatcagcatgacttcgagtgcctatcaaccccaaagattgttgaagcaagagatgttctcctacggaaacttgaagagaggaac[G/A]atatacatgctggagaatgcgacaattcaggtattatggatacacatcaagagggtagggaagacagtggagcagagagtatcacattagatcaaaacca

fava_c16981_176	fava_c16981	176	G/A	cttcaccttcttcatctcaatggagtagtattgattctaagggaacggcaaaagatgtgctcaaagaaaggtttaatgacaagactaagggttcaagaaa[G/A]tctcctaccaaagaaaatactaaaccacaagaattcaaactccacacacaagaaagagctgtcaaacgtgcaatattcaactatgaagtgacaactaaat

fava_c16982_1013	fava_c16982	1013	G/C	cgacatgatcaattagagaaatgccaataactgttccagtagcattcacaScagcaactatgagagataaaagaatagctaactgattggaattaaaccc[G/C]gccatttgaatgattgtggcggtgtagttcatgactatgccRataccggtgaactgcagaaaagcctgaagtccgcccccgacaaaaaaagcaattctga

fava_c16982_1055	fava_c16982	1055	A/G	cattcacaScagcaactatgagagataaaagaatagctaactgattggaattaaacccSgccatttgaatgattgtggcggtgtagttcatgactatgcc[A/G]ataccggtgaactgcagaaaagcctgaagtccgcccccgacaaaaaaagcaattctgatttcctttgatcggaaaacatccatgtatctaatgttcttcc

fava_c16982_1781	fava_c16982	1781	A/T	caaccaatgccatgcttacaatagtttcctgaaggaagctacttttgcggacatcatcaaaatcatctttaatatacagaagggcccctgatataacacc[A/T]gtRtcgtagccgaagaggagtccaccaaagccagcagccgcagttactccgagaatgtagggattcttgaaatatgatattttcctttccggatacaatt

fava_c16982_1784	fava_c16982	1784	A/G	ccaatgccatgcttacaatagtttcctgaaggaagctacttttgcggacatcatcaaaatcatctttaatatacagaagggcccctgatataacaccWgt[A/G]tcgtagccgaagaggagtccaccaaagccagcagccgcagttactccgagaatgtagggattcttgaaatatgatattttcctttccggatacaattcaa

fava_c16982_1885	fava_c16982	1885	A/G	tcgtagccgaagaggagtccaccaaagccagcagccgcagttactccgagaatgtagggattcttgaaatatgatattttcctttccggatacaattcaa[A/G]gtaacaggagcttcccggtgtcgcctgcggcatcttcttctcttttYgatggcgctgcttgaacccaaaagaaatggagaagaactgcaaatttttcttR

fava_c16982_1932	fava_c16982	1932	C/T	gagaatgtagggattcttgaaatatgatattttcctttccggatacaattcaaRgtaacaggagcttcccggtgtcgcctgcggcatcttcttctctttt[C/T]gatggcgctgcttgaacccaaaagaaatggagaagaactgcaaatttttcttcaaaataactttttaggacaaaaaatatctttaacaaccaatgtaaaa

fava_c16982_262	fava_c16982	262	G/A	taaccttcccactaacttattaatagaaacccactatttataacattaaagtttatttaacaaatttaacaataacagaacaaagatttaggaggccttc[G/A]gtattcatgataaacaaaccaataatgtatttgtaacgatacaaatctacaacataggatcacacaaaacactaccaccaccatcctgtaatgtggccgc

fava_c16982_478	fava_c16982	478	T/C	tgactatgattggtttcctcacttgcgttcaataatattgcagctcacactaagaattggcttcctttatcaagaagggtttgcctgttataatccttac[T/C]ccgggctctcttcttccatatgagagacacttcttcaaaagtcaatccttttgtctctggtacaaagaagagcacaaagaaaaaagcaaccacgcatata

fava_c16982_674	fava_c16982	674	T/A	atataacgccaagaatcatgaagctgccaccaaggccaacggaatcagaaactgaaagaaaactttcagacattatcacactgcaaatccagttaacagt[T/A]gctgacattcccccgcatacacctcggaactcttcagggtagatttctgagttcacagtccatggcaccaatcccattcctggagtgaaaaaaataatat

fava_c16982_898	fava_c16982	898	C/T	atccacccatagatcgtatttgaggagtcagatgaatgaaggtaagatcctgtagaaaggattgccagcgctatagttgcacctgacaagcttgagagag[C/T]caacttccttcgtccgacatgatcaattagagaaatgccaataactgttccagtagcattcacaScagcaactatgagagataaaagaatagctaactga

fava_c16982_963	fava_c16982	963	C/G	cagcgctatagttgcacctgacaagcttgagagagYcaacttccttcgtccgacatgatcaattagagaaatgccaataactgttccagtagcattcaca[C/G]cagcaactatgagagataaaagaatagctaactgattggaattaaacccSgccatttgaatgattgtggcggtgtagttcatgactatgccRataccggt

fava_c16986_1103	fava_c16986	1103	A/G	atcctcatgatacttgttgtgttttactcaatttttttgaataattattgttgtttggttacagatgaatagagaaagagagagagttgtgttgttgagt[A/G]tttgttctttaaaaRatgagatgtgaaatccgaaagtaatggcagaggaagtagaatgagggatttgagaggaaaggttgagaggaagcagagggataaa

fava_c16986_1118	fava_c16986	1118	G/A	gttgtgttttactcaatttttttgaataattattgttgtttggttacagatgaatagagaaagagagagagttgtgttgttgagtRtttgttctttaaaa[G/A]atgagatgtgaaatccgaaagtaatggcagaggaagtagaatgagggatttgagaggaaaggttgagaggaagcagagggataaatagagggaatttagt

fava_c16987_1147	fava_c16987	1147	A/G	gaatactattggatctttgatttctctcttcatggtaactgattgatcttgctataataagttttgaagaaaggcatcccatagtataagtgtatctaaa[A/G]aaacactttcacacctgtaattgatgcaaaacctcaaatcWacacctgcaaaagatacagaactaggatcaaaagagggtttaaggatccatgaatacta

fava_c16987_1188	fava_c16987	1188	A/T	attgatcttgctataataagttttgaagaaaggcatcccatagtataagtgtatctaaaRaaacactttcacacctgtaattgatgcaaaacctcaaatc[A/T]acacctgcaaaagatacagaactaggatcaaaagagggtttaaggatccatgaatactacaaagcagaatatttataggctttgacttattatggtagta

fava_c16987_183	fava_c16987	183	A/T	aaatttcacttgcattagcaatcaagtccaaaagaaaaaactcacctaaaaatgttgatggtaaattcaatgcatacaaacattgaagatgtgaatcaaa[A/T]tttcYcttacatttatatgttattcacaaaactcatctaaaagtatctttatgctctttcttaggtctgcaWgattccttcatcaatttcttcttctYcc

fava_c16987_188	fava_c16987	188	C/T	tcacttgcattagcaatcaagtccaaaagaaaaaactcacctaaaaatgttgatggtaaattcaatgcatacaaacattgaagatgtgaatcaaaWtttc[C/T]cttacatttatatgttattcacaaaactcatctaaaagtatctttatgctctttcttaggtctgcaWgattccttcatcaatttcttcttctYccacagt

fava_c16987_255	fava_c16987	255	A/T	catacaaacattgaagatgtgaatcaaaWtttcYcttacatttatatgttattcacaaaactcatctaaaagtatctttatgctctttcttaggtctgca[A/T]gattccttcatcaatttcttcttctYccacagtctctagaatttgtttcaaaatgttttcttcctctgcttcaagcttttgcatacactgttcaaaagca

fava_c16987_281	fava_c16987	281	T/C	aaWtttcYcttacatttatatgttattcacaaaactcatctaaaagtatctttatgctctttcttaggtctgcaWgattccttcatcaatttcttcttct[T/C]ccacagtctctagaatttgtttcaaaatgttttcttcctctgcttcaagcttttgcatacactgttcaaaagcagatactaactctggactaaatataga

fava_c16987_561	fava_c16987	561	C/T	gagtagattccatctgcaggaagccattctttaagactagcattggctggagattcaatattggatggaattctaagagattctagtctatttgctatag[C/T]cttcctcttatttcccttctcaagcattcttgttttatgactttgaaacacttcctcattcttggttaaccataatgSttgcattttctcgatggaagaa

fava_c16987_639	fava_c16987	639	G/C	gattctagtctatttgctatagYcttcctcttatttcccttctcaagcattcttgttttatgactttgaaacacttcctcattcttggttaaccataatg[G/C]ttgcattttctcgatggaagaagaatcttcatcctgaatgtccattttgattattgaaccatcatcatMatcttcttcattattaaactcacttggtcga

fava_c16987_708	fava_c16987	708	A/C	aacacttcctcattcttggttaaccataatgSttgcattttctcgatggaagaagaatcttcatcctgaatgtccattttgattattgaaccatcatcat[A/C]atcttcttcattattaaactcacttggtcgaacaatttgggattccttcaaccaaattctattaaccaYatcatcatattcttcaacggtgtgaaaactc

fava_c16987_777	fava_c16987	777	T/C	atgtccattttgattattgaaccatcatcatMatcttcttcattattaaactcacttggtcgaacaatttgggattccttcaaccaaattctattaacca[T/C]atcatcatattcttcaacggtgtgaaaactcctattacctagagcatccttagatcccaagtYatctttttcttcaaaaccagataRattctcctgttcc

fava_c16987_840	fava_c16987	840	C/T	acaatttgggattccttcaaccaaattctattaaccaYatcatcatattcttcaacggtgtgaaaactcctattacctagagcatccttagatcccaagt[C/T]atctttttcttcaaaaccagataRattctcctgttccaacttctcaataacctcagaactttctggctcaaYagaaatttttgaactagtgttggagctt

fava_c16987_864	fava_c16987	864	A/G	attctattaaccaYatcatcatattcttcaacggtgtgaaaactcctattacctagagcatccttagatcccaagtYatctttttcttcaaaaccagata[A/G]attctcctgttccaacttctcaataacctcagaactttctggctcaaYagaaatttttgaactagtgttggagcttaagctcttagaatgcaaattattg

fava_c16987_912	fava_c16987	912	T/C	ttacctagagcatccttagatcccaagtYatctttttcttcaaaaccagataRattctcctgttccaacttctcaataacctcagaactttctggctcaa[T/C]agaaatttttgaactagtgttggagcttaagctcttagaatgcaaattattggatactttgtttgctgtgcagagaagagaaaggtagtggtcaccatta

fava_c16990_1214	fava_c16990	1214	C/T	aaatgatatggtacaacaatgctcctcacacaaagcttgtagaatctaagggtcatcaaaactggatcaaagtttctggtgaatatcttacttttcctgg[C/T]ggtggaactcaatttaagtatggcgctcttcattacattgaattcattcagaattctcttcctaatattgcatgggggaagagaagtcgtgtgatattgg

fava_c16990_1325	fava_c16990	1325	C/T	aatttaagtatggcgctcttcattacattgaattcattcagaattctcttcctaatattgcatgggggaagagaagtcgtgtgatattggacgttgggtg[C/T]ggggtagcgagcttcggtggctatctgtttgaaaaagatgttctgaccatgtcgtttgctcctaaagatgtccacgaagcccaagtgcaatttgcattgg

fava_c16990_1553	fava_c16990	1553	C/T	tcatgggaactaaaagattaccattccccggttctgtctttgatcttctccactgtgctcgttgtagagtcccttggcacatagaaggtggcaagctact[C/T]ttagagctgaatcgtgtcttgcggcctggtggttactttgtatggtccgctactccagtttatcaaaaggatccagaaaatgccgggatttggaaagaca

fava_c16990_1885	fava_c16990	1885	C/T	ccaccaatgtgtagtgaatccgatgatccaaatgcagcttggaatgtatcattacaggcttgcatgcacaaagtgccagtaggtgcatcagagcgcggaa[C/T]aatctggccggaacaatggccactgaggctagagaaaccaccttactggttaaacactcagaccggtgtttWtggaaRatctgctccaKtagaattcacc

fava_c16990_1957	fava_c16990	1957	A/T	gtgccagtaggtgcatcagagcgcggaaYaatctggccggaacaatggccactgaggctagagaaaccaccttactggttaaacactcagaccggtgttt[A/T]tggaaRatctgctccaKtagaattcaccgccgattacaagcattggaaaaacgtaatttcacattcatatttgcacggaatgggtatcaattggtcttca

fava_c16990_1963	fava_c16990	1963	A/G	gtaggtgcatcagagcgcggaaYaatctggccggaacaatggccactgaggctagagaaaccaccttactggttaaacactcagaccggtgtttWtggaa[A/G]atctgctccaKtagaattcaccgccgattacaagcattggaaaaacgtaatttcacattcatatttgcacggaatgggtatcaattggtcttcagtaaga

fava_c16990_197	fava_c16990	197	G/A	ttgcttggattgcagatgaaggtgtgtttatcttgaaatacaagtaactaagaatcatggcattaggtaagagttcccgaggcgacggcaggaattcttc[G/A]aactactgttccacagttcccgtagctgtttttgttgcattttgtttagttggtttatggattgcaatgtcatccattgttccggttcaggattcagtca

fava_c16990_1974	fava_c16990	1974	G/T	agagcgcggaaYaatctggccggaacaatggccactgaggctagagaaaccaccttactggttaaacactcagaccggtgtttWtggaaRatctgctcca[G/T]tagaattcaccgccgattacaagcattggaaaaacgtaatttcacattcatatttgcacggaatgggtatcaattggtcttcagtaagaaatgtcatgga

fava_c16990_2178	fava_c16990	2178	G/A	gaaagctgtttacggaggtttcgccgcggcactcagagcgctgaaattgaacgtctgggtaatgaatgtagttccggttgattctccagacacattgcct[G/A]taatatacgagcgtggattaatcggtatttatcacgattggtgtgagtcgtttagcacctatcctcgatcttatgatcttctcaatgccgattctttatt

fava_c16990_2381	fava_c16990	2381	C/A	caaatcttaaagaaaggtgcaacatagtggcagcgattgcagaagttgatagaatgttaagaccagaaggatatttgattataagagacaatgaagaaac[C/A]attggtgagatagagaacatggtaaaatctctacattgggatattcgttttagttatgcaaaaRatggagagggtttactttgtcttcagaagacatttt

fava_c16990_2445	fava_c16990	2445	A/G	agaaggatatttgattataagagacaatgaagaaacMattggtgagatagagaacatggtaaaatctctacattgggatattcgttttagttatgcaaaa[A/G]atggagagggtttactttgtcttcagaagacattttggagaccaacaaaggttgaaacggttgtgtcagccattgcttaatttatgatgcttcaccttca

fava_c16990_308	fava_c16990	308	T/C	ccacagttcccgtagctgtttttgttgcattttgtttagttggtttatggattgcaatgtcatccattgttccggttcaggattcagtcatggaggtatc[T/C]gagacgattaatgaggtaaaaaatatagccaatcaaactgattctaggaagttcgaaaacagattaggtgatgtaccagatgaatcggaaacaagagata

fava_c16990_439	fava_c16990	439	A/G	aatcaaactgattctaggaagttcgaaaacagattaggtgatgtaccagatgaatcggaaacaagagatagccaaaccccagaatctcagagtgaaagcc[A/G]ttcagaatctgtagaaacagaaacagtgaatggtcaaattgatgataatcaactcaaaggatcggtggaaactttaggtgaaagtaaatctgacaagagc

fava_c16991_1005	fava_c16991	1005	A/C	cttgaatctgatagctcgtacgtattgccattggtgKtaSttgagcaaagccttgtattgaaggtgagacacaaatgggtattattgttaagctcagaaa[A/C]gtgattgtggatgaaaaaggtaccgcattttctttgggagaagcaattgaagcgttgtaacagaaactgatgacgagagaggggtgtggaattgaagtgg

fava_c16991_391	fava_c16991	391	T/C	attagccataaaacctacagccactccatatgattctgaaggcggaatatgagcatatgtaacaaactcaaaaccggagaccatgttttagaggaaaaaa[T/C]gattagSggataataaaagtttatacatttccattgatgatgaaatttggatgtcctcttaagtagcaaaattgcattcaggcccaaagtagtgtgatga

fava_c16991_398	fava_c16991	398	G/C	ataaaacctacagccactccatatgattctgaaggcggaatatgagcatatgtaacaaactcaaaaccggagaccatgttttagaggaaaaaaYgattag[G/C]ggataataaaagtttatacatttccattgatgatgaaatttggatgtcctcttaagtagcaaaattgcattcaggcccaaagtagtgtgatgaccaaaaa

fava_c16991_504	fava_c16991	504	C/T	ataaaagtttatacatttccattgatgatgaaatttggatgtcctcttaagtagcaaaattgcattcaggcccaaagtagtgtgatgaccaaaaatacaa[C/T]gagggccaatccaaaacagaatccaactattgccgcgaaagccttcccttgtgattgaaatttaaacttattagtaaaccaggttaatcctctgtcaaca

fava_c16991_730	fava_c16991	730	C/T	cggctcttagaagcttagtgacttgactaccggcccaaaccatcgccaacagtttgagaaatctttctacagctggacgataacccatctttcccggtgc[C/T]ggagcaatataaaaccacacaaaaagaaatgttgtcagatagtatgccgtgtttaaaagtccataggataggattccagaaactccatatctcttcagtt

fava_c16991_941	fava_c16991	941	G/T	ctcattgattgatagtgactttcctttcttactctctttgctttcttctgaaccttcatggctgcttgaatctgatagctcgtacgtattgccattggtg[G/T]taSttgagcaaagccttgtattgaaggtgagacacaaatgggtattattgttaagctcagaaaMgtgattgtggatgaaaaaggtaccgcattttctttg

fava_c16991_944	fava_c16991	944	C/G	attgattgatagtgactttcctttcttactctctttgctttcttctgaaccttcatggctgcttgaatctgatagctcgtacgtattgccattggtgKta[C/G]ttgagcaaagccttgtattgaaggtgagacacaaatgggtattattgttaagctcagaaaMgtgattgtggatgaaaaaggtaccgcattttctttggga

fava_c16992_195	fava_c16992	195	C/T	aattcccttttacaacagaatgagtggagagccacaaaattcttcatatttacatttcaatttataataactcaaaaatattacaagcaataagagcttg[C/T]cgccaaaaaattcccagcccttataaagaacttYaaattatgttgtacctatttgatggcgtacgaccgagtacacaccgagtcgagagcctcggtgaac

fava_c16992_229	fava_c16992	229	T/C	caaaattcttcatatttacatttcaatttataataactcaaaaatattacaagcaataagagcttgYcgccaaaaaattcccagcccttataaagaactt[T/C]aaattatgttgtacctatttgatggcgtacgaccgagtacacaccgagtcgagagcctcggtgaacaacgaagaaaaactggtcaatgcttgaaagactc

fava_c16992_389	fava_c16992	389	G/A	ggtgaacaacgaagaaaaactggtcaatgcttgaaagactccgggcaagcctgtctgaagattgttgagtgtcattgctcttgttacctctacggctttc[G/A]aatgcttcatcatttcatcttcaacgcgtctttggcaggttgctagttctagttttttctcagcaagtggatcgcacgcgtctaagccttgtccattatc

fava_c16992_493	fava_c16992	493	C/T	gcttcatcatttcatcttcaacgcgtctttggcaggttgctagttctagttttttctcagcaagtggatcgcacgcgtctaagccttgtccattatcagg[C/T]gcRgagtcaggaaggctaatccctaccaaagaatatgagctgtaaaactttctttctatattccKtacagacgaagcctttttctcgagttccttggaag

fava_c16992_496	fava_c16992	496	G/A	tcatcatttcatcttcaacgcgtctttggcaggttgctagttctagttttttctcagcaagtggatcgcacgcgtctaagccttgtccattatcaggYgc[G/A]gagtcaggaaggctaatccctaccaaagaatatgagctgtaaaactttctttctatattccKtacagacgaagcctttttctcgagttccttggaagcgt

fava_c16992_558	fava_c16992	558	T/G	ggatcgcacgcgtctaagccttgtccattatcaggYgcRgagtcaggaaggctaatccctaccaaagaatatgagctgtaaaactttctttctatattcc[T/G]tacagacgaagcctttttctcgagttccttggaagcgttctcggttcgcttctttatcttgagttcttcggcttgcttggaagatatcacatggacaaca

fava_c16992_697	fava_c16992	697	A/G	tctcggttcgcttctttatcttgagttcttcggcttgcttggaagatatcacatggacaacattgataaagctcttgatggcttcagacgcaacggtatc[A/G]gggacacggtcaagtgcaagcttccagtcatcaaaaaacatgtaggcgtcggagtgttccattctgttgttgacgttatcgttatcaaccggaWtaagac

fava_c16992_791	fava_c16992	791	A/T	ggtatcRgggacacggtcaagtgcaagcttccagtcatcaaaaaacatgtaggcgtcggagtgttccattctgttgttgacgttatcgttatcaaccgga[A/T]taagactaagcttaaaccaaccatgtaaggagagaataaagtcccgttgaaattttataaggcgacaaaaactcgagtgccaagccgagacggcggattc

fava_c16994_1091	fava_c16994	1091	T/C	tgtcgaatccaaaactgaaattggactaggttcatatccctcatggaagttaccgacatgatctgcatcagccccggtggtaagtgaatacacaaagtct[T/C]cgtgaaattccaatatctcctgttgctttgaagcatgacttaaataatcacggtctccgtgcatcgttacagatgccattgattcagattgcttcatgga

fava_c16994_1239	fava_c16994	1239	C/G	tcacggtctccgtgcatcgttacagatgccattgattcagattgcttcatggatggattattatcagaccaaccaacaaccttagtcttcacatctgaca[C/G]tgagaaatccactgatgaatcaagagagaaRgcaattttcttagacatgttactgtcttcattgttcttcacatcttcgttcattaagcatgtctgctgc

fava_c16994_1270	fava_c16994	1270	G/A	ttgattcagattgcttcatggatggattattatcagaccaaccaacaaccttagtcttcacatctgacaStgagaaatccactgatgaatcaagagagaa[G/A]gcaattttcttagacatgttactgtcttcattgttcttcacatcttcgttcattaagcatgtctgctgcgagatttgattgtctacgaaaccaggagaag

fava_c16994_1575	fava_c16994	1575	C/G	gatcgatttgcggatacattcttacttaaaccccaatatttctgaaacaagtcatccttagtcacagttccagaatcatcagcaagacgttttgtcattg[C/G]cacttttctggaaaagctataaccaaacccaaccttagatctaatgttgcttttaatcttaccatttccttccttttcaggatttgctttcgaaacagaa

fava_c16994_1753	fava_c16994	1753	C/T	caggatttgctttcgaaacagaaattctcgagtcaaaagttaaatccttccgatccaattgaaatttcgaaaaccgaaatgcttcacgcaatccatcttt[C/T]ccatggacaacacttcgcgacaaattatacattttctgtcgaggcagcaaatttccctgctcagatttcacactcgttttctgtggcaccacatcttcta

fava_c16994_188	fava_c16994	188	A/G	tctttcctttattcttgttttggatacaaatcacaaacactttcctttctttcttttttcgttttcggatacaaatcaaatacaatattgtaccatataa[A/G]tcaataatcaaacaatacctaaattctaatgcaaactctataacctctcaaaatcaacctgaaaaaaatcgactcaaaatcaacctcgccaattcaaact

fava_c16994_1887	fava_c16994	1887	C/T	ttctgtcgaggcagcaaatttccctgctcagatttcacactcgttttctgtggcaccacatcttctaccttagttcgaaacagcaataaattctcttttc[C/T]atttcctttcgacgagttatgattcttttctctccttactaatgtttcaaccaccaYcataacatcgtcagaaccgtccttttcatcagtacYcatttct

fava_c16994_1944	fava_c16994	1944	T/C	acatcttctaccttagttcgaaacagcaataaattctcttttcYatttcctttcgacgagttatgattcttttctctccttactaatgtttcaaccacca[T/C]cataacatcgtcagaaccgtccttttcatcagtacYcatttctaaagaaccacgctgacaagacaaacgcttcttcctgacaccaattgaagcaactttc

fava_c16994_1980	fava_c16994	1980	C/T	tcttttcYatttcctttcgacgagttatgattcttttctctccttactaatgtttcaaccaccaYcataacatcgtcagaaccgtccttttcatcagtac[C/T]catttctaaagaaccacgctgacaagacaaacgcttcttcctgacaccaattgaagcaactttctgcaaataatcctccgacaaaactttcggtctatct

fava_c16994_2088	fava_c16994	2088	T/G	aaagaaccacgctgacaagacaaacgcttcttcctgacaccaattgaagcaactttctgcaaataatcctccgacaaaactttcggtctatctctaacag[T/G]ttcatatcgggaagccactttatcaagtcccatcaatttcaaaatcacgctcgacccttcgtatttcgactccattttgcaacaactattctgtgagaat

fava_c16994_329	fava_c16994	329	C/A	taacctctcaaaatcaacctgaaaaaaatcgactcaaaatcaacctcgccaattcaaacttactcatgaattgctcctccacacacaaagctagagtttt[C/A]aaaaattctctaacctaactatatcatctacaagctcctctattaacacattaactacctctctaacaaaaccttcaatatcttcaactaattctatcca

fava_c16994_453	fava_c16994	453	C/A	tcatctacaagctcctctattaacacattaactacctctctaacaaaaccttcaatatcttcaactaattctatccaccgaatttcccctccaagcatat[C/A]gtctattgactccttaccRgctttcttttcttgcgaaacaagcaatccccacatcttgtcctcaatcatatcctgtctcaattcagtattcaatcttctt

fava_c16994_472	fava_c16994	472	A/G	ttaacacattaactacctctctaacaaaaccttcaatatcttcaactaattctatccaccgaatttcccctccaagcatatMgtctattgactccttacc[A/G]gctttcttttcttgcgaaacaagcaatccccacatcttgtcctcaatcatatcctgtctcaattcagtattcaatcttcttgacacaggcgtttcccaca

fava_c16994_637	fava_c16994	637	C/T	cagtattcaatcttcttgacacaggcgtttcccacattggaacgtataaatatggatgtagaatatctgacaaacctaaattaatacgatcgaaaagaag[C/T]cgcctttcagatcttttccaaagctgctgctcgccgaatttcttttctagggtatcgaaaaSRtaagggctaataggacattccgcagagtgccatgttg

fava_c16994_699	fava_c16994	699	C/G	atatctgacaaacctaaattaatacgatcgaaaagaagYcgcctttcagatcttttccaaagctgctgctcgccgaatttcttttctagggtatcgaaaa[C/G]Rtaagggctaataggacattccgcagagtgccatgttgaaaaatctctgagcaagctcgcattggacatacctgcttcggttaagacctcgacaacatag

fava_c16994_700	fava_c16994	700	A/G	tatctgacaaacctaaattaatacgatcgaaaagaagYcgcctttcagatcttttccaaagctgctgctcgccgaatttcttttctagggtatcgaaaaS[A/G]taagggctaataggacattccgcagagtgccatgttgaaaaatctctgagcaagctcgcattggacatacctgcttcggttaagacctcgacaacatagg

fava_c16996_330	fava_c16996	330	T/C	gcactcttagcaaaactacagcaactgatccaggtggtgccgccccaatctccgtcatgcctccagatgttattcaaactcatatcctcacgagtctcga[T/C]ggtccgtcgctcgcttccgcagccagcacgtgctctcaactcaatatcctctcatcgcatgagcatttatggtgcaaaatttgcaactccacgtggcctt

fava_c16996_681	fava_c16996	681	C/G	ttgatttattccagcgggaaaaggttgttttatcgagggtggtggagactgaaaccgagtctggttggtttcggtgttctccgtttaggattgatattct[C/G]gacccgaaggattctgttgagacgagcatggagtatccgagagaggaggaagcgtgtaggaatctggaagagaaattgagtttgagttggatagtgatcg

fava_c17002_762	fava_c17002	762	G/T	atgacaatgggagattttcgaagatgaagattgaaagcgattctggcgatcgatcaaggttcttggagaattatgcggccggttgaataagaaagagatt[G/T]ctggttttgttggacagctttgggggaagcacataatggagaaaacgcctttgctgattcattagaagcatttggaggaggctacgacgaccctgttagt

fava_c17004_1179	fava_c17004	1179	A/T	cataattgtggataggctttataatggaccttcacaatacacatctcctgttaatatgaaagagttacttcatgtatcaattgccaaatcactttttccc[A/T]tcaattttttcattttattgagtttgatggttacttgttatcaaattaaagtaaagtaaaacatggaaagaaagaaattgtattacaatatgagttgtca

fava_c17006_130	fava_c17006	130	A/T	atatagcaaatcacataattcagactactgttacatgaactgtattttgcatgtcatttccaactaaactatctagactcattcacagtatcacaagcct[A/T]caaatttgttacatcgcaagcttaccatttttaaccacaacgacgtcaacaacattcgttactgtcaaaattttcatccaggggatacaaaagaacatat

fava_c17006_305	fava_c17006	305	T/C	catccaggggatacaaaagaacatatataactttaagtaactagcaaaacaaaattttagggtggggggacctctctggtacactagttgtccattctct[T/C]actgcgtaagctaaccatacaaacagttcccattaagtgctatgcccacatgcagctgctagaatgtagggtttacgcatgcaacttcaagcccaataat

fava_c17006_633	fava_c17006	633	G/A	caaaaactcagaacacaatttggagaggggtggcaacatatgtgatgacccactaaatatggtttcccaaggccgcctatttgatgcagaagagtaaaca[G/A]atgaagttttgttcctttttaaatcaaacttaggcaactctccataaattgatgtatatccaaattgtccattttcttcaagcccaacaaaatcatcatt

fava_c17007_1088	fava_c17007	1088	C/A	cRggcttcattcctacatcaaccggtgttgtgttcacatagagcttaggacccatgaaactgaactgtaaagatgaagaactatgcgacttcctatcagg[C/A]ccaagaacaagctgaccaaatacaggggcccattgccttggaagctggaactccaaaaattgatgcagttcatcaattgtcggtttatatcttaaataga

fava_c17007_124	fava_c17007	124	A/G	ttgtctgcatgaaagaactctaacacagaaatacattaaaaaacagacaaaaaaactaaggtgttgttttgttacagaaacttttctcaacaccacatca[A/G]ggaattctcccaaaacatctgttgtgtagaYatacatacatacacaaataataatgacaacatcaaatagaaacacctataatgaatagagaaaatgtac

fava_c17007_1529	fava_c17007	1529	G/A	taaacttttctttcttattaaatgtcccatcattattgttatattgtccagcctggcgcaggaagaattcgtctgccaagtctttcaattttttttgtac[G/A]tcagaaggttggagaggagacgaatacagctgcttagcataaattatatcggttccccctattttaaccccgactacagcatgggttccatatttctcaa

fava_c17007_155	fava_c17007	155	T/C	tacattaaaaaacagacaaaaaaactaaggtgttgttttgttacagaaacttttctcaacaccacatcaRggaattctcccaaaacatctgttgtgtaga[T/C]atacatacatacacaaataataatgacaacatcaaatagaaacacctataatgaatagagaaaatgtacaacagaaaagaatcagacactattatgtaca

fava_c17007_1763	fava_c17007	1763	G/T	aaggaacagcttgtttgacatgatcactcagaaccacatgtgctttatcaagtgctatgttgtaaagtgtaatggagacaccatcaaatgcaagactctt[G/T]gtattggccgcatctctttgccaaactcctgaaaactgaaacgcagaattgaaatggcccgtcggaatcttacccgacaacgacacctccaggttgaact

fava_c17007_473	fava_c17007	473	T/C	attttactttaagcgatattttacccttctccacaaaaagccttgccccagacacaacccagtagccaggcaaatcttgtggtcctcttgtcatttctgt[T/C]gtgtcaacaaacttcagaagtttaggtgattgagcaggtacaggaggacctccagggtataaagcagagttgacatttacgtccgatggctgtggcggtg

fava_c17007_989	fava_c17007	989	A/G	cctcaagttggaaagtcttggggagggaggagagatgctgtaaatggattgccaaacaattacttttctttccttccaaatgcaatcgaagacctgtcac[A/G]ggcttcattcctacatcaaccggtgttgtgttcacatagagcttaggacccatgaaactgaactgtaaagatgaagaactatgcgacttcctatcaggMc

fava_c17011_186	fava_c17011	186	T/A	ccaaatccccaactcacacaacttcaaacaaaaacctctcattctatataattcttcatcatctagtttaatttatttctattttatttttcaattttgt[T/A]aaattttcccaaattcatggctacagaagcgagagcgaagttccaaaatccagattaccggcccgattttcacaccttgacgccgtcaactcacgacggt

fava_c17011_622	fava_c17011	622	C/T	tctcggtttatgagacttgtaaaaagaaattctcgcaagggaaccctaataattcggtagctcatgctgcttcgggtgtttgtgctacggttgcgagtga[C/T]gcggtgtttacgccgatggatatggtgaaacagaggttgcagttgagtaagagtgcttataagggggtttttgattgtgtgaagagggttttgagtgagg

fava_c17013_1113	fava_c17013	1113	G/A	gtgccggaaaccgctgaaggaagtcgttcttctttttctctacgccagaaggttgaaactcacctaacaagactgaatccaccattaattctttgattct[G/A]tgttgttttgttattaacttgaacttaggttttcaaaaaaRcttttRtagatcttgtgcaattatttgatttYctttgaaaattaRttggagatttRgac

fava_c17013_1154	fava_c17013	1154	G/A	acgccagaaggttgaaactcacctaacaagactgaatccaccattaattctttgattctRtgttgttttgttattaacttgaacttaggttttcaaaaaa[G/A]cttttRtagatcttgtgcaattatttgatttYctttgaaaattaRttggagatttRgactgMggagacaaaaccgaacacggtgatatgaaaaattttcg

fava_c17013_1160	fava_c17013	1160	G/A	gaaggttgaaactcacctaacaagactgaatccaccattaattctttgattctRtgttgttttgttattaacttgaacttaggttttcaaaaaaRctttt[G/A]tagatcttgtgcaattatttgatttYctttgaaaattaRttggagatttRgactgMggagacaaaaccgaacacggtgatatgaaaaattttcgaaactg

fava_c17013_1186	fava_c17013	1186	T/C	tgaatccaccattaattctttgattctRtgttgttttgttattaacttgaacttaggttttcaaaaaaRcttttRtagatcttgtgcaattatttgattt[T/C]ctttgaaaattaRttggagatttRgactgMggagacaaaaccgaacacggtgatatgaaaaattttcgaaactggaaatttcacggcggcgtcgtcgtga

fava_c17013_1199	fava_c17013	1199	A/G	aattctttgattctRtgttgttttgttattaacttgaacttaggttttcaaaaaaRcttttRtagatcttgtgcaattatttgatttYctttgaaaatta[A/G]ttggagatttRgactgMggagacaaaaccgaacacggtgatatgaaaaattttcgaaactggaaatttcacggcggcgtcgtcgtgaggcttcaccagcc

fava_c17013_1210	fava_c17013	1210	A/G	tctRtgttgttttgttattaacttgaacttaggttttcaaaaaaRcttttRtagatcttgtgcaattatttgatttYctttgaaaattaRttggagattt[A/G]gactgMggagacaaaaccgaacacggtgatatgaaaaattttcgaaactggaaatttcacggcggcgtcgtcgtgaggcttcaccagccgccgccgtgag

fava_c17013_1216	fava_c17013	1216	C/A	ttgttttgttattaacttgaacttaggttttcaaaaaaRcttttRtagatcttgtgcaattatttgatttYctttgaaaattaRttggagatttRgactg[C/A]ggagacaaaaccgaacacggtgatatgaaaaattttcgaaactggaaatttcacggcggcgtcgtcgtgaggcttcaccagccgccgccgtgagtacttc

fava_c17013_284	fava_c17013	284	A/T	aatcacaaagatttaagttcaacatcaattctatacatgaattcaagttctaaacctaaaactccaagttatatcatcaattctacatgttacaaacatt[A/T]caagtttcctacacctaaaatgatatggacatcacaaggctttgatttttcttcttcaaccttggactttccaagttcttctaagccacattttcatctt

fava_c17013_570	fava_c17013	570	G/C	catgacgtcaagagaagaattgaattagagttaacaagaatccaacaaattcattccaaatctctcaacaacaaaacaaataaacaacaaagtcactgct[G/C]aattcaagccctcgattcatacccaaagtttcaacaagtgcaataaaaacaattcacaagttcaaacaagttcaataatcattccaattcaacaaccagt

fava_c17013_700	fava_c17013	700	T/A	ttcaacaagtgcaataaaaacaattcacaagttcaaacaagttcaataatcattccaattcaacaaccagtccaacaaaatgattaatttcaggcccaag[T/A]ttaagatgagattatcatgaaaaagattgcaaacaaagttgaattattggactaatacaagcaattttcgttaggttgtgaagagagaaaatgagcgttt

fava_c17013_918	fava_c17013	918	T/G	ggcaacccaactcagatttcacactaactttgttctatcctaagccctaacaatctcacagtatttaaacaatcatcagaggcactgaatagagggattt[T/G]tcagacatatttcaacaacacacaaaaacaStagagaaaaggagaacattacctgaaccgccaccggaacccctgatcggaacattcaacctccgtgccg

fava_c17013_949	fava_c17013	949	G/C	gttctatcctaagccctaacaatctcacagtatttaaacaatcatcagaggcactgaatagagggatttKtcagacatatttcaacaacacacaaaaaca[G/C]tagagaaaaggagaacattacctgaaccgccaccggaacccctgatcggaacattcaacctccgtgccggaaaccgctgaaggaagtcgttcttcttttt

fava_c17014_197	fava_c17014	197	T/C	aataagtattttcaaattttatcctttctctctctatcttttcttatcaaataattttcttatcaagtttagtagatatgtagatttgtagatctggatc[T/C]aaaaaaataattcagYtttgYtaattttctgttgaattttcagatctgatggggataaagaaggttcttcaagaYaaaaggtgggaggattcttgttcct

fava_c17014_213	fava_c17014	213	T/C	ttttatcctttctctctctatcttttcttatcaaataattttcttatcaagtttagtagatatgtagatttgtagatctggatcYaaaaaaataattcag[T/C]tttgYtaattttctgttgaattttcagatctgatggggataaagaaggttcttcaagaYaaaaggtgggaggattcttgttcctgatttagggttcttga

fava_c17014_218	fava_c17014	218	C/T	tcctttctctctctatcttttcttatcaaataattttcttatcaagtttagtagatatgtagatttgtagatctggatcYaaaaaaataattcagYtttg[C/T]taattttctgttgaattttcagatctgatggggataaagaaggttcttcaagaYaaaaggtgggaggattcttgttcctgatttagggttcttgatttgg

fava_c17014_272	fava_c17014	272	C/T	atatgtagatttgtagatctggatcYaaaaaaataattcagYtttgYtaattttctgttgaattttcagatctgatggggataaagaaggttcttcaaga[C/T]aaaaggtgggaggattcttgttcctgatttagggttcttgatttgggYggtttgtgatactttaYtggaatagagaagaaaKatgagaggattcttgttc

fava_c17014_320	fava_c17014	320	T/C	aattttctgttgaattttcagatctgatggggataaagaaggttcttcaagaYaaaaggtgggaggattcttgttcctgatttagggttcttgatttggg[T/C]ggtttgtgatactttaYtggaatagagaagaaaKatgagaggattcttgttcctgatttagggtctttaacaagtatttatgataaRtgaYgattatgta

fava_c17014_337	fava_c17014	337	C/T	tcagatctgatggggataaagaaggttcttcaagaYaaaaggtgggaggattcttgttcctgatttagggttcttgatttgggYggtttgtgatacttta[C/T]tggaatagagaagaaaKatgagaggattcttgttcctgatttagggtctttaacaagtatttatgataaRtgaYgattatgtaattttttttgttttctc

fava_c17014_354	fava_c17014	354	G/T	aaagaaggttcttcaagaYaaaaggtgggaggattcttgttcctgatttagggttcttgatttgggYggtttgtgatactttaYtggaatagagaagaaa[G/T]atgagaggattcttgttcctgatttagggtctttaacaagtatttatgataaRtgaYgattatgtaattttttttgttttctcaagacatgagaattgtt

fava_c17014_407	fava_c17014	407	G/A	ttcttgatttgggYggtttgtgatactttaYtggaatagagaagaaaKatgagaggattcttgttcctgatttagggtctttaacaagtatttatgataa[G/A]tgaYgattatgtaattttttttgttttctcaagacatgagaattgttctattatttaaaaggaggacaagttgatWatggtgaagaatatagcaaggagt

fava_c17014_411	fava_c17014	411	T/C	tgatttgggYggtttgtgatactttaYtggaatagagaagaaaKatgagaggattcttgttcctgatttagggtctttaacaagtatttatgataaRtga[T/C]gattatgtaattttttttgttttctcaagacatgagaattgttctattatttaaaaggaggacaagttgatWatggtgaagaatatagcaaggagtgaga

fava_c17014_483	fava_c17014	483	A/T	gtctttaacaagtatttatgataaRtgaYgattatgtaattttttttgttttctcaagacatgagaattgttctattatttaaaaggaggacaagttgat[A/T]atggtgaagaatatagcaaggagtgagaacactcacaatttggaagaacctacgaacaaggtttggacatttttttaatattggagcatgggttggaaat

fava_c17017_341	fava_c17017	341	C/A	gctctatgcactagtcactaaatgatagcaccatttttttatgaacatctccaataatagcatgtgatataaaaatcagcatgtataattacatgtaaat[C/A]aaaaagtgaatgaaccaaacataagagattcttgcaaaatcatcatgaacaacaaggagSctgtttagttgcagtgacttcatcagctaaataaatctct

fava_c17017_401	fava_c17017	401	C/G	catgtgatataaaaatcagcatgtataattacatgtaaatMaaaaagtgaatgaaccaaacataagagattcttgcaaaatcatcatgaacaacaaggag[C/G]ctgtttagttgcagtgacttcatcagctaaataaatctctctcccattcaaaagcctaagtggtctttcattccttttgacttccaaacttttctggctt

fava_c17017_671	fava_c17017	671	T/C	gtttccataaaacacaaaccttccttctctgcaaactctttaccttctttttcctcaacgtgtcttgattcaactagatcacttttgtttccaaccagaa[T/C]aaccaccatgtcttcgccaccaaattctcttagctccactaaccagtttcttacattctcaaaactctttcgcctcgttatgtcatacactaacaatgct

fava_c17017_888	fava_c17017	888	T/C	aatatgaacttgtgatagctttaaacctttcttggcctgcagtgtcccatatttgagctttgatgaacttatctctaactctgatgtttctgtaagcaaa[T/C]tcaactcctatagttggttttgaatccaacctgaattcatcttttgcaaatcttgataacagatttgattttcccactccagaatcaccggttagtactg

fava_c17021_347	fava_c17021	347	G/C	cactataacattgtataaccaattggagcattaatactcatttcttggaaaccacttcttacttctgataagcctttccttgcaatcgcatactgtgctt[G/C]tgaagcagccaattcatccctaagcttcttcatttttatatccaaagcaacaactttatcctttagttttttcaatttctttgctctctctatatgagat

fava_c17021_609	fava_c17021	609	A/C	tctcaagttcctcccacaaacttctaattttctcgacatcatcttcatccatatctttcactttcttcgtcttaagaaaatgcaatacttgacctaacga[A/C]gttaatgYccattgtcgaaacaatggagWttttttcatcaaaStctctatcaaagacggacgccacgtgcaaatttcatctaaaagtggaacaaaacatt

fava_c17021_617	fava_c17021	617	T/C	tcctcccacaaacttctaattttctcgacatcatcttcatccatatctttcactttcttcgtcttaagaaaatgcaatacttgacctaacgaMgttaatg[T/C]ccattgtcgaaacaatggagWttttttcatcaaaStctctatcaaagacggacgccacgtgcaaatttcatctaaaagtggaacaaaacatttttcttcg

fava_c17021_638	fava_c17021	638	A/T	ttctcgacatcatcttcatccatatctttcactttcttcgtcttaagaaaatgcaatacttgacctaacgaMgttaatgYccattgtcgaaacaatggag[A/T]ttttttcatcaaaStctctatcaaagacggacgccacgtgcaaatttcatctaaaagtggaacaaaacatttttcttcgggttttaaattttccaaatct

fava_c17021_652	fava_c17021	652	C/G	ttcatccatatctttcactttcttcgtcttaagaaaatgcaatacttgacctaacgaMgttaatgYccattgtcgaaacaatggagWttttttcatcaaa[C/G]tctctatcaaagacggacgccacgtgcaaatttcatctaaaagtggaacaaaacatttttcttcgggttttaaattttccaaatctagaagttctcttag

fava_c17021_758	fava_c17021	758	A/C	atcaaagacggacgccacgtgcaaatttcatctaaaagtggaacaaaacatttttcttcgggttttaaattttccaaatctagaagttctcttagtgata[A/C]atccgaactagatgttcttggactcgaagtagaagccgatgttcttggacaaaccgtttcccttgcatgggaggattgatcagtagaatttggRttcaaa

fava_c17021_852	fava_c17021	852	A/G	gtgataMatccgaactagatgttcttggactcgaagtagaagccgatgttcttggacaaaccgtttcccttgcatgggaggattgatcagtagaatttgg[A/G]ttcaaagaattgatgtcatgaattttgggtgtaaaattggagacacaaatatgagcttcaattatgcatgtgtcccttacaagataaccttgtttatgat

fava_c17022_158	fava_c17022	158	C/G	gagacttaagttttaattatatgaataacataattaataaaaataatgattacagaaaaaatatatagtacaacgctaaaagactaactaacttaaaatt[C/G]taaaaggctcaatagtatgaatccaaaaaactagttgattttcttctaattattagaagaaatgggaattattatacttttagtcatagcttggatgtaa

fava_c17022_292	fava_c17022	292	C/A	gttgattttcttctaattattagaagaaatgggaattattatacttttagtcatagcttggatgtaagctacacgccaaagcaaagcctcgagtaggagt[C/A]aatttatcaatttgaacaaagtcttcaatagctggttgtaagctacatcgccaaagctcaagaaaccatgttatctataaatagtgcagcatttcctata

fava_c17024_1083	fava_c17024	1083	T/G	tggttggatcacttcaccctacacctgttgcaacactcccttttctctcaccacattcggcggttgtggcattctgtgaagggattttaaagcacggcac[T/G]gcatgggaagcactgagaaatgaccttgcaactgagaaagttgtaatgactctcgaggaggtgagagaacggatgagaaatgtgctgtcactcacagatg

fava_c17024_1281	fava_c17024	1281	A/G	atgtcacacgctttccaattcctaaaaaccctgatgctgtaatccttgtctccgccactgatgacggatacattccgaaacactcggtcctagaactgca[A/G]aaagcttggccaggttctgaggtgagatgggtcactggcgggcatgtctcatcattccttctccataatgacgagtttcgaagagccattgttgacggcc

fava_c17024_1420	fava_c17024	1420	T/C	cgggcatgtctcatcattccttctccataatgacgagtttcgaagagccattgttgacggccttgatagattaccatggaaagaatctccattgtaataa[T/C]acagttgttgcgcgYgaagaagatatttattttgtaccattaattgattgatcccgtggtcttttccgaaagcttgtaacatgcatagttaatccctctg

fava_c17024_1435	fava_c17024	1435	T/C	attccttctccataatgacgagtttcgaagagccattgttgacggccttgatagattaccatggaaagaatctccattgtaataaYacagttgttgcgcg[T/C]gaagaagatatttattttgtaccattaattgattgatcccgtggtcttttccgaaagcttgtaacatgcatagttaatccctctgaactccttccccaaa

fava_c17024_1537	fava_c17024	1537	G/A	aagaagatatttattttgtaccattaattgattgatcccgtggtcttttccgaaagcttgtaacatgcatagttaatccctctgaactccttccccaaac[G/A]aagagggaaagtgttgtacattatcatctacatgtacattaatgatgtttctgaataagataaagctggatgggaattcttatgtccttataaaatcctc

fava_c17024_216	fava_c17024	216	A/T	gcttctctctctttctcatcctcgtccgatccaatctaatccataacgtcacaatcaccctcctcttccgccattctttccgctctccgatttttcattc[A/T]cttttctaataccctttStgtttttctcaactgggtttaacaWattttccccattttttcgttgggtcattcccaagtttcaaactttaatctKtttaca

fava_c17024_234	fava_c17024	234	C/G	tcctcgtccgatccaatctaatccataacgtcacaatcaccctcctcttccgccattctttccgctctccgatttttcattcWcttttctaatacccttt[C/G]tgtttttctcaactgggtttaacaWattttccccattttttcgttgggtcattcccaagtttcaaactttaatctKtttacaattttgggaaaatagtta

fava_c17024_259	fava_c17024	259	T/A	taacgtcacaatcaccctcctcttccgccattctttccgctctccgatttttcattcWcttttctaataccctttStgtttttctcaactgggtttaaca[T/A]attttccccattttttcgttgggtcattcccaagtttcaaactttaatctKtttacaattttgggaaaatagttatggtgacggtgaatctgggaatgct

fava_c17024_310	fava_c17024	310	T/G	tcattcWcttttctaataccctttStgtttttctcaactgggtttaacaWattttccccattttttcgttgggtcattcccaagtttcaaactttaatct[T/G]tttacaattttgggaaaatagttatggtgacggtgaatctgggaatgcttcattatgtattagaccatgtttatggagcattcatgcatagaactaagat

fava_c17024_411	fava_c17024	411	C/T	tttacaattttgggaaaatagttatggtgacggtgaatctgggaatgcttcattatgtattagaccatgtttatggagcattcatgcatagaactaagat[C/T]agcactccgtttttctcaagaggatggggtggtacaaagcttgatatgcttgagaagatgatcaaccaattgttccctgatttgggggcacagaattggK

fava_c17024_672	fava_c17024	672	T/C	gagaaggggtttttagaactccttgtgaggatcaacttcttggtgcattgccacctgagagtcacattgcaagggtggcttttctcatgccaaaaagtgt[T/C]ccgSctcatagaatgtcttgcgttgttcatcttgcaggaaccggtgaccatacatttgaaagaagactgcgtcttggtggtccattggtgaaggaaaaYa

fava_c17024_676	fava_c17024	676	C/G	aggggtttttagaactccttgtgaggatcaacttcttggtgcattgccacctgagagtcacattgcaagggtggcttttctcatgccaaaaagtgtYccg[C/G]ctcatagaatgtcttgcgttgttcatcttgcaggaaccggtgaccatacatttgaaagaagactgcgtcttggtggtccattggtgaaggaaaaYatcgc

fava_c17024_771	fava_c17024	771	C/T	tYccgSctcatagaatgtcttgcgttgttcatcttgcaggaaccggtgaccatacatttgaaagaagactgcgtcttggtggtccattggtgaaggaaaa[C/T]atcgcaaccatggtgcttgagagtccattctatggacaaagacgtcctgtgctgcagcgMggtgcaaagcttttgtgtgtaagtgacttgcttatattag

fava_c17024_831	fava_c17024	831	C/A	aaagaagactgcgtcttggtggtccattggtgaaggaaaaYatcgcaaccatggtgcttgagagtccattctatggacaaagacgtcctgtgctgcagcg[C/A]ggtgcaaagcttttgtgtgtaagtgacttgcttatattaggaagagcaaccatYgaagaggcgcgcagtctcttacactggttggactctgaggcaggtt

fava_c17024_885	fava_c17024	885	C/T	tgcttgagagtccattctatggacaaagacgtcctgtgctgcagcgMggtgcaaagcttttgtgtgtaagtgacttgcttatattaggaagagcaaccat[C/T]gaagaggcgcgcagtctcttacactggttggactctgaggcaggttttggcaagatgggtgtatgcggtcttagtatgggaggagtacatgctgcaatgg

fava_c17025_1295	fava_c17025	1295	G/C	aaatgaaactgaaattaataattgagaattccaacgacagacaagccacattctcaaagaggatatcataaatgggttgctcaagaaggctaacaatacg[G/C]tagattctaagatgatggagattttaagacaccaaattacaaagctagagacaaacaattaaaagaaaggttttatgaccttggaataatRaacctcaca

fava_c17025_1386	fava_c17025	1386	G/A	aacaatacgStagattctaagatgatggagattttaagacaccaaattacaaagctagagacaaacaattaaaagaaaggttttatgaccttggaataat[G/A]aacctcacaaaccaatgggatcgccagaaagtgtacgaggcatttaaccaaatgaagcactctccaaggctacatacatggctgttgttgaacgacattg

fava_c17025_1533	fava_c17025	1533	C/T	accaaatgaagcactctccaaggctacatacatggctgttgttgaacgacattggctttgcaagaagcagggtgttttgctgttgttctagaatgtgttc[C/T]tgctccggtggctgccgcaacaacagcaRcgcttcaaattccgacaattggaattggggctggaccctattgcagtggacaggtgcttgtttaccatgat

fava_c17025_1562	fava_c17025	1562	G/A	acatggctgttgttgaacgacattggctttgcaagaagcagggtgttttgctgttgttctagaatgtgttcYtgctccggtggctgccgcaacaacagca[G/A]cgcttcaaattccgacaattggaattggggctggaccctattgcagtggacaggtgcttgtttaccatgatcttcttggtatgttacaacaccctcacca

fava_c17028_1229	fava_c17028	1229	T/C	tgttacgccatgtttgaaggttgcaatcatagagaggaaagtgagtgcggaattgagggtgagagagtaagagaaaagaagagaaaggtgaagaagaagg[T/C]gagtgtttggagaYgaatgaaggagagaagatcaatggccagaaacctttgcaagtggatctgagactcatgaaggttttgagaggaaggaaagaRagaa

fava_c17028_1243	fava_c17028	1243	T/C	tgaaggttgcaatcatagagaggaaagtgagtgcggaattgagggtgagagagtaagagaaaagaagagaaaggtgaagaagaaggYgagtgtttggaga[T/C]gaatgaaggagagaagatcaatggccagaaacctttgcaagtggatctgagactcatgaaggttttgagaggaaggaaagaRagaatgttttcgaggatt

fava_c17028_1325	fava_c17028	1325	A/G	aaggYgagtgtttggagaYgaatgaaggagagaagatcaatggccagaaacctttgcaagtggatctgagactcatgaaggttttgagaggaaggaaaga[A/G]agaatgttttcgaggatttctggtggtagtttgctccaaatttcaggatccatgtttgatgtttgaggtggttttggattcttggtgattatgttaaggt

fava_c17028_210	fava_c17028	210	T/G	caactcgaaatcggattctctacaatcaatgtttccacaatcataacataacatttatggtcgttggatcttgatccagacactaaactttacaatcgat[T/G]atacattacatacaataacactagaatagaagaagcataaaacattcagtcttttcacccttcaaacctgagcatacaagttaggaacaaaagaaaacca

fava_c17028_427	fava_c17028	427	T/C	catttcaaagccaaagaagaacacctaggaagccaatgccaagtccttctagaaaccaaataatacaaaatctctggccaagtataacagcatatacaaa[T/C]cattccttcatgccaaaaacaatatacatgctcataattatgataacaaactgacacaaatttcctacacatcaaatcaggcaaactctgaatctcaacc

fava_c17028_619	fava_c17028	619	A/G	atctcaacccaattactcccttcttcattcatttcccataatttaacactccttgaaattccattgctcccaaccccaccaatcaaaaacagttttttac[A/G]atcatcaccatcatcatctttcatactaaccaatctcacaaaagtgagttcattaggcaaatcacaagcaggtctctcccattttccatttcccaaatca

fava_c17028_765	fava_c17028	765	C/T	gagttcattaggcaaatcacaagcaggtctctcccattttccatttcccaaatcaaaatacacaacagaaaaaggctctggtgtagcaaaatacaaacct[C/T]ctttaaagtaaacaccttgttgatgagggttatcactaagaatcggatcaaaaccRttgaatttcctccaagattcaacctttgagtttgagtttgactc

fava_c17028_821	fava_c17028	821	A/G	aatacacaacagaaaaaggctctggtgtagcaaaatacaaacctYctttaaagtaaacaccttgttgatgagggttatcactaagaatcggatcaaaacc[A/G]ttgaatttcctccaagattcaacctttgagtttgagtttgactcataaagataagcattgcttgaagatgattcagaagagagtataaaaatgaaatacc

fava_c17031_1132	fava_c17031	1132	G/A	tatactgggaaacaaacatggaatcactcatacaagattacagaaccgtcgacggaattcaaatagcccatgccgggaaaacaaaggtgaccttgtttag[G/A]tttggtgagaggcccgagactcgttcgaggacaagaatggaagaggtttggcaagttgaagaagttgattttaatattaaaggtttgtcaattgattgtt

fava_c17032_183	fava_c17032	183	G/T	ccattcatacctcgccgtgagtgaccaccacaatccaacaaacccagaccctaattctcatttctccccaatttttcattcctttccttcgtttcaccaa[G/T]attcaaccctaattctccatttcacccctcttgtcacatacacaacttcttatcttatttattttcctcttaattctttaattttctcttcttttttttt

fava_c17032_284	fava_c17032	284	C/T	attcaaccctaattctccatttcacccctcttgtcacatacacaacttcttatcttatttattttcctcttaattctttaattttctcttcttttttttt[C/T]cgacacccYcggtagatcaggtttcagaatccatgcYgacgattccgaaacgaatggtttagtgtataaatttttggaaggtacctacctatctgttgaK

fava_c17032_293	fava_c17032	293	C/T	taattctccatttcacccctcttgtcacatacacaacttcttatcttatttattttcctcttaattctttaattttctcttctttttttttYcgacaccc[C/T]cggtagatcaggtttcagaatccatgcYgacgattccgaaacgaatggtttagtgtataaatttttggaaggtacctacctatctgttgaaagaatgagt

fava_c17032_321	fava_c17032	321	T/C	atacacaacttcttatcttatttattttcctcttaattctttaattttctcttctttttttttYcgacacccYcggtagatcaggtttcagaatccatgc[T/C]gacgattccgaaacgaatggtttagtgtataaatttttggaaggtacctacctatctgttgaaagaatgagtagcagtgacgtagataaaaccttaaaag

fava_c17032_855	fava_c17032	855	G/A	caggcagcagtgaagctgatggtaaacctcatgaggcgaaggaaaaattaccaatcaaaagatcaaaaggaagtattggtagtttgaatatggttacagg[G/A]aaaaataatgaactcagtaaaacaccaggagcatctgccaatggaattcattccaaaagtggtgagagtgcaagcgaaggttctagtgaaggaagtgatg

fava_c17036_1523	fava_c17036	1523	T/C	tccagcccacaagtttgttgccggaacatccaaatccatacacaagcatgccaccagcgccgtttggcatgaaggcaccaacaccaaatgtcccctccta[T/C]acagttccttcaactgcaccgccttatggtcttgatggtgtctcaatggctcccaatggaaacctcaatcaatctatttctcatccgaattcatcacaac

fava_c17036_1627	fava_c17036	1627	T/C	gttccttcaactgcaccgccttatggtcttgatggtgtctcaatggctcccaatggaaacctcaatcaatctatttctcatccgaattcatcacaacaac[T/C]actagttatgccgggttattatgccccaataggtcccagcagcaacatcggtcaaggtggttctcatcctaacctgccagagccacgagttatgccgggt

fava_c17038_1039	fava_c17038	1039	A/T	ccactggtcattattctcRaccaaaattgccatccattaaaggaatggatacatggaaaagaaaacaaatgcacagtcacatttacaggactccagaacc[A/T]ttccacaatgaggttgtggtgttagttggaaattcctttagtggacaagatatagcaatagagctagtgggagtagcaaaggaaatccatataagttcga

fava_c17038_1245	fava_c17038	1245	C/T	cttaatactataactgaagggttatctaaagtcatatccaaacatgacaccttacatcttcacccacagattgacacacttgaagaggatggaagggtga[C/T]ttttaaggatggttcttggcttaaagcagacactattgtRtactgtaccgggtactcctatagcttcccttttcttgacaccaaaggaatggtggttgtt

fava_c17038_1285	fava_c17038	1285	A/G	aacatgacaccttacatcttcacccacagattgacacacttgaagaggatggaagggtgaYttttaaggatggttcttggcttaaagcagacactattgt[A/G]tactgtaccgggtactcctatagcttcccttttcttgacaccaaaggaatggtggttgttgatcatgacagagtagggcctttgtttgagcacacttttc

fava_c17038_1796	fava_c17038	1796	C/T	ggagacatatagagattcatgggatgatgatgagttgcttcaagaggcacttaaaagtcctcacttcactcaacttggacttgaagattgactcttttct[C/T]SgaatctgagatatgtagaaataaaatttaaataaaaaSttgatttaaataaatctgtaaaaggtttatgcatgtgttcaattatatttatctattgtat

fava_c17038_1797	fava_c17038	1797	C/G	gagacatatagagattcatgggatgatgatgagttgcttcaagaggcacttaaaagtcctcacttcactcaacttggacttgaagattgactcttttctY[C/G]gaatctgagatatgtagaaataaaatttaaataaaaaSttgatttaaataaatctgtaaaaggtttatgcatgtgttcaattatatttatctattgtatg

fava_c17038_1835	fava_c17038	1835	G/C	tcaagaggcacttaaaagtcctcacttcactcaacttggacttgaagattgactcttttctYSgaatctgagatatgtagaaataaaatttaaataaaaa[G/C]ttgatttaaataaatctgtaaaaggtttatgcatgtgttcaattatatttatctattgtatggtgtagtcactttccaacataaataataatattgtgag

fava_c17038_532	fava_c17038	532	A/G	cgacgaaccaatccaagaatgtgtgtgtaattggagccgggccaKccggcctagtagcggcgcgagagctaagaaaagaaggtcacaaagcaatagtctt[A/G]gaacaaaatcatgacattggaggacaatggttatacgacgataaaaatgtcgaaggagaggatcctttaggaagaaacccttttctaaaagttcatagca

fava_c17038_839	fava_c17038	839	C/G	tgttgaagtgcttaggtatctaaaggatttttgtgagtggtttgggttgagggagatgataaagttcaacacaagggttcaacatgttggaatgttggat[C/G]ataatggtgtttgtagaaatgaSttaaagtggattgttacaagtaaagaaaagaattgtgataagKttgttgaagaagtttttgatgcagttgttgttgc

fava_c17038_862	fava_c17038	862	C/G	aggatttttgtgagtggtttgggttgagggagatgataaagttcaacacaagggttcaacatgttggaatgttggatSataatggtgtttgtagaaatga[C/G]ttaaagtggattgttacaagtaaagaaaagaattgtgataagKttgttgaagaagtttttgatgcagttgttgttgccactggtcattattctcRaccaa

fava_c17038_905	fava_c17038	905	G/T	caacacaagggttcaacatgttggaatgttggatSataatggtgtttgtagaaatgaSttaaagtggattgttacaagtaaagaaaagaattgtgataag[G/T]ttgttgaagaagtttttgatgcagttgttgttgccactggtcattattctcRaccaaaattgccatccattaaaggaatggatacatggaaaagaaaaca

fava_c17038_957	fava_c17038	957	A/G	aatgaSttaaagtggattgttacaagtaaagaaaagaattgtgataagKttgttgaagaagtttttgatgcagttgttgttgccactggtcattattctc[A/G]accaaaattgccatccattaaaggaatggatacatggaaaagaaaacaaatgcacagtcacatttacaggactccagaaccWttccacaatgaggttgtg

fava_c17039_1016	fava_c17039	1016	C/T	tacatatgcagggcYgctaatgctgttagagtattatccacgttaggattcgttataacattggaaatcattcagggagtYattgatctaagcctttctg[C/T]gaatctagatatacacgacatgcttggttctgagttctgtgttttggtcgctgaaaaMgaggcagaaaggagaagctcaactaaaaagaggcggtaaatt

fava_c17039_1074	fava_c17039	1074	A/C	cattggaaatcattcagggagtYattgatctaagcctttctgYgaatctagatatacacgacatgcttggttctgagttctgtgttttggtcgctgaaaa[A/C]gaggcagaaaggagaagctcaactaaaaagaggcggtaaattgtggaagggtaggggtaaagaagaggaaaggaatcattctaattgtagcaagtcttta

fava_c17039_1218	fava_c17039	1218	C/G	tggaagggtaggggtaaagaagaggaaaggaatcattctaattgtagcaagtctttattcaacggcattgatactctgtccggggacactaagagttaga[C/G]aaacttcttttttatgttaaagttcttgcacctataggatatgtggaatgaattgtttttctKattggtggttgccaccttgctttccaggagtgataca

fava_c17039_1281	fava_c17039	1281	G/T	ggcattgatactctgtccggggacactaagagttagaSaaacttcttttttatgttaaagttcttgcacctataggatatgtggaatgaattgtttttct[G/T]attggtggttgccaccttgctttccaggagtgatacataccagctcatgtgtgtattcttatgcaataatcttStattgaaggttgtYtctggagctttt

fava_c17039_1355	fava_c17039	1355	C/G	ggatatgtggaatgaattgtttttctKattggtggttgccaccttgctttccaggagtgatacataccagctcatgtgtgtattcttatgcaataatctt[C/G]tattgaaggttgtYtctggagcttttgtatactgagttgtggYaaaatttgtaatgattttttcactatttttcattggaaacgtagactaaagttttat

fava_c17039_1369	fava_c17039	1369	C/T	aattgtttttctKattggtggttgccaccttgctttccaggagtgatacataccagctcatgtgtgtattcttatgcaataatcttStattgaaggttgt[C/T]tctggagcttttgtatactgagttgtggYaaaatttgtaatgattttttcactatttttcattggaaacgtagactaaagttttatgaaccaatttatat

fava_c17039_1398	fava_c17039	1398	C/T	ttgctttccaggagtgatacataccagctcatgtgtgtattcttatgcaataatcttStattgaaggttgtYtctggagcttttgtatactgagttgtgg[C/T]aaaatttgtaatgattttttcactatttttcattggaaacgtagactaaagttttatgaaccaatttatattgtagtggtctaagttgttttaatgcaca

fava_c17039_588	fava_c17039	588	C/T	acagtcaaaatgcttatctcatgctttctgctatgaatatgcctgatggtgtccagaggcgaaggacagctttagatgagttgaatatgcgttgtatttt[C/T]gaggaggcagatgtcatttgcgccgaagttcgtggttttcagcatgatggcctacaccttcaagcaagaagtaaaaaatatggaaagcttactactggtc

fava_c17039_750	fava_c17039	750	G/A	aagcaagaagtaaaaaatatggaaagcttactactggtcaactgattacggtcacaccgtatttagtgaagagacaaaaacaacatttccatcatctagt[G/A]cagtatggtattgatctaatacttggctgtaatggattcatatgggttggggaacatgtcgaagctagagatgacatgatagaagatcaagtgaaccaaa

fava_c17039_879	fava_c17039	879	A/G	gtaatggattcatatgggttggggaacatgtcgaagctagagatgacatgatagaagatcaagtgaaccaaactgatccacaagttttaagtcttgaaga[A/G]caagagaaaaactatacgtcactggagactagaaagtacatatgcagggcYgctaatgctgttagagtattatccacgttaggattcgttataacattgg

fava_c17039_930	fava_c17039	930	T/C	tagaagatcaagtgaaccaaactgatccacaagttttaagtcttgaagaRcaagagaaaaactatacgtcactggagactagaaagtacatatgcagggc[T/C]gctaatgctgttagagtattatccacgttaggattcgttataacattggaaatcattcagggagtYattgatctaagcctttctgYgaatctagatatac

fava_c17039_996	fava_c17039	996	T/C	cgtcactggagactagaaagtacatatgcagggcYgctaatgctgttagagtattatccacgttaggattcgttataacattggaaatcattcagggagt[T/C]attgatctaagcctttctgYgaatctagatatacacgacatgcttggttctgagttctgtgttttggtcgctgaaaaMgaggcagaaaggagaagctcaa

fava_c17044_662	fava_c17044	662	A/T	taaactcaggaagaatgaagtagttgatccgagatgaagcatcaaacgggttgatatagtcagcttccaagtcagataaaatcaaaacctggtagaaatt[A/T]gaagcgagaagagcRatRttggcggggaaagaaatcacaagccagaagaagaggttccaacccatttacagaagaaaagagtaaagtagggtttacgaaa

fava_c17044_677	fava_c17044	677	G/A	tgaagtagttgatccgagatgaagcatcaaacgggttgatatagtcagcttccaagtcagataaaatcaaaacctggtagaaattWgaagcgagaagagc[G/A]atRttggcggggaaagaaatcacaagccagaagaagaggttccaacccatttacagaagaaaagagtaaagtagggtttacgaaaaagaggatctgaagt

fava_c17044_680	fava_c17044	680	A/G	agtagttgatccgagatgaagcatcaaacgggttgatatagtcagcttccaagtcagataaaatcaaaacctggtagaaattWgaagcgagaagagcRat[A/G]ttggcggggaaagaaatcacaagccagaagaagaggttccaacccatttacagaagaaaagagtaaagtagggtttacgaaaaagaggatctgaagttgg

fava_c17046_220	fava_c17046	220	T/A	cccttttattcaattcttcaggacttattgatttttctctccttaaagaagctctatcattgaaagtttctgactttttcaattccctttgagcccaagc[T/A]actggatcatcatcgtcgtcaccgccaccaccaccaaaaccaccattgttacgaaatggctctgctttcacaatccttgcagtccaagtatcacttttct

fava_c17052_1013	fava_c17052	1013	G/A	ttatcaatctgtcgattctgaaagcatcgacgcctaagtggagcaataaatgggcaccttgtgcgtaWgggaaaaaYgttctgttcacggcgaagccagt[G/A]cagttacYgaccaccacaggagacttttgtatgactttaccaactgcaatcagatcaagaataacttgtgcagatgttttatttgttcgaacaatctcca

fava_c17052_1021	fava_c17052	1021	C/T	ctgtcgattctgaaagcatcgacgcctaagtggagcaataaatgggcaccttgtgcgtaWgggaaaaaYgttctgttcacggcgaagccagtRcagttac[C/T]gaccaccacaggagacttttgtatgactttaccaactgcaatcagatcaagaataacttgtgcagatgttttatttgttcgaacaatctccaaaagaggc

fava_c17052_139	fava_c17052	139	C/A	tggaccgcacaaattttcaaaaagtttgcattcaaaataaatgttgaggaccatgcattttcaaaatgtaacaagaaaagcataaaccttatgaaccccc[C/A]aaaaaaaaacatttattttcctgctaagtgtgtgggggaatatactaaaaggaaatataattttatttattatttaaaacttgaaaggaagaaaagatta

fava_c17052_1814	fava_c17052	1814	G/A	gtatcccatgttcaatcacatctacgcaagcttgttgctgaggcaaatgtggagcagtcttcttgacacgttgtctggcagttgccaacacctcacgtgc[G/A]tcggaaccaagtttgtctgtcctatgaagtgaacgaacccaaggtttgcgcttttcggcaatttcaagagcccacagcctagatacctttagtaactcct

fava_c17052_1939	fava_c17052	1939	T/G	tgaagtgaacgaacccaaggtttgcgcttttcggcaatttcaagagcccacagcctagatacctttagtaactcctcggaagatacaatagcatcaataa[T/G]tcctagtttctgcccttcttccgacgtgattggtttagatgtcagcatcatttcaactgctttagacgtcccaacaagcctcggaagacgctgtgtacct

fava_c17052_2342	fava_c17052	2342	T/C	caccggtttggtgaactttctgcataacactgatatcaaacccaccagaaaatctcccacctttaccagtcaaaactatagcttttacatcattcctcct[T/C]gccgcttcatcgaatttatatttcaacgccctgataattggaagagctaaagcattaacaggtggattacatatggtgatgacagcaaccccRtcatttc

fava_c17052_2435	fava_c17052	2435	A/G	tcctcctYgccgcttcatcgaatttatatttcaacgccctgataattggaagagctaaagcattaacaggtggattacatatggtgatgacagcaacccc[A/G]tcatttccaacctgaaaatcaaccttaacagatgccatctaagagataaatggRtRtKttttgaataaagaaagagtttttttggatgaatggagaagaa

fava_c17052_2489	fava_c17052	2489	A/G	ctaaagcattaacaggtggattacatatggtgatgacagcaaccccRtcatttccaacctgaaaatcaaccttaacagatgccatctaagagataaatgg[A/G]tRtKttttgaataaagaaagagtttttttggatgaatggagaagaaaatgagatgagtttatagtggcatagggaatgaatcagaggaacaagaagaggc

fava_c17052_2491	fava_c17052	2491	G/A	aaagcattaacaggtggattacatatggtgatgacagcaaccccRtcatttccaacctgaaaatcaaccttaacagatgccatctaagagataaatggRt[G/A]tKttttgaataaagaaagagtttttttggatgaatggagaagaaaatgagatgagtttatagtggcatagggaatgaatcagaggaacaagaagaggcgt

fava_c17052_2493	fava_c17052	2493	T/G	agcattaacaggtggattacatatggtgatgacagcaaccccRtcatttccaacctgaaaatcaaccttaacagatgccatctaagagataaatggRtRt[T/G]ttttgaataaagaaagagtttttttggatgaatggagaagaaaatgagatgagtttatagtggcatagggaatgaatcagaggaacaagaagaggcgtct

fava_c17052_267	fava_c17052	267	A/T	gtgtgtgggggaatatactaaaaggaaatataattttatttattatttaaaacttgaaaggaagaaaagattatgagctaaattccctatatcttagaac[A/T]tccaacttgaggaaaactttgtaagatcatatccttctcacagtcgcgccttcgtYgttggattagatgaagcRggtgcactcaaaggaatgccttttgt

fava_c17052_323	fava_c17052	323	C/T	aaaggaagaaaagattatgagctaaattccctatatcttagaacWtccaacttgaggaaaactttgtaagatcatatccttctcacagtcgcgccttcgt[C/T]gttggattagatgaagcRggtgcactcaaaggaatgccttttgttgctctttcttccaaataccttgatggtttataaaagttaccatataattgtgtcc

fava_c17052_341	fava_c17052	341	A/G	gagctaaattccctatatcttagaacWtccaacttgaggaaaactttgtaagatcatatccttctcacagtcgcgccttcgtYgttggattagatgaagc[A/G]ggtgcactcaaaggaatgccttttgttgctctttcttccaaataccttgatggtttataaaagttaccatataattgtgtccatttctttaggctgctat

fava_c17052_636	fava_c17052	636	G/A	agatgctcgaataacaactccttcttctaaaacacgacaagcctcgttcactattggaaagagtaccatctccacaatctcttggtcagagacagatacc[G/A]gctttccattgggagcaatRttgcaaattcttctagactcctcaatgattggtagtacggaaggatcgggttttggtttgctacccttttcataaatgta

fava_c17052_656	fava_c17052	656	A/G	cttcttctaaaacacgacaagcctcgttcactattggaaagagtaccatctccacaatctcttggtcagagacagataccRgctttccattgggagcaat[A/G]ttgcaaattcttctagactcctcaatgattggtagtacggaaggatcgggttttggtttgctacccttttcataaatgtagtaYcctctcccattatttt

fava_c17052_740	fava_c17052	740	C/T	ttccattgggagcaatRttgcaaattcttctagactcctcaatgattggtagtacggaaggatcgggttttggtttgctacccttttcataaatgtagta[C/T]cctctcccattattttttccgtttcgcccgcttttgatcaaaagatcaaccagtggacttttgaatgtgcggtcaggaaatgcaccagcaaattcttttc

fava_c17052_980	fava_c17052	980	T/A	gctggagaggacccatgggaaggccaaaattggttatcaatctgtcgattctgaaagcatcgacgcctaagtggagcaataaatgggcaccttgtgcgta[T/A]gggaaaaaYgttctgttcacggcgaagccagtRcagttacYgaccaccacaggagacttttgtatgactttaccaactgcaatcagatcaagaataactt

fava_c17052_989	fava_c17052	989	C/T	gacccatgggaaggccaaaattggttatcaatctgtcgattctgaaagcatcgacgcctaagtggagcaataaatgggcaccttgtgcgtaWgggaaaaa[C/T]gttctgttcacggcgaagccagtRcagttacYgaccaccacaggagacttttgtatgactttaccaactgcaatcagatcaagaataacttgtgcagatg

fava_c17053_143	fava_c17053	143	A/G	aattaggaaaaacaacaaaatagataaaacataaatttatttgtgatattcatgaattcttatgacaaggataaatgaaataaagtcaataaaccaatcg[A/G]aaaaagtttccatcttcaaattcattcttgcaaaaaagtgcttatatcatcggaaagcaaaacacgtttacgcagttcagcagatacaccgttaatcaga

fava_c17053_274	fava_c17053	274	T/A	caaaaaagtgcttatatcatcggaaagcaaaacacgtttacgcagttcagcagatacaccgttaatcagatttcaaatcttctcaatatcgtagcagaca[T/A]actcgaggtaattgtggtaatgatggggtgtttcccttcaacacccaagaaactggcaatgaccattgcaggttccctctctggagctgccgttttagct

fava_c17054_1018	fava_c17054	1018	C/T	tgacttccatttgtgttggtcgaatcgggaaggattcaggaSWtgaatccgatcagtataggataatgagtgtcgggttggatggttattttaaggtgtt[C/T]gattacggttctatgaaggttacttattcgatgaggtttcctgcgccgctMatgtctgttgggtattcaccggattgttctactagggtgatYgggactt

fava_c17054_1069	fava_c17054	1069	C/A	atcagtataggataatgagtgtcgggttggatggttattttaaggtgttYgattacggttctatgaaggttacttattcgatgaggtttcctgcgccgct[C/A]atgtctgttgggtattcaccggattgttctactagggtgatYgggacttcgaatggaataatttatgctgccaagaggaagataaacaagagtgagattg

fava_c17054_1111	fava_c17054	1111	T/C	aggtgttYgattacggttctatgaaggttacttattcgatgaggtttcctgcgccgctMatgtctgttgggtattcaccggattgttctactagggtgat[T/C]gggacttcgaatggaataatttatgctgccaagaggaagataaacaagagtgagattgcagagagtgagggtagtgagggtagtgcgttttggagaatta

fava_c17054_128	fava_c17054	128	T/C	tgaacatggtgtattgagtgttaagattttacagtgccgccacctcccaaaccttaaaccctaattcatcaaacgatcaaagacagtgaagggtttaggt[T/C]tagctgcatcaatggaggaaagcaaagtatcgaaaagcttcccagtaaaaccaaatctcaaaaccaaaccacgaactccaaagcaaacctccgaatccaa

fava_c17054_1444	fava_c17054	1444	A/G	atgataagcttttgaacaagtttaggcacggggaggctctagtttgtgtgttggaaggtaagaatccgggctatgtggttgctgtgatggaggaattggt[A/G]tctaggaagaaactgttgaggtgtgtttctgatttggatttggagaagctcgaactgcttttggctttcttgcataagtattgtactgtccccaagtatt

fava_c17054_1818	fava_c17054	1818	C/T	attgctggaagaagatgaaattttggccttttgccctcatctatgagttgtttttgttcacttgttctgttatattttagccttttatttgttttttttc[C/T]ccagaatttgtgtgccttgtaaagcaccctgaagtgttatggctaactattgagttattctctactgttttgtacgaaagtaaaggaaaattcttttcat

fava_c17054_703	fava_c17054	703	T/C	gggatgttgctgaggagactatgattcatcagttttacgggcataaggattatgtcagatgcggtgattcgtctccggttaattctgatacttttgttac[T/C]ggctcttatgatcatttagtcaagctttgggatgttagggttaaggattcgtccaaatctgcaatggattttaaccatggctctcctgtggaggatgttg

fava_c17054_959	fava_c17054	959	G/C	ttgggacttgattggtggtgggaagttactctattccatggagagtcataacaagactgtgacttccatttgtgttggtcgaatcgggaaggattcagga[G/C]WtgaatccgatcagtataggataatgagtgtcgggttggatggttattttaaggtgttYgattacggttctatgaaggttacttattcgatgaggtttcc

fava_c17054_960	fava_c17054	960	A/T	tgggacttgattggtggtgggaagttactctattccatggagagtcataacaagactgtgacttccatttgtgttggtcgaatcgggaaggattcaggaS[A/T]tgaatccgatcagtataggataatgagtgtcgggttggatggttattttaaggtgttYgattacggttctatgaaggttacttattcgatgaggtttcct

fava_c17064_619	fava_c17064	619	A/G	ggacaatcccgtttgcaagaaaccgaacaaccatcacaccaaaaaggttcattttgctcatattacttatctggagaattcgggtaaggaacttgcactg[A/G]ccgtgagaaatccaaacacttctttggaattgagctctaatgggaaaagtcctcttccccttgctcaacaagctgcgtcgacccagtcgactcactatca

fava_c17064_744	fava_c17064	744	G/A	tggaattgagctctaatgggaaaagtcctcttccccttgctcaacaagctgcgtcgacccagtcgactcactatcagttctttcagcatcagcagcagca[G/A]cagcaacagaggttgatgatgcagcagcaacagcaScagcagcaccagcagcagcagcaaatgaagcatcaagcagaaatgatgtttcgaaggaacatta

fava_c17064_780	fava_c17064	780	G/C	ttgctcaacaagctgcgtcgacccagtcgactcactatcagttctttcagcatcagcagcagcaRcagcaacagaggttgatgatgcagcagcaacagca[G/C]cagcagcaccagcagcagcagcaaatgaagcatcaagcagaaatgatgtttcgaaggaacattaacagcataaaccttagttttgacaacactactagcg

fava_c17065_1108	fava_c17065	1108	T/G	aattttcactaaactccaaagaaattaagaatgaaaacaaaatagaagacatagcaagtttaatacacatgcacatgaaagatgttagtgagaaagaaat[T/G]tggtcttctatagaatcgtttgagtcggaaaaagaatcgaYaccgaaatgcatgtatggttcttctgagttaacatgtgtttgcatggaagaggaagaga

fava_c17065_1149	fava_c17065	1149	C/T	atagaagacatagcaagtttaatacacatgcacatgaaagatgttagtgagaaagaaatKtggtcttctatagaatcgtttgagtcggaaaaagaatcga[C/T]accgaaatgcatgtatggttcttctgagttaacatgtgtttgcatggaagaggaagagacaRaatcgttgttgtatgaatcaatgtttggtgataatgaa

fava_c17065_1211	fava_c17065	1211	G/A	gtcttctatagaatcgtttgagtcggaaaaagaatcgaYaccgaaatgcatgtatggttcttctgagttaacatgtgtttgcatggaagaggaagagaca[G/A]aatcgttgttgtatgaatcaatgtttggtgataatgaaaaaccagctcatgtttcgtgtcgtattgggaatgttggtggtgaaggtttggttatagtgat

fava_c17065_1423	fava_c17065	1423	A/G	aaggagggtttgcaagaaatgtgatagtaatgttgcaagaagaacaagttgataaactatgtaaggatgaagaaatattgaagcttgaaccaacgattat[A/G]ttttctggttgaaataattaattactttacttgtgcttaaaattattattcaatctatttcaatattattgttgtttcagcgaatgaaatttattttaaa

fava_c17065_209	fava_c17065	209	G/T	agaaccaaggatgtcagattgtaaggtaacacttgattcaaaactaacggttgtttcaagtagacctgtgaaatcgggtatgacccataagttgctggga[G/T]tggaccatggcatgggttatcatacactgcatttgatattttactacaagaatgaagaaaagtggtttgagtcatttggattggatccattgagggaatc

fava_c17065_403	fava_c17065	403	C/T	gggaatcattgtctgaggttttgtctttatacccgacagtaacaggtcggttgggccgggagacggaagacggtggtgattgggaggttaggtgtaacga[C/T]gccggtgttcgggtcataaaggctaatgtggatagtactattgaagaatggctatcatcgtctaagggtgSctccgatgaagtgcagcttatcgcttggg

fava_c17065_474	fava_c17065	474	G/C	ggtggtgattgggaggttaggtgtaacgaYgccggtgttcgggtcataaaggctaatgtggatagtactattgaagaatggctatcatcgtctaagggtg[G/C]ctccgatgaagtgcagcttatcgcttgggatgatatgcctcttgatacaagtacatggtctcctttccgaattcagataaatagcttcaaggaaggtgga

fava_c17065_774	fava_c17065	774	T/C	atttctcacccacctttctatactccagtttcattcacttcttcaccaaacaacaagaatatgatgagtccgaattcgggaccaaaaccacaaacaaaca[T/C]aacctcggccactttcaaattctcaagttcaatcataaaacaattggcctcaaaatgtcctggtgccacggcttttgatatcctcgcagctttgttttgg

fava_c17065_881	fava_c17065	881	G/A	ggccactttcaaattctcaagttcaatcataaaacaattggcctcaaaatgtcctggtgccacggcttttgatatcctcgcagctttgttttggtctcgt[G/A]tKactggtttgRaggattctaaaatgtgtaaacWaaatcattctttatcgatctgYcgagactttagaaagcttatcaacccgaatctcccagttgatta

fava_c17065_883	fava_c17065	883	T/G	ccactttcaaattctcaagttcaatcataaaacaattggcctcaaaatgtcctggtgccacggcttttgatatcctcgcagctttgttttggtctcgtRt[T/G]actggtttgRaggattctaaaatgtgtaaacWaaatcattctttatcgatctgYcgagactttagaaagcttatcaacccgaatctcccagttgattatt

fava_c17065_893	fava_c17065	893	A/G	attctcaagttcaatcataaaacaattggcctcaaaatgtcctggtgccacggcttttgatatcctcgcagctttgttttggtctcgtRtKactggtttg[A/G]aggattctaaaatgtgtaaacWaaatcattctttatcgatctgYcgagactttagaaagcttatcaacccgaatctcccagttgattatttcggaaacgc

fava_c17065_915	fava_c17065	915	A/T	caattggcctcaaaatgtcctggtgccacggcttttgatatcctcgcagctttgttttggtctcgtRtKactggtttgRaggattctaaaatgtgtaaac[A/T]aaatcattctttatcgatctgYcgagactttagaaagcttatcaacccgaatctcccagttgattatttcggaaacgctttacatttttcgaaattttca

fava_c17065_937	fava_c17065	937	C/T	gtgccacggcttttgatatcctcgcagctttgttttggtctcgtRtKactggtttgRaggattctaaaatgtgtaaacWaaatcattctttatcgatctg[C/T]cgagactttagaaagcttatcaacccgaatctcccagttgattatttcggaaacgctttacatttttcgaaattttcactaaactccaaagaaattaaga

fava_c17066_1053	fava_c17066	1053	C/T	tggatgaagcataaaagcaatgtgatacttatggaaagataccattttttcgcttcaagttgtcggcaatttggcttcaattgcaaatctcttgctgaaa[C/T]gaagagcgatgaaaatgaaattgatggagcacttgcaaaaatacttgaagtgctaaagcaaatccatagcacattctttgataaacttcaaggagatctt

fava_c17066_1285	fava_c17066	1285	T/C	cagttcgaggtgaaatattaagtgaatgtgtgatcgttttcagtcgtattaatcatggtgcattgccaactcttcagagagttgcacagcaaatgggagc[T/C]acctgtttgacagaacttgaccccacagtaacacatgtggttgctactgatgctggaactgagaagtcccgatgggcagttaaagaaaaKaagtttYtgg

fava_c17066_1375	fava_c17066	1375	T/G	aaatgggagcYacctgtttgacagaacttgaccccacagtaacacatgtggttgctactgatgctggaactgagaagtcccgatgggcagttaaagaaaa[T/G]aagtttYtggttcatcctcgatggatagaagctgcaaattacttttggcaaaaacaaccggaggagaactttggtctaaaaaagccgactaacaactcgt

fava_c17066_1382	fava_c17066	1382	T/C	agcYacctgtttgacagaacttgaccccacagtaacacatgtggttgctactgatgctggaactgagaagtcccgatgggcagttaaagaaaaKaagttt[T/C]tggttcatcctcgatggatagaagctgcaaattacttttggcaaaaacaaccggaggagaactttggtctaaaaaagccgactaacaactcgtagtatag

fava_c17066_1615	fava_c17066	1615	A/G	aaaacttctctttttaacttcttattttggtagaagcaaaatgagaactttctcttgtgccaacacaagcttaggcttttattataattttgtagtttat[A/G]aaaccaaagaacgtggaaggatacaatgctattgcagttataacatgtacagtttttattggttcccattgttttttttaattccttgagtgttgtaaca

fava_c17066_378	fava_c17066	378	T/C	gatgtcagcaataaaagacgtaagtttgaaagtactgaagaaactgaggggtctacttcagaaggaattgtgaaacatgaatcagagtcatccgtgaagg[T/C]agatgtYtgtacacatcctggctcatttggaaacatgtgtatatgttgtgggcaaaagttggatgaggaatctggtgtgacatttgggtacatacacaag

fava_c17066_385	fava_c17066	385	T/C	gcaataaaagacgtaagtttgaaagtactgaagaaactgaggggtctacttcagaaggaattgtgaaacatgaatcagagtcatccgtgaaggYagatgt[T/C]tgtacacatcctggctcatttggaaacatgtgtatatgttgtgggcaaaagttggatgaggaatctggtgtgacatttgggtacatacacaagggactga

fava_c17066_616	fava_c17066	616	C/T	gtaatacagacatgaagaaatttttatacagtagaaaactttacttggttcttgacctggatcacacattgttaaataccactatccttagtcatttgag[C/T]ccagaagagttgcatttacataatgagacagattctctagaagacgtctccaaaggYagcctcttcaaattggaRcatatgtgtatgataaccaagttga

fava_c17066_673	fava_c17066	673	T/C	tggatcacacattgttaaataccactatccttagtcatttgagYccagaagagttgcatttacataatgagacagattctctagaagacgtctccaaagg[T/C]agcctcttcaaattggaRcatatgtgtatgataaccaagttgaggccctttgtccgtacgtttcttaaagaagcaagtgaaatgtttgagatgtacatct

fava_c17066_691	fava_c17066	691	A/G	ataccactatccttagtcatttgagYccagaagagttgcatttacataatgagacagattctctagaagacgtctccaaaggYagcctcttcaaattgga[A/G]catatgtgtatgataaccaagttgaggccctttgtccgtacgtttcttaaagaagcaagtgaaatgtttgagatgtacatctacaccatgggtgataggc

fava_c17066_801	fava_c17066	801	T/C	atgataaccaagttgaggccctttgtccgtacgtttcttaaagaagcaagtgaaatgtttgagatgtacatctacaccatgggtgataggccatatgcat[T/C]ggagatggccaagctccttgatcctcaaggagaatacttcaatggtaaagtgatttctcgagatgatgggactcaaaagcatcagaagggtctcgatatt

fava_c17069_1385	fava_c17069	1385	T/G	atcattctgttctgtttttgctctcacatggaatcataatagcgaatgaaactttttccatatttgtaaattttaaagttaagttaagggaaaagtaaat[T/G]gaaaaaagaagcaatagtgcaatgctatgagcatgaggcccatgaaaggtacctagaagctacaacacaaaagtgcaataccgtgagcagtgggaactaa

fava_c17069_363	fava_c17069	363	G/A	ttattttaaccacaacaaactaccaacaaccgttctttcctctgtcttttccatactttgccgtttctcgaaaaaacaagaccaaatttctgggtattgt[G/A]tccttaaagttttgtcttgtgaccattcagtacgtaacttttgacgctggagaacaaaaataaacactgtccttttttttcttcttggaaagtgatggaa

fava_c17069_649	fava_c17069	649	C/T	ttttggtttcggatcacataaacgcgtaccaatactcttctgagaaagtagatagttttgtggttgatatggattctttctcttctagcatcaataaaga[C/T]atcgtcaatgccaattcaagaacaacattgcagaggagcctttctaggaaaggatctcaacgttggggtgataggaatgtgaatggttgtaatggtgcag

fava_c17070_152	fava_c17070	152	A/T	gattttcattttcagctttttctcgttcattcaattcggttttcttcatgatgccgagtgtgaaccccgattcatctgacaaagactccgaggtattcgc[A/T]gaaactgatccaactggtMgataYggtagatacactgagctattaggctgcggtgcggtgaagaaagtgtaccgaggatttgatcaagaagaaggaattg

fava_c17070_171	fava_c17070	171	C/A	ttctcgttcattcaattcggttttcttcatgatgccgagtgtgaaccccgattcatctgacaaagactccgaggtattcgcWgaaactgatccaactggt[C/A]gataYggtagatacactgagctattaggctgcggtgcggtgaagaaagtgtaccgaggatttgatcaagaagaaggaattgaggtagcatggaatcaagt

fava_c17070_176	fava_c17070	176	C/T	gttcattcaattcggttttcttcatgatgccgagtgtgaaccccgattcatctgacaaagactccgaggtattcgcWgaaactgatccaactggtMgata[C/T]ggtagatacactgagctattaggctgcggtgcggtgaagaaagtgtaccgaggatttgatcaagaagaaggaattgaggtagcatggaatcaagtgaagc

fava_c17070_387	fava_c17070	387	G/A	ctgtgatgagcctgctatggtaaagaggctttatgctgaagtgaggttattagaaaacttaacaaacgaaaacatcattgaactttacagtgtttggacc[G/A]atgatgaacacggcacactgaattttatYacYgaggtttgtactagtgggaatttgagagagtacaggaagaaacataggcatgtttcgatgaaggcgtt

fava_c17070_416	fava_c17070	416	T/C	tttatgctgaagtgaggttattagaaaacttaacaaacgaaaacatcattgaactttacagtgtttggaccRatgatgaacacggcacactgaattttat[T/C]acYgaggtttgtactagtgggaatttgagagagtacaggaagaaacataggcatgtttcgatgaaggcgttgaagaaatggtcgaggcagattttgaaag

fava_c17070_419	fava_c17070	419	C/T	atgctgaagtgaggttattagaaaacttaacaaacgaaaacatcattgaactttacagtgtttggaccRatgatgaacacggcacactgaattttatYac[C/T]gaggtttgtactagtgggaatttgagagagtacaggaagaaacataggcatgtttcgatgaaggcgttgaagaaatggtcgaggcagattttgaaagggt

fava_c17070_956	fava_c17070	956	T/C	aggtcaaagattctgaggtgaaggacttcattgaaaagtgtcttgctcaaccaagggttaggccttctgctgatgagcttctcaaagatcctttctttga[T/C]gaacttgttgaYgatgacgatgacgaaaatgctgattcatactaaagaaaaacagttcttatcttagtttttttagtttggtttatttacctttttagtt

fava_c17070_968	fava_c17070	968	T/C	ctgaggtgaaggacttcattgaaaagtgtcttgctcaaccaagggttaggccttctgctgatgagcttctcaaagatcctttctttgaYgaacttgttga[T/C]gatgacgatgacgaaaatgctgattcatactaaagaaaaacagttcttatcttagtttttttagtttggtttatttacctttttagtttatatgttaatt

fava_c17073_738	fava_c17073	738	G/T	catgagagaatctcttgatgagtctaatgtttctgaagagaatgagagagaagttgaagttaatgcaagagaatagaatcagggttgtgatgatatgaga[G/T]agaggttatgaaggagtagttgtatattcttgagagaatagtgtttagaagattagtagtagtaatactcaatttgattatatcaatcatgatggaaatt

fava_c17075_179	fava_c17075	179	C/T	gataatcaacaatgaaccataaagcacatgaatattatgttattctcatactgggctgaaatccattcactgttagtcctccatcaacacaaataacctg[C/T]cctgtgatgtaagatgcagccggaagacaaaggaaagcgaccaaggatgaaacttcttgagcttctgcgatgcgtccaagaggagttcgagacaacattt

fava_c17075_312	fava_c17075	312	C/T	gaaagcgaccaaggatgaaacttcttgagcttctgcgatgcgtccaagaggagttcgagacaacatttcgtctacatattgcttgttgcgaagcagatgt[C/T]ctacaagtggtgtattagtggtagcaggaacaacacaattgctccttatgccatctttcgcccattcacaagccaaattttttgtaagctgattgattgc

fava_c17075_636	fava_c17075	636	A/T	atatgcagaatctaaattaattgccataatttctgaatatacttcagcagtgtactcaatggttggtttcctaaagtttgccccaacattgttcacataa[A/T]tgtgaagtttgccattgaagattgaggtgacttgttgaataagcttctccctttggtctcgagatgacacatcacacacagatccagaaaccaaaaagcc

fava_c17076_1511	fava_c17076	1511	G/A	gatgatctggttgtgaaacgaagacctgctactgggatttgagttcagcagccccagtttttgtcctacaatgggtgtctgttgtcctctaatgagccat[G/A]ctctagagagatatgttctatgcgcaagaaaatgaaccttgtgtagtgctgagaattgggacggagcaattgtatgcgggaaattatctgtgaaaatacc

fava_c17078_1917	fava_c17078	1917	C/T	aagttattgcagaaactatgatgaggagaaaatagaggaaaatgaatagatccctattgtaactcttttttgatgaagcaaagaaatccgtgcgatcaca[C/T]aaatagtagtaagccaaaagaagaccaaattcagagatggctctaagggtcaaacggttttcaataaggaaatgttcatccatcaagaggaatcggataa

fava_c17078_2028	fava_c17078	2028	G/A	aagccaaaagaagaccaaattcagagatggctctaagggtcaaacggttttcaataaggaaatgttcatccatcaagaggaatcggataattgatgggga[G/A]gtagataaacttcgagcttttggagttggggatactggttgttgagctcctgcttccaataaaactgctctgtcttcatctttaacaacatcactgctca

fava_c17078_2238	fava_c17078	2238	G/A	agtttatctcggagtgcctagatttcatcaaagaacttttccgatactccaatatctctgaataaatccatgctataataaccggaatgatcccgagcaa[G/A]aacgagacctgaccgggagtaaaaggagcgacgatatgcatttttgcaaaaatcacagaagcggcatYcagagaaaatagcaattcggaagggatccact

fava_c17078_2306	fava_c17078	2306	T/C	catgctataataaccggaatgatcccgagcaaRaacgagacctgaccgggagtaaaaggagcgacgatatgcatttttgcaaaaatcacagaagcggcat[T/C]cagagaaaatagcaattcggaagggatccactagatctggtgatgaagaaatgcgaaccctagatttgcgaaaacgataatgtgaatgcgaatgcgaatg

fava_c17078_475	fava_c17078	475	G/A	ttgcataactaataaaagacatgccctccactgccctcttctcacccattttttatacaactttctaaaattgcagcaactagcctattagaaagaaagg[G/A]ttccatcttccttttgtagttttttttgtggggggagttgtgaagactttgcatctttctctaaatacttgacaaatcaactgcaccagctgcagagcat

fava_c17078_589	fava_c17078	589	T/C	ttgtagttttttttgtggggggagttgtgaagactttgcatctttctctaaatacttgacaaatcaactgcaccagctgcagagcatttttcggtgtcca[T/C]tgaaacaggcacaagcttgcaacRtatactatacatgtttaaatactcaaaccaagacttgcggtatcgtaaggaaggcaaaggataatgaatataaaac

fava_c17078_613	fava_c17078	613	A/G	ttgtgaagactttgcatctttctctaaatacttgacaaatcaactgcaccagctgcagagcatttttcggtgtccaYtgaaacaggcacaagcttgcaac[A/G]tatactatacatgtttaaatactcaaaccaagacttgcggtatcgtaaggaaggcaaaggataatgaatataaaacaactgagattgttgtccctgtaat

fava_c17078_786	fava_c17078	786	G/T	aaacaactgagattgttgtccctgtaattatgttgtgtagcagacgcttgtcatctttgctaggcacaaatgcaattttcagtgtattggtcagctgaaa[G/T]agcctataggaaattgcaacatatatagaagtagtaagcaggaagttcaacattgggtagtctggaatcagggaaagcaacaacttgggttgaccatctg

fava_c17080_1018	fava_c17080	1018	A/G	agtcatgaaattgaagacgaaggagttgagcaactgcctctggattaatcttaacactttcagcaagagcttcagcaacgattttctcagcattgggaca[A/G]gtttggctatagaatcctaccttaagtttagctctaccatcagccgcagcatttgcaattagcttttcatcttcaaacaacttttcatttccctttccat

fava_c17080_1125	fava_c17080	1125	G/A	ctatagaatcctaccttaagtttagctctaccatcagccgcagcatttgcaattagcttttcatcttcaaacaacttttcatttccctttccatcgccaa[G/A]ctttgcatttccMttttcttcaccaaatactggtagtggttggacttgttgtttatcatcttgtgcatcatcattatttccaaaatgaaatccccagtca

fava_c17080_1138	fava_c17080	1138	C/A	ccttaagtttagctctaccatcagccgcagcatttgcaattagcttttcatcttcaaacaacttttcatttccctttccatcgccaaRctttgcatttcc[C/A]ttttcttcaccaaatactggtagtggttggacttgttgtttatcatcttgtgcatcatcattatttccaaaatgaaatccccagtcaagtccaaatggaa

fava_c17080_457	fava_c17080	457	T/C	ttgttggtgtctcatcgaaattcacagttgggtttctaaatagctgtgtcccagcattcttacagataccttgaaactcctgaacaactggatctgttag[T/C]gtagggtcaggcttcctcgtgttttcgtaattaaaaatcctttccataaaaacatcacaatgagccgaaccaattgaatgtgctccgagtaagataacca

fava_c17084_229	fava_c17084	229	A/T	ttatcaatcattgtaagagaaaatttccatacacgaaaatttaattagtggctatatacaatttagaataaagccaccaaacaaattagctaaatcactt[A/T]aatttctagctaaatagctttcacaactaatggactcttaacataatgcttcccatctgaccaagtcatcttcccaaacacataactctttgttgctttt

fava_c17084_477	fava_c17084	477	G/T	atgtttgaactccaatatacttggtttcacagaaacagttataccatttggattctgaacatggacaacgtatgttgctggagcaccaacatttttcagt[G/T]ttctttgaaccgtcactgatccagagagatttgggacagtgattgaagggtagtttaggttgaggagactaaaattcttatggcgatggtaagtagcatt

fava_c17086_1078	fava_c17086	1078	C/T	atgtgatcttctccatgagtgtaaatgaagattacctttttgtagtttgatgattatttttgtcattgttagggagtgaatgcagcaggagaatactgtt[C/T]ataattgtgatcacttgttagagaaaaagaaaattagattgtactcaaatttaattgtaattttgatgttacaatctaacctattcaaaaagaataaagg

fava_c17087_390	fava_c17087	390	G/A	tgacatgcatcaagtgatgtatgtatattccttcaacattatcagcctactgcaaaggtgtaccgccaccatcagacataagagatgcagcggcggcggc[G/A]gcagtaggaacaacaccagttgaaacgcctggtttggacagatgaagaagcggggcaaggtattcacaaaaagattgcatcccagcagcaacatcggtat

fava_c17087_935	fava_c17087	935	C/T	tctagatcacataaaagtcgaacctctgaggccactgtgccgagatttggaccgttcttaagacatatttcatggatcctcaatcgttctcttttaacac[C/T]gcatagcccctggagaagaatctcaagggcctcgacgtgctgtgtctcaatgatcccctgaacaacacattccatatctctgcttccttcagcggagttc

fava_c17092_214	fava_c17092	214	A/C	gcgctaactttcattgaaaaaagataaaagactaggccttatacttcacttcatcatggaaacattccatgggaggaagcaccgactttctaaattgcac[A/C]cactatttcaattcctatttccttgagcatgaaaatgacagtgggttgtgcttcaaactggaaatatcaaatagttcgatgaacctctggtctgagactc

fava_c17092_89	fava_c17092	89	T/C	ttattcaatctgcgtcttcaagtatattggtatgtttggcatccaacatttaaacaggaaaggaaataataagtaatgaagtagaaca[T/C]ctatttaaaaatcctactcatcctgcgctaactttcattgaaaaaagataaaagactaggccttatacttcacttcatcatggaaacattccatgggagg

fava_c17095_269	fava_c17095	269	A/C	accgcatttcaacgtatcccaattctttcgcgcctgattgcaactctctttttcttccattttcaagatttttgttgtaacctaccaagagttgacgatt[A/C]ttaaattgaagcagagattgttgttgtgttgtatatttgaagaaattgaggttgtttagagggaacaatgtctggtcggaatgaacgccaacaccatcac

fava_c17096_1363	fava_c17096	1363	T/C	aaaacctgaatgaggtgccaccagatgatgccatggcaatggctagaagtcatggtcaatggagcatagatcatggatcgcaggtagctgaaacggcaac[T/C]agctatacaaactctgttgaagtggaaaaccatggtgaactgtcgtctatgtcacagtctaatgacattgatggaagccttcaaagtaccaccgacccca

fava_c17096_1815	fava_c17096	1815	C/G	ttcacacaaattacaatatatagatgtcaaaggaaaactaatgattcaagttggtgaaaggttgtttcttaatgtcattttttttactgcgtgaaatcgt[C/G]tcattgtttttctgtaaataattttttctttttgataaacgtcttttcaatttatagtaatatttagcgcattctaagcatgtatcaaacaagaaaggaa

fava_c17097_146	fava_c17097	146	T/C	aattcaacattacaattccaactctcacaatgtttcttctcaagacatggagacaatctgcttttggtgtttatggttacctcaatttcaccaaacctgc[T/C]tttcttgagcattctaagaatttcaaaccagaggaaatggaaacacaaataccagggaagaattgcatagttacaggagcaaactctggaattggttatg

fava_c17097_406	fava_c17097	406	C/T	gagagaggagaggctgctctttctcaaattcaaaccaaaactggcaatcaaaatgtacatttggagatttgtgatttgtcatctgtctcagatgtcaagt[C/T]acttgcttccaggttttccaaaaagaatataccactacatgtgttggttaacaatgctggtgtgcttgagcaaaatcgagtgacaacatcagaggggttt

fava_c17097_648	fava_c17097	648	T/C	gactgaactgatggtaccactattggagaaagcttcccctgatgcacgcgtcattacggtttcgtctggtggaatgtatagtactcctttgaccaaagat[T/C]tacagtactctgaaagcaactttaatgggagtgaacagtatgctcgaaataagcgcgYtcaggttgcactgacagaaaattggggcgagacatataagaa

fava_c17097_706	fava_c17097	706	T/C	gtttcgtctggtggaatgtatagtactcctttgaccaaagatYtacagtactctgaaagcaactttaatgggagtgaacagtatgctcgaaataagcgcg[T/C]tcaggttgcactgacagaaaattggggcgagacatataagaacaaagggataggattttactcgatgcatccaggttgggctgaaactcctggtgtggca

fava_c17098_1150	fava_c17098	1150	T/G	tcatagcagcaccccatcacagctaccagatttggatgtcgaagctttgcaaccctgccaattgctgaattgaactcctttttcctctgaaagcttgacc[T/G]ctttattctcttaacagcgacctttgagccatcaggttggactccactgtacgtaccaccagtcttggtatccccagcaagacggtttccttcactgaaa

fava_c17098_125	fava_c17098	125	T/C	ctagtaaaaattctaccaagactagtagtagtagtagttaccttgtactattacaaaacatgtatgttccacagaatttacatactctaatccaaggtgc[T/C]gctgcctaataattaagcaaatctattactttaagaaaaaaattctcaagtgtgaagttaRgttatacatacaaaacctStaagtccaaactaaggtttt

fava_c17098_1337	fava_c17098	1337	T/C	ttccttcactgaaatttttcgtaattgaccttagttcctctttggtatagactttccatgatggaagtgaagtggcaggatcagatagttttctagaccg[T/C]cttctctttttgcctcgcttaaatacaagaagccagacaacaactgctaatgttgttgagagtatcaagccactcaccactgcaaggattataaggtatt

fava_c17098_1505	fava_c17098	1505	G/A	agccactcaccactgcaaggattataaggtattccctgtggcagtgcatatgattacatttcgtatctatgtccaggatacatagagaagcatgaggatt[G/A]tcgcatctctcactcatgagggattttgaattattagtcattacggcacataaattagatgtactgttcctaagacatgatcgtccggttccagtacaat

fava_c17098_1664	fava_c17098	1664	T/C	atgtactgttcctaagacatgatcgtccggttccagtacaattaagtggaacattagccaaaggaaatatggacttgttccattcagaattatccgacca[T/C]gtccactgaaaacccgttatagtgctgttgagtaatcttcctccaatccaacaactatttatggattcgccacaaagactttgagcaaaatgttgctctt

fava_c17098_186	fava_c17098	186	A/G	gtatgttccacagaatttacatactctaatccaaggtgcYgctgcctaataattaagcaaatctattactttaagaaaaaaattctcaagtgtgaagtta[A/G]gttatacatacaaaacctStaagtccaaactaaggttttaaattaaggtatgcagccgcgtagttaatatttttgggacctctacaaccgcagtcgcagc

fava_c17098_205	fava_c17098	205	C/G	catactctaatccaaggtgcYgctgcctaataattaagcaaatctattactttaagaaaaaaattctcaagtgtgaagttaRgttatacatacaaaacct[C/G]taagtccaaactaaggttttaaattaaggtatgcagccgcgtagttaatatttttgggacctctacaaccgcagtcgcagctgtatcagccaaatttgtt

fava_c17098_346	fava_c17098	346	A/G	gtagttaatatttttgggacctctacaaccgcagtcgcagctgtatcagccaaatttgtttccaattttttaatatcgaggatcgtgaggacctgagacc[A/G]caatttaaaaccttagtcatagaagctaaattagctttattttgcaggtggctgggcaaactgttgcagctgatgaacaacatgcgacattctaggccgc

fava_c17099_1048	fava_c17099	1048	T/C	cKacgccgacacagccaccgtctcgcggtggcggggtaaaccattttgatctttgggatcagagctgtgaagaggagcctgtgatggagagagtggagtc[T/C]ggtagggatataagggtcaagatgtttgagaaactgagcaaagagaattcttttaatggatccgggtctggtaaggttgatggggctccggatattggat

fava_c17099_1191	fava_c17099	1191	T/G	gagaattcttttaatggatccgggtctggtaaggttgatggggctccggatattggatgggtttcggagcttgtgtgactgatgttgatgttgatttcat[T/G]ttggagtcgtctgtggtttaacttttggacaagatttaatgttggaaaattgtattcttgtaaatatgtctttttctcaaggttttggggttgaaggaaa

fava_c17099_1299	fava_c17099	1299	T/G	cgtctgtggtttaacttttggacaagatttaatgttggaaaattgtattcttgtaaatatgtctttttctcaaggttttggggttgaaggaaaattaagt[T/G]aaaatatttaagccattttttttggaaggttttcaggaaatccagacgacctacaatttaatttcaaggttttcttgtatttatgtaattcaattaaaaa

fava_c17099_132	fava_c17099	132	C/A	tccaaatcaaaaatttcttccatcaccatgtcgatgatgatgtggggggaaccgccccatcgtacaaatccaaccgtccacgtggcaccggcaccatggc[C/A]ggtgatgaacaatccaacggcggagattttttctgatgattattctcagttttatttcgatgaagcgctKagtgctcttcaacaatatgttccgtcatcc

fava_c17099_202	fava_c17099	202	G/T	caaccgtccacgtggcaccggcaccatggcMggtgatgaacaatccaacggcggagattttttctgatgattattctcagttttatttcgatgaagcgct[G/T]agtgctcttcaacaatatgttccgtcatccatggaagaRgaagacgattctgactcggagatttttccgagtcatgactcggtggatgcgtatacgaatg

fava_c17099_241	fava_c17099	241	G/A	acaatccaacggcggagattttttctgatgattattctcagttttatttcgatgaagcgctKagtgctcttcaacaatatgttccgtcatccatggaaga[G/A]gaagacgattctgactcggagatttttccgagtcatgactcggtggatgcgtatacgaatgaccattttcgtatgtttgagttcaagattcggagatgtg

fava_c17099_426	fava_c17099	426	T/C	aagattcggagatgtgcacgtggtcggtcacatgactggaccgagtgtcctttcgctcatcccggcgagaaagctcgccggcgtgacccgaggagggttc[T/C]gtactccgggacttcgtgtccggagtttcggaaaggaagttgcaagaaaggcgattcgtgtgagtttgctcatggggtttttgagtgctggcttcatcct

fava_c17099_700	fava_c17099	700	C/T	ctgagcaactcagggctccgacgaatctacagagtccattgagtatgaactctgttgagtcgtacgacggttcgcctctccggcttgccattgaatcctc[C/T]tgtgYtaaaaagcttcggttcatgtctcctaccggttctgtttcgccgccggcggagtctcctccgtcattgggaaggtcaccgggttctgtgaatgaga

fava_c17099_705	fava_c17099	705	T/C	caactcagggctccgacgaatctacagagtccattgagtatgaactctgttgagtcgtacgacggttcgcctctccggcttgccattgaatcctcYtgtg[T/C]taaaaagcttcggttcatgtctcctaccggttctgtttcgccgccggcggagtctcctccgtcattgggaaggtcaccgggttctgtgaatgagatggtg

fava_c17099_863	fava_c17099	863	G/A	tccgtcattgggaaggtcaccgggttctgtgaatgagatggtggcttcgctgcgaaatttgcatcttgggaagatgaaatctttaccttcctcatggaat[G/A]ttcaaatgggttcgtgttcgggttctcctcgattcggatctccaagaggacccatgctccgacccggattctgtagcttaccaacKacgccgacacagcc

fava_c17099_949	fava_c17099	949	G/T	cttcctcatggaatRttcaaatgggttcgtgttcgggttctcctcgattcggatctccaagaggacccatgctccgacccggattctgtagcttaccaac[G/T]acgccgacacagccaccgtctcgcggtggcggggtaaaccattttgatctttgggatcagagctgtgaagaggagcctgtgatggagagagtggagtcYg

fava_c17100_1047	fava_c17100	1047	A/G	cagcatatcgagtagacatgcaattaggttccagttttgctgccttctccaagcacttgctggaacttcgatgatccccactaatagaaaatgcataggc[A/G]agatttgcccaaatatgggcYgattttccatctgctttaattgcagctaacaaacattccttagcgacattagcagccatggattgttcttccgaagcac

fava_c17100_1068	fava_c17100	1068	C/T	aattaggttccagttttgctgccttctccaagcacttgctggaacttcgatgatccccactaatagaaaatgcataggcRagatttgcccaaatatgggc[C/T]gattttccatctgctttaattgcagctaacaaacattccttagcgacattagcagccatggattgttcttccgaagcaccttcagaagcatttgcaccag

fava_c17100_107	fava_c17100	107	A/G	acacaaaagaaaaagaaaactatatacccaattgcttattttcaaagcatgtaagcattcaagtgcatgcctatttaatttcaaagattgttcatcttta[A/G]atatacaatttcattcctctctgatttgattaWtataaatggaggtgcagcaRaagagaggRactacatacaatatcattctttaagaatacatgaggaa

fava_c17100_1229	fava_c17100	1229	C/T	gattgttcttccgaagcaccttcagaagcatttgcaccagcacctggtacaactgaagcatatttacacaaaagaagggtagcataattgaccagagctg[C/T]aggatggttttgatcttttaggatcaactcttgaaaacacttagcagataattccaaatttcctatttgaagataagctattccaaggttaccgaggcag

fava_c17100_1381	fava_c17100	1381	T/C	ttccaaatttcctatttgaagataagctattccaaggttaccgaggcagtcatagttctctggtgcaacggctaacaaggatgacaaaactgagatagca[T/C]tctgcacacgcctagttttaagaaggataaaacccaatgtattccatacagccgcctgcctgatatctgattgcattgattctttcagtttRgaaagaat

fava_c17100_140	fava_c17100	140	T/A	gcttattttcaaagcatgtaagcattcaagtgcatgcctatttaatttcaaagattgttcatctttaRatatacaatttcattcctctctgatttgatta[T/A]tataaatggaggtgcagcaRaagagaggRactacatacaatatcattctttaagaatacatgaggaaaggcctttgcatagcttcgacattgtggcaacc

fava_c17100_1473	fava_c17100	1473	A/G	agatagcaYtctgcacacgcctagttttaagaaggataaaacccaatgtattccatacagccgcctgcctgatatctgattgcattgattctttcagttt[A/G]gaaagaatttcctcaagttcgtgaggttcaagttctttgtctgaactattttctgatgaactttctattatcaggcactgtgcatggtgaatctgaacta

fava_c17100_160	fava_c17100	160	G/A	agcattcaagtgcatgcctatttaatttcaaagattgttcatctttaRatatacaatttcattcctctctgatttgattaWtataaatggaggtgcagca[G/A]aagagaggRactacatacaatatcattctttaagaatacatgaggaaaggcctttgcatagcttcgacattgtggcaaccttaatagagttagacttggc

fava_c17100_1608	fava_c17100	1608	A/G	ctttgtctgaactattttctgatgaactttctattatcaggcactgtgcatggtgaatctgaactaaagaaaggaactccgatctatcaatctcaacctc[A/G]ggcctgagtaatatctcttcggccttctcataagccaatacRgccttttgcggttggtttaatctttggtacatcaagcctaatataaaatgagcatgag

fava_c17100_1650	fava_c17100	1650	A/G	actgtgcatggtgaatctgaactaaagaaaggaactccgatctatcaatctcaacctcRggcctgagtaatatctcttcggccttctcataagccaatac[A/G]gccttttgcggttggtttaatctttggtacatcaagcctaatataaaatgagcatgagcatttttgggcatcttcctcgcaacatgaaccaRtccagtct

fava_c17100_169	fava_c17100	169	A/G	gtgcatgcctatttaatttcaaagattgttcatctttaRatatacaatttcattcctctctgatttgattaWtataaatggaggtgcagcaRaagagagg[A/G]actacatacaatatcattctttaagaatacatgaggaaaggcctttgcatagcttcgacattgtggcaaccttaatagagttagacttggctgcatcaag

fava_c17100_1742	fava_c17100	1742	G/A	gccaatacRgccttttgcggttggtttaatctttggtacatcaagcctaatataaaatgagcatgagcatttttgggcatcttcctcgcaacatgaacca[G/A]tccagtcttcatgctgctaactttttcttcccgtgaagaaggaggaccttgaacatgttgatcaccatccgcatcaactccacagtcaatagaagaatcc

fava_c17100_1872	fava_c17100	1872	C/A	cccgtgaagaaggaggaccttgaacatgttgatcaccatccgcatcaactccacagtcaatagaagaatccgtcttactcgatttggaacggcattttgc[C/A]actttgttcaatttcttaccttctccttcattactatcggtgtctttggaSaacaaactcctatcttgactgctttcatcgtttatcaacctggtgattt

fava_c17100_1923	fava_c17100	1923	G/C	cacagtcaatagaagaatccgtcttactcgatttggaacggcattttgcMactttgttcaatttcttaccttctccttcattactatcggtgtctttgga[G/C]aacaaactcctatcttgactgctttcatcgtttatcaacctggtgatttcagtgtgtggaggaggggggagggaagaatcgtcKtcgtcggcttcaggag

fava_c17100_2007	fava_c17100	2007	G/T	tatcggtgtctttggaSaacaaactcctatcttgactgctttcatcgtttatcaacctggtgatttcagtgtgtggaggaggggggagggaagaatcgtc[G/T]tcgtcggcttcaggaggatcgacgtttaggtcagccagaacgacgagtttgggaggggtagggttaggtttgttcaagcttgacctttctactgaacctc

fava_c17100_335	fava_c17100	335	C/T	gcaaccttaatagagttagacttggctgcatcaagcattggatctgattcgagtgctttctcaaaacaatacttggcctcttctaacagaccctcagcaa[C/T]aaaaacaagacccagattgttgaacgcgggagcatacccaggttttagttcaagcgactttgtaaacatagccttcgcaagttgatattttttctgatgg

fava_c17100_681	fava_c17100	681	G/T	aacctttgtcacgggcaacagcagctttgaggtatttttgtgatgttttgaattgcctagcacaaagattgtgaaggccaagttgatgccactgcacaac[G/T]tcatccggatcttctgttatggcctgctttagactggaaacagcacgctcttccatttccttcagcccgtcctgttcacattcgtatgcagctgatatct

fava_c17103_1095	fava_c17103	1095	T/C	aacttaaaggggatagttctcaactttatgatagtaagagaactgtttttgtgggtaatctcccatttgatgtaaaggatgaagaactttatcagctatt[T/C]ggtggtatacccaatctagaatcaagtgtagaagctataagagttgttcgagatcctcatcttaatgttggaaaRggaattgcttatgtgctatttaaaa

fava_c17103_1170	fava_c17103	1170	G/A	aggatgaagaactttatcagctattYggtggtatacccaatctagaatcaagtgtagaagctataagagttgttcgagatcctcatcttaatgttggaaa[G/A]ggaattgcttatgtgctatttaaaacaagggaagctgccaattccattgttaataaRcgatccttgaatcttcgagatcgagcattgagactcagtcgtg

fava_c17103_1227	fava_c17103	1227	A/G	aagctataagagttgttcgagatcctcatcttaatgttggaaaRggaattgcttatgtgctatttaaaacaagggaagctgccaattccattgttaataa[A/G]cgatccttgaatcttcgagatcgagcattgagactcagtcgtgctaacgcaaatgctggtgctaccccatctaaaaggctacactctccagcRgcacgac

fava_c17103_1320	fava_c17103	1320	G/A	ttaataaRcgatccttgaatcttcgagatcgagcattgagactcagtcgtgctaacgcaaatgctggtgctaccccatctaaaaggctacactctccagc[G/A]gcacgactcagtcatggcaaaccagacgcWactccatctaaaaggccatacactccagctgcacaagctcatagtacccctgcaaagaagttttcatctR

fava_c17103_1350	fava_c17103	1350	T/A	gagcattgagactcagtcgtgctaacgcaaatgctggtgctaccccatctaaaaggctacactctccagcRgcacgactcagtcatggcaaaccagacgc[T/A]actccatctaaaaggccatacactccagctgcacaagctcatagtacccctgcaaagaagttttcatctRcttcaaagtctccttcaagcagtaaaaacg

fava_c17103_1420	fava_c17103	1420	G/A	RgcacgactcagtcatggcaaaccagacgcWactccatctaaaaggccatacactccagctgcacaagctcatagtacccctgcaaagaagttttcatct[G/A]cttcaaagtctccttcaagcagtaaaaacgggtcaaacagaaaacctactgcatcttatcaRggtctgcgtgcaaccaaatctgatgtccacaagaaaag

fava_c17103_1482	fava_c17103	1482	G/A	cacaagctcatagtacccctgcaaagaagttttcatctRcttcaaagtctccttcaagcagtaaaaacgggtcaaacagaaaacctactgcatcttatca[G/A]ggtctgcgtgcaaccaaatctgatgtccacaagaaaagtcatggtggagaaaaaccaaaagagcgcacaaagaaaagaccaaccgtagctgccaggaagg

fava_c17103_1589	fava_c17103	1589	C/T	cgtgcaaccaaatctgatgtccacaagaaaagtcatggtggagaaaaaccaaaagagcgcacaaagaaaagaccaaccgtagctgccaggaaggctaaaa[C/T]aaaagttcagcaggagggtggtacacccaaacaagcggggtcaaaaggtacaccaaaacaagcRgggtcaaaaggtacaccaaatcaagcRgggtcaaaa

fava_c17103_1653	fava_c17103	1653	A/G	agaaaagaccaaccgtagctgccaggaaggctaaaaYaaaagttcagcaggagggtggtacacccaaacaagcggggtcaaaaggtacaccaaaacaagc[A/G]gggtcaaaaggtacaccaaatcaagcRgggtcaaaacgcaagtttgatagccgtactcctgatagctcccggcgagttatgaaagctcaaaagaagcggt

fava_c17103_1680	fava_c17103	1680	A/G	aggctaaaaYaaaagttcagcaggagggtggtacacccaaacaagcggggtcaaaaggtacaccaaaacaagcRgggtcaaaaggtacaccaaatcaagc[A/G]gggtcaaaacgcaagtttgatagccgtactcctgatagctcccggcgagttatgaaagctcaaaagaagcggtagaatgtttctaatggcatcaaSgatt

fava_c17103_1776	fava_c17103	1776	G/C	aagcRgggtcaaaacgcaagtttgatagccgtactcctgatagctcccggcgagttatgaaagctcaaaagaagcggtagaatgtttctaatggcatcaa[G/C]gattaaattttgttttcttgtcataattttStagctctaaaggacatggaggaaagagtttgacactgatgattttatgcagtcgggtggcttcagtgtc

fava_c17103_1807	fava_c17103	1807	C/G	tactcctgatagctcccggcgagttatgaaagctcaaaagaagcggtagaatgtttctaatggcatcaaSgattaaattttgttttcttgtcataatttt[C/G]tagctctaaaggacatggaggaaagagtttgacactgatgattttatgcagtcgggtggcttcagtgtcagttgaattttcacagttaaaaaagcagtag

fava_c17103_1935	fava_c17103	1935	C/T	tttgacactgatgattttatgcagtcgggtggcttcagtgtcagttgaattttcacagttaaaaaagcagtagacaggggatagaaattagctcttgtat[C/T]gtttaaatcattttacactRttgtgtaagttcYattatagaacttatataaggtttgttgtggcactYtgtcttagaaattgaaattggccatcaatttt

fava_c17103_1955	fava_c17103	1955	A/G	gcagtcgggtggcttcagtgtcagttgaattttcacagttaaaaaagcagtagacaggggatagaaattagctcttgtatYgtttaaatcattttacact[A/G]ttgtgtaagttcYattatagaacttatataaggtttgttgtggcactYtgtcttagaaattgaaattggccatcaatttttgttcctgaacttctgttta

fava_c17103_1968	fava_c17103	1968	C/T	ttcagtgtcagttgaattttcacagttaaaaaagcagtagacaggggatagaaattagctcttgtatYgtttaaatcattttacactRttgtgtaagttc[C/T]attatagaacttatataaggtttgttgtggcactYtgtcttagaaattgaaattggccatcaatttttgttcctgaacttctgtttatcaaagttttagt

fava_c17103_2003	fava_c17103	2003	T/C	agtagacaggggatagaaattagctcttgtatYgtttaaatcattttacactRttgtgtaagttcYattatagaacttatataaggtttgttgtggcact[T/C]tgtcttagaaattgaaattggccatcaatttttgttcctgaacttctgtttatcaaagttttagtaaagctgatttcgcctaaaaaaaaaaa

fava_c17103_292	fava_c17103	292	C/A	cgttttcgatgtcctcttcggcaacgcgccggaacagataaccggcaccgccgcttctctcttttccgatgacaacccctataagagaaagcctccacaa[C/A]ccgctcttatttccgacgttaccgatcacactccaaacccagaaRacggcgtcgtagatgagaagaagcgaaagaggaacaaagagaaaagccccgtgct

fava_c17103_337	fava_c17103	337	A/G	caccgccgcttctctcttttccgatgacaacccctataagagaaagcctccacaaMccgctcttatttccgacgttaccgatcacactccaaacccagaa[A/G]acggcgtcgtagatgagaagaagcgaaagaggaacaaagagaaaagccccgtgcttgattcaaattctgcaattcaagtttcagagaagaagaaaaagaa

fava_c17103_594	fava_c17103	594	G/C	aagcgattgggacgaagaagaagaggaagagggatgaggttgagaaagagtgggaggagaagaagtatgggattgttcagggaggagatagagatgaaag[G/C]gaagggtttgaggaggaaaaggttgggagcaagagaaaagtgcgtgataatccagcagatattatggtttcaaaagaaggttttgatgacgaagataagc

fava_c17103_843	fava_c17103	843	C/T	agactttgttgaaggagtttaagaattttggtgaggttgaatctgttaggattcgctctgttcctttacaagatactaaaaagcctagaaagggagccat[C/T]cttgcgaagaagatcaatgaatcggctgaaagtgtcaatgcttacattgttttcaaaactgaggaatctgcccaggcttcattgtcccacaacatgacta

fava_c17104_1276	fava_c17104	1276	T/C	gaaattttgacattttgtgcatgtctggctcttacttgcctactgagagtggcagcttaatggaccgaactggtggaataagtgttatgcttagtaatcc[T/C]gatggcagtctctttggtggaagagttgctggagtgattgctgcttctggtcctgtccaggtgatggttgggacctttctcattggaagattgaagggaa

fava_c17104_1509	fava_c17104	1509	G/A	gcagaagtggctgctgaatcatctgagcgaggagctcgcaatcctggcgccctgaacaacacttcacctaatcagaatctgactccaacctcatcctcaa[G/A]cccttggtcagcagcagcgtcgcgtccaatggagatgcgtgattccaatgctgacattgacttaatgcgcggttgacgcgtattgtgtatatctgtttca

fava_c17104_1720	fava_c17104	1720	C/G	agtaagtaccaacatcaggatagagctacctgactgataaatctcacttagctacccttcacccatgatttcacttggtgttgtcatgatttttaggtgt[C/G]aggaattttaatgtgatggtttcaattggtaatgataataataaatggcctgcttgaataaaatagctttgagaatgcaagtgtgtcaattttatttgag

fava_c17104_323	fava_c17104	323	T/A	aaggacttttgtgtagctcatacactacactggatagttagtgagagaaaaaaagcattcttttttttccattttaataataatcctttttctcttttcc[T/A]ttgttgctgagtaatttgcactcttcacttctccaagtgcagaaaaatcatagcttttttgatattttttacaatcattaggttcaatttcttcatMggt

fava_c17104_420	fava_c17104	420	C/A	tccWttgttgctgagtaatttgcactcttcacttctccaagtgcagaaaaatcatagcttttttgatattttttacaatcattaggttcaatttcttcat[C/A]ggtggaattggtgatgtgcaataacMYacccttttgatcttgcctacaaaatagcttagtgataggtttttagtatatttgaatatcggaggtcaaagtt

fava_c17104_446	fava_c17104	446	A/C	cttcacttctccaagtgcagaaaaatcatagcttttttgatattttttacaatcattaggttcaatttcttcatMggtggaattggtgatgtgcaataac[A/C]Yacccttttgatcttgcctacaaaatagcttagtgataggtttttagtatatttgaatatcggaggtcaaagtttcttgtgaaagaaggcattttgaaag

fava_c17104_447	fava_c17104	447	C/T	ttcacttctccaagtgcagaaaaatcatagcttttttgatattttttacaatcattaggttcaatttcttcatMggtggaattggtgatgtgcaataacM[C/T]acccttttgatcttgcctacaaaatagcttagtgataggtttttagtatatttgaatatcggaggtcaaagtttcttgtgaaagaaggcattttgaaagt

fava_c17104_773	fava_c17104	773	C/T	acaatttcaatccagcattggtggtggtggcagtaacattggatccacattacctttagaatcttcggcaatttcatctcaaggtgtcaatgtgagtggt[C/T]ctaccggggtacagtctggagagactggaaaaaggaggagaggaaggccgagaaaatatggcgctgatagagttgtgtcattggctcttagtccaagtcc

fava_c17106_2296	fava_c17106	2296	C/T	cggctttttttccttctctcccaatgataaattctgtgattacatgtttcttctgattctgtaattatgttgtacatctgcatagatggaagtacaaact[C/T]agagagcaagattgtatatttgttaggggtttatttgaaagacttcttgtgttttaaacgaccgagcttggaaaaagagtaattctatgtgcattattta

fava_c17108_1343	fava_c17108	1343	G/A	tctgcaaatgtggctcatggttcttttgtgaaaactccagtaaaaactagttactcgcgaggaagaggcaggtatactcgtggttgaggccatagtgctc[G/A]acaaaattcttctcatgatgtaagWcaacaagctgcaacagacaatagacctacttgtcagctatgtaataaatttggacattttatacttgattgtcag

fava_c17108_1368	fava_c17108	1368	T/A	ttgtgaaaactccagtaaaaactagttactcgcgaggaagaggcaggtatactcgtggttgaggccatagtgctcRacaaaattcttctcatgatgtaag[T/A]caacaagctgcaacagacaatagacctacttgtcagctatgtaataaatttggacattttatacttgattgtcagcataggttcgatgagcaatttgttc

fava_c17108_552	fava_c17108	552	T/C	attagttattcttaattgtatgttacatgtgatactcggagtttcatagattcaagaatggaagatctcgaatattcatcgaatctggtgtactaactaa[T/C]tcacctggggcaccattgcaccactcccaaaattcgatgtcgcatgtgagttttacgggatcttgtccttttttagtcctaaactctccatcaaattgca

fava_c17108_878	fava_c17108	878	G/C	tgtggtgtttgaagaatatgaagtatgggtggttcaagatcaagctcttttcacatggctatttcccgccatctctgaatctgtgttgcctcatgttctt[G/C]cttgcaagcatgcacatgaagtgtgggYcaaagtccacaaacatttcaactctcaaatgaaagcacaattttatcaattgcgatatgagttaaagactag

fava_c17108_906	fava_c17108	906	C/T	gtggttcaagatcaagctcttttcacatggctatttcccgccatctctgaatctgtgttgcctcatgttcttScttgcaagcatgcacatgaagtgtggg[C/T]caaagtccacaaacatttcaactctcaaatgaaagcacaattttatcaattgcgatatgagttaaagactagcaagaaaggtatcagatccatctctaag

fava_c17109_1107	fava_c17109	1107	A/G	gagcagtggtaatagccattggattttacacagttatgtgggcacaatcaaaagagggaaatgtgaatggcctagaagttgatggatcattatcatcctc[A/G]tctgccgaagagagtcctctactagggtctcagtgaaagcaagcacacatccaatgactacagtctattaagtgtgtttggtcaaatataaaatcaggtg

fava_c17109_1287	fava_c17109	1287	C/A	ggtcaaatataaaatcaggtggcaaaaaatataccttgcagagttcaataacagtgcattcacctccacatcatggaaactaacatgttataattctttt[C/A]tttttcctatgtgtggaaacaaacatgctatagttcattttKKtcaatttgtatggaaacatgcatgctaaaattcatttttgtcaatttattgagacaa

fava_c17109_1329	fava_c17109	1329	T/G	gttcaataacagtgcattcacctccacatcatggaaactaacatgttataattcttttMtttttcctatgtgtggaaacaaacatgctatagttcatttt[T/G]Ktcaatttgtatggaaacatgcatgctaaaattcatttttgtcaatttattgagacaacatgtattggcaatgaaaacatttcaattttcaggagtatag

fava_c17109_1330	fava_c17109	1330	G/T	ttcaataacagtgcattcacctccacatcatggaaactaacatgttataattcttttMtttttcctatgtgtggaaacaaacatgctatagttcattttK[G/T]tcaatttgtatggaaacatgcatgctaaaattcatttttgtcaatttattgagacaacatgtattggcaatgaaaacatttcaattttcaggagtatagc

fava_c17109_429	fava_c17109	429	T/A	gcatcactataatgcagaattgtgtattcactggcataagctacagctctcccacccttggatctgctatgagcaacttgactccagcaatcacttttgt[T/A]ctagctgtcattttcaggatggaaaagttggatgttggaagttcaataagccagatcaaaatggtggggacagtactgtctatctcaggagcattgctag

fava_c17109_876	fava_c17109	876	T/G	taattgcagtcaaagatcgaaatgcttggaatctcagtgctgatattgagcttatctctataatatattcagcagtttttggaagtgtggtgacattttc[T/G]gtactgacttggtgcatagataggaaaggacctgtatttgtttgcatgttcaaacctgtaggaattgccattgctgcttttatgagtgctgccttccttg

fava_c17112_1048	fava_c17112	1048	C/T	atggtaatcctgaaacctctggtgctgtagaatcatctcccaatgcaagtaatgcaagagaacaaacttctagttctgctggcactagtaagatatcaga[C/T]gctgatgaagcgaagtcaaaagagcagggcaacagaagaggaagcagacagaaactgcttgcctctRttgataacaaatgcaagttagttgacttcggta

fava_c17112_1115	fava_c17112	1115	G/A	ttctagttctgctggcactagtaagatatcagaYgctgatgaagcgaagtcaaaagagcagggcaacagaagaggaagcagacagaaactgcttgcctct[G/A]ttgataacaaatgcaagttagttgacttcggtaatgcttgctggacatataaacagtttacaaatgatattcagactaggcaatatcgatgcccagaggt

fava_c17112_1928	fava_c17112	1928	G/A	aaagagctatttccagtgtaattgtatttattgtctgcgcatgcccaatggtgtctgtgtgaattgttttgggggcatgttttctgaactgctggatttg[G/A]acggagttgcacttgtttgaagctactgccaggctaggggctagctagttccttcaagtctgctatacaacaccgcaaggatactccgtgagctatatat

fava_c17112_349	fava_c17112	349	T/C	gcacagaagattaccgacgcggcggctaccacgccgtccgaatcggcgatactttcaactccggtcggtatgtcgttcagagtaagcttggttggggcca[T/C]ttctctactgtctggctcgcttgggatactMatcattctagatatgtggctttgaaagttcaaaaaagtgctcagcattacaccgaggctgccctagatg

fava_c17112_380	fava_c17112	380	A/C	cgccgtccgaatcggcgatactttcaactccggtcggtatgtcgttcagagtaagcttggttggggccaYttctctactgtctggctcgcttgggatact[A/C]atcattctagatatgtggctttgaaagttcaaaaaagtgctcagcattacaccgaggctgccctagatgagataactattttgcaacagattgcggaggg

fava_c17113_1048	fava_c17113	1048	G/C	attggaacagtagccagagttacgttttaaccaaaggctggagccggttcgttaaggagaagaatctcaaagccggtgatgcggttcggtttttcaggtc[G/C]accggaccggatagacagctctatatagactgcaaagccaggagcattagtgttgtcggtgtcagtgttggtgaaaccaatgataatacttgtggtttgt

fava_c17113_115	fava_c17113	115	A/C	acctattctctcattcttcttcttcctcttttcttcatcttctctctttccttcacttttttStaactaacttgatagttagttataactgaataacaaa[A/C]caaagttagtttataatagataacaaaaaacaaatggagttaggaggaggtagttgcacagatgaaaccaccacaagcaatggttctctttccgtatctc

fava_c17113_1160	fava_c17113	1160	C/A	tagacagctctatatagactgcaaagccaggagcattagtgttgtcggtgtcagtgttggtgaaaccaatgataatacttgtggtttgtttatcccggtc[C/A]gaccggtagttgaaccggttcaaatggttcggcttttcggggtaaatattttgaaaatacctggttcaagttctgatggtgttagctgcaatgggaagag

fava_c17113_77	fava_c17113	77	G/C	acacaacacctaccacctattctctcattcttcttcttcctcttttcttcatcttctctctttccttcactttttt[G/C]taactaacttgatagttagttataactgaataacaaaMcaaagttagtttataatagataacaaaaaacaaatggagttaggaggaggtagttgcacaga

fava_c17113_835	fava_c17113	835	C/T	agcagttgtttgagaaaacggttacgccaagtgacgttggaaagttgaatcggttggtgataccgaaacagcacgcggagaaacactttccgttgaatgc[C/T]gtggccgtaactgtctctggcgatggaacMtcgccgtctgtgacggcggcgaaaggtttgctattgaacttcgaagacgttggagggaaagtgtggcgat

fava_c17113_865	fava_c17113	865	C/A	gtgacgttggaaagttgaatcggttggtgataccgaaacagcacgcggagaaacactttccgttgaatgcYgtggccgtaactgtctctggcgatggaac[C/A]tcgccgtctgtgacggcggcgaaaggtttgctattgaacttcgaagacgttggagggaaagtgtggcgatttcgttactcttattggaacagtagccaga

fava_c17114_1134	fava_c17114	1134	G/A	ggtgtctccggttgctgccaacgatcggaactgatagatcgaatcttcctctcattctttgttcggcgcactggcgagtatagacggagaggaaaccgag[G/A]atgaagaacacgacaactaggatgactagtacgatggccatggacttgtcaaatttgagggcggagatgttgccgggcggtggtggtgggaggaatttcg

fava_c17114_235	fava_c17114	235	C/A	tcacctgtatttccaactttttacgataaataaaaacaataaatagtaaataaaaaaaactattttcatttttaccaaacaaaacctatcattctaaccc[C/A]gattaccggcaaacaaacgatcagaagaccgttctcccacatcatccaatttcccaggtgacttattcttcaaccaacttgaaccggttcgacccataaa

fava_c17115_286	fava_c17115	286	C/A	acaatcaaatttaaaccttttcatcattctgattctgttccaattccacctcagaaacctctgttacccttcttctacacaaagaaaccatgaaaatcca[C/A]ataacacctttcacaattctcaccattccaattgtaacagttaaataatacaaccaccatccaacaaattcctctattccaacatccatcaattgttctt

fava_c17115_418	fava_c17115	418	A/T	ttgtaacagttaaataatacaaccaccatccaacaaattcctctattccaacatccatcaattgttctttttctgtttcacctataacacccaaattctc[A/T]atctcctctctcaacttccaccaccacaatgttcctaaacaaccagcaacaaccactttctcaacccatagtcYctctctttttccattaccaaaaacag

fava_c17115_492	fava_c17115	492	C/T	gtttcacctataacacccaaattctcWatctcctctctcaacttccaccaccacaatgttcctaaacaaccagcaacaaccactttctcaacccatagtc[C/T]ctctctttttccattaccaaaaacagtgtcatccacYactggtctcacaacaacctttgaccaaatttgcgtcgtttcgtgtaaccccaacagaaatatc

fava_c17115_529	fava_c17115	529	C/T	tcaacttccaccaccacaatgttcctaaacaaccagcaacaaccactttctcaacccatagtcYctctctttttccattaccaaaaacagtgtcatccac[C/T]actggtctcacaacaacctttgaccaaatttgcgtcgtttcgtgtaaccccaacagaaatatcattctgcttaaaaagcttctctcaacaacatcttcaa

fava_c17116_840	fava_c17116	840	G/A	gtcgatcaggagccccatatgccttgtgaatctggaacctttggggaccagagataccttgcaaggctttaaaaccctccaaaggaacctttgacgttcc[G/A]gtcacaaattgcagtaatctggccatgtcttccttgttgaaagatttgaccacctcccaaaaccattgaacaacatttgatgccacRgtgtaggctgtat

fava_c17116_927	fava_c17116	927	A/G	cctttgacgttccRgtcacaaattgcagtaatctggccatgtcttccttgttgaaagatttgaccacctcccaaaaccattgaacaacatttgatgccac[A/G]gtgtaggctgtatactcggtgtttgccttcaaatcgtccaagtcaatttctggaagaccacttatgagtagctcaagctctttgtcattaaagattgata

fava_c17117_1814	fava_c17117	1814	A/G	tgatatcagtgaaagaagataagcataaaaaagaagtggtgctgttatcagttaaccgaaaacatgctgattattcatccttcaagcctgaaaaaaggcc[A/G]gtggagaaaattgagcagtccagtgtccaatctgtcaatgaagttcgttcatccaaaactctggaagtggctgaaatctacaagccaagtgtacatgtca

fava_c17121_169	fava_c17121	169	T/A	gtattccactccaccaaaaatgcactcataaccatttttcaccaatcctacaaaatcatcatgcccacagattcccctgttgcgtctggctctagatcaa[T/A]cggtgaatgcatttacgcggcgctcatcgccgccgtcgagactttcgtcttcgtctttgcaggttgtttcaactctgaYcgtccagtttttcattttcac

fava_c17121_248	fava_c17121	248	T/C	ttgcgtctggctctagatcaaWcggtgaatgcatttacgcggcgctcatcgccgccgtcgagactttcgtcttcgtctttgcaggttgtttcaactctga[T/C]cgtccagtttttcattttcacaaatcttcctattctgctaccgatttcactcgccttgctcaagaaactagattcactgttaatgaagtggaggcattgt

fava_c17125_1594	fava_c17125	1594	G/C	tcggcgactttggtttggcaaagttagtagatgttcgaaagactaacgtgacaactcaagttcgtggaacaatgggccacatagctcctgaatatttgtc[G/C]acSggaaagtcctcagaaaggactgatgtttttggctatgggattatgcttctggagcttgttacaggccaacgtgcaattgacttctcacggttggaag

fava_c17125_1597	fava_c17125	1597	C/G	gcgactttggtttggcaaagttagtagatgttcgaaagactaacgtgacaactcaagttcgtggaacaatgggccacatagctcctgaatatttgtcSac[C/G]ggaaagtcctcagaaaggactgatgtttttggctatgggattatgcttctggagcttgttacaggccaacgtgcaattgacttctcacggttggaagagg

fava_c17125_1846	fava_c17125	1846	G/A	aaagactagaagttattgttgatcgcaacctaaacaaaaattacaacatacaagaggtagaaatgatgataaaagttgcattactgtgcactcaagcaac[G/A]ccagaagaccgtccactaatgtcggaggttgtaagaatgctagaaggagaaggattggctgaaaggtgggaagaatggcaacacgtggaggtgaaccgga

fava_c17125_1981	fava_c17125	1981	T/C	gaatgctagaaggagaaggattggctgaaaggtgggaagaatggcaacacgtggaggtgaaccggagacaagaatacgagagattacaaagaagatttga[T/C]tggggagaagattcagtttataatcaagatgccattgagttgtctggtgggagatgagaagatgcatagatttgaaacaattatcctttttttgataggt

fava_c17125_241	fava_c17125	241	A/G	cccagctattgatgaagatttcaaatatgattgttgattgtcatggtcattgaataggtggttgatgccagcaagtagatatggacttgttatttgtatt[A/G]ttgctgttgggttgcttctgttcttttgtgttgtctgatcagcaaggagatgcactgattgcgttgaagctatcactgaatgcttcRagtcaacagctta

fava_c17125_328	fava_c17125	328	A/G	tgttatttgtattRttgctgttgggttgcttctgttcttttgtgttgtctgatcagcaaggagatgcactgattgcgttgaagctatcactgaatgcttc[A/G]agtcaacagcttactgactggaatgaaaatcaagtcaacccatgtacttggtcaaatattaactgtaatcaaaacaacaatgttcttggagtttcgttgg

fava_c17125_548	fava_c17125	548	G/A	atacttgacccctaggatagaagctctaaaatatctccaatttatttctttgcaaggaaatgacatcactggtgacataccaaaagaattcggaaacctg[G/A]ctagcttggtcaggttggatttggaaaacaacagattaaccggtgaaataccgtcttcccttggtaatctcaaaaagctccaattcttgacattgagtca

fava_c17125_880	fava_c17125	880	G/A	gcggaaataatttgaattgtggcatgagttatggccaaccttgtgctaacaataattcagatcaaggttcatcacataaaccaaccggcctcataatcgg[G/A]tttagcatagcatttgtagctatccttgttattggtggcctcctatttttttggtgcaagggWagacacaagggctacaaacgtgaagtttttgtagatg

fava_c17125_943	fava_c17125	943	A/T	aaggttcatcacataaaccaaccggcctcataatcggRtttagcatagcatttgtagctatccttgttattggtggcctcctatttttttggtgcaaggg[A/T]agacacaagggctacaaacgtgaagtttttgtagatgttgcaggtgaagttgatcggcgaattgcatttggtcaattaagaagatttgcatggagagaac

fava_c17127_1156	fava_c17127	1156	T/C	aagatgatgcttatggaaatcagcttgtggctttgcctatgtacataggaggtggatttgctggacttcaagggattatatatgcaggaacaaattgttt[T/C]catagaagaaRagttatttatggtctttctcctaatcttgacattcaaaatgcaaacaaggatcatggcttcaccaaaggaacattgttatcagaaaaag

fava_c17127_1167	fava_c17127	1167	A/G	tatggaaatcagcttgtggctttgcctatgtacataggaggtggatttgctggacttcaagggattatatatgcaggaacaaattgtttYcatagaagaa[A/G]agttatttatggtctttctcctaatcttgacattcaaaatgcaaacaaggatcatggcttcaccaaaggaacattgttatcagaaaaagaaacagtgcaa

fava_c17127_1495	fava_c17127	1495	T/A	atactgcttggggtaaacaggtgggatggttatatggatcgacatcagaggacgtactaactgggcttaagtttcacacaaaaggatggagatccgagct[T/A]tgttcaccagatccgatagcatttatgggctgctcaccgcaagataacttgggccaaatggcccaacataagagatggtcctcagggttgtttgatatct

fava_c17127_1621	fava_c17127	1621	C/T	tgggctgctcaccgcaagataacttgggccaaatggcccaacataagagatggtcctcagggttgtttgatatctttcttagcaagcattgtccgatttt[C/T]ggtaccctatttggtaaactccaattcagagagtgtttggcatatatttggattactaattgggccttaagatctctccctgaaatttgttatgcccttc

fava_c17127_1771	fava_c17127	1771	A/G	ggattactaattgggccttaagatctctccctgaaatttgttatgcccttctacctgcctattgcatcctttctaactccagtttcttgcctaacaagga[A/G]ctcggtatgtggatccccgtRactctatttgcgatatacaacatatctactctctccgagcatataaaatcaggattatcgttccgaacatggtggaaca

fava_c17127_1792	fava_c17127	1792	G/A	gatctctccctgaaatttgttatgcccttctacctgcctattgcatcctttctaactccagtttcttgcctaacaaggaRctcggtatgtggatccccgt[G/A]actctatttgcgatatacaacatatctactctctccgagcatataaaatcaggattatcgttccgaacatggtggaacaatcaaaggatgggaagaataa

fava_c17127_257	fava_c17127	257	A/T	gcttcttggttacagattttcatcacatgagaatcacagtttcccttggtttgttgcatttctatgtgaatcatggttcaccatcacttggattaccacc[A/T]tgtccaccaaatggactccggctcgtaccaaaacattcctccaccgtctcgcacatagggtatctgaatctgagcttccagcattggatttgtttgtgac

fava_c17127_484	fava_c17127	484	A/C	caatcatcacagtgaacactgtcttgtcattactggcacttgattacccttctaacaagctagcttgctatgtttctgatgatgcttgttcacctcttac[A/C]ttYtatgcccttcaagaagctRcaaaatttgctgaaatttgggtacctttttgtaagaaatacaatgtacaatgtagagcaccttttcgatacttctgcg

fava_c17127_487	fava_c17127	487	C/T	tcatcacagtgaacactgtcttgtcattactggcacttgattacccttctaacaagctagcttgctatgtttctgatgatgcttgttcacctcttacMtt[C/T]tatgcccttcaagaagctRcaaaatttgctgaaatttgggtacctttttgtaagaaatacaatgtacaatgtagagcaccttttcgatacttctgcgatg

fava_c17127_506	fava_c17127	506	G/A	cttgtcattactggcacttgattacccttctaacaagctagcttgctatgtttctgatgatgcttgttcacctcttacMttYtatgcccttcaagaagct[G/A]caaaatttgctgaaatttgggtacctttttgtaagaaatacaatgtacaatgtagagcaccttttcgatacttctgcgatgaagccgtagataacaaaga

fava_c17127_610	fava_c17127	610	G/T	aatttgctgaaatttgggtacctttttgtaagaaatacaatgtacaatgtagagcaccttttcgatacttctgcgatgaagccgtagataacaaagattt[G/T]ccagaattcaaacaagattggttgaaaatgaaggaagagtatgaacaacttagcggtaaaattaaaaatgcagctgaaaagtcgattccgtgtcagctta

fava_c17128_850	fava_c17128	850	A/G	tagcatagagtgaagttcaaatgcacaaggagactttgaagttttgaagaaactttcttgtagttttggatttagtattagaaacttgaaatattttctt[A/G]tgaatWgatagaaatttgagcactcttgtaagttRaatgtgagttgaagttttctttgaaccttgagaagaacttgccttgaaatttctttgaatctctc

fava_c17128_856	fava_c17128	856	T/A	agagtgaagttcaaatgcacaaggagactttgaagttttgaagaaactttcttgtagttttggatttagtattagaaacttgaaatattttcttRtgaat[T/A]gatagaaatttgagcactcttgtaagttRaatgtgagttgaagttttctttgaaccttgagaagaacttgccttgaaatttctttgaatctctcttgatg

fava_c17128_885	fava_c17128	885	G/A	ttgaagttttgaagaaactttcttgtagttttggatttagtattagaaacttgaaatattttcttRtgaatWgatagaaatttgagcactcttgtaagtt[G/A]aatgtgagttgaagttttctttgaaccttgagaagaacttgccttgaaatttctttgaatctctcttgatgaaaaagacttgtgatgttcttgaaagatt

fava_c17130_1148	fava_c17130	1148	G/A	tgttatcttctctattatcactcaacagccatcctgaaacctgactcaacaaattcctcttcgaattaacaaagtcatccacaaaaaaagaaataatatc[G/A]aaaggatgatccgagacttcaccatccgcaccatcgtcaaagataacWgtctcagcaacaaacgactcacttctttgYgtaagataatcaatcatttctt

fava_c17130_1196	fava_c17130	1196	A/T	acaaattcctcttcgaattaacaaagtcatccacaaaaaaagaaataatatcRaaaggatgatccgagacttcaccatccgcaccatcgtcaaagataac[A/T]gtctcagcaacaaacgactcacttctttgYgtaagataatcaatcatttctttcctcaaatcatccgcaactgaaccagcagtgttaaagggatctccag

fava_c17130_1226	fava_c17130	1226	C/T	ccacaaaaaaagaaataatatcRaaaggatgatccgagacttcaccatccgcaccatcgtcaaagataacWgtctcagcaacaaacgactcacttctttg[C/T]gtaagataatcaatcatttctttcctcaaatcatccgcaactgaaccagcagtgttaaagggatctccagttgtgttatcaacacaagcYgaggctaaac

fava_c17130_1316	fava_c17130	1316	T/C	cacttctttgYgtaagataatcaatcatttctttcctcaaatcatccgcaactgaaccagcagtgttaaagggatctccagttgtgttatcaacacaagc[T/C]gaggctaaacctggcaaaaaMatctgagcaagcttgtgaactatttctgtatcatgtaaatcacaatggaacattggacctccctctctctcattttcaa

fava_c17130_1337	fava_c17130	1337	C/A	caatcatttctttcctcaaatcatccgcaactgaaccagcagtgttaaagggatctccagttgtgttatcaacacaagcYgaggctaaacctggcaaaaa[C/A]atctgagcaagcttgtgaactatttctgtatcatgtaaatcacaatggaacattggacctccctctctctcattttcaatcttctccggcgaaaggtcca

fava_c17130_262	fava_c17130	262	T/C	cttctcaactaagaatttgtatgtcatcctacaagttatcatttctaatatccttgttcctcattcattctctgcattacccttttttttccattttcct[T/C]atgctcactatcttcactaYtcaccacattctctgcaccattcttattcagtgtagcattcacttcagctttatcttatgaagttgaagcttgtgcacca

fava_c17130_282	fava_c17130	282	T/C	atgtcatcctacaagttatcatttctaatatccttgttcctcattcattctctgcattacccttttttttccattttcctYatgctcactatcttcacta[T/C]tcaccacattctctgcaccattcttattcagtgtagcattcacttcagctttatcttatgaagttgaagcttgtgcaccacccttggtttcggtgccaat

fava_c17130_548	fava_c17130	548	C/T	aaggccctaacttagcttcaagatctctgacaataacttttagagatctaacatcgcgagcttctgctaacctattgcttgatgatgcctgtaaaattcg[C/T]tctactcgtcgactaagatctccgccagatgcatccttgtggatccctttaagcaagtgcaataagtgSgaWtgaagatcatctgagcaatgttttccaa

fava_c17130_617	fava_c17130	617	C/G	acctattgcttgatgatgcctgtaaaattcgYtctactcgtcgactaagatctccgccagatgcatccttgtggatccctttaagcaagtgcaataagtg[C/G]gaWtgaagatcatctgagcaatgttttccaacgctacattgtgcaatggccgatcgacaaccgactgcatagaaaggacaattcacaagcttcatcggac

fava_c17130_620	fava_c17130	620	A/T	tattgcttgatgatgcctgtaaaattcgYtctactcgtcgactaagatctccgccagatgcatccttgtggatccctttaagcaagtgcaataagtgSga[A/T]tgaagatcatctgagcaatgttttccaacgctacattgtgcaatggccgatcgacaaccgactgcatagaaaggacaattcacaagcttcatcggacaaa

fava_c17134_368	fava_c17134	368	A/G	ggcttttctgtggattacgcctttggctgaaatttttgtaaacaaaaactatagtgcaaccacgagtggtgttggtaatgctgagagattggtggggaat[A/G]tagggtttctgccgtcggattttgcgaactttagacggtggtgtttgttgggatcgagtttgttgcagattgtggctttaaggcctaacctgcaaatgta

fava_c17134_742	fava_c17134	742	A/G	atggtccttcctttggaaactttccattggcattgcctagctctgtttttttcaaggaggccgcattgtttttggcttggtgggtgacttttctgcaggc[A/G]atatattcttcagcRatccttttgctacaccggcattgtattttgtatgtttcttgaacctttgatttctaaagcacaatttcatatggcgggccttttg

fava_c17134_757	fava_c17134	757	G/A	gaaactttccattggcattgcctagctctgtttttttcaaggaggccgcattgtttttggcttggtgggtgacttttctgcaggcRatatattcttcagc[G/A]atccttttgctacaccggcattgtattttgtatgtttcttgaacctttgatttctaaagcacaatttcatatggcgggccttttgttgtaagctgaacat

fava_c17137_147	fava_c17137	147	G/A	gtgagtatttaaagcaactaagtagcaatacaaaaaagtaggcaaattcaaatatacaaagaaagatcttctaagaacttgctcccttacaacaaagacc[G/A]tttagttagaatctttccaataaggagcagtggatgtagaggtttgKMtcattcatgtcatcattattctggtacaacacgtagaggtagaagcagagaa

fava_c17137_194	fava_c17137	194	T/G	tcaaatatacaaagaaagatcttctaagaacttgctcccttacaacaaagaccRtttagttagaatctttccaataaggagcagtggatgtagaggtttg[T/G]Mtcattcatgtcatcattattctggtacaacacgtagaggtagaagcagagaaagagagagtgttctcttacttctggaatatttgtacgagagtttgcc

fava_c17137_195	fava_c17137	195	C/A	caaatatacaaagaaagatcttctaagaacttgctcccttacaacaaagaccRtttagttagaatctttccaataaggagcagtggatgtagaggtttgK[C/A]tcattcatgtcatcattattctggtacaacacgtagaggtagaagcagagaaagagagagtgttctcttacttctggaatatttgtacgagagtttgccc

fava_c17137_541	fava_c17137	541	C/A	tctatcccacccagatggcctccgcctttctctaggcttggaactactgtttgtgttgttctgaccaggatttactccaggactttgactggtggaattt[C/A]cggctgatgtagcaggattgtatgaagaagtttgctgatgagtatttatatcgcctccaattgatccaccagatacaaagccagcgagtgctggtctctt

fava_c17137_759	fava_c17137	759	G/A	gattattaccgagtcctccattttgggagtttgatgatgcagcaacgaagctactcctaaattgggacatcatccctgttcgcagagaatttacagcggc[G/A]gaccgatttggaactgagttagatggtgcattgtttgactctgaactatatccaatgcccaagccaaaatccaccccacgcacgcctttgccacccacac

fava_c17138_1253	fava_c17138	1253	T/G	ggtgcggactttgggcttttcttaggaatattcgaaatgggtactccttcttcaagaccctccatgagttcccaagcattgattttgataacagtttctg[T/G]ttctttgtctctctgtgttggtgatgggtttggtctSgacactgtttctgSttctgtttcagaaacttttgaaacactggaatcggaagagtcattggaa

fava_c17138_1290	fava_c17138	1290	C/G	tgggtactccttcttcaagaccctccatgagttcccaagcattgattttgataacagtttctgKttctttgtctctctgtgttggtgatgggtttggtct[C/G]gacactgtttctgSttctgtttcagaaacttttgaaacactggaatcggaagagtcattggaatccgaagagtcgtttctgttgctgctgttgttgttct

fava_c17138_1304	fava_c17138	1304	C/G	tcaagaccctccatgagttcccaagcattgattttgataacagtttctgKttctttgtctctctgtgttggtgatgggtttggtctSgacactgtttctg[C/G]ttctgtttcagaaacttttgaaacactggaatcggaagagtcattggaatccgaagagtcgtttctgttgctgctgttgttgttcttgtctagactgagt

fava_c17138_254	fava_c17138	254	T/C	aataatcaaatcaactacaaactagggactaaaacactctttagaaccctactcctacaaacatgacacaacaaacaacacatcaagcatcattgcttgc[T/C]taaaccatcccaaaatacaaactcaacttttcaacaacatatagaacaacgaataattccattctcattacaataaccacacttgacacttttcttcatc

fava_c17138_639	fava_c17138	639	T/C	ccggaacttgaacttgtttcatttccattaacttcttcaattcctccttaaacccggaatccatagacacatcgcgctcgcgcaaacacacacaataaga[T/C]tcaatgatcgatctaactttattacaatcttcaaaagtctttctgattcctcttaacgtggttgtgtaaatcacaacagaattttcccctcccggaggac

fava_c17138_834	fava_c17138	834	T/C	gaggacatttgctctcatacaaattcaaaagtgtgattgagtcgagagacttcctctctcttctagttttcgcagtagcaaaaacaactctcttgatttg[T/C]tcttcctcttctgaaagttccggttcaaacaaaRgactagaaatggaaccggtactctttctctcattcccaaaactgtttcttttagcagaaaacggag

fava_c17138_868	fava_c17138	868	A/G	gattgagtcgagagacttcctctctcttctagttttcgcagtagcaaaaacaactctcttgatttgYtcttcctcttctgaaagttccggttcaaacaaa[A/G]gactagaaatggaaccggtactctttctctcattcccaaaactgtttcttttagcagaaaacggagaacctttattcttaggagatgaatttgcggtgga

fava_c17139_1025	fava_c17139	1025	A/T	acgaatcatactcataatgaattcatgtttcagcataactttccccagcaaaatcaaattgacaatgaacaaaatcaaccaatgagcaatatgacaggat[A/T]tgtaaacccagatgtaaaMggagcattcaaaagggtcaactatcagRatatttctcatgctgatagggacctatcttctttccgacatggttctattgat

fava_c17139_1044	fava_c17139	1044	A/C	aattcatgtttcagcataactttccccagcaaaatcaaattgacaatgaacaaaatcaaccaatgagcaatatgacaggatWtgtaaacccagatgtaaa[A/C]ggagcattcaaaagggtcaactatcagRatatttctcatgctgatagggacctatcttctttccgacatggttctattgatcaaattggtaSacRagaga

fava_c17139_1072	fava_c17139	1072	G/A	gcaaaatcaaattgacaatgaacaaaatcaaccaatgagcaatatgacaggatWtgtaaacccagatgtaaaMggagcattcaaaagggtcaactatcag[G/A]atatttctcatgctgatagggacctatcttctttccgacatggttctattgatcaaattggtaSacRagagagaactagtgacagaacatttgtaaatgg

fava_c17139_1136	fava_c17139	1136	G/C	gatgtaaaMggagcattcaaaagggtcaactatcagRatatttctcatgctgatagggacctatcttctttccgacatggttctattgatcaaattggta[G/C]acRagagagaactagtgacagaacatttgtaaatggcaatagcaacatgtatcaaccttcacatgagcatgacgagactgcKtcctctgtctctgaggat

fava_c17139_1139	fava_c17139	1139	A/G	gtaaaMggagcattcaaaagggtcaactatcagRatatttctcatgctgatagggacctatcttctttccgacatggttctattgatcaaattggtaSac[A/G]agagagaactagtgacagaacatttgtaaatggcaatagcaacatgtatcaaccttcacatgagcatgacgagactgcKtcctctgtctctgaggatggc

fava_c17139_1218	fava_c17139	1218	T/G	ctattgatcaaattggtaSacRagagagaactagtgacagaacatttgtaaatggcaatagcaacatgtatcaaccttcacatgagcatgacgagactgc[T/G]tcctctgtctctgaggatggccctggcattgagaattttcaaatttgtggtgatgctattcctggagaaaaacttcttggatgtggatatccagtccgtc

fava_c17139_1473	fava_c17139	1473	C/T	agtacattgaaggagccacaaatccagagtatgtagttacagctgatgatgttgataaattgatagcggttgagtgtattccaatggatgataaaggccg[C/T]cagggagagatagtgagactttttgctaatgaccaaaacaaaataacatgcgacccggaaatgcagcatgagattgacacattcctgtctaaaggagaag

fava_c17139_157	fava_c17139	157	A/C	ttttcatccttctccatctttctctttggattttgctttttcacacccaacaacctcgtacttgctgtcggtgcatctcttggttaggaacgactcatac[A/C]aaaaccttgatttggacctaaataMggggatgatcagacgagacaaaatgcagaaatttattgggaagaatcgactatgattcaattacaagttgtttgt

fava_c17139_182	fava_c17139	182	A/C	ttggattttgctttttcacacccaacaacctcgtacttgctgtcggtgcatctcttggttaggaacgactcatacMaaaaccttgatttggacctaaata[A/C]ggggatgatcagacgagacaaaatgcagaaatttattgggaagaatcgactatgattcaattacaagttgtttgtaagaggacattgtgcaagccattct

fava_c17139_289	fava_c17139	289	T/C	gatcagacgagacaaaatgcagaaatttattgggaagaatcgactatgattcaattacaagttgtttgtaagaggacattgtgcaagccattctatgtct[T/C]ctgttaaaagcccttcgcacgggcttaaaaatgatgagattcRgggtcaagcctctgaaatcttaggcaggcacaatcttgagaaacaactggctcaaag

fava_c17139_332	fava_c17139	332	G/A	ctatgattcaattacaagttgtttgtaagaggacattgtgcaagccattctatgtctYctgttaaaagcccttcgcacgggcttaaaaatgatgagattc[G/A]gggtcaagcctctgaaatcttaggcaggcacaatcttgagaaacaactggctcaaagcaatttcaaaagcaatgacgcactcaatcaagttgaKgaccta

fava_c17139_426	fava_c17139	426	G/T	agattcRgggtcaagcctctgaaatcttaggcaggcacaatcttgagaaacaactggctcaaagcaatttcaaaagcaatgacgcactcaatcaagttga[G/T]gacctagaaactatggaactttattcacgagcaaggaggcaagaagaagagatcctctccctccgtgaacagattgcggcttcctgtttgacRgagttac

fava_c17139_519	fava_c17139	519	G/A	aagttgaKgacctagaaactatggaactttattcacgagcaaggaggcaagaagaagagatcctctccctccgtgaacagattgcggcttcctgtttgac[G/A]gagttacagcttttgaatgagaaatgcaaactagagagagaattttcggaattaagaatggcaattgatgacaagcaaaatgaagccatcacatctgcct

fava_c17139_634	fava_c17139	634	C/A	gaatgagaaatgcaaactagagagagaattttcggaattaagaatggcaattgatgacaagcaaaatgaagccatcacatctgcctctaatgatctggct[C/A]gaaggaaaggttatctagaagaaaatttaaaacttgcccatgatttgaaagttgcggaggacgagagatatgtctttatgtcatctatgcttggattgct

fava_c17140_175	fava_c17140	175	C/A	tttgaagttccataagtgctctaggcagtgaaaagtgaaaacacaagaaaagagcaaaattcaaacgatgcaagctatatctatcatcactctttcacat[C/A]tttttcaKttgtttttttactttctgtgcgYaYaaattcacagatatacaatttaaaatgttcaattatgcatcattttcctttcaaacgcaagaacctc

fava_c17140_183	fava_c17140	183	T/G	tccataagtgctctaggcagtgaaaagtgaaaacacaagaaaagagcaaaattcaaacgatgcaagctatatctatcatcactctttcacatMtttttca[T/G]ttgtttttttactttctgtgcgYaYaaattcacagatatacaatttaaaatgttcaattatgcatcattttcctttcaaacgcaagaacctcttgggaga

fava_c17140_206	fava_c17140	206	C/T	aaagtgaaaacacaagaaaagagcaaaattcaaacgatgcaagctatatctatcatcactctttcacatMtttttcaKttgtttttttactttctgtgcg[C/T]aYaaattcacagatatacaatttaaaatgttcaattatgcatcattttcctttcaaacgcaagaacctcttgggagaatacattctgaaaactctatagg

fava_c17140_208	fava_c17140	208	T/C	agtgaaaacacaagaaaagagcaaaattcaaacgatgcaagctatatctatcatcactctttcacatMtttttcaKttgtttttttactttctgtgcgYa[T/C]aaattcacagatatacaatttaaaatgttcaattatgcatcattttcctttcaaacgcaagaacctcttgggagaatacattctgaaaactctataggct

fava_c17140_650	fava_c17140	650	G/A	ttgaagaacaagagtgcttgcataattgaagcatgttatatccagcagctgaagatgaaacatacagcaagatcttgagagcatccacgtctttataagt[G/A]actttcttttcaatagctaaaaagacaaccttggtttgagtatcaaaagcaattaaacaaactgttaataccaagaacaatattgctgtgactcttagaa

fava_c17141_1054	fava_c17141	1054	C/T	ttgaaccttgttctcgccacgatgattagcttttttgtatctattttctagtacgggtggagaaactgaatcattcctgtcgttaacacttttatctgat[C/T]tatcagccgagaatagaaattgcttgttaccatgagaaatcagaggctcttgagtttcaaactgatattcaggagaactctgattcttatggttaataga

fava_c17141_1344	fava_c17141	1344	C/T	tcttagccggtccatatttcagatgtctgactatcatataagcttctttttttgtcaaatgaggtccactttccacaatacagacatcttctatcagagc[C/T]gaaagacgggatattgagtctaacatggcctgatctgcgttgcctgttgccttgaccttcacccgataacccttatccattaactttcgaaccatgtcag

fava_c17141_1536	fava_c17141	1536	A/G	ccatgtcagctttcatcttaagatcttttgattcggttttttcagaaaatcgtacttccttacattcctttcgcattgtcatctcagacttgcttttagc[A/G]cgttccttgtcattttcttttttcttgtacatttccttgtggaagtccatgattttacaaacaggtggcttggcatttttatcaacctcaactaaatcaa

fava_c17141_1674	fava_c17141	1674	C/T	tgtggaagtccatgattttacaaacaggtggcttggcatttttatcaacctcaactaaatcaaatttaagcttcctggcatgttccaatgcttcaaacct[C/T]gacactactgaatgtccatcatccatgacaagtctgacaaatttagctttRatttgatcattcaaccgtggcccatctgaatcgtcctcctcattcttag

fava_c17141_1725	fava_c17141	1725	A/G	caactaaatcaaatttaagcttcctggcatgttccaatgcttcaaacctYgacactactgaatgtccatcatccatgacaagtctgacaaatttagcttt[A/G]atttgatcattcaaccgtggcccatctgaatcgtcctcctcattcttaggcttaacctgaaactgaacgggaacagcgaaaaacctgacggcattgaaaa

fava_c17141_1836	fava_c17141	1836	T/A	tcaaccgtggcccatctgaatcgtcctcctcattcttaggcttaacctgaaactgaacgggaacagcgaaaaacctgacggcattgaaaacaaaagaggg[T/A]ttgtgatggaaaaggggaatagggggagttttgggggtggaattggagagaRaagaatggggaagatgatgatgagtgtaagatctgtggaattgggagt

fava_c17141_1888	fava_c17141	1888	G/A	ctgaacgggaacagcgaaaaacctgacggcattgaaaacaaaagagggWttgtgatggaaaaggggaatagggggagttttgggggtggaattggagaga[G/A]aagaatggggaagatgatgatgagtgtaagatctgtggaattgggagtataaggttttgaggttagagttacggattcggtgccaaagcgccataacctc

fava_c17141_377	fava_c17141	377	A/G	acaccccttctcaagagtcaaacaaaatgaatatagaaagaaaaacaatacaaatttgtgtacttgcatccaatattagattatgcaaaatccctagtaa[A/G]ctaagctaaataaacaacattttcatgaatcagtaaaaggtaaaatacagttgcaggactgaggagtaactgattcattgcttcattttYcatcaaaatt

fava_c17141_467	fava_c17141	467	C/T	tccctagtaaRctaagctaaataaacaacattttcatgaatcagtaaaaggtaaaatacagttgcaggactgaggagtaactgattcattgcttcatttt[C/T]catcaaaatttcttaattgaatagtgtgtttaatgggaccaaaattgtccttgtaatatgcttatttgccactctaatctagctcactttcataccaact

fava_c17141_666	fava_c17141	666	G/A	ctgaaagattcattctctattgaatctctgctgaaaagttgtgaagttgaaatactgagtgttaccttctatgttcctatgcatccctactaaaaagacc[G/A]tagccgggactttttgtattcggacccctaggattcgggtggatattgtcacggggaggcatcgggcttcttgatccaggaggactatcaattttgggat

fava_c17144_185	fava_c17144	185	G/C	tgttgatttttttcatctgaatcagtaacatggatgtgaagttcaagtctctttctgaaggtgaacatgaagaagaagaaaaagaaaacatacactttct[G/C]aaatctcttcaggagttaagagaaYtgagRtctcagcttcactatgctgctgaKtactgtgaaacaacattcWtgaaaagtgaaaagaaaaaagatgtga

fava_c17144_210	fava_c17144	210	T/C	taacatggatgtgaagttcaagtctctttctgaaggtgaacatgaagaagaagaaaaagaaaacatacactttctSaaatctcttcaggagttaagagaa[T/C]tgagRtctcagcttcactatgctgctgaKtactgtgaaacaacattcWtgaaaagtgaaaagaaaaaagatgtgatggaaaacacaaaagagtatatatg

fava_c17144_215	fava_c17144	215	G/A	tggatgtgaagttcaagtctctttctgaaggtgaacatgaagaagaagaaaaagaaaacatacactttctSaaatctcttcaggagttaagagaaYtgag[G/A]tctcagcttcactatgctgctgaKtactgtgaaacaacattcWtgaaaagtgaaaagaaaaaagatgtgatggaaaacacaaaagagtatatatgtagag

fava_c17144_239	fava_c17144	239	T/G	ctgaaggtgaacatgaagaagaagaaaaagaaaacatacactttctSaaatctcttcaggagttaagagaaYtgagRtctcagcttcactatgctgctga[T/G]tactgtgaaacaacattcWtgaaaagtgaaaagaaaaaagatgtgatggaaaacacaaaagagtatatatgtagagctatggtgactgtggttgatcatc

fava_c17144_258	fava_c17144	258	T/A	agaagaaaaagaaaacatacactttctSaaatctcttcaggagttaagagaaYtgagRtctcagcttcactatgctgctgaKtactgtgaaacaacattc[T/A]tgaaaagtgaaaagaaaaaagatgtgatggaaaacacaaaagagtatatatgtagagctatggtgactgtggttgatcatcttggaaatgtttcttccaa

fava_c17144_398	fava_c17144	398	T/C	aagagtatatatgtagagctatggtgactgtggttgatcatcttggaaatgtttcttccaatcttgaaggacttatttctcacactaattcattttctga[T/C]gcYcagataaaaattcaatgcctcaaacaaagacttttctcgtgcgaacaatatgcggataaactttccatctcaaatatgcaatggaaggagaaatttc

fava_c17144_401	fava_c17144	401	T/C	agtatatatgtagagctatggtgactgtggttgatcatcttggaaatgtttcttccaatcttgaaggacttatttctcacactaattcattttctgaYgc[T/C]cagataaaaattcaatgcctcaaacaaagacttttctcgtgcgaacaatatgcggataaactttccatctcaaatatgcaatggaaggagaaatttccaa

fava_c17144_534	fava_c17144	534	G/A	tttctcgtgcgaacaatatgcggataaactttccatctcaaatatgcaatggaaggagaaatttccaagattccatacaagatatctatcaccatctcct[G/A]tccttgaaaaaacacaagagaatttgaagccagccattgctacaaataacaagcacaacaatttggcttttgtgatgcctgttcgcgacggtttgaaagt

fava_c17144_638	fava_c17144	638	G/A	ttgaaaaaacacaagagaatttgaagccagccattgctacaaataacaagcacaacaatttggcttttgtgatgcctgttcgcgacggtttgaaagtgtt[G/A]gcaaaagtttcaaatcctaaatttcattttcaagctactccaaaggtcagaccgcgtcatagaagatcggtgcacggaagcgatattttatggctcatta

fava_c17145_236	fava_c17145	236	T/A	ctagatgataaaatagtaaagctgccaaacaaaaaatctttgccatctctgcatttctcaagatactaacttaaatttctccattgaatcaaagagattc[T/A]gctaagcccgtcaMtattatagaaaatgaaatattacatctcaatataacaaagaaaattcaccaagtactccaatcggtgtttcttttcttaatttaaa

fava_c17145_250	fava_c17145	250	A/C	agtaaagctgccaaacaaaaaatctttgccatctctgcatttctcaagatactaacttaaatttctccattgaatcaaagagattcWgctaagcccgtca[A/C]tattatagaaaatgaaatattacatctcaatataacaaagaaaattcaccaagtactccaatcggtgtttcttttcttaatttaaaaacccttttgaagt

fava_c17145_987	fava_c17145	987	A/G	ctctttcaagcgcactcaccgccaaaagcggatcccaattaaccaactcaagaacatgctttccgtcattgttgttatctacaacaacgcggaaatagtt[A/G]gggtatttcacaacgcggtcgcgaaaatcatcaggtaaaccaaataagattctacagtgatgcaatttactaagcggaatacggtgattcgctgacatca

fava_c17146_292	fava_c17146	292	T/A	cttcgaataggtaaaacttgccgatcaaaacatttttttccagatcaataacaaaacttgttttagttactagaatttgcttcataaggttccttcccat[T/A]ggaatgagcttcggatgatttgaattccgtttaaattataatgcatgactWtatgaactaatttgtttggtctcatacaatttcgttggaatcatgatcc

fava_c17146_343	fava_c17146	343	A/T	caaaacttgttttagttactagaatttgcttcataaggttccttcccatWggaatgagcttcggatgatttgaattccgtttaaattataatgcatgact[A/T]tatgaactaatttgtttggtctcatacaatttcgttggaatcatgatcccggtactaactgatctcgagtagaactttttgaagtgcttctgttgttaga

fava_c17146_512	fava_c17146	512	T/C	gtagaactttttgaagtgcttctgttgttagagggatgtgcaagcaatcttgaccgtcagaatgcctcttggttttgaccagctcgtggtctttaaattc[T/C]gtcaaatgagaattcagggtaacctggctgctaacaaggaaacgttcacgacagactgagtaaagatcactgattggcattccttcatcgggatgagaaa

fava_c17151_1252	fava_c17151	1252	G/C	cgtcatccttctcattgccagccctcaaagctaatttgtcaataaaatatgtagcaacagctatttgctgcttcttaatatctatatttgtgaaatcttt[G/C]gtatatgcagccctgatgttccctatataactcttcaacatccttgctttctcgtacttttccttgtcgctttgacccttccaagaactactagctgcca

fava_c17151_1432	fava_c17151	1432	T/C	tccaagaactactagctgccagaaacacgtatttgaataactttggattgattgggtcattccagtaggctaaccaagtaactgtattatcatgtcttat[T/C]tctccccagctttccccaggaagaggacattcaggaattggtgcatcctttccaatattaattacgacatcgtttggagtaatacgtcttttcactttgc

fava_c17151_2562	fava_c17151	2562	T/A	gtgcaaggaatcagtcttatctagtggtctcttaacagacagtgtagatggcctttcctgtttaacactagcatcggaaccatattgatgtcctttcaga[T/A]cttttRgaaatggtttcttctgatcggcgtcattataattagaactagattttcccaaattattattctgtgacacccttgctgacaagggaacatcatc

fava_c17151_2568	fava_c17151	2568	G/A	ggaatcagtcttatctagtggtctcttaacagacagtgtagatggcctttcctgtttaacactagcatcggaaccatattgatgtcctttcagaWctttt[G/A]gaaatggtttcttctgatcggcgtcattataattagaactagattttcccaaattattattctgtgacacccttgctgacaagggaacatcatcatcatc

fava_c17151_2689	fava_c17151	2689	T/G	gcgtcattataattagaactagattttcccaaattattattctgtgacacccttgctgacaagggaacatcatcatcatcagagtcttcattagatttct[T/G]tacaacaatattagttgctttcttagagtcttcattagatttctttaMaacaataggagttgctttcttaRagtcttcattagatttctttacaacaata

fava_c17151_2737	fava_c17151	2737	C/A	acccttgctgacaagggaacatcatcatcatcagagtcttcattagatttctKtacaacaatattagttgctttcttagagtcttcattagatttcttta[C/A]aacaataggagttgctttcttaRagtcttcattagatttctttacaacaataggagttgctttcttagagtcttcattagatttctttacaacaatagga

fava_c17151_2760	fava_c17151	2760	G/A	atcatcatcagagtcttcattagatttctKtacaacaatattagttgctttcttagagtcttcattagatttctttaMaacaataggagttgctttctta[G/A]agtcttcattagatttctttacaacaataggagttgctttcttagagtcttcattagatttctttacaacaataggagtagctttcttagcactgtcatt

fava_c17151_3003	fava_c17151	3003	T/C	gtcctcagaatcttcatatttgtcttctttaagatgtttggtagaattattttcctctttagcttgaacaggaattttcttttctaaaagagactctttt[T/C]tatttccaaacgatggaattttatttgctgaaggggacctcacataggttttgagaattgacggtgatgcctttgttacactcacagaagattcagcggc

fava_c17151_3584	fava_c17151	3584	A/G	gattactcttcgacacagaacttctcttaaaaactaccggcccatcatcatcatcatcatcaaattcacgcgacatctttaaattaccagaagcctccac[A/G]gtcatttatcaactacgaaatgtcatagcacgtacatcacagcttgtttgaaaattgatgaaaatggaaggtgaatccttgggtttttggtttagctatg

fava_c17151_505	fava_c17151	505	A/G	gagttttactgaaaatcttttcaataggaacctcatgccgcttgcaccaggcaactgttatcctgggatctaagtagttgatcttggacgtgccaagtgc[A/G]acggtttttagatcttcttttgtcctcatatcacgttgcattttatcaattttcgcacttgtttgatccatctttttctctatcgcttcaggactcaagt

fava_c17153_1112	fava_c17153	1112	C/G	taacatgggttcttaagtacttgcatgacaatctcaatctcttagaaaccgtcacgaaagaacatgaaagaataagaaacaaacttgttaatgaaaaccg[C/G]ggtctttcatgggaggatacaaggcaaatgccattttctaccaRggtgatccaagagacattaagaagtgcaagtatattatcatttacatttagagaaR

fava_c17153_1156	fava_c17153	1156	A/G	gaaaccgtcacgaaagaacatgaaagaataagaaacaaacttgttaatgaaaaccgSggtctttcatgggaggatacaaggcaaatgccattttctacca[A/G]ggtgatccaagagacattaagaagtgcaagtatattatcatttacatttagagaaRcagtacaagatgtggaacttgaaggttacactattccaaaaggt

fava_c17153_1212	fava_c17153	1212	G/A	SggtctttcatgggaggatacaaggcaaatgccattttctaccaRggtgatccaagagacattaagaagtgcaagtatattatcatttacatttagagaa[G/A]cagtacaagatgtggaacttgaaggttacactattccaaaaggttggaaagtgcttccactgtttagatccattcatcattcacctcatttcttccctca

fava_c17153_128	fava_c17153	128	C/A	tactcaaattttcattccttttccctctataaatacctaactaaccgccctatcttaccttattgctttgtgccttaaatcattacacaatgcaaatttt[C/A]acatcacttcttaattcttgtttaaattcttctctctacttcattatatttctcctctttttctcttctttttctcttctccttcaatggtggaaacacc

fava_c17153_1479	fava_c17153	1479	G/A	aagtggagtccactcttgtccaggtagtgagcttgctaagcttgaggttcttgttcttcttcatcatctcacccttaaatacaggtgggaagttgttgga[G/A]atggagatggaattcaatacggtccttttcctgtgccaaaaaacggattgccactaaagattactctaaacaagagatagatatactttctcataaactt

fava_c17153_1641	fava_c17153	1641	A/G	tactctaaacaagagatagatatactttctcataaacttggaatttgaaaccaaagtggggccagccaatttgtaacatgtcagtacaatcaatggattt[A/G]tttttcttttgttttttcttgtatagattcttttcccatttatagccattatgagacaaattttagttcacattggttatactatgcatgacccatgtat

fava_c17153_608	fava_c17153	608	C/A	aaggtgcttatcactccatgctcaagaagatggttcaagcctcttttttaccttctacaattaaaaactctgtcccttttgtggaccaaattgtacaaaa[C/A]atggtcccatcttggactaatacaaccatcaacacattggaagagatgaaaaagtatgcttttgaagtggctgcaaattcagcttttggtgaaataaagg

fava_c17153_754	fava_c17153	754	A/G	atgaaaaagtatgcttttgaagtggctgcaaattcagcttttggtgaaataaaggaaatggaaatggaagaaataagagagttgtatcattgcttagaga[A/G]aggctacaattctcatcctttaaatcttcccggaactttctattggaaggcaatcaaggcaaggaaggttttgaatgagattataaggaagataatagaa

fava_c17156_118	fava_c17156	118	T/C	cttcgtcgataattgaagacgaatcccaattcctccattgttccatttcagtagctccataatcgaaagacctccacagtctccggcgattcggaatttt[T/C]cggaaacaatgaggcgacaggggcagtatggtgatgcatctgcgaatagttatggagcttcgcagatgcatcatcatcatcttcatatggctggtcaaag

fava_c17156_336	fava_c17156	336	A/G	gtaattttgaagggcggctagaggcattcactccagagagagataatcagtatccgaattctaagccagagggtcagtggagatgggaattggatgaatc[A/G]aagatgtctagtaatcctatgatgtcgaacatgtttaatgaaggtcaagagggtggtgatgcttcaaggtcctattttcaagggcagaggcctgatccta

fava_c17156_930	fava_c17156	930	T/C	cagcttctggtggagaaccccaacgaggctattccgctgatcactttgatccctacagagagcaatctcgatttcttgggaatgcaagagatcaaggatt[T/C]gaacctagaggtccttatcctgggggacgtgtctatgacaccggttcacgttactacaactgagttttgactaaaagttcacctctcttgttcggtttgt

fava_c17158_1198	fava_c17158	1198	C/T	tggctaaaggtttatcagatattttatctggagacatgcaaaagggtattaagcttcagacaaagcacctggaggctttgattgaattacacaatatcac[C/T]ggatcctttgcaaggaatattcagcacttattttctggttctgacgtacaagttttaatggatgtgctgaaagccgtgtacttgccatatgaatcgttta

fava_c17158_1324	fava_c17158	1324	T/C	acttattttctggttctgacgtacaagttttaatggatgtgctgaaagccgtgtacttgccatatgaatcgtttaaacagaggtatggacaaatggagca[T/C]gccatcctttcctctgagattgcaggaatagatttaagaggagctgtaattcgaggggtaggagcccaaggagttgaactcagtgaaactgttcgtagga

fava_c17158_1600	fava_c17158	1600	G/T	ctgaggcagatgagctgattcttgcccttgatgacataatgttacaatacatttccactctacaagaaactctcaggtcactaagaactgtgtgtggtgt[G/T]gattatggtggcgatggtactggaaagaaagacagcgagaagaaagatgcaagtcaaaatgctcggagagttgatttggtctcaaatgaggaagaatggt

fava_c17158_502	fava_c17158	502	A/G	ttgctaaggtcgatgtagttaagcagaggatggaagctgcttatgaaactttgcaggatgctgctggattaacccaattaagttccacagtggaggatgt[A/G]ttcgccagtggtgatcttcctcgtgctgctgaaactctcgctaatatgaggcattgcttgtctgctgttggggaggttgctgaatttgccaacataagaa

fava_c17160_1192	fava_c17160	1192	A/G	tgtctttcgatcccattattgctcgagaatccccaatatatcccatgaagagatttgaaccctgcttcacaatagttacagatgtgctcccactacagaa[A/G]caRtctagatttgaatgagacttgagctctctgtccatggccttgtatgctttcgtgaaagcttctctccaagtggagttcagtttatcctcagcagcag

fava_c17160_1195	fava_c17160	1195	A/G	ctttcgatcccattattgctcgagaatccccaatatatcccatgaagagatttgaaccctgcttcacaatagttacagatgtgctcccactacagaaRca[A/G]tctagatttgaatgagacttgagctctctgtccatggccttgtatgctttcgtgaaagcttctctccaagtggagttcagtttatcctcagcagcagagc

fava_c17160_1701	fava_c17160	1701	T/G	aaggatgataaatggtgtagagaaataacatgatcagagaacgttctccttgctctcttttgtccacagaatccaacatctaagcaggatggagtgatca[T/G]gtctccgttgctcccactgctacaagtacttcgactgctcgttgagacacaacagcccattttcttcagttccagaatgcacacattagatggtggtgac

fava_c17161_1078	fava_c17161	1078	C/T	atggtacacttggctcaaattgttagacatggagttctagccaaattcaattatgaaagaccagattataacattgtgcactatggacaagtagtcccac[C/T]aatWtataacctatcaaatattccacatgatctccctctcttcattagctatggaggacaagatgcactttctgatgttcgtgatgttgagaatttgctt

fava_c17161_1082	fava_c17161	1082	T/A	tacacttggctcaaattgttagacatggagttctagccaaattcaattatgaaagaccagattataacattgtgcactatggacaagtagtcccacYaat[T/A]tataacctatcaaatattccacatgatctccctctcttcattagctatggaggacaagatgcactttctgatgttcgtgatgttgagaatttgcttgata

fava_c17161_1187	fava_c17161	1187	C/G	acctatcaaatattccacatgatctccctctcttcattagctatggaggacaagatgcactttctgatgttcgtgatgttgagaatttgcttgataagct[C/G]aagtttcatgatgtcgataaaaRaagtgttcaatttgtRaaagaatatgctcatgctgattacataatggggtttaatgctaaggacatagtgtataatt

fava_c17161_1210	fava_c17161	1210	G/A	ctccctctcttcattagctatggaggacaagatgcactttctgatgttcgtgatgttgagaatttgcttgataagctSaagtttcatgatgtcgataaaa[G/A]aagtgttcaatttgtRaaagaatatgctcatgctgattacataatggggtttaatgctaaggacatagtgtataattcagttatttcatttttcaattct

fava_c17161_1226	fava_c17161	1226	G/A	gctatggaggacaagatgcactttctgatgttcgtgatgttgagaatttgcttgataagctSaagtttcatgatgtcgataaaaRaagtgttcaatttgt[G/A]aaagaatatgctcatgctgattacataatggggtttaatgctaaggacatagtgtataattcagttatttcatttttcaattctcaaggtaatacttgat

fava_c17161_309	fava_c17161	309	C/G	tgatggtatatgtgcttcttctattattgtttatggatacaaatgtcaagaaattcaggttgtaactaaagatgggtatattttaagcctacaaagaatt[C/G]ttgaaggtaggaataaagctaaaaatgggagcatgaaaaaacagcctgtaatattacaacatggagtattagtggatggtatgacatggcttatgaactc

fava_c17161_494	fava_c17161	494	C/T	catggcttatgaactctccagaacaagatttgcctctaattttagctgataatagttttgatgtttggattgctaatactagaggaaccaaacatagtcg[C/T]cgacatgtctcattagacccctctaacccggtcttttggaattggtcttgggaYgaacttgttacatatgacttaccagctgtgtttgattatgtgtaca

fava_c17161_548	fava_c17161	548	T/C	gttttgatgtttggattgctaatactagaggaaccaaacatagtcgYcgacatgtctcattagacccctctaacccggtcttttggaattggtcttggga[T/C]gaacttgttacatatgacttaccagctgtgtttgattatgtgtacagtcaaacgggacaaaagattaattatgttggtcattctttgggaacattaatag

fava_c17161_932	fava_c17161	932	T/A	atgcctttctgaagcttctttgtgattatcctggaatagactgctacgacttaatgactgaattaactggtaaaaattgttgtctcaattcttcaattat[T/A]gaccgattcttgatgaatgagcctcaatcaacatcaacaaaaaacatggtacacttggctcaaattgttagacatggagttctagccaaattcaattatg

fava_c17165_193	fava_c17165	193	A/T	agaaaatttggtgatcgaattcgaagtgaaagaagatgaaggtacgttcagaaaggggaaaaaccaaagtagtgattcgccatttgcctccttccctaac[A/T]gaatccgatctcattcaacaaatccataaccgcttcgcttctcgctaccgctggttcgttttccgtcccggtaacaccagtcacaggaatcagaggtatt

fava_c17165_2081	fava_c17165	2081	T/C	ttaaccatgttgatgggttaggcccttgactggtttgtgtctttctcaattgttgaatatttcggttttgagaaatgtttgtgtttggtgtttaactgaa[T/C]agtcttggtatttgccatgagatagtatatgctgggaagagtgaatcatttatagtccagttcattggacatatatatagttgaatagtgtatgaacgac

fava_c17165_548	fava_c17165	548	C/T	ggatggccgtgaagggactatatataaagatccagactatcttgagttcctcaaactcattgcaaagcctcaggaacatcttcctagtgcagaaatacaa[C/T]tggaaagaaaggaagctgagcaagctggggctagcaaggaagcacctattgtaacacccctgatggaatatgttcgtcagaaacgtgctgttggcagtgg

fava_c17165_895	fava_c17165	895	C/T	actccaaagacaaatcagcctttactgtggtccgcaggcatgatgatcagtctgctgaatcaagtttaaaaggaatttatgaaattgaatctttgcacgg[C/T]gttgaaggttctgtctctggaataccactgacttctgaatctggaaagaaaaaaatcctccttctgaaaggaaaacaacgcgaagttcccaaagccactg

fava_c17166_1014	fava_c17166	1014	G/T	gccaaactctaagcgagaagggatacaatgcRtttgccttctccactccttcagggaaggagtttctgtcaatatttagtcttgcYgctcctgtatttgt[G/T]acattgatgttaaaggtggttttctactctctaattatatattttgctacatcaatgggtacacataaaatcgctgctcatcaagtcatgcttcagatct

fava_c17166_1125	fava_c17166	1125	C/T	taaaggtggttttctactctctaattatatattttgctacatcaatgggtacacataaaatcgctgctcatcaagtcatgcttcagatctacatgttatg[C/T]gcaatatgtggtgaacctctttcccaaactgctcaatcatttatgcctgagttgatgtatggagtaaatcggagtttggcaaaggctcagtcactgctaa

fava_c17166_1282	fava_c17166	1282	T/A	gtatggagtaaatcggagtttggcaaaggctcagtcactgctaaagtctcttttaactattggagctgtatttgggttgcttttggggatttttggaaca[T/A]ttgttccWtggttattcccctacatttttacacccgatcagatggtcatcaaRgagatgcataagattctgatYccatactttttagcgctagtggtgac

fava_c17166_1290	fava_c17166	1290	T/A	taaatcggagtttggcaaaggctcagtcactgctaaagtctcttttaactattggagctgtatttgggttgcttttggggatttttggaacaWttgttcc[T/A]tggttattcccctacatttttacacccgatcagatggtcatcaaRgagatgcataagattctgatYccatactttttagcgctagtggtgacacctgcaa

fava_c17166_1335	fava_c17166	1335	A/G	taactattggagctgtatttgggttgcttttggggatttttggaacaWttgttccWtggttattcccctacatttttacacccgatcagatggtcatcaa[A/G]gagatgcataagattctgatYccatactttttagcgctagtggtgacacctgcaactgtcggcctggagggaacattgctggctggaagggatctaagat

fava_c17166_1356	fava_c17166	1356	T/C	ggttgcttttggggatttttggaacaWttgttccWtggttattcccctacatttttacacccgatcagatggtcatcaaRgagatgcataagattctgat[T/C]ccatactttttagcgctagtggtgacacctgcaactgtcggcctggagggaacattgctggctggaagggatctaagatttataagtttgtcaatgagtg

fava_c17166_1602	fava_c17166	1602	G/T	atggtttgctaggctgctggtttgcccttgctggatttcaatgggctcgattttcaatggcccttctgcgccttctatctcccaaaggcgttttatactc[G/T]gaagatacaagccagtatgagYtgcaaaagttgaagactgcatagtgttggagcttcgtttagttcatgatacattatacaaaaagaagcatgttttatg

fava_c17166_1624	fava_c17166	1624	C/T	tgcccttgctggatttcaatgggctcgattttcaatggcccttctgcgccttctatctcccaaaggcgttttatactcKgaagatacaagccagtatgag[C/T]tgcaaaagttgaagactgcatagtgttggagcttcgtttagttcatgatacattatacaaaaagaagcatgttttatgttgcttgcaaatcttgataaat

fava_c17166_1729	fava_c17166	1729	T/A	aaagttgaagactgcatagtgttggagcttcgtttagttcatgatacattatacaaaaagaagcatgttttatgttgcttgcaaatcttgataaatacgt[T/A]acaagataggcattcatttcaatgtcgatacaattcagtgcagggaaactactggtgctgagttgtatcRgaaaccaagtggggaaatggggatgtgttg

fava_c17166_1799	fava_c17166	1799	G/A	tatgttgcttgcaaatcttgataaatacgtWacaagataggcattcatttcaatgtcgatacaattcagtgcagggaaactactggtgctgagttgtatc[G/A]gaaaccaagtggggaaatggggatgtgttgtggggaaacYgaatccatcaccaccaccRccgcgctgaacgtatcttttttatcaagtgaggggctactg

fava_c17166_1839	fava_c17166	1839	T/C	gcattcatttcaatgtcgatacaattcagtgcagggaaactactggtgctgagttgtatcRgaaaccaagtggggaaatggggatgtgttgtggggaaac[T/C]gaatccatcaccaccaccRccgcgctgaacgtatcttttttatcaagtgaggggctactggggtaaaggtaatcggaaaacctttctattttaatggtgg

fava_c17166_1858	fava_c17166	1858	A/G	tacaattcagtgcagggaaactactggtgctgagttgtatcRgaaaccaagtggggaaatggggatgtgttgtggggaaacYgaatccatcaccaccacc[A/G]ccgcgctgaacgtatcttttttatcaagtgaggggctactggggtaaaggtaatcggaaaacctttctattttaatggtggattttaacacggatcgata

fava_c17166_291	fava_c17166	291	T/G	gcgtagctcaaaatcacgacgtcattgatgagtctgaacacaccgaacaaatccgcgacggttttgatgtggaccgagatgaagaagaagtgaatgaact[T/G]gttgagcagagtatatggattcagatgaaggaaattgttttgtttactggacctgctattggactttggttgtgtggacctttcatgagtctcattgata

fava_c17166_945	fava_c17166	945	A/G	taggctatgggattgctggggctgcatgggctacattggcatcacaagttgttgctgcgtatatgatgagccaaactctaagcgagaagggatacaatgc[A/G]tttgccttctccactccttcagggaaggagtttctgtcaatatttagtcttgcYgctcctgtatttgtKacattgatgttaaaggtggttttctactctc

fava_c17166_999	fava_c17166	999	T/C	ctgcgtatatgatgagccaaactctaagcgagaagggatacaatgcRtttgccttctccactccttcagggaaggagtttctgtcaatatttagtcttgc[T/C]gctcctgtatttgtKacattgatgttaaaggtggttttctactctctaattatatattttgctacatcaatgggtacacataaaatcgctgctcatcaag

fava_c17167_1038	fava_c17167	1038	A/G	ttcgaagtaagaaaRtgatttgaagactaagtatcggttaatgtaaatagcaggattataaaacaatgaatgtgtttctgctctttgcaaatgtgagcgt[A/G]tgtaaatatctggatggatatattttgtgctcgttgtctggatggatacatttgaactaaataaatataagggatttaaaataaaaaaattatgattaat

fava_c17167_952	fava_c17167	952	A/G	gccttcgctgattatgacattgaagttaatatagaaggggaagccataactgagtacctctctatgattgagtcagcagacactatttcgaagtaagaaa[A/G]tgatttgaagactaagtatcggttaatgtaaatagcaggattataaaacaatgaatgtgtttctgctctttgcaaatgtgagcgtRtgtaaatatctgga

fava_c17168_1029	fava_c17168	1029	G/A	acgcaataaccctactaacagttatattaacaacattcctcataacctcagtcgtccacatcaaactcaccaccaacccgatgataaccaaaggcggaaa[G/A]taaaagttcaaagctccaataacagctaaaatccatagagatatccataagaacccaatcgcaagcatattgtaagtaggtcgattcaaattgttgaatt

fava_c17168_1635	fava_c17168	1635	A/T	ataagaatataaacaaggatattctgttggtatatcttcttgaattcaatgtcaccggttgcggttgtggtggtggttgtggagtttgaattggtgctgg[A/T]gctggtgctggtgctggtgctggagctggtgttggtatttgtgttggaacagaacgattatgagtatgagacctttgagattgagcaggaggagaaggtc

fava_c17168_318	fava_c17168	318	C/G	aaacagaaaaaaattagagacgaaaaattaatccgagagacaatttttgtcgttaagatcttgaggtatatctccgaaatcctcttggtgtaggtatagc[C/G]gcttcgcgaccgttttttaacaaagcaagacgatctttgattgttttatcgaacaatctgttttctggattctcggcatagcatacatagtaacaactca

fava_c17168_812	fava_c17168	812	G/T	tttctgaaaatcgattccatgacgcccaaacaacaacgagcacaacaaaacatgaactcgtcttctccctcgagcaaattcaaccccctagcaacaattc[G/T]cagtgcttcaattgtcggcacaaatagMgaacccaaacaagcacttccaagattgcgagtcaaYgctctcagaaagcaaaattgagtgctagattgcatt

fava_c17168_840	fava_c17168	840	A/C	aacaacaacgagcacaacaaaacatgaactcgtcttctccctcgagcaaattcaaccccctagcaacaattcKcagtgcttcaattgtcggcacaaatag[A/C]gaacccaaacaagcacttccaagattgcgagtcaaYgctctcagaaagcaaaattgagtgctagattgcattcctcgcaagtaatacaacgcaataaccc

fava_c17168_876	fava_c17168	876	T/C	ctccctcgagcaaattcaaccccctagcaacaattcKcagtgcttcaattgtcggcacaaatagMgaacccaaacaagcacttccaagattgcgagtcaa[T/C]gctctcagaaagcaaaattgagtgctagattgcattcctcgcaagtaatacaacgcaataaccctactaacagttatattaacaacattcctcataacct

fava_c17170_1040	fava_c17170	1040	T/C	atcatcttctccacaaccatactcttcaatctcattcaccgtggttcatcttcaccaaaattctacaaaaatatctctttgatctctctctgaatttcat[T/C]atcttcatcacaatgtatcaaccttcaacaacaataactgcatctcgacagttcaactatagcacctcactgcgtcacttctgcattttcgatctgcatc

fava_c17170_1182	fava_c17170	1182	A/C	atctcgacagttcaactatagcacctcactgcgtcacttctgcattttcgatctgcatcacaaacctcacaaattcgcgtaacagaaaagtgcagaaatg[A/C]gataaagatgaaatgaattaaaagaagaaatgcagagtaatcgaaaatatggatgacatYgtacctgaatcgaagaaactccagatttctctccgatagg

fava_c17170_1242	fava_c17170	1242	C/T	caaacctcacaaattcgcgtaacagaaaagtgcagaaatgMgataaagatgaaatgaattaaaagaagaaatgcagagtaatcgaaaatatggatgacat[C/T]gtacctgaatcgaagaaactccagatttctctccgataggtaagacttaatcttcgatttcttccatcttttccaagttttcttaagcgcRtagatcgga

fava_c17170_1333	fava_c17170	1333	G/A	ggatgacatYgtacctgaatcgaagaaactccagatttctctccgataggtaagacttaatcttcgatttcttccatcttttccaagttttcttaagcgc[G/A]tagatcggagttgtggatccatggtattcgatgttcgcctgatggattgttctgaagatgaaacaaaaagatagagatggagaacgatggaggagatgag

fava_c17170_314	fava_c17170	314	C/A	atatttataacattattctggactgggttatgcttcaaaaccgcaacagtgaggctgcacaaagtctgcaaaatcttctccaagaaaataggtcttcaat[C/A]attagtttcYtaaaatgtctccatatttagaaaccatgcagatcttcgaaaaccaacataaaatattYtgatctgtcaatcttRgatgccacataagctW

fava_c17170_324	fava_c17170	324	C/T	cattattctggactgggttatgcttcaaaaccgcaacagtgaggctgcacaaagtctgcaaaatcttctccaagaaaataggtcttcaatMattagtttc[C/T]taaaatgtctccatatttagaaaccatgcagatcttcgaaaaccaacataaaatattYtgatctgtcaatcttRgatgccacataagctWactgaatatt

fava_c17170_382	fava_c17170	382	C/T	caaaatcttctccaagaaaataggtcttcaatMattagtttcYtaaaatgtctccatatttagaaaccatgcagatcttcgaaaaccaacataaaatatt[C/T]tgatctgtcaatcttRgatgccacataagctWactgaatattccttaaatattcagtaactcacaaatcagctgaaacatcactaacaactactgtagac

fava_c17170_398	fava_c17170	398	G/A	aaaataggtcttcaatMattagtttcYtaaaatgtctccatatttagaaaccatgcagatcttcgaaaaccaacataaaatattYtgatctgtcaatctt[G/A]gatgccacataagctWactgaatattccttaaatattcagtaactcacaaatcagctgaaacatcactaacaactactgtagacccgatgtcctgtctcc

fava_c17170_414	fava_c17170	414	A/T	MattagtttcYtaaaatgtctccatatttagaaaccatgcagatcttcgaaaaccaacataaaatattYtgatctgtcaatcttRgatgccacataagct[A/T]actgaatattccttaaatattcagtaactcacaaatcagctgaaacatcactaacaactactgtagacccgatgtcctgtctcctRgttgagacatcttt

fava_c17170_500	fava_c17170	500	G/A	atgccacataagctWactgaatattccttaaatattcagtaactcacaaatcagctgaaacatcactaacaactactgtagacccgatgtcctgtctcct[G/A]gttgagacatctttactcgacatgttgaaaactgcaagttgaaacatctttttcaacatattgcttccaagttggttttaccaaaatgtatgcaatcata

fava_c17171_1180	fava_c17171	1180	G/A	caaaggatcagcaccaagcatgataacatgaccacctttgagtccatcttgtttgtaaagcatagcatactcagctgctgatttatcagactcaaaattg[G/A]taattggatctgacccatcatcccctttcaaatctttaagtgttgacccaacaatttgtttcactttccccttgtgaatgtcgatgcaggggcggaattg

fava_c17171_1385	fava_c17171	1385	A/G	gcgcattgaattgaaggtaggtattttctggaaggggaagatgaaggggggttgagggttagaaaacgaagatgattggagcggtgaagtggagaagatg[A/G]gaaagctcttatggaacaagaggaagtgagattgcgcattgagtgagtcgaaaagaagctagtgaggaacagagggaactaatgtggtgatggaactt

fava_c17171_481	fava_c17171	481	G/A	agattgatggaaaaatcactagattcaaggttagtactaagagaagcaatggaacaaaccttgctaagagctcacccaccccggcctccaacaattctca[G/A]cgatgcttacttaagccacaaagaccttttgttggttatgccaagctacaacatctttgtaagccaagtttcccccgaaaatgtccaaggcacttccgac

fava_c17171_632	fava_c17171	632	C/T	acatctttgtaagccaagtttcccccgaaaatgtccaaggcacttccgacggtaacgtccacattttccattccagctgttcttatcctctctaaatcat[C/T]catcacagtcacaccaccagcgtaagtcacaggaatcggtgaatagttgccaagcaaagcaacgagctcttcatcgattcccaacttctttccttcaaca

fava_c17172_793	fava_c17172	793	C/T	accaagaacattcacgtaaaaatccattgattcttcaactgatttgcatatgagtgaaatatgattcacagatttcaagtgcaaagggtttccaacactt[C/T]ccttcatgttttttctaaacaaaattaacaccaattgaacaaaataacagagattaacgtatcaagaaatgattaaacaaaaactttagagaaaaaaatt

fava_c17178_1097	fava_c17178	1097	A/G	gtgaaaactgggttgaaccaccaagaagggagcggaagcgcaattactcagagtctgaatacttcaagcaaaccttgcgccaaggtgcccccactaaacc[A/G]aaggaRcctcgcattcctaggatgccacagttgcatgatttccagtttttcaacactcaaagactgagtgagctgtatgaaaaggaagtgcgttatctca

fava_c17178_1103	fava_c17178	1103	G/A	actgggttgaaccaccaagaagggagcggaagcgcaattactcagagtctgaatacttcaagcaaaccttgcgccaaggtgcccccactaaaccRaagga[G/A]cctcgcattcctaggatgccacagttgcatgatttccagtttttcaacactcaaagactgagtgagctgtatgaaaaggaagtgcgttatctcatgcaag

fava_c17178_1571	fava_c17178	1571	T/C	aattaaatgattatgatagaattatcaaaaacatagagaggggagaggctagaatttctcggaaggacgagatcatgaaagctatcggaaagaaattgga[T/C]cgctacaagaatccttggctagagttgaaagtacagtatggacaaaacaaaggaaagctatacaatgaagaatgtgaccgattcatgatatgcatggttc

fava_c17178_1896	fava_c17178	1896	G/T	catccggttggtagaaaaggaaaaccaggaatatgatgagagggaaagacaagctcgcaaagagaagaagcttgccaagaacacgactccaacaaagcgg[G/T]ctttggcaagacagactgagagtccRtcttcYgcaaagaagcggaagcaatcaactatggatgattatgctaactcgggtaagaggagaaaataaatgga

fava_c17178_1922	fava_c17178	1922	A/G	aggaatatgatgagagggaaagacaagctcgcaaagagaagaagcttgccaagaacacgactccaacaaagcggKctttggcaagacagactgagagtcc[A/G]tcttcYgcaaagaagcggaagcaatcaactatggatgattatgctaactcgggtaagaggagaaaataaatggacattgtaaaggagtggttggggtagg

fava_c17178_1928	fava_c17178	1928	T/C	atgatgagagggaaagacaagctcgcaaagagaagaagcttgccaagaacacgactccaacaaagcggKctttggcaagacagactgagagtccRtcttc[T/C]gcaaagaagcggaagcaatcaactatggatgattatgctaactcgggtaagaggagaaaataaatggacattgtaaaggagtggttggggtagggtttga

fava_c17178_503	fava_c17178	503	G/T	tagatggcaatactggtggggatgatcgtgatgcctccattgaggcttacaacaaaccgggaagtgagaaatttgtcttcttgttatcaactcgagctgg[G/T]ggtcttggtattaatcttgctactgctgatgtcgtcattctgtatgatagtgactggaacccacaagttgatttgcaggctcaggatcgtgctcatagga

fava_c17178_860	fava_c17178	860	A/G	tgcttcaaatggttagatttggagctgaaatggttttcagttccaaggatagtacaattacagatgaggatattgatagaattattgccaaaggagaaga[A/G]gcaactgcggagcttgatgccaagatgaagaaatttactgaagatgcgatcaaatttaagatggatgacactgctgagttatatgactttgatgatgaca

fava_c17179_1138	fava_c17179	1138	C/G	gatgttacagaagataagtcaagcatggaaaaagatccacagaaaagggcaagagcttagatacacaagtaatggtgtaaaggatacttattctcaatgg[C/G]tcaaggacagggttaaagtgatcaagttaccctgggttggaacgtctaaaatagttgaagaaacaccagttcaatctgttaacattaatgatgaagtaga

fava_c17179_1522	fava_c17179	1522	A/G	ttctaatgaacaattgaaaaatttgttaaaggtttcaaagatagctgaaatgaatatgcaaagagagcttgaagttttgaaagaacaactcaagaaggtg[A/G]cagctgagtacgagagtcaagtcaaatatgagaggcagcaaaatgaagaaatgcaaggaatacttacagagtaccaagatgctctaagaaaagcagtgga

fava_c17179_1828	fava_c17179	1828	G/C	ccaagcttttggtgaatggtaaattcactctgcagacatctatcatctggtgcattggatggaggagcggaagtgtgaaaatggcaaagtactatcgttt[G/C]atgtagcagagatatcttatgcacaattacttccaacatgcaatgtcaagcccgatttttctcgttacatttctgaatattggaacctacactcacacac

fava_c17179_342	fava_c17179	342	G/A	tggcccactactacgaccctcctcttagatgcttcacttttagagattttcaattagctccaacgttggaagagttcgcactaatcatgggttattcctt[G/A]gacaaaggaaatccattccgaggaattggacaattccctacaccacaaagaattgataaactactgaatgttgacataaccagaatggtgaaaaaaggaa

fava_c17179_444	fava_c17179	444	G/T	acaaaggaaatccattccgaggaattggacaattccctacaccacaaagaattgataaactactgaatgttgacataaccagaatggtgaaaaaaggaac[G/T]attgatggattttcattttcaagaaaatatctagaacaaaaggctcaagaactggccggtgaaaaccaatgggatgatttcatggatttgttggctctcc

fava_c17180_1017	fava_c17180	1017	A/T	gatgattgatgatgatgggaaaatataataaaataaaaaagKgtggaattggaatcatttgggttgagtgaaagggtagtaaataagaaataatgatggt[A/T]gtagtgtgtgattaggggaaggtaatgcttggtgagaaatggtgaagtacactactcattgaaaatgacttagtggttggtactatgtcactgStcacta

fava_c17180_1111	fava_c17180	1111	G/C	gatggtWgtagtgtgtgattaggggaaggtaatgcttggtgagaaatggtgaagtacactactcattgaaaatgacttagtggttggtactatgtcactg[G/C]tcactacttctWctttgtccattaccatgagggggaatattttggtgactcaaatctatatatttctctatgttgtttttttattatcatattttgctta

fava_c17180_1123	fava_c17180	1123	A/T	gtgtgattaggggaaggtaatgcttggtgagaaatggtgaagtacactactcattgaaaatgacttagtggttggtactatgtcactgStcactacttct[A/T]ctttgtccattaccatgagggggaatattttggtgactcaaatctatatatttctctatgttgtttttttattatcatattttgcttactgtaaaattct

fava_c17180_381	fava_c17180	381	T/A	agtttcaattcatatattatcatgtaataatcttactactattaattaattagttaatttttgagataacatcttaattagggtttttttttttcgagtt[T/A]tttttgttgttgttattgttgttgtaagtgtagtagcttttgattgatggcttgttcgggtggaaattcttctgcttcaacgaaattcgggagttctgga

fava_c17180_616	fava_c17180	616	C/T	gaaaaaacaagaggaaacaatcgaatcgggaatcagcgaagagatctcggatgaggaagcagaagcatgttgatgatatgatgaatcaggtatctgatct[C/T]acaaaagagaataatgagattgtgcataagataaatatcacaacacagcactatttgaatgttgaggctgataattcaattttgagggcccaaataggtg

fava_c17180_958	fava_c17180	958	G/T	ataataaccagcccattaccacttctgcagatgtttttaagtggtgatgagtgatgattgatgattgatgatgatgggaaaatataataaaataaaaaag[G/T]gtggaattggaatcatttgggttgagtgaaagggtagtaaataagaaataatgatggtWgtagtgtgtgattaggggaaggtaatgcttggtgagaaatg

fava_c17184_123	fava_c17184	123	G/C	ctgccgcctctctctccaactccaaacatctcgttcaccgtagctgccgYctctcttgtttttcttcatctacttcacctctgtgtccaaatcgaaggtt[G/C]tggcaatgtcgaaagatgattcagagagttacaaacctcatcacgagtttaagcaagatgtcaaggaaccacttgttgatcttaacttgactgaatccaa

fava_c17184_293	fava_c17184	293	G/A	cacttgttgatcttaacttgactgaatccaatgagctttggctgcttagattgccgttttctaatgatttgttagctgacattgacgggcaggaattatc[G/A]ctcaaacttgataaggacggcaccttggcaagctttgagggtgcatctgggaaagcgtatgattttgtcagctttgcttccatggagcctgacgaaacag

fava_c17184_679	fava_c17184	679	G/A	agactgaagagctcattatctgaattttctgaacgatcaaatactccaaaaagaagaccgcatgggaacaaatctaatgcaaaacctcctgaattatccc[G/A]tggtcacaactccgtcgcttctgcaatctcttctgatcattctcatggaggaaagtcaaacgcgtctgcgatctcttctgatcattctcatggaggaaag

fava_c17184_72	fava_c17184	72	C/T	atcctctcactcaccgccatagctgccgcctctctctccaactccaaacatctcgttcaccgtagctgccg[C/T]ctctcttgtttttcttcatctacttcacctctgtgtccaaatcgaaggttStggcaatgtcgaaagatgattcagagagttacaaacctcatcacgagtt

fava_c17184_915	fava_c17184	915	C/T	tggaggaaagtcaaatgcttccacaatctcttctgatcattctcatggaggaaagtcaaagagaagaaaacatacggagtaatgtttccttcacttgata[C/T]atactttctcaggaccgcattaatatctgaaattaatacacttgcaagtacttgagctatgttaccttttctgtttcttttcttctttttcattttttca

fava_c17186_1193	fava_c17186	1193	C/T	acggtgtaggtggtggtgatggttcctgagatgatgatgacccttcagagcttccatggttataactcactggtgaccagagagttgaatttggtgaaac[C/T]gttaagtcccatggcttcatgaaaccgagtgactcgttatcttcttgaagaatgaagtgagacatgtggtgagttaactcagtgatgaaatcgtcttctt

fava_c17186_322	fava_c17186	322	A/G	ttaagagagaagcgaattacaaaacaagacacatatttgtcatactgagtgcgacttaaaaaagattccctctttatattcatgaatcgaatctcttgca[A/G]ggagtttcttttatttgtttatttcactagtctagtagtaaaaagttttgccaaaacaaaattattttcatcaccattattatttattataatgataatg

fava_c17186_473	fava_c17186	473	G/A	ccaaaacaaaattattttcatcaccattattatttattataatgataatgatgattatgatttattaccaatatcacgtgagtgtgtgcgtgtaaacaga[G/A]gacttagtgttaaaacttgcaaatgcatgtgactctatagtattggattaacttcaatatgtccactcttgtggcagaatcatatcatcttggacatctt

fava_c17186_815	fava_c17186	815	A/G	cttgcacaactcttgctggtataaggacagtggagcaacctttccctttctttttgtttgattccgatggagcaacggttccggaacgaggcaagaaaac[A/G]ccggtcccgtttctcgaaccgggtccgccaataaaaaccgctcgcataccggtctgcaatggagtcggtctaggtggacgaggacgaacccggttcagta

fava_c17190_244	fava_c17190	244	C/G	ctaatatgataagatcaatgacctatttgtctatttcctactgcaaaacaatggatctggatctcaaaacaatcaccataagatatcaggcaaaaatcca[C/G]tgttactatttgttgtcatattccaaacatcagaaaaatcYttaaatttctctcccatagaatacaacgaaggagaatcacttgttatttcaccaattga

fava_c17190_285	fava_c17190	285	C/T	tgcaaaacaatggatctggatctcaaaacaatcaccataagatatcaggcaaaaatccaStgttactatttgttgtcatattccaaacatcagaaaaatc[C/T]ttaaatttctctcccatagaatacaacgaaggagaatcacttgttatttcaccaattgatgccaaatccttcaactcagactcatccaaaccttttgctt

fava_c17190_636	fava_c17190	636	G/A	acatacttctcagctcctcgtaattcactttcagaaatattatacttgggtttgattctgctatttgacatatttttgggtgaagagctttgcagcctcc[G/A]caaccaggtgagtagaaatccagcacaaccaacgcatcaccggcgctgaccaaacaatcaacaagttcttgagctgagtggatctcaatcatattactct

fava_c17195_105	fava_c17195	105	A/G	aagaaaacttactcaccccactcccactctctctctctctaagtttcaattattctctgcctcttSaactccccttcttcatctcttttgcaaatttgca[A/G]tctccacataaaccaaatcctaagtgaRaaaatgactcaacagaatgtggtagtgtcggagacgaaaaccggaatcaacggtggtaatagtagtattacc

fava_c17195_133	fava_c17195	133	A/G	ctctctctctctaagtttcaattattctctgcctcttSaactccccttcttcatctcttttgcaaatttgcaRtctccacataaaccaaatcctaagtga[A/G]aaaatgactcaacagaatgtggtagtgtcggagacgaaaaccggaatcaacggtggtaatagtagtattaccgtggcgcagaagccaccggcagcacccg

fava_c17195_1351	fava_c17195	1351	C/A	gacattgtacatctctaccagctataaaatacatgaccttttttaatattttatttttgttttgtttagaataggacatagagcaaaaatgtatgaaaaa[C/A]agaaaagaaagacaaaagtgtttttctgagtttgaagctcgaatgtctataaattgatgtaattaggtagtaattagtacggggaaaagtatatgtacta

fava_c17195_472	fava_c17195	472	C/T	aatacaattcttggattcttcatcatccatctgcattagacatgtttgagcagattatggatgcggctaaggggaaacagattgtcatgtttttggacta[C/T]gatggtactttatctcctatWgtcgacgaccccgaccgggcttttatgtcggaatcgatgaggaaaactgttaggaaacttgcaaggtgttttcctactg

fava_c17195_493	fava_c17195	493	T/A	atcatccatctgcattagacatgtttgagcagattatggatgcggctaaggggaaacagattgtcatgtttttggactaYgatggtactttatctcctat[T/A]gtcgacgaccccgaccgggcttttatgtcggaatcgatgaggaaaactgttaggaaacttgcaaggtgttttcctactgcaattgtcacYggaagatgca

fava_c17195_583	fava_c17195	583	T/C	tatctcctatWgtcgacgaccccgaccgggcttttatgtcggaatcgatgaggaaaactgttaggaaacttgcaaggtgttttcctactgcaattgtcac[T/C]ggaagatgcattgacaaagtgtacaattttattcgcttggctgaactatattatgcgggaagccatggtatggatattaaaggcccagcaaaagagtcta

fava_c17195_687	fava_c17195	687	T/A	agatgcattgacaaagtgtacaattttattcgcttggctgaactatattatgcgggaagccatggtatggatattaaaggcccagcaaaagagtctaaat[T/A]caccaaaaatgacaaagcagaggaaattctttttcaacctgcaagtgagtttgttcctatggtaaatgaggtgtatgaacaactagttgagaaaacaaaa

fava_c17195_70	fava_c17195	70	C/G	actgaagaaaacttactcaccccactcccactctctctctctctaagtttcaattattctctgcctctt[C/G]aactccccttcttcatctcttttgcaaatttgcaRtctccacataaaccaaatcctaagtgaRaaaatgactcaacagaatgtggtagtgtcggagacga

fava_c17201_1062	fava_c17201	1062	G/A	cataaaattttccatccataaatacaccagaacacatcttccttggttttttcatactcgggagtagctcccatgtttgcgcctccgagttgtatagttc[G/A]gctgagtcgagtatacgtccctcggaatcacaaccacctgctaatatcgcaatctcaccgaggcttgcagagccgaacaaacatcttggagcattcattc

fava_c17201_1341	fava_c17201	1341	G/A	gaagttcagttcctacagctaaagattccttatcagaacacatgaagcattcattcgaagccatcctcggcaaaatcatccatctttggcgaatcggatc[G/A]taggcttcccattcaaggagggcacatgagaaataaacccaatgttccatgataccattcagtctcctccatgtgtagagctcgccactacggattatgt

fava_c17201_1464	fava_c17201	1464	A/T	cacatgagaaataaacccaatgttccatgataccattcagtctcctccatgtgtagagctcgccactacggattatgttccgaaagcttctattaagaga[A/T]gcaagYgaaccataatcagaccttgaacagcggctgagacaagcaatcgagctgtctcggttcatccgcggtaaaagtgaaccagaatccgacgaatctc

fava_c17201_1470	fava_c17201	1470	C/T	agaaataaacccaatgttccatgataccattcagtctcctccatgtgtagagctcgccactacggattatgttccgaaagcttctattaagagaWgcaag[C/T]gaaccataatcagaccttgaacagcggctgagacaagcaatcgagctgtctcggttcatccgcggtaaaagtgaaccagaatccgacgaatctccagcct

fava_c17201_1833	fava_c17201	1833	C/T	tatgacaagattccaacatcttagtacacttcttaggatgaagctcatcctcatcctcagcatcatcaatttccatcggtctcttgccattcttaatatc[C/T]aaatccagcatgtacttcatgtaagaccaatcgttctccgtttcacaactattacaaaacaacctcggaacaacacaggaacgaccctccaacatctctc

fava_c17201_2049	fava_c17201	2049	C/T	caaacctcaatccacaacagcaaaatccaaaacagtgttaatcaaaatcagaaaaaagtccaattgatgaacaaattctgaaattgaacaagaatcttct[C/T]tttctcttcaactaatttttttctgtctcattttgtatataataaaaataaaatctattgcatgttagaaataatcaatattcaattcttctcttgagtt

fava_c17201_513	fava_c17201	513	A/G	tagtaccaattagcaagaatccagtgatgatgaatcatagtttgcttcataatgtgtagtttttgcagcattcaatggttcaacaacccatcacagcaca[A/G]ttataaacaaagttaccggatcgttgttttcgatcRagtagacgccattgtggaggtccttcactaggcacccaagaattgagctcaatgaaWccctcac

fava_c17201_549	fava_c17201	549	G/A	atagtttgcttcataatgtgtagtttttgcagcattcaatggttcaacaacccatcacagcacaRttataaacaaagttaccggatcgttgttttcgatc[G/A]agtagacgccattgtggaggtccttcactaggcacccaagaattgagctcaatgaaWccctcaccatgagtccttggtccaccgataacaataagccgat

fava_c17201_606	fava_c17201	606	T/A	cagcacaRttataaacaaagttaccggatcgttgttttcgatcRagtagacgccattgtggaggtccttcactaggcacccaagaattgagctcaatgaa[T/A]ccctcaccatgagtccttggtccaccgataacaataagccgatttccacatgctcgaaatgcaagaccccagccattcatcgagaccgctcgttccggaa

fava_c17201_777	fava_c17201	777	A/G	agccattcatcgagaccgctcgttccggaagtctcccaatggtattccacagttttctttccttgtcatactttttaacttccatgtcagcataatcagc[A/G]gcgtacaattcgttatttacaacggcaacaagaggYggtgcctcggttgtagcaggcatatcagaacctctactacttcgtccgggtgacatgttaggaa

fava_c17201_813	fava_c17201	813	C/T	caatggtattccacagttttctttccttgtcatactttttaacttccatgtcagcataatcagcRgcgtacaattcgttatttacaacggcaacaagagg[C/T]ggtgcctcggttgtagcaggcatatcagaacctctactacttcgtccgggtgacatgttaggaatctccgtccatgttctagtctgtagattgtactctt

fava_c17202_1067	fava_c17202	1067	G/A	gtaacttgagttcacaggcagataacgatgaagaagaaaagaaagattgtcaaaatcttgttagattttttggtttacaacattctgtatcaagattttt[G/A]tcaaactttcgtgtYactaggaagagacacgatacataaggtgaaactgtcgtattttgtagtcaatggcttctgactcttYtcgacatWtgtatcaaag

fava_c17202_1082	fava_c17202	1082	T/C	aggcagataacgatgaagaagaaaagaaagattgtcaaaatcttgttagattttttggtttacaacattctgtatcaagatttttRtcaaactttcgtgt[T/C]actaggaagagacacgatacataaggtgaaactgtcgtattttgtagtcaatggcttctgactcttYtcgacatWtgtatcaaagtgtttttgtatcttt

fava_c17202_1149	fava_c17202	1149	C/T	ttctgtatcaagatttttRtcaaactttcgtgtYactaggaagagacacgatacataaggtgaaactgtcgtattttgtagtcaatggcttctgactctt[C/T]tcgacatWtgtatcaaagtgtttttgtatcttttctatagataagcctgaatatttcagaaaacaccactacaaaatatagatacatgttagcataaatt

fava_c17202_1157	fava_c17202	1157	T/A	caagatttttRtcaaactttcgtgtYactaggaagagacacgatacataaggtgaaactgtcgtattttgtagtcaatggcttctgactcttYtcgacat[T/A]tgtatcaaagtgtttttgtatcttttctatagataagcctgaatatttcagaaaacaccactacaaaatatagatacatgttagcataaatttacttgtt

fava_c17202_176	fava_c17202	176	A/G	tggcttcttcttcttcgcccgaggagcaatgtggagctgaggtttctgataattatgagacgacatgctggggttgtggactgcgcgttatgcttccatc[A/G]tgtgcatccattttcaagtgtggttggtgtggggccattacggatcataacaaaaagaaacgtgacgaaaagtgccttgggtggagagtactgcgtgatc

fava_c17202_494	fava_c17202	494	G/A	ttacaacaattttgtccatagctacaatttcctttttcagccttgcagcatttagatgtgctggaacgccaccaaatatattatggggaagctaccccac[G/A]gtagggaaagatgaccttgaaaattatacattctgtcactactgcttgaagccaaagtcacctagagcacatcattgtcgttcatgtggaaaatgtatac

fava_c17202_953	fava_c17202	953	G/T	ctagaggacttcttcttgtatacctgtttatcgctagtgtttcgttgcagataggattgagtgttcttttatggcagcagctctcttttatctatcaggg[G/T]aaaacttttctgagtaacttgagttcacaggcagataacgatgaagaagaaaagaaagattgtcaaaatcttgttagattttttggtttacaacattctg

fava_c17203_1432	fava_c17203	1432	G/A	tttatgatccaattggtggagagattctttttgatggagttgctattcataagttgcaactcaaatggcttagatctcaaatgggattggtgagtcaaga[G/A]cctgctttgtttgcaacaagtattaaagagaatatactYtttggaagagaggatgctacatatgaagatgttgttgatgctgctaaagcttctaatgctc

fava_c17203_1471	fava_c17203	1471	T/C	ttgctattcataagttgcaactcaaatggcttagatctcaaatgggattggtgagtcaagaRcctgctttgtttgcaacaagtattaaagagaatatact[T/C]tttggaagagaggatgctacatatgaagatgttgttgatgctgctaaagcttctaatgctcataatttcatttcattgttgccacaaggatatgatacWc

fava_c17203_1570	fava_c17203	1570	A/T	tYtttggaagagaggatgctacatatgaagatgttgttgatgctgctaaagcttctaatgctcataatttcatttcattgttgccacaaggatatgatac[A/T]caggttggagagagaggagttcaRatgtcaggaggacaaaagcaaaggattgccattgcaagagctataataaaaatgccaaaaattctattgcttgacg

fava_c17203_1594	fava_c17203	1594	A/G	atgaagatgttgttgatgctgctaaagcttctaatgctcataatttcatttcattgttgccacaaggatatgatacWcaggttggagagagaggagttca[A/G]atgtcaggaggacaaaagcaaaggattgccattgcaagagctataataaaaatgccaaaaattctattgcttgacgaagcaacaagtgcactcgattctg

fava_c17203_2872	fava_c17203	2872	A/G	tttcatctcaagatagaatcctcaaaatacttgaaaaagcccaacaagggccaagtcaagaaagcattagacagtcttggtttgcaggtattgggcttgc[A/G]tgttcacaaagcctcaatttttgtacttgggctttggacttttggtatggtggaaaacttgtgtcacaaggttatatttcgggtaaggcgttgttcgaga

fava_c17203_4004	fava_c17203	4004	T/C	acatgaaatcaattaaccaaacatgataaagtttacatgtttgatgggagaagctacaaagaataattaatgatagagtcgatgtgatcatagatgaaat[T/C]gaaaagaaaattgaagtacaYcggcacatcatagaYacacaatcagtttctcttgaagtacctataaactataaattagcttataactagtgatggagag

fava_c17203_4025	fava_c17203	4025	C/T	catgataaagtttacatgtttgatgggagaagctacaaagaataattaatgatagagtcgatgtgatcatagatgaaatYgaaaagaaaattgaagtaca[C/T]cggcacatcatagaYacacaatcagtttctcttgaagtacctataaactataaattagcttataactagtgatggagagagccaacatgatgtcaRagca

fava_c17203_4040	fava_c17203	4040	C/T	atgtttgatgggagaagctacaaagaataattaatgatagagtcgatgtgatcatagatgaaatYgaaaagaaaattgaagtacaYcggcacatcataga[C/T]acacaatcagtttctcttgaagtacctataaactataaattagcttataactagtgatggagagagccaacatgatgtcaRagcagattggctctctccg

fava_c17203_4121	fava_c17203	4121	G/A	tacaYcggcacatcatagaYacacaatcagtttctcttgaagtacctataaactataaattagcttataactagtgatggagagagccaacatgatgtca[G/A]agcagattggctctctccgtttaggtttgtccYgtttttaaYccatcagaatcagggtgaggtggacaactcaaatgatcgagttccaacactcacccca

fava_c17203_4154	fava_c17203	4154	C/T	ctcttgaagtacctataaactataaattagcttataactagtgatggagagagccaacatgatgtcaRagcagattggctctctccgtttaggtttgtcc[C/T]gtttttaaYccatcagaatcagggtgaggtggacaactcaaatgatcgagttccaacactcaccccactcttagatgggttagcttgtcaaacaaacttt

fava_c17203_4163	fava_c17203	4163	C/T	tacctataaactataaattagcttataactagtgatggagagagccaacatgatgtcaRagcagattggctctctccgtttaggtttgtccYgtttttaa[C/T]ccatcagaatcagggtgaggtggacaactcaaatgatcgagttccaacactcaccccactcttagatgggttagcttgtcaaacaaactttatttttatt

fava_c17204_1009	fava_c17204	1009	T/C	ttttacacgaggcactactgtaccatcttactcgtaccgatccaaggcataacttctcaatctatttctatcacatatatcttcattatgggcacgatat[T/C]tcggtggtggaaaagcttgtctcctttttaccacagtttttggttcagcttgttcttattttcagctttgcaaaggatttgccattttgcctatttgtgc

fava_c17204_1221	fava_c17204	1221	G/A	tttgttgccttcaataaggtgattactgcacagtactttgtttggttctttagcttgttgcctctaatactcccatggagtaaaatgaagctaaaatggg[G/A]tggattgtcttgcattcttctgtggattggagctcagactcattggttaatgtggggctaccttctcgagttcaagggtaaaaatgtcttcttacagctt

fava_c17204_138	fava_c17204	138	G/C	aaannnnnnnnaagatgctgaatttttctcacacttgtcctactctttcacatgacaataaacatacgagaagtgtaacaaaagaatccctaatcagcag[G/C]ttgaatctaaScRatggcatggttagatatccgttcccttctgagattctcggcaattttccgcgtaattctaatcctatacggagaatggcaagattct

fava_c17204_1437	fava_c17204	1437	C/T	atgtttcttgctgccaacatattcatccttgtcgtgattattcgtcaacataaatattcttctgttttcaaagcttttgagtatactcactccaaacata[C/T]aactaagctcgagtgagtgactgtgactatcgccaacaagtttctattttcaccctcaccattgttacacattgcccttgtcgcgcWtcagttttggaag

fava_c17204_149	fava_c17204	149	G/C	aagatgctgaatttttctcacacttgtcctactctttcacatgacaataaacatacgagaagtgtaacaaaagaatccctaatcagcagSttgaatctaa[G/C]cRatggcatggttagatatccgttcccttctgagattctcggcaattttccgcgtaattctaatcctatacggagaatggcaagattctcacatggaagt

fava_c17204_151	fava_c17204	151	A/G	gatgctgaatttttctcacacttgtcctactctttcacatgacaataaacatacgagaagtgtaacaaaagaatccctaatcagcagSttgaatctaaSc[A/G]atggcatggttagatatccgttcccttctgagattctcggcaattttccgcgtaattctaatcctatacggagaatggcaagattctcacatggaagttc

fava_c17204_1524	fava_c17204	1524	T/A	cactccaaacataYaactaagctcgagtgagtgactgtgactatcgccaacaagtttctattttcaccctcaccattgttacacattgcccttgtcgcgc[T/A]tcagttttggaaggttaattttgcaaggtcactactacagcaaccaatgattatgttgaaacttgagttgttttcagaatgttaacaataggtgaatcaR

fava_c17204_1624	fava_c17204	1624	A/G	Wtcagttttggaaggttaattttgcaaggtcactactacagcaaccaatgattatgttgaaacttgagttgttttcagaatgttaacaataggtgaatca[A/G]tatattcKtaatagttttgtaacaccagataccKctttttttttgtagtacagaatctttaagcaatgagtttatttttattgctttcttatctttctgt

fava_c17204_1632	fava_c17204	1632	G/T	tggaaggttaattttgcaaggtcactactacagcaaccaatgattatgttgaaacttgagttgttttcagaatgttaacaataggtgaatcaRtatattc[G/T]taatagttttgtaacaccagataccKctttttttttgtagtacagaatctttaagcaatgagtttatttttattgctttcttatctttctgttatttatc

fava_c17204_1658	fava_c17204	1658	T/G	actacagcaaccaatgattatgttgaaacttgagttgttttcagaatgttaacaataggtgaatcaRtatattcKtaatagttttgtaacaccagatacc[T/G]ctttttttttgtagtacagaatctttaagcaatgagtttatttttattgctttcttatctttctgttatttatctgcacatgtagaatttgaactcaagg

fava_c17204_340	fava_c17204	340	T/C	acatggaagttcgatacaccgacgtcgattacatcgtgttttccgacgccgcttcactagtcgcttctggttcttctccttacaacagaaccacctaccg[T/C]tattcacctctactcgcttttcttcttgttcctaattcactacttcatcgctcYtggggaaagtttctcttctcttctgcagatatacttgtgggatatt

fava_c17204_394	fava_c17204	394	C/T	cactagtcgcttctggttcttctccttacaacagaaccacctaccgYtattcacctctactcgcttttcttcttgttcctaattcactacttcatcgctc[C/T]tggggaaagtttctcttctcttctgcagatatacttgtgggatattttatctattacattctaaagctgcagaaggttcctgaaaacctctgcaactact

fava_c17206_1018	fava_c17206	1018	T/A	caatgttggatgacattttaaatgaatgatgRttcacagggttaacaagaatagatggtgttctaaattttgtgtagtgaaatttgtttatgaagagatt[T/A]gttgaagcatgattttctttctctgttgccatgtatgcatagtccacatcatttgaaatgaaccattcttctaatgttgttactaggcttgaaccaattc

fava_c17206_949	fava_c17206	949	A/G	aaaactctgtggaccccatgaatcttctatagagtgactcagcttgttcaattttcaaccttgatatttcaatgttggatgacattttaaatgaatgatg[A/G]ttcacagggttaacaagaatagatggtgttctaaattttgtgtagtgaaatttgtttatgaagagattWgttgaagcatgattttctttctctgttgcca

fava_c17207_1043	fava_c17207	1043	T/G	tctagtcggagttatctggaaagggacMttatatattgctaatcttggagactctcgtgccgtgatcggttcaacgggtggacattctaagaagattgtt[T/G]ctgaacagttgacgaaagagcataatgcctccagagaggaaatcagaagagaactaaaggctttgcatcccgatgattcgcagattgttattatgaagca

fava_c17207_970	fava_c17207	970	C/A	aggacggatttctaactcttgttcgcaggagttttggaataaagccactgatagcagcaatgggttcttgttgtctagtcggagttatctggaaagggac[C/A]ttatatattgctaatcttggagactctcgtgccgtgatcggttcaacgggtggacattctaagaagattgttKctgaacagttgacgaaagagcataatg

fava_c17210_1214	fava_c17210	1214	G/A	aagctatactttcagtaatcttctgcttgctataagttagcaaggccgggaaacggtaaatcaccttcgcccattgtttcttgtcaacacccaaagattc[G/A]aagaattccattgtcggcttaagattttcagatagactgattccacataattgaggccttttcgagatgataataagaattttttcttgtggcactccaa

fava_c17210_1451	fava_c17210	1451	A/G	catccaagctatagtttgcgaaagctgggaacttccgtataatagtcctaatctggtcaatttccattccaagttgcatgagataaacaaaatgaggacg[A/G]agattccccgcagcaggatctaccccactcacttctgacacggctttaagtttcttggactcaaccactagactaggattaggagggtgaatcggttcgg

fava_c17210_256	fava_c17210	256	C/T	aagtacacaataaatactccaattactatatggactcaacacaatcacatatacaatcacaacttgcaagtagagctgactagtgttatttgtgtcaaaa[C/T]cctgcaaaaaataattggttgcaggtgctaggttgttcaaattcacttcaaagtcacttcaaaacaacttaaaagttgtaatttgtaatgccatgttcca

fava_c17210_482	fava_c17210	482	G/A	catttcctgaagctagaacgttctagcagtactacaacttttcacttcatatatgaattaaaagttgtaatttgtaatgccgttgatccaaaaaaacaaa[G/A]cagtcaaaaccatcttctgacctgaaacgttgcctgaaagtctcaattttagttatctgattgaacaaccttcattttctttattttacagtttcttcat

fava_c17210_694	fava_c17210	694	G/C	tgcagtttcttcaatttctttttaagaacctcttcaaacttggtggttgatagtgaaagaacctgattcagcaataatttcaccccataacttttcacgc[G/C]Wgcatatctcgcatatctcggtttaatccttcgttccaaattgtatccaaaaaattgagggaacttaaccaactcagatttagggtaacccatagtcaaa

fava_c17210_695	fava_c17210	695	T/A	gcagtttcttcaatttctttttaagaacctcttcaaacttggtggttgatagtgaaagaacctgattcagcaataatttcaccccataacttttcacgcS[T/A]gcatatctcgcatatctcggtttaatccttcgttccaaattgtatccaaaaaattgagggaacttaaccaactcagatttagggtaacccatagtcaaaa

fava_c17210_917	fava_c17210	917	A/G	aattcccagtcaagctgaaagtgtacaatggtccgtatctcgaaatcatagtgccaatttcttccgaagtgtatcccctctccaagaaaaactcggttac[A/G]ggctttatattagcctcaatgctgagaccaaaattttgtggacacttgaatagaagaaggccaacatcaacccccaaggaacggaagtacattgctgtcg

fava_c17211_341	fava_c17211	341	T/C	cattgagtttaacaaggactagttaagaagataaacacaagattatcgccctagttaaaaagatgcaattataagtgcttacaacatagacaataattat[T/C]cgtccttagtcgcaccagtccaaccaagtccgggcctgcttacttctccctgcggttcctcatagtctcgtgcatcRcggattgaagcttcRaagtcagt

fava_c17211_418	fava_c17211	418	G/A	cttacaacatagacaataattatYcgtccttagtcgcaccagtccaaccaagtccgggcctgcttacttctccctgcggttcctcatagtctcgtgcatc[G/A]cggattgaagcttcRaagtcagttgaatcacctgcaacagggagaaagctaccgatatcatctctagcaccaaatcccccagcccggataacatcatcaS

fava_c17211_433	fava_c17211	433	G/A	ataattatYcgtccttagtcgcaccagtccaaccaagtccgggcctgcttacttctccctgcggttcctcatagtctcgtgcatcRcggattgaagcttc[G/A]aagtcagttgaatcacctgcaacagggagaaagctaccgatatcatctctagcaccaaatcccccagcccggataacatcatcaSgtgttatggatgcct

fava_c17211_518	fava_c17211	518	G/C	RcggattgaagcttcRaagtcagttgaatcacctgcaacagggagaaagctaccgatatcatctctagcaccaaatcccccagcccggataacatcatca[G/C]gtgttatggatgcctccatgttaacatccccagatagatctttttcctttggacaattttctcggccttYcgagttacctgcctttgtctcttcaatatc

fava_c17211_588	fava_c17211	588	C/T	ccaaatcccccagcccggataacatcatcaSgtgttatggatgcctccatgttaacatccccagatagatctttttcctttggacaattttctcggcctt[C/T]cgagttacctgcctttgtctcttcaatatcgccagaggctcttgtaaaatgttttaaaacatctttKccgtcatcaatcttagaaagattggtgtcagtR

fava_c17211_655	fava_c17211	655	T/G	gatctttttcctttggacaattttctcggccttYcgagttacctgcctttgtctcttcaatatcgccagaggctcttgtaaaatgttttaaaacatcttt[T/G]ccgtcatcaatcttagaaagattggtgtcagtRgataaatcctttactggtgKagtgctaccatttttgtcttgcatcattttatctgattggtattcaa

fava_c17211_688	fava_c17211	688	G/A	YcgagttacctgcctttgtctcttcaatatcgccagaggctcttgtaaaatgttttaaaacatctttKccgtcatcaatcttagaaagattggtgtcagt[G/A]gataaatcctttactggtgKagtgctaccatttttgtcttgcatcattttatctgattggtattcaattgcattcctgtttcagttatgaaatccttgac

fava_c17211_708	fava_c17211	708	T/G	tcttcaatatcgccagaggctcttgtaaaatgttttaaaacatctttKccgtcatcaatcttagaaagattggtgtcagtRgataaatcctttactggtg[T/G]agtgctaccatttttgtcttgcatcattttatctgattggtattcaattgcattcctgtttcagttatgaaatccttgaccttaaataaatgattgaaag

fava_c17211_840	fava_c17211	840	G/C	tctgattggtattcaattgcattcctgtttcagttatgaaatccttgaccttaaataaatgattgaaagtgaagggaatatgagagaagaagaagcgtga[G/C]cgaaggaaaaatcagattctggaattgctgtgagtgtgtatgtgtcaagttcggcgacagaacaaaacacgacacgtgtgtcctacactgcgttttggca

fava_c17213_516	fava_c17213	516	A/G	atttctagatctccaaatcacccacttctgtaaatatcacaccaaagctaagctatcattgaatcttgaatcttaaatctaaccatagtgcaaaaagggg[A/G]aaaaaacccgcaaaacatccaaagaggtcacaaagtaactggcggaaccaccagaaaaatRcaataccaccacactcaaagaaaatcacaggtaacaaaa

fava_c17213_577	fava_c17213	577	A/G	aatcttgaatcttaaatctaaccatagtgcaaaaaggggRaaaaaacccgcaaaacatccaaagaggtcacaaagtaactggcggaaccaccagaaaaat[A/G]caataccaccacactcaaagaaaatcacaggtaacaaaaagatgtaccgcttcgcttactcgagYgaggcctcaaaccaMaaaggtggaagcctcgatat

fava_c17213_642	fava_c17213	642	C/T	ggtcacaaagtaactggcggaaccaccagaaaaatRcaataccaccacactcaaagaaaatcacaggtaacaaaaagatgtaccgcttcgcttactcgag[C/T]gaggcctcaaaccaMaaaggtggaagcctcgatatccYgaataatccWtcgatggagacatgtttgaaacagcaatttgacatYatcgactgtgtggctg

fava_c17213_657	fava_c17213	657	C/A	ggcggaaccaccagaaaaatRcaataccaccacactcaaagaaaatcacaggtaacaaaaagatgtaccgcttcgcttactcgagYgaggcctcaaacca[C/A]aaaggtggaagcctcgatatccYgaataatccWtcgatggagacatgtttgaaacagcaatttgacatYatcgactgtgtggctgtgatttgatccaatc

fava_c17213_680	fava_c17213	680	C/T	ataccaccacactcaaagaaaatcacaggtaacaaaaagatgtaccgcttcgcttactcgagYgaggcctcaaaccaMaaaggtggaagcctcgatatcc[C/T]gaataatccWtcgatggagacatgtttgaaacagcaatttgacatYatcgactgtgtggctgtgatttgatccaatccaaccaaacatKgattgcatttc

fava_c17213_690	fava_c17213	690	T/A	actcaaagaaaatcacaggtaacaaaaagatgtaccgcttcgcttactcgagYgaggcctcaaaccaMaaaggtggaagcctcgatatccYgaataatcc[T/A]tcgatggagacatgtttgaaacagcaatttgacatYatcgactgtgtggctgtgatttgatccaatccaaccaaacatKgattgcatttcattctgataa

fava_c17213_726	fava_c17213	726	C/T	gcttcgcttactcgagYgaggcctcaaaccaMaaaggtggaagcctcgatatccYgaataatccWtcgatggagacatgtttgaaacagcaatttgacat[C/T]atcgactgtgtggctgtgatttgatccaatccaaccaaacatKgattgcatttcattctgataagtcaacacttttgtcatatgcaaagaataaccattt

fava_c17213_769	fava_c17213	769	T/G	cctcgatatccYgaataatccWtcgatggagacatgtttgaaacagcaatttgacatYatcgactgtgtggctgtgatttgatccaatccaaccaaacat[T/G]gattgcatttcattctgataagtcaacacttttgtcatatgcaaagaataaccattttttcaatattatcatcatcaccctccttgttacactgcagctt

fava_c17213_885	fava_c17213	885	A/G	tgataagtcaacacttttgtcatatgcaaagaataaccattttttcaatattatcatcatcaccctccttgttacactgcagcttctgcgccccattcat[A/G]tcaaattggagggccRgccttagaacttcagacaacacaacttacgaactcgatcaaaccttcccaKgctttattaataggagctacctatacatatata

fava_c17213_901	fava_c17213	901	G/A	ttgtcatatgcaaagaataaccattttttcaatattatcatcatcaccctccttgttacactgcagcttctgcgccccattcatRtcaaattggagggcc[G/A]gccttagaacttcagacaacacaacttacgaactcgatcaaaccttcccaKgctttattaataggagctacctatacatatatatacacatatatactta

fava_c17213_952	fava_c17213	952	T/G	cttgttacactgcagcttctgcgccccattcatRtcaaattggagggccRgccttagaacttcagacaacacaacttacgaactcgatcaaaccttccca[T/G]gctttattaataggagctacctatacatatatatacacatatatacttatagatataactaagtattagttgattaactagaagatgatgagtttgcaga

fava_c17214_1423	fava_c17214	1423	G/A	tgaacggtaaaatgtatcaactagtttaccctggaaaatcagggattctaggtgattcactctgcatggaaaattcactggatccgaaacaagtaacagg[G/A]agaattgtagtgtgtgatagagggagtagccctagagtagcaaaagggcttgttgtgaagaaagccggtggcgttggaatgatcctcgcaaacggaatct

fava_c17214_1623	fava_c17214	1623	C/G	tcaaacggcgaaggactcgttggagatgctcatcttcttcctgcttgtgctgttggttccaacgaaggagatataatcaaagcctatatcacttcatcgt[C/G]gaacccaactgctacgattgatttcaaagggactatcctcggaatcaaaccggctccggttctcgcttctttctcagcaagaggacccaacggtttgaac

fava_c17214_1963	fava_c17214	1963	C/G	cacctcatgtgagtggcgctgccgcgttgctgaaatctgctcatccagattggagtcccgccgccgtgagatccgccatgatgaccaccgcttctgtttt[C/G]gataaccggaacctccctatgttggatgaggctacagggaataattccacaccttatgatttcggctcgggtcatctcaatttgggtaatgctatggatc

fava_c17214_2380	fava_c17214	2380	T/G	atgtgggagcggtgaactcagtttaccacgtgagtgtggagtcacagattaagggggttacggtgacggtgaagccgtcacggctcgtgttttcagaggc[T/G]gtgaagaagaggagttatgtggtgacggtaacggcggatacacgaaatctgaaactggatccttctggagcggttttcgggtcgcttacgtggacggatg

fava_c17214_448	fava_c17214	448	A/G	tcccaacccattatcactggtacaccgctgaattcactgaacaaacccatatccttcatgtttacgacactgttttccatggtttctctgctgtgctcac[A/G]agacagcaagctgcttccgtagggaaccacccttcgatccttgcggtttttgaggatcgtcgtagacagttacatacaactcgttcaccccagtttctgg

fava_c17214_748	fava_c17214	748	T/C	tctctgatgtgaacttgggtcctattccacggcggtggaaaggggtttgtgaatctggtgaaaaattctcacccagaaactgcaacaaaaaactcatcgg[T/C]gctagattcttctccaaaggtcatgaagcaggttctggtttttccggtcctttgaaccccatcaatgaaaccgttgagtttcgatcccctcgtgacgccg

fava_c17221_1060	fava_c17221	1060	G/A	acctctctaacaagcttgggtgaacgtaatacttgtctaaagagtcgtattcatcttcattatcttcccctgaagttctagaaatgtcattggaaacacc[G/A]gtcgctgaagcctcaggttcaacatttgtRtcattgctaagattctttaatacaagaaacttctcaataggttgRcatgactgSaacaaattgtgtctat

fava_c17221_1090	fava_c17221	1090	G/A	acttgtctaaagagtcgtattcatcttcattatcttcccctgaagttctagaaatgtcattggaaacaccRgtcgctgaagcctcaggttcaacatttgt[G/A]tcattgctaagattctttaatacaagaaacttctcaataggttgRcatgactgSaacaaattgtgtctatcctcgagtgataaatcatcttccttagcat

fava_c17221_1135	fava_c17221	1135	A/G	ttctagaaatgtcattggaaacaccRgtcgctgaagcctcaggttcaacatttgtRtcattgctaagattctttaatacaagaaacttctcaataggttg[A/G]catgactgSaacaaattgtgtctatcctcgagtgataaatcatcttccttagcatatgcRttgaaaggctcaaataatggcttaggagactgaatcagct

fava_c17221_1144	fava_c17221	1144	C/G	tgtcattggaaacaccRgtcgctgaagcctcaggttcaacatttgtRtcattgctaagattctttaatacaagaaacttctcaataggttgRcatgactg[C/G]aacaaattgtgtctatcctcgagtgataaatcatcttccttagcatatgcRttgaaaggctcaaataatggcttaggagactgaatcagctggcggctca

fava_c17221_1195	fava_c17221	1195	G/A	tgctaagattctttaatacaagaaacttctcaataggttgRcatgactgSaacaaattgtgtctatcctcgagtgataaatcatcttccttagcatatgc[G/A]ttgaaaggctcaaataatggcttaggagactgaatcagctggcggctcaaaaattcatttccaaaagatgacggttttctcttagccagacaaaagtaac

fava_c17221_1353	fava_c17221	1353	T/C	tttccaaaagatgacggttttctcttagccagacaaaagtaacgaggcctagaatatggaattccaaactgtaaagggctgagaatgaactcttgtgtaa[T/C]aaaatttgttttctccagtatttcaatcagttttgcatgtgtatcagatgtctcaaacccgacaacattttccacaaacaacataYttggaggccgcaat

fava_c17221_1439	fava_c17221	1439	C/T	gaactcttgtgtaaYaaaatttgttttctccagtatttcaatcagttttgcatgtgtatcagatgtctcaaacccgacaacattttccacaaacaacata[C/T]ttggaggccgcaataggaatgggatgagctcaagaatctgaagaaaagaaaacgcccgtgcatccccagtgtccttctggagaccttgtcgtgtgtaagg

fava_c17221_252	fava_c17221	252	T/C	ataaattaattatcagcatgcaagtgtactcattaatacattattcttattacaaacatgtataccagatcctatcctttgatctgcaagatacttggca[T/C]gcacattttaactctacaaacttcaaattacattttWtgcactcccttctcagtcagttYtaaaaataatgatcacataattttacactagaattatgaa

fava_c17221_289	fava_c17221	289	T/A	acattattcttattacaaacatgtataccagatcctatcctttgatctgcaagatacttggcaYgcacattttaactctacaaacttcaaattacatttt[T/A]tgcactcccttctcagtcagttYtaaaaataatgatcacataattttacactagaattatgaattctgcagtgaaaatattcaaactcgagtttgattac

fava_c17221_312	fava_c17221	312	T/C	tataccagatcctatcctttgatctgcaagatacttggcaYgcacattttaactctacaaacttcaaattacattttWtgcactcccttctcagtcagtt[T/C]taaaaataatgatcacataattttacactagaattatgaattctgcagtgaaaatattcaaactcgagtttgattaccattgttgaatctacRattcacg

fava_c17221_405	fava_c17221	405	G/A	gtcagttYtaaaaataatgatcacataattttacactagaattatgaattctgcagtgaaaatattcaaactcgagtttgattaccattgttgaatctac[G/A]attcacggcattgtgttacaacttacaaggtctcactattgttgcatgataggcacaggtagaaacaatctgcttctgttgaatgggatagtgtgattga

fava_c17221_557	fava_c17221	557	C/A	ggcacaggtagaaacaatctgcttctgttgaatgggatagtgtgattgaaaaatcactaagcttcagtaaaaagataatgaagtaagggagcaacaactg[C/A]tatgcttaaactgttccccagcaatgcataccgttgtttgagactgatgtgttctggaaacgtgaaatcttcaggaaaagaatgtaggtttgcaacctct

fava_c17223_1456	fava_c17223	1456	C/T	tctatctcttcaaaggcgtctccaaagaaatccttttgtccctcgttgcgaaaatgaatgcggaatacataccaccaaaagaggatgttataatgcaaaa[C/T]gaagcagcagatgatgtttacatcatagtgtccggagaagtagagatcatagatagtgttattgagaaagagagagttttagggactttaacaacaggag

fava_c17223_184	fava_c17223	184	G/A	ttcttcattttaggatctcttctcaaacttgttgaatctctcataacaatccttcacttagaattttgttcaagtagcaagataatggagaacaatttcg[G/A]tagcttgaatagaaaacatcatcatcatacatcatcttcatctttcaatctaaggaatgtttcaaaactcattcttccaccacttggtgtttctagagag

fava_c17223_1840	fava_c17223	1840	A/T	tgagtatcaaagatttaattgtggagaatgtggaagaagaagatcctaacatggctgtgaatttgttaactgtggctggcacaggtaatgctgcttttct[A/T]gaggagcttctaagagctggtttggatcctgatattggagactctaaagggaaaactccattgcacatagcagcatcaaatggacatgaagagtgtgtta

fava_c17223_2128	fava_c17223	2128	A/G	actcaatcttcagaatcctctatcagctatctgcactttctgatccatacacagcaggaaatctcctatgtctagcagccaaaagaaacgatttaaccgt[A/G]atgaaMgagcttttaaagcaaggactaaacatcgactcaaaagatggacacggcatgaaacctatacaaattgccatgacagaaaatcttgttgacatgg

fava_c17223_2134	fava_c17223	2134	A/C	tcttcagaatcctctatcagctatctgcactttctgatccatacacagcaggaaatctcctatgtctagcagccaaaagaaacgatttaaccgtRatgaa[A/C]gagcttttaaagcaaggactaaacatcgactcaaaagatggacacggcatgaaacctatacaaattgccatgacagaaaatcttgttgacatggttcagt

fava_c17223_2612	fava_c17223	2612	G/A	gctgcctgattcattagaaaaactcaaaattatcgcaggtgaaaaatttgagtttgatgcaagagatgcaatggtgacaaatgaagaaggagcagaaata[G/A]atagtattgatgtaataagagataatgataaactttatattgttgaataggctattattgagtttggttgttcatgtgagaaggaacaYaggttatcatt

fava_c17223_2701	fava_c17223	2701	T/C	gagcagaaataRatagtattgatgtaataagagataatgataaactttatattgttgaataggctattattgagtttggttgttcatgtgagaaggaaca[T/C]aggttatcattttcacaattttgccatgcagtaacattaatattaatagtgaggcttctgttgaagtttcaaatgcctttatagatgtgattgcttgggc

fava_c17223_547	fava_c17223	547	T/C	attattgatcttttctttgccattgacattgttatgactttctttctggctttcattgatggaactactcatcttcttgtcagagattccaaaaaaattg[T/C]tStYaggtacttgtcaacatggttcataatggatgtggcatcaacaataccatatgaagcaataggctttattttaactggcaaacacaaattaggcctc

fava_c17223_549	fava_c17223	549	G/C	tattgatcttttctttgccattgacattgttatgactttctttctggctttcattgatggaactactcatcttcttgtcagagattccaaaaaaattgYt[G/C]tYaggtacttgtcaacatggttcataatggatgtggcatcaacaataccatatgaagcaataggctttattttaactggcaaacacaaattaggcctccc

fava_c17223_551	fava_c17223	551	C/T	ttgatcttttctttgccattgacattgttatgactttctttctggctttcattgatggaactactcatcttcttgtcagagattccaaaaaaattgYtSt[C/T]aggtacttgtcaacatggttcataatggatgtggcatcaacaataccatatgaagcaataggctttattttaactggcaaacacaaattaggcctccctt

fava_c17223_745	fava_c17223	745	C/G	ctcccttacttcctcttgggaatgctcagattttggagaatcagacgtgtcaaacaattcttcacaaggttctcaatcatcttccaatctttaatcatat[C/G]attgtgtatgttaacttcatttatttggcttgccacaggcttgagaaagacataagattcagctatttttgggtcagatgtgctaggcttctttctgtaa

fava_c17223_940	fava_c17223	940	A/G	ctgtaacacttttctcaattcattgtgctgggtgtctctattacatgctagctgatagataccctcatcaaggaaatacatggattggagctgtcattcc[A/G]aatttcagagagactagtcctagaaccagatacatttcagccatctactggtccataactaccatgacaaccgttggttatggcgatcttcatgctgtca

fava_c17227_1113	fava_c17227	1113	T/A	agggttaaggagatgtcatgaaatggtgaatcaacaacatcttcttgttcatgggaaccgaattcgaatgtgtgtgtgatattttaggaaggacttatgt[T/A]ttcaattttctactatttttttattaataatcatcaaattttgaagtattcttatgtttcagagagaacaaaaaacgaacatattaggctatgtttcctt

fava_c17230_242	fava_c17230	242	A/C	ctggtttattgggcttgatgtgaagcatattgacgagacaaagttttgttgctcgtcttggtcgacaggagcaatttgtgcgggtgtttgagtttattcc[A/C]gtgcataattgtgagatgactaccagtgtaactgtaatacacaagagccatcagttgttagacgcttYtccacagatcttttaactctaatgaaaactga

fava_c17230_310	fava_c17230	310	T/C	gagcaatttgtgcgggtgtttgagtttattccMgtgcataattgtgagatgactaccagtgtaactgtaatacacaagagccatcagttgttagacgctt[T/C]tccacagatcttttaactctaatgaaaactgagtttttcctggaaagttgcctcaagatttgaagtgctccttacattaacttgtaagcacggttggcaa

fava_c17230_491	fava_c17230	491	T/A	cttgtaagcacggttggcaaattgatcggttactagcagtcattacttaaggcatctggcatagtttactccaaaagatttacctctacctataagttgt[T/A]ttatgtcattttatggttctttcattcgttcattgtataggagaggaatatatcgatcggcagaagaacttcctaaatgtattttaaagtaggagggaca

fava_c17230_706	fava_c17230	706	A/T	gagaattcgtattttttgtacatagcatagtaaatgaaatgagtcatgattatgttttgggcgaagactagggtgaacaagtgcctcatgtggtccacca[A/T]ggtcaaaggtttttttgtagtactattttgaagttcttttgtttttttcttatagttttcccatctcaaaatcccaaatcctaaatttacatattcttca

fava_c17235_139	fava_c17235	139	T/C	acattgtcttcttcaaccacatggtgataattgaactctatcccaaattcttcttctctcgaaacccaattcctaaacccttcaaaaaccctaatttcaa[T/C]ccaagaaacatcgttMaattttcccaccaaccaataaccactcatccaaaccgtggcataatcttccgagaaaaagttctataccttgaaaaactcaaag

fava_c17235_155	fava_c17235	155	C/A	ccacatggtgataattgaactctatcccaaattcttcttctctcgaaacccaattcctaaacccttcaaaaaccctaatttcaaYccaagaaacatcgtt[C/A]aattttcccaccaaccaataaccactcatccaaaccgtggcataatcttccgagaaaaagttctataccttgaaaaactcaaagtaaacccagaaaaagc

fava_c17235_604	fava_c17235	604	G/C	gtgttgagaatcgattgagacctgtgctttggtttttgaggggattagggtttgttggaccgcattctctcacgtgtcaaacgacattgcttttggtttc[G/C]agtgtKgaggaYacccttttgcctaaagtWgagtttttgatggggttRgggctcacacgtgtggaggtttcaaatatggttgttagatctccggggttgt

fava_c17235_610	fava_c17235	610	T/G	agaatcgattgagacctgtgctttggtttttgaggggattagggtttgttggaccgcattctctcacgtgtcaaacgacattgcttttggtttcSagtgt[T/G]gaggaYacccttttgcctaaagtWgagtttttgatggggttRgggctcacacgtgtggaggtttcaaatatggttgttagatctccggggttgttgactc

fava_c17235_616	fava_c17235	616	C/T	gattgagacctgtgctttggtttttgaggggattagggtttgttggaccgcattctctcacgtgtcaaacgacattgcttttggtttcSagtgtKgagga[C/T]acccttttgcctaaagtWgagtttttgatggggttRgggctcacacgtgtggaggtttcaaatatggttgttagatctccggggttgttgactcttagtg

fava_c17235_634	fava_c17235	634	T/A	ggtttttgaggggattagggtttgttggaccgcattctctcacgtgtcaaacgacattgcttttggtttcSagtgtKgaggaYacccttttgcctaaagt[T/A]gagtttttgatggggttRgggctcacacgtgtggaggtttcaaatatggttgttagatctccggggttgttgactcttagtgtggcgaataatttgggac

fava_c17235_652	fava_c17235	652	G/A	ggtttgttggaccgcattctctcacgtgtcaaacgacattgcttttggtttcSagtgtKgaggaYacccttttgcctaaagtWgagtttttgatggggtt[G/A]gggctcacacgtgtggaggtttcaaatatggttgttagatctccggggttgttgactcttagtgtggcgaataatttgggaccgaaagtggagtttttct

fava_c17237_1125	fava_c17237	1125	T/C	atcactttattagctacagaaaccttaccgccatgttgcttgatctcagccaaatcagcaacatatccccttttcatttcatcatttattcgagctcgct[T/C]cttcgcaatttcttccttattgctaaactggggcRaatcgatgaagcgtttggSggtgaggcgagccaaatgatgagaagggagtgaatgatggaaatta

fava_c17237_1160	fava_c17237	1160	A/G	ttgcttgatctcagccaaatcagcaacatatccccttttcatttcatcatttattcgagctcgctYcttcgcaatttcttccttattgctaaactggggc[A/G]aatcgatgaagcgtttggSggtgaggcgagccaaatgatgagaagggagtgaatgatggaaattatcgggttgaatggYgggtatggaagccatgatcga

fava_c17237_1179	fava_c17237	1179	G/C	tcagcaacatatccccttttcatttcatcatttattcgagctcgctYcttcgcaatttcttccttattgctaaactggggcRaatcgatgaagcgtttgg[G/C]ggtgaggcgagccaaatgatgagaagggagtgaatgatggaaattatcgggttgaatggYgggtatggaagccatgatcgaatctctgagatgggaacgg

fava_c17237_1239	fava_c17237	1239	C/T	tccttattgctaaactggggcRaatcgatgaagcgtttggSggtgaggcgagccaaatgatgagaagggagtgaatgatggaaattatcgggttgaatgg[C/T]gggtatggaagccatgatcgaatctctgagatgggaacggcgtcgtatcaattgcttcaaagcttgcattttgaaaagttcgattttgatgtgaatgcgt

fava_c17237_397	fava_c17237	397	T/C	cagaaaagtctcatccaatcttgtttatcacatcccaaaatttctgaaatacagaacaattttcctcctctcccacaaggtagttgctccataaatgtct[T/C]aacacacaatttaaaacagaaaagtctcatccaatcttgtttatcacatSccaaaatttctgaaatacagaacaacagtgtagtcttgcacctcattacg

fava_c17237_447	fava_c17237	447	C/G	acagaacaattttcctcctctcccacaaggtagttgctccataaatgtctYaacacacaatttaaaacagaaaagtctcatccaatcttgtttatcacat[C/G]ccaaaatttctgaaatacagaacaacagtgtagtcttgcacctcattacgcatctagaagaagtgatgtgcaagaaagaagggaagaaacttcatcttct

fava_c17237_904	fava_c17237	904	A/G	cttttgagttactaaatgttttagcaaaaggtacgctccaagaatcgatcattttctcggaatatcctcggaatgaaagacaaaccaaagatgcctttgg[A/G]acagatgatttgtcggaatcaactgcattatcagaaatacgaatYggaagcttcattgttttaccatctgggaagctgactttgatatcaggaaatttca

fava_c17237_949	fava_c17237	949	C/T	cgatcattttctcggaatatcctcggaatgaaagacaaaccaaagatgcctttggRacagatgatttgtcggaatcaactgcattatcagaaatacgaat[C/T]ggaagcttcattgttttaccatctgggaagctgactttgatatcaggaaatttcacagccatcatcgccggtataatcactttattagctacagaaacct

fava_c17241_1177	fava_c17241	1177	T/C	cattaattcaggaactcccaaagttcatgttaaaggctgttcctactgattgcagtgaatgccccatctgcttagaagagttccgagttgggaatgaggt[T/C]cgaggcttgccttgtgcacacaattttcatgttgaatgcattgacgagtggctcaggctgaatgtcaaatgtcctcggtgccgttgttcRgtgttcccaa

fava_c17241_1267	fava_c17241	1267	A/G	ggaatgaggtYcgaggcttgccttgtgcacacaattttcatgttgaatgcattgacgagtggctcaggctgaatgtcaaatgtcctcggtgccgttgttc[A/G]gtgttcccaaatctYgatcttagtgccctgtccaatctccgttcagattYtgagcgatcttctgccagtgttgtgacaacaactagatatgtgagaggcc

fava_c17241_1282	fava_c17241	1282	T/C	gcttgccttgtgcacacaattttcatgttgaatgcattgacgagtggctcaggctgaatgtcaaatgtcctcggtgccgttgttcRgtgttcccaaatct[T/C]gatcttagtgccctgtccaatctccgttcagattYtgagcgatcttctgccagtgttgtgacaacaactagatatgtgagaggccaaccttccagtcaaa

fava_c17241_1317	fava_c17241	1317	C/T	attgacgagtggctcaggctgaatgtcaaatgtcctcggtgccgttgttcRgtgttcccaaatctYgatcttagtgccctgtccaatctccgttcagatt[C/T]tgagcgatcttctgccagtgttgtgacaacaactagatatgtgagaggccaaccttccagtcaaagttacctgttaaggttacagggacttcttcgccca

fava_c17241_1528	fava_c17241	1528	C/T	aaattgcagggcctgttggtgagtcagataatgctttgcaaaatgctgagaatggtgttgtacctattgtgatacaaaatgcaccaaacagagtccaggt[C/T]tcttctgtggaatgcatgcctgttaatcattcctcttcacaaagttagattatacagtttcggagcttccgatatatttctagctcattctagaggtgaa

fava_c17241_296	fava_c17241	296	T/C	tttcttctctctcacaattgtattgttgtgtcactctttcgctttttccattactgtttcttcattcactatcacactctacttctttttactccttttt[T/C]ccccccttttctaaacctcaatttccttaaatgcattcacaatccaattccgattccgttaatgcattttctccttaagcgcgatccttctattccacac

fava_c17241_469	fava_c17241	469	T/C	ccttaagcgcgatccttctattccacacctcttcactcgttagggtttccttcaaatcgattcccatggctatcagaggcgttgatttcaagtggtacga[T/C]ggatttttcttgtccatgctcgccaccagcgtagttattgttgcaattaattggaagcgttaccattcttgtacatacccattgcacatatggattgtgg

fava_c17241_844	fava_c17241	844	T/A	tagttggtaccatgtggttcagcactgccaagagctgtttgcctgaagagggtcagaaatggggatttctcatctggttactttttagttactgtggact[T/A]ctttgcattgcttgcatgtcagtgggaaagtggttgactcgacgacaagcacacttagtgcgtgcccaacagggtatcccagtgtctgaatatgggattt

fava_c17243_352	fava_c17243	352	A/G	gattcctcaaaacttgctcacttgcacgatataaaaaacggcggcattcaaacacaaaaaaataattaattacacagttcagtacatgttctaaagagat[A/G]aggctgccaaaacataactaatttgtgcatacacattagcagaaaatcagtggtttcaaaaccaatttgccagctgatccttaagaatataagcagaaaa

fava_c17245_1182	fava_c17245	1182	T/C	atggtaaaataaatgaagaactcatacttgtaccagaaaaatggaaaggacagatagagaaggatctgccgcggacatttcctggtcatcctgctttgga[T/C]gaggatggtagaaatgctttgagacgactacttactgcatatgctcgacacaatccctctgttggttactgccaggccatgaatttttttgctggcttat

fava_c17245_1567	fava_c17245	1567	C/A	tgcatgggttactggaccatggtttctttccatttttgtgaacatgcttccttgggaaagtgttcttcgggtgtgggatgtgcttctttttgaaggaaac[C/A]gagtcatgctatttagaactgcagttgctttaatggagctatatggtccagcattggtaacaacaaaggatgctggagatgcagtaactttacttcagtc

fava_c17245_2148	fava_c17245	2148	C/T	tcagagcagatgagcttgaaacagcattaatggaaattgttaagcaggacaataggcgggaactgagcgccaaggtagagcgattagaagaagaggtcgc[C/T]gagcttagacaagcactttctgataaacaagaRcaggaagctgcaatgcttcaggttttgatgcgggtggagcaagaacaaaaggtaacagaggatgccc

fava_c17245_2181	fava_c17245	2181	A/G	aaattgttaagcaggacaataggcgggaactgagcgccaaggtagagcgattagaagaagaggtcgcYgagcttagacaagcactttctgataaacaaga[A/G]caggaagctgcaatgcttcaggttttgatgcgggtggagcaagaacaaaaggtaacagaggatgcccgcaggtttgctgagcaagatgcagctgcacaaa

fava_c17245_387	fava_c17245	387	G/A	atctgcaaagatatcgggaatacgccaacatatacaaggaggaagaagaggaaaggtcagataggtggaagtcatttttagataggcaggcagagacgga[G/A]tcttctgaattggcctcaaacagatcagccgtgggagacgatgagaaagtttctggtgatgaggttgttgacgcgagtttagaaaagggtgttgatggac

fava_c17245_506	fava_c17245	506	G/A	aacagatcagccgtgggagacgatgagaaagtttctggtgatgaggttgttgacgcgagtttagaaaagggtgttgatggacatcaaacaagtgcaaata[G/A]tacacttgaaaatggaagtcaaaaggaggagttaccggaatctgaggtgacagggattcatagagtccagttatggtcaaccataagatcatctcttcat

fava_c17245_927	fava_c17245	927	T/C	gtgttgatggcttgagtacctctgcaaatgggattgctgttgatgctgcaccactagaggttccgtgtccttggatagaagagttggaagttcttgttcg[T/C]gggggagtgccaatggcacttaggggagagctctggcaagcttttgtgggtgtcaaagcaagacgggtagagaattattatcagaatctactagcctcca

fava_c17246_1051	fava_c17246	1051	A/G	gccttccagatgataacctgctactgattcttcctctgtccaggtcccttcctctgtccaagtcccttcctctctctaagtccctctctaagtgaccctc[A/G]ccaccattcggtggaagtgttattcttcctcggaaacgattattgacggatctctcattcgaagataagtggagcgaatctttccgagagaatcgacgac

fava_c17246_1189	fava_c17246	1189	C/T	gattattgacggatctctcattcgaagataagtggagcgaatctttccgagagaatcgacgactttgctcttcgtcatgagtgacaacagatttcagacc[C/T]ttcctgcgcttggataaacgatgtcggagatctgagacctcaacactgtcaggactgcgagaactgttacgagtcctttctgtatgggttgatgccttcc

fava_c17246_237	fava_c17246	237	G/T	aatatccactcaaatctgatttaaaaactgtaaaaaataatacaagaaaataaattgagattcacttccccaattcagttctacaggcaaatcttaagta[G/T]agaaatgctgctaagttaaagagagacaatctaaggtcccacaaRagatgcttatgataattatgaaagaacaacaccaatcttcttggcaaaatcatca

fava_c17246_282	fava_c17246	282	G/A	gaaaataaattgagattcacttccccaattcagttctacaggcaaatcttaagtaKagaaatgctgctaagttaaagagagacaatctaaggtcccacaa[G/A]agatgcttatgataattatgaaagaacaacaccaatcttcttggcaaaatcatcaccatcttcatcttcttccatatattcttgctcttgattttccccc

fava_c17246_466	fava_c17246	466	G/A	gctcttgattttccccctcatcaacttgttcataatcatactcctcgtcaccttgctcatattcatagtctcccccttcctctccttgatcatcgccttc[G/A]tactcttgttcttcgacagcttcatcgtatattattccatcctcgggcaactgatgagttacattagtgttttcaacctctcttccaacaacatcagcag

fava_c17246_569	fava_c17246	569	T/C	ctcttgttcttcgacagcttcatcgtatattattccatcctcgggcaactgatgagttacattagtgttttcaacctctcttccaacaacatcagcagcc[T/C]gattcttggattcctcttccttggtctctaacaaYgcKccgttttcagttattccttcattcttattattataggctgatttgctgctctgagaagaggc

fava_c17246_604	fava_c17246	604	T/C	catcctcgggcaactgatgagttacattagtgttttcaacctctcttccaacaacatcagcagccYgattcttggattcctcttccttggtctctaacaa[T/C]gcKccgttttcagttattccttcattcttattattataggctgatttgctgctctgagaagaggctacaScagctccaactcctttcttctcYttcagaa

fava_c17246_607	fava_c17246	607	T/G	cctcgggcaactgatgagttacattagtgttttcaacctctcttccaacaacatcagcagccYgattcttggattcctcttccttggtctctaacaaYgc[T/G]ccgttttcagttattccttcattcttattattataggctgatttgctgctctgagaagaggctacaScagctccaactcctttcttctcYttcagaattt

fava_c17246_674	fava_c17246	674	C/G	cttggattcctcttccttggtctctaacaaYgcKccgttttcagttattccttcattcttattattataggctgatttgctgctctgagaagaggctaca[C/G]cagctccaactcctttcttctcYttcagaatttcactRagaggctttggaccttcaaatggaacatcatcttcatattgttggtagcttttcctcttYcc

fava_c17246_697	fava_c17246	697	C/T	ctaacaaYgcKccgttttcagttattccttcattcttattattataggctgatttgctgctctgagaagaggctacaScagctccaactcctttcttctc[C/T]ttcagaatttcactRagaggctttggaccttcaaatggaacatcatcttcatattgttggtagcttttcctcttYcccaatgattgttgctctgtacttt

fava_c17246_712	fava_c17246	712	G/A	tttcagttattccttcattcttattattataggctgatttgctgctctgagaagaggctacaScagctccaactcctttcttctcYttcagaatttcact[G/A]agaggctttggaccttcaaatggaacatcatcttcatattgttggtagcttttcctcttYcccaatgattgttgctctgtactttcagggtttctcccat

fava_c17246_772	fava_c17246	772	T/C	caScagctccaactcctttcttctcYttcagaatttcactRagaggctttggaccttcaaatggaacatcatcttcatattgttggtagcttttcctctt[T/C]cccaatgattgttgctctgtactttcagggtttctcccatatttaagYtcagcaagacttttaggtgcagagaacccacttcggtctcccaaagatcgac

fava_c17246_820	fava_c17246	820	C/T	ttggaccttcaaatggaacatcatcttcatattgttggtagcttttcctcttYcccaatgattgttgctctgtactttcagggtttctcccatatttaag[C/T]tcagcaagacttttaggtgcagagaacccacttcggtctcccaaagatcgacctctgaagtttctcctYtcatcatctggcaacctgtctctcattctat

fava_c17246_889	fava_c17246	889	C/T	ctgtactttcagggtttctcccatatttaagYtcagcaagacttttaggtgcagagaacccacttcggtctcccaaagatcgacctctgaagtttctcct[C/T]tcatcatctggcaacctgtctctcattctatcgtggatccttccctcgtgaggagggtgcagccttccagatgataacctgctactgattcttcctctgt

fava_c17247_1292	fava_c17247	1292	A/G	ttataatctggttttttaagaaccatgggttcaggggactggatttcaatttcatctttactatgtatctgaaatttcctgtgatcaaacccattcctgg[A/G]gtctccattaacaggacttagttctggtgaatcaggtgggctgatcagagacaccttcagcttattttcttctacatatctcactccgtctttgacaaac

fava_c17247_1448	fava_c17247	1448	C/T	ttcagcttattttcttctacatatctcactccgtctttgacaaacaaactggcagtaacatcttcgctggttatttccgcatcaaccaatgtgctttgaa[C/T]taagaacttgtccttgcagaccaaatcttcaggagctacccgttgagcttgcattgtaactatgaattcaccactagattttggcgcgaKaactccaaca

fava_c17247_1538	fava_c17247	1538	G/T	gtgctttgaaYtaagaacttgtccttgcagaccaaatcttcaggagctacccgttgagcttgcattgtaactatgaattcaccactagattttggcgcga[G/T]aactccaacatttggtcgcaccgaatatttcttaggcgacgtcgttttaaccttaaaagcaacatagtgataagtattattcgtcacctgaaccgaacaa

fava_c17247_1752	fava_c17247	1752	G/A	tcaattcaaatatgaatttgagttccctcggttcaatttcgagaatcggtgtcttgttcatgattacaccagaaagagtaatgaaaatcgaatcaacggt[G/A]tttctgtgaaaacgctcgattcgaggagaagaagagggaagaacgagtcaactcgagtgaactcggagtcagagattttttttattttgtttctaatttc

fava_c17247_185	fava_c17247	185	A/G	aaaagaactctcaaatccattcatagttcgctatcaaagaaacatgtgcagtgcaagtgcaattgtttttccaaaggcagtttctgttgtactttcgagt[A/G]ctctcaacaatcaaattggatgctctatcacggcttgtattcactaatgccacaattcaacaaaatcactgatcaacttcaactccacagagaaattttR

fava_c17247_84	fava_c17247	84	A/G	ttgcacttctcaataataacaacaaaagtcatggtaaagatatccgtgatattcaaaattatggtttcaaatgccatggttca[A/G]aaaagaactctcaaatccattcatagttcgctatcaaagaaacatgtgcagtgcaagtgcaattgtttttccaaaggcagtttctgttgtactttcgagt

fava_c17247_920	fava_c17247	920	C/T	gcattgtttactgtggtcaaatgatcttttgacaccatcaactctgcttcttttcctggtttcagttcttctgcattctttattctgattgaacccagat[C/T]ttttgactccttcatctctgcttcttttccaggtttcagttcctctgcattctttactgtgattgaatccagatgtttgggcacttctacctctattttt

fava_c17250_1100	fava_c17250	1100	G/A	gttttggtggtaatgaagaagctccaagttctgtagagacatcatgtgttgaatcgaaaggagatgtttgtgatgaagagaatgaaaatggttgggtcag[G/A]aaggtttcgtccacattggctattcctagtaataggaaacttgttcttgatgtgactgggagggttacgtttgagaccgttagatctttcatggagttta

fava_c17250_305	fava_c17250	305	C/T	ccacttacggttgttacaaaacttatcacgcacctttcatagctcaaaagcgaaagaatttttctaaaattcttaattctttcgtttctgtagcagaagc[C/T]gtttccgattctgcagataccattggtattgtcaccaaagacgtgaatgatttcttacaatccgattctgatcgagttccgaatagtttgaatcaaatct

fava_c17250_584	fava_c17250	584	G/T	gttatcaaaccgtgaaccggagtgatgaaattcatccgtccggttctggttcaatcattgcggacaaggtgtttgataaaatgtttacaccggctggttc[G/T]ggctttgcttctgttgttattggaagctttgctaggaacttagttattggtttttMttcRgatgccggtgggaaatttagtggagaatcgagttcgagta

fava_c17250_640	fava_c17250	640	C/A	attgcggacaaggtgtttgataaaatgtttacaccggctggttcKggctttgcttctgttgttattggaagctttgctaggaacttagttattggttttt[C/A]ttcRgatgccggtgggaaatttagtggagaatcgagttcgagtaatgcagatgggagtttgaattctgatcgtgttgttccggaatgggttgatgttgtt

fava_c17250_644	fava_c17250	644	G/A	cggacaaggtgtttgataaaatgtttacaccggctggttcKggctttgcttctgttgttattggaagctttgctaggaacttagttattggtttttMttc[G/A]gatgccggtgggaaatttagtggagaatcgagttcgagtaatgcagatgggagtttgaattctgatcgtgttgttccggaatgggttgatgttgtttgtg

fava_c17250_800	fava_c17250	800	G/C	tgaattctgatcgtgttgttccggaatgggttgatgttgtttgtggtgataaatgtggggaattgattgggaatttagttcaagtgtttgttagtacgtt[G/C]gttgctgtttatcttgacaaaaccatgcatatcaatacttatgatgatttcttttcgggattRactaacccgaaaaatgaaactagagttagagaaatgt

fava_c17250_863	fava_c17250	863	A/G	tgattgggaatttagttcaagtgtttgttagtacgttSgttgctgtttatcttgacaaaaccatgcatatcaatacttatgatgatttcttttcgggatt[A/G]actaacccgaaaaatgaaactagagttagagaaatgttggttgatgtttgtaatggtgccatagagagtcttgtcaaaacatctcaccaggtgtttaata

fava_c17250_985	fava_c17250	985	C/T	agagttagagaaatgttggttgatgtttgtaatggtgccatagagagtcttgtcaaaacatctcaccaggtgtttaatagctcaaacacggatgatagtt[C/T]gggtgcagtttcttgttttggtggtaatgaagaagctccaagttctgtagagacatcatgtgttgaatcgaaaggagatgtttgtgatgaagagaatgaa

fava_c17251_384	fava_c17251	384	T/C	ggattgcggataacatacttcgacaggccgacatctttcgcgcagagaagaacattattgacctatatgtcatgcttttaagattttctagtttggcatc[T/C]gagacaataccgcgtcaccgagagtataaatcatctccacaaaccaagaaacaatcattgaaaaagagattgctgatttccttgaatgaattRgagaagc

fava_c17251_477	fava_c17251	477	G/A	tggcatcYgagacaataccgcgtcaccgagagtataaatcatctccacaaaccaagaaacaatcattgaaaaagagattgctgatttccttgaatgaatt[G/A]gagaagctgaaaccattggcccaacagaagatcaacgagcttaacagcagaaatgctttccgacaaaatggacggggaaattttcattcaaataataacg

fava_c17251_847	fava_c17251	847	G/A	aaaggaggaaactctctctagacattctatccttggtccgcatgggcttaatgggcagtggtggtcacctactattgacacaggggtcaggtatccatcc[G/A]acatagatctgtctccagttgaactgccaagcttgcaacgccaattggaagatgtgtctctgagtaataaagataataatagcattgcagaactccaaaa

fava_c17252_1431	fava_c17252	1431	T/C	ctgaggctgttggcttggaggattatggccacctagaggcaggcaggatcttccatgctgcccgactagttgctattcttgaagcatatgcactatatga[T/C]cctgaaattggctactgccaaggtatgagtgacctgctatctcctatagttagtattatatcagaggatcacgaggctttttggtgtttcgtcggattca

fava_c17252_1719	fava_c17252	1719	A/G	atgcccatctatttaagcatttggagaagcttcaggctgaagattgcttctttgtgtatagaatggtggtggtactgtttcgaagggaattgacatttga[A/G]cagactatttgcctttgggaagtaatgtgggcagatcaagctgcagtcagggcaggtattgggatttctgcatggaacaaagtcagaaagcgagcaccgc

fava_c17252_390	fava_c17252	390	G/C	tcttcacggccctcgccggcgtcgctatcgtcgctgtcattttctacggtgccagtaggaaaagaaaacatgccctttcacctcagcaatggaaagtctt[G/C]tttacagaagatggtagactYcgtgatggtggaattaggctcctgaaaagagtgcgcagtggaggtgttggtcctagaataagagctgaagtctggccat

fava_c17252_411	fava_c17252	411	T/C	tcgctatcgtcgctgtcattttctacggtgccagtaggaaaagaaaacatgccctttcacctcagcaatggaaagtcttStttacagaagatggtagact[T/C]cgtgatggtggaattaggctcctgaaaagagtgcgcagtggaggtgttggtcctagaataagagctgaagtctggccattcttacttggagtgtataact

fava_c17252_621	fava_c17252	621	T/C	caaaggatgaaagagatgctgtgagaactcaaaatagaaaggaatatgaaaaactccgtcgacaatgcaggaaactcataaaaaaaagcaatgaaagcag[T/C]aagttaaatgaagttggtgaaattagctatgaaggaggtgatggaagtcttgttcaagattctggttctgatgatgcagccagcgccaatgagttccttt

fava_c17257_877	fava_c17257	877	G/A	tctggtcttgacactggatccctctggtctagacagccttttgagcagtttggtgttgctgataaaattcataatgttgcaatggaaggagcaggaagaa[G/A]gccgaatgcaattgttcaagaaactacatttgaagatacagaagggaaactacttcaatctctcagactttgcattatgaagctcttaaaactggagggg

fava_c17259_276	fava_c17259	276	T/C	tctcaccgtcgtctcaactatcgctgcttcgatcgcctcatcgatctcatttttcacataatttgcttctaagaatgccggagcgagacgcaggttggga[T/C]ttttacctccgtactctatctaccagtgccagagattccaacaccgccaacgatcctgcttccgatccttctcttattcaatccgtgaagaagcttcatc

fava_c17260_1178	fava_c17260	1178	T/G	attgttcaataagattgagattgagattcccttcaatttggtcaatcaataaaactagtagcattgttggccaaatttggagcaacaagagtgctagtga[T/G]ccaaactacttcaagatgataacatttWggaatgatcataattatcgtatcgggggRaaagacttgaagtatgagtatagcaagcttgatagagttaagc

fava_c17260_1206	fava_c17260	1206	T/A	cccttcaatttggtcaatcaataaaactagtagcattgttggccaaatttggagcaacaagagtgctagtgaKccaaactacttcaagatgataacattt[T/A]ggaatgatcataattatcgtatcgggggRaaagacttgaagtatgagtatagcaagcttgatagagttaagcaatcatgtttaacaaacaaggttgtcgc

fava_c17260_1235	fava_c17260	1235	G/A	gtagcattgttggccaaatttggagcaacaagagtgctagtgaKccaaactacttcaagatgataacatttWggaatgatcataattatcgtatcggggg[G/A]aaagacttgaagtatgagtatagcaagcttgatagagttaagcaatcatgtttaacaaacaaggttgtcgcgattcaaggaaaaaggtatccagatgcat

fava_c17260_1444	fava_c17260	1444	T/C	gacatgaaactcgatatgtcaattagagagtccaaaaaaagagtagcttggggttcttcttctcccttatttgttgatgatgattcctatgagtcagatc[T/C]atctacaataagtagtggatttgattatggaatcttgaacaacaataatggtagcttgtttaacattagctacaggattaggctctacatgatgtcttct

fava_c17260_153	fava_c17260	153	T/G	acttatcttagttttcactttcttctccttcaaccctttctcattagcctcaaaatccacttacaatgaccaatgtgcttctataatcccagaaacatat[T/G]SaacaaccaaactcaaattcaattctttccccttaagtgataagaacacaggttactacRtaggaggtgatagtatcatcaatgttgatactacttggaa

fava_c17260_154	fava_c17260	154	C/G	cttatcttagttttcactttcttctccttcaaccctttctcattagcctcaaaatccacttacaatgaccaatgtgcttctataatcccagaaacatatK[C/G]aacaaccaaactcaaattcaattctttccccttaagtgataagaacacaggttactacRtaggaggtgatagtatcatcaatgttgatactacttggaac

fava_c17260_1644	fava_c17260	1644	G/A	tccgccatttgaaaagaattccttgtttaacatgtcttactcttcggtgaagatttctgcagaaggaatttatgactcaagaaatggaaccttatgtatg[G/A]taggttgccgcaaccttgattcaaaccatggaacaccaacatcacattctttggactgtgagattttgatgaaatttcaattccctcctttagatacaat

fava_c17260_213	fava_c17260	213	G/A	ttacaatgaccaatgtgcttctataatcccagaaacatatKSaacaaccaaactcaaattcaattctttccccttaagtgataagaacacaggttactac[G/A]taggaggtgatagtatcatcaatgttgatactacttggaaccaattctccttatattttccaccaagaaatacacatgcaacttcacaccctcatctttt

fava_c17260_2405	fava_c17260	2405	G/A	acgatagaaactcgattacatcgatgtaccgccaacatgtttcgtcttgggagaacataaaatcttacggtggtttcatattagatggatttcttgtgcc[G/A]caagtcattctaaacttgttttcaaatacaaaggaaaatgctctttcaaatccattttactttggaactacttttgtgagactcttgccacatgcttatg

fava_c17260_2576	fava_c17260	2576	C/T	cttttgtgagactcttgccacatgcttatgatctttacaggactcgcagttacgatgaccaagatagtaactcatacttttatgcggatccaagcgaaga[C/T]ttttactcaacttcttgggatattttcattccattgctatgtattgtattatctatcattatctacttgcagcagcattttggtgctcaatRttttctgc

fava_c17260_2668	fava_c17260	2668	A/G	agcgaagaYttttactcaacttcttgggatattttcattccattgctatgtattgtattatctatcattatctacttgcagcagcattttggtgctcaat[A/G]ttttctgcctcgtagattcaaaggatcaaagggatatgcaaaggtgcctaaatcagaaggagaagtagaaacaaccaacatgtaaaaaggcccttttctt

fava_c17260_885	fava_c17260	885	A/G	ttcaaagaattgcactcctatagttagtggttctagtgatcaattgccatcacacatgtctttgaaaggaattgagtgctcttcccatattaagaaacat[A/G]Rggttagggttttggttgaattttcaaatagtgttgattactattggaataagaataaccaaagcttgaataccaaaactatgttaataggggaaggatg

fava_c17260_886	fava_c17260	886	A/G	tcaaagaattgcactcctatagttagtggttctagtgatcaattgccatcacacatgtctttgaaaggaattgagtgctcttcccatattaagaaacatR[A/G]ggttagggttttggttgaattttcaaatagtgttgattactattggaataagaataaccaaagcttgaataccaaaactatgttaataggggaaggatgg

fava_c17262_295	fava_c17262	295	A/C	tgttgtacaatggggtgtcatggttccttttaggatgttgaagaagatcattatggacattgcaccaaagggacactgggttgaggcttattgttggtct[A/C]tgccgcttctgcgtccccaactattccccctttgttaaatacaatatccaaattgtgaaaacacaaaagagtgtgtaagttgtaatcagaactttccatg

fava_c17263_123	fava_c17263	123	T/A	ttatggttctgaaatgcagtgatgaacaatactctgtctctgagtcgggaagatgcatggattctccactgttgacggctttgtggagataagcgagtgt[T/A]tggcagagatgattaaatacgtagcaaatgaaccatctgctggWcttttcttcatccaacagcatactcaRaatgcagttcccaatgtcattaagcttaa

fava_c17263_167	fava_c17263	167	A/T	tcgggaagatgcatggattctccactgttgacggctttgtggagataagcgagtgtWtggcagagatgattaaatacgtagcaaatgaaccatctgctgg[A/T]cttttcttcatccaacagcatactcaRaatgcagttcccaatgtcattaagcttaagaagaaggttattgacaagtctcatgaaacgcgtttgcacacag

fava_c17263_194	fava_c17263	194	A/G	ttgacggctttgtggagataagcgagtgtWtggcagagatgattaaatacgtagcaaatgaaccatctgctggWcttttcttcatccaacagcatactca[A/G]aatgcagttcccaatgtcattaagcttaagaagaaggttattgacaagtctcatgaaacgcgtttgcacacagaagatttggaggattctgtcacaaYgg

fava_c17263_292	fava_c17263	292	C/T	caRaatgcagttcccaatgtcattaagcttaagaagaaggttattgacaagtctcatgaaacgcgtttgcacacagaagatttggaggattctgtcacaa[C/T]ggtgaaatcaatgaaggagtgtggattcccaataattgatgacatgattggagacattaaaaaaactctggccactatgacatcaaagcaaccaaaacga

fava_c17263_698	fava_c17263	698	A/G	agctgtaccctaatgtgcctttgtctgtcacatatgcaagcagttgtacatctacagacaagttgccactgccaagagaggttgaagatgaaactctgcc[A/G]gaacagagtgatattgggattaatttattgtcagYaacagaaaaatatgatgacttcaaagctagtaaacaagctaagctggaggagtggctcgaaggaa

fava_c17263_733	fava_c17263	733	C/T	gcaagcagttgtacatctacagacaagttgccactgccaagagaggttgaagatgaaactctgccRgaacagagtgatattgggattaatttattgtcag[C/T]aacagaaaaatatgatgacttcaaagctagtaaacaagctaagctggaggagtggctcgaaggaactaataatcatgattgYgggtaaagtgatgaaaat

fava_c17264_1347	fava_c17264	1347	C/T	agataactgctgaagcaattgacatcctcgcccgaactttgttggtaggtgcatctcgggaagcctgcatccatgcccgtctaagaagcaaccagaattg[C/T]ttccaccatcctcctttcctttttgctatagtcctcttgctgattctctttctactttttgagaggtcgtctatagtaattggagttgcatgtattgttg

fava_c17264_1501	fava_c17264	1501	G/A	actttttgagaggtcgtctatagtaattggagttgcatgtattgttgtggatagtttttgtgagagggactcaacaagcccatcgatcctttttttagaa[G/A]aatggacactRtcagaagaactgtagtctatggatataagatcggccaaaaattctgcaggatttacatgatctgggcagtggtacccgaatttggagaa

fava_c17264_1512	fava_c17264	1512	G/A	ggtcgtctatagtaattggagttgcatgtattgttgtggatagtttttgtgagagggactcaacaagcccatcgatcctttttttagaaRaatggacact[G/A]tcagaagaactgtagtctatggatataagatcggccaaaaattctgcaggatttacatgatctgggcagtggtacccgaatttggagaagtactccaacg

fava_c17264_273	fava_c17264	273	C/T	actgctaaaaattaaccatgatcactgtttattattacagctgggtaggagtaagaatctgtgaacataagctggtgtttttgatgtccaataatggcat[C/T]ggggatatcagtgatagtggtcttgttctataggaaacgcatcctggtaattgaaggtcatccattgcaaagatatcactctactgagcaccttctagga

fava_c17264_387	fava_c17264	387	A/G	atagtggtcttgttctataggaaacgcatcctggtaattgaaggtcatccattgcaaagatatcactctactgagcaccttctaggataaaagagccaac[A/G]gggtctacttcaggtgactcaaggggctcgtcggaaccaacttggctgacMtctggtgcttcatatgtctgatcagctagttccgaattgaactcctcaa

fava_c17264_438	fava_c17264	438	C/A	ttgcaaagatatcactctactgagcaccttctaggataaaagagccaacRgggtctacttcaggtgactcaaggggctcgtcggaaccaacttggctgac[C/A]tctggtgcttcatatgtctgatcagctagttccgaattgaactcctcaagttttaaatgcaggttttcgtggtcgggaggggttgtttcaagttgctggt

fava_c17264_627	fava_c17264	627	C/A	caagttgctggtatttgggcttgtttttctcaaggagaaggtaggtggtacaataccaaaataacaatattctattttgcgctagcagcgtatctctaat[C/A]gtactttttccaaaggaaagacgctctagtgcctgttctccagtttgaatatcaaatgaatgctggtgatcaaattgaagaccacgaaattcattgatgc

fava_c17264_777	fava_c17264	777	G/A	tatcaaatgaatgctggtgatcaaattgaagaccacgaaattcattgatgcaaagtccttgaaaggcccatcttatcagagaaacactaggaatccagcg[G/A]aagataatcggggtattctccSggttaacatagtagccaccaaatactataaaaactgtcataagagaggggcctactgccattgctgcttcagtagttg

fava_c17264_799	fava_c17264	799	G/C	aaattgaagaccacgaaattcattgatgcaaagtccttgaaaggcccatcttatcagagaaacactaggaatccagcgRaagataatcggggtattctcc[G/C]ggttaacatagtagccaccaaatactataaaaactgtcataagagaggggcctactgccattgctgcttcagtagttggaaccatagcaccaacagtaag

fava_c17266_396	fava_c17266	396	A/G	atatagatattatattagtcctttgtctcttaacacttccagaagcagattcatctccaacactcttctccatcacagctttaagtgcttcttgtattga[A/G]ctaatgcaatattcatgatcatgatcttcaccagtgacgaacaaaacattcttaacacgtcctccaagtgttgttatatcagctttcactgttctcaacc

fava_c17266_581	fava_c17266	581	C/T	ttcactgttctcaaccttaatgctttcaatgtcttgattagttcaggtaaaagatctgacctatcatcacagcagagtgaagctttaagaataaacttgt[C/T]tccgtttgcatattcttcatcatcatcatttgcagcagcatccactgttaactcatcacattccgttggaacttgacatgtttctgatattaatgatgtt

fava_c17270_1472	fava_c17270	1472	A/G	cagttaatgcagaatcagccaatgaatcaaacaaatgtcagcaagttagaaagtggccgtcagtcaggagataaatgattatttgatggttacaatcaag[A/G]Rctgcttattattgtatagctgttaccactagctatcaaaatggtactactagtattattttatattttctgctacccatctacagtatgccaagttttg

fava_c17270_1473	fava_c17270	1473	A/G	agttaatgcagaatcagccaatgaatcaaacaaatgtcagcaagttagaaagtggccgtcagtcaggagataaatgattatttgatggttacaatcaagR[A/G]ctgcttattattgtatagctgttaccactagctatcaaaatggtactactagtattattttatattttctgctacccatctacagtatgccaagttttgt

fava_c17270_1594	fava_c17270	1594	T/C	gttaccactagctatcaaaatggtactactagtattattttatattttctgctacccatctacagtatgccaagttttgtctgtaacggaaggaactttt[T/C]atcagttctcagttgcaagctaataatatatgcattacaccgctaacttgtaaattattgtaacatRgttttctctRgttttttgtcctccctcatcgac

fava_c17270_1661	fava_c17270	1661	G/A	tgccaagttttgtctgtaacggaaggaacttttYatcagttctcagttgcaagctaataatatatgcattacaccgctaacttgtaaattattgtaacat[G/A]gttttctctRgttttttgtcctccctcatcgacacttttactttttacaaattaatttggtacgctcgatcataggttgaagtgtccatcgagtacctct

fava_c17270_1671	fava_c17270	1671	G/A	tgtctgtaacggaaggaacttttYatcagttctcagttgcaagctaataatatatgcattacaccgctaacttgtaaattattgtaacatRgttttctct[G/A]gttttttgtcctccctcatcgacacttttactttttacaaattaatttggtacgctcgatcataggttgaagtgtccatcgagtacctctatgtatgcaa

fava_c17270_758	fava_c17270	758	G/A	ggcttgacggcgcgttcgacgacgtttgacgaaccggatgggtcgagtagcgatgtagaaccggttcgggagatagcggagcagggccggaagcggaagg[G/A]aagggaggcggaggaatggaagtatcaaaacgaggatgttgattttgaatctgcggaagccaaagataaattcaggggatcatcaacaaagagatctcgc

fava_c17271_1191	fava_c17271	1191	C/G	ttcttttttcttttcctattccacttcatccttctttctcatcttcttccttctccatcttcttccttatccatctttggtggaaacttacttttgagaa[C/G]atatctYtggtagaaacttacatttgagaagatctcttttgagaaactgatatgtggttgcgttattcgaaattgtttcggtcgagctgaggttccgagc

fava_c17271_1198	fava_c17271	1198	C/T	ttcttttcctattccacttcatccttctttctcatcttcttccttctccatcttcttccttatccatctttggtggaaacttacttttgagaaSatatct[C/T]tggtagaaacttacatttgagaagatctcttttgagaaactgatatgtggttgcgttattcgaaattgtttcggtcgagctgaggttccgagctacgtca

fava_c17271_2429	fava_c17271	2429	T/C	attgtgctttcaacctgcaaattttttattaaaagtatttcttagatctatatttacaaatttcagggagaatatgatcatggaaaagacaaatatgatg[T/C]ggtatgtttatttgacacgaaaacgatattactgtcacaattctctgcattcatagcattactatataaaatcgtttttctgatgtactgtatgtgaaat

fava_c17271_2620	fava_c17271	2620	G/T	tatgtgaaatttgtgcgatatagtaacaaggagtttgtggttttcatcatgtaaatcgggtcataatgagatgcaagagtgagtgggcagaatttcaaac[G/T]cgcctcatcaattctgataaagggtatcttacaggatttagaYgaagtatgacgagttaacatatactgtttccaattctaatgtacgtgcattacttat

fava_c17271_2663	fava_c17271	2663	C/T	tcatcatgtaaatcgggtcataatgagatgcaagagtgagtgggcagaatttcaaacKcgcctcatcaattctgataaagggtatcttacaggatttaga[C/T]gaagtatgacgagttaacatatactgtttccaattctaatgtacgtgcattacttatattgatagactgcatataagtatatttatcataggaaagaaag

fava_c17271_2864	fava_c17271	2864	C/T	ttacatcttatatgtatggctaattatcataaactacttgacttctgcacccaattttgaaccaaagcatgctcacaataaaggattttggagaagtagt[C/T]tgcatgtgattcaatatttgaatggttttgtatctaaatagtgcaagagtttcaatctgctttcagtcatccacagttagagtcaatgtaatgaagaaat

fava_c17271_553	fava_c17271	553	C/T	gcaatgccgctcaattgcctttgtgcaacaccatcatcaactccaatgtcaacaacttcatcaggcttggtattgccaccgacactctcatcaacaacaa[C/T]gtcaccagccttctcagtaataacaatgtcacccggttgctcatgtgtaacattatcatcaactccaacctcaactccagtgtcaacaactttactaggc

fava_c17271_875	fava_c17271	875	G/A	tcacaacaggagcaggagcaggagcaggagccacaacctcatctttcacttcatccccaacaacagcaacagctccatccaccaaaggattaacttgaac[G/A]ctatcaacaacaacatctcctccaccactggtaaactccgcatcatcaacaaccctatcaccattctcaacaacaccaacaccagaattatcgccagaaa

fava_c17272_186	fava_c17272	186	T/C	taaaactaatgcatctaaactaataaaaacctagcaacataagttttatcaacctgcataacaaaacgccatataccatatttacaaacaacaaactaga[T/C]caactcatttcttaaatatgttacataattctcatgaccgtctatgaagtaYatccctgtaacgtaRaatggaatcaagcctttccaacctaagttgctg

fava_c17272_238	fava_c17272	238	C/T	cctgcataacaaaacgccatataccatatttacaaacaacaaactagaYcaactcatttcttaaatatgttacataattctcatgaccgtctatgaagta[C/T]atccctgtaacgtaRaatggaatcaagcctttccaacctaagttgctgcttaaacctttttataaaatcatctgcctttgcgtccacctcctcctcttcc

fava_c17272_253	fava_c17272	253	G/A	gccatataccatatttacaaacaacaaactagaYcaactcatttcttaaatatgttacataattctcatgaccgtctatgaagtaYatccctgtaacgta[G/A]aatggaatcaagcctttccaacctaagttgctgcttaaacctttttataaaatcatctgcctttgcgtccacctcctcctcttccatttctgatccaact

fava_c17272_522	fava_c17272	522	A/G	cgacggagctcttgttagcggattctggatcaagtcatccatgccttgattcatttctgttttacaaaggcttgcgtcaatcgcctttgtggaagcaata[A/G]cctgatcgagttggcccaggccttgattttgaatcggctcgatttcagtttctaccgatgctgctggtggtttgaatttacagcaacctagtccaaacga

fava_c17272_727	fava_c17272	727	C/A	actcgttcaaaaaacgacgatgttggagggactatttgctgtggcgagtcggattgattttttctctgagctgctaaacgagaggttataacaatggtgc[C/A]gatgacaaggtttacgactaggaatatacatgaaggacttataagccaacttgcaaccatatcatatgctgaaactgaatctgcttcacccatactgata

fava_c17273_1096	fava_c17273	1096	G/A	agatagatccaacctgcagcttcacgaatggaggaggaccacctgtatcacgatcctctagctgccttaaagatgaccgagagaagcctgcatatttttg[G/A]ttcatctgccattcttcatgtagtgattgaacaacaccccctaatacagtcaatactttgggatttaagcaagctatactgctacgaaccggaactttat

fava_c17273_1213	fava_c17273	1213	C/G	catgtagtgattgaacaacaccccctaatacagtcaatactttgggatttaagcaagctatactgctacgaaccggaactttattttccaaacgaatctt[C/G]gtgccgggaacaacattgtcaggtaagaatggaatgtgagagtattctatagcagttatctcggagtgaccatctgtgagacacaatctcagaacgcgtc

fava_c17273_1654	fava_c17273	1654	A/G	gatcgtcggtgtcttgcaaataccaacccttatttcttagggtttctatcaccattgcattcgaactcccctgcattttccctctttcaaattcacttca[A/G]aatccagcttctctcgggtgtgtattgtatataggaccaaatacacttcgagcatactattttactttgtttaggttactctctccttttaggtctcttt

fava_c17273_981	fava_c17273	981	T/C	tccataatatcttctttcagatttcggtcttgctggataccagttggcctcatctcatctctaccagtaccagcaatgggcttcctacttctagagttct[T/C]atctgcataacctgagatagatccaacctgcagcttcacgaatggaggaggaccacctgtatcacgatcctctagctgccttaaagatgaccgagagaag

fava_c17274_1327	fava_c17274	1327	A/C	aattttgtaaacataaaagtaaaaataaaaaagccaaagaggttcttcccctgaaaactttgagccaatctttgagtattataatatcttgaatttcttc[A/C]cgacctttgaaatcaaaatatcatcRtagtaaaaaaaataatgaggaaagtagcaattagaagaaattttttgtgtcaatttgaatgattttgaagcatc

fava_c17274_1353	fava_c17274	1353	G/A	aaaaaagccaaagaggttcttcccctgaaaactttgagccaatctttgagtattataatatcttgaatttcttcMcgacctttgaaatcaaaatatcatc[G/A]tagtaaaaaaaataatgaggaaagtagcaattagaagaaattttttgtgtcaatttgaatgattttgaagcatcaaaatgtgttgaattcttcagcatcc

fava_c17274_1697	fava_c17274	1697	T/C	ggtttccacatgccctaaatgcaagaccccaaccgttcatcgacgcagctcgctccggtaatctcccgatcgtgacccataattttcttcccttgtcgta[T/C]tttttaacctccatgtcggcgtagtccgcggcgtatagttcgttatttacaaccgcgactagaggtggtgcctcggccgtagcaggcatctcggatcccc

fava_c17274_2072	fava_c17274	2072	G/A	ggagtgtttcccatgtttgattctcggagttatagagctcagcggagtcgagtatatgtccgtcagagtcacacccgcctgctagaatcgcaatctctcc[G/A]aggctagcggatccaaacaagcatctcggaaaattcattctcattccagattcccacgagtttgtcaaaagactgtatctgtatatgacatgagactgca

fava_c17274_2257	fava_c17274	2257	T/G	atgacatgagactgcaactccctcccgaacacaagaagctcggtaccaaccgccaaggactccttatccgaacacatgaagcattcattagaagccattc[T/G]RggtaaatgcatccacctttcgcgaatcggatcataagcctcccattcgaggagggcacaYgaaaaataaatccagtgctctatgatatcattcagtctc

fava_c17274_2258	fava_c17274	2258	A/G	tgacatgagactgcaactccctcccgaacacaagaagctcggtaccaaccgccaaggactccttatccgaacacatgaagcattcattagaagccattcK[A/G]ggtaaatgcatccacctttcgcgaatcggatcataagcctcccattcgaggagggcacaYgaaaaataaatccagtgctctatgatatcattcagtctcc

fava_c17274_2318	fava_c17274	2318	T/C	ccttatccgaacacatgaagcattcattagaagccattcKRggtaaatgcatccacctttcgcgaatcggatcataagcctcccattcgaggagggcaca[T/C]gaaaaataaatccagtgctctatgatatcattcagtctcctccacgcatagagctccccgctccgtattatgttgcggaagctcctattcagcgaggcaa

fava_c17274_2560	fava_c17274	2560	G/C	attgagctgtcccggttcatccgcggcaaaagtgaactcgaatctgatgaatctccagcatgatgctccttctgctcatccgatggctcatcaagctgca[G/C]ctgtttcttctgatgttcatcgccgaactttgacaacaacatgaattgYggcgattgtgattgtgattgtgattgttcagccgaaaactgaacagaaaag

fava_c17274_2609	fava_c17274	2609	C/T	aatctccagcatgatgctccttctgctcatccgatggctcatcaagctgcaSctgtttcttctgatgttcatcgccgaactttgacaacaacatgaattg[C/T]ggcgattgtgattgtgattgtgattgttcagccgaaaactgaacagaaaaggaaacaaccttttcaactatttcacaatcattcaaccgctcgaaatcct

fava_c17274_2896	fava_c17274	2896	C/T	gactgcggttcatcgtcatcgccattggcacccaacagtctctttccattttttatctccaaatccggcatgtatttcatgtaagaccaattattctccg[C/T]caaacaagaactcgaaaacaaccccggaaccacgcaggatcgaccttccaacataRtcttcccaacMacaaaaactcaactttcaatcaaccaacctcac

fava_c17274_2952	fava_c17274	2952	G/A	ctccaaatccggcatgtatttcatgtaagaccaattattctccgYcaaacaagaactcgaaaacaaccccggaaccacgcaggatcgaccttccaacata[G/A]tcttcccaacMacaaaaactcaactttcaatcaaccaacctcacagacacagagatgaacaatctcagcacagaaacaaaagcgggtaagatttccgaaa

fava_c17274_2963	fava_c17274	2963	A/C	gcatgtatttcatgtaagaccaattattctccgYcaaacaagaactcgaaaacaaccccggaaccacgcaggatcgaccttccaacataRtcttcccaac[A/C]acaaaaactcaactttcaatcaaccaacctcacagacacagagatgaacaatctcagcacagaaacaaaagcgggtaagatttccgaaacagaaaacaaa

fava_c17275_133	fava_c17275	133	C/A	aatcattcaaagcaaaagactattctctttattgtatcaactcttatcaaagcacaacaaatcaaaggatcatcctcttgaatgtggtgcatcctatgaa[C/A]atttaactcttcatattaatgtcttactcaaacaattcacccacgtcatatttttctcacattagttttacatctttaaacatcctcattgttagaaaat

fava_c17275_286	fava_c17275	286	G/C	tttctcacattagttttacatctttaaacatcctcattgttagaaaattcatgcaccactaatccttactccaagtctgtcttgagatgatagatcagtg[G/C]aRaagtaaatattcaaagggtcacatttcgtctctgcactttatcgtcgtgctttgttttacttgagtgatgatttaattcgttccttcScttcaccttg

fava_c17275_288	fava_c17275	288	A/G	tctcacattagttttacatctttaaacatcctcattgttagaaaattcatgcaccactaatccttactccaagtctgtcttgagatgatagatcagtgSa[A/G]aagtaaatattcaaagggtcacatttcgtctctgcactttatcgtcgtgctttgttttacttgagtgatgatttaattcgttccttcScttcaccttgct

fava_c17275_376	fava_c17275	376	G/C	tagatcagtgSaRaagtaaatattcaaagggtcacatttcgtctctgcactttatcgtcgtgctttgttttacttgagtgatgatttaattcgttccttc[G/C]cttcaccttgcttttgtatcacggtttttcggttatgtgttgatcgaagatagagaccctStataccacggtggtttggatttacactgtagatttgttc

fava_c17275_437	fava_c17275	437	G/C	gctttgttttacttgagtgatgatttaattcgttccttcScttcaccttgcttttgtatcacggtttttcggttatgtgttgatcgaagatagagaccct[G/C]tataccacggtggtttggatttacactgtagatttgttcatatcatgccatctttgttgtccttgttgtgtgcaagattcagtttttcttcataaagctt

fava_c17275_978	fava_c17275	978	C/G	actttctctttccacctggctttgactactgttcttttggcatgcaatgctattgagaaagttgattgtgaaggattaaaccccaaatcctttaattcct[C/G]aaccaacttcagaagttcatttgtgttgagtaccttgctattgacttgaagcaatcttgcaatgcaggaatctggaactccatactcaatcaacattcta

fava_c17276_354	fava_c17276	354	C/A	catcaactcagcatcagttccgatacactcaaaccccttccaaggttcttcaccttcgcaaccttccatgggagtgttcagaagaggaactcgttgaact[C/A]tgtagtcccttcggtaggatcgtcaacaccaaatgtaatgtcggcgctaatcgcaatcaggcctttgttgaattcgtggatcaaaatcaagcaatttcaa

fava_c17276_888	fava_c17276	888	C/T	cggatcatgttggttcttgtaatttgcgtatttcatactcagcacataaagatctgaatatcaaatttcaatcaaatcgcagcagggactatacaaatcc[C/T]atgcttcctgtcaatcatagtgctattgagggggcagtgcagcctactataggtcctgatgggaagcggatagaaccggagagcaatgtacttttggctt

fava_c17278_1338	fava_c17278	1338	C/A	cagagaagcagacttgcaacaatcttctatctatatggctggagtatacttaatgctactaattcagtttcttgaaaacaaagtggccatagagctcaca[C/A]ggacagagttcgctgaagcacaagaggcattggtacagatgaaaaattggttcatgcgatttccgactatattacagccacgtgaatgcatcattgagat

fava_c17278_1478	fava_c17278	1478	C/T	tgaaaaattggttcatgcgatttccgactatattacagccacgtgaatgcatcattgagatgcttagaggccaatatgctcattctgttggctgttataa[C/T]gaagccacctttcattatattgaagcagtaaagcttacagacagcaaatccatgcaaactatgtgccaggtttatgcagctgtttcttatatctgcattg

fava_c17278_149	fava_c17278	149	C/T	gggttaacagtaaccaaaaaaaatcaacaatggaagaagtagcagaaggactatggggtttatcagactaccacgaaaatcgcggcgaaatcgcaaaatc[C/T]gtaaaatgcctagaagcaatttgccaaagcgaggtttcgttttccccaatcgtcgaagtcaaaacgcgtctccgaatcgcttcaatccttcttcatcatt

fava_c17278_2039	fava_c17278	2039	C/G	ataaagaattaggagaaaagggaagccaaatggagaatgatgagtataaaacaaaaatatcaaaagatttgcagaagaaactggctgaagcacaagcatc[C/G]atttatcatattgaattaattgacaaagcaagatttgaagttcagcaggaacatgggttgaacatcaagcgtaaaatggatgcccaatctgcaggtgtca

fava_c17278_764	fava_c17278	764	G/A	aatgcaatgagatttgggagtcaattcagccggataaaagagaacaatgccctggcttgctcttttataatgaattactgcatatattctatcgaacgcg[G/A]ctatgtgattacaagaatgctgcgccccatgtagacaatctggatgctgctgtgaaggctgaaaggaagcaaacacagcatatgcaagagctagtgaagg

fava_c17278_913	fava_c17278	913	G/A	gctgtgaaggctgaaaggaagcaaacacagcatatgcaagagctagtgaaggagctcagtgctttagatcaaagtctttccaggtctgatcttcaccaca[G/A]agaaagggcagcactgtctgagaagcaagcaatgattcaagaacagctgagaaacatgaatgggttcagttcaactgggcgggaatctcttgaacctgtc

fava_c17279_1110	fava_c17279	1110	A/G	ccaatagtagcccacttttaaaaacaccatctcctatggaaaagcaagctgcaagtctttataccagaaagatattcatgaagttccaagacgagttagt[A/G]gagacaatggcaaatcctgctaccaaaattgatgatgcaggaactatcaccacttaccgggtcgccaaRtttggggaaaaccaaacatctcaYattgtca

fava_c17279_1179	fava_c17279	1179	A/G	agatattcatgaagttccaagacgagttagtRgagacaatggcaaatcctgctaccaaaattgatgatgcaggaactatcaccacttaccgggtcgccaa[A/G]tttggggaaaaccaaacatctcaYattgtcacttttaattcttctgagatgaaagctagttgcagctgtcagatgtttgaatattcaggaatcatttgca

fava_c17279_1203	fava_c17279	1203	C/T	agttagtRgagacaatggcaaatcctgctaccaaaattgatgatgcaggaactatcaccacttaccgggtcgccaaRtttggggaaaaccaaacatctca[C/T]attgtcacttttaattcttctgagatgaaagctagttgcagctgtcagatgtttgaatattcaggaatcatttgcaggcacgtattagcagtcttcagag

fava_c17279_1326	fava_c17279	1326	G/A	agatgaaagctagttgcagctgtcagatgtttgaatattcaggaatcatttgcaggcacgtattagcagtcttcagagctaaaaatgttcttactcttcc[G/A]ccttattatgtgttgaaacgctggacaaggaatgcgaaaactggtgtgttgttagacctacgcgcatctgaactgcctagtagttctcgtgagtctacga

fava_c17279_1582	fava_c17279	1582	C/T	taaatcagtccaaacatatcatgttgcaatgaaagctttgcaagaagctgctaataaggtttgtattctgaaaaaagagagcactggaacagcagaggga[C/T]ctacaatagcgaacggaggcaggagtgagttgcttacaggagatgaagatgtgtcaagctatcaatctgtggtggagaagcaaaagaaaatccgagagct

fava_c17279_786	fava_c17279	786	C/T	ttgaaactgaattcatcaaatgcgtccatcacagtgaaactattgatgagtttgagtcttgttggcactctctattggataggtactgtatcatggataa[C/T]gaatggcttcactcaatttacaatgcRcgRcaccattgggtcccKgtctacttgcgcgacactttttttggagatgtatctttaaatgctggaactgaat

fava_c17279_813	fava_c17279	813	A/G	atcacagtgaaactattgatgagtttgagtcttgttggcactctctattggataggtactgtatcatggataaYgaatggcttcactcaatttacaatgc[A/G]cgRcaccattgggtcccKgtctacttgcgcgacactttttttggagatgtatctttaaatgctggaactgaatgtttgaactttttctttgacggacatg

fava_c17279_816	fava_c17279	816	A/G	acagtgaaactattgatgagtttgagtcttgttggcactctctattggataggtactgtatcatggataaYgaatggcttcactcaatttacaatgcRcg[A/G]caccattgggtcccKgtctacttgcgcgacactttttttggagatgtatctttaaatgctggaactgaatgtttgaactttttctttgacggacatgtga

fava_c17279_831	fava_c17279	831	T/G	atgagtttgagtcttgttggcactctctattggataggtactgtatcatggataaYgaatggcttcactcaatttacaatgcRcgRcaccattgggtccc[T/G]gtctacttgcgcgacactttttttggagatgtatctttaaatgctggaactgaatgtttgaactttttctttgacggacatgtgaatgcatccaccacat

fava_c17282_1493	fava_c17282	1493	T/C	aacgcttgcttgagggtaaaggtaagagaagtagtcaacggctgtaatgaatgagttccatgggaataagtaaccgattccaagtgtgaagtagattata[T/C]atgctaagttccatgtgtcttgtggtggttctttctttgtacttgaatatgaaggtagaagaagggttgattctatgattttgttgctgttattgtcttg

fava_c17282_533	fava_c17282	533	G/A	ctgcaacttcacagttttaggagccaaaatcatcaaaacactagtcagatatccatttgtaagccctaaaagacaagtcagaattgtcaccggaatctcc[G/A]tccggaagaatttcggaccgcgcaagcatccaaagaaaagagggaaaaacaacagtcttgctatgcaacagcctactgcaatttttgcattctctaataR

fava_c17282_633	fava_c17282	633	A/G	Rtccggaagaatttcggaccgcgcaagcatccaaagaaaagagggaaaaacaacagtcttgctatgcaacagcctactgcaatttttgcattctctaata[A/G]gtgtactgcagtaagactcttgccaacaaagtcgaacacattaaagcaggtaatcaagagaatcggataccaatcgttaagaaccttagagtggacatcc

fava_c17282_878	fava_c17282	878	G/A	aagcgagatgccaaatccataccacttgattgttcttacagtatcccatacagttgacctccatacaaatccggtcaaaggaccggtctcttcctcggca[G/A]cgattgcttgaatcttaagttccttatagtacttcataacgggaagtttttgtgctagattgtataaaactatacatattaacataatcacaattgatac

fava_c17288_1120	fava_c17288	1120	G/A	ttgaccaggaaggcatatgggagatctgggagaagtaagaatgaagagcgacaccgacgatgcaggcgcgtttggtcgatttaacagcgagatgaggttc[G/A]agaggaggagagataacgccRacggatggagtagggtttggggaaagggttttacggaggggagtatggtagatggagggaatggacagatcggggatgg

fava_c17288_1141	fava_c17288	1141	G/A	agatctgggagaagtaagaatgaagagcgacaccgacgatgcaggcgcgtttggtcgatttaacagcgagatgaggttcRagaggaggagagataacgcc[G/A]acggatggagtagggtttggggaaagggttttacggaggggagtatggtagatggagggaatggacagatcggggatggtaacaaggagaggcttaccgg

fava_c17288_349	fava_c17288	349	C/T	aatagcacaaccagaaccacgagataattttactgagaaacgaaattaaaacagacaactaatctacaacataaatatacataaacttaaaaacaataac[C/T]acggggcaagaacccacagctataaagaatctataatccaaacaggaactatgcttacagctagagtataaagctagagcagataaaacaatatcRaaac

fava_c17288_445	fava_c17288	445	G/A	taacYacggggcaagaacccacagctataaagaatctataatccaaacaggaactatgcttacagctagagtataaagctagagcagataaaacaatatc[G/A]aaacactagtacccatatactatacagagatacagcaagggttatatcttatSttataatcatcactttgaaactagaagcatttccggtttcttggYtt

fava_c17288_498	fava_c17288	498	C/G	ctatgcttacagctagagtataaagctagagcagataaaacaatatcRaaacactagtacccatatactatacagagatacagcaagggttatatcttat[C/G]ttataatcatcactttgaaactagaagcatttccggtttcttggYtttggtaggtgtgttgacatttgaagcagcagcagaagaagctgatttcgcaRca

fava_c17288_543	fava_c17288	543	T/C	tcRaaacactagtacccatatactatacagagatacagcaagggttatatcttatSttataatcatcactttgaaactagaagcatttccggtttcttgg[T/C]tttggtaggtgtgttgacatttgaagcagcagcagaagaagctgatttcgcaRcatccctggtcctgttatcaagctgaatgagacttcttgtagcaagt

fava_c17288_596	fava_c17288	596	A/G	atSttataatcatcactttgaaactagaagcatttccggtttcttggYtttggtaggtgtgttgacatttgaagcagcagcagaagaagctgatttcgca[A/G]catccctggtcctgttatcaagctgaatgagacttcttgtagcaagtattgcttgaggcactaaatcatgagaagctcttgcatacaaacacctaacagc

fava_c17293_273	fava_c17293	273	G/T	tctaatgaaacccttgttggttaactgtaaccacaatacatccaccactatacccctcttccattttttttatgtttgccctagtcctaaagaaacatcc[G/T]acagtaaataaagaaacaaggaaatgaaagggtgRgagagcaaaaaggaacacaggaaagaacagtgcaatcttcgcccRagggagatgaaagaaatgca

fava_c17293_308	fava_c17293	308	G/A	atacatccaccactatacccctcttccattttttttatgtttgccctagtcctaaagaaacatccKacagtaaataaagaaacaaggaaatgaaagggtg[G/A]gagagcaaaaaggaacacaggaaagaacagtgcaatcttcgcccRagggagatgaaagaaatgcatcagtaaacctccatctaaaagggagaacagcttg

fava_c17293_353	fava_c17293	353	G/A	ctagtcctaaagaaacatccKacagtaaataaagaaacaaggaaatgaaagggtgRgagagcaaaaaggaacacaggaaagaacagtgcaatcttcgccc[G/A]agggagatgaaagaaatgcatcagtaaacctccatctaaaagggagaacagcttgtctagtgacaaagaccactttctttctagagttaggttgcgtgat

fava_c17293_459	fava_c17293	459	G/C	gatgaaagaaatgcatcagtaaacctccatctaaaagggagaacagcttgtctagtgacaaagaccactttctttctagagttaggttgcgtgattgact[G/C]gattctctccaagcagatcatcatcRtaggaaacaggtatccttaatctatcatgaacagatatagacttttcttYggaMgtaactggttcatgttgatt

fava_c17293_485	fava_c17293	485	G/A	ccatctaaaagggagaacagcttgtctagtgacaaagaccactttctttctagagttaggttgcgtgattgactSgattctctccaagcagatcatcatc[G/A]taggaaacaggtatccttaatctatcatgaacagatatagacttttcttYggaMgtaactggttcatgttgatttcgtatttctgatgcatgaaatgcac

fava_c17293_535	fava_c17293	535	T/C	tagagttaggttgcgtgattgactSgattctctccaagcagatcatcatcRtaggaaacaggtatccttaatctatcatgaacagatatagacttttctt[T/C]ggaMgtaactggttcatgttgatttcgtatttctgatgcatgaaatgcactgttggaaacatttccctgatcactgccagcaaatgaaaaggtagctcct

fava_c17293_539	fava_c17293	539	A/C	gttaggttgcgtgattgactSgattctctccaagcagatcatcatcRtaggaaacaggtatccttaatctatcatgaacagatatagacttttcttYgga[A/C]gtaactggttcatgttgatttcgtatttctgatgcatgaaatgcactgttggaaacatttccctgatcactgccagcaaatgaaaaggtagctccttgag

fava_c17295_104	fava_c17295	104	T/C	ccaaaaaaacaaattaaaatacatcgagcctgcaaaacttgagttacccctttcgtttaacctaaccccagacattgaaatcaagcgcgacgtaggtaca[T/C]tgagatactcaYcatagagagtagttgaggtWtattctacccatatactgatggaacgtccaaatcatgattatgcagccgcgtctgccatggcatatgc

fava_c17295_116	fava_c17295	116	C/T	attaaaatacatcgagcctgcaaaacttgagttacccctttcgtttaacctaaccccagacattgaaatcaagcgcgacgtaggtacaYtgagatactca[C/T]catagagagtagttgaggtWtattctacccatatactgatggaacgtccaaatcatgattatgcagccgcgtctgccatggcatatgctcagcagcagcg

fava_c17295_136	fava_c17295	136	T/A	caaaacttgagttacccctttcgtttaacctaaccccagacattgaaatcaagcgcgacgtaggtacaYtgagatactcaYcatagagagtagttgaggt[T/A]tattctacccatatactgatggaacgtccaaatcatgattatgcagccgcgtctgccatggcatatgctcagcagcagcgacccgctgcaaatatgcaac

fava_c17295_1828	fava_c17295	1828	C/T	gtctcattcctggaatggttagaaagatgcagatcggaagtggtgtcccatattctcctatgagccctttggatatcccaactatgataccaccatcaac[C/T]gtaccagcatcagaaattcttcaaagggtgtcgaagttttttaaagaaattggagaagtaaatccttctgagggtcctatgagttctgatgcgagagatg

fava_c17295_1929	fava_c17295	1929	A/G	gtaccagcatcagaaattcttcaaagggtgtcgaagttttttaaagaaattggagaagtaaatccttctgagggtcctatgagttctgatgcgagagatg[A/G]agatgatgaatacgatggagaatatgagagagaatctcaagtacgaaagggaggtgcttgcatacctccacccgcaaacttgcagcaaattgatccagaY

fava_c17295_2047	fava_c17295	2047	C/T	gagaatatgagagagaatctcaagtacgaaagggaggtgcttgcatacctccacccgcaaacttgcagcaaattgatccagagacagggacctatgctga[C/T]gggagtgtagacagaaagccaggatcaagtggctccggaagattgggacttggggccactgctgatccaaatgaagtgagtcaatatgatgatgtatata

fava_c17295_2331	fava_c17295	2331	G/A	gtttcgcattgttggatcacttgggtgtttagactaggtcacgttggccccgtctttgttgatgtttatctgaaactgatatgtttgtttgttatgatcc[G/A]atgttctttgtcatgattcatgaatgattggatgtatctttctttgatggaaactgaagttcctgcaaacttgaaacaacactaggtgatgggataacag

fava_c17296_1682	fava_c17296	1682	G/A	ttgatgccgctaaaggaacttatgttgatatggtgaaggccggaattatagatcctgttaaagttgttagaacagctttggtagatgctgccagtgtatc[G/A]ttgttactgacgacaaccgaagcatctattgtggaaaatatgagtgataaaaacaaacctcctcaaagggtggcggatatggatgatttggactactaga

fava_c17299_1095	fava_c17299	1095	G/A	gctatagagaagatcttttcaaagacgataacaacaacaacaacaacgaaagcggtggcgatggttcttcttctccaaaaaatttcaaaacaaatcacca[G/A]caagacggtatgggtgaaggagaaggtggtggagttggagcctcttcatcttcttctcctggtaattctatttctggacttcttcgaccgtcgaatatgt

fava_c17299_618	fava_c17299	618	C/G	catccacagccgtcacggtagcaaatcctcccaaacgatccaccaaggacagacatactaaagtcgatggccggggacgaagaattcgaatgccggcgac[C/G]tgtgcagccagagtttttcagttgaccagagaattaggtcataaatccgacggtgaaacaattgaatggcttctccagcaagcggagccggctattattg

fava_c17306_1874	fava_c17306	1874	C/T	ggccattactaataaagctgagtcattaaaattggtccttcctctcacaaaagggcagatagttgaaccaggttttggaaccttggcagaaatagatggg[C/T]gtttggtagcggtcgggtctctagaatgggttcgtgaacgtttcaagactaaaatgaacccatctggtttggtgaatcttgaacttgctttgataaatca

fava_c17307_1025	fava_c17307	1025	C/A	aacttgcccaggctgcgcagcctctggtttaggtgcgggctctcctccgggctgttcaaagtccctaaagatagactccccgcttatgcccggggaattt[C/A]tttcgagcgcaccaggaagccgctgcccgactccctgagaaggctgcgaatttatttcttccccaggttcggcggtattttgaaaccagccagagagacc

fava_c17307_2659	fava_c17307	2659	C/A	ccatccatcccattgcattccatcgtctcgtattgtactgtaccgtaccagatacatctattgagtgagagaaaggtagcttctctatagatatattatt[C/A]attttttctattgatagggatccccagattcccgtcactacggattgattgtctctacctaactacatggtagtggtctagggagcgcaattgctagagc

fava_c17309_1031	fava_c17309	1031	T/C	gcctgtaaaaatattagattggccattgttttattttgtaatacttttctgaattttacatgagacaatcctagtttgttagtgatgtataacttaaaaa[T/C]tcagaacatgtatttgttatagttaagtatagagaaagctcaatgttaatacaaaattgtacttatggtccatggagttcgacatatttatcagattggt

fava_c17309_168	fava_c17309	168	T/C	gaactcaattgtgataattaaggaaactgcattattgctcaatcaactaatatagcaaaagcttttgcacttataccaagcttccacctagccaaaaaat[T/C]tcattcatgaagtttctttttggaaaagaagtgttaaagatcaactgatgcaaagtgaatcatgtttgaatcttaggtgttgtgagatattggatattat

fava_c17309_517	fava_c17309	517	G/A	attagaattatatagttgaaaaccctataagaataagttacatgaatgaattacctggttctgtgggcacaggtgtcagcttctgtctgagattaggcca[G/A]actttgttctcatctgcttcccttctctttatgtctttgggagttgaattctacagctacacggctttctgctatttggtgacaatcatgggtctggtta

fava_c17311_1583	fava_c17311	1583	C/T	attttcaagagacgagacaactatttattttttttgaaaggatcgagactgtttttatttccaggctaagcttggtattgagctccttgtcatacatttt[C/T]ggaatcttttttattcgatttgtacatttgaagcctgaaaattcatgatgatacaattgatatgttaataatgtcatgtactatatttatgtatttgatg

fava_c17311_694	fava_c17311	694	T/C	agcgtgttaatagagggatgaagattgaagaattcaagttcatatattggatggaatatggacatcgtatgtgggggagggcattgggattaatgtttgc[T/C]ttaccgtattcatattttctccataaagggtatattacWgttagattaggactgagactctctgttttgtttggSctgggtgccgggcagggtctcatcg

fava_c17311_733	fava_c17311	733	A/T	tcatatattggatggaatatggacatcgtatgtgggggagggcattgggattaatgtttgcYttaccgtattcatattttctccataaagggtatattac[A/T]gttagattaggactgagactctctgttttgtttggSctgggtgccgggcagggtctcatcggctggtggatggttaaaagtggtttagaggaaccagcaa

fava_c17311_769	fava_c17311	769	C/G	ggagggcattgggattaatgtttgcYttaccgtattcatattttctccataaagggtatattacWgttagattaggactgagactctctgttttgtttgg[C/G]ctgggtgccgggcagggtctcatcggctggtggatggttaaaagtggtttagaggaaccagcaactgaatattctcagccaagagtaagcccttatcgtc

fava_c17313_148	fava_c17313	148	G/C	atcatttccctctctctctgcgcgcgccgctgcttctcttcgtttcgaagcaacgatggagttattcgaaggagaccaactgaaggctgcagagaagctc[G/C]agtcggagaaattagctaagatcaatttcaaccctaYgcgcatagttgatttagatgcacttctttgtgatacctatgataaacaatgtccaaagccagt

fava_c17313_185	fava_c17313	185	C/T	cttcgtttcgaagcaacgatggagttattcgaaggagaccaactgaaggctgcagagaagctcSagtcggagaaattagctaagatcaatttcaacccta[C/T]gcgcatagttgatttagatgcacttctttgtgatacctatgataaacaatgtccaaagccagttcattatgacaacaggagagatttgattcgcatcttc

fava_c17314_552	fava_c17314	552	C/A	ccatgacacaaaacaaggctatcttgtgaacaacacatgcatcattgaagctcatatcaatgttcaagcttcatcacaaatagaatcctcaaaacaagaa[C/A]aaggtgttatagaaacatcagatgaaagtagtacaMcagaatcatttgactccaatgaaacaggaacaccagatttgacactaagggacctcttagattt

fava_c17314_588	fava_c17314	588	A/C	atgcatcattgaagctcatatcaatgttcaagcttcatcacaaatagaatcctcaaaacaagaaMaaggtgttatagaaacatcagatgaaagtagtaca[A/C]cagaatcatttgactccaatgaaacaggaacaccagatttgacactaagggacctcttagatttggagagtttaggcaatgaagaagcaagttatgttcc

fava_c17315_1045	fava_c17315	1045	T/G	tatacctccctagaagtccctgaatggagggctgtgatcaagtccgagtccacagctgatatagcggaaatctacggtgagctagaatactaccaattaa[T/G]taaatcagattcccacaaagggtatagataatgttgttttgtaRcataaattYttttcaagacactacccaatttttcaagacactaRagtttgagatta

fava_c17315_1089	fava_c17315	1089	G/A	cgagtccacagctgatatagcggaaatctacggtgagctagaatactaccaattaaKtaaatcagattcccacaaagggtatagataatgttgttttgta[G/A]cataaattYttttcaagacactacccaatttttcaagacactaRagtttgagattaacctacatgtttatgatggagggagctagctagacaaagccaac

fava_c17315_1098	fava_c17315	1098	T/C	agctgatatagcggaaatctacggtgagctagaatactaccaattaaKtaaatcagattcccacaaagggtatagataatgttgttttgtaRcataaatt[T/C]ttttcaagacactacccaatttttcaagacactaRagtttgagattaacctacatgtttatgatggagggagctagctagacaaagccaacatctgatag

fava_c17315_1133	fava_c17315	1133	A/G	actaccaattaaKtaaatcagattcccacaaagggtatagataatgttgttttgtaRcataaattYttttcaagacactacccaatttttcaagacacta[A/G]agtttgagattaacctacatgtttatgatggagggagctagctagacaaagccaacatctgatagagaatttgttcatgaaactgtaattttattttcat

fava_c17315_347	fava_c17315	347	T/A	tcaacgaactccttggatccattgattttgtggagaaagggacggaaagtgttgtctttagaacctgtgatcacgaaaaagataaagttgtgtttcaaat[T/A]ggcacatctgatgctgccagggccctaactgctgctcagttggtgtgtaatgatgttgctgcMatagatgttaacatgggatgccccaagccgttttctt

fava_c17315_410	fava_c17315	410	A/C	cctgtgatcacgaaaaagataaagttgtgtttcaaatWggcacatctgatgctgccagggccctaactgctgctcagttggtgtgtaatgatgttgctgc[A/C]atagatgttaacatgggatgccccaagccgttttctttgagtggtgggatgggtgctgctctgttgtccaaaccagagcttattagtgatatcttaacaa

fava_c17315_855	fava_c17315	855	G/A	acgcattaaatctgctacaggtgcctcgtcggtgatggctgcaagaggggcactttggaatccttcaatattttcaccagaaggagaagtttcttacgag[G/A]ttacacaaaaaSaatatattagaaagtgcatcttgtgggataatgatatYagaagcaccaaacatacattaagagatatgaagtcacattatacctccct

fava_c17315_867	fava_c17315	867	C/G	tgctacaggtgcctcgtcggtgatggctgcaagaggggcactttggaatccttcaatattttcaccagaaggagaagtttcttacgagRttacacaaaaa[C/G]aatatattagaaagtgcatcttgtgggataatgatatYagaagcaccaaacatacattaagagatatgaagtcacattatacctccctagaagtccctga

fava_c17315_905	fava_c17315	905	T/C	cactttggaatccttcaatattttcaccagaaggagaagtttcttacgagRttacacaaaaaSaatatattagaaagtgcatcttgtgggataatgatat[T/C]agaagcaccaaacatacattaagagatatgaagtcacattatacctccctagaagtccctgaatggagggctgtgatcaagtccgagtccacagctgata

fava_c17317_1004	fava_c17317	1004	T/C	gattcttcacatgggatgttttggacaagcttggtgcagatacatttggatctctgcatcttaaaccggatggcatgtttccgaatcatattccaaaccc[T/C]gaggacaaaattgccatggcaatgacacgagcagcagtgttagaaaactcggccgatcttggaatagtctttgataccgatgttgacagaagcggtgtag

fava_c17317_1499	fava_c17317	1499	C/A	tggtggtgaaaattataattgaaatggtgagaatgaagcttggtggatccgatgaaggaattggtagtcttattaaagatcttgaagagccttgtgaatc[C/A]atagaattaaggataaatatcatttctgaacctcgagacgctaaagcaaaaggtctcgaggcaatcgaaacatttcgaaactacatcgaggaaggcatgc

fava_c17317_1714	fava_c17317	1714	T/C	ttagattcatgtggtgattgttgggttagtgaagggtgccttgttgatacaaatgatactccagctcacattgatgctcaaatgtacagggcaagagtat[T/C]aaatgatgaacatggacaacatggttggatacacatgagacaaagtattcacaatccaaacattgctgtgaacatgcaatcatctgtccaaggagggtgt

fava_c17317_250	fava_c17317	250	T/A	tctttgcacaacaaacttggtttctcatcttttatgcaattcaataaatttgtaaaaagtttgaaatcttatggagctgctaagtatgatggagtggtgg[T/A]ggatgaagaattggacaagattcggagacttcagaatggttcagatgttagaggagtcgcgttggaaggtgagaaagggagaacggttgaccttactccg

fava_c17317_419	fava_c17317	419	A/C	gtgagaaagggagaacggttgaccttactccgcctgctgtggaggcaatatcagagagtttcggcgagtgggttatcaaaggcttagagaaggaaaaagg[A/C]tatctggtagagaatgtgagcgtgtctcttggacgcgacccgcgaattacagggtctaaactgagcgttgcagtgtttgcaggtcttactcgtggtggtt

fava_c17318_1188	fava_c17318	1188	C/T	ctttcagctccgcttcattttttatggcctttgctagagaaacaggggataatttctggacggcaatgggtacatccgagttctcatttgattttttgtt[C/T]ctagtcttgtgcttactctcgtagttctgataatgtttgttgcaggcagctttgtaggcatttacaatagcagcattaatagaggaagtatccaaccaaa

fava_c17318_1297	fava_c17318	1297	C/G	gtgcttactctcgtagttctgataatgtttgttgcaggcagctttgtaggcatttacaatagcagcattaatagaggaagtatccaaccaaagggcggca[C/G]cRcgtgctgccaaattttcaatctctgatataatcgaattgtatggcctgatctctatgtttgctttcttcaagtggtcatccacctcttcagtaacttt

fava_c17318_1299	fava_c17318	1299	G/A	gcttactctcgtagttctgataatgtttgttgcaggcagctttgtaggcatttacaatagcagcattaatagaggaagtatccaaccaaagggcggcaSc[G/A]cgtgctgccaaattttcaatctctgatataatcgaattgtatggcctgatctctatgtttgctttcttcaagtggtcatccacctcttcagtaacttttg

fava_c17318_1611	fava_c17318	1611	A/G	caagctcggagacaataattgcagatgaaccagccttgataagttcggacctcagagttgacagcttcgacgcaacatctaaaccagcatactttaagtc[A/G]tgcactctaaccggcttaWttggagattctggcctagcttctttccatatttcatccacaagatttgaattgtacaaataaacaagctcatggtttttct

fava_c17318_1630	fava_c17318	1630	A/T	tgcagatgaaccagccttgataagttcggacctcagagttgacagcttcgacgcaacatctaaaccagcatactttaagtcRtgcactctaaccggctta[A/T]ttggagattctggcctagcttctttccatatttcatccacaagatttgaattgtacaaataaacaagctcatggtttttctgagaaatgacctgcttaag

fava_c17318_2051	fava_c17318	2051	C/T	ccagtaaaaccagatatgtattctcgtctagcgtaacattcagcaatgaactcgctctggtgagcgtcttgagaagggatgatgtatgcgtcaatggaga[C/T]atcgggtttgaagaagagttggcggagggcagtgagtttgaggtcgggggtggagtttgggggacggtttttgcggaagtgggaagagggtttggctgtg

fava_c17318_882	fava_c17318	882	G/A	ttcgtgtaatgtcggttgtaccatcaacatattgagcaccactatccaataagaataatttattggcatccacaatactacaactctctggttctggttt[G/A]tagtgtattatagcaccgtttggaccggaaccacttatcgtgtcaaagctagtatccaagaaaccagcttgttttgtgcgaaactcaataagtttgtctg

fava_c17319_456	fava_c17319	456	T/G	acattgttcatgtggtctaagagtttggcaatgaaggatagacatattcatgaggagaaagggttacgccaataagattgaagaccttgggattgttaat[T/G]ataatgcaaagaagattttaatttcgatttcatgaaaatatttgtgtatagtgcagtgattttgtggaacaatgtattggtcagaagggatctaattatc

fava_c17321_269	fava_c17321	269	G/A	cttgccctaacagagtctctacctataaactacagatatccaattgcttgcaaagagctaagctttattctcagggaagcttttcatcaattcccaaaga[G/A]tttgcaaaccatcgttttccaagatactctctcttccttccgtctccttccacaaatgcagactgagaatgctgtttccgccgtccatttactcctccaa

fava_c17321_961	fava_c17321	961	C/T	taccaaacctgtaacgcaattccaggttgtggaatatcttctacatgattctctatccatagcatcttcttcagacagtgacactgattcagaaggagga[C/T]cagtttctaagttttgggatatacatttaagaaaatactagggaagcagcttttacattaactcagatctatgttcattctattcttgcgtgatatttta

fava_c17322_164	fava_c17322	164	C/A	atattttctcagatgctggacctgatgttagatgggtaggaaatgaacaagggatggcgggggacacgtgttggtctaccattaatcgaagttctcttgc[C/A]attggaagtgcggRcatagagcagtatttaaacacgggcgatccaagaggaactgattggctaccagcagaatgcgatgtttcaattcgtaaaggatggt

fava_c17322_178	fava_c17322	178	G/A	gctggacctgatgttagatgggtaggaaatgaacaagggatggcgggggacacgtgttggtctaccattaatcgaagttctcttgcMattggaagtgcgg[G/A]catagagcagtatttaaacacgggcgatccaagaggaactgattggctaccagcagaatgcgatgtttcaattcgtaaaggatggttttggcacaaatca

fava_c17322_356	fava_c17322	356	T/C	aaggatggttttggcacaaatcagaatcaccaaaaaaactaagtgagctacttgatatttattacgcatcagttggtagaaattgcgtgttaaatctcaa[T/C]gtaccacctaacacaaccggtcttatatctgaaaatgatgctMgtagattaaaagaatttagaaSagcaatcaatacaRtttttcacaagaacatagcta

fava_c17322_399	fava_c17322	399	C/A	tgagctacttgatatttattacgcatcagttggtagaaattgcgtgttaaatctcaaYgtaccacctaacacaaccggtcttatatctgaaaatgatgct[C/A]gtagattaaaagaatttagaaSagcaatcaatacaRtttttcacaagaacatagctaaaggttgttatgttaaagttagtaStcaaagaggaggaaaaga

fava_c17322_421	fava_c17322	421	C/G	gcatcagttggtagaaattgcgtgttaaatctcaaYgtaccacctaacacaaccggtcttatatctgaaaatgatgctMgtagattaaaagaatttagaa[C/G]agcaatcaatacaRtttttcacaagaacatagctaaaggttgttatgttaaagttagtaStcaaagaggaggaaaagagggagggtttggaccagaaaac

fava_c17322_435	fava_c17322	435	A/G	aaattgcgtgttaaatctcaaYgtaccacctaacacaaccggtcttatatctgaaaatgatgctMgtagattaaaagaatttagaaSagcaatcaataca[A/G]tttttcacaagaacatagctaaaggttgttatgttaaagttagtaStcaaagaggaggaaaagagggagggtttggaccagaaaacatgttggatRgtga

fava_c17322_481	fava_c17322	481	G/C	atatctgaaaatgatgctMgtagattaaaagaatttagaaSagcaatcaatacaRtttttcacaagaacatagctaaaggttgttatgttaaagttagta[G/C]tcaaagaggaggaaaagagggagggtttggaccagaaaacatgttggatRgtgaccatttgtggtcatactgggccccaagggaagatgataagaaaaag

fava_c17322_531	fava_c17322	531	A/G	tacaRtttttcacaagaacatagctaaaggttgttatgttaaagttagtaStcaaagaggaggaaaagagggagggtttggaccagaaaacatgttggat[A/G]gtgaccatttgtggtcatactgggccccaagggaagatgataagaaaaaggaggaccattgggttgaaatttgggacaatgatggagatttaaggtttaa

fava_c17322_786	fava_c17322	786	G/A	aatttatgtggatggtaaattgttgatccaagggacaacagttggttacaagaggcttcataggcttgatggagatgttgtgcatgctcaagttgtgagg[G/A]ttagattcatagaggctagaggagttcctcttatttcttctattggtttgtattttgatcctttttggcattcaaggtttaaggcggcataaagaagata

fava_c17325_1640	fava_c17325	1640	G/T	aagacacatatttcgagaggctgttgtatgtggtctttaccggagcgggaaaatatacttccacccaaatgattacgagactctgcagaaaactgacaaa[G/T]tattgttcattggatcccttcagtatactaaaaggccacaagttgtaatcccgaaggtgaaagaaggaaaagatggaattcataatgacgaattacttga

fava_c17325_1918	fava_c17325	1918	T/G	ccaaggcatcggacgggaatgtaggaccaaaagaatgcattcttttagtagggtggcgacctgatgttgtagaaatgattcaagagtatgataactatct[T/G]ggtcctggaagtgttttggaagttttatcagagacgccattaaatgacaggattactagagaagccaacatcattggtcgtagcaaactcaagaatgttc

fava_c17325_2296	fava_c17325	2296	G/A	tacttgcagaaaatatctgcgacaaacttggaatcaaggtgcaaaatctagttgcagaaattgtcgattcaaagttggggaaacaaattgctagaatcaa[G/A]ccatcggtgacctacgttgcagccgaggaaatattgagccttgtaactgctcaagttgccgagaacagtgaactgaatgaagtttggaaggatatactaa

fava_c17325_2464	fava_c17325	2464	G/A	gtgaactgaatgaagtttggaaggatatactaaacgctgagggagatgaaatatatttgaaggatattagcctttacatgaaagaaggagagaatccatc[G/A]ttttccgagctttctgagagagcgtatttaaggagagaagtagcgataggttatgtgaagaagaacaagaatatgatcaacccaattccaaagtcagaac

fava_c17325_251	fava_c17325	251	T/C	atcatcattatcatcatcatcaatcaacaagaattccaggaggtttatgccttgtcatttcggttggctcaagtcttcaccattacatggacatggggga[T/C]gggaggttgactctcacagactagcttttaagtccaattccaacactagtaaacttgttccagaaagttcgagcRccaagttcattttgacagagaaaaa

fava_c17325_2571	fava_c17325	2571	C/T	gagctttctgagagagcgtatttaaggagagaagtagcgataggttatgtgaagaagaacaagaatatgatcaacccaattccaaagtcagaacccctct[C/T]tcttgaattaaccgactcattgatagtcatttcggagctggaaggtgaacaacccatcattttgtaatactgaggctttaatcattctgagaagagaata

fava_c17325_2720	fava_c17325	2720	G/A	acaacccatcattttgtaatactgaggctttaatcattctgagaagagaatagtgtgtaggaaaatgctgacccctaaatttttgttattgcactgtggc[G/A]atttggatgaatgttattcagaataaattgtaacaaaatgatacttaaatgtaaaatatggaaatggaaacttaatttactttcttgcactaccaaaacg

fava_c17325_326	fava_c17325	326	A/G	ttcaccattacatggacatgggggaYgggaggttgactctcacagactagcttttaagtccaattccaacactagtaaacttgttccagaaagttcgagc[A/G]ccaagttcattttgacagagaaaaataatcatagttcSctgcatctacaggctaaacacatgctagaatacatgccgttgtacttggtactgaaactgac

fava_c17325_364	fava_c17325	364	G/C	ctcacagactagcttttaagtccaattccaacactagtaaacttgttccagaaagttcgagcRccaagttcattttgacagagaaaaataatcatagttc[G/C]ctgcatctacaggctaaacacatgctagaatacatgccgttgtacttggtactgaaactgacacatacaaagtttgttgatctggtgataaacgttgtcc

fava_c17328_1596	fava_c17328	1596	A/G	atgaagatggacaagatatggtagacatgaatcatatagctcatgcagtacagaaacaacggagtgcatccattagtgagtttgagatttttcatgatag[A/G]Rgttcaaatgtttggagtgtagttggatctggattgcaacgttttgttcagatgacaaattggcggtatgttgattcagagaaaagattccaaaatgttc

fava_c17328_1597	fava_c17328	1597	G/A	tgaagatggacaagatatggtagacatgaatcatatagctcatgcagtacagaaacaacggagtgcatccattagtgagtttgagatttttcatgatagR[G/A]gttcaaatgtttggagtgtagttggatctggattgcaacgttttgttcagatgacaaattggcggtatgttgattcagagaaaagattccaaaatgttct

fava_c17328_1877	fava_c17328	1877	G/A	atgatatggcatgactcctcaaacaaaaccagtgcatcaaagatgaagatatccacagattcaattaagtggcctgaacagaagtaatgccactctatct[G/A]ggtgcttctagatccatgttgcaactaaacgatagttcatgtaggaagcaagtttggtcaaaatgtgatgatatagacgagggcaaaagttgtgaaaagc

fava_c17328_2059	fava_c17328	2059	T/C	ggcaaaagttgtgaaaagctgcctcgagaagcacaacgttaaatcatacaaattacaaacttgttcttgatagaatgatgctagttgcattcacaatttt[T/C]caaattcttttatttgccaggaagctgaggtcttcaactgYgcacaaKtgagttaagcaagRtacagttgtaatcgtaagggtgtattatttgtaaccaa

fava_c17328_209	fava_c17328	209	T/C	cgcacYaaaactaaacctcctccaaaccctaaactcaacaccaagcatacatctccgcctgaaccgctactcctctcagccgccgaacccaccacctaca[T/C]ccgtctccctctcaaagaagactattcctcRYccttctctccttcctcccccgaaatcaaactctccgaatcagcaccaattcaccaatttctccacaaa

fava_c17328_2100	fava_c17328	2100	C/T	aatcatacaaattacaaacttgttcttgatagaatgatgctagttgcattcacaattttYcaaattcttttatttgccaggaagctgaggtcttcaactg[C/T]gcacaaKtgagttaagcaagRtacagttgtaatcgtaagggtgtattatttgtaaccaaaggaagaRaatctagtttcttgtgtaacttatgatggtgtg

fava_c17328_2107	fava_c17328	2107	T/G	caaattacaaacttgttcttgatagaatgatgctagttgcattcacaattttYcaaattcttttatttgccaggaagctgaggtcttcaactgYgcacaa[T/G]tgagttaagcaagRtacagttgtaatcgtaagggtgtattatttgtaaccaaaggaagaRaatctagtttcttgtgtaacttatgatggtgtgttgtaac

fava_c17328_2121	fava_c17328	2121	A/G	gttcttgatagaatgatgctagttgcattcacaattttYcaaattcttttatttgccaggaagctgaggtcttcaactgYgcacaaKtgagttaagcaag[A/G]tacagttgtaatcgtaagggtgtattatttgtaaccaaaggaagaRaatctagtttcttgtgtaacttatgatggtgtgttgtaacaattttgttattag

fava_c17328_2167	fava_c17328	2167	G/A	ttttatttgccaggaagctgaggtcttcaactgYgcacaaKtgagttaagcaagRtacagttgtaatcgtaagggtgtattatttgtaaccaaaggaaga[G/A]aatctagtttcttgtgtaacttatgatggtgtgttgtaacaattttgttattaggtgtaagatgtcccatattgtctcatcattatatttttacaggatg

fava_c17328_2283	fava_c17328	2283	C/G	gtaacttatgatggtgtgttgtaacaattttgttattaggtgtaagatgtcccatattgtctcatcattatatttttacaggatgctcttatgtaggttg[C/G]atagttcggtgaattttgagtttcacctgaaatcattgtataagtgttctattttacctcgagtttctaatgtatctgaatagagatgacaattatgcta

fava_c17328_240	fava_c17328	240	G/A	aactcaacaccaagcatacatctccgcctgaaccgctactcctctcagccgccgaacccaccacctacaYccgtctccctctcaaagaagactattcctc[G/A]Yccttctctccttcctcccccgaaatcaaactctccgaatcagcaccaattcaccaatttctccacaaagatgaaattgaagatgatggtgaagagatca

fava_c17328_241	fava_c17328	241	T/C	actcaacaccaagcatacatctccgcctgaaccgctactcctctcagccgccgaacccaccacctacaYccgtctccctctcaaagaagactattcctcR[T/C]ccttctctccttcctcccccgaaatcaaactctccgaatcagcaccaattcaccaatttctccacaaagatgaaattgaagatgatggtgaagagatcaa

fava_c17328_636	fava_c17328	636	T/C	ggaacggatcaatggcattccggcgcgagaaatatgtgccgtttggagggccgtccggaggggatggagggagaggtggtaatgtgtatttggaagttga[T/C]agagcaatgagttcgttgttaccgtttcggaatgggattcattttcgggctgggagggggttacatgggcaaggaaggaagatgattggtgctaaggggg

fava_c17328_815	fava_c17328	815	A/T	aagatgattggtgctaagggggaagatgtggtggtcaaggtgcctcctgggacggtggttagagaggctgggagtgatgtggtgctgttggagatggtgt[A/T]tcatggRcagaaagcgttgttgttgccgggtggtagaggtgggagagggaatgctgcttttaagtctgggaataataaagctcctaagattgcggagaat

fava_c17328_822	fava_c17328	822	A/G	ttggtgctaagggggaagatgtggtggtcaaggtgcctcctgggacggtggttagagaggctgggagtgatgtggtgctgttggagatggtgtWtcatgg[A/G]cagaaagcgttgttgttgccgggtggtagaggtgggagagggaatgctgcttttaagtctgggaataataaagctcctaagattgcggagaatggtgaag

fava_c17329_134	fava_c17329	134	A/T	ttggggggatgattctaatactaattctaatcaaaacaacaatttctcttttttttttccttttgattctaaataggaaaacaaaacacaaaaaaattat[A/T]aaaaaatggaagagatctatcaagaaaaccctcgatttcggatagcaaactaaaaaacgcaccaaaaacttcacggagtttcttgcgaggaagaatccag

fava_c17329_241	fava_c17329	241	C/A	tggaagagatctatcaagaaaaccctcgatttcggatagcaaactaaaaaacgcaccaaaaacttcacggagtttcttgcgaggaagaatccagttataa[C/A]aataaacgaaattgaaaaccaaaaatagaaagaaaacagcaagactataatcgaatcagatgaagaaaaacaaatcgtattttttttcttccagaaatga

fava_c17329_449	fava_c17329	449	C/T	aacaacgaaattttttgtagcaatattttttgttgtttttttttagtggtgatgattgggattgaaaaagaaaagatgataaaagagaatcgaagaaaaa[C/T]gagagcgttagttgagaaatagaatgaagaggaatgaggagaggaaccctaattaagatgagggaatgagaatggaaagggtgcgaaagagaagggaaat

fava_c17330_1216	fava_c17330	1216	G/A	cttattgaattggctcttggattagaagaaaccaaaatgccatttattttggttattagggatggaactaataaaactcatgagttggaaaagtggatca[G/A]tgatgaaaattttgaggaaaggaacaaagggaaaggcctcttaataagagggtgggccccacaRatggtgatattatcacactcctctattggtggattc

fava_c17330_1280	fava_c17330	1280	A/G	gaactaataaaactcatgagttggaaaagtggatcaRtgatgaaaattttgaggaaaggaacaaagggaaaggcctcttaataagagggtgggccccaca[A/G]atggtgatattatcacactcctctattggtggattcttaacacattgtggttggaattcaacacttgaagggataagttttggtgtgccaatgataactt

fava_c17330_1385	fava_c17330	1385	T/A	tgatattatcacactcctctattggtggattcttaacacattgtggttggaattcaacacttgaagggataagttttggtgtgccaatgataacttggcc[T/A]ttatttgctgaccagtttttgaatgagaagcttgtgactcaagttttgaggattggagtgagtcttggtgttgaggttccaatgaagtggggtgaggaag

fava_c17330_1518	fava_c17330	1518	A/C	tgtgactcaagttttgaggattggagtgagtcttggtgttgaggttccaatgaagtggggtgaggaagagaagataggtgttgttgtgaagaaggaagtt[A/C]ttaaggaggtaatatgcaatgtgatgaatgaaggagtagaaagtaaagagagaagagaaagagctagtgaactcagtgagatgactaagagagccgttga

fava_c17330_434	fava_c17330	434	T/C	ttaccattttcactacgccaaagaatgcgtcacgtttttcttctgttctttctcgtgctgtttcctctggccttcaaatcaatcttgtaccactcaattt[T/C]ccaKcWaaacaagctggattaccagacggatgcgagaatttggacatggttgatatttcaaaagatatgatgcatagtttgtttcaagcRatttccttgc

fava_c17330_438	fava_c17330	438	T/G	cattttcactacgccaaagaatgcgtcacgtttttcttctgttctttctcgtgctgtttcctctggccttcaaatcaatcttgtaccactcaatttYcca[T/G]cWaaacaagctggattaccagacggatgcgagaatttggacatggttgatatttcaaaagatatgatgcatagtttgtttcaagcRatttccttgctcca

fava_c17330_440	fava_c17330	440	T/A	ttttcactacgccaaagaatgcgtcacgtttttcttctgttctttctcgtgctgtttcctctggccttcaaatcaatcttgtaccactcaatttYccaKc[T/A]aaacaagctggattaccagacggatgcgagaatttggacatggttgatatttcaaaagatatgatgcatagtttgtttcaagcRatttccttgctccaaa

fava_c17330_524	fava_c17330	524	G/A	cactcaatttYccaKcWaaacaagctggattaccagacggatgcgagaatttggacatggttgatatttcaaaagatatgatgcatagtttgtttcaagc[G/A]atttccttgctccaaaaaccagcagaggaactgtttgatatactgtcgccaaaaccaagttgcattatctctgatttttgcattccttggacttctcgaa

fava_c17333_1346	fava_c17333	1346	G/A	acccctgtctgaaatagatttaaatgatgttgtaaactatgaagagtttttgagaatcttgactaatgaagagcagcagcaattactgaagtttcttccc[G/A]tggttgatactgctaaacttcctgatagccttaaggtcatgttcgagagctctcaattcaaggagaacttaacttattttcagcagcttctttcggaagg

fava_c17333_168	fava_c17333	168	C/T	ttttctctcttcaattggattctacactttctcatcttcatcaatcccttcaattatacttcccatctgtgaatttttctctgaattttaactaaaaaaa[C/T]aaataaataaataaaaatacttttctgggtttattgacttctgtttattctgcaaactatcatttaggtttcctgcagaaaacaaaacccttttaatata

fava_c17334_1038	fava_c17334	1038	G/T	attttaaaccaaaactttctgatttcggactcgctagggagggaccggtagcgggtgatactcatgtttcaactgctgtaatgggaacacatggttatgc[G/T]gctccggattacattgaaacaggccatctcacttccaagagtgacgtRtggagtttcggtgtcgtgctataYgaaatactcaccggWaggcgttcKttgg

fava_c17334_1086	fava_c17334	1086	G/A	tagcgggtgatactcatgtttcaactgctgtaatgggaacacatggttatgcKgctccggattacattgaaacaggccatctcacttccaagagtgacgt[G/A]tggagtttcggtgtcgtgctataYgaaatactcaccggWaggcgttcKttggcaagaaaccgKccRaaaaccgaacaaaaactattggaatgggtgaaga

fava_c17334_1110	fava_c17334	1110	C/T	ctgctgtaatgggaacacatggttatgcKgctccggattacattgaaacaggccatctcacttccaagagtgacgtRtggagtttcggtgtcgtgctata[C/T]gaaatactcaccggWaggcgttcKttggcaagaaaccgKccRaaaaccgaacaaaaactattggaatgggtgaagaactatccatctgatagcaaaaatt

fava_c17334_1125	fava_c17334	1125	T/A	cacatggttatgcKgctccggattacattgaaacaggccatctcacttccaagagtgacgtRtggagtttcggtgtcgtgctataYgaaatactcaccgg[T/A]aggcgttcKttggcaagaaaccgKccRaaaaccgaacaaaaactattggaatgggtgaagaactatccatctgatagcaaaaatttcgacacRataatgg

fava_c17334_1134	fava_c17334	1134	G/T	atgcKgctccggattacattgaaacaggccatctcacttccaagagtgacgtRtggagtttcggtgtcgtgctataYgaaatactcaccggWaggcgttc[G/T]ttggcaagaaaccgKccRaaaaccgaacaaaaactattggaatgggtgaagaactatccatctgatagcaaaaatttcgacacRataatggatccaagac

fava_c17334_1149	fava_c17334	1149	G/T	acattgaaacaggccatctcacttccaagagtgacgtRtggagtttcggtgtcgtgctataYgaaatactcaccggWaggcgttcKttggcaagaaaccg[G/T]ccRaaaaccgaacaaaaactattggaatgggtgaagaactatccatctgatagcaaaaatttcgacacRataatggatccaagacttgaaagacagtatt

fava_c17334_1152	fava_c17334	1152	A/G	ttgaaacaggccatctcacttccaagagtgacgtRtggagtttcggtgtcgtgctataYgaaatactcaccggWaggcgttcKttggcaagaaaccgKcc[A/G]aaaaccgaacaaaaactattggaatgggtgaagaactatccatctgatagcaaaaatttcgacacRataatggatccaagacttgaaagacagtattcaa

fava_c17334_1218	fava_c17334	1218	G/A	tcaccggWaggcgttcKttggcaagaaaccgKccRaaaaccgaacaaaaactattggaatgggtgaagaactatccatctgatagcaaaaatttcgacac[G/A]ataatggatccaagacttgaaagacagtattcaatcaacgcagcgagaaaaatcgcaaagctcgctgatcattgtctgcgtaagagcgcgaaagataggc

fava_c17334_127	fava_c17334	127	G/T	tcctttgttttttccttctaaattctctgatttccgtgacacaaagagaaccaaaaaagcaaagaaacaaaagacaaagttgacaaagtagacaattacg[G/T]gagtgtgaaccttctctttcccttcagcaaatcacaagcaaacctctagaaggtgttgtagcctaatagccatgcttctttgtgttcttctatatacctg

fava_c17334_228	fava_c17334	228	T/C	gagtgtgaaccttctctttcccttcagcaaatcacaagcaaacctctagaaggtgttgtagcctaatagccatgcttctttgtgttcttctatatacctg[T/C]tgattggtgagagtacatgagaatcatagataattgaattgtgtgaatgaaatgaagtgcttttacttcaagtttacactcaagggtaggggtcagagaK

fava_c17336_1224	fava_c17336	1224	G/A	acatggaaatagtgatgcagctatcagcagctgagtttgtctcttaactcctaaaacttgtcattttttataaaaaccggagagctttaccgctcttacg[G/A]tctatttagcYtggcagatttctcacagttgtgtgtagagggttcttagtttatactcaactttttKttgggtctagtaggctagtggctgtaattttac

fava_c17336_1235	fava_c17336	1235	T/C	gtgatgcagctatcagcagctgagtttgtctcttaactcctaaaacttgtcattttttataaaaaccggagagctttaccgctcttacgRtctatttagc[T/C]tggcagatttctcacagttgtgtgtagagggttcttagtttatactcaactttttKttgggtctagtaggctagtggctgtaattttacctcttaaagga

fava_c17336_1291	fava_c17336	1291	G/T	ttataaaaaccggagagctttaccgctcttacgRtctatttagcYtggcagatttctcacagttgtgtgtagagggttcttagtttatactcaacttttt[G/T]ttgggtctagtaggctagtggctgtaattttacctcttaaaggaagtgggatgccgcaaattctaacctatgccactccagttggatgtttgtttctgtg

fava_c17336_233	fava_c17336	233	C/T	ggctgagatgagactactagccgaaaatcaacatcatctttgggctcaacgatttgaatgctgctgctatttctgcacctccttccttttcaattgaaag[C/T]cggcttctcagagagatttgaaacataaacgtagaattgaaatagagttaaRttgccatgtcaagaacatcgagcagcaaacctgttggttttaccgcta

fava_c17336_285	fava_c17336	285	A/G	tttgaatgctgctgctatttctgcacctccttccttttcaattgaaagYcggcttctcagagagatttgaaacataaacgtagaattgaaatagagttaa[A/G]ttgccatgtcaagaacatcgagcagcaaacctgttggttttaccgctactaaagatatatttgtttcagctattcgctttgctgtgtctattgaRgaacc

fava_c17336_380	fava_c17336	380	G/A	gttaaRttgccatgtcaagaacatcgagcagcaaacctgttggttttaccgctactaaagatatatttgtttcagctattcgctttgctgtgtctattga[G/A]gaaccMtgtttYccatttggtgacaagctaagaatctctgctcaagagcaagttgactacatgctMggagaagacgaagatattacaaacattatggctg

fava_c17336_386	fava_c17336	386	A/C	ttgccatgtcaagaacatcgagcagcaaacctgttggttttaccgctactaaagatatatttgtttcagctattcgctttgctgtgtctattgaRgaacc[A/C]tgtttYccatttggtgacaagctaagaatctctgctcaagagcaagttgactacatgctMggagaagacgaagatattacaaacattatggctgacgatR

fava_c17336_392	fava_c17336	392	C/T	tgtcaagaacatcgagcagcaaacctgttggttttaccgctactaaagatatatttgtttcagctattcgctttgctgtgtctattgaRgaaccMtgttt[C/T]ccatttggtgacaagctaagaatctctgctcaagagcaagttgactacatgctMggagaagacgaagatattacaaacattatggctgacgatgaagtaa

fava_c17336_446	fava_c17336	446	C/A	ttgtttcagctattcgctttgctgtgtctattgaRgaaccMtgtttYccatttggtgacaagctaagaatctctgctcaagagcaagttgactacatgct[C/A]ggagaagacgaagatattacaaacattatggctgacgatgaagtaaaatctgtagtaagaatcggtgtttacaatatcattcactcgttcgaggcagact

fava_c17336_720	fava_c17336	720	A/G	ggatcttgaatggatgtgtaatgtacttccaaagatggatttgatgaaatcttttgtttctaattggatcgcaatttctagtaaaattctggtgatcatc[A/G]aagacgaaaagtttgataatatcatgtggggtttgaaagtaaaggtgattgaactaacatgtaagRttttggaagcagttagttatggaagtgtgattgt

fava_c17336_786	fava_c17336	786	G/A	gatcgcaatttctagtaaaattctggtgatcatcRaagacgaaaagtttgataatatcatgtggggtttgaaagtaaaggtgattgaactaacatgtaag[G/A]ttttggaagcagttagttatggaagtgtgattgttccagctccgagccgggttcagttgctaaaaRcgtggtttccgtacgttcaaaaaatgaagccgtt

fava_c17336_852	fava_c17336	852	G/A	tttgaaagtaaaggtgattgaactaacatgtaagRttttggaagcagttagttatggaagtgtgattgttccagctccgagccgggttcagttgctaaaa[G/A]cgtggtttccgtacgttcaaaaaatgaagccgttgcttgattcaaaagctgttgcaaaaactgattttYgttacgtaatggatgaggatctgtgtcaggc

fava_c17336_921	fava_c17336	921	C/T	tccagctccgagccgggttcagttgctaaaaRcgtggtttccgtacgttcaaaaaatgaagccgttgcttgattcaaaagctgttgcaaaaactgatttt[C/T]gttacgtaatggatgaggatctgtgtcaggctattgagggagcgattgtgtcgttggtattgacgttgccttcgaacgatcaagcagatattttggccga

fava_c17338_655	fava_c17338	655	A/C	gcctccagcccctctttccaagctgaaatgaggcgtttgcgactgagccactgaagattatttatgtagaaaaaaaagttaacctggctggaagatccaa[A/C]gataagcaatacgtatctgaggtattttgggtgtatttatttctctttataacaaatctagctggccggctttcttcagtgttctagttgaaaatctaga

fava_c17341_1025	fava_c17341	1025	T/C	accggcagttacaacgcacgagtagtttgattgtgttaccaagagaaagtagctcgaggcatgggtatcgaaYcggtgaaggaagtagtaaagggaaaag[T/C]tcgaggtggWtggaccggagtttaaaatcggaccggtgggtttttacYagggcaccgtcgtttttggctagggcgttgtcgtttaggtcaccRagRccaa

fava_c17341_1035	fava_c17341	1035	T/A	acaacgcacgagtagtttgattgtgttaccaagagaaagtagctcgaggcatgggtatcgaaYcggtgaaggaagtagtaaagggaaaagYtcgaggtgg[T/A]tggaccggagtttaaaatcggaccggtgggtttttacYagggcaccgtcgtttttggctagggcgttgtcgtttaggtcaccRagRccaaaggttaataa

fava_c17341_1073	fava_c17341	1073	T/C	gtagctcgaggcatgggtatcgaaYcggtgaaggaagtagtaaagggaaaagYtcgaggtggWtggaccggagtttaaaatcggaccggtgggtttttac[T/C]agggcaccgtcgtttttggctagggcgttgtcgtttaggtcaccRagRccaaaggttaataatagtgacgacgatgaaggaacttcttctgctactgctc

fava_c17341_1118	fava_c17341	1118	G/A	ggaaaagYtcgaggtggWtggaccggagtttaaaatcggaccggtgggtttttacYagggcaccgtcgtttttggctagggcgttgtcgtttaggtcacc[G/A]agRccaaaggttaataatagtgacgacgatgaaggaacttcttctgctactgctcctatcatgccgtcatctgccgttgactctgcacgcYgttaaatct

fava_c17341_1121	fava_c17341	1121	A/G	aaagYtcgaggtggWtggaccggagtttaaaatcggaccggtgggtttttacYagggcaccgtcgtttttggctagggcgttgtcgtttaggtcaccRag[A/G]ccaaaggttaataatagtgacgacgatgaaggaacttcttctgctactgctcctatcatgccgtcatctgccgttgactctgcacgcYgttaaatcttaa

fava_c17341_1209	fava_c17341	1209	C/T	taggtcaccRagRccaaaggttaataatagtgacgacgatgaaggaacttcttctgctactgctcctatcatgccgtcatctgccgttgactctgcacgc[C/T]gttaaatcttaagaaaYcggctgcctttaatttaMaccgtccaatactgccgttaagttatggcgaatatgtgcattggagttggactattagccacgga

fava_c17341_1226	fava_c17341	1226	T/C	aggttaataatagtgacgacgatgaaggaacttcttctgctactgctcctatcatgccgtcatctgccgttgactctgcacgcYgttaaatcttaagaaa[T/C]cggctgcctttaatttaMaccgtccaatactgccgttaagttatggcgaatatgtgcattggagttggactattagccacggagagatacaggtctgaaa

fava_c17341_1244	fava_c17341	1244	C/A	acgatgaaggaacttcttctgctactgctcctatcatgccgtcatctgccgttgactctgcacgcYgttaaatcttaagaaaYcggctgcctttaattta[C/A]accgtccaatactgccgttaagttatggcgaatatgtgcattggagttggactattagccacggagagatacaggtctgaaatttgactgtttttttaat

fava_c17341_353	fava_c17341	353	T/C	tcgccataatcatagtcatcttagtcgccgctcttttcctcatgggctttttctccatctacatccgccgctgctccgattcatctccctccaacaccgg[T/C]ctccctatcatcaacggccgcttaggtatcacatctcgcggactcgattcctcagtaattgaaaccttcccggttcttcaatactccgaagtcaagatcc

fava_c17341_635	fava_c17341	635	G/C	agtgtgaccacgtttttcacactgactgtatcgacgagtggctatctttgcacacaacatgtcccgtttgtcgtgcgaatctcgttccagaacccgctga[G/C]tcagttcacgctaattctaatcccaatcccgagtcgcaacaacaagatattgaagctcaaaacgacacggctcaaacaccaaacgcggagSctgattcgg

fava_c17341_726	fava_c17341	726	G/C	acccgctgaStcagttcacgctaattctaatcccaatcccgagtcgcaacaacaagatattgaagctcaaaacgacacggctcaaacaccaaacgcggag[G/C]ctgattcggttYtggtttctccggaagtgatttcgctggagaaaacgctgaataggaatcgaacgcgtggatctcagtcaaaccgggcgcgtcggtttcc

fava_c17341_738	fava_c17341	738	C/T	agttcacgctaattctaatcccaatcccgagtcgcaacaacaagatattgaagctcaaaacgacacggctcaaacaccaaacgcggagSctgattcggtt[C/T]tggtttctccggaagtgatttcgctggagaaaacgctgaataggaatcgaacgcgtggatctcagtcaaaccgggcgcgtcggtttccgaagtcgcactc

fava_c17341_997	fava_c17341	997	C/T	ccttttgcggtgaggaaagagttaatgaaccggcagttacaacgcacgagtagtttgattgtgttaccaagagaaagtagctcgaggcatgggtatcgaa[C/T]cggtgaaggaagtagtaaagggaaaagYtcgaggtggWtggaccggagtttaaaatcggaccggtgggtttttacYagggcaccgtcgtttttggctagg

fava_c17342_278	fava_c17342	278	A/G	gaaagaaaaacaattagctaggggtgagtcttgaaactaggtgttgcttcagaaaattgccaagcccactctgaatgagttcaatgagatatttttgttc[A/G]gtctacttcaagtcaccaaaaatgctacgggcccagcaagaatgtccagccttaaagcccaacgaaatcaatagtcgacctgcaacatctcttgatacaa

fava_c17342_655	fava_c17342	655	T/C	caaccttcttcgaatcatcaatggcgatcgattctcttctagtcgctgattttcctcttccacctattcgttttctccgaatttcgaaacagagccaatt[T/C]cgcctcatctttcgtgaacgactccgtctttcttccgattctgatcttctccgtgaagcttcatcgcaagtccaccttcatctctgattcaaccttcttc

fava_c17342_762	fava_c17342	762	T/C	atctttcgtgaacgactccgtctttcttccgattctgatcttctccgtgaagcttcatcgcaagtccaccttcatctctgattcaaccttcttcgatccc[T/C]cgKRttctacgtgtttttMgtttttagtttgtgttgcaattttttgttgtgtactgaagatcgacgtgtgatgttattgctatgatcacaggttggtgtg

fava_c17342_765	fava_c17342	765	G/T	tttcgtgaacgactccgtctttcttccgattctgatcttctccgtgaagcttcatcgcaagtccaccttcatctctgattcaaccttcttcgatcccYcg[G/T]RttctacgtgtttttMgtttttagtttgtgttgcaattttttgttgtgtactgaagatcgacgtgtgatgttattgctatgatcacaggttggtgtgttg

fava_c17342_766	fava_c17342	766	G/A	ttcgtgaacgactccgtctttcttccgattctgatcttctccgtgaagcttcatcgcaagtccaccttcatctctgattcaaccttcttcgatcccYcgK[G/A]ttctacgtgtttttMgtttttagtttgtgttgcaattttttgttgtgtactgaagatcgacgtgtgatgttattgctatgatcacaggttggtgtgttga

fava_c17342_781	fava_c17342	781	C/A	gtctttcttccgattctgatcttctccgtgaagcttcatcgcaagtccaccttcatctctgattcaaccttcttcgatcccYcgKRttctacgtgttttt[C/A]gtttttagtttgtgttgcaattttttgttgtgtactgaagatcgacgtgtgatgttattgctatgatcacaggttggtgtgttgatgtgttKtgttgatR

fava_c17342_873	fava_c17342	873	G/T	gtgtttttMgtttttagtttgtgttgcaattttttgttgtgtactgaagatcgacgtgtgatgttattgctatgatcacaggttggtgtgttgatgtgtt[G/T]tgttgatRcttactcagaaatttgaacgcagaacgttgatacgatactgttgttgttgtgaggttgaaaagcagtgatgatgaatattagcggtggttta

fava_c17342_881	fava_c17342	881	G/A	MgtttttagtttgtgttgcaattttttgttgtgtactgaagatcgacgtgtgatgttattgctatgatcacaggttggtgtgttgatgtgttKtgttgat[G/A]cttactcagaaatttgaacgcagaacgttgatacgatactgttgttgttgtgaggttgaaaagcagtgatgatgaatattagcggtggtttattgaggat

fava_c17343_1539	fava_c17343	1539	A/T	ggagaagatttagggacttgttccattaatgaactacaaaaaatagagcaacagttggagaagagtataaccaaaattcgagcaaaaaagactcaagtct[A/T]catggaacaaatagatcagctaaaagaaaaggaaaaagccctagttgctgaaaatgtcatgctctYggagaagtatgatagttattcatctcagcaagca

fava_c17343_1605	fava_c17343	1605	C/T	ataaccaaaattcgagcaaaaaagactcaagtctWcatggaacaaatagatcagctaaaagaaaaggaaaaagccctagttgctgaaaatgtcatgctct[C/T]ggagaagtatgatagttattcatctcagcaagcaaaagaagaWgatagagaaaatatagctgatgttgaagcttatgcagatcaaagtagtccaagYtca

fava_c17343_1648	fava_c17343	1648	T/A	aaatagatcagctaaaagaaaaggaaaaagccctagttgctgaaaatgtcatgctctYggagaagtatgatagttattcatctcagcaagcaaaagaaga[T/A]gatagagaaaatatagctgatgttgaagcttatgcagatcaaagtagtccaagYtcagatgttgaaactgaattattcattggtcttccagaaacaagaa

fava_c17343_1702	fava_c17343	1702	C/T	tctYggagaagtatgatagttattcatctcagcaagcaaaagaagaWgatagagaaaatatagctgatgttgaagcttatgcagatcaaagtagtccaag[C/T]tcagatgttgaaactgaattattcattggtcttccagaaacaagaacaaggaggatttctccaatgttgaggaYYaattaatcYctctgtctatgcaaaa

fava_c17343_1776	fava_c17343	1776	C/T	gcttatgcagatcaaagtagtccaagYtcagatgttgaaactgaattattcattggtcttccagaaacaagaacaaggaggatttctccaatgttgagga[C/T]YaattaatcYctctgtctatgcaaaagataataaaatttgttaatcattttgatatttgttaaaactcaaacaactacatatatgaaaaggtaattctat

fava_c17343_1777	fava_c17343	1777	C/T	cttatgcagatcaaagtagtccaagYtcagatgttgaaactgaattattcattggtcttccagaaacaagaacaaggaggatttctccaatgttgaggaY[C/T]aattaatcYctctgtctatgcaaaagataataaaatttgttaatcattttgatatttgttaaaactcaaacaactacatatatgaaaaggtaattctatg

fava_c17343_1786	fava_c17343	1786	T/C	atcaaagtagtccaagYtcagatgttgaaactgaattattcattggtcttccagaaacaagaacaaggaggatttctccaatgttgaggaYYaattaatc[T/C]ctctgtctatgcaaaagataataaaatttgttaatcattttgatatttgttaaaactcaaacaactacatatatgaaaaggtaattctatgagtacaata

fava_c17343_366	fava_c17343	366	A/C	gtgtttacatttttattattagcatatagcattacatcagatacaataaagctagagtatttgcagcagcaacagcaaatgtgttgaggtgagggacata[A/C]aaacttcacaaagaaattcctaaggRtatcaactaatttttaacccctgatgttatggtagctaattctgcacctttgaacatgaagagatacctgtctc

fava_c17343_392	fava_c17343	392	A/G	tagcattacatcagatacaataaagctagagtatttgcagcagcaacagcaaatgtgttgaggtgagggacataMaaacttcacaaagaaattcctaagg[A/G]tatcaactaatttttaacccctgatgttatggtagctaattctgcacctttgaacatgaagagatacctgtctcattatatgactgttcccaattcttcc

fava_c17343_618	fava_c17343	618	G/T	tgtcatcgttccggggtcaacacacctgaatgtaacagaaaccccatctgggtttgagcgtggataatagaacgaagttataccacatattttgcaaaac[G/T]tgtgcttcgcagtatgagtgccaaacgtataagttgtaagaaactcagcagaatttcccaaaagctcaaatttatcagcaggcacaatgaattgagtatt

fava_c17344_1086	fava_c17344	1086	A/C	accggtgatattctcgccaaagattgcctcgaagacactccaattagctttgtcgaaagaaaaccatttgcttggttcgagtgcgaatgggaagtactat[A/C]aaactgttcttgagaatggtctcataattgcggtgaaaaagcttacaccgttcgagagcaattcRcaagagagtaggagaaaatcSgtgaagaggaagat

fava_c17344_1151	fava_c17344	1151	G/A	atttgcttggttcgagtgcgaatgggaagtactatMaaactgttcttgagaatggtctcataattgcggtgaaaaagcttacaccgttcgagagcaattc[G/A]caagagagtaggagaaaatcSgtgaagaggaagatacagatggagctagagcttcttgcaagcttaagacataggaatttgatgagtttgagggcttaYg

fava_c17344_1172	fava_c17344	1172	G/C	atgggaagtactatMaaactgttcttgagaatggtctcataattgcggtgaaaaagcttacaccgttcgagagcaattcRcaagagagtaggagaaaatc[G/C]gtgaagaggaagatacagatggagctagagcttcttgcaagcttaagacataggaatttgatgagtttgagggcttaYgttcgcgaaaacgatggattct

fava_c17344_1250	fava_c17344	1250	C/T	cRcaagagagtaggagaaaatcSgtgaagaggaagatacagatggagctagagcttcttgcaagcttaagacataggaatttgatgagtttgagggctta[C/T]gttcgcgaaaacgatggattctcattggtttatgattatgtgtcgaccggtagtcttgcagatgtgatgaatagggttagagaaaatgagttgcagattg

fava_c17344_1628	fava_c17344	1628	C/T	atagagggacttctctcaacacccctcccgaatgtttccacaatcgcagcaggtacacagaaaagagcgacatattcagcttcgggatgatactaggagt[C/T]ttactaacYggtaaggaccctacggatgcattctttggagaagcggtgagaggtggaagtttgggatgttggctgcgacacttgaagcaagcgggagagg

fava_c17344_1637	fava_c17344	1637	C/T	cttctctcaacacccctcccgaatgtttccacaatcgcagcaggtacacagaaaagagcgacatattcagcttcgggatgatactaggagtYttactaac[C/T]ggtaaggaccctacggatgcattctttggagaagcggtgagaggtggaagtttgggatgttggctgcgacacttgaagcaagcgggagaggaacgagaag

fava_c17344_442	fava_c17344	442	T/C	ctaaactatggagtagttctttgttattgttcttatttgtgtaaaggtggaaattttatgggtgagtgaagagaataaaaagatgaggttttctagaaaa[T/C]gataaggattctatgaaggcttgtttgtgaagattttggtttgaaaaatgaaaagtttgtggaatatgcttatgcttatgcttattcttatgtttgtgct

fava_c17344_827	fava_c17344	827	G/T	caaataagttgttggtatttcagcttcttgttttgttgttgtttagtttgcatcatgacaatactgtgcaatgtcatgggagattgagtaaacatgtgtc[G/T]tatgagtctSggtcgggggcggaatatagggatgattcWaagaagattattgttagtgtagttttaggagtaatcactggattaatcggttcRattctgt

fava_c17344_837	fava_c17344	837	C/G	gttggtatttcagcttcttgttttgttgttgtttagtttgcatcatgacaatactgtgcaatgtcatgggagattgagtaaacatgtgtcKtatgagtct[C/G]ggtcgggggcggaatatagggatgattcWaagaagattattgttagtgtagttttaggagtaatcactggattaatcggttcRattctgtttgtggttgt

fava_c17344_866	fava_c17344	866	A/T	tgtttagtttgcatcatgacaatactgtgcaatgtcatgggagattgagtaaacatgtgtcKtatgagtctSggtcgggggcggaatatagggatgattc[A/T]aagaagattattgttagtgtagttttaggagtaatcactggattaatcggttcRattctgtttgtggttgtgattcgttgYRttgttcagtatctgaata

fava_c17344_920	fava_c17344	920	G/A	atgtgtcKtatgagtctSggtcgggggcggaatatagggatgattcWaagaagattattgttagtgtagttttaggagtaatcactggattaatcggttc[G/A]attctgtttgtggttgtgattcgttgYRttgttcagtatctgaatagaacgccYatcctcaaaggaccggtgatattctcgccaaagattgcctcgaaga

fava_c17344_947	fava_c17344	947	C/T	cggaatatagggatgattcWaagaagattattgttagtgtagttttaggagtaatcactggattaatcggttcRattctgtttgtggttgtgattcgttg[C/T]RttgttcagtatctgaatagaacgccYatcctcaaaggaccggtgatattctcgccaaagattgcctcgaagacactccaattagctttgtcgaaagaaa

fava_c17344_948	fava_c17344	948	G/A	ggaatatagggatgattcWaagaagattattgttagtgtagttttaggagtaatcactggattaatcggttcRattctgtttgtggttgtgattcgttgY[G/A]ttgttcagtatctgaatagaacgccYatcctcaaaggaccggtgatattctcgccaaagattgcctcgaagacactccaattagctttgtcgaaagaaaa

fava_c17344_974	fava_c17344	974	T/C	ttattgttagtgtagttttaggagtaatcactggattaatcggttcRattctgtttgtggttgtgattcgttgYRttgttcagtatctgaatagaacgcc[T/C]atcctcaaaggaccggtgatattctcgccaaagattgcctcgaagacactccaattagctttgtcgaaagaaaaccatttgcttggttcgagtgcgaatg

fava_c17345_1114	fava_c17345	1114	T/A	gcgcgtttttcagagactctaaacacttgtaatcagaaactctccaaatcttgaatgttctatcccacgaagcggagtaaagaaaaaacccgtctttgga[T/A]agagctagtgaagaaactgcgtcgatatgattcacccatgtaaacttcttgtgtcttcgagcttcgatgtagtttttcgataaaaaaagcttcgagaaac

fava_c17345_515	fava_c17345	515	G/C	cagattggttttcaaaacagtgaaaatattacaatcaaaagcagaacaaaatcaaaacaagggaattcttatttgccaaactttgatgtcacaatcaaga[G/C]caccactgtaaacaagataagaactacaatcatcaccattttcacgacttttggaatcaacttccatagccaaacacttcacaggtttcctatgaccctg

fava_c17346_336	fava_c17346	336	C/T	tgaagacatggcttctgatagtttaagatgtgttgccatcgcatatagaccatatgaggatgcagttccagatactgaggaagaacttgctcactggtct[C/T]tgccagaggatggacttgttttactagccattgttggtatcaaggatccttgtcgacctggtgtcaaagattcagtacagctttgccaaaaagccggggt

fava_c17346_764	fava_c17346	764	C/T	agggccatgttgttgctgtaaccggggatggaacgaatgatgctcctgcacttcatgaggcggatattggtcttgctatgggtattgctggcacagaagt[C/T]gccaaagagagctctgatatcattatattggatgacaactttgcttcagttgtgaaggttgtcagatggggtcggtctgtttatgcaaatattcagaaat

fava_c17349_1490	fava_c17349	1490	A/T	cttttgatttacatcacggttattcacattctccactttaggtaaagattgtggcggtggaactgatgaaacattagcatcaatttctttctttttctct[A/T]caccaactgYSttgtttagatctggagaatcctttattggtattgaatcaatcaactttctaacaaaagaagattcaacaccatgaaacacgattagaga

fava_c17349_1500	fava_c17349	1500	C/T	acatcacggttattcacattctccactttaggtaaagattgtggcggtggaactgatgaaacattagcatcaatttctttctttttctctWcaccaactg[C/T]Sttgtttagatctggagaatcctttattggtattgaatcaatcaactttctaacaaaagaagattcaacaccatgaaacacgattagagaaaacaataat

fava_c17349_1501	fava_c17349	1501	G/C	catcacggttattcacattctccactttaggtaaagattgtggcggtggaactgatgaaacattagcatcaatttctttctttttctctWcaccaactgY[G/C]ttgtttagatctggagaatcctttattggtattgaatcaatcaactttctaacaaaagaagattcaacaccatgaaacacgattagagaaaacaataata

fava_c17349_1619	fava_c17349	1619	C/T	atcctttattggtattgaatcaatcaactttctaacaaaagaagattcaacaccatgaaacacgattagagaaaacaataatagtttcagtaacgtgtgt[C/T]ttcccattgtgatgcagttccaatacgttcaagttcacgtggcagaggcaaattgagaagaaacgatacagaaagtgaaatcaagagaaaccctttggcg

fava_c17349_1775	fava_c17349	1775	G/A	agaagaaacgatacagaaagtgaaatcaagagaaaccctttggcgagaaaatggattgaagattgaaaagaacaaattctgaaggtgtggggttgtgatt[G/A]tgaggaagaaatggttgttgggattgaaaattgggaaatatgtggaatcttgttgttgtggaaatttggattgtgatggaaagtttctgaattgggattg

fava_c17349_411	fava_c17349	411	C/T	gtatctttgctaggatatactttaacttaagccctagcacagccacattcgtcacatccctccctaactatgtttgctttttctataacatcattcccag[C/T]ctttgaagtttatattcctttcccttatttctttccccctttttaagactaatcatcccagttatcttcccatccatctttgtttaaagatctagcagta

fava_c17350_1133	fava_c17350	1133	G/A	aatttgtttgcccactctggttttctgctggcgcaaaggctttgatacataaaattttggatcccgatcctaaaacacgtgttaaaataaaagaaataat[G/A]caagatccatggttcaagaaaaactattctcctgtcacactcagagaagacgagaaagtgaatttggatgacgttctggctgtttttaatgatattgagg

fava_c17350_1754	fava_c17350	1754	C/T	agactagcccagcttctggtttgcttcgacaaatgacattctgatccagctccagcccttatgatgcaaatctaaataaaacaacaccagctgctggtgc[C/T]cccaggaaaattggaacggaaatggatatcgctgtcaaattagagcatgacttgatttcttaggttaMatacgtattgcaatgcatgatgcaatgaacca

fava_c17350_1822	fava_c17350	1822	A/C	aatctaaataaaacaacaccagctgctggtgcYcccaggaaaattggaacggaaatggatatcgctgtcaaattagagcatgacttgatttcttaggtta[A/C]atacgtattgcaatgcatgatgcaatgaaccaatttgttatctaaataaatagtgtttggttcgggcggaaattgtataaaatatatttatttgtaccaa

fava_c17350_247	fava_c17350	247	T/C	tttttccgatcattttcttcttcttcatcaaacaccacaacctctactttcctttccgaggatcgcgtccaagttattagaatttcaacttggatttttt[T/C]ctctttctattaagatttcaagttgaaaaatcgcgactgagttcgtgattctaggtcattttttgaattgcgaaWtcgtgaaagggaacgacgaatgagc

fava_c17350_322	fava_c17350	322	T/A	attagaatttcaacttggattttttYctctttctattaagatttcaagttgaaaaatcgcgactgagttcgtgattctaggtcattttttgaattgcgaa[T/A]tcgtgaaagggaacgacgaatgagcgttgaaaaaatgaagaaggtgaggagaaagatcggaaagtatgaggttggtcgaactatcggtgaaggtacattc

fava_c17352_1188	fava_c17352	1188	A/T	tctcacctccttcacataattcaaccaaacaaattctacacactgcctcctcttgagctatatcttggccatcatcagcatcttcgatttcagtatcctt[A/T]gtcggagacgtgctcaacaattcatttccttcctttactcgaggagtagaagaaacaacgcgaaaaaacgaatccatcctctttaggcttttatccttct

fava_c17352_1482	fava_c17352	1482	G/A	ttccaatttcatctaatggcaatgatgaagttcttttcatcctaggagtaaatatcttactaagtgacagtgatctcgagatcaaaggcttttctcgatg[G/A]cctgaagaggaaacctccggagttgatatgttcatcttgtcattatctgccggtgtcctactcctgaagctcaatttcggtagcatgtttcttaacgatg

fava_c17352_1724	fava_c17352	1724	C/T	ggagcatcaaccgaccgagaagtcatcagaaaattcactctcttaggagtaggactcggtgtcatcggaactttgatagccacataatcttgtgaacact[C/T]ttctcgtgYtcttgatggtatttccaacagaagattctgtttcttccgcggaggaacatgtgagatttcttcaattatccccgtcgaaccatcgttatcc

fava_c17352_1733	fava_c17352	1733	C/T	accgaccgagaagtcatcagaaaattcactctcttaggagtaggactcggtgtcatcggaactttgatagccacataatcttgtgaacactYttctcgtg[C/T]tcttgatggtatttccaacagaagattctgtttcttccgcggaggaacatgtgagatttcttcaattatccccgtcgaaccatcgttatccttgtgagca

fava_c17352_202	fava_c17352	202	C/A	atccttgtctctcactttgaccaaaaccaaaatcattgtttcaccaaggctacaaaagaagaaggccaaaacccttctagttttgatgtggtgaaatatt[C/A]atcaatacaaataaacaaacagaaattccatagcaagaaaagctatattcataaactgtacaatgtagtctcgtgtagacgtgtagtaaattagtaaaat

fava_c17352_320	fava_c17352	320	A/G	aacagaaattccatagcaagaaaagctatattcataaactgtacaatgtagtctcgtgtagacgtgtagtaaattagtaaaatggcctgctctatacata[A/G]taagttatgcagccactagctttgtcatgagaaagaagcacacaaatatcaattcaactttgatttgagtcttgttgattttgcactacagattgattgt

fava_c17352_474	fava_c17352	474	T/A	tcaactttgatttgagtcttgttgattttgcactacagattgattgtggttggattcacctgatcgtggcgtgtttgcaggccgaggttgttgtcctact[T/A]gtgttatcggtagagggccatggaatagctctgatgaagcctgctgtctccttctccatctaaaaagctcaacaaggaaagaacttacactcattaccac

fava_c17352_792	fava_c17352	792	T/A	cataaatccatatgaacctgctcttcaccatggttgatgatgtcatggaggagagcaggcctagtacacaggaaaatggaagggatatggcaattgcacc[T/A]gttcccatttttgtaaccaacagttgctcaagaaaacagaaatatgccaacatgctaacaatgacaagcactggaacttcctgccaaaccccgtttacat

fava_c17357_231	fava_c17357	231	A/G	aaaatattcacattttcacactcatatgattttcaatgcgatcattaatattagcgccgcgaaccacgccggaaatccccgtcgatttccgtgcaccacc[A/G]ccatctccRgtagcatccggccgtagatcaaccgtaacaaacaacgagtttctgaccgagtttttggaaacctcgcttcgagttccagatctagtgttac

fava_c17357_240	fava_c17357	240	G/A	acattttcacactcatatgattttcaatgcgatcattaatattagcgccgcgaaccacgccggaaatccccgtcgatttccgtgcaccaccRccatctcc[G/A]gtagcatccggccgtagatcaaccgtaacaaacaacgagtttctgaccgagtttttggaaacctcgcttcgagttccagatctagtgttaccgaacaaga

fava_c17357_612	fava_c17357	612	A/T	aggcagccagaggaatatttcttgtgccgccgggaaagcgaaaagtagtatcgagatcgccggagaagctttggggtttcgaggtgtatcatgaaggaga[A/T]gatgagggaagtgaattgaatgMagaatttatttggtgcaaagatgaagagttgaagttgaaattgaagggaatttggcctattggatatcctaatttta

fava_c17357_635	fava_c17357	635	C/A	gtgccgccgggaaagcgaaaagtagtatcgagatcgccggagaagctttggggtttcgaggtgtatcatgaaggagaWgatgagggaagtgaattgaatg[C/A]agaatttatttggtgcaaagatgaagagttgaagttgaaattgaagggaatttggcctattggatatcctaattttagcaagaMgatagaagttctaggg

fava_c17357_719	fava_c17357	719	C/A	ggaagtgaattgaatgMagaatttatttggtgcaaagatgaagagttgaagttgaaattgaagggaatttggcctattggatatcctaattttagcaaga[C/A]gatagaagttctagggtcacgtgtggagaaggtggcagagaaattatttcctatcatattgaaaaatgttcctaagaaattagcaagtgatggtggtata

fava_c17358_1044	fava_c17358	1044	T/C	tttctgatttctctggtgttgctggacccccatggagatagagtgtagatgattttacattagaatgtgatgtttgcaggacacgtgcagcattaaatcg[T/C]gcctgtgcccgcacaagYgattgcatccgtttgagccactctgctgttcttttcctctcaatgtgccctctcacaagagcctggagcttcaccagccctt

fava_c17358_1062	fava_c17358	1062	T/C	ttgctggacccccatggagatagagtgtagatgattttacattagaatgtgatgtttgcaggacacgtgcagcattaaatcgYgcctgtgcccgcacaag[T/C]gattgcatccgtttgagccactctgctgttcttttcctctcaatgtgccctctcacaagagcctggagcttcaccagcccttttaatgcccgcagtgcct

fava_c17358_111	fava_c17358	111	A/C	ttcatctctcagcgtttataatcaaatataagagtactctaatcctgacacaaaaacgaactttcataacccaacaaaatacaaccagttagttagatag[A/C]taaatgttactaagagacttaaacaacactcaaaataaaaatataataataatacYctcttaacagaaattagcattaacatcaagatcagtatcttatc

fava_c17358_167	fava_c17358	167	C/T	cgaactttcataacccaacaaaatacaaccagttagttagatagMtaaatgttactaagagacttaaacaacactcaaaataaaaatataataataatac[C/T]ctcttaacagaaattagcattaacatcaagatcagtatcttatctataccccacaggcattccaagcttatccaaacgaccagaacctggatacccttta

fava_c17359_1190	fava_c17359	1190	C/T	ccatatccttaaaagcaacactctgatgataccgatccaacatcaacaatgaagtataatccaagtcaaaaacatacaccggaataaccctcccgaaatc[C/T]tcctccggtatcccaccaattttcctcatctcatccgcagaatcagacaaaatctgatgcaacctcttagaatccaaatactcactcacaatcaaagtat

fava_c17359_1295	fava_c17359	1295	A/G	ccggtatcccaccaattttcctcatctcatccgcagaatcagacaaaatctgatgcaacctcttagaatccaaatactcactcacaatcaaagtataatt[A/G]tcaaacaaaaacctcgaagtataggaattcgtcgacctagaaacggcaaaagaacaaatcggacactcatcatacttaacatcatRaaccttaaaactca

fava_c17359_1381	fava_c17359	1381	A/G	atcaaagtataattRtcaaacaaaaacctcgaagtataggaattcgtcgacctagaaacggcaaaagaacaaatcggacactcatcatacttaacatcat[A/G]aaccttaaaactcaaactctgctcccctaacaaaatcttcccattactctcatctctaaaactttcctcaatcaatttccaatccaaccccttcctatcc

fava_c17359_635	fava_c17359	635	A/G	atgtgagtgatgcctcaatttcttgagaggcatgatacacgatggagtgaatggcataaagatcatgatcagaagacctcgcgtaaaacagagccatctc[A/G]taatccagatgcgacattgcagacaccgccttgttaagcttgtacttgaaaaggttccatctctgaacaaactcatcatgctgcttctgcttgagcatat

fava_c17360_1072	fava_c17360	1072	T/C	ttgttctttatgaaatgatttcggcRaaggaagcaattgtcaagaccagcgaatctgttgctgcagactcaaagggacttgtatctatgtttgaaggagt[T/C]cttagtcagcccgatcctaccgaagatcttcgtaaaatagtggatccaaggcttggggataactaccctgtagattcagtgcgaaagatggctcaactag

fava_c17360_1355	fava_c17360	1355	C/G	tacagacgattgggatattggctccttttacgaaaatcaaaaccttgtgaatctgatgtcaggaagataggattatacgaatgcatcgattttgtatgta[C/G]aatgtttggagttagatttttgacattgcaacaattgttgaaactaaagtgtttataaaatggattagcaagtaggaatttgtacattgtaatagctgta

fava_c17360_592	fava_c17360	592	G/A	ctgcaataaaaaagatggatatgcaagcaacaaaggaatttctcgctgaattgaatgtcctgacacgcgttcatcatcttaacttggtgcggttgattgg[G/A]tatagtattgagcgctctcttttccttgtctatgaatacatcgagaatggaaacttgagtcaacatctgYggggttcaggtagagatccactgccatggg

fava_c17360_662	fava_c17360	662	C/T	tcatcatcttaacttggtgcggttgattggRtatagtattgagcgctctcttttccttgtctatgaatacatcgagaatggaaacttgagtcaacatctg[C/T]ggggttcaggtagagatccactgccatgggctacacgtgtgcagattgctttggattctgccagaggtcttgaatatatccatgagcatacagtgcctgt

fava_c17360_997	fava_c17360	997	A/G	catttggatacatgccaccagagtatgctcaatatggagatgtttctcccaaagtagatgtttatgccttcggagttgttctttatgaaatgatttcggc[A/G]aaggaagcaattgtcaagaccagcgaatctgttgctgcagactcaaagggacttgtatctatgtttgaaggagtYcttagtcagcccgatcctaccgaag

fava_c17361_2394	fava_c17361	2394	A/G	ttctcacaagtaaagaaggattagaagcaataatagatccatctataggactcgacgcgacttttgatagcgtggccaaagtagcagccattgcttcaat[A/G]tgcgtgcaggcagtagtatcgaaccgtccattcatgagtgaagttgttcaggctttaaaacttgtatgcagtgaatgtgaagaagcaaaagaagatgatg

fava_c17361_2712	fava_c17361	2712	G/T	gaagaaaatcatattcaggtcctatgggaaatggaagaagcaaacaattgtggcagataatgagaaggctttctggtggtaatgttagtgaacatggaac[G/T]atatgaatcgagtaatgacaaggttactatagagaaatagcatcttttttaattgtctactttgtggatattgtttttcatgtatattaaaaataaacta

fava_c17361_495	fava_c17361	495	A/G	ggccattgtcacctcctgcatcagctccaactcctcaaaagagtaaagaaactgaaccatttatatctccaacttcaagttctatagcattatcaccaca[A/G]tatgaagcttttccttcaccaacaacagtgcaagggaatatgccaccatctgtagctcctgcagcacaaaaggcaagacaattacatcatgctcctcagc

fava_c17361_888	fava_c17361	888	C/T	ccaagacactgccacacccacctcctaatgaggattgtttatcaactttatgcacagagccttatgtaacttctcctcccggagcaccttgcagatgtgt[C/T]tggcctattcgagtgggtcttcgccttagggtatctctttataccttcttccctttggtctcggagctggcttctgagcttgccagtgggatttttatga

fava_c17362_1096	fava_c17362	1096	A/C	gcttgttcttattgtgcaggaaggtggatatcatgtaacatattcagcatattgtttacatgcaacacttgaaggtgttctcaacctatcacttcctcta[A/C]tacaagatcctgttgcctattatcccgaggatgagacattttctgtcaaagttatagaagccattaaaagttacgtaaaagataaaatgcccttatggag

fava_c17362_1253	fava_c17362	1253	A/G	gaagccattaaaagttacgtaaaagataaaatgcccttatggagaacaggttaacttttgagattaaactttctccaagaatgttacaagttcaaattgc[A/G]cacRaattgagcataggttgactgtaatgtttatgtgtactaggactgataatgactattattattagttgggtaaatgctccacattcaaactaaggca

fava_c17362_1257	fava_c17362	1257	G/A	ccattaaaagttacgtaaaagataaaatgcccttatggagaacaggttaacttttgagattaaactttctccaagaatgttacaagttcaaattgcRcac[G/A]aattgagcataggttgactgtaatgtttatgtgtactaggactgataatgactattattattagttgggtaaatgctccacattcaaactaaggcatggc

fava_c17362_141	fava_c17362	141	G/C	ttgtgatggcaaggaatacagacgcagcatcagaatcttggacggcgaaccaaatagccgttttctggcatgaaggtatgctcaatcatgataccggcaa[G/C]ggagttttcgacacaggaattgatcccggttttctagacgtgttggaaaaacatcctgaaaactcagacagagtcaaaaacatggtttccatcctcaaaa

fava_c17362_1433	fava_c17362	1433	T/A	ctccacattcaaactaaggcatggcacactgcaataagtggaggcttaaaataaacatcaactttgtgatgattgtcattgggagcaaacataaattggc[T/A]caattgttgagtaacaaatgttatgtttggtctagtggtgatgaggtacaacaatcttcctatatgatgacgWtaagYacctaaatctataatcatttga

fava_c17362_1506	fava_c17362	1506	A/T	tgtcattgggagcaaacataaattggcWcaattgttgagtaacaaatgttatgtttggtctagtggtgatgaggtacaacaatcttcctatatgatgacg[A/T]taagYacctaaatctataatcatttgaccatcattaagatgagtcttgattgaaggatccaagagagtgctcacatKtttgctccaccttgattgaagga

fava_c17362_1511	fava_c17362	1511	C/T	ttgggagcaaacataaattggcWcaattgttgagtaacaaatgttatgtttggtctagtggtgatgaggtacaacaatcttcctatatgatgacgWtaag[C/T]acctaaatctataatcatttgaccatcattaagatgagtcttgattgaaggatccaagagagtgctcacatKtttgctccaccttgattgaaggatccaa

fava_c17362_1583	fava_c17362	1583	G/T	aacaatcttcctatatgatgacgWtaagYacctaaatctataatcatttgaccatcattaagatgagtcttgattgaaggatccaagagagtgctcacat[G/T]tttgctccaccttgattgaaggatccaagagagtgccaaaatcttcaagcaactcaaggaatggcagtagtatctggWtcagatgatagatcaatggtca

fava_c17362_1661	fava_c17362	1661	T/A	ggatccaagagagtgctcacatKtttgctccaccttgattgaaggatccaagagagtgccaaaatcttcaagcaactcaaggaatggcagtagtatctgg[T/A]tcagatgatagatcaatggtcaagtgtcattgataatgtattaggttgtgtgacaagaattctatttgtggatagttgaggttgggttggatttgtagga

fava_c17362_250	fava_c17362	250	A/G	cgacacaggaattgatcccggttttctagacgtgttggaaaaacatcctgaaaactcagacagagtcaaaaacatggtttccatcctcaaaagaggccct[A/G]tctctcctcatctttcttggaacattggaagacacgctctMatccatgagctcctttcatttcacactcctgagtacataaatgaactggtagaagctga

fava_c17362_291	fava_c17362	291	C/A	aacatcctgaaaactcagacagagtcaaaaacatggtttccatcctcaaaagaggccctRtctctcctcatctttcttggaacattggaagacacgctct[C/A]atccatgagctcctttcatttcacactcctgagtacataaatgaactggtagaagctgataaagaaggcggaaagacgctttgtaccggcacRttcttga

fava_c17362_384	fava_c17362	384	A/G	acgctctMatccatgagctcctttcatttcacactcctgagtacataaatgaactggtagaagctgataaagaaggcggaaagacgctttgtaccggcac[A/G]ttcttgaaccctgggtcatgggatgctgcacttctagctgctggcacaacactgtcttccatgaagcatttgctagatggacatggaaaaattgcttatg

fava_c17362_801	fava_c17362	801	G/A	catggggtccgtctcatccgcagaatggttctgttgatgagttaggtgaaggagaaggttatggttataaccttaacatacctctaccaaatggaactgg[G/A]gacaaaggatatgtatatgccttcaatRagttggttgttccgtYgatcgataaatttgagccggatatgatagtgttggttataggacaagactcaagtg

fava_c17362_829	fava_c17362	829	G/A	ttctgttgatgagttaggtgaaggagaaggttatggttataaccttaacatacctctaccaaatggaactggRgacaaaggatatgtatatgccttcaat[G/A]agttggttgttccgtYgatcgataaatttgagccggatatgatagtgttggttataggacaagactcaagtgcattcgatccaaatggaaggcaatgctt

fava_c17362_845	fava_c17362	845	C/T	ggtgaaggagaaggttatggttataaccttaacatacctctaccaaatggaactggRgacaaaggatatgtatatgccttcaatRagttggttgttccgt[C/T]gatcgataaatttgagccggatatgatagtgttggttataggacaagactcaagtgcattcgatccaaatggaaggcaatgcttgacaatggaaggatat

fava_c17362_993	fava_c17362	993	C/T	actcaagtgcattcgatccaaatggaaggcaatgcttgacaatggaaggatatagagaaatcgggcggatagttcatgatcttgcgacgagacatagtga[C/T]gggcttgttcttattgtgcaggaaggtggatatcatgtaacatattcagcatattgtttacatgcaacacttgaaggtgttctcaacctatcacttcctc

fava_c17366_1427	fava_c17366	1427	C/T	aaccaagtcttcagctagcagcagcagtgctttctgaggttaaacttcctgcatcagagattgcggcaactattaatgaattcagatcccgccatctggc[C/T]gagcttacagaactatctgaaacaagtggaagttctcttggatatggatatggatataatagaattatgagcaaacccaaaacccaatctcctgattcaa

fava_c17368_1109	fava_c17368	1109	T/G	aggtacaatgttcgtagcacagacaagtcgaccggccaaacagcgcggaaagcagaaggtggtggttcacctgttgtacaataatattaggaaaatagat[T/G]Ragggaaagttctccatttaactcattttgttgcagtgaacataactaggaaatcaatatgaaggaaccaggcaccttttatcatctgtcatttttagtt

fava_c17368_1110	fava_c17368	1110	G/A	ggtacaatgttcgtagcacagacaagtcgaccggccaaacagcgcggaaagcagaaggtggtggttcacctgttgtacaataatattaggaaaatagatK[G/A]agggaaagttctccatttaactcattttgttgcagtgaacataactaggaaatcaatatgaaggaaccaggcaccttttatcatctgtcatttttagtta

fava_c17368_1231	fava_c17368	1231	G/A	ctcattttgttgcagtgaacataactaggaaatcaatatgaaggaaccaggcaccttttatcatctgtcatttttagttaaatttcttatcctccatttt[G/A]tttgggctggttcctgttacatagtatttgttgtactgtagtgtatataaaatcaacgatataacttcctattttcttggctcttttttgtttcccctgt

fava_c17370_2034	fava_c17370	2034	C/T	ttctgtaaagatatgttattctttattttaaaatgttctgttggaaaaaggaattaaaggcttctgttgaaagatttttcagagaaaagaagaataacat[C/T]taagggtaggcagtttatataggaacatattccactgtttatttgtggtttatcgctacaagttttgtattctttttagttctgcaggagaaattttgat

fava_c17370_753	fava_c17370	753	A/C	ggcaatgatgaaagtcaattgagctcaaatattgagggtgtcatccacaatatgtactatgccatttcacatgtcccctttcttgaaaaacctgatgata[A/C]atctgattttgaaatgtggagtgtatcagagagtgatcataaagtgctttctggagatctggatgataagcagcttaagtccaagaagcataaaagtaat

fava_c17373_688	fava_c17373	688	T/C	ctaagccactctgagattgatgatggtggtttctttgtgggagaaatgtgttgcttagagcattagcctcgtttaggacttggtatattaccatgtcttg[T/C]gaatcgttttcgtttaaaggtagttctgtttgggattgggatgatagaaccatggctctgttttttctctgtttttgctctgtttttcttgctttgtttc

fava_c17377_1075	fava_c17377	1075	G/C	ctattacaactatgttgaatagcggctgtagagcaatgaagcatagctgaatttacataaatggctgttttctctgagctgcaattggaaatactggttc[G/C]attgaagctagattttgggttgaggtttaagaatggatagtgaaattgagttgattSaagttgaattgaggatagtgaagttgaattaggattttgttat

fava_c17377_1132	fava_c17377	1132	G/C	taaatggctgttttctctgagctgcaattggaaatactggttcSattgaagctagattttgggttgaggtttaagaatggatagtgaaattgagttgatt[G/C]aagttgaattgaggatagtgaagttgaattaggattttgttatactgctatcatgttatgttatgcgtagcagctgcgttcaggggttgcagtgctttat

fava_c17377_1350	fava_c17377	1350	C/A	ctggctgctgttgatggtatgtttctgctctgaaaatctgaaccttttcagtgtgtaaatgaacaagttaagttcatagatgcaatattttctgttttca[C/A]tgacttataagtttctagatttgtgtcttaatagcttttaatttgtatgtaaatgtgagcacttttagatttagtctatggaagtactttacaaaaataa

fava_c17377_735	fava_c17377	735	G/A	ggatttgtggttgaagaaggagatgatgagtggagagattggttggttggttttggtttcggatgatcagggattttcggatatgttgaggaaggttagg[G/A]aagtggatttSaagacggttgttgttggtgattattggggtagggatttggggaagaatgctgatttgtggcttccttggattgttgttgagaatgggaa

fava_c17377_746	fava_c17377	746	G/C	tgaagaaggagatgatgagtggagagattggttggttggttttggtttcggatgatcagggattttcggatatgttgaggaaggttaggRaagtggattt[G/C]aagacggttgttgttggtgattattggggtagggatttggggaagaatgctgatttgtggcttccttggattgttgttgagaatgggaaggttgagggaa

fava_c17377_861	fava_c17377	861	A/G	tggtgattattggggtagggatttggggaagaatgctgatttgtggcttccttggattgttgttgagaatgggaaggttgagggaatgggtttaaaggga[A/G]taataagagcagtaacagaggatttggatgRtgaattggtttagggttttaagataaatagcggctatggWggtgctacaatgatattgcattgYgKWat

fava_c17377_892	fava_c17377	892	G/A	aatgctgatttgtggcttccttggattgttgttgagaatgggaaggttgagggaatgggtttaaagggaRtaataagagcagtaacagaggatttggatg[G/A]tgaattggtttagggttttaagataaatagcggctatggWggtgctacaatgatattgcattgYgKWatttgaaacaaataactattacaactatgttga

fava_c17377_932	fava_c17377	932	A/T	ggaaggttgagggaatgggtttaaagggaRtaataagagcagtaacagaggatttggatgRtgaattggtttagggttttaagataaatagcggctatgg[A/T]ggtgctacaatgatattgcattgYgKWatttgaaacaaataactattacaactatgttgaatagcggctgtagagcaatgaagcatagctgaatttacat

fava_c17377_956	fava_c17377	956	T/C	agggaRtaataagagcagtaacagaggatttggatgRtgaattggtttagggttttaagataaatagcggctatggWggtgctacaatgatattgcattg[T/C]gKWatttgaaacaaataactattacaactatgttgaatagcggctgtagagcaatgaagcatagctgaatttacataaatggctgttttctctgagctgc

fava_c17377_958	fava_c17377	958	G/T	ggaRtaataagagcagtaacagaggatttggatgRtgaattggtttagggttttaagataaatagcggctatggWggtgctacaatgatattgcattgYg[G/T]Watttgaaacaaataactattacaactatgttgaatagcggctgtagagcaatgaagcatagctgaatttacataaatggctgttttctctgagctgcaa

fava_c17377_959	fava_c17377	959	A/T	gaRtaataagagcagtaacagaggatttggatgRtgaattggtttagggttttaagataaatagcggctatggWggtgctacaatgatattgcattgYgK[A/T]atttgaaacaaataactattacaactatgttgaatagcggctgtagagcaatgaagcatagctgaatttacataaatggctgttttctctgagctgcaat

fava_c17378_308	fava_c17378	308	G/C	tcaagctatcaaagtgcggttatgacatcagaggaatagaaaacaaccgtatattcattggcaggaccaaagatcaatgtctcagctatttctggcaatc[G/C]gcggtcagcaaagtggggttagcatgctactgccgcactgccggtgattcatgtttcctctagaatctcatcatgaactgggtgtttgattctctccaaa

fava_c17379_1051	fava_c17379	1051	G/A	agagaaatattttacgtagaggaacattcttcagaaatccattataaagtccaacaccaagcagagatgcaatcgaggtaacgagcttgacacgtcctaa[G/A]aactcaggggtaaaaccaagagaatttgtgctaaagtagaacatagcagagtcagactgtggggttgcttgccacaagaaaataaataatgtgggaagaa

fava_c17379_1621	fava_c17379	1621	C/T	gtatgcaaatagcagcactatatttgttgtcaacagaagtagccatcagactccatgagagtgcaccaagcagccctgataaaactaggtatgaccttct[C/T]cgRtaaccaaaaagggggatagaatcgctaataaaaccatacagaggtttgacaagccatggaaacgcagaaattccagaaatcacagctgcctcggcag

fava_c17379_1624	fava_c17379	1624	A/G	tgcaaatagcagcactatatttgttgtcaacagaagtagccatcagactccatgagagtgcaccaagcagccctgataaaactaggtatgaccttctYcg[A/G]taaccaaaaagggggatagaatcgctaataaaaccatacagaggtttgacaagccatggaaacgcagaaattccagaaatcacagctgcctcggcaggat

fava_c17379_2033	fava_c17379	2033	G/C	tcccgcaccggtacgagaacctaataatgcgtcgctatcatcggtgctgagtcggaaagctgatgcggacctcggaacggtgccggacatttcagttgcc[G/C]gcagtttccgatgagttctccggcggtattgcggtgaacgagcaacgatcggtgtggagcgtaggttggaggtgtagaaattgggaaatttagagggtaa

fava_c17379_313	fava_c17379	313	T/C	tgtcgttggtggctggcattgtcttgtctcaatccgaagcatattgtacaacattgtctcaaggttcataacattgcactttcgccacaaccaaatgttc[T/C]gaagtgaattttgaYcttgtgttactaaaacatataatcaagatggtttaaaacaaggcccttgcggcaactttagcatcatactatatacaacaagact

fava_c17379_328	fava_c17379	328	C/T	gcattgtcttgtctcaatccgaagcatattgtacaacattgtctcaaggttcataacattgcactttcgccacaaccaaatgttcYgaagtgaattttga[C/T]cttgtgttactaaaacatataatcaagatggtttaaaacaaggcccttgcggcaactttagcatcatactatatacaacaagactctatctgcgcgtgga

fava_c17379_667	fava_c17379	667	A/G	cagggttgtcgccggggagaagaccaagaagtggcagaggcaataatgagctaagattgcagaggattatcaaagctgccaagttatcaaatttgtcctt[A/G]gtaattccaaatacttgagtcaggcctgcacctatcaatcccccaaggacactccctccattagatacagacatgagagttgcaaaaagagttgcttcca

fava_c17380_1108	fava_c17380	1108	C/T	agctttccatgctttggtgcactgaaacttgtttttactttcttttttgtaggcatttcaagggaagcatcgagactagcatgatcacttccttcttccc[C/T]atctgcagctgaatcagcatcaatgatattccatttttgcctcgagaggttcaatacttcaacttcaccRtcgtcataaacMaccttatgcttctttttt

fava_c17380_1178	fava_c17380	1178	G/A	cgagactagcatgatcacttccttcttcccYatctgcagctgaatcagcatcaatgatattccatttttgcctcgagaggttcaatacttcaacttcacc[G/A]tcgtcataaacMaccttatgcttcttttttataggatcaaaacgttcaatgacacctttgtaaaactcacgatccttaggccaccatacttcaacccgta

fava_c17380_1190	fava_c17380	1190	C/A	gatcacttccttcttcccYatctgcagctgaatcagcatcaatgatattccatttttgcctcgagaggttcaatacttcaacttcaccRtcgtcataaac[C/A]accttatgcttcttttttataggatcaaaacgttcaatgacacctttgtaaaactcacgatccttaggccaccatacttcaacccgtagaccaacaaggt

fava_c17380_1337	fava_c17380	1337	A/G	tgtaaaactcacgatccttaggccaccatacttcaacccgtagaccaacaaggttttcaccgtattccttgatcccagatccattttttcttcctgaggc[A/G]ggtttcctctttgcacttgttttgggagtctcctctaacttttcatcttctaaggcgggtttcctctttgcacttgttttgggagcctcctctaactttt

fava_c17380_1448	fava_c17380	1448	G/A	ttgcacttgttttgggagtctcctctaacttttcatcttctaaggcgggtttcctctttgcacttgttttgggagcctcctctaacttttcatcttctaa[G/A]gcgtgtYtcctctttgcacttgtcttggtagtctcttctaacttttcattttcggaggatttggtatcggaccttggagaagaaaYcatttccttatcaa

fava_c17380_1455	fava_c17380	1455	T/C	tgttttgggagtctcctctaacttttcatcttctaaggcgggtttcctctttgcacttgttttgggagcctcctctaacttttcatcttctaaRgcgtgt[T/C]tcctctttgcacttgtcttggtagtctcttctaacttttcattttcggaggatttggtatcggaccttggagaagaaaYcatttccttatcaacacctac

fava_c17380_1534	fava_c17380	1534	C/T	ttttcatcttctaaRgcgtgtYtcctctttgcacttgtcttggtagtctcttctaacttttcattttcggaggatttggtatcggaccttggagaagaaa[C/T]catttccttatcaacacctacactagaagactttgctgcacccgtctctgagttggcttccccccaccccaactttttttcatctacaagctgtctggaa

fava_c17380_227	fava_c17380	227	A/G	ctgccagtattcaagactagcaccaaagcattctttcaattgaggctgctaaccaaatggttttaactgttctcaaaaatccatccatctagtctagaag[A/G]aataatggaaaaaagaaataaaattattactctatagacccaaaatactactaaactagtcttaaggataacaatgctagcatagcaacaatgagctgag

fava_c17380_971	fava_c17380	971	A/G	taaactttctgctaacttcatcctcagaatcgctaatagtattggattctttggatttgtttccatccttagacttctgaccagacatcaaaacaccctt[A/G]gatatacttgatccagatgccccaccactcgaggatagctttccatgctttggtgcactgaaacttgtttttactttcttttttgtaggcatttcaaggg

fava_c17383_1290	fava_c17383	1290	T/C	ttagatttggtagaagtatgctcttggttatcagcaccactttcagattgatttgctaatgcatttccagtggctttcccctttttcttctttggccctt[T/C]atcagcatgcttgttggagccactgtcacttgccatttcactggacacactcaacctgcttgaatcataccctgcttttacttcattggctattggtaaa

fava_c17385_1836	fava_c17385	1836	A/G	acccaagtagcagatgggtcaaacggctcaagttatgctcatcagaacatgctcttggcaccaagagtgaaaatattggagaaacttcacatgtaaaact[A/G]actaatattttcagcaaaactaatttggaagaggaaacggaatataatgatgaaggtcacgctgaagccgaaggacagacggtactagaaccattatcaa

fava_c17385_2269	fava_c17385	2269	T/A	gggacatgtgatagtttggaacatgaaggggctttgaagtttttggccactgctaatataaatatctgagaagtcttggtataagtttgttaggaatata[T/A]atggtgtgtaatgggagtagcRtatcatactagtggagcaacatctaaataaagatattatattatgtatgggacttaatagtttRaattcgaaacacaa

fava_c17385_2291	fava_c17385	2291	A/G	atgaaggggctttgaagtttttggccactgctaatataaatatctgagaagtcttggtataagtttgttaggaatataWatggtgtgtaatgggagtagc[A/G]tatcatactagtggagcaacatctaaataaagatattatattatgtatgggacttaatagtttRaattcgaaacacaaatgtgaatgcaacatatataac

fava_c17385_2355	fava_c17385	2355	A/G	ttgttaggaatataWatggtgtgtaatgggagtagcRtatcatactagtggagcaacatctaaataaagatattatattatgtatgggacttaatagttt[A/G]aattcgaaacacaaatgtgaatgcaacatatataacataaaagtgttcatacccaaactactatggaatttattcctcccaatgacagttcacaaacaac

fava_c17385_3262	fava_c17385	3262	A/T	acctccattttagaagcttgagtaaaacatcggtcttgaatacgtaaacccccatagatgcaataaaaggcgacgttattgcatcttgaggtgacaatcc[A/T]aatagagaggtatctacttgcattgctttaagatcagctcccttaggtttttctgaaaattggatgatgcggcctctgctatctactttgactaatccat

fava_c17385_3477	fava_c17385	3477	C/G	ctgtcaccaacagcagcgcatgatactgttatatctgcatttctgtctatgtgactctgcacaagatccatgtaatccattctgtatagatgatctccag[C/G]caagattattacattctcaatgtttgtgttctttgcatcctcaaatacccatgtaaattgcctcacagcatctgctgttccttgaaaccaattcttcccg

fava_c17385_3918	fava_c17385	3918	C/T	tgaacaccaggacctcctcccaatatgatggaaacaacttttttagggtcagcttttcttctcaaaaaagatggaacctgaaatgtcttagactcagtgg[C/T]atcatttgatgtaagaacagcagacacaacaccatgttttaacttcttaaccctctctttggttttcaaacatatttccatctgattaataatccatgga

fava_c17385_4092	fava_c17385	4092	A/G	atttccatctgattaataatccatggactgtaattcacccccactttgattctctcacctagaaaaccattatcttgatgaaaaatattgtctttcctca[A/G]gtatgttatatgtgtattggtcttaaagttcacacaagtagacaccattgtgatggtttcaatgctgtgaagaaatgccacaaattcaagtcctttttaa

fava_c17389_1584	fava_c17389	1584	T/C	ctcaaaacacgcgcccatgggatggacctttcctttgcttcagttgccaggagaagaaagaagccatggaaggaaaaagatcatcagatagacttagcag[T/C]ggaagtgacgaatgaactctcgtgtacatttgcattttgtgagagtgatcaaaattgttcattccacgggaacttcgaacctcctagcaacagtgtctcc

fava_c17389_927	fava_c17389	927	C/T	caaatgaagaggagagggttcgtatcacttctagtgggggtgaggttggtcgtttaaatacagggggaggtgcagaggttggtcccttgagatgttggcc[C/T]ggtggcttgtgcctttcacgatctattggagatatggatattggtgaatttgttgtYcctgtaccacatgtaaaacaagtgaagctgtccactgctggag

fava_c17389_984	fava_c17389	984	C/T	atacagggggaggtgcagaggttggtcccttgagatgttggccYggtggcttgtgcctttcacgatctattggagatatggatattggtgaatttgttgt[C/T]cctgtaccacatgtaaaacaagtgaagctgtccactgctggaggccggcttgttatctgcagtgacggtgtttgggattctttacctgcagaagtggctc

fava_c17390_374	fava_c17390	374	G/A	ttgccaatttaatatcccaccatccaaaccatggtggctccacctcaaaccgccactcccacggctcacaccaccaattcacttcctcaagggagtatga[G/A]tttagctgtagcaacactcccaaccacttYttctcaataggaaagcgtcaccacaaccacaagcacaatactcaagcaaaacttacacatgatgatgagg

fava_c17390_404	fava_c17390	404	C/T	atggtggctccacctcaaaccgccactcccacggctcacaccaccaattcacttcctcaagggagtatgaRtttagctgtagcaacactcccaaccactt[C/T]ttctcaataggaaagcgtcaccacaaccacaagcacaatactcaagcaaaacttacacatgatgatgaggtgatgactatSagttcaatgaaggcggtgg

fava_c17390_485	fava_c17390	485	G/C	gcaacactcccaaccacttYttctcaataggaaagcgtcaccacaaccacaagcacaatactcaagcaaaacttacacatgatgatgaggtgatgactat[G/C]agttcaatgaaggcggtggtggagatgctttacaatgatcaagcgattgtagaagcgtctccaggtttccctttaagagatgatgatgagaaggataatc

fava_c17390_702	fava_c17390	702	T/G	tgaagatttcataaaaaggttttacagtcaattgaggaagcaagattgattgattaatattgattcaatttactttgtttaatggataatcaagtagcca[T/G]atttYtctctctttattttgcttattatcatggtatatacctattagtgagggYttttgattcattttaattatgtatttgcagatgattaMtttaaYtg

fava_c17390_707	fava_c17390	707	T/C	atttcataaaaaggttttacagtcaattgaggaagcaagattgattgattaatattgattcaatttactttgtttaatggataatcaagtagccaKattt[T/C]tctctctttattttgcttattatcatggtatatacctattagtgagggYttttgattcattttaattatgtatttgcagatgattaMtttaaYtgctgct

fava_c17390_756	fava_c17390	756	C/T	taatattgattcaatttactttgtttaatggataatcaagtagccaKatttYtctctctttattttgcttattatcatggtatatacctattagtgaggg[C/T]ttttgattcattttaattatgtatttgcagatgattaMtttaaYtgctgcttaacaataaggtaataaattacataattttttaaagagagtattagtgt

fava_c17390_794	fava_c17390	794	C/A	agtagccaKatttYtctctctttattttgcttattatcatggtatatacctattagtgagggYttttgattcattttaattatgtatttgcagatgatta[C/A]tttaaYtgctgcttaacaataaggtaataaattacataattttttaaagagagtattagtgtatatttctgtcttgagctttatcatgtaatggactttc

fava_c17390_800	fava_c17390	800	C/T	caKatttYtctctctttattttgcttattatcatggtatatacctattagtgagggYttttgattcattttaattatgtatttgcagatgattaMtttaa[C/T]tgctgcttaacaataaggtaataaattacataattttttaaagagagtattagtgtatatttctgtcttgagctttatcatgtaatggactttcaatctg

fava_c17392_513	fava_c17392	513	A/C	gtgatccgtgtccatgaataagctcggttgttatggggtggttggattcactatttggaaatggacgcagattcatcaaaaggaaagacagtgatgcagg[A/C]gaatcaggtaaagcgctggaagaactaagatcttctttgtacaatgagctccgaacatcagaaggagccaagcgacaacaacaaagatattgtggaccag

fava_c17404_1002	fava_c17404	1002	C/T	gtggcgaacaaagcaagaagtggaaagaggatggagggtttaagtaatgatgaattttcatcttttctaggggctcgtataagacgtgattccYgtccat[C/T]tttgtggtttgttatgcacatagcctcaccagggtagaaattcggtgcccttgaacttaatttaaacgggcctagatttctatatacatttgatggagct

fava_c17404_1423	fava_c17404	1423	C/A	aaacaagttcatattggtagcgacactttgcacaagatactatctgaataccttgctgtggctgtgcaactgcaacagctatccgacaactaggacacct[C/A]actatctttccaaagggRacctcttcagaaggagacacgcccacctcacatatatacttaaccacactaagagcatctgaaaccctcaaagatgcagcca

fava_c17404_1441	fava_c17404	1441	G/A	agcgacactttgcacaagatactatctgaataccttgctgtggctgtgcaactgcaacagctatccgacaactaggacacctMactatctttccaaaggg[G/A]acctcttcagaaggagacacgcccacctcacatatatacttaaccacactaagagcatctgaaaccctcaaagatgcagccaaaccctgaatcaacattg

fava_c17404_1872	fava_c17404	1872	A/C	gcggtggctgcgattcgatgaagaagctgttgatccaacaggttgttggagttggaggtttcgttttggctaacggaagctttgaggttggggaatttgg[A/C]tggtttgtgaagcgtggaagtggtgaaaatgcgattggaacaagtttgattgagtttgaaagaagagagaKgaagtgaaggagtagaaaggtttagaatc

fava_c17404_1943	fava_c17404	1943	G/T	aacggaagctttgaggttggggaatttggMtggtttgtgaagcgtggaagtggtgaaaatgcgattggaacaagtttgattgagtttgaaagaagagaga[G/T]gaagtgaaggagtagaaaggtttagaatcatggctgaaaattgaagcgaaatgcaaaaagttagttagatta

fava_c17404_253	fava_c17404	253	G/A	ttgttctaacataacaaatttcaaaatttgcctgctaaaaagatacatgtgacaggaagctgtgctctttttgctccgtcaagaacagagcattgaacag[G/A]aaagtRWggcttcaactaatgatgtagtggtatgaaccggatacatccataaatctttaaacttcatccaagggcaggggctgggagctaagaagtcttt

fava_c17404_259	fava_c17404	259	A/G	taacataacaaatttcaaaatttgcctgctaaaaagatacatgtgacaggaagctgtgctctttttgctccgtcaagaacagagcattgaacagRaaagt[A/G]Wggcttcaactaatgatgtagtggtatgaaccggatacatccataaatctttaaacttcatccaagggcaggggctgggagctaagaagtctttcggcct

fava_c17404_260	fava_c17404	260	T/A	aacataacaaatttcaaaatttgcctgctaaaaagatacatgtgacaggaagctgtgctctttttgctccgtcaagaacagagcattgaacagRaaagtR[T/A]ggcttcaactaatgatgtagtggtatgaaccggatacatccataaatctttaaacttcatccaagggcaggggctgggagctaagaagtctttcggcctc

fava_c17404_995	fava_c17404	995	C/T	atctccggtggcgaacaaagcaagaagtggaaagaggatggagggtttaagtaatgatgaattttcatcttttctaggggctcgtataagacgtgattcc[C/T]gtccatYtttgtggtttgttatgcacatagcctcaccagggtagaaattcggtgcccttgaacttaatttaaacgggcctagatttctatatacatttga

fava_c17406_1257	fava_c17406	1257	A/G	tcgaaggtatactcagagtccgacatattgttacaatatctacactacacatacacatgtaactacattcaaattttatgttattttttgaattattacc[A/G]gtgtcgatatKtctggtgtatgattgagagataaaatacaaatKtgttaagataagaagtagttgtttctttttcagattcttcatgtgattgatggtgg

fava_c17406_1268	fava_c17406	1268	G/T	ctcagagtccgacatattgttacaatatctacactacacatacacatgtaactacattcaaattttatgttattttttgaattattaccRgtgtcgatat[G/T]tctggtgtatgattgagagataaaatacaaatKtgttaagataagaagtagttgtttctttttcagattcttcatgtgattgatggtgggattattatga

fava_c17406_1301	fava_c17406	1301	G/T	ctacacatacacatgtaactacattcaaattttatgttattttttgaattattaccRgtgtcgatatKtctggtgtatgattgagagataaaatacaaat[G/T]tgttaagataagaagtagttgtttctttttcagattcttcatgtgattgatggtgggattattatgattctaatgtaaagatagttaccctttgaaagca

fava_c17406_373	fava_c17406	373	G/A	tttgtccgattgggccgttactcggatctcacgaacgcaaccgaagtgatcgaaatccagaaaggaagtggtagaaagtggactctggagagggatttgg[G/A]tcacttgagagaKgttaaagatggttcatttKatgactatgaggtggttaagattgttgggaaaaacccttttttgtggcatgatcattggcctgttatg

fava_c17406_386	fava_c17406	386	T/G	gccgttactcggatctcacgaacgcaaccgaagtgatcgaaatccagaaaggaagtggtagaaagtggactctggagagggatttggRtcacttgagaga[T/G]gttaaagatggttcatttKatgactatgaggtggttaagattgttgggaaaaacccttttttgtggcatgatcattggcctgttatggattacgctaagg

fava_c17406_405	fava_c17406	405	G/T	gaacgcaaccgaagtgatcgaaatccagaaaggaagtggtagaaagtggactctggagagggatttggRtcacttgagagaKgttaaagatggttcattt[G/T]atgactatgaggtggttaagattgttgggaaaaacccttttttgtggcatgatcattggcctgttatggattacgctaaggttttcgagtgcttggtttt

fava_c17406_556	fava_c17406	556	A/G	gatcattggcctgttatggattacgctaaggttttcgagtgcttggttttggttgatgagatttcgcgagaagctgatagagttgtgtctatgattagag[A/G]agttggaaacaatgatagatatgttgctgttcacatgagggtagaaattgattggatgattcattgcaagaaattggagcaaagRttgaacacaaatcaa

fava_c17406_641	fava_c17406	641	A/G	tgtctatgattagagRagttggaaacaatgatagatatgttgctgttcacatgagggtagaaattgattggatgattcattgcaagaaattggagcaaag[A/G]ttgaacacaaatcaaatatgtagtagcaagaatgagataatcgaaagagttgggaacattgaaggtcttaataagagtcctccaattgttctttatcttg

fava_c17406_743	fava_c17406	743	G/A	tgaacacaaatcaaatatgtagtagcaagaatgagataatcgaaagagttgggaacattgaaggtcttaataagagtcctccaattgttctttatcttgc[G/A]gtggctgataaacttcttcaaaactcttcaatacttgaaggttggaaacaaggttttttaccttatgagaagaagaaacttggtgttgatagaatctatg

fava_c17407_1022	fava_c17407	1022	A/C	ttctcttcagatgaggccacaccgcgacaacggaaggttggttttagaagctgtgtctcttccttcattgaacaacttctgtgttcaacgccaagatggt[A/C]gactSgtcctcacttttgcaaatcaagatgaagaaaWtggcgagaatgatgaggtaggtgagttggaggaagagtttgaaggattcgaagaagatgaagt

fava_c17407_1027	fava_c17407	1027	C/G	ttcagatgaggccacaccgcgacaacggaaggttggttttagaagctgtgtctcttccttcattgaacaacttctgtgttcaacgccaagatggtMgact[C/G]gtcctcacttttgcaaatcaagatgaagaaaWtggcgagaatgatgaggtaggtgagttggaggaagagtttgaaggattcgaagaagatgaagttgaag

fava_c17407_1059	fava_c17407	1059	T/A	ttggttttagaagctgtgtctcttccttcattgaacaacttctgtgttcaacgccaagatggtMgactSgtcctcacttttgcaaatcaagatgaagaaa[T/A]tggcgagaatgatgaggtaggtgagttggaggaagagtttgaaggattcgaagaagatgaagttgaagatgaggaatatgagagtgtgataggaaaagga

fava_c17407_1492	fava_c17407	1492	C/T	atgcagcaactgcttcattcaattttaattcttatcaacattactggaggaccacaaatccaacatcaaccaaaggtggtaattatccacttgaccgtta[C/T]caagagaaaaacaacaccaacgacaacaacaacaacgacaacaacaagtgtaacaaRattgtcgtgtctgcaaaaggtgagatgaagaacatgaacatga

fava_c17407_1549	fava_c17407	1549	G/A	atccaacatcaaccaaaggtggtaattatccacttgaccgttaYcaagagaaaaacaacaccaacgacaacaacaacaacgacaacaacaagtgtaacaa[G/A]attgtcgtgtctgcaaaaggtgagatgaagaacatgaacatgaaccgtgtttcaaatgagaagcaacagcttttggttcttaaaggaaagaatggtgatt

fava_c17407_1746	fava_c17407	1746	A/T	gattatttggttcataatttgaagaattgcaaggattcaagaaggtcttttctcttttgggaaccttattgcattgctacctcatgaatcacaaacctaa[A/T]catcactcacaaatatttactactatttcgaattaattaatccacaacaacaacatccattgctttMatctccgtcYctttttttataattWctcgtttg

fava_c17407_1813	fava_c17407	1813	C/A	attgcattgctacctcatgaatcacaaacctaaWcatcactcacaaatatttactactatttcgaattaattaatccacaacaacaacatccattgcttt[C/A]atctccgtcYctttttttataattWctcgtttgcattttgcaaacgaRtttatattYagRgacggatRtaatatttattcctctatatttYctttttatt

fava_c17407_182	fava_c17407	182	C/G	ttgagacctctgagtttgttgctgtactttgctctttcaagctaagtgcaattttaagatgtcaatttgtatgagcatgaagatagaagaagacagtgtg[C/G]tSaatcaaaagcaagggatagtcactattttgaactgtaacactgacactaatttcacaccaccttctcttagaagaactctctcagcagacatgtcttc

fava_c17407_1823	fava_c17407	1823	C/T	tacctcatgaatcacaaacctaaWcatcactcacaaatatttactactatttcgaattaattaatccacaacaacaacatccattgctttMatctccgtc[C/T]ctttttttataattWctcgtttgcattttgcaaacgaRtttatattYagRgacggatRtaatatttattcctctatatttYctttttattatgtctacta

fava_c17407_1838	fava_c17407	1838	T/A	aaacctaaWcatcactcacaaatatttactactatttcgaattaattaatccacaacaacaacatccattgctttMatctccgtcYctttttttataatt[T/A]ctcgtttgcattttgcaaacgaRtttatattYagRgacggatRtaatatttattcctctatatttYctttttattatgtctactaagttcaaatccttcg

fava_c17407_184	fava_c17407	184	G/C	gagacctctgagtttgttgctgtactttgctctttcaagctaagtgcaattttaagatgtcaatttgtatgagcatgaagatagaagaagacagtgtgSt[G/C]aatcaaaagcaagggatagtcactattttgaactgtaacactgacactaatttcacaccaccttctcttagaagaactctctcagcagacatgtcttcca

fava_c17407_1861	fava_c17407	1861	A/G	atttactactatttcgaattaattaatccacaacaacaacatccattgctttMatctccgtcYctttttttataattWctcgtttgcattttgcaaacga[A/G]tttatattYagRgacggatRtaatatttattcctctatatttYctttttattatgtctactaagttcaaatccttcgtgaaggtgtaatgtagtgtagtc

fava_c17407_1870	fava_c17407	1870	T/C	tatttcgaattaattaatccacaacaacaacatccattgctttMatctccgtcYctttttttataattWctcgtttgcattttgcaaacgaRtttatatt[T/C]agRgacggatRtaatatttattcctctatatttYctttttattatgtctactaagttcaaatccttcgtgaaggtgtaatgtagtgtagtcaaataaaag

fava_c17407_1873	fava_c17407	1873	A/G	ttcgaattaattaatccacaacaacaacatccattgctttMatctccgtcYctttttttataattWctcgtttgcattttgcaaacgaRtttatattYag[A/G]gacggatRtaatatttattcctctatatttYctttttattatgtctactaagttcaaatccttcgtgaaggtgtaatgtagtgtagtcaaataaaagaaa

fava_c17407_1881	fava_c17407	1881	G/A	aattaatccacaacaacaacatccattgctttMatctccgtcYctttttttataattWctcgtttgcattttgcaaacgaRtttatattYagRgacggat[G/A]taatatttattcctctatatttYctttttattatgtctactaagttcaaatccttcgtgaaggtgtaatgtagtgtagtcaaataaaagaaaagggaatt

fava_c17407_1904	fava_c17407	1904	C/T	cattgctttMatctccgtcYctttttttataattWctcgtttgcattttgcaaacgaRtttatattYagRgacggatRtaatatttattcctctatattt[C/T]ctttttattatgtctactaagttcaaatccttcgtgaaggtgtaatgtagtgtagtcaaataaaagaaaagggaattaagaggaaagggtagtaagagtt

fava_c17407_322	fava_c17407	322	C/T	acactgacactaatttcacaccaccttctcttagaagaactctctcagcagacatgtcttccaagaaatggctttctcataatggttcttccaacaccat[C/T]aagaagatttcatcatctgagaatctgtttggacaagatgatggtgagttcaaagaagctaagttgggtgatgatgaagatgaagctgagagagagaggc

fava_c17412_1032	fava_c17412	1032	A/G	tttttgactcttgttcWcagctaaaggtgttaaagttacaggcatgcaaatatctcactgactcatcacttgaacctctttacaaagggggtgctttacc[A/G]gctcttcaggagttggacttgtcttatggaactttgtgtcagaaagccatagaagagcttctttcttgctgcactcaccttacacgtgtgagtttgaatg

fava_c17412_948	fava_c17412	948	T/A	tagagggactttgttccctgcactggctcccaaatttagctcttcttgacttatcatacactttcttggtgaacctgcagcctgtttttgactcttgttc[T/A]cagctaaaggtgttaaagttacaggcatgcaaatatctcactgactcatcacttgaacctctttacaaagggggtgctttaccRgctcttcaggagttgg

fava_c17413_300	fava_c17413	300	G/C	aaactacaacagagttagcagcagcagcagcagtagtaaaggatcatcatcatcatcttgccatggaaaatgcagaggatttaggctgaacctcacaaga[G/C]tttactactcgcggctacgcaaacgggtcgatttctttctcagaatctttgataagctgaaactatcacatgcacaagcttttcagttgttgaagaaagt

fava_c17416_1917	fava_c17416	1917	A/T	tatcattactcttacttgcactatctgcatcagtttgactgtctgattccctctttgacttatgttttgatcgcttatctaatttccttcgcttcttttc[A/T]cggcgtttatctcttctttttccatgtctatgtttatctttcctatatctctttctcttcttccgcctgtcatcactagaagagcttgtgtaagatgacR

fava_c17416_2017	fava_c17416	2017	G/A	Wcggcgtttatctcttctttttccatgtctatgtttatctttcctatatctctttctcttcttccgcctgtcatcactagaagagcttgtgtaagatgac[G/A]aggacaaatccgagtcagagtcactgtcactttctgaagattctgtgtctgaatctgaggaactgtctgattctgatgagtaatgttttcKcttttttct

fava_c17416_2108	fava_c17416	2108	T/G	taagatgacRaggacaaatccgagtcagagtcactgtcactttctgaagattctgtgtctgaatctgaggaactgtctgattctgatgagtaatgttttc[T/G]cttttttcttctgtctactgaagattttttgtgttttcccttcctccgcgcttcatgactgatggcttcagaagatccattccttccaattttKgacttc

fava_c17416_2202	fava_c17416	2202	G/T	gttttcKcttttttcttctgtctactgaagattttttgtgttttcccttcctccgcgcttcatgactgatggcttcagaagatccattccttccaatttt[G/T]gacttctgtgtcttcttaccatcctcgttatgttcaccactattaattattttaacagttacggatgggagtccttcatcgcccacatcttcaattttct

fava_c17416_702	fava_c17416	702	C/T	caggtgacctggatcgagatggactcctgctgagggatgatgacatgcgcctattcacatgaggtgaacgatggcgaaccgggctacggcttgggctata[C/T]cgtcgagaacggtaaggtgggctgcgtgatacagatggtgtcctgcttctcctactccggtaccttggtggagttcttctccgtggcggtggactcctat

fava_c17419_1238	fava_c17419	1238	G/A	ctcgaagtgtggtgagaagaaacttggttctatcttttctagatacattttccccaatataatgctttggtatctctctaccattagctttttgctcaga[G/A]ggaatagattcagatttggtttgcgaataacatggttgggacaacatttgactgtaacatcggttgacatcactagtgttcactttactcaatctattaa

fava_c17419_293	fava_c17419	293	A/G	ccatgcaacccatacccacacactgaagacttgacgaatagtcaattttccagtagactgtccttgatactgctgttttttaaattatatgacctcgtac[A/G]tatggcccgggtgcatggtgtKttgcttctatgacaagctcgggaaaaaactgcttcYattacaagctcgggaaaaaacgaaacaaatgtttatattatc

fava_c17419_315	fava_c17419	315	T/G	ctgaagacttgacgaatagtcaattttccagtagactgtccttgatactgctgttttttaaattatatgacctcgtacRtatggcccgggtgcatggtgt[T/G]ttgcttctatgacaagctcgggaaaaaactgcttcYattacaagctcgggaaaaaacgaaacaaatgtttatattatcctctacctaaagttggttgatg

fava_c17419_351	fava_c17419	351	T/C	tgtccttgatactgctgttttttaaattatatgacctcgtacRtatggcccgggtgcatggtgtKttgcttctatgacaagctcgggaaaaaactgcttc[T/C]attacaagctcgggaaaaaacgaaacaaatgtttatattatcctctacctaaagttggttgatgctgttaaaaccaatataacaattaattcctacatcc

fava_c17419_462	fava_c17419	462	C/T	cgggaaaaaacgaaacaaatgtttatattatcctctacctaaagttggttgatgctgttaaaaccaatataacaattaattcctacatcctgctgcaaat[C/T]atggaagcttgatttaaggattcacgtcccacagtcaacaaattagacagcctcaggttctatcttaataaaattcagcttaccgctacttttcctcaga

fava_c17419_585	fava_c17419	585	T/A	cacgtcccacagtcaacaaattagacagcctcaggttctatcttaataaaattcagcttaccgctacttttcctcagagtcctgtatagattcattgggc[T/A]gccttttcttctttgttgcactcgactttcttccacctttcctgactggtcttggtccttcctctatgaagagatgactgWatttttccattaYtctctc

fava_c17419_666	fava_c17419	666	T/A	ctgtatagattcattgggcWgccttttcttctttgttgcactcgactttcttccacctttcctgactggtcttggtccttcctctatgaagagatgactg[T/A]atttttccattaYtctctctttctcttcaatcaagcctaacatctcataagcatttgccactttcttaatgattaatttatcacgaggtttacgatcaaa

fava_c17419_679	fava_c17419	679	T/C	ttgggcWgccttttcttctttgttgcactcgactttcttccacctttcctgactggtcttggtccttcctctatgaagagatgactgWatttttccatta[T/C]tctctctttctcttcaatcaagcctaacatctcataagcatttgccactttcttaatgattaatttatcacgaggtttacgatcaaaagcttccagtccc

fava_c17430_1402	fava_c17430	1402	C/T	caataacctcggcactctctttaacccgagcatagcctggattagacttagccttctgatatttagcctccatttcctgctcagaggcaggaagaaatcg[C/T]ggaacattcttccatggcccaatagtcctctctcgtccaaaaattttaaaagggcgtttgatgtctccgtaaatgttatctcccatcaatataaatattt

fava_c17430_1602	fava_c17430	1602	T/G	tgaggattgaagtctgccactgcatcccatataggctgaggagcactctggttagaacatgatccaaaggcaattctcgacacaaggttttcgcggtttt[T/G]gtgactgtcggaagcggttacggtggttgaaacggttgttatttcgatgaataaaaggaagcaccgccatagccagcgccgcggacattcatcgacaccc

fava_c17430_209	fava_c17430	209	T/A	tcaaaggatcaatactaaaagttgtataaatattttaaatcaaaaatcaaacacactaactgtaacaactagtgaaaacagtagaagaatactctatata[T/A]cttattaacctataataatcagtagtaatatggttaaatctataaagggaaaaatatcacacatgctccctttagctgattcagtattcacctaactata

fava_c17430_529	fava_c17430	529	C/T	gatatcttttaatccacggcaaactagcttcaagagtgcaatgcctctgattatctcctgtttttactttgtcaagagagtctgattttgatggatgtaa[C/T]tccgttaacgMaacattgacacctgtaacagcgaccccattcttgtcccgaacttcgaatttcacactcacggggtgagattcccaatcgatttcaatgg

fava_c17430_540	fava_c17430	540	A/C	atccacggcaaactagcttcaagagtgcaatgcctctgattatctcctgtttttactttgtcaagagagtctgattttgatggatgtaaYtccgttaacg[A/C]aacattgacacctgtaacagcgaccccattcttgtcccgaacttcgaatttcacactcacggggtgagattcccaatcgatttcaatggttccgaagttt

fava_c17430_904	fava_c17430	904	A/G	agtccgaagcacagtcatatcttgttatttcgccaaagtgaacatctccgcttataaataatactccacctcgcttactatcggctattaatttgaaaag[A/G]cggtctctttcctttggaaaacgaccccatgattccattgcgaccaacggttgaagtgttgccgatagatttgatataacctgaatagaagatccaatta

fava_c17435_1255	fava_c17435	1255	A/T	ttaaggagcatttgctttcggtagcatcttactacacaaaagccaagtacttactagtggaaaagaaaggaatcgttaacgttattgttcataaagattc[A/T]agtagtgctgatgttctgaagtcatttattcatgctcttgtcctcgcaaataatgcttataaaagcaaatctttgcattcagacagccaaacatggatgg

fava_c17435_1470	fava_c17435	1470	G/A	gtatttattcagaaggtcaagtcattagggtggaaaacagaacgcctcctatcatcacctatcgtatggagagcaaactggatacaccaaccgtcggccg[G/A]aaaaattgattaggctcatctccagttgtgatacattaacactggccaaggggaaactaatggaaggaccggatggactgagttatttatgattgtacaa

fava_c17435_161	fava_c17435	161	T/C	cctgttacaacctgcgaagtgatcaaggtttgtctcgtaattcaactatttcacttcacttcatgttctcagcatcatggaatcagtttcaccttcttct[T/C]cacccgtcatctcctccaacaaaactccatcattaacattggaagaatggaacggttcatctcccactaaRctctccaagactttcaccatcaaagcctc

fava_c17435_232	fava_c17435	232	A/G	gcatcatggaatcagtttcaccttcttctYcacccgtcatctcctccaacaaaactccatcattaacattggaagaatggaacggttcatctcccactaa[A/G]ctctccaagactttcaccatcaaagcctcttcttcctctttctccatacacagatctggtgcccgMtttacccatgtttggagacgctttcttcaagcat

fava_c17435_298	fava_c17435	298	C/A	cattggaagaatggaacggttcatctcccactaaRctctccaagactttcaccatcaaagcctcttcttcctctttctccatacacagatctggtgcccg[C/A]tttacccatgtttggagacgctttcttcaagcatttgttcctgaggggtttccaagcagtgttactccggattatgttccttttcaaatctgggatttat

fava_c17435_640	fava_c17435	640	C/T	gaatcttattcacattctaccagggatcaaatcttgatagtaatgcaaaaatgtggcgtttggttgcagatctcatgaatgatctcggtatgctaatgga[C/T]ctcatttcaccattatttccatcagcttttgtttttattgtttgcttaggaagcatatcaagatctttcaccggggttgcgagtggagctactagagcag

fava_c17435_781	fava_c17435	781	T/C	tttgcttaggaagcatatcaagatctttcaccggggttgcgagtggagctactagagcagctttgactcaacattttgctcttcaggataacgctgcaga[T/C]atatctgctaaggaaggaagtcaagaaacagtggctacgatgattggcatggcactgggaatgcttgttgctcgcattactattggacatccactagcaa

fava_c17435_906	fava_c17435	906	C/G	gaaacagtggctacgatgattggcatggcactgggaatgcttgttgctcgcattactattggacatccactagcaatttggttttcttttttatctctga[C/G]cctgtttcatatgtatgcaaactaccgagctgttcgatgcttggcactgaactcactaaaccctgaaagaagctctattcttttgcagcattttacggag

fava_c17443_1085	fava_c17443	1085	C/T	tgcgttggtcaccatattcatccgaagcagatcttctgaaacaatttcaagacatgggatcgtacggtactaaagttattgtctacaatttgtggttcaa[C/T]gatgaggggatYacggagttagattttgatacagatcccaaggatattcgcattgcttgggatgtcaRgaagatcagtactatacctacacggaagagaa

fava_c17443_1097	fava_c17443	1097	T/C	catattcatccgaagcagatcttctgaaacaatttcaagacatgggatcgtacggtactaaagttattgtctacaatttgtggttcaaYgatgaggggat[T/C]acggagttagattttgatacagatcccaaggatattcgcattgcttgggatgtcaRgaagatcagtactatacctacacggaagagaatccaagaggaac

fava_c17443_1153	fava_c17443	1153	A/G	actaaagttattgtctacaatttgtggttcaaYgatgaggggatYacggagttagattttgatacagatcccaaggatattcgcattgcttgggatgtca[A/G]gaagatcagtactatacctacacggaagagaatccaagaggaacatattgcaaaaacatttcgttactcattacgtgtgtacttgtccatattgtacttg

fava_c17443_1806	fava_c17443	1806	A/C	attgcaaaaatcacttggatatcataataagaggaagacagatgaaccgatagaccttcagaagaggaaaaaacatgcaagtgaagaatatgtaactggt[A/C]ttggtttcRgtcagaataaacagatcaccacaactcctgcagacgaggttgtaaacccagagactataaatttgatgcaagaaaataagaaactacatac

fava_c17443_1815	fava_c17443	1815	G/A	atcacttggatatcataataagaggaagacagatgaaccgatagaccttcagaagaggaaaaaacatgcaagtgaagaatatgtaactggtMttggtttc[G/A]gtcagaataaacagatcaccacaactcctgcagacgaggttgtaaacccagagactataaatttgatgcaagaaaataagaaactacatacagaatgttt

fava_c17444_1027	fava_c17444	1027	G/A	acatgcatcaaggtcagcctaatgtggggaattcttttttaagtggtgtaaagcagcgattgctttcattcatattccagggattatggaatgaagaaac[G/A]gatgataccttgaggatccaaagacttaggaacaagttttacttccactttcaagattatgttgatttggttatctggaaggtgcagtttttggatcgac

fava_c17444_1285	fava_c17444	1285	T/C	accatccagcttttgttgctgtatacaacatggatacaactgaaattatatctttttatcacaattcagcagatgagctttatctgttgtttgagaagtt[T/C]ggtgatcactttcatgtaacctcaaggaatttaatgtatatgaatttcatatcttctcattcaaataatattcatgcgctggaacagctatggagcataa

fava_c17444_1769	fava_c17444	1769	G/A	atttttgccctttgctctctctattcagcagactttgttcttgcagccttccgttgtaaatattcactttcgaagatgagttttctctccactgtaaact[G/A]ttttcactgaatctagaggagtcaacgcatgtcaattcatgtttgttttttttttataaaaaaattcctctggtgtagttttagtgaattttattaagca

fava_c17444_280	fava_c17444	280	C/T	aaatttgcactcctgctcctggcactagtgttcatcatgcccggcgattctatgagaatttggtgccaagttacacagtatatgaggttgagttccctga[C/T]cattcatttcgtaaattcacagatgatggtcaataccttgtaagtttcagcagaaatcatcaggagttgattgtttatagacctagatggctttcttttt

fava_c17445_113	fava_c17445	113	A/G	ttacaataataacctgattattatcgttgttgggaaaaggtgatcatcggaagctctttacaacacttctcgtcgtcttctcttcaattcttttcctctc[A/G]caaaacttgtaatcgtttcggacatgtcaagtggatctgtgagacgggtttctcgggaagatatYcagcttgtRcaaaatctcatagaacgatgtctgca

fava_c17445_1249	fava_c17445	1249	T/A	gcagtgatgggaaattaaattttaaagggtttgtatgtactcaataaataataaatctctcttattttggtttgtttttcctcctgtatccgcacgggta[T/A]atagctttgcatagtggttttttctactttgtttatataccagctctattgtgttttgatgatgttctttttggcatagaatccatgaaactgtatttta

fava_c17445_178	fava_c17445	178	T/C	cttctcgtcgtcttctcttcaattcttttcctctcRcaaaacttgtaatcgtttcggacatgtcaagtggatctgtgagacgggtttctcgggaagatat[T/C]cagcttgtRcaaaatctcatagaacgatgtctgcaattatatatgaaYccaaaagaagttgtSgaaactctactagctcaagcaaaaattgagcctggtt

fava_c17445_187	fava_c17445	187	A/G	gtcttctcttcaattcttttcctctcRcaaaacttgtaatcgtttcggacatgtcaagtggatctgtgagacgggtttctcgggaagatatYcagcttgt[A/G]caaaatctcatagaacgatgtctgcaattatatatgaaYccaaaagaagttgtSgaaactctactagctcaagcaaaaattgagcctggttttaccgaac

fava_c17445_226	fava_c17445	226	C/T	tcgtttcggacatgtcaagtggatctgtgagacgggtttctcgggaagatatYcagcttgtRcaaaatctcatagaacgatgtctgcaattatatatgaa[C/T]ccaaaagaagttgtSgaaactctactagctcaagcaaaaattgagcctggttttaccgaacttgtttggcaaaagcttgaagaggaaaatcaagagtttt

fava_c17445_241	fava_c17445	241	C/G	caagtggatctgtgagacgggtttctcgggaagatatYcagcttgtRcaaaatctcatagaacgatgtctgcaattatatatgaaYccaaaagaagttgt[C/G]gaaactctactagctcaagcaaaaattgagcctggttttaccgaacttgtttggcaaaagcttgaagaggaaaatcaagagtttttcaaggcttattatt

fava_c17445_667	fava_c17445	667	T/A	tgttcaataatggcgggtcatcactgaacacaagtatacgcgatttgactcagatatctgctcgtggcaataggattagcagcccaccaagcatgcttcc[T/A]tctcaaaactcaagcttgggtttgatacaaggaattaatggtgggatgataaaatcagagccYggatattcgggtagtcccccttacatttttggtcatg

fava_c17445_730	fava_c17445	730	T/C	gtggcaataggattagcagcccaccaagcatgcttccWtctcaaaactcaagcttgggtttgatacaaggaattaatggtgggatgataaaatcagagcc[T/C]ggatattcgggtagtcccccttacatttttggtcatgatggcactgtcttggaagcatgYccaactattggtgatgcagcggttacatcatttaatagtg

fava_c17445_790	fava_c17445	790	C/T	tgatacaaggaattaatggtgggatgataaaatcagagccYggatattcgggtagtcccccttacatttttggtcatgatggcactgtcttggaagcatg[C/T]ccaactattggtgatgcagcggttacatcatttaatagtgttgactcaaactcccattctatgaatggagcactcattgacccagatatttcatcatttg

fava_c17447_1055	fava_c17447	1055	A/C	ttttgagctgctaatactagaagaagactcaactatgtagcccgtaatcgtatgatcaatcttggtctctgggttgtattctgtgctcgttgagattgtt[A/C]cctcggaattgttaggacgatagcttgtatgtacatcgRcatttccctcaattggatgaacaagttccaaacgatttacgaatcccatttctctRtcgtt

fava_c17447_1094	fava_c17447	1094	G/A	gcccgtaatcgtatgatcaatcttggtctctgggttgtattctgtgctcgttgagattgttMcctcggaattgttaggacgatagcttgtatgtacatcg[G/A]catttccctcaattggatgaacaagttccaaacgatttacgaatcccatttctctRtcgttatggcccttgccgccactagctttggaggatctttttgt

fava_c17447_1150	fava_c17447	1150	G/A	ttgttMcctcggaattgttaggacgatagcttgtatgtacatcgRcatttccctcaattggatgaacaagttccaaacgatttacgaatcccatttctct[G/A]tcgttatggcccttgccgccactagctttggaggatctttttgttttatcctttgagttaactatgggatacactggaagaacattagatgatgcattat

fava_c17447_1699	fava_c17447	1699	A/G	gaccaaaatcacctagccgaatattgttgtccttggtgaggaatatgttggaacacttaagatcccggtggattacacggttagaatgcaagtaatctac[A/G]gctatcaaaagttgagttagccacttgcagaccttctcctctggaaaaaacgatcctcgagctttcttaatgctctcggccatgtcacctccttcacaat

fava_c17447_289	fava_c17447	289	A/G	cagggttaaacatttgagcagatatgaggctctttgtcaaccatattgctgtttctcttgatgatacagcttctttcccaaatggtcttagcacacctgc[A/G]agttcttcgagtttatcttgtttaagtaactgagcacataactcgagcagagattccaatgcatcggctctttcctggcatggaccatccaacaatgcat

fava_c17447_466	fava_c17447	466	G/T	ggcatggaccatccaacaatgcatcgtcgattgctttgtcgccctcatcaccaccattcattgttacattatcatcttctttctgtgtggagcaaataat[G/T]tgWagttttttgacgccatcctccttaacggaaagtatatcgctatgatYgttgctctctgacgacaagcagttcgtatgcaagactttttcKgtaaaag

fava_c17447_469	fava_c17447	469	A/T	atggaccatccaacaatgcatcgtcgattgctttgtcgccctcatcaccaccattcattgttacattatcatcttctttctgtgtggagcaaataatKtg[A/T]agttttttgacgccatcctccttaacggaaagtatatcgctatgatYgttgctctctgacgacaagcagttcgtatgcaagactttttcKgtaaaagaat

fava_c17447_516	fava_c17447	516	C/T	ccaccattcattgttacattatcatcttctttctgtgtggagcaaataatKtgWagttttttgacgccatcctccttaacggaaagtatatcgctatgat[C/T]gttgctctctgacgacaagcagttcgtatgcaagactttttcKgtaaaagaatcacggttttcatttttctctgagtgttctctggattcagtactgttt

fava_c17447_559	fava_c17447	559	T/G	aaataatKtgWagttttttgacgccatcctccttaacggaaagtatatcgctatgatYgttgctctctgacgacaagcagttcgtatgcaagactttttc[T/G]gtaaaagaatcacggttttcatttttctctgagtgttctctggattcagtactgttttgttcaacccttagaggatgcataWtaMgcgatgagctttcct

fava_c17447_641	fava_c17447	641	A/T	cgtatgcaagactttttcKgtaaaagaatcacggttttcatttttctctgagtgttctctggattcagtactgttttgttcaacccttagaggatgcata[A/T]taMgcgatgagctttcctcgttaatgcaaatatttttgtcagtgctgctattgctcacactgcaagtagttgattttctcaggtcatcaagtttggcatc

fava_c17447_644	fava_c17447	644	A/C	atgcaagactttttcKgtaaaagaatcacggttttcatttttctctgagtgttctctggattcagtactgttttgttcaacccttagaggatgcataWta[A/C]gcgatgagctttcctcgttaatgcaaatatttttgtcagtgctgctattgctcacactgcaagtagttgattttctcaggtcatcaagtttggcatcttc

fava_c17447_749	fava_c17447	749	A/G	tgagctttcctcgttaatgcaaatatttttgtcagtgctgctattgctcacactgcaagtagttgattttctcaggtcatcaagtttggcatcttccctc[A/G]gtgctttRgcattatcaaaacctttacttgagaaggtaccttcgacttcggtagtcgctacagcaaatctttgttctttttcatgcagagaatcgtgtat

fava_c17447_757	fava_c17447	757	G/A	cctcgttaatgcaaatatttttgtcagtgctgctattgctcacactgcaagtagttgattttctcaggtcatcaagtttggcatcttccctcRgtgcttt[G/A]gcattatcaaaacctttacttgagaaggtaccttcgacttcggtagtcgctacagcaaatctttgttctttttcatgcagagaatcgtgtatgctttttg

fava_c17453_1216	fava_c17453	1216	T/C	ggaaaaaacttgtgttattcagtttgtctggtgttattttgtcacttgttgttttgactgttgtgtttcatcagagttcgactcactcgccaatgatcag[T/C]gctggcgaaacctcacatttcaataatacttgccctgattacagtactgctatggactctggtggatgggattgcatgaagtgccttaaggcttctccag

fava_c17453_1831	fava_c17453	1831	G/A	ttccaatggaagaggtggagaatatgcttgaaagaaggtctcttaattttaaattttggcagggaagttctgattctggttaagacacagaagactctca[G/A]tcaatttaaaatgttaatattaaaatgtgggtgttaatttgttaggcaatgtagacaatttgtcaaggaaaaaaaaacgtttatgtgtctataatatggt

fava_c17453_595	fava_c17453	595	C/T	ttggtgttggaatggcttcaatggcatcaccattatacatctcagaagcttctccaactcgagttcgaggtgccttagttagtctcaatggatttctcat[C/T]actggtggacagtttctctcttacctcatcaacttggccttcacaaatgcaccaggaacatggaggtggatgctaggagtagcagcggtaccagctctat

fava_c17453_713	fava_c17453	713	A/G	ctcttacctcatcaacttggccttcacaaatgcaccaggaacatggaggtggatgctaggagtagcagcggtaccagctctattacaattcgcgctgatg[A/G]taatgcttcctgaatctccgcgttggttgtttcgaaaaggaagagaagaagaagctaaaacaattttaaggagaatttatccacccgaagaagccgaagc

fava_c17453_862	fava_c17453	862	T/C	aagaagctaaaacaattttaaggagaatttatccacccgaagaagccgaagccgaaattaatactctaaaggaatcagttgaattagaaatcaaggaagg[T/C]gaatcttctgataaactcagcataatcaaaatgctKaaaaccaaaacagtgaggagaggtttatatgcaggaatgggccttcaaattttccaacagtttg

fava_c17453_898	fava_c17453	898	G/T	ccgaagaagccgaagccgaaattaatactctaaaggaatcagttgaattagaaatcaaggaaggYgaatcttctgataaactcagcataatcaaaatgct[G/T]aaaaccaaaacagtgaggagaggtttatatgcaggaatgggccttcaaattttccaacagtttgtaggaatcaacactgtcatgtactatagtccagcaa

fava_c17459_382	fava_c17459	382	A/G	agagaagtgtgatggatctacaaggtgaagtaatggcatgtggatatgaagatgttcaagttatgtggtcaattttggataggactaaatccacagccac[A/G]gaacagaatgcgacataacatcttctatgtgaatttactgaaatgcccttttcttccatttcataaattagcgaatgttttttatatgctaattaatgtt

fava_c17462_744	fava_c17462	744	A/G	ttttcagcataaatctttttacttgcggtgaaaatgaagatttcaaacatctttgatactctctccaaaaactcgtggaggaagggtctttttctcacat[A/G]aacggtgtactccttatcaagtaaaatttgaactgtgaaatcagcaccactatcatattgtgtcattgtagagtggatcagtgtttcatccaaatcaaga

fava_c17462_856	fava_c17462	856	G/A	ccttatcaagtaaaatttgaactgtgaaatcagcaccactatcatattgtgtcattgtagagtggatcagtgtttcatccaaatcaagaacaagggtaac[G/A]cgcttggttctacttgtctccctgctcactagagctggcaacaagttagactcgtcagatacctccaagaagttttttatcaataactcttgatcagcat

fava_c17466_1329	fava_c17466	1329	G/A	tcatcctctcctgccaaagaaacacatcctcatcttttgcaggatggaaataagtaggataaggaatcccaaaatcattcgcattccaaggactagattc[G/A]acaacaagcatagacatattcttagcagctggtaaaaacaaaagcttgttcccccaatccgattcctcctcagacaatctcctaaaatcccaagttatcc

fava_c17466_1463	fava_c17466	1463	T/A	aaaaacaaaagcttgttcccccaatccgattcctcctcagacaatctcctaaaatcccaagttatcctacccgcaacaagaaaatgatctctcccgttca[T/A]gaYcccccactggggcYgcttggtcaaccaatcaacaagatcaagcgaagcagcatctctcatagaaatattataaccccaaagataacgagcaatgtca

fava_c17466_1466	fava_c17466	1466	T/C	aacaaaagcttgttcccccaatccgattcctcctcagacaatctcctaaaatcccaagttatcctacccgcaacaagaaaatgatctctcccgttcaWga[T/C]cccccactggggcYgcttggtcaaccaatcaacaagatcaagcgaagcagcatctctcatagaaatattataaccccaaagataacgagcaatgtcaaag

fava_c17466_1480	fava_c17466	1480	C/T	cccccaatccgattcctcctcagacaatctcctaaaatcccaagttatcctacccgcaacaagaaaatgatctctcccgttcaWgaYcccccactggggc[C/T]gcttggtcaaccaatcaacaagatcaagcgaagcagcatctctcatagaaatattataaccccaaagataacgagcaatgtcaaagccagcgtaaaacgg

fava_c17466_405	fava_c17466	405	C/A	atagtacaagttctgcaacatgttgatagcatgatccctctaactattttatagttgcaattcttgttctccctaattaagggaaatctttagaatcttg[C/A]gctagacagcttttcagaaaccagcagccagaagatgcagatttttacctcaccatggcaaatcattattgagttctttgctcatttttccaagaatttt

fava_c17466_865	fava_c17466	865	T/C	atacactaatctaggaatgagactaatcaccttctctctcatgaccctcacttgctcctcaggtatctgcctaagcctctgctctatagtaacactcttc[T/C]ttcgaatatcatcctcgggaatgaatacagaatacttagtataatccttaggtagatgccaggtatattgcgtatacgcggaacccggatggaaaaacac

fava_c17467_1015	fava_c17467	1015	G/A	gtcaggagcatccgctgttggccaacctgccttcaccacaaaaccatccttcttcaaaatctctctccaaataaactctgcataatgtggacagataggt[G/A]ctagaaggcgtgtctgcacatccataaaacgccacaccaaatcacgatttaaaccgccaaccccacatgacaatatgtactcatccttagcagcctgaag

fava_c17467_1168	fava_c17467	1168	T/C	accgccaaccccacatgacaatatgtactcatccttagcagcctgaagatcatgaaagccagtcttgagggcatctcgaaacatatagttggagtagttt[T/C]gctcagttgttctgacagcaatgttaatgtcattagcaaaaacacgatcagcgtaaatagacggcggaccagttctcatagaagattctgcaggcaaaac

fava_c17467_1578	fava_c17467	1578	C/A	tgcatctaaacccacgaggccagtgatgtttgggaaaaattgcagtatggttgtaaatgcaaaaagtaaggtgattttggataagatctttaccagaaac[C/A]cgaagatcaaatggataccagtactcaaactcRagcttcattctttctaaaacggatgatgaaatatcggtggccttagggagagctccatcatagaaaa

fava_c17467_158	fava_c17467	158	A/C	caaagtgttccttacagcaaactgataaacaaaacaaaaacattgttgactggaaataaatcatgataaattaagttggtcagtgtaatgcaactgtact[A/C]cgatcattataattgtaaaagcacgaggttcttgaagatacaatgtaacaaaattccggcctgttttcacaaggatttctgccaacRgagataaacttgc

fava_c17467_1611	fava_c17467	1611	G/A	gaaaaattgcagtatggttgtaaatgcaaaaagtaaggtgattttggataagatctttaccagaaacMcgaagatcaaatggataccagtactcaaactc[G/A]agcttcattctttctaaaacggatgatgaaatatcggtggccttagggagagctccatcatagaaaatataatcccagaYatcatcagtcagttgttgag

fava_c17467_1691	fava_c17467	1691	C/T	ggataccagtactcaaactcRagcttcattctttctaaaacggatgatgaaatatcggtggccttagggagagctccatcatagaaaatataatcccaga[C/T]atcatcagtcagttgttgaggtttgatagatgattcaccggttccatacatgtccccatcctgcaaataatgtgcaatagtgtaataagccatgtaaatg

fava_c17467_245	fava_c17467	245	A/G	atgcaactgtactMcgatcattataattgtaaaagcacgaggttcttgaagatacaatgtaacaaaattccggcctgttttcacaaggatttctgccaac[A/G]gagataaacttgcccgctgcggttccaatcttaaccttaagattcacaKcttcgtttcgtagagatcccagcagctacttattgaggaaagaagatggga

fava_c17467_294	fava_c17467	294	T/G	agatacaatgtaacaaaattccggcctgttttcacaaggatttctgccaacRgagataaacttgcccgctgcggttccaatcttaaccttaagattcaca[T/G]cttcgtttcgtagagatcccagcagctacttattgaggaaagaagatgggagtaggttttccaggggacggaggattttcctttagtatagttaYaagag

fava_c17467_389	fava_c17467	389	C/T	tcacaKcttcgtttcgtagagatcccagcagctacttattgaggaaagaagatgggagtaggttttccaggggacggaggattttcctttagtatagtta[C/T]aagagatccagctttagctacagaatcaggatctgctgcaaatgcaatgtccacatcttccacaagcacatcttttgaacttacctttctcttaatcaag

fava_c17467_708	fava_c17467	708	G/A	gaatacgtctaaattcagtagattgacctacagaactctgttgtaaagcctcctttatttctgaatccggaggaaaagtacgagtatctttgttaaagct[G/A]ttttggagaatactaaggcactcagcttcccaaccRtcatattgctcattcacatatatcaagcattttaccttggtttgtcccggcattacaactggag

fava_c17467_744	fava_c17467	744	G/A	tctgttgtaaagcctcctttatttctgaatccggaggaaaagtacgagtatctttgttaaagctRttttggagaatactaaggcactcagcttcccaacc[G/A]tcatattgctcattcacatatatcaagcattttaccttggtttgtcccggcattacaactggagcagatttcttatttcctttctttgagcctgaaagtt

fava_c17468_439	fava_c17468	439	C/G	gttccttttggagagttccgtgtctcttgatattctacctcgttgctatgcttgtgcttattatactcctcgtttgtttggttgtttttgtttacatggt[C/G]actgttcgtggccatggtatgattgaaccaaatcgggcttatttggaataccgtttggatgatttttcggggtttcttaagaggagagtgagaagctctt

fava_c17468_654	fava_c17468	654	C/G	attagaagttgtttgagtgagactaatatgtgtgctgagttgaatcagaattttcgtatggctcaggacttctttaatgctcgtcttacgccaatgcaga[C/G]Yggatgctgcaagccaccaacacaatgtgcatacacatttgtgaacccaacctattggattagtccaataaacaatgcagcagatatggattgtcttcaa

fava_c17468_655	fava_c17468	655	C/T	ttagaagttgtttgagtgagactaatatgtgtgctgagttgaatcagaattttcgtatggctcaggacttctttaatgctcgtcttacgccaatgcagaS[C/T]ggatgctgcaagccaccaacacaatgtgcatacacatttgtgaacccaacctattggattagtccaataaacaatgcagcagatatggattgtcttcaat

fava_c17468_898	fava_c17468	898	G/C	gcaaagctggtctgttggcaaatctcaggaaggaatggaaaagagcaaatgtgatattgatcatcagtgttgttgttttaattgtggtttatttgattgg[G/C]tgctttgcttttaggaatgccaaaactgaagatctctttcgcaaatacaaacaaggctacacttgatcatcacaacattaattggaggctcattatgttt

fava_c17469_249	fava_c17469	249	A/G	gggaataaaaataaataaaaaaagtaacaaactaaatcaggagataaaaaccatcttaaattgataggaagcaactccagaagaagaatgcaaaagtgga[A/G]RRaaaaaaatacataaaaaaagaaaacaaaacgacaattgtactaaatttctatgcctttttgatcaggctgagaacatcagcttctgtgggaagagctg

fava_c17469_250	fava_c17469	250	G/A	ggaataaaaataaataaaaaaagtaacaaactaaatcaggagataaaaaccatcttaaattgataggaagcaactccagaagaagaatgcaaaagtggaR[G/A]Raaaaaaatacataaaaaaagaaaacaaaacgacaattgtactaaatttctatgcctttttgatcaggctgagaacatcagcttctgtgggaagagctgg

fava_c17469_251	fava_c17469	251	A/G	gaataaaaataaataaaaaaagtaacaaactaaatcaggagataaaaaccatcttaaattgataggaagcaactccagaagaagaatgcaaaagtggaRR[A/G]aaaaaaatacataaaaaaagaaaacaaaacgacaattgtactaaatttctatgcctttttgatcaggctgagaacatcagcttctgtgggaagagctgga

fava_c17469_759	fava_c17469	759	C/G	attcaagctccacatcactgaccttgatgaggtcagccttatcccatatactgagtatttgcttacgagcttcctcaggtgagggccacaaaggtagccg[C/G]aggtttggatcatacgagagtaagcaccctgcttctttggcaacttccatcgccttcagatgtgctgttctgcaYggctccacaatcaaacttattgatc

fava_c17469_834	fava_c17469	834	C/T	caggtgagggccacaaaggtagccgSaggtttggatcatacgagagtaagcaccctgcttctttggcaacttccatcgccttcagatgtgctgttctgca[C/T]ggctccacaatcaaacttattgatccataatggaacacttttgcagatctgatgagttcaagattgagatcttctggagttagaagcatatcggcgctag

fava_c17470_1021	fava_c17470	1021	T/G	cacatgctacacggtggtgctcctctagccacctcgcatattccacatcaaacatcgcaccctctgagctaatgctattcattgaaataggaaggttttg[T/G]tctcctcccaaaactgcacctccaccaaggaacatgccttgatttctagcacgctgtagctcttgttccatttgactaagcctgattctgcttgactcaa

fava_c17471_434	fava_c17471	434	T/A	ttcaacaccatcaatctttcactaacaaaaacctgcaaaccttcaaacaaaatctaaataaaaaaataccttcacattatcatcaccttaatccaccaca[T/A]gtaacaaacatacacgattccacacacaattatttttgtctttttttttttgcagagaaagaatgaaatagagaaaggatattcaagtgaagtcacaacc

fava_c17471_614	fava_c17471	614	C/T	tattcaagtgaagtcacaaccacatcaactaccgaatcaacaatcaaagaaaagaggaggaaaatacaatttctgctgcaaaaaacttcgacgaattcga[C/T]ggaggaagccacttctcccatgcatctccgcaaccggatgcaactttgccatcgcgaattaacactattagctgcttcaccatcgcttctggatcccggc

fava_c17477_1237	fava_c17477	1237	C/G	catatggttacatggacccggcatacatttccacaaacaagttaacaaccaagagtgacatatacagttttggtatcatactttttgagctcatcacagc[C/G]atccatcctcatcaaaatttgttggaatatgttaaccttgctgcaatggaacatgatggtatagatgagatacttgacaagcaacttgttggaaaatgca

fava_c17477_1507	fava_c17477	1507	C/T	cctctataagtgaagtttctcaaagcatatcaagaataaagcaaagaagattgcgtcatgttatggaagataacttgtcatttgcaaatagtaataactt[C/T]tcaagaacttcgagtcgattagatgatcgacaagttgaattaagtaggatagttaccatgaccatcaaagaaactgtatgaactaaattactccaatatg

fava_c17477_526	fava_c17477	526	A/G	ttacacgatctgcgaacgagcctagtttgacaactctccccatacgaacaaatggagttgaagcaagtactgactttagtgcatcgatcactagttccgt[A/G]accacgtcaaggtcagaaaatctacaaagaaattccaatttctcttggtggaatcatcaaaacaaagaccggtttgcttcagcatcaggcattctaaagt

fava_c17477_994	fava_c17477	994	T/C	gaagtttagcaagtattttatatggtgaagaaaagaagttaagttgggatgaaaggctgcaaattgctattgatatttcacatggaatagagtatcttca[T/C]gaaggagcagtcccaccggttatacatcgtgatttgaagtctcccaacatattgctagatgacacaatgagagcaaaggttgctgattttggcctctcaa

fava_c17478_1423	fava_c17478	1423	T/A	attggaggatgtggaatatgcctgtgcataaatggatggttcgccacgtatattttccctgtataaggtttggtataccaaagggtgcttctgctttgat[T/A]gctttcctggtttctgccgtcttccatgagttatgcattgctgttccttgccgcatgttcaagatgtgggcttttattggaattatgtttcaggttcctt

fava_c17478_388	fava_c17478	388	C/A	gaaataacagcgaagaggtcaaggatcgaaaaacagatcatgcaggaatcggtaatgacgttcagaatgacgtcattgatcgggaaaaagttaccgatat[C/A]aaattcacttaccgtccttcagttcccgctcaccggagaatcaaagagagtcctcttagctccggcaacatttttagacagagtcatgcaggactgttca

fava_c17478_704	fava_c17478	704	G/A	ttggcccctcttcatgtgttgtcttagtcttgcaatatttccactagctgcctttgtagtcgaaaagttagcacaacaaaagagtatttctgaaccagtt[G/A]ttgttctacttcatgttgtaattaccacagttgcaattatctatccagttttagtaatcctctggtgtgactctgcatttttatcaggggccacgttgat

fava_c17481_1311	fava_c17481	1311	G/A	cattcatcagatcaagtattttctttggaacagccttctccaagccgagttggttaacagcatagacaaatttcttatgcagatcaacagaccaaaccac[G/A]cggggtttcttttgtgatgacgggtcatcctcatctccagtcccttcaccttcttcRtcttcttctccctcctcgtctcggtccttccttcttttactga

fava_c17481_1368	fava_c17481	1368	A/G	cagcatagacaaatttcttatgcagatcaacagaccaaaccacRcggggtttcttttgtgatgacgggtcatcctcatctccagtcccttcaccttcttc[A/G]tcttcttctccctcctcgtctcggtccttccttcttttactgatccttttattctgatcaccattgttttccaatatgttttgactattYccccccaaaa

fava_c17481_1458	fava_c17481	1458	T/C	caccttcttcRtcttcttctccctcctcgtctcggtccttccttcttttactgatccttttattctgatcaccattgttttccaatatgttttgactatt[T/C]ccccccaaaatRttagcgaccctatcctcattggaagccttattttgataattggaataggtttttttctttcgaaaaacatgttgccatatgttcttga

fava_c17481_1470	fava_c17481	1470	A/G	cttcttctccctcctcgtctcggtccttccttcttttactgatccttttattctgatcaccattgttttccaatatgttttgactattYccccccaaaat[A/G]ttagcgaccctatcctcattggaagccttattttgataattggaataggtttttttctttcgaaaaacatgttgccatatgttcttgagctcttcaagtc

fava_c17481_155	fava_c17481	155	T/C	ttattgtaccatgataaacaacttaaaaataagtacatccaaagaaaaactacacagggaaataaactacatggggataggataggagtaagcttcagga[T/C]tttagtgtttctggattcttgttgaatcaaaagcaatcaaatacatgatccaatagggtaagcatcataaaatcccatttctccatcgattaatgtcatc

fava_c17481_1875	fava_c17481	1875	C/A	gcgcatcaattgcatttgtagatgtagtaacatgatatttgcattgacgaaggagtttatccaaaaccatgagacaagttgggttgtcatcaacggcaag[C/A]acacgcataccgacaggaaaacggtcaatgacctcatcttcatccctcaaacaacccctttgattctccacaaccatcttcagagaagaacaaaaattgt

fava_c17481_399	fava_c17481	399	A/T	tgtaatccagtttatgttcttcccacctttgttgcggagcataaccagaaggctgcataatatttccaataaagccgccttcttggcacctcgtcaaaac[A/T]Yctctggcatccttcaaagtaacattgattgcttctctagatgaaaagttaactggactattaccaatcggaggtatttggttaaaagcagtaatgctag

fava_c17481_400	fava_c17481	400	T/C	gtaatccagtttatgttcttcccacctttgttgcggagcataaccagaaggctgcataatatttccaataaagccgccttcttggcacctcgtcaaaacW[T/C]ctctggcatccttcaaagtaacattgattgcttctctagatgaaaagttaactggactattaccaatcggaggtatttggttaaaagcagtaatgctagt

fava_c17481_516	fava_c17481	516	G/T	aagtaacattgattgcttctctagatgaaaagttaactggactattaccaatcggaggtatttggttaaaagcagtaatgctagtaggaggcatttggtt[G/T]gtattgaaagacctgcataccggcaaagaactggcaggaaacttaggcaacgcttcacttttcaagttttcagtacactgattagaagaaccacaaatgt

fava_c17481_983	fava_c17481	983	T/C	agtttgctcaacgagctgttgatgttttgactctgaacaggccgaatcagtgttgaaggacttattcctctcatgttacagctggctggagaattcagcc[T/C]accgaaaagtccacttgatgaatatgatggcaaggtggtactcgagatccttctagatccaggcgaggtgcaaatatctgcatatccatctattccactc

fava_c17485_226	fava_c17485	226	T/A	gcatgtttactactacttcttcaactttacctattcctcatcagaatcaaagcttgctcttccccttttcaacctcctcaagctcttttgacacaaccac[T/A]tgttcatcttccaatgtttattactaYggacaagagtttaaaacaatggaggaacaggaacaggaagatgaacctataattcccaacacaaagccatggg

fava_c17485_253	fava_c17485	253	T/C	tacctattcctcatcagaatcaaagcttgctcttccccttttcaacctcctcaagctcttttgacacaaccacWtgttcatcttccaatgtttattacta[T/C]ggacaagagtttaaaacaatggaggaacaggaacaggaagatgaacctataattcccaacacaaagccatgggagacaccgataccaataccaataccga

fava_c17486_1038	fava_c17486	1038	T/G	cgcaacagcagagtaacttgaacaatcccttcatgagtccacaagagtcaatatttgcgtcaacgtcaataaatggaattgatcttcaaactctttcacc[T/G]gctggccagctctcgaatccaagtctagccaagcttcaagcagctggacttttcagttctgagaacttaaaactaagatttggagaagggcaactgcaac

fava_c17486_122	fava_c17486	122	C/A	tatgtgagagacaacaaaactattgtgttttgttttgtttcacgttctttgttttttgttgtcagtttttcgatctctcaactctcagccatggaactca[C/A]caacgacaaattttccatcccaaccccagcttctacttcaacttcatcatcaagactcKccgaccttgtctccgaccagtttccggcgggtctcagagta

fava_c17486_1761	fava_c17486	1761	C/T	gggttaaaagtgaaagaattcctaatccaagcttcgagattaaccctttctctgagcaattcggccaagaggatctcatgagtgcgtttctgaatcagca[C/T]caaggcgttggtcacgttggaaatgagtttgacttggatggatattcgctagacaacattccggtctataacagatcctgttagttatcttcctaattgt

fava_c17486_181	fava_c17486	181	G/T	tgtcagtttttcgatctctcaactctcagccatggaactcaMcaacgacaaattttccatcccaaccccagcttctacttcaacttcatcatcaagactc[G/T]ccgaccttgtctccgaccagtttccggcgggtctcagagtacttgttgtcgatgatgaccccacctgtcttatgatccttgagaggatgctccggttttg

fava_c17486_456	fava_c17486	456	G/T	acattgttttaagcgacgtgcatatgcctgacatggatgggttcaagcttttagaacatattggattggagatggatcttccggttattatgatgtcggc[G/T]gatgatggaaagaatgtggtaatgaaaggtgtgaaacatggtgcttgtgattatctgatcaaaccggtgagaattgaggctttgaagaatatatggcagc

fava_c17495_712	fava_c17495	712	T/C	tcacttgaataccctggaattgtcaaatcccaatttctctctaggtgtagcaataaatccttggactccaaagttgatgattttctatgttttgccaaaa[T/C]gcaaccatgtgtagttgcagtatcaatgaagtcatcagcaagctctaaaagaagatctataacggaagggtccagcttaccttggggatccacctgtgca

fava_c17495_898	fava_c17495	898	T/C	ggatccacctgtgcaactaaatcctgtatctttctctttccaagtacttggtttgttgctgcttcagttccttggcttgaacttccaccaggtgtagtcg[T/C]accagatgcagtagcatctggctgtgatcctgttaaacttagtgttttttggcctgaaggtcctggcatcctcggagactgctgctgttgctgttgatga

fava_c17497_1334	fava_c17497	1334	A/T	gccagactggatttgatttcgatgaggaagatctcttagagttggatcagtaccatgatatcattgttgcgtctgccatatttggaaactatgatgtaat[A/T]cagcagcccaggaacatcagtagagaagcaaggagaaacattcctttctatatgtttattgacgaagagacagaaatgtatatgagaaatgccagtattt

fava_c17497_1565	fava_c17497	1565	A/T	gaatcattgttgtccggaatattccatatgctgattctaggcgtaatggaaagattccaaaacttcttcttcacagaatcttccccaatattcgatattc[A/T]atttggattgatgggaagcttgagctcgttgttgacccatatcaagttcttgaaaggttcttgtggcgcccaaatgctacRtttgccatttcaagacact

fava_c17497_1646	fava_c17497	1646	G/A	tccccaatattcgatattcWatttggattgatgggaagcttgagctcgttgttgacccatatcaagttcttgaaaggttcttgtggcgcccaaatgctac[G/A]tttgccatttcaagacactataggcgctttgatgtctttgtcgaggctgaggctaataaagtagctggaaaatatgaaaatgcttccattgatcgccaag

fava_c17497_2262	fava_c17497	2262	G/A	tcatcgtaaagtagtagacaattttcttatgaactcggggttctaaattgtaaatgcattctttgttgcaaattatacatcaaagagttctgaaaaactg[G/A]taccatttcaaaaatgttgctccctttttatgttttattttgtaaatatctgtctttgacaagatccttgcttacttttttcaaaggggaaagaaaaaag

fava_c17499_138	fava_c17499	138	C/T	tctctcttctcttgtcaactgcaaacgcttcagagaaaaatccagagacataaccaaatcaaacttataaaaatcatcactctcaatctgttaaagtcca[C/T]aagcagttccaacaatactaaacacgaattacacccaaaactccatctgacttgaatccaaaaaaacaaaaaagtgagaagaaatctatcagtcagagtc

fava_c17499_476	fava_c17499	476	C/T	tggaatatggtaccaattagagccatgacagagtcgctcgcggttataggttggaacatcatgggctgtcggagttgttggtggatcgcagttgagattg[C/T]cggcgagacggtgactcgttggagcttggcagcgtgggttgaagccgaatgcgcgtgggtttctatctactttctctatttttctaYagagagaaaaaaa

fava_c17499_563	fava_c17499	563	C/T	gcagttgagattgYcggcgagacggtgactcgttggagcttggcagcgtgggttgaagccgaatgcgcgtgggtttctatctactttctctatttttcta[C/T]agagagaaaaaaagaaagcaaaattctgtacaaatttaatgctgatgttgttattgRttttgtgttagtatggtgaatKttagtgataagtgttgaaaat

fava_c17499_620	fava_c17499	620	A/G	gccgaatgcgcgtgggtttctatctactttctctatttttctaYagagagaaaaaaagaaagcaaaattctgtacaaatttaatgctgatgttgttattg[A/G]ttttgtgttagtatggtgaatKttagtgataagtgttgaaaatattcacaggtatatgcaaattggacgcagtgatgaatttgtgaaaaaaatttatgtt

fava_c17499_642	fava_c17499	642	T/G	tctactttctctatttttctaYagagagaaaaaaagaaagcaaaattctgtacaaatttaatgctgatgttgttattgRttttgtgttagtatggtgaat[T/G]ttagtgataagtgttgaaaatattcacaggtatatgcaaattggacgcagtgatgaatttgtgaaaaaaatttatgtttgaatgttataaattttggtgt

fava_c17500_1018	fava_c17500	1018	C/T	acaaggaagcctcctccccaccactccctgctcgtacctccaaaatgcaatctctttcatcagcatcatccttMggaagtaatgacttaagcagcatatt[C/T]tgcagttttttttcttcYtctatggcttggttcatttcctctgttgccatattaagcatgtctttgtcatcagaacattcagttattagtgacttcaagc

fava_c17500_1036	fava_c17500	1036	C/T	caccactccctgctcgtacctccaaaatgcaatctctttcatcagcatcatccttMggaagtaatgacttaagcagcatattYtgcagttttttttcttc[C/T]tctatggcttggttcatttcctctgttgccatattaagcatgtctttgtcatcagaacattcagttattagtgacttcaagccatcgatttccttctgtt

fava_c17500_406	fava_c17500	406	G/T	catcttctatgttgtgataagtaataccaactctatgatcagttacgcggccttgaggaaaattgtatgttcggatacgttcagatctgtccccactacc[G/T]atctgctcYgatcggagctttgatcgactactatgaagtctRatcctttccatttcatatagctttgcacacagtaccttgagagctttggccttattca

fava_c17500_415	fava_c17500	415	T/C	tgttgtgataagtaataccaactctatgatcagttacgcggccttgaggaaaattgtatgttcggatacgttcagatctgtccccactaccKatctgctc[T/C]gatcggagctttgatcgactactatgaagtctRatcctttccatttcatatagctttgcacacagtaccttgagagctttggccttattcatatgctgag

fava_c17500_448	fava_c17500	448	G/A	ttacgcggccttgaggaaaattgtatgttcggatacgttcagatctgtccccactaccKatctgctcYgatcggagctttgatcgactactatgaagtct[G/A]atcctttccatttcatatagctttgcacacagtaccttgagagctttggccttattcatatgctgagaacgctcatcttgtatagcaaccacaatcccag

fava_c17500_880	fava_c17500	880	T/A	caaatacaccaactcctacaattgctgcactagcctccttgaatcccttcatatcagattgagctatatctaccacttcaaacttccagcctttgttgtg[T/A]gcatacttctcgtacatcttaaggatgttcattgcaaacaaggaagcctcctccccaccactccctgctcgtacctccaaaatgcaatctctttcatcag

fava_c17500_991	fava_c17500	991	C/A	cgtacatcttaaggatgttcattgcaaacaaggaagcctcctccccaccactccctgctcgtacctccaaaatgcaatctctttcatcagcatcatcctt[C/A]ggaagtaatgacttaagcagcatattYtgcagttttttttcttcYtctatggcttggttcatttcctctgttgccatattaagcatgtctttgtcatcag

fava_c17501_1030	fava_c17501	1030	G/C	aatcttttcctgtgacattgctttRacttctttggcaacaatgcccatagattttcctagtcgcttacctaaaacactaaagtcaggctcggcgcgtaag[G/C]ctgcatactttaaagtatcattacatggaacaagggatcgtatattgagttcctcaagtacatactccttcaactttccattaatgtcttcaaggaagtc

fava_c17501_273	fava_c17501	273	A/G	tgcaaccagataccatataatctggataatgaagaacccgaaatgaagctattgaacaaaattagacagattttgccgcaacatagtaatcccctacagt[A/G]agaaatacatgctcaccaagtaccacttcggccgcaggttgatgttcaatggagtctacaattttctttccatttccttcctgaaactctgacttcaaat

fava_c17501_3099	fava_c17501	3099	T/G	ctatataaactttaccagtatacttattgcgtaccttcaaataggtaaaattagcattgatgcatagagcaagattgctaggaagagtccatggcgttgt[T/G]gtccaagccacgaaagatgcaccatctggatcgcccaacaacggaaacgtcatgaaaacttcagggtcagatacatccttataattctgRccagcctcaa

fava_c17501_3189	fava_c17501	3189	A/G	atggcgttgtKgtccaagccacgaaagatgcaccatctggatcgcccaacaacggaaacgtcatgaaaacttcagggtcagatacatccttataattctg[A/G]ccagcctcaaaattagatagtggagttttgcagccagtactatatggcatgaccttgaaacctttataaacaaggtttttctcataaagcttagaaaaaa

fava_c17501_507	fava_c17501	507	C/T	aattgtgtgcaaatttggagtcacctgaaaatagtgaaagaatggctttttcattgaacatcgacgcgggtctcgccaaggtgatggcaaacgacatgct[C/T]gaaattccatggaaactctcttcaccaatgataactgcatgtgcaggcatcaaagaaaattgaagcaatggagaaccaatagcctctctgatataggatt

fava_c17501_903	fava_c17501	903	G/A	ccccagcagcatcaatctcattatccgccataccatcaggacgtttaaaatcacggagaaccttaatatcagacagctgcaaacagtgacttgcaatgac[G/A]acttctccagccttttcaaaagccaaaatcttttcctgtgacattgctttRacttctttggcaacaatgcccatagattttcctagtcgcttacctaaaa

fava_c17501_954	fava_c17501	954	A/G	cacggagaaccttaatatcagacagctgcaaacagtgacttgcaatgacRacttctccagccttttcaaaagccaaaatcttttcctgtgacattgcttt[A/G]acttctttggcaacaatgcccatagattttcctagtcgcttacctaaaacactaaagtcaggctcggcgcgtaagSctgcatactttaaagtatcattac

fava_c17503_2002	fava_c17503	2002	A/G	caaacgccggaagaggtgacaatgaactatgagaaggcactctcatcaagtaaactctatcattctccttgacagccctttcaaggtttctattaatatt[A/G]ccctctaacttgttaatagcatcaggaagctgcgctgcagcacccctggagtttttctttgcYtcagttagcacactaacagcactcttcagccgcgata

fava_c17503_2065	fava_c17503	2065	T/C	tctccttgacagccctttcaaggtttctattaatattRccctctaacttgttaatagcatcaggaagctgcgctgcagcacccctggagtttttctttgc[T/C]tcagttagcacactaacagcactcttcagccgcgatatctcctctgctatttcctctttctcatgcagctctaatccatacctataacaagcttctgcat

fava_c17509_1069	fava_c17509	1069	G/A	tctcaccatcaagacgttcttgatgcccttcccttttggcagttagccacgtcgaataatcggcactgtttgttctaactgaagctgctttgtatttgcc[G/A]cccacaggtgaaacaccatttgagaaagcaacatgaaatctaatgtttccagaagacctggctctgtcctgaRcaaatacaagagcaacagcttctatga

fava_c17509_1142	fava_c17509	1142	A/G	tctaactgaagctgctttgtatttgccRcccacaggtgaaacaccatttgagaaagcaacatgaaatctaatgtttccagaagacctggctctgtcctga[A/G]caaatacaagagcaacagcttctatgaaccctttactacaacaagcatttctggaatcatttccgcatctgaatacagagcagcctctctcatcccccac

fava_c17509_1543	fava_c17509	1543	C/T	caactttcaatcctggcaagtagccaattgtcaaaactatgccttcattaatatgtcttgcatattcatcgtcaccaatgccactaaaacgggcacccca[C/T]ttgacctctttaaattcatccgtaagagcgtcccttccaataataccattattgaccgtgacaataatcggaacattgaaccctaattttcgtttaataa

fava_c17509_1752	fava_c17509	1752	A/G	atcttgttggtgtcgaatccacatccaacattgacaatagcaaaatgaggtcgaattggctcagatagaaccttgttaacaacctcctccacagcctcgc[A/G]aagcaaagggttgagagaaagtgcggaagagagcttagggagagaaatgatgcgactgcagatggaattccaagatttgttaacgcaggatgcggaagcg

fava_c17509_1857	fava_c17509	1857	A/G	aaagggttgagagaaagtgcggaagagagcttagggagagaaatgatgcgactgcagatggaattccaagatttgttaacgcaggatgcggaagcgaacg[A/G]tagggcggggagtctggtgaaaatattgtggagaagatcgtcgttcagcaaggtaataccgatgttgagatcgattgaggattttcctttggtgattgat

fava_c17510_1992	fava_c17510	1992	G/A	cagcaactgggatatcgaatggagttctaatccagaaaattttctgaagcatgaatttgcttttgtggaaatcgtatcagatttttctgaaaatgttggc[G/A]ttgtagtagcttgcctaggcaaagtggaaggctttacaagtctctatgagaaaactggaaagaatggagagaacacattttgtattctgccaaaagagct

fava_c17510_2370	fava_c17510	2370	T/G	taaaaaagtgggggaaaacctttttcctcaagttatttttttggttaaatccattccttaaagatggtactttttcttcctacataccatgtttagtcta[T/G]aatttatccYagtcttgtgtttttttagtactaatcttttattgtgactccgtgttccttaattgtgtttatgtactatagtttcccgatttgcctaagt

fava_c17510_2380	fava_c17510	2380	T/C	ggggaaaacctttttcctcaagttatttttttggttaaatccattccttaaagatggtactttttcttcctacataccatgtttagtctaKaatttatcc[T/C]agtcttgtgtttttttagtactaatcttttattgtgactccgtgttccttaattgtgtttatgtactatagtttcccgatttgcctaagttcattttcac

fava_c17510_416	fava_c17510	416	C/G	tgaagtttgctaacaaggcgaaaagactgtaccctgatgttgaaaacattgctcagattcttgcagtctgtgaggttcacagtgctgcattgaacaaact[C/G]tatagggctgaaatggactggtatggaattcttcagacagagaaactctcggaagaagctgtcataagaaaacagtacagaaagcttgcactgttacttc

fava_c17510_695	fava_c17510	695	T/G	aagcaaagcggattttacatgatatgaaagttagagcccatttacaagagatgaaagttaaagtccatttgaaaactgcagtgcctaacacctcatccca[T/G]cactcaaatgggaacatgtttgttgcaaaccaagctccaaatgcaacaaaatatcagaaaaatgtttcttcaaatatcccttgtgatcccaatctgaaac

fava_c17513_1105	fava_c17513	1105	C/T	cttgattgatcaacttgggaaaaaatactagaaatttgtttacttgattttccttgtgaagattcataggttcttgcaacttgcaagttttatttaaaaa[C/T]gtttgtgaattgtatgattcttgtaaagtcctgtaattaccaaatacaagtgatttgtttgcaaatactgtgcctagttgtataacatctttgaagaata

fava_c17513_155	fava_c17513	155	A/G	gtaagaacacacacaccctcacaaagttatatatccatggtagcactttcaggtcagaaatttgcaaaactctacaacaagctggagaatcatcatcatc[A/G]tcatcaccatgaagctgaggaggagttatcagcatccctgatggcttttcgatcccaagtttcgaaattgatccatgagttagcatcaaatttgaaacct

fava_c17513_499	fava_c17513	499	G/C	ctacagtttgtgtttgctagaccttttcaattcaatttcttcttctctttctcatcttgataaggctaagctttctctgattcatgccttgaacgtgttt[G/C]agaatSagaattcaccatctttcactacaagtcatcRtcatctcagagcaattccacctagctgtttgaatacaaaatttggtgaRaagctgtgtgagaa

fava_c17513_505	fava_c17513	505	G/C	tttgtgtttgctagaccttttcaattcaatttcttcttctctttctcatcttgataaggctaagctttctctgattcatgccttgaacgtgtttSagaat[G/C]agaattcaccatctttcactacaagtcatcRtcatctcagagcaattccacctagctgtttgaatacaaaatttggtgaRaagctgtgtgagaaaaatga

fava_c17513_536	fava_c17513	536	A/G	tcttcttctctttctcatcttgataaggctaagctttctctgattcatgccttgaacgtgtttSagaatSagaattcaccatctttcactacaagtcatc[A/G]tcatctcagagcaattccacctagctgtttgaatacaaaatttggtgaRaagctgtgtgagaaaaatgataaagtaaaSttttttactgggaaggagtta

fava_c17513_585	fava_c17513	585	G/A	ccttgaacgtgtttSagaatSagaattcaccatctttcactacaagtcatcRtcatctcagagcaattccacctagctgtttgaatacaaaatttggtga[G/A]aagctgtgtgagaaaaatgataaagtaaaSttttttactgggaaggagttagttgttaatgaaggtgtgaaggaattgaagagaattggtttttgggtgt

fava_c17513_615	fava_c17513	615	G/C	catctttcactacaagtcatcRtcatctcagagcaattccacctagctgtttgaatacaaaatttggtgaRaagctgtgtgagaaaaatgataaagtaaa[G/C]ttttttactgggaaggagttagttgttaatgaaggtgtgaaggaattgaagagaattggtttttgggtgtgtgggattttgttatctggtttgtgtagtg

fava_c17514_104	fava_c17514	104	A/T	acaaaagataattggatttttatatataataagagtagtagatgacaatagtgagataataatacaaaggaataagtattcactcgtggtcctagcctca[A/T]ggaaaggaacatataaaatgtacaatgctacaatgttggtagggtaacaagcatcgaaaacctaatagagcctagaggaagaagaaaccataaaaaWttg

fava_c17514_201	fava_c17514	201	T/A	tcaWggaaaggaacatataaaatgtacaatgctacaatgttggtagggtaacaagcatcgaaaacctaatagagcctagaggaagaagaaaccataaaaa[T/A]ttgaaaacagtcctattaacaatgcccttgtacttcaattgtgtgtgaactaccgaaagaaataacttaattcagatccaaacccctccaaattcaatac

fava_c17514_369	fava_c17514	369	A/G	taattcagatccaaacccctccaaattcaatacacatgtatatagctagtaggtagcatgtggtgtgtaagctaataactaattgaacttataattaatg[A/G]ggttgaacttgaatttgtaggcctttRggaaaatcgacaaaagggtaggcaaaagcttgatcattattatcaattaactcccaatggtagttgaggatga

fava_c17514_396	fava_c17514	396	A/G	caatacacatgtatatagctagtaggtagcatgtggtgtgtaagctaataactaattgaacttataattaatgRggttgaacttgaatttgtaggccttt[A/G]ggaaaatcgacaaaagggtaggcaaaagcttgatcattattatcaattaactcccaatggtagttgaggatgagatagtggatgaaaacagccatttcaa

fava_c17514_561	fava_c17514	561	A/G	tgaggatgagatagtggatgaaaacagccatttcaagcttagctaattctgatcctgcacatagtcgtggtcctcctccaaacggtaagaaattattatt[A/G]ttactgcttgcgtttgagcaatttccattgttctgccatctccatggattgaagtgttgaggttggtcaaaaagtaatggatccaaatgcacggctgcaa

fava_c17516_244	fava_c17516	244	C/G	aatagcatctcctcctaagtggtagtgcactatatagccttgatccaaattgaagcattaaaattctagttcttttgtcatagcattacgagacaatgga[C/G]aaccctaccatattacacacctataaatttacaattcatgagacatggattatggattatggaccctaccaaatagatttccagattggtatgaattttg

fava_c17518_1461	fava_c17518	1461	T/C	ctattctcacatgaatccttaggaccacttctttcatttggagacgcattaccatcagcgttgtcgagaagatttttactctgtggatttgttgagacac[T/C]gctctcaccttttgaactatcctcttcctccttaatcagaggttttgttggttgaagagaattttcggtattcaaattctcacatgaagtttcgacaaat

fava_c17518_1814	fava_c17518	1814	G/A	ttcgttatcactaaacttagacaccttatctaaatctatcgacatggactcaacggattctttgacaggtttttgcggttcagacatagcacttttatcc[G/A]cattgaaggccttgtgaaccacatgctgccaaatatttttcagcttatcctcagagagcggtttagttaggaactcaactgcaccaagcgcaatgcactt

fava_c17518_2014	fava_c17518	2014	C/T	tcatcattgtattcaagcaatgaatgttcgaagtcataatggttggcaagtccttagcattctcgagaaacttgaaacctccatcaggacagcttgtact[C/T]acctcgactacRgcaacatggaagcctttgggactactcgaKatccccgacaaagcttcgttcacattgtagaagatagaaacattatagtccatagatt

fava_c17518_2026	fava_c17518	2026	G/A	tcaagcaatgaatgttcgaagtcataatggttggcaagtccttagcattctcgagaaacttgaaacctccatcaggacagcttgtactYacctcgactac[G/A]gcaacatggaagcctttgggactactcgaKatccccgacaaagcttcgttcacattgtagaagatagaaacattatagtccatagattcaagctttgttc

fava_c17518_2056	fava_c17518	2056	G/T	ttggcaagtccttagcattctcgagaaacttgaaacctccatcaggacagcttgtactYacctcgactacRgcaacatggaagcctttgggactactcga[G/T]atccccgacaaagcttcgttcacattgtagaagatagaaacattatagtccatagattcaagctttgttcttatttcagaagcagaattgttgtctcctt

fava_c17518_431	fava_c17518	431	G/A	catgtcctttttatctctttgttgcatgcatcattcatcctatgtcatttcccagaagctacatacatagggtgtccgtgttatgggggggtccacaggg[G/A]aaaaataattggttggaatctgagtgagagggtaagtctgagagacacaagattattacaacagtccacatggcattggcccatctatgtctgccacagc

fava_c17520_1741	fava_c17520	1741	G/A	aaagtattagtactacacctaatgccagtgcttaatcacaggttaatatcaattgttgataatgtatttttactgaatcaactttaagcaatgattgtta[G/A]ttgaaacatgaaaatgagYgagtacaatgtatcattctcgtctcatccgtttgtatagatggtaatacgatgattattttacgagacattatggtcaggY

fava_c17520_1760	fava_c17520	1760	C/T	taatgccagtgcttaatcacaggttaatatcaattgttgataatgtatttttactgaatcaactttaagcaatgattgttaRttgaaacatgaaaatgag[C/T]gagtacaatgtatcattctcgtctcatccgtttgtatagatggtaatacgatgattattttacgagacattatggtcaggYaaaaatctgagatctagat

fava_c17520_1841	fava_c17520	1841	C/T	RttgaaacatgaaaatgagYgagtacaatgtatcattctcgtctcatccgtttgtatagatggtaatacgatgattattttacgagacattatggtcagg[C/T]aaaaatctgagatctagatggaagtacatgtaattatcataaaaagaggtacagaaataaaacaaaaaatggaaaattttgggcacaatgcttgctatgc

fava_c17520_823	fava_c17520	823	G/A	acgaacacaactatagaagagtagaacagtttatgaatgaagttaatatcctgacaactttacgccataaaaatctcgtgtccctctatggctgcacatc[G/A]cggcacagccgtgaactactgcttgtgtacgaatatatctcaaatggcaccattgcaagtcatctccatggtgaattggccgagcccaacttactaccgt

fava_c17524_1316	fava_c17524	1316	C/T	tcattaaaaagtaggcactagaaattggtccaaataatttgttgcaccaatcatggtcaaattttggcatctttccataggtggcatatgcttcttgatg[C/T]ctcagaagtttcaacaaggcttcagtttgtaaagcataKacctttggagctgagtctacacctaagtatattgcagactttgtttctgtcaatataagtt

fava_c17524_1355	fava_c17524	1355	G/T	tgttgcaccaatcatggtcaaattttggcatctttccataggtggcatatgcttcttgatgYctcagaagtttcaacaaggcttcagtttgtaaagcata[G/T]acctttggagctgagtctacacctaagtatattgcagactttgtttctgtcaatataagtttccaatcattgacttttcttgcttcactgcatttgttaa

fava_c17524_1491	fava_c17524	1491	T/C	agactttgtttctgtcaatataagtttccaatcattgacttttcttgcttcactgcatttgttaaggtgaatttgaagagcatgtgctttgaaagcaaga[T/C]ctgaatcaaagtatggaatttctttattgcaatttaatgccttttctgYttctcccaacctgaaatatattgttgctaaacggctatgagctctagcata

fava_c17524_1540	fava_c17524	1540	C/T	tcactgcatttgttaaggtgaatttgaagagcatgtgctttgaaagcaagaYctgaatcaaagtatggaatttctttattgcaatttaatgccttttctg[C/T]ttctcccaacctgaaatatattgttgctaaacggctatgagctctagcataggaaggttgaatcctaatagcttcctcacactcaacaattgcttgtYga

fava_c17524_1638	fava_c17524	1638	T/C	tgYttctcccaacctgaaatatattgttgctaaacggctatgagctctagcataggaaggttgaatcctaatagcttcctcacactcaacaattgcttgt[T/C]gaaaccttcccaaaccaattaaagcagcactcttgttacaaYgatatgttgccttattagaatcaatagcaatagctctttcatacaaagccaaagcctc

fava_c17524_1680	fava_c17524	1680	T/C	agctctagcataggaaggttgaatcctaatagcttcctcacactcaacaattgcttgtYgaaaccttcccaaaccaattaaagcagcactcttgttacaa[T/C]gatatgttgccttattagaatcaatagcaatagctctttcatacaaagccaaagcctcataaaatcttccttgcttataagcttcatttcccattgattt

fava_c17524_1783	fava_c17524	1783	G/A	tatgttgccttattagaatcaatagcaatagctctttcatacaaagccaaagcctcataaaatcttccttgcttataagcttcatttcccattgatttca[G/A]cacctcaggatcaactactctgttacttctaggacttcgaaattgcgacacaccatcaccacttttccttattatattccccataacaccgttactcaca

fava_c17524_2129	fava_c17524	2129	T/A	gacgactagtagtgcttattcttgcaagtcctgaggaattaccattaacctctctctgttgaattgagcttctagcagaatctaaaggtgtgtgattttt[T/A]actggttgaggctctgttgaaacttttgagatgtgatttggtgaccctttggattcatttagttgWgWattgttaaatgttttgatgggtagtgaatgaa

fava_c17524_2195	fava_c17524	2195	T/A	agcttctagcagaatctaaaggtgtgtgatttttWactggttgaggctctgttgaaacttttgagatgtgatttggtgaccctttggattcatttagttg[T/A]gWattgttaaatgttttgatgggtagtgaatgaacagatgatgaattttttggcttgttggctttgagtttgaagattttggccatgaaactacagccta

fava_c17524_2197	fava_c17524	2197	T/A	cttctagcagaatctaaaggtgtgtgatttttWactggttgaggctctgttgaaacttttgagatgtgatttggtgaccctttggattcatttagttgWg[T/A]attgttaaatgttttgatgggtagtgaatgaacagatgatgaattttttggcttgttggctttgagtttgaagattttggccatgaaactacagcctaat

fava_c17524_423	fava_c17524	423	T/C	aatcattcctgtagtgtatgtcactatgttgttgacatttgtagaaaattttgtaaaatattttcagctgctatataatttaattgatttttctaacaac[T/C]catggttgttgccagaaatttcttttacccttgatccattcttgtatattttgaaagctggaatggtgtttacaccttctgattttgttaagtaaggatg

fava_c17524_581	fava_c17524	581	C/T	ctggaatggtgtttacaccttctgattttgttaagtaaggatggtcctcaatctccaccttaaggaaattaactgatgggaatctcttgcaggtttgctc[C/T]aacaccaacaaaatttgattatgagttRatttgcccttgttgcaaaaaagcaccacagacataccaggtgaagttacataatgtctaaacttatcattgc

fava_c17524_609	fava_c17524	609	A/G	tgttaagtaaggatggtcctcaatctccaccttaaggaaattaactgatgggaatctcttgcaggtttgctcYaacaccaacaaaatttgattatgagtt[A/G]atttgcccttgttgcaaaaaagcaccacagacataccaggtgaagttacataatgtctaaacttatcattgcttgagatgcaaaccaaatttgaaccaaa

fava_c17524_860	fava_c17524	860	G/A	tctcaaacaaagctctagcaacttcctcatcccctggcttttctctaatcaacatttcatagtcttgaattgcagcctcccatctttccaacttggcatt[G/A]caatcagcgcgacgcaacattgccttgctataactaggctgaactttaagagctacactacaatcttcaattgctttctcatattgacctagcttagaac

fava_c17525_344	fava_c17525	344	G/A	ataacaataacaagatggttattacttctgatgagaacaaatccaatgcagtcgtgcccagaaattgtcaaggtggtggaagaaatgctgggcagaatag[G/A]aggactctgagtgtgattaatcagaatcttgtgcagggtcgaccttacccttgtgttgtgaacaagagggccttggtttctgataaacatgagatttgtg

fava_c17525_986	fava_c17525	986	G/C	ttgactggcttattgaggtgcatgacaaatttgacctcattcaagagacattgtttcttacaattaatctcatagacagattcttggcaaagcaaaatgt[G/C]gtaagaaagaaacttcagctggttggtcttgttgccatgcttttggcctgcaaatatgaggaagtctccgtccctgtcgtctcggatctaatccatatct

fava_c17528_1139	fava_c17528	1139	T/C	ccttagtttcttcaactgggtctgattgatgcttagactcatctgtgtcattgatgccattaacatccctgtgtccgtctcttagtactgtagttaattc[T/C]gtgcagtcatcttgggtacctttggttttctgaacagctacatatccctgattctgttcgaggataatattagatggatcaggcaacacagactcaggtt

fava_c17528_1835	fava_c17528	1835	G/A	aactatgacttccataacctaaatcagcacttgaacctactccagacttcttttccagcagccatttctttgcctccataacagcagcgctacagagttc[G/A]ggtgagccacttgcaagagttgtgttggatatatcccttggtctcgtaaccccatcgtcatttgaaggagagtccacaactcttgatcttattattttaa

fava_c17528_2311	fava_c17528	2311	A/G	aaaacagaggacagaatcttcccggcaccggaagcaatgaaacgagaaggagagataacgaaacgcgaaagccagttagggctttgaggctgaggctgag[A/G]ctgaggctgagacggtgatggaacgggtcgggtgtacggagtacggtttctggcagcggcggtacggcggagtcggaccaatttgcctcccgattgagat

fava_c17528_267	fava_c17528	267	A/G	ctataccttctgttgcctggtgaaggtgcatgttctaaccgcccacgcctgcatctttgttggcttgtgatggaggagcctcatggagcctgactgtctc[A/G]agggccagttttatcattcaggtcgcacatgcttgtgcatgcagatccatgtcaattagttaaacaggaaaagaaaaacttacatgtctctaattaaacc

fava_c17528_800	fava_c17528	800	A/G	cctcatactgtacactggagctgttctgcgactgcgaaccggtggcaacaccatcatctttggaagcaacagtaccgtctacactcacctcaggcacctc[A/G]gtagatccgcgcaacattttacaagtctcttgctcaagaacaccctgagctatcctctcctgacttgaatcaacagtacttggttcattatttgattttt

fava_c17529_1153	fava_c17529	1153	T/G	aaggttctttgtttactcgttggattcattcgaagtcctttggttgatgagatttattccaacggagagattcctagtattgttgggtttttgttgtcga[T/G]Rgatttgtgtgtttgtgttgcgggtttggattgtgttcttgaggttggttatattgggagaagggaagttattgaagctatgatggggcagaacttggtg

fava_c17529_1154	fava_c17529	1154	A/G	aggttctttgtttactcgttggattcattcgaagtcctttggttgatgagatttattccaacggagagattcctagtattgttgggtttttgttgtcgaK[A/G]gatttgtgtgtttgtgttgcgggtttggattgtgttcttgaggttggttatattgggagaagggaagttattgaagctatgatggggcagaacttggtga

fava_c17529_762	fava_c17529	762	G/T	acgccaaatcatcgcatacaagatttcccaagcttcctcatctctagaatccgaaattcaaatctacttagaccgtgtcatcatcataaacctcgttaaa[G/T]cacttcaagaagaacaaaacgaagaagaacagctccaggctattatcgagtttcaacagagattatccaaaggttttgatttggagtttcaagacttgat

fava_c17530_169	fava_c17530	169	A/T	aaaagttgtatgcttctcatatataattaatacatatatagattggcttccataaataactagcaaaagattaaaaaaaaaaatggttaaaattgtcaaa[A/T]gacagactatctgcacataggactatgaggttgatgataataaccaagctggtttgaggaacagtatcccatcattttttcaataccaaaactacatttc

fava_c17531_1027	fava_c17531	1027	C/T	cagttcatgctcttgtaagcaactggggtcactcgcatattgtagttgcaattggatacgaggtgttaacggggattctatatgccacaggtgttgtgtt[C/T]tatgtgaccagaataccagagagatggaagccYggagcatttgatattgcaggacatagtcatcagatatttcatgtttttgttgttcttggggcacttg

fava_c17531_1060	fava_c17531	1060	T/C	cgcatattgtagttgcaattggatacgaggtgttaacggggattctatatgccacaggtgttgtgttYtatgtgaccagaataccagagagatggaagcc[T/C]ggagcatttgatattgcaggacatagtcatcagatatttcatgtttttgttgttcttggggcacttgcacatagcactgccacacttgttatcatggatt

fava_c17531_391	fava_c17531	391	C/T	accggtcggaatggccggtgaaggattctttgttgagcgttttcgcgtggcacaacgagacgctgaatgtttggagtcatttaggaggctttttgatatt[C/T]gcggcgatggcggtcatgagtttttcggcggtgagggaactcggaggaggttttctctcaaaaatttacaggaacagagtaggaSccatgggaacgcaga

fava_c17531_476	fava_c17531	476	C/G	aggctttttgatattYgcggcgatggcggtcatgagtttttcggcggtgagggaactcggaggaggttttctctcaaaaatttacaggaacagagtagga[C/G]ccatgggaacgcagatcaacggttctgaatctcaagataaagcctttcaggattggcatttgagacagatattggaagtaaccatctttgatggagtgac

fava_c17531_712	fava_c17531	712	T/C	tagttggaggaatgtgttgcttagcttgcagttctgtttcccatcttctagcatgccactccaaacgctttaacctctttttttggcgtctagattatgc[T/C]ggaatatcaatcatgatagtatcctctttctatgcgccWatttactacgtatttttctgcaacccacacgcaaggttactctatttgacatcaatatcag

fava_c17531_751	fava_c17531	751	A/T	cccatcttctagcatgccactccaaacgctttaacctctttttttggcgtctagattatgcYggaatatcaatcatgatagtatcctctttctatgcgcc[A/T]atttactacgtatttttctgcaacccacacgcaaggttactctatttgacatcaatatcagtccttggagtaatagcaataataacccttctttccccga

fava_c17533_1064	fava_c17533	1064	C/G	atgaacgaaatgcattttgaatcctagtagcagcaatattgtcaatcaacctggatggaaccgtcctgtccatcatcaaaccttcattcagagtgatact[C/G]gactcttcaccagggctttgcttcccattagaggaYtcatttgatatttcaccagtagattgtaccttttctKgtttggatttatcttccttatgtttct

fava_c17533_1100	fava_c17533	1100	T/C	tattgtcaatcaacctggatggaaccgtcctgtccatcatcaaaccttcattcagagtgatactSgactcttcaccagggctttgcttcccattagagga[T/C]tcatttgatatttcaccagtagattgtaccttttctKgtttggatttatcttccttatgtttcttccttctgagtagtatcttaaaccatttctttggac

fava_c17533_1137	fava_c17533	1137	T/G	catcaaaccttcattcagagtgatactSgactcttcaccagggctttgcttcccattagaggaYtcatttgatatttcaccagtagattgtaccttttct[T/G]gtttggatttatcttccttatgtttcttccttctgagtagtatcttaaaccatttctttggacccattttggttgagaaataaaagatgtgaaattggag

fava_c17533_262	fava_c17533	262	C/T	tcaaccaacactagggaattcatgatacatacaaaagcaaataacaagttaaagtcttctaacgagacacaatacaaaccaaatggtagattctcacaag[C/T]caaatatagaaaaattaaacttcaaaagaagatgaagaaaccaatatccatctaattttctgccccagcattttttggcaacccttatgttttgtttttc

fava_c17533_935	fava_c17533	935	C/T	cttgtattctgctccatgaatgtatatagttaagtgtagttgatgtttgctctctggccatgtgatcttgaattaaagcttcaaaattctctgctcccct[C/T]agatgttgaaatgttcttcttgccatgaatgaacgaaatgcattttgaatcctagtagcagcaatattgtcaatcaacctggatggaaccgtcctgtcca

fava_c17534_1054	fava_c17534	1054	G/A	tttaagaaacaaagcaacagaatgaagagtagaagcaatcatggaaggacccttataagtaaccaaatcaccttttccaacaaccataacattcctaaaa[G/A]caccaaaaaccctctcggatttcacatactttgcaacctcaagcctctctgcatcagaagcttctagatcaaggtgaagaaggtagtagtttcttggatg

fava_c17534_318	fava_c17534	318	T/A	atagtttcaacatgagtttctcaagaatctttgatcttaaactaggtttaacaacatctggatttccataaacatcacaaggatttttctccttaagaga[T/A]tcgccaacgcaccaagcaccacgagtaaagtgactatccgatcttcccagcagttctttgtcaattttatcaagaaccgggtcgtccttgtcaaacctcc

fava_c17535_1368	fava_c17535	1368	T/C	tatggaagtcagtttatccaaagggaattcatgacctgttgctctatattaaaacaaagtacaacaatcctttgatatacatcacagaaaatggtataga[T/C]gagctcaatgatccaacactctcacttgaagaagcccttgcagatacgcctaggattgattactattatagtcaccttcgttatcttcaaattgcaatca

fava_c17535_168	fava_c17535	168	G/A	catttgaagtaaatcaaatctggtgatctatagttatgacgttgaatattcttcttcttgctagctttttgcttattctttgttcagtggttattactga[G/A]gcYgctaccgtattcgccaacggtattagtcccccgctcaaccgtagtaRttttccagatggtttcattttcggaacagcttcatcagcttaccagtgtg

fava_c17535_171	fava_c17535	171	T/C	ttgaagtaaatcaaatctggtgatctatagttatgacgttgaatattcttcttcttgctagctttttgcttattctttgttcagtggttattactgaRgc[T/C]gctaccgtattcgccaacggtattagtcccccgctcaaccgtagtaRttttccagatggtttcattttcggaacagcttcatcagcttaccagtgtgaag

fava_c17535_1728	fava_c17535	1728	G/A	tacaaaagaagaaagatatgcatagtgatttgcgctaagaaggtgaacttcattaaatatgatttgttggcgatattgtctcaaggtataagtttatttt[G/A]caacgatgagtcttgatatttggtcattctatgttaacctcctcctcaattttggtcatttcctcaaatatttggactcaaatttgatgttgtaggacac

fava_c17535_218	fava_c17535	218	A/G	cttcttcttgctagctttttgcttattctttgttcagtggttattactgaRgcYgctaccgtattcgccaacggtattagtcccccgctcaaccgtagta[A/G]ttttccagatggtttcattttcggaacagcttcatcagcttaccagtgtgaaggtgcggcaaatattggtggcagggaaccaagtatatgggacacttac

fava_c17535_351	fava_c17535	351	A/T	catcagcttaccagtgtgaaggtgcggcaaatattggtggcagggaaccaagtatatgggacacttacagtcataattatccagaaaagatattcgggag[A/T]agcaatggagaYgtagccatcgatgaatatcatcgttataaggaagatgttgagatcatgaaggacatgaacatggacgcttatagattctccatctctt

fava_c17535_363	fava_c17535	363	T/C	agtgtgaaggtgcggcaaatattggtggcagggaaccaagtatatgggacacttacagtcataattatccagaaaagatattcgggagWagcaatggaga[T/C]gtagccatcgatgaatatcatcgttataaggaagatgttgagatcatgaaggacatgaacatggacgcttatagattctccatctcttggtcaagaatac

fava_c17535_634	fava_c17535	634	G/C	acttctagacaaaggaattcaaccctttgtgaccctttttcattgggatcttcctcaaatacttgaagaagaatatggtggctttctaagtcctaatata[G/C]tgaatgattttcgagactatgcagagctttgctttaaggaatttggaRatagagtaaagcattggatcacattaaatgagccatggaccgtaagcaaatt

fava_c17535_682	fava_c17535	682	A/G	tcttcctcaaatacttgaagaagaatatggtggctttctaagtcctaatataStgaatgattttcgagactatgcagagctttgctttaaggaatttgga[A/G]atagagtaaagcattggatcacattaaatgagccatggaccgtaagcaaattcggttatggtgatggatgggcagcacctggRcgatgttcaccctggct

fava_c17535_765	fava_c17535	765	A/G	gctttaaggaatttggaRatagagtaaagcattggatcacattaaatgagccatggaccgtaagcaaattcggttatggtgatggatgggcagcacctgg[A/G]cgatgttcaccctggcttaacaaaaattgtactggtggtgattcagctactgaaccctatatagtKgctcacaatcagttacttgctcatgcaactgctg

fava_c17535_831	fava_c17535	831	G/T	aattcggttatggtgatggatgggcagcacctggRcgatgttcaccctggcttaacaaaaattgtactggtggtgattcagctactgaaccctatatagt[G/T]gctcacaatcagttacttgctcatgcaactgctgtcaaagtctacaaaaccaagtatcaggcatctcaaaagggtttgataggaataacgctatcgtgtg

fava_c17541_1158	fava_c17541	1158	T/C	aggttaaaaactccccaacaatatatgagtactacaaaacccttcaacattctggtatgtacaggatttagaaatttattgaaggttgaactattcaaga[T/C]ggtctcaaattttgtttcaagatgcataattatcacattcttttatttattttacatttgtggcacctattacaatttctttaagctatagcaatgctag

fava_c17541_238	fava_c17541	238	T/G	ctgagaatactgatttcactgtgattggagttattggatctcccggtgttggaaaatctactattatgaatcaactttatggttttgattcaagttctcc[T/G]gggatgctacctccttttaccatacaatctgaagagattagagctatggcaagacattgttctattggcattgaaccaaggatatcatctgaacgcatta

fava_c17541_729	fava_c17541	729	A/G	tttcccagagttctaactcagggcatgataatgataacaaagtttctgaatgtgaatacatggctactcctgtttttgtacataccaagttacaagatca[A/G]gagttgactcctaaaaattgtttgcagctgaggaaggccctcaagcagtatctcaagccaacttcttttgttaaagaaaatgttagaaacaaacccgacg

fava_c17542_523	fava_c17542	523	C/T	cagtattgttgttgctttcccttagtgcagattgtgcatttctgatagtaggaccagattgttcttctccgatatcttttttaggtttcttttgttccaa[C/T]ggggatgaataccacaaattctttgtaggttcatttaaatttgtctgcattttggaaggctttgggttatttgaactctttggagtgggtagcacatatg

fava_c17544_191	fava_c17544	191	A/G	ttattgaatttgtaataacaaagtgtaccaaaacactaaagtttttcaaggcacagttgaagttcccttgttttacatcaatccgatcttattcaatttt[A/G]acatttgatagcctttttaccaattcattgaaatcatcatccatgccaagcatgctaaccctaacaaactttgaatcagcaccaaatctctccccttctc

fava_c17544_346	fava_c17544	346	C/G	ctaaccctaacaaactttgaatcagcaccaaatctctccccttctcttgaagaaatattcaattttcttagatagttatgaccattttctttgccctcct[C/G]gcatttcagccaagcaaaactagggtatgtttcagatatctcattggtgaaattacagtaagcatttggatacttRactaccccaaaaaccatgctttgc

fava_c17544_422	fava_c17544	422	A/G	tatgaccattttctttgccctcctSgcatttcagccaagcaaaactagggtatgtttcagatatctcattggtgaaattacagtaagcatttggatactt[A/G]actaccccaaaaaccatgctttgctcaacagcttctccgagtttctcccacctttcYttcatgagacgcttgctatattcaaagaagagttcagattcWt

fava_c17544_479	fava_c17544	479	T/C	cagatatctcattggtgaaattacagtaagcatttggatacttRactaccccaaaaaccatgctttgctcaacagcttctccgagtttctcccacctttc[T/C]ttcatgagacgcttgctatattcaaagaagagttcagattcWttggacttgagattttggtaaccatcacaaatcactccaatgatcttagcagctcgtg

fava_c17544_521	fava_c17544	521	A/T	tRactaccccaaaaaccatgctttgctcaacagcttctccgagtttctcccacctttcYttcatgagacgcttgctatattcaaagaagagttcagattc[A/T]ttggacttgagattttggtaaccatcacaaatcactccaatgatcttagcagctcgtgtttgggattctttcgacacccccattgaactcgactgcacaa

fava_c17544_826	fava_c17544	826	G/T	tcgtcagccttgtgagtaatcggagtaaattgcggccaataatacgccaaatcataaataaacttcccttcagctacagagttcaccactggctcacgaa[G/T]agtcccgtcagggttgttcggagaggtcaccatctcaatgtaaggttcatttttatcatacacagcagcatcaccaccccattgaaacatcctcgaatgc

fava_c17544_990	fava_c17544	990	T/C	agcagcatcaccaccccattgaaacatcctcgaatgcaatatattaatcgcactcttgtattccgagtaataaggagcagccgcaacaacattgacagga[T/C]gatcgggaacatctaaaggagacagagcaaacaaagaagcttgaaagagctgagaagaaccatttccaaccactatgtatctatctttggtcacagcatt

fava_c17546_1482	fava_c17546	1482	G/T	gcatcagaataaatcttagtcataatcgggtcaatggtcaaaattcttgtcaatggtcaacagtttgatcaaaagtcaatcgttgacccaaatgtcaact[G/T]tagtgttttcttcaattttcctatttttttgtaatgacttgtatttgaaccttgttgtatatattgtgatgactctgcagtatcttgtagttcaagcaaa

fava_c17550_167	fava_c17550	167	C/T	taaaaccatacaacagtgattttttttttactaatacgaaatcacatggtaccctgaggtctacaccacacactttgtgtagtaattatttgcgttagac[C/T]tcaatggtaccaccgtgtaacactaactatttacatgcaaacagagttttatgaattacatattccatttcttaatacatgtttttccattaaaatgaca

fava_c17550_478	fava_c17550	478	T/C	ttccaaactcagtgaaaagtaaagcaaaacagtcataaaagcaactcctgtatcaagtgctgctgatagaacatagttgtacctctgccaccatttcttt[T/C]tgtaacggaacacaaagaagttgaaaactgttccgaagaaaatccaagcattgtaRttcaatggtgttgctggtggcatcgatccggtagctccaaggag

fava_c17550_534	fava_c17550	534	G/A	gtgctgctgatagaacatagttgtacctctgccaccatttctttYtgtaacggaacacaaagaagttgaaaactgttccgaagaaaatccaagcattgta[G/A]ttcaatggtgttgctggtggcatcgatccggtagctccaaggagaactggaaggttaatcaaaggaatccatgattgtttaggaaaggctttatgcaata

fava_c17550_765	fava_c17550	765	A/G	caaggaaaaaccaattcattacactgtagtttcctttagaaccaaatatcctttttggtcctaccagaccccaaataaccgaagcatcgaagaaaacacg[A/G]tcacctgggcatgtccaaggactaccttctggaaggagatcatcatgacatatattctcaatagagtttagcaaccaccatgctaccccgatgttgatgg

fava_c17552_1054	fava_c17552	1054	A/G	tccaaaaacaacatcctcaaaatcaaatgtgatgcagtaattgttggttctggctgtggaggaggagtagcagcttctgtgctttcaaaagcgggccaca[A/G]aatcattgttctagagaaaggaaactactttgttcctaaagattattcatcgctagaaggtccttcgatggatcaacaatacgaatgtggaggcatgtta

fava_c17552_604	fava_c17552	604	G/C	ttagtaaagagaggtttaacagaagcagtgatattgattagagtgattctatggcttctgtccacgaggtttgggacttttttgatttgtggttttctct[G/C]tcttagtaagaaatggccatttatcaacaatttttcaagcttgtctttggatcaaagagaaaagattgtgcagaggtgtctcaagcttaggttcttaact

fava_c17559_507	fava_c17559	507	A/T	taaaaaattccatctataaacttcctggtggccgtttgaggccaggtgaatcagatactgatggattgaagcgcaagctggcaaagaagctttctcttga[A/T]gaagatgaggcacaatgggaggtaggagagtgccttggaatatggtggaggtctgattttgaaacattgatgcaccctttcttaccacctaatgtcaaac

fava_c17559_660	fava_c17559	660	G/A	ctgattttgaaacattgatgcaccctttcttaccacctaatgtcaaacatccaaaggagtgtacaaaactcttccttgtaaagcttccggaaactcaaag[G/A]ttcactgtgccaaagaacatgaagttgctttcagttccattgtgccaaattcaagaaaatcacaagatatacgggccaattgtatctgcagtcccacagc

fava_c17559_775	fava_c17559	775	T/A	gaacatgaagttgctttcagttccattgtgccaaattcaagaaaatcacaagatatacgggccaattgtatctgcagtcccacagctactctcaaagttc[T/A]ctttcaatatgatcggRatttaaaccttagccgcaacttggtagcaatcttgttaggcatccagttcagtccaatttttattaggatgccaaaataagga

fava_c17559_792	fava_c17559	792	G/A	cagttccattgtgccaaattcaagaaaatcacaagatatacgggccaattgtatctgcagtcccacagctactctcaaagttcWctttcaatatgatcgg[G/A]atttaaaccttagccgcaacttggtagcaatcttgttaggcatccagttcagtccaatttttattaggatgccaaaataaggaaggacttgtaaatatta

fava_c17559_916	fava_c17559	916	G/A	gtagcaatcttgttaggcatccagttcagtccaatttttattaggatgccaaaataaggaaggacttgtaaatattatgggtttttatacaacattgatt[G/A]tactaaaatgttgaaaacagaaagatatttgacttgaatggtgttttggcttgagatatacaagtagttgatggtagtttgattattggtttgttacaag

fava_c17562_1003	fava_c17562	1003	G/A	aatatgtgatttttgttgttgcgaatgtgatgtaaaaatatcRgcgtcgatatcggcaYtttcWgataMRgtWttgtctcaagatcaagtattgattgaa[G/A]ttagtaggtgagagatgagatgtgctggaacagttaacgagaagaagtgagaatcaggagaagctaaactatagctatagcttatcttagctaggtatct

fava_c17562_1201	fava_c17562	1201	T/A	tctgtgtactttgatcaccattcaccattatatcttgttaatttccaagaaagcagaagaaaaagcagatagattatagatagatatatgtatgaggaac[T/A]tccattgtgtgatgcaatgaagattagttcagatagatagatatagccatagatgtatctgagctgatgcaaaagtaaacctcaaaatagagagtactgt

fava_c17562_1311	fava_c17562	1311	G/T	tgatgcaatgaagattagttcagatagatagatatagccatagatgtatctgagctgatgcaaaagtaaacctcaaaatagagagtactgtcatgtttct[G/T]gatWtttttctgtttcaatgtatttgtatgtgttttagtgtaatgcatttatgaaggttttaaagttgttgaaaattcagaaaccatgttaggaaaaagc

fava_c17562_1315	fava_c17562	1315	A/T	gcaatgaagattagttcagatagatagatatagccatagatgtatctgagctgatgcaaaagtaaacctcaaaatagagagtactgtcatgtttctKgat[A/T]tttttctgtttcaatgtatttgtatgtgttttagtgtaatgcatttatgaaggttttaaagttgttgaaaattcagaaaccatgttaggaaaaagctttg

fava_c17562_188	fava_c17562	188	A/C	gttcattcttaattgtatgaactataaggcttcggaccaggcttcattcccctcaaaatctttattattccatcttcttcatcttatatgtgttctatag[A/C]aatatcaaagaaacgcaagagttaggttgagaggaacgttgtctggctcgaggtcaccgagattgatgagtacttataactctcaactcaatctcggacc

fava_c17562_496	fava_c17562	496	G/A	gaattttctactctaataataatcatcaacttatcttctactaagcagtgtcaatgaatgaattccaagcaagaaagaagaacagtaccatttttctaag[G/A]agttcattttatgcagtactttttcgtaatggttaaaagttaaaaccgctttaaaagttctggatctgaaacttttcagttattagattatgtgatttaa

fava_c17562_601	fava_c17562	601	T/A	cattttatgcagtactttttcgtaatggttaaaagttaaaaccgctttaaaagttctggatctgaaacttttcagttattagattatgtgatttaaaaag[T/A]gtaacagtgcgcaaatctctctagttttctggtataataataatttatggctaatattgtgaccacaccaggcccctacctctccctcaaatgcagcagg

fava_c17562_834	fava_c17562	834	T/C	atcaacagtgtcaaaacctatcacaagtgaaattctttcttttcatctgtaaacccaaaaccctagctaggtctttcaataacactctgagttataacta[T/C]aacagtattataacccttcttcatttgYcatttYacattttatattttactattaagtgagcattaacaatatgtgatttttgttgttgcgaatgtgatg

fava_c17562_862	fava_c17562	862	T/C	gaaattctttcttttcatctgtaaacccaaaaccctagctaggtctttcaataacactctgagttataactaYaacagtattataacccttcttcatttg[T/C]catttYacattttatattttactattaagtgagcattaacaatatgtgatttttgttgttgcgaatgtgatgtaaaaatatcRgcgtcgatatcggcaYt

fava_c17562_868	fava_c17562	868	C/T	ctttcttttcatctgtaaacccaaaaccctagctaggtctttcaataacactctgagttataactaYaacagtattataacccttcttcatttgYcattt[C/T]acattttatattttactattaagtgagcattaacaatatgtgatttttgttgttgcgaatgtgatgtaaaaatatcRgcgtcgatatcggcaYtttcWga

fava_c17562_945	fava_c17562	945	G/A	taacccttcttcatttgYcatttYacattttatattttactattaagtgagcattaacaatatgtgatttttgttgttgcgaatgtgatgtaaaaatatc[G/A]gcgtcgatatcggcaYtttcWgataMRgtWttgtctcaagatcaagtattgattgaaRttagtaggtgagagatgagatgtgctggaacagttaacgaga

fava_c17562_961	fava_c17562	961	C/T	gYcatttYacattttatattttactattaagtgagcattaacaatatgtgatttttgttgttgcgaatgtgatgtaaaaatatcRgcgtcgatatcggca[C/T]tttcWgataMRgtWttgtctcaagatcaagtattgattgaaRttagtaggtgagagatgagatgtgctggaacagttaacgagaagaagtgagaatcagg

fava_c17562_966	fava_c17562	966	T/A	ttYacattttatattttactattaagtgagcattaacaatatgtgatttttgttgttgcgaatgtgatgtaaaaatatcRgcgtcgatatcggcaYtttc[T/A]gataMRgtWttgtctcaagatcaagtattgattgaaRttagtaggtgagagatgagatgtgctggaacagttaacgagaagaagtgagaatcaggagaag

fava_c17562_971	fava_c17562	971	C/A	attttatattttactattaagtgagcattaacaatatgtgatttttgttgttgcgaatgtgatgtaaaaatatcRgcgtcgatatcggcaYtttcWgata[C/A]RgtWttgtctcaagatcaagtattgattgaaRttagtaggtgagagatgagatgtgctggaacagttaacgagaagaagtgagaatcaggagaagctaaa

fava_c17562_972	fava_c17562	972	A/G	ttttatattttactattaagtgagcattaacaatatgtgatttttgttgttgcgaatgtgatgtaaaaatatcRgcgtcgatatcggcaYtttcWgataM[A/G]gtWttgtctcaagatcaagtattgattgaaRttagtaggtgagagatgagatgtgctggaacagttaacgagaagaagtgagaatcaggagaagctaaac

fava_c17562_975	fava_c17562	975	A/T	tatattttactattaagtgagcattaacaatatgtgatttttgttgttgcgaatgtgatgtaaaaatatcRgcgtcgatatcggcaYtttcWgataMRgt[A/T]ttgtctcaagatcaagtattgattgaaRttagtaggtgagagatgagatgtgctggaacagttaacgagaagaagtgagaatcaggagaagctaaactat

fava_c17563_1556	fava_c17563	1556	G/T	gagctgattacactgcatatccattcaacacgcgcccagtcagccgaaatgtttgtcagaaaccgtttatttattatttgtctaatgcaattcaagatga[G/T]gatacagatgagactgctagtcgatatgtccgaactcaaacgaatccggattgcaagtggaaaatggaggatccaactcaaatcaaagtggtaatggttt

fava_c17563_1850	fava_c17563	1850	C/T	atgtaggggaatgcagagaagatgaagttgttgaattgtaattatagagctccaacttgcttgttgatagttcttgactcaatgatttattttatatata[C/T]ataattgtttacaaaattctttacttgctgatttcttttggaggcaaatgcaaacaagaatgagttgtaaaagtccaaattactttattttctactatag

fava_c17563_380	fava_c17563	380	T/C	aagacaacacacaaaaaaacccatttttctaaaccatgaagaacactttcaaagacccagatcaccacaatctccactcatcaataaccttctcaaagct[T/C]ataactttcStcttcctcttaatctcaatcKcatacctcttctactccctcagattcgttacWcataactccaattgtgaccaaaaccYacccattattc

fava_c17563_390	fava_c17563	390	G/C	acaaaaaaacccatttttctaaaccatgaagaacactttcaaagacccagatcaccacaatctccactcatcaataaccttctcaaagctYataactttc[G/C]tcttcctcttaatctcaatcKcatacctcttctactccctcagattcgttacWcataactccaattgtgaccaaaaccYacccattattcataacaccca

fava_c17563_411	fava_c17563	411	T/G	aaccatgaagaacactttcaaagacccagatcaccacaatctccactcatcaataaccttctcaaagctYataactttcStcttcctcttaatctcaatc[T/G]catacctcttctactccctcagattcgttacWcataactccaattgtgaccaaaaccYacccattattcataacacccacacaagtttccaacctcaaac

fava_c17563_443	fava_c17563	443	T/A	accacaatctccactcatcaataaccttctcaaagctYataactttcStcttcctcttaatctcaatcKcatacctcttctactccctcagattcgttac[T/A]cataactccaattgtgaccaaaaccYacccattattcataacacccacacaagtttccaacctcaaacacaYaacccatttcaagaaaaacaacaagagt

fava_c17563_469	fava_c17563	469	T/C	ttctcaaagctYataactttcStcttcctcttaatctcaatcKcatacctcttctactccctcagattcgttacWcataactccaattgtgaccaaaacc[T/C]acccattattcataacacccacacaagtttccaacctcaaacacaYaacccatttcaagaaaaacaacaagagtctcctaccaacatctcacacatagtg

fava_c17563_515	fava_c17563	515	T/C	acctcttctactccctcagattcgttacWcataactccaattgtgaccaaaaccYacccattattcataacacccacacaagtttccaacctcaaacaca[T/C]aacccatttcaagaaaaacaacaagagtctcctaccaacatctcacacatagtgttcggaatcggcgcatcagcSaagctctggaaaaagcgaaaagagt

fava_c17563_590	fava_c17563	590	G/C	acacaagtttccaacctcaaacacaYaacccatttcaagaaaaacaacaagagtctcctaccaacatctcacacatagtgttcggaatcggcgcatcagc[G/C]aagctctggaaaaagcgaaaagagtacatcaaactctggtacaaaccaaaccaaatgcgcggcattgtStggttagaacaaaaggtgaaaccagatcctg

fava_c17563_659	fava_c17563	659	C/G	cacacatagtgttcggaatcggcgcatcagcSaagctctggaaaaagcgaaaagagtacatcaaactctggtacaaaccaaaccaaatgcgcggcattgt[C/G]tggttagaacaaaaggtgaaaccagatcctgaagacaaaaaccttttaccaattctaaaaatttccgaagacacaacgagtttcaagtacaaaaactcca

fava_c17566_1345	fava_c17566	1345	A/G	atctagatgcataaatttttaacgcatcgccaatgaaatttgaaggaaccttgccaccagatttgattgctatcattgctctccaataaagatctatgct[A/G]agttctgctaaatcttcagcccaccatcctttgcttcctgatttggttctcaaactttctgtatcattgcaagatatatcatctctcactcttctagaat

fava_c17566_1672	fava_c17566	1672	C/T	ctatggtatccttccatccgtagaggatgcaagaattgaagaaaacttcaagcttgtgaatcaggtttcctttctcaacttcctcagtcatttggagata[C/T]tcagcagcacatcttgcggctacaatgttgtaaggacttagagtgatggtgattccatagcagaacttagcacacatctcaaatgcttctactccaccag

fava_c17566_1798	fava_c17566	1798	G/A	tgttgtaaggacttagagtgatggtgattccatagcagaacttagcacacatctcaaatgcttctactccaccaggaaaatcaggaagttgaactatttg[G/A]tgatgagatgaatctggaggtgattctgagcataatctttgcaggcataagcattttgacaacaaaggaaacttatgaagaagatatctacttcccctta

fava_c17566_2023	fava_c17566	2023	A/T	cttcagaagagattgaccttatggactcagaagtgtagaaagtatctggacgagatccaagtttcatgaacttcatggctttaagtttcttaccctcaaa[A/T]tcatgtgttaaccaaaMacactacttgcataattcagtatgtKaagtaagagaaaaagagaaaagaagtacatcccaaataccaagatgaaagaatattt

fava_c17566_2040	fava_c17566	2040	C/A	cttatggactcagaagtgtagaaagtatctggacgagatccaagtttcatgaacttcatggctttaagtttcttaccctcaaaWtcatgtgttaaccaaa[C/A]acactacttgcataattcagtatgtKaagtaagagaaaaagagaaaagaagtacatcccaaataccaagatgaaagaatatttgtttatttgttagcaat

fava_c17566_2066	fava_c17566	2066	T/G	atctggacgagatccaagtttcatgaacttcatggctttaagtttcttaccctcaaaWtcatgtgttaaccaaaMacactacttgcataattcagtatgt[T/G]aagtaagagaaaaagagaaaagaagtacatcccaaataccaagatgaaagaatatttgtttatttgttagcaatggatttttctgactagccaagctcaa

fava_c17566_241	fava_c17566	241	A/G	gcattttttttggttgtgtaggaagagcacaaatggatttaaatctagagtttcttccgattccactgtcattcaccaaaccgttttcagcgaactcgtc[A/G]tgatcttcggctagtttcattcttagagttgtcgaccttagtttcggtgacttgaaggcagaaacgctccagttatcctcgccttgaatgcttgtagtag

fava_c17566_834	fava_c17566	834	G/T	tccacaagctttgccactttcaattttgagctgtgagaacctgaagaggatctcctactctcttgtaattcgaagttaatattctcggccgaacgagacc[G/T]tctcctttcaaaggtcttcaaggatcttgaaggacttgttggtggactttgtccttgtaacatgaactgttccaatatagtcatcaccaactcaacatcg

fava_c17566_946	fava_c17566	946	G/A	aggtcttcaaggatcttgaaggacttgttggtggactttgtccttgtaacatgaactgttccaatatagtcatcaccaactcaacatcgtagacagtatc[G/A]tttgtataagaaagtgtaggtattaatagatcattcactgtggcttcctccaattgaagtcctacccttcttgccaattccatctttgaagatgaagaag

fava_c17567_1109	fava_c17567	1109	A/G	gtttcactaaaagtatgtccaaagtcacaagcagatcctgataatgatgtttctgatgctaaagttgaagactcaaaatgtttggtccattatgtacatc[A/G]tgagaaagcggaagagctgattaaatcacctggcaagcaggaatctcaatctgaaaatgagagtgacgaatcagatgttgtggaacatgatgtgaaggtg

fava_c17567_533	fava_c17567	533	A/G	aatgagggtaatgtacattctcttagtagcagagcttgtgaaaacttgcagcttgatgtcagtgaaacaagtaatatgctcagtattaattcaaatcatg[A/G]ttctctatctgaaaatgccgagagtagacagatcttgatgaataagtatcaggattccaagcatttagaaggtcatgatgacaacacatcttgtatcagt

fava_c17567_822	fava_c17567	822	A/T	attttggggcggaacaatctgtaattgcttctaaagatggagacactgatcatagttcacctatggtacaaaggcagcatggacaatctcaaaatgataa[A/T]tctctttcagataatccaagcttaatacacatSgaaagggacttgaattctcagataccagaaaaaatttcagaatgctctatagagaattgcagttcat

fava_c17567_855	fava_c17567	855	G/C	aagatggagacactgatcatagttcacctatggtacaaaggcagcatggacaatctcaaaatgataaWtctctttcagataatccaagcttaatacacat[G/C]gaaagggacttgaattctcagataccagaaaaaatttcagaatgctctatagagaattgcagttcatcattaaccaaagagagtgcacccgttgttgttt

fava_c17569_949	fava_c17569	949	T/C	cttcactggtgtcacactgattgtggattacgagaatcttatattagaaatgggcttactactggaatcaatgggacgaccgagatgcagtttcactgca[T/C]tgcttgtgatcacccttctgagatgtttggctttgtcaaagaggtgtttcaaagctttgcgaaagagtggtcggtcgaatctcttcacaaggaacttgaa

fava_c17570_1953	fava_c17570	1953	A/G	cgatgtatatgttgatgttcctgatatcatagatatcagtcatatgcgtagcaaaggccatcaacctggagaagagcttctaccagatggagtacccacc[A/G]aggatgattcggaaacacctttagccaatgaagacattgtttcacagctgaattctatgggctttcaccttattcattgtcaaaaagctgctataaatac

fava_c17570_2258	fava_c17570	2258	T/C	accagtcaaaagttgatactctaatatcatttggatttcaagacgacgttgctcgaaaggcactgaaagcatcgggtggtgacatagagaaagcaacaga[T/C]tggatctttagcaatcccgatgcatctgtttccaacatggatgcaactgcatcaaacactacttctggtacaaatgatggtgacttacccgatggaggag

fava_c17570_2387	fava_c17570	2387	T/C	tttccaacatggatgcaactgcatcaaacactacttctggtacaaatgatggtgacttacccgatggaggaggaaaatatcgactaatgggaattgttag[T/C]cacattggaacctcgacgcattgtggtcactatgttgctcacatcttaaaagatggtagatgggttatttttaacgacaacaaggtaggggcttcaatca

fava_c17570_2548	fava_c17570	2548	C/T	tgggttatttttaacgacaacaaggtaggggcttcaatcaatcctcccaaggacatgggatatctttacttttttgagaggcttaactcttgaattagat[C/T]gtgtgtcaagaatcgaactctgaatatttcaactgtgttgagagtgagaggcacactgttctaaaatggcacaacctgttatctacttgtgctgatggga

fava_c17570_825	fava_c17570	825	T/G	tgggaggaaaaattgggatgggagtggtggaaacaatcatgctgttgaacattataaaaagaccggctacccacttgctgtaaagcttgggactattact[T/G]ctgatctggaagcagcagatgttttctcttatccggaggatgagaatgtattggacccacttttagcacaacatctggcattttttgggattgatttctc

fava_c17572_1059	fava_c17572	1059	A/T	attctgtaggattgagtagttctaaccattcttgagcgcctgccggctcccatgctgccaaacctccttttttactctgaagtgatattaggagattgac[A/T]gaatcaaataacctttcaggttccatcttttcccccacaatttctggagccaacaatgataaaagtagacaacactttaaaccttctgcggtgcaatcgg

fava_c17572_1746	fava_c17572	1746	T/A	gatcttgtatcaaaggatgaggatagtaaatatcttcctttgcacatagatgacgtgctttcgtccaattaattttttcataaggttgaatatggagttc[T/A]tctcggagctgtaagatgagtggtgtgattggacccacaaatctttttccatataaataagacataggcatgtataccaatcgacaataacaccacattt

fava_c17572_1956	fava_c17572	1956	T/C	gcataggaagaaatgaaggaaggatccaaaattcaggaggcattggattacttcccaaccaatcaaatacaccaagtatcgaaagccaagtttttcccca[T/C]gaaggtatatgtgtgacaccaccgtgttcacgaatccaatttcttgctctaacacaagcattgtcttSaccaccattaggcccttctccaagWattcgca

fava_c17572_2024	fava_c17572	2024	G/C	acaccaagtatcgaaagccaagtttttccccaYgaaggtatatgtgtgacaccaccgtgttcacgaatccaatttcttgctctaacacaagcattgtctt[G/C]accaccattaggcccttctccaagWattcgcatgcatatatagttgagtgcagtacaaaacatagtactRtgaccctcaatgtgaagcccccaccctcca

fava_c17572_2049	fava_c17572	2049	A/T	ttccccaYgaaggtatatgtgtgacaccaccgtgttcacgaatccaatttcttgctctaacacaagcattgtcttSaccaccattaggcccttctccaag[A/T]attcgcatgcatatatagttgagtgcagtacaaaacatagtactRtgaccctcaatgtgaagcccccaccctccatcttcattctgatgacaatatatat

fava_c17572_2094	fava_c17572	2094	G/A	aatttcttgctctaacacaagcattgtcttSaccaccattaggcccttctccaagWattcgcatgcatatatagttgagtgcagtacaaaacatagtact[G/A]tgaccctcaatgtgaagcccccaccctccatcttcattctgatgacaatatatatagcgaagaatctctctgcggtgttcttgtgggaataccgaatcaa

fava_c17572_286	fava_c17572	286	C/G	gctaagatgaaagaaaaaaaaagtaatataaagaatgaaataggcttgactgcctaacttgatttgttttatttgagcgattctttctttaattatatgg[C/G]ttgctcctttttgtgttgatagatttttatcaagttaaaccacgatagaaggcaaaggaactcgtctacgatactcggctagagcccaSagtggataaat

fava_c17572_375	fava_c17572	375	C/G	taattatatggSttgctcctttttgtgttgatagatttttatcaagttaaaccacgatagaaggcaaaggaactcgtctacgatactcggctagagccca[C/G]agtggataaatatctctatacattggataatgcaacatgcaatttttcatgaatactcctgtgatttcctgttggggccaatcgccttgttctaattggg

fava_c17572_570	fava_c17572	570	T/C	attgggaattaatgagtaattttgctgcacgatgaagaggagtgggatctctctctgactgtccagtatgaattaaacccataagagcccacgcagtatg[T/C]acgacatttgaccgatttccttcaagcggtacatatatcttttttgggcttgaaagatagctctccccccacccgccRtcctctctctgtgttgtgagaa

fava_c17572_648	fava_c17572	648	G/A	ccataagagcccacgcagtatgYacgacatttgaccgatttccttcaagcggtacatatatcttttttgggcttgaaagatagctctccccccacccgcc[G/A]tcctctctctgtgttgtgagaagaaatttaacacctttgcgaatagcagcacaattggtataagtctttccagcagctgctaaaccaccaagagcgaacc

fava_c17572_878	fava_c17572	878	A/G	tttccataccatgaaccatcatctgtttgcgtatcttcaaggaacccaactgcattcaaaatgaaattttctatctctttcttcctatgacccggatata[A/G]cttcttaaacaaaactaaagcttgaattgctgatcctgtgcactcaacatattcatgttcaactacaatgtccgcaaaaaattctgtaggattgagtagt

fava_c17574_692	fava_c17574	692	A/C	taagcatccgcaattctaccaactctgcacaaagccctgacaaacgtagcataagaagccatatcaagcttcagagcatccgatttcatcgtatcataca[A/C]ttccatggccttatcaaacagcctagacttgcacaacccatgtagcaacgtgttatacgtgcacgaatttggactgcagcccttcatctccatctcttcc

fava_c17574_997	fava_c17574	997	T/C	caacaacttcttagcttccgtgacccgaccggatttcgataacccataaatcaacgtgttataagtaacaagatcaggctccaccccattctctttcatc[T/C]gattataaacctcaatcgcctcactccctttactcagcatacaataacctttcataattgtattataaacaaagcagtccggcttaaaaccttcctcatg

fava_c17575_161	fava_c17575	161	A/G	ttaaataatactagtttatactgagaaatttcatataaaaaaaaacactataataatatgtttttggtatgactaaaataattattaaatcatcttagct[A/G]tagatgcactaaaaatgaaagttacattcatatattatYacaaaacaaagtagttacaatcacctttctgatagtgttgcatcaaaatcatgtaaagatc

fava_c17575_200	fava_c17575	200	T/C	aaaaaacactataataatatgtttttggtatgactaaaataattattaaatcatcttagctRtagatgcactaaaaatgaaagttacattcatatattat[T/C]acaaaacaaagtagttacaatcacctttctgatagtgttgcatcaaaatcatgtaaagatcctctcccactagtaaatttcttcatagcatccaatgatg

fava_c17575_308	fava_c17575	308	A/G	aaagtagttacaatcacctttctgatagtgttgcatcaaaatcatgtaaagatcctctcccactagtaaatttcttcatagcatccaatgatggaggaac[A/G]ctactaaccatagcYgatgtcttcttttcccggccttgtttccttcYctcgcaacttcccttagaaatccaaaccagaaccttcttttcatcattcgttt

fava_c17575_323	fava_c17575	323	T/C	acctttctgatagtgttgcatcaaaatcatgtaaagatcctctcccactagtaaatttcttcatagcatccaatgatggaggaacRctactaaccatagc[T/C]gatgtcttcttttcccggccttgtttccttcYctcgcaacttcccttagaaatccaaaccagaaccttcttttcatcattcgttttggttgaaacaacct

fava_c17575_355	fava_c17575	355	C/T	aaagatcctctcccactagtaaatttcttcatagcatccaatgatggaggaacRctactaaccatagcYgatgtcttcttttcccggccttgtttccttc[C/T]ctcgcaacttcccttagaaatccaaaccagaaccttcttttcatcattcgttttggttgaaacaacctgctgaacctctttatgcaatttcttttccttt

fava_c17575_623	fava_c17575	623	G/A	ttggactaaaactaattccacgtttctgctttcttctagcttccttagggaataatgaaacaacatgtgtcataggtgatcgacaaggactgagagttgg[G/A]ttctgaaatgttactaatgatatagacacgggttgttttgacataaacctcgagatcgtggttttgattttacgtagtttctccatctagagattatatg

fava_c17583_503	fava_c17583	503	C/T	caattatcaagctcacatcagtacaacactaaaagaaaacctgcataaatggccattttccaaacaagttcagtatctatatataatcaagccaattcaa[C/T]ttgaagtacaaaaatcaggacatacctacaattacaccaagtgtcacgtacctaaRMcaaaccagtttatcacactcataacacctacactacactcatg

fava_c17583_559	fava_c17583	559	G/A	tttccaaacaagttcagtatctatatataatcaagccaattcaaYttgaagtacaaaaatcaggacatacctacaattacaccaagtgtcacgtacctaa[G/A]Mcaaaccagtttatcacactcataacacctacactacactcatgactgaattgaccacacacaaccctatatgcttcaggttttcagtcgagttaagctg

fava_c17583_560	fava_c17583	560	A/C	ttccaaacaagttcagtatctatatataatcaagccaattcaaYttgaagtacaaaaatcaggacatacctacaattacaccaagtgtcacgtacctaaR[A/C]caaaccagtttatcacactcataacacctacactacactcatgactgaattgaccacacacaaccctatatgcttcaggttttcagtcgagttaagctga

fava_c17587_1573	fava_c17587	1573	C/T	cagctccataagcatcacttcttttgagtctgtcacctaagaattcatggaatgagattcttatgtaagggtagaagtagtccatgattctatgactgta[C/T]ttttcaaagaaacgtaggagttggtatggacagtattgccttatgatagcccaaatgaacatgaagcttgctaaggttgacccaattgttgtccacatct

fava_c17587_289	fava_c17587	289	C/G	ctttctcatcatcatcaacaaactcaccattttccttaataacactactaacacattccacttcctcatcatgttccttaatcaaaccactcttccctct[C/G]acttcctcaagagcctcaatcaaattcaaaagacacttatcagcatcatccattggtgacttaggcataagattctcagcaacatcagcaggagtaatct

fava_c17589_127	fava_c17589	127	A/T	gttgcaattctttattgttgaaaagaacaaaaccagcgcacaaagaaaaaaaatcccaaaattcgaaaatgacgaaagaaatcgaaactctcacagaaga[A/T]gaaagacgcgcacttcgcggaagcaaattcgctcctcttcccacccttcctcactcSaacaagcccaaacccagattggctcatcccggtggacctgttg

fava_c17589_184	fava_c17589	184	C/G	aaaattcgaaaatgacgaaagaaatcgaaactctcacagaagaWgaaagacgcgcacttcgcggaagcaaattcgctcctcttcccacccttcctcactc[C/G]aacaagcccaaacccagattggctcatcccggtggacctgttgccacgaataaagcagcggctttggctaagtttttggagaggaagttgaagggtccta

fava_c17592_839	fava_c17592	839	T/C	aaaacaacccagaagaagattgtgttgattttgtggccgcaacaactgctgctcttggagttccttctcctagtctttcgaaagctatgtcttttgatga[T/C]tctccacattatgtttctgatcagacaccaagaaaaggtgatcttgaagaagaagaacagttgttgagagccttgaagatgtctgaagcgtcaatgagtg

fava_c17596_1099	fava_c17596	1099	A/G	tctacataagcctcattagaaaaagttaacattgagcttttgctacagataaattgttgagactattattttgatagtacaacacaaattgttggtatgc[A/G]cgagtgttgtaaactctagactatcaagtttaattcctattactttttttagaaaagtagttgagattatttaatttcatgttgaccattaatttgtaaa

fava_c17596_256	fava_c17596	256	A/C	aacacgaaaaaccattttccatttcagaaacatagagatggattcgaactcgaactcgaagaacgctggttcatcattggatgcattgatgtcttccttc[A/C]attctcgcatactagagcttcaagaattcgttattgctagaagcatgtatccagcgagcagtattccagatctttccgatgttgatgctgYtgtgagtgc

fava_c17596_347	fava_c17596	347	C/T	tcttccttcMattctcgcatactagagcttcaagaattcgttattgctagaagcatgtatccagcgagcagtattccagatctttccgatgttgatgctg[C/T]tgtgagtgctatggagcttcaggtgcaaacccttaaggacaaccttcRcaatgagatggaagctattcctaaagcRaagaaatttattgatgcattgctg

fava_c17596_395	fava_c17596	395	A/G	agaagcatgtatccagcgagcagtattccagatctttccgatgttgatgctgYtgtgagtgctatggagcttcaggtgcaaacccttaaggacaaccttc[A/G]caatgagatggaagctattcctaaagcRaagaaatttattgatgcattgctgcagcagcaaaagaaactgcagaatatgtctctttacgttccttcccaa

fava_c17596_423	fava_c17596	423	A/G	cagatctttccgatgttgatgctgYtgtgagtgctatggagcttcaggtgcaaacccttaaggacaaccttcRcaatgagatggaagctattcctaaagc[A/G]aagaaatttattgatgcattgctgcagcagcaaaagaaactgcagaatatgtctctttacgttccttcccaaacggctgatagaatgacactgtcaaatt

fava_c17596_570	fava_c17596	570	T/C	atatgtctctttacgttccttcccaaacggctgatagaatgacactgtcaaattcagaaactagtagagctttgtttcctgaattttctggactagactc[T/C]ggatcaWttgaggctttaaaacttgatgcggagcctcctgcattgcctaaggagaaaaaaggccgcggtcccccaccaacgtggtatgtcactggaagtg

fava_c17596_577	fava_c17596	577	T/A	tctttacgttccttcccaaacggctgatagaatgacactgtcaaattcagaaactagtagagctttgtttcctgaattttctggactagactcYggatca[T/A]ttgaggctttaaaacttgatgcggagcctcctgcattgcctaaggagaaaaaaggccgcggtcccccaccaacgtggtatgtcactggaagtgaactaga

fava_c17598_1363	fava_c17598	1363	T/C	taggcggcctcctcaccggactcttggccgagccggccctatgtcgattagtattgccggtgaccaattcaagaggtgcattctatggtggaagtggtgg[T/C]gtacaattcctaaagcaattagtggcagccatgtttgttattggatggaacatagtctccaccacaatcattctccttgttatacaattgttcatacctt

fava_c17598_1570	fava_c17598	1570	T/C	tgcccgacgagcagctcgagatcggagacgatgcggctcacggtgaggaagcttatgctctttggggtgatggagaaaaatatgatccaactaggcatgg[T/C]tccttaaatcctggtaccactggagtttcaccttatgttaatggtgcaagaggtgtgacaataaatttgtgagtcatagctataggaaatttattgtgtg

fava_c17598_565	fava_c17598	565	T/C	ctttaggacagaagtttctggttagaagagcaagagtacctgcgagtactcattattacaagaatggtcgtgttgagacagagatggaagaaccgttttt[T/C]cctatggcttcgcttgtgtattttcagtttacttttgcggcgattactatgattttgttggcgggttcggttctYgggaggatgaatattaaagcttgga

fava_c17598_640	fava_c17598	640	C/T	agacagagatggaagaaccgtttttYcctatggcttcgcttgtgtattttcagtttacttttgcggcgattactatgattttgttggcgggttcggttct[C/T]gggaggatgaatattaaagcttggatggcttttgttcctctttggcttgttttttcttatactgttggtgcttttagtctttggggtggggggtttcttt

fava_c17600_1296	fava_c17600	1296	G/A	gtcctccattggcagcaattagtttacagccaatagggttgtcgttagtcaaattcataagcaccttaacagcagtaaggaggcaatcacttagaaggct[G/A]gaatcttcttcataactgatgtcagaactggaacaatcgatgttccMgtcacttgattcttgttgactcattttggtttgcaattgatgttcaatttctt

fava_c17600_1343	fava_c17600	1343	C/A	gtcaaattcataagcaccttaacagcagtaaggaggcaatcacttagaaggctRgaatcttcttcataactgatgtcagaactggaacaatcgatgttcc[C/A]gtcacttgattcttgttgactcattttggtttgcaattgatgttcaatttcttgactcattttgatttgtgattgatgttcattttcttgactcattttg

fava_c17600_729	fava_c17600	729	G/A	gtgaaatcatgtccaaagacagatgaaattccacaaatctgtccaacacaggtacaagactagccaagttgtgatctggaagattgtcagcaatagattc[G/A]cggatgctcttgctttcagtggaaagaaatgctagaagcaacgcagagtaagcttcgacaatcattttctcagcttctttttcaccttgtagaacagctg

fava_c17601_1362	fava_c17601	1362	T/C	ttccaaacccaccttggccaatgacacagctttcgctgaaatttttagtcgcaagttggagttcgcgcaaagaaaactttcttaattggccaaaataaac[T/C]ttggtctcatcttcacctaaaacatctacaaaSacatcatttttgtgtctgtccttttgatgatatcgatatgtaaagatggccccgagaatcagaagtg

fava_c17601_1395	fava_c17601	1395	G/C	cgctgaaatttttagtcgcaagttggagttcgcgcaaagaaaactttcttaattggccaaaataaacYttggtctcatcttcacctaaaacatctacaaa[G/C]acatcatttttgtgtctgtccttttgatgatatcgatatgtaaagatggccccgagaatcagaagtgcaaaagcaccacaacttgcataRcgcacgactt

fava_c17601_1485	fava_c17601	1485	A/G	catctacaaaSacatcatttttgtgtctgtccttttgatgatatcgatatgtaaagatggccccgagaatcagaagtgcaaaagcaccacaacttgcata[A/G]cgcacgacttttgccaattttgatttgttggttgaatctggatgatcagatttagaaacacaagRctgctcgaagctagagccgccgcaaccaaggcgcg

fava_c17601_1550	fava_c17601	1550	G/A	agaatcagaagtgcaaaagcaccacaacttgcataRcgcacgacttttgccaattttgatttgttggttgaatctggatgatcagatttagaaacacaag[G/A]ctgctcgaagctagagccgccgcaaccaaggcgcgtatccgaaaagttaaacatcggaactgaaaagagttgtgttgggatagttccggtaagatcatta

fava_c17601_1728	fava_c17601	1728	G/A	ggatagttccggtaagatcattagacgaaagatccacattgttgagattagatagttgaccccatgtagctggtattgaaccgttgaaattattgttagc[G/A]agatttagatattgtaggaatgtYaagttggcaatgtaatcaggtaaagggcctgaaagattgttgttctgcaattccaagtcaaccaaatattccaatc

fava_c17601_1752	fava_c17601	1752	C/T	acgaaagatccacattgttgagattagatagttgaccccatgtagctggtattgaaccgttgaaattattgttagcRagatttagatattgtaggaatgt[C/T]aagttggcaatgtaatcaggtaaagggcctgaaagattgttgttctgcaattccaagtcaaccaaatattccaatctggtaattgagggagaaagtgttc

fava_c17601_339	fava_c17601	339	G/A	tctttagagcatagtgaaagtacatatatattctatgttgcaagcaacagcattttcaaaattgacttcaatagttatgttattcctatcttgctcttga[G/A]agttgtatagcttcttgatcaagagttgattcatcactccaaggaaattgatgagtcatgagtgacaattctatttcttgatttctagcctcttcaagtt

fava_c17601_441	fava_c17601	441	C/T	gttgtatagcttcttgatcaagagttgattcatcactccaaggaaattgatgagtcatgagtgacaattctatttcttgatttctagcctcttcaagttg[C/T]ttccagtccgcccatctatctgctaatccgactccctgcaacatttttacgacctcggacatcgtcggacgatcctctggatagcattgtgtgcaaagta

fava_c17601_543	fava_c17601	543	T/C	tccagtccgcccatctatctgctaatccgactccctgcaacatttttacgacctcggacatcgtcggacgatcctctggatagcattgtgtgcaaagtaa[T/C]gcgacttgaagaatggtttcgacttcttttggatgatatgtgtccaaattgttatccactatgtcctccagcctattttctcttaatagattttttacat

fava_c17608_1122	fava_c17608	1122	C/T	acttcattgatcaagatcataatcaagtaatagaagacattaaatggtctgaatatctaaacactactccattttttctcggaaacaacaacaacaacag[C/T]aRcaacaacaacaacgacaacacaattcaacagcatcatagtacaacaatctacagtgatgatgtgattaaacaagaaatgagattcattcataattcag

fava_c17608_1124	fava_c17608	1124	G/A	ttcattgatcaagatcataatcaagtaatagaagacattaaatggtctgaatatctaaacactactccattttttctcggaaacaacaacaacaacagYa[G/A]caacaacaacaacgacaacacaattcaacagcatcatagtacaacaatctacagtgatgatgtgattaaacaagaaatgagattcattcataattcagat

fava_c17608_459	fava_c17608	459	T/G	gatgcggtaagagttgtagattgagatggataaattaccttagacctgatttgaaaagaggagcattctcacaagaagaagaaaattctataatcgaact[T/G]catgcacttctcggYaacaggtggtcScagattgcagctcagttacctggaagaactgataatgagataaagaatctatggaattcaaatttgaagaaga

fava_c17608_474	fava_c17608	474	C/T	gtagattgagatggataaattaccttagacctgatttgaaaagaggagcattctcacaagaagaagaaaattctataatcgaactKcatgcacttctcgg[C/T]aacaggtggtcScagattgcagctcagttacctggaagaactgataatgagataaagaatctatggaattcaaatttgaagaagaagctgaaacaaaaag

fava_c17608_486	fava_c17608	486	C/G	ggataaattaccttagacctgatttgaaaagaggagcattctcacaagaagaagaaaattctataatcgaactKcatgcacttctcggYaacaggtggtc[C/G]cagattgcagctcagttacctggaagaactgataatgagataaagaatctatggaattcaaatttgaagaagaagctgaaacaaaaaggaattgatccaa

fava_c17608_753	fava_c17608	753	C/T	aaagcaataatcagactcaaaaaagctctgttggatccaatgaagtgaatctttttgaaacatcaaagaaaaattcaattcctatggataataactgtaa[C/T]tatccacttSaaatgaattcttcaactatttcaaatatggttggattaggaggatcatcatcatcagcatatctctcttttcaacaacttaactatggaa

fava_c17608_763	fava_c17608	763	C/G	tcagactcaaaaaagctctgttggatccaatgaagtgaatctttttgaaacatcaaagaaaaattcaattcctatggataataactgtaaYtatccactt[C/G]aaatgaattcttcaactatttcaaatatggttggattaggaggatcatcatcatcagcatatctctcttttcaacaacttaactatggaatcaattcacc

fava_c17609_1977	fava_c17609	1977	C/T	ataagggcttacggtgacaacgaattttatcaaggtgcaattgaccttttcaatcagatgagatcgaatggtttttctccaaatcacttcacctttggag[C/T]cattctatctatatgtgaaagagctggatttgtcaatgatgcaaataaaatcttcaatttaatgcctaaatatgaaattgaggcatctaaggaacacttc

fava_c17609_2132	fava_c17609	2132	C/T	caatttaatgcctaaatatgaaattgaggcatctaaggaacacttcgatgtaatggtccgacttcttactcgatatggacaactaaagaaagctcagaga[C/T]ttgtacagatgagttctttcttgtagtaaaggaaagaccacatttttcctttgaatggttcataaattgtcaaagattcctgaatacttYcatgtaactt

fava_c17609_219	fava_c17609	219	C/T	atggaaaactctgtctcttctctctccctacatttcccactaaaccacaaacccaacattttccaactcttcaacttcaaaccttcctcacaaaccccaa[C/T]aacccatccaagaaacctacccaaattcaccatcaccaagaagaaattcgcagaagaagatgcatttccatcttccttaccactccacagcaaaaaccca

fava_c17609_2222	fava_c17609	2222	C/T	agctcagagaYttgtacagatgagttctttcttgtagtaaaggaaagaccacatttttcctttgaatggttcataaattgtcaaagattcctgaatactt[C/T]catgtaacttttggtttagaacatttcactagtaacttRtttataacttgtaatcattctttcagtaaaaacaatcgataaatcagattttccttgagtg

fava_c17609_2261	fava_c17609	2261	A/G	aaggaaagaccacatttttcctttgaatggttcataaattgtcaaagattcctgaatacttYcatgtaacttttggtttagaacatttcactagtaactt[A/G]tttataacttgtaatcattctttcagtaaaaacaatcgataaatcagattttccttgagtggttgccgaatttcatgtgtttaagatctcagctaaggtc

fava_c17609_493	fava_c17609	493	C/T	acgttgaccaacaaggtattccagtcaacgccaccactttctcttctctcatagcttcttgcatccgcacaaactcgttgtctataggaaaacaagttca[C/T]acccatatcagaatcaatgggctcgaaaaaaacacatttttgctcacgaaacttgttcaaatgtacacttcttgtggctctttaggtgatgcattgaagc

fava_c17615_1182	fava_c17615	1182	C/T	gtatcgaggcatctggcgggtatgtctatgatggatatcttaacggacaacttagtgtcactcgagctattggtgactggcacatggaaggaatgaaagg[C/T]aaagatggcggtccactcagtgcagagcctgaactcatgacgacaaaacttactgctgaggacgaatttctcatcataggctgtgatgggatttgggatg

fava_c17615_1547	fava_c17615	1547	T/C	ttggtagcaccgcgcgccagagtgcacaggagcttctctgctgaaagtttgaaggagttgcaaagctttttggatagtttgggaaaatgagttttttgca[T/C]gtctttttggttccttcttgttttattttcttttctgtacccctttctgagaatYgagattcatttgtaactgtaggtgttaggtgtagtaggatgagag

fava_c17615_1602	fava_c17615	1602	T/C	agttgcaaagctttttggatagtttgggaaaatgagttttttgcaYgtctttttggttccttcttgttttattttcttttctgtacccctttctgagaat[T/C]gagattcatttgtaactgtaggtgttaggtgtagtaggatgagagagaactttctgactgtatcatatgcggcagctgacattttaatttaaattggttc

fava_c17615_267	fava_c17615	267	C/T	tctctctacccaacccaacccaaccactcctttcttcaaaccctttccaacgaatctctacattcctgaaaccacctttgaatcaccgacatataaccac[C/T]tcaacctaacacacaaaaaacctaatttttacactttctgtaaccacccaatagcttttttcaatctgggtcgaaccagtttcaaatttcggttactggg

fava_c17615_492	fava_c17615	492	T/C	tatggttaagaatggaagaaactgaaggtgtgaattgttgtagtgaaggtagaagctctagtgagagtcgacctcctaatcctaatccatctattgcttc[T/C]gcgtatcggaaatgcaaacgcaatgatcctgttgttttgccatgcagaaactcccttgttcgtcacgcttctcttcaaacgaaaatgaagttgtctgatg

fava_c17616_165	fava_c17616	165	T/C	tttttttatttattttaattttctcttctttttagtttcttaattttttcaaaaaagtttcaaccatgtcttctattcaaatgatgtctgttaacaaact[T/C]ggttaccaaaagctgagacatgaagttgggtctgatgatgaaagggagagaatcaccattcagagaccaaggaattttataaggttccgaaagattccta

fava_c17620_998	fava_c17620	998	T/C	tgttgaaacttgatcataattttccttctaaggcagcatcaataatggaaccagaaacacctctagattggtgggggaactcgatgaatgttcttagtct[T/C]gaattcttcgaacgagttgtatccgttatgaagtcgaagggactaaaacaagacttgatcagcaagattttggtaaactatgcacataattctcttcaag

fava_c17621_1034	fava_c17621	1034	G/A	atatccacattgacaaagaacatcYctaacaacagccaaattaagatcacaatcattcccaagcatggaataaaggaactgctccgcctcatccttatca[G/A]cRacctcattcccaaacctcttcttcctcccactacccctcctcacatactcatttcccaaaatcgtcgaaacagttccYgtagcagcaacaatcttctt

fava_c17621_1036	fava_c17621	1036	A/G	atccacattgacaaagaacatcYctaacaacagccaaattaagatcacaatcattcccaagcatggaataaaggaactgctccgcctcatccttatcaRc[A/G]acctcattcccaaacctcttcttcctcccactacccctcctcacatactcatttcccaaaatcgtcgaaacagttccYgtagcagcaacaatcttcttct

fava_c17621_1114	fava_c17621	1114	T/C	gctccgcctcatccttatcaRcRacctcattcccaaacctcttcttcctcccactacccctcctcacatactcatttcccaaaatcgtcgaaacagttcc[T/C]gtagcagcaacaatcttcttctgcttctgtttctgtttctgctgatgcWtaaccccttcaaaacccaccacatcctccctacaaatcaggtccaaaaacc

fava_c17621_1163	fava_c17621	1163	T/A	cccactacccctcctcacatactcatttcccaaaatcgtcgaaacagttccYgtagcagcaacaatcttcttctgcttctgtttctgtttctgctgatgc[T/A]taaccccttcaaaacccaccacatcctccctacaaatcaggtccaaaaacccatccgaagaacccgaagtggaaccgaaatcaaacccagaaaaaacccc

fava_c17621_1435	fava_c17621	1435	G/T	cgaccaaacccctccttaagatctcagaagccttggttggatcaccgtttgcaacgtcgtatgcaacaaccgcttcttcgattgaagataaagagaaagc[G/T]tcaaccaacgattgtaaaactagattttttttctcttctttttcaactacccctttggattctttgttcgattccacgttcttgggttgtttttgcttct

fava_c17621_382	fava_c17621	382	T/A	tagcaacaaatatgtcatggcttgccttagtgtctgctttctgagctactttagtttgttctttaccctgatcagaaagatatgcggcatatcctcgatc[T/A]ccattagtataagctgttgccgctttctgaaagtaacatttcacagaatcccactgctgtcttgcaccttccctaaatgcatgatattcctctcctttac

fava_c17621_958	fava_c17621	958	C/T	aattagaatttctatcattactggatctctcattggcagctaggtcaagcaatacatccgaggccttttcaacatcatatccacattgacaaagaacatc[C/T]ctaacaacagccaaattaagatcacaatcattcccaagcatggaataaaggaactgctccgcctcatccttatcaRcRacctcattcccaaacctcttct

fava_c17622_1491	fava_c17622	1491	T/C	ttgcaaagcaagtctaatgctccttgggcgatttcaagtagttggtagtctacaaaaaggatctttgcttgtgaatgctctaataggattgaaaccatag[T/C]tgcgtctaggcgagaattaagggtacagagaattgctccagccattggtactgcaaaatgcagctcgtacattgcaggaacattaggtgctagtgtggca

fava_c17622_1615	fava_c17622	1615	T/C	tacagagaattgctccagccattggtactgcaaaatgcagctcgtacattgcaggaacattaggtgctagtgtggcaaccacatctcctcgagaaattcc[T/C]aatttggtaagagaagaagcRaatttgagacaacgttgatgggtttgaccccaattatagctcaaKgaaccataaacaagagatgttctatctctgcaaa

fava_c17622_1636	fava_c17622	1636	A/G	ttggtactgcaaaatgcagctcgtacattgcaggaacattaggtgctagtgtggcaaccacatctcctcgagaaattccYaatttggtaagagaagaagc[A/G]aatttgagacaacgttgatgggtttgaccccaattatagctcaaKgaaccataaacaagagatgttctatctctgcaaacctttgctgctctctccaaga

fava_c17622_1681	fava_c17622	1681	T/G	ctagtgtggcaaccacatctcctcgagaaattccYaatttggtaagagaagaagcRaatttgagacaacgttgatgggtttgaccccaattatagctcaa[T/G]gaaccataaacaagagatgttctatctctgcaaacctttgctgctctctccaagaagttgataggagataaaggaactgaatttgcagagcaatgaagaa

fava_c17622_388	fava_c17622	388	G/T	aggcaattatctcttgagcatctgcatcaaacccttctctcagcttcaaaaaagcacaaggagtttgaccccaatgatcatccggtcgggcaacaaccgc[G/T]gcctcatcaacagccggatgactatacaaaacggtttctacctcgaccgagctgatgttttctcctccagagactattatatccttcaatctatccttaa

fava_c17622_741	fava_c17622	741	G/A	gccggaacactttccatagtttcagaatctttgacatcaatgtcctccaatcctacatgaggcacaccttgtctagctttcatcttcgatctttcttccg[G/A]aggcaacatgtcccactcaggtctccacgcgcaaaaactccccggcccgtatgtttctgtcaaaccatagagatgagaaacgctaaaaccaatctcttcc

fava_c17624_608	fava_c17624	608	A/T	agttgtctatgtgtataataataacataaaatgattaacatcacaatcatgtaatagtaaaaaccgtgacagggacaaaataatattggaccatggaaca[A/T]Matagtcatatcattgtatgggcggcataaatggcttcatttgatttgtttcaaggtatagaaagttgaagtcagcactctacagtgacatggcatgcaa

fava_c17624_609	fava_c17624	609	C/A	gttgtctatgtgtataataataacataaaatgattaacatcacaatcatgtaatagtaaaaaccgtgacagggacaaaataatattggaccatggaacaW[C/A]atagtcatatcattgtatgggcggcataaatggcttcatttgatttgtttcaaggtatagaaagttgaagtcagcactctacagtgacatggcatgcaat

fava_c17626_1082	fava_c17626	1082	G/A	ctattctccaacacgtgctaagcctaaattcaagaaatatcagtgtgtattatgggtcctctccttgatcattttaatcgttgggctttctgttggactg[G/A]ttgcactgctccgaggtgttgatgcaaatgatcactgctcMtggtgccattatctgtcttgtgttccaacttcaaaatggagctgtcataccgaagcagc

fava_c17626_1123	fava_c17626	1123	C/A	agtgtgtattatgggtcctctccttgatcattttaatcgttgggctttctgttggactgRttgcactgctccgaggtgttgatgcaaatgatcactgctc[C/A]tggtgccattatctgtcttgtgttccaacttcaaaatggagctgtcataccgaagcagcatattgtttgtcaaaccagcttggcaaccagctgaatgtaa

fava_c17626_1237	fava_c17626	1237	C/T	tgtcttgtgttccaacttcaaaatggagctgtcataccgaagcagcatattgtttgtcaaaccagcttggcaaccagctgaatgtaacatgctcaagcaa[C/T]ggtaaatccagtacatacttcatgcaagatccaactagttcgaagatccagcaattgtgtactcaaatttgtaattgagttttgaccaaatgggatgatg

fava_c17626_481	fava_c17626	481	A/G	gtccgagtaattcggtttcttgtatcgcgaggttcctccgtcggttctcgtttcagcctcttaaagaaaatcctcttcttggtccttcttccttgacgct[A/G]cagaagatgggagctcttgatgtgaataaagtgattcacatgcaccagggttggcgtctcgtcacttgtatgtggctacatggtggRgttttccatctat

fava_c17626_568	fava_c17626	568	A/G	cttccttgacgctRcagaagatgggagctcttgatgtgaataaagtgattcacatgcaccagggttggcgtctcgtcacttgtatgtggctacatggtgg[A/G]gttttccatctattagcaaatatgttgggtattctattcattggaattcggcttgagcaagaatttggctttgtgcttattggactgctatttgtcatat

fava_c17626_940	fava_c17626	940	A/T	ttatcaatcttgcaatcgggattctcccgcatgtggacaactttgctcatattggaggctttcttactggatttcttctcgggtttgtgtttttgatacg[A/T]cctcagtttggatgggttaaccaacgatatgctcgtgtagactattctccaacacgtgctaagcctaaattcaagaaatatcagtgtgtattatgggtcc

fava_c17627_1209	fava_c17627	1209	A/C	atgggagtgtagaggcaatttatttgaaaataagttgagacaatgtgaagctgaaaatttggagcaaaaggagctgtatgctgcacaactattggttata[A/C]aaagtgaaataagttctttaaaggaagaacttcgtcagagaagagatgacgtggaggctgtgaataaggagtttgacaggcataaacaaaaatttgacat

fava_c17627_1498	fava_c17627	1498	G/C	aaaagagagcttttccagctaagcacacagcagcttgagctgaccttagaatctgaaatgagaatgactttaatagatgagctgaaactgaaagtggaga[G/C]tcagaatgctgtgatctctgatcgRaatgaggagaagagggaggcactgactttagtaggtgagttgaaactgaaattggagagacagaatgttgagatc

fava_c17627_1523	fava_c17627	1523	G/A	cacagcagcttgagctgaccttagaatctgaaatgagaatgactttaatagatgagctgaaactgaaagtggagaStcagaatgctgtgatctctgatcg[G/A]aatgaggagaagagggaggcactgactttagtaggtgagttgaaactgaaattggagagacagaatgttgagatctccgataggaatgaggagaagaggg

fava_c17627_386	fava_c17627	386	C/T	tgaaggaaggctctggatcaccttcctcgccctctttggattcagaacctgaatcttttacccagtctgtaatccgtaaccagggcacgccggtgaatac[C/T]gatgatgaagaaaccctggaaggtaagctgaaagaagacattcctgatatggaaagaccgagccagtttcttggggacggagaaaatagaagttatgatg

fava_c17627_525	fava_c17627	525	A/G	cattcctgatatggaaagaccgagccagtttcttggggacggagaaaatagaagttatgatgagttgctcaagaagtttataaaaaatgaggaagagcta[A/G]Kagttgccaatgtaaaacttcagctctcagaagaagagattatcaagttgaagaatcaaattgagaaaagcgaggatcaacttgataatgtgttgaaaga

fava_c17627_526	fava_c17627	526	T/G	attcctgatatggaaagaccgagccagtttcttggggacggagaaaatagaagttatgatgagttgctcaagaagtttataaaaaatgaggaagagctaR[T/G]agttgccaatgtaaaacttcagctctcagaagaagagattatcaagttgaagaatcaaattgagaaaagcgaggatcaacttgataatgtgttgaaagaa

fava_c17631_1178	fava_c17631	1178	C/T	gttccagcgccaagttctatggcagtaacttggcgaaactcagatgaacacaatgttttatacaatataaaatcagttaacaccaattcagctctccaaa[C/T]ttgcaatccaacatttgaaattgacgaagtgatattatgctgaattctcagacaatacctattatcacatgatgcatttgcatcggtgcgacgacgaggt

fava_c17631_320	fava_c17631	320	G/A	aaacaataaaaggaatctgaaccaaaactagacaacaagtgatcatgataaggccacagaaacattctcatcaaactcaaacattggcactcttacagat[G/A]attttcaaattataaaattgcctaaaagtcctctaacaatattcttatgcagttgaataaaatatctgcactttaattaattttataagattgagcaaaa

fava_c17631_945	fava_c17631	945	C/A	caatatctatggaagtccaagaatatctatgtgtgcaagtagcttctcctactctgactctgggaggccaggaatcaaaccaatcaaggtcacgtacata[C/A]actgtagctggattattcaataatccacggttaagcagaacattcttcacacagttgtcaagtatttgattcccatgatctgttataaacactttttttg

fava_c17633_1260	fava_c17633	1260	A/T	gggcttccatgtcttgcagttcttgatttaaagatgagcattcaacctcgaggagtgccaccttctgctcaagttctgtggcctttgttgtcctttcatc[A/T]gctattctctgtatcctcgcaagggctgtagatgactcaactgctctatgttccaattccacttctctttccatagcagccaatttggtagcattatgtg

fava_c17633_1575	fava_c17633	1575	C/T	gcagggaagctagatttccttctgacaatgcagcctgccttttaactgcttccacagaacttagaactgcctcgatttcagcatttttggctgccaaggc[C/T]tcacccatatttgattcRactcttgtaacttcctttttactttcagacaaatccttttgtagctgctttatgctagcctcatatgatttgctcagctctc

fava_c17633_1593	fava_c17633	1593	A/G	cttctgacaatgcagcctgccttttaactgcttccacagaacttagaactgcctcgatttcagcatttttggctgccaaggcYtcacccatatttgattc[A/G]actcttgtaacttcctttttactttcagacaaatccttttgtagctgctttatgctagcctcatatgatttgctcagctctctctctgcagtaagaagtt

fava_c17633_1707	fava_c17633	1707	A/G	cctttttactttcagacaaatccttttgtagctgctttatgctagcctcatatgatttgctcagctctctctctgcagtaagaagttcctctaactgtgc[A/G]ttttcggatttgtattcttgaaggcgagatgatagtccagcacagacccgagctagccttgcctctttagactgaccagtagattttgttgttttgagca

fava_c17633_1896	fava_c17633	1896	C/T	ttgttttgagcaatccttgggcctcttcaagctgatcctgtactttttcaggggatatgtcagttttatgatcctcttgactcttttgattcactatagg[C/T]tcagcttttatatcagaatctttagggatggtatcactattaggagctacatcatcagccacagattcaggtttttcttggtccttatattggatggagt

fava_c17633_2333	fava_c17633	2333	C/T	cctattatgggtgatgttgcatcagtaggttgctgctcctttggttgatttgtagaaatggaatcactctcatcatgtttggctaccggatcaatttttt[C/T]gcttggagttgttacatccaatgttgctgctggagatccagttttctccttagtagtatcactgactatagttgatgggtcggacagtctcttttgagcc

fava_c17633_2505	fava_c17633	2505	A/C	ttgatgggtcggacagtctcttttgagccttagcttttaatttcttccttttgccttgagatccttgcccatttgaagctaattctggagacttggaatc[A/C]gattgttcttctgcaatgtcactggcaaccagcttcgctcttcgatcaacaacttcaaataaatcttcagcggctttgagccacgaYgccattgtaagaa

fava_c17633_2592	fava_c17633	2592	C/T	gagacttggaatcMgattgttcttctgcaatgtcactggcaaccagcttcgctcttcgatcaacaacttcaaataaatcttcagcggctttgagccacga[C/T]gccattgtaagaaaaacgaatcagattcagagattcaatagtagttgagcttcaatcataatacgacgtcgctttgatttcagaatccacacaaaacact

fava_c17635_1450	fava_c17635	1450	C/T	aatgctcaagactctaggaacaattcatctgcggatgacagagatcctttacctgaaaaggattctaatcatagccaatcaaatgcttgagttgattcaa[C/T]ctcatactactatactgatgctctcaaaattaacttgccaagacagaattgtggttgtcatcatcatgtgtcaaaagttgcaatgtttagtggtaggtgt

fava_c17635_374	fava_c17635	374	C/T	aattgttctgagttttttagtgtgattagggtgtgaaaacgtttgaatcttgaatggttagggtttaaaccaaatttagaagtgttgggtttcttagttt[C/T]tccatgatttgcataaaaatggaggaaagagagaatttcggtgctgtgaatgcagtggcgagtaatcaagttccagtgagcttcgttgcagctcccagga

fava_c17635_512	fava_c17635	512	T/G	tcggtgctgtgaatgcagtggcgagtaatcaagttccagtgagcttcgttgcagctcccaggattgaaaacaatttggatttttccatggcaacggtgcc[T/G]gtgtcgatgccggtgacggcggtgaagaagaagagggggaggccaaggaagtatgggccagatgggaagcctgctcctggggctgtgaccgcgctttcgc

fava_c17635_974	fava_c17635	974	G/A	tatcgcgtgctatatgcattctctctgcaactggcacaatttcaaatgttacacttcgtcaatcgacttcttcagggggtactttaacatatgaggggcc[G/A]tttgagattctttccttgtctggttcctttatgccaactgagaatggaattacaagaagcagatctggtgggatgagcgtctctttggcaggtccggacg

fava_c17639_1059	fava_c17639	1059	C/T	caaactgagttatgcagtaagacaactgcgggtggtacataacaacggatttaggtttatgcagaggtaatagcgccctaacaagaaacaacttatgctc[C/T]tctttcatgggcaaagcaaacccattaatgatgctaccaagaatctccaaaagctccccaataccacagtgtctctcagtctcatatataaaccgataaa

fava_c17639_1407	fava_c17639	1407	G/C	tgtaacgtttggcaaccttggtgtcagtatcagaagaaaccacatatctaaggagaagctcataaacaagctgcaagtgaggccaagctggctccaagct[G/C]ggctcatcctcttccggatcagtaggctcttgaccggtgttctcatgagaMgccggaggcaagcatcggaaaatattgatagatatcattctaatcatct

fava_c17639_1458	fava_c17639	1458	C/A	ggagaagctcataaacaagctgcaagtgaggccaagctggctccaagctSggctcatcctcttccggatcagtaggctcttgaccggtgttctcatgaga[C/A]gccggaggcaagcatcggaaaatattgatagatatcattctaatcatctcttcctgactactctcagatatcttccccgaacctgattgaataaactcca

fava_c17639_2087	fava_c17639	2087	C/G	gagaaacacgattcatcggaagctgttaatccatggataatttcctcgattttcgaaccaattttcgaatcgacggaaacggatctgaaagcgaaaaccg[C/G]tatgaggaaggtgMagatggcgcttgattggtagagatggaaagctatggatcatagtgaggaatcatcggagagggatcgccggaggaaaaagatcggt

fava_c17639_2101	fava_c17639	2101	A/C	atcggaagctgttaatccatggataatttcctcgattttcgaaccaattttcgaatcgacggaaacggatctgaaagcgaaaaccgStatgaggaaggtg[A/C]agatggcgcttgattggtagagatggaaagctatggatcatagtgaggaatcatcggagagggatcgccggaggaaaaagatcggtcggagatgttccgg

fava_c17642_1106	fava_c17642	1106	C/T	tatgtcattgcccactatgttctggttttctgcgttattctggaggaatgtaccggtgtagcggttacatttccgagtggagtaaatgttcctactctac[C/T]tgtgaaccaaaacgtactgaagggaagtggaaaatcccagaggaaacagataatcagtatcttaaaaagtggttcaaatctcaaaaagggaagaaaccaa

fava_c17642_1500	fava_c17642	1500	G/T	agatactagctgcttggtggtcagtggagcgctcaaggacgaagctgagatgcagaaggcaaggaggatgaaaataccaatagtcagagaggactatttg[G/T]ttgactgtatggaaagaaaaaagaagcttccgtttgatatgtacaaagttgaaatgattggtgagacttctagcatggtcaccgtcaaagtgaaggggca

fava_c17642_307	fava_c17642	307	G/A	ttgaagaaatccaatcagattaaattagttgatgatgttgaaaacctagacacgcttcgatgggaagatcaacagaagattagaacgtacattgagagcg[G/A]tggcggtggtggttctacttctaaatccaagtcgaagtcgagtgctgcatcagcgtcKaagtcSagtgcgagcaagaatgttgaatgtggtattgaagtt

fava_c17642_365	fava_c17642	365	T/G	gatgggaagatcaacagaagattagaacgtacattgagagcgRtggcggtggtggttctacttctaaatccaagtcgaagtcgagtgctgcatcagcgtc[T/G]aagtcSagtgcgagcaagaatgttgaatgtggtattgaagtttcacaaacttcccgtgctgcttgcaagcattgtggtcagaaaattattaaaggagagg

fava_c17642_825	fava_c17642	825	G/A	aaagcgggggaaagatgcagagggtgagcagaagtcaaaagctgctaaggtcaagggagacgtgtctgtgggtagggaagcggcagtgaatggggaggca[G/A]gtggtcttgagaatagactagaggcgcagagcaaagaactgtgggctttgaaggatgaccttaaaaaacacgtgtcaaacgcagagatgcgtgaaatgct

fava_c17644_1445	fava_c17644	1445	C/T	tcttttgatcttcttcagatagtggagaccctaaagccgccggggttagccgggtagtggactcgccggaaaccttacgtcgatttaaaccaggcaagta[C/T]gttttcgcatagWaagcatgagaaccagcaataatttcctgcctaatgccccgagattccatgacagctatcagcctcttataaattggtaaactcaaat

fava_c17644_1458	fava_c17644	1458	T/A	ttcagatagtggagaccctaaagccgccggggttagccgggtagtggactcgccggaaaccttacgtcgatttaaaccaggcaagtaYgttttcgcatag[T/A]aagcatgagaaccagcaataatttcctgcctaatgccccgagattccatgacagctatcagcctcttataaattggtaaactcaaatttgcgacatcctc

fava_c17644_1559	fava_c17644	1559	A/G	aagcatgagaaccagcaataatttcctgcctaatgccccgagattccatgacagctatcagcctcttataaattggtaaactcaaatttgcgacatcctc[A/G]taccaccaatctaaacttgtattctttggtcttgccccggtacttattccattccacaaaacacttccaccaggactctgcaagggaccaccgcgctctW

fava_c17644_1904	fava_c17644	1904	C/T	gaagaaccacttgattgaaaaagccttcggcttgcgaaataagattactttcaccatattcctcggtcatttcaagatgctcagcagcacagcgtaggct[C/T]actacatttgatgccgttagttcaagtttcacaccgtagcagaatttcgctacaagttcaaatgttttggccccaccgggaatatcgtgaaggggtatgc

fava_c17644_485	fava_c17644	485	C/G	acccttcttgagcactacacatctgagactttatcttaaacccaagtttcttcgacaccgctccccaagcgcttgtcgtcgaaccttttgtcttaccgcc[C/G]agtttctcgatctcttgcctcatgtttgaacactctttctcgagctcggataccctcatcctcatgttatccatcccgactttcaatacctggttttcct

fava_c17644_758	fava_c17644	758	A/G	ctgaaactagaaagcaaccggcgattgatgttcgaagctggagctgctcgaaaaatagaacttgaactatgattctaatgggtagcctctcgttttgtgc[A/G]gcRtgggtgcatgcttctagtgagagcttctggcagtccattagcctgcataactcctctctctctgactccaccaaccatggatgagacttcaaataaa

fava_c17644_761	fava_c17644	761	A/G	aaactagaaagcaaccggcgattgatgttcgaagctggagctgctcgaaaaatagaacttgaactatgattctaatgggtagcctctcgttttgtgcRgc[A/G]tgggtgcatgcttctagtgagagcttctggcagtccattagcctgcataactcctctctctctgactccaccaaccatggatgagacttcaaataaatat

fava_c17646_1569	fava_c17646	1569	C/T	accgtcttcgcggtgaagttgaacttaagcatgtggacttctcgtatcccactagaccagatatgccagtttttcgcgaccttaatctccgtgtccgagc[C/T]ggcaaaactcttgcccttgttggacctagtggctgcggtaagagctcggtgatttcacttatacagagattctatgatccaacgtctggccgggtcatga

fava_c17646_1767	fava_c17646	1767	C/T	tgatcgatggaaaggacattagaaaatacaacctcaagtccttaagaagacacatttctgtggtacctcaagagccatgcctattcgctactactattta[C/T]gaaaacattgcatacggccatgactctgcaacagaaaccgagataatcgaagccgcgacacttgccaatgctcacaagttcatatcttctctaccagacg

fava_c17650_1094	fava_c17650	1094	G/T	agttattagcatttgattttgaaattggctcattccaatcttatcttcctcaccaacaattccactcatgagtagttggctccaagattccggaagctct[G/T]gatgattatcataccaagaaggaaaaggaagggaagaagaagaatttggtgaaaaattagggatgagaaagttagaattaggagtagaaaagaaaggagg

fava_c17650_432	fava_c17650	432	A/G	agggcaggtgcccaataatcagctccattgtcactcccaacttgaagtgtgcatgacactggaacaagacataagcctctactttttaggtccttctttg[A/G]ttcttcttSagaaacctcttcaactgctttcctcttcaaactatgttcatgtagcagctgaccagggtcttcaggaaataaacaattcttatctccttga

fava_c17650_441	fava_c17650	441	G/C	gcccaataatcagctccattgtcactcccaacttgaagtgtgcatgacactggaacaagacataagcctctactttttaggtccttctttgRttcttctt[G/C]agaaacctcttcaactgctttcctcttcaaactatgttcatgtagcagctgaccagggtcttcaggaaataaacaattcttatctccttgaactgaatgt

fava_c17650_568	fava_c17650	568	C/G	tcaaactatgttcatgtagcagctgaccagggtcttcaggaaataaacaattcttatctccttgaactgaatgttgtcttgtgtttccagatccattgct[C/G]aggtatggtaagctaagagcctcaatttgtgtctgaaggaatctgatataaccaatagcttctaacaagacagaggctgtgtcagtctttccaaatgggg

fava_c17654_1234	fava_c17654	1234	A/T	cctggtcttagctcaagatatgattcatattatactaaacatagttctcaagatactcatatggaagaagatgatgactttggttccttgcgtgatgcgg[A/T]tgttcatgataaacaagataaggaacctctccatggtacaaaacaccgacaagcttttcagagtaRtgccactcgtgttcctgccaggaactcttccttg

fava_c17654_1300	fava_c17654	1300	A/G	gaagatgatgactttggttccttgcgtgatgcggWtgttcatgataaacaagataaggaacctctccatggtacaaaacaccgacaagcttttcagagta[A/G]tgccactcgtgttcctgccaggaactcttccttggatttgtatgaagactctaccggggaagctgcttataatatcactaagaatcataggaaagacacc

fava_c17654_1822	fava_c17654	1822	C/T	gcaaaaaagcaatttcgcgatacgggtggagcagcgatgctttcaaatgaaactatggttgcaaaacgacttaaaccaaatacgtttctaccatacaacg[C/T]gaatcaattttccgcttccgaaatagaaccaaggaaaattcagaggtctggtgcagagatgccgtctagctttagcgaggacgaaaccggcgatacaagt

fava_c17654_737	fava_c17654	737	C/T	ggtatctggatcctaaagaagttgagagtcatggtctctataatcgtgatttgtccgttgctgatatgacctatggacgtaggaatcatttttctgaaaa[C/T]gttagtggaatggacgatacgtcgtctgagagtagctcttatttgcaagaacggttgtatcctcaaggScagggtccgtatgagatggagccttctagat

fava_c17654_806	fava_c17654	806	C/G	cctatggacgtaggaatcatttttctgaaaaYgttagtggaatggacgatacgtcgtctgagagtagctcttatttgcaagaacggttgtatcctcaagg[C/G]cagggtccgtatgagatggagccttctagatatctagcatctggtaaatcacttccacccttaaaaccaaagggtgcaatgaacatgggatataaaccgt

fava_c17656_1026	fava_c17656	1026	T/C	aggcacaagtacttgactttctcaggttcttgcgaaaaaatcagagagcatttttcctttcttcttatcacgtccaggaagatgttgagaacagcagcga[T/C]aaacgattttgcacataatatcacatagcatccgcRgttgccgaatgcttgttaaaaatgctgtttactaacaaatcaaaatgttgtcacattagatttg

fava_c17656_1062	fava_c17656	1062	A/G	aaaatcagagagcatttttcctttcttcttatcacgtccaggaagatgttgagaacagcagcgaYaaacgattttgcacataatatcacatagcatccgc[A/G]gttgccgaatgcttgttaaaaatgctgtttactaacaaatcaaaatgttgtcacattagatttgttaagtgttacttgtagtagtggtaatccaaccttt

fava_c17656_1166	fava_c17656	1166	G/T	gccgaatgcttgttaaaaatgctgtttactaacaaatcaaaatgttgtcacattagatttgttaagtgttacttgtagtagtggtaatccaaccttttta[G/T]ggtaaatgaagaggaactatttaaaggatgcgactgaccctcatttttaaatgtcagttgtatctctgaaatttattttagattgtctgttgttgaatga

fava_c17656_198	fava_c17656	198	G/C	aagaagaaaaataactagaaataaaaatgggaagttcgaaaacaagcagtgatgtttccgatagcagtagctctcaaaccgaaatcgagaatcacatttc[G/C]gcttctccatacgctgaaggagagaaagtccttgctttccacactgcatgtctttatgaagccaaggttaagcaaattgagtacaagcgtaaacaatgga

fava_c17656_519	fava_c17656	519	C/T	gcaatgaaaagaatgcgagaaagcctcgtgcgtcatccaagggttctaatggagcgagaggcaggaaacgaaagaatgattctctggttaaggaaaaaac[C/T]gcggttcttcctgataaacttgtcaaYtttcagattccaccaacactaaggaagcaattagttgatgattttgagtttattacgcatctcggcaagcttg

fava_c17656_546	fava_c17656	546	C/T	gtgcgtcatccaagggttctaatggagcgagaggcaggaaacgaaagaatgattctctggttaaggaaaaaacYgcggttcttcctgataaacttgtcaa[C/T]tttcagattccaccaacactaaggaagcaattagttgatgattttgagtttattacgcatctcggcaagcttgttaaactcccacgtactcctaatgtga

fava_c17656_701	fava_c17656	701	C/G	acgcatctcggcaagcttgttaaactcccacgtactcctaatgtgaacgatatattcaagacttattttgattacagatcgaagaaaagtggaccgatag[C/G]tgattcggttgaagaaattatgaaaggactgagttgttactttgacaaagcactgccagtgatgctcttgtacaataatgagcgtcagcagYaccgagaa

fava_c17656_793	fava_c17656	793	T/C	accgatagStgattcggttgaagaaattatgaaaggactgagttgttactttgacaaagcactgccagtgatgctcttgtacaataatgagcgtcagcag[T/C]accgagaagcttgtgcaaatgatattgttccttctgctatatacggtgctgagcatttgttacgtctcttcgttaaattgccagagttattgttccatgc

fava_c17660_372	fava_c17660	372	A/T	caacagtgttcttcgatagtgttgttaaagccaaccttagtttcggacagcttaaggttttggagggtatgtctcgtgaggagcttttcctatggctacc[A/T]gttaaagatatcattgtcacYgatcaaWcttctggactaattctcattgatattggttttgcctataaacgactctctttctctcgttttgatgaacccc

fava_c17660_393	fava_c17660	393	C/T	ttgttaaagccaaccttagtttcggacagcttaaggttttggagggtatgtctcgtgaggagcttttcctatggctaccWgttaaagatatcattgtcac[C/T]gatcaaWcttctggactaattctcattgatattggttttgcctataaacgactctctttctctcgttttgatgaacccccaatttgtagatctcatcaag

fava_c17660_400	fava_c17660	400	A/T	agccaaccttagtttcggacagcttaaggttttggagggtatgtctcgtgaggagcttttcctatggctaccWgttaaagatatcattgtcacYgatcaa[A/T]cttctggactaattctcattgatattggttttgcctataaacgactctctttctctcgttttgatgaacccccaatttgtagatctcatcaaggtctgtt

fava_c17660_501	fava_c17660	501	T/G	cttctggactaattctcattgatattggttttgcctataaacgactctctttctctcgttttgatgaacccccaatttgtagatctcatcaaggtctgtt[T/G]tttcgtatggttggaaggaagggttttggatttRgagataagagatgatgagattattcaatttgtatgaaacgggatggctttactctattcaattttW

fava_c17660_535	fava_c17660	535	G/A	ctataaacgactctctttctctcgttttgatgaacccccaatttgtagatctcatcaaggtctgttKtttcgtatggttggaaggaagggttttggattt[G/A]gagataagagatgatgagattattcaatttgtatgaaacgggatggctttactctattcaattttggggtttatctgaatgaaaaactatatgtatgtac

fava_c17664_1147	fava_c17664	1147	A/G	tctccctcttcgttggctctataatgctagtgttatgagagggtcttcatacgtttcattagattacagaagttcgaattgacaataaaactgggtggta[A/G]aaaagtgcccaaaatacctttaacactctggctaagaatYatttttgcttcatattattcattgtatacgagatgttagctcagtgataagagcKtKaca

fava_c17664_1187	fava_c17664	1187	C/T	gggtcttcatacgtttcattagattacagaagttcgaattgacaataaaactgggtggtaRaaaagtgcccaaaatacctttaacactctggctaagaat[C/T]atttttgcttcatattattcattgtatacgagatgttagctcagtgataagagcKtKacattgccaactcaaagaacaaagttcaaatctcatatgagac

fava_c17664_1242	fava_c17664	1242	T/G	tggtaRaaaagtgcccaaaatacctttaacactctggctaagaatYatttttgcttcatattattcattgtatacgagatgttagctcagtgataagagc[T/G]tKacattgccaactcaaagaacaaagttcaaatctcatatgagacagttagaaaatagttcgaatctcaggttatgatttatgagtgtctattttcctta

fava_c17664_1244	fava_c17664	1244	G/T	gtaRaaaagtgcccaaaatacctttaacactctggctaagaatYatttttgcttcatattattcattgtatacgagatgttagctcagtgataagagcKt[G/T]acattgccaactcaaagaacaaagttcaaatctcatatgagacagttagaaaatagttcgaatctcaggttatgatttatgagtgtctattttccttaga

fava_c17664_191	fava_c17664	191	T/C	ttccatttttcaattcctacccacaaaccctcaatcttggattctccgagcttaacaagatggatgaaggtagcagaggcaccatcatgatcacaaatga[T/C]gatggaatcgacgcacctggcctcagagctttggttaccgctctcgtYgccacMaatctYtacaatattcaagtttgcgctcctgattctgagaagtcag

fava_c17664_239	fava_c17664	239	T/C	agcttaacaagatggatgaaggtagcagaggcaccatcatgatcacaaatgaYgatggaatcgacgcacctggcctcagagctttggttaccgctctcgt[T/C]gccacMaatctYtacaatattcaagtttgcgctcctgattctgagaagtcagcagttagtcattctattacttggattcatcctatagctgccaagaaag

fava_c17664_245	fava_c17664	245	C/A	acaagatggatgaaggtagcagaggcaccatcatgatcacaaatgaYgatggaatcgacgcacctggcctcagagctttggttaccgctctcgtYgccac[C/A]aatctYtacaatattcaagtttgcgctcctgattctgagaagtcagcagttagtcattctattacttggattcatcctatagctgccaagaaagttcaca

fava_c17664_251	fava_c17664	251	C/T	tggatgaaggtagcagaggcaccatcatgatcacaaatgaYgatggaatcgacgcacctggcctcagagctttggttaccgctctcgtYgccacMaatct[C/T]tacaatattcaagtttgcgctcctgattctgagaagtcagcagttagtcattctattacttggattcatcctatagctgccaagaaagttcacattSatg

fava_c17664_348	fava_c17664	348	C/G	tctYtacaatattcaagtttgcgctcctgattctgagaagtcagcagttagtcattctattacttggattcatcctatagctgccaagaaagttcacatt[C/G]atggaaccactgcctttgcagtttctgggactccagctgatagcacttctctgggaatttccaaagcactttttcctatagtagctgatctggtaKtaag

fava_c17664_444	fava_c17664	444	T/G	cattSatggaaccactgcctttgcagtttctgggactccagctgatagcacttctctgggaatttccaaagcactttttcctatagtagctgatctggta[T/G]taagtggcataaacatgggtaacaactgcggttatcacattgtttactcgggcacagttgctggagctcgagaggcattcttctatgatataccttStat

fava_c17664_541	fava_c17664	541	C/G	gtaKtaagtggcataaacatgggtaacaactgcggttatcacattgtttactcgggcacagttgctggagctcgagaggcattcttctatgatatacctt[C/G]tatctccatttcRtatgactgggctaaggcaacaagtaacctacacgacttcacccttgccgcgcaagcatgcttacctatcataagtgctttaStggct

fava_c17664_554	fava_c17664	554	A/G	taaacatgggtaacaactgcggttatcacattgtttactcgggcacagttgctggagctcgagaggcattcttctatgatataccttStatctccatttc[A/G]tatgactgggctaaggcaacaagtaacctacacgacttcacccttgccgcgcaagcatgcttacctatcataagtgctttaStggctgatataaagaacc

fava_c17664_636	fava_c17664	636	C/G	accttStatctccatttcRtatgactgggctaaggcaacaagtaacctacacgacttcacccttgccgcgcaagcatgcttacctatcataagtgcttta[C/G]tggctgatataaagaaccaaagataccctcaaaagtgctttttgaatatagatattccaagcaaagttgctaatcataagggatacaagttgactaagca

fava_c17664_836	fava_c17664	836	T/C	agggtaagagtataatcaaaatgggatggaagcaagtcgcctctgagacggaaggacgaaaaatgtcatctgatatgacctctacagacatagcagcgcg[T/C]tcagattgtaatgcgtcatctatatcacctgaaaatcttttatttgcgagagaagtaagaggtgctcaacatgatgatgatgatgatgatgatactgatc

fava_c17664_960	fava_c17664	960	A/G	atcacctgaaaatcttttatttgcgagagaagtaagaggtgctcaacatgatgatgatgatgatgatgatactgatcacaaatgtttgcaggaaggatat[A/G]ttactgtcacccctcttgctgctatctctcaggcggaggtagattgtcaaaactattttaaggattggctacaaaatgtctcggaatctccctcttcgtt

fava_c17666_206	fava_c17666	206	T/A	gtagttagttacaactgtcagacacgaccacatcattggcatatttttttttacaacaatacatgtttgttatttctaagctgtcaacaatgatttattt[T/A]tgtgcccaccccttttcttcttttatcttgttcttttctctcttccaaacaagggctcatattcactgtatcatttctggtttagaatgtctgtggaagt

fava_c17666_420	fava_c17666	420	T/C	cggaaaacaactgtgaaggtgagatgttttgtttacggttccagcaggaagacaacaaacccaacattcctgtttctacaggctgtccgatgtccctttc[T/C]cgcttgtgtagcactctgatgaattcgtctgtacttaggtttccgtctttatttgtatcgaacaaatgaaaaacaatctcaaccacattgtccgagagag

fava_c17675_103	fava_c17675	103	A/G	ttggcgtacaaaatatatttattttctctaattttctcgcgcttcatattaccaatagtaacataagatcgcagaaccataaacctaacataagatcgca[A/G]aatctctcgcacatttctccaccgattcttcatcgagcattcgtgctgaccttccgctactctcgcagttttcactgagYgcttttcaattcgattgttg

fava_c17675_1079	fava_c17675	1079	A/G	gagcaaagctctactttagagcaagcaaaaaatcagaaaacaatgaggactactgtagacactgctgggagatccattctcaaacctgatcaaaggaaac[A/G]Rcaatcattgacaactgaaagttcaatcccagtggcaggatctgctcaaatacaacattctactttgaagcaagcaaaaactcagaaaacaatgaagact

fava_c17675_1080	fava_c17675	1080	A/G	agcaaagctctactttagagcaagcaaaaaatcagaaaacaatgaggactactgtagacactgctgggagatccattctcaaacctgatcaaaggaaacR[A/G]caatcattgacaactgaaagttcaatcccagtggcaggatctgctcaaatacaacattctactttgaagcaagcaaaaactcagaaaacaatgaagacta

fava_c17675_1241	fava_c17675	1241	C/T	actttgaagcaagcaaaaactcagaaaacaatgaagactactataggaactgctgggagagccattcctaatcatggtcaaaggaaacaacaatcattta[C/T]ggctggaagttcaatccaagtggaaggatctgctcaaagcaggccagaggaccacagtcttttcggtggattaagcaatcgatcttctgctcagttccaa

fava_c17675_183	fava_c17675	183	T/C	aaacctaacataagatcgcaRaatctctcgcacatttctccaccgattcttcatcgagcattcgtgctgaccttccgctactctcgcagttttcactgag[T/C]gcttttcaattcgattgttgagattggatttttgtcacaatgagagtaaacaagggaaagaaaggtccacagcctggggcaaagaagaaaaaaagtaaca

fava_c17675_549	fava_c17675	549	C/T	gatgttcttcaaagcaggggattgagcaaagttccaccttaaagcaagctaaaaatcagaaaacaacgatgaccactgtggacaccgccattctcaagca[C/T]ggtcaacggaaacaacaatcattgatacctggaagtccaacccaagtgggaggatccgcacaaatacaacattcaactttaaagcaggcaaaaaatcaaa

fava_c17675_820	fava_c17675	820	A/G	atttacagctggaagttcaatccaggtggcaggacctgctcaaatacaacataaaaagcaagattacaggccagagataaagaagtccagagatgtttac[A/G]ttagtccacaactcaaacttcccatgctaccttctagtggagaaccgttagtagatgttccagacagtataaaatccaatgctgaattgtatcgaggcaa

fava_c17675_937	fava_c17675	937	A/T	acttcccatgctaccttctagtggagaaccgttagtagatgttccagacagtataaaatccaatgctgaattgtatcgaggcaagttgagaagttctaaa[A/T]cactgaagagaagcatgcaatgttttccaaagcaggggatggagcaaagctctactttagagcaagcaaaaaatcagaaaacaatgaggactactgtaga

fava_c17677_1163	fava_c17677	1163	T/C	atatacaccataaaaaacagcactagaaggagccatgcttataagtgacggtactagacccttgtatagagaaaagatgccttcagttcgtatcatgtgt[T/C]ggaaagcaccaattacaccacccaaagtttcaccgccaggtgccactatcttggtccgaatagtgtcaagtggtaggcaaataatagtagcagtgagccc

fava_c17677_1322	fava_c17677	1322	C/A	aatagtgtcaagtggtaggcaaataatagtagcagtgagcccagcagcagcacccgcaataaacctttcaaaattggtagtttcttcatttccagagaat[C/A]taagtagctgttttcgataagtatcatacgcgctaaaattaacagctttaaacggagccgtccgcagaatatttagaaggttccctttccaaaaacctct

fava_c17677_1492	fava_c17677	1492	T/C	tatttagaaggttccctttccaaaaacctctgaacccttgagaagtagcaattgatttaataacctcaaagatattcctcttctcaccacgaaccatata[T/C]tccagcttaagtctttcaagtggagcaacacaggttctagaaaccatggcagatatagcaccagaccaaagatgcttagtggtattattgatagcaccgc

fava_c17677_2071	fava_c17677	2071	G/C	tattggttaggaggtgttgtaggttatagttctgagatagcttcaactttcgttaaaactaatagtttatcggaaatgggaattgaatagcgaagtgtgt[G/C]ttctcgttgccggaaaactgctcaactccatcgataactcatgctactaaaaactttatttcatttttgtttgttaccagcaatttttttcagcctgctg

fava_c17677_331	fava_c17677	331	A/C	gtagaatatcctgttgggtgtaaaaaaatgtccaagaataatggataagctactttaagagttacaacctaaagcaaagggacttgcttgcttggactga[A/C]catgctgataatcaagtccactgccagaaaacaaaatgttaacaaaaatgtgWagacggaacaatcataagatttaggaMcScgcagttgctattaattt

fava_c17677_384	fava_c17677	384	A/T	tttaagagttacaacctaaagcaaagggacttgcttgcttggactgaMcatgctgataatcaagtccactgccagaaaacaaaatgttaacaaaaatgtg[A/T]agacggaacaatcataagatttaggaMcScgcagttgctattaattttttttgcagacttgaggcttttgataatcaactgtaaagccagtccaaaacgg

fava_c17677_411	fava_c17677	411	A/C	gacttgcttgcttggactgaMcatgctgataatcaagtccactgccagaaaacaaaatgttaacaaaaatgtgWagacggaacaatcataagatttagga[A/C]cScgcagttgctattaattttttttgcagacttgaggcttttgataatcaactgtaaagccagtccaaaacggtaatatgtgtgatgtccWtaacatgat

fava_c17677_413	fava_c17677	413	G/C	cttgcttgcttggactgaMcatgctgataatcaagtccactgccagaaaacaaaatgttaacaaaaatgtgWagacggaacaatcataagatttaggaMc[G/C]cgcagttgctattaattttttttgcagacttgaggcttttgataatcaactgtaaagccagtccaaaacggtaatatgtgtgatgtccWtaacatgatgg

fava_c17677_502	fava_c17677	502	T/A	gatttaggaMcScgcagttgctattaattttttttgcagacttgaggcttttgataatcaactgtaaagccagtccaaaacggtaatatgtgtgatgtcc[T/A]taacatgatggcatcggcattagtatggaatggtagaccattatggttttactttaaccttgagagatcaaatatagcccattgaagttggaaattacca

fava_c17677_639	fava_c17677	639	A/C	accattatggttttactttaaccttgagagatcaaatatagcccattgaagttggaaattaccattccacgacagtatcagatgctcacttaaatcagga[A/C]tactgcttatccatataaaattgaagatctcatctattcattgccaactcactctactttgaggacaatcttcatgaactcatacacaaagtaactaatt

fava_c17681_1783	fava_c17681	1783	T/A	ttcgagggagccaagttgtttttcaatatcaacacgttgcacttgcaaactttcaagctcctgctcacttttacgcagctccatttccagagctgcaata[T/A]ttttttccgcaacctgatatcgtttcacagcatccatcattcttYggtgaattgctttcaatttaYcagtcaatccagacaattccctctcagcacttgc

fava_c17681_1828	fava_c17681	1828	C/T	caaactttcaagctcctgctcacttttacgcagctccatttccagagctgcaataWttttttccgcaacctgatatcgtttcacagcatccatcattctt[C/T]ggtgaattgctttcaatttaYcagtcaatccagacaattccctctcagcacttgcaagggctttatcagacacatttggatctcgaacagatgtgcccat

fava_c17681_1849	fava_c17681	1849	T/C	acttttacgcagctccatttccagagctgcaataWttttttccgcaacctgatatcgtttcacagcatccatcattcttYggtgaattgctttcaattta[T/C]cagtcaatccagacaattccctctcagcacttgcaagggctttatcagacacatttggatctcgaacagatgtgcccatcttcccactcttgggcttacc

fava_c17681_286	fava_c17681	286	T/C	tcatttatctctgatgtgtgctgacactagtaaattagtaacaattaatatgccagatcaaactgatacaagaaactagttgtttctatgcattctataa[T/C]ttaacatctaagcacactgacagatttatcatgcaccctKctcgcaaacgacaaaagtgcacggatcaatagtgatgctctttgtgcaattatcagtttt

fava_c17681_326	fava_c17681	326	T/G	acaattaatatgccagatcaaactgatacaagaaactagttgtttctatgcattctataaYttaacatctaagcacactgacagatttatcatgcaccct[T/G]ctcgcaaacgacaaaagtgcacggatcaatagtgatgctctttgtgcaattatcagttttataaataccaacaagtcgatcagccaattcaaacatattg

fava_c17681_427	fava_c17681	427	T/C	ctcgcaaacgacaaaagtgcacggatcaatagtgatgctctttgtgcaattatcagttttataaataccaacaagtcgatcagccaattcaaacatattg[T/C]tccttagactgatgataatgaactgagcatccttagtcctgtctttcacataatgccccacgatagatacatttttgaagtcaagggcggcatcgatttK

fava_c17681_816	fava_c17681	816	T/A	ctccaagtgtaatcatctgatacatttcctttaactttaatgatattgcattaaatccttccataaattcatccaatctcttctttctcaattcatcata[T/A]tgtttctttatatcatcccgctcctgagtgactgcattaagttcttcaactcgttcattgtacaatgcgaccttcttccggtactctgcaattgaatcaa

fava_c17682_1044	fava_c17682	1044	T/A	taacggcatccattttgatgctttgaagtctgtctggtggttgatggaggcgtgaaggccagttagtaacattcactccatatccattattaggcaattg[T/A]gttatgcatgccttaagaccaacataccaaacatcatctgcatcatcattggacttgcataatggaggacgcacagatatattacgactaaggtagcagc

fava_c17682_1500	fava_c17682	1500	T/A	tcgggtacatcaaacgatgtgtagcaaatactgctacatgggcaggcacgccacgctcaagagcaaactgaatctggttttcatgaacatcttttggtgc[T/A]attgaaagagtggtcacattacgttgcatcaaaaatgcaccaaaacttgccactccacagccaatatccaatgcaactcttgtgttgtaaccaaatgtaa

fava_c17682_2003	fava_c17682	2003	G/C	tcttgaacattatccaaacaaggtatataatccaccattttcacatcatcacaaatcttatacttgttcaccaccacatcaacctccttccccaccagaa[G/C]tgattcattgttgttgctgctgttccaattccaattgagcagtgagtcgtcgtttccgacagtgaaattgtctgtcatgacgccattttggtcaattaga

fava_c17682_215	fava_c17682	215	A/G	aaagtgtcatcttagcatctcaggtacactgaagaatttaatttgtatggacatgatataaaaatgattgtacacatttcatttcatcacacatttcacc[A/G]tttaagtatttgtataaactcttctagaatggtatgaagtatagaactagctcttcaaatttctttttgcatgaacaaatatatgaatctccctgttact

fava_c17682_348	fava_c17682	348	C/A	tatgaagtatagaactagctcttcaaatttctttttgcatgaacaaatatatgaatctccctgttactctacaatttagaagcactttttataatgaatt[C/A]aagtttaaaccagttggaccccagagctcaccagatagaccgagcagtcgttagcggcttcagaagtgcttctcacaccttaatatcttccaacttgcat

fava_c17682_938	fava_c17682	938	T/G	attacatttcttaagttgagcttcttccaaccgaaagcatgaacataactctcaattatttcattccaatattttgattcggccctaaagagttcctttc[T/G]ggatataacggcatccattttgatgctttgaagtctgtctggtggttgatggaggcgtgaaggccagttagtaacattcactccatatccattattaggc

fava_c17689_952	fava_c17689	952	C/T	catctttgttggatagccattcaagctcttcttctgcacactcagacaatgaatttggatcgaccaggtctaatgtatcaagcgacagttgatcaccgct[C/T]ttcggttcaagtgaaggaaaggttttctttgtgttgtcatcatcatcctcgcctatgtcagatgagaaatccaaaaggtcatccacagaaccaagggctt

fava_c17690_152	fava_c17690	152	A/G	ctacttggttggttttgattttctgtattgttcaacattttgcataaagtttcaatttttattttctgtgaaaataatgtggggtaggtggggttgttcc[A/G]gtgggggttttaggtcattgagcaaaagggtgtcgtctattgtgaatctaactcatggttatggcgaaaagcttcatccttttggattcggtcctcattt

fava_c17691_1065	fava_c17691	1065	G/A	tgaaacctattatatggtgttcaacatatcctttttcaaatatttcatttacacctgagaattcaaaaccccgggctccttcagaatctacaagtacaaa[G/A]agggagggaccgcctgctgctYgtagtaaacccagttctgaaccaagccttgctgagtacttggcttccgcgttcaatgaagctgccaacactgaagaat

fava_c17691_1087	fava_c17691	1087	C/T	aacatatcctttttcaaatatttcatttacacctgagaattcaaaaccccgggctccttcagaatctacaagtacaaaRagggagggaccgcctgctgct[C/T]gtagtaaacccagttctgaaccaagccttgctgagtacttggcttccgcgttcaatgaagctgccaacactgaagaataatgtatgatgaagactgcatt

fava_c17691_1357	fava_c17691	1357	T/C	caggaatgtgttaggttactaacatgaatgattagtggcagatagaaggtccagcacgatccgtgtagaaactgttaaaactgtgcaaccacattttgag[T/C]gttgtggctgtggttgattaatgtttaagtttaataaatgctttggcataagctgacatactgaacttgagaagcatataatgatgacctcgatgatcag

fava_c17691_223	fava_c17691	223	T/C	agctgatcttgctgaggattctggtggtggtggcggtggtggaggttctggtacgcagggtgttctttcgaatccattggctgaagctccttcgactatg[T/C]taccatcggatgctgctccaagatttaactattatacagatccaatgaatgcgttctctKctgataaaaggagtactgacaatgttagggcacaagggcc

fava_c17691_283	fava_c17691	283	T/G	tgttctttcgaatccattggctgaagctccttcgactatgYtaccatcggatgctgctccaagatttaactattatacagatccaatgaatgcgttctct[T/G]ctgataaaaggagtactgacaatgttagggcacaagggccYSaatatttgcctccccctcctcctttgRattttggtggatctcctatggcacagttttc

fava_c17691_324	fava_c17691	324	C/T	taccatcggatgctgctccaagatttaactattatacagatccaatgaatgcgttctctKctgataaaaggagtactgacaatgttagggcacaagggcc[C/T]SaatatttgcctccccctcctcctttgRattttggtggatctcctatggcacagttttcgtcgYcgcatacagaatcaacaaatccacagatgtctcctY

fava_c17691_325	fava_c17691	325	C/G	accatcggatgctgctccaagatttaactattatacagatccaatgaatgcgttctctKctgataaaaggagtactgacaatgttagggcacaagggccY[C/G]aatatttgcctccccctcctcctttgRattttggtggatctcctatggcacagttttcgtcgYcgcatacagaatcaacaaatccacagatgtctccttc

fava_c17691_352	fava_c17691	352	G/A	ctattatacagatccaatgaatgcgttctctKctgataaaaggagtactgacaatgttagggcacaagggccYSaatatttgcctccccctcctcctttg[G/A]attttggtggatctcctatggcacagttttcgtcgYcgcatacagaatcaacaaatccacagatgtctccttctcccactcaagcattgccagcacccta

fava_c17691_388	fava_c17691	388	T/C	taaaaggagtactgacaatgttagggcacaagggccYSaatatttgcctccccctcctcctttgRattttggtggatctcctatggcacagttttcgtcg[T/C]cgcatacagaatcaacaaatccacagatgtctccttctcccactcaagcattgccagcaccctatagaaactcagtttggaatggaccaagaggccctcc

fava_c17691_809	fava_c17691	809	G/A	agtccaaatcattctgacggatatattccaaatccttcttcagggtacagtccaaaccattctccagcattcaggaacagtcctaacactagtcgagggc[G/A]aggcagaggcttctggcataataccagaggccctgtttcaggacgtggtggtagacaaggatcaggttctcatggtcgctggtcaaatgaagacagaagt

fava_c17692_1020	fava_c17692	1020	T/C	atttaaaatcacagaattggaatgtcaagaaagcgtctcaaatgttgaagcagagcctgaaatggagacgggagtacaaacccgaagagattcgctggga[T/C]gatgtttctaatgaagcaaagattgggaaaatgtatagagcgaattattgtgacaagcatggaagaccggtgattataatgagaaccgatcgccagaatt

fava_c17692_1224	fava_c17692	1224	C/T	agtcgttaacagaagagattaagcattttgtttactgcatggagaatgcagttttgaatctaccacctcttcaagaacaggtgatctggctggttgattt[C/T]catggtttcaatttatcaaatatatcgttcaagatgacgcgtgaaattagccatatattacaaaaatactatccgcagcgcctcggattggcaatcatgt

fava_c17692_1503	fava_c17692	1503	A/C	agtttgtttatcctaacgaccaaaacacaaagaagattatggagagtttgttcgatatggatcaacttgaacctgcatttggtgggaacgacgacacaga[A/C]ttcgatatgaataaatatgctaagagaatgaaagaggaagataacaagatgcactYcttttggacctcacacaatRttccttcttctgattcaacagggt

fava_c17692_1559	fava_c17692	1559	T/C	atggatcaacttgaacctgcatttggtgggaacgacgacacagaMttcgatatgaataaatatgctaagagaatgaaagaggaagataacaagatgcact[T/C]cttttggacctcacacaatRttccttcttctgattcaacagggtcagaagcagattctgatgcttccaacaatgagaaaacacttggttcctcacttcct

fava_c17692_1579	fava_c17692	1579	A/G	atttggtgggaacgacgacacagaMttcgatatgaataaatatgctaagagaatgaaagaggaagataacaagatgcactYcttttggacctcacacaat[A/G]ttccttcttctgattcaacagggtcagaagcagattctgatgcttccaacaatgagaaaacacttggttcctcacttcctaaccctgagaaatcacttga

fava_c17692_1692	fava_c17692	1692	G/C	attcaacagggtcagaagcagattctgatgcttccaacaatgagaaaacacttggttcctcacttcctaaccctgagaaatcacttgaagtatgagctat[G/C]atatcctagtttgaaacctacatttaagagacatggtgtaaacttYatcttggatttgtaatgataaatgaYtttacctttaaaaataacagacagaata

fava_c17692_1738	fava_c17692	1738	T/C	aacacttggttcctcacttcctaaccctgagaaatcacttgaagtatgagctatSatatcctagtttgaaacctacatttaagagacatggtgtaaactt[T/C]atcttggatttgtaatgataaatgaYtttacctttaaaaataacagacagaatacatggagcaatgcttgtttattgttgaaaatgcttcaatagttagt

fava_c17692_764	fava_c17692	764	T/C	aaaaaccaaactaaactatatcctttaattcacttttctttcttcaaaacttttgcttctgcaagtgttcactgttcatttttagattgtttttatacag[T/C]tattactatgagtgaggatctgaagaaaactggttctgttggccatgagaaaatgtcacaagagcaacatgcaaagatcattgaggtgagagagatgata

fava_c17693_1279	fava_c17693	1279	T/C	aacttgtctatgaattgctgcttaaatttatctcttcttcgtgcctcgatgctaaggtagcgaaaaagtatatcgatcattcgttcatttcgaagttgct[T/C]gaactgtttgattctgaggatccaagagaaagagactgtttgaagacgattctgcatagggtttatgggaagttcatggtgcatagaccctttattcgga

fava_c17693_1468	fava_c17693	1468	G/C	cctttattcggaaaacaattaacaatatattctatcggtttgtgattgagaccgagaaacataacgggattgctgagttgttggagatttttggaagtgt[G/C]atttgtggatttgctttacccttgaaagaggaacacaaaatcttcttgtggagagtgttgatccccttgcacaagcctaaatctttgggggtgtactttc

fava_c17693_730	fava_c17693	730	C/T	tggtggatttctgcttctgggttgcaatgctgaagcagcttttcaacaagcttcctcggaaatcttcaaaaatcgatgaagatgagtcgacccgggttgt[C/T]gaatccccgcgtgctgctggtaaatgccacaatcggcagcaaggcggtggtgctgtgttgaaacgagcttcttcgtcggctgtgtttccagctagtatgg

fava_c17695_500	fava_c17695	500	G/A	gtgccaagggaatgggaatgggaatggaaaggggaaagaaatggaggtttggtgcgctgagattgatgtgaacaagaacaaagatggggagttgtggggt[G/A]aagtttgttggttgaataaagttctctctgttccaaaaggatcctccattgttcattgttcttctgttgccttgtgattattgtgcctcatcaggtcact

fava_c17696_1577	fava_c17696	1577	T/C	gagtgtttgcacaaaatctgtttttgttgggaagccagaaactgcagtgcttagtctccatattgctttctaggtagcttccttcaccaatcaaaatgct[T/C]acagcctcgaattaaggttttcagtagattttctcataggtgcacatacgaaggtcagccaagtcaccagggcaattcaatgaattagcRgtctaactgc

fava_c17696_1667	fava_c17696	1667	G/A	tcaaaatgctYacagcctcgaattaaggttttcagtagattttctcataggtgcacatacgaaggtcagccaagtcaccagggcaattcaatgaattagc[G/A]gtctaactgccagaaacgtagtcgaacaaggaaactgaggSaacgaagagttcgacgaaaacgacgccggcgatggaacctttccacagagacgttgatt

fava_c17696_1708	fava_c17696	1708	G/C	ttctcataggtgcacatacgaaggtcagccaagtcaccagggcaattcaatgaattagcRgtctaactgccagaaacgtagtcgaacaaggaaactgagg[G/C]aacgaagagttcgacgaaaacgacgccggcgatggaacctttccacagagacgttgatttggagtgtgcagaaaacgggggaaacaaagcaccgcctaaa

fava_c17696_998	fava_c17696	998	C/T	tgaccgtagaaacaccatcatccttccccttaaccggttcctcacatgttgactgtttcctactaagagaattctttaatacaccaaaattttccaaatt[C/T]cccacaatagatgcctgctgcattatatgtttctcctgaaacggcaatttcctttcttcttctttgcttttaatccaaagactgcaaacatctggcatca

fava_c17697_1104	fava_c17697	1104	G/A	atcatagtgacccctcccaatttctgctagcatttgcaggaagtagtggttacaatataaacctatgccaaaagtacatatacgtggtgtgcggattgat[G/A]gcccatttgttacatagcttttcacaaattcacatatctctctctcatcctcaacagccccatcagttacaagaaaaataagagggactgaatggcttga

fava_c17697_1414	fava_c17697	1414	G/A	gaaactgttaccatcgatggcgagaataaataggcctccccattgaaagctataatgttaaatgtatcttgctggtttaatttaaagagtgaagctagta[G/A]cgcgttctttacgttttggagaggagttcctttcatgcttgcacttatatctactacgaacaccatatcctttttaaaaaccattgtatcctgaaaattt

fava_c17697_1602	fava_c17697	1602	G/C	atcctgaaaatttccaggataaagatacaagcaaaatatctctctttcatcgaaatcacgtaggaaaggcgattgtaagagaacacctccaaataagtca[G/C]ttgaagaaaccttgtacgagaaactgaagtctgtatttgaccaagttgatacctctgcttcataagataaacttaactttccagcttggcgcgttagtcc

fava_c17697_2341	fava_c17697	2341	G/C	taaacatacggttgataactccgaatgtcaggattatctactacatcaggatcaggtatcaccgcatagcacatcggcgccgacggtagataatcctccg[G/C]tgatcttgtcatttccggcaccggtgcgggagccgatcctttgaagtggtgaattctcttggagaggttgagtccgaagtcgacggaactggaaaagtgt

fava_c17697_764	fava_c17697	764	G/T	ctaataaccaggcacgagcagtgactaggtctatatgtctctttgaaaagacattagtgagaagcatatccttttctcttctcactttaaggtcaacttc[G/T]aagctcgtcctatcagccaaagtaccagtaaccttaactagttcaggaaaagctccattgtatctgcctgatatgatcaatggacttccaaatgataggt

fava_c17698_1594	fava_c17698	1594	C/T	actattggagcaaaatcaagaacctcgagttctaagaaaaggaagcagttgtctgagccgcaggcatctgaagcaggattagctcctaacgacccaaccc[C/T]accaaaagttgaaacaggcaagaaaacaaagaaaaagaaagggaaatgatatataattgcctttttttcctaaggcgcaaagattagcatccctcctttt

fava_c17699_1028	fava_c17699	1028	C/T	gaagtgaattgtaagatctgatataagctctYtcagacattccactgagtttcactgccgaagatcgatcaaagagaacaccgagcctcgtcgctgcaat[C/T]tccatacagatgatagctttggcaacttcgccgacgccgatgatcgaggaatcgaagtgtacatcgcaKaggcgacggagttcagcggcttttcggatga

fava_c17699_1097	fava_c17699	1097	T/G	caaagagaacaccgagcctcgtcgctgcaatYtccatacagatgatagctttggcaacttcgccgacgccgatgatcgaggaatcgaagtgtacatcgca[T/G]aggcgacggagttcagcggcttttcggatgaggagttttgaatcggagagtccgagtttttttgcgatttctgagatatccatttttcttgtttaggttt

fava_c17699_529	fava_c17699	529	C/T	acagaagatttccacttctcatagtcatgtgtttccatctgtttcttttttttataacatgaaacctctactccatcatcagatggatagccaccatcct[C/T]agcttttcttttgcttggcaatacatcaagcagatctctattggtctttaattctttagggtctttcttttctttagctaccccaaaaacatcatagcac

fava_c17699_758	fava_c17699	758	C/G	aaaattccgattcagaagtaccacaaagttcaattagcttaatcttatcaactttaagcttttgtttctttgcagataaataaaaagcaacagccatgaa[C/G]accggacgagtaaaatcagcactagcacggcgagcagccggcaacgaagMaagaaacctatccttataaagcttgagaccgtcRcgaacataaggtatga

fava_c17699_808	fava_c17699	808	C/A	actttaagcttttgtttctttgcagataaataaaaagcaacagccatgaaSaccggacgagtaaaatcagcactagcacggcgagcagccggcaacgaag[C/A]aagaaacctatccttataaagcttgagaccgtcRcgaacataaggtatgatcctcacacaaccaaatcgaatagccaattctctcacatcaagcttcaac

fava_c17699_842	fava_c17699	842	G/A	aagcaacagccatgaaSaccggacgagtaaaatcagcactagcacggcgagcagccggcaacgaagMaagaaacctatccttataaagcttgagaccgtc[G/A]cgaacataaggtatgatcctcacacaaccaaatcgaatagccaattctctcacatcaagcttcaacttgacaccaatgccattgtgaagtgaattgtaag

fava_c17699_959	fava_c17699	959	C/T	tcctcacacaaccaaatcgaatagccaattctctcacatcaagcttcaacttgacaccaatgccattgtgaagtgaattgtaagatctgatataagctct[C/T]tcagacattccactgagtttcactgccgaagatcgatcaaagagaacaccgagcctcgtcgctgcaatYtccatacagatgatagctttggcaacttcgc

fava_c17700_1101	fava_c17700	1101	T/C	caccacgagtagcattcttggaccatcaacgtttgattcttaatgtcgacaacagtgccttaaagcaaaggattgcagcattggctcaagacaagctttt[T/C]aaagatgctcatcaagaagcattaaagaaggaaatagaaaggttgagacaaatctaccaccaacaaaacatacataagatggggaattcaatcaataata

fava_c17700_1370	fava_c17700	1370	T/C	cacttggaatgcaaagacaaagagcaacttctcagttaaaaggaaaaaaagtggatgatgttattatgttccttggtgctaacatgtgatatgttcctat[T/C]aaagttgatgtgggaccttgacacacatttatgagtaattggagggtaggaagttgacatgtggaattggttctaaatagattggtgttttttttttgtt

fava_c17700_223	fava_c17700	223	C/T	tctccctctggttttagaaacccaagaaaacaagattttgaagtaaagatttgtggttgtttgtgtcttgtaacactttgacacataacatagcacaaac[C/T]caataaattcggtgagaattctctatatttggggtcacatacatacacatttgggtttaatggcacagttacctcctaaaataccaaccataccccaaaa

fava_c17700_352	fava_c17700	352	T/C	ttggggtcacatacatacacatttgggtttaatggcacagttacctcctaaaataccaaccataccccaaaattggcaacatttttctcaccaaaaagta[T/C]catcatcctccRcaatgtcaaatatcacctccaccaccaccacYactgatcatcaacaaataccgtcatggatcgacgaattcctcgacttctcctcggt

fava_c17700_364	fava_c17700	364	A/G	acatacacatttgggtttaatggcacagttacctcctaaaataccaaccataccccaaaattggcaacatttttctcaccaaaaagtaYcatcatcctcc[A/G]caatgtcaaatatcacctccaccaccaccacYactgatcatcaacaaataccgtcatggatcgacgaattcctcgacttctcctcggtaagacgaggagc

fava_c17700_396	fava_c17700	396	C/T	ctcctaaaataccaaccataccccaaaattggcaacatttttctcaccaaaaagtaYcatcatcctccRcaatgtcaaatatcacctccaccaccaccac[C/T]actgatcatcaacaaataccgtcatggatcgacgaattcctcgacttctcctcggtaagacgaggagcccatcggcggtccgccagtgactctatagcct

fava_c17700_72	fava_c17700	72	G/T	acaacacataccataatctaagaaagaaaaaaaaacatatcataattgtctacatgacacaatattgtgta[G/T]tgtaggtcttattgcctctcaatcatctctttattaccctctctcttatgtctccctctggttttagaaacccaagaaaacaagattttgaagtaaagat

fava_c17703_1279	fava_c17703	1279	G/C	caacgatgatttgagagaattgccttgttcacatcttttccacaaagattgcgtagacaggtggttaaagataaatgcattatgccccctttgcaagagg[G/C]aagttggcgggaatgaaacagaaccagtcatcgaggaaaatgccagccaacaaaggggcgagaccgggtttgagaatagtcctgccaataccttgtcata

fava_c17703_473	fava_c17703	473	A/C	ttaggacgtgtttcccggactccattgagttcaggcttttgtgtttctttggaatttgtttttacttttactcagattattgcttctgttattgttctta[A/C]ttcctcgagaaatgaacatccacgtgctcccttgtttgcttggattgttggttatgcttctggatgtgttgtttcgcttcctttgttgttgtggagatat

fava_c17703_696	fava_c17703	696	T/A	ctcgaacctccaccacggttgatgatccttccgggattcttctttcgaattcgagtacaatacttggggaagaagatgttgttcctatttcatccaatgt[T/A]tcatccaatcaagcttcgtcggtattcaccaatatgagaaccaagatacttRtggaatacatcaaaatcgtcctagatttattttttgcatcatggttta

fava_c17703_748	fava_c17703	748	A/G	gagtacaatacttggggaagaagatgttgttcctatttcatccaatgtWtcatccaatcaagcttcgtcggtattcaccaatatgagaaccaagatactt[A/G]tggaatacatcaaaatcgtcctagatttattttttgcatcatggtttattgttggaatcatatggatatttggagggcattcatccgctaatgaagctcc

fava_c17704_1057	fava_c17704	1057	C/T	ctcgacgcttcaggaatgctcaacaacctgaaatgaaccaacaaagtggagaatcttctgtttcatgcagctcatgtggagcaccaggtgttttgcgaac[C/T]gcaaatacagcaaataacaggggcaggaggttctacacatgtcaatcacaggagtgcagcttctttgtatgggaggatagcctcaataatggcggtggag

fava_c17704_1211	fava_c17704	1211	G/C	gtgcagcttctttgtatgggaggatagcctcaataatggcggtggaggaagaggttctgcacgttcaaatagtattccaacatccacccgtggtcgaaat[G/C]gtggtcgtggacctcgtggtaggggtcggagtggatctaattcttctagcgggacatttgtgacagccactggggatcctatttctggtagaaggtgctt

fava_c17704_1371	fava_c17704	1371	T/G	gtgacagccactggggatcctatttctggtagaaggtgctttacctgtggtgatccatcccactttgctaacgcctgccccaatcgtggaaactgatgtc[T/G]tactgattctcatcagccagaaggaagttttggtRggagatccagaacctctattttgcaYgtgtttaagaagcaYcgatacatcaactatgttgaatat

fava_c17704_1406	fava_c17704	1406	A/G	gtgctttacctgtggtgatccatcccactttgctaacgcctgccccaatcgtggaaactgatgtcKtactgattctcatcagccagaaggaagttttggt[A/G]ggagatccagaacctctattttgcaYgtgtttaagaagcaYcgatacatcaactatgttgaatatagcatgtattacgatactttgtactggttctaata

fava_c17704_1432	fava_c17704	1432	T/C	actttgctaacgcctgccccaatcgtggaaactgatgtcKtactgattctcatcagccagaaggaagttttggtRggagatccagaacctctattttgca[T/C]gtgtttaagaagcaYcgatacatcaactatgttgaatatagcatgtattacgatactttgtactggttctaatatgccaaYgttaactgttttattcatt

fava_c17704_1447	fava_c17704	1447	T/C	gccccaatcgtggaaactgatgtcKtactgattctcatcagccagaaggaagttttggtRggagatccagaacctctattttgcaYgtgtttaagaagca[T/C]cgatacatcaactatgttgaatatagcatgtattacgatactttgtactggttctaatatgccaaYgttaactgttttattcatttgatcattgttcatg

fava_c17704_1513	fava_c17704	1513	T/C	ccagaacctctattttgcaYgtgtttaagaagcaYcgatacatcaactatgttgaatatagcatgtattacgatactttgtactggttctaatatgccaa[T/C]gttaactgttttattcatttgatcattgttcatgtctggcataaaggaacttgtagaatttgcttattgaatattatgaatcattaaagattcattttca

fava_c17704_1631	fava_c17704	1631	T/C	tttgatcattgttcatgtctggcataaaggaacttgtagaatttgcttattgaatattatgaatcattaaagattcattttcagtttgatctttttaggt[T/C]agggtggagatttaatccccgtatcccatcacttatgggatacatgtatgccctgaagccatttgatagggtttgtgatctatcaactgattcccgtcag

fava_c17704_878	fava_c17704	878	C/G	tggctgtgatgagattcttacacagttgacagaaatatgtggcactggctctcgcatgccagcaagagctcgaggacctactccagcaaccggaaacacc[C/G]atcataccaacccaaggcagtcagcttgtttgcactgtcaagaaacagggcactcttctaatgattgtccttcacagactcgacgcttcaggaatgctca

fava_c17705_1476	fava_c17705	1476	A/G	aagtagaggtagctgcagcatcagccagtgaacaaccaatacgctcatttgacattctataaacatcgacctcattgaaaccggcttgatcaaggatggc[A/G]cgaacattttctgacataaagtccacacctagcactgttatgaatttgcatccagcttttgccattttgactgcagaatctgccatgaccagtgaatcag

fava_c17705_467	fava_c17705	467	T/G	tctggaagctttttagtagcctgaaagttcctcatatgcaaaattggttcacatcctacgtctgccactgattgaccatttggagtctgcaacttgaatc[T/G]ctctgctttgtatgcagacaacctattttcttcgtcgggtaggtgatggcaaactgtcaggagtgagctaagagaattcatcttcatgtaaggacaagat

fava_c17706_1800	fava_c17706	1800	G/A	cagctgtttcaactggcttgaaccttggatgacataatggtgaagggatcaacatcgaagatgcagaattcactgaagaatcaggttggtatgtttcccc[G/A]ggccttcctactaaatcaatcatatactccctgtcagaaccaaattgcactatacaagaagcacYtgtgtcctttctacaatacttgcagcccttagcaa

fava_c17706_1865	fava_c17706	1865	C/T	gaattcactgaagaatcaggttggtatgtttccccRggccttcctactaaatcaatcatatactccctgtcagaaccaaattgcactatacaagaagcac[C/T]tgtgtcctttctacaatacttgcagcccttagcaattcgacacggtaggttgattaaatcKgctaacactttaaacagcaatgctcgatggacacaaagR

fava_c17706_1926	fava_c17706	1926	T/G	actccctgtcagaaccaaattgcactatacaagaagcacYtgtgtcctttctacaatacttgcagcccttagcaattcgacacggtaggttgattaaatc[T/G]gctaacactttaaacagcaatgctcgatggacacaaagRccgacaggtaRgcttccaataggaagaatgatagactgtagacagcttttaagaagttcag

fava_c17706_1965	fava_c17706	1965	A/G	YtgtgtcctttctacaatacttgcagcccttagcaattcgacacggtaggttgattaaatcKgctaacactttaaacagcaatgctcgatggacacaaag[A/G]ccgacaggtaRgcttccaataggaagaatgatagactgtagacagcttttaagaagttcagagcattccttccagtgtgtacctaaattatcttcgtcgg

fava_c17706_1976	fava_c17706	1976	G/A	ctacaatacttgcagcccttagcaattcgacacggtaggttgattaaatcKgctaacactttaaacagcaatgctcgatggacacaaagRccgacaggta[G/A]gcttccaataggaagaatgatagactgtagacagcttttaagaagttcagagcattccttccagtgtgtacctaaattatcttcgtcggaagatcctcct

fava_c17706_522	fava_c17706	522	A/G	atatgagagtgtgagtaatgatattgtatatcatgtatataacattaaaaggtagtctgctgctctctaaaccaccagtataattcagctcttcagcaag[A/G]tcctgaataaactRtaatctaagtataatactgttcaatattgatattatcattcatatagtggtagatgtttatStgcgggaactagttcacactttcg

fava_c17706_536	fava_c17706	536	G/A	gtaatgatattgtatatcatgtatataacattaaaaggtagtctgctgctctctaaaccaccagtataattcagctcttcagcaagRtcctgaataaact[G/A]taatctaagtataatactgttcaatattgatattatcattcatatagtggtagatgtttatStgcgggaactagttcacactttcgcgtcagcaattatt

fava_c17706_598	fava_c17706	598	C/G	agtataattcagctcttcagcaagRtcctgaataaactRtaatctaagtataatactgttcaatattgatattatcattcatatagtggtagatgtttat[C/G]tgcgggaactagttcacactttcgcgtcagcaattatttgcagcaaatatttcataacgtaggagaaagaaggccgcctccttggatccgcagaccaaca

fava_c17706_723	fava_c17706	723	G/A	cgtcagcaattatttgcagcaaatatttcataacgtaggagaaagaaggccgcctccttggatccgcagaccaacagagctcaatcaaggcagccgcttg[G/A]ggattcacatgccttggaatttctagccttttgcccataaaaccaacagctgcaacaacctgtgaaggatttaactgcctccatggttgttgaagggtca

fava_c17706_990	fava_c17706	990	G/A	tgacttctggtgccatccattcaggagttccagcagctgttttagaagaaagatatgtatttgcttttgtacgtgaaaggccaaagtcacataccttaac[G/A]gtgtatgaatcatcaaccaaaagatttggagacttcaaatctctatgaacaattggaggtttcatttgatgaagataattcattccacttgccacgtcat

fava_c17709_337	fava_c17709	337	T/C	aggctatctggttgaagtttcacaagaaacgacgacaactatggctatcgcttcgcccaccgttaatcttcatttcatcaaattccccattcatcgttct[T/C]tctcgcatcaaccttcatctcgattacgattacgctatcgcctcgctgctagtagaggattgaaccttagtaaatactaccacagttgtgtctctcacag

fava_c17710_1018	fava_c17710	1018	C/G	aactctcgggaagtcgagcatgggaaagagctaatgaggcagaccagagcgatggtggctatgatattattttaatgacagagattccgtactccgttac[C/G]tctttgaagaagctgtatgctctcattaagaagtgtataaggcctcaatatggagtcgtatatctagctccgactaagaaacactatgtMggctttagta

fava_c17710_1108	fava_c17710	1108	C/A	actccgttacStctttgaagaagctgtatgctctcattaagaagtgtataaggcctcaatatggagtcgtatatctagctccgactaagaaacactatgt[C/A]ggctttagtaatggagtgaaacagctgagaaatatggtggatgaagagggcatttttggagttcatttagttaaggatttggctgacagagatatctgga

fava_c17710_147	fava_c17710	147	C/T	cttttcttgcttacatacacacattccattttagtctttacacatctttgaaaaaacttcttcaatcgcatttctcattcatcaccaaatctaactttca[C/T]catcttctctctttctcttcttccttctgttaggatgcgtgcaccggcgatactcgcgcaatgcttgcctggcctagtgcctcatgatcgtggaagtctc

fava_c17710_358	fava_c17710	358	C/T	cctccatttttgagaaagatattcatcttgcatcaccagcagtggagattcttccttccaaggcatttcacactgacaaggaaggcggcgaaaatatcga[C/T]cattttaagggaatagtaagtgttgctgatatcatagggttcggtggttcagagacaatatcctcaaaacaggatggctatctaaaRtcctgggccagtt

fava_c17710_445	fava_c17710	445	G/A	gcgaaaatatcgaYcattttaagggaatagtaagtgttgctgatatcatagggttcggtggttcagagacaatatcctcaaaacaggatggctatctaaa[G/A]tcctgggccagttccattgatcttgtcagtgttcttaagaatgagattcgcgatgggcaactgtccttcagaggaaaaagagtacttgagctcaattgta

fava_c17710_632	fava_c17710	632	G/A	tgagctcaattgtagctatggacttcctggaatctttgcttgcttgaagggagcttctatggtgcattttcaagatcagaatgcagagactataagatgc[G/A]caactataccaaatgtccttggtaatctgaagcaagctcgtgataggcagagtcgacagccagaaactccccttactccttcaagacagacKctggcgcc

fava_c17710_724	fava_c17710	724	T/G	taagatgcRcaactataccaaatgtccttggtaatctgaagcaagctcgtgataggcagagtcgacagccagaaactccccttactccttcaagacagac[T/G]ctggcgccatcagttaacttttatgctggagactgggatgaattacctgttgtattgtctactgtgaaaaatgatggatatgaagtgacacctggaatga

fava_c17711_1236	fava_c17711	1236	A/T	gcgagaagccgaaagatactctcggaatggttttctccttgtgagaatataggaatcgcagctgagcacggttactttctgagaatgaagcgagacgaca[A/T]atgggaaacctgtgttcctgcaacYgattgtagttggaaacaaatcgcagagcctgtgatgaaactttataccgaaacaactgatggatctaccatcgaa

fava_c17711_1261	fava_c17711	1261	C/T	aatggttttctccttgtgagaatataggaatcgcagctgagcacggttactttctgagaatgaagcgagacgacaWatgggaaacctgtgttcctgcaac[C/T]gattgtagttggaaacaaatcgcagagcctgtgatgaaactttataccgaaacaactgatggatctaccatcgaagataaggagactgcacttgtttggt

fava_c17717_1152	fava_c17717	1152	T/C	agaacctgattgacatgcagtttggtgcactgcagaaggcactgaagatacctcataaggcctcagaagcacgtttgatggtatccaaaaagttccttac[T/C]ctattcagggctggtaaactaggaccttttattcttgatgatgtccctgatgccaaacttgtataaatatatacacgacccctcttatttgtatcaYgtg

fava_c17717_1249	fava_c17717	1249	C/T	tacYctattcagggctggtaaactaggaccttttattcttgatgatgtccctgatgccaaacttgtataaatatatacacgacccctcttatttgtatca[C/T]gtgtattatggcgaatttgactgtaacttgcaaatatcgatggttttagaatcgagcctttcctgtggatgtgtgtaataagtcacattccactatattc

fava_c17717_254	fava_c17717	254	C/T	acaccggcggaggacttatcaactggttcccaggacacatggctgctgccacacgcgccatccgtaaccgccttaaactcgccgacctcgtcatcgaagt[C/T]agagacgctcgtattccgttatcctccatcaatgtcaatcttcaacctcacctttccgctaaacggcgcgtcatcgcactcaacaaaaaagacttagcaa

fava_c17717_809	fava_c17717	809	G/C	ccaagtatctcagacatagagacagggttaaagcttgctctcgcagggtctgttaaagattcagtggtgggcgaggagcgaattgctcaatactttttgg[G/C]agttttaaatacccggggtactccgtttcactggaagcacctcaataataggagagttgatgggattgcaRaYgaaaccgaggaaaatcatagatacgac

fava_c17717_880	fava_c17717	880	G/A	cgaggagcgaattgctcaatactttttggSagttttaaatacccggggtactccgtttcactggaagcacctcaataataggagagttgatgggattgca[G/A]aYgaaaccgaggaaaatcatagatacgacctgaaaaatctaaagcaaaggaaaggtcagcctaacagatccgacttggtatatgtggaggatattgtgtt

fava_c17717_882	fava_c17717	882	T/C	aggagcgaattgctcaatactttttggSagttttaaatacccggggtactccgtttcactggaagcacctcaataataggagagttgatgggattgcaRa[T/C]gaaaccgaggaaaatcatagatacgacctgaaaaatctaaagcaaaggaaaggtcagcctaacagatccgacttggtatatgtggaggatattgtgttgg

fava_c17721_530	fava_c17721	530	C/A	aaaaacccatttcaaaagaagaatcaagaaacaagaatcacaaatccaagaagaaaagatggtggaaaaacgctctttttttcttcaaatggaaatgggc[C/A]cacccccaccaccgttgcgatgatcatcgctacgacgacggcgacagtgatgacattcatcaagcccgggctaaggcctttcgggccacgatttccgggc

fava_c17722_207	fava_c17722	207	C/T	ggtgtacttgtctagaaaagtacacactatgtcaagatggtatgtatcacaagcaagaccaatttgggcgcctgctgccaacattcgttcagcttatcaa[C/T]tttaccgagcaagaagaatctgaatactgggctgttgtatctgctgtttttgagggcaagcaagtggatagtattgcatcccagtcaaaatttgacttga

fava_c17722_433	fava_c17722	433	A/G	catcaataataaggtacatcttttgagaacaatgctttcctgggaagagcaattgcggtctgaagttagtatcagccaagcaaagcatgatgcaaagttt[A/G]atgatgctcggagtcctcttaaccttggtgaagtgttaattatatgttggcctcatggtgaaattatgaggttaaaagctggtagcagtgctgttgatgc

fava_c17723_194	fava_c17723	194	T/G	aataattgtacaaagcgaatgaagaaaaatggctagggaaaaaagccacatatcataaccaatatctcttttgtagtagatttaatgactaaattcatgg[T/G]gagactaaaccctatatataaataatagtccctgaccaatgccataaaatgaaaagcttctaagaccattcaaaaaccgttctattgcaactgtaacttt

fava_c17723_627	fava_c17723	627	A/G	acgtcgatgtcttcttgcacgagccacctccacaatcaacccaacggcaagcatcaaatattgactcattggcttttcttgctttctctggagcaggggc[A/G]ggtgctttggagcatgagtagtccaatgtacaattaggaatgatgcaaggaagaaacttcaagccaccaccatcattagaatcaaaagctttcttccaac

fava_c17724_219	fava_c17724	219	A/G	cttccaaaacaacacaaccatggacggactaaatattccttacaatcctcgtactgtcgacgaagtctttcgggatttcagaggccgcagaactgcatta[A/G]tcaaagccctatctactgatgtcgacgagttcttccggcagtgtgatcccgataaggaaaacctttgtctttatggatctcccagtgagcagtgggaggt

fava_c17724_467	fava_c17724	467	T/C	tgggtattaactttgcgagagatgggatgctagaaaaggattggctgtctttggttgctgtacacagtgatgcttggttattatcagtggctttttattt[T/C]ggtgctaggtttggattcgataaagctgataggaaaaaactatttgatatgataaatgatcttccaacaatctttgaaactattgcgggggttgtcaaga

fava_c17724_716	fava_c17724	716	C/T	aatctgaagcaaagcccaagcaaggttctgaacctcaggcaaaattttcaaaaccaatggcaatgcaaatgcaaatgcaaatgcaatcaaaggaaaatga[C/T]gaggacggtctaggcgtggacgaggaagatgaggaagacgagSaagacatggaagatgacgaagagcacggagatactttgtgtggggcatgcggagaga

fava_c17724_759	fava_c17724	759	G/C	attttcaaaaccaatggcaatgcaaatgcaaatgcaaatgcaatcaaaggaaaatgaYgaggacggtctaggcgtggacgaggaagatgaggaagacgag[G/C]aagacatggaagatgacgaagagcacggagatactttgtgtggggcatgcggagagagctatgaaactaaccagttctggatttgctgtgacatttgtga

fava_c17725_211	fava_c17725	211	T/C	cgagccagttcgacggtaacgccgccttctccggcggcggtttctcggttactcagaccactccgagcggcgactctcccttcactccctccaagaatcg[T/C]gatgcYcaggcYttgcttcctctgacgatcaagcagatcaacgatgcttatcaMgcaagtgatgataaaaccaatctgactattgatggtgttgaagttg

fava_c17725_217	fava_c17725	217	C/T	agttcgacggtaacgccgccttctccggcggcggtttctcggttactcagaccactccgagcggcgactctcccttcactccctccaagaatcgYgatgc[C/T]caggcYttgcttcctctgacgatcaagcagatcaacgatgcttatcaMgcaagtgatgataaaaccaatctgactattgatggtgttgaagttggcacaa

fava_c17725_223	fava_c17725	223	C/T	acggtaacgccgccttctccggcggcggtttctcggttactcagaccactccgagcggcgactctcccttcactccctccaagaatcgYgatgcYcaggc[C/T]ttgcttcctctgacgatcaagcagatcaacgatgcttatcaMgcaagtgatgataaaaccaatctgactattgatggtgttgaagttggcacaatttctc

fava_c17725_265	fava_c17725	265	C/A	agaccactccgagcggcgactctcccttcactccctccaagaatcgYgatgcYcaggcYttgcttcctctgacgatcaagcagatcaacgatgcttatca[C/A]gcaagtgatgataaaaccaatctgactattgatggtgttgaagttggcacaatttctcttcttggtagggtgtgcagcaaagctggacaaatcactgatg

fava_c17725_544	fava_c17725	544	T/C	tggagagtattttaaatggaatgtatgtgcgggtttatggacacttgaaaggctttcagggtaaaaaaaacttgaatgttttctcttttaggcctgtgac[T/C]gacttcaacgagattgcccaccatttcattgaYtgcatatatgttcatctatataattcgaagtttcgggctagcaatcccaatcagcaacatgttccta

fava_c17725_577	fava_c17725	577	C/T	tttatggacacttgaaaggctttcagggtaaaaaaaacttgaatgttttctcttttaggcctgtgacYgacttcaacgagattgcccaccatttcattga[C/T]tgcatatatgttcatctatataattcgaagtttcgggctagcaatcccaatcagcaacatgttcctaattccacacaaatcactcccacgagaggtcatc

fava_c17728_180	fava_c17728	180	T/C	acagtgaagaactgataaaataagcagaagttacataactagaagttaatcaatcaccatacatgagttcttgagatctcatacccaaacactaattagt[T/C]ttttctttcatcatacataaatgcttatacaactcacatgtcaacttaatatattgttttacatagaggaactaacatttaatcaaRcagcacatcatta

fava_c17728_267	fava_c17728	267	G/A	aacactaattagtYttttctttcatcatacataaatgcttatacaactcacatgtcaacttaatatattgttttacatagaggaactaacatttaatcaa[G/A]cagcacatcattatggtgctcttggagtgtgacgaccatttgggccagatggtggataatcatggagttctaaatccatcctttcagtaatgtttaactc

fava_c17729_1194	fava_c17729	1194	T/C	taattcgaagctcttgaacactaatattgaggtttccaagtctagcttttgcagaagcaatagcgtccatcaaagacattcgtttcgcctctttgcggat[T/C]ttttctgcgagtactttaacgagttcatcttcgacttccttcagggtggctttaatcagttctagttgatcttgttttcgacaagtttgttgagctgtga

fava_c17729_510	fava_c17729	510	C/A	agttgtgcttgcaagagcacctctgggagttcagcacatattatcaaatgaggtggcaaacatcatatgtattttatttcctaacctttaaaggcagaga[C/A]tggcgaacagatgaggcagaatcagaagataaaactgccaaatttttccttgtgcgggtgttttgtttctttaaaagtgcactaccRtcaSagaattcca

fava_c17729_597	fava_c17729	597	G/A	ttaaaggcagagaMtggcgaacagatgaggcagaatcagaagataaaactgccaaatttttccttgtgcgggtgttttgtttctttaaaagtgcactacc[G/A]tcaSagaattccaattgatgttcattttctgtggttgtagaatcacttctaccggatacacctggagcagatgaagagataaaggctgattcttcaggYt

fava_c17729_601	fava_c17729	601	C/G	aggcagagaMtggcgaacagatgaggcagaatcagaagataaaactgccaaatttttccttgtgcgggtgttttgtttctttaaaagtgcactaccRtca[C/G]agaattccaattgatgttcattttctgtggttgtagaatcacttctaccggatacacctggagcagatgaagagataaaggctgattcttcaggYtcaac

fava_c17729_696	fava_c17729	696	T/C	cRtcaSagaattccaattgatgttcattttctgtggttgtagaatcacttctaccggatacacctggagcagatgaagagataaaggctgattcttcagg[T/C]tcaactgttgttcgaactgagctcccttgagccaatgttttttgaaatttgctccttataactctaacaaacttaaatagatcatttgttttgtttaatt

fava_c17729_810	fava_c17729	810	G/A	gaactgagctcccttgagccaatgttttttgaaatttgctccttataactctaacaaacttaaatagatcatttgttttgtttaattcatggaccaactc[G/A]tcaataccatcaagggaMggttcacaacttaacaatttgtaggtRtttttatcatggtacacggtaagagagtactcctcattcggaYtMttcaaatcta

fava_c17729_828	fava_c17729	828	A/C	ccaatgttttttgaaatttgctccttataactctaacaaacttaaatagatcatttgttttgtttaattcatggaccaactcRtcaataccatcaaggga[A/C]ggttcacaacttaacaatttgtaggtRtttttatcatggtacacggtaagagagtactcctcattcggaYtMttcaaatctatattcgtgaaSgtgaact

fava_c17729_855	fava_c17729	855	A/G	taactctaacaaacttaaatagatcatttgttttgtttaattcatggaccaactcRtcaataccatcaagggaMggttcacaacttaacaatttgtaggt[A/G]tttttatcatggtacacggtaagagagtactcctcattcggaYtMttcaaatctatattcgtgaaSgtgaacttaaccccacgtccccctttgacatgaa

fava_c17729_898	fava_c17729	898	T/C	atggaccaactcRtcaataccatcaagggaMggttcacaacttaacaatttgtaggtRtttttatcatggtacacggtaagagagtactcctcattcgga[T/C]tMttcaaatctatattcgtgaaSgtgaacttaaccccacgtccccctttgacatgaaaaccgagtacagtgttataccaagaaatggcttgttgtgtttc

fava_c17729_900	fava_c17729	900	C/A	ggaccaactcRtcaataccatcaagggaMggttcacaacttaacaatttgtaggtRtttttatcatggtacacggtaagagagtactcctcattcggaYt[C/A]ttcaaatctatattcgtgaaSgtgaacttaaccccacgtccccctttgacatgaaaaccgagtacagtgttataccaagaaatggcttgttgtgtttcat

fava_c17729_921	fava_c17729	921	G/C	caagggaMggttcacaacttaacaatttgtaggtRtttttatcatggtacacggtaagagagtactcctcattcggaYtMttcaaatctatattcgtgaa[G/C]gtgaacttaaccccacgtccccctttgacatgaaaaccgagtacagtgttataccaagaaatggcttgttgtgtttcatcagtatgatcaatgctcctcc

fava_c17730_360	fava_c17730	360	A/G	ggttgtcctctttgcttgctatcatgattcgatgctcatagtaacgcttcaccgtctccgtcgatctcgcgttatcctctgtcgtcttgcttcaaattcc[A/G]atgaaaactccaccatgccaagcctctattcaccgcacgcgattgcttYtgttttgctcaaaatttgttcaacgttcttcaattcgacatttatgaagaa

fava_c17730_409	fava_c17730	409	C/T	caccgtctccgtcgatctcgcgttatcctctgtcgtcttgcttcaaattccRatgaaaactccaccatgccaagcctctattcaccgcacgcgattgctt[C/T]tgttttgctcaaaatttgttcaacgttcttcaattcgacatttatgaagaaattcgtttcaattttatcgtaagaggatccgaacWaaatcatcgcaagc

fava_c17730_495	fava_c17730	495	A/T	gcacgcgattgcttYtgttttgctcaaaatttgttcaacgttcttcaattcgacatttatgaagaaattcgtttcaattttatcgtaagaggatccgaac[A/T]aaatcatcgcaagcagatccaaacaaaatcaacgcaagcatattcaaacaaaatcaatgcaagcagattcatagagttcacaacacaaagataatatgaa

fava_c17735_1800	fava_c17735	1800	T/G	tggtacctgaagtcgtggttccgaaatggggtgctgtatatatcccttcaattatcactcttctcaatgctgttggaactccaaggtcactccatttact[T/G]gtattttggattctgtttgagaatgtgatgtctctacatcgaacgaaagcaacWattgttggtcttttggagactagtcgagtgaatgaatggattgtca

fava_c17735_1854	fava_c17735	1854	A/T	tcactcttctcaatgctgttggaactccaaggtcactccatttactKgtattttggattctgtttgagaatgtgatgtctctacatcgaacgaaagcaac[A/T]attgttggtcttttggagactagtcgagtgaatgaatggattgtcactgagaagctcggagacgctctcaaggttaaagcaggtacaaaagcactcaaga

fava_c17735_2010	fava_c17735	2010	A/G	tcggagacgctctcaaggttaaagcaggtacaaaagcactcaagaagccccggttccgaatcgaagacaggattcacttgttagaacttggtgttggaat[A/G]tatctcctctttatcggctgctacRatgttatgttcgggaaaaaccatttcttcatctttctctttatccaagcgatcgcctttttcatcatggcatttg

fava_c17735_2035	fava_c17735	2035	G/A	aggtacaaaagcactcaagaagccccggttccgaatcgaagacaggattcacttgttagaacttggtgttggaatRtatctcctctttatcggctgctac[G/A]atgttatgttcgggaaaaaccatttcttcatctttctctttatccaagcgatcgcctttttcatcatggcatttggatatgttggaaccttcattcccaa

fava_c17735_2193	fava_c17735	2193	T/C	ttttcatcatggcatttggatatgttggaaccttcattcccaactcctagaaggacttagtaacaatttcatcagtcaatcgcgttgtaatatattgtgt[T/C]ttcagcattgttagtttaaaggaaacggttgttattctttgaggttttgcataagggtgagtgactcttgaagaRtttagttttcttgtgaatattcaac

fava_c17735_2268	fava_c17735	2268	G/A	tcaatcgcgttgtaatatattgtgtYttcagcattgttagtttaaaggaaacggttgttattctttgaggttttgcataagggtgagtgactcttgaaga[G/A]tttagttttcttgtgaatattcaacaaaaacataaagaaattatattaagtccttctcatggaaggtaagtcctcagaataagaacaaggcagcaatcta

fava_c17735_261	fava_c17735	261	C/A	acacaaacagaacattatttgacttttcacacaagtcttgttttgtcacaaaccttctttatttccatttcagaacaaacatacttagccttatgaaaag[C/A]taacaaaaattgtttgttatttttgagatctagctatggtttttgttctctctctagagtaactcgcattgttttgcgtggaccagaagcaaaacataac

fava_c17735_645	fava_c17735	645	A/G	atcgtttttcttctactactactataccttttgcttttcatggagctaaagatgacatcaccatgcaattttctttgatttggactcaaatcaaagcacc[A/G]ttgatagttccattgctaagaatatcagtgtttttgtgtttgataatgtcagtgatgatggttattgagagggtttatatgggtattgtcattagtcttg

fava_c17735_882	fava_c17735	882	G/T	aatgggaacctattaaggatgatattgagttgggaaactcttgttatccaatggttcttgttcaaatccctatgtataatgaaagagaggtctatcaatt[G/T]tcaattggagctgcatgtggcctttcttggccctctgatagRatcattatacaagttcttgatgactcYactgacccaacRatcaaggagttggtgcaag

fava_c17735_924	fava_c17735	924	G/A	gttatccaatggttcttgttcaaatccctatgtataatgaaagagaggtctatcaattKtcaattggagctgcatgtggcctttcttggccctctgatag[G/A]atcattatacaagttcttgatgactcYactgacccaacRatcaaggagttggtgcaagtagaatgtcaaagatgggcaaacaaaggtgtgaacataaagt

fava_c17735_951	fava_c17735	951	C/T	ctatgtataatgaaagagaggtctatcaattKtcaattggagctgcatgtggcctttcttggccctctgatagRatcattatacaagttcttgatgactc[C/T]actgacccaacRatcaaggagttggtgcaagtagaatgtcaaagatgggcaaacaaaggtgtgaacataaagtatgaagttagagacaatagaaatggat

fava_c17735_963	fava_c17735	963	G/A	aaagagaggtctatcaattKtcaattggagctgcatgtggcctttcttggccctctgatagRatcattatacaagttcttgatgactcYactgacccaac[G/A]atcaaggagttggtgcaagtagaatgtcaaagatgggcaaacaaaggtgtgaacataaagtatgaagttagagacaatagaaatggatacaaagcagggg

fava_c17741_1081	fava_c17741	1081	T/C	agaaaagtgatagatacgcttgagagatatcgtcccaaatgtcaagtatccctccactccactttggtctattttcaacagtccttcgtttatctttcgg[T/C]gcaaatgaatatttcctctctaatgatgtcactctcatttgctcttgttttttatcagcagtaatttgaacctgtgattcttcgtccgatccagattcat

fava_c17741_1737	fava_c17741	1737	T/G	cacgcaaaaagcgccatgttcatcggattcctaatccattccatcgacatcttcttaatcgaattccaatcaagtttcttagaaaatataccactgaatc[T/G]ttctctcaagctatgcgaactcggagaaggcacagactgcgacgcctggtccatcaaagtagtaagccgcttgaatttcgcagacgccgaaccaaatttc

fava_c17741_1882	fava_c17741	1882	C/A	cctggtccatcaaagtagtaagccgcttgaatttcgcagacgccgaaccaaatttcaagaagttcatcctattaggaataacactcatcttactcgggaa[C/A]tttctctcgggattttcaccggtcaccaatgttcttYgagaactcgaaatatctatggaagtaaactcttctactttactcatactcccattattattat

fava_c17741_1919	fava_c17741	1919	C/T	agacgccgaaccaaatttcaagaagttcatcctattaggaataacactcatcttactcgggaaMtttctctcgggattttcaccggtcaccaatgttctt[C/T]gagaactcgaaatatctatggaagtaaactcttctactttactcatactcccattattattattacactcctcaatttcttctttaccattatcaacaga

fava_c17741_212	fava_c17741	212	C/A	gaatgaatgaactgtacaatgctctccacaatcatatcaatggatgtaaagaaagcctcaaacaagaatgaaagtactttgtttttgtcacagacatcat[C/A]taaatcaattacatgctgctgctgRtaatcaRcagctaaattgtgcctcctcaaatacacgttcgtcggtcgatataaaaactacgaKcaggttgaaagg

fava_c17741_237	fava_c17741	237	A/G	ccacaatcatatcaatggatgtaaagaaagcctcaaacaagaatgaaagtactttgtttttgtcacagacatcatMtaaatcaattacatgctgctgctg[A/G]taatcaRcagctaaattgtgcctcctcaaatacacgttcgtcggtcgatataaaaactacgaKcaggttgaaaggattctgagtctttttcttcaggaac

fava_c17741_244	fava_c17741	244	A/G	catatcaatggatgtaaagaaagcctcaaacaagaatgaaagtactttgtttttgtcacagacatcatMtaaatcaattacatgctgctgctgRtaatca[A/G]cagctaaattgtgcctcctcaaatacacgttcgtcggtcgatataaaaactacgaKcaggttgaaaggattctgagtctttttcttcaggaactaagcag

fava_c17741_300	fava_c17741	300	T/G	cacagacatcatMtaaatcaattacatgctgctgctgRtaatcaRcagctaaattgtgcctcctcaaatacacgttcgtcggtcgatataaaaactacga[T/G]caggttgaaaggattctgagtctttttcttcaggaactaagcagattctctttgtatcaatggcggagttggtggagtcattgttacatctttatctttt

fava_c17741_442	fava_c17741	442	A/G	cagattctctttgtatcaatggcggagttggtggagtcattgttacatctttatctttttcagaaggttcttcttttagagtagatagtgaaccatctgg[A/G]ctatgatgttcggtaaagaaactgctagaacttagaggactcgacggtttgaatgtaggagacgaattgacaccgagaggggaactcgtaccYgatttgg

fava_c17741_535	fava_c17741	535	C/T	catctggRctatgatgttcggtaaagaaactgctagaacttagaggactcgacggtttgaatgtaggagacgaattgacaccgagaggggaactcgtacc[C/T]gatttggttgaagccacaccctcacggcgcaaaggtgaaattggtggttggtcaacagtgctaatttctttgctgaagaatttgtcgtctacaatatgat

fava_c17749_691	fava_c17749	691	C/T	agcgagccccgtgaggtcctcaccggtggccatggtccagatctcgcgccacctcacttcatccctccggccgattctttccgtcgccacctctctttcg[C/T]cttcgatcagtacatgatgttttgtgttggattaattaatttttgcttgagttttactgttatgacagatttgcaaaaagtgaagaccatgactttgttc

fava_c17751_1054	fava_c17751	1054	A/G	tatgatcatcagtccttctatgctctcttttccaatacttggagtgtcgatcatccacattgcgatcaagtggaatgtctttcattaacagcctaatttt[A/G]ggttttttcctccatgtattatggtcaagcatgctctcttccttaagttctgtcaccttttttcKggtatcaatttttggatatgagccaacatccaaag

fava_c17751_1119	fava_c17751	1119	T/G	tcaagtggaatgtctttcattaacagcctaattttRggttttttcctccatgtattatggtcaagcatgctctcttccttaagttctgtcaccttttttc[T/G]ggtatcaatttttggatatgagccaacatccaaagccgagtatccagttccttccgtggtaagatttttctgcctgacgcgtcttttaagttcttccatc

fava_c17751_210	fava_c17751	210	G/A	tttaactgatattctcttatttcttaagataaaactaagttaagcattaataagctagttttagaaacatttcaaatgaataatgcacttacaagttaca[G/A]cattaattttaaatttctgactcagtcctcaYtagttgatggctttgaacatccacaaacacgcagttttcttcgccttttgctgtttaYccttccaatt

fava_c17751_242	fava_c17751	242	C/T	aactaagttaagcattaataagctagttttagaaacatttcaaatgaataatgcacttacaagttacaRcattaattttaaatttctgactcagtcctca[C/T]tagttgatggctttgaacatccacaaacacgcagttttcttcgccttttgctgtttaYccttccaatttggataaactccctcagcaatatacctgtttt

fava_c17751_300	fava_c17751	300	C/T	acaagttacaRcattaattttaaatttctgactcagtcctcaYtagttgatggctttgaacatccacaaacacgcagttttcttcgccttttgctgttta[C/T]ccttccaatttggataaactccctcagcaatatacctgtttttccagagattctgtttttcactttgatgttgttctcctcggccaatttcagcaatatg

fava_c17751_912	fava_c17751	912	C/A	tcaacacctaattccttctctatgtccaattctgaggagtaatttaaatatcttcctgaattgtctgaaagatggcatattataacatcctcgattggag[C/A]agatactggttcatgatcattaRtttcgcataactctaacatatgatcatcagtccttctatgctctcttttccaatacttggagtgtcgatcatccaca

fava_c17751_935	fava_c17751	935	G/A	gtccaattctgaggagtaatttaaatatcttcctgaattgtctgaaagatggcatattataacatcctcgattggagMagatactggttcatgatcatta[G/A]tttcgcataactctaacatatgatcatcagtccttctatgctctcttttccaatacttggagtgtcgatcatccacattgcgatcaagtggaatgtcttt

fava_c17754_1206	fava_c17754	1206	T/G	tacatgtaattgtaaaaattgaaagtgcagactctataccgaacttgcattcaattattaccgcttctgatggggctatggttgcaagaggagatcttgg[T/G]gcagaactccctattgaggaggttccgcttttgcaggaagagataattaggatatgccgtagcatgggaaaagctgttattgtggcaacaaatatgctcg

fava_c17754_2021	fava_c17754	2021	C/T	gcctccgatacttttataagttcacattctctttttggtgtgatagtgataccaatatattttttttgcttcataggtttcatcttttcttcatttattg[C/T]cttgttttcattttttgtttcttctgtaaactcttttatggttcaatagaacgtgtcagatgttggttcatttcagagaaagttttgatgagaaatcaaa

fava_c17754_546	fava_c17754	546	G/T	gtggaatgtggtctaaaccagtagttagaagaaagactaagattgtttgtactattggtccttctactaatacaaaggaaatgatttggaagttggcgga[G/T]gctgggatgaatgttgcgcgtatgaatatgtcgcatggagatcatgcttctcataagaaagttattgatttggttaaggaatataatgcacaatcaaaag

fava_c17754_693	fava_c17754	693	G/A	cttctcataagaaagttattgatttggttaaggaatataatgcacaatcaaaagataatgtaattgcaatcatgcttgacaccaagggtcctgaggttag[G/A]agtggggatttacctcaaccaattttgttaaaatctggacaggagtttacttttactatccagagtggtgttggaactgcagattgtgttagtgtgaact

fava_c17757_1752	fava_c17757	1752	T/C	aagattggggtaatggtgatataccaaagagtggcggaattactagcacacctagtatgagaggaaccgtttgttacattgctcctgaatatggcggcgg[T/C]ggacaattatctgagaagtgtgatgtgtatagtttYggYgttctgcttttggttttggttgctggaagaagaccacttcaagtaacagcatcaccaattt

fava_c17757_1788	fava_c17757	1788	T/C	gaattactagcacacctagtatgagaggaaccgtttgttacattgctcctgaatatggcggcggYggacaattatctgagaagtgtgatgtgtatagttt[T/C]ggYgttctgcttttggttttggttgctggaagaagaccacttcaagtaacagcatcaccaatttcagaatttgagagagcaaatctaatttcatgggcta

fava_c17757_1791	fava_c17757	1791	C/T	ttactagcacacctagtatgagaggaaccgtttgttacattgctcctgaatatggcggcggYggacaattatctgagaagtgtgatgtgtatagtttYgg[C/T]gttctgcttttggttttggttgctggaagaagaccacttcaagtaacagcatcaccaatttcagaatttgagagagcaaatctaatttcatgggctagac

fava_c17758_288	fava_c17758	288	A/G	atagaggaattttcgagtttttcggttgggtttatcacttgggagtgaattcaattggtcgtgagtattgtcatctccgattccttttcattaggggaaa[A/G]tatgtttctatgtacaagcgtgatcctcatgaaaaccacgccattaaacctattaggaaaggagttgtaggacccacactaatggtggaggagctaggtc

fava_c17759_1128	fava_c17759	1128	G/A	gttcagtgaaacatgtgaagttgatgaagatgatgccattgctatggtcccctgaaattcatgctcatcagttcgagcctttgtaattatctgagcagtt[G/A]ctatttatgtaagttgcagcaggttatttatttcaYgaacttcatttgtatatgattttgagcatcattgcagtatctacaatgcccatatgagaggctR

fava_c17759_1164	fava_c17759	1164	T/C	cattgctatggtcccctgaaattcatgctcatcagttcgagcctttgtaattatctgagcagttRctatttatgtaagttgcagcaggttatttatttca[T/C]gaacttcatttgtatatgattttgagcatcattgcagtatctacaatgcccatatgagaggctRtgctgtaatttatttctactgaacttttccaagact

fava_c17759_1228	fava_c17759	1228	A/G	RctatttatgtaagttgcagcaggttatttatttcaYgaacttcatttgtatatgattttgagcatcattgcagtatctacaatgcccatatgagaggct[A/G]tgctgtaatttatttctactgaacttttccaagacttcttttttattttgttgtgtcatctgagcactatgttgctcattttctttcacgggttgtcaat

fava_c17759_703	fava_c17759	703	C/G	tcgttcctaaatcgtgctctggagaacgagatgtctccgatattagttgttgctaccaacagaggcatcacaactattcgtggcacaaattacaaatctc[C/G]acatgggattcctattgatctacttgatcgtcttctcattatctcgactcaaccttataccgaggatgaaattcacaagattttggatattagatgccaa

fava_c17759_941	fava_c17759	941	A/G	tgttaaccaaaataggtgtagaaacatccctgcgatatgccattcatctgatcacagcagctgcactggcctgccaaaagcgaaagggaaagacggtgga[A/G]ttggatgatataaaccgggtttacaatttgtttttggatgtcaaaagatcgacacagtacttgatggaatatcaaagtcaatacatgttcagtgaaacat

fava_c17760_1019	fava_c17760	1019	T/C	tggacactacgcgtttccagtattcataatcagcagcaaaatcttcgggagcaggctttccataaactccaacttcagagtttgataaccagtaaggtga[T/C]tggccgagtcttgctggccatttctcaggccactgtgacccacgttctgtagaactcactggcgctttgtgcatgcatgtttgcaatggaacgttccatg

fava_c17760_1151	fava_c17760	1151	A/G	actgtgacccacgttctgtagaactcactggcgctttgtgcatgcatgtttgcaatggaacgttccatgcagcattaggatcatcagagtcttgacatag[A/G]gaaggctcattcttggaacgtttctcataacactcattagaagttggcttcctgtatacagctataccaactccattaagtttatccttgctgatattta

fava_c17760_2180	fava_c17760	2180	T/C	cctgagttgaaaaagaaccattttgagttgtactttcattcagaagctctgactgggtcccagaagggagtacctcattagagccctggttgttggtatt[T/C]tcttctgttttcttctcattagagctttcattagacccctggttatcactttgatctaccttttcttctgttttattatctgttgcttcattcgagtcat

fava_c17760_2441	fava_c17760	2441	G/C	gtcctttaccatctgattcagatttcttctcactctcatcagaataggacttcttctcactcacatcagaatccaacttcttctcactcacatcagaatc[G/C]Racttcttactgtcatcagaatcgaacttcttttcgccctcatcagaatcagatttcttgttatcctcatcaaaaccaggtttgttattgctatcatctg

fava_c17760_2442	fava_c17760	2442	G/A	tcctttaccatctgattcagatttcttctcactctcatcagaataggacttcttctcactcacatcagaatccaacttcttctcactcacatcagaatcS[G/A]acttcttactgtcatcagaatcgaacttcttttcgccctcatcagaatcagatttcttgttatcctcatcaaaaccaggtttgttattgctatcatctga

fava_c17760_2585	fava_c17760	2585	T/C	cagaatcagatttcttgttatcctcatcaaaaccaggtttgttattgctatcatctgaatcagatgacttcactttctgagttgaatcggcatataccag[T/C]ttcgtatcttcaggagacttatcagaaataatttcctgtttttctggtataacaggaWtgttttcggatgagacactgctatcctttttgatttcatcct

fava_c17760_2643	fava_c17760	2643	A/T	atcagatgacttcactttctgagttgaatcggcatataccagYttcgtatcttcaggagacttatcagaaataatttcctgtttttctggtataacagga[A/T]tgttttcggatgagacactgctatcctttttgatttcatcctgaggcagatctccctgcttatcttcaaactgctgagcattactattaccggtatcact

fava_c17760_2816	fava_c17760	2816	G/A	gctgagcattactattaccggtatcactagtggcttcacttgcctgatccttcacctcactctgttctttcacctcattcttattattcaccgaagactc[G/A]tctacattttgcacaggaactaccgaatacgatgtcatcatccatagcccaaccaagcatagagccacaaacacaacaatagtcaccgtcgaacagtaac

fava_c17760_396	fava_c17760	396	T/C	ccaagagaaaaaacaaatgcctaacttaacaatacatgaataatggcagaaacacacttacacaagaattttgagcaaccaaaaccaaaaatcacctaac[T/C]cctgttggcatatcgaccatacttaagcaatagcatactctacagtttccaattctgtaggccgccacatggacttctggatgtataaaaagccctctgt

fava_c17760_542	fava_c17760	542	C/T	tttccaattctgtaggccgccacatggacttctggatgtataaaaagccctctgtttctttagagtaggtcatgcgaacctcccactgcatagacttcag[C/T]aagctctgaagctcattaatgatctcagcagtgtctcggacaataagctttccgccaggcctgagaattctatcaacctcggccactacagcttcaaact

fava_c17766_1166	fava_c17766	1166	T/C	agattaataaaaaccaagttcatgattttgttcttgtaggagggtctactagaattccaaagattcaacaactattgaaggaaatgtttagagtcaacgg[T/C]gaaatcaaagagccttgcaaaagcattaatcctgatgaagctgtggcttatggtgcagcagttcaagcagcaatattgaatgatgaaggagacaagaaaa

fava_c17766_1603	fava_c17766	1603	A/G	acacctaggaaggttccaaatatcaatatttgttttgatgttgatgtgaatggtattttagaggttactgctgaagataagacacaagggttgaaaaaga[A/G]gatYactatcatcaacaaggagggaaggttgagttgtgaagagatgaggaggatggtgagggatggagagaagtataaggtagaagatgaggaggcgagg

fava_c17766_1607	fava_c17766	1607	T/C	ctaggaaggttccaaatatcaatatttgttttgatgttgatgtgaatggtattttagaggttactgctgaagataagacacaagggttgaaaaagaRgat[T/C]actatcatcaacaaggagggaaggttgagttgtgaagagatgaggaggatggtgagggatggagagaagtataaggtagaagatgaggaggcgaggaaga

fava_c17769_472	fava_c17769	472	C/T	tcctatactcctctcagtacccaagacccaggtccctcctctagtgatgcgtttactgtgggtatgccgccgtcttgggtggatgattctgaagaaatag[C/T]gacgaatatacagagtgcaaaggttaaaatgtctgagttaacgaaagctcatgcaaaggctttgatgccttcctttggtgatggtaaagatgatcagcgt

fava_c17769_641	fava_c17769	641	T/C	cttcctttggtgatggtaaagatgatcagcgtcagattgagacgctaactcgggaaattacagctctcattagaaaatctgaattgaggctaaaaaaact[T/C]tctgctggtagaggatcttctgaggaatctaatgtcagaaaaaatgtgcagcgttcgcttgctacagaccttcagagcctgtcaatggacctccggcgga

fava_c17771_385	fava_c17771	385	C/T	tcggaatggaatcttggtttggtgaaggaaaacaagaaggttctacagccacggccgcataggaaggttagaaggaaaaggcaggacaaaagggaagaga[C/T]gggaattgatggagagtttgatggtgtgtttgtggcatctgatgatagtagcgattggtgttttttgtctgattcggaatccgatgactcggattggtct

fava_c17771_506	fava_c17771	506	T/G	atggtgtgtttgtggcatctgatgatagtagcgattggtgttttttgtctgattcggaatccgatgactcggattggtctattgggtggttggagcctct[T/G]ggttctgattttgaaagcagtgatgatgatgatgagtctggtcgtgatagttttgctgttttggttccttgttattcacctggctgcgaagagcttgaag

fava_c17771_650	fava_c17771	650	T/A	gtgatagttttgctgttttggttccttgttattcacctggctgcgaagagcttgaaggaacaaacaatgtgcttttgagtgctcttaagaatctccccat[T/A]gaatttRcttcagatgggaagagttacatggaacaatggttggcttctcttgagggtttcggagcttagaatgaaaaaaaacaacttcttcacattgatg

fava_c17771_657	fava_c17771	657	G/A	ttttgctgttttggttccttgttattcacctggctgcgaagagcttgaaggaacaaacaatgtgcttttgagtgctcttaagaatctccccatWgaattt[G/A]cttcagatgggaagagttacatggaacaatggttggcttctcttgagggtttcggagcttagaatgaaaaaaaacaacttcttcacattgatggtccttg

fava_c17772_247	fava_c17772	247	T/C	gaagtgatctatacacgaataataaaatgaattggttaatcggtcagctatttcctttccataatataaaaagtaatgaaaccacagagggttttattca[T/C]caaacagtatatRagaaacatttagttggaggaaatgagtagaagccattttcccccaaagccaatttcctttagtcagcactcgccagagaagatgagt

fava_c17772_260	fava_c17772	260	A/G	cacgaataataaaatgaattggttaatcggtcagctatttcctttccataatataaaaagtaatgaaaccacagagggttttattcaYcaaacagtatat[A/G]agaaacatttagttggaggaaatgagtagaagccattttcccccaaagccaatttcctttagtcagcactcgccagagaagatgagtcaRcgtcaggttt

fava_c17772_350	fava_c17772	350	G/A	aacagtatatRagaaacatttagttggaggaaatgagtagaagccattttcccccaaagccaatttcctttagtcagcactcgccagagaagatgagtca[G/A]cgtcaggtttcttctcctcggcaagYtctgtgttgttatcttcgtcgcttccattctctagaagaaccgcctgctgtctgagttctttRactttaaggaa

fava_c17772_376	fava_c17772	376	C/T	gaggaaatgagtagaagccattttcccccaaagccaatttcctttagtcagcactcgccagagaagatgagtcaRcgtcaggtttcttctcctcggcaag[C/T]tctgtgttgttatcttcgtcgcttccattctctagaagaaccgcctgctgtctgagttctttRactttaaggaattcatcataatgggcccgtcgaagct

fava_c17772_439	fava_c17772	439	G/A	aagatgagtcaRcgtcaggtttcttctcctcggcaagYtctgtgttgttatcttcgtcgcttccattctctagaagaaccgcctgctgtctgagttcttt[G/A]actttaaggaattcatcataatgggcccgtcgaagctctttaaaactacaacctgcatattcatcaatttgttctgtttcRtcttcctcgtcctcagatg

fava_c17772_520	fava_c17772	520	G/A	cctgctgtctgagttctttRactttaaggaattcatcataatgggcccgtcgaagctctttaaaactacaacctgcatattcatcaatttgttctgtttc[G/A]tcttcctcgtcctcagatgatgaccagccgtcagattgcctagttttttttctgctgcaacaggccgcattatcaaaagcagtttcttcaRcttttgctg

fava_c17772_611	fava_c17772	611	G/A	ttctgtttcRtcttcctcgtcctcagatgatgaccagccgtcagattgcctagttttttttctgctgcaacaggccgcattatcaaaagcagtttcttca[G/A]cttttgctgagtcgcttttatgatcatcacattcatcataccctcctcttccaggagatggagagctgtcatcttcgatcataggatgatacggagtctt

fava_c17775_1032	fava_c17775	1032	T/C	ataaaaggtggttgtttacacattagtgagaacataagctgtaaattttagacataagaatccactagattgttgggtaagtgcatcagcaaaggtttta[T/C]tacagcacctcattgttgcaaaaacttgggaagtcctgtaaccctgaagtgtgtcaggtgtccgtcccgacaaatatgattgtattcgattatatttttt

fava_c17775_332	fava_c17775	332	C/T	tactcactttttctcccctaactgttcccatgactacctcgccgcaccgttccggcggcgaaagcgacggcaacggcgacaaaagaccaagacacttcga[C/T]acaaagacaaagaccatctgctggacaaaggcagatactgtacccggccgccacccagaaaggtggcgtaaagacgccgccggtaacgttgtctgcaagc

fava_c17775_745	fava_c17775	745	A/G	gctgtttatggtgatgtaatccggccaggaaaccagtgtagatgcagaactgttgatgaaatgcttggaaaatttaaggcaaaagcaaaagacaacacag[A/G]tgcttgcaagttgccataggaggatgaacctataataaataaattttcaagaYRacgtgcattagtaaaagaggacgaatcttttacttcactctctttt

fava_c17775_798	fava_c17775	798	C/T	tgatgaaatgcttggaaaatttaaggcaaaagcaaaagacaacacagRtgcttgcaagttgccataggaggatgaacctataataaataaattttcaaga[C/T]RacgtgcattagtaaaagaggacgaatcttttacttcactctcttttcttatctatcagacaaaacattacaattctcactttccKtcattggttgtgta

fava_c17775_799	fava_c17775	799	A/G	gatgaaatgcttggaaaatttaaggcaaaagcaaaagacaacacagRtgcttgcaagttgccataggaggatgaacctataataaataaattttcaagaY[A/G]acgtgcattagtaaaagaggacgaatcttttacttcactctcttttcttatctatcagacaaaacattacaattctcactttccKtcattggttgtgtaa

fava_c17775_884	fava_c17775	884	G/T	ataaattttcaagaYRacgtgcattagtaaaagaggacgaatcttttacttcactctcttttcttatctatcagacaaaacattacaattctcactttcc[G/T]tcattggttgtgtaaagattgacatttagacagtYtatactcgaYgaataaaaggtggttgtttacacattagtgagaacataagctgtaaattttagac

fava_c17775_919	fava_c17775	919	T/C	gacgaatcttttacttcactctcttttcttatctatcagacaaaacattacaattctcactttccKtcattggttgtgtaaagattgacatttagacagt[T/C]tatactcgaYgaataaaaggtggttgtttacacattagtgagaacataagctgtaaattttagacataagaatccactagattgttgggtaagtgcatca

fava_c17775_929	fava_c17775	929	T/C	ttacttcactctcttttcttatctatcagacaaaacattacaattctcactttccKtcattggttgtgtaaagattgacatttagacagtYtatactcga[T/C]gaataaaaggtggttgtttacacattagtgagaacataagctgtaaattttagacataagaatccactagattgttgggtaagtgcatcagcaaaggttt

fava_c17783_430	fava_c17783	430	C/A	gtcgcagcacctttaccggattccgaacaccggaacccgccgccgaatgaccacccggcagtggtggttaacagagccatgaggaatgaccctcctccaa[C/A]cgcaacgaatctaggccatRccgtccccggaggaggccgtgaagacggcggaggaaggagcagaccaaggacaaccacgccgacgactaccgcgtggtcg

fava_c17783_450	fava_c17783	450	A/G	ttccgaacaccggaacccgccgccgaatgaccacccggcagtggtggttaacagagccatgaggaatgaccctcctccaaMcgcaacgaatctaggccat[A/G]ccgtccccggaggaggccgtgaagacggcggaggaaggagcagaccaaggacaaccacgccgacgactaccgcgtggtcgaaaagtgatgtaacgacagg

fava_c17784_2231	fava_c17784	2231	C/T	tggcctcttccaacaaagcaacagagagatcatccttttctttgtttagaatagcagccatcttttctgcctctctagcacgtgtttgagatgctttcag[C/T]gccttaagaagctctctcttatccttttcgtcatcaccattttcgagtccacagtgtttactatcttgctctttttctttgYctgaaatattacagacct

fava_c17784_2313	fava_c17784	2313	C/T	tgtttgagatgctttcagYgccttaagaagctctctcttatccttttcgtcatcaccattttcgagtccacagtgtttactatcttgctctttttctttg[C/T]ctgaaatattacagaccttattcatagataatatgacagttttatctgaacctagaaaaaccttggaaggtggaggaagatcacagttctgcataaggtc

fava_c17785_113	fava_c17785	113	A/T	tccttcaccaatcaaactcacaagaaagaaagaaaaaaaacattctcaatggggagcacaagaagtgaatattgtgtcttcatgaactatgatcgagaat[A/T]caatcgacttcaaaataatcgaactgagagaggggcaattgagcttgatttgtaccttagtagaaagcatgatgaggttttagcaagaagtttagaggca

fava_c17785_292	fava_c17785	292	G/C	tttagcaagaagtttagaggcaggaagctacaacaagataacatctttggctattgttgatgctttcatagtggaaatcagtgaggaacaagctaatgtg[G/C]ttagatcagcaaatggRgtgagagttgtggagaaaaatgaagtggtatagttaagcaggttcaagaaagagattcagaagttgttttggatgaagaactt

fava_c17785_309	fava_c17785	309	A/G	aggcaggaagctacaacaagataacatctttggctattgttgatgctttcatagtggaaatcagtgaggaacaagctaatgtgSttagatcagcaaatgg[A/G]gtgagagttgtggagaaaaatgaagtggtatagttaagcaggttcaagaaagagattcagaagttgttttggatgaagaactttgttttagaataattgc

fava_c17788_1096	fava_c17788	1096	C/T	ctcttgcgagctttaatgcaaacttcagatcatttctcgccacaagccttggactgcagtattcaaattttctttggagtagattcacctgattaactaa[C/T]gtagcaatcttccgctgtcttgcaggccattttccacttaaaaattggaaaagtggatggtaatccccaaaataaacaaSacgcttcaaatccaaagcaa

fava_c17788_1176	fava_c17788	1176	C/G	agattcacctgattaactaaYgtagcaatcttccgctgtcttgcaggccattttccacttaaaaattggaaaagtggatggtaatccccaaaataaacaa[C/G]acgcttcaaatccaaagcaataacagcattgaaagcttcaatcaaagccttaagctccgcagcaattttactagttccattcccaaccaaagatttcgaa

fava_c17788_1663	fava_c17788	1663	A/G	cctcgcgcattactctctcgctctgttctctatcgttcatctcgatttccattctagcgagctcttccaattgcagagacggtgctttgaaaacgtcgcc[A/G]tcgtMgaggggaggttctacgagcatagcgtccatggaggaggagggatgatcagtgagggaagcSgcgagggcttcctgtagctggagatggaaggcga

fava_c17788_1668	fava_c17788	1668	C/A	cgcattactctctcgctctgttctctatcgttcatctcgatttccattctagcgagctcttccaattgcagagacggtgctttgaaaacgtcgccRtcgt[C/A]gaggggaggttctacgagcatagcgtccatggaggaggagggatgatcagtgagggaagcSgcgagggcttcctgtagctggagatggaaggcgaagtcg

fava_c17788_1729	fava_c17788	1729	C/G	ccaattgcagagacggtgctttgaaaacgtcgccRtcgtMgaggggaggttctacgagcatagcgtccatggaggaggagggatgatcagtgagggaagc[C/G]gcgagggcttcctgtagctggagatggaaggcgaagtcgagatcggattccatggattccgctgccgtgagttcgcggcgttgcgctgttaataagaagt

fava_c17788_205	fava_c17788	205	A/G	aactaacttttccaatgtaaaaagaaacatttacaagtaataatccaaaccgaccgttcaaaaaaaaaaatcaaacatacggcaagccagttaagttgtc[A/G]gtttcctaataaaaatcatcttcaaaatcagagtcttcttcatattcctcgacatgatgWtcttctaaccaaatattttcctcttcccaaagtggacaag

fava_c17788_265	fava_c17788	265	T/A	aaaaaaaaaatcaaacatacggcaagccagttaagttgtcRgtttcctaataaaaatcatcttcaaaatcagagtcttcttcatattcctcgacatgatg[T/A]tcttctaaccaaatattttcctcttcccaaagtggacaagaacatgttgcctttttgtccttccactcagctccacatttatagcaaaattcgtaactac

fava_c17788_436	fava_c17788	436	G/A	ctccacatttatagcaaaattcgtaactacatctgcaagtcatatggtagcagccttcagcaagttcaatcatatggctacacttcacgcactgtctcca[G/A]agactccttgatgccagagacttcaacctaatatcttctgctggagaattaggattcaaccttttgtaagtataacatgtcataccactatgccaaggga

fava_c17788_619	fava_c17788	619	G/A	taccactatgccaagggaccttgcagccgaaacagaaaagaccatgacatttcacgcatttcttgggtaatgaatgttcagagcccataagactttgaga[G/A]taatctaaaacttcactttttgacattaatgcagagcatcttggataaggacagtaKattttctcagtaggcggaattgaagcttccaattttcgttgct

fava_c17788_676	fava_c17788	676	G/T	atttcttgggtaatgaatgttcagagcccataagactttgagaRtaatctaaaacttcactttttgacattaatgcagagcatcttggataaggacagta[G/T]attttctcagtaggcggaattgaagcttccaattttcgttgctgcatggtttcaaYtaacttagaagtcaagaatttccggcaactctcaattagaagtt

fava_c17788_732	fava_c17788	732	C/T	tcactttttgacattaatgcagagcatcttggataaggacagtaKattttctcagtaggcggaattgaagcttccaattttcgttgctgcatggtttcaa[C/T]taacttagaagtcaagaatttccggcaactctcaattagaagttcattcttgcatccatcgtgagggcactttggcaccattccatgaagcaacttcacc

fava_c17788_939	fava_c17788	939	T/C	tattgcttcatgcaagaaaagcaatacctgtgttgacagtcatcaactgaaaaaatttgactaggatcagtatcctccatacaaatgacacaagtttcat[T/C]cagactgtttaaactgctagattcagcaggcctattggactgagaatcaattgcatctcttgcgagctttaatgcaaacttcagatcatttctcgccaca

fava_c17789_2141	fava_c17789	2141	G/T	ttagtaaagcttctacattgcttctcgatttgaaatccgagttaaatgcctatatggaatctaagtcaaaccaagaaggcggcgatgaagaaggagtctc[G/T]aaagcggaactggagaaaaaatcacacaacgaaatacaagaagcaattgcatcggcgaaaaaggaacttgaggaagtaaagcttaacatagagaaagcta

fava_c17789_652	fava_c17789	652	T/C	gctgaagattctgaacctgaagccttagaagatataaataacagacaacaagatggtggttcaactgctaccgctagcggcaacgttgataatcttgtta[T/C]ggagcYctcaacatcctcttctggaacaaacgagttgccgattgaacctatagattcacctcgagacggagttattgatgcccctctagaaaatttagtt

fava_c17789_658	fava_c17789	658	C/T	gattctgaacctgaagccttagaagatataaataacagacaacaagatggtggttcaactgctaccgctagcggcaacgttgataatcttgttaYggagc[C/T]ctcaacatcctcttctggaacaaacgagttgccgattgaacctatagattcacctcgagacggagttattgatgcccctctagaaaatttagtttcaacc

fava_c17790_1404	fava_c17790	1404	T/G	tcatattcggaaacgcggctgcagtacttcaaagctgtaacatctacgcaagaaaaccgatgaaaaatcagaagaatgctgtaacggcacaaggaagaac[T/G]gatccaaatcaaaacacaggaatctcaatacaaaattgtaggattgatgcRgcgccagacttagcggaagatatgaattcgactaagaattatttgggaa

fava_c17790_1455	fava_c17790	1455	G/A	gaaaaccgatgaaaaatcagaagaatgctgtaacggcacaaggaagaacKgatccaaatcaaaacacaggaatctcaatacaaaattgtaggattgatgc[G/A]gcgccagacttagcggaagatatgaattcgactaagaattatttgggaagaccatggaaagtttattcaaggactgtttatttgcagtcatatattggtg

fava_c17790_1614	fava_c17790	1614	A/T	aagtttattcaaggactgtttatttgcagtcatatattggtgatttgattcaaccttctggatggttagagtggaatggaacagttggattggatacact[A/T]ttttatggtgagtttgataatcatggacctggttctatgaccaataatagggttcaatggcatggttataatttgttgactcctaatcaagcttggaatt

fava_c17790_338	fava_c17790	338	A/G	tcctctccgaccgacccctaccacttaggcaaattctcaataaaacaaagcctaaaacaagccaaaaggctatccaaagtattcaaagactttctcaaaa[A/G]ccatcaccaatcatctccccttaaccacgccgagttcgcggccctagacgattgtcgcgaactaaaccaacttaacgtagactacttagagttagtaaaa

fava_c17790_546	fava_c17790	546	C/T	ttaaatcagccgattcatcatctttcaataacaccgatgtgttggtggaaaaagttgaaacttatcttagtgccgtggctacaaatcactatacttgtta[C/T]gacggtttggttgtgacgaaaagtaacattgctaattcttttgctgttcctttgaStaatgttacgcaattgtatagtgtttcacttgggttggctatgc

fava_c17790_602	fava_c17790	602	G/C	gaaacttatcttagtgccgtggctacaaatcactatacttgttaYgacggtttggttgtgacgaaaagtaacattgctaattcttttgctgttcctttga[G/C]taatgttacgcaattgtatagtgtttcacttgggttggctatgcaggctttgaataaaaatattaagaaacgtaagactcgtaaacatggtcttcctact

fava_c17790_735	fava_c17790	735	A/G	ggttggctatgcaggctttgaataaaaatattaagaaacgtaagactcgtaaacatggtcttcctactaagacttttaaggtcagacagccacttcaaaa[A/G]ctcattaagcttcttagaacaaaattcagttgcaacaaatcatcSaactgtacaagaaccgaaaggattatagaagaaagtgaaaacaaaggaattctac

fava_c17790_780	fava_c17790	780	C/G	ctcgtaaacatggtcttcctactaagacttttaaggtcagacagccacttcaaaaRctcattaagcttcttagaacaaaattcagttgcaacaaatcatc[C/G]aactgtacaagaaccgaaaggattatagaagaaagtgaaaacaaaggaattctactcaaagaatatgttattgtaagccttgatggaagtgaaaacttta

fava_c17792_162	fava_c17792	162	T/G	ataaccataacataataaYcctcaRgaagagtatcaatgctccaatgcagaatctaccaaatgtaaaaccctaaaatgtcattcaaaacaaaaatctaga[T/G]aagaaaacagaaaattagggattcgaaattcgaaattcgaaYacgagatctaatggaacaatctccatttataatcccaatcacactcagtaacttgaaa

fava_c17792_204	fava_c17792	204	C/T	caatgcagaatctaccaaatgtaaaaccctaaaatgtcattcaaaacaaaaatctagaKaagaaaacagaaaattagggattcgaaattcgaaattcgaa[C/T]acgagatctaatggaacaatctccatttataatcccaatcacactcagtaacttgaaacgcattactcaaagaacttccattgtttgatttcagaggaac

fava_c17792_751	fava_c17792	751	T/C	ttgtttttggtgaaattgaattgagaaagagatgcatcggtggcggtgaagtggaccacgttaggacggacgatgagccagaagaggaagccgaggatta[T/C]gacgatgatgaggattgtggtgagRattttgcagatgatgctcaatatgcagttgaagatgcagccgcaacagccgcggaagcagccgcagaggcagctg

fava_c17792_776	fava_c17792	776	A/G	aaagagatgcatcggtggcggtgaagtggaccacgttaggacggacgatgagccagaagaggaagccgaggattaYgacgatgatgaggattgtggtgag[A/G]attttgcagatgatgctcaatatgcagttgaagatgcagccgcaacagccgcggaagcagccgcagaggcagctgcagaagccgcaggaggagtcgttgc

fava_c17792_80	fava_c17792	80	C/T	aaaggaaacacttttagatcaacaccagccatactaatattattaatatcaagataaaatcataaccataacataataa[C/T]cctcaRgaagagtatcaatgctccaatgcagaatctaccaaatgtaaaaccctaaaatgtcattcaaaacaaaaatctagaKaagaaaacagaaaattag

fava_c17792_86	fava_c17792	86	G/A	aaaggaaacacttttagatcaacaccagccatactaatattattaatatcaagataaaatcataaccataacataataaYcctca[G/A]gaagagtatcaatgctccaatgcagaatctaccaaatgtaaaaccctaaaatgtcattcaaaacaaaaatctagaKaagaaaacagaaaattagggattc

fava_c17794_276	fava_c17794	276	T/C	tatgcaaaacttatccatcttcactctaattcttctaattccacgaccaatcttcatgcgtattacttcatgacttaaccatgcctttgccacttctcaa[T/C]ttccaaacctattcaagcataaaggaaaaagaggtgattaatatcaagactgccttaattggagaaaaattaagtaacctgctttccctttaaaattcat

fava_c17798_321	fava_c17798	321	T/G	gcttttcggccttcgtttctaactctattcttctttcaatttctgcagtctgtgcattttcttcagctttggcatcattgtaacggttacttttgctaca[T/G]agtaagcccatattttctgttaaaaaacacagcatagaagatgactactcaagctcccacagaaatcacagtatttcaatgcccttctctaatatgtata

fava_c17800_1354	fava_c17800	1354	T/G	aagttgtgaaaaacagaacaatacataataaaagtgccgctggtaagattgcatagaaacatggtattttactctttgattcagcaaccaaaaccgtccg[T/G]gacggcgacggcttcatgtctggcattgcaagattcagaaaacagaagaacagaataagcaacgtgcaattgtattaggaagaagaagaataattaggga

fava_c17800_511	fava_c17800	511	C/T	cattttttgtccgatcaccctgagagcagtagccaagttgcatatctagtccccatgcatggaccaagtcattctgaatcatgtaccaaacacaacgcca[C/T]gcagcttttgagaaaacaggagccatYacttctatccacccSgtgcaaggaggagcagtgctattttcatcacagcctttaccatcactacccgcaactt

fava_c17800_538	fava_c17800	538	T/C	agtagccaagttgcatatctagtccccatgcatggaccaagtcattctgaatcatgtaccaaacacaacgccaYgcagcttttgagaaaacaggagccat[T/C]acttctatccacccSgtgcaaggaggagcagtgctattttcatcacagcctttaccatcactacccgcaactttaaatgttctcctgtgcacttccgacc

fava_c17800_553	fava_c17800	553	G/C	tatctagtccccatgcatggaccaagtcattctgaatcatgtaccaaacacaacgccaYgcagcttttgagaaaacaggagccatYacttctatccaccc[G/C]gtgcaaggaggagcagtgctattttcatcacagcctttaccatcactacccgcaactttaaatgttctcctgtgcacttccgacctcctcccaagagctg

fava_c17803_164	fava_c17803	164	T/C	aagcaaaaaccctttgctttcgaagttccaccgaactgaaccgcccgtaaaaccctagaccacgccggaaaacgcaatcggacttcttcgtctgccgccg[T/C]caatcacagttaacatgccttcattcttagggtttagggttcataaaattgaagggttaatttcccttttgaattccaaacaatatttgccaccgtggat

fava_c17803_405	fava_c17803	405	A/G	ggcaccgtcaaacaggcggcagtgggagaatctgatgatgaattttcccgagacacgagttttaatgataatggtagcactagaaatgaagaaggatttg[A/G]tattggacaacatagggttttgagaggtcagaggccgataaatcagcctcctttgcattctcaagaatctaatcgaggtggccgtcgtccgagattcgat

fava_c17803_514	fava_c17803	514	T/C	aacatagggttttgagaggtcagaggccgataaatcagcctcctttgcattctcaagaatctaatcgaggtggccgtcgtccgagattcgatggaaatcg[T/C]ggcaataaatcttctcaaattgatcttggttttcagggtagaaatgtggctgaaactggtagagatgcgggtcaattaggagatagttttctcgataaat

fava_c17803_693	fava_c17803	693	T/C	ggagatagttttctcgataaatttaaacttggttttgatgataagttagggaatccgtcagaggttcaagcaaacagccaatctggagaagcgacaaatt[T/C]gtcaagttccagtcaactagctgtagattctatgcctcaagatgttgatgagatYttcaagaagatgaaggaaactggtcttatccctaatgcagtggct

fava_c17803_748	fava_c17803	748	C/T	cgtcagaggttcaagcaaacagccaatctggagaagcgacaaattYgtcaagttccagtcaactagctgtagattctatgcctcaagatgttgatgagat[C/T]ttcaagaagatgaaggaaactggtcttatccctaatgcagtggctatgcttgatggtttatgtaaagatgggaatgttcaagaagctttgaagcttttcg

fava_c17803_871	fava_c17803	871	A/G	gtcttatccctaatgcagtggctatgcttgatggtttatgtaaagatgggaatgttcaagaagctttgaagcttttcggtttgatgcgggaaaagggaac[A/G]attccggagattgtcatttacacagctgtagtggagggatacactaaggcccataaggctgatgatgctataaggattttcaggaaaatgcagagtaatg

fava_c17804_1170	fava_c17804	1170	A/G	acgatgaaggggaaagatctccgttggttggtaatgcgaatcgtgaggaggaaaggtctccactagttgaggtgaataggactgagagaaatatggcgac[A/G]aggagagaagaggaagaactttcggagagaggtattaagcttggacttggtgattttgttttctacagtgttcttgtgggtagagctgcaatgtatgatc

fava_c17804_1374	fava_c17804	1374	G/A	tgactgtctatgcttgttatcttgcgattatatctggacttggctgcactcttattttgttatctgtgtgtcgtcatgctctgcctgcgctcccgatttc[G/A]attgcgttgggtgtgcttttctatttcttgactagattgctaatggaaccgtttattgttgggactgccacaaatttgatgatgttttgagtcttatgaa

fava_c17804_568	fava_c17804	568	C/A	cctctactactacaactgcacatccttcctccgtcactacgtccgcttctccgctttcttcgtcctcgcttccatgggcgggtcgattctcctgtcgctg[C/A]ttcagcggttctctattcccttagattccattacttgtttcatttttctcttcaattttactattgttggtgttctatcggttttcgcttccgcgattcc

fava_c17807_1128	fava_c17807	1128	G/T	gatgatttggcgaaagtgtgttgcacttgttcggagttgtcttaccttgcgtcgaatgatgagttatggaggaagaagtttgaagaggagtttggagata[G/T]gatgaatgggatgaagttttttaagaacttgtttgcgcattattgggcaactaagaagaaatcaaaacaaaaaccgccattttttccaactcctcggtct

fava_c17807_1350	fava_c17807	1350	T/C	aggagaagaggtcctcatccttttggagtgcctccaatttggggtggtgagtatgatctgcagccaaattttggtgtgaatctcccccttgcatacacac[T/C]gaggcgaactttctttcccccatgtcatttttaggagaattttaatgaatgatctgacgagtcgaagtgtgggactatatcatccttgtactgaaacaac

fava_c17807_151	fava_c17807	151	T/C	ccaaaaaaaaacgacaaaatccaaatcagatcRagaacaccatgaaactcaggctcagatctttcgaatccaaagaaaccctaaaaatcgaagttcccga[T/C]ccatgttctcttcaacaactcaaatccaaaatttccaccatcctctcttcctcttcctctcttcatctttccctcaaccgaaaagacgaaatcacccctc

fava_c17807_1628	fava_c17807	1628	C/T	cttccttttggttatacattttgaatagaatccatgtatgtatggtgttgggaggaaataattgttctcaatgtgtttggttccagtgtttaggtaaaaa[C/T]aatttctgttcagcacaagagcatgtagatgatgatgtttccgaacatttttttaccaataatgcaaatggaactgtttttcatttatcatctctgttgc

fava_c17807_324	fava_c17807	324	C/T	ctcaaccgaaaagacgaaatcacccctccctttcccaccgattccctccactcactcggcatcgccaccggcgacctcatcttctacaccttcaacccca[C/T]cgccttctcctctcaaaccctcccccacaaacccgccgcagaaaaccctccgactttaccagaaaagcccgcagaaccgcaaaccctagaaatggcggat

fava_c17807_551	fava_c17807	551	T/A	caattcagaacctgattttgtgaaaaggattctcaaagaagcgcttggtgatgatgttaccgatttcaaactcttggtatttgcggttcatgctgttatt[T/A]tcgaatctggRtttgttcgtgttgatcgagattcggStaaggcgattgatgacttttcgtcttcttcaatgatatctttgaggtatacacttcctgagaK

fava_c17807_562	fava_c17807	562	G/A	ctgattttgtgaaaaggattctcaaagaagcgcttggtgatgatgttaccgatttcaaactcttggtatttgcggttcatgctgttattWtcgaatctgg[G/A]tttgttcgtgttgatcgagattcggStaaggcgattgatgacttttcgtcttcttcaatgatatctttgaggtatacacttcctgagaKtgttactaaag

fava_c17807_588	fava_c17807	588	C/G	gaagcgcttggtgatgatgttaccgatttcaaactcttggtatttgcggttcatgctgttattWtcgaatctggRtttgttcgtgttgatcgagattcgg[C/G]taaggcgattgatgacttttcgtcttcttcaatgatatctttgaggtatacacttcctgagaKtgttactaaaggtttatctcatgctgtggatttaaag

fava_c17807_651	fava_c17807	651	T/G	WtcgaatctggRtttgttcgtgttgatcgagattcggStaaggcgattgatgacttttcgtcttcttcaatgatatctttgaggtatacacttcctgaga[T/G]tgttactaaaggtttatctcatgctgtggatttaaagattcaaactKtagggaattttgtgaatgtttatggttctttggttgatgatgttgggtctagg

fava_c17807_698	fava_c17807	698	T/G	tgatgacttttcgtcttcttcaatgatatctttgaggtatacacttcctgagaKtgttactaaaggtttatctcatgctgtggatttaaagattcaaact[T/G]tagggaattttgtgaatgtttatggttctttggttgatgatgttgggtctagggttcatagggtttatttagataaatttaggtttgctaagcctttaga

fava_c17807_83	fava_c17807	83	A/G	aggaccgacttatttccatttaacagcttcttaacaactgagaattcagaccaaaaaaaaacgacaaaatccaaatcagatc[A/G]agaacaccatgaaactcaggctcagatctttcgaatccaaagaaaccctaaaaatcgaagttcccgaYccatgttctcttcaacaactcaaatccaaaat

fava_c17811_1188	fava_c17811	1188	G/A	aacagtcgacaacccgtagctgtctcgactttgttgtctttatcaacttgatcaagcacacgatcaatgttcagtatagacgattgagactgtgaaacag[G/A]ccaagctgagacggttttgctatcatcgattgtgtcgtgaagcaaatgagaaggacatgcagaacggggagacgataaccaacttccttccgtatgattc

fava_c17811_2158	fava_c17811	2158	C/T	taaatatgtgcttaaagcgccattcataaccatgaagatccttagcaaccaattcttgagttggtgttgtttgagacatgtccaaaggaggaaggcattc[C/T]gttgcatgttttcttagaacggagaagccaccatgggtgctcgtatccgaagcagttaagatcttgcagaacgagtgaattttcggcctcaRaggttcat

fava_c17811_2250	fava_c17811	2250	G/A	aggcattcYgttgcatgttttcttagaacggagaagccaccatgggtgctcgtatccgaagcagttaagatcttgcagaacgagtgaattttcggcctca[G/A]aggttcatcggtacacggatcagggactgtaggctcgtcttgattagattccggcaccaaagttatctgYgcataaacttcatcggtttcttgttcagcc

fava_c17811_2320	fava_c17811	2320	C/T	tcttgcagaacgagtgaattttcggcctcaRaggttcatcggtacacggatcagggactgtaggctcgtcttgattagattccggcaccaaagttatctg[C/T]gcataaacttcatcggtttcttgttcagccagtaaatggatattcacgatgcgacaaagaatcttcgtaggaagtttcaacaaaggactcctctgattca

fava_c17811_2580	fava_c17811	2580	G/T	ctttgaccaacacaaggaacatccaccaattgtcctgcaattgtcttccatagttcttcatctgaacaaccactcacttcagccgaaacatttgacccac[G/T]attcaacatcataatcaMcacaagtcaacaaaaaaaatcaaaaaacaaaaacagaaacacgttttatgaaccaatKaaaatcctctttgtttcaacaaca

fava_c17811_2598	fava_c17811	2598	A/C	acatccaccaattgtcctgcaattgtcttccatagttcttcatctgaacaaccactcacttcagccgaaacatttgacccacKattcaacatcataatca[A/C]cacaagtcaacaaaaaaaatcaaaaaacaaaaacagaaacacgttttatgaaccaatKaaaatcctctttgtttcaacaacaacaaaaaaKagaatatca

fava_c17811_2656	fava_c17811	2656	T/G	cttcagccgaaacatttgacccacKattcaacatcataatcaMcacaagtcaacaaaaaaaatcaaaaaacaaaaacagaaacacgttttatgaaccaat[T/G]aaaatcctctttgtttcaacaacaacaaaaaaKagaatatcaagaacaataacgagatcttgttttttttttggacagaaactgagaattagaatagaat

fava_c17811_2689	fava_c17811	2689	G/T	tcataatcaMcacaagtcaacaaaaaaaatcaaaaaacaaaaacagaaacacgttttatgaaccaatKaaaatcctctttgtttcaacaacaacaaaaaa[G/T]agaatatcaagaacaataacgagatcttgttttttttttggacagaaactgagaattagaatagaatcagaatcaagagaagaataagaagatgaatggt

fava_c17811_491	fava_c17811	491	G/A	tgtttcacatctaaataataataacatgaaaaactcacctagctaagaaaatgaaactaacttcaaccataaaatcaacagtttggtgctgagaaacaaa[G/A]aaaaaagtgaaaaggacgagaaaataatgtcgatgtttcatYgttMggWtgtgtctgagcttattacggtcccttcttcgattgaagagatcggtaattt

fava_c17811_533	fava_c17811	533	C/T	ctaagaaaatgaaactaacttcaaccataaaatcaacagtttggtgctgagaaacaaaRaaaaaagtgaaaaggacgagaaaataatgtcgatgtttcat[C/T]gttMggWtgtgtctgagcttattacggtcccttcttcgattgaagagatcggtaatttgcttcctgatttcatcttcttYacatcttggcttgaacaaat

fava_c17811_537	fava_c17811	537	C/A	gaaaatgaaactaacttcaaccataaaatcaacagtttggtgctgagaaacaaaRaaaaaagtgaaaaggacgagaaaataatgtcgatgtttcatYgtt[C/A]ggWtgtgtctgagcttattacggtcccttcttcgattgaagagatcggtaatttgcttcctgatttcatcttcttYacatcttggcttgaacaaatgaag

fava_c17811_540	fava_c17811	540	T/A	aatgaaactaacttcaaccataaaatcaacagtttggtgctgagaaacaaaRaaaaaagtgaaaaggacgagaaaataatgtcgatgtttcatYgttMgg[T/A]tgtgtctgagcttattacggtcccttcttcgattgaagagatcggtaatttgcttcctgatttcatcttcttYacatcttggcttgaacaaatgaagatt

fava_c17811_613	fava_c17811	613	C/T	aaataatgtcgatgtttcatYgttMggWtgtgtctgagcttattacggtcccttcttcgattgaagagatcggtaatttgcttcctgatttcatcttctt[C/T]acatcttggcttgaacaaatgaagattcttcttaccatgttacagaattcaggccatggatcgtctccgacaagcatcatatctccttcRtcgtcggtga

fava_c17811_703	fava_c17811	703	G/A	tcatcttcttYacatcttggcttgaacaaatgaagattcttcttaccatgttacagaattcaggccatggatcgtctccgacaagcatcatatctccttc[G/A]tcgtcggtgaagacgatttcccacttgttcctgtgttgaagctgtccctttatgtcgaacaactcttctaattcgccgataagttgatcgtacccattca

fava_c17812_1369	fava_c17812	1369	C/T	tagtatgaatcttttttccatttcatatacacgttcacataactatagctctgaatgtgtgagaaaggaagaccagggatcttgtagctataatacaaga[C/T]tgatacttcaaaaggcttgagcaactttctaattgttttggctgcagatttttccaagttgaataacatctagtaaaaaaaactttgacgcagcagtttc

fava_c17812_177	fava_c17812	177	T/C	agatcggtttagattagaaaagatctgtagacatattttcctttccacagtgtgagtgatacatagtgtcttactgcagataggccctttctactaatgc[T/C]tattgctcctcaactgctttgagttatttctctgtaggtaaaaacctctcatctacgcctttttgataaatcaatttgactctaatgtgagtttttcact

fava_c17812_604	fava_c17812	604	C/T	aaatacttcttgagacaaaagagctttcttctttcggctgaatgatcgataaatagggaaagcagttcgtgaagaacgccaataagtcttttctctgcta[C/T]gttgctgtttggatccaaattcgagaaatcttcatagggtgaaatatacggtagcttctgaaattcatKtagccagttttcaatcttgcttttcatcctt

fava_c17812_673	fava_c17812	673	T/G	tgaagaacgccaataagtcttttctctgctaYgttgctgtttggatccaaattcgagaaatcttcatagggtgaaatatacggtagcttctgaaattcat[T/G]tagccagttttcaatcttgcttttcatccttaRagcctttgatggaaaaaaaggaaattcaattacatcagtggcWcttttcatggcattcttctccatt

fava_c17812_706	fava_c17812	706	A/G	ttgctgtttggatccaaattcgagaaatcttcatagggtgaaatatacggtagcttctgaaattcatKtagccagttttcaatcttgcttttcatcctta[A/G]agcctttgatggaaaaaaaggaaattcaattacatcagtggcWcttttcatggcattcttctccattatagaataaactttctctccgctttcaatagcc

fava_c17812_749	fava_c17812	749	A/T	tatacggtagcttctgaaattcatKtagccagttttcaatcttgcttttcatccttaRagcctttgatggaaaaaaaggaaattcaattacatcagtggc[A/T]cttttcatggcattcttctccattatagaataaactttctctccgctttcaatagccagccccttcagtccatcttccatttctacaaacctgaaagatt

fava_c17816_1144	fava_c17816	1144	T/C	cttttcttcctgaaggagaagacagagaccatcatgcgtgtaacccttttggtcccaagggtcgaagccttgaaggggttgtgtttcctaaaagcctcgt[T/C]gtggttgctggtttggatcttgttcaggattggcaattgggttatgcaaaagggctcgagaaagctggacaaaatgtgaagttgctctttctggagcaag

fava_c17816_287	fava_c17816	287	T/C	aaaaccactcaaatgcttcatggatttgtgctattcgtgctgtgaaaaacgcttttgatcgtgaagaaacctcgttacggatctgcactgaaaacttcat[T/C]actgtttcaatccaatggcgggaagtaaccaactcaaccctagcgattcaaagatggtggttcctttgaacatgtgggttttaatctcgaatttcaagtt

fava_c17816_550	fava_c17816	550	C/T	atcggaaagttccggcgaatgcaaaccctgtggatggagtgttttcttttgatgtgattgttgacagggaaacgaatcttcttactcgaatttatcgtcc[C/T]gttgagggagatgaaagtgtgaacattgttgatcttgagaagcctgttacctctgaggttgtgccggttctgatgttcttccatggaggaagttttgcgc

fava_c17816_652	fava_c17816	652	T/C	ttgagggagatgaaagtgtgaacattgttgatcttgagaagcctgttacctctgaggttgtgccggttctgatgttcttccatggaggaagttttgcgca[T/C]tcttcagcgaatagtgcgatatatgatactttgtgccgtcgattggttggtatttgtaatgctgttgttgtgtctgtgaattataggcgtgcaccggaga

fava_c17816_975	fava_c17816	975	T/C	gggaacattgtgcaccatgttgctttgaaaggtatggactctgggattcaggtgttggggaatatacttctgaacccattgtttggcggggaggaaagaa[T/C]ggaatctgaaaagcgtcttgatgggaggtactttgttagagtgaaagatagagactggtattggagggcttttcttcctgaaggagaagacagagaccat

fava_c17817_1211	fava_c17817	1211	T/C	taacttgtctttgaaacctctcaccattttcttaatttctttgtatgaatatccaatgggcatgaggttgttttgttgctgtagatatacttcgatatat[T/C]caaacattgacaaatgccttttcctccatttgtatatcaacaaaccaataaataatgtcatcacaaacataaatctagccactaaaattgacggtagaac

fava_c17820_1300	fava_c17820	1300	T/C	gtatcgtcaatgtcagagaatgaagtgattcttgtaaaagaagatatagaagtgcttgttgagaagtttatggatcttgaaagtcaagagggaaaagaaa[T/C]tagagatagagcaagagaacttaagactatgtgtcataaagctattggtaaaggtggatcttcgtataagaacatYgacgcattttttaatgacatttca

fava_c17820_1376	fava_c17820	1376	C/T	ttgaaagtcaagagggaaaagaaaYtagagatagagcaagagaacttaagactatgtgtcataaagctattggtaaaggtggatcttcgtataagaacat[C/T]gacgcattttttaatgacatttcatttcaaggtcactgatactaggagtttaatggaagtagttgatgcacaatttgtgtgagagttgaatactccatac

fava_c17820_1481	fava_c17820	1481	G/C	cattttttaatgacatttcatttcaaggtcactgatactaggagtttaatggaagtagttgatgcacaatttgtgtgagagttgaatactccatactaat[G/C]agtttgattccttcttatatactttctgggtattaaaataaatgttacattttaataaagtatYtattctaaaataagtgtggtttttagtttctaatgt

fava_c17820_1545	fava_c17820	1545	C/T	cacaatttgtgtgagagttgaatactccatactaatSagtttgattccttcttatatactttctgggtattaaaataaatgttacattttaataaagtat[C/T]tattctaaaataagtgtggtttttagtttctaatgtaaatttcattttcttttttccactgtatccttcaattaatgttgtatatattattttcaattta

fava_c17820_448	fava_c17820	448	T/C	tttgagcggcttcttgatcagcttgtatctccggtggatgtgatcgtcggcgacgtcgagctgcggtggcctgttgctgttgcgaatcggaggaatattc[T/C]agtggctgcgttttggaccatgtcggcgtcgttttactctatgctacatcaccttgatgttttctcacgtgaccaRcacttaacgattggtacacttggt

fava_c17820_524	fava_c17820	524	A/G	ctgttgcgaatcggaggaatattcYagtggctgcgttttggaccatgtcggcgtcgttttactctatgctacatcaccttgatgttttctcacgtgacca[A/G]cacttaacgattggtacacttggtgagaaaacagagaacatttcaggWatttcttcacttcatatggaaggccttttaactgttgttcacaaaacggatg

fava_c17820_572	fava_c17820	572	T/A	cggcgtcgttttactctatgctacatcaccttgatgttttctcacgtgaccaRcacttaacgattggtacacttggtgagaaaacagagaacatttcagg[T/A]atttcttcacttcatatggaaggccttttaactgttgttcacaaaacggatgaccgagcaatgcagcttgcattagaatgcatatttWcagcRtccaaag

fava_c17820_660	fava_c17820	660	A/T	gaacatttcaggWatttcttcacttcatatggaaggccttttaactgttgttcacaaaacggatgaccgagcaatgcagcttgcattagaatgcatattt[A/T]cagcRtccaaagcaaattatcttttgttcaccacagttcaagaattagaggctgaaacaattgatacccttaaagatatattctcctttcccgtctatcc

fava_c17820_665	fava_c17820	665	A/G	tttcaggWatttcttcacttcatatggaaggccttttaactgttgttcacaaaacggatgaccgagcaatgcagcttgcattagaatgcatatttWcagc[A/G]tccaaagcaaattatcttttgttcaccacagttcaagaattagaggctgaaacaattgatacccttaaagatatattctcctttcccgtctatcctatcg

fava_c17820_802	fava_c17820	802	A/G	gaattagaggctgaaacaattgatacccttaaagatatattctcctttcccgtctatcctatcggtcctgcaataccgtatttagaactcgaaaacacgg[A/G]tSRtagYcatgactatataaaatggctagattctcaacccgaYggatcagtattatacatttcSttaggtagtttcctttcggtatccaatgcYcaaatg

fava_c17820_804	fava_c17820	804	C/G	attagaggctgaaacaattgatacccttaaagatatattctcctttcccgtctatcctatcggtcctgcaataccgtatttagaactcgaaaacacggRt[C/G]RtagYcatgactatataaaatggctagattctcaacccgaYggatcagtattatacatttcSttaggtagtttcctttcggtatccaatgcYcaaatgga

fava_c17820_805	fava_c17820	805	G/A	ttagaggctgaaacaattgatacccttaaagatatattctcctttcccgtctatcctatcggtcctgcaataccgtatttagaactcgaaaacacggRtS[G/A]tagYcatgactatataaaatggctagattctcaacccgaYggatcagtattatacatttcSttaggtagtttcctttcggtatccaatgcYcaaatggac

fava_c17820_809	fava_c17820	809	C/T	aggctgaaacaattgatacccttaaagatatattctcctttcccgtctatcctatcggtcctgcaataccgtatttagaactcgaaaacacggRtSRtag[C/T]catgactatataaaatggctagattctcaacccgaYggatcagtattatacatttcSttaggtagtttcctttcggtatccaatgcYcaaatggacgaaa

fava_c17820_81	fava_c17820	81	G/T	acactacaagaatccaccaaccactcaactttcaccaacccatttctccggcaatgagtttcaccggcgacggcagcaaa[G/T]tatgccacgttttagctatgccatttccgggaagaggacatatcaaccccatgctaagcttctgtaagatcctagcatcacgaagaccagaaaaaattct

fava_c17820_845	fava_c17820	845	C/T	cctttcccgtctatcctatcggtcctgcaataccgtatttagaactcgaaaacacggRtSRtagYcatgactatataaaatggctagattctcaacccga[C/T]ggatcagtattatacatttcSttaggtagtttcctttcggtatccaatgcYcaaatggacgaaattgttgaagcgctaaacaatactggaattcgatacc

fava_c17820_866	fava_c17820	866	G/C	gtcctgcaataccgtatttagaactcgaaaacacggRtSRtagYcatgactatataaaatggctagattctcaacccgaYggatcagtattatacatttc[G/C]ttaggtagtttcctttcggtatccaatgcYcaaatggacgaaattgttgaagcgctaaacaatactggaattcgatacctatatgttgctcgcggYgaag

fava_c17820_896	fava_c17820	896	T/C	acacggRtSRtagYcatgactatataaaatggctagattctcaacccgaYggatcagtattatacatttcSttaggtagtttcctttcggtatccaatgc[T/C]caaatggacgaaattgttgaagcgctaaacaatactggaattcgatacctatatgttgctcgcggYgaagcatcgcgattaaaagacaagtgtggagata

fava_c17820_962	fava_c17820	962	C/T	tttcSttaggtagtttcctttcggtatccaatgcYcaaatggacgaaattgttgaagcgctaaacaatactggaattcgatacctatatgttgctcgcgg[C/T]gaagcatcgcgattaaaagacaagtgtggagataaaggcatggtgataccgtggtgtgatcaattaagagtattgtcacattcttctataggtggatttt

fava_c17824_1071	fava_c17824	1071	A/G	tagaaccctctttctctaagtcaaccctaccctttttcaaaatcacaccactctttaattttcttaaacttctcatacttcttctcctagaaatttccac[A/G]tgtttaggaacccagcaatccacacatacagaaaactcagaacccaaacaagaataagaccaaggagcaacatctctgaacttaaagaaacaatttttgt

fava_c17824_131	fava_c17824	131	T/C	acaagagaataattatgtaccaattccgttttatgacagatctctgaaaatatcagctttcaaaagctaattattataaaggagcattacatgaatgaca[T/C]gaagcattggatatgtattctcaattgtacagaacaatcgaatggaatctacacagtcaaatcttgattctcaaggctattaagagttggtcttaaacaa

fava_c17824_532	fava_c17824	532	G/C	ttctcggcttaagtttattagccggtcagaacaacttccctccccttccttcaatgcctcttccccaatctcttcaacaccaaatgcagctgtgcattca[G/C]cacctttagggaaagctatcatgtcactccctcccatgcagggacaactcagatcagttgaagaatcactatccaacatattatcagaaccactagcatt

fava_c17828_180	fava_c17828	180	C/G	aaaaatacatttagtaataattcataatacatctaattgtacatgttttcattaagtaatgaatttagaaatgaacaagaaatggcatggcccaaaaacc[C/G]attatcatctttaaagcagYgaatttcaatggtgcagcattgctaggagaaggtgcaggtacattggaagctggaacagtaggaggagaRgccaaagctg

fava_c17828_200	fava_c17828	200	C/T	ttcataatacatctaattgtacatgttttcattaagtaatgaatttagaaatgaacaagaaatggcatggcccaaaaaccSattatcatctttaaagcag[C/T]gaatttcaatggtgcagcattgctaggagaaggtgcaggtacattggaagctggaacagtaggaggagaRgccaaagctgaagaatatggagtaggtgaa

fava_c17828_270	fava_c17828	270	G/A	cccaaaaaccSattatcatctttaaagcagYgaatttcaatggtgcagcattgctaggagaaggtgcaggtacattggaagctggaacagtaggaggaga[G/A]gccaaagctgaagaatatggagtaggtgaagaagctaStggagaaactttgagtacattaatatcaactttctgtcctgcttgacagtgtccaggtacac

fava_c17828_308	fava_c17828	308	C/G	aatggtgcagcattgctaggagaaggtgcaggtacattggaagctggaacagtaggaggagaRgccaaagctgaagaatatggagtaggtgaagaagcta[C/G]tggagaaactttgagtacattaatatcaactttctgtcctgcttgacagtgtccaggtacaccacagaagaaaaagtggtgaccatgagtggttatcttt

fava_c17828_665	fava_c17828	665	G/A	ttgtagtcaatattaccaagtgtagtccagccagaagagtcaccaaccttgtaaatagctgcatatgaaaatgaaatctgcatagttaccaacactaaag[G/A]caaaaccatagatctttctaccaaagccatgacaataggctctgcaagagatccttgtctttggttttttgtgttggtatttgtgtttttgttgaggt

fava_c17829_134	fava_c17829	134	C/A	caactaaactcagttacatgtctttgttaatccattcgtgttctctaaccctaattcaacctaagtcaaatccaattcaataccctttcttcagattcag[C/A]aaatctaacatagatttctgctaaattccctctttttcagatcYaacaatagctcaacaccactcttcaaattSaatggcggttcaacaaaaaggcaagt

fava_c17829_1592	fava_c17829	1592	A/G	atgtgagagtcggggtgcctcggccgattttctcctcaagcatcgatgagcttaggtcctcattcttgaataatccgaccccgagtttggatgacgaaac[A/G]aaagtgatgataccgttgatagctagtgaccgcgaaaagcttgtYatcgttggacttggaggcggttacgttggaatgtatcattccaatctacctatct

fava_c17829_1637	fava_c17829	1637	T/C	atgagcttaggtcctcattcttgaataatccgaccccgagtttggatgacgaaacRaaagtgatgataccgttgatagctagtgaccgcgaaaagcttgt[T/C]atcgttggacttggaggcggttacgttggaatgtatcattccaatctacctatctttaaaggcgagtataataccatgatgtggacYagtccggtattgt

fava_c17829_1724	fava_c17829	1724	T/C	gcgaaaagcttgtYatcgttggacttggaggcggttacgttggaatgtatcattccaatctacctatctttaaaggcgagtataataccatgatgtggac[T/C]agtccggtattgttcttcgtacttttccttttcggggcttggcattttttctccaaaaagaaggaagcacttacatcatggggaccggacgaccccttag

fava_c17829_178	fava_c17829	178	T/C	ctaaccctaattcaacctaagtcaaatccaattcaataccctttcttcagattcagMaaatctaacatagatttctgctaaattccctctttttcagatc[T/C]aacaatagctcaacaccactcttcaaattSaatggcggttcaacaaaaaggcaagttttttatcttctgtttgcttatctttttctcttctgYtcatgta

fava_c17829_208	fava_c17829	208	G/C	attcaataccctttcttcagattcagMaaatctaacatagatttctgctaaattccctctttttcagatcYaacaatagctcaacaccactcttcaaatt[G/C]aatggcggttcaacaaaaaggcaagttttttatcttctgtttgcttatctttttctcttctgYtcatgtatcttgtaaccctattacccctgaatcatgt

fava_c17829_271	fava_c17829	271	T/C	tcagatcYaacaatagctcaacaccactcttcaaattSaatggcggttcaacaaaaaggcaagttttttatcttctgtttgcttatctttttctcttctg[T/C]tcatgtatcttgtaaccctattacccctgaatcatgttcRatacaatcaccgtccaaatccaatgatgaatctgtttctgaRtacaaaacaagtaatgat

fava_c17829_311	fava_c17829	311	G/A	tggcggttcaacaaaaaggcaagttttttatcttctgtttgcttatctttttctcttctgYtcatgtatcttgtaaccctattacccctgaatcatgttc[G/A]atacaatcaccgtccaaatccaatgatgaatctgtttctgaRtacaaaacaagtaatgatcaattgaatcaacaagttttgttgaataagcttgaagaRt

fava_c17829_353	fava_c17829	353	A/G	ttatctttttctcttctgYtcatgtatcttgtaaccctattacccctgaatcatgttcRatacaatcaccgtccaaatccaatgatgaatctgtttctga[A/G]tacaaaacaagtaatgatcaattgaatcaacaagttttgttgaataagcttgaagaRttggtgagaaatYtaagtgatatagttgcgagattagagtcaa

fava_c17829_410	fava_c17829	410	A/G	cRatacaatcaccgtccaaatccaatgatgaatctgtttctgaRtacaaaacaagtaatgatcaattgaatcaacaagttttgttgaataagcttgaaga[A/G]ttggtgagaaatYtaagtgatatagttgcgagattagagtcaaaacaactgcagcatgagcttcctaaggttgctcaagaaaaaagtaggtttactcagg

fava_c17829_423	fava_c17829	423	T/C	gtccaaatccaatgatgaatctgtttctgaRtacaaaacaagtaatgatcaattgaatcaacaagttttgttgaataagcttgaagaRttggtgagaaat[T/C]taagtgatatagttgcgagattagagtcaaaacaactgcagcatgagcttcctaaggttgctcaagaaaaaagtaggtttactcaggtaaagggtggtgg

fava_c17829_599	fava_c17829	599	T/G	ggtttactcaggtaaagggtggtggtgatgatggaagatctagtgatagtagagtagttgttgaagatggtgaatttgaggggaaaactcaaaatggtga[T/G]agagctaagggaatgtctgtgaccaagtatRctcctttttggtcagagaggtttcaatttgcatctgctgtgaaattggattctgatgcaacttgtataa

fava_c17829_630	fava_c17829	630	A/G	tggaagatctagtgatagtagagtagttgttgaagatggtgaatttgaggggaaaactcaaaatggtgaKagagctaagggaatgtctgtgaccaagtat[A/G]ctcctttttggtcagagaggtttcaatttgcatctgctgtgaaattggattctgatgcaacttgtataaatgttttgccttttaaagatcacgaagggtt

fava_c17830_103	fava_c17830	103	T/C	gttggataccagagcatcatagtatatacactaaaaattctctcataaaattttcattttagtatattttacaatttacaacaacttgccaatcaaatat[T/C]acatagattactattgttttatactcgcttgattaccctggcatttcaccttgaaagaatgactaactacatatttttacaatagtttctttccttttta

fava_c17830_1261	fava_c17830	1261	G/A	ccgaaactattccagatactccagattttgcaccactttcatgattaacagctgatcttgaaaaggatcctgtggtggggtatgctgaaaagaatgagcc[G/A]agaacattggaaacaccaagaccgaataactcttgatttgaatccagctcatacccattctttgcagctaatgccttagcaattcctacagattccagta

fava_c17835_379	fava_c17835	379	T/C	tgtgatgcgaaattccacatctttaaagatgaaaagtttacaatgcaaaggcagaagtcacggcaatatctgtatataagccattgtcacacgaataact[T/C]tcccacaaagttgagctccagcagttagcttcccctttagactaccaagccctttcaacctcaataaaaatcgttaactaacatgaagtgtggaattcga

fava_c17836_1072	fava_c17836	1072	T/C	tgcaaggaatataggaacgtcgagttcagttgtctccactttgcttcagggtgtttcacatgaacttcccgggaacaatgtaccctccgaaaactcagat[T/C]tgttggtgacttttccattggatcagcgacagaagttgaacagaattcttaatacacagcagcagagagtggccacggcaaaaacagatgttgaagatct

fava_c17836_123	fava_c17836	123	T/A	atccatatccatatcccgaagaactcgacgacaatggcagcaacgatctcctcgacgacgacgccgtatcagaaacatctccgcttccgttgtgtatgga[T/A]tggagtcctccgcctcctaattgggacggtcgcaacactctttggccgcacgctttRtcttcttggactttctcttcaacMattccttcRtggactcttg

fava_c17836_1275	fava_c17836	1275	A/G	tagcaagattgaatcaagaagtggctgcaagacaatatcttgcgacaaaggtcaaagatttggaagttgaacttgaaacaactcgactgaactgcagaga[A/G]aacatgcagcaggctgtcttaactgaaaaggaaaggtttacacaaatgcaatgggatatggatgRacttYggaggaagtgcctggagaaagaaacgaaat

fava_c17836_1340	fava_c17836	1340	A/G	gttgaacttgaaacaactcgactgaactgcagagaRaacatgcagcaggctgtcttaactgaaaaggaaaggtttacacaaatgcaatgggatatggatg[A/G]acttYggaggaagtgcctggagaaagaaacgaaattgaagtttgaagaggatgaaaggttgctagcagaatcaacaaaagcgtYcattattcaggagaag

fava_c17836_1345	fava_c17836	1345	C/T	acttgaaacaactcgactgaactgcagagaRaacatgcagcaggctgtcttaactgaaaaggaaaggtttacacaaatgcaatgggatatggatgRactt[C/T]ggaggaagtgcctggagaaagaaacgaaattgaagtttgaagaggatgaaaggttgctagcagaatcaacaaaagcgtYcattattcaggagaagcaaat

fava_c17836_1424	fava_c17836	1424	T/C	caatgggatatggatgRacttYggaggaagtgcctggagaaagaaacgaaattgaagtttgaagaggatgaaaggttgctagcagaatcaacaaaagcgt[T/C]cattattcaggagaagcaaatgttgcagcRagagctagatatggctagagaacaacttaagcacttgcagaaacgtcatgatgagtttgagatgaaatca

fava_c17836_1454	fava_c17836	1454	A/G	tgcctggagaaagaaacgaaattgaagtttgaagaggatgaaaggttgctagcagaatcaacaaaagcgtYcattattcaggagaagcaaatgttgcagc[A/G]agagctagatatggctagagaacaacttaagcacttgcagaaacgtcatgatgagtttgagatgaaatcaaagacagatatgaagttgctagtaaaggaa

fava_c17836_1653	fava_c17836	1653	G/C	aagtgaaatccctcagaagttctcagttagaactaaaacagcagctcagcgaattgacggaggaaaaagtagatgcggagaaaactcttcagaaggaaaa[G/C]gaaagaatgcagctttcacataatgttaatgcaaagttgttgcatgagtgtgcaatccttcagaggaggctacaggagtgcagtgtaaattttcttgttg

fava_c17836_180	fava_c17836	180	A/G	acgacgccgtatcagaaacatctccgcttccgttgtgtatggaWtggagtcctccgcctcctaattgggacggtcgcaacactctttggccgcacgcttt[A/G]tcttcttggactttctcttcaacMattccttcRtggactcttgttcctcaatcatcaccttcttctgatcctgttgtgttttttagggttcaagttggtg

fava_c17836_1881	fava_c17836	1881	C/T	cttcaacatctgacgctttggatttgttagcaacatcagataatcggattggtctcttgcttgcagaggcacagctcttggcacaagatgtagaaaatgt[C/T]gtggatgctgtggaaagaaatacaactactgatgataccgggacaactaatgaagaattaaggaaaatgctggcaaatatatttgtagacaatgccagtt

fava_c17836_204	fava_c17836	204	C/A	cgcttccgttgtgtatggaWtggagtcctccgcctcctaattgggacggtcgcaacactctttggccgcacgctttRtcttcttggactttctcttcaac[C/A]attccttcRtggactcttgttcctcaatcatcaccttcttctgatcctgttgtgttttttagggttcaagttggtgttcgatccccggaagctatcacca

fava_c17836_213	fava_c17836	213	G/A	tgtgtatggaWtggagtcctccgcctcctaattgggacggtcgcaacactctttggccgcacgctttRtcttcttggactttctcttcaacMattccttc[G/A]tggactcttgttcctcaatcatcaccttcttctgatcctgttgtgttttttagggttcaagttggtgttcgatccccggaagctatcaccactacgagaa

fava_c17836_2177	fava_c17836	2177	T/C	actttaataagcaagttcttagagagatgacatgttcaatgctatgtaatgtatgtaccctatgtatgttaccaagttacttctgtcaatattatcttta[T/C]gatagtgcgtagtttcactactgtaataatataactatttcaaaaacaataatgcctcaatcataaatccatcatcacatttagcaaacaaaccaaccat

fava_c17836_889	fava_c17836	889	T/C	aagtgttgatcatgctgcatctaaccaagactttatttatgaggataatagtagtagtacagacaaggttgctgagaattctggtgatgctatagctctt[T/C]gtctagatggagctgactttacaccagctaatgctcatgttgagagactgtcaacagaaagtattgggagtgacttaagttctgcaaggaatataggaac

fava_c17837_687	fava_c17837	687	A/G	cagacaWcatcaaggtcatcagcatcttctggtattggtttgtcatttgaagacaataatggttccttgtttcttgacagtaactcttgctctcagaata[A/G]tggagattacaggaacaggtatgtgtatggacaaaaagaagaggtggatgagtatgcatttttcaaagaaccatctggtagcactactatgaaaagcttc

fava_c17839_408	fava_c17839	408	C/G	ttcgaaaattgagaaatgttgctgaagagataggtacagaagtcaagacaaataaagattttctcgaacagatgcaaatgactatggcaaaagctcaagc[C/G]ggggtgaagaataatttaagaagattgaacaagagcatcgtgcgcaatggttcaaatcatattgttcatgtgatcgcttttgcattgatttgtttcttga

fava_c17839_654	fava_c17839	654	C/T	gggtctactcctcccagcattgtagttgaaaagatttcatccaaaaatgactcacaatgatgcctaactttgttgtatagcgaaactttgtaatatactt[C/T]ctgggtaacatactaacaKtcatggatcgaagtaggtttgtgtgaagaaaaRagataatatggtgtttagcagtttgcatgtcacattttcatttggtaa

fava_c17839_673	fava_c17839	673	G/T	ttgtagttgaaaagatttcatccaaaaatgactcacaatgatgcctaactttgttgtatagcgaaactttgtaatatacttYctgggtaacatactaaca[G/T]tcatggatcgaagtaggtttgtgtgaagaaaaRagataatatggtgtttagcagtttgcatgtcacattttcatttggtaaaagttaccatcattcaaaa

fava_c17839_706	fava_c17839	706	G/A	cacaatgatgcctaactttgttgtatagcgaaactttgtaatatacttYctgggtaacatactaacaKtcatggatcgaagtaggtttgtgtgaagaaaa[G/A]agataatatggtgtttagcagtttgcatgtcacattttcatttggtaaaagttaccatcattcaaaaagaggagaaagattttctgaatatacgtaaaaa

fava_c17841_1072	fava_c17841	1072	A/G	tttaatgatgtgaaacatgatgatggtgagagaagatcagtttcagaaatcattggtgtttctagatttagagaatttgatacgaagaaaagggtgtttg[A/G]agattcttcattggttgaaaatgatgttaaagaggaaagaatgaggaagctttggtttccaattgcaagaagaacaRctgaatggtttgtgaatagagaa

fava_c17841_1149	fava_c17841	1149	A/G	tgatacgaagaaaagggtgtttgRagattcttcattggttgaaaatgatgttaaagaggaaagaatgaggaagctttggtttccaattgcaagaagaaca[A/G]ctgaatggtttgtgaatagagaaaaaagttctcaaSaaaaatcatttttagatgtataatccaattttcaccttcctaagtatcagcttcacaaccctaa

fava_c17841_1185	fava_c17841	1185	G/C	ggttgaaaatgatgttaaagaggaaagaatgaggaagctttggtttccaattgcaagaagaacaRctgaatggtttgtgaatagagaaaaaagttctcaa[G/C]aaaaatcatttttagatgtataatccaattttcaccttcctaagtatcagcttcacaaccctaattgcagccacatcaaatattttcatgtccattttgc

fava_c17841_554	fava_c17841	554	T/C	cttttcatattgattgtattgatcattggcttgaaaaacattcaagttgtcctatttgtagacataaagtcaacattgaagatgaaacaacttttgcata[T/C]tcaaatagtttaaggatgttggtaaatgatgaatcaaacatagagatctttgtcgaaagagaagaagaaaaagaaaatcgacatggttcatcgtcaagat

fava_c17841_691	fava_c17841	691	A/C	aacatagagatctttgtcgaaagagaagaagaaaaagaaaatcgacatggttcatcgtcaagatttagtattggaagtagctttagaaaaattgggaaaa[A/C]tagtatgaaggaSgaagaattgcttatacaaaacggatcaggatcagaagaaaaatataatcacatgtgccagtgtcgtaatagtgatgatactggtaac

fava_c17841_704	fava_c17841	704	C/G	ttgtcgaaagagaagaagaaaaagaaaatcgacatggttcatcgtcaagatttagtattggaagtagctttagaaaaattgggaaaaMtagtatgaagga[C/G]gaagaattgcttatacaaaacggatcaggatcagaagaaaaatataatcacatgtgccagtgtcgtaatagtgatgatactggtaacgtttttaataaga

fava_c17845_1219	fava_c17845	1219	G/A	tgcagccgtcaagtttccagcatttggcccgtgtaaccagaagagtataaacgtacaatgacactcttcatttttacgagaatgcggacgttcttgcacc[G/A]cttcaacagtggcgtcgtagaagcgaaggtcgtcggggatgaaatgctgagaggcacagaccttaacacctggaacgagtttccggcactcgtgatcctg

fava_c17847_1138	fava_c17847	1138	C/G	acatatttaatgtctacaggatccatttcagcaccgccacctttgataaatccacagtgcaccttcatatttgactttagtttgaaactctcctccctct[C/G]tttccaactaagatgRcctccaaatagaggagacgactcggaactatcatttggaacatcatcttcgataacatacgataaatttttgacaattYtgtcg

fava_c17847_1154	fava_c17847	1154	G/A	caggatccatttcagcaccgccacctttgataaatccacagtgcaccttcatatttgactttagtttgaaactctcctccctctStttccaactaagatg[G/A]cctccaaatagaggagacgactcggaactatcatttggaacatcatcttcgataacatacgataaatttttgacaattYtgtcggaagttcttccagtag

fava_c17847_1233	fava_c17847	1233	T/C	cctctStttccaactaagatgRcctccaaatagaggagacgactcggaactatcatttggaacatcatcttcgataacatacgataaatttttgacaatt[T/C]tgtcggaagttcttccagtaggggtaactattttatcagggtcatcagaaacaggaatcggacatctgtgaggttttgttacaagtcccatagtgtctaa

fava_c17847_154	fava_c17847	154	T/C	ttaattaggataaaaaaattcttgaaataaaaaatacatataacaaatatgttactacaaaataaacaaaaatgtcatccagcttttgatgttcttgtta[T/C]ttgtggcagaacaactgaattaagatccccaggataagatgtaaacaagcctaatcctcccctactttcttttaaattgctgttctttttaagttgatcc

fava_c17849_1296	fava_c17849	1296	C/T	gcaagggattttgttaagagattacttaataaagatccacggaaaagaatgagtgcagcacaggcattaagtcatccatggataaaacactatgaggata[C/T]gaaagtacctttggatattttaatattcaagctcatgaaggcaYacatgcgttcttcttctctacgaaaagctgctttaagggctctgtccaagacatta

fava_c17849_1340	fava_c17849	1340	C/T	aagaatgagtgcagcacaggcattaagtcatccatggataaaacactatgaggataYgaaagtacctttggatattttaatattcaagctcatgaaggca[C/T]acatgcgttcttcttctctacgaaaagctgctttaagggctctgtccaagacattaactgtggatgagctgatttatttgaaggagcagttttcactttt

fava_c17849_1447	fava_c17849	1447	T/C	gttcttcttctctacgaaaagctgctttaagggctctgtccaagacattaactgtggatgagctgatttatttgaaggagcagttttcacttttagagcc[T/C]aacaaaaataacaccattagcttggaaaacataaaaacggcctttacggtaaatgcaacagatgctatgaaggagtcacgcattgccgactttctggcat

fava_c17849_191	fava_c17849	191	C/T	tcagtttaactaacaaaatttcaattcaatgggtctctgcacttcaaaacccactcactcctctcacaacgaaacaaactccgtcacagttaacgtcact[C/T]ccaaacctcaaacaaactccgtcaatggcgaaaaccctaacgaaaccgaaaacgttaagagatctccgtttttcccgttctacactccgagtcctgctca

fava_c17849_292	fava_c17849	292	C/T	ccaaacctcaaacaaactccgtcaatggcgaaaaccctaacgaaaccgaaaacgttaagagatctccgtttttcccgttctacactccgagtcctgctca[C/T]cagttcttctctaagatgtctccggccaggaaatttttcaagcggccgccgtctccggcgaagcatataaagtcgcttcttgcccggcggcatggctcgY

fava_c17849_481	fava_c17849	481	C/T	ggcatggctcggtgaagcctaatgaagcttcgataccggaagggggtgaggaggaggcggtggtttcgcttgataagaatttcggattctgcaagcattt[C/T]ggaagtaagtatgatgttggagatgaggttgggagaggacattttggttatacctgtgctgctaagttgaagaaaggagatcttaaaggtcaacaagttg

fava_c17849_690	fava_c17849	690	G/A	gtcattcctaaagccaagatgacatctgctattgctattgaggatgtgagaagggaagtgaaaatattgaaagctttgaatggacataaaaatctagttc[G/A]attccaYgatgcttatgaagatcatgataacgtgtatatagtaatggaattgtgtgaaggtggagagttgttggacagaatattatcaagaggtggMaaa

fava_c17849_697	fava_c17849	697	T/C	ctaaagccaagatgacatctgctattgctattgaggatgtgagaagggaagtgaaaatattgaaagctttgaatggacataaaaatctagttcRattcca[T/C]gatgcttatgaagatcatgataacgtgtatatagtaatggaattgtgtgaaggtggagagttgttggacagaatattatcaagaggtggMaaatacacag

fava_c17849_787	fava_c17849	787	C/A	ttcRattccaYgatgcttatgaagatcatgataacgtgtatatagtaatggaattgtgtgaaggtggagagttgttggacagaatattatcaagaggtgg[C/A]aaatacacagaggaagatgcaaaagcYatccttagacaaatactgaatgctgctgcattttgccatctacagggtgttgtgcatcgtgatcttaaaccgg

fava_c17849_814	fava_c17849	814	T/C	atgataacgtgtatatagtaatggaattgtgtgaaggtggagagttgttggacagaatattatcaagaggtggMaaatacacagaggaagatgcaaaagc[T/C]atccttagacaaatactgaatgctgctgcattttgccatctacagggtgttgtgcatcgtgatcttaaaccggagaacttcttgtttgcatccaaggacg

fava_c17850_1284	fava_c17850	1284	A/G	atttcaaggaaaatgataccatggtgaaatgttcagactctatgattgacttagaattcacacttggaagaccaaattgggaaaaagaaaatggtgaatc[A/G]tcaagagaattacctcttcttaagtgttaatcaaaaactaattaattaattaattaatttttaatttctttgttgttacattgctaggaaatattccatg

fava_c17851_1062	fava_c17851	1062	A/G	taccatgcgctatatttttggagggtttcaagtttactccttcgtatctgaaatctgcggcaagccagggattgaatgttagagagttatgcataagatc[A/G]gttttggctgcattctgcttccatgcataccagcaggtttcatatgggatattagagaaagtgtcccctgtgacacactcagttggaaattgtgtgaagc

fava_c17851_1188	fava_c17851	1188	C/T	cataccagcaggtttcatatgggatattagagaaagtgtcccctgtgacacactcagttggaaattgtgtgaagcgtgtggttgtcatagtctcttctgt[C/T]atcttcttccaaMctcctgtctcRcctattaatgcacttggaactgctattgcacttgttggtgttttcttgtattcaagagcaaagaggattaagccaa

fava_c17851_1201	fava_c17851	1201	A/C	ttcatatgggatattagagaaagtgtcccctgtgacacactcagttggaaattgtgtgaagcgtgtggttgtcatagtctcttctgtYatcttcttccaa[A/C]ctcctgtctcRcctattaatgcacttggaactgctattgcacttgttggtgttttcttgtattcaagagcaaagaggattaagccaaaaacaaatacagc

fava_c17851_1212	fava_c17851	1212	A/G	tattagagaaagtgtcccctgtgacacactcagttggaaattgtgtgaagcgtgtggttgtcatagtctcttctgtYatcttcttccaaMctcctgtctc[A/G]cctattaatgcacttggaactgctattgcacttgttggtgttttcttgtattcaagagcaaagaggattaagccaaaaacaaatacagcataattactta

fava_c17851_127	fava_c17851	127	C/A	ctcctgtcacaaattcatgcaaacaacaactatgtctccttactttctctcttcaaacccaaaaatccatactctctctatgttaacttctcattcaacc[C/A]tacaaattYcaaaccacataaccatacccaagtttcacccttttcaattctctcattctcaccatttttcttcttcaccacttagactcRYcattgataa

fava_c17851_136	fava_c17851	136	T/C	caaattcatgcaaacaacaactatgtctccttactttctctcttcaaacccaaaaatccatactctctctatgttaacttctcattcaaccMtacaaatt[T/C]caaaccacataaccatacccaagtttcacccttttcaattctctcattctcaccatttttcttcttcaccacttagactcRYcattgataaattttcatg

fava_c17851_217	fava_c17851	217	A/G	tcattcaaccMtacaaattYcaaaccacataaccatacccaagtttcacccttttcaattctctcattctcaccatttttcttcttcaccacttagactc[A/G]Ycattgataaattttcatgcccatcttcttctttctttcaaactcttagaaagtcttcaccttttctcatttccactccaaaaatccattcttttagaat

fava_c17851_218	fava_c17851	218	C/T	cattcaaccMtacaaattYcaaaccacataaccatacccaagtttcacccttttcaattctctcattctcaccatttttcttcttcaccacttagactcR[C/T]cattgataaattttcatgcccatcttcttctttctttcaaactcttagaaagtcttcaccttttctcatttccactccaaaaatccattcttttagaatc

fava_c17851_379	fava_c17851	379	C/A	ttttctcatttccactccaaaaatccattcttttagaatccttgctgcttctctaccagagggacaaagtcatgaaccaactcaaccttctggtgttttt[C/A]agtctctacaacttggtttcatgtttgctacttggtatctcttgaatatttacttcaacatttacaacaaacaggttctaaaagtctatccatttccagc

fava_c17852_1182	fava_c17852	1182	T/C	tccagggagagaggtttcaacttttttcaatggtggtggtctgcaaactcggaaagcagaatttccagggaggagggtttcaacttttttctgtggtggt[T/C]tgatgaaaacatcggaggaagtatgagtttcaactttttccaatagtggtcggcaaacttgggaggaagaatgagtttcaacttttttcaatagtggcct

fava_c17852_1363	fava_c17852	1363	T/A	acttttttcaatagtggcctgcaagaggcagaatttccagggagctgatttgtgttctcaacttttgtgacaccctgcagtggcctttgaacttttcttg[T/A]ttcctgagtagttgaatctgaaattccaacacttcttgaagaagaacccaattgaaaccctcgtttgacttcctcaggttctttatgtttcctcaatgga

fava_c17852_1657	fava_c17852	1657	T/C	tcttgaggagaagagacaggtttgtcaagttcctccgtaatatcactcttctcaacatgttcattctcataatatttacccttttgcaacttcacattgt[T/C]ttttggttcctttWcttcattcacaagcttgagctttgaatctttctgagtctcttctttctcccttttccttttctccttcctctccctcttcttctcc

fava_c17852_1671	fava_c17852	1671	A/T	gacaggtttgtcaagttcctccgtaatatcactcttctcaacatgttcattctcataatatttacccttttgcaacttcacattgtYttttggttccttt[A/T]cttcattcacaagcttgagctttgaatctttctgagtctcttctttctcccttttccttttctccttcctctccctcttcttctccttcctcaacttcct

fava_c17852_658	fava_c17852	658	C/G	aaccaatcttgatccaatgaatcaaaacaatcatactttatcggtggaatggtaagtaaggagttatataaagattccatccttcgactttcttcattaa[C/G]tgattcatttgctgttggagcaacaacaggaaccaaattcttcaatggcttggaaggagcatcaacaggaaccaaattattcaatgctttgcaaactttg

fava_c17856_680	fava_c17856	680	T/C	atcttacccttgtcacaaaattgagcctttgcagagtaaaatctcttgccgaaaacatgttctctcttagcaataaaaaccgcatcagaattataagcac[T/C]aacattgttgtcataatcaatcttcttgcatgcttcctttttcagatctcctaaaagcaaaaccatctttttgttaaacaaaacaactaaatagtaccct

fava_c17857_1838	fava_c17857	1838	G/C	cgaatacgtattccctcacaacaacccgaggaaacttttagccccagcctgaacttccgacttcttatccagctagagttgtccgtaaaggtaagatcag[G/C]cagtgttcctacaccctccttcagggtcacttgcagatcMccagtcagaagaggtcgttttccttctcgctctttcacaacatggctctcaaattcttct

fava_c17857_1878	fava_c17857	1878	C/A	gccccagcctgaacttccgacttcttatccagctagagttgtccgtaaaggtaagatcagScagtgttcctacaccctccttcagggtcacttgcagatc[C/A]ccagtcagaagaggtcgttttccttctcgctctttcacaacatggctctcaaattcttcttgactccaagcatcgtcatcctcattattgaaatcccctt

fava_c17857_2075	fava_c17857	2075	T/C	ccttcaagtacaataacatctagtctgacacatgacgctggaccggatgtgacaacacggcctgtgtttgcatcaatcaagacaatatgtatggcagtgc[T/C]cttctcgccctccactttcccaccagtaaagaggggaagagacagcctggtcctaaattgtaactgtaaatttcctccatctggactttctatgcgcttt

fava_c17857_2201	fava_c17857	2201	T/C	gtaaagaggggaagagacagcctggtcctaaattgtaactgtaaatttcctccatctggactttctatgcgctttggagaagatctctcagtaagattgg[T/C]agagcctaattttgctaaagcgcgctccacttcttcgctaacaactcttcgtagaataggctccagtgatgagcaaagtttctgcagactgtcgaccttc

fava_c17857_2447	fava_c17857	2447	G/A	ggctgatcttcctcagtcgaagtcgaatctaagcttcgtttctccctcgccatactattcgatctctccatatacctcgtttgcattctcacttccaaaa[G/A]cactgtttgcttgtagaaaaagtgaaRcgaaagaaaataatcttacagcaacaactcttggcagttaaataaaacttcaacttctttttccttcgttaac

fava_c17857_2474	fava_c17857	2474	G/A	tctaagcttcgtttctccctcgccatactattcgatctctccatatacctcgtttgcattctcacttccaaaaRcactgtttgcttgtagaaaaagtgaa[G/A]cgaaagaaaataatcttacagcaacaactcttggcagttaaataaaacttcaacttctttttccttcgttaacggattccgccgttaaatcctttaaaaa

fava_c17858_1006	fava_c17858	1006	A/G	ttggtgtggaattgtcatcacattttaaagagagatcaaagtctgtgagcatgatgtggccatctgatctaactaacacgttctctggtttgagatcacg[A/G]tatactattcccaacatgtgaagatattcaagtgccattaacacctctgatgcatagaatctcacggcggattcagtgaagcgtttattctgctgccgtt

fava_c17858_149	fava_c17858	149	C/G	ataatatgttttgtgtgctcgagcagagcactcgttaatttaatttgtcatcaatcttaattaacatagtttggttcacaaaacctattaaatataaaga[C/G]aagagcatgtgacaaattaaatttaaaaaaagaaagagaaatataacWgaattttcttaaacttagtaactacctagctaactgtactYttccttctctc

fava_c17858_197	fava_c17858	197	A/T	catcaatcttaattaacatagtttggttcacaaaacctattaaatataaagaSaagagcatgtgacaaattaaatttaaaaaaagaaagagaaatataac[A/T]gaattttcttaaacttagtaactacctagctaactgtactYttccttctctcttattgtgatcaataataatcaacaggagtttgtggacaactttcatc

fava_c17858_238	fava_c17858	238	T/C	aaatataaagaSaagagcatgtgacaaattaaatttaaaaaaagaaagagaaatataacWgaattttcttaaacttagtaactacctagctaactgtact[T/C]ttccttctctcttattgtgatcaataataatcaacaggagtttgtggacaactttcatcagaaacatcttctttatctttagtataaggtggtggaacat

fava_c17858_479	fava_c17858	479	G/A	ttggaaaaatgagtggtgtttgattgtagaagctcccattattgatcccaacctccttgctgggtccttaatcagtaactgtgagataagatcttttgcc[G/A]tggttggcacggtgggttccttggggaactccaaagctcgagccacgatgtttgctagagtgagttcattatccaagcctctaaaaggtgtgacgccgta

fava_c17858_742	fava_c17858	742	T/C	cagagactatttcaggagctaagtattcgtgtgtcccaacgaatgacatggaccgaacatcgataggttcagcaacaaactcagggccgtggagatgagt[T/C]tgtttcttctttcgcttgcgttttggttggaaacaggatactgctgggactatacagttgggtattatgcatgaagatgaggcRaattgagagggttcgg

fava_c17858_826	fava_c17858	826	G/A	ggccgtggagatgagtYtgtttcttctttcgcttgcgttttggttggaaacaggatactgctgggactatacagttgggtattatgcatgaagatgaggc[G/A]aattgagagggttcggagtggggatttttctgtggggctacttgtggagagtcttggctggagattatgatctgggctgttggtgtggaattgtcatcac

fava_c17859_364	fava_c17859	364	A/T	cacctcttgttcatgattttgtccatagaccaacaagtaattctcacactgtttcagttataaatcctcttcatgttgaactttcactttcaccgtggtt[A/T]accaaattaaccacagaaacattggaattaggcaccgttWttgatactatagatgactcttctttgaatcaagttactttcagtgttgttctcagtgcag

fava_c17859_404	fava_c17859	404	T/A	ttctcacactgtttcagttataaatcctcttcatgttgaactttcactttcaccgtggttWaccaaattaaccacagaaacattggaattaggcaccgtt[T/A]ttgatactatagatgactcttctttgaatcaagttactttcagtgttgttctcagtgcagccattgctgtgttattctttcccaccatccaacgcaggat

fava_c17859_967	fava_c17859	967	T/G	aagaaagtagtgaaagcaaaataatggagcagacagttctatcaagttcatcggatgaagatcaaagttcaaataattcacagtaatcaaagttcatttt[T/G]gggacattttggtttttaacaagtgtttagggtgtgctgtttttatacatgtgcataatgtttgtaagtaaagaaactatagcatttatactagtttcca

fava_c17860_1202	fava_c17860	1202	A/G	ctctcataagtagcttcaatcaaatcaagagcacaagtctcagaagcaaaaacaacagcaccattacttcttctccccataaccaaaggccgaaacccta[A/G]aggatctctgacagcaacaagcttatcctcagtaacaaacacaatagaataagccccttcaagcttctcacacgcatcaacaattctcaaaataaaaggt

fava_c17863_1091	fava_c17863	1091	C/T	tatcttacataaatttcggcggctacaaacttcatccatatcctctgttcgtcctttcaaaacacctgatgatagaggaatcggatttggaatctttttg[C/T]cattcttgcggtcttgagacatgtatagacgaatctggaaataagttacttcaaacaaagcaaactaccttcagtcctcccaaaggtggtttccagaaat

fava_c17863_1199	fava_c17863	1199	T/C	gcggtcttgagacatgtatagacgaatctggaaataagttacttcaaacaaagcaaactaccttcagtcctcccaaaggtggtttccagaaatatagctt[T/C]ttgggcttgcaagattatgaaagRtagtttttgtaacatcagttcagattggaacaaaaatggtcaaaggattgtaaagaacaaaaatggaataaatagt

fava_c17863_1223	fava_c17863	1223	G/A	aatctggaaataagttacttcaaacaaagcaaactaccttcagtcctcccaaaggtggtttccagaaatatagcttYttgggcttgcaagattatgaaag[G/A]tagtttttgtaacatcagttcagattggaacaaaaatggtcaaaggattgtaaagaacaaaaatggaataaatagtatttacattaactaacatatttat

fava_c17863_127	fava_c17863	127	T/C	gggaatgggaaccgtcgctcttcgttttcccaacaacggcgctaccaattccctttYaatgctacccaacaatccaaagtttcaaacttttctcaaaggg[T/C]cactcctcctcctaccactggtagtaaaagtaacgcctctacctcYgatttcaatgaaaattcacaaggatcttccaattttgggggtttacttggaact

fava_c17863_173	fava_c17863	173	C/T	aattccctttYaatgctacccaacaatccaaagtttcaaacttttctcaaagggYcactcctcctcctaccactggtagtaaaagtaacgcctctacctc[C/T]gatttcaatgaaaattcacaaggatcttccaattttgggggtttacttggaacttgttcttatatgtgcccagaaagagagaggatccagcgtgagRaac

fava_c17863_270	fava_c17863	270	G/A	ctcYgatttcaatgaaaattcacaaggatcttccaattttgggggtttacttggaacttgttcttatatgtgcccagaaagagagaggatccagcgtgag[G/A]aacttcgagatttggcagtttttgagaggctaaatggtaatcctggaaaRtcatctccagctcttgctgtcaagaagttttgcagaactatatccatcaa

fava_c17863_320	fava_c17863	320	A/G	ttggaacttgttcttatatgtgcccagaaagagagaggatccagcgtgagRaacttcgagatttggcagtttttgagaggctaaatggtaatcctggaaa[A/G]tcatctccagctcttgctgtcaagaagttttgcagaactatatccatcaaagaagttcaagcatctgatatgcgacctcttaatgtattagaagatactt

fava_c17863_424	fava_c17863	424	A/G	tctccagctcttgctgtcaagaagttttgcagaactatatccatcaaagaagttcaagcatctgatatgcgacctcttaatgtattagaagatactttaa[A/G]ttatctattgggcttgctggattctaaggagcatccgttcgaagtagttcatgacttcatctttgataggacaaggtctatacgtcaagatcttactatg

fava_c17863_613	fava_c17863	613	C/T	gatcttactatgcaaagtattataaacaagagagctattttcatgtatgagggaatggtgaaatttcatgttatatcacatcgcaagctctggtgttcta[C/T]ggRtgatccRaacatcgcatccatgcatcatctcaacatggaacagctcacaaagacaYtgtcttctttattcaatctctacgRagcaaatagaaattca

fava_c17863_616	fava_c17863	616	G/A	cttactatgcaaagtattataaacaagagagctattttcatgtatgagggaatggtgaaatttcatgttatatcacatcgcaagctctggtgttctaYgg[G/A]tgatccRaacatcgcatccatgcatcatctcaacatggaacagctcacaaagacaYtgtcttctttattcaatctctacgRagcaaatagaaattcaaat

fava_c17863_623	fava_c17863	623	A/G	tgcaaagtattataaacaagagagctattttcatgtatgagggaatggtgaaatttcatgttatatcacatcgcaagctctggtgttctaYggRtgatcc[A/G]aacatcgcatccatgcatcatctcaacatggaacagctcacaaagacaYtgtcttctttattcaatctctacgRagcaaatagaaattcaaatgatttac

fava_c17863_672	fava_c17863	672	C/T	gaaatttcatgttatatcacatcgcaagctctggtgttctaYggRtgatccRaacatcgcatccatgcatcatctcaacatggaacagctcacaaagaca[C/T]tgtcttctttattcaatctctacgRagcaaatagaaattcaaatgatttacatgaaaatgaagctgagttccattcattctatgtgcttcttaatcttgg

fava_c17863_697	fava_c17863	697	A/G	aagctctggtgttctaYggRtgatccRaacatcgcatccatgcatcatctcaacatggaacagctcacaaagacaYtgtcttctttattcaatctctacg[A/G]agcaaatagaaattcaaatgatttacatgaaaatgaagctgagttccattcattctatgtgcttcttaatcttggttctcacagcaaaccaatgggagaa

fava_c17863_83	fava_c17863	83	C/T	aagaaacaagaattatgatggagagtgggaatgggaaccgtcgctcttcgttttcccaacaacggcgctaccaattcccttt[C/T]aatgctacccaacaatccaaagtttcaaacttttctcaaagggYcactcctcctcctaccactggtagtaaaagtaacgcctctacctcYgatttcaatg

fava_c17863_864	fava_c17863	864	C/A	taatcttggttctcacagcaaaccaatgggagaacctctttctttatggtttagtcatgtatcaactccaatattgaagtcaaaagaaatgcgttttgct[C/A]ggagaatcKtacgctcgttccgaatgggtaattatatggatttcttccgaactgtagcagctggtgcatcatatctacagtactgcattatggagcctta

fava_c17863_873	fava_c17863	873	G/T	ttctcacagcaaaccaatgggagaacctctttctttatggtttagtcatgtatcaactccaatattgaagtcaaaagaaatgcgttttgctMggagaatc[G/T]tacgctcgttccgaatgggtaattatatggatttcttccgaactgtagcagctggtgcatcatatctacagtactgcattatggagccttatatcgatga

fava_c17871_1114	fava_c17871	1114	T/C	ttcagattgaagggcttcaaaggcatccaagatacggagctcgagttgagcatcgagcatacagtaatatgtatgatgccgtgcataggatatttcagtc[T/C]gagggttgggctggactatacaaggggataattccatcaactattaaagctgcacctgctggtgctgtcacatttgttgcatatgagttggtatcagatt

fava_c17871_379	fava_c17871	379	G/A	ttgattcatctgctggagcaatttctgggggaatttctcgtactgtaacatcacctcttgatgttattaagattagatttcaggttcaacttgaaccaac[G/A]tcatcatgggctttactgcgaaaagatttggttttaaccgcgccgtcgaagtatactggtatgctgcaagctagtaaagatattcttagagaagaaggct

fava_c17871_682	fava_c17871	682	T/C	gttcttccaggacagaaaaccacactaatttgagcccttatctatcctatgtcagtggcgccctagctgggtgtgcagctactgttggttcatatccttt[T/C]gatcttctccgaaccatattagcttctcaaggcgagccaaaggtgtatccaaacatgaggtcagcatttatggacatcatYcagactcgtggattccaag

fava_c17871_763	fava_c17871	763	C/T	ctgttggttcatatcctttYgatcttctccgaaccatattagcttctcaaggcgagccaaaggtgtatccaaacatgaggtcagcatttatggacatcat[C/T]cagactcgtggattccaaggcatgtatgctggattatctccaactctagttgagattgtaccttatgcaggtttacaatttggaacatacgatactttta

fava_c17872_476	fava_c17872	476	T/C	ctggctaccaaatgaagaaggtgtgtctggtgtttgacacgtgtaatattgtcgaatatcgacacgatatacccatgtatagttcgtagttagattcaat[T/C]acttttagtttctcaaatcaattacttatgtgtatggttaattgagggcagattgtgaactctgtaatatttttcatataataattttcatgaagtctgt

fava_c17873_1146	fava_c17873	1146	G/A	tcagcctcatcagttactcaaaccttgagtagagaagtgctcgagggtcagagaaagctaatgtcgcttgcaacctcaagaacaagctcaggcacgttga[G/A]taccctgcccatccagctgaacaatggtccattacttcatgaaaaggttgaggcacctctagatcccacaaaggagctagcaaggttgatttctgaacgg

fava_c17873_1513	fava_c17873	1513	A/G	acgacatgtcccgaaagatcgcatggatgacagatgtggctactgccattaatccctcagatcccatgataacaatgcatgtccggcccatattcgaaca[A/G]gtctaccaaatactgaatcatcaacgtagcttgcctacaatcaccggtgctgatctttcaagtacccgccttttattgcatgtcatcaactccatgctta

fava_c17873_1653	fava_c17873	1653	A/G	atcaccggtgctgatctttcaagtacccgccttttattgcatgtcatcaactccatgcttacgacgtgtaaatgatttatttatagaactgtgcaaattt[A/G]tctttgaattcttctaatacgattagaattttgtacaaagctgtgagtttcattgaaagggtcaaaatcagtaaagtttaagctgtcaattatgtcaatt

fava_c17873_326	fava_c17873	326	C/A	aaaaggtaaaaatcctcaaccaacaggtccgtcttccccatctccaagtgtatgcaattcaactgattcgtccaatgagccaaatggaatttcaaatctt[C/A]catctactgaaaatggtttgcctcaaattatggcaatgcaagattcactcaatcagttattgacaatgcaaaaggagatgcagaaacaaatgacaatgat

fava_c17873_940	fava_c17873	940	G/A	caactgccaaacaggcgcttcaggatgctctgaaatccagttttgaaacatcagtggttcctgcatttgagatctcctgcaaaaccatgtttgagcaagt[G/A]gatgctacattccagaaaggcatggctgagcattcaaatgcagtgcagcaacggcttgaatccgggcctacttcattggcgatgactctaagggattcca

fava_c17879_281	fava_c17879	281	G/C	agtgtgtagtaggaacatcttgtatatgtgaaatgtggtataccagattagtagggacaagttgtcaaacaatggttagtgaaatggaaaagtaaagtag[G/C]tgaagatactacgtaggaagatKaagacttgctgaaaagtcagtttccttgcatcaaccttgaagacaaggctgcagttgtagttgtagggggactgcat

fava_c17879_304	fava_c17879	304	T/G	atatgtgaaatgtggtataccagattagtagggacaagttgtcaaacaatggttagtgaaatggaaaagtaaagtagStgaagatactacgtaggaagat[T/G]aagacttgctgaaaagtcagtttccttgcatcaaccttgaagacaaggctgcagttgtagttgtagggggactgcatgacaatgagaatgttgaagtccg

fava_c17880_1525	fava_c17880	1525	A/C	agtttcagattctccgtgtttgggctcctttacatgacagggttgccggtcaacaacggaaggaacctgtttgcccgtctttacaattcagcataatggg[A/C]caaaagctatatgtcagtcaagaacagattacagttggacgtagaccagtaaccggcttacgtctctgtctcgaaggaagcaaacagaatcggctgagtg

fava_c17880_1960	fava_c17880	1960	A/G	gggatttcggatcacggaacgtcctttacattaagctcttatattcaaggttacccggttgcaccatcagaagatcactttgggatcacgtaccaaacac[A/G]ccgcaaaaaactgcaatttccggaaactcttcgaatgcagccaacacaacgagctcaggtttgagagaaaacaacgccRcaacgaacaagttagcgaagt

fava_c17880_2039	fava_c17880	2039	G/A	ttgggatcacgtaccaaacacRccgcaaaaaactgcaatttccggaaactcttcgaatgcagccaacacaacgagctcaggtttgagagaaaacaacgcc[G/A]caacgaacaagttagcgaagtacgttgatttgtccgaaatgagcaaaggacctgaagatcctcctggtcactggttagttactggcggaaagctcggtgt

fava_c17880_2326	fava_c17880	2326	G/A	tttagatttctcttgtggttaatgtgaaaaccattttccagtttgatgaaatgagttgaataggaaacttttatatggtgatatctcataggtgttactt[G/A]gaaccattttcaaaccatgcYaagttaWagtaatgttaatattgttatagtactattgagaatacaaaatgacactgttctttattttctttctgttata

fava_c17880_2347	fava_c17880	2347	C/T	atgtgaaaaccattttccagtttgatgaaatgagttgaataggaaacttttatatggtgatatctcataggtgttacttRgaaccattttcaaaccatgc[C/T]aagttaWagtaatgttaatattgttatagtactattgagaatacaaaatgacactgttctttattttctttctgttatactcgggggttttccttaacc

fava_c17880_2354	fava_c17880	2354	T/A	aaccattttccagtttgatgaaatgagttgaataggaaacttttatatggtgatatctcataggtgttacttRgaaccattttcaaaccatgcYaagtta[T/A]agtaatgttaatattgttatagtactattgagaatacaaaatgacactgttctttattttctttctgttatactcgggggttttccttaacc

fava_c17880_362	fava_c17880	362	G/T	atgctcacatagctaaaagcttcaatttttttgaagttttcgtaggatttaatcatctgggtcgttgtgatttgcttcgattgatgagataatcatctgg[G/T]tttctctctgattgcataaaggaagagtttttattgaaagggtttgaagggattttgttttttgatgagatatggatgagcatgtagcagctttgcacac

fava_c17880_719	fava_c17880	719	G/A	ggtttcaagggatattagagcctttggtgaatctatgggaaggcagagttctggtgtctgcagttttcaagagatggtagattatttcaatcataaggca[G/A]atatacccgggagtaatcctctcgggagcttcaactctgcatttagctttactggttcaaaacatgttgatgctgcagctacaaaaaccttgtcttctga

fava_c17881_1445	fava_c17881	1445	T/C	acttgaatatgcgagtccctggttcgtcaacacagggaaatcccttattgatgcagatgccacaacaatcccaacctagaggtcagatgctaagtgaaaa[T/C]atcagtccYcgtattcccagactaccaaacctcttgggRcagtctactgtatcaaatggaatttccgacggtcttttagggagaaacggcatagctagta

fava_c17881_1454	fava_c17881	1454	C/T	tgcgagtccctggttcgtcaacacagggaaatcccttattgatgcagatgccacaacaatcccaacctagaggtcagatgctaagtgaaaaYatcagtcc[C/T]cgtattcccagactaccaaacctcttgggRcagtctactgtatcaaatggaatttccgacggtcttttagggagaaacggcatagctagtagcagcagag

fava_c17881_1484	fava_c17881	1484	G/A	atcccttattgatgcagatgccacaacaatcccaacctagaggtcagatgctaagtgaaaaYatcagtccYcgtattcccagactaccaaacctcttggg[G/A]cagtctactgtatcaaatggaatttccgacggtcttttagggagaaacggcatagctagtagcagcagagggccttcatttaattctgttcaacagagct

fava_c17881_166	fava_c17881	166	C/T	ttcactttcactccagcttcaaattcagattgaaaattgaaatgaaattaactatgtgaaccaacaccaacgtttgtgtttgttgttttggatctctcag[C/T]tatgaatcttactaacggtaaaggatccatttctacggttactactacgactgtttctatgaaatccggcgaaggttccgatcagtttccggcgggtctc

fava_c17881_1697	fava_c17881	1697	T/C	attctcccatgaatcaaagcactgaaatgtctgttactaattttcctctcggaagcactccaggtatatctagtatcacaaccaaaggcacatttcaacc[T/C]gaagttaattcgggtattaaaggatcagctggRtttccaagttatgaYatttttaacgaactgaaccatcagaagcctcgKgattggggaatgacaaacc

fava_c17881_1730	fava_c17881	1730	A/G	ttactaattttcctctcggaagcactccaggtatatctagtatcacaaccaaaggcacatttcaaccYgaagttaattcgggtattaaaggatcagctgg[A/G]tttccaagttatgaYatttttaacgaactgaaccatcagaagcctcgKgattggggaatgacaaaccccggtctaacatatgattcctcgcagcatgcaa

fava_c17881_1745	fava_c17881	1745	T/C	tcggaagcactccaggtatatctagtatcacaaccaaaggcacatttcaaccYgaagttaattcgggtattaaaggatcagctggRtttccaagttatga[T/C]atttttaacgaactgaaccatcagaagcctcgKgattggggaatgacaaaccccggtctaacatatgattcctcgcagcatgcaaatcctttacatggta

fava_c17881_1778	fava_c17881	1778	G/T	ccaaaggcacatttcaaccYgaagttaattcgggtattaaaggatcagctggRtttccaagttatgaYatttttaacgaactgaaccatcagaagcctcg[G/T]gattggggaatgacaaaccccggtctaacatatgattcctcgcagcatgcaaatcctttacatggtaacatcgatgtctcgccatcagttttagttcatc

fava_c17881_2324	fava_c17881	2324	C/T	gattgcaattacaagtgtaaatagttgctgttacatatgcatatcattctgcaattagctattttcaaaccgagtctaaattatctagacaaaagaattc[C/T]gttgatgtcaaatattcaaattctgggagtagtgtactatattggttttagtcttttgacgttctttatcaatcaagatgaatcgactacaaaactcgac

fava_c17881_2768	fava_c17881	2768	T/C	ggttgttcaagatatttgttctgactattcttttccagatgtttatgtttgtgtcgatcagtagttttgtgctttaatgtttcagctgtgtctagtctgc[T/C]ctttgattatgttatggattctttgtacatgaacatgatttagcacttgtttgggattttatgttatagctctcataagagtgaatgaataaata

fava_c17881_347	fava_c17881	347	G/T	atcagtttccggcgggtctccgagttcttgttgtggatgatgatcccacgtgtctgaaaatcctcgagaagatgctaaggtcgtgttattatgaagttac[G/T]cagtgcaatcgagccgaagctgcactttcgctactcagggagaacaagaatggatttgacattgttatcagtgatgtccatatgccagacatggatggat

fava_c17882_1595	fava_c17882	1595	C/T	accatgatattgaagccattgatcctgactatttcaaaaatttgaaatggatgcttgagaatgatatcagcgatattttggatcttacttttagcattga[C/T]gcagatgaggaaaaactaatcttatatgaacggacagaagtgactgattatgagttgattcctggtggacggaatatcaaRgttactgaagagaacaagc

fava_c17882_1676	fava_c17882	1676	A/G	atcttacttttagcattgaYgcagatgaggaaaaactaatcttatatgaacggacagaagtgactgattatgagttgattcctggtggacggaatatcaa[A/G]gttactgaagagaacaagcatcagtatgttgatttggttgctgagcatcggttgacaactgctattcgacctcaaataaatgctttcttggaagggttca

fava_c17882_2063	fava_c17882	2063	T/C	aggcccggcttttgcaatttgtgactggcacatccaaggtgcctttagaaggttttagtgctctacaaggaatttcaggttcccagaagtttcagataca[T/C]aaagcataYggaagtcctgatcacttgccttctgctcatacttgtttcaatcaattggatttgccggagtatccatctaaacaacatttggaagagaggt

fava_c17882_2072	fava_c17882	2072	C/T	ttttgcaatttgtgactggcacatccaaggtgcctttagaaggttttagtgctctacaaggaatttcaggttcccagaagtttcagatacaYaaagcata[C/T]ggaagtcctgatcacttgccttctgctcatacttgtttcaatcaattggatttgccggagtatccatctaaacaacatttggaagagaggttactgctag

fava_c17886_1208	fava_c17886	1208	G/A	atcggattaaattagcaatctcgtaattcacagttcccgtttcttcaaccccaagatgaactgacggcaacttttgtggaataaactccctctggtctcc[G/A]tgaccaatgtttgccctcggtcgaccaagttctaaagacattaaccttggctgctgataaccaacagctagcaagtcctgaagccatgccgcggtaagtt

fava_c17886_1505	fava_c17886	1505	T/A	ccaaatacttgattaaacgaccgacattcacgtgaagatccttctcctccgaaaaaggataagcctcaaccctgtcgtacctatggacagtagggggata[T/A]acRgcaacataacccccaacttcccaaagaagccKctgtccccaataacctctcaaaacatcagaagccatcctgctaaccgacgccggaagcatcaaag

fava_c17886_1508	fava_c17886	1508	G/A	aatacttgattaaacgaccgacattcacgtgaagatccttctcctccgaaaaaggataagcctcaaccctgtcgtacctatggacagtagggggataWac[G/A]gcaacataacccccaacttcccaaagaagccKctgtccccaataacctctcaaaacatcagaagccatcctgctaaccgacgccggaagcatcaaagccc

fava_c17886_1540	fava_c17886	1540	T/G	agatccttctcctccgaaaaaggataagcctcaaccctgtcgtacctatggacagtagggggataWacRgcaacataacccccaacttcccaaagaagcc[T/G]ctgtccccaataacctctcaaaacatcagaagccatcctgctaaccgacgccggaagcatcaaagcccaaaacgcaggagaatggtacattgtattaaaa

fava_c17886_1979	fava_c17886	1979	C/T	aacgctgtccgaaatgaacataaggattcacaacggatcgattcggattatcatgactatactgcaacaaaacctcttgcctagcagcctccccaaccaa[C/T]tcaacatcaaaatgtttccccaaatctccatcaatgacttcccctctatcatccgcatcaaaaatcttcctagccccatgttgcattgcaaacaaatacc

fava_c17886_2219	fava_c17886	2219	G/A	ccacaaccctaaaccccaaattagcctgttcatccaaagacaaaaagatcgcacctttcaaattccaatccgccggcgtcctggaatcaccaatcgccac[G/A]acctgccaccctttcaacttcaccagcttcttcaaagcatcactcgggtaatccgaaaccgaaacgacgatccatttctcggagcgaaaattggcaaact

fava_c17886_254	fava_c17886	254	A/T	ggtgagagaaaaaaaaggttaaaaaaccaccctagaaaatttagaagataaaaacaaaaatgaacaagactttataacactaatttttgattgagaagag[A/T]tcgtgtagaagttttatccaagtgttacatgtttctcggttctctattctcggctagtatcaagaaacagtttttacctttcaaaccaattccattagga

fava_c17886_365	fava_c17886	365	T/C	gttttatccaagtgttacatgtttctcggttctctattctcggctagtatcaagaaacagtttttacctttcaaaccaattccattaggagtggatctcc[T/C]tcagccattattctgataagcttaataaactcttgctcacttgatacactccatggatgaacagcagggactttagccgaataaatagttgaggaattag

fava_c17886_545	fava_c17886	545	G/A	aataaatagttgaggaattagtcggaggttttttcttgtaaattgttgtatcaagaataggatcaaaattctgtggagaatccatggagacaaagaacat[G/A]ggaattgcaaccttttgatggatctctagattgtctactaggttaacaagctcaacaaaatcactggtcaagcgctggggaacgtaaaatatctcggaac

fava_c17886_686	fava_c17886	686	A/G	ggttaacaagctcaacaaaatcactggtcaagcgctggggaacgtaaaatatctcggaactgcataacaagaggttcttgtcattgctacttgtttcttt[A/G]taatttacttgaaagtgagctggcatcatgcttacaatcttctgtaccatgcttgcttgttgagataaccagtcagcattgttgtcctcggttataacag

fava_c17887_1964	fava_c17887	1964	G/T	atttgaccgaagaaattttcaccaagtggctgctgaaggtattcaaaatatgttgatgaaagagatattttagttagttggattcccttgttgcaatcca[G/T]ttttcagtttcctgtatagctttgtcaactgaccctcatttgtagttgccatcactcactcttctttcagaattgttttcatttctgtagtttgagtatt

fava_c17887_2149	fava_c17887	2149	A/G	ctgtagtttgagtattggtcctgtatcaatatcaatgctgcatatattagtcatcagtgattatactagccatccctttggagggattcaaatttgtgtt[A/G]ggcaaatcaattatatgcctctctagttttaagcacagtagttgttaattgttaacgatttgccatgggcgtaatcttcatgattcattgcttatgttta

fava_c17888_923	fava_c17888	923	G/A	gaaacaaatggataatgttttccctggattatggttagtgggtataactgctgatgcttatagggtagctgatacaccatcaaagagatagagaagctgc[G/A]aagtgaRttgcaccttgtaaatggggtatggaacttgaggtataaagttgaagaattttgtaaagaaggagatattggaaggagtgaattgaagatgcta

fava_c17888_930	fava_c17888	930	G/A	atggataatgttttccctggattatggttagtgggtataactgctgatgcttatagggtagctgatacaccatcaaagagatagagaagctgcRaagtga[G/A]ttgcaccttgtaaatggggtatggaacttgaggtataaagttgaagaattttgtaaagaaggagatattggaaggagtgaattgaagatgctaattccct

fava_c17890_222	fava_c17890	222	T/C	gaaattggtggttatatatcccataggaaaaagttataactaaaaagtaaatgaactattttctactatattttaaatcatctattaagaaaaacattca[T/C]atgaatgccacctacaagtctacaactacaacatgttcacaatttccctgaaacaggaaactgaKgctgaagtaaagtggcaagagccatgaggacacta

fava_c17890_287	fava_c17890	287	T/G	ctatattttaaatcatctattaagaaaaacattcaYatgaatgccacctacaagtctacaactacaacatgttcacaatttccctgaaacaggaaactga[T/G]gctgaagtaaagtggcaagagccatgaggacactagtgcagatatgtattgactccaattcggtaatttagccttgagcctagactttgaatccaccata

fava_c17895_854	fava_c17895	854	T/A	tgcttcagttgtactcaatttctttggtggatgtatttcttgtattgtttatattaccttttggcattcttctcccatttcctgttggaatcaatgctct[T/A]tttagtcatggaccaaggcgttctgcaggccttgcacgtctatatgctctgtggaaccttacgtcctttataaatgttgtggttgcatttttatgtggat

fava_c17896_230	fava_c17896	230	A/C	cagagttaaaggaaaatgaacaagcatactttcttcatatttaccttcctggttttataaaggagagaataagtatcaaatttgtggcactttctcgaac[A/C]ctaagggtWataggacaaaggcaaataattggtagcaacaaatggagccaatttgatcaatcatatcctgttccagaaaattgtgaggttgagaaactcg

fava_c17896_239	fava_c17896	239	A/T	aggaaaatgaacaagcatactttcttcatatttaccttcctggttttataaaggagagaataagtatcaaatttgtggcactttctcgaacMctaagggt[A/T]ataggacaaaggcaaataattggtagcaacaaatggagccaatttgatcaatcatatcctgttccagaaaattgtgaggttgagaaactcgaaggaaagt

fava_c17896_745	fava_c17896	745	A/C	tataatgtagagtcttctagacctaaagtttttgatgaggaaaataatgtgttactaaaagtaaaaaacaatgcaataggtgcaaaaggtcaaaagggtc[A/C]agaggaaattgatcaaaaatccaaatttaaaatggaacctataaaccaaatagatgatgaaaaatttcaagatgaaattagaaaaaaggccatYttagaa

fava_c17896_839	fava_c17896	839	T/C	agggtcMagaggaaattgatcaaaaatccaaatttaaaatggaacctataaaccaaatagatgatgaaaaatttcaagatgaaattagaaaaaaggccat[T/C]ttagaaaaaattaaaaaacaactacgcgacaacgacaaaaacgatcaaaagaaaaaaagtgttgcaaataagaataaggaaattgaagatgataataaga

fava_c17899_124	fava_c17899	124	T/A	atttgtatatactattacataaataacgtgtttgtatctcatttgaagcttattatttttattatatcaaacttatcttaatagcaaaataacatatata[T/A]aMacataaactatacatcattgattgacatatcttctaacatgatatatacacaagtcttattcaaatcaatatatacatgatttcaccatgtcctttga

fava_c17899_1713	fava_c17899	1713	C/T	ggttgttttttcgatggtgtttttttgtcgtgacattgaatccaggaaagctggcttcctgtgtcaagaaccatttgttgaagctgtggtggtgttccaa[C/T]ggggagagttacgacaagaRccatagaatatttgaaagatgatttgaggtttaaagatgaagatgatgaaagggtagaaagttttgtgttKgtggaaagt

fava_c17899_1733	fava_c17899	1733	G/A	ttttttgtcgtgacattgaatccaggaaagctggcttcctgtgtcaagaaccatttgttgaagctgtggtggtgttccaaYggggagagttacgacaaga[G/A]ccatagaatatttgaaagatgatttgaggtttaaagatgaagatgatgaaagggtagaaagttttgtgttKgtggaaagtggaagagatgttaaagggaa

fava_c17899_1804	fava_c17899	1804	T/G	gtgttccaaYggggagagttacgacaagaRccatagaatatttgaaagatgatttgaggtttaaagatgaagatgatgaaagggtagaaagttttgtgtt[T/G]gtggaaagtggaagagatgttaaagggaaagagatggagaatgagtcgtttgcatggtgattattgggYtttgatgagaaacatgtgaagaaaaagaagg

fava_c17899_1873	fava_c17899	1873	T/C	aagatgatgaaagggtagaaagttttgtgttKgtggaaagtggaagagatgttaaagggaaagagatggagaatgagtcgtttgcatggtgattattggg[T/C]tttgatgagaaacatgtgaagaaaaagaaggatgagagaagaaccatgcagagtgaaacataggaaaacagagccattggaaaaagaggaaaaatgaaga

fava_c17899_608	fava_c17899	608	G/A	cttaattcgtatatcagtataatcattgaaataagattagatgatttaaaatttaagatattattatataaatcatctaatctaaattcaatgatcattc[G/A]gtcaaaagaatctaatatatttctccttttttgtattttatttagccgatttgctacaatcagcctcaccaaatccaactctccgattcgcaagatcgaa

fava_c17899_769	fava_c17899	769	T/C	cagcctcaccaaatccaactctccgattcgcaagatcgaattctatccataaattttgttgatgaatgttaccgataatgttattacccgcacccaatct[T/C]tctgacctaccaattgcaaaacaatggacaccaccttccacctcagcaagaaccctttctttaggaatcactatttgcacccctttctcaaactcaaaca

fava_c17902_543	fava_c17902	543	T/A	tcttgaacttgatcccgccatgttattatccttatagaaactactcttggaagaaggcggtgtcttcgtctccatcactgacggcagaaaattcgacggt[T/A]tcctattttctttcctcaatactgcagagaaatcaggcacagattgagcaacagcataagacccttttctcccctcactagctactccaccttgaaactt

fava_c17904_241	fava_c17904	241	C/T	caaaaaataatttgcatctacacactactttcatacattcaaaaagtgtcccaagattcacccttccccttaaaataagaggaaaattctatgtaagctg[C/T]atctaaggcgtgaccgattttccagattgctactgggggaaaatgaagttgcgatgttacttggttgcaaatcaatcctgagacgtggcagaatattcaa

fava_c17910_1152	fava_c17910	1152	A/C	tcatttttagaaatttgttcttgacctttttgagaaatttcgtccggacccttttgagctatagactcttttgtggcttcttgcgaggttggattctcat[A/C]atcttttgtaacttgatctttttgagaagtttcttctaaacccttttgaggagtagtcactttttgcgcatcttgtggagtagcatcttctttactttca

fava_c17910_901	fava_c17910	901	T/C	tttgaggcatagactcgtgtgttcctttcgatgttgaagtctcgtgatgagatttttcctttctttcaacttttgtaacttgagattccggaaaattttc[T/C]tgttgatcctttttatgtataaactctttYtgagaaatttctttccaaccctttcgaggtacatgttctttttgagaaatttcttctcgRctcttttgag

fava_c17910_931	fava_c17910	931	C/T	atgttgaagtctcgtgatgagatttttcctttctttcaacttttgtaacttgagattccggaaaattttcYtgttgatcctttttatgtataaactcttt[C/T]tgagaaatttctttccaaccctttcgaggtacatgttctttttgagaaatttcttctcgRctcttttgaggcacatgttctttttgagaaatttcttctc

fava_c17910_991	fava_c17910	991	A/G	gaaaattttcYtgttgatcctttttatgtataaactctttYtgagaaatttctttccaaccctttcgaggtacatgttctttttgagaaatttcttctcg[A/G]ctcttttgaggcacatgttctttttgagaaatttcttctcgatccttttgaggtacggactcatttttagaaatttgttcttgacctttttgagaaattt

fava_c17914_310	fava_c17914	310	C/A	tcttccaaatactaaaatcattacaagtttaaaacaaaactgtatacatacaaagaaaattacacaaaaatttcatattctaagctactgcatcttgcta[C/A]aagtcYatttcccatgagccaaagaagtatccattagtctttttctttatcatgcgttcacgatatagtcccttcttcaaatgttcatcctactttgcac

fava_c17914_316	fava_c17914	316	C/T	aaatactaaaatcattacaagtttaaaacaaaactgtatacatacaaagaaaattacacaaaaatttcatattctaagctactgcatcttgctaMaagtc[C/T]atttcccatgagccaaagaagtatccattagtctttttctttatcatgcgttcacgatatagtcccttcttcaaatgttcatcctactttgcacttcttc

fava_c17914_450	fava_c17914	450	T/C	tttttctttatcatgcgttcacgatatagtcccttcttcaaatgttcatcctactttgcacttcttcctttagagctccaagtttcttcattgaccttct[T/C]catgattcaagccaatttaaaacatagaccYttgacacaacattgccaaacctgcatgatgtattagcccannnnctgccgaatcataaccaaatatcac

fava_c17914_481	fava_c17914	481	T/C	ccttcttcaaatgttcatcctactttgcacttcttcctttagagctccaagtttcttcattgaccttctYcatgattcaagccaatttaaaacatagacc[T/C]ttgacacaacattgccaaacctgcatgatgtattagcccannnnctgccgaatcataaccaaatatcacaaaaaacagacatgcattaacgaattaacaa

fava_c17918_159	fava_c17918	159	G/C	aaagttctccttctccggtgaagcgccgtcatggaagcttgcaagccgccgcgctattctcgtaccttctttgaccgaaaatcgcggcgccgccgctctt[G/C]tcgtacctcttcgatcttcgccttgatctgagaagtcagttgcaaaatgaatgatgagattggaggtttatcatttggttctgattttgagttggcggct

fava_c17919_149	fava_c17919	149	G/A	aggtaagaattacaatcactacaaccttgtaaacttaatagcttttcaggattaataaattatatgaagagaggaactacagagacaattgtatacaaga[G/A]aatataagaacaggcttagctctcagcgtgaaaatgcctaaaagtacaagaatatggataatatacactataattctattaacctataaaacctttggag

fava_c17922_1011	fava_c17922	1011	C/T	gtgtgaaacaccgtcaggtgtcaaagatccagaacctagtcttgaaccttgacctgcaccatctggtgtcaatgatcctgatcccattcttgaaccccat[C/T]ttcgagtggagaaatgttcgaaacctaagatcttcggtgcttcccctctgttgaattcaagggatgaacgtctatcgggtaaaggggtagaggtgccaga

fava_c17922_1157	fava_c17922	1157	T/C	ctctgttgaattcaagggatgaacgtctatcgggtaaaggggtagaggtgccagatgttgaaataactgatccaggtgatatgagttgagcaccggggct[T/C]ccaggatattgctgataaggctgaaactcataattgtataatgcaaacttgtgtgttccattacttttacgagctcggtccagagaagatgctaacaatt

fava_c17922_1331	fava_c17922	1331	T/G	ctcggtccagagaagatgctaacaattgagcaaacggaacctcgggagacgacggtgttgtcatctgtacagattcaggtggtggagtaaaagaagcggt[T/G]gaaggctcagttgtgaagtttgaaaataccggaggtgaaaccaactgtgtctcataggcatacggccctatggtgaaaatcgatgcaggaccactagtag

fava_c17922_1553	fava_c17922	1553	T/C	aactaagcgaaagcaatccagccgccggtgagtgagtagaagacgggggatcagattggagaaacgatgccggagacgagggaggagcaatgaaaggcat[T/C]acaatggtagttgaaggattcggtgcagcattggcaaccggaaccgtcggtgcaacaggttcaggaacaagaacagcatggccgattcgcttgctgggtt

fava_c17922_3276	fava_c17922	3276	C/T	ccgatgatatcataaaggaagccgcagctaaaaccgatggattttccggaagggaaatagccaaactgatggcaagtgtccaagctgccgtgtatggaag[C/T]gataactgtgttcttgactcaagcttgttccgtgaagttgtagattacaaagttgctgagcatcaacagagaataaagttggcagcttctgataaaaact

fava_c17922_844	fava_c17922	844	T/C	ccgtagtcagagtctgcaacagacacgccgttggaaatctggttttgtacatggatgttgtctcgagtggttgggccggcaccatctggagtcaaagaac[T/C]ggaacctaacctRgagtctttccctagaccatcaggtgtagtgcacccggaacctaatcgtgatgcgtgtgaaacaccgtcaggtgtcaaagatccagaa

fava_c17922_857	fava_c17922	857	G/A	ctgcaacagacacgccgttggaaatctggttttgtacatggatgttgtctcgagtggttgggccggcaccatctggagtcaaagaacYggaacctaacct[G/A]gagtctttccctagaccatcaggtgtagtgcacccggaacctaatcgtgatgcgtgtgaaacaccgtcaggtgtcaaagatccagaacctagtcttgaac

fava_c17924_1008	fava_c17924	1008	T/A	tagacaatgcaggggctagatggattgtggaagactgtgattggcttcaaggtccaatggcagtagtccaggatgcactttgtgttatgagcaatggatc[T/A]atcatgaagcaagacaaagaagggaaaaagatcataagttcagcaacagattttaaacagagaattgggtttgcaatgattggactaggcggtgatttat

fava_c17924_561	fava_c17924	561	T/C	taagagatatctggattacccttcctgttcttccctcaaagatcaggcaccttgctcactttggtgccgtatctgtagctggaaaattatttgtcattgg[T/C]ggtggaagtgatgctgttgatccagcgaccggtgatcacgatggatgctttgcaaccgatgaagtctggtcatatgaccccataatccagcagtgggctc

fava_c17924_812	fava_c17924	812	C/T	aacggaaaaattgttgtcgccgggggattcactagctgcagaaaaacaatatctcaagcagaaatatatgaccccgagaaggatttttggactccaatcc[C/T]tgatcttcatcgcacacataattcagcctgttcaggaatagtgattggaggaaagatgcatgtactgcacaaggatatgtcaacggtgcaagttttagac

fava_c17925_404	fava_c17925	404	C/A	taagcttatcaccaatatgtgcatcaacaagaacaccagcaacacttctacaactgttagcaagagccctacccacttcacttgcatcatctggttgatt[C/A]actacaagaggactccctcctttgaaaacttcaagtttgttaatgRaaaatgaaccatcagcttcaaccacttccttaaagtcttgtaaacttggtgcat

fava_c17925_450	fava_c17925	450	G/A	tctacaactgttagcaagagccctacccacttcacttgcatcatctggttgattMactacaagaggactccctcctttgaaaacttcaagtttgttaatg[G/A]aaaatgaaccatcagcttcaaccacttccttaaagtcttgtaaacttggtgcataaaccggaatgttgaaatcatcccttttctcattgctaattaaccc

fava_c17930_382	fava_c17930	382	A/T	gattcatcatgcttttctttttctccttcaatttccaccttcatctatatccatgaattttacatgtttgtgtgtaataactctttaatgggatattttt[A/T]atttttKtaatttttttcttYatattttgagtttgtgttaggtgtWtgaagggttgatggaaaaatatttatatttgaactcatgtattatatttcattt

fava_c17930_389	fava_c17930	389	G/T	catgcttttctttttctccttcaatttccaccttcatctatatccatgaattttacatgtttgtgtgtaataactctttaatgggatatttttWattttt[G/T]taatttttttcttYatattttgagtttgtgttaggtgtWtgaagggttgatggaaaaatatttatatttgaactcatgtattatatttcatttgcttatt

fava_c17930_403	fava_c17930	403	C/T	tctccttcaatttccaccttcatctatatccatgaattttacatgtttgtgtgtaataactctttaatgggatatttttWatttttKtaatttttttctt[C/T]atattttgagtttgtgttaggtgtWtgaagggttgatggaaaaatatttatatttgaactcatgtattatatttcatttgcttattttctttttatatgt

fava_c17930_428	fava_c17930	428	A/T	atatccatgaattttacatgtttgtgtgtaataactctttaatgggatatttttWatttttKtaatttttttcttYatattttgagtttgtgttaggtgt[A/T]tgaagggttgatggaaaaatatttatatttgaactcatgtattatatttcatttgcttattttctttttatatgtaactgaagggtaataatcactaaaa

fava_c17931_233	fava_c17931	233	G/A	agcgaagcctgaaaaccgtcgatggttcagctctctccggcaaaacttccaaaactaaaaacctaaaacacgattcacagtcacaaccttcactctactt[G/A]actaacgctgttttcttcggtctcttcttctctgttgcgtactttcttcttcacaggtggcgtgagaaaatccgaacctctactcctctacacgtcctca

fava_c17931_686	fava_c17931	686	G/A	acgaggaagttgtccaggctgtcgtttcaggatctattccgtcttactcacttgaatccaaactcggagattgccggcgtgcggcggcgattcgtaacca[G/A]gcggtggagagagttaccggaaggtcacttgagggtctaccgatggaaaattttgactacgattctatattaggacagtgttgcgagatgccgataggat

fava_c17932_1491	fava_c17932	1491	C/T	ttccatcgccagttctcataatccttatcaggcctcccattaccctggcaattctgcgtctgtgtcaatataggacacgacttgtttgtgtataacggtc[C/T]tgatggatcatatatccaatttccatggtaaagatcacaacctttcacaattgaggaattgctaccaaccaccggaatacgtgcttttgaagccaaaccc

fava_c17932_1656	fava_c17932	1656	A/C	ccaaccaccggaatacgtgcttttgaagccaaacccgaagtttctaagccattcacctccttagttacaggaacaattttcggcgaagtagaatccctaa[A/C]aattacttgYgagtttctattattatcaatccgttctctcttgctaccatcactggacRcaacattagaaactatagaaggYtgcaaatcaaagagtgtt

fava_c17932_1666	fava_c17932	1666	T/C	gaatacgtgcttttgaagccaaacccgaagtttctaagccattcacctccttagttacaggaacaattttcggcgaagtagaatccctaaMaattacttg[T/C]gagtttctattattatcaatccgttctctcttgctaccatcactggacRcaacattagaaactatagaaggYtgcaaatcaaagagtgttttattctctc

fava_c17932_1715	fava_c17932	1715	G/A	cttagttacaggaacaattttcggcgaagtagaatccctaaMaattacttgYgagtttctattattatcaatccgttctctcttgctaccatcactggac[G/A]caacattagaaactatagaaggYtgcaaatcaaagagtgttttattctctcctaaacctaaactggcatccatgcgaacatcaacagaagttgtattcat

fava_c17932_1738	fava_c17932	1738	T/C	gcgaagtagaatccctaaMaattacttgYgagtttctattattatcaatccgttctctcttgctaccatcactggacRcaacattagaaactatagaagg[T/C]tgcaaatcaaagagtgttttattctctcctaaacctaaactggcatccatgcgaacatcaacagaagttgtattcattcgagtttgcgaactgtctacca

fava_c17932_1896	fava_c17932	1896	C/A	tcaacagaagttgtattcattcgagtttgcgaactgtctaccaccatagaaatgaccataaaggatgaactaatagggtatgatagaaaaaaccatgaag[C/A]aaaagttatgaatactaatgatactccaattgaaacaacaatccatgaaagtggtttcgaattcgaagaaccattaggggatgcaaccatccaacaactc

fava_c17932_330	fava_c17932	330	C/G	tctggactaaaaaacatcatcataatgcaccattcttacataccgataatttcgatctcttcgacaccaaaaaaatttaaagttcacacacatacataca[C/G]aacatacaYgagagaagatgaatgctctataatatgcttctatcagataggcatgtaacagcacaagtcgccctccgagcttagaagacgaatSatgttt

fava_c17932_339	fava_c17932	339	C/T	aaaaacatcatcataatgcaccattcttacataccgataatttcgatctcttcgacaccaaaaaaatttaaagttcacacacatacatacaSaacataca[C/T]gagagaagatgaatgctctataatatgcttctatcagataggcatgtaacagcacaagtcgccctccgagcttagaagacgaatSatgtttggtttccga

fava_c17932_424	fava_c17932	424	G/C	catacaSaacatacaYgagagaagatgaatgctctataatatgcttctatcagataggcatgtaacagcacaagtcgccctccgagcttagaagacgaat[G/C]atgtttggtttccgatgaaacaaataatttcatgatgtgctatcgctttcatattctttcttaatgatttcaaacacaatctcattccaagtatctacag

fava_c17932_526	fava_c17932	526	T/C	tgtttggtttccgatgaaacaaataatttcatgatgtgctatcgctttcatattctttcttaatgatttcaaacacaatctcattccaagtatctacagg[T/C]ccaggcatgcaccaatgcaagcaatcttgtggtggtggccttccatctcggcctcgttttgtaaccttRttaggatcagtgctcctatacggaccaggat

fava_c17932_595	fava_c17932	595	A/G	caaacacaatctcattccaagtatctacaggYccaggcatgcaccaatgcaagcaatcttgtggtggtggccttccatctcggcctcgttttgtaacctt[A/G]ttaggatcagtgctcctatacggaccaggatgaccatcgtgtcgatattgaaaggcttcagtaatatccatcaatatcatcttcgatccatttgtcgcct

fava_c17932_706	fava_c17932	706	C/T	tgctcctatacggaccaggatgaccatcgtgtcgatattgaaaggcttcagtaatatccatcaatatcatcttcgatccatttgtcgccttttttaccac[C/T]cggttaaaaactgtaatctgttgtttgtacatcRcagcattatacttattttccaccaattcgccgggcacaagaggcttaactttcccggtgcacgatc

fava_c17932_740	fava_c17932	740	G/A	atattgaaaggcttcagtaatatccatcaatatcatcttcgatccatttgtcgccttttttaccacYcggttaaaaactgtaatctgttgtttgtacatc[G/A]cagcattatacttattttccaccaattcgccgggcacaagaggcttaactttcccggtgcacgatccaccggtgttccatcctccaccctcgtaatggtc

fava_c17935_106	fava_c17935	106	T/A	gtgacgtaaaatagacaagtgaattcagaaaatgaaggaaaaaaaacttggtgcatatcatgaaactactagtgttttacttattccacctataatccct[T/A]actgaaacaaacatttcaaatKtttggtataaaacaactgatatgagcattgtgtgtgggtttctatggtcaaWgcaatgagctWaaatgatttttcaaa

fava_c17935_128	fava_c17935	128	G/T	attcagaaaatgaaggaaaaaaaacttggtgcatatcatgaaactactagtgttttacttattccacctataatccctWactgaaacaaacatttcaaat[G/T]tttggtataaaacaactgatatgagcattgtgtgtgggtttctatggtcaaWgcaatgagctWaaatgatttttcaaattaattcctacaagtatattca

fava_c17935_1386	fava_c17935	1386	A/G	tcacggaagaatatgcaaggcctaccctaccaccaacaagttcaccaagagttgcaccaaaccgtttattgaccgaaggaagggtacttgtgtactgccc[A/G]tcacgggagacatctccaaaacggtttagaagattctctcgaggaattctcacagagcggaatcttagagctccattatcaacaccattaagaccaactt

fava_c17935_180	fava_c17935	180	T/A	ttttacttattccacctataatccctWactgaaacaaacatttcaaatKtttggtataaaacaactgatatgagcattgtgtgtgggtttctatggtcaa[T/A]gcaatgagctWaaatgatttttcaaattaattcctacaagtatattcatcttcttcgcagctKttattttattcaaatccaacatcttgtgtgtactgag

fava_c17935_191	fava_c17935	191	A/T	ccacctataatccctWactgaaacaaacatttcaaatKtttggtataaaacaactgatatgagcattgtgtgtgggtttctatggtcaaWgcaatgagct[A/T]aaatgatttttcaaattaattcctacaagtatattcatcttcttcgcagctKttattttattcaaatccaacatcttgtgtgtactgagagtaagctcct

fava_c17935_243	fava_c17935	243	T/G	actgatatgagcattgtgtgtgggtttctatggtcaaWgcaatgagctWaaatgatttttcaaattaattcctacaagtatattcatcttcttcgcagct[T/G]ttattttattcaaatccaacatcttgtgtgtactgagagtaagctcctgattgcttggcaataggtgcccgtaatacatgatcaggaagatcaaatgcat

fava_c17935_441	fava_c17935	441	G/A	catcaacaagttcctttgcaatattcctcacttggaaacttagatattctgaaagtttgtggattgccttggctttgttgggagcaacatagtcaacatt[G/A]cggtaggttccaatgtcgttccatattcgatccagagcataaaggtcacatgcaagcttMagagcagcttgagcacttgggtcaggacagctctgSacag

fava_c17935_501	fava_c17935	501	C/A	ggattgccttggctttgttgggagcaacatagtcaacattRcggtaggttccaatgtcgttccatattcgatccagagcataaaggtcacatgcaagctt[C/A]agagcagcttgagcacttgggtcaggacagctctgSacagcttcaatgaatctagcaagaatttctgactcaatatgtgattctgcaagtgtcaatagat

fava_c17935_537	fava_c17935	537	C/G	cattRcggtaggttccaatgtcgttccatattcgatccagagcataaaggtcacatgcaagcttMagagcagcttgagcacttgggtcaggacagctctg[C/G]acagcttcaatgaatctagcaagaatttctgactcaatatgtgattctgcaagtgtcaatagatgatttaaacatctattccatgcaccaaagtccccaa

fava_c17935_669	fava_c17935	669	A/T	actcaatatgtgattctgcaagtgtcaatagatgatttaaacatctattccatgcaccaaagtccccaagagattttgaatgcttttgaagtcgaacagc[A/T]acgctatgaagtaatctagatgttcggtatctgaaagcatccaattgaaatttcggatttcgtagatggtcttgactttcccatctagcagtaactggat

fava_c17936_1863	fava_c17936	1863	G/A	gctctcatgcaacatatgatagtactgatcaacaaaggcatgaccaacaacatcgggtgccggagttttcgctggagctagctgctctgaagttgccatt[G/A]caatggtgtcaaaaagccaacaacaactgataggagaaggagtSgtgaaatgagagcgagtagaggaagcgtcttctcggtgctttctctctccctctct

fava_c17936_1907	fava_c17936	1907	G/C	caacaacatcgggtgccggagttttcgctggagctagctgctctgaagttgccattRcaatggtgtcaaaaagccaacaacaactgataggagaaggagt[G/C]gtgaaatgagagcgagtagaggaagcgtcttctcggtgctttctctctccctctctgcgaaggggtttgctagggtttggatcttacagagaggagcgaa

fava_c17936_398	fava_c17936	398	T/C	tccaattgctactgcaaattgttataaactatcaatcagtcagactcttagatgcatagcttcctcaagcagaggcaggaacacgattagcagcagtagg[T/C]ttgggagccgagttaacagaaggcccaccagtacgatttgtacggccaccattggttcccatatgatcgtttccctgatacccatcacctccacggcttg

fava_c17936_599	fava_c17936	599	G/A	aaaatcctccccgattaccattcctatatccatattcccctcttccattaaagtctctgcgcccgttatagtctctgcgcccgttatagtctccatggcc[G/A]ttatagtctccatggccattatagtctccatggccRttatagtctccacggccattaaagtctccatggccaaaacttcggctatttccatagtttcctc

fava_c17936_635	fava_c17936	635	A/G	cccctcttccattaaagtctctgcgcccgttatagtctctgcgcccgttatagtctccatggccRttatagtctccatggccattatagtctccatggcc[A/G]ttatagtctccacggccattaaagtctccatggccaaaacttcggctatttccatagtttcctcgccctcttgctacctcacccYtgaatccaggtaccc

fava_c17936_720	fava_c17936	720	C/T	atagtctccatggccRttatagtctccacggccattaaagtctccatggccaaaacttcggctatttccatagtttcctcgccctcttgctacctcaccc[C/T]tgaatccaggtacccttccagacgagaatcgacctctataacctcgattagttgatctcttttcctcgacaataacgttacgaccattgatcagaattgg

fava_c17937_2002	fava_c17937	2002	T/A	tctgtggattggtacaaaagatggttggatataatgtgacgagatcaaaagaaccaactgaatgtggtggattgctgtttctcttgttggaaggcagtcg[T/A]ctgggtttaagcctttacttgatatctttcctttgtgtctataaaaatttgtgaattttggtttgcacgggcattttgtcatgtgtcctgtataccttat

fava_c17941_152	fava_c17941	152	C/A	tggtgatattcagcaagagtacttgttgtttgagccattctgtaacatcactgatacgtagctttggagccaaccctattgtttatgagcttgataagat[C/A]acaaatgggtcgcagattgagagagagttgcttcaaatggggtgcaaaccaagtgtgcctgctgttttcataggacaacagttcagaggtggttctaaga

fava_c17943_177	fava_c17943	177	T/C	gcaaaatacattgaacaataattttgagtaaaaattgtgtttagaggcaaacactacaaatcacagcttcaaattaaaataaattctaattgagaatatc[T/C]aaacataccaaaatgaatgttaaatgtcacttttgcctcttccaaaaagtgattccaaacatgcattggttcatataaaacagacaaacaaatttattac

fava_c17943_621	fava_c17943	621	T/C	aatgaaacccaccttcacagtgacaagaatcacaatcaccgtcatcaccctcaaaaccaaaaccatccctttgttcctctgaaattcctagtagcatcat[T/C]aactcttcaatttcatcaacatcttcatttgcaattgtgatgtcatctgaaggagtggtttgaatagggttttgatgggtcagtgagaatccttgagaaa

fava_c17945_1012	fava_c17945	1012	G/A	gtaaaggRtaatYgcccattccacgagatgacacaacctccgacacacattcagagaaaaaacaaggcttgctaaattcagaggtatccaatttaagatc[G/A]cggagatatttccagagattcctccaccgtttcgacaaaacactagtcttaacaacatcttctatcRtaagatatgacattatatgaagcagaatctcat

fava_c17945_1079	fava_c17945	1079	A/G	cttgctaaattcagaggtatccaatttaagatcRcggagatatttccagagattcctccaccgtttcgacaaaacactagtcttaacaacatcttctatc[A/G]taagatatgacattatatgaagcagaatctcattgggcaagtcatcgaggctgtgcatcttcatcttcagcctatcagtcattccacttcaacctctctg

fava_c17945_1231	fava_c17945	1231	T/A	tgtgcatcttcatcttcagcctatcagtcattccacttcaacctctctgattattttcttctagtgagttgaacagaattgtgaatcgatagatggtttc[T/A]aagctatgcctgtgtgtggcgttgatgagtgacggcgatcggcggcggtgatgagattggcgctaatgtatgtgctcaaaccactctcatccgatttacc

fava_c17945_172	fava_c17945	172	T/C	ccgatccaaaccgaacgacgaacacccctacattaatcaactattaacataaatacaacaacgcatccttttaattaaagacatttaatgaacattaaca[T/C]gaatatatactactaatcagtataagtttgatcatcagaatagatYagagaWgcactgctctgctcaatattcaattaataagctttaggatattcattg

fava_c17945_218	fava_c17945	218	C/T	acataaatacaacaacgcatccttttaattaaagacatttaatgaacattaacaYgaatatatactactaatcagtataagtttgatcatcagaatagat[C/T]agagaWgcactgctctgctcaatattcaattaataagctttaggatattcattgcatgaaaaaccacttttctgtcacctgaagagatctggaggtgatt

fava_c17945_224	fava_c17945	224	T/A	atacaacaacgcatccttttaattaaagacatttaatgaacattaacaYgaatatatactactaatcagtataagtttgatcatcagaatagatYagaga[T/A]gcactgctctgctcaatattcaattaataagctttaggatattcattgcatgaaaaaccacttttctgtcacctgaagagatctggaggtgattgtattg

fava_c17945_706	fava_c17945	706	C/A	tacagctcgagaagggttcagcatgagcagtatcatcggcctgaatcccaacatgttcgagacgcaaacttcttaaacctggaagatcaagatgcttagg[C/A]agtctagttctttttttgagatcataccttgaaacagtgatataaatagatgtcaaagaatgacaagaaaagatagaatacggcaacccaatattgctcg

fava_c17945_811	fava_c17945	811	C/A	tagttctttttttgagatcataccttgaaacagtgatataaatagatgtcaaagaatgacaagaaaagatagaatacggcaacccaatattgctcgggac[C/A]gctatgttcagctgctgaagaccattcgacatagcatggtttatgagaccagtgaaaatctcgtgccgaaaagctccatggcgtttgaattccagactac

fava_c17945_919	fava_c17945	919	A/G	tcagctgctgaagaccattcgacatagcatggtttatgagaccagtgaaaatctcgtgccgaaaagctccatggcgtttgaattccagactacgtaaagg[A/G]taatYgcccattccacgagatgacacaacctccgacacacattcagagaaaaaacaaggcttgctaaattcagaggtatccaatttaagatcRcggagat

fava_c17945_924	fava_c17945	924	C/T	tgctgaagaccattcgacatagcatggtttatgagaccagtgaaaatctcgtgccgaaaagctccatggcgtttgaattccagactacgtaaaggRtaat[C/T]gcccattccacgagatgacacaacctccgacacacattcagagaaaaaacaaggcttgctaaattcagaggtatccaatttaagatcRcggagatatttc

fava_c17950_1000	fava_c17950	1000	C/T	ttactcgtgcctacgcccaccaaatatatgttgatgggtttttcaacggtgatcctcatccaggaaattttcttgtgagcaaggaatctccacatcgtcc[C/T]gttttactcgactttgggcttacaaagaaactttcaagcaccattaagcaagcacttgcaaagatgtttttgtcatctgttgagggggatcatgtggccc

fava_c17950_2425	fava_c17950	2425	T/C	tagctgatggtggcaagataccaccacctcattcttcttcttctaagccaatacttggaagtcatccccatatccccaaattaacttctaagaaagcccc[T/C]aaaaagcggaaatgtcttggaaggggggtggcaactttacYtgcaattaatagaagttatgaaaaagtctctagcaaagaagaatctgaggttacagaag

fava_c17950_2466	fava_c17950	2466	C/T	tctaagccaatacttggaagtcatccccatatccccaaattaacttctaagaaagccccYaaaaagcggaaatgtcttggaaggggggtggcaactttac[C/T]tgcaattaatagaagttatgaaaaagtctctagcaaagaagaatctgaggttacagaaggaagaagcaacatcRYagatagtagcagtagcgatgatgtg

fava_c17950_2540	fava_c17950	2540	A/G	tcttggaaggggggtggcaactttacYtgcaattaatagaagttatgaaaaagtctctagcaaagaagaatctgaggttacagaaggaagaagcaacatc[A/G]Yagatagtagcagtagcgatgatgtgggtagtagcaatgtggaaagtagtaatccacgaacccatgttcctggtaaggtctatcggaatcctaggatcat

fava_c17950_2541	fava_c17950	2541	T/C	cttggaaggggggtggcaactttacYtgcaattaatagaagttatgaaaaagtctctagcaaagaagaatctgaggttacagaaggaagaagcaacatcR[T/C]agatagtagcagtagcgatgatgtgggtagtagcaatgtggaaagtagtaatccacgaacccatgttcctggtaaggtctatcggaatcctaggatcata

fava_c17950_2657	fava_c17950	2657	A/G	cgatgatgtgggtagtagcaatgtggaaagtagtaatccacgaacccatgttcctggtaaggtctatcggaatcctaggatcatagacgagttcttgggt[A/G]caggagagtacgagaacttaactttgccaaatggaggttttggcttaggatttaagcggtttagttcaaaggatggatcgaccattgcgtttggtcactc

fava_c17950_2924	fava_c17950	2924	C/T	caaaatgtcgtttggaggagtcactggaaaaattgtccagcttgtctgttcagagttaaacattcctgtgccagacgatttcctaagatatgcagcagac[C/T]atacgggagatgcaaatccagggaggcctatgattaattaagggcggaaggttttgtacaatttttccaacactatttatgcataaattttcattttatc

fava_c17950_496	fava_c17950	496	A/G	gtacgatacagaaagagttggggaaatcaatggatgagctattttcggattttgtcaatgaacccttggcaacagcatcaatagcccaagtccaccgtgc[A/G]actctgctcaatggacaggaagtggttgttaaagttcaacaYgatggcatcaaaacagtcatactcgaggacttaaaaaatgcaaaggcaattgttgact

fava_c17950_538	fava_c17950	538	T/C	tttcggattttgtcaatgaacccttggcaacagcatcaatagcccaagtccaccgtgcRactctgctcaatggacaggaagtggttgttaaagttcaaca[T/C]gatggcatcaaaacagtcatactcgaggacttaaaaaatgcaaaggcaattgttgactggattgcatgggccgagccacagtataacttcaatcctatga

fava_c17950_745	fava_c17950	745	A/G	aatggtgcaaagaagctcctaaagaacttgacttcaatcttgaagcagaaaacaccagaactgtagcaaaaaatcttggctgcagaaacctacatgatgg[A/G]aattcaaatcccaatagagtggatgttttaatcccagatgtaattcaggctacagaaaagatccttgttttagagtacatggatggaattcggttgaatg

fava_c17951_420	fava_c17951	420	A/G	ctcgatgttgttgataattcatcggcccggacgaagcgtccgaagccattgaatcatcactttcttcttcatcatctttcttctttctcccatgcttttc[A/G]ccataaacttcaccatgattatgatgatatccatcattttcattgctgcatctagcatcatcttcttgcattggagaatcaatatacatagtccaaccag

fava_c17951_580	fava_c17951	580	T/G	atcttcttgcattggagaatcaatatacatagtccaaccagattcactactactacaaccttcaccccaaatttgtttaaaagaatccatatttttatga[T/G]gaagaagaaaagaatgaaagaaaatatatagatgatgaattaaaaaaaaaaactatatattaaaaatctttatcttgttgagcttcaaaagcataacttg

fava_c17953_1388	fava_c17953	1388	A/G	atcgacacatgattagagtaggggctgcaccatcctttttggtttgagaaaggagcatttgagctgcctccatgtcatccttcttctcactggccacaat[A/G]agtattgagaatgtgatgctgttagggcgcaatcctaaacctttcatttcagataaaacctccaaagctttctgaaatctatccccgttacaaagtgcgg

fava_c17954_1334	fava_c17954	1334	A/G	ttttatcaggtgctagaagttctatttctgcagctagaagttctggtgtgaatggtggtttgatgttagaatctggtaggagaagtggttacagtgaagc[A/G]gaaccaagaagaagtgatttctattatgaatatgagaatggtagaaagagtggagtaatggaagttgatggaagctacataaacagacgtgttttttcac

fava_c17954_716	fava_c17954	716	G/C	aggccatgatggacagaaacaaaggtgttgaagcttataccaatcacatggattgctactactctacttctgattttcttccatgtaagaaacatccttc[G/C]tcttcttcttcttctacttctgctggtggcggtatttgtgcttactgtcttaaggagcgtttggtgaagcttgtgtgttcagattgtggtgaacaaaggc

fava_c17954_869	fava_c17954	869	T/G	aggagcgtttggtgaagcttgtgtgttcagattgtggtgaacaaaggctttcttcttgttcttgttctgatgagatcacttcaaatagaaactcatgttc[T/G]gttgaggtgggaagtgttggcagggtttcttttcttattgagaatgagaagaatcagacaaatttgattcagcatttgagttctaaggccaagatgaatg

fava_c17957_1701	fava_c17957	1701	T/C	tactgtctcgtttttatgagccaacaagaggttgtataacagttggtggagaggatgtccgaacatttgacaaaagtgaatgggcccgggttgttgctat[T/C]gtaaatcaagaacctgttttgttttcggtgtctgttggagaaaatattgcatatggacttccagatgatgatgtttctaaagacgacgtgattaaggcag

fava_c17957_322	fava_c17957	322	G/A	accgctacatgtttacgtcacgggacccgcttccgacccgaatatcgctgatacggatccgaaactcgacgggttgcggcaagaggattcacccacacct[G/A]gggttgtaacgtgggagcttctctctatgcttctcatgaaacataaatttcgtcttgctgtttgtgtaggttctctcttggcatgtaccacctgtaccct

fava_c17957_708	fava_c17957	708	T/C	ttggaagactattgattcagaaggttgagttttttgacaaatacaaggtaggtgaaatcactggtttgttaacatctgatttgggttctcttaagaacat[T/C]gtcggtgagaatgtttcaagggatcgcggattcagggcattatctgaggtcactgggacaatattcatactttttactttatcccctcaactggctccaa

fava_c17958_1040	fava_c17958	1040	A/G	acaaaacaaatatgaggcttttgattgttgcttgtgaacataattgaattcaacgccctatatttctgattctttcacaaaatccataatgatgaaattt[A/G]tgaaaactctttaagcttctttgtcaWgtattgattgtttRctaaccatttccttgtttgttttcatgtgatgttgattaatgagctggaaagagcataa

fava_c17958_1067	fava_c17958	1067	T/A	ttgcttgtgaacataattgaattcaacgccctatatttctgattctttcacaaaatccataatgatgaaatttRtgaaaactctttaagcttctttgtca[T/A]gtattgattgtttRctaaccatttccttgtttgttttcatgtgatgttgattaatgagctggaaagagcataaaggaatacttgaagaagaaacttatgg

fava_c17958_1081	fava_c17958	1081	G/A	aattgaattcaacgccctatatttctgattctttcacaaaatccataatgatgaaatttRtgaaaactctttaagcttctttgtcaWgtattgattgttt[G/A]ctaaccatttccttgtttgttttcatgtgatgttgattaatgagctggaaagagcataaaggaatacttgaagaagaaacttatggaaaggtgtgagaac

fava_c17958_1239	fava_c17958	1239	T/G	aaaggaatacttgaagaagaaacttatggaaaggtgtgagaacaagaagcttgaagatggagaaagaagatttggtgggagattcaaaataaaagcaatg[T/G]aaagttctcttataaatcataggattaggcttgcaagattaagaatttcacttRagcttgtcttgtaaaagtttgagcactaagaccttggatcatgctt

fava_c17958_1293	fava_c17958	1293	A/G	agatggagaaagaagatttggtgggagattcaaaataaaagcaatgKaaagttctcttataaatcataggattaggcttgcaagattaagaatttcactt[A/G]agcttgtcttgtaaaagtttgagcactaagaccttggatcatgcttgtaacaaactttagagaaagtatgtatttgaatgaaaaatcttgcaatataaac

fava_c17958_437	fava_c17958	437	C/T	gaaataaggagattcgagggaggattcacaatctgagatgaaagataaaccttctggactgggacaattgaagtttgggagcgattttagagttcttcgg[C/T]ggtgttcagtgagttctMttcctctttctctctgattttgtaacgataacggttagYatctctYtctctctcagccttatgacYgtatctctcttgattt

fava_c17958_455	fava_c17958	455	A/C	ggaggattcacaatctgagatgaaagataaaccttctggactgggacaattgaagtttgggagcgattttagagttcttcggYggtgttcagtgagttct[A/C]ttcctctttctctctgattttgtaacgataacggttagYatctctYtctctctcagccttatgacYgtatctctcttgatttctgaagggtttgtgtagg

fava_c17958_494	fava_c17958	494	T/C	actgggacaattgaagtttgggagcgattttagagttcttcggYggtgttcagtgagttctMttcctctttctctctgattttgtaacgataacggttag[T/C]atctctYtctctctcagccttatgacYgtatctctcttgatttctgaagggtttgtgtaggaaaatgttgtggttgtggatgtgtgaacaatattctctc

fava_c17958_501	fava_c17958	501	T/C	caattgaagtttgggagcgattttagagttcttcggYggtgttcagtgagttctMttcctctttctctctgattttgtaacgataacggttagYatctct[T/C]tctctctcagccttatgacYgtatctctcttgatttctgaagggtttgtgtaggaaaatgttgtggttgtggatgtgtgaacaatattctctctcttgat

fava_c17958_521	fava_c17958	521	C/T	ttttagagttcttcggYggtgttcagtgagttctMttcctctttctctctgattttgtaacgataacggttagYatctctYtctctctcagccttatgac[C/T]gtatctctcttgatttctgaagggtttgtgtaggaaaatgttgtggttgtggatgtgtgaacaatattctctctcttgataactgtgYaataattgttta

fava_c17958_609	fava_c17958	609	C/T	cagccttatgacYgtatctctcttgatttctgaagggtttgtgtaggaaaatgttgtggttgtggatgtgtgaacaatattctctctcttgataactgtg[C/T]aataattgtttatatactgatgaaattagggttctcttgttgatggatctgagtaatttgttaataggcatttggtaaattttgtgttgggctttgcaag

fava_c17960_578	fava_c17960	578	A/G	ccccaatcctgttccaaagatcatgcttagagagaactcagtgaagaaacaaaattatcaagctattgaaccaagagaacttcagaaaacaccaacagat[A/G]atgtttttcaggtggttgtgatgagggttgcgattcactgtcaaggatgtgctggaaaagtgaagaaacatatctctaagatggaaggagttacatcatt

fava_c17960_766	fava_c17960	766	G/A	gagttacatcattcagtatagatgtagagtctaagagggtgacagtgatggggaacatttctccggtggaagttgttgatagcatttcaaaggtgaaaaa[G/A]gctgagctttggtgttaaagcacttSattaacaccataaaaggaatcataggagatgcagagatagagtcaatgaactttgaatttttcctttgtgtttg

fava_c17960_792	fava_c17960	792	C/G	gagtctaagagggtgacagtgatggggaacatttctccggtggaagttgttgatagcatttcaaaggtgaaaaaRgctgagctttggtgttaaagcactt[C/G]attaacaccataaaaggaatcataggagatgcagagatagagtcaatgaactttgaatttttcctttgtgtttgtggtcctcaatagttgttgggtagaa

fava_c17965_1346	fava_c17965	1346	C/T	gaagctgtcatcaaatgatttcacctcgcttcctattctaccattttcaacaagtgacaatagccttgcaacatcagtagaagttttctgcaagggctta[C/T]ttgcttcagctggtaacgacaatcttctaagtgaagatacaccagttcctggaacagaactgttcaacattctggtcatcttatctgaatgatcaacact

fava_c17965_1586	fava_c17965	1586	T/C	acctggccaccgatgctgatctatcaaccgagaatgcaaaccgtcaactggttttgagttctcagattgatcagaggcattcttcccttgaagaggactt[T/C]tccttctctccggcgtaggcttacgtgtgttttttgtttcaacctgcttgtgtgtcacgtttgaagttggccgcagagtacgatcagaagcactagtaac

fava_c17965_1714	fava_c17965	1714	T/C	tgttttttgtttcaacctgcttgtgtgtcacgtttgaagttggccgcagagtacgatcagaagcactagtaacaggcctctctttcttgctaacaggtat[T/C]gaaatggtatctgactgaaaagaaacactcaaacttcgcattgtggaaggccatagagtctctggcaaacggctactagctacctttttggataaatgca

fava_c17965_1817	fava_c17965	1817	T/A	aatggtatctgactgaaaagaaacactcaaacttcgcattgtggaaggccatagagtctctggcaaacggctactagctacctttttggataaatgcaca[T/A]cgtcgtggacaggcgtagacggRcttgttggagaaggaggggttgcaggccgattcctctcagctgattgagctctcttgggtaacaatttagatgatgc

fava_c17965_1840	fava_c17965	1840	A/G	cactcaaacttcgcattgtggaaggccatagagtctctggcaaacggctactagctacctttttggataaatgcacaWcgtcgtggacaggcgtagacgg[A/G]cttgttggagaaggaggggttgcaggccgattcctctcagctgattgagctctcttgggtaacaatttagatgatgcagttgttcttgtgaagctgtttg

fava_c17965_282	fava_c17965	282	T/C	gacatgtatgagggaagaggggaagaaaaaaaaacctatttaaaagatttacatactaatactacccattttgggtgcagcgcaagagactatttctcgt[T/C]ggcttggcaagtcaggctcatgtgtgtctaaatttctgagattggttacttttttacctccgaagcttgcttgatttgcatcacatgcgaacgaaggcta

fava_c17965_462	fava_c17965	462	A/C	atcacatgcgaacgaaggctatattcctctacctgcatagacactgaaaaagccagtagcgcttcacattcatcaagcatggctttctcttgtgctgata[A/C]gatagcaacttcagaaattaagttattcatggcttccacccgtgagagcaaagggcggattgcagatcccatagcttgcatgacatcaacagctgaacag

fava_c17967_1002	fava_c17967	1002	C/A	attcaaggatagatagtgttgaagttcatctcatctaaattattcaaaataaaatttggataaattggcctaattatattgtggcaaagatgttttccat[C/A]aWggaatgcaacaaagagaattccttttgttatgtttctatgatttctaaaattctgaagcattttaagattggtgtacctaatttacaatatatctcac

fava_c17967_1004	fava_c17967	1004	T/A	tcaaggatagatagtgttgaagttcatctcatctaaattattcaaaataaaatttggataaattggcctaattatattgtggcaaagatgttttccatMa[T/A]ggaatgcaacaaagagaattccttttgttatgtttctatgatttctaaaattctgaagcattttaagattggtgtacctaatttacaatatatctcaccg

fava_c17967_118	fava_c17967	118	A/T	aactacttatcctcatacatcatcaatatcacaacctttcatcctaaatcaaatttaactttcatacccattcatctcaaccaacaccactcttcatcaa[A/T]ctccttcttcaccaaaacccatcaccacccatttaaaaWctgctcatattttcaactccttccatatttgtgtgttttatatcagatttcttgtgtttgg

fava_c17967_1182	fava_c17967	1182	A/C	ctaatttacaatatatctcaccgggattagttaaaaagttcgacaaatgcaccatgaccaacatgggctgtcactgggatgataccaacaaggtatactt[A/C]ttcctctaaagaggtctactaagttcatttataactatgatgaccccactgaatttgatcaagtaaatgttgaagagcaactggtaaatgttgaagaaca

fava_c17967_157	fava_c17967	157	A/T	catcctaaatcaaatttaactttcatacccattcatctcaaccaacaccactcttcatcaaWctccttcttcaccaaaacccatcaccacccatttaaaa[A/T]ctgctcatattttcaactccttccatatttgtgtgttttatatcagatttcttgtgtttggattagtgggtactcctcatttctctcagacacatattcc

fava_c17967_875	fava_c17967	875	A/G	gaattcaattctcaaggcaccaaggttggatgataactttaaccaattttccacaagcctcttgaagcgagatgttcgcattttcaatggattgtttctc[A/G]tgtgttgagaccaaagaagggtggccattcaaggatagatagtgttgaagttcatctcatctaaattattcaaaataaaatttggataaattggcctaat

fava_c17972_1508	fava_c17972	1508	C/T	ctagaaaaagagtcatatgactagtaagattaaacggtgcagtaagccaaaagccaggtggcacattcaactgagcaccacccttatccccaaacttaga[C/T]acaacagaaaccgcYctttcaaaagcctgagtattaagagtaaccccatcSccMaccccaccaaaatcagtcaaattgaaaaccacRggcctcagcttcg

fava_c17972_1523	fava_c17972	1523	C/T	tatgactagtaagattaaacggtgcagtaagccaaaagccaggtggcacattcaactgagcaccacccttatccccaaacttagaYacaacagaaaccgc[C/T]ctttcaaaagcctgagtattaagagtaaccccatcSccMaccccaccaaaatcagtcaaattgaaaaccacRggcctcagcttcggtgactcccaaacct

fava_c17972_1559	fava_c17972	1559	C/G	agccaggtggcacattcaactgagcaccacccttatccccaaacttagaYacaacagaaaccgcYctttcaaaagcctgagtattaagagtaaccccatc[C/G]ccMaccccaccaaaatcagtcaaattgaaaaccacRggcctcagcttcggtgactcccaaacctgctccccactcaaaaccaaaaacccattaatcgcat

fava_c17972_1562	fava_c17972	1562	A/C	caggtggcacattcaactgagcaccacccttatccccaaacttagaYacaacagaaaccgcYctttcaaaagcctgagtattaagagtaaccccatcScc[A/C]accccaccaaaatcagtcaaattgaaaaccacRggcctcagcttcggtgactcccaaacctgctccccactcaaaaccaaaaacccattaatcgcattct

fava_c17972_1595	fava_c17972	1595	A/G	ccccaaacttagaYacaacagaaaccgcYctttcaaaagcctgagtattaagagtaaccccatcSccMaccccaccaaaatcagtcaaattgaaaaccac[A/G]ggcctcagcttcggtgactcccaaacctgctccccactcaaaaccaaaaacccattaatcgcattcttttgccacaccataaaaaccgaaccaagagctg

fava_c17972_317	fava_c17972	317	A/G	tgactgttccatctcctcacaaaaatgatcatgcagatacaatcagacttaaattgaggcctgataaatgatggaacagaagctagtagtttgcaatact[A/G]ttagatatacctcatatttcataatctatatctgttacattctgcgacacagcaggcttaaccaactctccttgctcgttgagctgatcaaaattccgac

fava_c17972_695	fava_c17972	695	A/G	tgaagcttatgtctctgagaattggaagtgcggtagggtcgtatccggcgtcagggtgttcgttgtaattggtcttgataacaattccaacacggacgtt[A/G]tggaaagttagatttcggtaagttatttggcgaacgtatccgcctcttccaggtgcggttttgatcctcatggcacgtctagattcccatacaagaatgt

fava_c17972_800	fava_c17972	800	A/G	aagttagatttcggtaagttatttggcgaacgtatccgcctcttccaggtgcggttttgatcctcatggcacgtctagattcccatacaagaatgttctc[A/G]atSgttacgttggatactccgccagacatctcRcttcctatggagatgccagcactgacattagaacgaacRatgaggtttcggattattatgttcttcg

fava_c17972_803	fava_c17972	803	G/C	ttagatttcggtaagttatttggcgaacgtatccgcctcttccaggtgcggttttgatcctcatggcacgtctagattcccatacaagaatgttctcRat[G/C]gttacgttggatactccgccagacatctcRcttcctatggagatgccagcactgacattagaacgaacRatgaggtttcggattattatgttcttcgaag

fava_c17972_833	fava_c17972	833	G/A	atccgcctcttccaggtgcggttttgatcctcatggcacgtctagattcccatacaagaatgttctcRatSgttacgttggatactccgccagacatctc[G/A]cttcctatggagatgccagcactgacattagaacgaacRatgaggtttcggattattatgttcttcgaaggctttccataagcaattccatattgatccc

fava_c17972_872	fava_c17972	872	A/G	gtctagattcccatacaagaatgttctcRatSgttacgttggatactccgccagacatctcRcttcctatggagatgccagcactgacattagaacgaac[A/G]atgaggtttcggattattatgttcttcgaaggctttccataagcaattccatattgatcccagccacttttaatcgcaattgcatcatcRcccacactta

fava_c17972_962	fava_c17972	962	G/A	tagaacgaacRatgaggtttcggattattatgttcttcgaaggctttccataagcaattccatattgatcccagccacttttaatcgcaattgcatcatc[G/A]cccacacttatgtaacaatcctctatcaacatatcctcgcaagaatctggatcaatgccatcggtattcggagcaagatatacgggagccaatatcgtaa

fava_c17974_342	fava_c17974	342	G/A	attagcctgtaaagcatctggagattcaaatgaatcatgtttctttccaagttgcttggtgttagaaaccttctctttttcaagcagcttgttcataaca[G/A]caYtcatgatgttaatttcttcatcatttattccaccagtttcagctgccgtattcatagataacttttcctttttagggataaaaggactacagtgttc

fava_c17974_345	fava_c17974	345	C/T	agcctgtaaagcatctggagattcaaatgaatcatgtttctttccaagttgcttggtgttagaaaccttctctttttcaagcagcttgttcataacaRca[C/T]tcatgatgttaatttcttcatcatttattccaccagtttcagctgccgtattcatagataacttttcctttttagggataaaaggactacagtgttcctc

fava_c17975_1036	fava_c17975	1036	G/A	acccccgtgaacgtctgggacatccgagcaccagtgccaaatactttctttgtaggattctcttttccgtgccattatcatctgaagccctggaagtttt[G/A]tcgcggaagatggaatgcagaagcgtagtacttgtctttgttcttcccatgacgagaattccagaagagtctctgtctagtctatgcaccagacgagggg

fava_c17975_646	fava_c17975	646	C/A	aatgaagatgtaaatgaggacgcttgtcagagatgctacccttgttcaagttaaggccaaaaggtagtgcctcttccttcagaagctcttcagttgagtc[C/A]tccaaattagacaaatcaaaatgtccccacttcttatgagcttgccatccatacttgtagtccccaactattggtgtacctaacacctcggcacaatgga

fava_c17975_778	fava_c17975	778	G/A	tcttatgagcttgccatccatacttgtagtccccaactattggtgtacctaacacctcggcacaatggactcggagctggtgttttctaccagttaaggg[G/A]RaaagctccaaccatgtataRccatgagatgatgatgcaatcactcggtactctgtaattgcatgttggggtgacagtgatgtagaattgtcaacgattg

fava_c17975_779	fava_c17975	779	G/A	cttatgagcttgccatccatacttgtagtccccaactattggtgtacctaacacctcggcacaatggactcggagctggtgttttctaccagttaagggR[G/A]aaagctccaaccatgtataRccatgagatgatgatgcaatcactcggtactctgtaattgcatgttggggtgacagtgatgtagaattgtcaacgattgt

fava_c17975_799	fava_c17975	799	G/A	acttgtagtccccaactattggtgtacctaacacctcggcacaatggactcggagctggtgttttctaccagttaagggRRaaagctccaaccatgtata[G/A]ccatgagatgatgatgcaatcactcggtactctgtaattgcatgttggggtgacagtgatgtagaattgtcaacgattgttattcgatctgattttccgt

fava_c17984_1216	fava_c17984	1216	G/A	gttattgatctttgagatgtcttaggagctgtcacggaaggctttgaagtaggcgttgaagccctcgaagcggatctagtaggagttgatgatctcaccg[G/A]tggagctgttgacttggtagaagctatcgtgactcgggaggtaggcgtagaSggtctagaYgatttggttattgaaggtaatgtcggccttccagatgga

fava_c17984_1268	fava_c17984	1268	G/C	gcgttgaagccctcgaagcggatctagtaggagttgatgatctcaccgRtggagctgttgacttggtagaagctatcgtgactcgggaggtaggcgtaga[G/C]ggtctagaYgatttggttattgaaggtaatgtcggccttccagatggagttgaagatcttgaagtagctggagatggacctcgtgacaatgaaattctct

fava_c17984_1277	fava_c17984	1277	C/T	ccctcgaagcggatctagtaggagttgatgatctcaccgRtggagctgttgacttggtagaagctatcgtgactcgggaggtaggcgtagaSggtctaga[C/T]gatttggttattgaaggtaatgtcggccttccagatggagttgaagatcttgaagtagctggagatggacctcgtgacaatgaaattctcttactgttac

fava_c17984_1568	fava_c17984	1568	A/G	ttaaagccgttggatgagaattgcgtgtctccgttttactctttactgaaatttgtgattccttgtccaaagtagggaaaagtggtgcatctggcggagt[A/G]agaagccattcataatcagatttgtcattctctgaattcaagaactcttcaaccactgtcttctttggtggaactgaagacaccRtctcagaaaccatag

fava_c17984_1653	fava_c17984	1653	A/G	tgcatctggcggagtRagaagccattcataatcagatttgtcattctctgaattcaagaactcttcaaccactgtcttctttggtggaactgaagacacc[A/G]tctcagaaaccatagagttctcattattggattccaaattggaaaaatcaagttcatcagagttttgaaagaggagaagactgttcttctcattctcttt

fava_c17984_179	fava_c17984	179	T/A	ttgtaacacaaacaaacttataactatatagtttcctacaacaaagttcatcccattttatcacaacacttgtagaccctattgcaaactttctaccatt[T/A]ccacattcacatggaaaataatatcacgttctatttgttccaccacatacaaataaaaatggcaaaagcattgataaccatgccttgacaatcatatgag

fava_c17984_315	fava_c17984	315	T/C	tgttccaccacatacaaataaaaatggcaaaagcattgataaccatgccttgacaatcatatgagtgctgatgattcaataaaccatcttctaaacatca[T/C]tttcttcaatctcgcttccataagatatggagctattatttacactcggttcagagctagcggtgctgcttgtcgcatgcggcgaatcagaaaaactcac

fava_c17984_998	fava_c17984	998	T/C	ctggcaaagacgtcttcaaattcgtaggagcttcattagaaaatccagcaatttgcgggggtttccaaggtctagattttacagctggagagatgccacg[T/C]gattgcaccggactcttcacaacaacaacagggcgagcttttgatgctgacgaaggtcgagtcggaggagcagatactccaaatgctcttgacggagttg

fava_c17995_169	fava_c17995	169	C/T	cccgcggaaccatgaaactcttcaaccatctccgaaacctaaacactcaccaccgcattctccgtaatcgatttcaccctctcttcaacaccaccaactt[C/T]ttctccgaatcttcattgccWcaagaacccaccactccacacgttcaacaaccttcacctccttcttccttgaaccgtaatgaactcgccaagtttgccg

fava_c17995_190	fava_c17995	190	T/A	tcaaccatctccgaaacctaaacactcaccaccgcattctccgtaatcgatttcaccctctcttcaacaccaccaacttYttctccgaatcttcattgcc[T/A]caagaacccaccactccacacgttcaacaaccttcacctccttcttccttgaaccgtaatgaactcgccaagtttgccgccattgctgaatcctggtggg

fava_c17995_479	fava_c17995	479	G/A	ggacccttacagtgctaagccccttgaaggacttaaaattgctgatgtgggctgtggaggtggaattctttccgagccattagctcggatgggagctact[G/A]tgacgggtattgatgctgtggaaaagaatatcaacattgctcgacttcatgctgaatcagatccagcaacttcaactattgaattttgttgcacaacagc

fava_c17995_993	fava_c17995	993	C/T	ccggtaacaggaagatggtcgttatcagatgatataagcgtcaatttcattgccatgggcaccaaaacaaacaacacataatagaatacagaatagaata[C/T]gatagataaaatattactaaaacttgttctttatttatttcatttgtaagaatagtattaaatttgtcttgaatgctaaataaacattgaaaattttaaa

fava_c17998_1166	fava_c17998	1166	T/C	cccagaaaaacccattaacaacctcatgaagagttccatcacctccaactgcaatgacagcatcagctccttcccttatagcctctcgcgttatgtcaat[T/C]gcatgacaagggcctgaagtcaaggattcgcatatattgcattctttaccaaggcgagacttaagatatggaactaacttcctccattctctaccagtcc

fava_c18007_178	fava_c18007	178	G/A	atgattgctatcccaaacatctaacagggtataataatacataatctaatctaacagaacaaaaaattgttcctctgcacaaaggcagggagaacaagaa[G/A]aaaaaaaaRgggggtgaaagtacaaggaaaaaagagacgaccctgtacatgtacatctatcttcgcctctttccaaaaaattaaatgggagaagaacccc

fava_c18007_187	fava_c18007	187	A/G	atcccaaacatctaacagggtataataatacataatctaatctaacagaacaaaaaattgttcctctgcacaaaggcagggagaacaagaaRaaaaaaaa[A/G]gggggtgaaagtacaaggaaaaaagagacgaccctgtacatgtacatctatcttcgcctctttccaaaaaattaaatgggagaagaacccctctaagaac

fava_c18008_1205	fava_c18008	1205	G/A	tcttttgcaattgaagtctcaccttagctgtatccaaaggaatagtacaaaactcggcgaaacaagcggcgaaagcgctgcaaaggaaggattgcgcgaa[G/A]gaaatacggttgggatctgagattgacattgttgagtgtaatgattaagaaagatggttgttgttgtagcaacagtagcggaatagcatcgaaatcaaag

fava_c18008_179	fava_c18008	179	T/C	taacatccactatcaacacaggcacgcagaaaacaaatataggaatacaatctgatgttttattcgattaagaacatatgactatgaggcgtgacttctc[T/C]gcattgcagaagtacgaaataattgtagaaatctttctcttgcaacaaatctaggagcagttgggtagctagcactcatccgagaaaaaaaggtatttct

fava_c18008_377	fava_c18008	377	G/A	tctgcttgctgaatctaatcatcatgaagagtaacaacgatttttatcctctaaaaactcctttagcttgttcaagggtaagaaacataatcacattcca[G/A]gctcctactcgaccaaaattaggaaggaagcctttgtaaaaggccaaaaatccctcattaaacaaagtcttgagaaaacagtcaaatgtgtttttgtagc

fava_c18009_169	fava_c18009	169	A/G	ttcattccattttcccacttacttcctttatcacattcagattcagaattgttgctgctgctataaagtttctgtgatcatcgatttggaattgttggag[A/G]aatttggggctttcaaaaaagcttggttttttggtatcaaatggccgccgtggcggcttgcaggcgattagggaaaatagggaacaattttccccttccg

fava_c18009_915	fava_c18009	915	C/G	gctagaagcagccaaatatgatgtgataaaatactgcataggaacacttgtttcaatatccgctgttggccttgccgttcttcgtatcttgatgtaaaaa[C/G]agaatgtattcagattaacactaacaaagtcgaggtgaaggaaactatagcagctttttggaagatgttgtatgttctgtaWgagaaacaagtatattgc

fava_c18009_997	fava_c18009	997	T/A	cgtatcttgatgtaaaaaSagaatgtattcagattaacactaacaaagtcgaggtgaaggaaactatagcagctttttggaagatgttgtatgttctgta[T/A]gagaaacaagtatattgcagtgctggtggagtggagctgaagtagtttccattagtcatcaaatgggccctttttttaaaagcccttattatttacaaat

fava_c18010_1351	fava_c18010	1351	T/C	gatcctgcttcctccaccgatgaagatgaaaaccagaccgactctgacgccaccaactgaaagttgaaatcacaaaatcccatttttttttggtgatatt[T/C]cagtccagttaagttgtgaatcctcaaatccatccacatggtctataaggttttcttgatttgtgcatattggttttattttctatgcaaccgaatctgt

fava_c18010_1465	fava_c18010	1465	C/T	ttgtgaatcctcaaatccatccacatggtctataaggttttcttgatttgtgcatattggttttattttctatgcaaccgaatctgtcgcttggttgtag[C/T]aattggtgaccatttattttcatgagttagatatagttatgacaataagcatcYacctctattgataaggYccggtacatatttctaaSagttttctgaa

fava_c18010_1519	fava_c18010	1519	T/C	tattggttttattttctatgcaaccgaatctgtcgcttggttgtagYaattggtgaccatttattttcatgagttagatatagttatgacaataagcatc[T/C]acctctattgataaggYccggtacatatttctaaSagttttctgaatttacaaaaataagtatactctaaaaatatattatgcatgagaatgaagtacat

fava_c18010_1536	fava_c18010	1536	T/C	atgcaaccgaatctgtcgcttggttgtagYaattggtgaccatttattttcatgagttagatatagttatgacaataagcatcYacctctattgataagg[T/C]ccggtacatatttctaaSagttttctgaatttacaaaaataagtatactctaaaaatatattatgcatgagaatgaagtacatatatacttgaacccttt

fava_c18010_1554	fava_c18010	1554	G/C	cttggttgtagYaattggtgaccatttattttcatgagttagatatagttatgacaataagcatcYacctctattgataaggYccggtacatatttctaa[G/C]agttttctgaatttacaaaaataagtatactctaaaaatatattatgcatgagaatgaagtacatatatacttgaaccctttatagtaaaagcagtgtaa

fava_c18010_164	fava_c18010	164	T/A	tctcaactcgttgYtgaattttgcagagaatggatgataacgcattgaaagatgaacagcttgttgtaaacgatcaacaagaaatctatgaatcctttgt[T/A]tgctgcgtttgcctggatcttttatacaagcccgttgtgctatcttgtggtcatgtatcatgtttctggtgtgtccataaatcgatgaatagatcgcgcg

fava_c18010_436	fava_c18010	436	G/A	cttcttaagacatatcctgcagcttacaagagaagggaaaatcagatacttgaggaagaacaggaaatggatttcttctcccctcaaattatatgtgatt[G/A]tccggggaattcRcctacctctactatcataaatcaggcatctaactcatccagcacagaatcttttgaaattatggWgcaatctatggagcaatcaggg

fava_c18010_449	fava_c18010	449	G/A	atcctgcagcttacaagagaagggaaaatcagatacttgaggaagaacaggaaatggatttcttctcccctcaaattatatgtgattRtccggggaattc[G/A]cctacctctactatcataaatcaggcatctaactcatccagcacagaatcttttgaaattatggWgcaatctatggagcaatcagggtcYgccaatcata

fava_c18010_514	fava_c18010	514	A/T	tcccctcaaattatatgtgattRtccggggaattcRcctacctctactatcataaatcaggcatctaactcatccagcacagaatcttttgaaattatgg[A/T]gcaatctatggagcaatcagggtcYgccaatcataaaggagatgaaggaatcatttctgaatactcgtctgacagaaaacctcaaattataggaacttct

fava_c18010_539	fava_c18010	539	T/C	cggggaattcRcctacctctactatcataaatcaggcatctaactcatccagcacagaatcttttgaaattatggWgcaatctatggagcaatcagggtc[T/C]gccaatcataaaggagatgaaggaatcatttctgaatactcgtctgacagaaaacctcaaattataggaacttcttctgttgagggaaaaagattgcctc

fava_c18010_77	fava_c18010	77	C/T	ctgattcgttcgttcgattcaatactgattcgttcgttcttcttcatctgttgttaaacccattctcaactcgttg[C/T]tgaattttgcagagaatggatgataacgcattgaaagatgaacagcttgttgtaaacgatcaacaagaaatctatgaatcctttgtWtgctgcgtttgcc

fava_c18010_956	fava_c18010	956	T/C	tagagaagcaatttctagaagagtacacccagagaagagatgcaattcaacttggccaaatcaaagtccaacctgagaccacatcttcttgttcattgcc[T/C]aaggacagcggagaaaagatagcttggtggtctaatcctgaactgttggttcatagaggagttggttgtgatttttgcgggatgtacccaataattgggg

fava_c18014_215	fava_c18014	215	C/T	ttctcttcattaggcagatacttgaaatgttcactagctagtcttagatttttggaataataataatacatgctttcggaacataagcagaaataaacat[C/T]gaSgcaaactcacatgccatgaaggtgaattatgcaaattaagattacagcaaggtgtgaaattgaaaatattggaagagatgcaccctgagaagaagga

fava_c18014_218	fava_c18014	218	G/C	tcttcattaggcagatacttgaaatgttcactagctagtcttagatttttggaataataataatacatgctttcggaacataagcagaaataaacatYga[G/C]gcaaactcacatgccatgaaggtgaattatgcaaattaagattacagcaaggtgtgaaattgaaaatattggaagagatgcaccctgagaagaaggagcc

fava_c18014_341	fava_c18014	341	A/G	tgaattatgcaaattaagattacagcaaggtgtgaaattgaaaatattggaagagatgcaccctgagaagaaggagccaaagctggcgatatagactcgg[A/G]ctcggacggtggaaaagctgaaccaggcccttgatgagYtgagatgtctggatttggagggctaggagttgaaggaattgttggYaatgaagaatcggat

fava_c18014_380	fava_c18014	380	T/C	gaaaatattggaagagatgcaccctgagaagaaggagccaaagctggcgatatagactcggRctcggacggtggaaaagctgaaccaggcccttgatgag[T/C]tgagatgtctggatttggagggctaggagttgaaggaattgttggYaatgaagaatcggatggagaaaaagcagctcctccgggagaaggaaacagtggc

fava_c18014_426	fava_c18014	426	T/C	gcgatatagactcggRctcggacggtggaaaagctgaaccaggcccttgatgagYtgagatgtctggatttggagggctaggagttgaaggaattgttgg[T/C]aatgaagaatcggatggagaaaaagcagctcctccgggagaaggaaacagtggctYcatatctggtgaaagtgtaggagaaaagagaggagtttctggta

fava_c18014_482	fava_c18014	482	C/T	gagatgtctggatttggagggctaggagttgaaggaattgttggYaatgaagaatcggatggagaaaaagcagctcctccgggagaaggaaacagtggct[C/T]catatctggtgaaagtgtaggagaaaagagaggagtttctggtaatRtagttggtgctgctgagattgcagaaacatacattatttttgcagaagccaat

fava_c18014_529	fava_c18014	529	G/A	tgaagaatcggatggagaaaaagcagctcctccgggagaaggaaacagtggctYcatatctggtgaaagtgtaggagaaaagagaggagtttctggtaat[G/A]tagttggtgctgctgagattgcagaaacatacattatttttgcagaagccaatgaacaattgcatgttattgttaaRgctatgaWgaatattgctgagaa

fava_c18014_606	fava_c18014	606	G/A	aaaagagaggagtttctggtaatRtagttggtgctgctgagattgcagaaacatacattatttttgcagaagccaatgaacaattgcatgttattgttaa[G/A]gctatgaWgaatattgctgagaatgaccaaaaggaagccataaatgatgatgccttaaaatcaagtgcttaatgagtgtgtttgagagagaaaaatgtgt

fava_c18014_614	fava_c18014	614	T/A	ggagtttctggtaatRtagttggtgctgctgagattgcagaaacatacattatttttgcagaagccaatgaacaattgcatgttattgttaaRgctatga[T/A]gaatattgctgagaatgaccaaaaggaagccataaatgatgatgccttaaaatcaagtgcttaatgagtgtgtttgagagagaaaaatgtgtatttggag

fava_c18018_131	fava_c18018	131	G/C	tttccattttcccccgatccaaatccaatttactcagaattattcttcaattttctttttcttcttctctttaccctgcgcttttcaattttcttcaatc[G/C]ttttcgttttctcttcgtgttttctgtagttttgcctttttatggtagagatggatggatttggtggcggggatgatgttggaggacttcccaataaact

fava_c18018_1465	fava_c18018	1465	A/T	tcaataatggatggccccgatttaggtctaggtatatgacgactgaggaacttgagaatatattcaggctgcagcttgcagcaacacatagtaatgatcc[A/T]tatgtagatgattattacaatcaaggttgtcttgcaaaaaaatcttcaggtgctaaattgaggcgtcacttttcaccagctcaaataagggaaatccctt

fava_c18018_3119	fava_c18018	3119	C/A	ctaggtttcacatgttgaaagtgaagcagcattgtgggtttggccaatttgaccattggacaagggtggaattggcatgtctaataataagttctgtcaa[C/A]tttttttcttggtgttaatggtcttagtttcatcttcgctagttttgtatgtccYgttctcagtcaattggagtattattggtctatctttcttgtcttt

fava_c18018_3174	fava_c18018	3174	T/C	ttggacaagggtggaattggcatgtctaataataagttctgtcaaMtttttttcttggtgttaatggtcttagtttcatcttcgctagttttgtatgtcc[T/C]gttctcagtcaattggagtattattggtctatctttcttgtctttgctcaaggtttttcttggatttcagttcatgtttaacaaataatctggcattttg

fava_c18022_1333	fava_c18022	1333	T/C	taatttttcctgcttcagatctctccactttataaagaataggcttttcattatccgaatacgcaaaaaacatccctgaattagcaagggcgctgcaaat[T/C]atcttacgagatgccttaatcttgaatcgcccaacactttcagcctttgcataRtctccagttctgcgaacatttttgagatgtagcaaacggacttcta

fava_c18022_1387	fava_c18022	1387	G/A	ccgaatacgcaaaaaacatccctgaattagcaagggcgctgcaaatYatcttacgagatgccttaatcttgaatcgcccaacactttcagcctttgcata[G/A]tctccagttctgcgaacatttttgagatgtagcaaacggacttctaaccaatgtgaagactgtagtaaaagcattggtgattgattgaaggaagtattaa

fava_c18022_1546	fava_c18022	1546	G/A	actgtagtaaaagcattggtgattgattgaaggaagtattaagtgctacttgaatgggtgttctctgaggcaaaggacagatacaatgaggtttaaaccc[G/A]gtgaattctttaacagcatatgcatagagttttgtgtcatctccagctgagataagcatgggatgccctgaatgagcccatttgtggtaactagaatcat

fava_c18022_2114	fava_c18022	2114	G/A	aacaagtgtcccagaccttaaatacagtagtgaccatatgcaaagttcatgtccactgtcaccacccagtctagctggaatcctatgggcttcaagaccc[G/A]atttggcattccagatacgaattatcccatcactacttcctgaaaatatgaacttagcatcgtcgctccaagccacacttaaaactcgtcctgtgacccg

fava_c18022_385	fava_c18022	385	C/T	tacatgattcttcatcccagcaatggaggagaaagtgaaacgacaaaatgggacatttatcaccatgatgaatcccctaaagctgagaacaattctatct[C/T]gaagcctatgtcccataaatatgtctgtggacaggttgaacttctaagttattgactacatcagaccatggtttttctatcataaagaagagactatttg

fava_c18022_553	fava_c18022	553	C/T	atggtttttctatcataaagaagagactatttgaaatatgggataggtataaaactcgatccttttctaacggcaaaacttcaaagttttcaaagttctt[C/T]ctaaatttagtctttttcttaccatttaaatttggcgaattcttgaccactgaatctcgagtatgtaacttttcaccttcatcttgattcacgggcaagc

fava_c18028_2679	fava_c18028	2679	C/T	gtggaacttttaaggaagcggattcacgagtaacgattcccatatccttcacgaagatatcaagtgctgaataaggtgtgtaatcattgtttagcatgtg[C/T]ggaacgcgattttcaaacatccaggatgttcccccactagttgtaataaaatcaaacaataatctggtattcaaacccaatcttgctgcaaatgccattg

fava_c18029_1660	fava_c18029	1660	A/T	gttaaccgaaaaatcgtctccttggttctgcgattccgatgctttacagtaatcaaaaggcaggcttactgacagcaacttgatgaatggttcttaaatt[A/T]acacattctttgaaccgaaaacgaaaagcactgagttcgacttattggcagttctacaaagacgtgcctaaagcagcaaaaactcatcatcaaataagtt

fava_c18031_240	fava_c18031	240	G/T	ccaacaacaaactatgttccacatccgacatatcaatctcttcattccttcacaacgaactctccggccaattccgacaagttttcaaactcatcgacac[G/T]aacaacgacggaaaaatctcatcaacagaactcactgaagtcctttcatgtctaggatacaacaactacacagctgccaaagaagccgaaagcatgatgc

fava_c18034_431	fava_c18034	431	G/T	actccaagatggagaaggaagactcagaagagagaaagcatagagaagcacagttcttgatatacaaagcattagaaaaagttgattctcgaagaaagcc[G/T]tcattcttgaggatYaaaatatttaagttgaagattaagttWgggaacagctgtaggaagatcaagaagagatttttgYtcaagtgtaataggagttgga

fava_c18034_446	fava_c18034	446	C/T	aggaagactcagaagagagaaagcatagagaagcacagttcttgatatacaaagcattagaaaaagttgattctcgaagaaagccKtcattcttgaggat[C/T]aaaatatttaagttgaagattaagttWgggaacagctgtaggaagatcaagaagagatttttgYtcaagtgtaataggagttggagcttgttttattaat

fava_c18034_473	fava_c18034	473	A/T	gagaagcacagttcttgatatacaaagcattagaaaaagttgattctcgaagaaagccKtcattcttgaggatYaaaatatttaagttgaagattaagtt[A/T]gggaacagctgtaggaagatcaagaagagatttttgYtcaagtgtaataggagttggagcttgttttattaataaacaagtttttgaaccatgaaattta

fava_c18034_510	fava_c18034	510	C/T	agttgattctcgaagaaagccKtcattcttgaggatYaaaatatttaagttgaagattaagttWgggaacagctgtaggaagatcaagaagagatttttg[C/T]tcaagtgtaataggagttggagcttgttttattaataaacaagtttttgaaccatgaaatttaaaagtctttcttcttcccttttattttcaaatgattt

fava_c18038_143	fava_c18038	143	G/A	accttcttcttttcaattactacttcactactattcatcgctagaaaactgaagttgaaataagttgtttttctccttctgataagtgattctgagtttt[G/A]aatcttcaacatggatatttccatttcacaaagaaagagaactttgcttaaggtcatcgtccttggagatagcggagttgggaagacatctctgatgaac

fava_c18038_372	fava_c18038	372	G/A	agtataaagccacaattggagctgattttgttacaaaggagctactagttgatgataaactagtaaccttacagatttgggatactgctggacaagagag[G/A]ttccatagtcttggagctgcattttatagaggggcagattgttgtgttttggtatatgatgtaaatatacaaaaaacatttgatacattaagcaattggc

fava_c18043_1565	fava_c18043	1565	A/G	tgggagtataaagagagggagcattcctgatatttgttaccttagtttgttgaagttggttgaataagttatcagcatcttggatgagatgtggcttttt[A/G]cctacgaaggcttgcgcaaccaatgcaagtgccaccccataggcctcaaaactttcggctggaatattactaggtgttgctacaaaaagttcgacctgtt

fava_c18043_219	fava_c18043	219	T/C	tatttgattaaatacccggcaagtaattacagcctggactacattggtgcttgaagaatgcacaaaacaataggtacatacaatacagtggagatagact[T/C]aagggagtgacactcaagtgtattaacctgacatatattactagttacaaaaacctaattatgactcaaggacagctccttcaacaattttccaccctga

fava_c18043_347	fava_c18043	347	T/C	ctgacatatattactagttacaaaaacctaattatgactcaaggacagctccttcaacaattttccaccctgagcctgaataagacatttcatatcttgt[T/C]gtgtagattctgctgttagaagcatcatgttgtggatgacctacagcagtgaggtgtatagattctttcagagttgtttccacgactgcacgttgtccat

fava_c18043_587	fava_c18043	587	G/A	agttgtagtcataggaccaaccaagctctgcaatctcagctgctcgatcagtccatatcttcaacatttcaccgtccaacacctcttgcaagcttactag[G/A]caatgatcaggYccaaaagcttgggatttgatatgttgccacttgcgaactagagcttctgcaacccttgcattcatttttggtaattgctcaactactt

fava_c18043_599	fava_c18043	599	T/C	aggaccaaccaagctctgcaatctcagctgctcgatcagtccatatcttcaacatttcaccgtccaacacctcttgcaagcttactagRcaatgatcagg[T/C]ccaaaagcttgggatttgatatgttgccacttgcgaactagagcttctgcaacccttgcattcatttttggtaattgctcaactacttcttcatctccta

fava_c18043_711	fava_c18043	711	T/G	ggatttgatatgttgccacttgcgaactagagcttctgcaacccttgcattcatttttggtaattgctcaactacttcttcatctcctacaggacctaaa[T/G]tgatagtatctgttgcaattgctgaaccaggcactttatgaaaaataggtctagcaggtaaaaacttcaaaccagctaaagttacgagtccaattactac

fava_c18043_973	fava_c18043	973	G/A	tttatctcagcagtaccttttattcccgaaacctcaacatcgactgaagtatcccgatctgaaaatatcaggggatcacctttttcagaaaagcttgaat[G/A]atccttctcaccattttctaaatgtgtcaagattttatcattggggctaacaggaaaaaccctcttcaatgcttttattgcactagcatggacatggcct

fava_c18044_1117	fava_c18044	1117	C/G	atttcatcccatctctgttcttgatgccatctttcgagcaggagttgaatgatctcttcccaaagaaagaaaaagttttccatttcttgggtagatatct[C/G]ttacaYccaacaaacaatgtgtggggacttgttgtcagatactataatgcatacttagctaaagttgatgagagaataggcatacaaatcagagtgtttg

fava_c18044_1123	fava_c18044	1123	C/T	tcccatctctgttcttgatgccatctttcgagcaggagttgaatgatctcttcccaaagaaagaaaaagttttccatttcttgggtagatatctSttaca[C/T]ccaacaaacaatgtgtggggacttgttgtcagatactataatgcatacttagctaaagttgatgagagaataggcatacaaatcagagtgtttgacaccg

fava_c18044_1320	fava_c18044	1320	G/C	accgatcccggtccatttcaacatgtactggatcaggtcttagcttgtactttgaaggagagtattttgcctcatgttaaccgcgagcagaatcttaatc[G/C]ttcatcaggaacaccaaagtcaaaagctgtactgataacatcgttaaRttctggttattttgaaaaggtaagagacatgtattgggaatatcctacagaa

fava_c18044_1368	fava_c18044	1368	G/A	actttgaaggagagtattttgcctcatgttaaccgcgagcagaatcttaatcSttcatcaggaacaccaaagtcaaaagctgtactgataacatcgttaa[G/A]ttctggttattttgaaaaggtaagagacatgtattgggaatatcctacagaaacgggagaagtcgttggcatttaccagccaagtcatgaaggttatcaa

fava_c18044_1630	fava_c18044	1630	G/C	ccgatgttctagttactagctcatggtctacttttggatatgtggctcaggggcttggaggtttgaaaccatggatactctacaaaccagagaatcgaac[G/C]gccccaaatccgccttgtcaacgtgccatgtcaatggagccatgttttcatgctcctcccttttatgactgcatggctaagagaggaactgacactggtg

fava_c18044_790	fava_c18044	790	G/C	ttctttatgctctcctcactgatcgcgttctactggttgaccctggagtggatatgactgatctattttgtgaaccatttccagatgcttcctggtttgt[G/C]cctcccgattttcctctcaacagccatatSaatagtttcaaccaggaatcaaatcagtgtcacggtaaaatgctgaaatcSaaatcaattacaaattcca

fava_c18044_820	fava_c18044	820	G/C	tactggttgaccctggagtggatatgactgatctattttgtgaaccatttccagatgcttcctggtttgtScctcccgattttcctctcaacagccatat[G/C]aatagtttcaaccaggaatcaaatcagtgtcacggtaaaatgctgaaatcSaaatcaattacaaattccactgtgccatcttttgtttatcttcatctag

fava_c18044_871	fava_c18044	871	G/C	cagatgcttcctggtttgtScctcccgattttcctctcaacagccatatSaatagtttcaaccaggaatcaaatcagtgtcacggtaaaatgctgaaatc[G/C]aaatcaattacaaattccactgtgccatcttttgtttatcttcatctagcccatgattatgatgatcatgacaagcttttcttctgtgatgaagagcaRc

fava_c18044_970	fava_c18044	970	A/G	cSaaatcaattacaaattccactgtgccatcttttgtttatcttcatctagcccatgattatgatgatcatgacaagcttttcttctgtgatgaagagca[A/G]ctttttctccaaaatataccttggttaattatgaaaacagataattatttcatcccatctctgttcttgatgccatctttcgagcaggagttgaatgatc

fava_c18046_1179	fava_c18046	1179	G/A	aagcattccacttcatcttcttaattgcataaacctgtccatcaatttcacctttataaacacacccttgaatcaaaaaactttcatcaaacccatcagt[G/A]gcttccaccaattcatcaaatccaaaaactctatacttatccaaacaatcagaMacattagccataaaattcacatccattcccttcttcttatactcat

fava_c18046_1233	fava_c18046	1233	C/A	tataaacacacccttgaatcaaaaaactttcatcaaacccatcagtRgcttccaccaattcatcaaatccaaaaactctatacttatccaaacaatcaga[C/A]acattagccataaaattcacatccattcccttcttcttatactcatccccYtttctccctttctctcccaaatagatatccctctctttcttctccttct

fava_c18046_1284	fava_c18046	1284	C/T	ccaccaattcatcaaatccaaaaactctatacttatccaaacaatcagaMacattagccataaaattcacatccattcccttcttcttatactcatcccc[C/T]tttctccctttctctcccaaatagatatccctctctttcttctccttcttagacaaaatctctctataaaaccaaatccccaaaatcaaaatcaacaaaa

fava_c18046_204	fava_c18046	204	A/G	attttaataaaatcacaaacaaaatgtccactaggcacactatatcactcacctcattaacactcatcacaaaattcatgcatccattatgatgagcaat[A/G]tctaacacacatatYacatacatccattatgatgagcttaactaatttattttaatgatcacattcttaaactaatcttaataccaaatagaccaacaca

fava_c18046_219	fava_c18046	219	C/T	caaacaaaatgtccactaggcacactatatcactcacctcattaacactcatcacaaaattcatgcatccattatgatgagcaatRtctaacacacatat[C/T]acatacatccattatgatgagcttaactaatttattttaatgatcacattcttaaactaatcttaataccaaatagaccaacacaataacctagttcacc

fava_c18046_429	fava_c18046	429	G/A	atctttgtgaactaaacccatcatcagatagctcaaatccattctcttcacatttagtaagtgaataaacaatatctataatactaggtctctttgatgg[G/A]tctctatgcaaacaagcaatRgcaacactaagcacacccatcaaactttccattgaaagtgtctccttcaaaatagccttatccaaccaatccttaagtc

fava_c18046_450	fava_c18046	450	A/G	catcagatagctcaaatccattctcttcacatttagtaagtgaataaacaatatctataatactaggtctctttgatggRtctctatgcaaacaagcaat[A/G]gcaacactaagcacacccatcaaactttccattgaaagtgtctccttcaaaatagccttatccaaccaatccttaagtctcMtagctttttcttgttcat

fava_c18046_532	fava_c18046	532	C/A	tctatgcaaacaagcaatRgcaacactaagcacacccatcaaactttccattgaaagtgtctccttcaaaatagccttatccaaccaatccttaagtctc[C/A]tagctttttcttgttcatttttcacttcaaatgtttttatagcactagcccacaaaacatttccctcctcatcaatcacttccttcccagaaatcaactc

fava_c18047_1232	fava_c18047	1232	C/T	agggagaatgttagcatcttgtgcttggttaagttatgatagatatataatattcacattttggtggtgctgtcaaaaccagccatattatgtttcttaa[C/T]gtcattctagttgcagtgttctgacatatagatcacagttcaaattctagaaaatggttctctggttgRatgaattaagtttgattgtatttttgaaatt

fava_c18047_1301	fava_c18047	1301	A/G	ctgtcaaaaccagccatattatgtttcttaaYgtcattctagttgcagtgttctgacatatagatcacagttcaaattctagaaaatggttctctggttg[A/G]atgaattaagtttgattgtatttttgaaatttgcttatttggaacttggaagccttttgtgtttaagtcacaattctggcctaaattactagttttatat

fava_c18047_359	fava_c18047	359	A/G	attccgtcatcaccttcttcaaaacccacggtttcttcactcaccacattcaaaccattatcctcaaggatcctgagcttctagtctcaaaccccatcaa[A/G]tcccttttgccaaagttccaatttttatcctccaaaggcgtttccccctcWgatatagtcgccaccgtcatcagaagccctcgtttcctccgtgtaagcc

fava_c18047_410	fava_c18047	410	A/T	aaaccattatcctcaaggatcctgagcttctagtctcaaaccccatcaaRtcccttttgccaaagttccaatttttatcctccaaaggcgtttccccctc[A/T]gatatagtcgccaccgtcatcagaagccctcgtttcctccgtgtaagccttgagaaacacgttatccctgcttttgaattggtaagaagcttctgtccct

fava_c18047_516	fava_c18047	516	A/C	agtcgccaccgtcatcagaagccctcgtttcctccgtgtaagccttgagaaacacgttatccctgcttttgaattggtaagaagcttctgtccctctgat[A/C]aaaaagctattgattcRatcattgtttgtcccacttcgatcagtgacggtcgaatgaaaccaaatgttcaatttttaattgattttggggtaaccccttc

fava_c18047_533	fava_c18047	533	G/A	gaagccctcgtttcctccgtgtaagccttgagaaacacgttatccctgcttttgaattggtaagaagcttctgtccctctgatMaaaaagctattgattc[G/A]atcattgtttgtcccacttcgatcagtgacggtcgaatgaaaccaaatgttcaatttttaattgattttggggtaaccccttcttgtatttatcatttcc

fava_c18047_728	fava_c18047	728	T/C	atttccttaggactagaccttctataatatgttctaatgatttgagaaatgctgtggaagaaattaaggaattagggtttgacccttccaaagtcagttt[T/C]ggtgtagcattattggctaaaagggctatcactaaatcccaatgggatgccaaatttgatgtcttgaagaagtggggttgctctgaagatgcaatttcta

fava_c18047_911	fava_c18047	911	C/T	ctgaagatgcaatttctaatgcatttaagaggcaacctaacttcatgcttaggtcgccgcagaaactcaatgcggtgatgagtttttgggtcgaggagct[C/T]ggttgggatccttctgtgctcttggctgcaccgacgctttttgggtatagtatagagaaaaggcttactccaagggctttgattgtcaagtatcttctgt

fava_c18047_97	fava_c18047	97	C/G	aggttcaacaaaaaacattgggttaaaatttgaagacaattttaaaaccgatgttcctctatccccgcctcagaggtcttctccattcccaaaccc[C/G]aaaatgttaccttgtccactctctaaagtccttctccacaacttcagattcgtctcaacaatccttcaccatatcctacctcatcaacaattgcggttta

fava_c18048_1129	fava_c18048	1129	G/C	ttttggttggatatgctcatgatttgttgtgatcaaacagataactgctccgacaaaacgatatcggtagatgctgataccagtggggaatttccaccac[G/C]aYgacSaatgcaatcaatgttgcaacacctgaaccgatagaccgaaatcRRccgaaattgcgaaatccattagtatgattcattttgatacttttgttgt

fava_c18048_1131	fava_c18048	1131	C/T	ttggttggatatgctcatgatttgttgtgatcaaacagataactgctccgacaaaacgatatcggtagatgctgataccagtggggaatttccaccacSa[C/T]gacSaatgcaatcaatgttgcaacacctgaaccgatagaccgaaatcRRccgaaattgcgaaatccattagtatgattcattttgatacttttgttgttg

fava_c18048_1135	fava_c18048	1135	G/C	ttggatatgctcatgatttgttgtgatcaaacagataactgctccgacaaaacgatatcggtagatgctgataccagtggggaatttccaccacSaYgac[G/C]aatgcaatcaatgttgcaacacctgaaccgatagaccgaaatcRRccgaaattgcgaaatccattagtatgattcattttgatacttttgttgttgttgt

fava_c18048_1179	fava_c18048	1179	G/A	cgacaaaacgatatcggtagatgctgataccagtggggaatttccaccacSaYgacSaatgcaatcaatgttgcaacacctgaaccgatagaccgaaatc[G/A]Rccgaaattgcgaaatccattagtatgattcattttgatacttttgttgttgttgttgttaatgttgatgatgttgtttagaaatttgtgattctttttt

fava_c18048_1180	fava_c18048	1180	A/G	gacaaaacgatatcggtagatgctgataccagtggggaatttccaccacSaYgacSaatgcaatcaatgttgcaacacctgaaccgatagaccgaaatcR[A/G]ccgaaattgcgaaatccattagtatgattcattttgatacttttgttgttgttgttgttaatgttgatgatgttgtttagaaatttgtgattcttttttc

fava_c18048_736	fava_c18048	736	T/C	tcaacacgggtgtgatggtgatggacatggtgaaatggagggaagggaattatcgaaagaggattgagaattggatggagttgcagaagaagaggaggat[T/C]tatgagctgggttctttgccaccttttttgcttgtttttgcagggaatgttgaagcgattgatcatagatggaaYcagcatggacttgggggtgataatt

fava_c18048_811	fava_c18048	811	C/T	tggagttgcagaagaagaggaggatYtatgagctgggttctttgccaccttttttgcttgtttttgcagggaatgttgaagcgattgatcatagatggaa[C/T]cagcatggacttgggggtgataatttgaatggggtttgtagatctttgcatcctgggcctgtgagtttgttgcattggagtgggaaagggaagccgtggg

fava_c18049_720	fava_c18049	720	C/T	cggggctgaaagaagcttctaatgtggagttgcatgagacaggcgtgacattatatgtcgataaaagtgtaccacggaatgagtcaagaacatggacatg[C/T]ccatctgcagttgttaagagaatgagtctcccatcgttactaaacttcacaacatttgcatcagaaatatctcccccaacagaaaatatctcaaagggac

fava_c18052_1120	fava_c18052	1120	G/T	taaagcaaaccaacggttggtttaaccttaggacggtagcgaaaggaaccttaaagctaaccgggtaaattcaaactcactacatactccatggaattga[G/T]aacccggcgtaattggttttctccagcggctctttatcctatattctttcagatggccctgctacgtctgcccactacagaacaggcctgactcccgtga

fava_c18053_1212	fava_c18053	1212	T/C	aatcaccatacacaacggatcaaaaacctccttacatccgagtctcgcaagcgacacaaaaccaactggaaacaattcttcccaaacagccctctgatgc[T/C]gtYtcccagccaaacaaacatttgccaaaacctgcaaaccccaccggaccaacatccgatcagaaccaacctccaacctcaaaatcctcgaaacaacaat

fava_c18053_1215	fava_c18053	1215	T/C	caccatacacaacggatcaaaaacctccttacatccgagtctcgcaagcgacacaaaaccaactggaaacaattcttcccaaacagccctctgatgcYgt[T/C]tcccagccaaacaaacatttgccaaaacctgcaaaccccaccggaccaacatccgatcagaaccaacctccaacctcaaaatcctcgaaacaacaataac

fava_c18053_721	fava_c18053	721	C/T	tcctcttcattacctctcgcactatcattagcacagatgtccctcagtatagtaagcgagtagccaagaacgtccactgcggtaacgccactcggaagac[C/T]agacttgcctctcactgcatgctcaagaaccctcRtagatttcttgaatattccataaacaaacaacgcaacatcattcgaaacagtaacatcttcgatt

fava_c18053_756	fava_c18053	756	A/G	gatgtccctcagtatagtaagcgagtagccaagaacgtccactgcggtaacgccactcggaagacYagacttgcctctcactgcatgctcaagaaccctc[A/G]tagatttcttgaatattccataaacaaacaacgcaacatcattcgaaacagtaacatcttcgatttgctcattcaaaaattctgataggatttgcagaag

fava_c18053_943	fava_c18053	943	G/A	aggatttgcagaagaaacgcttgttcagatgaaaactgatcatcttttgactcactatcttcaccctcgtgaatatcctttaaccgtaatttagaaaaca[G/A]tacaggRagttgagattcttcaagacaaattcttgagagaatcaactttatccaatcctcaccaaaactagcagaagaagcagttctcactaattccacc

fava_c18053_950	fava_c18053	950	A/G	gcagaagaaacgcttgttcagatgaaaactgatcatcttttgactcactatcttcaccctcgtgaatatcctttaaccgtaatttagaaaacaRtacagg[A/G]agttgagattcttcaagacaaattcttgagagaatcaactttatccaatcctcaccaaaactagcagaagaagcagttctcactaattccaccactacag

fava_c18056_1176	fava_c18056	1176	T/C	ggagctgcagcaatggcgtcattgatggttgtaaaatttccactcccatcttgactaaccgtcacaatatctctcaccacaacattctcaccaacttgat[T/C]actctggagaagctttctccgactaacggattcataaatggcgcgtgttttgctcgacattttaaggggtaatcgcccgttacgaaatgagggatgcaga

fava_c18056_1294	fava_c18056	1294	T/A	tccgactaacggattcataaatggcgcgtgttttgctcgacattttaaggggtaatcgcccgttacgaaatgagggatgcagattattatgaaacgaggt[T/A]tttggtttggttttggggacccatcctttggtgaagaaagctagagagacactgtagagtttcgtgtcgttagaaatagggaKtgtgactccattagtgg

fava_c18056_1377	fava_c18056	1377	T/G	ttattatgaaacgaggtWtttggtttggttttggggacccatcctttggtgaagaaagctagagagacactgtagagtttcgtgtcgttagaaataggga[T/G]tgtgactccattagtggaagaaatgtctttaagaccatctaaacatgtctgttggttggttagaatggcactaagtagtgtttggatgttgtcagcttgt

fava_c18056_1774	fava_c18056	1774	T/A	atcgagcgtaatcgtagacatttccgttttgaggagggagaacggatctgcaataggttggatccggtgtggatttgcatgcggttccgggtgaagtagt[T/A]gttattggatttggactaatggtgtctgagaatgctatagaggagaataagagaatcaaacatattgagagaggattgcataacttggaacccattttgg

fava_c18056_507	fava_c18056	507	T/C	cgccatccagaaccatcaacaacattatccataaatgtttgcatataaacagttctagaatattccttccacggtcttcctaggtaagtcgaaacagtgc[T/C]attgctggaagctaaatcgtcagaagctttgattgtacagttgtggattgaagtacctgtgtcttggtttggatcggttcggccttgcgctgttatcgcg

fava_c18056_691	fava_c18056	691	T/A	cttgcgctgttatcgcgttgaattgtccagtcattggtagacgaggatagaggttacagttttggaagacaacctttgcgtttccgaagatgaaatctac[T/A]gttccgtagatgtcgcattctcggtagaattgtcttagggaatgtgtgtatagtgtgtcttggtaaccttcgaaactgcagctgtagaaggttgataaat

fava_c18056_835	fava_c18056	835	C/G	gtgtgtatagtgtgtcttggtaaccttcgaaactgcagctgtagaaggttgataaatctgctccgttacgaagtgctactgcttggtgctttatagcacc[C/G]gcagtgttacggatcgtcatgttcacgccgacRaaaccttgcccaaccacagctaaagttggggaRccgaatgtggtccagccatcaacaacgctatgat

fava_c18056_868	fava_c18056	868	A/G	tgcagctgtagaaggttgataaatctgctccgttacgaagtgctactgcttggtgctttatagcaccSgcagtgttacggatcgtcatgttcacgccgac[A/G]aaaccttgcccaaccacagctaaagttggggaRccgaatgtggtccagccatcaacaacgctatgattaccggtgatgatagtcttgttgataccgtctc

fava_c18056_901	fava_c18056	901	G/A	tacgaagtgctactgcttggtgctttatagcaccSgcagtgttacggatcgtcatgttcacgccgacRaaaccttgcccaaccacagctaaagttgggga[G/A]ccgaatgtggtccagccatcaacaacgctatgattaccggtgatgatagtcttgttgataccgtctccgatcatcatcaaataagtctttttcttatcaa

fava_c18058_100	fava_c18058	100	G/A	aatgagcatcgagttgcaatcataagataagtaattaatcatggcaatattggcatctattcttcatgctcgtagcttcatgKtgattgcttatgcaat[G/A]atgttcagtttcatgggagaaccaacagtaactgctgtgacatttagacccagtaaatggtctcttgctcatgccacattttatggagatgaaactgctt

fava_c18058_241	fava_c18058	241	T/C	catttagacccagtaaatggtctcttgctcatgccacattttatggagatgaaactgcttctgaaaccatgggaggagcatgtggatatgggaatctgtt[T/C]acgaatggttacggaacagatacagtggctttaagctcaactttgttcaacaacggctatgcatgtggaagttgtttccagattaaatgctatcaagcga

fava_c18058_358	fava_c18058	358	T/C	cagatacagtggctttaagctcaactttgttcaacaacggctatgcatgtggaagttgtttccagattaaatgctatcaagcgagtgcatgtaacaaaaa[T/C]gtggccttcaccactgtcaccgccaccaatctttgtccacctaactggtccaagccctccaacaatggtgggtggtgtaatccaccacgagttcattttg

fava_c18058_83	fava_c18058	83	T/G	aatgagcatcgagttgcaatcataagataagtaattaatcatggcaatattggcatctattcttcatgctcgtagcttcatg[T/G]tgattgcttatgcaatRatgttcagtttcatgggagaaccaacagtaactgctgtgacatttagacccagtaaatggtctcttgctcatgccacatttta

fava_c18062_237	fava_c18062	237	C/G	gatagtaagaaatgtgtgaacaaaaaaaaggcatggtggaacacatgaactaacaattctaatacaaagtaaattaaaaaagaaaaagtaatatgatcat[C/G]actaaacatcaacagtgcaRctttgcaaaacctgaaacttaaccgccggagMaaSaactccgacaccattattccgatcaccgacgaacctaacataaSc

fava_c18062_257	fava_c18062	257	A/G	caaaaaaaaggcatggtggaacacatgaactaacaattctaatacaaagtaaattaaaaaagaaaaagtaatatgatcatSactaaacatcaacagtgca[A/G]ctttgcaaaacctgaaacttaaccgccggagMaaSaactccgacaccattattccgatcaccgacgaacctaacataaScaggacagttaacaaaaatat

fava_c18062_289	fava_c18062	289	C/A	acaattctaatacaaagtaaattaaaaaagaaaaagtaatatgatcatSactaaacatcaacagtgcaRctttgcaaaacctgaaacttaaccgccggag[C/A]aaSaactccgacaccattattccgatcaccgacgaacctaacataaScaggacagttaacaaaaatatggtacctccgagagatccaactcccaaccttc

fava_c18062_292	fava_c18062	292	C/G	attctaatacaaagtaaattaaaaaagaaaaagtaatatgatcatSactaaacatcaacagtgcaRctttgcaaaacctgaaacttaaccgccggagMaa[C/G]aactccgacaccattattccgatcaccgacgaacctaacataaScaggacagttaacaaaaatatggtacctccgagagatccaactcccaaccttccac

fava_c18062_336	fava_c18062	336	G/C	tSactaaacatcaacagtgcaRctttgcaaaacctgaaacttaaccgccggagMaaSaactccgacaccattattccgatcaccgacgaacctaacataa[G/C]caggacagttaacaaaaatatggtacctccgagagatccaactcccaaccttccacttaacccttccattaactttaacactaagcaacaccattccgtt

fava_c18062_554	fava_c18062	554	G/A	aagcgctgaatgtaaaaggcgaaaccggaacggcaacagcgtagataaacggcgaccaaactgtgaaatcgttatgcccttggtaagtatccggaagtgc[G/A]gttggaaatgagatctgctgggtttggtaggaRgcgtagacgtgaaggttttgatagtagattccgattttgttgttagggttgtgagtggagagtgtga

fava_c18062_587	fava_c18062	587	A/G	caacagcgtagataaacggcgaccaaactgtgaaatcgttatgcccttggtaagtatccggaagtgcRgttggaaatgagatctgctgggtttggtagga[A/G]gcgtagacgtgaaggttttgatagtagattccgattttgttgttagggttgtgagtggagagtgtgacttgcattgtgagggagagtgtgttgggtgtgg

fava_c18062_752	fava_c18062	752	G/C	tgacttgcattgtgagggagagtgtgttgggtgtgggaaaaggtgaggaagttggagaagagatgttgaaattgtagattgttgcgtcttggattatgaa[G/C]tgtggttttgttggtttgaggatgaggtatattatgaagatggttaggagtattaccaagattagtgatgctatcccggcgattattaagcggaggagtt

fava_c18063_407	fava_c18063	407	T/C	catggatctaagctaaccaccatcttggaaatagctgtcaggtatctttagacccaacagcaaacttagcaatgaagaatccaattgtatcaaggggagc[T/C]gatgggtcRaatctctgaacaaggttttcctcaccaggtcgtaaccattcctctacatatccatcaggaaactcgcagctaccaacaagaccaggccctc

fava_c18063_416	fava_c18063	416	A/G	aagctaaccaccatcttggaaatagctgtcaggtatctttagacccaacagcaaacttagcaatgaagaatccaattgtatcaaggggagcYgatgggtc[A/G]aatctctgaacaaggttttcctcaccaggtcgtaaccattcctctacatatccatcaggaaactcgcagctaccaacaagaccaggccctccaatttttg

fava_c18064_1124	fava_c18064	1124	A/G	tatgctaactcttaatttgttggaaattgttggaatgaatcttgatggaaacaaaagtgttgttgaatcttgtgtctttgagaaatcctggaatgaattt[A/G]ttggaaatatttggaattgcaatgttgaagtgtgtRtgctaattcttcctctttatttYttctttttcatgagttgataaatgagSttgaagaaagtgaa

fava_c18064_1160	fava_c18064	1160	A/G	gaatcttgatggaaacaaaagtgttgttgaatcttgtgtctttgagaaatcctggaatgaatttRttggaaatatttggaattgcaatgttgaagtgtgt[A/G]tgctaattcttcctctttatttYttctttttcatgagttgataaatgagSttgaagaaagtgaatggcgtgaagRggRaaccgtggcatagaggYaagat

fava_c18064_1183	fava_c18064	1183	C/T	ttgttgaatcttgtgtctttgagaaatcctggaatgaatttRttggaaatatttggaattgcaatgttgaagtgtgtRtgctaattcttcctctttattt[C/T]ttctttttcatgagttgataaatgagSttgaagaaagtgaatggcgtgaagRggRaaccgtggcatagaggYaagatggagaagcttggcactattgaga

fava_c18064_1210	fava_c18064	1210	C/G	cctggaatgaatttRttggaaatatttggaattgcaatgttgaagtgtgtRtgctaattcttcctctttatttYttctttttcatgagttgataaatgag[C/G]ttgaagaaagtgaatggcgtgaagRggRaaccgtggcatagaggYaagatggagaagcttggcactattgagatcggaaagcttaaagaatcgagacttt

fava_c18064_1235	fava_c18064	1235	A/G	ttggaattgcaatgttgaagtgtgtRtgctaattcttcctctttatttYttctttttcatgagttgataaatgagSttgaagaaagtgaatggcgtgaag[A/G]ggRaaccgtggcatagaggYaagatggagaagcttggcactattgagatcggaaagcttaaagaatcgagactttgtaacatcctaaagagaattaggca

fava_c18064_1238	fava_c18064	1238	A/G	gaattgcaatgttgaagtgtgtRtgctaattcttcctctttatttYttctttttcatgagttgataaatgagSttgaagaaagtgaatggcgtgaagRgg[A/G]aaccgtggcatagaggYaagatggagaagcttggcactattgagatcggaaagcttaaagaatcgagactttgtaacatcctaaagagaattaggcatag

fava_c18064_1255	fava_c18064	1255	T/C	tgtgtRtgctaattcttcctctttatttYttctttttcatgagttgataaatgagSttgaagaaagtgaatggcgtgaagRggRaaccgtggcatagagg[T/C]aagatggagaagcttggcactattgagatcggaaagcttaaagaatcgagactttgtaacatcctaaagagaattaggcatagaattttcaaaccatttt

fava_c18064_412	fava_c18064	412	G/C	cgcgaatttttccggtttcaaattttgtttatacctttgcgttcagtttcttcttctcgtttcaaatcttaaattcgttttgaaaaagatcaaaagaaaa[G/C]tgtagatctacaaacaaagtttcaaaWKttttttggttaaaacaacaataaacagtcacggaaagaagaaaataagggtggattcagtttcttgagagta

fava_c18064_439	fava_c18064	439	A/T	tttatacctttgcgttcagtttcttcttctcgtttcaaatcttaaattcgttttgaaaaagatcaaaagaaaaStgtagatctacaaacaaagtttcaaa[A/T]Kttttttggttaaaacaacaataaacagtcacggaaagaagaaaataagggtggattcagtttcttgagagtattgtcatccttttggtaagaagatgag

fava_c18064_440	fava_c18064	440	G/T	ttatacctttgcgttcagtttcttcttctcgtttcaaatcttaaattcgttttgaaaaagatcaaaagaaaaStgtagatctacaaacaaagtttcaaaW[G/T]ttttttggttaaaacaacaataaacagtcacggaaagaagaaaataagggtggattcagtttcttgagagtattgtcatccttttggtaagaagatgaga

fava_c18065_1195	fava_c18065	1195	C/T	gttatggcaattatgctgaagcacacgatataggagtacggaagagatacaaagtagctggtcaggacatggaagtgacgtatttccatacttatattga[C/T]ggcgtcgactttgtttttattgacagtccaatatttcgcaatctagagagtaacatatatggcgggaaccgattggatattctaaggcgcatggtattgt

fava_c18065_1804	fava_c18065	1804	C/T	acgactggaaatttcggggaattgtgaacggtgtcgacacaaaagattggaaccctcaattcgacgctttcttgacatcagacggctacactaactacaa[C/T]ctgaaaaccctacgaaccggtaagcctcaatgtaaagcagctttgcaaaaggagctcgggttgcctgtccgcgaggacgttccgataatttccttcatcg

fava_c18065_2226	fava_c18065	2226	A/G	ccgtcaagattcgagccgtgtggactgaatcaactctatgcgatgagttacggaacagttcctgttgtacacggggtgggcggactccgagacacagtgc[A/G]gccttttaatccgttcgatgaatcgggtgttggttggacgtttgatcgcgccgatgccaacaagttaatgacggcattatggaattgcttattgacttat

fava_c18065_2416	fava_c18065	2416	A/G	tattgacttatacggattataaaaagagctgggaagggattcaagaaagagggatgtcacaggatcttagttgggacaatgctgctcaacaatatgaaga[A/G]gtWcttgttgctgccaaataccaatggtgatttcttgacatgtactccatctttgtagtttgatcgaaatgaagggctttcatgctgaatttgtttcacg

fava_c18065_2419	fava_c18065	2419	T/A	tgacttatacggattataaaaagagctgggaagggattcaagaaagagggatgtcacaggatcttagttgggacaatgctgctcaacaatatgaagaRgt[T/A]cttgttgctgccaaataccaatggtgatttcttgacatgtactccatctttgtagtttgatcgaaatgaagggctttcatgctgaatttgtttcacgaga

fava_c18065_534	fava_c18065	534	G/A	aaaaaggttcttgctttgcaaagggaacttattcaacagattgctgaaagaaagaaactagtttcttctatagatagtgacagcattgctggattggaag[G/A]aaacggYgtttcttatttaagcagtgaaaaatctctgtcgagtgactcaaatccacggaaaggttcttctgccagcagcaacgctgttgaaaaccaaaat

fava_c18065_541	fava_c18065	541	C/T	ttcttgctttgcaaagggaacttattcaacagattgctgaaagaaagaaactagtttcttctatagatagtgacagcattgctggattggaagRaaacgg[C/T]gtttcttatttaagcagtgaaaaatctctgtcgagtgactcaaatccacggaaaggttcttctgccagcagcaacgctgttgaaaaccaaaatggcggca

fava_c18066_1102	fava_c18066	1102	C/A	gcaacggcctttctccaaacaaagaaagaggcattggtttgatgtggggtcccgattgtaccgaacaatttttgaagaagtatcaattgaagttaatcat[C/A]aggtctcatgaaggtcctgatgctagggagaagagggatggttttgagggaatggctgaaggatacactattgatcatgttgtagattctggaaagctgg

fava_c18066_1429	fava_c18066	1429	G/A	ctgtaactccaagaccaaaggcgaatccctattatgattatgaagaagtgatcgactctgacggagaattggacttggcctccatggtaacttcatgaga[G/A]atttttgtaagaagtaaatatttgtattatcatttttgagaaacagctgtgatcattaaattttagctgtttagtattcaccctatttattaagtttgtg

fava_c18067_1596	fava_c18067	1596	A/T	gcctcttattgtctcaggagctgatgaccgtcaagtgaaactttggaggatgaatggtaatatgaaaccttgttatttattttactctgcttgaatggta[A/T]ctatctgaaaatatacatggttttcgattttgtctattaaacttctgcttaccgggcttcYgtacaatgtcttgatgctatgcaagtaattacaaatttt

fava_c18067_1657	fava_c18067	1657	C/T	atgaaaccttgttatttattttactctgcttgaatggtaWctatctgaaaatatacatggttttcgattttgtctattaaacttctgcttaccgggcttc[C/T]gtacaatgtcttgatgctatgcaagtaattacaaattttcataaaacagtgtttagaggagcttgtgtattgttggcagaaaaatgttccgtgcaagaca

fava_c18069_2352	fava_c18069	2352	C/G	tttgaaagaaccttcattaaagaatgacgatcaactcgaaactagtgaaaacaagatggaaaataattcagaaatcctctcaccacatggaagtatggtg[C/G]tggtggagaaacaaaatcatacctttaaccatgaagatggacttccaaagaaaatggggagaaaggaaaggatgttaggtttggggaagaaaatggggga

fava_c18069_2528	fava_c18069	2528	A/C	taggtttggggaagaaaatgggggagaagttggaagagaaaagacgtcatattgaagaaaagagcaggcacattgttgagaagatgcgaggaccatgata[A/C]atgttgttcgtggtatgtttgtcttgtgcaaaacaatttctatccatacaatttagattcatgtttactctcatgtttgaggaatgtttcgttttagata

fava_c18069_311	fava_c18069	311	T/C	gcgatgagcttcatgtcgaacaagctcaactggaaactgaatcaaaccagcttgatcatcaacaatctctgcattttgcgtctcacaagcagggtgtggt[T/C]tgggtccttgaatctggaaaaattccaaaagaaccaaaaaggaaagggttattggaggtttcacctattaaattgtatggaacaatcaagggtgagtcac

fava_c18070_1114	fava_c18070	1114	C/T	ctctgcaccaaccatttatcaaaatattgaaagaagccgagctcaatggaatcgaatccttaaactcaataagaacattgtgagcaagttcaacattttg[C/T]cctttcaccaaagcatccaagagcttattcagtgcaattctatcctttttgacgccaaacYtgtcaaWtctgcgaaaagcttcaacagcgtcttcatgct

fava_c18070_1175	fava_c18070	1175	T/C	aaactcaataagaacattgtgagcaagttcaacattttgYcctttcaccaaagcatccaagagcttattcagtgcaattctatcctttttgacgccaaac[T/C]tgtcaaWtctgcgaaaagcttcaacagcgtcttcatgctKacgactcttMgcatacctcctcattaccttagtcattgtatccaatgtcacatacccttc

fava_c18070_1182	fava_c18070	1182	T/A	ataagaacattgtgagcaagttcaacattttgYcctttcaccaaagcatccaagagcttattcagtgcaattctatcctttttgacgccaaacYtgtcaa[T/A]tctgcgaaaagcttcaacagcgtcttcatgctKacgactcttMgcatacctcctcattaccttagtcattgtatccaatgtcacatacccttcaatcatt

fava_c18070_1215	fava_c18070	1215	G/T	cctttcaccaaagcatccaagagcttattcagtgcaattctatcctttttgacgccaaacYtgtcaaWtctgcgaaaagcttcaacagcgtcttcatgct[G/T]acgactcttMgcatacctcctcattaccttagtcattgtatccaatgtcacatacccttcaatcattgacatttccttcactaactcccacatgagatcY

fava_c18070_1225	fava_c18070	1225	C/A	aagcatccaagagcttattcagtgcaattctatcctttttgacgccaaacYtgtcaaWtctgcgaaaagcttcaacagcgtcttcatgctKacgactctt[C/A]gcatacctcctcattaccttagtcattgtatccaatgtcacatacccttcaatcattgacatttccttcactaactcccacatgagatcaaactccttga

fava_c18070_435	fava_c18070	435	A/G	ttcaacaccatctcctcaaagaatgcacaagcctccacaagttttccactcttgcacaaactaagcaccaaaagtgtataagttcccaaatcagggctca[A/G]atcatttttaaacatgtgatccaacaaaaacttaagcaccttcatcctcttctttttgcaacacatcttcaacaatggatgataagtccgaatatccggc

fava_c18070_811	fava_c18070	811	A/G	tgaagatcaatgagctataaaatggagtgtcagccacaataccatccttcttcattctttcatatgcctccaaagctttgctcaattgccccgccttacc[A/G]tagccgagcaacataatagtataagtYacagcattaggagaacaaccattctccctcatctcctccaaaacttgatcaactttgcgaaaatccttgttKt

fava_c18070_838	fava_c18070	838	C/T	tgtcagccacaataccatccttcttcattctttcatatgcctccaaagctttgctcaattgccccgccttaccRtagccgagcaacataatagtataagt[C/T]acagcattaggagaacaaccattctccctcatctcctccaaaacttgatcaactttgcgaaaatccttgttKtgacaataagcttcaatgaaattgttat

fava_c18070_910	fava_c18070	910	T/G	cRtagccgagcaacataatagtataagtYacagcattaggagaacaaccattctccctcatctcctccaaaacttgatcaactttgcgaaaatccttgtt[T/G]tgacaataagcttcaatgaaattgttatacgagaaaacatccggttcaaacccatgttgcttcctctcctccaaaaccttcagcgcattctcatattttc

fava_c18070_97	fava_c18070	97	G/A	tgtcaagaactaatttagcagtttattcattctttcaaaacattatggaatcaaaagtctaataacccacatgattttcaagtgcaacaattacat[G/A]cacactttgcatactttgaaataagggtgtagtattatctagtgactagtgtagacttagtctagccttgaactatggcttccaacactaagtgttatgc

fava_c18071_1297	fava_c18071	1297	T/C	caaagtgaccattcacataatttttgatatccaagtaataagaagtagtttcagagtcacaattttcaacgtaaaaaccgggtttagaatgttgtaggca[T/C]gcttgagcaaaatgggaagtgcgaatagtatttgcccatggtttgcaaacataccttgcagagttgagcaaacagttgagaggaagcaaagtaagtatag

fava_c18071_370	fava_c18071	370	C/G	gattaccaccggatatatactattcgaagaaacctaactatgttggagtacttggcaacgaaaataggctgttggtgtgaagccatacctcatgattgcc[C/G]atatcaatgccctcaatctttgtgacttgtctagtattgacattgtaactaaaatggatgctgtaacggcctctaggaaaaatctctggcgaactagcta

fava_c18071_596	fava_c18071	596	T/C	ccagtaacgaaatagaagaaaattaataataagttcacgaccgcaagcagccacaaaatcaaacggtcccatttcatgataaagagaccattttccagaa[T/C]caaaatccaaaatggagatttgatatgtttcatgaagaacaatactgacaatgcaagtaagttgatttcccctccataagaatgttgtaaaacgataagg

fava_c18075_1412	fava_c18075	1412	A/T	tgtttctgatgttaacaattctgatatggcgagctcttcctttttcaccaatatgcaacagcgattgtcaacaaatccgcagcatggaccgtcagccaac[A/T]tgcaaaccatgtctcaagccaatcaattacaagctgcatttgtttcagcaccccttccccctcagtatgtccaaccaactagtttcatggatggaggaat

fava_c18075_1537	fava_c18075	1537	T/C	aattacaagctgcatttgtttcagcaccccttccccctcagtatgtccaaccaactagtttcatggatggaggaattccttacggatacatgtcaaataa[T/C]ctaccacctccacctccttttcctccacatgctgcatcagggttatcaatgcccagcactcaaccagcacatcagtcaccgccaggaggattttacaggc

fava_c18075_1855	fava_c18075	1855	T/C	ggaatttccgtgagactgtataatgatgtaaattattagatatataaatctgtccagagttcatttgtatccttaaacacccattccttttttattttgc[T/C]tactacctaaattttgtgtggtaggcttttgacatcatagaggtatttgagatgtacttcatcagtgtcaaactactgtttgaaaggaagtacctagtgg

fava_c18075_1987	fava_c18075	1987	T/A	acatcatagaggtatttgagatgtacttcatcagtgtcaaactactgtttgaaaggaagtacctagtggggcctttactaatttttatgaggcatcacaa[T/A]gtccttgttatgatgtatatgaattcttgtttccatgaagctgcctatggggaaaaccataagcagctgatgttattcccctctagatgacagacaccat

fava_c18080_548	fava_c18080	548	G/A	ttaagagaactcatttcttcatgagttagaactgagtatagcagctccattggtagaagaaagtaaaacttgcgcctttcgagttcttcatcggctaata[G/A]tccttgaattcgatgtcctactttgaggtagcttgagtcacaaacaaactgtccgggatattctaccattaactctgcagcttttattgcttttgtgtat

fava_c18083_1233	fava_c18083	1233	A/G	tgaggattggaagctcaagagggattcagtatgcagtgttggcacttactttactgtgttttcatagcaaggaaatgcttttggtgaccctgcaagaagg[A/G]gttttggaaatctgtcatgggttgttagaggatgataatgagaaagttaggagaaattcctccaatttgattcgggttcttcgctgaaacaactatcacc

fava_c18083_1360	fava_c18083	1360	T/C	agaggatgataatgagaaagttaggagaaattcctccaatttgattcgggttcttcgctgaaacaactatcacctgatcagttgatgatggaaaggaaga[T/C]gtgtatttctctcccttgtgaggaggttagttttgcttatttggtttattgggtctStctKgttgggtggatcctttgtacaaaatgaaaagaagatcaa

fava_c18083_1417	fava_c18083	1417	G/C	ctgaaacaactatcacctgatcagttgatgatggaaaggaagaYgtgtatttctctcccttgtgaggaggttagttttgcttatttggtttattgggtct[G/C]tctKgttgggtggatcctttgtacaaaatgaaaagaagatcaaatttttgttgtgaatgggatagggttcttttgtatattgaataatgatgatgggcta

fava_c18083_1421	fava_c18083	1421	T/G	aacaactatcacctgatcagttgatgatggaaaggaagaYgtgtatttctctcccttgtgaggaggttagttttgcttatttggtttattgggtctStct[T/G]gttgggtggatcctttgtacaaaatgaaaagaagatcaaatttttgttgtgaatgggatagggttcttttgtatattgaataatgatgatgggctagcta

fava_c18083_786	fava_c18083	786	T/C	ttcttctcaaccttagcctcgacgacgacactaaaattggacttgtcgccgagggagcaatctcccgcgtcgtcgcttttctcctccgtgcagcctcgtc[T/C]gactgccgtgctttggctgcaactataatcacgagccttgctgtcgtKgaggtKaataaagcgactatcggggctttcccgggagcaatcggagctttgg

fava_c18083_834	fava_c18083	834	G/T	ccgagggagcaatctcccgcgtcgtcgcttttctcctccgtgcagcctcgtcYgactgccgtgctttggctgcaactataatcacgagccttgctgtcgt[G/T]gaggtKaataaagcgactatcggggctttcccgggagcaatcggagctttggtggcgattcttcgggatgggaaggggagagagaggaaagaagcggcta

fava_c18083_840	fava_c18083	840	T/G	gagcaatctcccgcgtcgtcgcttttctcctccgtgcagcctcgtcYgactgccgtgctttggctgcaactataatcacgagccttgctgtcgtKgaggt[T/G]aataaagcgactatcggggctttcccgggagcaatcggagctttggtggcgattcttcgggatgggaaggggagagagaggaaagaagcggctactgcac

fava_c18083_975	fava_c18083	975	A/G	tcggagctttggtggcgattcttcgggatgggaaggggagagagaggaaagaagcggctactgcactttacgcgctttgctgttttcgggataatcggaa[A/G]agggcggtggattgtggcgcggtgccgattttaatgagatgtgttgaatgtggacttgaaagaggtgttgaggttattggtgttttggctaagtgtaagg

fava_c18085_1384	fava_c18085	1384	G/A	aagatgaatccattcctgactcatcgtgaattgtgccctgcaaccagtgatctgcagaagaatccatattgcagacgtgaagagaagataaacccattca[G/A]aagccaagaattgagcagtgtagttggttaaactaaagtatcactcactcacactactatagtatatgactatgttaacaagaattcatagaRtaagaag

fava_c18085_1477	fava_c18085	1477	G/A	ccattcaRaagccaagaattgagcagtgtagttggttaaactaaagtatcactcactcacactactatagtatatgactatgttaacaagaattcataga[G/A]taagaagagaaagaaagaaaatgagagagaggttataaaatttagagtagtgtgttaagtgagaagaatttgcatgtgttagtgtggtttgcattgcatt

fava_c18086_1932	fava_c18086	1932	T/C	tcagtctgttcccattcatggggagaatcattccatccggatcatggattttggaaaagcagcttgaactcatttcaaacaacagcctcttatggactaa[T/C]tatggactacgctctcttgccaaaacaagttccatgtacatgaagcgcaacacagagcacgatcttccatattggagaggtcccatttggataaatatga

fava_c18088_1197	fava_c18088	1197	C/T	tgtgacccaaggtaacgggtttaacaggggttgaccagttttcaaataatggcttcaaagagtaagatgcgattttgtaaggtttttcaaaatcggaaaa[C/T]acccattgttgagtggtcatgcgtgagatKtgaagatgRttctttgcgaattgggaatcggagattagggttttccatgatttgcagacgcacttaaatt

fava_c18088_1227	fava_c18088	1227	T/G	ttgaccagttttcaaataatggcttcaaagagtaagatgcgattttgtaaggtttttcaaaatcggaaaaYacccattgttgagtggtcatgcgtgagat[T/G]tgaagatgRttctttgcgaattgggaatcggagattagggttttccatgatttgcagacgcacttaaattgaacaagatacttcaccggaagccgcaaga

fava_c18088_1236	fava_c18088	1236	A/G	tttcaaataatggcttcaaagagtaagatgcgattttgtaaggtttttcaaaatcggaaaaYacccattgttgagtggtcatgcgtgagatKtgaagatg[A/G]ttctttgcgaattgggaatcggagattagggttttccatgatttgcagacgcacttaaattgaacaagatacttcaccggaagccgcaagagaatctctg

fava_c18088_1341	fava_c18088	1341	G/A	ttgcgaattgggaatcggagattagggttttccatgatttgcagacgcacttaaattgaacaagatacttcaccggaagccgcaagagaatctctgtgat[G/A]agttcttcMggtaagataggcggtggaatgaattgttccggtgagaaaggcggcggaattcggagaataatggcagtggaaaccgtgtcgttttgagagt

fava_c18088_1350	fava_c18088	1350	C/A	gggaatcggagattagggttttccatgatttgcagacgcacttaaattgaacaagatacttcaccggaagccgcaagagaatctctgtgatRagttcttc[C/A]ggtaagataggcggtggaatgaattgttccggtgagaaaggcggcggaattcggagaataatggcagtggaaaccgtgtcgttttgagagtgatgaagat

fava_c18088_510	fava_c18088	510	C/G	tgaacaaatataggccagttagagatttctctgttctcatgaaaagaacactattttctgaaatgaacaaagggtcaacaaaatacaatagacttttctc[C/G]agaggaataatcatcaatttagtccatgactcaacaacgccatactctttcatcaaccacacaacaccgttacaatcccacacacaaagacaattcctca

fava_c18088_630	fava_c18088	630	T/C	tagtccatgactcaacaacgccatactctttcatcaaccacacaacaccgttacaatcccacacacaaagacaattcctcaaaacatatagattaggatg[T/C]gtgcggacMttatcaccatcattttgaggtaataacatttccctgtaagtttccttctctacatcaaaggaaaaaatattcatgagttgatcatcaatat

fava_c18088_639	fava_c18088	639	A/C	actcaacaacgccatactctttcatcaaccacacaacaccgttacaatcccacacacaaagacaattcctcaaaacatatagattaggatgYgtgcggac[A/C]ttatcaccatcattttgaggtaataacatttccctgtaagtttccttctctacatcaaaggaaaaaatattcatgagttgatcatcaatatcatcacttt

fava_c18088_840	fava_c18088	840	T/G	tatataccatccaattcagagtgccactcacaaattttcctatcctcctagttggcctaatggaaaaattctgaatgattttccaaatattttcaccaaa[T/G]gtataaattttgctcaaagattgagtaaaatcacgcttatgtcgcgcaactaatagcatcttgtacttgtcgttaacctgatcatatccaaagccatatt

fava_c18088_990	fava_c18088	990	T/C	ctaatagcatcttgtacttgtcgttaacctgatcatatccaaagccatattgcaagataagccaatcgtgtgacgaaacggctggtggagatttgttgga[T/C]ttgaagcggatagaagggttccacagtttgacagtattttgagagctatgaaagacacatagcagaccattgcatgaacctataatacatctcttgatat

fava_c18089_1067	fava_c18089	1067	G/A	cggcctgggccttcttcagcactattctctttggaaatattgtgatattctatgccgacagtattgcagaggagagattgcacatggttttctcctcacc[G/A]aggcatttgatggcagctagcgcatgcatcattgtcatggagatcgtgtacaagatggatttttctcttacaggcttcgtgatttgcagcttaatattag

fava_c18089_1169	fava_c18089	1169	G/T	ggcatttgatggcagctagcgcatgcatcattgtcatggagatcgtgtacaagatggatttttctcttacaggcttcgtgatttgcagcttaatattagg[G/T]tttgggatataYgaagcaacttcattggagcgaaatagaaaagattctacccgacaatcagatttattgaatggagaattYgataatccaatcgagatgt

fava_c18089_1181	fava_c18089	1181	T/C	cagctagcgcatgcatcattgtcatggagatcgtgtacaagatggatttttctcttacaggcttcgtgatttgcagcttaatattaggKtttgggatata[T/C]gaagcaacttcattggagcgaaatagaaaagattctacccgacaatcagatttattgaatggagaattYgataatccaatcgagatgtcatcgctaccta

fava_c18089_1250	fava_c18089	1250	C/T	tttgcagcttaatattaggKtttgggatataYgaagcaacttcattggagcgaaatagaaaagattctacccgacaatcagatttattgaatggagaatt[C/T]gataatccaatcgagatgtcatcgctacctacttgaatctgttttggagcatcaaatggatgattctattgacacataacatatgtccctttgttatatc

fava_c18089_1462	fava_c18089	1462	T/A	ttgatctctgaaaaattcattttgtgcactactagttgcaaaaatgccgttatcgacgacttccggggacttacagtggcaaaaaagttatcgacccaaa[T/A]tcttgctgattaatccgatttttagggaggttgaactgcacttttagcttacaaagttcatcgttttgtcttgtacatgatcattaattaatctaggagt

fava_c18089_408	fava_c18089	408	T/C	cttgaggatatggtcaatatactccatgttccgatacttatcgcagactggagcttcggttgttctttttctttttgcctgtctggctcctgcagctatc[T/C]tgtttttgattttgcaaaagccttggaagggaaggccacttKYtaatacacaggttgtgccttctataataaatggttttataacagcactgtatctggt

fava_c18089_450	fava_c18089	450	G/T	gcagactggagcttcggttgttctttttctttttgcctgtctggctcctgcagctatcYtgtttttgattttgcaaaagccttggaagggaaggccactt[G/T]Ytaatacacaggttgtgccttctataataaatggttttataacagcactgtatctggtcttatggggaaagggactgaaatcatgtggccctgttagagc

fava_c18089_451	fava_c18089	451	T/C	cagactggagcttcggttgttctttttctttttgcctgtctggctcctgcagctatcYtgtttttgattttgcaaaagccttggaagggaaggccacttK[T/C]taatacacaggttgtgccttctataataaatggttttataacagcactgtatctggtcttatggggaaagggactgaaatcatgtggccctgttagagcg

fava_c18089_695	fava_c18089	695	A/G	gagtgttgtatgggaggagaagcaatctgtggaaaaagataggtggcctaattgccatgtgtgcttcattttacctgctatctgaaggatgggccacagc[A/G]acattttctccgttctcgtggcaagatagagaggaatctgaggctcagaaagaacacgttttgggcttgaagcatatgttaattcctattattgctggta

fava_c18094_152	fava_c18094	152	G/A	accacaaccgcaaagctcgcgccgcttttcacattMgtttcctttcaatttcaacttctttttcttcctcacacttttcacttcttccctaagatttagg[G/A]tttatcaccatgagagccaaattgattgtcttccccattcgcgccagaaactggtgcttctccagatccattgatcgttctctccctcctccttcttcat

fava_c18094_648	fava_c18094	648	A/G	tcgtcgtagactaaggcatattgctatgaggggcacaattgtacacaggaaattcttttatggttctgtttcattgattccattgtcctctgcacttagt[A/G]ttttRcctttgcctaatgtcgcattcttctgggttttataccgatcgtactcgcattggagagcccttcagggaagtgagaaactgtttcaactagtctc

fava_c18094_653	fava_c18094	653	A/G	gtagactaaggcatattgctatgaggggcacaattgtacacaggaaattcttttatggttctgtttcattgattccattgtcctctgcacttagtRtttt[A/G]cctttgcctaatgtcgcattcttctgggttttataccgatcgtactcgcattggagagcccttcagggaagtgagaaactgtttcaactagtctcaaata

fava_c18095_331	fava_c18095	331	T/A	gaaaaggaattgtgatgtgagtagcttctactgcttcttatacttccactgctctataatcaagggttggtaacaaagccaatgcttcaccttcaatgac[T/A]agaatttctccattcttgaagcaccttgtcttaaactttgcaagatatcgatttttattttctcttagattagtagcttctacttcaccaacaatccgat

fava_c18095_563	fava_c18095	563	C/A	cgtttgagacacatacacagctgcaggaaaatgtgatgagatgatattcggaaaaagtgaaacaacaagcatcccatgtaccaatggaccttcaaatccc[C/A]catttcgagcagcagcggaatcggtatgcaaaggattagaatcatgactgactttggaatactgaagaacatcttcctcagtaaaaacccttgctttttt

fava_c18095_728	fava_c18095	728	T/C	aagaacatcttcctcagtaaaaacccttgcttttttcaaaacatcaccaggtttaagtacaagaggaggagctgcagatgaaaaacacctcatactcaga[T/C]actgaagtttcctactagaatgtaaactcctgaagagcattcttgcaaaggctcaactcaacaaaacaatgccctgatgctaatgctgaaaatgaaggtg

fava_c18095_97	fava_c18095	97	C/A	tttactcgcaattacagtacacttttattccaaaaccattagtgttaaagggtacaaaatgccaaaaattgatcatagtcatactgatagattata[C/A]cctttctcccatttgattcatagttatagaaagaactgagatattacaaaacatgaacagatcaacattaaataatcaagcatgagtcattatcaattac

fava_c18096_1927	fava_c18096	1927	C/G	tgttgtgattgaccgcatggaattacaaccaccattacatcgtagaaggcccatgcccatgcctagaaatcctatgcccatcatgcctagaaagcccatg[C/G]cgaacatgcctagaaagcccatgcctagttcaggtttaggaccaataggcttttaaaaaggaaagatgcacagttagttcttcttcattagaatctggtt

fava_c18096_2153	fava_c18096	2153	A/G	gaagagcaataaagaagcgtcaagaaactcaattatgctttgcacaaatggtggcattggtccaccagggacaccaaatgaagtttataggagtaaaatt[A/G]aattggatctcactagcttcaactttaagaactataccaaagggcaaaaggaaaatgcttaccttgtggatttgggaaccaactgtattaaaaccattgt

fava_c18096_621	fava_c18096	621	A/G	gtgatggctcaactacatctgttattcttgctcatggcttaatcaaagaaggggcaaaagttattgccgctggaatgaatcctgtccaaattgtccgtgg[A/G]attgagaagacagcaatagcacttgtttctgaacttagtttgatgtctagagaagttgaagatcatgaacttaaagatgttgcagcagtaagcgcgggga

fava_c18098_725	fava_c18098	725	C/T	agcgctctcggaattttaagctcttccgttgactttttaggctcatagttttggtctcttccctgtatggatgaaaacttggggtctgcttctttagggg[C/T]acattcaatactaattacacctttgYcaggctcaaccttatgatctatcctaacattagaagctgccgcatgtttctgtccgatttcttccttcttttgc

fava_c18098_751	fava_c18098	751	C/T	cgttgactttttaggctcatagttttggtctcttccctgtatggatgaaaacttggggtctgcttctttaggggYacattcaatactaattacacctttg[C/T]caggctcaaccttatgatctatcctaacattagaagctgccgcatgtttctgtccgatttcttccttcttttgctcaccttttcccatcacctcatcctt

fava_c18098_924	fava_c18098	924	C/T	gctcaccttttcccatcacctcatccttctcatcttcctgttcctcaaactcctcttctgactcaggtattggagtaagagacaactgtgtcttcaagcg[C/T]gcgtgaaggttttgatagacctcggaggatggattcaactccaagagtctgttgacatcaaaaagtgcagaatggtactccttcagggtcaccagtgttt

fava_c18101_2367	fava_c18101	2367	G/A	atatagcaaaagagattggaaacatgaaaaacttggaatctcttgatctatctcagaataaacttgttggagaaatccctgataccatttcagttttgtc[G/A]ttcttaagcttcttgaatttgtcaaacaataaccttgttggacaaatcccagtcggcactcagcttcagagttttgatatttcttgctatgctgggaatc

fava_c18104_1235	fava_c18104	1235	A/G	gaagaaatttcaattgaaagattattttgagaatttagaggttccgagttccaaatgacactctacatctatatttctacatctgatatctcttcaatac[A/G]tgtaataatcatactttagagaattgtttttttatcataagattgagacggttatttatatttaacaaagttgtgttaacattgcgtgagggaatacttc

fava_c18104_1519	fava_c18104	1519	G/A	aaaaagaaagaagctattatgtacaattctcaatgcatagagagatctttcaactgagataaataaattaacaaattgaattaaatttgtccttgagatc[G/A]gaatccattttcagaattgaacgtagaggctttgaatcttcttcttgatccatggaacttgatttttggaaggatttgctcctctttttgttctttttat

fava_c18104_374	fava_c18104	374	G/A	tcacggcgagtctcaaggaaaccttgacacgtcagcatacaccaccacacctgaccacagcattcagttaactccacaaggcatcgcccaagctcgcctc[G/A]ccggcaccaaactccgccgcctcgtttccggcgaaggctgctcccctgactggcgtctctacttctacgtctctccttacgcccgaacYcgatccacact

fava_c18104_463	fava_c18104	463	C/T	aagctcgcctcRccggcaccaaactccgccgcctcgtttccggcgaaggctgctcccctgactggcgtctctacttctacgtctctccttacgcccgaac[C/T]cgatccacactccgtgaagttggtaggtccttctcgaagaagcgcgtgatcggcgtcagagaagagtcgcgtgttcgagaacaggatttcgctaacttcc

fava_c18107_1417	fava_c18107	1417	G/A	aagacatggtaatcgaatgtgatcctataaaagaaagctgctcaagcctcgtaaatgagaacaaggaaacacccgataaaaaggaaagcaatactgagat[G/A]gatgaaagtcatgctgagttgaatgagaattctggtgctggagtcattgtattggatgagtaaacgcaattatctagctgacagatatttgttgtactat

fava_c18107_947	fava_c18107	947	A/T	gctcaatgctgagtaccagaaagaatactttggtgatccaaaagctggcgaagtaaccaatgaagaacttaaaagtgaactcaagtctaccagtgttcca[A/T]gttataaatcagtggatactaatacccaaatggttgatcaattgccacaagtagaaaccatagctccaggagatgtttcagttgcttgtgatagtgatcc

fava_c18110_221	fava_c18110	221	C/A	ctacttttttaatctaaaggatagctcccactttcttttgtcaaaaaaagaatacattaaattgatcaaaaagtgaaagtagggtttcactaaaacttaa[C/A]aaacatttgtggMttcttgatgattaccctttcatatagcacctatatatcttcttaattaacctgctgcttttttgcaaattgagagaaggacaactag

fava_c18110_234	fava_c18110	234	C/A	ctaaaggatagctcccactttcttttgtcaaaaaaagaatacattaaattgatcaaaaagtgaaagtagggtttcactaaaacttaaMaaacatttgtgg[C/A]ttcttgatgattaccctttcatatagcacctatatatcttcttaattaacctgctgcttttttgcaaattgagagaaggacaactagaactgctagaccc

fava_c18110_496	fava_c18110	496	T/A	tttcatcatagtaaccagaagcccaataccgagcataacactgtcccaagaagacatcagctgcagctgctgatccacacaagctcttcagctttccaac[T/A]gcatcaaccaagcaagttgaacaatctgtaacactcaaatcccccaaacactgcgcaaacccttgaactagtccagaactactaaccctaaatccRttMg

fava_c18110_592	fava_c18110	592	G/A	caacWgcatcaaccaagcaagttgaacaatctgtaacactcaaatcccccaaacactgcgcaaacccttgaactagtccagaactactaaccctaaatcc[G/A]ttMgcggtttgcaaatcttcaagaacatcgtctctacgtcgaaaaaactcgacatcactactaacacctttRctacatttcttaaacctaatacctgtat

fava_c18110_595	fava_c18110	595	C/A	cWgcatcaaccaagcaagttgaacaatctgtaacactcaaatcccccaaacactgcgcaaacccttgaactagtccagaactactaaccctaaatccRtt[C/A]gcggtttgcaaatcttcaagaacatcgtctctacgtcgaaaaaactcgacatcactactaacacctttRctacatttcttaaacctaatacctgtatcca

fava_c18110_664	fava_c18110	664	A/G	ctagtccagaactactaaccctaaatccRttMgcggtttgcaaatcttcaagaacatcgtctctacgtcgaaaaaactcgacatcactactaacaccttt[A/G]ctacatttcttaaacctaatacctgtatccaacttcccaagaaaatcaccactatgttcatatctcacataacaaccttcaaattgcaaagaagcaccaa

fava_c18111_2356	fava_c18111	2356	T/A	cagcagggtttttatcccttgaaactgctcagcagcagagatgtaccatgcttgatatattcattgaaattgacaggttacttcgcccagagggttggat[T/A]ataattcgggacacagttcctttaatagagtcagctagagctttaacaactcggctgaagtgggatgcaagagtcatagaaatcgaaagtgatagcgatc

fava_c18111_2509	fava_c18111	2509	C/A	ggctgaagtgggatgcaagagtcatagaaatcgaaagtgatagcgatcagagactcctgatctgccagaagcctttctttaaaagacaagcaaattaatt[C/A]attcaagtcctgaagaaatcaaaccatagtattcatatattcattttcataatgttcatttatttcataatggtctgccatagaaagtggggattgaaaa

fava_c18111_847	fava_c18111	847	C/T	ctttttggtggacagtatcgatttcaacctcatcaagaggtcatatttatcatggttatagaagactacaagagaaacttgtatcggaccttttggatat[C/T]ggagagatttcttatgctccctcgaaaacgaaagaattggaattctgttctcaggagtttgaaaattttgttccttgctttaacgcttcagacaatctag

fava_c18116_1046	fava_c18116	1046	A/G	attgctgcaggttttggtgctgctggacaaaggtgcatggctttaagtacagttgtttttgttggtggctcaaaaccatgggaaagtatactcgtagagc[A/G]tgccaaagctcttaaagtaaatgctggaactgaacctgatgcagaccttggtccaatcattagcatacaggcaaaggagcgaatacacaaattaattcaa

fava_c18125_187	fava_c18125	187	T/G	atcatttaaaaaaaccatacatcgccaccatgtattattacatgcataaaactcataaactaattccctagctagtggaaaaactgagctaatgcattct[T/G]catcaagtagtttgcaacaatgccatttgttttctctgttggatggaatgagtcccaaaatacatatctagaagcatccatacaactgaaaatactagcc

fava_c18125_359	fava_c18125	359	T/C	aagcatccatacaactgaaaatactagccctgctgcatgcatatcccatctcaaacattccagtagcacaacacgccatcgaagtcacttgaaacccata[T/C]tgtgctggctttttaacaacactgaacaaaatatcatacggattcgaaaacaccaatctgatcccaggaagacccttcttcagttttgtggtcaacttat

fava_c18126_1016	fava_c18126	1016	C/G	gaagctccagtccctccccattattgaggcctccaccgtcagggaatgcttcaaactcctttaacaactcaaatatatcatcaatagatgagcttgagaa[C/G]tttgccatgggcMaggtgcagagtaatgctRatagaagagcaaataccaaYactggtgagattaagcaaaactcagcggctaagacaaacaaagataaag

fava_c18126_1029	fava_c18126	1029	C/A	ctccccattattgaggcctccaccgtcagggaatgcttcaaactcctttaacaactcaaatatatcatcaatagatgagcttgagaaStttgccatgggc[C/A]aggtgcagagtaatgctRatagaagagcaaataccaaYactggtgagattaagcaaaactcagcggctaagacaaacaaagataaaggatctccagctac

fava_c18126_1047	fava_c18126	1047	A/G	tccaccgtcagggaatgcttcaaactcctttaacaactcaaatatatcatcaatagatgagcttgagaaStttgccatgggcMaggtgcagagtaatgct[A/G]atagaagagcaaataccaaYactggtgagattaagcaaaactcagcggctaagacaaacaaagataaaggatctccagctacaaaagtgaatcaatcaaa

fava_c18126_1067	fava_c18126	1067	C/T	caaactcctttaacaactcaaatatatcatcaatagatgagcttgagaaStttgccatgggcMaggtgcagagtaatgctRatagaagagcaaataccaa[C/T]actggtgagattaagcaaaactcagcggctaagacaaacaaagataaaggatctccagctacaaaagtgaatcaatcaaatggtgcggatgatcttgaat

fava_c18126_1389	fava_c18126	1389	A/G	atcacgatccccagccaatgcaaaaaaatcttcacccacaacatcttttgatgacttgtcattgatatttggcgcttccccatcatcggaatttgaggaa[A/G]tggaaggtgaaactgaagaaaggagaaaagcgagattgggacgtcaccagagaacccaagagcgagcgttgaaagcagtggctgatatgaaccagcgtga

fava_c18126_1796	fava_c18126	1796	C/T	aagccacattatgtattcatccagataaagtccaacagaaaggtgctaatcttgagcaaaaatatactgcggagaaggtttttgacatccttaaggaagc[C/T]tggactaagtttaatgcggaagagctttgctaaatctgtcacttgaggttatMacctcttcctaaatctgtcacttgaggtcatcgcgagtcaaYggatc

fava_c18126_1849	fava_c18126	1849	C/A	gagcaaaaatatactgcggagaaggtttttgacatccttaaggaagcYtggactaagtttaatgcggaagagctttgctaaatctgtcacttgaggttat[C/A]acctcttcctaaatctgtcacttgaggtcatcgcgagtcaaYggatccatcaRtgacttgcacYgttttggtttgggactttcacttgcctggttgatgc

fava_c18126_1891	fava_c18126	1891	T/C	gaagcYtggactaagtttaatgcggaagagctttgctaaatctgtcacttgaggttatMacctcttcctaaatctgtcacttgaggtcatcgcgagtcaa[T/C]ggatccatcaRtgacttgcacYgttttggtttgggactttcacttgcctggttgatgcctgatacatgtttttcttgtgcagtcgaagattattactctg

fava_c18126_1902	fava_c18126	1902	G/A	taagtttaatgcggaagagctttgctaaatctgtcacttgaggttatMacctcttcctaaatctgtcacttgaggtcatcgcgagtcaaYggatccatca[G/A]tgacttgcacYgttttggtttgggactttcacttgcctggttgatgcctgatacatgtttttcttgtgcagtcgaagattattactctgcacatatatat

fava_c18126_1913	fava_c18126	1913	T/C	cggaagagctttgctaaatctgtcacttgaggttatMacctcttcctaaatctgtcacttgaggtcatcgcgagtcaaYggatccatcaRtgacttgcac[T/C]gttttggtttgggactttcacttgcctggttgatgcctgatacatgtttttcttgtgcagtcgaagattattactctgcacatatatattacatggtgtc

fava_c18126_2211	fava_c18126	2211	A/G	tatttcagtcggcctgataaaatcgtgaaggggtagacttcatatatctggtgtatatttggattgacgtgagatcgatgagctccctgcctatctagac[A/G]tacatttatttaattttttgaWgaaaaatgcaaagttgcatacgggaatgaggataattcagttattacttgtaattaggcttttatttactaatagaaa

fava_c18126_2233	fava_c18126	2233	A/T	tcgtgaaggggtagacttcatatatctggtgtatatttggattgacgtgagatcgatgagctccctgcctatctagacRtacatttatttaattttttga[A/T]gaaaaatgcaaagttgcatacgggaatgaggataattcagttattacttgtaattaggcttttatttactaatagaaatgtgattttttttgttgcaaaa

fava_c18126_596	fava_c18126	596	G/T	gcggaaacggaaacggaaacgccagatcgaatgtcggcaagggtgttacccttgatgatgatctcatgttcggtggtttgagtcagtcggtttcgacttc[G/T]tcgtcgtcacaggtttatgtggatgatatattcggtggagggaatgaaataagtgttggtcttgatgatttgctaggtaagattggtggattgcatgcca

fava_c18126_779	fava_c18126	779	T/C	ttggtggattgcatgccacgagcaatagaagttcgatgaataaatcgcctgattttgatgatttgatggctgggtttggtggtagcagcgttaacaataa[T/C]gggaaagcaagcaaaccgactgccacatctcatgacgatccatttttagtatttgaaactttaagcacaacatcttcaggatcattcctggactcattag

fava_c18128_103	fava_c18128	103	G/A	gatctaccatctctaagaacttccatcttcaacttaatcaacaataaatccaaaccaaccgtaaattcaggattttgaggaacaccaaaagtacccctaa[G/A]aactagtccaccagatccacttattttcttctcaaaattctctttattaacagtctcaagaatttctctcaacttataaagtgtatccttaacatctttg

fava_c18128_437	fava_c18128	437	T/C	tttctttggtgagaagcttgatttcctccctaggtggattcaactgatcattatattgatcgatctggtgaaccagtggagacaaatcttttaaggttga[T/C]ctaaagaaacgcatggatgttttgtcatgttgaacttgtttgtatgtttgttgaagctgtgaggtaataacatctagttctgtggacttttccatgaatt

fava_c18128_576	fava_c18128	576	A/T	tttgtatgtttgttgaagctgtgaggtaataacatctagttctgtggacttttccatgaattataactaactaggtcgagaaatactaggttgtttaacc[A/T]ggatctagaactcaaagcttatgatatgaagaagaagaaaaaaagaaaaaagaaatgtttatttatttacctttgaaataacaaacggtggtttcaaagt

fava_c18130_1211	fava_c18130	1211	T/C	atcctagaaaggaaggagcatccaacatacaggagacttacaaatccttctgcactcgctttgtccgtctaaatggaatcctgttcactcgtacaagtct[T/C]gagaccttcactgaagttctctctcttattagYactggcctgcgcgagcttctgtcttcaggtcaagatgaagaactgaattttggccaggatgctcttg

fava_c18130_1244	fava_c18130	1244	C/T	agacttacaaatccttctgcactcgctttgtccgtctaaatggaatcctgttcactcgtacaagtctYgagaccttcactgaagttctctctcttattag[C/T]actggcctgcgcgagcttctgtcttcaggtcaagatgaagaactgaattttggccaggatgctcttgagaatggcctcRccattgtcagaatcatttcca

fava_c18130_1323	fava_c18130	1323	G/A	tgaagttctctctcttattagYactggcctgcgcgagcttctgtcttcaggtcaagatgaagaactgaattttggccaggatgctcttgagaatggcctc[G/A]ccattgtcagaatcatttccattattgtctttacagttcataatgcaaagaaggaatctgaaggtcaaacttatgcagaaattgtacagagggctgttct

fava_c18130_1517	fava_c18130	1517	A/G	ctgttctacttcagaatgccttcactgcagctttcgaattgatgagtattattatagaaagatgtgtgcagctgcaggatccttcttgtagttttcttct[A/G]ccgggcattttggtttttgttgagtggttggcatgttatcctgatcttgctgcaggaaatgatgttgatgagaatcaatcaactgttagatcaaaatttt

fava_c18130_235	fava_c18130	235	G/A	ttcacaccgattgagtttaattgataaatgtttgattgatggatgatgatagtagaaactgaaaagatgtctgctccttcaccgcgggagcgtgcacagc[G/A]cctttttgataagaatcttgaattggagaaaagacgccgtagatcagctcaggcacaggttccgtcagatcccaatatttggccacagcttcgtgagaac

fava_c18130_353	fava_c18130	353	C/T	ttgaattggagaaaagacgccgtagatcagctcaggcacaggttccgtcagatcccaatatttggccacagcttcgtgagaactttgaagcaataattct[C/T]gaggatcatgctttttctgagcagcacaatattgagtataatctttgggtattgcattataaaaggattgaagagttaagggcacatttcaatgctgcta

fava_c18130_760	fava_c18130	760	G/C	ggaaagaaatctgctgagatgaagaaaagtttgttatcttgtcatcgttgtttgatatacttgggtgatcttgctcgctacaaaggattgtacggtgaag[G/C]tgactcaaaaaagcgcgagtttgctgcagcttctagttactatttacaagctgcgtctatctggcctttaagtggaaatcctcaccatcagcttgctttg

fava_c18133_321	fava_c18133	321	G/A	aatgactttgtcatttacacccttttcattaatgtacatgtatgatgtatcaacaataagcaagataatatttttatagagaaggtaaaatccaaaaatt[G/A]aaaagacatgaagctgatgaaagaaaccaagacaaaaatgccatagcagcagacaattgatatctaccacataacgttgcagggcaacgtgatccaccag

fava_c18133_588	fava_c18133	588	T/G	ccatgtctccaaaaacgattatcattatgagccttctttgaatcggcaagcattgtatgaagacggagtaagcatctaccaagaataatgttatgctcca[T/G]ggaattgccaaacccattactgttaccaagaacgagaaagaagtgaaggagtagaaaccaacgtccaagcacatgaaRcaaagagaagcagatgagaaaa

fava_c18133_666	fava_c18133	666	G/A	ccaagaataatgttatgctccaKggaattgccaaacccattactgttaccaagaacgagaaagaagtgaaggagtagaaaccaacgtccaagcacatgaa[G/A]caaagagaagcagatgagaaaacggtttgacccaaacgaagagacaagctagcgctggtgccaaacgctccgggaacttcctccatcatagcgtagtata

fava_c18137_1512	fava_c18137	1512	C/A	agaatgaactaaaagaggtgaagcgatactttgaatacgacgaaccagacgggacagtgaatgagatttggcgccaagtttcgaaaaaactacccttgat[C/A]aaattgatggttaaccgcgagaaaaggctgtttgcaaaggaMgctgattgctcctgcatatgtacaaaccagactgccattataaccttatagcattcct

fava_c18137_1554	fava_c18137	1554	C/A	aaccagacgggacagtgaatgagatttggcgccaagtttcgaaaaaactacccttgatMaaattgatggttaaccgcgagaaaaggctgtttgcaaagga[C/A]gctgattgctcctgcatatgtacaaaccagactgccattataaccttatagcattcctctcRcaatcaaatttggcatcccatgaactctggctcggttt

fava_c18137_1616	fava_c18137	1616	A/G	ttgatggttaaccgcgagaaaaggctgtttgcaaaggaMgctgattgctcctgcatatgtacaaaccagactgccattataaccttatagcattcctctc[A/G]caatcaaatttggcatcccatgaactctggctcggtttgacgagacattttggggcgatgagaaaattaaggatggtgatggttgtaaatgtagtttctg

fava_c18137_1741	fava_c18137	1741	A/T	ctctggctcggtttgacgagacattttggggcgatgagaaaattaaggatggtgatggttgtaaatgtagtttctgtcatatgaaattcactttacaatt[A/T]aattcctRacacgcttgagttagatatgggagagaaattatgtgcaactatcattattcattagttactggggctggttggcatgagtgtgatattcaga

fava_c18137_1749	fava_c18137	1749	G/A	cggtttgacgagacattttggggcgatgagaaaattaaggatggtgatggttgtaaatgtagtttctgtcatatgaaattcactttacaattWaattcct[G/A]acacgcttgagttagatatgggagagaaattatgtgcaactatcattattcattagttactggggctggttggcatgagtgtgatattcagatgctggac

fava_c18137_759	fava_c18137	759	C/T	atgaagcggatttgttcttcgtgccgtttttctcgtcattgagcttgattgtgaacccggtccggcctcccggttcaggttcggagaaaactgcttatgt[C/T]gatgaggagaatcaagaggcattggttgagtggctgaagaggcagaagtattggcagagaagtaaaggaagggatcatgtgattgtggcRtctgatccga

fava_c18137_849	fava_c18137	849	G/A	ctgcttatgtYgatgaggagaatcaagaggcattggttgagtggctgaagaggcagaagtattggcagagaagtaaaggaagggatcatgtgattgtggc[G/A]tctgatccgaatgcgatgtatagggtaattgatcgggtcaagaactcggtgctgctggtatcggattttggacggttgcgacctgaccagggatctctgg

fava_c18139_476	fava_c18139	476	G/T	aaccttgcttttctaccttatcagtaacagctatattcggagactttacctgaataaaacggagacctataactactaggattcctagcttccagcttta[G/T]aaaatgttcgttattttttgaaaaccttggcaggatcatagctatcctccctcctatcttcttgactccttataagctattctcttactttacctgcttg

fava_c18140_193	fava_c18140	193	T/G	gaatctgaatggattcattgtaaacaatttttttaactcgtacacgtccacacaatctccatgtacagaattgtacctagagctctagattctttgctgc[T/G]aaactaaaaaYatttcctaaatcctcctttaccgtattatctgcaaatataaattcaacagcattctttgatagctcaaacatctccttccttccaaggc

fava_c18140_204	fava_c18140	204	C/T	gattcattgtaaacaatttttttaactcgtacacgtccacacaatctccatgtacagaattgtacctagagctctagattctttgctgcKaaactaaaaa[C/T]atttcctaaatcctcctttaccgtattatctgcaaatataaattcaacagcattctttgatagctcaaacatctccttccttccaaggccgaatgaagaa

fava_c18140_307	fava_c18140	307	G/C	ttcctaaatcctcctttaccgtattatctgcaaatataaattcaacagcattctttgatagctcaaacatctccttccttccaaggccgaatgaagaagc[G/C]gcgattttgtattcgttggagagactggtagagaacacgcccgagtcatcagtacacagaactacaggatgttttgctttatacaagtccacaaaatgat

fava_c18140_507	fava_c18140	507	C/G	tgagcatctattgatggaacggacaatgtcctaatgttcgatgtcaagcaaagttcaaccggaattttagaggacttcagctttctccagtgttcctcct[C/G]aaagaaacatgcatgtccaatcctcccgggaagaaagtcaagcatatcatgtatctctctcgaattaggtacctctccacagtgaagagttacataaaga

fava_c18140_628	fava_c18140	628	T/C	tcctcccgggaagaaagtcaagcatatcatgtatctctctcgaattaggtacctctccacagtgaagagttacataaagaccttgctctcgagcaaattt[T/C]aatgctggaaaatatgtaacccattcaccaacagctggattcccagagaggtcaattccaacgaccccaaaatgcctcatttccagcgcaagcatgacag

fava_c18140_837	fava_c18140	837	C/G	gctgcttctgttgtctctctacggtcaatgcttaagagaagcctaacaaagatttttttcctagtatcttcattacatttatcatttatggctgagactg[C/G]gagtatagggtttgaatgaatttgtaactccttgctgtgaggaataaaacacacatcaactgaactgacagctcttagaccttccagaacagcttcaata

fava_c18143_181	fava_c18143	181	G/A	cgtgacatcatcactttcaaaagaagcaacgtgcaattcaagagagagtttgttttgaatatatgaagttttctcattgatttcaaaatgcattacaaga[G/A]tacttttctactaatattcgagtttcctccacgaaattccacttcaccagcttcatatcttcgtcgactttacacaaccatgactatggtgtccgttgtg

fava_c18145_1341	fava_c18145	1341	C/G	gatcaccatcaaatgcaaatgcagatgcaaacacaatcctctcatgaacatgatcgaggatctgatgtgaaatctgaaccgcatgatggtaacgatgact[C/G]ggatgatcaagagcatcgttgggatttgatgaaatagtgcaaagScagtgtttggaaaaattgctaYgtttcacacatRtttgaacatggcaaacacgtt

fava_c18145_1386	fava_c18145	1386	G/C	gaacatgatcgaggatctgatgtgaaatctgaaccgcatgatggtaacgatgactSggatgatcaagagcatcgttgggatttgatgaaatagtgcaaag[G/C]cagtgtttggaaaaattgctaYgtttcacacatRtttgaacatggcaaacacgttacagtgcaatggattataaagtaacgtgtcgctaccattaacgtt

fava_c18145_1408	fava_c18145	1408	C/T	tgaaatctgaaccgcatgatggtaacgatgactSggatgatcaagagcatcgttgggatttgatgaaatagtgcaaagScagtgtttggaaaaattgcta[C/T]gtttcacacatRtttgaacatggcaaacacgttacagtgcaatggattataaagtaacgtgtcgctaccattaacgttgtgccagtgccattggacaagg

fava_c18145_1420	fava_c18145	1420	G/A	cgcatgatggtaacgatgactSggatgatcaagagcatcgttgggatttgatgaaatagtgcaaagScagtgtttggaaaaattgctaYgtttcacacat[G/A]tttgaacatggcaaacacgttacagtgcaatggattataaagtaacgtgtcgctaccattaacgttgtgccagtgccattggacaaggacgtggttggaa

fava_c18145_1691	fava_c18145	1691	C/T	agcaaagagtgtgaaagccatgcaaaagaaagttaatattgtcgacatcaattggttaaagaaagattatcaacacatgcaaatggatttgctaaatcaa[C/T]ggaaagtattcataaagttgtcttttttagacacgcgtgtggaatacatattcaacggtcaacactgtttgtggctgataatatgaattatttccaattt

fava_c18145_1817	fava_c18145	1817	C/T	tttagacacgcgtgtggaatacatattcaacggtcaacactgtttgtggctgataatatgaattatttccaatttgatacatttaccaggatgcttccaa[C/T]ttatggaccaagtttgtttctaagttacataattttagtacttttattataagataacgtggtcattttgatttgatatatgttttgaattagtttattt

fava_c18145_723	fava_c18145	723	C/T	gttagaatagtgtctccaacaacttccggtgatgaacaggtggtttcatcgaactcgtctcctattgtggcgcacaatagtactacagtgcaccgtagta[C/T]aagttacactacttcgccYgagttatYtgaagagaatgaaaggcttaagaaagagaatatgcaactgagtaacgagttatgtcaattgaaaggtctttgt

fava_c18145_742	fava_c18145	742	C/T	caacttccggtgatgaacaggtggtttcatcgaactcgtctcctattgtggcgcacaatagtactacagtgcaccgtagtaYaagttacactacttcgcc[C/T]gagttatYtgaagagaatgaaaggcttaagaaagagaatatgcaactgagtaacgagttatgtcaattgaaaggtctttgtaatagtatattatctatga

fava_c18145_750	fava_c18145	750	T/C	ggtgatgaacaggtggtttcatcgaactcgtctcctattgtggcgcacaatagtactacagtgcaccgtagtaYaagttacactacttcgccYgagttat[T/C]tgaagagaatgaaaggcttaagaaagagaatatgcaactgagtaacgagttatgtcaattgaaaggtctttgtaatagtatattatctatgatgagtaat

fava_c18146_1274	fava_c18146	1274	T/C	cctccaccggcgtattggggttgtgtgccacctccatggaatataccatgtctctcaccaaatagtccttctgttaaccaaagtgactctccgcagtatt[T/C]acctactttgggaaaacactcgagggatggaaacatcattacctcggtgaactcacgtcaagaacagcctgataaagaacacaatagtaccgaaagcggt

fava_c18146_1501	fava_c18146	1501	C/T	cgatgatccaaacgaagctgccaagagttccttatggtcaaagcttggaatcagtaatgacaaagccaactccctcaatggaggaagcatgttcaatgca[C/T]ttcaatccaagggaaaggacaataatcactcggttgaagcgtcgccgatgatgtatgccaaccctgcagctttatcgcggtctcgtaccttccacgaagg

fava_c18146_309	fava_c18146	309	T/C	tcatcaaacagaaaatgtcagaggttaaagaccctgcaataaagctatttggaagaacaattcctcttccactcatcatatctaatgactcttcatcttc[T/C]tctccttcttcttctccaccacaattcatctctgcaatacaacaagaggaaccatcaaggaaagaactatccacagcacaagatgaagaagcctttcaac

fava_c18146_483	fava_c18146	483	C/T	cagcacaagatgaagaagcctttcaacaaaccgcggaggatttaaaatcacttgcagcatcttcagtcatgcttgagaatcctccgaagactccctcagc[C/T]gaaaccgaaaattcattaccaaaagcttctacaaatggagaacaaagtgaggctagtgtttcagaagataaaaccctgaagaaacccgacaaaataattc

fava_c18146_648	fava_c18146	648	G/A	aagataaaaccctgaagaaacccgacaaaataattccatgtccgcgatgcaatagcatggacaccaagttttgttacttcaacaactacaatgtcaacca[G/A]ccccgtcatttttgcaagaaatgtcaaagatattggactgcaggaggaacaatgagRaatgttccggttggtgcagggcgccgcaagaacaagacctcgc

fava_c18146_705	fava_c18146	705	A/G	tggacaccaagttttgttacttcaacaactacaatgtcaaccaRccccgtcatttttgcaagaaatgtcaaagatattggactgcaggaggaacaatgag[A/G]aatgttccggttggtgcagggcgccgcaagaacaagacctcgcctaccgcacattatagacatctgatcattccggaaggagttaaggttaattccccta

fava_c18148_812	fava_c18148	812	C/G	ttgttctttgctaatagtaacttccttgagatactctctagccaagatgatctgtgtctttgcattgtctgtatcctcatcgaccattttaaatacttgg[C/G]cacagttatcctgaaattctgttgcaattccaacagcttcaacacggttttcaaaactcattggaagatctgcactcaaattaattgcaatgcggtctaa

fava_c18148_940	fava_c18148	940	A/G	ttccaacagcttcaacacggttttcaaaactcattggaagatctgcactcaaattaattgcaatgcggtctaaaagatgttcacgaacagaaccctcgtc[A/G]gggttataggtagcaatcagaaggggcctgcatgggtgcctaaagctgattccctctctttcaacaatatttactccttcagtcagtacattaaggagca

fava_c18151_353	fava_c18151	353	G/A	ctaggaacagagaaacatgttgttgataatggatggagtcctataggggccccactaagtgtacagagagatgaacaacaacaacaacattggagtaaca[G/A]caacaataatggtttcgatagttccgttaatgctgtctcttttggttttgtggctactgcaattcttatttccatgttcttactcatggctatttttgag

fava_c18151_483	fava_c18151	483	G/A	atgctgtctcttttggttttgtggctactgcaattcttatttccatgttcttactcatggctatttttgagaggtttcttgcaccttcttcacaagcttt[G/A]tttcctaatcgtcgccggaaccgtcgtgccggtgagtctccgatgaggaaactcagtcacccatcaccgaaaatgagtatttttactagctggatttcag

fava_c18152_1234	fava_c18152	1234	G/C	atttgcaacatcatgagaaggatgatcatcattcgcagacgagcccgtcaagtcaaaatacctcggctcaatcaccgtcaaggccggaagcttcccattt[G/C]cagcatttttcttaaaactagactcataccgatgaaacttggatatatacttcaacttccttagatttctatagaaaaaagtagtgggtatgttctcaaa

fava_c18152_231	fava_c18152	231	T/G	gtacaaaaggattgaagatgtcttctttgaaatcactcaccaaccaaaccaaaactccccaagtttactttgttttcctaagttcttccaatatcaaaca[T/G]agaaagacattccaYgcttgcatgattttctgacagcattctagccaaMatacacacacacacttcccctcccatatgagctaatgcattatatatttca

fava_c18152_246	fava_c18152	246	C/T	agatgtcttctttgaaatcactcaccaaccaaaccaaaactccccaagtttactttgttttcctaagttcttccaatatcaaacaKagaaagacattcca[C/T]gcttgcatgattttctgacagcattctagccaaMatacacacacacacttcccctcccatatgagctaatgcattatatatttcacagtcctaacaaaat

fava_c18152_280	fava_c18152	280	C/A	caaaactccccaagtttactttgttttcctaagttcttccaatatcaaacaKagaaagacattccaYgcttgcatgattttctgacagcattctagccaa[C/A]atacacacacacacttcccctcccatatgagctaatgcattatatatttcacagtcctaacaaaatctaacataataattcaaacatatccacacaactc

fava_c18152_494	fava_c18152	494	T/C	tttttttgggatgttgcatagttttaattatgagtagaagatctagtagtgagagaggatctcatatctacaatagcagactcatcagcccccaacttga[T/C]agcctctttgctagctcttatgaatctcgaaacagctcctgtcacataatcatgagcttccttcacattcatcttcttactcatttcattYgggaaactg

fava_c18152_585	fava_c18152	585	T/C	ccaacttgaYagcctctttgctagctcttatgaatctcgaaacagctcctgtcacataatcatgagcttccttcacattcatcttcttactcatttcatt[T/C]gggaaactgctcaagaagtggtctccatttagaacagctgccaactgaaccacctcactctgaaactcagaaaggctgccattttctttcgcttcagtgc

fava_c18153_2441	fava_c18153	2441	T/C	tattgaatgatttgttgttttttggaagattgtagatagttttcagattctcacacattataagaataaggccatgtggattggaattgctatgctatca[T/C]actaaggaaagataccaagtgtaataacttttccatgtttccttgtattgtaaatggtattgaaagttgtgatgtttgctttcatgttagtattctttaa

fava_c18154_1776	fava_c18154	1776	A/G	gaagtgtagtttgatttatttttctaccagcgccgtgataaaaagagggtcctgaatcgggtgtaggcttgaaagcaaaggatgacgcgtacatgtcacc[A/G]aagccattaagtttagatttttgctgatcatcagaagataattctgtgcYgcggatgtttccgtttgagacaaatggaaactttcctgttgttggRgatg

fava_c18154_1826	fava_c18154	1826	C/T	cctgaatcgggtgtaggcttgaaagcaaaggatgacgcgtacatgtcaccRaagccattaagtttagatttttgctgatcatcagaagataattctgtgc[C/T]gcggatgtttccgtttgagacaaatggaaactttcctgttgttggRgatggttgtgccaatgaattcgcaaggaaaacaggagaatccaaaagtgtagta

fava_c18154_1872	fava_c18154	1872	A/G	caccRaagccattaagtttagatttttgctgatcatcagaagataattctgtgcYgcggatgtttccgtttgagacaaatggaaactttcctgttgttgg[A/G]gatggttgtgccaatgaattcgcaaggaaaacaggagaatccaaaagtgtagtaggactaagaccaggcggtatagtcaaatacggagactgaatctcag

fava_c18154_2178	fava_c18154	2178	C/A	acttatgatcatttcgataactagcatcgctcaattgagtgcccgaatcaccatcttgcgcctcaattctaccactctcagattgttcagagatttgtct[C/A]gttacattatcactctcttcatctagcatcctagaaaatagggttctcggactcggattcggaagaccccattcaccattaagcccaacattttcatcaa

fava_c18154_413	fava_c18154	413	T/A	tcaggatttgtgtccatatttggttctccctttggtaacatgaagcccatttcatttgcggcacgctgttgttgttgttgctgatgttgttgttgcgcca[T/A]gaatggattcataggcattcgtgaagaggggcctaaaccagacattgcaaggttggagaggtgttgattcatgccaaaggagaagccgtgcggatgagct

fava_c18154_639	fava_c18154	639	G/A	atgagccgagtgaaggccttcccattccgttgtgaatttgagatggttcagttctatgagcatggttttgaagaagaccagaagcttgcccttgacctgc[G/A]ttagaagcgttgttYgcattgacgtgactgctggaacgagcagcggggacatcatgattgtgctttccctcgtaYgtggtgatcacagatttaaggtcat

fava_c18154_654	fava_c18154	654	C/T	gccttcccattccgttgtgaatttgagatggttcagttctatgagcatggttttgaagaagaccagaagcttgcccttgacctgcRttagaagcgttgtt[C/T]gcattgacgtgactgctggaacgagcagcggggacatcatgattgtgctttccctcgtaYgtggtgatcacagatttaaggtcatgYgatgctctctcca

fava_c18154_714	fava_c18154	714	T/C	gaccagaagcttgcccttgacctgcRttagaagcgttgttYgcattgacgtgactgctggaacgagcagcggggacatcatgattgtgctttccctcgta[T/C]gtggtgatcacagatttaaggtcatgYgatgctctctccacatgcttccttacagtgcaccctgcattggtgcacttgtagtaactccttgggttgggat

fava_c18154_741	fava_c18154	741	C/T	tagaagcgttgttYgcattgacgtgactgctggaacgagcagcggggacatcatgattgtgctttccctcgtaYgtggtgatcacagatttaaggtcatg[C/T]gatgctctctccacatgcttccttacagtgcaccctgcattggtgcacttgtagtaactccttgggttgggatttcctttgacaactttttgcccatatt

fava_c18162_1738	fava_c18162	1738	C/T	gacaacctactcatttctactcatctattcattccccttaaattacacaattttgaatcttttcaatgccaccaatgtcttcaactgctgtggttaaacc[C/T]Rgattaaatgcctcttgagaaatactattggagagacttccctaaagaagtgatttaccacaccatcaaattgacaaagcctttgacagcccctggttct

fava_c18162_1739	fava_c18162	1739	G/A	acaacctactcatttctactcatctattcattccccttaaattacacaattttgaatcttttcaatgccaccaatgtcttcaactgctgtggttaaaccY[G/A]gattaaatgcctcttgagaaatactattggagagacttccctaaagaagtgatttaccacaccatcaaattgacaaagcctttgacagcccctggttctg

fava_c18162_1864	fava_c18162	1864	A/C	tattggagagacttccctaaagaagtgatttaccacaccatcaaattgacaaagcctttgacagcccctggttctgatggcatgccagttttcttctacc[A/C]aaataactggaaagtgatgtctttacagtgatcctcgaggtttccaacaggggggctaatacttagatctgtaccgaaatcagtttgaaaagcaatgaag

fava_c18162_1969	fava_c18162	1969	C/T	aactggaaagtgatgtctttacagtgatcctcgaggtttccaacaggggggctaatacttagatctgtaccgaaatcagtttgaaaagcaatgaagatcc[C/T]taaatagtactattgcggaatcagaaacaaaccagtgggaattgatttgagatctagatgcagttgttgaaattgttatggcggcaatttgtggcggaaa

fava_c18162_398	fava_c18162	398	A/G	ttgaaggtcttggttttggtggcatataatacgaattaccacaatcaattggagtttgcataagcaaattcgatctgtcaaatattatgcggccacctct[A/G]ccaattcttccacgaaatgtatatggtggttgtgctgacaccccattttttgcgatagaatcaggtggcaaaatacctgccactgccaacttaccaggaa

fava_c18162_811	fava_c18162	811	A/G	cttgaaacaagcttggaagagaagggagtaaatccagacattgccaagttgtcttccaaaaattctgtttcatgcttgtacttcatttgcatcctctgta[A/G]tgtaacttcactctctattaYgtctctttttttctcttcccgcttgatcaaagcttccaacaagctcttggcttggtcaaggttgcgtctaacctggcga

fava_c18162_832	fava_c18162	832	C/T	aagggagtaaatccagacattgccaagttgtcttccaaaaattctgtttcatgcttgtacttcatttgcatcctctgtaRtgtaacttcactctctatta[C/T]gtctctttttttctcttcccgcttgatcaaagcttccaacaagctcttggcttggtcaaggttgcgtctaacctggcgaagcttttcaaacgactgcaca

fava_c18163_253	fava_c18163	253	A/C	gcttttgaggtgagttcagtgaggtttcctaaaaaatggatcttcaaggcaaaaactttgggaggggcagtagagaacttggaggtggtaacgatctcgt[A/C]agccaatataagctttggcccttctacaatttcttctgcaagaggtcagttccattgtcagagacacattatcttcgcaatgtggttggtgacactcaaa

fava_c18163_669	fava_c18163	669	C/T	aataaaaaggacaacaaatataggaagcacaaactacttcaaattaaaaatggaagttgtgttgctaataacacaattatacacaatgattctcatccct[C/T]gcaattgaagaatcaacaagacaagaaaagaagggctgaggcaagctacgacccttctactatcaagcgattaaatagtagacaRtgaggcagaattaga

fava_c18163_754	fava_c18163	754	A/G	atgattctcatccctYgcaattgaagaatcaacaagacaagaaaagaagggctgaggcaagctacgacccttctactatcaagcgattaaatagtagaca[A/G]tgaggcagaattagaatttagatattcttttttgttttggggaatgtataatcatYttagaactaaggggaatttaaaaaaactttatattttggggaat

fava_c18163_810	fava_c18163	810	C/T	gcaagctacgacccttctactatcaagcgattaaatagtagacaRtgaggcagaattagaatttagatattcttttttgttttggggaatgtataatcat[C/T]ttagaactaaggggaatttaaaaaaactttatattttggggaatagtaaactttatttttggtatagttaacatttgtgattctttgtataaaaaaaact

fava_c18164_128	fava_c18164	128	A/T	ctcctctctttctctttctctttcccattcctattcctctagttgaatgaaactcgaaagcgtgtaattgtaaacacaagacttttcttttcttcttctc[A/T]atctctaaaaatggaagaagaaagaggtagttacaagaacaagaagagtttgtttttactagggaagaagaaagaagatgtttatcatgtactttacaag

fava_c18164_640	fava_c18164	640	T/C	gatctacaaaggtgaagccttcaatgggaaactcactaaaactgcacgatctcatggcaagaagtttcaagtttctattaaacaagaaacttcaagatta[T/C]tgggaaacttgggttgggcctacatgcaaaagatgaactaYgtgatggcggaggcggtgtatcggaaagcccaaatgatcgaYccggattgtaacaaggc

fava_c18164_681	fava_c18164	681	C/T	ctgcacgatctcatggcaagaagtttcaagtttctattaaacaagaaacttcaagattaYtgggaaacttgggttgggcctacatgcaaaagatgaacta[C/T]gtgatggcggaggcggtgtatcggaaagcccaaatgatcgaYccggattgtaacaaggcgtgtaacttgggcctatgtctcattagacaagcccggtacg

fava_c18164_723	fava_c18164	723	T/C	aagaaacttcaagattaYtgggaaacttgggttgggcctacatgcaaaagatgaactaYgtgatggcggaggcggtgtatcggaaagcccaaatgatcga[T/C]ccggattgtaacaaggcgtgtaacttgggcctatgtctcattagacaagcccggtacgaagaggcccaattgattattgatgRaatattaaaaggagaac

fava_c18164_806	fava_c18164	806	G/A	aaagcccaaatgatcgaYccggattgtaacaaggcgtgtaacttgggcctatgtctcattagacaagcccggtacgaagaggcccaattgattattgatg[G/A]aatattaaaaggagaacttccaggctcagatgatatcaagactaggaaacgggcagaagatcttgtaatagagttgaaatcattgataccYccatcacat

fava_c18164_897	fava_c18164	897	C/T	ttattgatgRaatattaaaaggagaacttccaggctcagatgatatcaagactaggaaacgggcagaagatcttgtaatagagttgaaatcattgatacc[C/T]ccatcacatacattggatcttttagttcttgatgatgagtttatcaaaggcatagaacagttgatgaatgaatggggtccacttagatcaaagagacttc

fava_c18167_1467	fava_c18167	1467	G/A	aatgaacttgagcagtggaacaatcctatggagtttaaaccatctactgtgaataacatctatttcaataattttaattctattttgtattcaaatagca[G/A]atcaagttgattaccttgggtctgcatagggaaaaggttaacatgggtctgttaacaatcaatggaactaagaaaagttcaaaaaagaggagaagcattt

fava_c18167_617	fava_c18167	617	A/G	tataatggtgagctggaatcaaactgggagttgtttgggggatgaatgcagacctagacttgttggatttcttggatgcaaaaatgttagaatcttcaat[A/G]ttactctcaatcaacctgcttattggtgcttacacttggtgcggagtgaaaacatatccattcatgatattgcaatttatggagacttgaatataccaaa

fava_c18167_748	fava_c18167	748	C/T	tacacttggtgcggagtgaaaacatatccattcatgatattgcaatttatggagacttgaatataccaaacaacgatggaattgacattgaggactcaaa[C/T]aacacggtaatcactcgatgtcacattgacacaggagatgatgcaatctgtccaaagtcatccactggccctgtttacaatttaacagtcacagattctt

fava_c18167_968	fava_c18167	968	C/A	tgcaatcaaactaggcagtgctagttggtttgaattcaagcattttgtttttgataatatcactattgttgattctcatagagggcttgcgttccagata[C/A]gtgatggaggaagtgttgatgacattatcttctcaaacataaatataagcacaagatactatgatccattatggtggggtagagccgagcctatctatgt

fava_c18168_1045	fava_c18168	1045	T/C	tatccactcgattcaccgaaaagatccttacaagtactgcagtacttcactacagtaactcacaaggatctctttcggacgtcattcctggtggaccaac[T/C]gagatcgattggtccatccaacaagcgcgatcaatcaggacgaatcttactgcgagtggaccaaggccaaatccacaaggttcataccactatggtacaa

fava_c18168_1176	fava_c18168	1176	A/G	tcaatcaggacgaatcttactgcgagtggaccaaggccaaatccacaaggttcataccactatggtacaataaacattagtagaacaatcaagctagtga[A/G]ctcagctgcacttgtgaatggtaagcaaagatacgcggtcaacggtgtatctttcgtaccggcYgacactcctctgaagttagctgattacttcaagatt

fava_c18168_1240	fava_c18168	1240	T/C	gtacaataaacattagtagaacaatcaagctagtgaRctcagctgcacttgtgaatggtaagcaaagatacgcggtcaacggtgtatctttcgtaccggc[T/C]gacactcctctgaagttagctgattacttcaagattgacggaRttttccatgttggaagcatccccgatagYccttccggtaaatcaactcatcttgata

fava_c18168_1283	fava_c18168	1283	G/A	tgcacttgtgaatggtaagcaaagatacgcggtcaacggtgtatctttcgtaccggcYgacactcctctgaagttagctgattacttcaagattgacgga[G/A]ttttccatgttggaagcatccccgatagYccttccggtaaatcaactcatcttgatacttcagtcatgggtgctgattttcgagcYttYgttgaaatcgt

fava_c18168_1312	fava_c18168	1312	C/T	cggtcaacggtgtatctttcgtaccggcYgacactcctctgaagttagctgattacttcaagattgacggaRttttccatgttggaagcatccccgatag[C/T]ccttccggtaaatcaactcatcttgatacttcagtcatgggtgctgattttcgagcYttYgttgaaatcgtgtttcaaaatcgggaaaatatagtccaaa

fava_c18168_1369	fava_c18168	1369	C/T	tcaagattgacggaRttttccatgttggaagcatccccgatagYccttccggtaaatcaactcatcttgatacttcagtcatgggtgctgattttcgagc[C/T]ttYgttgaaatcgtgtttcaaaatcgggaaaatatagtccaaagttggcacattgatggatactcattttgggttgtaggaatggacggtggtatgtgga

fava_c18168_1372	fava_c18168	1372	C/T	agattgacggaRttttccatgttggaagcatccccgatagYccttccggtaaatcaactcatcttgatacttcagtcatgggtgctgattttcgagcYtt[C/T]gttgaaatcgtgtttcaaaatcgggaaaatatagtccaaagttggcacattgatggatactcattttgggttgtaggaatggacggtggtatgtggacgc

fava_c18168_1652	fava_c18168	1652	G/A	aatttatatggcactagacaatgtgggaatgtggaacattaggagtgaattttgggcacgacaatatcttggacaacaattctatatgagggtatattca[G/A]atgctggatcccttagggatgaatatctaattccagaaaatgcacttctttgtggcaaagcattRagtagaaaaactacacYattatgagaaatgttaac

fava_c18168_1717	fava_c18168	1717	A/G	atcttggacaacaattctatatgagggtatattcaRatgctggatcccttagggatgaatatctaattccagaaaatgcacttctttgtggcaaagcatt[A/G]agtagaaaaactacacYattatgagaaatgttaaccaacaacatagctatatattagggaataacttgttccattctttgcaaattcttatgtaatttct

fava_c18168_1734	fava_c18168	1734	T/C	tatatgagggtatattcaRatgctggatcccttagggatgaatatctaattccagaaaatgcacttctttgtggcaaagcattRagtagaaaaactacac[T/C]attatgagaaatgttaaccaacaacatagctatatattagggaataacttgttccattctttgcaaattcttatgtaatttctttgYattctttattagt

fava_c18168_1821	fava_c18168	1821	C/T	agaaaaactacacYattatgagaaatgttaaccaacaacatagctatatattagggaataacttgttccattctttgcaaattcttatgtaatttctttg[C/T]attctttattagtactacatgttcatcatcacattgtgataataagttagtttccccttattattttattttttgtgaattaaataattttYcgtactcg

fava_c18168_1913	fava_c18168	1913	C/T	tttctttgYattctttattagtactacatgttcatcatcacattgtgataataagttagtttccccttattattttattttttgtgaattaaataatttt[C/T]cgtactcgatgtatcagcatgttgttgctttaatgttacaaaaatgaattgaaatgaaatgaagcagactagttcaatctgaatgtatacacaacatgaa

fava_c18168_499	fava_c18168	499	T/C	ttcaagatggagtttatggcacaacatgtcccattcctcccggtaaaaacttcacatacaaacttcaagtcaaagatcaaattggaagcttcttctattt[T/C]ccatctcttgctttccacaaagcagctggtggttttggatccatcaaaatccttagcagaccattgattcctgttccattccctaatccagctgatgatt

fava_c18168_859	fava_c18168	859	C/G	gaatatcgaatgttggattagaaaacacactcaactttaggattcaagaccatactatgaaattggttgaagtggagggaactcacactttgcaaacatc[C/G]tattcRtctatcgatgttcatgttggacaatcttactccgtgcttatcacagcKgatcaagcacctaaggatttccacattgttgtatccactcgattca

fava_c18168_865	fava_c18168	865	A/G	cgaatgttggattagaaaacacactcaactttaggattcaagaccatactatgaaattggttgaagtggagggaactcacactttgcaaacatcStattc[A/G]tctatcgatgttcatgttggacaatcttactccgtgcttatcacagcKgatcaagcacctaaggatttccacattgttgtatccactcgattcaccgaaa

fava_c18168_913	fava_c18168	913	T/G	ctatgaaattggttgaagtggagggaactcacactttgcaaacatcStattcRtctatcgatgttcatgttggacaatcttactccgtgcttatcacagc[T/G]gatcaagcacctaaggatttccacattgttgtatccactcgattcaccgaaaagatccttacaagtactgcagtacttcactacagtaactcacaaggat

fava_c18171_1142	fava_c18171	1142	T/C	aggcttctggtgatagaaagaatgagggggaacctgctgttgatcctatggttgctgatgttcgtggcaatgagtttcgaaatgacactgtggaggaagc[T/C]gctcctttgcaaagagatgaagttgctcaccattctcttggagatgctgccggtactcagccactgaacaaaggggacaaggatgctcatggtgcatcac

fava_c18171_70	fava_c18171	70	C/A	tcacattccatacttttcattttcttcttccacactattatccattttccctctttcttcttctctaag[C/A]gccattttcatcttcctttctcttYtcactcgtttcttccacaccgaaaaccatgaacattgaacccaaacccgaacccggtttaccagcgtggtccatt

fava_c18171_726	fava_c18171	726	C/G	ggaggtggaggttttagggttgagggagatgaagggaaaatgggaggaagatagcaatgaacttggtgtgttgagaaagatggttcatgatttggagaat[C/G]agattacggagttgaggaaatcgaagaaaaaatggttggatgatagtgttgcgctcgatgtggttaagaggaaggttcgtgtgttggaggStgagaaaga

fava_c18171_817	fava_c18171	817	G/C	ttggagaatSagattacggagttgaggaaatcgaagaaaaaatggttggatgatagtgttgcgctcgatgtggttaagaggaaggttcgtgtgttggagg[G/C]tgagaaagatgctttggttaaacttgagattaagaaagatgaattggagggatcagtgaagaagaatttgggaactataagtgagttgaggaaggaaaac

fava_c18171_95	fava_c18171	95	T/C	tcacattccatacttttcattttcttcttccacactattatccattttccctctttcttcttctctaagMgccattttcatcttcctttctctt[T/C]tcactcgtttcttccacaccgaaaaccatgaacattgaacccaaacccgaacccggtttaccagcgtggtccattcccacaaaccctaacaacttcgaaa

fava_c18172_1459	fava_c18172	1459	T/C	atgacaattgactcagaaattcgataccacatccctagctggaatcctagtgaaactcccgacaatagttcttaccatgattcaggccatacccctgaaa[T/C]tatgcccgaggcttctcccataacaaatgactctcctggcagccttgctttggaaatattaccttcaggtcgtaagtactggtcagactcacctaagggt

fava_c18172_632	fava_c18172	632	T/A	tgcgccaataccggaagaaacataagcatgttgatttgagagctttgaagaaatggtctaggcagattttggagggcctttcgtatcttcatagtcataa[T/A]ccacctgttattcatcgagaccttaagtgtgacaatatttttgtcaatgggaatcaaggggaggtgaaaattggtgatttagggttggctgccattcttc

fava_c18172_737	fava_c18172	737	G/A	ctgttattcatcgagaccttaagtgtgacaatatttttgtcaatgggaatcaaggggaggtgaaaattggtgatttagggttggctgccattcttcaaca[G/A]gccagttcagctcacagtgttataggtacScctgagtttatggccccagaactttatgaagaggagtacaatgagcttgttgacatatatgcttttggca

fava_c18172_767	fava_c18172	767	C/G	atatttttgtcaatgggaatcaaggggaggtgaaaattggtgatttagggttggctgccattcttcaacaRgccagttcagctcacagtgttataggtac[C/G]cctgagtttatggccccagaactttatgaagaggagtacaatgagcttgttgacatatatgcttttggcatgtgcttgctagagttggtaactgttgagt

fava_c18173_1069	fava_c18173	1069	C/T	aaagtcacaatcaactttcgtacctcaaattcaactcgaatgtatccgaaacatcaacaaaaatctctttcaaatgagcccaaacaaaaaccaaacaaca[C/T]taaatttcactcaacccaacaacacRgtttgcaaccagaggcatagctRacaattagaaacaaaRcggaagcaSatMttgacgcaaacgaaatgatacta

fava_c18173_1095	fava_c18173	1095	A/G	aaattcaactcgaatgtatccgaaacatcaacaaaaatctctttcaaatgagcccaaacaaaaaccaaacaacaYtaaatttcactcaacccaacaacac[A/G]gtttgcaaccagaggcatagctRacaattagaaacaaaRcggaagcaSatMttgacgcaaacgaaatgatactaacatcagaaattaccacaggatttct

fava_c18173_1118	fava_c18173	1118	G/A	aacatcaacaaaaatctctttcaaatgagcccaaacaaaaaccaaacaacaYtaaatttcactcaacccaacaacacRgtttgcaaccagaggcatagct[G/A]acaattagaaacaaaRcggaagcaSatMttgacgcaaacgaaatgatactaacatcagaaattaccacaggatttctcagatcgaaccaaatttccagaa

fava_c18173_1134	fava_c18173	1134	A/G	tctttcaaatgagcccaaacaaaaaccaaacaacaYtaaatttcactcaacccaacaacacRgtttgcaaccagaggcatagctRacaattagaaacaaa[A/G]cggaagcaSatMttgacgcaaacgaaatgatactaacatcagaaattaccacaggatttctcagatcgaaccaaatttccagaacttgacagtaacacaa

fava_c18173_1143	fava_c18173	1143	C/G	tgagcccaaacaaaaaccaaacaacaYtaaatttcactcaacccaacaacacRgtttgcaaccagaggcatagctRacaattagaaacaaaRcggaagca[C/G]atMttgacgcaaacgaaatgatactaacatcagaaattaccacaggatttctcagatcgaaccaaatttccagaacttgacagtaacacaaaacgctcaa

fava_c18173_1146	fava_c18173	1146	C/A	gcccaaacaaaaaccaaacaacaYtaaatttcactcaacccaacaacacRgtttgcaaccagaggcatagctRacaattagaaacaaaRcggaagcaSat[C/A]ttgacgcaaacgaaatgatactaacatcagaaattaccacaggatttctcagatcgaaccaaatttccagaacttgacagtaacacaaaacgctcaaaac

fava_c18173_1285	fava_c18173	1285	T/C	acaggatttctcagatcgaaccaaatttccagaacttgacagtaacacaaaacgctcaaaactatcaaaccaattactcacacaatatttatcacaggac[T/C]ttctRcatcacgWcataaaacagcattgcaattataaaatagtgttgagaattaccgaacaaaagctggaatcgccgttgcttctgaatcgtcgggaaat

fava_c18173_1290	fava_c18173	1290	G/A	atttctcagatcgaaccaaatttccagaacttgacagtaacacaaaacgctcaaaactatcaaaccaattactcacacaatatttatcacaggacYttct[G/A]catcacgWcataaaacagcattgcaattataaaatagtgttgagaattaccgaacaaaagctggaatcgccgttgcttctgaatcgtcgggaaataggct

fava_c18173_1298	fava_c18173	1298	T/A	gatcgaaccaaatttccagaacttgacagtaacacaaaacgctcaaaactatcaaaccaattactcacacaatatttatcacaggacYttctRcatcacg[T/A]cataaaacagcattgcaattataaaatagtgttgagaattaccgaacaaaagctggaatcgccgttgcttctgaatcgtcgggaaataggctgaactgat

fava_c18183_1101	fava_c18183	1101	A/T	atcgtctgaaggtggtcttgcaagtccagactacacgttcttccgccatgtcagcagttacgacaatatggaagcaagataagttaaggggttttttccg[A/T]ggtaatgggttgaacgttgtcaaggtagcaccagaaagtgccatcaaattctatgcttttgaaatgctgaaaaatgtgatcagagatgctcaaggcaaca

fava_c18183_1212	fava_c18183	1212	C/T	tgaacgttgtcaaggtagcaccagaaagtgccatcaaattctatgcttttgaaatgctgaaaaatgtgatcagagatgctcaaggcaacaagtctgatat[C/T]ggtgcatctgggagacttttagcaggtggcgtggctggtggaattgcgcagactgcaatctatccgttggatcttatcaaaactaggctacagacttgtg

fava_c18183_1527	fava_c18183	1527	A/G	cttatgctggcattgatctcgctgtttatgataccttaaaagatatgtccaggagatatattattcatgataatgatcctggtcctctaatacaactagg[A/G]tgtggaacaatttctggMacccttggagctacttgtgtctatccgttgcaggttattcgtactaggctgcaggcacaatcttcaaacagttcggatgcgt

fava_c18183_1545	fava_c18183	1545	A/C	tcgctgtttatgataccttaaaagatatgtccaggagatatattattcatgataatgatcctggtcctctaatacaactaggRtgtggaacaatttctgg[A/C]acccttggagctacttgtgtctatccgttgcaggttattcgtactaggctgcaggcacaatcttcaaacagttcggatgcgtacaaggggatgtttgatg

fava_c18183_1683	fava_c18183	1683	T/C	ttcgtactaggctgcaggcacaatcttcaaacagttcggatgcgtacaaggggatgtttgatgcattttgcagaacttttcagcatgaaggctttagagg[T/C]ttctacaaaggactccttccaaatctactcaaagttgtaccagctgctagcattacttacatggtctatgaaagaatgaagaaaaatcttgaKctagagt

fava_c18183_1776	fava_c18183	1776	G/T	ttagaggYttctacaaaggactccttccaaatctactcaaagttgtaccagctgctagcattacttacatggtctatgaaagaatgaagaaaaatcttga[G/T]ctagagtagagtttatatattacctggttttcgcacacRcattgagctacattaagtgttggtttagctctgtcctcatggttgagctattatatttatt

fava_c18183_1815	fava_c18183	1815	G/A	aagttgtaccagctgctagcattacttacatggtctatgaaagaatgaagaaaaatcttgaKctagagtagagtttatatattacctggttttcgcacac[G/A]cattgagctacattaagtgttggtttagctctgtcctcatggttgagctattatatttatttttttgatgtaattgagctaatgcaaaatacattagtaa

fava_c18184_1428	fava_c18184	1428	T/C	acattgttgctgacatgcggaaagtattagccaaaaatcctcaagttgagcagcagaagttgcacaggagagtgtttttggacaatataaaccctgaaaa[T/C]caggctctcatgattctgatttcttgttttgtcaagacctcgcattttgaagaatatttgtgtgttaaggaaactatactattggatcttcttagagtta

fava_c18184_1533	fava_c18184	1533	C/T	ctctcatgattctgatttcttgttttgtcaagacctcgcattttgaagaatatttgtgtgttaaggaaactatactattggatcttcttagagttatcag[C/T]catcaccgagcccggcttgcaacaccagtccgtaccctaYtgaaagtatatagcgatgcYgacttggacaacataccatacgcagattctggaacagtat

fava_c18184_1573	fava_c18184	1573	C/T	ttttgaagaatatttgtgtgttaaggaaactatactattggatcttcttagagttatcagYcatcaccgagcccggcttgcaacaccagtccgtacccta[C/T]tgaaagtatatagcgatgcYgacttggacaacataccatacgcagattctggaacagtatctaaccgcccattattagtgattgaatcgccttacaaaaa

fava_c18184_1593	fava_c18184	1593	C/T	ttaaggaaactatactattggatcttcttagagttatcagYcatcaccgagcccggcttgcaacaccagtccgtaccctaYtgaaagtatatagcgatgc[C/T]gacttggacaacataccatacgcagattctggaacagtatctaaccgcccattattagtgattgaatcgccttacaaaaacaatgcagatgataaaacaa

fava_c18184_1938	fava_c18184	1938	T/C	tcaaagtcatggcatctacctctgatgcaaatggaaactttaaacttgattccgaagtgggtgaaaacaagccactgagatcaaatttaaacaaggcaaa[T/C]gtggaggtgccagaaatgttttMtaattctaactccaaagtaactggtttagaattagataattcatcccagaaggatatccatgtgaaacaatctaagg

fava_c18184_1961	fava_c18184	1961	C/A	gatgcaaatggaaactttaaacttgattccgaagtgggtgaaaacaagccactgagatcaaatttaaacaaggcaaaYgtggaggtgccagaaatgtttt[C/A]taattctaactccaaagtaactggtttagaattagataattcatcccagaaggatatccatgtgaaacaatctaagggtcaaaccattaaaaacatcaag

fava_c18184_2488	fava_c18184	2488	G/A	tggttagttgtttgaaagcttagtcttcaagcttttaagtacacactgcagaaaagtttgaaccacattcccatccctgagaagctgtatatattacctt[G/A]tataattcatactcccttcaaagttcttgagctatattgtaacatcaattcactatttttgctgtttcagcaattcatatatgatttctattatactgtc

fava_c18184_274	fava_c18184	274	A/G	ggctgtcaattttgattgaataatggcttttcctggttccttgcaactgtccaatggattgggcatttgcgcgaacctgaacctgagctccaataaagat[A/G]tgagggtaacRgggagatacaagttacaattattcaaKgaatatccctYgYattcagttttgttcatgaaacaggactgcaggggtcttcgacacctccg

fava_c18184_285	fava_c18184	285	G/A	ttgattgaataatggcttttcctggttccttgcaactgtccaatggattgggcatttgcgcgaacctgaacctgagctccaataaagatRtgagggtaac[G/A]gggagatacaagttacaattattcaaKgaatatccctYgYattcagttttgttcatgaaacaggactgcaggggtcttcgacacctccgtcgtgtaaaca

fava_c18184_312	fava_c18184	312	T/G	ccttgcaactgtccaatggattgggcatttgcgcgaacctgaacctgagctccaataaagatRtgagggtaacRgggagatacaagttacaattattcaa[T/G]gaatatccctYgYattcagttttgttcatgaaacaggactgcaggggtcttcgacacctccgtcgtgtaaacaagtcaacacatagattgtcttgcagat

fava_c18184_323	fava_c18184	323	C/T	tccaatggattgggcatttgcgcgaacctgaacctgagctccaataaagatRtgagggtaacRgggagatacaagttacaattattcaaKgaatatccct[C/T]gYattcagttttgttcatgaaacaggactgcaggggtcttcgacacctccgtcgtgtaaacaagtcaacacatagattgtcttgcagatcacgatctttt

fava_c18184_325	fava_c18184	325	C/T	caatggattgggcatttgcgcgaacctgaacctgagctccaataaagatRtgagggtaacRgggagatacaagttacaattattcaaKgaatatccctYg[C/T]attcagttttgttcatgaaacaggactgcaggggtcttcgacacctccgtcgtgtaaacaagtcaacacatagattgtcttgcagatcacgatcttttaa

fava_c18184_817	fava_c18184	817	C/T	ttgcagatcatttgagtcagtaattctacctaccgaaactagccaggttgttaaggaacggcttctgagttttgttaaatcattgtcaactgtagtggct[C/T]ttgcctattgtttatcaagtgcaattcaacaagcacagaaatttgtagcagagaatgcagatgcaagtgagacaagaaatatggggttccaatttgcagg

fava_c18185_400	fava_c18185	400	T/C	gatgatgttgacgctaaactcaaagcgctcaagcttaaatactcttctaagaacccaaacccaaaccctaacaatgttaagctttatcttcataactctg[T/C]tgagtccaaatgggtgatttatgagaaattaacgacttactcgtttgttaaaagttgtagtgttgagggagagagtgatgaagacgaggaagatgaggac

fava_c18186_1551	fava_c18186	1551	G/T	ttcccatcagaaaaccagtttatagaagtatcttcgccagctacacgccctcctcgattatcccaacgaaagctccacgtcggtgaacaccgaatattcc[G/T]atgtgaattctcattagtcgatccactctgtacagttttatctctagcagcaacaaaacaagcagcacccattatgaagtaacaacagctttcaccttct

fava_c18186_1665	fava_c18186	1665	A/C	ttagtcgatccactctgtacagttttatctctagcagcaacaaaacaagcagcacccattatgaagtaacaacagctttcaccttctcacactaaaacag[A/C]caccaaatcaccaattgcatccgaaaacRcagatctagaccacaactatcaacctattgttgaaatcggcgagtttacggagagatatgaagaagaagaa

fava_c18186_1694	fava_c18186	1694	G/A	tctagcagcaacaaaacaagcagcacccattatgaagtaacaacagctttcaccttctcacactaaaacagMcaccaaatcaccaattgcatccgaaaac[G/A]cagatctagaccacaactatcaacctattgttgaaatcggcgagtttacggagagatatgaagaagaagaagaagatgatgatgatgagagtgtttggga

fava_c18186_471	fava_c18186	471	T/C	gaaccttttgatccaaatgtaaagtgtcgtctcaaaaaaggtttcccaaaggagcttcttctcccacgagaactggagtccagccgaggacctttacctt[T/C]acctataggttctttccctttaatgctatcaaacgcaatagaatcatcgttttcaatatcactgtactcaacctgattctccaRtgtctggtttctagcc

fava_c18186_555	fava_c18186	555	G/A	cgaggacctttaccttYacctataggttctttccctttaatgctatcaaacgcaatagaatcatcgttttcaatatcactgtactcaacctgattctcca[G/A]tgtctggtttctagccttcaaatccatttcaactttcagaacttttttggacagctccatagtttgtttctcaccgaagctgcagactgggcaagctggg

fava_c18186_665	fava_c18186	665	C/T	tctagccttcaaatccatttcaactttcagaacttttttggacagctccatagtttgtttctcaccgaagctgcagactgggcaagctgggtcgtactta[C/T]taatatcagctgtcatattctccaaacattcagcatgatagacatgtccacagataagaacggcaactacYgaaaggtcattacttgtatatatcttttg

fava_c18186_736	fava_c18186	736	C/T	tgcagactgggcaagctgggtcgtacttaYtaatatcagctgtcatattctccaaacattcagcatgatagacatgtccacagataagaacggcaactac[C/T]gaaaggtcattacttgtatatatcttttgggtactccaaggagaYttctcctttaaaacctttgagcaaaagccacatgttctcaaatcaacaggMgagg

fava_c18186_781	fava_c18186	781	T/C	tattctccaaacattcagcatgatagacatgtccacagataagaacggcaactacYgaaaggtcattacttgtatatatcttttgggtactccaaggaga[T/C]ttctcctttaaaacctttgagcaaaagccacatgttctcaaatcaacaggMgaggctgaaaaccaactaccggatctagctatcctttcgtaattagaac

fava_c18186_832	fava_c18186	832	C/A	ctacYgaaaggtcattacttgtatatatcttttgggtactccaaggagaYttctcctttaaaacctttgagcaaaagccacatgttctcaaatcaacagg[C/A]gaggctgaaaaccaactaccggatctagctatcctttcgtaattagaaccaaaggactcactatccaatgaccatctttgtctgtggtgagctcccatta

fava_c18188_1112	fava_c18188	1112	G/A	gtgtagctatgttcctcatgtatggttcggcgtgcgcttctaaataaggaagtcctttgatcttcaatatttgatcatagacacctttgttgaaaatgtc[G/A]gccacggatttcgagattaataatgccaacatcacgagtggaagtaacaagagatcattagtgagttcRagtaatataacacaaatcgagaccgtcattc

fava_c18188_1181	fava_c18188	1181	A/G	tttgatcatagacacctttgttgaaaatgtcRgccacggatttcgagattaataatgccaacatcacgagtggaagtaacaagagatcattagtgagttc[A/G]agtaatataacacaaatcgagaccgtcattctcattgtgccgccgaggaaagaggcagctcctaggagagaaaagagtcctcgatcaagttttgtgatcg

fava_c18188_1499	fava_c18188	1499	G/A	caaagaagatgaacaaacttgaaatgtgaaactctttgtttatcttaggactgaatagattacgaatagcatcgtcattggtgtttagaaagagggaagc[G/A]agatcattgtagtagcccggcggacattggaagaYtttgtattctccggattcgtctactgaggggcaggctaYcgtcccatctgtagggcaagggagac

fava_c18188_1534	fava_c18188	1534	T/C	ttgtttatcttaggactgaatagattacgaatagcatcgtcattggtgtttagaaagagggaagcRagatcattgtagtagcccggcggacattggaaga[T/C]tttgtattctccggattcgtctactgaggggcaggctaYcgtcccatctgtagggcaagggagacagtttgcaatccatggtaggaaataataacaacat

fava_c18188_1573	fava_c18188	1573	T/C	tcattggtgtttagaaagagggaagcRagatcattgtagtagcccggcggacattggaagaYtttgtattctccggattcgtctactgaggggcaggcta[T/C]cgtcccatctgtagggcaagggagacagtttgcaatccatggtaggaaataataacaacatgatgtcaaaagagaaactgtaattgcaagagagattttg

fava_c18188_267	fava_c18188	267	A/T	gaaaaaattattgaccgtatacttcaacactgattggtgttgttgactgatacctgtatagtttcaaaagcattcaaattgacttcatgaagaaaaaaga[A/T]aataataataataacaaaacatatattacacggttgctaaatacacccccatcaaaactatgtagcaactgcatctcttagcagcattttgtttttgctt

fava_c18188_2794	fava_c18188	2794	T/C	tatgtcatgttcctttcagaaaactgtgaccaatatcttccgatttcttcgccgtttccatcaaagctaccgtcattttctatatcaagcacatcatcac[T/C]atggtttgcattttcttctttctccatcttctcRgtaacttcgttcgatgatgtcacaggaacactcattataaattgcagcttttgaactcaaactcaa

fava_c18188_2828	fava_c18188	2828	G/A	atcttccgatttcttcgccgtttccatcaaagctaccgtcattttctatatcaagcacatcatcacYatggtttgcattttcttctttctccatcttctc[G/A]gtaacttcgttcgatgatgtcacaggaacactcattataaattgcagcttttgaactcaaactcaaactcaaaatcttcaaagctttagtttgcaataac

fava_c18188_2940	fava_c18188	2940	C/T	cgatgatgtcacaggaacactcattataaattgcagcttttgaactcaaactcaaactcaaaatcttcaaagctttagtttgcaataaccaataataatg[C/T]Ytctgaagatcacaaataaatgaaagttacttctcaatcatcactttgtaaagtcaaagaactctctatactatgatggatacaacaatctcaaaaggac

fava_c18188_2941	fava_c18188	2941	T/C	gatgatgtcacaggaacactcattataaattgcagcttttgaactcaaactcaaactcaaaatcttcaaagctttagtttgcaataaccaataataatgY[T/C]tctgaagatcacaaataaatgaaagttacttctcaatcatcactttgtaaagtcaaagaactctctatactatgatggatacaacaatctcaaaaggaca

fava_c18188_616	fava_c18188	616	T/C	ggcatgaaatcgtggcgcgtaagaatcccgacgataggaggtctctcttggctcttcggaacaacacacatgtgccgtagtccttgttgacggaaaataa[T/C]agcggctttagcaagtgacattgtctcaactacggtgtacggtgatgtattggttatYggatgaagatcaacgtacatatccaaatcttcRtctccgata

fava_c18188_674	fava_c18188	674	C/T	gaacaacacacatgtgccgtagtccttgttgacggaaaataaYagcggctttagcaagtgacattgtctcaactacggtgtacggtgatgtattggttat[C/T]ggatgaagatcaacgtacatatccaaatcttcRtctccgatatctaaatcttctaatttgactccttttcccgatcccgcctttccaaaatccagcaccg

fava_c18188_707	fava_c18188	707	G/A	ggaaaataaYagcggctttagcaagtgacattgtctcaactacggtgtacggtgatgtattggttatYggatgaagatcaacgtacatatccaaatcttc[G/A]tctccgatatctaaatcttctaatttgactccttttcccgatcccgcctttccaaaatccagcaccgaaatattttctaagatacccgcatccgcgaaaa

fava_c18189_233	fava_c18189	233	G/A	ataccactccgcatttcgtgctatatatttatatatgttacaatctatcatttacctatacaaaaaaaagcccatccaacttgcatcccttggcaatcca[G/A]gctcaaWaaggttgcaagaaaacaaaacaaccagcagatctcttatacaaggatttcggtgtttataagacgcaagcaaMacatgcagaccgtaaccaaa

fava_c18189_240	fava_c18189	240	T/A	tccgcatttcgtgctatatatttatatatgttacaatctatcatttacctatacaaaaaaaagcccatccaacttgcatcccttggcaatccaRgctcaa[T/A]aaggttgcaagaaaacaaaacaaccagcagatctcttatacaaggatttcggtgtttataagacgcaagcaaMacatgcagaccgtaaccaaacataaaa

fava_c18189_313	fava_c18189	313	C/A	ttgcatcccttggcaatccaRgctcaaWaaggttgcaagaaaacaaaacaaccagcagatctcttatacaaggatttcggtgtttataagacgcaagcaa[C/A]acatgcagaccgtaaccaaacataaaactatgttcatagtgcatactcttccaaagtataacctagtaaagattaatcaaggacgagtaacacgggagtg

fava_c18191_1329	fava_c18191	1329	T/C	cacttgataaaacacttgttttcggaaaaatcgttgtctgtgaaagaggaattaatggtagaaccgaaaaaggcgaggtagtaaaaaaatcaggcggata[T/C]ggaatgatactactcaactccgaaaatcaaggcgaagagcttctcKccgacgctcacgtcttaccaggtacttcattaggtgcttcagcaggtaaagcca

fava_c18191_1375	fava_c18191	1375	T/G	aggaattaatggtagaaccgaaaaaggcgaggtagtaaaaaaatcaggcggataYggaatgatactactcaactccgaaaatcaaggcgaagagcttctc[T/G]ccgacgctcacgtcttaccaggtacttcattaggtgcttcagcaggtaaagccataagaatctacctcaacactactaaaaaaccaacagcttcaatttc

fava_c18191_1746	fava_c18191	1746	C/A	aaagaagagtcttattcaacataatttcaggtacttcaatgtcttgtcctcacgttagcggcatcgcagcattgattaaatcagttcataaagactggtc[C/A]cctgcaatgatcaagtcatctcttatgacRacagcttacacattgaacaacaaaaatctccccatttccgatcttgcttcaaataacaactcatcttctg

fava_c18191_1776	fava_c18191	1776	G/A	gtacttcaatgtcttgtcctcacgttagcggcatcgcagcattgattaaatcagttcataaagactggtcMcctgcaatgatcaagtcatctcttatgac[G/A]acagcttacacattgaacaacaaaaatctccccatttccgatcttgcttcaaataacaactcatcttctgctaacccttttgcatttggttcaggacatg

fava_c18191_1893	fava_c18191	1893	T/C	acaacaaaaatctccccatttccgatcttgcttcaaataacaactcatcttctgctaacccttttgcatttggttcaggacatgttaatcccgaaagtgc[T/C]tctgatcctggattagtctatgatatgaacaccaatgactatttgaattacttttgcagccttaactttacatcttcagagattgctatattgacaaaaa

fava_c18191_879	fava_c18191	879	C/T	gaatgaggcgtacgtcaagaatcgcggcttataaagtttgctggctttctggctgcgcgaattccgacctattagcagccatggaccaagcagtttcgga[C/T]ggtgttgatgtactttcWctatctttaggaagtattccaaaaccattttataatgacagtattgctatagcttcattcggagcaatgaaaaacggcgttt

fava_c18191_897	fava_c18191	897	A/T	gaatcgcggcttataaagtttgctggctttctggctgcgcgaattccgacctattagcagccatggaccaagcagtttcggaYggtgttgatgtactttc[A/T]ctatctttaggaagtattccaaaaccattttataatgacagtattgctatagcttcattcggagcaatgaaaaacggcgtttttgtttcttgctcagcag

fava_c18192_292	fava_c18192	292	T/G	gaatgaggaataacaagttcaagctttcagacatgattcccaatgcttggttttacaagctaaaggaaataggcaaaccaaaaaaccaaacatcaacaac[T/G]acctcaaagaacagaaaacattcatcttccttaccctcaaaaacaacctcaatatcctcaaaaccaaatcaacccaaccaaaaccaatacattcctagaa

fava_c18194_1732	fava_c18194	1732	G/A	cctcaggggcgtactttgggtggcttccgggccggtatttcagttcctagtttggtgcctgagcgtcctttaacttctaaatcaacattgcaaaacttca[G/A]gaacccaatatccaatccgtctcatggattcgatggaacaatgatgctgaagggagcaagtaacgacccgttactgctttcagcttccaatacgtccatt

fava_c18194_2008	fava_c18194	2008	C/T	tcagaagctcgaaataggtcatttctgcaaatagaaatgccaaattcagatactgaaacaagcagtaatggaaggaaaccagtgatacctccgcaagaac[C/T]ggcgtcatctgcacctaaatctccaaggcagcatgcacagaatctgacttatacaggagagtctatcttttttggtggtccatttttgagtgtggctgct

fava_c18194_2528	fava_c18194	2528	A/T	ctcgactccatcaactaagcggctgtttatcaaatcaacatttaggtttgacatctgtcatctgacagcctgtattttgagctacatctcaatgctactg[A/T]gcttttgaaagaatgagctccattcttccttctgctaaacatgccttggatatagacgaagttagttatgtatgtatgtatactattgttattttagcaa

fava_c18195_161	fava_c18195	161	A/G	aatctcaaaagaagccagttgctctgttcaacaagtctRgactattgaagagcagggctctctaatgagttatgatacatctattggttaaaatatttca[A/G]aaagaaaaaactcaacagttaaccatctttacaaaattcacatattgaacagaaaacctcaacaagaattacatagccgctcattgtctattctagctat

fava_c18195_1759	fava_c18195	1759	T/C	aactgagtcaatgtatcttcagaatgttgaggaagcatccacttttccggcgtgtaagaaacttgttgatgaggtgccttagaagtagttttatcaaaat[T/C]tccagattttacatYaagaactgtgtggttaaaactgctggaagcactttcagaatttccatcattgttagaagacaaactaagcttatcactttcttcR

fava_c18195_1774	fava_c18195	1774	C/T	tcttcagaatgttgaggaagcatccacttttccggcgtgtaagaaacttgttgatgaggtgccttagaagtagttttatcaaaatYtccagattttacat[C/T]aagaactgtgtggttaaaactgctggaagcactttcagaatttccatcattgttagaagacaaactaagcttatcactttcttcRactttgggcagtgca

fava_c18195_1859	fava_c18195	1859	G/A	YtccagattttacatYaagaactgtgtggttaaaactgctggaagcactttcagaatttccatcattgttagaagacaaactaagcttatcactttcttc[G/A]actttgggcagtgcagacaaacgactgaagtattctttgcttgattggataccatttgtaccattttttgaagaatcYatttttttcgtatcatctcctt

fava_c18195_1937	fava_c18195	1937	T/C	aactaagcttatcactttcttcRactttgggcagtgcagacaaacgactgaagtattctttgcttgattggataccatttgtaccattttttgaagaatc[T/C]atttttttcgtatcatctcctttgtccttYggcaaaaatgataaactgtttaaagtttcaccaaatgttttcatatcaatttctacatttttggaaagct

fava_c18195_1967	fava_c18195	1967	T/C	gcagtgcagacaaacgactgaagtattctttgcttgattggataccatttgtaccattttttgaagaatcYatttttttcgtatcatctcctttgtcctt[T/C]ggcaaaaatgataaactgtttaaagtttcaccaaatgttttcatatcaatttctacatttttggaaagcttgttttgagccacactaccctgcttgctcg

fava_c18195_2279	fava_c18195	2279	A/G	tgtctttcaaacccacttttgaagaatgtaagccagtatgaaccacgtcatctggagatggaacgtcaaacttgaaaggatctgttttgttactgttgga[A/G]ggtccattgtaaaccaagggatgacgtacaacgccacaaatatcacacgaagtcatactctcatcgttgtcataggtgcaaatggggcattgccaaactc

fava_c18195_2498	fava_c18195	2498	A/G	gctttgcaacaggtccttcttctgctccatagttttcagcttcaacatcataatcatagtcatcatagtcctcatagtcatcgtcgtagtaatcgattcc[A/G]tagttaactttacgaggcatggttttcacagcacactgagcatacacacctaattgcttcttattatcacgaaagagttaaactgcaaaacctaattgct

fava_c18195_300	fava_c18195	300	C/T	cacatattgaacagaaaacctcaacaagaattacatagccgctcattgtctattctagctataaattatatggcaaacaaagatgtgtgtacttgaacaa[C/T]aacatacatggtatactgggaacctcttcaatgttattatcggctgatcttttcgaagaaataacatcatctaatcttgttcttcgattattcttgtcac

fava_c18195_787	fava_c18195	787	C/T	aactccaaatgcttcgcaactgcaacaggaaagtcgggatggcataatacacctccagccaatacatgacttccatcaacaccatgcaacgttacagcta[C/T]gttatctccagctcttgcaacagagcaaacattagagtcacgctccaatgtgcgtactgttccaacaacatctgaaggcataactagtacctttgttcca

fava_c18195_99	fava_c18195	99	G/A	cgaaaaatcctaaccaatgaaggatggaattaaatcccatcaagtgaagcatttaaaagtaatctcaaaagaagccagttgctctgttcaacaagtct[G/A]gactattgaagagcagggctctctaatgagttatgatacatctattggttaaaatatttcaRaaagaaaaaactcaacagttaaccatctttacaaaatt

fava_c18197_1334	fava_c18197	1334	A/C	ttggaagttgagagctgagactgaacagaaattaaatagactagaagttataagtttgaaagaagggtgcaaaatttaagaataatatatgttttgtaag[A/C]attacYaaagcttaaYccattggcacacggttcgcaattcgcttcggtacatagtatgaattcggatacggaattcacaactctcatagaattaggtacg

fava_c18197_1340	fava_c18197	1340	T/C	gttgagagctgagactgaacagaaattaaatagactagaagttataagtttgaaagaagggtgcaaaatttaagaataatatatgttttgtaagMattac[T/C]aaagcttaaYccattggcacacggttcgcaattcgcttcggtacatagtatgaattcggatacggaattcacaactctcatagaattaggtacgcgaggg

fava_c18197_1350	fava_c18197	1350	C/T	gagactgaacagaaattaaatagactagaagttataagtttgaaagaagggtgcaaaatttaagaataatatatgttttgtaagMattacYaaagcttaa[C/T]ccattggcacacggttcgcaattcgcttcggtacatagtatgaattcggatacggaattcacaactctcatagaattaggtacgcgaggggtatacatag

fava_c18197_788	fava_c18197	788	C/T	acatcaacatctcattgattggaatccatgatttacggtccagactgagcataattcctcaggatccaacaatgtttgaagggactgtaaggagcaacct[C/T]gacccRctRgaagagtacacagatgaacaaatttgggaggctttaRatatgtgccaactaggagatgaagtgaggaagaaagaaggaaagctcgactcaa

fava_c18197_794	fava_c18197	794	G/A	acatctcattgattggaatccatgatttacggtccagactgagcataattcctcaggatccaacaatgtttgaagggactgtaaggagcaacctYgaccc[G/A]ctRgaagagtacacagatgaacaaatttgggaggctttaRatatgtgccaactaggagatgaagtgaggaagaaagaaggaaagctcgactcaacagtta

fava_c18197_797	fava_c18197	797	G/A	tctcattgattggaatccatgatttacggtccagactgagcataattcctcaggatccaacaatgtttgaagggactgtaaggagcaacctYgacccRct[G/A]gaagagtacacagatgaacaaatttgggaggctttaRatatgtgccaactaggagatgaagtgaggaagaaagaaggaaagctcgactcaacagttagtg

fava_c18197_834	fava_c18197	834	G/A	gagcataattcctcaggatccaacaatgtttgaagggactgtaaggagcaacctYgacccRctRgaagagtacacagatgaacaaatttgggaggcttta[G/A]atatgtgccaactaggagatgaagtgaggaagaaagaaggaaagctcgactcaacagttagtgagaatggagaaaactggagcatgggtcaaagacagtt

fava_c18199_145	fava_c18199	145	G/T	gcaacaacagtttcccactttgcttcactcaaataacacaacaatcttctcctcatcactttgcgtttcgtatggcttatgttgacgaggatggttcttc[G/T]ggttctggagatgatgtgaacatgttagacggacaaaagcgtcaggctggtgtttcggctagtggtggtcggaagagaggccgaaaagctactggtgatg

fava_c18199_348	fava_c18199	348	T/C	atagtggatgccatgatgcagatagcagctgcttcgaaaatgagggcaaatgcaattatgaagaatgaggatagattttcaataagcaagtgcatcaatg[T/C]gttggatgagatgcaaggtgttgatgaaaggatttacttttttgctttgggtttgtttgaggatcaccctaatgcccgggaggttttcatctctcttaag

fava_c18201_1416	fava_c18201	1416	C/T	aatgaaattgctgtcaaagttgtcattggtggtcttatgcagaaactttccagcaatttatcccctagagatttgcttcgagttcctgctaagttggaga[C/T]gaaggagcgtggtttattttcctttactgttgaaaatgtagtcaatcagcaggatggtcatgacaaagggaaatcttcatcagataaatcRattaagttt

fava_c18201_1507	fava_c18201	1507	A/G	agttggagaYgaaggagcgtggtttattttcctttactgttgaaaatgtagtcaatcagcaggatggtcatgacaaagggaaatcttcatcagataaatc[A/G]attaagtttcaaagtcgtcgcagagttgatcatgttatttctgacattgctaagtttattggcaaggaaaatgtacctgcaaaggtgctgaaagaggtag

fava_c18201_2543	fava_c18201	2543	A/T	tattgtagagctgtagtgttcacatggcattcttcatcatggtatccttcatcaatttgtatagtggtccaattttttcttatatgttatattcccatgt[A/T]tcatagctacataatcatgcttagtctaatgtgttcagggtttcattgttcattctgttaagatgaatgaaataagttcattacaagtggcattatttat

fava_c18201_757	fava_c18201	757	A/T	ctgctgctggtgttcctctatttgagttttttcttacaatggataaaaaaggtgacttcaagtcgcaggctgtgtatctaaagcgaaaagggtcctaccg[A/T]ggccccccaacaatgtcctctaaaacatcggatgctagtgagctgtttgttgtgagtactgaagattcagaaagtggtgatgataggagtgatggctctg

fava_c18202_1089	fava_c18202	1089	T/C	accattaaataaggtgatgtttagtactgatgcttatgcctttcctgaactcttctacttaggtgcgaaaaatgcccgcaaagttgttttcacagttctg[T/C]gtgactcgtgcattgacggcgatctcactgtttcagaggctgtggaagctgcaaaagacctctttgcacgaaattcaatcaatttctataagattaccag

fava_c18202_1227	fava_c18202	1227	G/A	ggctgtggaagctgcaaaagacctctttgcacgaaattcaatcaatttctataagattaccagttaacattgttgttatgttcacatgattattgtgtta[G/A]aagactttatgttgggtggttattactttttcattaaggttctattttaggactatagttttttatcagttatctagtagttgtcaagtgttggttttta

fava_c18202_1336	fava_c18202	1336	G/A	atgttgggtggttattactttttcattaaggttctattttaggactatagttttttatcagttatctagtagttgtcaagtgttggtttttagatatgtt[G/A]tttttcaatcatatgaagttattttttttcttcaataggtgaaacgctagtgccatttttatcattctgtgcttttgatgtatagtaagaaactttgtta

fava_c18202_381	fava_c18202	381	A/G	ttctggattagaatctatcgcctcaacatgcttcaaagctgcaagaatctctacagtaatcttcgatgatggaatagacttggacaaaataattgatacc[A/G]aatggcacaagacttttactcctcatgttgctacactcgtaagaattgaacgccttgctgagaaaatcctcaatcaaggtttggcagatggatattcttg

fava_c18202_574	fava_c18202	574	G/T	tattcttggacactggactcattcactactgcatttctatccaagttggaatcagttgctggtgagatagttggtttgaaaagcatagcagcatactatt[G/T]tggtttagatatcaatacaaatgtcaccaaaatggaagctgaagaggctctacaacaggtgttatctgtttggaagcccgtcttggtagcaaacaaaaac

fava_c18202_893	fava_c18202	893	T/A	atctccacaatattcttgaggacaagagatttgcaaagtgtaggattgttcttttacatacatcatatccattctcaaaggaagcatcatatctagcttc[T/A]ctctactcccaggtttatcttgattttgggttggctattccaaagcttagtgttcatggcatggtttctgcagtgaaagatcttttagagcaagcaccat

fava_c18206_1144	fava_c18206	1144	A/G	aaacgctgttgatgcaaacgacgccgaagtcgatgatcttaatcgaggatctggatcgtttattagcgggaaaatcaacggctgttaatttatcaagtgt[A/G]ttgaacttcatggatgggataatgagttgttgcggtgaagagcgtgtgatggttttcacRatgaacggtacgaaagatgaagttgatcaagcggtgttga

fava_c18206_1204	fava_c18206	1204	A/G	tattagcgggaaaatcaacggctgttaatttatcaagtgtRttgaacttcatggatgggataatgagttgttgcggtgaagagcgtgtgatggttttcac[A/G]atgaacggtacgaaagatgaagttgatcaagcggtgttgagacctgggagagttgatgttcacatacacttccccttatgtgatttctccacctttaaga

fava_c18206_1333	fava_c18206	1333	G/A	aagcggtgttgagacctgggagagttgatgttcacatacacttccccttatgtgatttctccacctttaagattctggctaatagttatttagggttgaa[G/A]gagcacaagcttttccctcaagtggaagaggtttttcagaccggtgcgtggctcagcccggcggaaatcggtgagattatgatatccaaccggaattcgc

fava_c18206_1438	fava_c18206	1438	C/T	acaagcttttccctcaagtggaagaggtttttcagaccggtgcgtggctcagcccggcggaaatcggtgagattatgatatccaaccggaattcgccgag[C/T]MgggccttRaaaacggttataaccgctatgcaggtgcaatccaatggtcaggggcagaggttgagtaatagtgggtcgggtcggagctccgaagaggtta

fava_c18206_1439	fava_c18206	1439	C/A	caagcttttccctcaagtggaagaggtttttcagaccggtgcgtggctcagcccggcggaaatcggtgagattatgatatccaaccggaattcgccgagY[C/A]gggccttRaaaacggttataaccgctatgcaggtgcaatccaatggtcaggggcagaggttgagtaatagtgggtcgggtcggagctccgaagaggttag

fava_c18206_1447	fava_c18206	1447	G/A	tccctcaagtggaagaggtttttcagaccggtgcgtggctcagcccggcggaaatcggtgagattatgatatccaaccggaattcgccgagYMgggcctt[G/A]aaaacggttataaccgctatgcaggtgcaatccaatggtcaggggcagaggttgagtaatagtgggtcgggtcggagctccgaagaggttagtgatgcaa

fava_c18206_1585	fava_c18206	1585	G/A	gtcaggggcagaggttgagtaatagtgggtcgggtcggagctccgaagaggttagtgatgcaaatggtgtgatatgtagagagagtgttcatacggtgag[G/A]gagtttaggaagctgtatgggcttttgcgtttgggaagtaggaggaaggaggagtctggttattcgggtcccatacagaaagaaccgccgcgcatcgagg

fava_c18206_1709	fava_c18206	1709	A/G	tttgcgtttgggaagtaggaggaaggaggagtctggttattcgggtcccatacagaaagaaccgccgcgcatcgagggtcgggtcggataaaatggatcc[A/G]atttaaatatWtttttttcttggaaaaatgtaaagtggcttgacagttatttgctagctgctactaacaattattgcagtttctttttttttatttgtcg

fava_c18206_1720	fava_c18206	1720	T/A	gaagtaggaggaaggaggagtctggttattcgggtcccatacagaaagaaccgccgcgcatcgagggtcgggtcggataaaatggatccRatttaaatat[T/A]tttttttcttggaaaaatgtaaagtggcttgacagttatttgctagctgctactaacaattattgcagtttctttttttttatttgtcgataattcacat

fava_c18206_305	fava_c18206	305	C/T	atcagttttatttattctcaatcaaaatatggtttggatttgggccagttccatgacaatctcttccatcttcttcattttggctctctctttcttaatt[C/T]tccgtttcttccgtaaaacctctgcacttcatatcctgaaacgctggtggctttcgtttgaaagccgtcttcatcttcaccaatcctttaaaattccttt

fava_c18206_409	fava_c18206	409	T/C	gtttcttccgtaaaacctctgcacttcatatcctgaaacgctggtggctttcgtttgaaagccgtcttcatcttcaccaatcctttaaaattcctttata[T/C]gaccataatttccgggaaaatcagctttaccggaaaatcattacWtaccttgaYtcattaccttccgttcaagactctgatttcaccaacctcttttccg

fava_c18206_454	fava_c18206	454	A/T	ggctttcgtttgaaagccgtcttcatcttcaccaatcctttaaaattcctttataYgaccataatttccgggaaaatcagctttaccggaaaatcattac[A/T]taccttgaYtcattaccttccgttcaagactctgatttcaccaacctcttttccggtgctaatccctctgatatcttcctccatctagatgctaaccaaa

fava_c18206_463	fava_c18206	463	C/T	ttgaaagccgtcttcatcttcaccaatcctttaaaattcctttataYgaccataatttccgggaaaatcagctttaccggaaaatcattacWtaccttga[C/T]tcattaccttccgttcaagactctgatttcaccaacctcttttccggtgctaatccctctgatatcttcctccatctagatgctaaccaaattgttcatg

fava_c18206_604	fava_c18206	604	C/T	tttccggtgctaatccctctgatatcttcctccatctagatgctaaccaaattgttcatgatactttcttaggagctaagctttcctggaccaacaacac[C/T]gtcgccggtgattctgcgtcagcgctcgtgcttagaatgaaaaagaaagataagcgtagggtttttcagcagtatttccagcatattctctcggttgcag

fava_c18210_1300	fava_c18210	1300	T/C	aaagaagggagctcaatttcctcctttggaaaaatatgcaaattcaatagacggacattcagttacgaagactatcgtcagtcttggcgatagcgataga[T/C]gcatggatgtttcataccttagttctcatgtgatttctaattacagtaaagaacttcagaatcaaggttcgactgctgcttctgaaatggagatagagaa

fava_c18210_665	fava_c18210	665	G/A	ctggtattttctgaagatgactcacttctaatatctggttttaaaaacggaagcgtacgagtttggtctctcttaatgatatttgatgatgtgagaagac[G/A]ggaagcaagccagatttacgagtatagtttttcagatcacactttgtgtgtaaatgatgttgtgattggttctggagggtgcaatgcaattataRtttca

fava_c18210_760	fava_c18210	760	A/G	aagacRggaagcaagccagatttacgagtatagtttttcagatcacactttgtgtgtaaatgatgttgtgattggttctggagggtgcaatgcaattata[A/G]tttcagcatcggatgatcggacgtgtaaggtatggagcttatctaatggaatgctacaaagaaatatagtattcccttcaaagattaaagctattgcatt

fava_c18212_202	fava_c18212	202	T/C	actgtgttttcaagagtactgatacaatacattagaggaatatagaaagatgtcttttttatctctctacaactttgtacccttaatctcaaacctaact[T/C]aactctaaaccacatattaacattgaaattacaagccaccaaaaagacaaaaatgagcaaaagtgaagatctatttgctttaccctttcagcttttgctc

fava_c18214_149	fava_c18214	149	C/G	caaacttcagataaccagattcatcatgaatcaagtttcaaaatctttattttctcaatttcaactcattccacacacacaaaaatagaacgattgaaga[C/G]ttaccttaaggatgcggcYgtggcgatgatagagaKgatcgacgttggttgtcgtttgtggctctgcagttgtcaaaggctggatggtgttgagattgct

fava_c18214_168	fava_c18214	168	T/C	ttcatcatgaatcaagtttcaaaatctttattttctcaatttcaactcattccacacacacaaaaatagaacgattgaagaSttaccttaaggatgcggc[T/C]gtggcgatgatagagaKgatcgacgttggttgtcgtttgtggctctgcagttgtcaaaggctggatggtgttgagattgctgcgcaggggtggtagtcgc

fava_c18214_185	fava_c18214	185	T/G	ttcaaaatctttattttctcaatttcaactcattccacacacacaaaaatagaacgattgaagaSttaccttaaggatgcggcYgtggcgatgatagaga[T/G]gatcgacgttggttgtcgtttgtggctctgcagttgtcaaaggctggatggtgttgagattgctgcgcaggggtggtagtcgcttggatctgtggcggct

fava_c18215_1004	fava_c18215	1004	A/G	acaaccactgttgtttgatctctcaattggaatgcaagaagatcaaggttttgtccagatttaacactgcaaaagactggctggtatccccagtcatgaa[A/G]cctagccttccaggaatccaaggtctctttatccacaagctcagtcttgttaagagctagagtgagaggaaYcccagtggactcagcttcaaccaaaaac

fava_c18215_1076	fava_c18215	1076	T/C	aagactggctggtatccccagtcatgaaRcctagccttccaggaatccaaggtctctttatccacaagctcagtcttgttaagagctagagtgagaggaa[T/C]cccagtggactcagcttcaaccaaaaaccttgtaagcgtaaacgtctcaagctttggttgatcaagcgagaagagaaccagtaaatggtccacgttagcc

fava_c18215_1206	fava_c18215	1206	C/T	ttgtaagcgtaaacgtctcaagctttggttgatcaagcgagaagagaaccagtaaatggtccacgttagccacgggagggtcaagcatttcagaatttcg[C/T]tgaaacacgttttcaatcattcccctccgatccacccaatcaacagaacctactataaccttatccccaaccataaccctacgctttatcttcttcagca

fava_c18215_157	fava_c18215	157	G/A	atccagaacagtgtgtttgttagttaatgtcattcacacaaactctatacaaggttgaaccaaagtgacagaagactctaatcgaactaagagaaaaagg[G/A]aactcggttgacccaaagatacaagccgatatttacaatcagtattatggggattaatggaaatttaaggattttcattccttaaagcatttaaaaaggg

fava_c18215_774	fava_c18215	774	T/G	cgggagtgtcagcaagataaccccctccagttaatggaagcagagagacatgacgagtagtatgcttccccctgccacttcttgttgaaacctccgcaac[T/G]cgctgttcctctaaccacctgctacctaaaacaggttcaaaccaattctccccatctgcagtatcacaagtacgatggttgcttctaagagcattgatca

fava_c18216_1039	fava_c18216	1039	C/T	agattcttcagcaacgcattttaagagtgatgagaatagtggaagcgttggagggaaaaggctgccgtattccattaacaaaaggggaagctgaactcgc[C/T]gagaaattagccacaataactagacagttgaaaggatctggagcagaattatctcgaagggtacaaaatctactcggtgtatcccgagtcaaggctaata

fava_c18216_1186	fava_c18216	1186	T/C	aattatctcgaagggtacaaaatctactcggtgtatcccgagtcaaggctaatagcaatgaatttggtaactctgtttatcttccaggctcaaccaaaat[T/C]catgaacagagtctgactgatctgcaagaggttttacaccagcagatggatgccatagcaagacttggcagtgttctaaagcgagatgcgcggaatatgg

fava_c18216_1385	fava_c18216	1385	G/A	ggagattatgatggcagaggacacagaaacaacagaaaatggaaatttaatgtaaacatgcctgagaaaatctgtagttttctcgcttcatgtttgcatt[G/A]agttatatatttttatgtatcttSatttggaaatatagttggcacaatagtttcttctaggggacacctttttggcMtatcggttgagtagtttaacttg

fava_c18216_1409	fava_c18216	1409	G/C	agaaacaacagaaaatggaaatttaatgtaaacatgcctgagaaaatctgtagttttctcgcttcatgtttgcattRagttatatatttttatgtatctt[G/C]atttggaaatatagttggcacaatagtttcttctaggggacacctttttggcMtatcggttgagtagtttaacttgacccacgagcgttgtttccgtggt

fava_c18216_1462	fava_c18216	1462	C/A	ttttctcgcttcatgtttgcattRagttatatatttttatgtatcttSatttggaaatatagttggcacaatagtttcttctaggggacacctttttggc[C/A]tatcggttgagtagtttaacttgacccacgagcgttgtttccgtggttattttaacatgctatgttattgaagagttcgactagtttagcaagggtatgt

fava_c18216_433	fava_c18216	433	A/G	ctgcttttggggcttcttctactcctgctttcggcggtacttcccttttctcttcgccgttctcttctcagcaacagcaacaaccacagcagcagcaaca[A/G]acttcactctttcaacaatcaccttctaccggtttcggatttcagaattctatgcaagcgccacaagctacgccgttcccgaattctcaattgactactc

fava_c18216_799	fava_c18216	799	C/T	atatcatgtgggcagaagctatggggaagcttgagggtatggaaagtgccgatagagaacgtctgtggccacagcaagttcaaggttttaaggatctttc[C/T]caacgccttaagctccaagatgaagtcattgtttctgatgcagagagattaagtgtaacccaaagcaatgttaaaatgctccaaagacattttcaagctg

fava_c18217_1013	fava_c18217	1013	T/C	agtctatctttaagtcccaaatcagcagctgaggagattgggtacactttcctgccatgtgtgctagctggcctcagtaaagctccgcaatacttggtca[T/C]gcattctgaatcaatggaaaagggttgcatattggcaagtgatgtggattctgtagtccttcctaaagatgcttgtggaggggaagggactcttgctttt

fava_c18217_1176	fava_c18217	1176	T/C	ctaaagatgcttgtggaggggaagggactcttgcttttgcaagaaatgaaaaacataagccacttatcattactgtggaagaaaatgaaactgttctgga[T/C]gatactgcatataaacttggttttgaagcgctgcatgtctcaaattattgggaggctattggagtaatggctgctcacaaagcgggRatagatccatttt

fava_c18217_1263	fava_c18217	1263	G/A	aaactgttctggaYgatactgcatataaacttggttttgaagcgctgcatgtctcaaattattgggaggctattggagtaatggctgctcacaaagcggg[G/A]atagatccattttcRctgagaagaaataaaattcctaacatcggttgcacttcttctaagccggtcaatggtcgctacaatacaagggaaagagctatct

fava_c18217_1278	fava_c18217	1278	A/G	atactgcatataaacttggttttgaagcgctgcatgtctcaaattattgggaggctattggagtaatggctgctcacaaagcgggRatagatccattttc[A/G]ctgagaagaaataaaattcctaacatcggttgcacttcttctaagccggtcaatggtcgctacaatacaagggaaagagctatctactgatataaatttt

fava_c18217_1387	fava_c18217	1387	A/C	aaataaaattcctaacatcggttgcacttcttctaagccggtcaatggtcgctacaatacaagggaaagagctatctactgatataaattttgagagctc[A/C]atgctagtatataatttttaattttgttatattaacaccattagaggaaatagtatattgagttctggattttaggtcttgtagccttttgcatttctca

fava_c18217_542	fava_c18217	542	G/A	tatgctcttgacagatttgcagaagggtcatgggctttacaacctgttcaccaaaatagggttggaatagttcttgatgccggtatagaggaagatctcc[G/A]gattcgccagttacaagtagccgatgctgcacgagcttcccttggattgcctgttgtggaatatattgtcacagacactccacttgaggtagagaaatgg

fava_c18217_735	fava_c18217	735	G/C	agaaatggattgatcccaaaactggaaaatcaacagggaggattaagcaccctgattctttacttagagctgtgcagaccttagtgagtaggtcaaaagt[G/C]aatgctgttgctgttgtcggacgtttcccagacgatgatattgatgatgttgatgaatatcggcagggattgggagtagacctattggcgggagttgagg

fava_c18220_1153	fava_c18220	1153	G/A	agcaagtggtacaagtaatggttgtctcggtgtcccattcctggtagagaagcaagactgaatggtgccaatggggatcataggataataagtgaggttt[G/A]ggttttcagatttaggtttcgttaattcttttctcaacaactgttgagccctaattcggcctcggcgttcggaggtgcattgatcggaggaagatttgag

fava_c18220_185	fava_c18220	185	A/T	tttctcagaatttgtaagagaaaaaaatacatttctatcaaaaaaagaaaatcaaatttaataaggtttaaaatgtaattaggcaggtaattatttttta[A/T]aaaaataatcaggtgcaacaatagaaatcactgcatcctgtctttccatgtcaagtaattgcagggactaatctttttgtcaaagacagcagaggttgat

fava_c18220_372	fava_c18220	372	A/G	cagcagaggttgataccttttctacagtgacattaccatcctgagtaattctatagtggacattgagtccctccaagaagaggtgataaactacttcggc[A/G]ggatttagagaattctgctctctttcatggacaataRctgtttcatctaggtgctcatctacatcagaagtatcatccgagagttcattcttttctttca

fava_c18220_409	fava_c18220	409	G/A	atcctgagtaattctatagtggacattgagtccctccaagaagaggtgataaactacttcggcRggatttagagaattctgctctctttcatggacaata[G/A]ctgtttcatctaggtgctcatctacatcagaagtatcatccgagagttcattcttttctttcatcgggatagcttcatgaggtctatttcgctgactcag

fava_c18220_516	fava_c18220	516	G/A	catctaggtgctcatctacatcagaagtatcatccgagagttcattcttttctttcatcgggatagcttcatgaggtctatttcgctgactcagtgaccg[G/A]atatcccatgatagaacctgctttattaagctttggaattcatYtggtgatgcatagagtgacttcgtttctgccattatccagcaattttcaagtgatg

fava_c18220_560	fava_c18220	560	C/T	ttcttttctttcatcgggatagcttcatgaggtctatttcgctgactcagtgaccgRatatcccatgatagaacctgctttattaagctttggaattcat[C/T]tggtgatgcatagagtgacttcgtttctgccattatccagcaattttcaagtgatgaagtgaagtcctctgaaaagctaacagaagctactgaaagcaag

fava_c18220_670	fava_c18220	670	A/G	atagagtgacttcgtttctgccattatccagcaattttcaagtgatgaagtgaagtcctctgaaaagctaacagaagctactgaaagcaagttatccccc[A/G]taagccagttWggcaccgcagcatcttggatgctgtcacaaaagggtatataaggcttgacatcaagtaccggagttccatcaaccagatccacaccaga

fava_c18220_681	fava_c18220	681	A/T	tcgtttctgccattatccagcaattttcaagtgatgaagtgaagtcctctgaaaagctaacagaagctactgaaagcaagttatcccccRtaagccagtt[A/T]ggcaccgcagcatcttggatgctgtcacaaaagggtatataaggcttgacatcaagtaccggagttccatcaaccagatccacaccagaaagcaaaacca

fava_c18220_984	fava_c18220	984	A/T	tagggaccttcacctttgccttgattcctgatttagatggatgtttccacatcttttcaagattggtatttaaatgaaatacatatataatccagcaatg[A/T]gaatattcggatagaccctcaagagacgctggaggaacacggcttgtattgaaaagcaaacatgctctagcaagtggtacaagtaatggttgtctcggtg

fava_c18221_662	fava_c18221	662	G/A	tgatacaaatcattcaatctagggatgctgataaatagtgcaactacaagaaatgcaactataagggggaatactggtgccggagtcaactcctgcttca[G/A]ctgatcttcaatcctgaatttcagtggtcccacgtggatttgaacaYttttgtccaagtcgacctcaattatatcagattcaaatctaagtgagcttgat

fava_c18221_709	fava_c18221	709	T/C	aagaaatgcaactataagggggaatactggtgccggagtcaactcctgcttcaRctgatcttcaatcctgaatttcagtggtcccacgtggatttgaaca[T/C]ttttgtccaagtcgacctcaattatatcagattcaaatctaagtgagcttgatgggagaccagatcgaagctgtagaaggtgtcttccctttgataagcc

fava_c18223_1601	fava_c18223	1601	T/C	ttcgctgtttagtggacgttgatatccctctcaatcaaggctgtctggctccagtgaagattcttattccagaaggctcatttctctctcctagtgatac[T/C]gcagctgtggtaggaggtaatgtccttacatctcagagaatcactgatgttgtatttacagcatttcaggcatgtgcttgctcacagggttgtatgaata

fava_c18223_1727	fava_c18223	1727	C/T	ttacatctcagagaatcactgatgttgtatttacagcatttcaggcatgtgcttgctcacagggttgtatgaataatctcacatttggagatgatacttt[C/T]gggtactatgaaaccattggaggtgggagtggtgctggaccttcatgggacggaaccagtggagtacagtgccatatgacaaatacaagaatgactgatc

fava_c18225_329	fava_c18225	329	A/G	atgggttcgcgatggtgtggtcgtcgggttcaattcctcggagtcgcgtctgattttctttgttcttcttctcctttgattttgttttgcctttggattc[A/G]ttgcttttaattttattctgataaaatctcacgatgtctgagtttRacggcggagatcggattcacagtgatgttgcccgctgcgtgggtacgaattcga

fava_c18225_375	fava_c18225	375	G/A	cgtctgattttctttgttcttcttctcctttgattttgttttgcctttggattcRttgcttttaattttattctgataaaatctcacgatgtctgagttt[G/A]acggcggagatcggattcacagtgatgttgcccgctgcgtgggtacgaattcgatggaagctctggctggggcatgacgttgatgatacaccggcgatct

fava_c18226_322	fava_c18226	322	C/T	accttttacattagttacaattgttttagaaaatacattacgattattttcacaaaataaacacaactttacatttacaagtggcgaaacttaacattta[C/T]aagtgggaaagaaaacacaactacagagaggctttttgatagtacaatatgaaattacattattgaacacgcatttgggttatcatcattaaaaagcacc

fava_c18226_475	fava_c18226	475	T/G	aattacattattgaacacgcatttgggttatcatcattaaaaagcaccatcactttcatctcaggagacttagggtcacccacagcttgaggaacattcc[T/G]gacaaaaccagttggtgctttcatgtcatatgccccttgagcataaggataagtcactaaattgtatggaacaaatggccaattgtcagctgtttttcct

fava_c18226_702	fava_c18226	702	C/T	ctttgcatctacatttccagtcacggttaccctttgtagcttttgatttacagtaaatgactccaccccttccatgtctttcactgcatttttaactttc[C/T]ttacacaaccatcacaatccattttcactctgacatgaaccgtcttcttaggaatacgtttYtctataggtcttgaacattcaatgaaacattctgaaag

fava_c18226_764	fava_c18226	764	C/T	ccaccccttccatgtctttcactgcatttttaactttcYttacacaaccatcacaatccattttcactctgacatgaaccgtcttcttaggaatacgttt[C/T]tctataggtcttgaacattcaatgaaacattctgaaagatggcRaagaatacccatttatatatgatccaaggcaataaaatgtgacaagaaaacttatt

fava_c18226_808	fava_c18226	808	A/G	caaccatcacaatccattttcactctgacatgaaccgtcttcttaggaatacgtttYtctataggtcttgaacattcaatgaaacattctgaaagatggc[A/G]aagaatacccatttatatatgatccaaggcaataaaatgtgacaagaaaacttattactgaaaggagagaatttgatgaaacttggttatacttgtttgt

fava_c18229_1057	fava_c18229	1057	A/G	gtatgcctgaaacccaaactggtagaactcttattaagcatttcacttcttcaacttgttgtatactgcatagattccatggatcagatgcagaaccatc[A/G]gggtttatgttgtcttctcgagttataatagctgctttgtcaagaaacctgaattgagKtgtaYgaggaagtttgcaattgatagagtgaggagatatat

fava_c18229_1116	fava_c18229	1116	T/G	tgtatactgcatagattccatggatcagatgcagaaccatcRgggtttatgttgtcttctcgagttataatagctgctttgtcaagaaacctgaattgag[T/G]tgtaYgaggaagtttgcaattgatagagtgaggagatatataRctaaaaagtgaaaccaatgaaaactcatcctcttgtagattgagtttccttttcttt

fava_c18229_1121	fava_c18229	1121	C/T	actgcatagattccatggatcagatgcagaaccatcRgggtttatgttgtcttctcgagttataatagctgctttgtcaagaaacctgaattgagKtgta[C/T]gaggaagtttgcaattgatagagtgaggagatatataRctaaaaagtgaaaccaatgaaaactcatcctcttgtagattgagtttccttttctttatagc

fava_c18229_1159	fava_c18229	1159	G/A	ggtttatgttgtcttctcgagttataatagctgctttgtcaagaaacctgaattgagKtgtaYgaggaagtttgcaattgatagagtgaggagatatata[G/A]ctaaaaagtgaaaccaatgaaaactcatcctcttgtagattgagtttccttttctttatagccacagaaataacttgaacaatRctYgttacaggactac

fava_c18229_1243	fava_c18229	1243	A/G	agtgaggagatatataRctaaaaagtgaaaccaatgaaaactcatcctcttgtagattgagtttccttttctttatagccacagaaataacttgaacaat[A/G]ctYgttacaggactaccagttgctttaactttcacgtaatatttacttcctaagaaataaacaatacatgaaaacagcatcaaagcagcaggaattccaa

fava_c18229_1246	fava_c18229	1246	T/C	gaggagatatataRctaaaaagtgaaaccaatgaaaactcatcctcttgtagattgagtttccttttctttatagccacagaaataacttgaacaatRct[T/C]gttacaggactaccagttgctttaactttcacgtaatatttacttcctaagaaataaacaatacatgaaaacagcatcaaagcagcaggaattccaagac

fava_c18229_1729	fava_c18229	1729	G/A	ttttttttatccatgctgttaattgtataacaagtaaccccaaaaaggaagcgaacgtggagaatccaagtgttttgtagcgaccaaaataagtgtcaga[G/A]agaaaagctccaagcaaagtagctaaactagcactaccattaaagatattgacaatatttgtggcttcaatactgctaaggttgaagattgttgtaagat

fava_c18229_248	fava_c18229	248	T/C	acctccatttcaaagttcaaacatcatcaaatgaaaacttaatcactaaacaccattaatctttgtttcatgttgtttagtcacttgatcaagttcagta[T/C]cactacctccattaccatcctcccctttgtacYtataccaccttgagcacaaaacaaagtaacccaaattaacaacttccataatacaaatcatataata

fava_c18229_281	fava_c18229	281	C/T	aaaacttaatcactaaacaccattaatctttgtttcatgttgtttagtcacttgatcaagttcagtaYcactacctccattaccatcctcccctttgtac[C/T]tataccaccttgagcacaaaacaaagtaacccaaattaacaacttccataatacaaatcatataataaaagtaatccaatcttcctttgttcaaatcYtg

fava_c18229_379	fava_c18229	379	C/T	acYtataccaccttgagcacaaaacaaagtaacccaaattaacaacttccataatacaaatcatataataaaagtaatccaatcttcctttgttcaaatc[C/T]tgtggtaaccaattcccatttgcagatttatccgtcgaccgatgaataatcgaaatcaagagactactaagataacttgacaatgccaaaccacagaaaa

fava_c18229_622	fava_c18229	622	G/A	tgtagtaaaattcaacttgtcctaccaaagtaaatgcatcagagaatccagctagaattaactgaggaatcaaccacattgcagacatagaagaaatcgc[G/A]ccttttcttggttcaaatcctttaggatttgtaatagccatRgttcttcttttccattctacaacaccagaaactagcatgcatagtatagagataaaca

fava_c18229_664	fava_c18229	664	A/G	agaatccagctagaattaactgaggaatcaaccacattgcagacatagaagaaatcgcRccttttcttggttcaaatcctttaggatttgtaatagccat[A/G]gttcttcttttccattctacaacaccagaaactagcatgcatagtatagagataaacattccaattcccattctttgtagaagcgtgatcccgccttctt

fava_c18229_772	fava_c18229	772	G/A	ttttccattctacaacaccagaaactagcatgcatagtatagagataaacattccaattcccattctttgtagaagcgtgatcccgccttcttttcgggt[G/A]actcttctgatcgttggcacSattattcgatcgtagattggtagccatattgttaggcttaacatggtgaagattgtgaaagaagccgcagggatcttga

fava_c18229_793	fava_c18229	793	G/C	aaactagcatgcatagtatagagataaacattccaattcccattctttgtagaagcgtgatcccgccttcttttcgggtRactcttctgatcgttggcac[G/C]attattcgatcgtagattggtagccatattgttaggcttaacatggtgaagattgtgaaagaagccgcagggatcttgaaatctgtgttgaaaaaKgttc

fava_c18229_889	fava_c18229	889	T/G	gcacSattattcgatcgtagattggtagccatattgttaggcttaacatggtgaagattgtgaaagaagccgcagggatcttgaaatctgtgttgaaaaa[T/G]gttcggtcggattgaagtgcttggaaaactaacattgtgctttgttggactattgcgacgaagtagagtatgcctgaaacccaaactggtagaactctta

fava_c18233_2087	fava_c18233	2087	T/C	cgcgagcccatcagggtcttgcgcgtgttcattttcttaaaaacgacaaggctgctgcttacaaggaaatgactgaacttattgagaaggcaaaaagcaa[T/C]gcgtcggcRtatgagaagagatccgagtacggtgatcgtgatcttacaaaggcggatctagcaatggtcactcgattagatccacttcgtgtgtatcctt

fava_c18233_2096	fava_c18233	2096	A/G	atcagggtcttgcgcgtgttcattttcttaaaaacgacaaggctgctgcttacaaggaaatgactgaacttattgagaaggcaaaaagcaaYgcgtcggc[A/G]tatgagaagagatccgagtacggtgatcgtgatcttacaaaggcggatctagcaatggtcactcgattagatccacttcgtgtgtatccttatcgatatc

fava_c18233_2219	fava_c18233	2219	C/T	gtgatcgtgatcttacaaaggcggatctagcaatggtcactcgattagatccacttcgtgtgtatccttatcgatatcgagcagcagttttgatggacaa[C/T]cataaggaacaggaagccattgccgaactatcacgagcaattgcatttaaagctgatttgcacctcttacatttacgcgcagcattccatgaacacaaag

fava_c18233_2369	fava_c18233	2369	C/T	aagctgatttgcacctcttacatttacgcgcagcattccatgaacacaaaggcgatgtcctaagtgcgttaagggactgtcgtgctgcactctcggtgga[C/T]ccaaaccaccaagaaatgttggaacttcacactcgtgttaacagccacgaaccgtgaggagtcaagttcgggaagtaaggacagcatttgtacaatcgtc

fava_c18234_1239	fava_c18234	1239	C/T	ataagcattgattaaagaaccctgcatgccctttgttcgtgtccattgatcttatttgctttttggaaagcactgctacttcgtcgtgaacatgtttctt[C/T]atttcagatgatttatatgaactcacaaggatactaattgcttcttctgtcgatatttctatctcggtatcctgatcaaaatccttgtttatagttttaa

fava_c18234_1355	fava_c18234	1355	G/C	tatgaactcacaaggatactaattgcttcttctgtcgatatttctatctcggtatcctgatcaaaatccttgtttatagttttaagtaaatgatctggta[G/C]actttggagaggagaacaaggaaatccattcaaagcaaaaaactcacatgcggcagaagcaggtccaaaataaacattttcaccRgaagacagaagacaa

fava_c18234_1440	fava_c18234	1440	G/A	gtaaatgatctggtaSactttggagaggagaacaaggaaatccattcaaagcaaaaaactcacatgcggcagaagcaggtccaaaataaacattttcacc[G/A]gaagacagaagacaaaggttatcaaaaagttgaaaaacttcagaactaggttgatggatagaagtgatgatagtcctttgaattccatctttatgatcca

fava_c18234_158	fava_c18234	158	A/G	catataaatgtgaagttgaccctacttctccatttcttacRcaaccaaacaacaataaataagaagttcaatagactcatacagttcacatctataaaat[A/G]caaagttgttatttcttcactaaYtacctaaaaagttataattaataaacctttctaagattacacaacgtgctcattcaaaggagtagcatcaggcttc

fava_c18234_1713	fava_c18234	1713	A/T	gaagtctaggacgggttagaatctcaatgcaaatgctaactcttcttttctgacccccacttatgcctttaacaccccatcctccaattcttgtgttagt[A/T]gcatcttgtagacccatttctttgattgtaaaatcagctctttctttcttctctgatttggacattgtgttaggtaattgaagttgagctgaatagtaca

fava_c18234_182	fava_c18234	182	T/C	cttctccatttcttacRcaaccaaacaacaataaataagaagttcaatagactcatacagttcacatctataaaatRcaaagttgttatttcttcactaa[T/C]tacctaaaaagttataattaataaacctttctaagattacacaacgtgctcattcaaaggagtagcatcaggcttctcaacatgtactactgtcttggaa

fava_c18234_326	fava_c18234	326	T/C	acgtgctcattcaaaggagtagcatcaggcttctcaacatgtactactgtcttggaagactctcctatgctagataacattgatccaacaataggtttca[T/C]cttctctgtacctttaatgataaaaagaaacaaaacacgatacaaaacaatcatcccaagcaaaattgcaagatcaacccactttgaataactcaagtcc

fava_c18234_485	fava_c18234	485	C/T	agcaaaattgcaagatcaacccactttgaataactcaagtccatttgccaagtatctgtcaaaacgacttcaccagtaatcgaacggtttacgccttgta[C/T]atttggaatatSaaatcttagtccttcaaactcattcttgaacaatccctcatttacataggtatgaaatgcaacataatgaaatggatatttccaaaat

fava_c18234_497	fava_c18234	497	C/G	agatcaacccactttgaataactcaagtccatttgccaagtatctgtcaaaacgacttcaccagtaatcgaacggtttacgccttgtaYatttggaatat[C/G]aaatcttagtccttcaaactcattcttgaacaatccctcatttacataggtatgaaatgcaacataatgaaatggatatttccaaaatatgttgggaata

fava_c18234_806	fava_c18234	806	C/G	atgagactctcaactagcattagacttgaaaataaaacactagcaaagaagaaaaagtgctcatatcctttttgaagaccacataggtaataagttataa[C/G]tgctggaattattgaaaYcacgaaaacaaatggaactgatgaaaatgtgttcccaatcacatatgctacaacaccataatgtccattttgtctctctagt

fava_c18234_824	fava_c18234	824	T/C	attagacttgaaaataaaacactagcaaagaagaaaaagtgctcatatcctttttgaagaccacataggtaataagttataaStgctggaattattgaaa[T/C]cacgaaaacaaatggaactgatgaaaatgtgttcccaatcacatatgctacaacaccataatgtccattttgtctctctagttgaaataccttcatgtct

fava_c18234_98	fava_c18234	98	A/G	ttttttttcgtcaaaacaaagtactttaaattatctattagactattacatccaacccatataaatgtgaagttgaccctacttctccatttcttac[A/G]caaccaaacaacaataaataagaagttcaatagactcatacagttcacatctataaaatRcaaagttgttatttcttcactaaYtacctaaaaagttata

fava_c18237_1157	fava_c18237	1157	C/T	aaaactgattggctccaacccagcaatcatccctcctttcgaaaagcttgagctttgggaatttccttccgtgctgtgtgcaagatcatcgggttggtcc[C/T]tattataggaatcRcgtgaatagaggacagaaattgcatatctagcaaacaagcctcctaaagaRtgggccagaaaggagattctttttagactttctgt

fava_c18237_1171	fava_c18237	1171	G/A	ccaacccagcaatcatccctcctttcgaaaagcttgagctttgggaatttccttccgtgctgtgtgcaagatcatcgggttggtccYtattataggaatc[G/A]cgtgaatagaggacagaaattgcatatctagcaaacaagcctcctaaagaRtgggccagaaaggagattctttttagactttctgtttttctgacaactt

fava_c18237_1222	fava_c18237	1222	A/G	cttccgtgctgtgtgcaagatcatcgggttggtccYtattataggaatcRcgtgaatagaggacagaaattgcatatctagcaaacaagcctcctaaaga[A/G]tgggccagaaaggagattctttttagactttctgtttttctgacaacttgcaaaacttcatcggctaatcgctttccagctccatcaatcccagtaaagg

fava_c18237_1339	fava_c18237	1339	T/C	ttctttttagactttctgtttttctgacaacttgcaaaacttcatcggctaatcgctttccagctccatcaatcccagtaaaggtcttggtataagtatt[T/C]gatgaacttacaaaaattagaaagttctttccaaggcgccttttcaactctgcttctgcataagtccagtccgcagtgctagccaagataccatgaacaa

fava_c18237_259	fava_c18237	259	A/T	tgataatgaatccttatactaagtcgtataataattacaattaagtaaaaactactacatgttttcctttttttttttataaaatcataatctttcatga[A/T]tatatgattataaggaattgttgtaaaaatattggtgacaSagaataaaaatcctgtattatgcagttaggcgtatcWcacaggcatagtgaagttacat

fava_c18237_300	fava_c18237	300	C/G	taagtaaaaactactacatgttttcctttttttttttataaaatcataatctttcatgaWtatatgattataaggaattgttgtaaaaatattggtgaca[C/G]agaataaaaatcctgtattatgcagttaggcgtatcWcacaggcatagtgaagttacatacactagttacaagctagcggtcaatattgaggatgcttct

fava_c18237_337	fava_c18237	337	T/A	ataaaatcataatctttcatgaWtatatgattataaggaattgttgtaaaaatattggtgacaSagaataaaaatcctgtattatgcagttaggcgtatc[T/A]cacaggcatagtgaagttacatacactagttacaagctagcggtcaatattgaggatgcttcttgttgtctcaagctatcagccacatgagcaattactc

fava_c18237_627	fava_c18237	627	C/G	acttttctccatcccaactgttgtaatccttgtatgacctcctcttcgatgatttcatgatattcaacagtcttctgggtaccaggtgcattttgtgctt[C/G]ttcctttgctctcactgcttcttgagaaaattgagggccatcagaagggactggagggcaatattcaacatcaaccacgtgctggtatccatctaaagat

fava_c18243_1134	fava_c18243	1134	T/A	atcacgaagaagtctacctaggaaacttgaagaggttccaattaagagaactacagattgctactcacaacttcagcaacaaaaacatattaggaaaagg[T/A]ggtttcggcattgtctacaaaggaattctttcggatggtacacttgttgctgtaaagaggcttaaagacggaaatgccaagggaggagagatacaatttc

fava_c18243_1374	fava_c18243	1374	A/G	accttctcaaactctatggattttgtatgacttcatcagaaaggcttttagtttatccttacatgtccaatggcagcgtcgcttctcgtctcaaagctaa[A/G]ccagtgttggattgggggacaaggaagcaaattgcattaggagcagcaagaggactattatacctacatgaacaatgtgatccaaagataatccatagag

fava_c18243_1485	fava_c18243	1485	C/T	attgggggacaaggaagcaaattgcattaggagcagcaagaggactattatacctacatgaacaatgtgatccaaagataatccatagagatgtgaaagc[C/T]gcaaatatattacttgatgattattgtgaagcagtagttggtgattttggtttggcaaagcttttagaccacaaagattcacatgtaacaactgcagtta

fava_c18243_1677	fava_c18243	1677	T/C	ctgcagttaggggcacagtaggacatattgcacccgagtatctttccacaggacagtcttctgaaaagactgatgtttttggatttggcattttgcttct[T/C]gaactgattacaggtttgagggcccttgaatttggaaaggctgccaaccaaaaaggagccatgctggattgggtaaagaaaattcatcaagagaagaagc

fava_c18243_1833	fava_c18243	1833	G/A	gagccatgctggattgggtaaagaaaattcatcaagagaagaagctagatttgcttgtagacaaagatctcaagaacaactatgacaagattgagcttga[G/A]gaaatagttcaagttgcacttttgtgcacacaatatcttccaagtcacagaccgaaaatgtccgaagtcgtacgcatgctcgaaggcgacggtctagccg

fava_c18243_217	fava_c18243	217	T/G	aatataatttctttcttattttgaaaataaaacatttgagagttgttatcaacttctatcctttacaacaataatcttaatggaagtttcaagaggagaa[T/G]attttgtatgttttgtgacattgttgttgtttttgtgttctacaaatgctttgctttctcctaagggaattaactttgaagttcaagctttgatgagtat

fava_c18243_812	fava_c18243	812	A/C	acttttcttgatttgtcattcaacaatcttagtggcagtgtgcctagaatcttggccaaatcattcagtattgttggaaaccctctggtctgtgctacag[A/C]aaaagaaacaaactgccatgggatgaaactcatgcctatgtcaatgaacttaaacaatactaaccatgtattaccatcaagcaaaacaaaagctcacaaa

fava_c18243_969	fava_c18243	969	C/T	atactaaccatgtattaccatcaagcaaaacaaaagctcacaaaaaggtcattgcctttggtttgagtcttggatgcctttgcttgttagtccttggttt[C/T]ggatttgtactatggaggagacacaWgcataatcaacaagcattctttgatgttaaagaccgacatcacgaagaagtctacctaggaaacttgaagaggt

fava_c18243_995	fava_c18243	995	A/T	aaaacaaaagctcacaaaaaggtcattgcctttggtttgagtcttggatgcctttgcttgttagtccttggtttYggatttgtactatggaggagacaca[A/T]gcataatcaacaagcattctttgatgttaaagaccgacatcacgaagaagtctacctaggaaacttgaagaggttccaattaagagaactacagattgct

fava_c18244_1115	fava_c18244	1115	G/C	tctcggttactcttttgacatccacggtggagggattgaccttgtatttcctcaccatgaaaatgaaattgctcagagttgtgctgcttgtatcaaaagc[G/C]atattagtgtatggatgcacaatggatttgtcaccattgactctgtgaagatgtctaaatctctgggaaactttttcacaatacggcaggttgtagatgt

fava_c18244_1226	fava_c18244	1226	C/G	atggatgcacaatggatttgtcaccattgactctgtgaagatgtctaaatctctgggaaactttttcacaatacggcaggttgtagatgtttaccatcca[C/G]tggctttgagatattttttgatgagtgcccattatagatctccgatgaactactctaatatacaccttgaaagtgcttcagaccgtgttttttatatata

fava_c18244_1392	fava_c18244	1392	T/C	cttgaaagtgcttcagaccgtgttttttatatatacgagacattacatgcatgtgaaagcttgctgaaccagcatgatcagacagttaggaaggattccg[T/C]cccatcggatactttgagtattattgataacctgtataatgtttttctgacctcaatgtctgatgatcttcacactccagttgtattggccggactatct

fava_c18244_1612	fava_c18244	1612	C/A	acgacttactgaatactcgcaaggggaaaaaacaacaatttcgaatagaatcacttgcactgttgaagaaaagcattgaggacgtccttactgttatagg[C/A]cttatgccttcaagttacaatgaggttttgcaacagcttagggaaaaagcgctgaagcgtgcaaacgtgacagaagatgaaatcttgcagaagattgaag

fava_c18244_1715	fava_c18244	1715	C/A	tatgccttcaagttacaatgaggttttgcaacagcttagggaaaaagcgctgaagcgtgcaaacgtgacagaagatgaaatcttgcagaagattgaagaa[C/A]gggctgctgctagaatacaaaaagacttcaccaaatccgatgacataaggaaagatttggctgttcttggtattgctcttatggatagtccaaatggcac

fava_c18244_1935	fava_c18244	1935	G/C	cctcttccgcttcaagagcagctctagtcaaaataagcatcagttgtccaacatttgcaaagcaaaatcaaaggaaatcgctcttcctcaatccttggtg[G/C]cttaaagctatgaattaaaatttttgtttcgataaaaaaaagtgtcacgaggttagtgctattgtcttggtggtcactattgaattaaatattttttaac

fava_c18244_286	fava_c18244	286	C/G	aatgctacacacccttctcctccatgctcttcacccactcccctcgaacccttctctccggccaccgcgccgccgtcttcagaaaaaataactttccttc[C/G]tttcgcgccgtctcgtcttatcaaccRttgacgScggagaagaattccaacgacggtgaaagctcttcaaaaattccatcgccggaagtatggctttaca

fava_c18244_313	fava_c18244	313	A/G	tcttcacccactcccctcgaacccttctctccggccaccgcgccgccgtcttcagaaaaaataactttccttcStttcgcgccgtctcgtcttatcaacc[A/G]ttgacgScggagaagaattccaacgacggtgaaagctcttcaaaaattccatcgccggaagtatggctttacaacacaatgagtaaaaagaaagagcttt

fava_c18244_320	fava_c18244	320	G/C	ccactcccctcgaacccttctctccggccaccgcgccgccgtcttcagaaaaaataactttccttcStttcgcgccgtctcgtcttatcaaccRttgacg[G/C]cggagaagaattccaacgacggtgaaagctcttcaaaaattccatcgccggaagtatggctttacaacacaatgagtaaaaagaaagagcttttcaaacc

fava_c18244_574	fava_c18244	574	C/T	tcagccatattggtcatgctcgtgtttacgtcaattttgatcttctttatagatattttaagcacttgggattggaagtttgttatgttcgcaattttac[C/T]gatgtagatgacaagataattgctagatcaaaggagttaggagaagatccaatcagtttgagtcgacgctattgtgaagagttYtgtcatgacatggtaa

fava_c18244_658	fava_c18244	658	C/T	atgttcgcaattttacYgatgtagatgacaagataattgctagatcaaaggagttaggagaagatccaatcagtttgagtcgacgctattgtgaagagtt[C/T]tgtcatgacatggtaactcttaactgtttgcctccaaccgtggaaccaaaagtctctcagcacataccccaaatcattgatatgattgagaagattctta

fava_c18247_304	fava_c18247	304	C/T	ttaattttttgttctacaatctgcatgcaatggctcaaaaggatggcatgaattcaaagccttggttcaaaagagctccggccacccatagcttcaaatt[C/T]tgaagattcatttcttgttcagaacctttcacattcaatgtaaatgtaagtagaaaagtgtcagaaacggtagctaagtttgaattttgaacattgaaat

fava_c18247_892	fava_c18247	892	A/G	tatcggttttagccctagatttggcatcatcgaccgagttcgtggttgttgtttccggagaatcgtcaccattattttctccttctcccttagtctcctc[A/G]tcataacaagagaaggagccaattgttgaagagcttggatcatacacattcacaatattataaatattgttgttgttggaatcaaataatgggtttagtg

fava_c18248_1098	fava_c18248	1098	T/C	atactggactttttgatgattcctatttgagctgtggatgggttaattcgactgttttttcgtttctcagcaactcgactacttgtcctttcaaagaacc[T/C]gatgatatcgttgatccaacagtttttgattttggaatatttattgatggtctaRagtctcgtgtcgtcggttcgactacagactttcatcataaattct

fava_c18248_1153	fava_c18248	1153	A/G	tttttcgtttctcagcaactcgactacttgtcctttcaaagaaccYgatgatatcgttgatccaacagtttttgattttggaatatttattgatggtcta[A/G]agtctcgtgtcgtcggttcgactacagactttcatcataaattcttctactgtttttggtggggtttgcgcaatttgagttctgttgggcagaacctgaa

fava_c18248_1299	fava_c18248	1299	C/T	tctactgtttttggtggggtttgcgcaatttgagttctgttgggcagaacctgaagacaagcacttatattggggagataatctttgcgatcttcatcgc[C/T]gtctttggattggttctattcgcattacttattggaaacatgcagaaatatctacaatctacaactgttagagttgaagagatgagaatcaaaaggaggg

fava_c18248_1671	fava_c18248	1671	G/T	tacttaaaaaagtgcctattttcggcaacatggataaacagttgctggatgcaatgtgtgataaactcaaaccagtcctttataccgagaaaagttatgt[G/T]gttcgcgaaggtgatccggtggacgaaatgctcttcatcatgcgcggaaaacttaagacYgcaacaacaaatggtggaagaactggtttctttaactcgt

fava_c18248_1731	fava_c18248	1731	T/C	ataaactcaaaccagtcctttataccgagaaaagttatgtKgttcgcgaaggtgatccggtggacgaaatgctcttcatcatgcgcggaaaacttaagac[T/C]gcaacaacaaatggtggaagaactggtttctttaactcgtcggagcttaagtctggtgatttttgtggagaggagcttctaacatgggcattggatccta

fava_c18248_2339	fava_c18248	2339	G/A	ctaccactcttgcctaagaaaccagctgagccaaatttcattactcaaaagaactaacagtgtgttgtagaaatagtcttcttgtcaagtttagtttttt[G/A]gYaaaccagactcaagaagtgtgcatgctcaagatcattatctatagcaaatgatgttggtttgattgagaatttgtaaaactatgtatgtgtgcatata

fava_c18248_2341	fava_c18248	2341	T/C	accactcttgcctaagaaaccagctgagccaaatttcattactcaaaagaactaacagtgtgttgtagaaatagtcttcttgtcaagtttagttttttRg[T/C]aaaccagactcaagaagtgtgcatgctcaagatcattatctatagcaaatgatgttggtttgattgagaatttgtaaaactatgtatgtgtgcatatata

fava_c18251_271	fava_c18251	271	C/T	aacttcaaatttcaaaccaaacaccacattcacagaatttgaaattgagaaacacgcaaattatacccaaaatgcccattgcaacaaagcttcacgttcg[C/T]tctaaaccgcgagctattctgaaaccaggcggaaacatagttccggttcatgaagaaacaactaaacataacaagaagaaaacacagcagggtccaagaa

fava_c18251_394	fava_c18251	394	C/G	aaccaggcggaaacatagttccggttcatgaagaaacaactaaacataacaagaagaaaacacagcagggtccaagaaaacaggtggcggaaatttctga[C/G]tcacttgttgttgtcggaagtaacgtgtgtttggatttggacagcacatgttcctcggattcctcttctgtcaaaaaggtcaactctgcgaatggaaatg

fava_c18251_538	fava_c18251	538	A/G	gcacatgttcctcggattcctcttctgtcaaaaaggtcaactctgcgaatggaaatggaaattcaaagatgaagcgcaacgggtttaagctgccggtgag[A/G]gatgctgYccacgtgtctctgccttataagggtgagccaccgtccaagacatgcgattggatcacaccSaattcagaccctgtttatacttctttccatg

fava_c18251_546	fava_c18251	546	C/T	tcctcggattcctcttctgtcaaaaaggtcaactctgcgaatggaaatggaaattcaaagatgaagcgcaacgggtttaagctgccggtgagRgatgctg[C/T]ccacgtgtctctgccttataagggtgagccaccgtccaagacatgcgattggatcacaccSaattcagaccctgtttatacttctttccatgacRaagaa

fava_c18251_607	fava_c18251	607	G/C	tgaagcgcaacgggtttaagctgccggtgagRgatgctgYccacgtgtctctgccttataagggtgagccaccgtccaagacatgcgattggatcacacc[G/C]aattcagaccctgtttatacttctttccatgacRaagaatggggtgttccagttattgatgatgatacaaagctatttgagcttctagtaKtttcacaag

fava_c18251_641	fava_c18251	641	G/A	tgctgYccacgtgtctctgccttataagggtgagccaccgtccaagacatgcgattggatcacaccSaattcagaccctgtttatacttctttccatgac[G/A]aagaatggggtgttccagttattgatgatgatacaaagctatttgagcttctagtaKtttcacaagcattagcagaacacacttggccaaccattctYaa

fava_c18251_698	fava_c18251	698	T/G	gatcacaccSaattcagaccctgtttatacttctttccatgacRaagaatggggtgttccagttattgatgatgatacaaagctatttgagcttctagta[T/G]tttcacaagcattagcagaacacacttggccaaccattctYaaccacagaaacattttcaggaaatttttcgaaWattttgatccatcctcaattgcaca

fava_c18251_739	fava_c18251	739	T/C	acRaagaatggggtgttccagttattgatgatgatacaaagctatttgagcttctagtaKtttcacaagcattagcagaacacacttggccaaccattct[T/C]aaccacagaaacattttcaggaaatttttcgaaWattttgatccatcctcaattgcacagtttaatgagaagaagttgctaacaccaaaaataaatggca

fava_c18251_773	fava_c18251	773	A/T	tacaaagctatttgagcttctagtaKtttcacaagcattagcagaacacacttggccaaccattctYaaccacagaaacattttcaggaaatttttcgaa[A/T]attttgatccatcctcaattgcacagtttaatgagaagaagttgctaacaccaaaaataaatggcaRcaaccctttgttatccgaacaaaaggttcgcgc

fava_c18251_840	fava_c18251	840	A/G	aaccacagaaacattttcaggaaatttttcgaaWattttgatccatcctcaattgcacagtttaatgagaagaagttgctaacaccaaaaataaatggca[A/G]caaccctttgttatccgaacaaaaggttcgcgccattgtggaaaacgcWaaacaattacttaaggttcaacatgagtttggttcattcagccattactgY

fava_c18251_889	fava_c18251	889	T/A	caattgcacagtttaatgagaagaagttgctaacaccaaaaataaatggcaRcaaccctttgttatccgaacaaaaggttcgcgccattgtggaaaacgc[T/A]aaacaattacttaaggttcaacatgagtttggttcattcagccattactgttggaaattcgttaacaacaaaccgataaaaaatgaattccgatatggac

fava_c18257_1905	fava_c18257	1905	T/C	agtacactatgcaggcctcggtacttgaaatatacaacgagacgataagggatttgttagcaccaacagaaaatcctggaaaaaagtacaatattataca[T/C]gatgccaatggaaaaaacacatatgttcctgacctgactattgtggacgtgtgcggcgcagatgagatttccactctcctacaacgggctgcacaaagca

fava_c18257_2058	fava_c18257	2058	C/A	gcggcgcagatgagatttccactctcctacaacgggctgcacaaagcagatcagtaggaagaacacagatgaatgaacaatcgtcacgaagtcactttgt[C/A]tttactctgcgcataagcggcattaatgagaacaccgagcaacaagttcaaggtgtgctaaacctgatcgatcttgctggaagtgaaagactttcgaaaa

fava_c18257_2238	fava_c18257	2238	G/C	gaagtgaaagactttcgaaaagtggagcaactggggaccgtttgaatgaaactaaggcgataaacaaaagtctttcctgtttgagtgatgtcatttttgc[G/C]ttggcgaaaaaagacgatcatgtccccttcaggaattctaagctaacaacccttctccagccatgtcttggtggagattcaaagactctgatgtttgtaa

fava_c18261_1768	fava_c18261	1768	C/T	agagaatcggatatgtgggacaggaaccaaagctattccggatggatattagctcgaacatcagatatggatgtacccgagatgtaaagcaggaggatat[C/T]gagtgggctgcgaagcaggcctatgctcacgactttatctcagcacttcctaatggttatgaaacacttgttgatgatgatcttttaagtggcggacaaa

fava_c18261_2050	fava_c18261	2050	A/T	ctgaaagtgaacataacgtgaagggtgttcttcgatctgttagaagtgattctgcatcaaggagaagtgtcatagtcattgcacacaggctttctaccat[A/T]caagctgctgacaggattgtggttatggataggggtcaaattgttgagaatggaagtcacagggaacttctcttgaaagacggtttatatgctcggttga

fava_c18261_2173	fava_c18261	2173	G/A	ttatggataggggtcaaattgttgagaatggaagtcacagggaacttctcttgaaagacggtttatatgctcggttgactagaaaacaagctgattcgat[G/A]gagtgatctttgtttccacagcagatattcaatttcttttaaaaggaagtatagtgggtcacacaacaatttttttactgctgacccatgtattcataat

fava_c18261_2635	fava_c18261	2635	T/C	ttggtcaattggagggggtatagtcaaaatgctaatatttgtcattctagtagagtgaccaaaatagactttttttaatccatgtttgatgattcttcat[T/C]gtcaagctgaagtctaaacaatgagttttaagaactcttcaatatttatagtcttatttccattgatcgagaacttttaatgYtaaggaagagctattac

fava_c18261_2718	fava_c18261	2718	T/C	gtttgatgattcttcatYgtcaagctgaagtctaaacaatgagttttaagaactcttcaatatttatagtcttatttccattgatcgagaacttttaatg[T/C]taaggaagagctattacatggaatgtgtttattacttttccagggcaacactttttatatttgttgtgttgtgagttggagaaatacagaaaggcaactt

fava_c18261_325	fava_c18261	325	C/T	actctaagcgcattctcttaccatctccgttaaagtcagcatccatcaacgaagtctccgtccagaataatccagaagcttccagtatttctgatgaagt[C/T]ctcggcagaatacgagaattggttaagttccttccttcaatttttcccggaggagcctggtggaacttctccgatgaggtggatgcttcgattttcggtc

fava_c18267_107	fava_c18267	107	G/A	attatcaaattcattctcacaaaagcattaacaaaatctggaaaaaagtttcaacaaagaaaaaaaaaagccagaaaacaatcaaataagtacaagtgaa[G/A]tggtgcatgtgtgataggtgtttctgtatatgcatgacaccaaatcaatgaagagattcataaaaaagctacctcagaaaatttctcatatataaataag

fava_c18267_1097	fava_c18267	1097	T/C	aaatctcatcaattcatgaccaaaacgacaccaaccaatccactgaaatcctgcgttttggcaaccacaacattctatccccacctccacaacatcctct[T/C]cacacccctccattcaatttcatcactcaacaacaaccaaaccataattttcatcatgaacaacattctcaaacattccaaggactaatatcattgtctg

fava_c18267_1583	fava_c18267	1583	G/A	tcactagagattttcttggagttggtcaaatactaatgagaaacatgaatggaggaggagtttcacaaagagaacaacgaaacttcaacgtggaagctga[G/A]aaaaacgctgcagcttttggagggaactttgagtgattgtgtgaaYctttSttggacatgcaattgaaaaaaaaatacaaaaggatatgtctacttgttc

fava_c18267_1629	fava_c18267	1629	T/C	gaatggaggaggagtttcacaaagagaacaacgaaacttcaacgtggaagctgaRaaaaacgctgcagcttttggagggaactttgagtgattgtgtgaa[T/C]ctttSttggacatgcaattgaaaaaaaaatacaaaaggatatgtctacttgttccaaaagattatgactcttataagtccatctaattcagtttttggga

fava_c18267_1634	fava_c18267	1634	G/C	gaggaggagtttcacaaagagaacaacgaaacttcaacgtggaagctgaRaaaaacgctgcagcttttggagggaactttgagtgattgtgtgaaYcttt[G/C]ttggacatgcaattgaaaaaaaaatacaaaaggatatgtctacttgttccaaaagattatgactcttataagtccatctaattcagtttttgggagcaat

fava_c18267_1768	fava_c18267	1768	A/G	atatgtctacttgttccaaaagattatgactcttataagtccatctaattcagtttttgggagcaatattggggaatcaaattggagttaaaccatgtat[A/G]tataaagctagcatgaatagaaagttatggatgcaatggaagttggaagcacatgaaaataRtattgggatttcaaacaactttctgtaaaattttcatg

fava_c18267_1830	fava_c18267	1830	A/G	gcaatattggggaatcaaattggagttaaaccatgtatRtataaagctagcatgaatagaaagttatggatgcaatggaagttggaagcacatgaaaata[A/G]tattgggatttcaaacaactttctgtaaaattttcatggcttaacKtaatagattttgttgggagatgatttaatatgtatattggagtttaggtcatag

fava_c18267_1876	fava_c18267	1876	T/G	ctagcatgaatagaaagttatggatgcaatggaagttggaagcacatgaaaataRtattgggatttcaaacaactttctgtaaaattttcatggcttaac[T/G]taatagattttgttgggagatgatttaatatgtatattggagtttaggtcatagttctctatagtatcatatatagaaatagtatatcatcatatgtata

fava_c18267_1999	fava_c18267	1999	T/C	atttaatatgtatattggagtttaggtcatagttctctatagtatcatatatagaaatagtatatcatcatatgtatatggttgttgaatcctcattttg[T/C]ttattgagagataggtctatgtcctctaattctttaattgtaagggtttaaagatcctgctactctttctatctataatctgttctcattcaaaaagaaa

fava_c18267_267	fava_c18267	267	G/A	ataaaaaagctacctcagaaaatttctcatatataaataagctaacatgagagaaaaaaaatcatattaggttttttcttttcctttcaaatttctagag[G/A]tttttcatgatatggatgcatcattttcagcagcatctttctttacaagaactgaagaggatcagagtgagatgaatcaMcaaaattcatcatcatcaac

fava_c18267_347	fava_c18267	347	C/A	ttcctttcaaatttctagagRtttttcatgatatggatgcatcattttcagcagcatctttctttacaagaactgaagaggatcagagtgagatgaatca[C/A]caaaattcatcatcatcaacaaStccaWctgcaccaccacctcagaagaaaagaagaaaccaacctggaacaccatatccagattctgaagtgataaYac

fava_c18267_370	fava_c18267	370	C/G	tttcatgatatggatgcatcattttcagcagcatctttctttacaagaactgaagaggatcagagtgagatgaatcaMcaaaattcatcatcatcaacaa[C/G]tccaWctgcaccaccacctcagaagaaaagaagaaaccaacctggaacaccatatccagattctgaagtgataaYactttctccaaggagtctaatggca

fava_c18267_375	fava_c18267	375	T/A	tgatatggatgcatcattttcagcagcatctttctttacaagaactgaagaggatcagagtgagatgaatcaMcaaaattcatcatcatcaacaaStcca[T/A]ctgcaccaccacctcagaagaaaagaagaaaccaacctggaacaccatatccagattctgaagtgataaYactttctccaaggagtctaatggcaacaaa

fava_c18267_445	fava_c18267	445	C/T	caMcaaaattcatcatcatcaacaaStccaWctgcaccaccacctcagaagaaaagaagaaaccaacctggaacaccatatccagattctgaagtgataa[C/T]actttctccaaggagtctaatggcaacaaacagatttgtatgtgaagtgtgtcaaaaagggtttcaaagagagcaaaatttgcaacttcatagaagagga

fava_c18267_608	fava_c18267	608	G/A	ttcaaagagagcaaaatttgcaacttcatagaagaggacacaatctaccttggaagctaaagcaaaagagtaataaagagccaaagagaaaggtttatct[G/A]tgtcctgaaccttcttgtgttcaccatgacccttcaagRgcacttggagacctaactggRatcaagaagcacttttctagaaagcacggtgagaaaaagt

fava_c18267_647	fava_c18267	647	A/G	acaatctaccttggaagctaaagcaaaagagtaataaagagccaaagagaaaggtttatctRtgtcctgaaccttcttgtgttcaccatgacccttcaag[A/G]gcacttggagacctaactggRatcaagaagcacttttctagaaagcacggtgagaaaaagtggaaatgtgaaaagtgttctaagaaatatgctgttcaat

fava_c18267_668	fava_c18267	668	G/A	agcaaaagagtaataaagagccaaagagaaaggtttatctRtgtcctgaaccttcttgtgttcaccatgacccttcaagRgcacttggagacctaactgg[G/A]atcaagaagcacttttctagaaagcacggtgagaaaaagtggaaatgtgaaaagtgttctaagaaatatgctgttcaatctgattggaaagctcattcta

fava_c18267_779	fava_c18267	779	G/A	acttttctagaaagcacggtgagaaaaagtggaaatgtgaaaagtgttctaagaaatatgctgttcaatctgattggaaagctcattctaaaacttgtgg[G/A]actagggagtatagatgtgactgtggcactctcttctcWagaagagacagtttcatcacacatagagccttttgtgatgcacttgctcaagaaagttcaa

fava_c18267_818	fava_c18267	818	T/A	aaaagtgttctaagaaatatgctgttcaatctgattggaaagctcattctaaaacttgtggRactagggagtatagatgtgactgtggcactctcttctc[T/A]agaagagacagtttcatcacacatagagccttttgtgatgcacttgctcaagaaagttcaagacaaccacaacaattaccaaccttaatcaccaacactt

fava_c18269_470	fava_c18269	470	C/G	agaccaaacccctctggaggaggactcagtggtaatgacactgatgaatatgactgtgaaagtgaggagggtattgaagtattgacagaagaagctccaa[C/G]aaagacYgttggttctaggagttcttcaaagagaagtagagcagcagaagttcataatttgtctgaaaagagaaggaggagtaggatcaatgagaaaatg

fava_c18269_477	fava_c18269	477	C/T	acccctctggaggaggactcagtggtaatgacactgatgaatatgactgtgaaagtgaggagggtattgaagtattgacagaagaagctccaaSaaagac[C/T]gttggttctaggagttcttcaaagagaagtagagcagcagaagttcataatttgtctgaaaagagaaggaggagtaggatcaatgagaaaatgaaggcct

fava_c18269_582	fava_c18269	582	A/G	gttctaggagttcttcaaagagaagtagagcagcagaagttcataatttgtctgaaaagagaaggaggagtaggatcaatgagaaaatgaaggccttaca[A/G]aatctaatacctaattctaacaagactgataaggcttcaatgctagatgaagctattgaataccttaaacagcttcagcttcaagtacagatgttgtcat

fava_c18269_811	fava_c18269	811	C/T	gtgctatcccgaaggcttgcagcctctgtcactgtctcggttgggcatggaattgagtgacggaaaccggtctacccctttaaacatgacatctactctg[C/T]ctcatccccaagacaacaactctttgctctatgcatccaatcttcctaacaaaaacacattaacaagtcagtcatcaatgccctcctatcctacatatgt

fava_c18271_310	fava_c18271	310	C/G	acgcactcgcgacgccgtcataaaccgcttgatcgaaaccttttccacctcctcaattctctccaaacgctacggcacattatcccttgacgaatcctcc[C/G]tcgccgcaagccagatcgaggccgagtccttctccgtcgccgatgtttcctcttcccccgacgacgacagcattcagatcctcgaggtttactcgaaaga

fava_c18273_1059	fava_c18273	1059	T/A	acatacaattgattggactgtagagctcttaaaaaccgatactcttcgagcgtcgatgaggatcaactggtatggattttttgggaagaaaatcaaagat[T/A]tattcggaaacattttcggacccgaacttagtggattagtcggtcttaaggagccacgagatcatggagttccYtattcacttaccgaagaattcactag

fava_c18273_1133	fava_c18273	1133	T/C	gattttttgggaagaaaatcaaagatWtattcggaaacattttcggacccgaacttagtggattagtcggtcttaaggagccacgagatcatggagttcc[T/C]tattcacttaccgaagaattcactagtgtttatagaatgcacgcgcttctacctgaggagattgttctccgaaaactcaagcctgcaacaggcgcgaaca

fava_c18273_1364	fava_c18273	1364	T/C	tggcagaacttataggaaaacaaggggaaaaaagattgtcaaaaataggaatggagcaaatgttactgtcaatgggtcatcaatcatgcggtgcagttgc[T/C]ttatggaactttccttcatggctaagaaacctcatttcacatgatattgatggagaagaaagacctgatccagttgacatggctactatggaagtKtata

fava_c18273_1460	fava_c18273	1460	T/G	ttgcYttatggaactttccttcatggctaagaaacctcatttcacatgatattgatggagaagaaagacctgatccagttgacatggctactatggaagt[T/G]tatagagatagagaaaggggagttccgaggtataacgaattcagaagaaacttattgatgattccgattagcaagtgggaaaatttaaccRatgatgtag

fava_c18273_1551	fava_c18273	1551	G/A	tatggaagtKtatagagatagagaaaggggagttccgaggtataacgaattcagaagaaacttattgatgattccgattagcaagtgggaaaatttaacc[G/A]atgatgtagaggttattRaaggtctcaaagaaatatatgatWatgatgttgagaagttggacttgattatcggtcttcatgcagagaagaagataaaagg

fava_c18273_1569	fava_c18273	1569	G/A	tagagaaaggggagttccgaggtataacgaattcagaagaaacttattgatgattccgattagcaagtgggaaaatttaaccRatgatgtagaggttatt[G/A]aaggtctcaaagaaatatatgatWatgatgttgagaagttggacttgattatcggtcttcatgcagagaagaagataaaaggattYgctattagtgaaac

fava_c18273_1593	fava_c18273	1593	A/T	taacgaattcagaagaaacttattgatgattccgattagcaagtgggaaaatttaaccRatgatgtagaggttattRaaggtctcaaagaaatatatgat[A/T]atgatgttgagaagttggacttgattatcggtcttcatgcagagaagaagataaaaggattYgctattagtgaaaccgctttctttatctttgtgataat

fava_c18273_1655	fava_c18273	1655	C/T	atgtagaggttattRaaggtctcaaagaaatatatgatWatgatgttgagaagttggacttgattatcggtcttcatgcagagaagaagataaaaggatt[C/T]gctattagtgaaaccgctttctttatctttgtgataatggcttcaaggaggctagaagctgaccgtttcttcacaacgaatttcRatgcgaaaacataca

fava_c18273_1740	fava_c18273	1740	A/G	gaagataaaaggattYgctattagtgaaaccgctttctttatctttgtgataatggcttcaaggaggctagaagctgaccgtttcttcacaacgaatttc[A/G]atgcgaaaacatacacaaatgaagggcttgaatgggtgaacaaaacagaaaatttgaaggatgttattgatagacacttccctagaatgactgaaaatat

fava_c18273_644	fava_c18273	644	T/C	ttcctgatgaaggttttgcaagtggttgtcctttgaagactttcaagtttttcaagactaagaaattccaaactggctcttcgcataccaagtttggctt[T/C]caaaacaccaggactccgtggtgggatggaagtgtgatatatgggaacaatgagaagggaatgagtagagtgagaacattYagagagggaaagttgaaga

fava_c18273_725	fava_c18273	725	C/T	cgcataccaagtttggcttYcaaaacaccaggactccgtggtgggatggaagtgtgatatatgggaacaatgagaagggaatgagtagagtgagaacatt[C/T]agagagggaaagttgaagatMtcagaagatggacttcttgagcatgatgagaagggtattcctgtgtcgggcgatgttcgaaacacatgggcgggttatt

fava_c18273_746	fava_c18273	746	C/A	aaaacaccaggactccgtggtgggatggaagtgtgatatatgggaacaatgagaagggaatgagtagagtgagaacattYagagagggaaagttgaagat[C/A]tcagaagatggacttcttgagcatgatgagaagggtattcctgtgtcgggcgatgttcgaaacacatgggcgggttattcccttctRcaggctttgttta

fava_c18273_833	fava_c18273	833	G/A	gaaagttgaagatMtcagaagatggacttcttgagcatgatgagaagggtattcctgtgtcgggcgatgttcgaaacacatgggcgggttattcccttct[G/A]caggctttgtttatcaaagagcacaatgcagtatgtgacatgctaaaaaaacactatcccgattttgatgatgagcaactctataggtatgcaagattgg

fava_c18276_749	fava_c18276	749	G/A	gacgggagaattcttccaccgtctgttgttgacgaaagatcgtccaccgcgggaagtatttgtggtagatttccggtttcgatcaccgggatctgtagca[G/A]tggtgctggatgacatcaagtttgattttctatttttaacaaaattatactgaaacataaaaataactgtattttctttttgtgattgaaatttcatgga

fava_c18279_1310	fava_c18279	1310	G/T	gggttctgctactaatcttgcaccatggatgcttacagttgctgctagctcaactgatagggatttcacttctgatatcttgttaggaaacggtgcaaaa[G/T]tcacgggtgaaagtctgagtctgtttgaaatgaatacatcatcaaggataatttcggcttctcaagcatttgcgggatacttcactccttatcaatccag

fava_c18279_1462	fava_c18279	1462	A/G	tttcggcttctcaagcatttgcgggatacttcactccttatcaatccagttactgtttagagagttctttgaataaaacaaagaccaaagggaaggttct[A/G]gtttgtagaaatgtagagagttcaacagattcaaaggtgaaaaagagcaaaatagttaaagaagctggtggagttggcatgatacttattgatgagacag

fava_c18279_2143	fava_c18279	2143	T/C	accctgcaagagttcttgaccctggtctcgtttatgattccaaaccaacagattttatcgcattcctatgctcactcggttacgatcaaagatcacttca[T/C]ctagtaactagagacaacagcacatgtaatagcattagtacattcaacactgcatcagacctcaaccatccatccatttcagttcctaatctcagagaca

fava_c18279_2408	fava_c18279	2408	T/A	ttcttcaccacctggtgtcaatgtcaccgttacgccgaaccggttagttttcacaagaatgggacagaaaatcaagttcagtgtgaatttcaaagtaact[T/A]ctccttcaaaaggatacaaatttggattcttgtcatggaaaaataaaagattacaagttactagtcctttggttgtaaaggttggttagagagccatgct

fava_c18279_2519	fava_c18279	2519	A/G	aggatacaaatttggattcttgtcatggaaaaataaaagattacaagttactagtcctttggttgtaaaggttggttagagagccatgcttgtaaatatc[A/G]gtatattagcttaaaaagctttgaattaatactcctaaaattgtgttaggtatatgtttggataacaaacaattacttaaaatagaatccaaagaaaata

fava_c18281_1314	fava_c18281	1314	T/A	ttccagatcgagcacccagtgatatgcatctctataccaccttcatagggggaactcgaaatcgggaacttgctcaagcttcaactgatgagcttaagaa[T/A]atcgtcacttctgacctgRgaaagctgttgggagcagagggggaacccacattcgtcaaccatttctactggagtaaaggctttcctttatatggtcata

fava_c18281_1333	fava_c18281	1333	A/G	tgatatgcatctctataccaccttcatagggggaactcgaaatcgggaacttgctcaagcttcaactgatgagcttaagaaWatcgtcacttctgacctg[A/G]gaaagctgttgggagcagagggggaacccacattcgtcaaccatttctactggagtaaaggctttcctttatatggtcataactacgggtcagttcttga

fava_c18281_1562	fava_c18281	1562	A/G	cctggatttttctatgcaggtaaccacagaggtggactctcagttggtagagcaattgcctcagggtacaaagcagctgatcttgtaattgcctacctca[A/G]caatgcctcagacaacactgtctgataaatgatcgtatagatagatgtaggaactgcacgttttctgtttactagatttctttgtttcagcatctgtaaa

fava_c18281_213	fava_c18281	213	T/G	accaaagatctgttaaaagagttgctgttgttggtgctggggtaagtgggcttgctgcggcttacagattgaaatcacatggtctggatgtaactgtatt[T/G]gaagctgaaggaagagctggagggaggctaagaactgtttctcaagatgggctagtttgggacgagggagctaatacaatgactgaaaatgaaacagagg

fava_c18281_982	fava_c18281	982	G/A	ggttttgacactagcttacagtcatgatggaagttccccatcagaaaactggtcaattacttgcacttctaacaaaaaaacacaagacgttgatgcagta[G/A]ttatgacggctcctctaggtaatgtcaaagacatccagatcacaaaaaagggaatcccctgttcacttaattttcttcccgaggtaacctatttgccact

fava_c18282_168	fava_c18282	168	T/A	gtacaaataagagtgttcatagtttctaaaactaaacaactcatacagttatgtaaaatactacctattgactaccaacctcttctttttacctaaacaa[T/A]tttgtttatcaaagtaaactaatcttatattagtatatttaattttaacatggtgggttcatcccgatccatcaaaaataaaagtatgtcRggtcgtctc

fava_c18282_1682	fava_c18282	1682	T/C	ctgaatcatgcaaaggttgagaagtttgagaaataagttgatagaagataaaatcatcatgcttaatcaaacaacttggagcaagaacttgccaaccaat[T/C]tttgtcccacaacatttaggaactttctctaacattgcctccaagcattcgctacattcattctcactaaggtccctactacattgcaccaatccatacc

fava_c18282_1903	fava_c18282	1903	C/T	aactctctcattgcatacaatttaccactctccaaagtagcttcttttattaaactttgcatgttatcctcacctttctcaacttcgtgtggattggtga[C/T]gttttttgtccccaaaatataccatgaaggtgttgttgttagcttcccaaagaaattatggtttgaatacctgagaatgcaataattgtaccatataaca

fava_c18282_2030	fava_c18282	2030	G/A	aaggtgttgttgttagcttcccaaagaaattatggtttgaatacctgagaatgcaataattgtaccatataacagccgaggctctattaggacagtgttg[G/A]agaatatctgaggcagcagtagagacacaaaattgacaaaaagaacctgtgacatcacccctacaatcatagagaccatagacaccatcaactgtgttac

fava_c18282_259	fava_c18282	259	G/A	cctaaacaaWtttgtttatcaaagtaaactaatcttatattagtatatttaattttaacatggtgggttcatcccgatccatcaaaaataaaagtatgtc[G/A]ggtcgtctcgaaaagatatatagaatctcttttttaccgtctatctcatacagaaatttaaagtcaatgaatcagtctgatagatcaaattcattttaac

fava_c18282_369	fava_c18282	369	G/A	gaaaagatatatagaatctcttttttaccgtctatctcatacagaaatttaaagtcaatgaatcagtctgatagatcaaattcattttaacaactctagt[G/A]atatcgactatctaggaaagacatttgatattgtaacttcatttatagaaacattatgggaagattttgacgttgattcatcttcaactacttttcttcc

fava_c18282_483	fava_c18282	483	T/G	aggaaagacatttgatattgtaacttcatttatagaaacattatgggaagattttgacgttgattcatcttcaactacttttcttccaactgaaaatgca[T/G]gttgattaggagtgggaagtgtcattgtctcacttgccaacatgacaacaaYattggacatggttggtctatcRattgcatcttcttgtacacacaataa

fava_c18282_535	fava_c18282	535	T/C	tttgacgttgattcatcttcaactacttttcttccaactgaaaatgcaKgttgattaggagtgggaagtgtcattgtctcacttgccaacatgacaacaa[T/C]attggacatggttggtctatcRattgcatcttcttgtacacacaataaaccaatgtgtacacacttcttaacttcattttctatgYatgtctcttttagt

fava_c18282_557	fava_c18282	557	G/A	ctacttttcttccaactgaaaatgcaKgttgattaggagtgggaagtgtcattgtctcacttgccaacatgacaacaaYattggacatggttggtctatc[G/A]attgcatcttcttgtacacacaataaaccaatgtgtacacacttcttaacttcattttctatgYatgtctcttttagtattgcatccaaaagttccatac

fava_c18282_621	fava_c18282	621	C/T	caacatgacaacaaYattggacatggttggtctatcRattgcatcttcttgtacacacaataaaccaatgtgtacacacttcttaacttcattttctatg[C/T]atgtctcttttagtattgcatccaaaagttccatactttttccttcacaccaaagattccaactatatataagaagactttgaccatgctctgcaagata

fava_c18287_127	fava_c18287	127	C/A	gggacacctcacccacccactcacccttcccctccctctcctatctacccccgttccgttctccaccttcaacaccaccgtctctcaaaaatttcactca[C/A]ttcatcaatttctctttctagaatctaatctaatctaaccgtaacttcccttctttcattcgaatccatctgaatccaaaaaatttcattctctcccaca

fava_c18287_359	fava_c18287	359	A/G	tccaacactcctttttccataaattgagttcccaaaccaaacatatcgctttttcattttttctctctctttttttcctttgtgaaatttcagatttgtc[A/G]tcggaacatgtcatcacctatgatctcaaactcaacaagtgaccgMtcacacagaataaagaaaaagaagaaagccctatcaaaaatccaacaacatgcc

fava_c18287_405	fava_c18287	405	A/C	cgctttttcattttttctctctctttttttcctttgtgaaatttcagatttgtcRtcggaacatgtcatcacctatgatctcaaactcaacaagtgaccg[A/C]tcacacagaataaagaaaaagaagaaagccctatcaaaaatccaacaacatgcccaagaacaagaacaaagaataaccaaatggagatcacaagctcaac

fava_c18295_354	fava_c18295	354	G/A	atctcggaggcggcgctgaagcgcacgcgcggttcaagcagtacgagtacagggccaactccagtctagtcttaaccaccgattctcgtccacgtgacac[G/A]cacgaacccaccggagaaccagagtcgctgttcggaagaatcgatccgaaaaacttcggtgaccgtgtttcccacgaccgtccaccggacctSagcgaga

fava_c18295_447	fava_c18295	447	G/C	gtgacacRcacgaacccaccggagaaccagagtcgctgttcggaagaatcgatccgaaaaacttcggtgaccgtgtttcccacgaccgtccaccggacct[G/C]agcgagaaactcgatgccgcgaagaggaagaagaaggaaagagagagggatccgattgattcggtttcttctcgtcggagcaagcgccgccgtcttcagg

fava_c18299_225	fava_c18299	225	A/C	tttgtgagtttatataagagttgattgctaagataaaaaatccttgatagaaattcattcatagttacataatcccataatcagaagagggattctccct[A/C]actgtcttaaacaagtgcaagttaaagaaaatattaccctttagttcctcaaagggtttattcagttgcaacaaaaaacagaaaaacatatttttcagaa

fava_c18307_233	fava_c18307	233	C/T	agcaacattttatatacagaacaaatgtccttttccaatatggccagcaacagcaccaaacagtggtcaaccaattatagcagatggtggattctatctt[C/T]cttcaagccaaacaaagaaaattctagcaccatggtcatggagtggtagaatttgggcaagaacaggttgtaattttgcttcaaatacttggaaaccatc

fava_c18308_1256	fava_c18308	1256	T/G	aaactaaagttgtttgcggaaattctagtgatagaaaatttgatgttgctggtggagaagatagtctttccattcaaggagttgtgccaagaaagcttcc[T/G]gatagagattttcttttggagccaattataatcaaccctaaaattgagttagcttcagtgatgcagaatatggtaaacactgcatacgatggactagcta

fava_c18308_1538	fava_c18308	1538	C/T	ctgtttttaaacctattgatgaagagccgatggctgtgaataaccctcgaggtcttccattatcattaaatggcgaaggtttgaaaaagggtacaagagt[C/T]ggccagggagcgttcagggaRgttgcggcttatgttttggatcatccaataagtggacgccgcaatttatttggtgacgtgaagggttttKctggcgtac

fava_c18308_1559	fava_c18308	1559	A/G	aagagccgatggctgtgaataaccctcgaggtcttccattatcattaaatggcgaaggtttgaaaaagggtacaagagtYggccagggagcgttcaggga[A/G]gttgcggcttatgttttggatcatccaataagtggacgccgcaatttatttggtgacgtgaagggttttKctggcgtacctccaactttgatggtaaaat

fava_c18308_1629	fava_c18308	1629	G/T	tacaagagtYggccagggagcgttcagggaRgttgcggcttatgttttggatcatccaataagtggacgccgcaatttatttggtgacgtgaagggtttt[G/T]ctggcgtacctccaactttgatggtaaaatgcttgcataaaggatttaaccatccaggagacatgattgctaagattggatcaatgcagatgtttgtgaa

fava_c18308_2235	fava_c18308	2235	G/A	aattggaaacttgatgtgcagggaatcattgaacaaagaatctgtgatagaggaaatagttgaagaagcactggattctgttcttccgggcacaagtgaa[G/A]ctacgttgctggatgctgtttctcaaatcatggatcagcgtcttcataatattttcaactctcctttttaataagagctgagttctgcaatagttgcagg

fava_c18308_2346	fava_c18308	2346	T/C	ggatgctgtttctcaaatcatggatcagcgtcttcataatattttcaactctcctttttaataagagctgagttctgcaatagttgcaggtatataaatg[T/C]gtgtgtgcagtagtacatatatgtatagctaagtaattggattttaattcWtaacaaaggaggtttgttttctttggttggaatttcactccattctctg

fava_c18308_2397	fava_c18308	2397	A/T	tcctttttaataagagctgagttctgcaatagttgcaggtatataaatgYgtgtgtgcagtagtacatatatgtatagctaagtaattggattttaattc[A/T]taacaaaggaggtttgttttctttggttggaatttcactccattctctgctatagaggttcataggtttttcttatttttgtagtttccttccgataaca

fava_c18308_307	fava_c18308	307	A/G	tgattcagacttgtttttcgtttcatctttggttggaattggtctctttgatcattttcttcaaaagatctgtgtttgtttgttgttcaattcttgacct[A/G]agctaggtatgagtatgatgatcaattgatcattattaccaattgttgacctaggtaccaatttgttgaagtataatattggttgagctatgatggttat

fava_c18309_1026	fava_c18309	1026	G/A	taccctttcctaaagcagcctctaggagttgcccRggagtttgacgtgagggaaatgcatgtggatttatgacaatatcaggaacaataccttgttttgt[G/A]aaaggaaaattttcttgagactccagaaatcctaaaacacccttttgtccatgcatactagagaacttatctccaagaacaggacttcgaacctgtctta

fava_c18309_241	fava_c18309	241	C/A	gnnnnnnnntacaagggcagatatgtatatcaagatgcatgtcatgaaatactcctggctgttatatcaaggaaacatccaggaacccgacagatatgta[C/A]accaagttcaagagactagtctagggaaagcattagataagtgactgttaaatagtagttgtctagcaatcaaaagataggcgatatgttaaatagtagg

fava_c18309_585	fava_c18309	585	A/G	tccggccaccagataccggccgcaagatcacatttgcaacatttttgcatttactacaaatgtgtatctgagacgaatcactcagggtgaagagacgttc[A/G]tacaagttggaagatgctccatgggcaatcaggcaatcacgctccatttcaccaaacttgatacctccaaatcgcttcctgtccgcaactggctgtcggg

fava_c18309_705	fava_c18309	705	G/A	catgggcaatcaggcaatcacgctccatttcaccaaacttgatacctccaaatcgcttcctgtccgcaactggctgtcgggttagcgggtgaactgggcc[G/A]gtgttcctgaacttcactttgtcctcagacatgtggtgtagtcgctggtagaatgttgggcccataaatattagtgattgaaccatctcaccagtcctcc

fava_c18309_960	fava_c18309	960	A/G	cagtaatggcttcaacagacggagtggagaaaggggtagcatttctcaatgaaccaacacaagcaataccctttcctaaagcagcctctaggagttgccc[A/G]ggagtttgacgtgagggaaatgcatgtggatttatgacaatatcaggaacaataccttgttttgtRaaaggaaaattttcttgagactccagaaatccta

fava_c18310_446	fava_c18310	446	A/G	aagaagaagaactctcaagctgtaaattctcgcatcttcttgcttccaaggatcgtgattttcttctatcaccaactggagctcaggtaaaagtttcgga[A/G]ctcgaaggaaaagtagtgggactattattcgctgccaactggtacccaccatgtcgaggattcacccaattgctcattggaatataccaacaactcaaaa

fava_c18310_746	fava_c18310	746	C/G	tctctgatttagaaacaaagaaagcgttaaacagaaagtacgacgtcgaaggtattccgtgtttggttatgttgcaacctgatgatagtaatggcgaagc[C/G]acactccgggatggtgttgagcttatttatcgatatggtgttcaagcttttccgtttagtaaagagaggttggaggaattgcatgaggctgagagagaga

fava_c18310_878	fava_c18310	878	A/G	gatatggtgttcaagcttttccgtttagtaaagagaggttggaggaattgcatgaggctgagagagagaagcttgagaatcagactctagctaatttgct[A/G]gctaataatcatagagattatgttttgagtcacacaagtggattactcactcaggtacctgttgcttctttagtaggcaaaactattggactatatttct

fava_c18312_484	fava_c18312	484	G/A	aacagctccagtccatgataggatagtaggatgaaacctgaacttgaaaaggatatactcagaattatgtactggaaggatgattttgacaaagctcggc[G/A]ctcagcatatgatgaagaagtgcatcaaccctctggcttcgtgttttcgttccaagagttcgtcgcctcggatatctatctcctgcaactcccttgtcat

fava_c18313_2200	fava_c18313	2200	G/A	tctaaaaaataacgtgtccactttgtttcttgtagttgccatttatgagagattccttatcagtatatagctaaaaggtttctgtaaacagttaccattc[G/A]ctcaagatgcaacatacaagtaataagtgtcaacgagcttatacctttactcaatatataagcttgtttataactttgtgcagcttatacctaactcaat

fava_c18315_1873	fava_c18315	1873	T/C	ccacatacaatgctcttctaaacgctcttgctaggcgaggtgattggaaagcagcagaatctatcatttttgacatgcagaataagggctttaagccgaa[T/C]gaaacttcgtactcacttttgcttcattgttactctaaggctgggaatgtcaggggattagagaaggttgggaaggaaatctatgacggtcatgtctttc

fava_c18315_2567	fava_c18315	2567	G/T	cattagatatatgattgaacagggttgcatgcctaatgagttatcttacaagattgtaattgatggttatattagagataaaaagcataaggaagcaatg[G/T]attttgtgtctaggattaaagagattgatatctcttttgatgaccaatctttgaaaaagctagcttcttgtattagggagagtttggggtcttgactttg

fava_c18317_665	fava_c18317	665	A/G	ggaactcactatttccaaaattgttgagaagttcgaattgttcaactgtccaccgaaaccacctcataaggaaaactcgagaagccagcccatgtgacac[A/G]attatcagattcaaatcgtttgaagggttatgattaagcctgttcatgtcaatgtccctccatagagattcaagaaaactcgaaacgcggtcgaaaacgt

fava_c18317_959	fava_c18317	959	G/C	aattcccaaaatcctgttcacgaatgcggcactcttctctcacaccaataacacgtttcttagagaaagatctggcgagttcacggagagtggatcgggt[G/C]cgagcatacggcgaggcgtagaagtaaacgcgccaatcaggtgaagaagattcagaagcgatgaggtggcggattttggaaccggcgagacgcgcttgcg

fava_c18320_383	fava_c18320	383	T/C	atttcaaacgccgacaaatattctagacaatggccggtggtcgattccgcgagctcctgaaaaagtacggtaaagtagcgatcggcgttcactgttccgt[T/C]tcagcagcttccgtcaccggcctctaYgtcgccataagaaacaacgtcgacgtWgaagccatcctcgagaagttccatttgagcgccgcttcgtcggaag

fava_c18320_410	fava_c18320	410	C/T	caatggccggtggtcgattccgcgagctcctgaaaaagtacggtaaagtagcgatcggcgttcactgttccgtYtcagcagcttccgtcaccggcctcta[C/T]gtcgccataagaaacaacgtcgacgtWgaagccatcctcgagaagttccatttgagcgccgcttcgtcggaagaaaaccctaaccctaactctgattccg

fava_c18320_437	fava_c18320	437	A/T	tcctgaaaaagtacggtaaagtagcgatcggcgttcactgttccgtYtcagcagcttccgtcaccggcctctaYgtcgccataagaaacaacgtcgacgt[A/T]gaagccatcctcgagaagttccatttgagcgccgcttcgtcggaagaaaaccctaaccctaactctgattccgactcttccgccgtgccggttaagagcc

fava_c18322_213	fava_c18322	213	C/G	tctaggccagatcaacaacaaatgacaatcaacattaaaaacaaactcgattctcacctctcacaaacaaattcacaaaacaaaataacaaagtccacac[C/G]aattatccaaatctggaaaaaaacctcaagaacacactaaccctaaataatatatctactctgtttgtggcaattaggaaacattagcatgtaatttcat

fava_c18322_457	fava_c18322	457	T/A	aggtggaaaatgaaaacctaggatttttgtaatttccaaaatccgatgaaggtttgaagattccgatgaattttctggcataactttgaattttccggcg[T/A]ggttgaagattgcgatgagttttccggcgagctatgaattttgctgccaacttttccagattccggcgacgtactccagcgagttaaaaaatgattctca

fava_c18323_1487	fava_c18323	1487	A/T	gaggaatcgaaccacgaatctgaacaaatgaggcagtatacccattgaactgcataagttgttctgtttccacaaaattggcaacatatccatctggatc[A/T]gctcctcttctccacattcgagttccatttcttcttgtgcatctcctcgcaatcaaagtgacatcaataatatctttcccaatggctgcttgaaagtaat

fava_c18323_1757	fava_c18323	1757	A/G	aattgttccatagaaatcgaggctctgcctgtctccaaagaggaagcaatctagactcatcacccaggtcattcaaccgctgggcactcagagttaaatt[A/G]gtttcatatgagaagaacaggccagaagttttctcagcaacatttagtaggccagaaaattccatctccacctttttctgttcagcaggggtacttttaa

fava_c18323_1931	fava_c18323	1931	T/C	ttttctgttcagcaggggtacttttaagtgaatgatcacatggaaaaaccttcatggatgtaattttgaaaattggatgccccaagtaagatccagcaca[T/C]tcacgctcagttatgaccatcaaatatgatccagccaaaagcctcactatcccaacaacaccataaatcgtaaaaatcttaggaactcggagagtggtac

fava_c18323_335	fava_c18323	335	C/T	tgcaaattagtaataaataacaaaagtatgataagcagcaaaagaggggcttaaagattgaattgtttaagtgatttaaatgtagaactaaatcccctga[C/T]ggtgactccttttgcagaagctgaatacttcacaaaattctaattccccaagttgatttgtgtagaagttaacgatggggattgtttaggcgRggtctgt

fava_c18323_428	fava_c18323	428	A/G	cccctgaYggtgactccttttgcagaagctgaatacttcacaaaattctaattccccaagttgatttgtgtagaagttaacgatggggattgtttaggcg[A/G]ggtctgttgcagaaaacccgtccattggccctcacaaaagctgctaaaccgacactWatgccRgcccacattagtgaaaagaaggcgtgcctaaaatcat

fava_c18323_485	fava_c18323	485	A/T	aagttgatttgtgtagaagttaacgatggggattgtttaggcgRggtctgttgcagaaaacccgtccattggccctcacaaaagctgctaaaccgacact[A/T]atgccRgcccacattagtgaaaagaaggcgtgcctaaaatcatattgaacttgtctcaatgacatggttgcaaaaaaaaatccagttaaaaccagtScaa

fava_c18323_491	fava_c18323	491	A/G	atttgtgtagaagttaacgatggggattgtttaggcgRggtctgttgcagaaaacccgtccattggccctcacaaaagctgctaaaccgacactWatgcc[A/G]gcccacattagtgaaaagaaggcgtgcctaaaatcatattgaacttgtctcaatgacatggttgcaaaaaaaaatccagttaaaaccagtScaaaagcca

fava_c18323_582	fava_c18323	582	G/C	actWatgccRgcccacattagtgaaaagaaggcgtgcctaaaatcatattgaacttgtctcaatgacatggttgcaaaaaaaaatccagttaaaaccagt[G/C]caaaagccagaggaaacgaagctatagcttcaattcctcctttctgagaagaggcagctgtatctcggccaacagatactatataatgtccttgcaggag

fava_c18323_929	fava_c18323	929	C/T	ggatacttacatcatccccatgattagcccacaagatcttaaacctttcatcaagatttggatgagaactgatggtttcttcagccccaaagactccaag[C/T]cttctaagctggtattctaacatatttcggcctatcatgctctgagttacatttgttcggtcgagacaatcaatacagttggtcctaacaactccaagtt

fava_c18326_1068	fava_c18326	1068	T/C	agccagttatgcagttatatacagaaactactgatggttctaacatagagtccaaagagagtgcacttgtttggaattacgaatttgcagaccgagattt[T/C]ggttcatgccaagctaaggagcttcttgatcatctagaaagtgttctcgccaatgagcctgtttcagttaaaagtggcccaaacattgtggaagttaaac

fava_c18326_1194	fava_c18326	1194	A/T	ttgatcatctagaaagtgttctcgccaatgagcctgtttcagttaaaagtggcccaaacattgtggaagttaaacctcagggtgtgagtaagggtattgt[A/T]gcggaacgtcttctgacaacaatgcaacaaaagggtgtgattccggatttcgttctatgcattggtgatgacagatccgatgaagacatgtttggagtaa

fava_c18326_1313	fava_c18326	1313	C/T	acaatgcaacaaaagggtgtgattccggatttcgttctatgcattggtgatgacagatccgatgaagacatgtttggagtaataacaagtgcaagggcat[C/T]gctatcgcctattgctgacgtctttccttgtactgttggtcagaaaccaagcaaggcaaagtactacttggaagacacaagtgagattttaaggatgttg

fava_c18326_705	fava_c18326	705	C/T	ttcgagtaattgctttggatccaaactttagaaaactatctgtggaacatattgcttccgcttataagaggactaagcaacgggcgattcttttggatta[C/T]gatagtactatggtgcagcctggatcaattagtacaacacctaatgctgaatctgttgccatcttgaacagcttgtgcagtgacaccaagaattgtgttt

fava_c18326_894	fava_c18326	894	C/T	agaattgtgttttccttgtaagtggaaaggagagaaagactcttactgaatggttttcttcttgcgagaagcttggacttgctgcagagcacggctattt[C/T]gtgaggacaagtcatactgaagaatgggaagcttgtgtttctgtgYctgattttgactggaaacagatcgctgagccagttatgcagttatatacagaaa

fava_c18326_940	fava_c18326	940	C/T	tgaatggttttcttcttgcgagaagcttggacttgctgcagagcacggctatttYgtgaggacaagtcatactgaagaatgggaagcttgtgtttctgtg[C/T]ctgattttgactggaaacagatcgctgagccagttatgcagttatatacagaaactactgatggttctaacatagagtccaaagagagtgcacttgtttg

fava_c18331_1921	fava_c18331	1921	T/C	gatccaaacactcgcctgcatggtaggtagaggcccataacaaggggttttccttttcactagcaaaacctctcatcatgtgcctaatagaatcagttga[T/C]acaactgttgtgattcccaaccgacttcccagYaatgcagacaatgtggatttgccacaaccgctagtgccacacagcagcactgtcacagattccttcc

fava_c18331_1954	fava_c18331	1954	C/T	cccataacaaggggttttccttttcactagcaaaacctctcatcatgtgcctaatagaatcagttgaYacaactgttgtgattcccaaccgacttcccag[C/T]aatgcagacaatgtggatttgccacaaccgctagtgccacacagcagcactgtcacagattccttccgttctcggatcctgcaagctagcatcaagtctg

fava_c18331_2208	fava_c18331	2208	A/C	tctagaaaagtttctctcttaacaaatacagtcgtgcgctttttgtacaactcaaatggcacgctcttactttcgtcatcttttcctaaaagtaagcaag[A/C]agaagcttcctgacaatcKttttgtgcatttcctttagaagtatcaaaRcctgataaaaccattccttcggacttgaaaggctcagtagaactcccactc

fava_c18331_2227	fava_c18331	2227	T/G	taacaaatacagtcgtgcgctttttgtacaactcaaatggcacgctcttactttcgtcatcttttcctaaaagtaagcaagMagaagcttcctgacaatc[T/G]ttttgtgcatttcctttagaagtatcaaaRcctgataaaaccattccttcggacttgaaaggctcagtagaactcccactccttgtgagttcaaaaaccc

fava_c18331_2257	fava_c18331	2257	G/A	actcaaatggcacgctcttactttcgtcatcttttcctaaaagtaagcaagMagaagcttcctgacaatcKttttgtgcatttcctttagaagtatcaaa[G/A]cctgataaaaccattccttcggacttgaaaggctcagtagaactcccactccttgtgagttcaaaaaccctttggctgatcttgaaagcatgacgagctt

fava_c18331_241	fava_c18331	241	A/G	caaatagttttgatataaactcaagagaccttactataaagacagcggggtcgacttaaaacacaaacgaatttctaaaatcaaaaggcatagcagaaaa[A/G]gttgggatagttgtcaagaattcaactaaagacagagtgtcatgctacctttgaggagctccagagggaatgggatcctggattgttgaactacccttcc

fava_c18331_2437	fava_c18331	2437	A/G	tcttgaaagcatgacgagctttacatcccataagctgcagtgtactctgtagaaccggacgcgtataacgaaacgactccttctccttcaacttcccctt[A/G]tctttcttctcgccttcatccaccaccaccaagtacaatatcttcgacacctcaaccatacctgaaacgttgttttgcgttggtagaaaggttttggatt

fava_c18331_772	fava_c18331	772	C/T	cctcatgaatatgtttgtcaccgtcgtcactgaaatcatcatcacttcccgcctctaattgatcatcaatctcttcatcactaccatgcactgaaaggtc[C/T]tccttaagctcctttgaaggtccatcggacatccttggtgaactacagcaggaggaaacatatctactacctgtgtcagtcccRtcagcttttgactcat

fava_c18331_856	fava_c18331	856	G/A	catgcactgaaaggtcYtccttaagctcctttgaaggtccatcggacatccttggtgaactacagcaggaggaaacatatctactacctgtgtcagtccc[G/A]tcagcttttgactcatcaacacttccagcacggcttgttccaccatcggagggccaagcactgatgccataaaaaccaaactgaaggtttacaggttctg

fava_c18333_1836	fava_c18333	1836	C/T	ttgtcctcttattagatgctacgactcccccatttgttgtatgagaagataactcagggctagtatgagtacttacaatttcttgatcttcgccatctgt[C/T]ctagtagaaatattctccaataagcgtatatggtaaacagggcgcttatcggggtctcttgccaaatcaagaagattccgatgaagtaaaacatcttcag

fava_c18333_2236	fava_c18333	2236	C/G	aagcaaatccataagtgatttttttgttctaggttcaagattgatgcatggtgtagaagacgaagaaatagaaagcgagttcaattgaatagaaaatttt[C/G]gcgtgcggtgagaaataagaaagagagagaggatctttgactctggtagaggagaaaggttgagttgggagtggaaaagaagtaaagggtgttttcttc

fava_c18333_468	fava_c18333	468	G/A	gaagtccttttttcccttggagtaaaagggagcgtgaaaaactctacagctatttgaatagcgaaagaccaccccaaccgtatgaaaataagtcgaatat[G/A]ataaatagtatgagcttgaaatcttaagcaccattaRcttctgaattattctccacttctagcaaaagattttcaagttcaactcttatctcattgaaga

fava_c18333_505	fava_c18333	505	G/A	aaaactctacagctatttgaatagcgaaagaccaccccaaccgtatgaaaataagtcgaatatRataaatagtatgagcttgaaatcttaagcaccatta[G/A]cttctgaattattctccacttctagcaaaagattttcaagttcaactcttatctcattgaagaatggacgactgcttggaattgcttcccaacatctttg

fava_c18333_885	fava_c18333	885	G/A	ttatgacctctggtgccatccatctataagttccagtctctgcagtcatcactcccccctgaataaggaaccgagcaactccaaaatctgccactttaac[G/A]acattgtKagtatccatcagcaaattggctgtcttcagatccctgtgaattatgttatttccatgcaaatactccattcccttgcagacatcaattgaaa

fava_c18333_893	fava_c18333	893	T/G	tctggtgccatccatctataagttccagtctctgcagtcatcactcccccctgaataaggaaccgagcaactccaaaatctgccactttaacRacattgt[T/G]agtatccatcagcaaattggctgtcttcagatccctgtgaattatgttatttccatgcaaatactccattcccttgcagacatcaattgaaaacttgagc

fava_c18334_1033	fava_c18334	1033	T/C	acacctgttattctaattttcgggggtccttcaggccggcgagcaagagcctgaattaggctaacccactgagttccttgtgcaatttgaaaatcaatta[T/C]gtgtatttcactttcatccttcatagcttcagcaatcgctccatttgccgacatatacccaaacttaaagtacggacatatttcataaagcacatgcatg

fava_c18334_1451	fava_c18334	1451	A/G	gcatttctttcaagtcccctcgagatacgatctccgtgtttctcttccagttttcagcctctaacgagaacaaagcagcctcttctcgaatttcagtatc[A/G]tacatatctagcatatcctcgttcggtccgagcataacagatttgagctcttgtatcttatgcctgaggtcatcaaaatcatgtgtcaagtaagaatctt

fava_c18334_1553	fava_c18334	1553	G/A	acatatctagcatatcctcgttcggtccgagcataacagatttgagctcttgtatcttatgcctgaggtcatcaaaatcatgtgtcaagtaagaatcttc[G/A]cccYcaggcgaaccgttaacaaattcaagagaattttgttggcttaacacgcatgaatttgactctagcttcgacaccggactattgttccgagagaaac

fava_c18334_1557	fava_c18334	1557	T/C	atctagcatatcctcgttcggtccgagcataacagatttgagctcttgtatcttatgcctgaggtcatcaaaatcatgtgtcaagtaagaatcttcRccc[T/C]caggcgaaccgttaacaaattcaagagaattttgttggcttaacacgcatgaatttgactctagcttcgacaccggactattgttccgagagaaactgag

fava_c18334_2016	fava_c18334	2016	A/G	atacacgagcactaccggaccgattgattttctagcacaaacaccctagaaattttccaatagcctttctttttaaacaagatatgtcagtaacttcgtc[A/G]tccacaaatgaattcaaacttttctggcaaaaRaagtataccgaaaaacgagctatattcttaagaagagtaccaaaMaggaaaatcggatctgaaacca

fava_c18334_2049	fava_c18334	2049	A/G	agcacaaacaccctagaaattttccaatagcctttctttttaaacaagatatgtcagtaacttcgtcRtccacaaatgaattcaaacttttctggcaaaa[A/G]aagtataccgaaaaacgagctatattcttaagaagagtaccaaaMaggaaaatcggatctgaaaccagcttagaagaagttgaattgaaggtgaatatag

fava_c18334_2094	fava_c18334	2094	C/A	aagatatgtcagtaacttcgtcRtccacaaatgaattcaaacttttctggcaaaaRaagtataccgaaaaacgagctatattcttaagaagagtaccaaa[C/A]aggaaaatcggatctgaaaccagcttagaagaagttgaattgaaggtgaatataggataaggatgttRttatgttgttatgtggttggagaaaaacttgt

fava_c18334_2162	fava_c18334	2162	G/A	aaacgagctatattcttaagaagagtaccaaaMaggaaaatcggatctgaaaccagcttagaagaagttgaattgaaggtgaatataggataaggatgtt[G/A]ttatgttgttatgtggttggagaaaaacttgtgagtagatggtaaaacacgaaggttgttttggttttggtttggtccactttgaagtc

fava_c18334_335	fava_c18334	335	G/A	gtcatcagattcttcatctccaagcacaagaaacaataagaggttgattcatccaaccaagatatagtgcaccatctctctcttcaagagtgtaatgtcc[G/A]cggtagctgtcgagaagattcttgattgaagagttgatgaacgagctcaacggatattgcgtgaatccagccattatgaaacgcgtttgccacttagtta

fava_c18334_521	fava_c18334	521	A/G	tttgccacttagttagaagctcgtgacgttctactcgttcttctccttcgcaggctactaagttgacaacttcacgagccaaacagtgctgttcgacgtt[A/G]atcctctctttgtgatcccttggaagaacaacgtcgattgattcgtagacggctgagtagtagttcattgtctcgacaaaacgcggcaagaacggagcgt

fava_c18334_761	fava_c18334	761	C/T	ttggagcgaaatacttagccaaatgcaataacctgtcgcgatgattatgaatgtttacggtctcgtctggcacgtggtgtagcataatggcgaaattcac[C/T]gccacggcttcgtaaggtcgaagttcaaagtcttcgagttggacttcagaagcagaaactctaacagcatggaactcaaaaggaacatgacatgattgtg

fava_c18334_896	fava_c18334	896	G/A	cgagttggacttcagaagcagaaactctaacagcatggaactcaaaaggaacatgacatgattgtgcaagtgccgataacctttcccccactatatcaac[G/A]cccccgccgcgggcgtaagctgaaaacgtgtcatcaacacctgttattctaattttcgggggtccttcaggccggcgagcaagagcctgaattaggctaa

fava_c18335_1233	fava_c18335	1233	C/T	cgtccggtgagggattgaatatgaatatgaacgcacaaatggactctgctgatcatgctgtactgggaacatggcttgaacagatgcagcttgaccatct[C/T]gtggctcagcaaaactgatagcaaaactgaaatcagttttgctgacctcgaggcaaggcagttaacaactgaaaggttcgagtatatactatatatgtta

fava_c18335_1362	fava_c18335	1362	G/A	gaaatcagttttgctgacctcgaggcaaggcagttaacaactgaaaggttcgagtatatactatatatgttatttttttccagaatttttgttgttgatg[G/A]aattgaaagatggttacattaactaagaaaggaagcgcaaagataaagaatgagaggaaaaattcattcacaagtttagtgttacaaaaaggtaacttta

fava_c18337_1953	fava_c18337	1953	G/A	tcaaaactagaagcttctatacttatattcctcattcgagggggaatatcaacttggcaacacccaatacccgaacatgttccatgatcaatcgatcttg[G/A]atcaccaaaagatcttgttaagcaccctgttgagtacttactgccatcatcaccgaaactgttaagataaccgtaactgttatcaccaacagttagtaac

fava_c18337_2086	fava_c18337	2086	G/C	agtacttactgccatcatcaccgaaactgttaagataaccgtaactgttatcaccaacagttagtaacttgttatctttgcttgaaatggtaaaagactt[G/C]gaggtaagcgatgggttattactattattcttgccgcaatacttggagacaattaacgacagttcaatttgagatgtggagatgtttatgcttaatactg

fava_c18339_1198	fava_c18339	1198	T/C	agaagcctccaggtgcaatgattagtggctttggctcaactcctctatatacatcttgaactatatctcttaaaaccataacatcagaagcgtatcgatc[T/C]gcagtaacactggctccaattgcatgtccacacaattcattaccaaattcccaaccatgaattgtatacttcttggcaaccgtataacgtataagagact

fava_c18339_1601	fava_c18339	1601	T/C	tggaatacaagattccatgttatctttagtcccatatgtcacaatatcttgcaaggtgccacctaatctaattttcaagggtgaaaaagctcttactgca[T/C]ttaacaaaattttgttattgaggtccagattaagcagagaagcatggccccaRctacattttccccagtcacatttctcaggtggccaccaatcaatagt

fava_c18339_1654	fava_c18339	1654	A/G	aggtgccacctaatctaattttcaagggtgaaaaagctcttactgcaYttaacaaaattttgttattgaggtccagattaagcagagaagcatggcccca[A/G]ctacattttccccagtcacatttctcaggtggccaccaatcaatagttgcacaaacaaaatcattatcaatccttccaataaaagctttgccatgaatga

fava_c18339_1860	fava_c18339	1860	G/T	acacctttatcatcagcagaaaaatttcctataccattcactccaatgaaactgaagcatatcaagtaaaaccatagaaacatacccagaagcttatttt[G/T]ataacccatttccagaatcaataaaagttaagatctttatggtttttgcataaatgggtcagcagaaacaagaaagagttgaggttttgttactgcagca

fava_c18339_494	fava_c18339	494	C/T	agccgaatttacacttagaatgtttccatttagtaacactatttggctatgcaaatttccatcttgggctgttaaatgatactctcttctctgtaacttt[C/T]ccgcaataggtggcgaaggtttcttcgcataatttaaggtcactcgtgcatttacagtggcgctgtcgtccaaattcagaaacagtaccgtgattccctt

fava_c18339_676	fava_c18339	676	A/T	acagtaccgtgattcccttagattcctttgcgcaatgagcataacttctgatcttctttgttccgtagaaggtagttgacagaacgcgtgatcccatgag[A/T]cggtgccaaagaagagcactgtaatagtctggatttggcatgaaagtagttgtattcaataaaccatagtttcctcctatcaaagtctgtctgcaatatg

fava_c18343_2127	fava_c18343	2127	C/T	gtatatgaaactggtgctacggttgcatttagctggtagagatatgaatatgctatcagagttcagtttttgaacacttaagtttccaattttgatttga[C/T]tgttgctgcaggttaacttgattttgcaagcttctgagaatccgaatgggtagttcacggtttgttctccgcatctttggttgcagtggttgttgttttg

fava_c18343_503	fava_c18343	503	C/T	catttccattataacgaggtgatgaacacccggatccaaccgatgagcctaagcatatagtttcctccattgtcgcccatccgctacgtctgatatgatc[C/T]aactcctcagcaacttcaatcatactaggcctcgattcRctatgaaaagcaaggcatctaaatgcaagctcagctaccttgttaacagaatagagtgtcc

fava_c18343_542	fava_c18343	542	G/A	ccgatgagcctaagcatatagtttcctccattgtcgcccatccgctacgtctgatatgatcYaactcctcagcaacttcaatcatactaggcctcgattc[G/A]ctatgaaaagcaaggcatctaaatgcaagctcagctaccttgttaacagaatagagtgtccatgcatctctattaagatcaaggaaaggatctatgatgt

fava_c18345_1181	fava_c18345	1181	G/A	cttccacctgatcaacactcaaaaccctagcaccacaaagcttcactctctcatactcatctctcctaaacggtgtctgatcagaagacaaatcctcagc[G/A]acaattctattcccatctttaacggccagcaccgccctcgaatcaccaacattcgcaacataaagcgtatcccctataaccagcaccgtaatSgccgtag

fava_c18345_1274	fava_c18345	1274	C/G	cctcagcRacaattctattcccatctttaacggccagcaccgccctcgaatcaccaacattcgcaacataaagcgtatcccctataaccagcaccgtaat[C/G]gccgtagtaccactcatacaatcatcaatctcattcttatgcaagtcatcattRgtatccaaaaacgccgaattataagccttaacaggatcctctaaca

fava_c18345_1328	fava_c18345	1328	A/G	caacattcgcaacataaagcgtatcccctataaccagcaccgtaatSgccgtagtaccactcatacaatcatcaatctcattcttatgcaagtcatcatt[A/G]gtatccaaaaacgccgaattataagccttaacaggatcctctaacaaSgcaRaatcatttgacagtttctctaccaacctaYtcttaacaaaattagaac

fava_c18345_1376	fava_c18345	1376	G/C	ccgtagtaccactcatacaatcatcaatctcattcttatgcaagtcatcattRgtatccaaaaacgccgaattataagccttaacaggatcctctaacaa[G/C]gcaRaatcatttgacagtttctctaccaacctaYtcttaacaaaattagaacactgSccaccaaattcaccatgtccatcaaaaacaccaaagaaatgaa

fava_c18345_1380	fava_c18345	1380	G/A	agtaccactcatacaatcatcaatctcattcttatgcaagtcatcattRgtatccaaaaacgccgaattataagccttaacaggatcctctaacaaSgca[G/A]aatcatttgacagtttctctaccaacctaYtcttaacaaaattagaacactgSccaccaaattcaccatgtccatcaaaaacaccaaagaaatgaacatt

fava_c18345_1410	fava_c18345	1410	T/C	cttatgcaagtcatcattRgtatccaaaaacgccgaattataagccttaacaggatcctctaacaaSgcaRaatcatttgacagtttctctaccaaccta[T/C]tcttaacaaaattagaacactgSccaccaaattcaccatgtccatcaaaaacaccaaagaaatgaacattagggttatcttgcaattgagtcttgacaca

fava_c18345_1433	fava_c18345	1433	G/C	ccaaaaacgccgaattataagccttaacaggatcctctaacaaSgcaRaatcatttgacagtttctctaccaacctaYtcttaacaaaattagaacactg[G/C]ccaccaaattcaccatgtccatcaaaaacaccaaagaaatgaacattagggttatcttgcaattgagtcttgacacaataacaatcttgattctccttat

fava_c18345_162	fava_c18345	162	G/C	agaccaagagctcccatttgagccacaaagctacacgataatacattcaccaacataattcaatgtaagtggtagtcaagtttagactttcttatttttt[G/C]aagttctttatacatatccacttttaaagaaggtgcagttaagttcaggtgtattctgaagtgcttctgtgtagttaaatgacatgttaacttagaatta

fava_c18345_1785	fava_c18345	1785	A/G	tgatggtgcagggtaacgactacaacacgttccatgaacacaacccatgtttgaaatagaatctaaactggtaaaaaacacaaactgaatctcaaaaaat[A/G]caatcctgggttagaatcataagcttacaaggttctaaaaaacagcattgtagttcaaaatcagattttgaagaacatgaataaacaaacaaacaaagaa

fava_c18345_393	fava_c18345	393	A/G	tctaaaagtccaagagaagaagtccttcagctgataagataatgcaccttctaccttcagcagcctaacaaaattgagtcgacatttcgactcaatgaat[A/G]atatatYggtgatgcagaaggctcaaaaagatagaactttagatatgtaccacctaaaagagcatctttccaattacaattcaactggactttgacatgt

fava_c18345_400	fava_c18345	400	C/T	gtccaagagaagaagtccttcagctgataagataatgcaccttctaccttcagcagcctaacaaaattgagtcgacatttcgactcaatgaatRatatat[C/T]ggtgatgcagaaggctcaaaaagatagaactttagatatgtaccacctaaaagagcatctttccaattacaattcaactggactttgacatgtgggcgaa

fava_c18349_1711	fava_c18349	1711	A/T	ttcatggtgtggaagaagatattgcagaagacaggcttcagttttggattgcccgcagtggacagtcaccaacttcacatgatgctgttgatgttgagcg[A/T]gatttgttggagttgaggaagctgggtatagaacagcaactttgggaagcttctcggaagggtattgatcagccttcagggttgtcgacacttgacaata

fava_c18352_571	fava_c18352	571	A/G	aagaattgaacaagtgatgaagatgatggaagagggagcagagaaaggctgttgaagattttggtgattcttctgtgtgttatagaatggtgaaaatgaa[A/G]cagagaatcgaaagagtgccgaggagaatggaccgtYtagatgtttggatatgggacttacttcacgaaaacttaatgcagcaaccctggatctgttaaa

fava_c18352_608	fava_c18352	608	C/T	gcagagaaaggctgttgaagattttggtgattcttctgtgtgttatagaatggtgaaaatgaaRcagagaatcgaaagagtgccgaggagaatggaccgt[C/T]tagatgtttggatatgggacttacttcacgaaaacttaatgcagcaaccctggatctgttaaagttctgattatgcaggtttatgtgtttggcatagcct

fava_c18352_987	fava_c18352	987	G/C	aattgaatatttcattgttcgtttgaggaccgtgatattattttgttgcacgtaattattattgccataatattttataaactcttttattgatgcatgt[G/C]tactgttcgatgaaatgccactgaggaataatgtcacgtggacgggctgcttcaggtcatttagaatcaaaagtatcgttttgaatgttgtaaatatttt

fava_c18353_229	fava_c18353	229	C/T	cacaaatacaataccatataataatataagtgtgaaactagaaaaacatcaaatattttattttccataataaaaagaaatatcttacttattctgtata[C/T]gaaaaactgggcatgtactaatttagtatttacaaggaggaaactcataatagggaacccgacaatactgcttcgtactgcactaatctcagtcatttgt

fava_c18353_338	fava_c18353	338	T/A	gggcatgtactaatttagtatttacaaggaggaaactcataatagggaacccgacaatactgcttcgtactgcactaatctcagtcatttgtcaattctc[T/A]SWgtcattcactYaYctttttcactcttctggacttttttttggaKgtagaagagagaaaagaggattggttggggggaaggggaaaaagggactcctac

fava_c18353_339	fava_c18353	339	C/G	ggcatgtactaatttagtatttacaaggaggaaactcataatagggaacccgacaatactgcttcgtactgcactaatctcagtcatttgtcaattctcW[C/G]WgtcattcactYaYctttttcactcttctggacttttttttggaKgtagaagagagaaaagaggattggttggggggaaggggaaaaagggactcctacg

fava_c18353_340	fava_c18353	340	A/T	gcatgtactaatttagtatttacaaggaggaaactcataatagggaacccgacaatactgcttcgtactgcactaatctcagtcatttgtcaattctcWS[A/T]gtcattcactYaYctttttcactcttctggacttttttttggaKgtagaagagagaaaagaggattggttggggggaaggggaaaaagggactcctacgg

fava_c18353_351	fava_c18353	351	C/T	tttagtatttacaaggaggaaactcataatagggaacccgacaatactgcttcgtactgcactaatctcagtcatttgtcaattctcWSWgtcattcact[C/T]aYctttttcactcttctggacttttttttggaKgtagaagagagaaaagaggattggttggggggaaggggaaaaagggactcctacggtatgatacagt

fava_c18353_353	fava_c18353	353	T/C	tagtatttacaaggaggaaactcataatagggaacccgacaatactgcttcgtactgcactaatctcagtcatttgtcaattctcWSWgtcattcactYa[T/C]ctttttcactcttctggacttttttttggaKgtagaagagagaaaagaggattggttggggggaaggggaaaaagggactcctacggtatgatacagtga

fava_c18353_384	fava_c18353	384	G/T	gaacccgacaatactgcttcgtactgcactaatctcagtcatttgtcaattctcWSWgtcattcactYaYctttttcactcttctggacttttttttgga[G/T]gtagaagagagaaaagaggattggttggggggaaggggaaaaagggactcctacggtatgatacagtgaaRatggcacagttgagctagtagaataatag

fava_c18353_455	fava_c18353	455	G/A	tttttcactcttctggacttttttttggaKgtagaagagagaaaagaggattggttggggggaaggggaaaaagggactcctacggtatgatacagtgaa[G/A]atggcacagttgagctagtagaataatagcctagctttacatgaaatagctcaaagatgtctttttccttgatccaccttctccatagaggtcattccat

fava_c18356_314	fava_c18356	314	C/T	tggacaagatgcaagtaatattcctttgcaactgatatgaatgagaaccaacctagggaagcatgaggcagctgcacacatactccacttcagggtttcc[C/T]attcctccttcagttcctacttccctttctcttgccctcccaattattttcctctaagagaaccaatttaccttagaaaagtaatgtaggaataagtcct

fava_c18361_1020	fava_c18361	1020	G/C	ccaagcaattctgagatccttttcattagtaagatgtcgagttttatcctctttgggtgaggctggagcaggcaaagtaccaagaaatcctttagggtct[G/C]tagaagcactagtagcRtacaagagtggctgtttgtgtaatagtccctgagacataaaatttctcaacagaagcatgtgatggggagcttcYgcatcttc

fava_c18361_1037	fava_c18361	1037	A/G	ccttttcattagtaagatgtcgagttttatcctctttgggtgaggctggagcaggcaaagtaccaagaaatcctttagggtctStagaagcactagtagc[A/G]tacaagagtggctgtttgtgtaatagtccctgagacataaaatttctcaacagaagcatgtgatggggagcttcYgcatcttccataatcatcacaaggc

fava_c18361_111	fava_c18361	111	A/T	atccaaacttatcttattttctctaattagatgtgcaattcatgaagcttagattaagaaaccaactcacagatactaacataaaatttcttctaggact[A/T]MaaatataacccgttatcaaaaRaataaagactacaacattcaactactgcYaagttattttggttaacacaMgcattaaagttatatataaggtgtatt

fava_c18361_1112	fava_c18361	1112	T/C	tagggtctStagaagcactagtagcRtacaagagtggctgtttgtgtaatagtccctgagacataaaatttctcaacagaagcatgtgatggggagcttc[T/C]gcatcttccataatcatcacaaggctaccaagagaataaccaccacctaaaatcttgtcgagatctggaatcccagacgatataaatgttgtcccattag

fava_c18361_112	fava_c18361	112	A/C	tccaaacttatcttattttctctaattagatgtgcaattcatgaagcttagattaagaaaccaactcacagatactaacataaaatttcttctaggactW[A/C]aaatataacccgttatcaaaaRaataaagactacaacattcaactactgcYaagttattttggttaacacaMgcattaaagttatatataaggtgtattc

fava_c18361_134	fava_c18361	134	A/G	taattagatgtgcaattcatgaagcttagattaagaaaccaactcacagatactaacataaaatttcttctaggactWMaaatataacccgttatcaaaa[A/G]aataaagactacaacattcaactactgcYaagttattttggttaacacaMgcattaaagttatatataaggtgtattctaaaaatcaagtgaccctgctt

fava_c18361_163	fava_c18361	163	T/C	attaagaaaccaactcacagatactaacataaaatttcttctaggactWMaaatataacccgttatcaaaaRaataaagactacaacattcaactactgc[T/C]aagttattttggttaacacaMgcattaaagttatatataaggtgtattctaaaaatcaagtgaccctgctttagttgacccagaacaactcccagatgtg

fava_c18361_184	fava_c18361	184	A/C	tactaacataaaatttcttctaggactWMaaatataacccgttatcaaaaRaataaagactacaacattcaactactgcYaagttattttggttaacaca[A/C]gcattaaagttatatataaggtgtattctaaaaatcaagtgaccctgctttagttgacccagaacaactcccagatgtgttatatgaactcccgcttgaa

fava_c18361_353	fava_c18361	353	C/T	tcccagatgtgttatatgaactcccgcttgaaccgtcaaccggggcttgatttaaacattctagaaccaaatacctccttttttgcaattttatcgagaa[C/T]gttgttgcctcgagaatcacaggaacctgtgtatttaaccttgcgactttatgtacattaagaagtccaaccatgtcttggtaacctgtgaggagtctKg

fava_c18361_452	fava_c18361	452	T/G	aYgttgttgcctcgagaatcacaggaacctgtgtatttaaccttgcgactttatgtacattaagaagtccaaccatgtcttggtaacctgtgaggagtct[T/G]gccatttctttgtcctcatctggaattgctctgacagaaagtaaggtgtctgccatatgttgcaatctttttgaacaagatggagaaagaagtgaaggtg

fava_c18361_662	fava_c18361	662	G/A	caacaacaacagcatttgaagaccgcgtcatggcttttagggacctaataaaagatagcatatgccactccatgtttgcatatttgcattgcggagaaca[G/A]aatgattgaattgcaatacggccagcagaggaaatattggcttcatttcttgagaaKtgagatagaaatccagcacattgatcttgaagagaaStgagRt

fava_c18361_719	fava_c18361	719	T/G	gcatatgccactccatgtttgcatatttgcattgcggagaacaRaatgattgaattgcaatacggccagcagaggaaatattggcttcatttcttgagaa[T/G]tgagatagaaatccagcacattgatcttgaagagaaStgagRtttggagaatcttggatgctgacacaatctgcattcttgccactataaaaatgtcgct

fava_c18361_756	fava_c18361	756	G/C	agaacaRaatgattgaattgcaatacggccagcagaggaaatattggcttcatttcttgagaaKtgagatagaaatccagcacattgatcttgaagagaa[G/C]tgagRtttggagaatcttggatgctgacacaatctgcattcttgccactataaaaatgtcgctccagaggcttccgcaagtcaaaatcattgcagtaatc

fava_c18361_761	fava_c18361	761	G/A	aRaatgattgaattgcaatacggccagcagaggaaatattggcttcatttcttgagaaKtgagatagaaatccagcacattgatcttgaagagaaStgag[G/A]tttggagaatcttggatgctgacacaatctgcattcttgccactataaaaatgtcgctccagaggcttccgcaagtcaaaatcattgcagtaatcatggt

fava_c18362_1470	fava_c18362	1470	C/G	gaaaaggaaccagagttttacagtagagtgatagctgtgttaaaacgagctgaaaatgactttggattatgacattagaggattataaccattccatttg[C/G]tataatagtgttgtaKgtctaatagtttttatttatttattataatgcaaRagtcaatttgtttttgatgcagattgttttaggactaatgagtatggtc

fava_c18362_1486	fava_c18362	1486	T/G	tttacagtagagtgatagctgtgttaaaacgagctgaaaatgactttggattatgacattagaggattataaccattccatttgStataatagtgttgta[T/G]gtctaatagtttttatttatttattataatgcaaRagtcaatttgtttttgatgcagattgttttaggactaatgagtatggtcaaggttatattcttaa

fava_c18362_1521	fava_c18362	1521	G/A	gaaaatgactttggattatgacattagaggattataaccattccatttgStataatagtgttgtaKgtctaatagtttttatttatttattataatgcaa[G/A]agtcaatttgtttttgatgcagattgttttaggactaatgagtatggtcaaggttatattcttaatagtaactgttatgtgatttttttttgttggaatt

fava_c18362_179	fava_c18362	179	A/T	tttggctttacttggaggtggtgctgttttaggctctctctcaaccttccttcttctcagacttctccaaactcaaaaaagagctgttcgaccgaaatac[A/T]ctgagaatgRcactactgaattgaatggttttgagggacgcactattagtggaaagaagagcgataaggtggttagtgatgatcttttgaaagatgagat

fava_c18362_189	fava_c18362	189	A/G	cttggaggtggtgctgttttaggctctctctcaaccttccttcttctcagacttctccaaactcaaaaaagagctgttcgaccgaaatacWctgagaatg[A/G]cactactgaattgaatggttttgagggacgcactattagtggaaagaagagcgataaggtggttagtgatgatcttttgaaagatgagattgtttctgaa

fava_c18364_196	fava_c18364	196	A/G	cttatatttcaaaaagttaagtactttattgagagtcccccaccttatgaactagttttacactactaaacataatacacaaaatggctaatgatctatc[A/G]tgcattagcacatggcaattccctttttttaatatttttttccatctggctgttaaaagaaagctagaaattacaatatgaatcactcacattcaagatt

fava_c18364_534	fava_c18364	534	G/C	ttgtagttcactcattacgctgtcaagtgctggagctttaagtccaagtggtagcggcaaccatggctttgataaaacgtctccaatagctgcatctatg[G/C]tctcattYgagggatggaagtcgaagaagggaccgggaccgggggcggcaatactgtgatctgctttgtacataccatgtggtgggatgctggggagttg

fava_c18364_542	fava_c18364	542	C/T	cactcattacgctgtcaagtgctggagctttaagtccaagtggtagcggcaaccatggctttgataaaacgtctccaatagctgcatctatgStctcatt[C/T]gagggatggaagtcgaagaagggaccgggaccgggggcggcaatactgtgatctgctttgtacataccatgtggtgggatgctggggagttgacatccaa

fava_c18367_1206	fava_c18367	1206	G/C	gtttttcgccatcgattcgtccttggaatgatattccaatggcggattcgtctgttgatcatcataatcagaaatctcagcaaccgattcaccatggttc[G/C]atctttggtagcaggtttttgtctgatgctttgacaggtttctgtattcctgctagaattcaaggagaWgatgaaagccatggagttggctcggaacagc

fava_c18367_1275	fava_c18367	1275	A/T	agaaatctcagcaaccgattcaccatggttcSatctttggtagcaggtttttgtctgatgctttgacaggtttctgtattcctgctagaattcaaggaga[A/T]gatgaaagccatggagttggctcggaacagccttcctcatcttctaattcgcatcactaaccagaaaaatcaactgcataatKttScaatcttcattttc

fava_c18367_1358	fava_c18367	1358	G/T	gctagaattcaaggagaWgatgaaagccatggagttggctcggaacagccttcctcatcttctaattcgcatcactaaccagaaaaatcaactgcataat[G/T]ttScaatcttcattttctatcaggggcatattgtaccagatatgaaaacatgggaaggttatgaagacacaaagattgtctggttggattcaWgtttttt

fava_c18367_1361	fava_c18367	1361	G/C	agaattcaaggagaWgatgaaagccatggagttggctcggaacagccttcctcatcttctaattcgcatcactaaccagaaaaatcaactgcataatKtt[G/C]caatcttcattttctatcaggggcatattgtaccagatatgaaaacatgggaaggttatgaagacacaaagattgtctggttggattcaWgttttttgta

fava_c18367_1451	fava_c18367	1451	T/A	gcataatKttScaatcttcattttctatcaggggcatattgtaccagatatgaaaacatgggaaggttatgaagacacaaagattgtctggttggattca[T/A]gttttttgtaactttatttattttctgttattgcMaccattgacattggtgtttttttaatgtgttttaagaaactgaaagtgtgttcactacttctttt

fava_c18367_1486	fava_c18367	1486	A/C	atattgtaccagatatgaaaacatgggaaggttatgaagacacaaagattgtctggttggattcaWgttttttgtaactttatttattttctgttattgc[A/C]accattgacattggtgtttttttaatgtgttttaagaaactgaaagtgtgttcactacttctttttatatatgtaagcattatatgtagtattttacgtt

fava_c18367_288	fava_c18367	288	T/G	actttaaatcaagtggcatcgaatttcacacattggagaaatatcaaatatcagaaacacaatatagaaccattgttttgaaattcataaggtagagaaa[T/G]Waattcatgggaatgaagagtactggtggagaaatagttcaagttcaaggaggtcacattgttagagcaacaggtagaaaagatagacatagtaaggtat

fava_c18367_289	fava_c18367	289	T/A	ctttaaatcaagtggcatcgaatttcacacattggagaaatatcaaatatcagaaacacaatatagaaccattgttttgaaattcataaggtagagaaaK[T/A]aattcatgggaatgaagagtactggtggagaaatagttcaagttcaaggaggtcacattgttagagcaacaggtagaaaagatagacatagtaaggtata

fava_c18367_396	fava_c18367	396	G/A	tgggaatgaagagtactggtggagaaatagttcaagttcaaggaggtcacattgttagagcaacaggtagaaaagatagacatagtaaggtatacactgc[G/A]aaaggccctcgcgatcggcgcgttcgattatcagcgcataccgcgattcaattctacgatgttcaagatcggctcggctacgataggccgagtaaagctg

fava_c18367_874	fava_c18367	874	T/C	tgattccatgaattttcaaagctactcttctgatataatctcaagaacaaacaactcaacacaggatcttggtctttcacttcattcattccaagataat[T/C]cagtttcaaatgatcaaacacttttctctggatcaaatcatgttggatttgatgctaactatcataggattgtgaattggaacaatgatcatcatggaac

fava_c18379_1289	fava_c18379	1289	C/T	accctccgaattgcattgaattttgaattgtcaacatcaccagcttccgtgatcggggcatcataatcgtatgaagtgagatcaggcttgtagtcagcct[C/T]gtctgcaccagtatttgctccattataaaatccgaagtttgaaccaccatgtgccatatataggactgcagaaccattttttttcaaaatttcttcaaga

fava_c18379_1786	fava_c18379	1786	C/T	tttgtcatctccatacgaaccatattcattttcgacctgaaccattattatagggccaccattgtcatataggagaggaacaaattttggaagcagcttg[C/T]cccaccatctttcaaccaggttaagaaaagcaggatcagatgatcttagtttgggggatgggttcatggaagataaccaacttgggaaaccaccccagtc

fava_c18379_77	fava_c18379	77	G/C	aataggtaacgcgccaatattagaaagttattgataaccagatattaatgttattacatacaaaccacaacttttt[G/C]ataagcctggtaaaaaaaaccgattgaatcaacttgtcatgaagttttgcttcacatgttgtcttacaagcataacaacaatggctataagaggtttctt

fava_c18379_990	fava_c18379	990	G/A	ttggtcttgcaccccttccttcagaagaacatgagataaatacttgggctctgtcatgcacctttggtatggacaaaattcttctccctctccgtgcttc[G/A]tattcagtgacatacaataaaaaaccaaaaaactggcccacattctccattgacatcggattttttgattcaaaagcattggaagaattggtgaaatcaa

fava_c18380_715	fava_c18380	715	C/T	taacaccacgttcatgaaacctattagaaccagcatcacttacctcaccaaaactcccagaattatcatctataccaccatcctgcgaactgaaagacag[C/T]gaactcctccggttagtactacatctgctaacagcgttaggcgggatagtattacaaatcagcacatgacgaaactcatcctcgagcctcgacatagcaa

fava_c18380_868	fava_c18380	868	G/C	tacaaatcagcacatgacgaaactcatcctcgagcctcgacatagcaagctggatcgcgctctcagcgcgatccaaaatgatccgatcagttttcgcggt[G/C]gaatgatcaggcggcgagatcgagaaatgttcgatccagtgaatgatctcatcaacagcgagaaaatactcgaccggatcattaggaggatcRaaaagcg

fava_c18380_961	fava_c18380	961	A/G	tcgcggtSgaatgatcaggcggcgagatcgagaaatgttcgatccagtgaatgatctcatcaacagcgagaaaatactcgaccggatcattaggaggatc[A/G]aaaagcgaagtcgattggcgagaggactcggtgtatgtagaagagtcatcgagtatgattttctccgcggcttcgaaacggtcaagctcttcgtcttcgg

fava_c18381_167	fava_c18381	167	C/A	atgcttctgatgacgattcttgcgRcataacctcagggctcttgctcagggagtggtaaactatgcaccggaatgaagaagacgactaacggatggaggt[C/A]aagtgagtttcgaggattatctgccggagcaaattccggtgattcatcgatagatctgccggagcaaattccgagagtatgatgagagatgaagaaagtg

fava_c18381_285	fava_c18381	285	A/G	tatctgccggagcaaattccggtgattcatcgatagatctgccggagcaaattccgagagtatgatgagagatgaagaaagtggttcgtgattgtggttt[A/G]tggagtcgcagagagaaagggagaagatgaaggtgagggtgagaggatgaagaagatgatggtgaatggtgataaatctggaaaaaaaaatgatcgagag

fava_c18381_395	fava_c18381	395	G/T	agagagaaagggagaagatgaaggtgagggtgagaggatgaagaagatgatggtgaatggtgataaatctggaaaaaaaaatgatcgagagaagctgaat[G/T]aagatggatgaacggttatatagttgtggaggtttgttagaattcaaYYaggacttgattgatttgagccattggattgagaaagtcggttatgagatag

fava_c18381_443	fava_c18381	443	C/T	gatggtgaatggtgataaatctggaaaaaaaaatgatcgagagaagctgaatKaagatggatgaacggttatatagttgtggaggtttgttagaattcaa[C/T]Yaggacttgattgatttgagccattggattgagaaagtcggttatgagatagaggggctggccatggactgtttggtgcattactggtcagaggagtatg

fava_c18381_444	fava_c18381	444	T/C	atggtgaatggtgataaatctggaaaaaaaaatgatcgagagaagctgaatKaagatggatgaacggttatatagttgtggaggtttgttagaattcaaY[T/C]aggacttgattgatttgagccattggattgagaaagtcggttatgagatagaggggctggccatggactgtttggtgcattactggtcagaggagtatgg

fava_c18381_91	fava_c18381	91	A/G	aaggtctaattaattcaatctaaagaaaaccgaaagaagaaacctaagtattctaactacaattaaatgcttctgatgacgattcttgcg[A/G]cataacctcagggctcttgctcagggagtggtaaactatgcaccggaatgaagaagacgactaacggatggaggtMaagtgagtttcgaggattatctgc

fava_c18384_1339	fava_c18384	1339	G/T	tgcaagtataacattttgaaaagtagtagcctcggcgaagaggaagtgtagatgatctcagagaagtttctagatgccattttgagaaatctgcatactt[G/T]tcgatcatgcataataggctcaacatgtcaatacagttcaatacaggcctatgcatttgtccttgttgctgctgtgctttgtgctgtaatcatgtagcga

fava_c18387_1057	fava_c18387	1057	A/G	tagagatcctaaatcaaattcatctgaaaagaaatcaagacactccccggttagtaagagaataggttcacctgcaaaatttaatgacgaggatgagttt[A/G]atcctgaaagggtagctagtcatctggcttctgagtataatcattatgatagcaatgaaaggagtaagaaaggccaggatattaaatgtgatgaatcctc

fava_c18391_1171	fava_c18391	1171	C/T	gtgcagacgagcgagcactatcccatacaatgacatgactatccgggtagaacccctgatccaagtacaccgtaactttcttaaagaaagccgaaaactt[C/T]gggtacaaatgYttttcccttttcgaaatccctgccaccacaggatccttaccgtcttcttccaatatcctcccagttatcctgagcgaccaagacgggt

fava_c18391_1183	fava_c18391	1183	C/T	gagcactatcccatacaatgacatgactatccgggtagaacccctgatccaagtacaccgtaactttcttaaagaaagccgaaaacttYgggtacaaatg[C/T]ttttcccttttcgaaatccctgccaccacaggatccttaccgtcttcttccaatatcctcccagttatcctgagcgaccaagacgggtcctcaacatcac

fava_c18391_1355	fava_c18391	1355	C/G	gagcgaccaagacgggtcctcaacatcaccatccaatttggtctgattcgaaaacgtattatacacataaacgcgaagagttttcctaaacgaaagagga[C/G]ttttaacagcctcctgcacatcaatctttttcctattcaaagccgcatctatctgagcctcgagttcaagtaRccgagtataaagagcagactcaggaac

fava_c18391_1428	fava_c18391	1428	A/G	cgaagagttttcctaaacgaaagaggaSttttaacagcctcctgcacatcaatctttttcctattcaaagccgcatctatctgagcctcgagttcaagta[A/G]ccgagtataaagagcagactcaggaacaagcttagcYaccttttcaggtatcaaattatcaggtaaattcctctttttcctcataggagcaggagttaac

fava_c18391_1465	fava_c18391	1465	C/T	cctcctgcacatcaatctttttcctattcaaagccgcatctatctgagcctcgagttcaagtaRccgagtataaagagcagactcaggaacaagcttagc[C/T]accttttcaggtatcaaattatcaggtaaattcctctttttcctcataggagcaggagttaactccatagttttaaaggcagaggtttgattaccactcg

fava_c18391_1869	fava_c18391	1869	A/C	tgttgctgttgtgccattacgtggttttgaggttgtgatagctgaaaatgaccggggaagggagaagatgaagcaccttgcatttggggctgagaatgag[A/C]gagaagatgaattggttgtgggtttacaggaattgatgaattgctcaaaaacgacggtggcgccgctgtattattgggtggattgttgttgttgttcatc

fava_c18391_343	fava_c18391	343	A/G	aaaaatgaaggatggaatataaaatttagcgaggctggaacaaggctgattgatgtccaaaaaattaaccacctccccgtctcacaattttccatcaaat[A/G]ctttaaggagcatcagttcgagcaattttccggttcagatagcgaatcacagcatcctcaacccatggttgattgtagaattcagaacgttgttcatttt

fava_c18391_462	fava_c18391	462	C/T	cgagcaattttccggttcagatagcgaatcacagcatcctcaacccatggttgattgtagaattcagaacgttgttcattttcggcattatgactggcat[C/T]tccagcaaccagctttagatccttgctctgagaagctatcaatgcgttaataaactctccaggagactgactaaaaccaagaaggaaagctcgtctctta

fava_c18391_655	fava_c18391	655	C/A	gtctcttaagatgatcatggatcttctttacggaagcgcatatcacttcatcaaaagcatcaatctctttgtgcttctccatacttgttaagaatgcaga[C/A]atatctttctctagtggaagaggcacatcaacttgcacatcataacatgcagttccggctggacagtttccagaaagtttgattttatgctccaggtgta

fava_c18391_943	fava_c18391	943	C/T	aagcatcacacatgaaaaatgaagggtcattcggactctgcagtttcctgaacttcacatagttccaaagagcagccattatcctagggcgggtatcaaa[C/T]tcaatccctaaaactttagacaacggtgccgaaaccataaacttctcSggcgagtaattcactcttaacctaataaccgcagtgaactctttgtctcctt

fava_c18391_991	fava_c18391	991	C/G	tgaacttcacatagttccaaagagcagccattatcctagggcgggtatcaaaYtcaatccctaaaactttagacaacggtgccgaaaccataaacttctc[C/G]ggcgagtaattcactcttaacctaataaccgcagtgaactctttgtctccttttctcttcacctcaaaaccatcctgttgtgcagacgagcgagcactat

fava_c18393_1355	fava_c18393	1355	A/G	tgttatctacaaagcaaccaggttgtccacagttgtacatgtatagctctgcagacagagttattccggccgattcagtggagtcgtttgtcgatgcaca[A/G]cgtaaggctggacatgatgtgagggcttgcaattttgtctcttcaccgcatgttgaccacttYcgaaatgacccgaaWttgtatgcttctcagctcaatc

fava_c18393_1418	fava_c18393	1418	T/C	ttccggccgattcagtggagtcgtttgtcgatgcacaRcgtaaggctggacatgatgtgagggcttgcaattttgtctcttcaccgcatgttgaccactt[T/C]cgaaatgacccgaaWttgtatgcttctcagctcaatcagtttttagaggagtgtgttgttgaccatagtaaaattcgctaattcattgtttggctcactc

fava_c18393_1433	fava_c18393	1433	A/T	tggagtcgtttgtcgatgcacaRcgtaaggctggacatgatgtgagggcttgcaattttgtctcttcaccgcatgttgaccacttYcgaaatgacccgaa[A/T]ttgtatgcttctcagctcaatcagtttttagaggagtgtgttgttgaccatagtaaaattcgctaattcattgtttggctcactcatacaRcatatagtt

fava_c18393_149	fava_c18393	149	A/T	cattcctcgttcctttgtttcttcacacgcttttccaacagggctgtcttctcaaaacgacatcgtcctgtgtcgttttcgttgacagccccattcacat[A/T]ttcacattcagttgtgataatattaagggtcgagRgttgaaggtttctgcaatgagttattttcctggaccaattcagagacctcttgtggctgctgctg

fava_c18393_1524	fava_c18393	1524	A/G	tgacccgaaWttgtatgcttctcagctcaatcagtttttagaggagtgtgttgttgaccatagtaaaattcgctaattcattgtttggctcactcataca[A/G]catatagtttttgatttttttttWaaattRttttttagaatagtcattccattcattgatgatggttaggattttcaaatgttcatccctttaggggatc

fava_c18393_1548	fava_c18393	1548	T/A	gctcaatcagtttttagaggagtgtgttgttgaccatagtaaaattcgctaattcattgtttggctcactcatacaRcatatagtttttgattttttttt[T/A]aaattRttttttagaatagtcattccattcattgatgatggttaggattttcaaatgttcatccctttaggggatcMtataattgttcactgtcaactta

fava_c18393_1554	fava_c18393	1554	A/G	tcagtttttagaggagtgtgttgttgaccatagtaaaattcgctaattcattgtttggctcactcatacaRcatatagtttttgatttttttttWaaatt[A/G]ttttttagaatagtcattccattcattgatgatggttaggattttcaaatgttcatccctttaggggatcMtataattgttcactgtcaacttatcacca

fava_c18393_1625	fava_c18393	1625	A/C	catatagtttttgatttttttttWaaattRttttttagaatagtcattccattcattgatgatggttaggattttcaaatgttcatccctttaggggatc[A/C]tataattgttcactgtcaacttatcaccaattcattgatattgcaaatgaaaggcattgcaaattgcaaatttctgttgtaaataatgtatttatattcR

fava_c18393_184	fava_c18393	184	G/A	caacagggctgtcttctcaaaacgacatcgtcctgtgtcgttttcgttgacagccccattcacatWttcacattcagttgtgataatattaagggtcgag[G/A]gttgaaggtttctgcaatgagttattttcctggaccaattcagagacctcttgtggctgctgctgctgttgcggttgcYtctttctctactgatgcatct

fava_c18393_263	fava_c18393	263	T/C	gtgataatattaagggtcgagRgttgaaggtttctgcaatgagttattttcctggaccaattcagagacctcttgtggctgctgctgctgttgcggttgc[T/C]tctttctctactgatgcatctgataagttttcattccttggatcgtcgtctcgtgattgttccacttccaatttggaatgttcttcaagttttaactctt

fava_c18393_806	fava_c18393	806	G/T	agcagaagcatttaaagaagtatgcagaatggtatacttcgaagggatatcatgtcattacctttacgtttccgatgagtgaggtaatgagttatcagcc[G/T]ggaggaaaagcggaRcaaaatgttcacatgcttgttaatcacttggctgattggttagaagaggagaatgaaaagaatcttgtgtttcatactttcagca

fava_c18393_821	fava_c18393	821	G/A	agaagtatgcagaatggtatacttcgaagggatatcatgtcattacctttacgtttccgatgagtgaggtaatgagttatcagccKggaggaaaagcgga[G/A]caaaatgttcacatgcttgttaatcacttggctgattggttagaagaggagaatgaaaagaatcttgtgtttcatactttcagcaacactggatggttaa

fava_c18399_1203	fava_c18399	1203	G/A	taggaactccgaagatcacttttggtccttctttttgaagaccagaccttgccaatctgtgtgcatctgagttcttttcattcttgatgttcctaccaac[G/A]ttgaacactttatgatcttcattcaaattaagtaacctcgattcactgggattagctgattctgaagcataagtgccttttgaggtcttgtcttttcgcc

fava_c18399_695	fava_c18399	695	C/T	acttcaactttaccgcaccttccatcagcagctgtttgttttcctttacttactcttgatgtgggtggctttttagtggggtgagaattggttgaagatg[C/T]acgtgaggaatttgaggtttgccttgctcctggggtttcagaataggatgccctttccatctgattttcacgctgagatgcattactagcatgacttgaa

fava_c18399_969	fava_c18399	969	A/T	ggatcactctacccaaaacctgaggctttccagatttagaagtgggcttgcagtcagctcctaatttttctttggtgtgttttattgaactatttttcca[A/T]ccgcgtgatcctgaagcatgtggtaacaaagaatctgcaatcttatcggaatcatttccatcattaatcctacttgatcccccagcaacataatgtttgc

fava_c18404_732	fava_c18404	732	T/C	tgcaatgtaatgtttctttctcatcctcatctgcttgaccaattttccaaccatttcttgtattggatcagtaaatcttagagcttggtaattaactctg[T/C]atttcaacttctgatactcagtacccaaactgtttgcaagcctgtagtcaaacttgttgagttgaacaacacgctttttcacgagtacaggcagtatacg

fava_c18406_1198	fava_c18406	1198	A/G	atttctcctcgacttgctgtatattgtatttgttggaagtaattcatattcaaaagatgttattattcagaagcagtagaacttctgaggatgttagcca[A/G]aaactgtaagcttttgagactgcttagttagaaatagtagtatgatataatcataagttgttattaatcagaagcagaagcttYcaaggttaMgttcaga

fava_c18406_1282	fava_c18406	1282	C/T	ctgaggatgttagccaRaaactgtaagcttttgagactgcttagttagaaatagtagtatgatataatcataagttgttattaatcagaagcagaagctt[C/T]caaggttaMgttcagaagtatgcttaacttctggKgttctggattctgagtttgttagcgtagctgttaagtttagtatttYYtaatttgtaccttttaa

fava_c18406_1291	fava_c18406	1291	C/A	ttagccaRaaactgtaagcttttgagactgcttagttagaaatagtagtatgatataatcataagttgttattaatcagaagcagaagcttYcaaggtta[C/A]gttcagaagtatgcttaacttctggKgttctggattctgagtttgttagcgtagctgttaagtttagtatttYYtaatttgtaccttttaacttagtcat

fava_c18406_1317	fava_c18406	1317	G/T	actgcttagttagaaatagtagtatgatataatcataagttgttattaatcagaagcagaagcttYcaaggttaMgttcagaagtatgcttaacttctgg[G/T]gttctggattctgagtttgttagcgtagctgttaagtttagtatttYYtaatttgtaccttttaacttagtcattaagtttaccttttattagggtctac

fava_c18406_1364	fava_c18406	1364	C/T	aatcagaagcagaagcttYcaaggttaMgttcagaagtatgcttaacttctggKgttctggattctgagtttgttagcgtagctgttaagtttagtattt[C/T]Ytaatttgtaccttttaacttagtcattaagtttaccttttattagggtctacatgacaatttactttgtgtataaatagactagtggtagctgttattt

fava_c18406_1365	fava_c18406	1365	T/C	atcagaagcagaagcttYcaaggttaMgttcagaagtatgcttaacttctggKgttctggattctgagtttgttagcgtagctgttaagtttagtatttY[T/C]taatttgtaccttttaacttagtcattaagtttaccttttattagggtctacatgacaatttactttgtgtataaatagactagtggtagctgttatttg

fava_c18408_401	fava_c18408	401	T/A	ccatgggtctcggaggttatttggcggcaaaaggtgaagctgatcattacaatagagaattgaaaagggaacaagatgaaatcatcgccgtaccagaaac[T/A]gaagctgctgaggtgggagaaatattggcggaatatgggatagaggaacatgaatatggacctgtggtgaatgctctcagaaagaatcctcaagcRtggc

fava_c18408_497	fava_c18408	497	G/A	aaacWgaagctgctgaggtgggagaaatattggcggaatatgggatagaggaacatgaatatggacctgtggtgaatgctctcagaaagaatcctcaagc[G/A]tggctcgatttcatgatgaaatttgagctgggactagagaagccagatccaaggagagcattgtatagtgctatgacaattgctgttgcttatgtattgg

fava_c18408_844	fava_c18408	844	A/T	ttggaaactactcttatcggcgccgttgcctctgcagctgcttatggtttggcaaaggctttccatccatagttggtagtagtgaacactagctcatttt[A/T]gaattWttgtttccttctttttggctattagctcttttggtggcaacttttcatgaacaacttttaacttttggcttcagcttttatggttgatgatatc

fava_c18408_850	fava_c18408	850	T/A	actactcttatcggcgccgttgcctctgcagctgcttatggtttggcaaaggctttccatccatagttggtagtagtgaacactagctcattttWgaatt[T/A]ttgtttccttctttttggctattagctcttttggtggcaacttttcatgaacaacttttaacttttggcttcagcttttatggttgatgatatcacttta

fava_c18413_279	fava_c18413	279	T/G	gggaagagagtttgtggccggtggaattggaggcacggcgggtatcgtggcgagttatcctttcgacactcttcgtgtcatgcaacagcagtccggcagt[T/G]catctgctgttggcatctttagaaacttgttggctaaggaaggtcgctttgctctctaccgtggcatggctctKcctttggtttctgttggttttcagaa

fava_c18413_353	fava_c18413	353	T/G	gtgtcatgcaacagcagtccggcagtKcatctgctgttggcatctttagaaacttgttggctaaggaaggtcgctttgctctctaccgtggcatggctct[T/G]cctttggtttctgttggttttcagaatgctataatttttcaaagttataccatttttacgaggatttttagcccttatggttcttccaatggacctcatt

fava_c18413_629	fava_c18413	629	T/C	ttaaaattcgcattcaacttcaaaaaaatattgagaatctttcaaaaaacaaaaagagcactcctttgatattggctaagaagatttggaaaaatgaggg[T/C]gtttgtggcatttatcgaggatttggcatcactgttcttagagatgcacctgctttggcctcctactttgtaRcatatgagtacacgagggagaaactcc

fava_c18413_702	fava_c18413	702	A/G	ggctaagaagatttggaaaaatgagggYgtttgtggcatttatcgaggatttggcatcactgttcttagagatgcacctgctttggcctcctactttgta[A/G]catatgagtacacgagggagaaactccaccctggttgtagagaaagttgtcaagaaagcgtgcatactatgtttatagcaggaggagtagcagggattgc

fava_c18413_890	fava_c18413	890	G/A	tagcagggattgcgagttgggtttttaactatcctacagatgttataaagactagattgcaggctcaaacatcatcttcattgaaatacaaaggtatctt[G/A]gattgtaccttaaagataatcaaagaagaaggttcaattgtgttatggcggggattaggagcaacacttgttcgagcttttgttatgaatagtgctattt

fava_c18415_1133	fava_c18415	1133	G/A	agacattgttgatatatgttgaatttccgagactattaatccaaaggcgaaaagctctttcttctctagagtcttcggtgtcatcaggcaaggcttgaag[G/A]agtgaaatctgttttgtctggtctgtaagtccattcctgtgctggaaaatatgtgcaacaaaggcaagattaagatttggggaaccttccacgatgtctt

fava_c18415_1604	fava_c18415	1604	A/G	tatcatcaagcaactcaagaagttgaggagttttctttagattgagatcggccaatagttgtatcttaataatctgtgatatcactccaagcactagatg[A/G]cgccttccttcaatgaagtcttgtgtgccaatgttgactacggtacatccaatggctttagcagaattgagacaaagtgtgtgattttcattcctttccc

fava_c18415_1719	fava_c18415	1719	C/A	gaagtcttgtgtgccaatgttgactacggtacatccaatggctttagcagaattgagacaaagtgtgtgattttcattcctttcccatggattgagtaat[C/A]ttttcgtattgattgcacgttcatcaatagtccccggaactgccacattgataagcttgcagagaagaacaccgtcttttgcaacttcgaagagctcatt

fava_c18415_545	fava_c18415	545	T/C	taagaatcatcttttgattcacctcagtgatgtcttctggaagaaggaatatagaacagcctagctttcttgcaatactgatgatgtagctggcattcat[T/C]tttttttcttcatctgttactcctttagttactagtccccaatttacagYtcttggctgcactgaactaagaagctcaaggaaaaatattccatccgata

fava_c18415_595	fava_c18415	595	C/T	atagaacagcctagctttcttgcaatactgatgatgtagctggcattcatYtttttttcttcatctgttactcctttagttactagtccccaatttacag[C/T]tcttggctgcactgaactaagaagctcaaggaaaaatattccatccgataaacgtttgtccttaaaactgttcatactgctttgacttcctgtgctgcta

fava_c18415_806	fava_c18415	806	C/A	tagcccattccaaaatatcagcatcagttatttccttcccgcgagcatgaaatcgcaagttctttagaagttgtaggatgttgtatcgcatcaattgcca[C/A]agataagcgagtattaactttttatttccYtgcacaatgtcatttccagcaacatttaccaatgagaatttaagttgttttcctattttcacaacttgat

fava_c18415_836	fava_c18415	836	C/T	tttccttcccgcgagcatgaaatcgcaagttctttagaagttgtaggatgttgtatcgcatcaattgccaMagataagcgagtattaactttttatttcc[C/T]tgcacaatgtcatttccagcaacatttaccaatgagaatttaagttgttttcctattttcacaacttgattgcaattctcaacttttttaaacggcatct

fava_c18419_1346	fava_c18419	1346	A/G	ttggcaatcagtcctgattacaataccttcaatattctaatcaaatacttttgtagtaaaaacatgtatctattagcttatcgaaccgtggaggacatgc[A/G]tagtaaaggctatcaaccaacggaggagctctgttcttctttaatattccatcttggtcaagcaaaagctcattcagaggcgttttctatttacaatatt

fava_c18419_614	fava_c18419	614	A/G	ctagttccggatcttgttacctatagcacccttattgcaggctgtgttaaagtaaaagatggatatcctaagacactagagctaattcaggaattgcagg[A/G]Maataatctgcgaatggatgacgtaatttatggggcagttctggcagtatgtgcttccaatggtaaatgggaagaagcagaatgctattttaagcagatg

fava_c18419_615	fava_c18419	615	C/A	tagttccggatcttgttacctatagcacccttattgcaggctgtgttaaagtaaaagatggatatcctaagacactagagctaattcaggaattgcaggR[C/A]aataatctgcgaatggatgacgtaatttatggggcagttctggcagtatgtgcttccaatggtaaatgggaagaagcagaatgctattttaagcagatga

fava_c18422_1554	fava_c18422	1554	T/A	aaaccaggcattttccctgcccaacaaaagaacctaaaagcaggcttcctagcatgtttaaaccgatgcaacacatcaacaaccaaatcacgtgataaca[T/A]aactccacactcatctagaaccgcctccatgttcctatccaaagcaaacaattcatcaatcaccttgcataccctatcaacctcatccggatctgcacat

fava_c18422_1801	fava_c18422	1801	T/C	cactaccaaacgacctataattatccaagaacataaactttccttgaaaactagaaatgggtaacttaaaaatcagcactttttcttgtaaaaccatgaa[T/C]gaagaggtgtgtgacaaaggaaatggaatttgaaaacaagaatgaaggaaagaacaattaagagaagaatgaaacaaacgagacaagggcatagagctac

fava_c18422_216	fava_c18422	216	A/G	aaaataaagagcttaataataattttttcaaagaaaatgattgacacaaggggtttttgctaacctgcaagtaaatgaatcgagtaaagtaatcataaaa[A/G]ctacctataagattatgtagccactgttctctttgagtttttcttcatcatatcaacccaactggctaagacattagacacttcaaacttaccagcaaaa

fava_c18422_527	fava_c18422	527	C/T	ttcttcaatataattgcatgcctcatctggcctgccttgtcttataagcccgccgatgaataccgtgtacgagttatcatcaggacaacaccccttgtga[C/T]gcatctcatcccaaacagctcggcccatctcgtaattctttgttgcaaaataggatttcatgatcatattgtatgtgtgaattgttggtYcgatgccact

fava_c18422_617	fava_c18422	617	C/T	ccccttgtgaYgcatctcatcccaaacagctcggcccatctcgtaattctttgttgcaaaataggatttcatgatcatattgtatgtgtgaattgttggt[C/T]cgatgccacttcgaatcattttcttgtatactctcactgcatcatctggcatgtgttggcttaccatcagttttatcaaagcattgtatgtccttccgtc

fava_c18422_775	fava_c18422	775	C/T	ggcttaccatcagttttatcaaagcattgtatgtccttccgtcaggtggataacctctttcgcgcatttccttcagtagatcgtataccgtatccatttt[C/T]ctctgcctcccaaacccggtgatcaaacaagtgtacaatgccgcgtcaggttgacacccgcgatcaaccatttcRttaaaataatcaacagcttccYgca

fava_c18422_850	fava_c18422	850	A/G	gtagatcgtataccgtatccattttYctctgcctcccaaacccggtgatcaaacaagtgtacaatgccgcgtcaggttgacacccgcgatcaaccatttc[A/G]ttaaaataatcaacagcttccYgcatcatatttcgtttgcacaagtcctgtatcagaatcgtataactacgaacatcgggcRacggaccttttgctttca

fava_c18422_872	fava_c18422	872	C/T	tttYctctgcctcccaaacccggtgatcaaacaagtgtacaatgccgcgtcaggttgacacccgcgatcaaccatttcRttaaaataatcaacagcttcc[C/T]gcatcatatttcgtttgcacaagtcctgtatcagaatcgtataactacgaacatcgggcRacggaccttttgctttcatgacttcaaataacttaatgct

fava_c18422_932	fava_c18422	932	G/A	cccgcgatcaaccatttcRttaaaataatcaacagcttccYgcatcatatttcgtttgcacaagtcctgtatcagaatcgtataactacgaacatcgggc[G/A]acggaccttttgctttcatgacttcaaataacttaatgctatccgacttcttctgacattttaacaacccttgtagcataatattatgcgcaacaatatc

fava_c18424_498	fava_c18424	498	C/G	aacgtaggaagttctcacagtcgtcggagacacggtggtggcggtggaagtggccgacgtgttcatccgccggtgactcctcctctacaagaagctgcgg[C/G]taatcagtttgctcaccctgccgctgctcaaccgtatcagaattacccgaataactatcctccgccggcgagtgttccgatgcctttaccggcgccgtat

fava_c18431_1028	fava_c18431	1028	G/C	gcattccctctacacttaacactccatcctttagtactgataaaatgattgagaaaaaRcagttgttgcgagaatcggttcgaaaagaaatgggattaac[G/C]gataatgatatgcttgtgatatctctgagtagcatcaaccccgcaaagggccagctgctgcttcttgaatcagcaagctcRgtagtagaacatatacagt

fava_c18431_1109	fava_c18431	1109	G/A	gaaaagaaatgggattaacSgataatgatatgcttgtgatatctctgagtagcatcaaccccgcaaagggccagctgctgcttcttgaatcagcaagctc[G/A]gtagtagaacatatacagttggaagatggtaaaaaaatgaaaaaattatcaaatataaaggaaggcctgtctactcaggcaagaagacatcgtgctagaa

fava_c18431_986	fava_c18431	986	G/A	ttgtgcctttgtctgttaatgaggaactggcatttgtagctggcattccctctacacttaacactccatcctttagtactgataaaatgattgagaaaaa[G/A]cagttgttgcgagaatcggttcgaaaagaaatgggattaacSgataatgatatgcttgtgatatctctgagtagcatcaaccccgcaaagggccagctgc

fava_c18432_1060	fava_c18432	1060	T/C	ttaaggttccaccaacaccgcatgttccggcgaggaatagtaagatcatatctatgctgaaaagtgcttgaagtattgtgaaatcttcacctttttttgg[T/C]ggtgagaaaatattttcccaccatctagtttctttttcttcggtcgtcgtcgtcatcgttgtcatcgtggtcggtggtggtgagatattatcaccttgat

fava_c18432_1295	fava_c18432	1295	C/G	cggaaccttaaccttttgaatcttcttttgttcaacaaaaacaacagcaagtggcagaaacaagagaataagaacaacagaagcacttcctatatattca[C/G]tttgtttaaacgacactttttgctgtagtataatcataaccaacaaaaaactagcaagaccaagtgaaatatacagaaacttataaaaaacattaagttc

fava_c18432_1438	fava_c18432	1438	A/G	acaaaaaactagcaagaccaagtgaaatatacagaaacttataaaaaacattaagttcgttgttctgtttaaccggtttcatgtaccgaatggttcgaag[A/G]aaaagaaaagaaattgcagcaggaagccaagcaatgagaagaatcaaagcttttgggtcattgaagtaaatagcattataaagctgtgtgatgatagcac

fava_c18432_1650	fava_c18432	1650	C/T	acatagccttttagaatccccaacacaacaccgcgagtttcagggaaattcttaacacatgtaacaagtgaacccgtgtttgaaaaagactgagaattcg[C/T]acctatgcatatgtaaagacacatttgccaaacttttggttttggtatttttttggatactgctaaccatatcatgaagtagccgaagaaattaagaagg

fava_c18432_1876	fava_c18432	1876	G/T	gtggtgttatttcgttgattaacccggagaagacaccaaggtttcctcctaagtctttgaagaaactaaggaggttgagagttgtttggtcgtagcctaa[G/T]gagttttttatcacgccggagtatagaccgaacatgtacgtggctccggaggctgacatgattagaaaggaagcgaacaccacgaaccaccgtcctgtga

fava_c18432_276	fava_c18432	276	C/T	aagacccaaaaatataaagttgaacaccaaccgaagaatatgtatccattaaattggattaattgataggtgtgcatacactaacgaagagttttttctt[C/T]ttgttctctctcgccaccgttatgaaccaccgccaactgtgccgtatctcctccaacctccaccgcttccgaccggtacctcttatatatatccccttta

fava_c18435_1039	fava_c18435	1039	G/T	tggctgatgttgtagtcgaagcYgagtataataaaatggatgcaagaaatattgcaatggttttcgctccaaatatgactcaaatgtctgatccattaac[G/T]gctcttatgcacgcggtccaagtgatgaaYttacttaaaacactgatattgaagacacttcgagaYcgcgaagaaacatcRacagaagaatattcatcaa

fava_c18435_1069	fava_c18435	1069	T/C	ataaaatggatgcaagaaatattgcaatggttttcgctccaaatatgactcaaatgtctgatccattaacKgctcttatgcacgcggtccaagtgatgaa[T/C]ttacttaaaacactgatattgaagacacttcgagaYcgcgaagaaacatcRacagaagaatattcatcaatgtcatctcattcatcagatcRtcaatcag

fava_c18435_1105	fava_c18435	1105	T/C	ctccaaatatgactcaaatgtctgatccattaacKgctcttatgcacgcggtccaagtgatgaaYttacttaaaacactgatattgaagacacttcgaga[T/C]cgcgaagaaacatcRacagaagaatattcatcaatgtcatctcattcatcagatcRtcaatcagaagtagatgactatgacagtcaacaagaaatggata

fava_c18435_1120	fava_c18435	1120	A/G	aaatgtctgatccattaacKgctcttatgcacgcggtccaagtgatgaaYttacttaaaacactgatattgaagacacttcgagaYcgcgaagaaacatc[A/G]acagaagaatattcatcaatgtcatctcattcatcagatcRtcaatcagaagtagatgactatgacagtcaacaagaaatggatactaatggtgaatcca

fava_c18435_1161	fava_c18435	1161	A/G	gtgatgaaYttacttaaaacactgatattgaagacacttcgagaYcgcgaagaaacatcRacagaagaatattcatcaatgtcatctcattcatcagatc[A/G]tcaatcagaagtagatgactatgacagtcaacaagaaatggatactaatggtgaatccaaaagagccaagttagattatgatgatcatgatgactacagc

fava_c18435_1282	fava_c18435	1282	A/G	atgacagtcaacaagaaatggatactaatggtgaatccaaaagagccaagttagattatgatgatcatgatgactacagccacaacattgaagaggcagc[A/G]tctaactataatgtggaatctgttatatcagttactgatacaaaaactgaggattcttgtttgagttccagttctagagacgactcgacaacaactgaag

fava_c18435_1385	fava_c18435	1385	T/A	taactataatgtggaatctgttatatcagttactgatacaaaaactgaggattcttgtttgagttccagttctagagacgactcgacaacaactgaagga[T/A]cgaatgatgatacaacaagtagtccatcRattgaaagtaaagaaaccaatgatgtggagatggtaaataaatgtggtgattgtgtttcatctagctaaga

fava_c18435_1414	fava_c18435	1414	G/A	ttactgatacaaaaactgaggattcttgtttgagttccagttctagagacgactcgacaacaactgaaggaWcgaatgatgatacaacaagtagtccatc[G/A]attgaaagtaaagaaaccaatgatgtggagatggtaaataaatgtggtgattgtgtttcatctagctaagaacatgagtttctggttatgagaaatgttt

fava_c18435_352	fava_c18435	352	T/C	gagcagtagtggtgagtggtggatgcggcagtggtgtgaaggggagaagaggagggcgtgacagcggcgatgaagtgtcgccggcggcgctaatgttggc[T/C]gctttgaagaaatcaatggtggcttgtagtgtggagagtcctgacgatgtgatttcgactgttcatcatcctatggagattggatggcctaccaacgtta

fava_c18435_856	fava_c18435	856	C/T	acattgatgttcattgtttggctggtttgataaaagcatggtttagggaacttccttgtggagttttggatggactttcacctgaggaagttcttgaatg[C/T]aacacagaagaagaatctgttgagcttgtgaaggagctgaaaccggttgaatcggctttgcttagttgggctattgatctcatggctgatgttgtagtcg

fava_c18435_961	fava_c18435	961	C/T	cagaagaagaatctgttgagcttgtgaaggagctgaaaccggttgaatcggctttgcttagttgggctattgatctcatggctgatgttgtagtcgaagc[C/T]gagtataataaaatggatgcaagaaatattgcaatggttttcgctccaaatatgactcaaatgtctgatccattaacKgctcttatgcacgcggtccaag

fava_c18436_1198	fava_c18436	1198	T/C	tcattcagaggaacttggctctgttcttctccggcggtgacaggaaggagttgaaacttcgagttaccggaaggatttggaaagaacaatgaatgtagtt[T/C]tgcttgattcatgtttatgtagcaattatattaaataggatagtttgcaatgcacctatctcttgtagtagtatggattttctggtcatgtattgtgttt

fava_c18436_334	fava_c18436	334	G/T	aatttacattacagggtttgctggtgtttggacgtgcaagtatggcatctagtaatccgttttttgatgatatacggggcagatcaggggttgatcctcc[G/T]caaactgaagagtcRacagaaattcctgaactcataaatgatcctatacagacYgtcgttaaacctaatggaactgtttcgagcagtgttcgtgagcttt

fava_c18436_349	fava_c18436	349	A/G	gtttgctggtgtttggacgtgcaagtatggcatctagtaatccgttttttgatgatatacggggcagatcaggggttgatcctccKcaaactgaagagtc[A/G]acagaaattcctgaactcataaatgatcctatacagacYgtcgttaaacctaatggaactgtttcgagcagtgttcgtgagcttttagagtgccctgtgt

fava_c18436_388	fava_c18436	388	C/T	atccgttttttgatgatatacggggcagatcaggggttgatcctccKcaaactgaagagtcRacagaaattcctgaactcataaatgatcctatacagac[C/T]gtcgttaaacctaatggaactgtttcgagcagtgttcgtgagcttttagagtgccctgtgtgcttaaatgctatgtaccctccaatccaccagtgctcaa

fava_c18436_655	fava_c18436	655	C/T	agcttggaaatattagatgtttggcattggagaaggtggctgcatcgttagaacttccttgtaaatatcaaggttttggatgcataggcatataccctta[C/T]tacagcaagctaaaacacgagtctcaatgtgcatttagaccttataactgtccatatgctggttcggaatgtgctgtcgtgggtgaYatacggttcttgg

fava_c18436_742	fava_c18436	742	C/T	gcatatacccttaYtacagcaagctaaaacacgagtctcaatgtgcatttagaccttataactgtccatatgctggttcggaatgtgctgtcgtgggtga[C/T]atacggttcttggtggcacaccttaaagatgatcacaaagtggacatgcatagtggttgcacttttaatcatcgctatgtcaaatcaaatccacaagaag

fava_c18437_1267	fava_c18437	1267	T/C	ttatctattgaagtagaagagtaaaaatattcaacattgtggcattttcacttggtgctatcattgctaatatagagatcttgccttgctgtggaagatt[T/C]gagtttgtatgtgaagaagcatctccaaactgtccaagctatacatgcaatgaaaaggtggYtattatccatcttaatcggccagattgaaatattttct

fava_c18437_131	fava_c18437	131	A/T	agctccgtcaaaaagagattcaaggaaaacacgagaagattccgacgtagacacagactccaacagaaagcacagaggttcgcggaggaaatcgcgcgac[A/T]gttcggattccgacggcgataaacgaagcaggaagaagaggaattcacgtaagcgatacagtagtgattctgaatctgattcggattctgaaggacgcag

fava_c18437_1329	fava_c18437	1329	C/T	attgctaatatagagatcttgccttgctgtggaagattYgagtttgtatgtgaagaagcatctccaaactgtccaagctatacatgcaatgaaaaggtgg[C/T]tattatccatcttaatcggccagattgaaatattttctgatcctttaagcaaaaatttcaattgtaatgatgttcttgcatgtatcttttttatttgaat

fava_c18440_1420	fava_c18440	1420	A/T	ttttattggccaagtgcccataatcaggactggttgcaaaatattcacgttccttgcgcctagcaacaatcatatcaacatttttattgatgtctgcctg[A/T]gaccgatttactatgccaacccaaggatgttgcagatggtaagatcttccctccaggacatccaacgcattagttcctttgtccatcaaatctagctttg

fava_c18440_159	fava_c18440	159	T/A	tgctatttttttatggacattaaatgtcaaaattgatttgtcctatactatcaataaatattcagcttgtgtaataataacaaagtagaagatttgaaat[T/A]tgaaaagctgatagttgaagcattgaaacaaagaaaggaatgttgagcgtagaagcaagccaacaataaaataataacaaatatgtaaattacttatggg

fava_c18440_1716	fava_c18440	1716	G/A	gctagaatgatggaattaggcttctcaacataagatcggaccatagcttcaatatcttgaacaatactctcagactgtccttctacagccactttagtca[G/A]accgggtaaatcaatcaaggttaggttgacaacattcggggaataaatactgagatgaataggaataggagatatttgttttgtcttccctgttactcga

fava_c18440_351	fava_c18440	351	C/T	acttatgggtcaaggcaacccataattgttatcagttagtccaaggcgaggaagacatccggctacgcttatcgcacccaagatacagaatcaatttcat[C/T]ccttgctgctttatatagttccagcctttttgcacactgaattctcctctccatcaatgcaggctcttcatccaacatttgagatagatgtttaccctct

fava_c18440_742	fava_c18440	742	T/C	ttccagctttctccacttcctgaggtagtctccggaagaaatccacagtgagatacgaagattccatatctacaagccggagagttgtcttcttactttc[T/C]ccacgaaacctttctaaagcctcatttgctgctgaagctagttcagcttggaaagttggaaatcgtttcagttcctgagtttcagctattgattttctca

fava_c18441_191	fava_c18441	191	C/T	tcgaataatattgagtgacttgtacaattagttgaacacacggaaatctacataaaatccctatatttatactagaactatgtaaccgtcgaggagttgt[C/T]ttgctctaagatgaggacaagtgtcctctgcatatgcacttcgacttctgtccttatgccacgttgctttcttcaactRccaggtgaactgcttttccct

fava_c18441_270	fava_c18441	270	A/G	atgtaaccgtcgaggagttgtYttgctctaagatgaggacaagtgtcctctgcatatgcacttcgacttctgtccttatgccacgttgctttcttcaact[A/G]ccaggtgaactgcttttccctcMtttcagattcaaaatcacttgttgctgatacgaagtttcctggtcgaaagacttcttggtcgaacaccccttttaac

fava_c18441_293	fava_c18441	293	C/A	tgctctaagatgaggacaagtgtcctctgcatatgcacttcgacttctgtccttatgccacgttgctttcttcaactRccaggtgaactgcttttccctc[C/A]tttcagattcaaaatcacttgttgctgatacgaagtttcctggtcgaaagacttcttggtcgaacaccccttttaacttagaaaatttatctaagtattt

fava_c18441_674	fava_c18441	674	T/C	aatatcattccaaacatgcacaatacaaccttcaagttcaagacccattgcttgaagcagtttagtgcttcggctaaaacaccaaatttgaaatacctac[T/C]tcactgttaacacatatactagtagaaatatccacgttacttcaaactagatcacaaattcacatattgcaaacaatttccagcggtagctatttatctt

fava_c18442_1536	fava_c18442	1536	G/A	ttttcttaggcttgtcaattcctgaatacttcagagaattcactatcagagcccttcatggtcctgctcataccaaagctggatggttcaatgactttct[G/A]aataccattttctattcttcaccaactgttgcattgataattgctgtattcttggacaacactcttgactataaggacagtgccaaagatagaggaatgc

fava_c18442_1797	fava_c18442	1797	G/T	ttccattcaatctcaaccgtttcttccctccttcataagaaaaattccaaaatacatgttagaggaaaatcatgtaatatatggttttgagagtgaattt[G/T]attattttcatgtaataatcgaacaaaaggtcattttgttcttttaatttcaaacatattagtcttattttttcattcattttatcacttttagtggttg

fava_c18442_729	fava_c18442	729	A/T	tcttcagcccacttggaatggttccagtaattacattagttggttttggattatttgacagaggctttcctgtggtggggacttgtgttgaaattggaat[A/T]cccatgctaatcttgtttataatcttctcccagtacttgaaaaattttcagacaagaaatgtaccgatactagagcgatttgcgctgctcatatcgacca

fava_c18442_73	fava_c18442	73	C/A	agagagagtcacaaaatttctaacataacagttgaagaaaccagaactgaagcaagtgctagagtagaagtg[C/A]aaattctcttgctcagttttttttctcaagctaagtaggatttggaaggtgaaataataatttaagttgcaaagttggaagtgattatgggagatataaa

fava_c18442_873	fava_c18442	873	T/G	attttcagacaagaaatgtaccgatactagagcgatttgcgctgctcatatcgaccacggtgatatgggcatatgcacatctattgacagcgagtggagc[T/G]tacaaacaccgaccggatttaacgcaacataattgcaggacagatagggctaatctcatttcttctgctccttggataaagattccataccctcttgaat

fava_c18443_768	fava_c18443	768	T/C	aagataataataataataataatagtaatcatcagcataaggctgccacaccttctcatgatcttaaccggcggatacaagggtttgggaatacaaacta[T/C]gaagtgccatctgaagataagaagtcttttattagtgaggtagttggtattggaagtgcatcaattaagcaaggacttcataccttaacacagggacatt

fava_c18448_1293	fava_c18448	1293	T/C	ggttcatctgcaagccggttgccattgttgccggcgattatgtcactacgacagaatcccggcgagaattgatatcccggtggaaaccctagctcgtcgg[T/C]ttRgggaagcttRacgcgggatgcatagccgtttaactttcgtgagataatggaagacggacagtggagaatcaatatgcagaagagattgataaaaatg

fava_c18448_1296	fava_c18448	1296	G/A	tcatctgcaagccggttgccattgttgccggcgattatgtcactacgacagaatcccggcgagaattgatatcccggtggaaaccctagctcgtcggYtt[G/A]gggaagcttRacgcgggatgcatagccgtttaactttcgtgagataatggaagacggacagtggagaatcaatatgcagaagagattgataaaaatgcgc

fava_c18448_1306	fava_c18448	1306	G/A	gccggttgccattgttgccggcgattatgtcactacgacagaatcccggcgagaattgatatcccggtggaaaccctagctcgtcggYttRgggaagctt[G/A]acgcgggatgcatagccgtttaactttcgtgagataatggaagacggacagtggagaatcaatatgcagaagagattgataaaaatgcgcgagaatttgg

fava_c18448_386	fava_c18448	386	A/T	tgaaaaaatttaatttaaaattgattgatgatttccaaaagatacctccataaagcattacttcgactttaatatcatccaagatatcgagggtggtagg[A/T]aactctWgtaagatgtaagaattaatcagaatKttatccagtcctaacatacaaaagcctcctggttggagcYggaggSgccaatcctgggatatctcta

fava_c18448_393	fava_c18448	393	T/A	atttaatttaaaattgattgatgatttccaaaagatacctccataaagcattacttcgactttaatatcatccaagatatcgagggtggtaggWaactct[T/A]gtaagatgtaagaattaatcagaatKttatccagtcctaacatacaaaagcctcctggttggagcYggaggSgccaatcctgggatatctctaatatctt

fava_c18448_419	fava_c18448	419	G/T	tccaaaagatacctccataaagcattacttcgactttaatatcatccaagatatcgagggtggtaggWaactctWgtaagatgtaagaattaatcagaat[G/T]ttatccagtcctaacatacaaaagcctcctggttggagcYggaggSgccaatcctgggatatctctaatatctttgttgtgtggattcacaatctttaca

fava_c18448_459	fava_c18448	459	C/T	atcatccaagatatcgagggtggtaggWaactctWgtaagatgtaagaattaatcagaatKttatccagtcctaacatacaaaagcctcctggttggagc[C/T]ggaggSgccaatcctgggatatctctaatatctttgttgtgtggattcacaatctttacaacaatgatggcaattacaccgcacacgataaggaacagaa

fava_c18448_465	fava_c18448	465	C/G	caagatatcgagggtggtaggWaactctWgtaagatgtaagaattaatcagaatKttatccagtcctaacatacaaaagcctcctggttggagcYggagg[C/G]gccaatcctgggatatctctaatatctttgttgtgtggattcacaatctttacaacaatgatggcaattacaccgcacacgataaggaacagaaaaagca

fava_c18448_579	fava_c18448	579	G/A	tatctctaatatctttgttgtgtggattcacaatctttacaacaatgatggcaattacaccgcacacgataaggaacagaaaaagcatgatgcacttatc[G/A]gtagctacctttcttccgatctccttcacaagctgggaggccttcttaattgagaactgaattgaatcgagctcgttaacaatacgacccatctgatcag

fava_c18448_708	fava_c18448	708	G/A	caagctgggaggccttcttaattgagaactgaattgaatcgagctcgttaacaatacgacccatctgatcagtttggcctttcaaggtggtggcagtttg[G/A]gtacctacttcaattgtttgatgtacaacctgtttcgatctttctatagcctgatcagtttcatccattgtttttgtcccagtatcgataagttcttgat

fava_c18450_224	fava_c18450	224	C/T	aaatgggtccactcgtgctgagccaattagccaccggtctaagcgtcctagccggagcggttctcgttaaatcggttttggaccaaaaacccatggcggg[C/T]caatttcccagatgccccagctgcaacggaacYggYcgagtcccttgtctctgctcacgctggtctgacggtgatgtcggttgtagtacgtgttccggtt

fava_c18450_257	fava_c18450	257	C/T	ccggtctaagcgtcctagccggagcggttctcgttaaatcggttttggaccaaaaacccatggcgggYcaatttcccagatgccccagctgcaacggaac[C/T]ggYcgagtcccttgtctctgctcacgctggtctgacggtgatgtcggttgtagtacgtgttccggttcgggtcgcatgggttgcagcagttgtggtgggt

fava_c18450_260	fava_c18450	260	C/T	gtctaagcgtcctagccggagcggttctcgttaaatcggttttggaccaaaaacccatggcgggYcaatttcccagatgccccagctgcaacggaacYgg[C/T]cgagtcccttgtctctgctcacgctggtctgacggtgatgtcggttgtagtacgtgttccggttcgggtcgcatgggttgcagcagttgtggtgggtccg

fava_c18451_417	fava_c18451	417	T/A	cggccagagtaagaattggtgctacagaggtcattgctagtgtgaaggctgaacttggaaaaccaggcatggggcaacctgacaaagggaaagtgtttat[T/A]tacattgactgtagttcgactgctgagccagcttttgagggtagaggaggcgaggaattgtcagcagaactctcaactgctcttcatcgctgtctcttgg

fava_c18451_871	fava_c18451	871	A/T	atctggaatccctgctatagttacactgactaaggttgggaggcactatattgtggatgccacacctgaagaggaatcacaaatgagctctgcagtttct[A/T]tctctattaataggaaagggcacgtctgcggtataaccaaacgaggaggKgcaggactggatccaagtgtcgttcttgatatggtatctgtggctaagca

fava_c18451_921	fava_c18451	921	T/G	ttgtggatgccacacctgaagaggaatcacaaatgagctctgcagtttctWtctctattaataggaaagggcacgtctgcggtataaccaaacgaggagg[T/G]gcaggactggatccaagtgtcgttcttgatatggtatctgtggctaagcatgtaagcgagcagttaatgaataaattggattcagaaatagcttctgctg

fava_c18453_1423	fava_c18453	1423	G/A	ttgagcccagcacatatcaaggagagatgaaatctgcattcatcaatgatggcagcagtccaaatcttgatctcaatttgggaatagccaaaccaggacc[G/A]ggtcccaaagaaaattgggagaaatttcactttccctctgtttcttacacaagttcaaggatggaaaccaatgtttcaggaatcggtaattcatctttaa

fava_c18453_1570	fava_c18453	1570	G/C	acacaagttcaaggatggaaaccaatgtttcaggaatcggtaattcatctttaaaaaggttattagtaactgaagagcatcattctttacggaatgacac[G/C]tatcccggtttctttcccaatgaggaaagagcagatggtatcaacaaagctcctttaaaaggactccccatttgggcgtggcaaactaatggtcaggtca

fava_c18453_1778	fava_c18453	1778	C/T	cccagtggcacgggtctctgcagcagcatcatcaggattctcaatttcagctcccttttcacctactcccttatttccaccaagatctatgaactcagtt[C/T]cccacagtctctattttacttcgcccaatgcacgtcacagcaacacatttcaataatattatcagggtaagtccctgcaagcaccgctctagcctatcat

fava_c18453_436	fava_c18453	436	A/T	tcaccggcgacgttgaaagttagttcatcttgtttcaactccaatctaactaacttcaataaaaaatattaataaaataagaatagaaagaaaattaaag[A/T]gttcagttaagctgaagataaagcattatgttagatcttaatctatacgatgcgaactcgtccgattcgactcaggacattgactcgccgatgatatcgg

fava_c18456_1037	fava_c18456	1037	G/A	ggattgttgctggttcgagtgttggatcgattatgtgtgctattgctgctactagatcgtggccggagcttcagagttttttcgaggattcgttgcattc[G/A]ctgcagtttttcgatcaaatgggtgggattttcgcggttgtcaagagggttgcaacgcgcggggctgttcatgagattaggcagttgcaaatgctgttga

fava_c18456_141	fava_c18456	141	T/G	aacacacatcttgtgaaattccactttaatttcagtttctcttatacataatcttttatagtttttagctttgagtattttgatttgtgtgaaatttttt[T/G]attaagttgaaatggatcatattagtaatgaagctagggttgaccgttttccaattggtccatcagacattcttggcaaaacaatagctttcagggttct

fava_c18456_1916	fava_c18456	1916	G/A	aagcaaattgtgaaattgagcttgctcttgatgaatctgtggcaattctcaatcacatgagaaaactaaaaagaattgccgagagaacttctgcttcagc[G/A]tctcatggtttgtcaagtactgtcagattcagtggttcaagaagaattccttcatggaattgcatggcacgggagaattcgacaggttctcttgaagacc

fava_c18456_2162	fava_c18456	2162	T/C	ctgaaaatattgattcgatttcttggaccagatctggtggccctttgatgagaaccgcttcggcaaatatgtttgttgactttctccaaaacttagaggg[T/C]gttgttgatactgaactaaatagaggcRcggtggctcattccagtcctcttgattttcagtatcataatttcagactcacaggaccagataggaactcgg

fava_c18456_2190	fava_c18456	2190	A/G	cagatctggtggccctttgatgagaaccgcttcggcaaatatgtttgttgactttctccaaaacttagagggYgttgttgatactgaactaaatagaggc[A/G]cggtggctcattccagtcctcttgattttcagtatcataatttcagactcacaggaccagataggaactcggaatccgagcagaaggtaatYgacaacag

fava_c18456_2282	fava_c18456	2282	C/T	atagaggcRcggtggctcattccagtcctcttgattttcagtatcataatttcagactcacaggaccagataggaactcggaatccgagcagaaggtaat[C/T]gacaacagggttgttaacggatctagcttactgataaccgaaggtgatattttgcagactgaaaagattcataatgggattgtgttaaatgttgtcaaga

fava_c18459_161	fava_c18459	161	G/T	gagtaatgataaatacaattacatatagtaatgacaaagaattgtaatatgattctcctttgacacaacaaaataatcataaaacttatctaactctaga[G/T]gacatgaaaaataaacatataacaatatccaaaatcaatcttttgtgtaactaaaaacaaaccatattaatattgtttggtttggtctttacaaaatgaa

fava_c18459_495	fava_c18459	495	T/C	tataagtctcttcaatgttctcacacgtctctgaattccataggagcctcttctacaaccaacccttctcttcatggagattcttcttgacctcttcacc[T/C]ttttcttggcttgtccatccaataccttgctccacaagtatgatgaattgttacttccttgaagagcttctttcaaagtgattgcatcaaaagaagtttg

fava_c18461_1248	fava_c18461	1248	C/T	ccactaggctaagtgcctgctcaccacaatctggctgtgagagaagcaagttctcggtatttacgccaatagattcagcaagtgacttatcaagtgcatg[C/T]tcagcatcaataaaggcacaatagcctcctagtttttgtgcctctgcaatcacatgcaaagcaagagttgttttcccagaagcctctggaccgaatattt

fava_c18461_126	fava_c18461	126	T/C	tgaatatccgcaaaatatgagttttgttttcatgcctagttcaataaaaaatacactgagaaacatatgcaccaatactgtaatccagcttgtagattaa[T/C]tatccttataaataactcttgacatagcattgacatttaatttctaactagatagWcacaaaacttgattccKcctcatccacaaaactttggaatgcca

fava_c18461_1644	fava_c18461	1644	C/A	ttgagtcacttccatctgatttagaccgcttcttacctttacaagttgaaaagctcaaattttgagatgagcccattactccatgtttgaaacaagagcg[C/A]gtYggaagRccgtggttaWagaaagagcggttgaagagaaaagtgttagcgtttcgaagtagcctcgccatggagtagaatcacactcttctattgaagt

fava_c18461_1647	fava_c18461	1647	T/C	agtcacttccatctgatttagaccgcttcttacctttacaagttgaaaagctcaaattttgagatgagcccattactccatgtttgaaacaagagcgMgt[T/C]ggaagRccgtggttaWagaaagagcggttgaagagaaaagtgttagcgtttcgaagtagcctcgccatggagtagaatcacactcttctattgaagtgtg

fava_c18461_1653	fava_c18461	1653	A/G	ttccatctgatttagaccgcttcttacctttacaagttgaaaagctcaaattttgagatgagcccattactccatgtttgaaacaagagcgMgtYggaag[A/G]ccgtggttaWagaaagagcggttgaagagaaaagtgttagcgtttcgaagtagcctcgccatggagtagaatcacactcttctattgaagtgtggaacgt

fava_c18461_1663	fava_c18461	1663	A/T	tttagaccgcttcttacctttacaagttgaaaagctcaaattttgagatgagcccattactccatgtttgaaacaagagcgMgtYggaagRccgtggtta[A/T]agaaagagcggttgaagagaaaagtgttagcgtttcgaagtagcctcgccatggagtagaatcacactcttctattgaagtgtggaacgtgaagagaagg

fava_c18461_182	fava_c18461	182	T/A	tgagaaacatatgcaccaatactgtaatccagcttgtagattaaYtatccttataaataactcttgacatagcattgacatttaatttctaactagatag[T/A]cacaaaacttgattccKcctcatccacaaaactttggaatgccacgtgactatgtcaatcataaaattaccaaaaaaacatctaatttacaaaggcaatg

fava_c18461_199	fava_c18461	199	T/G	aatactgtaatccagcttgtagattaaYtatccttataaataactcttgacatagcattgacatttaatttctaactagatagWcacaaaacttgattcc[T/G]cctcatccacaaaactttggaatgccacgtgactatgtcaatcataaaattaccaaaaaaacatctaatttacaaaggcaatgtgccacgcgactgtaag

fava_c18461_392	fava_c18461	392	G/C	actgtaaggaatgatgtcgattcaaaatccaaacaagcataggaagcaccagttgcattttagagccaccttcatttgagtgcattaattgtaagtcaca[G/C]ccttgggagtgtRaagaaaactttaWggttttatgcataaattcaagcttctgccacaacagtagcttcttcatcagtagaatcagatgataaagtttct

fava_c18461_405	fava_c18461	405	G/A	atgtcgattcaaaatccaaacaagcataggaagcaccagttgcattttagagccaccttcatttgagtgcattaattgtaagtcacaSccttgggagtgt[G/A]aagaaaactttaWggttttatgcataaattcaagcttctgccacaacagtagcttcttcatcagtagaatcagatgataaagtttcttcSgggatgtctc

fava_c18461_418	fava_c18461	418	A/T	atccaaacaagcataggaagcaccagttgcattttagagccaccttcatttgagtgcattaattgtaagtcacaSccttgggagtgtRaagaaaacttta[A/T]ggttttatgcataaattcaagcttctgccacaacagtagcttcttcatcagtagaatcagatgataaagtttcttcSgggatgtctccaactatcacctc

fava_c18461_495	fava_c18461	495	C/G	ttgggagtgtRaagaaaactttaWggttttatgcataaattcaagcttctgccacaacagtagcttcttcatcagtagaatcagatgataaagtttcttc[C/G]gggatgtctccaactatcacctccgattctgaaactgtttccgtctcggcattgagaagcttttccctaagctttgttgttaattcttgcagaccgtcgc

fava_c18461_732	fava_c18461	732	G/A	ggaaattcttgccattatattcgtaaaacgaaccgctcttttttatgagtttgtgttttatgctcaactctatgacctcagcttctttgcatatcccctt[G/A]ccaaactcaagctcaaactgtgcagttttaaacggaggggcgtgtttgttcttcacaactttgacaagaatttggcttcctaaagtctcttcacccttct

fava_c18461_845	fava_c18461	845	C/T	tcaaactgtgcagttttaaacggaggggcgtgtttgttcttcacaactttgacaagaatttggcttcctaaagtctcttcacccttcttgataaacccta[C/T]tcttttgatatttagcctcacggaagcatagaacttcaatgcattaccaccacaagtaacttcagtagggccagcgaatcccccaaacgtagaaatcttt

fava_c18464_403	fava_c18464	403	G/C	aagccttaagagccttcaacttacagtgattataataagcaacacccaaaaaggcaagtccataaccaatcaaattaaggggtgtaacagtgtccttaat[G/C]acagaccaagaaaaagcaataagcaaccaatccttcacaacaccagcaacattcatagtcaaagctgaagttttcccaacaagcaaaaacacagcaagat

fava_c18464_508	fava_c18464	508	A/C	accaagaaaaagcaataagcaaccaatccttcacaacaccagcaacattcatagtcaaagctgaagttttcccaacaagcaaaaacacagcaagattcaa[A/C]gcaaaagcacacaaagaattactaccaaaaacccataaatctaattccaaacttgacttatcccttaataaaggatactccatcacaaaccaaggaagtg

fava_c18464_721	fava_c18464	721	C/G	aacaacaaggagcaatgtagtaaagtgatgtaataggattcaattttatccctttagaattcaaaagaatctgaattaaaaccaatcgcgtggcttcaaa[C/G]gcYacagccatgagctgtagcgtaacaccaaagacatcaaacttggcttcgccgtaggcagctacagcRacgccaagcgaaatagacaccatgttgatca

fava_c18464_724	fava_c18464	724	T/C	aacaaggagcaatgtagtaaagtgatgtaataggattcaattttatccctttagaattcaaaagaatctgaattaaaaccaatcgcgtggcttcaaaSgc[T/C]acagccatgagctgtagcgtaacaccaaagacatcaaacttggcttcgccgtaggcagctacagcRacgccaagcgaaatagacaccatgttgatcatgg

fava_c18464_790	fava_c18464	790	G/A	tctgaattaaaaccaatcgcgtggcttcaaaSgcYacagccatgagctgtagcgtaacaccaaagacatcaaacttggcttcgccgtaggcagctacagc[G/A]acgccaagcgaaatagacaccatgttgatcatggtgtcttttttgaaagactcttttttaaacgaaacgccgatggagtaaactgcgacgggcataagtg

fava_c18464_934	fava_c18464	934	G/A	tgaaagactcttttttaaacgaaacgccgatggagtaaactgcgacgggcataagtgctttaagcatttgaatgaatgaaacagagaggtaaatgtaagc[G/A]gagtttgagaaccaaagggagagagaatagagKgcaccaatagggacaactgatttaaggtaaatgtcgaaagacattgaaattggttcgacaagtttga

fava_c18464_967	fava_c18464	967	T/G	agtaaactgcgacgggcataagtgctttaagcatttgaatgaatgaaacagagaggtaaatgtaagcRgagtttgagaaccaaagggagagagaatagag[T/G]gcaccaatagggacaactgatttaaggtaaatgtcgaaagacattgaaattggttcgacaagtttgaagattttgacgaggatgtaagcaagggaggaac

fava_c18471_1150	fava_c18471	1150	C/T	agcttcagatgtcgtagatccatagacaccacttgggttggttatggaatggcctttccatttatcaagaaatggtgtaatcatattcgccgtgtcctga[C/T]atgtattttttaagtcgtgtagcttgtcttataacaaaacaaaaacttcccagaatcacatattggaagttcacagtacttatacctctgtgccattgaa

fava_c18471_1262	fava_c18471	1262	T/G	aagtcgtgtagcttgtcttataacaaaacaaaaacttcccagaatcacatattggaagttcacagtacttatacctctgtgccattgaagatcacacctg[T/G]catctcatgtacaatgaacagaaatccagctgagttcaccattttagaaaatgggaatgtctatatgatcagaggagaggatctcaaatttttaaatagt

fava_c18471_1420	fava_c18471	1420	G/T	tgtctatatgatcagaggagaggatctcaaatttttaaatagtattccaaaaaaaaacaatggacataactccatggcaaaacaaaaaaaaaagaataac[G/T]taagtcttcattctacaRccacaccataattaggaaattcgatttttttcagttaactcaaaaattcgactcttcttaatcaaatcaaaataacactaca

fava_c18471_1438	fava_c18471	1438	A/G	agaggatctcaaatttttaaatagtattccaaaaaaaaacaatggacataactccatggcaaaacaaaaaaaaaagaataacKtaagtcttcattctaca[A/G]ccacaccataattaggaaattcgatttttttcagttaactcaaaaattcgactcttcttaatcaaatcaaaataacactacagatttctcaattacaaaa

fava_c18471_1812	fava_c18471	1812	G/A	cttatctttcatcaccacggtttggaaatcgcgtggagcgacgctggaaaggtagaaggaaatacttggcgacgaagacagcgacgacgcgacaggggtt[G/A]aRtgtgcgcgattttggagWaactgcggcggtgaagcatatgagagagcaatttcgcacgcgcttgaaatggaaaagattgaaatcttctccgtctacct

fava_c18471_1814	fava_c18471	1814	A/G	tatctttcatcaccacggtttggaaatcgcgtggagcgacgctggaaaggtagaaggaaatacttggcgacgaagacagcgacgacgcgacaggggttRa[A/G]tgtgcgcgattttggagWaactgcggcggtgaagcatatgagagagcaatttcgcacgcgcttgaaatggaaaagattgaaatcttctccgtctaccttt

fava_c18471_1832	fava_c18471	1832	T/A	tttggaaatcgcgtggagcgacgctggaaaggtagaaggaaatacttggcgacgaagacagcgacgacgcgacaggggttRaRtgtgcgcgattttggag[T/A]aactgcggcggtgaagcatatgagagagcaatttcgcacgcgcttgaaatggaaaagattgaaatcttctccgtctacctttaatcagatctactaattt

fava_c18471_496	fava_c18471	496	G/C	atctgaaaacgtagaaacatagagaaatacaaattatccatatattgatgcaatatcaaggtgatttcatgactcaatagaattgtcatcttattgctcc[G/C]ttgaaaagctctcaaatatgcagcaattgcttccaactccttRagcataaagctatttaattagctaagaaaataagattaagtgacaaaaccaaaaact

fava_c18471_539	fava_c18471	539	G/A	attgatgcaatatcaaggtgatttcatgactcaatagaattgtcatcttattgctccSttgaaaagctctcaaatatgcagcaattgcttccaactcctt[G/A]agcataaagctatttaattagctaagaaaataagattaagtgacaaaaccaaaaactcttgttcatttggtgttgggttaattgagaatatatattgatc

fava_c18471_882	fava_c18471	882	C/T	tgcatttatcacggtaacaaacagtgtagcaacaacacctttaagaaaagacatgtcagtgtcagtatctcaacaaccttggtaccatcaacaacattgc[C/T]tctttcctcacttcaacactcaatttcttttcctttagctttgtctcaatttgtgcaccaacctatatgacagccacgccaccatacagttttgcattcc

fava_c18473_1156	fava_c18473	1156	T/C	caacgcttggtggtctaaccacaaaagtccttcttcttggtgtggtgtattttgtggcatcagaagcacttgagctggtggaacatctcggcaatattaa[T/C]gacttctctggaaaagcaaggcttttcctggtgctgcctgtggctctgttggatgcttgctttatcctttggatcttttcatccttatctaaaaccttgg

fava_c18473_121	fava_c18473	121	T/C	ctcacttttttaccatcattcagatcagcgacgaaatggcttacaccgatcacggcggcgatcggagaagataccggagcccccgctcttcaaacgctca[T/C]tgccgcattgttttcctcttcctcactttccattcaWtcaYcaacctcgtcggtgcttccattcacgactaccaaaacgaaaccttcatccgtcgagcta

fava_c18473_1507	fava_c18473	1507	G/A	gagcttgggtgatcccagctttctggactctgctttcttatgctctcctgatactgatctgcattctctgggtcccatctcgaaatccaaccaggtattc[G/A]tactcRgaagaaacaggggatgactttgatgacgaagctgtcactgttgttggaagtggagtgaaggtctctggagaaatggctaccatgctagaaagaa

fava_c18473_1513	fava_c18473	1513	G/A	gggtgatcccagctttctggactctgctttcttatgctctcctgatactgatctgcattctctgggtcccatctcgaaatccaaccaggtattcRtactc[G/A]gaagaaacaggggatgactttgatgacgaagctgtcactgttgttggaagtggagtgaaggtctctggagaaatggctaccatgctagaaagaaaagata

fava_c18473_158	fava_c18473	158	A/T	ggcttacaccgatcacggcggcgatcggagaagataccggagcccccgctcttcaaacgctcaYtgccgcattgttttcctcttcctcactttccattca[A/T]tcaYcaacctcgtcggtgcttccattcacgactaccaaaacgaaaccttcatccgtcgagctaattcgtttttcttccatggtggtagtgaaggtctcta

fava_c18473_162	fava_c18473	162	T/C	tacaccgatcacggcggcgatcggagaagataccggagcccccgctcttcaaacgctcaYtgccgcattgttttcctcttcctcactttccattcaWtca[T/C]caacctcgtcggtgcttccattcacgactaccaaaacgaaaccttcatccgtcgagctaattcgtttttcttccatggtggtagtgaaggtctctatgct

fava_c18473_469	fava_c18473	469	C/T	ctgctcagatggcaagtgcaatgcagcaaaagacagggttggttgaagctataataattagggtgagagataggaataacattgggggtgtttctttgaa[C/T]tctgatgctatatgctgcactccagagcttgctaaagaaggttcttgtaagttaggagaggttatcatccgtgagaacaccgatgaaccgaatgggccaa

fava_c18473_850	fava_c18473	850	G/T	agatgacgccattgatgacattttatggtttgatgtctttggcttacctttttctcggtcttgtctggtttctctggtttgtacggtattggaaagatgt[G/T]atccagttgcattaccacattactgcagtcattggccttggcatgtgtgaaatggccttgtggtattttgagtatgcaaatttcaattcaacaggaagca

fava_c18473_969	fava_c18473	969	T/G	attactgcagtcattggccttggcatgtgtgaaatggccttgtggtattttgagtatgcaaatttcaattcaacaggaagcaggccgatgataattacag[T/G]ttgggtcgtgactttcacagctgtcaagaagacagtgtcgcggcttcttcttttggttgtttctatgggttacggagttgtccgtccaacgcttggtggt

fava_c18474_1098	fava_c18474	1098	C/T	tccgtgtcagcattgcaacatacaagtgttttcctctggtttcaaagctagctaatgaaattgcagcgagtgctatgctgaaccatacgcaagttcgtat[C/T]gtgggagctgatgcaactaatcagcaactagagaaaaccgatattatcattaacctggtaccaaaaggagtaaaatttgatgataccgtcgcattattaa

fava_c18474_1926	fava_c18474	1926	G/T	gtgagttcttggcagaagttgagatgcttggccgcctacaccatagaaatttagttaaactgataggtatatgcatagagaaaaagactcgttgcctagt[G/T]tatgaacttgtccctaatggaagtgtggaatcccacttgcatggtgctgacaaggaaagtgatccattggattggagtgcccggRtgaaaattgctcttg

fava_c18474_2011	fava_c18474	2011	G/A	gactcgttgcctagtKtatgaacttgtccctaatggaagtgtggaatcccacttgcatggtgctgacaaggaaagtgatccattggattggagtgcccgg[G/A]tgaaaattgctcttggtgcagctcgaggattggcttatctgcatgaagattccaatccgtatgtcatacaccgggacttcaagtccagtaacattttatt

fava_c18474_2587	fava_c18474	2587	G/A	agtatctcaacggcctttcatgggggaagttgttcaagccttgcagcttgtatgcagtgagtttgaggaaacaaattatataaaatcaagtaactttcac[G/A]aaggagatcttgtagtaactaatacggaagggaagtttttggagRtttcagatgagagagtggagttttcggagtgtcaaaaatcaatttatggctatca

fava_c18474_2632	fava_c18474	2632	G/A	gcttgtatgcagtgagtttgaggaaacaaattatataaaatcaagtaactttcacRaaggagatcttgtagtaactaatacggaagggaagtttttggag[G/A]tttcagatgagagagtggagttttcggagtgtcaaaaatcaatttatggctatcaatccggtgaagaaaatgtaagattatcagcttctgagctgctcag

fava_c18475_1061	fava_c18475	1061	A/G	ttcaggaagaaagttcatccagaaaattcaacaactggacacaaatctggtaaacataaaaagaatgagaacaagaagaaaataatgaatgatggaggtt[A/G]caacaaagggtacctcgttcatccagatgaagattcatcttcttacagggagcattggatcaaaacagatgcagattatttagttctagagctttgaaaa

fava_c18475_138	fava_c18475	138	C/G	gttttttttctcttcttcaatatcaaaacatgaagttacttggctggatgcataggaagtttcgacagaataatagtgcagaaccattcaaagatttggt[C/G]attgggaattcatgcaattgcctctcaagccaaScRtcYctagatgaagaacacaactatcatcaaaaagcaaatttgggaatcaaattcaSSaaaaaac

fava_c18475_172	fava_c18475	172	G/C	gttacttggctggatgcataggaagtttcgacagaataatagtgcagaaccattcaaagatttggtSattgggaattcatgcaattgcctctcaagccaa[G/C]cRtcYctagatgaagaacacaactatcatcaaaaagcaaatttgggaatcaaattcaSSaaaaaaccacaaaScaacacgttcagaaaatcattcggggg

fava_c18475_174	fava_c18475	174	G/A	tacttggctggatgcataggaagtttcgacagaataatagtgcagaaccattcaaagatttggtSattgggaattcatgcaattgcctctcaagccaaSc[G/A]tcYctagatgaagaacacaactatcatcaaaaagcaaatttgggaatcaaattcaSSaaaaaaccacaaaScaacacgttcagaaaatcattcgggggtc

fava_c18475_177	fava_c18475	177	C/T	ttggctggatgcataggaagtttcgacagaataatagtgcagaaccattcaaagatttggtSattgggaattcatgcaattgcctctcaagccaaScRtc[C/T]ctagatgaagaacacaactatcatcaaaaagcaaatttgggaatcaaattcaSSaaaaaaccacaaaScaacacgttcagaaaatcattcgggggtctag

fava_c18475_230	fava_c18475	230	G/C	gatttggtSattgggaattcatgcaattgcctctcaagccaaScRtcYctagatgaagaacacaactatcatcaaaaagcaaatttgggaatcaaattca[G/C]SaaaaaaccacaaaScaacacgttcagaaaatcattcgggggtctagaatcaacccgSgaacactacggagaagagttcggaagagagtcccaagacgac

fava_c18475_231	fava_c18475	231	C/G	atttggtSattgggaattcatgcaattgcctctcaagccaaScRtcYctagatgaagaacacaactatcatcaaaaagcaaatttgggaatcaaattcaS[C/G]aaaaaaccacaaaScaacacgttcagaaaatcattcgggggtctagaatcaacccgSgaacactacggagaagagttcggaagagagtcccaagacgaca

fava_c18475_245	fava_c18475	245	C/G	aattcatgcaattgcctctcaagccaaScRtcYctagatgaagaacacaactatcatcaaaaagcaaatttgggaatcaaattcaSSaaaaaaccacaaa[C/G]caacacgttcagaaaatcattcgggggtctagaatcaacccgSgaacactacggagaagagttcggaagagagtcccaagacgacacaatgttcgatctc

fava_c18475_288	fava_c18475	288	C/G	aacacaactatcatcaaaaagcaaatttgggaatcaaattcaSSaaaaaaccacaaaScaacacgttcagaaaatcattcgggggtctagaatcaacccg[C/G]gaacactacggagaagagttcggaagagagtcccaagacgacacaatgttcgatctcttccccggcttcctcgccatcggaaccctaggatccgacacac

fava_c18475_459	fava_c18475	459	G/A	tcgccatcggaaccctaggatccgacacaccaacatttccaaacagctcggtgcaaaccataaccgaaaacgaagacgaagtaaccgagaacgatctcaa[G/A]ctcatcaacgacgagctagagaaagtccttgtcgctgaaacaaaagacgatgtaataagctacgattcatcSagccgaaacagccatgttagcacgggga

fava_c18475_531	fava_c18475	531	G/C	aagacgaagtaaccgagaacgatctcaaRctcatcaacgacgagctagagaaagtccttgtcgctgaaacaaaagacgatgtaataagctacgattcatc[G/C]agccgaaacagccatgttagcacggggagaagcagccaYgtaagcataaYaacaataagtggaaaaccaactgaagtagcaggaacaacaacaagagatt

fava_c18475_570	fava_c18475	570	C/T	acgagctagagaaagtccttgtcgctgaaacaaaagacgatgtaataagctacgattcatcSagccgaaacagccatgttagcacggggagaagcagcca[C/T]gtaagcataaYaacaataagtggaaaaccaactgaagtagcaggaacaacaacaagagattcatattcaaatgctgtttgtcctttacaaggctaYttgt

fava_c18475_581	fava_c18475	581	C/T	aaagtccttgtcgctgaaacaaaagacgatgtaataagctacgattcatcSagccgaaacagccatgttagcacggggagaagcagccaYgtaagcataa[C/T]aacaataagtggaaaaccaactgaagtagcaggaacaacaacaagagattcatattcaaatgctgtttgtcctttacaaggctaYttgtttggaacaact

fava_c18475_666	fava_c18475	666	T/C	gccaYgtaagcataaYaacaataagtggaaaaccaactgaagtagcaggaacaacaacaagagattcatattcaaatgctgtttgtcctttacaaggcta[T/C]ttgtttggaacaactgttgaacattctgaaacagcagctgctagtgttggtgggaaYaagaaagaacacaggacttcacttggtgagctgtttcagagaa

fava_c18475_723	fava_c18475	723	C/T	caagagattcatattcaaatgctgtttgtcctttacaaggctaYttgtttggaacaactgttgaacattctgaaacagcagctgctagtgttggtgggaa[C/T]aagaaagaacacaggacttcacttggtgagctgtttcagagaagtaaaatggctgaggagaatttttttgggaaagatgaaaagagagaYgctgagaaat

fava_c18475_813	fava_c18475	813	T/C	ttggtgggaaYaagaaagaacacaggacttcacttggtgagctgtttcagagaagtaaaatggctgaggagaatttttttgggaaagatgaaaagagaga[T/C]gctgagaaatattctgctatcaatctcatgaaagagaaagtcaagaaaagaatgttccattcttgttctaagaattcttcttctatcattgctggaaatg

fava_c18477_1120	fava_c18477	1120	G/A	ttcctgtaattatatcatgacctccaaacctcatgtatagttctatgtggagacaaaatcactaaatgaatggttttcgctgttgttactttgtcaagtc[G/A]gcgaggttggcttgtatctttgaacgaagaatgagtagaggcaaaaattgtctcgaaaccttgtcaactagcagaaaactatcaaacatgagtttaagaa

fava_c18477_1323	fava_c18477	1323	C/G	aacctctattgtgttggtcatggttactgaatgtctcccatgcatgttatacatatcatcagcattacattctagttcaaagagtaattctttatagact[C/G]aagtgatctatttgatcccattctgttgagagggggggtctaagtgtagattgtactgtatttaaaaattgggatttcctcttgtaaaaactttagataa

fava_c18480_405	fava_c18480	405	T/G	tcaaataaaacatgtatcatgtgagaacagtcacagtttttcatactacacctacctacgtgtaagcaattaaaacttaaaggagagagagagagagaga[T/G]agagagagagatatggatagggagcaaaaattaaaagagcaagagagctccttatcgccatgccgatgctactgtgttgattcctcgccaaaaacctcag

fava_c18480_780	fava_c18480	780	G/A	tcgaggttggaagtgtatcaataagttcttgggaaagacctcggctttgagttccaactgcttcccctaggtccagaagttcctcataagtcatattgtc[G/A]ggRtcaatgttgtcttgccatatgacctggctactactggactcttgatgattgggactacattcttcagggatagtctgcatgctaactccatctcttc

fava_c18482_902	fava_c18482	902	C/T	cattctttctcatgacagcgaatccacctgccaaattcatttggtcatacagtcaattgggttctctgtgatcgtctctctaagtttgcacacttcattg[C/T]cctgccatcccacttctcaacRaaggaccttgcagcttgcttcttggttgagattttttgtctgcacggaaacccaaagcatattgtctctgaccgtgat

fava_c18482_924	fava_c18482	924	A/G	tccacctgccaaattcatttggtcatacagtcaattgggttctctgtgatcgtctctctaagtttgcacacttcattgYcctgccatcccacttctcaac[A/G]aaggaccttgcagcttgcttcttggttgagattttttgtctgcacggaaacccaaagcatattgtctctgaccgtgatgctttgtttctcaccaactgag

fava_c18483_1891	fava_c18483	1891	C/T	aaaattgcatttcctgataccgacttccgagacgcaagaagtgcactctcttttgctggtccagtttcggcctcaaccaccgtatcgtctccaaggcaag[C/T]ggctgttgggcaaggcaagaggcacaagctgatgaacgaggtggaacccgattcacacccttcacagagaccactgcaacgcggatcagcttctagcgga

fava_c18483_2101	fava_c18483	2101	T/C	aggattagagtccgtatgccacctaaggaatcaaaaaccggaaatgctagtggcagcaatatacgagagcaacctcgaccgcaggatgattctccaccac[T/C]acttactcatcctggtgaactagtcgtgtgcaagaaaagaagaaacgagagggagaaatcaacggtgaagactaggactggccctgtttcgccggctatg

fava_c18486_1096	fava_c18486	1096	C/T	taattgaaattcaggacgcgtgccattttcgatagaggaaacaatgatgccttgttcgttgggaatgctcttgccatgtctcagaatccaatatttgttt[C/T]tcaataccaaagacattcttgttttcaattacttcaccctttccctttctcctcttctattctattctactagtgtgcttgtactct

fava_c18486_368	fava_c18486	368	G/A	tcttctttaccgtgctctagtttgtagatctgatctcaaacaaaaattattggataaatccttcaaaaaagttttggatatgataataaacaaaataatc[G/A]aataggagatctaatcMaaccaaaacagtaaattgYatttatggWagacctcaaatttcaaatgactctcctgagttctccagtattcagtgcgWattgc

fava_c18486_385	fava_c18486	385	C/A	tagtttgtagatctgatctcaaacaaaaattattggataaatccttcaaaaaagttttggatatgataataaacaaaataatcRaataggagatctaatc[C/A]aaccaaaacagtaaattgYatttatggWagacctcaaatttcaaatgactctcctgagttctccagtattcagtgcgWattgccgatgctgggacaagat

fava_c18486_404	fava_c18486	404	T/C	caaacaaaaattattggataaatccttcaaaaaagttttggatatgataataaacaaaataatcRaataggagatctaatcMaaccaaaacagtaaattg[T/C]atttatggWagacctcaaatttcaaatgactctcctgagttctccagtattcagtgcgWattgccgatgctgggacaagattggtgccacctgaactgcc

fava_c18486_413	fava_c18486	413	T/A	attattggataaatccttcaaaaaagttttggatatgataataaacaaaataatcRaataggagatctaatcMaaccaaaacagtaaattgYatttatgg[T/A]agacctcaaatttcaaatgactctcctgagttctccagtattcagtgcgWattgccgatgctgggacaagattggtgccacctgaactgccgctaatatt

fava_c18486_463	fava_c18486	463	T/A	taatcRaataggagatctaatcMaaccaaaacagtaaattgYatttatggWagacctcaaatttcaaatgactctcctgagttctccagtattcagtgcg[T/A]attgccgatgctgggacaagattggtgccacctgaactgccgctaatattgaaacYaagtcatcagaagtcagttccttatgattatKagctgcatgcag

fava_c18486_519	fava_c18486	519	T/C	tcaaatttcaaatgactctcctgagttctccagtattcagtgcgWattgccgatgctgggacaagattggtgccacctgaactgccgctaatattgaaac[T/C]aagtcatcagaagtcagttccttatgattatKagctgcatgcagaactgtttgcaagatttgcagaggatccccgtggctgacaatcaaaattgcacatc

fava_c18486_551	fava_c18486	551	G/T	gtattcagtgcgWattgccgatgctgggacaagattggtgccacctgaactgccgctaatattgaaacYaagtcatcagaagtcagttccttatgattat[G/T]agctgcatgcagaactgtttgcaagatttgcagaggatccccgtggctgacaatcaaaattgcacatccttcaaaWtcYgattcaataacgtccattgtt

fava_c18486_627	fava_c18486	627	A/T	cagaagtcagttccttatgattatKagctgcatgcagaactgtttgcaagatttgcagaggatccccgtggctgacaatcaaaattgcacatccttcaaa[A/T]tcYgattcaataacgtccattgttcttgaaagcctaccagcMacatccttaacgctttcacctccttctggcccaacaaaaggatctttcgcatctaatt

fava_c18486_630	fava_c18486	630	C/T	aagtcagttccttatgattatKagctgcatgcagaactgtttgcaagatttgcagaggatccccgtggctgacaatcaaaattgcacatccttcaaaWtc[C/T]gattcaataacgtccattgttcttgaaagcctaccagcMacatccttaacgctttcacctccttctggcccaacaaaaggatctttcgcatctaattccc

fava_c18486_669	fava_c18486	669	A/C	tttgcaagatttgcagaggatccccgtggctgacaatcaaaattgcacatccttcaaaWtcYgattcaataacgtccattgttcttgaaagcctaccagc[A/C]acatccttaacgctttcacctccttctggcccaacaaaaggatctttcgcatctaattcccaaatctctgcatatttatcatgagataaaagctcaaagg

fava_c18486_829	fava_c18486	829	A/C	ccaaatctctgcatatttatcatgagataaaagctcaaaggaaggaccaaaataccgctctcgaagatcatcgatcaccttacactgagatgctgcatca[A/C]attcaaaaggaatgttcaattcagtagcaacaacttttgccgtatgacttgttcttgaaaaaggggagtaacatatgcgcacattagcaagaggtatatt

fava_c18486_968	fava_c18486	968	G/C	gccgtatgacttgttcttgaaaaaggggagtaacatatgcgcacattagcaagaggtatattatcagtctctagttcctttcgaaatgaaattccagcga[G/C]ctgtgcttgattaactccgtcagaggctaattgaaattcaggacgcgtgccattttcgatagaggaaacaatgatgccttgttcgttgggaatgctcttg

fava_c18490_686	fava_c18490	686	C/T	tatatattttgtaaacatgatatgctgggcgtatgtgtttttttatagtcactgtcctgcatgtatttgaaccttgtaaaatttctgtgctccttatatt[C/T]ggcaagtctggggaatgtgtaaaagtagtaggcatttttacttttatcctaaatccaatctggatactgatatacaattttacatgcatcttgtattaat

fava_c18493_198	fava_c18493	198	C/G	cttgttttgtccttcaaaacaaccttaattattgtcatggaaaaccaaattgaagtagaaaatcaacaacagaaaccttcacaaccatcttcacaatctt[C/G]ttctccaactcatgaattctccttctcgatctcacataacacattcaacgaatcattcctaaatagcaacaataaatcaaaatcatcttcaccaaattcc

fava_c18493_403	fava_c18493	403	T/A	cagcccttgatttatcaccagctgatgttattttcttcaatggtcatttacttcctctccaaatcctctctaacttcccttcttcgatatcgcctcgcgc[T/A]tcgacgaattcaatggacagtttcacactccctataagagaattattctcagaagaagaagatgatgaaaatttccccacaaaggagagcaWaatcaaga

fava_c18493_495	fava_c18493	495	T/A	cctcgcgcWtcgacgaattcaatggacagtttcacactccctataagagaattattctcagaagaagaagatgatgaaaatttccccacaaaggagagca[T/A]aatcaagaatatggaaccatcaaaaaaggtagagggtaagttcaagtttgcattttcattatttagttcaacaaaagggacaaaagggtgtcacaaagag

fava_c18493_759	fava_c18493	759	G/A	ttaagaatggtgaaaccacttttcaaaaaagagaaaattagagttcatgataaaaagtgttattcttcttcatattcaggaaatttaacacctagaaata[G/A]acaagagttgatgaaaagttggaaattaggacaatattcatcagcWccgKcatcgatgagaacttctccatcaaatagtggtgttctgtttccaactaca

fava_c18493_805	fava_c18493	805	T/A	atgataaaaagtgttattcttcttcatattcaggaaatttaacacctagaaataRacaagagttgatgaaaagttggaaattaggacaatattcatcagc[T/A]ccgKcatcgatgagaacttctccatcaaatagtggtgttctgtttccaactacacctttaactcctgttagtgatagctccatggaagaattgcagaatg

fava_c18493_809	fava_c18493	809	G/T	taaaaagtgttattcttcttcatattcaggaaatttaacacctagaaataRacaagagttgatgaaaagttggaaattaggacaatattcatcagcWccg[G/T]catcgatgagaacttctccatcaaatagtggtgttctgtttccaactacacctttaactcctgttagtgatagctccatggaagaattgcagaatgctat

fava_c18494_1457	fava_c18494	1457	A/G	tgaaaaaatcatgtctatgccattgtaagagagtttttgtgtgccatgtgatggaggtctactagatacaccatggtcatgatctttgagaatttgttga[A/G]ctagttgtggtgtggaaataacaatagctttcttgaaaccaatttgaagggagaaaagagggccataaaWttttgagagattcaagaattgaatatggag

fava_c18494_1527	fava_c18494	1527	A/T	ccatggtcatgatctttgagaatttgttgaRctagttgtggtgtggaaataacaatagctttcttgaaaccaatttgaagggagaaaagagggccataaa[A/T]ttttgagagattcaagaattgaatatggagatttgatatgtcaagttgatggaggtttccaattatgggaaggccttttggaccttttggaaaggttgat

fava_c18494_1629	fava_c18494	1629	A/G	tttgagagattcaagaattgaatatggagatttgatatgtcaagttgatggaggtttccaattatgggaaggccttttggaccttttggaaaggttgatg[A/G]tgagttcttgatattggttttgtatttcatgaaaaggaataacaccaataaaggaagggctagaagtgtgtgaagaaatgataccattgttgataagagc

fava_c18494_171	fava_c18494	171	C/G	aaagtaaccaagaaaaaatgtaatgagtaaaaaaataacaagaaaggattgaaccctcaagtccttcaccaacataatagttccttaaggagaggtctaa[C/G]agacacatacattacttaaaaaacattgccttgtaacattcacatatgattcttggcaacaaggcagagatgattcttcttgtgtctagcaagtcctggc

fava_c18494_303	fava_c18494	303	C/T	tgtaacattcacatatgattcttggcaacaaggcagagatgattcttcttgtgtctagcaagtcctggcaacccttcaatatctatatcttcctttctca[C/T]accttcaggcatttcccaatcaaacgaattgagaagattcgcagttaccatttcttgcatcgcgattgccaWtggtatgcccgggcaaattcttcgccca

fava_c18494_375	fava_c18494	375	A/T	ccttcaatatctatatcttcctttctcaYaccttcaggcatttcccaatcaaacgaattgagaagattcgcagttaccatttcttgcatcgcgattgcca[A/T]tggtatgcccgggcaaattcttcgcccaccaccaaacggaatcaactcaaaatctaatcctttaaaatcaacatcattgtttaagaatctttcgggataa

fava_c18494_537	fava_c18494	537	G/C	ttaaaatcaacatcattgtttaagaatctttcgggataaaactcgtctggatctttccatgtctcgggatccctatgaatggcccaaatgttcacataca[G/C]taatgtttttggttctattttatatccatttagaatgaaacttttgttggcctctcttggagccagtggtactggtggataatatctcagcgtctctttt

fava_c18494_697	fava_c18494	697	G/C	gagccagtggtactggtggataatatctcagcgtctcttttatcacagctttgagatatactagcttttgaatatcatcttcatctataaactctttttt[G/C]ccacaYatgtttctaatttcttcttgagctttcttcattgctcttggattcttcattagtgcagtcatcacccaaaccgatgtggctgcacttgtgtccg

fava_c18494_703	fava_c18494	703	C/T	gtggtactggtggataatatctcagcgtctcttttatcacagctttgagatatactagcttttgaatatcatcttcatctataaactcttttttSccaca[C/T]atgtttctaatttcttcttgagctttcttcattgctcttggattcttcattagtgcagtcatcacccaaaccgatgtggctgcacttgtgtccgtagctg

fava_c18494_970	fava_c18494	970	T/C	gattttttagctcaagcaaaacatcaactatatccttctcttcttcttctctcttcctattattaggatcaagatgttccttaagcacttgttcaaaaaa[T/C]gcatccaaagacttaaaagtgttgtcaactcgagcaatcaacccggtaagtttatcaatccatcccatgaaaggaatataatctgaaacataaaaggaaa

fava_c18498_267	fava_c18498	267	C/A	ctttgtctcaaagctttcaattcgcagcgttgtctcagttcgcgaccgtcgattcgtttaacatcttagatcgctcaccatcaccaattcaatctatata[C/A]aactaaagcctcaacaacaccatgaaatcgcaaaacaaaaacYaaagattacctatcggaagaactgtcaggtgcttcgccgacctcgcatcaggtacaa

fava_c18498_310	fava_c18498	310	C/T	gaccgtcgattcgtttaacatcttagatcgctcaccatcaccaattcaatctatataMaactaaagcctcaacaacaccatgaaatcgcaaaacaaaaac[C/T]aaagattacctatcggaagaactgtcaggtgcttcgccgacctcgcatcaggtacaaattatgtatgttctgtaatgggatcacgagtagtattatccgc

fava_c18498_641	fava_c18498	641	C/T	agttatgtgttgggagaactaaatgttgtgtcctcatcgaaacatatagtcacgtaaatctctagtacagtaggatttttatttttttcaaaaggaaatt[C/T]gatatcttgaatgaggccttggaattttagcatgtattttggtaatgaatcatattgtaatgggaactcatatgtaatgaattggatatttgatgtacac

fava_c18499_1173	fava_c18499	1173	T/C	ctggatcatattatgcaatgcatagtgactcaccatcagaatcatttgcatcatatggtgtaaggcatagtgacttgcctcccttgtcaactgcaaaatc[T/C]atctcgcctccgtcagaaccatctgcatcatttactgcaacaaggcatagtgactcagctccctctttatcaactgtaaaacccgaagctaaggacttct

fava_c18499_1594	fava_c18499	1594	C/A	tacaacagaaagggatcatttcactgagcaaaatgctgcaagtaaccctgatggcaatgttaaggatcatgatattcactataatcattatgattccaaa[C/A]gctctcattcatcaagttttccttcatttgacacacacaaggaagatKttgattcttctctacccactgatcatggtttggatgatagatccaRtagtca

fava_c18499_1642	fava_c18499	1642	T/G	tgatggcaatgttaaggatcatgatattcactataatcattatgattccaaaMgctctcattcatcaagttttccttcatttgacacacacaaggaagat[T/G]ttgattcttctctacccactgatcatggtttggatgatagatccaRtagtcaccagcctaagagattaccttcaatggaagacgacccatatttctcata

fava_c18499_1688	fava_c18499	1688	A/G	tccaaaMgctctcattcatcaagttttccttcatttgacacacacaaggaagatKttgattcttctctacccactgatcatggtttggatgatagatcca[A/G]tagtcaccagcctaagagattaccttcaatggaagacgacccatatttctcatatcccaacttattcacctctcaaaactcaaatgttggatctcacact

fava_c18499_1820	fava_c18499	1820	T/C	gaagacgacccatatttctcatatcccaacttattcacctctcaaaactcaaatgttggatctcacactcattcagataatagccgcaccacttatgaca[T/C]gtgatggggaattatcattcaaccacacattttcgtcagtccgaactctgaaatcttgcttgtgagtgtgttttatgctgttgaagaatccaccatattt

fava_c18499_2136	fava_c18499	2136	G/A	caaaagcctctgtttgtgctttgtggggctgatacaatgtgcaaatctgagagtttcagaaaaaaaccattcagcttcgattttctttacttacattata[G/A]gaggatacaggacatgtaatacaacttcatttgtaattttattgtaatattgttgcatattgccgtgtgaatgttatcgctttacttgcatctgatttat

fava_c18499_570	fava_c18499	570	C/T	aatttgcaaccaaatatgggaaggaatttatttctgctgctactgagctcaggccagattgcggtgttaatcgacagttaatagagctgctgtcaattcg[C/T]gctccttcaccagaaaagaaactcaatcttctaaaagacattgctactgaacatgagctagagtgggatccagctacttctgaaatcgaaatgtttaaaa

fava_c18499_828	fava_c18499	828	C/T	ttcctgaagaaaaacatgaggagcagttgtattccactcatgatacacccaaaaaagagcaatttgattctgattcggactctgacatgttggactttcc[C/T]gaagttccaaaggtatctgtccagccaagtgcaaattttgccacagctccYgatatggttatacctccagcagctatgccacaccctaaagttgattttc

fava_c18499_879	fava_c18499	879	T/C	aaaaagagcaatttgattctgattcggactctgacatgttggactttccYgaagttccaaaggtatctgtccagccaagtgcaaattttgccacagctcc[T/C]gatatggttatacctccagcagctatgccacaccctaaagttgattttcattcatcaagccattctggagaatttgcagatatgaagcaggaacacattg

fava_c18500_1304	fava_c18500	1304	C/G	cctcaggtaatttccaagtcaagcctccaatactaaatatagttccactcgccccaccagaatcggaatcatcactgcaaccacaaactgcttcttggtg[C/G]ccatttaatttcttagtatcttttgatggaaacatatatggagatggaacatcagcaaagcttatgtcagatctgcaagatgttgatgattctaacagcg

fava_c18500_1487	fava_c18500	1487	C/T	ttgatgattctaacagcgctgttctaatctgttgtattgaatgtgcatactccagcccaaaaagaaccatgataggcttgttattttccagcgcatactg[C/T]gMcacactttcaacacaatcagctaYgccaatggaagccttaccaaaaacaaaaaaagccggtagactcgttgtcgggctaaaacacgtatgtccataat

fava_c18500_1489	fava_c18500	1489	A/C	gatgattctaacagcgctgttctaatctgttgtattgaatgtgcatactccagcccaaaaagaaccatgataggcttgttattttccagcgcatactgYg[A/C]cacactttcaacacaatcagctaYgccaatggaagccttaccaaaaacaaaaaaagccggtagactcgttgtcgggctaaaacacgtatgtccataatgt

fava_c18500_1513	fava_c18500	1513	T/C	atctgttgtattgaatgtgcatactccagcccaaaaagaaccatgataggcttgttattttccagcgcatactgYgMcacactttcaacacaatcagcta[T/C]gccaatggaagccttaccaaaaacaaaaaaagccggtagactcgttgtcgggctaaaacacgtatgtccataatgtaggacacagtcagcattaatgtgt

fava_c18500_162	fava_c18500	162	T/C	atgacgactattattaattgtgtgattgcattgttatgtttatcattaatacagatatataaataaatgctccagccaacactgaccagacaacttacac[T/C]tatttaagtcaactgattgcatcttgatgtttatgtttacaagcacaattcattgccgcctttctagcacaaatggcatacatgacagttgattctaact

fava_c18500_1628	fava_c18500	1628	A/G	taccaaaaacaaaaaaagccggtagactcgttgtcgggctaaaacacgtatgtccataatgtaggacacagtcagcattaatgtgtgatgctccaacttc[A/G]tcaacacagcaactgccataggctgtatcagccatgacatacaatccaatgtctgtagcatccgattcYcgcaacgaatgaagcttctttcgcagaacac

fava_c18500_1697	fava_c18500	1697	T/C	agtcagcattaatgtgtgatgctccaacttcRtcaacacagcaactgccataggctgtatcagccatgacatacaatccaatgtctgtagcatccgattc[T/C]cgcaacgaatgaagcttctttcgcagaacacggacaagtctcgttgaatcctttagaagatcatcaggaaattgtaaagcaactctggtgaatttgtgag

fava_c18500_1810	fava_c18500	1810	C/T	agcttctttcgcagaacacggacaagtctcgttgaatcctttagaagatcatcaggaaattgtaaagcaactctggtgaatttgtgagtgcgaatgaatt[C/T]ggcagtggcggcaatatcgtaatttgattcgaaatccattgctacagaggaagaccaatagtgaaaccaaaaacaaaaaaaaagctatctcctttcctga

fava_c18500_282	fava_c18500	282	T/C	catcttgatgtttatgtttacaagcacaattcattgccgcctttctagcacaaatggcatacatgacagttgattctaactcaccagcctcacagaacaa[T/C]caatatgctcttctttccctttcccatattctcatccattacaatcaaatgcaaaacaaagaatacatcattgagccagttgtcataaatttcaatgttt

fava_c18500_533	fava_c18500	533	G/A	atggctgtggggtggtgttctcgtcgggtatactcagaccttgataagttcgatttgcgaaaaattctgctcctgatttagcgtccccttttattaggat[G/A]ttacagttgtcccgcaactgcagtgccttctcagttgcatttgctaaagcaagatcttcttgttcgtcttcaacattttcttgattttcaaaatcttgaa

fava_c18500_764	fava_c18500	764	C/T	cttcttcctcgtttccatcctgtacttgaggaagattaattaaatctccaaatcccattacataggcacctgtccattggcttcccctgttgaaagcaat[C/T]gttgcttcaaagggagtaataactggagctagatactctttgctatccagtaaagcagtttgggcacaagagacRtataggaaaacatcacactcaggaa

fava_c18500_839	fava_c18500	839	G/A	attggcttcccctgttgaaagcaatYgttgcttcaaagggagtaataactggagctagatactctttgctatccagtaaagcagtttgggcacaagagac[G/A]tataggaaaacatcacactcaggaaagttagcaagtttagctgggtttggttttcccataaccaaagtgtaggcctttttgcctgccgaagtaattagct

fava_c18500_957	fava_c18500	957	G/A	ctcaggaaagttagcaagtttagctgggtttggttttcccataaccaaagtgtaggcctttttgcctgccgaagtaattagctccatcatctgattaatc[G/A]cgtgcagataaccagcaactccaagtgttccaaccaaaattccaacaatattagcatccttagccttctccaccaggtaatatctacgtttaagaatcct

fava_c18502_320	fava_c18502	320	C/A	gacaaatttgcataaaaaatctttaaccaaatccgggtaaatataaccaaagcttgaagatggaaaaaaggcaggattgaacaaagcaggaaaatcaaag[C/A]ttgaatatgaaaaagaggcaggattatcatacttagagggtgtcaatggacgatttctgatgttgaatagaaacctccttgacattaaaKtaggttccac

fava_c18502_410	fava_c18502	410	G/T	aaaatcaaagMttgaatatgaaaaagaggcaggattatcatacttagagggtgtcaatggacgatttctgatgttgaatagaaacctccttgacattaaa[G/T]taggttccacaatctcaattttagaatcttgagctgggttagaagaactttcaccaatatttgctttgcctttatctttcccttgaagagccattgatgg

fava_c18505_263	fava_c18505	263	G/C	acgagttcatgctcaaatctatcaaccaattgtctaaatggtgaatggccctgcaaaacctgagaaggtgagacgcttaaacccaagagttgactaagct[G/C]agaggctcttttagcttcaggaataccaccaccattggtaaggaaaacgtaagggattttcattctaccttgtgtgtcgtatagttttctcaaagctgcc

fava_c18505_504	fava_c18505	504	C/G	cgtcgatgtcgaaagcaatgccgaatgatggcctgctaaaccgttcggatttgtgagaaaaagagcggggaagtatgtgagaaaaggcggtgtggttttt[C/G]gattgggtcYgttgtcgccatgctttggttaagagacggaagctcattgtttgatcggaatcaactcagtgctaaccgtcgaatgtcgatagcaaaagtt

fava_c18505_514	fava_c18505	514	T/C	gaaagcaatgccgaatgatggcctgctaaaccgttcggatttgtgagaaaaagagcggggaagtatgtgagaaaaggcggtgtggtttttSgattgggtc[T/C]gttgtcgccatgctttggttaagagacggaagctcattgtttgatcggaatcaactcagtgctaaccgtcgaatgtcgatagcaaaagtttgttgctctg

fava_c18508_177	fava_c18508	177	C/T	cttcctcacgcgtttgaggtttttattgatcttgttcttcttcgaggcctctgcgctcttgttgctcatgctttccttggccacaacaccgtttttctca[C/T]ctgcgtgactatttaggatcttttctcctcctttttgaaccttRtttgtataagcattgctgcagatgaagatacccttcacaatctgaccagttcctcc

fava_c18508_221	fava_c18508	221	G/A	aggcctctgcgctcttgttgctcatgctttccttggccacaacaccgtttttctcaYctgcgtgactatttaggatcttttctcctcctttttgaacctt[G/A]tttgtataagcattgctgcagatgaagatacccttcacaatctgaccagttcctcctgcaataagcttggacagaaaatgattgtaatcttcgaccctcg

fava_c18508_362	fava_c18508	362	C/T	tctgaccagttcctcctgcaataagcttggacagaaaatgattgtaatcttcgaccctcggagcaaactccttccaatcaagatcattcttgttactcaa[C/T]tttaagccagaaaagcaagagtgttctttcaaaaatgagtctagaaaactcaaacttccacaactatcttccgaaaaggttacaccatagctaagaggct

fava_c18508_683	fava_c18508	683	C/T	cattctcctccaatgttttgatgaccatgaaatggccttgagcaagttcaaaagcctctccttcatccatcagatgaactttacatcctggtatttgtat[C/T]agaacttcttgtttgaggttcttggatcctgcatatttttcgacagtacaacttcccggtatagaggtttccattggaggcatagctgtggagcaatgga

fava_c18509_1967	fava_c18509	1967	A/G	tcctgcactgctgtagtttgtcacaagtcatagctttttaaggcaggtagatgcagttctacattttacttttcttcctatattatttttgtttcaattt[A/G]gcttacatattctttatgtattattatttgtaacacattaagttttcaatgtaaggcttaagatggaccggaattaaacagggagatgaattttgtgatg

fava_c18509_410	fava_c18509	410	C/G	tacattgatcatttcgctgtttcagataatacttcttctgttgatttgaaactgagtgaatcgcgtaggattttggagtttgtggatgggtttgagtgga[C/G]atttggagaaaaggttgtgcattatagaactgggaatgtggggttgcaggcacagtggttggaggcgtggtggccgggttcggatgatgagtttgctttt

fava_c18509_549	fava_c18509	549	C/T	tggggttgcaggcacagtggttggaggcgtggtggccgggttcggatgatgagtttgcttttgttgtggaggatgatttggaggtttcgccgctttacta[C/T]gagtttgttaagatggtgattgtgaatttctattataatgcttccaattatagtccttccgtttttggtgttacgttgcaacgtgcaaggtttgttccag

fava_c18512_1719	fava_c18512	1719	G/A	agaaggactataatgatgtgctcctttatattctcggattgcttgtaaagggtgctattgattttgaagaaattgttgctggatctacttctagtttgac[G/A]catgtagaagtcaaagtagatgatctctccatgaaggctcaagagcatgatatatatgatctgaagccgttcttcaacagcagccagttttcaaggtcca

fava_c18515_2682	fava_c18515	2682	C/T	agaagataacgcagaggatgcagttgatgccgaaattgttaaggttgaaagagaagttgttagagacaaactagaagaagatgcagacaactgagaatct[C/T]ggcagttttcacttgtcaaccatccaatggatgagatgagtaggtttttggagataattgRaagagaaatcaagagaaaactttgaagaaRttattggga

fava_c18515_2743	fava_c18515	2743	A/G	agagacaaactagaagaagatgcagacaactgagaatctYggcagttttcacttgtcaaccatccaatggatgagatgagtaggtttttggagataattg[A/G]aagagaaatcaagagaaaactttgaagaaRttattgggaatgaattcctttcgggaaaataaagagatctatttggagagggattgatgcatattcaact

fava_c18515_2773	fava_c18515	2773	A/G	tgagaatctYggcagttttcacttgtcaaccatccaatggatgagatgagtaggtttttggagataattgRaagagaaatcaagagaaaactttgaagaa[A/G]ttattgggaatgaattcctttcgggaaaataaagagatctatttggagagggattgatgcatattcaacttctctctaaatagagctctccatttactcg

fava_c18518_278	fava_c18518	278	A/G	accacagcgcgtctctttgctaatgaaggcacgcgtgtggttgtgatcgccgatatccaagatgagcttggtgaacaagtagctgcatcgattggtaacc[A/G]aaggtgcatctatgttcattgtgacgtgacggacgaagatcaagttcgaaatctcattcaatcaactgttaatacttatggacagatagatataatgttc

fava_c18519_499	fava_c18519	499	T/C	gcaacgtaacgtctaagaacaacgccaagaaactagcaacaaatggttcagacgcaaacggaacattgatcatgtcattgaaccatcttgcgcgcgtgtg[T/C]acaggaccatagcctttaaatgctgtgtattcgttgaaatattgcggtatagaaaatcccatgaaaatagagaagcctaagatgaattttgttctgaaac

fava_c18522_106	fava_c18522	106	A/G	accaaatatctttatttaattccattacatcaagtaatacctcaaaattattgtggtacaatacaagctacacatccaacaaaacatgaaaatattgaag[A/G]atatatctacaagaagttacaattgaaaggcatgtgttttgtttctataaccaaactctcctaataaatcagaagcagaagaagccataatagtagcaaa

fava_c18522_394	fava_c18522	394	A/C	tttagccatagctccagccaattcagcagctgaaatataaccatttccatcgcgatcaaagctcttaaacacacctatgagttgttcttgattcacaaat[A/C]cttcttcattcatcatgtttggcattatgactcttactagttcatcaaactcaacagaaccgtttctgtttgagtccatttttgataacatSacttgaac

fava_c18522_486	fava_c18522	486	G/C	tcacaaatMcttcttcattcatcatgtttggcattatgactcttactagttcatcaaactcaacagaaccgtttctgtttgagtccatttttgataacat[G/C]acttgaacttggtcacctaatggtttgagacctaatgaacggataagtgctgctaactctaacattgttaggcttccatctgaatccatgtcgaagcgtg

fava_c18522_614	fava_c18522	614	C/G	agacctaatgaacggataagtgctgctaactctaacattgttaggcttccatctgaatccatgtcgaagcgtgtgaagatttctcttagttgattaagtt[C/G]atcaacttYaacctttgacataataattactaatgaagatagagcaaagttgaagtgaaaagatgggaagagaagtgtggcttattttggtttcaatttg

fava_c18522_623	fava_c18522	623	T/C	gaacggataagtgctgctaactctaacattgttaggcttccatctgaatccatgtcgaagcgtgtgaagatttctcttagttgattaagttSatcaactt[T/C]aacctttgacataataattactaatgaagatagagcaaagttgaagtgaaaagatgggaagagaagtgtggcttattttggtttcaatttgatgtgcttt

fava_c18523_730	fava_c18523	730	T/C	gctagagaaacaagcagtgatagcagcagtggctatcctcatgaaagaggaacaaggtgtgtagttaatggcatttcgaaccagcctagcttttcagatg[T/C]acacagtcatggtctggaaagagtttcactagaaagtaaacctgactcatgtccatcaagtgatgagaacggaagctgcggtgctcttggtcagcttgta

fava_c18524_1330	fava_c18524	1330	T/C	gcagactgggaaccagcaagaagattggtttgtggagaattattgttgcacggaatcttccttatacagatgcaagacgtacagggaagattccaaagct[T/C]ctattgcatagaatggctccaaatgcccgctactctatatggcttgatggaaagcttgagcttgttgttgatccatatcagattcttgaaaggtttttgt

fava_c18524_1564	fava_c18524	1564	C/T	attatagacgctttgatgtatttgttgaggccgaagcaaataaagctgctgcaaagtatgataatgcctccattgacttccagatcgatttttacaaaaa[C/T]gagggactgactccgtattcggaggccaaatttcctcttataagtgatgttcctgaaggatgtgtaataatacgagagcatgttcctattagcaacctct

fava_c18524_1881	fava_c18524	1881	T/C	gattgtgaaagacgcaacttcgtagttcagaaataccataggggtgtattagggcggctagctgcgccagtggttgttgctctcaaccctccaccgccgc[T/C]cttgtctccccctccYccactgcttgaaaatacacaagaaagagttgttattccaataactaggagaggccctggaaggcgcggaaaagataagagaccg

fava_c18524_1897	fava_c18524	1897	T/C	acttcgtagttcagaaataccataggggtgtattagggcggctagctgcgccagtggttgttgctctcaaccctccaccgccgcYcttgtctccccctcc[T/C]ccactgcttgaaaatacacaagaaagagttgttattccaataactaggagaggccctggaaggcgcggaaaagataagagaccgggttctaagcgccacc

fava_c18524_821	fava_c18524	821	G/A	tcctccggggcatccgtgtaatagttttacactccctccaccaccagctgataaaaagcgtactgggccacgaccatgtccggtatgttaccttcctgtg[G/A]atgaagccattagactcatgccaaagtctccctcaccgtctccagtgcttaagaatttgacgtttacctacgaggaaaatctaagcagagatgcagaatt

fava_c18531_907	fava_c18531	907	C/T	tggtttcccttatgatgtttggtgacggtgaaacMgcaaggaagctacagcaaatgggtgaaaactttcaattgtctcagatggctgcagtcaaattagc[C/T]gaggagacaatatcaaccgaaaccataaatgagtatgcacacactttggagcaatatacccgaaaagtcaggaatgaaattcacaactctgaggatttgt

fava_c18532_264	fava_c18532	264	A/G	taatctgctcatttgaaaacactttaaagtataataatgtctcgatgaataaaaaacacgaatgatacattctttttgacaacatatgatgaaaactcag[A/G]gctcttcatccatcaacgcttcggacctgctctattttgaagttctgttacctagcttatgatcaaaatgcatccctttctgcatgtaatttatttcttt

fava_c18534_1114	fava_c18534	1114	G/T	gtttagatgacagtggtagagatggtgaattgtgcggtgtggatggaattggtggagagaaaaactacatgattttgagaaggaacggggtggaaccacc[G/T]cataaggagttattggaggaagtaacaacgttgtctttggaggaatgtgaagggttatgtgagaaaaactgtagatgttggggagcgttgtataataacc

fava_c18534_1397	fava_c18534	1397	G/A	ggtgaggaaaggtgcacggaagaggaatagggttggggttataattggaattgtggtagttttttttgttggggttattattattgggggtgtgatttgt[G/A]tattgaggtggagaaagaaaaaagtgagtatgaaggaggaagataattgggcttcgtccggcccatataagaatctgagatccaaaagttttagttcaat

fava_c18534_1540	fava_c18534	1540	G/C	ataattgggcttcgtccggcccatataagaatctgagatccaaaagttttagttcaattgaaatgtcgaatagtgcacaataaataaatgaggaagtgac[G/C]taacgggtgacgtcattgaaaggttgacaRagagattttgaattgattatggatWtattttggagacactttagtataatggaatagaatctatctaaat

fava_c18534_1570	fava_c18534	1570	A/G	atctgagatccaaaagttttagttcaattgaaatgtcgaatagtgcacaataaataaatgaggaagtgacStaacgggtgacgtcattgaaaggttgaca[A/G]agagattttgaattgattatggatWtattttggagacactttagtataatggaatagaatctatctaaatctaatatttctcctgagtagtggttggtgc

fava_c18534_1595	fava_c18534	1595	T/A	aattgaaatgtcgaatagtgcacaataaataaatgaggaagtgacStaacgggtgacgtcattgaaaggttgacaRagagattttgaattgattatggat[T/A]tattttggagacactttagtataatggaatagaatctatctaaatctaatatttctcctgagtagtggttggtgcactttagcacgtgatatgaccttgt

fava_c18534_318	fava_c18534	318	G/A	ctccgccacaaccaaaacgaccttcaactagccgtcgttcacacaccttccttagaaccactatggatcgccaacccaacccaattagtatcatgggccg[G/A]caccacRcgcctctttttcaatggcagcttagttttgtcggatcctaaaagaagcatcacgtggtcaacgaacacagacggagactgcgtggtgcttctc

fava_c18534_325	fava_c18534	325	G/A	acaaccaaaacgaccttcaactagccgtcgttcacacaccttccttagaaccactatggatcgccaacccaacccaattagtatcatgggccgRcaccac[G/A]cgcctctttttcaatggcagcttagttttgtcggatcctaaaagaagcatcacgtggtcaacgaacacagacggagactgcgtggtgcttctcaacacct

fava_c18534_748	fava_c18534	748	G/A	actggaaacgcactgcattacaagcaaaagctaaggtaataatcggtgcaggacctatttctgcaagagtaagcatagagggttacatcggaatgtatca[G/A]acaagttcaaaaccgattgacgttcagaaattcaacagcttccaacaaacatcatcaacgtcgtttctcttggttcgtttagaatctgacgggaatctca

fava_c18534_904	fava_c18534	904	A/T	caacgtcgtttctcttggttcgtttagaatctgacgggaatctcaaaggttattattgggattcaacaaagtcaacctggctacttaactatcaagctat[A/T]acagaatcctgcgggcttccgaatccttgcggttcgtatggtttgtgtacgccaggagagtctagttgttcttgtttagataaccaaaccagtttcaaat

fava_c18536_1263	fava_c18536	1263	G/A	gctcttcacaagaagtggcgaataaagccttacaagaggggcttcaggaatctcaaactcgaatatatggtttgcctataaggccttctttcgctcgcgc[G/A]gttcttgttaaggctcaactaagagaagaacttgagatggatcctgacttgccagcagttttgctgatgggaggtggtgaaggaatgggtcctgtcaaga

fava_c18536_1770	fava_c18536	1770	C/T	atggagctggtgtcttcacgcgtagttctaatgaaacagcgcgaattgtggcagaatggttcagtacaaaacaagatgatctaaagaaaatgtcagagaa[C/T]gcacttaaactagcgaatccagaggccgtgtttgagatcgtgagggacattcatgaacttgcaaagcaacgcgaaccaggagcttttccttacatgttga

fava_c18536_2031	fava_c18536	2031	A/C	agctttttttccgatcatttccattagaattgacatgtacagataattcttcaagttacattattctttatcgcagtggcgtggtttttactgtcatata[A/C]ggcaaaagtctgtggttttagcagactttgggggtttatttaggatgtttgttggaagataagattggtttgttttgtaactgtgtaatggtttcacatt

fava_c18536_408	fava_c18536	408	T/C	acactttcttttctccgccggagaaactttgtttcggtggcgggagtggtttcttcatccgtttgaccgttaggtgtttgctgtattgtttgtatcgtat[T/C]atattatattatattcttcttcctctgtgccttttcgaagacatggaggtttcagaattgccaccgggaaagtttgccgatgtgttgccggaaaagtcta

fava_c18537_1449	fava_c18537	1449	C/A	gaacgatgtgaaagtaaacgacattccaaatgacagagttagtggagttgtacaaaaaggcaagaggagtgtgtatatccgctcagaggtggaacaggct[C/A]tagaaacattagacaaggccatttcaatggttagggaataccgattacgttccccgattgccacttctagctttgccaatgaagagtctccttgcatgaa

fava_c18537_1563	fava_c18537	1563	A/G	caaggccatttcaatggttagggaataccgattacgttccccgattgccacttctagctttgccaatgaagagtctccttgcatgaaaaaatatggtaga[A/G]ttgattcattttccataaaaactgttaaaccttgtgataacaatgaggttagtgttggttcatattctttggaaaatggagcaattctggaccaagcaac

fava_c18537_2083	fava_c18537	2083	G/A	ttaggtagtcaaatatacgtatacaaagttctccttatacaaatcattattttcatatttattgttctactaatcatgtaatatatgtagactatcttgt[G/A]tttgtcaatctatcttcatgatacttcttcatataattagaactggcatggaaatattcactataacttcttttgtaaattattttttatgtgtaacatt

fava_c18537_314	fava_c18537	314	A/G	cccgaatatatgcgtaaatggagaaaaaacggaagatagtgcagtatagggagaggctggataggactcttgctttacctgatcttacaaatgttgagat[A/G]ctcaaaaatcttgttaaaagtcaactcatacctttatcagaacaagaagatgaaggatataaagagaaattaatagaacataagactgcagggatatcta

fava_c18537_710	fava_c18537	710	G/A	ggcaaaccactctttataaaaaatggtggcctcagtctactattcctactttcaaaatcttatcatgtgaatgtttagagcaggcccagattggggaaca[G/A]atatcactagtgaggatgaaggtttcgtggcctctgtctatgagggaagcWgtagtacactattatctgtttgagtactttcaagaggacttggttgttg

fava_c18537_761	fava_c18537	761	T/A	tcaaaatcttatcatgtgaatgtttagagcaggcccagattggggaacaRatatcactagtgaggatgaaggtttcgtggcctctgtctatgagggaagc[T/A]gtagtacactattatctgtttgagtactttcaagaggacttggttgttgttcttacgaattcggttcctgattcgagtgtcgcggggaccctttatggtt

fava_c18542_98	fava_c18542	98	G/T	tggattctgaagtaaaccatcgcagccgtctcttgcgttaagcctcaaccaaaacctctctccggcgttttccaccgcaaccgctcgattccgatca[G/T]cacgcggcggtttccgatcggcgttggattattatggattcaaattttgattcaaagcctccggcacgaagaaaggttaaatatgcaccaaaagctccac

fava_c18549_199	fava_c18549	199	C/G	ggcaacaatctgggtgcttctcccttgagaaaaagcattttatgggtcgagttcaccaataatccccacatgaacatgaacctttgtcaaagtggtggaa[C/G]cggttccggtccctcattacaatttgacgccgggtgatcctagatatcatcttcatcacagcatgacaatctacacaaacccgaaggttctttacaatct

fava_c18549_840	fava_c18549	840	C/T	caaagtaaagttccccaaagcacaggatcaggatcgatcttcatacacctcacaagatcataagcttcttgcaaatgcccggctcgaccaagtagattaa[C/T]catacacccaaaatgctcaacccttggttccatctcatactcattcttcatcaaattgaaaatctcccatcctttggtaaccaaaccagaatgtccacaa

fava_c18550_260	fava_c18550	260	T/A	gtagcagcaacagccttctcagcagctcgattttcatccgcaattctagtcaggttgttaattatcagtttcgaattgaaagtcagctctgcaagcgcac[T/A]cttatactgcgccaccagctcctgaaacggcagcggctgaggctggtaaccaccgtcatcattctccacgtctctatcattttgtctgaaccgtgaagca

fava_c18550_525	fava_c18550	525	C/T	ctttcattaatctaggtctcttcaaaatcggttctttggatctatcaaacggtcttcgcaaattctccatagcagataaagcaaaatacaaaccctaatc[C/T]aaatcctaaacctagtttacaaattacagataattgatatagatattgatgaaactagattacaggattggaaatgaatacatcgatcaataatgatatt

fava_c18551_1062	fava_c18551	1062	C/T	acaagacaaagcactttcacatgaacaataattcaattagcggccaaatcccaccggaactctcgcgattaccaaagcttgttcacttccttcttgacaa[C/T]aacaatttatcaggatttcttcctcctgagctatccaaacttccaagcctacttatacttcaactagacaataataacttcaaaggaaatagcattccag

fava_c18551_2970	fava_c18551	2970	C/T	tgtttcttgaacttttgaccggaatgcaccctatctcccatggcaaaaatattgttagagaggtcaatctttcataccaagccggtgtaatattttcaat[C/T]atcgacgagcgaatggggtcttacccatccgagcacgtagagaaattcttgacattggccctcaagtgttgcaacgacgagccagataatcgacctaaaa

fava_c18551_452	fava_c18551	452	A/G	gcccacgtttatgcctctttcactctctttgggagagatatttaaagggctgtgttgtggtgatttctatatatttcaaggatgtttctttcggaaggtt[A/G]caagcatcaagttgttttcgttttatggttttgttgctatttggtacttattgctgcacaggagaatatcactgaccctaccgaagttgaagcattaaaa

fava_c18551_575	fava_c18551	575	A/T	ttatggttttgttgctatttggtacttattgctgcacaggagaatatcactgaccctaccgaagttgaagcattaaaagccataaaagaaagattgattg[A/T]tcctaacggaaatttgagcaattgggaacgcggagacccgtgtacatctcgctggacaggagttttatgtttcaacgaaacattagttgatggctattta

fava_c18552_1817	fava_c18552	1817	G/A	agcttagattcaccagtcattgagaataagagtacttctgtgactggtaaaagaaaagagaggtctcatccttcatgatagatggttttatttgacatta[G/A]tttattagaggaccttgtgacattatttaattcatgttgtcaacccatcttatatggaaaaggcattggtggttttttttataaccatatattttctgaa

fava_c18552_465	fava_c18552	465	T/C	ttgcattagattatggatttcgtatattcgatcatatgtcctcccaaagtatcactcctaatgaagcgactgttgcgaccgtagctagactcgctgctgc[T/C]aatggtgatggtgaccgcgcgtttgaattggtgaaaaaaattggtgattataatgttgcacctaggttgaggacttatgatccggcgttgttttgttttt

fava_c18555_1351	fava_c18555	1351	T/C	ttgatgagttagagagtgatgtttcatttgttcgtgtaatatgggtcgataattcaggacagcaaagatgccgtgctgttcctaaaaagcgtttcaatga[T/C]gttgttacgaaaaatgggcttggtttagcgtttgYggttatgggaatgacttcatttttggatggacctgcacctggttctggtttaggttcagttggtg

fava_c18555_1386	fava_c18555	1386	T/C	gtaatatgggtcgataattcaggacagcaaagatgccgtgctgttcctaaaaagcgtttcaatgaYgttgttacgaaaaatgggcttggtttagcgtttg[T/C]ggttatgggaatgacttcatttttggatggacctgcacctggttctggtttaggttcagttggtgaagcaagattaacacctgatttgtctactataagg

fava_c18555_1990	fava_c18555	1990	A/G	gcgaaaccattagatctattgctacaaaacacggtttgcttgcaacttttattccaaagtatgcattagatgattttggttctggatgccatgttcatct[A/G]agcttgtggcagaatggtcgaaatgtatttatggcatctgatgaatcatcaaagtatggaatatcaactttgggaaaagaatttatggccggagttcttc

fava_c18555_2095	fava_c18555	2095	C/T	tgtggcagaatggtcgaaatgtatttatggcatctgatgaatcatcaaagtatggaatatcaactttgggaaaagaatttatggccggagttcttcatca[C/T]cttccttcaattttgccatttgtagcaccgcttcctatcagttacgatcggctYcagcccaacacatggagtggcgcatacttattctggggaaatgaaa

fava_c18555_2149	fava_c18555	2149	T/C	gaatatcaactttgggaaaagaatttatggccggagttcttcatcaYcttccttcaattttgccatttgtagcaccgcttcctatcagttacgatcggct[T/C]cagcccaacacatggagtggcgcatacttattctggggaaatgaaaataaggaggctccactgcgagctacatctccgcccggaactcgtagcggtttta

fava_c18555_2302	fava_c18555	2302	C/T	aggctccactgcgagctacatctccgcccggaactcgtagcggttttacaagtaattttgagattaaatcatttgatggctccgcaaacccgtacctcgg[C/T]ttagctgctataattgctgctggcattgacgggcttcgcaggcatctttcccttcctgaacctgttgatacaaatccaaatccaaaaaaccttcagcgat

fava_c18555_2438	fava_c18555	2438	G/A	tcgcaggcatctttcccttcctgaacctgttgatacaaatccaaatccaaaaaaccttcagcgattgccaaaatcactttcggaagctttggaagctctc[G/A]ataaggctgacttccttgaggaatttattgatgttaagttgctgactgcgataaaagcaattcggaaggctgaaagtgatcattacttggagaacaagga

fava_c18555_2602	fava_c18555	2602	T/C	ggaaggctgaaagtgatcattacttggagaacaaggaagcatacaagcaactcatacatcgttactgattatatattttaatggtttgaatttacatttc[T/C]gtgtttaStgattgtagttaaattaagaataattgtatcttaaaactgaggatcaataatcgccataaaaaaatatgttatcgataatcagaaaaatgtc

fava_c18555_2610	fava_c18555	2610	G/C	gaaagtgatcattacttggagaacaaggaagcatacaagcaactcatacatcgttactgattatatattttaatggtttgaatttacatttcYgtgttta[G/C]tgattgtagttaaattaagaataattgtatcttaaaactgaggatcaataatcgccataaaaaaatatgttatcgataatcagaaaaatgtctgtgttta

fava_c18555_405	fava_c18555	405	A/C	gaatccacgtgttcgacatgtttcaaagctgcaggaatctctgccatacttattgatgatgggctagaattggacaaaaagcatgatatagaatggcaca[A/C]gagttttacgccttttgttggtagaatcttaagaatcgaaaaagtggctgagaaaatccttgatgaagatgtttcggttggatctcgctggacattggat

fava_c18555_830	fava_c18555	830	A/G	atcctatgacttgccaatgcagatacacacagggtttggggataaagatttggatatgcgactgtcgaatcctcttcacctccgttcagttttcgaggac[A/G]agagatattcaaaatctcggattgttcttttgcacgcgtcctatccattctcaagggaagcatcatatctagcatctgtgtactcccaggtttatcttga

fava_c18555_943	fava_c18555	943	A/G	aatctcggattgttcttttgcacgcgtcctatccattctcaagggaagcatcatatctagcatctgtgtactcccaggtttatcttgattttgggttagc[A/G]attccaaagcttagtgtacatggcatgatttcatcactgaaagagcttttagatctggctccaataaataaggtgatgtttagtaccgatggctacgcat

fava_c18557_681	fava_c18557	681	A/T	aagtcaaggaggctttaagagcacagatggaagtgcaaagcaaactacatttactagtggaggcagagaagcacctgctaattcgccaagatgcagagcg[A/T]agatatatgaccatgcttgagagagcttgtaagatgttggctgatcagtttattggcgacacaactatagacacagacatccaaaaatttcaagaactac

fava_c18560_525	fava_c18560	525	A/C	ttgattcagtcgctacctatgcgaggaagcgtcaacgagggatctgtgttcttagtggtagtggaactgtcactaacgtgatcctgcgacagcctgctgc[A/C]gcaggatcagttgtgacacttcaaggaagatttgagatattatctttgtctggatcgtttctaccgcccccggctccaccgggggcaaccagtttgagtg

fava_c18562_527	fava_c18562	527	C/T	gctttgtaattctgactcctatgttttgagagttgtgtttcagaactggggttgtgattcttgtatgtggttttgaatgtttcaggaacggctggagctt[C/T]aggatcatgcgtagatggagtatcaggaagctggtgaggctcaaggaagtcgtacggcttttgtgaaggaaKgggtttgaaatcaccagttggattagaa

fava_c18562_599	fava_c18562	599	G/T	ttgaatgtttcaggaacggctggagcttYaggatcatgcgtagatggagtatcaggaagctggtgaggctcaaggaagtcgtacggcttttgtgaaggaa[G/T]gggtttgaaatcaccagttggattagaaccgaaatagggtctgagaagcgatacatgaaccacatgatggatacgtgaggaggatggaagatccaacata

fava_c18563_1222	fava_c18563	1222	T/C	ctgcttcggagatccttaaccgtttgattcagttagtgaatccattgctgaattttgatggtagattaacctattgtatggtctcagcgctgaaatcccg[T/C]atgaatccttgcgaaattccgcctccaatagcggaattgttcagcaaggaacacgctgagtcaactcagcttctgtttgataattctctctgctttaagg

fava_c18563_1519	fava_c18563	1519	C/T	tggtggattttgatattggccatgggcaaagatacattaaccttctccatgagctacacgcgcgtatcaaaagttcaggttcaccggcaactttgaagat[C/T]acagctgttgttgaYaacatcaacgacgagaggttgaagatggtgtgtgaSaatttgagttYacaagctgagcgtttaacaatcgggtttgaatttaaag

fava_c18563_1534	fava_c18563	1534	C/T	ttggccatgggcaaagatacattaaccttctccatgagctacacgcgcgtatcaaaagttcaggttcaccggcaactttgaagatYacagctgttgttga[C/T]aacatcaacgacgagaggttgaagatggtgtgtgaSaatttgagttYacaagctgagcgtttaacaatcgggtttgaatttaaagtggtgacactgaaaa

fava_c18563_1570	fava_c18563	1570	C/G	agctacacgcgcgtatcaaaagttcaggttcaccggcaactttgaagatYacagctgttgttgaYaacatcaacgacgagaggttgaagatggtgtgtga[C/G]aatttgagttYacaagctgagcgtttaacaatcgggtttgaatttaaagtggtgacactgaaaatcgctgagctaactcgcgagtctttgggtttcaact

fava_c18563_1581	fava_c18563	1581	C/T	cgtatcaaaagttcaggttcaccggcaactttgaagatYacagctgttgttgaYaacatcaacgacgagaggttgaagatggtgtgtgaSaatttgagtt[C/T]acaagctgagcgtttaacaatcgggtttgaatttaaagtggtgacactgaaaatcgctgagctaactcgcgagtctttgggtttcaactcggaggatatt

fava_c18563_403	fava_c18563	403	A/C	tcaataataacaatcaattctcaaccgcaaccaccaccaccaccacccagaattttcataaccctttccaccgagcccaaatccaagaaaaccgatcatc[A/C]aaccttatcagaagataccttgaccctgcttcgcaaatcgcacatcccctaaattccaccctcgtccgaaaacgtaccctctctgaatttcaaacccaaa

fava_c18563_802	fava_c18563	802	T/C	ctatagccacgcttcttcgctccaaccctagtggtcaaaactcaatccccgtgaataattccaccccccaaaacacgaattttcaatatcaaaattccaa[T/C]tttgatgagattcgaacaacactccctgacccacaaaagaaaatgatggaggattgcttaatggagttggaaaaacagcttcttgatgacaatgacgaag

fava_c18563_994	fava_c18563	994	A/G	atgacgaagaagtagaacccgacccaaattctgtgcttacaaacagtgaatggtcggaaacaattcagaatttgattagcttcgagcaaaacccgattct[A/G]atacccgtttcatctcccgcttcatccaacaattcatcttcttcctctgttgtttcacctgctatgttctcttccaagcaatcgttagtggaagcggcta

fava_c18567_194	fava_c18567	194	G/A	ggcaacaacggccacaattctcgaacatgcaacgacggttgcaacgacggaggaggagagaacagcatcatgcttttcggtgttcgactcaccgggggaa[G/A]caacacagccaccaccaaccaacacaacccgttcagaaaaagcgccagcatgaccaatctctctcaatacgatcaatcaacgcctcaagattccMacccc

fava_c18567_289	fava_c18567	289	A/C	gggaaRcaacacagccaccaccaaccaacacaacccgttcagaaaaagcgccagcatgaccaatctctctcaatacgatcaatcaacgcctcaagattcc[A/C]accccgccgacgccggttatgtctccgatgacatcgttcatgcctccggtagatcccgtgaacgcaagcgaggtgttccttggacggaagaagaacacaa

fava_c18567_421	fava_c18567	421	A/C	catcgttcatgcctccggtagatcccgtgaacgcaagcgaggtgttccttggacggaagaagaacacaagttgttcttgttgggattgcaacaggttggt[A/C]gaggtgattggagaggaatttctagaagctttgttaaaactcgcacaccaactcaggtggcaagYcatgctcagaagtatttcctccgccgtcataacca

fava_c18567_486	fava_c18567	486	T/C	acaagttgttcttgttgggattgcaacaggttggtMgaggtgattggagaggaatttctagaagctttgttaaaactcgcacaccaactcaggtggcaag[T/C]catgctcagaagtatttcctccgccgtcataaccagaaccgccgccgccggagatctagtctctttgatatcaccaccgatacagtgattgaatcttcta

fava_c18567_57	fava_c18567	57	G/A	ttatgttccttactagtggatttcattgagcattgctgagggagtaagtgtgtgaa[G/A]gcgaagatgtctctgtcccgcacgtgctcgcagtgcggcaacaacggccacaattctcgaacatgcaacgacggttgcaacgacggaggaggagagaaca

fava_c18567_608	fava_c18567	608	A/C	cgccgtcataaccagaaccgccgccgccggagatctagtctctttgatatcaccaccgatacagtgattgaatcttctactatgatggaagagcaatttc[A/C]gcatgaaacggcagtggcgctgcctccacctagccccaccttatatccgtcgtcacattacggtggcatcccggggacaccttttccgatgggactcagt

fava_c18567_799	fava_c18567	799	C/T	gggactcagtccggtagcaatgccggtgatgagtggtgaaagagcaggcaagccagttaggccaacaccaatgttgcctccttcttctaagatggctaat[C/T]tgaatttgagagagaaagcttcttcttcttcttccattgagccattgccRttgtcattgaaactgcaacctccatcaaaggaacattcgccggatagtag

fava_c18567_849	fava_c18567	849	G/A	agccagttaggccaacaccaatgttgcctccttcttctaagatggctaatYtgaatttgagagagaaagcttcttcttcttcttccattgagccattgcc[G/A]ttgtcattgaaactgcaacctccatcaaaggaacattcgccggatagtagcggccaYtcgtcaccgtcgtcttcatcgtcggcttttaaaactatatctg

fava_c18567_906	fava_c18567	906	T/C	tgagagagaaagcttcttcttcttcttccattgagccattgccRttgtcattgaaactgcaacctccatcaaaggaacattcgccggatagtagcggcca[T/C]tcgtcaccgtcgtcttcatcgtcggcttttaaaactatatctgcagggaagtatagtggtgggggtggggatagcattatcagtgttgcttgaaaatata

fava_c18569_1068	fava_c18569	1068	G/A	taggagatgcttttcaatgggttcttaccaatacgtagttgctgattcagatttggtagtggctttgtgtccaaagagaggagaaggaagaggaaatagt[G/A]ctagtatgagacaattgaaaggagggcttactcaaaatgggaattgttccattgatgtagatgttgttgaagggaagaaaatcaatgttgctaggaaagg

fava_c18569_1227	fava_c18569	1227	T/C	agatgttgttgaagggaagaaaatcaatgttgctaggaaaggtgaaagcttttctatctcaaagatttggttatggtctaggaaggataaggtggcaagt[T/C]tatcacaatcccatttggttaattctaatgtctcttcaactttgccatggatgaatagagaactcaaggaagcaacttagtgtttatgtgttatagacaa

fava_c18569_216	fava_c18569	216	G/C	aactcaaccatgtaagttctaactaccaccctatactactactcttcattaacctacacaaatttaattctttttacaatgtatgatttgggttgaatct[G/C]aaaacaacccaaacaccatcactttgaacaaatgtattgggttcttctgaatcttctgctgattacaagaaactgttagaatgagaatgaccccttcaat

fava_c18569_545	fava_c18569	545	T/C	tgtctgaacctgatccttatcagagacagttacagcaactcttcaatcttcatgattcgggtctagatcaagcttttattgatgctctgccggtttttct[T/C]tacaaagagataataggtttgaatgaggtgtttgattgtgctgtttgtctttgtcaattcaatgaacaagacatgttgagattgttgcctctttgtaatc

fava_c18569_764	fava_c18569	764	C/T	gcatagatacatggcttctttctaactcaacttgtcctctttgtagagggagtctttatgaacatggatttgcttttgaaaatccggtttatgatgaatt[C/T]gagagtctRagtgaagaaaatatggtttctggtaatgttgttggtgagggtaactctgttaacaagcatgcagagaatcatataatgagtgggaaaaggg

fava_c18569_773	fava_c18569	773	A/G	catggcttctttctaactcaacttgtcctctttgtagagggagtctttatgaacatggatttgcttttgaaaatccggtttatgatgaattYgagagtct[A/G]agtgaagaaaatatggtttctggtaatgttgttggtgagggtaactctgttaacaagcatgcagagaatcatataatgagtgggaaaagggttttttctg

fava_c18571_1257	fava_c18571	1257	A/T	ggattgttgcctcactgactgcctcttcacgcaaagaggataagtttggggctgctcagctttctgggagtaatgctgctgtggtttcgacgctgatatc[A/T]tgccttcttgctgttgagaatttcatgggaaagaaaacaaacctgcaatcaccaaatcaattgttgggatcttcgggtatcaaatgggcgacagctaata

fava_c18571_210	fava_c18571	210	C/T	aaaacctagaaacaacaacaaccatgtctccaacaccatcaaaaccagagattctcttaaacaacaggttcctaagctacctcatatggcaatcaatacc[C/T]tccactctcatcttcatcttctcctttttccttctcccccaaacccctttttggtctttcctcccYttcctcactttccagttcctcttctccgtttccc

fava_c18571_276	fava_c18571	276	T/C	ggttcctaagctacctcatatggcaatcaataccYtccactctcatcttcatcttctcctttttccttctcccccaaacccctttttggtctttcctccc[T/C]ttcctcactttccagttcctcttctccgtttccctatccttcgtctcttcaccttcctcccaccccatttttctcaacttcaagctctcaMtcactttYc

fava_c18571_367	fava_c18571	367	C/A	tttcctcccYttcctcactttccagttcctcttctccgtttccctatccttcgtctcttcaccttcctcccaccccatttttctcaacttcaagctctca[C/A]tcactttYcttctcttcatcaccgcctcagccttttccggttccgttgcggcgctttctttatccggatttaacgattctggaagagttgggttaagggg

fava_c18571_375	fava_c18571	375	C/T	cYttcctcactttccagttcctcttctccgtttccctatccttcgtctcttcaccttcctcccaccccatttttctcaacttcaagctctcaMtcacttt[C/T]cttctcttcatcaccgcctcagccttttccggttccgttgcggcgctttctttatccggatttaacgattctggaagagttgggttaaggggtttccttg

fava_c18571_579	fava_c18571	579	T/G	gtttgatttttgggtttcattatgtttttaagcgtcgttgggttttggagtttcccattattcagcgtcctcctttcttcaacttcaagatggggattcc[T/G]ttggctgcYaaacgtgcttttaagctttcMactgttgctttcattttttctgctgttttgttagagattattcctcacccttttaagtgcagcattgcta

fava_c18571_588	fava_c18571	588	T/C	ttgggtttcattatgtttttaagcgtcgttgggttttggagtttcccattattcagcgtcctcctttcttcaacttcaagatggggattccKttggctgc[T/C]aaacgtgcttttaagctttcMactgttgctttcattttttctgctgttttgttagagattattcctcacccttttaagtgcagcattgctacacagaaat

fava_c18571_609	fava_c18571	609	A/C	agcgtcgttgggttttggagtttcccattattcagcgtcctcctttcttcaacttcaagatggggattccKttggctgcYaaacgtgcttttaagctttc[A/C]actgttgctttcattttttctgctgttttgttagagattattcctcacccttttaagtgcagcattgctacacagaaattcttcggtgaacagattgtgt

fava_c18573_1365	fava_c18573	1365	A/C	gtcccatcttttactggaaatgtaatgcttaaggttcccccaggcacccaacctggtcaaaaggtggttttgaaaaggaaaggtataaggaaaggaacaa[A/C]tagtttcaaatatggggaccaatatgttcattttaatgtcagcattccagcaagcctgacagagagacagcatggattgatcgaagagtttgccaaggaa

fava_c18573_1518	fava_c18573	1518	A/T	agcctgacagagagacagcatggattgatcgaagagtttgccaaggaagagcgagaggaatcggataaacggaaagctgcttcggcttctagttaaaaac[A/T]tttgcagagtctccacttctcattattttattWaatacttccaagccatgttcaactaggtgggaaggatcccacttgctgatgaagttttgtaggtgca

fava_c18573_1551	fava_c18573	1551	A/T	gagtttgccaaggaagagcgagaggaatcggataaacggaaagctgcttcggcttctagttaaaaacWtttgcagagtctccacttctcattattttatt[A/T]aatacttccaagccatgttcaactaggtgggaaggatcccacttgctgatgaagttttgtaggtgcattcatcaaggttcactgttttgtttggccMcgt

fava_c18573_1648	fava_c18573	1648	C/A	attWaatacttccaagccatgttcaactaggtgggaaggatcccacttgctgatgaagttttgtaggtgcattcatcaaggttcactgttttgtttggcc[C/A]cgtccacttagaaagtatgaatttttcgaatcataagtgatgttcacaattcaaattataaaatttagaacttagaaatcaatattatcatatgcttgtg

fava_c18573_188	fava_c18573	188	C/T	agggttttttctagggttttactattcatcccaaaaaatcccaattctcctccaaacaaaacttcctaatttcttagtcaattcaattatccaaatggtt[C/T]gctctagtgtcaccatcctctttacccaccttgctcgtcgatcactctttcacgctaacgattctgtatatcaattagttttccaaaggggttataggac

fava_c18573_685	fava_c18573	685	C/T	aagtcacagtggcatatgaggttttgaaggatggggaaaaacgtcaacagtatgatcaggttggacatgatggctatgtaaaccaacaaagcactggttt[C/T]ggaggcgagggtggcttcaatcctttcgagcatgtgttcaatggcttcaatcctttcaacaacattttccgtcaaagtgctgatgtYaagactgtcattg

fava_c18573_772	fava_c18573	772	C/T	aaagcactggtttYggaggcgagggtggcttcaatcctttcgagcatgtgttcaatggcttcaatcctttcaacaacattttccgtcaaagtgctgatgt[C/T]aagactgtcattgaactatcttttaccgaagctattgaagggtgcaccaaaactctggcatttcaaacagatgtcctttgtaatgcttgtggtgggagtg

fava_c18574_1816	fava_c18574	1816	A/G	caagtaggaaaggccaaacttcagctcttatacttggatcaacacctccactacgaactcttttcaagaacttaagtccaccatcgcgaattttcccgtc[A/G]tgggtaaacaagtttttccattgttgaggagaaagaacgcgttttcttttccggcgtgaccatggtgatttaagacggcctcgatcagaggatgaacaag

fava_c18574_322	fava_c18574	322	C/T	ccagcactattgaaactaaagactgtaaataagcagtggaaagtttcagcaagcagcaagattcattttgataaacccagcatttgcagtgcttattgtc[C/T]ggcgcaggaggagatatccagccgcctgaaactaaagatcttttgaagacaatataataaagtagtttatgtatccgctgcagtcattggaatgatgcct

fava_c18574_886	fava_c18574	886	T/C	gcttctgcaagtgcctaaacaggtgggaatccttaaactttatgatttttgcaactatatcaagttgccttctgatacccacctcatcaagcctgaaatt[T/C]tgcctagctttcttcataaatccaacaaaacaccagaatgcctcgtgatcttctgatataacactaattataggagataacagatcactcataccctggc

fava_c18575_1175	fava_c18575	1175	T/C	taacgaggggatgtgaaaaattgaagtagttcctaaaatgtttagacattacccaatcatcttcacctctgcaagtttccacggcgccatttacctttcc[T/C]tcaaggtYaatttcccaaagtggagacaagtcgcgctgaatcacaacatcatcatccaaaaagaccaccttctcaaggtttgggaaaagctctggtaggt

fava_c18575_1183	fava_c18575	1183	C/T	ggatgtgaaaaattgaagtagttcctaaaatgtttagacattacccaatcatcttcacctctgcaagtttccacggcgccatttacctttccYtcaaggt[C/T]aatttcccaaagtggagacaagtcgcgctgaatcacaacatcatcatccaaaaagaccaccttctcaaggtttgggaaaagctctggtaggtatattcgc

fava_c18575_2495	fava_c18575	2495	A/T	cagattcagatccaccaatcgtcctcagatcgacgcagtttttgattttcctcttggtaatttcggtcttctgcaagaccacatttggggattttgaacg[A/T]ccaaattagggttatgaaaaaaaattaaatttttttctggattggggaattgaggttaagtataattaactggaatgaagattttgaagatttgtttgta

fava_c18577_222	fava_c18577	222	A/T	ttggaaactattccttttaggtaacattcagcactttggtactatgaacagaccaatgtctagatctacatcatttgcacaatgttgggatcaatttatt[A/T]gtcccataatgataatacctcttggtttatatacaaatttacctRcaactcagatactttatattcttagatagtgtcaaagttctagtagaaatgaaca

fava_c18577_267	fava_c18577	267	G/A	gaacagaccaatgtctagatctacatcatttgcacaatgttgggatcaatttattWgtcccataatgataatacctcttggtttatatacaaatttacct[G/A]caactcagatactttatattcttagatagtgtcaaagttctagtagaaatgaacaagtgttgtttattctctacttgaagctataagtaatattcatcat

fava_c18577_452	fava_c18577	452	C/G	aagtaatattcatcatgttatgaagaactcagtattattttttaagtacttttattcctagttcaatggcataccatttgtaacagtaatagtgagatat[C/G]Waaatgttaaatctccaaccaaaagatagaggatttagtttatttattgcagaaaataattttatttttcattcattaatatcatatattttgcttgctt

fava_c18577_453	fava_c18577	453	T/A	agtaatattcatcatgttatgaagaactcagtattattttttaagtacttttattcctagttcaatggcataccatttgtaacagtaatagtgagatatS[T/A]aaatgttaaatctccaaccaaaagatagaggatttagtttatttattgcagaaaataattttatttttcattcattaatatcatatattttgcttgcttg

fava_c18579_453	fava_c18579	453	T/C	tcgtgtcatgagaaaagcccctggagttggtcttgctgctccacaaattggtatccccttaaggataattgttttggaagataaagaagaatatattagt[T/C]ataacacagaggaggagaccaaattacaagatagaagaccttttgatcttttggtgattctgaaccccaagctaaagaacaagagcaataggactgcctt

fava_c18579_637	fava_c18579	637	A/G	agcaataggactgccttcttttttgaaggctgcttgagtgttgatcgatatcaagctttggtagagcggtatcttgatgttgaggttgttggttttgatc[A/G]ttatggcaagccaatcaaaataaaggcttctggatggcaggctcggattttacagcatgagtgtgatcacttggatggaacactatatgttgataagatg

fava_c18582_348	fava_c18582	348	T/A	catcctgtttattttgtactccatcttccatcagatttcatctccatgggaactgcggcatcatgcttacttcatgaaggagatatactcatggatgatt[T/A]tatctgcagattgggtggctattttagcattctcatttgccatcaaaggactgcattacgagtcaatgcatcacagaagatggatctggtactcatggta

fava_c18582_518	fava_c18582	518	C/A	atcacagaagatggatctggtactcatggtatattagcgctgttctggcagttttctggttatactacatgtcgaggttggcaaagttccgttgggatat[C/A]atatggcttccatttgggcctcttagtgcatctactatctgtctctacgtggatcacttactaaccgagtcatccgaagaagtaagaaagcttcatggat

fava_c18592_1075	fava_c18592	1075	A/G	aaaaagaatgactggtggaacttctcaagaaggttgtttattgagcgtatgactccttcaatcaaccttccaccacggagaataaattcattacgactgt[A/G]actgRtcattttataaggagaaacttttggtgaaatttcaagagtgattgcgtcaacttgataatcacggaaggcaccatgaggaccagtgggaacaccc

fava_c18592_1080	fava_c18592	1080	A/G	gaatgactggtggaacttctcaagaaggttgtttattgagcgtatgactccttcaatcaaccttccaccacggagaataaattcattacgactgtRactg[A/G]tcattttataaggagaaacttttggtgaaatttcaagagtgattgcgtcaacttgataatcacggaaggcaccatgaggaccagtgggaacacccaaccc

fava_c18592_1481	fava_c18592	1481	G/A	cgagataattaacaatattgatgaggtcgaggtttggcatctgcccatttgacgcctcagggtagatattaagaatgtcttcatgacggttaccttgctc[G/A]gcaacttttattacaagagctgcagccatagttccagcacgYctaaaaccactgtataacttcctatcggaagaaggattctgtccaacttcattRatag

fava_c18592_1523	fava_c18592	1523	C/T	gcccatttgacgcctcagggtagatattaagaatgtcttcatgacggttaccttgctcRgcaacttttattacaagagctgcagccatagttccagcacg[C/T]ctaaaaccactgtataacttcctatcggaagaaggattctgtccaacttcattRatagcattgcaagtttcactattatctatgtcagcttcacggaatg

fava_c18592_1577	fava_c18592	1577	A/G	gctcRgcaacttttattacaagagctgcagccatagttccagcacgYctaaaaccactgtataacttcctatcggaagaaggattctgtccaacttcatt[A/G]atagcattgcaagtttcactattatctatgtcagcttcacggaatgcaggRgcatggtattctcttagccaagcatccactgccgaatattctccgtatt

fava_c18592_1628	fava_c18592	1628	G/A	aaccactgtataacttcctatcggaagaaggattctgtccaacttcattRatagcattgcaagtttcactattatctatgtcagcttcacggaatgcagg[G/A]gcatggtattctcttagccaagcatccactgccgaatattctccgtattgtgaatctgccacaagccatatgacgtcctttgccaaccaagtaacccgtg

fava_c18592_1817	fava_c18592	1817	A/G	aagtaacccgtgaaagcagtgagaacaccgagtaagcaatgcccaaagacaaagcctctcccggaccaactttccttggattgtaaggcgttaccaagac[A/G]atagattccttgccatcaccccgtggKgctctaataattccagcaatgttgattccaagtgaYgaacagcttatattttttgagactatacccgaatcgg

fava_c18592_1844	fava_c18592	1844	T/G	ccgagtaagcaatgcccaaagacaaagcctctcccggaccaactttccttggattgtaaggcgttaccaagacRatagattccttgccatcaccccgtgg[T/G]gctctaataattccagcaatgttgattccaagtgaYgaacagcttatattttttgagactatacccgaatcgggacttgtgaaaaagtgcagtggattaa

fava_c18592_1880	fava_c18592	1880	T/C	gaccaactttccttggattgtaaggcgttaccaagacRatagattccttgccatcaccccgtggKgctctaataattccagcaatgttgattccaagtga[T/C]gaacagcttatattttttgagactatacccgaatcgggacttgtgaaaaagtgcagtggattaaactgattcagttgaggatggaacttgtgataagtaa

fava_c18592_440	fava_c18592	440	T/C	cataagtatcttcaaatgcgccttttattaaaaaatatgctaccggaggaaacacaacaagacctagagcaagattacatgtggccctcaataaggttct[T/C]aaacttctggatYgaatatcaagcttcaacggtYgagccatcaaacaaattggRacaatgagtaatgccccgatttcagctgtagcaaagttaatgactg

fava_c18592_453	fava_c18592	453	C/T	aaatgcgccttttattaaaaaatatgctaccggaggaaacacaacaagacctagagcaagattacatgtggccctcaataaggttctYaaacttctggat[C/T]gaatatcaagcttcaacggtYgagccatcaaacaaattggRacaatgagtaatgccccgatttcagctgtagcaaagttaatgactgacatgagagataa

fava_c18592_474	fava_c18592	474	C/T	atatgctaccggaggaaacacaacaagacctagagcaagattacatgtggccctcaataaggttctYaaacttctggatYgaatatcaagcttcaacggt[C/T]gagccatcaaacaaattggRacaatgagtaatgccccgatttcagctgtagcaaagttaatgactgacatgagagataacccaatgaaagtggtagctat

fava_c18592_494	fava_c18592	494	A/G	caacaagacctagagcaagattacatgtggccctcaataaggttctYaaacttctggatYgaatatcaagcttcaacggtYgagccatcaaacaaattgg[A/G]acaatgagtaatgccccgatttcagctgtagcaaagttaatgactgacatgagagataacccaatgaaagtggtagctattgttacagactttaagctag

fava_c18592_965	fava_c18592	965	T/C	cttcggaatagagagatcccgcaactattggaagtggtgcaacaagtaatgcaaatggaatcatgtaaactcctacagacacaaatttactagaagatgt[T/C]aagaggtacaaaaagaatgactggtggaacttctcaagaaggttgtttattgagcgtatgactccttcaatcaaccttccaccacggagaataaattcat

fava_c18595_648	fava_c18595	648	A/T	aaattagaaggaactgatattaggcttgttaattgggccaggatggtgaaggaacggaacactgaaatggagatgttggatgtaaatattccaagagaag[A/T]agaaaatttgaaggagagtgttagggaatatgtacgcattgcttgtatgtgcactaatgagttacagaaagataggccccaaatggcagaagttgtcaaa

fava_c18595_759	fava_c18595	759	T/C	aaggagagtgttagggaatatgtacgcattgcttgtatgtgcactaatgagttacagaaagataggccccaaatggcagaagttgtcaaattattggatt[T/C]aaYgcctctttgaacaattcaatcctacttgccatagtatgaagcatgatttttgagaattgatcatcatattcatgtaaatacatacattcaggcMaaa

fava_c18595_762	fava_c18595	762	T/C	gagagtgttagggaatatgtacgcattgcttgtatgtgcactaatgagttacagaaagataggccccaaatggcagaagttgtcaaattattggattYaa[T/C]gcctctttgaacaattcaatcctacttgccatagtatgaagcatgatttttgagaattgatcatcatattcatgtaaatacatacattcaggcMaaacaa

fava_c18595_856	fava_c18595	856	C/A	attYaaYgcctctttgaacaattcaatcctacttgccatagtatgaagcatgatttttgagaattgatcatcatattcatgtaaatacatacattcaggc[C/A]aaacaaatactatggcaaataggattgaattgatgtaattaaatgtattactacaaaattaattagattagttttcatttggttgataaatgctatccat

fava_c18597_1704	fava_c18597	1704	T/C	cgtgtctcaccttttccaactccaagccaaacacactagtagaaaagaaaagaaaagaactttttttgttactttatagaatcacttgttgtagagagtc[T/C]acagaccatggtgaaaatgataagaaagaaacgagggatagaactgagttgccaagtgttaaagtcatagtttggggatttgcttttgctcttttatatt

fava_c18597_765	fava_c18597	765	G/A	tcaattttccggttgaagtatggttggtttaacatacatgaacctacatcattgggtatatcctccatgctggttccctgtgagtttggcgtacagtctg[G/A]aggaacagatacaggttgcattgctaatttagaggctttatgtgatgagcgggacaagattgaggcaactgcagaatacaaaacaactgacgtgggcaat

fava_c18598_224	fava_c18598	224	C/A	ctattattctgtactacctacattagtaccaaaacaacactatgataaccatttggagaggcaaaacatataacttaagagtgtgcaaatcaaaactcat[C/A]aatcattagccagataaacacatgaaaagtgggatgatctcattttctccatccctttgatttttctactgaaaattgaagtacctagtcaacaatcact

fava_c18598_410	fava_c18598	410	C/G	tagtcaacaatcactctagcagttcatagattacaccagttatccaaattgagattaaaaaaaaagctattaccaccaaaaaaaaacttttaacaattag[C/G]cacatacatacttccaccattttttaatagcatatccataaattaagctataagtaatcaccctcagagaagacttagtaaaccttcgaaatccagatcc

fava_c18600_1030	fava_c18600	1030	G/A	tcctaaaatgatcatcccatatcccgccatgcgtaaagtccttacaaaatcataagattttgatggttgccactcaatagtctgggaggataaatcagcg[G/A]cggtataaataagtgcagaagtaatacttttagtgagaataggatgtgacttaatcatccccaaataccaacctacaaaaccaactttcgaaacggaagc

fava_c18600_1217	fava_c18600	1217	A/G	ttcgaaacggaagctgttgaagaagaagacgaagaagaaaacaaagaccccggagaaatttcatgttgtttgggtttcttgacaaaatccggtaatcgaa[A/G]gtaaggtttggaatgaagcttgttgcagagaagagaatgaagtctaattggattacaggaatggtttatgaacattgattgtcgttgcaacatgatgaaa

fava_c18600_1335	fava_c18600	1335	T/G	gcttgttgcagagaagagaatgaagtctaattggattacaggaatggtttatgaacattgattgtcgttgcaacatgatgaaatgggaaacgttagagcg[T/G]ttcttgagaacaataccgctcatgtttttggtttcagggttttaggtttttaggttcggaaagtaagtataaaaatgtgtcagagttccgggttcgtggg

fava_c18600_462	fava_c18600	462	A/C	acaatacaattgaggaacaaaactaaaaaataaaaaaggtttcaaccgtgggtcctgatcagtagtagtagtagtagtatagccatagtgtgagatcgga[A/C]atctctgtagacacccaaaagatttgtttgtatactgcagcgcagcttccagataaactgtaagagtatagtcagtccgaatctcagtcgtgaactaccR

fava_c18600_562	fava_c18600	562	G/A	Matctctgtagacacccaaaagatttgtttgtatactgcagcgcagcttccagataaactgtaagagtatagtcagtccgaatctcagtcgtgaactacc[G/A]acgacgatcagctagttatctcRgctttctctaggcctgccatataggtcatataaatggtccacaagtacgcgaatgaattgctgactaatggctgtaa

fava_c18600_585	fava_c18600	585	G/A	tttgtttgtatactgcagcgcagcttccagataaactgtaagagtatagtcagtccgaatctcagtcgtgaactaccRacgacgatcagctagttatctc[G/A]gctttctctaggcctgccatataggtcatataaatggtccacaagtacgcgaatgaattgctgactaatggctgtaaatggacaggaatgaatctaaaag

fava_c18602_1046	fava_c18602	1046	G/A	acatgatgcccgagggaagtcaaagaggagatctccgtctctacagagcagttaactttcctttgcagtggaaattggaaaaagtcattataaaaaagcc[G/A]cttattgattcctttatcatYgactatgatggaaagtattggctttttggctcggatcatagtggttttggcaccaagagaaatgggcagttagagattt

fava_c18602_1490	fava_c18602	1490	T/G	agagtcgaaatgcttggaatggtgctagataccatcacttagatgcccaacgtcttccgtctggtggttggattggtgtaatggatggggatcgtgttcc[T/G]tctggagatcctatccgacggctcatggttggttgtgcttctgttgtagtcgctgctatactcattgtgctattgggcgtgttactcgggtttgtaaatt

fava_c18602_1901	fava_c18602	1901	T/C	tgatgtgcataggattcaaaaacatttatggtggtaacggttcagaagaaccttatccattgaaaggtcgatactcgcagttcacattattaacaatgac[T/C]tacgatgctcgtctctggaacttgaagatgtatgtgaaacattactcaagatgctcttccgtgtccgaaatagttgtagtgtggaacaagggaatccctc

fava_c18602_2291	fava_c18602	2291	C/T	ggattgtcggattctatccccggcttatcgctgggagtccattaaagtacagaggagaaaaatacgcgcggaggcataaaggctacaacatgattcttac[C/T]ggtgcagcctttatcgattcaacactagctttcaagaggtactggggggaggaagcaaaacaagggagggaaactgtggacaagttatttaactgcgaag

fava_c18605_191	fava_c18605	191	C/A	ttctaccattcctttaggagttcaacacacgatatactatggttcatacctttggaattgaagaagcatgtgaataaaaaccagagaaaattacattcgc[C/A]tccccctaaggtttgttaaaaagacactgcacttcctttggtaatatggttctaatgaagatcatagcattatggtgaatgtgacagtgagttagcgggg

fava_c18610_1404	fava_c18610	1404	T/C	cttgattaatatatcataacttatcacaacagttttgatcctttgtgagtaatggaatcatagctgccacactaatgggtaggttggagaagcaaaacaa[T/C]gtagcagggtcttgaacattatcattcaacatttttttgtatggacatagtgaaacttttaggaaacagattatctgtggttaggaatcattgataaaga

fava_c18610_1557	fava_c18610	1557	A/T	aaacttttaggaaacagattatctgtggttaggaatcattgataaagattggacggttaaagttttgagttcttcttgtaagtcatctgatctctatcta[A/T]cgatttagattgatgtattgataacagaaggaaatttgtacctttgggagtttgaattcattcttttagcaccttagactttgtggtgacaagtaaatgt

fava_c18610_308	fava_c18610	308	C/G	tctaatatgtgaattcgaatgtgttttggttattgattgtttggaacagatcatgatggaaagggaaaactggaacgagaataacgacaatggttgttat[C/G]agaatcaattgttgtgtgataatgatcgggattcagttatgatgaattcttccatgtcttcgtctcctccgaagcttggatatattgaacatcatgtgtc

fava_c18610_922	fava_c18610	922	C/T	tggaaaaggatggtacagcaccagctggagaaacctgggaacttaactctgataaatggaatgacaaatttgcacagagacgtcagaaatctgtagctga[C/T]tttaccaggataccggagttgattctgagggaggatgttggcagcagtggcggtcttccttcaagaacaggaaagggtttagctctgagacagaaagcag

fava_c18613_1034	fava_c18613	1034	G/A	gcgtaaacttaactactagaggaRttgttgtttatgatggtgcctcccaaacacaaacacctataatgccgattttacctgattcacatgataccccaac[G/A]gctcacaagttttacagcaacattacagggttgacaagtggtMgRttttgggttccggttccacgcaatgtcgatgaacgtatgttcattacaattggaa

fava_c18613_1077	fava_c18613	1077	C/A	ctcccaaacacaaacacctataatgccgattttacctgattcacatgataccccaacRgctcacaagttttacagcaacattacagggttgacaagtggt[C/A]gRttttgggttccggttccacgcaatgtcgatgaacgtatgttcattacaattggaattggtttRgaccaatgcattaatccaggacctggtcgttgtct

fava_c18613_1079	fava_c18613	1079	A/G	cccaaacacaaacacctataatgccgattttacctgattcacatgataccccaacRgctcacaagttttacagcaacattacagggttgacaagtggtMg[A/G]ttttgggttccggttccacgcaatgtcgatgaacgtatgttcattacaattggaattggtttRgaccaatgcattaatccaggacctggtcgttgtctag

fava_c18613_1142	fava_c18613	1142	G/A	agttttacagcaacattacagggttgacaagtggtMgRttttgggttccggttccacgcaatgtcgatgaacgtatgttcattacaattggaattggttt[G/A]gaccaatgcattaatccaggacctggtcgttgtctagcgtttaaatcacgactttccgctagcatgaataatgaatcttttgttcttcctcagggaagag

fava_c18613_1798	fava_c18613	1798	C/A	tttgaggttgagaatggacctactcctgaaactagtgtgcctcctccaccggctgatcttcccaagtgttgagcgcatttaatgcattcaaaagtaaaga[C/A]ttRgttttgttacYattttgtaataatcctaatataatttgtttgattaataaaagtgtaataaaggtgaaaaggttagcctattctttcaaaatagcta

fava_c18613_1801	fava_c18613	1801	A/G	gaggttgagaatggacctactcctgaaactagtgtgcctcctccaccggctgatcttcccaagtgttgagcgcatttaatgcattcaaaagtaaagaMtt[A/G]gttttgttacYattttgtaataatcctaatataatttgtttgattaataaaagtgtaataaaggtgaaaaggttagcctattctttcaaaatagctacct

fava_c18613_1812	fava_c18613	1812	T/C	tggacctactcctgaaactagtgtgcctcctccaccggctgatcttcccaagtgttgagcgcatttaatgcattcaaaagtaaagaMttRgttttgttac[T/C]attttgtaataatcctaatataatttgtttgattaataaaagtgtaataaaggtgaaaaggttagcctattctttcaaaatagctacctgMtagtctagg

fava_c18613_1903	fava_c18613	1903	C/A	ttttgttacYattttgtaataatcctaatataatttgtttgattaataaaagtgtaataaaggtgaaaaggttagcctattctttcaaaatagctacctg[C/A]tagtctaggtcataccttgtacacaatacttattgatttggtattatgaataaattatttttttataagaaatatttgttgtagtgtct

fava_c18613_269	fava_c18613	269	C/T	gattgtgcagtccacaggtgatcacagctgtgaatggaagcttgccaggtccaacactttatgctcgagagggtgacactgttatcattcatctagtcaa[C/T]aaatcaccatacaatatgactattcattggcatggaatcactcaatttttaactccttgggcagatggtccagagtatataactcaatgtccaattccMt

fava_c18613_368	fava_c18613	368	C/A	aYaaatcaccatacaatatgactattcattggcatggaatcactcaatttttaactccttgggcagatggtccagagtatataactcaatgtccaattcc[C/A]tcaggtggaaaWtatacttataaattcaacataacaggacaagaaggaacactttggtggcatgctcacacttcttacctacgtgctactgttcatggtg

fava_c18613_380	fava_c18613	380	T/A	acaatatgactattcattggcatggaatcactcaatttttaactccttgggcagatggtccagagtatataactcaatgtccaattccMtcaggtggaaa[T/A]tatacttataaattcaacataacaggacaagaaggaacactttggtggcatgctcacacttcttacctacgtgctactgttcatggtgccattatcatta

fava_c18613_590	fava_c18613	590	A/G	taggaaactcgtacccctttcctagtgtttatcaagaagtccccatattgcttggagagtggtggagtactaatgttgtagaagttgagagaaatgcaac[A/G]gatactggtaacggcccacaagaatcaaatgcgacctcaattaatggctttcttggtgatttgtataattgcactcaagatccgctttacacgctgaRag

fava_c18613_800	fava_c18613	800	C/A	gaaaaacgtacttacttcgtatgatcaacgctgcactcgatgaacatcatttttttaagatagcaaaccatagtttcacagttgtggctttggatgctgt[C/A]tacacagaccaRtatgaaactgatSttattgttctcgcgcctggtcaaaccgctgatgtcctcttaaaaaccaatacagctgttggttcttattacatgg

fava_c18613_812	fava_c18613	812	G/A	tacttcgtatgatcaacgctgcactcgatgaacatcatttttttaagatagcaaaccatagtttcacagttgtggctttggatgctgtMtacacagacca[G/A]tatgaaactgatSttattgttctcgcgcctggtcaaaccgctgatgtcctcttaaaaaccaatacagctgttggttcttattacatggttgccacaccat

fava_c18613_825	fava_c18613	825	G/C	caacgctgcactcgatgaacatcatttttttaagatagcaaaccatagtttcacagttgtggctttggatgctgtMtacacagaccaRtatgaaactgat[G/C]ttattgttctcgcgcctggtcaaaccgctgatgtcctcttaaaaaccaatacagctgttggttcttattacatggttgccacaccatatcgttccgcaag

fava_c18613_957	fava_c18613	957	G/A	tgtcctcttaaaaaccaatacagctgttggttcttattacatggttgccacaccatatcgttccgcaagcgtcagaagcgtaaacttaactactagagga[G/A]ttgttgtttatgatggtgcctcccaaacacaaacacctataatgccgattttacctgattcacatgataccccaacRgctcacaagttttacagcaacat

fava_c18615_1102	fava_c18615	1102	G/C	attccgaatgggtggtgctgcaattcttttatcaaacaaaaaatcagaacacaaaagagcaaaatatagattactccaagttgttagaacacacaaaggc[G/C]gagacgataaagcttaccgttgcgttttcgaagaagaagaccgagaaggcaatgtaggaatttcactttcaaaagatcttatggctatagcaggagaagc

fava_c18615_1777	fava_c18615	1777	C/G	aatctatggaagaagaatcatatatatgccgcaaagaaatatagattctttgtggtttggttaatttgatttaccatgttccttgtgtagaagctgatta[C/G]atatttgtcatttctctattatacttgttactcaaacatcttggtattttttttcaccttatgtcaacattgaaaagtaattggaatttacatatacaaa

fava_c18620_426	fava_c18620	426	G/A	aaaaccttggttgtgttcaaagttctatatttagaagcatccatggaaatatgattatatggtatggagcatggcataagcaatccaacaaagaaaaaga[G/A]cttctaacttcaaatctgaaatcaatgttaaccaaagtatcaaccatggctaaattaattgaccataatttccttgaagcatatgctggagaatcaagag

fava_c18620_639	fava_c18620	639	C/T	ctgcaaaactaacaactggagacataatatctataaacacagctgacacggttagtcgaaaggaccttgataatctttcctacgccgtgttagctatttt[C/T]cgatcacgctttgcaaaaatggaaggtgtaacttcaggtctatgtttgaagggacaaactagaccaattgttgtttgcattcatgtgtggaagtctcttc

fava_c18620_981	fava_c18620	981	G/A	tttctcaacatcagatgttagaaaatgggaaagggagtatagaaagacaggttgtgcaaaattgacatttctgtgaaagatttcaagttttgaatcatca[G/A]ctttggttaagttttttcttcggtgtaaatattgttgactactttgtgaaaagcaaatttataaagagaagttaatgaaagaattgataaatattaagtt

fava_c18625_1351	fava_c18625	1351	G/A	gatgcccaccttgtccgtgactatgcacatgctcatgtgccattgcttggctaaccatttcaggggcagctgagaacaatcccgtcaaaatatcaacagc[G/A]tgccacgcgatgccacccccagtggccaaaagcatacaagagattcccaaagctcctagagtctcaaatttaccatgtccatatggatgttctttatctc

fava_c18625_138	fava_c18625	138	C/A	cataaatctactgttataagaggatcagttccagccccttcattatttgacaatattcaataaatttacatagctccaagggaactaatctgtgttctct[C/A]ctgtgctaacagaaatagttttcataacatacaaacaaaactcgtccaatgggcgcaaataaattatttcgagtttttaacagatctcctggacctatcg

fava_c18627_426	fava_c18627	426	T/G	acttaccttgctggtatgagacccatagtcaccactactactcgtgaaacgcatgtcactgccatcatcactcgcaaactttggatattgtatcattttc[T/G]cactttttcccttttcttgagtcccRccaaacttactatcctcctcactcatctccaactgcaatgcaaactctaacgccccaacaacatcactcatcga

fava_c18627_452	fava_c18627	452	G/A	agtcaccactactactcgtgaaacgcatgtcactgccatcatcactcgcaaactttggatattgtatcattttcKcactttttcccttttcttgagtccc[G/A]ccaaacttactatcctcctcactcatctccaactgcaatgcaaactctaacgccccaacaacatcactcatcgacatcctctgatttccatcatcatgca

fava_c18627_698	fava_c18627	698	A/T	ttatagaatcctttatgaatggatctaccatctcttcaattatcacaccttctttataacatctttgaaaacgaacaaccaaactcacgtttttcttatc[A/T]agagaacgcattaaaggaggtctcgcacacaacacctcaagaagcactacaccgaaagagtaaacatcagacttaagagtcaaYctttgacgtaagtagt

fava_c18627_782	fava_c18627	782	T/C	tcacgtttttcttatcWagagaacgcattaaaggaggtctcgcacacaacacctcaagaagcactacaccgaaagagtaaacatcagacttaagagtcaa[T/C]ctttgacgtaagtagtactccggatccaaatacccgagactacctttcaccacagtgctgacatgggtcatcgacattccagttggaccaacttttgata

fava_c18628_255	fava_c18628	255	A/G	cgtataacaaagagtaatgaaattttcttatcaaaagaatttataaattaatggaatttaaaaggccatttatacagtgctaagtttctatctaagctcc[A/G]ctgtatttacaaatattcaaagctaatctatgactgcaaaacttgtttttttctgtcYctctgagaactcaaagatggagtctaatacaccactttgtta

fava_c18628_313	fava_c18628	313	C/T	taaaaggccatttatacagtgctaagtttctatctaagctccRctgtatttacaaatattcaaagctaatctatgactgcaaaacttgtttttttctgtc[C/T]ctctgagaactcaaagatggagtctaatacaccactttgttatctcatgttgcagcatagaggacttgtttgccgctgcaatttttcccaaaggcacatc

fava_c18630_1879	fava_c18630	1879	C/A	acttccccaaaaccatattcacccattcaaccgactctttcctctgcaaatccttctccaaaaacaaagaaaaactagtaggcacttgaggccacacacc[C/A]cgaaaactatcctcgctactactaactttgtttctcctcttcctaaaattccacagcttatcaaaaacaacaccaacaaagaaaaaaaacacaaacaaac

fava_c18630_2157	fava_c18630	2157	G/C	ggatcttcttgaaaagtagtgaaatttgaaggcaattgaacttgtgaatcttgcaacttaccttcatcaagttcatcatttgaaatcctattatcaagta[G/C]gaaagttttagcagcccttgtagtgRaattggcgttccaattagtggaaccttgtttgtgaagtgaagaggagtaaagaatccatttttttcttcggaat

fava_c18630_2183	fava_c18630	2183	G/A	tgaaggcaattgaacttgtgaatcttgcaacttaccttcatcaagttcatcatttgaaatcctattatcaagtaSgaaagttttagcagcccttgtagtg[G/A]aattggcgttccaattagtggaaccttgtttgtgaagtgaagaggagtaaagaatccatttttttcttcggaatttgtgatatgagtaataatttgaaat

fava_c18630_2318	fava_c18630	2318	G/T	aagtgaagaggagtaaagaatccatttttttcttcggaatttgtgatatgagtaataatttgaaatgaagcgtttcttcatggttttggaaagggggtga[G/T]aattagaaggaaagccattgttgttgttgcagggacatagagagaagtgaaaagtgggagaagcttgttgaagaatcatgaatggattgagaagaggaag

fava_c18630_757	fava_c18630	757	C/T	acatggcatctcttacacccttttgatcagtgacatttgcttcttcggtatcagacaactcgtcatctgaagcatctgtatcaatggaatcctcttcggt[C/T]ttgtcatcttcctcatcctccacataggctttatatgttagtctaagcaatatctccccagatgagccctttcctctaaaaccccaacccccttgtaaaa

fava_c18631_1079	fava_c18631	1079	G/A	gttccaagggagatggatgaggtttccaactggcttagttcaaggttggggcttgaaggattctcatcttaaaatgacaaaactatagtatgaatgaatc[G/A]gttaatgtttgtagaataacatataaacatgagatttaattttctgggtccctttttgtcatgttggtgaggaataaatatatatgaatgtgtttactaa

fava_c18631_1240	fava_c18631	1240	A/G	atgttggtgaggaataaatatatatgaatgtgtttactaagtatttatctctttattatattattgtagaatctaggtgcttgattagtagtattattgg[A/G]gagatttttagtttttgttttcgttagtactgtgattgtaacaaaacagggatcttggactccttgtttttcaatttcaataaactttctattttgggtg

fava_c18631_454	fava_c18631	454	G/C	cgtgaggcctaagggaaaacaccaagctactatagtttggttgcatggacttggtgataatggtttgagttcatctcaattgttggaatcacttccactt[G/C]caaatataaaatggatttgtcctactgctcctacccgtcctgtggcgatactcggcggttttccctgcactgcatggtttgatgtgggagagctttcaga

fava_c18631_726	fava_c18631	726	G/T	cagctgatgtgaaagttggtattggagggttcagtatgggtgcagcaatagcattatactcttcaacttgttatgccatgggaaggtatggaaatggaat[G/T]ccatatcctgtctgtttaagagcagttgttggactcagtggttggcttccaggatcaaggagcttagggaacaagatagaagtgtcacacgaatcacgaa

fava_c18635_1358	fava_c18635	1358	T/A	aggttttcaacttctacaatgagctccatgcttacttagcttcatgtggagtagacggtgtgaaggtagatgttcaaaacattattgaaacccttggtgc[T/A]ggacatggtggccgagtctcacttacacgcagctatcatcacgctcttgaagcttccattgctcgtaacttttccgacaatggatgcattgcgtgtatgt

fava_c18635_1553	fava_c18635	1553	T/C	gtatgtgccacaacactgatggactttacagtgctaagcagactgctgttgtgagagcttccgatgatttttacccgcgtgatcctgcttctcacacgat[T/C]catatttcatctgtcgcatacaactccctttttctgggagagttcatgcaaccagattgggacatgttccatagtttacatccagcagcagagtatcacg

fava_c18635_1797	fava_c18635	1797	C/T	taagccaggcaaccacaattttgatcttcttaagaagctggttctctctgatggttcggttctccgtgctcagttacctggaaggcctacgcgtgactct[C/T]tatttgttgatccggcgagagataggactagtctgcttaaaatatggaacatgaacaaatgtactggagttgttggtgtatttaactgccaaggtgctgg

fava_c18635_2093	fava_c18635	2093	G/A	aagtagctggtgctgaatggcatggggagactattgtttatgcctacagatcaggcgaggtgattcggctgcccaaaggtgtttcgattcccgtgacact[G/A]aaggttctggagtttgaacttttccatttctgtccaattcaagaaattgtgtccagtatatcttttgcagcaataggtctaatggatatgttcaacactg

fava_c18635_2210	fava_c18635	2210	G/A	aacttttccatttctgtccaattcaagaaattgtgtccagtatatcttttgcagcaataggtctaatggatatgttcaacactggaggagctgtggaaga[G/A]gtcgaaattcatagagagactgacaacaaacaagaactatttgaYggWgaggctgtatccgaacYgataacttctctaggtcctaaccgaacaaYgactg

fava_c18635_2255	fava_c18635	2255	C/T	cttttgcagcaataggtctaatggatatgttcaacactggaggagctgtggaagaRgtcgaaattcatagagagactgacaacaaacaagaactatttga[C/T]ggWgaggctgtatccgaacYgataacttctctaggtcctaaccgaacaaYgactgcaactattactttgaaagttagaggaagtggaaagtttggagttt

fava_c18635_2258	fava_c18635	2258	A/T	ttgcagcaataggtctaatggatatgttcaacactggaggagctgtggaagaRgtcgaaattcatagagagactgacaacaaacaagaactatttgaYgg[A/T]gaggctgtatccgaacYgataacttctctaggtcctaaccgaacaaYgactgcaactattactttgaaagttagaggaagtggaaagtttggagtttact

fava_c18635_2275	fava_c18635	2275	C/T	atggatatgttcaacactggaggagctgtggaagaRgtcgaaattcatagagagactgacaacaaacaagaactatttgaYggWgaggctgtatccgaac[C/T]gataacttctctaggtcctaaccgaacaaYgactgcaactattactttgaaagttagaggaagtggaaagtttggagtttactcctcgcagcatccactg

fava_c18635_2305	fava_c18635	2305	C/T	gaagaRgtcgaaattcatagagagactgacaacaaacaagaactatttgaYggWgaggctgtatccgaacYgataacttctctaggtcctaaccgaacaa[C/T]gactgcaactattactttgaaagttagaggaagtggaaagtttggagtttactcctcgcagcatccactgaagtgcgcggtggatgggactgaaactgac

fava_c18635_2589	fava_c18635	2589	G/A	tcaagtttaagtcatcgacatttggcattaagtgaaatgtgtagggagaagggttttgtcataacaataactacacatttggtatcatgtagctatatct[G/A]ggagatggggaaaatgggggtcgagggttSttattgtaacttgtaacgtatgtagctgagagagtttcaaatcatgtcattagtcggggagaggaatgat

fava_c18635_2619	fava_c18635	2619	G/C	agtgaaatgtgtagggagaagggttttgtcataacaataactacacatttggtatcatgtagctatatctRggagatggggaaaatgggggtcgagggtt[G/C]ttattgtaacttgtaacgtatgtagctgagagagtttcaaatcatgtcattagtcggggagaggaatgatgttcttttcccatggatgtagtatgttttg

fava_c18638_1860	fava_c18638	1860	T/C	aaatgtttgtaaattacaactagaagaaaataaacaattttgaaaagcttgatagttacagaaacaacacatacagtgtatagtccaacacctttacagg[T/C]tattctttttactttcttaggattaactcaaataacctagaggttattctttttcctttcccttgagaaagtgttgtttttgtgtatgtatacttaaagt

fava_c18641_168	fava_c18641	168	T/G	gtctctgtctattggctatcccgttaaaattccaacggttgaaactgtaaaacaaaacacttgttcttttacaatgattagaattgtaaataaaaacgtt[T/G]aaatatagcatcagaaatgaattaatagagagataatctgagctccaaatctggttcttgagtttcactcgattccaaatccaaagaatcacttctatgc

fava_c18641_283	fava_c18641	283	A/C	aaatgaattaatagagagataatctgagctccaaatctggttcttgagtttcactcgattccaaatccaaagaatcacttctatgcatattcatgtcttg[A/C]gagtcatatagatttagaaatggttgagttatgtttgtttgaccaggagagcagtggttgatattgccatgttgagaacctgcatatctgaagtttgatc

fava_c18641_549	fava_c18641	549	A/G	ggcatttgcagtagtcttggatcttcattgtagttgaaataaggaggaggatgaggtggttgatgatgcaattgagaatgcaatattctgattttctcca[A/G]ctgagcaacaccaagtcctctttgtggttgctttcttggtttactattttctgaattattgttcttctttccttttcttgatgatgaagaagaagaagat

fava_c18648_1011	fava_c18648	1011	A/C	caatctcatttatatgatccatcagaagctgaaatgtgcagttcgatggctcaaccaccatgacacccgagttaaacagagtggcgttgttaccgatagc[A/C]gtgatttcYggcatcccaaatagaaaatctatgtttctgagtataagtaaatctgcatcgatgaatattatcttgtcgtaatctgtcagctgccataacc

fava_c18648_1020	fava_c18648	1020	T/C	ttatatgatccatcagaagctgaaatgtgcagttcgatggctcaaccaccatgacacccgagttaaacagagtggcgttgttaccgatagcMgtgatttc[T/C]ggcatcccaaatagaaaatctatgtttctgagtataagtaaatctgcatcgatgaatattatcttgtcgtaatctgtcagctgccataacctgaatttgc

fava_c18648_1787	fava_c18648	1787	T/C	ctgggagatacaagactcggtagagaagggcaaacgggaacttcagtttcttcttcttcatcaatccattctggatatagagactcccatgtcacattgt[T/C]tgcagcacgctccagatgcagaacaacatgtgtggcatcttgaaaattatgcttccagtRggaaatctcagtcttgttgaaatttacaagtccaatgcct

fava_c18648_1847	fava_c18648	1847	G/A	tcaatccattctggatatagagactcccatgtcacattgtYtgcagcacgctccagatgcagaacaacatgtgtggcatcttgaaaattatgcttccagt[G/A]ggaaatctcagtcttgttgaaatttacaagtccaatgccttgaaattcattttttcctgtcagtttttctgtgattttcactatatcatcccaatcggta

fava_c18648_2283	fava_c18648	2283	G/A	tcttgtataaatcttcaatcgatgctggtaatcgttgtcttgtttcaacagaacgaggagaagttcccattgttcctctcatattcaccaagctatagtt[G/A]gtttagtacatgctgctccaacacaatcctcagaagacaaagtgtgattacaatattgttgttgttgttgttgttatgaagaaaacacagatactgataa

fava_c18648_259	fava_c18648	259	C/T	aatctgttttcataagatcagagcaagtagaataaaccatttttaagcataaaaaatgcatacaaaggaattttcttcaattatcccttttgtctaacgc[C/T]gatcccaactagtSggataaattttgKttgttgtcgttgtttgttatgaaacgtatggcaggcaaggaaagaaaaaaaYgaatctggcagttcataatgc

fava_c18648_273	fava_c18648	273	C/G	agatcagagcaagtagaataaaccatttttaagcataaaaaatgcatacaaaggaattttcttcaattatcccttttgtctaacgcYgatcccaactagt[C/G]ggataaattttgKttgttgtcgttgtttgttatgaaacgtatggcaggcaaggaaagaaaaaaaYgaatctggcagttcataatgcaggaagagatgatg

fava_c18648_286	fava_c18648	286	G/T	tagaataaaccatttttaagcataaaaaatgcatacaaaggaattttcttcaattatcccttttgtctaacgcYgatcccaactagtSggataaattttg[G/T]ttgttgtcgttgtttgttatgaaacgtatggcaggcaaggaaagaaaaaaaYgaatctggcagttcataatgcaggaagagatgatgtYgtaactgtggg

fava_c18648_338	fava_c18648	338	T/C	attatcccttttgtctaacgcYgatcccaactagtSggataaattttgKttgttgtcgttgtttgttatgaaacgtatggcaggcaaggaaagaaaaaaa[T/C]gaatctggcagttcataatgcaggaagagatgatgtYgtaactgtgggaggtgttggagtaaaagactcatcatctgtccagttactctctccccaatgc

fava_c18648_375	fava_c18648	375	T/C	gataaattttgKttgttgtcgttgtttgttatgaaacgtatggcaggcaaggaaagaaaaaaaYgaatctggcagttcataatgcaggaagagatgatgt[T/C]gtaactgtgggaggtgttggagtaaaagactcatcatctgtccagttactctctccccaatgccgcaacatgcttttccagttacacagRttatctatac

fava_c18648_465	fava_c18648	465	A/G	gagatgatgtYgtaactgtgggaggtgttggagtaaaagactcatcatctgtccagttactctctccccaatgccgcaacatgcttttccagttacacag[A/G]ttatctatacacttcttcaagcgtcgatctttaactttgattcgccaatgcccgtttgtgtagtttgctatttcagcttgtctccgatcccactccaact

fava_c18650_1080	fava_c18650	1080	C/T	ctgcttggagaccttattttgatgcttctatttgcatttttatacatgcaccttcaagtagtcgtcaactcctttatgacggagaaaagccatgttttac[C/T]aatccgcaatgtgccagaaatatttctatgattgtccgcaggcccactctcagggaagcaaaacgtgtatatggccagttgaccctagtatcctatgaag

fava_c18650_1193	fava_c18650	1193	A/C	gccagaaatatttctatgattgtccgcaggcccactctcagggaagcaaaacgtgtatatggccagttgaccctagtatcctatgaagcagatgagaagg[A/C]aattttacaatgcaatcaacaggacgcggtgccaccaattcgtattgctaaaggaaatggcgccccacctgcYagcaaagYggacatggctggattggat

fava_c18650_1266	fava_c18650	1266	T/C	tagtatcctatgaagcagatgagaaggMaattttacaatgcaatcaacaggacgcggtgccaccaattcgtattgctaaaggaaatggcgccccacctgc[T/C]agcaaagYggacatggctggattggataaaaacaacaaagccaaagctggttccagtaaccaaaatgatgaacctcttatatcgagtaacgacgaaagtg

fava_c18650_1274	fava_c18650	1274	C/T	tatgaagcagatgagaaggMaattttacaatgcaatcaacaggacgcggtgccaccaattcgtattgctaaaggaaatggcgccccacctgcYagcaaag[C/T]ggacatggctggattggataaaaacaacaaagccaaagctggttccagtaaccaaaatgatgaacctcttatatcgagtaacgacgaaagtgagaatgac

fava_c18650_1851	fava_c18650	1851	T/C	ctacaggacaatcacagccaaaaagtggtgtgaaattatcaaaagaggaggaaatgaaaagagcacaggatgaagaaagagaaaagagagcagctgctgc[T/C]gaaagaagaatggctgccctcaaaatccaagctgataatagcatatcagaggctaataaaagtggattatctggtgacatagtctgttcctgttgtaatt

fava_c18650_250	fava_c18650	250	C/T	gatgacaccgtctcaagaaaaacgccaccattccatctttgaacttcccccaaactacttcgactcttgtcgtcttctcccttcaccccactcttcagtt[C/T]ccgacctctacgcaaatcacgaaaccctccaattaccctctaacgacgccgtcgttttgggcactaggttaacctgcaatacctgtaaggctcagttcga

fava_c18650_354	fava_c18650	354	C/T	acctctacgcaaatcacgaaaccctccaattaccctctaacgacgccgtcgttttgggcactaggttaacctgcaatacctgtaaggctcagttcgattc[C/T]cttcaagaccaacgttcccacttcaaatccgatattcatcgtttYaatgtgaagctgacaattgctgggaaacatattgtgaaagaagatgattttgaag

fava_c18650_399	fava_c18650	399	C/T	ccgtcgttttgggcactaggttaacctgcaatacctgtaaggctcagttcgattcYcttcaagaccaacgttcccacttcaaatccgatattcatcgttt[C/T]aatgtgaagctgacaattgctgggaaacatattgtgaaagaagatgattttgaagttctgacatctgattttgttaaagactatgatgtgtcaagtatat

fava_c18653_1071	fava_c18653	1071	A/G	gtgcagatagggactcttggcctagttatgagacaggtcacagctatccactcttaatcagaatcaccctttgaaattccttctatatattctgcatggg[A/G]cttcctttcattgtttggttgactgttaaacaaaagtaaaaatctattcctccaactaggaaatgtctaagtccagacacttaccaattgcacacatatg

fava_c18653_313	fava_c18653	313	G/A	acgcatttcaagaagaaccagtcacggattcaacttgataccctctcatgtaatcgacgatgaacatagttcaaatcaatgcaatgtttcttctaccaaa[G/A]atgctcgagtttgttatactttgccaatccctggcgctcctcaactgtttttgaggcaaagacttgacaaccatgctgatctcagtgattttcagacatg

fava_c18655_243	fava_c18655	243	C/G	aaaaaagaaaaaaaagtatatgtgatttgtataaattatagtcgttgactataaatgtgacgatggtctttcacctagacctaaataaccaaaacggatt[C/G]ggcatgaaacgcttgattggaacaaaaccatctacttcatcatgctgtgaactgcaccYattattcatctcatSaatcacYtctcttccYtcacatatga

fava_c18655_302	fava_c18655	302	C/T	acgatggtctttcacctagacctaaataaccaaaacggattSggcatgaaacgcttgattggaacaaaaccatctacttcatcatgctgtgaactgcacc[C/T]attattcatctcatSaatcacYtctcttccYtcacatatgattttaacatcacatctttcttctacactttttaacaccatctctgcagtagaaatttgt

fava_c18655_317	fava_c18655	317	C/G	ctagacctaaataaccaaaacggattSggcatgaaacgcttgattggaacaaaaccatctacttcatcatgctgtgaactgcaccYattattcatctcat[C/G]aatcacYtctcttccYtcacatatgattttaacatcacatctttcttctacactttttaacaccatctctgcagtagaaatttgtcttcttgacacatgt

fava_c18655_324	fava_c18655	324	T/C	taaataaccaaaacggattSggcatgaaacgcttgattggaacaaaaccatctacttcatcatgctgtgaactgcaccYattattcatctcatSaatcac[T/C]tctcttccYtcacatatgattttaacatcacatctttcttctacactttttaacaccatctctgcagtagaaatttgtcttcttgacacatgttttctta

fava_c18655_333	fava_c18655	333	C/T	aaaacggattSggcatgaaacgcttgattggaacaaaaccatctacttcatcatgctgtgaactgcaccYattattcatctcatSaatcacYtctcttcc[C/T]tcacatatgattttaacatcacatctttcttctacactttttaacaccatctctgcagtagaaatttgtcttcttgacacatgttttcttagattagatt

fava_c18655_463	fava_c18655	463	T/C	ttctacactttttaacaccatctctgcagtagaaatttgtcttcttgacacatgttttcttagattagattgtgtagttccaatcaccaattttgtaata[T/C]ttagattcgtaacagtctctacaacggcttcaccaacgttgtcaccctcaataagcaaaatctccaccttaacttttgagtcaatacataggtcaatgaa

fava_c18655_810	fava_c18655	810	C/G	taaaattagggttcaccaagtgttttaaggtccacgaaagtgcttccatgcttgaaaaagtttctccaacagccacataaaccacatcagaatcagataa[C/G]ttatcgttgtggatatcaagagagaagacagtactgctttcacaatcttcttctttgattgaatccaatggttcttccttcttaagatttatctcaaaca

fava_c18655_918	fava_c18655	918	A/C	tgtggatatcaagagagaagacagtactgctttcacaatcttcttctttgattgaatccaatggttcttccttcttaagatttatctcaaacaaatcatc[A/C]tcttcttctatctcgctattgaaattgtaatcaaacacttggtttttcttgaacccaatcaaaccacaaccacatcttatgccgatgtcttcatcggagt

fava_c18657_1059	fava_c18657	1059	T/C	atcccataactccggtaaatattatcagcttgataaattgacgtaacacccaaccctttcgaataaaaggggtgcgaggatatcttggctggtaacataa[T/C]gtaggagcaaccataaagtaagtcaggcttttgaagctcacgtcgtatggatagtccatattcagagtactgggaagagcttctcccttttcaattgaat

fava_c18657_1664	fava_c18657	1664	G/A	tgactctgcaactgacggaaaatgttatcggagctgagaggactttccttgattttccgatgagccggaacagaaggacggtacgtgaattggattgcag[G/A]aacatYattgactcggttttgatcaggattaccgtcaatattctgatcaccgctgacagagtcgtcggaactagaagaatccttgactaactcctcaccg

fava_c18657_1670	fava_c18657	1670	T/C	tgcaactgacggaaaatgttatcggagctgagaggactttccttgattttccgatgagccggaacagaaggacggtacgtgaattggattgcagRaacat[T/C]attgactcggttttgatcaggattaccgtcaatattctgatcaccgctgacagagtcgtcggaactagaagaatccttgactaactcctcaccggaactc

fava_c18657_349	fava_c18657	349	C/T	ctattgttccttggcagttatcaatgtcagtgcattgctcaaaatttacatgttgacagaattgccagagatgttgaattagtaatcaagatgtagtgcg[C/T]gtataatgatgtaatgctagctttggtcaagtttgctttttctRttcatcaagtcatggtagtatagtagtacacacataggttgaccaagaatacagaa

fava_c18657_393	fava_c18657	393	A/G	tttacatgttgacagaattgccagagatgttgaattagtaatcaagatgtagtgcgYgtataatgatgtaatgctagctttggtcaagtttgctttttct[A/G]ttcatcaagtcatggtagtatagtagtacacacataggttgaccaagaatacagaatgagaaccaaaaaaacatatttccaaccattgagttgctgaatt

fava_c18657_582	fava_c18657	582	T/G	agttgctgaatttattttgcaggtagttagttatcaagaccaaaggaacctgaaatataattccactaaaagcccacaacttgaatatgtggcaaggaac[T/G]gcaatgcacagctcatggaacaaagcagaaaccaggaaggcaattaaaacagcagcacccttgggtataccactccttaaacatggaaaatataggtgac

fava_c18657_756	fava_c18657	756	C/T	tccttaaacatggaaaatataggtgacgaatcatccatttgtgaacaggcatattccacatcctccaatactcttcaagagttctggcattccaccaatc[C/T]ttgtaaaattcgcgatcaccaaatcgaagaagctctgccagtatatttaaccaaaggtggaaaaagcagtagaacatgcagagccacacatatagatttg

fava_c18657_905	fava_c18657	905	T/C	aaccaaaggtggaaaaagcagtagaacatgcagagccacacatatagatttggaacagacagcttcagaactctttcgatggcataaagaaggtttccct[T/C]aaaaggatgctgggagttttgtacaataggattgatatattgttcaattataaatcccataactccggtaaatattatcagcttgataaattgacgtaac

fava_c18659_408	fava_c18659	408	C/G	tttgagagcagcaatgtgaagaatcttatctgtgtagttcaagttgaaatcgaagagagcgttgttattagcatgatctaacagctccaaatccctatta[C/G]caccatgatctaacagctccaaatccctaacaccttccaccaacatgttgagataagatggggagtttgatgcgattgaatcggattcctccaatgagag

fava_c18671_149	fava_c18671	149	T/A	ccagaatcttgtagttcaataataattcagtattgcaatgtcagtcgaaaaccaaaccggtattttacataactttgaccattgcaactagtaagaactg[T/A]tggtatatttaaaaacaacaccagtaagccctgatttaagttacttctttttcttaaaaaaacctgaatacaacgtcagactcatactgtatttgggtac

fava_c18671_291	fava_c18671	291	G/A	tacttctttttcttaaaaaaacctgaatacaacgtcagactcatactgtatttgggtacaacaaaattggcaagacaggggaatctggatctgctcactt[G/A]gatcattttaataggtattctccRctcctttcgctgtgtctgtctttataatacctggttgttcaaccaccaggcatatttcagtacttcacacctgttt

fava_c18671_315	fava_c18671	315	A/G	gaatacaacgtcagactcatactgtatttgggtacaacaaaattggcaagacaggggaatctggatctgctcacttRgatcattttaataggtattctcc[A/G]ctcctttcgctgtgtctgtctttataatacctggttgttcaaccaccaggcatatttcagtacttcacacctgtttttcccaatttatggttttccaagt

fava_c18671_768	fava_c18671	768	C/T	gcttaaacagttgaagtaatcaacttcgatagtaggcttatctggatttgtatttttcacatgctaagctgagctttttccagacacaattctttggtga[C/T]taactgaagcagtacttgcaccagctcccgtattcttaaKtctcttgatgtaacaagctcacaaaaggaaggtaataacacaaacagatgggaacggttc

fava_c18671_808	fava_c18671	808	G/T	tctggatttgtatttttcacatgctaagctgagctttttccagacacaattctttggtgaYtaactgaagcagtacttgcaccagctcccgtattcttaa[G/T]tctcttgatgtaacaagctcacaaaaggaaggtaataacacaaacagatgggaacggttcccgtgcttctccttgtcctctactaggtcagttctcaagc

fava_c18674_132	fava_c18674	132	G/A	tcggtggactatttctatccttctcttttatagatattatatttgtcttgaaaaaaattgaaattcaacatattcatcacagatacaaacttttccaccc[G/A]tgtaaagtactttacaaggagaactaatgcattaataaccatagattttctgatcaggtacaagagctcttgttggatcattaatcttgtgtaacttccc

fava_c18675_129	fava_c18675	129	G/A	aaaaggaaagaagaatcttgtcatataagaaacgggaaaataaatctttctactgataaagaagatcaattacaacagattaatctgaaacaaatcaaat[G/A]caaagaatgtMtggtcaaaaaacagtggtggtgaactattggcagaagacatggtcgatgcaaacaatgaattgtcggaggagaaaacaatggacacgcR

fava_c18675_140	fava_c18675	140	A/C	agaatcttgtcatataagaaacgggaaaataaatctttctactgataaagaagatcaattacaacagattaatctgaaacaaatcaaatRcaaagaatgt[A/C]tggtcaaaaaacagtggtggtgaactattggcagaagacatggtcgatgcaaacaatgaattgtcggaggagaaaacaatggacacgcRggtcaacatca

fava_c18675_229	fava_c18675	229	A/G	RcaaagaatgtMtggtcaaaaaacagtggtggtgaactattggcagaagacatggtcgatgcaaacaatgaattgtcggaggagaaaacaatggacacgc[A/G]ggtcaacatcaatagctgtgaattgtccatgatctctcaccctttatctctgtttgcatcttctctgtcacccaagaatccccattagtctcaaataaaa

fava_c18675_352	fava_c18675	352	G/A	ttgtccatgatctctcaccctttatctctgtttgcatcttctctgtcacccaagaatccccattagtctcaaataaaaaagaagtgaaacagataatcat[G/A]gcaagggcaKaatcaagttagtattgactatgctaaacaattccatctctgttatttattctgacaacaattcgtcttccatgtctgcaatgactatgtc

fava_c18675_362	fava_c18675	362	T/G	tctctcaccctttatctctgtttgcatcttctctgtcacccaagaatccccattagtctcaaataaaaaagaagtgaaacagataatcatRgcaagggca[T/G]aatcaagttagtattgactatgctaaacaattccatctctgttatttattctgacaacaattcgtcttccatgtctgcaatgactatgtcttctgcaatt

fava_c18675_525	fava_c18675	525	G/A	cgtcttccatgtctgcaatgactatgtcttctgcaattagttcattgtctacctttgatttgactatgtcttcaactcatgttgaccatgattgaaggtc[G/A]tggaaagatctgtaaacaataacgcatagtagaagaacctgcatatagtagtttaaactttaaacatccctctcattcatcactcagcagtgtgatatca

fava_c18678_154	fava_c18678	154	T/C	caaaatgctcgaagggcctgaacaaaatatgatgtattctcattactagtatattaagggaagtacacaacagaacaatgggagctaatgttatatgcat[T/C]ttacaatttatacagtcctcaactcatacacaactatccgacagatttaaacaccccccacaattgtcctgtaacctaatatgttatatgtatagagtac

fava_c18681_2226	fava_c18681	2226	T/A	aggttacgtccaatttgaaaagcacaccattcaatccctgtctatcccctgcaaaatattgaagttgtgcattgtttatgttatgacagtatttttagtg[T/A]aatccaatctagagatatcataatcactattcctgatactcaaaatgttagtggttgcctatgaattaagtttttttgcactatgccattcctccctagc

fava_c18683_607	fava_c18683	607	G/A	gcgcaacacttttccctgaagttttgcaagtttggttcgattttggccatgaccaggtgttcgcgtaccttctattatcggcgagtgcggcggggacggc[G/A]atggcgaggacgcttaaggatatggacacgtgtacggtgagcaattcgttYtgcgtgcaaacggacatagcgatttcgttaggatacgcggcgtttttgt

fava_c18683_658	fava_c18683	658	T/C	accaggtgttcgcgtaccttctattatcggcgagtgcggcggggacggcRatggcgaggacgcttaaggatatggacacgtgtacggtgagcaattcgtt[T/C]tgcgtgcaaacggacatagcgatttcgttaggatacgcggcgtttttgtttctagggtttacttcgctgttatctggattccgtctcgtttgtttcataa

fava_c18683_832	fava_c18683	832	T/C	ctggattccgtctcgtttgtttcataatcaacggttctcgttttcatctctgatgaatcttttctgagaagttttgcgtggaaacgacgtcgtagatgaa[T/C]gaatgccggggaatatttgtgtggggtcaggagcattcttttcttggttctctcaattggatgtgatttgatttggacggacatgattgcagaagaactt

fava_c18684_531	fava_c18684	531	G/A	cagcgttccaccagtgagaaactcgacgataacacaacatgccctagaaggaagggtctcttgattgtcagtagagttttttgaaggaatcttaagattc[G/A]aagttcccattcaagctccaacaaaMtgcgtttacttcaaccatgcagRaaaattttccatcagtctcctcatcagattcaatgacaattgcacagtctc

fava_c18684_557	fava_c18684	557	A/C	cgataacacaacatgccctagaaggaagggtctcttgattgtcagtagagttttttgaaggaatcttaagattcRaagttcccattcaagctccaacaaa[A/C]tgcgtttacttcaaccatgcagRaaaattttccatcagtctcctcatcagattcaatgacaattgcacagtctcttgctcccattatctgttaatggatg

fava_c18684_580	fava_c18684	580	G/A	ggaagggtctcttgattgtcagtagagttttttgaaggaatcttaagattcRaagttcccattcaagctccaacaaaMtgcgtttacttcaaccatgcag[G/A]aaaattttccatcagtctcctcatcagattcaatgacaattgcacagtctcttgctcccattatctgttaatggatgagcaccttttccatttccttaaa

fava_c18688_1022	fava_c18688	1022	A/G	gatgtcttcttatatattccatctccatattactaaaccttccacttccaattccattcattttttcacacccaattcaaaacgcaccgttttcacctcc[A/G]aatgaaactttgttatgcaaaagctttgaataaaacgtgtggatttcagaaaaagagtgtgtgattggatattcagggttactataccagagatcgtgga

fava_c18688_139	fava_c18688	139	C/T	ctgttacaagaattaacttcaactatgcaattaaaaacttcgactgaccattaaaaacaaatctacataccatgtttatatacaataaagagcacaaccg[C/T]gaaaacaaaaaccWtMgaaattatcaagagcacctgatatctatcacttgttactgcactggatcgaatcacatctctcaaatactgttgttcttttact

fava_c18688_153	fava_c18688	153	A/T	aacttcaactatgcaattaaaaacttcgactgaccattaaaaacaaatctacataccatgtttatatacaataaagagcacaaccgYgaaaacaaaaacc[A/T]tMgaaattatcaagagcacctgatatctatcacttgttactgcactggatcgaatcacatctctcaaatactgttgttcttttactacagaaaagagaaa

fava_c18688_155	fava_c18688	155	C/A	cttcaactatgcaattaaaaacttcgactgaccattaaaaacaaatctacataccatgtttatatacaataaagagcacaaccgYgaaaacaaaaaccWt[C/A]gaaattatcaagagcacctgatatctatcacttgttactgcactggatcgaatcacatctctcaaatactgttgttcttttactacagaaaagagaaacc

fava_c18688_408	fava_c18688	408	A/T	tggaagaagcgtctcaatcgccagtccgtctcctaggcctccgtaaggaaaactgctgaagcttcatccgtggcagcaatgtctttcgcaacaacttatt[A/T]taagatcctaaatccgatcaatttgttcagtgcggtcttgcaaagcaatcccttctgagacatcagcaagtgatttaagattgcacttaataacagcttc

fava_c18691_513	fava_c18691	513	G/C	aacaaccccagaatccaactcaacgaacgaaacacacagcaacaacgaacacaaacacacagagacatcgaagaagacggaaccaaccccgaatggaact[G/C]tgatatgcgtttcgatttagcttttctttcctcgcattcagatcccgccgagtttttcctatgtttcgagtttcgtcacgacggcgtcatcctcggcgac

fava_c18692_548	fava_c18692	548	G/A	atgtttcaccatttccactagacccaagagctaccttataagaaccatccggaagaacttcttttataactccaacagattcttctccagccctgtgtac[G/A]ttgaccaatatgtctggcataagacaaggcccttcattgtccccacctaataccggagacatcatgtctagaccgccagttccgggagtcataggctgtc

fava_c18693_213	fava_c18693	213	G/C	ttgttacaaatgtgcatgtattttatcacattatcaaaggttacttatattctaaagtacacttcctaactttagaggaacttaaacttaatgcaaactt[G/C]ttaactgttattctgaatattccacctgcttttcccatttcaaattcttaagaagtggagcatcatcataaatatcaacaatcttctccRcattagcttg

fava_c18693_303	fava_c18693	303	A/G	atgcaaacttSttaactgttattctgaatattccacctgcttttcccatttcaaattcttaagaagtggagcatcatcataaatatcaacaatcttctcc[A/G]cattagcttgcggatctctgcaaccgatcaatatcttcaagacttccttcattgatggtctactagctggcagtgttgaagtgcatataaccccgagttt

fava_c18693_431	fava_c18693	431	A/G	tcaatatcttcaagacttccttcattgatggtctactagctggcagtgttgaagtgcatataaccccgagtttgaagatgttgatcatttcaatcaagtt[A/G]ctaggttccacagcgtcttcgtccagaagctcttctatatcggttcctatctgaatatggcgccacgcccattctgcaagcgacgagtattcgtctccat

fava_c18695_1180	fava_c18695	1180	A/G	ccgttgattcatcaagaataggaatttccagagtttgaggccaaagatagaggcttgcaacttgcttttttattgtttcctgaacaaatctataaagacc[A/G]ggtattgacattatgtcccctcctgatatcgccatcccaaaatccacgtgaggcttctccatcaaagacaccacaatctttgcaaaacatggaagtgtcg

fava_c18695_1507	fava_c18695	1507	T/G	cttgttccatgaccaattccttttcatttgtttccaaaactttcataccgcatacaataggaggaagagtaccaaggctcagttgatcaaactcaattcc[T/G]ttgatctgatactttccaatgtattcagcaaatattggttctactgtagttctaatcatcccacaaattgccttctctaagaaaggccacatatccaata

fava_c18695_643	fava_c18695	643	A/G	gtatcacaacggaaagcaaacctgctccttcttggacctcatcatcagatatactatcgattccactatcctttctaacatatccttccgaagatccacc[A/G]aatttcatactatcttctttgaatggcacaaaagtcaagtccaccacaatttgtcctctgaacttcttattcggagtttcgttgatatttgtgtccttaa

fava_c18695_847	fava_c18695	847	A/G	aatcaagcataaattctttattctcgtacggcttaagcagctttagcgggactaactgcattcccagcttgtcatgtgcaccaaccttgtcccaatcata[A/G]acttgtaattggagaacttgagattgagggtccttcacaacaatcttgaacttctcgttccactgtggattcaaattctttctcttgatagtagttttct

fava_c18697_1199	fava_c18697	1199	G/A	tcatggaggtaaaaacatgtaggtaagtcaggcagatgaaattgatttatgagaagaaaggtttgataatcagaatcagaaccgtgacgcgatatgaatt[G/A]gttggtgtagattttcatgaaatcagggttttcaaataaattggaccatgatttgcgcacgcatccaaagcgcttcaatgattttatgggcagttttgaa

fava_c18700_1628	fava_c18700	1628	A/G	cggatctttacttcaagaccggaaaattaagagagtcaatgtcttattgtgaaaacgcattgagaatatatagcaagcccttacctggaataaccgccgg[A/G]gagattgcgagtggcttgaccgaaatctcggcaatctacgaagctctgaacgagcccgaggacgcRttgaagctcttgcagaaggcggtgaaactgttgg

fava_c18700_1694	fava_c18700	1694	G/A	tatatagcaagcccttacctggaataaccgccggRgagattgcgagtggcttgaccgaaatctcggcaatctacgaagctctgaacgagcccgaggacgc[G/A]ttgaagctcttgcagaaggcggtgaaactgttggagggtatccctggacaatataggacagtggcaggaatagaagctcaaatgggagtgctgttctaca

fava_c18700_1804	fava_c18700	1804	G/A	ttgcagaaggcggtgaaactgttggagggtatccctggacaatataggacagtggcaggaatagaagctcaaatgggagtgctgttctacatggtcggac[G/A]gcatgtggatgcttggaaatcttttgataatgctgtcgtcaagctgagagccggcggggagaggaaatcagcatttttcggatttgtgttgaatcagatg

fava_c18700_1947	fava_c18700	1947	G/A	gctgagagccggcggggagaggaaatcagcatttttcggatttgtgttgaatcagatgggcttggtttgtgttcagctctataaaatagaagaggctgct[G/A]cacattttgaagaagctagagagatattggagagggaatgtggaaactatcatttggacactcttggagtgtatagcaacctcgccgcaacttatgatgc

fava_c18700_2190	fava_c18700	2190	T/A	actgaaaatgcgagaagaaaaacttggaactgcaaatccagatgttgatgaagaaaagaaaagattgtttgagcttttgaaagaagcgggaagagttcga[T/A]gtagaaagggaaaatctcttgaaaagcttattgattcaaactcgttgaagatgaagaaggaaggaaRaaggagatggggtgcatttagttttagaacKta

fava_c18700_2257	fava_c18700	2257	G/A	tttgagcttttgaaagaagcgggaagagttcgaWgtagaaagggaaaatctcttgaaaagcttattgattcaaactcgttgaagatgaagaaggaaggaa[G/A]aaggagatggggtgcatttagttttagaacKtagagatgttttgttcttacatcaacataaatggtcttttttgttttgcacttgtgagaatttgtgaac

fava_c18700_2288	fava_c18700	2288	T/G	gaWgtagaaagggaaaatctcttgaaaagcttattgattcaaactcgttgaagatgaagaaggaaggaaRaaggagatggggtgcatttagttttagaac[T/G]tagagatgttttgttcttacatcaacataaatggtcttttttgttttgcacttgtgagaatttgtgaacgtttggtactgaagatgcttccttgttcttc

fava_c18701_1197	fava_c18701	1197	T/C	tttggttgatgaaggattatggtgtgagagagtcttgggttaagttggtgacaattccttatttgcctaatcctgaggacttttcatattcagggcctta[T/C]tgtgtttcagagaatggtgaagtgttgttgatgtttgagtttgatttgattgtgtataRtccgaaagatcattcgtttaggtatccgaggattgaaggtg

fava_c18701_1256	fava_c18701	1256	A/G	tatttgcctaatcctgaggacttttcatattcagggccttaYtgtgtttcagagaatggtgaagtgttgttgatgtttgagtttgatttgattgtgtata[A/G]tccgaaagatcattcgtttaggtatccgaggattgaaggtgggaagggttggtttgatgcagaggtttatgttgaaagtttggttacaccaRtgaaggat

fava_c18701_1348	fava_c18701	1348	A/G	tgtgtataRtccgaaagatcattcgtttaggtatccgaggattgaaggtgggaagggttggtttgatgcagaggtttatgttgaaagtttggttacacca[A/G]tgaaggattaaggttgatgtgcttgtgctagctatgcaactgaagtataaggaagaataaaatgatgtcaagtatttgaaatctttctggtttttttgtt

fava_c18707_1159	fava_c18707	1159	A/G	gagtttgaattgcaggtttctccatgcagtgttgttcgtctaacagcaacgaatgaatcacctctattatccattttcttcttcttcttcaacaatatct[A/G]ctactatagcatcatcaacaccgacSggtagttcattttttaaccgtaaaatctcgccggagaaatccgaagatgccggaaatcggaggtgacggaaacc

fava_c18707_1185	fava_c18707	1185	C/G	cagtgttgttcgtctaacagcaacgaatgaatcacctctattatccattttcttcttcttcttcaacaatatctRctactatagcatcatcaacaccgac[C/G]ggtagttcattttttaaccgtaaaatctcgccggagaaatccgaagatgccggaaatcggaggtgacggaaaccggtgagagatagagtggagRagacga

fava_c18707_1279	fava_c18707	1279	A/G	accgacSggtagttcattttttaaccgtaaaatctcgccggagaaatccgaagatgccggaaatcggaggtgacggaaaccggtgagagatagagtggag[A/G]agacgagaggagttagggtttgattggttgagatctgagaggaatggaataatataacaagagttttggaatggaaaacggttacagttagaaatggaga

fava_c18707_376	fava_c18707	376	T/C	aaagttgactttgtcttgctgaaatgttactgtatgctgaatgccaggcatgaagattatagaagcagcaaaaggccatcaatgctataactctaggaga[T/C]tgacaggtttcttgaactgaWtcctatcgggaaagtagtttgtatgagggtaagtcttcaagacgcaatacttcttcttgaagaagctctttctctagct

fava_c18707_397	fava_c18707	397	T/A	aaatgttactgtatgctgaatgccaggcatgaagattatagaagcagcaaaaggccatcaatgctataactctaggagaYtgacaggtttcttgaactga[T/A]tcctatcgggaaagtagtttgtatgagggtaagtcttcaagacgcaatacttcttcttgaagaagctctttctctagctcacgccgtgtagatttcatca

fava_c18708_268	fava_c18708	268	T/C	gtgcccgtggaaacaaccgagcttcgacgccaaacaaagacaattaccatgctactctcaaagctcttaactccaaaggcagatcccctagaaaatctct[T/C]ggccagaattatatgctgaactctgacataaatgagcaacttgttggtgtggctggtgttgaggaagatgatgtggtgctggaaattggaccgggaaccg

fava_c18708_697	fava_c18708	697	G/A	ctttttatatcagtacaaatgtgatcaaactactacttccaatgggtgacattttctcagaagtggttctattacttcaagaggagactgccatgcgttt[G/A]gtagaaccatcactcaggactcctgagtatcggcccatcaatatatttgttaatttctactcagatcctgagtacaaatttaaggtcgaaagaacaaatt

fava_c18709_1370	fava_c18709	1370	C/T	aggctatcctatttattcatgaagctttgctctgtattgaaaatttcccctattgttactccattagacaataggcaattggttccagagctcgaagact[C/T]cgctttccatccatgctcagccaatgtccaagaatcatcatgtaacgaagatgttactgtgtggaccaataacacaaaaaaaatcaattgtgaacatttg

fava_c18709_1869	fava_c18709	1869	T/C	agagagccttggagagttctctcagtgatgaaaagattagtagtgaaagaaaaccttctgagattcaatggtgtaatgacaatgtacttaactttgatga[T/C]ctttaatgttgttaacttattctttgatgttagttctattgattgcaaagaataaggcaattttcttcacaatattttttgannnnnnnnnnnnnnnnnn

fava_c18709_579	fava_c18709	579	C/T	cgtatttgtccagagcacaagagtcaattgctaaaattcgtttagggactggttctccctctcttggtgtgatgaaatcttcttcaccccctactgttgt[C/T]gatacagtttttatcaagaggattagatcacggattaaacattggagaaatacatgtaaaaactctggcacaaagacaagcaaacacgaagaaattatcg

fava_c18709_811	fava_c18709	811	T/G	acagttcaaatcaagaccatgcttgaatatagatgtttgcagtgaacgccaagtgcagctaattctgcagatgatagcggacttctccgatgacttcgtt[T/G]ctttggtgatcccttcaaagggtgggaYcactcaggcagtacgcgttgttttggcgaattcaagagaagataaggaattgttggagaaagagcttcaaag

fava_c18709_839	fava_c18709	839	C/T	atagatgtttgcagtgaacgccaagtgcagctaattctgcagatgatagcggacttctccgatgacttcgttKctttggtgatcccttcaaagggtggga[C/T]cactcaggcagtacgcgttgttttggcgaattcaagagaagataaggaattgttggagaaagagcttcaaagtcttgaagatgaggagagcaagaaaatt

fava_c18709_991	fava_c18709	991	A/G	gttggagaaagagcttcaaagtcttgaagatgaggagagcaagaaaattcgtggatttcgagaggtgatcgatttgatttccgcttcagagaaacctgtt[A/G]tctcccacaattgccttaatgattgtacacttattcattcaaaattcattgcaccacttccatcagaagtagatgattttgtgagctccttgtgcaagtt

fava_c18711_1297	fava_c18711	1297	T/G	gttgtcgcggtgaagccaagaatggcaacgataaccatgataagatttttgaatgtccattttgttttaaggtgtttggttctggtcaagcacttggtgg[T/G]cacaagagatctcatttgattccttcatcatctaacaccacacctaatSttaatgttaatctttcagctacattcaaagagactttcatagatctcaatt

fava_c18711_1346	fava_c18711	1346	G/C	tgaatgtccattttgttttaaggtgtttggttctggtcaagcacttggtggKcacaagagatctcatttgattccttcatcatctaacaccacacctaat[G/C]ttaatgttaatctttcagctacattcaaagagactttcatagatctcaatttgccagctcccgttgaagaagaYgatcttagcgttgtttcatatgccta

fava_c18711_1420	fava_c18711	1420	C/T	cttcatcatctaacaccacacctaatSttaatgttaatctttcagctacattcaaagagactttcatagatctcaatttgccagctcccgttgaagaaga[C/T]gatcttagcgttgtttcatatgcctaattgtgtggctaattatcattagttcttaaattgttggaattgggtWaaatggttttagtttttatttttattt

fava_c18711_1493	fava_c18711	1493	A/T	caatttgccagctcccgttgaagaagaYgatcttagcgttgtttcatatgcctaattgtgtggctaattatcattagttcttaaattgttggaattgggt[A/T]aaatggttttagtttttatttttattttttaaattgatagatcaaatgctttttgattctataatggtagatgaatattagttatgattttcaccaactg

fava_c18711_529	fava_c18711	529	T/C	tgagagagaaaaagagagagaataagaaagaaagaaagaagtaagagaaactaaaattgttgcagttggtgataatggagagacacaaatgcaagctatg[T/C]tcaagaacatttagcaatggaagagcacttggtggacacatgaaggctcatcttgccatagctaagtctcagaaacaacaaactgttttgttttcgagct

fava_c18714_1719	fava_c18714	1719	G/C	gatgctgctaaaatcttggaaatgtttggggtgcctcatgaggtaagaatagtttcagcacatcgaactccggatctgatgtttgattatgcctcgtctg[G/C]tcataaacgaggcattcaagttatcattgctggtgctggtggtgcagctcacttgcctggaatggtggctgctatgactcccttgcctgtagttggtgtt

fava_c18714_2103	fava_c18714	2103	G/C	caaaaagaaagcgtattgacgaaaggagagaagttagaaaaaattggttggcaatcttacttaaatgatagtaattagttgaacaacccattgattttgt[G/C]ctcctttttggtttagtctcaattttggacaagtagagttttgatagacattttgatatagagggtgtttagaccccggtgtatcaataagtagttaact

fava_c18714_2238	fava_c18714	2238	A/G	agagttttgatagacattttgatatagagggtgtttagaccccggtgtatcaataagtagttaactggagagatgaagatatagtctagcagaatgagtc[A/G]gtctttgcatctatttgttattccactccttattgtaatgtttttttaaaatgtttttgttggttaaccataggattgaatcaatgtaattgaggcttaa

fava_c18714_291	fava_c18714	291	T/C	actgtgtctttagggcacacttccacttcactctttcccttcaaaccttctcacccttctccttcttcaactgccttctacatggagcaatctcctcttt[T/C]atctttcaacttaaagctgtctaaccaaccccgcatcacttgtcaggcatcattacccaaacacgaagcaaatgcagtttctccaaggaatgaagaatca

fava_c18714_895	fava_c18714	895	T/A	aaacaggggaactttttggctatcctcttatgatcaagagtaggagactagcttatgatggacgaggaaatgcagttgcaaaaagtgaagaggaactggc[T/A]tctgccgtggatgcacttggaggatttgttcgtgatttatatgctgaaaagtgggcaccttttgtcaaggaactagctgtcattgtcgcgagagggagag

fava_c18717_321	fava_c18717	321	T/C	atacatctaagattaagtatatacttagaagagggcagccatacctttgggtgccagaaaatgatttgcataatatgaacaatatcattgacgagcgtgg[T/C]tcatttgctgtgactagtccctttccaggccgactaggagtcatcttgaaatcattgaaaaagttaccagcaagggttgctttatctggagatgttcttc

fava_c18717_810	fava_c18717	810	T/G	tttcagcaaagctgattgacgggattaaccaaagtgagactaggcgtagagccctcgtgcttttatgttttgtttacatgaatgccaatgcaaaggatgc[T/G]tacgtaacatcggttgatcgcaaggggttYgatgtgctRgcaaaagttactggtccagtttcgaaggaYggtgttggcgagtaccaatggaaagagttca

fava_c18717_840	fava_c18717	840	C/T	aaagtgagactaggcgtagagccctcgtgcttttatgttttgtttacatgaatgccaatgcaaaggatgcKtacgtaacatcggttgatcgcaaggggtt[C/T]gatgtgctRgcaaaagttactggtccagtttcgaaggaYggtgttggcgagtaccaatggaaagagttcaggttcatgttcgagcgagaggcaaacgatg

fava_c18717_849	fava_c18717	849	G/A	ctaggcgtagagccctcgtgcttttatgttttgtttacatgaatgccaatgcaaaggatgcKtacgtaacatcggttgatcgcaaggggttYgatgtgct[G/A]gcaaaagttactggtccagtttcgaaggaYggtgttggcgagtaccaatggaaagagttcaggttcatgttcgagcgagaggcaaacgatgttgaaatgt

fava_c18717_879	fava_c18717	879	C/T	ttgtttacatgaatgccaatgcaaaggatgcKtacgtaacatcggttgatcgcaaggggttYgatgtgctRgcaaaagttactggtccagtttcgaagga[C/T]ggtgttggcgagtaccaatggaaagagttcaggttcatgttcgagcgagaggcaaacgatgttgaaatgttctgcaatcaactagttgaaatggaagagg

fava_c18719_108	fava_c18719	108	T/G	ttgaacaaaattaccaagtatattatgtatccttgacatttgaagatatgattctcatcaataaatgaggaaaactgggcaagaagtggagtagtagtac[T/G]ctgtacatccattaccctatatcattttgttcttttaaatgttctattacatatacaaacttaaatcacatgtKacaatttgagatcaaggaactactct

fava_c18719_1090	fava_c18719	1090	C/T	acataaaatatttgtgttgctgtttgctccgccgaagccatgagcctttttcttcttgatcccgaatgttttggaagaaaaccaacaaatgtaaattcat[C/T]Sgtggataaacctgacgccgaaagagcagataccaaagcacaaggacctgggattggaacaacaggaacattttcactaacacataattttgccaactcc

fava_c18719_1091	fava_c18719	1091	C/G	cataaaatatttgtgttgctgtttgctccgccgaagccatgagcctttttcttcttgatcccgaatgttttggaagaaaaccaacaaatgtaaattcatY[C/G]gtggataaacctgacgccgaaagagcagataccaaagcacaaggacctgggattggaacaacaggaacattttcactaacacataattttgccaactcca

fava_c18719_1384	fava_c18719	1384	C/T	tgtgattcattgaatttgtgataactcatcaggggtgttttgatattgtagtgatgaagcaattttccagagtgcctcgtgtcttccgatagtataacat[C/T]tgccgagttcaaaacacgaagagccctgaatgtKatgtcttcgagatttccgatcggtgttccgacgagataaagaccgggtttcagatgctgcgggcga

fava_c18719_1418	fava_c18719	1418	G/T	gtgttttgatattgtagtgatgaagcaattttccagagtgcctcgtgtcttccgatagtataacatYtgccgagttcaaaacacgaagagccctgaatgt[G/T]atgtcttcgagatttccgatcggtgttccgacgagataaagaccgggtttcagatgctgcgggcgacgattatccggttcgtgttcaggtttatctggat

fava_c18719_182	fava_c18719	182	G/T	ctgggcaagaagtggagtagtagtacKctgtacatccattaccctatatcattttgttcttttaaatgttctattacatatacaaacttaaatcacatgt[G/T]acaatttgagatcaaggaactactctcatggcaatatcacctaaYgactgactcccttagtcaagtgttggggaaggaacatctgggatgagtacaagaY

fava_c18719_227	fava_c18719	227	C/T	atatcattttgttcttttaaatgttctattacatatacaaacttaaatcacatgtKacaatttgagatcaaggaactactctcatggcaatatcacctaa[C/T]gactgactcccttagtcaagtgttggggaaggaacatctgggatgagtacaagaYgtaggagatgaagtttgcttatcttagaattatcctttttggatt

fava_c18719_282	fava_c18719	282	T/C	KacaatttgagatcaaggaactactctcatggcaatatcacctaaYgactgactcccttagtcaagtgttggggaaggaacatctgggatgagtacaaga[T/C]gtaggagatgaagtttgcttatcttagaattatcctttttggatttaacaacctgcgactctatggtacttcttctatttctacatctcaagtgatcaag

fava_c18719_461	fava_c18719	461	C/T	ttctacatctcaagtgatcaagtttgtacaatcaattttgctgtcatattgccgagtgttgccttcaagatctaaactgtgggaagccaaggcagcagtg[C/T]aaatgctagtgcagcaacccatccatatatgatcaacagttgttcaacaacaaatggtatactattgtttcctaatYgaaggattgagcaacagatacat

fava_c18719_538	fava_c18719	538	C/T	tgtgggaagccaaggcagcagtgYaaatgctagtgcagcaacccatccatatatgatcaacagttgttcaacaacaaatggtatactattgtttcctaat[C/T]gaaggattgagcaacagatacatgtacagttttacatgatgccggtctctccaggaaaatctgctgctaactcaaatcatcttccacttcaagttgctta

fava_c18719_897	fava_c18719	897	A/T	tgcttgcccttcaaccaatatggtaagttctccttttggcagacgagtagaaaatacatggttggcttcaccaagtgatccgcgccaaaactcttcatga[A/T]acttYgtcatttctcgagcaataacacatttccttgtatcaccaaaaattgatgaaccctcttcaagaaactgagaaagcttgtgaggaggaacataaaa

fava_c18719_902	fava_c18719	902	C/T	gcccttcaaccaatatggtaagttctccttttggcagacgagtagaaaatacatggttggcttcaccaagtgatccgcgccaaaactcttcatgaWactt[C/T]gtcatttctcgagcaataacacatttccttgtatcaccaaaaattgatgaaccctcttcaagaaactgagaaagcttgtgaggaggaacataaaatattt

fava_c18730_1022	fava_c18730	1022	T/G	cagccttggtttcttgccgaagagtttgatcatagtcactccccttcaaaattgagggcttcttgggctttagtctattagtatcaattagtctcttaat[T/G]gctcttaaactcacccacatgtttgttgtatctatgagtttgaatYtgtccttcaagtgtttatcttggttttgagcaatctcctgaagcttaaaattca

fava_c18730_1068	fava_c18730	1068	T/C	caaaattgagggcttcttgggctttagtctattagtatcaattagtctcttaatKgctcttaaactcacccacatgtttgttgtatctatgagtttgaat[T/C]tgtccttcaagtgtttatcttggttttgagcaatctcctgaagcttaaaattcattggagttaagatcaaattagatgaatggcttggtgtcacctccat

fava_c18730_1209	fava_c18730	1209	G/T	aagcttaaaattcattggagttaagatcaaattagatgaatggcttggtgtcacctccatgcaataatcaatagtatttgtcatcaaatgatttaatata[G/T]ttggatcaacaattgttgccacattgtcggacttcatcacaaggatatactcctttccctgtgataataataaatcaagggttccatctatcattagtga

fava_c18730_1316	fava_c18730	1316	A/T	caacaattgttgccacattgtcggacttcatcacaaggatatactcctttccctgtgataataataaatcaagggttccatctatcattagtgaacggaa[A/T]atatcaccatcgtccactgtttgcaSttcYtctttgctgcaatgtYcgcctgataatgttaactctggatgttcRccctgcttaaaagtgtgcacatcaa

fava_c18730_1342	fava_c18730	1342	C/G	ttcatcacaaggatatactcctttccctgtgataataataaatcaagggttccatctatcattagtgaacggaaWatatcaccatcgtccactgtttgca[C/G]ttcYtctttgctgcaatgtYcgcctgataatgttaactctggatgttcRccctgcttaaaagtgtgcacatcaatgcttgattccaaatacttttccaaa

fava_c18730_1346	fava_c18730	1346	C/T	tcacaaggatatactcctttccctgtgataataataaatcaagggttccatctatcattagtgaacggaaWatatcaccatcgtccactgtttgcaSttc[C/T]tctttgctgcaatgtYcgcctgataatgttaactctggatgttcRccctgcttaaaagtgtgcacatcaatgcttgattccaaatacttttccaaaacct

fava_c18730_1362	fava_c18730	1362	C/T	ctttccctgtgataataataaatcaagggttccatctatcattagtgaacggaaWatatcaccatcgtccactgtttgcaSttcYtctttgctgcaatgt[C/T]cgcctgataatgttaactctggatgttcRccctgcttaaaagtgtgcacatcaatgcttgattccaaatacttttccaaaacctttaaagtactatcgtg

fava_c18730_1391	fava_c18730	1391	A/G	ttccatctatcattagtgaacggaaWatatcaccatcgtccactgtttgcaSttcYtctttgctgcaatgtYcgcctgataatgttaactctggatgttc[A/G]ccctgcttaaaagtgtgcacatcaatgcttgattccaaatacttttccaaaacctttaaagtactatcgtgagtgtcatctttgttaaaaataaaYaatg

fava_c18730_1487	fava_c18730	1487	C/T	gttcRccctgcttaaaagtgtgcacatcaatgcttgattccaaatacttttccaaaacctttaaagtactatcgtgagtgtcatctttgttaaaaataaa[C/T]aatggaacctggcatccatacttggagttgagggtctcaatctggttaatgatcaagtcaagaaatgtctttccatcaccaatatctattgcagatttgg

fava_c18730_1646	fava_c18730	1646	G/C	caagaaatgtctttccatcaccaatatctattgcagatttggggttttcaaaacccatgttacttccggagtcatgattgaccttcaaaacaacaagctt[G/C]tccaaaagtttcttagtttcttcKatatcttcaggtaagggtgctaaatcatcgtagggtataacagtgaagccaaccttagccaatttatttttgcgct

fava_c18730_1670	fava_c18730	1670	G/T	tatctattgcagatttggggttttcaaaacccatgttacttccggagtcatgattgaccttcaaaacaacaagcttStccaaaagtttcttagtttcttc[G/T]atatcttcaggtaagggtgctaaatcatcgtagggtataacagtgaagccaaccttagccaatttatttttgcgcttcttatcttcaattctgatagctt

fava_c18730_1907	fava_c18730	1907	A/G	agttgttcttctcgagaagcaatgttttggtaattaaattacaaaacagatagacaaactgaacagagggattaacaggaataggataagcaatgcggct[A/G]aaagtatgaagaggcatagcagaatcgacgagagccacaatgggaatctgaagtttataagcctcgtcgataacagaagatttactctcagtatcaacaa

fava_c18730_2348	fava_c18730	2348	A/C	cgctacgcatgcagattaaggtcttatcggaatcgataatggccattcggttgcggaaaccgtatgtgtagtctttgaaatggtgagtagcgacttgacg[A/C]ccgatgtgggcgttggtgctcagtagcttctgaatcactgcagaatgaatcgtcatgatgacgatgatgatgattctcgattgattggagttgaagtaga

fava_c18730_807	fava_c18730	807	C/T	actttggaaatcatcaatctttgcaaattccggtcccaagtcaatcacaggatttaaagggttggttctagagggattacgagttaaaacaccttctcta[C/T]aagtgtatagatctgactgtagaagcagtaRatcYgaggttgcgtccaagggaagaaaacgagactcRgRtgttgagacaccgattacRttatcaaagaa

fava_c18730_838	fava_c18730	838	A/G	ggtcccaagtcaatcacaggatttaaagggttggttctagagggattacgagttaaaacaccttctctaYaagtgtatagatctgactgtagaagcagta[A/G]atcYgaggttgcgtccaagggaagaaaacgagactcRgRtgttgagacaccgattacRttatcaaagaactgtactgccgattcagccttggtttcttgc

fava_c18730_842	fava_c18730	842	C/T	ccaagtcaatcacaggatttaaagggttggttctagagggattacgagttaaaacaccttctctaYaagtgtatagatctgactgtagaagcagtaRatc[C/T]gaggttgcgtccaagggaagaaaacgagactcRgRtgttgagacaccgattacRttatcaaagaactgtactgccgattcagccttggtttcttgccgaa

fava_c18730_875	fava_c18730	875	G/A	tagagggattacgagttaaaacaccttctctaYaagtgtatagatctgactgtagaagcagtaRatcYgaggttgcgtccaagggaagaaaacgagactc[G/A]gRtgttgagacaccgattacRttatcaaagaactgtactgccgattcagccttggtttcttgccgaagagtttgatcatagtcactccccttcaaaattg

fava_c18730_877	fava_c18730	877	G/A	gagggattacgagttaaaacaccttctctaYaagtgtatagatctgactgtagaagcagtaRatcYgaggttgcgtccaagggaagaaaacgagactcRg[G/A]tgttgagacaccgattacRttatcaaagaactgtactgccgattcagccttggtttcttgccgaagagtttgatcatagtcactccccttcaaaattgag

fava_c18730_896	fava_c18730	896	G/A	caccttctctaYaagtgtatagatctgactgtagaagcagtaRatcYgaggttgcgtccaagggaagaaaacgagactcRgRtgttgagacaccgattac[G/A]ttatcaaagaactgtactgccgattcagccttggtttcttgccgaagagtttgatcatagtcactccccttcaaaattgagggcttcttgggctttagtc

fava_c18731_1518	fava_c18731	1518	T/C	agattgaggagtcatttattgaatgcacagaagatgataaagatgcccatttgtattatattctcactgtccatggacaaggacgcacaatagttttttg[T/C]acttcaattgcagctttgcgccatatttcttccatattgcgcattcttggcgtcaatgtttggactcttcatgctcaaatgcaacagcgagcgcgtttaa

fava_c18731_162	fava_c18731	162	A/C	gcttcactgcattttcaaccatggcagaagcagcaacgtcttcaacgaaaaaggctaaaaggaaaaacggaaacctaaaaagtcaggatcctgaactcga[A/C]cgcctcgaatcacttccatggaactcatctctccctcaccacgacgaacaagacgacaccactttttcactcttcaccggctccaacgaactcgaaggag

fava_c18731_1698	fava_c18731	1698	C/T	tgcaacagcgagcgcgtttaaaggccatggaccgctttcgcgaaaaggaaaatggcatccttgttgccacagatgttgctgcaagaggtcttgatattcc[C/T]ggtgttaaaactgttgtccattatcagcttccacattcagcagaggtttatgttcatagaagtggaagaacagctagagcttctgctgaaggttgtagca

fava_c18731_294	fava_c18731	294	C/T	tccctcaccacgacgaacaagacgacaccactttttcactcttcaccggctccaacgaactcgaaggaggatttctttcacttgaggagattgatgaagc[C/T]gagtacggtttaaatattcccgatcctgaaaaccatgacaggaaccacaattctaatcagagcaagaagttgagtaagcagaagcaagatggtacttgta

fava_c18734_1487	fava_c18734	1487	A/G	ccaatggatccttatttagccaaaaagagaaaactttgtccaatacctgtccttgttctatcatattataccacactggtgtgactcttccaactgaaat[A/G]tcctggaatccaagtccaagtataccatcaaatggaagagctaaaagtgcgaaatttccttctcttgtaatctcagaaaattcttgatctttgataataa

fava_c18734_1595	fava_c18734	1595	A/G	atccaagtccaagtataccatcaaatggaagagctaaaagtgcgaaatttccttctcttgtaatctcagaaaattcttgatctttgataataatatcccc[A/G]agttttacattgtcttgactgaagaatccataaatgtatccttgatcataaggaatttcacaaggtactccaatttctttatatgtgctagatatctttg

fava_c18734_1735	fava_c18734	1735	C/T	ccttgatcataaggaatttcacaaggtactccaatttctttatatgtgctagatatctttgagctatatttggaatgaaaatagcaagcaattgagaaaa[C/T]gcatttggaagatggaatccaRagattSgagctaccagtgtcaaaaacaacattgaagtactgWggtggtgtaccaataccaatttcaccaaaatattgt

fava_c18734_1757	fava_c18734	1757	A/G	aaggtactccaatttctttatatgtgctagatatctttgagctatatttggaatgaaaatagcaagcaattgagaaaaYgcatttggaagatggaatcca[A/G]agattSgagctaccagtgtcaaaaacaacattgaagtactgWggtggtgtaccaataccaatttcaccaaaatattgtacatcaaagtaattcttaacat

fava_c18734_1763	fava_c18734	1763	G/C	ctccaatttctttatatgtgctagatatctttgagctatatttggaatgaaaatagcaagcaattgagaaaaYgcatttggaagatggaatccaRagatt[G/C]gagctaccagtgtcaaaaacaacattgaagtactgWggtggtgtaccaataccaatttcaccaaaatattgtacatcaaagtaattcttaacatacacaa

fava_c18734_1799	fava_c18734	1799	T/A	tatatttggaatgaaaatagcaagcaattgagaaaaYgcatttggaagatggaatccaRagattSgagctaccagtgtcaaaaacaacattgaagtactg[T/A]ggtggtgtaccaataccaatttcaccaaaatattgtacatcaaagtaattcttaacatacacaacaacctctttttcagaaccattttgatcaaaactct

fava_c18734_1907	fava_c18734	1907	T/C	taccaataccaatttcaccaaaatattgtacatcaaagtaattcttaacatacacaacaacctctttttcagaaccattttgatcaaaactcttaaaatc[T/C]ttgagatgaattgcctctttgattcttgcagaattaagactttcaaagtccaaatttctcctttttagagtaattctcatcaacccatcatcagaaattg

fava_c18734_772	fava_c18734	772	C/T	gaaacaatttgcttccaattgtgaatgtaatatttggcatatcagagacactgctgcagtttataaatgattgtccaaccggattcgggagcctctcgca[C/T]agctcgtccacgtgttttaaYactttttcctttacKttgctttgcgtaatctgaacttgaatccaaaggacaatcatattgcaaaaactacaaaaggggc

fava_c18734_793	fava_c18734	793	T/C	tgaatgtaatatttggcatatcagagacactgctgcagtttataaatgattgtccaaccggattcgggagcctctcgcaYagctcgtccacgtgttttaa[T/C]actttttcctttacKttgctttgcgtaatctgaacttgaatccaaaggacaatcatattgcaaaaactacaaaaggggctctcctttgtttgcgatccat

fava_c18734_808	fava_c18734	808	G/T	gcatatcagagacactgctgcagtttataaatgattgtccaaccggattcgggagcctctcgcaYagctcgtccacgtgttttaaYactttttcctttac[G/T]ttgctttgcgtaatctgaacttgaatccaaaggacaatcatattgcaaaaactacaaaaggggctctcctttgtttgcgatccatcgcgagtttcattat

fava_c18734_924	fava_c18734	924	T/G	tgaacttgaatccaaaggacaatcatattgcaaaaactacaaaaggggctctcctttgtttgcgatccatcgcgagtttcattatgcaccactgtttcaa[T/G]aacatcgttcgatctttgaaattcgttatttgaacaaagtccaatgtccgcacaYaagatatcaggatttaatcctgaaatcaaggattcccatatcgaa

fava_c18734_979	fava_c18734	979	T/C	ttgtttgcgatccatcgcgagtttcattatgcaccactgtttcaaKaacatcgttcgatctttgaaattcgttatttgaacaaagtccaatgtccgcaca[T/C]aagatatcaggatttaatcctgaaatcaaggattcccatatcgaatttccgtagttatggacaatgtttttacactcaaaactaacatatccttctgttc

fava_c18735_1671	fava_c18735	1671	C/G	gtgttacttccaagtttgatgttatgaagtttgttggattcatttttctcatactaggatcttcgattgggtgttcaatttattggtctcaaactactgg[C/G]tggattggttacactatactagtaccgatttggtttgtagggacattcggcatctggttttttgttccgcttgcaaagaaaccgaagatttggggtgtac

fava_c18735_1960	fava_c18735	1960	A/C	tgtttggactgcaattttggtggtatattacttgcttgtaggagtacatgcatcttatgacatagcaaaggcacaaaaggagaaggaaaaacttgagact[A/C]agattgagtcaaagatggatgaagaaaatggtgtttccaaaaatgaaaatcatacatagttgaattattaacttatatactttctattgatagttatgtg

fava_c18735_570	fava_c18735	570	A/C	ggtttggcatgggcagtgtcattggctcaggcatatttgttctaacaggacttgaggttagagacactgtgggacctgctgtggtgatatcctatgctgt[A/C]tctggcctttctgccatgttgtctgttttttgttacactgaatttgctgtggaaatccctgtggcgggtggttcctttgcttacctaagaattgagcttg

fava_c18735_999	fava_c18735	999	T/C	gttcctcgcgtttaaactacattgcatcaatatcacatctcattatcctaatgttcatcatagttgctggtttatcgaaagccaatggtgaaaattattc[T/C]gacttcacaccttttggaacgagaggaatattccaatccgcggcagttttgtttttcgcttatgtaggattcgacgcggtttccacaatggccgaggaaa

fava_c18736_1336	fava_c18736	1336	C/G	ggcaagctaagtattcactaaaccttggacttggaaacaaggaactaagaaatgcgaggaatgttgagtatcttccatctagatggtgtgtggcgaaacc[C/G]tcgagtgatctgacgaacgtagtaaaccatatgaaaattgcttgtagtgttgcagactgcacgacacttaactatggaggttcgtgtaatgaaattggcg

fava_c18736_1588	fava_c18736	1588	A/C	aagattcacaaagctgtgaatttgacgggcttggtatggttactttcctagacccttctgttggtgattgccattttcttgtcggagttatagataaagg[A/C]ttgaattcatctgcatctcaaacaacctatcagtggtgggttctagtattatttctaactttacattggagttttttgctttcgatgtgatttagatgtt

fava_c18736_446	fava_c18736	446	G/A	agtggttgatcttttgagagacaacaggatctcaaaagttaagctttttgaagctgatcctgatgtgcttaaagcattgatggggagtggcattcaggtt[G/A]tggttggaatacctaatgaaatgttgtctcttttgggttcttcttctgtggctgctgatttttgggttcggcagaatgtttctgcttatatcactaaagg

fava_c18736_666	fava_c18736	666	C/T	gttgcggtaggaaatgaacccttccttacaaattataacggtcaatttgcgaaccttgttatgcctgcagtaaccaatatacaacagtcactagtcaaag[C/T]gaatcttgctggttatgtaaagatagttgtcccttgcaatgcagatgcctacgagtcctcgggaggacttccttcccaaggggtgtttcgttctgaactg

fava_c18737_1552	fava_c18737	1552	C/G	agaagttatccggtgctgttgaagcattctcccataggaaccgcccaagttctactcctcatcatcatcacggtgaacatgtcagacatagaacttatga[C/G]gaggcagcaatgcacaaggaaatgcactatgcccaacacaattcaactcgatatggcagcaattcgaaaagagcaatggtaacatcatcaaacagRacga

fava_c18737_1648	fava_c18737	1648	G/A	atgaSgaggcagcaatgcacaaggaaatgcactatgcccaacacaattcaactcgatatggcagcaattcgaaaagagcaatggtaacatcatcaaacag[G/A]acgaattcctcaggcgaccatactaacaggatggcctcaggtggtgggggccgtccatctgccgcacatagagttcaacctgtttacgagaccagacaag

fava_c18737_1934	fava_c18737	1934	G/A	tcaagggtctcattttgttgtcgtttctaaatttcacctgagatttcaagaagccttatgaggtcatggataatactgaattacctatgaggtcacaatc[G/A]catgacctcttgatatcatttctggatccccccagattgcttcagaagatttatctcagcaactgtaatttgttcttataacatgtcgaagatataattc

fava_c18737_442	fava_c18737	442	A/G	gaaaccttgttgtggagaatctatttggtccttgttccttcctttgtttggatttttccacaatcaagtggtgaattgtgcttaaatttgaaggtgattg[A/G]ttttatatcatggatcatgtgattggtgggaagtttaaactaggaaggaaaattgggagtggctcttttggagagctctatttagctgttaatatacaaa

fava_c18737_577	fava_c18737	577	G/A	ttaaactaggaaggaaaattgggagtggctcttttggagagctctatttagctgttaatatacaaactggagaggaggttgctgttaagctggaatctgt[G/A]aagacgaggcatccacagcttcactatgagtcaaaattgtatatgcttcttcaaggaggaacggggattccccacctcaaatggtttggagttgagggag

fava_c18737_742	fava_c18737	742	C/T	ggattccccacctcaaatggtttggagttgagggagagtacaatgtcatggttattgatcttcttggaccaagtctagaggatttattcaattattgtaa[C/T]cggaaatttacattgaagtcagtgttgatgctYgccgatcaattaattaatagagttgaatacatgcattcacgaggctttcttcaccgtgacataaagc

fava_c18737_775	fava_c18737	775	T/C	gagagtacaatgtcatggttattgatcttcttggaccaagtctagaggatttattcaattattgtaaYcggaaatttacattgaagtcagtgttgatgct[T/C]gccgatcaattaattaatagagttgaatacatgcattcacgaggctttcttcaccgtgacataaagccagacaattttctgatgggcctcggacgtaaag

fava_c18743_315	fava_c18743	315	T/C	gaaattgagcagcctaggctctcaacagtttgatgtctaatccataggtgttcagaactttctagaacatggattgcaaacaagaggtattgattttcat[T/C]aacgtattcagcaatacactttccaggggatcatgaaagtagtagttagctgaaagcttttcaacacacaaataacggcaatcgccatgaagcttgagca

fava_c18743_432	fava_c18743	432	G/A	cactttccaggggatcatgaaagtagtagttagctgaaagcttttcaacacacaaataacggcaatcgccatgaagcttgagcatcaataaaccaaaggt[G/A]caatttcgttcattgacaaaaccttggccgatatttttgtgacagggtctctgatactaattagtttcaagtaacggattcttatagatcagaaagggca

fava_c18744_310	fava_c18744	310	G/A	caagagtgtttactatagtaaactattgcaaagagactctttggccagctgtaatacctggagaaaatttcaacggaggaggattcgtccttaaacaagg[G/A]caatcctccattttcaacgctccagtcagttggagcggtcgaatttgggctcgaacaggctgcaacttcgatcaaaacggagatggacaatgccaaactg

fava_c18744_910	fava_c18744	910	C/A	catgcactggaacagattatgtcattgctttctgtgcagacaggaaaaaaccgatgtgcacttatcatgatcatgaccttaagtgtagtggatcacaagg[C/A]ttgaaKttRttgattggaWgctggtggatgggaatattcataatRgtgttttcagttcttggattctagaccctaaaactatgaatcttgttaagttgaa

fava_c18744_916	fava_c18744	916	G/T	ctggaacagattatgtcattgctttctgtgcagacaggaaaaaaccgatgtgcacttatcatgatcatgaccttaagtgtagtggatcacaaggMttgaa[G/T]ttRttgattggaWgctggtggatgggaatattcataatRgtgttttcagttcttggattctagaccctaaaactatgaatcttgttaagttgaaatcgag

fava_c18744_919	fava_c18744	919	G/A	gaacagattatgtcattgctttctgtgcagacaggaaaaaaccgatgtgcacttatcatgatcatgaccttaagtgtagtggatcacaaggMttgaaKtt[G/A]ttgattggaWgctggtggatgggaatattcataatRgtgttttcagttcttggattctagaccctaaaactatgaatcttgttaagttgaaatcgagtga

fava_c18744_929	fava_c18744	929	A/T	tgtcattgctttctgtgcagacaggaaaaaaccgatgtgcacttatcatgatcatgaccttaagtgtagtggatcacaaggMttgaaKttRttgattgga[A/T]gctggtggatgggaatattcataatRgtgttttcagttcttggattctagaccctaaaactatgaatcttgttaagttgaaatcgagtgaatctcatgag

fava_c18744_955	fava_c18744	955	G/A	aaaaaccgatgtgcacttatcatgatcatgaccttaagtgtagtggatcacaaggMttgaaKttRttgattggaWgctggtggatgggaatattcataat[G/A]gtgttttcagttcttggattctagaccctaaaactatgaatcttgttaagttgaaatcgagtgaatctcatgagtttgtataaatggaaactcttttcat

fava_c18745_1582	fava_c18745	1582	C/G	caacctatggaaaacgcacagcccttcaagttatcgaccgtttgttttacatgggccaagctgatccagtcgaagggttgaagaaaggaatcaagatact[C/G]gaagaccgattacacaagaaccccgagtcatgtatcctccacctatctgacaatcctacaagaccataccaYgcgatcagcatggaacttccatccacac

fava_c18745_1654	fava_c18745	1654	T/C	aagggttgaagaaaggaatcaagatactSgaagaccgattacacaagaaccccgagtcatgtatcctccacctatctgacaatcctacaagaccatacca[T/C]gcgatcagcatggaacttccatccacaccaattcatcgattccatgtaggattcggcttcggcacttcaagtggatttgtSatgcaagagtttgaggaat

fava_c18745_1735	fava_c18745	1735	G/C	atcctacaagaccataccaYgcgatcagcatggaacttccatccacaccaattcatcgattccatgtaggattcggcttcggcacttcaagtggatttgt[G/C]atgcaagagtttgaggaatttttggccaaaatgttaggaggcattgttagggaaatccaactaagaatatgcggagccggcgaggaaggtggaaatggaa

fava_c18747_522	fava_c18747	522	G/T	gaaccgacggtgaagatggtaacagaggggaaggaaaatgggggcattggatgaaaggacagttggctagagcaccttctgtttcgtcgtcttctggttc[G/T]tcgtcttcttcgggtttagcttgcaagaaatccgatctgagattgcttcttggtgtgctgggtgctcctcttgctccggttcatgtttgcactactgatc

fava_c18749_958	fava_c18749	958	C/T	caggcaggtcaaccaattcacagggaagattaaggtgggttgatacacgactcagaagagatatggctgtggatgttgatagacatcgaatgattataac[C/T]ccggtcttgagattagtcttttcattaacttccttggttgatacatcagattttatggagatgaaaaataagattgttcgtgaagtgattgattttgtca

fava_c18753_1240	fava_c18753	1240	A/G	gcgcctgcaaagtataatattatgatgaaagtgcaaggtttgtctggtcctgtcaattataagattttacaggggcattagagaatcaaacaacaactta[A/G]accataatcggacaccctatcatttggggaagaaatttgagatgatattattttcatctgtggtttcctgcataatttgagattgtctgggatatctgat

fava_c18753_202	fava_c18753	202	A/T	cctccctccaattcatctctcaactacagattaccattattgcagacgcgccgcgcccctcaaccatatcccaagcattgttgtacatttaacaaggatc[A/T]ttcaatggcKttcagaaagacgatcttgaacgatcttcttctatacgaaaaactgggcgggtattactttggaaacttgggcagattctatgctaccaaa

fava_c18753_212	fava_c18753	212	T/G	attcatctctcaactacagattaccattattgcagacgcgccgcgcccctcaaccatatcccaagcattgttgtacatttaacaaggatcWttcaatggc[T/G]ttcagaaagacgatcttgaacgatcttcttctatacgaaaaactgggcgggtattactttggaaacttgggcagattctatgctaccaaagtggacttga

fava_c18755_276	fava_c18755	276	C/T	tcagcaacagcagttctactaggagctcttgcacccggtgttaatggagctacatggaatacgttaaaatctgcgtttttaatgttgggtctatgtctcg[C/T]tgttatgttgggtttagctttctcttctagtgattcgtggttggtgcttcatgttgcgtttcttgttctaatttgtgttaccctcttctttcttcttagc

fava_c18765_1272	fava_c18765	1272	A/G	aaaagtttggtcaaaaggaacacagacaacagtcttgtggaaatgatagcagccaaaggtacttgtttttatcaaaaacggaacttcagccagagctttg[A/G]acatgattaacttggttttaggtccttagatttcactctcccttttgttaatagcaaaaatttgaatttagatagactttagtgcctcattgattcacaa

fava_c18765_277	fava_c18765	277	A/G	cggccatcatatccaagagtgcagtagccccgattgagagagtgaagcttttattacaaaaccaaagtgaaatgattaaaagaggacagctcaaaagacc[A/G]tacctcggcgtggctaatagctttaagagggtcttcgccgaagagggtctcgttgctttctggagaggtaaccaggccaatgttataagatatttcccca

fava_c18765_440	fava_c18765	440	G/A	gagaggtaaccaggccaatgttataagatatttccccacgcaggctttcaattttgcattcaaaggttacttcaaaagcatctttggatattccaaagag[G/A]atgacgggtacattaagtggtttgctgggaatgttgcttctggcagtgctgccggagccaccacttcaatgcttttgtatcatttggactttgcacgtac

fava_c18765_553	fava_c18765	553	G/T	ttaagtggtttgctgggaatgttgcttctggcagtgctgccggagccaccacttcaatgcttttgtatcatttggactttgcacgtacaagattggccac[G/T]gatgctgtcgtgtgccatgttactggtcagcgtcagtttaaaggWctaattgatgtatatcgtaaaaccttatcMagtgatggaattgctggcttgtaca

fava_c18765_598	fava_c18765	598	A/T	ccaccacttcaatgcttttgtatcatttggactttgcacgtacaagattggccacKgatgctgtcgtgtgccatgttactggtcagcgtcagtttaaagg[A/T]ctaattgatgtatatcgtaaaaccttatcMagtgatggaattgctggcttgtacaggggatttggggtttcaatattgggaatcaccatgtatcgaggga

fava_c18765_628	fava_c18765	628	A/C	actttgcacgtacaagattggccacKgatgctgtcgtgtgccatgttactggtcagcgtcagtttaaaggWctaattgatgtatatcgtaaaaccttatc[A/C]agtgatggaattgctggcttgtacaggggatttggggtttcaatattgggaatcaccatgtatcgagggatgtactttgggatctatgacaccatgaagc

fava_c18766_1124	fava_c18766	1124	G/T	agtctcctcgattgaagtataaatgttgccactgcgaacaattccagttgatacccacattttcccattcttctcaataacctctgccattcccatctct[G/T]caYtccacctgcattttgatttcacattcctgaactgcaatttgggcaaggaaStggatgtgaagtgaaggtcctcttcatcacttgcattttgcaaata

fava_c18766_1127	fava_c18766	1127	C/T	ctcctcgattgaagtataaatgttgccactgcgaacaattccagttgatacccacattttcccattcttctcaataacctctgccattcccatctctKca[C/T]tccacctgcattttgatttcacattcctgaactgcaatttgggcaaggaaStggatgtgaagtgaaggtcctcttcatcacttgcattttgcaaatagac

fava_c18766_1178	fava_c18766	1178	C/G	ccacattttcccattcttctcaataacctctgccattcccatctctKcaYtccacctgcattttgatttcacattcctgaactgcaatttgggcaaggaa[C/G]tggatgtgaagtgaaggtcctcttcatcacttgcattttgcaaatagacttgatcattatcatcatcactatcttcatttgaaggatattcccacacttt

fava_c18766_574	fava_c18766	574	T/C	caaactatccagataaaacaggaagttcaaccttgtcaaaggaaaagaaactgacccttccttctgtgacctggcagattttcaacggaattcccccgca[T/C]tgtctctcaagggcttcaatttcagctctagatggcagattacccctcgacaagtacagcataaaatttggatcacctggagaagattttctaaaYctgc

fava_c18766_670	fava_c18766	670	C/T	cgcaYtgtctctcaagggcttcaatttcagctctagatggcagattacccctcgacaagtacagcataaaatttggatcacctggagaagattttctaaa[C/T]ctgctgtcaggaagataaacatcaaaatccggcttcaaatcSttaatcttcaagtcaccaaatagcttacagatagaaagctcgagtttggaacctatgt

fava_c18766_712	fava_c18766	712	G/C	gattacccctcgacaagtacagcataaaatttggatcacctggagaagattttctaaaYctgctgtcaggaagataaacatcaaaatccggcttcaaatc[G/C]ttaatcttcaagtcaccaaatagcttacagatagaaagctcgagtttggaacctatgtctactacttgcgtgctttcttctgtaacttcaagagcaactt

fava_c18768_259	fava_c18768	259	C/A	tgggattctttgttgttctactcttgttgagttcatctacttgtttagcttggttcggtagtagtaaacacaaaacttcaactgcaagtgaacaatcctc[C/A]tcatcatcatcatcatcatcatcaccttcgttcttgaaccgttttcgttctggctcatcggtcgtgttcccggttcacggaaatgtttaccctgttgggt

fava_c18768_574	fava_c18768	574	C/T	agacgccccatccgctttaccggcctagcaatgacttagtcccttgcaggcatcccctctgtgcatccgtgcatcagacggataactatgactgtgaagt[C/T]cagcatcagtgtgactatgaagttgagtatgcagattattactcatcccttggtgtacttgtcaatgaYgtctatgttctcaactttacaaatggagtcc

fava_c18768_643	fava_c18768	643	T/C	tgcatcagacggataactatgactgtgaagtYcagcatcagtgtgactatgaagttgagtatgcagattattactcatcccttggtgtacttgtcaatga[T/C]gtctatgttctcaactttacaaatggagtccaacttaaagttcgtatgggacttggatgtggatatgatcagatttttccagaatcttcctaccaccctg

fava_c18768_979	fava_c18768	979	G/A	atgattcgtctcgattggcttggactccaatgtcatcaaatgatcacaaacattactcagcagggccagctgaactcatttttggagggaagaagactgg[G/A]tgtggaaatcttttagctgtttttgacactgggagttcttacacttacttcaactccaacacttatcaagcactaatttcttggttgacaaaagaattag

fava_c18769_1125	fava_c18769	1125	T/C	ctgggacaaacatggttagaggaaggattatgcaaaataaagtgaacgagtaaaagggaaggattaatttccttagcagaaaaaatagcagaattaagtt[T/C]gctttcttccatggtgataccttagatttcaaaattgctgggagacacagtctRaaaagttgcataggcccggaatgccagcggtgttgttgctttctgt

fava_c18769_1179	fava_c18769	1179	G/A	agggaaggattaatttccttagcagaaaaaatagcagaattaagttYgctttcttccatggtgataccttagatttcaaaattgctgggagacacagtct[G/A]aaaagttgcataggcccggaatgccagcggtgttgttgctttctgtaagcttcatatgactcgggaacttcacaaagtacctttacatcatttaggaaga

fava_c18769_439	fava_c18769	439	G/A	tgaagaagaccagactttgtttgctcattgaagcactgatacaaacacggacacaacactgatactgaaatgttgccacaaataataattgaatgcaacc[G/A]catgtgtttgtgccgcgtcagcatagacctttttttctcttctgttattaacgaaaactaactagctcatttgttctccaatcaaatcaaggcctacgag

fava_c18769_849	fava_c18769	849	C/A	ggatcttttgttgttctattgacttagtgtccctttctgcagcagcaagtaaatcagactctgacgaccttccagctttctttgtgacaatccattcata[C/A]gagcttcccaattggaaaagaccagataccattgcattgaatttagtaacagacatggtgttttcaaaaaggaggtagggaacaataaaaggaaaagatt

fava_c18769_956	fava_c18769	956	C/T	cccaattggaaaagaccagataccattgcattgaatttagtaacagacatggtgttttcaaaaaggaggtagggaacaataaaaggaaaagatttaggtg[C/T]gggaagaatgttcaggattgacatgaataccggaacatagcagattaaccataaagggagttcagcctctgggacaaacatggttagaggaaggattatg

fava_c18770_1007	fava_c18770	1007	C/T	ttcaaatgatgactagtgactatgtatggcttgtcacagattggcttgctgctaccctacattcatttctgccatYgaatcagaatgctcttagcattgt[C/T]gaaggagttgtcggtcttcgtcaacatattccMgattctagtaaaagaagagattatatttctcggtggaaaaaaatgcaaaaggaaggtatagcaaata

fava_c18770_1040	fava_c18770	1040	A/C	tcacagattggcttgctgctaccctacattcatttctgccatYgaatcagaatgctcttagcattgtYgaaggagttgtcggtcttcgtcaacatattcc[A/C]gattctagtaaaagaagagattatatttctcggtggaaaaaaatgcaaaaggaaggtatagcaaatactagcttgaactcttatggattYctcgcttacg

fava_c18770_1130	fava_c18770	1130	C/T	aacatattccMgattctagtaaaagaagagattatatttctcggtggaaaaaaatgcaaaaggaaggtatagcaaatactagcttgaactcttatggatt[C/T]ctcgcttacgatacaatttggacagttgcgcattcaattgataaatttttgaaagtatataaaaatattacattctcgcttcatgacaacaatatggtag

fava_c18770_146	fava_c18770	146	G/C	ccctcacctcggtaccaatcaaaatgtaatctttcttccatttcaatgacttttaagtgttttcaaatgatgggcatgaacaagtttcgatctttactct[G/C]aaaRctttgttgggtgcgttagaatttgagtaaaaaatgaagcattttatggttttRcaccttgtgatttggattcatgtaatcctcctttttggtattg

fava_c18770_150	fava_c18770	150	G/A	cacctcggtaccaatcaaaatgtaatctttcttccatttcaatgacttttaagtgttttcaaatgatgggcatgaacaagtttcgatctttactctSaaa[G/A]ctttgttgggtgcgttagaatttgagtaaaaaatgaagcattttatggttttRcaccttgtgatttggattcatgtaatcctcctttttggtattgctgg

fava_c18770_203	fava_c18770	203	G/A	tgttttcaaatgatgggcatgaacaagtttcgatctttactctSaaaRctttgttgggtgcgttagaatttgagtaaaaaatgaagcattttatggtttt[G/A]caccttgtgatttggattcatgtaatcctcctttttggtattgctggtagtgaaaggcctgaaagtgtgaaaattggagcagttttcaccttcgattctg

fava_c18770_2135	fava_c18770	2135	G/A	gtggtcctcctaagagacaaattgtaacaatgtttatgttcagcctctcaacattgtttaagacaaataataataccgtaagctcactttcgaaaatggt[G/A]ttgatagtctggcttttcctactgatggtgatcacagctagctatacagcaagtttgacttcaattctcacagtggaacagctttcgtcgccgatcacag

fava_c18770_2897	fava_c18770	2897	C/G	tggaaccgtcggacagtcgttgttcccgggttgtcgttaacttctttaacttcattgataaaaaggaagatgctatcaagaaaatgttcactcaaagtga[C/G]aatcctcataaccccaactcagaattatgatagtgataaacaataatgatWatcatgtacatagtatttgtagaagcacaaatatttgtctagtttttaa

fava_c18770_2948	fava_c18770	2948	A/T	tcattgataaaaaggaagatgctatcaagaaaatgttcactcaaagtgaSaatcctcataaccccaactcagaattatgatagtgataaacaataatgat[A/T]atcatgtacatagtatttgtagaagcacaaatatttgtctagtttttaacaatggtttgctacattattcgaatgaaatgtaataagttaataataacat

fava_c18770_982	fava_c18770	982	T/C	agaatcttttccatcgctcgcaaacttcaaatgatgactagtgactatgtatggcttgtcacagattggcttgctgctaccctacattcatttctgccat[T/C]gaatcagaatgctcttagcattgtYgaaggagttgtcggtcttcgtcaacatattccMgattctagtaaaagaagagattatatttctcggtggaaaaaa

fava_c18773_361	fava_c18773	361	G/C	tttatatatgtaaagaacactactttagaggccttaactaatttgtgtcggtatctatataatgaagggcaaatcaccccaaaaacagaacctgattcca[G/C]tgtagtttcaaagaagatataattttctaaacaccatcattaccacctttcttgttaggtttgcggtatttttcctcgaattccttggccgttaacaaga

fava_c18779_208	fava_c18779	208	T/C	ccggttccatagcgcgatggggagattcttttgaggtttctatgagcaaatggaatgaaaatgatcacacagaagcttggggtgcaaagtacaaaacaag[T/C]cttaaaaatcgcacttgtggttttaatctaaaagtaaccaacgacaacaatggtattgacagcatacattttggtttactcgacactgctaacaacaaaa

fava_c18779_375	fava_c18779	375	G/A	cattttggtttactcgacactgctaacaacaaaacggaacttgctgtgaatatctcacgaattgatcatcacactcttcgtggaggaataacaggactga[G/A]ttcttctgtttctccaaaatctttcacgaggaccaaaccagattRcttWatagagactactgttgtttcctacaattgttcccacagagaaggtcttttt

fava_c18779_420	fava_c18779	420	A/G	gtgaatatctcacgaattgatcatcacactcttcgtggaggaataacaggactgaRttcttctgtttctccaaaatctttcacgaggaccaaaccagatt[A/G]cttWatagagactactgttgtttcctacaattgttcccacagagaaggtctttttgttcttgaaatgaagaaaaaggRaaactctgaaaatgcttgcata

fava_c18779_424	fava_c18779	424	A/T	atatctcacgaattgatcatcacactcttcgtggaggaataacaggactgaRttcttctgtttctccaaaatctttcacgaggaccaaaccagattRctt[A/T]atagagactactgttgtttcctacaattgttcccacagagaaggtctttttgttcttgaaatgaagaaaaaggRaaactctgaaaatgcttgcatagtga

fava_c18779_498	fava_c18779	498	G/A	ttcacgaggaccaaaccagattRcttWatagagactactgttgtttcctacaattgttcccacagagaaggtctttttgttcttgaaatgaagaaaaagg[G/A]aaactctgaaaatgcttgcatagtgaatctagcacattattatgtaaccaaagatgttggtctttctgttagggctaaaatttWtcgaaacaaaggtaat

fava_c18779_582	fava_c18779	582	T/A	gaaatgaagaaaaaggRaaactctgaaaatgcttgcatagtgaatctagcacattattatgtaaccaaagatgttggtctttctgttagggctaaaattt[T/A]tcgaaacaaaggtaataRttttgttgYcgaagttgaaggtccatttaaacatcctaStgtWgatttacgtaaggttgttgttaaaacatgtagcagtgga

fava_c18779_600	fava_c18779	600	G/A	aactctgaaaatgcttgcatagtgaatctagcacattattatgtaaccaaagatgttggtctttctgttagggctaaaatttWtcgaaacaaaggtaata[G/A]ttttgttgYcgaagttgaaggtccatttaaacatcctaStgtWgatttacgtaaggttgttgttaaaacatgtagcagtggagtttggtcaccSggtgct

fava_c18779_609	fava_c18779	609	T/C	aatgcttgcatagtgaatctagcacattattatgtaaccaaagatgttggtctttctgttagggctaaaatttWtcgaaacaaaggtaataRttttgttg[T/C]cgaagttgaaggtccatttaaacatcctaStgtWgatttacgtaaggttgttgttaaaacatgtagcagtggagtttggtcaccSggtgcttgttctcac

fava_c18779_639	fava_c18779	639	G/C	tatgtaaccaaagatgttggtctttctgttagggctaaaatttWtcgaaacaaaggtaataRttttgttgYcgaagttgaaggtccatttaaacatccta[G/C]tgtWgatttacgtaaggttgttgttaaaacatgtagcagtggagtttggtcaccSggtgcttgttctcactgtaaaggaacaaaaacaaaggctagcacK

fava_c18779_643	fava_c18779	643	T/A	taaccaaagatgttggtctttctgttagggctaaaatttWtcgaaacaaaggtaataRttttgttgYcgaagttgaaggtccatttaaacatcctaStgt[T/A]gatttacgtaaggttgttgttaaaacatgtagcagtggagtttggtcaccSggtgcttgttctcactgtaaaggaacaaaaacaaaggctagcacKggtg

fava_c18779_694	fava_c18779	694	C/G	gtaataRttttgttgYcgaagttgaaggtccatttaaacatcctaStgtWgatttacgtaaggttgttgttaaaacatgtagcagtggagtttggtcacc[C/G]ggtgcttgttctcactgtaaaggaacaaaaacaaaggctagcacKggtgtaaaaaatgaagattctaactcaatgtcgaaaggtcaaactcgtgttcaac

fava_c18779_739	fava_c18779	739	T/G	StgtWgatttacgtaaggttgttgttaaaacatgtagcagtggagtttggtcaccSggtgcttgttctcactgtaaaggaacaaaaacaaaggctagcac[T/G]ggtgtaaaaaatgaagattctaactcaatgtcgaaaggtcaaactcgtgttcaacattctagttctttaatgttgaaaggtgaatctcatgttcaacagc

fava_c18779_856	fava_c18779	856	A/G	attctaactcaatgtcgaaaggtcaaactcgtgttcaacattctagttctttaatgttgaaaggtgaatctcatgttcaacagcagaataatggaaaggt[A/G]gtaggtgaagttgttaaacatggtttcttctcgagtactgttRgagagcaaattaacactgggcttattaattcaactggatatacttctggtactttaa

fava_c18779_899	fava_c18779	899	A/G	tagttctttaatgttgaaaggtgaatctcatgttcaacagcagaataatggaaaggtRgtaggtgaagttgttaaacatggtttcttctcgagtactgtt[A/G]gagagcaaattaacactgggcttattaattcaactggatatacttctggtactttaaataactctatcatttttgtagaatgtaatttttaaatgcacat

fava_c18780_1330	fava_c18780	1330	G/A	taaaagtcatgagagtaatcccaaaggaaccgttccacattccatcacggcagaaaaagtaaaagcttttgtccctcttgaatcagaaggaaaaaatgcc[G/A]cattccaaagcaccatgtcRacgacacaatattcttggactgaaattccaaaaacacagctaaagaaagaacaaaagccaattgttcatccagaggataa

fava_c18780_1350	fava_c18780	1350	A/G	ccaaaggaaccgttccacattccatcacggcagaaaaagtaaaagcttttgtccctcttgaatcagaaggaaaaaatgccRcattccaaagcaccatgtc[A/G]acgacacaatattcttggactgaaattccaaaaacacagctaaagaaagaacaaaagccaattgttcatccagaggataacttcaacctattgcccttac

fava_c18780_1976	fava_c18780	1976	A/T	gatccagggcttctcacagaagttccaaaccacttatatgattcaacagattattcagtaattgatcaaggtctattcatagcatgataagctctgatat[A/T]tcctgcttatatccagcaaaaatcagaatttcatgtatgaaatgacagatttaatgtttgtagtagccagctatatttatctattattgaacaattataa

fava_c18782_1094	fava_c18782	1094	G/A	ccatctttgaagtgaatatagtaaggctgcttctttgaaccatcttttttccactcataaaaaccttctactgcaacaaggcatctgtttttaggaagca[G/A]gcgacggaatgaagctttctcatcaatggattcagatcgagcattaaacatcttgtagtgatcaggtttgtccgttttcttagtgaaactggggattaaa

fava_c18782_1287	fava_c18782	1287	C/T	ggattaaaccccatttcatacaatgaacaacatggccttcactctccgctgcttcatctctacgaacaacaggcatattgaatcccggcgacacattatg[C/T]gacggcacataccggtcgatatggagaaggcgggtgggagcggtggtacggtggcaggctctcggaatgtcatcggctcttagactgcaacgagttcttc

fava_c18782_494	fava_c18782	494	A/G	ttttcctttttctttgccgggtttgcacttgacttatcattatttgctaaagagggtttcaaatctgatgaaatagcaacatactcacgcttgattggga[A/G]tttggttacattttgagaaggggaagaaccactgggttttaagtcactttcaccctcctctgttttagcttcttcgctgaggtctttggcttgttcggtc

fava_c18782_669	fava_c18782	669	C/T	cgctgaggtctttggcttgttcggtcttgacaggttcatgaaatagtgttttatgctctggctttgtgtcttcatcttcagccacctttcttgaaaagaa[C/T]ttagatattggagtgtttccttctgtctttacttgaatctcctttatacactcaggtccattaaatgatggcttgcccatggcaggtgttacagggtacc

fava_c18785_1478	fava_c18785	1478	C/A	gtatatttgtgttacgtcatatgctagttttcatgaacaataccccaaattggaaatacattgttcattgcatctcatttttgcaattcacaaatttata[C/A]atttgattagcagcatcaagttgtttggtatttactatttatgttgacaatctttcaccaaatgtagttctctaatcccaagtatagatggagaagtttt

fava_c18785_525	fava_c18785	525	A/G	caaaagatccatatttcatgcgcaatcatctcggtagctacgagtgcaaactctgtttaactctccacaacaacgaaggaaactacctcgctcatacaca[A/G]ggcaaacgccatcaaaccaatctcgctaagcgtgctgcacgtgaggctaaagatgctcctactcaacctcaacctcataagcggaagcttaaccttaaga

fava_c18785_852	fava_c18785	852	C/G	gatttatgtcctcctatgagcagagggtgcaaccatttgataaaagatatcagtatctcttgtttgcagctgaaccatatgaaacaatttctttcaaggt[C/G]cctagcacagagattgacaagtccactccaaagtttttctcgcattgggacccagattccaagatgtttacgctgcagttgtattttaaaatcaagccac

fava_c18788_197	fava_c18788	197	A/C	ttactttgacacttggtcactagaatacacataaaattacaccaattaaaagaaattaaatatttactatgtagcatttccaagatttttggtttgctat[A/C]aattttcttagattttgctcaccaagaacaactcattgaaatactatctaaataaaactcaacacaagcttcaatttgagcYcatggtattttcttgtca

fava_c18788_279	fava_c18788	279	T/C	agatttttggtttgctatMaattttcttagattttgctcaccaagaacaactcattgaaatactatctaaataaaactcaacacaagcttcaatttgagc[T/C]catggtattttcttgtcacatgcccaaatatagttacttattcatgaatatgctaatgttgattttcctttgtccctatagtttctatctcattgaaata

fava_c18788_675	fava_c18788	675	G/C	atcactacagagactatctagcaaaagagattcttctccacaagaattttctgtagggggtgagcttgaattagtgttttctgagttcagaattccatca[G/C]tttccttcacttcatggctattgtcaggctgtgacaaattctctgcaggggatgaagtatcattggatgatgcttgtttgttcagaggctcatgagtaat

fava_c18789_1130	fava_c18789	1130	G/A	catattcaaatgagaaatcagagtcccgaaaagttgaggaagtgtcagaaacaagctcatctacttcaagccagaattctaaggaattaaacgagtaaac[G/A]tgtcgggaaaaaactatatataggttcacgacagttacaagggtcaccaaccttgcaaaagcatttgatgcattggcttaaaactagaagaatctcaatt

fava_c18789_1360	fava_c18789	1360	A/T	agcaatggtgacaattatagaaaatacaattatactggttcaggttaaatacaattatagaccatctgttgcatccttcaacaatgatgatctgttcagg[A/T]tttaattcatcacttRcatatatcatttgtatatgtaagcatatttgcSatagttgaattgtgttttacttctttaggggtatattcaattagcttatca

fava_c18789_1376	fava_c18789	1376	A/G	atagaaaatacaattatactggttcaggttaaatacaattatagaccatctgttgcatccttcaacaatgatgatctgttcaggWtttaattcatcactt[A/G]catatatcatttgtatatgtaagcatatttgcSatagttgaattgtgttttacttctttaggggtatattcaattagcttatcatctgtaattgtgtata

fava_c18789_1409	fava_c18789	1409	G/C	tacaattatagaccatctgttgcatccttcaacaatgatgatctgttcaggWtttaattcatcacttRcatatatcatttgtatatgtaagcatatttgc[G/C]atagttgaattgtgttttacttctttaggggtatattcaattagcttatcatctgtaattgtgtataaagagcatactatgcctgaatatatgaaaaacc

fava_c18789_302	fava_c18789	302	A/G	tccttcccttctctaagcctcacaaaacctctgctcctatctcttcccaatccccaaaacgatgtcgtatcatcacctccgtttcagaggatcgtaaacc[A/G]atcccggtggtgtcaaatgacagaaaagtccttcagccttctccgccttccgaagacaacaacacagagattgttaacgacactgatatcctcaccggac

fava_c18789_632	fava_c18789	632	C/T	ggattgcctatgagcaatctctgaaaacaaatccagttttagctaaaatggcaataagtggagttgtctactcaattggagattggattgctcagtgtta[C/T]gaaggaaagcctatttttgaRtttgatcgggcgcggctattcagatcRggtcttgttggatttactctccatggatcactttctcactattactaccaac

fava_c18789_653	fava_c18789	653	G/A	tgaaaacaaatccagttttagctaaaatggcaataagtggagttgtctactcaattggagattggattgctcagtgttaYgaaggaaagcctatttttga[G/A]tttgatcgggcgcggctattcagatcRggtcttgttggatttactctccatggatcactttctcactattactaccaactgtgtgagactctttttcctt

fava_c18789_680	fava_c18789	680	G/A	tggcaataagtggagttgtctactcaattggagattggattgctcagtgttaYgaaggaaagcctatttttgaRtttgatcgggcgcggctattcagatc[G/A]ggtcttgttggatttactctccatggatcactttctcactattactaccaactgtgtgagactctttttcctttccaagaatggtgggttgtccctgcta

fava_c18789_878	fava_c18789	878	T/C	ctaaagttgcttttgatcaaactgtgtggtcagctatttggaatagcatctactttgtggttttgggcttgttgcgtttcgattctttgactaacattta[T/C]ggtgaactcaagtcaacatttgtgcccatgcttactgcaggttggaagctttggccttttgcccatttgattacttatggtgttattcctgtggaacaaa

fava_c18794_1046	fava_c18794	1046	G/C	cgtcacaaacaccttgagtgaaataaagagcagcatcagcaatgaaacaagaaacaggaacagaaccatcatgatgatcaccatcctgtgacgacgacaa[G/C]aacgtagccaagaattccttcaaaggctgaacgcatctgatattgatgatgtcagtgagatgaaccgcgtccacctttgaagcatcggtttccgataaag

fava_c18794_221	fava_c18794	221	C/T	ctctctttagcttactctctaattgcaatccaaccttccaaacatcactagcatactttgcattgatcttttggtctccaaaagacggcatacaaatcat[C/T]ggaacaccttcacatacactctccaaagttgaattccaaccattatgcgtccaaaatgctccaactgcaggatgcttcaacacttgttcttgaggagccc

fava_c18794_416	fava_c18794	416	C/T	gagcccatttcacaatgtaacctcttcctcccaaattctcaaggaaatcattcggcaatggttcaagccattctgagtcgcggattgtcccgggtcgaat[C/T]gcccacaagaatggttgattgctgttggctaaaccccaagctatctccaagaactcagcctcgcatattgatacaatactcccaaaactcacataaacaa

fava_c18794_533	fava_c18794	533	T/G	gattgctgttggctaaaccccaagctatctccaagaactcagcctcgcatattgatacaatactcccaaaactcacataaacaacacttttatgttcttg[T/G]ttgtctaaccaagcgatgcaactttgatctggtgttaataagctagtagaagttgatccaggaagagagtatttgtgaaaagggcctataggatatattg

fava_c18794_637	fava_c18794	637	C/G	tctaaccaagcgatgcaactttgatctggtgttaataagctagtagaagttgatccaggaagagagtatttgtgaaaagggcctataggatatattggca[C/G]agaaaaatcttggcgcaattttgttaWcgcggatgattccaactcttcataagtgttccaaataatcccggacgatttcttgcattcatcaataaagcga

fava_c18794_664	fava_c18794	664	A/T	ggtgttaataagctagtagaagttgatccaggaagagagtatttgtgaaaagggcctataggatatattggcaSagaaaaatcttggcgcaattttgtta[A/T]cgcggatgattccaactcttcataagtgttccaaataatcccggacgatttcttgcattcatcaataaagcgacaaactagtKtgtaaaatgcctcaggt

fava_c18794_747	fava_c18794	747	T/G	ttggcgcaattttgttaWcgcggatgattccaactcttcataagtgttccaaataatcccggacgatttcttgcattcatcaataaagcgacaaactagt[T/G]tgtaaaatgcctcaggttcttttgactgaaacacgggaaggtctttcactctgagaggtggaagatccacaacagcttcctccaatcgagactcttgaac

fava_c18794_875	fava_c18794	875	G/A	gaaacacgggaaggtctttcactctgagaggtggaagatccacaacagcttcctccaatcgagactcttgaacaggaaaatagcccttttctttgaggag[G/A]ggaaacgaagcaaaaatcagaaaagacgatgcaccaccagtcctaagcacaagacgaggaattcgaaactcgtcacaaacaccttgagtgaaataaagag

fava_c18796_1401	fava_c18796	1401	A/T	tataagagaatgtggccttcctattgttcaagaaggctcagagacatgagatggcatagtcgcctcgccagtgacttttattcaattttggaaactgaag[A/T]atgaagatcattaatgagatgttgctgaaatggggctttggctaaaagctgcaggaccattggattggtgataatttatatgctgctagataaatactag

fava_c18798_162	fava_c18798	162	T/G	gtacgaaataaaattaactcaaggaaaaggctaaagtagattcgcatattaaaaaaacaaagtactactacgtgtaccttctgaaacaaaaaacaagaaa[T/G]agagaaaaaaacaagacaagtaacatcgaacagcgaatcaagcatgaaacaaattcaaacactgccaccgtcactgcccctgttccaccagcacggaatc

fava_c18799_823	fava_c18799	823	C/G	aggtcatcgctggcacattttctattgaacccaaggacactggtgccagtataaaaggcaatacttctaccagcaagttaccatcaccggttggtgatcc[C/G]acatcaggtttaggtttccgcccagcacttaactcttcaaacgggaatgcaatcccgggaaacaaagagcatcaagctattggaggaagtcatttcatgt

fava_c18801_1122	fava_c18801	1122	G/A	tgaagccatcaaggtcagtcttatgctttcctaataaatctatcaactccttatatccctttgcccataccatctttcccaagaaatatgccccttttgt[G/A]aaagccYtctgccctatctctctctctgcagccatcatttctccaatttttaagaacttgggattcacaccatgaacRttgcaaattatagagttgggta

fava_c18801_1129	fava_c18801	1129	T/C	atcaaggtcagtcttatgctttcctaataaatctatcaactccttatatccctttgcccataccatctttcccaagaaatatgccccttttgtRaaagcc[T/C]tctgccctatctctctctctgcagccatcatttctccaatttttaagaacttgggattcacaccatgaacRttgcaaattatagagttgggtaaatcctg

fava_c18801_1200	fava_c18801	1200	G/A	ccaagaaatatgccccttttgtRaaagccYtctgccctatctctctctctgcagccatcatttctccaatttttaagaacttgggattcacaccatgaac[G/A]ttgcaaattatagagttgggtaaatcctgagtagcagctgagagacgaagaaccttgttacagtatgctcttgtaacccagttgttgatgtgtttcacga

fava_c18801_2262	fava_c18801	2262	G/C	tcttaaccccatcaaaaatttccacagaagaactccgcttctcaaactctctaatcctagttttcaacgctcgaataggttcccaatcactagttccact[G/C]tcagtctctcctttcaacccttcccaaaaccctaaccttctccccttaccccctctctcaaaatccacaaccccgtcgccaagctcctccgacacaatgt

fava_c18801_283	fava_c18801	283	G/T	gctacaaaacttaaggattatgacaggcacctaattacaaactataaacaagcagaaagcagaaactacaaatttgaagccctagagctattcatcagca[G/T]tataacttttttacaatgctgcttgtcatgatctggcatcacaWatatttcttagccatccccatattcaaactagtcaatctatttaccagccatagat

fava_c18801_327	fava_c18801	327	T/A	taaacaagcagaaagcagaaactacaaatttgaagccctagagctattcatcagcaKtataacttttttacaatgctgcttgtcatgatctggcatcaca[T/A]atatttcttagccatccccatattcaaactagtcaatctatttaccagccatagataggattttccacctgtgggggcaagagatgaaggtcttcacagt

fava_c18801_587	fava_c18801	587	C/T	ttcccagtgagacagtagtgagcaaatgctaaacctccattagctagttctgaaagattaggcattgatgctgacttgccaatcagctttcctttatctc[C/T]acttggttttacgccatccttttccatgttcaggatttggtctagatcggaatattccataaatctttgagttgcagcttcccaagagagtttatatctt

fava_c18801_699	fava_c18801	699	G/C	cgccatccttttccatgttcaggatttggtctagatcggaatattccataaatctttgagttgcagcttcccaagagagtttatatctttgttcaggtgt[G/C]agaggataaggctcattttctaatgcttcttttacttttgctacaaagtcttcaggtgtcttgtaaatcaaacaattgggaaatgagttgaagaactcat

fava_c18802_125	fava_c18802	125	C/A	attttgtgcaaagaggagttatgggtctgacgaatttcgtactgacggtggccggcgtaagcgccgtcgtccttcttctaaggagcgacgtgaagcaatc[C/A]gcgtcgattttcaaacgcaacgtcaaacaaattcgccactggctagaagaagaaaccgccaattcttcaaagaaaatggagcagactaaaaaggaacttg

fava_c18802_274	fava_c18802	274	A/G	gaagaaaccgccaattcttcaaagaaaatggagcagactaaaaaggaacttgaatcgaaagttaaagacattcataaggacgataagcactaatgtttat[A/G]tagttaatgtgatgggtggtttagtgtagtaatttttctgtttctgtgctcacctgttactgctgWgagccatctgattgtaactttgaaaatgttaact

fava_c18802_340	fava_c18802	340	T/A	gacattcataaggacgataagcactaatgtttatRtagttaatgtgatgggtggtttagtgtagtaatttttctgtttctgtgctcacctgttactgctg[T/A]gagccatctgattgtaactttgaaaatgttaacttctgRacttctgtttgtgtcattactctaaggtgatgaaatattcaacttgaaatgcattggtctt

fava_c18802_379	fava_c18802	379	A/G	taatgtgatgggtggtttagtgtagtaatttttctgtttctgtgctcacctgttactgctgWgagccatctgattgtaactttgaaaatgttaacttctg[A/G]acttctgtttgtgtcattactctaaggtgatgaaatattcaacttgaaatgcattggtctttcaagaagttcttttagtgcattttttgcttgtggagta

fava_c18803_1047	fava_c18803	1047	C/A	gctttatcgaccccctcgtacttctcattgttctatatgcaataactgtgtccagagattcgatcatcactgtccctgggttggccagtgcatcggaaga[C/A]gaaactatcggtacttctttatgtttattacaacatcaacctttttgtgcctatatgtctttgtgttttcttgggtcaatctttccaagactcggaatga

fava_c18803_1589	fava_c18803	1589	T/A	tgggatcaatgcgtgcagaccatggtggttttccagttcctgaacttttgcggaattttgattttgataatttcgaggatgacatgaagtttggagatga[T/A]gaggagggacgagcctcatttgatcccttctacagtgccgatgaaaacataaaggactcggcccgaacatctgttgccaccgttttgaatttccatagca

fava_c18803_1958	fava_c18803	1958	G/A	gcatagaggtcctctcttttgattactggttttaccttgaggattgaagtggtatggaggatagttgccttccgtattggttcagcaatcattgatgctg[G/A]aaatgagggactgactttgcacttcctttctcattttggtataaatatttggcaagtgtgtttgttgtaagatgtgtaaactccacttgcattcaaaata

fava_c18803_2091	fava_c18803	2091	A/G	attttggtataaatatttggcaagtgtgtttgttgtaagatgtgtaaactccacttgcattcaaaataatgcagaaagatttttctggcaaagttttatg[A/G]taggtactgttctaggtgtgtctgtttctttcacttagtctcttgttaaattctagattgattgattttttatttactaatagatatttagaatttctgt

fava_c18803_713	fava_c18803	713	T/C	taattgctgcacctgcaattacattttgtgtcaagatgtatcttaaagccaagaacgagaatgccagtagtggtcatggtttttggtgtcccgtagtgat[T/C]gtgggtctaactctcactcttctggatttcctattccttctcttgacttctgggagagatcctggaattatccctagaaattcaacacctcctgaatgcg

fava_c18804_253	fava_c18804	253	C/T	ccgaaatgcgcaaagaagcttttgaaggaaacatttccagactccttcgtaacgaaattcaatacgagattcaatcctcttcttcttccaaaccccctgc[C/T]aacaagtttggttcctttttggtggatggccgaccMagtgagcgatgggttacactgaatagacaatttgccgatgaaaacattaaagttgaagtcacta

fava_c18804_289	fava_c18804	289	C/A	ccagactccttcgtaacgaaattcaatacgagattcaatcctcttcttcttccaaaccccctgcYaacaagtttggttcctttttggtggatggccgacc[C/A]agtgagcgatgggttacactgaatagacaatttgccgatgaaaacattaaagttgaagtcactatgtttgatggagctgctcctgctccgaaagtaagtg

fava_c18805_255	fava_c18805	255	G/T	taggctagtactaattaaatactactagtactcagcaactacttttatctactagaaaatcaatctttttaattactatcttagtggtggtatgaacgaa[G/T]aacagctccacaaaaagtgcaaacaatagctctccaagatttcgtataaaacggaacatagcaaaatcttgtagctgtcttcatatccgccacgctagcR

fava_c18805_355	fava_c18805	355	G/A	Kaacagctccacaaaaagtgcaaacaatagctctccaagatttcgtataaaacggaacatagcaaaatcttgtagctgtcttcatatccgccacgctagc[G/A]ccaccgccgcaccgtgagcaagttcccgccgccggcttgctccgctgcatctccttagtYtggtccaccagaaaacaaaagcagaccattcttccttctc

fava_c18805_415	fava_c18805	415	C/T	agcaaaatcttgtagctgtcttcatatccgccacgctagcRccaccgccgcaccgtgagcaagttcccgccgccggcttgctccgctgcatctccttagt[C/T]tggtccaccagaaaacaaaagcagaccattcttccttctctacgcggcgttgtttatttaatacagttacaaaaagtgatcccg

fava_c18810_183	fava_c18810	183	G/A	ctatcgttcatcctgcggtgccggcaactcaacactctgatgaactccatatcgctatcatcgtttccatttaacctctgccttcacgaacgctgtgact[G/A]aacgcttttattcatcatctYcgttcacctttcttctccctcatcgccatgttacactcatcatctctgtctcaatccttctaatgattcttaactttgt

fava_c18810_204	fava_c18810	204	T/C	cggcaactcaacactctgatgaactccatatcgctatcatcgtttccatttaacctctgccttcacgaacgctgtgactRaacgcttttattcatcatct[T/C]cgttcacctttcttctccctcatcgccatgttacactcatcatctctgtctcaatccttctaatgattcttaactttgtcgttcgcgcctccgtctcagt

fava_c18810_669	fava_c18810	669	T/G	agatttaactatgaaatgatgatgatgttatgcgttgtagttaaagattgaagatgataatcgatgtagaggagagatgaaacatgtgcattggatgaca[T/G]atgaagatgtagagatttctgcagattgaggatgatgattacagataaattttggtggattgaatcagagagagataaattttatgtggttttcttggtg

fava_c18810_833	fava_c18810	833	A/G	atcagagagagataaattttatgtggttttcttggtgattctccaaatgctgaagaatgagcatgattcagggcatttcttggacagagtaaagtgtatt[A/G]ttgaatcaattttggatctgatttctggtttcgactctttttgtcactttctgatttttgttgctatgagctgggcatgattcgggtttgatgtacattg

fava_c18812_1375	fava_c18812	1375	G/A	atatagtcaatgagttggatcgtgggaaggcggtagagaaaccctcaaatcatgatagtggcaatgatcgacttggcattgcaatgggaatgagtggggc[G/A]ttgtcccttcaaagaaatgtttatccattgacgccattcttgtctgcaccacttttgacagcctacaacactgttgatccactagtagatcctgttcttt

fava_c18812_1567	fava_c18812	1567	A/G	ctgttctttggacatctctcattcctatgcttcctgctggcctttctcgtacagctgaggttacaaagacagagactgattcaacgtacactatgtttca[A/G]ccagaggagtaattagaaatctgaatttctaattgaaaaaagccaaacacccgaaaactgagcttgtgtaccaaacaaacaacaatgtaaagttctaaaa

fava_c18812_1718	fava_c18812	1718	C/A	ccgaaaactgagcttgtgtaccaaacaaacaacaatgtaaagttctaaaatctgaagagctcaagcaaatcttaagtaatgtccccatggccttttgcaa[C/A]tatcttatggattgcWgaggtggaagaaaagaagttaccatagtcttacattatgaaatagatatataggattatagcacaaatatttacatttYaaacc

fava_c18812_1734	fava_c18812	1734	A/T	tgtaccaaacaaacaacaatgtaaagttctaaaatctgaagagctcaagcaaatcttaagtaatgtccccatggccttttgcaaMtatcttatggattgc[A/T]gaggtggaagaaaagaagttaccatagtcttacattatgaaatagatatataggattatagcacaaatatttacatttYaaaccagatatgtaccagttg

fava_c18812_1813	fava_c18812	1813	C/T	tgcaaMtatcttatggattgcWgaggtggaagaaaagaagttaccatagtcttacattatgaaatagatatataggattatagcacaaatatttacattt[C/T]aaaccagatatgtaccagttgatatctcgatcatgtatatgcagtcagcattagacaaattttgattttatgcggagtattctgtacagaataggagatg

fava_c18812_667	fava_c18812	667	C/G	cgcatctttgggataaaagtacatggaaccaaaatcagaataagaaaggaaagcaagtttacttgggcgcatatgatgatgaggaagcagcggctagagc[C/G]tatgatcttgctgctctgaaatattggggtcctgggactctaattaatttcccagtgactgattatacaagagatctcgaagaaatgcagaacatttcaa

fava_c18812_850	fava_c18812	850	T/C	aaatgcagaacatttcaagagaagaataccttgcgtctttgcgccggaagagcagtggtttttcaagaggactagcaaaatatcgtggactctccagtcg[T/C]tgggatccatcatatggccgaatgactggatcggattacttcagtagcatgcattatggtgcaggggatgattcagctgccgaaagtgaatatgttagtg

fava_c18813_1620	fava_c18813	1620	A/C	aagaaaccagaaattgctccagtaccatactgaattgcagcagaagtcccatttggcttgtaagtacttgattggctagacttgtacttggcatggaata[A/C]gcatgcaagcgagaaataacatttaaccgacggaacccatgtattcgagctaccagtatcaaaaatcaccgtaaacttctgaggaggagtaccaatggaa

fava_c18813_1889	fava_c18813	1889	G/T	aagaatatttttagacaataaagcttcaccacctttcaaccccaagaaatccttgggtcccaatttcacctttctcaaaccaatcctacgcaacccctca[G/T]ttggcacacaagaaacaaaacaaaacaagagggtccacacacacaaacataacgtcaccacattcaccttgtttcccattttcaccagttcttccaaatt

fava_c18813_929	fava_c18813	929	G/A	gcatacagaacatgtagcatcttgatgaccgccagatgatattctatcagtctgtggcacaacactctggatacccatactaacaccacgagtcccatca[G/A]acgtgcacaacccaatttgggagcatatcttctttggctgtgtttcagccaagagcaattgcaaaattgtttgtccatattgatcaacaacattcctgca

fava_c18814_1014	fava_c18814	1014	C/G	tattagatcgttttatgtatggaacatgggttctagtaccactgaattttctaaaattcaattttctttcctcgggcggagactattatggaactcacca[C/G]tggcactggtacttcactcagggatttacagtcatgatcttctctcatataccattttgtatagccggtatcatttattccaaacagtggaagtttactg

fava_c18814_1135	fava_c18814	1135	A/G	gggatttacagtcatgatcttctctcatataccattttgtatagccggtatcatttattccaaacagtggaagtttactggcctccttgcttgggtgttg[A/G]gtttctacagtatcttaggtcacaaggaattcaggtttgtcttgccagttcttcccatagctctgatgttctctggttattctttggctatgatagaaga

fava_c18814_1243	fava_c18814	1243	G/A	cagtatcttaggtcacaaggaattcaggtttgtcttgccagttcttcccatagctctgatgttctctggttattctttggctatgatagaagatctcggt[G/A]ccggttcttcgcagtataaagaaaaaaagttttcaaaagagcataacaaatgtcctcccaagatRacagctgctataWtatttttgcttgctaccaatat

fava_c18814_1308	fava_c18814	1308	A/G	ctggttattctttggctatgatagaagatctcggtRccggttcttcgcagtataaagaaaaaaagttttcaaaagagcataacaaatgtcctcccaagat[A/G]acagctgctataWtatttttgcttgctaccaatataccaatggctttgtatatgagtttggttcatcagagaggacctgaggatgtcatgaaccacctcg

fava_c18814_1321	fava_c18814	1321	T/A	ggctatgatagaagatctcggtRccggttcttcgcagtataaagaaaaaaagttttcaaaagagcataacaaatgtcctcccaagatRacagctgctata[T/A]tatttttgcttgctaccaatataccaatggctttgtatatgagtttggttcatcagagaggacctgaggatgtcatgaaccacctcgctaaagaagcttt

fava_c18814_1814	fava_c18814	1814	C/T	cacttcaaggtagatcgtgatcttcaagcttcaatagttgtatatgttcgaataagatagcatcatctgatctacagcaaatggcaagtactgtgacagt[C/T]cattgttggttgcttcaaatgttaattttggtaactaaggtctcttaaatgacttcatagtgattgtaaatgtttctatactatgttttagatgtcaaca

fava_c18815_71	fava_c18815	71	C/T	ggaagataaaattcaaggactatattcaaaagtagcacatatcctatgtacttatttactgagtttggac[C/T]tgtataacaataagactcattttcttcccattacaaaattgtatggtgtggtttactttgcaaactatattactccaacaataatggcttataataatat

fava_c18816_1105	fava_c18816	1105	T/C	agagtctcaaaggctgtgaaggaagcctatcataaaacaaacttttcctggaaaaaatcgcaagtggagaaggagaagaagaagaaaattgcgcggtttt[T/C]ttcttcttcttcgcgttcggaactataagcgataaaccagtttccgccatRgaaggttagagaaatggagaaagagaaagttcaggtttgtgtgaaaaat

fava_c18816_1156	fava_c18816	1156	G/A	aaaaaatcgcaagtggagaaggagaagaagaagaaaattgcgcggttttYttcttcttcttcgcgttcggaactataagcgataaaccagtttccgccat[G/A]gaaggttagagaaatggagaaagagaaagttcaggtttgtgtgaaaaatggagttaatttaaatttgagcgtaaaaataataaagtccattgatgagaga

fava_c18816_376	fava_c18816	376	T/C	tatacaatctcaatccaatacaattccacatgcgtgctgattgatgagtcaaaatttacaagggtcacagttttcatctgttttgagtcttacctaaata[T/C]tRttagtaccgtttccaaatagagtactgctgtctcttgccaaaacaaacaatccttctaaYctttctgaaactgccaagcaaacacctggaaattgKcg

fava_c18816_378	fava_c18816	378	G/A	tacaatctcaatccaatacaattccacatgcgtgctgattgatgagtcaaaatttacaagggtcacagttttcatctgttttgagtcttacctaaataYt[G/A]ttagtaccgtttccaaatagagtactgctgtctcttgccaaaacaaacaatccttctaaYctttctgaaactgccaagcaaacacctggaaattgKcgac

fava_c18816_438	fava_c18816	438	T/C	ggtcacagttttcatctgttttgagtcttacctaaataYtRttagtaccgtttccaaatagagtactgctgtctcttgccaaaacaaacaatccttctaa[T/C]ctttctgaaactgccaagcaaacacctggaaattgKcgacgggaacatctggttttgagtttttgtttttctYacaattgctagtggtgtggataacaaa

fava_c18816_474	fava_c18816	474	G/T	taYtRttagtaccgtttccaaatagagtactgctgtctcttgccaaaacaaacaatccttctaaYctttctgaaactgccaagcaaacacctggaaattg[G/T]cgacgggaacatctggttttgagtttttgtttttctYacaattgctagtggtgtggataacaaatctcccataaagccagtaatcttgttYtttgcaaca

fava_c18816_511	fava_c18816	511	T/C	tcttgccaaaacaaacaatccttctaaYctttctgaaactgccaagcaaacacctggaaattgKcgacgggaacatctggttttgagtttttgtttttct[T/C]acaattgctagtggtgtggataacaaatctcccataaagccagtaatcttgttYtttgcaacatgcacttctttagtgtgatccttgacctctgcattct

fava_c18816_565	fava_c18816	565	C/T	tggaaattgKcgacgggaacatctggttttgagtttttgtttttctYacaattgctagtggtgtggataacaaatctcccataaagccagtaatcttgtt[C/T]tttgcaacatgcacttctttagtgtgatccttgacctctgcattctcatcagaaccaatgttgtcgtcRtcatcgtcattgctgctattttctttctgtt

fava_c18816_634	fava_c18816	634	G/A	acaaatctcccataaagccagtaatcttgttYtttgcaacatgcacttctttagtgtgatccttgacctctgcattctcatcagaaccaatgttgtcgtc[G/A]tcatcgtcattgctgctattttctttctgtttgcagtagttcacttgtgacctcctgtgttgtttcaaatggaaaactcttttctttgaccgctttcttt

fava_c18816_742	fava_c18816	742	A/G	cattgctgctattttctttctgtttgcagtagttcacttgtgacctcctgtgttgtttcaaatggaaaactcttttctttgaccgctttctttgaacacc[A/G]aaattatttgatgcatggcggatgaaattgagctggtcaccatctttgacaccataatttctaagataatcctcctccttaactagcttatgttcatcat

fava_c18817_208	fava_c18817	208	T/A	gtcaaacaatcaaatcattttggttagcttccatctctagctatgttgcatgaataaaactcaagtacaaatcaaattaaaattcaaccaaacagaacat[T/A]aaaagggttcataaccaaaaccaagatactggactaggccattttgcagtcacacggaagcctctatcctcggctcgaacgaaaaagcagacataaactc

fava_c18817_387	fava_c18817	387	T/C	acgaaaaagcagacataaactccatgacttctccatttatgcaacattttggcaattcaacccatttgttggtcaccaaatcagacataacataagtgca[T/C]agctcaggagagttcaagcatataaaWatccgagaaccggcaccaacacagttgatatccggcttctttccgtaccattcgtgagacattgaggcaggca

fava_c18817_414	fava_c18817	414	A/T	cttctccatttatgcaacattttggcaattcaacccatttgttggtcaccaaatcagacataacataagtgcaYagctcaggagagttcaagcatataaa[A/T]atccgagaaccggcaccaacacagttgatatccggcttctttccgtaccattcgtgagacattgaggcaggcattgctgcaatctgttgccagcatcggt

fava_c18821_1004	fava_c18821	1004	T/A	atgagcctgtatctcattcttcaaaagatatcggatcatttatgatgtttgcaaatgataaagaatcggttaaaaagtccctagctgYKccaatYtccaa[T/A]YctSagcaagtcgataaatttcagaatccggtagatgtccatgtgaaccgcactgttgccggatgtgaaccagaaatggaagtttgttacgaagagaata

fava_c18821_1005	fava_c18821	1005	C/T	tgagcctgtatctcattcttcaaaagatatcggatcatttatgatgtttgcaaatgataaagaatcggttaaaaagtccctagctgYKccaatYtccaaW[C/T]ctSagcaagtcgataaatttcagaatccggtagatgtccatgtgaaccgcactgttgccggatgtgaaccagaaatggaagtttgttacgaagagaataS

fava_c18821_1008	fava_c18821	1008	C/G	gcctgtatctcattcttcaaaagatatcggatcatttatgatgtttgcaaatgataaagaatcggttaaaaagtccctagctgYKccaatYtccaaWYct[C/G]agcaagtcgataaatttcagaatccggtagatgtccatgtgaaccgcactgttgccggatgtgaaccagaaatggaagtttgttacgaagagaataStta

fava_c18821_1105	fava_c18821	1105	G/C	YctSagcaagtcgataaatttcagaatccggtagatgtccatgtgaaccgcactgttgccggatgtgaaccagaaatggaagtttgttacgaagagaata[G/C]ttaccatgttgtgaaggatatatgtgttgataaaggagtctttaataagcataaattcatgtttgaggaaaatgttgacagagccgSttataatttcttW

fava_c18821_1192	fava_c18821	1192	C/G	tacgaagagaataSttaccatgttgtgaaggatatatgtgttgataaaggagtctttaataagcataaattcatgtttgaggaaaatgttgacagagccg[C/G]ttataatttctttccattggagagtttcgatgataaccggaatccagaagacaacactggtatcaaggtgttaaatcaaccagaaactgatgattccgac

fava_c18821_1402	fava_c18821	1402	C/T	tctaatcacgatcagcacaaagacgacagtgagattgaagatcttattgataacttcaccaaagcgacggatttacgtgaagacacacaagatgctgtac[C/T]gactggtggcaaggatgaacagctttccgttgaacatgattcgcactcccagttgaaagacgcaaataatatggttgaggaagaagtattgacaagtYct

fava_c18821_1500	fava_c18821	1500	C/T	acYgactggtggcaaggatgaacagctttccgttgaacatgattcgcactcccagttgaaagacgcaaataatatggttgaggaagaagtattgacaagt[C/T]ctgctttaggatYaaccgtWgatgaaccgaacagtSatcatcattttgcgccttcagctcctgcggactgtgtcaaaaaggaacttcaccaattcggtgg

fava_c18821_1513	fava_c18821	1513	T/C	aaggatgaacagctttccgttgaacatgattcgcactcccagttgaaagacgcaaataatatggttgaggaagaagtattgacaagtYctgctttaggat[T/C]aaccgtWgatgaaccgaacagtSatcatcattttgcgccttcagctcctgcggactgtgtcaaaaaggaacttcaccaattcggtggctgtaactgcgat

fava_c18821_1520	fava_c18821	1520	T/A	aacagctttccgttgaacatgattcgcactcccagttgaaagacgcaaataatatggttgaggaagaagtattgacaagtYctgctttaggatYaaccgt[T/A]gatgaaccgaacagtSatcatcattttgcgccttcagctcctgcggactgtgtcaaaaaggaacttcaccaattcggtggctgtaactgcgatgaaactc

fava_c18821_1536	fava_c18821	1536	G/C	acatgattcgcactcccagttgaaagacgcaaataatatggttgaggaagaagtattgacaagtYctgctttaggatYaaccgtWgatgaaccgaacagt[G/C]atcatcattttgcgccttcagctcctgcggactgtgtcaaaaaggaacttcaccaattcggtggctgtaactgcgatgaaactcaacttactaccgttga

fava_c18821_2174	fava_c18821	2174	G/A	gtctgcctactgcatattcaatgctccgtcgtggtttcgagtcagtgatcttatatctctgctgcaagcaaaatgtattgattctagttttgcttgcatc[G/A]caagacttaagctacatactatctttggttatacatggttttattgattttttatttatttcaatggcaaacaattcttagtagataattgattatttta

fava_c18821_582	fava_c18821	582	G/A	aaccattcaattcaatttatgaaacacagttaccaacttgactagaaactcctgctttctcttcttctctagctttggactttggttcctgttgaatatt[G/A]tatgatMagattactagtccctttggttgaaagtaagaagaatagcgttcgttctggcggtttggaaactcacacatgtattcaaatattagaacctcat

fava_c18821_589	fava_c18821	589	C/A	caattcaatttatgaaacacagttaccaacttgactagaaactcctgctttctcttcttctctagctttggactttggttcctgttgaatattRtatgat[C/A]agattactagtccctttggttgaaagtaagaagaatagcgttcgttctggcggtttggaaactcacacatgtattcaaatattagaacctcatgaaacta

fava_c18821_991	fava_c18821	991	C/T	gtaaaggactcacatgagcctgtatctcattcttcaaaagatatcggatcatttatgatgtttgcaaatgataaagaatcggttaaaaagtccctagctg[C/T]KccaatYtccaaWYctSagcaagtcgataaatttcagaatccggtagatgtccatgtgaaccgcactgttgccggatgtgaaccagaaatggaagtttgt

fava_c18821_992	fava_c18821	992	G/T	taaaggactcacatgagcctgtatctcattcttcaaaagatatcggatcatttatgatgtttgcaaatgataaagaatcggttaaaaagtccctagctgY[G/T]ccaatYtccaaWYctSagcaagtcgataaatttcagaatccggtagatgtccatgtgaaccgcactgttgccggatgtgaaccagaaatggaagtttgtt

fava_c18821_998	fava_c18821	998	C/T	actcacatgagcctgtatctcattcttcaaaagatatcggatcatttatgatgtttgcaaatgataaagaatcggttaaaaagtccctagctgYKccaat[C/T]tccaaWYctSagcaagtcgataaatttcagaatccggtagatgtccatgtgaaccgcactgttgccggatgtgaaccagaaatggaagtttgttacgaag

fava_c18822_1046	fava_c18822	1046	T/C	atggcatctttgccgagtttgaRaagcttagcattggaatataacttaccaattgtctctataactgatttgataaggtatcggagaaagcgagagaaac[T/C]agttgaaagaacttatgtctctgatgttccaacaaaatggggtctatttcaagcatattgttatagttcaaagttagatggaactgagcatgtggctgtt

fava_c18822_1632	fava_c18822	1632	T/A	tggctgtgatagggcgtgttcctgtgctaacaccaatcactgaacaaaataagcggtacttagaaacaaaaaggaccaaaatgggtcatatttatggctc[T/A]gatatataaggatcattacaggaactcaatgattcaRttgtaaataatcgaaattaacatgtctYtggaactgcctttggaagccattcgagtccaaaag

fava_c18822_1669	fava_c18822	1669	A/G	cactgaacaaaataagcggtacttagaaacaaaaaggaccaaaatgggtcatatttatggctcWgatatataaggatcattacaggaactcaatgattca[A/G]ttgtaaataatcgaaattaacatgtctYtggaactgcctttggaagccattcgagtccaaaagacattttcatcccgaaaacaaagaagctacaatacta

fava_c18822_1697	fava_c18822	1697	T/C	acaaaaaggaccaaaatgggtcatatttatggctcWgatatataaggatcattacaggaactcaatgattcaRttgtaaataatcgaaattaacatgtct[T/C]tggaactgcctttggaagccattcgagtccaaaagacattttcatcccgaaaacaaagaagctacaatactaagcttctatgaattgctgactcaaacac

fava_c18822_248	fava_c18822	248	G/A	cctcgtcctttgatttccaacgtcatcacccacaccttgcatagttcatgcactactccacgtagtgcaagatttggtctatggaagaacaacctgagaa[G/A]cttggcattttatgctattggattctctaaagttggaaatgaagacatatttgatgatagtgatttgaagagaaatgaaaatggagcaatcttgggagct

fava_c18822_417	fava_c18822	417	T/C	agagaaatgaaaatggagcaatcttgggagcttttggtactgaaataacacaagaaaataatgatttctttgtgagtgaagccgaaggtgatcccgattg[T/C]ccttctaaaggatactcttccattgatcatgctcttaacgcattgcaccaaggaaagtttgtagtagttgttgatgatgaaaacaWtaatgttgaaggRa

fava_c18822_503	fava_c18822	503	A/T	ggtgatcccgattgYccttctaaaggatactcttccattgatcatgctcttaacgcattgcaccaaggaaagtttgtagtagttgttgatgatgaaaaca[A/T]taatgttgaaggRaatcttataaaggctgcatctcttattagtaccaaggatgtggcttttatgattaaacatggatcaggtattatttctgttggaatg

fava_c18822_516	fava_c18822	516	A/G	gYccttctaaaggatactcttccattgatcatgctcttaacgcattgcaccaaggaaagtttgtagtagttgttgatgatgaaaacaWtaatgttgaagg[A/G]aatcttataaaggctgcatctcttattagtaccaaggatgtggcttttatgattaaacatggatcaggtattatttctgttggaatgaaagaagaagatc

fava_c18822_636	fava_c18822	636	C/T	ctcttattagtaccaaggatgtggcttttatgattaaacatggatcaggtattatttctgttggaatgaaagaagaagatcttcaaagactgaatcttcc[C/T]cttatRtcacMagagactgaagaagaagattcatctgcccccacWtttactatcacagttgatgcaaaatatggaacttcctctggtgtatcggccgcgg

fava_c18822_642	fava_c18822	642	G/A	ttagtaccaaggatgtggcttttatgattaaacatggatcaggtattatttctgttggaatgaaagaagaagatcttcaaagactgaatcttccYcttat[G/A]tcacMagagactgaagaagaagattcatctgcccccacWtttactatcacagttgatgcaaaatatggaacttcctctggtgtatcggccgcggatagag

fava_c18822_647	fava_c18822	647	C/A	accaaggatgtggcttttatgattaaacatggatcaggtattatttctgttggaatgaaagaagaagatcttcaaagactgaatcttccYcttatRtcac[C/A]agagactgaagaagaagattcatctgcccccacWtttactatcacagttgatgcaaaatatggaacttcctctggtgtatcggccgcggatagagcaaaa

fava_c18822_681	fava_c18822	681	T/A	caggtattatttctgttggaatgaaagaagaagatcttcaaagactgaatcttccYcttatRtcacMagagactgaagaagaagattcatctgcccccac[T/A]tttactatcacagttgatgcaaaatatggaacttcctctggtgtatcggccgcggatagagcaaaaacaatacttgttctatcatctcRggagtctaaat

fava_c18822_770	fava_c18822	770	G/A	tctgcccccacWtttactatcacagttgatgcaaaatatggaacttcctctggtgtatcggccgcggatagagcaaaaacaatacttgttctatcatctc[G/A]ggagtctaaatctgaggatttgagaaaaccaggtcatgtttttcctctcaagtatagaaatggcggagttcttagaagagccggtcatactgaggcttct

fava_c18822_918	fava_c18822	918	T/C	tcaagtatagaaatggcggagttcttagaagagccggtcatactgaggcttctgtcgatttagtcacattggctggattgcctccgttttctgttctcac[T/C]gctcttgtcgatgttgacgatggaaatatggcatctttgccgagtttgaRaagcttagcattggaatataacttaccaattgtctctataactgatttga

fava_c18822_968	fava_c18822	968	G/A	tctgtcgatttagtcacattggctggattgcctccgttttctgttctcacYgctcttgtcgatgttgacgatggaaatatggcatctttgccgagtttga[G/A]aagcttagcattggaatataacttaccaattgtctctataactgatttgataaggtatcggagaaagcgagagaaacYagttgaaagaacttatgtctct

fava_c18827_1170	fava_c18827	1170	G/A	cttcgggctcctcgggttctcttcgactaaataaataaatcctatgctttttgaaagcacaacatagaagagtaggatctgtcaaaggctccttgctctc[G/A]ttagcatttgcagctgctgaaggaatctttctcacttttttgccacttcgttccccatgatataaagctattcttaagaatctatcattattctccacct

fava_c18827_1647	fava_c18827	1647	A/G	gtatcctacgatcaggtgaagtaatggaaaattgtttaccatctgggctgacctcaatagccgaaagagaagtcttgcattttactatttcgaagaggtt[A/G]gtatcacttctaaccttaaaaStcacctcatcctctgggaactggagtgttgtagggttccagtattcaatgaaaccctttgcatcagccgaaattacgg

fava_c18827_1669	fava_c18827	1669	C/G	aatggaaaattgtttaccatctgggctgacctcaatagccgaaagagaagtcttgcattttactatttcgaagaggttRgtatcacttctaaccttaaaa[C/G]tcacctcatcctctgggaactggagtgttgtagggttccagtattcaatgaaaccctttgcatcagccgaaattacggtatcatacacaggattgaactt

fava_c18827_2277	fava_c18827	2277	A/G	taaccacatcacgatgcatatagcttttctcatacatattagcggaaggtaaagcatcgagataagcttgctcgaactgaagaggtcgtttgagtcgagc[A/G]cgaggagctggaccgggtccaactatgggttcgtcttcgattgcattcgcgccattttcgtgctcctccattttcggattggttttgaatgaaaaagagt

fava_c18827_450	fava_c18827	450	G/C	aacaagacagcatcgttgagcttgaaacatagttcaagaatacggaaagagggaatctatgacagaaagagttgtgatgaatgaaggtgctgcactatca[G/C]cagtccggtatagacacggctgacttcatgaatcataaaaatcaagaccttcaagactttggtacagtgacatttaaaattttaacatcttgatatggct

fava_c18827_621	fava_c18827	621	T/G	catttaaaattttaacatcttgatatggcttggctcccttgtccgtcttcactttttctatcgcctggacaacatccattcctttcacaactctaccaaa[T/G]acagtgtgtttgttatccagccatggagtggccactgtggtgataaaaaactgagaaccgttggtatttggtcccgcatttgccattgacaatgtgaaag

fava_c18830_1525	fava_c18830	1525	A/T	ataaggatgacactgcagcatcccatttgtatctcgctctatcttcaaggttagctgcctcagtcgggattctacccaacccctccacaaaagcaaatca[A/T]cggcatctaatgcaactatgtctacttgtaaataatttttgtatgcctcaaaaaagacatacggcttaaaaagtgcactccattgtgatttgttaagctc

fava_c18830_217	fava_c18830	217	G/A	cttctaagccacttaaccaaaatgactatttgattacatttacattacaagatgtatggatgatccacatcacaagcaaacaactaagttcccattcctc[G/A]gcatgcaaatatgacaagttttatgagatgattcaagctccaacgatgaaatgagctcatgttttatacacaacagatgtggccggattttcaagcatcY

fava_c18830_317	fava_c18830	317	T/C	Rgcatgcaaatatgacaagttttatgagatgattcaagctccaacgatgaaatgagctcatgttttatacacaacagatgtggccggattttcaagcatc[T/C]atgtcatRctgttcatccatgtacatgcaacactcgccaaaagcttaatctgattagcttacattctaaccgtccagctaaagtctccccgcatgttcat

fava_c18830_325	fava_c18830	325	A/G	aatatgacaagttttatgagatgattcaagctccaacgatgaaatgagctcatgttttatacacaacagatgtggccggattttcaagcatcYatgtcat[A/G]ctgttcatccatgtacatgcaacactcgccaaaagcttaatctgattagcttacattctaaccgtccagctaaagtctccccgcatgttcatacaacctc

fava_c18830_517	fava_c18830	517	A/T	tacaacctcagtacagcaacagccctaatatagcataaagttttgcagcaatttccataattcacatccatgtgatgcatcccaaactaccctagctcca[A/T]ctttaatttatacactcctgagtctgacatcggtctcgcaattaaggttctggatgctggcatttggtgcataatctgaacattcagcccttgtgaaatt

fava_c18838_173	fava_c18838	173	A/T	aatgatatacccagcgtgagagagaactgagtaaaaatgaaatgaaaaatagatacgcgtttgattaacaacatttattgattgaaattaactgcaaaag[A/T]cgatttaaattcttcactgaaaaatactactgacagaagtatcatcatgaatacgccaaatagtttcacccaacaggtttgctacggtaagaatagttaa

fava_c18839_1004	fava_c18839	1004	A/T	gRaaaactaaccaatcgccagctaaggcgagaacatcctcgccggtgaatggttgccgRaaaatggagaattcaacaatggagaataaatggccatcaac[A/T]gcaaatgagtcactggaaaacccacttgtgtcattggaatgtttcatctttctctagtgaaattttcaatttacatctgatattttcggcgaaactgatg

fava_c18839_258	fava_c18839	258	C/A	gtagaaaacaaccttttccatgacacactaaccaaatttttacaaactatgatgtttgtgttttcaatagaaacaacaacatagtacgtagtttcaccaa[C/A]aacagtgtaaagccaaaatgggttgttgtgctagtagtaacagatcttcgttgtcaacaacaaagagcaatgattttcaggtatcaagatctagcatttc

fava_c18839_362	fava_c18839	362	G/T	agtgtaaagccaaaatgggttgttgtgctagtagtaacagatcttcgttgtcaacaacaaagagcaatgattttcaggtatcaagatctagcatttctca[G/T]gtgaaaggctcRgaaaacagagcaccccctcctgttccattggaagaagaaaccgtgaaggaagtgttgtcggaaacgtcaaaatggaagaagtatgaag

fava_c18839_374	fava_c18839	374	G/A	aaatgggttgttgtgctagtagtaacagatcttcgttgtcaacaacaaagagcaatgattttcaggtatcaagatctagcatttctcaKgtgaaaggctc[G/A]gaaaacagagcaccccctcctgttccattggaagaagaaaccgtgaaggaagtgttgtcggaaacgtcaaaatggaagaagtatgaaggtgaaaagccaa

fava_c18839_788	fava_c18839	788	G/A	ggacggcggggaagtcgccggcgagaaggtcagagcagtctccggttaaaagaaatgctgggtcggcaaggagagatcaaattggaaatgggggagtcaa[G/A]aacaatcttcaccggagagacgccggagaaaactccggccgacgatctagatcaccggccactcgcactgacaacggatcaacgagatccgttgcgggtc

fava_c18839_905	fava_c18839	905	G/A	gagacgccggagaaaactccggccgacgatctagatcaccggccactcgcactgacaacggatcaacgagatccgttgcgggtcgaagcttatctgcaag[G/A]aaaactaaccaatcgccagctaaggcgagaacatcctcgccggtgaatggttgccgRaaaatggagaattcaacaatggagaataaatggccatcaacWg

fava_c18839_962	fava_c18839	962	G/A	acggatcaacgagatccgttgcgggtcgaagcttatctgcaagRaaaactaaccaatcgccagctaaggcgagaacatcctcgccggtgaatggttgccg[G/A]aaaatggagaattcaacaatggagaataaatggccatcaacWgcaaatgagtcactggaaaacccacttgtgtcattggaatgtttcatctttctctagt

fava_c18842_198	fava_c18842	198	A/G	caccaaaataccctcacctaaagcccccaacattcaacaacaattgcaattgtagaaaagaaacaaaggtatgaagaacgacgacagcgacgaagatgca[A/G]cgacgaggaattgagagcgacaacgacgaatttgaggagcttgaaggcgacgacgtcggtcaccacagatctaggatttcagaaattaaaacccggatct

fava_c18842_299	fava_c18842	299	T/C	cgacgaggaattgagagcgacaacgacgaatttgaggagcttgaaggcgacgacgtcggtcaccacagatctaggatttcagaaattaaaacccggatct[T/C]aactcttctacttctcctttcgttcttcgtctgtcacaatttatcctcccttcttcttttagattatagagtttttatagtgttagagttagattaattR

fava_c18846_1035	fava_c18846	1035	G/A	ttcaaagcttctcttactcttcaccttcctcttctgaaattgaRccYtcaacaacaccagaagaacaagaattcgtgaccaaactcagaaaccctcaatc[G/A]aatatcgtagaagaagctctaatctctctcagaagaatcacaaaaacgaaagaRgaatctagggttcaactctgcaccaataggRttctctgYtctttgc

fava_c18846_1089	fava_c18846	1089	G/A	caccagaagaacaagaattcgtgaccaaactcagaaaccctcaatcRaatatcgtagaagaagctctaatctctctcagaagaatcacaaaaacgaaaga[G/A]gaatctagggttcaactctgcaccaataggRttctctgYtctttgcgttctttgattttgttcaaaaacgaggttgtaagagtcaacgctcttgcttcat

fava_c18846_1120	fava_c18846	1120	G/A	cagaaaccctcaatcRaatatcgtagaagaagctctaatctctctcagaagaatcacaaaaacgaaagaRgaatctagggttcaactctgcaccaatagg[G/A]ttctctgYtctttgcgttctttgattttgttcaaaaacgaggttgtaagagtcaacgctcttgcttcattagtcaacctttcgMtcgagaaagtcaacaa

fava_c18846_1128	fava_c18846	1128	C/T	ctcaatcRaatatcgtagaagaagctctaatctctctcagaagaatcacaaaaacgaaagaRgaatctagggttcaactctgcaccaataggRttctctg[C/T]tctttgcgttctttgattttgttcaaaaacgaggttgtaagagtcaacgctcttgcttcattagtcaacctttcgMtcgagaaagtcaacaaagtgaaga

fava_c18846_1204	fava_c18846	1204	A/C	actctgcaccaataggRttctctgYtctttgcgttctttgattttgttcaaaaacgaggttgtaagagtcaacgctcttgcttcattagtcaacctttcg[A/C]tcgagaaagtcaacaaagtgaagatcgtacggtcaggaattgttccaccgttgattgaggttttgaaatttggatccagtgaatcgcaggaacatgcttc

fava_c18846_1398	fava_c18846	1398	A/G	atgcttcgtgtgtgctctttagtttagcgcttgatgatgataataaaactgcgattggtgttttgggtgctcttttgcctttgcttcacgcgcttaaatc[A/G]gagagtgagaagacgcgtcatgagtcgagtttggctctatgtcatttgtctttagtgaagagtaatagggttaagatggttaaactcgggtttgtttcgg

fava_c18846_1506	fava_c18846	1506	A/G	agaagacgcgtcatgagtcgagtttggctctatgtcatttgtctttagtgaagagtaatagggttaagatggttaaactcgggtttgtttcggttctttt[A/G]gggatggttaagtcgggttatatgatggatcgagttttgttgattttgggtaatttggggtttgggttggatggtcgggctgcaatgttggatgcaggtg

fava_c18846_1765	fava_c18846	1765	G/A	tgagagatgtgttggggttttgtatacactgagtcatggcgggttgaggtttaaggcggtggcgaaaaagattagggttgtggaaatgttgcaaaagatg[G/A]agaaaatgaagagtgagaaaactaatgataaagtgaggaggatattggagattatgagcgagaaggaggtggaggaggaagaggtggattgggaggagtt

fava_c18846_478	fava_c18846	478	C/A	aaacaagtggaaacattttcacatggaaatttacaaaaacacaaacaagaaatggagaggttccaaaaaggaatcaaaatggaagaaaatgattttcttc[C/A]acacttcaaaaactacccaaaatccacctcaagaatttctctgccccatttctggatctctcatgtctgaccctgtaattgtctcctccggccattcctt

fava_c18846_585	fava_c18846	585	A/G	caaaaactacccaaaatccacctcaagaatttctctgccccatttctggatctctcatgtctgaccctgtaattgtctcctccggccattccttcgaccg[A/G]acctcaatccaagcctgtacaaatctcaactttacccctcaactccaagatggcaccacccccaatttcacaaccctaatcccaaatcttaacctcaaat

fava_c18846_744	fava_c18846	744	T/C	cccccaatttcacaaccctaatcccaaatcttaacctcaaatcctcaattctcaaatggacccaaacccaatcccaaaccaaaatccaaaccaaccaaaa[T/C]ctcaYcacaactgaaaacctcgtacgtacattaatgtcctcaaaaaaacatcaaaaaccccaaaacgacatcgtacaagaaaaagaaacactacaaatct

fava_c18846_749	fava_c18846	749	T/C	aatttcacaaccctaatcccaaatcttaacctcaaatcctcaattctcaaatggacccaaacccaatcccaaaccaaaatccaaaccaaccaaaaYctca[T/C]cacaactgaaaacctcgtacgtacattaatgtcctcaaaaaaacatcaaaaaccccaaaacgacatcgtacaagaaaaagaaacactacaaatctcaatc

fava_c18846_978	fava_c18846	978	G/A	gctcagaggaatccatagcaactgccacgtcatcatcaacaacaacaccaccacaatttcaaagcttctcttactcttcaccttcctcttctgaaattga[G/A]ccYtcaacaacaccagaagaacaagaattcgtgaccaaactcagaaaccctcaatcRaatatcgtagaagaagctctaatctctctcagaagaatcacaa

fava_c18846_981	fava_c18846	981	T/C	cagaggaatccatagcaactgccacgtcatcatcaacaacaacaccaccacaatttcaaagcttctcttactcttcaccttcctcttctgaaattgaRcc[T/C]tcaacaacaccagaagaacaagaattcgtgaccaaactcagaaaccctcaatcRaatatcgtagaagaagctctaatctctctcagaagaatcacaaaaa

fava_c18847_788	fava_c18847	788	T/C	tagaccaatcctgcatgaagttcaaatgtgaaaaatgtggatttcctctgaaaatttgagctgaaagaaacatatatacatctgcaaatgttggaagaaa[T/C]gagcttcattgttggctgatgaccaagttctgcatccaaatcatcttcgagcagcagtaaagtatttacttttagaaatgctggaaacaggccgaacaaa

fava_c18850_1014	fava_c18850	1014	C/G	attgtaaatgcccacaccggaaggattttcacgaatgatttgaattcttcgacttgtgtcactgtgcatacactccatcggtctttaattgtatcttttt[C/G]tgttacaattgcggcYttgtcaaaaaacttgtattgtagagtccttggaagcttgcgagcgccaacaatatcagaatgagtggtMtcgacctcatagaga

fava_c18850_1030	fava_c18850	1030	T/C	ccggaaggattttcacgaatgatttgaattcttcgacttgtgtcactgtgcatacactccatcggtctttaattgtatctttttStgttacaattgcggc[T/C]ttgtcaaaaaacttgtattgtagagtccttggaagcttgcgagcgccaacaatatcagaatgagtggtMtcgacctcatagagatgagtttcatcttcta

fava_c18850_1099	fava_c18850	1099	C/A	taattgtatctttttStgttacaattgcggcYttgtcaaaaaacttgtattgtagagtccttggaagcttgcgagcgccaacaatatcagaatgagtggt[C/A]tcgacctcatagagatgagtttcatcttctactactactcctctttgatgattctttacagaagctacaataacctgaagaaatctagtaaaagggcttc

fava_c18851_897	fava_c18851	897	T/C	tctgtgttgaattttaaaaaaaaacggttcgtcRattttggtgtgaattagttcgttatgcccctgttatgttgtaaatttcagtgacaagttctgaaaa[T/C]tggttcggtaggttcaattaaatccttatgctacgaaatttatgataaaattcggtttggcaatgcctacattgagtgtgtcaggattggttgaattact

fava_c18852_657	fava_c18852	657	G/T	ttctgccactgcctcttaattaacgattaagaaaacacctagagtggaggccacttttgctcttgcttttgtgcttttgaggttttggctttaccaaatt[G/T]gttgctttactaaatattatgtactatggggtagaaaaagaaaatcccattttcaattgatcatcaaattatcctctccactaatccactgacacgtgtt

fava_c18853_1414	fava_c18853	1414	A/T	aatcgctcaaggacttctggagtgtcactgataaaacaagagaagagaagaatcatttgatcatcttggattagttatatcctatttttattttgagatt[A/T]gctatttaatgcttgttttttgagatttttccttttgacattagctaattagtgcttgtttttgagattagctacttaatgcttgttttagagatttttc

fava_c18853_263	fava_c18853	263	G/A	ttcataggttttacacaaacacgcctcctttgttccctctgctctcgattggtttggattttgtcaggttagattctcaattcttttcaatcataggttc[G/A]attgttgttgttaWagttaacatgaaggcaagatatccaaaggaagaacaSgtacctagagaatttataaacctaaccaattcgagtgatttcacgaagg

fava_c18853_277	fava_c18853	277	A/T	acaaacacgcctcctttgttccctctgctctcgattggtttggattttgtcaggttagattctcaattcttttcaatcataggttcRattgttgttgtta[A/T]agttaacatgaaggcaagatatccaaaggaagaacaSgtacctagagaatttataaacctaaccaattcgagtgatttcacgaaggtatataacaKgctg

fava_c18853_314	fava_c18853	314	C/G	gtttggattttgtcaggttagattctcaattcttttcaatcataggttcRattgttgttgttaWagttaacatgaaggcaagatatccaaaggaagaaca[C/G]gtacctagagaatttataaacctaaccaattcgagtgatttcacgaaggtatataacaKgctgatgcttattaagatgaaaagcaatgagaagacatatt

fava_c18853_373	fava_c18853	373	T/G	gttaWagttaacatgaaggcaagatatccaaaggaagaacaSgtacctagagaatttataaacctaaccaattcgagtgatttcacgaaggtatataaca[T/G]gctgatgcttattaagatgaaaagcaatgagaagacatattgggaagcaatcggttcaacttataacaagcagttatgtatttatgtttgctgtatgatc

fava_c18856_125	fava_c18856	125	A/C	ttcatctccaacaacattcttaaacaacaattttcacctcaaaaattcacattcttcaacacaaaacaaaaatggtgtactccaattccaactacacccc[A/C]acttactactcMtcactccatgattcaatcacctcattctgcaaaaccattcttcctttcagcttcaagaaacgatctttacaatctgcagaacataagc

fava_c18856_1348	fava_c18856	1348	G/A	cgatatggtgatgttgtattggatgtggtgaagaaggaattcaaaaaccatgttgaagaaatggaaaacatgcggagcatagcaaaagaaaaacacagcg[G/A]gaactcgaggcggtggacaacatttgatgatgatgagaatattcatccgaatctttttgttcatcgtgacaattcagttagcagtagtaacatcaatcca

fava_c18856_137	fava_c18856	137	A/C	aacattcttaaacaacaattttcacctcaaaaattcacattcttcaacacaaaacaaaaatggtgtactccaattccaactacaccccMacttactactc[A/C]tcactccatgattcaatcacctcattctgcaaaaccattcttcctttcagcttcaagaaacgatctttacaatctgcagaacataagctctcaaagctac

fava_c18856_1667	fava_c18856	1667	G/A	ctgtagagtatgttcaacctctttgaactacgctttaagacctcatctggaggagtctaatactgttatgtttagactttagaagaccataaaaagactt[G/A]attactgtctgaattatcaataatactatatctcttttatgtgtatcaaacatctatggtgttacttcgtcatttctatttatgtttcatgtgtgcttta

fava_c18856_476	fava_c18856	476	G/T	cttctgaacaagagaatcctgttatattgagagacaagcttctgtttttgcaggaactgctttatgctaagtgtatttctgaagatgattaccattcttc[G/T]aagaggccgttgttacaaagattagcagtgcaaggagttcagattcaagctaaggatgtgattgcggggaagtcgaaagatgtgagagagaattMtgaag

fava_c18856_571	fava_c18856	571	A/C	tcttcKaagaggccgttgttacaaagattagcagtgcaaggagttcagattcaagctaaggatgtgattgcggggaagtcgaaagatgtgagagagaatt[A/C]tgaagaggagtggtctgtgattgatttgaaggatgataagagtttgatgaacaaggagaatttgaattctaggggaaaatcgaatcacgggttgggtttg

fava_c18856_923	fava_c18856	923	G/A	ggaaaccttttaggacattgtttcatagggagcagagagagggatcggtgaaaaagcaatgggggtttgatggatttaagaaagggaagaaaattgaatt[G/A]gatgatgatgaaacggctcctttgcctctgaatcagagatctgatagtgaggccttctcagcttcttctcagtcttttgcaagggatggaccagacacca

fava_c18857_899	fava_c18857	899	C/T	ctggacaagtaacaaggaagtgggatcaagaagccgtgttaagaagtgcagtggatgttttgaggaaaaacaaagaaattgtgataactgctgttctagc[C/T]ttctcctttgggatgtatgtatctagtaaRttccaatcttcagaagctctataggaagtcctttgacctgcaaccaggactgtatatccaccactaagtt

fava_c18857_929	fava_c18857	929	A/G	aagccgtgttaagaagtgcagtggatgttttgaggaaaaacaaagaaattgtgataactgctgttctagcYttctcctttgggatgtatgtatctagtaa[A/G]ttccaatcttcagaagctctataggaagtcctttgacctgcaaccaggactgtatatccaccactaagttcctttcatatgtatctatagaactaagtat

fava_c18861_194	fava_c18861	194	A/G	tctttctgtgtagataattccatctactttgaaccaaactctactctaactgaatcaaagagaaaaacaaaacctgtttgtatgtcatagttgaaggagg[A/G]tcatgatattgatctatcaaagctgttttatcaccaaaattctcaacagaagttctccatatatcaggaactgctttccaatcctctaaagccacagcac

fava_c18861_375	fava_c18861	375	C/T	atcctctaaagccacagcaccattaccagaactttctaataatggagaaaatcttcttgtctcagttttctctgtctgcatatacataaaacatttatgt[C/T]ggaacttcgagaaaaacaagtaacatgtatatgaatagtacttcagttcagacatgtttgttatagaagtagttaccttggattgagtttggcaaaagac

fava_c18862_1880	fava_c18862	1880	T/C	gaggctccatgcaatttgcttgcacatgatgatcagtctcgtgccagagttgaagaactgaaagacagtcttgggaaagaagcaaagtcagttttgagga[T/C]aWgtggctgggcagataaggttgatgagtatatgagaaaatggttgcaagaaaaMgcagagaatttaacgagtgatgttaaagggaagttaatacaagat

fava_c18862_1882	fava_c18862	1882	T/A	ggctccatgcaatttgcttgcacatgatgatcagtctcgtgccagagttgaagaactgaaagacagtcttgggaaagaagcaaagtcagttttgaggaYa[T/A]gtggctgggcagataaggttgatgagtatatgagaaaatggttgcaagaaaaMgcagagaatttaacgagtgatgttaaagggaagttaatacaagatgc

fava_c18862_1935	fava_c18862	1935	C/A	aactgaaagacagtcttgggaaagaagcaaagtcagttttgaggaYaWgtggctgggcagataaggttgatgagtatatgagaaaatggttgcaagaaaa[C/A]gcagagaatttaacgagtgatgttaaagggaagttaatacaagatgcccgtagtgctttcacatcacaggttcccgggtcacttagMaaagacctaattg

fava_c18862_2022	fava_c18862	2022	A/C	ggttgcaagaaaaMgcagagaatttaacgagtgatgttaaagggaagttaatacaagatgcccgtagtgctttcacatcacaggttcccgggtcacttag[A/C]aaagacctaattgaacgtctcttatcatttaaccaagaaaaagtacattttaaaggaccttgatcaaaacacgcatgttgatctgtcttgctagtgaaac

fava_c18866_1016	fava_c18866	1016	G/A	cgtttgaaacggcgaggtcagtgacggcgtcggcgtgttctatccagagacgcttatcgtggcgacggacagcgacgtagtttttcgggaggaggaagcg[G/A]cggagacggtcgttgacggtggggagagttgttagcttccggtggtgattgtttttccagacgcggatcttgcagtcttggtgagtggtgaaaaccatgt

fava_c18866_1442	fava_c18866	1442	A/T	agagattctgaagagaaggaactgaaggaagagtttgtattgagtagttgctatgaagagtactggatgtgctgtcatcagaggttattaatgaagaaga[A/T]gaagtacttgagtcaaatgcatgttgttgtttttgtgatgggaggagttcgacggttgagttgtgggtggcggtggcgcagttggcggaggagcaactgc

fava_c18866_797	fava_c18866	797	G/A	gcttctctaaagtcgcaactagaacgtgtcttttttctccaactggtttcgcccaaacgcgaattcgtctatcggatgatccagtgtagacggttccatc[G/A]ttagaaactgcaaccgcgttgaccgcgtcttcgtgagctttgacagattcaacgcaacgaagatcggagattctccatatcttaagcgttttatcccacg

fava_c18870_1410	fava_c18870	1410	G/A	ttccaaagagtgcattcaagccaaaagtcacaacggctcctgtgttgttgaaaaaatgattcagctcattccatcttttcatgcgtaaacatccttttga[G/A]tatccaaacaatccaccattcatcttttgaaatggctgacaagaaaacttcaaacttcctacatcatacaccacttgatcttgcaaagaacctccgattc

fava_c18870_1515	fava_c18870	1515	C/T	caaacaatccaccattcatcttttgaaatggctgacaagaaaacttcaaacttcctacatcatacaccacttgatcttgcaaagaacctccgattcttat[C/T]ctcaaaggcttcagggcttggattgcctttgcaagaataggattagacaagtccaaattcaaaagagatgaagatccccacgggcaataattgtagtcRc

fava_c18870_1614	fava_c18870	1614	A/G	tYctcaaaggcttcagggcttggattgcctttgcaagaataggattagacaagtccaaattcaaaagagatgaagatccccacgggcaataattgtagtc[A/G]catttatcacgaggccaccaatcaatagtagcacaaatgaaattatcatcattttcagctattgcttgaactccatctactataatcaaaccgttttcaa

fava_c18872_253	fava_c18872	253	T/A	ccaagccagtttctgctctccggcaggtgtatgatgccggtcaccgattcttcggcgagaattacgttcaggaaatcatcgataaagcacctcagcttcc[T/A]gaggatattgaatggcatttcatcggtaatttgcagagcaataaagtcaagcctcttcttgctggtgttcccaaccttgcttatgtagagactgtagatg

fava_c18872_496	fava_c18872	496	T/A	ttggcaggaaaccattgaaagtttttgttgaagtgaatacaagcggagaagcatctaaatttggtgttgaaccagcactgtgtctagaccttgtaaaaca[T/A]attgttacaaattgtccaaacctggaattttgtggcctaatgacaattggcatgctggattattcatccaccccagaaaattttaagacattRtccaaat

fava_c18872_589	fava_c18872	589	G/A	taaaacaWattgttacaaattgtccaaacctggaattttgtggcctaatgacaattggcatgctggattattcatccaccccagaaaattttaagacatt[G/A]tccaaatgtagaactgaagtatgtgaggcagttggaataYcagaagcacaatgtgagctatcaatgggcatgactgcagactttgagcaagctattgaga

fava_c18872_629	fava_c18872	629	T/C	tggcctaatgacaattggcatgctggattattcatccaccccagaaaattttaagacattRtccaaatgtagaactgaagtatgtgaggcagttggaata[T/C]cagaagcacaatgtgagctatcaatgggcatgactgcagactttgagcaagctattgagatgggaagtacaaatgttagggttggaacWgccatatttgg

fava_c18872_718	fava_c18872	718	A/T	cagttggaataYcagaagcacaatgtgagctatcaatgggcatgactgcagactttgagcaagctattgagatgggaagtacaaatgttagggttggaac[A/T]gccatatttgggcccagagagtatccacataaagaagagaagtagatgtcctcattttgctgtcttatttggagtaRtattgttgtgcttRtagattcac

fava_c18872_795	fava_c18872	795	G/A	agtacaaatgttagggttggaacWgccatatttgggcccagagagtatccacataaagaagagaagtagatgtcctcattttgctgtcttatttggagta[G/A]tattgttgtgcttRtagattcacatgtgaagttgtagagcagcagaaMaaattattttagctggaaataaatatgctcatgagcttgatggtagtagttt

fava_c18872_809	fava_c18872	809	A/G	ggttggaacWgccatatttgggcccagagagtatccacataaagaagagaagtagatgtcctcattttgctgtcttatttggagtaRtattgttgtgctt[A/G]tagattcacatgtgaagttgtagagcagcagaaMaaattattttagctggaaataaatatgctcatgagcttgatggtagtagtttacaagctccgtgag

fava_c18872_843	fava_c18872	843	C/A	ccacataaagaagagaagtagatgtcctcattttgctgtcttatttggagtaRtattgttgtgcttRtagattcacatgtgaagttgtagagcagcagaa[C/A]aaattattttagctggaaataaatatgctcatgagcttgatggtagtagtttacaagctccgtgagctgtgtcttcMgaaaataaccttcaaggccaaat

fava_c18872_920	fava_c18872	920	A/C	tgtgaagttgtagagcagcagaaMaaattattttagctggaaataaatatgctcatgagcttgatggtagtagtttacaagctccgtgagctgtgtcttc[A/C]gaaaataaccttcaaggccaaatactatatatattgttgcttgtaaaatgtaaaattYaaccttttttttaactgaactagtactaaggtagtctatttt

fava_c18872_978	fava_c18872	978	C/T	gcttgatggtagtagtttacaagctccgtgagctgtgtcttcMgaaaataaccttcaaggccaaatactatatatattgttgcttgtaaaatgtaaaatt[C/T]aaccttttttttaactgaactagtactaaggtagtctatttttgacaagaggactgtgataatcttgaaaagtcaaaaccttttatcatcattatagcaa

fava_c18875_1354	fava_c18875	1354	T/C	atcgatatagcgcagtaagaggagcacataaaaatttgaagcgggcagagaaagcacggattcttgtcagcaagattcaatctatggtagagaacttgac[T/C]gcaaaagtgaaagcatgggagacggaaaaaggaattcctttcttatatgaaaaggctccgttgctgcaaagcttggacgaatacaatgtacagcagcaat

fava_c18875_1920	fava_c18875	1920	A/G	cttttgtagcgttatacgaaagcattgctctctacaaatagagattgattttaacatggggggttgattttgaattcttacatgacagatgacaagactt[A/G]ttgtaaggcttgacacagaaatataagctgtgggctaatagaaataaatttcagaaaactcatccccactcatctattgtagtggttggaataggtatgt

fava_c18879_169	fava_c18879	169	C/T	accttcacacaccaaaataatatttgggatttagatctcactctatcccaattcttcttcttctgatgaatgaatggatacaataaaacctatatttcat[C/T]gttcaccattttgaaacattttccactgtttattttctgttccaaatcccacaccccactctcactctttctacaaagcaaccatcccagtccaagctct

fava_c18879_2287	fava_c18879	2287	C/T	cacgtgaagtttaaaacaccagcaacagagtgataattttgagcacatcattgcaaaaaatttgctttcatgtgttctaggtgttccctatatgtcgttc[C/T]tcttctctacgcYacaggtgtgagttacaatgaaagctcatcctgaatcatctttttgaacttatttgattttgaacacactatgtcaccctgttatcgt

fava_c18879_2300	fava_c18879	2300	T/C	aaacaccagcaacagagtgataattttgagcacatcattgcaaaaaatttgctttcatgtgttctaggtgttccctatatgtcgttcYtcttctctacgc[T/C]acaggtgtgagttacaatgaaagctcatcctgaatcatctttttgaacttatttgattttgaacacactatgtcaccctgttatcgtgattaggaaaatg

fava_c18879_294	fava_c18879	294	A/G	actgtttattttctgttccaaatcccacaccccactctcactctttctacaaagcaaccatcccagtccaagctcttgctttgtgattctcaactgcatc[A/G]cctaggtactttgtggaactYgagtcattaaatcgagggcttcctaggtgaactttgaatcatggcgagaatggctagaggaagagcagatgggaattca

fava_c18879_315	fava_c18879	315	C/T	atcccacaccccactctcactctttctacaaagcaaccatcccagtccaagctcttgctttgtgattctcaactgcatcRcctaggtactttgtggaact[C/T]gagtcattaaatcgagggcttcctaggtgaactttgaatcatggcgagaatggctagaggaagagcagatgggaattcaagaacacgattgatcatatct

fava_c18885_480	fava_c18885	480	A/T	attatggctggtgattcaaacccttcgtttttggctactccggtttttcctaaatcttcttctgtaatggatctgaattccgatgaagaggttccaaatc[A/T]tgaagcggttgagaaatcggagaaggaaacggaagttgcagatcatcggaatcaccaacagtgacttKatttgatttgcttaattaatttcgtttctcat

fava_c18885_548	fava_c18885	548	T/G	ggatctgaattccgatgaagaggttccaaatcWtgaagcggttgagaaatcggagaaggaaacggaagttgcagatcatcggaatcaccaacagtgactt[T/G]atttgatttgcttaattaatttcgtttctcatctggtttaatttcttaatttttgctttaaactctgttggattgtgttgcatgattcgggatgtgtaaa

fava_c18888_1921	fava_c18888	1921	T/A	cctcttaactcctcaggaacttcagttgagataccttgattttgtgccagtttgttatgtaacacccaaggggcacttcggtatgtagattctcggataa[T/A]agacttcagaatatttctactaaaaagaggcttcttttgcaccaaatcYtctgcagatatttcagttttttcactaatattcttgtttttgtcaagccag

fava_c18888_1970	fava_c18888	1970	T/C	gtttgttatgtaacacccaaggggcacttcggtatgtagattctcggataaWagacttcagaatatttctactaaaaagaggcttcttttgcaccaaatc[T/C]tctgcagatatttcagttttttcactaatattcttgtttttgtcaagccaggctacctcatatcgatatttgttgactccttcctggatgacttttagta

fava_c18890_603	fava_c18890	603	G/A	ccggagagacatgttgttctttacaatgtgttgtttaagctttttagagaaattaatgattttgaaaaagcagagaaggtgtttgaggaaatgctgcaga[G/A]aggagtcaagcctgatgttgttacgttttcgactttgattaggtgtgcttctgtttgttctctgcctcacaaatccgtggagttgtttgagaggatgcct

fava_c18891_2347	fava_c18891	2347	C/T	ttgtatcaacaaaatcagagtctacYgaagatcttaaagtaagggtgggagagttgaaaaacatgtatggttctggaattaaagctttggataatctagc[C/T]gaggaacttaaaggaaataaccaattaacttatgaagacttgagatctgaagtagctaagcattcatctgccttggaggatctttttaaaggaatcgcct

fava_c18891_2698	fava_c18891	2698	A/G	agacaatagacaggcatgcatcaagtttgacccaaattgttgaagaaacacaatttgttaatgatcagaaattgagcgagcttgaaaggaagtttgagga[A/G]tgcactgcttatgaagaaaagcaactgctggagaaagtagcagaaatgctagcaagttcaaatgctagaaagaaaaaactggttcaaatggccgttaatg

fava_c18891_2827	fava_c18891	2827	C/A	tggagaaagtagcagaaatgctagcaagttcaaatgctagaaagaaaaaactggttcaaatggccgttaatgatcttcgagaaagtgcaaactgtagaac[C/A]agtaaattgcagcaagaagcattaaccatgcaggattccacttcttctgtcaaggcagaatggatgRttcacatggaaacaacRgaatccaactatcacg

fava_c18891_2894	fava_c18891	2894	A/G	taatgatcttcgagaaagtgcaaactgtagaacMagtaaattgcagcaagaagcattaaccatgcaggattccacttcttctgtcaaggcagaatggatg[A/G]ttcacatggaaacaacRgaatccaactatcacgaggatacatctgctgtggaatctggaaagcaaaaccttgcagaggttcttcaaatctgtctcaacaa

fava_c18891_2911	fava_c18891	2911	A/G	gtgcaaactgtagaacMagtaaattgcagcaagaagcattaaccatgcaggattccacttcttctgtcaaggcagaatggatgRttcacatggaaacaac[A/G]gaatccaactatcacgaggatacatctgctgtggaatctggaaagcaaaaccttgcagaggttcttcaaatctgtctcaacaaggcagaagtaggttcac

fava_c18891_3581	fava_c18891	3581	A/G	acaagcaaatggagatgttaaacatattggttcttatgaagctactactcccactcaatcagtaagagattccagagttcctcttacagctattaattaa[A/G]acacgaaattctgtaaatagcacattgttcagatgtaattccataaaattatataatttatttattgttattatttttgggaaatagaggaaaaatatta

fava_c18895_679	fava_c18895	679	T/A	gttagcgggtgtttcgttttgtgagaatttgaagactttgaggtttgtgtcgtgtaaggggattgatgggaatccgggattggaggaacatttagggttt[T/A]gtgttgcacttgaaagtttgcattttcagaaatgtcagttgagggataagaatgcaatgggagctgttttttcggtttgtagggcggccaaggaggtYRt

fava_c18895_777	fava_c18895	777	T/C	ttWgtgttgcacttgaaagtttgcattttcagaaatgtcagttgagggataagaatgcaatgggagctgttttttcggtttgtagggcggccaaggaggt[T/C]Rttcttcaggattgttgggggttggatgacggtgttttccgtttggccgttgtttgcaggcgagtaaaactgtttgacatagaaggatgctcattgctta

fava_c18895_778	fava_c18895	778	G/A	tWgtgttgcacttgaaagtttgcattttcagaaatgtcagttgagggataagaatgcaatgggagctgttttttcggtttgtagggcggccaaggaggtY[G/A]ttcttcaggattgttgggggttggatgacggtgttttccgtttggccgttgtttgcaggcgagtaaaactgtttgacatagaaggatgctcattgcttac

fava_c18895_921	fava_c18895	921	T/C	tggccgttgtttgcaggcgagtaaaactgtttgacatagaaggatgctcattgcttacaaccgaaggtttagagtccgtcattgaatcatggaatgatct[T/C]gagtgccttagagttgtctcgtgtaaaaacataaaagacagtgatatctctcctgcactcgcaacgttatttacgaStctcaaagagttgaaatggaggc

fava_c18895_998	fava_c18895	998	G/C	gtcattgaatcatggaatgatctYgagtgccttagagttgtctcgtgtaaaaacataaaagacagtgatatctctcctgcactcgcaacgttatttacga[G/C]tctcaaagagttgaaatggaggccggatacaaaacatcttctgccatcaaaggaggtaaacatggggaaaaaaggcggtaaatttttcaaatgaacgcga

fava_c18900_1122	fava_c18900	1122	C/T	tagccggattcccttccttctcccacccttccaacaatatcgcaaaactatccccatccaaatcaaccttccccttaaccttctccaaaaactccacacc[C/T]gctgaagtctgattctcctcacgacaaattgcactaagcaatgaattaaccgccacaacattcttctcaacatcatacctatccatcacatcaaaactca

fava_c18900_1382	fava_c18900	1382	C/T	ggcaaagacagcacaccttcctgcttcatagtcctaacagcatcccacatgggctcgaaaagctggttccggcccaacaaatcaaccatcaagttccacg[C/T]gtgggcYgagtgttStctcaaacgcccagcccaccggaaaaacttgatggccgaagaagggtaattgtaagaaagcttaagaacctcgtgaacacattct

fava_c18900_1389	fava_c18900	1389	C/T	acagcacaccttcctgcttcatagtcctaacagcatcccacatgggctcgaaaagctggttccggcccaacaaatcaaccatcaagttccacgYgtgggc[C/T]gagtgttStctcaaacgcccagcccaccggaaaaacttgatggccgaagaagggtaattgtaagaaagcttaagaacctcgtgaacacattcttcagaag

fava_c18900_1397	fava_c18900	1397	C/G	ccttcctgcttcatagtcctaacagcatcccacatgggctcgaaaagctggttccggcccaacaaatcaaccatcaagttccacgYgtgggcYgagtgtt[C/G]tctcaaacgcccagcccaccggaaaaacttgatggccgaagaagggtaattgtaagaaagcttaagaacctcgtgaacacattcttcagaaggatgaatc

fava_c18900_1622	fava_c18900	1622	T/G	atctcctgaggggatgttcgggtgagtagattgcaaagggttcgagctgttgatgatacatttggggtatcaagatgggatggaaattcgttgttttgat[T/G]attaggtttagcaattgggcttgattgtctgttagggtttttcgatggatatttggaaacttttgttctagggttttttgacactggttccatcatgaga

fava_c18900_1778	fava_c18900	1778	A/T	gaaacttttgttctagggttttttgacactggttccatcatgagattgatgaattactcaaaattaatgggtttggaaactgaatcaacataaaaaaaaa[A/T]gctttttgaattgaaattgatKgtaaaatgtgaaatcgagtagagaaaaattgaagcacctgatgagagtgcgattttggttgatcaagttcagttgagt

fava_c18900_1800	fava_c18900	1800	T/G	ttgacactggttccatcatgagattgatgaattactcaaaattaatgggtttggaaactgaatcaacataaaaaaaaaWgctttttgaattgaaattgat[T/G]gtaaaatgtgaaatcgagtagagaaaaattgaagcacctgatgagagtgcgattttggttgatcaagttcagttgagtgatgaagaaattgaaacatgtg

fava_c18900_504	fava_c18900	504	A/G	tacaaaaaccaatgagcaacacatttgcgctcacctcaagtaccccgacatgagtttcaaccatgtaactccaaatctcaagtgctgcatcagggtcatc[A/G]caatcaaataacatttcaatggccgcagcacaattagaacctgtgggcagccactcattcttaatcatctcggcaaagaacctctccgtttcacgaacct

fava_c18900_900	fava_c18900	900	C/T	cagcatgagcagcatcatttcgcttaacaagcacatcaagagcattggtaaagaatttcaaacctggaaagcagtcattgtccttcaaaactttaagaaa[C/T]gtcacaacctcgtcaaactgcaaagctcgaaggagagtcatcaaaaatgcatcgtaagccgctacattatcctgactccaaccaacccgaatgaccatct

fava_c18902_379	fava_c18902	379	A/G	ttgggcaaaacgtcaagatgctgagggaaatggcaaaacaaaataattccatttctaagctgatgatcctgataaaagttaccaaaaaattacagaacca[A/G]actacaaactaatcattcttgaggcaacccctcttcaatttcactccactgctcactgttttcagcaaacttctccttgatttccttaataatctcttta

fava_c18905_129	fava_c18905	129	G/A	tttttcaattcaaccatcagcaatgtcctcggagttttcgacttcgtcatcagtttgcagttcggatggatcatctgtgtcttccaaggccaaggaagag[G/A]caggagctatggttctacgaggttgtcttcggtgtctcatgtatgttttgttatcagaggctgatcctaatccaaaatgtcccaagtgtaagagcactgt

fava_c18905_362	fava_c18905	362	G/A	ccagaaaagcaagcagctagcaatgtgcacatcaggaatatgaaatgtaacatgtatttgtgctattctatgtaagaaataattcaagttatatatagat[G/A]gttacRtttttctatgtattttaatattgcggtaaaacttttagaaaaacaaataagagttgtggagaaggaaattagtaagcgaaggaaaagttagagt

fava_c18905_368	fava_c18905	368	A/G	aagcaagcagctagcaatgtgcacatcaggaatatgaaatgtaacatgtatttgtgctattctatgtaagaaataattcaagttatatatagatRgttac[A/G]tttttctatgtattttaatattgcggtaaaacttttagaaaaacaaataagagttgtggagaaggaaattagtaagcgaaggaaaagttagagttacaag

fava_c18911_361	fava_c18911	361	C/T	gaagcttttgggtctccaagaaagctaaggaagagatttccaacattaccaacgatatatcttctttctcaaatacggttgaagacaaagctaaactttt[C/T]atcaacaagctcaaaggMaaaactcagaaagccttaccagatctcttacgagagcacaaccttcctccgggtctgtttccgcgtaacataacatgctatg

fava_c18911_379	fava_c18911	379	A/C	agaaagctaaggaagagatttccaacattaccaacgatatatcttctttctcaaatacggttgaagacaaagctaaacttttYatcaacaagctcaaagg[A/C]aaaactcagaaagccttaccagatctcttacgagagcacaaccttcctccgggtctgtttccgcgtaacataacatgctatgagtttgatgaatcaaaag

fava_c18911_799	fava_c18911	799	C/T	aatcaaggcctaaagatgcctatgaaatgcctcgtgaagctgttaaggttgaggaattttgattccaatgaatatacacataagcttattattcattgaa[C/T]aattcattaatttatatatgatagtatagtatacttggatataatatgttacactttatattgtgttgtgacctgtgttaagaagagcactatatgtctt

fava_c18912_1152	fava_c18912	1152	C/T	attacaacggttcaagagtttctcaaattatctgtaattgatcctcaacgactccgaaagatattaggcattgcaatgtcagagaaaatgtgggatgcaa[C/T]aatgaaacatgcaaagacttgtgtaatggaaaacaaagtttatgtgtaccgtgggcctcaatttaccattcatctgaatgcgatatgccaaatggttaga

fava_c18912_1451	fava_c18912	1451	G/T	gaatgagttggaagaaattgatgaagctttgaatgataacgttgctttgttaactcaaggtgaaagaatggaacaatttcaaaacaataataatcatcaa[G/T]tatcatatgatcagaatgacttttttgttgataaatctgggtgcatagaatggtcaccRtttgccacgtcaccgttggtRaatggaatagcttatagctt

fava_c18912_1510	fava_c18912	1510	A/G	gtgaaagaatggaacaatttcaaaacaataataatcatcaaKtatcatatgatcagaatgacttttttgttgataaatctgggtgcatagaatggtcacc[A/G]tttgccacgtcaccgttggtRaatggaatagcttatagcttctcaggagcaatcactgatgatggagccttaaggtggcattagaaaatattgtaatttg

fava_c18912_1531	fava_c18912	1531	G/A	aaaacaataataatcatcaaKtatcatatgatcagaatgacttttttgttgataaatctgggtgcatagaatggtcaccRtttgccacgtcaccgttggt[G/A]aatggaatagcttatagcttctcaggagcaatcactgatgatggagccttaaggtggcattagaaaatattgtaatttgacttgcagctgtatgtatata

fava_c18912_266	fava_c18912	266	A/G	tagcttcttcacttcagcatttcttatacacaaacatactatccaaacagctcctagattcttcatcaaaatggctgctaaacgtcttttggatgattct[A/G]atcaagacaaacacaaacgtatcagaactacaacaactccttttgcttcggcaataggagaaatagttatggtgaaaaacatgcaaaaccttttctctgg

fava_c18912_434	fava_c18912	434	C/T	tatggtgaaaaacatgcaaaaccttttctctggcttggaaccattacttagaagagtggtgaacgaagaagtggagcgagcaatgagacaatgttatcca[C/T]cgcgatcaataacaagatcaccttcattgagacttcaagcaatggaacatccatcaagttttcaattcattttcaaaaagaagctttcactttcaatatt

fava_c18912_760	fava_c18912	760	T/C	atggagattttccatctagtgaaaaggaatcatcatggactagtgaagaattcaatagtaatattgtgaaagaaagaaccgggaagaggccattgcttac[T/C]ggagaattgaatcttactatgagagatggaattgYaccaattggggatattgWgttcaccgataattcaagYtggactcgtagcagaaagtttagacttg

fava_c18912_795	fava_c18912	795	C/T	tggactagtgaagaattcaatagtaatattgtgaaagaaagaaccgggaagaggccattgcttacYggagaattgaatcttactatgagagatggaattg[C/T]accaattggggatattgWgttcaccgataattcaagYtggactcgtagcagaaagtttagacttggtgtaagagttgtgccagggactaatcaaaatgtt

fava_c18912_813	fava_c18912	813	A/T	aatagtaatattgtgaaagaaagaaccgggaagaggccattgcttacYggagaattgaatcttactatgagagatggaattgYaccaattggggatattg[A/T]gttcaccgataattcaagYtggactcgtagcagaaagtttagacttggtgtaagagttgtgccagggactaatcaaaatgttcaaattcRtgaaggcatg

fava_c18912_832	fava_c18912	832	C/T	aaagaaccgggaagaggccattgcttacYggagaattgaatcttactatgagagatggaattgYaccaattggggatattgWgttcaccgataattcaag[C/T]tggactcgtagcagaaagtttagacttggtgtaagagttgtgccagggactaatcaaaatgttcaaattcRtgaaggcatgactgagccttttgttgtta

fava_c18912_903	fava_c18912	903	G/A	ggggatattgWgttcaccgataattcaagYtggactcgtagcagaaagtttagacttggtgtaagagttgtgccagggactaatcaaaatgttcaaattc[G/A]tgaaggcatgactgagccttttgttgttaaggatcatcgcggagaattgtaYaagaaacaccatccaccaagtctaaatgatgaagtgtggcgtttagag

fava_c18912_955	fava_c18912	955	C/T	gacttggtgtaagagttgtgccagggactaatcaaaatgttcaaattcRtgaaggcatgactgagccttttgttgttaaggatcatcgcggagaattgta[C/T]aagaaacaccatccaccaagtctaaatgatgaagtgtggcgtttagagaagatcggaaaagatggagcattccacaaaaaattgactttggcaaagatta

fava_c18915_897	fava_c18915	897	A/C	tttaaatcccatctctcaattgcttctgtgaagagctctagttcttccaaggttccatacacatcataaacatcatcaattattgtaattaaagcattcg[A/C]ctttgttagaaYttttcgaaaatattcRaattctggatcgaaactcattcccacattccaaaYaaagttctctacaaacctgtctctaacaaatttcaac

fava_c18915_909	fava_c18915	909	T/C	ctctcaattgcttctgtgaagagctctagttcttccaaggttccatacacatcataaacatcatcaattattgtaattaaagcattcgMctttgttagaa[T/C]ttttcgaaaatattcRaattctggatcgaaactcattcccacattccaaaYaaagttctctacaaacctgtctctaacaaatttcaacttttctccaatt

fava_c18915_925	fava_c18915	925	G/A	tgaagagctctagttcttccaaggttccatacacatcataaacatcatcaattattgtaattaaagcattcgMctttgttagaaYttttcgaaaatattc[G/A]aattctggatcgaaactcattcccacattccaaaYaaagttctctacaaacctgtctctaacaaatttcaacttttctccaatttcagttcctttccacc

fava_c18915_960	fava_c18915	960	C/T	tcataaacatcatcaattattgtaattaaagcattcgMctttgttagaaYttttcgaaaatattcRaattctggatcgaaactcattcccacattccaaa[C/T]aaagttctctacaaacctgtctctaacaaatttcaacttttctccaatttcagttcctttccaccatctcgatgtatactttagttcttcttggtagatg

fava_c18917_1500	fava_c18917	1500	T/C	taaacaaatccttactacaaatggttttaggaaaaggtttataacatgcaaaatctactagatatacgttttttgagcatttctttaggtaaaaggttaa[T/C]atagatgttattaacataaaccatagtaagagtgagaaaatgttgcaagagtgtaatagtgtttggatatttagatactctccaatgatagagaagaaag

fava_c18917_501	fava_c18917	501	A/G	tccccttagcttcacaataagccaaaacataccaaacagaactacttgaagtatttccatatctataaagtgtcatccttgaaggctcaatttgccaatc[A/G]cttaatccaagaactttctgtatctcatcttgaaccgcttttccaccagtatgaatacaaaagtgctcaaaacacaacttaaaatcaggtgtataaacct

fava_c18917_987	fava_c18917	987	C/T	agattgctgcaccacctacacgaaatagacaatttgatacaagcattgatctgttgtttcctaagtaccatccactgttgatgttttctgtgctcactac[C/T]aacgcatatgagtttggatgaacctgtaggagctgtttggcaaggtcaatagctatgagtccagcactacaacccataccacttaaattataacacaaaa

fava_c18919_1115	fava_c18919	1115	G/A	tttaacattagaacaaatggaagaaatagcttgggggttaaaacaaagtgaagtaaatttcttatgggttttaagagaatcagagcaaggaaaattacca[G/A]aagggtttaaagaattgataaaggaaaagggtataattgtaacatggtgtaaccagctagagttattggcacatgatgctgttggttgttttgtgacaca

fava_c18919_1570	fava_c18919	1570	C/T	ctgttagtgaagggggtagttctgataagaatatcaatgattttgtggatcaattgatgaagaaagatagaaatggaaatttaagtggttactagaatgg[C/T]taYtctcatccatgaataatgtgaatttgcttagattgatttatgtgattgtatgactttatttgtccttttgaatcttgttagacttgctagaattctt

fava_c18919_1573	fava_c18919	1573	C/T	ttagtgaagggggtagttctgataagaatatcaatgattttgtggatcaattgatgaagaaagatagaaatggaaatttaagtggttactagaatggYta[C/T]tctcatccatgaataatgtgaatttgcttagattgatttatgtgattgtatgactttatttgtccttttgaatcttgttagacttgctagaattcttcca

fava_c18924_1002	fava_c18924	1002	A/C	tttttgtgaacgtttctatccactctagatcttctcctccatatagacatatgatttttccttctgtcatccagtctaaaactgcgggatcaatgccatc[A/C]acaagaagctcaaggctccaaatctcYtgcttccacaaggattcttctttctcacttgtgaaagggaatgccaaatttccccaaatccatatcatatgaa

fava_c18924_1029	fava_c18924	1029	C/T	gatcttctcctccatatagacatatgatttttccttctgtcatccagtctaaaactgcgggatcaatgccatcMacaagaagctcaaggctccaaatctc[C/T]tgcttccacaaggattcttctttctcacttgtgaaagggaatgccaaatttccccaaatccatatcatatgaagagcattttgggaggacaatctacctt

fava_c18924_1215	fava_c18924	1215	T/A	aggacaatctaccttgtggatcaagagctactagaatagctctctttgtgaaattccacacttctttgatatacttaatcactgatggttcgataatgaa[T/A]ggatcgcgaacactgaaccatggcattgatgattgaagatactcaaacttttgcttattcacttcattccatgttgctttgtccacaacaggaatccata

fava_c18924_1551	fava_c18924	1551	A/C	cccttaattttgaagttccatctataagtggagggatatcatcctttgcataaattagtgctctcatgattttcatgttgtctacatgaactgtctcgaa[A/C]agacgtatcagattgtgaaatgcctcaatatgcctcttttcatctatatattgatagcaaagagcaagttggtttttgaggtgctcgtatatactagcRa

fava_c18924_1650	fava_c18924	1650	G/A	aMagacgtatcagattgtgaaatgcctcaatatgcctcttttcatctatatattgatagcaaagagcaagttggtttttgaggtgctcgtatatactagc[G/A]actttgtgagccaaactagataattcccatgcctctgaagctgatgacatKgcctcatttctcatccctatgagacttgcaatttgtgaagcacaggcta

fava_c18924_1701	fava_c18924	1701	T/G	attgatagcaaagagcaagttggtttttgaggtgctcgtatatactagcRactttgtgagccaaactagataattcccatgcctctgaagctgatgacat[T/G]gcctcatttctcatccctatgagacttgcaatttgtgaagcacaggctacaatacttctaatgacccaataggtggcaataggaatatgagcacttgcaa

fava_c18924_1965	fava_c18924	1965	A/G	acattgtaacatcaagtgcagccttcacaagcttattgagtgcatcaaattgtggtctcaatgaaacagaattctctacaatgtcaggtagttgtttaag[A/G]agagccacagattttgcaagtgtgtttgaagcacttgattgagcaacaagccaaaactctccgaaaataacagcaaatgctgctaaggctaacacaactt

fava_c18924_2205	fava_c18924	2205	T/C	ccattgttgatgcatgtgcatctccacctcctgagcatttgcatgatagctcgcaagaaattttgttaataataaaagcgagtgcatcaagcatatcaaa[T/C]tcagccaaagcaacagcagtttccaatgtctcttgctcttttttcacatgttcatgaccctcgacgcttcgagcaatgacttgaatcaagatttcatcaa

fava_c18924_264	fava_c18924	264	C/A	aaacaagctatacttctaagaccttaatagcttatcagctagtaataacttaaacatttatatcccaaccttacataaacaacactacattacgacccta[C/A]aacaaaacataaaactctcaaaccaatcWtaatSatacataaatgaacacatgaagcagtattaataatgtgacatgaaatYtcctgtgtcactccacac

fava_c18924_293	fava_c18924	293	T/A	gcttatcagctagtaataacttaaacatttatatcccaaccttacataaacaacactacattacgaccctaMaacaaaacataaaactctcaaaccaatc[T/A]taatSatacataaatgaacacatgaagcagtattaataatgtgacatgaaatYtcctgtgtcactccacacaacaacgatacatgaaatatttctccatt

fava_c18924_298	fava_c18924	298	G/C	tcagctagtaataacttaaacatttatatcccaaccttacataaacaacactacattacgaccctaMaacaaaacataaaactctcaaaccaatcWtaat[G/C]atacataaatgaacacatgaagcagtattaataatgtgacatgaaatYtcctgtgtcactccacacaacaacgatacatgaaatatttctccatttgacg

fava_c18924_346	fava_c18924	346	C/T	cactacattacgaccctaMaacaaaacataaaactctcaaaccaatcWtaatSatacataaatgaacacatgaagcagtattaataatgtgacatgaaat[C/T]tcctgtgtcactccacacaacaacgatacatgaaatatttctccatttgacgaccgcattcagcgcaaacaactttctggggtattccaccagtactgcc

fava_c18926_337	fava_c18926	337	T/G	agatgcaccaccaccaccattatttctacttcctatgtaagcaatatccaaacccattccaacaggcaaaaaaaccgatctaaccacacgtgtgcctctt[T/G]caagaaccatgttccatttaaaccaagagaaatttgatctttgccatgcatttttgcatgccaaaactgcMcctctagtactaactttcgcaaccttaag

fava_c18926_408	fava_c18926	408	A/C	aaaccgatctaaccacacgtgtgcctcttKcaagaaccatgttccatttaaaccaagagaaatttgatctttgccatgcatttttgcatgccaaaactgc[A/C]cctctagtactaactttcgcaaccttaagaaccctagcaaaatccctaagtctacaatccactacgagaaaatccaacccgtcgaggcttttgatcaccg

fava_c18926_669	fava_c18926	669	G/A	ttgtgtagtctagtcttgtaccttcatctgggactatgcatacatgccttgcacccgtgttgcgagcagcaaccgctaggcctatactcgcggctatcgg[G/A]ccaccgtgagagtaacattctacgatgaactttgagttccatccggctgccatggatgatagaagctcagctaccccagattccttgcacttttcacatt

fava_c18928_622	fava_c18928	622	G/A	gctcttgacttttatcagaactaacacctactccactgactgtatagctggatggctgagcatcttgcggaacaaaaggagtgtaagctgtctccctctc[G/A]gaagcaagacgctgtgccttctgaaattctttcaacactgtctgaaaatctttggctaatttagcatctgctatcttcttggttgcattaacatcagcat

fava_c18930_1111	fava_c18930	1111	T/G	gctaacccatcagaaccttctgcatcagataaaaaatttatgggaaattgtacctgctgaagttggggacagatttctaagcatgcttccaccttggcat[T/G]catatgaaagagcttgtgagtattttattttcacatgcggtgttgagcaagtatacacaactgtgagaaacctgaaggatgatttgaaacgttaccaacc

fava_c18930_1973	fava_c18930	1973	T/C	cattctactgggcggagtcgcaactctagtggtgtggttgttgtggacggccgagctaaagacactattgtgctttcaacaggtgaaaatgttgaaccgg[T/C]ggaacttgaagaagctgccatgaggagtagcataattcaacaaattgttgttgttggtcaggataagcgacgtcttggagctatcatcgttcccaacaga

fava_c18930_2285	fava_c18930	2285	G/A	tcagaatctccatttcaaattggaccaatccttcttgtaaacgaaccattcacgattgataatggtctaataacaccgaccatgaaaattcgaagggata[G/A]agttgtggctaaatacaaggagcaaatagaaaatttatacaagtgaacactctaattttatcaagtctccaaataatttgactcctgaataaaattgtat

fava_c18930_906	fava_c18930	906	A/T	caggcttgagcgtgatttctgaagataacaaagatgttcaaatctttagtttcatggaagtcatagatttgggacgagagagtcgcaagacatggtctga[A/T]tctcatgaagccagtcaacaatatgtttatgaagcaatcaactcagaggacattgctactcttgtatactcaagtggaacaactggaaacccaaaaggtg

fava_c18931_1157	fava_c18931	1157	G/C	tagattcagattcccctaaatagaagatcatttgatgactgattttgatgatctttcaaagtgatctactgatctctttacaaagataacggaaaaatat[G/C]tttttttacttaacgtaccacttatttcttgataagttgtgtatatctagtttgacacagttaaaatttcctgtaattgctagtgagttttgatttttaa

fava_c18931_260	fava_c18931	260	C/T	actctctaggtcttcccatctctccccaaaccctcgacatcatgcttcgcgcactcaaagactcagatcagtttctccgccatcagaatgcgtcgctcct[C/T]gataaattgaaagaaaaagacgaactcatcgaacaaaccaagtatgaagcgtgtttgagtgctgtRgcgattaagaaatttgtggaggagaatcagaaat

fava_c18931_326	fava_c18931	326	G/A	atcagtttctccgccatcagaatgcgtcgctcctYgataaattgaaagaaaaagacgaactcatcgaacaaaccaagtatgaagcgtgtttgagtgctgt[G/A]gcgattaagaaatttgtggaggagaatcagaaattggcggtggagtgtgagaatcttgtggagtattgtctaaaattggagaaggagtttgtgctgtatg

fava_c18934_325	fava_c18934	325	A/C	cacaattgtgttgctaattcaaggtttaaaaagttctggtgaaggctcacttggaatatttcaacaagttgctgagatcataagagcatgttttgagttc[A/C]tccttcagttattaattggttcaataaattctatagtctctttagtgtttgatgctgttacagatataatcacaggRtcagtttctgccacggggtctat

fava_c18934_402	fava_c18934	402	A/G	tcataagagcatgttttgagttcMtccttcagttattaattggttcaataaattctatagtctctttagtgtttgatgctgttacagatataatcacagg[A/G]tcagtttctgccacggggtctatggctgctgaRcttgcagagaaattgaagacttcgtttgaagaatcattgaaacaggtKccagaactaWttgaggaag

fava_c18934_435	fava_c18934	435	G/A	tattaattggttcaataaattctatagtctctttagtgtttgatgctgttacagatataatcacaggRtcagtttctgccacggggtctatggctgctga[G/A]cttgcagagaaattgaagacttcgtttgaagaatcattgaaacaggtKccagaactaWttgaggaagtgtttgatatgatgtcaaatatggtcactgatt

fava_c18934_483	fava_c18934	483	G/T	ttacagatataatcacaggRtcagtttctgccacggggtctatggctgctgaRcttgcagagaaattgaagacttcgtttgaagaatcattgaaacaggt[G/T]ccagaactaWttgaggaagtgtttgatatgatgtcaaatatggtcactgatttRtggaacaattataaggaagctgtggggtatgttacaggaaatgttt

fava_c18934_493	fava_c18934	493	T/A	aatcacaggRtcagtttctgccacggggtctatggctgctgaRcttgcagagaaattgaagacttcgtttgaagaatcattgaaacaggtKccagaacta[T/A]ttgaggaagtgtttgatatgatgtcaaatatggtcactgatttRtggaacaattataaggaagctgtggggtatgttacaggaaatgtttgattttggtt

fava_c18934_537	fava_c18934	537	G/A	ttgcagagaaattgaagacttcgtttgaagaatcattgaaacaggtKccagaactaWttgaggaagtgtttgatatgatgtcaaatatggtcactgattt[G/A]tggaacaattataaggaagctgtggggtatgttacaggaaatgtttgattttggttgctatatgattttatattatttttatttttagtaacattaacat

fava_c18935_1235	fava_c18935	1235	T/C	tgattggtgatttctcacaaattgatgcgccggttgaggatttggtagaaaagaatgatgattggactgtggatttgcagaaccttgttgatcaaatgga[T/C]tttgaWccttaatcaaaatggttgtagaattttgatctttgttattttgtcatttcagttgcagctttctggtatgggatttgttgttattgWtgttatt

fava_c18935_1241	fava_c18935	1241	A/T	gtgatttctcacaaattgatgcgccggttgaggatttggtagaaaagaatgatgattggactgtggatttgcagaaccttgttgatcaaatggaYtttga[A/T]ccttaatcaaaatggttgtagaattttgatctttgttattttgtcatttcagttgcagctttctggtatgggatttgttgttattgWtgttattgtttta

fava_c18935_1328	fava_c18935	1328	T/A	aaatggaYtttgaWccttaatcaaaatggttgtagaattttgatctttgttattttgtcatttcagttgcagctttctggtatgggatttgttgttattg[T/A]tgttattgttttatgattgatgattaataaaagtttctgtgatgSttaattgatgtagaatatgaatggcttttatgcagcagggaagattgtagttgca

fava_c18935_1373	fava_c18935	1373	G/C	tttgttattttgtcatttcagttgcagctttctggtatgggatttgttgttattgWtgttattgttttatgattgatgattaataaaagtttctgtgatg[G/C]ttaattgatgtagaatatgaatggcttttatgcagcagggaagattgtagttgcagaggaggccaaaagatgtgtatcctttattgaacaacagtataag

fava_c18935_157	fava_c18935	157	G/A	caaccaagtgtaaataactacaccatttctaaaattggttctcacacacaagctttcttcatgaagggaattttagtgaaagaagaagaaacactagcat[G/A]taYaaatgttggttcttcaacaagttcatcttcctcttccaRttcaagtcttcaaccaaaaccaatggaagggttacacgaaacaggtccacctccattt

fava_c18935_160	fava_c18935	160	T/C	ccaagtgtaaataactacaccatttctaaaattggttctcacacacaagctttcttcatgaagggaattttagtgaaagaagaagaaacactagcatRta[T/C]aaatgttggttcttcaacaagttcatcttcctcttccaRttcaagtcttcaaccaaaaccaatggaagggttacacgaaacaggtccacctccatttctc

fava_c18935_1768	fava_c18935	1768	G/A	tcattgttcaggagggtgtataactttttcttagtaacatttcaatgttctttgacaatttcaaaatagatgggttaattgttcaccattaaaaatttaa[G/A]attcctgcatttttcagatgtttagacagagtaacatgttgacaggttggttaatgttggttttcttgtgactgaatatacatgatctacttataatgtg

fava_c18935_199	fava_c18935	199	A/G	cacacacaagctttcttcatgaagggaattttagtgaaagaagaagaaacactagcatRtaYaaatgttggttcttcaacaagttcatcttcctcttcca[A/G]ttcaagtcttcaaccaaaaccaatggaagggttacacgaaacaggtccacctccatttctcaccaaaacctttgatgtcgttgaagatccttccaccgat

fava_c18935_2517	fava_c18935	2517	A/T	ctgccaaaggaagagggtcttacaggactgagatgatgaactgatgaattcaagaaaggagcttagagaccccttttcccgagttgttttaggtgcggcc[A/T]tgtgtttatatccggctgcttaatggtgttttatttggtgatcctggtaactggttggattacttttccgatttcctggttgtattattactttttgatt

fava_c18935_531	fava_c18935	531	A/G	tggttttagaaaagttgatccggatcgatgggaatttgcgaatgaagggttcttagctggacagaggaacttgttgagaacgatcaagagaagaagaaat[A/G]tagcgcagtcgccgtctatgcagcgagaaagtggtggtgcttgtattgaattaggagagtttggtcttgaaggtgagattgagaggttgagaagagacag

fava_c18935_860	fava_c18935	860	G/C	caaaagcacttagtaatcactcttttattcagcaattggcaaacaacaaagagttgaaaggtgttgaaatgaagcgaaagaggaggctcactgctagttc[G/C]agtttggagaatttgcagaatgattcMgtgacgatgatggcagtgccgaYtgagtctgtggttgattgttctagtcaagaacaacaagagggtttgacta

fava_c18935_887	fava_c18935	887	C/A	ttcagcaattggcaaacaacaaagagttgaaaggtgttgaaatgaagcgaaagaggaggctcactgctagttcSagtttggagaatttgcagaatgattc[C/A]gtgacgatgatggcagtgccgaYtgagtctgtggttgattgttctagtcaagaacaacaagagggtttgactactattgagtccgagatagagacattgt

fava_c18935_910	fava_c18935	910	T/C	gagttgaaaggtgttgaaatgaagcgaaagaggaggctcactgctagttcSagtttggagaatttgcagaatgattcMgtgacgatgatggcagtgccga[T/C]tgagtctgtggttgattgttctagtcaagaacaacaagagggtttgactactattgagtccgagatagagacattgttaagtgcttatggtaatgaatca

fava_c18936_1204	fava_c18936	1204	C/T	aactttggcaccaaaacccgtcaattcctccacaatggcgtgcccaattccgcgagttccacccgtgacgagagctgtcatgccgttaagagaccaccta[C/T]ggtgtggtggggtgttggaatttaacatggtttggctcctaatccttgtcgtgtgtttggaYtgtgattgttttgtagggttgaaagaggtgtttgtagg

fava_c18936_1266	fava_c18936	1266	T/C	ccgtgacgagagctgtcatgccgttaagagaccacctaYggtgtggtggggtgttggaatttaacatggtttggctcctaatccttgtcgtgtgtttgga[T/C]tgtgattgttttgtagggttgaaagaggtgtttgtaggtgaacagaaaacgttgatgattgacYgtagtggaagagtagcagctggatttgggactgatg

fava_c18936_1330	fava_c18936	1330	T/C	catggtttggctcctaatccttgtcgtgtgtttggaYtgtgattgttttgtagggttgaaagaggtgtttgtaggtgaacagaaaacgttgatgattgac[T/C]gtagtggaagagtagcagctggatttgggactgatgcacagagacatggctgatgcatttttgttctgattattagttattaccgttYaatcgtgatttt

fava_c18936_1418	fava_c18936	1418	T/C	ttgatgattgacYgtagtggaagagtagcagctggatttgggactgatgcacagagacatggctgatgcatttttgttctgattattagttattaccgtt[T/C]aatcgtgattttgtagcattattattatcaatcaattggatcttgatttcaatcaagaataaacctcaggggttgcgatagataatggatttgtttgcta

fava_c18936_551	fava_c18936	551	C/A	ttgatagacattcctccatcaacacatataatctggccagtgatgtacgacgatgctggtaagcaaagaaaggcaacaacagaagacacttcagctggat[C/A]tcctaggcgttggagcggagttcgggagtacacctcttcYaaatactctttgttgctcagaacttgctctaccatcgaagttttgatgtaccaaggtgca

fava_c18936_591	fava_c18936	591	C/T	tgatgtacgacgatgctggtaagcaaagaaaggcaacaacagaagacacttcagctggatMtcctaggcgttggagcggagttcgggagtacacctcttc[C/T]aaatactctttgttgctcagaacttgctctaccatcgaagttttgatgtaccaaggtgcaactgcattactccttatgttgtcttttgcccactcacaag

fava_c18936_695	fava_c18936	695	G/A	tactctttgttgctcagaacttgctctaccatcgaagttttgatgtaccaaggtgcaactgcattactccttatgttgtcttttgcccactcacaagcta[G/A]acttcttgtaaattgattaattgccccttttgttgctccttgaacagacattgacttcaatgagacaaaaccagaaacagaagagataaaaacaacacta

fava_c18936_800	fava_c18936	800	C/G	cttgtaaattgattaattgccccttttgttgctccttgaacagacattgacttcaatgagacaaaaccagaaacagaagagataaaaacaacactaccca[C/G]tcccgacgcttttagaagtggatatgcaagttggcacatatgaaaggtggatcctaaattggtatcaatgagaYttgaaaactctgcagctgtgaaatct

fava_c18936_874	fava_c18936	874	C/T	agaagagataaaaacaacactacccaStcccgacgcttttagaagtggatatgcaagttggcacatatgaaaggtggatcctaaattggtatcaatgaga[C/T]ttgaaaactctgcagctgtgaaatctgtcatcggtttccgaatgtttgtccctacattgtttataaggatgttgagttttccatggaagacgKatgaaac

fava_c18936_967	fava_c18936	967	G/T	aatgagaYttgaaaactctgcagctgtgaaatctgtcatcggtttccgaatgtttgtccctacattgtttataaggatgttgagttttccatggaagacg[G/T]atgaaacatcctccatgagcatctccctctgttgtggaacagacacatcacaaatcgagccagtaacatcaaaacccaaatgattccattccttgagaca

fava_c18938_213	fava_c18938	213	A/T	ccttttatcatttgcatcgtacaaaattttcaacatcttatatttacatatatcttcaattacactctttcttaccctataaaggagcactaaaagaaga[A/T]aaataaattaagScatctctagacatgaacccaaaacttctactaWgttgtatggagcagtagcagagcaatagagaatccagattctttatcaaattgt

fava_c18938_226	fava_c18938	226	C/G	gcatcgtacaaaattttcaacatcttatatttacatatatcttcaattacactctttcttaccctataaaggagcactaaaagaagaWaaataaattaag[C/G]catctctagacatgaacccaaaacttctactaWgttgtatggagcagtagcagagcaatagagaatccagattctttatcaaattgtcaagttttgatct

fava_c18938_259	fava_c18938	259	A/T	catatatcttcaattacactctttcttaccctataaaggagcactaaaagaagaWaaataaattaagScatctctagacatgaacccaaaacttctacta[A/T]gttgtatggagcagtagcagagcaatagagaatccagattctttatcaaattgtcaagttttgatctccgtgatgtagtccgtcttatacaccttcaaaa

fava_c18938_558	fava_c18938	558	C/A	ttcctcctatgaacatcatggctagaaggcttaataacttgcaagagaaatgtcttcaaagaattagctttaacaaccatagatgatgatgacgacgacg[C/A]cgattcattcaaatgatgagaggtattggatttgaaggggttcttaggaatggatctttgaggcttgttcttgtgcatgctacatctaaaggaaccagga

fava_c18939_1462	fava_c18939	1462	A/G	aagtctctttatagaattcatcttcttgtctagatcctcaagcttgagctctgcctctcttttattccgtttatagtcatttgcgtctttttgctcatta[A/G]atgcatcattactaagtttctcaatatcagcttcaacagaactagttagacgaccatatcgctgcttcttatttggaggaagagttgtctgtaccggacc

fava_c18939_170	fava_c18939	170	A/G	gatttaaaatatggatgtttgcttcatcatgctctcgcccacaaaacacaattaaaagtaaaacatatataattatatatatgtaacatgatgaaacgca[A/G]ataaagttgcagatgaagaaccaaaaatgaagattttcataaatagagaatcacatgtgatcaagaacggggagctgccatttgcatcttctttactctg

fava_c18940_129	fava_c18940	129	C/T	tatattatgagttgcaatacaactttactgaaccaaatcatccaaataaatcatcttacatgataattattaaagtatcttcataaactatagaaaaaaa[C/T]aaactttttagctaacataacaaaaaactatttcgtccatctgaaMtctctattgaatgttgatcactgtccttgattgctgcgccattgcaaatgtcga

fava_c18940_175	fava_c18940	175	C/A	taaatcatcttacatgataattattaaagtatcttcataaactatagaaaaaaaYaaactttttagctaacataacaaaaaactatttcgtccatctgaa[C/A]tctctattgaatgttgatcactgtccttgattgctgcgccattgcaaatgtcgaccaggaattcttctagaagaatagttgcgctggggattagtatgag

fava_c18940_501	fava_c18940	501	A/T	tgtgaaactagaggcatccttgttgaagatgttctgcccctagggctcaactctctataataattttcaacaatttcactatgagcctgagtcattcttg[A/T]gggcggaatattttctctccatctggcagtacgatttgcctctggatcaYgggatgatgacgaaatggatctccatccacctctatcaaatctctccctt

fava_c18940_551	fava_c18940	551	C/T	ctctctataataattttcaacaatttcactatgagcctgagtcattcttgWgggcggaatattttctctccatctggcagtacgatttgcctctggatca[C/T]gggatgatgacgaaatggatctccatccacctctatcaaatctctccctttgatStgttgaaggtaaggtggtYctccgtctagctgattgatttgtgtg

fava_c18940_606	fava_c18940	606	G/C	ggaatattttctctccatctggcagtacgatttgcctctggatcaYgggatgatgacgaaatggatctccatccacctctatcaaatctctccctttgat[G/C]tgttgaaggtaaggtggtYctccgtctagctgattgatttgtgtgtgtattatttgataacccgtgcatcgattcagatcttctgtcRtatgatgacatt

fava_c18940_625	fava_c18940	625	T/C	tggcagtacgatttgcctctggatcaYgggatgatgacgaaatggatctccatccacctctatcaaatctctccctttgatStgttgaaggtaaggtggt[T/C]ctccgtctagctgattgatttgtgtgtgtattatttgataacccgtgcatcgattcagatcttctgtcRtatgatgacattctaggtgttggataaggca

fava_c18940_694	fava_c18940	694	G/A	tctccctttgatStgttgaaggtaaggtggtYctccgtctagctgattgatttgtgtgtgtattatttgataacccgtgcatcgattcagatcttctgtc[G/A]tatgatgacattctaggtgttggataaggcaaactattcaSaaatctatctgcttcagaggatgacatttcaggcaaggtgaaatgtgttgaagggaaaa

fava_c18940_735	fava_c18940	735	G/C	gctgattgatttgtgtgtgtattatttgataacccgtgcatcgattcagatcttctgtcRtatgatgacattctaggtgttggataaggcaaactattca[G/C]aaatctatctgcttcagaggatgacatttcaggcaaggtgaaatgtgttgaagggaaaacggatctcgttctagctgattgatttgtcagtgcatcattt

fava_c18941_389	fava_c18941	389	T/G	aactcaaagagcgtaagtaataacatcgaagcggaaaaaatggctagagtttagttttaggctttaatcctggaaggctctcccacaacgctctccattc[T/G]tgtgtaccactcaccaatacgtgtgtgctccaccatatccttaccagacctcaaatagcgtatgggccttagaaccccaaagacagccagatcggcaaag

fava_c18946_1002	fava_c18946	1002	T/C	tgttgacaaaagagtcgtaaaatgctggatcatcaactccttctaaaccatcctcgtaaaaattgtcaccagttgagatcacaaaatctatatttaaatt[T/C]tctcctacaattcccatctgatgagcaacaaaagattgattgtagtttccttttcttccccaatctccaacaaccaaaaagttgagagatgtttgttgtg

fava_c18946_1150	fava_c18946	1150	T/C	tccttttcttccccaatctccaacaaccaaaaagttgagagatgtttgttgtgcttttagacgatgttggaaccttggaagattctcagcaatggaagaa[T/C]gaatcgacaataagcataacaYaRtaatgRtagagataacaggaaataacatgcgttggtttaagcaccaaaccatcttccaatgccttaaaaattctct

fava_c18946_1172	fava_c18946	1172	C/T	acaaccaaaaagttgagagatgtttgttgtgcttttagacgatgttggaaccttggaagattctcagcaatggaagaaYgaatcgacaataagcataaca[C/T]aRtaatgRtagagataacaggaaataacatgcgttggtttaagcaccaaaccatcttccaatgccttaaaaattctctcaagtaagtttttgtcc

fava_c18946_1174	fava_c18946	1174	A/G	aaccaaaaagttgagagatgtttgttgtgcttttagacgatgttggaaccttggaagattctcagcaatggaagaaYgaatcgacaataagcataacaYa[A/G]taatgRtagagataacaggaaataacatgcgttggtttaagcaccaaaccatcttccaatgccttaaaaattctctcaagtaagtttttgtcc

fava_c18946_1180	fava_c18946	1180	G/A	aaagttgagagatgtttgttgtgcttttagacgatgttggaaccttggaagattctcagcaatggaagaaYgaatcgacaataagcataacaYaRtaatg[G/A]tagagataacaggaaataacatgcgttggtttaagcaccaaaccatcttccaatgccttaaaaattctctcaagtaagtttttgtcc

fava_c18946_456	fava_c18946	456	A/G	cccatggcttcacatctcccctccatgcctttgatccacctccacttgtcaaaaattgtattccactttctttgtcaatgatgtgctccaagcaatgatc[A/G]tgtccattgatgtatgcatcaaccttgtttgactttaagatgggaataagaagYtcttcaagttcttgagtattaccatgatgacctgctgttttgatgg

fava_c18946_510	fava_c18946	510	C/T	attgtattccactttctttgtcaatgatgtgctccaagcaatgatcRtgtccattgatgtatgcatcaaccttgtttgactttaagatgggaataagaag[C/T]tcttcaagttcttgagtattaccatgatgacctgctgttttgatggtatgatgacccaccactatcttccatttggctttggattgcaccaaagctgaat

fava_c18946_615	fava_c18946	615	T/A	caagttcttgagtattaccatgatgacctgctgttttgatggtatgatgacccaccactatcttccatttggctttggattgcaccaaagctgaatcaac[T/A]tccttcaagagctctgcaatgtaagattcgcgaggaagcacacctctccaatcataagtgtgtttttcaagctcctctgggtctgtaaagtacttttcta

fava_c18946_816	fava_c18946	816	A/G	caaatgggtttgtgtccacaaagaaaaactctacaatctctgcatcaagaataaaagatctcaagcagacccatctaccatctttttgtcttagaattgg[A/G]cttagttgtgcttcaacgtcacccctgtagtcatggtttcccaaaacagcgtaccaaatcttttgcaagcttggggcaatatagatgttgacaaaagagt

fava_c18948_224	fava_c18948	224	A/G	gttcatcaaacacaagaaatagaagattcattgattgctccgaaggacttgaagttagagagtttggacaaagatggcttcttttcatttctgttgttgc[A/G]caaaaggttcttgWtgcctcggaacccttgttgaaaatggtaggagacatgttggagttgtggctcaatcgtatagcaagtaatggaggattcattgggc

fava_c18948_238	fava_c18948	238	T/A	agaaatagaagattcattgattgctccgaaggacttgaagttagagagtttggacaaagatggcttcttttcatttctgttgttgcRcaaaaggttcttg[T/A]tgcctcggaacccttgttgaaaatggtaggagacatgttggagttgtggctcaatcgtatagcaagtaatggaggattcattgggctcttcttcaacatt

fava_c18948_371	fava_c18948	371	G/A	acatgttggagttgtggctcaatcgtatagcaagtaatggaggattcattgggctcttcttcaacattcttagaggaaagatgataacgccggaaaagtc[G/A]tcaccggagttcttatcRgtggtgggacaccttgacaccagaattgatttagacaaaaatattcatgaaaatgatccaaagtacaaaggattactatcta

fava_c18948_389	fava_c18948	389	A/G	tcaatcgtatagcaagtaatggaggattcattgggctcttcttcaacattcttagaggaaagatgataacgccggaaaagtcRtcaccggagttcttatc[A/G]gtggtgggacaccttgacaccagaattgatttagacaaaaatattcatgaaaatgatccaaagtacaaaggattactatctatcatggcttcaaaatttg

fava_c18948_582	fava_c18948	582	A/G	aaaatttgcttatgagaatgaacaatttattagcaccatggttacaaatcattggggtatggaacttttgggtcaatacagcttttggaacgattaccaa[A/G]aRcacgagtccacaaaagcaatgataattagagatacaaaatctgaacccaacttgattctggttgcatttagaggtacagttccatttgatgctgagca

fava_c18948_584	fava_c18948	584	G/A	aatttgcttatgagaatgaacaatttattagcaccatggttacaaatcattggggtatggaacttttgggtcaatacagcttttggaacgattaccaaRa[G/A]cacgagtccacaaaagcaatgataattagagatacaaaatctgaacccaacttgattctggttgcatttagaggtacagttccatttgatgctgagcaat

fava_c18948_778	fava_c18948	778	G/A	gagcaatggagaacagacatagatatctcatggtttgacatacccaacgtgggcaagatacatggtgggttcatgaaagcattgggccttataaagaacc[G/A]tggatggcccaaagaaatagatgaRagagacgcacatcgttatgcttactacacgattagagaggagcttagagcaatattgagagaaaatgaagatgca

fava_c18948_803	fava_c18948	803	A/G	tctcatggtttgacatacccaacgtgggcaagatacatggtgggttcatgaaagcattgggccttataaagaaccRtggatggcccaaagaaatagatga[A/G]agagacgcacatcgttatgcttactacacgattagagaggagcttagagcaatattgagagaaaatgaagatgcaaaattcatattgacagggcatagtt

fava_c18949_1078	fava_c18949	1078	G/A	aaacgtgccattagcagattttgctgtatcattccatgcaattgcagttgaagtgtagctttgtccttgaaacgtaatgtttggtttggtttttggaacc[G/A]tgactttctcatagtagaagccactgtttatccatattatggttcttttgaagctgaaatcaggaactgcattaacagcttgttgcacagttgtgaaatt

fava_c18949_1314	fava_c18949	1314	A/G	ttgtattggtgtttggaggtggaattcctggtggaaaatcatcacaaacagaatcattattctcattgtcagggtgttttctgtgtggcctatgatgatg[A/G]tggtggtttcttccaaagtaaatagatgatggtgacaaagttgtgaaaaactgtggatttggattgataagatacaatgatgcaaaaattgcagcaatag

fava_c18949_320	fava_c18949	320	G/A	atacaacttttacacattagaagaattgaaatggaaaaaggagttatcaatgatcaagaatagttatcttaatagtttcattcctaagtttccaaccata[G/A]atcaccatcaataaaacttgtaKtgaggaatggaRaagcttgtgtatcattcaatctctgtacataaggggccctggtattcacattagcccctggccca

fava_c18949_343	fava_c18949	343	G/T	aattgaaatggaaaaaggagttatcaatgatcaagaatagttatcttaatagtttcattcctaagtttccaaccataRatcaccatcaataaaacttgta[G/T]tgaggaatggaRaagcttgtgtatcattcaatctctgtacataaggggccctggtattcacattagcccctggcccagaacaattgtactctccataaaa

fava_c18949_355	fava_c18949	355	A/G	aaaaggagttatcaatgatcaagaatagttatcttaatagtttcattcctaagtttccaaccataRatcaccatcaataaaacttgtaKtgaggaatgga[A/G]aagcttgtgtatcattcaatctctgtacataaggggccctggtattcacattagcccctggcccagaacaattgtactctccataaaatatagtctggtc

fava_c18949_498	fava_c18949	498	A/T	tattcacattagcccctggcccagaacaattgtactctccataaaatatagtctggtctcttgtagggtcattgaagtcattccaaccttcaggtgcaat[A/T]atatcagacataactgtgaatgcaaaaatgacacgagaatatggtctccatgccctacctaaccatattctaccattgcctcctattgtactgttcacaa

fava_c18953_1135	fava_c18953	1135	G/A	agtcaaattgaaatcaataatcactatggttatgatagtctctaggccacacacatctcagtcggattgagtttcttagttcataatcagtttatttata[G/A]cgaaaatgaaagaWagaaaatcttgatagttagtatatttagctattttttacYggcaacctcatgcatcgtatttgtctggagtgacatgttttgtaaa

fava_c18953_1149	fava_c18953	1149	A/T	caataatcactatggttatgatagtctctaggccacacacatctcagtcggattgagtttcttagttcataatcagtttatttataRcgaaaatgaaaga[A/T]agaaaatcttgatagttagtatatttagctattttttacYggcaacctcatgcatcgtatttgtctggagtgacatgttttgtaaaggtacattgtgtag

fava_c18953_1189	fava_c18953	1189	T/C	atctcagtcggattgagtttcttagttcataatcagtttatttataRcgaaaatgaaagaWagaaaatcttgatagttagtatatttagctattttttac[T/C]ggcaacctcatgcatcgtatttgtctggagtgacatgttttgtaaaggtacattgtgtagaacagtaatggagtacctctagttatggattgtgagatat

fava_c18953_243	fava_c18953	243	G/A	ctatgaaactaaaaccattcaatctccgttcatttttcctctccttaagaacgattcaacctccattcatattctcccattcatcacacaaatgtttcac[G/A]actaaaccaccgcacttcttccacgacgtcgtaccagggacctgcgccgtttacaatcacgctctcaaattccagcgYccccctactatcaaatggaagc

fava_c18953_321	fava_c18953	321	T/C	attcatcacacaaatgtttcacRactaaaccaccgcacttcttccacgacgtcgtaccagggacctgcgccgtttacaatcacgctctcaaattccagcg[T/C]ccccctactatcaaatggaagccacacctaaaaaacaccgccaccttcatcggttccgtcacgcgtgaactcaagcgcgtgaacagtaaaaccggtagag

fava_c18953_465	fava_c18953	465	G/C	ccttcatcggttccgtcacgcgtgaactcaagcgcgtgaacagtaaaaccggtagagttgggtttcacactacgattcgtgttccaacttctaacaaacc[G/C]aatagctcatctttttgggtgcttcttatgatgtgggatgaaattgctgaatttgcttatcaacatgtgaaaccaaatcattttatttgtgtttcaggtt

fava_c18953_584	fava_c18953	584	T/G	gtgcttcttatgatgtgggatgaaattgctgaatttgcttatcaacatgtgaaaccaaatcattttatttgtgtttcaggttgtttggggtcttacattg[T/G]agagacgcRtttctgttataagctaagtgtgaatcagttagagtttgttgctcagagtccgggctacggagagcatgagaaagaaaataaatttgaagcY

fava_c18953_593	fava_c18953	593	A/G	atgatgtgggatgaaattgctgaatttgcttatcaacatgtgaaaccaaatcattttatttgtgtttcaggttgtttggggtcttacattgKagagacgc[A/G]tttctgttataagctaagtgtgaatcagttagagtttgttgctcagagtccgggctacggagagcatgagaaagaaaataaatttgaagcYggtaaccag

fava_c18953_684	fava_c18953	684	T/C	KagagacgcRtttctgttataagctaagtgtgaatcagttagagtttgttgctcagagtccgggctacggagagcatgagaaagaaaataaatttgaagc[T/C]ggtaaccagttatctgatgggaatcagcttcacctctggcaagtgtttttcgccagtccaaatgaatggtgggataacagaaagaccaagttaaatccaa

fava_c18953_892	fava_c18953	892	A/G	tgattttagacacaaggatacaggggaagctctctggctgagtaaacataatccaccatgggttacaagacaacttgaattacttgactcaaaatttgca[A/G]Kaggatttgcaggtcgtcggccacgtctcacatcttgggtttacgatgaatagctgaaattaattaaagaagacaactgtggcaattcacctttaatctt

fava_c18953_893	fava_c18953	893	T/G	gattttagacacaaggatacaggggaagctctctggctgagtaaacataatccaccatgggttacaagacaacttgaattacttgactcaaaatttgcaR[T/G]aggatttgcaggtcgtcggccacgtctcacatcttgggtttacgatgaatagctgaaattaattaaagaagacaactgtggcaattcacctttaatctta

fava_c18958_1053	fava_c18958	1053	C/T	cacactgtctatacatgacagacacaatacagtacaagaaggcttttctagtagtgatgatagtgaaactgggaatcctcaagaacttctttttcaatat[C/T]tggaacaaggtcatccttatagccgagaaccgctgactgacaagatactagatcttgcacgcgactatcctgccctcatatcattgagaagttgtgattt

fava_c18958_150	fava_c18958	150	A/T	agaaacagcgttcaacgttttcttcttctttctttctttctttcccttcacatatatattctcttcctcattccaatttcttctcccaaattcacactct[A/T]tcacttcccaattgatcctcttttcccccaactttttccgaatttcgttcttcaatcaaatttgggtcttgctgtttataataaccctaattaagcactt

fava_c18958_1536	fava_c18958	1536	T/C	actcttgcaagctgcagataaatggttaaggctagttcaggtgactcacccggatcatcagttttttaaatcacatggcacatactacaaatgagtttaa[T/C]gatatgatgacatgcMagttccatgcccttggttaaaactaagccttaattttgttccattggtgcaaactttaaaaagtcgatccatctgtagagattg

fava_c18958_1552	fava_c18958	1552	C/A	gataaatggttaaggctagttcaggtgactcacccggatcatcagttttttaaatcacatggcacatactacaaatgagtttaaYgatatgatgacatgc[C/A]agttccatgcccttggttaaaactaagccttaattttgttccattggtgcaaactttaaaaagtcgatccatctgtagagattgtgatgacatggtgtag

fava_c18958_2024	fava_c18958	2024	T/C	ctttaatttgtcgacataaatatttacacatgttgtgataatagaagaaatgctagatttcgctgcgtggcaaggcacagttttggtactatctgatgaa[T/C]atcttgttgctttgatgataggagagatggtgttgtaatattttgtacaattcaaataaatgttacaaaatgtttgtatggtagtatatgttggtgttat

fava_c18958_317	fava_c18958	317	C/T	tcttgctgtttataataaccctaattaagcactttcctccgttttctcggcgaccgaacggaggagaagaattttgaaataaagattggaaaatgctagg[C/T]actgcactgcagtttggaggtgttcgcggtggtggtggtgatgatcgggtttacattcctgttaaggcgaggaagaatcagaaccagcgtaagcaagctc

fava_c18958_512	fava_c18958	512	C/G	aagctcagaaagctaagaatggtgaagaggaaactgtagattcggcttttgatgatgataataattccaaacaatgtttggactcaccatcaactgttcc[C/G]tctgttgagtctgttagtaacattgataggtttttggattccactaagctattggtcccggctcagtatttctccaagacgacaatgagaggttggaaga

fava_c18959_156	fava_c18959	156	A/T	gaaaattttaactttgaaatttacaaactatgtatagaaaattgaaaaattacacgtttgaaccagcaccaaaacacaataaagagaaaagaaaaaacaa[A/T]gatgaacaaYgatgttttcttatgtagttttacaagcatagatcaacaatgaaaactcatctctatttatatYtttcacaYccaatttttcctStaacca

fava_c18959_166	fava_c18959	166	T/C	actttgaaatttacaaactatgtatagaaaattgaaaaattacacgtttgaaccagcaccaaaacacaataaagagaaaagaaaaaacaaWgatgaacaa[T/C]gatgttttcttatgtagttttacaagcatagatcaacaatgaaaactcatctctatttatatYtttcacaYccaatttttcctStaaccacaacctatta

fava_c18959_229	fava_c18959	229	C/T	acacaataaagagaaaagaaaaaacaaWgatgaacaaYgatgttttcttatgtagttttacaagcatagatcaacaatgaaaactcatctctatttatat[C/T]tttcacaYccaatttttcctStaaccacaacctattatccaaaccactcctagtcctaaacataaacacagcataaccagaaacacctctaaaaaaccaa

fava_c18959_237	fava_c18959	237	T/C	aagagaaaagaaaaaacaaWgatgaacaaYgatgttttcttatgtagttttacaagcatagatcaacaatgaaaactcatctctatttatatYtttcaca[T/C]ccaatttttcctStaaccacaacctattatccaaaccactcctagtcctaaacataaacacagcataaccagaaacacctctaaaaaaccaattatgaac

fava_c18959_250	fava_c18959	250	C/G	aaacaaWgatgaacaaYgatgttttcttatgtagttttacaagcatagatcaacaatgaaaactcatctctatttatatYtttcacaYccaatttttcct[C/G]taaccacaacctattatccaaaccactcctagtcctaaacataaacacagcataaccagaaacacctctaaaaaaccaattatgaacatcccaaagaaaa

fava_c18959_596	fava_c18959	596	G/A	aaatatcatgaaacgttccactttcacagaacttagtcttacttgtgtaatgaactgtgttacctgaaaaatattcacttcttgaaaaaagtgaaacttt[G/A]cctaaaaaagttctttttttcagcttctttgttaagKtttcttcaactttgtcacctagaataatcacaatttctgaatcaactaaaactgttagataaa

fava_c18959_633	fava_c18959	633	T/G	cttacttgtgtaatgaactgtgttacctgaaaaatattcacttcttgaaaaaagtgaaactttRcctaaaaaagttctttttttcagcttctttgttaag[T/G]tttcttcaactttgtcacctagaataatcacaatttctgaatcaactaaaactgttagataaaatccatcaacaggttcaggactagtttcatattttgc

fava_c18959_863	fava_c18959	863	T/C	ttgaatatccatgatgaatatgttccacttcaaccaacttgcttccttttttccttctgaaaagccttgagtttgtgttgagtctgaaaactcttgcagc[T/C]agtggatcttcttcttcattgaagactattgtgagtccttgatttgagtggcttttactccaagtaactgtgatcaacatctgttttttgttggagagga

fava_c18962_375	fava_c18962	375	T/G	gtacccctcttgtttccaacttcaccaacgtcgcattcgtctcaccaaacgcatgatcgacctcgttcatcaagaacaaaccatcaaagattcgcttgat[T/G]ctgttttcgcccagcgtctcgccaaacttctcatgctttcggttaataataccctcaatgtattaaaaatcaatgaaatcaagaacactttagggtttcc

fava_c18962_710	fava_c18962	710	T/G	cttcggctttgaaaaacggggttgacccgaatttcaaatgctgtttgccttctagttgggtgaaatcgttggagaagtttcgcgaatttgaatcagttcc[T/G]tatgtttcgccgtattcggatccSagagttttggtggacgggtccaaggagatggagaagaggaatgtgggtttggttcatgagttgctgtcattgactc

fava_c18962_734	fava_c18962	734	G/C	acccgaatttcaaatgctgtttgccttctagttgggtgaaatcgttggagaagtttcgcgaatttgaatcagttccKtatgtttcgccgtattcggatcc[G/C]agagttttggtggacgggtccaaggagatggagaagaggaatgtgggtttggttcatgagttgctgtcattgactctttggaagaaggtttcaattatga

fava_c18962_893	fava_c18962	893	T/C	agttgctgtcattgactctttggaagaaggtttcaattatgaaattgggtcattttaaaagagagttttttttgcctgataagttgaatgtgttgctgct[T/C]aagcatcctgggattttctatgtttctaataagtataggatttatactgYtttacttagagaagggtatgttgggtctcagttagttgataaggatcctc

fava_c18962_943	fava_c18962	943	T/C	cattttaaaagagagttttttttgcctgataagttgaatgtgttgctgctYaagcatcctgggattttctatgtttctaataagtataggatttatactg[T/C]tttacttagagaagggtatgttgggtctcagttagttgataaggatcctcttgtggttgttaaggagaaatttggggaaattatgcaggaaggacttcat

fava_c18972_1028	fava_c18972	1028	A/G	ctccaagcttccaaaagaagagagaaattagagttgtctgaaacacaggatgcatcatatttcagtttctccttaggacaatttgcttttaacttgtgat[A/G]ccttaatatggtagatgaaatggcctttgcagcaaggaggaatatatcatttgtttcattagMatgtttcacgaatttaaggagtgcctctctacgcctt

fava_c18972_1091	fava_c18972	1091	C/A	agtttctccttaggacaatttgcttttaacttgtgatRccttaatatggtagatgaaatggcctttgcagcaaggaggaatatatcatttgtttcattag[C/A]atgtttcacgaatttaaggagtgcctctctacgccttggatcagaactctcaccagtacagagcaaagaatgggaggattcccaatcagcctctgcacac

fava_c18972_1680	fava_c18972	1680	A/G	ccttgagaacaagttgatcctctttgaaatccattgcagcatacagaccttttccaaagttgtgatcttgtttgacggtgataccggagcagagtgttgc[A/G]gagaaaatgttgtgataatattcctgaagttggggaggtgagggaggagatagaagagYggatatctcagtggcgcaagacgaggagatagggcaaacgg

fava_c18972_213	fava_c18972	213	A/G	acctgtcatctaactaagtgtttggtttaaatcaaactctaaaagaccaatggttatttccacacgttcagtaagaacttaaaagacaagagatatttcc[A/G]tatgtttattatcattttaatgaggtacatttaaggggaaactgattttggttctacataaaattcRttgttaaaccttgcattcagcctctttatacaa

fava_c18972_280	fava_c18972	280	G/A	tcagtaagaacttaaaagacaagagatatttccRtatgtttattatcattttaatgaggtacatttaaggggaaactgattttggttctacataaaattc[G/A]ttgttaaaccttgcattcagcctctttatacaatggatactgtttatggctcttgttcaatgcactttggacatttacatctaaaaccgtaatctgcaag

fava_c18972_471	fava_c18972	471	T/A	aatctgcaagggatgcttgtctctcttcaaatggaaggtcttcatcaacatatgaaatggttatctcttctcctttacagatggacctcagtgcaattat[T/A]gttgcttggccgtctctatcctcatctcttttgaaagctttggYattaggacaacaggaatggttcatacagctttgcaaaggaaaaaatgcagttcctt

fava_c18972_515	fava_c18972	515	C/T	tcaacatatgaaatggttatctcttctcctttacagatggacctcagtgcaattatWgttgcttggccgtctctatcctcatctcttttgaaagctttgg[C/T]attaggacaacaggaatggttcatacagctttgcaaaggaaaaaatgcagttccttcRcaacaagtagaatagtcttcacctagagcatctagaattggt

fava_c18972_573	fava_c18972	573	G/A	ttgcttggccgtctctatcctcatctcttttgaaagctttggYattaggacaacaggaatggttcatacagctttgcaaaggaaaaaatgcagttccttc[G/A]caacaagtagaatagtcttcacctagagcatctagaattggttgtgtaattttctcagcttcttccttatcagggtttgtcatttcatcaatgtatagaa

fava_c18976_1433	fava_c18976	1433	A/G	aatgtttgcctgacactgcaagggattgctttgatgggatctacagagccattgatatctatcttgagtctcatccaaatgtcccatttgaggaaagatc[A/G]agattgtgtaaatgtcttaactacagtaaactcagctttggagctagtaaagatcttgccaagaaccccagaatacctccaacggttgcaatgcaggcac

fava_c18976_1817	fava_c18976	1817	T/C	aaatgaatggtcagatttccaatatgtttagccgtaatgtcttgctcactcctgctcgtgctagagcctcgccaagatattgttgaataccttcctcttg[T/C]gtgtctatcactatccattatacactggtattttattgtattgtaaaaagctgtatttttatttttatttttattttatattttgcttagaatttcaacg

fava_c18977_1703	fava_c18977	1703	G/T	ctattttcttgccagcccaacaattggtacaccacaatgacatctccaatatctcaaagtcttggaagactacggaagagccaattactatatggagtaa[G/T]agcagtttcactgcagaagatatatgggagcacttaaatgtaacaaaagatcagtctgactatctttggtattcaaccagaatatatgtttcggatggtg

fava_c18977_2315	fava_c18977	2315	A/G	agacatactttgatgtccctggtggtaatgatccagttgctcttgactttgaaagcatggggaaaggtcaagcttgggtgaatggccatcatattggaag[A/G]tactggactcgtRtttccccaaaaagtggatgtgaacgagtctgtgattatcgtggggcttatgattctgacaagtgcacaactaaKtgtggaaaaccaa

fava_c18977_2328	fava_c18977	2328	G/A	tgtccctggtggtaatgatccagttgctcttgactttgaaagcatggggaaaggtcaagcttgggtgaatggccatcatattggaagRtactggactcgt[G/A]tttccccaaaaagtggatgtgaacgagtctgtgattatcgtggggcttatgattctgacaagtgcacaactaaKtgtggaaaaccaactcaaactttgta

fava_c18977_2402	fava_c18977	2402	T/G	atcatattggaagRtactggactcgtRtttccccaaaaagtggatgtgaacgagtctgtgattatcgtggggcttatgattctgacaagtgcacaactaa[T/G]tgtggaaaaccaactcaaactttgtaccatgtgccacgatcatggttgaaagcagccaacaatttccttgttatattggaggaaactggaggaaatcctt

fava_c18977_2720	fava_c18977	2720	G/A	caaatgatttgatacctgaaatgcagttgcgctgtcgagatggacacgtaatctcctccataacatttgctagctttggaactcctaaaggtagttgcca[G/A]agttattctagagggaactgtcacgcacctagttcaatgtctatagtttccaaggcttgtatggggaaaaggagctgctccattaaaatctcaagtgctg

fava_c18981_1326	fava_c18981	1326	G/C	atatacaaatggtagagagcgaacgtactaagctgaagaaagaactagaagaacagcataaagatattaagtatcttacactagaatctaaaatgagtca[G/C]gagacagcacaaactgccataagagaagttgaagcattaaaacaagagcttatagaagagcgcaaaaatgttcaacgaattaaagcgaattttattagag

fava_c18981_1675	fava_c18981	1675	T/C	cagagactgtggatcaaaactatcaaagtgtccaatatgtcgtgagcagatcacgagtcatattaaattgttttctggctgaatcactgtttttgagtgc[T/C]tttgtgccaccaaatggatgttgtacatagattgtcatagtgctacaaagcaacatatagcttacaattaaaggttacaaatttgtatagtattgtactg

fava_c18981_180	fava_c18981	180	T/C	ttgtttatgtggcaacatcattggacctaagttggtttcttgggttgtttggttccactgaaaagaaagcacacatttgtgatcatagcaataaagtgga[T/C]gagtctaattcgtcacgaggttacatattattgataattgtgattgctcttgtgttgttgatgttctattggactggagaaaacaaagaaaacaattcca

fava_c18981_393	fava_c18981	393	A/C	tcgatgatgaggaagaagaacctgagtatcccgtggatgaacaacaactcattccccaggaatccgagcaccaaggtgcgtcttcaagagacgttattcc[A/C]tgtgccttctgtggcaattcaagtaccacaagatgtgctcgctgcaaggttgctaggtactgctcaggaggatgYcaaatagaacattggaggtcaggac

fava_c18981_468	fava_c18981	468	C/T	gtgcgtcttcaagagacgttattccMtgtgccttctgtggcaattcaagtaccacaagatgtgctcgctgcaaggttgctaggtactgctcaggaggatg[C/T]caaatagaacattggaggtcaggacatagatatgaatgcatccaattagagagtgaagaaggYgaaScaagaaatgctcatgaagaacaaactcatatgc

fava_c18981_531	fava_c18981	531	T/C	ctcgctgcaaggttgctaggtactgctcaggaggatgYcaaatagaacattggaggtcaggacatagatatgaatgcatccaattagagagtgaagaagg[T/C]gaaScaagaaatgctcatgaagaacaaactcatatgcttgtggacaagtatgataatgagaataacccaaatgggtctgtgagttccaatagtagcagta

fava_c18981_535	fava_c18981	535	G/C	ctgcaaggttgctaggtactgctcaggaggatgYcaaatagaacattggaggtcaggacatagatatgaatgcatccaattagagagtgaagaaggYgaa[G/C]caagaaatgctcatgaagaacaaactcatatgcttgtggacaagtatgataatgagaataacccaaatgggtctgtgagttccaatagtagcagtagtaa

fava_c18981_770	fava_c18981	770	C/G	caatgtggcagaccaagtaccaccaaatgttcacgatgcaaatctgtcagatactgctctacaaagtgtttgattaccaattggagatggcataaatata[C/G]ctgcattgcaggagatgtcaattcagctcaaccagaaacacctaRtagaaacgttgaattgctcaagaactcaaatgaggaggaagagaaaattcattca

fava_c18981_815	fava_c18981	815	A/G	gtcagatactgctctacaaagtgtttgattaccaattggagatggcataaatataSctgcattgcaggagatgtcaattcagctcaaccagaaacaccta[A/G]tagaaacgttgaattgctcaagaactcaaatgaggaggaagagaaaattcattcacctaggccactttatttggagcttcatcctgtcactactaccaag

fava_c18983_1019	fava_c18983	1019	A/G	aatgaacctgagttacatcaYccatatgagaatcttctagacaagcaatttgcttgttatttctccaatcccataacagtacctgcgaatcacaaccggc[A/G]gcaactaaacttccattaggccctccagtagagaaactaaagacttcttgagaaggaccggcRttgagaacagaaacctgctgaaatgatcttgtgtccc

fava_c18983_1082	fava_c18983	1082	A/G	tccaatcccataacagtacctgcgaatcacaaccggcRgcaactaaacttccattaggccctccagtagagaaactaaagacttcttgagaaggaccggc[A/G]ttgagaacagaaacctgctgaaatgatcttgtgtcccaagctcggatagtgccatctgaagaacacgagctaagaacatgaggattcgaaggacccgaaa

fava_c18983_1220	fava_c18983	1220	C/T	aagctcggatagtgccatctgaagaacacgagctaagaacatgaggattcgaaggacccgaaaacaaaatctggttgatagttccagagtgacccttgca[C/T]tccccataatactgacccaccactggtgaatacagtttcaccgcattcgttgacagtgaaaccgccattgcactccaatcatcctttgggattatttgga

fava_c18983_151	fava_c18983	151	A/G	catgataaatatgaggggtaatgtaaaacgccccattacaaatattacacgacataaaaatacatatttttcaaatcgaaatcaatcacatgcttagatc[A/G]acgcaccgagttcagtgaaagagaaaaaRaaaactaactaaatgaacaaaaagaatgcttcattggcatcaaactgtttaattaatggtaattgggctac

fava_c18983_180	fava_c18983	180	A/G	gccccattacaaatattacacgacataaaaatacatatttttcaaatcgaaatcaatcacatgcttagatcRacgcaccgagttcagtgaaagagaaaaa[A/G]aaaactaactaaatgaacaaaaagaatgcttcattggcatcaaactgtttaattaatggtaattgggctactggtattagtagggttgatgacgattttt

fava_c18983_299	fava_c18983	299	T/C	aaaaagaatgcttcattggcatcaaactgtttaattaatggtaattgggctactggtattagtagggttgatgacgatttttcttgcaagttctttctgg[T/C]ttcataaccaatgtacttgaaatccaagattgatttttttgaggggaatcatcggacaaccaacagcatagccttccatcttccccacccgtccatccaa

fava_c18983_484	fava_c18983	484	C/T	ccacccgtccatccaaaaatgcctgagctatgactactactactactaccaagttcactatgaattctggacatgggtagaacacttctaacaacacttg[C/T]atggccaccttcaagaattgcttctgcagctccaattgttgccgttccactgtaatttacaggaaaataacctaatgtacccgcatttgtgccaccaatc

fava_c18983_610	fava_c18983	610	A/C	gcagctccaattgttgccgttccactgtaatttacaggaaaataacctaatgtacccgcatttgtgccaccaatcaaccatagtttttcagcttctttcg[A/C]gtaRtgacagtcaacaaaatagtcaacatgatccaaattccaactttcagaagctaaagtacgagcttctgagaagtttacttcatttctaccatctttc

fava_c18983_614	fava_c18983	614	G/A	ctccaattgttgccgttccactgtaatttacaggaaaataacctaatgtacccgcatttgtgccaccaatcaaccatagtttttcagcttctttcgMgta[G/A]tgacagtcaacaaaatagtcaacatgatccaaattccaactttcagaagctaaagtacgagcttctgagaagtttacttcatttctaccatctttccaat

fava_c18983_860	fava_c18983	860	T/A	gataactctctccaaaaaaacccaccttggcaattgaagttcccacattaatcactgaatctagatgatcatcatcattgatatctccgctggtatcaaa[T/A]atgcagatcaacccatctactgaagccgaaatgagcttgcctggttcatcagggacaaaatgaacctgagttacatcaYccatatgagaatcttctagac

fava_c18983_939	fava_c18983	939	T/C	gatatctccgctggtatcaaaWatgcagatcaacccatctactgaagccgaaatgagcttgcctggttcatcagggacaaaatgaacctgagttacatca[T/C]ccatatgagaatcttctagacaagcaatttgcttgttatttctccaatcccataacagtacctgcgaatcacaaccggcRgcaactaaacttccattagg

fava_c18984_206	fava_c18984	206	C/T	ccccacaaaaatagtatacactaatcctaaccaactaattaaaagttgagttgatcttttagtttttctttgtttaagttgatcttttggttttgatcta[C/T]aaataatttcttcccaccatgaaaaacaacctcagaaattagaagattcctttgaaattgYatgatttttgagagtgaaatagtagagtaagtggaagtg

fava_c18984_267	fava_c18984	267	C/T	gtttttctttgtttaagttgatcttttggttttgatctaYaaataatttcttcccaccatgaaaaacaacctcagaaattagaagattcctttgaaattg[C/T]atgatttttgagagtgaaatagtagagtaagtggaagtggatattataggattatagcatggaagaaaacaagaagtggatgcataaagagactgaaaga

fava_c18984_573	fava_c18984	573	C/A	atgaactatatcaaccacctacaaatcaaggtgaagcagttagaagctaagaaggaagagttaacgaatatgtctagtttgaatacaaacgtccctcaaa[C/A]tgaatctactagtgctaccaatcttcaacccttKgttattgttcaacctttccctggaggaattgagattgtgtgcagttacagcttcaagaagtgtttc

fava_c18984_607	fava_c18984	607	T/G	agcagttagaagctaagaaggaagagttaacgaatatgtctagtttgaatacaaacgtccctcaaaMtgaatctactagtgctaccaatcttcaaccctt[T/G]gttattgttcaacctttccctggaggaattgagattgtgtgcagttacagcttcaagaagtgtttctttcctttgtcaaaagtgcttttcatgctggcta

fava_c18984_787	fava_c18984	787	G/C	aagtgcttttcatgctggctaaagaagggcttaatgtaataagctgtacttccaacaaaatagatgacagattcatatacaacatactatctgaggatcc[G/C]ataatgaccggcgttgattKctctgagctgcaaagaaaacttaccgaagcaKtatcatcttcaagtttgccagagagttcatctgaaccacaaaattggc

fava_c18984_807	fava_c18984	807	T/G	aaagaagggcttaatgtaataagctgtacttccaacaaaatagatgacagattcatatacaacatactatctgaggatccSataatgaccggcgttgatt[T/G]ctctgagctgcaaagaaaacttaccgaagcaKtatcatcttcaagtttgccagagagttcatctgaaccacaaaattggcaatatcttgagaaaaatgta

fava_c18984_839	fava_c18984	839	G/T	caacaaaatagatgacagattcatatacaacatactatctgaggatccSataatgaccggcgttgattKctctgagctgcaaagaaaacttaccgaagca[G/T]tatcatcttcaagtttgccagagagttcatctgaaccacaaaattggcaatatcttgagaaaaatgtatattttcaaagtttctgatctcatatcatatt

fava_c18984_963	fava_c18984	963	T/A	agttcatctgaaccacaaaattggcaatatcttgagaaaaatgtatattttcaaagtttctgatctcatatcatatttgtgcactcttaaattttctaga[T/A]gctagYttgaagaagattcaaattcgctgtggtatgcatgcaatactcattgagctaatggatatctttttcatttgaagttagaaacaattgctagtca

fava_c18984_969	fava_c18984	969	T/C	tctgaaccacaaaattggcaatatcttgagaaaaatgtatattttcaaagtttctgatctcatatcatatttgtgcactcttaaattttctagaWgctag[T/C]ttgaagaagattcaaattcgctgtggtatgcatgcaatactcattgagctaatggatatctttttcatttgaagttagaaacaattgctagtcatgcaaa

fava_c18985_313	fava_c18985	313	C/A	tgttttgttcaattttcaatttgctgtataacaggtggtgtgaatgttttctgttgtaatgatcacacaaaactgatctgaaattttgaactgattggga[C/A]ctttttgcYacttggattttctctagaaaggttcataaaatggttgggttcttatccaatattctagggaatgatttgaaatgtgaaagctactttattt

fava_c18985_322	fava_c18985	322	C/T	caattttcaatttgctgtataacaggtggtgtgaatgttttctgttgtaatgatcacacaaaactgatctgaaattttgaactgattgggaMctttttgc[C/T]acttggattttctctagaaaggttcataaaatggttgggttcttatccaatattctagggaatgatttgaaatgtgaaagctactttatttttagtgKag

fava_c18985_420	fava_c18985	420	T/G	gcYacttggattttctctagaaaggttcataaaatggttgggttcttatccaatattctagggaatgatttgaaatgtgaaagctactttatttttagtg[T/G]agaaactgatcttgggaataatgttaggatgtttttgtattttcttgctcttgcttattgttttattgggttatcggctataacggcgcgattctttcaa

fava_c18985_670	fava_c18985	670	G/C	ctgttactaaaggtgaaatcattacacatgagaaagtttggaattatactattgcagatattagcttgttggcgtttggaactagctttcctcagatttc[G/C]ttggctactattgatgctatacagaatttagggaacttgtatgctggaggtttgggacctggaacactcgttggttctgctgcatttgacctcttcccca

fava_c18986_455	fava_c18986	455	G/A	cagattccaacagcaagtggtagctacggcttatgctcccagaatgagagacactcctgttgagactatgttgatgcggcttctgcttcttgatggcatg[G/A]ataagatatggaatcaaaccttccattgcctcttcttttcttttctttcacgaacacaacctttgactatgatgataattgaagccttgaagtaaaattt

fava_c18987_1072	fava_c18987	1072	C/T	ggtacaggctgctgtgaattatttctcgggtcggctctatgctgagaggatcggaatctgtttttgggtcgtaaacccatgttcctccgacgcggttgtt[C/T]ttctcgaaaatcacgacggtgtgcccttgctcttgaagctccttggcggcggtgaggccggacacgccggctccgatcactgcaactttgaccgatcgag

fava_c18987_766	fava_c18987	766	G/C	tcggttctacaaaatgacccgaacacacaacaaccgcttcaaacacttcatgactcaccgactcacttccccgagtaagcctcgactcaaccacccactc[G/C]ttccctttccctttcctctcaaccttcactacctccgtttcaaatcgggtcaacccataaatcccaaactcacccgcaaaatcctccagaaaacggacca

fava_c18988_243	fava_c18988	243	T/G	atatatgttaaaaacagacaatactaagagttaattgttcttgttgatgaagtgtaattcctttgcagtgatgatgaatttgttagctacagttttattg[T/G]tagcaataacttctgtggctaatgcactgaccaaaagtgatttccctgctcacttcttgtttggtgcttcaacttctgcttatcaggttgaaggtgcagc

fava_c18989_1480	fava_c18989	1480	C/G	cacattatgtgcccacatcgagaattgagaattatatgccgagttctgctgctagttttctttatcctactgattcatctgatcgaaaacgtgagtgcct[C/G]attgaagggtgggatattgttgaacatcctactcttcctcctccgccatcacaaactgaggatgttgagcactggactcgagccatgttcatagatgcta

fava_c18989_472	fava_c18989	472	T/G	ttcgacacgaaaagcttgcaaggttgagggagaagctccaccatagtcaacaacaagttactcaaggacgagctaagaatgaggctatgtctgctgatct[T/G]aaacttaaatatggagtacttgattctgccctttctatgttggaaaagaatcgagtggagcaaYtggagaagttttaccctaatctaatatgcacccaaa

fava_c18989_536	fava_c18989	536	T/C	aggacgagctaagaatgaggctatgtctgctgatctKaaacttaaatatggagtacttgattctgccctttctatgttggaaaagaatcgagtggagcaa[T/C]tggagaagttttaccctaatctaatatgcacccaaagcttagggcatggggcgatcaccgctgagcgtcttcataaacagtctgtggtcataaaacagat

fava_c18989_691	fava_c18989	691	A/G	tcaccgctgagcgtcttcataaacagtctgtggtcataaaacagatatgcaagttgtttccactacgccaggtagtagtcaatatacagggaaaggatga[A/G]ttatatgatcaaatttgcaatgcactcttgccaagagcagttgatcccaagtctgttccaccagaagatctttccgtatctttaggatacatggtgcaac

fava_c18991_358	fava_c18991	358	G/C	cccacaaaggccacgtcaccatctccgtctcctccgtctccgtcgccctcctctcctcctcctcccaacccgaatcctcccatctcggcttcttcctcct[G/C]aacgaagaaaccctcctccaagtcctcatcgagatccaacaaaacccttccttctgcgtcctcaattcccactacatcttccgtctcttcaccttccgcg

fava_c18991_508	fava_c18991	508	T/A	ccttctgcgtcctcaattcccactacatcttccgtctcttcaccttccgcgacctttcacctcctccatccgcttccttcaaccgttcctaccccgtctc[T/A]tcccctaacgagtacagcctcttcttcgctaattgcgctccgcaaacctccgtctccatggtcgtccgcaccgagattttcaatctcgattccgacggct

fava_c18997_389	fava_c18997	389	A/T	aattaaaaatttatacacattgaggaactttctcagaacaaatgagtttatgattgtattgatatcaaagcaaagctaagctaagccagagtacaaacgt[A/T]tgtcgcatacagacatatataaattataataatatcaacatcaatgccagtcagctatRcctccaagtgtggtagatcccactccctcccatacttcact

fava_c18997_448	fava_c18997	448	G/A	tgatatcaaagcaaagctaagctaagccagagtacaaacgtWtgtcgcatacagacatatataaattataataatatcaacatcaatgccagtcagctat[G/A]cctccaagtgtggtagatcccactccctcccatacttcactgactcccaatgcctcattatcaacaccttccatgtcatagtcccaccagccgcagccat

fava_c18997_720	fava_c18997	720	C/A	cttgattcacctccaagaactaccacatgctttcgaacagtttggttctcatttccaccttcaaatggatttttccttttcctagctccttgaccatcat[C/A]tgccacagaatttcctaactcatcaagatctaacggaggaacaaagtcacatttgccctcttgcttgaacattcccaaaatcaacttctcatcagcagtt

fava_c18998_146	fava_c18998	146	C/T	ggaacataaaatcaaaattttccttttagttacactcttggtcttaattccttttcctttatcttcagggttagccgacgaagaattcagagaaaatata[C/T]ttcctactcatcacttttttgacaaggatggtataaagacaaactcaaggaagctattgtcccatgattttgtgttggattatgatgaggcaggacccaa

fava_c18999_1270	fava_c18999	1270	C/T	gaacctccttcagataatgtaagatttctccatgccaataaaggcggaatcaagggggctgacattaagccgctaccaggttttccaaggaagtttgaac[C/T]taatggcaagaacaaaacctgttatatttgtggtcaaagggggcatatttctaaatactgtagatggctgtaattaattgttctgaattatggtagttta

fava_c18999_863	fava_c18999	863	T/C	tgtgtcgtcgcaacatcttgatccatttagctgatagtctattcaacaagtatgcaaatagaaaaatgagtgctaaggagttatgggaagaattgagata[T/C]gtttatctgatcgaggaatatggaaccaagagatctcaggtgaaaaagtatattgaatttcaaatggttgatgaaaaagcagttgctgatcaaattcaag

fava_c19003_1428	fava_c19003	1428	C/A	ttctggtatgtcatgtatttattggtttacttgttaggatccatgtgtttccaaaggggtgtcatgggaagaaaatgcttgtttattactacctccgatt[C/A]tWtttataagagacaatttactttttaggttcattgaataatcaatgtatctagaaggaacggaggaagtatttatttctagttttaaaagcatgatgat

fava_c19003_1430	fava_c19003	1430	T/A	ctggtatgtcatgtatttattggtttacttgttaggatccatgtgtttccaaaggggtgtcatgggaagaaaatgcttgtttattactacctccgattMt[T/A]tttataagagacaatttactttttaggttcattgaataatcaatgtatctagaaggaacggaggaagtatttatttctagttttaaaagcatgatgatga

fava_c19003_312	fava_c19003	312	G/A	aagggaagggtgaacctgctgagcagatgcttgaacaagtgggggaaactaaaagctttagctatagctcattgagatctgctacgggtgattttcattc[G/A]tcYtgcaaaattggtggaggaggctatggagttgtctacaagggtgttttaagggacggcgcYcaagttgctatcaagtctctctctgtagaatctaaac

fava_c19003_315	fava_c19003	315	C/T	ggaagggtgaacctgctgagcagatgcttgaacaagtgggggaaactaaaagctttagctatagctcattgagatctgctacgggtgattttcattcRtc[C/T]tgcaaaattggtggaggaggctatggagttgtctacaagggtgttttaagggacggcgcYcaagttgctatcaagtctctctctgtagaatctaaacaag

fava_c19003_375	fava_c19003	375	T/C	atagctcattgagatctgctacgggtgattttcattcRtcYtgcaaaattggtggaggaggctatggagttgtctacaagggtgttttaagggacggcgc[T/C]caagttgctatcaagtctctctctgtagaatctaaacaaggaactcatgaatttatgacagaaattgagatgatatcaaatatacaacatccaaatcttg

fava_c19011_1319	fava_c19011	1319	A/G	attccccatcctccactctcggccctcttttcaaaacttccaacataagcctactagactccttagcataaatctgcacagcagaactcccatcaccatc[A/G]ggctcctcttcaaaatgctgggtcgcagtcacaaacgccgcctcctcaatctccttagcatcctccccagcctcttctttactaagaagtccatattttc

fava_c19011_244	fava_c19011	244	C/A	ttaaatccaggtttctgaacaacagcttcagccaccactttagctcccggccatgtgatcaaatttgtactaagatcaacttcatttaattggccatgac[C/A]ttcttccaatgccttaatgattaaaactgcaccttcatccttcaattcattttcagataaatttaacttggtgaggaactgctttgaagatatacaggca

fava_c19011_410	fava_c19011	410	C/T	acttggtgaggaactgctttgaagatatacaggcagccacagaaacagcagcttttgcagtaatgtcattcccagccatatccaaaatttccagtgatgg[C/T]gcagattccttcagagcatttgcaagggcttcagcaccatcatcctctaagttcaaataactgaggtatatctctgtaagatcaacaaacacaggtataa

fava_c19015_1738	fava_c19015	1738	T/C	tccgatctaaataccaagcgaaaccaaatgacagaagtaaacgtagtaaaaaggaagaggcaaaaaacaatgctcatcaagttgaggaaaatcagattgc[T/C]gctacacctgcaactgaKatggatggtgaaagcattagaacagataccacagcaacacctatggaattcgatgctgttcctatccctggttctcaaggtg

fava_c19015_1756	fava_c19015	1756	G/T	cgaaaccaaatgacagaagtaaacgtagtaaaaaggaagaggcaaaaaacaatgctcatcaagttgaggaaaatcagattgcYgctacacctgcaactga[G/T]atggatggtgaaagcattagaacagataccacagcaacacctatggaattcgatgctgttcctatccctggttctcaaggtggaacctcaacttccgacg

fava_c19015_2006	fava_c19015	2006	G/A	attggaagcagatgctgaagtcgaagccggaattatagatgagaatgatgcagatgcagatgctgatgctgatgttgatttagatgcaagtggctgagag[G/A]atttcacagatctactagctaagaatgaggatcattagaatagtggatattaacatcctttctttaaatgacaaagatagctgcaacattcttccgcgtt

fava_c19015_2121	fava_c19015	2121	G/A	ctagctaagaatgaggatcattagaatagtggatattaacatcctttctttaaatgacaaagatagctgcaacattcttccgcgttagatcaagttaaag[G/A]aatttttttagttatatttcaaatgacaggaaggagattatctttgaattgttgtaccatatcagttgaaagtgattctctcaatgaaaattgacaatat

fava_c19015_367	fava_c19015	367	C/T	tggcccaagatgagaatctgttcctagaaaacattctacggctgttacttcaagaattcgtgtcggcagcatttcagtgtggggagaaagtaatgcaata[C/T]gggcaatcaattgatgccaatgaaactgcccagggccacattcctcgccttcttgatatagtgctatatctgtgtgagaaggaacacattgaaggaggaa

fava_c19015_82	fava_c19015	82	C/T	cctcttaaacgcagcgttttcctgtttcactctcattcgctctgttccaattcgtctctgctggttaccgcgccagaatca[C/T]aatttctcaaaaaatcaagttttttcaactattctcgaaggctttgaaattcaattgtaataggttttgtgctcaaacttcatcatggaggtgtttaaaa

fava_c19016_1805	fava_c19016	1805	T/C	ctcaaaccagccaaacagaaaacctttctgttagtaaatccttggatgctccttcaaataatgaagtagcaagttcagaaggtagtgatagtttctttat[T/C]cagaatttgaagaggtcggtaagagaagctgcactgtcattgcagtcatacaatttagagtcatctctggatagccaYtctgaYggaaattctgagcact

fava_c19016_1883	fava_c19016	1883	T/C	aaggtagtgatagtttctttatYcagaatttgaagaggtcggtaagagaagctgcactgtcattgcagtcatacaatttagagtcatctctggatagcca[T/C]tctgaYggaaattctgagcacttctttgtccctctttcagagagtagtttttctcatttggatgcagaaaaaaaggccactccattgaggagcaaaaggt

fava_c19016_1889	fava_c19016	1889	T/C	gtgatagtttctttatYcagaatttgaagaggtcggtaagagaagctgcactgtcattgcagtcatacaatttagagtcatctctggatagccaYtctga[T/C]ggaaattctgagcacttctttgtccctctttcagagagtagtttttctcatttggatgcagaaaaaaaggccactccattgaggagcaaaaggttatttg

fava_c19016_2019	fava_c19016	2019	A/T	ttcagagagtagtttttctcatttggatgcagaaaaaaaggccactccattgaggagcaaaaggttatttgtatctccatcggatgatcctttgcttcag[A/T]gtcatgcatcagatggacaagagagcgtgttYgatgaatttccagatgctttgaatgatttggaagggctttccgattatgacaatgtaaatgggtttat

fava_c19016_2051	fava_c19016	2051	T/C	aaaaaaaggccactccattgaggagcaaaaggttatttgtatctccatcggatgatcctttgcttcagWgtcatgcatcagatggacaagagagcgtgtt[T/C]gatgaatttccagatgctttgaatgatttggaagggctttccgattatgacaatgtaaatgggtttatctcgtatacccgttcaaatgaaacacccaatc

fava_c19016_383	fava_c19016	383	T/C	agcttctgtatttcattctctcttctcttcgaggaccaattcaatcttccaaagatttcgataaagtttggccgattttcgattccgctcaatcgcgtga[T/C]tttcgtaaggttgtgcaagggattattagtgagcttgagtcacaaggagcacttcctagaagcaattcgagggtttcttcacttgctacttgttgtggac

fava_c19016_746	fava_c19016	746	T/C	tggcccttgaaagaaggaagtttttagaaagtgcagaaatggctgtacaacggcaggcgatgtggtctagtttggctcatgaaatgacttccgaatttcg[T/C]gggttatgcgctgaagaggcttatttgcaacaagagctagaaaaactgcacgacttaaggaataaagtcaaattggaaggagaacaatgggatgatcttg

fava_c19020_110	fava_c19020	110	C/T	cacaaaacttccacgactgcgcgcgccatgaaaaccccctttcctgcgaaaattcaccccaaaaaatcaaacatcaccaaaccctacaagtaaaatccaa[C/T]tcccaaatccataaccacaScatagatggatcattacaaggttctaggattacacaaaacagcatccaaggaagaaatcaaatccgcattcaRaaaactc

fava_c19020_130	fava_c19020	130	C/G	gcgcgccatgaaaaccccctttcctgcgaaaattcaccccaaaaaatcaaacatcaccaaaccctacaagtaaaatccaaYtcccaaatccataaccaca[C/G]catagatggatcattacaaggttctaggattacacaaaacagcatccaaggaagaaatcaaatccgcattcaRaaaactcgcgtttcaatttcatccaga

fava_c19020_203	fava_c19020	203	A/G	aatccaaYtcccaaatccataaccacaScatagatggatcattacaaggttctaggattacacaaaacagcatccaaggaagaaatcaaatccgcattca[A/G]aaaactcgcgtttcaatttcatccagataagcattctcaatcaccaaaagctgtacaagagaatgcaactcttcgMttcaaacaggtttctgaagcgtat

fava_c19020_279	fava_c19020	279	C/A	aggaagaaatcaaatccgcattcaRaaaactcgcgtttcaatttcatccagataagcattctcaatcaccaaaagctgtacaagagaatgcaactcttcg[C/A]ttcaaacaggtttctgaagcgtatgaaattctcatggatgatcggaaacgagccgagtataattaccggtggcgttccggtgccggtggtaatagcaatt

fava_c19020_824	fava_c19020	824	C/T	cactcaaatgcttcttcatgtttaataaatagtgaatgatggaagctttgagttttggggattgacttgatggctgtaatcattggtgcaccatgattta[C/T]gatattagtatgtattaatgtacgggatacttgattcttggcagtcacatggtctctctagtcacgtggtcgcagatttaggaggtttgatgaagatctg

fava_c19023_816	fava_c19023	816	G/A	taaacgtagtctaagtgttttattttaatgttaaattttcattaatacacaaacttacatatgttgaaaattgtgcaaacttaaatatttttgaagttac[G/A]acttttgttgggaaaaaaaacatcaataaacattgaaaaaggaatgaattgaaaaaaataWatattttccttattttggtttatagcatttgttatgaga

fava_c19023_877	fava_c19023	877	T/A	tgttgaaaattgtgcaaacttaaatatttttgaagttacRacttttgttgggaaaaaaaacatcaataaacattgaaaaaggaatgaattgaaaaaaata[T/A]atattttccttattttggtttatagcatttgttatgagaaatgagattgaagaaagtgtgacacaaagaaactcaccttttaattStcatgRacatgtaa

fava_c19023_963	fava_c19023	963	C/G	aattgaaaaaaataWatattttccttattttggtttatagcatttgttatgagaaatgagattgaagaaagtgtgacacaaagaaactcaccttttaatt[C/G]tcatgRacatgtaaaatttgttgatggatttggaagatgttcatgattgtaagggtttgggatgatgattcgataaaattagataagattggaagaaagg

fava_c19023_969	fava_c19023	969	A/G	aaaaaataWatattttccttattttggtttatagcatttgttatgagaaatgagattgaagaaagtgtgacacaaagaaactcaccttttaattStcatg[A/G]acatgtaaaatttgttgatggatttggaagatgttcatgattgtaagggtttgggatgatgattcgataaaattagataagattggaagaaaggagaaag

fava_c19024_325	fava_c19024	325	T/G	taggctgcttcaacagtacaaaatccttcaaatctttcatctctttcaaatcaaccgctcttctcatttcgccgacagctttcacgtccaccgcaccacc[T/G]tcgttttcttcgtcggtgtaccattccttgacgacggtaacaacgccgacgattcgacgagccttggtaccggagtggtagaagaagcagacatcgttga

fava_c19029_194	fava_c19029	194	T/A	catgcatgagtattacttgatacacacgttttgaatttataaattgctatacaagtattgacagtataaacaagcctataaataaataccccccaaaaaa[T/A]taaaaatctcctcaaacaattgcttagaggtatatctatgtttgtttcatgccactgtKacaaatgcagcaYtaatcaataaaaggtaaYatcggattca

fava_c19029_253	fava_c19029	253	T/G	gacagtataaacaagcctataaataaataccccccaaaaaaWtaaaaatctcctcaaacaattgcttagaggtatatctatgtttgtttcatgccactgt[T/G]acaaatgcagcaYtaatcaataaaaggtaaYatcggattcatcccaagccccaaccctcctScaaaactatctggctctacatgcttcaccctaccaaaa

fava_c19029_266	fava_c19029	266	C/T	agcctataaataaataccccccaaaaaaWtaaaaatctcctcaaacaattgcttagaggtatatctatgtttgtttcatgccactgtKacaaatgcagca[C/T]taatcaataaaaggtaaYatcggattcatcccaagccccaaccctcctScaaaactatctggctctacatgcttcaccctaccaaaatgtcgaatatggc

fava_c19029_284	fava_c19029	284	C/T	ccccaaaaaaWtaaaaatctcctcaaacaattgcttagaggtatatctatgtttgtttcatgccactgtKacaaatgcagcaYtaatcaataaaaggtaa[C/T]atcggattcatcccaagccccaaccctcctScaaaactatctggctctacatgcttcaccctaccaaaatgtcgaatatggcgcatcagccttgctagga

fava_c19029_315	fava_c19029	315	C/G	tgcttagaggtatatctatgtttgtttcatgccactgtKacaaatgcagcaYtaatcaataaaaggtaaYatcggattcatcccaagccccaaccctcct[C/G]caaaactatctggctctacatgcttcaccctaccaaaatgtcgaatatggcgcatcagccttgctaggaacggattgtgacatatagttgagctgtcaca

fava_c19029_726	fava_c19029	726	C/T	agcaacataaggggactgaactacaatagcttttgggctaacaccagcataaattaaggaaaaaacatgctgcaaaactatatcagcttctccttcatta[C/T]ataatgagccagttccagccggatctagatactcttcgcagccaactgataaacttccatacggcatWcgagtgtcaagcaatagcaaagggcactgtgt

fava_c19029_794	fava_c19029	794	T/A	gctgcaaaactatatcagcttctccttcattaYataatgagccagttccagccggatctagatactcttcgcagccaactgataaacttccatacggcat[T/A]cgagtgtcaagcaatagcaaagggcactgtgttatccatgtaggcttcacaaaaggagagtctacaagaagatgagaaaaaacagtctttgaggacttca

fava_c19034_2217	fava_c19034	2217	A/G	tctcaggagagattgaagaagccagccatgcagaagaatgagcagcaggagtgtcactggaataaatagcagaaaaaacgtagaaagacgaaacaacgat[A/G]ccgataataacaacagtgtagatcgtgatgaacagaggcttcgattgcgtcagttcctcacaagcgtttctccaagccattttcgtgaaatgtggtttca

fava_c19034_2373	fava_c19034	2373	G/A	cctcacaagcgtttctccaagccattttcgtgaaatgtggtttcacgataaacgacgtgttgatatcatcgtttcttcgttgattaagaagaagaagatg[G/A]tggtgatgaaaatggaaaacctagaattgagatttgagatgactgagagaggtttatgagaatgagattggtgtttgg

fava_c19034_587	fava_c19034	587	C/T	ccaataggtttatccgaaccacagcaccatatgccgacatactgtttaacgcgtcttctgaatctttcttccctttccttattagtgaaaccagtgaaag[C/T]gtcttgcactgaagacaacttaatgacttttacatctttgaatgatgagaatactttcataaacatttcttcactgctatgttttggaaatttaagtact

fava_c19034_691	fava_c19034	691	C/T	ttgcactgaagacaacttaatgacttttacatctttgaatgatgagaatactttcataaacatttcttcactgctatgttttggaaatttaagtactcct[C/T]cagcagtggtattatatgatccatcacaatcttgagttccttctttacagagctgaacatcaagccatgactttttcacctctgacggcagagaggggtt

fava_c19034_852	fava_c19034	852	T/C	caagccatgactttttcacctctgacggcagagaggggttatcaaatattgaatactctctaaaatctattccagggccaaagtcttctttggggagttt[T/C]ttcaacatgacattcacctcaaatacatggtccaaaggacaaagaaatggttgtctagtcatagacccctccaaaacaccaggatggggataccatagct

fava_c19039_1053	fava_c19039	1053	T/A	aaaaaMatgaacccaggcataagagccaggatctggccaatgctctttctgaagcaagttatgctacggcaaaaatggatacccctgaaatgattagttc[T/A]aatgaaaataRtgttaagagggggaRagcaggtgaagggctttgctgttcttgtagtaagaagtccttatgcaaaactacaaagtgcaaatgccgatcca

fava_c19039_1064	fava_c19039	1064	G/A	cccaggcataagagccaggatctggccaatgctctttctgaagcaagttatgctacggcaaaaatggatacccctgaaatgattagttcWaatgaaaata[G/A]tgttaagagggggaRagcaggtgaagggctttgctgttcttgtagtaagaagtccttatgcaaaactacaaagtgcaaatgccgatccaatggtgggagc

fava_c19039_1079	fava_c19039	1079	G/A	caggatctggccaatgctctttctgaagcaagttatgctacggcaaaaatggatacccctgaaatgattagttcWaatgaaaataRtgttaagaggggga[G/A]agcaggtgaagggctttgctgttcttgtagtaagaagtccttatgcaaaactacaaagtgcaaatgccgatccaatggtgggagctgtggaccatcatgt

fava_c19039_1194	fava_c19039	1194	C/T	tttgctgttcttgtagtaagaagtccttatgcaaaactacaaagtgcaaatgccgatccaatggtgggagctgtggaccatcatgtggctgctcactttc[C/T]aagtgcacaaatagggaactaWgcaccttgccagaaaatgagtcactaaaattagagaattcaggatgctctacaaacaaagatggYgaagctgtgatcg

fava_c19039_1216	fava_c19039	1216	T/A	gtccttatgcaaaactacaaagtgcaaatgccgatccaatggtgggagctgtggaccatcatgtggctgctcactttcYaagtgcacaaatagggaacta[T/A]gcaccttgccagaaaatgagtcactaaaattagagaattcaggatgctctacaaacaaagatggYgaagctgtgatcgcttctgaatgtgctaaactact

fava_c19039_1281	fava_c19039	1281	T/C	gctgctcactttcYaagtgcacaaatagggaactaWgcaccttgccagaaaatgagtcactaaaattagagaattcaggatgctctacaaacaaagatgg[T/C]gaagctgtgatcgcttctgaatgtgctaaactactccagaatgcacttgttcaaaaacctgccaaccaggagcctaaaaagaagccattgtgtgacattc

fava_c19039_1599	fava_c19039	1599	G/A	gtgttgaaacatacaaaattgtaacatgggaaaatagttcaatgaggttggactaagtgaagcaaccagctaggaaagagcaactgtggttgcgccttcc[G/A]ttagtgaaacagctgattttcaatattgtaagaagtccaactcaaagttaactttttgattaacttatcattttcacacccattcttctWttctgggggg

fava_c19039_1689	fava_c19039	1689	A/T	tgcgccttccRttagtgaaacagctgattttcaatattgtaagaagtccaactcaaagttaactttttgattaacttatcattttcacacccattcttct[A/T]ttctggggggataatataaagagctggaattgtgtatttgttattcctggtttttggcaactcttaccatgctgaattgttgagtgggatcttacattgt

fava_c19039_363	fava_c19039	363	C/G	aagattatcttcagaagctgaatttactggaagatcaggtatcagaattgaagaaaaaattgggatctcaatcccaattttcgactcataggaaaagagc[C/G]gatgaatcaactaggcaattgcagtatgagaatcagaatttgaaggctcaaaaggccaaactgcaatgtacgatgaagttggagtctgtgcgRtttaggt

fava_c19039_456	fava_c19039	456	A/G	aaagagcSgatgaatcaactaggcaattgcagtatgagaatcagaatttgaaggctcaaaaggccaaactgcaatgtacgatgaagttggagtctgtgcg[A/G]tttaggttaagcaaggccttacttgaaaaRgaagttcttcagcttaagaaggaactaagacggaatggaatcaagacYcacagtttgctgacttctaatg

fava_c19039_486	fava_c19039	486	G/A	agtatgagaatcagaatttgaaggctcaaaaggccaaactgcaatgtacgatgaagttggagtctgtgcgRtttaggttaagcaaggccttacttgaaaa[G/A]gaagttcttcagcttaagaaggaactaagacggaatggaatcaagacYcacagtttgctgacttctaatgagatgcttaaaatggtagtgcaacggaaga

fava_c19039_534	fava_c19039	534	C/T	cgatgaagttggagtctgtgcgRtttaggttaagcaaggccttacttgaaaaRgaagttcttcagcttaagaaggaactaagacggaatggaatcaagac[C/T]cacagtttgctgacttctaatgagatgcttaaaatggtagtgcaacggaagactgaggaagcttctgcttctatcaagcgtctaagagatatgatagctg

fava_c19039_683	fava_c19039	683	G/C	aagactgaggaagcttctgcttctatcaagcgtctaagagatatgatagctgcacgaaaagctatatcaaatcgatcatcaggtgccaaaaataaaagtg[G/C]tcaagtaattcaggatgctgagcatgaacttgaagtctcaactcatttgcacaagttatgttctcaatatgaatccaaaatcgcaaagatgactggggag

fava_c19039_825	fava_c19039	825	A/C	ctcatttgcacaagttatgttctcaatatgaatccaaaatcgcaaagatgactggggagattgcacagctgaaagaagaacttgaaatgcagaggcagga[A/C]aattcaaagtctgaattacaggaacaagaccttggcagcccggagaaggattatgatgatattcaagatttaaaggaacaagtaaacaaccttagtggtc

fava_c19039_937	fava_c19039	937	C/T	tgaattacaggaacaagaccttggcagcccggagaaggattatgatgatattcaagatttaaaggaacaagtaaacaaccttagtggtctgcttaaagaa[C/T]tacaattgcagaaggaaaaaMatgaacccaggcataagagccaggatctggccaatgctctttctgaagcaagttatgctacggcaaaaatggatacccc

fava_c19039_958	fava_c19039	958	A/C	tggcagcccggagaaggattatgatgatattcaagatttaaaggaacaagtaaacaaccttagtggtctgcttaaagaaYtacaattgcagaaggaaaaa[A/C]atgaacccaggcataagagccaggatctggccaatgctctttctgaagcaagttatgctacggcaaaaatggatacccctgaaatgattagttcWaatga

fava_c19039_99	fava_c19039	99	T/C	cattgaaaaccagttccatttcaaaatctctagtctctctcattttactttaacatcatgaagaagacaactcgcagatccaaacaaccaagtcccgt[T/C]gttgccgcagcttccgacgcaaacgaaaagcaacacttagaagaaaggatccgcgaacttgaacaagcgaacaaagcgtatcagactgagattgcggaat

fava_c19041_645	fava_c19041	645	G/C	acataatataagccataacttatttcatatccacccaatatttcgaatgaatccaacaagctccatacaaaataaccttttacatttgatccactcctta[G/C]tgcattagccatgcttccaatgtattcatgcaagtacttcaccctagaccaatcatctaatgatgaattactgaatgttttttggccattttcgtgtatg

fava_c19049_997	fava_c19049	997	G/C	attccgagagtgaacaatccaagaagagatcttcttcttctccaagggtgtttagagatgctaagtcagtatcgaagtcaccttctgtgtctaaggattc[G/C]gaaagtgcacagtccaagagtgttagtgttgaggtgaagaaaactgaagagttattgcaacaggttcagagtggaagcggtagcgaaatggaagaggggg

fava_c19050_1083	fava_c19050	1083	A/G	ctatccctagacacaatgcttttcttcttcttcttcttttcagtaacattatcgccactttcattccgttgatcttgattcacttttcccttatcctcac[A/G]ttttgctttggaatttaactttctctttgatgctaccttcttgtgatcaattttccccgcattgaccgcactaatgtcaccattttgtgtagaacaatta

fava_c19050_272	fava_c19050	272	C/T	ctactcccctggtaatggggatattgacatccaaYtttagtgcgtcgagttcaatctacaactaactaaacaaatcaactatagcaatattgtagaggaa[C/T]tttgatcaactagagtgagaatgcaaccatctaYattaattccttcaaacgcggctactattatatgtcgtacaataactaattcattgccttgccatga

fava_c19050_306	fava_c19050	306	C/T	YtttagtgcgtcgagttcaatctacaactaactaaacaaatcaactatagcaatattgtagaggaaYtttgatcaactagagtgagaatgcaaccatcta[C/T]attaattccttcaaacgcggctactattatatgtcgtacaataactaattcattgccttgccatgacctgtatattttttttcttgaatctacatatctc

fava_c19050_575	fava_c19050	575	C/T	catgttcattttgacttcgactagtgaactcatgggtaatctctaaactatctaagacagattttctcttctccagttctcctccttctccaaggttgtt[C/T]tcaacaatggacccaagaacacctccagcatgttttgagcaggatgatctatttgagatgtgcttgtaggaattacctcgccagctgttggagcaaaagt

fava_c19050_683	fava_c19050	683	T/C	tggacccaagaacacctccagcatgttttgagcaggatgatctatttgagatgtgcttgtaggaattacctcgccagctgttggagcaaaagtatataaa[T/C]cRtccctttccaaactagatgattttttgttctcacgcgaagaatcaagagtagttgctgcttccgttgtggccagcaattgccctttttgctcgccctg

fava_c19050_685	fava_c19050	685	G/A	gacccaagaacacctccagcatgttttgagcaggatgatctatttgagatgtgcttgtaggaattacctcgccagctgttggagcaaaagtatataaaYc[G/A]tccctttccaaactagatgattttttgttctcacgcgaagaatcaagagtagttgctgcttccgttgtggccagcaattgccctttttgctcgccctgtc

fava_c19050_793	fava_c19050	793	C/A	ccaaactagatgattttttgttctcacgcgaagaatcaagagtagttgctgcttccgttgtggccagcaattgccctttttgctcgccctgtctacctac[C/A]gcttcttgcatctccttgtccttgtaatctttgacatactgttcagcaatggccattaaatcttcaacagtcaactccacatcatcatcgtcactagacg

fava_c19053_1177	fava_c19053	1177	G/A	atcctcccttcaacacatcgatgagaaacggaacaaatcctgacctcacaatcctcatcttgttggatttctcaagcgaaaggttgaccaacgaagcaac[G/A]gcgttgacctgaaccacaacgtaacgYgagtcaatcaaagagcgcagcgacgaaagaatccgcggcgtgcataacgaaaccctagcttcctctctgctcc

fava_c19053_1204	fava_c19053	1204	C/T	acggaacaaatcctgacctcacaatcctcatcttgttggatttctcaagcgaaaggttgaccaacgaagcaacRgcgttgacctgaaccacaacgtaacg[C/T]gagtcaatcaaagagcgcagcgacgaaagaatccgcggcgtgcataacgaaaccctagcttcctctctgctcctcgtgatgctcctaagcgagataaccc

fava_c19053_313	fava_c19053	313	C/T	tgtttatagacaaaattaatccacaaaagttaaaacaaaatagcaagtttgaaatggccacctccatgatttacaacccaaatccaacaacaccaataaa[C/T]Rtttcagaaccacccacttgttcaattcatgccttttcctagagacctataccagcaaatttaaacctttgaaaaaataaatgggaaacctaaaatgtag

fava_c19053_314	fava_c19053	314	G/A	gtttatagacaaaattaatccacaaaagttaaaacaaaatagcaagtttgaaatggccacctccatgatttacaacccaaatccaacaacaccaataaaY[G/A]tttcagaaccacccacttgttcaattcatgccttttcctagagacctataccagcaaatttaaacctttgaaaaaataaatgggaaacctaaaatgtagt

fava_c19053_586	fava_c19053	586	T/C	aaagactatcaaactcaccatcttcgtcatcaccatcttcaaaacccctcatcttctgcaacacccttttggccttctccctcgccctctctgtcccggt[T/C]tcttcaattacccttaaaacctccacagctttcgcttccttcgccaaccctttaaacctcaagctcccgtgactcaacgcatacaRagcagcaacacaat

fava_c19053_672	fava_c19053	672	G/A	ctctctgtcccggtYtcttcaattacccttaaaacctccacagctttcgcttccttcgccaaccctttaaacctcaagctcccgtgactcaacgcataca[G/A]agcagcaacacaattctcacgagtcgcctcYgagtccaactcRtttcctctcaacaaactcaccagacactccactgcattggcatcaagcatagacgtt

fava_c19053_703	fava_c19053	703	C/T	aaacctccacagctttcgcttccttcgccaaccctttaaacctcaagctcccgtgactcaacgcatacaRagcagcaacacaattctcacgagtcgcctc[C/T]gagtccaactcRtttcctctcaacaaactcaccagacactccactgcattggcatcaagcatagacgttcttccctccacgcacatcgccagattacaca

fava_c19053_715	fava_c19053	715	G/A	ctttcgcttccttcgccaaccctttaaacctcaagctcccgtgactcaacgcatacaRagcagcaacacaattctcacgagtcgcctcYgagtccaactc[G/A]tttcctctcaacaaactcaccagacactccactgcattggcatcaagcatagacgttcttccctccacgcacatcgccagattacacagaatcaacaaaa

fava_c19053_823	fava_c19053	823	T/C	tcaacaaactcaccagacactccactgcattggcatcaagcatagacgttcttccctccacgcacatcgccagattacacagaatcaacaaaacccgact[T/C]gccattgtcccagtcatcaccattgaaatcaatgtaggaacaaccccaagtttcacaagcttgactctgttactctgaaccagtgtcaaatgataaagtg

fava_c19054_525	fava_c19054	525	G/A	tttacaccttctcccctcaaacttcaatacaagattttgcaaaatgaatcaagcagctgcaagacttgatgttcccctccagatcagcacctcaacagtt[G/A]tcaaagataggtgaatttgaattatttatgttttacttcatatatatgaattcattctcaaactatattccataattcagttattttgtatattatgtgt

fava_c19054_724	fava_c19054	724	C/G	gtttatgtttatgttttacatgtattatgttgacaggaaatcgattgaggatttacgtagtttgcgacttatcacagcggttaagactccgtatttacct[C/G]aaggaaaaattgatctggaagcatatgataacttggtgaatatacaaattgtgaatggcgttgaaggtattcttgttgctggctcaactggtgaaggcca

fava_c19055_485	fava_c19055	485	C/T	taaataaaaacaatcattataatgtgatggttacaattctcataacaagaacccatctcagcaccgatccagatccaaagcttgtgacaatatttttgat[C/T]atatggtgaacctccttcgatatagctccgattgWccatcgtcatctgcctacctacaaacaccgtaaacagcaccaaagactcaagattttgcactgtg

fava_c19055_520	fava_c19055	520	T/A	attctcataacaagaacccatctcagcaccgatccagatccaaagcttgtgacaatatttttgatYatatggtgaacctccttcgatatagctccgattg[T/A]ccatcgtcatctgcctacctacaaacaccgtaaacagcaccaaagactcaagattttgcactgtggagtcccctctcagccagctccgatctgcaccaaa

fava_c19055_626	fava_c19055	626	G/C	gtcatctgcctacctacaaacaccgtaaacagcaccaaagactcaagattttgcactgtggagtcccctctcagccagctccgatctgcaccaaatctga[G/C]aaagctccatcgcagaatcgcaactccaccacgacgcctccagcacggacaactgcttcacaccgtcgcccaaggagcaactgccgaacgaaactgcacg

fava_c19058_1041	fava_c19058	1041	A/G	ataccggcgtcaatttccgacggcgtattgttgggtaaagtgtctccgacgaaaggggtgctttgcaaaaggtgttgctgggatatggatataagagaga[A/G]ttctggaaacaaaKcgtatgaatcttggttggaatcatccatattctcttgttattgttattttccttcaaatccttgttaactacatttaaaatagaat

fava_c19058_1055	fava_c19058	1055	G/T	ttccgacggcgtattgttgggtaaagtgtctccgacgaaaggggtgctttgcaaaaggtgttgctgggatatggatataagagagaRttctggaaacaaa[G/T]cgtatgaatcttggttggaatcatccatattctcttgttattgttattttccttcaaatccttgttaactacatttaaaatagaatggggaggctcattt

fava_c19058_149	fava_c19058	149	G/A	tcattcaaatgtttgatatcatccctccttttaaatagttgaagtttatacatatgcattataataattaaacaaagaagacaagtgacttaatggagag[G/A]tattaaagttatgaacaaaggtctagggttcgaaaccacttacaaaatattcatagactaaaatcaaaatcctcctaacttacatggacgaaaacatgat

fava_c19058_250	fava_c19058	250	C/G	tattaaagttatgaacaaaggtctagggttcgaaaccacttacaaaatattcatagactaaaatcaaaatcctcctaacttacatggacgaaaacatgat[C/G]atcatcatcctctatggtctaacctgatcatcatcatcgaagttgaataatttacacgcataatgatctggccaccaactcggtctaggaaacgtaataR

fava_c19058_394	fava_c19058	394	G/A	tgaataatttacacgcataatgatctggccaccaactcggtctaggaaacgtaataccagcaagttttgcagcttccaacacagtaagaccatcactatc[G/A]tgatcttcatcttcatctttattgctatcagagagaactttcaatacatcaaaaacagaaatcttttgcttccctttatccttcccagattccaattcac

fava_c19058_505	fava_c19058	505	T/G	cttcatctttattgctatcagagagaactttcaatacatcaaaaacagaaatcttttgcttccctttatccttcccagattccaattcactctccggcat[T/G]SgcagcaatccagaaccttctacaacctcagcattWtgcatctcRacattttctgatactgagttttcagccaccagaatctctctgtttaaatcatcat

fava_c19058_506	fava_c19058	506	G/C	ttcatctttattgctatcagagagaactttcaatacatcaaaaacagaaatcttttgcttccctttatccttcccagattccaattcactctccggcatK[G/C]gcagcaatccagaaccttctacaacctcagcattWtgcatctcRacattttctgatactgagttttcagccaccagaatctctctgtttaaatcatcatt

fava_c19058_541	fava_c19058	541	T/A	catcaaaaacagaaatcttttgcttccctttatccttcccagattccaattcactctccggcatKSgcagcaatccagaaccttctacaacctcagcatt[T/A]tgcatctcRacattttctgatactgagttttcagccaccagaatctctctgtttaaatcatcattttttgcagcatctacattcacttgttcacatccca

fava_c19058_550	fava_c19058	550	A/G	cagaaatcttttgcttccctttatccttcccagattccaattcactctccggcatKSgcagcaatccagaaccttctacaacctcagcattWtgcatctc[A/G]acattttctgatactgagttttcagccaccagaatctctctgtttaaatcatcattttttgcagcatctacattcacttgttcacatcccacagcattga

fava_c19058_685	fava_c19058	685	T/C	tctctctgtttaaatcatcattttttgcagcatctacattcacttgttcacatcccacagcattgaaatttcccaaagaatgaaaacctaatttcaattt[T/C]aYctcaggaacttgacctaattcagaaatacccctttctagaaccctaaattcatcaaccagtccacctccatcaacacgttgcgagttctccggaaacc

fava_c19058_687	fava_c19058	687	C/T	tctctgtttaaatcatcattttttgcagcatctacattcacttgttcacatcccacagcattgaaatttcccaaagaatgaaaacctaatttcaatttYa[C/T]ctcaggaacttgacctaattcagaaatacccctttctagaaccctaaattcatcaaccagtccacctccatcaacacgttgcgagttctccggaaaccct

fava_c19059_271	fava_c19059	271	G/A	gaagtaacttcttcgacgtttcaatacgcatactaaattgttttcgatacacatgtatatgtcgaacaaacctgttttgacacaagaaattacaatctca[G/A]caaatttggttcgattactcaaataatattttYatctcataagtttctatgaacgcggtatttcccaacaataaactaccatccatataaaaccacaaat

fava_c19059_304	fava_c19059	304	T/C	taaattgttttcgatacacatgtatatgtcgaacaaacctgttttgacacaagaaattacaatctcaRcaaatttggttcgattactcaaataatatttt[T/C]atctcataagtttctatgaacgcggtatttcccaacaataaactaccatccatataaaaccacaaattaaaaaaaaaatgattcggaaaaaaaaagttta

fava_c19059_566	fava_c19059	566	C/T	cacgtatgcttaatccatcggatcccatcccggagcgtcgccttaaccttaccctccaccgcataaaccttatacctcgctattctctttctcctcttcg[C/T]tttcgcgtctccaaagctccaaggcttcgccaccgctgccgctccgccgtgactcgcctgagtcatctcgccagataactcactccgactcgcgtagtag

fava_c19060_160	fava_c19060	160	A/T	ttatatattactgtcgcttaaaccacaatctttacactaataaaaaaaaaaaacagcacatcaatttgtaagataccaatatggagtatgtggcaaatca[A/T]tgctctttattggaagtttttSataatttcgttgagagggtcaaatgggttggtgtcctcatcgtctctgtcatcatcgggaatatcttttctgcgatct

fava_c19060_182	fava_c19060	182	G/C	ccacaatctttacactaataaaaaaaaaaaacagcacatcaatttgtaagataccaatatggagtatgtggcaaatcaWtgctctttattggaagttttt[G/C]ataatttcgttgagagggtcaaatgggttggtgtcctcatcgtctctgtcatcatcgggaatatcttttctgcgatcttgactttctgctttgcatttgt

fava_c19060_415	fava_c19060	415	T/C	tcatagggcaagaaactgagagtggcttcattcgaggtgctgctgtcggtgctatgtctggagctgtcttctctattgaagtatttgaatattctcttaa[T/C]ctttggcgttctaatgaatctggaattgattgtctcttatacttgattgatgttattaccagtttaatgagtgggagacttgtacgtgaaaggataggtc

fava_c19060_676	fava_c19060	676	A/T	ttcaaaacttctatgatgttggcgatacaagaggtttaccggtagttttagttgaaaagatcccaacaatctctatcacaagtaacgatagttatgatac[A/T]tctggtgatagagtttcatgttcagtatgtcttcaggaYttgcaagttggagagagcgttagaaggctaccccgttgtcatcatatgtttcacctacctt

fava_c19060_715	fava_c19060	715	T/C	cggtagttttagttgaaaagatcccaacaatctctatcacaagtaacgatagttatgatacWtctggtgatagagtttcatgttcagtatgtcttcagga[T/C]ttgcaagttggagagagcgttagaaggctaccccgttgtcatcatatgtttcacctaccttgcattgataagtggctcatcaagcatggttcttgcccat

fava_c19060_892	fava_c19060	892	G/A	tcatcaagcatggttcttgcccattatgtcgaagagatgtataattattctgtccataccaccgtcagaatagcttctatatttgtttatactctcttag[G/A]atatactatggtaataattattgtattaaatttgccttaaagatttgcacaaattttagaaaataaaaaagataacaagaggtatttaatagagt

fava_c19061_1163	fava_c19061	1163	G/T	gtaagaggaaagcagagaaatgcagacggcttgcagaagctcaatcttcttacagaaccatgcttgagaagatgattagagacaccatgcaccagagtgt[G/T]atttataaggaacaggtaagattgaaccaggctgcgagtaatgcactaacggctagacttgaagcacagagatcaatttgtgataatgctgagaaagagt

fava_c19061_1527	fava_c19061	1527	A/G	tcacaaagagctcagagttttgttggaggaagaacaaagagcgtccgaagctggcctatctgcgaacgaagaggagctgaaaatcgaagctaataatgat[A/G]agtcggaagagaagctagaagagcatgctaaMtcatttgtggctttggatgaggaaaagttaattgcgcagagacttcagaagctagaaatcagtgaaga

fava_c19061_1559	fava_c19061	1559	A/C	aacaaagagcgtccgaagctggcctatctgcgaacgaagaggagctgaaaatcgaagctaataatgatRagtcggaagagaagctagaagagcatgctaa[A/C]tcatttgtggctttggatgaggaaaagttaattgcgcagagacttcagaagctagaaatcagtgaagaaaagagagctccaggtgtttctttccgYggtt

fava_c19061_1655	fava_c19061	1655	C/T	ctaaMtcatttgtggctttggatgaggaaaagttaattgcgcagagacttcagaagctagaaatcagtgaagaaaagagagctccaggtgtttctttccg[C/T]ggttttcatgagaaaaaagacgaggaaaatgatgaaaYgagRaaccaacgaggtaaagggaatgttgaaaaatggcttcaattgcttttagagaatggtc

fava_c19061_1693	fava_c19061	1693	C/T	gcgcagagacttcagaagctagaaatcagtgaagaaaagagagctccaggtgtttctttccgYggttttcatgagaaaaaagacgaggaaaatgatgaaa[C/T]gagRaaccaacgaggtaaagggaatgttgaaaaatggcttcaattgcttttagagaatggtcaagaagaagaaacaaatgaaagtgcctttggtaagaat

fava_c19061_1697	fava_c19061	1697	A/G	agagacttcagaagctagaaatcagtgaagaaaagagagctccaggtgtttctttccgYggttttcatgagaaaaaagacgaggaaaatgatgaaaYgag[A/G]aaccaacgaggtaaagggaatgttgaaaaatggcttcaattgcttttagagaatggtcaagaagaagaaacaaatgaaagtgcctttggtaagaatgaag

fava_c19061_1862	fava_c19061	1862	G/T	aagaaacaaatgaaagtgcctttggtaagaatgaagatataatcgaacaactgaacctgaagtttccacaaaaggagttgagaactgtaaagaaggagct[G/T]caagtccttcaagataagaagaaagaagccgatgaaatagagaaagcaaatgatagttttgataaagcaaaaattggaaaaaattggaaagaagagcaac

fava_c19061_344	fava_c19061	344	A/G	caattattgatccaaaagctaaaccgacaaccaaacctagaatctttgtcagtatcttgtggaaccagctcagtcatctagggaatgctggttttgatcc[A/G]ggggtcgttcgagttgatgcttatggcaatgtcttgtattatcatgctgattcagcttctcctcttgcttgggattttgatcattggtttccttgttcaa

fava_c19061_773	fava_c19061	773	G/C	tgaattcgcattcttttccgcaacacttcattggtttgagcgaagaagttggactagctcccgctgctatagtcgaatcgcgaagggatccctatgatgc[G/C]ttagctttacgacaacttgatcacaataagaaaacaaggcctatgtcttctgcgatagtggctgcaagaaaatcaaagggcaRtgcgccaaaagaaaatg

fava_c19061_856	fava_c19061	856	G/A	agggatccctatgatgcSttagctttacgacaacttgatcacaataagaaaacaaggcctatgtcttctgcgatagtggctgcaagaaaatcaaagggca[G/A]tgcgccaaaagaaaatgaagatccaaattttgttagaaccccctaYcaagcaattgtcatggctagagattctctaaagcaaaaagaagaagcttcaaaa

fava_c19061_902	fava_c19061	902	T/C	agaaaacaaggcctatgtcttctgcgatagtggctgcaagaaaatcaaagggcaRtgcgccaaaagaaaatgaagatccaaattttgttagaacccccta[T/C]caagcaattgtcatggctagagattctctaaagcaaaaagaagaagcttcaaaaatgcaggtggaaatgcagaagttagataatgaagtgaacgaaatga

fava_c19062_1281	fava_c19062	1281	C/G	ggggtgtcttgggaactttcctaggaggcttaggaggagtagcagattcattagagaaaccagaatggtgaccaaaaatcgaaggcgacgaagaaaacgg[C/G]gaagaaggtccagaatgatcggtcttaaaccgaagcatattcttgctaggactgatcataggcgaagcaggagcaccagcagcaccacaacctgcaggag

fava_c19062_1404	fava_c19062	1404	A/T	tcttaaaccgaagcatattcttgctaggactgatcataggcgaagcaggagcaccagcagcaccacaacctgcaggagaagaaggagaagcaaaatcagg[A/T]ccgaaaagttcagatttcaacaacctcgaataagcttcattactcccttctttaaccggtgacggtttttcgattaatccgaacgtgtggagccttgaag

fava_c19062_1506	fava_c19062	1506	G/A	cgaaaagttcagatttcaacaacctcgaataagcttcattactcccttctttaaccggtgacggtttttcgattaatccgaacgtgtggagccttgaaga[G/A]gatctacaaggtatgaatctatcactacatgaagaagttttagaaggtgatgatgatgagagattcgagattgcgcgaggcgaagcggaggaaggtggag

fava_c19062_903	fava_c19062	903	C/T	cacttgaaaccctcatgtcatgctgaagtatgttcctatccctactccccgaagccaaaattcgagaattccatgccaagacaccggttcttgtttgatg[C/T]cctgccattgttctgacctttttgcatttagtaccatcccaaatctgaacttgaccaaggtttgtaccaatagatatgaaagaaccctccttggtccact

fava_c19065_2652	fava_c19065	2652	A/C	tgtcgaagatattctttggaatttgcagtttgcagcacaagttcagaattcatggagaagagaaacaagtgattatagagactcacctaaatcaacctca[A/C]gagagacaaagttactatgaaattagcaagaactRttatggtgtatgctactatgtacattaagtttcattttatataKattgtataagcatgatYatta

fava_c19065_2687	fava_c19065	2687	A/G	cacaagttcagaattcatggagaagagaaacaagtgattatagagactcacctaaatcaacctcaMgagagacaaagttactatgaaattagcaagaact[A/G]ttatggtgtatgctactatgtacattaagtttcattttatataKattgtataagcatgatYattactattttgaaattatctttgaagagtgtcctacct

fava_c19065_2731	fava_c19065	2731	T/G	gactcacctaaatcaacctcaMgagagacaaagttactatgaaattagcaagaactRttatggtgtatgctactatgtacattaagtttcattttatata[T/G]attgtataagcatgatYattactattttgaaattatctttgaagagtgtcctaccttggataaaatatgtggcctcaaaattttgtttatgtatggttga

fava_c19065_2748	fava_c19065	2748	C/T	ctcaMgagagacaaagttactatgaaattagcaagaactRttatggtgtatgctactatgtacattaagtttcattttatataKattgtataagcatgat[C/T]attactattttgaaattatctttgaagagtgtcctaccttggataaaatatgtggcctcaaaattttgtttatgtatggttgagtttgaaaattttgtat

fava_c19065_522	fava_c19065	522	A/G	cttcaactcacacactttcttttcaacactttccactctttcaaccttgaaagtcctctctttggtttctcttggcttatggggccctctacctgaaacc[A/G]tagctgaattgtcctcattggagatactcaacattaRctcaaactacttcactggagaaataccagttcacctttcatatctcaacaatcttcaatcact

fava_c19065_559	fava_c19065	559	G/A	ctttcaaccttgaaagtcctctctttggtttctcttggcttatggggccctctacctgaaaccRtagctgaattgtcctcattggagatactcaacatta[G/A]ctcaaactacttcactggagaaataccagttcacctttcatatctcaacaatcttcaatcactagtacttgatgataacaacttcaatggtgaaattcca

fava_c19065_732	fava_c19065	732	A/G	tgataacaacttcaatggtgaaattccaaacttgttaacctctcttcaaggtttggttgttcttagcatgaagaagaattttcttacaggtaacttgcca[A/G]attcaatgaatgatcttgttactcttagagttttggatctgtcaaataatcaactgtttggtgaattacctcattttcataacttggttaaccttcaagt

fava_c19067_1020	fava_c19067	1020	A/G	tggcaacaaaactgtccaccttgccgcccttatgaagagaaagggtaggattatagcatgtgagctgaaaaaggaaaggattaagcgcttgaatgacacc[A/G]taaagctatcYggcgcttccaatatacaggttctcaatgaggatttcttgaacataaatcccaaggacccttcatattctaagttaaaagctattctttt

fava_c19067_1031	fava_c19067	1031	T/C	ctgtccaccttgccgcccttatgaagagaaagggtaggattatagcatgtgagctgaaaaaggaaaggattaagcgcttgaatgacaccRtaaagctatc[T/C]ggcgcttccaatatacaggttctcaatgaggatttcttgaacataaatcccaaggacccttcatattctaagttaaaagctattcttttagatccWtcct

fava_c19067_1127	fava_c19067	1127	T/A	tatcYggcgcttccaatatacaggttctcaatgaggatttcttgaacataaatcccaaggacccttcatattctaagttaaaagctattcttttagatcc[T/A]tcctgctctggttctggtactgccgcttctagactagaccatctgcttccgtctaaagcaRcaggtgatatYgacacagaaagattaaacaagcttgcag

fava_c19067_1188	fava_c19067	1188	G/A	cccttcatattctaagttaaaagctattcttttagatccWtcctgctctggttctggtactgccgcttctagactagaccatctgcttccgtctaaagca[G/A]caggtgatatYgacacagaaagattaaacaagcttgcagcttttcagagaaaagctcttcagcatgctttcctttttccagctttggaaagaattgtata

fava_c19067_1199	fava_c19067	1199	C/T	ctaagttaaaagctattcttttagatccWtcctgctctggttctggtactgccgcttctagactagaccatctgcttccgtctaaagcaRcaggtgatat[C/T]gacacagaaagattaaacaagcttgcagcttttcagagaaaagctcttcagcatgctttcctttttccagctttggaaagaattgtataYagtacttgtt

fava_c19067_1289	fava_c19067	1289	C/T	caggtgatatYgacacagaaagattaaacaagcttgcagcttttcagagaaaagctcttcagcatgctttcctttttccagctttggaaagaattgtata[C/T]agtacttgttccataaaccaaattgagaatgaagatgttgtgaaatctgttctgcccatRgccgagtcgtatggattccaactggcaaagccattYcccR

fava_c19067_1349	fava_c19067	1349	A/G	agcatgctttcctttttccagctttggaaagaattgtataYagtacttgttccataaaccaaattgagaatgaagatgttgtgaaatctgttctgcccat[A/G]gccgagtcgtatggattccaactggcaaagccattYcccgagtggcagtgtcgtggtcttccggtctttgaaggcgctgaaaacctgRttcgaacagatc

fava_c19067_1385	fava_c19067	1385	C/T	tataYagtacttgttccataaaccaaattgagaatgaagatgttgtgaaatctgttctgcccatRgccgagtcgtatggattccaactggcaaagccatt[C/T]cccgagtggcagtgtcgtggtcttccggtctttgaaggcgctgaaaacctgRttcgaacagatcctgccaagcatggagagggtttcttcattgccttgt

fava_c19067_1437	fava_c19067	1437	A/G	tgttctgcccatRgccgagtcgtatggattccaactggcaaagccattYcccgagtggcagtgtcgtggtcttccggtctttgaaggcgctgaaaacctg[A/G]ttcgaacagatcctgccaagcatggagagggtttcttcattgccttgtttgtcaagaaagatgctaacctttcRgtgaggtcaaataaaaatgttaataa

fava_c19067_1511	fava_c19067	1511	A/G	cggtctttgaaggcgctgaaaacctgRttcgaacagatcctgccaagcatggagagggtttcttcattgccttgtttgtcaagaaagatgctaacctttc[A/G]gtgaggtcaaataaaaatgttaataagactctccccaattctaccagaattagaaacgtgcgaaggaagataccgatgttcattcacactaacatgttca

fava_c19067_1624	fava_c19067	1624	A/G	aaaaatgttaataagactctccccaattctaccagaattagaaacgtgcgaaggaagataccgatgttcattcacactaacatgttcaaaatgtggttac[A/G]tggtcaaaattggagggatagaagatatttttcttgccagtgtaaaagccattcataaaatagactaaagcagttttgtttgcagcatttttttcaattt

fava_c19067_699	fava_c19067	699	C/A	tttgtcacggaagaatgtgacgactgttaaagacttgtttgctctacaagaggttcctgatatttctgtacctcgaaatgttcgtgtgaatacacttaaa[C/A]tggatgttgattctgccttagttgaacttcagaaaaaatactcggttcaaaaggatgatttactgcctgacttattagtgctccctccaggaaccgattt

fava_c19068_623	fava_c19068	623	T/C	actctgtggacagcccctcagcaatcggcttcatccatcctccgtcagccgttacatccatgttatcagcgtacaaccgatgaattcttatcagaaaaca[T/C]cgtgaaccactgccaccgttgcatcagcttcacttctccgcgtcgatttcccctttacggtaacattctatactctcttttgttttgtgaattgagaatg

fava_c19071_185	fava_c19071	185	A/G	acaataaaaatattactatagtttagtaacgttaacccacttgcatactaagaaacacaataatttaacccaaagataaaacttaagaacggggcagtgc[A/G]acaaaaaccgtttccaaaaaacgggtatgtaattgcagtttaaataaaaaaaatgttcaaagtagaaaaaaataataatatgctatatgacagtccaaat

fava_c19077_871	fava_c19077	871	T/A	tggtggtgaatttggtgtgactaatcgaagggctagtgaattattgagttctgatgattgcgtgaaacaaattttggatgagagacaacatggaaccgaa[T/A]attaaagcaatgttcaaacgaggaagcacgtcctgcatgattttgttgtctaatgtctcaggtagcatttccatgttctaatgtttgtttaaacttattg

fava_c19080_173	fava_c19080	173	C/T	cgattcaattttattcaaagtacagagttacaaattaccctttacattcaactgaatcttgcaagattacaccattgcaagttacaaagctgcacagtgc[C/T]ttgccaaaaatagtcatgccatgcttccaaatctgaagcatccttgcacacacatccttagccaaaccaatacagtccaccagttcacacaaccaaaccc

fava_c19080_389	fava_c19080	389	C/T	ccctacaatcatgaagaaaccgccctgcatcaatctcggaaaaaaaaccaaaagaagatcacaaaaaagaaccagtttcactcggttcgataatagaaaa[C/T]cagttcagccaatgccattcagaaaatcagaagttatatatcagaaaaagaggaaatccaaaaaattcaaaaatatcaggagcaactgtgaaactcggaa

fava_c19080_743	fava_c19080	743	T/A	tcatcatcatcatctctcaggtttaattagattcgcctcagctttcgatcatcttcatctttatcacgtaaacgcaacaaMgtttcttcaacaacaacga[T/A]ttcatcatcagctttatcttcgatccaYttccctgcaacaaatctgcataactttcaacgtaataataacacaactcaaggtgcatcgctgaaacgcaaa

fava_c19080_771	fava_c19080	771	T/C	agattcgcctcagctttcgatcatcttcatctttatcacgtaaacgcaacaaMgtttcttcaacaacaacgaWttcatcatcagctttatcttcgatcca[T/C]ttccctgcaacaaatctgcataactttcaacgtaataataacacaactcaaggtgcatcgctgaaacgcaaaaattgtgaagtagaagaagaagagagtg

fava_c19080_875	fava_c19080	875	G/T	cctgcaacaaatctgcataactttcaacgtaataataacacaactcaaggtgcatcgctgaaacgcaaaaattgtgaagtagaagaagaagagagtgaaa[G/T]cataagaaMgttcctgagttctctgcgacaaatccacKagttctcgtcgactctggcatcttttaagaagcaccggacttctccggtaggtaatccttga

fava_c19080_884	fava_c19080	884	A/C	aatctgcataactttcaacgtaataataacacaactcaaggtgcatcgctgaaacgcaaaaattgtgaagtagaagaagaagagagtgaaaKcataagaa[A/C]gttcctgagttctctgcgacaaatccacKagttctcgtcgactctggcatcttttaagaagcaccggacttctccggtaggtaatccttgaaactcgttt

fava_c19080_913	fava_c19080	913	G/T	cacaactcaaggtgcatcgctgaaacgcaaaaattgtgaagtagaagaagaagagagtgaaaKcataagaaMgttcctgagttctctgcgacaaatccac[G/T]agttctcgtcgactctggcatcttttaagaagcaccggacttctccggtaggtaatccttgaaactcgtttcaatcttcaatgtcctacgtcgctttctg

fava_c19083_1338	fava_c19083	1338	G/T	cagataatcttactgtaattgttatttgtctgtctcctgttgaaagcattgttgaatcatgtccacctcaaaggcgaagattcagagcttgctctctgtc[G/T]gaagaggctcgaaRcaaattaaggagcttgcttgaaagtaactgaatgtaaatacagttgtcatgattcatgcagaagttgattaatgtttgtaggctgt

fava_c19083_1352	fava_c19083	1352	A/G	gtaattgttatttgtctgtctcctgttgaaagcattgttgaatcatgtccacctcaaaggcgaagattcagagcttgctctctgtcKgaagaggctcgaa[A/G]caaattaaggagcttgcttgaaagtaactgaatgtaaatacagttgtcatgattcatgcagaagttgattaatgtttgtaggctgttgtaggagagttag

fava_c19083_450	fava_c19083	450	A/G	aatccgtttcaacagaaacgtctctctttggatcgactgcgagtcgctctgagatgataactgatactattatagaagaagctgttgcaaaatttacccc[A/G]attgttcgttctggtagctgtgctgatattggaccgaggggatcaatggatgatgatcatattcaaatagatgatttagctacccaccttggattcatgt

fava_c19083_597	fava_c19083	597	C/T	tggatgatgatcatattcaaatagatgatttagctacccaccttggattcatgttaaaatatcctacaccgagtgctttttatggagtttttgatgggca[C/T]ggaggtcctgatgcagctctgtttgttaagaacaatgctatgcaattattttttgaagacgctaaaatgctgcaatcKtatggcactgatgcacttctaa

fava_c19083_675	fava_c19083	675	G/T	tttatggagtttttgatgggcaYggaggtcctgatgcagctctgtttgttaagaacaatgctatgcaattattttttgaagacgctaaaatgctgcaatc[G/T]tatggcactgatgcacttctaatgaagaggttggaagattctcatcggaaagcttttctaggtgccgaccttgcaatggctaatgaaaagagtattagta

fava_c19083_855	fava_c19083	855	C/A	ctaatgaaaagagtattagtagttcttgtgggacaacagcactgactgctctgatacttggaaaacatttgttggttgccaatgctggtgattgtcgagc[C/A]gttctttgtaagcgtggagtagctgttgacatgtctcacgatcacagaccaagttatttacccgagcggaagagagtggaRgagttgggtggttatattg

fava_c19083_936	fava_c19083	936	A/G	atgctggtgattgtcgagcMgttctttgtaagcgtggagtagctgttgacatgtctcacgatcacagaccaagttatttacccgagcggaagagagtgga[A/G]gagttgggtggttatattgatgatggatatctcaatggttatctttctgtaactcgtgcccttggcgactgggacttaaaattacccattggttctgcct

fava_c19084_150	fava_c19084	150	G/A	aacctgcagaactttggcagcttgatgataaaacaccgcgtcctacaaagagattgcatggatcatctgtttacacactcaaggaaatggaagaggcaac[G/A]tgttctttcagtgaagaaaatcttcttggaaaaggaggatttggcaaagtctaccgaggtactttgcggtcaggagaggttgtagcaatcaagaaaatgg

fava_c19084_943	fava_c19084	943	A/T	caagaaactccgtaaggtgatagatccggagatggctcgaaattcttacaccattcagtctatagtcatgttagccaatctggcatcaagatgtgtacga[A/T]ctgaaagtaatgagcggccttcaatggcagattgtgtaaaagagatccaaatgattatctatacaaattcaaaaggcttgggaatggttatgcatagttt

fava_c19087_1296	fava_c19087	1296	T/G	ggaaaatattggacgaggtttctcctaagaagttagagaagatgaataagttattggtcgttaaggatgatgaaatagttaaacttaaggatgaaattaa[T/G]ataatgtctgctcattggaagcttaagactaaggaattcgagtcacagttggagaaacagcggcgtgctgatcaggatctgaagaagagagtgttgaagt

fava_c19087_1715	fava_c19087	1715	A/C	tgaaaccgttcctttgattataaggactcttgtatagatcctctagtttattgtaggagtatagataagttatagaatgcgtttcgtgccgtagaaacgc[A/C]gttttagagacaatctagctttagcttatgctatatgtagtagcatcaggatcaaaacacaattttgccgcggtatttataaaactagtctatgtagttg

fava_c19087_767	fava_c19087	767	C/T	caaagctttaggcgaaacgtggagtctttagcttctgagttgaaggagcttcggaatcgtttggcttcacaggaacagtcatatgttaaagagactcaaa[C/T]aagacaggaagcagagacaaatgccaagattatggaattggaaattagtagaatgcagaagaaattggaagaaaaaaatgagcagcttcaggcttcaaac

fava_c19088_136	fava_c19088	136	T/A	agaagaaatttgaacattgtttcatttagttttcaacaaactagaactagaattacWacgttggtacatcagtatgcctgccattaatcttttaaggcaa[T/A]ggtgttcctacttcaaagtaattgcagcaaggaaaggtatttaaaattggtaaMaaaatttcatacaatactgcctaactgttcaactcatcaacattga

fava_c19088_190	fava_c19088	190	C/A	acWacgttggtacatcagtatgcctgccattaatcttttaaggcaaWggtgttcctacttcaaagtaattgcagcaaggaaaggtatttaaaattggtaa[C/A]aaaatttcatacaatactgcctaactgttcaactcatcaacattgattagaaaagcaaaatcttcactttctaactcctcatggagcagattaaaagatg

fava_c19088_433	fava_c19088	433	A/T	acctctgtccctttttcagaacatagacaaaatagtgaaaggtttcaccaaaagtagtccactcaacagaccaggtgaagtctggtgcattaaatatagg[A/T]cgcctgaaatgtggctggccaaaagttatcgagataaatatgccatcttttttcaatactctgtgaacaccatttaatgtggacatcaccttggacatRg

fava_c19088_532	fava_c19088	532	G/A	gWcgcctgaaatgtggctggccaaaagttatcgagataaatatgccatcttttttcaatactctgtgaacaccatttaatgtggacatcaccttggacat[G/A]gtttccggctttggattccatgggtcaccgctatccacgaacaatacatccatagttcctttctcgataaccaaatcaaaacactcatcgtcaaaaggta

fava_c19088_634	fava_c19088	634	G/C	tttccggctttggattccatgggtcaccgctatccacgaacaatacatccatagttcctttctcgataaccaaatcaaaacactcatcgtcaaaaggtag[G/C]tctagcatatcagcttgcatcacttttatgtctttgaatccctgagaaagtaaacggttttgcatgttgttgactgcaacRtgtgaaaggtcaatgcaag

fava_c19088_715	fava_c19088	715	G/A	actcatcgtcaaaaggtagStctagcatatcagcttgcatcacttttatgtctttgaatccctgagaaagtaaacggttttgcatgttgttgactgcaac[G/A]tgtgaaaggtcaatgcaagtaatgttggttgttccatccttatacaaaccatcacacatttgagagtttccacatccaagctccagYacagaggaattgg

fava_c19088_802	fava_c19088	802	C/T	tgttgactgcaacRtgtgaaaggtcaatgcaagtaatgttggttgttccatccttatacaaaccatcacacatttgagagtttccacatccaagctccag[C/T]acagaggaattgggtgtgagatggggttgaataatgtgacggaaatgggaataatccttgaaccactcatatttctcctccttcgagaatctctcgtccc

fava_c19088_92	fava_c19088	92	T/A	ggtgctaaaagtaatggccttttgtttttccatatagaagaaatttgaacattgtttcatttagttttcaacaaactagaactagaattac[T/A]acgttggtacatcagtatgcctgccattaatcttttaaggcaaWggtgttcctacttcaaagtaattgcagcaaggaaaggtatttaaaattggtaaMaa

fava_c19089_1155	fava_c19089	1155	C/G	gtgatattgggttgcaagctatttcacattgttcgaatttggagattttgcatcttgtgaagacacctgagtgcactgatttgggacttgttgcaattgc[C/G]gaacggtgtaagttgttaaggaagcttcacattgatggatggaaagcgaatcggattggcgatgaagggttaatagcggttgctaagttttgccctaatc

fava_c19089_1473	fava_c19089	1473	G/A	ctgagatatcatgcattgctgccaagtgtttagctttaaagaagctttgcattaagagctgtccggtttctgatcatggaatggaagctttggcgaacgg[G/A]tgtcccaatttggtgaaagtgaaggttaagaaatgtaaaggtgttacccctgagggMggagattggttgaggctaactcggggatcagttgctgttaatt

fava_c19089_1530	fava_c19089	1530	C/A	gctgtccggtttctgatcatggaatggaagctttggcgaacggRtgtcccaatttggtgaaagtgaaggttaagaaatgtaaaggtgttacccctgaggg[C/A]ggagattggttgaggctaactcggggatcagttgctgttaatttggatactgttgaagcagaacttcaagatgctagtgatggtggagcgcaagacaatg

fava_c19089_1659	fava_c19089	1659	C/T	cagttgctgttaatttggatactgttgaagcagaacttcaagatgctagtgatggtggagcgcaagacaatgttatagagtttccgccaatgcccgtctg[C/T]gcagcagcatctacatccggaaatattgcgtcaagaagtactactcgatcggcttctttcaagcaaaggttaggacttttttctgggaggaaaatagtgg

fava_c19093_649	fava_c19093	649	A/G	cattcgagcaaattgaatccagctgtaccctttcatgaaatttataggcctcacaacatactatgactggtacccgagatgcatgagcaaccattgcaac[A/G]cttgcagtccccactcttgaatataccgttccgttagagagtattgattcagcacctagaaaaactcgactaacttcatgcattatgtaagaaacagcat

fava_c19097_369	fava_c19097	369	T/C	caaaagcttaaaccttttccatctgggtacctctaaatcccccatcttgcaaagtttgctttttcatccaattcattttcaatatgcacgcaaaaactga[T/C]tctgatgtcgcaagtttcgacccatcRtcacctacatcaacaaaacgagccgtttactatgttcagagtccttctagagactctcatgatggtgacaaat

fava_c19097_396	fava_c19097	396	G/A	tacctctaaatcccccatcttgcaaagtttgctttttcatccaattcattttcaatatgcacgcaaaaactgaYtctgatgtcgcaagtttcgacccatc[G/A]tcacctacatcaacaaaacgagccgtttactatgttcagagtccttctagagactctcatgatggtgacaaatcttcaactatgcaaccaagtcctatgg

fava_c19097_537	fava_c19097	537	C/T	gtccttctagagactctcatgatggtgacaaatcttcaactatgcaaccaagtcctatggattcaccttcacatcaatcctatgttcaccattcaagagc[C/T]tcttcttccagcagaatctctggtggttataactcttctttgggtaagaaagggaataggaaaggtaatgatgataaagggtggcttcaatctaaggtta

fava_c19097_661	fava_c19097	661	T/A	tggttataactcttctttgggtaagaaagggaataggaaaggtaatgatgataaagggtggcttcaatctaaggttatagaagaagaagatggtgatttt[T/A]atcatgagaggaatggaatttcaaRgagKtttcaattcttcattgctattgttggggttatgttggtttttggggtgttttgtttcatcatttgggctgc

fava_c19097_686	fava_c19097	686	G/A	aaagggaataggaaaggtaatgatgataaagggtggcttcaatctaaggttatagaagaagaagatggtgattttWatcatgagaggaatggaatttcaa[G/A]gagKtttcaattcttcattgctattgttggggttatgttggtttttggggtgttttgtttcatcatttgggctgctagcttgcattacaaacctcaactc

fava_c19097_690	fava_c19097	690	T/G	ggaataggaaaggtaatgatgataaagggtggcttcaatctaaggttatagaagaagaagatggtgattttWatcatgagaggaatggaatttcaaRgag[T/G]tttcaattcttcattgctattgttggggttatgttggtttttggggtgttttgtttcatcatttgggctgctagcttgcattacaaacctcaactcagtg

fava_c19097_812	fava_c19097	812	A/G	gttggggttatgttggtttttggggtgttttgtttcatcatttgggctgctagcttgcattacaaacctcaactcagtgtcaagagtttgacagtacata[A/G]cttctacttcggagaagggtcRgacataaccggagttccaactaagatgttgacagtgaattgttcaatgagaatgacagtgcataaccctgcatctttt

fava_c19097_834	fava_c19097	834	G/A	gggtgttttgtttcatcatttgggctgctagcttgcattacaaacctcaactcagtgtcaagagtttgacagtacataRcttctacttcggagaagggtc[G/A]gacataaccggagttccaactaagatgttgacagtgaattgttcaatgagaatgacagtgcataaccctgcatcttttttcggtatttatgtcagctcca

fava_c19101_1062	fava_c19101	1062	C/T	gcaccaaagtgcctccactgaaacccataaacaggtcctaagtcaccctcctccctttctttcaaaccaatgctatcaaggtattctctcgatgcatttc[C/T]gtcccatatatgaatgcctttttcctgcagcaccttggcatttgttgagccactgataaaccaaagaagttcttcaacaacccctcgccaaaatactttc

fava_c19101_147	fava_c19101	147	C/A	tgtgacaaagcaaagagaataatgtagcaagacaacatcccaaaagtcatatttaactctatcctcccttttttcggtttgagaccgagggtggtaataa[C/A]ttacagtcaaatagccaacacaaRgacaccattgatcttgaaacatgaagttgttgatcattcaaaattataccagcacgtctcaaccgtaaagatgtga

fava_c19101_171	fava_c19101	171	G/A	tagcaagacaacatcccaaaagtcatatttaactctatcctcccttttttcggtttgagaccgagggtggtaataaMttacagtcaaatagccaacacaa[G/A]gacaccattgatcttgaaacatgaagttgttgatcattcaaaattataccagcacgtctcaaccgtaaagatgtgaaaatgttaacctaaatcacctcag

fava_c19101_1979	fava_c19101	1979	T/C	aaacttttgatcagtaggcaatgtccatggtaatttcccatccttagaaatacccatatctcgggttgcaatcactactgcttggtaagtcctctgagga[T/C]tcggctgtgagttctcaatgccatttccattggcaattaccaaagaatcactatccataatttgattcaaaagcctagaaacgttctgcagaagctccca

fava_c19103_383	fava_c19103	383	A/G	tgcatgttttggcttccatttgtgacaccctttttccttctatccaattcaacaatgaaaatcaaccactctcatccttttacacaacttctggttctca[A/G]ttcccatttcctgatgagggtgcagagttattgatgttgttcaagaggagtgtaccagaagcattgcctttggtgaagtggattctaaggattctgtcat

fava_c19106_1261	fava_c19106	1261	G/T	acatttcctttttgtgtaacaaacacagattcatttggttcccttgacttgatctgtctaaagctataacttctctccccactctcacgggacttcaaca[G/T]ttctctgcctaacagggttccagctgatgtctccttgatcatattccaatcatcttgtgatttcctccggttgttagccataaaccccttaatatcatca

fava_c19106_1583	fava_c19106	1583	G/A	gcacctttacatcataaaatggagtcttccataattcaactcttggaatgtcttgtggaaccttcatcgatttcaaatgttggttgacaacatccatgac[G/A]aagagcagttcatgttccacctcttgttccgcaaatacacaagtggctttgacctctttYgcaagttgttgtatcacattctcagcattgccaaacctaa

fava_c19106_1643	fava_c19106	1643	T/C	ccttcatcgatttcaaatgttggttgacaacatccatgacRaagagcagttcatgttccacctcttgttccgcaaatacacaagtggctttgacctcttt[T/C]gcaagttgttgtatcacattctcagcattgccaaacctaatcatgagatcggaaccccgttcctgcaacgacttcctcaaatccttcaacgcaaacagaa

fava_c19106_2467	fava_c19106	2467	A/G	ttaattttttgaagtggttcaactttctgatggatttggctgtattttatgcaagaagtatgctacattggcagggggcttgaatatgactttaagaagt[A/G]attttgatgctKaatatcaaatttgtggaggttatatggtatatggaagttagtttaattggctattaagttgtggttaagacatctatacttagcctag

fava_c19106_2479	fava_c19106	2479	G/T	agtggttcaactttctgatggatttggctgtattttatgcaagaagtatgctacattggcagggggcttgaatatgactttaagaagtRattttgatgct[G/T]aatatcaaatttgtggaggttatatggtatatggaagttagtttaattggctattaagttgtggttaagacatctatacttagcctagttaggcattgaa

fava_c19106_356	fava_c19106	356	G/T	gcatttcatttatgagccaatcatctgctctttcgatcttagttggataacatttcttaacaagttcttgaattcttagcctcaagtaaaacagaataag[G/T]cgagaacctaaccatgtagccactgacgtttgtctatcactttgcctagacaatgatatggatgtatttcttgggataacattgccagcactgttgatca

fava_c19107_143	fava_c19107	143	G/A	accaaataatcgagtcagcaaacatttacaaacgcggatcaaaacaagtgcaaggttatttatccctcattgatacaggtgacagataaaaataaagggg[G/A]agatagaaacaacatgagaaacaatgaagagatYaatgttaatgaggagaataatctgtcagtaatttcatgatagattgaactatactaatttacaggg

fava_c19107_177	fava_c19107	177	C/T	cggatcaaaacaagtgcaaggttatttatccctcattgatacaggtgacagataaaaataaaggggRagatagaaacaacatgagaaacaatgaagagat[C/T]aatgttaatgaggagaataatctgtcagtaatttcatgatagattgaactatactaatttacagggattattaaactatgcattaggaactattatgaga

fava_c19107_2192	fava_c19107	2192	G/A	actcaactaaccatttgtcaaggtctgaaatttccgtatcttcagagtccagtccactcacagaaaaaccattagaatcggagatcgataattgatcaac[G/A]tgaactccatcagccaaaccatgtatcaaggtagcttgagttgatagtaggtttttgattgacgaaagctcgccttctaaatctgatatctccttggaag

fava_c19107_454	fava_c19107	454	C/T	ggttttccacttaatctttccatagcatcttgacatatttcattaaaccattcatcttcaggcagttccccatatggatccaaccctgtagcagaaaatg[C/T]cgtcattgctttcgttataatttcattgacgatccgttgcaaattccttgacaaataccgaccattggaagcaaaacacacaacaaacttcatatctaaa

fava_c19107_634	fava_c19107	634	T/G	acaacaaacttcatatctaaataaaactgctgtagaccaagaggacctaaaggccttggcccttcctcaatatcatcccaaaagctctgatcttctgaaa[T/G]ccacaaaataacagtttctgtaagtctcatcaagagcagcgtagcaaatctttctctccctacaaacatatctgctgctatattcgccattctgttcaat

fava_c19107_911	fava_c19107	911	T/G	tgtcagctgtaagaagactatctccttcctctgtgaaaatcaggcccaaagcatgttgccgacagaatgtatctttcaacttgtcaactgaacccacaag[T/G]cgtctcctccattcccgttgttcaggatggcgattttgcctttctgaRgttcttctacggttatcatccttgtagggatcttggtttatggaggagagct

fava_c19107_959	fava_c19107	959	G/A	aagcatgttgccgacagaatgtatctttcaacttgtcaactgaacccacaagKcgtctcctccattcccgttgttcaggatggcgattttgcctttctga[G/A]gttcttctacggttatcatccttgtagggatcttggtttatggaggagagcttcatggctgcacgtggaagtagttcatcagctagtaatgaggcattag

fava_c19108_1099	fava_c19108	1099	C/T	caagagggtcaagtttcaaccatacagatttctattcaatgatgggtcgtaattccaactttggtgcttctgatttgaagggaacaacaccaagaagttc[C/T]aactatgatcttcccacaacggggaatacttatcctgcgccgaatcctggaatgttctctcccaggaatgttgctgctaagaaggctaatagtaatggtc

fava_c19108_1311	fava_c19108	1311	C/A	aaaactgatgatcttcatatgtttgtttggagttcaagtggttcgccggtttctgatgtgttcggttctcatcttgaatatggaggaacaggaacaggaa[C/A]tggaaatggaactRctgttcaagttcatcatgatcacaaacaaStcaagttgaatctctctcccagaaaagtggagggtgaagaggagtacatagatgag

fava_c19108_1325	fava_c19108	1325	G/A	tcatatgtttgtttggagttcaagtggttcgccggtttctgatgtgttcggttctcatcttgaatatggaggaacaggaacaggaaMtggaaatggaact[G/A]ctgttcaagttcatcatgatcacaaacaaStcaagttgaatctctctcccagaaaagtggagggtgaagaggagtacatagatgagtctaggaatgagga

fava_c19108_1355	fava_c19108	1355	G/C	gccggtttctgatgtgttcggttctcatcttgaatatggaggaacaggaacaggaaMtggaaatggaactRctgttcaagttcatcatgatcacaaacaa[G/C]tcaagttgaatctctctcccagaaaagtggagggtgaagaggagtacatagatgagtctaggaatgaggaaagtggaaaggggaacccaaaagctatgcc

fava_c19109_1221	fava_c19109	1221	C/T	ttgatttttcaacaatggctctacattgtaaaatacaatttttggggtttttaaaatgaagacagaactgaaaaatactggataagcgattgaggttgta[C/T]tcaagaggtgagtctttgcaaattgaggatctaaaatgagcgttttccaagatttgcatacgcaccttaattgatggagggatcttacggaaaatctcag

fava_c19109_899	fava_c19109	899	A/G	tccaagaattttcaccagacgtgtaaattatggtagcaatttgactaacattctcgaaattgaaaacaaccatgagtaccttgtacttgaaattaacttg[A/G]tcataataaccaaacccatgatatgacataacactattctcRccatagggagaagcaaggggtgRagatatttcagatttcaaattcataaaagggttcc

fava_c19109_941	fava_c19109	941	A/G	gactaacattctcgaaattgaaaacaaccatgagtaccttgtacttgaaattaacttgRtcataataaccaaacccatgatatgacataacactattctc[A/G]ccatagggagaagcaaggggtgRagatatttcagatttcaaattcataaaagggttccacaatatgacaacatctcgttcttcgtctaatagacacaaca

fava_c19109_964	fava_c19109	964	G/A	acaaccatgagtaccttgtacttgaaattaacttgRtcataataaccaaacccatgatatgacataacactattctcRccatagggagaagcaaggggtg[G/A]agatatttcagatttcaaattcataaaagggttccacaatatgacaacatctcgttcttcgtctaatagacacaacaaaccattgcatgtgcctaaaata

fava_c19111_568	fava_c19111	568	G/A	atattaaaaatggcaatgaaacttcacttaatctttcttcaatttctgtagcaactctattaagttcatagcctgttctcaaacgtggttttcctttaaa[G/A]caatatttattagctttaatctgttctctaacttcaggaactttaaaagcaatatcaattatattttgagttgggattatttgccaagtKggagtcagtt

fava_c19111_658	fava_c19111	658	G/T	ttcctttaaaRcaatatttattagctttaatctgttctctaacttcaggaactttaaaagcaatatcaattatattttgagttgggattatttgccaagt[G/T]ggagtcagtttgcttagtgcacttaataaagtaatcaataatgtgYtgggttttatatcatctgcaatcttgcacatgggtgctgacaaaatagctccat

fava_c19111_704	fava_c19111	704	T/C	aggaactttaaaagcaatatcaattatattttgagttgggattatttgccaagtKggagtcagtttgcttagtgcacttaataaagtaatcaataatgtg[T/C]tgggttttatatcatctgcaatcttgcacatgggtgctgacaaaatagctccatcccaatattctggctttttccagtgcaaaagcacagccacagctcc

fava_c19111_869	fava_c19111	869	G/C	tggctttttccagtgcaaaagcacagccacagctcctcccattgattctcctaacaaatatctcatcttcttcttattttcacccttttcacatattttg[G/C]tgaaatgaStgaaacaatcatcaatgacatcatcaaaattcataacaagaccagctaatccatctgattttccatgtccttcataatctataccataaac

fava_c19111_878	fava_c19111	878	G/C	ccagtgcaaaagcacagccacagctcctcccattgattctcctaacaaatatctcatcttcttcttattttcacccttttcacatattttgStgaaatga[G/C]tgaaacaatcatcaatgacatcatcaaaattcataacaagaccagctaatccatctgattttccatgtccttcataatctataccataaactgcatatcc

fava_c19117_1173	fava_c19117	1173	T/A	aatgtgcttgatgccatgattgattccgcttatttttctatgaagaatcttaatgttactgatgttgttgttcttgttactgaaactggttggcctagta[T/A]ggctgattctaaagagccttatgctaccaaagataatgcggatacttataattcgaatttgattaagcatgtttttgatcacagcgggactcctttgcat

fava_c19117_1351	fava_c19117	1351	T/C	atcacagcgggactcctttgcatcctgaagctacttctagtgtttttatatatgagctttttaacgaggatttgaggtctccgccggtttctgaggcgaa[T/C]tggggtcttttttatgggaacaccactgctgtttatttgcttcatgtgtctggaattgggacttttttggcgaatgatacgacgaatcagacctactgca

fava_c19117_1585	fava_c19117	1585	C/T	aggctgcgttggattgggcgtgtggaccgggaagggctaattgttcggatattcagcctggtgagagttgctataaacctaacaatgttaagaatcacgc[C/T]tcttatgcttttgatagctattaccagaaagaaggcaagtctccggggagctgtgatttcaaaggagttgctatgatcactaccacagatcccagtcacg

fava_c19117_1747	fava_c19117	1747	T/A	aaggagttgctatgatcactaccacagatcccagtcacgggagctgtgaatttcctggaagcaagaacataagcaacaagacaaaggaagtggtgaattc[T/A]actcaattaagcaatgcaggagagaagttaaggttcagaaccatgaacagcatcaaaataaagacaattaacaacattttgcacattttcttaacatgtc

fava_c19117_655	fava_c19117	655	A/G	aatccttgtccggaaccaaaattcgtgtcatcatcagtgtccctaacaaccagcttctcgctattggttcttccaacaccaccgcagcttcatggatcca[A/G]cgcaacgtcgtcgcttattaccctcaaacattaatcaccggaatctccgtcggtgacgaggttcttaccactgttggttcttccgctcctcttcttcttc

fava_c19121_638	fava_c19121	638	T/G	gcaatgcgagctactttgtcgattacttcaggagcatcgctcaccacaatggttccctctggccgcagtatgcggtcaatttccaccatcaaatcaacaa[T/G]gttacatctgcttttaccagaagctggatcttttataagcgactcaatgctagccacatggatcagatcataagttcgagggtatgttgaaaaaggttca

fava_c19128_1073	fava_c19128	1073	G/A	ccttttttgaaagattttgatgttcaaaaaatccggttcaattgtgctaatttagtacctaaagtgaaccggttcgttggttcaattatctccgaccacc[G/A]agccgacaaaMaccaaaccaacatggatttcgttcatgttctgctctctcttcaagaacctgataaattgtctgaatccgacatgatcgctgtactctgg

fava_c19128_1084	fava_c19128	1084	A/C	agattttgatgttcaaaaaatccggttcaattgtgctaatttagtacctaaagtgaaccggttcgttggttcaattatctccgaccaccRagccgacaaa[A/C]accaaaccaacatggatttcgttcatgttctgctctctcttcaagaacctgataaattgtctgaatccgacatgatcgctgtactctgggaaatgatatt

fava_c19128_1249	fava_c19128	1249	G/A	atccgacatgatcgctgtactctgggaaatgatatttagaggaaccgacacggttgcggttttgatagagtggatactagcgaggatggtgctacatcct[G/A]acgtgcaaaggaaggtacagatggagcttgacgcggtggcgagtggaggtgcatgtggtgttacggaggaggacatggcggcgacggtgtatttacaggc

fava_c19128_1470	fava_c19128	1470	T/G	ggttgcatccaccaggcccactactttcgtgggcccgactggccatcaccgatacgacaattgatgggtatcacgtgccagctgggacgacagctatggt[T/G]aacatgtgggccatagctagggacccggaagtatggggggacccgcttgaatttaagcccgagaggtttatgaatgaagatggtgYtgagttttcggttc

fava_c19128_1556	fava_c19128	1556	C/T	acgacagctatggtKaacatgtgggccatagctagggacccggaagtatggggggacccgcttgaatttaagcccgagaggtttatgaatgaagatggtg[C/T]tgagttttcggttctcgggtcggatcttaggttggctccgttcgggtcgggtcgaagaagttgYcccgggaagaatttgggtttggctactgtgacttat

fava_c19128_1620	fava_c19128	1620	C/T	aatttaagcccgagaggtttatgaatgaagatggtgYtgagttttcggttctcgggtcggatcttaggttggctccgttcgggtcgggtcgaagaagttg[C/T]cccgggaagaatttgggtttggctactgtgacttattgggtggctaagcttttgcatgagtttgaatggaagccgttggatgaaatgaacggtgtggatt

fava_c19128_783	fava_c19128	783	C/T	ggcgaacccttcgaaaaatctcaaccaaccatcttttctcacccatgcaaatcaaatcatctgggccgcaacgtactgaaattgcgactcagatgattga[C/T]ttgttccgaagccgcgggttcggtccggttcgtgatgttttgaaaaaagcatcgctgaataacatgatgtgctctgtttttggacagaggtttaagattg

fava_c19131_281	fava_c19131	281	A/T	aattcaaaattcaatacaagtacaccaaggcacatcacacaaaaataagagttacatcaaatgtcctaaacctatgtgccaatcctcaaacttcatccaa[A/T]ttgtcttcaaacgcttctcttgatcgtattactttcatatgctcttcttgtaaccatttttaagaccaaccatactctatgagacatttcctttcatcta

fava_c19133_138	fava_c19133	138	A/C	tgttttttctataaaaatcttatctttattcatttcagaacattgggttgatttggaaaagcatcaatggaaactgggttgaactcagaatcaccaaaca[A/C]aagaggtgccggtgttgaacccaaagtgggaccttttgttccgaaaactgatcgcaacccaagagatttacgttcatgggccaagagaactggctttgtt

fava_c19133_2881	fava_c19133	2881	C/T	atttcatcaccctttcttcttatcttattgagttcttctatgacctttgcaatacttagatttaggtggggtttgcttttcatggacatgcaagtcatag[C/T]caggctcacaagttcttttgctagatccaatggccattgtcctgccatgtcatcaagaatgttaaccaatgtttctctatccatgtccattgtcatgagc

fava_c19135_1098	fava_c19135	1098	T/C	cttcttctagcaaatccgcacggaaaattttcagttggtcgccacctttccacagtgacaggaggtgtaacgacttttcaggatctctaactgtaacatg[T/C]acagtgcaacctYtttgaagaagagattcaacaagccatgaaccaatataacctgttgctcctgtaacacagtatttggcgYtaggaacactgtttatca

fava_c19135_1111	fava_c19135	1111	C/T	atccgcacggaaaattttcagttggtcgccacctttccacagtgacaggaggtgtaacgacttttcaggatctctaactgtaacatgYacagtgcaacct[C/T]tttgaagaagagattcaacaagccatgaaccaatataacctgttgctcctgtaacacagtatttggcgYtaggaacactgtttatcaccatattcactac

fava_c19135_1180	fava_c19135	1180	C/T	atctctaactgtaacatgYacagtgcaacctYtttgaagaagagattcaacaagccatgaaccaatataacctgttgctcctgtaacacagtatttggcg[C/T]taggaacactgtttatcaccatattcactactttttcctccatttcaattctttcacaaccaccaaaaacaccaactatttgtttattctctcttcttcg

fava_c19135_543	fava_c19135	543	G/C	cattgcatatatcttcaatgtgaactaaagcaattgaccccattcgcgcatttacagccgataatatcttgtagttttccgcttcacctgttagtggtga[G/C]agtagaactttaacacttgcaggtacattcgYagtgaagaaagggcctgcaacagtggttgttataactgaaacaagatcaataccattctcgtttgcga

fava_c19135_57	fava_c19135	57	A/T	aaaagctgtgtttaaaactttgaattaagttaagaagagaccatgtatactatagt[A/T]ttgccttattattttccttatgagaagtctaacaaaaaaaaatacaaaatctcattatattttttgttacatagaagctcaactactgtgttgtattgaa

fava_c19135_575	fava_c19135	575	T/C	attgaccccattcgcgcatttacagccgataatatcttgtagttttccgcttcacctgttagtggtgaSagtagaactttaacacttgcaggtacattcg[T/C]agtgaagaaagggcctgcaacagtggttgttataactgaaacaagatcaataccattctcgtttgcgaatttaaacgccgcttcttctgtgagaagcttY

fava_c19135_675	fava_c19135	675	T/C	Yagtgaagaaagggcctgcaacagtggttgttataactgaaacaagatcaataccattctcgtttgcgaatttaaacgccgcttcttctgtgagaagctt[T/C]gaaagtgcataaacccatccacttgcttgtgtattccacagaatatcagtatgtatttgacaagattcatcaacaatagatttcaactttccattgatat

fava_c19135_930	fava_c19135	930	A/G	tggatttcaagcatgacttgagaagatttacggttcctttgattgcagggtcaattatatttgcttgaacaaaagcctcagtattttctttgtcagtaac[A/G]ttgaattgcattgaagctgcaacatggaacacaccaacacatccttttacggcctcatcgaaacttccttcttctagcaaatccgcacggaaaattttca

fava_c19136_1084	fava_c19136	1084	T/C	tcgactacctcctaagaactcggacggaatgtatcatcgattgaaagattctgaacttatcgttccaactgataaactaaggtttctgaataaagcttgt[T/C]tcgttcgaagtccagaaaaagatatagcctcggatccttggagtttatgcacaattgatcaagtagaagaattcaaagctattatcaaagttattccttt

fava_c19136_1542	fava_c19136	1542	C/T	tacacttggtgacagcggcaatagtcgagactataaggcgaagaagagcaatcggagaaggttacagtaataatcctaacgcggtgttgaacatgtctgc[C/T]atgtggctttttcctcaactYtgcttaggtggtatagctgaagcattcaatgctataggccaaaatgagttctactacaccgaatttccaaagactatgt

fava_c19136_1563	fava_c19136	1563	T/C	tagtcgagactataaggcgaagaagagcaatcggagaaggttacagtaataatcctaacgcggtgttgaacatgtctgcYatgtggctttttcctcaact[T/C]tgcttaggtggtatagctgaagcattcaatgctataggccaaaatgagttctactacaccgaatttccaaagactatgtcgagcatcgcttcgtctcttt

fava_c19136_1693	fava_c19136	1693	A/T	tgctataggccaaaatgagttctactacaccgaatttccaaagactatgtcgagcatcgcttcgtctcttttcggactaggaatggccgttggatacgtg[A/T]tatcttcatttttgttcagcattgtcgaaaggattacctcaaaaggcggaaaagatggttggatttcggataatattaacaagggtcgattcgataagta

fava_c19137_1922	fava_c19137	1922	T/C	tctgaaaatcccatgctttcaagtttgtgtggaactctcttccccgaaaacagccaatctgtgtggagtggtccgcactcgctaatacatcaaagaaatt[T/C]ggctcgcagtagttcaatccacgcggctattaggtcgttcatgaagtagttgtcttactttaatggattcactacttctagttaagtctagatttttaac

fava_c19137_612	fava_c19137	612	G/A	actcgtctgatgctccttcgcaaagtgatactgataggagtagtcagcaacatgggctggagagacatagtgtgaatggcgatagaattgaagatagtgt[G/A]agttcgttgcctcatattaagtttggtgcttcgtcaaagaaagctggtagcataaagattaagtcttcaaagggtttgggattaaattctgaccagacaa

fava_c19140_248	fava_c19140	248	G/A	gttcacaagtttgttgaaacaaaattatcagaaacagttcatcatccaaatcatccatcataagacagcaactataacctgcttaggttcggcggcgcag[G/A]tattatgaaacccattttcaggaacaggctgctcttgctgctgctgctgatcagcttcaatcttgaaaggtttacagcaattgatgagtaaattgggaac

fava_c19141_1185	fava_c19141	1185	G/A	cggaaacaagaggcttatgcctttcagaagagcaaactgtcaccagtgtacatagatggtcaaaaataggtggccatcgattaattgggatgcgaggtca[G/A]cctcaaaagttgattcgaaaatgtgaagttacagctattcttgttctctatggtcttccgaggttactcacaggtgctatcctcgcgcacgagttgatgc

fava_c19141_1312	fava_c19141	1312	T/C	agttacagctattcttgttctctatggtcttccgaggttactcacaggtgctatcctcgcgcacgagttgatgcacggttggttacgccttaaaggttac[T/C]gtaaccttgatccagcagtagaggaaggtatttgtcaggttctttcttacatgtggcttgacgcggaagttatgtcaggttctagaaccatgccatcgat

fava_c19141_1503	fava_c19141	1503	A/G	tgccatcgatatcagcggcttcttcctcctcctcatcctcatcctcatcctcctcctactcgtcaaagaaaggtgtgaaatccaaagtcgaaaataaatt[A/G]ggtgagtttttcatgaaccagattgctaatgattcttccccagcttatggtggaggctttagatcggctaatgcagcggttaataaatacggcttacgtt

fava_c19141_702	fava_c19141	702	G/A	cagattgctttcggtgtcactcttgccatcatcctattaccgagcgtgagttttctttgtcagggaagcatccatatcacaaatattgttttaaagagtt[G/A]agccatcctaaatgtgaagtttgtcatcattatatacctataaatggttctggtttgattgagtataggtgtcacccttattggaaccaaaagtactgtc

fava_c19143_1094	fava_c19143	1094	G/A	aattgcattggctacaaaatcttcaaccctttcttccatctcataagtattacacacaaatgcaagattctgatttgaactcaactcaaaacataacatc[G/A]atatctgtcagatgaattaggttatgcggacttttgagttttattatatgagttgaaaaatttgtttagcttaattaattatatcatatagttgtataaa

fava_c19143_154	fava_c19143	154	A/T	atatgcctaccaacattctaaccatgaagacaaacaatgattttgaacttgattgcaagaaaattcatgattctttaagtggtttaagccaactaattcc[A/T]tcacttgcttMcttaaccatccttgaaagaaaacaactaagggaaacatataaggaagtgtatggtgaagacttgataaaccaactacaaaaatatgatg

fava_c19143_165	fava_c19143	165	A/C	aacattctaaccatgaagacaaacaatgattttgaacttgattgcaagaaaattcatgattctttaagtggtttaagccaactaattccWtcacttgctt[A/C]cttaaccatccttgaaagaaaacaactaagggaaacatataaggaagtgtatggtgaagacttgataaaccaactacaaaaatatgatgaagatgatttc

fava_c19143_493	fava_c19143	493	G/T	taggtagaaaatcaagtcatgttggtctcatcacacaagcatatcataaaatgtttagaaggcaactggatcaagatattatgaatttggatcctccaca[G/T]ccatttcaaaagattcttatggcattgtcagcatcacataaagctcaccaagtagatataagccatcatatatcaaagtgtgatgcaaggaggctatatg

fava_c19143_941	fava_c19143	941	A/C	gaaaaagggcataggaactttggcaagaacacttgtaagcagggcagatatagacatggatgagattagaagtgttttcaaggaaaagtatgagaaagaa[A/C]ttggtgatgttatatttgaaagcattccatgtggggactatagagactttttaattgcattggctacaaaatcttcaaccctttcttccatctcataagt

fava_c19145_1160	fava_c19145	1160	C/T	tcaatggagaggattgcttcatacttaagctctgtactgatcctgaaacattgaaggctcggagcgagggtccagctgagattataagacatgtcttatt[C/T]ggctactttagccagaaaactggactactggttcacattgaggattctcatctgacccgcatccaatctaatggaggtgatgcggtttattgggaaacca

fava_c19145_1641	fava_c19145	1641	C/G	tcggaccaaagttgttgcactggagaaatcacacgagtgcagcattgacaagatgatgtggaagatggaaatctaatgcaattgttcatagcagcagtca[C/G]tagattttgactactaacgtttagattttagagtaggggtttcatttggttctttgtggaagtttttgtttatgcctctatatataatctgtaaatagag

fava_c19145_650	fava_c19145	650	A/G	tgagactcctgctcggtgtaatggctgctccattggctccgtttcatgtttgctccactgacccgcttcctcatctcagcatcaaggatactccaatcga[A/G]acatcgtctgctcagtacatattgcagcagtacatagcggcatctggggggcagaaattgcagagttcgatacgaaatgcatatgctatggggaaggtta

fava_c19145_818	fava_c19145	818	G/A	cgatacgaaatgcatatgctatggggaaggttaaaatggtagcgtctgaatttgaaactgcgactagggtggttaagaatcggaatgcttctaggtgtgc[G/A]gagtctggtggatttgtgctatggcagatgaatcctgatatgtggtatgtggagctttcggttgggggaagcaaggttcatgctggatgcaatgggaagM

fava_c19145_918	fava_c19145	918	C/A	Rgagtctggtggatttgtgctatggcagatgaatcctgatatgtggtatgtggagctttcggttgggggaagcaaggttcatgctggatgcaatgggaag[C/A]ttgtttggaggcatactccttggcttggtgctcacacggcaaagggacctgtgaggcctttgcgccgtgcacttcagggtcttgatcctagaaccactgc

fava_c19146_1335	fava_c19146	1335	T/C	agcagcttggtgtaatgagacaagcttggagacctcagaggggacttcccgaaagctctgtttccatactccgtgcgtggctcttcgagcatttccttca[T/C]ccttatcctaaggactcggagaaaattatgctggcaagacaaacaggcttgacccgaaaccaggtggcaaattggttcattaatgcgagggtgcgtctat

fava_c19146_1667	fava_c19146	1667	T/C	gatgagtctcatgacaagttaataaccattgatagcgctcaacaacaaggtcaaatcgcagggttgaagctggatcatgcatcctcttctgccaccagag[T/C]gacagaattggaaagaggaattcaaagtaatgatcattggacgaatgtgatggattctagaattgggaaaatgcagggtgatcaacaaaggtttaacatg

fava_c19146_1779	fava_c19146	1779	C/G	aaagaggaattcaaagtaatgatcattggacgaatgtgatggattctagaattgggaaaatgcagggtgatcaacaaaggtttaacatgaacaatagtcc[C/G]tattcgaacgcacctatctcgataaaccaaaatggtgatggYtgcttaatggattcaactcctactacttatgatgacttatcagagttgagtaactttg

fava_c19146_1821	fava_c19146	1821	T/C	attctagaattgggaaaatgcagggtgatcaacaaaggtttaacatgaacaatagtccStattcgaacgcacctatctcgataaaccaaaatggtgatgg[T/C]tgcttaatggattcaactcctactacttatgatgacttatcagagttgagtaactttgctgttggtggaaatgtctcacttgcattggagttgaggaact

fava_c19146_276	fava_c19146	276	T/C	ggtattcattaatcttgcttacaatgtatccgaaccaagcgttttcgtcgggatcttacgctgagatgatttcggggaacactttgttacctcataacta[T/C]agtgaatctgaaggaggacaaaatgagttgaagtttatcgcatctatggacgactcaatgaatatgcagtctattggtcaagggcattcgaatgctgcta

fava_c19146_390	fava_c19146	390	G/A	gaggacaaaatgagttgaagtttatcgcatctatggacgactcaatgaatatgcagtctattggtcaagggcattcgaatgctgctacgagcgatccaac[G/A]acgcaatttggactcgttgagagtgagcaaaatgtacaatgtcaaggcttgtctcttagccttggaacaatgatgccttcctttcaatatcagtatcccg

fava_c19147_456	fava_c19147	456	G/A	tccaaatcgtcatctgcttttggtaagagacctgcaacggcgaggaataaacgcgcttgaaacagttgttattcgatcagttttgagagaatggaaagag[G/A]agaaRaattgtaggtgtgaagggatcgatgtaggaggggatcggatcgatggaaatatcccctcctctatttgccttttggtattttttagtgttgagtt

fava_c19147_461	fava_c19147	461	G/A	atcgtcatctgcttttggtaagagacctgcaacggcgaggaataaacgcgcttgaaacagttgttattcgatcagttttgagagaatggaaagagRagaa[G/A]aattgtaggtgtgaagggatcgatgtaggaggggatcggatcgatggaaatatcccctcctctatttgccttttggtattttttagtgttgagtttgtat

fava_c19150_1207	fava_c19150	1207	C/T	gtagtccttagacaaatgtacaggctttgttgtagtgagaatgagattgactataaggcaaattcacaagaggtagacttcgatgataaaccttccaggg[C/T]cgtgcctgattctctgtttgaggaggatggtggcgcacaatgcgtcaagagaaaaggaattcgcggatatttctggaatagtcttaaacatattctagga

fava_c19150_1469	fava_c19150	1469	G/A	ttaggcatgctagacttcaagagtttcttagatcgagtgaatcgataaggctaaacttgaatgcctcaaatatgaaactatcctcttatagagcatggcc[G/A]aatatgcatgatagtaggtatgcactttacaagttacctttgcgagttcccggtgcYgaccaagaatatgctggattatggggaggtgcttttggttggc

fava_c19150_1526	fava_c19150	1526	T/C	tgaatgcctcaaatatgaaactatcctcttatagagcatggccRaatatgcatgatagtaggtatgcactttacaagttacctttgcgagttcccggtgc[T/C]gaccaagaatatgctggattatggggaggtgcttttggttggcctccagggaagccttccgaagacaaatctggaaaggctttgttttttcttctcctct

fava_c19150_1748	fava_c19150	1748	G/C	aacaacttctcattgcaaccaaaatattggaaggcacacactatgtcttgcatcctaatggttcagcaatgtttatagccaacatcaacgaatcttcatc[G/C]gaaccttttccttggaacactgatgcagattcattgccggtgaatatcaagcatgctttcaccggagagggtattgcaaatggttacgggtttagatatc

fava_c19150_2108	fava_c19150	2108	T/C	catatctgacaaagtcctactcgaatgtgtttgccggcattcccacctcttccaattctttgtcttcgccaaggaaaggacgatgctagtatattaagct[T/C]tgaaggYgcgaaattgtcattgctggtgttgctttgcttctggctgagcggaaaacacttcctgccgtggtgttttctagttgttgcttattggaaccaa

fava_c19150_2115	fava_c19150	2115	C/T	gacaaagtcctactcgaatgtgtttgccggcattcccacctcttccaattctttgtcttcgccaaggaaaggacgatgctagtatattaagctYtgaagg[C/T]gcgaaattgtcattgctggtgttgctttgcttctggctgagcggaaaacacttcctgccgtggtgttttctagttgttgcttattggaaccaaagacaga

fava_c19150_2265	fava_c19150	2265	G/T	acttcctgccgtggtgttttctagttgttgcttattggaaccaaagacagagagaggatacacgagtctatgcgaagtaatgtattatgaagtaaatttc[G/T]tgatgaattgcggtatcataatactctttgttgtagtatatatgggagaaagtatacaataaagttgttctctacactatgtataaattggcatgaatgt

fava_c19150_253	fava_c19150	253	A/T	tttttttttgtctttcatgtttcgtaaaaccaataattgaacctccaaaaccactttcacacacagattcagactattccactttcaacaaggtggatca[A/T]Yatgttacctgagatatctgattcaaacatgcttttatatttacctgatgatgtttttgctataatttctcgatctttttcacctagagatatatgtaat

fava_c19150_254	fava_c19150	254	C/T	ttttttttgtctttcatgtttcgtaaaaccaataattgaacctccaaaaccactttcacacacagattcagactattccactttcaacaaggtggatcaW[C/T]atgttacctgagatatctgattcaaacatgcttttatatttacctgatgatgtttttgctataatttctcgatctttttcacctagagatatatgtaatc

fava_c19150_606	fava_c19150	606	A/G	gaaagttcaacctttgattgggatatgggttcatcagaatccggagctagggaacgttgtgtatgtgatgcctggttttgtttctgttgttggttgtagg[A/G]ttattcctcaagaggttgggccgttaggtatggaggacggtccgattctatgggcgtctgtttttgaagttgttgctgattttgatggttcgacgatgtt

fava_c19150_729	fava_c19150	729	G/A	gttaggtatggaggacggtccgattctatgggcgtctgtttttgaagttgttgctgattttgatggttcgacgatgtttttccttcatggaagggagaag[G/A]gtgttgattatgtttatcctggtttggctaagtcgatcgataaattttgtaatgtattgttgctcgaggttgagccggagaaatataaaaaagatggttc

fava_c19153_2167	fava_c19153	2167	A/G	ttgtggaggagggaatgatttctggagaccattacaatgtagcgttgcaggcttttgaagaatcacttaataaggttcatgacatagaacagaggcatga[A/G]taacaatcaatgtactttagaaaactagtgttggtctaatatgttatatgctatgtatatatttatttctataacttctatagctaaatatatctctagt

fava_c19153_331	fava_c19153	331	T/A	aagatgaagatgatgtactaattggattagattgtcaaaatgatatttctgaaggaagacaggagtttctcgcacccgctgttggaatggaattcgagtc[T/A]tacgatgatgcttacaattattacatttgttatgccaaagaggtgggattttgtgtSagggtgaagaattcatggttcaagagaaatagcaaagaaaaat

fava_c19153_388	fava_c19153	388	G/C	gacaggagtttctcgcacccgctgttggaatggaattcgagtcWtacgatgatgcttacaattattacatttgttatgccaaagaggtgggattttgtgt[G/C]agggtgaagaattcatggttcaagagaaatagcaaagaaaaatacggtgcagtgctttgttgtagcagccaaggtttcaagagaattaaagatgttaata

fava_c19153_637	fava_c19153	637	T/C	ggttagtggagtctcggagatggaggatacgcgaggtaactcttcaacataaccacgtcttaggcgctaagattcataaatcagttagaaagataggaag[T/C]ggaactaaaaacaagttgttacctagttctgatgctcaRgtgcaaacaattaagttgtatcgcgcacttgtgatagaYgcaggtgatggtaattcgaatt

fava_c19153_676	fava_c19153	676	G/A	ctcttcaacataaccacgtcttaggcgctaagattcataaatcagttagaaagataggaagYggaactaaaaacaagttgttacctagttctgatgctca[G/A]gtgcaaacaattaagttgtatcgcgcacttgtgatagaYgcaggtgatggtaattcgaattccaatgcaagagacgacagagctttctctgaatattcta

fava_c19153_715	fava_c19153	715	T/C	aatcagttagaaagataggaagYggaactaaaaacaagttgttacctagttctgatgctcaRgtgcaaacaattaagttgtatcgcgcacttgtgataga[T/C]gcaggtgatggtaattcgaattccaatgcaagagacgacagagctttctctgaatattctaatgagttgaacctaagaaaaggtgacacgcaagccgttt

fava_c19153_851	fava_c19153	851	C/T	cgacagagctttctctgaatattctaatgagttgaacctaagaaaaggtgacacgcaagccgtttataattttatgtgccgtatgcaactgactaatcca[C/T]acttcttttatttgatggatttcaatgatgaagggcatttgaggaatgtattatgggttgatgcaagatctagggctgcatgtggttattttagtgatgt

fava_c19160_1228	fava_c19160	1228	C/T	gaacatttgcagctctgggaaactacaaagtagatggaaataaggaaatgatggcaatcgggtttatgaatgtggttggctctttcacttcctgctatgt[C/T]acaacaggtgctttctctcgctcagctgttaataacaatgctggagcaaaaacagcagcttcaaacatagtaatgtctgtgacagtgatggtKacactcc

fava_c19160_1321	fava_c19160	1321	G/T	gctatgtYacaacaggtgctttctctcgctcagctgttaataacaatgctggagcaaaaacagcagcttcaaacatagtaatgtctgtgacagtgatggt[G/T]acactccttttgcttatgccattgttccagtacacacctaatgttgtattgggtgcaatcatagtcactgcggtgattggccttgttgatatccctgctg

fava_c19160_172	fava_c19160	172	A/C	ctctcttcttctcttcaatggattctaataccttacattcttcacaatcacattgtgtacaattggaaattcaccaagttgttccaccacctcacaaaac[A/C]actttacacaaactcaaacttagactcaaagaaacttttttccctgatgatcctatgcgccaattcaaaggaaaatctcctaagatRaaacttatcttag

fava_c19160_1778	fava_c19160	1778	T/C	catcaatttcgcaaacatcacatatctcaatgacaggacaatgcgatggattgaagaggaagaggaagataacgaaaaggaacactcgagccttcgattt[T/C]taatcctagatatgtcagctgtgagcgccattgacacaagcggaatctcgcttttcaaggaattaaaggcaacaatggaaaagaaaggcattgagcttgt

fava_c19160_1948	fava_c19160	1948	G/A	caacaatggaaaagaaaggcattgagcttgtgttggtgaatcctctggctgaggtcatagaaaagttgaaaaaagcagaccacgctaataacttcattcg[G/A]gcagacagccttttcttgacagttggagaggctgtggcttcactttcatcaacaatgaagagtcaaacaaccatgacagaagaaacacaaaacagtgcca

fava_c19160_2049	fava_c19160	2049	C/T	gcagacagccttttcttgacagttggagaggctgtggcttcactttcatcaacaatgaagagtcaaacaaccatgacagaagaaacacaaaacagtgcca[C/T]gtaactgattagttaatcaagattgcatgataaatatgccgagataaaaaaaaagtagacaccacagaaacagaccgcatagaatttgtatcaaatcagR

fava_c19160_259	fava_c19160	259	A/G	cacctcacaaaacMactttacacaaactcaaacttagactcaaagaaacttttttccctgatgatcctatgcgccaattcaaaggaaaatctcctaagat[A/G]aaacttatcttaggagcgaaatacatgttccctattcttcaatggggttcgaattataacgtgaaactcttcaaatccgatcttgtttccggcctcacta

fava_c19160_916	fava_c19160	916	G/A	catggcaaacaatagtgatgggaatttgcttcttggtgatactactcatagcaagacacgttagcataaggaaaccaaagctattctgggtctctgctgg[G/A]gctcctcttatgtgtgtcatcatctctactcttctggcttttgcaatcaaggctcaaaatcatggcatcagtgtaattggaaaattacaagcaggaataa

fava_c19164_1103	fava_c19164	1103	T/C	atgcgatacgcgcacttatatttctctttgagtgtgagaatRctaatggaaggtatatttgttcctcggatcaaatatcatttcatcaaatgtatgaatt[T/C]cttcatcaaaaatatccagaatatcacataacaataccaatttcaaaggagacaaaaaatgataatgaaaagtttagtgttttatcatcaagaaagctct

fava_c19164_413	fava_c19164	413	C/T	gcacagtttgtgtgacaggagcaacaggttatatagcatcatggttgattatgaagcttcttcaacatggttacaatgttcatgctactgttaggtcaca[C/T]cattttaaagaaaatacaaacttggattatcttacaagcttaccaaaagcaacaatgaagctaaaaatcttccatgctaatcttgacgattcaactagtt

fava_c19164_863	fava_c19164	863	G/T	gtagaagtagtaatcttgttggatcttcttatttggtgtctaaaattttgactgaaaagagtattttagagtttggaaaagtgaatggattggaagttgt[G/T]actttgattcttcctttggttgttgggccctttatttgtcctactataccatcatctgtttatatagcactagccatgatatttggtgatcatagtcgat

fava_c19166_2361	fava_c19166	2361	G/A	cactacaagtattatcttgtttctgcttatttaacttcttgttctgattagaattgtgtttcctgtcatgattttcaggatcgggaatatttagaccata[G/A]tccgcttcatcaatctcctcgagtgaaagaaaacctccttcgagttcattggagccagtgaagagtgaaaaagagttgtcgtcttgttcatcgtgtKgag

fava_c19166_2458	fava_c19166	2458	T/G	ataRtccgcttcatcaatctcctcgagtgaaagaaaacctccttcgagttcattggagccagtgaagagtgaaaaagagttgtcgtcttgttcatcgtgt[T/G]gagggagagatgagttccacggaagagattccaggcggtcgagctcagggtcctgagtttttaggcttcggttcttccttttggtctttctcatcgaaga

fava_c19168_1009	fava_c19168	1009	C/T	ttgctggtgctggactacaggcttttcagcaatttataggtattaacacagtgatgtaYtacagtccaacaattgtccaaatggctggctttcactctaa[C/T]gagttggctcttcaactctctctcatagttgccggcttgaatgctgctggaaccgttcttggcatttaccttattgatcatgcagggcggaaaaaattgg

fava_c19168_1417	fava_c19168	1417	T/C	gaggaatgtgcggaggcatgtcagctactgtgaattgggtttcaaatttgattgtggtacaatcatttctttctattgcagatgctgccgggactggtcc[T/C]actttcttgcttctcgcagttatagccgtggttgcatttctgtttgtggttttgtttgttccagaaaccaaaggattgacatttgatgaagtggaactgt

fava_c19168_1574	fava_c19168	1574	G/T	tgttccagaaaccaaaggattgacatttgatgaagtggaactgttgtggagggagagagcatggggtaaaaatcttgaaactaaaacccttcttgagcgt[G/T]gaactcaatcatcctagaagctacatccatttaaatatgttgttattctatgaataaaactcagctaataaaggtcgaattgttgcaaatatgctatgag

fava_c19168_784	fava_c19168	784	C/T	cagctcagttttttctcatgctcttgctgcctgaatctcccagatggctcttcataaatcgtagagaaaatgacgccattattgtgcttggtaagatata[C/T]ggctccgatcgtttagaggatgaagttgttttgcttacagctcagtctgaRcgagaccgccKaaaaagagatgatgtcacagtcctgcatgtttttaaat

fava_c19168_835	fava_c19168	835	A/G	tcataaatcgtagagaaaatgacgccattattgtgcttggtaagatataYggctccgatcgtttagaggatgaagttgttttgcttacagctcagtctga[A/G]cgagaccgccKaaaaagagatgatgtcacagtcctgcatgtttttaaatcaaaagagatcagacttgcattccttgctggtgctggactacaggcttttc

fava_c19168_846	fava_c19168	846	T/G	agagaaaatgacgccattattgtgcttggtaagatataYggctccgatcgtttagaggatgaagttgttttgcttacagctcagtctgaRcgagaccgcc[T/G]aaaaagagatgatgtcacagtcctgcatgtttttaaatcaaaagagatcagacttgcattccttgctggtgctggactacaggcttttcagcaatttata

fava_c19168_967	fava_c19168	967	T/C	tcctgcatgtttttaaatcaaaagagatcagacttgcattccttgctggtgctggactacaggcttttcagcaatttataggtattaacacagtgatgta[T/C]tacagtccaacaattgtccaaatggctggctttcactctaaYgagttggctcttcaactctctctcatagttgccggcttgaatgctgctggaaccgttc

fava_c19169_1361	fava_c19169	1361	C/T	tgtcaaaaagatctctccaacccatatcatttggaaggaatctattaaaagaatgtcgcatcattttgtctgtgtaatgaaagtccgagttggtaataag[C/T]acaagcttttttccagcctccttctgatccaaaagtgccaaaggtaattctggatcaggctccacaaactgttcRggcttagacatgatctcgctcttaa

fava_c19169_1436	fava_c19169	1436	A/G	aatgaaagtccgagttggtaataagYacaagcttttttccagcctccttctgatccaaaagtgccaaaggtaattctggatcaggctccacaaactgttc[A/G]ggcttagacatgatctcgctcttaagacgaccttcaacatgagcccagaaaagagcttttccaacagccttataaagacctttataatcaagcgggccaa

fava_c19169_1808	fava_c19169	1808	G/T	catgcatggctctttttacataaccaaatcgatcagctttaaccaagttgcctctctctttatctatgacaaggcctctaataaccaggtcaggatcaaa[G/T]gcaagtccgtcaacagggaagcccatattcttaaggttttccatacagtagtcataagcccgtccttcccaagccttcacattataatggataagggtat

fava_c19169_1952	fava_c19169	1952	C/T	tacagtagtcataagcccgtccttcccaagccttcacattataatggataagggtatagtccatgtcatatccaatggcactaattgaccgcagattcaa[C/T]gtacgggtgcagaatattccacggggcgaatttctggatgaagaggggctcggaatgcccaagtctctaagcaaatcttctgcctctctagcctcagatc

fava_c19169_316	fava_c19169	316	G/C	aattacgaattataaaactcaaatgataccaatttcagtcatttggaaacaaaattcctagtctctagtacactatttatttccgtttttgcttgggtct[G/C]ttctctcctctaaccaggccaaagcaaaagtgctacttcagttttttgaggacttgatatcagcttaaataataYaaggtatcatcatcatcatcatcat

fava_c19169_391	fava_c19169	391	C/T	tttatttccgtttttgcttgggtctSttctctcctctaaccaggccaaagcaaaagtgctacttcagttttttgaggacttgatatcagcttaaataata[C/T]aaggtatcatcatcatcatcatcatcacaatgtttaaatattgttacaatgatacatctacttataatgtcaaatgcagattctttatttctgatattta

fava_c19169_608	fava_c19169	608	G/T	tccattgtctctaggaagaagcttcattattgatttgagaacaatagtgcgtgtatgagtcgtgtgcgaggttctgttcctgagaacggaaatacatgaa[G/T]ggtgtataatataaaaagtttgacaccctagacgtgtaaatatcagcatatttctcaatttgtctcatcaagtggctcttatcccacaatccagcacgag

fava_c19174_572	fava_c19174	572	T/C	ttgatgctcgttttgatcgctctgatgacatgtttcagattagagctgcagagctaaggaagatattggtggagcttgggccggcatatatcaaaattgc[T/C]caggctatatcatctcgttccgatttgataccaccatcatatctagatgagctctcactgctgcaagatcgaataagtccattctccactgaagttgctt

fava_c19174_722	fava_c19174	722	A/T	agctctcactgctgcaagatcgaataagtccattctccactgaagttgctttcaacatgattgaacaagaacttggtttatccttagttgaaattttctc[A/T]gagatttcacctgagcctgttgctgctgcttccctcggtcaggtttatcaagctaggcttcgcagaacagggcaggttgttgctgttaaagtgcaaagac

fava_c19178_1069	fava_c19178	1069	T/A	ctacaccagaacccattccaattcccacaccagaacccgttccaattcccaccccacctaggttcaacccaagattctttcttgctgccctactttcaca[T/A]gttatagcactcattgcaacaagcaaagctacaacacctaaaatcctcatgaaagacattgtgataaaactcaaagatatcgatcagcacaaggttagaa

fava_c19178_250	fava_c19178	250	A/G	acatacatatatgaattacactatgtctattgaaccattgagttatatttgttgcttaatgaagttttatttatttatttaatacatatgagagattgta[A/G]tttcagtttcctgatccagcatgKgaacctgcatatgaaccagctcctgcttcagaatacccagacccagatccagcatgagagcctgcatatgaaccgg

fava_c19178_274	fava_c19178	274	G/T	gtctattgaaccattgagttatatttgttgcttaatgaagttttatttatttatttaatacatatgagagattgtaRtttcagtttcctgatccagcatg[G/T]gaacctgcatatgaaccagctcctgcttcagaatacccagacccagatccagcatgagagcctgcatatgaaccggcttcggaacctgcttcagaaccgg

fava_c19180_1153	fava_c19180	1153	A/T	gtaggcctttcctaacagttctcattcgatgcttatcttcgggattatcatcaggactgctgctgtcattgttcaaagatccagatacggttggattatt[A/T]aagttgccagatatatcattaagttccccgtgaatctctgtRtcSaggcgtcgatccttcccttttcttggctcccaggtttgggaaacctcacttcctg

fava_c19180_1195	fava_c19180	1195	A/G	gattatcatcaggactgctgctgtcattgttcaaagatccagatacggttggattattWaagttgccagatatatcattaagttccccgtgaatctctgt[A/G]tcSaggcgtcgatccttcccttttcttggctcccaggtttgggaaacctcacttcctgggtggtgaacccaaactcctgtcttcttttgccctttaattt

fava_c19180_1198	fava_c19180	1198	G/C	tatcatcaggactgctgctgtcattgttcaaagatccagatacggttggattattWaagttgccagatatatcattaagttccccgtgaatctctgtRtc[G/C]aggcgtcgatccttcccttttcttggctcccaggtttgggaaacctcacttcctgggtggtgaacccaaactcctgtcttcttttgccctttaatttcta

fava_c19180_1456	fava_c19180	1456	A/C	aacttcttttggtggtctcatttacaaaagaatctattctatcttgaatgtctattggttcctggtcgtgtgtaggatcagcttccttttccttatcttc[A/C]aaaactgttattcgtaaatgcaatctccccattttaacattctttagtggcagccacttatcatatttttgtccatccctaaaatcattgatattcacag

fava_c19180_1930	fava_c19180	1930	A/G	taaaccaaggctcttgctctggtgatacaaatttctcaacatcaacaaccagcatattaggctcaacaagggtctgttcaaaagcaatggacagaagctt[A/G]tctagccagccagcaattcctggaagttctgtcacgtcaagcccgtgagtaaaaataggtttgacagtcatctgaaaatatggaggctcgacaaagcata

fava_c19180_2152	fava_c19180	2152	T/C	gccatgtcttgagaaactttacccccaccaagaccttcccttcaacatgcattcccgttatatgcagctttgttgacattccaaagcccaatctttttct[T/C]agtttcacagcaagtattgcactcatatcatccgctgtgagaaaattcattcctaactccataaccaagtggtcatcatcactctggcgaagtaccctga

fava_c19180_297	fava_c19180	297	A/C	agtacaacaccaacagcactcgtggcataaacgtgttttatgaaaacgaaacctatcactactatacaacaaaaactactctagatgtaaccagagtata[A/C]cRtaacatataataacaaaaatattaactYgaaaaactgtacagtatataaacgtgtctgacaactatatttcggtgtcgtgtttgatatctgtgtgctt

fava_c19180_299	fava_c19180	299	A/G	tacaacaccaacagcactcgtggcataaacgtgttttatgaaaacgaaacctatcactactatacaacaaaaactactctagatgtaaccagagtataMc[A/G]taacatataataacaaaaatattaactYgaaaaactgtacagtatataaacgtgtctgacaactatatttcggtgtcgtgtttgatatctgtgtgctttg

fava_c19180_327	fava_c19180	327	T/C	acgtgttttatgaaaacgaaacctatcactactatacaacaaaaactactctagatgtaaccagagtataMcRtaacatataataacaaaaatattaact[T/C]gaaaaactgtacagtatataaacgtgtctgacaactatatttcggtgtcgtgtttgatatctgtgtgctttgtatagaWtgcggccgttgacttgattaa

fava_c19180_406	fava_c19180	406	A/T	ataataacaaaaatattaactYgaaaaactgtacagtatataaacgtgtctgacaactatatttcggtgtcgtgtttgatatctgtgtgctttgtataga[A/T]tgcggccgttgacttgattaaacattcttcacttcagcaacactgctaacaacccttttatcagccaccatttcctcattttcaccagacttaacaaatt

fava_c19180_602	fava_c19180	602	T/G	aaattcttcattagacctttcaggggagcatgccttttcaggcttggtgttggatggaacactatacacctcactcaaattggatttgggggaaccactt[T/G]tgggagcaatggcctgggaataaactggagttcttattacatctatttgtggcgaaagaaattcatcattagaagagtcagattcattttctctttctgg

fava_c19180_739	fava_c19180	739	C/T	ttacatctatttgtggcgaaagaaattcatcattagaagagtcagattcattttctctttctggaagggttgccactgaatcacctttagacttccttgc[C/T]ttaccagaaagaacgtgttttaagctacgagcYgatttttctgcattttttaaaacatgctttgccatgtccttgacatctctctttgccgggctttcag

fava_c19180_772	fava_c19180	772	C/T	tagaagagtcagattcattttctctttctggaagggttgccactgaatcacctttagacttccttgcYttaccagaaagaacgtgttttaagctacgagc[C/T]gatttttctgcattttttaaaacatgctttgccatgtccttgacatctctctttgccgggctttcaggaccgcaaccactcccttcagtcgatccacctt

fava_c19180_888	fava_c19180	888	T/C	tttaaaacatgctttgccatgtccttgacatctctctttgccgggctttcaggaccgcaaccactcccttcagtcgatccaccttctacctgaatcttac[T/C]ggtcggaaagccagcaatattatcatccataacaaacttcacaccgacccctttagaggcatttactgacctaatgttatcatgtggagattgaacgtcc

fava_c19181_470	fava_c19181	470	A/G	cttgagaatcctcaaagtgagctccgaggctgcggattatgcagatgttccttgagagcaagtacaaattcagcttgctgagaaattgtaaggagacgac[A/G]aaattgatgcaagaattcttggcgaagattatcagcctctttaaagaagcWtagttgYtgaggtacatacgtcaattgaaactcttcgccatttgctgtt

fava_c19181_521	fava_c19181	521	A/T	ttgagagcaagtacaaattcagcttgctgagaaattgtaaggagacgacRaaattgatgcaagaattcttggcgaagattatcagcctctttaaagaagc[A/T]tagttgYtgaggtacatacgtcaattgaaactcttcgccatttgctgttgttgatttatcaaaattttgcttgagttttaccatgccttgtgcaagagca

fava_c19181_528	fava_c19181	528	T/C	caagtacaaattcagcttgctgagaaattgtaaggagacgacRaaattgatgcaagaattcttggcgaagattatcagcctctttaaagaagcWtagttg[T/C]tgaggtacatacgtcaattgaaactcttcgccatttgctgttgttgatttatcaaaattttgcttgagttttaccatgccttgtgcaagagcatattctg

fava_c19181_797	fava_c19181	797	T/C	tgaggcagaatcacctgaagaatttgagaaggacgaaagcctccaatccaccattgattgctttcacatgttatggccgaaccaacattcaagacatcta[T/C]ataatgtttcatgattgtgtttataactaactgtagcttgtcatcctcaataacatggttgtgatcatttagagctttatgtatcacagaaataagcttg

fava_c19181_993	fava_c19181	993	T/G	gcttgttctgttcttcagacgaaagtgcatactttttcacaggaattatgtttccattactcttgaagacttctccttgagcatttgtctctttcatggg[T/G]ttgtttacaagtagccaaaacttgtttgaattggaactcaagatttcctttgaatagagaggcaatcacaagcacttaatttcataatgcaccaat

fava_c19182_707	fava_c19182	707	A/T	ttgtccataatggttttaccacaccagagatatggtgcacaatgatgtcaacatcctcttcctgagtaagggcttgaatttcccttgttaaccaagtgcg[A/T]agccagttatttggctgattatacttacgagatttccaatagagtgatatattaaatatgtcttctaagaaaccttgttgagtataatagcattgtaacc

fava_c19182_996	fava_c19182	996	T/C	aatgatagaaaagttatctgtcttacaaagggggcattttacggaagaaggtggagtttggtgcttggaggaaacaactttggtccagcggagaatgcaa[T/C]tgaagcaaaatttatgcaaacaatggtcgagataggaatcttgagttaagggtccaaggcagattgggcaaatcgaatcgtgtgaattgctggccgattc

fava_c19183_1009	fava_c19183	1009	C/G	gaaaatgttgcaggtacaactttagacaagatagctcccactaaagacctgcctttgtcctcagaaagtctataagctgaaatcaccgcatcgtctccca[C/G]agccactgcacaataataacgttgactcgactgatattccatcagcggtggaggcattatgccagtaatggccgcatcagcgcaagtaccatacttacca

fava_c19183_1352	fava_c19183	1352	T/A	aacgagcaatcaccccgggcataatgagacaaaactcttctgatgaactatcttgaatcaagtctttctttgttccacgaactctcaacttcaaaactcg[T/A]ccaggataaatcatctgcttatgaatcaRttcggcgcggagggagtaaatgagcaaatatccagaggaggtaccagcgacgatgacgcggatgtcgtcaa

fava_c19183_1381	fava_c19183	1381	G/A	caaaactcttctgatgaactatcttgaatcaagtctttctttgttccacgaactctcaacttcaaaactcgWccaggataaatcatctgcttatgaatca[G/A]ttcggcgcggagggagtaaatgagcaaatatccagaggaggtaccagcgacgatgacgcggatgtcgtcaaagRccaaccactcgacagcggagatgaaY

fava_c19183_1481	fava_c19183	1481	C/T	RttcggcgcggagggagtaaatgagcaaatatccagaggaggtaccagcgacgatgacgcggatgtcgtcaaagRccaaccactcgacagcggagatgaa[C/T]tcggcatcaatgggggacaaatcgggtcggatcctgagtggagattggtgggaatcggaccatgagaggaggaggatggtggagcggttggcgaggagga

fava_c19183_276	fava_c19183	276	T/A	cagtcaagatcattttactgcataaaattaaaatcgacatctgtaccgtaatcgcatatcaatggataaaattcattacagtgaatcagtgtgatttccc[T/A]tcaYgRgggaatcctagtctgtttggYctcgcRgggaaacatatatgaaatcctatcaaaactacaagttccgatttaaaactgatatctgaccagagtc

fava_c19183_280	fava_c19183	280	C/T	caagatcattttactgcataaaattaaaatcgacatctgtaccgtaatcgcatatcaatggataaaattcattacagtgaatcagtgtgatttcccWtca[C/T]gRgggaatcctagtctgtttggYctcgcRgggaaacatatatgaaatcctatcaaaactacaagttccgatttaaaactgatatctgaccagagtcccca

fava_c19183_282	fava_c19183	282	A/G	agatcattttactgcataaaattaaaatcgacatctgtaccgtaatcgcatatcaatggataaaattcattacagtgaatcagtgtgatttcccWtcaYg[A/G]gggaatcctagtctgtttggYctcgcRgggaaacatatatgaaatcctatcaaaactacaagttccgatttaaaactgatatctgaccagagtccccatt

fava_c19183_303	fava_c19183	303	T/C	ttaaaatcgacatctgtaccgtaatcgcatatcaatggataaaattcattacagtgaatcagtgtgatttcccWtcaYgRgggaatcctagtctgtttgg[T/C]ctcgcRgggaaacatatatgaaatcctatcaaaactacaagttccgatttaaaactgatatctgaccagagtccccatttaacaagaaaacttctagagg

fava_c19183_309	fava_c19183	309	A/G	tcgacatctgtaccgtaatcgcatatcaatggataaaattcattacagtgaatcagtgtgatttcccWtcaYgRgggaatcctagtctgtttggYctcgc[A/G]gggaaacatatatgaaatcctatcaaaactacaagttccgatttaaaactgatatctgaccagagtccccatttaacaagaaaacttctagagggacata

fava_c19183_542	fava_c19183	542	A/T	agggttgcaacattttgctaccttttgcacatgtaattgttctaagacgtggtccagttctcatttgccatatctcaataattccttttctgggagcatg[A/T]atagccaaacacaagcagtaatcactcttcattggttcataataagtggagcttgatgagactgtatctttgtttaccaacatctccatgaataaacagc

fava_c19191_1243	fava_c19191	1243	T/C	ggtgagagcatttatctggatgaactaatagtgatatcttccagtactttttctttatgttaccatcagacatattatggttaactcctagaacatcgta[T/C]gggctgtcgatctcaacgtccattatcctggtaacctcctcaaaacgttctgcctcgtttgctgattctgcttcagaaaccatagcaggaggtggaggtc

fava_c19191_1492	fava_c19191	1492	G/A	tctgtgcctctgttaacttagcagcagcagcgagtaattcagcagatggcattgcaggaccaataaccctttttctaggaccaacagaatgatcctcggg[G/A]ggttttttYgaaccgtcgtctcccatctgttctgtcggaaccgaacatgtttcaggcattgcagcggaaggatcagcttgatcattcccttgatgcataa

fava_c19191_1501	fava_c19191	1501	C/T	ctgttaacttagcagcagcagcgagtaattcagcagatggcattgcaggaccaataaccctttttctaggaccaacagaatgatcctcgggRggtttttt[C/T]gaaccgtcgtctcccatctgttctgtcggaaccgaacatgtttcaggcattgcagcggaaggatcagcttgatcattcccttgatgcataagagattgaa

fava_c19191_1632	fava_c19191	1632	G/A	accgaacatgtttcaggcattgcagcggaaggatcagcttgatcattcccttgatgcataagagattgaatcagagggccaacaacatctaaagtagggg[G/A]agctttagaaggcagtaaaaaaactctatccccattttcctttagattcaaggaaagaaagagattcttcaaatgcttcactagagacttttctgatata

fava_c19191_262	fava_c19191	262	C/T	tacatgtaatactaccatttttacactttaaagtgagattttcagtttatagcagttcaatcctatgtagaggagggaatcagcttatgatagaattaat[C/T]tagaagataaaataaaaaacaatatacatgactgagaaaatataaatctggaaaatgaataaatcagtttctctaaaaaactcacttgacatatctttga

fava_c19193_747	fava_c19193	747	A/G	gaagaggaaaacaacatagaagatccaaaagctgatcactgtctataggttcatcaatgcacgacaagcgttgccatgcttcagcagaaaaacgtgcgac[A/G]cgaacaacaacgtgagtgttgaacaccattttctgtcggatgagtttgatttcctcacacgaatattgtttgtgaatctatctgatatctcgtcgtcatc

fava_c19194_1306	fava_c19194	1306	T/C	ggaaagaaggatttggataatagctacaataaggattttgaatttgattagtactttgttttaaaccaaaaggaaaagtgatatcaatatcaccgcatga[T/C]aaggtgttgcatattgtttgtttggctttagaagtttgaaggtggagaataatgaagatgtaaaatataagaggtgttagagttgaagaagccattagta

fava_c19194_197	fava_c19194	197	T/A	ctacattaaatacattaaatatatacgacttgaagcatgtctaattcatcgtgaagatgtcaatggtgaagagtttggggataataacaatgaagaaaaa[T/A]aaggagtcgtRgaagaatatgcctctggtgaRttcctacatatagggcaagtgccattcattttaagccactcatcaatacaagcagcatgaaaataatg

fava_c19194_208	fava_c19194	208	A/G	acattaaatatatacgacttgaagcatgtctaattcatcgtgaagatgtcaatggtgaagagtttggggataataacaatgaagaaaaaWaaggagtcgt[A/G]gaagaatatgcctctggtgaRttcctacatatagggcaagtgccattcattttaagccactcatcaatacaagcagcatgaaaataatggttgcattcag

fava_c19194_229	fava_c19194	229	A/G	aagcatgtctaattcatcgtgaagatgtcaatggtgaagagtttggggataataacaatgaagaaaaaWaaggagtcgtRgaagaatatgcctctggtga[A/G]ttcctacatatagggcaagtgccattcattttaagccactcatcaatacaagcagcatgaaaataatggttgcattcagggatacttctcaatgtctctt

fava_c19194_482	fava_c19194	482	C/T	caaacgtccactctctccaatgagaattttaggatacttctcaatagttgcaccatcgaggccagttacaaaagaaggaagtggctctaaggaaattgta[C/T]ttgaaggatgatttgagcttctttgattatgaggtgagattctcctcatcttaccacgtataaagcagaaaattataactagacatagaagtcctggtag

fava_c19194_687	fava_c19194	687	C/T	aatcctattgatatggagtatttggcacttgtagacaaacctctattggaaggattagggaagcattcaacttgaagaccatttatcttatcatcagact[C/T]agagaatccacaaacttgaccattaagtgcacatttctcacatccaggtttgttccaaaccaactcaacatcacgattaagatcaacccaaaaaggccac

fava_c19197_1518	fava_c19197	1518	G/A	tgtgcggtagattgtgctccccaagctggaaatcatgctgcaggaaaagtggatgccaaactcattctgtaacctgtggagctggtactggagtgtttct[G/A]ttaattaagagaactacaatcctactacagagatctgcacgtcaggccccttggccttctccttacttggatacttttggtgaggaggattttgaaatga

fava_c19197_1898	fava_c19197	1898	C/T	tgctaatttatcatgttctacacttccccctggctaatcaaaagagctttagtccatgctatcatgtctctgtggaaattgtacagcactttcagaatga[C/T]ttacttggtgcataaagctttagatcttagctcgtgtggggatgatctacctgctggcagttgctgtacagaatttccatgctaaaagaattgcaaRctt

fava_c19197_1995	fava_c19197	1995	G/A	tgaYttacttggtgcataaagctttagatcttagctcgtgtggggatgatctacctgctggcagttgctgtacagaatttccatgctaaaagaattgcaa[G/A]cttaattgtgcttcatgcttagtatgtagatctttattaaattgatgtacatatgtgtacatatatcatcagactataatgtataatgtggaattacgag

fava_c19199_1079	fava_c19199	1079	G/A	aaataagggagatcatgagaagtcttccagagaagcatcaaacactgctgtttagtgcaacaatgcccgtggagatcgaagcattagcaaaggagtactt[G/A]gctaacccagttcaagttaaggtgggaaaagtgagcagcccaacaactaatgtttcacaaactcttgtaaaggtttctgaaagtgagaagattgaccgac

fava_c19199_821	fava_c19199	821	G/A	ctagagaacttgctcaacaaatagaaaaagaggtccaagcatttagcagatctctcgagtcattaaaaactgccatagttgttggtggaactaacattga[G/A]aagcagaggtctgaactaagagcaggagttgaaatagcagttgccacccctggaagattcattgaccacttRcaacaaggcaacacttccctctctagaa

fava_c19199_893	fava_c19199	893	A/G	ccatagttgttggtggaactaacattgaRaagcagaggtctgaactaagagcaggagttgaaatagcagttgccacccctggaagattcattgaccactt[A/G]caacaaggcaacacttccctctctagaatttcttatgttgttctagacgaggcagatagaatgcttgatatggggtttgaaccacaaataagggagatca

fava_c19202_276	fava_c19202	276	C/A	taagtaattaagtacaagatatcatgtaaaattatcgaataaaatgcgaatcagagaagatgttgccagaaaattaacacacaaacggagggggactcag[C/A]tgtggccggaatcagYggaaatgaagggaggcgacgcagttcgtcRgaaaatcgtaaagaggaggcggggggaatttccatggtggccgaaggcggctag

fava_c19202_292	fava_c19202	292	T/C	agatatcatgtaaaattatcgaataaaatgcgaatcagagaagatgttgccagaaaattaacacacaaacggagggggactcagMtgtggccggaatcag[T/C]ggaaatgaagggaggcgacgcagttcgtcRgaaaatcgtaaagaggaggcggggggaatttccatggtggccgaaggcggctaggcggtggcttcggtca

fava_c19202_322	fava_c19202	322	A/G	cgaatcagagaagatgttgccagaaaattaacacacaaacggagggggactcagMtgtggccggaatcagYggaaatgaagggaggcgacgcagttcgtc[A/G]gaaaatcgtaaagaggaggcggggggaatttccatggtggccgaaggcggctaggcggtggcttcggtcactgtcagttgatcggaggaatatttgcaac

fava_c19205_519	fava_c19205	519	G/A	gcattcgtctatcttccaaagttttgacatattccttctgaaatgccgatggagattcaaggaagagagcatttctgagaccatatatgagaggaacacc[G/A]ggtatctcttgcaacttcttccgtatgtcagcatcttgactagcaacaaagaagtgctcggaattgttttctccgacaacctccatgatgcatagatcgc

fava_c19205_685	fava_c19205	685	G/A	gttttctccgacaacctccatgatgcatagatcgcccctcacacatttttcatgctcacatcttgcaactataagattctgagctgccgcaagagcctcc[G/A]aatacgatttaccaagccgtttcaactcagctagaacacatcttgttgtgtagagcttgacagtagaactaagaatactggcaagagccgtatcagcggg

fava_c19211_1395	fava_c19211	1395	T/C	tcataaaactcatcatcggcatcatttagactaacaaggcctacatgttccgattgtgttggatctacttcttcaattgagttatcaggatttctctcat[T/C]tgcctgtatttgggtatcggaattggcatattcttccttcagactattccttctgtttcttgtcaactctcccgatgaataatcagacaaagtgcaattc

fava_c19211_323	fava_c19211	323	A/T	cagaaccaacctattcatcctaacattgctcacagcaccataaactgctgcgtgattcaaataaaagattgaacaaatgatgatatttctgaaagcaggt[A/T]tacggagacatgaatctagaatttcaatttcagttatctacatttgcaacttggatttcccttttcaaaatcaaggcattagacaaactgatttggaggc

fava_c19213_1082	fava_c19213	1082	C/T	cagaaactgttgcatcagcaatggaatggtctatgtcggagttactaagaaatccagaagaactcaagcgcgtgcaacaagaactcgccgccgtggttgg[C/T]ctagaccgacgcgtggaagaatcagacatagagaaattgacatacctaaaatgcatcgtcaaggagacgcttcgtcttcacccgcccattccgctactcc

fava_c19213_1364	fava_c19213	1364	T/C	ttggaagagatggactttcatggaaggattcagaagagtttcagccttcaagatttttaaattcaagtgcgcctgattttaaagggagtaattttgagtt[T/C]attccgtttggatcgggtcgaagatcttgccccggaattcaattgggcttgtatgcattggacttggcgttggcccatttacttcattgctttatttggg

fava_c19213_422	fava_c19213	422	G/A	tacagctgaatgacaatatcttctcaaatcgcccagcgacgatagccataagctatctcacttataatcgagcggacatggcgttcgctcactacggtcc[G/A]ttttggcgccagatgaggaagatttgtgtcatgaaactcttcagccgtaaacgcgcggagtcgtgggaatctgttagagatgaagtggacctcgttatca

fava_c19213_541	fava_c19213	541	A/T	aagatttgtgtcatgaaactcttcagccgtaaacgcgcggagtcgtgggaatctgttagagatgaagtggacctcgttatcaccaacgttaacgataatc[A/T]gggaaaatctgttaacgttggtgagttggtttttaatttaacaaagaatattatctatcgtgcggcttttgggtcgagttcaaaRgaaggacaagatgag

fava_c19213_626	fava_c19213	626	A/G	acgttaacgataatcWgggaaaatctgttaacgttggtgagttggtttttaatttaacaaagaatattatctatcgtgcggcttttgggtcgagttcaaa[A/G]gaaggacaagatgagtttatttctatacttcaagagttttcgaaattgtttggtgcttttaatattgcggattttgtaccttgtttgaggtgggttgatc

fava_c19213_774	fava_c19213	774	G/C	gtttggtgcttttaatattgcggattttgtaccttgtttgaggtgggttgatcctcaaggtttgaatgctaggcttgttaaggctcgtggtgcgttggat[G/C]gctttattgataaaatttttgatgaacatgttgagaagaagaagaggaatatcagtggtgatgaagatagtgatatggttgatgaattattggcttttta

fava_c19213_935	fava_c19213	935	C/T	atgaagatagtgatatggttgatgaattattggctttttacagtgatgaggctatagtgaataatgaatcggatgatcttcataactctatcaaactcac[C/T]agggataacatcaaagccatcatcatggatgtcatgttcggaggaacagaaactgttgcatcagcaatggaatggtctatgtcggagttactaagaaatc

fava_c19214_1202	fava_c19214	1202	A/G	ttcttaggtctggtatcaacaacatcttcccggaaccgtttgccacttccggaagcatcatcgggcttcaagaaccctcccgcaccctggtccaagctca[A/G]gcccaaatggtagaccggtgcatgaaatcccgctccgggagcaagatgactagcaccgtcgccggagttaagctgcagcatcatcggagaagctaaYgaa

fava_c19214_1299	fava_c19214	1299	T/C	tcaRgcccaaatggtagaccggtgcatgaaatcccgctccgggagcaagatgactagcaccgtcgccggagttaagctgcagcatcatcggagaagctaa[T/C]gaagaagcgtcggttccgtcggcggaggtgaaattcgggagRccgagaatttgctccaggaaatcgtctgcgggggtatcggagggatgattcgacatga

fava_c19214_1341	fava_c19214	1341	G/A	gagcaagatgactagcaccgtcgccggagttaagctgcagcatcatcggagaagctaaYgaagaagcgtcggttccgtcggcggaggtgaaattcgggag[G/A]ccgagaatttgctccaggaaatcgtctgcgggggtatcggagggatgattcgacatgactcggtttggactcggttgagtttggaaaacgagttgagata

fava_c19214_374	fava_c19214	374	T/A	ttatacaaaaaggaaaagcatatcaaatgaactttgacaagttttttccctttacaataaattttctaagacaagataaggtctgcttgcatttttaggt[T/A]tcacagtaaaccctgtattgacaaaagctaaaacaacctcaaaaggaaaggactatggcatcactgactaaaactaaggagattaggaacatgcaaataa

fava_c19214_556	fava_c19214	556	G/A	attaggaacatgcaaataaattgtgagtcccagaagtaatatgggtgcgcttgatgggaccactggatattaccgaaggtatgtctaagaaggaggagta[G/A]tttcgggcttaactatactggagttgtctggtggttgtgattggtatattgcggatgccagtgagattggcatgatRcagagtgccttggattgaagaaa

fava_c19214_633	fava_c19214	633	G/A	ggtatgtctaagaaggaggagtaRtttcgggcttaactatactggagttgtctggtggttgtgattggtatattgcggatgccagtgagattggcatgat[G/A]cagagtgccttggattgaagaaattgcatcgcagccccaacattttcttccatgagcttaactacttgcttttctgtgccatcattagaccacttctccc

fava_c19214_846	fava_c19214	846	T/A	ttctcccactctcacttccttcttcctcgactgacgataatgggatgtcagttactagtggtgccaccgcaccggctccacccaatctactcatgctcaa[T/A]accttcacttgaagccttaagaatttgacataatccacaatttcgtcgagcattgcagctcgatctgtcttgttgacactgggaacaagttcttgcaaag

fava_c19215_1054	fava_c19215	1054	A/C	tagtttgttatggattggtggtgctatgKagttacgtgataatgacggttttttattgtgagtgtagaaaacggcattcaattagggaatttcatcaaat[A/C]gatgaagaagttcaagatcaagaaactcaactttctgtactatagcaatgtactatttttgttgttgtacttgttgtcttgttctttctttttgtaagaa

fava_c19215_317	fava_c19215	317	T/C	cgccgccaccatctggcgctgcttctccccattcaagattctcttcacttccctcacccttctatccaacaacaaactcctcttcacaaccctcttcatc[T/C]tcaccatcctccctctctcaaccctcactatccaccaatccatcttcacacaaccactcacctctcaaatccgccacctcgaagccttgtctcacttcgc

fava_c19215_607	fava_c19215	607	C/T	cttcttacatcatctccctcatctccactctctccgccgtacactcctctctcctcgccgcccatggtatcaccccgacactccactcggccgtcgcctc[C/T]ttcaaacccaacatcatgcgactcctcgtaacttccatctttgtctacgccatcctctttgtgttttcctctttgcctctcttctcaaccgcactcgggt

fava_c19215_787	fava_c19215	787	C/T	tcttctcaaccgcactcgggtcgaatactttcgtcttctttatcgggtcgggtctcgaggtttacttaatggcggtgttgggcgtcggtctggtggtgtc[C/T]gtggcSgaagagagattcggatgggatgctattagaacaggatccggtttaatggaagggaaccgggtttgcgggtgggttctttcgggtttatttatgt

fava_c19215_793	fava_c19215	793	C/G	caaccgcactcgggtcgaatactttcgtcttctttatcgggtcgggtctcgaggtttacttaatggcggtgttgggcgtcggtctggtggtgtcYgtggc[C/G]gaagagagattcggatgggatgctattagaacaggatccggtttaatggaagggaaccgggtttgcgggtgggttctttcgggtttatttatgtgggttt

fava_c19215_982	fava_c19215	982	T/G	ttatgtgggtttcgagtttgatcgggtcgaggatagacggtgaggattcgtttgaagataaagcagttgtaatagtttgttatggattggtggtgctatg[T/G]agttacgtgataatgacggttttttattgtgagtgtagaaaacggcattcaattagggaatttcatcaaatMgatgaagaagttcaagatcaagaaactc

fava_c19237_1012	fava_c19237	1012	G/C	ttttgtgggagtgaatatacaaatttYggttggtatttaggatcgtcccaaattatattgtggcctcggacatcaatgttgtgattttttgcaaattgta[G/C]cattccatcggcatcRttgtagttttccttaccttgttcatattcatttgtgtaccacttcatttcgttttcaaatgtggtaacggtaaacctagatgca

fava_c19237_1028	fava_c19237	1028	G/A	atacaaatttYggttggtatttaggatcgtcccaaattatattgtggcctcggacatcaatgttgtgattttttgcaaattgtaScattccatcggcatc[G/A]ttgtagttttccttaccttgttcatattcatttgtgtaccacttcatttcgttttcaaatgtggtaacggtaaacctagatgcaaaccaatcttgRtatc

fava_c19237_1124	fava_c19237	1124	G/A	catcRttgtagttttccttaccttgttcatattcatttgtgtaccacttcatttcgttttcaaatgtggtaacggtaaacctagatgcaaaccaatcttg[G/A]tatcctttattgttgaggatgtttttgttcattgaacatccaaatggaaaacctcctctattcaatgtaacagatatacttgcatttgataaagggtttc

fava_c19237_1544	fava_c19237	1544	G/A	cataaattgcaccaccaaacttatatgctttacttgttttcaccaatgctgttaccgcaacattttcctccgtcgttcgaatccaagctgataaagtata[G/A]tgtaatccctttcttagataaatcttctgggagacactatcatacggttgttttctgctatRcgctaccaaaaatttgttgcctaatgattctcgatgtt

fava_c19237_1606	fava_c19237	1606	A/G	ttttcctccgtcgttcgaatccaagctgataaagtataRtgtaatccctttcttagataaatcttctgggagacactatcatacggttgttttctgctat[A/G]cgctaccaaaaatttgttgcctaatgattctcgatgttcaatctttgcttccccaaacgttgtccaaccttgtaatccatcattgagttgtggattttga

fava_c19237_1709	fava_c19237	1709	T/G	ctaccaaaaatttgttgcctaatgattctcgatgttcaatctttgcttccccaaacgttgtccaaccttgtaatccatcattgagttgtggattttgaat[T/G]attcctccattatatagaggcttttctggatgtgctaaacattcaatgctggcactgtaatcataggataacgattcagcttcaaaccctgtggtaaata

fava_c19237_878	fava_c19237	878	A/G	tgaaatcatttccaagtttattttcaaagtatgagaaatgcatattttcgttaacaacatcccaaccaataacttgacccttgtatctttgaacaatgga[A/G]tttaccctcttactaactgcagggtaaagctggttttgtgggagtgaatatacaaatttYggttggtatttaggatcgtcccaaattatattgtggcctc

fava_c19237_938	fava_c19237	938	C/T	cccaaccaataacttgacccttgtatctttgaacaatggaRtttaccctcttactaactgcagggtaaagctggttttgtgggagtgaatatacaaattt[C/T]ggttggtatttaggatcgtcccaaattatattgtggcctcggacatcaatgttgtgattttttgcaaattgtaScattccatcggcatcRttgtagtttt

fava_c19238_288	fava_c19238	288	C/T	tttattcaagaccactcttctatccaataactaaaataaagaatttaagttacaatacaggatgttgggatattgctctcgctcttcttatcccaacgaa[C/T]acaggcatgataaccatatataaatgttacactataaactatataacaggcttatccaagtacaagtgaaaattttgaacccccctcccccaaaaaaata

fava_c19239_1002	fava_c19239	1002	T/C	ttgcttatttctttgttaccgagtccccRaggtggctagttatgcagggtcgagagaaagatattttgaaaatgctcaaaagggtttcttcaSaagaaac[T/C]gctcatgaYagtttacctaaaccacYcaccaaagaaaacttttcaatttttcagctttactcatcggtaggagagttgtttcataaaagatgggctgtta

fava_c19239_1011	fava_c19239	1011	T/C	tctttgttaccgagtccccRaggtggctagttatgcagggtcgagagaaagatattttgaaaatgctcaaaagggtttcttcaSaagaaacYgctcatga[T/C]agtttacctaaaccacYcaccaaagaaaacttttcaatttttcagctttactcatcggtaggagagttgtttcataaaagatgggctgttataagaatgg

fava_c19239_1028	fava_c19239	1028	C/T	ccRaggtggctagttatgcagggtcgagagaaagatattttgaaaatgctcaaaagggtttcttcaSaagaaacYgctcatgaYagtttacctaaaccac[C/T]caccaaagaaaacttttcaatttttcagctttactcatcggtaggagagttgtttcataaaagatgggctgttataagaatggtagccgtcatgattctt

fava_c19239_1143	fava_c19239	1143	G/A	tttcaatttttcagctttactcatcggtaggagagttgtttcataaaagatgggctgttataagaatggtagccgtcatgattctttcttttgggattgg[G/A]atggtttattttggtatgccactagcYgttggaaacttgggattcaacatttacttgtcagtggttttcagtgcttccatggaatttccatcctacgtag

fava_c19239_1170	fava_c19239	1170	T/C	taggagagttgtttcataaaagatgggctgttataagaatggtagccgtcatgattctttcttttgggattggRatggtttattttggtatgccactagc[T/C]gttggaaacttgggattcaacatttacttgtcagtggttttcagtgcttccatggaatttccatcctacgtagcaacatatttcttagaaaactataaaa

fava_c19239_1444	fava_c19239	1444	A/T	agtaccggtagctaaagtggtgttagcaatggttgcattctttgggtgttgtacagcttataatgtgttcctgatatacactatagagctatttccgaca[A/T]gtgttcggaacacaacaacatccttggtgagacaagctattatgtttggctgcatattctgtccatttctgatatctgctgggaggaagaacaatatatt

fava_c19239_1586	fava_c19239	1586	T/C	atgtttggctgcatattctgtccatttctgatatctgctgggaggaagaacaatatattctcttatggcgtgtttggagttgttataatgttatccaata[T/C]tacattgttgtatttgcctgagactatagggattgttctttgtgatactatggaacaacaagagaagaaagaaatagctctgtctcatgccttgaatcaa

fava_c19239_672	fava_c19239	672	C/T	atacatctgttggtagaaaaaacatgttaatattttcttgtgtctctatgtccataacatccatgctcataatcttctccaccaacgtttggatttactc[C/T]gccttgaaattcttgatcggtttctggcgctcctcgattggaacRtgcgtccttgttttacttacagaaaaagtgagcgctgaatggcgattccgagttg

fava_c19239_717	fava_c19239	717	A/G	ctatgtccataacatccatgctcataatcttctccaccaacgtttggatttactcYgccttgaaattcttgatcggtttctggcgctcctcgattggaac[A/G]tgcgtccttgttttacttacagaaaaagtgagcgctgaatggcgattccgagttggaattgttgagtatttaactttcacaatgggttacatgtctttac

fava_c19239_930	fava_c19239	930	G/A	atataacccgaaactcttcctggaaatctctttacctttcctcctccattcctgctataatttactctctcgttgcttatttctttgttaccgagtcccc[G/A]aggtggctagttatgcagggtcgagagaaagatattttgaaaatgctcaaaagggtttcttcaSaagaaacYgctcatgaYagtttacctaaaccacYca

fava_c19239_994	fava_c19239	994	C/G	ctctctcgttgcttatttctttgttaccgagtccccRaggtggctagttatgcagggtcgagagaaagatattttgaaaatgctcaaaagggtttcttca[C/G]aagaaacYgctcatgaYagtttacctaaaccacYcaccaaagaaaacttttcaatttttcagctttactcatcggtaggagagttgtttcataaaagatg

fava_c19242_682	fava_c19242	682	G/C	aattgatcactttttgtttcgcaaattgaagtttgtgacgggaaagaattgtcttcaaactctcgacatgcacatcaggtataaaatctacaacaagtct[G/C]cctttgtaacccgaactaaacagaaagcgagcaccccaagcagacaaacgaagaatagccggtccactgagtccccagtgagtaactaacatcggaccga

fava_c19243_343	fava_c19243	343	A/G	aataaccattacaaaactcaggctgtatgcataaaattcagaaactcgtacgagaaaatttttctcccttcctatcagttgtgtgtccttcttacagtga[A/G]gttatgataacagcttccgaaggaagcacacactactagggggYtcaaccaaatggggaaggaactgggccaactgacccagtgagtgtatctgatcttt

fava_c19243_387	fava_c19243	387	C/T	ctcgtacgagaaaatttttctcccttcctatcagttgtgtgtccttcttacagtgaRgttatgataacagcttccgaaggaagcacacactactaggggg[C/T]tcaaccaaatggggaaggaactgggccaactgacccagtgagtgtatctgatcttttgatgcttcccattccacagattaaacatccaggagcattcaca

fava_c19243_529	fava_c19243	529	G/A	gtgtatctgatcttttgatgcttcccattccacagattaaacatccaggagcattcacagctgaaaatttggatccacatagatcacatacatcatatcc[G/A]atggttgacagtctgcttaatgtggcagcacagaactgcgaaggatcttcctgtggatcaatggatctgttagtcaaacctctctgaacacacaagttca

fava_c19243_972	fava_c19243	972	C/T	agaagagtgactgttatcttgtattgagcacagattgttgcttgagcttttatatcacttccgcgagattgttccttagctaatgcaagaaaagcttcat[C/T]aaagcaagacaaagcatctggaagattattgagctcaaggtgagcaagtccagtcttgaaacatacagaggcagcagcctcttgaggaacaaaatcttca

fava_c19246_1209	fava_c19246	1209	A/G	ggaaaactcgctttgagagacatttaaaaggggaaggaatggagtgcggacgtgtgcaggatgaggatacatccgatggtggattgtctcgagataactc[A/G]acggctgggattgcgtgagagcatagtgatagatcaaaatctgagagcatgatgtgaccgtctgatctgactaacacgttttcaggttttagatctctgt

fava_c19246_776	fava_c19246	776	G/T	acctccggcggcgttaccatccgtatcaacgccaaattaagcccaacaaaaaacggatgcatcttaacgtccgccgcgccacgctttgacccgagtctac[G/T]attcgggtctttgttaagcaaacccgaaataaggtcccgcgcgtgcatttcaagcgcactggaaggcgtagcagtgggaaaactaagcggYttttttata

fava_c19246_867	fava_c19246	867	C/T	cgagtctacKattcgggtctttgttaagcaaacccgaaataaggtcccgcgcgtgcatttcaagcgcactggaaggcgtagcagtgggaaaactaagcgg[C/T]ttttttataatgttgcgcaacgttacttcattcgaYggacccgcaaacggtgttcttccgtacaccatctcgtaKatgaatatcccaaacgaccaccaat

fava_c19246_903	fava_c19246	903	T/C	aaataaggtcccgcgcgtgcatttcaagcgcactggaaggcgtagcagtgggaaaactaagcggYttttttataatgttgcgcaacgttacttcattcga[T/C]ggacccgcaaacggtgttcttccgtacaccatctcgtaKatgaatatcccaaacgaccaccaatccacagcgttaccgtgggagtttccagaagctactt

fava_c19246_942	fava_c19246	942	G/T	gcgtagcagtgggaaaactaagcggYttttttataatgttgcgcaacgttacttcattcgaYggacccgcaaacggtgttcttccgtacaccatctcgta[G/T]atgaatatcccaaacgaccaccaatccacagcgttaccgtgggagtttccagaagctacttctggtgaaacgtactcgtgtgttccgacgaatgaacaag

fava_c19247_127	fava_c19247	127	C/G	agttaggcaagtggaccaactttcattgtcaatagagcttactgtgtccaagactcttagtaccagatgtaaattatgtacaagacactgaaatcaagaa[C/G]aatatacacactaataattactttgataatgtattaatacatatttgcagatgcctctaatctgaaactatgctgcacaagctaaaactagctgttttgc

fava_c19247_340	fava_c19247	340	T/C	ttttctgtggttaaatatacagttagtacggttagtaaattgaagccatttagaagattgaatgactcgtattttgagttggtgttctaagtcccatttg[T/C]taaattgtaatatgagtgacttaacacggcttcaatttacgaaccaggatgatgactgccacaggatgatcctttttgctagaaaaatgcgtaagcaagt

fava_c19247_558	fava_c19247	558	T/C	atcatgttgctagctgtctttgttgtgtttggcttgaagatgaatttgtgttgagatctgacacggcaaagcaatcactggtatcagaaaatggatctag[T/C]tttcttattctctgtgctcgaggatgctgaatgattaccagaacctcatttttggagtgttcatcatgagaagtggaagttggcaacccaatgtgccatt

fava_c19247_726	fava_c19247	726	C/T	gagaagtggaagttggcaacccaatgtgccattcatgactactaaattctttaaatgttgctgcatccatttcaatactatagatgggaggcactagagt[C/T]tcgcctttagactttgtggccggaataacagagaagtgtggtgctgaatcttgtgatctgaaaatccatccgatgtacacaaagatgcaaaattgtgccc

fava_c19248_409	fava_c19248	409	A/G	ggctgataaaactacaaatggaaagaactatgctacataattaataccatgaatctcatcgttgggcctgatcagcagtgtggttgctagttgcagacga[A/G]gatggtgccttaccattcaagactccgttgtccataggaggagaagatccccacttaccctctgattcacttggttctatcactttgacagacccatcac

fava_c19248_589	fava_c19248	589	A/T	tcactttgacagacccatcactcaacccaacagcaaactggttaggttgaagtggatgagccgcaatcacaagaggatacacagcttggcttctatttaa[A/T]gcggctgatgacaagcatattgatggagcaatacgacatcttagtttcaagctctccgcatcaaaaaccccaatgtttgcatcacagaatgaagcaaata

fava_c19262_1049	fava_c19262	1049	C/G	gcagaggttgaggcgttaaaggctataccgcttgaaattgtcaaagaagcgtttgagttatgaatagattgaaaacagaatcaaagtcagacataggtta[C/G]ataatKactggagtgtttatggtggctctatggtgaaaccgatgatgaagagtttgggaYgaaggggaaaaacatcgcaaaggaatagatgacgctgaca

fava_c19262_1055	fava_c19262	1055	T/G	gttgaggcgttaaaggctataccgcttgaaattgtcaaagaagcgtttgagttatgaatagattgaaaacagaatcaaagtcagacataggttaSataat[T/G]actggagtgtttatggtggctctatggtgaaaccgatgatgaagagtttgggaYgaaggggaaaaacatcgcaaaggaatagatgacgctgacacagagg

fava_c19262_1109	fava_c19262	1109	T/C	tgaatagattgaaaacagaatcaaagtcagacataggttaSataatKactggagtgtttatggtggctctatggtgaaaccgatgatgaagagtttggga[T/C]gaaggggaaaaacatcgcaaaggaatagatgacgctgacacagaggagttagtcgatcgaacctgatgaagcttgtcggagtagagaatgggttaacggc

fava_c19262_917	fava_c19262	917	T/G	tctctatcccaatcgataccgatgatgtcgcgtatcagattttctcttgaatttctttggatttcatgaagaacgcttcttcggttgaaggatcggaaca[T/G]cgacgagtcttccttgaatcatgaacagacggcagaggttgaggcgttaaaggctataccgcttgaaattgtcaaagaagcgtttgagttatgaatagat

fava_c19266_229	fava_c19266	229	T/A	aaaacgtgatattgttgtggaggttacggaacgttaatcaacaacaacgtttgtattcttgttgcatctacgctacttcatgtttgttgtgttttgattt[T/A]ttttattttatttgggtgattccagagtcgaatggaattggatctattagctcagatcaattcattcttgcctgtttttgattggtttgaatgtttcatg

fava_c19266_338	fava_c19266	338	A/G	tatttgggtgattccagagtcgaatggaattggatctattagctcagatcaattcattcttgcctgtttttgattggtttgaatgtttcatgtgggaaaa[A/G]ttgaaattttcatgattcaaattttgtgcccacctagtgtttgttattgggtctgagtctttttgttagatattggataagtaaagtatcgttttttttt

fava_c19266_735	fava_c19266	735	G/A	cgatatcatcagctgcatcagcttcagcaccagcagcaccagcaccagctccaccacctcctgttgtttctaaggttcagaagaatccttcaacaaataa[G/A]cagagatctgtgtctcctttgcctgaaacctatctccctgaaacctttaaggaagccaagcttgaacagaagaggttctctactccaggtcgtaggagga

fava_c19267_146	fava_c19267	146	C/T	cgttaatttaccgaacataaattaatagtagaaaggaagaacattagcataataaagtgaaagaaaataagaaccacaatcttcttaagacaaatactag[C/T]atatgacatcaaaatggctatgctatacttaaaaagaaactaRagagtataagaccaacattgaatgcagctacacaatattccaatctagttgaactag

fava_c19267_189	fava_c19267	189	G/A	ttagcataataaagtgaaagaaaataagaaccacaatcttcttaagacaaatactagYatatgacatcaaaatggctatgctatacttaaaaagaaacta[G/A]agagtataagaccaacattgaatgcagctacacaatattccaatctagttgaactagaagacttgttgtcatgaaattctcctgaacttggtgttggtgc

fava_c19272_1062	fava_c19272	1062	C/T	tgttggaaagatcttcgatttcgtcgaactcgtggtggtggaactcgattttgggagggttgcgcgatttgagaatggttcgatcgagaaatggtgggag[C/T]tttaagtctaaacctctggctgtttgagcccatgcattaacaaactccatggcagatagtccatctttcatgcaatggagcatgttgagtcctaatgtga

fava_c19272_1285	fava_c19272	1285	C/G	tacctgaacggtcataggaggcatctccaatatactcctagcaccaggaacatcataaaccaatttcccaagcttatcaggatctggttttttcaaatca[C/G]caatctcatctacaccacaatcagcttcagcctcaacaaaaactgcaccttctcctgtattatccactatcaacttcccttctgaacatataatcaactt

fava_c19272_302	fava_c19272	302	G/C	aatttaagaaaacacttctatatatatcttggcaaactgttattacacataaaacatatataggtatttatatatatgtatgcagataatgcagtatatg[G/C]tatatgaaaaattgattttcataccatattaatcaccaattcttcaaaacgttccatagcagaagcaggcaaaccaagaagaacacttataYttttcttt

fava_c19272_394	fava_c19272	394	C/T	agtatatgStatatgaaaaattgattttcataccatattaatcaccaattcttcaaaacgttccatagcagaagcaggcaaaccaagaagaacacttata[C/T]ttttcttttcttgaccatgtgacaagaacaaaacaacttctttatctggcaatgtaacaggaccagaacaaagtggttccccccaaccaaaatctgttgt

fava_c19275_897	fava_c19275	897	C/T	ctttgcttaacaaagattgcgagcttgggcgtggaggatttggtgctgtgtaccaaacagtactaggagacggtcgctcagttgctataaagaaactcac[C/T]gtgtcgagccttgttaagtcacaagaagatttcgagagggaagttaagaaattgggaaaagtcagacaccaaaatcttgttgaactcgaaggctattatt

fava_c19277_1259	fava_c19277	1259	G/A	aagatttgcagcaagcataaacaacaactcttttgttcttcctagaacaacttcattaatgcaagcttattatcaaggaattcctggcgtttttaccaca[G/A]attttcctccggttcctcccatacagttcaattatacaggaaatgttcctaggggtttgtggactcctagaaaaggaactaagttgtttagagtgaaata

fava_c19277_1459	fava_c19277	1459	G/A	atggttcaaatattcaaattgttttgcaggatactagtatagtaacaattgaggatcatcctatgcatgttcatgggttccatttctttgtagttggttc[G/A]ggttttggaaattttaatccaagaacagatccRgcaaggttcaacctggtggatccacccgtgaggaacactattggcacgcctccgggtggatgggtcg

fava_c19277_1492	fava_c19277	1492	G/A	ctagtatagtaacaattgaggatcatcctatgcatgttcatgggttccatttctttgtagttggttcRggttttggaaattttaatccaagaacagatcc[G/A]gcaaggttcaacctggtggatccacccgtgaggaacactattggcacgcctccgggtggatgggtcgccattcgtttcaaggctgataatccaggaattt

fava_c19277_169	fava_c19277	169	A/T	ctatcattttttcctttgcttctgctgctgcagaggatcattaccatcaatttgttattcaaactgcaacggtgaataggctgtgtaaaacacgaaacat[A/T]ctcactgtaaatggacagtttcctggcccaacaattgtagctagagatggagattctatggcgatcaaagtaacaaatgctggaccttacaatatctccc

fava_c19277_745	fava_c19277	745	C/T	gtcaaggaactatacgttttcaagtaaatgcaggagaaacaattctcttaagaatcatcaacagtggactcaatcaagaactcttcttttctattgcaaa[C/T]cacagaatgacagttgttgcaatggatgctgcttacacaaaaccttccacaaccggagtcctcatgatcggaccaggccaaacaatcaacgtcctcgtca

fava_c19279_1091	fava_c19279	1091	C/A	cagtgacagctccaatgagggaaactccagcaatggaattcaagtttggcaactgagacaaaaccacagcagcacatgtaaaaaccaagtaccattccac[C/A]gtggtcattggttttgcagtgcatgaWtctccacatacaacttggtagaatgttcttgctgttgaccctcctatgatgatcaatgtagtgcatgttcctg

fava_c19279_1118	fava_c19279	1118	T/A	cagcaatggaattcaagtttggcaactgagacaaaaccacagcagcacatgtaaaaaccaagtaccattccacMgtggtcattggttttgcagtgcatga[T/A]tctccacatacaacttggtagaatgttcttgctgttgaccctcctatgatgatcaatgtagtgcatgttcctgctgataggtataatattggaaataatg

fava_c19279_1289	fava_c19279	1289	G/A	ctgctgataggtataatattggaaataatgctagtagtttccccagtttctcaccgaatgtggcgaagctgagttgaaggtaacggctgtagcgaacacc[G/A]ttctcSacggattcgtgaagatgtacgaggagccataaggtgtatagttgccatatgaatgctattgttaaaRttattattccccaagtccaaccaagaa

fava_c19279_1295	fava_c19279	1295	C/G	ataggtataatattggaaataatgctagtagtttccccagtttctcaccgaatgtggcgaagctgagttgaaggtaacggctgtagcgaacaccRttctc[C/G]acggattcgtgaagatgtacgaggagccataaggtgtatagttgccatatgaatgctattgttaaaRttattattccccaagtccaaccaagaatagtga

fava_c19279_135	fava_c19279	135	T/C	caattatgctagatacacatattttttcagtttgcttggtgtgtggaggtcaagttacaactacaaactgaggtatacaattttaaaaagacaactataa[T/C]cttcctaatttccttcatacataagtttcatagtaagtcttttcctctttgcttgagactatttagggtcaaaaaagctcacattaacaccagtatcaac

fava_c19279_1362	fava_c19279	1362	G/A	ttgaaggtaacggctgtagcgaacaccRttctcSacggattcgtgaagatgtacgaggagccataaggtgtatagttgccatatgaatgctattgttaaa[G/A]ttattattccccaagtccaaccaagaatagtgaaagcaacaggaagaataagagcttgaataccaatYccagaacaaagagtatgaaaagcagcataata
[truncated: 24,513,911 more chars]
